# Novel Insights into the circRNA-Modulated Developmental Mechanism of Western Honey Bee Larval Guts

**DOI:** 10.3390/insects14110897

**Published:** 2023-11-20

**Authors:** Yiqiong Zhang, Xiaoxue Fan, He Zang, Xiaoyu Liu, Peilin Feng, Daoyou Ye, Leran Zhu, Ying Wu, Haibin Jiang, Dafu Chen, Rui Guo

**Affiliations:** 1 College of Animal Sciences (College of Bee Science), Fujian Agriculture and Forestry University, Fuzhou 350002, China; zhangyiqiong1121@163.com (Y.Z.); imfanxx@163.com (X.F.); zanghe321@163.com (H.Z.); liuxiaoyu2000@163.com (X.L.); fengwangman@163.com (P.F.); 18080164357@163.com (D.Y.); leran2001@163.com (L.Z.); dfchen826@fafu.edu.cn (D.C.); 2 National & Local United Engineering Laboratory of Natural Biotoxin, Fuzhou 350002, China; 3 Apitherapy Research Institute of Fujian Province, Fuzhou 350002, China; 4 Jilin Institute of Apicultural Research, Jilin 132013, China; wy569703@163.com (Y.W.); jhb18047513706@163.com (H.J.)

**Keywords:** honey bee, *Apis mellifera*, larva, gut, CircRNA, regulatory mechanism

## Abstract

**Simple Summary:**

Current understanding of the regulatory part of circular RNAs (circRNAs) in the honey bee gut development is very limited. Here, 1728 circRNAs were detected in the larval guts of *Apis mellifera* workers. Seven among the most highly expressed 10 circRNAs were common among the 4-, 5-, and 6-day-old larval guts (Am4, Am5, and Am6 groups). Overall, 43 and 73 differentially expressed circRNAs (DEcircRNAs) were detected in the Am4 vs. Am5 and Am5 vs. Am6 comparison groups, respectively. These DEcircRNA parental genes were associated with an array of functional terms and pathways relevant to growth and development such as biological regulation and metabolic process. Novel_circ_000838 in the Am4 vs. Am5 comparison group potentially targeted ame-miR-6000a-3p, further targeting 518 mRNAs involved in 31 functional terms and 38 pathways, including five developmental signaling pathways and five immune pathways. The back-splicing sites and expression trends of six circRNAs were verified using molecular approaches. These results provide novel insights into the circRNA-modulated developmental mechanism of *A. mellifera* worker larval guts.

**Abstract:**

Circular RNAs (circRNAs) are a class of novel non-coding RNAs (ncRNAs) that play essential roles in the development and growth of vertebrates through multiple manners. However, the mechanism by which circRNAs modulate the honey bee gut development is currently poorly understood. Utilizing the transcriptome data we obtained earlier, the highly expressed circRNAs in the *Apis mellifera* worker 4-, 5-, and 6-day-old larval guts were analyzed, which was followed by an in-depth investigation of the expression pattern of circRNAs during the process of larval guts development and the potential regulatory roles of differentially expressed circRNAs (DEcircRNAs). In total, 1728 expressed circRNAs were detected in the *A. mellifera* larval guts. Among the most highly expressed 10 circRNAs, seven (novel_circ_000069, novel_circ_000027, novel_circ_000438, etc.) were shared by the 4-, 5-, and 6-day-old larval guts. In addition, 21 (46) up-regulated and 22 (27) down-regulated circRNAs were, respectively, screened in the Am4 vs. Am5 (Am5 vs. Am6) comparison groups. Additionally, nine DEcircRNAs, such as novel_circ_000340, novel_circ_000758 and novel_circ_001116, were shared by these two comparison groups. These DEcircRNAs were predicted to be transcribed from 14 and 29 parental genes; these were respectively annotated to 15 and 22 GO terms such as biological regulation and catalytic activity as well as 16 and 21 KEGG pathways such as dorsoventral axis formation and apoptosis. Moreover, a complicated competing endogenous RNA (ceRNA) network was observed; novel_circ_000838 in the Am4 vs. Am5 comparison group potentially targeted ame-miR-6000a-3p, further targeting 518 mRNAs engaged in several developmental signaling pathways (e.g., TGF-beta, hedgehog, and wnt signaling pathway) and immune pathways (e.g., phagosome, lysosome, and MAPK signaling pathway). The results demonstrated that the novel_circ_000838-ame-miR-6000a-3p axis may plays a critical regulatory part in the larval gut development and immunity. Furthermore, back-splicing sites of six randomly selected DEcircRNAs were amplified and verified by PCR; an RT-qPCR assay of these six DEcircRNAs confirmed the reliability of the used high-throughput sequencing data. Our findings provide a novel insight into the honey bee gut development and pave a way for illustration of the circRNA-modulated developmental mechanisms underlying the *A. mellifera* worker larval guts.

## 1. Introduction

As a representative social insect with crucial ecological, economic and scientific value, the western honey bee (*Apis mellifera*) is widely reared and applied in the apicultural industry in considerable countries around the world [1]. In addition, *A. mellifera* has been applied as a research model for development, social behavior, epigenomics, gene regulation and host–pathogen/parasite interaction [2]. As early as 2006, the first version of *A. mellifera* genome assembly (Amel_4.0) was published by the Honey Bee Genome Sequencing Consortium (2006). Recently, based on PacBio, 10x Chromium, BioNano, and Hi-C [3], Wallberg et al. reconstructed the *A. mellifera* reference genome (Amel_Hav3.1), which lays a solid foundation for dissecting the biology of *A. mellifera* at the molecular level.

Circular RNAs (circRNAs), a type of single-stranded, covalently closed, and endogenous non-coding RNAs (ncRNAs), are capable of producing a closed-loop structure connecting the 5′ and 3′ ends by a non-canonical “reverse splicing” process [4]. This unique structure makes circRNAs more stable than linear RNAs and highly resistant to degradation mediated by the exonuclease RNase R [5,6]. Hereafter, following the revolution and development of RNA sequencing (RNA-seq) technology and related bioinformatics, more and more circRNAs have been discovered in various animals, plants, and microorganisms, such as *Drosophila* [7], *Bombyx mori* [8], *Homo sapiens* [9], *Mus musculus* [10], *Oryza sativa* L. [11], *Triticum aestivum* L. [12], *Ascosphaera apis* [13], and *Nosema ceranae* [14]. CircRNAs have been demonstrated to exert diverse and vital functions in numerous life activities such as gene regulation and development through various manners, including interaction with RNA polymerase II to facilitate the transcription of host genes as well as action modulation of the expression and activity of target miRNAs [15,16,17,18].

The gut tissue of insects is a major tissue for the digestion of foods, absorption of nutrition, and immune defense against various pathogens or parasites [19,20,21]. Both miRNAs and long non-coding RNAs (lncRNAs) have been demonstrated to participate in regulating the growth and development of the insect gut. For example, Foronda et al. [22] reported that miR-305 was involved in adjusting the balance between differentiation and self-renewal of *Drosophila* gut stem cells by regulating Notch and insulin signaling pathways, enabling adaptive homeostasis in the gut to respond to changing environmental conditions. Wang et al. [23] discovered that lncR26319 was able to modulate *Endophilin A* (*EndoA*) through the competitive absorption of miR-2834, thereby increasing the endocytic activity in the vitellogenin (Vg) uptake, which gave rise to the normal progression of *Bombyx mori* egg production. However, little advancements in the regulatory function of circRNAs in the developmental process of insect gut have been achieved.

Accumulating studies have shown that circRNAs were vital regulators in many aspects of honey bees, such as ovary activation and oviposition [24], brain aging and division of labor [25], immune response [26], and host–parasite interaction [27]. Based on deep sequencing and transcriptomic investigation, our group discovered that circRNAs were likely to regulate the responses of both *A. mellifera* and *Apis cerana* to infections by two widespread fungal pathogens including *A. apis* and *N. ceranae* [27,28]. By analyzing the expression profile and modulatory part of circRNAs in the midgut of *Apis cerana* workers, Chen et al. [29] discovered that circRNAs were potentially engaged in modulating the development of *Apis cerana cerana* workers’ midgut tissues through diverse ways like the ceRNA network as well as the regulation of neighboring gene transcription. The developmental stage of honey bee larvae lasts for six days. The gut tissue of adult honey bees can roughly be divided into the foregut, midgut, and hindgut; however, it is hard to precisely distinguish different sections of the honey bee larval gut [30,31]. At present, the modulatory manner and role of circRNAs in the *A. mellifera* larval guts is still completely unknown.

Previously, the worker larval gut samples of *A. m. ligustica*, a subspecies of *A. mellifera* widely used in the beekeeping industry, were prepared followed by the deep sequencing of cDNA libraries [32]. Our data could provide not only a new perspective into the honey bee gut development but also a foundation for elucidating the developmental mechanism of the larval guts.

## 2. Materials and Methods

### 2.1. Bee Larvae

*A. m. ligustica* worker larvae were extracted from three colonies kept in the apiary of College of Animal Sciences (College of Bee Science), Fujian Agriculture and Forestry University, Fuzhou city, China. These colonies with strong populations were free of seven common viruses (BQCV, SBV, KBV, IAPV, CBPV, ABPV, and DWV) and two fungal parasites including *N. ceranae* and *N. apis* [33].

### 2.2. RNA-seq Data Source

The gut tissues of *A. m. ligustica* worker 4-, 5-, and 6-days-old larvae (Am4, Am5 and Am6 groups) were previously dissected using the established method by our team, which was followed by the isolation of total RNA, construction of cDNA libraries, and RNA-seq on a Illumina HiSeq^TM^ 2500 platform [34]. The quality control of raw reads was also previously conducted using fastp (version 0.18.0) software [35]. In brief, the produced raw reads from RNA-seq were aligned to the ribosome database with Bowtie2 software (version 2.2.8) [36], allowing a mismatch rate of 0 to remove the aligned reads. TopHat2 (version 2.0.3.12) [37] was employed to map the remaining reads to the current reference genome of *A. mellifera* (Amel_HAv3.1). After removing the mapped reads, the unmapped reads were then extracted, and the two ends were further intercepted (default 20 bp) to gain anchor reads, which were mapped again to the *A. mellifera* reference genomes. Then, the mapped reads were finally subjected to the find_circ software (version 1.2) [10] for the screening and identification of circRNA. The raw datasets from RNA-seq could be acquired from the NCBI SRA (Sequence Read Archive) database (http://www.ncbi.nlm.nih.gov/sra/ accessed on 17 September 2023) under the SRA number PRJNA406998.

### 2.3. sRNA-seq Data Source

Previously, the gut tissues of *A. m. ligustica* worker 4-, 5- and 6-days-old larvae were prepared followed by library construction and sRNA-seq on an Illumina MiSeq^TM^ platform [38]; strict data quality control of the generated raw data was then performed following procedures: (1) the sRNA-seq-generated clean tags were aligned to the GenBank database and Rfam database (version 11.0) [39] after removing rRNA (ribosomal RNA), small intranuclear RNA (snRNA), transport RNA (tRNA) data, small cytoplasmic RNA (scRNA), and small nucleolar RNA (snoRNA); (2) Bowtie2 software (version 2.2.8) [36] was used to compare the unannotated tags with the sequences annotated in the current reference genome (assembly Amel 4.5), and the location information of related tags on the reference genome was obtained, which was mapped tags. The raw datasets from sRNA-seq were uploaded onto the NCBI SRA database and linked to the SRA number: SRP221700.

### 2.4. Identification and Expression Level Calculation of circRNAs

Find_circ software (version 1.2) was utilized to perform the detection of circRNAs following the method reported by Chen et al. [27] with screening conditions set as follows: (1) breakpoints = 1, only circular RNAs with one clear breakpoint are retained; (2) anchor_overlap ≤ 2, and the overlap of two anchor reads of each read on the genome should not exceed 2 bp; (3) edit ≤ 2, only 2 bp mismatch is allowed; (4) n_uniq > 2, and more than 2 uniq reads; (5) best_qual_A > 35 or best_qual_B > 35, where one of the anchor reads of each read was more than 35 points higher than the second best mapping result; (6) n_uniq > int (samples/2), the number of uniq reads supporting the circRNA was greater than half of the total number of samples; (7) the length of circRNA is less than 100 k. The sequences of the identified circRNAs in the present study are displayed in Table S1.

The expression level of each circRNA was normalized to the mapped back-splicing junction reads per million (RPM) mapped-reads value on basis of the following formula: RPM = E × 10^6^/T (E represents the circRNA counts and T represents the total circRNA counts) [40].

### 2.5. Screening of DEcircRNAs

Following the thresholds of |fold change| ≥ 2 (|FC| ≥ 2), *p* < 0.05, and false discovery rate (FDR) ≤ 1, DEcircRNAs in the Am4 vs. Am5 and Am5 vs. Am6 comparison groups were screened by using the edgeR software (v.4.2) [41]. Venn analysis of the DEcircRNAs was then carried out utilizing the corresponding tool in OmicShare platform (https://www.omicshare.com/ (accessed on 18 July 2023)).

### 2.6. Parental Gene Prediction and Annotation

Anchor reads at both ends of each circRNA were aligned to the current reference genome (Amel_HAv3.1) with the Bowtie 2 software (version 2.2.8) [36], and the same gene aligned to both ends was identified as the parental gene from which the circRNA originated. The parental genes of circRNAs were then annotated to the GO (https://www.geneontology.org accessed on 17 September 2023) and KEGG (https://www.genome.jp/kegg/ accessed on 17 September 2023) databases utilizing the Blast tool, with default parameters, to gain corresponding functional terms and pathways.

### 2.7. Analysis of ceRNA Regulatory Network

Four software, including Mireap, Miranda software (version 3.3a), TargetScan software (www.targetscan.org accessed on 17 September 2023) [42], and miRTarBase sofeware (version 6.1) [43], were employed to predict DEcircRNA-targeted DEmiRNAs and target DEmiRNA-targeted mRNAs on the basis of the standard of *p* ≤ 0.05 and free energy ∆*G* ≤ 35. The DEcircRNA–DEmiRNA–mRNA regulatory networks were constructed followed by visualization using Cytoscape software (version 3.8.2) [44] following default parameters. The target mRNAs were mapped to the GO and KEGG databases to obtain corresponding annotations. The sequences of DEcircRNA-targeted DEmiRNAs and corresponding DEmiRNA-targeted mRNAs are shown in Table S2.

### 2.8. PCR Validation of DEcircRNAs

Six DEcircRNAs, including novel_circ_000027, novel_circ_000231, novel_circ_000438, novel_circ_000653, novel_circ_001617, and novel_circ_001729, were randomly chosen for PCR verification. By utilizing the Primer Premier 5 software, specific divergent primers (Table S3) over the back-splicing sites of these six DEcircRNAs were designed and then synthesized by Sangon Biotech (Shanghai) Co., Ltd. (Shanghai, China). The total RNA was isolated from the 4-, 5-, and 6-day-old larval guts, which was followed by removal of the linear RNAs with RNase R to enrich the circRNAs. Next, the resulting cDNA were gained via reverse transcription with random primers and then as templates for PCR reaction, which was carried out on a T100 thermocycler (Bio-Rad, Hercules, CA, USA). The system and procedure of the PCR reaction were set according to the report by Ye et al. [27]. The amplification products were examined by 1.8% agarose gel electrophoresis using GoldView staining (Accurate, Beijing, China) and detected by a Bio-rad ChemiDoc XRS system (Shanghai Peiqing Science & Technology Co., Ltd., Shanghai, China).

### 2.9. RT-qPCR Detection of DEcircRNAs

The RT-qPCR assays of these 6 DEcircRNAs mentioned above were carried out according to the protocol of the SYBR Green Dye kit (Vazyme). The prepared cDNA in Section 2.8 was utilized as templates for DEcircRNAs and the internal reference *actin* gene (GeneBank accession number: LOC406122). The reaction system and procedure were set following the documentation by Zhu et al. [28]. There were three replicates for each reaction. The relative expression level of each DEcircRNA were calculated with the 2^−ΔΔCt^ method [45]. Student’s *t*-test of the qPCR data was conducted with Graph Prism 7 software [46].

## 3. Results

### 3.1. Highly Expressed circRNAs in the Gut Tissues of A. m. ligustica Worker Larvae

**Figure 1:**
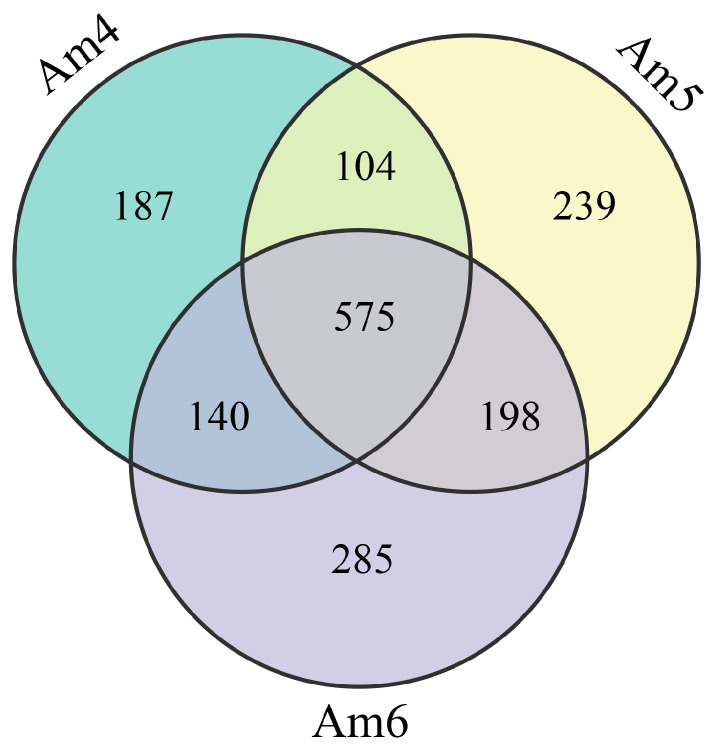
Venn diagram of circRNAs found in the *A. m. ligustica* worker 4-, 5-, and 6-day-old larval gut tissues.

In the 4-day-old larval gut, the top three circRNAs with the highest expression levels were novel_circ_000069 (RPM = 54,161.61966), novel_circ_000027 (RPM = 46,028.72469), and novel_circ_001830 (RPM = 30,974.21699); novel_circ_000069 (RPM = 67,741.05436), novel_circ_000438 (RPM = 50,942.36547), and novel_circ_000115 (RPM = 36,328.87189) were the most highly expressed three circRNA in the 5-day-old larval gut; novel_circ_000069 (RPM = 58,471.61079) was the most up-regulated circRNA in the 6-day-old larval gut, which was followed by novel_circ_000438 (RPM = 56,778.41743) and novel_circ_000115 (RPM = 28,897.16672). Details of the circRNAs identified in the 4-, 5-, and 6-day-old larval guts are shown in Table S4. Venn analysis showed that 575 circRNAs were common among these three groups, while the quantities of unique ones were 1006, 1116, and 1198, respectively (Figure 1). Additionally, among the top 10 highly expressed circRNAs in these three groups, seven (novel_circ_000069, novel_circ_000027, novel_circ_000438, etc.) were shared, as shown in Table 1.

### 3.2. Dynamic Expression Profile of circRNAs in the Developmental Process of Larval Gut Tissues

**Figure 2:**
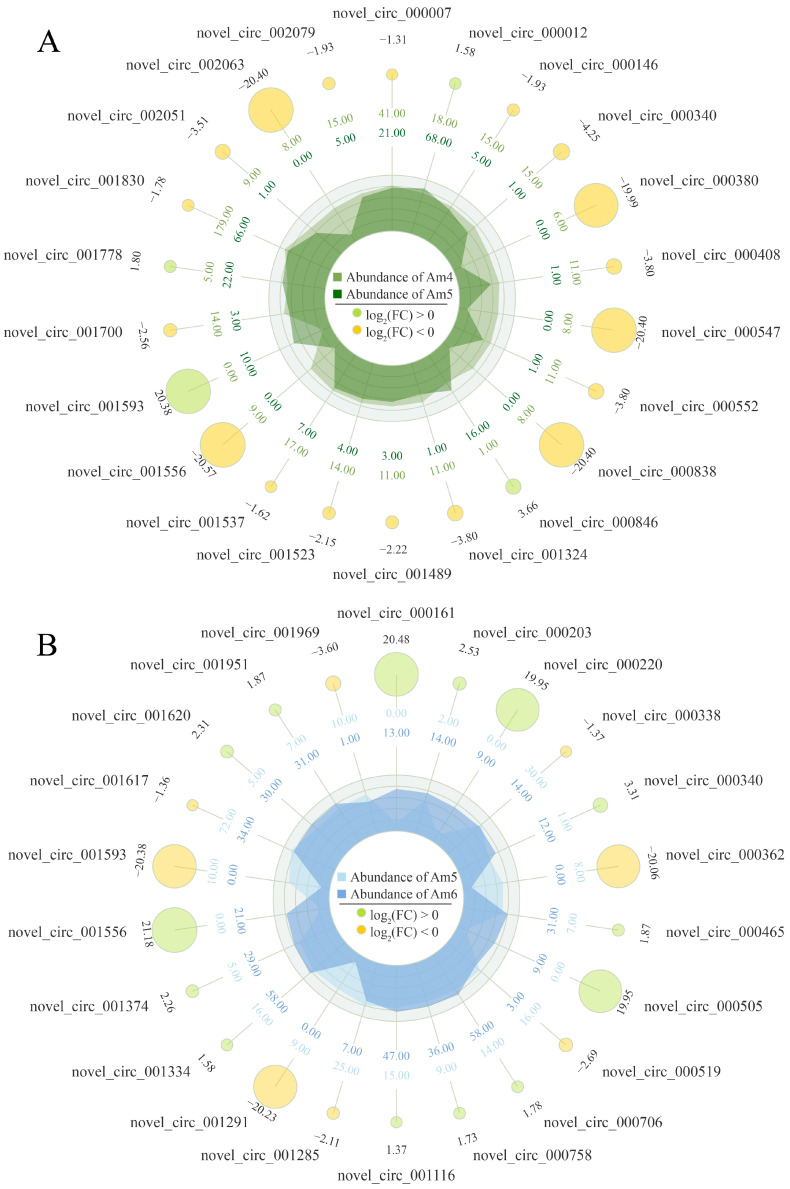
Radar maps showing the top 22 DEcircRNAs in Am4 vs. Am5 (**A**) and Am5 vs. Am6 (**B**) comparison groups. Yellow circles indicate down-regulated circRNAs, while green circles indicate up-regulated circRNAs. The larger the circles, the greater the difference. From outside to inside, the numbers adjacent to circRNA ID indicate the log_2_(FC) value of corresponding DEcircRNAs; the second circles represent the average expression of Am4 (**A**) or Am5 (**B**); the third circles represent the average expression of Am5 (**A**) or Am6 (**B**).

In total, 43 circRNAs were observed to be differentially expressed in the Am4 vs. Am5 comparison group, including 21 up-regulated and 22 down-regulated circRNAs (Table S5); novel_circ_000012 (log_2_FC = 1.58, *p* = 7.83 × 10^−6^), novel_circ_000846 (log_2_FC = 3.66, *p* = 5.21 × 10^−4^), and novel_circ_001778 (log_2_FC = 1.80, *p* = 9 × 10^−3^) were the top three up-regulated circRNAs, whereas the top three down-regulated ones were novel_circ_001830 (log_2_FC = −1.78, *p* = 1 × 10^−20^), novel_circ_000340 (log_2_FC = −4.25, *p* = 2.76 × 10^−4^), and novel_circ_000007 (log_2_FC = −1.31, *p* = 9.15 × 10^−4^) (Figure 2A, see also Table S5). Comparatively, 46 up-regulated and 27 down-regulated circRNAs were detected in the Am5 vs. Am6 comparison group (Table S6); novel_circ_001556 (log_2_FC = 21.18, *p* = 1.93 × 10^−6^), novel_circ_000706 (log_2_FC = 1.78, *p* = 6.07 × 10^−6^), and novel_circ_001334 (log_2_FC = 1.58, *p* = 2.65 × 10^−5^) were the most up-regulated three circRNAs, while novel_circ_001617 (log_2_FC = −1.36, *p* = 4.57 × 10^−6^), novel_circ_001285 (log_2_FC = −2.11, *p* = 3.28 × 10^−4^), and novel_circ_000519 (log_2_FC = −2.69, *p* = 7.32 × 10^−4^) were the most down-regulated three ones (Figure 2B; see also Table S6). In addition, nine DEcircRNAs, including novel_circ_000340, novel_circ_000758 and novel_circ_001116, were found to be common among these two comparison groups, whereas the quantities of unique ones were 34 (novel_circ_001830, novel_circ_000012, novel_circ_000552, etc.) and 64 (novel_circ_001617, novel_circ_001620, novel_circ_001374, etc.), respectively.

### 3.3. Analysis of DEcircRNAs’ Parental Genes

**Figure 3:**
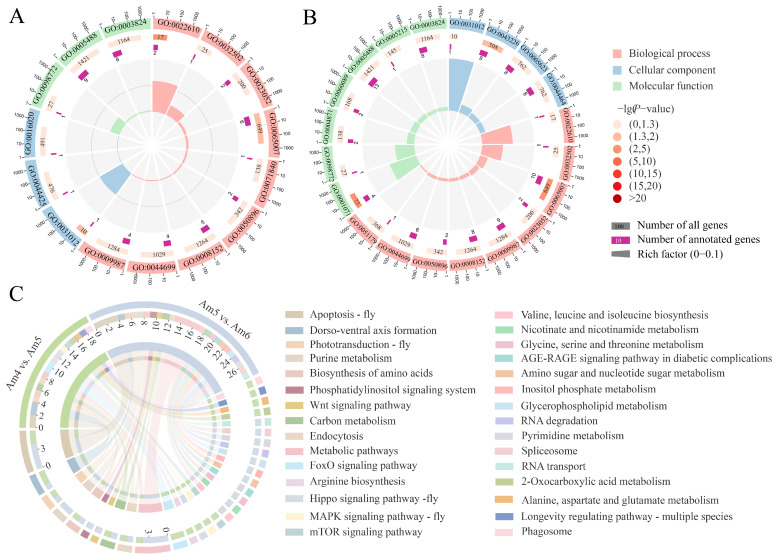
Functional terms and pathways enriched by parental genes of DEcircRNAs. (**A**,**B**) Loop graphs of parental genes of DEcircRNAs in the Am4 vs. Am5 and Am5 vs. Am6 comparison groups. From outside to inside, the first circle represent the enriched terms with GO numbers in the rectangles, different colors correspond to different GO categories; the next circles indicate the number of parental genes enriched in different GO categories in contrast to the background genes and corresponding *p*-values, the darker the orange color, the larger the number of parental gene and the smaller the *p*-values; the third circle indicates the ratio of up- or down-regulated parental genes, the dark purple rectangles represent the ratio of up-regulated parental genes, whereas the light purple rectangles represent the ratio of down-regulated parental genes; the fourth circle represents the set criteria for the quantity of parental genes, different colors represent different GO categories; rich factor means the number of foreground parental genes enriched in GO categories divided by the number of background genes. (**C**) Chord diagram of parental genes of DEcircRNAs in the Am4 vs. Am5 and Am5 vs. Am6 comparison groups. Various colors represent various KEGG pathways; the quantities inside the chord diagram represent the parental genes annotated to corresponding pathways.

Fourteen parental genes of DEcircRNAs screened in the Am4 vs. Am5 comparison group were found to be associated with 15 GO terms, including nine biological process-relevant terms such as developmental process and metabolic process, three molecular function-relevant terms such as catalytic activity and binding, and three cell component-associated terms such as extracellular matrix and cell membrane, as presented in Figure 3A (see also Table S7). Comparatively, 29 parental genes of DEcircRNAs in the Am5 vs. Am6 comparison group were relative to nine functional terms related to biological process like response to stimulus and localization, seven molecular function-relevant terms, such as catalytic activity, and molecular function regulator, and six cell component-associated terms, for example, cell and extracellular matrix (Figure 3B; see also Table S8). In addition, we observed that the parental genes of DEcircRNAs in the Am4 vs. Am5 comparison group were enriched in 16 pathways, such as dorsal–ventral axis formation, apoptosis, the Hippo signaling pathway, endocytosis, and the metabolic pathway (Figure 3C; see also Table S9), while those in the Am5 vs. Am6 comparison group were involved in 21 pathways, e.g., apoptosis, the Wnt signaling pathway, the metabolic pathway, purine metabolism, and inositol phosphate metabolism (Figure 3C; see also Table S10).

### 3.4. Investigation of DEcircRNA-Engaged Regulatory Networks

**Figure 4:**
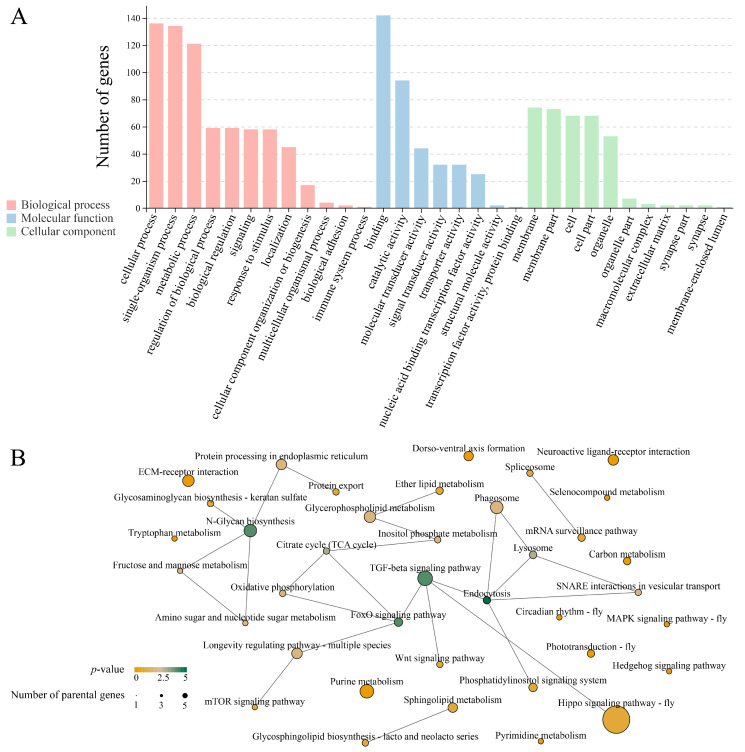
Functional terms and pathways annotated by the targets within the DEcircRNAs-engaged ceRNA network. (**A**) Functional terms annotated by the target mRNAs; (**B**) KEGG pathway network of target mRNAs. Nodes of different colors and sizes indicate various KEGG pathways, the dimension of the node represents the quantity of genes annotated to certain KEGG pathways, the gradient color of the node indicates the *p*-value of certain KEGG pathways; the solid line is indicative of a linkage between two pathways or between a pathway and a parental gene.

DEcircRNAs in the Am5 vs. Am6 comparison group were not observed in any DEmiRNA. Comparatively, in the Am4 vs. Am5 comparison group, one DEcircRNA (novel_circ_000846) was detected to target 1 DEmiRNA (ame-miR-6000a-3p) and further target 518 mRNAs, as shown in Figure S1 (see also Table S11); these target mRNAs were enriched in 31 functional terms, including 11 biological process-associated ones such as cellular process and metabolic process, 9 molecular function-related terms like binding and transporter activity, as well as 11 cell component-relevant terms like the membrane part, cell, and organelle (Figure 4A; see also Table S12). Also, these targets were relative to 38 KEGG pathways in total, namely inositol phosphate metabolism, dorsoventral axis formation, endocytosis, Hippo signaling pathway, and spliceosome (Figure 4B; see also Table S13). Further analysis demonstrated that a subseries of target mRNAs could be annotated to three lipid metabolism-related pathways (sphingolipid metabolism, glycerophospholipid metabolism, and ether lipid metabolism) and four carbohydrate metabolism-related pathways (amino sugar and nucleotide sugar metabolism, inositol phosphate metabolism, glycerophospholipid metabolism, and fructose and mannose metabolism) (Figure 4B; see also Table S13).

### 3.5. Analysis of DEcircRNA-Involved Sub-Networks Relative to Developmental Signaling Pathways

**Figure 5:**
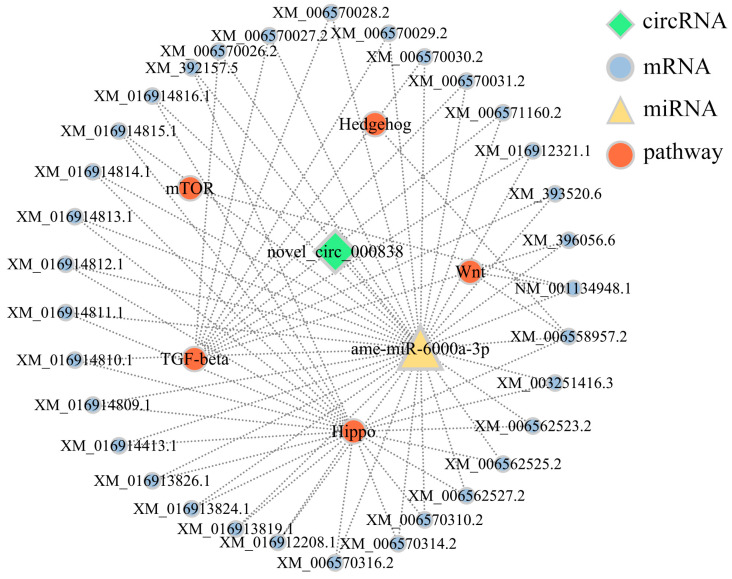
DEcircRNA-involved sub-networks relative to 5 developmental signaling pathways. The lines indicate the potential targeting relationships among novel_circ_000846, ame-miR-6000a-3p, target mRNAs, and pathways. The same is shown below.

ame-miR-6000a-3p targeted by novel_circ_000846 was detected to target 33 mRNAs involved in five developmental signaling pathways like Wnt, TGF-beta, Hedgehog, and Hippo (Table S13). Further analysis indicated that among these targets, XM_006558957.2 was annotated to both Wnt and Hedgehog signaling pathways, while NM_001134948.1 was annotated to both mTOR and Wnt signaling pathways (Figure 5; see also Table S13).

### 3.6. Investigation of DEcircRNA-Involved Sub-Networks Associated with Humoral and Cellular Immune Pathways

**Figure 6:**
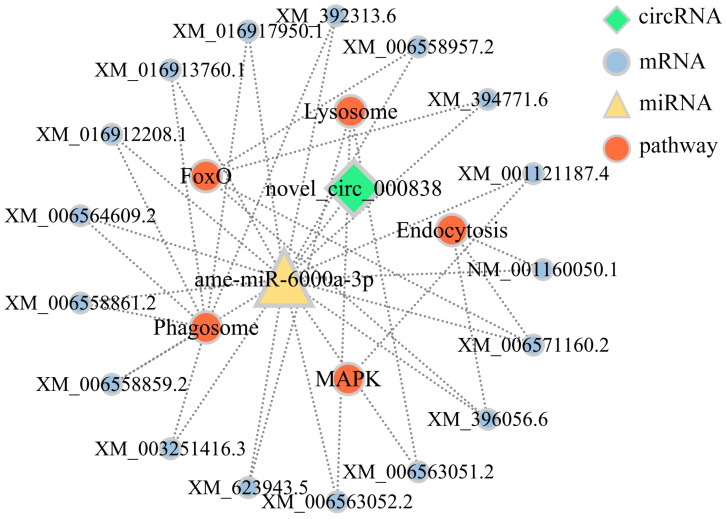
DEcircRNA-engaged sub-networks relevant to humoral and cellular immune pathways.

Additionally, 17 mRNAs targeted by ame-miR-6000a-3p were involved in two humoral immune pathways including MAPK and FoxO signaling pathways; these targets engaged in three cellular immune pathways, namely the phagosome, lysosome and endocytosis (Table S13). It is observed that both endocytosis and the FoxO signaling pathway were enriched by two target mRNAs (XM_006571160.2 and XM_396056.6) (Figure 6; see also Table S13).

### 3.7. Validation of DEcircRNAs Based on PCR and RT-qPCR

**Figure 7:**
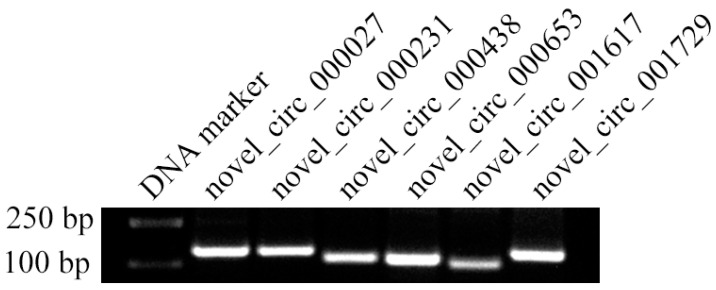
PCR-based molecular detection of six DEcircRNAs.

The agarose gel electrophoresis indicated that the fragments that could be amplified from six randomly selected DEcircRNAs matched the expected size, which confirmed their back-splicing sites (Figure 7).

**Figure 8:**
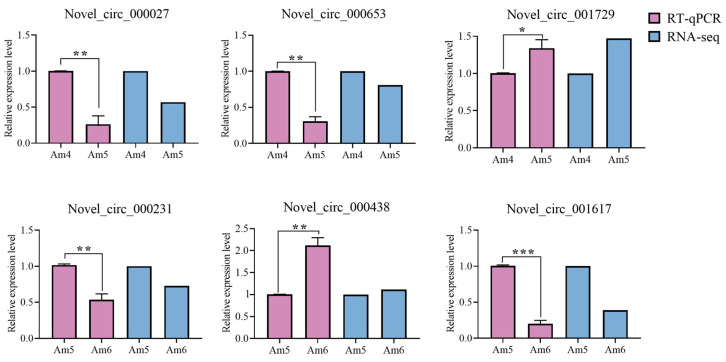
RT-qPCR validation of six DEcircRNAs. The qPCR data were presented as mean ± standard deviation (SD) and subjected to Student’s *t* test, ns: *p* > 0.05; *: *p* < 0.05; **: *p* < 0.01; ***: *p* < 0.001.

Furthermore, RT-qPCR assay of DEcircRNAs showed that their expression trends were in accordance with those in the transcriptome data, proving the confidence of the RNA-seq datasets analyzed in this work (Figure 8).

## 4. Discussion

Currently, following investigating expression levels, we detected that among the top 10 highly expressed circRNAs, 7 (novel_circ_000069, novel_circ_000027, novel_circ_000438, etc.) were shared by the *A. m. ligustica* worker 4-, 5-, and 6-day-old larval guts (Table 1), which is indicative of the great importance of these seven common circRNAs during the process of larval gut development, thus deserving further investigation. Additionally, 43 and 73 DEcircRNAs were, respectively, identified in the Am4 vs. Am5 and Am5 vs. Am6 comparison groups (Figure 2; see also Tables S5 and S6), which suggested that the development of larval guts was accompanied by the dynamic change in the overall expression profile of circRNAs. In other animals such as *Aedes albopictus* (Diptera: Culicidae), *Bombyx mori*, and *Drosophila*, the change in expression pattern of circRNAs was also detected in the developmental process. For example, Liu et al. [47] found that circRNA can act as hub genes, manipulate chitin metabolism, and further promote the growth and development of *Aedes albopictus* (Diptera: Culicidae). Wang et al. [48] reported that the expression level of circEgg in the *Bombyx mori* midgut dynamically changed during the developmental process and circEgg was potentially involved in regulating the homeostasis of midgut tissue. These findings reflected that circRNAs are possibly involved in the regulation of the development of honey bee and other animals. Interestingly, two up-regulated circRNAs (novel_circ_000758, novel_circ_001116) were observed to be shared by the above-mentioned two comparison groups, suggesting the potential part of them in the development of larval gut tissue. These DEcircRNAs are believed to be candidates for further functional investigation using our recently established RNAi-based methods [49].

Several lines of evidence have demonstrated that circRNAs exert regulatory functions in diverse aspects of vertebrates such as growth, development, metabolism, and immunity through *cis*-acting effect [50,51,52,53]. For instance, Weigelt et al. [51] previously detected the continuous up-regulation of the circRNA transcribed from the *sulfateless* gene (circSfl) in the brain and muscle of insulin mutant flies, and the circSfl overexpression prolonged the host lifespan. Zhang et al. [52] discovered that the interference of circRNA2030 was capable of suppressing the expression of its parental gene *phospholipid-transporting ATPase* (*PTA*), and it can enhance the infectivity of RBSDV for *Laodelphax striatellus* (Fallen) midgut tissues; thus, the authors speculated that circRNA2030 was likely to control RBSDV infection via *PTA* regulation. Here, the parental genes of DEcircRNAs were relative to several vital functional terms and pathways associated with growth, development, metabolism, and immunity, such as biological regulation, catalytic activity, and multicellular organismal process (Figure 3A,B; see also Tables S7 and S8). The results indicated that corresponding DEcircRNAs potentially affected the aforementioned functional terms and pathways of great importance during the gut’s developmental process. Additionally, it is noticed that novel_circ_000758 was differentially expressed in both Am4 vs. Am5 and Am5 vs. Am6 comparison groups, and the parental gene (ncbi_413021) of novel_circ_000758 was involved in the metabolic process, catalytic activity, and extracellular matrix (Figure 3A,B; see also Tables S9 and S10), indicating the key role of novel_circ_000758 in the gut development. Thus, it is worth further study in the near future.

Papilins are secreted extracellular matrix proteins, homologous among various species ranging from nematodes to humans [54]. Papillin is an extracellular stromal glycoprotein and is associated with the thin stroma layer during gastrula formation, the stroma associated with phagocytic blood cells, the basement membrane, and the space-filling stroma during drosophila development [55,56]. In this study, the differential expression of novel_circ_000758 was detected in the Am4 vs. Am5 and Am5 vs. Am6 comparison groups, and the parental gene (GeneBank accession number: LOC413021) of novel_circ_000758 was annotated as the papilin protein. It is speculated that novel_circ_000758 played an essential role in regulating the gut development of the larval gut by regulating the transcription of genes encoding papilin.

Apolipophorins are carrier proteins by binding lipids in animals, and they mediate the transfer of lipids between tissues [57]. Also, apolipophorins are engaged in stress response and lipid transport in insects [58] such as mosquito [59] and *Locusta migratoria* [57]. In this current work, the parental gene (GeneBank: LOC408961) of novel_circ_001116, a DEcircRNA in the Am4 vs. Am5 and Am5 vs. Am6 comparison groups, was annotated as apolipophorins protein, which suggested that novel_circ_001116 may be a modulator in the lipid transport and immune response of *A. mellifera* worker larval gut through affecting the transcription of apolipophorin-encoded genes. Further work is needed to unclose the functions and molecular mechanisms of novel_circ_000758 and novel_circ_001116.

It has been suggested that circRNAs are crucial regulators in immune defense of insects against pathogens or parasites [60]. Hu et al. [61] investigated the circRNA expression in the gut tissue of *Bombyx mori* following BmNPV challenge, and the results showed that circRNAs potentially regulate the genes annotated to ubiquitin, apoptosis, and endocytosis signaling pathways by the ceRNA axis. The antiviral defense system of honey bees has been suggested to include hemocyte-mediated mechanisms of cellular immune (e.g., endocytosis, melanization and phagosome), and conserved signaling pathways (e.g., MAPK, and FoxO signaling pathways) [62]. Here, we detected that two parental genes (ncbi_551176, ncbi_100577393) of novel_circ_000838 and novel_circ_000861 in the Am4 vs. Am5 comparison group were associated with phagosome and endocytosis, which are two insect cellular immune pathways of great importance; one parental gene (ncbi_727312) of novel_circ_001933 in the Am5 vs. Am6 comparison group was enriched in endocytosis (Figure 3C; see also Tables S9 and S10). For insects like the honey bee, the gut is a critical immune organ that fights against pathogenic microorganisms through cellular and humoral immune pathways [63,64]. Here, in the Am4 vs. Am5 comparison group, one (ncbi_100577393) and two (ncbi_725827 and ncbi_100577393) parental genes of novel_circ_000861, novel_circ_000400, and novel_circ_000861 were observed to be, respectively, enriched in the MAPK and FoxO signaling pathways (Figure 3C; see also Tables S9 and S10). Based on the findings mentioned above, corresponding DEcircRNAs were speculated to participate in the adjustment of immune responses in the process of the gut’s development.

Increasing studies have demonstrated that circRNAs containing miRNA response elements (MREs) could act as “sponges” to bind to target miRNAs and indirectly affect the downstream gene expression [65,66]. Gao et al. [67] documented that circRNA-407 was capable of promoting the expression of its target gene *Foxl* via targeting aal-miR-9a-5p and eventually regulated the *Aedes albopictus* ovarian development; the siRNA-mediated knockdown of circRNA-407 leads to a depression in follicle number and follicle size during the stage of ovarian development. In the Am4 vs. Am5 comparison group, novel_circ_000838 that significantly down-regulated was detected to putatively interact with ame-miR-6000a-3p, further targeting a subseries of mRNAs relevant to not only catalytic activity, the metabolic process, the immune system process and biological regulation but also three developmental signaling pathways (Wnt, TGF-beta, and Hedgehog) and two cellular immune pathways (phagosome and endocytosis) (Figure 4B; see also Table S13). The results indicated that miR-6000a-3p may be not only a pivotal regulator in diverse processes in the honey bee such as gut growth and development but also a hub to bridge other ncRNAs like circRNAs and genes. The functions of the majority of miRNAs in the honey bee larval guts, including ame-miR-6000a-3p, are currently unknown. Therefore, more efforts for the functional dissection of *A. mellifera* larval miRNAs should be undertaken. Recently, our group established the feeding-based method of functional study on miRNAs in the gut tissue of *A. mellifera* larvae [68], offering a platform for investigating the function of ame-miR-6000a-3p and continuous investigation of other miRNAs. Additionally, the technical platform was recently established for the functional dissection of bee larval circRNA by our research team [49]. Together, these platforms pave the way for exploring the functions as well as mechanisms of circRNAs, miRNAs, and ceRNA axis in the honey bee larvae.

## 5. Conclusions

**Figure 9:**
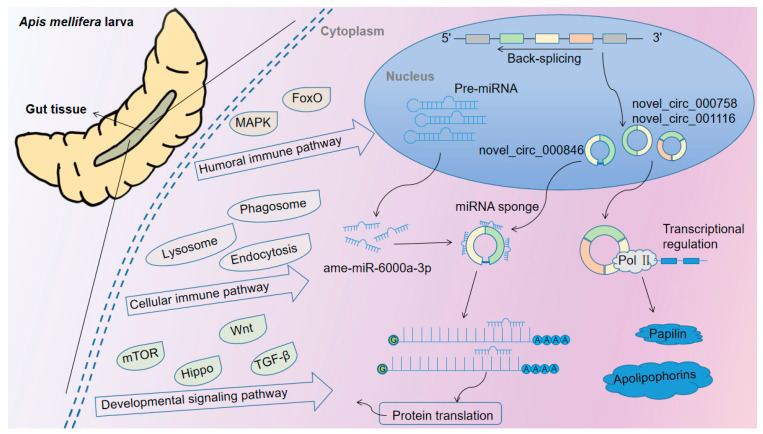
A hypothesized working model of multiple regulation of circRNAs during gut development of *A. mellifera* larvae. According to the results obtained in this study, the differential expression of circRNAs (DEcircRNAs) potentially regulates developmental signaling pathways as well as cellular- and humoral-immune pathways through promoting the transcription of host genes to regulate the expression of parental genes or competing endogenous RNA (ceRNA) networks in the larval gut.

In conclusion, circRNAs are abundantly and differentially expressed in the larval gut tissues of *A. m. ligustica*, DEcircRNAs potentially participated in multiple growth and immunity-associated signaling pathways in the larval gut development through regulation of the parental gene transcription and action as ceRNAs to absorb miRNAs, and novel_circ_000838 putatively served as a “miRNA sponge” to directly or indirectly regulate development and immune response by competitively binding to ame-miR-6000a-3p (Figure 9).

## Figures and Tables

**Table 1:** Details of 7 common highly expressed circRNAs among the *A. m. ligustica* worker 4-, 5-, and 6-day-old larval guts.

CircRNA ID	Am4 Group RPM	Am5 Group RPM	Am6 Group RPM
novel_circ_000069	54,161.61966	67,741.05436	58,471.61079
novel_circ_000027	46,028.72469	26,222.34362	21,108.47725
novel_circ_000438	29,589.89445	50,942.36547	56,778.41743
novel_circ_000115	28,378.61222	36,328.87189	28,897.16672
novel_circ_000131	19,207.47534	19,393.6083	15,125.86071
novel_circ_000630	16,957.9512	12,564.87299	14,900.10159
novel_circ_000231	13,497.14483	17,071.8383	12,416.75133

## Data Availability

All the data are contained within the article.

## Supplementary Materials

The following supporting information can be downloaded at https://www.mdpi.com/article/10.3390/insects14110897/s1, Figure S1: DEcircRNA–DEmiRNA–mRNA networks in the Am4 vs. Am5 comparison groups; Table S1: The sequences of the identified circRNA in the *A. m. ligustica* 4-, 5-, and 6-day-old worker larval guts; Table S2: The sequences of DEcircRNA-targeted DEmiRNAs and corresponding DEmiRNA-targeted mRNAs; Table S3: Detailed information about the primers of DEcircRNAs; Table S4: The expression level of each circRNA in the *A. m. ligustica* worker 4-, 5-, and 6-day-old larval guts; Table S5: Results of DEcircRNAs in Am4 vs. Am5 comparison group in the *A. m. ligustica* worker larval guts; Table S6: Results of DEcircRNAs in Am5 vs. Am6 comparison group in the *A. m. ligustica* worker larval guts; Table S7: Functional terms enriched by parental genes of DEcircRNAs in the Am4 vs. Am5 comparison group; Table S8: Functional terms enriched by parental genes of DEcircRNAs in the Am5 vs. Am6 comparison group; Table S9: Pathways enriched by parental genes of DEcircRNAs in the Am4 vs. Am5 comparison group; Table S10: Pathways enriched by parental genes of DEcircRNAs in the Am5 vs. Am6 comparison group; Table S11: DEcircRNA–DEmiRNA–mRNA in the Am4 vs. Am5 comparison group; Table S12: GO terms annotated by the target mRNAs in the DEcircRNAs-engaged ceRNA network; Table S13: KEGG pathways annotated by the target mRNAs in the DEcircRNAs-engaged ceRNA network.
