# Supplementary material for: Novel Insights into the circRNA-Modulated Developmental Mechanism of Western Honey Bee Larval Guts
Source: Insects. 2023 Nov 20;14(11):897. doi: 10.3390/insects14110897 (PMC10671861; doi:10.3390/insects14110897)
Supplement: Supplementary file 1 [file insects-14-00897-s001.zip › Figure S1.pdf]

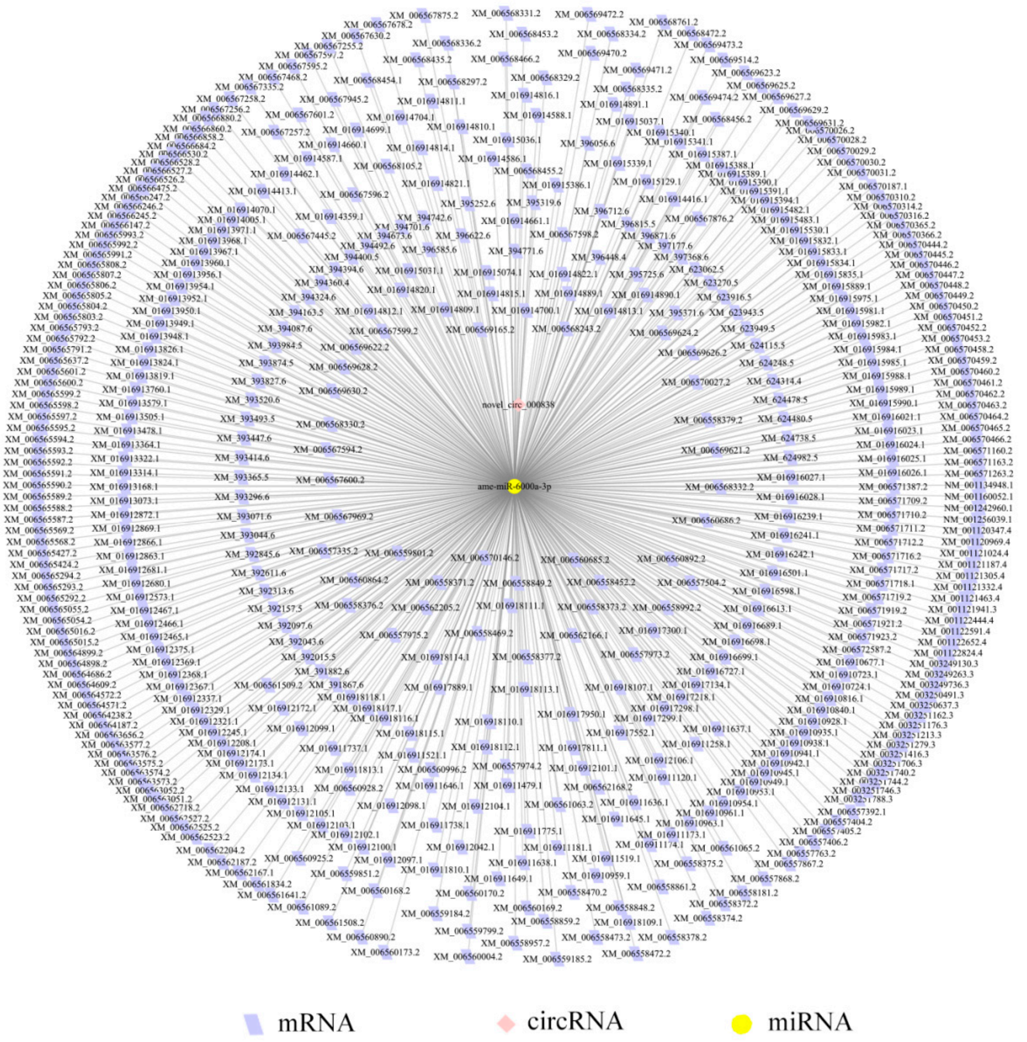

Figure S1. DEcircRNA-DEmiRNA-mRNA networks in the Am4 vs. Am5 comparison group. The purple quadrilateral represents mRNA, the pink diamond represents circRNA, and the yellow circle represents miRNAs. Grey lines indicate potential targeting relationships between DEcircRNAs, DEmiRNAs and mRNAs.
