# Supplementary material for: Novel Insights into the circRNA-Modulated Developmental Mechanism of Western Honey Bee Larval Guts
Source: Insects. 2023 Nov 20;14(11):897. doi: 10.3390/insects14110897 (PMC10671861; doi:10.3390/insects14110897)
Supplement: Supplementary file 1 [file insects-14-00897-s001.zip › Table S1.pdf]

Table S1. The sequences of the identified circRNA in the *A. m. ligustica* worker 4-, 5-, and 6-day-old larval guts.

>novel_circ_000001
ACAATTGTATTATAAATTTGATCATTGCTAATTCATGATCCTGGGGATCTTAATTCTATTC
GAATAATAAGTCTCATTGATGATCCTAGTATTCCAGATCATAAAGCTAGAATAATATACA
AGATCCCAATATTTTTATGATTGGTTGATATGAATCACTTTATTATT

>novel_circ_000002
GGAATACTAGGATCATCAATGAGACTTATTATTCGAATAGAATTAAGATCCCCAGGATCA
TGAATTAGCAATGATCAAATTTATAATACAATTGTTACTAGTCATGCATTCCTAATAATTT
TTTTTATAGTTATACCATTTTTAATTGGAGGATTTGGAAATTGGCTTATTCCTT

>novel_circ_000003
GTGAAGAATGATATAAATATGCTGATAATGGTGGATATACTGTTCATCCAGTTCCTGGTC
TTGGATAAAATAAATTTCTTAATAAAAGTATAAATAATGAGGGAGGAAGTAATCAAAAT
CTAATATTATTTATTCGGGGGAATGCTATATCAGG

>novel_circ_000004
GAGCCGGAAGCGGCACTGGTGGCAGTGGAGGCAGCAATGGCGCGGCAAACGGTGCT
GGAGGTGGTGGTTGCGGCACATCGACAGCCCTCGAGAGTAGCGGCAGCAATTATACCG
GTGGCGGTGGTGCCGGTACCGGTGGTGGAGGAGGAACGGGTGGCGGCGGTGGTGCG
GGTGCCGCTGGCGGTAGTGGGCCCACCGCAGGGAATGCCTCTGCTGGTGGTGGCGGT
AGCGGTAGTGGTGCGGCCAGCGGTGCAAGTGGCGGCGGATCCGCTACGGTAAGCGGC
AATGTCGCGATACCAACTACCACCTACTCATTACCAACTGGTACCCTCTGTCATCCCGG
ATTGGGTCAGGTCGGGGTTGGTGTCGTGACCGGCTCGATACCGTGTCCGTCCGAATTC
CCCGATACCAAGGACATCATCATAGAAGAACTCTGTCCGGTCTGCGGCGACAAAGTTT
CCGGATACCATTACGGGCTACTCACTTGTGAATCCTGCAAAGGTTTCTTCAAACGCACC
GTCCAAAACAAAAAGGTCTACACGTGCGTCGCCGAGAGGTCCTGTCACATTGACAAA
ACGCAAAGAAAGCGGTGTCCCTACTGCCGTTTTCAGAAGTGCCTCGAAGTCGGCATGA
AGCTTGAGG

>novel_circ_000006
CTCATGTGCAAAATGGCGCCCCTCATTGGCAAAGAGATGACGGAAAAGATTTTTCTGG
ATAGATACATCGCTCTCTGCAAAGACAAAGAATTTTATACGAGAAAAATCTGCGCGTCG
CACTTTGGCAAACTTTGCTCCGCTGTTGGGAGGAAGACGCTTTTCCGAAAATTGTTCC
CGGTATTTGTCGATCTGTGTTGCGATACAGTGTGGGGCGTCCGAAAAGCGTGCGTGGA
CGTTATGATGCCGGTTTCGTGCTGCATGACCCTTCAACATCGTCGATTACTCTTGGCAGA
TCTTCTTGCCACGCATCTTAACGACGACTCGAAATGGGTGCGAATGTCTGCCTTTCAAA
TTTTAGGACCATTTATATCTACGTTCGCGAAACAGTTTACCGAAGTGACTTATAATCAAC
ACGGTGAATTAGTATTTACCAGCCAACAAGATACTCGTTTCAGTATAAGGTATTTGTACG
AAGGAATATTCCCCACTAAGTGCGCGATACGGAGTCATACACTCGACGCGGAGGATAAT
AATTCTAAGGATAACTTCAATTTATCTGTGATAATACAAAAACCAATTTTCGAAGAAGA
CGATGACGAGGACGAGTCTCAGGAAGATCATATTTCCAGAATGAGAGCTTATAAATCG
AAG

>novel_circ_000007
ACACGCAAGTTCGGTTGGACCTGGTGGAGCATATACCTCATGTGGCGACGATCTGCCA
AGAATCTCCTCATCTATTCGACAATGTCGTTCACGATCATTTGCTCGGTATCATTATCAAA
TATCTACAGGATCAAGATAACCAGGTACGAATGACAGCCCAGACAGCGGTGTTAACGT
TAACGAAGAAGGGAGTTCTCGACAACATCACGATCGAGGTCAAACTGTGTCCCGTAAT
AGAAACCCTCTGTAGGTCCGTCGATTTCCTTAGCTCTGGCATCTCC

>novel_circ_000008
ACAAAGAATCGGAAGGATGTTGGTCGAGGTATTTCGCACTGCTGCAAACAATGGAACG
AAACTTAACGTGAATGAAGTGATGCAATCGGTGCAACAGATTGTCGAAGATCCTGACA
CGCAAGTTCGGTTGGACCTGGTGGAGCATATACCTCATGTGGCGACGATCTGCCAAGA
ATCTCCTCATCTATTCGACAATGTCGTTCACGATCATTTGCTCGGTATCATTATCAAATAT
CTACAGGATCAAGATAACCAGGTACGAATGACAGCCCAGACAGCGGTGTTAACGTTAA
CGAAGAAGGGAGTTCTCGACAACATCACGATCGAGGTCAAACTGTGTCCCGTAATAGA
AACCCTCTGTAGGTCCGTCGATTTCCTTAGCTCTGGCATCTCC

>novel_circ_000009
GTAAGTCGAATCGAGCTACTATCCATCCACTGTTGCGAGCGATCGTTTCCGGTAAGTGT
GTTTCCCTTACCGATCATCATTCGCCTCGTTCGATCCGAACGCGTTTTTCCACGAACG
AGAAAGAAGAAAAATGAAGAGAGAGAGAGAGAGAAGAGAGGAGGATACGTAAAAG
AGACGACATTCGTGGACGGAGATTTCGGGACGGTTGCGGTTCTCGTAGTCGCGCGCGT
TCGGCCCGGACAAGGAAGCGAAGAAGCCTACGACGCAGTTGCTCCCGCG

>novel_circ_000010
TGACAACGGCTGGAACGACACGTGGATACGCGAAGAAATCTTGGGGTTACTTTGTGCG
ATTAAAAAGCAATGGAGATTGATGAAGTCGACGACGATGATGTTGATTATAATCTGAGT
CCCGACGCTTCCGGAAACGATATTTGCAATATTGGAGCAAGTGGCGACGAAGGTGGAA
AGGACGAACTGCTACTGAAATTGCAACAGTACGTAACCTCGGACGCAAACTTCACACG

>novel_circ_000011
CGGCAGGGGATTGGAGCAAGGTGGGAAAATCCGTGGAAACGGGTCGTGATGGTCAAC
GATGCCGCACGTCGTCATGGATGTAATTGGACGTTTCCGGTCGAACGAAGGAAAATAA
CGACGAGGACGACTTGGACGTAGTCGAATATCGGGGGAAATCGCGAATGATCGTCGAA
TGACGAGCGGTGAAAAAACGGCTCGGTCGGAGATAAGTGGTTGATCCCCGGTGCAAA
TATGACCGACAGGATTACGTAGTGGCGCGTGGCGTGTCAGACCAACTGACACGGGACT
TCCAAGTGCGTCCTCTTCGATTTCGTTGCTTTCGGAAGCTGGATATATCGAGGAGATAG
CATTATCGGTCGCGGGACATATATCGCAGGCATGTCGAGACGCGGGAATTCGGGTCGTC
GACTCGACGTGGAATCAGCTGACATCGACTCGGCGTGAGGCGAGCCGACGTGTTCGA
GAAGAAGAGAAGACGAGAAGAGAGGGAGAGAGAGAGAGGGAGGGGGGGATAGATC
GAGAAAGATGGAAGCAAGGAGATTCGTGTTGGGAAGCGCTATCCTCGGACTTTTTTGG
AGCGGATTGTTCAAGGGTGCCCTGACCAATTCGATGAACAGATGCGACTATCCTCCCTG
CTCCTGCGATTCTCACGGCAGGCTGACCTGCGACTGTAAAGAGGAAGGAGAGAGGTG
GAAAAGATATTCGCGGACGTATATTTGAGGAGAACTCGTTCTCGAGGGAAAACCTTA

GCTAACAACGAGGACAAAGGGGGAGGAGAAGGAGGAGAGGAAGGTGGCAAAGGAG
GCGAAGAAGAAGAAAGGGGAAAAAAAACGAGGAGAAAAAGAGGCTGAAAGTTCTAT
AACGTATAGCGAGTTCTGCCGTGGATTTCGCATAATACAGCCGGCATTTCACGAACACG
CTCAGCCAATTTGTCGCGCCGTCAACTCGTGACTACGAATATATCGAGAGAGACTGGA
CGTCTTTCCCTCCTTCTCCGTTCCCCAGCAAAGTTCGAACCCTACGCTCTCCCTTCCCC
CCCCTTATCCCTCTTTTCCATTCTCGACGAGGATTTCATCCAAGTTTCTTATTCCGATCTA
TTATCCGCTTCGTGGCAACGAGACGACGCCGCTTGGCAATACGTAAAAATCATTACGTC
TGCGCGCTTATTTACGGAAGAGAAAAATAAAAAAAAGGGAGAGAAAGAAAGGAAAG
AAAATTTCTATCTACTTTTTCTTCTTCCCTCTTATCCCGATACGCGCGAATATTTATTTCTC
CTCCCCGTTTCGAAACAGCTGCGTTTTCCACCACCCTATTCTCCGAGGCAAACGTTTCG
ATGCCGAACAGTCAACGCAGAGAGCAAAGTTGTCAAGCGCGCCGGCATCCCTTGAAT
AATTGACGTCACCCTTGAATAATTGAGCAAACCTTTTAGCGGATACAGCCTTAAACCCG
GCTCAGTTTCCCGTAACGAGAGACGCAGGAAGGAAGGCAAAAGGGGAAGAAAAAAA
TACGGAATAGGGAACGCGGTTTGGACCCTGCCAAAGTGCAACGGCGATATCGCCACGC
CTAGAAAATTAGTTACGGATAAATACGTTAATCGACTCGTTCGCATCTTATTTCGTTCGT
TTCACCGATCGCTTTCCACGCTCGAGTACTTTTCTTTATATATGGAAAAATGCACGTGGA
ACGAGTCTCTTTTCCATCGACGTGTATTTCCACGCGTTAAAAAGAGGGATTGGGAGAGT
AAACGACGGATAAAATTCCTCTTCTTACGTTCACCAATACTCGTCTCTGATCGATGCGC
ATTCTGATTAAAATTACGCTATATCGAGGAATATTAAAAGAGTATAACGCGTCGTATCAA
CGCGAATCCATCCGTCGTTCCATACTACGAATCAATTGGAAGAAAACTTTCATCACCTC
CGTTCGAGAATCACGATTCAAAGTCCCAAGTGACGTAGCACGCTCGCCTCTCGTTTTTT
TCCGTACTTGAAAACGGCGTCCTATCTCGAGAGAGAGAATATATAAAGTTCGAAGAAAT
AAAACTCGGATTCCCTCCTCTTTGCTCGATGATTTTCGGAAGAGGAATATTCACGATTA
CGCGATCCGAGTCGAGTGACTCGGACTAAAAACACGTTCGACGAGAGCAAAAACGAG
AGAGAGAATGAAGAGGAAAGGAGATAGAGAGAGCATCGGAAGGAAACGAAAACGTT
GGCTCGATAATCATTGGCGGAACGAGGCGCGTAGACGCGAGGAGACAAGCATTTCGG
GTGTGGTAATCTTTTCTATGGGCGGGGAGAGAGAGAGAGAGAGAGATTGTGCGTCGG
GAATTGACCAAGAACAATGAGACGGGAACAAAGGTCGGGACAGACAGGAAGGGACG
CGAGGAGGAGAGATCGGTTCGGGGGCGAGACGACGGTGAAACGCGCGAGCTGGTAC
CCGAGAAGAAAAAGGGGCACAAAGGTTTCGCTTTTATCGGCCACGCACTACACGTGG
GCCACACGGTCCCTTCTTTATTCCTCCCCCCGCATTCACCACGCACGCCCTTCCTTCAC
GGCTAATCTCGCGTCCAAACTCCTTCACGATTCTCATCCCGCCTCTCTATTCTTTATATAA
CGTCGAGAAAAAAAAATTTCTGCTCGAAATTATTTCAACGCGTGCACTCGTTCGTTCGT
TCGTTTATTCTACGCGTAGATCTGTATCGAGAACGACTCGACGCATGGAATAAGGCGCG
GAGCAACGTCGCATTTTCCACGTATCACTCTCGTTATCGGGTGTTCGTTAAGAGAGAGA
GAGGGAGAGAGAAAGAAAACGCGATAACCTCGCGAAACCTCGTATTTGCGCGGACGA
TATCTCGATCGTGTCTATGGTTGGTAGGAGGCAGGTATGCGGTTGGCATTTCGCCATCGA
TTGTTTAACAAACAAGGAGCCGCCGAGTGGCATCGCGCGCGTCCCTCCTCCTCCTCTC
CTTTCGCTCTTCCTTCTTCGCTCGAGTCTATTAATAAAAAACGGAGCGAGCCTTCCCCA
ACTTTTCCTCCCTTTTCTCTTTTCGATTAACGAGCTGTTGCGACAAACAATTTCTTCTCG
GTTTATCGAAACGTCGGAGAAAGAGACGACCCTTTCACCTTGGACGAGAAAGGCCCT
TTCGAAAACTTTCGAAAGAAAAAGAAGAAGAGGAAGAAAGAAAAATGGCGATAATC
GGGCCGGTTAATAGCTGGAGCTGGTGTTAACCACGGACGGGGACAAAAGGTTGAGCC
CGCACACGAGCCGGATAGTGGTGAGCAATTGCTCGTCCGTGATCCTGGCCAACTCGAG

CCTCGCCTCGATGGTCGGGCTCCGCGCGATCGACCTGATCAACGTGGCCAACCTGACC
CTGGTCAGGCAGAGTTTCGAGCTCTCCCCCCAAAGCACGCGCACGTGGATATCGGTGA
GGAACTGCAGCATCGACACCATGCCAAGTTTCGTGTTCCGGGGGGACGTGGAGGCGA
TCATCCTGGAGAACGTGAGGATCGGCCAGTTGAGCGCGTTCTCGTTCGCCAATCTCGT
GCACACGGAGAAACTTAGGCTGGAGAATTGCCGCATCGAGAGCATCGAGGCGCAAGC
GTTCAAGAAGTTCGACGTGGGCCACTTGCGAGTGATCGGTACTAGGTTCGGGGATCAG
GTGCCGAGCAGGACGATGAACGACGTCGAGGTTTACCACACGTTCATGTTGGACGGTG
TGAGGATGGGCACGGTGAGGAGCGAGGCGTTCATCGTGAGAAGCCCGCGGACAGTGG
CGATACAGAATTGCGTGGTGGAGAGTTTGGAGAGCGAGGCGTTCGACGTGACCGCGA
GGGGGCCGGTGATCGTGAAGAACAACACTTTCGGCAGCCTCTCCGTAGGCGCCTTCCT
CGGCGTACGGGCGGACGCCGAAGCGAGGACGCAGCCCTCTCTCCACGACCTTATCTTT
AAGAATAACAGCGTGGTCAACTTCGAGGAGGGCTCGATCATGTTCGACCGAAATAGCT
TTCGCCTCGAGCTCGACAACCTGTTGGTCGGCCGATCGTGCGACTGTCGAATGTTACC
CGTATGGAAGAACAATATCCTCAACTACACCAATCTCTACGCCAGGTTTTACAAACAAA
ACACCGCCTCCTCCTCCTCCTCCTCTTTTTCTTCCTCCTCCTCCCTTTTGCTACCAGTCC
TCGTATCGAGCCAAGACACCCCGATCAACGAAACCCCGGAGACGTTCCTCTGCTACGA
CGGCGAGACGTCGAACGGCGTGGCGATCAGTTTCGTCGAGTACGAGACGCGACATTG
CTCCCTCGGCGGTTCCGTGCTGGTGTTCGTCCTCGTCATCGCGGTCCTCCTCCTCCTATC
GATCCTGATCGCCTGCCTGATCGTGTGGTGCTGCAGGAGGCGACGCAGAAACAGCAG
GAAAAAGTGGATCAGCGTGCCGATGAACGCGCCCGACGTCGTGTCGAAAAAGAACGG
GGTGATCGGCAGGGAGGCGGCCGCCTCGGCCACGCCGGTCGACAGCCGGATAACGAT
GGTCGTGCCGGACGGGAGACTGTACAGGGAGACGGAGTTTCACGTGATCGTGGAGAA
GGCCGAACCGTTGACCACGGAATTGTAACGAGAAGCGTGGAACGGCTCGACCAGCAG
GCAGGCTGCCCGCTCGCCGATGCCTCGAGTCGGCGAGCAACGATACGAGCGGTGCAA
CGCTTCGAGCCGTTTTCGCGTCTACGAGCTGTGAACGAATCGATGTAACATTCGACTAA
GAGCGGACTACTCTCTCTCTCGAAAGAACTCGAGAAAATTTCTCACCCAACGATTATC
AACTCGCGTTGACGAAGAAACGGAACGGGAGGAGATCTCGGAGCATCTCGAGATCTC
TCTCTCTCTCTCTTGTTTTCAATTTAATTCTCCTAGTACAAGGCAGTTCGTAAGCTTCCA
TTTACATATCAAGAACGAGCATCGATTATCCATCCAATCCCCCTTTCCTCCTCTTTTCTTT
TCGTCTTCTCTCCGCGTGGTCACGTACACTTGTATGTAATCGAAGAGTTTCCTTTTCGTT
TCTCTTCGAGCAGGTCGCGTGGATGGATAATCGATGCGCGTTGTTAGTTAAGTTTAAGT
TAAGCGTTAGTTAAGTAGCTTCCAAGAGGGGAAAAAAAAAGAAGAGGAAAGAGGGC
CAGTGTCGTTTCGCGAAGATCGGAGATCGAAGCGATACGAGAGAGGCGACGAAAGAA
GAGGCGAGTACAAAGGGGAGTGATCGAAACTGATAACGGACTCGGTTGACGTCGCGT
TCGAGATTTTCTTATCGGCGATGATCCGTCGAACAAGCGTCGGGAAAATGCGATGATCG
CACGTTCGATCGGAAACCAGCGTGCCATTCTTCCTCAGTGTAGCCATTCGCTTCCGTTT
GGAAAAATGGGGGCGAGCGAAAGACGAAGCGCCGAATTACTCGTCGAAGAAAATTGT
TCGAAGCGAAAATTGTTCACTCCCTACACCGAGAATCCCTGTATTATATATCCCTCCTTC
TGATCGCGTTTCTAATCGTGCTTGTAAACACAATAAAAAAAAAAACAGACATATTACCT
GACACCCCTTGACAATACGAGATAAGAGGAATAACGAAGAATATATCTGCGAAATTATT
ATTACCGAGCGAGTGGAGAAGGATGATGAACGATAACGTAGAGAAGACTTCTTGTACA
CGAATCCATACGAAATGAAACGTATCCACGTTACACGCAAAATTTGTATTCCGTTCCCG
AACCCGTTCGTGAGCGTGACAACAACTCGCGACACCCTTCTCGAGTGTTCACAAAACT
CTTTTCTCCTTTAATTTATTGTATTATTTCACCACGATTATTTCATGTTTATTTATGAGCTCT

GGACTATTGCCTATAATAAATTGTTGTTACTGTTATCATTCCGATCGGTATCTAATTTTGA
CAAATGCGCAAACGGGGGATTTTCCCCTCGACGAGGGTAATCGATTCGCCACGGGGGA
AACCTCGTTCGCGTTTACGAATTCGGCCGGTAATCTCGAAAACGGGGGACGGGGAGA
GGCTTCTCTCTCTCTCTCTCTCTTTCTCTGTCTATCTTTCGGGGAGTAATACGCGCAATA
GCGTTTAGCGTAAAACCGCAACCGACTGCCGCCACTCGAGTATCGTTCCGCGCTTTCG
AGCTGCTTACGGTGATTAATAGACCCGATATAATTCGTCCTCCTCTCCGTTTCATCAAGG
TAAGCCATCGAGATATTTTACCCGTCGAACGAACCGACGACGATCCCAGTGAAAGGGA
AGCTGAAAGGAATTCGAGAAACGAGGAAACGTTGAGCAACAACGAGTATGACGATAG
ATTCGAAGCTGTTTCTTTTTTTATTTTTTAAAGTACAACGAAGAGAAATACCGACCGCC
TCGTACTCTCGCGTGTCGACCTATCTCGCGCACGTGTTACCCATGCATTATTGATACACC
CGCCATTTCCTCTCCACGCAACGAGTCCCGCGCGGCATCGTTTCGTTTTATCCCCCCAA
GAGCGGCGAACAGCGGAGATCGATCCTCCCGAGTTCGTTCGAGATTGTTCAACGATCT
TCCCTCCGAACATTTCATCCGAGGCACGCCCGATTAGAGATCGAGCGTGAAAAAAAGT
GGCGGAAGTGGGAAGTGGCTGCGATATTTCGTCTAACGGAATCTCGCGTGTTCGCGCG
AGACGCGTCTACGATTATCGTGCGTCGGAAAATTAAGCATTTCTAAGGACGTTTTAGTT
GTAATTATCGTCAGACTGTGATATAACGACAGCCGATTGTATAATTTATTTTACATCGAA
AGAGACTTCGTACCGTCGAGATAATAAAAAAAAAAAAGATAATAAAGAAGCAAATTGA
TGCATACAGTACACAACTGACGACCACTAGATATTCCCGATCGATATTCCCAAAATACC
ACCTTTCATACCTTTTCAACCTCCTTGAATAAACCGCCTCTTTTTTCTCCCCCCCTTTCC
TTTACTTAATGAATATGAATACACGCGTGAAAAAGGAAAAAAAAATTTGCGATATATCG
TCGCGCGATCGATCATTGCGGTTATTCGCAGTCGCTCGCGCGTAGAAACGTGGAAACG
TGAAAGCTACCGGGTCTATTAATTCTTGCGGAGCATAATGTACCGATTCATAAACGGTTT
CAATAACGCGATTATAACCGGGTATGATTCATTTTTAATGAATCTGTTCGTCGATTTGCC
GTTTAATCGGATCTAATCGAACGTTTCGACGGAATTGAAACGTTGGAGAGGAAATAAC
GGAAAAATTTTTATCCGTCGTATTAACCAATACTTGGCTGATTGACCAAGTATTCTATTT
AATCGCGGATAATATTCGTATCGTCGATGAAGATCGACCACTCCATTTCAACCTTCAGTT
CTCGCGACGAGCTCGAGCTCTCAAGTGTGCGTGTGAAAAAAAGGAAACACTTTGCAC
TCACCTGAACGGAAGAACGCCATCGAGGGCCTGGGAAACTGTAGGGGGTGAAGCTAG
AGCGAATCCTCGTCCAAGTAAGGTCGAAT

>novel_circ_000012
CGGCAGGGGATTGGAGCAAGGTGGGAAAATCCGTGGAAACGGGTCGTGATGGTCAAC
GATGCCGCACGTCGTCATGGATGTAATTGGACGTTTCCGGTCGAACGAAGGAAAATAA
CGACGAGGACGACTTGGACGTAGTCGAATATCGGGGGAAATCGCGAATGATCGTCGAA
TGACGAGCGGTGAAAAAACGGCTCGGTCGGAGATAAGTGGTTGATCCCCGGTGCAAA
TATGACCGACAGGATTACGTAGTGGCGCGTGGCGTGTCAGACCAACTGACACGGGACT
TCCAAGTGCGTCCTCTTCGATTTCGTTGCTTTCGGAAGCTGGATATATCGAGGAGATAG
CATTATCGGTCGCGGGACATATATCGCAGGCATGTCGAGACGCGGGAATTCGGGTCGTC
GACTCGACGTGGAATCAGCTGACATCGACTCGGCGTGAGGCGAGCCGACGTGTTCGA
GAAGAAGAGAAGACGAGAAGAGAGGGAGAGAGAGAGAGGGAGGGGGGGATAGATC
GAGAAAGATGGAAGCAAGGAGATTCGTGTTGGGAAGCGCTATCCTCGGACTTTTTTGG
AGCGGATTGTTCAAGGGTGCCCTGACCAATTCGATGAACAGATGCGACTATCCTCCCTG
CTCCTGCGATTCTCACGGCAGGCTGACCTGCGACTGTAAAGAGGAAGGAGAG

>novel_circ_000013
GTTGCGTATTCGGTACGTGATGCCGGCGGCGCACCTGACGCTGCGCGAGGAGGATGTG
GTACGACTAACGTTGGAGTTCCTCCACAGCCGAGACCTTCACATCTCGCAGCTCTCGT
TGGAGCGAGAGACGGGGGTGATAAACGGCCAATACAGCGACGATGTCCTGTTTCTTCG
CCAGCTGATCCTCGACGGTCAATGGGACGACGTGCTCGAGTTCATCCAGCCGTTGGAG
GCGCTCCCGGACTTCGACATGAGAAAGTTCACCTACTCGATCCTCAGGCACAAGTACG
TGGAGCTGCTCTGCATCAAGTCCGAGGCGAACTTCATCGCCGCTGGGACGAACGGCA
GCGTGGACAACGCGGTCGAGGAGGTGGTCAAGGTTCTGAGCGATCTCGAGAAGGTCG
CCCCGTCCAAGGAGGAGTACAGCAGCTTGTGCCTGCTGTTGACCCTTCCTCGGCTCAC
CGACCACCTCCAGTACAAGGATTGGAATCCGAGCAACGCCAGGGTTCAGTGCTTCAGG
GAGGTGCACCCGTTGGTGGAGAAGTTTCTGCCCGGGGACAGGAAGAGCACCGATCCG
GCTCACCCGGTCACCTCCGCGAAAAACGATCGGCTGATACAGCTGATAATCAAGGGGA
TTTTGTACGAGTCGTGCGTCAATTACTGCCAGGCCAAAGCTACCGGCTCCAAGGAGAG
CGAACAAGTGGAAATGAATTTCTCCAGGCTGTTGGACGGCTCGGTCGGTTTCAGCGAT
TCCGACTTGAGCCTACTCTCTTGGCTGCAAAGCATACCGCCCGAGACGTTCGCGGTGC
CTTTCGCGCAGCGCACGTTGAACGTGGACGTGGAACGGTTGGAGAGACCGTCGTTGG
AGACCTCCTGGACCGAGCACATGTTGATCACCCCCATAAAGCCCAAGACTTTCCCGCA
CAGCGCGATGCCGTTCACGAGGCCGCGGTCCGCCGCCGACATGATGTCCCGCAGTTTA
GTGCCGGCATTGGAAGCTGGACTCGGACCAAGGAGTCCCGGGCCTAAAAACACCACG
ATACCATCCGCGGCGCTAATGGCTCTGTCCACCGGCGATATAAACCCGATGTCGAGATC
CTCCTTCGCCAGCTTCCATTTGACCGGTTTCAAGAACAACAAACTGATGAACACGAGC
GTGGACAGACTGTTCGAGAACGAGGGCGACGTCTTCCTCAGCTCGAGCTACGCCGAG
TTCCAACAACTGCCTTCCATACAGGAGACCACGGCCAACAATCAGCCGAAGGCTCCTC
CCAGAACGAGGGGACACTCCAAGAGTCCCGAGACTATCAAAGCCGATCCGTCTGCGG
CCTCCACGCCGGAGCGTAGAGTCGCCGGCAGAGAATCGCCGGCCCCGTCGACCGCCA
GAAGCTCGAGAAGGGATTCCTTGGCGGAGAAGCCAACCGGTGTCGCGGCAAAGATCA
CGGAGCCAACCCCGATCCCTGTCCGAGCCTCGCTGGGTGGAGATCATCTGGAGCAGAG
TTACAACGGTGACCTGTTCAAAGAATATCAAAAGCAGAAGCAAAGGCTGCAGGAAAA
GGTTCAGCAGAAGGAAAGGGAGTGGGACGATTTGCTGAGGCAATTGTCCGCTCCGTT
ACCTGCACAAGTGGTCGATAACAGACTTCAGGGAGACGG

>novel_circ_000014
GTCCTCCACTCGCGCAGTTTCCTCGAACGAGCCTCCTCTCGAAGAGAAGGAGGAGGA
GGAGGAGAGGAGGTCTCCGGCCCCGTTTCGATTTTTGCCGTGGCTGGGTATGCGGGC
ACACCTACGTCAAATCGTAAATACATTTATTCATGTTAGGCAGCCGCGAGTATTGTATTC
CGTCGTGGCCCCGGTGTTAGACAATGAAACGGGGGCGTTTTGCCCGTTCCCGCTTGCG
CCGCCGCATCTCGGCTGAGACGGGACGCGTTTCCAACCATCTCCATCCACTACTCGTG
AAAAAGAATTTTATTTCGAGAAGTAGCTTCTTCGGTTTCGCTCTCGCGAATAAAAGAGG
AAAGGAGCAACAGGGATATACGTGCACATTGTAGACGAACCGGACACCGGTTTGCAC
GTCGCCTCGCCATTCGTTCCTCCTGCTTACGTGCCGATACACGTAACTCTCCGCAAAGC
GGGCGGCTGGTTAAAAGCTGGCGGCGGACGGACGGCGATTCGCGCGACAGTTGACGC
GGTGGTCGATGCGCGCGCATCCTCGCTGCCTCGCGCGGCAGAGAAAGGTGCACCGTG
CAAGGTTTATGGCCGCGGAGGGATGCGGCGCGTCTGTGGACAGGGATACTTATACGCG
GTTACTTATACGGGTGGACCGGCGAATAAATATAATGCCTGGCGTGGGCGGCCATTGTT

CGCACGCGTTAAAGTTGAAACGCGAAACAGGCCGACCGTAAATCCAAGATTTCCAAG
TTAATGGCTCCGCCGTAAGGAAAGGCTCTCCTCTCGCCCGTGAGAACTCTGGGTTTCTT
GGACGACTTTTTCGGACGCATTTGCATTTGAACACTTATCGCGCGAATTTTTCCATCTCT
ACGAAGAGGAGGAAAATGAGAGGAAGGAAAGACAGGTTGGAGGAACGGAAGAGCC
GGTTCGACGATGGTTGTACGAATGACCGCGTGAGGATGGATCAGGTGGCGGAAGGAA
CGAGTTACGAGAAGAGGCGCATCGATCGCCTCGTTGGATGGATGTGCACGGTACACGC
GTGTTCGCACGCGAACGGCGGACGACCTCGGGCCGTGTACTTTGCTCGTAACGCACGG
AACCCTGTGCTCCGGGGAGCCCCTTATTACAGATGCGGAATCAATTTCAATTTCGCGCC
ACCTGCGATCTCGCCCTATTACCCTTGCAACTGCTTCGCGTCATTCTACGAAGTCCAGC
TTTCCATATTGGGTATGTAATGCGTTTATTATTAGGATGAATTTGAGACTCTTTAACCCTC
GACGGGTTACATTATCCGTTCAAATCTATGTAAGTGGAGTTAAATGGAATCTCCTTAAA
AATTCCTCGTAAGATTTTTATTCTTTCTTTTTTATTTTAAAATTCTATCAGGATGGCTCGT
AGCCATTGAAATTTTTATGGAGAAAAATTTAAATTGCGCCTTCCCTTACGGAAGGAGGG
AAATGAAAGGGGGCGAGACGAAGTTGATCGAGGCTCGTGGGTGGCCCCTGGCATCGA
CGCTACCTGGTAATACTCTTAATCACGGTCGAGCCTTTCACCATCCCGCTTAATAAATGT
GATTTTGAACGAATACCAGAAGCATCGTGGTCGATGTAATTGCTGGTAGACGTGATATA
TCTCGTGGAGTATATATTGCGATATCGATGATAGAAGACAAAGACACGCCGAAATTTTT
CTTCTACAATTCTTATGGTTAATTTGTTTAACTCATTGTTAATCATTTGAATGGAACAAGG
CTGTTTGACAAGTGGACTATGGCTGGTAATGGATCGAGTTTGGATCGAATATATGATTTT
TAAATCGATAGACGAGCAATAATGATAATGGTAATGATCAGTCAGATAACGATCGGCGA
AAACAGAAACAAAACTGCACGGTCGATATCGGCGAGCAGTATAATAAGGAATGCGTAT
TTGCTCGTTTTTTTTTTTTTCGGCTACTCGACGAGATATTTACGGGCGGTTGCTATCTTC
CTTCTTATTTGCCCGTGTCTGATCGTGGCCATATTGAAAAGAAAAGAAAAAAAAACTCT
TATTTGCGATATTAACGTCTCGACGACGTATTAATAACATGTAAATGTATTTTGTACCGGC
CACTCGTATTTTCTACAACCATCAATTTTATATCTGATTCCTCGTATCGCGAACGTATTAA
CCAGTATTCCGTCCAGTGGTATAAATCCTATAATTTCTCGCAAAATCATTTTACAAAATC
ATTTTCCCGTGTCGTCGAACTCTCGCGTTTCTTTCTCCTTTTTTTTCTTCGACGAACTCC
GTCCGTCGTTTGTCACGAAAAAAAAAAAAAAATTGTGTAAAGACATTACAAAATCATAC
CTCTGCAATTCTTCTATTTTTCTTTTTGTTTTTCCTTCTGTTCTCTCCTCTACGCTCGACA
ACAGCGTAACTTTTGTTGATTCACTTTCACCAAGTTCGGTGAAGAGACGAGAGTATCG
ACGCGGGTAGAATTCCTTTGTACGAGCGAGCATATAAGCGTTTGTGAAATTCAGCCTAA
CTTTGTTCAAACGTTGCGAACGTTTAGCAAAGTCAGGGGAATGCCTGGTGAACGTGTT
ACACACGGAGAACGAAACAAATTAATGGTTTTCAAGTTGATTTTCGTGGTGGTTTCGT
AACGGGGACGTAACGAGCAAACGAGCGTTCGCGTACGTTTTCAAAAAAAAAAAAGAA
AAGAAAAGAAAAGAAAAATGGTATAAGCAGTTTACAATTGAATCTCTCTCCGGGAACA
AAAACTAATTTGCTTAAGAGGAAAAAAGAGAAAGAAAAATTCGAGATTGCACAATGC
GATAATTCAACGTGATAATTCGGAATTGAAATCTGAAAGAGAAACTGGGAGAATCGAG
GGAGGAAAAAAATTTCTGCATTTTTCTGCATTATTCGATCGGAAACTGGAACGTTTCTG
GCCCAACCATTGCCATTATTTCGATCGATTTTATTAGCATCGTAAGCTGAATGGCCCTATA
ATTCTATCGTACGAAACGTTTACTAACTATTTCACGCGGGTTACCGTTTTTTCCGTGTTA
CACGAATCCAAAGGTAAGCACCGTTTCGAGCGCGAACAGTAGCGCTGAATTGTTTTCA
ATCGGTGGACGATTGGCGGAGAAACGGCGGCCCATTAATAATTACCCGCCGCTTTCCCC
TGGAAAACCAGGAAAGCCCGAGGGACAGAAACGGACAGGAGAGCACGTACGATTCC
GATTGAGCCGATAACGCGAAACGTTCGAATTACCATTCGTCCTTTCACGTGTTGCCAAT

CCCTGCGCACGCGGCCGCTCTGCGCAACGCTTTAATTTATCACATCGCATTGTGACTGT
ACTGGGGTAGTAATTTCGCGATATAAATCGTTTCGGCTCTCTCGCGCGCAACGCAATTC
AGCTCGCAGTTTCGCCAATCGCGAATCCCGATTCTATCGAACGAAATCGGACGAAGAA
AAAAAAAAGAGAGAAAAATTTCGACTTATAAGGAAAGAGACACGTGGTGGTTTATCG
CGGCAAGCATCGCGCGAAAGTTCGATAATTTTACGGTTTACTCATTGAGGAATCGCTCC
CGTGGAAGAAACAGCGGTGGCAGTCGAGCAGCATTTAAAGAGAAAAGACTTCATTAT
GCATCTCGGGAACGAGTAACGATCTCGCGTAACGGATACGCTGATTCACCGGTGATCG
GCCGATTTATTAATCGCCCGAGGCTTCCCTCCCTTCCCTTCCCTTCCCTCCGTAAAAATA
TACGAAAAGTGTTTCGCGCGGGTGTTATTATTGAGATCGGACTCGTATCGATAAAAGAA
TCGTCGAATGACTTAAACGTCACGCGAAAATTACGTTCATTTAGGCGGAGATTAAATCC
CGCTCTCGATTTTGTACTCTCATCTCTTTTAATTAACCTCTCGATCGATGATCTCGTAAAT
GCATAAAAAGCGCAGCTCTCGATAATCGTGCAAGTAGTGGTGGATAAAAGAGAAGAGT
TAGGAAAAAGAAGAATAATAGAGCGATAGGAGGAAGAGATGGAATAATTGAAAGAAT
AAAGGAGAGGGAAGAGAGAGAGAGAGAGCGGATGAGTTCCCCCCCGTGTTGCTGCG
GACAGGCATGTAGCAGGTAGGTTCGGGCGAGCGCGCATGTGCGCGTGCCGCGGGTAG
GCGGTAGCGAGCGCGCATGCGCGACGCGGTTCATGTAAATCGACCATGCGCAGTGGCG
TTAGCGGTGGCGCGTTCGTGCTGTTGCAGACCGTACGTGTCGCGGCACGTCCTTGTAA
GGCCACTTGCCTCCCGTCTAGTGGTGCGCACGCGTGCACACTAGCGTTGCGCGAACCG
TGTCTAAGCGGCGCGATGCATGGTGCGCGCGCGCGCGCGTGTGCGTGTGCACACGTGC
CGTCCAAGTGGCGCTGCGCCGAGGTTTCGCGGCTTTCTCTCTCTCTCTCTCTCTCTCTC
TCTCTCGGTGTAATGGTGTGTGCCGCCGCTGTTCACCGGGGGACTGACGCAATGCGCC
GTGTACACGCGTTTGCTCGTAAATCGATTCCGAGGGAACGAACGAGAAGGAAGGGGC
AAGTCGAGGCAAGAAACTGAGCGAGAGCTCGCCTTAAGCAAAACACCTCTCCTAGAA
GACTAGCTCTGCCTCCCGTAACGACGGGTATTGTCGTAATGAATGCTGTTATCGCGACG
ATCGTTCGCTTCTTCCGTGCAAGGAATTTTCTATTGAAAATTCGGTTCGCCCGAATTTAA
GTATAATTAGGGAGGGAAGGCAATGATTTTTCTTTTTGCAATGTTCGAATCGTATTTTTT
TTTTTATATAAGATTATATAAAGGAAAGACTCGAGTACGTTACAAATTACGTTCTTTCGA
ATCGTAATCGAATTACGAGATAATCTTCCAATTGAACGAAAACACGGGTGTTCGAAATC
TAACTTTCTCCATCGAGCTCTTATCTCTTCTCCTTCGTTTCGAGCGTAACTCGTTAAAGA
TTCCTCCCAGGACGAAGAGAGAATCTATCGCATCTTTTTTTTTTTATTTTCCTCTCGCGT
TTCGTATCGAAGCTAAGCTAGTCTATCCTTTTCGCCTCGTCTCTTTCCTCTTGGCGATAC
GCTCCTAGCCACAGGCGGGGAACCGAAGAGACTGTCGAAAGTGAAAGGAGCCGAAG
AAATCAAGATGGATCGTGCGTTCGTTATAACGCGGCTTTTCTCGCTGCGTGCTGCACGC
ACCTCCACCTTCCACCTTCTCTTCTTCCACTGGTTCAAGGACTCGCGGCGAGCGTGGC
AGCGTCGATATATACCCAAGGCATCGATACCATCTCGTGCCGCTCGTGGATGACGAGGT
GGTCGGGAGAGGGGAGGAGGGGAGAGAGACAGACGAGTCGGCCGGTTGTCCCGATC
AAGTGAACCGTGTTAACGTCAGCCCTGTTAACGGGGAGTCGAATTAGTATCGACGAGG
GTGAATCGATCGATCCCCGTCCCTCCTCTTTTTTTTTCCTCTTTCCCTCTCTCTCCTCGTTT
CCAATTCCAGCTCTCGGCTGTCGAGGGCTTGACCCCGTGCACGGCCCTCGGTGCACTT
GTTGCGAGCCGGTGCCGTTAGAAAATACTGCACCGTTCCGGCAAATGGCGTCCGCGCT
TTCGGTATTCCGGGGAGAGGTGGAGGAGGAGGAGGAGGAGGAAGGAGGCCTCC
GCGCCGATTCCGTTATGTTTCCTCTTCGTAAACCCTAAACGACGCTTTTTACAACCCGA
CGTGCACGATTTGACCGCTCCTTTCGCCTTTTCTCGCGTTTCATCTCGGCTTGTTATTAG
GTTCCCCCGAAACGAAGAGACCGCGCGGTTCTCCTCCATCGGTTAGACTGGTTTCTCG

CGGGGTATGCGAAACGGTCGTTAAGAGAAAGTACGTAGGTGTGTACATATGTGTGTTA
GTGTGTGTGTGTTCGTGCGTGCGTGTGTGTGTGTATACGCTTGTCCGTAGGATCGTT
GGATCTTTATAACGCGAAGATCGGAGAATTCCACGTTTCACGTGGCCCTCGCCGACGGT
ATCTTTACACTCGAACGGAGGATAGAGAGGGACGAGGTGAAAGCAGGAAGCGAGCCA
CGTACGCTCGGATCCCTCTATTCATCGATCGAACTATTATCCGTTGCTTCATGGTTCAGG
TATTATGAACTCGTCCATCTTTTTTCTTTTCTTTTTGCGCGGAATTCCGAACGGAAAAGA
AACGAGATAAAAGTTGCGCTTAGAATTCGTCCAACATTCCCCTTGCTTTGTATCGCCTC
GCTTCCATCGAACAGGGAGGTTTCCGTCGTGGTTTCAAATTGCGACAGTTCGATTAAAA
TTTAATCGATCGTTCGCAACTTGGATTATGATAAACCGTTCGGATTCTTGGCAACATTCC
GACCGGGATGGAATCCATGTGGAGGAAGAACGATCCTCCTCCTTCCCTTCTCCCTCTCC
TCCCGCGCACAGGTCGACCTGAAAATCGCGTCGACCCAGAATGTCGCCGCGTCGTTTT
TTGTCCCGATCGATTGTATCGGGACGATCTCGCGTCCTCCGTTCCGTTCGAAAACGCGT
GGGACGGGGTCCGCGGCAGGCGTGTTACTCAACGTTATTACAGGTTGTTAGCTACTCCT
TTCGCGGCGTGACGAATTTGATAACTTTCAGGCGCTAATTTATTCGGTCAAGGTCCATTA
TGCTCTCGGTTATAACGGCCGGGCCACTCATTATGCGGATCCGTTAGCCTGGGATCGTT
GACGCGTCAAAAAAAAAAAAAAAAAAAAAGGAAAAGAAACAACTTTAACAACTTCTC
CTTATATCAATTTATATCGAATATCGATATCGCGGCGTTCGAAGAAATCTGACGAACCGA
TTAAAAAACGATATGTTTTCACGATTGTGAAAAATCGTGATATCAGCGAAATTACAATTT
GGATGAAACGAAATGAAAAAGTTTTAATAACAAATTTTTTTAAAGTCCGCGTATAAGTC
GAGGATAATGATAACTAAAATAAATCGGAGATAATTTTAAAATCGTTTCATCCTTTCTTT
TTTTGCACGCCCGTTTAAAATGTTTTAAGACGGAGTAAAAGGCGAATCGAGCGCGGAG
AATAAAGAGGGTTTAACGATTCCGCCGATTTTTTCGGAATCTTTATTATTTACACGCCAA
TTTAATCAGTTTCCGAAGAAGGCGTTCTCGGAAGGCGTTCAACCTCGTACAAGTAAAA
ACACCCGCACAATTATGTCATAAAGCGGCCAATAATTTTTTTTCACCATCGACAAAGTT
GCCGACTCGGTGTTCGCCTCTCGCTTCTATCCTTTCCCGAACCGCTCCTTCTCCATCCGA
TATCCCCGAGTTCCCGATTATCGAGTTCAACAAATTGAAGTAACACGGTATCTTAATTAA
ACTTTGTTTGATAAACGTTGCCGATCAACAACGGCGTTCGACTTTTTTTCTTTTTCTTTT
TCAGCCAAGCGGACGTGAAAGGGTGATGGGCACGGTTCTTGAGAGTTTCGAAAAAAA
AAAAAAAAAGAAAGGAAAAGAAAAAAAAGGAGAAAAGAAAGGAAGAATTTTGTAC
GCTGCCCACGGCGCGGCATCGGGGGACTATCTGAAAGAGAGTCCAGAAATCAGGGAG
AAGATAATTCGATGTTTATG

>novel_circ_000015
GACGAGGTGGGAGGCAGTACGGTGGTAGCCGAGGGTGTCGTAGGCGGCATAAAGGGC
CCAGGCGGCGGGTGCCGCGCATCCTCGATGACGATGGCCGAGAGCGCCGTGGAGGAC
GCTGCAGCCGCGCCCCCGGGTCCGGCCAAGCCGCCACCGCCCTCCTGCACCAACAAC
AACACCCTCGCGGCGACGGCCAACCGGAACCATCGCAACCTCCGCCGCCGGAAACGA
CTCGACCAGGTCCTCGACATCCTCCAACACCGGAGCTCGCCCTCCGAGGGCGAGGTGT
TGAGGTTCGAGGGTAGCGGCCCCGCGTCCGAGGAGGAGGAGGACGTGTTCGGCTCCC
CGAGGTCGTCTTCCAGATCGGCCGCCGACCCGCCGTCCGGCATCAGCCTTCTACCCCT
CAGGTTGAAGAGCGAGGAGCCCGCCACGCCTACCGGCGAGGACCAAGAGGACGGGT
CGAGCGCCAACCAGTCGACCACCGCCACCACCGCCACCACCACCATCACCACCACCA
TCACCACCACCACCACCACCACCACCACCGTCACCACCAACAACAACAACAACAGCA
GCAACAGCGGCAGCAGCAACAACAACAACGACGACGAGGAGGGCGACAACAACGGC

GAAGGGACGCAGCAACAGCAGCAACCGCAGCAGCAGCAACAGAATCATCACTCGCA
CCACCACTCGTCGCACCACCACCGCCACCGGCACAGGCATCACCACAGGAACAACGC
CAACCACCTGGCTTACGACCACCTCCACCCCCCTCATCAAAGATCCTCGGCACCCAAA
TACGCGGGCTGCACCTGCGTCCAGTGTTCCCAGTCCCCCACTCCCCCCATGGTGCTCCC
CTCGCCCACCTCCCCGCTCACCCCCCTCACGCCCCTCACCCCGTTAACCCCGCTCACCC
TGCCCCCGTTAACGCCAGGATCTCTCTCGTCGTACAGTTTCGAGCACTACATGCACACC
AAGTACCTGCCGGATATATTCAGGGGACGGAGCCACTCGGACTCGGACCTGGTGCCGG
GATGGGAGCAGAAGCCGCCGCTCTCCACCGCCTCGTCCGTGTTCGTCAACCACCCGTT
GAGGACCGGTTTCTTGGAGAGCGACACGACCAGCCCCCAGGAGTCCCCGCTCGACCT
CTCGATGAAGAACCTGATGGCGTCGGCCGCGGCGGCAGCCGCGGCGGCCGCTGCGGC
CGCCGCCGCAGCCGCCGCCGCCTCGGGCCGCCCGCCCGCCCTCCAGCTGCTCCCGCCC
GGCATCGTGTCGAGGGGCTCGGTGAGCGTGCCCGTGGTCAAGGGGGACGTGGCGTCG
CCCACCACCAAGGAGAGCGTCGCCGTCAGATACAACCTCGAGGTGTCGCCCGTCGTG
GAGGAGATGCCGCCGGGGGCGGACGTGGCGTACGTTTGCCCCGTGTGCGGGCAAATG
TTCAGCCTGCACGATCGTTTGGCCAAGCACATGGCGTCGAGGCATCGCAGGCAGGGGC
CCCAGGACGCGTCCGCCAAGGCCTACCTGTGCGACGTGTGCAAGAGGAGTTTCGCGA
GGTCGGACATGCTCACCAGACACATGAGGCTGCACACGGGGGTAAAACCGTACACGT
GCAAGGTATGCGGCCAGGTATTCAGCAGGTCGGACCATCTGAGCACCCACCAGAGGA
CGCACACCGGCGAGAAGCCGTACAAGTGTCCCCAGTGCGCGTACGCGGCCTGCCGCC
GGGACATGATCACCAGGCACTTGAGGACCCACGCAAGATTTCCCGACGTTCAGACGCC
TAAGAGCGAGCCCGGCCTCCTCCCAGGCGAGGACAGCCCCACGTTCGCTCGGGAGAT
GGCCTCCCCCTCGGCAACGATCAAAGCCGAATGACGACGGCCAGGTGGATGACCGCC
CCGTTTCTCGCCGCTCCCCAGCGGATTTCGAGGAGGAGGAGGAGGTGGAGGAGGAGG
GAGATCAACTTCGACGAACGGTTTCGGGATCTGGGAGGTGGAGGAGGTTGGAAGGTC
CCGATGACGACCACGAAGACGAAAGAACGAGAGGAGGACAACGATGGGAG

>novel_circ_000017
ATAGAGAGCACTCTTCCTCCGCCGGCGATCGAAGGTAATTTGATCGGGCGCCTCCGAG
ATCCAGCTGGGAATCCCCGAAAGAAACTCAGAGGCGCGAGTTATTGATCGACTGTGGC
CAACGCCAGACAGAGAAAGAGAGAGAAGGAGAGGGCGAGGGAGAGGGAGAGGGAG
TGAGAGGAGATCGTGCCCGACGGGGGAGCCGTAAGTGACGGGGAGGGGAGGAGTAA
ACTTGTGAACCGGAGAGGGGAAAAAGTGTCGTGTGCGGGTACATAAAAGAAAAAAA
AAAAGAAAAAAGAAAAAAGAAAAAAGAAAAAAAGAAAAAACAAAAAAAAAACGTG
GAATCAAGAGGAAAGGGGGGAAGAAGAAAATTTCGAGAGATAATCGAGATAGCGTAC
AATAGTTGGGCAGTGACGAAAGTGTCGAATGTTTTTCGTGTCGGAGAGAGAACGTTGC
TCGGTGATCGGATGTATAATTGAAGAAGAAAGGAAGAACGAGGCTTTTGGGTTCCTTT
CCTTCCATGGACAAGCGCGGTAGAAGATAATCGAGCGCACCTCGAGCCAGGACCGCC
GACATCCAAG

>novel_circ_000019
ATAGAGAGCACTCTTCCTCCGCCGGCGATCGAAGGTAATTTGATCGGGCGCCTCCGAG
ATCCAGCTGGGAATCCCCGAAAGAAACTCAGAGGCGCGAGTTATTGATCGACTGTGGC
CAACGCCAGACAGAGAAAGAGAGAGAAGGAGAGGGCGAGGGAGAGGGAGAGGGAG
TGAGAGGAGATCGTGCCCGACGGGGGAGCCGTAAGTGACGGGGAGGGGAGGAGTAA

ACTTGTGAACCGGAGAGGGGAAAAAGTGTCGTGTGCGGGTACATAAAAGAAAAAAA
AAAAGAAAAAAGAAAAAAGAAAAAAGAAAAAAAGAAAAAACAAAAAAAAAACGTG
GAATCAAGAGGAAAGGGGGGAAGAAGAAAATTTCGAGAGATAATCGAGATAGCGTAC
AATAGTTGGGCAGTGACGAAAGTGTCGAATGTTTTTCGTGTCGGAGAGAGAACGTTGC
TCGGTGATCGGATGTATAATTGAAGAAGAAAGGAAGAACGAGGCTTTTGGGTTCCTTT
CCTTCCATGGACAAGCGCGGTAGAAGATAATCGAGCGCACCTCGAGCCAGGACCGCC
GACATCCAAGGGGGTCCAACGCGTTACTTTCCTCGGGATAATGCGAAGCCTGGAGAGG
TTATCGGGTAGCGATCAAGACTGTGATCAGGAGATGTATATCGACCTGGACGACGCTTC
CGTCGACTCGCTTACCGGAAAACGAAGTCGCCATCATGTCGTTGGCGCGGAGGTGGGC
GAGGAGAGCGACAGCAGCGGGAGCGAAAGAAGCCCTAAACAGGCTAAAAGAAGGAA
TCGTGGTGGCCCACCGACGACGAGGAGGCGAAAGAGCGGGATCTCTGCGCGGGAAA
GGAACCTGAGGAGGCTCGAGAGCAACGAGAGAGAAAGAATGAGAATGCATTCCCTAA
ATGACGCTTTCGAGCAACTACGCGAGGTGATACCGCACGTAAAGATGGAGAGGAAACT
CAGCAAGATAGAGACGCTCACGTTGGCCAAGAATTACATAATGGCCCTGACAAATGTC
ATATGCGAAATGCGTGGGGAGGAGCAACCGTACAC

>novel_circ_000020
GGGGTCCAACGCGTTACTTTCCTCGGGATAATGCGAAGCCTGGAGAGGTTATCGGGTA
GCGATCAAGACTGTGATCAGGAGATGTATATCGACCTGGACGACGCTTCCGTCGACTC
GCTTACCGGAAAACGAAGTCGCCATCATGTCGTTGGCGCGGAGGTGGGCGAGGAGAG
CGACAGCAGCGGGAGCGAAAGAAGCCCTAAACAGGCTAAAAGAAGGAATCGTGGTG
GCCCACCGACGACGAGGAGGCGAAAGAGCGGGATCTCTGCGCGGGAAAGGAACCTG
AGGAGGCTCGAGAGCAACGAGAGAGAAAGAATGAGAATGCATTCCCTAAATGACGCT
TTCGAG

>novel_circ_000021
GGGGTCCAACGCGTTACTTTCCTCGGGATAATGCGAAGCCTGGAGAGGTTATCGGGTA
GCGATCAAGACTGTGATCAGGAGATGTATATCGACCTGGACGACGCTTCCGTCGACTC
GCTTACCGGAAAACGAAGTCGCCATCATGTCGTTGGCGCGGAGGTGGGCGAGGAGAG
CGACAGCAGCGGGAGCGAAAGAAGCCCTAAACAGGCTAAAAGAAGGAATCGTGGTG
GCCCACCGACGACGAGGAGGCGAAAGAGCGGGATCTCTGCGCGGGAAAGGAACCTG
AGGAGGCTCGAGAGCAACGAGAGAGAAAGAATGAGAATGCATTCCCTAAATGACGCT
TTCGAGCAACTACGCGAGGTGATACCGCACGTAAAGATGGAGAGGAAACTCAGCAAG
ATAGAGACGCTCACGTTGGCCAAGAATTACATAATGGCCCTGACAAATGTCATATGCGA
AATGCGTGGGGAGGAGCAACCGTACAC

>novel_circ_000022
GTGGGCGAGGAGAGCGACAGCAGCGGGAGCGAAAGAAGCCCTAAACAGGCTAAAAG
AAGGAATCGTGGTGGCCCACCGACGACGAGGAGGCGAAAGAGCGGGATCTCTGCGCG
GGAAAGGAACCTGAGGAGGCTCGAGAGCAACGAGAGAGAAAGAATGAGAATGCATT
CCCTAAATGACGCTTTCGAGCAACTACGCGAGGTGATACCGCACGTAAAGATGGAGAG
GAAACTCAGCAAGATAGAGACGCTCACGTTGGCCAAGAATTACATAATGGCCCTGACA
AATGTCATATGCGAAATGCGTGGGGAGGAGCAACCGTACAC

>novel_circ_000024
GTCACAGTGGCGTGAATCAGCTCGGGGGTGTGTTCGTCGGCGGTAGACCGCTTCCAGA
CTCGACCAGGCAAAAGATCGTCGAGTTGGCTCATTCCGGCGCGAGGCCCTGCGACATC
TCCAGGATACTGCAGGTCAGCAATGGCTGCGTCTCCAAGATCCTTGGAAGCAGGTACT
ACGAGACCGGGTCGATCAGGCCCAGAGCCATCGGCGGATCCAAGCCCCGCGTCGCCA
CCGCCGAGGTCGTGAGCAAGATTTCCCTCTACAAGAGCGAGTGCCCCTCCATCTTCGC
GTGGGAGATCAGAGACCGCTTGCTCCAAGAAGGCGTGTGCACGAACGACAACATTCC
CAGT

>novel_circ_000027
AGGGGCCTGTCGGTAAGCCTTACTGATGAGTCCTATGGCAGGCCCCAGGACGAAACGC
TTTCCCTTTCCTATTCTCCCTTCTATCGTCTCTCTCCTTCTTCTTTCAAGTTGCACCGAGG
CACGAGAATACACGAGAGGGAGAAGGGGGACCATCATCAACGGTGAAATGTGGACAT
AGATTGTCATTACTCGCAGGAATTCGTAATTCCTCTCCTAGGATGGTCAACGGGGCCAA
ATACCACGACGACACGAGTTCTTCACATCCATCAAAACACACCAATATTCCAAATTGGA
CCGAGATGAAGACCAGGCTAGGACAGTTTAAATACGACTGCTTTATTTGCCGCAAACT
GTGGAACCGCGTATGGACTTTACGAAAGAGATCTGAAAATCGCTACAAGTTGGCACTT
CAGCATATATTCTCAATAGAGGTGTGTTATACACTCGTGTCGCATTGTTTCTACTTCTACG
GCTTACTTTACTACGCGTCTTCTGCAATTAAGGCAATTTGAATGGCACTCTTTCTTACTA
TGTTCCATCACCGTCATGGGGGAAATAAAACAGTCTTTCTTGTGGAATGCCTCGTGGAA
GGCGAGAGAGAGAGAAGTACTGTGTATTCGCCCACTTAGGCAGATTTGGAACGAACA
AGGACTTCGTGCAAATATACCTGCAACTAATTCCTCTATTCCCTCCAATCGTCTAAGGC

>novel_circ_000028
TAGAATGGACGGGCCGGCGGTATTATCGATGCCGCCGATAACCGCGCCCCAAAAATCG
GCACAATCGCCGCAGCCGCAGTCGCATTCGAAATCCAATCAGGAGTTGCAGGTGGGCA
CCGTGTCCCTTTACGGAATCCACATCGTGTCGCTGGTGATCGAGGGCCAGGAGAGACT
CTGCCTGGCCCAGATCAGCAACACGTTGTTGAAGCAGTTCAGCTACAACGAGATCCAC
AACAGGAGGGTCGCGTTGGGCATCACCTGCGTCCAGTGCACCCCCGTCCAGTTGGAA
ATCCTCCGCCGTGCCGGTGCCATGCCCGTCTCGTCGAGGAGGTGCGGGATGATTACCC
GGAGGGAAGCCGAGCGTCTCTGCAAATCGTTCCTCGGCGACAATGCGCCCCCCAGAC
TGCCGGAGGACTTCGCCTTCTCGGTGCACCACGAGTGCGCGTGGGGTTGCCGGGGCG
CGTTCCTGCCTGCCCGTTACAACAGTTCCCGCGCCAAGTGCATAAAGTGCGCCTACTG
CGGCCTCTTCTTCTCGCCGAACAAGTTCATCTTCCACTCGCACAGGATCGGCCCCTCGG
ACAAGTACGTGCAACCGGACGCTGCCAACTTCAACTCGTGGCGGCGCCACATGAAGC
TGTCCGGGAATCCGCCGGACGAGGTGGTGCACGCGTGGGAGGACGTGAAGGCGATGT
TCAACGGGGGGACAAGGAAGCGATTGCTCAACAACCCGAGTTCGAGGGAGTCGCCGA
GCCCCGCCAAGCAGCCGAGATCGTCGCCCACCGCTCAGATACCCACTCTCGTCCCCTC
CCCCACCCATCCACGGGTGCCCCCGTTCCCGGAGTTACCTCTGCCGCTGTCGAGGAGC
CTGGTGATGGATTACGTGTGGCACCAGCACCAACAGGCCGCCGCTGTCGCGGCGGCA
AAGACGCCCGGCTTCCCATTCTCCCCGTACGCTTTGCCGTGGCTGGCCAAGAGGGGAC
CGGTCCTCTTTCCCGGGCCTCTACCACCGCCGATAAGCGGCTCCAGCCCCACGGAACC
GCTCATACCAACGGTCAACCCGTCGTTGCATCAATCCGCCTTTCGCCCCGTGATCCGCG
GCCCGCCGATCGTCGAACCCGTCGCCCAGGAACACCCCTCCGAGCACACCCCCGCGT

CCCACAAAGAGGACAACTCCGACGACGAGGTGGACATCGAGACGACCGAGGACGATT
CGGTCACGCCCCTCAACATCGCCCCGCAGAACCTTCATCATTCCGTCTCGAGTTCCGCG
ACCGGCGGAACCCACAGCGGCCGCAACTCGCCCCAGTGCTGGAGCCCCCCCCGAGAA
ACC

>novel_circ_000029
TTCCGGTCGATCAGCCGGATCGCGACACACGAAGAAAACCACGAAAACTTGTTCTCA
CTTTAACAACGCTGGCCCGGATGTGTGCTCAACTGCTGCACTGTTCCAACTCATAATCA
GTTTACTCGAACACAAGAATTCCGTTTTATGTTCGCTCGCGCTTTCCCTTCGATACATC
CGCTGCTAAAAACCACGCGCGACTAGACGACGATTGCAAGAGCAATAACGACGACGT
ACTGCGTGTGCGACGATGACGACGGCAGTAGCAGCAGCGGCGACGGCGGCGGTGGCG
GCAGCAATGGCGACGACGAACAACGACGAACGACGTCGGAGGGACCGGCTGATGGA
ACGGGGCAAATATATGCAGCTGACGATTTATAAGAGGGTGAAAAG

>novel_circ_000030
AACAGCAGAATCCGGAGTTCGACCGCGGGAGGAACCAGGAGTGCGCCGAATAAATGA
CGTAAATCCGAGGAACGGAAACGCAACGTCACTCGCTAACTCGCAATTCCCCCTCTCC
CTCCTCCGAACCTCCTCTCCCTCCCTCTTCTCTCTCTCTCTCTCTCTCTTTCTTTCTCTCC
TCCCCCTCCTCTCTCTCTCTCTCTCTCTCTACCTGTCTCTCCTCGCTTTTCCACCGTCTCT
CCTGCCCCGCCCTCCCCCGCCCGCCCCTTCTCGCACCTCCCCCACGCCTCCCCCTTCGG
CACACCACCGCCTCTGACCCCGGAACCACTTACGATCCACGATCCACGCAACCGGACC
CCCGCCTGCCCCCCCGCCTCTTCCCAGGCCCCGCTTACTCACGTCGCCCACTCTCGCCT
TCTCTCGTGCTTACTCTCGAAAAACCTCTGCCCCCTCCCCGTCTCTCTCGTCCGCCGGA
GAGACGACCCCACCGCTCCCTTTCACCTCAGCAGTGAAGCCGCATCTCTCGTCCCTCC
TTCTGCTTCTCCTTTCTCTTCCTCTTATTCCTCTTCTTCTTATTCCTCGAAGAGGTACAGT
TCGGGTAACCCTCTTTCTTCTTCCCCTCCCTCCCAATTCGAGAGAGAGCTACCTCGCCT
CCAGAATCAACCTTCTTCTCTTACGGCCTACCGCTACTACTAGTTACTACTAGTTACTAC
TACTACTACTACTACTACTACTGTTACTATTAGTACTACTACTACCTCTTCCCGGAGTCGT
CCATCCTCGTCCTCGTAGTAGAGTGCCACGAGACACCACGCGTTAGCTTGAGAACCGG
CTTGAAGGACACCACGACCGGCTTTTGAGACGCATATATATATATATATATATATACGCG
TATGTATATGTAACAATAGAAAAGTCGAGCATCGGCAGCAACAGCAGCGACAGTCAGC
CTACTACATATATATATGTATATATATATATATTGTACATCTGTAATTATTCGATAAACGAAA
CGCGCCTAAGAGAACAGCATACTGTACACACACGTATACACACATAAACACACGCATT
ACTCCTCCGTGGTCGACGTTCAGCTTCTCTCTCGGAAGGCTTTCAAGCTTTTTCGCCC
AACTGTACCATCGCTCTGCGTTGCTAAAGCGCGCGCTCCCTCTGTAAAGCTGTTCCGAG
TGCATCGCGCGAGGATAAAAGGAGGAATATCGAAGGAAGGATCGAAGGATTTACCACT
ACGCCTATTATTATATATACCTCGTCTCGTAGAGACAAAAGAGAAAGAAAAGCAGAGA
AAGAGAACAACAATAACAAACAGCATCGCCTAAACGAAGCCTAATATCGCTGACAATA
TATACATAAATATATATACATATATATACACACACACAAAAGAGAAGGAAAAAAAAACGT
ACGTTTGTTTGGTTGTTGCCAACCTTTGATACTACTCCTGTTTCACTTTCTTCTCACCAA
CCTCACGTGACAATTCTTCTTCTTCTTTCGCCAATCGCTACCGCAAATTTCCCCCCACCC
CCGTCGCCCCACCGATCTCTCTAACCTCGCACCTCGAGGCGTCGAGACGCGAACACCT
TCCCCACCTGGAGACGACTTTAAAAAGTCGGTGGCGAGGATAGAAAGGAGGAGAGAA
AAAGGAGAGAAAAAAAAGAAAGGAAAGAAAAGGAAGCTAGCTCTCTTTCTCCACGT

TAACCACGCTTAACACAGCTTAACCGGCAAACAGCGCTAAGAGCCGCGCACACTGGT
CTCTCACCCGACTAAACGGCTGCGATCGATAACGAGTGGTGTTTGATAAAGAGCAACC
GAGGCGATAACGGTGTGTGTGTATGTGTATATGATCGTCTAAAAATAGAGTATAGAGAA
TTGGCCGTACGTACATTTGCCGGTGACAATCGTAGCAAGCCTTTCACGTACACACGAA
CACGGGTGTTCGATAATAATAATATTAATAATAATAATAATAATAATATTAATACGCGACA
ACGAAGGTAGCGAGGCGAATCGAAAAGAAGGAAGGAAGGAAGGAAAATTAAGAGGA
AGAGAGAGGATTTTTCGAGAGGGTTCTGAAAACTCGGGCGAGTTGGAGAGAAGATAA
GTTCTTCGCACGGTTCTTTGCCGCGTGTTCTTTTTTTTTTTTTTTTACACCTACGTGATTG
TACGCACCACCACGGAAGATAGAAAAGCTGGAGGCCTGAAGATAGAGAAACTCTACA
CGGAAGATAGAAAAAGATCGAAGATCCGCAGACTCGCCACTTGGGACCCGTTCATGG
GACAATAAGTTACTCCTCGCTCCTACTCAAAGAAACACTTCTTGCTCGAAAAAGACGG
TGCGAGTGAACGCGACCGTGAACAGGCTACGTGGGGATCGAGAGGAAGAGGAGAGAT
AAAGTTGAAAAGTCGGGTCGAGTAGCGCACCGGACTTTTGTGTTATCCTTCGCTCGAA
GAGGAAGTTATAAGTCGCACGGTCTTCCAGGAATTGGAATTATGGCAATGGTCAACATG
AATAACCTGCTGAACGGCAAGGACTCCCGGTGGCTGCAGCTAGAGGTTTGCAGAGAG
TTCCAGCGCAACAAGTGCACCAGGCCCGACACGGAGTGCAAGTTCGCCCATCCACCG
GCTAACGTCGAGGTGCAGAACGGACGGGTCACCGCTTGCTACGACAGTATCAAG

>novel_circ_000031
GGCCGATGTAATCGGGAGAAACCCCCGTGCAAATATTTCCATCCACCGCAACATCTGA
AGGACCAGCTTTTGATAAACGGGCGCAATCATTTGGCCCTGAAGAACGCGCTGATGCA
GCAGATGGGTCTCACACCGGCCCAGCCACTAGTGCCTGGCCAGGTACCAGCAGTGGA
GGCACCAGCACCTCCGGCACCACACCATCACCTTCAACAGCAAATCCAGCAGCAACTT
CTCGCTACCCACGCCTTTATGGCGACGAATCCGTACCTGACCGGTATGCCGCAGGTTGG
CAACACGTACAGCCCGTATTTCGCGCCCAGCCCCATCATGCCAGCGATCATGGGGCCC
GCGGACCCAACCGGAGTCGGAAGCCCGTTGGGCGTTGTCCCGCAGACGGTGGCGATG
CCGCAGAAGATGCCACGTACCGACCGCCTCGAG

>novel_circ_000032
GCGACGAATCCGTACCTGACCGGTATGCCGCAGGTTGGCAACACGTACAGCCCGTATT
TCGCGCCCAGCCCCATCATGCCAGCGATCATGGGGCCCGCGGACCCAACCGGAGTCGG
AAGCCCGTTGGGCGTTGTCCCGCAGACGGTGGCGATGCCGCAGAAGATGCCACGTAC
CGACCGCCTCGAGGTATGTCGCGAGTTTCAACGCGGGGCGTGCAAACGCGGCGAGAC
GGAGTGCCGGTTTGCGCACCCCCTCGAGACGGTGCAGGCGAACGAGGACGGCTCTGT
GACGGTCTGCATGGACGCTGTCAAGGGCCGATGCAATCGGGACCCCTGCCGCTATTTC
CACCCCCCTCTGCACCTTCAAGCCCACATTAAGGCCGCACAATCTCGGGCTAGCATTGC
GACGGCGACTATGCCGACGAACGTAGCGGGAATGGGCGCGTTGGCTGGCGGAGTGTC
AGGAGTGCCGGCAACTGCGGTTCCCGGAGGACTTCAGAGCGCCAGTATGGCAAGCAT
CGAACTCGGCAAGAAGCGGATGCGCGACTCCAATGATGATCTGCTAATGGTACCACGG
CTTATGCATGAAATGGACATGAAGTCTGTCGGATCGTTCTACTACGAGAACTTTGCCTT
CCCAGGAATGGTTCCGTATAAACGACCGGCGGGCGACAAGTCCGGCATGCCGGTCTAT
CAGCCTACCGGCGCGACGACCTATCAACAGTTAATGCAGCTGCAGCAGCCCTTCGTGC
CTGTGTCAT

>novel_circ_000033
AGTGCAATATTCGTGATTATATATGATAGGATGGACGCAGTCGAGTGGTCGTCGAAGAT
CGGCGAGAGAAACGTACCACTCGAGTGGTAGCCGGCGGTACACGAGGTCTTCCTTGAT
CCTGCCTTTTCTTTTCTCTTTTTCTTGCCTAGTTTCCTTAACACTTCCACGAAAAAGAGC
CAAAACAAGATACATCGAATAACCAAATTTTTTTTTTTTTTCAAAGTATCTCTTCGTTT
AATCGTCTGCACAGACATTTCTCTTTCTCATCGAGCTCTATTTCGGTCGATTTGAAATTC
ACTCTCCGTTATTAATCACTATTTTGCTTCAATATT

>novel_circ_000034
GTTATGCGTTCTTGTACAAAGGTATCAGCTCGACAGTGTTACGTGCGTTTCCTATGAATG
CTGTCACGTTCACTGTGGTCAACTGGACGTTCAAATTGCTTGGCGAAGATGAGAAGAA
GAAGTCGAAAACCACAGAGTCTATCGGTGAGATTTCAGAACCGTGCGAAATACTTTTG
GAAAAATGGAACAATTTCCTAACGAGCGCATCCCGAAGTATGGTTATTCATAGGTTACC
GTATTCCACGTTGTCGTTGACATCGATGAACGAACCGAAGCTCGAGTGGGGCGTGCGC
AATAGGCGAATGAAGGAAG

>novel_circ_000035
GTACTACTTGATTGGGCTGTACTCGAACTACCTGGTTGTTCTGTGCTACCTGAACTGGA
TTGTTGTGTATTAGTGCCAGATCCTCCTGTACTACTTGAGGATGGCAGTCCTGTACTAGA
CGATCCTCCTGTGCTACCTGACGTGGATGGTGTACTAGAGCCAGGTGGTCTTTCTGTGC
TACCTGAACTTGGTTGTTCTGTACTAGCACTAGACGATCCTCCTGTACTACTTGATTGGG
CTGTACTCGAACTACCTGGTTGTTCTGTGCTACTTGAACTGGATTGTTGTGTATTAGTGC
CAGACGATCCTCCTGTACTACTTGAGGATGGCAGGCCTGTACTAGACGATCCTCCTGTG
CTATCTGACGTGGATGGTGTGCTGGAGCCTGATGGTCTTTCTGTGTTCCCTGAACTTGG
TTGTTCTGTACTAGCACTAGACCATCCTGCTGTACCACTTGATTGGGCTGTACTCGAACT
ACCTGGTTGTTCTGTGCTACTTGAACTGGATTGTTGTGTATTAGTGCCAGACGATCCTC
CTGTACTGCTTGAGGATGGTAGTCCTGTACTAGACGATCCTCCTGTACTACTTGATTGG
GCTGTACTCGAACTACCTGGTTGTTCTGTGTTAGTTGAACTGGATTGTTGTGTATTAGTG
CCAGACGATCCTCCTGTACTGCTTGAGGATGGTAGTCCTGTACTAGACGATCCTTCTGT
GCTACTTGATGTGGATGTACTAGAGCCTGGTGGTCTTTCTGTGCTACCTGAACTTGGTT
GTTCTGTACTAATGCTAGACCATCCTCCTGTA

>novel_circ_000036
TACAGGAGGATGGTCTAGCATTAGTACAGAACAACCAAGTTCAGGTAGCACAGAAAG
ACCACCAGGCTCTAGTACATCCACATCAAGTAGCACAGAAGGATCGTCTAGTACAGGA
CTACCATCCTCAAGCAGTACAGGAGGATCGTCTGGCACTAATACACAACAATCCAGTTC
AACTAACACAGAACAACCAGGTAGTTCGAGTACAGCCCAATCAAGTAGTACAGGAGG
ATCGTCTAGTACAGGACTACCATCCTCAAGCAGTACAGGAGGATCGTCTGGCACTAATA
CACAACAATCCAGTTCAAGTAGCACAGAACAACCAGGTAGTTCGAGTACAGCCCAATC
AAGTGGTACAGCAGGATGGTCTAGTGCTAGTACAGAACAACCAAGTTCAGGGAACAC
AGAAAGACCATCAGGCTCCAGCACACCATCCACGTCAGATAGCACAGGAGGATCGTCT
AGTACAGGCCTGCCATCCTCAAGTAGTACAGGAGGATCGTCTGGCACTAATACACAAC
AATCCAGTTCAAGTAGCACAGAACAACCAGGTAGTTCGAGTACAGCCCAATCAAGTAG
TACAGGAGGATCGTCTAGTGCTAGTACAGAACAACCAAGTTCAGGTAGCACAGAAAG

ACCACCTGGCTCTAGTACACCATCCACGTCAGGTAGCACAGGAGGATCGTCTAGTACA
GGACTGCCATCCTCAAGTAGTACAGGAGGATCTGGCACTAATACACAACAATCCAGTT
CAGGTAGCACAGAACAACCAGGTAGTTCGAGTACAGCCCAATCAAGTAGTAC

>novel_circ_000037
AAGCGAGAGAGGTCACCGGGAAAAAGAATGGACCGACTTCGACGAAGTTTTCGCGAT
AGCTTTCGACGGCGGAAAGATCCTCATGTACCGGAATCGAGTAAACCGCATCAATGGC
AAGCGGACGAAACGGCTGTCCGCTCGGCCACATGTGCTTTCCATGTTAAGTACCTGGG
ATGTGTCGAGGTGTTCGAATCAAGGGGTATGCAAGTGTGCGAGGAAGCTCTAAAAGTA
CTTAGGAACTCGAGGAGGCGGCCGGTACGCGCGGTGCTGCACGTATCGGGAGACGGT
TTGCGGGTGGTTGAAGATGAGACAAAAGGGCTGATCGTCGATCAGACGATAGAGAAA
GTTTCCTTTTGTGCACCGGATCGAAATCACGAGAAAGGGTTCAGTTACATCTGTAGAG
ACGGCACGACCAGACGGTGGATGTGCCATGGATTTTTGGCATTGAAAGAATCGGGAGA
ACGTTTGAGTCATGCAGTGGGATGTGCTTTTGCTGCGTGTCTGGAAAGAAAGCAACGA
AGGGACAAGGAATGCGGTGTCACGATGACGTTCGATTCGAAAACTTCAACCTTCACGA
GGAGCGGCAGTTTCAGGCAGCCATCTTTAACCGAGCGATTGCAGGATTCGCGCGAACG
CGCCGTAGATGTACCACCTATAAAGCAAGTCTACAATCCTTTTGCGATAGAGAGACCAC
ACGCCACCCCGTCGATGCTCGAAAGACAAGGTTCCTTCCGGGGTTTCACTCAATTGAA
CCAAGCATCACCGTTCAAAAGGCAGCTTAGTTTACGGATTAACGATCTTCCTAGCAATC
TTGAACGCACACGTAGCCATAGCCTTGAACCGACGGATTTGTCACGGATGCCATCTGCT
ATGTCTCACATCGTACCTCTCAAGCCACCAG

>novel_circ_000038
AAAAAAAAGAAAAAGACAACACTCCTCGAACGAATCCATCCACCTACGAGCTACCTC
CATGTTTTATTTCGCGTACACGCGCACCGTTTCCGCGTATGGTCGCCACTGCTTGGTCTG
TTCCACATTTAACCGAGGATAGATGGCAGCACACTGATTGTCAATCGTGGCGCGTTACT
CTGTGAACGCTCTGCTTCGTAAATACAAAAAAACGATATTAAAAGA

>novel_circ_000039
GGTCCACCGGAGGTAGCCTGAGTCCTGTCACGTCCCTAGCGCTCAATTCCTTAACGGG
GACACCTTTGAACGGGTTGCAGGACTCGTTGAGCAACGCCTACTCGAGCTTACAACAG
TACGCAGCTCTCATTGGAAAGACGACGGTGACCGTGGAAAAGAAAGAATTACATAGAT
TTCCCGCGTTCACAGCGGCGGCGGCGGCGGCGGCGGCCGCTCAAAAGGCGAAAT
TCACTAACACGGATAAGCAGATAGAGGGTCCAGAAGGTTGCAATTTATTTATATATCATC
TACCGCAGGAGTTCAGCGATACTGACCTTGTCTCAACGTTTCTGCCGTTTGGAAATGTC
ATCTCGGCGAAGGTTTTCATCGATAAGCAGACGCAATTGAGTAAATGTTTTGGCTTCGT
GTCGTATGACAATGTGGCGAGCGCACAAGCAGCAATCCAAGCGATGAACGGCTTCCAG
ATCGGCATGAAGCGGCTGAAGGTCCAACTGAAACGATCCAAGGACGCATCGAAACCC
TACTAGCCGACATCGTCCACCGTTCGTAG

>novel_circ_000040
CAAATGCGGGAGTATCGCCGATGAGTATGCAAAACCTGGTTACCCTGGCTGCCATGAC
AGGCGGAAACGGCAACCTTCAAGTGTCGCCAAACACGTTAAGCGGACTGGCGAATTC
GACAGCAGGTATGTCGCTATCTGTCCTTCTTGGAAAGAGCGGAAACACAGGGTCCACC

GGAGGTAGCCTGAGTCCTGTCACGTCCCTAGCGCTCAATTCCTTAACGGGGACACCTT
TGAACGGGTTGCAGGACTCGTTGAGCAACGCCTACTCGAGCTTACAACAGTACGCAGC
TCTCATTGGAAAGACGACGGTGACCGTGGAAAAGAAAGAATTACATAGATTTCCCGCG
TTCACAGCGGCGGCGGCGGCGGCGGCGGCGGCCGCTCAAAAGGCGAAATTCACTAAC
ACGGATAAGCAGATAGAGGGTCCAGAAGGTTGCAATTTATTTATATATCATCTACCGCAG
GAGTTCAGCGATACTGACCTTGTCTCAACGTTTCTGCCGTTTGGAAATGTCATCTCGGC
GAAGGTTTTCATCGATAAGCAGACGCAATTGAGTAAATGTTTTGGCTTCGTGTCGTATG
ACAATGTGGCGAGCGCACAAGCAGCAATCCAAGCGATGAACGGCTTCCAGATCGGCA
TGAAGCGGCTGAAGGTCCAACTGAAACGATCCAAGGACGCATCGAAACCCTACTAGC
CGACATCGTCCACCGTTCGTAG

>novel_circ_000041
GGATGCTCGTCGCCGCTGGTTGTGAAATTTGCAGACACGCAGAAGGAAAAGGACCAG
AAGAGGATGCAACAACTTCAGACGAATCTTTGGAATATAGCAGGAGTCAATATGGCGC
CGCATTACCTGACCAACGATACGCCAGCTTTGGCTCCCGCCTCGTTACAGCTGTTGCAA
CAACTTCAAGCGACAACAAGCGCCGCGACTCCAGTTGCGGCAGGTGCTGGCGCAAGC
GCGCTGTCGAGCGTACAGCATCAATTGTTGCTCCAACAGCACCTCGGTCTCGGCGCAG
CAACTCCTGCGCATGCGGCCGCGCCAGCCGTTCCGAGTCCGCCGGAAATCAACCCTGC
GAATCTGCAGGGACTGGCGACGCTCGCGTCTTTGAGTAACACCGCCGGTGAGTATGCA
AATGCGGGAGTATCGCCGATGAGTATGCAAAACCTGGTTACCCTGGCTGCCATGACAG
GCGGAAACGGCAACCTTCAAGTGTCGCCAAACACGTTAAGCGGACTGGCGAATTCGA
CAGCAGGTATGTCGCTATCTGTCCTTCTTGGAAAGAGCGGAAACACAGGGTCCACCGG
AGGTAGCCTGAGTCCTGTCACGTCCCTAGCGCTCAATTCCTTAACGGGGACACCTTTG
AACGGGTTGCAGGACTCGTTGAGCAACGCCTACTCGAGCTTACAACAGTACGCAGCTC
TCATTGGAAAGACGACGGTGACCGTGGAAAAGAAAGAATTACATAGATTTCCCGCGTT
CACAGCGGCGGCGGCGGCGGCGGCGGCGGCCGCTCAAAAGGCGAAATTCACTAACAC
GGATAAGCAGATAGAGGGTCCAGAAGGTTGCAATTTATTTATATATCATCTACCGCAGG
AGTTCAGCGATACTGACCTTGTCTCAACGTTTCTGCCGTTTGGAAATGTCATCTCGGCG
AAGGTTTTCATCGATAAGCAGACGCAATTGAGTAAATGTTTTGGCTTCGTGTCGTATGA
CAATGTGGCGAGCGCACAAGCAGCAATCCAAGCGATGAACGGCTTCCAGATCGGCAT
GAAGCGGCTGAAGGTCCAACTGAAACGATCCAAGGACGCATCGAAACCCTACTAGCC
GACATCGTCCACCGTTCGTAG

>novel_circ_000042
AACGTAAATTGTTCGTTGGTATGCTGTCCAAAAAGTTTACCGAGAACGACGTTAGAAA
CATGTTCAGCGTGTATGGAACCATCGAGGAATGCTCGGTGCTGAGGGACAGCACCGGG
AAAAGTAAAGCCTGTGCCTTCGTTACCTTCGCTAGTAAACAATACGCCATCAACGCCAT
TAAAGCTCTTCATCACTCGCAAACGATGGAGGGATGCTCGTCGCCGCTGGTTGTGAAA
TTTGCAGACACGCAGAAGGAAAAGGACCAGAAGAGGATGCAACAACTTCAGACGAA
TCTTTGGAATATAGCAGGAGTCAATATGGCGCCGCATTACCTGACCAACGATACGCCAG
CTTTGGCTCCCGCCTCGTTACAGCTGTTGCAACAACTTCAAGCGACAACAAGCGCCGC
GACTCCAGTTGCGGCAGGTGCTGGCGCAAGCGCGCTGTCGAGCGTACAGCATCAATTG
TTGCTCCAACAGCACCTCGGTCTCGGCGCAGCAACTCCTGCGCATGCGGCCGCGCCAG
CCGTTCCGAGTCCGCCGGAAATCAACCCTGCGAATCTGCAGGGACTGGCGACGCTCGC

GTCTTTGAGTAACACCGCCGGTGAGTATGCAAATGCGGGAGTATCGCCGATGAGTATGC
AAAACCTGGTTACCCTGGCTGCCATGACAGGCGGAAACGGCAACCTTCAAGTGTCGC
CAAACACGTTAAGCGGACTGGCGAATTCGACAGCAGGTATGTCGCTATCTGTCCTTCTT
GGAAAGAGCGGAAACACAGGGTCCACCGGAGGTAGCCTGAGTCCTGTCACGTCCCTA
GCGCTCAATTCCTTAACGGGGACACCTTTGAACGGGTTGCAGGACTCGTTGAGCAACG
CCTACTCGAGCTTACAACAGTACGCAGCTCTCATTGGAAAGACGACGGTGACCGTGGA
AAAGAAAGAATTACATAGATTTCCCGCGTTCACAGCGGCGGCGGCGGCGGCGGCGGC
GGCCGCTCAAAAGGCGAAATTCACTAACACGGATAAGCAGATAGAGGGTCCAGAAGG
TTGCAATTTATTTATATATCATCTACCGCAGGAGTTCAGCGATACTGACCTTGTCTCAAC
GTTTCTGCCGTTTGGAAATGTCATCTCGGCGAAGGTTTTCATCGATAAGCAGACGCAAT
TGAGTAAATGTTTTGGCTTCGTGTCGTATGACAATGTGGCGAGCGCACAAGCAGCAAT
CCAAGCGATGAACGGCTTCCAGATCGGCATGAAGCGGCTGAAGGTCCAACTGAAACG
ATCCAAGGACGCATCGAAACCCTACTAGCCGACATCGTCCACCGTTCGTAG

>novel_circ_000044
GGATGCTCGTCGCCGCTGGTTGTGAAATTTGCAGACACGCAGAAGGAAAAGGACCAG
AAGAGGATGCAACAACTTCAGACGAATCTTTGGAATATAGCAGGAGTCAATATGGCGC
CGCATTACCTGACCAACGATACGCCAGCTTTGGCTCCCGCCTCGTTACAGCTGTTGCAA
CAACTTCAAGCGACAACAAGCGCCGCGACTCCAGTTGCGGCAGGTGCTGGCGCAAGC
GCGCTGTCGAGCGTACAGCATCAATTGTTGCTCCAACAGCACCTCGGTCTCGGCGCAG
CAACTCCTGCGCATGCGGCCGCGCCAGCCGTTCCGAGTCCGCCGGAAATCAACCCTGC
GAATCTGCAGGGACTGGCGACGCTCGCGTCTTTGAGTAACACCGCCGGTGAGTATG

>novel_circ_000045
AACGTAAATTGTTCGTTGGTATGCTGTCCAAAAAGTTTACCGAGAACGACGTTAGAAA
CATGTTCAGCGTGTATGGAACCATCGAGGAATGCTCGGTGCTGAGGGACAGCACCGGG
AAAAGTAAAGCCTGTGCCTTCGTTACCTTCGCTAGTAAACAATACGCCATCAACGCCAT
TAAAGCTCTTCATCACTCGCAAACGATGGAGGGATGCTCGTCGCCGCTGGTTGTGAAA
TTTGCAGACACGCAGAAGGAAAAGGACCAGAAGAGGATGCAACAACTTCAGACGAA
TCTTTGGAATATAGCAGGAGTCAATATGGCGCCGCATTACCTGACCAACGATACGCCAG
CTTTGGCTCCCGCCTCGTTACAGCTGTTGCAACAACTTCAAGCGACAACAAGCGCCGC
GACTCCAGTTGCGGCAGGTGCTGGCGCAAGCGCGCTGTCGAGCGTACAGCATCAATTG
TTGCTCCAACAGCACCTCGGTCTCGGCGCAGCAACTCCTGCGCATGCGGCCGCGCCAG
CCGTTCCGAGTCCGCCGGAAATCAACCCTGCGAATCTGCAGGGACTGGCGACGCTCGC
GTCTTTGAGTAACACCGCCG

>novel_circ_000046
ATGACACTCGGCAGGCCGTCGTCGTAGGGTCGACGCAGCTTTCCACCTCCCTCCACTC
GCCGAGAAAATCACCATGATCATCGAAACGCTCGTAAGAATATCATAATCCATCCCCGC
GACAACAACAACAACAACAATATCCCGACACCGCAACAAGTAGCCAAACTATATAGCT
TATATATATATAAAACCCAACCTAATCGGCGAGTCTAATAAAAAAGCTCGTAAAAGAGA
GAGAAAAAAAAAAAAAAGGAGGAAAAGAACTCGAAGAAGAAGAATAAGAGGAAAA
AGAAGATAAGGATCAAAGGCGAAGGAGGAAGAGGAGGAGAGAGAAAAAAAAATAAA
TAAATAAAAAAAAGGAAGCGCGGAGAAGCGTGTGCGTGCGTGTGTGTCAAAGTTGGA

AGCGGGCTGAAGAAGGAAAGTGGAGCGTAAATGCTCGCAGAGGCGCTTAAGCGGGCT
TACGCGCGAGCAAGCGTATCGTGAGCCGGCTTCGTAACGTGAAAACGTGTGAGGGATG
CGGCCCATGGGCGAAGGGTGGGGTCTTTGGGCACCGATAAGGCTTATCCGAAGATACG
ATGGCCGGCTGCTCGGCTCTTGTCGCCAGTCGTAGTGCTCAGGTGGCCGAGGCGGGAT
CGTCGTGCGCTTTAATTCACGCTACGATATCGCGGCTACGCGTCTATTCCGAGTATCACC
ATCGCTATCGCATCGGGCTGGCCGGGATCGCCGATCGTTCGATCGAAGATCGGAGTCAC
GCGTGAGGACCACACGGGAACCAGGTGGCCAAACGGGTGTGCTCCGCAGGGGGTGA
CGATTGGCCGTGGATCGACCGACAGAAACAGGGTGAACGAGGCCGAGGGAAGGAGG
CACCGAGATCCGGCCGCCGGGAGGAGGACTCCTATGAAAATGCTTCAATCGTTGAACG
CTCTGGCGGGTAAGATCTCGCCGGGCTCGCCGACCGACAGCAACGCGAACAAGGTGC
ACATGCACAACAATAACAACAACAATAACAACAACGTGGTGCATCATCATCATCATCCG
TACTCGAAGCAACAGGCGCAGCAGCCGTCGCCGTCGTCGCAGTCGAGCAACGGCGAC
CAGAAGGAGAACAC

>novel_circ_000047
ATGACACTCGGCAGGCCGTCGTCGTAGGGTCGACGCAGCTTTCCACCTCCCTCCACTC
GCCGAGAAAATCACCATGATCATCGAAACGCTCGTAAGAATATCATAATCCATCCCCGC
GACAACAACAACAACAACAATATCCCGACACCGCAACAAGTAGCCAAACTATATAGCT
TATATATATATAAAACCCAACCTAATCGGCGAGTCTAATAAAAAAGCTCGTAAAAGAGA
GAGAAAAAAAAAAAAAAGGAGGAAAAGAACTCGAAGAAGAAGAATAAGAGGAAAA
AGAAGATAAGGATCAAAGGCGAAGGAGGAAGAGGAGGAGAGAGAAAAAAAAAATAAA
TAAATAAAAAAAAGGAAGCGCGGAGAAGCGTGTGCGTGCGTGTGTGTCAAAGTTGGA
AGCGGGCTGAAGAAGGAAAGTGGAGCGTAAATGCTCGCAGAGGCGCTTAAGCGGGCT
TACGCGCGAGCAAGCGTATCGTGAGCCGGCTTCGTAACGTGAAAACGTGTGAGGGATG
CGGCCCATGGGCGAAGGGTGGGGTCTTTGGGCACCGATAAGGCTTATCCGAAGATACG
ATGGCCGGCTGCTCGGCTCTTGTCGCCAGTCGTAGTGCTCAGGTGGCCGAGGCGGGAT
CGTCGTGCGCTTTAATTCACGCTACGATATCGCGGCTACGCGTCTATTCCGAGTATCACC
ATCGCTATCGCATCGGGCTGGCCGGGATCGCCGATCGTTCGATCGAAGATCGGAGTCAC
GCGTGAGGACCACACGGGAACCAGGTGGCCAAACGGGTGTGCTCCGCAGGGGGTGA
CGATTGGCCGTGGATCGACCGACAGAAACAG

>novel_circ_000048
AATGGTGGAGCCAAAGATGGCTACGACATGAATCTTGGCCCGGCGTGGCAGAAAGGTT
ACACTGGTAAAAGTGTCGTCGTCTCGATCCTCGACGACGGAATACAAACGAATCATCC
GGATTTGGCGTTGAATTACGATCACCAAGCCAGCACAGACATCAACGACAATGACAAT
GACCCGATGCCAAGGGACAATGGCGACAACAAGCATGGAACTCGCTGCGCCGGTGAG
GTCGCTGCCGTCGCTTTCAACCAGTATTGCGGCGTTGGAGTCGCGTATAATGCCAGCAT
AGGAGGCGTTCGAATGCTCGATGGCCCGGTTAACGACGCCGTCGAAGCCAGGGCGTT
GGGATTGAATCCTGATCACATAGACATATACAGCGCTTCTTGGGGCCCGGAGGACGATG
GCAAGACGGTGGACGGTCCCGGTCCTCTTGCCAGGAGGGCGTTTATCTACGGAGTAAC
CAGCGGGCGCAAAGGCAAGGGTTCCATCTTCGTATGGGCGTCCGGGAACGGTGGCCG
CCACACGGATTCTTGCAACTGCGACGGTTACACGAACAGCATCTTCACCCTGTCCATAT
CTAGCGCCACCCAAGGCGGCTACAAACCTTGGTACCTGGAGGAGTGCAGCTCGACGC
TCGCCTCGACGTACTCGTCCGGCACGCCCGGCAACGACAAGAGCGTGGCCACCGTGG

ACATGGACGCGAAACTGAGGCCGGACCACATATGCACGGTCGAGCACACGGGGACGT
CTGCCTCGGCGCCCCTCGCGGCCGGTATCGCCGCCCTGGCCCTCGAGGCCAACCCGAG
CCTCACGTGGAGGGACATGCAGTACCTGGTGGTGCTCACCTCGAGGTCGACCCCCCTC
GAGAAGGAGTCGGGCTGGATCTTGAACGGGGTTAAGCGGAAAGTCTCCCACAAATTC
GGTTACGGGCTCATGGACGCGGGCGCTATGGTGAACCTGGCCGAGCAGTGGACCAAC
GTGCCTCCCCAACATATCTGCAAGTCCGATGAGATCAACGAGGAGAGGCGCATCGACC
CGACCTATGGTTACACGCTGAGCGTGTACATGGACGTCACTGGTTGCGCCGGATCCGT
GAACGAGGTCCGGTTCCTCGAGCACGTGCAGTGCAAG

>novel_circ_000049
GTACGATGGTTCCAACAGCAGCACGAGAAGAAACGCAAGAAGAGGGATTTCATGCCC
TCGTCGTTCTCGTTCGATTATCCGACGTTTTTCGACGAGTTCGGCCCACGTCCCAGGCA
GACCACGTACCATCGGCAAAGGAACAGAGGTGTGCCTCTTCAAAACCTGTTCACGGAT
CCTCTCTTTAAGGAACAGTGGTACCTGAATGGTGGAGCCAAAGATGGCTACGACATGA
ATCTTGGCCCGGCGTGGCAGAAAGGTTACACTGGTAAAAGTGTCGTCGTCTCGATCCT
CGACGACGGAATACAAACGAATCATCCGGATTTGGCGTTGAATTACGATCACCAAGCC
AGCACAGACATCAACGACAATGACAATGACCCGATGCCAAGGGACAATGGCGACAAC
AAGCATGGAACTCGCTGCGCCGGTGAGGTCGCTGCCGTCGCTTTCAACCAGTATTGCG
GCGTTGGAGTCGCGTATAATGCCAGCATAGGAGGCGTTCGAATGCTCGATGGCCCGGTT
AACGACGCCGTCGAAGCCAGGGCGTTGGGATTGAATCCTGATCACATAGACATATACA
GCGCTTCTTGGGGCCCGGAGGACGATGGCAAGACGGTGGACGGTCCCGGTCCTCTTG
CCAGGAGGGCGTTTATCTACGGAGTAACCAGCGGGCGCAAAGGCAAGGGTTCCATCTT
CGTATGGGCGTCCGGGAACGGTGGCCGCCACACGGATTCTTGCAACTGCGACGGTTAC
ACGAACAGCATCTTCACCCTGTCCATATCTAGCGCCACCCAAGGCGGCTACAAACCTT
GGTACCTGGAGGAGTGCAGCTCGACGCTCGCCTCGACGTACTCGTCCGGCACGCCCG
GCAACGACAAGAGCGTGGCCACCGTGGACATGGACGCGAAACTGAGGCCGGACCAC
ATATGCACGGTCGAGCACACGGGGACGTCTGCCTCGGCGCCCCTCGCGGCCGGTATCG
CCGCCCTGGCCCTCGAGGCCAACCCGAGCCTCACGTGGAGGGACATGCAGTACCTGG
TGGTGCTCACCTCGAGGTCGACCCCCCTCGAGAAGGAGTCGGGCTGGATCTTGAACG
GGGTTAAGCGGAAAGTCTCCCACAAATTCGGTTACGGGCTCATGGACGCGGGCGCTAT
GGTGAACCTGGCCGAGCAGTGGACCAACGTGCCTCCCCAACATATCTGCAAGTCCGAT
GAGATCAACGAGGAGAGGCGCATCGACCCGACCTATGGTTACACGCTGAGCGTGTACA
TGGACGTCACTGGTTGCGCCGGATCCGTGAACGAGGTCCGGTTCCTCGAGCACGTGCA
GTGCAAG

>novel_circ_000050
GCCGGCACAAAGTTATGTAAACATCAAGAGCAGCAGTCCTGTGTCTCCAAGAACGATA
GGAGCAGGAGTGACGTACGTGCAGGGTGGTGCGGCTGCATCTTCAGCGGCGGCGGCG
GCGGCGGCGGGGACGACCGGAAGTGGAAGTGGCGGAGGTCCTTCAATCGGAGGAGG
CGGCGGCGGTGGCGGAGGAGGTGGCGGTGGTATCAACAACGCCGGTGGCGCGAATAG
TGGAAGTGGAGTCGTTGGGACTGGAGCTGGTGGCGGTGCCGTTGGAAACGTAGCATC
GGTCGGAAATGGTAGCAACAACGGTACCAATAACAGTGCCGGCACGGGGAACAACAG
TGGTGGAAACGGTGGTACAGTGGGTAGTGCGCCAGGGGGTTATGTTGCCGTTCCCATG
GCTGGCGGTCTGCGGTACATCCATCCGTATCCATCGACGCCAACGTCGGCATCGGTGCC

GGCTGTAGCGACAATGGTTACCTCAGCCTCGTCGGCACCGACTCCAGTGCCAGTTCCA
GCACCCGCAGCCGCGCCTGTCAATCCACCTACAGTTAGTCAAACGCCACCACCATCGC
TTTCGGGAACTGCTTATGCCGCGAGAGACGCATATCGCAGCGCCACCACGAATGTGAA
CGAGAGGATTGCCATCAGCGTGAGCAGTAGTCCTTTGTCGCAGATTGGAGTCGATGTT
GGAGTAGTTGCGGCCGAATACGCGGCAAATAGCGGTATAAGTAGTGGAAGAGGGGAG
AGACCGAGGATATCTCTTTTACCACCTGCTTCGGTAACGCCGACACCTTCACCAACAGC
ACCAGGCTATCAGCACAACATGTCTGGAGTTAGAAATTCACGAATAGCCCTGATGCCC
GTCCAGCCGGATCAGTATTGCAATCGTACAGCCGTTGAAATGGCTAATCTGCAGGAGG
CGCCGCCCTTCAAGAAGATACGCCTCAGTCAACCGACCCAGATTCAATTGCAGTCCCA
AACGCAATCTCGTTGTCATAGTTTATCACCCGCTGATCCGTGTGTTAAGCAGGAGCACG
TGGTGCAGTTGCAACAACCGCTTAGGATAGATACTAGG

>novel_circ_000051
GCCGGCACAAAGTTATGTAAACATCAAGAGCAGCAGTCCTGTGTCTCCAAGAACGATA
GGAGCAGGAGTGACGTACGTGCAGGGTGGTGCGGCTGCATCTTCAGCGGCGGCGGCG
GCGGCGGCGGGGACGACCGGAAGTGGAAGTGGCGGAGGTCCTTCAATCGGAGGAGG
CGGCGGCGGTGGCGGAGGAGGTGGCGGTGGTATCAACAACGCCGGTGGCGCGAATAG
TGGAAGTGGAGTCGTTGGGACTGGAGCTGGTGGCGGTGCCGTTGGAAACGTAGCATC
GGTCGGAAATGGTAGCAACAACGGTACCAATAACAGTGCCGGCACGGGGAACAACAG
TGGTGGAAACGGTGGTACAGTGGGTAGTGCGCCAGGGGGTTATGTTGCCGTTCCCATG
GCTGGCGGTCTGCGGTACATCCATCCGTATCCATCGACGCCAACGTCGGCATCGGTGCC
GGCTGTAGCGACAATGGTTACCTCAGCCTCGTCGGCACCGACTCCAGTGCCAGTTCCA
GCACCCGCAGCCGCGCCTGTCAATCCACCTACAGTTAGTCAAACGCCACCACCATCGC
TTTCGGGAACTGCTTATGCCGCGAGAGACGCATATCGCAGCGCCACCACGAAT

>novel_circ_000052
TACACAATGCGCTAGGAGTGATGGAGACAGTGAGCAAGCAAGTCCAGGTAGTAAGCG
GACTGGGGGTGGGGGGTCCGGTGGGCCCTGTGGGTCCAGTGGGGGCAGATTTGCATC
ACCATCATCATCCTCACCCCCACTCCCATCCTCCACACCCACACGCCCACCCCCACGGT
CATCCTCACGGACCCCATGGCCACTCACTGGTCGGTGTAACGTCGACCCCTGGCAGGG
GTCAGACACAACCACCGGTCTCGCATAACCAGCATTTACCCGCGAGGTCTACTCCGGT
ACAGCTGCATAGTATATATGAACGGTTATGTAGTGTATTTTAATGAAATTATTAAAGTGA
AATTACAAATTTCGAAAACGCGATGTACCAGATGTGCAAATGGAGAATGTATGCAGAA
AGGAAAGATTTCTTTGAAACTCAAGATTGTCCGTTTGCATGATGAAGCCGGCACAAAG
TTATGTAAACATCAAGAGCAGCAGTCCTGTGTCTCCAAGAACGATAGGAGCAGGAGTG
ACGTACGTGCAGGGTGGTGCGGCTGCATCTTCAGCGGCGGCGGCGGCGGCGGCGGGG
ACGACCGGAAGTGGAAGTGGCGGAGGTCCTTCAATCGGAGGAGGCGGCGGCGGTGG
CGGAGGAGGTGGCGGTGGTATCAACAACGCCGGTGGCGCGAATAGTGGAAGTGGAGT
CGTTGGGACTGGAGCTGGTGGCGGTGCCGTTGGAAACGTAGCATCGGTCGGAAATGGT
AGCAACAACGGTACCAATAACAGTGCCGGCACGGGGAACAACAGTGGTGGAAACGGT
GGTACAGTGGGTAGTGCGCCAGGGGGTTATGTTGCCGTTCCCATGGCTGGCGGTCTGC
GGTACATCCATCCGTATCCATCGACGCCAACGTCGGCATCGGTGCCGGCTGTAGCGACA
ATGGTTACCTCAGCCTCGTCGGCACCGACTCCAGTGCCAGTTCCAGCACCCGCAGCCG
CGCCTGTCAATCCACCTACAGTTAGTCAAACGCCACCACCATCGCTTTCGGGAACTGC

TTATGCCGCGAGAGACGCATATCGCAGCGCCACCACGAAT

>novel_circ_000053
GAATACTGCCGGGACTATTGCCGTTGCTGCCATTATCACTACACAAAAATATAAGAAGA
ATGATATCCAAAGATAAAATGCTTCGATAGATGAAGTAATTTGCGCAACAACATAAAAA
GATTAGAAAGAAACAGAATTATTAATTTCAAATGTATGTATTATTAGAAAGTTTGTATTA
AAAAACGAATGGAAACGAAACGAAATAAATACATAAAGGAAGCAGAAGAAGATTAAT
GATAGTGAAAGAAACAATAGAATGATATATACTTTAAGAAGAAAAGAATATTGATTTGA
ATATTTTTCGCACGACCAGTTTTGTGATGATTCGCAAATGTGCGTATGTGTGTACGTGAT
TGTGTATACGTTAAAACTGTGCGCGTATGTTTCTCGTTCTGCCATCTTGAAAGTCATCCA
ATTTCTTTCTCTTGGTGGCGCTGGCTCACGTGTGCGGGAAGCGTGCACGGTGCGTGCA
TCGTACACGCACTGTGCTAAGTTGCTAATAAACGAAGAAATCTCATTTTGAAAATAACT
GCAAAACAAAGATTTATCGATAGCGATATAGTAAAACTGCGCGATGAAAGCGAGTTTGT
CGATAAATTTATTTCTTTGTGGGAGTGATAACGAGTGTCCTAACAATATTAATAGTGAGA
GCAGTATCGGTAGCAACAACAATCTCGGCAACAGTACACATGACATTAGTAACAGTAA
GAAAAACGGATATGACAATGGTTTTAACAGCAACAAGAAATCACAAGTGGAATATAAT
GTGCAAGTTAAGTGTAAGCCATATGGCAATGATGAGCAGTTGCATTTCTGTGAGGTGTA
CTCGTACTGATTGTGAGTGCTAGTAGAGAAAGAGAAAGCGGGAGACAGAGAGATAGA
AGCCACCAAGACTCTCGTGGAGAGCGGGGCACTGAAAGATATGCGGACCTTTGTGCG
AACAGCTGAAGCGGAAGTTGTGAGAACAAGTGGAGTGCAACGCGCTCCACCTAATCG
GTGATAGGGTCAGTCGGTTAAAAGAAGACAAAATTGGCGTTCACAGCGACGGCAACG
CGGCATCTTTCGCGATCACCTTTTCCTCATCCCTTCCTTTTTCCTACTTCATTTGTTTCA
CTTCGTTTCCTCTCTCTTTTTCTCAAACTCTGACGCAATTCATTCGTTTATTTATATATTT
TTCTTCTCTTAGTTATCGATAGAGAGAAAAAAAAAAAAAAAAATCTCGTCTTTTATTTC
GATTGAAACTTTTGTATAATGATACGCAATTAAAATGTTGGAAGAATAATGCAAATGAG
AATTCGCCGTATAGAAGCCGCGTCGCGTTTCCTTACAATAATAAAGAAATCGTCGCTGG
AGCAACAGCGGAAGAAAGAAATTTGAAAAATCGGTTAGATTGTGTTCCAAGTGTATAA
TATATCGAGTGATATGGAACGCATGAAAGTGTACGTAAGCTAGCAAGAAGATAAAAAG
CGGCGAAAGAGAAAAGAAGAGAAAAGAGGTAGCGTAGAGAAAAACGAACAACACG
CAACAACGAAGAAACGAAGAGAAGACAGTAGTAGTAGTGTAAAGGAAACAGAGATG
ATAAAGAAAAAAAAAATCTACGGAAGGGAATAGCTTGTGAGAAGAAAAGCAAAAGTT
TGCGCGACGTACAGTATAATGTGGAGTAGCGTGTTTCGCGAAAGTTTTAAAAGTCGAT
ATAGAATTTATAAATAGATCGGTTGGTATACTCACATATGTGCACGCGTACGCATGTGTG
CGTGATTACGCGCGCACATGTACGTGATTTTCACTCGAGACTGAAGCGGTGAATTATTC
TCGCACGGATCGACTACTCGTGGGGGAAATTCGAGATCCGGCTTACCTTCCACAGCTG
ACGTCATTACCGTACACAATGCGCTAGGAGTGATGGAGACAGTGAGCAAGCAAGTCCA
GGTAGTAAGCGGACTGGGGGTGGGGGGTCCGGTGGGCCCTGTGGGTCCAGTGGGGGC
AGATTTGCATCACCATCATCATCCTCACCCCCACTCCCATCCTCCACACCCACACGCCC
ACCCCCACGGTCATCCTCACGGACCCCATGGCCACTCACTGGTCGGTGTAACGTCGAC
CCCTGGCAGGGGTCAGACACAACCACCGGTCTCGCATAACCAGCATTTACCCGCGAGG
TCTACTCCGGTACAGCTGCATAG

>novel_circ_000054
GAATACTGCCGGGACTATTGCCGTTGCTGCCATTATCACTACACAAAAATATAAGAAGA

ATGATATCCAAAGATAAAATGCTTCGATAGATGAAGTAATTTGCGCAACAACATAAAAA
GATTAGAAAGAAACAGAATTATTAATTTCAAATGTATGTATTATTAGAAAGTTTGTATTA
AAAAACGAATGGAAACGAAACGAAATAAATACATAAAGGAAGCAGAAGAAGATTAAT
GATAGTGAAAGAAACAATAGAATGATATATACTTTAAGAAGAAAAGAATATTGATTTGA
ATATTTTTCGCACGACCAGTTTTGTGATGATTCGCAAATGTGCGTATGTGTGTACGTGAT
TGTGTATACGTTAAAACTGTGCGCGTATGTTTCTCGTTCTGCCATCTTGAAAGTCATCCA
ATTTCTTTCTCTTGGTGGCGCTGGCTCACGTGTGCGGGAAGCGTGCACGGTGCGTGCA
TCGTACACGCACTGTGCTAAGTTGCTAATAAACGAAGAAATCTCATTTTGAAAATAACT
GCAAAACAAAGATTTATCGATAGCGATATAGTAAAACTGCGCGATGAAAGCGAGTTTGT
CGATAAATTTATTTCTTTGTGGGAGTGATAACGAGTGTCCTAACAATATTAATAGTGAGA
GCAGTATCGGTAGCAACAACAATCTCGGCAACAGTACACATGACATTAGTAACAGTAA
GAAAAACGGATATGACAATGGTTTTAACAGCAACAAGAAATCACAAGTGGAATATAAT
GTGCAAGTTAAGTGTAAGCCATATGGCAATGATGAGCAGTTGCATTTCTGTGAGGTGTA
CTCGTACTGATTGTGAGTGCTAGTAGAGAAAGAGAAAGCGGGAGACAGAGAGATAGA
AGCCACCAAGACTCTCGTGGAGAGCGGGGCACTGAAAGATATGCGGACCTTTGTGCG
AACAGCTGAAGCGGAAGTTGTGAGAACAAGTGGAGTGCAACGCGCTCCACCTAATCG
GTGATAGGGTCAGTCGGTTAAAAGAAGACAAAATTGGCGTTCACAGCGACGGCAACG
CGGCATCTTTCGCGATCACCTTTTCCTCATCCCTTCCTTTTTCCTACTTCATTTTGTTTCA
CTTCGTTTCCTCTCTCTTTTTCTCAAACTCTGACGCAATTCATTCGTTTATTTTATATATTT
TTCTTCTCTTAGTTATCGATAGAGAGAAAAAAAAAAAAAAAAATCTCGTCTTTTATTTC
GATTGAAACTTTTGTATAATGATACGCAATTAAAATGTTGGAAGAATAATGCAAATGAG
AATTCGCCGTATAGAAGCCGCGTCGCGTTTCCTTACAATAATAAAGAAATCGTCGCTGG
AGCAACAGCGGAAGAAAGAAATTTGAAAAATCGGTTAGATTGTGTTCCAAGTGTATAA
TATATCGAGTGATATGGAACGCATGAAAGTGTACGTAAGCTAGCAAGAAGATAAAAAG
CGGCGAAAGAGAAAAGAAGAGAAAAGAGGTAGCGTAGAGAAAAACGAACAACACG
CAACAACGAAGAAACGAAGAGAAGACAGTAGTAGTAGTGTAAAGGAAACAGAGATG
ATAAAGAAAAAAAAAATCTACGGAAGGGAATAGCTTGTGAGAAGAAAAGCAAAAGTT
TGCGCGACGTACAGTATAATGTGGAGTAGCGTGTTTCGCGAAAGTTTTTAAAAGTCGAT
ATAGAATTTATAAATAGATCGGTTGGTATACTCACATATGTGCACGCGTACGCATGTGTG
CGTGATTACGCGCGCACATGTACGTGATTTTCACTCGAGACTGAAGCGGTGAATTATTC
TCGCACGGATCGACTACTCGTGGGGGAAATTCGAGATCCGGCTTACCTTCCACAGCTG
ACGTCATTACCG

>novel_circ_000055
ATAAAATACATTCGTATATGATGGCGTCCGCGAAAAGAAAGCAAGACGAGAAGAATTT
GAAAATATTACGCGAACTGGTTTCGCAATCTGGTAATAAAGAATGTTTTGACTGCCATC
AACGAGGCCCAACTTACGTCAACATGACAATCGGTTCATTCGTCTGTACCTCTTGTTCT
GGTATGCTACGAGGTTTAACACCACCACATAGAGTAAAGTCTATTTCTATGGCAACATTT
ACACAAGAGGAAATAGATTTTATAAAGGAACGTGGTAATGAATATTGCAGAAGAATATG
GTTAGGCTTGATGAATCAAAATTCATCTCAAAATTTAGATACAAAAGATGAACAAAAAA
TGAAAGATTTAATGAGTGCAAAATATGAACTTAAAAGATATTATTTGGATCCATCCATGG
CAAATCAAAACTCAAATCAAAAGTCACAATCATC

>novel_circ_000056

ATGATATGATTAAAGAATTAAGTACAAGTTTATGGGGTGTGAAAAATGAGCAAGAATAT
ATGCAGGTTAGAGATCGAAATCATAGAGCTATTAGTGAAAGCACAAATTCTCGTGTAGT
TATGTGGGCATTTTTTGAAGCTATGGTTTTGGTTTGTATGACAATAGGACAAATTTTCTA
TTTAAAACGATTTTTTGAAGTCAGAAGAGTTGTCTAATTAAAGATATACAATAAAATAAT
ACATTGTACATTCACATTCATTGTACATTTTTTAT

>novel_circ_000057
AATTATCAGTAAATGCGTCCACACAACAATCGGAAGTAATAGCGAGGTTACTGCCTATC
AACGGGAACCCCGAGATCACAGGATGCAAAGATCGACCTCATATTTGTGGAAAAGAA
GCGGCATGTCGTAGCTTTAAAGACAACACATCCAAATGCGTCTGTCCGCACGATCTATC
CCCACCTACAACAGATTTGAAATGTCCAAACCGTCTAATAGTTCCCCTAACACCTCGAC
CTATACCCAACATAATCCCTCCAAATGGTAATTCTACCAACAGCACGACTGCTCTTCCA
GAAGCAGAACAGTATTTTCATACTGTTCAACAGGCAGAGCGTGTGAGACAAAAGGTAC
CTGAAATAATAGGAATCACCATAGCATTCGTCGCGGTATTAGCAATATTGTTGAGTATAG
TCTACTGCGTGAAGAAAAGAAGTTATAACGTGAAATCGCAGAGGAATTCCCTGGATGG
AACACCGATGAATCTGAAAAAGGGATTGCTGCTAGCGAATAAGTACACACCTAACCCA
CAGTATTTCAGTTGCGCCTCGCCGGAAGTGTCGATTCTGCAACGTGAGTCTCTTGCATT
TTTGCAAGAAATTGGCGAAGGGTGTTTTGGAAAAGTATACAAAGGAGAATTATGCATA
GGGGATTCGAAAGAGATCGTCGCTATAAAAGTTTTGAAAGAGACTGCACCGCGAGAA
GCTGAAGAAGATTTTATGCGAGAAGTCGACATCATGTCCACATTTGGGCATAGAAATAT
TTTGTCGTTGAAGGGTGCAGTGCTTCGTGAAGGTAATAGTAGTCCATGGATGGTTTTCG
AATACATGCCGTATGGAGATCTCGCTGAAGTTTTACGATCGAACTCTCGACAATTTAATA
GATCGCCGAAACCTGAGATGCAACCTTTAACTGAGGAATCTTTACATTGGATAACAATC
CAAATTGCAGCTGGTATGACATATTTATCGGGTCAAAGATTCGTTCATAGAGATTTGGCA
TGTAGAAATTGTCTAGTTGGTTATGATTTCATTGTTAAAATAGCAGATTTTGGGATGTCA
AGAGATGTTTATACTTGCGATTACTATAAGATAAAGGGCTCTAGACTTTTACCAATCCGT
TGGATGTCACCGGAAAGTGTAATGTACGGGCGATTCACATTGGAAAGTGACGTATGGA
GTTTTGGCGTTGTTCTATGGGAAGTCTATTCCTTTGGCAAACAACCATATTATGGACATA
ATAATGAAGAA

>novel_circ_000058
ATTGCAATATGATTATAAATAAAGAATGTTAAATGATTTTGTATTAGATCTTTTCCAGATA
CTCGTAAATCTACTGGATACCAATAATTAAATTCACGTCGCATATGATCTAATGCCTCTTT
CTTTATTTTTGTTTTGGGAAACTTCGTATCTTTAAAAAAAATATAATCCCATACTTCAGAT
GTCATTTCATCAGGCCTAAATATAAAATATTCTATATGTAAAATATTTTGCATTAATAAAA
ATAATTTAACTAATTTAATAAGAAATTAATTTTACAGCAAATAATTGCACAATTAATAATT
ATTTAAAATTTGACTTTGAAATTTTACTTTATGTTATAAGTATTTGGTTTATCGCCTTTAAA
TGTTCCTCCTTGTAATAAATGCGCCACAGTATAATAAGCCATATAAATTGTTGAATCTGA
GAGTGATTCTATTAACCAATTTTCATCCCATGGTAATTTAGTACCTGGAATTTATATCTAT
AAAAATATATGATACAAAAATTGTCATATCTTTTATTTAATCATTATCTTACCAAGTCCGT
AAGTTCTTGAACATGCATACTCATGTAACCAATTAAGACAAGCCATAAAATTTTTTCTC
ACTTCATCATGAAAAGTATTAAGGTTATTTAAGGCTTCTATTGCTTCTTTTTTCCAAGTTT
CTTCTCCATAGTCTAAATACCATTGATTACATAAGGCTACTACACATTCATCATTTGATCT
TGAAATTATA

>novel_circ_000059
GATTCTACGCTAGAGGAGAAGAATCGTCGACTTCGTTTTCTCGATGATCGACATCAATT
CGAGTGCAATAATCGAAGCTTCGAGATGAAAAATGGACGACAGTCGCGCAAGTGTGTT
TCGTTTACGTTTCTTCAATTCGTTCGTGGTCGATGAAACGAGTAAGTGAATCGGATATAT
TCGGTTATTAAAGAAACGGAGGACCAATAAGAGAACAGTGTCATAACGCGTGTGAAAT
GGATGGTACGCCAAAGAAAATCGAAGCAGTGTTAATATATGGATGCCCATGGGTGGAAT
TTGGCCGAAATGTGCCAGCGTTACCATCACGCTGTACCGGCAGCTCAAAATTCTTCGCC
AGCTAGGGATCCCTCAGCTGCCATTGTGCAAGGCTTGTCAACGGCTACGCACATCGAT
CAGGCTAGCCCTTCTTATTCCCATTACACGATGCTGGATAATTATGACAGGGGTGAGGT
GACACCACCGCCAGTGAGCAGTTATGGCGGTTCCCCTGGACTCTTGGCACTTCATTCCT
CGCTTTATGCGACACCTACGTCGAGGGCCTACGATATCGAGTCGAACATCGATGGCAAC
GACAGTTCCATGCCCATCGATACCCAAATCATCTCGATACCGGGAATGTTGAGCGGCCC
AGCGCAGCAGCGGTTGGAGGACATGTCCTGGCTCGCCTCCGGCCCGTCGTTGGAGTAT
CAGCAAGGCATCTCGCCGATTCCCGCGGGAGTGAAGATGTGCCATCCAGGCAGCACG
GCCCAGAGGAGCCTGAAGGAGCAGCGGATACGTCGCCCGATGAACGCGTTCATGGTC
TGGGCAAAGGTCGAGCGCAAAAAATTGGCCGACGAGAATCCTGATCTCCACAACGCC
GATCTCAGTAAGATGCTCG

>novel_circ_000060
ATCCACGAACACATGACAAGGGAAGAGATGTTATCGGTATTTCACACAGGACACGAGT
CTGTACCAATGTACGAGATTGTGCCTGTGTTGCATTCGGTGCACAAAAGGAGTACAAG
GGAGCCGGAGATACACTTGAAGGCCTTCGGGAAGGACATCAGTCTGTCGCTAAAGCCT
ACGGAAGGGTTGCTCAGAGGAAATCATTTGCCCATGTGGACCGTCACGAAGAATGCGT
CGCAACCTGATGGCCTCCACTACGAGAAGGTGACGAAT

>novel_circ_000061
CATATCTGGGAAAAACTGTCAGTAATGCCGTGATCACTGTACCTGCTTACTTCAACGAC
TCGCAGCGTCAGGCAACTAAGGACGCTGGTACCATCTCGGGCTTAAATGTTTTACGTAT
TATTAACGAGCCCACCGCTGCTGCCATTGCTTATGGCTTGGATAAAAAAACTACCAGCG
AACGCAACGTCCTTATCTTTGATCTTGGTGGTGGCACCTTTGATGTCTCAATTCTGACCA
TTGAAGATGGCATTTTCGAGGTCAAGTCAACAGCAGGAGATACCCATCTTGGCGGCGA
AGATTTTGATAACCGTATGGTTAATCACTTTGTACAAGAATTCAAACGGAAATATAAAA
AAGATTTAACTGCAAATAAACGCGCTCTTCGTCGTTTGAGAACAGCTTGTGAGCGTGC
GAAACGCACTCTGTCTTCGTCTACTCAAGCTAGCATTGAAATTGATTCGCTCTATGAGG
GAATCGATTTTTATACATCGATCACCAGAGCTCGATTTGAAGAACTCTGTGCTGATCTCT
TCAGAGGAACACTCGAGCCAGTAGAAAAATCATTACGCGATGCTAAAATGGATAAAGC
TCAGATTCACGATATCGTTCTGGTTGGTGGATCCACGCGAATTCCTAAGATTCAAAAAC
TTCTGCAAGACTTCTTCAATGGAAAGGAATTGAATAAATCTATCAATCCTGATGAGGCT
GTGGCATATGGAGCTGCTGTGCAAGCTGCCATTCTTCATGGTGATAAATCTGAAGAAGT
ACAAGATCTATTACTTCTTGATGTAACACCTCTATCTCTTGGTATTGAAACTGCTGGTGG
TGTTATGACTGCTCTTATTAAGAGAAATACTACCATTCCAACAAAGCAAACTCAAACCT
TCACTACATATGCTGATAATCAACCAGGTGTATTGATTCAAGTCTATGAAGGAGAACGT
GCTATGACGAAAGATAACAATCTGTTAGGTAAATTTGAGCTCAGCGGCATTCCTCCAGC

TCCTCGAGGAGTTCCTCAAATTGAAGTCACCTTTGATATCGATGCCAATGGTATTCTCAA
TGTCTCTGCAGTTGATAAATCTACTGGTAAAGAGAACAAAATCACTATTACCAACGATA
AAGGTCGTCTCAGCAAAGAAGATATCGAAAGGATGGTCAACGAAGCTGAGAAATATC
GTAGCGAAGACGAAAAACAAAAAGAAACTATCGCTGCTAAGAATGGTCTTGAGTCTTA
CTGCTTCAACATGAAGA

>novel_circ_000063
ATTCTTTAATTTCTAACTCTATTTCTTTGATTTTCTGTTCTTGAATTTCTGGCTCTTTAATT
TCTGATTCTTTTATTTCTGGTTCTTTAATTTCTGATTCTTTCATTTCTGGTTCTTTAATTTC
TGGTTCTTTTAATTCTGGTTCTTTTAATTCTGGTTCTTTAATTTCTG

>novel_circ_000064
GCAATGTCCAGCAAGTTTACAAGCATATTTAGTTCATCAAGTGATACAAGAAATTAAAT
CTATGTGCAAAAAACAACCAGAAGATTGTGGATTTAAATCCCAAGAAAAAACATATAC
TTCATTAAAATTAATGCAAGCTATTATTAATAAAGTTAATGAAATTTGTACCCGTTATCTG
GACAATAGTAGATTGGCATTATTACCACCTCCTCCTTCAACTCCATTACCACAAATAACA
GCAGGTGGAAGCAAAAATTGTAGAAGAAAAATGGAAGACCGTTATGTGATTTTACATG
ACTTACATACTACGTTTGGTATTGAGGATGATTCTATAGCAAATTATTATGCAGTATTTGA
TGGACATGCAGGACAAGATGCAGCTGTATATTGTGCTGCTCATCTACATCAATATTTAAC
AGAAAGCATATATTATCCTACAGATCCAGAGCGTGCCTTACGCGATGCTTTTCTTACTAC
AGACAGACAATTTATAGAAAAATCAAAGACACAAAAACTATGTGGTGGGACTACTGCA
GTTTGCACTTTAATATTAAATAAAAGATTATATGTTGCCTGGGTAGGAGATTCAACAGCC
ATGCTGATAAAACGTGATAGTGTTGTTCAGCTAGTGAATCCTCATCGACTTCATCGCGA
AGATGAAGTGCAAAGGATACGAAAAGCAGGAGGAGTAGTGATGCCAAGTATGGGAAC
AATGCGTGTAAATGGAGTATTAGGTGTTTCAAGGGCAATAG

>novel_circ_000065
CCTGAGAACCGAGCAACCGAGTGGCCTCGACACGGGCGGCGTACGCTTCGAGCTGAG
CAGGGCTCTCGATCTGTGGGCGAGGAACTCGAAACTCACCTTTCAGGAAGTTAACAGC
GACAGAGCGGATATTCTGGTTTACTTTCATCGTGGTTATCATGGAGATGGATATCCATTC
GATGGCAGAGGCCAAATTCTAGCACACGCATTCTTTCCTGGCAGAGATCGAGGAGGAG
ACGTACATTTTGATGAAGAAGAAATATGGTTGTTGCAGGGCGACAACAATGAAGAAG

>novel_circ_000066
CAAAATTATCTCATGAAATTCGGATATCTACCCCAGTCGGATCTAGAGACCGGAAACCT
TAGAACGGACGATCAGCTTACGGACGCCATTAAAAATTTACAGATGATGAGGAATGTC
GACAAAGATTCGGTGGCATTCCTGTAACTGGTGATATCGACGAAGCTACTATGAAATTA
ATGAGGCTTCCGCGGTGCGGACTTCCGGATAAAGTAGATCCTAGATACACTAGGGTCA
GGCATAAACGGTACACGATCCATGGGCAACAATGGCCACATCGGAATCTCACTTGGAG

>novel_circ_000067
GAAGATTTTTACCTACACCAGGTAAATCATGAATAACCGGAACACCAACAGCATTCAAT
TCTTCCCGAGGACCGATACCACTGTTCAAAAGTATTTGTGGTGAATTAACGGCACCTAA
ATAACAAAAATATTACGCATATTTATGTAATATTTTAATCATAAATAAAAAAAACTACTTT

TGTTAATAATTAATATTGAGAAAGAAACTCATTGTATATATTTATATATACATTTCCATTATT
ACAATTATAATTATTTATGATATATTTAGCTATATTAATTACAAGTTTTAATATTAAGTCTGT
TTTCTAGTAATAGAAATAAATATAGTATTCGCGTATTTCACAAATCATTCTATGTAATTATC
CAACTGTATTTTACAAAAAGAAATCGCAATTTGAACAGGATATGGTCATTGATCGATCG
CACAAAAGAATAATAATATCAAAGGACTGACTAATCAGAAAAATATGTACATAGAATAA
AAGCTTAAAAACGACCTTGTAACTAATATATAATTATCAATGAGTCCTTTCTAACCTCAT
CAACAATATATTAACCAAATGTCCTCTTCCGTAGCAATTTTTATTTCCTTTGTATTCCGAA
AGGCACACAATTTCTATTATTCGCGTGATTATAGTTATAAATCACTATGAATCATGAGTCA
TTTTGATGATAAATAATTATTTTAAAATTATTCTATGATTGTCTAAGGACATTCAATATTTT
TCAATATTTTTGATAAGTTGACTTATAATAATTATATAAAAATATTAATGTTTTTATATTTTA
TTTTTTATATGTATTAATGTAATATTATAATTATAATTATCATGTTAATAATTTTTATAAATTA
TACGAACGTAAAATGCTATATAATTAAATCTTAATAAAAAAAAGTTAATAATAATAATAA
AAGTTAATAAAATAAATAAAAGTTATTGATTTTAAATATTGGATATTATATACTTTTACATA
AATTACTATTAAAAATATGAAGAATTTTTTAATACGAATAAATATTGTTTACCAATTAACT
TGAATGTAGGTTATAGAAATATAGAAATGTAGATTGTTTATGAAATATACGGACTAGCAA
ACTAAAATTGGAAATGAAAAATTAGATTTCCATGTATGATCATGCAATTTAATGGTATAA
CATAAACCATACAATCATATGAAAAACTAATAAACCATGAAAATTGATTATTCAAATACT
TTGATAATGAGTATTTTTTTCTGTGAGAGAAAATTATTTGTTCTTTCTCAATAGTGCAATT
TATTTCTTCCTACTTTCCTTTTTTGATGGGTCTGAAATTTGATTGACAGCTATCATGACAT
ACGTTAGATGATATTACATGATGTTTAAATTATTTTTGATATATTTTTGATATATGTATTTAC
ATTTTAATGCATTATCAATTACAACATATATTAATATACATATATTTATTTTTTCAAATATAAT
AATTTTAAATTAATATGTTAAATTTTTGAAGAATTTTTTAAATTTTATTTTATATTTGTTTTT
TTTAAATTGTTTGTATATAGAAATGCAATTAAATAATTTATTTATTATTTATAATTTATTATT
AATTATTTATAAATTATTATTATTAATAATTTATTTATTTATTATTTATTCTGAAGTCTAATTA
GTTAAAGAAATTTATTTAAATTACTTCATGAATATGAAATCATTAATGAATATAAATTTAT
GTAAAAAAATATATTATAAAGTATAATAATACTATTATAGAACATAAAATAATAATAATAAT
AAAAATATAAATTTATATTTAAAATGTGTATAATTCTACTGTGTTTAAAATTATCTATTATT
ATCATAATATTTAAATGTCAATCAATTAATTTAAATATTTTTATTTATTATACAATATTTCTT
CATGTTATATTACAAATAAAAATCAGTTTTTTGTATCAATGTAAATTAATGCATATCTTAG
CAATATGATATAAATGCATGTTTAACATTGAACATGATAAGTTCAATCTTAATATAAACTT
ACTAACTATTATAATCTAATAATCTAATATAATCTTGTAAAAGAAATCATTATCCATTTTTA
TTTAAAAAAATACATCAATATTGCAAATAGTTCTATATAATAAAAAGAAATATTCATTGAT
AAAATCTATCGTTATTTATTACAGTTATTTATAGATATTTATGCAAATATTTCCAATTTTTTC
TTTTAATAGAATATAAAATGATTTGTAAAATGATTTAAAACGTTTTAAATCGAATTATAAA
ATGAAACGATTTTAAAATTTCATTTATGTAAATAAATTTCACTTGTAAAAATAAGAATAA
TCCAAATCAGAATAATTTGAAAAAAAAAAAAATAATAGCTAAAATTCAAATCACAAAAAT
AACACCTAACAAGCATAATATCTTATAAATATTAAATAATTCGGTTTAAGTTCGAATCTG
CAAACAAATGGTTTTTTAAAATGCACAATCTTTCTTTATTTCTCTAACTGAGAATAATGA
AGACAAATTGTGCAATAAAAAATCGTTCATATGCATTCTTAAATTTATATTATCTTTGTAC
AGAATTTTACAGCAAAGAAAGAAAAAAGCAAAGAAAGTACTGCTTTATATTTAACTTT
ACTTGATATTGAAATATTAATATTAAAATAAATACCTCCACTTATGACTACTTCTTTAGCC
ACAGAGACTCGATGGATCTTTCCATCATGAACAAATTCTACACCAACTGCCCTTTTGTT
GTTGTCAAACAATATTCTAGTGGCCGTGGAGTTGAGCATTATATGAAGATTTGGACGAT
TTTTAGCTGGTCGTAAAAATGCCCGGGCAGTGGAAAGTCGTGAACCATTTCTGGAGGT

CGTTTGGGCTATAGCGAATCCAGTGTGAGTTCGTCCATTTAGATCCGCGATACCATAACC
TGTAAATAATTTGGAGAAATATTACAATTAAATTTAATTCAAAATTGAAATTTAAAGAAA
AGATTTACAATAATATAAAGATTCGAATCCAAATCCTTTGAGAGATCTCAAAAGAATTG
AAATTTAAAACTTTAGAATAATTTTTTTTCTTATAAGTATTCTCATCAATTTTTTTATTA
AAATTTAAAATTTTAATAAGTTAAATTGTTTGTTCTGCGTTGGAATTCGGAATCGATTAC
ATACATCGATGAAATGCTAGTATTATTTGTAAATTAGTATAACAAGGTCGATACGCGTTC
ATTCATCATCAAAATGATGTATTATTATTGGGAACAAAAGCCAACAGTGAACACAATGT
CGCAATTAGCAGAAGTGATCGAATTACATAACTAGTTTTTCGATGAATATATCGAAACG
AGTCATTAGACTCATGCGAGCAATAACAACGATGTAAAGAAGAAACGAGTTCCTCGAC
GTCTCGTAATAACAGGTTAAATGCAATTCGAGTTAAGTAGAACGACTTGTTGTTACTAA
AGTATTTTCCACTTTTTTCCCCAACTTTCTCGCGTATTTATTTTTATTGCAACCGGAATAC
TTATAATGAATATTAAAAAGAAGTTGTTTTGAACAGAATTTTACCAAGCCATTACTTAAT
TGGTAATCTTCTTTTGAGAGAAATGATTATCCTTTTTAGAATCGAATTCTTTTAGGAAGT
ATCAGGAAAATTCTTTTTCACCATTTAATTAAAACGTAACAGATATATCTTAATATCTTAA
TATTAAACGTTAGTATTAATTAGATGTCAGTTTGTCTAAAAAAACAGAAAATTAATCCAAT
TAGTTTGTGCATGTACTGATAATTTGTTTAATCATTGATAAATAAAAAAAAAATTAAAAA
TAAATTTACCAAGTTCTTTTCCAGCTTCGAGAATAGAATAACTCAAGGGCGGATGATAA
GGGAACTGCGTGACCGTGAGTGGCCCACCAACACCGTGATAACCATAATCCATGGTGT
TTGCTTGAAGATTATCCTCGCTCCTGATGAAATATGGAAGGACGTCTTGATAAGACCAT
CCTATGTTACCTAGCCTCGCCCAGTCGTCGTAATCCTTTCGCGAGCCCCTTATATACATC
ATGCCATTCATAACGCTGGTTCCACCCAGAACCTAAATAATATTTAATTATCCATTCGTT
TGCTTTCTGTCGGCTATTCTTGTACGTTATTACGGCAACAGTTGAAAGAGGATTATATGA
AATATAATACGGAATAATTAATAGTAATAATTAGAGTTGAAATAAATAAAAAAAATAATA
ATGAAATAATAATAATAAATAATATTGCAATTTACCTAATTTAAATATTTTAAATAAGTAAT
AAATAAGTTTTTTGTTTCTTTTAATGTGTTCTTCATAATTAATGATTTTATATGTTTGTACA
TTTTTTGTTTTATACATGTTTTTACAGCTGCTATAAAAAATAAATATTTTTTTTTTATTGAT
AATTCATTTATTTTTTTTTATTAATTATATATATATGAAATATCATATTGATTAAATGATAATT
TGTAAGTGTTCTCTTAATATTTTTACGTATTTCTTTTATTAATTTTTTGACGAAATTATTAT
TTCCAAATAAAGTGTACACATACTTTCCCCCTGGGCCAATAACATTTTCGATCATCTTTA
TTTAGAC

>novel_circ_000068
GCTTTTCGTCGCGAATCACGCGTACTAAAATAATCATTAAAATATTGATAAGGGTTAGAT
AATTAGAATCAAACATCGTCAACGTCGATCACCGTTGCGATATAAAAAAAAAACATAAC
TATCGACAGTGAAGATGGGAAACAAATAAAGAAAGACTTAAGAAAAATGCTCGAAAA
ATGCTCGAAACATAGAACACAGCAGCGTCTGTATAATTGTACATTTGACAGGGAAGAAT
TTAAATTTAAAAATCGGATGGATTCAATTTTAATCTATTGCTATTGAAAATTTATATGTTTA
TACGTGTTTTTTAAAATTATATTTTAACGATCGAAATGAATTTTAACGA

>novel_circ_000069
TCGAGCTAAAGAATTGGTATTATCAATTGTAAATCAAAGAAGTAGAACTGAAGGAATTG
GCGATATGAGTGGAAGCAGTGGAGGAATGATGGGTCATCCAGGATTTGTTGAAATAAT
GATTCCTGGACCAAAAGTTGGTTTAATTATTGGAAAAGGAGGAGAAACAATAAAACAA
CTTCAAGAAAAATCTGGAGCAAAAATGGTTGTTATACAAGAAGGCCCTTCTCAAGAAC

AAGAAAAACCTTTAAGGATAACTGGTGATCCTCAAAAAGTTGAATATGCCAAACAATT
AGTATATGAATTAATAGCTGAAAAAGAAATGCAAATGTTTCATAGAGGTTCAAGAGGGA
GTGATAGGAGTGGAAATTATTCAAATGATAGTAATTTTAATCATGGTTCTGGAACTACTG
ATGGAGTAGAGGTACTTGTTCCAAGAGCTGCTGTAGGTGTTGTTATTGGTAAGGGAGG
AGATATGATTAAAAAAATACAAGCAGAAACTGGAGCAAGAGTTCAATTTCAACAAGGA
CGAGAAGATGGTCCTGGGGATAGAAAATGTATAGTATCTGGAAAGCATCAAGCTGTAG
AACAAGTGCGTCAACGTATTCAAGAACTTATTGATAGTGTAATGAGAAGAGATGATGGT
AGAAGTAATATGGGAACAAGAAGTGGTCCAAGAGGCAATGGATTTAGCAATAATCGAA
ATCCAAATGAATATGGTGGATGGGATAGACGTCAAGGAGGTCCTATGCAAGATAAAATA
GAAACGACATTCACTGTTCCATCTTCAAAATGTGGTATCATTATTGGAAAAGGTGGTGA
AACCATTAAACAAATTAATCAGCAGACTGGAGCACATTGTGAATTAGATAGAAGAAAT
CAAAGTAATGAAAATGAGAAAATTTTCATAATTCGTGGAAATCCCGAACAAGTGGAAC
ATGCAAAAAGAATATTTAGTGAAAAATTAGGCATGGCTCCGGCTAATACTTCATTTACT
GGTACACAAGGAGCAATTGGATATAATCCAACATGGAATGCAGGAACAGCTTATCAAG
CATGGCCAAGTCAACCTCAATCTACTGATACAAATACTGCAAGTCAAACACCTGTTCAA
GTAAATCCACAAACGGGACAACCGGATTATAGTGCACAATGGGCAGAATATTATAGATC
ACTAGGTATGCATAGAGAAGCAGATATGATTGAACAACAAGCAAAACAAGGAAAACA
AGATCATAATCAACAATCAGGAAATACACAGAATCAATCTAATACATCAACACCTAATG
CTGCTGGATCTACCCAACAACAACAACAACAAACACAACAACAACAACAGACAGGAG
CAACACAGAATGGCGGTCAAGCCGATTATAGTGCACAATGGGCAGAGTATTATAGAAG
TATTGGTAAAATAAAAGAAGCAGAAGCTATAGAAGCTCAAATGAAAGCAGGCAAGTTG
GGGATACAAAACAATCAAATTGCTCAACAACAAATACAACAAAGTATACAAAGTCAAT
CCGGTCCAACAAGTGGGACTCCTAATACATTTCCACAAACTTATGGTAGTTATCCAACA
ATAAATGCTAGTTCAACTCCTACGGGTTATTATGCAGCAGGAGCTGGATCTACCACACA
GCCACAAACTGGTCAACAAAATCCGTCTTTCCCAACGGGCTATCAGAACTATCCTTATT
CACAAACGAATACAGAAAGTTAAACTTCCATCAGGAAAAATATTAGCT

>novel_circ_000070
GTGGTAACCGAACGGAAAATACAAATACATCAAATACTACTTCAAGGTCATCGAGTTTA
CAACAACGAGGGTCAAAAGGTCGCTGGACTAGTAAAAATGGGGAATCGGCAGTCCAG
GCATCTATTAATCGTAAAGAAGATTTTCGAATGCGACAAATGCTCGTGAATGGATACGA
TCTCAATACGAAAGAAACAAATGATATTACAGGTGTGAGTTCACCTGATGAAAGTACA
ACAGTTGCTCCTGATAGCCCATGCTCTCCGGCTGAAACAAGCACATCAGCATCATCACA
CGAACAACAAGAACAGGAACAACAGCAGCAACTTTACGAACATCACCATTCTCAATTT
CAACAACAGCAGCAACGTACGACCGGAAGTGCGCCAGCCGCTGCACTGCACGTTGCT
GAGGACAGTGGAGCCCCCTGTGAGATGCGGAGGACACCACCGCAAAATAAAG

>novel_circ_000071
ATTACCGTAACCACGATCGGATACGGTGATACCGTCCCACAAACGTGGATGGGAAAAAT
AGTCGCCTCGTGTTTCAGCGTATTCGCTATATCCTTTTTCGCACTTCCAGCCGGAATATT
GGGCTCCGGTTTCGCGTTGAAGGTGCAGCAAAAGCAACGGCAGAAACACTTCAATAG
GCAGATACCTGCCGCGGCAATGTTGATACAGTGCCTGTGGAGGTGTTACGCCGCTGAC
AAAGCCATCAACAGCGTGGCCACGTGGAACATTTACATCAAGGATCCGGCACCGGCCG
GCCAACCCTCCACGCCATTGGGAAAG

>novel_circ_000072
GAGAAAGTTAATACTTCACCAACGGGTATAGTACAGCAGCCGCACCAGCTGCACTTAC
CGATCAACGCTCAGATATTGAACACGAGTCAAGGTTTACCAACTATCGTCCCAACGCTT
CAACACATCGGGCCGAGTTTGAGGGTGATACCCGGTGACACAAGGCAACTTCTGGTTA
CTCACACAGCTGGCAATAACGAGTCCAGGCCGCTCACGTTAGCTGTGCAAAACTCGTC
GGATCAATCGAGGCCGCTCATTGCCGTGCAATCGAACACTGGAAGTGAGCCAAGGCCC
GTAGCGCTGGTCGTTCACTCGTCCACGGCCAACGACAACAGGGTAACGATCGTGCATT
CTAATCTATCCAACAACGACAGGCCGTTGGCCCTAGCCGTGCAGTCGTCCGCCAATGA
CGTCAGGCCAGTTACATTTGTGCACTCTGCAAACGAGGGAAGGCCGTTAGTTCTTGCT
GCGCATTCTCCTGCATTGAATGTGTCAA

>novel_circ_000074
AGGAGGAGCCGTTGGAAAATAATAGGACGAATGGGAATCACACGGCGAGCTCCCGAG
GTAGCCCCGTGATCTCGAGCACGCGAGTAACGCCACCCTCGGCGAGCGCCAACTCGG
CTCATCTGACCGCCCTGCACTCCGCGTCGCACCACCACCACCACCTATCTCATCACCAA
CAGCAGCAGCAACAACAACCGGCGCAGCAGCAACAACAGCAGCAACTGCAACACCA
ACAACAGCAGCAACAACAACAACAACAGCAACAGCAGCAACATCACCATCACCATCA
CCATCACAACAATCACAACAGTACCCATAATCCTTACGTGGAAAATCATAATTCGAATC
ACGCGCAACAGAATGCTGGCAATCTCGTCGTCGACGTTGAACCGAGCCACGACAATAA
GAAACATCGAAATGGACCGTGCGTAATCCGTTCTGGCACCAGAGAAGTGCATAACAAA
TTGGAGAAAAATCGAAGGGCACACCTGAAAGAATGCTTCGAATTGTTGAAGAGACAG
CTTCCCTCGCAAGAAGAGAAAAAATCCTCGAATCTTTCGATTCTTCATGCTGCGATCAG
GCATATACAC

>novel_circ_000075
AGGAGGAGCCGTTGGAAAATAATAGGACGAATGGGAATCACACGGCGAGCTCCCGAG
GTAGCCCCGTGATCTCGAGCACGCGAGTAACGCCACCCTCGGCGAGCGCCAACTCGG
CTCATCTGACCGCCCTGCACTCCGCGTCGCACCACCACCACCACCTATCTCATCACCAA
CAGCAGCAGCAACAACAACCGGCGCAGCAGCAACAACAGCAGCAACTGCAACACCA
ACAACAGCAGCAACAACAACAACAACAGCAACAGCAGCAACATCACCATCACCATCA
CCATCACAACAATCACAACAGTACCCATAATCCTTACGTGGAAAATCATAATTCGAATC
ACGCGCAACAGAATGCTGGCAATCTCGTCGTCGACGTTGAACCGAGCCACGACAATAA
GAAACATCGAAATGG

>novel_circ_000076
GTGAGGAAAATGCAAGACAGGGTGCAGAAGACGAAGGAAGAAGTTCAAAAGGCGAA
AGAAAAGTACGAAGCGGCGTTGCAGGAGATTAATCAGTACAATCCTAAATACATGGAG
GATATGACCCAGGTGTTCGAAAAGTGTCAAGAGATGGAGGCGCAACGGTTACAGTTCT
TCAAAGAGGTCCTATTCGGCATTCATAAATGCCTGAATATATCTCAGGATCCGATATTAC
CACAAATATACGAAGAATTCTATCACACAATTAATAACGCGGATCATGAGAAAGATCTG
AAATGGTGGTCGAACAACCATGGTGTAAACATGGCAATGAATTGGCCACAATTCGAGG
ACTACACAGAGGAGTTTCGTGACATCGCCAAAGGCTCGAAGTCAAAGGAGGCACTTC
CGGCTGGCTCCATCACGCTCATCAATCAACGTCCGGTCGGCGAAGATGTGCAT

>novel_circ_000077
GTGAAAGGAATTTCTCGACTTAGGCTAGGATGTCACATCACAGCGACGACAACATGCT
GATAGCAACCTCGGACTCCTTCTGGGAGCCGGGTAACTACAAACGAACGACCAAGAG
GATCGAGGACGGGCACAAATTGTGCGACAGCTTGGTGACTCTGGTCCAGGAAAGAGC
GGAGATCGAGAAGAGTTACGCGAAAGCGTTGAAGAATTGGTCGAAAAATTGGAACGA
CAAGATCGAGAAGGGGCCGGAATATGGAACCACCGAGGCTGCCTGGAAAGGTGTCCT
CGTCGAGTCGGAAAGGCTGTGCGATCTTCATCTCAGGGTCAAGGAGAATCTCTGTAAC
GACATCATTCAGCAGGTGAAAACGTGGCAGAAGGAAACGTATCACAAATCGATGATGA
CGCTGAAGGAACGGAAGGAGATGGAGGACGCGTTCAAGAAGGCGCAGAAACCGTGG
GCGAAGCTTTTGCAAAAGGTGGAAAAAGCGAAGTCCGAGTATCACAACAGTTGCAAA
ACCGAAAGGACAGCGGCCAACATGGAAAGGAACGCATCGGCGGACAGTTCGCTTTCC
CCTGACCAG

>novel_circ_000078
AAAAAAAAAAAAAATTAACAAGAAATACACGTACACAACACGCGTAGTCTGTCCGCG
CGAGGTTTATTTTAAAGACTACATCATTATATAATGATTACCATGTAATGATATTTAATTGA
TAAGGTAACATATATTCTATACTCTTACACGCTAAGTACTCAATAAATATATCTATAAAATT
TC

>novel_circ_000079
GGTGTTTGTTGGACAATTCGGCGCCGCGGGGCCCGCCCGATGTGCCTCCGCGTAATCCT
ACAATGAGTCGCGTCAACGGAAGATTGCCAGGAAGTCACGCAGCCAGCGACCACGAG
CGTGATCCCGACCTCCAGCCATCCTGCCTCGTACGCACCTCGTCCGGCGGCTTTTACAG
TATACCAAAGATACCGAAGAATGAGTACAACAACAAGAACCAGTCCACGGGGAATAGT
CCGATAAAGGTCGAGCTGCAGAACAACATGGACAGGGTACCTCTGCCGTATGGCCACG
CGTCTTCGATGATCCCGATGAGGAGGCAAAGCATTCGTTGCCATTTCGTGAAAGGGGT
CGACTGGTGCAGCTGGAAATTGATCGCCATGATACTGCTTATGGTCTCGCTGTGCATAA
CGGCGGCCCTAGCCTACGTCGTGGTGTCGAGCATAGTGAACCGGTCGTACCAGGGCAC
GAAAGCGTGCGCGGTACTGGTTAGCGAGAGCGCGGACAACACGAAGCCGTTGTCCTC
GGACGGGAACAAGACCTCTTCGTCGGCGAGCGCCTCGTCGTCGCAGTCGAGCGGTCG
AACGCGGCAACAGGCATCCTCTGGAG

>novel_circ_000081
AAGCATCGGAACAGTGGAGGAGGGCTCGGTGGCCACGAGCGTGACGATCGCCGAGGT
CGTTGATTCGCAAGAGCAACAGCAACAGCAATCGAAGAGCAACGGCGCAGTATCTCG
CCGTGTTGCTGGTCAAAACGTTGCGTTGAGCAACGGAAATAGTACCAGCAACGTCGGC
CGTGTGACACCGCGAAGCCACATGGAGCAGGAGAAGCGTATGCGGCGAGAAATCGCC
AACAGCAATGAACGACGTCGGATGCAGAGTATTAACGCCGGTTTCCAATCGCTGAGGA
CGTTGCTGCCGCACCACGAGGGCGAGAAACTTTCTAAG

>novel_circ_000082
GTAAGTTGAACGAGTGATCTAGAGGCATCTTTTAGCAATGAAATTACAGTTTAGTTTTA
AATTTGTCAAGACACATGGACAAGGTGAGATCGATTCTGTAAGGCAGTTCTATCGTTCT

AGGCCAATTGTAAAACGAAACTATCCAAGTTTATGCGTACTATCTACATCTTTGTCAAAT
CATGGTTTGTTCCCCGTGCTTTTGCAAGTGGCCCATTTACAGGGTCGGCACAGTTTCGA
GAATCGAGGATCTTATCGAGGAAAAGGGGGAGGCAGACGAGCAGATCGACGT

>novel_circ_000083
GCTGCGATACTTCAGCAGACTGCCGAATATATATACCAGCTGGAACAAGAGAAGACCC
AACTGTTGTCACAGAATTGTCAATTGAAACGGTTGGTGAATCAGCACGAGGGTGGCGA
TGTTCCGATCAAGAAACGCAAACCAGATAATCAAGGAGGCGTCGTTGTTAGCCTACCG
ATGCACGTTAGCGAGAGCGGTGACGAAGGATTGGGCAGCATGTCCCCTGAACCATTGT
CGGTAATAACGGTAACCACGGAGGGACATTCCGTGGTCAACAACGAGGTGGTAGAGC
TGAGGCGACAGTTGGAGAGGGAACGTCACGCGAGGTTGCGTCTAGAGAAGCAAATGA
GAGTTATACAGAATCAACTGTATCCGGAGAGATTTAGAGATAATCAGCTAATCACTTAC
CAACCTCACGAGGTGATCGAACACACGGACAACGTGATCGCTCAAGAGACGGAGGAC
GCGGTCGCCGCGCTTCAGGTGGTTTCCGTGGTGTCTTTGGCACAGGTTGGCACGACGC
AGACCGTGGAAACGTCTAGTCCGGATCCAAGTTCACCGCCCCTGTCCCCACAGCCCGC
GGACATGGTACCAGAGGAGATGAAAGAGGAGGAGGCGGCCCGGGCTCGCCAAAGA
TCGTGACGTTCGGCCAGAACCAGGTGTTCTCCGCGATCGACGAGCAGACTACCGGCG
ACTCGTTCTCCTCCTCGAGTCGCGTCACGTACACCACGAGTACGGGGGAGTTCAAGAA
CGCGTCACCCCAGTACATCACCACCAGCCCCACCAGACCGTTCTCGCCTGCCGCGGAA
CCGCAGCGGCTACCCAGCATCCTCGAGGCCGCGATGAAAGCGGAGCCGAAGGTTGAG
GTCGAGAG

>novel_circ_000086
AGTCTGGCCACAGGTTGGACAGCGGCTCCCCGTTCTGGAGGATTCAGCTCGATCAGGA
TCTTCTGGACACCATCAGCGCGATATATCCGGAGTGGTTTAAGGACTATACCAACACCC
GAGCCTCGACTTCCTCCTCGTCGAGTACTTCCGGTGAACTACAGTTGAACACCGAGGG
CATCGTGGCGGCCGACACCAGCGCCGTTCTCGAGCACAAGGCCGTCAAAGGTGATAC
CAAGTCCAAGTCCAAGGCGAAGAAGGTGGACTGCTTAAAGGAGAAGGAAAGGGACA
GGAAGGAGGGCAGGCGAGAATCCAAAGACGAGAG

>novel_circ_000087
AGTCTGGCCACAGGTTGGACAGCGGCTCCCCGTTCTGGAGGATTCAGCTCGATCAGGA
TCTTCTGGACACCATCAGCGCGATATATCCGGAGTGGTTTAAGGACTATACCAACACCC
GAGCCTCGACTTCCTCCTCGTCGAGTACTTCCGGTGAACTACAGTTGAACACCGAGGG
CATCGTGGCGGCCGACACCAGCGCCGTTCTCGAGCACAAGGCCGTCAAAGGTGATAC
CAAGTCCAAGTCCAAGGCGAAGAAGGTGGACTGCTTAAAGGAGAAGGAAAGGGACA
GGAAGGAGGGCAGGCGAGAATCCAAAGACGAGAGGGACGCTGGAAACGGTAAGAAA
GGGACGAAGCCCTCGCCGCAGCAGGATAAAGCGGCGGACGTGGCAGGAAGGAAGGC
TGCGGGTGTTCCAGGATCGGGCGCGGAGATGGCCGAGGGTAATCAGTCGCCGCCGAA
GGCGGAGGTCGACGACTCGTCCCTTCAACACGCCATGGATCGCAACAAGGAAT

>novel_circ_000088
CATTGAAGACGCCACCGGCGTTCCACGAGCAGAGGAAGCAACTGGAACGGGCGAAG
ACCGGTGATCTTTTGAAGGCGAAGATCCAGCAGAGACCGGGCAGGGAGGAGCTGGTC

AGGCAGCATATCCTCGAGGATGTGGGTCACGTGGACCCAAGCCTGGCCGAGAGGCAA
CGGATGCTGAAGAAGGCCAGGCTGGCCGACCAGCTGAACGACCAGCTCAGTCATCGA
CCCGGACCGCTCGAGCTCATCCAAAAGAATATTCTCCACACGGAGGAGCCTATTGAAA
GGGCGGTCAAAGAGGGTCACATTCCCTTCAAGGCCACTTGCGAGGGCCAAGTAACGA
AGCCCCAACACCCGGACCACTACATCACCTTCGAGGACGACTCCCAGAGCTCGGAGG
GGGCGCCCTCGCCCCAGCTGGAGTCCCGGTCGGACGTTCTGGAAACGGCGGCTGCCT
CGGCTGGGATAGTGACCGTCTCCCTCAGTATTCCGACGACAGGCGGTGCCGTCGTCGT
CTCGTCCACGTCGCCAGTGTTCCAACAAGCGTCCAACGAGTCGACGATACAAACCTTC
GCCGAGCTGTGCAACACCGTGGTCGGCTCGCAGCAGCAGCAGCAGCAACAGCAACA
ACAGCAGCAACAGCAGCAACAGCAGCAGCAATCGCATCAACAGGCCCAGCATAATCA
GCAACAACATGCTTCCACGCAGGCGAGTCAGACAAACGGTCCGTCGACGACCCTCCT
TCCACTGGCGCCGGCGCCGAGTCCGATGTCCTTGGGCTCGACCACGTCCAGCCTGAGC
CCCCTTTCCAATATCTCGATAGCGTCGCCACCGGCACCACCGCCGGCCGCCATCACCCC
CAGGCCTCAGGCGAGCCCCATTGCGGCCGCGTCCACGTGTCAGAGGAGCGACGCGCC
CGGCAAGGATAAGAACAGGAAGAAGTCCAAGACCAAGAGTCAACCGAAGACGCGCA
CCATCAAGTTTCACGAGTACAAA

>novel_circ_000089
CGTGTGGCGAACCAGGCGCTTACCGTTTCGCCACAGCCGGCAGTAACGGTGGAGGGG
CCGGTGTCCAAAATGTCTCCACCAACGAACGACGTGACCAATGGTGTGGTAGCGCCAC
CCAACACCCTGGCGCCCCGGGTTTCACCGACCACGAACCCGGCCCTCACCACCATCAA
CCCGCCAACGCACAAGCGGACTCCCAAGGACTTCATCTTTGGCAAGGCGATCGGCGA
GGGGAGCTTCTCCACCGTTTACCTGGCCAAGGATATCCACACCGGGAAGGAGTACGCG
ATCAAGGTGTGCGACAAACGTCACATCATCAAGGAGAAGAAGACGGAGTACGTGAAG
CGAGAGAAGGAGGTGTTGAACATGCTCGCGGGCGCGAAGCACTCGTTCGTGCGCCTG
TTCTGCACCTTCCAAGACGTGGAAAGACTCTATTCGTCCTGTCCTACGCGAAGAACG
GTGAGCTGTTGCCGTACATCAACAAGGTTGGCTCGTTCGACATCGAGTGTACCAAGTT
CTACTCGGCGGAGATCCTTCGCGCGCTCGAATACCTCCACGGCCTGGGGATCATCCATC
GCGATCTCAAGCCGGAGAATATATTGTTGGACGAGAAGATGCACGTGCTCATCACCGAT
TTCGGTAGCGCCAAGATCCTCAAGGACCCGGAGACGGTGACGCATCCCGCGGCCGAC
GAGTCGCAGCAACAGCAGCAGCAGCAACAACAACAACAACAACAGCAGCAGCAACA
ACCGTTCCGAAGAGAACGCAGGGGCTCGTTCGTTGGCACGGCCCAATACGTGTCGCCC
GAGCTGTTGACCGATAAGACGAGCAGAGCGTCCGATCTCTGGGCCCTCGGCTGCATAA
TCTACCAGATGGTCGCCGGCCTGCCCCCCTTCCGCTCCAGGAGCGAGTACATGATATTC
CAAAAAATCCTGAAGCTCGAGTACGAGATCCCGGATGGATTTTGCGAGCTGGCGAGAT
CCCTCGTCAGTCGGCTGCTGGTGCTCGAGCCATCGCAGAGATTGGGCGCGCAGGACG
AGCACGGAGCCGGTTACCCGAGCATAAGGGCGCATCCGTTCTTCGAGGGTGTCGACTT
CGAGACCCTTCACGAGCAGACACCGCCCCCGATATACCCCTACCTACCCGGCACGTCG
GAGAACGAGGAGCTGAGGTCGCAGTACAGGGTTCCCGACCACCTGGAGCCCGGCCTC
GACGACAAACAGTTGACGAGGCTTCTCGGCCTGGGAATCGGCGAGGACGACGACGAC
GACGACGACGAGGACACGGCCACGACCGAGCGCGAGAGACCCGCGCAGACGAGCAC
CGTCGTGTGCGAGAAGCCGAGGAGGACCAAGATCACGACAAGCAGCGACGGTAACAT
CGCCGACCTGTCGCCGGAGGAGATCAGGAACCGATTGGAGAAGCAACGCGCCTCCAA
CCAGTGGGACGGTTTCGTCGAGGGAAATTTGATATTGAAGCAAGGGTTCGTCAACAAA

AGGAAAGGGCTGTTCGCAAGACGGAGAATGCTCCTGCTCACCACCGGGCCACACCTT
TACTACGTTGATCCTGTGAACATGGTGCTCAAGGGGGAAATACCCTGGAGTCCGGAGC
TTAGGGTCGAGCCAAAGAGCTTCAAAATTTCTTCGTCCACACGCCGAACAGAACGTA
TTATCTGGAAGATCCCGAGGGATTCGCATTGGAATGGTGTAGAGCGATAGAGAATATGC
GAGTCCATTACTACGGCCTGCAGGAGTCCACCGCTTAAACAGAAGACTGATGAACGGA
AATCGTTTCGGATGGAACAGAGAGCGAATCGTTTCACCGATTACG

>novel_circ_000090
GTTCATTTCGGGATTCGATTGTTCAATTGCGAAACAGAAAAGGAGGAGAATAGTAAAG
GATATTTTTCTTAAAAGTATTCTACGCAAATTTAAAATCAGAAATAGATTTTTCGAAATA
GATAAGACTTCTGTGAGACTTTTGTGACTGATTGTATATATTTGGTGAACAGCATTTTGT
GACATGGGTATCGGATATAACTTGAAGATCTCGGTGAGATCGGATGGGCTCCTTTTATAT
TTGTCCCTCTGAATATCTTGCTATGGGGTTTAGTACGTGATTGATAAATCCGGATGAGCC
GAGCCGCCTGCCTTCAAACGAATTTCCTTTAATATCTTGCTCTCCGGATCGAGAAGAAA
AGAGAGAGAAAAAGAAACAGAGAGAGAGAGAGAGGAATGGCGATGCAATTTCTCAT
CAGCCCCCGCGAGAATCGCTTTCTCTTCTAGGGGAGTAAATTAGAGAATCGTGTTACGA
GGAATCGGCGCTGGCTAAGTGCTCTCTTCACGATTTATTTTCCAATTGAAACAGCTTCA
TCTGACTTTTTGAAATTCTTGTTTCAGAATGTTTGCGCGATCTCTGTTTCTGGGCAATGA
TTATGGATATAGTTTAAGTTTCGAGGAGAATTCTTGGGAACATAAGTGTTGGCAAAATTT
ATTCGTTTCGTATATACATGAGGAAGAATGATTTAAGAATGGACGGAAGAAGAAATTTA
TTGGTAATTTCTTGATACATTCTGTGTTACATATATGTTTGATTTTGTTAAAATTTACAAG
AAAATGTAAGAGTAAATTCACCATTCATTTTTGTTGCTTCTTTAATCGTTTAACAATTTT
CTTTGAAATTTGTTAAACATATAACACGCGTTCAAGGATATCCATAATATATTTCATATAA
TTCGAGAAGATTTCATTGAACCACTCATCAGAAAATATTAAAAATTGTATTGAATTTGGA
TTTCGTAGATCCATTATAATAAGATTACAAACTATTCGATAGAAAAATCCATTGAACGAT
AGATTATTCTATATTTAATATTTGCAACATTACCGTTGAACAGTTACTGTACTTATGGAAT
TATGGCAGTAGCTTTCGAAGAAGGGCATCCGAAGTTTGATGATCCGTAGGTCGTGAAA
GGGGATAATGAAGGAAGGATGACGAAGTGCAACTTTCGAAACTGTTGTACACAGAATC
GAAGCGGTGCACT

>novel_circ_000091
CTCCACTATCTGTCAAGCGTTTTACTATTTGTGCAACAGTAGATCTCTTCTGACCAATAG
CAACATAAATACAATACAATTTTTTCTTCTCCTCTCCAGCATCATTAAATCGTTTTTGATT
AATAATTGTATCAATAGCAAGAGCAGTTTTTCCAGTTTGCCTATCTCCAATAATTAATTC
ACGTTGACCACGACCAATAGGTACTAAAGAATCTACAGCTTTAATTCCAGTTTGCATAG

>novel_circ_000092
CTTCTGATGCAAAATTGAAACAAGTTGTTACTGACTTCCTTGCTTCTTTTTCAGGCTAA
ATGTCAGTATAAAGTAATTAATAATTCTCTGCTATGCAAGAGCCACATGCAAATAATATT
CATTATTATATAATTTTTAATGTATAATATTTGCACATTATAAATAACATTAAAGAATTCCAT
ATTTACATATTAAAACACAATAGTCATACAAATATTTATAAATATTACATGTTGACTCTTAT
TATCAAGAATAATTTTGGTAATTAAAAAAATACTTCTTCTGAAGAAAGTTTCATAGCAG
AAATAGGAGAA

>novel_circ_000093
GCATGTATCAATGATGATCTGGATATGGTCGAATTTTTAGTGGAAAAAGGAGCTGATATC
AATTGTGGAGATAATGAAGGATGGACACCATTGCATGCAACTGCATCTTGTGGATTTATA
TCTATTGCTAAATATTTAATAGAAAAAGGATGTAATTTGGCAGCAGTTAATTATGATGGT
CAATTGGCTCTTGATATTGCTGAAAGTGTTGAAATGGAAGATATGCTGCAACAACATAT
AAGTAAAGCTGGTATAGATTGTGATCAAGCTAGAAGTGAAGAAGAAAGATCAATGTTA
AATGATGCCAGAGCATGGCAATCAGGAGTAGCTGGTAAGGATTCAATCCATCCTAAATC
AGGAGCTACTGCTCTTCATGTTGCTGCTGCTAAAGGTTACATTGATGTCATGGAAATATT
ACTTCAAGCAAGATGTGATGTTAATGCTCAAGATTTTGATGGTTGGACACCTTTACATG
CTGCAGCACATTGGGGACAATTAGAAGCTTGTGAACTTTTAGTGGAAAACTTTTGTAAT
ATGGACATTAAGAATTATGCTGATCAAACTGCATTTGATATTGCTGATACAAATATATTAT
CAGCCCTGGAGGAATTAAAAGCAAAGCAACAGTTGTGGATGAAAGACCATCCACAAA
TTATTAATAAAAAACAATCAACTGTACCAAAGAAACGTATATCAACAAATAATGAAAAT
ACTATAACCACACAAGAATCTGTAGAAACATTTGAAGAAGAAACACCAAATAAAGTTA
AAAAGGTTGAATTAGAAATTCAATCTGATAAAGAAGATTCTAGTGGAACAAATAGCGA
CGTTGCAATTTGTATTTCTTGTAACAATGCTGCATGGACAGAAGCGACACGTGAAACA
GATATGGAAGAGAGTGATGGTGAAGGTGAATCTGAGACTAGCTCAGACTCACATTCTT
CCACTTACTCCAATCAATCTGATAAGTCTAACGAATCAAACCACTCTCCATGTCTTACA
GATGATGAAAAGAAAAATAGAGCAAACAGAGAGGAAACATCATCACGTAACACTTCA
TCAACTATTGATACTAATAAAGTTCCTAATCAGGCTCCAATAATGCCTCCAAAACAACA
GACTGATAATGAAGAAGGAATTATACCATCTTGGCGACGATCTGGTTCTTTTAGAAATA
GGGTACAAAATACAGAGACAACTCACACTTTATCTTCTA

>novel_circ_000094
GATCAACGTGTCTTTCTCGTACCCTGGATTATCGTCGTCATCACCACCTGTTTCGTCGAT
GTGGCCCACTCCTTATATTTGTTTATCGTTGCTTCCACTTTTGACCCGACAAAGGTGATG
CTATATACGTTGAACTTCTTCCTTTTCTGCCTGAACATATATTCCGTGTTGTGCGTGATCT
CCCAGTATCAAGAATACGTGGCCGGCCGAGGCACTGCCGCGGACGACTACGAATACAG
GGTCAGTATTCTGAGACTCTCTCAACGGAGACAGGAACGAGATAAGGGACGAAAAGT
TCCAGCAGTGAGATATGCCGTTCAGCCGCCAACTACAACGGCTACGTCTTGCCTGAGT
TCTCGTAGGGCGGCAACCAACAACGAAACCAAAGCAACCGCAACGCCAACCCAGAG
CCCTACCGCGGCTCAGAACACCCTCTCCTTGGATAAAAGTCCTGTCACGGGACGATCG
AGAAAACACGTTCAGTTCCCTGACACAACGAGATCCGATAATCAGAAGGGAAAATCC
G

>novel_circ_000096
GTCGAACAAAGATCGAACGAACGTTTTGATGTTGTACACGAAGAGAGTGAGGCATTTT
ACGAAATATAAAATAAACTCGGTGGATATTTTTCTCGAAACAACACGCTTCTGGATGGG
AAACAGTGTTATTTACAGTCTTAGAGCAACTTAACCGATACGGTTTTGCGTTGGAACCT
TAGGCTGGAAGTGACTTAGAGAGAATATTTGTCACTTGTCTGAATACAGTGAAACAAA
GTACAAGAGATTCGAGCAAGAAGTAAGGACGATCAAAAATTACTTTTTCTTTTTCTACG
ACGATATTTCATGAATTAAAAATCGTATCGAAATAAATAAAAAAGAGTTTTAAAGGGAA
AGAAAAATACATTGATAAAAGTGTAATATCATATAATTACCAAAATATGCATATGAGTTT
CATTCACTGAAATTAAGAACACGTTTGTAATCCAGTTCTGGTGCAGCACGTGAATAGAA

TCGGTTGGCAAATTCCGTAGCATCACGATTGTATGTTTCTTATGAGCATATGCTGCTCCT
TGTTTTCTTTTATTGCACGCGATATTACATTTGAATGCGTGAAATTTAAATTTCCCTAAAA
CCATCTCGTTTTCAACGTATGTTAACAAAATTGCATCATTGAATCTTGATCGTCCAATAA
AAAGACAAGAGAATTGCCGGATAATGGAACGCAGATCTTAAACGTAAAAGTGAAACA
CGTTTATTTATAACAAAATTAGGATATCTCCAATTTGCACAAACTCTTG

>novel_circ_000097
ACAGCTACCAGTATCAGCTGCAATCCATGTGGCAGAAATGTTGGAACACCAATCAGAG
CCTGGTCCACAATCTGCGCTTTCGCGAACGTGGCCCGCTCAAATCTTGGAGACCGGAA
ACGATGGCGGAGGCGATCTTCAGCGTCCTGAAGGAAGGATTATCCTTGAGCCAAGCCG
CTAGGAAATACGACATCCCTTACCCGACGTTCGTCTTGTATGCCAATCGAGTACATAATA
TGCTAGGTCCGAGCGCCGATGGCGGAGCTGATCTTCGCCCAAAAGGTAGAGGAAGGC
CGCAAAGGATTCTACTAGGCGTTTGGCCGGACGAGCACATTCGCGGGGTGATCCGAGC
CGTGGTGTTCCGCGACGGTCATTCCCCGCACATGGTGAAGGAAGAGCCAACCATGTAC
CCTGGTCTGGCGAATTACGCAGCTTGCAACGGTCCCGAAGGGGCGGTCAGTCCCGGA
GCGGCAGCCGCGGCCGCGGTTGCCGCAGTCGCTCAAGGACTGCGGCATCAAATGTGC
AGCATGGTAGCGGCGGCGCACTCGCAACAGCACGACATGATGGCGACGAATCTCTCCA
CGAACCTGTCCACGAGCCTCACGTCGAACCTACAGGCGGCGATGCAAGCCAGCCAGC
ATCACCACAACAACAGCGGGCCTCTGCTCGGCAACAACGGCGGGAACACCGGCAACT
CGCCGTTGAACCTAGGCCTGGGTAGCCCGGGCAGCGGTGGCCCGCCCGAGAGCCCGA
TGGAGAGCCCCCTCGCCAGCCCCCTCGGCCCCCTCATGGATCCCGGCCACATACCGAG
CAGCGTGGAAGTCGGAATCGGCGTGAGCGGAATGACCTACAAACCCGCACGAAGCTT
CGTCAGCCCCCGGCCGGAGACCCTTTTTCAGGAGGATATCGCGGACCTCGTGAAGAGT
CCGCACGGGCTCAAGGACAAGGACAAGATCCCCGTTAAGCTCGAGCCGTTGACGGAC
TCGCGTTGCGAATAGTAAGCAAGGCAGGCTCGAGACGGAACGAGCTAATTACCATTGG
ATCGGCGGAACAGCGTCGTCGTCGTCGTCGTCGTCGTCGTCGTCGTCTCACTTCCATCG
CGATCCTTTTAACGGGATTCTGCCATCGTCGCAATCATCGACGGAGCATCGCGCAGAGC
CGAGTCCACGGGGAGGCGACGCTCGACTGTACGGATTATTCGAAG

>novel_circ_000098
GGGTTGGAATGACGAAAAAAGACGAGGGGATGATAAAGGGAATCGAGGACAATTTGA
CGAGAGAGAAACGATGAAATGATCTGTTTGATCTGTTCGATCGTAAAAAAAAAAAAAA
CAAATTTTCGGACGATATTGCTTATTTAATCTTCCGTTATCTCTTTACAAAATGAAAAAT
ATTTAATAATATAGAGTCGAGTGCAACAGTTGCGCGAGATCCGATTAAAGGCTTACTGT
CGCGTACGCGCCAACACTGATGGTTCATGGATGCCTCTACTACGGCCCGATACTTGTAA
GAACTTTTTTTTTTTTTTTCAGAGAACATCGCGATTCATGAATGACGCGTTTCACTACGAT
AAAGGATAGGGTTGAAGAAAATTGTGATGCGATTAAAAGAGAATCGTTACAAATTCTT
GTTATTGTTGTTGGGTCAACGATACGATAGATTACGACAGATTTTTATTTTCTAATTAAAT
ATTTAAAAAAATGAAACGTTCTTTTAACGTTTAAATTGTAATAATAATAATACAAGGGAT
TGATATTATATTTCGAATTAGTGGAGCGGAGGAAGAAGAAGAAAAAGAAAAATGTTTG
CCCATATTACCGATGAATTTTACAAGTGTTGCATTCGAGCCACTATCCTCCGATATTAAAT
TTTAACGCTTATTTCTTACGTTTTTCTTAAAAGGATCGAATATATTTTTCCTCCTCGTGTT
ATCTTCTCTTTAATAATCGCGATCGACCATCAAATGACCGATTTATTCTCGTATTCTCGTC
GTCGGATATATATATTTCATTTTTGTTTCAATAAGAGGAACAATAAAAAAAAAAAAAAAT

CTATTCTTGCCCAATTTTCATCCGTATACGCGATACACGTTCGAAAGAGAGAAAGAGAG
AGAGAGGAAAAAAAGGGGTGCAACGTTCACCAGGTCATGCAAAATCGATATAACGCG
ATATACATTTCATGCGGAGTATCCTATAGGCCCTGCTATTCTCTCTCTCTTTCTCGCGATC
TCGTGCAGGAAACGAAGTAGTAAGCGCGAGAGTTGCAAAGTAACGTAATTCAACAAC
AACAACAATTTAGATTTTATACCGGTGATTATATATGGAGTGGGGGGGAGAGAGAGGTA
TCGTTTCCTTGCCATTTACAATATCGCCCGTGTCGAGGAGGCAGAAAAAAGGAAACAA
GAAGGGAGAATCCGTGGAAATAAAAAAGTCGTCGTCCCGCGAAACTACCCCCGTTTTC
GAGAAATTGCATCGCGTTTTCCATTAACGTTGCAACATATTAAGAACTGAGACGATCGA
ATAATTGCGGCTTTATCCCGGTGTATCGTGTTGATATCAACCGTGAATAAACTCGTCGAC
GAAACACATTCTCCTTCTCTCTCTCTTCGAAGTCCAAGAAATAGAAGAAGAAGAAGAA
GAAGAAGGACGAACACGATAATGGAACGATGCGCCGTTGCGTATTACGAGCGCATTAC
GAAATTACAATTTAAGCGAGGTTGAGCGGAAGAGCGATCGGAATGGCGCGAAAAGTG
GGGGATGAGAGAGGCCTGGTTGACGCTGGGCGGAAGAAACAAGCAAGGGAGTAGGA
GGAGGAGGAGGAGGAGGAGGGTGGGAGGGCTCGGAGGTGGACGCGGAGGAGGAGG
AGGAGGAGGTGGAGAGGAGGGTGGCGTAGCGCGCGAGCTTCCGATTTGTCCCCGGCT
TTCCAATATTCTTCTCGGTCGCCGATTGGAGATCGAATTGTCCGTTAATTGAAATGATTA
ATTGTCTCACCTCTGGCGGGGCCCTGGTCGAACGCGTTCGCGTTCCCGTGATTGCTTAT
TTACAAATAGGAAATTCGAAAATGTGCAGAATCGCGCGCGGCGACTGAGCGGGAACG
CGCGCGACGAGCAGAGAGGGAGAGAAAGAGAGAGAGAGAGAGGGTGAGCGAGAAA
GGAGGGGGGGACACTCGACACTCGTTTCTCGTGCAACAGAGATCGAACACGGAAACA
CGAGGGTTAGAGGCGTGAACACAGAAAGAGAGGGAGAAAGAAAGAGACAGGTTGTA
TGATCGTTCGTGAGACACAGATACACGTATATATATATATACGTATATACGCGTTAGTAGT
GGTGGTGATGGTGGTGGTGGTGGTATTCGTGGAAAGGGGGTGGTGTAACTCGGATACT
CGCTCGCTCACCGAGTTTCTACCGGTGGGGGTGGCCCGAGGGGGAGGGGGAGGCGAG
ATGGAGGCCATACGAGGGCCGCAATATCGTAGGATATTACGAATTTGCTATTTTCCGCGG
CGGCCGAATTGAAACTGGGATTGTTAATTACTCGGTGATTATCGCAGGCAGAATCGAAG
TCGCGCCGAAATTGCAGAAATCGAAATTACTTTCACGATCCTTCTTCTCTTTCTCTCTCT
CCCCCTCTTTCTCTCTCTCTCTCACACTTTCGTGCGCGAACACGATAATACAAGCGTATT
CTCTTTCTCTCTCTCGCTCTTCGTCCCACACTTTTCGACACCGCTCTCTCTCGCTCGTCT
TAATCGTACCTCGAAACACAGTTTCTAAACGCACTCTGTGCGTTTAGTAGACGGTCAAA
AAAGAAAGAGGAGAAAAAAGATTGAATTGAGAGAGAGAGAGAGAGAAGAAAGAGC
GGGCTAGGAAACGAGGAGAGAAAGAGAGAGAGATAGAGAGAGAGAGAGAGCGAGC
GGGCAAATTGAAATTCGTAGATGCGGTGCGGGTGTACGCTCGGCCAATTGGAATGCAG
AATCATACCGGAAATGCCGAGTAATGCACCAATCAGAATCTGGGAATAGTTAGAGGCC
CCGGATGATTAGGAATGCGCGCAATAACCTCTGATTAATGTGATCAGTGTATGATATATG
CCGTGTAGCGTGGCACGGCGGCGTCGGTGGCGTCGGCGGCCTCGGTGGCGTCGGTGG
CTCGTTGCTCCATCCTTTGGCTCCGCTTACATGAATACCACCCCCTTCCTCCCTCTCTCC
TCCATTTCGCTCGCAATCCTTCTCTCTCTATCTTGGCTCGCTTCCGTCCGAACGTACTAC
TACTGCTAGCACCACTACTCCTCCTCCTCCTCTCATCTTTCTCTATCTGCCACGAATTTG
CGATCCGTTCCTCTCCCTCGATCCTTCGATCCTGGTTGAACGCATCCATCCTCGTTGCGA
ACGCGAACAGGAGTTGCTGCTCCTACTACCACCTCCACCACCCTTCTACTACGATTATA
CGCGCCTTACGCGCATCTACGAACGTGTGTGTTCACGCAAATTCCGCGCGTGTGTGTGT
GTAAATACACGCGTCCTCCTCCTCCTCCACCTCTTCCTACGTATGTATACCTAGAACGAA
TAACAGGGAGAGAGGGAGGGAGAGAGAGAGAGAGAGATTCATATTGCTATGTGCGCG

CGTATAATATATCTCCGTCCACGAACGAGGAGGAGGGGGAGGGGAAAGGGGGCGAGTA
TAGTTTGTGCAAGAGCTTGGTCGGTCGCAAACGAGAGAACACCGAGCGTTGGGGTTC
GTAGTTGAATCAGGGGGAGAAGGAGGAGGGGGGGGGGTAAGGGAAACAGGGTGCTG
CGCGCGTGTGCTGCTTGCCGCCGCCGCTGCACCCGCGCTTGGATGGAATAGGGGGTAG
GGGGTAGGGGATAGGGGGAGGGTAGTATGCATTTACATAAATACATAACGCGGGTGCAT
ACAATGTTAGTGCTATGCTGTCAATTCTACCCTTGCAGCGAGGCAATTCTGAGTTATGC
CAGAACGCCATGCCCTATGACGGGCCTTCCCTCCGTAGTATGCGGCTGGGTACCCTTCG
CGCGCGCTTACCCGTATGTTTGTGCCTACCCCGTGTGCTCTCACCTCTCCCGTCTCCCCC
CTCCTCCCTTCCCTTATATCTCGCCCTTAGCCCCGCTCTCTCGCGTCTCTCCCTTCTTCCA
CCCTCTCCTCCGCCCTTTCTCTCTCTCTCTGTCTCTCTCACCGTGATTGCAACATTCATC
TTTAAAGTTTCCTGGGCGAGATACGAGACGGGGATGGATCTAGCCAGTCTTCCCGGTTT
TTCCTACCTTTCCAAGAGCGAACGACGAACAGGCTCGGATTCCAATGGATGGAAATGA
ACGTAGATTATTATTTATATGTTATTATTGTTGTTAGGTCGAAGGAAAGAGTTACGATATA
TTGTTAAGCGAATTCGATGAAGATTGTGAAAAAATATGTATATATATATATATCGTGTTTA
TCACGCGCGGTTTATATGCACACCGATCTAGTAAGATACTAATCGAAACTGTTATCGAAT
GAGTAGTGTACATGAATGAACGATTCATGTACAGCTATCGCGTGAATGCTGTCATTTGT
CTCGATGTTTCTTTTTTTTAAACCACCATCACGATTCCGAGGAAAAATCGTAAAAGAGA
GACCGTTCCGTGCAATATAATACAATATAATCGTCTGAATCCCGAGGAATCTTCGACCCT
CCCTTTCCTCCTCCTGCTGCTCGAAAAAGAATTCGCGAGAAATTAAACGATTGGAACG
CGCAGGCCGGTTTCGGCCGGTTTTAAAAAAAGGAACGGGGAAAGGGGAGGGCAGCC
AACGCGCGCGGCTTAATCGAAACGCGGGGAATTGAAAGGCCGGGGGTACCTTCGACT
CTTTGCCGGGGACCGCAGAGCCCATTAAACGCGCCGCCACTTACGCTTACATAATAATA
ATGGTGAATAACGAGAAAGCGTCTTTGTCCCGTTGCTTGTTTTTTTCCCATAGTTGGCC
GGGCGCAAGGGGTCGGGGTGTGGGGAGAGGGGGTAGTGGGTGGCACCGCCGCGAAA
TTAAAACTACTAAATCTCTTTTGGCGAGCGCTATAAAGCGATGTCGTGTCCCCTACCTG
GGCTATATTTCTCGCCGGTGACGATAGAGAAGAACAACCGTGGCGTTTTGCATTTGATG
TATTTACAGATACGCGCGTGTTTATCGTTAATTCATTCTCGTTACATTTTTTTTTTGGAATA
TATTTTCTGGACATCGATGCTGAATATAGATGTAATCGAGAGGATTGATCGTTATCTAGG
GTAGGAAGGAATTGGTAATTAATTTGTTAATAGTAAATGGTAACCGATAACTTGATATCG
TTGGGAAAAAATGTAAATTTAATGAGCGAGGGAAGAATTTTCGAACATCTCGATCCGTT
GAAAATTCTTTTATAATTGAATCTTCCTCCTCTTTATTTTTCTCTTTTCTTTCAAAAGATAT
CACGAAGATATCGTATCGATGTCAATTATTTTTATATACATCTCACGCATTCGAATCGAAT
CACGTTTTTTTCCTTTTCTGTTTCAGGTGAGTAGCTTTGTTCGTGCGATGACAACGTGTT
GCGTTCTCGCGTGCACGCGTCTCCAGATCTCCAACGAAGCTTACTTTAG

>novel_circ_000099
ATGCGGCCTCGCTGTTGAGAATTCGTTGGTGACCGGTCGGTCGAGAGAGGGAGAGGC
GAGAGAAGTTTCGACGACCGAAAAACGGAGGGAAGGGTGAAAGCGAAAGAGGAGG
CCGCGGAACGCGTAAGGAAGCGCGCCAAAAGGCCGAAGGAGGCGGGGGAGGCGGAG
GGGGAGGAAGAAGAGGAGGAAGAGGAAGAGGAGGAAGAGGAAGAAGAGGAAGAG
GAGGAGAAAGAGGAAAGCGGAAGAAGAGGAAAAAGAAGAAGGAGGAGGAGAACTA
GCGAGGAGGAGGAGGAGGAGGAGGAGGAAGGTGGAGAGGAGCGAGTACGAGAGCG
AGAACGAGGAGGAAGAGGAGGAAGAGGAGGAGGCGGAGGAGGAGGAGGAGGAGG
AGGAGGTCGGGGCGGAGGAGGAGGGAAGCGGGGTGGGAGGAGGAGGAGGAGGAAG

GGGAGAGAGGAGCGGAAACGGAAGAGGAAGAAAGGAGCGAGGCCGGAGGACGAGA
TTTCTCGACGCGGCAAACATGACGCAGCAAAGCGGGGCTGGGTCGCCCCAGCAATTC
TGCCTACGGTGGAACAATTACCAGACGAATCTGACCAACGTGTTCGACCAGCTTCTCC
AGAGCGAGAGTTTCGTTGACGTGACCCTCGCCTGCGACGGGCACAGCGTGAAAGCGC
ACAAGATGGTACTGTCCGCGTGCAGCCCCTACTTCCAAGCCCTTTTCTTCGACAATCCA
TGCCAGCACCCGATCGTCATCATGAAGGACATCAAGTGGCCCGAGCTCAAGGCCGCGG
TCGAGTTCATGTACAAGGGGGAGATAAACGTGTCCCAGGAGCAGATCGGCCCCCTCCT
CAAGGTGGCGGAGAGCCTCAAGATACGGGGCCTGGCCGACGTGAACAACGAGCACG
AGTTGGCGTCGAGATCGAACCTCGAGGAGGCGGCCAGCGCCGCCCTTCACAGGAAGA
AGAGGCGCCGAATATCGGGCGAGAGGTCGCCCCCCGCGTGCAGCCCCGACCGGATCC
CCGGCTCGGCCAGCATCCCCGACGACGGGGAGCCGAGCCAGGTGGGGGGCGGGGTGA
TCGTGCCCGACATCCACGGCATGCTGCCGAGCAGCTCGACCCCGCGCTCCCTCGGCTC
CCCGGGCACGCCCAACGTCTCTGTCACCCCGCAGATCAACCTCCAGGAACTTCCGGTA
TCGCTGCCCCTGCCGCCGCCGCCGCCGCCGCCCCCTCAGCCCGGCCAGCCGAGCTCGC
ACTCCATACCCGCTCACCACGTGCCTCCCCACGTGACTTCCGGTCCCCACGCGCCCGT
CAACCACCTGACCGCCCATGGCCAGCAGCTCGCCGTGCAGCAGCAGCAGCAGCAGCA
ACAGCAGCAGCAACAGCAGCAGCAACACCACCCCCAGCCAGGACCTATCGTCGTCCC
GCCGAACCCCGGGGACGATCTCGAGATCAAGCCTGGCATCGCCGAGATGATTCGCGAG
GAGGAAAGG

>novel_circ_000100
GTCTTTCGACGACGCGGAAGATCGGGCGAGGAACGGGCGAGGAGGGCATTCTGAAAA
AGTCTTCCGCGTTTCTGTCCAATTTCGCCCATCCGGCGGGCAAAATCAGGGAGAGGAA
ATTGAACGATCCGGGGGAAGGGTCGGAGTCGAGCTCCAGGTATCCGAGTTTCAAGGGT
TACATCTCGTTGCTGAACAACCATCTCGACCCGTTTCAACGAGCTGGCTCCCCAACAA
CGTTGCTGGCCGAGCGGAGGAAAACGAACGGCGTAAAGAAATCGGTGTCCTTCAGTT
CGGACACTAGCTTCGAGGAGAAACGCGCTCCTTATAAAAGAGCCGCCGTTCACGAGGT
CAAGGTGTATCACAAGGGTGTTCTGCAAGATCGTAGCGTACGCGAGGTGGATATCGTC
ACGAAAGTGCCGTCCTCGTGGGAAGAAACCTCCGAGGGCCCAGCGGCCCTTCTGAGG
GCAGCAAGAGACGCCGACGATGCGGTCCTCAACGAGATCGCAGTTCAAGTGCGAAAA
TTTGGATTTGGGAGCATGGACGTCAACATGGCTGATAGCAGCGGCAGA

>novel_circ_000101
TTTCAGGCTGGCTAGTTCGAAGATTTTACAACAGCGATGTCTCATCATCATTCACGATG
GATTATGCGAAACATCGTATATCTCTGTCAACGGTACAACGCGCGATTTTGACAGTTGG
TGCTGCTGCGATATCACTTCTCAATCCTTATCGAGGTGACATGATCGCATGCTTGGCAG
AAACAACCGGTACTGATGCTCTTTCCTATTGTCATCGGCAAATGCTTGCAACCTCTGAG
GGATGTCGCATTCTTGCCGAAAAACCACGCATATCCTCTTCAACAGTTGATTTTTCCGC
GTTAAAACGATTACCAGCAGGAACTCTTGGAAAAATTTATTATGATTTCTTGGAAGTAA
ATAATGTATCTCCCGATTCAAGAACTATGGTCAAATTCGTCCAAGATACTGAATTGGCGT
ATGTAATGCAACGATATCGAGAAACACATGATATTTATCATGCAATACTTTTGATGCCAA
CTACAATGCTTGGGGAAGTATCAGTTAAATGGATAGAAGCATTGCAATTACGTTTGCCA
ATGTGTTTAAACGGAGCGATATTTGGAGCATTTCGATTGCATCCAAGGCAAAGAAAATT
ATATCTTGATCATTATCTTCCATGGGCTATTAATACAGGAATTAAAGCAAAATTTCTTTG

GGTATCTATTTTGAGGAACGTTGGGAACAACCTCTTGTTGATTTCCATCGAGAATTAAA
CATCGTTCCTTTAATATCGATGGAAAATATTTATAATGATATGTAAAGTCTTATCTCAAAC
GCTTTTTTCTCTGTTATATAATAACAAGAATAGAAGTTATTAGGTAGTATATAACAATTTT
TTTATTCACCGTAACGTTGGAAATATGAAGAATATAATTTTTAATGGAATACGTATGATTT
GTATTACAAATATTTCGATCGCTATAATCAGAAATTTTAAAAAATTTATATTCCTGTGTATA
TATATAGTGCATATACGCGCGATACGATGAAAAGTTGATTCATTTACAAGCAATCAACAA
TCTGACTAGCATCATAATGGACATAATATTCTAGTTTTCGGTAAGTATTAGTAAATTGATC
AATAATTACATTTTGAGATCACATATCGCATAATATGAAACGATATTTGTAATATTGATTG
TCCCATTTGGTTTGTGGTAACGCGCACAAACTATTACGAATGACTAAATTTTGATATAAA
CAGAATATATACTTAAATAATAATTATTCATTTGGCAGTTTTATCGATTATAATATTTGCAG
TGTAACATATTTTAAATTTTCCATATATATATAAAATATAATATCTTTCATGTTAATTATTCT
GTTGAATTTTTGAATTCTAAATCAAATTCGTTATAAAATTAATATTATGTATTTTTGTCCAC
CTTTTGTTGCATGCTTCTCCAAACTGATATAAATTTTATTAATTTATTTATACAATTTCGCA
TTCATATAGAGAGAAAGCATATTAATATTAGTTATGAAGAGTCAATATTTAATTGAAGAAT
ATTTTGTAGATTATAACCAATAATTGGAAAAAAATAAGATAATAACTTTTATGAAAAAGCT
TTGCGTTTCTTGTTCACAGGAAATATATCCAATAAGTTCTCTCTCTCTCTCTCTCTCTCTC
TCTCTCTCTCTCTCTCTCTCTCTCTCTCTCTCGCATGTACATTATGTATTTTTATACATATAT
ATATGCGCAAAGTTGATTCATACATAAACCTAATTGAGCATTAAAAGTTAATGGCCAGA
GCATCCTGATGTTTTTAATAGAAAGGAGTAGTTTTATGATATATTTTTGATATAAGCTATAT
TAGGAATATATTAATAAAAAATGGACGAAGCAGAAGAAGAATAAATCAACATAAAAAG
CTGAAATTTATGTACAGGATGCTTCTTGGCTGATTGTAATTGTTTGGTCCATACTACTCA
ATTATGAGTACCTGGAACATTCAATCGAAGGTTAATTTCTCGAGATATAGATGAATTTCC
TCGAAACTAGGTCTCTCTTCAACTCGTGTGCTCCAACATTTTTTCATTATACGATAAAGA
TTATCCGGACAATGATCAGACTGTGAAGGAATTCGAGGTTGATTTCTTTTATCATTATTA
TCATCATATTCCTTATCGATGCTCGTTTCTCTTTGATACCAATGACCATAGTTTTCTAAAA
TTTGTGCGACTGTTAAATCTGCATATGGTATTTCTTTACAATTTCGAAAAATTTCCCAAA
TTGTTACAGCAAATGACCAAACATCGGCACGGCAACTACGTTTACCCTGAGAAACAGA
AAAAATGAATGCAATATGTAGAAACTAGAAAATATCTATCTGAAGAACAAAGGAACAA
ATATATATATTATATTCATAATATTAAAAAAGAAATAAGAAGAAAATAAGAAACGTTAAAT
AATATTTACCAATAAAACTGCTTCCCATGCCATCCAACGAAGGGGAATTTTTATATTATG
TCCATCGATGAAATAATGATGATCATATTTATTACAATATATAGCATGATCGGATACCTTTA
TTTTCAGATCGAGACAAACGATGCAATTTCTAGAATTTATATTGATATTAAATTAATTTGT
TTTCTTTTATTTTATTTTTTACAAATATTTTATTACCTAGCCGCTAAATCACAATGTGCAAT
ATTCATGGATTCCAGATATTTCATACCAGATGCAATTTGGATAATAAAACTTAAACGGCT
GCCATAGCTGCGAAGAATAGACACGAATTGAAATACGAAGAAATGACATCTTGATACAT
CATTAAG

>novel_circ_000103
AGTCAAGATGGACATGGCATTGATGGGCATTCTGGTATGAGTGGGAATCCTGCTGCGGA
TTATAGCGAAGGTGCGTCTCATGTATTGGCAGTGATCCATCAAATTTTGGGTCATCATAG
AGCGTTGGAAGCTCGTTGGCATGCTCGAAAAGTCAAACTGCATCAACGACTCGCGTTA
AGATTATTTCAAGAAGATGTGAAACAAGTTTTGGATTGGTTAACTAATCACGGCGAAGT
TTTTATTAGAAAGAATACAGGTGTTGGTCGTAATCTTCAAAAAGCGAGAGTATATCAGA
AAAGTCACGAACATTTTGAAAATGTCGCACAGAATACATATACGAATGCTACTAAACTA

CTTACTGCTGCACAAGAATTGGCGCATACTGGTGAATGTGCTGCTGATGAGATATATGC
TGTAGCGCAAGAATTAGAGGCTCACGTCAGTAGTTTTGCGGCAAGAGTCGAACAACGT
CGTCGAAGATTAGATTTAGCAGTTGTATTTTATACACATGAAAAAGAGTTAAGTGGTTG
GGTTGACGAATTACGACAAGAATTACAACAAGACGAAGTGGCGGAGAATTTAGAAAC
AGCGGAAAGATTGTTGGAACAATGTGCACAACATCGAGCCTCTTGCATGGAAGCATGT
GCATCGACTATTGTTCAGGGAGAAGCATTGCTTCGAGAATTGCGAGAATCTACTGATGC
TCCTGATACTACTGGCTCTATATCTGCGGTAGAAGCTGCATTAGATAGGTTAGCGAGCTT
AAGACAGGAATTAGAAGATTTATGGGCTACTAGAAAATTAAGGCTAGAATTATGTTTAC
GACTACGAGTATTTGAAAGAGATGCTCTAGAAGCGAGCGGTCAATTAGAGATGTGGGC
ACAAGAATTACAAGGCCCATCTCGAGAAGGTTCTCCTGAACAATTATTACGTGCTCATA
ATGATGGTGTTGCTCATATGCAGAATACTGCATTTCAAGTTCTGCAACAAGGTCAAGAA
CTTGCTCAGGTGTTGGAACAAGCAGGAGTTTGCATAATGGCAGATGGACAACATAGTG
CTGCAACTAGAGTACAAGTGCTTCTTGAATTTTTAAACGAAAGAGAAATGGATGCGGA
AGATTTAGCGGAGATGAGAAGAGTTCGTTTAGAACAAGCTTCTCAACTGGTTCAATTA
CAAACCGATGCTACACATGTTGCAAATTGGATTCGTAATGGAGAAGCTATGTTATTAGC
TTCTTTGAGAGTTCCGGAAAATCTTCAAGATGCAGAACAGCTTCGTTTAGAACATGAA
CAGTTTCAAGTCGCTATAGAAAAAAACACATACTTCAGCTGTTCAGGTAAAACACAGAG
CAGATGCGCTAGTGAGTGCTAATCATTATGACCCAAAGAGTATAAGAGAGGTAGCTGA
GGATGTAACTAAAAGATGGCAACAACTTGTGACGTGTGCAGAGGAAAGACATAAACT
AGTAACAGCTAGCATTAATTTTTACAAAACGGCAGAACAAGTCCGTTCCGTATTAGATA
GTCTTGAACGTGAATATAAAAGAGACGAAGATTGGTGTGCTTCTGGTGAAAAAGCTAC
ACAAGTTCCAACACTTGTTGGCAAACATCAAGAACAAAAAGAAGCATTTTTAAAAGCA
TGCACATTAGTACGGCGAACTGCGGAAACATTTCTTAAATATACGAATCGTAGTCTTCA
ATTTTATAGTTATCAGGCAAATAGTGCTGGCTCTGAGAATAAAGTTAAAAATATTTTGGA
GGAGTTACTTAGTAAAGAGAATCGTGTGCTCGAATACTGGACGCAACGTAAGAAACGA
TTGGATCATTGTCATCAGTATGTTCTGTTTGAGCGTAGTGCTAAACAGGCTTTAGAATG
GATTAGAGAAACGGGTGAATTATATCTAGCAACACATACCAACGTTGGTAAAAATCGTA
TCGAAAACGAACAATTATTGCGCGAGCACAATGAATTTAAAGGTGCTGCGAAGGAAAC
ACGAGAAAGAGTAAAGCTGTTGATTCAACTTGCTGATAATTTAGTCGAGAAAGGACAT
GCTCATGCAGTGGCAATCAAACAATCTGTTGCTGAAGTGGATCAACGATATAAGGATTT
TAGTACGCGTATGGATTGTTATAAAACACAAATTGAAGAAGATCTTGGAATTCAATCAG
ACGATGGTCAAAAGGATCTTTCTATCGATCGCAATTCCGATCCGTTACTCGAGGAAAAG
ATCAAAGGAAAAGATTTAAAAGAATTAAACGAAGAAAAAAGAAGATCAGCGCGGCGA
AAGGAATTTATAATGGCTGAACTGCTACAAACAGAACGAACTTACGTGAAAGATTTAG
AAACCTGTATTCGATGTTTTTTGGAAGAAACACGTTGTGGAAAAGGAAATGTTCCATCT
GGATTGCAAGGACGAGAATCTATAATTTTTAGTAATATGGAAGAGATTCATCAATTTCAT
AGTAACATATTTCTTCGTGAACTTGAAAAATACGAAACTATGCCCGAAGATGTTGGACA
TTGTTTTGTAACATGGGCACCTAAGTTTGATATGTATGTGACATATTGCAAGAATAAACC
AGAAAGTAATCAATTGTTAGTTACTCATGGTGGGACATGGTTTGAAGAATTACAAAGA
AAACATCGAGTTGAACATCCGATTGCTGCATACTTAATTAAACCCGTACAAAGAATAAC
AAAATATCAATTATTATTAAAAGATCTTCAGGCCTGTTGCCAAGAAGGACAGGGCGAAA
TAAAGGACGGCCTTGAAGTAATGTTAAATGTGCCTAAGAAAGCAAACGATGCCTTACA
TTTAAGTATGCTGGAAGGTTGCGATGTAAGAATAGATACATTAGGTGATGTAGTATTACA
GGATTCTTTTACGGTATGGGATCCCAAACAATTGATTAGAAAAGGCAGAGATCGGCACA

TATTTCTATTTGAATTATATCTGCTCTTTAGCAAAGAAGTGAAAGATTCGGCTGGGAAGG
TGAAATACATTTACAAAAGTCGTTTAATGACTTCTGAGTTGGGTGTAACTGAACATATT
GAAGGTGACGAATGCAAATTCGCAGTATGGACAGGTCGTGCACCGACTAGCGATACAC
GCGTTGTTCTTCGAGCCAATTCAATGGATGCTAAGCAATTATGGGTGAAAAGATTACGC
GAAGTTATTCAAGAAACATATTTCAGTTTAAGTATGCCCAAGAGTCCTGCGAAAAAAA
GTTCAAGTCAACGATCCAGTAGAGATCTTGAAGAATGTACTTCTTTGGATGATAGTGTC
GAGAATTTGGATAGAAATTCTTTGGCATCTTTTGGCTCTACTAATACTACAGACTCAGAT
AAAACTGGCGTAGCCGAGGTGACCTGGGTGATTGCCGATCACTCTGCAGCACCGGGTT
CGAAAGAGTTGACGGTAACGAAAGGCCAACAAGTTGAAGTATTAGAAAATGGAAGTA
ACATCAGCGGTGTGAATACATCCGAGTGGACTCACGTGCGTTTACTAGTTGCTCCAGG
ACAAGTTGATCCTCCTCCAGAAGGATTAGTTCCTACTAGTGCGCTTAAACAACCCCCTC
CAGTTTCTAGTAAAACTTCACCATCTAGGAAAGTTCCAGGACAGCAGCAGCAGCAACA
ACAACAACAGCAGCAACAGCAACAGCAGCAGCAGCAGCAACAACAACAATTACATTA
TCAACAACAATCGAGTAGTCAAGTTCAAACTGTCGCATCCAGTGGAGGATTTGCAACT
GCGTCCTCTGCTGCTGGAGTAGCAGGCTCTTCGTCGACTGTTGCAACAAGTGTAACAC
CTGTGTTACCAATTCAAAATGTTCCTGTGTTGCCGGATGAAACTGAAAACGTTGCAAA
CGATGGAAGTACTCCTGCGAACACAAATTCTCCAGGAAACAAAAGGCGTGGCTTTAGC
GGGAGAAAGTGGTTACCTCCACCATTGCGTAAACTTAGTCAAGGTAAAGTCGAGAAAT
CGCCACCGACGACGACAGCGACAGCGACATCGATGACATCGATTGTACCGCCATCAGG
TGATCGGTTGAAAAAGAATGTCTCGGAAAAACGATTCAAATTACCGACTGGTGCGGAA
CAATCTCGACCGTCCAGAACTGCTTCTATTTCTATCTCCGCGTTAACAACAGCGACTGC
CTCTATGCGTGTGTCTGCTTCTGAAGCGGACCTTGACACTGAACTTCAACCAGGAATC
GAAGGAGAAGAGGACGAAGGAGAAACGGAACAATCGGAACCAGAATTGGAAGAAGA
TGTACCGGAACCCGATAACACAGAAGCTTTAACTTACTCGGAACAAAATGGAGCGGAC
GATGTCGAAGACGAATTAGAACTTCCTCCGCCGATGAAACCCATCACTGAACCGATAC
TTGTAGGAACTGCGAATGGAGCATCGGGATCAGCAATTACAACAGAAGGCTGTGGAA
AATCTCGAACATCTGAACGTTCAACGAAGATTCTTGACGGTGCCACGACGGTTGATTT
GGCTGAAATTGAACAGATCGTAAAAGAAAGAATGGAACAACATACGGAAAACCAAGA
AAGACAAAGTCTGATGCGAACACCTAGTGGTAAAAATTCAAGCATTGGTGGCAGTGG
GGATAACGATTACGACAAAGATGCAGTATCGAGCGCAACTATTGCTACTACGACGATTG
CTTCATCGACGACGATAATGACAACAGCAGCGACGGCCACTGTTGCGACGGGGATAAC
GATGACGACGACGACGACTAAGATGGGAACAACGAGTAGCCCTGCTCACGGGAATTC
GGAGGAATACGACTTGGAAAGCGCGGTGCTAGTAAAACGACAATTCGTTATTCGAGAG
TTGGTCGAGACGGAAAAGGATTATGTAAATGATTTGAAGCAAATAGTCGAAGGGTACA
TGGCATTGATGCGAGATTCTGAATCTCAAGTGCCATTGCCAGATGATTTACGCGGTGGA
AAAGACAAAATGGTTTTTGGTAACATAGAGGCAATCTACGAATGGCATAGAGATTTCTT
TCTGAAAGCTTTGGAACGTTGCTTGGAACGGCCGGAAGAGCTTGGGCCGTTGTTTAAG
CGATACGAAAGGAAGTTACATATGTATGTTGTTTATTGTCAAAACAAACCAGTCTCCGA
GTATATTGTTTCTGAGTATATAGACACCTATTTCGAGGATTTGAGGCAAAAGCTCGGAC
ACCGATTACAATTATGCGATCTTTTAATCAAACCGGTTCAAAGAATCACAAAGTATCAA
CTTCTTCTTAGAGAGGCATTAAGACTTACCGAACGAACTCAAAGAATTTCGGAAATCG
AAGGACTTAGAGCAGCGGTTCATGTAATGCGTATTATTCCAAAAGCGGCCAATGACATG
ATGGATGTTGCAAGACTTCAAGGTTTCGACGGAAAAATTACAGCGCAAGGAAAATTAT
TATTGCACGGACCACTTTTAGTCTCAGAATTTTCTTCGAATTTACCTAGTAAGGAAAAA

GAATGGCAAGTCTTCCTGTTCGAACAAAATATTATTTTAGTGAAGCTGTCGGAAAAAA
GACACAATTCACCAATCCTGTCTATATTTACAAGGCTCATATTCAGGTGAATAAAATGAG
TTTAGAAGAATGTTATGATGATTCGGAGAAGTTTATAATTCGATCAACAGATCCTCGAA
AACCTGGCCTTGGATTCTCTTGTAGCGCAGTAGAAGAAAATGGGCCACGGAAGCAGG
AGTGGGTAGATACGATCACTGCCATCCTGCAGACCCAACGTGACTTCCTTAAAGCGATA
CAGTCACCAATTGCCTACCAAAAGGAACTTACCAAAGATCCATTTCGTGGCGTGAGTC
CGGATTCTCCGTGTCGAGGGAGTGTACTTTCATCAACAATACCGAACATGTCTAAAACG
AACGAAGAACGGAGAAATGAAATCGCTGCTACTACCTCTTCAAAAGCATTAGCTACAG
CTGCAGCAGCGACAGCTACGGCAACATCGGTTTTACAGCGACCTCGTACTGGAGGCTT
GGATCAGGATTCTTCGCAATCGCCAAGTCCTAGCAAAAGCAGACTGAACTTTCTCGAA
GGATTCAGAAGTACTCTACGCCCACGATCACCTGTTCGCAACAACTCTATTCCG

>novel_circ_000104
ACTGGCGTAGCCGAGGTGACCTGGGTGATTGCCGATCACTCTGCAGCACCGGGTTCGA
AAGAGTTGACGGTAACGAAAGGCCAACAAGTTGAAGTATTAGAAAATGGAAGTAACA
TCAGCGGTGTGAATACATCCGAGTGGACTCACGTGCGTTTACTAGTTGCTCCAGGACA
AGTTGATCCTCCTCCAGAAGGATTAGTTCCTACTAGTGCGCTTAAACAACCCCCTCCAG
TTTCTAGTAAAACTTCACCATCTAGGAAAGTTCCAGGACAGCAGCAGCAGCAACAACA
ACAACAGCAGCAACAGCAACAGCAGCAGCAGCAGCAACAACAACAATTACATTATCA
ACAACAATCGAGTAGTCAAGTTCAAACTGTCGCATCCAGTGGAGGATTTGCAACTGCG
TCCTCTGCTGCTGGAGTAGCAGGCTCTTCGTCGACTGTTGCAACAAGTGTAACACCTG
TGTTACCAATTCAAAATGTTCCTGTGTTGCCGGATGAAACTGAAAACGTTGCAAACGA
TGGAAGTACTCCTGCGAACACAAATTCTCCAGGAAACAAAAGGCGTGGCTTTAGCGG
GAGAAAGTGGTTACCTCCACCATTGCGTAAACTTAGTCAAGGTAAAGTCGAGAAATCG
CCACCGACGACGACAGCGACAGCGACATCGATGACATCGATTGTACCGCCATCAGGTG
ATCGGTTGAAAAAGAATGTCTCGGAAAAACGATTCAAATTACCGACTGGTGCGGAACA
ATCTCGACCGTCCAGAACTGCTTCTATTTCTATCTCCGCGTTAACAACAGCGACTGCCT
CTATGCGTGTGTCTGCTTCTGAAGCGGACCTTGACACTGAACTTCAACCAGGAATCGA
AGGAGAAGAGGACGAAGGAGAAACGGAACAATCGGAACCAGAATTGGAAGAAGATG
TACCGGAACCCGATAACACAGAAGCTTTAACTTACTCGGAACAAAATGGAGCGGACGA
TGTCGAAGACGAATTAGAACTTCCTCCGCCGATGAAACCCATCACTGAACCGATACTT
GTAGGAACTGCGAATGGAGCATCGGGATCAGCAATTACAACAGAAGGCTGTGGAAAA
TCTCGAACATCTGAACGTTCAACGAAGATTCTTGACGGTGCCACGACGGTTGATTTGG
CTGAAATTGAACAGATCGTAAAAGAAAGAATGGAACAACATACGGAAAACCAAGAAA
GACAAAGTCTGATGCGAACACCTAGTGGTAAAAATTCAAGCATTGGTGGCAGTGGGGA
TAACGATTACGACAAAGATGCAGTATCGAGCGCAACTATTGCTACTACGACGATTGCTT
CATCGACGACGATAATGACAACAGCAGCGACGGCCACTGTTGCGACGGGGATAACGAT
GACGACGACGACGACTAAGATGGGAACAACGAGTAGCCCTGCTCACGGGAATTCGGA
GGAATACGACTTGGAAAGCGCGGTGCTAGTAAAACGACAATTCGTTATTCGAGAGTTG
GTCGAGACGGAAAAGGATTATGTAAATGATTTGAAGCAAATAGTCGAAGGGTACATGG
CATTGATGCGAGATTCTGAATCTCAAGTGCCATTGCCAGATGATTTACGCGGTGGAAAA
GACAAAATGGTTTTTGGTAACATAGAGGCAATCTACGAATGGCATAGAGATTTCTTTCT
GAAAGCTTTGGAACGTTGCTTGGAACGGCCGGAAGAGCTTGGGCCGTTGTTTAAGCG
ATACGAAAGGAAGTTACATATGTATGTTGTTTATTGTCAAAACAAACCAGTCTCCGAGT

ATATTGTTTCTGAGTATATAGACACCTATTTCGAGGATTTGAGGCAAAAGCTCGGACAC
CGATTACAATTATGCGATCTTTTAATCAAACCGGTTCAAAGAATCACAAAGTATCAACT
TCTTCTTAGAGAGGCATTAAGACTTACCGAACGAACTCAAAGAATTTCGGAAATCGAA
GGACTTAGAGCAGCGGTTCATGTAATGCGTATTATTCCAAAAGCGGCCAATGACATGAT
GGATGTTGCAAGACTTCAAGGTTTCGACGGAAAAATTACAGCGCAAGGAAAATTATTA
TTGCACGGACCACTTTTAGTCTCAGAATTTTCTTCGAATTTACCTAGTAAGGAAAAAGA
ATGGCAAGTCTTCCTGTTCGAACAAAATATTATTTTTAGTGAAGCTGTCGGAAAAAAGA
CACAATTCACCAATCCTGTCTATATTTACAAGGCTCATATTCAGGTGAATAAAATGAGTT
TAGAAGAATGTTATGATGATTCGGAGAAGTTTATAATTCGATCAACAGATCCTCGAAAA
CCTGGCCTTGGATTCTCTTGTAGCGCAGTAGAAGAAAATGGGCCACGGAAGCAGGAGT
GGGTAGATACGATCACTGCCATCCTGCAGACCCAACGTGACTTCCTTAAAGCGATACA
GTCACCAATTGCCTACCAAAAGGAACTTACCAAAGATCCATT

>novel_circ_000105
AGTCAAGATGGACATGGCATTGATGGGCATTCTGGTATGAGTGGGAATCCTGCTGCGGA
TTATAGCGAAGGTGCGTCTCATGTATTGGCAGTGATCCATCAAATTTTGGGTCATCATAG
AGCGTTGGAAGCTCGTTGGCATGCTCGAAAAGTCAAACTGCATCAACGACTCGCGTTA
AGATTATTTCAAGAAGATGTGAAACAAGTTTTGGATTGGTTAACTAATCACGGCGAAGT
TTTTATTAGAAAGAATACAGGTGTTGGTCGTAATCTTCAAAAAGCGAGAGTATATCAGA
AAAGTCACGAACATTTTGAAAATGTCGCACAGAATACATATACGAATGCTACTAAACTA
CTTACTGCTGCACAAGAATTGGCGCATACTGGTGAATGTGCTGCTGATGAGATATATGC
TGTAGCGCAAGAATTAGAGGCTCACGTCAGTAGTTTTGCGGCAAGAGTCGAACAACGT
CGTCGAAGATTAGATTTAGCAGTTGTATTTTATACACATGAAAAAGAGTTAAGTGGTTG
GGTTGACGAATTACGACAAGAATTACAACAAGACGAAGTGGCGGAGAATTTAGAAAC
AGCGGAAAGATTGTTGGAACAATGTGCACAACATCGAGCCTCTTGCATGGAAGCATGT
GCATCGACTATTGTTCAGGGAGAAGCATTGCTTCGAGAATTGCGAGAATCTACTGATGC
TCCTGATACTACTGGCTCTATATCTGCGGTAGAAGCTGCATTAGATAGGTTAGCGAGCTT
AAGACAGGAATTAGAAGATTTATGGGCTACTAGAAAATTAAGGCTAGAATTATGTTTAC
GACTACGAGTATTTGAAAGAGATGCTCTAGAAGCGAGCGGTCAATTAGAGATGTGGGC
ACAAGAATTACAAGGCCCATCTCGAGAAGGTTCTCCTGAACAATTATTACGTGCTCATA
ATGATGGTGTTGCTCATATGCAGAATACTGCATTTCAAGTTCTGCAACAAGGTCAAGAA
CTTGCTCAGGTGTTGGAACAAGCAGGAGTTTGCATAATGGCAGATGGACAACATAGTG
CTGCAACTAGAGTACAAGTGCTTCTTGAATTTTTAAACGAAAGAGAAATGGATGCGGA
AGATTTAGCGGAGATGAGAAGAGTTCGTTTAGAACAAGCTTCTCAACTGGTTCAATTA
CAAACCGATGCTACACATGTTGCAAATTGGATTCGTAATGGAGAAGCTATGTTATTAGC
TTCTTTGAGAGTTCCGGAAAATCTTCAAGATGCAGAACAGCTTCGTTTAGAACATGAA
CAGTTTCAAGTCGCTATAGAAAAAACACATACTTCAGCTGTTCAGGTAAAACACAGAG
CAGATGCGCTAGTGAGTGCTAATCATTATGACCCAAAGAGTATAAGAGAGGTAGCTGA
GGATGTAACTAAAAGATGGCAACAACTTGTGACGTGTGCAGAGGAAAGACATAAACT
AGTAACAGCTAGCATTAATTTTTACAAAACGGCAGAACAAGTCCGTTCCGTATTAGATA
GTCTTGAACGTGAATATAAAAGAGACGAAGATTGGTGTGCTTCTGGTGAAAAAGCTAC
ACAAGTTCCAACACTTGTTGGCAAACATCAAGAACAAAAAGAAGCATTTTTAAAAGCA
TGCACATTAGTACGGCGAACTGCGGAAACATTTCTTAAATATACGAATCGTAGTCTTCA
ATTTTATAGTTATCAGGCAAATAGTGCTGGCTCTGAGAATAAAGTTAAAAATATTTTGGA

GGAGTTACTTAGTAAAGAGAATCGTGTGCTCGAATACTGGACGCAACGTAAGAAACGA
TTGGATCATTGTCATCAGTATGTTCTGTTTGAGCGTAGTGCTAAACAGGCTTTAGAATG
GATTAGAGAAACGGGTGAATTATATCTAGCAACACATACCAACGTTGGTAAAAATCGTA
TCGAAAACGAACAATTATTGCGCGAGCACAATGAATTTAAAGGTGCTGCGAAGGAAAC
ACGAGAAAGAGTAAAGCTGTTGATTCAACTTGCTGATAATTTAGTCGAGAAAGGACAT
GCTCATGCAGTGGCAATCAAACAATCTGTTGCTGAAGTGGATCAACGATATAAGGATTT
TAGTACGCGTATGGATTGTTATAAAACACAAATTGAAGAAGATCTTGGAATTCAATCAG
ACGATGGTCAAAAGGATCTTTCTATCGATCGCAATTCCGATCCGTTACTCGAGGAAAAG
ATCAAAGGAAAAGATTTAAAAGAATTAAACGAAGAAAAAAGAAGATCAGCGCGGCGA
AAGGAATTTATAATGGCTGAACTGCTACAAACAGAACGAACTTACGTGAAAGATTTAG
AAACCTGTATTCGATGTTTTTGGAAGAAACACGTTGTGGAAAAGGAAATGTTCCATCT
GGATTGCAAGGACGAGAATCTATAATTTTTAGTAATATGGAAGAGATTCATCAATTTCAT
AGTAACATATTTCTTCGTGAACTTGAAAAATACGAAACTATGCCCGAAGATGTTGGACA
TTGTTTTGTAACATGGGCACCTAAGTTTGATATGTATGTGACATATTGCAAGAATAAACC
AGAAAGTAATCAATTGTTAGTTACTCATGGTGGGACATGGTTTGAAGAATTACAAAGA
AAACATCGAGTTGAACATCCGATTGCTGCATACTTAATTAAACCCGTACAAAGAATAAC
AAAATATCAATTATTATTAAAAGATCTTCAGGCCTGTTGCCAAGAAGGACAGGGCGAAA
TAAAGGACGGCCTTGAAGTAATGTTAAATGTGCCTAAGAAAGCAAACGATGCCTTACA
TTTAAGTATGCTGGAAGGTTGCGATGTAAGAATAGATACATTAGGTGATGTAGTATTACA
GGATTCTTTTACGGTATGGGATCCCAAACAATTGATTAGAAAAGGCAGAGATCGGCACA
TATTTCTATTTGAATTATATCTGCTCTTTAGCAAAGAAGTGAAAGATTCGGCTGGGAAGG
TGAAATACATTTACAAAAGTCGTTTAATGACTTCTGAGTTGGGTGTAACTGAACATATT
GAAGGTGACGAATGCAAATTCGCAGTATGGACAGGTCGTGCACCGACTAGCGATACAC
GCGTTGTTCTTCGAGCCAATTCAATGGATGCTAAGCAATTATGGGTGAAAAGATTACGC
GAAGTTATTCAAGAAACATATTTCAGTTTAAGTATGCCCAAGAGTCCTGCGAAAAAAA
GTTCAAGTCAACGATCCAGTAGAGATCTTGAAGAATGTACTTCTTTGGATGATAGTGTC
GAGAATTTGGATAGAAATTCTTTGGCATCTTTTGGCTCTACTAATACTACAGACTCAGAT
AAAACTGGCGTAGCCGAGGTGACCTGGGTGATTGCCGATCACTCTGCAGCACCGGGTT
CGAAAGAGTTGACGGTAACGAAAGGCCAACAAGTTGAAGTATTAGAAAATGGAAGTA
ACATCAGCGGTGTGAATACATCCGAGTGGACTCACGTGCGTTTACTAGTTGCTCCAGG
ACAAGTTGATCCTCCTCCAGAAGGATTAGTTCCTACTAGTGCGCTTAAACAACCCCCTC
CAGTTTCTAGTAAAACTTCACCATCTAGGAAAGTTCCAGGACAGCAGCAGCAGCAACA
ACAACAACAGCAGCAACAGCAACAGCAGCAGCAGCAGCAACAACAACAATTACATTA
TCAACAACAATCGAGTAGTCAAGTTCAAACTGTCGCATCCAGTGGAGGATTTGCAACT
GCGTCCTCTGCTGCTGGAGTAGCAGGCTCTTCGTCGACTGTTGCAACAAGTGTAACAC
CTGTGTTACCAATTCAAAATGTTCCTGTGTTGCCGGATGAAACTGAAAACGTTGCAAA
CGATGGAAGTACTCCTGCGAACACAAATTCTCCAGGAAACAAAAGGCGTGGCTTTAGC
GGGAGAAAGTGGTTACCTCCACCATTGCGTAAACTTAGTCAAGGTAAAGTCGAGAAAT
CGCCACCGACGACGACAGCGACAGCGACATCGATGACATCGATTGTACCGCCATCAGG
TGATCGGTTGAAAAAGAATGTCTCGGAAAAACGATTCAAATTACCGACTGGTGCGGAA
CAATCTCGACCGTCCAGAACTGCTTCTATTTCTATCTCCGCGTTAACAACAGCGACTGC
CTCTATGCGTGTGTCTGCTTCTGAAGCGGACCTTGACACTGAACTTCAACCAGGAATC
GAAGGAGAAGAGGACGAAGGAGAAACGGAACAATCGGAACCAGAATTGGAAGAAGA
TGTACCGGAACCCGATAACACAGAAGCTTTAACTTACTCGGAACAAAATGGAGCGGAC

GATGTCGAAGACGAATTAGAACTTCCTCCGCCGATGAAACCCATCACTGAACCGATAC
TTGTAGGAACTGCGAATGGAGCATCGGGATCAGCAATTACAACAGAAGGCTGTGGAA
AATCTCGAACATCTGAACGTTCAACGAAGATTCTTGACGGTGCCACGACGGTTGATTT
GGCTGAAATTGAACAGATCGTAAAAGAAAGAATGGAACAACATACGGAAAACCAAGA
AAGACAAAGTCTGATGCGAACACCTAGTGGTAAAAATTCAAGCATTGGTGGCAGTGG
GGATAACGATTACGACAAAGATGCAGTATCGAGCGCAACTATTGCTACTACGACGATTG
CTTCATCGACGACGATAATGACAACAGCAGCGACGGCCACTGTTGCGACGGGGATAAC
GATGACGACGACGACGACTAAGATGGGAACAACGAGTAGCCCTGCTCACGGGAATTC
GGAGGAATACGACTTGGAAAGCGCGGTGCTAGTAAAACGACAATTCGTTATTCGAGAG
TTGGTCGAGACGGAAAAGGATTATGTAAATGATTTGAAGCAAATAGTCGAAGGGTACA
TGGCATTGATGCGAGATTCTGAATCTCAAGTGCCATTGCCAGATGATTTACGCGGTGGA
AAAGACAAAATGGTTTTTGGTAACATAGAGGCAATCTACGAATGGCATAGAGATTTCTT
TCTGAAAGCTTTGGAACGTTGCTTGGAACGGCCGGAAGAGCTTGGGCCGTTGTTTAAG
CGATACGAAAGGAAGTTACATATGTATGTTGTTTATTGTCAAAACAAACCAGTCTCCGA
GTATATTGTTTCTGAGTATATAGACACCTATTTCGAGGATTTGAGGCAAAAGCTCGGAC
ACCGATTACAATTATGCGATCTTTTAATCAAACCGGTTCAAAGAATCACAAAGTATCAA
CTTCTTCTTAGAGAGGCATTAAGACTTACCGAACGAACTCAAAGAATTTCGGAAATCG
AAGGACTTAGAGCAGCGGTTCATGTAATGCGTATTATTCCAAAAGCGGCCAATGACATG
ATGGATGTTGCAAGACTTCAAGGTTTCGACGGAAAAATTACAGCGCAAGGAAAATTAT
TATTGCACGGACCACTTTTAGTCTCAGAATTTTCTTCGAATTTACCTAGTAAGGAAAAA
GAATGGCAAGTCTTCCTGTTCGAACAAAATATTATTTTTAGTGAAGCTGTCGGAAAAAA
GACACAATTCACCAATCCTGTCTATATTTACAAGGCTCATATTCAGGTGAATAAAATGAG
TTTAGAAGAATGTTATGATGATTCGGAGAAGTTTATAATTCGATCAACAGATCCTCGAA
AACCTGGCCTTGGATTCTCTTGTAGCGCAGTAGAAGAAAATGGGCCACGGAAGCAGG
AGTGGGTAGATACGATCACTGCCATCCTGCAGACCCAACGTGACTTCCTTAAAGCGATA
CAGTCACCAATTGCCTACCAAAAGGAACTTACCAAAGATCCATT

>novel_circ_000107
ATCGCGATCTGGATACGATTCTAGTAGCGGCGGAGAATCTGACAATGAATACAATGCTA
TTGCATGTCGTGGAGCTGATACTCAATCTCAAAATCAGAACACAAATGCAAGCAGTCA
TGGGCAGGGAAAGCATATAAACAAAGGAAGATGGACCAAAGAGGAGGATGCTGTATT
AAAACAGTTAGTGAGCAATGCTGAACAACTTGGGACTGGGCTTCGATGGGACGCCATA
GCCAGCCACTTTCCGGATCGAAGTGATGTTCAATGTCAACAGAGATGGGCGAAGGTCG
TAAACCCGGAATTAGTCAAAGGACCATGGACAAAAGAG

>novel_circ_000109
TATATGGATATCGGGGAAGATATTGGTGTCCCCGAGGATTTTACAACTGGTGAGATGGT
CTCCGGAATGTGGTGGCGACATTTGGTGTCCGGTGGCGTAGCAGGTGGTGTATCACGC
ACATGTACTGCACCTTTAGATCGTATTAAAGTTTATCTACAGGTGCATGGAACACGACAT
TGCAAAATCAAAAGTTGCTTCAGATATATGCTTCGTGAAGGTGGTTCTCTTAGTTTATG
GAGAGGAAATGGTATAAACGTACTTAAAATCGGACCAGAAAGCGCTTTGAAATTTATG
GCCTATGAGCAAATTAAAAGAGCGATTAAAGGAGATGATGTTAGAGAGCTTGGACTCT
ACGAACGATTAATGGCGGGATCATTAGCTGGCGGAATTAGTCAATCAGCTATATATCCAC
TTGAGGTACTCAAAACTAGATTTGCACTTAGAAAAACAGGAGAGTTTTCAGGTTTAGT

AGATGCAACAAAAAAGATATATAAACAAGGAGGTTTAAAATCTTTTTATAGAGGCTACA
TACCTAATTTAATGGGAATAATTCCATATGCTGGTATTGATTTGGCTGTATATGAAACTTT
GAAAAATAGGTATTTACGAACACATGATAAAAATGAACAACCACCCTTTTGGATACTAT
TATTATGTGGAACAGCTTCAAGTACAGCCGGTCAAGTGTGCTCATATCCTTTAGCATTAA
TTAGAACACGATTACAAGCAGATATATCACCAGGAAAACCAAATACTATGATTGCTGTT
TTTAAAGATATTATTAAAAATGAAGGAATTCGCGGTTTATATAGAGGTTTAACTCCAAAT
TTTTTAAAGGTGGCACCAGCTGTATCCATTAGTTATATAGTATATGAAACTGTTAGAGAT
TTTTTGGGTGTTAATATGACGTGATGATTATTTATTTTAG

>novel_circ_000110
AGCACAATTACAGTATATTTCACAAATCGTTTAAACATTCATATATAATTATACGAATCTT
CGCATATGACGGAAAGGCGATTTGATCAATTAGGGGAAGATTATTTCGTAAAAACAATG
TATTTTTAAGGACAGGAAAACATTAGGAGATTGAAGGGCTTTGCGAAAATGGCTTCATC
TGGCAGACTGCCATGCCAGTTTGGATAGCTGGCTTAGAAATTGCGAGGCCTATCTCTCT
TTGCTGTGAGAGGTTCTTTCCTAACTTTTTTAGTTTTTTTTTTTAG

>novel_circ_000111
AGATACATTTAAGTACCAGCCGACGCATGCCTCAGATAAAATCCACGACATTGCGAATA
TTACAAAGTACCAAAAACTATTTTATCAATTTAGGCATTCGAGAAAAGTATAAGATTGAT
TATTGAGAACATCTTGGACATCATTAAATATACTGTATTGATAAAACAGGTAATAATAAG
GAAGTTTTGAATATCCTTCGAAGTTGCAAATACCGTATAAATATAACTGTGTGACGCTAT
GGCCAAAAATATCACACGACTGTGTTAATATCGCAACCAAACGGCGATGAATGTGCAC
GT

>novel_circ_000112
AAATGTCGAAAAACAATGAAAAAGAAGCGCATTAATTAGCCAATAATCGGAACGATGC
ATCATGGCTTCGTCTTTAGGGGGACCAGGAATGGCGTCGGGTGTTCCGACACCGCGAA
GAGTCAACCTGGAGTTTCCACCACCTCCACCTTACCCACCGCCTCACAGTCAGGTATAA
TTTGCGATGGCCTGCACTGGATTAGTGGATCCTGGTCCTGTGGCGTCCGCAATGACGAA
TCCAACTCACCAGCATCCCCTTCAGGATTATTCGTATGCTTACTTCGAACCTGGTCCGA
GTCGGCTGCACGGCACATATCCGGCGGAAGTGGGCCTGAATCGTGTACAGCAGCAGCA
ACAACAACAAGTGCGTCCGGATTCATTCAAAAGGCATACATACATGACACGATACGGC
ACGGAGGAGAATATTTACGAGGAGATATCGGAAATCAA

>novel_circ_000113
AAATGTCGAAAAACAATGAAAAAGAAGCGCATTAATTAGCCAATAATCGGAACGATGC
ATCATGGCTTCGTCTTTAGGGGGACCAGGAATGGCGTCGGGTGTTCCGACACCGCGAA
GAGTCAACCTGGAGTTTCCACCACCTCCACCTTACCCACCGCCTCACAGTCAGGTATAA
TTTGCGATGGCCTGCACTGGATTAGTGGATCCTGGTCCTGTGGCGTCCGCAATGACGAA
TCCAACTCACCAGCATCCCCTTCAGGATTATTCGTATGCTTACTTCGAACCTGGTCCGA
GTCGGCTGCACGGCACATATCCGGCGGAAGTGGGCCTGAATCGTGTACAGCAGCAGCA
ACAACAACAAGTGCGTCCGGATTCATTCAAAAGGCATACATACATGACACGATACGGC
ACGGAGGAGAATATTTACGAGGAGATATCGGAAATCAACAGACAATGTCATCGAGCGT
TGCACGGTTCGAGAAGATCCCTGGTGGCGGAGGAAGTGCGACGTGTTCAGTCACGGC

ATCGTCGAGTATTAGGAGAATTGAACCTGAGCGTGGAGGCGATGTTAATGCCCACGGT
GGTCGATAACAACAACGAGGACGAAGCACAGGATCAACGCGGAGCATCTACGGAGGA
ACTGTTATCCTCGGTCAGTCCCACCGATGACCTCCTTTCGCCTGTTGGCTGTGACATGG
ACAGTGGCTTCAGTGGAAGCTCCAGTGCAAGTTACAGGTCTGGACTAGGATCGTTGAG
GAGAGGAATGGGCAAGACTAGTACTCCGGAATTAGCAGGCGGGCAACGTAAAACGAA
GGCAGTTATGATCTGGAAAAAGGGTTGGAAAGGTTGGAAGAAACTGCATTCTTTCGGA
AACAATAGCAACAAGACTG

>novel_circ_000114
GATTTACTGAAACATACACCGCAAGGTCATCATGATAGAATGTCGCTGCAATTGGCATT
AACTCAATTAGAAAGTTTAGCAGAAATGTTGAATGAAAGAAAACGGGAAGCAGAACA
ATTCCAAGCATTTAAAGAAATGCTTCGGCATGTATCTGGAAAACTATCTCACCGTCCTC
TTTCATCTTCATCTAGATATTTAATAAGAGAAGATAATGTTACACAGTTGGAATTTAATCA
AAATGGCATGATAACAAAATCAAAAAGGAGAAGGTTATTACTTTTAAATGATCTCGTAG
TATGCGTTTCGGTGACCCCAAGATCTGCAGAAGATTTTAGTGGTAGTGAAAGACTAACT
TTGAAGTGGACATATCCTGTATCAGATATTGAG

>novel_circ_000115
ACTAATGAGAGAAAGTACATTTGTATGTACATGAATTAATGATCTGACCATGACAAGTA
ACGTAGCAGCAATTAAATTAGAAGAGGGGCAAGAATCCGTAACAGAGATACAGACTTA
TTTAGAAACATTTAATAAAGAAATAGAAGGAGGTCAGGGAGAGCAATTACAACATGTA
CAATTACAACAAGTGGAAGGATTATCAGGTGGAGAAGAGGGTGGGACTTATTTTGTTG
ATCAATCTGGACAATATTATTATCAAGCAAATAATGATGAAACACCTGTGATGACACAG
GTACAAATTCAAGAAGTAGAAGAAACAGATGTACAAAATGATGGAGAAGCAGCTCAA
GAAGAACAATATAATGAAATTGAAGAATTAGAAAATGTTGATGGTGATGAAGATAGTCA
TGTAACTAACGGTGGAAATAATCAAGTTGTAATCAATTCTGGAGATGCATATCAAACAG
TTACTATTGTACCATCTGATACAAATCCAGGAGAAGTTAGTTATGTATTAATAGTTCAGC
AACCTGATGCTGAAGACAAAGAAAGCAGACCTGTAGAACGAGAGGGAACAGAAGGA
GAAGAAGGGGAAGGGGAACAAGATCTTACAGTTTATGATTTTGAAGATAATGAAGATA
ATGAAGCACCTGTAGAATCAGAAGCAGAAGATGACAAAACAAAAATTATTAAATTTTT
ACCTAAAAAGTCTCAAACTGTTACTCAAGCTCATATGTGTAATTATTGTAATTACACAAG
CCCAAAAAGATATCTGTTATCACGGCACATGAAATCACATTCTGAAGAAAGACCACATA
AATGTAGTGTTTGTGAAAGAGGATTTAAAACATTAGCTTCACTTCAAAATCATGTTAAT
ACACATACTGGGACCAAACCACATCGTTGCAAATTTTGTGATAGTGCATTCACAACTTC
TGGTGAACTTGTTAGACATGTTCGATATAGACACACTCATGAAAAACCACATAAATGTC
ATGAATGTGATTATGCATCTGTTGAATTATCTAAGCTCAAGCGTCATATACGATGTCATAC
AGGGGAACGTCCATATCAGTGTCCACATTGTACATATGCAAGTCCTGATACTTTTAAATT
AAAGCGACATTTGCGCATACATACCGGAGAAAAGCCATACGAGTGCGATTTTTGTCAG
GCAAGGTTTACCCAATCTAATAGCTTAAAAGCTCATAAATTGATACATAATGTTGGTGAT
AAACCAGTATTTCAATGTGAATTATGTCCAACCACTTGTGGAAGAAAAACAGATCTCA
GAATTCATGTACAAAAATTACATACCTCTGATAAACCTTTGAAATGTAAACGTTGTGGA
AAAACATTCCCAGATAG

>novel_circ_000116

GTGATACCGGCAGAGCGGCGTGGGCCAGAGAAGGAAGAGACCTGGACCCTGGCCCCG
GACCAAAGCGGGGTGGACGCGTGTCCGTTCCTCGACGACGTTCTATCCGACATAAAAT
CGAAACCGATCCAAGATGACAATCCCCAACCCCGTATCCACGATCCTGGCGATACCCA
AAGCCAAAGCAACCAACAGTTGGAGCATCCTGACTGTGGAACGTTGCCGCGCAGCCA
GGAGACGAGGAAGAGCAAGACGAAAAAGGTGAAGAGTTACCTGAGGAAGTGCAAAG
GCGCTCTGTCGAAAGCCGACGAGGCGTCCACGGAGAAGAAACGCCAGGAGCACTGC
ACCTCTTGGTATCTGGACGAGTCTCATCAGGAGGAGGATAATCCTGTGATGGAGATTCA
GAAACAGAATTCGGAATTGCTCGAGGAGAGGATCGCCGAGGAGGAGGCCTCGCCGCT
CGAGGACGGAAGAGGAGAGGATCCCTCGAATCTCTCGAAGGAGGAAAGTCGGGAGG
ACGAGGAGAGTGTCTCGAGGCTGGCCGAGGATCTGAGGAGGAGCCGGACGTCCTTGT
ACGAGGACGCCAGGGATTCCATGCACGACAGAGAGTGCGCAGCGAGCGAGGAGGGC
TGCTGCAAGCGGGACGACGCCATCTCCCTCGAGACACTGACCACGGAGGAGACGACG
AATTTGAACAAGTGCGACTCGAACGACACGCTGATTGCGTGTGAAGTGGCGGCCCCC
AACGGCCCGCCGCCCCTCGTCGAGGAGGGAAAGGAGGGAGAGGAGGCGGACGGCGA
GATGCAGGCCCTGTCTCTGTTTGGG

>novel_circ_000117
GGGAACGAGTCGGTGATACCGGAAGGTTGGCCTCTGACGTTGGGCAGACGTGGCCTC
GTCCTGCAGCTAGGAGAGCTCGAGGCCTCCGCGCTACGTCTCGGGGAATGTTACCTTT
GCATACGCAGCGCAGCTGCAGTTGCAACCACCTCAGGGAGCGGCGAAGTTGCCGCTG
GTGACGAGCGGAATACCGTCGAGATTGCAGTAGTTTGGCGAACGACGTCGATGAGGG
CCCCAGGTTTCCTCGGCTGGGACCGCGAGGAGGAGTTCATGGACTGGCGGGAGGTGT
CTCGAAAGGGGCAGGGGAGCAGCGACGCGGCGAGCGGCAGCCTGGCCGGGCCGCAA
TCGTCGGGCACGGCGAAGAGCATGCGGCCCAGGCGGCGTTCGCGCGAGGAGGCTCGC
TCGCGTACGCGCGAGCAGGGCTTCGTGCTGGACCACCGCCGCGAGGAGACCCGTTCG
ATCGATCGTCTCGAGTGCAACAGCTCGAACGGGGGGGGCGCGAGGGGGGAGGAGCG
GAGGAAGAAGAACGAGATGGACGGCGGCCACGCGGTGATGAAGTCGAGATCTACGTC
GAGCGTGGGCCAGTGGAAGGCGGAATTGGAATCTTCGCTGGGCGAATCGAACGGGGC
GAGCAACCGCTCGAACCAATCTTCTAGTCTTTCCGGCGACGAGAGCCGCGCCAAAGTG
AAGAGGTGCGCCAAGGTCGTCTCCTCGATGACCATGGAGAAGTGGAAGGAGACGGCT
AGGTGCGAGACCAGGAGCAAACCCCTAGCGCCCGACGACTTGAAGACACCGTCGAGA
CTGTTGGACGTTGAGGCTGCCCTCGAGAAGAAGCGAGTAGGGGCCGCGTCGGGGGAA
GGCGAGAACAGGTTGCACGGGTCGAGGAGCGTGGACGAAGTGGAGGATGACGAGCT
GCCGGCTTTCATCGGCAATCTTTTGGTCTCCGTGGAGAGGAAAATCGAGAGAGTTTCC
CTGGACGATTTGACGTTCCCTTGCCCAGCGTGCAGGAATATAGACACCGACGATCCGTT
GCTGGGTTGTAGGTGCGCCGGTGACGATTGCAGCTGCGGCGAGGATACCTGCGATTGT
TCGCCCTCGGCCAACGCGAGGAAGGAACAATCGACAAGGAGCTTCGAGATCGCTGGT
ATCAAGCACATAGACAGCGACGACGAGGAGGAGGTGCGGATGGGATTCG

>novel_circ_000118
TGAGAAAAGCAAAAATTCCGAAGTATTCAAGGCTTTCATAGCAGAAGTATCTTCGTCTA
AATATTTTGATTTTTACAACAGTATTTTTGAGAATAAACATTATAAGAATTTGGAAAACG
AGGCCGTAAATTTGGGCGTAACTAGAAATGATTTCTATACTTTTTCTCCCATCTTTGTTT
TCGTTACAGCAGCATTCAATTTGTAAATAACAATGATTTTGATTAATTAATTTATAAAAAA

TAATTCATTTTATTCTTATGTAGAAAATTTTATGTTGTTTTCTTTTTCTTAAAAAAAAATAA
AATATATTTATATTAGATTAAAAAAAATTAATTTTTCTATTTTTTATTTATCCTATTTATCTAT
TCTATTTCTTTTACACTTACAGGATTAATTTTTTTTTAAAATTGAAAGTAATTATTCTTCTT
TGTCATTTATTTCCCACAGAAATCTATTCTTTTTCTATATTTTTTTATATTTTTGTTAAATGT
ATTAAAATTAAGATTTATTTGGAAAGATATCATTGTAGAAAAATATATTCATTAATTAATTA
TTTAATAGTATTATATTATATATATGCAATATAAAAAATTGAATATGAAAAAACTGAATATG
AAAAAATTGTGAACTTAATATGAAAGAGTTGTTAGTAAAAATTTTTCTTCTTTTCTGAA
ATGTTATATATATATTTGAAAATCAATTGTTTTAGAAAAATTTTATAATAGCTTAAAAAAC
TATATTATAGAAATTTTTTTATTATTTTAAATTCTAAAAGCTATGTTGTACATGAATTAAAA
ATTAGAAATATTAATAAATTAGAAATTTTTAAACAAAAAAATATATTTATATAATTACTTTT
CATGAAAAGAAAATGTATAAATTTATTCAAAATTCTAAGACATTCGTAATGTTTTGCGAA
TAATTTCATTTAATTTACTTTTTTATATTTGAAATTTATGTAAATTATATTGCAAATAAAAA
TAATTACAGATTGACATATATTGCATTAATTTAAAATGAAATAATTTAAAATGAAACGCA
AAAATTAAATTGTTTGCAGATTAAAGCTATGATTACTTACTTAAATATTATTACCTGATTC
TAAAATACAATTTAAAAAATATCAAAGGAAATTGTATAAGATTTTAGTTCTTATACGATT
TTTTTAGAAAAAAAAGAAGAAATTAGAGAAAATAAGTATTCTAATTTTTAATAATAAAA
TTTTATAAAGATTCGGCTTTGAATTTTATAAATTTGATTCATATTTCATACTACTTTTAATA
TTTTTAAATCTTAATTATTATTTGAAAATTGTGAAATTGATGTATATACTATAATGTATATA
CTAAAATGATAAGATGGCTTATATTTCTTAAAATGATTAGTATATTATATTATTTTAATATTT
TAATTTTAATATTCAATTTAAATAAAAAAAAATATATTTGTAAACATAAATACATAAATATTT
AGAATATTAAAGAACAAAAGAAAAAATAATTAAAATAGATTTTGAAAAAAAAAAATATA
GTTAAATTTTATATATCTATTTATGAATTCTAAATATTTTGTTTCGATCAAAATATTCGATT
CTGTTCAAAGTGTGATATAATGTTATTAGTCTAATAAAAAAAAAATAATAAAAAAAATTATT
TTTTTTTTTCATATGAAATATTTTCACATTCTTAACACTATATTAAAAAATTCTGAATATCC
TTAGTGAAATCAAGTCAAATATTTATCTTGTATATGATGTACCTGATATTTAACGCATTTA
ATATTATTGTGCTTTTCTCCTGAAGTATTATAATTTTCTTATTTTGAAAAATCATTTAAAAA
ATTTGCATCGGAAATAAATTTTGCATTAACTATTAAAATTTGTATATTAACTATAATAACTA
TTTTTTATCTATTCAATTTTTTTAAACATTTTGTCTAAAATTCAATTAAAGACTTCTTTTTT
CAATAATTCTTTCTTTATCAATGTTTTTAGAGAATATTAAAATAATAACAGTTTGTTTTCT
ATATTTTCTGATTTGTTCTTATTCTATAAGTTTTATTCCATAAAATAAAAATCGAGCTATCG
ATCCAAAAATCAATAATTATTCTCAGTTTTCTTTTTTTAATAACCATACAATAATTATATAA
TATTTATATATTAATATTATTATATAATTATATTAATAATTATATAATAATATTCCATCATAACA
TAAATATGATTTCTAAATATTATATATTATAACACATTAATACATTCTGAAAAAAAATCATT
TTCTGATAAAATGTAAAAAAATTGTAATTTAAAAGATACGTAAATTTTCGGGTGTATTTT
TAAGTATATTATTTATAGCATAATATATAATATCATTTTTATATTACATATAGTTTATCTGATT
TTTAAATTGAAAATAAACAAAAATAACAGTTAATATATTTTATAGGAAATATACAATATAT
AAAAATAATAAAAAATCAGAACATTTTATAGTTTTTACGATCATCTCATAAAATTATCCTA
TGCATATTTTATTTTATTTTAACATCTTAATATTAAAAAATATTTCAGAATAAAATTAAATA
TCAAAACATGCATATTTGGATGTTTTTAATTATTTAATGAATGGATACGTAAAAATATATG
AAGGTCAATTTTGTTTTTTTAAATAGAAATAATAGAATATATTTCTATATTTTTTAATGTAC
TAATTGATGCGATTCGATTTTTTATAAAAAAAAAATATTAATTCATATATACTTAAAAATTA
TTAATTTGGCAAATATTTTAATTTAAATTTTATAAAAATTATTAAAATCTTTAAAAGAAAA
TAAAATATTTTATTTTATGAATTTCTTGTTTTTCTCATAACAAATTTTACAAAATTTAAGTC
AAAATATTTTTTAAATTAATAATTTTTAGGCATATATGAATTAATAAAATTTTTAAGAAGA

AGAAGAAATATAATATTCCTCGTTTAAATGTAATATAATATCATTTAAATATTTCGTCTCTC
TATCTTGATATAAATTGAATTTTTCCAAAAATAAATCTGAATCTAAAGTTGAATTTGAAA
AATTTTTCAAATGTTACTATTAAAAATTAAAAAATTTAAAGCAGATTTTAAATATTAATTA
AAATTTTTTTTGTTTTTGTTTTTGAGTAATAATACCTGTAACAAAAATAATATGTATTCAC
GGAATAAATACGTACAAAATCTATTACTATTTTACAAGAGTAATACGCAAATGTTTTCAT
TTCCAATATGAAGTTGATTATAATATAAGATACTTGAAATCAATACGATGTAGTAGATGA
GATATCTGACAAAAGTACCTGTTGATAATATAATGAGATAACGCATATAATCACAAAAGT
TGATTAATCTTATCGAAAAATTTTTATCATATTTAAAGCTAGTATATAAAATGCAATGAAA
AACGATTCATAGGTATAGTTTATATTAAGAAGAATAACAGAAATAATTAATTGCAATTATT
TCACGATGAAATTTGCTTCGGCAATTTTTGTTATCCTGATTGCAGCCAGTCCCCTGAAC
GCTTATAAAATTCCAGCCACTGGTTCAGGTGCTCTCGCCAAGGAACTCCAAGACTTTAT
AGACTTGATACCGCTCGACCAAATAATCCAAGTGACGAAGACGTACTATGCCCAGGAT
ACGCAATTCCGTAACATTATTCAATTGATGAAAACTGAAGAATTAAAACAATGGATGCT
AGATATCGAATCGGCTCCGGAATTCAAATTACTGATAAATTACGTTCAGAAAAATGGTC
TCGATATTTATTACTTGGTTAATGAATTCAACAAATCTTTAAATCTTCCGCCTCTTGTTAG
TGGAATTAAAGCCTTTTTCGGTTCTTTCGACAATAAAATCACTGGTGGTTTTATCGGTTA
TTTGATTGATATTGCTGCTCTTGTACCGCTAGAGAAGTTGTCTGATCTGTTCAC

>novel_circ_000119
ATAAACATTATAAGAATTTGGAAAACGAGGCCGTAAATTTGGGCGTAACTAGAAATGAT
TTCTATACTTTTTCTCCCATCTTTGTTTTCGTTACAGCAGCATTCAATTTGTAAATAACAA
TGATTTTGATTAATTAATTTATAAAAAATAATTCATTTTATTCTTATGTAGAAAATTTTATG
TTGTTTTCTTTTTCTTAAAAAAAAATAAAATATATTTATATTAGATTAAAAAAAATTAATTT
TTCTATTTTTTATTTATCCTATTTATCTATTCTATTTCTTTTACACTTACAGGATTAATTTTT
TTTTAAAATTGAAAGTAATTATTCTTCTTTGTCATTTATTTCCCACAGAAATCTATTCTTT
TTCTATATTTTTTTATATTTTTGTTAAATGTATTAAAATTAAGATTTATTTGGAAAGATATC
ATTGTAGAAAAATATATTCATTAATTAATTATTTAATAGTATTATATTATATATATGCAATATA
AAAAATTGAATATGAAAAAACTGAATATGAAAAAATTGTGAACTTAATATGAAAGAGTT
GTTAGTAAAAATTTTTCTTCTTTTCTGAAATGTTATATATATATTTGAAAATCAATTGTTTT
AGAAAAATTTTATAATAGCTTAAAAAACTATATTATAGAAATTTTTTTATTATTTTAAATTC
TAAAAGCTATGTTGTACATGAATTAAAAATTAGAAATATTAATAAATTAGAAATTTTTAA
ACAAAAAAATATATTTATATAATTACTTTTCATGAAAAGAAAATGTATAAATTTATTCAAA
ATTCTAAGACATTCGTAATGTTTTGCGAATAATTTCATTTAATTTACTTTTTTATATTTGAA
ATTTATGTAAATTATATTGCAAATAAAAATAATTACAGATTGACATATATTGCATTAATTTA
AAATGAAATAATTTAAAATGAAACGCAAAAATTAAATTGTTTGCAGATTAAAGCTATGA
TTACTTACTTAAATATTATTACCTGATTCTAAAATACAATTTAAAAAATATCAAAGGAAAT
TGTATAAGATTTTAGTTCTTATACGATTTTTTTAGAAAAAAAAGAAGAAATTAGAGAAA
ATAAGTATTCTAATTTTTAATAATAAAATTTTATAAAGATTCGGCTTTGAATTTTATAAATT
TGATTCATATTTCATACTACTTTTAATATTTTTAAATCTTAATTATTATTTGAAAATTGTGA
AATTGATGTATATACTATAATGTATATACTAAAATGATAAGATGGCTTATATTTCTTAAAAT
GATTAGTATATTATATTATTTTAATATTTTAATTTTAATATTCAATTTAAATAAAAAAAATAT
ATTTGTAAACATAAATACATAAATATTTAGAATATTAAAGAACAAAAGAAAAAATAATTA
AAATAGATTTTGAAAAAAAAAATATAGTTAAATTTTATATATCTATTTATGAATTCTAAAT
ATTTTGTTTCGATCAAAATATTCGATTCTGTTCAAAGTGTGATATAATGTTATTAGTCTAA

TAAAAAAAAATAATAAAAAAATTATTTTTTTTTTCATATGAAATATTTCACATTCTTAA
CACTATATTAAAAAATTCTGAATATCCTTAGTGAAATCAAGTCAAATATTTATCTTGTATA
TGATGTACCTGATATTTAACGCATTTAATATTATTGTGCTTTTCTCCTGAAGTATTATAATT
TTCTTATTTTGAAAAATCATTTAAAAAATTTGCATCGGAAATAAATTTTGCATTAACTATT
AAAATTTGTATATTAACTATAATAACTATTTTTTATCTATTCAATTTTTTTAAACATTTTGT
CTAAAATTCAATTAAAGACTTCTTTTTTCAATAATTCTTTCTTTATCAATGTTTTTAGAGA
ATATTAAAATAATAACAGTTTGTTTTCTATATTTTCTGATTTGTTCTTATTCTATAAGTTTTA
TTCCATAAAATAAAAATCGAGCTATCGATCCAAAAATCAATAATTATTCTCAGTTTTCTT
TTTTTAATAACCATACAATAATTATATAATATTTATATATTAATATTATTATATAATTATATTAA
TAATTATATAATAATATTCCATCATAACATAAATATGATTTCTAAATATTATATATTATAACA
CATTAATACATTCTGAAAAAAAAATCATTTTCTGATAAAATGTAAAAAAATTGTAATTTAA
AAGATACGTAAATTTTCGGGTGTATTTTTAAGTATATTATTTATAGCATAATATATAATATC
ATTTTTATATTACATATAGTTTATCTGATTTTTAAATTGAAAATAAACAAAAATAACAGTT
AATATATTTTATAGGAAATATACAATATATAAAAATAATAAAAAAATCAGAACATTTTATAG
TTTTTACGATCATCTCATAAAATTATCCTATGCATATTTTATTTTATTTTAACATCTTAATAT
TAAAAAATATTTCAGAATAAAATTAAATATCAAAACATGCATATTTGGATGTTTTTAATTA
TTTAATGAATGGATACGTAAAAATATATGAAGGTCAATTTTGTTTTTTTAAATAGAAATAA
TAGAATATATTTCTATATTTTTTAATGTACTAATTGATGCGATTCGATTTTTTATAAAAAAA
AAATATTAATTCATATATACTTAAAAATTATTAATTTGGCAAATATTTTAATTTAAATTTTAT
AAAAATTATTAAAATCTTTAAAAGAAAATAAAATATTTTATTTTATGAATTTCTTGTTTTT
CTCATAACAAATTTTACAAAATTTAAGTCAAAATATTTTTTAAATTAATAATTTTTAGGCA
TATATGAATTAATAAAATTTTTAAGAAGAAGAAGAAATATAATATTCCTCGTTTAAATGTA
ATATAATATCATTTAAATATTTCGTCTCTCTATCTTGATATAAATTGAATTTTTCCAAAAAT
AAATCTGAATCTAAAGTTGAATTTGAAAAATTTTTCAAATGTTACTATTAAAAATTAAAA
AATTTAAAGCAGATTTTAAATATTAATTAAAATTTTTTTTGTTTTTGTTTTTGAGTAATAAT
ACCTGTAACAAAAATAATATGTATTCACGGAATAAATACGTACAAAATCTATTACTATTTT
ACAAGAGTAATACGCAAATGTTTTCATTTCCAATATGAAGTTGATTATAATATAAGATAC
TTGAAATCAATACGATGTAGTAGATGAGATATCTGACAAAAGTACCTGTTGATAATATAA
TGAGATAACGCATATAATCACAAAAGTTGATTAATCTTATCGAAAAATTTTTATCATATTT
AAAGCTAGTATATAAAATGCAATGAAAAACGATTCATAGGTATAGTTTATATTAAGAAGA
ATAACAGAAATAATTAATTGCAATTATTTCACGATGAAATTTGCTTCGGCAATTTTTGTTA
TCCTGATTGCAGCCAGTCCCCTGAACGCTTATAAAATTCCAGCCACTGGTTCAGGTGCT
CTCGCCAAGGAACTCCAAGACTTTATAGACTTGATACCGCTCGACCAAATAATCCAAGT
GACGAAGACGTACTATGCCCAGGATACGCAATTCCGTAACATTATTCAATTGATGAAAA
CTGAAGAATTAAAACAATGGATGCTAGATATCGAATCGGCTCCGGAATTCAAATTACTG
ATAAATTACGTTCAGAAAAATGGTCTCGATATTTATTACTTGGTTAATGAATTCAACAAA
TCTTTAAATCTTCCGCCTCTTGTTAGTGGAATTAAAGCCTTTTTCGGTTCTTTCGACAAT
AAAATCACTGGTGGTTTTATCGGTTATTTGATTGATATTGCTGCTCTTGTACCGCTAGAG
AAGTTGTCTGATCTGTTCACTGAGAAAAGCAAAAATTCCGAAGTATTCAAGGCTTTCAT
AGCAGAAGTATCTTCGTCTAAATATTTTGATTTTTACAACAATATTTTCGAAG

>novel_circ_000120
GATTAAATCTCGAACCACTTAGAGATTTGATTGAGAATATTATTGGCATTCATCTCCCTA
GACCTTGAATCTATTCTAAATTTGTTATACGATAATAATAAATTTTTAATTTAAGTTTGTTA

TAAAATTATAAAAAATCTCTAATCGTTTAAA

>novel_circ_000121
ATCCCAACATCCTGTTGAAGTCAGCGAAAACAGCAGAGTTAGCCACTCTCTCCAGGTA
GAGGGACTTGAGCTGGTCCTGAGGTATCAAAGCTTCGACTTCATCGAGGAAACCACGG
ATTCCACCAATATTTTTGGTAGCATGGGAACTTGGTTTGAGCGATGGCAA

>novel_circ_000122
ACATGCTACGAGATGGAGAGGTACCTGAAGGACGAACCGAAACTGCAATCGTACAAA
AAGTTGCCCACAGAGTTAGATACAGCACCCTGGAATCTCTTCAGGCCTCCTATTTCGTG
GACAGCGACCACTGGCACCGCCAACGCACAATTCGAATCACCAATCAAAATGGAGGT
GACGACGTCGTCGGAAGACAGAGAAACGCTCTGCTTGGACGATATTAAGTTCAGTCCT
GGTGATCACAACCACAATGGCCGTGATAGGGATCTTCTGGACCGGCACCTCGATTCCTT
ATCGATGTCGTCATCGAGTTCGGCGTGTTCTTGGGATAGTTCGCCTTCTGTTTCGTGCA
CGGCGTTCATTCTCAAGAAAGAGCCCAGCGAGGACCTCGAAGAGGACGAGGAGCAC
GAGGAAATCGAGGAGGAAGAGGACAGCGGATGCGAAAGCGAAGGTCAAATCCTGAC
ACCACCATCGAGTCCGGGCTCGGGTCAAGGTCATTCCAACTCCAGTCACTCGTCGAGC
GGGAGTTCTATGCTCGATGTACAGAACCTCAACCTTCACGGGACGGGTGGCAGGAGCG
CTATCGTCAGGGTTACCACTAGCAACGCACAGGGTGTCGCAAGACTGATCTCCGTCAC
AGCGAATGGCTACCCCGCAGTTGCAGGCACGCAACATGCACATGCAGGCACAACTGC
GGCTGCGAGCACCGTGGCTAATAGGCACCATGCGAGGAGCCATGAGCACAGTCCACCT
GATACGAAGCGCAGGATACACAAATGCCAATTTCCGGGTTGCAAGAAGGTATATACCA
AGAGCTCACATCTGAAAGCTCATCAGAGGACGCACACAG

>novel_circ_000123
ACATGCTACGAGATGGAGAGGTACCTGAAGGACGAACCGAAACTGCAATCGTACAAA
AAGTTGCCCACAGAGTTAGATACAGCACCCTGGAATCTCTTCAGGCCTCCTATTTCGTG
GACAGCGACCACTGGCACCGCCAACGCACAATTCGAATCACCAATCAAAATGGAGGT
GACGACGTCGTCGGAAGACAGAGAAACGCTCTGCTTGGACGATATTAAGTTCAGTCCT
GGTGATCACAACCACAATGGCCGTGATAGGGATCTTCTGGACCGGCACCTCGATTCCTT
ATCGATGTCGTCATCGAGTTCGGCGTGTTCTTGGGATAGTTCGCCTTCTGTTTCGTGCA
CGGCGTTCATTCTCAAGAAAGAGCCCAGCGAGGACCTCGAAGAGGACGAGGAGCAC
GAGGAAATCGAGGAGGAAGAGGACAGCGGATGCGAAAGCGAAGGTCAAATCCTGAC
ACCACCATCGAGTCCGGGCTCGGGTCAAGGTCATTCCAACTCCAGTCACTCGTCGAGC
GGGAGTTCTATGCTCGATGTACAGAACCTCAACCTTCACGGGACGGGTGGCAGGAGCG
CTATCGTCAGGGTTACCACTAGCAACGCACAGGGTGTCGCAAG

>novel_circ_000125
AACATTAGGCCAAGGGAAATAAAAACCGGTTTTATATGATTTTTCCTGAAAAGTTCTAT
CAAAGCACCCTCTGTAGCGATACGTTCACTTTCGATATGCATTTTTTGAATGGAATCTAA
TTCCTCGCTAATATCGGCAGTCTTGCCCCGCAACCATTGCAACGATTTTCGTGCCTCTTT
TATTTTCCCTTTCGAAAT

>novel_circ_000126

GAACGAACTGTTCTACCAAAATACTATACGAGAGTGCCTTCGGTCGCAAGATCGTCGA
CGAACAGTTCAGGCATCAATCTAGATTGTTCATCGAATACGACACTCGCAACAATCGCG
AGTCCGTTCAACAGTCAAACAGCCCTAATCGCGGAGAAAAAAGTGATTCCTTCTGGCA
ATATTGTCATTGCTAACCATTACTATGCACAGAACTTGAGGAACAATCATCAGAAGACT
CTAAACGGGTCTCACATATACGACCCTCTTGACAAGGGTTCTGATCACGATTACAAGCA
AATCGATCCATTATTGGTCGGCGAACCTCCGTACATGGGGGTCGAAAACACCAAGCAA
ACCATGAGCTCGCAAAATATAAAACCGGCCAAGGACTCGGACGATGTTTTGCATACAC
AGTTCAAAGAAGTGAAACGATCACCTATGCGCTATACTATGCAG

>novel_circ_000127
AGGCCCTGGCTAGTATGGGATATACAAGAAGTGAAATAGAAGATTCCTTAGGACAAGC
AAAGTACGATGACGTGTTCGCCACGTACTTACTCTTAGGAAGAAAGACAACTGATCCT
GAATCAGATGGCTCGCGATCAGGCAGTTCGTTGTCATTGCGCAACATTCCACCGCAAG
CTGGTTCAGGTGGTGGTGGAAGTGGTGGTGGAGGAGGAAGTGGAGGAGGCACTGGT
GGTGCTGTGCAGAGTCCGTCGCATAGAGGTGTTCATAGAAGTATCTCTGCTAGCAATCC
AAAACCGAGTAGACGAGTTTCATCCGGTCTCGAAACGCTTCGAGCAGCGCCGAGCCC
AGGAAATGCAACGAACCATAATCACGCAACTCCTACGACTGGTGGAACTGTAACTGGA
AACACTGGTAGTAATTTTAAGAGACAAAATACCGTGGATGCGGCCACGATCAAAGAGA
ATACTGCTCGCGTTTCTGCGAGCCGACCCTCGGCTCCTAAAAACAGTCCTGGCCAATTA
GATACTATGACAATGTTCATTCTAGGCGTGGGTACAAGTCCCGGTGGTAGAGCATGGGC
AGGAAAGAGCAACACAATGAATGCAGGGGTAGGTAGTGGTCGGCCTCTTACTTCACCT
GCCCCTGGTTCTGTTGGTCGACGTAGTACCATCTCTTATGATCAAGCGAAAACGTCCTC
TTCATCTACCGAAAGAACCAACGATACACCCAG

>novel_circ_000128
ACAGGGGATGGCGTGGGAGTGTCGAGTAGCGGTGGTCGACTCTCTTCTCGAAGTCGA
ACCTCGGAGGAACCCCACATCGGCAAGTACAAATTACTTAAAACAATTGGCAAAGGTA
ATTTTGCCAAGGTAAAACTTGCCAAACATGTACCTACCGGAAAAGAAGTGGCTATCAA
AATTATTGACAAGACTCAACTAAATCCCGGTAGCCTTCAAAAATTATTCCGAGAAGTAA
GAATAATGAAGATGCTCGATCATCCGAATATAGTGAAGCTTTTCCAAGTGATCGAGACT
GAGAAAACATTGTACCTGGTAATGGAATACGCCAGTGGTGGCGAAGTTTTTGACTACT
TGGTCCTGCATGGTCGGATGAAAGAAAAGGAGGCTAGAGCAAAGTTCCGGCAAATTG
TCTCTGCTGTGCAATACTGCCATCAAAAAAAGATCATCCATAGGGATTTAAAGGCTGAA
AATTTACTACTCGATAGCGAGATGAACATCAAAATCGCCGATTTTGGTTTCAGCAATGA
ATTCACACCAGGCAACAAGTTGGATACTTTCTGCGGATCACCTCCTTATGCAGCACCTG
AACTTTTTCAAGGTAAAAAGTACGATGGACCCGAAGTAGATGTGTGGTCGTTGGGAGT
AATATTATACACTTTAGTGTCTGGATCACTGCCCTTCGACGGTTCGACACTGAGAGAAT
TGAGGGAACGAGTATTACGAGGAAAATATCGAATTCCATTTTACATGTCCACGGATTGT
GAGAATCTTTTGAAAAAGTTTTTGGTGCTTAACCCGACGAAACGGGCATCCTTAGAGA
CTATTATGAAGGACAAATGGATGAACATGGGATACGATGACGACGAATTGAAACCGTA
CTTAGAACCTGAACCTGATTATAAAGACCACAAGAGGATAGGTGAGTCTGCCAAGGCC
CTGGCTAGTATGGGATATACAAGAAGTGAAATAGAAGATTCCTTAGGACAAGCAAAGT
ACGATGACGTGTTCGCCACGTACTTACTCTTAGGAAGAAAGACAACTGATCCTGAATC
AGATGGCTCGCGATCAGGCAGTTCGTTGTCATTGCGCAACATTCCACCGCAAGCTGGT

TCAGGTGGTGGTGGAAGTGGTGGTGGAGGAGGAAGTGGAGGAGGCACTGGTGGTGC
TGTGCAGAGTCCGTCGCATAGAGGTGTTCATAGAAGTATCTCTGCTAGCAATCCAAAAC
CGAGTAGACGAGTTTCATCCGGTCTCGAAACGCTTCGAGCAGCGCCGAGCCCAGGAA
ATGCAACGAACCATAATCACGCAACTCCTACGACTGGTGGAACTGTAACTGGAAACAC
TGGTAGTAATTTTAAGAGACAAAATACCGTGGATGCGGCCACGATCAAAGAGAATACT
GCTCGCGTTTCTGCGAGCCGACCCTCGGCTCCTAAAAACAGTCCTGGCCAATTAGATA
CTATGACAATGTTCATTCTAGGCGTGGGTACAAGTCCCGGTGGTAGAGCATGGGCAGG
AAAGAGCAACACAATGAATGCAGGGGTAGGTAGTGGTCGGCCTCTTACTTCACCTGCC
CCTGGTTCTGTTGGTCGACGTAGTACCATCTCTTATGATCAAGCGAAAACGTCCTCTTC
ATCTACCGAAAGAACCAACGATACACCCAG

>novel_circ_000129
AGAAATCGAGCTGCCGCCCATCCGCCCGTAGACGCCTGATCCATCGACGAGGGGGTCC
GACTATTGAAGAGTTGGCAATATTGATCGAGGTTTGAACGGAATTATGCGGAGAACGAT
CGAAATCTGACCGTTTCCTTCCCTCTTCCGCGAATACAAAATCGATACGTAAGAAACTC
GACGACGCGCGCGATAATTATTTGGAAATATAATCGAAAATCTTGTGTAATGGAGAATC
AAGAGAGATCGTTGTTCAGTCGGATGCAACCGAGCAGGGATTCGATCCGATTGCAAAT
CGGGCGGAAAGGGTTCGAGGCGGGGGGGAAGGAGAGCGGGAAACAAATCGGTGAAC
GCCACTCCGTGAAAGAGCAAATCGGTCCATCCGCTTCTCCCGGAAAGTATGCGGCTAC
GCCTCGTCCTGTCGTGGACAATCTGAAGCAATGGGAGTTGGTAGAGTGCGGTCAGAGT
TTGCGAGTGGTGGAAAAGAGGATCGGCGTGAAATCTTGTCTCCCTCGGGAGGAGTCG
TCGGACGTTGAACGTGCGCCGAGCCTCGTTGGGAGGAAAACTCCCCCTTCGGAGAGC
AGCGTGGCCATCGACGCGAATCTCGAGGAGAAGTTGAAAGCTTTCAAAGACTCGAAG
ATCGTTCAACCTATTGTCTCGTCTCAACGCGAACGCACCGACGACGGGAAAGTCCCGA
TCAATCTCGAACTGATCCTGAAACAGCCTAGGAAAGGCTCGGCTATCGAGAGCACGGA
TATGCTTCAACGGTTAGAACAGCAGGTCAAGGCCATCGAAATGGACACTCTTGCAGCT
AGAACGCGCCAGTTGGAGTTTAGCTCGGATTTCGAGCCGGAAATTCGATACGCGGACC
TTCACCGATACAGGGAACACGAGGATCTACTTCGAAACGTGACGGATAACGAGGCCGA
GGGGTCTCCGTTACACCGAGGAGATGCGACGAGGAATTCGACGGGAAATGCAGGGAT
CGTTACGAAGAAAGGTTCGACGAAATTGTCAAGGACGGCCTCGGACTCGAGGCGGGG
ACAGGAAGCGGAAGTACTCCTTCGAGCGCAAGCTAGGGTGACACAAGCGCGTGTATT
GATACAGGCTAACAACGCAACAAAGAAACATCAACGTCAACAGCAACAGCGACAGAG
CAGTGCGGTCAGACCGTTGTCAACCGTAACGCAGAAGGGTGCGCATAATGAGAAGGA
GACAGGGGATGGCGTGGGAGTGTCGAGTAGCGGTGGTCGACTCTCTTCTCGAAGTCG
AACCTCGGAGGAACCCCACATCGGCAAGTACAAATTACTTAAAACAATTGGCAAAGGT
AATTTTGCCAAGGTAAAACTTGCCAAACATGTACCTACCGGAAAAGAAGTGGCTATCA
AAATTATTGACAAGACTCAACTAAATCCCGGTAGCCTTCAAAAATTATTCCGAGAAGTA
AGAATAATGAAGATGCTCGATCATCCGAATATAGTGAAGCTTTTCCAAGTGATCGAGAC
TGAGAAAACATTGTACCTGGTAATGGAATACGCCAGTGGTGGCGAAGTTTTTGACTAC
TTGGTCCTGCATGGTCGGATGAAAGAAAAGGAGGCTAGAGCAAAGTTCCGGCAAATT
GTCTCTGCTGTGCAATACTGCCATCAAAAAAAGATCATCCATAGGGATTTAAAGGCTGA
AAATTTACTACTCGATAGCGAGATGAACATCAAAATCGCCGATTTTGGTTTCAGCAATG
AATTCACACCAGGCAACAAGTTGGATACTTTCTGCGGATCACCTCCTTATGCAGCACCT
GAACTTTTTCAAGGTAAAAAGTACGATGGACCCGAAGTAGATGTGTGGTCGTTGGGAG

TAATATTATACACTTTAGTGTCTGGATCACTGCCCTTCGACGGTTCGACACTGAGAGAAT
TGAGGGAACGAGTATTACGAGGAAAATATCGAATTCCATTTTACATGTCCACGGATTGT
GAGAATCTTTTGAAAAAGTTTTTGGTGCTTAACCCGACGAAACGGGCATCCTTAGAGA
CTATTATGAAGGACAAATGGATGAACATGGGATACGATGACGACGAATTGAAACCGTA
CTTAGAACCTGAACCTGATTATAAAGACCACAAGAGGATAGGTGAGTCTGCCAAGGCC
CTGGCTAGTATGGGATATACAAGAAGTGAAATAGAAGATTCCTTAGGACAAGCAAAGT
ACGATGACGTGTTCGCCACGTACTTACTCTTAGGAAGAAAGACAACTGATCCTGAATC
AGATGGCTCGCGATCAGGCAGTTCGTTGTCATTGCGCAACATTCCACCGCAAGCTGGT
TCAGGTGGTGGTGGAAGTGGTGGTGGAGGAGGAAGTGGAGGAGGCACTGGTGGTGC
TGTGCAGAGTCCGTCGCATAGAGGTGTTCATAGAAGTATCTCTGCTAGCAATCCAAAAC
CGAGTAGACGAGTTTCATCCGGTCTCGAAACGCTTCGAGCAGCGCCGAGCCCAGGAA
ATGCAACGAACCATAATCACGCAACTCCTACGACTGGTGGAACTGTAACTGGAAACAC
TGGTAGTAATTTTAAGAGACAAAATACCGTGGATGCGGCCACGATCAAAGAGAATACT
GCTCGCGTTTCTGCGAGCCGACCCTCGGCTCCTAAAAACAGTCCTGGCCAATTAGATA
CTATGACAATGTTCATTCTAGGCGTGGGTACAAGTCCCGGTGGTAGAGCATGGGCAGG
AAAGAGCAACACAATGAATGCAGGGGTAGGTAGTGGTCGGCCTCTTACTTCACCTGCC
CCTGGTTCTGTTGGTCGACGTAGTACCATCTCTTATGATCAAGCGAAAACGTCCTCTTC
ATCTACCGAAAGAACCAACGATACACCCAG

>novel_circ_000130
GCCCTGGCTAGTATGGGATATACAAGAAGTGAAATAGAAGATTCCTTAGGACAAGCAA
AGTACGATGACGTGTTCGCCACGTACTTACTCTTAGGAAGAAAGACAACTGATCCTGA
ATCAGATGGCTCGCGATCAGGCAGTTCGTTGTCATTGCGCAACATTCCACCGCAAGCT
GGTTCAGGTGGTGGTGGAAGTGGTGGTGGAGGAGGAAGTGGAGGAGGCACTGGTGG
TGCTGTGCAGAGTCCGTCGCATAGAGGTGTTCATAGAAGTATCTCTGCTAGCAATCCAA
AACCGAGTAGACGAGTTTCATCCGGTCTCGAAACGCTTCGAGCAGCGCCGAGCCCAG
GAAATGCAACGAACCATAATCACGCAACTCCTACGACTGGTGGAACTGTAACTGGAAA
CACTGGTAGTAATTTTAAGAGACAAAATACCGTGGATGCGGCCACGATCAAAGAGAAT
ACTGCTCGCGTTTCTGCGAGCCGACCCTCGGCTCCTAAAAACAGTCCTGGCCAATTAG
ATACTA

>novel_circ_000131
AGGCCCTGGCTAGTATGGGATATACAAGAAGTGAAATAGAAGATTCCTTAGGACAAGC
AAAGTACGATGACGTGTTCGCCACGTACTTACTCTTAGGAAGAAAGACAACTGATCCT
GAATCAGATGGCTCGCGATCAGGCAGTTCGTTGTCATTGCGCAACATTCCACCGCAAG
CTGGTTCAGGTGGTGGTGGAAGTGGTGGTGGAGGAGGAAGTGGAGGAGGCACTGGT
GGTGCTGTGCAGAGTCCGTCGCATAGAGGTGTTCATAGAAGTATCTCTGCTAGCAATCC
AAAACCGAGTAGACGAGTTTCATCCGGTCTCGAAACGCTTCGAGCAGCGCCGAGCCC
AGGAAATGCAACGAACCATAATCACGCAACTCCTACGACTGGTGGAACTGTAACTGGA
AACACTGGTAGTAATTTTAAGAGACAAAATACCGTGGATGCGGCCACGATCAAAGAGA
ATACTGCTCGCGTTTCTGCGAGCCGACCCTCGGCTCCTAAAAACAGTCCTGGCCAATTA
GATACTA

>novel_circ_000132

AGAAATCGAGCTGCCGCCCATCCGCCCGTAGACGCCTGATCCATCGACGAGGGGGTCC
GACTATTGAAGAGTTGGCAATATTGATCGAGGTTTGAACGGAATTATGCGGAGAACGAT
CGAAATCTGACCGTTTCCTTCCCTCTTCCGCGAATACAAAATCGATACGTAAGAAACTC
GACGACGCGCGCGATAATTATTTGGAAATATAATCGAAAATCTTGTGTAATGGAGAATC
AAGAGAGATCGTTGTTCAGTCGGATGCAACCGAGCAGGGATTCGATCCGATTGCAAAT
CGGGCGGAAAGGGTTCGAGGCGGGGGGGAAGGAGAGCGGGAAACAAATCGGTGAAC
GCCACTCCGTGAAAGAGCAAATCGGTCCATCCGCTTCTCCCGGAAAGTATGCGGCTAC
GCCTCGTCCTGTCGTGGACAATCTGAAGCAATGGGAGTTGGTAGAGTGCGGTCAGAGT
TTGCGAGTGGTGGAAAAGAGGATCGGCGTGAAATCTTGTCTCCCTCGGGAGGAGTCG
TCGGACGTTGAACGTGCGCCGAGCCTCGTTGGGAGGAAAACTCCCCCTTCGGAGAGC
AGCGTGGCCATCGACGCGAATCTCGAGGAGAAGTTGAAAGCTTTCAAAGACTCGAAG
ATCGTTCAACCTATTGTCTCGTCTCAACGCGAACGCACCGACGACGGGAAAGTCCCGA
TCAATCTCGAACTGATCCTGAAACAGCCTAGGAAAGGCTCGGCTATCGAGAGCACGGA
TATGCTTCAACGGTTAGAACAGCAGGTCAAGGCCATCGAAATGGACACTCTTGCAGCT
AGAACGCGCCAGTTGGAGTTTAGCTCGGATTTCGAGCCGGAAATTCGATACGCGGACC
TTCACCGATACAGGGAACACGAGGATCTACTTCGAAACGTGACGGATAACGAGGCCGA
GGGGTCTCCGTTACACCGAGGAGATGCGACGAGGAATTCGACGGGAAATGCAGGGAT
CGTTACGAAGAAAGGTTCGACGAAATTGTCAAGGACGGCCTCGGACTCGAGGCGGGG
ACAGGAAGCGGAAGTACTCCTTCGAGCGCAAGCTAGGGTGACACAAGCGCGTGTATT
GATACAGGCTAACAACGCAACAAAGAAACATCAACGTCAACAGCAACAGCGACAGAG
CAGTGCGGTCAGACCGTTGTCAACCGTAACGCAGAAGGGTGCGCATAATGAGAAGGA
GACAGGGGATGGCGTGGGAGTGTCGAGTAGCGGTGGTCGACTCTCTTCTCGAAGTCG
AACCTCGGAGGAACCCCACATCGGCAAGTACAAATTACTTAAAACAATTGGCAAAGGT
AATTTTGCCAAGGTAAAACTTGCCAAACATGTACCTACCGGAAAAGAAGTGGCTATCA
AAATTATTGACAAGACTCAACTAAATCCCGGTAGCCTTCAAAAATTATTCCGAGAAGTA
AGAATAATGAAGATGCTCGATCATCCGAATATAGTGAAGCTTTTCCAAGTGATCGAGAC
TGAGAAAACATTGTACCTGGTAATGGAATACGCCAGTGGTGGCGAAGTTTTTGACTAC
TTGGTCCTGCATGGTCGGATGAAAGAAAAGGAGGCTAGAGCAAAGTTCCGGCAAATT
GTCTCTGCTGTGCAATACTGCCATCAAAAAAAGATCATCCATAGGGATTTAAAGGCTGA
AAATTTACTACTCGATAGCGAGATGAACATCAAAATCGCCGATTTTGGTTTCAGCAATG
AATTCACACCAGGCAACAAGTTGGATACTTTCTGCGGATCACCTCCTTATGCAGCACCT
GAACTTTTTCAAGGTAAAAAGTACGATGGACCCGAAGTAGATGTGTGGTCGTTGGGAG
TAATATTATACACTTTAGTGTCTGGATCACTGCCCTTCGACGGTTCGACACTGAGAGAAT
TGAGGGAACGAGTATTACGAGGAAAATATCGAATTCCATTTTACATGTCCACGGATTGT
GAGAATCTTTTGAAAAAGTTTTTGGTGCTTAACCCGACGAAACGGGCATCCTTAGAGA
CTATTATGAAGGACAAATGGATGAACATGGGATACGATGACGACGAATTGAAACCGTA
CTTAGAACCTGAACCTGATTATAAAGACCACAAGAGGATAGGTGAGTCTGCCAAGGCC
CTGGCTAGTATGGGATATACAAGAAGTGAAATAGAAGATTCCTTAGGACAAGCAAAGT
ACGATGACGTGTTCGCCACGTACTTACTCTTAGGAAGAAAGACAACTGATCCTGAATC
AGATGGCTCGCGATCAGGCAGTTCGTTGTCATTGCGCAACATTCCACCGCAAGCTGGT
TCAGGTGGTGGTGGAAGTGGTGGTGGAGGAGGAAGTGGAGGAGGCACTGGTGGTGC
TGTGCAGAGTCCGTCGCATAGAGGTGTTCATAGAAGTATCTCTGCTAGCAATCCAAAAC
CGAGTAGACGAGTTTCATCCGGTCTCGAAACGCTTCGAGCAGCGCCGAGCCCAGGAA
ATGCAACGAACCATAATCACGCAACTCCTACGACTGGTGGAACTGTAACTGGAAACAC

TGGTAGTAATTTTAAGAGACAAAATACCGTGGATGCGGCCACGATCAAAGAGAATACT
GCTCGCGTTTCTGCGAGCCGACCCTCGGCTCCTAAAAACAGTCCTGGCCAATTAGATA
CTA

>novel_circ_000133
GCCCTGGCTAGTATGGGATATACAAGAAGTGAAATAGAAGATTCCTTAGGACAAGCAA
AGTACGATGACGTGTTCGCCACGTACTTACTCTTAGGAAGAAAGACAACTGATCCTGA
ATCAGATGGCTCGCGATCAGGCAGTTCGTTGTCATTGCGCAACATTCCACCGCAAGCT
GGTTCAGGTGGTGGTGGAAGTGGTGGTGGAGGAGGAAGTGGAGGAGGCACTGGTGG
TGCTGTGCAGAGTCCGTCGCATAGAGGTGTTCATAGAAGTATCTCTGCTAGCAATCCAA
AACCGAGTAGACGAGTTTCATCCGGTCTCGAAACGCTTC

>novel_circ_000134
AGGCCCTGGCTAGTATGGGATATACAAGAAGTGAAATAGAAGATTCCTTAGGACAAGC
AAAGTACGATGACGTGTTCGCCACGTACTTACTCTTAGGAAGAAAGACAACTGATCCT
GAATCAGATGGCTCGCGATCAGGCAGTTCGTTGTCATTGCGCAACATTCCACCGCAAG
CTGGTTCAGGTGGTGGTGGAAGTGGTGGTGGAGGAGGAAGTGGAGGAGGCACTGGT
GGTGCTGTGCAGAGTCCGTCGCATAGAGGTGTTCATAGAAGTATCTCTGCTAGCAATCC
AAAACCGAGTAGACGAGTTTCATCCGGTCTCGAAACGCTTC

>novel_circ_000135
AGAAATCGAGCTGCCGCCCATCCGCCCGTAGACGCCTGATCCATCGACGAGGGGGTCC
GACTATTGAAGAGTTGGCAATATTGATCGAGGTTTGAACGGAATTATGCGGAGAACGAT
CGAAATCTGACCGTTTCCTTCCCTCTTCCGCGAATACAAAATCGATACGTAAGAAACTC
GACGACGCGCGCGATAATTATTTGGAAATATAATCGAAAATCTTGTGTAATGGAGAATC
AAGAGAGATCGTTGTTCAGTCGGATGCAACCGAGCAGGGATTCGATCCGATTGCAAAT
CGGGCGGAAAGGGTTCGAGGCGGGGGGGAAGGAGAGCGGGAAACAAATCGGTGAAC
GCCACTCCGTGAAAGAGCAAATCGGTCCATCCGCTTCTCCCGGAAAGTATGCGGCTAC
GCCTCGTCCTGTCGTGGACAATCTGAAGCAATGGGAGTTGGTAGAGTGCGGTCAGAGT
TTGCGAGTGGTGGAAAAGAGGATCGGCGTGAAATCTTGTCTCCCTCGGGAGGAGTCG
TCGGACGTTGAACGTGCGCCGAGCCTCGTTGGGAGGAAAACTCCCCCTTCGGAGAGC
AGCGTGGCCATCGACGCGAATCTCGAGGAGAAGTTGAAAGCTTTCAAAGACTCGAAG
ATCGTTCAACCTATTGTCTCGTCTCAACGCGAACGCACCGACGACGGGAAAGTCCCGA
TCAATCTCGAACTGATCCTGAAACAGCCTAGGAAAGGCTCGGCTATCGAGAGCACGGA
TATGCTTCAACGGTTAGAACAGCAGGTCAAGGCCATCGAAATGGACACTCTTGCAGCT
AGAACGCGCCAGTTGGAGTTTAGCTCGGATTTCGAGCCGGAAATTCGATACGCGGACC
TTCACCGATACAGGGAACACGAGGATCTACTTCGAAACGTGACGGATAACGAGGCCGA
GGGGTCTCCGTTACACCGAGGAGATGCGACGAGGAATTCGACGGGAAATGCAGGGAT
CGTTACGAAGAAAGGTTCGACGAAATTGTCAAGGACGGCCTCGGACTCGAGGCGGGG
ACAGGAAGCGGAAGTACTCCTTCGAGCGCAAGCTAGGGTGACACAAGCGCGTGTATT
GATACAGGCTAACAACGCAACAAAGAAACATCAACGTCAACAGCAACAGCGACAGAG
CAGTGCGGTCAGACCGTTGTCAACCGTAACGCAGAAGGGTGCGCATAATGAGAAGGA
GACAGGGGATGGCGTGGGAGTGTCGAGTAGCGGTGGTCGACTCTCTTCTCGAAGTCG
AACCTCGGAGGAACCCCACATCGGCAAGTACAAATTACTTAAAACAATTGGCAAAGGT

AATTTTGCCAAGGTAAAACTTGCCAAACATGTACCTACCGGAAAAGAAGTGGCTATCA
AAATTATTGACAAGACTCAACTAAATCCCGGTAGCCTTCAAAAATTATTCCGAGAAGTA
AGAATAATGAAGATGCTCGATCATCCGAATATAGTGAAGCTTTTCCAAGTGATCGAGAC
TGAGAAAACATTGTACCTGGTAATGGAATACGCCAGTGGTGGCGAAGTTTTTGACTAC
TTGGTCCTGCATGGTCGGATGAAAGAAAAGGAGGCTAGAGCAAAGTTCCGGCAAATT
GTCTCTGCTGTGCAATACTGCCATCAAAAAAAGATCATCCATAGGGATTTAAAGGCTGA
AAATTTACTACTCGATAGCGAGATGAACATCAAAATCGCCGATTTTGGTTTCAGCAATG
AATTCACACCAGGCAACAAGTTGGATACTTTCTGCGGATCACCTCCTTATGCAGCACCT
GAACTTTTTCAAGGTAAAAAGTACGATGGACCCGAAGTAGATGTGTGGTCGTTGGGAG
TAATATTATACACTTTAGTGTCTGGATCACTGCCCTTCGACGGTTCGACACTGAGAGAAT
TGAGGGAACGAGTATTACGAGGAAAATATCGAATTCCATTTTACATGTCCACGGATTGT
GAGAATCTTTTGAAAAAGTTTTTGGTGCTTAACCCGACGAAACGGGCATCCTTAGAGA
CTATTATGAAGGACAAATGGATGAACATGGGATACGATGACGACGAATTGAAACCGTA
CTTAGAACCTGAACCTGATTATAAAGACCACAAGAGGATAGGTGAGTCTGCCA

>novel_circ_000136
AGAAATCGAGCTGCCGCCCATCCGCCCGTAGACGCCTGATCCATCGACGAGGGGGTCC
GACTATTGAAGAGTTGGCAATATTGATCGAGGTTTGAACGGAATTATGCGGAGAACGAT
CGAAATCTGACCGTTTCCTTCCCTCTTCCGCGAATACAAAATCGATACGTAAGAAACTC
GACGACGCGCGCGATAATTATTTGGAAATATAATCGAAAATCTTGTGTAATGGAGAATC
AAGAGAGATCGTTGTTCAGTCGGATGCAACCGAGCAGGGATTCGATCCGATTGCAAAT
CGGGCGGAAAGGGTTCGAGGCGGGGGGGAAGGAGAGCGGGAAACAAATCGGTGAAC
GCCACTCCGTGAAAGAGCAAATCGGTCCATCCGCTTCTCCCGGAAAGTATGCGGCTAC
GCCTCGTCCTGTCGTGGACAATCTGAAGCAATGGGAGTTGGTAGAGTGCGGTCAGAGT
TTGCGAGTGGTGGAAAAGAGGATCGGCGTGAAATCTTGTCTCCCTCGGGAGGAGTCG
TCGGACGTTGAACGTGCGCCGAGCCTCGTTGGGAGGAAAACTCCCCCTTCGGAGAGC
AGCGTGGCCATCGACGCGAATCTCGAGGAGAAGTTGAAAGCTTTCAAAGACTCGAAG
ATCGTTCAACCTATTGTCTCGTCTCAACGCGAACGCACCGACGACGGGAAAGTCCCGA
TCAATCTCGAACTGATCCTGAAACAGCCTAGGAAAGGCTCGGCTATCGAGAGCACGGA
TATGCTTCAACGGTTAGAACAGCAGGTCAAGGCCATCGAAATGGACACTCTTGCAGCT
AGAACGCGCCAGTTGGAGTTTAGCTCGGATTTCGAGCCGGAAATTCGATACGCGGACC
TTCACCGATACAGGGAACACGAGGATCTACTTCGAAACGTGACGGATAACGAGGCCGA
GGGGTCTCCGTTACACCGAGGAGATGCGACGAGGAATTCGACGGGAAATGCAGGGAT
CGTTACGAAGAAAGGTTCGACGAAATTGTCAAGGACGGCCTCGGACTCGAGGCGGGG
ACAGGAAGCGGAAGTACTCCTTCGAGCGCAAGCTAGGGTGACACAAGCGCGTGTATT
GATACAGGCTAACAACGCAACAAAGAAACATCAACGTCAACAGCAACAGCGACAGAG
CAGTGCGGTCAGACCGTTGTCAACCGTAACGCAGAAGGGTGCGCATAATGAGAAGGA
GACAGGGGATGGCGTGGGAGTGTCGAGTAGCGGTGGTCGACTCTCTTCTCGAAGTCG
AACCTCGGAGGAACCCCACATCGGCAAGTACAAATTACTTAAAACAATTGGCAAAGGT
AATTTTGCCAAGGTAAAACTTGCCAAACATGTACCTACCGGAAAAGAAGTGGCTATCA
AAATTATTGACAAGACTCAACTAAATCCCGGTAGCCTTCAAAAATTATTCCGAGAAGTA
AGAATAATGAAGATGCTCGATCATCCGAATATAGTGAAGCTTTTCCAAGTGATCGAGAC
TGAGAAAACATTGTACCTGGTAATGGAATACGCCAGTGGTGGCGAAGTTTTTGACTAC
TTGGTCCTGCATGGTCGGATGAAAGAAAAGGAGGCTAGAGCAAAGTTCCGGCAAATT

GTCTCTGCTGTGCAATACTGCCATCAAAAAAAGATCATCCATAGGGATTTAAAGGCTGA
AAATTTACTACTCGATAGCGAGATGAACATCAAAATCGCCGATTTTGGTTTCAGCAATG
AATTCACACCAGGCAACAAGTTGGATACTTTCTGCGGATCACCTCCTTATGCAGCACCT
GAACTTTTTCAAGGTAAAAAGTACGATGGACCCGAAGTAGATGTGTGGTCGTTGGGAG
TAATATTATACACTTTAGTGTCTGGATCACTGCCCTTCGACGGTTCGACACTGAGAGAAT
TGAGGGAACGAGTATTACGAGGAAAATATCGAATTCCATTTTACATGTCCACGGATTGT
GAGAATCTTTTGAAAAAGTTTTTGGTGCTTAACCCGACGAAACGGGCATCCTTAGAGA
CTATTATGAAGGACAAATGGATGAACATGGGATACGATGACGACGAATTGAAACCGTA
CTTAGAACCTGAACCTGATTATAAAGACCACAAGAGGATAG

>novel_circ_000137
GCAATAACAACGGTGGATATTAAAGAGGCAGAGGGAGGGAGAAGAAGGTGAAGGAG
GAGGAGGTAATAACCTCCTGGCGAGAGAAAGTAGAGAAAGGGCAGAGATCGGGGAAT
CGCGAGAAATACGTGCTACGAAGGAAGGAAGAATTATCGACTGTCAAGTTTTTTAATC
AACGTCCCCGCGACCGAAGAAGACACGCAGCTTGCAAATTAACCGCAAAGTTCCAGT
TCGATAGGCCGGCCTGGGATATTCGTTTCGACGAATTCGTTTTGGAAAAACTTTAATCA
TTTAATTAAACGAGCAAATTCGAGATAACCTCCGGAGCTTCCCACGAGTTAACCGGAG
TTTCAACCGGCGAAACGATAGATTCGGGAGAAGTTTGAGGGAAGCGGTGGATTGGAA
AAGCGATTATCGAGCGACCGGCCTATTAATTACCGGAATTTTCAACGGGAGAAGAATCT
ATCTAAGGGGTCAGTCGAAAGGAAACACGTTTATGGATGAACGTCCCGGCGAGACGAT
TCGACGACAGCGTTAGAAGATGTTAAGGAAAAATTGTCTCGCATTGTTCTCAGGGGAG
AAGAAAAACATCGAGGGGAATCCTCCTGTTTGAGGGATATCGCACGTGCGGTGTGAAG
AGAGAACCTGTATATATATATAGATATATAAAAGTGTTGGTATATTTCAAAAAAATCGAG
GAAGAGAGGGAAACACGTTGAAGCAAGTTATTAGCGAACGACGTCGTGTTTCTAGTGT
GGAAAGAGTGTGGAAAGAGTGTGTTAATCAACAGCAGCATCGTCGGTGGGATATAATG
GCGGTTCGCTGAGCGAAGGAAACGTACGAGGGGAAGGTCACGACGTTCGTTCACGAC
GAGACGGCCTGGTATGACGTGACGAGAGGAAACCGCTTTGGTGCACTTCCATCGCGA
GTATTCCAGAAAAGAAATCTACCATGAGAGCGTCTCGAGTAGCTGCGCTGTTGATCCTC
TGTTACGCCATCG

>novel_circ_000139
AATATGTGAAAGGCAAAGAGATGTTGATAGAAATGAAGATAGAGGAGAGGACGAGGA
TGAGGATGCTAATCCATTGGGAAAATGGCCACATGCCCTTAGTCCAGCTTTAGCATGTC
TTAGTTGCACCCTTGGTCTTTTTAATATAAGTAGATTTTCTATTCTCAGTATACAATTTGG
AGCAAATTTCATTGTACAATTTTTAATTTTATCTTTGATATTGGGTATTCCACTTTTGACA
TTACATGTATGTCTCGGTCAAAGATTGGCGGCTGGATCTGTAAATATGTGGAAAATTTC
ACCTGTTTTTCAAGGTGTTGGAATAGCTCTTCTAATTGCACAAGCTTTTATTGGTATTTAT
AGTGTTGTTGGCATATCTTGGATGTTCGTATACTTTAGAGATTCGTTCATAACGAAACAG
GATAAGTATCGATGGGCAGAACCATTTTCTCTATACAGGGATGATAAATTTATACAAAAT
AATAGTAATTTGCATAAATTATATGAAACAGTACCAGATTATTTTAGCGGAGTTGTCTTG
CAAAGACATCATTTAAATGAATCAGATCCTGGTGTTATAACATTAAAGTTTCAATTAGCT
TTCAATTTAGCCGTTGTATGGATGATTGTATTTGTATCATTAAGCAAAGGATTAAAATCTT
ATGGAAAAGTAGTGTATGTTTTTACACTAGTACCTGTATTTGGTACTTTAGTTCTTTGTA
CAAAATTACTTGGTCTTACGCCTCCAGGTTCTATACATCAACTATTTCCAGCCACTGTAT

GGACCGAATTTTTATTAATGGTAAATCGTGGTTAGCTGCATCTATTGAAGTTTTTCTTA
CATGGGGATTATTTGGTGCAGCTGCTATGCAGATAGCTGCTCATAATAAACATAAACATT
TATTACAACGAGATACAAGTCTTGTAATTGTATTAACAATTGCAGTTTTACTTCTCGCTG
CTTTTCTAGCAAATACTTGTGTACAAGTTCTCAGACATCACGGATATATTTATATACCTAG
TTCATTTGAAAAAATATCATTATATATGTTTATGAGACCCGTCAGTCAACCTGCACCATC
AGGATATAGTAGTACACCAGAAAGATTTATGGCACATACTTCTTTTATTGTTGGAGAACG
CGTAACTCGTCCTGGTGCAGATTTTAGCATTGAATCTGGTTATCAAGTTTTAAGATTATC
GACTGAATTGGTTCCCGCAACATTAGCATTATTAGGTACTGAGCAAGTATCGCCATTTTG
GGCAATTCTCTTTTATTTCATTCTTATTCTATTTGGAATAGCTCAACAGCTGGCAATATGG
CATTGTGTGATAACTGGTATTATGGCAATCAATACAAAAATGATGAAGTTGTGGGAAAC
TACTGTTACATTCTTCAGTTGTGCTTGTGCTTACATACTAGGTTTACCTATGGCTACAGA
A

>novel_circ_000140
AATATGTGAAAGGCAAAGAGATGTTGATAGAAATGAAGATAGAGGAGAGGACGAGGA
TGAGGATGCTAATCCATTGGGAAAATGGCCACATGCCCTTAGTCCAGCTTTAGCATGTC
TTAGTTGCACCCTTGGTCTTTTTAATATAAGTAGATTTTCTATTCTCAGTATACAATTTGG
AGCAAATTTCATTGTACAATTTTTAATTTTATCTTTGATATTGGGTATTCCACTTTTGACA
TTACATGTATGTCTCGGTCAAAGATTGGCGGCTGGATCTGTAAATATGTGGAAAATTTC
ACCTGTTTTTCAAGGTGTTGGAATAGCTCTTCTAATTGCACAAGCTTTTATTGGTATTTAT
AGTGTTGTTGGCATATCTTGGATGTTCGTATACTTTAGAGATTCGTTCATAACGAAACAG
GATAAGTATCGATGGGCAGAACCATTTTCTCTATACAGGGATGATAAATTTATACAAAAT
AATAGTAATTTGCATAAATTATATGAAACAGTACCAGATTATTTTAGCGGAGTTGTCTTG
CAAAGACATCATTTAAATGAATCAGATCCTGGTGTTATAACATTAAAGTTTCAATTAGCT
TTCAATTTAGCCGTTGTATGGATGATTGTATTTGTATCATTAAGCAAAGGATTAAAATCTT
ATGGAAAAGTAGTGTATGTTTTTACACTAGTACCTGTATTTGGTACTTTAGTTCTTTGTA
CAAAATTACTTGGTCTTACGCCTCCAGGTTCTATACATCAACTATTTCCAGCCACTGTAT
GGACCGAATTTTTATTAATGGTAAATCGTGGTTAGCTGCATCTATTGAAGTTTTTCTTA
CATGGGGATTATTTGGTGCAGCTGCTATGCAG

>novel_circ_000141
GGCCCACTTTGGAACCGCCATTGCCACGGCGAAAGAAATGTCCGGAGCTTGCGTACAA
AACGCACGAATTCATCACGGCAGCCGATGGGGGTGGTATCGATGCCGTCGATTCCAAC
GTTGTAGCTGACAAAGTTCGAGAAGTTACAACGCAGTCGGGTTTACCACCGAGTCAAT
TGAGCAAAGCAATCTCACGGATCAGCACCGAGTATCGGTTGCAGTTTGCCTGGCCTAG
AAGACCCCAACTGACGAATGGCGAGACAGTGGCACCAGGAGTGACGGCCGCCGGAC
TCCCTGCAGGTACGACAGGACCACCTAGAAAGTCTCTCAGCATGGGAGCTCTTAAGCA
GGGTATCGCTCCTACAGGGCCCGCCCCGGTACACAAGAAACGTCCCGGTGATGTCGAT
CACAAGCGTGATGGGATGCAAGCATCGGAGCTAGAACCTTTGGTCGGAGGAACTGGA
ACGGACACTATCGACGGAGTGGTGCCCGAGGGCGATGAGCGCGAGGAAGACATGTCG
GACTTGAAAATAACTTTCAGGGGTAAAAAATCAGGTAGGACCGTAAGGATATCGGATG
ATAAAGGGCTGAGGGAGAAACCGCAGGAGAAACCGTTTCCCACTGCTGAAGTCATCG
AGCTTAGACGACTCGCTGACGAGTACAAG

>novel_circ_000142
GTTCGCGTGACGGTTCTAGACAAGAATGACGTGGCACCGACCTGGGGAACCGGCACC
TGGAAATTCAAAATCTCCGAGGAGGCGCCACCCAACACCGTTGTCACGGTCCTGAAA
GCGTTCGATCCGGATACCATCGGGACCTTGACTTACACCCTGGTACCGCATCATTGCCC
AGGCCCCGCCGATCCGATGCCCAGCCAAGACGCGGCCCAAGGCGAAGCCCAATGCCA
GTTCAGGCTTCACCCGACCACGGGACAACTGACACTCGCGGAGGCCCTTGACAGAGA
GACCAGAGAGAAATATGTCCTGAAAGTTCGAGCCGATGATGGATTGCAACACACGGAC
ATCGCGTTAACCATTCAG

>novel_circ_000143
ATCTTTCACAGAAGAATATGAGTTTTCAAACGCTACTTCGGAGATTTCGATATACCTCA
AGAGTTCAATTTTTCTCCACAAAACCGGACTTCACATCGGATAGATACAAAGTAGAAA
GAGGGCCGTATGCAAATATTTGCAATGATCATGTAACATTTTTCAATAACTTGCTTGGAC
AAAACAGAGTTATCACAGATGCAGAAGAATGCGAGGGATATAATATAGATTACTCAAAA
ATTGTCAGAGGAAAAAGTAAAATTGTATTGAAACCGAAAACTACCAACGAAGTATCTG
CCATATTAAAATATTGTAACGAAAATCGTTTAGCTGTATGCCCTCAGAGTGGAAATACAG
GTCTCGTTGGCGGTAGCGTGCCAGTTTTTGATGAAATTGTTATTTCTATGAAACTTATGA
ATAAAATTATTGAAACTAATGAACTAGCAGGAGTGCTGACTTGTGAAGCTGGCTGCGT
GCTCGAAGATCTGGAAAATCATTTAGCGACAGTAGATTTAATGATGCCGATTGATCTCG
GCGCTAAAGGTAGTTGTCTGATCGGTGGCTGCGTATCTACAAATGCTGGTGGTTTAAGA
CTTCTTCGTTATGGTAATCTTCATGGAAACATTTTGGGTCTCGAAGCCGTAAAAGCAAA
TGGGAGTGTGGTGGATTGTTTAAACACACTGAAAAAAAACAATACCGGTTATCATCTA
AAGCATTTATTTATCGGATCTGAGGGAACGCTAGGGATTGTAACGAAAGTCGCAATTCA
ATGTCCACCTCTTCCTACAGCTGTCAACGTTGCGTTTTTAGGATTAAACAATTTTGACA
AAGTATTGAAAGCGTTTCGTCTAGCTAAAAAAGAATTGGGAGAGATTCTGTCTTCGTTC
GAAATGATGGACAAATTGTCATTAGATGTTTCCATTGAAACTTTTGGTTTAAAAAGTCC
ATTGACAACAAAGATCGACGGCCATGACTTTTATGTGTTGATGGAAACTTCTGGTAGCA
ACGTAAATCACGATGAAGAAAAACTTACTTCTTTTGTGGAAAAGGCGCTTGCCGACGA
TATCATTGAAGACGGTACTCTGACTTCTGATCCTACTAAAATTAAA

>novel_circ_000144
AGCAAGCAAATCACCTGAACTTGTTCAACAACCGGCAGCAACCGAAGAAGAAGCGCA
AGAGTCGAACAGCATTTACGAATCAACAGATCTTCGAATTGGAGAAACGATTCCTATAC
CAGAAGTATTTGAGCCCGGCCGATAGGGACGAGATTGCTGCGCAATTGGGACTGAGCA
ACGCTCAGGTGATCACGTGGTTCCAGAATAGGCGAGCGAAGCTAAAAAGAGACATGG
AGGAGTTGAAAAAGGACGTGCAGACAGTGAATCCTCTGTTAGTCGCTCAACATCACA
AGACGTTTTTAGAGAACGTTCAAGATCTTGGAATCCTGAAGAAACGACCCTTGCCGAG
CATGGACGAGAAGTCGTAGGGAACGAGACGCCTCGATGCTCGTCGTTGAGGGAAAAT
CGTGATTTCTTAAAACTAGAGGCTTACACAGGAAAAAAAAAACTCTATTGATCTAGAG
TGATCGTTTGTCGATTGTAGCAAGCGTTTTCTATAATGCAATCGTAAAATTACGCAAAAG
TGAAGTCATACTTGTTCTTTTTTTTCTACGTGAATCGACTCGAACGAAAGATTTTGAGG
TTAGGATTTTCGTAGTTCTGTTTTCCGTAGTATACGATATTACGAGTATAATATTGGGGGA
AAATAGGAATTGTGATCGTTGTGACAGCGTGTTTTTTTTTTTCTTTTTTTGGAAATATTT
GATACATTATAGAAAATTGTGGATAGAGATGGAGGATACGACTAGGCATAGTACAAAGA

AAGCGTCTTCTTCGACAATATATCGCGGGAAACGAAAGGAGAAACAATTGTTTCGACG
AAACAATGAAATAGGCGGAATTCGTTCATAGAAATTACGCATTGATTTCTGTCGGTTATC
CGTGATGTCGATACGTCGAGAAGATGTACAAAGACTGAGAGAGTCTGCAAAAGTCTG
ACGGATGATAAGGCGACGTTGATGAGGCGATTGAAAGTGATCGTGGCATGGCGAGGTG
AGAATATTAAAATGAATCTTTTAATTGTTTGTAAATTAAAGACTGCCAAATATATTCACA
GCTGGCCCTCGTCGACCTCCGGTCCATCGAATGACATTTTCGAAATCAGGATCGGATCA
CCCCGTGTTCTCAGCCATTTACACAAAAATTTTTATCAGGCGAGGCGATGTCAGACGG
AAGATATAAATTCTACGTTACACGAAAGAAAGGAAGAGAAATACGTGTTCTGACCAAA
ATGTTAAACGGATTTTACGTTTCCTTTAACGAATACGTAGGACGACTATTCCTCGGACA
CCTGTATCATATTGTATTTATTTGCGGAAAGACGAGATGAAGGGAATCGCGATGAATAA
AAATCTAAAAGTGATCCAGAAGAAGCATCGATGATTTAT

>novel_circ_000146
CCCAGATCGAAATAATCCCGTGCAAGGTGTGTGGCGACAAGAGCAGCGGTGTTCATTA
TGGTGTGATCACCTGCGAGGGCTGCAAAGGCTTCTTCAGGCGGTCCCAGTCGTCGGTC
GTCAACTATCAATGTCCGCGGAACAAGAATTGCGTGGTCGACCGTGTAAACAGAAACC
GGTGCCAGTACTGTCGGTTGCAAAAGTGCCTACGCCTCGGCATGTCTAGAGATGCGCG
CGCGCGTACTCTTCCTGTTGTCATCTCTCTCTCCCTCCCTCCCTCCCGCACCTCTCTCTC
GAGCGCGCAGCCACGCTCCTTCGCCTGCCTCGTCCCGTCCTCGTCTCGCAGCCACGAG
CCCCTGTCCGCCCTCTCTGTCCCTCGTTCGCCACGTTCGGTCGATCACCCTCCGCTTCT
ACTCCTCTTCGTCGTTCGACCGGCCGCTCTTTCTCTTTCTCTCTCGTTCGCGCGCGATCC
CGATAGCGGTTTCAGCTGGCAGCTGACGGTCGCGAGAGACGCTCGCCTGTCTCTCTCT
CTCTCTCTCTGTTTCTCCCCCTTTTCACCAGTTTCTCGTTCACAAACCGTTGTCGGATCT
CTTCCTCGCGCTGCCTGCTAACCCTCCTCTCTTCTCTCCTCTCCTTTATACATCACGGTG
GCATGAAGTCGCGTGCGTGAAGTCACGCGTGTGTCGCAGCGAGCAAACGGACCGAGA
TCCGAGATTCGTTGCGTTGAGAGTTTCAAGGAAGGGCGGAAACTGAGCTGCCCTCTGC
CTGTGGCGCCAGGATTCGGTTTTTGGAGCGTGAAGAGGGAGGGATGTTGAGTTGGGAT
TTGATTAATTGCGATGCGGATTTTGGGGGGATTGGGGGAAGCCGTTAAATTCGGGCGCA
TGTCGAAGAAGCAGCGGGAGAAGGTCGAGGACGAAGTTAGGTTCCACAAGGCACAA
ATGAGAGCGGCCTCCGAGACTGCGCCCGACAGCAGTGTGTTTGAACATCAGACCCCA
AGCAGTTCGGATCAACATCATCCTTTCAATGGCGGGTATACGTATGGCGGTAGTGAATA
CGCTTCCCCTGGCCCCGGAACGGGCGGGTCGGGTTATTACAATCATCAAGCGATGACG
AACTACGAGCTTAGCGCCGACTACGTCGACAGCACGACGACCTACGATCCACGACCGA
CGCAGACGCAGGTCGCGGACACTGTCGCCCCTGATACACCCTCCGCTGGAGTAGCAGC
TGGAGTACTTCCCAGCGTGGTCACCACCG

>novel_circ_000147
CCCAGATCGAAATAATCCCGTGCAAGGTGTGTGGCGACAAGAGCAGCGGTGTTCATTA
TGGTGTGATCACCTGCGAGGGCTGCAAAGGCTTCTTCAGGCGGTCCCAGTCGTCGGTC
GTCAACTATCAATGTCCGCGGAACAAGAATTGCGTGGTCGACCGTGTAAACAGAAACC
GGTGCCAGTACTGTCGGTTGCAAAAGTGCCTACGCCTCGGCATGTCTAGAGATGCGCG
CGCGCGTACTCTTCCTGTTGTCATCTCTCTCTCCCTCCCTCCCTCCCGCACCTCTCTCTC
GAGCGCGCAGCCACGCTCCTTCGCCTGCCTCGTCCCGTCCTCGTCTCGCAGCCACGAG
CCCCTGTCCGCCCTCTCTGTCCCTCGTTCGCCACGTTCGGTCGATCACCCTCCGCTTCT

ACTCCTCTTCGTCGTTCGACCGGCCGCTCTTTCTCTTTCTCTCTCGTTCGCGCGCGATCC
CGATAGCGGTTTCAGCTGGCAGCTGACGGTCGCGAGAGACGCTCGCCTGTCTCTCTCT
CTCTCTCTCTGTTTCTCCCCCTTTTCACCAGTTTCTCGTTCACAAACCGTTGTCGGATCT
CTTCCTCGCGCTGCCTGCTAACCCTCCTCTCTTCTCTCCTCTCCTTTATACATCACGGTG
GCATGAAGTCGCGTGCGTGAAGTCACGCGTGTGTCGCAGCGAGCAAACGGACCGAGA
TCCGAGATTCGTTGCGTTGAGAGTTTCAAGGAAGGGCGGAAACTGAGCTGCCCTCTGC
CTGTGGCGCCAGGATTCGGTTTTTGGAGCGTGAAGAGGGAGGGATGTTGAGTTGGGAT
TTGATTAATTGCGATGCGGATTTTGGGGGGATTGGGGGAAGCCGTTAAATTCGGGCGCA
TGTCGAAGAAGCAGCGGGAGAAGGTCGAGGACGAAGTTAGGTTCCACAAGGCACAA
ATGAGAGCGGCCTCCGAGACTGCGCCCGACAGCAGTGTGTTTGAACATCAGACCCCA
AGCAGTTCGGATCAACATCATCCTTTCAATGGCGGGTATACGTATGGCGGTAGTGAATA
CGCTTCCCCTGGCCCCGGAACGGGCGGGTCGGGTTATTACAATCATCAAGCGATGACG
AACTACGAGCTTAGCGCCGACTACGTCGACAGCACGACGACCTACGATCCACGACCGA
CGCAGACGCAGGTCGCGGACACTGTCGCCCCTGATACACCCTCCGCTGGAGTAGCAGC
TGGAGTACTTCCCAGCGTGGTCACCACCGGGAAGTCGCTGTCTGCCTCGACGACCGGA
AGCAGCACGGGGGGTGGTGGTGGAGGCGGTGGTGGTGGTGGTGGTGGCGGTGGTGGT
GGTGGTGGTAGTAGCGGCGGCGGAGGCGGTGGTGGCGGTGGTAATGGCGGTGGTGGA
TCCGGTGGTGGTGGCTCGGGTGGTGGAGGGAGCGGTTCTGGCACCGGGGGCGGTGCG
GCCGCCGGTGGCGGCGGTGGTGCCGGTTCCCTGAGCGGGGGTGGAATCGTTGCTGTTA
AGCAAGAGACCACCGTCGAACTGGCCAGCTTGGGCCACGGCTCGTACACGATGGTCG
ACTCGACGACCTTTCTGAGCGGTCAGCAACAGAGGGTCAGCAATCCATCCGAGGACG
ACGAAGTGCCGAGTCTGCCCAGTATGTCCCATGCGAGATATCCGGCACAGATCAGCGA
GTTGCTCTCAAAAACGATAGCGGATGCACACGCAAGAACGTGTCTGCTGTCGACGGA
ACAGATCCAGGAATCTTTCCGGAAGCCGCATGATCTCTCCAGATTGATCTACTACAAGA
ACATGGCGCACGAGCAGCTTTGGCTCGAGTGTGCCCAAAAACTAACCACCGTTATCCA
GCAGATCATCGAGTTTGCTAAGATGGTTCCTGGATTCATGAAGCTCTCGCAGGACGACC
AGATTGTTTTATTAAAGGCTG

>novel_circ_000148
CCCAGATCGAAATAATCCCGTGCAAGGTGTGTGGCGACAAGAGCAGCGGTGTTCATTA
TGGTGTGATCACCTGCGAGGGCTGCAAAGGCTTCTTCAGGCGGTCCCAGTCGTCGGTC
GTCAACTATCAATGTCCGCGGAACAAGAATTGCGTGGTCGACCGTGTAAACAGAAACC
GGTGCCAGTACTGTCGGTTGCAAAAGTGCCTACGCCTCGGCATGTCTAGAGATGCGCG
CGCGCGTACTCTTCCTGTTGTCATCTCTCTCTCCCTCCCTCCCTCCCGCACCTCTCTCTC
GAGCGCGCAGCCACGCTCCTTCGCCTGCCTCGTCCCGTCCTCGTCTCGCAGCCACGAG
CCCCTGTCCGCCCTCTCTGTCCCTCGTTCGCCACGTTCGGTCGATCACCCTCCGCTTCT
ACTCCTCTTCGTCGTTCGACCGGCCGCTCTTTCTCTTTCTCTCTCGTTCGCGCGCGATCC
CGATAGCGGTTTCAGCTGGCAGCTGACGGTCGCGAGAGACGCTCGCCTGTCTCTCTCT
CTCTCTCTCTGTTTCTCCCCCTTTTCACCAGTTTCTCGTTCACAAACCGTTGTCGGATCT
CTTCCTCGCGCTGCCTGCTAACCCTCCTCTCTTCTCTCCTCTCCTTTATACATCACGGTG
GCATGAAGTCGCGTGCGTGAAGTCACGCGTGTGTCGCAGCGAGCAAACGGACCGAGA
TCCGAGATTCGTTGCGTTGAGAGTTTCAAGGAAGGGCGGAAACTGAGCTGCCCTCTGC
CTGTGGCGCCAGGATTCGGTTTTTGGAGCGTGAAGAGGGAGGGATGTTGAGTTGGGAT
TTGATTAATTGCGATGCGGATTTTGGGGGGATTGGGGGAAGCCGTTAAATTCGGGCGCA

TGTCGAAGAAGCAGCGGGAGAAGGTCGAGGACGAAGTTAGGTTCCACAAGGCACAA
ATGAGAGCGGCCTCCGAGACTGCGCCCGACAGCAGTGTGTTTGAACATCAGACCCCA
AGCAGTTCGGATCAACATCATCCTTTCAATGGCGGGTATACGTATGGCGGTAGTGAATA
CGCTTCCCCTGGCCCCGGAACGGGCGGGTCGGGTTATTACAATCATCAAGCGATGACG
AACTACGAGCTTAGCGCCGACTACGTCGACAGCACGACGACCTACGATCCACGACCGA
CGCAGACGCAGGTCGCGGACACTGTCGCCCCTGATACACCCTCCGCTGGAGTAGCAGC
TGGAGTACTTCCCAGCGTGGTCACCACCGGGAAGTCGCTGTCTGCCTCGACGACCGGA
AGCAGCACGGGGGGTGGTGGTGGAGGCGGTGGTGGTGGTGGTGGTGGCGGTGGTGGT
GGTGGTGGTAGTAGCGGCGGCGGAGGCGGTGGTGGCGGTGGTAATGGCGGTGGTGGA
TCCGGTGGTGGTGGCTCGGGTGGTGGAGGGAGCGGTTCTGGCACCGGGGGCGGTGCG
GCCGCCGGTGGCGGCGGTGGTGCCGGTTCCCTGAGCGGGGGTGGAATCGTTGCTGTTA
AGCAAGAGACCACCGTCGAACTGGCCAGCTTGGGCCACGGCTCGTACACGATGGTCG
ACTCGACGACCTTTCTGAGCGGTCAGCAACAGAGGGTCAGCAATCCATCCGAGGACG
ACGAAGTGCCGAGTCTGCCCAGTATGTCCCATGCGAGATATCCGGCACAGATCAGCGA
GTTGCTCTCAAAAACGATAGCGGATGCACACGCAAGAACGTGTCTGCTGTCGACGGA
ACAGATCCAGGAATCTTTCCGGAAGCCGCATGATCTCTCCAGATTGATCTACTACAAGA
ACATGGCGCACGAGCAGCTTTGGCTCGAGTGTGCCCAAAAACTAACCACCGTTATCCA
GCAGATCATCGAGTTTGCTAAGATGGTTCCTGGATTCATGAAGCTCTCGCAGGACGACC
AGATTGTTTTATTAAAGGCTGGTTCCTTCGAGTTGGCTGTGCTACGTATGAGCAGGTAC
CTCGATCTCCAGCAAAACTGTGTCCTCTACGGTGACACTATGTTGCCTCAGGACGCGTT
TTACACGACAGATACAGCGGAAATGAAGCTTGTCTCTTGCGTGTTTGAGTTGGCCAGG
AGTATTGCCGAATTGAAGCTTACGGAAACAGAGCTGGCTCTTTACAGTGCAGCGGTTC
TCCTTAGCCCCGATCGGCCGGGGCTGAAAGGGCTCGCGGAAATCACGAGACTGTCCCA
GGCGGTGATCAGGGCGCTCAGGTCGGAGCTGGATCGTAACCACGTGTCGCCCATTAAG
GGCGATGTGACGGTGTGCGACGCGATCCTCGCAAAGATACCGCAGCTCCGCGAGATCT
CCCTGTTGCACATGGACGCCCTGGCCAAGTTCAAACGGTCCCAGCCGCACCTCGAGTT
CCCGGCCCTTCACAAAGAGCTTTTCAGCGTCGACAGCTGAACGATCGACGCGGCGCA
GGGCGTCCCGACGCTGACGAACAAAGCGGTACGCGAGTAGCGCCGGGCCTTGCTCTG
TCAAG

>novel_circ_000149
CCGTTAAATTCGGGCGCATGTCGAAGAAGCAGCGGGAGAAGGTCGAGGACGAAGTTA
GGTTCCACAAGGCACAAATGAGAGCGGCCTCCGAGACTGCGCCCGACAGCAGTGTGT
TTGAACATCAGACCCCAAGCAGTTCGGATCAACATCATCCTTTCAATGGCGGGTATACG
TATGGCGGTAGTGAATACGCTTCCCCTGGCCCCGGAACGGGCGGGTCGGGTTATTACAA
TCATCAAGCGATGACGAACTACGAGCTTAGCGCCGACTACGTCGACAGCACGACGACC
TACGATCCACGACCGACGCAGACGCAGGTCGCGGACACTGTCGCCCCTGATACACCCT
CCGCTGGAGTAGCAGCTGGAGTACTTCCCAGCGTGGTCACCACCG

>novel_circ_000150
GTCGTAGAAATGGCAAACAACCTAACACGAAAATGCAGGAGGTCTTGAAGAAAACGA
TCGAGGAGGCACGAGCTATGGTTTCGAAGAAATTGATACAGCAAGAAAAATTGGTGAC
GCAGAAAACAGTTCAAGAGGCATTGGACATTCTCAGAGGTGCTGTGATGATCGTTTAT
CCGATGGGACTTCCGCCTCATGATGTTATTCAACAAGAATTCGAAAATACGGAAGATTT

GAGCGGAACTCAAGCATCACTTGAGGTTATCGACGTGCAACTGGCACAACTTTGGTTC
TCAGGAAAAGAAATGATAATAGGAAAGAAATTAAAGGATTATTTGGGGATGAACGAAA
AGACAAAAATCATTGTGAAACTGCAGAAAAGGGGAGCTGGAAAACCTGCTCGTGAAC
CATTGATGTCAGAAGAAGAACGAAAACAACTAATGCTGCATGCCTACAGACGTCAAGA
ACAGCTCAAG

>novel_circ_000153
GAAAGTCTGCCGCCATTGAACTCGCGGGAAGAATCGCAGGAGAGGCGTCGAAAGAGG
AGTTCCAGGGAGAGCGACTCTCGCGAGAGACTGACCGATCGGTGTCCAGAGAACTCG
GCCGGTCTCGAGCTGAGCACGAGCATCGCGGATTGCTGTTGCATAGGTCCACGTTCGC
GTCTACCGTTGCCGAGGATCCCGCCGTCGACGACGATCGCCTCTTCGTCCTCTCCGTGC
GTAACAGGCGGAGGTGGTGTAGGTGGAGTTGGAGGTGGCGGTGGAGGAGTTAGCTGT
GGACAGTCTCCTTTTCCTGGGCCTCCCACGTCTTCGAACAGCCGTGTTGCCGGCGTGG
AACAGCCGGCGACGCTGACGACGCCAACGACGACGTCGGCGCAACCGACGTTGGTG
AACTCTAATAATAACCGAGGGATGCTGCAAATGTCGGTGGGGAACAGCACTGGCCAAC
AGCACCATCAGGCACAATCGTCTGCGGCTCTAACGGCGGCGGTACCGTCGGTATCCAC
CTCCACGTCGTTCAACACGTCGTCCACCGCCGTCCCGACGCTGCAGTCCGTCTCGGTA
CCGCGACAGTTAGCACAGTGGACCAAGCATCAGACGCTGAAGCTGCAG

>novel_circ_000154
GTGACGTACCCAATGATTGTACGTTGCATGCTGGTGCTCTGGTTGGTCGCCCTGGCATT
GACAAGTATGCCAATGATGGGTTTTGGCGTGTACTACAAAGGTGAACGATGCGTACGAT
ACAGGGAAGCCACCGAGCCAGCTGACATCGCTTACGCGTATGTTTGGTTCGTTTTCGGT
ACGGGTCTCTGCCTGTCGATAGTTTGGTGCAATCTGGCCGTTTCCAGGGCGCTTAGCAG
GCTAAGTCGTAAAGCTGGCGTCCTCAGGCGAGTAACGAGATCCTCCTCGAGGGCGAA
ACCACTTTTGACAGTGACGGGGCCAGCGCCGGAAATCGTCACCACCGCCGAGGAAAG
GGCATTCGCAAGATTGATGGCCGTGCTATCGATATCGTTCGTCGTATGCTGGATGCCGCA
AATGATTTCCATTCCATTGGCACAATTCGCGATGCAGTTGCCAGAGAAGGCGATACTCG
CGCGAAAATTGATTCGTATATTTCACCTGGCCGCCGACGTGCTCCTTTGCATTCACTTCA
CCCTGGATCCGTACATTTACGTGTTGTTAAGGATGCCACGGCCGAGATTCCGCCTTCTG
AAACCCCTGTGCAAGATCTGTTGGCCAGCAAGAAGCCGGTCGAGCTCGTTCAATG

>novel_circ_000157
GAACCGATCGTTATCGAGAAAGCGGGCGACGAGGAGGCGGCGGTGATTTGCCAGCCG
AGCCAGCCAGTCGAACACGAGGAGCACGAGGAAAAGGAAGCGGCGATGAACGTGAA
GATCGAGACGTCGTACGAGGCACCGGCACGGATCGAGGAGTCGATCGACGTGCACGA
TACACCTGAGACCGTCCCTGCGCCTGAAAGACCGACGACCTTGACCTTTGGCAAACA
GGAAATGGCCACGGTGGACACGATCAGCCTACTCAGCCCCATATCGCCCATCTCGAAA
CAGATTTTGCCACTGAATCTATCCAGTGACGAGGAAATAGTGACAGGGTTGGCGTTTC
CCCTGGGGCCGCCGACCAGCGTGGAACCCCCGAAGGAGAAACCACCGCCGCCTCCGG
TAGACGTCAGCGACGAAGAGAATCTTCCAGTGGAACCTCTCAAGAGATTGAACTCCAC
CCGCAGAATAAAGAAGGAACTACGCACGAGACGCTCTGATTTCCTTGGAATCGAGGG
GGTAAACGATGACGAGCTGGAACGCGAGTTGACCCTGACAAAACCGCCAGACATGGC
GGCGATCCTTGCAGAGGAGAGACGCATCGAGCAGCTTCATCGTCGCTCCTACGATACG

GACAGCAATTACGAGCAAGACTCAAGCCACGAGAGAGATTCGGGCGTGGAATTGGGC
CACGCCGAAGATTGGACTAAACAACCAGTTAGCCCTGATATGTCTCAGCACAGTCGCC
AAAGCAGCGAGCCGTTCGGCGCCAGTGTCACCTCCTCCGAAGAGGACGAAATCACGA
AGAAAGAGAAGGAGATCATCGAAGTCCTCGAGAAAGAGGAGCAGTGGCGGTACGAG
AACAATCGTGAGATGAACAGTGACTTGGGCGAAAAATTGGCGCACAAGCTGCGCGAA
CTCGAAGAGGAGAAGATGCAGTTGGAGAGGGAGCGCGCCCAGGAAGCGGCCCTCCG
CCGACAGGAAGAGACGCTGCGCAAAAACAACGAGGACAACATACGGAAACAGGAGG
AGACGGCGAGACAGCAGGAGGTGGCGCGGGCTGAGGCAGAGGCGAAACGCGCCGAG
GAGAAGAGGCAGGAAGAGGAGCTGAGGGCGCAGGCAGAGAAGCTGAGGATTCAGAA
TGAAATCGAGGAGGAGAGAAGGGTTGCCGATGCGCGACGTGTCGAGGAGAAACGCAT
TAGGGACATGGAAAACCAAATTCGAGAACAAGAG

>novel_circ_000158
GAACCGATCGTTATCGAGAAAGCGGGCGACGAGGAGGCGGCGGTGATTTGCCAGCCG
AGCCAGCCAGTCGAACACGAGGAGCACGAGGAAAAGGAAGCGGCGATGAACGTGAA
GATCGAGACGTCGTACGAGGCACCGGCACGGATCGAGGAGTCGATCGACGTGCACGA
TACACCTGAGACCGTCCCTGCGCCTGAAAGACCGACGACCTTGACCTTTGGCAAACA
GGAAATGGCCACGGTGGACACGATCAGCCTACTCAGCCCCATATCGCCCATCTCGAAA
CAGATTTTGCCACTGAATCTATCCAGTGACGAGGAAATAGTGACAGGGTTGGCGTTTC
CCCTGGGGCCGCCGACCAGCGTGGAACCCCCGAAGGAGAAACCACCGCCGCCTCCGG
TAGACGTCAGCGACGAAGAGAATCTTCCAGTGGAACCTCTCAAGAGATTGAACTCCAC
CCGCAGAATAAAGAAGGAACTACGCACGAGACGCTCTGATTTCCTTGGAATCGAGGG
GGTAAACGATGACGAGCTGGAACGCGAGTTGACCCTGACAAAACCGCCAGACATGGC
GGCGATCCTTGCAGAGGAGAGACGCATCGAGCAGCTTCATCGTCGCTCCTACGATACG
GACAGCAATTACGAGCAAGACTCAAGCCACGAGAGAGATTCGGGCGTGGAATTGGGC
CACGCCGAAGATTGGACTAAACAACCAGTTAGCCCTGATATGTCTCAGCACAGTCGCC
AAAGCAGCGAGCCGTTCGGCGCCAGTGTCACCTCCTCCGAAGAGGACGAAATCACGA
AGAAAGAGAAGGAGATCATCGAAGTCCTCGAGAAAGAGGAGCAGTGGCGGTACGAG
AACAATCGTGAGATGAACAG

>novel_circ_000159
GAACCGATCGTTATCGAGAAAGCGGGCGACGAGGAGGCGGCGGTGATTTGCCAGCCG
AGCCAGCCAGTCGAACACGAGGAGCACGAGGAAAAGGAAGCGGCGATGAACGTGAA
GATCGAGACGTCGTACGAGGCACCGGCACGGATCGAGGAGTCGATCGACGTGCACGA
TACACCTGAGACCGTCCCTGCGCCTGAAAGACCGACGACCTTGACCTTTGGCAAACA
GGAAATGGCCACGGTGGACACGATCAGCCTACTCAGCCCCATATCGCCCATCTCGAAA
CAGATTTTGCCACTGAATCTATCCAGTGACGAGGAAATAGTGACAGGGTTGGCGTTTC
CCCTGGGGCCGCCGACCAGCGTGGAACCCCCGAAGGAGAAACCACCGCCGCCTCCGG
TAGACGTCAGCGACGAAGAGAATCTTCCAGTGGAACCTCTCAAGAGATTGAACTCCAC
CCGCAGAATAAAGAAGGAACTACGCACGAGACGCTCTGATTTCCTTGGAATCGAGGG
G

>novel_circ_000160
ATCACGCACGAGCCGCCAAAATTAAAAACGACGCTGAAAACGCCTCCGAAACAAGCG

ACCAATCCTCTTCAATTCGTGAAAGTCGGGCCGTGCTCTTTGTATCGCACAGCTCAGGA
ACAATTGCAAAAAGTTCAAGAGGTGAAGAAAATAAAGCAAGAAGTCCGCGATGATCC
CGAAGATTGGCAATCGAATTTAGACAACTGGAAGAGCAGTCGAAGGAAGCGCCAGGA
GCACATCATCGAGCGAGTGGTCGAGGTGAAGAAACTCGAGCTGGAGGAACACGATCG
GCAGCGTCGCCGGAGCAAAACTTTCTCGGAAATGATGGAGGAGAGGGGCAACAGGG
GTCGGAAATTGAGCATCAGCTTGGCCATGTACCACGAGGAGGATGCCAATGATCTGAG
CGATCTTGGTATAGGCACAAGTAGCGGGAAGAGTTCGGTTAGTGGAGATACTCATGAC
GACACACATAGCGTTTTGAGCGATAGAGACAGCGAAATAGAGAAAAATCACTCGGAC
GTGGACAACGTGCAAAGCGAGAGCGCGATCCACAGCGATATCACGACGTCGTCGGCG
ACCACGACTGCGACCACGACAACGGCGACGAAGAAATCTTTCGGCAACGGATTCCAC
ACCAATCACAGTCACAACAATCAGGAGTACGATTCGGCGACCACGGCGACGACCAGC
TCCCCGGAGCCGGAGGAGTACACGTACGAGGGTGCGATTCGAGGTTACGTGTCGCGA
GTGTCGCAAAACATACCGAGGCGATCGTTGGTGAATCTGGATTCGAAGCTTGACAACG
TCGCGAAATCGTCGACGAACGGCTCGAAGCTGAGCCTGGCCGACGAGGCGAAATCCA
CCATACCCGTGGTAAAAGTCGATATCCTGAAGAGGCGGGAGATATTCGAGAAGGGATC
GCAGAAGAGCAACGAGACGAAAACGAACAACAGGCTCTCCGGCGATTTCACCGGGA
CGAAATCTATCAAGGAGCGGCTCTCGAACCTGGAGAAGCAGAAGCACGAGGCGGAGA
ACAGCGAGAAGGCGTTGGGGAGCAAGGCGTTGAACAGATTATCGGGCGACATGAGCT
CGATCCGCGAAAGATTGTCCCATCTGGAGAAGCAGGCGTTGGAGAGGGAGAAGAGTT
GTTGCGCTCATCGTAAACTGAGCACCGAAGAGTTGGAGACGGTGAGACCGTTGCGGG
ACAGATTGTCCACCCTGGAGAAATACAGCAGCAGCGACGAGTCGTCGTCGGCGCCTG
GCGCGGCTCACGAGTCACGACACAACGGCGAGCTGTCCGCCAGATCGATAAAGGATC
GTCTCAGCGCCTTGGACGCGGCCAGGAACAAAGAGACGGCCGACAAGAGGTCCTCCG
TGGGCAAGCACTCGCTCTGCTTCCGGGATCAGGAGAACAGAATCGACACCTCGACTCC
TAGCGAGAGGAGCTCGTCCCCAGACTCGGAGTATCGCGTGCCGAGGGCCGCCTTCCAC
AGAAGCCTCGACTCCTTGGACGCGGACGCGTCCAGCGGCCCCGACACGTTCGAGCGC
GTGCAAAGCTTGGAGGAATTGGATTACGGGAGGCGTTATCCGGCGTCCTCGTCGTCCG
CCGAACTGCTGAACGACACCGATCGCGAGGATTCGGGCATTCACACGGCCGACGTGA
GCTGCTCCGTCAGCCAAGCCGACGAGCCGGTCGACGAGGACATCGTGCACTCTAACA
CGATCATTGAGAGGCAGGAGGTGATTGAGGAGAAAGGCGAGGAGCCGCGCGAAGAA
ACGGCGAACGAGAAGGTCGGTGAACAACAACAACAGCAACAGCAACAGCAACAACA
ACAACAACAACAGAGCACTGCTGTATCCGTGAACGAGATCTCGACGACTTCTGCCAGC
CAACCGGACGCAGTAACGGCAAAGAAG

>novel_circ_000161
GGACGAGGCGTAGGATGGGCCCGACCGCGCGAGCTGTCGTGCTACACTGAAGAGGAG
GAGCTCCCCCGAAGAAGAGAGAGAGAAGGAGGAAGGAACGAAGGATGCCCGCCTCG
CAGACCGCCGCCACGGAGCGGCGAAACGAGAGGATGATCACGCACGAGCCGCCAAA
ATTAAAAACGACGCTGAAAACGCCTCCGAAACAAGCGACCAATCCTCTTCAATTCGTG
AAAGTCGGGCCGTGCTCTTTGTATCGCACAGCTCAGGAACAATTGCAAAAAGTTCAAG
AGGTGAAGAAAATAAAGCAAGAAGTCCGCGATGATCCCGAAGATTGGCAATCGAATTT
AGACAACTGGAAGAGCAGTCGAAGGAAGCGCCAGGAGCACATCATCGAGCGAGTGG
TCGAGGTGAAGAAACTCGAGCTGGAGGAACACGATCGGCAGCGTCGCCGGAGCAAA
ACTTTCTCGGAAATGATGGAGGAGAGGGGCAACAGGGGTCGGAAATTGAGCATCAGC

TTGGCCATGTACCACGAGGAGGATGCCAATGATCTGAGCGATCTTGGTATAGGCACAA
GTAGCGGGAAGAGTTCGGTTAGTGGAGATACTCATGACGACACACATAGCGTTTTGAG
CGATAGAGACAGCGAAATAGAGAAAAATCACTCGGACGTGGACAACGTGCAAAGCGA
GAGCGCGATCCACAGCGATATCACGACGTCGTCGGCGACCACGACTGCGACCACGAC
AACGGCGACGAAGAAATCTTTCGGCAACGGATTCCACACCAATCACAGTCACAACAAT
CAGGAGTACGATTCGGCGACCACGGCGACGACCAGCTCCCCGGAGCCGGAGGAGTAC
ACGTACGAGGGTGCGATTCGAGGTTACGTGTCGCGAGTGTCGCAAAACATACCGAGGC
GATCGTTGGTGAATCTGGATTCGAAGCTTGACAACGTCGCGAAATCGTCGACGAACGG
CTCGAAGCTGAGCCTGGCCGACGAGGCGAAATCCACCATACCCGTGGTAAAAGTCGAT
ATCCTGAAGAGGCGGGAGATATTCGAGAAGGGATCGCAGAAGAGCAACGAGACGAAA
ACGAACAACAGGCTCTCCGGCGATTTCACCGGGACGAAATCTATCAAGGAGCGGCTCT
CGAACCTGGAGAAGCAGAAGCACGAGGCGGAGAACAGCGAGAAGGCGTTGGGGAGC
AAGGCGTTGAACAGATTATCGGGCGACATGAGCTCGATCCGCGAAAGATTGTCCCATC
TGGAGAAGCAGGCGTTGGAGAGGGAGAAGAGTTGTTGCGCTCATCGTAAACTGAGCA
CCGAAGAGTTGGAGACGGTGAGACCGTTGCGGGACAGATTGTCCACCCTGGAGAAAT
ACAGCAGCAGCGACGAGTCGTCGTCGGCGCCTGGCGCGGCTCACGAGTCACGACACA
ACGGCGAGCTGTCCGCCAGATCGATAAAGGATCGTCTCAGCGCCTTGGACGCGGCCAG
GAACAAAGAGACGGCCGACAAGAGGTCCTCCGTGGGCAAGCACTCGCTCTGCTTCCG
GGATCAGGAGAACAGAATCGACACCTCGACTCCTAGCGAGAGGAGCTCGTCCCCAGA
CTCGGAGTATCGCGTGCCGAGGGCCGCCTTCCACAGAAGCCTCGACTCCTTGGACGCG
GACGCGTCCAGCGGCCCCGACACGTTCGAGCGCGTGCAAAGCTTGGAGGAATTGGAT
TACGGGAGGCGTTATCCGGCGTCCTCGTCGTCCGCCGAACTGCTGAACGACACCGATC
GCGAGGATTCGGGCATTCACACGGCCGACGTGAGCTGCTCCGTCAGCCAAGCCGACG
AGCCGGTCGACGAGGACATCGTGCACTCTAACACGATCATTGAGAGGCAGGAGGTGAT
TGAGGAGAAAGGCGAGGAGCCGCGCGAAGAAACGGCGAACGAGAAGGTCGGTGAA
CAACAACAACAGCAACAGCAACAGCAACAACAACAACAACAACAGAGCACTGCTGT
ATCCGTGAACGAGATCTCGACGACTTCTGCCAGCCAACCGGACGCAGTAACGGCAAA
GAAG

>novel_circ_000163
CCTTGTTATTAGGCACTTTGAAGACCTACCAGCGAGCAGCCTGCGACGAAGAATCGAT
GACGCTCAAGTGTCCACTAGGTACCACCATCTCCATCGTTCTGGCTCAATATGGAAGAG
CGGGACCAAATGGTACCGACAGATGCAATACCTATTCAGAACCATCGATGTATCTCGGG
GACAGACAAACGAATCAGACTTGCATCTGGCCTCAGTCGCTACAGACGGTGGTGGAA
GTATGTCACAAGAAGAGGCAGTGCAAGTTCAACACCAGTCCGAAAACCTTTCAGGGC
GATCCGTGTCCTGGATTGCCGAAATACATCGAGGTCGCCTATCAATGCCGTCCTT

>novel_circ_000165
GCACTTTGAAGACCTACCAGCGAGCAGCCTGCGACGAAGAATCGATGACGCTCAAGT
GTCCACTAGGTACCACCATCTCCATCGTTCTGGCTCAATATGGAAGAGCGGGACCAAAT
GGTACCGACAGATGCAATACCTATTCAGAACCATCGATGTATCTCGGGGACAGACAAA
CGAATCAGACTTGCATCTGGCCTCAGTCGCTACAGACGGTGGTGGAAGTATGTCACAA
GAAGAGGCAGTGCAAGTTCAACACCAGTCCGAAAACCTTTCAGGGCGATCCGTGTCC
TGGATTGCCGAAATACATCGAGGTCGCCTATCAATGCCGTCCTT

>novel_circ_000166
TACTCACTGTTGCAGACGGTGGTGGAAGTATGTCACAAGAAGAGGCAGTGCAAGTTC
AACACCAGTCCGAAAACCTTTCAGGGCGATCCGTGTCCTGGATTGCCGAAATACATCG
AGGTCGCCTATCAATGCCGTCCTTATGAGTTCAGAAGTAAAGTTGCCTGTGAGAATGAT
GTCATCAATCTTGTCTGCCATCCAGGACAGAGGGTTGCCATTTTCAGTGCATCGTTCGG
TAGAACGGAATACGAATCCCTTCAATGTCCACAACCCCACGACGTAAAGGAGGAAACT
TGCATGGCATCGTACGCGACAGAAACCGTGATGAATTTGTGCCATGGAAAACGACGCT
GTTCGGTGGTTGCAAACAGTTCGACATTCGGTGAACCCTGTAGCCCGGAAAGCAGAA
CGTATTTGAAAGTTGTTTACACGTGTG

>novel_circ_000167
ATACGTTTTTTTTCTCGTGAACGAGTGAACAAACCAAACCGCCACCATGGACGCGATC
AAGAAGAAGATGCAGGGAATGAAGCTGGAGAAGGACAACGCGATGGATCGCGCGTTG
CTCTGCGAACAGCAAGCTCGTGATGCGAACGCGCGAGCCGAAAAGGCTGAAGAAGA
GGCGCGCGCCTTGCAAAAAAAGATCCAGACCATTGAAAATGAACTCGATCAAACTCA
GGAGGCTCTTATGCAGGTCAATGCCAAACTTGAAGAGAAAGATAAAGCGCTACAAAA
C

>novel_circ_000168
GTGGCACCCTTCCCTCCACTTGCTCCACCCCGCCCCCGCCCTTTGACGATGCAGTGCG
CTCAAACGACCAAACCTTGGCTTCTGGTGGTCAACTTCAACAGGGTGCACCGACCAC
AATGTTAGCGAGCAGCCTGAGCACTGCTCAATCGAAACAGAATAAGGTTGTTCACCAT
GTCACTCTTACAAAGACTTATCACGATGCCGTGAGCATATCTGACGTGCATTTGGAAAG
CAGCGGTAGCGGAAGCGGAAGCGGCGGAAGGGAAACGAAGTCGTCGTTGAGCGTGG
AAAGTGGTGGAAGCAGTCGAGGAGGTGGTGGATTCACCTGGACACCACCCGCGGGCA
GCGCCGAGGGCTCGACGCCCACCTGGACAGAGGGCACACCGTCCTTCACCGAGAGTT
CCAGCAGCGGTGACGCAG

>novel_circ_000169
AAGATGAACGGGATGGAGCAGATCCCTGAAGGTTGAGAGCGAGCGGATCCTCGATATG
ACCGGCTGCTGCAGAATGCGGCCGACTGCATCGGCGCTCGGCGTCATCGCCATTGTCT
CCGTCATGATCACTGCCACATCGTCAGAAGCGGGGAACGGCGCCGTGGAGACCAGCA
TCAATCACCCCGTAGTGGACGAGGAGGACCAACGCGTGCGAGGGGACGAGGCGACCG
CGAACGGAACGGTGGCCGATGACCCGGTCACGCTCCACGACCCCACCGACGAGGCTC
CAATTGCCAGCAGCAAACCTACCCAGTCGCCCTTGGAGAAACCCGCGAACGTCTCATC
CGAGGAAATCATCGTGGAATCACTGCAGAGGGATCAAAGCGACCTGCTGGTGTCCGTC
GCGGTGGCGGTGAACGACGAGGTGGACGGCGATCCGTCAGAGTCGAGGTCGGGGGA
GCGTGAGAGTGGTGGGAGGAGCAGGGGCGGGGTGCAGCAAGCGAGCAAGGATTTCG
TCGCGTCGACCCGGCCGAATCGAGTGGAAGTCGCGTCGAGTGTCGTGGACCAGTTGG
ACGGCGTCCAGGCGGAGGACGAGGGTTTGTCGAACGTTGTGGAGCGCGGGGAGAGC
AACGTGGACAAATGGCGGAGAGGAGGTGGCTCTTTCACGACCGCGTCCGTCCGCGAG
ACGATCTCGACGGAGGTGGCGAGGCCGATCGAGCGGAAGAACGTGGAGGACATCAGG
GCGATCTCGTTCCAAGTGCCTGCGGTTGTCGAGGGGGTGGAGGAGGCCGTGGACAGG

CAGGGCAACGAGCAGGCGAGGAAGGATCATTCGAGGCCGGTGAACAATCTCCTGTTG
GCCGGTGGCAAGTCGACGAAGGCGTTCTACGAGATAAAACCGTCGATCAAAACCGAG
ATGGAGGGTGATCAGGTGAGCCCGACGATCGCCACCCAAAGGCCCCTCCACGGAAGG
AAGTTCGTGTTGCCCAGCGTGGACAGCGCGTTCGGCAAATTTGGCCCCTATTTCGAGG
ACGGGGATCAGGAGATGAATATCACTGCCAGGATAGGGAGCACCATGCTGTTGGATTG
CAAGATCGGCATGCTCGGTAATAAGGAG

>novel_circ_000170
GCTCATCGACACTCTGCAGACGAAATCCAGAAAGGCGGACGAGGACTTGGAGGCGTT
CGAAAGCACCGAGCGTGAGATCGATCAGCTGAGGAAGCGTTTGAACGAGGCGCGCGA
ACGCGCCTCGAACTTGTACATATTCGGGCCGGATCAGGACGCGACCGAGGAGGAGCTT
GACGAGCTGCGTTGGGCTGTCGAGCAGCTCCTCGAGAGCGGGAAAAAGTTCTCAGGA
AGCACTAAGGCGCGGTACCAAGCTAGCCAGCAGCTGGTCCCCAGCGATCTTGCCCAAC
ACCTGACCGCCCTCGAACTTTGCGCCGAGGCGACAGCGCAGGCCATGGAGGAGAAGC
AGAGGGAGCAGAAGCGCGCGAGAACCGTCAGATCGGATTATCTGACCGATCTGGACG
AGGTTCAAGCGTGGATCAGGCAGGCCGAGTTGAAGGTTCAGGACCGATCGATCGAGC
CTGTACCTCTCAAGGATCAATTGAGGCAGGTGCAAGAGGAGTTGGGCACGATCAGCG
ACAAGCTCGAACGATTAACGAGAAATGGCCGTACGATTGCGGAGAACACGAGGGACG
ACACGGAGAAACAATTGATCGACAGCACCGTTCACAACGTGACCGAACAGTTGAACC
AAGTGAGGAATTGGTTGGACGAGAGGAAACAAGTGGTGGCTGATACCATTGACGCTT
GGCAGAGATTCTTGTCGTTGTACGAAGCCGTGAGGACGTGGACCGAGGAGAAGAGAC
AATTCCTGGTTGAGCCGTTGAAGCTCAGCACCCTCGTGCAGGCTAGGCAGAGGCTGCA
CGAATATTCGACAGCCGTGAAAAGCTGCAAACAGATCAACAAGAATCTCAGTGACATG
GGTAAGGAGCTCGAGAGTATCGGTCAGGTTTGCAGCGTGGGCGACTTGCCCGAGAAA
CTGCTCGAGGCTGAAGAAGCTAAGGTTCAAGTCGAGGGTCAACTGTTGGAGAGG

>novel_circ_000171
GCTCATCGACACTCTGCAGACGAAATCCAGAAAGGCGGACGAGGACTTGGAGGCGTT
CGAAAGCACCGAGCGTGAGATCGATCAGCTGAGGAAGCGTTTGAACGAGGCGCGCGA
ACGCGCCTCGAACTTGTACATATTCGGGCCGGATCAGGACGCGACCGAGGAGGAGCTT
GACGAGCTGCGTTGGGCTGTCGAGCAGCTCCTCGAGAGCGGGAAAAAGTTCTCAGGA
AGCACTAAGGCGCGGTACCAAGCTAGCCAGCAGCTGGTCCCCAGCGATCTTGCCCAAC
ACCTGACCGCCCTCGAACTTTGCGCCGAGGCGACAGCGCAGGCCATGGAGGAGAAGC
AGAGGGAGCAGAAGCGCGCGAGAACCGTCAGATCGGATTATCTGACCGATCTGGACG
AGGTTCAAGCGTGGATCAGGCAGGCCGAGTTGAAGGTTCAGGACCGATCGATCGAGC
CTGTACCTCTCAAGGATCAATTGAGGCAGGTGCAAGAGGAGTTGGGCACGATCAGCG
ACAAGCTCGAACGATTAACGAGAAATGGCCGTACGATTGCGGAGAACACGAGGGACG
ACACGGAGAAACAATTGATCGACAGCACCGTTCACAACGTGACCGAACAGTTGAACC
AAGTGAGGAATTGGTTGGACGAGAGGAAACAAGTGGTGGCTGATACCATTGACGCTT
GGCAGAGATTCTTGTCGTTGTACGAAGCCGTGAGGACGTGGACCGAGGAGAAGAGAC
AATTCCTGGTTGAGCCGTTGAAGCTCAGCACCCTCGTGCAGGCTAGGCAGAGGCTGCA
CGAATATTCGACAGCCGTGAAAAGCTGCAAACAGATCAACAAGAATCTCAGTGACATG
GGTAAGGAGCTCGAGAGTATCGGTCAGGTTTGCAGCGTGGGCGACTTGCCCGAGAAA
CTGCTCGAGGCTGAAGAAGCTAAGGTTCAAGTCGAGGGTCAACTGTTGGAGAGGAAC

GCGCTGCTGCAAGAAACTAGCGAGGAATGGGAACAATGCGAGCGAAAGATGAAAGA
AGTGAAAACGTGGATCGAGAAAGCGAAACAAAGTCTCGAGTCGCCGCAGAACAAAA
AGAAACCGTTACGGGATCAGCACAGCATACGGGAGAAAATGCTGAGCGATATCGCGAT
ACAGAAAACGAAAATCGGTATATCGATGGAGAAGCTTCAGGTTCACTTCCGGTCGGGG
ATCGGCGGCGATAGCCGAATAGGCGAAACGGTTGACGAGCTACTGGCCGAGTTAGACA
ATCTTCACGCCAACGTCAAGGAACAGACCACAGCGTTGGAGGGTTGTTTAGCTCAGAT
TGATCAATATCAACAGGAGATACAACAGTTGAGGCAACAGATTATGCAGGTAGAGCAA
CAATTGAGGACAGTGTTGAGTCCGACATACCTGTCCACCGACAAGGAGAAGGCGTTG
CAGGAGCAACAG

>novel_circ_000172
GACATCATACAGGAACGCCTAATGGTTTTACAATTGAGTCTTTCGAACTTAAAAGGAAC
TTATTTACCACGAGATAACATAGACACTATGCTCTCTGCACTCACGAATTTGATAACGGA
GCTTCAAAGTTATAATCAAGAGACGCATCACTTGGAAGAGGAATTGCATAAAATACCA
GCCGATTTAGAGGCTCAAAAGCTTCTATCCAATCTAGAAGAAATTCAAAGTAAAATTAC
AACCCTGTTGACGCAAGCGGAACAAGGGCGTGCCACCCTTGAGGGTGCACGTGGACG
TCAAGAGCAACGTGGCCATGATATTCATGCGTATAAAAAATTCTTGGATGAAACGGATA
GTTGGTTGAAGAATATCGTGGCTAGTATGAAACAGGAATCACCGATTGCTACGAACAA
GGCTTTACAGGATGAGTTGAGCAGTCACGCGCAGCGGGTATCGGAGCTTGAATCTTTG
CCAGAGATCAGCACATTGGCAAAAGTCATGAAGAATGCGCTGTTGGAGATGATGTCGC
GTCTGCAGCAAAGGCAGCAG

>novel_circ_000173
GTGACGACTTTCAACGTTGAACAACTGGGCGTTCGTCCTGTGGGAGACAGAGAGCAG
AAATAAGGATGGCCTTTCATTGGGATAAGCTCTCGCCAGCGGAATTTCAACAACTCCA
GGATTTAGCAGCTTATTCCACACGAAAGCTACAGGATGTCCTCACTGAGTTTTGCGGAT
CAAATACAACACCAAGCGGTGCGCCCAAATATCATCCAGATGGTGACATTGATTACGAG
GGATTTCGTAAATTTCTGGACACCTATCTCGAAGTGACAACCCCTGACGAACTTTCACG
ACATCTTTTTTTGTCGTTCGTTAAAAAGGTGCCACGAGGAGTCGATGGGAAAGCTTTTA
AGATTTATCAGAGATAGGAATCTTTGCATCAGGAAATGGCCGTGCTCTCGTCGACAACC
GCTTGTGCCGCCATTACGTCTCACACAACGTCCAGCAGCAACGTGAACAGCACAGGTC
TACCTGGTGGAACATCGTCGGAGGGTGGACACGGTTCCTCTTTGGCTGACAAAATTCA
CGGTCTCACAGAGAAATTGCAGGCGTTGGGACATCATAGAACGGACAGTGAAGTCTCC
ACCAGAGCACGCACAGGAAGCGTTCATCCCATATTGACGATACAACAGCACACGTCGT
ACAGTTGCCATGACGTGATAGAGAAAAAGAGCACGGACAGCAGCCCGAGCCATAGCC
AAATGTCGCGGAATTCGTCCAGGAAGTCGAACAATTCCCTCCTCATCAACAACGGGAA
ATTAGAAG

>novel_circ_000174
TGCTCGAAAACTTGTGGAGGAGGTGTGAAGAAACGAAAAGTGAGTTGCGAACAAATA
ATGGCTCAGGGGCGTAAACAAAATCGAAAGGATAACGAATGCCCGTCTCAAAAGCCA
GCCTCGGAGAAGCCTTGCAACACCAGAGCATGTCACGATATGGATGCAGGTTCCCTGC
CCATAATTACCAGTCAAAATACAACATATAACCAGACAGACATGAACGCGAAGGTAGAT
TTAAAGATCGGCGGTATTGCGAAGATTTTTCAAGGCACGTCCTCTATAAAAATTCGTTG

CCCCGTGAGGAAATTTGACAAAGCACAGATCACGTGGACCAAAGATAATAAAGAATTA
AGGAAATCGAGGAAATATAAAATTAGCAGGAAGGGGGCGTTAAAGATAAACGACATAA
CATCCTCGGATGCTGGTGTTTACGCGTGTATAGCCGGAAATTCTCATGCGGAGACACAA
TTGATAGTGAAATTTCGTTCAAAGGAACAGATAAGTAGCGAGGAGTATTTACGATTAAG
CAATTCCGTCCATCGACAACGAAATGCAAATTTGGACTCGGCGCCTGCTAATTCAGGC
GAAAGTCTCTACACTGATCCAGCAGTTGATTTTCTAATAGCCGCGTACGGAAACCGTTT
TGTTCCCATCGACAGCGAAGATTTAAGTCATGAACGAGCAGTCCCTAGTAATCCTACGA
GAAAGCCACATCAGAAAAAACAGAAAACTAGCCCAACGCCGCCGGATTCTACGATGA
TGCACAAAGAACACGTTGTAACATCGTTGAATCAG

>novel_circ_000175
ATTTTGCCAACCACCCACTCGCTTGTACTCAGTATGCGGAAGGCGCCCGTGGTGCTGTT
CTCGGGTAAGTAACGAGGCGAGAGATAGACGAACGGTCTTACAACGGAAGAGGTTAG
ATGGTGAAGAAACTCGCGAGGAAAGGCGAGGAACAGAAAGAGAAAAAAATGCGGGG
TTAGACGACAAGAGGAGAGAATGTGTAGAAAGGTGGAAGCGCGTCACAAGGAAGAG
ATCCTGAGAGGAGTGCAGAGGAAGAAATGGAAGAGACGAAGGAAAATGTTTATGGCG
ACAACAACAGCAACCGCAGCACGACAGCAGCTTGGTTGAACGGTGACAGTTTATAGT
GCAACAACATCGCAAAATACTTCTCTGTTCTCGCAAAACGCGTCAACGATAACGTTTC
ACCAGTAACTGTATAATGAAATTTTCTACACTTGGCTATGAATTGTTTTCAGCTGCAATA
AATAATTTTCTAATTTGAGCTAAATCTTCCATTATAATAATAATGGAAATAAGAAACGAG
CTTTGTACGTACAATGTTACTTCAACGTTTTTTCCAATATAATTTGTCAGCATTCCTCGAC
GCCGCGATGAAACGATGAGAAGAAACGACGAAATGTTTGAACAACAGACGCGCCTCG
TCTATCCGGAAGTTTCAACCAGTTTCAACAGAGAGGGAATACCGATTGCATTGCGAAA
GCCTTTATACGATGCTTTCTTTGCGAGGCAAATTCCGTGATCCCTTCCGTGAACCATT

>novel_circ_000176
GACCTAAAGAAGCGCGGTTCCCACACCTGTGACATGCAGGCCCTACAGCCACTTCCGG
TCCCAACCACCACAACCACCAACACCGCGCCTGCCATGACTATTTCCGGTGGCCAGCC
ACCGCTTCCGGTATCGGAGACCGCTTGAGTCGATCACCGAGACCAATCACGATCACCA
TATCCATAGTTATTATGAACAAAAAAAGAAAAGAAAAGAAAAGAAACGAAAAAAAAA
GAAAAAAGAAAAAAGAGAAAAAATAATAATAACGTCACCTTTAACAAAAAAAAAAAA
AACACCATCACCGTCCTCCTCTTCTACTCGATGAAAGAACAATGGCCGACCGGCCGGA
AGAACG

>novel_circ_000177
GTACGGTGACATGGTTCCGAAAACGATCGCGGGAAAAATCGTGGGGGGTGTATGCTCT
CTCAGCGGTGTCCTTGTGATCGCTTTACCGGTGCCTGTTATCGTCAGTAATTTCAGCCG
AATATATCACCAGAATCAGCGGGCCGATAAGCGAAAGGCGCAGAGGAAAGCAAGATT
GGCACGGATCAGGATCGCGAAAGCGTCGAGCGGGGCGGCCTTCGTGAGCAAAAAGA
AGGCGGCCGAGGCACGTTTGGCCGCGCAGGAGAGCGGCCTTCAGCTGGACGACAATT
ACAAGGAGGAGGATATCTTCGAGCTGCAGCATCACCATCTGCTTCGTTGTTTGGAGAA
GACCACGGATCGCGAGTTCGTGGAGATGGAGGTGCCCTACAACGGTGCCCCGAAGAG
GCCGGGCTCCCCCTCGCCCCTCACCTCTCCCGCGCACAGCACTGCCTCGAGGGGCGGC
CTGCTGCACGCCTGCTGCGGCCGCTGCTACCCCCAACGTTACCAG

>novel_circ_000178
ATTTATCGAAGAACCGTTTCGCCGAATTGCCGGAAGAGGTGACGGAATTCCTGTTCCTC
GAAAAGCTTCATCTCTACCACAATGCCATAAGAATAATACCGGAAACTGTGGTGATGCT
ACAGTCCCTCAACTACCTCGATCTTAGTCGCAACCAATTGACTTCTCTACCTCGGGAGA
TATGCAGGCTACCGCTGCAAACCTTGTTGGTCGCTCACAACAGATTGGCATCTCTGCCG
GACGAATTGGGCAGGATGTCGGCGTTGGCCGAGCTCGATGCGGGATGCAACGAAATC
ACGAATCTGCCACCCCGCATGGGCGATCTTGCGCGACTTAGGTCCTTGGATCTGAGGA
GCAATATGATCGTCCACCTGCCTATAG

>novel_circ_000179
ACCGTGGATGCAGCCGGGTATCGGAGGTGGAGGTGGCGCGGCCCACACAGGTGGGAC
AGGTGACGACGAAGCTCCGAAAGATCCCGGTGTTCGGATCACTCATCAACCTTTCACC
GAGAAAGACTACGAAGGTCACAGAGCGCACACGGTGTATGTGGGCGTGCATCTACCA
GGCGAAAGGAGACATCGTCGTCATCACAAGCACCATCATTCTCAACGTCAAACGTACA
GTGCCGATAAGGATAATGTCGACAACGATAGGCCCA

>novel_circ_000184
GGAAAAAAGAAGAAGAAAAAAACTATTAAAGAGAAATATACAGAAGATGAAGAATTA
AACAAAACAAAACCTATCTGGACTAGAAATTCTGATGATATTACACAAGAAGAATATGG
TGAATTTTATAAATCATTGACAAATGATTGGGAAGATCATTTGGCTGTTAAACATTTTTC
TGTAGAAGGTCAATTAGAATTCAGAGCTTTACTTTTTATTCCAAAACGTATGCCATTTGA
TTTATT

>novel_circ_000186
GTAAAACATTCTGCCAGTCTTGTAAGAAGAAGTGCAGCGGGGAGGTACTACGAGTGC
AGGACAAGTATTTCCACATAGGGTGTTTCAAGTGTGCTCAATGTAACGCGAGCTTAGC
GCAGGGTGGTTTTTTCGCGCGCGAGGGCTCTTACTATTGCACCAAGGATTACAGGGAA
CGGTGGGGAACGAAGTGCGCAGGTTGTGGAGAATACGTGGAAGGCGACGTGGTCACT
GCCGGAGACAAGCATGCCTTCCATCCCAACTGTTTCCACTGCCAACGATGCAGACAAC
CGTTACTCGGACAGGGAACCAAGGTTTCCCTCGTCCAAGGTCAAGCTCTTTGCCACCG
ATGCGTCGGTATCCCTGTGCGAGAGGCCTCGACGCCAATCGGGAATAGCTCCGCCACC
AGAGGATCCGGAGATGGGCCCTCCGACCCCGGTGCCTGCGCTGGTTGCGGAAACCAA
TTACGAGAAGGTCAGGCTTTGGTAGCTCTCGATCGACAGTGGCACGTATGGTGTTTAA
AATGTCACAGCTGTGACACCGTCCTTCATGGAGAGTATATGGGAAAAGACGGGGTGCC
TTATTGCGAGAAGGATTACCAGAAGTTGTTCGGCGTGAAATGCGCCTATTGCAATCGTT
ACATCAGCGGAAAGGTGCTGCAGGCAGGTGACAATCATCATTTTCACCCGACTTGTGC
CCGTTGCACCAAGTGTGGCGATCCTTTCGGCGATGGAGAGGAGATGTACTTGCAGGGC
GCTGCCATTTGGCATCCCCGCTGTGGCCCGGGCCCTAGCGGACCCAATGGTATCGTGA
ACGGCCACGGAGAAGCTCACACGCCACAGCATCGAGAATCGGAACGGATTTCCAGCA
GCGCATCGGAGATGCAG

>novel_circ_000187
TTTTCATTGCGGTCACGCACGCCAAGCCTGAACGGATCACTCTGCAGCCCTTACAGCA

GCCTCAGTCGCAAGTATTATCCCGCGCGAACTGGAAGTCCCGGATTGATATTGCGAGAA
TACGGACGTGGTCCATCCGAAGATGTGTCTAGGATTTACACTTACTCGTATTTGACCGA
AACGCCGACCCAAGGATACTTGAGACGTCCGATACAGCCGTACGACAAACCTCCGACT
AGCCCACACTTTCATAGACCTAGCTCGTCACGTTCGATAAGAAGCAGCGGCGGACGCA
GCAGCCGATCTGGAATGCGTGCTCTGGTCGATGCTCTCAGCGAGACCAGACCAAAGTC
ACCGGCCAGTCAAGTAGATAATGACGAGCCAATAGAGCTGGCGCATTATCCGGACGCC
ATGAAACCTCCTCCTGGAACCAAACCGCCGATCGAGAGAGATGATTTTCCCGCTCCAC
CTTATCCTTATACAGATCCTGAAAGACGTAGACGATGGTCCGACACTTATAAGGGAGTA
CCTGCATCCGACGACGAGGACGAAGTGGATAACAAAACTTATATAAAGGAGGTGGAG
GAGAAGTTGAAGAAGGAACAGGACGAGCTAAGCAAAATCGATACTGGAATAGCGAAA
GTGTTTTTGCAAGATCGTGAAAAGGATCGAGAGAATTTGAGACACAGAGCCGCGAAC
GTTGATCCTAGAAATGCGTCGAGAACGCCATCAGCTGCCAGAGAACCGACTTATAGAC
TGCGATACGAGAGTCCAGTTGGCGCTTCGCCTTCGAGGAACATAGATCACGCCAGACC
GTGGGAGGACGATGATGGTTTCAGTTATAGATCTAGCGTAGGACCTAGTTACAACG

>novel_circ_000188
TATTATCCCGCGCGAACTGGAAGTCCCGGATTGATATTGCGAGAATACGGACGTGGTCC
ATCCGAAGATGTGTCTAGGATTTACACTTACTCGTATTTGACCGAAACGCCGACCCAAG
GATACTTGAGACGTCCGATACAGCCGTACGACAAACCTCCGACTAGCCCACACTTTCAT
AGACCTAGCTCGTCACGTTCGATAAGAAGCAGCGGCGGACGCAGCAGCCGATCTGGA
ATGCGTGCTCTGGTCGATGCTCTCAGCGAGACCAGACCAAAGTCACCGGCCAGTCAA
GTAGATAATGACGAGCCAATAGAGCTGGCGCATTATCCGGACGCCATGAAACCTCCTCC
TGGAACCAAACCGCCGATCGAGAGAGATGATTTTCCCGCTCCACCTTATCCTTATACAG
ATCCTGAAAGACGTAGACGATGGTCCGACACTTATAAGGGAGTACCTGCATCCGACGA
CGAGGACGAAGTGGATAACAAAACTTATATAAAGGAGGTGGAGGAGAAGTTGAAGAA
GGAACAGGACGAGCTAAGCAAAATCGATACTGGAATAGCGAAAGTGTTTTTGCAAGAT
CGTGAAAAGGATCGAGAGAATTTGAGACACAGAGCCGCGAACGTTGATCCTAGAAAT
GCGTCGAGAACGCCATCAGCTGCCAGAGAACCGACTTATAGACTGCGATACGAGAGTC
CAGTTGGCGCTTCGCCTTCGAGGAACATAGATCACGCCAGACCGTGGGAGGACGATGA
TGGTTTCAGTTATAGATCTAGCGTAGGACCTAGTTACAACG

>novel_circ_000189
GGTGATTATTCGTTCAGTGGGATGGGAGACAAGACGCACAGCACTGATTTCTCATCTG
GCAAATCAGATATATCGACAGGAAGCATCACGGATGTGGATCGACGGGCATTGGTATGT
ACCACAGCCCCATACTACTCCCGGCGAATTAGCATG

>novel_circ_000190
AATGATGGTGGAATGCTGCCATCATCTACCACGTATACAGGTGGCCTAGGTTCGGTAGT
TGGAAGTCACGGGGGCCATCACGTGAGGAGATCACTGCCAGACATGGGGACAGCACC
GTCCGAACCGCCGAAACTCTATCCTTATCATTTACTTGTCATCACAAACTACAGGCTGC
CCGCTGACGTGGATCGTTGCAACCTCGAAAGACATCTTTCCGACGCGGAGTTTGAGGC
AGTACTTCAGTGTACTCGTGCCGAGTTCTACAGATTGCCGCAATGGCGTCGTAACGAAA
TCAAAAGACGTGCCCGGCTGTTTTAACTGGCAGCTATCACGCTACGTGTTCTACTTATT
ACCTTCATCGTGTCACGACTGTGTTACCCTGCTATAGAGTGTACATTCCTTCGGCGTG

>novel_circ_000191
CAAACCTTTGTTGCGTGTCAGCCGGTGCTATGTTAGGATGGACCAGTTCCGTGATACCT
CTTTTAAAGGACGAAGAAGCTGTCAATAATGGGTACAATCCTCTGGGTAGGATAATCGA
CAACGAGGAAGATTCATGGATTTCCTCGTTGGTTTCCATAGGAGCGATAATAGGAAGTT
TCGTGGCTGGATATTTGGCTGAAAGATATGGTAGAAAGATGACACTGCTGTCAGCCGTA
GTACCTTTCCTGATCGGTTGGGTTTTAATCGCAACAGCCAAAGTCGTGATCCAATTGTG
CGTGGCCCGAGTGATCCTCGGTTTCGCCTTAGCGTTCGCCTTCACGGTCGTTCCCATGT
ATTGCGGTGAAATCGCCGAGATATCCGTGAGAGGGGCTCTGGGATCTTTCCTGCAGTTG
TTCGTCACCATCGGTCTCTTGTATTCGTATTCTATCGGCCCCTACGTGTCTTATCTGGTAT
TCTGCATAGTCTGCGCCATTGTACCTGTCGTGTTCGTCGGCTGTTTCATCATGATGCCCG
AATCGCCGTATCAATTGCTCAAGATCGGCAAAAAACAAGAGGCGCTCGAGTCTTTGGC
TAAATTGCGAAGCAAGACCATCGCCAGCGTGCAAAAGGAAGCAGATGAAATGCAGGC
TTCCATCGACGAGGCGTTCAAAAACGAGGCGAAATTGTCGGATTTATGGAAAGTGAAA
GCAAATTTGAAAGCTTTGGTGTTTACGTGTGTTCTCGTCGCCTTCCAACAAGCCAGTG
GTATCAACGTGGTCCTCTTTAATATGGGAACTATCTTCACAGCTGCAAAATCATCGTTAA
ATAGCTCGGTGGCAACTATAATCGTCGGTACAGTTCAAGTAATTACTTCCGGAATCACG
CCATTGGTCGTCGACAGACTTGGCAGAAAAATACTGCTTATTTTCTCCGGCGTTGGCGA
GATCGTCTCCCTG

>novel_circ_000192
CAAACCTTTGTTGCGTGTCAGCCGGTGCTATGTTAGGATGGACCAGTTCCGTGATACCT
CTTTTAAAGGACGAAGAAGCTGTCAATAATGGGTACAATCCTCTGGGTAGGATAATCGA
CAACGAGGAAGATTCATGGATTTCCTCGTTGGTTTCCATAGGAGCGATAATAGGAAGTT
TCGTGGCTGGATATTTGGCTGAAAGATATGGTAGAAAGATGACACTGCTGTCAGCCGTA
GTACCTTTCCTGATCGGTTGGGTTTTAATCGCAACAGCCAAAGTCGTGATCCAATTGTG
CGTGGCCCGAGTGATCCTCGGTTTCGCCTTAGCGTTCGCCTTCACGGTCGTTCCCATGT
ATTGCGGTGAAATCGCCGAGATATCCGTGAGAGGGGCTCTGGGATCTTTCCTGCAGTTG
TTCGTCACCATCGGTCTCTTGTATTCGTATTCTATCGGCCCCTACGTGTCTTATCTGGTAT
TCTGCATAGTCTGCGCCATTGTACCTGTCGTGTTCGTCGGCTGTTTCATCATGATGCCCG
AATCGCCGTATCAATTGCTCAAGATCGGCAAAAAACAAGAGGCGCTCGAGTCTTTGGC
TAAATTGCGAAGCAAGACCATCGCCAGCGTGCAAAAGGAAGCAGATGAAATGCAG

>novel_circ_000193
GAAGAAATTGAAATAGATGAAGATGAAGATGAAAGTAATATAGAAGATGAGGAAGATG
ATGATGAAGATGAGGAAGATGAGGAAGATGAGGAAGAGGATGAGGATAATGATGAGG
ATGATGAAGATGATGATGATGATGATGATGATGATGATGATGATGATGATGATGATGATG
ATGATGATGATGATGATGACGACGATGACGATAATAATGATATAGAAGAAAAAGATAAA
AAAAATGAGAATAATAAGCAACATGAAGATAATAAAATTAAAAAAAGTATACAAGATTT
A

>novel_circ_000194
AAGATTCAGCTTCAATTTCTTGTTTAAGACGTTTAACATTTTTCTCATTTTTTAAAGAAT
CTGTAGCTTTTCTCTTAGATTGTTTGTCAAGTAATTGTGGTACCTGCTCATCTTCATCTTC

TTCTTCTATTTCATCTAAATCCAAATCCTCATCGG

>novel_circ_000195
ATACTCCATCGTTCGCGATAACGCGTTTACCTGGTTTTGGAATCCCGATCGTCGAGGGG
ATGTCCGTCAGCTTGAAATGCGAGGTGGACAGCAATCCGGCCAGCACGCCCATTTGGC
AGCGTGACAATGGACCGCCCGTCGAGCAGAGCGACGACGGATGGCTCAACTTCACAA
AAATCAGCCGCGCCGAGAGCGGCTGGTACAAATGTTACACCAGGCACATGCTCGGCAT
ATTCACCAGCATCGGTTATTTTCTCAACGTGCGCTACGATCCAGACATGGAAACGGAAG
CGGAAGCGTTGAACGGCGAGGCAACCGATTCCCGGAAGATGGAAGTTCAAATCGGGG
GTGCGGTGACCCTGGAGTGCGACGGTGGTTGTTGGGGACACGGTCCTGGAATGGATC
CAGTTGGTGGTCCCGGTCCACTGGCTTTGAGTCGAGTCGTTTACCAGGAAGCCGGCGA
ATATCGATGCGTCGCGCCCGACAGGAAAATGCAAGACACCTGGCGCGCCCAATTGCCG
TATCACATCAAGATCACCG

>novel_circ_000196
AGGCACCCCGTGGGACGTACCTGCTGATAGATGGCCGTCGACTGGACCCCGGCAACCA
GTTCGTCCCTGTGAAGGAGGGGTCCGAATTGACGTTGGAGTGCGCCGCCGAGGGTGG
GAACCCGCCACCCGATTTGGCATGGGGGATGACTCTGTCTCAGGCCACTTTGGACGGT
CCCGAGCAACCGCCGGACAATCTGACCGTGTTGCCCTCGACATCCGGTAGACGCAGCG
GGGCACACCTCAAGGTTCTCAGGGGTCACCACAACGCGACCATCGCCTGCGTCGCCC
GCCACATTACCCTCACCATACCCATGAACGCGAGCATTCTGTTGGACGTGCAATATACT
CCATCGTTCGCGATAACGCGTTTACCTGGTTTTGGAATCCCGATCGTCGAGGGGATGTC
CGTCAGCTTGAAATGCGAGGTGGACAGCAATCCGGCCAGCACGCCCATTTGGCAGCGT
GACAATGGACCGCCCGTCGAGCAGAGCGACGACGGATGGCTCAACTTCACAAAAATC
AGCCGCGCCGAGAGCGGCTGGTACAAATGTTACACCAGGCACATGCTCGGCATATTCA
CCAGCATCGGTTATTTTCTCAACGTGCGCTACGATCCAGACATGGAAACGGAAGCGGA
AGCGTTGAACGGCGAGGCAACCGATTCCCGGAAGATGGAAGTTCAAATCGGGGGTGC
GGTGACCCTGGAGTGCGACGGTGGTTGTTGGGGACACGGTCCTGGAATGGATCCAGTT
GGTGGTCCCGGTCCACTGGCTTTGAGTCGAGTCGTTTACCAGGAAGCCGGCGAATATC
GATGCGTCGCGCCCGACAGGAAAATGCAAGACACCTGGCGCGCCCAATTGCCGTATCA
CATCAAGATCACCG

>novel_circ_000197
GCTCGGTGAACGAGGCCGGTGTTTTCACCCTTCGTAGCAGGCAGGAGGGTGGACGGC
GTGCTAGAAGAACCACGACGAGCACCACCAGCACAACGGCGTCCAGCTCGACGTTGA
TTTCTCTGTCGGACGAGGTGGCGCAGCCCGCGGCCCTTTTAGCCCCCGAAGAGGACTT
ATGGTCTACCAGTTCTTCCGCTACAACCAATGTTCCAGTCACCTTAGATACAGCTGCCG
CTTCGTCAAAGCCACCCTCGGAAATGGTGGTGAAACCTCACAGCAAGGTCATACTACC
GTGCGACCTGGAGGAAAATTACTCGAGGCTGCTGTTGCCCGGGGTCAGGAGCTACAG
AATACGGCCAGCGACATGGTTGCACGAGGGAAGTCCTGTAGATATGATTACGATCAAC
ACGAAGACGGAGATGAGCGGGACCGGACATAGATATATCGGCGACTCCATGACTGACG
CGTTGCACATAGACAACGTGAGATTAGAGGACGACGGTATATGGGGTTGCAGGCTCGA
GGACGATCAAGGGAAGATACTTTCCGGCAGGCCTGTGAAACTCGTCGTCCTCGAGGCA
CCCCGTGGGACGTACCTGCTGATAGATGGCCGTCGACTGGACCCCGGCAACCAGTTCG

TCCCTGTGAAGGAGGGGTCCGAATTGACGTTGGAGTGCGCCGCCGAGGGTGGGAACC
CGCCACCCGATTTGGCATGGGGGATGACTCTGTCTCAGGCCACTTTGGACGGTCCCGA
GCAACCGCCGGACAATCTGACCGTGTTGCCCTCGACATCCGGTAGACGCAGCGGGGC
ACACCTCAAGGTTCTCAGGGGTCACCACAACGCGACCATCGCCTGCGTCGCCCGCCAC
ATTACCCTCACCATACCCATGAACGCGAGCATTCTGTTGGACGTGCAATATACTCCATCG
TTCGCGATAACGCGTTTACCTGGTTTTGGAATCCCGATCGTCGAGGGGATGTCCGTCAG
CTTGAAATGCGAGGTGGACAGCAATCCGGCCAGCACGCCCATTTGGCAGCGTGACAAT
GGACCGCCCGTCGAGCAGAGCGACGACGGATGGCTCAACTTCACAAAAATCAGCCGC
GCCGAGAGCGGCTGGTACAAATGTTACACCAGGCACATGCTCGGCATATTCACCAGCA
TCGGTTATTTTCTCAACGTGCGCTACGATCCAGACATGGAAACGGAAGCGGAAGCGTT
GAACGGCGAGGCAACCGATTCCCGGAAGATGGAAGTTCAAATCGGGGGTGCGGTGAC
CCTGGAGTGCGACGGTGGTTGTTGGGGACACGGTCCTGGAATGGATCCAGTTGGTGGT
CCCGGTCCACTGGCTTTGAGTCGAGTCGTTTACCAGGAAGCCGGCGAATATCGATGCG
TCGCGCCCGACAGGAAAATGCAAGACACCTGGCGCGCCCAATTGCCGTATCACATCAA
GATCACCG

>novel_circ_000198
GCTCGGTGAACGAGGCCGGTGTTTTCACCCTTCGTAGCAGGCAGGAGGGTGGACGGC
GTGCTAGAAGAACCACGACGAGCACCACCAGCACAACGGCGTCCAGCTCGACGTTGA
TTTCTCTGTCGGACGAGGTGGCGCAGCCCGCGGCCCTTTTAGCCCCCGAAGAGGACTT
ATGGTCTACCAGTTCTTCCGCTACAACCAATGTTCCAGTCACCTTAGATACAGCTGCCG
CTTCGTCAAAGCCACCCTCGGAAATGGTGGTGAAACCTCACAGCAAGGTCATACTACC
GTGCGACCTGGAGGAAAATTACTCGAGGCTGCTGTTGCCCGGGGTCAGGAGCTACAG
AATACGGCCAGCGACATGGTTGCACGAGGGAAGTCCTGTAGATATGATTACGATCAAC
ACGAAGACGGAGATGAGCGGGACCGGACATAGATATATCGGCGACTCCATGACTGACG
CGTTGCACATAGACAACGTGAGATTAGAGGACGACGGTATATGGGGTTGCAGGCTCGA
GGACGATCAAGGGAAGATACTTTCCGGCAGGCCTGTGAAACTCGTCGTCCTCGAGGCA
CCCCGTGGGACGTACCTGCTGATAGATGGCCGTCGACTGGACCCCGGCAACCAGTTCG
TCCCTGTGAAGGAGGGGTCCGAATTGACGTTGGAGTGCGCCGCCGAGGGTGGGAACC
CGCCACCCGATTTGGCATGGGGGATGACTCTGTCTCAGGCCACTTTGGACGGTCCCGA
GCAACCGCCGGACAATCTGACCGTGTTGCCCTCGACATCCGGTAGACGCAGCGGGGC
ACACCTCAAGGTTCTCAGGGGTCACCACAACGCGACCATCGCCTGCGTCGCCCGCCAC
ATTACCCTCACCATACCCATGAACGCGAGCATTCTGTTGGACGTGCAATATACTCCATCG
TTCGCGATAACGCGTTTACCTGGTTTTGGAATCCCGATCGTCGAGGGGATGTCCGTCAG
CTTGAAATGCGAGGTGGACAGCAATCCGGCCAGCACGCCCATTTGGCAGCGTG

>novel_circ_000199
AGGCACCCCGTGGGACGTACCTGCTGATAGATGGCCGTCGACTGGACCCCGGCAACCA
GTTCGTCCCTGTGAAGGAGGGGTCCGAATTGACGTTGGAGTGCGCCGCCGAGGGTGG
GAACCCGCCACCCGATTTGGCATGGGGGATGACTCTGTCTCAGGCCACTTTGGACGGT
CCCGAGCAACCGCCGGACAATCTGACCGTGTTGCCCTCGACATCCGGTAGACGCAGCG
GGGCACACCTCAAGGTTCTCAGGGGTCACCACAACGCGACCATCGCCTGCGTCGCCC
GCCACATTACCCTCACCATACCCATGAACGCGAGCATTCTGTTGGACGTGCAAT

>novel_circ_000200
GCTCGGTGAACGAGGCCGGTGTTTTCACCCTTCGTAGCAGGCAGGAGGGTGGACGGC
GTGCTAGAAGAACCACGACGAGCACCACCAGCACAACGGCGTCCAGCTCGACGTTGA
TTTCTCTGTCGGACGAGGTGGCGCAGCCCGCGGCCCTTTTAGCCCCCGAAGAGGACTT
ATGGTCTACCAGTTCTTCCGCTACAACCAATGTTCCAGTCACCTTAGATACAGCTGCCG
CTTCGTCAAAGCCACCCTCGGAAATGGTGGTGAAACCTCACAGCAAGGTCATACTACC
GTGCGACCTGGAGGAAAATTACTCGAGGCTGCTGTTGCCCGGGGTCAGGAGCTACAG
AATACGGCCAGCGACATGGTTGCACGAGGGAAGTCCTGTAGATATGATTACGATCAAC
ACGAAGACGGAGATGAGCGGGACCGGACATAGATATATCGGCGACTCCATGACTGACG
CGTTGCACATAGACAACGTGAGATTAGAGGACGACGGTATATGGGGTTGCAGGCTCGA
GGACGATCAAGGGAAGATACTTTCCGGCAGGCCTGTGAAACTCGTCGTCCTCGAGGCA
CCCCGTGGGACGTACCTGCTGATAGATGGCCGTCGACTGGACCCCGGCAACCAGTTCG
TCCCTGTGAAGGAGGGGTCCGAATTGACGTTGGAGTGCGCCGCCGAGGGTGGGAACC
CGCCACCCGATTTGGCATGGGGGATGACTCTGTCTCAGGCCACTTTGGACGGTCCCGA
GCAACCGCCGGACAATCTGACCGTGTTGCCCTCGACATCCGGTAGACGCAGCGGGGC
ACACCTCAAGGTTCTCAGGGGTCACCACAACGCGACCATCGCCTGCGTCGCCCGCCAC
ATTACCCTCACCATACCCATGAACGCGAGCATTCTGTTGGACGTGCAAT

>novel_circ_000201
GCTCGGTGAACGAGGCCGGTGTTTTCACCCTTCGTAGCAGGCAGGAGGGTGGACGGC
GTGCTAGAAGAACCACGACGAGCACCACCAGCACAACGGCGTCCAGCTCGACGTTGA
TTTCTCTGTCGGACGAGGTGGCGCAGCCCGCGGCCCTTTTAGCCCCCGAAGAGGACTT
ATGGTCTACCAGTTCTTCCGCTACAACCAATGTTCCAGTCACCTTAGATACAGCTGCCG
CTTCGTCAAAGCCACCCTCGGAAATGGTGGTGAAACCTCACAGCAAGGTCATACTACC
GTGCGACCTGGAGGAAAATTACTCGAGGCTGCTGTTGCCCGGGGTCAGGAGCTACAG
AATACGGCCAGCGACATGGTTGCACGAGGGAAGTCCTGTAGATATGATTACGATCAAC
ACGAAGACGGAGATGAGCGGGACCGGACATAGATATATCGGCGACTCCATGACTGACG
CGTTGCACATAGACAACGTGAGATTAGAGGACGACGGTATATGGGGTTGCAGGCTCGA
GGACGATCAAGGGAAGATACTTTCCGGCAGGCCTGTGAAACTCGTCGTCCTCG

>novel_circ_000202
GGTTGCGACAACGGTCCGGTCCTACCGACACGCCAAGAAGCGGAGGCTCGGTGAACG
AGGCCGGTGTTTTCACCCTTCGTAGCAGGCAGGAGGGTGGACGGCGTGCTAGAAGAA
CCACGACGAGCACCACCAGCACAACGGCGTCCAGCTCGACGTTGATTTCTCTGTCGGA
CGAGGTGGCGCAGCCCGCGGCCCTTTTAGCCCCCGAAGAGGACTTATGGTCTACCAGT
TCTTCCGCTACAACCAATGTTCCAGTCACCTTAGATACAGCTGCCGCTTCGTCAAAGCC
ACCCTCGGAAATGGTGGTGAAACCTCACAGCAAGGTCATACTACCGTGCGACCTGGAG
GAAAATTACTCGAGGCTGCTGTTGCCCGGGGTCAGGAGCTACAGAATACGGCCAGCGA
CATGGTTGCACGAGGGAAGTCCTGTAGATATGATTACGATCAACACGAAGACGGAGAT
GAGCGGGACCGGACATAGATATATCGGCGACTCCATGACTGACGCGTTGCACATAGAC
AACGTGAGATTAGAGGACGACGGTATATGGGGTTGCAGGCTCGAGGACGATCAAGGG
AAGATACTTTCCGGCAGGCCTGTGAAACTCGTCGTCCTCG

>novel_circ_000203

TTACGAAGCTGAGGGAGCACTGGGACGAGATGAACTCGAAAATGATGCAGAGGAAGA
CGGAGTTGGACGCGATGCTCGGGGACAGCCAGCGATACGAGGCGAAAAGGAACGAG
GTGGAGGTCTGGTTGGCGCGAATGGAAACTCGGCTGGAGAAAATGCGGGCCGTCGGC
CACACCGCCGACGTTCTCGAGGCCCAGCTCCGGGAACAAAAGTCGTTCCACGCCGAG
CTCCACCAGTACAAGCATCAGATCGAGCAGTTCAATCAGCTCACGCAGAAGCTGATCG
CCGTTTATCAGGAGGACGACACGACCCGCGTGAAAAAGATGACCGAGACCATCAATC
AGAGATACAACAACCTCAACACAAGCATCATCAATCGAGGAAAACTGCTTCACTCCGC
GATGAACTCCCTCCACAACTTCGACCGGTCCCTGGACAAGTTCTTGGCTTGGTTGAGC
GAAGCTGAGTCCTCGATGGAGGGATTGGAAGCGGAGGCCGATCGGCTAGGCGGGCGA
CGAGACCAGGGTGCGCTCAGACGACCGCAACATCAGCTTAAG

>novel_circ_000204
GACGATCATCGCGGGTTCAGGAGGCAGCTCGAGGACAAACGACCGATCGTCGAGAAC
AATTTGCTGAGCGGACGGCAGTACATCGCCAACGAGCCGCCCCTCTCTGACACGTCGG
ATTCCGAAGCCGGAAGAGAATTGGATGGCGATTCGAGGGGCTACAGGAGCGCGGAGG
AGCAGGCACGCGAATTGACGCGCAGCATTCGCCGGGAAGTGAACAAACTTTCAGAGC
AGTGGAACGCCCTAATCGAACGTAGCGATGCGTGGAAGCGGAAACTCGACGACACCG
CTAAC

>novel_circ_000205
GATCGAGCGCGCACAAAAACGACGCGACAGGAAATGGAAGGCCGGCTCCCCTTTTGG
CGGAACCCAGATACGGCGCCGCGCCACAGCCATACGGATCCACGCCACAGGTAACTTT
CCCACCTGGTGCCGGTCACGATCGTTACGAGGAGAATTTCAATGGACCAGCCACGTAT
GCGGAGCCGTACGTGGAGCGTTACGGTATTAAGAGCGATTTCAGCGGTCGAGAACCAT
TACATCCTACGCCACCTCGACCTGGTTACGTGCCCACCTTGGGGCAGACGCCACCTTC
GAGCACGCAGGGTTCCGTGATGATGGTCTACGGGCTGCAACCGGATAAAGTCAACACG
GACAAACTGTTCAACTTGTTCTGCCTGTATGGAAATGTAACCAAGGTGAAGTTCTTGA
AGACGAAAGAGGGCTGCGCAATGATCCAGATGGGCGACAGCATAGCCGTGGAACGTT
GCCTACAGAATCTGAACAACGTGACGATCGGCACAGACGGAAGGTTGCAGCTCGGTT
TCTCGAAGCAGGCTTTCCTCTCGGACGTCACCAATCCCTACATTCTGCCCGACAAGAC
GGCGAGCTTCAAAGATTTCACAGGAAGCAAGAATAACAGGTTCCTAAATCCAGCGATG
GCCAATAAAAATAGAATACAACCACCCTCAAAGATAGTCCACTTTTTCAACACGCCCCC
GGACTTAACCGAGGAGACCGTGCATCGCGTGTTCGTGGAACGCGGTATCGAGGCGCC
GACGACGGTAAAACTGTTCCCCCTTAAATCGGAACGATCGTCGTCCGGCCTCATCGAG
TTCAGCAGCGTCGGAATCGCGGTAGCCGCCATCATGGAATGCAATCACACAGCATTGG
AAAACAGCAATGGCAAATTCCCATACATAATGAAACTGTGCTTCTCGTCGTCCCGCACC
ATACCAACGAGTTTCCCGCCCAGCAGCGGCAGCACGTCGAGCAATTCCGCCGGTAATG
GCCACGTCAAAATGCAGCAGGACTCGGAATAGAACGGCGAGGTCCAGGGCCAGCAGC
GTCAGCGTCAGCGCCAGCGTCCCTCTCTCGCCCTGCACCCGTTGTGTGCCCCGACGGG
CACGGCCCTGCACCCAGCCGCAGCGATTACACTCTCCCCGGTCTTACCGCCGCCCGTG
CCGCCTCCCCTGCCGCCCACCTGCGTTCTGATCGCCACTCCAACTCTCTATCCTACCGT
GCCACCGCCACCACCGCCTCCGTTGCCTACGTTCTGGCCGTACTGATGAGAAGAGCAG
CGGGCCTACTCGCCGGACGAGGCAGCAAAAGACGGAAAAAGAAAAGAAAAAAAAAA
ATATATATATATATATACATATATACATATACATGTATGTATACACATATGTGTGCATATATGT

GTATATATGTGTGTGTGTGTGTGTACGTGTGTGTGTATGTGTGTATGTATGTGCGTATATA
CGCCACGCGCTTGTCCTCGAATATTCATATTCTACTACTGCTGCTGCTGCTGCATACTGA
ATATCCTTTTATTATCGACTACCGTCGCTATTCCTCCTCGCCGCGTGGTGACCAAATCTCT
GGCAAAGATCAGACGCTGGTCGCTGGAAGAAGAATCGGTTCCGTTCGGGGGTCAGCG
GGCCGGAACTCGGCCTCGATCGAGGGTCGAGAG

>novel_circ_000206
GATCGAGCGCGCACAAAAACGACGCGACAGGAAATGGAAGGCCGGCTCCCCTTTTGG
CGGAACCCAGATACGGCGCCGCGCCACAGCCATACGGATCCACGCCACAGGTAACTTT
CCCACCTGGTGCCGGTCACGATCGTTACGAGGAGAATTTCAATGGACCAGCCACGTAT
GCGGAGCCGTACGTGGAGCGTTACGGTATTAAGAGCGATTTCAGCGGTCGAGAACCAT
TACATCCTACGCCACCTCGACCTGGTTACGTGCCCACCTTGGGGCAGACGCCACCTTC
GAGCACGCAGGGTTCCGTGATGATGGTCTACGGGCTGCAACCGGATAAAGTCAACACG
GACAAACTGTTCAACTTGTTCTGCCTGTATGGAAATGTAACCAAG

>novel_circ_000207
ATGCAGCCTACTCGTTGTTCACTTGACGGTGATGATGGATTCACGTGGAACCGAGTCA
GGGCCCGGTGAGAATGTGGGGCTTAGGCAGGTCTACGGCTCTTACACCCTCTCCCCCT
ACTACTGTCTGCGAAGAGTGAGAGTGATAGTCATCGGTCTCTGTGAGAACGCGGACAT
CAAATCGTCCGTTCTATCTAGAGTTCGGAGAGGAACGGGGTGATCGAGAGAACACAG
AACGCGGGGTTGATCACGGTAAGTCAGTTCATAAAACAAGTGTCTGTTCATAAAACCA
GTTTCTACCAGAACAGTTTTTTTTTTCTTTCTCTTATACCTTTCTCTCTTTCTCTTTCTCT
CTCTCTATATATATATATATATGTATATATGTACGTATATACGTATATATATATATATTTATTTCT
CTCTTCTCTTTTCGAATTCTAACTGTCCACCACCATCCATCCTGTGCCCGCGAGAACGG
GAGACTTAAATTTCCCGGATATAAATACCAGTTTGGAAAATCCTTTCCTCCGTTTTCACC
CGAGCGAATCTTGTTCGCGCTTCGACGAAAGGTGGAGTCGATTTGTGCTCTCTGATCC
GAGAGTGGCCGACCACCGAGTGAATACCGATTCGCGAATACCGCAGACACGGGAAAG
CAGTTCGGGATTTATCGGGGTTTTTTAAGTTGAGAATCGTGGAGAAGAAAGGTGGTC
GAGTCCCCACCGCACAAGGATTAAACGGAAGAAGTTATCGCGATTTAATAGATTTTTTT
TCAAGATTCGATATTTCTTTTTTTTTTCTTTCTTTTCTTTTCTTTTCAAACCGATTCGAAA
AGAATTAACCAGTAGCAAGAAATGCTACACGCTACCCACTCTTTTTCAACGACGCGAA
CAATAGCTCAAGTGGGAAAGCAATGGTGAGTTGGAAGAGTTACGGAATCGTGGAACG
TGACGTTTCGAACAAGGATTTTTCGCGATCGATAATAACCAGCAGTGAAAACCGGGGA
GGAAGGAAGAGAAAAGTCCTTCTGTGTTGCATATACACCCCTATATCGTGCTCGATAAG
CATCGCTGGAACTGATCACCGTTTTTTTTTTTTTTTTTTTTTTTTACCCTCGGAAACAC
GGTGATCGATGGCGCGTCAGTTTGTTTGCAAGAGCGATGGCCGAAGCACGATTAAAAA
CTCGGGATTAAGAAAGTTCGATCCTGTTCGAGCTTTTTTAAAGGTCCCTGGGGAATTGC
TATCTTTTTCCTCTCTTGTCGTCTCCGATCCAACGACCGTGATATTGGTTCTCTTTTTTTT
TTTTTTTTTTTTTTTGCTTTCGAACGTGACAAAATTTCCTGTGTTTCAATTTAATTTTGTT
GATCGAGAGAGAAAAACGGAGAACGTCCGAGAGGAATGAATATCGTTGCGCGGTGAT
GCTAACGCGATTGGAATGCCGGTGAACGTCCAATAATCAGTCAAAGCGTGAAGACCGT
AAGTAGAAATGCTTGGATAACAGGCGCAAAGAGAACGGATTGAGAAACGAATGTAGT
TGTGCTACTACACGATATACGTATGAACGCGTTCCACGTATATATACGTGGTTATTAATTA
ACGTGGCTACGTCACGATTGGTCACATTGTGTAAAGGATTATACGAAACGAAGCAATAT

TGCATTTCTAATTGCTCGCCGTTCATACGAAACACACGATAAACGATTCACCAGGGAAT
CGTTTCCAGATTCTATAAATATCACTGACAATCATTAAAAGCGTATTTTTCTTTTTTTTTT
TTTTCTTTCCCCTCCTCCTTTTTTTCCATTCCTCGAAATTTTATTGCGTAAAGTGAATTCG
TTACTCGTTTAATTATTGGAGGCGTGTGATTCATCAGAGCCGCGATAAAACGAAGTTTC
ATGCCTCGACCTTTCACCTTTCCTCGAACGCGTCCATACTGATATATTCGCATCGCATTTA
TAAGCCCTTTCTCGAACTGATTCCTGGAAATGAATCATTGGAAATAAATTTCTCGTCCTT
TTATTTTTTTTCCTTTTCTTATACAATTGCGCCTCGATAGTGGATACGTATTTTGCACTTG
ATACTAGAGATAAATTATTATTATTTATTCTCTTCATTTTCTCGAACGAGCCTCTGCGCTT
CTTTATTTTTATTTCGTTCAAATTTATCGTGTCGCACTCTTGTTAAAGAATCATCAACATC
GAAAATCTCCAAGACACTTATCCTCCTCGTTATAATTCCAATATGACATCTGTGCGTCAA
ATCACACTGTGATACTTCGTTATCTGAAAATTTCGTCGAAAAAAAAAAAAGAATTAATAA
TTAATATAAGAGATCCGATGGAAAACAAGCACGTTCCAATATTAGGTAATGCTTTATTCA
TGTAACGTGGCATCATATGCGTTATAAAAATGTAAAATAAAATAGAAGTAACAATACAG
AGGAGATTAAAATTAACATACACGTGTCGTCCATAAATTTGTTTTCCAATAATAAAAAA
AGAAAAAAAATAAACTCTTAAACTCTTTAACCATCGAGAGAGAAAAATGCACGCGAA
CATTTTATCTTTAGCTAAGATGCTCTTACTGTTCCTTTGAATACCGTCGAAATTAAACATT
CCCTCTGAAACATCACACTAGAATCAGAAATTCCGTTCCCCTTCGTTTGAACCTGTTGC
TCCCCCTTTCTCTTTAAACCGGCAACGGAAAACTTACCTGCACAACCACCTCGAAAAA
TCCTCCACGGTGACGAGCCGCCGTTACTTTACGATTATCACTGGGAGGAGATTCTTTCG
AACGGCGGTTGAAACGTTAGATTCAACGTTGATAAACGTTGGCTTGCGGGGCAACAAT
TACGAATGCATTAAATTCCAAGATGCGGCCAAGATGTGTTTAAGAAGCGACCCTAATTT
TCGCCCGTCACAAACTCTGCAACACTTCCTCTGGAATGGAGGCGATAACTCATCCCCC
GCAAACTTGCGTAATCGCAACTTTTATCGCGCTTGTATTCGCAGCGTGGGACGTTCCCC
GTGTTTATCCTAGAGGGAAAGAGGATGGTGGCCTAGGGGGTGATTTAAATTTCTTTCGA
GCTTAATTTTCATTCTCGTCTCTTCGTGATGATTTTTTTCCTCCCTTGGAAAAAGGCTTT
TTTAGAGGGTTGGGTTGTACAGAGGAGGAGGAGGAATCGATGTGACGATCACGGGTT
GAATTTGTCTCTGTTGTGTGTCGATTTGTTAACGCATTTTTTCCTTCCTGGAAAATCACA
TGAATTAGTTTCTCGAATAATATTAAACATTTTTATCTCGCAAATAAGCGAAAGGTATTTT
TAGAAAAATAAAAATTCCGGTATAGAAGTGAGAAGTGGTAGATTGAATTGTAGATCTGG
AGTATGTAGAGTGTGAATTTTAAAAATTCCATTTTCCATTCTAAAAAATGTAATTCGTTT
TGAAAAAAAGAAAAATAATAATAATAACGTGAATATATCTCTGACCTATCGCGATTAATC
ATATACACGAGTCGTTCTCGTATATATATATACACTCGTGTATGCAAATATAATAGTTTCG
AAGCGAAATGAAATATTAATAATTTTATGGAGTCGCGTAATCTAGTTTTCACGGTGAAAA
TATACCGCGTAACTATATTTTCTTTCGTGGTAAATAAATTGACATAATGGAACGGGGGAC
AACGGGAGATATGGGATAATTTTCCCGACTAAATGAAACATTCTCGTACATCTGGTGAA
ACGGCGGCCATAATAACGGAAACAAGTAGATTTCAGCAACATTATTAATCTTGCGCAAG
GTGTTTTAGCCCCTTTAGGTGATGCGATTAATCCAACCGTGAAATTTATTGTCATTATCTT
AATCCTTAAGCGTCAGCATTAATCAACTTCTGATATTCATCATAACTCTCTAATAATTCGA
ATTCGAAGCTCGAGATTGCCAAGGCAAATCCAGCTTTCCCTTAACTAATTTCTCCCGAG
ATAATGGGTATCCGTTTAACAATTTCTCGACAAAACCAGATTTCTCATTTTGCAATTTTG
CAATTCTTTCTCTGGAATAATGAAACGTAATATTTAAAAAAAAAAATTTCAGAGATGGAT
ACTCGACGAGAACAAAATTATTCTCGTTTGAAATTGAGCTTATCAATGCTCATTCCAGT
TCTCTGATAAAATTTCAAATATTCTTCAAGTATCTTCTATCAAAGATTATTATTATTACAAT
GTTTGACATAAAAAGTCACGTCAGAAATATCGGAAGGGTTAAAAAAACAAAAAGACT

CGACACTTTTCACCTCCCTCTATGTTATACCCTCGCAGATATCGACCAGGAATAACTAAC
CAAAACCATCGTGCACGCAATCAGGAACCGCATACGCTCGATCACTCACGATCATCCA
ATCCGTTTATAGCTTGCGTTCACGGTTTTGGGCGATGGGCTAACAGCCATCCATGTGGT
CGGTGTTCTCCTCGACCGAGGTAATTTTATGGGGTGCGCGTTGCCAGTCAGCATTGTAG
GTCGATACATAGGGGGCTCATAGTTGTTGTTGTTGGCCCGATGGAAACGCGTTTGAAGA
GAGAGATTGCACTCGTGGTGCAGCCTCGAAAAGAAACAAAGGGATAACGACACGACG
TTTCTGTCGTGACACGGCGCGGCCCCTCGTTGCACGCAACTGGATCGTTTGTAATTGTC
GTTTCGGTGCAAAAAAAAAAAAAAGGAAAAAAAAAAGGAGAAAAGGGGAAAAGAAGA
AAAAAAAGAGAGAAACGTTTTCATTACGCGTTGCCGTGGTTGTCCACTTCCAAGTACG
AGTCGCGTTTTTATTCCCCGTCGTCGATGATTGAAAATTTTGGAAACTAGCGGAGTTTC
GGGGTCACGAACTGTAACTTTAAAAACTACCCTCGAGCGTTGAAAAAAAAAAGCACT
CGTGTTTCAGTTTGTACGAGTTTTTCGAGAGAATCGGTCAGATGCACTTGAGAATGAGT
TTTTTTTTTTCTTTCTTTCTTTCGTTTTATGTAAAACTTGTTTGCCATCATATTTTCATCAA
TGTAAATTACAATTCCTGGATGTGATTGAATTGAATTTTTTTTCAACAGTTTTCTAATGT
GACTCTTCAATTTTTATTAGATACAGATTAATCTTTAAAAAAAAAAAAATTCCAAGGAAAT
TCTCTAAAAGCTGGAATCGTTAGAGTCAATTTACCATGCAACCTCATTTTAATCAGTTAA
AAAGAGCAATTGATTTTTTTTTCCTTTTTCTTTTTTTTTTTGGTCCAACGTTTCCCTCGTT
TCCAACGTTAAGCTGTTCATAGAGTAATACGCTCGTTATTCTGGCGCGTGTTAGAATTTC
GTCAACCTATCCATCGGTTCCAATACCTGATTATAATAGTCCTCGAAGAGAGGCTTGCGT
AGGGCAATTTATAAAGTTACAAGCCACGTTATGAAGGGTAATGGATCGTTAGTCTCGTG
GTGGATGGTCGAATCACGTCGCGGAACGTGTTGACGTTTACGCTGATGGATGCCATCTT
CCAACTCCTCCTTCCGTCATAACGCTCCTCTTTCGTCGGATCAACTCATTAGAATAGATG
GACGAAAATTGACAATTTGCAGCTGGATTAATAGAGATTTAATAATAGTTAAACCGGTT
AAATTCGTACAATCGCATTAATCCGCCGAATAATCCGTTTATTTAACGCGCATTTCATTAC
ACGAGAATAACACAATGACGCCTACGCAATGACGCGAGCACAATTCTTTGTTACGCGG
GTTATTACCGTCCTTAACAGTTCCCCCTCCCGTTTTTCGTTACACATCGCTGGAAAAAA
CGCGGAAAAAGACATAGGAGCGCGCGTTCGAAAATCCAGCGCAACACGAATAGCTCG
TGTTCCCAATTGAAAGTCGGGAAGTGCTCGTCGATCGAGGCGCAATAGAGGAACGTTT
TTGTCCGTCGGACACGCGATATCCAAAATCGCAATCTCCCAAGGTCCTCCGCGAGACCT
ACAAGTGCCAATATCGAGTTTCATGGACTTGTAAAAGCTTCCATATGACCCGGATGTAT
CCATCCCCGTATAGGAGCGAGCCCTAGCCTAAATTTTGCTTCGATGTTTCTCTCGTTATT
AAAAATATCCCCCGCCCTCTCAGACAGCGTAGATTTCGCCGTCCCGAAATTTTTACACC
ATTTCTCCTTCTCCCATCGACGACACGCCGATGAAGAGAATTCACTTTAATGAAAAAAG
TAATATCGGATTCTTCGACGGGGCGAGAAAAAAAGAAGAAGAAGAAGAAGAAGAAG
AAGAAGAAGAAGTAGAAGAGGAGGGAAAAAAAAATTACATTCCCATCCTTTCCAAAG
ATTTTCGATGGATATTCCAAGAATTTAACACTGTCATATTACACCGTCCTGAATTATACAC
CACAACTACACCCTATCCTATCCCCTATCTATTATTCACGTATCAACGTGACCGTTTTGA
AAACTTTGCGCGACACTCTCGATCTTGGTAAAATTATCCATCAAACCGTCAGATCGCTC
GTTCCGAACTTCATCGTATATATAACTTCACCCTACTTTCAATCTTCCATCCAGTTAACCA
ACATGCAAACCGAGTTAACCCACCACACCCCCTCCACGTCGTCCCCTAACGAACTTAA
CTCTCGAAACTCGTAAATTATCCGAAACAGACTGATCTCTCCTCTTTCTTTTCTTTTCTT
TTTTTCTTTAACGAAATATCAAACGTCGATTATTTATATGTGTTATACGAGAATAAGCGCG
GCAACTTTCACAACAGACTGGCAAAAGTTATCCGTGTCTTTTCGCGGAAGATTGCAGC
CGAAAGTTTCGCGTGGGGGAGGAACGGAGATCGATACGGCCATCACGACACGTAGGA

GATTCTCTGGCCAACGGGTAATAGCATGTTTAAATCTAAGAGGAGGACGGATGGAACC
GATTTTTGAAGGAGGAGTATCTCTCGTGTCTCTAACCTTTTACTTGGTCCAACCAAGCG
GATAAGTATTCGCGAATCGAATCACGATCGACGAGCCATTGATCCATCGATCGTCCGGT
TGATTTCGAATCATCGCGATCGTTGACCCTCCCCCAAAATTTTCTTATTACACTCCCGTT
TCCGGTTGTTGGGGAAAGGAAAGGAGCGGCCGCCTCTCGAATAGCTCGAGGCTCACG
TCATTGCTACGATTATTTGCTCGGCAGGTAAGTGTTTGTTTTCACGAGTTTGATAATCGA
ATCGTTCGGTTATGTAATTCCTCGGCCCGTTATGTATCGCCACTTTATCCCAACGGAAGT
TATCGGGAACGGAATTTATACGCCGCGAATTTATCGACCGTGCGACCGTGCTTCCCCGA
GTTTCGCTTCGCGTTAAATAGTTAGAATTAAATCCTTGGCCGGTTGTCGAGAAGATACG
ATTCCTTGAATTTAATCTAATTGACGTAATTGTAACGACGTACAAATTTTCCCTATTCTAT
GCAAATACGCGAATTATAATAATTAATTATAATAATGCGATGCATTGCTGAAATTGTAATT
TAATTAATACATTTGTAAAGACGTTTAAGGAAAACTTTAGATTAAAATTATCTCGGTCAT
AGGTCGTATTTCCTTTCTTCGCGTTAATTGAAAAATAATACATAATTCAAAATTCGCGAG
AAAAGTGCAACGTCGAAGGTGGCTTGCGTTTCGTAATTTCTGCCACGTTTAAAAAAAC
GTTACGTTCGCCGTTTATCGCCACCATACAGCGGTTGATTTCCCAGACGTCTTGATTTTC
CAGACGTATCTTTAGTAAAAGAGCGACGCGACGACTCAAGACAACACTCGGTCGAGT
GTTTTGATACGAATCTTCTTTTGTTACTTACTCTCTACTTGTCTCATTCCCGAGACATTGT
TCAAGAAAAGTATCTTTTATTCCAAGAAATTTTTCAAGTTCTGATGATGCATCACCGCTA
TCCTGCAAAAATATAGAACGAAACAATTATTTCTTCGAATCATCTTTAATTTTAGAGTCT
GTATATTTCCTCCCCAATATATTTTTTTTACAAAAGGAAGATTTCTCTCTTTTCCTCTTTC
TCTCCTTCGAGAAAAAGAATTTATAAATCAAAAATTCAAAAATCAAGATGAATAAATCG
TCGAGAAAAGAATCATCTTAAAATCTCCAGAAATTTTCAACGTTTCGATACTCGACAAA
ACAAGCCGATTGACTTCTCAAGTAAAACTTTGCTCACGATCTCGTCACCTGCCCTCCAA
TTTAATTTCAACATCCAAACGCAAACGTACAACGACGCGGTGACCGATACATTAATCAC
AACGCGTTACGCGTCTCTTTCCTCGCTTGGGTGAGGAAAAATCGTCGCAGGAAATCAT
CAAGGTGGCGGCGGTTTAACGACCGGAAATCCCTCCCCTTCTCCGTCGAATGCGAACG
AACGAAATAAACGGAATGGATCGTTCCGCGTATGGATCTATCCATTGGGATGAAGATGC
ATCGTCGTTCTCGGCGGGCCGTGTGCACATTGGTGTGTGTCGGCACGCGATATCGAATA
TTCGAAATCAATATACAGGTTGCACGGAGGGGGGAGGAGAAGGGTGCATCCCTCGATG
CAACGTGTGCGCGTGCACGCGCGACGTATATACAACTGTAATGCGGTTTAATATAGACC
AATTGTATATGCCGCTTGGTTTCGGCGAAGAGGCGGTGAGATTATATCTCGAGGCGACA
GGCGACAACAACGAAGGAAAACAAGTGACGATGGGCCTGTCTCTGCGCGCGCAATTG
TGTATGGTAAAATGAAGCGTGGCCGGAAGCGGGAGCGTTTTTTCGAGAGGACGGATAA
ACGGAATTGGGAATCGTTTCGTTAGATGGAATTTTTATGAATATTGTTATTAATTTTGCCC
GCGAAATTGGAACGTTAATGTTTATATCGCGTGTTATACCAAATTTGCCGTTTATTTTATC
GGCTTGTTTCGAGCGATTGTGATTCGGGAAGAGAATATATTTGAGGAAATATATAGGAG
AGGTATATATGGGAGAGAGGACGCTTTTGATTTATTATTAAACGGTTTTTCTTTCTCTCT
CTTCGAAAGAACGCTTTTTGATGAAACAGCGTAGGGGGGAGAGGTAAATTGATTAAGT
CGATCGAAGCAATATTTTCGCGCTGAAATAAAAAAAAAAAAAAAATAAAACGTCCAACT
CCATCGGAGGCGATATTTTCGCGTTAAATTGAGGATTCTTCGAAAAGCGAAGTTGATCA
CGATTCATAATGATTTTACTTGCGATATTTATAACGTTTTACTTCTCTAAAAAAATATATAA
TTAAAATTTCCTATTAATTCCCTCCTATTTATCACTTTGATTTACGTCACCGGTATACGGT
AGCTGGCAAGAATTAAGCAGCCCTCGTGGTAAAGGAAATTCAAACCCCCTCCTCCCTC
GAACTTTCCAACCACCTCGCTGCAACTCTTCGTCCACGGAGACCACCAAACACTCCCT

TTAAATCCTCGAGGACCAGGCCAGAGTTAGTTTGGAATTTTTCGATCCTGACGTGGATC
CTGACCTATCAAGAATCAGAGTTTCGTTCGAACCCTTTGCGGCCGTGTTTGTATCCCGG
GAGACCGTCCCTCCCTCTCTCTCCCCCCGTAACTGCCGCGCTAAACTGGCCCCAAACT
GTCAAAATCTCGCGTGTCTCCCCGGAATATCCGGCTATATCTTTGACTCATCCGAGTTAT
TCCCCTTCCCTCTCCAAGGAGATCGAGGTTGACACCACAGATGGCAGATTTCGCACAA
CACGAGCCGTGTTTCACTAGTCGGCTTGTTTCTGACGATCCGGACGCGAGAGATGATC
CCCCGAAAGGGGATCGCGCGGGAATTGATTAATCCGCGCCACGTCGCGTCCGTCCGTT
AGCTTTGCTAACTTATTCACAGGATTCGTTCTACGGTTTGCGCGCCGCCAATCGAGAGG
AAAACCGATTCAATCCGTAGATTGAATTTCGCGCAATTCCGCTTGGCGAAACAAATTGC
GCCTCGGCCAATCCCGATACACACACCGGAATAAAATAAAACCTGGGGGGAGGACCTG
CTCGTTCCGTATACTATTTCTATAAATGGAAACCGAGGAAGGAGGGGAAGGAAAAAAA
AAAAGAACGGGAGACAAGAAGATCCCTGATCGATATCGATCGATCGATTCCAGAAGTT
TTGCTTGCTTGGTTGCCAGCTTGCTTGCGATCGTTCGGAAACAAAACGATGGATAGTAT
AATTGAGATGGTTTTTTTCTCTTTTCTTTTTTACAGGATCGAGCGCGCACAAAAACGAC
GCGACAGGAAATGGAAGGCCGGCTCCCCTTTTGGCGGAACCCAGATACGGCGCCGCG
CCACAGCCATACG

>novel_circ_000208
GGCATGGCTGAGAGGAGCGATCGTCCTTGTCTTTCTGCTGGGACTCACCTGGACCTTC
GGCCTTCTCTACTTGAACCAAGAATCCGTAGCGATGGCGTACATCTTCACCATATTGAAT
AGCCTGCAGGGGCTGTTTATCTTTGTGTTCCATTGCGTTCAGAACGAGAAGGTGAGGA
AGGAGTACAGGAAGTTCGTGCGCCGCCACTCCTGGTTGCCCAAGTGCCTCAGGTGCTC
CAAGACGGTGGCCGGAAGCTCCGGTGGCTCTTCCGGCACCAGCGGCGGCGGTGGTGG
CGCGAATGGCGGCAAGGACTTCGGCTCGCACAACACCTCGTCGAATCCGTCCGCGCCC
ACCACGGACAGCTCCGGATTGTCGCCGCACGCCGCCTCCAATGTGGGTACAACAACGA
CCACGTCACCGCCCAACAATCACAACAACCTGACCGCCGCCTGTAACAATACGAATCT
TTCGATAAACGTGAACCTGAACAATTTGTCGCTGAGGTCGCCGGTCGGGGCACACCAG
ATGCACATGATGGCACCTAGCAACCACAATAGGCATCCCGCTCAGCAAG

>novel_circ_000209
CGGCAACGACCACGACAACGTCCCGTCCGAGTCCTCCGTGGTTCATGACTTCTCAGCC
GAGCGTTTGGAGCACGGCTAAGCCGACTGTCAGGCCTCCCTCGAGGATTACGACACCC
TCGGCTCAGCAACCGCCGCAGCCACCAGCACCGTCCACCCCGGCGCTCCCAATAACCA
CCACTCCAAGTCCTACGTCGACGAGGTCGCCGCTCGATATGGAGGTCGAAGTGGACCA
AGACTTAATATCTCCTGCGAACGTTCCGTCTGCGAATGCACCGGCGATGCCTCCGATCG
TTCCCGAGACCACTCAGGCCACAACCACGTCGACCTTTGCCCCTTGGAGAACGAGTC
GAAGGACCACTGTGCCGAGTACCTTATATCCGGAGTCGAGCTCGAACGGGCAGTGGTA
CGACTACGGCAGACAAGTGTGTCCCCCGGTGATAGCCAGGAACCTGTCGTGGAACAC
GACCAGGGCGGGCGACGTAGCTGTTCAGAGTTGTCCGGGCGGCGCGAACGGTTTGGC
GCGTTGGAGATGCCTGGCCAGGGGAGACACGGCCTTCTGGCACAGGGACAGCCCGGA
TCTGAGCGAGTGCCGCTCCGTTTGGCTGACCACGCTCGAGAACAGGGTGACGGAGGG
CGACGTGATCCTCGGGATCAGCCGCGAGCTGTCGCAGGTGACCAACAACAGCAGAGG
GTTGTACGGTGGCGACATGATGATCACGACCAAGATCATCAAGAACATGGCGGAGAAG
ATGGCGCAGGATATAAGAACGTATCAGGACGCGAATCAGAGGGAGGTGAGCGTGACC

GAGCTGCTCCAAGGTGTCGTGAGGACGGGTAGCAACCTGCTCGACAAGGCGCAAATG
GCGTCCTGGAAGGATCTGAGCCATCAGGAGCAGATGAGAGTCGCGACCTCTTTATTGA
TCGGGCTCGAGGAGAACGCGTTTTTGCTCGCCGACACGTTGATGCACGAGAAAACGA
TCACTCACGAGGGAAGGAACATACTGATGGAAGTACGCGTGTTGGATGCACGGAACAT
CGGTGACGATTTAGAGATATTCCCGACAGAGGCGGCTCAACAGAGGTGGACAGCCTCG
AACGATCGTGTCGAGTTAACTAGAGGAGCTTTGCTAGAGAACAGCGAGGGTGGCATTG
TCCGTTTGGTCTTCATGGCCTTTGACAGGCTCGAGGAGATCTTGCAGCCGCAAGCCGA
GGTGTCATCGTTGGTCATGAACGACGAGCAGCAACCATTACCGAAAAGGAACACGAC
CAGGCTTCTAAATAGCAAAGTGATCTCCGCGTCTTTAGGCAAAGGAAGGCATATTCAG
CTTAACGAGCCTGTCAGAGTCTATTTCAAGCATCTATCTATAGAGAACGTTACCAATCC
GACTTGTGTGTTCTGGGATTACATCTTAAGCGCTTGGTCAGAGGAGGGTTGCGAAATAC
GGAAAACCAACGAGACTCACACAGTGTGCGAGTGCAATCATCTTACGAATTTCGCCGT
TCTGATGGACGTTCACGCTGTCAGATTGGACATAGCTCACCAGGTCGCGTTGCAAATCA
TCACATACATCGGCTGCATCATTTCCGTGGTTTGCCTGGTTCTAGCAATTTTGACCTTTC
AATTATTTCGCGGACTGAAGTCGGACAGAACGACCATCCACAAGAATCTCTGCGTGTG
CCTGTTGATCGCGGAAATTTTATTCGTTTGCGGAATCGGGCAAACGAACCAGAGAATT
GTCTGCGGTATCGTCGCTGGATTATTGCATTTCTTTTTCCTCTGCGCATTCGCCTGGATG
TTCCTCGAAGGCTTCCAATTATACGTGATGCTGATCGAGGTGTTCGAAGCGGAAAAATC
AAGGTTACGCTGGTATTATCTGATCGCCTACGGTGCTCCGTTACTGGTCGTGGCCATTTC
TTGTATAATCGATCCTCTCAGCTACGGAACCGATCGATATTGCTGGCTACGGGCGGACA
ACTACTTCATCTTCAGCTTCGTTGGACCAGTGATACTCGTTATATTGGCGAACTTGGTAT
TTCTTTCGATGGCGATTTATATGATGTGCCGGCACGCGAACACAACCGTAGCGATGAAG
AGTAAGGAGCACTCACGATTGGCCAGCGCAAGTGGAAAGGAAGAGAATGCTCTTCCC
AACAAATTGCAAGCGCACTTGGCATGGCTGAGAGGAGCGATCGTCCTTGTCTTTCTGC
TGGGACTCACCTGGACCTTCGGCCTTCTCTACTTGAACCAAGAATCCGTAGCGATGGC
GTACATCTTCACCATATTGAATAGCCTGCAGGGGCTGTTTATCTTTGTGTTCCATTGCGT
TCAGAACGAGAAGGTGAGGAAGGAGTACAGGAAGTTCGTGCGCCGCCACTCCTGGTT
GCCCAAGTGCCTCAGGTGCTCCAAGACGGTGGCCGGAAGCTCCGGTGGCTCTTCCGG
CACCAGCGGCGGCGGTGGTGGCGCGAATGGCGGCAAGGACTTCGGCTCGCACAACAC
CTCGTCGAATCCGTCCGCGCCCACCACGGACAGCTCCGGATTGTCGCCGCACGCCGCC
TCCAATGTGGGTACAACAACGACCACGTCACCGCCCAACAATCACAACAACCTGACC
GCCGCCTGTAACAATACGAATCTTTCGATAAACGTGAACCTGAACAATTTGTCGCTGAG
GTCGCCGGTCGGGGCACACCAGATGCACATGATGGCACCTAGCAACCACAATAGGCAT
CCCGCTCAGCAAG

>novel_circ_000211
CGGCAACGACCACGACAACGTCCCGTCCGAGTCCTCCGTGGTTCATGACTTCTCAGCC
GAGCGTTTGGAGCACGGCTAAGCCGACTGTCAGGCCTCCCTCGAGGATTACGACACCC
TCGGCTCAGCAACCGCCGCAGCCACCAGCACCGTCCACCCCGGCGCTCCCAATAACCA
CCACTCCAAGTCCTACGTCGACGAGGTCGCCGCTCGATATGGAGGTCGAAGTGGACCA
AGACTTAATATCTCCTGCGAACGTTCCGTCTGCGAATGCACCGGCGATGCCTCCGATCG
TTCCCGAGACCACTCAGGCCACAACCACGTCGACCTTTGCCCCTTGGAGAACGAGTC
GAAGGACCACTGTGCCGAGTACCTTATATCCGGAGTCGAGCTCGAACGGGCAGTGGTA
CGACTACGGCAGACAAGTGTGTCCCCCGGTGATAGCCAGGAACCTGTCGTGGAACAC

GACCAGGGCGGGCGACGTAGCTGTTCAGAGTTGTCCGGGCGGCGCGAACGGTTTGGC
GCGTTGGAGATGCCTGGCCAGGGGAGACACGGCCTTCTGGCACAGGGACAGCCCGGA
TCTGAGCGAGTGCCGCTCCGTTTGGCTGACCACGCTCGAGAACAGGGTGACGGAGGG
CGACGTGATCCTCGGGATCAGCCGCGAGCTGTCGCAGGTGACCAACAACAGCAGAGG
GTTGTACGGTGGCGACATGATGATCACGACCAAGATCATCAAGAACATGGCGGAGAAG
ATGGCGCAGGATATAAGAACGTATCAGGACGCGAATCAGAGGGAGGTGAGCGTGACC
GAGCTGCTCCAAGGTGTCGTGAGGACGGGTAGCAACCTGCTCGACAAGGCGCAAATG
GCGTCCTGGAAGGATCTGAGCCATCAGGAGCAGATGAGAGTCGCGACCTCTTTATTGA
TCGGGCTCGAGGAGAACGCGTTTTTGCTCGCCGACACGTTGATGCACGAGAAAACGA
TCACTCACGAGGGAAGGAACATACTGATGGAAGTACGCGTGTTGGATGCACGGAACAT
CGGTGACGATTTAGAGATATTCCCGACAGAGGCGGCTCAACAGAGGTGGACAGCCTCG
AACGATCGTGTCGAGTTAACTAGAGGAGCTTTGCTAGAGAACAGCGAGGGTGGCATTG
TCCGTTTGGTCTTCATGGCCTTTGACAGGCTCGAGGAGATCTTGCAGCCGCAAGCCGA
GGTGTCATCGTTGGTCATGAACGACGAGCAGCAACCATTACCGAAAAGGAACACGAC
CAGGCTTCTAAATAGCAAAGTGATCTCCGCGTCTTTAGGCAAAGGAAGGCATATTCAG
CTTAACGAGCCTGTCAGAGTCTATTTCAAGCATCTATCTATAGAGAACGTTACCAATCC
GACTTGTGTGTTCTGGGATTACATCTTAAGCGCTTGGTCAGAGGAGGGTTGCGAAATAC
GGAAAACCAACGAGACTCACACAGTGTGCGAGTGCAATCATCTTACGAATTTCGCCGT
TCTGATGGACGTTCACGCTGTCAGATTGGACATAGCTCACCAGGTCGCGTTGCAAATCA
TCACATACATCGGCTGCATCATTTCCGTGGTTTGCCTGGTTCTAGCAATTTTGACCTTTC
AATTATTTCGCGGACTGAAGTCGGACAGAACGACCATCCACAAGAATCTCTGCGTGTG
CCTGTTGATCGCGGAAATTTTATTCGTTTGCGGAATCGGGCAAACGAACCAGAGAATT
GTCTGCGGTATCGTCGCTGGATTATTGCATTTCTTTTTCCTCTGCGCATTCGCCTGGATG
TTCCTCGAAGGCTTCCAATTATACGTGATGCTGATCGAGGTGTTCGAAGCGGAAAAATC
AAGGTTACGCTGGTATTATCTGATCGCCTACGGTGCTCCGTTACTGGTCGTGGCCATTTC
TTGTATAATCGATCCTCTCAGCTACGGAACCGATCGATATTGCTGGCTACGGGCGGACA
ACTACTTCATCTTCAGCTTCGTTGGACCAGTGATACTCGTTATATTGGCGAACTTGGTAT
TTCTTTCGATGGCGATTTATATGATGTGCCGGCACGCGAACACAACCGTAGCGATGAAG
AGTAAGGAGCACTCACGATTGGCCAGCGCAAGTGGAAAGGAAGAGAATGCTCTTCCC
AACAAATTGCAAGCGCACTTGGCATGGCTGAGAGGAGCGATCGTCCTTGTCTTTCTGC
TGGGACTCACCTGGACCTTCGGCCTTCTCTACTTGAACCAAGAATCCGTAGCGATGGC
GTACATCTTCACCATATTGAATAGCCTGCAGGGGCTGTTTATCTTTGTGTTCCATTGCGT
TCAGAACGAGAAGGTGAGGAAGGAGTACAGGAAGTTCGTGCGCCGCCACTCCTGGTT
GCCCAAGTGCCTCAGGTGCTCCAAGACGGTGGCCGGAAGCTCCGGTGGCTCTTCCGG
CACCAGCGGCGGCGGTGGTGGCGCGAATGGCGGCAAGGACTTCGGCTCGCACAACAC
CTCGTCGAATCCGTCCGCGCCCACCACGGACAGCTCCGGATTGTCGCCGCACGCCGCC
TCCAAT

>novel_circ_000212
TGATGGAAGTACGCGTGTTGGATGCACGGAACATCGGTGACGATTTAGAGATATTCCCG
ACAGAGGCGGCTCAACAGAGGTGGACAGCCTCGAACGATCGTGTCGAGTTAACTAGA
GGAGCTTTGCTAGAGAACAGCGAGGGTGGCATTGTCCGTTTGGTCTTCATGGCCTTTG
ACAGGCTCGAGGAGATCTTGCAGCCGCAAGCCGAGGTGTCATCGTTGGTCATGAACG
ACGAGCAGCAACCATTACCGAAAAGGAACACGACCAGGCTTCTAAATAGCAAAGTGA

TCTCCGCGTCTTTAGGCAAAGGAAGGCATATTCAGCTTAACGAGCCTGTCAGAGTCTAT
TTCAAGCATCTATCTATAGAGAACGTTACCAATCCGACTTGTGTGTTCTGGGATTACATC
TTAAGCGCTTGGTCAGAGGAGGGTTGCGAAATACGGAAAACCAACGAGACTCACACA
GTGTGCGAGTGCAATCATCTTACGAATTTCGCCGTTCTGATGGACGTTCACGCTGTCAG
ATTGGACATAGCTCACCAGGTCGCGTTGCAAATCATCACATACATCGGCTGCATCATTT
CCGTGGTTTGCCTGGTTCTAGCAATTTTGACCTTTCAATTATTTCGCGGACTGAAGTCG
GACAGAACGACCATCCACAAGAATCTCTGCGTGTGCCTGTTGATCGCGGAAATTTTATT
CGTTTGCGGAATCGGGCAAACGAACCAGAGAATTGTCTGCGGTATCGTCGCTGGATTA
TTGCATTTCTTTTTCCTCTGCGCATTCGCCTGGATGTTCCTCGAAGGCTTCCAATTATAC
GTGATGCTGATCGAGGTGTTCGAAGCGGAAAAATCAAGGTTACGCTGGTATTATCTGAT
CGCCTACGGTGCTCCGTTACTGGTCGTGGCCATTTCTTGTATAATCGATCCTCTCAGCTA
CGGAACCGATCGATATTGCTGGCTACGGGCGGACAACTACTTCATCTTCAGCTTCGTTG
GACCAGTGATACTCGTTATATTGGCGAACTTGGTATTTCTTTCGATGGCGATTTATATGAT
GTGCCGGCACGCGAACACAACCGTAGCGATGAAGAGTAAGGAGCACTCACGATTGGC
CAGCGCAAG

>novel_circ_000214
CGGCAACGACCACGACAACGTCCCGTCCGAGTCCTCCGTGGTTCATGACTTCTCAGCC
GAGCGTTTGGAGCACGGCTAAGCCGACTGTCAGGCCTCCCTCGAGGATTACGACACCC
TCGGCTCAGCAACCGCCGCAGCCACCAGCACCGTCCACCCCGGCGCTCCCAATAACCA
CCACTCCAAGTCCTACGTCGACGAGGTCGCCGCTCGATATGGAGGTCGAAGTGGACCA
AGACTTAATATCTCCTGCGAACGTTCCGTCTGCGAATGCACCGGCGATGCCTCCGATCG
TTCCCGAGACCACTCAGGCCACAACCACGTCGACCTTTGCCCCTTGGAGAACGAGTC
GAAGGACCACTGTGCCGAGTACCTTATATCCGGAGTCGAGCTCGAACGGGCAGTGGTA
CGACTACGGCAGACAAGTGTGTCCCCCGGTGATAGCCAGGAACCTGTCGTGGAACAC
GACCAGGGCGGGCGACGTAGCTGTTCAGAGTTGTCCGGGCGGCGCGAACGGTTTGGC
GCGTTGGAGATGCCTGGCCAGGGGAGACACGGCCTTCTGGCACAGGGACAGCCCGGA
TCTGAGCGAGTGCCGCTCCGTTTGGCTGACCACGCTCGAGAACAGGGTGACGGAGGG
CGACGTGATCCTCGGGATCAGCCGCGAGCTGTCGCAGGTGACCAACAACAGCAGAGG
GTTGTACGGTGGCGACATGATGATCACGACCAAGATCATCAAGAACATGGCGGAGAAG
ATGGCGCAGGATATAAGAACGTATCAGGACGCGAATCAGAGGGAGGTGAGCGTGACC
GAGCTGCTCCAAGGTGTCGTGAGGACGGGTAGCAACCTGCTCGACAAGGCGCAAATG
GCGTCCTGGAAGGATCTGAGCCATCAGGAGCAGATGAGAGTCGCGACCTCTTTATTGA
TCGGGCTCGAGGAGAACGCGTTTTTGCTCGCCGACACGTTGATGCACGAGAAAACGA
TCACTCACGAGGGAAGGAACATAC

>novel_circ_000215
CGGCAACGACCACGACAACGTCCCGTCCGAGTCCTCCGTGGTTCATGACTTCTCAGCC
GAGCGTTTGGAGCACGGCTAAGCCGACTGTCAGGCCTCCCTCGAGGATTACGACACCC
TCGGCTCAGCAACCGCCGCAGCCACCAGCACCGTCCACCCCGGCGCTCCCAATAACCA
CCACTCCAAGTCCTACGTCGACGAGGTCGCCGCTCGATATGGAGGTCGAAGTGGACCA
AGACTTAATATCTCCTGCGAACGTTCCGTCTGCGAATGCACCGGCGATGCCTCCGATCG
TTCCCGAGACCACTCAGGCCACAACCACGTCGACCTTTGCCCCTTGGAGAACGAGTC
GAAGGACCACTG

>novel_circ_000216
GTAGTGGAATGTACAACTCGGATGCAGAAGCGTGGACATTTTTGTATAGATTATCTTTC
GTGTAACGTTATCAGTTCGCAATGGAATGGAATAGAATAGTAAACGCTCTCGAAAACA
GAAACAATTTTTCAACATGAACATCGGAGCTACGAGTTATCCAGCCGGTCCGCGCTTCC
AGACAGACGTATAGATCGCGGCCCATAAATAACAAGGAAGGGAAGGGCCCTCGTCTTT
AATACGATCCCCGTTTCCCGAACTCATAACCGTTGATCTTCCGCTGGAAATTCACGAGG
AAAATGCGACAAAAGTTCACCGCGCAAGAAGATGCACACGATGTAACATTCAACAAA
CACCGGTAGACAATGTTCCAAACTATACAACATACATTTTGACAAGTTCCAGTTTCGCT
TCGCAAGCAGCCGGAAATTGAACACCGGCTGTGTTTCCTGTATCCACGGCGGCGAGCC
AGATTCCGCGATGGTAGTTTTACCGCGCTTTCAAGACAAACCCTGAGACGATGAAACG
GATTTACACGGTAAGGCAGACACGGTTCACGCCACTCTTAAGTGGCGGCCATGATACT
TTCGGTAGTTCATCCTGTTCCTTCTTTGGATTAAATATTTCAAATTCTAAGTCTGTGTTAA
ATAGTCGGATACTATGCAATATGTGGTAATTTTTATTGGTGACAAACTCTTTAATTCTTTA
ATTAATATGAAATGATCTTTTAGATAAAATTATCAAGAAAGAAAATAAGAAGATCGAAA
TTGAAAAATTCATCACATATCGAATATATACACGTTCATGGATCATCTTTGAAAGGATTCT
AGAAGCAA

>novel_circ_000217
GTAGTGGAATGTACAACTCGGATGCAGAAGCGTGGACATTTTTGTATAGATTATCTTTC
GTGTAACGTTATCAGTTCGCAATGGAATGGAATAGAATAGTAAACGCTCTCGAAAACA
GAAACAATTTTTCAACATGAACATCGGAGCTACGAGTTATCCAGCCGGTCCGCGCTTCC
AGACAGACGTATAGATCGCGGCCCATAAATAACAAGGAAGGGAAGGGCCCTCGTCTTT
AATACGATCCCCGTTTCCCGAACTCATAACCGTTGATCTTCCGCTGGAAATTCACGAGG
AAAATGCGACAAAAGTTCACCGCGCAAGAAGATGCACACGATGTAACATTCAACAAA
CACCGGTAGACAATGTTCCAAACTATACAACATACATTTTGACAAGTTCCAGTTTCGCT
TCGCAAGCAGCCGGAAATTGAACACCGGCTGTGTTTCCTGTATCCACGGCGGCGAGCC
AGATTCCGCGATGGTAGTTTTACCGCGCTTTCAAGACAAACCCTGAGACGATGAAACG
GATTTACACGGTAAGGCAGACACGGTTCACGCCACTCTTAAGTGGCGGCCATGATACT
TTCGGTAGTTCATCCTGTTCCTTCTTTGGATTAAATATTTCAAATTCTAAGTCTGTGTTAA
ATAGTCGGATACTATGCAATATGTGGTAATTTTTATTGGTGACAAACTCTTTAATTCTTTA
ATTAATATGAAATGATCTTTTAGATAAAATTATCAAGAAAGAAAATAAGAAGATCGAAA
TTGAAAAATTCATCACATATCGAATATATACACGTTCATGGATCATCTTTGAAAGGATTCT
AGAAGCAAGTACAAAA

>novel_circ_000219
GTAGTGGAATGTACAACTCGGATGCAGAAGCGTGGACATTTTTGTATAGATTATCTTTC
GTGTAACGTTATCAGTTCGCAATGGAATGGAATAGAATAGTAAACGCTCTCGAAAACA
GAAACAATTTTTCAACATGAACATCGGAGCTACGAGTTATCCAGCCGGTCCGCGCTTCC
AGACAGACGTATAGATCGCGGCCCATAAATAACAAGGAAGGGAAGGGCCCTCGTCTTT
AATACGATCCCCGTTTCCCGAACTCATAACCGTTGATCTTCCGCTGGAAATTCACGAGG
AAAATGCGACAAAAGTTCACCGCGCAAGAAGATGCACACGATGTAACATTCAACAAA
CACCGGTAGACAATGTTCCAAACTATACAACATACATTTTGACAAGTTCCAGTTTCGCT
TCGCAAGCAGCCGGAAATTGAACACCGGCTGTGTTTCCTGTATCCACGGCGGCGAGCC

AGATTCCGCGATGGTAGTTTTACCGCGCTTTCAAGACAAACCCTGAGACGATGAAACG
GATTTACACGGTTGATTGAAGAGGAGTTATACAGTATCGACGTCAGCGACAATGCTAGG
CCGAAAGTTGTAGCGGGCGATGTCGGCAAATTGGTGAAAGGGTTGGCTTCAAACTGA
AATTGAGAGGGGTTGGACGTCGCCGGTACCCAACATCAGAAGGGCAGAGGCGGGATA
CGACGCTTCTGGGCTGTCGTGAACCCTGGGCTGGAGCCACCGGTGTACTTGGCAGAG
AGTGCTTTTCATCTCCGTAAATGGACCGGCGGTAAAGCAATTTTGGTACATTACCTATAC
CTGCTTTAACCCCTCCCCCGCCTTTTCTGTCAGACTTCCCGACCACCCCCATCGTCTTTC
ATCTTGCATCATCCGACTCTCGATCGCGGGGAACGTTTGATTTCGATGGGAAACGTCGT
AAAGGCATTAAAGGGGGTGGGGGAGGTATAGGGGTACAGGATCGGTGGCGGTGGTGG
TGGCCGCCAGACCCGTGAAACAAAGAAGCGATTTCGCTGTTTCCATCCACGTTGGCGC
CGGCGACGGCCTTTTTGTAAATGACCGTAATCGCGCGCCACCCTCCCTCTTTGAGAGA
AGTTAAAACCCTCTTCTCTCTCTCCCCTTCTCGCTCTTTCTATTTCTCTCGCTCTCTGCG
ACGAGTGACGTTGATCGAGCGGTTGCTCCAACTTTAGCGGAAATTTCAACGCGGCGTC
CAACAGTTTCTCAAACAATCGATCCGTAATGTGGCACCGGCGGCGGCTATTTTTAGTTG
CTTCTTTCTTCCTTCTGGACCCGGCTAACTTCTTCTCTATAATCGTGCCGTACTTTTAACC
TTTCACGTGAAGAACGCCAACGCGTCTCGTTGTGTTCCGGTTCGTCTATCGCGAATTTT
CTAAATATTTCTTTTCCCTTCTCTTTTTCCTCTCTTTTCTTTTCTTTTACTTCGTTTCAATC
TACGAGTTGATTCGTGTGTATAAAATTTTTACGTTCGATCTCTTTTTCTATCGACTATCTA
ATACGCGTGGAACATTTATAAAACTCGTTTATTCTTTTATAGATTGATGGAAATAACAAA
AGGAGAAGGGGATGGATGGTAAATTTTCAAAGGCTGCAACATCGATCTTAACGAGCAT
GAAAACGCGTACCGACGTTATTTCGGGCGACCTCGCGAGATATCCAATTCAAAAATCTC
GTTCGCAATCGAATCGTTGTTTAAATGTCGGCGACCCTCGTTGACCCAGTTCGCTTGCC
GTAGCGTTAGATTTACCGCAGCGAGAAAAAAGGAATGGAGAGAAGAATAAGAGAGAG
AGAGACAGAGAGAGAGAGAGAGAAAGAGAGGGAGAGGGAGAATTGGCTCGTAGCTA
GAAAGTATCAACGTTTAATCCAGCCTGATTGCATTCGGTCGTGCGACGATTTTACGAGC
GCGCTCAAAACGGCCTGGCAAACACATTTTCCTTTTCGCGAATTTTTTTCCTGCAATCA
CGACCGAGATGAGCAAACGTTCGTAAACGTTCGTGCATCGTTCCCTAGTTCGCTTCTG
ACCTGGCAACTAATTTTTGTCCACCAGGAGAAGAAATCCAGCCGAAACGAAGTGCCTG
GGGAGAGTTGACGAATTTATCGATTCGTGTACGGGGTAAAAATTGCACGCGTGCACAC
GAGAGATTTGAAACAGGTTTGTCGGAAATTGGAATATGCCTCGTGCGTATTTAATGAAA
ATATGATATTTTTAAATCGAATTTTTTTACTTTTTTTCTCTCTTTTTTTTTTTTTTCGCCAT
CGTAACATGTTTTGAAACATTTAGTGCTAAAAGCAATGCGCGAAATGTAATTAATCCTC
CCATGCGCAACATTTTCCGATTTTTCGTCTATAATAATATTGTTTTTTTTTTTTTATTATTT
CAGTTTATTCGATTATCGAAACTATAAGATCAAAAGCATCGATTTCGTTCAACAATATAT
TTTAATATTTGATTATTGTCGTTGGTTCACGCGGGAGGAGAAACCAATGAGACGCGTAA
AAATGAAAGATTTTTTATCGAGTCATAACAATCAATTCGATCAATTCTATATCGTTTATAT
ATCGGTTATTTAATTATTTAAATTCTTTTCTGTTTAAATTATACGTTTATCAACCTCGAATT
GAAAGTACGATAAAAATTTCTATTTTAAATTAATTAAACAAGAAAACTTCTAAATCTTCT
CAAGTTAATTAAAAAAAGAAAAACAAGTTTCTCACATAAATCTTTATTTACAAAGATTC
TATCGATTTCAAGAACAGGCAATCCATACTTCCAACTATCTCAGAAAGTGTCGCAAAAA
TCAATCGAATAGATGCGTAGCGAATTCATTTATCACAGAGTGAAATTTAGTTACGGATTC
TCATCGAGGCAAAGAAAAAGACGGCTAGACACGACTAGAGACAGAGAGAGAGAGAG
TAACTTTCCTCCGCGCGCTTTGCGGCCGTGAAGAAGGGGGTTGTGCCGCTTTTGTCGT
GCTGCACAGACGCGTGGAAAAGAAGAGGCCGATGGGCAGCCTCTTTAAAGAGTAATT

ACGCCTACACTCGATGTATCCTGCGCCCCGTGATATTCTTCATCGTTTTTTTTCAG

>novel_circ_000220
TGGAATGTACAACTCGGATGCAGAAGCGTGGACATTTTTGTATAGATTATCTTTCGTGTA
ACGTTATCAGTTCGCAATGGAATGGAATAGAATAGTAAACGCTCTCGAAAACAGAAAC
AATTTTTCAACATGAACATCGGAGCTACGAGTTATCCAGCCGGTCCGCGCTTCCAGACA
GACGTATAGATCGCGGCCCATAAATAACAAGGAAGGGAAGGGCCCTCGTCTTTAATAC
GATCCCCGTTTCCCGAACTCATAACCGTTGATCTTCCGCTGGAAATTCACGAGGAAAAT
GCGACAAAAGTTCACCGCGCAAGAAGATGCACACGATGTAACATTCAACAAACACCG
GTAGACAATGTTCCAAACTATACAACATACATTTTGACAAGTTCCAGTTTCGCTTCGCA
AGCAGCCGGAAATTGAACACCGGCTGTGTTTCCTGTATCCACGGCGGCGAGCCAGATT
CCGCGATGGTAGTTTTACCGCGCTTTCAAGACAAACCCTGAGACGATGAAACGGATTT
ACACGGTAAGGCAGACACGGTTCACGCCACTCTTAAGTGGCGGCCATGATACTTTCGG
TAGTTCATCCTGTTCCTTCTTTGGATTAAATATTTCAAATTCTAAGTCTGTGTTAAATAGT
CGGATACTATGCAATATGTGGTAATTTTTATTGGTGACAAACTCTTTAATTCTTTAATTAA
TATGAAATGATCTTTTAGATAAAATTATCAAGAAAGAAAATAAGAAGATCGAAATTGAA
AAATTCATCACATATCGAATATATACACGTTCATGGATCATCTTTGAAAGGATTCTAGAA
GCAA

>novel_circ_000221
ATTTTTCAACATGAACATCGGAGCTACGAGTTATCCAGCCGGTCCGCGCTTCCAGACAG
ACGTATAGATCGCGGCCCATAAATAACAAGGAAGGGAAGGGCCCTCGTCTTTAATACGA
TCCCCGTTTCCCGAACTCATAACCGTTGATCTTCCGCTGGAAATTCACGAGGAAAATGC
GACAAAAGTTCACCGCGCAAGAAGATGCACACGATGTAACATTCAACAAACACCGGT
AGACAATGTTCCAAACTATACAACATACATTTTGACAAGTTCCAGTTTCGCTTCGCAAG
CAGCCGGAAATTGAACACCGGCTGTGTTTCCTGTATCCACGGCGGCGAGCCAGATTCC
GCGATGGTAGTTTTACCGCGCTTTCAAGACAAACCCTGAGACGATGAAACGGATTTAC
ACGGTTGATTGAAGAGGAGTTATACAGTATCGACGTCAGCGACAATGCTAGGCCGAAA
GTTGTAGCGGGCGATGTCGGCAAATTGGTGAAAGGGTTGGCTTCAAACTGAAATTGAG
AGGGGTTGGACGTCGCCGGTACCCAACATCAGAAGGGCAGAGGCGGGATACGACGCT
TCTGGGCTGTCGTGAACCCTGGGCTGGAGCCACCGGTGTACTTGGCAGAGAGTGCTTT
TCATCTCCGTAAATGGACCGGCGGTAAAGCAATTTTGGTACATTACCTATACCTGCTTTA
ACCCCTCCCCCGCCTTTTCTGTCAGACTTCCCGACCACCCCCATCGTCTTTCATCTTGCA
TCATCCGACTCTCGATCGCGGGGAACGTTTGATTTCGATGGGAAACGTCGTAAAGGCA
TTAAAGGGGGTGGGGGAGGTATAGGGGTACAGGATCGGTGGCGGTGGTGGTGGCCGC
CAGACCCGTGAAACAAAGAAGCGATTTCGCTGTTTCCATCCACGTTGGCGCCGGCGAC
GGCCTTTTTGTAAATGACCGTAATCGCGCGCCACCCTCCCTCTTTGAGAGAAGTTAAAA
CCCTCTTCTCTCTCTCCCCTTCTCGCTCTTTCTATTTCTCTCGCTCTCTGCGACGAGTGA
CGTTGATCGAGCGGTTGCTCCAACTTTAGCGGAAATTTCAACGCGGCGTCCAACAGTT
TCTCAAACAATCGATCCGTAATGTGGCACCGGCGGCGGCTATTTTTAGTTGCTTCTTTCT
TCCTTCTGGACCCGGCTAACTTCTTCTCTATAATCGTGCCGTACTTTTAACCTTTCACGT
GAAGAACGCCAACGCGTCTCGTTGTGTTCCGGTTCGTCTATCGCGAATTTTCTAAATAT
TTCTTTTCCCTTCTCTTTTTCCTCTCTTTTCTTTTCTTTTACTTCGTTTCAATCTACGAGTT
GATTCGTGTGTATAAAATTTTTACGTTCGATCTCTTTTTCTATCGACTATCTAATACGCGT

GGAACATTTATAAAACTCGTTTATTCTTTTATAGATTGATGGAAATAACAAAAGGAGAA
GGGGATGGATGGTAAATTTTCAAAGGCTGCAACATCGATCTTAACGAGCATGAAAACG
CGTACCGACGTTATTTCGGGCGACCTCGCGAGATATCCAATTCAAAAATCTCGTTCGCA
ATCGAATCGTTGTTTAAATGTCGGCGACCCTCGTTGACCCAGTTCGCTTGCCGTAGCGT
TAGATTTACCGCAGCGAGAAAAAAGGAATGGAGAGAAGAATAAGAGAGAGAGAGAC
AGAGAGAGAGAGAGAGAAAGAGAGGGAGAGGGAGAATTGGCTCGTAGCTAGAAAGT
ATCAACGTTTAATCCAGCCTGATTGCATTCGGTCGTGCGACGATTTTACGAGCGCGCTC
AAAACGGCCTGGCAAACACATTTTCCTTTTCGCGAATTTTTTTCCTGCAATCACGACCG
AGATGAGCAAACGTTCGTAAACGTTCGTGCATCGTTCCCTAGTTCGCTTCTGACCTGGC
AACTAATTTTTGTCCACCAGGAGAAGAAATCCAGCCGAAACGAAGTGCCTGGGGAGA
GTTGACGAATTTATCGATTCGTGTACGGGGTAAAAATTGCACGCGTGCACACGAGAGA
TTTGAAACAGGTTTGTCGGAAATTGGAATATGCCTCGTGCGTATTTAATGAAAATATGAT
ATTTTTAAATCGAATTTTTTTTACTTTTTTTCTCTCTTTTTTTTTTTTTTCGCCATCGTAAC
ATGTTTTGAAACATTTAGTGCTAAAAGCAATGCGCGAAATGTAATTAATCCTCCCATGC
GCAACATTTTCCGATTTTTCGTCTATAATAATATTGTTTTTTTTTTTTTATTATTTCAGTTTA
TTCGATTATCGAAACTATAAGATCAAAAGCATCGATTTCGTTCAACAATATATTTTAATAT
TTGATTATTGTCGTTGGTTCACGCGGGAGGAGAAACCAATGAGACGCGTAAAAATGAA
AGATTTTTTATCGAGTCATAACAATCAATTCGATCAATTCTATATCGTTTATATATCGGTTA
TTTAATTATTTAAATTCTTTTCTGTTTAAATTATACGTTTATCAACCTCGAATTGAAAGTA
CGATAAAAATTTCTATTTTAAATTAATTAAACAAGAAAACTTCTAAATCTTCTCAAGTTA
ATTAAAAAAAGAAAAACAAGTTTCTCACATAAATCTTTATTTACAAAGATTCTATCGATT
TCAAGAACAGGCAATCCATACTTCCAACTATCTCAGAAAGTGTCGCAAAAATCAATCG
AATAGATGCGTAGCGAATTCATTTATCACAGAGTGAAATTTAGTTACGGATTCTCATCGA
GGCAAAGAAAAAGACGGCTAGACACGACTAGAGACAGAGAGAGAGAGAGTAACTTT
CCTCCGCGCGCTTTGCGGCCGTGAAGAAGGGGGTTGTGCCGCTTTTGTCGTGCTGCAC
AGACGCGTGGAAAAGAAGAGGCCGATGGGCAGCCTCTTTAAAGAGTAATTACGCCTA
CACTCGATGTATCCTGCGCCCCGTGATATTCTTCATCGTTTTTTTTTCAG

>novel_circ_000222
ATTTTTCAACATGAACATCGGAGCTACGAGTTATCCAGCCGGTCCGCGCTTCCAGACAG
ACGTATAGATCGCGGCCCATAAATAACAAGGAAGGGAAGGGCCCTCGTCTTTAATACGA
TCCCCGTTTCCCGAACTCATAACCGTTGATCTTCCGCTGGAAATTCACGAGGAAAATGC
GACAAAAGTTCACCGCGCAAGAAGATGCACACGATGTAACATTCAACAAACACCGGT
AGACAATGTTCCAAACTATACAACATACATTTTGACAAGTTCCAGTTTCGCTTCGCAAG
CAGCCGGAAATTGAACACCGGCTGTGTTTCCTGTATCCACGGCGGCGAGCCAGATTCC
GCGATGGTAGTTTTACCGCGCTTTCAAGACAAACCCTGAGACGATGAAACGGATTTAC
ACGGTTGATTGAAGAGGAGTTATACAGTATCGACGTCAGCGACAATGCTAGGCCGAAA
GTTGTAGCGGGCGATGTCGGCAAATTGGTGAAAGGGTTGGCTTCAAACTGAAATTGAG
AGGGGTTGGACGTCGCCGGTACCCAACATCAGAAGGGCAGAGGCGGGATACGACGCT
TCTGGGCTGTCGTGAACCCTGGGCTGGAGCCACCGGTGTACTTGGCAGAGAGTGCTTT
TCATCTCCGTAAATGGACCGGCGGTAAAGCAATTTTGGTACATTACCTATACCTGCTTTA
ACCCCTCCCCCGCCTTTTCTGTCAGACTTCCCGACCACCCCCATCGTCTTTCATCTTGCA
TCATCCGACTCTCGATCGCGGGGAACGTTTGATTTCGATGGGAAACGTCGTAAAGGCA
TTAAAGGGGGTGGGGGAGGTATAGGGGTACAGGATCGGTGGCGGTGGTGGTGGCCGC

CAGACCCGTGAAACAAAGAAGCGATTTCGCTGTTTCCATCCACGTTGGCGCCGGCGAC
GGCCTTTTTGTAAATGACCGTAATCGCGCGCCACCCTCCCTCTTTGAGAGAAGTTAAAA
CCCTCTTCTCTCTCTCCCCTTCTCGCTCTTTCTATTTCTCTCGCTCTCTGCGACGAGTGA
CGTTGATCGAGCGGTTGCTCCAACTTTAGCGGAAATTTCAACGCGGCGTCCAACAGTT
TCTCAAACAATCGATCCGTAATGTGGCACCGGCGGCGGCTATTTTTAGTTGCTTCTTTCT
TCCTTCTGGACCCGGCTAACTTCTTCTCTATAATCGTGCCGTACTTTTAACCTTTCACGT
GAAGAACGCCAACGCGTCTCGTTGTGTTCCGGTTCGTCTATCGCGAATTTTCTAAATAT
TTCTTTTCCCTTCTCTTTTTCCTCTCTTTTCTTTTCTTTTACTTCGTTTCAATCTACGAGTT
GATTCGTGTGTATAAAATTTTTACGTTCGATCTCTTTTTCTATCGACTATCTAATACGCGT
GGAACATTTATAAAACTCGTTTATTCTTTTATAGATTGATGGAAATAACAAAAGGAGAA
GGGGATGGATGGTAAATTTTCAAAGGCTGCAACATCGATCTTAACGAGCATGAAAACG
CGTACCGACGTTATTTCGGGCGACCTCGCGAGATATCCAATTCAAAAATCTCGTTCGCA
ATCGAATCGTTGTTTAAATGTCGGCGACCCTCGTTGACCCAGTTCGCTTGCCGTAGCGT
TAGATTTACCGCAGCGAGAAAAAAGGAATGGAGAGAAGAATAAGAGAGAGAGAGAC
AGAGAGAGAGAGAGAGAAAGAGAGGGAGAGGGAGAATTGGCTCGTAGCTAGAAAGT
ATCAACGTTTAATCCAGCCTGATTGCATTCGGTCGTGCGACGATTTTACGAGCGCGCTC
AAAACGGCCTGGCAAACACATTTTCCTTTTCGCGAATTTTTTTCCTGCAATCACGACCG
AGATGAGCAAACGTTCGTAAACGTTCGTGCATCGTTCCCTAGTTCGCTTCTGACCTGGC
AACTAATTTTTGTCCACCAGGAGAAGAAATCCAGCCGAAACGAAGTGCCTGGGGAGA
GTTGACGAATTTATCGATTCGTGTACGGGGTAAAAATTGCACGCGTGCACACGAGAGA
TTTGAAACAGGTTTGTCGGAAATTGGAATATGCCTCGTGCGTATTTAATGAAAATATGAT
ATTTTTAAATCGAATTTTTTTTACTTTTTTTCTCTCTTTTTTTTTTTTTTCGCCATCGTAAC
ATGTTTTGAAACATTTAGTGCTAAAAGCAATGCGCGAAATGTAATTAATCCTCCCATGC
GCAACATTTTCCGATTTTTCGTCTATAATAATATTGTTTTTTTTTTTTTATTATTTCAGTTTA
TTCGATTATCGAAACTATAAGATCAAAAGCATCGATTTCGTTCAACAATATATTTTAATAT
TTGATTATTGTCGTTGGTTCACGCGGGAGGAGAAACCAATGAGACGCGTAAAAATGAA
AGATTTTTTATCGAGTCATAACAATCAATTCGATCAATTCTATATCGTTTATATATCGGTTA
TTTAATTATTTAAATTCTTTTCTGTTTAAATTATACGTTTATCAACCTCGAATTGAAAGTA
CGATAAAAATTTCTATTTTAAATTAATTAAACAAGAAAACTTCTAAATCTTCTCAAGTTA
ATTAAAAAAAGAAAAACAAGTTTCTCACATAAATCTTTATTTACAAAGATTCTATCGATT
TCAAGAACAGGCAATCCATACTTCCAACTATCTCAGAAAGTGTCGCAAAAATCAATCG
AATAGATGCGTAGCGAATTCATTTATCACAGAGTGAAATTTAGTTACGGATTCTCATCGA
GGCAAAGAAAAAGACGGCTAGACACGACTAGAGACAGAGAGAGAGAGAGAGTAACTTT
CCTCCGCGCGCTTTGCGGCCGTGAAGAAGGGGGTTGTGCCGCTTTTGTCGTGCTGCAC
AGACGCGTGGAAAAGAAGAGGCCGATGGGCAGCCTCTTTAAAGAGTAATTACGCCTA
CACTCGATGTATCCTGCGCCCCGTGATATTCTTCATCGTTTTTTTTTCAGGTGAATACGT
GTTTGTTTGCCATTGTTCCATCCTCGAGGAGCAGCCACTTGAAGTCCCATACGCGTCGA
ATCCTTTAGAAAACTGCGACGAGAGGCGCGGGAGGATCGAAGAGACGAGACTTCATA
CGAGACTTCG

>novel_circ_000225
CTAAAATGGCGTATTCCGCGCCGAAGAACGTGGTCTCGTTGGCCGAGGGTATGCCGAA
CGAAGAAACCTTCCCTTTCACGGAGATCTCCATCAAACTTAACGATGGATCGTCTTTCA
CCCTAGACGAACGAGAATTGGGCGCAGCTTTGCAATATATTCCCACTCAGGGTTACCCG

CCGCTTCTTCAGACCCTGAGGGAGTTCCAGAGAAGGGCGCACGCACCTCCCTTATGGG
AGAGCCGCGACATCATAATCGTCTCCGGTTCTCAAGATGGATTAAGCAAAACTCTCGA
GGCCATACTCGAACCTGGCGACTCGCTCTTGGTGCACGATCCTTTCTATCCAGGCGTGG
ACATTGTG

>novel_circ_000226
CTAAAATGGCGTATTCCGCGCCGAAGAACGTGGTCTCGTTGGCCGAGGGTATGCCGAA
CGAAGAAACCTTCCCTTTCACGGAGATCTCCATCAAACTTAACGATGGATCGTCTTTCA
CCCTAGACGAACGAGAATTGGGCGCAGCTTTGCAATATATTCCCACTCAGGGTTACCCG
CCGCTTCTTCAGACCCTGAGGGAGTTCCAGAGAAGGGCGCACGCACCTCCCTTATGGG
AGAGCCGCGACATCATAATCGTCTCCGGTTCTCAAGATGGATTAAGCAAAACTCTCGA
GGCCATACTCGAACCTGGCGACTCGCTCTTGGTGCACGATCCTTTCTATCCAGGCGTGG
ACATTGTGGTCTCGCCGTATAAGATAGAGTTGATCCCGATCCCTCAGGATGAGCACGGT
TTGATTCCGGATATTTTGAGGGAAACGTTAAGAAACAGAAAGCTGTCCGGGAAGAAAA
TGCCGAAAATAATGTACATAAATGCCACCGGAATGAATCCCACCGGTGTCGTTATTCCT
TTGGAAAGACGAAAGGAGATCTATAGGATAGCTTGCGAATACAACTTCTTGATCCTGG
ACGATGATCCTTACCACTTCTTGCACTTCGAGGAGGTGGAGCCAAAATCGTTCTTATCA
CTGGACACCGAAGGAAGGGTAATTAGGCTTGACTCCTTCTCTAAAGTGGTCAGTAGCG
GTATCAGATTGGGCTTCATCACGGCGGCGGCTCCATTGATAGCCAGTATAGAGCTCCAC
TTGCAAAGCAGCCATCTCCACGCCCCTACGTTATCCCAGGCGATACTGTACAGGCTGAT
GAAATTATGGGGATACGACGGGATGATAAACCATTTCAAATGGATAAGGCATTTCTATA
AGAAACGGAGGGATATTATCGCTAGGTTCGCTGAAAAACACTTGAATG

>novel_circ_000227
GTGTGGTTTCAAAATCGTCGAGCAAAGTGGAAAAAACGGAAGAAGTGCGGCCCTGTT
TTTCGTTCCCCACCGGGTCCATTGCTGCCATCGCACGGGTTGCCCCCGTTTGGGCCGAT
GAGCTCCGATCTCTGTGGTGCTGGTATGTTTAGCGGACCTCCTGAAAGATGGACAATG
GGAACAGGTCTTGGACAACTGGGACAAGGAGGAATGGGAATGAGTGGTTTTGGCCAA
AGTTTAGGACAACTTGGTCAGCAAAGTACGGGGAGCCTCGGCTCGAGCTTGGGTCTTG
GTAATTCTGGACTCCCGATCAGCAGTCCGTCGCAAGCCGTCTATCAAGCCAGTTATGGA
CTGAATTCCCTTG

>novel_circ_000228
ATTATAAATAGTTATCAGAAAAAAGAAATATGCTAACGCGTCTCTTTGAAATTCACGGC
CGGTTTTGCGCTGGACATCCCCTAGAAGTCATTGTGACGACCTTCACACTTACGGCATG
CATTCTGAACATGGAGACAGGAAACGGACATCCAAGTGTTCCTTTGACTGCACATTGT
GGACCTGGTAGATGTAATTCTGATGATTTAAATGCAGCTGATGTTATAGTCATGACTATA
ATACGATGTTTAGCTATATTGTTTACTTATCATCAATTTCGCAATTTGCAAAAATTGGGTT
CAAAATATATTTTAG

>novel_circ_000230
GTGGCGTGGATCCACGTGGGTCGTCAGATGTTGTTGACCATCCACAAGCACGTGGTCG
TGAAGATACCGAGGTTCTCGGTGTCCCACGACAATCAGAAGACCTGGCTGCTTCACAT
CAACAACGTGCAACAAGACGACCGGGGCTATTATATGTGCCAATTAAACACGAACCCG

ATGATGAGCCAGGTCGGCTTCCTTCAAGTCGTCGTGCCGCCCAACATTTTGGACTCGTT
GTCCACCGAAAGCACGGTGGCGGTTCGTGAGAATCAGAATATCACGCTCACGTGCAAG
GCGGACGGATATCCAACACCGAAACTGATGTGGAAGAGGGAAGACGGGCAGAACATA
AACATCAATCGACATAAGAAAG

>novel_circ_000231
AAGAATAGTATGTATAAAGTACTTCACAATCAAGACATAAAACACGAGTGCTCGCGGTC
AGGGGGACTGCACGCTTATCCTGATCATTTCAGGGAACCTAAAAAGCTTTACCTCTAAA
CCAGCCGTCTGCTAAATGATCATTTAAAGGTTCCTGTCCTCTGGTAGGACTTTGTATATA
GTGTAAATATCGCTTTTCTAACAAAGGATCTCGCTCAAATAAAAGAAAAAAAAAAAAA
AAAAACGAGCAAGCTCGAAGAATCGAAACAAGTCACGCGGGGATTTCAAGGAGTCG
AGAGAATTCGAAAGATTGCACTATTCCAAACCCCATATATAATATCCGACGCATGCATCA
ACCTAGAACGCACTGACTCAGTCAACTAGTTCGCAAAACGCAACAAAAGAAATAAAG
GAAAGAGGAAGATTGTTGGGAGAGAAAGAACGAGTGAAAAGAACGAGAGCACAAGA
TGAAGCAACAAGCGTCGGAAGAATTCCCTTTATGGATAAAGTTACTTTCCGCAGGCAC
AGCAGCCTGTATCGCTGATTTAGCGACGTTTCCTTTGGACACCGCCAAAGTCAGAATG
CAGATTGCCGGAGAAAGTCGTCCTCTTCTATTGGCGACTACGGATGGCTCCATGCTCGC
GATGCGAAATACGCAACCAGGATTATGGAGGACAGTAAAGAACATCGTCAGACTCGAA
GGAGCAAG

>novel_circ_000232
AAGAATAGTATGTATAAAGTACTTCACAATCAAGACATAAAACACGAGTGCTCGCGGTC
AGGGGGACTGCACGCTTATCCTGATCATTTCAGGGAACCTAAAAAGCTTTACCTCTAAA
CCAGCCGTCTGCTAAATGATCATTTAAAGGTTCCTGTCCTCTGGTAGGACTTTGTATATA
GTGTAAATATCGCTTTTCTAACAAAGGATCTCGCTCAAATAAAAGAAAAAAAAAAAAA
AAAAACGAGCAAGCTCGAAGAATCGAAACAAGTCACGCGGGGATTTCAAGGAGTCG
AGAGAATTCGAAAGATTGCACTATTCCAAACCCCATATATAATATCCGACGCATGCATCA
ACCTAGAACGCACTGACTCAGTCAACTAGTTCGCAAAACGCAACAAAAGAAATAAAG
GAAAGAGGAAGATTGTTGGGAGAGAAAGAACGAGTGAAAAGAACGAGAGCACAAGA
TGAAGCAACAAGCGTCGGAAGAATTCCCTTTATGGATAAAGTTACTTTCCGCAGGCAC
AGCAGCCTGTATCGCTGATTTAGCGACGTTTCCTTTGGACACCGCCAAAGTCAGAATG
CAGATTGCCGGAGAAAGTCGTCCTCTTCTATTGGCGACTACGGATGGCTCCATGCTCGC
GATGCGAAATACGCAACCAGGATTATGGAGGACAGTAAAGAACATCGTCAGACTCGAA
GGAGCAAGGAGCCTGTACGGAGGTCTGTCCGCGGGACTCCAGAGACAGATGTGTTTC
GCCAGCATACGGTTGGGCCTCTACGACGGGGTGAAGTCACGCTACGCTGGAATCATCG
ACG

>novel_circ_000233
ACGTTGCCGTATCTCATGCAAAAAATATCTCCAGCGTTCGCATTGCCACTGTGCTCGTT
CAAGGTCGAGAGGCACAAGGAGTCCACCGCCAGGGTAGCCATATGGCGGCAGTTAGG
TGTTTCGAGAAGTTACACGATGGAGAGCAGTTTCTGCGGATGCGACCAGGGATTGCTC
GCGGGTCTGCATCTGGACACGAAACACTTGAACGCCATCGGCCAGGATTTCTGTCAGG
CTCTGTCAATGATGAAGGATTATACCGAGAATTGGAGTATTGACAAGTCGTTGAAAGAG
GAGTGTTGCCCGCTAAAATGCATTTCGAAGAAATCAAAAACCGTTGCTAAATGTATTCA

GGATAAGCCTGCGGCCCTTCATCTTGCCGCAATTCCTTAACGAATGATTAAGCAAAACT
TGATTTGAGGATTCCTATGGGAGAAGGTATTCCCGAGGAGTCAGGATGCATGGAAGAT
GACATTTCGTCTAGCTGCGAGTCAGACGAATCTGATTTTGAGACTTAAATAAATTTTAAT
TTACAACGATTCGACAACGATATCGACGATATCATAGCTTCTTTATAAATAGATCGGAAG

>novel_circ_000234
GTGTGGGCGAGGCTCGAGGAAGGGCACCTCGCGGAGGATTCTCGGGCGCAGCGAGG
GAGATCGTAGCACAAGAGTGGAAATAGGACAGACGAAAGATGGCGGCGTTCCCGGGG
AGGAGGAGGAGTAGCGGCGAGGGGTCGGCGAGGATGGGATGGTGGACGGTGTCGCA
CCAGCAGCAGCTCCGGCTTCTCCTCTCGTTCCTGGTCCTGTTGTCGCCCCGACTCCCCG
TCCGCTCGACACCCGCGGACATAGTGCAAAGGTTCCACGACCCGGAGGTGAAGAGGA
TGAACCATTTGGTGGTGGACAAGAACACGGGGCGGGTGTACGTGGGGGCGGTGAACC
GGCTTTACCAGCTCTCGCCCGACCTGAGCGTGGTCGTGAAGGAGGTGACCGGGCCGA
AAGGGGACTCGAACGCTTGCTCCATGATCGACTGTCCCAGGGAGACCCCGACCCGGC
TCGTCGACAACGTGAACAAAGCCCTGGTGATCGACTACACGACCACCAGGCTCATCTC
GTGCGGCAGCCTGTCCCAGGGCACGTGCAGGGTGCGCAATCTTCACAACATCTCGGAC
GTGGTGCAGGAGGTGAAGGAGGCCGTGGTCGCCAACAACGCGACCGCGTCGACGGT
CGCGTTCATAGCCCCGGGGCCCCCCAACCCCCCCGTCTCCCAGGTGATGTACGTGGGC
GTCACGTTCACCGGCAACTCGCCCTACCGATCCGAGGTGCCCGCGGTCAGCTCGCGCT
CGCTCGACAAGGACAGGATGCTCAACATCGCGGAGGCCGCCGTCACGACCGGGACCA
GGATGTACGTCAACTCGTTGTCGCGCGAGAGATACCCCATCAACTACGTGTACGGGTTC
AGCAGCGGCGGTTTCAGCTACTTCATGACCACGCAGATGAAGAACACCGATACGGCCG
TGTTCATATCCAAGTTGGTGCGGGTCTGCCACGACGACGAGCATTACTACTCGTACACC
GAGATCCCGATCAACTGCACCAACGACGGCACGTACTACAATCTGGTGCAGGCGGCCT
ACGTGGGCAAGGCCGGCTCGGTCCTCGCCGGGGATTTGGGCATCACCGCCCAGGACG
ACGTGCTGTTCGCCGTTTTCTCCGAGAGCAACACCACCAGCAGCGACATACCCAAGAA
CCATTCGGCGCTGTGCGTGTACTCGCTCAAGGCCATCCGAAGGAAGTTCATGACCAAC
ATACAGAAGTGCTTCAGCGGCGAGGGGCACAGGGGATTGGAGTACATCTCCCCGAGC
CACAAGTGCATCTTAACG

>novel_circ_000237
GTCGTGGTTGTATCTGTTTTGCACGCGATGCCACGGTCGCGACGACACCCCATCGTGG
GAGCCACCCGGTTGGAGGAAAGCCTGCCCCCAACCGTTTTGCCCGAGCTACAGAAAA
TTCGCCCGGCTGTTGTGCCTGTTCCTGCTGGGCCTGTTGCTGTGGGGCATCGTGTACAC
GATACTCGGCGAGGACGCCGCCCCGGGCGGCCAGCTTTTCGGCCTCGCCGTCCTCTGC
ATCGCGGCCCACTTCGGGGGATGGCTCTTCTCCTTGACCACCCTCCCCGCCCTGATCGG
TATGCTCATCACCGGGATCGTGATGCAGAACATCGGCATGGTGAGCATCGACAGAAAC
TACAACATCGTCGTCTCCAACTTGCG

>novel_circ_000239
AAAAAAACATTAAAGTACAATTGAAAAGACAACCTGCTGAACCAATGCCTGCACATAT
TGTATCAGGACGTGAAAATCCTATTTCACTTGCACCTATACCCTATGAATTTATTGCTTAA
AATTTAAATAAAAATGTTCTGAAAAAATTAA

>novel_circ_000240
AAAGGAAAAAAACATTAAAGTACAATTGAAAAGACAACCTGCTGAACCAATGCCTGC
ACATATTGTATCAGGACGTGAAAATCCTATTTCACTTGCACCTATACCCTATGAATTTATT
GCTTAAAATTTAAATAAAAATGTTCTGAAAAAA

>novel_circ_000242
ACTCAGAAAAGAAGCAAAGGAAAAAAACATTAAAGTACAATTGAAAAGACAACCTGC
TGAACCAATGCCTGCACATATTGTATCAGGACGTGAAAATCCTATTTCACTTGCACCTAT
ACCCTATGAATTTATTGCTTAAAATTTAAATAAAAAT

>novel_circ_000243
GTTGTCTTTTCAATTGTACTTTAATGTTTTTTTCCTTTGCTTCTTTTCTGAGTCTTTCATTT
TCTTTTACTCGTTTCAAAAAATCTTCTCTACATTTAGAATGAGTTAAATGTTCAATACGA
ACATTTATTCTTTTTGCAATAATTCGTCCAC

>novel_circ_000244
GAAAAAGAATTCGACTGGGATTCAAAAACACAAGGACTAGTTTTAAGTTCTTTTTTCTA
TGGATATATAAGTACTCAATTACTTGGAGGATTGCTTAGCACGCGTATTGGCGGGAAAA
AAGTTTTTGGTTTTGGAATTGCAATTACTGCTTTTCTTACAATTATCACACCACCTTTAG
TTAAAGTGAGCGTTTACATTTTGCTCGCTTTGCGAATTATAGAAGGAATTTGTGAGGGT
GTAACATATCCTTGTATACATGCAATATGGGCACAATGGGCACCTCCATTGGAAAGATCA
AAATTAGCAACCATTACATTCTCCGGAAGTTTCTTTGGAACTGTATTTGCCATGCCAGTT
GCCGGATTGATGACTGAATATCTTGGTTGGCCTTCTGTTTTTTATGGTTTTGGAGGAGCT
GGTCTTATTTGGTTTTTTTATTGGTGGATAGTCATCAAAGATAGACCAGAGGACGATAAA
TCTATTTCAAAAGCTGAACTTGAATATATTAAAAATAGTCTTGGAAATTTGAAAGAAGA
AAAAATTAAACATCCATGGAAAGCTATGCTTACATCTCCACCAGTTTGGGCAATTATCGT
AGCTCATTTTAGCGAAAATTGGGGTTTCTATACTATGCTTACTCAGTTACCAACATTTAT
GAATGATGTACTTGATTTTAAATTGGACAAAACTGGATTTTTATCTGCATTACCATATTTA
GCTATGACTTTCGTCGTTCAATTTTCTGGACATTTAGCAGATTATTTAAGAGTGAAAAA
AATTTTAACGACTACACAGGTGCGTAAATTATTCAATTGCGGGGCTTTTTTATTTCAAAC
TATATTTATGACCTGTACAGGTTTCATTTCAACAACAGCAGGGGTTGTTATTTCTATTACT
ATAGCTGTTGGTCTTGGTGGCTTTGCATGGTCCGGTTTTGG

>novel_circ_000245
GCCAAACAACGTGCCTCCGTAAAATGGCTCTTGTCGAAGGCGTACAACAACCGAGTG
CCAGAAAACCTTCGCGAGCCGTATTATGTCGATAATGAGAATCAAGAACACCTGAAGC
CGCAGATCGTCCACGCACTTTCCAATGCGGAGCTGTACTGTTTGGCGCTGGCGAACAT
CTATTCGGATCCAAATTATCACAATCAGAATCACTGCGGCATCCTGCAGGCCCTCGCCA
GGAAAGGTGTTTACCTGGCGGAGCCGAACAACACCCAGCTCACCGAGACGATCCTCA
TTCAAAATTCGCCGCTCAAGATGTCCGCTCATATGGCTGTGATAGAGGGCCTGATGGTG
TTGTACGCGAAGGAAGTGGTGACGGGCGATCGAGTGGTGTCGGCGATCCGGCGTTTCG
ATCCCCAGGCGGAGGTGGAGGTGCCGGGGGACCACGAGAAGGGCCTCCTCCTGTGGA
TCAGTCACGCGTCGCACGCGCTCATCGCCAAGATCCAGGCGGAGGAGGGCGGCGGCG
ACAAGACGCGGCTCCCAGAGCTGCCCGCTGCCAAGGATTTCCAATCGTTGTGCGACGG

CGTGGGCCTGGCAGCGGTCGTCGCCTTCTACTGCCCCGGCGAGCTCAACTGGATGGAC
ATCAGGGTGTCGAAGAGACCCTCGGTCGCCGACGCGCTGCACAACTTGTCGCTGGTCC
ACGCCTTTTGTAACAGATGCTTACCCTATTCCATTTTCCACATGCAACCCGAGGACGTG
ACGTACATGAGGGGGTCTATGAAGCAAAATTTAGTCGTTTTCCTGGCGGACATGTACAA
CGTGTTGGAGATCCATCCTGCGAAGTGTGTACGTTATCCGGGCGAGGAGAGGGCGATG
CAGTTCTTAGATGCCTGCCCGCGCAATAGTCATGGCGTAGCTCATAAAAGAAGTCTGCC
ACAGTCTATAGCTCCGATACCTGATCTGAGAAGCAACCTCTCCGTATCCGCGCCAGGCT
TCACAGTTGCAAAAGCATCGTCATCTTCCTCCGTCAAGAAGTCTCAATCATTACAACAA
ACTGCCGAAAATTATTCTCACGACGACAGACGAGCGGGGAGCGAAGAAAGCTTCGTG
GTCCATCGCGGAAAAGGCATTCCTACGTTAAGCTCCGTGGCGGACGAGAAATCCGTGA
CCAGAGCGGACGCCGCTGGCCGGCCGAGCAACTGGGAAGATCAGAGGAGAAGCTCGT
ACGCTGGCCGACGATCCAGGCGTAACAGCGTTTCGGACGACTCTCAGTTGACAATAGA
GAACTTTGGCGGATCCCAGGATAATTTACACAACTTCGGCAGAAATCCAGACAAGGAG
GTCGGGGCGCACATCGGGAAAAGGAGCACCACGGAGCCGACGTTACCGGCGAGATCG
AGCGTTCAGGACGTGTATGGTAGCGGGGTGCAGCACATTTTGTCGGACAACGGATACG
ACAAGGAAGAGCCGCCAAGATTGAGAAGGCAGACGTCCAACTCGAGCTTGGACAAC
GTCGCGCTCAAGCAAATTTTACATTCCAGCGAGAACGTCAATTCGGAAGGTGACACGT
CCAAATTGGCCAGCTTCGCGAATCTGAGCAGGCAAAGCTCGGAGAAGGGGATCAACT
TGACGTACACGGAACAGGATCGAGATGACGGGAAATCGAATATGTCCGGTAAGAAGTT
TGGCCAGACGAATGGTAATGGGAACGGTAATGAGAAGAAGACTACGTTCGCCACTCTG
CCGAACACGACCACGTGGCAACAGCAGAGCAGCCAGCAATCCCAACAGGTGGAACA
ACATTCTGTTGATGAAAACGGTGGTAACACCATTATGGCCTCGCAACTGAATAACATTA
GATTGAAGCTGGAGGAGAAACGTCGGCACATAGAGAACGAGAAGAGGAGGATGGAG
GTCGTGATGTCGAAACAGCGTCAAAAAGTTGGCAAGGCTGCGTTCCTGCAAGCTGTC
ACGAAG

>novel_circ_000246
AATCAAGAACACCTGAAGCCGCAGATCGTCCACGCACTTTCCAATGCGGAGCTGTACT
GTTTGGCGCTGGCGAACATCTATTCGGATCCAAATTATCACAATCAGAATCACTGCGGC
ATCCTGCAGGCCCTCGCCAGGAAAGGTGTTTACCTGGCGGAGCCGAACAACACCCAG
CTCACCGAGACGATCCTCATTCAAAATTCGCCGCTCAAGATGTCCGCTCATATGGCTGT
GATAGAGGGCCTGATGGTGTTGTACGCGAAGGAAGTGGTGACGGGCGATCGAGTGGT
GTCGGCGATCCGGCGTTTCGATCCCCAGGCGGAGGTGGAGGTGCCGGGGGACCACGA
GAAGGGCCTCCTCCTGTGGATCAGTCACGCGTCGCACGCGCTCATCGCCAAGATCCAG
GCGGAGGAGGGCGGCGGCGACAAGACGCGGCTCCCAGAGCTGCCCGCTGCCAAGGA
TTTCCAATCGTTGTGCGACGGCGTGGGCCTGGCAGCGGTCGTCGCCTTCTACTGCCCC
GGCGAGCTCAACTGGATGGACATCAGGGTGTCGAAGAGACCCTCGGTCGCCGACGCG
CTGCACAACTTGTCGCTGGTCCACGCCTTTTGTAACAGATGCTTACCCTATTCCATTTTC
CACATGCAACCCGAGGACGTGACGTACATGAGGGGGTCTATGAAGCAAAATTTAGTCG
TTTTCCTGGCGGACATGTACAACGTGTTGGAGATCCATCCTGCGAAGTGTGTACGTTAT
CCGGGCGAGGAGAGGGCGATGCAGTTCTTAGATG

>novel_circ_000248
GTGTATCAAATAGAATACGTGATAGTGAAGGCGGCGAATTCACCGAGGCCGGCCGCGT

GGATTTTGGAGAAATCTGTGGACGGGGAAAATTTCCAGCCGTGGCAGTATTACGCGCC
CAGCGACGAAGAATGTTGGACGCGTTACTCCGTCCCTCCAGTGACGGGGAAACCCATT
TTCATCGGTGACGACGACGTGATATGCACCAGCGTCTATTCCAGACAGACACCTATGGA
AAACGGAGAGATCCATACCCATCTGGTGAACGGTAGACCCGGTGCCCTGAATCACAGC
ACGACCCTACAGGAATTCACGCAGGCGAGATACGTGCGGCTTCGTCTTCAGGGTGTTC
GACGTAACGGGGAGACGATCGGTGACAAAAGGCGCGCTTTCTACTCGATAAAGGAGA
TCAACGTCGGCGGAAGGTGCCTGTGTTCCGGCCACGCTGGCAGATGTCGCTACAGCGT
CCAACACGGG

>novel_circ_000249
AGCACACTTGCAAGGACTTCCGATGCACCCAGAACGAATTTTGCATCGAGAGGGATCT
CCTTTGTGACGGCGTGAATCACTGCGGCGACGAGTCCGACGAGGCTACCTCCACCCTC
TGTGCCAATTCCGAGGCATCGACGATCCTGGGTATGCAGACGATCTGGTTCGTGATCGC
GCTGGTTTTCCTTATCCTGAGCGTGGCAGGCCTGGTCACGGCGGCGGTCCTCTGCTTCT
GCAGGCAGCGTGCAGCAACCCCCAGGCACCCCCACAACGCGCACAACGCCCAGACTC
ATCCTCCGGTCAGCTTCCCAT

>novel_circ_000251
GCGATCCATTACAACTTGGACCAGTTGTACAAAGTGGAATAGCAAAAAATTTTGGTTTA
GACGAGTCATTTTTATTGAGATTATTACGTCATTTTCCTTATCAAAGAGATCCTAATGGAT
TTGAAACACATTACGATCCTCGACTTGTTACAAAACTTATTGTAAATTATCGAAGTTTAC
CAGAAATACTCGAATTATCAAGTTCATTATTTTATGATTCTGAATTAAAAGCACAGATAT
CTTCTAAAAAAAGTAAAGAAGCAAAACTTTTACAAACATTAGCTTCTGAATTGCCAAA
AAGAAAAGGTATTCCACCAGCTATAGTTTTTCATGGTGTAAATGGTGAAAATTGTAAAG
ATAACGATAGTCCAAGTTGGTATAATCCTGAAGAAGCAACTCAAGTATATCTTTATTTGT
TAAAATTATATAAATGCGGTCTTTCACCTGATGATATTGGAATTATAACTCCTTATCAAAA
ACAGGTTCTTCAAATTCGTGAATTACTCATGGAATTAAATATAGAATTACCAAAAATTAG
TAGCGTTGAAGGATTTCAAGGTCAAGAGCGCAATGTTATCATTATTTCAGCT

>novel_circ_000252
GAGCAGATAGCGAAAGAGAAGCAGGTGACGGGCAGGATACAGATACAAGTCTCGTAC
GAGGCGGATCGCAAGGAGCTGATCGTCTCAGTTTTCATGGCGGACGACTTGTGCGCAA
GGGAGGACACCGGATACGGCACGTTACCGGAGGCGTATGCCAAATTGGCCCTGGTTCC
GATCGGCGCCGATCAAATTACATTGAAAACGGTGGTCGCGGAGCCAAGTCAAAAGCC
AATTTGGAATGCAACGTTGCTTTTCTCCGGGGTTGACGGGGAGAGCTTAATGAAGCGG
GCAATCGAGGTGACCCTGTGGGATTTCTGCCCCGACGGCGATAACGTTTTTCTCGGCG
AGTGCACCGTCGATTTGGAACGGGCCCTCGAAAACGACAGGGCAGTTTGGTATCGACT
GGAGGACCCCCGTGGGCTTCGCGCGAGCAAATCGCCCTACTGTTCCCCCCGAGGCTCG
CTCTCCATGGAAATTGCCCAAAGGTTACTGCGAAAGACGGAGCTGCGCGAAAGAAGT
TACAGCGACGACACGCAAAGCGACAGCGGAAGTCCGGAGCTCTGTTTTCTCCACCCG
GATCACGCTTGGCACGCGAATTCGAGAAGAGGCTCCAGCCAATCCGAGCAGCTAGAA
GTCGAACCGTACGAGCTTAACAGGGATTACAGTAGATCCTTGCCGGGCAGCCGAAGGA
GCAGTTTCCAGAGCCAAGGTGGCACGGACAGTAAACGCGGAAGTATGGGGGAGACGG
AGATGCCGACGATACACTTCAACCGTGATCGGCGACGGAGCTCGTTCACCAGGCCGAT

GAGGGACCCGGAGGAGATTCTCGAGGGTCTACGATCGTTGAAAGCGGCCAAGAGGGA
GCTTGGCAGGACGATGAGTCTCTCGGGAGACAAAAGGCGCGAGAGCC

>novel_circ_000253
ATTTACGATAATTTAATTATATTTAACGTATCAACGAATCTTTATACATTTTATTTAACAAC
TTTCTAACAAAATTTTAGCCGCTAGTTTTTGAACGATATTCCACAAATAATAATGCATATT
AATAATTTATTTATTAGTTATTATATGTAAGATGTGATTCCTTAGATGTTGGATCACTCCAG
GTTTTTTATTCTTTATTCGTCATATTCG

>novel_circ_000254
GAAGATTTAAATGAGAACAACACGATGATCGTACACCCGTGCAACTGTAGCACGTTCT
TCGTGTGCACCACGGATCCACCAATTCCCATGGATTGCCCGGCTGGTCTTCAATTCGAC
GAGAAGAAACAAGTTTGCGACTTCAAATGGCGCGTGAAATGCAAACCGAGAAAGGAA
TGCCCG

>novel_circ_000256
ATGACATTGTAGGGGTTCATTGCACTCACGGTGTAAATCGTTCCGGATACCTAATTTGTA
GGTATCTCGTACAGCAATTAGGTTGGGAGCTGGAGAATTGTTTAAAAGCGTTCGAAAC
GGCCCGTGGATATCCCATCGATCGTAAAATCTATATAAACGCGTTGCAGAAAGTGCCGC
GCGAGAAGATCGACACGAGCAAAATCAGCGTGGATTCGAGGGGTACGGCAGGATCGG
AGGCGGCCAATAGTAGAAGAGTGATGCGCGTGTCGTTCAAGTATCCAGGGGGCAGCCC
GTACACGATGGGTCCGCCAGGTTTCGGAGCAACGAGACGCGGTTTCGCGAAAGACGG
TCCATTTTCACCGGCGCATTTCGGTTACGCCGGCCCTCCGCCAGGTTTCCGTCCTATGC
CACCTCCGCCCGGCATGCCACCGGTACCACCGCCACCAGTTCCTCCACCTATGTACGG
GCCGAGGCCGTTCAG

>novel_circ_000257
GCATGTACACCATGCGCATGCTAGATAATGGTAATAACAACGCTTCTGGTCCAACGGGA
GCAGCAGCCTTGTCAGGAGTTCAGACAGCTGGTGGTACAACCTCTTCGGCAACAGGA
GTTACGACACTTCCGGGTGTAACGGATGAAAGGATGGATGCATCGAGCGATAGTGCTG
TCAGCAGTATGGGCAGCGAACGTGTACCATCCCTTTCGGATGGCGAGTGGATGGAAAC
TGGATCTAATTCCAGCCATACACAAGCGGATTCTCATTACACTATGGATTATGCTAGCAA
ATACCGCATGTCGTACGATTGTAGCTATTCCGTTTCCGGAAGAAATGCTGGCTCTCCGA
GATGTCAAACGGAACGTACGATGCCTCCGGTTGCTCAGAAAAAGCATCAAATGTTTGC
AAAACGATATTTTCAGGAACAAGGAACTGGTTCGCCACTCGGAGCTACAGCGCATCCG
ACAACTCCAATGAAATACGAATATGATTCTCATACAGTTGGTGCTGGAGCACCTGGAAA
TGCTTATTCCGGACCGATCGAGGGCGCAACTGGACCTCAACCTGAAATAAAATATAGTT
GTAGTGTAGATTTCAGTCGGCATCAATCAGGACGTTCAGCTATAGAACATGTTCATCATA
ATCATACGTATCATTTACCTGCAGAAAGTTCTGGATCTCTTCAGCGTCCAGTTTCTCGTG
ATAAAAAAG

>novel_circ_000258
GGTCGCATGCCATTCATGCGTGCAATGAGCATGGAACAACGGTGGCAAGATCTGGCAT
CTCTTTTATCGCTACCTGGTGCACCAGAACATTTTCCACATACACATCCTGGATACCCAG

GGCACGGTATAAGCCACAGTCATTATGAAGCACAACGTAACGTGTTGCTTCACAATGC
CACTCTGGCTCCACCGGTGGGTGATCTTAATTCGACCAGTCCTTACCACAATGTAGGTG
GATCGTCGAATTTGGGCTCGGCTGTAGCAACGTCGATGAATTTAACGAATAGTAGCGAA
CCGATGGGTGCGGAAAGTGGAGCAGCTTATAAATCCGAGCCGGCAGATATGATGTACTA
TCACACACCAACATCCGATTCGATAAATCAAACTACCGATGGTTTTCTTTCGTCTCTACT
TAACGACGAGGATTTACATCTGATGGACATGGCTATGAACGATG

>novel_circ_000259
ACACACACATGAATGACGTGCAGCAGTTTGTCGTTAAGCCTCATTCACAGAGCATCGA
ACAACAATATCAAGCTACTTCAGAATGTTGGAATCTGGTTGTGGACGAAGGTTTGTTAC
AGGTATATATATACATATACATATACATATATTTCTCGTACATCAAGGATAATTATATTATAT
AATGAGATAAAAGACGTAAAAGAAAAATATCAATTTACGAAGGAATTAAACGAATTCG
AAGCTGGAAAAGTAGTTTGTTCTATGGTTAAATTATCTGTAACGAAGGTAGATGCTACT
CGCTTATTTTGCATATGCGCTTATTACATATAATAGGATAATTTTCGAAAGTATCGAACGT
TATTCTTATCGACGTTATTATTTTTTTTTTTCTACGACACAACGGTAATTATAACGCAATAT
CTCTATCTGAAATAATCAATGTTACACCGCGTTGACCACGCATCGCGCTTTTTATTTTTA
CAAAAAACACCCCGTTTCTACTCTAATCCTTCTTCTTTCCATATCTACAATCTCTACGTAT
AAAACACACGAACACGCGTACACGTTACGTTCGATGAAAGAGAAGGAAGAAAGTTCG
GTTCGGGAAGATCGGTAAGAAAGAGAAAAAAGACGTATATGTAGCGATGTCGCGACCT
TGCCTCTAATATGGAAGCGGATAATTGGTATACGGATACGTATAATAGCGTCGAACAGCG
TCCACAGGACTCTCAATACGTAATGAGCGAAGTAAATTAATACATATGTGCAATACGGG
CGGGTGGCTTTTGCCCAAGAGAGAAGGATGCGCGTATTGTACGAAGGGATTGCGCAAC
GAGACACGAGTGTCGCCGTACGCGAACCGTATCATTTTTGGAACTCTTCTCTCACGCG
ACCGTCGTATTAACACTGACCTCACTCACCTGTAATTGCTACTTGTTAGCAACCGTACC
ACCAAGACGCGGGGCGACGTATGTAATACCCGGCCTATTATACCATAGCCACGCTATGC
TCTTACGTCTATCGTCGGATTTTTATTCCCCGCCAAGGAACAGCCTTGAACGTGTCGCG
CCACCATCGAAGGAATCGGCTGTATTCCGCGATTCCATTCCACTCCGTTTTAGTATTATT
CCTAATGGCGTACTTTGTTCTCGATAACGCCAAATTTAAATAATTATCGCCAAATGAATG
CGATCCAACGTAATACCGTGGAAAAAAAGAAATATCTAAACGCGAAGATGCGTTTAGA
TATACCTCGTTTCGATACCAGACAATTCTACGAATTTAGATATATTTTTCTTTCCATCGA
GTTTTTCTAACTTCTTCTCCGCGTAGGTTTCCATCTTTTTCGCTTGGAAAAGGCTTGAA
AATTTCCGCAGTTTTGACCCTACGCTACTTAAAAATTTGAAATCGTAATTAAGCCACCA
ATCTTTATTACTCGTCGCTTATCCTTCTTACTTATCTTAATCGTCTTTGTACTAATTTAACC
GCGTTTAAACCGGTTTAAACATCATCGTCGATAACATTGCACTATAAATTTGATATCGATA
TCGTAAAACTATCGTTAAGTATGCCGGATGAAAATCTTTATTTCTTCGCTTTCCTATCGA
AAGAACAAACGCATTTTCGTTAGAATCGTTCGATTTATTCAATGATCGATTTATTCGAAA
GATGGAAATTGTGGGGAAGATAGTGTACGAGGTAGAGATAAAGATTAAAGATAATTTG
GTAATAATAAAAATGTAAGGAATAATAAAATGTTTGAATTATGCGCGATAATAAAAAAGT
TGGTCTGGCTGTATATATCGAGCGGGTTTGAAAAGAATAGAGAATAATTAAAAATAAAA
GGAGGATGGTATGGCGCGCTCTGTTCGCGGATAACGAACGAGAAAAAAAACGAGAAA
GGAGTGGAGGAAGAGTTCCGCGCGAGAGAACGAGAGAAGCAGGCTGAGCGCATCAG
AATGTAATAGTAATTACAGCCGCAACGTTTATCCTGCGGTGCTGATTACATAGTTGGAAG
GATTAGTAATGCAAAACAATTTAATCTCCGCGCTAATAAGTTCAAAAGACGGTGATTCG
CTCAATGTCGAAATTTTAAATACGCGCGCTTCTCGCAAATACGACAAATATTTTTCTTTC

TTCTTTTTTTTTTTTTTCTTTTACACGAGTATCCTTAGAAGTATCCTAGATTTCAGGATT
TTTCACAAATTTCATTCGTTGGAATTCTTAAAGAGAACGATATAAAAATGGATTTTGTTG
ATTTTGATTTGATTTTCGGTTCAAGGATCAAAAGATAATTCGATCTCGCGGAAGAAAAT
TTTCGAAGGAGATTTGATTCGAGAAAATGAACTCGTAAATGAGATGATAAATCACCTTA
TTTTAATAATTGAATTAAGCTTAATATTTTTCTTTTTTTTTCTTTCTTGAGTATCTTAGAAT
CCTAGATTTCAGGATTTTTCACAAATTTAATTCCTTGGAAATCTTAAAGAGAACGATATA
AAAATGGATTTTGTTGATTTTGATCCGATTTCGATCCAAGAATCAAAAGATAATTGCGA
TGGTGGAACGCGATCTTGCGGAAGAAAATTTTCGATTCGAGAAAATGAACTCGTAAAT
GAGATGATAAATCATCTTATAATCCATAGCTCCCCGTTTTAATAATTGAATATCAGAAGA
AAAAGATGCAAGATCGACCGAACGCAGCTTAATATTTTTCTTTTTACATGAGTATCTTAG
AAGTATTCTAGATTTCAGATATTCATTCGTTGGAAATCTTAAAGAGAACGATGCAATGG
CGTAACGCGATCTCGCGGAAGAAAATTTTCAAAGAAAAATTTGATTCGAGAAAATGAA
CTCGTAAAAAAGATGATAAATCACCTTATTTTAATAATTGAATATCAGAAGAAAAAGAG
CAAGCTCGACCGATCGCATCTTTTGAATTTCGTCCGCATTTCGTAATAAAGTCGAGCGG
GTATCGATCAATATCGAAGGACGATCTTTGGCCGTGATTGACGGACATTGGTTACGTCC
ATTGCACTTCTGATATTTGTTTAAAATACGTTGAGTTGTAAAAAGAAAAAAAGAAGAA
GAAGAAGAAGAAGCAAGAAGAAGCAAGAAGCAATCCGAGTATCCTTGGACTTTCGAT
AATATTTACACGCAACATCGAGTTACGATTGAAAAGAGAGAAGAAAGCGATGCGCGGA
GATAAAGAGATGGGGATGGAGATGAAATAAGGGGAATGAAGGAACGGGAAGAAGGA
ACGGGAAGATCGTGAGAGAGGGCGATCCTCGGGAAGCCGCGGAGGCTGGAGGGAAG
GCGGAGGTTGGATCGCGTTGGATCGCGAGCGAGAAGAGTCGAGTAAGACGAAATTAAT
AAGAGGAACGAAGTATGTAATATGGAAATGAGAGGCCGGGGGAAAGACTGAGACCGA
CCGAGACATTAGGATGCACTTGTAACGATACTCGAAGGACTCGCCGCGAGCCCGCCGC
GGTAACCCGTGCCAGTGGTGCATTAATCTGATGCCTATCTGAATGAATCATAATTAATAT
CTTGGCCCATTAGCCATCACGGTGCACCGCCGCACTGTATAGGTAATGCGCATACTTTCT
CCGCGTATCTTTAATTACTTGTATTTTCTCCGTAATATTCCTCGCCGCGAGATTATTGTATT
CGGTTAACCAATCCATTTTTCCATTTAAATTTACCACTCCAAATGTATAATTTCAAAAGA
GATTCGTCCTCCACGAATTTTCCTTGCGAGACAATTTTTCTATTTCTCTCTCTCTCTTTGT
TTCGACGATTTTATAAAGAAAATCCAGTTTTTAGAGTAACCAATCCATTTTTTCATTTAA
ATTTACCGCTCCAAATGTATAATTTCGAAAGAGATTCGTCCTCCACGAATTTTCCTTGCG
AGACAATTTTTCTATTTCTCTCTCTCTCTTTGTTTCGACGATTTTATAAAGAAAATCCAG
TTTTTAGAGTAACCAATCCATTTTTCCATTTAAATTTACCGCTCCAGATATACATTCGAGT
AGAAAAATATTTAATTTTTCTCGTCCTCTACGAATCTTCCTTGCGAGACAATTATTTTCG
TCTATTTCTCTCTCTCTCTTTGTTTCGACGATTTTATAAAGAAAATCCAGTTTTTAGAGTA
ACCAATCCATTTTTCCATTTAAATTTACCGCTCCAAATGTATACATTCGAGTAGAAAAAT
ATTTAATTTTTCTCGTCCTCTACGAATCTTCCTTGCGAATTATTTTCGTCTATTTCTCTCTC
TCTCTTTGTTTCGACGATTTTATAAAGAAAATCCAGTTTTTAGAGTAACCAATCCATTTT
TCCATTTAAATTTACCGCTCCAGATATACATTCGAGTAGAAAAATATTTAATTTTTCTCGT
CCTCTACGAATCTTCCTTGCGAGACAATTATTTTCGTCTATTTCTCTCTCTCTCTCTTTGT
TTCGACGATTTTATAAAGAAAATCCAGTTTTTAGAGTATTCTCAAGGCACGTACGATCA
AGCAATTTTCACGAAGAACGAAGCGACGCGACGGGGCGTCACAATTCCTTTCCGTTCG
TCGGGGAGATTCCAAACAGCTGGTGGACTTATGCACTGTGAAATACATAATGCGTCGC
GGTATGAGCTATCTAAGATGTGTGAAACGAATTTACCTCTTTGCGAAGCGATCATCAAA
CTTCTCCGTCAGCTTACTGTAAACAGTAAATACAATAATGCGATAATCTTTGAAACGCG

AATGTAATTAATTACAATTTCGATCCTCTCCGTTTCTATTTGCGTTGCGTTATATGTACTTT
CCTCGACGAATGACGCTTATCTTGAGAAATTTCATTCACCGTAAAATATAAATATCTATAT
CGTGTGCGTCGTATTAGTTTTTGGTTAACGAATGAATTTAATCCGCGATCCGTATGCGGT
AAACGTAAACAATTTCAAATATATATATATATATATATATATATGAGAAGAAACGAGGGATAC
GAGAGACAAAATATTTTTTTATAGGCTGTAAAGTGTGAGGGATTGAATCGGTATCCGTG
GATATTTTGATTCACAACTTTCGTGACAGAAACGAGCTTTACCGGCAGTCACGTACGAA
AACACGAACGATGCGCCGAGGTAAAAAGTAGGTAAGCGATCGAGGATCGAAGGGACG
TTCGTAGAGTAAACGATGCTGGCAGGTATCGAATACCCGGTTGATCGCTGTCGTACTTC
TCGATCAATTGCATTCGCATCGATGTGTACAAATGTATTGGAAAATTTAGAGAGGAAGA
AGAGATGCGAAATAAAAATTATTAATCGAAGAGATTTTGAAGGATTTGGAAAGAGAGG
AGGATTTTTAGAAGAGGGAAAAGGATAAATATTCTTCTTAAGAATACGCGAATCCAAGA
TTCCGATTTATTATTGCGACGATAATAGTTGGCAAAAAGATGAAAAGCGTGGTAAAGAT
AGAAGTGGAAGAAAAGAAAGGGGAGGAAAGAGGAATGGAAAACAGATATAAACGGA
CAAAGGAAGAAGGAAGAGAGAGCTCGGTTAGAAACTGGCAAGTCCTCGACAGCTCG
TTTCAAACATCTGTTCGCTCCGAGAGATCTCTTTCCTCCCAGTCCCGCGATCTCGCAAA
TTCATACGGCCTGTGACGCGAGCCAACATCGTATACGCCTTCGCGCGCGCGCATATTAC
GTGTATATGTGTTATACGTTTTTCGAATTTCTCCGATGCGCGGGGAAGAAAGAGCGGTG
ATCCAAAAATCGGAACCGTAACCAAACGGTACGCGGTGGATAATGTGGATAGAGAGCT
GGTGGGAAAATGATTGGTGGAAAGTGCAAGAACCGGGGAATGGAAATAACCGAGATC
TCCAAAACTGTGTATAGCGGAAAGACCATGAGAAATTGTTTTAGCACGGTGAACTGGC
AACTGGTGTGCGCGTCAAATTCCGAGAAGCGTTCTTGGAAACGGTATAAACGCGCCGA
GTTTTTTTTCCCGTCCACGGGGCTCAATCATGAGCGTTGCATCGAAACGGCGAACGAG
CGTAAAGTTGCGCGCCATTAGAAATCCGCGATTCGGTATATGGAGGACAAATGGTCGCG
GAACAAACGTCTCGTCCTACATTTGGCGGCCTACACAGCCGACAGATGCGGTGACAAG
CGAGAATCACCTTAAAGGCGTCGCGCTCCACAGATTGCACCGACCGCTGATTGATGCG
AGCGAGCAACAATCGCAAGCCAGTTTCTCTATTCATCTTCTTCTTGCGCTCCATGCATTA
GCGACAATGCTAACCTGCATTAAGCGCCACTGACCACTACCGCACTTACGCGTGTACTC
CTTTCCTCCTCTCTCCTAACCTGCCCGTTCCTTTCCCACCATTTTATCATTTCAACACTTG
CGTAGATTTATGGCCTTGAATATTAGAGCGAATCTTGTTGATAGCGAGTTTGATAACGCT
TCGTATACGTTTTTAGATCGTTATCCGTGTAAATTCCGATCGAAACGATCTACTACTCGAT
CTACCTCGATTCCATTATTGACCAAATCTTTATTGATAAACGCTCTTGTAAATAATCAAC
GATCGTGTTGCGCGTACGCTTGGTTGAAAGAGTTCGCGTCGTTTTCGCTGTTCGAATAA
AAATTATTCTCTCCGATATAATTTCCATATTCGACGCCCAGTTTGACGTTTGATCATGATC
AACGCGTTCTTCCTACACGGTTCAAAGGATACTCGTGACACGCTTGATACCAAAATCAT
TAATATCTCGACCGTCCTTACGTGTGGCCAACACCTACGCGATCAGCAGGGTTTCCGAC
GATCCATCCAACGAGTCGAGATTTGACAGGTTCGATAACCGCGGCATTCTGTAGACGT
GACACTGGATAGATATCTTATAACGAAGCAAGAAAGAAAAAGGATCGTAATTGTAAAG
ATAGGTAGATTTTATTTTATCGATTGGTAGAAAATAAAATTGAAACATGTTCGGAATGGC
AGATTCGGAATAAAATTATTAAAATTTAAACGTTAGGAAAAGTTTGTGTTAATCCGTATA
ATTTATACATATTTCAGGGTCGCATGCCATTCATGCGTGCAATGAGCATGGAACAACGGT
GGCAAGATCTGGCATCTCTTTTATCGCTACCTGGTGCACCAGAACATTTTCCACATACA
CATCCTGGATACCCAGGGCACGGTATAAGCCACAGTCATTATGAAGCACAACGTAACGT
GTTGCTTCACAATGCCACTCTGGCTCCACCGGTGGGTGATCTTAATTCGACCAGTCCTT
ACCACAATGTAGGTGGATCGTCGAATTTGGGCTCGGCTGTAGCAACGTCGATGAATTTA

ACGAATAGTAGCGAACCGATGGGTGCGGAAAGTGGAGCAGCTTATAAATCCGAGCCGG
CAGATATGATGTACTATCACACACCAACATCCGATTCGATAAATCAAACTACCGATGGTT
TTCTTTCGTCTCTACTTAACGACGAGGATTTACATCTGATGGACATGGCTATGAACGATG

>novel_circ_000260
TTTCCAGTTGGCGGAGGAGGTTGTTTGGTAGGCTGGCAGGTTACGCGCGGTGATCGT
AAAAGCCGGCGTGGTCTCGCTCGCTCGCCGCGAGAAAAGTCATGTTCGTTAGCCGAG
GAGGAGGACGATCGTGACAATGACCTACCGAGATACATAGTCCTCGGACGAGGAGCGC
GAGGCTCGCAAAACGTGACGCAATGCGTGGTTGCATGCACGTGAATAAGCGTAGCTGA
AAACTGCCGCCAATCAGAGAACACGGGGACCCCTACCATGATCGTTCACCGTGCACGA
TCGTGATCGCGTTGTATTCCTGCCACTCGCGGACGACATGCGGCTCGAGGTAAACGTTA
AACCTTGTGGAAAATTAGTTTGCCGTTTTTCCATCGCGTTGAAAAACGCTTTGTCCCGT
TAAGTCGTTACGCCAATGTCCCGGGATAAATGCTTCGTTTCGATAAAACGTGATTGTGC
GACTTGGAAAAGTGTTTGTGTTTCTCTACCCTCTCTCGTCTACCCGCTGATCGTCAATTT
CCTTCAGGGGGGAGGGGAGGAAAAAAAGAAAAAGAGAGAAAAAAAATTGAGCGGA
ATGTGTGATTCGGTACGCGTTTATCCTCTCGTGGGATAGATAGTTTCTTCGCATTTTTTT
TTCTTTCCTCTTTCCTTTTTTATTTTTTAAGAAATAAGAATAAAATGATTCGCGGCAGAA
GGACGTTATTATCGTTGCTCGTCTAAGGACAATTATCTCGATATTCCGAGTATCGCGAGT
TTCGTAAAAGTGCGCGTTTCTCGTGTATTCGATCTTGTTATTTTTTAATACAGCGACGAT
GATCGATCTTATTTTCGCGCGGTGGCAAACGTTCCGTACGATCGTACGTCTCGTATCGA
CGAGTGGCGTTTGTTTTTGATCAACTTTACATCCCCGCGAATCCTTTGATCCGTTCGAG
GAAAATTGAAGGAAGAACGCAGAGAGAGTCGAGTCGATTTTTTTTTCCTTTTTTTTTTT
TCGTTGTGTGTCAAATCGTATTCCGATAATACACAAATACGTCGAGCGAATGAATATATT
GGTTAATAGACACACACATGAATGACGTGCAGCAGTTTGTCGTTAAGCCTCATTCACAG
AGCATCGAACAACAATATCAAGCTACTTCAGAATGTTGGAATCTGGTTGTGGACGAAG
GTTTGTTACAGGTATATATATACATATACATATACATATATTTCTCGTACATCAAGGATAATT
ATATTATATAATGAGATAAAAGACGTAAAAGAAAAATATCAATTTACGAAGGAATTAAA
CGAATTCGAAGCTGGAAAAGTAGTTTGTTCTATGGTTAAATTATCTGTAACGAAGGTAG
ATGCTACTCGCTTATTTTGCATATGCGCTTATTACATATAATAGGATAATTTTCGAAAGTAT
CGAACGTTATTCTTATCGACGTTATTATTTTTTTTTTTCTACGACACAACGGTAATTATAA
CGCAATATCTCTATCTGAAATAATCAATGTTACACCGCGTTGACCACGCATCGCGCTTTT
TATTTTTACAAAAAACACCCCGTTTCTACTCTAATCCTTCTTCTTTCCATATCTACAATCT
CTACGTATAAAACACACGAACACGCGTACACGTTACGTTCGATGAAAGAGAAGGAAG
AAAGTTCGGTTCGGGAAGATCGGTAAGAAAGAGAAAAAAGACGTATATGTAGCGATGT
CGCGACCTTGCCTCTAATATGGAAGCGGATAATTGGTATACGGATACGTATAATAGCGTC
GAACAGCGTCCACAGGACTCTCAATACGTAATGAGCGAAGTAAATTAATACATATGTGC
AATACGGGCGGGTGGCTTTTGCCCAAGAGAGAAGGATGCGCGTATTGTACGAAGGGAT
TGCGCAACGAGACACGAGTGTCGCCGTACGCGAACCGTATCATTTTTGGAACTCTTCT
CTCACGCGACCGTCGTATTAACACTGACCTCACTCACCTGTAATTGCTACTTGTTAGCA
ACCGTACCACCAAGACGCGGGGCGACGTATGTAATACCCGGCCTATTATACCATAGCCA
CGCTATGCTCTTACGTCTATCGTCGGATTTTTATTCCCCGCCAAGGAACAGCCTTGAAC
GTGTCGCGCCACCATCGAAGGAATCGGCTGTATTCCGCGATTCCATTCCACTCCGTTTT
AGTATTATTCCTAATGGCGTACTTTGTTCTCGATAACGCCAAATTTAAATAATTATCGCCA
AATGAATGCGATCCAACGTAATACCGTGGAAAAAAAGAAATATCTAAACGCGAAGATG

CGTTTAGATATACCTCGTTTCGATACCAGACAATTCTACGAATTTTAGATATATTTTTCTT
TCCATCGAGTTTTTCTAACTTCTTCTCCGCGTAGGTTTCCATCTTTTTCGCTTGGAAAAG
GCTTGAAAATTTCCGCAGTTTTGACCCTACGCTACTTAAAAATTTGAAATCGTAATTAA
GCCACCAATCTTTATTACTCGTCGCTTATCCTTCTTACTTATCTTAATCGTCTTTGTACTAA
TTTAACCGCGTTTAAACCGGTTTAAACATCATCGTCGATAACATTGCACTATAAATTTGA
TATCGATATCGTAAAACTATCGTTAAGTATGCCGGATGAAAATCTTTATTTCTTCGCTTTC
CTATCGAAAGAACAAACGCATTTTCGTTAGAATCGTTCGATTTATTCAATGATCGATTTA
TTCGAAAGATGGAAATTGTGGGGAAGATAGTGTACGAGGTAGAGATAAAGATTAAAGA
TAATTTGGTAATAATAAAAATGTAAGGAATAATAAAATGTTTGAATTATGCGCGATAATA
AAAAAGTTGGTCTGGCTGTATATATCGAGCGGGTTTGAAAAGAATAGAGAATAATTAAA
AATAAAAGGAGGATGGTATGGCGCGCTCTGTTCGCGGATAACGAACGAGAAAAAAAA
CGAGAAAGGAGTGGAGGAAGAGTTCCGCGCGAGAGAACGAGAGAAGCAGGCTGAGC
GCATCAGAATGTAATAGTAATTACAGCCGCAACGTTTATCCTGCGGTGCTGATTACATAG
TTGGAAGGATTAGTAATGCAAAACAATTTAATCTCCGCGCTAATAAGTTCAAAAGACGG
TGATTCGCTCAATGTCGAAATTTTAAATACGCGCGCTTCTCGCAAATACGACAAATATTT
TTCTTTCTTCTTTTTTTTTTTTTTTCTTTTTACACGAGTATCCTTAGAAGTATCCTAGATTTC
AGGATTTTTCACAAATTTCATTCGTTGGAATTCTTAAAGAGAACGATATAAAAATGGATT
TTGTTGATTTTGATTTGATTTTCGGTTCAAGGATCAAAAGATAATTCGATCTCGCGGAAG
AAAATTTTCGAAGGAGATTTGATTCGAGAAAATGAACTCGTAAATGAGATGATAAATCA
CCTTATTTTAATAATTGAATTAAGCTTAATATTTTTCTTTTTTTTTCTTTCTTGAGTATCTT
AGAATCCTAGATTTCAGGATTTTTCACAAATTTAATTCCTTGGAAATCTTAAAGAGAAC
GATATAAAAATGGATTTTGTTGATTTTGATCCGATTTTCGATCCAAGAATCAAAAGATAA
TTGCGATGGTGGAACGCGATCTTGCGGAAGAAAATTTTCGATTCGAGAAAATGAACTC
GTAAATGAGATGATAAATCATCTTATAATCCATAGCTCCCCGTTTTAATAATTGAATATCA
GAAGAAAAAGATGCAAGATCGACCGAACGCAGCTTAATATTTTTCTTTTTACATGAGTA
TCTTAGAAGTATTCTAGATTTCAGATATTCATTCGTTGGAAATCTTAAAGAGAACGATGC
AATGGCGTAACGCGATCTCGCGGAAGAAAATTTTCAAAGAAAAATTTGATTCGAGAAA
ATGAACTCGTAAAAAAGATGATAAATCACCTTATTTTAATAATTGAATATCAGAAGAAA
AAGAGCAAGCTCGACCGATCGCATCTTTTGAATTTCGTCCGCATTTCGTAATAAAGTCG
AGCGGGTATCGATCAATATCGAAGGACGATCTTTGGCCGTGATTGACGGACATTGGTTA
CGTCCATTGCACTTCTGATATTTGTTTAAAATACGTTGAGTTGTAAAAAGAAAAAAAGA
AGAAGAAGAAGAAGCAAGAAGAAGCAAGAAGCAATCCGAGTATCCTTGGACTTT
CGATAATATTTACACGCAACATCGAGTTACGATTGAAAAGAGAGAAGAAAGCGATGCG
CGGAGATAAAGAGATGGGGATGGAGATGAAATAAGGGGAATGAAGGAACGGGAAGA
AGGAACGGGAAGATCGTGAGAGAGGGCGATCCTCGGGAAGCCGCGGAGGCTGGAGG
GAAGGCGGAGGTTGGATCGCGTTGGATCGCGAGCGAGAAGAGTCGAGTAAGACGAAA
TTAATAAGAGGAACGAAGTATGTAATATGGAAATGAGAGGCCGGGGGAAAGACTGAG
ACCGACCGAGACATTAGGATGCACTTGTAACGATACTCGAAGGACTCGCCGCGAGCCC
GCCGCGGTAACCCGTGCCAGTGGTGCATTAATCTGATGCCTATCTGAATGAATCATAATT
AATATCTTGGCCCATTAGCCATCACGGTGCACCGCCGCACTGTATAGGTAATGCGCATAC
TTTCTCCGCGTATCTTTAATTACTTGTATTTTCTCCGTAATATTCCTCGCCGCGAGATTATT
GTATTCGGTTAACCAATCCATTTTTCCATTTAAATTTACCACTCCAAATGTATAATTTCAA
AAGAGATTCGTCCTCCACGAATTTTCCTTGCGAGACAATTTTTCTATTTCTCTCTCTCTC
TTTGTTTCGACGATTTTATAAAGAAAATCCAGTTTTTAGAGTAACCAATCCATTTTTTCA

TTTAAATTTACCGCTCCAAATGTATAATTTCGAAAGAGATTCGTCCTCCACGAATTTTCC
TTGCGAGACAATTTTTCTATTTCTCTCTCTCTCTTTGTTTCGACGATTTTATAAAGAAAAT
CCAGTTTTTAGAGTAACCAATCCATTTTTCCATTTAAATTTACCGCTCCAGATATACATTC
GAGTAGAAAAATATTTAATTTTTCTCGTCCTCTACGAATCTTCCTTGCGAGACAATTATT
TTCGTCTATTTCTCTCTCTCTCTTTGTTTCGACGATTTTATAAAGAAAATCCAGTTTTTAG
AGTAACCAATCCATTTTTCCATTTAAATTTACCGCTCCAAATGTATACATTCGAGTAGAA
AAATATTTAATTTTTCTCGTCCTCTACGAATCTTCCTTGCGAATTATTTTCGTCTATTTCTC
TCTCTCTCTTTGTTTCGACGATTTTATAAAGAAAATCCAGTTTTTAGAGTAACCAATCCA
TTTTTCCATTTAAATTTACCGCTCCAGATATACATTCGAGTAGAAAAATATTTAATTTTTC
TCGTCCTCTACGAATCTTCCTTGCGAGACAATTATTTTCGTCTATTTCTCTCTCTCTCTCT
TTGTTTCGACGATTTTATAAAGAAAATCCAGTTTTTAGAGTATTCTCAAGGCACGTACGA
TCAAGCAATTTTCACGAAGAACGAAGCGACGCGACGGGGCGTCACAATTCCTTTCCGT
TCGTCGGGGAGATTCCAAACAGCTGGTGGACTTATGCACTGTGAAATACATAATGCGTC
GCGGTATGAGCTATCTAAGATGTGTGAAACGAATTTACCTCTTTGCGAAGCGATCATCA
AACTTCTCCGTCAGCTTACTGTAAACAGTAAATACAATAATGCGATAATCTTTGAAACG
CGAATGTAATTAATTACAATTTCGATCCTCTCCGTTTCTATTTGCGTTGCGTTATATGTAC
TTTCCTCGACGAATGACGCTTATCTTGAGAAATTTCATTCACCGTAAAATATAAATATCT
ATATCGTGTGCGTCGTATTAGTTTTTGGTTAACGAATGAATTTAATCCGCGATCCGTATG
CGGTAAACGTAAACAATTTCAAATATATATATATATATATATATATGAGAAGAAACGAGGG
ATACGAGAGACAAAATATTTTTTTATAGGCTGTAAAGTGTGAGGGATTGAATCGGTATC
CGTGGATATTTTGATTCACAACTTTCGTGACAGAAACGAGCTTTACCGGCAGTCACGTA
CGAAAACACGAACGATGCGCCGAGGTAAAAAGTAGGTAAGCGATCGAGGATCGAAGG
GACGTTCGTAGAGTAAACGATGCTGGCAGGTATCGAATACCCGGTTGATCGCTGTCGTA
CTTCTCGATCAATTGCATTCGCATCGATGTGTACAAATGTATTGGAAAATTTAGAGAGG
AAGAAGAGATGCGAAATAAAAATTATTAATCGAAGAGATTTTGAAGGATTTGGAAAGA
GAGGAGGATTTTTAGAAGAGGGAAAAGGATAAATATTCTTCTTAAGAATACGCGAATCC
AAGATTCCGATTTATTATTGCGACGATAATAGTTGGCAAAAAGATGAAAAGCGTGGTAA
AGATAGAAGTGGAAGAAAAGAAAGGGGAGGAAAGAGGAATGGAAAACAGATATAAA
CGGACAAAGGAAGAAGGAAGAGAGAGCTCGGTTAGAAACTGGCAAGTCCTCGACAG
CTCGTTTCAAACATCTGTTCGCTCCGAGAGATCTCTTTCCTCCCAGTCCCGCGATCTCG
CAAATTCATACGGCCTGTGACGCGAGCCAACATCGTATACGCCTTCGCGCGCGCGCATA
TTACGTGTATATGTGTTATACGTTTTTCGAATTTCTCCGATGCGCGGGGAAGAAAGAGC
GGTGATCCAAAAATCGGAACCGTAACCAAACGGTACGCGGTGGATAATGTGGATAGAG
AGCTGGTGGGAAAATGATTGGTGGAAAGTGCAAGAACCGGGGAATGGAAATAACCGA
GATCTCCAAAACTGTGTATAGCGGAAAGACCATGAGAAATTGTTTTAGCACGGTGAAC
TGGCAACTGGTGTGCGCGTCAAATTCCGAGAAGCGTTCTTGGAAACGGTATAAACGCG
CCGAGTTTTTTTTCCCGTCCACGGGGCTCAATCATGAGCGTTGCATCGAAACGGCGAA
CGAGCGTAAAGTTGCGCGCCATTAGAAATCCGCGATTCGGTATATGGAGGACAAATGG
TCGCGGAACAAACGTCTCGTCCTACATTTGGCGGCCTACACAGCCGACAGATGCGGTG
ACAAGCGAGAATCACCTTAAAGGCGTCGCGCTCCACAGATTGCACCGACCGCTGATTG
ATGCGAGCGAGCAACAATCGCAAGCCAGTTTCTCTATTCATCTTCTTCTTGCGCTCCAT
GCATTAGCGACAATGCTAACCTGCATTAAGCGCCACTGACCACTACCGCACTTACGCGT
GTACTCCTTTCCTCCTCTCTCCTAACCTGCCCGTTCCTTTCCCACCATTTTATCATTTCAA
CACTTGCGTAGATTTATGGCCTTGAATATTAGAGCGAATCTTGTTGATAGCGAGTTTGAT

AACGCTTCGTATACGTTTTAGATCGTTATCCGTGTAAATTCCGATCGAAACGATCTACT
ACTCGATCTACCTCGATTCCATTATTGACCAAATCTTTATTGATAAACGCTCTTGTAAATA
ATCAACGATCGTGTTGCGCGTACGCTTGGTTGAAAGAGTTCGCGTCGTTTTCGCTGTTC
GAATAAAAATTATTCTCTCCGATATAATTTCCATATTCGACGCCCAGTTTGACGTTTGATC
ATGATCAACGCGTTCTTCCTACACGGTTCAAAGGATACTCGTGACACGCTTGATACCAA
AATCATTAATATCTCGACCGTCCTTACGTGTGGCCAACACCTACGCGATCAGCAGGGTT
TCCGACGATCCATCCAACGAGTCGAGATTTGACAGGTTCGATAACCGCGGCATTCTGTA
GACGTGACACTGGATAGATATCTTATAACGAAGCAAGAAAGAAAAAGGATCGTAATTG
TAAAGATAGGTAGATTTTATTTTATCGATTGGTAGAAAATAAAATTGAAACATGTTCGGA
ATGGCAGATTCGGAATAAAATTATTAAAATTTAAACGTTAGGAAAAGTTTGTGTTAATC
CGTATAATTTATACATATTTCAGGGTCGCATGCCATTCATGCGTGCAATGAGCATGGAAC
AACGGTGGCAAGATCTGGCATCTCTTTTATCGCTACCTGGTGCACCAGAACATTTTCCA
CATACACATCCTGGATACCCAGGGCACGGTATAAGCCACAGTCATTATGAAGCACAACG
TAACGTGTTGCTTCACAATGCCACTCTGGCTCCACCGGTGGGTGATCTTAATTCGACCA
GTCCTTACCACAATGTAGGTGGATCGTCGAATTTGGGCTCGGCTGTAGCAACGTCGATG
AATTTAACGAATAGTAGCGAACCGATGGGTGCGGAAAGTGGAGCAGCTTATAAATCCG
AGCCGGCAGATATGATGTACTATCACACACCAACATCCGATTCGATAAATCAAACTACC
GATGGTTTTCTTTCGTCTCTACTTAACGACGAGGATTTACATCTGATGGACATGGCTATG
AACGATG

>novel_circ_000261
GAAACGAAACCGGAAGTGAAGAAGAAGGAAAAAGATAGCATCGAAGAATCGACGAG
CGAAGCAAAGGACGACGAGTCGCCGATTATAAGCAGCTCGAGCAGCGTCGAGGTCGC
GAAGTCGTCGAGGTGCGAGGATCCTGAGACCGGCGACATGATTCACACACCGCAATTT
CATCATCCCCATCATCCTCACCATCGTTCCTTCCAGGGTCGCATGCCATTCATGCGTGCA
ATGAGCATGGAACAACGGTGGCAAGATCTGGCATCTCTTTTATCGCTACCTGGTGCACC
AGAACATTTTCCACATACACATCCTGGATACCCAGGGCACGGTATAAGCCACAGTCATT
ATGAAGCACAACGTAACGTGTTGCTTCACAATGCCACTCTGGCTCCACCGGTGGGTGA
TCTTAATTCGACCAGTCCTTACCACAATGTAGGTGGATCGTCGAATTTGGGCTCGGCTG
TAGCAACGTCGATGAATTTAACGAATAGTAGCGAACCGATGGGTGCGGAAAGTGGAGC
AGCTTATAAATCCGAGCCGGCAGATATGATGTACTATCACACACCAACATCCGATTCGAT
AAATCAAACTACCGATGGTTTTCTTTCGTCTCTACTTAACGACGAGGATTTACATCTGAT
GGACATGGCTATGAACGATG

>novel_circ_000262
GTGAATATATACTCAATTCTGGAAATCAAGAGGGAGACGGGAACAACGCGATCGAAGA
GGACGATCGCCTCCTTAGAGAAGCGTCGCTAGATTTAGACAATCACCCGTTGGCTGGAT
TAACCGATGATTCTTTGGGATTAACCGACACTTTAGAACTCGAGAATGATCTTCCTTCG
GACTTGCTCGGAGGTAGTCTCTTAGCGAGCGCAAACGTCGAAAGTCTTCTTAACAACG
ATAGTTTGGATCTACCCGACGGATTCAATCTCGAGGAGGCACTCCAACTCGTTGGCCTC
GATGAGGCGCAATCAGAGGAAACGAAACCGGAAGTGAAGAAGAAGGAAAAAGATAG
CATCGAAGAATCGACGAGCGAAGCAAAGGACGACGAGTCGCCGATTATAAGCAGCTC
GAGCAGCGTCGAGGTCGCGAAGTCGTCGAGGTGCGAGGATCCTGAGACCGGCGACAT
GATTCACACACCGCAATTTCATCATCCCCATCATCCTCACCATCGTTCCTTCCAGGGTCG

CATGCCATTCATGCGTGCAATGAGCATGGAACAACGGTGGCAAGATCTGGCATCTCTTT
TATCGCTACCTGGTGCACCAGAACATTTTCCACATACACATCCTGGATACCCAGGGCAC
GGTATAAGCCACAGTCATTATGAAGCACAACGTAACGTGTTGCTTCACAATGCCACTCT
GGCTCCACCGGTGGGTGATCTTAATTCGACCAGTCCTTACCACAATGTAGGTGGATCGT
CGAATTTGGGCTCGGCTGTAGCAACGTCGATGAATTTAACGAATAGTAGCGAACCGAT
GGGTGCGGAAAGTGGAGCAGCTTATAAATCCGAGCCGGCAGATATGATGTACTATCAC
ACACCAACATCCGATTCGATAAATCAAACTACCGATGGTTTTCTTTCGTCTCTACTTAAC
GACGAGGATTTACATCTGATGGACATGGCTATGAACGATG

>novel_circ_000263
GACATGGATTTGATCGAAGTGCTTTGGAAGCAAGACGTGGATCTGGGATTCACGTTGG
TGGAACCGACTACCACGGCGACCAAGAAGTTGTCCACGGTGGAGAAGGGAAGCGAC
GATGAGATCGAAAAATTAAAAGCTTTAGAAGCGATCAATGGAAGCAACGAAGAGAAG
GATACAAAGGAATACGACGAAGCGCAAGACGATCCATGGGCAGGCCTCCCTTACACCA
TTGATCTCGAGACCGGTGAATATATACTCAATTCTGGAAATCAAGAGGGAGACGGGAA
CAACGCGATCGAAGAGGACGATCGCCTCCTTAGAGAAGCGTCGCTAGATTTAGACAAT
CACCCGTTGGCTGGATTAACCGATGATTCTTTGGGATTAACCGACACTTTAGAACTCGA
GAATGATCTTCCTTCGGACTTGCTCGGAGGTAGTCTCTTAGCGAGCGCAAACGTCGAA
AGTCTTCTTAACAACGATAGTTTGGATCTACCCGACGGATTCAATCTCGAGGAGGCACT
CCAACTCGTTGGCCTCGATGAGGCGCAATCAGAGGAAACGAAACCGGAAGTGAAGAA
GAAGGAAAAAGATAGCATCGAAGAATCGACGAGCGAAGCAAAGGACGACGAGTCGC
CGATTATAAGCAGCTCGAGCAGCGTCGAGGTCGCGAAGTCGTCGAGGTGCGAGGATCC
TGAGACCGGCGACATGATTCACACACCGCAATTTCATCATCCCCATCATCCTCACCATC
GTTCCTTCCAGGGTCGCATGCCATTCATGCGTGCAATGAGCATGGAACAACGGTGGCA
AGATCTGGCATCTCTTTTATCGCTACCTGGTGCACCAGAACATTTTCCACATACACATCC
TGGATACCCAGGGCACGGTATAAGCCACAGTCATTATGAAGCACAACGTAACGTGTTG
CTTCACAATGCCACTCTGGCTCCACCGGTGGGTGATCTTAATTCGACCAGTCCTTACCA
CAATGTAGGTGGATCGTCGAATTTGGGCTCGGCTGTAGCAACGTCGATGAATTTAACGA
ATAGTAGCGAACCGATGGGTGCGGAAAGTGGAGCAGCTTATAAATCCGAGCCGGCAGA
TATGATGTACTATCACACACCAACATCCGATTCGATAAATCAAACTACCGATGGTTTTCT
TTCGTCTCTACTTAACGACGAGGATTTACATCTGATGGACATGGCTATGAACGATG

>novel_circ_000264
GTGAATATATACTCAATTCTGGAAATCAAGAGGGAGACGGGAACAACGCGATCGAAGA
GGACGATCGCCTCCTTAGAGAAGCGTCGCTAGATTTAGACAATCACCCGTTGGCTGGAT
TAACCGATGATTCTTTGGGATTAACCGACACTTTAGAACTCGAGAATGATCTTCCTTCG
GACTTGCTCGGAGGTAGTCTCTTAGCGAGCGCAAACGTCGAAAGTCTTCTTAACAACG
ATAGTTTGGATCTACCCGACGGATTCAATCTCGAGGAGGCACTCCAACTCGTTGGCCTC
GATGAGGCGCAATCAGAGGAAACGAAACCGGAAGTGAAGAAGAAGGAAAAAGATAG
CATCGAAGAATCGACGAGCGAAGCAAAGGACGACGAGTCGCCGATTATAAGCAGCTC
GAGCAGCGTCGAGGTCGCGAAGTCGTCGAGGTGCGAGGATCCTGAGACCGGCGACAT
GATTCACACACCGCAATTTCATCATCCCCATCATCCTCACCATCGTTCCTTCCAG

>novel_circ_000265

GACATGGATTTGATCGAAGTGCTTTGGAAGCAAGACGTGGATCTGGGATTCACGTTGG
TGGAACCGACTACCACGGCGACCAAGAAGTTGTCCACGGTGGAGAAGGGAAGCGAC
GATGAGATCGAAAAATTAAAAGCTTTAGAAGCGATCAATGGAAGCAACGAAGAGAAG
GATACAAAGGAATACGACGAAGCGCAAGACGATCCATGGGCAGGCCTCCCTTACACCA
TTGATCTCGAGACCGGTGAATATATACTCAATTCTGGAAATCAAGAGGGAGACGGGAA
CAACGCGATCGAAGAGGACGATCGCCTCCTTAGAGAAGCGTCGCTAGATTTAGACAAT
CACCCGTTGGCTGGATTAACCGATGATTCTTTGGGATTAACCGACACTTTAGAACTCGA
GAATGATCTTCCTTCGGACTTGCTCGGAGGTAGTCTCTTAGCGAGCGCAAACGTCGAA
AGTCTTCTTAACAACGATAGTTTGGATCTACCCGACGGATTCAATCTCGAGGAGGCACT
CCAACTCGTTGGCCTCGATGAGGCGCAATCAGAGGAAACGAAACCGGAAGTGAAGAA
GAAGGAAAAAGATAGCATCGAAGAATCGACGAGCGAAGCAAAGGACGACGAGTCGC
CGATTATAAGCAGCTCGAGCAGCGTCGAGGTCGCGAAGTCGTCGAGGTGCGAGGATCC
TGAGACCGGCGACATGATTCACACACCGCAATTTCATCATCCCCATCATCCTCACCATC
GTTCCTTCCAG

>novel_circ_000266
GTGAATATATACTCAATTCTGGAAATCAAGAGGGAGACGGGAACAACGCGATCGAAGA
GGACGATCGCCTCCTTAGAGAAGCGTCGCTAGATTTAGACAATCACCCGTTGGCTGGAT
TAACCGATGATTCTTTGGGATTAACCGACACTTTAGAACTCGAGAATGATCTTCCTTCG
GACTTGCTCGGAGGTAGTCTCTTAGCGAGCGCAAACGTCGAAAGTCTTCTTAACAACG
ATAGTTTGGATCTACCCGACGGATTCAATCTCGAGGAGGCACTCCAACTCGTTGGCCTC
GATGAGGCGCAATCAGAG

>novel_circ_000267
GACATGGATTTGATCGAAGTGCTTTGGAAGCAAGACGTGGATCTGGGATTCACGTTGG
TGGAACCGACTACCACGGCGACCAAGAAGTTGTCCACGGTGGAGAAGGGAAGCGAC
GATGAGATCGAAAAATTAAAAGCTTTAGAAGCGATCAATGGAAGCAACGAAGAGAAG
GATACAAAGGAATACGACGAAGCGCAAGACGATCCATGGGCAGGCCTCCCTTACACCA
TTGATCTCGAGACCGGTGAATATATACTCAATTCTGGAAATCAAGAGGGAGACGGGAA
CAACGCGATCGAAGAGGACGATCGCCTCCTTAGAGAAGCGTCGCTAGATTTAGACAAT
CACCCGTTGGCTGGATTAACCGATGATTCTTTGGGATTAACCGACACTTTAGAACTCGA
GAATGATCTTCCTTCGGACTTGCTCGGAGGTAGTCTCTTAGCGAGCGCAAACGTCGAA
AGTCTTCTTAACAACGATAGTTTGGATCTACCCGACGGATTCAATCTCGAGGAGGCACT
CCAACTCGTTGGCCTCGATGAGGCGCAATCAGAG

>novel_circ_000268
GTAATTTAATCTCGTTGTATTCGATTTCTGTGCAAGATCTATCGCTTAAGCGACGGATTTA
TTCGATAATTTTATTATTCTTCGAATTTTGAGAAGAAGCAAGCACATTTATATAAAACAA
ACCTGTGAGCAAGATTTTCACGATTGGAATAAAGAGGAG

>novel_circ_000270
CCTCAGCACAAAGGAAGGTATATTCTCGAATATGTATGCAAGCAACTCAATATCTTGGA
AACGGATTATTTTGGGCTTCGATATGTGGATCATTGTAGGCAAAGGCATTGGTTAGATCT
GGCAAAAACAGCGATTAAGCAAGTAAAAGACATGGACCCGATTCTGTTTAGTTTTCGC

GTGAAATTTTATCCACCGGATCCTCTGAGGCTGAAGGAAGAAATAACTAGGTATCAAGT
TTACCAGCAGCTTAAGAGGGACCTTCTTCATGGACGACTATATTGCAGTCCAGGTGAA
GCAGCGCTGCTGGCGGCGTGCATCGTGCAAAGCGAATTAGGGGATTATGATCCTAAAC
TTCACGAAGGAAACTACATTTCCGAGCATAAATTATTGCTTAAACAGACGGAAGCTATT
GAAGAAAAGGCTATGAAACTGCATCAAACAGAATTGAAGGGATTCACTCCACAGCAG
GCAGAAACTCATTTCCTTAGGTTAGCATCACAGTTGGATACATATGCTGTTGATCCACA
CCCCGTAAAGGACCAAAAAGGTGCGCAACTATATCTTGGTATTAATCATTGTGGAATTC
TGACATTCCAAGGATCTCGTAAAACACATCATTTCCGCTGGCCGGAGGTTCAGAAAAT
CAACTACGAAGGAAAAATGTTTATTGTACATTTAACAATAAGTGAG

>novel_circ_000271
GAAGCCAAACAAACTAAGTGTTGTATGGACAAGACGTAGTAGAAGAGTTAGTTCAGA
ACCATTAGATTGGGAACCCAGTTTAAGTGATCCATTAAAAGGTGTCATTAGTTGGGCTG
TTCCTGATAATCATACAGTTTCAGTAACTTTGTTTAAAGATCCAAGAACTCATGAATTAG
AAGATAAAGATTGGACATTTGTAATTGAAGATGTTTCATCTACTGGTAAAAGACGACAT
GTAGCTGCTACAAACATAAACATGAAAAAGTATGCTACTTTAGAATCTAGTCAACAACA
ACTTAAATTGGATTTAAAACCTACATCAAAAAAAATTGTTAATGCTACATTAGAATGTAC
TTTATCTTGTGTCTTTTTAAGAGAAGGCAAAGCAACGGATGAAGATATGCAGAGTATGG
CTAGCTTAATGTCAGTTAACAACAATAGTGATATTGCACCTTTAGATGATTTCGATAATG
AAGATATACCAGAAGATGTTGAAGAAGTATCTGGAAAAAACCTTGATGAAATTTTAGAT
ATCTCTGCTCAACTTGATCTAATGACAAGTAGTCTTACTGAAAGTGAATTACCTAGTACT
CCAATAAGTGTGGCAAGTTTATCAAAAGATGATACCACTCCTGTAAATGATAGCGATCA
TATCATCCGTGATGTTAGTTTGTCAGGCATCGGTGATTCCTTTAAAGAAAAATCACCTTT
AAAGGATACTATTGCTGTTGAAAACAATAAAAATATTGAGCATACATTGATAAGATTACC
ATTGCAACCATTAGAATTAAAAAAGAATGATGCCAATATTCCTAGATTAAAAGAAATTA
CTCCAGGTCAGGATTTATTAGAATGGTGTAAAGAAGTAACTAAAGATTATTCTGGAGTG
AAAGTCACTAATCTTACAACATCTTGGAGAAATGGAATGGCATTTTGTGCAATCATACA
TCATTTTAGATCAGATCTTATAGACATTGATTCGTTATTGCCTCATGATGTAAAGGGTAAT
TGTAAAAAAGCATTTGATGCTGGAGAAGCTCTTGGGATACCTAGAGTAATAGAACCAG
CAGATATGGATATATTGACTGTACCTGATAAATTAGCTGTGATGACTTACTTGTATCAATT
GAGAGCACATTTTACTGGTCACGAATTAGAGGTACATCAAATAGGTAAAACAACAGAT
GAATCATCTTATATGATTGGCAGATTTAATACAGACAATAATTCAGATGTAAGTGTGCAA
TTATTTGGTCAGGAAATAATTAATTTGCGCAAAAAAGATCAAATGGATCATAAAAATAAT
AAAAGTGATAATAACAGACGATCAAATCCATTTGATAATAAGAAAGAAGATGTTAATAT
TGATGCTTTAAAAAATAAATTACATTTAAGTTTGAACATAGAAAATCAGGATCATGATCA
ATGCAAAGATAAGTCACCATCAAGTGTTAAAGATGTTAAAGATATTATATTAGCTAGTTC
AAAAAGTATTTTAGAGAAAGTATTATCACCAACGAAAGAAAAATATTTGTCTAGAGAA
AAGAGTAAATCTCCACCAAGAATATCGCAAACACAGCAACGACCTATATTAATGACTCG
TCGACAATTAACAGATCCTTTTGGATCTGATGAAGAAGAAGAAAATATTCAAATAATTG
ACGAAAAATGGTCTCAATCAATTTCGATTAATAAATCAGAAACGCCAATTAATAATGATT
CAATCAATATAAGTGACAGAGGAAATCGTACTGAATATCAGACAATATCACCACAAGGA
GAACAACGTTGTCAAAATCCATTAGTCAGTCGACATGACGAATTAAGACAACGTGCCA
GACAACTATTAGAACAGGCTAGGAATCATACAAAGTCAACTGGTCTTATTTCTACTGCT
GCTTGTCCTATTGAGAGTCAAAATGATGATGAAAGACAGCAACAATTACGTGAAAGAG

CAAGACGTTTAATAGCAGAAGTGAAAATGGGTGTGAACATTACTTCAAATCAAATTAAT
GATGACAATAATAGTGATAGACGTTCAATAGATGATCAAAATAATAGTCCAAGACGAAG
TATTACACCGTCTACTCCGGGTGATAAATTTAGTACAAAG

>novel_circ_000272
CGTGTGACTGCCATCCGATCGGCGCTTCCGGAAAAACTTGTAACCAAAGCACCGGCCA
ATGCCCGTGTAAAGACGGCGTAACTGGCACTACTTGTAATAGATGCGCCAGAGGTTAC
CAACAGAGTAGATCGCATATCGCACCTTGCATCAAAATACCGAGAGTTGTGCAGACTC
AAGGCACGGCCGGCGAGGAGAGCGGGGAGCACGAAGACGACGACGACGATCGCGGT
TACGACGGACGAGCCG

>novel_circ_000273
CCCTTCGAAAATTTCTTCGGGCCGATCTTCCTCGAGGTAAACGCGCAAGGAGAGGCGA
AGGCAGCGGGCGAAAAGAGGAGAGAAGGGAATATCGTCTGCTGGTCCAGTGGTCGGC
TGGTTTCCGGTAGCGCGGCGCGGCTGCTGGATCCTCGATCCAGTGTGGCGGAACGCGA
GTAGTGGGGTCGCGGCGAGTAGTGGCGCCCCCGGATTGGCCGAGCAGGAATTGGTCTT
GGACGAGAGGATGCGAGCCGGCGGTCAAAGGGGAAAGAGAGGGAGACTCGGGCGAG
CGGAGGAAGAGCGGTGGTGGGGATGGCTGCGAGCTAGAGAGAGACTCGGAACAGTT
CGGATGCACACCGTGCAAGGACGGCGATTCAGTTAGCGGCGAGCCGCCGTCCGTGGA
GGATCGTTGATCGTCGATTTTTCTTCCTTCTCCTCGGGTCGCATCGCTTCGGCCCGAGC
GGCGTGCCGCGTACCGTGTCTTTTCGAGGTATACACACGTTTCAACGATTCGCGTTCGA
GCGAAACTGTCGCGTGTAACGCGCGCACGCGCCCCTACCGCATCTCGTTACGATCGTA
CCGTACTCGCTCAGGAATGAAATTGACACGCGCGTTCCGCGTTCTGAACATCGAATAC
ACCTGAGTGAATACACCTCTCGATGAAATTTATCGAAGCTGCTTTCCCGGCAATCGTCT
CGACGGGCAACAACAACAACAACGTTCGAAAATATTCGAGAGTATTGAATCGAAGAG
GCGAGCTTTCGAGAAGAATTCGCGAAATAATGCAAGTAAAGGAAGAACGATCGAAGG
AAGCGGGCTCGCTAGTCGGCATCGACGCGTTGAACGAATCGTAAAATCGATCGCGTCG
CGAGATGACGACCAGCCGAGAAGCGTAAGATCGAGAGTTCTCTGTTGGAGAGAGGAT
CGAACGGGATCGCAACCCTCGTCGTTCTTCCGTTATCGGGAGAAGAACGGAGCGAGG
AAATAATCGTCGGACGAAAGAAGGACGGTTTGCGCGCGTAGGTGACACAGTAAGGGG
CTGTGCGGGTAAGAAGGGACGAGGAGAGGCGAGGCAGAGCGAGAGGGAAAGGGGG
AAGGAGGGCGGGAAGGGAGAAGGGAAGGGAGAAGAGAGGAGATAAAATCGGCCGG
CGCGCGAAATAACACCACGGTCGAAGATGAGGAGCGGATCGAGGGACGCGCGATTCC
TCGTCGGGATCATTTGCGGAGTGATCGTGGGTATCGATCGCGGCTACGCGTTGGGGGG
GAGCGAGATCTACGCGAAAACGTTCCCCAGCCAGCAGCACCCATCGGATCCGTGCTAC
GACGAGGACAGGCCGCGACGGTGCATACCGAATTTCGTGAACGCGGCGTTCGGCGCG
GCGATCGAGGCCTCCTCGACCTGCGGGAGCGGCGGGCCCACGAGGTATTGCGACGTG
GTCGAGCAGCCGGGAGGTAGCACCGGCATAGGTCAGTGCCACATCTGCGACGACAGC
ACGCCGCGGCGCCGTTTCCCCGCGAGCTACCTCACGGACGTGAGCAACCCCAACAAC
GTGACCTGCTGGCGGAGCGAGCCGCTCGTCACGTCGCAGAGTTTCTCCGCGCCGCCC
GACAACGTGACCTTGACCCTCTCCCTGGGCAAGAAGTACGAGCTGACCTACGTCAGCC
TGCAGTTCTGCCCGAAGGCGGCCAAGCCTGACTCGATCGCGATTTACAAGTCGATGGA
CTACGGCAAGACCTGGCAACCTTTCCAATTCTACTCGTCCCAGTGCCGCCGCGTCTACG
GGAGGCCGAACCGGGCCACCATCACGAAGGCGAACGAGCAGGAGGCGAGGTGCACG

GACAGCCATCGGTACACGGGCGGCAACGGGCTCGCGCCCGTCGGCAGGATCGCGTTC
AGCACGCTCGAGGGCCGGCCGTCGGCCTCCGACTTCGACAACTCGCCGGTGCTGCAG
GACTGGGTGACGGCGACCGACGTCCGCGTCGTGTTCAACCGCCTGCACATGCCCCAA
CAACCGTCCCAGGAGCAAGTGCTGCAGCTGGGCGGCGGCGACGAGCACGGCCTCGGC
CTCGAGGAGCTGGCGAAACGCGAGAGGGAGCGGGAGGAGAGGCTGAAGAGCCAGA
AGAGCCGGGGCTCGCTCGACCCCCTGGCCACCGCTCTGCCCTCGGTGCACCAGGTGAT
CGCCCCCCAAGAGATGCAGTCGAACGACGCGGTGGAGGAGGGCGGGGGGGGCGCTT
TCGCGGCCGTCACCGTGTCCACCACGACGAGCAGCACCCTCGCAGCAGCCGGCCTCG
GGACGGGCACGCTCGCCCATCACTACGCCGTCTCCGACTTTGCCGTCGGCGGCAGGTG
CAAGTGCAACGGCCACGCGGCGAGGTGTATCCAAGGCAAGGACGGCGAGGTCGCCTG
CGAGTGCAGGCACAACACCGCCGGCAGAGACTGCGAGAGATGTCGGCCGTTCCACTT
CGATCGACCCTGGGCACGGGCCACCGCCAGGGATGCCAACGAATGCAAAG

>novel_circ_000274
CTTTATAATTTATAACTGATGATATGGCAGAAGCACATTCAGCTGTAGCATTTAGTTTTTC
TATTACTCATGAGGGATGGGATGTTAATTTTGATAGAGAAGTTTTACATTTAGTATGGCA
ATCAGGTATCCGTTCTTGGAAGAAAAGATTTTTTAGATTTGTTAACAATTTGAGAAGTG
GTGTATATCCTGCTTCTTTAGAAAGTTTATGGTTTACTATAGCTGTAGTGACTGCTATACA
TTTTGCTGGTTTTAAAGTACCTTATGATCTTGTTGGAAAAACTGCACCATATCTTTCAGC
ATCTTCAGTATTGGCTCATTTAGCAGGATCTTTTATAATTGGATTGCTTTTATGGTTGATG
GTAATATATGTAATTCGATATACTTTTAAATTGTTACTTATGTACAAGGGATGGATGTATG
AATCAAGAGAAAAAGGATGTAATGCATCGAGGATCACTAAAACATGGACCACATTAGT
GAAATTGTTCTTTGGGTGGCACAAACCAATGCTTTATAGTTTCCAAGGATCATTGCCAC
GGCTACCATTACCCTCAGTAGTAGATACAATGAAGCGGTACCTACGAAGTGTTCGACCA
TTGCTCGATGATGAAAATTATGCTCGAATGGAGACATTAGCTAATGAATTTCAAAAAGG
AATTGGTGTGAAACTTCAGCGATATTTAATTTTAAAGTCTTGGTGGGCTACTAATTATGT
TTCTGATTGGTGGGAAGAATATGTGTATTTACGTGGACGATCTCCTATTATGGTTAATTC
AAATTTTTACGGTATCGATGCTATTCTTATGTATCCTACTAATATACAAGTTGCTCGTGCT
GCAAGTGTTATTTATTCGTGTCTACAATATCGACGGCTCATTGAACGACAAGAATTAGA
ACCGATATTAATTCAAGGATTGGTACCTCTTTGTTCATGGCAATATGAAAGAGTATTTAA
TACTACAAGAATACCAGGACGTGAAACAGATAAAATTGTACATTATCAAGATTCTAAAC
ATATTGTTGTATATCATAAGGGAAAATATTTTAAAGTTCCTATTTATCACAAAAATAGAAT
TCTTCAACCTTCTGAAATTGAAAT

>novel_circ_000275
GGTGGATTCGCGAAAGTTTTTCTGATGACTGATGTCAATAATGGAAATGAATACGCATG
CAAGATTATCAGGAAAAACCGAATGCAAAAAATCCATATGCAAAAGATCGCACGTGAG
ATTATGATTCACAAAGAGCTGAATCACATGAATGTTGTCCAGCTGCATCACTACTTCGA
GGATAATCTTAACGTGTACATGCTTCTGGAAGCCTGTCCTCGAAAGAGTTTGATGCACG
TGCTGAAGTACCGTGGCAAAGTCACGGAGCCGGAAGCACGGTATTACATGAAACAAAT
GGTAACCGGTGTTGCGTATATTCACTCTCAGAAAGTTGTCCATCGGGATTTGAAGCCAG
GTAACATGTTCCTATCGGACCGTATGATCGTCAAGATCGGAGACTTTGGACTGGCGACA
AGGCTTGACGGACAGTGCAGACGAGT

>novel_circ_000276
ATAAACGTCGTAATAAGGAAATTATGGAGACATATGCCACCAAATTTCCAGACTACACA
TTCCTTCGAACAAAATTCGATACAAATCTTTTTCGAACTAATGAAACATTTTGTGTGGA
AATTAAACCAAAACAAGGCTATTTGCAAGATATTGATCAAAAATTCCAAAAGTGTCCTT
ATTGTTTGACACAATATTATAAGTTAAAGAAGAAGATGATCACATGTCGCAGTACTTATT
GTCCATTTGATCTCTTTTCTGGAGTAGAAACACGCATGAAATCGGCACTAAAAGGATTA
TTAAAATCACCTCAAAATAATTTAAAAATTTTTAAGAATGGTATTCTAGTCTATGATCAA
GAATCATCTTTAAGCGATCTTGAATGTGCTTTATCTGATTGGTTTCGTAATTCTATTAATT
CCACAAAAAAGGAATACATTGATAAATTCTGTAATTTGATTTGTACTGCTCTTTTACATC
CATTTATTTTAGAAGAATATAAGTCTAACATATTTTCTTCTTATGAATTATACACATCTGAT
GCACATGATTTAAAAACAACTTCCTATATTAATACAGATCTAATTGCAAAAACTAAAAAA
TTTTTGTATTTCGCTGGAGAG

>novel_circ_000277
AATCGAAGTGCCAGAGTAAGCGTGAGCAATGAAAACAATATATTACTGGACCCAGAAG
TATTGACAGATTTTTCTATTCAAACTTTAGTTTTAACCGTGTTGGTAACTTTAGTTAAAA
ATTCAACGGACGAAAATGAACAACGGATTCTTTATGAATATCTTGCAGAAGCTTCGGTG
GTCTTTCCAAAAGTTTTTCCTGTAATTCATAATTTACTTGATGCAAAAATTAATAATGTAT
TATCTTTCTGTCACGATCAATGTATATTGAATTCTGTACAAGCAATTATACAGAATATGAT
CGCATGTGAGGATACAAGTCAACAGCAATTACATTACTTGCAAAGTTGTGGATTTGGAG
GATTATGGAGATTTGCAGGTCCATACACAAATTCTAATTGCACAGCTGAAAGTGCTCAA
TTATTTGTTAATTGTCTGGAAGCTATGGTGGAAACGTGTTTACCAGTAGATGAACCTGA
AATTTTAAGCACTGAAATCCCAAGTGAAAAATTTAAACGATCCGATGGTCATTATTCAG
ATATTGCACAAAGAAGCAAATCTTCAACTCATGGAACTGATCGATCAAGTGCCCGCATT
TTTTCGGATAGAATGGATGAAGCTTCTCATAATGCTTCTTTCGTTCACAGAGATCGCAGT
ATAGAAAGTAGAGATGTTTCATTGGATTCAAGCCAAGGAGAAAATATTACTAATAATAG
TAACAATTCATTACCTTAGCGATTAATATGTGAATCCAAAAAACAATGCAGTAAACATG
GACGAAAAGGACAAGTAGGTCGAAAATTACAAAGACAATTAAG

>novel_circ_000278
ATGGTTGGAATAGAACAACTAATTCATATAAAAAATTAGATTATGGAAAATGGGAATTAC
ATTTACCTCCTAATGCTGATGGTAGTTGTCCAATAAAGCATCTTTCAGAAATTAAGATAA
TTGTAAAAGATCAAAATAATGAATTATTGGAACGATTAAGTCCTTGGGCTACATATGTCA
CACAAGATAAATCTGAAAGTGCTACATATAAACAACGTATATGGTATCCATTACCTGAAA
ATGTTTATAAATTTAAATATTCCAAACAGAAAAAACCAAAAAGTCTTAGAATTTATGAAT
GTCATGTTGGTATTGCAACTCAGGAATTAAAAATTGGCACATATCTAGAATTTGCTAAA
AACATAATACCTCGTATTGTAAAACAAGGCTATAATGCTATACAATTAATGGCTATCATG
GAACATGCTTATTATGCTAGTTTTGGATATCAGGTAACTTCATTTTATGCTGCTTCATCTC
GTTATGGTACACCAGAAGAATTAAAACTATTGATAGATACAGCGCATCAATATGGTTTAT
ATGTTTTATTAGATGTGGTACATTCACATGCATCAAAGAATACTTTAGATGGTTTAAATAT
GTTTGATGGTACTGATGGTTGTTTTTTCCATTCTGGCAATCGTGGTCATCATCCACTGTG
GGATAGTAGACTTTTTAATTATGGAGAATATGAAGTATTACGATTTCTACTTTCCAATTTA
CGTTGGTACATTGAGGAATATAATTTTGATGGATTTAGATTTGATGGAGTTACATCAATG
TTGTATCATTCAAGAGGATTTGGACAAGGTTTTACAGGTCATTATGAAGAATATTATGGA

CTTAATGTTGATGTTGAAGGTGTAGTTTATTTAATGCTTGCAAACTATATTTTGCATTATT
TATATCCAAATATGATTACCATAGCAGAGGATGTTAGTGGAATGCCTGGAGTTTGCAGG
CCAATTACTGAAGGTGGTTTAGGCTTTGATTATCGTTTAGCCATGGCTATTCCTGATAAA
TGGATTAAACTTTTAAAAGAAATAAAAGATGAAGATTGGAATGTTGGGGATATTTGTTG
GACATTAACTAATCGAAGATGGATGGAGAAAACAGTAGCTTATTCAGAATCTCATGATC
AAGCTCTTGTAGGTGATAAAACAATTGCATTTTGGCTTATGGATAAAGAAATGTATTTTC
ATATGAGTACACTAAGTCCACCTAATGCAATTATAAATCGTGGGATTGCTCTTCATAATC
TGATTACATTAATCACACATGCATTAGGAGGTGAAGCTTATTTAAATTTTATGGGTAATG
AATTTGGTCATCCTGAATGGTTGGATTTTCCACGAGCTGGTAATGCAGATAGTTATCATT
ATGCTAGAAGACAATGGAATTTAGTAGATGATGAATTACTTAAATACAAATTTATGAATA
ATTGGGATCGGGCTGTAAATACTCTTGAAGCAAAATATGGATGGCTACATGCTGAACCT
GGATATATAAGTTTGAAACATGAAGAAGATAAAATAATTGTTTTTGATCGTGCAGAACTT
ATATTTGTTTTTAATTTTCATCCAATCAAATCATTTCCTGATTATACTATTGGTGTAAAAA
GTGCAGGAACTTATAAAATTCTTTTGTGTAGCGATGATAAAAATTTTGGTGGAGAAAAC
CGTGTTGATACTAATATACAACATTTTACCAAACCAGAATCTTTTTCTGATTATTCAAATA
GTATGATGATTTACATTCCTTGTCGCACAGCTATTATTTATATTCGAGAG

>novel_circ_000279
ATGGTTGGAATAGAACAACTAATTCATATAAAAAATTAGATTATGGAAAATGGGAATTAC
ATTTACCTCCTAATGCTGATGGTAGTTGTCCAATAAAGCATCTTTCAGAAATTAAGATAA
TTGTAAAAGATCAAAATAATGAATTATTGGAACGATTAAGTCCTTGGGCTACATATGTCA
CACAAGATAAATCTGAAAGTGCTACATATAAACAACGTATATGGTATCCATTACCTGAAA
ATGTTTATAAATTTAAATATTCCAAACAGAAAAAACCAAAAAGTCTTAGAATTTATGAAT
GTCATGTTGGTATTGCAACTCAGGAATTAAAAATTGGCACATATCTAGAATTTGCTAAA
AACATAATACCTCGTATTGTAAAACAAGGCTATAATGCTATACAATTAATGGCTATCATG
GAACATGCTTATTATGCTAGTTTTGGATATCAGGTAACTTCATTTTATGCTGCTTCATCTC
GTTATGGTACACCAGAAGAATTAAAACTATTGATAGATACAGCGCATCAATATGGTTTAT
ATGTTTTATTAGATGTGGTACATTCACATGCATCAAAGAATACTTTAGATGGTTTAAATAT
GTTTGATGGTACTGATGGTTGTTTTTTCCATTCTGGCAATCGTGGTCATCATCCACTGTG
GGATAGTAGACTTTTTAATTATGGAGAATATGAAGTATTACGATTTCTACTTTCCAATTTA
CGTTGGTACATTGAGGAATATAATTTTGATGGATTTAGATTTGATGGAGTTACATCAATG
TTGTATCATTCAAGAGGATTTGGACAAGGTTTTACAGGTCATTATGAAGAATATTATGGA
CTTAATGTTGATGTTGAAGGTGTAGTTTATTTAATGCTTGCAAACTATATTTTGCATTATT
TATATCCAAATATGATTACCATAGCAGAGGATGTTAGTGGAATGCCTGGAGTTTGCAG

>novel_circ_000280
GTTTATAAATTTAAATATTCCAAACAGAAAAAACCAAAAAGTCTTAGAATTTATGAATG
TCATGTTGGTATTGCAACTCAGGAATTAAAAATTGGCACATATCTAGAATTTGCTAAAA
ACATAATACCTCGTATTGTAAAACAAGGCTATAATGCTATACAATTAATGGCTATCATGG
AACATGCTTATTATGCTAGTTTTGGATATCAGGTAACTTCATTTTATGCTGCTTCATCTCG
TTATGGTACACCAGAAGAATTAAAACTATTGATAGATACAGCGCATCAATATGGTTTATA
TGTTTTATTAGATGTGGTACATTCACATGCATCAAAGAATACTTTAGATGGTTTAAATATG
TTTGATGGTACTGATGGTTGTTTTTTCCATTCTGGCAATCGTGGTCATCATCCACTGTGG
GATAGTAGACTTTTTAATTATGGAGAATATGAAGTATTACGATTTCTACTTTCCAATTTAC

GTTGGTACATTGAGGAATATAATTTTGATGGATTTAG

>novel_circ_000281
GCACCAGATGGCGAAACACAACCAAGTACAGAAGTTGACTTATTCATCTCAACAGAAA
AAATAATGGTTTTAAATACGGATTTGAAAGAAATTATGATGGACCACGCGTTAAGAACA
ATATCATATATAGCAGATATTGGTGATGTAGTAGTCCTAATGGCACGAAGACGCTTTGTA
CCGCACGAAATGGAAGAAGCACCAAAAATCAACAGAACTCCAAAGATGATTTGTCAT
GTTTTTGAAAGTGAAGAGGCTCGATTTATTGCACAAAGCATTGGACAAGCATTTCAAG
TAGCATACATGGAGTTCCTTAAAGCAAATGGAATAGAAGATCATAGTTTTGTAAAAGAA
ATGGATTATCAGGAGGTACTCAATTCTCAAGAAATCTTTGGTGATGAATTACAAATGTTT
GCCAAAAAAGAAATGCAAAAAGAGGTTGTAGTGCCTAAAGCAAAAGGTGAAATCTTA
GGTGTCGTAATTGTCGAATCTGGATGGGGTTCTATGCTACCAACTGTTGTAATTGCAAAT
CTGGCACCAGCTGGTGCGGCTGCTCGTTGTGGACAATTAAATATTGGTGATCAGATAAT
AGCTATAAATGGTGTTTCTTTAGTTGGCTTGCCTCTCTCTACTTGTCAGACTTATATCAA
AAACTCAAAGAATCAGACAGTTGTCAAGTTAACTGTCGTTCCGTGCGCGCCAGTAGTT
GAGGTCAAAATCAAAAGACCTGACACCAAATATCAGTTAGGATTTAGTGTACAAAACG
GAGTAATATGTAGTCTATTGAGAGGTGGAATCGCTGAAAGAGGAGGTGTTCGAGTGGG
TCACAGAATTATCGAAATCAACAATCAAAGTGTCGTTGCTGTACCGCATGAGAAAATC
GTCAATCTTCTTGCCACATCTGTTGGCGAG

>novel_circ_000282
AATTATAGAAATTGCTGGGGGACGAGAAGTTTTTTCTCAAAGCCGAGGTAAAGTAGCA
GCTAGAAAATCACGATTTCTTGCAGCATCAAATTCTTTAAATAATGAAGATATTCAAATA
GACAACAAGCTATCAGTTAAAAGAAATAATAAATCTCCAAATACTCAAACTATATGGGA
GTTAAGAAGTCGATGTGGTGATACAAGAAAACAGCGTTCTAATAGAGAAGTTACAGAA
ACTGTATTTTCTTCAGAAATACATTCAGTGCGACATAATCCAGCAAAATCTATTGATTAT
GATTCCCCTAAAAGCAATCACAGCAATCATATTATTAATTATGACTCAATTTTGAATAGC
AATAATGTAGAATATGATGTGTCAAAAAATACTGCAGATTTAGATTATGGATTATCAAAA
AACTGCATAGATTATCAATCAAATCATACTACACCAGCAAGAAGCATGGCTATTGTTAGT
GATGGTGAAGTAGTTGTTTTTGATGATATTGATGATAATTGGCAAAATTTGCCACTAGAT
TTAACTAATAATACTAATCAAGTTACATCAACTAATTTAAGTCTTGAGACTGAAGAATCT
CAAAAAGGTCGCATTCCTCCAGAACCTTTAAGTTCTAGCATTGAAAGTACTCCAAGTC
CAACAACAGCGTATCATCGTAATACTTCGGAATTTTTTAAGGTGATTCCAGCTAGTGATT
GTGAAATAGAATCACCATCTTCAGAACGAAATCATAAAGTTACAAGAGTAATTGGTGAA
TTACCAATAGCACAGTATTCTGAAAGTCCAAGACGTTATGGAATACGTGATACTCAACT
TCCATGTCTTCTGTCATCGCCATCTGTATATATGACACCAAGACCTGGTTTTCCACAAAG
AGTATTACCAACAACACCAAATCATAATGAG

>novel_circ_000284
ATTTTATTATTAAAATGGCTCCGACTATTTACTTGTCGGAGATACCTGTTGTAAGAACAT
CTACAATCCCATGTTCACGGCGAAGACAAAGGATTCATAATGGAGCTGTTGCAAAACA
ACAAAGGCTAGAAATTAGACGATTAGAAACGAATGAGTCCTCACGCGATAATGGGTCT
TTGACACCACCGGAAAATTCTCTCTGGGAACCAGAAAATTCCAATGACTCCTGTGATC
CGACAATCGTTAGTTCTTCCTTTCCAAATGTTTCTGCTAGAGTTCCAGTGGACACAAAG

GAACACTGGAAAGTGTTTCTAGCGAGACGAAAAGGTCTTCTAGACATTGTCGTACGTA
CTATGGCTCTGATACGGCGAAACAACATTCTTCAAGAAAGAGTAAACGCCCTGCGCGC
AGAGACTCGAGACTTTATTCATTCAGTATTGAACAATCCCGAAAACAAGTGTATACAAC
AAAGATTGGATATTATTGACTGCAAGGAAACGGATGTGACCTCGTCGACGGAGAAAAC
TATACCAAGACCATCGCTTCCGCCCACACCAAACTCGATAAATTCCTCGTCAAACGACG
TCACGTCAACCTGTAGTAACAACGAAAACGACTCTGAACAATCCGACATCGAATCGTA
ATGTTTGAGAATCATGGCGTAAGAAAAAGAG

>novel_circ_000286
AGTCGACGAGCGACGTGACGGAAGGTGTTTACGCCGGCGAACGTGCCCGATTGGTTT
ACGGGGTGTTCACGACCCCTGTGAATTCAATCGGGGGCTCGGCCGTCTGCGCGTTCTC
CATGAAGAACGTCCTCGAAGTCTTCCAGGGTGCGTTCAAGGAGCAGGAGACCATAAA
CAGCAATTGGCTACGTGTACTTCCGGAAAAGGTGCCCGAGCCAAGGCCTGGTGCATGC
GTGAACGACTCCAGAACGCTTCCAGACGTGACCGTAAACTTTGTGAAAGCGCACCCC
CTCATGGACGACGCGGTCCAATCCTTCTTCTCATTACCTGTGATAACCAAAGTCAGCTT
CAATTACAGATTTACCAAAGTCGCCGTGGATCCTCAAGTGAAGGCGTTGGATGGCAAG
GCCTACGACATTCTGTATGTCGGGACAGACGACGGCCGAGTTCTCAAAGCGCTCAACA
CTCGTGCACCGGATTCACTGAGCAATGTTAGCCAGGTCATCGTAAGCGACGTTCAAGT
GCTGAAGTATGGACAAGCGATCAAGGATCTGTTGGTGGTCCACCTGGCCGGCGAGGCG
AGCAAGTTGGTCGTGTTTGCTGACTCCGAAATTCGCGCGGTACCACTGCATCATTGCG
ACGCCCCTGCGGCGGCCACCTGCGCGTCTTGCGTCGGTCTTCAGGATCCTCACTGTGC
CTGGGACGCGACCAAGAACCTGTGTGTGGCCGTGTCGACGAAACTGCACGACAGCGA
CGCCGACAAAACCCTGTTCCAGGACATCGTGAACGGGAGACACAAGGGATGCGGCTA
CGAGCCAG

>novel_circ_000288
ACTTGTCTCCGGTGCGATGGGCTCGCCGGAAGTCGTCGAGCTCCGAGTCGGAGAGGA
ACTGCTACAACGTGCAGACGTCGCAGGTGACCACCGAGCGGAGTCCCACCAAGAAGA
ACAAAAAGAGAATCCAGAAAGAGAGGCGGAGGGTATGCGAGAAACGAAACCCGTTG
TTGGACCGCGTGAAAAATACCAGGTTGAGCTTCTTCAAGGATCGAGATTCGGACGAGG
AGGAGCAAGAAGAGGACGAAGAGCCGGAGAAGCTGCAATTTCGTTCCATGTGCGAGT
TGAACCAGCACAGCCTCACCAGAAACGAGTTTCGTAGATCGGTCTGTGAATCAGATTT
GAAAGAGATCATCGAGGAGGAGAAAACTCAGGCAAAGAGGAAAAGACGGTCGAAAT
CGCAGAGCAGATTACCTCATCGAAAGCAGCAGGAGGATCGCTCCTCGTTCCTGGGTCT
GCGATCATCCGCTGGCCAGAAGATGGCGACTTTGTCCGACGACATAATCGAGGAAACC
GTAGGTCAACAGAATCGAAGGTGGCGGAAGAGTAGAGAGTTCGTTCAGGATCATCAG
ACACCGAGACGGTCGTCCATGGATCACGTTCTAGAAGGTTCTACCAGCACCGAGGATG
CAACGTTCAAAGATGGCTCTCAGTTTGACAGTATAACACCGTCGAGCTGTTTGGCAGC
CAAGTTTCGGGCAATGCAGGACAAGTATCTGAAAAGTTCCACCAGCAAGTTAATAGCG
AAGATCTACAAGAAGGAGGGTAAGGACAAGGAAAGAAGAAGACTGAGGAGTTTCTC
GTACGGCACTTTGCCCGGCCTCGAGGAGCTCAGAACGAATCCCCTTTACGAGGAACAG
GATCAGGATGACAACGATTCCGGTATCCTGGACAACGACTCCGCCACCAGCTCTTTGC
TCGACGACAGGTGCAGCAGTAGCGCCTCTGGACTGTTAACCACGCTCAACGATTCGTC
CTTGAATTCACCACCCCAGTTACCACCGAGGAAACCTTCGTCCTCCATCGTGGACGTT

GAAAGAACAGCGAAGATGTTCTCCAGTTTGGACGTCTATTGTGGAAGGAACGAGGGG
AGAGGTTGCAGAAAGGACATGTCCAGGTTTAACGCTCTTGAAGATTCCTCGAGCCAGA
TCTGTCAAGCTGCGAACAACGAACTGATAGATCGACAGGATCGTGACAGCTCGAAATT
GTCCGAAGAGAAGAACGTGATCGAGAGGTTGAACAGTAAAGACTGTTTAAGGTCGGA
GAACTATCAAAGCTCCAGGCTTTCTGTTATCAGGAACAGGTCTTACACCACGGAGACG
ATGGTCGTGAAGCTTCCTAGAGAGAGTTCGGATCAGTGTCTGGGCATCTTCATCGCGA
AAACAGCGGAATCCAGTCCCGGTTACCTGGTGGCTCATGTAGTGCCTAATGGGTTGGC
CGATAAAGAAGGCACTTTGAGGATAGGCGACGAAATTTTGATAGTTAATGGGAAGAGG
CTACGAGGGTTGAGCATGGCCGAGGCTAGGAAGATTCTTGGAAGCGGAAATGGACCT
GGCGACGTGGACATCGTCGTCTCCAGGTACTCGGCTGTTGACCAGTCCCCTAAGAAGC
TGACAGAGAGCAGCGTTGACTACGAGAACGTTCATATGGAGAATGGCCACGGTGTCAT
CGTGGAGAATTCACCTGGTACGCACTTCAGAAAACATCAGACGAAGCATCATCGGGAC
AGGAAGAACGAGTGTAACAGGACTGGCTCCTCGGATAAGGCTGTCGTGGAGAATGGT
ACGAGCTCTCAAAATGTTTCGAATTTTTGCACGCTGCCAAGGAGGCCTAGGAACACCG
TCTCGACGTTCCTCACCGTCGTGTTTCAGAAGGGTCCTGGAAAGAAATCGCTGGGATT
CACTATTGTGGGTGGAAGCGATAGTCCTAAGGGAAGCATCG

>novel_circ_000289
GGAAGAAAAAGAAATTATTTATTGTTGAAGAAGGTGGTCGAAAGTGAGTCGAAATTAA
AGGTTGAAAATTTGCCAGAGGTTCTGAGAATTTCTCGAGGTGAACGTGAAGGGGTTGC
GTGCATTGGGCGCAAGAGGGTGCGAATTAAGGGGACGATAATGCACGAGAATGAACG
CGAGGGAG

>novel_circ_000290
GCATGCTATGCGTCAGAAGACTGGAAACAGATGTTCTCGCGAAAGATGACAGGGTTAA
GTAGATAAAAGGAGCAGCTGCTGGTTCCAACGACGATTCATCGCGTTTTCCATGCAGT
GCACAATGCTCTGCCATCGCGCCCTTGTCGTTCTCTTTGCTGTCAACGTGCCAATCGGT
AAGTGAACGGGTTATACGGTGTCGTTTGCCTTCACTGTCCCGTTTGTACTCATCTTGTTC
GTGAGAGCAGAATCGGGCTCGACTCGATTTCAGAATTTTACTCGATATCGGCTGCCTTC
GTCTCATTGAATTGCTCCGCGTTCAGGAATTAATTATTCCTGGGAAAAATTAAGTGTTCT
AGGAACAGGAAATTTTTTTATCGATATTCAAATAACTCAATGAAATAAAGAAATAATAC
AGATATGTTAATTGGGCCTGAAGAATGGAAAGAGGATATTAATTTCGTGAATCGATTTTA
TAAGTGATTGTAATCGTTCCTTTTTATAAGAGATACGTTTGTTCGAAGTGATCGATTTTT
AACCGAGGATAAAACGAATCAATAATTGATGATCGTCGTTTTCATCCGTGGTGAAATCG
ATCCGAGGTGAAATCATCGGACTCAATAGAGATATATGGACGCTGTACAGCGTTTAAAC
AATTGCTCGATAAAAGATGGAGGATCTTTTGTTCCGTCGAAACGGTAAAAACGAGCGT
ATCCTATGCACGTGTCGTTATCCCCGATTTTCAGCTGTTAAGCTTTTGTATAGATGGGAA
AAGCGGCGGATATTTCCGGAAACGGAATATATCTCTTACAAACCGTTTCGATTCGATTG
GTTTCTCATCAGATCTTAAATAAATTCCGTGAGAACGATCGAGAACGAGAATTTGAAAT
TGCATGCACAGAGGGAGCCGCGGATGAGAAATTCAACTTGTCACGTGCTATCGATATT
GGAATCATTTTTTATTAAATTCCCTTACACGCCGCCAAGAGTTTCATATTTCATAAGAAA
TAGTTTTGTGATTCTCAAAGGGGAATGAATAGAGGCGTGGTTTATGAATTTCAAATAAA
ATTACGATTACGGTGCTTCAGTGCAGTGTATTTTTCCGAGTACGTGTCTCTCTCTTTTTT
TTTTTTTAAGTGGAGAAGAATTGTTATTACATTCTCTTTTATTTATTCTATGTTAAATGTG

AATTTTCTTTTTTTTTTTTTTTTTGTTATATTTGCGTGTCACTTATGAAATTCTTGTTGCGT
CCATGCGTTTAAAAAAGTTATTCGTAAAATTATAAAGGACGCTTCTTACCACGTGCCTG
ATTATTAATCTGTCTCTATTCATTGCTTTGTATTATCTTTTTTTTTAACACTTTAAATCAAT
AATATTTACTTTATTTTGTATTCTAAATTAATTTTAGATATAATTGTTTCGCAATTATTAAA
GTATTTCAGCAATAATTTGTAATTTGTTTTACGAGAAAATGTTTCAAAAAATCAAAAATC
GCATAATTTCATTAAAAAAAAAAAAACAAATTTAAATTTCATTCATGTCCACTTACGGA
AAAATTAATAACTTTCATATGAAACATTTTTTCGAGTTATTTCGACTAGTCATATCGTTCT
TTAAAGATTCATCTTCTATATATATTTCCTCTCTATTCACAATTTTTTTTCTTTAACTCTCT
GTCTCTCTCTCTTATAAATTTCAATTTCATTCTAGGATGATCAGAATTACCCCATCCTCGA
CTAAAAAACTCCATCCCAGAAAGACGAAGAAAAAAATACAACCCCTCTCTTCTTCTTC
GAGAATAAAGAAAAAAAAGGGGAACGGATGCGAACACGCCGCGGATGGAAGCACACT
CCCTCCCTCTCCCCCTCCCAAATTCCGCTCGAAGTCAATCATCAAACAGTTCTCTTCGG
GGTAAACTCGGGAATTGGGGCGCAATCGGTCGGCGCAAAAGCAGTTAGAGCCCGCTA
ATTTCGCCGAGGCCTCCCCGCCGAATTGGATATCAATTCGTATATCGCGCCGATTCGCGG
ATTGCGATCGACACAGTTCGGGTTTGTACAGCGAAATCAAACCCTCCCCTCCTCCTCCT
CCTCCACCGTCACCGCGACGTCCGATGCAGATGCAAGGGAGGGGGGCCGGGAGGAAG
AGAGAAGGGCGCGGAGAAACGAGATTTGGAAACCTGTGCCAAACACGCGGGGAAGG
TTTGGATGAATTCCGGATATGCGCCGGGATCCGGAAACCGACATCCACGAGCGCCAAC
GAGAGGAGGACGAACCCGGTCAACTTCTGCCGTTGATGTTTATTCGCCGAGAGCAACG
GGGGTACGGCGACTGGGCTTGTCGTTCGTCGCGAATTAATTCACCGTTTCCGGGTCCC
GAACCTTTACGGCTCTTCGACGAGATTCGAGGCTCGTCGTACGCCCTTCGATTCCACCC
TTCGGAATTCTAGCAGGAATCCGGCTGGATCGGAATTCTCCTCCTAAAAAATAAATTG

>novel_circ_000291
GCATGCTATGCGTCAGAAGACTGGAAACAGATGTTCTCGCGAAAGATGACAGGGTTAA
GTAGATAAAAGGAGCAGCTGCTGGTTCCAACGACGATTCATCGCGTTTTCCATGCAGT
GCACAATGCTCTGCCATCGCGCCCTTGTCGTTCTCTTTGCTGTCAACGTGCCAATCGTG
ACGGGACTCGAGCCAGACTTCGTATACCCGTTGGAGAACTTAACGATCTCGCAGGGTC
GAGACGCAACCTTCACCTGCGTGGTGAGCAACCTCGGTGGATACCGGGTGAGTCCTTC
CTCCTCCGTGAGCGGAGATCACTCTGGCGGCGCCAAGGCCCGG

>novel_circ_000292
GCATGCTATGCGTCAGAAGACTGGAAACAGATGTTCTCGCGAAAGATGACAGGGTTAA
GTAGATAAAAGGAGCAGCTGCTGGTTCCAACGACGATTCATCGCGTTTTCCATGCAGT
GCACAATGCTCTGCCATCGCGCCCTTGTCGTTCTCTTTGCTGTCAACGTGCCAATCGTG
ACGGGACTCGAGCCAGACTTCGTATACCCGTTGGAGAACTTAACGATCTCGCAGGGTC
GAGACGCAACCTTCACCTGCGTGGTGAGCAACCTCGGTGGATACCGGGTGAGTCCTTC
CTCCTCCGTGAGCGGAGATCACTCTGGCGGCGCCAAGGCCCGGGTGGCGTGGATCAA
GGCCGACACCAAGGCCGTCCTGGCGATTCACGAGCACGTGATCACGAACAACGCCCG
TTTATCAGTTACACACAGTGACTACAATACGTGGACGTTGAACATTCGTGGGGTACGCC
GAGAAGACCGGGGCATTTATATGTGTCAGGTCAACACGGATCCTATGAAGAGTCAGAG
TGCGTTTCTCGAAGTGGTGATACCGCCGGATATAATATCCGAAGAAACCTCGAACGACC
TGATGGTACCCGAGGGTGGGTCTGCAAAACTAGTATGCAAAGCCCGCGGTTATCCAAA
ACCTGATATCGTTTGGAAACGGGAGGATGGCGCCGAGATTATTTCCCGTGCCGGATTGT

CTGGTGGCAAAACGAAAT

>novel_circ_000293
TGACGGGACTCGAGCCAGACTTCGTATACCCGTTGGAGAACTTAACGATCTCGCAGGG
TCGAGACGCAACCTTCACCTGCGTGGTGAGCAACCTCGGTGGATACCGGGTGAGTCCT
TCCTCCTCCGTGAGCGGAGATCACTCTGGCGGCGCCAAGGCCCGGGTGGCGTGGATCA
AGGCCGACACCAAGGCCGTCCTGGCGATTCACGAGCACGTGATCACGAACAACGCCC
GTTTATCAGTTACACACAGTGACTACAATACGTGGACGTTGAACATTCGTGGGGTACGC
CGAGAAGACCGGGGCATTTATATGTGTCAGGTCAACACGGATCCTATGAAGAGTCAGA
GTGCGTTTCTCGAAGTGGTGATACCGCCGGATATAATATCCGAAGAAACCTCGAACGAC
CTGATGGTACCCGAGGGTGGGTCTGCAAAACTAGTATGCAAAGCCCGCGGTTATCCAA
AACCTGATATCGTTTGGAAACGGGAGGATGGCGCCGAGATTATTTCCCGTGCCGGATTG
TCTGGTGGCAAAACGAAAT

>novel_circ_000294
TGACGGGACTCGAGCCAGACTTCGTATACCCGTTGGAGAACTTAACGATCTCGCAGGG
TCGAGACGCAACCTTCACCTGCGTGGTGAGCAACCTCGGTGGATACCGGGTGAGTCCT
TCCTCCTCCGTGAGCGGAGATCACTCTGGCGGCGCCAAGGCCCGGGTGGCGTGGATCA
AGGCCGACACCAAGGCCGTCCTGGCGATTCACGAGCACGTGATCACGAACAACGCCC
GTTTATCAGTTACACACAGTGACTACAATACGTGGACGTTGAACATTCGTGGGGTACGC
CGAGAAGACCGGGGCATTTATATGTGTCAGGTCAACACGGATCCTATGAAGAGTCAGA
GTGCGTTTCTCGAAGTGGTGATACCGCCGGATATAATATCCGAAGAAACCTCGAACGAC
CTGATGGTACCCGAGGGTGGGTCTGCAAAACTAGTATGCAAAGCCCGCGGTTATCCAA
AACCTGATATCGTTTGGAAACGGGAGGATGGCGCCGAGATTATTTCCCGTGCCGGATTG
TCTGGTGGCAAAACGAAATTAGCAACCGCCGAAGGGGAAACGCTGACTCTGTCGAAA
GTAACCAGAAGCGAGATGGGCACTTACCTGTGCATCGCGAGTAACGGAGTACCACCAT
CAGTCAGCAAACGGATGATGTTGCATGTGCACT

>novel_circ_000295
AAATTATGCGTGAAAGAGAGGAAGAGGAAGAGGCGAAGGAAAGGAAAAAAAACGAA
AGAAGAGCAAGAGAAAAAGAAGCAGCTTATCAAGAACGTTTGAGAGCATGGGAAAC
ACGGGAACGTCGTAAACAAAAAGAATATGAAAAAGAAACGGAAAAACGTCGAGCAA
AACGGGAAGCACGAGAAAGAGAAGCAAAACGATTGAAAGAATTTCTCGAAGA

>novel_circ_000298
GAGAACATCGCGGGGGCGTCATAGCCGGAAACGCGCGTAGTAGTAGCGAAAGAGGAC
GCATTTCCGCTTACAGCGGAGTCTCCGATCACGCCGATAGTATGGACAATCCTTCCTCG
CGGGAAGATATTTCAGAAATGGTGCAGCAAAATATTTCCAGTAAGCCAAAGACGTCAA
ACATAAACAGGTCTGAAAGTTACAAGGAGCGGATACATCACAAGCGACAGCTACGAG
AGAAGAGAAAGACCAGCGACCCAAATCTCTCTAAAACGAA

>novel_circ_000300
ATGCCACAGACGGTACCTACCGCAGCCGGCTACTCGCCGTCGTTCGATTACAGCACATT
CCAATTCGATTTATCCCTTCTTTCGGACGATTATGCCGCGACGAACGCGCAAAACACGT

TGAAACCAGCTCAAATCAAACAGGAAGCGCAATCGCCGCGTCCCGGCTCACCGACCA
CGTACTCGAGCTCTCCTTACCCCGGCGCCGGCGGTGAACTGTCCCCTAATTACTCGCAC
GAGGGTTCACCAGGATACCCGACCAGTCCCTACTACGTACCCAGAAGTCCTCAAAGTG
CTCAATTCTACCCGACAAGCCCAGAACCCTCCGCGAAACAGCCGGTTAAAAGGGAGA
AGAGCCTCGATCTTCTGGCCATCTTGCAAGAGTCCAGACTTCTGGCCGAGAGTCTGGG
ATACAGGGAGGACTCGACCGACTGCACAGTGCCGTGCACACCGCCAAGAACGGAGGA
GGACATTTACAACAGCCTTGACGCGGACTTCTTCGCGAAGAAAGAGGATACCGCAGTG
CAAGGACACCCGTTGCTGAAGGAGATCCTCTTCAAGTCGGAGCCACGGTCGAGCTGC
AACAGCAGCAACAGCAGCACAACGAGCAACACCAGCGATTCCTCGTCCACGTTGTCG
TCCTCCCCGTCGAGCGCTTCCTCCTCGCCCGACCCCGTGCAACTCGAGAACAGCTCGC
CGTTGAGGAGCTTGCTGTTCAAGGGTGCCAGAAAGGATCTCGCGGACGGAGCGAGAA
CAAATGTTCTGAAACTCGAGAGAATACAGGACGAAGTGAGGAATCCTCTTCAGAAGG
AGGAGGAGCTGTTCAAGGACGGGCAAAAGAGCGATCACCAGTTGCTCAGGGAGGTTC
TGAGGGACACCAGCTTTCAGAGGAAGTACAATCTGAGACCCGTGGACCTGGGCAGCG
TGGGGACAGGGTTCGTCGAAGACATGGAAGCCGGCGAATGCGTCGGTGACCTGACCA
GGGAACAGATAGAGCCTGTCCTCAGCCTGGCCATACAACAATTGCAAAAAGACTTTGA
CAACACCTGTGTCGCGCTTGGTATTCATCCTG

>novel_circ_000301
ATGCCACAGACGGTACCTACCGCAGCCGGCTACTCGCCGTCGTTCGATTACAGCACATT
CCAATTCGATTTATCCCTTCTTTCGGACGATTATGCCGCGACGAACGCGCAAAACACGT
TGAAACCAGCTCAAATCAAACAGGAAGCGCAATCGCCGCGTCCCGGCTCACCGACCA
CGTACTCGAGCTCTCCTTACCCCGGCGCCGGCGGTGAACTGTCCCCTAATTACTCGCAC
GAGGGTTCACCAGGATACCCGACCAGTCCCTACTACGTACCCAGAAGTCCTCAAAGTG
CTCAATTCTACCCGACAAGCCCAGAACCCTCCGCGAAACAGCCGGTTAAAAGGGAGA
AGAGCCTCGATCTTCTGGCCATCTTGCAAGAGTCCAGACTTCTGGCCGAGAGTCTGGG
ATACAGGGAGGACTCGACCGACTGCACAGTGCCGTGCACACCGCCAAGAACGGAGGA
GGACATTTACAACAGCCTTGACGCGGACTTCTTCGCGAAGAAAGAGGATACCGCAGTG
CAAGGACACCCGTTGCTGAAGGAGATCCTCTTCAAGTCGGAGCCACGGTCGAGCTGC
AACAGCAGCAACAGCAGCACAACGAGCAACACCAGCGATTCCTCGTCCACGTTGTCG
TCCTCCCCGTCGAGCGCTTCCTCCTCGCCCGACCCCGTGCAACTCGAGAACAGCTCGC
CGTTGAGGAGCTTGCTGTTCAAGGGTGCCAGAAAGGATCTCGCGGACGGAGCGAGAA
CAAATGTTCTGAAACTCGAGAGAATACAGGACGAAGTGAGGAATCCTCTTCAGAAGG
AGGAGGAGCTGTTCAAGGACGGGCAAAAGAGCGATCACCAGTTGCTCAGGGAGGTTC
TGAGGGACACCAGCTTTCAGAGGAAGTACAATCTGAGACCCGTGGACCTGGGCAGCG
TGGGGACAGGGTTCGTCGAAGACATGGAAGCCGGCGAATGCGTCGGTGACCTGACCA
GGGAACAGATAGAGCCTGTCCTCAGCCTGGCCATACAACAATTGCAAAAAGACTTTGA
CAACACCTGTGTCGCGCTTGGTATTCATCCTGAGCCACGACGTTGGAGCGCCGCGGAC
GTAGCAGCCTGGATCCAATGGGCTAGAAGGCAACTGCAATTGCCATCTGTCCCACTGG
AGAGCTTCAACGTGGATGGCGCTACATTGGCTTCCCTCACGGAAGAGGAATTCTGTCA
GCGTGCCCCTCAG

>novel_circ_000302
CTGGTTCGGCAGCCGAGAAGGGATACTTTGGTCCGTGGAGGCAGTGCAAACTTCTGTT

ATACGGTAGAGAAAGATGCGGCCAAGGGGTTTCCAGGTTTCAACCAGTGTTGGCTGTG
TGGGTGGCAGGATTAGCTGCTGCGACCGCCTCCGCCCTTTTGGCCATCCTGGTGGGTCT
TGCCGTGCTACAACTTGCGATGGCCTCCTCGGCAAAGCGAGTTATTATCTCGTACAGCA
CCGCTTTGATAGGGAAAGTGGCACTCGCCACATTGGCCACTTCGTTGGCGATCGTGGC
GGCGAGTCTGTTCGCTTTACAGACCGATGACAGAGCCAGCAGCTTCGTTGTCACGAGG
GGAGAAGCGTTTTATATGCAG

>novel_circ_000303
GTAAGCACTTGTTTATTTGTCTCGTTGAAGCCTTGCCTTGGATACCCTGCCGTGAGTAAT
GGTTTGATATTAACTAAAAGAGGATTGGAAGTTGTTGAACGATTAAGTCGCTTAGTCAT
ACCGCGAGCGCAAAGTAGGTTGTTCGGGAAACGTTCAAGTAGCGGATGGGGTGGCTT
TTGTTGAAACAAAGGGATCATTTGTTTTGTTTTCTCCAATGTTTTATCTGATAATACAGG
AAAGAGAATGCAAGATATCGTTTAATAATAACGACGAGATGATTCCTTTAGTTTTAGAC
TTCTTTTCTTTTCTTTTTTTTTTTTATCGTAAGTTTTTATCATACCTCGCTTCTCCAACGAA
TTTTAATTTTAATTACAATGAAACGTATATCTGTATATGATACGCGTAAGATAAATTTAAC
ATCCTCGGACGGAATCGATTTATTATTTATTTGGAAACGCGTAAAAATTTCCCGTTTAAC
AAAAATATTTATCTTTAAAATTTTGAAATTAATATATATATATTCTTCTATATCTTTATTTCA
AACGAAGGAGAAACGTATTTACTCTCGTACCCTTTGATTTCAAATTTTAATTAATTCGAG
TCACGCCATAAATAAAATACACGACGTAGGAAAAACAATGGACGGCGAAGCGTCGCC
GGATTACGTTTAGGAAGAAGGGAGGAGGAAAACGGGACGGGATCGAAGATAAACGTA
AGGCAACCGAACGTAAATCGCGAGGCAATCAAACTGAATTTTAATTCGAAAGGATATC
GAGCGCGCGATATTGGGGAAAAAATCCAACGCGAAGGGGGAAGAAGAGTGGAGGAG
AGATAGAGTTGGGGGGGAGGAGGATAGAGAGGGACGCGGATATCGTTTCGTAATCGAA
TCGCGTTCCACGTGGATGGAGGACACAGAAGGGAGATCGCAATCCCCAGTCTGCTTTC
TGTTCAACGGGGGATGGTCAAGCCCGCTTTCACGGTCCGTGCCGCGCTTCGTTTCGAT
TGTTCGACGTATCGATACTCGACATCGGCCATGCCCGTCTATTATTCGCCACGGCCAATG
GCCACCGTTTTATATATATATATATATATATATATATATATATATATATATATATATATATATAT
ATATATATATATATACTACAGCCTTTATGATTTGTTACCCTGTTTTCGATGTGTGTTGTTAT
GACCAACGAACTTCGGGATTATCCTTCTCCTCGAGCTTCCTGTTTTTCAACTTCCCTTTC
ACACTTATTTGAGCGATAATGCTCACGTTTTTCTGACGCGGCCGAGGGATAATTATTTCT
AGTAATGGTCGAACGAATGGATAGCTTTATGCGATAAATTTTGTGAGAAAGGGGGATGA
AACAATTCCTGGTTACATCTGTTCTTTGACAACGAGTATATTTTCAAGGAGAATTAGAC
GTACAGTTTTTATATCCTTCAGATTTCATTTTATCAGTATCCCTTTTTTTGCAGCATAAAC
AATCTTTAACAATTTTCCACTTAATAATCGGGAAATAAAACGTGCAGAAATAGAAAGAT
TCCTTTCGATTAATTATTTAATCAATTGTTCCGCGAGTTGCATTCGAGTTGAGGGTAAAA
CGTTAAAGAGACACGCATGTTTATAAGCACAACCATGAATACGCGCGATCTTCAGCTTC
GTAATCTTTTCCCGTGTTAATTCAAATAATTCTTCCTCCGAACCTTTCATCCCCCGATGA
ATTTGTTCGTTAACTCGAGCCGAGGATGCCGATCGTTATCGCGTCTAATTACGTTCAATT
ATGAGTCCCCTACGATCCCCTCTCGCGTCGTTGAATGCCAACCACGTTCGCCGACAATT
TACACAAATTTAATCGGGGCGCGATAAGCCGCGCGGTTCCATCTCCAGCCGAGTGTTCG
AATCCAGAAACTCTGAGCACAACCCGAGAGCAAGGCGAAGAGGATCGTACCAGGTGC
AACGTACCATTACGAAGAATCAATGGAGGATCAAGGGGAGGGGAGGGGAAGGAAGG
GGAGAAACGATACGGGAACAACGGCCTTTTCGCGCTTGGCATATCCTATAATCCTCCGT
TTCTCCCATTACCCTGTTGTTTCCTCCGTCCGCAATTTATTATATTTCCCTCTTTCTCCCT

GCTCCTCTTTCGTTTCCTCTCTCCTCCCCCCGTATTCGACGCTGCGATCGAGAAGAGGA
GGATGGGGAGAGGGGGAGGGATCGTTGCCAAGGTGCGCTACAGGTGTCGACGAGGAG
ATACAGGTGTTGCTAGGATAACTGTTACCCCGCTGCACCAGCAGGGTGAGAGCGGCCT
CCTTCGCTCCAAGCCACCCTTTCCGATGTTTCGAGCGGCATGCAACGTCACGAACCCC
TCTTCTCTCTGGATTCCATCGAACGAGGGCGAAAGTTTCGAAGATCGGAGGGCTTGGC
GGATGCGTCGAGGATAAATATTGAATTCGTTATTCATTATTCTTTAAGAAACTTTGGGAA
AAATTTGGTTGTCAAACTCGAATCCAAGCCGGGTTAATTATTTCTGATAGATCGGGGGA
TAGAAATTCGAGTTTTAAGGGATTAGGGGTTAGGGATCCGGAGTTTTGGGATGTTTGTT
TACGCAGCTGGGACGATGGATTATTTTTAGGATGATTAATTAGATTGAAGTTGATGTTCT
TCGAAGGCCTATCGAGGCAATCCTTTATTATATTTTGAGTCCTTCGTGCCAATTGTAATA
ATAATTGGAAATCACGTGATTTGACAATAGACAAAAATTTCCAAAATTGTTCAGGATAC
TCAAGAAGACGACTAATAACTCTTATTTATATTTACTCGAGATAATCCTGTTGTAATAAAT
TTTCAGTTCACGGATACGTTAAACTGACATAAATTTCTGCACGCTTCTGCACACCCCCT
GCTCAATTGATACATAATGTTTCGCAACATCGCAATCGATTACGAGAGTAATCAACGAG
GAACATCGACAATAAATTATCCCGATATTATCGTCGTCGTCGCCGTCGTCGCAGACGAA
TTCCCTATGAAATCGAGCCCGAGGCGCGAGATTTTCACGGGAACGGATAAATTGGACG
ACGAAATTGGCGGCGGGGATGATGGTCGTTGGAGATCGGGGAGAGGGTGAATGGGAG
AAAAGCGAGCGTGTGGTTGAAGGGACGAAATGTTGCTAGGGCTGACGCTATAATTGCC
ACGTGATGTATCGCCGAAGGCGTCGCCGCCACCCCCTGTAAAAGTTCCAGATTTCGTTT
TATCGCTTCGAAATCCCATCCCCCCGTCACCGAGTTCAGAACGCGACGATGCCGTATGA
TTTTTATCACAACTCCACTTCTTTTTTCCCCCTCTCTACGTTATCACCATTCAAACCGGTT
TCTATCCTTTCGAAATTTTCAAATCGATCGTGAAACACCGTGAAGCTGAACCTGAGAA
GTTTTCAGAATAATCCGTTTCCAATTTTCGTAACCGAACATGAACCGGTGTGCATAGAA
TGAAGAAATAAGACGAGCGTCGAAGAATGTCGGGAGGGAGGGGGGGGAATTCCGAGT
GAAACGGTGTTCGACTTTTACATCCGGAAGAAGTTTCGCAGAAATTTCGCGAACCAAT
TAAATCGTGAATTCAGACAGGCTCAAGGCTCTCCTCGATACGCTCGTTTAGAAACTTTA
AACGAAACCGGGATTTCGCTTTGGGAATTTTTTTAATTTCACTCGAATACTCCCCCTCC
CCAGTTTCCAGGGAATACCGAATTCCCTCCCTCCCCTACATTCGTTCAATGTATCAACAT
CCAATTTAAACATTTTTCTTTTTCTCTTATATACAATATTCTCGACAATAAAAAGGTCGAG
CTACGTTTCTGAATTCAATTACCGAGATCTGACATCCGGCGACACTGTCCGATAAGATT
CTTTTGACGAGAGAAAGAAAGAAAGAGAAGAAGGAGAAGAAAGAGAAATCTCTGAT
AAATTTCGATGCTCGTCAATCGAAAGTGTAATGAAAACGAGAGATGGATAAACGACGG
GGAAAGACACAAGACGGAACGAAGCCGTGTTTGAATGAGATCCGTGTCGGCCGCCAT
CTTGATAAAGGAGCTTCGCGTATAGCCTACCACCCTCGACATACTCTTCCTATGAAACA
GCGTCAACCTCGCATCCCACTGGAGATTGGATTCTCCAGTACGGTTCTTGTTCCCTTAT
CTACCATGTCCTTGTGCCGAGCGACAAGAGCAAGTAGCACGACGACACATTCTACTCT
CCTACGATACTTGACGTTAACGAAGCTCATCCAGTAATAAAAATCTTCATAAAGCAGTC
GAGGGTTGGCCAGAGGGAGCCAGGCCAACAACGGAGCCGAGGAGTATTTCGTG

>novel_circ_000304
ATTTCGAATAGACAACACAGACCATATAATAGACGTGAACAAGAACAACGCGGCGTTC
GAGTACGATCAAGTGAACATAATATGTCCTGTGTACCAGCCAGGAACGTATGACGAGG
ACGCGGAGAAGTATATCATTTACAATGTGTCCAAAGAGGAGTACGAAACATGTCGGAT
AACAAATCCGAATCCGCGAGTGATAGCGGTCTGTAACAACCCGTATAAGACTATGTACT

TCACGATCACCTTCAGGCCGTTCACACCGCAACCCGAAGGCCTGGAGTTCCTGCCAGG
CCACGATTACTATTTCATCAGCACTTCGTCCAAGGACGACCTCCACAAACGCATCGGTG
GACGATGCACAAGTAACAACATGAAGGTGGTCTTCAAAGTGTGCTGCCGCAACGAAG
CTGACACCTCGTCGTCATCAGCGACATCGCGTAACAATT

>novel_circ_000306
ACTCGACAACCTATATTCGTTCAATTACTTCATGCGGCTTTTAAAGTTTCACAATGCTCA
TGGCTAAATGCTGGTCAAAGATTTAATGTTGAAAATTGTATACGAACATTGTCGGATGT
AGGTAAGTTTGTTTGAATTGCTTGTTTGAATTGAATTCATTGTATGAATTTAAGACTTTC
CTGGATATTCATTTTATATTTTTAAAATGTTATTTAAATCATCAGCTAAAGGGAGAGGTA
TAGCCATTCCAACAGATTTAGAAAGCCAAGTTGCTTCTATGTTTAATAAAGCAGCAATG
CTTAGTAGACAAACAAGTAAATGGCTTCAAGCTGCGAAACAACCAAAGATAGAACGTA
CACAAAGTCAGGTAATTTTTCTTGAAAGACTTATTCCAAAAATCAGTTAATTAAAATTT
TTTTTATTTTTATCAATTTTTATATTATATATTGAATGATTGTAATCCATTTATAATTGATATG
ATCTTTGAATGTATAGAGTGACTTAATTTGTGAGGAAGATAGGGAAGAAAAAGATCAG
GTATAAGTCACTCATAAAAATTTGAGTTTAGTAGCTTAATATGATTTTAGTGCTTTAGTGT
AGTTCAATATTAGTGAACCCTAGTTTGCGAATAGTATCCACAAAACTACGAACAAATAG
GATATTGTATGTATAATAAAATAATAAAATTATTACTAGATATATAATATATTAGAAAAAT
ATTTAATTATTATGATATTAAATATTTTGTTAATCATTATGAAATAATATATCTTTATATAGCT
GATGCGTTTGGATCGAAGTATCATAGAAGGTTTACAAGATATAGTATCGTTATTAGAAGA
ACAATTGAAACCACTAGTTCAGTCAGAATTGTCTTTATTAGTGGATATACTCTATCGACC
AGAATTACTATTTCCAGCTGCGACTGACGCTAGAAAACGATGCGAAAATGGCGGCTTTA
TCAAAAGGTGAGTAATTTTCTACAATTGATATTATTTTAATATATTTTATCATCAATAATTC
TATATTGTTTAGATTAATCAAACATACTGAAAAATTACTTGAAGAGAAGGAGGAAAAAT
TATGTGTAAAGGTGTTAAGGACTCTTAGAGAAATGATGGCTATTGACCCTGAATATGGA
GAAAAA

>novel_circ_000307
CGCCTTACGAGGGTGGAATATGGAAGGTGAGGGTTCACTTGCCCGAGCACTATCCCTT
CAAATCTCCTTCGATCGGCTTTATGAACAAGGTATACCACCCTAACATTGACGAGGTCT
CAGGCACAGTGTGTCTAGACGTAATAAACCAGGCCTGGACTGCCCTTTACGATTTGTC
GAATATATTCGAGTCTTTCCTGCCCCAATTGTTAACATATCCTAATCCAATCGACCCCTTA
AATGGCGACGCCGCGGCCATGTATCTACATAAACCTGAGGAGTACAAGAAGAAAGTAG
CAG

>novel_circ_000308
CTGGCCTTCCTCTGTGCAATTATTCTTTGGATATCGAGTTTTGAGTTACGATTAGAAACA
AATTCTTATTTTATTCGACGACTCGGAACTCGCACAGTTTATCCATAACTCACCACTACC
GGCTGTATTACTCTGAAAATTCTTCGGAAGGCGATGCTTTCTATCTTCGTTCAACGATTC
GATTAAAAATCTTTTGTTAAAAAGCTACACAAATGCCGAAATCGGCAACGAAGTGGAC
ATTTTGGAGTCATTTACGTGACGTATTCACAAGTGTCAACACGAAATATGCTATTATTCT
AAAGTTTCTCTTATTAACTCGTATCATTTAAATTGTATTTTTCCATATTTCTCATGAAGTG
CTTCAATTTTTTTCGTATATTTTTTATATTAAAGTGCAAGAGACCTGTTGGCGAAGAGAA
CGAAGAGAATAAGAAAGGAAAACGGAAACGAGCAGCGGAAACAGAAATCGTGGTAT

CGACAAAGGAAAAAGAGGTTTCGGCTGTGGAAACGAAACGGAAAACCATGGAACAC
ACGCTAACAAGATGCGACACTCGTTTACCAAAAATCACACCAATTATTGATGACTATGA
GATCAGTAATCACGTGCTCGGTCTTGGAATAAACGGAAAAGTTGTTCAATGTTATGACA
AAAATACCAGGGAAAAATATGCGCTCAAG

>novel_circ_000309
CATGGTAGAAGATAAAAGATATGAAATTGACAGTCGAATTGAGTGTGATGGACATCAA
GGTACATTAAAGTACATTGGTCCTGTTGGTGAAACTAAAGGTTTGTGGCTTGGTATAGA
TTGGGATGATCCAACTCGTGGAAAACATAATGGTACATATGAAGGAATAAAATATTTA
AAGCTAGATATCCTACATCAGGTTCATTTATACGTCCTGGTAAAGCAAGATTTGGAATTT
CTTGTCCTGAAGCTATTAAAATTCGTTATGGTTTCATCAATGATGAATTAGCTGGTATTGA
TAGAGATACATTAATAAGTTTACAAAAAGAAATTAATGCTCCTTTTTTGGAAGTTGTTG
GTTTTTCTAAAGTAAATAAAAAACAAAGCAAATTTGATCAATTAAAAATAATATGGTTA
AGAGAACAATGTGTTAGCACTGCGGGTGATTCTGGTGAATTAAAAGAATTATGTCCCAA
TTTGGAAGAATTAGATATATCTAAAAATTTAATAAATAGTTGGCAAATTGTAGCAAATAT
TTGTTGTCAGCTTCATTGTCTTGTTCGACTTAATGTGAGTGAAAATTATTTGCCGATTGA
AAAAAATATGGAAATATTAAAAAATTCATTTTTTATGGTTAAATATTTAACTATGGCAAA
AATGAATTATAATTGGTTTGATATTCAACAGTGTATGTGTATGTTCCCATTTCTACAAGAA
CTTTCAGTTTCTTTCAATATTGTTAATATTATACAAAGACCATTAAAAGATGATAATTTAA
TGAAAATATGTAAATTAACACTTGAAGGAAATTTGATATCCAATTGGGATGAAATTCTTA
AATTGGATTCCCTTCCATG

>novel_circ_000310
TAAAGTGGAAGAAGTCTCACTGGAAGGAAACGCGTTCCATTTTCATTCTACCATGATCT
GAAGCTCGATCCTAAAGAGAATTGTAAGTTTAATAGGTGGTGGAATAAAAGGGGACGT
GGAGAGGTGTCTTCCGAAGGCGAGCGATTTTCTTCTTGCGAACAAAGAGAGTCGAGG
CAATATCGTTTTGGACGGAGATTTGGCTACATGTAATTGGACACATCGAATTTGCATCGG
GTTTAGATGCAAGCGGCCGACATCCTTGCGATTCCACCGTTTATAAAC

>novel_circ_000311
GAATTCGCGTTTACAAGAATGTGGACGAGGAGAGCGGGAGAGAATGGAGATCCACGG
GGCGTACGAGGTCACGGAGGAGACGAGTTAAACAGCATCCAACATCCTCCTCCCCCTC
CTCCTCCTCCGTATATGCGCACGGAGGCTATTCTCCGTCGGAATGCATCGAGCGGCAAA
TGTTCTCCTTTTCTTCCTCCAGTGTGGAGGAGGGGAGGGGAGGGCGATTGTTCAGCAC
GGCGAAAATTCGGCGATTCCCCGAAGTAAAGTGGAAGAAGTCTCACTGGAAGGAAAC
GCGTTCCATTTTCATTCTACCATGATCTGAAGCTCGATCCTAAAGAGAATTGTAAGTTTA
ATAGGTGGTGGAATAAAAGGGGACGTGGAGAGGTGTCTTCCGAAGGCGAGCGATTTT
CTTCTTGCGAACAAAGAGAGTCGAGGCAATATCGTTTTGGACGGAGATTTGGCTACAT
GTAATTGGACACATCGAATTTGCATCGGGTTTAGATGCAAGCGGCCGACATCCTTGCGA
TTCCACCGTTTATAAAC

>novel_circ_000312
AGAGAGAGGAAAGCCATCATAAAAAGGAAGTGAACGCAGTTTTCTTGGAAAAACTGA
TGATACGTTCAAAATTTTCAACAATTGAGAAGAAACTTTAGCGACAGTTTTTGAAGAG

AATTTTAAGATCAAGAAAGAACTTGTGTATCCAATAAAATGTTGAGTGTAATCAGTGTA
ATCGAACAGTGTTGAACAATTTTCCAAGTGCTCGATCCATGCAAGAATCTTTCCAATGA
TCATGCACTCTTCAAGATACATGTATGCAAAACATTCATCTTGTCTCGATGAATCCAGTT
GATGAAGTTTGGACGATATGTTCAAGGGGAAAAGATTGCGCATCGAAGTGAGAAGCTG
GTTCGAGAATGGGTTGCAAGAGTAGCGTTTTGGAATGTGTAAAGGGTACAGGGGAGG
AAAAGAGAAAAGAATCGGATGTGATAGAGCAAGGATTCCTATTTGGAAAGACAGAGT
ACGATATTTTGGGAAGAAAATTGTCGTTCGAGGATGTTGGCGATGAAAAAAATGCAGA
TTATTCGACGTTGATAGGATCCCACGATACTGAACCCCATCCGTTAACGAACAGTATTCC
TAGTCTCATAAAAATATTACTGGTCTTCCCCAAAGATGATCAACAAATGGATATCCTTGC
CACTACTTCGAGGCGTTTGGGATGGAGCGTGTCAGTTGCGAAAGACGCGGAAAAAGC
ACTCGAATTCTTCCAGGCTCGAGGCCACGAGTTAGTGATCATCGATCATCGGGGGCAA
CGCGCTGCGGAAGCTGACATTATTTGTAGAGCAATCAGGGCTAGTCCTTTTTATTATAAT
TCCATAATTATAGCTCTTGTAAAAAAATCGTATCTCATGCATAGCGAAAGTGAAAAAATC
GTAACATTGGATTTAATGGAAATAGGATACACAAAAACTTTAATGGAATGCGCTCACGA
AAGAATCTTAATAAACGAATTAGTAGGAATTTATACTAGCGGAATATTACCGAGGACAC
AACTGGTTGCTGTCAACATGCTGTATATGGCTCTTGACAGATGTCGTGACATGATTCAT
GTGACGGACGATAAATATATTATACAGTTTGCCAATAAAATTAGTGAAAAATTATTAGGC
TACAGGATTAATGAACTTTGGGGCAAAAACATTACAGATTTAATTTTGTATGATAATTTT
ATTCAAATGGAGCAACATATAGTCAAAGGCCGAGAATATGATGGGAATATAAACTGTAT
TCGAAGAAACAATCAAATGTTAACAATTAATTGCAGGGTGATACCTTTCGCCTATTTCA
A

>novel_circ_000313
ATAAAAGGTGAACGATTATCTGATCACGATGCCGTATTACAATAGTGGACACAGTCCTT
CCACCTATAACAAAGATGGAGGATCGAGTGGTCCATCGAGCACATCTGGTGGCCGTGT
GAGTAGTTCCGAGCCGACATACATGATGGAGCATTTGGCCACGTTCACGGTCACGAAG
GAAACCGGTATTGTTTATCCAGCGGACGGTATGCGGCGACTTTTGCAATTGGAGAAGA
GTAACGGCATCTGGAGTCAAAAGATGCAGCTCCGTCTGGAACGGAACTGGGTACTGAT
TATGGACAATGAGACAGGGGCCGTCATGGAGCGTTTCCCAGCCTCGTTGATACAGGAA
CCGACCGCGTTCACGTCGAGGGATCCTATGGAGATGTATAACAACATTCTCGTCTTCAC
AGTGGCGGATGACAGTGGTTCCGGTCAACGGGCCGAAATGCATATATTCCAGTGTCAA
AGTGTCTCGGCACAAGATCTCGTCGAGGATCTGAAGATGCTGCAGATGGGTAAACTGG
TACCCGGTGGTTCTCCAAGAGGTCCAAGAGGACGTATTCCACCGCCTCCCACTTTACC
ACCGCCAGAACCGCCCTTAAACGGGGTCAATGTCCGAGAACAAGTATCTGCATTTAAT
GCTAACAATGCCGATAGTCAAAATGATATATCTCGCGAGGAGAACAATGACGAAGTGT
CCTCGACATCTTCCGAAAAGTATGAACGCGACGTGACGATCCTGAATCACTGTTTCGAT
GATATTGAAAAATTTATCGCACGTCTGCAATACGCGGCGGCCGCCTCTAAAGAATTGGA
ACGTCGAAGGCGCAATCGAAAGTCGAAGAAAAGAAATCTCGGCGACGGGATGCTGAC
AATGAGAGCCAAACCACCGCCAGAGATGGAATTCATCGATATCTTTCAAAAATTTAAGC
TGTCGTTCAATTTGCTGGCTAAGCTGAAGGCTCACATTCACGACCCCAACGCCCCTGA
ACTAGTGCATTTTCTCTTCACGCCGCTTGCTTTGATCGTGGACGCATCCCACGATACTAA
TTATGATCCTAACTTGCCGAGCAAAGTTGTCTCGCCGTTGTTGACTAGAGAAGCGGTAA
ATTTGTTGATTAACTGCGTCACAAGTAAAGAGACGGAGCTCTGGCATTCATTGGGCGA
CACGTGGTTAATACCACGCGATCAATGGAAAGGTCACGTTCCCCCCTATCACCCTATCT

TCATGGATGGTTGGTCCCCCGAGTATCCAATACCAGAAGACAGGGACCACGATCATCTT
GCTTCGTTATTGAACGCTGACAAGCAGAAGAGGGACGAGCTCCCTGAACAACAGAAC
GATTCGTACTATAATCATCGAGAAGTTGACGAATCTCATTACAGCAGCGATTATTTGGAG
TACGAGAGCAGAGAAGAGCGTGCCGGCAACGAATATTTTGATAGAAATTACGGCACCG
GTTCAGAGTTGTACGGCAGAGAAGAAAGAGTGGTCGATCAAACGAGAGCGCATAGCG
ACATATCCGTTGATTCCATCGAAAGAACGCCGAGAACTGCCGGTATGGAAAGGGCCCA
GGAAGCTTGGTTGGACGATTTAGTTGCTAGACACGCGAAAATCGTACAAGTTACTTATC
CGAGGACGGCAAACAACGATAAAGAATTGACAGTTGTTAGAGGTGAATACCTAGAGAT
TCTCGATGATAGCAGGAAATGGTGGAAGGCAAGAAATTCCAGAGGCCAGGTTGCGCAT
GTGCCACACACCATTGTCACACCTCACAATCCTTCCCATTCTAACGACAACGATGTTTT
CAACAATCCTTTGTACACGAGTCGATATCCGAGGCAGGGGCACAGCTATAATTACGAGG
ATTCTGAAATCGAAAGGACCAGCACAAGTCCAGGACCTGAAGCCACTCATCGAACGC
ATGCGATTCCACCTCCCGCACCTGCAGATTGGGTGAGAAAAGAAAGACTTGGGAAAA
AAG

>novel_circ_000315
AATGGTGAGGATGCCGGGAGACTGAGCGAGGAACAGCGGTGAAGCGGATAAGATCGA
AAAGAGTTGGTGGAGAAATGGCTTTGACCGTCGTACCCTCGGCCCGCGTTTTCAATGG
AAACGTGGGGACGCAGGCCTACGTTTCCGGTTCAACAGGGTCGAAGTGCCTTTGCTGC
CCTTACGGATACCACATCGATCTGGACTTCGTGCGTTACTGCGAGGCGGTCGCAGCGG
GAAGCGCCGGGGACAGGTCGAGCATAGAGAGGCGCAAGAAAAGGGAACGTCGAAGG
CAATGCCAGTCGATGGAGGTTCTTCTCGGTCTGGTGAGTCCAGCCCTGGTTGGAATAG
AGGCCGAACTGCCGAAAATACCGCAGGAAGCTGGGACGAATGGCGCGACGTCGTCCT
CTTTGACGAGCCACGATAGGCGAGGAGGATGCACGCAGGAATCGCCGGCCCTCGATCT
CAGCGACGTCGTGGGCGACTTCGAGGCGACCCTGAAGCGATCCACCAGGCCGAGCAA
AACTTGCGATAAGCAGGAACGCACGGAGAACG

>novel_circ_000316
ATGGAACGAACACGGCAACACCGGTGCAAAACAATGGGCAGAGCGAGCACGCGGCT
GGTGATTTTGACAACGCGAGTGTCGGAAGCGGAAACTCGAATCTCAGCACTGGCGCG
CTACAGAACATCAGAGAGCAAATGGCCGCGTCGTTGGAGCGGATGAGGGAGCTGGAG
GAACAGGTCAAGGCAATTCCCATGTTGCAGGTGCAAGTGTCGGTGCTGAAAGAGGAG
AAGCGTAATCTGCTGCGGCAAGTGGACGAGCTGAGCAAGGCGAGCGAATGGAACGAG
AGCGGGTTGCACAGGTACCGTAGCCAGTCGTTCTCGGAACAGCGTGGCTCCCAGCGA
AATCTGAGGAACGCGGCTACGACGCCGACGCGGGACATGGGAACGATGTGCGGCGCG
ATGACACGGGACGTCGGTGTTTCGCACCAGCAG

>novel_circ_000317
AGATTGGTAGCCGAGCTGAACAGAGAACAGGAGGCTTTCATTGCGAACAAGCACACC
GAAAAGGGTGAGGCTGTCGCACCGTGGGTCGGTGCGCCTAACGAGGACATACTCCGC
GAGGAATGCTTATCTCTCTCCATGGATCAACGCAATTTCTTAAAATCGCCGCCAAAGGA
AGTCGATTTCCCATGGGACTTCGAGGCCGTACAACCGATGGCGCAAGCCACTCTCGCC
TTGGATCCGAATTTGGAGAACATGAGGTTCCAACTGGTGCCGAAAGTAATATCAGAGG
AGAATTTCTGGCGGAACTACTTTTACCGGGTGTCGGTACTCCGCCAGAGCCACGAGCT

GAACACAATGGCCAACCAGGCAGAGAACAACCTAAATCAAACATCCGCAGGAACTGT
GGATCAAGCTGAAG

>novel_circ_000318
AGATTGGTAGCCGAGCTGAACAGAGAACAGGAGGCTTTCATTGCGAACAAGCACACC
GAAAAGGGTGAGGCTGTCGCACCGTGGGTCGGTGCGCCTAACGAGGACATACTCCGC
GAGGAATGCTTATCTCTCTCCATGGATCAACGCAATTTCTTAAAATCGCCGCCAAAGGA
AGTCGATTTCCCATGGGACTTCGAGGCCGTACAACCGATGGCGCAAGCCACTCTCGCC
TTGGATCCGAATTTGGAGAACATGAGGTTCCAACTGGTGCCGAAAGTAATATCAGAGG
AGAATTTCTGGCGGAACTACTTTTACCGGGTGTCGGTACTCCGCCAGAGCCACGAGCT
GAACACAATGGCCAACCAGGCAGAGAACAACCTAAATCAAACATCCGCAGGAACTGT
GGATCAAGCTGAAGTTCAAGTGACCACTGGCCAGGAAAGCAGCGGAACCGTGATGAC
CGGGAGTAACAGCGCGTTGGCCGATTCGCCGGGCCACGAATTCGTGTCCGATAGCATG
AGGGTCTCGGACACGGACCTCGAAGAGGTCCGGGAAGGCATGAAAAAGCTGGGGAT
GCAGCCGCCTAAAG

>novel_circ_000319
GATCAACGCAATTTCTTAAAATCGCCGCCAAAGGAAGTCGATTTCCCATGGGACTTCG
AGGCCGTACAACCGATGGCGCAAGCCACTCTCGCCTTGGATCCGAATTTGGAGAACAT
GAGGTTCCAACTGGTGCCGAAAGTAATATCAGAGGAGAATTTCTGGCGGAACTACTTT
TACCGGGTGTCGGTACTCCGCCAGAGCCACGAGCTGAACACAATGGCCAACCAGGCA
GAGAACAACCTAAATCAAACATCCGCAGGAACTGTGGATCAAGCTGAAG

>novel_circ_000320
GATCAACGCAATTTCTTAAAATCGCCGCCAAAGGAAGTCGATTTCCCATGGGACTTCG
AGGCCGTACAACCGATGGCGCAAGCCACTCTCGCCTTGGATCCGAATTTGGAGAACAT
GAGGTTCCAACTGGTGCCGAAAGTAATATCAGAGGAGAATTTCTGGCGGAACTACTTT
TACCGGGTGTCGGTACTCCGCCAGAGCCACGAGCTGAACACAATGGCCAACCAGGCA
GAGAACAACCTAAATCAAACATCCGCAGGAACTGTGGATCAAGCTGAAGTTCAAGTG
ACCACTGGCCAGGAAAGCAGCGGAACCGTGATGACCGGGAGTAACAGCGCGTTGGCC
GATTCGCCGGGCCACGAATTCGTGTCCGATAGCATGAGGGTCTCGGACACGGACCTCG
AAGAGGTCCGGGAAGGCATGAAAAAGCTGGGGATGCAGCCGCCTAAAG

>novel_circ_000321
GTGACAACGCGTCGTTCAAATAACACGAGGCTCGACGGGTGTATATCGGTCGCGAGAT
GCAGGTAGTCGCGCATTGTTTGTATGTACACACAGTGAGGGTCCACGCCTTTCCGCGA
CCATTGACTCATCAAGGCGAAAGGCATCGAAGGGATTCAGCGAAATATCAGGCAGGCC
CCAGATACACCGCGGGTCCTAACCCAAGGGACCTAACCCGAGGGCCGACCGACCCTC
CCCCGACCGATCAACTCCCTCGCCGAGTTGACTCGAACGCGGTGTGTGACGGACCTAC
AGCACCAACAACAGGAAACGAACCCGGATGACTCACCGTCTCGCTCCGAGGAAACGA
AGCTACGAAGCATCGTGCACAGAAACTGGAACGCAACCAACTCTTACCGCGGTTACGC
GCATCGCATAATCTCGTTACCGTCTTCTCCTCAGCCGGTCACGCAATCGTCCACGAAAT
TCACGTGCCTTTCGTGGCATCACGTGTCGCAATCCGTCCGTGCAATCCTTGACAAATGG
TATCAAAAGTGGACAGGAAGTTTCGATTCTTGATTGAGAGTTGATTAACCCCCTTTCGG

TTAAAAATTCGATTGGCTATCCGTCGTATTTGAAAATTTTTTCGGGTATAAGTAATGAATT
ATTATATCGAATTATCAGTAAAGTTTCTCGTTTTCTCGGGTACAACGATCGAGCAAAAAT
GCATCCTCCAAGTTCCTTAACCTCGCGTTTCGATTATGGGGCGATTATGTGTCTGTTGTT
CTTCGCGGAAACAGGCGTCAGTTTCTGGTCGAGAAAATTCTCTCCGAAGAGGAGGAG
TGCTTCGACCCCCACGGGAAGGAGTTTCGTTTTTGTTAACAAGCCCGCGTGCGTTTCG
GCCGGAAGGGGCCGGAAGCGAGCGAATAATTTCGCAATAAGGTGTTTCTAATCGCCTC
GCAATCTCGCGTATCCTCCTCGGCATTTTCGTGCACGTGGAATCGTAATTAAGAGGAGA
GGAGAGGAAGTGGCCGGTCAGGAAACGAGAGGACGATTTTTCACGGCCCTCTCTCAT
TGTTTGTTGGCGATAAAACCGATTATTCTCGCGATCTTTTTTCGCGCGAGGAGGGAACG
AAGGGAAGACGAAGCGGCACAGGCCGACAATTCGCTTAAATGGAACCAAGAACACCA
TTTACTCGTTTATCGAGCGTTCGCCGTGCACAACTCGTGCGATCTATTGTATTACCCGAC
CGATGAGAAAATTTCATCGTGTTATGGTGCCGTTGCGTGCCACGCCGGCCTCAATCCTT
TTAATGGCCATCCCCTATCATTTAAATCCCTCACCGGGAATGGTTTATGGTTTATAAATGA
AGATTGTCTTCGCCTCCAACTTCGTTTCTAATATTCCTCGCAGAAGTGAGAAGACCGAT
CGATTCAATTTCAATCTCTAATAATTCAGGGAATTTTTAATTTTTACAATTCCATAAGAAA
CAAAGCAATCAATATTTAACGTTACGAGGAATCGTTTTCATTTTGCAAAAATGATTACG
ACGCGAAAGAAAACAAAAAAAAAATAAATAAATTTTCCAGAATCGAAGGGGCTAGAT
CTTCGACGATCTTCGTCGATTACGTCGAAAGAAAGTCGCGAGAGTAGAAAGATCCATA
AATCGCTGCGACAGCGGCGCGACTCGCAGGCCGGACAGGCATAGAGAGCCAGACAGT
CGCCAGGCAGACGGACACCAGACGGAGACG

>novel_circ_000322
ATCGGTTGCCACGACGAAGCGGTGACGACCGACAACTGGTGGTCCTGGTGGACTCTG
ATCTGTCCCGATTGCCGCTGCCCGATCTTGTCCTGTCCGCCGTGGAACAACTTCCGCCT
CCGACGCCCCAGGTGACGGTGTCCGCGCCCAGCAGCCCGACCAGGGACGCGGCATTC
CAGTTTCCGGAGTGCAGCGGGGACGAGAACGGTGCGGCACCGGTGCCGAGCCCGAG
GCCACGGCCGCGAAGACGCAGGCTCGCCTCGGAGGGTGGTGGCGCTACTATGAAGAA
AGGGCCCGCCACAGAGCGAATCTGCAAGAAATGCTGCGGCCACTTGGGACAGGCCTT
CAACGAGAGCCAGTGGGCGAGCGTAGGCGCCAACCTCAGGAACATCGCCGACGACTT
TCATGCGTCACAGACCAAG

>novel_circ_000323
GCCGCCGAGATCGAAAAGTCCCGACTGCCCGCGATGGACCCGAGCTTCGCAGATTCCA
ACAACACGAGCGGCTCAACCGACAGTATCCTGTCGCTGCTGATCCCGGCCCCCCTCCG
CGAGACTCTGTGGACGACAGTGGCCTTGTACCTCGGATGGAGGCTGGTCTCCCGTCTT
CGATAGACGCGACGGAATCGTCGTTGCGTCGACGCCACGACGATCGACCACGATGCCT
CGAGAACGTTAAGAATCATCATCACCGACGCTTGGAACCGACCCAACAGGGACAGCC
AGTTAGCCAGCGAGCCGTCACTTGGTAAACCAGCGACTGGTCCTCCTCCTTGGCCCTT
TGCACGCCGCACGAACCGACGGACGCCGCGCCCGTCCGTTTCCAACGTTTCGAACCA
GCACACGGAATCCTTCTTGCTGGACGACTCCTGCAGCAACGTCGAACGCGATCCTCTG
TTCTCGCGCCAACATCTGTTCTCCGTGCACGCGCGTCCCTCGCCGCGCGCGATCGAGA
ATGCGCTCGCATCGCGCACCCTTCGCTCCTTCGACGCGGATCCGTCGCGCCTCGAGGA
CGAGGACGAGGATTCGAGGGAGGAGGAAGAGGAGGAACTCGTGGGACGCGACGAAT
ACGAAG

>novel_circ_000324
GAATAACAATTATCGAAATTCGACAACCAGATCCTGTCTTCAAATCGTCTCCCTGTCGA
TTGGCAATTTTTCACACGATTCCAATCCTCGGAGAGACGGGCGGGACAACGCGAGTCT
GCACACAAAGGCGCATCCTCGCGGGGAAATTTATCGCGACAGGACGACGACGACGAC
GACGTCGTCGACGGCGGTGAAAAGCGGCCGAAGAAAGAGGATAGAAACGAGAGGGG
GAAGGGAGGGAGGGAGCGCACGAAGGATCCGGCAATCTCGTGAATAGCCCCGCGCCT
TCCACAATCGTCGCGAATCAATTCCCAGATTGATTTTGCGCCGGGTCACCGATTTCTCG
GCTTTCCCCGATCGAAAGACTCTCGCGCCGAAGATTTCTTCTTCTCCACGGAATATTTT
CCCGCTTGCCGAACAAAAAATGGACCGACAGGAATGCAATATGCGACGGATG

>novel_circ_000325
ACACGAAGCCAGAGACATCGTCCGACACGCGGTGGGAGCTGGAGAGCAGGATGCGGT
TCTTTTCACGGGTCAAGGCACGGCGGCTGCCCTCCGCGCGCTTCTGCGACACCTCGAC
CTCTCCAAATCAACTGTGGTCTTCGTTGGACCGTTCGAGCATCACGCGAATCTCCGCCC
TTGGCGGGAGCATGGAGTCAGGATAATACGAGTGTCGGAGACCCGGGAGGGCTTCTTA
GACTTGAACGATCTCGATCGAAGCTTGATCAAGATGCGATCCGAGGGTGTTACGCAAA
TGATCGGATGCTTCAGCGCTGCCAGTTGCATAACCGGGGTTTTGGCAGATGACGTCGC
CACGACCCTCCTCCTCCATCAGTATGGGGCGCTCAGTATCTGGGATTACACCACAGCAG

>novel_circ_000326
ACACGAAGCCAGAGACATCGTCCGACACGCGGTGGGAGCTGGAGAGCAGGATGCGGT
TCTTTTCACGGGTCAAGGCACGGCGGCTGCCCTCCGCGCGCTTCTGCGACACCTCGAC
CTCTCCAAATCAACTGTGGTCTTCGTTGGACCGTTCGAGCATCACGCGAATCTCCGCCC
TTGGCGGGAGCATGGAGTCAGGATAATACGAGTGTCGGAGACCCGGGAGGGCTTCTTA
GACTTGAACGATCTCGATCGAAGCTTGATCAAGATGCGATCCGAGGGTGTTACGCAAA
TGATCGGATGCTTCAGCGCTGCCAGTTGCATAACCGGGGTTTTGGCAGATGACGTCGC
CACGACCCTCCTCCTCCATCAGTATGGGGCGCTCAGTATCTGGGATTACACCACAGCAG
CCCCGTACGTGCAGATCGACATGAACCCACATCTGCCCGGCGTCGGTGAGACGACCGT
GCACAAAGACGCGATTATTTTCGCCGGGCACAAGTTCATCGGCGGCGTACAGTCGCCC
GGAGTGCTCGTGACCAAACGATCCCTTCTCAAAGATAAAATTGCCACGGAGGACATGA
GGGACTCGCATCACTACCACCGGGACGCGGAACTGCGCGAGGAGAGCGGGACCGCGG
GTGTCGTGGAGGCCATCAGATGCGGACTCGCGGTTCAATTGAAGGAGAACGTGACGC
CTCGGGCGATCGTCGCTCGTCAAGATAAGATATCGAG

>novel_circ_000327
ATAATACGAGTGTCGGAGACCCGGGAGGGCTTCTTAGACTTGAACGATCTCGATCGAA
GCTTGATCAAGATGCGATCCGAGGGTGTTACGCAAATGATCGGATGCTTCAGCGCTGCC
AGTTGCATAACCGGGGTTTTGGCAGATGACGTCGCCACGACCCTCCTCCTCCATCAGTA
TGGGGCGCTCAGTATCTGGGATTACACCACAGCAGCCCCGTACGTGCAGATCGACATG
AACCCACATCTGCCCGGCGTCGGTGAGACGACCGTGCACAAAGACGCGATTATTTTCG
CCGGGCACAAGTTCATCGGCGGCGTACAGTCGCCCGGAGTGCTCGTGACCAAACGATC
CCTTCTCAAAGATAAAATTGCCACGGAGGACATGAGGGACTCGCATCACTACCACCGG
GACGCGGAACTGCGCGAGGAGAGCGGGACCGCGGGTGTCGTGGAGGCCATCAGATGC

GGACTCGCGGTTCAATTGAAGGAGAACGTGACGCCTCGGGCGATCGTCGCTCGTCAA
GATAAGATATCGAG

>novel_circ_000329
ACGGCGCTCCAGCCGAAATCACGATGACGATGGACGTGAGCAAGTCGGAGCCGAGTA
AAAATGGCGCGACGCAGCACGAGTGCGCTGGTTGTGGGAAACCGATCACGGAAAGGT
ATCTCCTCAAGGCCATGGACCTGTTCTGGCACGAGGACTGTCTGAAGTGCGGGTGCTG
CGACTGTAGGTTAGGAGAGGTCGGTTCTAGTCTGTTCACCAGGGCCAACCTTATCCTCT
GTAAGAGGGACTACCTCAGGCTGTTCGGGAATCCGGGACATTGCGCGGCTTGCAACAA
GCAGATACCGCCGTTCGAGATGGTTATGAAGGCCAGGACGAACGTCTATCACCTGGAT
TGTTTCGCCTGCCAACAGTGCACTCATCG

>novel_circ_000330
CTGGTGTAGTGGTGTCACGTGACGGAGGGAATCTGGAGAATCGTTCTCGGCACAAGGC
ATCCGTGGAAACTGGCTCGAGCTTCGAACTCTGTTAGACGGCGCTCCAGCCGAAATCA
CGATGACGATGGACGTGAGCAAGTCGGAGCCGAGTAAAAATGGCGCGACGCAGCACG
AGTGCGCTGGTTGTGGGAAACCGATCACGGAAAGGTATCTCCTCAAGGCCATGGACCT
GTTCTGGCACGAGGACTGTCTGAAGTGCGGGTGCTGCGACTGTAGGTTAGGAGAGGT
CGGTTCTAGTCTGTTCACCAGGGCCAACCTTATCCTCTGTAAGAGGGACTACCTCAGGC
TGTTCGGGAATCCGGGACATTGCGCGGCTTGCAACAAGCAGATACCGCCGTTCGAGAT
GGTTATGAAGGCCAGGACGAACGTCTATCACCTGGATTGTTTCGCCTGCCAACAGTGC
ACTCATCG

>novel_circ_000331
AAGCAAGTTGTCCAGCGGCTGCGCAAAGAGGTCGAAACATTTAAAGCGGAGAATGCA
AACGGGGCAAAAGTTTCCATCGCATCGCTTCTTGGCTTGAATAACAATAATCATATTAC
AAATAACAATGAAAGTAAACTTAAAACCGGCAATGATTTTTCTACGAAGAATTCGAAA
GAGGACTACGAAGAGATTGTTAGAAATGGAGATGTTCCTTCACCCACCCGCAATCTCG
CACCTACCAAGGAACGATCCAGTTCAGTGGAGACGATTTCCGGTGAGGATGACGTTAT
TGAGGTTTCCATGGATGAAAATTCCAATGAAAAATCCGAAAAAGAAGAATCAGAGGAA
TGTAAAATAGAGTCGACTGAAAAACTTAATTTCTTAAATGCTCTTGGTCTTATTACTACT
ACAACATGTGCCGAATTACAAAACAAAAGAGCCGAAAGAAAACGTCGTAGCACTGCA
AATCCACAATTTGTTTATTCCAATCTTGAAGTGCCAACGAAACGAAAACGACACTCGT
ACCTGCAATCTGGAAATGTTCCGCAAACTCGTCAAACAACCGCGCGTATGAATGGTCC
ATCGCCACCTCCTGTAAAAGTTGTGCCACCAAAATCCATATCACCGCCAACGACTAGG
ACTGTGATGAAATCTTTAATTCCGGTGCAAAAGTCCACCACGCGACCGAATATACTTAG
AAATGTTACGGAAAGTAAAGTTTTTCCTAATAAAAACAAGATTGAAAACGGACCCACA
CAAATTCAGTTACCTGTATCTACTGTAAAGTCCGTTCAATCGATTGGCAACAAAGCAGT
TCACATACCAGGTTTACCTTCCAGTCTGACTATCGAGAGGATCAGCAGTGATTCAGCAG
TCTGCATAAGTTGTAGAAATCCAGGTACATTGACAGTGTGTGAAAATTGTGCATCAAAT
TACCACGTATCTTGCCATTCTGTATCTCCGGCGCCACCAAGAATATGTCCAAAATGTGC
GTTAATAGAAGAAGAAGAAATTGATGACGGGGACGAAGGAGAGGAAGAAGGACGTC
CATCGTTTAAAAAAGATGAGGAATTCG

>novel_circ_000332
ATCAAGGCTGACCGAGATAGGTAGGTCCGACGTGAAAGTATCTCAAGTCGATCCCGGA
CTTTGCCAATTTCGTAGAGGATACTACAAGATCGTCCGCCAACACCGAACCCGATATGG
TCGTTTCTCCGGTCTCGTCGACTGAACCGGCGCTGTACAACGTGACTGATATAAAATTA
AACGACTGCGCGGATCACAATGAGCATCTAGCAAACAAACTTAATAACAGGGGAGTTA
AGGATCACGCGTTCACCATACAGCGAGACAATATGTCGAACGGAAAGGATAATTATGC
CTTAGATTTCGACTGCATAGAGAAGGCGATCGATAACAAATTAAATTCATCCGCCGACA
TCAAAGCGGAGCCCGATATCGTGAAGAAAGTGGCAG

>novel_circ_000333
GAGGAAGCTGAATCGACGTAACGGTCTACGCCAGGATGGCCAGTTGGCCGCTGAGAA
TCGTGCTTCTGTCCAGCACCCTCATCCTTGTCCGCGGTAAGTAAGAACACAAATCCTGC
CGGTTCGTCGGTGGCGTTCGTTGGAAAATCATGGCCGCGCCCGACCGTTTCGGTTTCG
TCTCGTCGTGTTTCGACCGTCGGCCATTTCATCGGTCCACGGCTGGGCAATACCGGCTG
GGGGATATGCCTGTTTCAGGAGGAGGAGGAGGAGGGCGAGGGAAAGGGAAGTGCGT
TTAGTCGAGTAAATATTTGCGTCGCTGAGCAACGCGAAGCGAGAAAATGAGAGGGACC
CACCGACGAAAAGTATTTGACCAATAAAGTGACTTCCTCCAGGGAGAAATGATTTCCT
CGGTTTGCTCTTTTTTTAAGGGACGAATTTGATTTCCGCCTCGGAGTATAAATGCCATAA
AATCCCTTCTCTTTTACCTCCGGTAATACGTATATACGTATATACGTGCGTATATACGCGTA
TAATATACGTGGAGGCGTTACGGAGTAAATGTTTCTCCCTCGTTTCGTTATTCTCTTTTAT
TTCTTCTTTCCTCTCTCTCTCTTTTTTTTTTTTTTCTTGAGAAAAGTGGTAAAAAGTGTA
TGCGATATTGAAAACTTTTAATATCGCATAGTTACCTCGTCCCGGTTTACAGGGACGGTA
TTTGCCTCGTTTTCGAACTATCTCTCTCTCTCTTTCTCTCTTTTCTCGACGAGCAGGGAC
GAGCCCAAGTGTCCGGTGATTGATTTCTGTCACTCGACGATCCGTTCATCCGTTGCTC
AATCACGTGACGAAAACACTACGGGTGTCCATTTAAATTATGAACGTCGGTTTTATTTAT
TTCGAAATCTATTATGTTACTCAACTTCTCGTGTAATTTGTAAGCCAATACAAGAGAGGA
GTAAGTAACTTCGTACTCGGATATCCGTTTTCCTTCCAACTTCCATCATTAATAACTTTCT
CTTCCCTCGAAATGACGGAGGCGAGTCGTTCGAGTCGTACGGTAATCGATTAATAACTC
TTTGGGGAAAAAGGGAAAAAAGAAAAAGAATTTCGAGGTAGCGATAATAATCAGATTT
AAACGGACGCGTCTCTGGCAAATAAAAGTTTGTTACGAGAAATTGGTACCGTTACGTTA
TTTAAATGCTGTCGTATCGACGATGGAAACTTTTGTGCTCGAATTCTCAAAAGTAATTTC
GTTTCGCGGCGATCGAACCACGCTGTGATGTCTTTTTCATCCGCTGGCCAACTTTTTATT
TATCGCAATTCCATAAACGTCCCCGGGGATATTAAATCGTCGATAAATTCATTGTATTTCG
TCCAATGACAATCTCTCGAACTTTGCTTGAACAAGATATTCCGTTCTGCGAACGATACT
TGTTGCCGATTGAATTTTCACTTTCGACCTTTCCTTATCTTTTTCTTACTCTTTTACACCC
CTTTATTTTTCGTTTTAATCCCTCTCCTCTCGACTTCCCTTTCGCATTTCTTCTCTTTTTCC
TTTTTCTTCTTCTTCCTCTTCTTCCTTTTTTTTCCTTTACTCTTCGGTTATTATCGTCGAGT
TTTCTCAAGAAGTATTAAAGTAATGCCACGCTCTTTCGCGTAATTAAATGCCGTACTCG
GTGAACTGAAACAATATAAACTTTCGAGCGTCAAGTTTACGAGCGTTGAAAAACTTGC
TCCGATATTGAATATCATTGCGAATGTGTTTCAACGTTGCCTTCTTTTCCTCTCCTCCTCC
TCTCCTTCCCCTTCGACGCTCGTAAAAACTTTTAATCGACAGTGTACTCGCGAGTACA
CCGTTCTCTTCTTTTCTTCTCGTCTCTTTTTTCCCGCCCGTCTTCGCCGGGAAAACGAA
GAAGGGGGAAGTGGAGACGCTTGCCACGCTTATCGCTTCCCCTCTATTGGACCTCTATC
GAGGCAGAAACGCTCCTCTCTCTCTTTTTTTCTCGAGAAATCGGTGTCGGAGTGAACA

TCGAATTCGTGTTATCCGAGCCGCGAACTTCGTCTTTCAGCAGCGCGAGGGAGGAGGA
TCGGTCGTCCCCGCCAACTTTCTCTCACCCTTTCTCACCCTCGAAAGAGAGAAAGAGA
GAGAGAGGAGGAGGTGGAGGAAGGGAAAGGAGGAGATATACAAGCGGCGCGCGCGC
CCTCTCCGTTCCAACCATAGCTCGTTCGGAATGCAGAGTACGTGCACGGGCTGCGGTT
GCCGGTCGGTTTCCGGTCGTAAGCTATCCGCGAAGGGGAAAATAGCGGGGAAAAGAG
CGTGGAGTGATTTGGAAGGTAGTTGCGGCCTCGTCCAGGGGAGAATGGGGGGAGTAG
AGAGAGAGAGAGAGAGAGGGAAAAGAATGGAGCGAGAGGGAGAAAGGAAAGGAGA
GCGAAAGGAGGAAGGAAGAAAGCGGGGTGGGTGGCGGGGTGTTAGCGGCGGTGGTT
TAAACGAGGGAGGAGGGGCGGATGCTTTCTTAAAGATGTGGAGTCGGGGCGCACTTG
TTGCGCGGAAAAGGAGTACGCGGGCGATGCCAAGAAAAGAAGGAAGGAAAGAGGGA
AGGAAAGATAAAGGAAGGAAGGAAGGAAGGAAGGAAGGAAGGAAGAGGCGTGTGC
AGCCTCGGAAGCGTGCAAAAAGGCGTGGAGAGGGGGAAGTGAAGAAAAACGACGGC
GAGGGTAGGGTGGGGGAGATTTGACGGCGACGAAGTGAGAGTTTGAGGGGTGTAGGC
GAGGAAATGATAGGGGTTTGAGAGGGAGGGGGCGAAGATACGGCTGGGGAATCGAAT
TGATTCTCTTTTACGGGCTCGCGTTACTTTTCTTCACCGCCTTTTGCCATCCTTGCCTCT
TGACCGTGGCGCTGCTCCTCTTCCAATCTTTCCTCTTCTCTCTTTCTCTCTCTCTCCTTC
TCTCTCCCGCTCTTTCAATTTCCTCCAAGCCTTTCCTTCTCGCCGGTGTTATTTATTTATC
AGCTCTCGCACGTAGCTTTCCTCAAGCTCGTGTAAAGGAGCAGACGAGGCACCACTTC
GACCGGACGGACCACCTTCTTTATCCGGACCATCTCTCTTTCTTTCTTTTCGTTCCGACC
ACGGTCTTGGCCTTTTTTCCTCGCCTCTCGTCGGATCCTCCCCTCCCGTTGTACCACAC
GCTCTCTCATTTCATTTGCCGGTCGTTGGACGGTGAAAGGTAGTTCTTTCGCGTCGAGG
GGGGCAAGAATCGGCACTCGCCAATTGTGACGGGAGGATGAGAAGACTTATTGGGATT
CCTTGCGGACAACGGACGACCTTCCACGATCGATCGATGGACGCGCGACCAATCACGA
CGATCCATTGGTCACGGTGAATATCGTATATCGGAGGAAAGTAATAGCCTCGAGGATGA
TCGATAAGGAGTGGACAATTCTTCCTTTGCTTCCTACCATATATATATATATATATACGGAAG
AAACTGTGAACGCGTCGTCATAGAGAGAGAAAGAGAGAGAGAAAAAAAGAAATTCA
CCGAGCAATAATTTATTAATTCAAGATGGATTAAAGCGATGGAGAAGGGCGATGAGAG
AGATGGAGAGAGACAGAGAGAAGAATAGTGCTTTTAAGTTTATTAACCGTGAAACAAA
AGACGAAGCGTGAACAGGTATAGCCAGGTCCAAGGTGGTGTGTAGAAGTCTATTGAAC
TTCCGGCTTGAGAGAATTCACCACGTTACTTATTTATCAGCATCTACACGTAGCGCGGT
ACGGAGTATCACCTCATGGGAAAAAGGCACTTTCCGCGCGAGTAGATAGAATCAACCG
GCCCGGATTGGATTGGAGCCTCGAGGAAAAACTTATTTGGATTCCTTACGGATATAAGG
GGTTTCCGGGGCTGCTTTTACTCGGCCAGCTTGGACGCGAATTGATCGATATATCGAAG
GGAGAGGAGAGTGGTGGCGGCGGCGTTACATTGACCGTACAGAAATCGAAATTACGC
GTATAATTTGTTGCTAGTTTTTCTCCAAAGGCTCGCCCTTTAATCGGGAAGATTAAAAG
GGTAAAAAGGGCGCGCGCGCGCTTTCCCGAGTTATTCAACTTTTCCCGAGAAACTT
GATTCTCCGAGGTATGGTCGATGCGCGTCGTTGAATAACGAGCGTTAAGTAACAACATA
GAATGCCAGTAGTTGCGAGCTATCTGTATTTTTTTTTCCCTCCTCTCCTTTTTCTCTCCTC
GTAACGAGGAGAGAAAAACGAAGGTGAAACATTTCCTCTCTTCGTGTCTCGAGCGTGT
TTCGCAAAGAAAATTCTTTCAGCAAGCCAACCTATGCTTTACGGCTCTTAAACTTTATC
TCTTTCTCTCTTTCTCTCTACGATATCGATATTTCTCCGTGGAAACACACACGCGACGCG
AATATCGACGGGATATTGACTTTTTCTTTTCCCAACTCGTTGGAAAAAGAAGCTTAACC
TATTCTCTCTCGATTCGATGACTCTTGGTTGCATCCTTGGCCAATAAATAATAACTGTATC
TTGGAATAACTATACTTTTCCAGGCGGTCCATCGTAAATAACGTTTGATCGAGAATTGG

GGAAGACTTGGCCACGAGAGAGAAATTCGATGTAGCGACACCGATGTATCGATTCCAA
AATTATCGCACAAACGTCATCGTAGCAGTAGGCGAGGTTATTAGATTGGCCCGACAGAG
AGGAGAATAAGACAGGCGAACTCAAGATATTGTATTC

>novel_circ_000334
GAGGAAGCTGAATCGACGTAACGGTCTACGCCAGGATGGCCAGTTGGCCGCTGAGAA
TCGTGCTTCTGTCCAGCACCCTCATCCTTGTCCGCGGGCGGGGAGTGGTGCTGGACAG
TCAGGGGCCTGTGTTGATCTCGGAGCCACGCTCCTCGGTTGAATTCTCGAACGAGACC
GGGGCCATGATCCATTGCTCGGCCCAGGGCAGCCCGTTGCCGAGGATTGATTGGCTCA
TGGGGGACGAGTCGCCTGTCCTCCCTATACCTCACATACGCGAGATGCTGGTCAACGG
TTCCATGTACTTTTTGCCGTTCGGAGCGGAGACCTACCGGCACGACGTCCACTCGGCC
GTCTACAGATGTCAAGCCTCGAACAGCGTCGGTAGGGTATTGGGCCGTGAAATCACGG
TAAAGGCCG

>novel_circ_000335
GAGGAAGCTGAATCGACGTAACGGTCTACGCCAGGATGGCCAGTTGGCCGCTGAGAA
TCGTGCTTCTGTCCAGCACCCTCATCCTTGTCCGCGGGCGGGGAGTGGTGCTGGACAG
TCAGGGGCCTGTGTTGATCTCGGAGCCACGCTCCTCGGTTGAATTCTCGAACGAGACC
GGGGCCATGATCCATTGCTCGGCCCAGGGCAGCCCGTTGCCGAGGATTGATTGGCTCA
TGGGGGACGAGTCGCCTGTCCTCCCTATACCTCACATACGCGAGATGCTGGTCAACGG
TTCCATGTACTTTTTGCCGTTCGGAGCGGAGACCTACCGGCACGACGTCCACTCGGCC
GTCTACAGATGTCAAGCCTCGAACAGCGTCGGTAGGGTATTGGGCCGTGAAATCACGG
TAAAGGCCGTCGTGCGTCAAAAGTACGAGGTTCAAGTGCGAGACGCCTACGTCCTACC
TGGAAATACCGGTGTGCTCAGGTGCGAAATACCGACTTTCGTTAAGGAATACGTGGCA
GTCACGTCCTGGGTTCGAGATTCCGCCTACAACATCTTTCCAACGCCGAAAAGCG

>novel_circ_000336
GAGGAAGCTGAATCGACGTAACGGTCTACGCCAGGATGGCCAGTTGGCCGCTGAGAA
TCGTGCTTCTGTCCAGCACCCTCATCCTTGTCCGCGGGCGGGGAGTGGTGCTGGACAG
TCAGGGGCCTGTGTTGATCTCGGAGCCACGCTCCTCGGTTGAATTCTCGAACGAGACC
GGGGCCATGATCCATTGCTCGGCCCAGGGCAGCCCGTTGCCGAGGATTGATTGGCTCA
TGGGGGACGAGTCGCCTGTCCTCCCTATACCTCACATACGCGAGATGCTGGTCAACGG
TTCCATGTACTTTTTGCCGTTCGGAGCGGAGACCTACCGGCACGACGTCCACTCGGCC
GTCTACAGATGTCAAGCCTCGAACAGCGTCGGTAGGGTATTGGGCCGTGAAATCACGG
TAAAGGCCGTCGTGCGTCAAAAGTACGAGGTTCAAGTGCGAGACGCCTACGTCCTACC
TGGAAATACCGGTGTGCTCAGGTGCGAAATACCGACTTTCGTTAAGGAATACGTGGCA
GTCACGTCCTGGGTTCGAGATTCCGCCTACAACATCTTTCCAACGCCGAAAAGCGATG
ACAGACATCACATGCTGCCGACTGGGGAACTCCTCGTGTTCTCGGTGACCTCCTCCGA
CGCCCAGTCCAGTTATCGTTGCAGGACCGTGCACCGCGTCACCGGCGACACCGTGGAA
AGTTCCTCGTACGCGAGGCTCGTGGTCACCG

>novel_circ_000337
GAGGAAGCTGAATCGACGTAACGGTCTACGCCAGGATGGCCAGTTGGCCGCTGAGAA
TCGTGCTTCTGTCCAGCACCCTCATCCTTGTCCGCGGGCGGGGAGTGGTGCTGGACAG

TCAGGGGCCTGTGTTGATCTCGGAGCCACGCTCCTCGGTTGAATTCTCGAACGAGACC
GGGGCCATGATCCATTGCTCGGCCCAGGGCAGCCCGTTGCCGAGGATTGATTGGCTCA
TGGGGGACGAGTCGCCTGTCCTCCCTATACCTCACATACGCGAGATGCTGGTCAACGG
TTCCATGTACTTTTTGCCGTTCGGAGCGGAGACCTACCGGCACGACGTCCACTCGGCC
GTCTACAGATGTCAAGCCTCGAACAGCGTCGGTAGGGTATTGGGCCGTGAAATCACGG
TAAAGGCCGTCGTGCGTCAAAAGTACGAGGTTCAAGTGCGAGACGCCTACGTCCTACC
TGGAAATACCGGTGTGCTCAGGTGCGAAATACCGACTTTCGTTAAGGAATACGTGGCA
GTCACGTCCTGGGTTCGAGATTCCGCCTACAACATCTTTCCAACGCCGAAAAGCGATG
ACAGACATCACATGCTGCCGACTGGGGAACTCCTCGTGTTCTCGGTGACCTCCTCCGA
CGCCCAGTCCAGTTATCGTTGCAGGACCGTGCACCGCGTCACCGGCGACACCGTGGAA
AGTTCCTCGTACGCGAGGCTCGTGGTCACCGGCGGTTTACGTACGTATGTGACGAGCA
ATTACGCGTCAATATTTAAATCGAAACGATATTAATGTTTACGGACGCGTTGAAATCGTA
ACGATGTCCCGAACTCGCGGAGGACTCGATGAGTTTTAAATTTTCTTCCGGGATAAACG
TATCCCGGATTAATAATAAAGATTCGTACGTGCGATTGTCACGGCGTGTAATTAACGCGA
TAAAGTAATGGGGGAAAAATGTAGAATGTAAACGGAAGGGGAGAGCGGGAATAAATG
TAAATTTGTCGAGAGCGTAGCGGTTCGAATAATACGTAACAGCGACAAAAGGAAGGTC
TTTCACGGTTGTGAAAAATTGATACCTTGAGATCTTTGCGACCCGTTTCAACGAAATCT
CCGCGGAGATTTCTTTCCGAGATATTCGAAATGCATTTTCGAGGTATCAAGGGGGAAGA
GGATCGTGGAAAAATTTATGAAAGATGAAAAAGCAATTGAAATATAAGAACGGTAACC
CTCATACGCCAGTTCCGCCGAGATTCAACGAACGAGTGAGTCCAGCGCCTATAAGGAC
GGGGGAGACGATCGTCCTTTCCTGCATCTCGCAGGGGATCCCGCCACCCATGTATCTAT
GGTTTCGCGAGTCCGTGACGGGGACTGCGATGATCTTCAATTCGGAGAGGATCTACGC
GAGAGCAGGGGTCCTCGTGTTGCAGTCGGCGAGGGCGGAGGACGCGGGGCGATACGT
GTGCCACGCCAACAATACGGCCGGTTCCGAGATGGTCGAATTGGAAGTCTCCATTATAA
GCAGCCTCTCCATCCACCTGGTGCCTCAACAG

>novel_circ_000338
GAGGAAGCTGAATCGACGTAACGGTCTACGCCAGGATGGCCAGTTGGCCGCTGAGAA
TCGTGCTTCTGTCCAGCACCCTCATCCTTGTCCGCGGGCGGGGAGTGGTGCTGGACAG
TCAGGGGCCTGTGTTGATCTCGGAGCCACGCTCCTCGGTTGAATTCTCGAACGAGACC
GGGGCCATGATCCATTGCTCGGCCCAGGGCAGCCCGTTGCCGAGGATTGATTGGCTCA
TGGGGGACGAGTCGCCTGTCCTCCCTATACCTCACATACGCGAGATGCTGGTCAACGG
TTCCATGTACTTTTTGCCGTTCGGAGCGGAGACCTACCGGCACGACGTCCACTCGGCC
GTCTACAGATGTCAAGCCTCGAACAGCGTCGGTAGGGTATTGGGCCGTGAAATCACGG
TAAAGGCCGTCGTGCGTCAAAAGTACGAGGTTCAAGTGCGAGACGCCTACGTCCTACC
TGGAAATACCGGTGTGCTCAGGTGCGAAATACCGACTTTCGTTAAGGAATACGTGGCA
GTCACGTCCTGGGTTCGAGATTCCGCCTACAACATCTTTCCAACGCCGAAAAGCGATG
ACAGACATCACATGCTGCCGACTGGGGAACTCCTCGTGTTCTCGGTGACCTCCTCCGA
CGCCCAGTCCAGTTATCGTTGCAGGACCGTGCACCGCGTCACCGGCGACACCGTGGAA
AGTTCCTCGTACGCGAGGCTCGTGGTCACCGGCGGTTTACGTACGTATGTGACGAGCA
ATTACGCGTCAATATTTAAATCGAAACGATATTAATGTTTACGGACGCGTTGAAATCGTA
ACGATGTCCCGAACTCGCGGAGGACTCGATGAGTTTTAAATTTTCTTCCGGGATAAACG
TATCCCGGATTAATAATAAAGATTCGTACGTGCGATTGTCACGGCGTGTAATTAACGCGA
TAAAGTAATGGGGGAAAAATGTAGAATGTAAACGGAAGGGGAGAGCGGGAATAAATG

TAAATTTGTCGAGAGCGTAGCGGTTCGAATAATACGTAACAGCGACAAAAGGAAGGTC
TTTCACGGTTGTGAAAAATTGATACCTTGAGATCTTTGCGACCCGTTTCAACGAAATCT
CCGCGGAGATTTCTTTCCGAGATATTCGAAATGCATTTTCGAGGTATCAAGGGGGAAGA
GGATCGTGGAAAAATTTATGAAAGATGAAAAAGCAATTGAAATATAAGAACGGTAACC
CTCATACGCCAGTTCCGCCGAGATTCAACGAACGAGTGAGTCCAGCGCCTATAAGGAC
GGGGGAGACGATCGTCCTTTCCTGCATCTCGCAGGGGATCCCGCCACCCATGTATCTAT
GGTTTCGCGAGTCCGTGACGGGGACTGCGATGATCTTCAATTCGGAGAGGATCTACGC
GAGAGCAGGGGTCCTCGTGTTGCAGTCGGCGAGGGCGGAGGACGCGGGGCGATACGT
GTGCCACGCCAACAATACGGCCGGTTCCGAGATGGTCGAATTGGAAGTCTCCATTATAA
GCAGCCTCTCCATCCACCTGGTGCCTCAACAGGTGACCGTCGACCTGGGCAAGGACG
CGGAATTCCAATGCTCGGTCACCGGCCAGCCAATCCCCGTCATATTCTGGACCAAGGA
CGGGCTTCCCGTGGGAGAGAGCGCCTCTGGTAGAAGCAAGATCACAGGCAACGACGG
ATCCACGCTGCGCATTTCCTCGGTCGTGAGGGACGACAAGGGGATGTACCAATGCTTC
GCGAAGAACGACTACGAGATGGTCCAAGCGACCGCCGAGTTGCGTCTGGGGG

>novel_circ_000339
GAGGAAGCTGAATCGACGTAACGGTCTACGCCAGGATGGCCAGTTGGCCGCTGAGAA
TCGTGCTTCTGTCCAGCACCCTCATCCTTGTCCGCGGGCGGGGAGTGGTGCTGGACAG
TCAGGGGCCTGTGTTGATCTCGGAGCCACGCTCCTCGGTTGAATTCTCGAACGAGACC
GGGGCCATGATCCATTGCTCGGCCCAGGGCAGCCCGTTGCCGAGGATTGATTGGCTCA
TGGGGGACGAGTCGCCTGTCCTCCCTATACCTCACATACGCGAGATGCTGGTCAACGG
TTCCATGTACTTTTTGCCGTTCGGAGCGGAGACCTACCGGCACGACGTCCACTCGGCC
GTCTACAGATGTCAAGCCTCGAACAGCGTCGGTAGGGTATTGGGCCGTGAAATCACGG
TAAAGGCCGTCGTGCGTCAAAAGTACGAGGTTCAAGTGCGAGACGCCTACGTCCTACC
TGGAAATACCGGTGTGCTCAGGTGCGAAATACCGACTTTCGTTAAGGAATACGTGGCA
GTCACGTCCTGGGTTCGAGATTCCGCCTACAACATCTTTCCAACGCCGAAAAGCGATG
ACAGACATCACATGCTGCCGACTGGGGAACTCCTCGTGTTCTCGGTGACCTCCTCCGA
CGCCCAGTCCAGTTATCGTTGCAGGACCGTGCACCGCGTCACCGGCGACACCGTGGAA
AGTTCCTCGTACGCGAGGCTCGTGGTCACCGGCGGTTTACGTACGTATGTGACGAGCA
ATTACGCGTCAATATTTAAATCGAAACGATATTAATGTTTACGGACGCGTTGAAATCGTA
ACGATGTCCCGAACTCGCGGAGGACTCGATGAGTTTTAAATTTTCTTCCGGGATAAACG
TATCCCGGATTAATAATAAAGATTCGTACGTGCGATTGTCACGGCGTGTAATTAACGCGA
TAAAGTAATGGGGGAAAAATGTAGAATGTAAACGGAAGGGGAGAGCGGGAATAAATG
TAAATTTGTCGAGAGCGTAGCGGTTCGAATAATACGTAACAGCGACAAAAGGAAGGTC
TTTCACGGTTGTGAAAAATTGATACCTTGAGATCTTTGCGACCCGTTTCAACGAAATCT
CCGCGGAGATTTCTTTCCGAGATATTCGAAATGCATTTTCGAGGTATCAAGGGGGAAGA
GGATCGTGGAAAAATTTATGAAAGATGAAAAAGCAATTGAAATATAAGAACGGTAACC
CTCATACGCCAGTTCCGCCGAGATTCAACGAACGAGTGAGTCCAGCGCCTATAAGGAC
GGGGGAGACGATCGTCCTTTCCTGCATCTCGCAGGGGATCCCGCCACCCATGTATCTAT
GGTTTCGCGAGTCCGTGACGGGGACTGCGATGATCTTCAATTCGGAGAGGATCTACGC
GAGAGCAGGGGTCCTCGTGTTGCAGTCGGCGAGGGCGGAGGACGCGGGGCGATACGT
GTGCCACGCCAACAATACGGCCGGTTCCGAGATGGTCGAATTGGAAGTCTCCATTATAA
GCAGCCTCTCCATCCACCTGGTGCCTCAACAGGTGACCGTCGACCTGGGCAAGGACG
CGGAATTCCAATGCTCGGTCACCGGCCAGCCAATCCCCGTCATATTCTGGACCAAGGA

CGGGCTTCCCGTGGGAGAGAGCGCCTCTGGTAGAAGCAAGATCACAGGCAACGACGG
ATCCACGCTGCGCATTTCCTCGGTCGTGAGGGACGACAAGGGGATGTACCAATGCTTC
GCGAAGAACGACTACGAGATGGTCCAAGCGACCGCCGAGTTGCGTCTGGGGGACGCG
GCGCCGCAGCTTCTGTACAAATTCATCGAGCAGACCATCCAGCCCGGCCCTTCGGTGT
CGCTCAAGTGCATCGCCACCGGGAACCCGACGCCGCACTTCTCCTGGACCCTGGACG
GTTTCCCTCTTCCGCAGAACGACAG

>novel_circ_000340
GAGGAAGCTGAATCGACGTAACGGTCTACGCCAGGATGGCCAGTTGGCCGCTGAGAA
TCGTGCTTCTGTCCAGCACCCTCATCCTTGTCCGCGGGCGGGGAGTGGTGCTGGACAG
TCAGGGGCCTGTGTTGATCTCGGAGCCACGCTCCTCGGTTGAATTCTCGAACGAGACC
GGGGCCATGATCCATTGCTCGGCCCAGGGCAGCCCGTTGCCGAGGATTGATTGGCTCA
TGGGGGACGAGTCGCCTGTCCTCCCTATACCTCACATACGCGAGATGCTGGTCAACGG
TTCCATGTACTTTTTGCCGTTCGGAGCGGAGACCTACCGGCACGACGTCCACTCGGCC
GTCTACAGATGTCAAGCCTCGAACAGCGTCGGTAGGGTATTGGGCCGTGAAATCACGG
TAAAGGCCGTCGTGCGTCAAAAGTACGAGGTTCAAGTGCGAGACGCCTACGTCCTACC
TGGAAATACCGGTGTGCTCAGGTGCGAAATACCGACTTTCGTTAAGGAATACGTGGCA
GTCACGTCCTGGGTTCGAGATTCCGCCTACAACATCTTTCCAACGCCGAAAAGCGATG
ACAGACATCACATGCTGCCGACTGGGGAACTCCTCGTGTTCTCGGTGACCTCCTCCGA
CGCCCAGTCCAGTTATCGTTGCAGGACCGTGCACCGCGTCACCGGCGACACCGTGGAA
AGTTCCTCGTACGCGAGGCTCGTGGTCACCGGCGGTTTACGTACGTATGTGACGAGCA
ATTACGCGTCAATATTTAAATCGAAACGATATTAATGTTTACGGACGCGTTGAAATCGTA
ACGATGTCCCGAACTCGCGGAGGACTCGATGAGTTTTAAATTTTCTTCCGGGATAAACG
TATCCCGGATTAATAATAAAGATTCGTACGTGCGATTGTCACGGCGTGTAATTAACGCGA
TAAAGTAATGGGGGAAAAATGTAGAATGTAAACGGAAGGGGAGAGCGGGAATAAATG
TAAATTTGTCGAGAGCGTAGCGGTTCGAATAATACGTAACAGCGACAAAAGGAAGGTC
TTTCACGGTTGTGAAAAATTGATACCTTGAGATCTTTGCGACCCGTTTCAACGAAATCT
CCGCGGAGATTTCTTTCCGAGATATTCGAAATGCATTTTCGAGGTATCAAGGGGGAAGA
GGATCGTGGAAAAATTTATGAAAGATGAAAAAGCAATTGAAATATAAGAACGGTAACC
CTCATACGCCAGTTCCGCCGAGATTCAACGAACGAGTGAGTCCAGCGCCTATAAGGAC
GGGGGAGACGATCGTCCTTTCCTGCATCTCGCAGGGGATCCCGCCACCCATGTATCTAT
GGTTTCGCGAGTCCGTGACGGGGACTGCGATGATCTTCAATTCGGAGAGGATCTACGC
GAGAGCAGGGGTCCTCGTGTTGCAGTCGGCGAGGGCGGAGGACGCGGGGCGATACGT
GTGCCACGCCAACAATACGGCCGGTTCCGAGATGGTCGAATTGGAAGTCTCCATTATAA
GCAGCCTCTCCATCCACCTGGTGCCTCAACAGGTGACCGTCGACCTGGGCAAGGACG
CGGAATTCCAATGCTCGGTCACCGGCCAGCCAATCCCCGTCATATTCTGGACCAAGGA
CGGGCTTCCCGTGGGAGAGAGCGCCTCTGGTAGAAGCAAGATCACAGGCAACGACGG
ATCCACGCTGCGCATTTCCTCGGTCGTGAGGGACGACAAGGGGATGTACCAATGCTTC
GCGAAGAACGACTACGAGATGGTCCAAGCGACCGCCGAGTTGCGTCTGGGGGACGCG
GCGCCGCAGCTTCTGTACAAATTCATCGAGCAGACCATCCAGCCCGGCCCTTCGGTGT
CGCTCAAGTGCATCGCCACCGGGAACCCGACGCCGCACTTCTCCTGGACCCTGGACG
GTTTCCCTCTTCCGCAGAACGACAGGTTTATGATTGGGCAGTATGTGACGGTGCACGG
GGACGTGATATCGCACGTGAACATCAGCGTGGTTCACGTGGAGGACGGAGGGGAGTA
CCGGTGCACAGCGGCGAACCGGATGGCGAAGGTACACCATTCGGCTCGTCTCAACATT

TACGGCCTGCCCCACGTACGCCCGATGGGGAACTACGCCGCTGTGGCCGGCGAGACCA
CCGTCATCAAATGTCCGGTCGCTGGTTTCCCAATAGCGAGCATCACTTGGGAGAAAGA
CGGGAAGGTGTTACCGACGAGTCGACGTCAGGAAGTGTTCCCGAACGGTACGCTGGT
GCTGCATCACGTCGACAGAAGCACCGATCACGGCGCATACACGTGCACGGCGAAGAA
CAAACAAGGTCGCTCCGACTCTCAGACGGTCCACATCGAAGTGAAAG

>novel_circ_000341
GGCGGGGAGTGGTGCTGGACAGTCAGGGGCCTGTGTTGATCTCGGAGCCACGCTCCT
CGGTTGAATTCTCGAACGAGACCGGGGCCATGATCCATTGCTCGGCCCAGGGCAGCCC
GTTGCCGAGGATTGATTGGCTCATGGGGGACGAGTCGCCTGTCCTCCCTATACCTCACA
TACGCGAGATGCTGGTCAACGGTTCCATGTACTTTTTGCCGTTCGGAGCGGAGACCTAC
CGGCACGACGTCCACTCGGCCGTCTACAGATGTCAAGCCTCGAACAGCGTCGGTAGG
GTATTGGGCCGTGAAATCACGGTAAAGGCCG

>novel_circ_000342
GGCGGGGAGTGGTGCTGGACAGTCAGGGGCCTGTGTTGATCTCGGAGCCACGCTCCT
CGGTTGAATTCTCGAACGAGACCGGGGCCATGATCCATTGCTCGGCCCAGGGCAGCCC
GTTGCCGAGGATTGATTGGCTCATGGGGGACGAGTCGCCTGTCCTCCCTATACCTCACA
TACGCGAGATGCTGGTCAACGGTTCCATGTACTTTTTGCCGTTCGGAGCGGAGACCTAC
CGGCACGACGTCCACTCGGCCGTCTACAGATGTCAAGCCTCGAACAGCGTCGGTAGG
GTATTGGGCCGTGAAATCACGGTAAAGGCCGTCGTGCGTCAAAAGTACGAGGTTCAAG
TGCGAGACGCCTACGTCCTACCTGGAAATACCGGTGTGCTCAGGTGCGAAATACCGAC
TTTCGTTAAGGAATACGTGGCAGTCACGTCCTGGGTTCGAGATTCCGCCTACAACATCT
TTCCAACGCCGAAAAGCG

>novel_circ_000344
GGCGGGGAGTGGTGCTGGACAGTCAGGGGCCTGTGTTGATCTCGGAGCCACGCTCCT
CGGTTGAATTCTCGAACGAGACCGGGGCCATGATCCATTGCTCGGCCCAGGGCAGCCC
GTTGCCGAGGATTGATTGGCTCATGGGGGACGAGTCGCCTGTCCTCCCTATACCTCACA
TACGCGAGATGCTGGTCAACGGTTCCATGTACTTTTTGCCGTTCGGAGCGGAGACCTAC
CGGCACGACGTCCACTCGGCCGTCTACAGATGTCAAGCCTCGAACAGCGTCGGTAGG
GTATTGGGCCGTGAAATCACGGTAAAGGCCGTCGTGCGTCAAAAGTACGAGGTTCAAG
TGCGAGACGCCTACGTCCTACCTGGAAATACCGGTGTGCTCAGGTGCGAAATACCGAC
TTTCGTTAAGGAATACGTGGCAGTCACGTCCTGGGTTCGAGATTCCGCCTACAACATCT
TTCCAACGCCGAAAAGCGATGACAGACATCACATGCTGCCGACTGGGGAACTCCTCGT
GTTCTCGGTGACCTCCTCCGACGCCCAGTCCAGTTATCGTTGCAGGACCGTGCACCGC
GTCACCGGCGACACCGTGGAAAGTTCCTCGTACGCGAGGCTCGTGGTCACCG

>novel_circ_000345
GGCGGGGAGTGGTGCTGGACAGTCAGGGGCCTGTGTTGATCTCGGAGCCACGCTCCT
CGGTTGAATTCTCGAACGAGACCGGGGCCATGATCCATTGCTCGGCCCAGGGCAGCCC
GTTGCCGAGGATTGATTGGCTCATGGGGGACGAGTCGCCTGTCCTCCCTATACCTCACA
TACGCGAGATGCTGGTCAACGGTTCCATGTACTTTTTGCCGTTCGGAGCGGAGACCTAC
CGGCACGACGTCCACTCGGCCGTCTACAGATGTCAAGCCTCGAACAGCGTCGGTAGG

GTATTGGGCCGTGAAATCACGGTAAAGGCCGTCGTGCGTCAAAAGTACGAGGTTCAAG
TGCGAGACGCCTACGTCCTACCTGGAAATACCGGTGTGCTCAGGTGCGAAATACCGAC
TTTCGTTAAGGAATACGTGGCAGTCACGTCCTGGGTTCGAGATTCCGCCTACAACATCT
TTCCAACGCCGAAAAGCGATGACAGACATCACATGCTGCCGACTGGGGAACTCCTCGT
GTTCTCGGTGACCTCCTCCGACGCCCAGTCCAGTTATCGTTGCAGGACCGTGCACCGC
GTCACCGGCGACACCGTGGAAAGTTCCTCGTACGCGAGGCTCGTGGTCACCGGCGGT
TTACGTACGTATGTGACGAGCAATTACGCGTCAATATTTAAATCGAAACGATATTAATGT
TTACGGACGCGTTGAAATCGTAACGATGTCCCGAACTCGCGGAGGACTCGATGAGTTT
TAAATTTTCTTCCGGGATAAACGTATCCCGGATTAATAATAAAGATTCGTACGTGCGATT
GTCACGGCGTGTAATTAACGCGATAAAGTAATGGGGGAAAAATGTAGAATGTAAACGG
AAGGGGAGAGCGGGAATAAATGTAAATTTGTCGAGAGCGTAGCGGTTCGAATAATACG
TAACAGCGACAAAAGGAAGGTCTTTCACGGTTGTGAAAAATTGATACCTTGAGATCTT
TGCGACCCGTTTCAACGAAATCTCCGCGGAGATTTCTTTCCGAGATATTCGAAATGCAT
TTTCGAGGTATCAAGGGGGAAGAGGATCGTGGAAAAATTTATGAAAGATGAAAAAGC
AATTGAAATATAAGAACGGTAACCCTCATACGCCAGTTCCGCCGAGATTCAACGAACG
AGTGAGTCCAGCGCCTATAAGGACGGGGGAGACGATCGTCCTTTCCTGCATCTCGCAG
GGGATCCCGCCACCCATGTATCTATGGTTTCGCGAGTCCGTGACGGGGACTGCGATGAT
CTTCAATTCGGAGAGGATCTACGCGAGAGCAGGGGTCCTCGTGTTGCAGTCGGCGAGG
GCGGAGGACGCGGGGCGATACGTGTGCCACGCCAACAATACGGCCGGTTCCGAGATG
GTCGAATTGGAAGTCTCCATTATAAGCAGCCTCTCCATCCACCTGGTGCCTCAACAG

>novel_circ_000346
GGCGGGGAGTGGTGCTGGACAGTCAGGGGCCTGTGTTGATCTCGGAGCCACGCTCCT
CGGTTGAATTCTCGAACGAGACCGGGGCCATGATCCATTGCTCGGCCCAGGGCAGCCC
GTTGCCGAGGATTGATTGGCTCATGGGGGACGAGTCGCCTGTCCTCCCTATACCTCACA
TACGCGAGATGCTGGTCAACGGTTCCATGTACTTTTTGCCGTTCGGAGCGGAGACCTAC
CGGCACGACGTCCACTCGGCCGTCTACAGATGTCAAGCCTCGAACAGCGTCGGTAGG
GTATTGGGCCGTGAAATCACGGTAAAGGCCGTCGTGCGTCAAAAGTACGAGGTTCAAG
TGCGAGACGCCTACGTCCTACCTGGAAATACCGGTGTGCTCAGGTGCGAAATACCGAC
TTTCGTTAAGGAATACGTGGCAGTCACGTCCTGGGTTCGAGATTCCGCCTACAACATCT
TTCCAACGCCGAAAAGCGATGACAGACATCACATGCTGCCGACTGGGGAACTCCTCGT
GTTCTCGGTGACCTCCTCCGACGCCCAGTCCAGTTATCGTTGCAGGACCGTGCACCGC
GTCACCGGCGACACCGTGGAAAGTTCCTCGTACGCGAGGCTCGTGGTCACCGGCGGT
TTACGTACGTATGTGACGAGCAATTACGCGTCAATATTTAAATCGAAACGATATTAATGT
TTACGGACGCGTTGAAATCGTAACGATGTCCCGAACTCGCGGAGGACTCGATGAGTTT
TAAATTTTCTTCCGGGATAAACGTATCCCGGATTAATAATAAAGATTCGTACGTGCGATT
GTCACGGCGTGTAATTAACGCGATAAAGTAATGGGGGAAAAATGTAGAATGTAAACGG
AAGGGGAGAGCGGGAATAAATGTAAATTTGTCGAGAGCGTAGCGGTTCGAATAATACG
TAACAGCGACAAAAGGAAGGTCTTTCACGGTTGTGAAAAATTGATACCTTGAGATCTT
TGCGACCCGTTTCAACGAAATCTCCGCGGAGATTTCTTTCCGAGATATTCGAAATGCAT
TTTCGAGGTATCAAGGGGGAAGAGGATCGTGGAAAAATTTATGAAAGATGAAAAAGC
AATTGAAATATAAGAACGGTAACCCTCATACGCCAGTTCCGCCGAGATTCAACGAACG
AGTGAGTCCAGCGCCTATAAGGACGGGGGAGACGATCGTCCTTTCCTGCATCTCGCAG
GGGATCCCGCCACCCATGTATCTATGGTTTCGCGAGTCCGTGACGGGGACTGCGATGAT

CTTCAATTCGGAGAGGATCTACGCGAGAGCAGGGGTCCTCGTGTTGCAGTCGGCGAGG
GCGGAGGACGCGGGGCGATACGTGTGCCACGCCAACAATACGGCCGGTTCCGAGATG
GTCGAATTGGAAGTCTCCATTATAAGCAGCCTCTCCATCCACCTGGTGCCTCAACAGGT
GACCGTCGACCTGGGCAAGGACGCGGAATTCCAATGCTCGGTCACCGGCCAGCCAAT
CCCCGTCATATTCTGGACCAAGGACGGGCTTCCCGTGGGAGAGAGCGCCTCTGGTAGA
AGCAAGATCACAGGCAACGACGGATCCACGCTGCGCATTTCCTCGGTCGTGAGGGAC
GACAAGGGGATGTACCAATGCTTCGCGAAGAACGACTACGAGATGGTCCAAGCGACC
GCCGAGTTGCGTCTGGGGG

>novel_circ_000347
GGCGGGGAGTGGTGCTGGACAGTCAGGGGCCTGTGTTGATCTCGGAGCCACGCTCCT
CGGTTGAATTCTCGAACGAGACCGGGGCCATGATCCATTGCTCGGCCCAGGGCAGCCC
GTTGCCGAGGATTGATTGGCTCATGGGGGACGAGTCGCCTGTCCTCCCTATACCTCACA
TACGCGAGATGCTGGTCAACGGTTCCATGTACTTTTTGCCGTTCGGAGCGGAGACCTAC
CGGCACGACGTCCACTCGGCCGTCTACAGATGTCAAGCCTCGAACAGCGTCGGTAGG
GTATTGGGCCGTGAAATCACGGTAAAGGCCGTCGTGCGTCAAAAGTACGAGGTTCAAG
TGCGAGACGCCTACGTCCTACCTGGAAATACCGGTGTGCTCAGGTGCGAAATACCGAC
TTTCGTTAAGGAATACGTGGCAGTCACGTCCTGGGTTCGAGATTCCGCCTACAACATCT
TTCCAACGCCGAAAAGCGATGACAGACATCACATGCTGCCGACTGGGGAACTCCTCGT
GTTCTCGGTGACCTCCTCCGACGCCCAGTCCAGTTATCGTTGCAGGACCGTGCACCGC
GTCACCGGCGACACCGTGGAAAGTTCCTCGTACGCGAGGCTCGTGGTCACCGGCGGT
TTACGTACGTATGTGACGAGCAATTACGCGTCAATATTTAAATCGAAACGATATTAATGT
TTACGGACGCGTTGAAATCGTAACGATGTCCCGAACTCGCGGAGGACTCGATGAGTTT
TAAATTTTCTTCCGGGATAAACGTATCCCGGATTAATAATAAAGATTCGTACGTGCGATT
GTCACGGCGTGTAATTAACGCGATAAAGTAATGGGGGAAAAATGTAGAATGTAAACGG
AAGGGGAGAGCGGGAATAAATGTAAATTTGTCGAGAGCGTAGCGGTTCGAATAATACG
TAACAGCGACAAAAGGAAGGTCTTTCACGGTTGTGAAAAATTGATACCTTGAGATCTT
TGCGACCCGTTTCAACGAAATCTCCGCGGAGATTTCTTTCCGAGATATTCGAAATGCAT
TTTCGAGGTATCAAGGGGGAAGAGGATCGTGGAAAAATTTATGAAAGATGAAAAAGC
AATTGAAATATAAGAACGGTAACCCTCATACGCCAGTTCCGCCGAGATTCAACGAACG
AGTGAGTCCAGCGCCTATAAGGACGGGGGAGACGATCGTCCTTTCCTGCATCTCGCAG
GGGATCCCGCCACCCATGTATCTATGGTTTCGCGAGTCCGTGACGGGGACTGCGATGAT
CTTCAATTCGGAGAGGATCTACGCGAGAGCAGGGGTCCTCGTGTTGCAGTCGGCGAGG
GCGGAGGACGCGGGGCGATACGTGTGCCACGCCAACAATACGGCCGGTTCCGAGATG
GTCGAATTGGAAGTCTCCATTATAAGCAGCCTCTCCATCCACCTGGTGCCTCAACAGGT
GACCGTCGACCTGGGCAAGGACGCGGAATTCCAATGCTCGGTCACCGGCCAGCCAAT
CCCCGTCATATTCTGGACCAAGGACGGGCTTCCCGTGGGAGAGAGCGCCTCTGGTAGA
AGCAAGATCACAGGCAACGACGGATCCACGCTGCGCATTTCCTCGGTCGTGAGGGAC
GACAAGGGGATGTACCAATGCTTCGCGAAGAACGACTACGAGATGGTCCAAGCGACC
GCCGAGTTGCGTCTGGGGGACGCGGCGCCGCAGCTTCTGTACAAATTCATCGAGCAGA
CCATCCAGCCCGGCCCTTCGGTGTCGCTCAAGTGCATCGCCACCGGGAACCCGACGCC
GCACTTCTCCTGGACCCTGGACGGTTTCCCTCTTCCGCAGAACGACAG

>novel_circ_000348

GGCGGGGAGTGGTGCTGGACAGTCAGGGGCCTGTGTTGATCTCGGAGCCACGCTCCT
CGGTTGAATTCTCGAACGAGACCGGGGCCATGATCCATTGCTCGGCCCAGGGCAGCCC
GTTGCCGAGGATTGATTGGCTCATGGGGGACGAGTCGCCTGTCCTCCCTATACCTCACA
TACGCGAGATGCTGGTCAACGGTTCCATGTACTTTTTGCCGTTCGGAGCGGAGACCTAC
CGGCACGACGTCCACTCGGCCGTCTACAGATGTCAAGCCTCGAACAGCGTCGGTAGG
GTATTGGGCCGTGAAATCACGGTAAAGGCCGTCGTGCGTCAAAAGTACGAGGTTCAAG
TGCGAGACGCCTACGTCCTACCTGGAAATACCGGTGTGCTCAGGTGCGAAATACCGAC
TTTCGTTAAGGAATACGTGGCAGTCACGTCCTGGGTTCGAGATTCCGCCTACAACATCT
TTCCAACGCCGAAAAGCGATGACAGACATCACATGCTGCCGACTGGGGAACTCCTCGT
GTTCTCGGTGACCTCCTCCGACGCCCAGTCCAGTTATCGTTGCAGGACCGTGCACCGC
GTCACCGGCGACACCGTGGAAAGTTCCTCGTACGCGAGGCTCGTGGTCACCGGCGGT
TTACGTACGTATGTGACGAGCAATTACGCGTCAATATTTAAATCGAAACGATATTAATGT
TTACGGACGCGTTGAAATCGTAACGATGTCCCGAACTCGCGGAGGACTCGATGAGTTT
TAAATTTTCTTCCGGGATAAACGTATCCCGGATTAATAATAAAGATTCGTACGTGCGATT
GTCACGGCGTGTAATTAACGCGATAAAGTAATGGGGGAAAAATGTAGAATGTAAACGG
AAGGGGAGAGCGGGAATAAATGTAAATTTGTCGAGAGCGTAGCGGTTCGAATAATACG
TAACAGCGACAAAAGGAAGGTCTTTCACGGTTGTGAAAAATTGATACCTTGAGATCTT
TGCGACCCGTTTCAACGAAATCTCCGCGGAGATTTCTTTCCGAGATATTCGAAATGCAT
TTTCGAGGTATCAAGGGGGAAGAGGATCGTGGAAAAATTTATGAAAGATGAAAAAGC
AATTGAAATATAAGAACGGTAACCCTCATACGCCAGTTCCGCCGAGATTCAACGAACG
AGTGAGTCCAGCGCCTATAAGGACGGGGGAGACGATCGTCCTTTCCTGCATCTCGCAG
GGGATCCCGCCACCCATGTATCTATGGTTTCGCGAGTCCGTGACGGGGACTGCGATGAT
CTTCAATTCGGAGAGGATCTACGCGAGAGCAGGGGTCCTCGTGTTGCAGTCGGCGAGG
GCGGAGGACGCGGGGCGATACGTGTGCCACGCCAACAATACGGCCGGTTCCGAGATG
GTCGAATTGGAAGTCTCCATTATAAGCAGCCTCTCCATCCACCTGGTGCCTCAACAGGT
GACCGTCGACCTGGGCAAGGACGCGGAATTCCAATGCTCGGTCACCGGCCAGCCAAT
CCCCGTCATATTCTGGACCAAGGACGGGCTTCCCGTGGGAGAGAGCGCCTCTGGTAGA
AGCAAGATCACAGGCAACGACGGATCCACGCTGCGCATTTCCTCGGTCGTGAGGGAC
GACAAGGGGATGTACCAATGCTTCGCGAAGAACGACTACGAGATGGTCCAAGCGACC
GCCGAGTTGCGTCTGGGGGACGCGGCGCCGCAGCTTCTGTACAAATTCATCGAGCAGA
CCATCCAGCCCGGCCCTTCGGTGTCGCTCAAGTGCATCGCCACCGGGAACCCGACGCC
GCACTTCTCCTGGACCCTGGACGGTTTCCCTCTTCCGCAGAACGACAGGTTTATGATTG
GGCAGTATGTGACGGTGCACGGGGACGTGATATCGCACGTGAACATCAGCGTGGTTCA
CGTGGAGGACGGAGGGGAGTACCGGTGCACAGCGGCGAACCGGATGGCGAAGGTAC
ACCATTCGGCTCGTCTCAACATTTACGGCCTGCCCCACGTACGCCCGATGGGGAACTAC
GCCGCTGTGGCCGGCGAGACCACCGTCATCAAATGTCCGGTCGCTGGTTTCCCAATAG
CGAGCATCACTTGGGAGAAAG

>novel_circ_000349
GGCGGGGAGTGGTGCTGGACAGTCAGGGGCCTGTGTTGATCTCGGAGCCACGCTCCT
CGGTTGAATTCTCGAACGAGACCGGGGCCATGATCCATTGCTCGGCCCAGGGCAGCCC
GTTGCCGAGGATTGATTGGCTCATGGGGGACGAGTCGCCTGTCCTCCCTATACCTCACA
TACGCGAGATGCTGGTCAACGGTTCCATGTACTTTTTGCCGTTCGGAGCGGAGACCTAC
CGGCACGACGTCCACTCGGCCGTCTACAGATGTCAAGCCTCGAACAGCGTCGGTAGG

GTATTGGGCCGTGAAATCACGGTAAAGGCCGTCGTGCGTCAAAAGTACGAGGTTCAAG
TGCGAGACGCCTACGTCCTACCTGGAAATACCGGTGTGCTCAGGTGCGAAATACCGAC
TTTCGTTAAGGAATACGTGGCAGTCACGTCCTGGGTTCGAGATTCCGCCTACAACATCT
TTCCAACGCCGAAAAGCGATGACAGACATCACATGCTGCCGACTGGGGAACTCCTCGT
GTTCTCGGTGACCTCCTCCGACGCCCAGTCCAGTTATCGTTGCAGGACCGTGCACCGC
GTCACCGGCGACACCGTGGAAAGTTCCTCGTACGCGAGGCTCGTGGTCACCGGCGGT
TTACGTACGTATGTGACGAGCAATTACGCGTCAATATTTAAATCGAAACGATATTAATGT
TTACGGACGCGTTGAAATCGTAACGATGTCCCGAACTCGCGGAGGACTCGATGAGTTT
TAAATTTTCTTCCGGGATAAACGTATCCCGGATTAATAATAAAGATTCGTACGTGCGATT
GTCACGGCGTGTAATTAACGCGATAAAGTAATGGGGGAAAAATGTAGAATGTAAACGG
AAGGGGAGAGCGGGAATAAATGTAAATTTGTCGAGAGCGTAGCGGTTCGAATAATACG
TAACAGCGACAAAAGGAAGGTCTTTCACGGTTGTGAAAAATTGATACCTTGAGATCTT
TGCGACCCGTTTCAACGAAATCTCCGCGGAGATTTCTTTCCGAGATATTCGAAATGCAT
TTTCGAGGTATCAAGGGGGAAGAGGATCGTGGAAAAATTTATGAAAGATGAAAAAGC
AATTGAAATATAAGAACGGTAACCCTCATACGCCAGTTCCGCCGAGATTCAACGAACG
AGTGAGTCCAGCGCCTATAAGGACGGGGGAGACGATCGTCCTTTCCTGCATCTCGCAG
GGGATCCCGCCACCCATGTATCTATGGTTTCGCGAGTCCGTGACGGGGACTGCGATGAT
CTTCAATTCGGAGAGGATCTACGCGAGAGCAGGGGTCCTCGTGTTGCAGTCGGCGAGG
GCGGAGGACGCGGGGCGATACGTGTGCCACGCCAACAATACGGCCGGTTCCGAGATG
GTCGAATTGGAAGTCTCCATTATAAGCAGCCTCTCCATCCACCTGGTGCCTCAACAGGT
GACCGTCGACCTGGGCAAGGACGCGGAATTCCAATGCTCGGTCACCGGCCAGCCAAT
CCCCGTCATATTCTGGACCAAGGACGGGCTTCCCGTGGGAGAGAGCGCCTCTGGTAGA
AGCAAGATCACAGGCAACGACGGATCCACGCTGCGCATTTCCTCGGTCGTGAGGGAC
GACAAGGGGATGTACCAATGCTTCGCGAAGAACGACTACGAGATGGTCCAAGCGACC
GCCGAGTTGCGTCTGGGGGACGCGGCGCCGCAGCTTCTGTACAAATTCATCGAGCAGA
CCATCCAGCCCGGCCCTTCGGTGTCGCTCAAGTGCATCGCCACCGGGAACCCGACGCC
GCACTTCTCCTGGACCCTGGACGGTTTCCCTCTTCCGCAGAACGACAGGTTTATGATTG
GGCAGTATGTGACGGTGCACGGGGACGTGATATCGCACGTGAACATCAGCGTGGTTCA
CGTGGAGGACGGAGGGGAGTACCGGTGCACAGCGGCGAACCGGATGGCGAAGGTAC
ACCATTCGGCTCGTCTCAACATTTACGGCCTGCCCCACGTACGCCCGATGGGGAACTAC
GCCGCTGTGGCCGGCGAGACCACCGTCATCAAATGTCCGGTCGCTGGTTTCCCAATAG
CGAGCATCACTTGGGAGAAAGACGGGAAGGTGTTACCGACGAGTCGACGTCAGGAAG
TGTTCCCGAACGGTACGCTGGTGCTGCATCACGTCGACAGAAGCACCGATCACGGCGC
ATACACGTGCACGGCGAAGAACAAACAAGGTCGCTCCGACTCTCAGACGGTCCACAT
CGAAGTGAAAG

>novel_circ_000350
GGCGGGGAGTGGTGCTGGACAGTCAGGGGCCTGTGTTGATCTCGGAGCCACGCTCCT
CGGTTGAATTCTCGAACGAGACCGGGGCCATGATCCATTGCTCGGCCCAGGGCAGCCC
GTTGCCGAGGATTGATTGGCTCATGGGGGACGAGTCGCCTGTCCTCCCTATACCTCACA
TACGCGAGATGCTGGTCAACGGTTCCATGTACTTTTTGCCGTTCGGAGCGGAGACCTAC
CGGCACGACGTCCACTCGGCCGTCTACAGATGTCAAGCCTCGAACAGCGTCGGTAGG
GTATTGGGCCGTGAAATCACGGTAAAGGCCGTCGTGCGTCAAAAGTACGAGGTTCAAG
TGCGAGACGCCTACGTCCTACCTGGAAATACCGGTGTGCTCAGGTGCGAAATACCGAC

TTTCGTTAAGGAATACGTGGCAGTCACGTCCTGGGTTCGAGATTCCGCCTACAACATCT
TTCCAACGCCGAAAAGCGATGACAGACATCACATGCTGCCGACTGGGGAACTCCTCGT
GTTCTCGGTGACCTCCTCCGACGCCCAGTCCAGTTATCGTTGCAGGACCGTGCACCGC
GTCACCGGCGACACCGTGGAAAGTTCCTCGTACGCGAGGCTCGTGGTCACCGGCGGT
TTACGTACGTATGTGACGAGCAATTACGCGTCAATATTTAAATCGAAACGATATTAATGT
TTACGGACGCGTTGAAATCGTAACGATGTCCCGAACTCGCGGAGGACTCGATGAGTTT
TAAATTTTCTTCCGGGATAAACGTATCCCGGATTAATAATAAAGATTCGTACGTGCGATT
GTCACGGCGTGTAATTAACGCGATAAAGTAATGGGGGAAAAATGTAGAATGTAAACGG
AAGGGGAGAGCGGGAATAAATGTAAATTTGTCGAGAGCGTAGCGGTTCGAATAATACG
TAACAGCGACAAAAGGAAGGTCTTTCACGGTTGTGAAAAATTGATACCTTGAGATCTT
TGCGACCCGTTTCAACGAAATCTCCGCGGAGATTTCTTTCCGAGATATTCGAAATGCAT
TTTCGAGGTATCAAGGGGGAAGAGGATCGTGGAAAAATTTATGAAAGATGAAAAAGC
AATTGAAATATAAGAACGGTAACCCTCATACGCCAGTTCCGCCGAGATTCAACGAACG
AGTGAGTCCAGCGCCTATAAGGACGGGGGAGACGATCGTCCTTTCCTGCATCTCGCAG
GGGATCCCGCCACCCATGTATCTATGGTTTCGCGAGTCCGTGACGGGGACTGCGATGAT
CTTCAATTCGGAGAGGATCTACGCGAGAGCAGGGGTCCTCGTGTTGCAGTCGGCGAGG
GCGGAGGACGCGGGGCGATACGTGTGCCACGCCAACAATACGGCCGGTTCCGAGATG
GTCGAATTGGAAGTCTCCATTATAAGCAGCCTCTCCATCCACCTGGTGCCTCAACAGGT
GACCGTCGACCTGGGCAAGGACGCGGAATTCCAATGCTCGGTCACCGGCCAGCCAAT
CCCCGTCATATTCTGGACCAAGGACGGGCTTCCCGTGGGAGAGAGCGCCTCTGGTAGA
AGCAAGATCACAGGCAACGACGGATCCACGCTGCGCATTTCCTCGGTCGTGAGGGAC
GACAAGGGGATGTACCAATGCTTCGCGAAGAACGACTACGAGATGGTCCAAGCGACC
GCCGAGTTGCGTCTGGGGGACGCGGCGCCGCAGCTTCTGTACAAATTCATCGAGCAGA
CCATCCAGCCCGGCCCTTCGGTGTCGCTCAAGTGCATCGCCACCGGGAACCCGACGCC
GCACTTCTCCTGGACCCTGGACGGTTTCCCTCTTCCGCAGAACGACAGGTTTATGATTG
GGCAGTATGTGACGGTGCACGGGGACGTGATATCGCACGTGAACATCAGCGTGGTTCA
CGTGGAGGACGGAGGGGAGTACCGGTGCACAGCGGCGAACCGGATGGCGAAGGTAC
ACCATTCGGCTCGTCTCAACATTTACGGCCTGCCCCACGTACGCCCGATGGGGAACTAC
GCCGCTGTGGCCGGCGAGACCACCGTCATCAAATGTCCGGTCGCTGGTTTCCCAATAG
CGAGCATCACTTGGGAGAAAGACGGGAAGGTGTTACCGACGAGTCGACGTCAGGAAG
TGTTCCCGAACGGTACGCTGGTGCTGCATCACGTCGACAGAAGCACCGATCACGGCGC
ATACACGTGCACGGCGAAGAACAAACAAGGTCGCTCCGACTCTCAGACGGTCCACAT
CGAAGTGAAAGTACCGCCTAAAATCGATCCGTTTAGCTTTCCTGCCAATATACAAGAAG
GCGCGCGGGTGCACGTGGTGTGCGTGGTCAGCGAGGGCGATTCACCGTTGAAGATCA
CCTGGCTGAAGGATGGCAGGCCATTGAATTCCACGGAGACGACCACCCATCATATCGG
CGAATACGATCTCACGTTGATGATACAGAGCGCGACCACGGCCCATAATGGCAACTATA
CGTGCGTGGCGAGCAACGATGCCGCCGAAACGTCTCGCACGGCATCCCTGTTGGTCCA
CG

>novel_circ_000351
TCGTGCGTCAAAAGTACGAGGTTCAAGTGCGAGACGCCTACGTCCTACCTGGAAATAC
CGGTGTGCTCAGGTGCGAAATACCGACTTTCGTTAAGGAATACGTGGCAGTCACGTCC
TGGGTTCGAGATTCCGCCTACAACATCTTTCCAACGCCGAAAAGCGATGACAGACATC
ACATGCTGCCGACTGGGGAACTCCTCGTGTTCTCGGTGACCTCCTCCGACGCCCAGTC

CAGTTATCGTTGCAGGACCGTGCACCGCGTCACCGGCGACACCGTGGAAAGTTCCTCG
TACGCGAGGCTCGTGGTCACCG

>novel_circ_000352
TCGTGCGTCAAAAGTACGAGGTTCAAGTGCGAGACGCCTACGTCCTACCTGGAAATAC
CGGTGTGCTCAGGTGCGAAATACCGACTTTCGTTAAGGAATACGTGGCAGTCACGTCC
TGGGTTCGAGATTCCGCCTACAACATCTTTCCAACGCCGAAAAGCGATGACAGACATC
ACATGCTGCCGACTGGGGAACTCCTCGTGTTCTCGGTGACCTCCTCCGACGCCCAGTC
CAGTTATCGTTGCAGGACCGTGCACCGCGTCACCGGCGACACCGTGGAAAGTTCCTCG
TACGCGAGGCTCGTGGTCACCGGCGGTTTACGTACGTATGTGACGAGCAATTACGCGT
CAATATTTAAATCGAAACGATATTAATGTTTACGGACGCGTTGAAATCGTAACGATGTCC
CGAACTCGCGGAGGACTCGATGAGTTTTAAATTTTCTTCCGGGATAAACGTATCCCGGA
TTAATAATAAAGATTCGTACGTGCGATTGTCACGGCGTGTAATTAACGCGATAAAGTAAT
GGGGGAAAAATGTAGAATGTAAACGGAAGGGGAGAGCGGGAATAAATGTAAATTTGT
CGAGAGCGTAGCGGTTCGAATAATACGTAACAGCGACAAAAGGAAGGTCTTTCACGGT
TGTGAAAAATTGATACCTTGAGATCTTTGCGACCCGTTTCAACGAAATCTCCGCGGAGA
TTTCTTTCCGAGATATTCGAAATGCATTTTCGAGGTATCAAGGGGGAAGAGGATCGTGG
AAAAATTTATGAAAGATGAAAAAGCAATTGAAATATAAGAACGGTAACCCTCATACGC
CAGTTCCGCCGAGATTCAACGAACGAGTGAGTCCAGCGCCTATAAGGACGGGGGAGA
CGATCGTCCTTTCCTGCATCTCGCAGGGGATCCCGCCACCCATGTATCTATGGTTTCGCG
AGTCCGTGACGGGGACTGCGATGATCTTCAATTCGGAGAGGATCTACGCGAGAGCAGG
GGTCCTCGTGTTGCAGTCGGCGAGGGCGGAGGACGCGGGGCGATACGTGTGCCACGC
CAACAATACGGCCGGTTCCGAGATGGTCGAATTGGAAGTCTCCATTATAAGCAGCCTCT
CCATCCACCTGGTGCCTCAACAG

>novel_circ_000353
TCGTGCGTCAAAAGTACGAGGTTCAAGTGCGAGACGCCTACGTCCTACCTGGAAATAC
CGGTGTGCTCAGGTGCGAAATACCGACTTTCGTTAAGGAATACGTGGCAGTCACGTCC
TGGGTTCGAGATTCCGCCTACAACATCTTTCCAACGCCGAAAAGCGATGACAGACATC
ACATGCTGCCGACTGGGGAACTCCTCGTGTTCTCGGTGACCTCCTCCGACGCCCAGTC
CAGTTATCGTTGCAGGACCGTGCACCGCGTCACCGGCGACACCGTGGAAAGTTCCTCG
TACGCGAGGCTCGTGGTCACCGGCGGTTTACGTACGTATGTGACGAGCAATTACGCGT
CAATATTTAAATCGAAACGATATTAATGTTTACGGACGCGTTGAAATCGTAACGATGTCC
CGAACTCGCGGAGGACTCGATGAGTTTTAAATTTTCTTCCGGGATAAACGTATCCCGGA
TTAATAATAAAGATTCGTACGTGCGATTGTCACGGCGTGTAATTAACGCGATAAAGTAAT
GGGGGAAAAATGTAGAATGTAAACGGAAGGGGAGAGCGGGAATAAATGTAAATTTGT
CGAGAGCGTAGCGGTTCGAATAATACGTAACAGCGACAAAAGGAAGGTCTTTCACGGT
TGTGAAAAATTGATACCTTGAGATCTTTGCGACCCGTTTCAACGAAATCTCCGCGGAGA
TTTCTTTCCGAGATATTCGAAATGCATTTTCGAGGTATCAAGGGGGAAGAGGATCGTGG
AAAAATTTATGAAAGATGAAAAAGCAATTGAAATATAAGAACGGTAACCCTCATACGC
CAGTTCCGCCGAGATTCAACGAACGAGTGAGTCCAGCGCCTATAAGGACGGGGGAGA
CGATCGTCCTTTCCTGCATCTCGCAGGGGATCCCGCCACCCATGTATCTATGGTTTCGCG
AGTCCGTGACGGGGACTGCGATGATCTTCAATTCGGAGAGGATCTACGCGAGAGCAGG
GGTCCTCGTGTTGCAGTCGGCGAGGGCGGAGGACGCGGGGCGATACGTGTGCCACGC

CAACAATACGGCCGGTTCCGAGATGGTCGAATTGGAAGTCTCCATTATAAGCAGCCTCT
CCATCCACCTGGTGCCTCAACAGGTGACCGTCGACCTGGGCAAGGACGCGGAATTCC
AATGCTCGGTCACCGGCCAGCCAATCCCCGTCATATTCTGGACCAAGGACGGGCTTCC
CGTGGGAGAGAGCGCCTCTGGTAGAAGCAAGATCACAGGCAACGACGGATCCACGCT
GCGCATTTCCTCGGTCGTGAGGGACGACAAGGGGATGTACCAATGCTTCGCGAAGAAC
GACTACGAGATGGTCCAAGCGACCGCCGAGTTGCGTCTGGGGG

>novel_circ_000355
ATGACAGACATCACATGCTGCCGACTGGGGAACTCCTCGTGTTCTCGGTGACCTCCTC
CGACGCCCAGTCCAGTTATCGTTGCAGGACCGTGCACCGCGTCACCGGCGACACCGTG
GAAAGTTCCTCGTACGCGAGGCTCGTGGTCACCGGCGGTTTACGTACGTATGTGACGA
GCAATTACGCGTCAATATTTAAATCGAAACGATATTAATGTTTACGGACGCGTTGAAATC
GTAACGATGTCCCGAACTCGCGGAGGACTCGATGAGTTTTAAATTTTCTTCCGGGATAA
ACGTATCCCGGATTAATAATAAAGATTCGTACGTGCGATTGTCACGGCGTGTAATTAACG
CGATAAAGTAATGGGGGAAAAATGTAGAATGTAAACGGAAGGGGAGAGCGGGAATAA
ATGTAAATTTGTCGAGAGCGTAGCGGTTCGAATAATACGTAACAGCGACAAAAGGAAG
GTCTTTCACGGTTGTGAAAAATTGATACCTTGAGATCTTTGCGACCCGTTTCAACGAAA
TCTCCGCGGAGATTTCTTTCCGAGATATTCGAAATGCATTTTCGAGGTATCAAGGGGGA
AGAGGATCGTGGAAAAATTTATGAAAGATGAAAAAGCAATTGAAATATAAGAACGGTA
ACCCTCATACGCCAGTTCCGCCGAGATTCAACGAACGAGTGAGTCCAGCGCCTATAAG
GACGGGGGAGACGATCGTCCTTTCCTGCATCTCGCAGGGGATCCCGCCACCCATGTATC
TATGGTTTCGCGAGTCCGTGACGGGGACTGCGATGATCTTCAATTCGGAGAGGATCTAC
GCGAGAGCAGGGGTCCTCGTGTTGCAGTCGGCGAGGGCGGAGGACGCGGGGCGATAC
GTGTGCCACGCCAACAATACGGCCGGTTCCGAGATGGTCGAATTGGAAGTCTCCATTAT
AAGCAGCCTCTCCATCCACCTGGTGCCTCAACAG

>novel_circ_000356
ATGACAGACATCACATGCTGCCGACTGGGGAACTCCTCGTGTTCTCGGTGACCTCCTC
CGACGCCCAGTCCAGTTATCGTTGCAGGACCGTGCACCGCGTCACCGGCGACACCGTG
GAAAGTTCCTCGTACGCGAGGCTCGTGGTCACCGGCGGTTTACGTACGTATGTGACGA
GCAATTACGCGTCAATATTTAAATCGAAACGATATTAATGTTTACGGACGCGTTGAAATC
GTAACGATGTCCCGAACTCGCGGAGGACTCGATGAGTTTTAAATTTTCTTCCGGGATAA
ACGTATCCCGGATTAATAATAAAGATTCGTACGTGCGATTGTCACGGCGTGTAATTAACG
CGATAAAGTAATGGGGGAAAAATGTAGAATGTAAACGGAAGGGGAGAGCGGGAATAA
ATGTAAATTTGTCGAGAGCGTAGCGGTTCGAATAATACGTAACAGCGACAAAAGGAAG
GTCTTTCACGGTTGTGAAAAATTGATACCTTGAGATCTTTGCGACCCGTTTCAACGAAA
TCTCCGCGGAGATTTCTTTCCGAGATATTCGAAATGCATTTTCGAGGTATCAAGGGGGA
AGAGGATCGTGGAAAAATTTATGAAAGATGAAAAAGCAATTGAAATATAAGAACGGTA
ACCCTCATACGCCAGTTCCGCCGAGATTCAACGAACGAGTGAGTCCAGCGCCTATAAG
GACGGGGGAGACGATCGTCCTTTCCTGCATCTCGCAGGGGATCCCGCCACCCATGTATC
TATGGTTTCGCGAGTCCGTGACGGGGACTGCGATGATCTTCAATTCGGAGAGGATCTAC
GCGAGAGCAGGGGTCCTCGTGTTGCAGTCGGCGAGGGCGGAGGACGCGGGGCGATAC
GTGTGCCACGCCAACAATACGGCCGGTTCCGAGATGGTCGAATTGGAAGTCTCCATTAT
AAGCAGCCTCTCCATCCACCTGGTGCCTCAACAGGTGACCGTCGACCTGGGCAAGGA

CGCGGAATTCCAATGCTCGGTCACCGGCCAGCCAATCCCCGTCATATTCTGGACCAAG
GACGGGCTTCCCGTGGGAGAGAGCGCCTCTGGTAGAAGCAAGATCACAGGCAACGAC
GGATCCACGCTGCGCATTTCCTCGGTCGTGAGGGACGACAAGGGGATGTACCAATGCT
TCGCGAAGAACGACTACGAGATGGTCCAAGCGACCGCCGAGTTGCGTCTGGGGG

>novel_circ_000357
ATGACAGACATCACATGCTGCCGACTGGGGAACTCCTCGTGTTCTCGGTGACCTCCTC
CGACGCCCAGTCCAGTTATCGTTGCAGGACCGTGCACCGCGTCACCGGCGACACCGTG
GAAAGTTCCTCGTACGCGAGGCTCGTGGTCACCGGCGGTTTACGTACGTATGTGACGA
GCAATTACGCGTCAATATTTAAATCGAAACGATATTAATGTTTACGGACGCGTTGAAATC
GTAACGATGTCCCGAACTCGCGGAGGACTCGATGAGTTTTAAATTTTCTTCCGGGATAA
ACGTATCCCGGATTAATAATAAAGATTCGTACGTGCGATTGTCACGGCGTGTAATTAACG
CGATAAAGTAATGGGGGAAAAATGTAGAATGTAAACGGAAGGGGAGAGCGGGAATAA
ATGTAAATTTGTCGAGAGCGTAGCGGTTCGAATAATACGTAACAGCGACAAAAGGAAG
GTCTTTCACGGTTGTGAAAAAATTGATACCTTGAGATCTTTGCGACCCGTTTCAACGAAA
TCTCCGCGGAGATTTCTTTCCGAGATATTCGAAATGCATTTTCGAGGTATCAAGGGGGA
AGAGGATCGTGGAAAAATTTATGAAAGATGAAAAAGCAATTGAAATATAAGAACGGTA
ACCCTCATACGCCAGTTCCGCCGAGATTCAACGAACGAGTGAGTCCAGCGCCTATAAG
GACGGGGGAGACGATCGTCCTTTCCTGCATCTCGCAGGGGATCCCGCCACCCATGTATC
TATGGTTTCGCGAGTCCGTGACGGGGACTGCGATGATCTTCAATTCGGAGAGGATCTAC
GCGAGAGCAGGGGTCCTCGTGTTGCAGTCGGCGAGGGCGGAGGACGCGGGGCGATAC
GTGTGCCACGCCAACAATACGGCCGGTTCCGAGATGGTCGAATTGGAAGTCTCCATTAT
AAGCAGCCTCTCCATCCACCTGGTGCCTCAACAGGTGACCGTCGACCTGGGCAAGGA
CGCGGAATTCCAATGCTCGGTCACCGGCCAGCCAATCCCCGTCATATTCTGGACCAAG
GACGGGCTTCCCGTGGGAGAGAGCGCCTCTGGTAGAAGCAAGATCACAGGCAACGAC
GGATCCACGCTGCGCATTTCCTCGGTCGTGAGGGACGACAAGGGGATGTACCAATGCT
TCGCGAAGAACGACTACGAGATGGTCCAAGCGACCGCCGAGTTGCGTCTGGGGGACG
CGGCGCCGCAGCTTCTGTACAAATTCATCGAGCAGACCATCCAGCCCGGCCCTTCGGT
GTCGCTCAAGTGCATCGCCACCGGGAACCCGACGCCGCACTTCTCCTGGACCCTGGAC
GGTTTCCCTCTTCCGCAGAACGACAG

>novel_circ_000358
ACCCTCATACGCCAGTTCCGCCGAGATTCAACGAACGAGTGAGTCCAGCGCCTATAAG
GACGGGGGAGACGATCGTCCTTTCCTGCATCTCGCAGGGGATCCCGCCACCCATGTATC
TATGGTTTCGCGAGTCCGTGACGGGGACTGCGATGATCTTCAATTCGGAGAGGATCTAC
GCGAGAGCAGGGGTCCTCGTGTTGCAGTCGGCGAGGGCGGAGGACGCGGGGCGATAC
GTGTGCCACGCCAACAATACGGCCGGTTCCGAGATGGTCGAATTGGAAGTCTCCATTAT
AAGCAGCCTCTCCATCCACCTGGTGCCTCAACAGGTGACCGTCGACCTGGGCAAGGA
CGCGGAATTCCAATGCTCGGTCACCGGCCAGCCAATCCCCGTCATATTCTGGACCAAG
GACGGGCTTCCCGTGGGAGAGAGCGCCTCTGGTAGAAGCAAGATCACAGGCAACGAC
GGATCCACGCTGCGCATTTCCTCGGTCGTGAGGGACGACAAGGGGATGTACCAATGCT
TCGCGAAGAACGACTACGAGATGGTCCAAGCGACCGCCGAGTTGCGTCTGGGGG

>novel_circ_000359

ACCCTCATACGCCAGTTCCGCCGAGATTCAACGAACGAGTGAGTCCAGCGCCTATAAG
GACGGGGGAGACGATCGTCCTTTCCTGCATCTCGCAGGGGATCCCGCCACCCATGTATC
TATGGTTTCGCGAGTCCGTGACGGGGACTGCGATGATCTTCAATTCGGAGAGGATCTAC
GCGAGAGCAGGGGTCCTCGTGTTGCAGTCGGCGAGGGCGGAGGACGCGGGGCGATAC
GTGTGCCACGCCAACAATACGGCCGGTTCCGAGATGGTCGAATTGGAAGTCTCCATTAT
AAGCAGCCTCTCCATCCACCTGGTGCCTCAACAGGTGACCGTCGACCTGGGCAAGGA
CGCGGAATTCCAATGCTCGGTCACCGGCCAGCCAATCCCCGTCATATTCTGGACCAAG
GACGGGCTTCCCGTGGGAGAGAGCGCCTCTGGTAGAAGCAAGATCACAGGCAACGAC
GGATCCACGCTGCGCATTTCCTCGGTCGTGAGGGACGACAAGGGGATGTACCAATGCT
TCGCGAAGAACGACTACGAGATGGTCCAAGCGACCGCCGAGTTGCGTCTGGGGGACG
CGGCGCCGCAGCTTCTGTACAAATTCATCGAGCAGACCATCCAGCCCGGCCCTTCGGT
GTCGCTCAAGTGCATCGCCACCGGGAACCCGACGCCGCACTTCTCCTGGACCCTGGAC
GGTTTCCCTCTTCCGCAGAACGACAGGTTTATGATTGGGCAGTATGTGACGGTGCACG
GGGACGTGATATCGCACGTGAACATCAGCGTGGTTCACGTGGAGGACGGAGGGGAGT
ACCGGTGCACAGCGGCGAACCGGATGGCGAAGGTACACCATTCGGCTCGTCTCAACAT
TTACGGCCTGCCCCACGTACGCCCGATGGGGAACTACGCCGCTGTGGCCGGCGAGACC
ACCGTCATCAAATGTCCGGTCGCTGGTTTCCCAATAGCGAGCATCACTTGGGAGAAAG
ACGGGAAGGTGTTACCGACGAGTCGACGTCAGGAAGTGTTCCCGAACGGTACGCTGG
TGCTGCATCACGTCGACAGAAGCACCGATCACGGCGCATACACGTGCACGGCGAAGA
ACAAACAAGGTCGCTCCGACTCTCAGACGGTCCACATCGAAGTGAAAG

>novel_circ_000360
GTGACCGTCGACCTGGGCAAGGACGCGGAATTCCAATGCTCGGTCACCGGCCAGCCA
ATCCCCGTCATATTCTGGACCAAGGACGGGCTTCCCGTGGGAGAGAGCGCCTCTGGTA
GAAGCAAGATCACAGGCAACGACGGATCCACGCTGCGCATTTCCTCGGTCGTGAGGG
ACGACAAGGGGATGTACCAATGCTTCGCGAAGAACGACTACGAGATGGTCCAAGCGA
CCGCCGAGTTGCGTCTGGGGGACGCGGCGCCGCAGCTTCTGTACAAATTCATCGAGCA
GACCATCCAGCCCGGCCCTTCGGTGTCGCTCAAGTGCATCGCCACCGGGAACCCGACG
CCGCACTTCTCCTGGACCCTGGACGGTTTCCCTCTTCCGCAGAACGACAGGTTTATGAT
TGGGCAGTATGTGACGGTGCACGGGGACGTGATATCGCACGTGAACATCAGCGTGGTT
CACGTGGAGGACGGAGGGGAGTACCGGTGCACAGCGGCGAACCGGATGGCGAAGGT
ACACCATTCGGCTCGTCTCAACATTTACGGCCTGCCCCACGTACGCCCGATGGGGAACT
ACGCCGCTGTGGCCGGCGAGACCACCGTCATCAAATGTCCGGTCGCTGGTTTCCCAAT
AGCGAGCATCACTTGGGAGAAAGACGGGAAGGTGTTACCGACGAGTCGACGTCAGGA
AGTGTTCCCGAACGGTACGCTGGTGCTGCATCACGTCGACAGAAGCACCGATCACGGC
GCATACACGTGCACGGCGAAGAACAAACAAGGTCGCTCCGACTCTCAGACGGTCCAC
ATCGAAGTGAAAG

>novel_circ_000361
ACGCGGCGCCGCAGCTTCTGTACAAATTCATCGAGCAGACCATCCAGCCCGGCCCTTC
GGTGTCGCTCAAGTGCATCGCCACCGGGAACCCGACGCCGCACTTCTCCTGGACCCTG
GACGGTTTCCCTCTTCCGCAGAACGACAGGTTTATGATTGGGCAGTATGTGACGGTGC
ACGGGGACGTGATATCGCACGTGAACATCAGCGTGGTTCACGTGGAGGACGGAGGGG
AGTACCGGTGCACAGCGGCGAACCGGATGGCGAAGGTACACCATTCGGCTCGTCTCA

ACATTTACGGCCTGCCCCACGTACGCCCGATGGGGAACTACGCCGCTGTGGCCGGCGA
GACCACCGTCATCAAATGTCCGGTCGCTGGTTTCCCAATAGCGAGCATCACTTGGGAG
AAAGACGGGAAGGTGTTACCGACGAGTCGACGTCAGGAAGTGTTCCCGAACGGTACG
CTGGTGCTGCATCACGTCGACAGAAGCACCGATCACGGCGCATACACGTGCACGGCGA
AGAACAAACAAGGTCGCTCCGACTCTCAGACGGTCCACATCGAAGTGAAAG

>novel_circ_000362
GTTTATGATTGGGCAGTATGTGACGGTGCACGGGGACGTGATATCGCACGTGAACATCA
GCGTGGTTCACGTGGAGGACGGAGGGGAGTACCGGTGCACAGCGGCGAACCGGATGG
CGAAGGTACACCATTCGGCTCGTCTCAACATTTACGGCCTGCCCCACGTACGCCCGATG
GGGAACTACGCCGCTGTGGCCGGCGAGACCACCGTCATCAAATGTCCGGTCGCTGGTT
TCCCAATAGCGAGCATCACTTGGGAGAAAGACGGGAAGGTGTTACCGACGAGTCGAC
GTCAGGAAGTGTTCCCGAACGGTACGCTGGTGCTGCATCACGTCGACAGAAGCACCG
ATCACGGCGCATACACGTGCACGGCGAAGAACAAACAAGGTCGCTCCGACTCTCAGA
CGGTCCACATCGAAGTGAAAG

>novel_circ_000363
GTTTATGATTGGGCAGTATGTGACGGTGCACGGGGACGTGATATCGCACGTGAACATCA
GCGTGGTTCACGTGGAGGACGGAGGGGAGTACCGGTGCACAGCGGCGAACCGGATGG
CGAAGGTACACCATTCGGCTCGTCTCAACATTTACGGCCTGCCCCACGTACGCCCGATG
GGGAACTACGCCGCTGTGGCCGGCGAGACCACCGTCATCAAATGTCCGGTCGCTGGTT
TCCCAATAGCGAGCATCACTTGGGAGAAAGACGGGAAGGTGTTACCGACGAGTCGAC
GTCAGGAAGTGTTCCCGAACGGTACGCTGGTGCTGCATCACGTCGACAGAAGCACCG
ATCACGGCGCATACACGTGCACGGCGAAGAACAAACAAGGTCGCTCCGACTCTCAGA
CGGTCCACATCGAAGTGAAAGTACCGCCTAAAATCGATCCGTTTAGCTTTCCTGCCAAT
ATACAAGAAGGCGCGCGGGTGCACGTGGTGTGCGTGGTCAGCGAGGGCGATTCACCG
TTGAAGATCACCTGGCTGAAGGATGGCAGGCCATTGAATTCCACGGAGACGACCACCC
ATCATATCGGCGAATACGATCTCACGTTGATGATACAGAGCGCGACCACGGCCCATAAT
GGCAACTATACGTGCGTGGCGAGCAACGATGCCGCCGAAACGTCTCGCACGGCATCCC
TGTTGGTCCACG

>novel_circ_000364
ATTAAAAATCGAATCGGCGGAAGGGGTGTACGGTTTGACCGAGGGGCGTGGTGAAAA
GGAAAGACGCTCCTAGCTAAAAAGGAAGAGAGAAGTTTCTCGAAGGTTTGCTTCGTT
TGCCGCTTCTGCTCTGGATCCGCTCGAAAAGGAGAAGCACGGCGGAGTTCTCGTTCGC
GACAAGGCGAGAAAGAAACCGATTCGGGCGCGTCATCTTCGAGAAAGAGAGACGGA
CACGCGTTAACCTGGAACAGATTTAGAAGGCGTGCATCGAGTAAAACTCTAAAGGGAA
GATCGAGTGGAGGGAAGAGGATCAAAGAGAAACAGGGAAAGGAAGAAGAAGAAGA
AGAAGAAGAAAAAAAAAAGGGGCGAGCTATTGTCGAAGCGAGGGATAAAAACAAGG
TGTTCAACGACGGAGCTAGGAAATATAAAGGACGGGACGATAATAGAGAGGAAGAGA
AGACACGAGTGTATACCATTGGTGACGAAGATGACTAAGAGCAAACCGTTGACCGCGC
CTGAACGAAGAACCAACGACGTGTTTGACGAAGAATCGATCGCGGTTCGATCGAGAT
GGAGAAACCTCGCCGCCTTCTGGATACTCGGCCTCTGCAACAACTACGGATACGTGGT
GATGCTTAGCGCGGCGCACGATATCCTCGAGAGCAAGTTTGGAACCACG

>novel_circ_000365
CCCTCTTTCTCTTTTCAAATGGTTCACAGCTTTGCGTAGTGGTGTAAAGATTCAGAACG
TTTTTCGGGATTATTATATATGTTAGTTATTGGTTCTTTGTTTTGAGTAAATGAGACCCCC
CCCCCTCAAAGAGGGGGCTTTAAAGGGTATTTTGGTAGGATGCAGTCAGGTCTCGAAA
ATTACTTTTCTTCAAAATGACTCGAGTAGCGAAAAATTATTAAAATTTTGTAACAAAAG
AAATGTTTT

>novel_circ_000366
AAAAAAAAAAAAAAGAAGACTGATGATACTTATGTGACACGATCCATTTTTCGGATTTA
AATGAGAAGAAAAGTCGTCCTTTGAATATTACAAGATATTTGGCATCGTTTGTTAAACG
GAACAGAAAATCCTTTCGTGAATTAAAAAGAAAAAAA

>novel_circ_000367
AGAAAAAAAAAAAAAAAGAAGACTGATGATACTTATGTGACACGATCCATTTTTCGGAT
TTAAATGAGAAGAAAAGTCGTCCTTTGAATATTACAAGATATTTGGCATCGTTTGTTAA
ACGGAACAGAAAATCCTTTCGTGAATTAAAAAGAAAAAAA

>novel_circ_000368
CGCGAGGACGGGAGGCGTGGCAAGATCAACGCCGTCTATAATCTGGTTGTCGTTGCTA
CTGACACTGGGAACGAGGACAACGGCGTCAGGGACGAACATGACAAACGTGTCACC
GCTCGCCTCGACGGTGAGGCCGATCACGACCACGGAAGTAGTTTATCAGAGATTCGCG
ATCGAGCCGATGGATCAGACGGCGGTGATTGGCAGCCGGGTGACGCTTCCTTGCCGGG
TCCTCGATCAGAAGGGACCTATTCAATGGACGAAGGACGACTTCGGGCTTGGCGCTGT
TAGAAACCTCACGGGATACGAGCGTTACGCCATGATCGGCAGCGACGAGGAGG

>novel_circ_000369
ATCACGTGGATCGACGGGTTGGGGAACGTGCTGCACAGAGGCATAAAAACGACCAAG
GAATTGTTCGACGACGGCCCCTTGTACACCGTGAAATCCGTGCTGAGGGTGATGCCGC
GCAAGGACCACGACAACACGACGTTCACGTGCCAAAGTCAGAACGCGGCCGACCGC
ACGCCGCAGAACGCGAAACTGCGCGTCGAGGTCCGCTACGCGCCAAAAGTGTCTCTG
AGGATCCGGTCGGGGTTGGGCAAGAATGGGCGCATCGTGGAGGGCAGCGAGCTCCGC
TTCAAGTGTCGCGCGGAAGCGAACCCGCCGAACGTCGAGTACAG

>novel_circ_000372
CTGGAGCCGCTTCTGAGGAAACTAACCAAAGGTCCAGTTCTGCACACAATCGCCACTC
TCTCACGAAGGAGCAAACGATGCACTGGTTCGCCCAAATCGGAGGTGACGAGGCCTC
TCCCGATTCGTCAGATGTGAAGCGCCGGCAGAGTCTCTACGACGAGGACTCCCTCGAT
GGCGATCATCCTAGCGAGAACGAAGGACGGGAGTCGACGGGAAACGCGAAAGACGT
GGACAGGGCGAAGCTCGTCCGTGAGAGACAGAACGAAGAACGGCAGCGGAAGTTGG
AAGAGCTGAGACAGCAGGCGTTCGCCGCGCAACGTTTCCGGGAGCAACGGGAAGAG
GAACGTCGAAGACGCATCGACGAGCTCAGATCGCGTGACAATGACAGACGAAACCAA
GTGGAGGAGAGGAAACGGTTGATATGCGAGGCGGAAAGGGAACGAAGGGAGGCGAT
CCTCCGGAAGAACCAAGAGAGAGAGGCGCGTATCGAGGCGAAGAAGAAGAACGAGA

GGTCGCACATCGTGTTCGCTTTCGGAAGCTCGACACCGAGGATGCTGGAGCCGGCCGA
CACCGGCGGCTCCACTTTCTGGGGTACCCGTCGAGCAACGTCCACCACCAATGTCATG
ATGTTCTCCGCCGCTCAACCGTTGACCAGGAGGTCTTCCGAAAGAGAGCTCGATGGAA
GCAAGAAACGAGCCACGTCCGCTGGTGGGCTCGATCGGAAACCTGGCGAAG

>novel_circ_000373
ATGTGAAGCGCCGGCAGAGTCTCTACGACGAGGACTCCCTCGATGGCGATCATCCTAG
CGAGAACGAAGGACGGGAGTCGACGGGAAACGCGAAAGACGTGGACAGGGCGAAG
CTCGTCCGTGAGAGACAGAACGAAGAACGGCAGCGGAAGTTGGAAGAGCTGAGACA
GCAGGCGTTCGCCGCGCAACGTTTCCGGGAGCAACGGGAAGAGGAACGTCGAAGAC
GCATCGACGAGCTCAGATCGCGTGACAATGACAGACGAAACCAAGTGGAGGAGAGGA
AACGGTTGATATGCGAGGCGGAAAGGGAACGAAGGGAGGCGATCCTCCGGAAGAACC
AAGAGAGAGAGGCGCGTATCGAGGCGAAGAAGAAGAACGAGAGGTCGCACATCGTG
TTCGCTTTCGGAAGCTCGACACCGAGGATGCTGGAGCCGGCCGACACCGGCGGCTCC
ACTTTCTGGGGTACCCGTCGAGCAACGTCCACCACCAATGTCATGATGTTCTCCGCCGC
TCAACCGTTGACCAGGAGGTCTTCCGAAAGAGAGCTCGATGGAAGCAAGAAACGAGC
CACGTCCGCTGGTGGGCTCGATCGGAAACCTGGCGAAGCGTTGAATGGTTTGTTTGCA
GATATGAGAATGTCCTCGTCCATGTACGAGGTGTTCAATTGGAATTCTAGCCCTGATCCT
CCCTTAACCCCCGCCAAACATAAGAGAGCCAGCCTCTCTCTGCCTCCTACAACTGACAT
CTTTGCCATCGATGATAAGTCTGATAGCGACACTAGGCGTCCGATGATTCAGCGGGCTG
CCAGTGGTGAAGAAAGCGACGGAACGCCGGGAACCCCTAGCTCGGTTTATCTGCGGG
TGAACAGGAGGCGCACCGATCTGATGCCAACGATACCGTCGCCACGTGACGGACCGC
CGACATCCGGGCGTAGCTCGAGCGCGAAGGCCTTCACACGTTCGCCAGGTAGGACTTA
CTCCATGTCCAGGCTGGACCAACTGGCGCAGCCTAGGAAACGTCCGACAGAACTTAG
CACATTGACGGAACAGCAAAGCCAGCCTCTGAGCGCTTCTAGCATGAGTCGCAGCATG
TCACATTTAGCTGCGTCCGGGGGCAAGAGCCTGAAACGCTCAGATAACTCTCGTAGCA
TGGGTACATTGCCAGGCGCGGTTCCGATGCCGAGACCAACGAGGGCCGAGAGACTCC
GTCGCAAAGCCCGCGAGCATCAGAACCAACAGCAACAAG

>novel_circ_000374
GACGGGAGTCGACGGGAAACGCGAAAGACGTGGACAGGGCGAAGCTCGTCCGTGAG
AGACAGAACGAAGAACGGCAGCGGAAGTTGGAAGAGCTGAGACAGCAGGCGTTCGC
CGCGCAACGTTTCCGGGAGCAACGGGAAGAGGAACGTCGAAGACGCATCGACGAGC
TCAGATCGCGTGACAATGACAGACGAAACCAAGTGGAGGAGAGGAAACGGTTGATAT
GCGAGGCGGAAAGGGAACGAAGGGAGGCGATCCTCCGGAAGAACCAAGAGAGAGA
GGCGCGTATCGAGGCGAAGAAGAAGAACGAGAGGTCGCACATCGTGTTCGCTTTCGG
AAGCTCGACACCGAGGATGCTGGAGCCGGCCGACACCGGCGGCTCCACTTTCTGGGG
TACCCGTCGAGCAACGTCCACCACCAATGTCATGATGTTCTCCGCCGCTCAACCGTTGA
CCAGGAGGTCTTCCGAAAGAGAGCTCGATGGAAGCAAGAAACGAGCCACGTCCGCTG
GTGGGCTCGATCGGAAACCTGGCGAAG

>novel_circ_000375
ATATGAGAATGTCCTCGTCCATGTACGAGGTGTTCAATTGGAATTCTAGCCCTGATCCTC
CCTTAACCCCCGCCAAACATAAGAGAGCCAGCCTCTCTCTGCCTCCTACAACTGACATC

TTTGCCATCGATGATAAGTCTGATAGCGACACTAGGCGTCCGATGATTCAGCGGGCTGC
CAGTGGTGAAGAAAGCGACGGAACGCCGGGAACCCCTAGCTCGGTTTATCTGCGGGT
GAACAGGAGGCGCACCGATCTGATGCCAACGATACCGTCGCCACGTGACGGACCGCC
GACATCCGGGCGTAGCTCGAGCGCGAAGGCCTTCACACGTTCGCCAGGTAGGACTTAC
TCCATGTCCAGGCTGGACCAACTGGCGCAGCCTAGGAAACGTCCGACAGAACTTAGC
ACATTGACGGAACAGCAAAGCCAGCCTCTGAGCGCTTCTAGCATGAGTCGCAGCATGT
CACATTTAGCTGCGTCCGGGGGCAAGAGCCTGAAACGCTCAGATAACTCTCGTAGCAT
GGGTACATTGCCAGGCGCGGTTCCGATGCCGAGACCAACGAGGGCCGAGAGACTCCG
TCGCAAAGCCCGCGAGCATCAGAACCAACAGCAACAAGGCATCCGCAGCGGGGAGGT
GACACCGAACAGCCCATCACGACCGCACAGCTCCATGAGTCAGCAAAGCGCCAGCAG
TGTGGGCAGCAGTAACGTCAATCTGCGTCCCCGTACAGCTGCCCCGCGTCGACCGCGA
CCTGCTTCTATTGCCGGTACCGGTGTTTCGGTCACAGAACGACACAATCTAGTGAGCG
AGACAAAGTCGCCGAAAGATTCGAAGCCGCCACTGCCAAAGGTCCATAGCACTCCAA
AGAAAACGTCTGCACCGAAATCGGCGGAGATGAAGAAGCCGGCCGAGAAGCTGGTG
AAGAACGCGAAGGCTTCGCCTCGGATAACCCCGAAAGTGACACCGTTGCAGAGTCCT
GGCGCCGAGAACGCTCCATTGATCCGCGGTAGTACCGGGGAGATAATAAAAAGCGAGG
GGAAGGAGGATAAATTGGAGGATAAGCACGAGGAGAAGAAAGATCAAGGCAGCGAG
AAAACGCAGCACGAGATAAGCGCCGCGGAACAGGGTAACGACGAGGTGAAGAAGCC
GGCCGTTATAGAGCAATCCAATATCGTGTCCAAGCAGGCGAAGGACGAGACTAATTTG
AATCAGGAGAATCAATCGGCTGTGCGATCTCAAGAAACGCCGAAAGCTGCCAAGAAG
GAAGCCGAGGCCAAGTCCGAGAGCAACGTGGAGGAACAGGTCGATATGTCTGCATCG
ATGATAGCGAAGATCCGGATCACTACGGAAGAGGAAGCTAAGGCAGCTATAGCCGAAC
GTAGAAGATTAGCTAGAGAGCAGGCTGAACGGGAAGCCGAACTTGAACGCCAACGAC
AGGAGGAGGAAGCCCGTTTAGAGGCCGAGAGATTGCGCGCCGAAGAGGAGGAACAG
CGGCGTTTGGAGGAGGAAACGCTTCGTTTGGCTAACGAAGCTCGAGAAGCTGAGGAA
CAGAGACTGAGACTGGCGATAGAGGAAGCGAAACGTCGCGAGGAAGAGGACAGGAG
AAGAAGGGAAGAGGAGGCTCGTCAGAAACAGGAGAAAGAGGAGGCTGAACGGAAA
GCAAGGGAAGAAGCGGAAAGACAACGGATCGAAATGGCAGAACGCGTTAAGAGAGA
AGAGGAAGAGAGACTCGCTAGACGTAAACGCGTCGAGGCAATTATGCTTAGAACCCG
TGGAAAAAATCAGAGTAACACACCTACGAAGGGTGAAGGTGGTGATGGCGATAAATT
GAAAGAAGACAGTCCTAACGATGAAAATAAAACAGCACCAGTTAATAAAAGCGAGGA
TGTTATGACAGCCAGTCTGATATCCGAGGCGACTCAACAATTCATTAGCGGAGAGCAG
CGTGCTCATCATACGGAAAACAATACTACTACGGACATTGTGCATAACGGCACGCATAG
TAATGGCATTAATGAAAATAAAATTGTATTGGATAATAATCAGGGTAATGTGGAAGGAG
AACTGAATGGTCATCATACAAATCATGGAAACGGTATCAACAGTCAATCAATTACACTG
GATAATGCCACTGT

>novel_circ_000376
GTGAAGAAAGCGACGGAACGCCGGGAACCCCTAGCTCGGTTTATCTGCGGGTGAACA
GGAGGCGCACCGATCTGATGCCAACGATACCGTCGCCACGTGACGGACCGCCGACATC
CGGGCGTAGCTCGAGCGCGAAGGCCTTCACACGTTCGCCAGGTAGGACTTACTCCATG
TCCAGGCTGGACCAACTGGCGCAGCCTAGGAAACGTCCGACAGAACTTAGCACATTG
ACGGAACAGCAAAGCCAGCCTCTGAGCGCTTCTAGCATGAGTCGCAGCATGTCACATT
TAGCTGCGTCCGGGGGCAAGAGCCTGAAACGCTCAGATAACTCTCGTAGCATGGGTAC

ATTGCCAGGCGCGGTTCCGATGCCGAGACCAACGAGGGCCGAGAGACTCCGTCGCAA
AGCCCGCGAGCATCAGAACCAACAGCAACAAGGCATCCGCAGCGGGGAGGTGACAC
CGAACAGCCCATCACGACCGCACAGCTCCATGAGTCAGCAAAGCGCCAGCAGTGTGG
GCAGCAGTAACGTCAATCTGCGTCCCCGTACAGCTGCCCCGCGTCGACCGCGACCTGC
TTCTATTGCCGGTACCGGTGTTTCGGTCACAGAACGACACAATCTAGTGAGCGAGACA
AAGTCGCCGAAAGATTCGAAGCCGCCACTGCCAAAGGTCCATAGCACTCCAAAGAAA
ACGTCTGCACCGAAATCGGCGGAGATGAAGAAGCCGGCCGAGAAGCTGGTGAAGAA
CGCGAAGGCTTCGCCTCGGATAACCCCGAAAGTGACACCGTTGCAGAGTCCTGGCGC
CGAGAACGCTCCATTGATCCGCGGTAGTACCGGGGAGATAATAAAAAGCGAGGGGAA
GGAGGATAAATTGGAGGATAAGCACGAGGAGAAGAAAGATCAAGGCAGCGAGAAAA
CGCAGCACGAGATAAGCGCCGCGGAACAGGGTAACGACGAGGTGAAGAAGCCGGCC
GTTATAGAGCAATCCAATATCGTGTCCAAGCAGGCGAAGGACGAGACTAATTTGAATC
AGGAGAATCAATCGGCTGTGCGATCTCAAGAAACGCCGAAAGCTGCCAAGAAGGAAG
CCGAGGCCAAGTCCGAGAGCAACGTGGAGGAACAGGTCGATATGTCTGCATCGATGAT
AGCGAAGATCCGGATCACTACGGAAGAGGAAGCTAAGGCAGCTATAGCCGAACGTAG
AAGATTAGCTAGAGAGCAGGCTGAACGGGAAGCCGAACTTGAACGCCAACGACAGG
AGGAGGAAGCCCGTTTAGAGGCCGAGAGATTGCGCGCCGAAGAGGAGGAACAGCGG
CGTTTGGAGGAGGAAACGCTTCGTTTGGCTAACGAAGCTCGAGAAGCTGAGGAACAG
AGACTGAGACTGGCGATAGAGGAAGCGAAACGTCGCGAGGAAGAGGACAGGAGAAG
AAGGGAAGAGGAGGCTCGTCAGAAACAGGAGAAAGAGGAGGCTGAACGGAAAGCA
AGGGAAGAAGCGGAAAGACAACGGATCGAAATGGCAGAACGCGTTAAGAGAGAAGA
GGAAGAGAGACTCGCTAGACGTAAACGCGTCGAGGCAATTATGCTTAGAACCCGTGG
AAAAAATCAGAGTAACACACCTACGAAGGGTGAAGGTGGTGATGGCGATAAATTGAA
AGAAGACAGTCCTAACGATGAAAATAAAACAGCACCAGTTAATAAAAGCGAGGATGTT
ATGACAGCCAGTCTGATATCCGAGGCGACTCAACAATTCATTAGCGGAGAGCAGCGTG
CTCATCATACGGAAAACAATACTACTACGGACATTGTGCATAACGGCACGCATAGTAAT
GGCATTAATGAAAATAAAATTGTATTGGATAATAATCAGGGTAATGTGGAAGGAGAACT
GAATGGTCATCATACAAATCATGGAAACGGTATCAACAGTCAATCAATTACACTGGATA
ATGCCACTGT

>novel_circ_000377
GCATCCGCAGCGGGGAGGTGACACCGAACAGCCCATCACGACCGCACAGCTCCATGA
GTCAGCAAAGCGCCAGCAGTGTGGGCAGCAGTAACGTCAATCTGCGTCCCCGTACAG
CTGCCCCGCGTCGACCGCGACCTGCTTCTATTGCCGGTACCGGTGTTTCGGTCACAGA
ACGACACAATCTAGTGAGCGAGACAAAGTCGCCGAAAGATTCGAAGCCGCCACTGCC
AAAGGTCCATAGCACTCCAAAGAAAACGTCTGCACCGAAATCGGCGGAGATGAAGAA
GCCGGCCGAGAAGCTGGTGAAGAACGCGAAGGCTTCGCCTCGGATAACCCCGAAAGT
GACACCGTTGCAGAGTCCTGGCGCCGAGAACGCTCCATTGATCCGCGGTAGTACCGGG
GAGATAATAAAAAGCGAGGGGAAGGAGGATAAATTGGAGGATAAGCACGAGGAGAAG
AAAGATCAAGGCAGCGAGAAAACGCAGCACGAGATAAGCGCCGCGGAACAGGGTAA
CGACGAGGTGAAGAAGCCGGCCGTTATAGAGCAATCCAATATCGTGTCCAAGCAGGCG
AAGGACGAGACTAATTTGAATCAGGAGAATCAATCGGCTGTGCGATCTCAAGAAACGC
CGAAAGCTGCCAAGAAGGAAGCCGAGGCCAAGTCCGAGAGCAACGTGGAGGAACAG
GTCGATATGTCTGCATCGATGATAGCGAAGATCCGGATCACTACGGAAGAGGAAGCTA

AGGCAGCTATAGCCGAACGTAGAAGATTAGCTAGAGAGCAGGCTGAACGGGAAGCCG
AACTTGAACGCCAACGACAGGAGGAGGAAGCCCGTTTAGAGGCCGAGAGATTGCGCG
CCGAAGAGGAGGAACAGCGGCGTTTGGAGGAGGAAACGCTTCGTTTGGCTAACGAAG
CTCGAGAAGCTGAGGAACAGAGACTGAGACTGGCGATAGAGGAAGCGAAACGTCGC
GAGGAAGAGGACAGGAGAAGAAGGGAAGAGGAGGCTCGTCAGAAACAGGAGAAAG
AGGAGGCTGAACGGAAAGCAAGGGAAGAAGCGGAAAGACAACGGATCGAAATGGCA
GAACGCGTTAAGAGAGAAGAGGAAGAGAGACTCGCTAGACGTAAACGCGTCGAGGC
AATTATGCTTAGAACCCGTGGAAAAAATCAGAGTAACACACCTACGAAGGGTGAAGGT
GGTGATGGCGATAAATTGAAAGAAGACAGTCCTAACGATGAAAATAAAACAGCACCA
GTTAATAAAAGCGAGGATGTTATGACAGCCAGTCTGATATCCGAGGCGACTCAACAATT
CATTAGCGGAGAGCAGCGTGCTCATCATACGGAAAACAATACTACTACGGACATTGTGC
ATAACGGCACGCATAGTAATGGCATTAATGAAAATAAAATTGTATTGGATAATAATCAGG
GTAATGTGGAAGGAGAACTGAATGGTCATCATACAAATCATGGAAACGGTATCAACAG
TCAATCAATTACACTGGATAATGCCACTGT

>novel_circ_000378
GTAGTGGGCATCGAGCCGTTGCCAAGGTCACGAGGGAGGAGCTGAGGTCATCGCTGC
ACCTATGTTGTGCGGCGAACTCCTGAACGGTGGCTTCGTGAGTGGTGGAGCGGTACCG
GTGATTCCCTACGGCCTCTTAGGATTCTGGTTGCCCGGCTGTTTACGCGCTACCAGCCC
GACCTCTCTCAACTCGTCTTATCTGACCCTGTTGGCCGAACTTCTGGCCACGGGCCACC
AGGATCGTCGAAACGAGCCGATGGACCTCGCGAACAGGAGCCACCAGGCTCCCACGA
ACGGGGCGCTCGACCTTGACCTCGACCTGCACGCCGACGACATCGACGAGAACGTGT
TTCTCG

>novel_circ_000380
AACCGTACACCGAGTACGAGATATGGGTAAAAGCTTACACGTGGAAGAACGAGGGCG
AGCCTTCGGAGCACATCATTCGTCGGACAGATATTTCAGGGCCGAGCGCTCCGTTGATC
TTGAACGTCACTTGCCCGACCCACGACTCCGTCTACATCCAATGGGCCAGGCCGAACA
TCTTCTGGGGCTCGATCGACTACTACTTCATCAATTATAGGATCGAGAATGACAATCGTT
ACGAAGAGATCGAAGTGATGACGGAGAAGAATCATCTCGAGAGCGCGATGATCATACC
CAACCTGACGATGAACAGCGTATACGAGATCGTGGTACGCGGAGGCACGCGGAGCGCT
ATCGACAATCGGCTGATTATACGCGGAGAAGGCTCTGCTCCACAGAAGGTGCGCGGCA
TTCGCGATTGCAACAGAGCACCACCGTTATTGCCACACACCACCATATGGTTCAGCAG
CGGAGTGATCGCCGGGATGATCTGCGCTTGTCTCGCGGTGATGCTGATGATCACCTCCT
TCGTGCTGTGGAA

>novel_circ_000381
GTGGTACTACAATAGGAACCAGAGGGAGGAGCCGGTCGAGGATTCGTCCGGGCATTAC
GTGGTGCGAGACGGTTCGTTGATCATACAAGGCGTCCAGGAGAGCGACGGTGGATCGT
ACATGTGCACCGCTAGTAATTCCGAGGGCAGCGAGTCTATGGAAGTGAGGTTAACCGT
GTCTGCGCCATTAAACGTGCACGTCCAACCATCGATCCAGACCGTTGATCTCGGGAAA
GCCGCTCATTTGACGTGCAGCGCAAGTGGATTTCCGCAGGCGGCGTTGTACTGGCTGA
AGGACGGCCAGCCATTGAGAACGGGCGCTCGCATAAGGGCGGTTTCCAGGGAACGTA
TTTCCGTGATGTCGGTCGCAAGAGAGGATCGCGGCATGTACCAGTGCTTTGTGAGGAA

CGAGTATGAAATGGCTCAAGGAATCGCGGAATTGCGGCTGGGCG

>novel_circ_000382
TGGTGAATCAGCGTTACGATCCGGAAGTGCAATGTCCCGGCGGTTTCCTCGGCAACAA
CGTGCTTATGCGATGCAACGTGCCCAGTTTCGTCCGCGATCACGTCACCATCACATCTT
GGCTTCAGGAGCCGTCTTTCAACATCTACCCCTCCACGATGGGAGATGGGAAATACCA
CATGCTCTCGAGCGGGGAGCTCATGATATTGAACATCACGCGAGAGGACGCGGAAAGG
ACTTATCGGTGCAGGACGCATCACCGTTTGACGCAGGAAACGGTCGTCAGCAGCAAC
GTCGGCAGACTTCAGTTGACCG

>novel_circ_000383
ATTATCGTGCTCGTTAATGGCTGCCAGGGTCGTCGGCTAGAGGCCGTTCAGCGAAGTTT
TCGAGCCGCCAGCGCGATAACACAGCCCGTACCCGATGAGTGTTAGATCGGCAGGCGG
ACACATGGTGAAGCAGAGGAGGTTCATCGGCAATACGAATAACAAGATGATCAACATA
CGAGAATATTTATAGGTTTTTTTAG

>novel_circ_000384
ATTATCGTGCTCGTTAATGGCTGCCAGGGTCGTCGGCTAGAGGCCGTTCAGCGAAGTTT
TCGAGCCGCCAGCGCGATAACACAGCCCGTACCCGATGAGTGTTAGATCGGCAGGCGG
ACACATGGTGAAGCAGAGGAGGTTCATCGGCAATACGAATAACAAGATGATCAACATA
CGAGAATATTTATAGGTTTTTTTAGATTTCAGAGAACGACGGGGAGAAAGGATAAAGA
GAGAGAGAGAGAGAGAGAGAGATATACCGCGTCCATGCTATGGTCAGCCTTTGCAACG
ACCACGCGACGACCCGATTTCCCGTTTTTTACGTAATCCTTAGCGAGATGTTTGATTTTC
ACGCACAGATCCAATTTTCGTGCCGACGTTCGTGCGAGAGAGGACGAGTTTGAGATTG
ATTTATCGGCGGAAAAGTGATATAAAATAATCATCGTGGACGGAGGAATAGAGAAAGG
GAACGGAGTGGAACGGAAACATGATTTCTCCTTAACGCGGCGAAAACCATCGATCAGA
GATTGATAGATACACATAGGTTGTATCTTCGTTCCACTCGTATCCGCAGCCATATTCGAG
TTGGCGTTTCGTTGGAGTACAGGTTTTTCTTAGGTTTACAGGGATTGTTCTAGTATTGGT
ATCGAAAAGAGATAGAGGATAAGCCAAAGCGAACCAGTGATATTTTTTATGCAGTATTT
TTCTTCCATTATTACGAGCAAATAAAGCGAGCAAAAGAGAAAGAAGGAGAAAACGAG
AGTATTTTGATGAAAAATATAAAATAAGGACGAAAGAATTGATATTTATATATACATACAT
ATATATATATATATATATATATATATATACATATATTGGACGAATTTATAAACAGAGAGAGAG
AGAGATTCTTTTCTTTTTTTTTAACTTGTTTCTACTCGACTCGAGAGCCGACATCGGCA
GGTTTTCTTCAAAATG

>novel_circ_000385
TTTCGATTTGCAACATGCGGGGAGCTGATGCTGATCTTCGGCGGCCTGATCATGGGAAC
CCTGACAGGCCTGTGCATCCCCATCTCGACGATACAATACGGCGAGTTCACCACGTTGC
TGGTGGATCGAAACATGAAGAATCACACGAGCACGCCGACCCTAATAATGAAGTGGTT
CGGTGGAGGAAAGGTCTTAGGATCTAATTCGACGTACAAGGAGAGGATGGAGGCGCTT
TACGACGACTCGGTCGCGTTCGGCGTTTCATCCGCAGCGTTGTCCACGTTCCAATTCGT
GTTTGCCGTGTTCACGGTCGATTTGTTGAACGTAGCTGCATCCAGACAAATCGTTCGGG
TACGCAAGATGTTCCTCCGCTCTGTCCTCAGACAGGACATGACGTGGTACGACATCAA
CACGTCCACCAACTTCGCCAGCAGGATCACCGAGGATTTGGACAAGATGAAGGACGG

CATAGGGGAGAAGCTGGGCGTGTTCACTTATCTGATGGTCTCCTTCATTTCCTCCATCAT
CATATCGTTCGTCTACGGATGGAAGCTGACCCTGGTCGTGCTGAGTTGCGCGCCGATCA
TCGTGATCGCGACCGCCGTGGTCGCCAAAGTTCAGAGCTCCTTGACGGCCCAGGAGTT
GACCGCTTACGGGCAGGCGGGGAGCGTGGCCGAGGAGGTGTTGGGCGCCATCAGGAC
CGTGATCGCGTTCAACGGCGAGCAGAAGGAGGTGAACAGATACGCGGAGAAGTTGAT
CCCCGCGGAAAAGACCGGGATCAAGCGCGGTATGTGGTCGGGCGTTGGTGGCGGAGT
TATGTGGTTCATCATATACATCAGTTACGCCATCGCGTTTTG

>novel_circ_000386
GGATTTGGACAAGATGAAGGACGGCATAGGGGAGAAGCTGGGCGTGTTCACTTATCTG
ATGGTCTCCTTCATTTCCTCCATCATCATATCGTTCGTCTACGGATGGAAGCTGACCCTG
GTCGTGCTGAGTTGCGCGCCGATCATCGTGATCGCGACCGCCGTGGTCGCCAAAGTTC
AGAGCTCCTTGACGGCCCAGGAGTTGACCGCTTACGGGCAGGCGGGGAGCGTGGCCG
AGGAGGTGTTGGGCGCCATCAGGACCGTGATCGCGTTCAACGGCGAGCAGAAGGAGG
TGAACAGATACGCGGAGAAGTTGATCCCCGCGGAAAAGACCGGGATCAAGCGCGGTA
TGTGGTCGGGCGTTGGTGGCGGAGTTATGTGGTTCATCATATACATCAGTTACGCCATC
GCGTTTTGGTACGGCGTCCAATTGATATTGGAGGACAGGCCGAAGGAGGTGAAGGAGT
ACACGCCCGCGGTGCTGGTGATCGTGTTCTTCGGCGTGTTGGCAGGCGCCCAGAACAT
GGGCCTCACGTCCCCCCATCTGGAGGCGTTCGCCGTGGCGCGGGGCTCGGCCGCGGC
CATTTTCCAGGTGCTCGATCGCGTGCCCACGATCGACAGTTTGAGCAAGGAGGGGCAG
AAGCTTCCTGCCGTGAACGGCGAGATCGAGTTCAAGAACGTGCACTTCCAGTATCCGG
CCAGAAAGGACGTGAAGGTGCTGCAAGGCTTGAATCTGACCATCAATCGGGGCGAGA
CCGTGGCCCTCGTCGGAGGATCCGGCTGCGGCAAGTCCACCTGCCTTCAATTGATCCA
ACGTCTCTACGATCCTCACAAGGGACAG

>novel_circ_000387
GTCAATAGATCCGCCATCTTGAAATCCTACAACCAATCGAAAGATCGGACAAGTAACA
CGTGGTCCTCGAAGTACGCGCAGAGTGTAGTTTACGAACGGTAAACGAAACGCGAGG
GACATTCTGTTGGCCTCCGTCGTTCCTGCGTTCTCTCCCGGGTTTCCTTAGTTTATAAAT
AAACGACGTACTCGATGGGCTTACCTGGTTGCAAATTTGGGAAAATCCATCAAGTGCC
GTTTGAAGATTAATTTGCAGCATCAAAGAAAATAAAAAAGTCATTTAAATAAAATTGAT
GAGGGTTGATTCGTAAGACGTATTAACCACTTGTTAATAGTGTTGTCTAATTGTTACCTT
ACCGAATGGATCTTAATAATGTCATTACATTTAACTTACAAAATATAGTTATTTAATTCAT
GATTTAACGTTTTTTCGTGACATGCGTTTACGTGAGTGTCAATTATTTTTCAATTCACTTT
ATCTTTTTTGTTTTTTTATGGAAAACGATGCATCGTGTATCTAGTTAACCGAATATAACAT
CGGTACGGTGAATGGAAAGTTAACAGATCACGCGACAGTGATGCAGTGCCGTAGTGTC
AGTGCCAAGTGCCACGTGCCCATTGCTTGGATTACTTGAAGATGCCGTCGTCCGGTGG
ATACTATGACGACAGGAATCCTTTGCTCAAGGGCACCTTATCGAG

>novel_circ_000388
GTGAATCCGGACGCCCGGGACAGCACGACGCAGCAGATTCAATCAAAATTAGGCAACT
ACTCGTTGGTGAAACATCTACTGGACGAGCCTAAACGGCTGATCGGCATCGAGGGTGT
ACCGCCGAGCCCTGCCCCTCCGCCGCCGACCTCCTCGTTCCTTCGAATGAGTTCCAGT
TCGGTCGGCGCCAATTCCAGGAGCTCGCCCTCCTCCCAAGAATTCAAGAAGCCGGGA

GGGCCTCGCCCCGACGCTTCCTCCTCCTCCTCCTCCTCCTCCTCCTCCTCTTCCTCC
TCGACGTCATCTTTGTCTTCGTCGTCGTCTTCGTCGTCGTCGTCGTCGACTTCGACGTC
GTCATCGAGTCATCAACGCGGCGGTTTCGTGAAGCCAGCCGACGGGAAGCCGCCGTA
CGGGGGCAGGGGTGGTTACCCCGGCCAGCCGGTGAAGCACGGGGGCAACAGCAACG
ATCATAGGAGCCACGGTTTATTACCCGCCAAAGGACCGCCCACGAATTCGCCCGGGAA
CGGAACGCTCGCTGGCAATTCCAGTATCGGGAATTCCAGCGGCAGCAGGCTTCACTGT
GTCGGCAGCAGACTGTCCAGGTTACCTCTCGATAAC

>novel_circ_000390
GAGCTCAGAAGACAATGCCAGGACTTCGCCACCGCCCTGTTGGATCACACGAGAAGT
TCGTACGAATTGGAGGTGCTGTTGAACCACGATCCGACCGGGCCTGCGTTCGAGCACG
GCGAGAGGATGCATCTGAACCGGCTCAAGCTCGCCATCAAACTTCGACAGAAGAAGT
TCGTGGCGCATCCTAACGTGCAGCAGTTACTCGCATCGATTTGGTACGAGGGTCTTCCG
GGCTTCCGGAGGAAAAACATGGTTCTCCAGGCGTTGGAGATAGTCAAGATCGGCGTTC
TATTCCCTTTCTTCAGCGTCGCGTACATCATCGCCCCCCACTGCGTGGTCGGCCAGACC
ATGAGGAAACCGTTCATCAAGTTCATCTGTCACTCGGCCTCCTACTTCACCTTCCTCT

>novel_circ_000392
ATATACGTGGTGGAGGCCGAGGATGACCGAGGGGGCGAGGAGAGGGCCGACAGGGA
CAAGAGGAAGATCGCAGTGGCGAAGCTGGGCGTATCCAACGGCATAATCAATTTCGTC
TTCAACAAACTGGATTCGTTGATCGACGCCAAGACGAAAGCTCTGAGCGATCTGGACG
AGACGAATCGAATAAAGAACAAGGCTTACGGGATCGACGTGAACAAGTCTGCGACCA
GCAAGTTCATCAGCGAGCTGGTGAACGCGAAAATTCAATCGGCGACAGGGAGCATCG
GGCCGGTGGTGAGCACTGCCCAAAACTTCTTCTCGAACACGAAGAGCGGATTGACGA
ATGCGTTCGTCTCGAAACTGGCCCCCCTCAGCTCCATCGCCACCGGTTTGAGCTCTCCG
CCCTCCACTGGGGACAACAAGGAGAATTACGAAACCAAAACGTCGAACGCACTCAAC
TTTCTAGGAAACATTTTGAACGCCCAGCTTGGCGTGTTGTCGAACTCGAAGGGTCTTT
CTAGCGATTCGCCGTTTTCCGAAGGG

>novel_circ_000393
GATAAAAGAATCGAGAAGCTCAAAAAGGGAGAGAGAGAAGCAAAATGAGGATAACTT
GCTTACTCTTTGGCCTCTCGTTGCTGTTGGTTTACGTGCACTCCAATCCGATCGTCAAG
AGGGAGACGGAGCCCGTCGAGCTCAATCCATTGAACGAGATATACGTGGTGGAGGCC
GAGGATGACCGAGGGGGCGAGGAGAGGGCCGACAGGGACAAGAGGAAGATCGCAGT
GGCGAAGCTGGGCGTATCCAACGGCATAATCAATTTCGTCTTCAACAAACTGGATTCGT
TGATCGACGCCAAGACGAAAGCTCTGAGCGATCTGGACGAGACGAATCGAATAAAGA
ACAAGGCTTACGGGATCGACGTGAACAAGTCTGCGACCAGCAAGTTCATCAGCGAGC
TGGTGAACGCGAAAATTCAATCGGCGACAGGGAGCATCGGGCCGGTGGTGAGCACTG
CCCAAAACTTCTTCTCGAACACGAAGAGCGGATTGACGAATGCGTTCGTCTCGAAACT
GGCCCCCCTCAGCTCCATCGCCACCGGTTTGAGCTCTCCGCCCTCCACTGGGGACAAC
AAGGAGAATTACGAAACCAAAACGTCGAACGCACTCAACTTTCTAGGAAACATTTTGA
ACGCCCAGCTTGGCGTGTTGTCGAACTCGAAGGGTCTTTCTAGCGATTCGCCGTTTTCC
GAAGGG

>novel_circ_000395
ATCGACCGAGGAACAACTCTGAACAAGAGATGCAGCGGTGTCCGTATCTCCACGAGAT
GAAGGAGCGCCTGCTCTCCCAGCCGACACCGACAGACAGCCTGGACATGGAACGACT
CGACGGCACTCCCGTCAAGGAGCCCAACCTGCACACCGATTATCCTGCCCACACCAAC
CCTGGGGTGATACCGGATCCCCCGGAAATGCCGCCCGACTCGCTCATCGGCACGGTCG
TCAAGCTGAATTCGGATGGTTACGGCTCGCATACGTCGCCGGCGCACGCGAGGCAGCC
TTTAGTGCCTCAAAACGCCAACAACCCACCCCTCTGCCTCACCCCTACGCACTCCGGC
CCGGCGACTGGCACGCTGCCAAAGAACGCAAA

>novel_circ_000396
GCGTGTGCAGCAACGCGACGGCCCCATCGGTGAGCAGCATCAACCGAATCCTACGGA
ACCGCGCGGCCGAACGGGCGGCCGCCGAGTTCGCGAGGGCGGCCGGCTACGGGTTGT
ACGCGGCGGGCCCTCACCCGTACTTCAACAGCGGCCACCAGCACCCAACGACCAGCC
ACCACTTGCCCGCCGGTTGGCCGGCGCCGGGGGCGGCTGCCCACCCCTGGATGCTGCC
ACCGTTGGCTACCGGCATCTCGGGCGCCGCTTCCGCCCTGCTCCTTCCGCCGTCCTTGA
GCCCAGGCGCGGCAGCCGCGGCCGCTGCGGCCGCCTCCGCCTCCGCCGCCGGCGCGC
CTGACCACTCGCTGCACGCGGACGCCATCGCCCGGGGCTACTTGCAAG

>novel_circ_000397
ATTCATGCGATAAAGAGGCCGTGATGGTTTCTTAAAGCAACAACTGTTAAAATGTGTAT
ACGTCAGTGTGGCACATGAGTCATTGGAGAGGAAACGAGGACGGGGCGCTCGATGCG
CCCACAATATCGTCGTCCCTGCGCGGAAAGTGAGCGTAAATCGCCGAAGCGATCCGAA
GATGGCCGAAGTAAGGCCGTCGTGCGGCAGTCGAGCCCGAGCGATCCCCCGATCGTG
GTCGTGCGTGTTGGAACGACAGGTACGGGCAGTCAGTAGTGGATTAGCGGCAACGGG
AGCAGCAGCAGCAGCGTCCACGGCGGCGGCGGTGGTGCAGCGCTAACAACAGAACG
AAACGAAGAGGAGGTATGATCGCTCGAATCGACGGAAGACATCGCAAAACGGGTAAA
TAACATCGCATTTACCCTGTCCCGGAAACGTCCATTGAGTGAAACGAAGGTGCGCGCA
AACGCTGGCTGCTCCACGGAATATGTATGTGACGGAGGTGGGCGAGGGCCGTGCGAA
AAAGTGGCGAAAACTGTGAGAGAGGTGCAGTGCCTCCACGTCGGTGGCAGTGGCGGT
GTTGGTAGTAGTGGTGCTGGTGGTGTGGTGGTCGTGGCGGTGGTGGTGGCGGCGGTGT
GGCGTTGCTGGTGCTACTTCGGTGTATATGCGCGCGTGTATTGGTGGGACACGCACTTC
GGCGGGGCCGTGGTTCTCGCTCTCTCTCCTGTTACATCTCATTCCCTCTTTCTCTCGAAC
CCCTCTATTCACCGCGACTATCCTCTTTGTCGCCCTCACGGAGGCCATCGTACCGAAAG
CGACCGAGCCAGGCCAGGCACTACGCAAGATGGCTGTGTGTCGCTAACGAGAGGCCG
ACAAACCAGACGGGGAAAAGACAACCCGGGGATAAACGGAACATGCTGCTACGTGAT
CGTCGGGCCCGTCCCTGAACAGAGAGACACTGGCCAAGAAGCGAGAG

>novel_circ_000398
CTACTCGGAGTGTCAACGCGCAGAAACCTGATATTCGGTAGATGCCTTTTGAGAGCCG
CTGGCCCGAATCAGCGTGGAACCATACAAAGGCACATGGAATGTGTGACATGTTCATC
CAAACACAGCAGACGAGTAGCGTCAACGGCAGCAGCAGCAGCAGCAGCAGCAGCAG
TAACAACACCGTTCACCACCACAGCAAGAAACGCAAGTTGGAGTACAACGTGAGTCA
GCCGGTGATCCAACACGCATTGGTCCAATCGACCGGCGACTACCAATTAGACAATACC
GGTCTGCAACAACGGTACTCCGTGAACGGTGCTAATACCGCATTTAGCTCGCTGCACA

ACAATAATGCGCTGCAGAAGAGTAGCCCGAACCAACAGACCCTGGTACGAGCCTCGA
CGATCAAGCTCTTAGACACGTACCAACGCTGTGGCCAGAAG

>novel_circ_000399
GGAATGTGTGACATGTTCATCCAAACACAGCAGACGAGTAGCGTCAACGGCAGCAGC
AGCAGCAGCAGCAGCAGCAGTAACAACACCGTTCACCACCACAGCAAGAAACGCAA
GTTGGAGTACAACGTGAGTCAGCCGGTGATCCAACACGCATTGGTCCAATCGACCGGC
GACTACCAATTAGACAATACCGGTCTGCAACAACGGTACTCCGTGAACGGTGCTAATA
CCGCATTTAGCTCGCTGCACAACAATAATGCGCTGCAGAAGAGTAGCCCGAACCAACA
GACCCTGGTACGAGCCTCGACGATCAAGCTCTTAGACACGTACCAACGCTGTGGCCAG
AAG

>novel_circ_000400
GATTGTTGTTCGTCGATGGTGGGAAGATCGAGGAGAAGAGGAAAGCGTTTTGGGACG
CCTCGCGTGTAAGGAGAGAATAACCGAGAGGCGAGGGGGAATAAATCACGTTTGCAC
AGAGAGAGAGAGAGAGAGAGAGAGAGAGGGAGAGAGGGAGGAAGGGAGAGGATACGT
AGCGGTCAGGACAGGTAGCGAGGATTCACGGTTCGGCTGTCATCGAGTTCGACTCGAA
AGACGCGATCGATGGCGGCACACGAGGCACGCAGCCATCGGTGTTGTCATTTCTTCGC
AACGAGAGACTGATATTTGGCTCATTCTCGACACGGGATTGGGCGATAGTACAACACC
GCGGGAAGAGATGTCTCTTCTACCGACGTTCGCGTCCCGAGCTTGCGTCCCCGACCGC
TCCTGAGTCTAGTCGATCTTCCTCGAATCTTTGTCCACCTTTGCCTCTTTCTTCGAGTCT
TCTTCTTCTTCTTCTTCTTTCCCTTGCCCCTCTTCTTCCTCCTCTCCTTCGTTTTCCATTC
TTCGAGGAGGGAAAGGGGAAAGGAGGACGGTCGGTCGTGGAACGAGGAACCGGAAG
ACTGGATCGAGTTCGAGGAAGATGCACGACGAGGCGACGTCCGCCGAGACCAGCCCG
AGGACGAGCACCACGAGGACCATCGCCGCGACGACGAGAGACGGTCGGATGTGAGG
AGGAGGAGGAGGAAGTGTCCGGATTATCGAGATCTTGGGACAAAGGCGGCGAGAGGA
ATTGGCCGTGGTGTGTGATCGGGTGCACGAGGAAGGCAGGCATGGAGCCGTCAACGT
GGTGCACGCGCAGGCTCGGCGGCGGCGTAGCGACGTCCGCGGAGTCGACGATCGCCG
TGACTCTCGCGAAACTTGGCGCGACGACCACCTCCTCCCGTCTCTCGAGCGACGCTTC
CGCGATCATCCCCCAACCCGACCCCTGCCCCTCTTCCTCGCCGCAGCACCACGACCAC
GGACCTTTCCCGTCGCTCTGCGACCTGTGCGAGCGGCTTCGCGGCGGACGCCTCGTCT
CGGGCGGACGGTCGGAAACGGACGAGGAGGAGGAGGGGGGCCGCGGTCGGATGACG
AGACGCGGGCGATCGGTGGCGGATCGCGGCCACGCTTCCCCCGCAGGGGTCGTGTCG
GCGAAGGATCGACGAAGGCCCGACGACGGCCCTTCCTCGAGCGGCTGCGTGTCCAGC
GTGGTGCGGTGGAACTCGACGAGGTCGGCCAGATCTCTGCCCGCGACCAGCCACGAG
CAGCAGACGTTAACCTGGACCATTTTGTTCCTATTCCTGGTACTCGTGTTCTCCGCTTCC
GCGGACCTGCAAAAGCCAACGTCCAGAGAACGTTCCTCCAAAGAAGAGGGAG

>novel_circ_000401
TGTGCCAAAGCATAGACATAAGAAACACCGTGCGTCAGTTCTCAAGACTGAAGGGATG
CCGACTAGTCGAAGGATTCGTACAAATTTTGCTGATCGACAACGCCGAGCCATCCGAG
TACACCAACATCTCTTTCCCGGAGCTGAGAGAGATCACCGGTTACCTGCTTCTCTATAG
GGTAAAAGGGTTGCGCAGCATCGGTCGCCTGTTCCCAAACCTGACCGTGATCCGTGGC
CACTCGCTGTTCATCAACTACGCCCTCGTCGCGTTCGAGATGATGAACCTGCAGGAGAT

CGGGCTGCACAGCTTGACGACCATCGTCAGGGGCTCCGTCCGCTTCGAGAAGAACCC
CGCGCTCTGCTACGTGGACACGATCGACTGGGACCTGATCGCCAAGGCTGGCAAAGG
GGAGCACGTGATCTCG

>novel_circ_000403
GGAGTGGCCAGCATTCCGGACTGTAAAGATCAGCGGGCCAGCTACGAATGGAACTGTT
GGTCGACGACGATCCGGAAAATGGATGTCGCCAAGAGTGGAACAGAGGACTTCGTGA
CGGTGGTGAGCGTGGAGAGCGTTCATCATCCCCATCCCCAGGAGAATTTCGTCACGGT
GCTCTCGATCGGGAGCAGGAAGGAGGAGGAGGAGGAGGAGGCTGCGAGGTTGCCGC
TCGAGGAGGAGGTGGAGGTGTACAGGTTGCCCGGGGAGCGGTTGGGCTTCGGCCTCA
AGTTCGAGGGTGGGAACAAGACGTCGGAGAGGGTGAGACGGTTGTTCGTGCAGAGCT
GCGCGGAGCAGAGCCCTGCGAGCAGGGCGAGATGCTCTTGGGGCACGCTGGGGGAG
GGCGACGAGGTGCTCTCGATAGACGGCGTGCCGGTGACGCACATGACTCGGCTGGATT
GCGTGAGGCGGTTGAAGGAGTCGCAGCTGGTGATCAAGCTGATGGTGAGGTGCAGAG
GGGCGCTGAGGCCGGAGGTGGTGAGCGCCGAGAAGAAGACTTGCCCGGAGAGGGAC
AAGGTGCCGCCCGAGCTGCCCTCGGCGCCCCCCCCGGTCCCCCCCCGCAAGTTGAGA
CTGGCCCGAGGCCTGGCCGACGGCGAGGCGAACCCGAACCCGGCCAAGCGGTCGTG
GAACGCGGCGAGGGCCAATTCCCAGAACGCTTCCCCCGACCACTCGCCGACCGCCGC
GGCCGAGGCGAGCAAATCGACCTCCTACGAGAGCTGCGAGTCGTCGCCCAGAAGCGG
ATCGAGCCCCAAAGGATCCCCCCCGAAGGAACGTTCCCCGGAACTGGCCAAATCGGG
GGTGAAACAGGAGCCGCCCCCGGAGGCGATGGTGTACGTGGACGCGAGGTCGCAGTG
CGGCTCGACGCACGGGAGCACGTCGGACGACACGGGTAGCTCGATGTCGACGGTGAT
CGACCGGTTCTCGAGCTCGGACCGGGTCTCGACCATCTCGACCGCGTCCACGGCGTCG
ACGGCGTCCGAGCAGCCGGGCGACTTCTCGCGGATCGACCAGGATTTCGAGAGATCGT
CGAAACCGCCCGCCACGTCCGCGATCTGTCCATTCGAGGGGGTGGACGACTATTACGC
GTCGAACCCGCCCGACTACCTGCTCCGCCGTTTGGCAAGCTCCGAGGCGGTGACCCAC
GTGGAGTCGCGGGGCGAGGTGGAGAAGATCACGGCCGTGGTGGCGCCCAACACGGT
GCTGATCGAGGAAACGATCACGTTTCAACCGCCGTTGAGCTTCCAGGACGCGCCGCTG
AGCTACGGCCACGAGGCAAGGCCCGACCTCTTCTACACCGCCGACCTCGCCGCCGACT
CGACCACCCACTTCAGGCCGATCAAGGACGACGTGGAGCTGGTCGAGCGCGTCAACA
GCGCGCCCGACTCCTCGGACAGGACGCCGCCTCCCCTCCCAGCGAGGAACCACGTAA
ACAGGATCACGGTGAATCGGCCGTCGTCGTCGAGCAGCCTCGACTCGCGCCCTCCTCC
TCCCCGTTCCACCAACGAAGGCGCTCACGCGCCTCTCCTGCCCCCCAAACCGTTGCCC
AGAAGGGACGTCAAGATCAGGAGGAAGAGGCCACCGCCGCCGCCGCCGCCTCCGCC
GCCCGCCATCCTCCCAAGAACCGAGACCAAATCCTCCGTCGTTTCGTCCTCCGCTTGTT
CATTGCCAGAGTCTCGGCAGACGAGCTTCGAGAACGAGGACGAGAACGAGAACGAG
GACCAGCTGTGCCTGGAAACGATCGCCGCCGATCAATTGGAGGAGGCCAGCCTCGCG
AGAGCGTGTCTGGAGAACGGCAGAGCACGGGAGGAGAAGGAGGAGGATGGAGGGTT
GAACGGGGTTGAGGAGAGGAGGAACGAGGAGACGGACGACACCGAGTCCATGGAGA
ACTACGAGGTTGGGGAGGGGTACAGGACGAACAAGGTGTTGGAGATCATCGAGATAA
GGAAGGCGAGAGCGTTCCCTCCCCGAAGGGGGGCCAACGTTTCTCCCTCGAGCGAGA
AGAACGAGGAGAAAAGATTGTTAGGGGAGGCAGAAGGGACGAAGGAGGATGATTTTT
GCCAGCCGGAGGAGGCGGGAGGGATGGAGTCGGACGAGGAACAGGTGACCAAGTTC
AACGAGCCGGTGGACGAACTGGAGCAAGGGGGAGGGAAGGAGGTCGGGCGAGAGA

GGGAGGGCGAGGAGAGCGAGGGCTCGTTTCGATCGTGCGCCGATCTAACCTCGGCTAA
TCGTGGCGGCGAGGAAGCGGTCGTGGAGGATGACACGACGAGCGAGGAGAGCGACG
AGGGGGATTACTATTGGCAGAGCAACCTGGCCACGATCGGGGAGGAGGAGGAGACCA
ACTCTCTGGAATACGCGAACCCGAACAAAGAGGCGGGCGAGAGCGAGGCGATCGAAG
CGCAGGGGAAAACGGAGCGACAAGACTCGGCGGGCAAGGAAGACGCGTTCGATGCA
AATG

>novel_circ_000404
GATCCGCCGTACCGCAACAACGAGAGGGGAAGGAGCATGGGGGTCGGGCCGATGCGG
TCGCGGCCCATGGAGAAGAGGCACGATACCTCGCCGTACAGCGGCGTCCCGTATCTGT
CGCCGCCGCCCCCGGACACGTGGAGGAGAACCAATTCGGACTCGGCACTCCATCAGA
GCGCCAACGAAGCGTGCCAATCGACCACCACTATCCCCCATCGGCGAGATCAACACCC
CATAAGCGGATCCGGGAGCGAGAACAGAAACTTGCATTACGGATTCATCGAACGGCCG
AGATCCTCCTGCGAGATACCCCGAGTGCCTGGAAACAA

>novel_circ_000405
GATCCGCCGTACCGCAACAACGAGAGGGGAAGGAGCATGGGGGTCGGGCCGATGCGG
TCGCGGCCCATGGAGAAGAGGCACGATACCTCGCCGTACAGCGGCGTCCCGTATCTGT
CGCCGCCGCCCCCGGACACGTGGAGGAGAACCAATTCGGACTCGGCACTCCATCAGA
GCGCCAACGAAGCGTGCCAATCGACCACCACTATCCCCCATCGGCGAGATCAACACCC
CATAAGCGGATCCGGGAGCGAGAACAGAAACTTGCATTACGGATTCATCGAACGGCCG
AGATCCTCCTGCGAGATACCCCGAGTGCCTGGAAACAACATAGATCCGAGCTCTCAGC
CAGCGGGGCAGCAAATTCCCATAGGGAACACGCGATCGTTGCCCGACCTGGCCCATTT
CCAGTCGGATCAAGAGGATCACTCGAGCAGCACGACGCCGTTCAGCAACAACCATTTA
TCAGTGCCTGTTAACTCTAGGTTTCTTCACACGTGTAAGGGTGTGGCGTTGGAGAACA
GCACAAGTACTTCGCAACAGGATCTTCAGAGCTACTCGCAACAACCGCCGATGGCCCA
GCCGCCGACGGCGACCACCCACAGCCCGGCCGGCCACCACTACATTTATCAGCAACCG
CACAGTCCTGTGCCACCGCAAAGTCCGAAGTCGTCGCAAACGCAGCAACCGCAGCTC
AACTCGTTGGGCTCGTACAGATCCTCGCAGCAGGTGAACAGGCCGTCGCCGCAGTCGT
CGCCCGGGCTAACGATTCAAGGGAGCCCGTTGAGCTACAGCAATAATCCATCGGCGCC
TCCCAGCCCCACGGGTCACCCGGGTCCACCGAACTTGACGTCGGAGAGCATCGACCA
AAACAGTTATTTCATGAACCAGGCACAAGCGGCGGCGCTTCAACAGGATTTCGAGCAG
TTCACGATG

>novel_circ_000407
ATTATATAACAAACACAGATATGTTTGATCTTGAAAATGATCTACCTGATGATCTATTGTC
ATCGGGGTCTTGGGGTTCTGCAACTGAAAGTGCTAAACCACCAGCAACAGGTCCAGG
TCCAGGGCAACAAAATGGTGCTCTTGATTCAGAACTTAGACAGCATGTACAACAACAA
CAGCAACTTTCGCATCATCTCATACAACAACAAGGCAACAAAAACTTGGTTGCCAATT
CATTAGCAATGGCTGCTGGAACTTTGGGTAATAAAAGTCCAAATATGCAATCTCCACCA
AATGTTTCTGTCTCTAAAGTTGTTGATCCACAAATGGTTGTGAGTTTGGGAAATTTACC
AAGTAGTATAGCCAGTTCCTTGGCAAATAATCAAATGTCCATTGCTAATTCGATGGGAG
GCCTTCAATCGTCAATGAGCATGGCTGGAAGTAATCCTACCATGTCAATGCCAGGAGG
AATTAATTCTGCTCTAGTTATGACCAGCAGTGCTAGTGGAAATAACACTATGGGTGGAA

TGGCAGGAGGAAGTTTAATTGTAACCAACAGTTTGAATAAACAACCTATAAATACTGTA
ACTATGATGGGCCCTAATACTCAAGGAATTCATCATCCTAGTGGACCACATGGTGTTGC
CCAAATGCAAAATGGGCCTGGAATAATGAATACTAGGGCTGTAACAATGCAACAACAA
CAACAAGCTCATATGGTTGGTCCAGCTCGAGGTCAAAGTCCACATCAACAAGTACATC
AAGTTGGTATTGTTGGTCCTGGTCAAGGAGGTCCAAGAATCCAAGCTCCTCCAAATAT
GACAAGTATGCCAAATATGGGACAAATAGGTGCATCAAGTCCTTATTATGGATCTCCAG
CTAGTGCGCAAGGACCTGGTGTTACAGTGTGTACTAATAGTCCTGTTGGAGTTGTTGCA
CCACAACAAAAAGGAGTGGGAACAAATATGACTGCTATGCAAGGTAGCAGATTTGGA
GGAACTGGAGGTCCTATTGGTTCTACAAATGTTGTGGGTGGGCAAGAAGGTGGTATGG
CTCAACAAGCTCAACCACCTGCACCAAGTCCAGCCCAACCTCAGTCTGGTGCACCAA
GTGGAGGTCAACCTGGTCCACAACAAGCTACTCAGGGTCAAATGCCTGGCACTGGTG
CTCCAACTGGTACTACAAAATCAACTGCTGATCCAGAAAAACGAAAACTTATCCAACA
ACAATTAGTTCTTTTACTTCATGCACATAAATGTCAACGACGTGAAAGTCAAGCAAATG
GTGATGTCTGGCAATGTTCCTTGCCAGATTGTAAAACTATGAAAAATGTATTAACTCATA
TGACTGCTTGTCAAGCAGGGAAAAATTGTACGGTACCACATTGTAGTTCTTCAAGACA
AATTATTAGTCATTGGAAACATTGCAATCGCAATGATTGTCCTGTATGCCTACCTTTAAA
GCAAGCAAATAAGAATAAAACGACAAATACTGCTGCAGCATCTACAACTCAACCAAAT
AGTCAACCAAATCCGAGTCAAACCGAAATGAGAAGAGCGTATGACGCATTAGGAATTC
CATGCCCTACTACAACGCCTGGTTTAGTAGCAAGTCAATGTGTTACTAGGAGAATGCCA
ACACCAGGCATGCAAGGAACACCCGGAACTATAGGAAATGTCCGATTAACTCAACCTC
AAACTCAGACTGCACCAGGTCAATCCATAGTTGGTGCCGGGCAACAAGTTGTTGCTCC
AAATGTTTCTCTTCCTTTAAATCCAGATCCTAATACAGTTGGTGTAGCTAGTAATCAAGC
AGCACCTACCACCGGCCCCACGCCTGCTGCTGCAGCAACTGCTGCAAACATACAACAA
TCTGTTAATATGCAACAATTATTTGGTTTAAATGATTCAGGACAACTTAGTGTTCCAAAT
GAAAATAGATTGGCTAATCTTCAACTTCCAGCTGGACTTCAACCAGGTCAAGTAACAG
CAACATCTGTACAGGAATCAAAAGATTGGCATCAAACTGTAACACCTGATCTTAGAAAT
CATCTTGTTCATAAGTTAGTTCAAGCAATATTTCCAACTCCTGATCCACAGGCTATGCTT
GATAAAAGAATGCATAATCTTGTTGCATATGCAAGAAAAGTTGAAGGTGATATGTATGA
AATGGCCAATTCGAGATCAGAGTATTACCATTTGCTTGCCGAAAAAATTTACAAAATTC
AAAAAGAATTGGAAGAAAAACGACAAAAACGAAAGGAACAACAGCAACAGTTACAT
GCTCAACAACATCAGCAGCAACAACCTCAACCCGCAGGAACATCTGGTCCAGGTTTAA
GGCCATGTGCTCCTCCAGGTGTTGGTACTGTCCCGCCATCACGACCAGTTGGAACAGT
TACCCCTAGTTTACGTAGTCATTCACCAAGTATGGGACAACTTGGATCTTTACCTACAAT
GGGCATTCCTCAACATAATAGAATGCAATTTCCACAACAACAACAAACGCAACAACAA
GTACAAGTTCAGGCCCAAACTAATGTCCAAGCTCAGGCTCAAGCACAAGCTCAAGCC
CAAATGCAGCAACAACAACAACAACAGCAACAGCAGCAAGGGATTTTAGTTGGTCCA
TCTGGTCCTAGTCCAAATGGACAGTCGAGTTCCAATCCTAATGTGGTTCCAAATCCTGG
TCTTAGTCCTTTTGGACAAGCACAAATGTCACAAGCAAGTTTAACCACTACTACTACGT
CTGCAACAACTAGTCAATTCCCAACCTCAAATGGTACTTCCGGTTTACCTAACAGTCCT
TTACAAAATCAACATCAATTTCCTGATATTATGAAAGCTAGACTGACTCAAGCAGCAAT
TGTGCATCAACATCAACAACAACAACAGCAACAGCCATCACAACCTCAATCTCAACAA
CAACCAAGTCAGTCGCAATTACAGCAACCAACAAGTACTTCAAATCAAAGCGCTACAA
CAACGTCAATGCCACAAGCGCCATCACCTTTTAGTGGTATGCAACAACAAAGTAACCA
ACAACAAAATCAATTTAATAATAGTCGGCCTCTATCAGTTTCAACACCTAATGATAATGG

CATCAGCACATCAACTCCACAAACAATACCACCTCCTGCTTCTAGTGGACCTAGTCCCG
GTCCGGTAACAACGACAAACGGACCTCAATCTACAACTTCCACACCTAACACACCACT
AGTTCCTTCATTAATGACTCCAAATCAGACAGTTTCTTCCGCTAATCAAACTCCACCTC
ATCCTGCTACTACACCATCTCCTGCTGGTCTTGCAAGTCTTGGAAAAGGTATGACATCT
CAAGAAAGAGCTGCATTAAATGCACCGAGAACTTCATCCATGTCTTCGCAAATGGCTG
CTATTACAGCTGCTTTAGATCGTGATAATTCTCCTAGTCCACCAATGAATAATAATAAGG
GAAAATTGGATTCCATTAAAGAAGAAAATATAAAAATGGAAATCAAAACTCAAGAAGA
TAGTTCTGAGAATCATAGAATGGATGGAGGTAAAAGTGTTAATAATGATGTATCTATTAA
AACGGAAAATAAAATGGAACCAATGGAAGAAGGATCCAGTGAGAGTATCATAAAAGA
AGAACCCACTGGTATTAAAGAACCTGTAACTCCAATATCCAGTCAAGATACCACAGACA
TTAAACCATTGGTTCCTGAACCAATACAACCAAGCGGAACAACGGACAAAAAAAGGT
TGTGTTTGTTTAAACCTGACGAATTACGTCAAGCATTAATGCCAACATTAGAAAAACTT
TATCGTCAAGATCCGGAATCTATACCATTTAGACAACCTGTCGATCCTCAAGCGTTAGG
AATTCCAGATTATTTCGATATCGTTAAAAAACCAATGGATCTTTCAACGATCAAAAGAA
AATTAGATACTGGACAGTATAGTGATCCATGGGAATATGTAGATGATGTATGGATGATGT
TTGACAATGCGTGGCTTTACAATCGCAAAACTTCACGCGTTTATAGATATTGTACAAAG
CTTTCTGAAGTCTTTGAGCAAGAAATAGATCCTGTAATGCAAGCCTTGGGATATTGTTG
TGGAAGAAAATACACATTTAATCCACAAGTACTATGTTGTTATGGAAAACAACTTTGTA
CAATACCAAGAGATGCGAAGTACTACTCCTATCAAAATAG

>novel_circ_000408
TACCGTTTAAAGGGCCGCCACGCTACAACCCGAAACGGCGCTCGGTGGCGTTCGGAG
GCACGTCGACGATGACCACTAGCTGCTCGGCCAGCAACTTGGGCACCCTGACCAGGAT
ACCGTCCGCCGAACACCAACACGCCTCTGGCGGAACAGGAGGAGGAGGAGGAGGAG
GGGGAGGTGGTAGCCTGACCAGAACGATCTCAGTGGAGCAGTACGCGGTGACGAGAG
TACCGTCCGCCGAGCAATACGCGACCGGCAACCTGACCAGAGTACCGTCCACGGAAC
AATACGCCAGCCCTATCGCGACCGCGACCTCCACCACCGCCAATAACAACAACAACAA
CAGCAATAACAGCACAGTGTCGAGGCTGTCCACGGGGGATCATCATCACCATCATCAC
CACCACCATCATCACCATCAATACCAACCGGCAACGAGCGCCACCGGTACGCTTACCA
GAGTGCCGTCCGCCGAGCAATACACGACCGGCACCCTCACCAGAATACCGTCCGCCGA
GCAGTACGCGAACAGTATCGCGAGCAGGACGGCGGGGGAGCCGCATCATCACGCCGC
CGCCCTCGCCCCCCGAGTACCGTCCATCGAACCGACGACGACGACGACGACGACCAG
AAACGACCAGATACAGAGAATACCGTCCTCGGATTACCAGAGCCCGAATTCGCTGAGG
GATCCTAACGCGAGATTACCGACCACCGCGGAGAACTATCAGAACGTAAGAATGGAGG
GACTGACGAGGCTGCAAGAACACCGGCTCGAGCTTCAACACCGTCTCGACATGCACA
GAACGATGGAGATGCCGCCCTCGCCCTCGCCGAGCAGCAGGAGTCTAGCTCCTTTCAG
CTCGGCCAGCTCGAGCCTATCCGAAGGCAGCAATCAACCGTCCGGTGAAGATTCAGCC
AGCATCGTCACAG

>novel_circ_000409
CTCAAGTACAAAAGACGGGTCTACAAGATGCTGCACCTGGATGAAAAACAGTTGAAG
GCGATGCACACGAGGACGAACTTGCGAAGGTTGCTCGAGTACGTGGCAAACAGCCAG
GTCGAGAAGATCGCCAAGATGTGCAGCAAGGGCCTGGATCCTAATTTCCACTGCCAGG
AAACCGGAGAGACTCCCCTCACCTTGGCCACCACGTTGAAGAAACCCTCGAAGGTGA

TAATAGCGCTGGTGAACGGTGGCGCGTTGTTGGATTATCGAACGAAGGAGGGTTTGAC
GGCGATGCATCGCGCCGTGGAGCGGAACAGTTTGGAAGCTGTGAAGACGTTGCTCGA
GCTCGGCGCGAGCCCCAATTACAAGGACACGAAGGGCTTGACGCCGCTCTACTACAGC
GTCATCTACAAAACCGATCCGATGCTGTGCGAAACGTTGCTCCACGACCACGCGACGA
TAGGCGCCCAGGATTTGCAGGGATGGCAGGAAGTGCATCAGGCCTGCCGCAACAACC
TGGTCCAACACCTGGATCACTTGCTCTTTACGGTGCCGACATGAACGCGCGTAACGC
GTCCGGCAACACGCCGTTGCACGTATGCGCTGTGAACAACACGGACTCGTCGTGCATA
CGCCAGTTGCTGTTCAGAGGCGCGCAGAAGGACAGCCTGAATTACGCGAACCAGACT
CCCTACCAGGTGGCGGTGATCGCTGGGAACATGGAGCTGGCCGAGGTCATTAAGAATT
ATCAGCCGGAAGAAGTTG

>novel_circ_000410
TGGCGTGGCCGTGTCCTGCGGGGAATGCAGCAGCAGCGGAACCAGCGACATCACGGA
GCCCGGTTCCCCGTGCAGCACGAGCAGCGACGAGGGGGTAGCAGCCGTGAGGGGCGC
ATCCGCTAGTCCACTGCCGTCCCTACCCAAGCTGGCGCCCTCCTCGCAGATCAGCACC
AGGCCCCAGTGGCCCTGGACCCCGCCCCTCGCGGCCACCGCGTGCTCCACCTACAAG
AGGCTGAAGGGCGAGCCCGAGGCTGGCGCCCTGCCGAGGTCCGGGGCCGCGGATCCC
GGGTTCAGGGGGAGCGGCAAGTCGGCCGGCAAGCCGAGCGCCGCCGGCCATCATCAT
CACCACCACCACGAGTCCCAGGGGAAGATCACCGAGTACTTCAAAACCCAGATCAAA
CCCCAGCAACCCAAGATCAAGAAGAGCAGCGAGGCGATGTCGGTGCTGGTCGCGAAG
AGTTCGTCGGAATTTCGCAGGCCGGCTGCCACTGTGCAGAGGAGCAAGGCGGGTCTG
GCCAAGTACCTGGGCGCCGTGTCGGCGTCGGGCAACGATTGGGAAGTGAGAAGCCTG
GCCATGTCCCCGCCGCCCACCAAGAAGCCGCCGTTGGTCGTGGTGCCGAGGGCGAAC
GGCAAGATGGAGAAGAAATTGCCTAAACCGGTGCCCAGCCTGCCCATCTCGAACGAG
GTGTTGAAGAGCATCCCCAGCTGCAAGATCTCCTCGCTCAGGCTGACGTCGGACGCGA
GCTCGAACGCGAAGAACGGCGTCTCGTCCCCGCCCGCGACGCCCGCCCCCGCCCTGG
GCAACGTGGAGGCGGCCGCGCTCGACTTCAAGGGTTGCATCCTTTCGAAGCCGCACG
TGAACGAGAACAACAATCTGACGAACGGGTGCGGGGGGATCCTGGGCTCGTTGAGGG
CGTCGCAGGGTGGGCAAGACCCGTTGAAGAGATTCCCGCCCGAGGAGGGTGGCAAGG
AGGGCAAGGAGAGGCAGGTGAGGCCCGCGGACGAGGACGACGTCGAGGAGGAGGA
GGAGGACTCGAGGAGCAGCGAATCGAAACCACCCCTATCGCCGATACTCTCCGCGCCG
ACCACCATACGATTCCCTGCACGGGAGCCGGAGAAGGACAGGCAACAGGCGACGGAC
AGCGGCGTGTGCCGATGGGACAAGTGCGAGGAGAGCTTCGAGTCGAGCGGCGAACTT
CTCGAACATTTGCAGGTAGCTCATATCAACACCCAGACAGGGAGCGACAACTTCGTAT
GCCAGTGGCAGGGTTGCAAGGTGCAGGGGAGGAGTTCCTGTTCGAGGAGGTGGTTGG
AACGGCACGTGTTGTCTCACGGTGGGAACAAGCCCTTCCGGTGCATAGTCGACGGCTG
CGGCAGCAGATTCAGCTCTCAG

>novel_circ_000411
TGGCGTGGCCGTGTCCTGCGGGGAATGCAGCAGCAGCGGAACCAGCGACATCACGGA
GCCCGGTTCCCCGTGCAGCACGAGCAGCGACGAGGGGGTAGCAGCCGTGAGGGGCGC
ATCCGCTAGTCCACTGCCGTCCCTACCCAAGCTGGCGCCCTCCTCGCAGATCAGCACC
AGGCCCCAGTGGCCCTGGACCCCGCCCCTCGCGGCCACCGCGTGCTCCACCTACAAG
AGGCTGAAGGGCGAGCCCGAGGCTGGCGCCCTGCCGAGGTCCGGGGCCGCGGATCCC

GGGTTCAGGGGGAGCGGCAAGTCGGCCGGCAAGCCGAGCGCCGCCGGCCATCATCAT
CACCACCACCACGAGTCCCAGGGGAAGATCACCGAGTACTTCAAAACCCAGATCAAA
CCCCAGCAACCCAAGATCAAGAAGAGCAGCGAGGCGATGTCGGTGCTGGTCGCGAAG
AGTTCGTCGGAATTTCGCAGGCCGGCTGCCACTGTGCAGAGGAGCAAGGCGGGTCTG
GCCAAGTACCTGGGCGCCGTGTCGGCGTCGGGCAACGATTGGGAAGTGAGAAGCCTG
GCCATGTCCCCGCCGCCCACCAAGAAGCCGCCGTTGGTCGTGGTGCCGAGGGCGAAC
GGCAAGATGGAGAAGAAATTGCCTAAACCGGTGCCCAGCCTGCCCATCTCGAACGAG
GTGTTGAAGAGCATCCCCAGCTGCAAGATCTCCTCGCTCAGGCTGACGTCGGACGCGA
GCTCGAACGCGAAGAACGGCGTCTCGTCCCCGCCCGCGACGCCCGCCCCCGCCCTGG
GCAACGTGGAGGCGGCCGCGCTCGACTTCAAGGGTTGCATCCTTTCGAAGCCGCACG
TGAACGAGAACAACAATCTGACGAACGGGTGCGGGGGGATCCTGGGCTCGTTGAGGG
CGTCGCAGGGTGGGCAAGACCCGTTGAAGAGATTCCCGCCCGAGGAGGGTGGCAAGG
AGGGCAAGGAGAGGCAGGTGAGGCCCGCGGACGAGGACGACGTCGAGGAGGAGGA
GGAGGACTCGAGGAGCAGCGAATCGAAACCACCCCTATCGCCGATACTCTCCGCGCCG
ACCACCATACGATTCCCTGCACGGGAGCCGGAGAAGGACAGGCAACAGGCGACGGAC
AGCGGCGTGTGCCGATGGGACAAGTGCGAGGAGAGCTTCGAGTCGAGCGGCGAACTT
CTCGAACATTTGCAG

>novel_circ_000412
ATGTGCCAGCATGTCCTCGGAAGCCGGTGCGCCAGAAGCAGCGAGCAAGATCGACAG
GCAGGCGTCGGTTTACGTGTTCCCGGGTCCGGGGTGTCAGCATTACACCGCCTCGTCG
ACACAATCGGCCGACGCCTCGAGCTCCGCCTCAGCGATAGTCCCGAATGTATTCGCAA
AGGGGGGGAAGTTGGAGAACGACAACGCGGAGAACGAGGAGCTCGGGGGGCGAGG
AAGGCGAAGGCCGTTTCCGAGGACCCATGCGAGATCGGCAAGCCACGGTGGAATGTT
GGCGGAGTGTCTGACGAGCACCACCGGATTCCAGCCTTACGCGGGATCGCATCTCGGC
CCTTTGTCCGCGATCGGTGGGACGAGGCCCTCGGCCTTGAAGAAGCCCGGCCACCAG
AGAGCATTCAGCCAGGGTCAGGTGGTCGAGGTTCAGGGGCACGCGACTGTCACGGGC
CACAGCCGTGTCGGGTCGAAAACGGATTTTATCCTCCCGCCTGGCCACAAGGAGGACT
CGAAGCCGCCCACCGCCGGGAAAGTGCCCTCTTTTCGTGGCCACTCGCGACAAGCATC
CAGATCGGAGTCGATATATACGATAAGACGTAGCGTTGAACCCCCCTGGTGGAGAAAA
GTTTGGGCCCGATACTTTGGCCCTCTACCGGAGGAGCCACGTTTAAGGACAATAGTACC
GAATCATTTAGTCCCGCCAAAAACACCACCGAGTCAACATCCCAACGGCAAGAGAGT
GAATAATAGAGTGCGTACAACAAAGTACACCTTGTTGAGCTTCCTACCTCGCAATTTTT
TCGAACAATTCCGCCGTGTGGCCAATATATATTTGTATTTATCGTTTTGCTTAATTGGGT
GCCGGTCATAAATGCTTTCGGCAAGGAGATATCCGTGATACCCATCATTTTCGTGCTTGG
TGTTACGGCGTTGAAAGATTATTTCGAGGATCATCGGAGGCTTATCAGCGATCGTCGTG
TAAATAATTCCACCTGTCGTGTCTACGTTCG

>novel_circ_000414
ATGTGCCAGCATGTCCTCGGAAGCCGGTGCGCCAGAAGCAGCGAGCAAGATCGACAG
GCAGGCGTCGGTTTACGTGTTCCCGGGTCCGGGGTGTCAGCATTACACCGCCTCGTCG
ACACAATCGGCCGACGCCTCGAGCTCCGCCTCAGCGATAGTCCCGAATGTATTCGCAA
AGGGGGGGAAGTTGGAGAACGACAACGCGGAGAACGAGGAGCTCGGGGGGCGAGG
AAGGCGAAGGCCGTTTCCGAGGACCCATGCGAGATCGGCAAGCCACGGTGGAATGTT

GGCGGAGTGTCTGACGAGCACCACCGGATTCCAGCCTTACGCGGGATCGCATCTCGGC
CCTTTGTCCGCGATCGGTGGGACGAGGCCCTCGGCCTTGAAGAAGCCCGGCCACCAG
AGAGCATTCAGCCAGGGTCAGGTGGTCGAGGTTCAGGGGCACGCGACTGTCACGGGC
CACAGCCGTGTCGGGTCGAAAACGGATTTTATCCTCCCGCCTGGCCACAAGGAGGACT
CGAAGCCGCCCACCGCCGGGAAAGTGCCCTCTTTTCGTGGCCACTCGCGACAAGCATC
CAG

>novel_circ_000415
ATGCCTGCATTGTGCCTGTGTCTGTCCCTGTCTGTCACTTAGACTCTAGACAACAACAA
CAACAACAACAACCATCACTAATGAACAACCAACAACATCAACTCCAACATCAACAA
AACAATCCACCGCATCTAGCGTACCAACAAGCGAATCACCACAGCTACACGACTGTAA
CCACGTCCAATCAACATGGGCCCACACCTATGGGTGCCCATCAACAAGGTCCCGTACC
ACCCCACCTTCAACAACAG

>novel_circ_000416
GAATATAAGAAGAAACCCCGATTTGTAAGGATTTTATCATAAAATAATCCCCCTGATATA
AACATTAACGGTCCCTTTTTTTGAGAATAATTTCACTAATTTCATGCACATATATTTATATT
TTGTTATCAGTCTTGTTCATTCTCTTGACACGTTTTTTTCTCGTGTATCATGTATGTAGAT
CATAGAACGATTTTTCAGTGATGTCACAGGTACGAATATC

>novel_circ_000417
AAATAGAGAAGATTGCGGAGAAGTTGCGTGATTAAATTTGGCCAACGTAATAATAATTT
TCTTTATCCATTTGCATCTGTAATCTCTTTTAAACTTTAAAAATTACGTTTTTAATCGAGA
TAGTAATTTTCTTAGAAATTTGTAGATTAATCTTATATACATATATATATGTATTTTTTCGTT
ATTTCTCAAATAACACGTAAAG

>novel_circ_000418
CTTCTGGGACATTCCGAGCGGTTGGTGGCAGGCGAGCACGGTCACCATGGGGATCGG
GGTCGCGATCGCGGTGATCGGGGCCCTCACCCTGCTCGCCGCCGCCTCCACCTTCCTC
CCGCACATCCTCAAAACTCCGAGGCACACCAGGGTGCTTGGCTCTCTCCAGTTGCTCG
CCGCAGCGATGATATGCGGCGGTCTGGTCATGTATCCGATAGGATGGGACAACAGGGA
GGTGCGGGAGTCTTGCGGGAAGGGTGCTAACGTGTACAATCTAGGAAAGTGTTCCGTT
TCATGGTCGAGCTATCTGCTGGTAGGCTCGGTGGCGCTGTTGATGCTATGCTTCGGGCT
GAGCTTCTGCGCCGCGCGACACAAGCCGTCCAACCCGCACACCGATCCTCTGCGCATT
TGACAATCGAGATACTCTCAATCGATTCACGCCTATGCCTCGCCGAAGACGACCACTCT
CTCCTTCGTCACGGTCTGTTGAGCCGTGAGGAGGGCGAGGAGAAGGAGGAGGAACA
GGAGAGAAACAGCAAGGAGGAGAAGGAGGAATAGAAGCATCGAGAATCACCTCTAC
GAGCGTCTCATCCGG

>novel_circ_000419
CCCGGCACCCCCTCCTCGGGGGGCCACGACAGTGGCGTAGGCATGAGTCCCGCCGGT
CAAACGCCTCCGCCGAGATCCTCCGTTCTTCCCGACGTAGGCGAAGAGACGAATCAAC
CGCCCGACAACTCGCCCACCTCTTCCACGACCACCAACGTTTCCGGCCAACTCGAGTC
GGACTCGAGCCCCCGAACGATGCCGATCCAACGGCGAAACCAGCAGAGTCAATCGCC

CAAGAAATCCCCGTCCCCGTGTTCCGCCGTTGTCGCGACGACGACCGCCGTCACCACC
ACAACGAGCTCGGGGATCTCCTCGACGAACATCGCGTCCGAACAGTTGCAGATCGTTT
GCAAACCGGACGGTACGTATCAATTGGCCTCCGTGAATCCGAACGTGGTGTCGAATCA
GAGGAACGTTCTCAATCTGCAAAATATCGAGGACGGAGGAGGCGGTGGCGGAGGAGG
AGGAGGAGGAGGATCCAACTTTTCACGGCAAAATCAGAGAGGCGAGAACGGCGGGA
ATGGCAGGAACACGTACGAGAGATTGACGAAAGGGGGCGCGCAAAATTCCGGGCAGA
ACGCAGGCTTGCCGAAATTTCAACAGGCCTTCGGTAAAACTATATACACGCTTTCCACA
GACTCGTCGAGCGCGAGCGGCGTGTCGACAGGTGGCGCCGCCTCGGCGGAAACTCTG
GTACAATCGGTCTCTCCCCAAAAGGCGAGTAACCTCTCGAAAGCTGTTCAAACGTCGG
TGCCCAACGCGCAACAGGCGAGCCCGTCTACGGGGATAAACGTGCAATCCATCGCGA
ACATTCCAAACACGTTACAATTATCGACCACGAGTAGACAGATATTGAATATCGTTCAA
AGCTCCGGGAATAACGCGAACGTGGTGGCAACCAGCCCGAACGCGAGTCAAACGTTG
ACGCAGTTGGTGCAAAGTGTTCAGAACGCCTCCCCCGGTGTCATATACACGCACAAAA
TCCCTGTCACCATATCGACCACGAATCCAAGCCAGTTGAACATCATACCTACGATATCG
CACGCGAATTCGATACCGACCGGAAGGACACCTGTGGTCAAATTGAACATCATCCGAG
CTCCTTTGAGGCAACAAAATATCACGGGAGCTATACAAGCCGTACTCACCACACCAAG
ATTGCAACAACAACAGCAGCAACAGCAACAGCAGCAGCAGCAACAACAGCAACAGT
CCACGGTTCGAACGGACAGTTTGCTCGGTTCCCCCAT

>novel_circ_000420
ATGACAGTGGTACAGCGGGCAACGACGCTGGGGTACAGTTGGTAGTGGCGTGGAGGG
CGCACCGCGTGAGAGGAGCGGAAGATAACCCGGAAGAGGAGGGCGGTGAAGAACGA
GGGAGGAGGAGGCGGAGGAGGAGGAGGAGGAGGAGGAGGGGGTGGAGGAGGAGG
AGGAGGAGGAGAAAGAAGAAAGAGGGAAGACTGGCGGAGATAAGAAAACGGAAGA
AAGGGAGGAAAGTAGTAGGCAGAGGGAAGGGGAAGGGGAAGGGGAGAGGGAAGAG
AGGAGGAGGAGGCCGGTCTCGTAGAGGGGCATCGTCCACGGATTTTCCGGTGATCGG
GGATAATATCGTCGAACTTTGGGGGGCTGCGAAGGTCGCTGATGTCCGCTACGAGCCC
TGCCCCTACCCCTGCCGCCAGCTCACCGCCACCTACGGACGAACAACAACAACAACG
GAGGAGGAGGAGAAGGAGGAGGAGGAGGAGGAGCAGCAGCAGCAGCAGCAGCAGC
GGTGAAGGAAGAGGATCGGACGCCGCCGCGCCGCGAGCGACGCCGAGGAAAGTCGC
GTCACTACACCTTGCCTTATCCTTTGATCCGGGAAGAAAGAATATCTTTTACCAGCCTC
GCCGACCACCACCACCAAACGTTCTCTCCATCCATACTTCGCTATTACTGCTGCTGTATC
ACCGATCAGCTCGACCACCCCTCTGAATGTCTTCGCACATCCAAAAGTCGTGCGTG

>novel_circ_000421
CGAAACAGCAGAGCGAGATGGAGAGCACGGACCAATTGGAGGGCGACGAGGAAGGA
GTGCAACAGGAATCTCTGTGGAGAAGGAAAAAGCTGGATTTCGACAGAGTGAACAGA
AGGATGAGAAGGGCCTGCAGGAAAGCCGTCAAGTCGCAAGTTTTCTATTGGTTGATCA
TAGTATTGGTTTTCCTGAATACCGGCGTTCTTGCGACCGAGCACTACAATCAACCTCAT
TGGTTGGACGATTTTCAAGAAATCACCAATATGTTTTTCATCGCGTTGTTTACCATGGAA
ATGATGCTGAAAATGTATAGCTTAGGATTCCAAGGTTACTTCGTCTCGTTGTTCAATCGT
TTCGATTGTTTCGTCGTGATCGGTTCGATCACCGAGATGATCCTGACGAACACTCACGT
GATGCCACCGCTCGGCGTTTCCGTTCTCCGTTGCGTCCGGTTACTCAGGGTATTTAAAG
TGACAAAATATTGGAGGTCGTTGTCGAATTTGGTGGCTTCCCTTTTAAACTCTATACAAT

CGATCGCGTCTCTGTTGCTACTGCTCTTCCTTTTTATCGTGATCTTTGCCCTGCTGGGTAT
GCAG

>novel_circ_000422
CGTCGTCGTCCGAGCACGGACGTCCGAAGACCCAGCGTGGCGGACCTCGAGAATAAG
ATCAATCAGCCGAGCACACCTCTTCGAGATGTCGGACCACCGGGACCACCTCAAATCA
TCGATGTCCAGGAATCGTATTCCGCGGTGGAAGACTCGACGGCGTACCTCACGGTCGG
AGTTGAGGGTACGCCGGCGCCTACCTTCAAATTCTACAAAGGAATCACCGAGATCATC
GAAGGTGGCCGATTCAAGTTCCTTACGGACACAGAGACCAATACGATCACCCTCTGCA
TGAGGAAGACGAAGCCCAACGACGAGGGCACGTACAAGATCGTCGTGAGCAATATCC
ATGGCGAGGATTCCGCCGAGATGCAGCTTTACGTTTCCGACGCCAGTGGTATGGACTTC
CGTGCTATGTTGAAAAAGAGAAGGTATCAAAAATGGGCAAGGGAGGAGCAAGAACAG
GAAAAGGTAGATTTGAAGGAAGTGGAGAAACCGATACCAGCTTTGAAGAAAGTGGAA
AAG

>novel_circ_000423
AATGAAAAATTTGCTATTGTAGTCAATGTTGTCGACCCGTATCGTGTCGTCCGACACGA
TCTCCTTGTCTTGGAAGAACCAGGTTACATTTGGTGGCGGTTCACCAATGATATTCACA
TCCAAAGTGACGGTAAGACCGACTTTTACGGTTATCGGTTGCAAATTGGTTCGATCGAT
GAACGGTTTCACTGTAAACGATAAAATGAATTAAAACGCAATTTTTCCAACATGCCCAT
TCGACTCGAGAAATCGTATTATACTCACGCCAACGAGCTTTGGCAGTATGAGGATTGGT
AGCCTCGCTAGGTTCTCCGGGACCTGCTTTGTTCACAGCTCTTACTCTAAACTGATATT
GATTTCCTTCCTCCAATCGTGTCACTGTTCCCGTACACACTCGTCCTTCCACCTCGGCA
GCCTTCACGAACGTGGTGCCAAATCTGTCTTTCATTTCGATTATGTAACCTGTGATGGG
TGCACCACCGTCTCGTATCGGTGGTTCCCATTTCAATTTCACCATGTTTTCGGCCCAGTC
GATGATTTCTGGTAGACCAGGCGCAGATGGAGGATCTAAAAGTCGAGTTTAAACGAGT
TATAATGTTTCTCGACATAAGCCATCGAATGTACATATAATATAGTATAATATAGTATACAG
TATATATAGTATATGTATGGTTCACTGCTGGAAAGACTTCTGATTGGTTAATGTAGAAGG
AAACGCCTACTTGTTTCTAGCGCGGTATTTGTTACATATACTAATCCGACTCGAAGCTGA
GTGTAAACAAAGCGTTCGAAGTGGGGATAGACTTCTTGACTTTGTGCTGTCCCCGGAC
TTCTTTCCACCAGTAAACAGTATACATATATATATATACATACCATAAGGATTCTTGGCGA
TAATAGGAACGTCCGTTTGCAACGGTTCCGATTCTCCCTCTTCGTTTACAGCTTTCACG
CGGAATTCGTATCGTTTTCCAGGTTCCAAACCAGTGATAGTCTGTTCCGTTTTGTCTGG
ATCAACTATGCCAACTGGTACCCATCGGCCGGTCGTGACATCCATTTTCTCGACCAGAT
AACCGGACAACGGTAAACCGCCATCGTCCCTCGGTTTGTTCCACTTCAATTTGCATCCT
TCCTTGTGGATGTCCGTCACTTCCAGAGGACCTTCCGGTTTTCCGGGCTTATCTTGAAT
GATAATCTCCACGTCAGCTTCGTCCTGTCCAGAATCGTTCACAGCTTTGATGGTGTATTT
GCCACTCAGTTTGCGGCTAGTTTGGGTCAAATGAATTTTGGTTAGGTAATCCTCGTTATC
GATTTTCACGTTGGCACTTGTTTGAAGGACCGCGCCTCCAAAGTTCCAGGTAACGGTT
GGAGGTGGTTCACCTTCGATGTCGACTTCTAGTTTCACCATTTGACCCGCTCTTACTTTA
ATCGTTTTCAATTTATCGCGATTTATATGTGGTTTCACTGGAAAATGAAAAACGTGTATAT
ATATAAATGTATATATATCGAAATTGTTGAAGTATTGTTAGAAAGAAGTTTCGAAAAAGG
AATCGTAAATTTGATTGGATAAATTTGGAAAGAAACGCCTATTTTCATATATACTGATCC
AGAACATCAATTCGTAATTAAATCTCGATTAGTGTAGACATCTAGAAAATCTAACGTATA

ATATAAAAATTATTTCTCAAAATATGTGTAGGCTAATTATGATTTGTAATATTAGTTTAATT
TATAAATATAATTTTGTATTCGATTAAAATAAAAATAATTAAAATAAGAATTATATATATAC
AAATATATTATACAAATATATATAGATATATTAATATTATATCGAAATCAGTCTTAGATCCTA
ATGTAGCTTCATACAAAATAATTTTTACGACTTTGAATATTCAAATTATAGCTTTTCTATT
ACTAGTGTAGTGCGTGGTATATCAACAAAGCGTTTGAAGTGGGGAGAGGCGGTCCTAG
CGAAAACGCCCCTGTCTTTGTGCCTTCCCACCAAACTTGTTTCCAGCAGTGAACAGTA
CCGTTATAATCCTTCGTTAGGACCGCCTACTTTCATTTCAGACGCTTTGTTAACAATCCC
TTGACATCGCACACTACTAGTGGTGTAGACAAACTATAATTTGAATATTCAAAATCATAA
AAATTATTTTTCAATTATAAAATCACTTTAGAATAATCATTTAAGAAATTGAAATATTTTC
CAAATTATCAAGTTATTTTAAACTATTATAAATCATAATTAATCTACACATGAGAAATAAT
AAAATTTTTACATTATATCAATAGAATTCTTTGGATTTTATGTTTAAGTTAACGCAACGAG
ATCATTGTTTCCGATTAGTATAAGTACGGCAATGAACAGTACTCACGATGTCGCGCCTTT
GCCACGTGCGGCTTGGTTGGATCGCTGGGTTCCGATGGCCCAGCTTTGTTAACGGCAC
GCACGCGGAATTGGTACACGGAACCTTTCTTCAAACCAGTGACACGCCCTTTTGGTTG
GGGCGAGTCTGTTACAGTGGCCTCTTGCCAATTCGGCGACGGCTTCTCCTTCATTTCGA
TTATGTAACCCGTAATAGGTGCGCCTCCCGTACTTTTCGGCGGTTCCCATTTCAAATCGA
CACGATCCACGTCCCAATCTTCGAATTCCGGCACACCAGGCTTACTGGCTACGTCTAAA
AATTATTGTTATTCAAACTTATCAGTGAAAACTTTCTTATAGAAATATTCTTTTTATACGG
GCAATTTTTCAATGGTTTTCTCACCATAAGGATTCTTTGCAATGGTCGAACTGTCGGTG
ACCAATGGCTCGGATTCCCCTTCTTCGTTAATCGCCTTCACCCTGAATTCGTATTCGTGA
CCCTCTTGAAGACCTTTAACGTCCATTTCGGTATCCGAAGTACGCCCGACGGGGACCCA
TCTGCCGGTCGCTTTGTCCAGTTTCTCAATTTGATAACTGGTAATGGGTAAACCGCCAT
CGTCTTCCGGTTTTTCCCATTTCAATTTCAAACCGCTCTTTGTAACATCGTATACCTTCA
AAGGACCCTTTGGTTTTCCAGGAGCAGCTGTTAATAATTTATGAATCGAATTAGTAATAA
TAAATAAATAATAAGTTGTACAATATCAATTTCAGAGATTTTCAATATCATTGGGCTTACA
TAGTATCGTAACTTCGACCTCAGCCTCGTCTTCACCGTGAGGATTTACAGCCTTTATTTT
CCACACGCCGGTATTTTTTCGCACGCAGTCGGTAATATTGAGTTTCGTATTGTAATCGAC
GTTGACAATTTCTATATTACCACCGCTCTGAACCGCCTGATTCGCGTGATACCACGTGAT
CTCTGGAGGCGGTTCACCACGAACGTCCACGTCGTATTTGATTGGCTTGCCAGCACGG
ACCGTGATTGGTTTCAAGTTCGTACGATCGATTCTTGGTTTCACTGTAGGAATAAAAGA
AGAAATGAAAGAATGACTCGATCGAAACTTTACTCGAATACGTATACGTATTACGACAA
ATAAAAAGATGAAATGAAATACGATATTTACGCGCTTTGTGTTTGCATATTTGCATCTTT
GAAGCGTCGGATGGCGGAGATGGGCCGGCTTTGTTCACGGCGATGATCCTGAACTGAT
ATTCCCCGCGTTCCTTCAAGTCCCCTACTTTGGCCTCCAGTTGATTTCCAGGTACCTCCA
TCGCCTTCTCCCAATCTGGTTTGTAACGATCCTTCTTCTCGATTATGTACTTCTCGATAG
GCGCACCGCCGTCGAAATCAGGAGCGGTCCATTTCAAACTTATCGACTCGTTGTCATAG
TCGGTTATCTCAGGTGTTCCAGGTTTATGCGGCTCGTCTTAAAGAAACAAATCATCGAA
ATTAGATTAATTATAACGCATGGAAGGAGCAAAATTTAAAACGAATCTGGAAGAAAGTT
CTTTCCTTCTCGTCTTACCGTACGGATTTCTGGCGATGATTCCCCGCTCGCTCTCCAAAG
GTTCGGAATCACCTTCGTCGTTGACCGCTGTAACGCGGAATTTGTACTCGCTTCCCGGA
TTCAATCCCGTCACCTCGAATTCCGTGAGCGGCGCATTGCCCGGAACCTTTCCCACTCT
CACCCACTTGCCAGTTTTCGTGTCCAATTTCTCAATCTCGTATTCTTTGATCGGAACGCC
GCCATCGTCCTCCGGCTTCTCCCACTTCACCTTGGCCGACGTGGCTGTAATATCCGTCA
CTTCCAACGGACCTTTCGGCGCGTCTGGCTTGCCTGAACGAAGCAAAACGATACAAAA

CGTTTACTCGTTTCTTCATTTCATATACGTATATACCATGGATCGATAAAAAAGATAATCG
ACAATTTACCGAGAACAGTAAGTTCCACGGTTTCCTCGTCCTTTCCGTTAACATTCTCC
GCGATGAGGGTATACTTTCCAGTGTCTTTACGCATCGCGTTTACGATGGTAAAGTCTGT
GTGATAATCCACGTTCTCGATCTTGATACGTTCGGTAGTAGTAAGAGGTATGTTGTCCCT
CCAAATCCACTTAACTTCTGGAGGCGGTTCGCCTATGATGTCTACCGACCATTTGTGCG
TGCGTCCAGCCTTGATGACTATAGACTTGAAATTCGTTCTGTCGATTCTCGGTTTAACTG
AAATTTCACATCGAGATGCAATTATATATTTATATAAAACGAGGTTTTGTTTTCAAGAGCT
AACTTACGATTCTTGTGCTTGCAAAGATGATTGTCGGTGGGCTCGCTAGGCTGACTGGG
GCCCGCTTTATTGTGGGCCCTAACTCGGAACTGGTAGATCATCTTTTCCTTCAAACCTTC
GACCTTTGCCTCAGGAGTGGCGTCGGTCGTTTTCGTCACTTCCACCCAGTCGACAGCG
AATTTGTCCTTCATCTCCACGGTGTAATGAGTGATAGGTCTACCGCCGTCGTTCTGAGG
CTTCTCCCACTTCAAGGATACGCTGACGTTATCGTAGTCGAAGATTACAGGTTTGCCAG
GCTTGTCAGGCTCGTCTGGTTAAAAAAGAGAGCAAAGTGAGCAGACCAAAAAATGAT
AATTCGAAGGGAACAAGATTGTAACAAGATTCACCATAAGGATTCCTGGCAAGAATCG
CTTCCTCGACTTCGAGCGGTTCCGATTCGCCCTCCTTGTTCACAGCTTTCACGCGGAAC
TTGTACTTCTTCTTCGGCGTCAACCCGGTGAAGTTGAAGCTAGTTTCGTCCGGACCCAC
TTCGCCGGCAGGGATCCATCGTCCCGTATCCATGTCCATTTTTTCCAACACGTAGCCGGT
AATATCGGTACCTCCATTATCCTCGGGCTTTTCCCACTTTACCTTCACATGGTCAGCGCG
AACTTCTTCCACTTTCAACGGGCCGGTCGGTTTCGAAGGTTTGTCTGGAAAATAAAAT
AACGATTCGTGAAAGATCAATTGATAAAAAAGTGGAGATAAGTGAAAAGTGAGGAGAT
ATACCGAGAACAACCACGTCACCGACGCTCTCGCAAGTTCCAGAGCTATTGGACAGAA
CAAGCTTGTATTTTCCAGAATCAGGCCTAGTTGTCTTCCTAACGGTGAGCACCGTGTTT
CTCTCGTATTTATCGATCGTGATCCTCTCGGCTGTATCTTGAACCAACTCTTTCTCCCCA
AGAAACCAATGAACCTCCGGTTCAGGTTCACCGGCATACTTGATATCGTACTTGATTAT
CTGCCCCTTTTTGACGATTAAGTTCGTCAGACCGTCCCCGACGATGTAAGGCTTCACGA
ATCGGCACTTGGCTATGATCGGTTTCGTGGCGTCGGACGGTTCACCGGGGCCGGCTTT
GTTCACGGCTCGGATCCTGAACTCGTATTGGGTACCCTCCTTCAACCCGTCTATGGTCG
CCTCCGTTATATCTCCCTCGATTTCCTTCCCCTTCACCCACTCTTT

>novel_circ_000424GTGGCGATCAAATGCGAGACCCCGGTGGACCCGTACTCGTTCGTGG
ACGAGGAAATGTCGATGGGCGGCATAAGGACGGCGAGCAGTCCGGTCGGGAATCCCG
AGAACATGACGTGTCCAGGTGGTGGGCAAGCGGTGCTGGCTACACCTACGTCGACGC
CGCCCGGCTCGCTCTCCGCGCCGCAACATCTTCAAATGAACCTAGGCGTGATGAACGG
CAACGTGAATCCCCAGTTGACGGCGCACCAACCGAAAAAGAGAGGCCGCAAGAGGA
AATCCGAGATGATTCTCACCCCGGAAGAAGCGGAGCTAGCCGAGGCGAAGAAACGGG
CAAAGACGTACAAAGAGAGGAAGAAGCACGACAGATTCGACGGTATGCCGGAAGAG
GAAGTGAGCAAGCGCGTGCTTCCGGATCATCTTACCAACAATTTGGACATTATCATA

>novel_circ_000425
GAGTGATGGTAAAGCAGGAAGCGAGGGATGACGGTTACGAGACAAGTCCGGACCTCG
AGATGAGGAGCACCGGTGGTGACAGCAGTCAGCAGTACCACACGCCGCTGACACCGC
ACACACCGCACACACCGCACACGCCGCACACACCCCTCACGCCGATATCGGCGACAG
CGCATCAACCCCAGCAACCTGCCTCCGCCGCTTCCACCCTCGGACAGGTGGCGATCAA
ATGCGAGACCCCGGTGGACCCGTACTCGTTCGTGGACGAGGAAATGTCGATGGGCGGC

ATAAGGACGGCGAGCAGTCCGGTCGGGAATCCCGAGAACATGACGTGTCCAGGTGGT
GGGCAAGCGGTGCTGGCTACACCTACGTCGACGCCGCCCGGCTCGCTCTCCGCGCCGC
AACATCTTCAAATGAACCTAGGCGTGATGAACGGCAACGTGAATCCCCAGTTGACGGC
GCACCAACCGAAAAAGAGAGGCCGCAAGAGGAAATCCGAGATGATTCTCACCCCGGA
AGA

>novel_circ_000428
GGAAGATGCATCCCTGTTCTCGCAGCTGAGACCGCCCCGTAGACTGCGCCAGTCGGCC
CCTCCTCGTCCACGAGCAATCACTAGAAAGTAAGAAAAGCTCTAATATCGTCTTGCGTC
TCGTCGTTAATGCAATCGCAAATCACGATTATCAATTATATCGCATAAGATAAATAGAAG
CGAGTGCAAGACTAAATAAAAAAAAAAAAAAAAAAAGAGCTTAAGCGCGAGAAAGG
GGGGTGAAAAAGAGTGAGAGAAAGGGGGAAAGAAAAGGGACGCAGCAAGCTACAA
GGAGAAGAAAACGGGATGTTTTTATCCAAAAAAAGTGGACAGGATGCATAGACACTAT
ACATAAATTTAGTATATATATATATATATATATGTATATATTTTATCGCAAAATACTCGATTAA
AAAAAAAAAAACAAAAAATAAAAAAAGGATTCGCTGCGTGCGCGCGCGCACACGCAT
CCGTTTTTTCGAAATTTAGTAAAGCTAAGAGGTAAACCTAAAAAAAAAAAAAAAAGAA
GAGGAAGAAAACGGAAGAAGAAGAGGAGAGAAATAAGAGGGGAGGAGGAGAAAGA
CGAAAAAGAAAGGAAAAGAGAAGAAAAAAGTAAGAGAGAGAGAGAGACACTCGAC
TCGAGAAAAGAGAAAGAGGGAAGAGAAAGAGATCGCGGGAGATCATCGGTGAAGAG
AAAATGGCCTCGAGCTGTTCCATCCGGTGTGGTTCGAGAAATATCCTGGTGAATGCTGC
CGCTACGTTCCTCGCGCTCCTCGTCGTTCTTCGATGTTTCGCCAACGCCGAGAAACCAT
CTCCGTGCCAGAG

>novel_circ_000430
GTAAATGGCCAAGATGTTTCAAATTCGAGCCACGAGGATGCGGTACGGTGCTTCCAAT
CGGCCCAAGAGCCGATCATCGTGGAAGTATTGCGGCGTCAGCCGCAACAACAACAAC
AACAACAACAACAGCAGCAGCATCATCACGGTCACCGAGTACATTGTGCTCAAGAGG
AAAAAGAGTCGGAGAGACGAGAGCACGGAACGAGCAACGCTTGGCCGAATCTCGTC
TCGACCGCTGTGCAGACCGATTGGGCCGGACTTATCGAGACCGAGGAGGAGGTCGTG
GACGAGCAGCCGAACGACAGTTTCGAAGATTTTCTCGCGCACGACATCGACTTTGAGG
AGGTCACGTTGCGGAAGACGGGCAGCGCCGAGAAGCTGGGGCTCACCGTCTGTTACA
GTTCCGGTTCGGGCAGCGAAGACGCCGACACCGAGGTTTACATCTCGGAAATAGTTCC
TGAAAGTTTGGCGGCGCGCGACGGCCGATTGCGCGAAGGCGATCAAATCCTCAGG

>novel_circ_000431
TCCACCTACGTTTAAGACTGCAACACCCACCTCGCGTACGCAACTGAAACTTCAGCTG
ATGCGAGAACAGCTGCAAGAGCAGGAAAGGCGAGAAGCTGAATTTCGTCAAAATTTA
CAGCAACAGAGACCAACGGCTGCTCCTCCCAGGCCAGTACCTTCTACCTCACTCTCGA
CTATCGGTGTAGACGTACCACCACAAGTCTTGCAAGTACGGACATTACTGGAAAATCC
GACGCGCTACCATGTCGTTCAAAAGCAGAAGAATCAAGTGCGGCAATACCTTCACGAA
TCGTTTCGCGGTAACGGAACGATCAGCGCTGGAGACGGAAACGTTCTCGGTAGAAATT
CGGTGGAAGGTGCGCCGACATCCGCGCCGATGATAGTGCAGAGCGCACCACCCGGCC
CGACGGTGCATCATCCAAAGCCGCAGCATCCTCATCTGGCCTCGTATCCACACGGTCCC
ACGGTATTGCCGCTCGGTAATCAGGTTGCGGCCAGTCCAGATCCTACGACTGGTGCCAT

GTCACCAGGTCTTTCCAGCGTCGCGACCAGCAATTCTGAG

>novel_circ_000432
GCGGAGGATCTTTTAGACGACATACTGTCGTTCGAAGCCAACTCTCTGGGTGATAATTT
AAAGGACAGCCAGTCGGGAAGTCTGACCAATATTCCCGAGTTGCAAATCAAACCGGA
ACCTTTGTTACTCACGGAGGCGGAGATACATGCGCTTGCCAAAGATCGACAAAAGAAA
GATAATCATAATATGATCGAGAGACGACGACGGTTCAACATCAACGACAGAATCAAAG
AGCTCGGCACTCTTTTACCGAAAACCAACGATCCGTATTACGAAATCGTTCGGGACGTT
AGGCCGAATAAAGGCACGATACTCAAGTCATCGGTGGAATACATAAAATTGTTGAAAA
ACGAGTTGACGAGAATGAAACAGAACGAGGTGAGGCACAAGCAATTGGAGCATCAG
AATCGCCGACTCCTTTTACGAGTTCAAGAGTTGGAATTGCAAGCAAAAGCGCATGGCC
TTCCGGTTTCTGATTTCAATTGGGCATCCACTTCCGGTAGCATGTTAAACGCCTTTGCTC
GAAACAAACTCGAGCATCGCAAG

>novel_circ_000433
ATAAATATGCGTGTGCAAAATTACACGAATCGAACGAATCGAAAATAATTTAACGTACC
TGTACGCGCACGCTTGAATAAATATGATAAAAGACTTGAAGAAAAGCAAAGATGAAAG
AAACCAAGGATAAAAATTTGCGATCGATAAAAAAGAATTAACTCGATTAAACTCGAAA
GCTAAGAATTTAAGAAAAATCGTTGAAAGAAACGATTTCAGGATTGGACGAATTGCTC
GTGGATATCTCGTGGACGATTATATCTCGTGGTAAGATTACGAAACACAATCATTATCAT
TTCCAGCCAATCGAACGTAATTTTTCCATTATCTCGAATCGTTTTTCGAATACTCTTCCA
ACAATGCTTTCCTCTTTCTCTCGTTTAAAATTTAAAATTGCTCGAGATTATCGTTGTTAC
AGATCGATCGTCGATTTCAAATTGATCCTTCCGAGCCGTGAGCTAAATAAAATGGATAC
GGATGGATAAACGTGGAACAAGGATCGAACGAATAAATATGCAAACTTTTGCAAATTA
A

>novel_circ_000434
ATAAATATGCGTGTGCAAAATTACACGAATCGAACGAATCGAAAATAATTTAACGTACC
TGTACGCGCACGCTTGAATAAATATGATAAAAGACTTGAAGAAAAGCAAAGATGAAAG
AAACCAAGGATAAAAATTTGCGATCGATAAAAAAGAATTAACTCGATTAAACTCGAAA
GCTAAGAATTTAAGAAAAATCGTTGAAAGAAACGATTTCAGGATTGGACGAATTGCTC
GTGGATATCTCGTGGACGATTATATCTCGTGGTAAGATTACGAAACACAATCATTATCAT
TTCCAGCCAATCGAACGTAATTTTTCCATTATCTCGAATCGTTTTTCGAATACTCTTCCA
ACAATGCTTTCCTCTTTCTCTCGTTTAAAATTTAAAATTGCTCGAGATTATCGTTGTTAC
AGATCGATCGTCGATTTCAAATTGATCCTTCCGAGCCGTGAGCTAAATAAAATGGATAC
GGATGGATAAACGTGGAACAAGGATCGAACGAATAAATATGCAAACTTTTGCAAATTA
AGTAA

>novel_circ_000436
ATTTACACGTTCCACGAACATGAAACGAGTACTCTTATTATCAATCTGTTCACTGATGGG
ATTTCTCCAGTTTGCCAACCCTCTACCTCGAAACGCGACCGAATACGAAGATATGTCCT
ATTGGCTAAAATCTGGCCAAGAGAATCTTCGAAGGATCCTAGCTCATCGTAATAATGAA
AATCGCGCGAAGAACATTATTATTTTCATCGGTGACGGGATGGGAATTTCTACGATCAC
GGCTGGTCGAATCTACAAAGGCCAAATCAAGGGTAACACCGGCGAAGAATACAAATTG

GCGTTCGAAATGTTTCCCAACGCTGGATTCGCCAAGACTTACAACACGGACAAGCAAG
TTCCCGATTCGGCTGGAACAGCTACCGCGATATTTTCCGGCGTGAAATGTCGTTACAAG
GTTATCGGCTTGGACACGAGATCTTCGTTCAATAAATGCGACAAGTACATCGATCAAGC
GAGCAAACTTACAACCGTCGCCGATTGGGCTCAACAAAGTGGCATGGGCACGGGATTC
GTAACCACGACTCGGGTCACCCATGCCACTCCGGCAGGATTGTACGCTCACGTGAACA
ATCGCGACTGGGAATGCGACACCTCGATACCGAAACAATACAAGGATTGCGTGAAGGA
TATAGGAAGACAGTTGATGGAGGATGAACCGGGAAACAAGTTTCAGGTAATAATGGGC
GGTGGAGCGCAACACTTAGGTTTACCTATGGAACCGATAGATCCCGATACGTGCGTCAG
GGGAGATGGGAAAAATTTGGTGGAATATTGGGAGGGAAACAACCCTGACGGGAAAGT
GGTAACCAACACCGAACAACTCTTCTCCGTCGATATCGCCAACACGTCGAAAATTCTC
GGAATCTTCGCCACCAATCACCTCCCCTACCACGCCGTCAAAACCGAAAATACGCCAA
GTTTGGCCAATATGACCAAACAAGCTATCAAATTGCTCCGGAAGAACGACAATGGTTT
CCTTCTGATGGTCGAGAGCGGAAGAATAGACATGGCCCACCATCACAATTACGCAAAG
TTAGCATTGCGAGAGTTATCGGAACTCGAGGAGGCGATATTGGCCGCCCTTCAACTGGT
CAAGTTGGAAGAAACATTGGTGATCGTGACTGCCGACCATTCCCACGCGTTCACCATT
AACGGTTACCCGAAGAGAGGGAACGACATCCTCGGTTTCGCCAACGATCCAGCCAAG
CCGAACGTGCCTGCCTACGAGACGCTCAGTTACATCAACGGTCCAGGCTTCTTCTATCA
TCGACGAAACGACAGCAGCAACGTTAACGAAACGTGGAGGCCCGTCGATCTGGATCA
GACGCGCAATGATCCCTACTACACTCAAATGGCTGGGATATATTTGGAGGACGAGACGC
ACGGAGGCGAAGATGTCGGAGTTTACGCGATAG

>novel_circ_000437
CATTGGGTCGTGTTCGAGAAAGTGAGCGAACTCATCATTCTAGTCCCAGCGTTATTGGG
TTTGAAAGGAAATTTAGAAATGACTCTTGCATCCCGTCTTTCGACTCAAGCGAATCTCG
GCCATATGGACACGCCGAAACAACAATGGTACATGATCGTTGGAAATCTCGTCTTGATT
CAAAAAATCAGTTCGTGTTGGCAGTGCCAGGCGATAGTGGTTGGATTTCTCGGCTCTG
TGGTGGCAATCGTAATGGGAGCATCTCGAAACGGTACCATCTCGTTCGATCATGCATAT
CTATTGTGCGCGAGCAGTTTGGTTACCGCATCCCTGGCTTCGTTCGTCCTTGGATTGATA
ACTGCAGGTGTGATCGTTTTCTCCCGACATTGTCACATTAATCCGGACAACGTCGCGAC
ACCGATCGCAGCCAGTCTCGGAGATATCACCTCTTTGGCTTTACTCTCGTGGATATCTAC
CATTCTTTACGAATCAATAAATAAACAAGATTGGGTCGCACCCCTCGTGATCGCTTGCT
ACATCCTGATTACACCTCTCTGGGTATGGATCGCCAAGAGAAACAAATACACAAACGAT
GTCCTTTACTCTGGATGGACTCCTGTCATGATCGCTATGCTGATCAGCAG

>novel_circ_000438
CTTTGCTTCACACGCATTACGTACCGAATCTTAATCACGATCACGATGAAATTCTCGAG
AATTTGACGCGATACTGCGGAAAATCAGTTTATAATTATACCGAAGGCGATTATCCCCTT
AAATGGGATTTTTATAATAGTTTTTATTTCGCTTATACCGTCGTCAGCACAATAGGTTACG
GGAATTTGGCTCCAACGAACAGGCTCAGTCGCATCCTGATGATATTTTATGGTCTGATA
GGCATACCTATGAATGGAATTTTGTTGACACAATTGGGAGAATTTTTTGGCCGTGTATTC
GTTAAAGCTCATCAAAAATATAAATCTTATAAGCATGGCCGTGACAATTATTATCCTAGA
AAATTAACAACATTCGAAACGGGAAAAGCTGGATTGGCTGCACAAATATTTGCACACT
TGTTGCCAGGTTTCGTCATGTTCATCTTTTTCCCAGCATTTCTTTTCTCTCATTACGAGG
GTTGGAGTTACGAAGAAGCTGTTTATTACGCTTTCGTCACATTGACCACCATTGGTTTT

GGAGATTACGTGGCAG

>novel_circ_000439
AGAATACTTTGGACGAATCAACTTTTCATATTGTACATATCGTGACTGTCGTTTTGGGAT
CATTGTTACTAACTGTGCTTGCAATATCGATCGCAATATTGATCAGAAAGAAGTTACTTA
AGAAGAGAATGTCTAAAGGTCCGTACAAGATCATTTTAACTACATCGGATTTCGTATTT
CCTCAAGTGCCTCAAGTAGATTCTAGAAGAGTGGACGAGGGTATCGAAACAATGTTAT
GCTGTTGGTTGCAACAACTGCAAGAGTTTGGTGGACCTGAAGTGGAAAAGCCGGATTT
ACTTCAATTGGGAAGCGTATCTTCTTTGAAACCATGTTTACGAACTAGCACGGGAAACC
TGGCACGTCATAATTTCTTCAAAGATCCTCGCGCAAGATACAAT

>novel_circ_000440
TTATATCATTATTAGTCACAATTATTGGTATAACAACTTGCTTTTCGACCGAAATATTACA
ATCTACAAATTCCTACTTAACTACAGCTGATAGAATTCATCTGAAAAAAATACTTGAAC
CAGGTTTAACATCAAATGATGTTACATTTATGTATTATGCAATTCATGGATATACATTTCTT
GGTGAAGTTTTATCAAACAAACAAGATATTTGTAACTTTATGATAAAACTTATAAAAAAT
GAAAATAACATTACAACTGAAAAAGCTTTTCATATTGCATCTATATGGCAAACCATAGG
AAATTGTCAAGCCAATTCATTATCTAATATTATAAAGATATTTACAAATACAATAGAGAA
AGAAACTTCAAGTATGATGGAAATATATTATGCAGTAAATGGTCTGAATATTTTATTGGA
AAAATTGTCACGTGATAAAATTGACAATATAATAAAAACAGTACAAAATATGCTACGAA
AGGATGACAACTTATGGAACTTGGGTTATGCATTTCACATTGCTTCGGATTTTGGAACA
AGTGGTATGTTCGCTTTTGATCGCATTGAAGACGCTATTATTCAAGCTGATGAAGTAGAT
GGCCAATATTTACAATTTGAAGGTGGTCTTTCAATAACTA

>novel_circ_000442
CAAACAACAAAATATCGAATTACCAGAATTTGGCCATTATGCTACTGGTATTCTGTTTCT
TGATAAAAATTCACATCAACAAGCTGAAGCTGCATTTGAAAAATTAGCTGAAGAATGC
TCTTTGAGGGTAATTTGTTGGCGAGATGTTCCAACAGACAATATACGAATTGGTCAAGT
AGCACGAAAATGTGAACCTTACATGCGTCAAGTCTTTGTTGCTGGTAATGAAGATATTA
ATATTAATCTTGAACGACAA

>novel_circ_000443
GTAATTTGTTGGCGAGATGTTCCAACAGACAATATACGAATTGGTCAAGTAGCACGAA
AATGTGAACCTTACATGCGTCAAGTCTTTGTTGCTGGTAATGAAGATATTAATATTAATC
TTGAACGACAAGTTTTTATTTTGAGGAAAAGATCGTCCCATTCTATACCACAATTAGGA
ATTAGATATTATATATGCTCACTTTCTATACGAACAATAGTTTACAAAGGACAATTGACG
GCGGATCAATTATGGCTTTATTTTCTAGATTTGAAGTCTCCAAATTTTGAAACCTATTTG
GCATTAGTTCATACAAGATTCTCCACAAATACATTTCCTAGCTGGGAAAGAGCGCATCC
TCTTCGATTATTAGCGCATAATGGTGAAATTAATACTTTAAGAGGTAACGTGAATCTAAT
GAAAGCCCGTGAAGGAGTGATGAGTAGTAAAGTTTTTGGGGAACAATTGAAACAGCT
TTATCCTGTAGTAGAGCCGAATCTCTCGGATTCGGGAGCCGTTGATTGTGTTTTAGAATT
TTTGGTAATGGTGGGTCAACGATCTTTGCCAGAGGAAATCTGAAGAAAATATATATTTA
GGCAGTAATGACAATGGTACCAGAAGCATGGCAAAACGACTTAACAATGGCAACAGA
GAAACGAGATTTTTATCATTGGGCTGCATGTGCGATGGAACCTTGGGATGGTCCTGCTC

TTTTAACTTTTACGGATGGACGCTATGTTGGTGCTATTTAGATAG

>novel_circ_000445
GAATGGAAAGCATAATTATACAGAATTGGGTCACGGATCATCTATTTATGAAGGTGTTG
GCGAATGTATGGAAATGGAGAAGAGGTCTAGGGATCGTGCCTTATCAATTTGTCAAACT
TATGTTTGTAGAACATCACGTTATAATTTCGTTAAACAGCTAAATAATCTCGGTTCTAGA
ATGGACAAACATTGGTTTATAGTCAGAGATACTTCTTTAAAAACGGATCGATTGATTAC
GTTAGTTCCATTGACTAAAAACTGTTCATTGAATATAACTCCAATGACGAAGAATGTTTT
GAATGATTTATTCTTAGCCCTGCAACATCCATACATATGCCCTATATTTGATATCGATTTTC
TTGAATATGATAATAAAAATTATGTAATTGTAGTTCAGCCAATAAGTCAAGGTAGTCTGA
AAGATTTGATATATGGAATCGAAAGAACAGGTTGGAATGAAGATTGGAATCAAAAATAT
AGTTCTCGTGGTAAAGGACTTCCTTTACCTCAAGTTCAACAAATGGGACGTCAAATATT
GGAAGCATTAATTTTTTTAAAAGAAAGAGGATTTCCGACTGTAATCCATTTGCATTCGG
GAAATGTGCTCATTCAAAATGGAGTCGCACGTCTCGCTGGCCTTGAAAATACGCTTCTT
GGTTTTACCAGTCGAATACATCCATTAATTATTTCTCGAATATCACAAACTATTTCAATTG
ACATGACATGTTTTGGTCATATGTTATTTGAGATGTGCGCTGGTTACGAATTACCATCAT
TTAAGCCAAATTCTGTTCATCTATCGGATATTGAAATGTATCCTCAAGTCATTGAATTATT
CAAATTCATATTTGATGAATTATCTAATCGTCATAAAATTATCGAGGAAATTCTTGTTCAT
GATTTATTCAGAAATATTGATCTTCGTGAAATGCGAAGTGCTCCCGTTACGTTATTTCGT
CCAATCTTAACGCCGCCCATTGTAAACTTTCTCGACAATATTAAACGACAAAATGCTAAT
AAGAG

>novel_circ_000446
CAGTGACAGTGTCAACGCAATTGTCTATACTGCAGTTATCCGCAGTGCAAACGGATCG
GTTGAACTGGCTATTTTAAATTTCTTGTCCAGAGAGAAACTATTTAGCCAATAAGCCAG
GAAAATTCATCGGAACACTTGCAAGAGCAAACTTTAAAGCGAATGCAAAGCCTTTGCA
TGTGCACGAGATATATCATATGCTCTACGTAAAGCTTATACGAGAGAATAATAAACAGCA
TCAAGATTATATGAGAAAGTAGAATGGGCATTTGCAATCCATGTTGACGCAAAGAAAGA
TGACAACATTCGGATCACAGTGATATGAAAAATAATAGATTATGCTATCACTATATAGTA
GATGCATATTCTCATTTGTCCGCTGATGAGAAATTCAGTTATGCACTTAAATTAAATACC
GATATATGATCTGATCATTGATCCAATCAAAGTGTTTATAAAGCAGCAAATATCCCGGCA
ATCTGGCAGCAACGTATTGAAAAATCATTCAGAGAAAAAGTTGGAAAAAGTATGTATT
TTTCAACGATATATTTTTCAACGCTATAACCTGCGACCATCGTATTGATGCCAAGGGATT
ACACAAATCGGATAAATATATAGAGGCAATTCGTGATGCACCAAAACCTACTACTGCTG
AACTATTACAGCTATTCCTGGAGAAAGCAATATATTACAATATCTTCATTCTCAATCTATC
CACGTTGGATCGATCACTATACACAAATCAATTCATATGGACACTAACTGCAGAAGAGG
CTTGATGACAATCAAGAATATTTTGATCTCTCAAGTTCTAATGCCATATGATCCGACTCT
ATTTTTATGACTAGCAACAGATGCAAGTAAGACAGAGCTTGGAAAAATCTTATTATATAT
ATTCCCTAATGATCAAAAATCCAAGAACCAACGAATCAATTTTTTATGCGATTAAAATAG
TGTTGAATGAAACAAAGCAACACTCAGATAAACAAGGAAGCATTAGCGATTTGGACAA
TCCAAAAGTTCTTCTATTATGTTTATGTCCATCATTTTATTTTTGTAACAGATCATAAACC
ATTGACAAAGATATTATATCTGGAAAAATTTCTTCCGATTCTGTATATCAATAGAATGGCC
AACATGCTGATCGCATTTCAACTTTGAAACTGTTTTCAAAATTTCTACTAAGGAAAACA
TAAATGCCAACTACTGTTCACGAACATCACTGCCTGTGAGTAGAAAAACGATTCATGC

ACTGAACGATCATAGCATAAAAGTAGTACAAAGTGATGAATTTGACAAAAATTTGTGCT
GCACCAAATTAAGTAGTTGCTTATATGTGCTAATAACATCAGCAAGAAAACGAGGAAC
GATACAGATCTAAGAAAAATAATCCAATTACTTGAATTAAGTGAAAATTTAAAAAAACA
TGCTTATATAGCACCAAAAGCTTCAGCAGTAGCATGTTTAGTATATGAGTATCAAGTTAT
TCCCGATACGCTGCGCAATCATATACTAGCTGAGTTGCATGAAGAACATTTGGGAATTG
TCAAGATGAAAGGGATGGCAAGATTGTAAGGCCAAATACAAATAATATCGAAAAATAA
CGGGCATTATTTCGTCCTTTTTTCAGAAGCTTCGTGTCATCATAACCTATTTATATCAGG
CAACCCAAGAATGGATAAATGGATATCTGTAATATTGCCACGACATTCGGAAATCTCCAT
TACGAGATTAATTACTGAGCTAATGGATCAAACGCTATGCAGATCAGGACAGTGTCAAC
ACAACACAGACACTGATCAATCATAGGATCAATACATCCACTCTGAACGAAAGTTCGC
TTTTATAGATCTTTAAAGCTTACAATGTTTACTACCAAGTTTAAGCAGGCTACATCGGGT
GCGTCCGCAGCAGAAGTTATGACATCTATGAGTGTCATATGAATGCCAAATAGGTCACC
ATATTTACGGCCTTGCTCCGACGATTTAGTAGAAATACAGCGGTTTCAACAAACCTGTA
CATTGCTATACTTATCCAATTACGGAGATAGAAGAGAATGCG

>novel_circ_000447
CAGTGACAGTGTCAACGCAATTGTCTATACTGCAGTTATCCGCAGTGCAAACGGATCG
GTTGAACTGGCTATTTTAAATTTCTTGTCCAGAGAGAAACTATTTAGCCAATAAGCCAG
GAAAATTCATCGGAACACTTGCAAGAGCAAACTTTAAAGCGAATGCAAAGCCTTTGCA
TGTGCACGAGATATATCATATGCTCTACGTAAAGCTTATACGAGAGAATAATAAACAGCA
TCAAGATTATATGAGAAAGTAGAATGGGCATTTGCAATCCATGTTGACGCAAAGAAAGA
TGACAACATTCGGATCACAGTGATATGAAAAATAATAGATTATGCTATCACTATATAGTA
GATGCATATTCTCATTTGTCCGCTGATGAGAAATTCAGTTATGCACTTAAATTAAATACC
GATATATGATCTGATCATTGATCCAATCAAAGTGTTTATAAAGCAGCAAATATCCCGGCA
ATCTGGCAGCAACGTATTGAAAAATCATTCAGAGAAAAAGTTGGAAAAAGTATGTATT
TTTCAACGATATATTTTTCAACGCTATAACCTGCGACCATCGTATTGATGCCAAGGGATT
ACACAAATCGGATAAATATATAGAGGCAATTCGTGATGCACCAAAACCTACTACTGCTG
AACTATTACAGCTATTCCTGGAGAAAGCAATATATTACAATATCTTCATTCTCAATCTATC
CACGTTGGATCGATCACTATACACAAATCAATTCATATGGACACTAACTGCAGAAGAGG
CTTGATGACAATCAAGAATATTTTGATCTCTCAAGTTCTAATGCCATATGATCCGACTCT
ATTTTTATGACTAGCAACAGATGCAAGTAAGACAGAGCTTGGAAAAATCTTATTATATAT
ATTCCCTAATGATCAAAAATCCAAGAACCAACGAATCAATTTTTTATGCGATTAAAATAG
TGTTGAATGAAACAAAGCAACACTCAGATAAACAAGGAAGCATTAGCGATTTGGACAA
TCCAAAAGTTCTTCTATTATGTTTATGTCCATCATTTTATTTTTGTAACAGATCATAAACC
ATTGACAAAGATATTATATCTGGAAAAATTTCTTCCGATTCTGTATATCAATAGAATGGCC
AACATGCTGATCGCATTTCAACTTTGAAACTGTTTTCAAAATTTCTACTAAGGAAAACA
TAAATGCCAACTACTGTTCACGAACATCACTGCCT

>novel_circ_000448
GAGACCTACAGGCAGCAGGCTGAATAATAAAATCTCAGAACATATCTCAGCATCAGTA
ACACCCCTCCATGTTTGTCTGGAGGAACAAAGAGGACCAGAACTGGGCTCTTGTGAC
GCCTCGGCTTATAAGCCACAGGCAAAAAAGTTATTTGAAAAGCAGAAAACAAAAGAA
AGGAGTAGTAAAGAAGAAGTGGAAACTGAAATTATTGCAATCAAAGCAGGAGATTTG
AAATTCAGTCAGCCAAGTATGTCCAATACTATCAGTAATATGAAGATGACCAACCGTATT

AAAGGAATGGTGAGCAAACGTCGTAGGCGTTTCACAGAAGATGGTTTTGATCTTGATC
TCACATACATAAGAGATAATTTAATAGCAATGGGATTTCCAGCTGAAAAGTTAGAAGGA
GTATATAGAAATCACATAGATGATGTTGTCAAATTGTTGGAATCTAGACATAAAGATCAC
TATAAAATTTACAATCTATGTTCAGAAAGATCTTATGATTTTAAAAAATTTAAACAAAGG
GTAGCTACATATGCGTTTGATGATCATAATCCACCAATGTTGGATCAAATTAGACCATTT
TGTGAAGATGTACATGAATGGCTTTCTCGGCATCAAGAAAATGTAGCTGTAGTTCATTG
TAAAGCGGGTAAAGGTCGAACAGGTGTAATGGTGTGTTGTTACCTTCTTCATATTAAAC
AATTTCCCACTGCCACAGAAGCCCTTAATTACTATGGAACTAAAAGAACGCACGATAG
AAAGGGTGTAACAATACCTTCACAGAGGAGGTATGTGGATTATTATGCTACTCTTGTAC
AAGAAGGATTAAATTATCAACCTGTTACATTATTGTTACGGAAAATACAATTGGATCCGG
CACCCATTTTCCATGGAGGTCAAGGATGTAAGAAAAATAAAATATTCAAAATATATTCA
AAAAATAAAATACAATTATATATGTTTATATATAATTTTTATATTTTAGACTTACATTGTGTA
ATATCTGAATCTAAAAAGAAGATATTTAGCTCTGAAATAGTAGAAGTACGCAAAGGAAT
GCATACGATTAGTATTCCTTTAAAACACAGTGTAGCATTGACAGGAGATATACGAGTGG
ATTTCTTTAACAGACCAAAAATGAAACGAAAGGAAAAATTGTTCCATTTTTGGTTCAAT
ACATTTTTTGTACGGGATTATTTATCGCCAGAATATGATAACGGAGAGTTACCAGTTGAG
CGTTCAACTAGGGCATTGAGTTGCGATGGTACAACCATGGAATTACCAATGGTTATGTC
GCATATGAAATCCCGGGCCGGATCGCTGGCTAGTCTCGGACCTATGCCACCTACTCTTG
TTTTAAGTATAGATAAATGGGGTTTGGATGACGCACATAAAGATAAACATCACAAAGTA
TATAGTGCTGATTTTAAAGTTAGTTTATTTATGCATCGAATGGGTGGTACTATATCGCAAG
CTGTATCAGCAACAACAAATAGAACAGGAGAGGGTATACAAATAGGAATGGGAGGAC
AAGACACTCCGAGCGAATCAAGTGAAGCGGACAGCAGTGAATGCGATACGACCGGAG
ATACAACGGGTGATGAAGATGGGGAATCTG

>novel_circ_000449
ACGAATCCGGCGCGCATAAGAAGCGAGGAGCAAAGCGGCGGGGCGGCGGCGGCGTC
GTCCGGAAGCGAGGAGCAGAGAAGAAGCGGCGTGGTGCCGGCGCCGGTCCCGACAC
CGGTCCCGGTGCACGTGCCCCTCGGGCTGACGAGAAAACAGGGGCAAGAGGCCACGT
TTCCTTTCGGCCAGGTACGCTCGCACTACGGTCCAAAGTGGCTAAGAGCGTAAGTCCT
CATCGAGTGCACCAGGAGTGCTCGAGCGGAGGGGAGGTGCAGGTACAGGTGCAGCAG
GAGAGACGAGCACCGAGGCGCATGCCTTACCTGGGGCCTGATATGACAACCCGGCTAT
CCGCCATGTTTACACGGTCGAAGTCGGGGACACCAGATTCACTATTCTCAAGCGGTAT
CAGAATTTGAAGCCGATCGGTTCCGGTGCGCAGGGAATCGTTTGCGCGGCGTACGACA
CGGTGACAGCGCAGAATGTCGCGATCAAGAAACTGTCGAGACCTTTTCAAAACGTTAC
GCACGCGAAGAGGGCCTACAGGGAATTCAAGCTGATGAAGCTGGTCAATCACAAAAA
CATAATCGGTTTGTTGAACGCGTTCACGCCACAACGGTCCTTGGACGAGTTTCAAGAC
GTTTACTTAGTGATGGAACTTATGGACGCGAATCTATGCCAGGTAATCCAGATGGATCT
GGATCACGAACGTATGTCTTATCTGCTTTATCAAATGCTGTGCGGCATCAAACACTTAC
ACTCGGCCGGTATCATTCACAGGGATTTAAAGCCGAGCAATATAGTGGTAAAGGCCGA
CTGTACATTGAAGATTCTCGATTTTGGCCTGGCCAGAACTGCCGGGACCACTTTCATGA
TGACGCCATACGTTGTGACACGATATTATAGGGCGCCAGAGGTGATCCTCGGGATGGGA
TACAAGGAGAACGTGGACATTTGGTCGGTCGGGTGCATAATGGGCGAAATGATTCGGG
GTGGCGTCCTCTTTCCCGGGACGGATCACATCGACCAATGGAACAAGATAATCGAGCA
ACTGGGAACGCCGGCGCAGGAATTCATGCAGCGGCTCCAGCCTACGGTCAGGAATTAC

GTGGAGAACAGGCCACGGTATCCGGGATATCCGTTCGACAGGTTATTCCCGGACGTTCT
GTTTCCATCGGATTCGTCGGAACACAACAGGTTGAAAGCTAGTCAAGCCAGGGATCTA
TTGTCGCGCATGCTCGTGATCGACCCCGAACGACGCATCTCCGTCGACGATGCTTTACT
ACATAATTACATAAACGTGTGGTACGACGAGGGCGAAGTCAATGCC

>novel_circ_000450
ATACACGGATCGTTCCTACAGACATCATCCACGTGGGTTGATCATATACGGGCCTGTAA
CGGTGTCGAGCATACTGGGTAACAACAATGCCACCCTGCTTTGGGGTGGTAAGGATAA
TTCCAACTTCTCCAAGCTGGATGGTGACGACTCCATTCGAAACACGTACTTCGTTGGA
AGTTCCCAGGAATCTTTGACCGCAAATTCGACAAACAATACCGTGATACCGCGATGTCC
GCTTGTCTCGCCTCATCTCATTGGCCCACTGACTGTTAGCAAATCACCACCGGAGTTGG
AGGTGATGGAGAAGACGTTCACCGAGGTGAAGCCCGGCGGCAAAGGTTGTCCAACAA
ATTGCACGGCCAGGCATCGAGTAGCCATCATCATTCCGTTTCGCGATCGACCGAAGCAT
CTCCAAACTTTGCTCTACAATCTTCATCCGATGCTGCTTCGCCAACAGATCGATTATCAA
ATATTTGTGATCGAGCAGAAAG

>novel_circ_000451
CTACAATACAAGATCAAACTAGTGATTCAAGAAATAAACGCGAGGAAGCTGTTGGTGC
ATCAATGCGCGAGAGTCAAGGAATTTCTAGTGCATCGAATCGTAGAGATTCTTATGAAG
ACGAAGATGATGAGGTTTCTGGAAATAGTAGAGATGATAGTAGAGAAAGACGACGTGA
TAGCAGTTCGGAACGTCGAAGATATGAATACGAACGAAAAAGTGCATATTATGATCGTG
AACGAGAATATGATGATGATTATTACTATGATCGTCGTCGTGGAGGAGAAAATGATCGA
ACATATAATACACGCGATGAATTCGATCGTCGAGAAATTCCTTATCGAGAAGATGATCGT
AAGCATCATAGCCGAGACGATCTTGATAGACATGCAAGAGAAGAAATAGATAGAAGAA
ACAGAGCTAAAGAAGATATAGATGATAGAGATATTAGAAGAAGACCAGACGACCGTAG
AAAAGATAGAGTTGATGATGGGATGCGACGTAGAGAAATTAGAGACTATGATTTACGAT
ATTCTAGAGATCGTGATTATCTTGATCGTGATAGAAGAAGAGACGATAGACGACCAAGA
CGATATGATGATTATGATATAAGAGATCCATATAGAAGAGAATATTATGATGATCCTTATA
GTAGAGGATCTAGACCATCCAGTAGATCCTCTTATAATGATAGAGATCGAGAATATTATA
TGCGAACAAGAGATCCTTATTATCCTTATAATGGATATGGTGTTCCTGGATATGATTATGG
TGTTCATTATGCCAATAATTATTATGCATATATTGAAAATTTACGACGTACAAATCCTGCT
CTCTATTCAGAATGGTATCATAAATATTACGCTAACCAACATCAGCAACAACACATTTCT
CGTGGTGTTGGTAATTATCCAGAAGACAGAGCAAGTGTTCATTCTGGGCGTAGTTCTTG
CGACGAAAG

>novel_circ_000452
GATGTTGGGTGGCGTGGGAGGCCGGCACATGTCTACGCGTCGGCGTGGCAGCAGCCC
GCTGGTGCGTGGAGGTGCGGGCCTCACAGGGTATGCCGGACCAGGGGCCTCTGGAAA
TTCGAACGATGTTGCTGCAATACCACCGGATATGCAGAGATACGCTGGAAGAAGGCGC
AGAGCGGTTACAACTAGTGATCACAAGAGCTGCGCCCTTGTGCAGACACGTATTAAAC
TCGGCGATATCATTCGTTTTTGAGCGAGCGATCGAAGAGGAAGAGGAAACGGGATAAG
AAAGAGAATACGGGAGAATTTATCGAGCCAAGGAGGATAAGATCGAGGAAGAGAAGG
GAAAGAAAGGATATCGAAGAGAGAGAGAAAAAGTGTTTCCCCGTCTTTTTTTTTTTTT
TTTTTTTTTTTTTTCCTCTCCTTCCTTCCTCCTCTTTCGTTCCCTTCCGTTGTTCCCCGT

TGTTCTCGCCGGGGAAACAGAGGAGGGCTACTACTGTGTTGTTGTTCGCCTACCGTGG
AGGCGAAGCCTTTCGTCGAGTGTTAGTGTGGTTTCTTAAAGTGGTGCCTCAGCACGAA
GAGGGAATCGCCAGCAGGGCAGAGGCTCGAACGCATGTAGGAACATGGCCGCAACCA
GCCCCTGCCTCCCTCTAGAGGCCGCCAACACTACGATGATCATTCGGCTGGCCGTGGT
GGTGCTGGGGATCGTTCGATCGGCGTGCACGCGGTGGAGAAAAACGGGACGTGTGGA
AAAAGGGTTGGCAGCGCAAGAGGCGGCACAAAGGGACCCTGGATACGCGAGTCAGT
ACAGGAGAAGGCCGAGGTTGGCCGGACTGAGTTTCGGTGACTGGACTAGCTTTGGAG
GGGAGGTACCTGGTCTGGTGGACATGGCGCCAGGCCGGGATCAGGGCGGAGGGGCGG
GTGGGGGTGGGGGAGGCGGCAAGGGCACCACGTCGTTGTTCATCCTTTCGGAGGATA
ACTGCATAAGGAAGCATACCCGCTTCATAATCGAGTGGCCGCCCTTCGAGTACGCCGTC
CTGCTCACCATCATCGCCAATTGCGTGGTCCTCGCCCTCGAGGAGCACCTGCCCAAGC
AGGACAAAACCATACTCGCGCAGAAGCTCGAGGCCACCGAAATCTATTTCCTCGGGAT
CTTCTGCGTCGAGGCGAGCCTCAAGATCCTCGCCCTTGGCTTCGTCCTCCACCGTGGC
TCCTATTTGCGCAACATCTGGAACATCATGGATTTCTTCGTCGTGGTGACCGG

>novel_circ_000453
GATGTTGGGTGGCGTGGGAGGCCGGCACATGTCTACGCGTCGGCGTGGCAGCAGCCC
GCTGGTGCGTGGAGGTGCGGGCCTCACAGGGTATGCCGGACCAGGGGCCTCTGGAAA
TTCGAACGATGTTGCTGCAATACCACCGGATATGCAGAGATACGCTGGAAGAAGGCGC
AGAGCGGTTACAACTAGTGATCACAAGAGCTGCGCCCTTGTGCAGACACGTATTAAAC
TCGGCGATATCATTCGTTTTTGAGCGAGCGATCGAAGAGGAAGAGGAAACGGGATAAG
AAAGAGAATACGGGAGAATTTATCGAGCCAAGGAGGATAAGATCGAGGAAGAGAAGG
GAAAGAAAGGATATCGAAGAGAGAGAGAGAAAAAGTGTTTCCCCGTCTTTTTTTTTTTT
TTTTTTTTTTTTTTTCCTCTCCTTCCTTCCTCCTCTTTCGTTCCCTTCCGTTGTTCCCCGT
TGTTCTCGCCGGGGAAACAGAGGAGGGCTACTACTGTGTTGTTGTTCGCCTACCGTGG
AGGCGAAGCCTTTCGTCGAGTGTTAGTGTGGTTTCTTAAAGTGGTGCCTCAGCACGAA
GAGGGAATCGCCAGCAGGGCAGAGGCTCGAACGCATGTAGGAACATGGCCGCAACCA
GCCCCTGCCTCCCTCTAGAGGCCGCCAACACTACGATGATCATTCGGCTGGCCGTGGT
GGTGCTGGGGATCGTTCGATCGGCGTGCACGCGGTGGAGAAAAACGGGACGTGTGGA
AAAAGGGTTGGCAGCGCAAGAGGCGGCACAAAGGGACCCTGGATACGCGAGTCAGT
ACAGGAGAAGGCCGAGGTTGGCCGGACTGAGTTTCGGTGACTGGACTAGCTTTGGAG
GGGAGGTACCTGGTCTGGTGGACATGGCGCCAGGCCGGGATCAGGGCGGAGGGGCGG
GTGGGGGTGGGGGAGGCGGCAAGGGCACCACGTCGTTGTTCATCCTTTCGGAGGATA
ACTGCATAAGGAAGCATACCCGCTTCATAATCGAGTGGCCGCCCTTCGAGTACGCCGTC
CTGCTCACCATCATCGCCAATTGCGTGGTCCTCGCCCTCGAGGAGCACCTGCCCAAGC
AGGACAAAACCATACTCGCGCAGAAGCTCGAGGCCACCGAAATCTATTTCCTCGGGAT
CTTCTGCGTCGAGGCGAGCCTCAAGATCCTCGCCCTTGGCTTCGTCCTCCACCGTGGC
TCCTATTTGCGCAACATCTGGAACATCATGGATTTCTTCGTCGTGGTGACCGGGTCGAT
GACCGTGTTCGCCGAAACAAACGTGGACGTCGACCTGCGTATGCTACGTTCCTTCAGG
GTCCTCAGACCGCTAAAGCTGGTTTCGAAAATTCCAAGTTCATCACGGCGTTCTCGCA
AGGCATAGAACTGGATATGGATCTGCGCACGTTGCGTGCGATACGCGTGTTGCGACCG
CTCAAACTTGTCTCCGGCATACCGAGTCTCCAGGTGGTGCTCAAGTCTATTATCAAGGC
AATGGCGCCGCTTCTGCAGATCGGCCTGCTGGTACTTTTCGCCATCGTGATATTCGCCAT
CATCGGCCTCGAATTCTATTCGGGGACCCTGCATAAAACGTGTTACAGCATTAGGGATA

TCAATGTAATCGTGAAGGAGGGCGAACAGGCGAGCCCGTGTAACACGGACAACAAAT
CCGAGGCGCCATTCGGGGCCCACGTGTGCGACGCGAACATCTCGACGTGCATGGATCA
CTGGGAGGGACCAAACTTCGGCATCACCAGCTTCGACAACATCGGATTCGCCATGCTC
ACTGTCTTCCAGTGCATCACTATGGAGGGCTGGACTGCCATATTGTACTGG

>novel_circ_000454
GATGTTGGGTGGCGTGGGAGGCCGGCACATGTCTACGCGTCGGCGTGGCAGCAGCCC
GCTGGTGCGTGGAGGTGCGGGCCTCACAGGGTATGCCGGACCAGGGGCCTCTGGAAA
TTCGAACGATGTTGCTGCAATACCACCGGATATGCAGAGATACGCTGGAAGAAGGCGC
AGAGCGGTTACAACTAGTGATCACAAGAGCTGCGCCCTTGTGCAGACACGTATTAAAC
TCGGCGATATCATTCGTTTTTGAGCGAGCGATCGAAGAGGAAGAGGAAACGGGATAAG
AAAGAGAATACGGGAGAATTTATCGAGCCAAGGAGGATAAGATCGAGGAAGAGAAGG
GAAAGAAAGGATATCGAAGAGAGAGAGAAAAAGTGTTTCCCCGTCTTTTTTTTTTTTT
TTTTTTTTTTTTTTTCCTCTCCTTCCTTCCTCCTCTTTCGTTCCCTTCCGTTGTTCCCCGT
TGTTCTCGCCGGGGAAACAGAGGAGGGCTACTACTGTGTTGTTGTTCGCCTACCGTGG
AGGCGAAGCCTTTCGTCGAGTGTTAGTGTGGTTTCTTAAAGTGGTGCCTCAGCACGAA
GAGGGAATCGCCAGCAGGGCAGAGGCTCGAACGCATGTAGGAACATGGCCGCAACCA
GCCCCTGCCTCCCTCTAGAGGCCGCCAACACTACGATGATCATTCGGCTGGCCGTGGT
GGTGCTGGGGATCGTTCGATCGGCGTGCACGCGGTGGAGAAAAACGGGACGTGTGGA
AAAAGGGTTGGCAGCGCAAGAGGCGGCACAAAGGGACCCTGGATACGCGAGTCAGT
ACAGGAGAAGGCCGAGGTTGGCCGGACTGAGTTTCGGTGACTGGACTAGCTTTGGAG
GGGAGGTACCTGGTCTGGTGGACATGGCGCCAGGCCGGGATCAGGGCGGAGGGGCGG
GTGGGGGTGGGGGAGGCGGCAAGGGCACCACGTCGTTGTTCATCCTTTCGGAGGATA
ACTGCATAAGGAAGCATACCCGCTTCATAATCGAGTGGCCGCCCTTCGAGTACGCCGTC
CTGCTCACCATCATCGCCAATTGCGTGGTCCTCGCCCTCGAGGAGCACCTGCCCAAGC
AGGACAAAACCATACTCGCGCAGAAGCTCGAGGCCACCGAAATCTATTTCCTCGGGAT
CTTCTGCGTCGAGGCGAGCCTCAAGATCCTCGCCCTTGGCTTCGTCCTCCACCGTGGC
TCCTATTTGCGCAACATCTGGAACATCATGGATTTCTTCGTCGTGGTGACCGGGTCGAT
GACCGTGTTCGCCGAAACAAACGTGGACGTCGACCTGCGTATGCTACGTTCCTTCAGG
GTCCTCAGACCGCTAAAGCTGGTTTCGAAAATTCCAAGTTCATCACGGCGTTCTCGCA
AGGCATAGAACTGGATATGGATCTGCGCACGTTGCGTGCGATACGCGTGTTGCGACCG
CTCAAACTTGTCTCCGGCATACCGAGTCTCCAGGTGGTGCTCAAGTCTATTATCAAGGC
AATGGCGCCGCTTCTGCAGATCGGCCTGCTGGTACTTTTCGCCATCGTGATATTCGCCAT
CATCGGCCTCGAATTCTATTCGGGGACCCTGCATAAAACGTGTTACAGCATTAGGGATA
TCAATGTAATCGTGAAGGAGGGCGAACAGGCGAGCCCGTGTAACACGGACAACAAAT
CCGAGGCGCCATTCGGGGCCCACGTGTGCGACGCGAACATCTCGACGTGCATGGATCA
CTGGGAGGGACCAAACTTCGGCATCACCAGCTTCGACAACATCGGATTCGCCATGCTC
ACTGTCTTCCAGTGCATCACTATGGAGGGCTGGACTGCCATATTGTACTGGACAAACGA
CGCTCTTGGCAGCACGTACAACTGGATTTACTTTATACCATTGATCGTCCTTGGCTCATT
CTTCATGCTGAACTTAGTTCTCGGTGTTCTGAGCGGGGAGTTTTCGAACGAAAGAACA
CGCGTCGAGAGGCGGGCGGCTTATCAGAAAGCAAAGAGAAAGCGTCTATTCACGACT
GCGTTCAGTGCCTACTTAAAGTGGATCACTCAAGCAGAGAGTTTGCGAAGGAGAGAG
AAAAGGTGGAGAACCGGCAGTCCTTCCTCAAATTGCGAAGACAGCAGCAGCTCGAGC
ATGAACTGTACTGTTACCTGAATTGGATCTGCAAAGCAGAGGAGGTAATACTCGCGGA

AGAAAGGACCACAGAAGAGGAGAAGAAGCACATATTAGAAGGAAGAAAAAGAGCCG
AGGCGAAGAAGAAGAAGCTCGGCAAGAGCAAAAGTACGGACACGGAGGAAGAGGA
AGGCGATGACGACCAGGACGACGGTGAGTTGCTTTTCGAGATCCTCCTCGACGAAAG
AGAAGGGACCTTGCAAGCAGTTCTGGCTGGCCGAGAAAAGATTCAGGTACTGGATAC
GAAAATCCGTGAAATCGCAAAAATTCTACTGGTTCGTCATCGTTCTCGTGTTCTTCAAC
ACCGTCTGCGTAGCTGTGGAGCATTACGGTCAGCCCCAATGGCTGACCGACTTCCTCTA
CTTCGCAGAGTTCGTGTTCCTGGCTCTGTTCATGTTGGAGATGTTCATCAAGGTGTACG
CGCTCGGCCCTCGCACATACTTCGACTCGAGCTTCAATCGTTTCGACTGCGTGGTCATC
TCGGGATCGATCTTCGAAGTGATCTGGTCCGAGGTGAAGTCCGGCTCTTTCGGCCTCTC
CGTCCTACGTGCCCTTAGACTCCTGCGAATTTTTAAGGTCACCAAATACTGGAAGAGCC
TGAGAAACCTCGTAATATCGCTGCTCAGCTCGATGCGTAGCATCATCTCCCTGCTCTTCC
TGCTCTTCCTCTTCATTCTCATATTCGCGCTGCTCGGTATGCAGCTGTTCGGCGGCCAAT
TCAACTTCGATTCAGGCACGCCGCCCACCAACTTCAACACCTTTCCCATCGCGCTGCTC
ACTGTCTTCCAAATCCTGACCGGAGAAGATTGGAACGAAGTGATGTACCAGGGTATAG
AATCGCAAGGTGGTCACAAGAAGGGCATGATCTACTCGCTCTACTTCATCGTGCTGGT
GCTGTTCGGCAACTACACACTGCTGAACGTGTTCTTGGCCATCGCCGTCGACAATCTG
GCAAACGCACAAGAGCTGAGCGCCGCCGAGAACGAGGAGGAAGAGGAGGACAAGC
AGAAGCAGGCGCAGGAGATCGAGAAAGAGATCCAATCCCTGCAGAATCCGAAAGACG
GTGGCGCGCCAAAGGTAGAAATCTGCCCTCCGAGTCCGAACCAGAATTTCAAAGACG
GTAAAGGTGGGAAACAGTCATCCGAAGAAGAGAAAAAGCAGGATGAAGACGATGAC
ACGGGACCAAAACCAATGCTGCCATACTCCTCCATGTTTATACTGTCCCCTACGAATCC
AGTAAGAAGAGCCGCCCATTGGGTCGTCAACCTGAGGTACTTCGACTTCTTCATAATG
GTGGTGATATCGCTGTCGAGTATCGCGTTGGCCGCCGAAGATCCCGTATGGGAGGACTC
GCCGAGGAACGAAGTTCTCAATTACTTCGATTACGCGTTCACAGGTGTCTTCACCGTC
GAGATGATACTGAAGATAATCGATCTCGGTATCATACTTCATCCGGGCTCGTATCTGCGC
GAGTTCTGGAACATTATGGACGCGGTTGTTGTGATATGCGCCGCTGTTTCGTTCGCCTT
CGACATGACGGGCAGCTCGGCCGGGCAGAACCTTTCCACGATCAAGTCGTTGAGGGT
GCTCCGCGTGTTGAGGCCGTTAAAGACGATCAAGAGGGTGCCGAAGTTGAAGGCGGT
TTTCGACTGCGTGGTGAACAGTCTTAAAAATGTCATCAACATTCTCATAGTGTACATATT
GTTCCAATTCATATTCGCTGTGATCGCGGTGCAGCTGTTCAACGGCAAGTTCTTCTACT
GCAGCGACGAGAGCAAATATACGCAACAGGATTGCCAGGGTCAATATTTCGTTTTCGA
GGACGGCGCCCTGTTGCCAGAGCCAAAGAAACGCGAATGGCAGTCGCAGTTTTTTCA
CTACGACAACGTGATGGCCGCCATGCTTACGCTGTTCGCGGTACAGACTGGCGAGGGT
TGGCCCCAAATCCTGCAGAATTCTATGGCGGCCACGTACGAGGACAAGGGCCCCATAC
AGAATTTTCGTATAGAGATGTCCATTTTCTACATCGTCTACTTCATCGTATTTCCATTCTT
CTTCGTCAACATCTTCGTGGCCTTGATTATCATCACTTTCCAGGAGCAGGGAGAGGCCG
AGTTGCAAGACGGCGAAATCGACAAAAATCAGAAATCTTGCATAGACTTCACGATACA
AGCTCGCCCTCTGGAAAGGTACATGCCGAAAGAGCGGAACAGCGTAAAGTACAAAAT
CTGGAGAATAGTGGTATCGACGCCTTTCGAATATTTTATCATGGGTCTCATCGTATTAAA
CACAGTACTGCTCATGATGAAG

>novel_circ_000457
TTGTGTTTTTCCGCATCATCTGGTATACGATATACGTCCGCGAGTGTTGTGTGAACAAAT
TCACTCTTGCGACGGAAACAAAAAAGAAGGAAAGAAACGGCGACGAATAATATTCTT

AGTGCTGTAGTCTACAGTTGATGGATGAAACTAGCAGAATGCTGCAACACGGAGGATC
AGGTGTGGGTCTGATGGGAGGAAATCAGGGTCAGGGAGCCCAAAATACTGCCGGATAT
GGTAGTATGGGACCTGTGGATGGAACTGCAACACAAGGTCCAGATCAGGCTGTTGAGG
CTAGAAAACAGGATATCAGTGAAATTTTGCAACAAATTATGAACATCACCGATCAAAGC
TTGGACGAGGCACAAGCGAGAAAACATACTTTAAACTGTCATAGAATGAAGCCAGCCC
TGTTTTCAGTTTTGTGTGAAATTAAAGAGAAAACAGTGCTATCTTTAAGAAATACTCAG
GAAGAGGAACCGCCGGATCCCCAATTAATGAGGTTGGATAACATGTTGATCGCGGAGG
GTGTTGCTGGACCGGAAAAGGGTGGTGGTGCTGGAGCGGCGGCCTCTGCAGCGGCTG
CAGCCGCTGCTGGAGGACCACCTGGACAACCTGACAATGCGATCGAACATTCGGATTA
CAGAGCCAAGCTTGCCCAAATTAGACAGATTTATCATCAGGAGTTGGAGAAATACGAG
CAGGCGTGTAACGAGTTCACAACTCACGTAATGAATTTATTGAGGGAACAAAGTCGTA
CCAGGCCAATCACGCCAAAAGAAATCGAGAGGATGGTTCAGATTATTCATAAGAAGTT
CTCCAGCATTCAAATGCAACTGAAACAGTCTACCTGTGAGGCTGTGATGATTTTGAGG
AGTCGATTCCTCGATGCAAGGCGTAAAAGACGAAACTTTAGCAAACAAGCCTCTGAAA
TTCTCAACGAGTATTTCTATTCACATCTGAGTAATCCATATCCAAGCGAAGAAGCTAAA
GAAGAACTCGCACGAAAATGTGGAATCACCGTGAGTCAGGTTTCTAATTGGTTCGGCA
ACAAGAGGATACGCTACAAGAAAAATATAGGTAAAGCACAGGAGGAGGCGAACTTGT
ACGCAGCCAAAAAGGCAGCTGATTCTTGAGAAAAGAATGAAGAAAGAAAAATTCCAT
GGATATACCCACTGAAAATTCTTCGCATTCGCACAACTTGCAATTACGCGTACTTTTCTT
AACTACACCGACGTCTCTTTATTTTAGAGGGCAAGGCGAGATATTTTTAACTTACTAATT
ATACTGAGAAAGAGGGAGCGAAAAATGAGAAGACGAGGATGAGGATACATGGGAAG
GGCGATGAGATTCTCGCGTTTCTTTCGACACCTTCCCTGTGTTTGTCTTTCCTTAGCAAT
GCTCTTTCTGGTAATAACGCAGTCTTCTCGTAATTAAAGAAACGGAATTCCAACGATAG
TGGCGCTAACACTTTGGCGAATTCCTCGATATTTTGTTTCAAATAGCGAGAATGTTTTCA
CGTTTCAAAGTAAAAATTGCTCCTATTATGCTGGCACATAACTAAGCTAATTTCCACTGA
TATTGTTTCGTGTACTTTGAATAATTTGAAAGTTGATTAAAAATTCGCTTTCAACCGATA
ATTCGTTCATTTTCTGCGGTACAGATTGAAGATGATCCAATGATACATTTGTCGATTTGA
TTGATCAGTGGCGTCTGTAATCAAAAATTTTTACACTTTTATACATTATTAACTCTTTCTG
GAAAAATTTTTCTTTTGATCATGTTGCAATTAATGACGATATTGCACAGCTTTTGCAGGA
GCGTCGCCGTACAGTATGGGCGGTGCCAGCCAAGGCACCCCGACGCCGATGATGTCGC
CGGCGCCACCTGGTGGCCCGCAAGATATGGCCGGATACGGGATGGGCATAAACGGTAG
CGATTATGGCTCGCAACCGTACAACGACGGCTCGATGGGCTACGACCCTATGCACCAG

>novel_circ_000460
TCTCGACGACGAGGATGGCGTGCACGAATAATTGCCGTTTCTGAGCATCGAATAAAGG
ATCGAAGGAGGTGACGCCCGTGTGAGAAAGCGAGACAGAGTGAGCAGGGATGTCGCT
GAAGCTTCGAACAAGATCCGCCCAGCCGGCTTCAAGTGTTCAGCTTCGTCGCTCGAAA
TTGACCAACCTCTAAAACGAATCTAAACGAATAAATATACATATATATATACATAATATATA
TATATATATATGTATATATATATATATTATATATATATATAAAAGAAAATCGTTTGACGATTCG
ATAGAAGAGAAAGGAGGAAACGGGAGAAAAAGAGAGGAGAGGATCGTGGAACTGCC
TTTTTTTTTTTTTTATTTTTCGTTAGCAGAATAGAATAAAAGATATTTTGGTAAAAAGAGT
AATTAGTGAAAATAATAGCAGAGGCGCGAGACATAGTTGCTGCTCCCCGCGAAAGAGC
CAGAGTTAAATGGTCGCATTTAGTGCACAATTTCCGCTTTGCTCGCGTGTCGCGCCCCC
GTGTACCGTGCACCACGTTCTCCGCAAGAAATCGCCGAGGATTTTCAATGAGATGGCC

TTCATGATGAAGAAGAAGAAGTACAAATTCTCGGTGGAGGTGGACCTCGAGGAGCTC
ACCGCGGTGCCATTCGTGAACGCAGTGCTGTTCGCCAAGCTCAGACTCCTCGACGGTG
GATCCTTCGTCGATCATTCGACCAG

>novel_circ_000461
AAATCCTTCCAGTGGTTCGGGTCTCAGCGAAACAGGATCTCTGGATCGAGCAAAAGCC
GCGCTGGAGCGCAGAAAAAAGGCCGAGGATGGAACCGGAAATTCGATTTTGTGCAGA
GTTGAAGTGACGAGGCCAAATCCGGATTCCCTGATCGATGAGCTAATCAAAGCAACGA
ATTTGGAGCAAACTGATCTCGAGAGTTCTGAGACTACTGGACTTCAGCTGTTCATTGCG
AAAGACGGGACGACCGCGCTCGGCAGCCACGAGATCAAAAGTCAGATGCCCGCGGGT
GTGTTCAAGCAGGTGGTAATGGAAGAGAATAATAGGTAACACGAAGCTATCGGGACGA
GAAGACAGTACGCAAGACAAGTCAGCGCCACGTTCTCGTTGGCCCTGGTGCCAGAGC
TCGTCTACGAGGCCTACGAGCCCCGCTAGTTCACGAGATCAACGATGCTGGACGAGGG
AATCGTCGAGAAAAGACG

>novel_circ_000462
CTACTGGACTTCAGCTGTTCATTGCGAAAGACGGGACGACCGCGCTCGGCAGCCACG
AGATCAAAAGTCAGATGCCCGCGGGTGTGTTCAAGCAGGTGGTAATGGAAGAGAATAA
TAGGTAACACGAAGCTATCGGGACGAGAAGACAGTACGCAAGACAAGTCAGCGCCAC
GTTCTCGTTGGCCCTGGTGCCAGAGCTCGTCTACGAGGCCTACGAGCCCCGCTAGTTC
ACGAGATCAACGATGCTGGACGAGGGAATCGTCGAGAAAAGACGGTGAACACGGCG
ACGAAACGCGAACGCCATCTTTCATCTCTCAAAAAAGGAAAAAAAAAAAAAAAAA
GAAAAAAAAAGAAAAAAAAAATCCTCCCTAATCTAAAATTATCCTTTTTCGGTTGCCTA
TAACTGTCTTTCCGGCGTTCGGTGCTTCACGCTTCTCGTACAATGGATCGTCGATTTAA
ACGCTCAAAAACGCTGCTAAATGTTTCTTTTATATATATACATATATATATATATATATCAAA
TTATGGGAAGCAGTCTTCGTTGAATCGTGACACGCATATATTTTAAAGAGGGGGAACCG
TCGATTTCTAGGATGATTCTGTGGCAACGAGATGCAACATACGCACCGAAACGCTTCGT
TAAGAAAGAAGAAGAAATTTGTACAGAGCGCTTCGAAAGAGAG

>novel_circ_000463
AAATCTGTATCGAATTCTAATAGCAGAATAAAAGAGTTCATCTCGGGAGGAGACAGAG
GAAGAAGACTAGAAGATCAAACAAATTTCGTCTTATCTCTCTCTTGTTCCTGTCAGTAT
TGTCTTTGCCAAAGACCGCATCGAATCTGATTTTTTCCATTATTTTTCTTCCATCTTCAA
GAATTATATTATTTTGTTTTGATGTAATTGCTCGCTAATCCAACGATATTCGTCGCTGAAG
AAACTGGAACAGCCGTTGTCCATAAGTAGTGATCAGACCTTGCCAACTCGTTCTTGTAA
TCGAAACATGTGTCAGTCACGCTCTTAAGCCACGTATTGTCCAAATATCATATCTTTAGC
TTGTGCGGTGGAAAGTGGCCGCAGAAGGGTTGAAAAGAATAGAAATTTCTCTATAAA
TCGATCATACTATGGAGAAAAAATCTTGGTCGATTAATGAATCGAATTCGATAAAGTTGA
TCTGAATATTATTTCTTGATTAAAGAGTTCGCTCTTGAGGATAATAACGAGTTCAACGAG
TGAACGAATGTGCAAAGCAATAGAGTGGGAGAATGAACGATTGGCAGCTTTGGACATG
GGATAGATGATAGATCGCAAATAATGGGGTGGTAAAGTGAACAAGAAGTGCAAAATGG
ATTGGGTGCCTCGGCTCAGGTAGCCAGAGACGACAAAGTCGCGGGCACCAGGGCGAA
CGAGCCCCGAGCTGCTAGCAAAGCTTTTTCTTTTCTCTGTCTATCTTTTGTTCAAGAAA
AAAAAAGATATCGCATGGACGTCATTGATGGGCGTATTTTTCTGAAAGACTGTTTTTTAT

TTTTTGTCATTTACCGATCAGTGTTATGGAAAAAAAAAAAAAAAGACTGAACGTGATAAT
CAATAAATCAACCAAGGAATTAATTCGCAAGCTTTATCATTGTCGGTGAAACGTATTGG
AGGGAAGATTGAACTGATTAATCGATGCAACAGATCCCGGATTTATTCTAAGAAGGAG
AAGAAACTTGATACGAATTTACACGTGATCTTCGATCCAACAGAACGCTCGCGTCTGA
CTCACAAAAAGACTGAAGAACATATGTTGATTCATTTGTCGAAAAATATTTGCATATCG
CGTTCTGTAGAATATATACTGGCTTTCATTTGTTGCGTCACACGTACATATCTTTCGGGAT
CTCATGTAATTGTTATACATACACACGTGCATGTACACATACAGCTTGTCATACCAGCTAT
ACGAGCTTTTGAGAAATTTCTGACGAACATATACACAAAACATTATACGCGTGTCGTAA
CACGCTTTTTCGACTATTATATATACTGCAAATATATTACGTACATGTACATGCGTATAGA
AATAATATATATATACATATATATACATATATAAAATACAAAAAATATATATATATGTATATGT
ATCTATGTACATGTAAAAAATCTCTTTCGCTGCCTAATAGAAGTATTCGTGGATAAAGTG
GCGCATATAATACGGTAAAGGATTAACTTCCCTATTGATATATTTGTCCTTTTTTCTATGA
AAAGAATAATTTCTTTGTGCCAGAAGATATTTTTTAATTTCAAAGAAGGTATGTTTCTTC
ATTTTTTGTTTTTCTAACGAAACACGTATTGAGAATAACGCGTGCAACGAAAAACTAAT
AAAGAAAAGTACTCAGAGTAGTGAGAAGTGTAGTAGTTGAAGTGTGATCGTTGCACG
GGCTCGCAACAGATCAGGATGGGTTGTGGACAGAGCAAAATCGGCAATATCTATCCAA
AAAACAAGAAGAACAAAAACAACACTGGCAAGAAAAACGGAGATTCTATCG

>novel_circ_000464
AAATATAGTGTGTGGATACTGTGTTTGTATAGATGTACAGCCGCGTGCAATCATACACAC
GTATGCACGTGTTGTGTACAAATGTGTGTTTGTATATATGTGTACCTCTCCCTGAGCTCG
CTCTTTAAAACTTTTTAACATCTTTATCATTTCCGCACTATCATATTTTTTT

>novel_circ_000465
TGGACACGAGGGAAGTTCAGCCACACGATGCGAGCGAGAAGGAACAACTGGGCCTG
GCCAGCGTTAAAGCGGAACCAGGATCGACCGGTACGACGACGACCAGCACGGGGACA
CGTCTGCTGCACGGTATTCTCTCGCAGCATCCCCAGCAGCACGGTCTAGGGGTGCAAA
ATGGATACGGCCGCCACTTGCCCGGCCACGCTCAAATGGGCCAACCGTCCTACACCAC
TGCCACCATGGCCACCACAAGCACGCCAGGAAGCGGATCACTGCCAGCGAGCCCCGC
GGACAGCGGAGTCAGCGACGTGGAGTCCAGCACGTCCTCGGGCGGTAACGAGGACGC
GAATCTCTTATTGAAAGCAAGGCTCAATCCCAATTCGTCCCTTCAGCCAAGCCTGGCGT
CTCATCATTCTCATTTGTCGTCAGCAGCGCTCGGCAGGTCGGCCTGTCACAGTCCTGGT
GTTTATCCGTCGACGGCGGGCTTCCTGCCGCCCTCCTATCATCCTCATCAGCATCATCCC
TCCCAGTACCATCCGCATAGAGGGAGCTCGCCTCACCATCAACACGGGAACCACACGA
TGGGTCCCACCATGGGACCGCCCCATCATCATCACCATCATCAAACGCAGAGTCTACAA
CACTTGCATTATCGTCAGCCTCCTACAT

>novel_circ_000466
TGGACACGAGGGAAGTTCAGCCACACGATGCGAGCGAGAAGGAACAACTGGGCCTG
GCCAGCGTTAAAGCGGAACCAGGATCGACCGGTACGACGACGACCAGCACGGGGACA
CGTCTGCTGCACGGTATTCTCTCGCAGCATCCCCAGCAGCACGGTCTAGGGGTGCAAA
ATGGATACGGCCGCCACTTGCCCGGCCACGCTCAAATGGGCCAACCGTCCTACACCAC
TGCCACCATGGCCACCACAAGCACGCCAGGAAGCGGATCACTGCCAGCGAGCCCCGC
GGACAGCGGAGTCAGCGACGTGGAGTCCAGCACGTCCTCGGGCGGTAACGAGGACGC

GAATCTCTTATTGAAAGCAAGGCTCAATCCCAATTCGTCCCTTCAGCCAAGCCTGGCGT
CTCATCATTCTCATTTGTCGTCAG

>novel_circ_000467
GGGAATCAAAGTAGCGAGTAAATCGAAAGAAAGAAATCTGAGGAGCAGAAAAAGAA
GGAGGCGAAAAGTGGGGACGTTTCAAAGGTGGTTTGGATGTTAGAAAGCGCGGGGG
TGGCTAGAGAGGCCCCGCCGCAGGGTACGGGTCCCGATGCCGGTCCCCGTATCGCAAA
CCCTTACGCTGACCTCCACCCTCGTGCCCAAGGAGGAGTCAAACATGCCCTTCATAGA
CGACGAGCTTCTTTGGTGCCCTGACAATGACGGCAAAATGGTCGACTTAACGCAATGT
CTCCAGGGTTTATCGTTTTATCAGGAAAGCAGTACAGGGCAGTCGGTAGAATTTTCCCC
GATGGAATTGAATGCCCTTGTAGGGACGCCGGCTGCTCCAAATATGCCGGCAGAAGAG
GGCGAAGGAATGGCTGGTGTCACAGGGGAAGAGCCCTTTGACACGTTGGACACATTC
CTTCGAGAATTGCAAGCTGATTTGGCTGAAGCCAGTCAACCCACGTCCACTACAACTT
CCGTAACTCCTTCCTGTAGACGGCAGAGGTACAATATCGCAGCCGCAAATCCTTTGTTA
GCAGAGAAATTGGCAGCTCCTTCGTCGCAAGCATCTCCAACTTCTATTCCGTACGCAAC
AAGGGCGGAAATTAAAACGGAGAGTATTCAGCCTGAAACAACGAAAG

>novel_circ_000469
AGTGGAAAGCTCAGCGTTGCAGTTGGTGTACCCGCTGGATTCTCGTCCGGTGCAGCTA
ATCTTCCGGGGGTTGCAGGTGGTCAAGGAGAAACGATCCCTCCTCCGGGATGTCTCGG
GCGTTGTTAAACCCGGCGAACTTTTGGCCGTCATGGGCCCCTCAGGTTGCGGCAAAAC
GACGTTATTGAACTGCCTGTCAGGCCGCGTGGGCTTGGACGGGGGTGAAATCTGGCTG
AATCGTGAAAGGTTGACAAAAAGATGGCGCAGAAGAATCTGTTACGTGCAACAGCAG
GACGTTTTCTTTCCCGATCTCACGTTACGACAGACACTCGAG

>novel_circ_000471
GACGTGGCGGCACGAGGATTGGACTTGCCAAAAGTGGACTGCGTGATTCAATACACG
GGGCCGACGTCGGCCAGGGATTACGTGCATCGAATCGGTAGAACAGCGCGAGCTGGC
TCTTCCGGTTCAGCGACTATCCTTTTAACACCGCCAGAAATTGAATTCGTACGAATGCT
CGAGTCTAGGCGTATCAGAATCAGGCAGGAGAGCATGGACGACGTTCTGGATAGGCTG
ATGGGCCCGTTGTCCAAGCATTCATCCGCTCATAACGCGGCTGTCGCGTTGCAGAACGA
TTTCGAGAATTTGATACTCGAGGACACGAAGCTACGCGAGAGAGCGTGCAAAGCGTAC
ACGTCCTGGATACGTTTCTACTCGAGCTACCCTCGCGACATGCGGCAGATTTTCAATCG
GAGGGACCTGCACTTGGGCCACTACGCGAAAAGCTTCGCGCTCAGGGAGACCCCCCA
ACGAATCGGCGGGATAGGGAGGAGACTGCACGAGAAGGAATGCACGAGGAAGCGGC
AGTTGAACGACAACAGGTTGACGGGAAGCGGGAA

>novel_circ_000473
TGTCGATAGTATTTCCATGAATTCTACGATTACTATCGAAGATACCGACGAAGAGGTGAT
TGATTTGGACTCGAGCCAAGATGCCATCAATGTTACTGACAATGATCAAAGTGAATATT
CGATTGAAAAAAATTCATTATCGAACAACAATGCGAAGAAGACGAATATAATTAGTGAT
TATGTGGATAATAATAAGAATACATCTATGAGAGTAACATGTGATAGCGCAAGTACATCA
AAACCACTTTTGAAGGTTATATTTCGAGATGAAAGTATTTCACGGCAATATAATAAAAA
AATTAAAGAATTTCTAGAAGAATTAGAATCTTGCAAAATTAGATGTCAAGAAAATGATA

ATGGTTTAACATTAGAAGTTTGGGATAATGACACTGATTCTATAAGTCAGTCTATTGAAA
TTACTACAGAAAGTGAAGATAGTCAAGATATAATATGTGATACATTATTTACTGTAGATA
AACAACCAAAACTTAAAGATGATTTTGATGTTCCAACTTATGGAAAG

>novel_circ_000474
ACGCATCAATGACGAGTCTGTCGTCCACGGTGTCCCAGAGGGATCTCAGAATAGGTGA
CAGGGTAATAGTATCATCCAGCCAAGGCAGCAAAACCGGTGTCCTCAGGTACTACGGT
ACAACAGAGTTTGCAGTCGGTGAATGGTGTGGAGTGGAATTGGATGATCCTATTGGAA
AAAACGATGGTTCCGTCAACGATAAAAGGTACTTCGAGTGTCGACCCAAATACGGTCT
GTTTGCACCCGCGCACAAGGTCAGTCGGTCACCAACCAGCAAAAGGTCATCATGTATG
GTGCACAGGCCGACAGGCGCCGCCTTAAACACGTCCCTAAGGAAGACCGGTTCCAGA
GAGTCATTGGTCTCAATCTCAAGCATAGTCAGCACCACCAGTACAGCTACTAGAACTG
GTGTTAGTGCCGCGAGG

>novel_circ_000475
ATGTCGGAGTTCCCAGTGGAGGGAGGAGAAAACGGTGACATGCACACTGTCATGGAG
AATTTTCAAAAGAAGAACATGGTGACCGGAAGGAGCCATGTTACTCATCATCCACAGG
TCACTTCGTCATCACAGATGCTGACGACGAACCAGAGCCAGAGCAGCAGCAGTTCGA
GCAGGGTGGCCACCAAATCCTCGCAGAGGATTCTTACCTCGTCCTCGTCGAGTGAGAT
GAAAGCAAGCTCAATGAAGAGCGACCTAAGAGAACTTCAACGCGGCATATCCGAGAT
GAAGAACAATATTTCGACGAATTTTTCACAACGACTGCGCAGCAGCATGGAAAACCTC
GTGGACAGGGACACGAATGGATCGGAGGAAGCCGATCTGACGGAGCCCTTGGTCACG
TTTCCGGATCCGGATACGCCGCCACCAGCCACAGGGACAGGAGGATCACCCTCCCAAT
TGACCTCCCTGAACTCGTTGAACAATCTTCACACCATGAGCCCACAGATCAACATGTC
GAACATTTCGAACATGACGAACCTTCCTGCTGGACAGGAAACGATGAAGTTCGAGCA
GAAGAAGATGACAAGCGCGTCCAAGACCAAGGTCGTCACGGACGGATTCAGCGCCGA
GAAAGCGACGGCGAACAGCGCCGAGATGAGAGCTTTACAGACTGGCGACGTCTCGTA
CAAGGAGCAAAGCGCCGCGACGGCGGCCAGGGCCCGCGTCGAACTCGATGGCGTCTC
CGCCGAGAAAAGCGTGGCCGCTGCAAGG

>novel_circ_000476
ATGTCGGAGTTCCCAGTGGAGGGAGGAGAAAACGGTGACATGCACACTGTCATGGAG
AATTTTCAAAAGAAGAACATGGTGACCGGAAGGAGCCATGTTACTCATCATCCACAGG
TCACTTCGTCATCACAGATGCTGACGACGAACCAGAGCCAGAGCAGCAGCAGTTCGA
GCAGGGTGGCCACCAAATCCTCGCAGAGGATTCTTACCTCGTCCTCGTCGAGTGAGAT
GAAAGCAAGCTCAATGAAGAGCGACCTAAGAGAACTTCAACGCGGCATATCCGAGAT
GAAGAACAATATTTCGACGAATTTTTCACAACGACTGCGCAGCAGCATGGAAAACCTC
GTGGACAGGGACACGAATGGATCGGAGGAAGCCGATCTGACGGAGCCCTTGGTCACG
TTTCCGGATCCGGATACGCCGCCACCAGCCACAGGGACAGGAGGATCACCCTCCCAAT
TGACCTCCCTGAACTCGTTGAACAATCTTCACACCATGAGCCCACAGATCAACATGTC
GAACATTTCGAACATGACGAACCTTCCTGCTGGACAGGAAACGATGAAGTTCGAGCA
GAAGAAGATGACAAGCGCGTCCAAGACCAAG

>novel_circ_000477

ATCTGCGACTTCGGTTTGGCCCGAGTGGAAGAACCGGATCAGAACAAGCACATGACC
CAGGAAGTGGTGACGCAGTATTATCGCGCGCCGGAGATCCTAATGGGGGCGCGACACT
ACACGGCCGCAGTGGACGTTTGGAGCGTCGGTTGCATATTCGGCGAACTTCTTCGTCG
AAGGATCCTTTTCCAGGCACAGAGCCCTGTGCAACAGCTGGAACTGATCACAGAATTG
CTGGGCACGCCAACCCTCGAGGACATGAGGTTCGCGTGCGAGGGTGCGAGATCCCAC
ATGCTGAGGCGCGCGCCGAAGCCACCCTCGTTGACGGCACTCTACACCCTTAGCAGCC
AGGCGACGCACGAGGCCGTCCATTTGCTCTGCCAGATGCTAGTCTTCGATCCTGACAA
GAGGATCACGGTGGTGGATGCACTGGCGCATCCCTATCTGGACGAAGGGAGACTGAGA
TATCACTCGTGCATGTGCACGTGCTGTTACACGACCAGCGGCGGTTTGAGACAGTACA
CGGGTGATTTCGAGCCGGCCACGTCGCATCCGTTCGACGATCTTTGGGAACGGAAGCT
CACCACTGTGCAGCAGGTGAAAGAGGAAATGCACAAATTTATAGCGGAACAGCTGAA
CACGTCGCGCGTGCCGCTCTGCATAAATCCGCAGTCCGCGGCGTTCAAGAGCTTTGCA
AG

>novel_circ_000478
ATCTGCGACTTCGGTTTGGCCCGAGTGGAAGAACCGGATCAGAACAAGCACATGACC
CAGGAAGTGGTGACGCAGTATTATCGCGCGCCGGAGATCCTAATGGGGGCGCGACACT
ACACGGCCGCAGTGGACGTTTGGAGCGTCGGTTGCATATTCGGCGAACTTCTTCGTCG
AAGGATCCTTTTCCAGGCACAGAGCCCTGTGCAACAGCTGGAACTGATCACAGAATTG
CTGGGCACGCCAACCCTCGAGGACATGAGGTTCGCGTGCGAGGGTGCGAGATCCCAC
ATGCTGAGGCGCGCGCCGAAGCCACCCTCGTTGACGGCACTCTACACCCTTAGCAGCC
AGGCGACGCACGAGGCCGTCCATTTGCTCTGCCAGATGCTAGTCTTCGATCCTGACAA
GAGGATCACGGTGGTGGATGCACTGGCGCATCCCTATCTGGACGAAGGGAGACTGAGA
TATCACTCGTGCATGTGCACGTGCTGTTACACGACCAGCGGCGGTTTGAGACAGTACA
CGGGTGATTTCGAGCCGGCCACGTCGCATCCGTTCGACGATCTTTGGGAACGGAAGCT
CACCACTGTGCAGCAGGTGAAAG

>novel_circ_000479
GCCTCAAGTACCTTCACTCAGCCAGAATCTTGCACAGGGATATTAAGCCGGGCAATTTA
CTCGTGAACAGCAATTGCGTGCTGAAGATCTGCGACTTCGGTTTGGCCCGAGTGGAAG
AACCGGATCAGAACAAGCACATGACCCAGGAAGTGGTGACGCAGTATTATCGCGCGCC
GGAGATCCTAATGGGGGCGCGACACTACACGGCCGCAGTGGACGTTTGGAGCGTCGG
TTGCATATTCGGCGAACTTCTTCGTCGAAGGATCCTTTTCCAGGCACAGAGCCCTGTGC
AACAGCTGGAACTGATCACAGAATTGCTGGGCACGCCAACCCTCGAGGACATGAGGT
TCGCGTGCGAGGGTGCGAGATCCCACATGCTGAGGCGCGCGCCGAAGCCACCCTCGT
TGACGGCACTCTACACCCTTAGCAGCCAGGCGACGCACGAGGCCGTCCATTTGCTCTG
CCAGATGCTAGTCTTCGATCCT

>novel_circ_000481
CCAGAGACAGTTCCTCAAGCGAGGATCGATATGACCCGTGTGTTAGAGGTTGCTGCTG
CCGAGGATATAACCGGACATCCGTACAGCCTGGCGATCACCTCGCCGGAAGGAGTTAC
CTTCGTGAAGGGCACGTGCCGCGAGGAAACCAGATGGTGGGCCGATGTGCTTCAAGT
CTACTCGAGGAACAAGGGCCGGCATAAGAGGAATGCGACGTTCCCCGGTGGACAGAC
CACCATTCTTCAGGTCACTCCTACGATCAGAAGTAACACGCCGAATCCACCTCGACCA

CGGTTCAACAGCTGCCGTTCAGAGCCCCGCAGCAACACATGGATCTCGGAAACGAGC
GTCCCCGCAGACCTTTGCGCGTCTGTCTTCTCCTCCACGCCGTCTCTGGTGACCAACA
GCGTGGTAACGACGACCAGCAACACGTCGATGAGCAACGGGAACGCGGTGGAGAAC
AGCAACGACCATCGTGTCACCGCCAACGTGTCACCCCTGAGAACCAGCACGCCACTT
GAGAACGGTGGCTCCACCTACGTAACTTCCGTTCCATCCACCTCGACCATGAACGGGA
GCGTGTCCAGCACCGTGTACACGACCGTGACCACGTCGACCACGACCACCGTGGTGT
CGTCCGTCTCCAACTCGTTGACGGAGAAGCCACCGATCGTGCCCCCCAACGAGGGTAG
ATCGAGCTACAAGGATCAACCGGCTAGCAGCGCCTCCCCCCCGACCAGAGACAAGCTT
CGAGCGGAAGACAAGGCCAGACGCAGGATGAATCAGCACGGAGAACGAACCGGGAC
CGCCTGCTCTGGCGAGAAACTAG

>novel_circ_000482
GGCCGGCATAAGAGGAATGCGACGTTCCCCGGTGGACAGACCACCATTCTTCAGGTCA
CTCCTACGATCAGAAGTAACACGCCGAATCCACCTCGACCACGGTTCAACAGCTGCCG
TTCAGAGCCCCGCAGCAACACATGGATCTCGGAAACGAGCGTCCCCGCAGACCTTTGC
GCGTCTGTCTTCTCCTCCACGCCGTCTCTGGTGACCAACAGCGTGGTAACGACGACCA
GCAACACGTCGATGAGCAACGGGAACGCGGTGGAGAACAGCAACGACCATCGTGTCA
CCGCCAACGTGTCACCCCTGAGAACCAGCACGCCACTTGAGAACGGTGGCTCCACCT
ACGTAACTTCCGTTCCATCCACCTCGACCATGAACGGGAGCGTGTCCAGCACCGTGTA
CACGACCGTGACCACGTCGACCACGACCACCGTGGTGTCGTCCGTCTCCAACTCGTTG
ACGGAGAAGCCACCGATCGTGCCCCCCAACGAGGGTAGATCGAGCTACAAGGATCAA
CCGGCTAGCAGCGCCTCCCCCCCGACCAGAGACAAGCTTCGAGCGGAAGACAAGGCC
AGACGCAGGATGAATCAGCACGGAGAACGAACCGGGACCGCCTGCTCTGGCGAGAA
ACTAG

>novel_circ_000484
TCGGAGGTGTTTCAGGCAGCAGTGGCGGCCGCGGCAGCAGCGGCCGCGGGAGGCGG
TGGCCAGCAACCCGCCCCCACACCCGCCGGCAACGAGAGCAACCCCGAGGGCGCCGT
CGAGGGCAGCGCCCTGGTACCTGTCACTTCAGGTGCAACCACACCTGCCACCGCAAC
ACAGGGTACAGACCTGAAAGGTCAACCGAAACGCCTTCACGTCAGCAATATACCTTTC
CGCTTCCGAGACCCTGATCTCAGGGCAATGTTTGGGCAATTCGGGCCGATCCTCGACG
TGGAAATCATATTTAACGAAAGAGGAAGCAAG

>novel_circ_000485AGAGTCGTTCTACGCGCGACACAGCGACAGACGAGAGTCGAGGCG
CGTGCGGGAAACACGTAGTAGAGATCGGATAGTACGGTGTAGAGAACGAGCGAGAAA
TAGAAAAATATATATATATGTGTGGAAGGCTAGTAAAACGAGATACCTATATAACCGATG
GTTAACGGACGGAATATTGAGAGAGCGAGCAAACGAGATAAGAAAAAAAAAAAAG
AAAAAGAAACGAGAAAGAAAGAGAGTATGAGAGAAAATTGTTGAAAGAATTTTGAG
GGCAAGTAACGGCGGGCGAGTGAAGGGACACTGAAGATGCCGGTCGAAAAAACGAT
CCCAGAAGTGTCCGAAAAGGTAAGGAAACCAGAAAACCATAAGCATCGAGAAAAAG
CGAAATAAATAGTCGAGAAAACTCTTGACGAGAGCGACGAGAGAGAGATGTCGTCTC
GCAATGCCCGCTCTCTCACCCCGATCGAAATACACACGGGAGTCGCACGAACGAATTT
TTTTCATCGTACGCGTACAAATTATATCGGCGATTAAACCACGTTATGTTCGTTTATTCGT
TTATTATTCGGCGGATTATATTGGCGTGGAATGGGAGGGGGAATTTTTTTCTCTTTCTTTT

TTTTTTTTTTTTTTTTTCGGGCGGATTTCGTGCCCGAGCTTCTCGCGATTTTTGCGAACG
ATTATGCGCTATGGAGTGTGTTTATCGTAATGGCCGCCAGATGGCGACCGATGGCCACT
CGCCTCGGATAATCTGATTAAAAGTTTTTTGGGAAAAAAGTTAGTTAGCGGGGAATTGG
CAACACGTTGCAGCAATAAAAAGTGGCAAAAAGCTTTTAGCTTCGTTAAAAGATCAAA
CCTTGCTCCCTGTAATTGCATAAAAATCAATGTTAAAATCAATGGGTAAACTGTGCCCG
AGGTAGCCATATTGGAACAAAACGTGGAATAGAACGTAGAGCGGGTAATATGATCAAA
TTTAAAATTGAGAAAGGGAATAAAACGAAATTAAATTTCTGTATCCTTCAACGAACGA
GTTGTAAACTGTCTAATATTATATTAACTGTCGATTCGTTGGTCGATCGTTGCTGAAGAG
GGCAGCACGATGGATTAAATTTCCAATTGTTCGTTTTTACTTCGATCGGATATCAGATTG
TTACGAATGTTTCTCGATGCGTTCTCTCGTTTTTTTTTTTTTTTTTTTTTCTTTTTTCACC
AGCGAATCACGTGGAAAATAGATCCGCGAAAATAAAAGGGATTACGTTCGAACGGAG
AAATTCGTCATTAAATTTACCTGTCAGGAAGCAAGCCAGAATCTAGCGTAACGAAAAA
CTCGGAACGACTATTTAATCTCCATTTTCCTCTCTTCCATTCGATTCTGCTCTTTTTCTAT
CTTCTCTCTCTCTCAAAAAAAAAAAAAAAAAAAAAAAAGAAAAAAGAAACGAACCGTT
CGAACGTCGTCTGATAAATCGTCCGACAGCCAAAAAATTTGGCAACGTTCGCAAAACT
CTCGTAGCTCTGGCACGACAAACATTTGCACGGATTTTTTTGCTGCGTATTCTCGTAA
CTACCCCTCCATGTTCCACGCTCGTACGATTAATTTTCGAAATATTCAAAAAAAAAAAG
AAAAGGAAAGAAAAGAAGAACATTATTTTTTCTTTTCTTTTTTTTTTTCCTCTTTTTTTT
TTCTCTTTTTTTTTCCAATTTGCTTCCCGGTTCCACGCGACATTACGGATTTTTAATCCTTA
AGCTAAATAAGTTATGGACGGACGTTCGCACGAGTACTTTCGAAGCTGGTGTAATTATT
CGAAACGAAATATATACATATATATATACATATATACACACGCGAATTTAAAGCGTGAATT
TTAATCATTAATTTAACGATTTTTAGATATTTAATTTCAGGAAATTTTCGCGATAGGAAAC
ATGGTGGATTCATAAGTGGCACGAAATGAGAGAGGTAAAATTTTGACGTGCCGCCGCG
ACTAATGGTGTCGAAGAAAATGGCGGGGAAAATCAGTGGTGAGGATGAAAATGTCGG
AGCGGAATAGAGCGCGTCAAATTAAACTTAACAAGTTTTAATGCCCAATTACGGAGTTA
CACCTACCTGGCTGGCTCTTATTTCTGTTGCCGTAATAAGCCACGGTAATTGAAATTTAC
GAGCGTGTTTTACGCGGGCCGGTTCGTAAACAGGGAAAAAGCGGTGTCCAGCCAACG
TTTGAGACGCGCTAATTCGACGAAGTAAGACGAATGAATTTTAAATTTCTCGTAACAC
TCGTGTATATATATATTCTGCCACCTATGCGTTTAACCTTTCTATATTTTTTCCTTCCGATA
AATTATTGGCAATGAAAATTTACTTTAGAAACTCTTTACGATGCGAGTAAAATGTCCAA
AAATATTACATGCGCATTTTTTTCAAACACTTTAATTAAAATGGGTAATAAATTTACCTTA
GGAATTTGATAAATTCAGGGGAAAATGTACGTTTACTATTTCCGTTCTTGCTGTTTATAT
CGCATTTTATTCTCAAAAAATTTAATTGATCAGAAAATATTGACAACAAAATTTCAATTT
TTCGCTCTCCCTTAAAACTGTTCATATTCCAATTTTTTTTATTGAAAAATAATACGATAAA
AAGATTGCTTTATTTTGCGTATCATCGTAAAAACCATTACCGATTAAACGATATCCTTCTA
CCAAAAGGTAAATAAACCGTGCGGGAAGAGGGGGAGAGAAACTCTGCAATTTCACCC
GATTTCACGAAAAGGTACAACTTCGTTAAAAAAAAAAAAAAGAGGAGAAAGATCTCCA
CGTATCCCCATCAAACAAATATAAACCCAGTCGAGTAATTTCAACTTTTCCGGCAAATT
AAAAACCTCCATTAACAAAATCACTGAGATGTCCGCCGCCAAACGAGGGTCAAATTCC
AACCCCTTGCGAATTACATTCGCTCCAGGGATCCGGCAAAGCTTCGACGTGGAGCCTC
TAACGAGAAGTTAAAGACCGAAATGGCCGAGGGTTAAAGGGAGGAGGACGCCGAGCT
CGCATTGAAAAGGCGAGAGTCCAACGATGATCTCTCCCTCCCCTCCCTTTTTTTCCACC
AGTGCGAGCTTTTTAATTACCGGAAGGATTAAGGGAGATTTCAAATTAGAACCGAGTTT
TTTTTTGTCTTGCGAGAAAAGAAAAGAAAAGGAGAGAAAAAAAGAAGAAAAAGAAA

AAGAAGAAGAAGAAGGGGGATAAAATGTTAATCCGCAAGCCGAACTCGACTTGAGAA
TCTGACGATTCGTCGAAAGTTTATCCGGATACCGGTTCCTCTTTCAAAAGGGAACACGT
GTTCTCTCTGTAATTACGTCGCCGAAGAAAGATATATCGTGGGATGAGGAAGGGAGGA
GGGAGTCCCTGTCAGACCAAAAAAAAAAAAAGGAAAAAACCGTTGCAACGCATCGATG
TCGAACGAATATCGCCTCGTTTTATTGGCCTCCTGCTTGCCAACCGGAGATTATATCGGT
TTCCGGAAAAAGAGGTTTTGTTTCGGGCTGTTCGATTGTGACTTCGATGATTAAAGTTG
GAAACGGTGGACGATTATTCGGATGAAAGAGGAATGGAGAAATTGGAATGGATGGGGT
TTTATGATTTCTCTAAATTTGAAATTTAGAGTATCATTTGGCAATATTTGGACGGGCAGTA
TTGAAATAATTAAAACAGAAATGATAATTTTCTTAAAATTATAATACTTTTGGAATTAAC
AATCAAGAGGATTATTTCTCTCAAATTTAAAATTTGCAACAGTGAGAAATAAATCAAAG
GTAGAAGAGATTAAACTGAAGAGGTTTGTAATTCCTTCCTTCCCAAAAAATTATAATAA
CCAGAGACGAAACAATCCATTTTCGAAACGTTTTGAATACCCTCGAATCGCTTGGATCC
ATGGATTAAAATAAACCATTTGAGATTAAACTGAAGAGCGAGCAATTTGACTGAGATAC
ACCACAGCTCGTGAAAATTCGATCATCGAAATTATATCGCGGCCATCCAAACCAAGTGC
CACCCTGGAGGAGCCACTCTTGCATATTTTATCCTCCAACAACAGTGTTTTCCAGCTGG
AACATTTTCGGTCAAATATTTTGCCGGCTGCTCAAACATTATCTTTTCGTATTTACCCGG
TGATCCCGCGACCACCAGTCTTCTTCCGCCAACGAGCAGAAGAGAGAGAGAGAGAGA
ATCCAACCGACGCTAATAACGCCGCTTTAATTACCCTCGAACCAGGCCAAGAGGATCG
AGGGAATTTCCTGGGAGACCCGTGTTCCACGTTAAATCGTATATCTCGATCCTAATCGA
AGAAATTACACGTTATACAACCCGGGGATTGGGGATATCGAATCAATTCTAATACAAAC
GTATCCCAACTTCGTTCCCAATCTCTCTAATTATCCAGCTTGTTGGGATAGAAGGATAAC
TGGAGATAGGAGATTCGAATGAAAGGGAATCCTTCCTCGAAAGAAGTGTTGGAGAGG
GATCAAAACGTCATCCAAAACGTTTATTTTTATTTCTGGATGGAAAGTAACTTGGACAA
GATTCAAGAATTCTTTTTAAAAATACTCGAAAGAGAGGAGGATAACCGCCGAGATAGA
AAAGATGCGAAAAGTTCGAAATTTTTTAGAGAAGCAAGGAGAATGGAAGAATTCTTTA
CTTTTTCGATAAGGAATTTCGATTTGTTCCCGATAAGATCCAGAGTCAATCTTCCAATGA
TATTTAATAAATCTCTATTTAATAACGCGACACACCTTCCTCCACGACGGAAATACGATC
TTTCTTCGAACGATATTCAATAGATTCCGTTCGATACCGTTCCGGAAACTGACCAAAGA
ACTAGCTTCTACTACCATTTCTCTCTCTCTCTTTTCTATACACAATCGACACACTCGTCG
GATCTCTTTTCCTCAAATTCGACGGATCCTTTCAACTCAGAATCGTGGTCAGAATCAGT
TCCTCTTTCTTCAGCCCCTCCAAAGAAATGTCGCGAAAAAGAAGGGATCCCTCCTTGA
AACGACGCTCGATAGAAGTTTCGGGGCGAAGTTCGATCTTTGCTCACCTTGGTAACGA
GTGAAAACACAATGCTCGATGACTACAAAGGCCGTTCGTTAAGATAATGGGCCAGTGA
ATGGGGAGGGGTGTCGCGAGGCCGCGCTCGCGTTTCGACCCTCCCCCTCCTTCCCTTC
CACCTCGTTCGCCGTTCGTTCAAAGCTCCATTCATTGGGGAGGGGAGGGGAGGGAAG
GGGGGAGTTTAAACTGGTTTAAGCACCGGCGGTAGTTAGCGAGCGGCCCGATACTCTT
CGATTATCGAAGGTCTTTCGGCCTCGTTAGCGGGCTTCAATTTTGCACGCTCCCGCTGC
GCGAGCATTGTTGATCGCCCCTCTAAAGAGGAGGGGGGGGGGGAAGGGTGCGACGAA
AGGGTTTGTTTGCGGAGATCGAAGGTGGTGGCAAGGCTCTCCCTTCATTCCTTTTTTCT
TTCTCTTCTCTCGAGAATGAGATTTTTTTTTCGATAATTTTTCTTCGGACGTTCTTTTTTT
TCATAGTCGTCATTTTGAATCGAAAAAGGAGTTTCTTTTTTTTTCGAAGAACGTTTTTC
GAAAGGAAAGTATCTATTTTCGAAACATTTAGAAACGATAGAACGATTCAGTTTTCGAT
GGACGTTCGAGGGAAACTGGGTTATTCGTGCGAAACTCTCGCGTGAACTTTTAGATAA
TCTGTGATATTGATTCGGCAACTTTTGCGCCGAATTTCAAATTCAATGCTTTTCGTAAGG

GTCAACATTTGTCTTTCGTCACTTGCAATGTGGAATTTCCACACTGAAAATAATATCACC
AGCGAGATATCGAAATCGGTCTCCGAATAATTTTGGAGGAAGGATATTTCGTCGTTACG
AATCATTCGAAATCGTGTCGATACGAATAACGTCGAAAGGATTGAAGAACAGAGAAAC
AGAGATAATTATTTTACACGGACAAATTAATCCTCCCAAGAAGATTCTCGTAGAGAGAA
AGAATCGTGTTTCTTTCGCTCGATTGATCAAAAGAAAGATTGACAAACCTCCACTTGAT
GGTTAATTAACACCAGTGAAGAAAAAATAAATCAAAAGAGGGAAAGAGGTAATAAAA
TTCCAAATTAACTTCGCTCTAATTATCCCTCGATTCGCGAGAAAACTCTTCAAGTATACC
TTTAACGCTTTTTCAATTCAATTTAAAATCGAAGAAGATAATAATGACGTATCGATGAGT
AGAAAAAGTATTTGTGAAACGTCAAAAGATGTAATGAAATTCAAAAACCATTATATATA
TATGTTATACATTTTATTCATGTGGAAAGCTGGCTATCTGAAAAACTCTTCTACGTTTCTT
GATAGAAAACCCATAACGTTACCATCCATCCACCACTCTTCTCCTCATAGAATAAAAAA
TCCTCGTGCATTCACGTCACTCGTTGACGCGTTGGAATACGTTAAATACGATGGCAAAT
AACGTCACGTTGGGAAAAAAAAGGAAACGCATTTACACATCCCCTATAGAACTATAGA
AACGCCACATCCAACCTCCGCAACCACCCACGATTCGTTATTCCAACGAATTCCTCCAA
ACGCTTCGATTCCACCCTTGGACATCCGAAAAGCCATCATCCGTTGATGAAAATTCGCC
CACACACCTCCTACCTCCCCTCTCTCGAATCAATAGCCAAACCGTGGCAAGAGGACCA
GGTGAAAAGGGTAGCAGGCCACTACGCGAAGGTGAGCGAAGGACGAAAACAAACTC
TTGTTCCGGCGGAACCGTGGCATTCCGGAAAGCAGGAAGTACGACCCAGTGGTGTCG
TCGTAGCTCATGCACGACCTGCACGTCGGTGCATAACGACAGGTGGCTCGAATTTTGC
AGCCTTAGCAAGTCAAATTTATGCAGATGCCGGGAGGGGGGAGGAACAAGGTTGCCC
GAAGCTGTGTGACGTTTGTCCGGCCAACATCTTGTACCATCACGCGACCGCCATCTTAT
CGATATCGTCGATATGCGGAATATAGACAGAATTCTCGAGGATTCGAGGAGTGGCGGCC
ATTAATTCGAGCGAGCGTCTCTTTTATTCTTCTGTTTCTTCTTTGTGCCCTTCGATGAATC
GAGACTTGGATTCCTCTTTTTTTTCGTGAAGTACAGAAGAGGTATTTTCGAGAAAACG
AAAAATCGGAAGATATCTGACAGTAATCGGTCCAATATTGTTAGACTTGTTAGATCAAA
GGTTAATATTCTCGCGAGATATTCTCAATAAAATAGTTCTCTCTCTTCGTTTTTGAAAGA
GAAAGGTGTTTAAATTAAAACGGAGAAATCTTTGGATCGATCCACGTGCTTCCAACAG
GAGAATATCGTGAACAAGCGTTATAAATTATACAGCGAGGACGGCGAAGCTAATGAAA
ATGATCAATCCATACGATCCATTCGACCTATGCCCTTTTCCCCAATTAAAAAGTAGATTC
CCTCCAATATCGATCGCGTCGAATAAGCCTCCTCTCAAACAACACGTATCCGCTTATTTT
GAATAATCCCGTTGAATCGAAAGATTCTATACACGCAATCCCCGTCGAGAAGAGGGGA
GGGGATGTGTGTCATTCAGGGTAGAAGGGCAGTCACGCGTAGATCGAAAAATCGATGG
CCGAGCTGCGTTCCAGGCCAAATCTTAACGAGCCACAATTTACAATGGGCCAAGATTT
ACTTACTTCCTGCATTTCAATTCAGATCGCGGTGATACGCCCGGATCGGCCGGAAATCG
TGGATCGTTCGTGTCGGCCACCGTTCGTGTCACGAAACTTGGCGTATGTGCCGTCTATT
ATCCTCTCCTTTCCCCTCTCCGCGACATCCCTCTTCCCTCCATTTACGTCGAGACACGAT
TATTTCTTTCTCTTTTTTTCCTCTTCTTCGCAATTACACTCGAAGAGATACCGCCGTGCA
GAGGCAGGCCAGCGTGGCTTCGATGTCAATCGAAGATTACAATGGTTCCCCTTAATCG
GATCGATATCGTCGTCCAACGGCTCTGTCCACGAAATTGAATCGAGAATCTGCTCGAGA
GTTAATAGCGGATAAAGGCTTGACTATCCGGGCCCCCGCTAGTTGATAGTTAGGGGGAG
TCTTTGCGGAGGGTGTGGAAGGGGAAGAGGGGAGGGAAGAGTCGATGATCGAGGAG
GAGGATGGCTCTGACCCTGGTCGACGTCGTTTTATCTGTCGGCAAACGAGGCGCTGAT
GGATTTATTGGTGTTTGCACGCGCAAATAGAGGTAAGCCTGGGCCGACGATAGAGATAA
TATTGTAACAACCGCGGAACAGGTTCTGTATCTGATAGGGGGGAAATCGGCGGCGATA

GATGAATGGATTTGGCTTTGACGCAGATTTCTGCCGAGATTTCAATAGATTTCGTTGGA
ATTCGATTACGTGTAAGGATTGGAGAGAGAGAAAGAATTCGAATGCGTTAAGAATTCTT
CTTCGTTCCGTGAAAAGATTGGTTAAAAATTAGAGGGGGATAGATTTCGTTTTCCTCTG
AATGAATCTGCAATAAGCCAGGGTTTGAGTTTAGGATTATATCGAAATTTTGTTTGTACG
AAGGATATCTAAGGAACGTGTAAATTTTATTATCGTGATCATTGTTTATTATTATTATTAGA
TTTGCAAGGAAGAATTCACAAAACTATGTAATACAATTTTAATTCTAATGATATTTCGCG
AGATAAGTCTGTGTTATGGGGGAATTAAAATAGGATCTAATAAACAACTTGGATCACAT
ACACGCGCGCGCGGAATAAAGTAAGATTGTACCTTCTTCCGTGGCAAATCTGGACCGC
GATAAATCACGTTACGTTAAGTCCGGTGCCAGGAGAGATCTAAGCTGCAGTATACCAG
GCTCTGCTCTAACTTATGGCAGATCGGGTAACTTGGTTCCACGGAAAATTCGATTCACG
GTAAATCGAATTAAACTGTCGATGCAGGGAAAAATGAAGTAGAAAAAATTCCAAATTG
TGCCAGACACAGTTTTCGAATGCTCGAAGTATTACGTATTTGATCAGCGTTACCATTTTT
GTATAATTTTTTTAACCGAATTTAGATTCATTTGCATTAATATCGGAGCGCCATTCGTAAA
TTTTGTTTCAACAATAATTCTATAAAAAGAATTTTTTAAACATTTCGTTTTTCCTCTCGCG
ATAAGAAAAAAAAAATATAATTTCTATACTTTGAAAAATGATATTTATTATCTTTTAATAA
ATCGTGATAAATAAAATGATATTTATACTATTCGAATAAATTGAAAAAACATTATTCCGAA
ACGCGTCAAAGTGTTTAAATTATTGAACGAAATGAATGATTAATATCGAATGAAAAAAA
AAAAAATATATATTTCTCTTTTGATAGAATACTTGAAACGTATATTATTAAAAATTTAACG
TCCCTCCTTTTGTTTATTTTAATGCCGTTTTCCGAAACCAATTATATTATAAAATAAATAA
ATCGATTAAAAAAATATCTTTCCTCCTCGCATACCGAAATTTTCAAATTTACATCATACCT
TCTCTTTCTCTCTTCCTATATATGTATATATATATATATATATATATATATATATATATATT
AATATAGAGTAATTATACACGCGATTCGATCGAAGAACAATGGCCAAACACACTTGTCC
CAATTTCTAAAACCGAAGTGGTTCTTCAAAATTGATCCGTTGCCAGAAAATCGCGAGA
ATTCAAATGAACTCACTCGAGTTTTTAACATCAAAGTTCAAGGGCCCGTTTCGATGGAA
ATTTTCGATAAGCTTGACGACGAGAAAATTCCACCCTCCTCCTTCCTCCTCGTACCTCC
CACCAATTTTAATCATCCAACGACCATCTCCTTCCTCTCTCTCGATCTTTCTCTTAATTTC
AATCTCGATCGTGAAGATAACGAGGGAGCGAAGATAATAAAATCAAAATCGGAGCAAC
AAAGAAGAAACAATCGGGAGTCGTACGCGTGGACGGTCTAATTTGCAAAACAATCGG
ATTTACAGAACGTACGCAGCCGGGGTGTTGGACACGATACACGTATGGCAAACAAAGA
TTTCGAGGTTCATCCGTGCTCGCATTGTTTGCCGTGCAACAAGGCGGGCGCGGGCATC
GATTCTGACAACGCGGTAGACGCCTCGATGTCCGCCCACGGTTTGTTTGCTCGTTTGTT
GCGTATCCCGCTCGTTCAATTTTACCTAACAATAAGATCCGACAGTTTAGTGTTTGCCTT
TCATTTACACGCGATGAATATTTCCCCGTGTTGGTCCTCGCCCCTGGAGGTGAGATTCG
ATTTATTCGATGATGAATTTTCTCGTCTATGATCCTTGTCCTCGTGATATTAGGGAATAGT
AATCGATAATTTAACGATCGGGTGGGTCCAAAAGTTTTTCGCCCAATTTCTGTAACGCG
ATCGTGTTATGTATTAATGAACAAGATAGAGTTCGTGCGTGGAAAACTTTCAATATTTAT
CCAATATTTGAAACTTTGTGTTGGTGTTAGAAATTTTAAATACGATGAATTGATAGATGA
GGAAAGTTGAAATTGTCTATTGTGAAAAGGAGGAAAGTCTGTCGTTAATTATTATTCGT
TAATTTCTGGTAACGACGAAATTTAACGTGTATCTTCGAAAGAAATATTTATATTTAATTG
TCGTATATTTTTTTACTTTTAAAGTAAAAAAATCCCCGCTAATAGGATTAAATTCTACGA
GAACGATGCTTTATGAATAATAATTGTAGCGATCTTCTTTTTTTCTCTTTTTTATGAATCT
AAAAAATAATTGAAATTGAAATTTTTAATCTCTTTCCTTCGGGTTTTTGCACGGGTTACA
AAATTTTTTCCAGCCATTTTAATATTTAACGCGAACGAAATAATAACATGGCGAAATTTT
TCGCCGTGTATATAACGGGTTGATGTTCCACCCGATATTTAATATTCACAGGTGAATAAA

AGATTATGATACGAGCCGCGAAACTTTGTCGCAGAATATAAAGGATCTTGCCCCTGACG
GGCAACATTTCATACTTGCTAAACGTTCCACGTCGATATTTAATATTCATGAGCGTATGA
AACACGTATAACTCTAAAAACGTCGAAACTTCTCCGTGAAGCGAGACGTTGATACACC
AAAATCGGATCGTTGATCAATATTCATAACCAGCGTGCGAGCGATGGTTTCTAATACAA
ATTGCGAGAATCTAATATAAATTTCGAATAAAATTTAATGCTGAACTTGGAATAATAATAT
AACCGATATAACAATACGTAAATAAAAAGAAGTTTGAAAAATTTACTTCCCCTTCTTCA
CAATTACACAAATAATAAAAATTCTTAGAGAGAACGTATAACGTTGCTAATTAAAATTTT
CCTTGATAATTTTAGACTTCCCCAAATTTTATCCGCAAAAAAATTCAAGAGGAATTCAC
ACTTTTTCTAAAGGACAAGAGAATTGAATATAAATTTTTCTCGAAGGGGAAGTTGCTCG
TCTAATTAACCAGTTTATCACGTGCATCGAGCGAGATCTAACTTCTAGTCGGTTGAATTG
AACACGATTGCTCGTTACGAGAAGGCGACGTTCGTTTAATCCGATCATAACTATATAAC
GCGGCGAACAAACCCCTCGATCGATGGGAACACGATCGTAAAATGCAAATCGGTCGAT
AACGCGGGCCGGGATTGGCCGGCCGTATCCTCTCGCGACAAACAAATACCGGGTGGCG
TAGATAAACCGCACGTTCGAGATAGGATGCCGGTTGTTGGATATCGAGGTTGTAGGAGT
ATCGAGAATGGCGAAAAATACGGGGATATTGTTTCGATTATATTGAGCGAAAGATTTTTT
TTTTTTTTTGTTTAGATAACAATTTGAAAGTGTTGGACGAGAGTATTGGAAATTGGGAA
TTGGGGATCGAATTGATAAATGAAGAATGGAAGAGTTTAAATATTTCTCGAATTGTTGA
AATAAAAAAGTGTGGGGATACGTGTATTATGGATTGGTTAATAAAGATTTTTGTCTCGA
GAAATTCTATTTTTAGGTTTTGCTTTTTCTTACTGGATTCTGAAATTCTGAAGAAAAAGT
AGATTAAAATTGTAGATCGAATCTCGCTTAATTTAATTACAGAATAACACGAAGATAATG
GGAAACGGAAAATAATGGGAACGGATGATAAGCATAAGCGTGGCAGAAATACAGAAAT
ATTTAAAAATCGGAGGATAAAATATGCAAATAAGGGAATGATGAATCGGTCACAATCTG
GATAAGAATCACGGCCAATTAAAATGGTAAATCGGTATATCCGTAAAGTTGGCCGGAAT
TAAATCGATAAATCGAGAGATTATAAAATTATCATGCAAATTAATGTAGGAAATTAAAGA
TCGAGGAATAAAGCGATGAGAAGTGATGAATGAATTAAAATTTCGAAGGAAAGAAATT
TCGTGCAAATCCAACCTGGAAAAAATAAGATAGAGAAATAAAATAATGTATCGTGCATA
AGAATCAACGAACGTTGAACAAAATCTCCGTTTAATCGGGGATAGAGATCTCGTTTTCT
CTCCGTGAGAAAAACGAAATACGAGAGAGGAAAAAACGGGGGAGAAAGACGGAGAG
GCGGAAGGCAAAGCAGCGGAAATGGGGGAAAAATCCGAAGAACGGTAAAAGCGCAC
GGTAAGAGGGATCCAGCTGATAAAGAGAAACGCCGAACCGAACGGAGAATTTAGCCT
CTTCTAGTTACTTTTTTCTCTTCTTTTTAATTTTACCCACCAGCGACGAAAGTACTTGTG
CGAGAGATCGAGATAATCGGAAATCGACGGCGACGGTCTCTTGATTTCTTTTTATAACC
ATCTCTTTTTTTTTTTTTCGATCGTCCGGAAAAGTGGAGCGTTTAACGACCAGAAGAAG
AAGAAGAAGAAAAAAAAGAAAAAGAAAGAAAAATTGTTTCTTTTTTCGCGCCTCTCA
CGATTGTTCATCTCCTTTTTTTTTCTTTTTTTTTTTTCACGGGAGGAAGTTTCGAGATTGA
TTTTCTTTTTTCGCTTATTTACCTACACTTTGCCGGATTTTCGCATCGAAAGTCGTCGATC
GATCGCTTTTCACGTATAGATTAATGGTAAAGTCGTCTTTTTTCTTTTTTTTTTTTTTTTC
AAGATTCTAAGCTTTAAACGTAAGAGTTTTTCTCCTTTTCGTATCGATGCAGTGTAAAAA
TGAAATAAATAAAAAAATTTAGGAAGAAAAAGAATAGTGTGATTGAAAATCGTAAACGT
GAAAACGATCGATCAGATATTATTCTTGGCCTCGAAGATGGAAAATTTCTTATAGTTAAA
AACGTTTCGTTCTTGTTCTTTTTCAATATATATATATTCTTTTCAAATTGTTAACATCATTT
CTTCTAAATTTTATAAAATTTCCGATGTTTAAATTTACTCGTTGTCGTTGTCATCGGGGCC
AAGGATTCAATTTGACGGGATTTTCAAAAGATTTCAAAAGGAGAATTCTTTTGAAGAG
CCCGCATCTTTTTTTCTCGACAGACAACGTCCAATTCATTTCTTGAATTTAGATAAGATT

TCAGAGAAAGGAAAGAGAATCGAATCAATTCTAAATTTGGCGAACGATAATTTAAATCT
TGAAACTGTGTAAAAATTTCCAAAATAATAATATTTCACGTCAGTTTCTCTTATCGCCAA
GTTAACGAATTGAGGATTACTCGAGAAAGAAAACGAAATATAATTGCAAGTAGCTGTG
CATCGTTAAATTTATATATAGATTTATATCATTCCAAGTTTCCAAATTTTTCGTAAAGCTGT
TTATTTAAAGCTTGAAGTTATAGTTAAGAAGAAAAAAAACAAAAAGGAAGAAAATATA
AATTAACGATACACAGCTGCAGTAATTTTCGCATTTGGCCGTTGATCGATGCGCAAAAC
CCCTTCCTGGTTTATCCGGGCCCTTTTTGTGCCGAATTTCGAGGAAAATGAAAATTGAG
AGGCTGATACCAGGAGAAAATTGCATTTACCAGTCTGATTGCGCATCCATCAATGTCCT
CGATCGCGAGATTCGTGCACGGGCCGCGAAATTGGCGTTGTTGGATCTTCAAACCATT
CGAGTCAATGTCCATTTGCTTCCTTGCTCGTCGAAAATAAAAAAAAAAAAATAAATAAAT
AAATTCCCTCCTGTTTAAAAATTTAAATTTCAGATATCTTCTCTTATCCTTTTAATAAGCA
TCTTAAATTTTCTTTTACCTTTCGAGTAAATTCTTTTCGTTGAAATTTTTGAATCATCCGC
CAGATTTATAATTTGAAATTTTTTGAAACGACAAAGCAATCCTTTTGCTTTCTTGACGA
AACAAATGGACGAGATGGAATTTTTCCATATTTCCATTCGATACGGGAAGAAGCGGCA
GCGCGAATCTCGCGATCGATCGAAAGGAATAAAATCGGTACCGGTAGTCAGATGTAATC
GAGAGATTATTAATATTCCATTATTTATTTGACAGGTGAACAACGCGACAGCGAGGGTG
CAGACGAAAAAACCACCGACGGTACCCAACG

>novel_circ_000486
TATGCGTACAATGGCCAGAGGGTAGGTGCTTCCACGACGTGCCGGAATCGGCACTGCT
GAATCTCTACTTCGAGATCCGTGAGAATTGCCACGGGTTCTCCTGAAGGCGATGCCAA
GAAAAAGGCGGCGACAGTGATGACGGCCGAGAGAATGAGAGAGACGATCAACGAAA
CTCATCGGCCGAGCCGACAACCGTGTGCTCGGAGTAATCGCGACAGACAGTGAAAAA
CCAAAAAAAGAAAAGAAAAGAAAAGAAAAGAAAAGTTTCACATTTTTCGTTTGTTTC
TTCCCAACAGCCGCCAACACACCACGACCAAGCCAACATATAAAAAAAAAAAAAAACC
GAAAGAAAAGACAGCCACTAGGGAGAGACTCGAGGGTCCCTCCCGAGGGGAGGGAC
AGTATCTGTATCTCCACGATTCACGATTGCTCTTATACTGCTGCTACTACTTTTTTTTTT
CCTCTTATTTTTTTCTTTTTTTTTTTTTTTCGATCGCTATTTTTCTCCCATCAACTTCGCTG
ACTCGTAGCCAGCGAGCGTTGTCTCTCTTAGAGAAAATGTGGTACTTTGCGTGTCGTAG
AGTCGAGTCTGTGGAATAGAAGAGACGAGAAAGAGTGGTGGAAAAAATTTTTTACGA
GTTGAGGAGAAGAGTGAGTTTCTAAAAGATATAATATTTAAATATTGCTCGACTCGGCT
CGAAGACACGATTTTCCACAACACACTTGTGAAAAATGTTAAACTTGCTTTTGTCGTG
CTCGTGTCGTTTTCACGGGTTACCACAGGTCTAGCACTGACCCTTAGATGGTCCTCGCG
AAAAAAGGCTCCGTTCTTGACCTCCCCTTCCCCACGCTCTGCCAGACCCTCTTTTTTTT
TTTTTTTAGGGTTCTCGATGATTCCAGCAGAACCAAGGTGGGTGTACAGACTTTATTTA
CCTACAACTAATTGCTCGTCGTTCAACCAGCTCGCGAACTACTGTCTTATTCTTATATAC
TTGTTGAAGTATTTATTCTCTCATATATATATTGTATTTTTTCTTCTTCTTTTTTTTTCTTTT
TTTTTTCTTTCTTTTTTTTTTTTTTTTTTTTTTTTTTCTCTCTCAAACTCTTCTACTCACTCG
TCTTCCCTTGACCCCTTGTGTACGAGGGAGAGCTCGTGCTCACACACCAGACCCTGAT
CCCACGTCTTTCTCTCTTACACATACACATACATATGTATATGTATACGCGCAAGCAAGC
ACGAGAGTGTTTGCTACATCGTGGAAAGGAGCAATTCGTGGTTTCTCGCCGAGATAGA
GGCGGAAAAAAGAAGCGGAAATCATTGAAAAAAAAAGAGAGAAATTCTGTTCTCCCG
GTGAACATGATCGAACAAGTTTAAAACTGCTCTGTCAGAGCATTTCTTTTTTTTTTTTTT
TTGTTTTTTCTACGCATATACATTTGATATTGCATACTTGATGCATATGAAATTAATTAGAA

TTAATCTTGCATATATTATCAAAAGATTAAAATATTTCGAAGAGAGTTTATATTAAAGAA
AATGTGCAACGACATTCTAAGATCAAAGAAACTGATAATTCAATGATCCGATATCCATAT
TATATCCAAAGGATATAAATTTATCATTTTAAGAAAAATTATTCTAAATTTTGTTTATATAT
TCTGATATATAAGCGAATTTAAAAAAGAAGAGAAAAAAAACAAACAAAATTTCATGTG
CATCAATCGAAATCAATGTATTCAGTACGAGATAAGATATATTAAAAATCGTGAATTAAA
ATAATAGAAAGAGAAAAAAAGGCCACGTGGGTAGCGTCTATCGGTCAGTGGTAGGTCG
ACCCGAGACAGAGCGAGCCTCTGCCCTTCCATAATCATTCCTCGTTCGCTGAATGAACG
ATTTTCCATCGCGGAAGAAACTTCAGCGGGAACATAGAGATACAAGTTAAACGGTTCC
ATTTGACCAACGATCGGTAAAGAAAGCGTCGAACGATCGATGCTGGAACGATCGATGT
TGAGAAACGTACGTCCTTGACGTCTCATTTTTGCTCTCGCTGACGTTTCCACGCGATCG
CCTTCGCGGATTCGATTAGAATTGTAAGGTTAGCTTTAATCACGGAACTTACAGGGTATT
GGAAAGTATTTTTACGACATTCCACGTTTCTCGTTTTAGCATTAATATCGTTTTTGTACTT
TGGACGTATTTTGTATTATTATGTATTACGTTTTAAATGGTTTTACGTAGAAATGATTCGA
TGCAGGGTATTATTTCTAGTGATCGTAAGAGAGTTACCTTTTATAGTGTACATGGACATT
CCACGCGTGTCTTTTTTTTTTTTTTTTTTTTTTTTTTAAGAGAAATCACTTGTTTTAATTA
ATAATAATAATAATAAATGTTCCTTGAATCGGGTGTGTGATTATAACCGCCAACGTGCCA
TGAGGTATGTACGCCGAGGAACTTTGTATCGAGTTTAAGGGAAAAAGAATTCGTCGCC
GATCTGGATGGCCTTAATGAGTTCCTAAGTGTTTTTACGAAGTATCGATTAATCTTTCTA
CGTTGTTATAACGGGAGAAGATTATGATGACAGTGTGCCAACGATAAAGGAAAAAAGA
GTCGGATTAAAGTTTTTTAATTTAAACAAGAGTTAAACTTCAACATATATATATATATATA
CATGGATATAAACATTAAATCTTTTTTAAATTAACACCATACATCCATTTTTATTTTGGAA
AAAATTCAGGAATTAAAAATTAATAAAGATATTTAAATAATATTGCAAAATTCTTTAAGA
AATAAATATAGTATTCTTAAAATTTGATCAATAGAATTAAACCAATTAGAATGTTTATTAA
ACCATTCGTAGAAACGCAAGCATCGATTGCGGATACAAAATACAATTCTCAAGTGTGTG
TAATCCACCAGATGAATCGACCAATTCTCTTTCATCATTCTGTACACGTTGGCCCGCGTT
CGTGACTCGTAGATCGTCGTTCCCTAGATGGAACGAGAACGAGTATTACTGTCGAGATG
ACAGATTTTCAAACAGCCTGAATCGAAACACGAGCGCGTAACACGTAAGAATGGAATT
GCGAAGTGGTGCATCGTTTCCTCTTTTCACACTGTATTCGCCTGTATCCCCCGTTTTTAT
CTCTGTCGAGAAGCGTGCACCGCTTCGCGTTTCCGAATGCGTCGATTCGCAGTTAAAA
AAAAACTGGGGGAGGAATCGGCGACGAGGTCGAAAACGATCGAAAGCGATGAGCTAT
AAGAGGGAAGAAGAAATGGCTGGAAATGGGTGGAAAGATGAGAAGTGACAATGGAG
AGAGCACGCAACGGAGAGAGAGAGGGAGAGAGAGAGGAAGATGGATGAAGAGAGA
AGCGACACAATAAGAAAGAATGTTGCGAGAAAGGAATTAAACGAAAATCGAACGAAT
TTTCTTAACTTAAGAAGTGATTGGTAGCCTTGGAAATTTGATCTCGATGCGTGCGAGTT
TGTTTCGAAGAAGATGGAAATTAGATGGAATTAGTTTTGCGAGAAATTTTGGAAATATT
ATTGCTTGACGATAAGAAGATTCCAGAAAGAATAAAAATTATTTGGGATATCTTATGGA
GTTTTTTAGAAAGTTTTTGAAATTCGAAATTTCAACGAAATTTCACGAGTTATGAATAAT
TGATAAATTTTTTTGCAAAAGCAAGGAATCTCTATCCGCGGATGATTTTTTTATCGTCAA
GAAATTCGAGAAACGGTATATTTATCGCGGGAAGGAAAGAACATGTTCATTTCGAAAG
AATGCCAAGACGATTTAATATGTTTTCCAGCCAGGCAATAAAGAAGAAGTTTTTCTACT
TTATCAAAAGAACACGAAGAATTCTCACTGTCCATTTTTCGAGATTCGATTACGTGGCA
TGCATCGCTCGTTCCTTCTCCTAAACTTTTTATCTTTCTTCTTTTATCCCCCCCCTCTCTT
TCGTTTCTCTTGATCCAATCTCGTCATTGGATCGAACTTTTGTACTCTTTATCCTTTCGTT
TCATTTATCTTCTTAAGCGAAATACACCTCCGATTCAATAATTTTTTTATTTCACATTTTTC

CCTGATTTCTTTTGTTCGTTATTCAAATCGAACCTTATTAAAAATAGGAGAGAAAGATCG
TGATTTCTTAATCACGAACGCGGAAAAATTATTATTTACTCCGTTTCCTTCCTTTTTTTAA
AGTTCCTTAAAATTCTTTTTTCATTATTTTTATTATCATTATTATTACTATTATTATTTTCCG
AAGGATCAATCGAAAATCGATTAAATTTCGAGGATATATACTGCACATATATATATGCGTA
TATATCCATTCTCCGTTGTTGTAGAAATTAATTTAATCAGGTCACGTGAAAGAGCATAGA
TTTCAAAGGAATGCATGCAGTAGGCTTTTTTTGGTCTTGATTTAGTGGCGTTTAGCTTTC
AGTGAATAACACTGCGACAAAATGAAACCCGAACTCGATTGTAACCCGGTAGATATTG
GGACCTTGTCGAGCGATAAATCGTCAACGAAAGTTTCCAATTCCATAAAAAAAAAAAG
AAAAAAAAAGTATTAAAAAACAAAAATGGATATATTTGGAAGCAACAACAACGCGTTG
TATCGTTCGACGAGAGCCCCCTGAAGCTTGTCCGACACGCTCGACAATTCGTATACCAG
AAGCCCTCCCCCTCGTTCACCGTTTGCATGCCACGTGTATACCACCTTTCTTCACACAC
GTAACGCATTTTTAAAAATCGTTGCAAAAAAGAAAAGAAAAGAAAAGAAAAAAAAG
AATCGCGTCTTTCTTCCGTGTTCTCACGTTCTCTCTGTGTCGTTTCCTGCTCTCTATCGA
CTCTTACGATTCACTGCTGCTACAAGCCACGATCGTTGTTTGCTGTGCATCGTCGATGT
GCACGTGCCCATCCAGGAACACGTATACAACAACACACCCGTGTGTGGAAGGAACCG
GGAGGAGGGAATACGAAGAAGCTGTCTTCGCCTGCGAGTTGGTAGCTTCGTGCATTCG
ACAAAATCGCGAGATTTTATGTTGCTATCGCTGTTCAATAATATTATTCTCGAAGAGAAT
ATATCGAGAATGTGAAAAAAAGAAAAAAAAGTATTCGTGCGATAAAACGATAAGTGTG
CATGGTGAAAAAGAAAAGAAATAGTTTCATTTTAAATTTTAAATTTATTGAACGAAATC
GTGAGATTTTCGCGTTTTCGTTTTCCAACGCGAAAATTTGTCGAGGTTCTTTCCATGAG
TTTTCTTGGGAGGAGGGAAGGGGAATGAAACGCGATATCGAACGCACGAAGCTATTCT
CGAGGAAAGGATGGCTCGTTCGCGCGGCAAAAGGTCAAGAGCGGTGGAAAGTGGCT
GAGAGGTTCGAAAGAAGAGCGCAAGGAGGACTCGTGGAAAATGGAAGATGGTTATCG
AAGGGCGAAAATTGTCAAGAACCGGAAGCTGGACGTCTTTCCCACATACCAGAAGCG
GTATCGAGCGTGAAG

>novel_circ_000487
GCCGCGGCAGCAGCAGCTGCAGCGTCACCATTGCTGAAGACGCCCCTCTCGACGGCG
CAGCAGGCCACCTACGCAGCTGCAGCGACCTACACGGCAGTGGCTGCGCGCGCATAC
AGCGCAGCTGCAGCTGCGGCACAACCGGTCGCAGGATATGCCGCAGTCGCGGGTTATG
GGCGTGAATACGCTGATCCCTATCTTGGACATGGTATTGGACCAGTAGCCGGATATGGG
GCGACCGTGTACAGGGGCGGCTACAACAGGTTCACGCCATATTAAAAATGGTCAAATA
TCGAAGCGCGAGCCTTCCAAGTACACCGTCGTTGTACTCAGCTGAATGTGACCAGAAC
AAACAGAAATCGACACAATCATCATCGTCATCCGCCATCGATCACGATCACCCCACGA
AGCGATGCCTGGATCCTCCGCGCATGCTGACGCTAGCCTTCTTCGTAGCAGTACGAGAT
CGTCGACTTTGCCTCTCATCCACGCGGAAAAGAAGCAAAATTTTCCCTAAAAGGAAAC
ATCCAGCCATCGTGGACGAAGCTCCAATCAAGTCCAAGTTACCAAGGTGTCGACTAAT
CACGAAGAACTACCATTCAGAAGCAAAGAAATTGGAAGAAGGAAATGGATACAGGCC
GCGCACGGTTCCAGGATCTTCGAGAGGAAGAGAAGAAAAATTGGCGAATTATTGTGGA
TGAGAGTAATCGAATAGATGGACGATTGATAGAGAAAAGGGTTATGAAGATAATGAAA
GATGATAATAGAATGGTTTGAATTTAGATGTAAAATTTCTAGTTACTCCTCGATTAGGGT
TGCGAAACGGTCGACTAGACGACAAAGGTCCATCGTCGTCGAACTTTCGAACCCTGAT
CGATAATCGGCGTAGAGAAGAGAGGGATGGTAGAGACGGGGGAAATTGGTAAACTGA
ATTAGCAGACAAAGAGGAACCGGTTAAATTGAGTTTCGCTCGAAACATGCTAAACATT

GGAAACAGATTCGAATCGATCGTTTTAAAAAAAAAAAAGGGGAGGGGGGAGGAAAGA
AATAAAAGTGGTAAATGGTGATGCTGGATCAGAGTTGTAAGAGTTTATTGTATGAGAGA
AATTCTTTCGAAACATTCTTGAGAATTGTTTAGTCGAAATTCACGTGAGGGAACCGTGT
GTAAAGGCATCGCGTCGTTAAATTTATCCATTCGTATATACGAGTTTCAGACCCGGCAGC
CATTTCGATTTATACGAAGTTGAACGACAGACAGAGGGGGGGGGAGGGGGGAGAGAA
CGTGTCGAATATCGCCGGTGTACGTGTATATATATTTGTGTATATACATATATATATATATAT
AAATAAATAGATATAGATAATTGATCTCTCTAGAAATCAATTGTAATTATGCAATTCGTTG
CTCGTGCACGTTTCTAATAATCGTTGGGGGGGGGGAGAGAAAACTTTTAAAACGCGTT
TCCGGGCAAGGATTTTTATCACGAAATGAAAAATGGGGGTAATTTTTAATCTCATTACAT
GTATACGTTACTCGTCATTATTGTACGATAATTTCACGCGAAATCCGAAAGAGGAGGAA
GGGAAATGTTGAACACAGCATATAAACACAAGGAATTTTGTACATAATTCATAATTGATT
TCGTGGTTGGGGAAGCGGGATACACGACGACGCAACGATCACAACACAGAGAGAATT
TTGAATCAAAGAAGCAAAGAGCGATCGATCGTTGCTTCTCTCATATATTTCTTTTCTTTG
TTCCGTTTTTTTTCTGTTCTTTTCTTTTCTTTTCTTTTGTTGTGCATATATATACATACATAT
ATATATATATATATATAAAGGAATCAAGTTTTTCTTTTAATCAAGTTTTAATCGGAAAATC
GAGGATTCGAGGATTCTCGCGGGATCTTTTCTAATTACGAAAATCGGGAGAACGCACC
GATTGTATCAAACGCGATGATAAGAAGGGGGGAGGAATTTCGCGCTAATGGCTGCCGG
AAAGGAACTCTTCGCTTCGTGTCGGCGAGGTGAACGCGACTTCCGGTGACAAACGCC
AGTCGACATTGCTCGGTGATCAGCGAGGATACGCGAGACCCTTCTTAGGTAGCTAGCC
GAGGGAAAAGTTTAAGGAGAGGGAGAGAGGGAGAGAGAGAGAAAGAGAGAGAGAA
ACGACGAGAGCGACGAAGCAGTTAGTTACGCGTGACCGGTAGGGTTTCTATGCCAAAT
ATAATGTGTGTGTGTCTATGTTGCGCTTGTGGGCGCGCGCGTGTGTGTGCGTAGATTCG
CGGCTATAGAGACGTAGAATAGTTAATATGGCGGAGGGGAGAGAGAACCCTTTGAAAC
GAACTCTGAGAAAGCAAGATTCTGGGAAACAGGATCTAGTCAAATCAGTCTCTACCGG
TTCCTAATCGAGATTACGTCAATGTAAAACGCAGCTTTTTTTTTTTTTTATGAATACTCA
AGGCTTCGTCGATGCTCCGATGGGAAAGATGAGAAAGGAAAGAACGTGGCAGCATTC
TTTTTTTTTTTTTAGATATGGATATTCTTTTGTTCTGTCTTTTTATTATTACTATTACTATTAT
TATTATTATTACTATTATTAATATTATTATTATTATTATTATTATTATTATAATATTATTATTA
TTATTATTATTATTACTATTATCTTTTTTTAAGGAATGGTCAGTTTTTCGAGGAAAACGGA
GCTTGTCGATCGAGGTTTCTACGCTCTCTTATTTTCTAGCTACTTTTTGGTGGAGGAATT
GGAATAGAG

>novel_circ_000490
CTCCAAAAGAACCACCTACATCGCTGAGACCTTGCAGTCCTTCACCGTGTGGAGTCAA
CGCTTATTGCAGAGAAAAACAAAATACAGCGTATTGTGAATGTTTGCCAAATTTCCGTG
GAAATCCTTATGAAGGGTGTCAACCTGAATGTTTGACCAACACTGACTGTCTGAAATC
GCAAGCTTGCGTCAAGACTAAGTGTCAGGATCCTTGTCCTGGTACTTGTGGCGTTGGT
GCGATCTGTACAGTTTCTAATCACATTCCTATTTGTTCTTGTCCTCTACCTACTATCGGCG
ATGCGTTTACGATCTGTCAAGTCATTCCTGAAGGTCCAAAAGAAAGAGATCCTTGTTCT
CCATCACCATGCGGTCCTAATACCATTTGCGAAAAAATCGGCGATACGGCTGTCTGCAA
ATGTTTACCTGGTCTCCAAGGAATTCCTTCTAGTATAACAGGTTGTCATCCAGAATGTGT
GATTAGTTCAGATTGTCCGGGAGACAAAGCTTGCATCGATAATAAATGCGTGAATCCTT
GCACACAAAATGTGTGCGGTATCAAGGCCACATGTAAAGCCATCAACCATAGTCCTCTA
TGCAGCTGTCCACCTCCTTTAATTGGCAATCCATTCGATGAATGTATTATCCAAATTGAA

ACTGATCCTTGTAGTCCATCTCCATGCACTTATAATGGCGAATGTAAAGTTCGAAATGGC
ATTGCGATATGCATATATCCAGAATGTGTTATTAATTCCGATTGTCCACGAGAAAAGGCT
TGTTTCGGCCAAAAATGTAAAGATCCTTGCATCGGTGCTTGTGGTATCAATTCATTGTGT
CAAACAGTGAATCACAAGCCTGTTTGTTCATGTCCAATTGGATTCACTGGAAATGCTCG
TATACAATGTACTATTCCAACGGTCGAAGAACCAGTTCCAGAATGTATTCACAATTCCG
AATGCCCGAACGATAAGACTTGCTACAACCAAAAATGCATTGATCCTTGCACACTCAA
CTCCTGCGGTTTTAACAGTCGTTGCCACGTACAATTACATCGAGCTGTATGCGTATGCAA
TGAAGGATTCACTGGCAATCCTCAACAATATTGTGGTGAAA

>novel_circ_000492
AACCGGAACACTTCGACAATCTGAGGAATTGCCTGGAGCCGAAGGACTTCCTCAAGA
GGGCACGGTCCCTGATGGTGGTCGTGTCGACCCCGCCCACGCAGAATTACGAGGAGTT
CACCAAGAAGGTCAGAGAGTACAGCAGCAAGGAGCCGTTCAACTTTGTCATCCCCGA
GTTGCTGAGGAAATACGAGAAGTACGTGTCGATCCACGCCGCGTATCTCTACGATTCCG
TGAAATTGTACGCGAGAGCCCTGGATCAACTTCTCAGGGATCAGCCGGAGCACACTCT
CGAGGAGATCGCGAGCAATGGCACCCAGATCATCGAGACGATTATCAGAAATCACACG
TATCAGAGTGTCAGCGGAGCGACCATCAAATTCGACAAGTTCGGCGACTCGGAGGGG
AATTTCTCGGTGCTGGCCCTGAAGAAGGATCCGTTCCGTTTCAACAACTTCTCGTGCG
ACTTTCAAATGAAGCCTGTAGGCCAATTCCAACAGGGTGAAACTCTA

>novel_circ_000494
GATTTAAATTTCAAGAGAATCTTTTACGTACGATGATACAAATAGAGAATACGTTCTTCC
AGCTGGGTGACAGCTGTACAAGAAATTGTCTTATTATTTGTGATCGTGGTGCCATGGAT
GCTTCTGCATTTATTTCAAAGGATAAGTGGGAATTACTGATGGCTTCGAACGGATGGAA
TAGTGTCGAACTTCGAGACAATAGGTATAATCAAATCATCCATATGGTCTCAGCGGCAA
ATGGTGCTGAAGAATTCTATTCGACGGAAGAACATGCATGTCGATCGGAAGGTGTCGA
ATTGGCTCGGGAACTCGATTACAAAGCGGCCGCCGCCTGGGTCGGACATCCTTATTTCG
ATGTAATAGATAACTCGCAAGATTTTGAGACAAAGATCTGTCGTATGATCGAATGTGTG
TGCCAAAAACTGGGTATCGATACTGGAGATAGATTACGAGCGAGTAGCAGAAAAGTCA
AGTTTCTTGTCAAAGATCCTTTGCCACCAGACTCTGAGTTTCCACCGTTTCAGGACTTT
GATGTTGTCCATAACTACCTCCAAAGTAACAATCCGAAGATGCAAGCTCGTCTTCGTAA
ACGTGGCCAAAAAGGTCACTGGTCTTATATTCATACGATTCGACGTCCAAAAATGTGTG
GCCAAGTGATTGAAGTTAAAACACAACTGACTCATCGAGATTATTTGAACATGCTCGCT
CAACGCGATGATTCACATTTCACCATTTTCAAACGTCGACGTTGTTTCCTCATCAACAA
TCAGTATTTCCAATTAGATATATATAGAGAACCCGCTCATCCAAGATGTCGAGGATTGAT
GCTGCTTGAAACGTACACAGCATTATCAGGAGATGAACTTAAGAATATATTACCACAGT
TCCTGACAATCGAAAAAGAAGTCACTGGAAATCCAGATTACAGTATGTTCAATCTTAGT
CTACGAGAAGAATGGAATAATACCAATAAATATTGCCATAATTTACACG

>novel_circ_000495
CTACCTGGAGTGTCTCCTGGAGCTATTTCATCCGTGGTTGCACCCGTGTTATTAAAGTAT
CGGGTCTGCAGACCGAGATTGTTGCTCGCCGGTTCTCGAGCCGAGGTCGATCCAGCCG
CAGATGTGGGATTGTTGCACGGGGAAGTTTTGTTGATCGAGGACTCGGCGAGACAGTA
CGATCCGATAGGAGATACGGACAGGAGATACAGAGCCACGGAAAAGTTGTTGCATGCA

GAAGAGGAGTATCACGAAGCTTTGTGCAGCGCGAAAGAATTGTACGCACGGCCCCTG
GCGCGAAATTATCCCGAATTTCACGATGTCATCTTTCAACCGCTCGCGGATCTCTCCGT
GGTCACTTCTGAACATTGCCAGAGG

>novel_circ_000496
ATCCGCAGCGCACTCGAGAATTGGGACGGCGGTACCTTCAAATCGAGTGATTTGTTTC
CTGCCTCGTTTTGGAACTCTTACTGGGAATACCTGGAGAGCTATGGGGAGGCCAGGAG
GATTCTTGACGAATTGAGGGCATTCGAGGATCCCCTACTCCACTTCCTCGGTCTTAGGC
AAGCTGCGGCCAGGCATTCCCCGTCCTCGTTACTTCTTTTACCG

>novel_circ_000497
GAACCACCTGTGGATTTCGCGCGAAGCATCGAGTGACGGAAAGTGCATACGTACACAC
GTATAACTGACTAACTGTCCCCGCGGTGGAGGAGGAGGAGAAACACCCCGTGCATGTT
GGCACGAGGTGTTGAACTCGAAGGGCCGGGCCGTGGCGGACGATCATGCCGATCAAG
TCCGAGTGAGGGAGTATCGTTTCGATCGCGGGCAAAAGAGGTCGAATGATGACCATGT
TCGGGACCCTCGTCCAGAGGCTCACCTCGCCGGTGACCTCACCGACGAGCCCTCGAC
GCCGTTTCAAATCTCCGTCCCGTTGGGAGGTGCAGAGCGACCCGGAGGACGATCCGG
CCGCTCCGGTCCGCAAGTCGAGGGCCAAGCATTTGTTGGATAAGCGCAAGAGCAAGA
GCAGGGCGCGAGCTTTGAACGTGCATTCGGAACAAGTGCAGATGTGCAACTCCGGAT
GGCACGACGCCGCGTTTCCGCCGCTTCGTGGCACCAAGAGTCTCGGCAGGCTCGACG
GTATGAAAGAG

>novel_circ_000498
GTCCAGAGGCTGGCCGAATACGAGAGGTACCTGGGCCAAGAGGCGAGGAATCTAGTG
GAGAACGGAAACGGAAGCGTCGGGAGTGGCGCCGTTCTCCAACGTGGCCAACTGGA
GGGAGATCTTCATCGTATCCAGGAATTGTTTCCTGGCGATAATCTCCGGCTGCACGAGC
GAGATGTCCTCACTACGAAAATGAAGAGTCTTATTCGCAAGAGGAACAGTGGAAATCC
TGGAAGGCTCACGAGAGTGTTGTCGCAAAAATCATTGAACGTGTCCAACAACTCACC
AGCCCCGAAATCTCCGCCGAGAACCTTCCTCTTGGAGACACCGGTACAATTCACCACC
GGTGTGCAGTCCCAGGACAGACATCTTTTTCTCTTCTCGGATCTGTTGCTCATAGCAAA
GGCACGAAACGGAGGTAACTTCAAGCTGAAACAATCCGTAAGGATGAGCGAGTTGTG
GCTAACTGCCGGGCACATAGAGGACGTGGCCGAGACGAGCAAATCTCAGGAAACGAG
CTTCGTCCTGGGTTGGCCCACCACGAACGTCGTCGCCACTTTTATGACTGCTGCAGCC
AGGGATCTCTGGTGGGGACGTTTGACCGAACTCGTACGAGAGGAGAGTATGAAGGAG
CCGCCGGACACGAACATTCAAGTCGTGTATCACGACAGTGACACGAACACGGAATAC

>novel_circ_000499
AAAATGAAGAGTCTTATTCGCAAGAGGAACAGTGGAAATCCTGGAAGGCTCACGAGA
GTGTTGTCGCAAAAATCATTGAACGTGTCCAACAACTCACCAGCCCCGAAATCTCCGC
CGAGAACCTTCCTCTTGGAGACACCGGTACAATTCACCACCGGTGTGCAGTCCCAGGA
CAGACATCTTTTTCTCTTCTCGGATCTGTTGCTCATAGCAAAGGCACGAAACGGAGGTA
ACTTCAAGCTGAAACAATCCGTAAGGATGAGCGAGTTGTGGCTAACTGCCGGGCACAT
AGAGGACGTGGCCGAGACGAGCAAATCTCAGGAAACGAGCTTCGTCCTGGGTTGGCC
CACCACGAACGTCGTCGCCACTTTTATGACTGCTGCAGCCAGGGATCTCTGGTGGGGA

CGTTTGACCGAACTCGTACGAGAGGAGAGTATGAAGGAGCCGCCGGACACGAACATT
CAAGTCGTGTATCACGACAGTGACACGAACACGGAATAC

>novel_circ_000500
GTACAAAGCGAATCGTTGCGTGAGCGGGGGCGGTGGCCGCGGGAACGGCGCGACTGG
CACCGGAGGCGGTCTAGGTAACGGAGGCAGCGGCCGCGGGGGCGGCGGGGGCGGTA
GCCCCGGCAGGGGCGGCGGCGGAGGCGGCGTCGGAGGAGGAGGCGGAGGTAGGAAC
GGAGGAATAGACGGTGGTGCGATACCCGCTACACCACCGACGATCAGCCGTTACCACC
ACCGCTACCATCACCACCATCACCACCATCATCGTAATCACGACCACGTCACCACGACC
ACCACGGCGGACGCCACCACCATCACTGGCGTCGCCTGGCACCTCAGGCACAAGCTA
CCCCTTGGAAAGCAGTGCCTCGAACACCGTCTTCAACATCGGCATCACCATCGGTCCC
CCTATCGGGCCTTAAACATGGGCCAAAAGATTTCAG

>novel_circ_000502
GAAGCAGCGACGAAGGCGAGTCGACGTCGGGCACGCCAGCCGGGGGAGGCACGGGG
GGTAGCGGGGAGAGCCAGGAAATGGAGGAGAACGGTACGATCCGTGGCGGAAGCAA
CAATTCCGCCGGCAATCTGGTGTACAGGGATCTGAAGGCCCTTCACCCGTCAAGCCAC
GACGTGTCCTCCCAGGATTACAATCCGCAATCGAGGCCGTCGTACCAGCGTGGCTACT
CGCCGGCCATGGACAAGCCGAGTTACGAGAAGGAGCAGCACAGACCGCCCTCGTCCA
GGGACGAGGAGAAACCTTCCTCGTGGGAGTACGGGGAGAAGACCGAGAGGAAGGAG
TACTCGAGATCGCGGGATAGCTTGTACAGGAGCGAAGCGTATCAGGACGCGACGAAGC
AGGAGCAGAAGGAGCACGCGAACCGCGAATGGGTATCGCCGGTGCCGGAAGTGGAG
GTGGGCGGCTCGGCGCCGGGCGGCCCCCAGGTACGAGCGCGGAAGGACATTCCAAGG
GGCGCCAGATTCGGCCCTTTCCTCGGCAAATGGGCCAGCGAGCCTTTCAACCCCCGTT
ACGCGTGGGAG

>novel_circ_000503
GTTCGTACGGCCGGAAGCGGGGTGAGGGGGTGGTTGGACGCATCCCACGAGACGAAT
AATTGGTTGAAGTACGTTCGAAGCACTAGCAGCCCGCACGCGGTTAACATGCGACATG
TGCTCATCGGTGGACAGATGGTGTACGAGGCGGTGAGGGACATTGCGGCGGGTGAAG
AGCTTCTTCTCGGTGTTCGAGAACCTCTTCAGCTTCAGGACATGCTCGGCGAGAATAC
GACCGAGGATCGAAGCGACCGAGAGACCG

>novel_circ_000504
TTGACTAGCAATAGATTGAAGAACACGAGGGGTACCATACATTGCACCCAAACAGCTG
CTAAATGATGAAACATAAAGACCTGCTAGTAGCAATACTGAAATTGCTGATACTGTTGA
TGCAATCATAAAATTCGTAAGCAGTGCCTTTCGAGTACAAGTTGCCGCGAGAAATATTG
AAAAACATAAATATAAAAATGTTCTGAAATTAAATTACAAAATTGTAATTAATTTTTTAA
TGTTGAATGTAATATAAATACTTTCAAACTATTATTTTTACCCAGTTCCCACAGCTGCTA
ATGTGCCATTAGGAATATCAGTAGATGGATGTTTTAAATCTCCACTCATATTAATACCAGC
TAAAACACCAGTTACTGTAGGAAAAAATACGC

>novel_circ_000505
AATGTGGACCATAAGATGAAATTCCGTTCCAAGAAAGGCGCGTTACCATTTGTCGAGCT

AAATGGTGAGGAAATCGCGGATAGCACGATTATCCTTCGTGAGCTTAGCCAGAAATTTG
GTAAGGATCTGGACGCTGTTCTTACGTGCGAGCAACGAAGCGTATCCCACGCAATGATA
TCGATGATTGAAAACCACTTAGTCTGGGTGGTCATGTGCTGGCGAACTAAGAATCTTGA
TCAGGTTTTTAAGGGTTACAAGGTGAATCTTCAGCACGTGCTGGGTACTCGTATCCCAA
ATGGCATTCTCAATTTCTTTTTCAAACTCACGGTCGGTCGTAAGGGCGCAAAGAAAGT
GAAGGCGCAGGGTATGGGTGTACATACTCCAGAAGAGGTGTCCCAATTCGGTTGCGCC
GATCTTAAAGTTCTTTCTGACATGCTTGCAGACAAGCCTTTCTTCTTTGGAGACGAACC
GACTACGTTGGACGTTGTAGCATTTGCACATCTTGCCCAAATTCTCTATATCGATAAAGA
CACGCCATACAGTCTGCGAGACTATATGCAGGAGAATTGTCCAAACTTGGTGGGACAC
TGTTCACGCATGAAGGAACGATGCTTCCCGGATTGGGATGAGATTTGCTCCACTCTTGA
TATGAATACTCATCTACCGAAACCGGAAAAACAGGAAGAGAAGGAGGGCAAGGAGGA
TGCAAAGGAGTCGAAGGAATCAAAGGAAGAGAAAGAGGGTGACAAAGAAAAGGCG
GATAAAGAGGAGAAGGAATTGGAGAAAGACAAAGAAGTTGATGAAAACAAAGAAAA
GGAGAAAGAAGGGAAATAAGCTCTCTTGAAAGACAAACAAGTCGTTCAGAGAAAGG
GAAAAAGAAGATTTAAAAACTTGAGAAAAGAAAATTGAGGGTGTGATCTG

>novel_circ_000507
GGTACAAACGGTCCGAGACCGACGTATGTAGGTGCTAACAATGGAAATGCCGGTGGCG
CCGGTGGTGGCGGCGGTGGAGGCGGTGGAAGCGGCGGTGGTGGCGGTAGCGCTACGG
GTGGTGGCGTCGGTGTAGGTCCAGCGAGTGGTGGCGGCGGCGGCGGAGGTGGTGGTG
GTGGTGGTGGTGGCGGTGGTGGTGGTGGAGGCGGGCACGGATTGAAGGGGAACACTG
GTGGCGGGCCACGAGGCGGGAACCCGGTGCAGTATCGTGCGAGCGGTGCCGCGGCTT
GGGGCGCAGCGCCGCCCTCTCATGCGGCTGCGGCTGCGGCTGCGGCGGCAGCGTATCC
ACCGTACACGCGGTATCCAAGCGCAGCGGCTGCAGCTGCGGCCGGCCAGATGCCCGTC
GGAGCACAGCAACTACCGCCTACGGCGAATCCTTACGCCACCGCCACCTACGCACAGC
ACGCGTCG

>novel_circ_000508
GGTACAAACGGTCCGAGACCGACGTATGTAGGTGCTAACAATGGAAATGCCGGTGGCG
CCGGTGGTGGCGGCGGTGGAGGCGGTGGAAGCGGCGGTGGTGGCGGTAGCGCTACGG
GTGGTGGCGTCGGTGTAGGTCCAGCGAGTGGTGGCGGCGGCGGCGGAGGTGGTGGTG
GTGGTGGTGGTGGCGGTGGTGGTGGTGGAGGCGGGCACGGATTGAAGGGGAACACTG
GTGGCGGGCCACGAGGCGGGAACCCGGTGCAGTATCGTGCGAGCGGTGCCGCGGCTT
GGGGCGCAGCGCCGCCCTCTCATGCGGCTGCGGCTGCGGCTGCGGCGGCAGCGTATCC
ACCGTACACGCGGTATCCAAGCGCAGCGGCTGCAGCTGCGGCCGGCCAGATGCCCGTC
GGAGCACAGCAACTACCGCCTACGGCGAATCCTTACGCCACCGCCACCTACGCACAGC
ACGCGTCGTATGGTGGACAAAGGGTGCCTACAGCCTCCTCTCCAGCTAATACTAATAGT
AGTTCTAGCAGTGCTACCGGCAGTCGAAGTGGGACAATGAGCACAAGTCTTAGCAATA
ATGCTATACAAGGACAGCAAACAGAACAGTTGTCAAAAACAAATTTATATATTCGTGGC
CTCAACCAAAACACTACTGACAAGGACCTCGTTAACATGTGTTCTCAATATGGAACTAT
TACGTCTACAAAGGCAATCTTGGATAAAAATACAAATAAATGTAAAGGTTATGGATTTG
TAGACTTTGAATCACCAGTGGCGGCAGAGGGTGCTGTGAAAGCACTGGTCGCCAAGG
GCATCCAAGCGCAGATGGCGAAAGTGGGTATCTGGTTGCTCCCTAGACTGCCCAGT

>novel_circ_000509
GGTACAAACGGTCCGAGACCGACGTATGTAGGTGCTAACAATGGAAATGCCGGTGGCG
CCGGTGGTGGCGGCGGTGGAGGCGGTGGAAGCGGCGGTGGTGGCGGTAGCGCTACGG
GTGGTGGCGTCGGTGTAGGTCCAGCGAGTGGTGGCGGCGGCGGCGGAGGTGGTGGTG
GTGGTGGTGGTGGCGGTGGTGGTGGTGGAGGCGGGCACGGATTGAAGGGGAACACTG
GTGGCGGGCCACGAGGCGGGAACCCGGTGCAGTATCGTGCGAGCGGTGCCGCGGCTT
GGGGCGCAGCGCCGCCCTCTCATGCGGCTGCGGCTGCGGCTGCGGCGGCAGCGTATCC
ACCGTACACGCGGTATCCAAGCGCAGCGGCTGCAGCTGCGGCCGGCCAGATGCCCGTC
GGAGCACAGCAACTACCGCCTACGGCGAATCCTTACGCCACCGCCACCTACGCACAGC
ACGCGTCGTATGGTGGACAAAGGGTGCCTACAGCCTCCTCTCCAGCTAATACTAATAGT
AGTTCTAGCAGTGCTACCGGCAGTCGAAGTGGGACAATGAGCACAAGTCTTAGCAATA
ATGCTATACAAGGACAGCAAACAGAACAGTTGTCAAAAACAAATTTATATATTCGTGGC
CTCAACCAAAACACTACTGACAAGGACCTCGTTAACATGTGTTCTCAATATGGAACTAT
TACGTCTACAAAGGCAATCTTGGATAAAAATACAAATAAATGTAAAGGTTATGGATTTG
TAGACTTTGAATCACCAGTGGCGGCAGAGGGTGCTGTGAAAGCACTGGTCGCCAAGG
GCATCCAAGCGCAGATGGCGAAAGTGGGTATCTGGTTGCTCCCTAGACTGCCCAGTGT
ACGTTGGTTGTGCATGCAA

>novel_circ_000510
TATGGTGGACAAAGGGTGCCTACAGCCTCCTCTCCAGCTAATACTAATAGTAGTTCTAG
CAGTGCTACCGGCAGTCGAAGTGGGACAATGAGCACAAGTCTTAGCAATAATGCTATA
CAAGGACAGCAAACAGAACAGTTGTCAAAAACAAATTTATATATTCGTGGCCTCAACC
AAAACACTACTGACAAGGACCTCGTTAACATGTGTTCTCAATATGGAACTATTACGTCT
ACAAAGGCAATCTTGGATAAAAATACAAATAAATGTAAAGGTTATGGATTTGTAGACTT
TGAATCACCAGTGGCGGCAGAGGGTGCTGTGAAAGCACTGGTCGCCAAGGGCATCCA
AGCGCAGATGGCGAAA

>novel_circ_000511
TATGGTGGACAAAGGGTGCCTACAGCCTCCTCTCCAGCTAATACTAATAGTAGTTCTAG
CAGTGCTACCGGCAGTCGAAGTGGGACAATGAGCACAAGTCTTAGCAATAATGCTATA
CAAGGACAGCAAACAGAACAGTTGTCAAAAACAAATTTATATATTCGTGGCCTCAACC
AAAACACTACTGACAAGGACCTCGTTAACATGTGTTCTCAATATGGAACTATTACGTCT
ACAAAGGCAATCTTGGATAAAAATACAAATAAATGTAAAGGTTATGGATTTGTAGACTT
TGAATCACCAGTGGCGGCAGAGGGTGCTGTGAAAGCACTGGTCGCCAAGGGCATCCA
AGCGCAGATGGCGAAAGTGGGTATCTGGTTGCTCCCTAGACTGCCCAGT

>novel_circ_000512
TATGGTGGACAAAGGGTGCCTACAGCCTCCTCTCCAGCTAATACTAATAGTAGTTCTAG
CAGTGCTACCGGCAGTCGAAGTGGGACAATGAGCACAAGTCTTAGCAATAATGCTATA
CAAGGACAGCAAACAGAACAGTTGTCAAAAACAAATTTATATATTCGTGGCCTCAACC
AAAACACTACTGACAAGGACCTCGTTAACATGTGTTCTCAATATGGAACTATTACGTCT
ACAAAGGCAATCTTGGATAAAAATACAAATAAATGTAAAGGTTATGGATTTGTAGACTT
TGAATCACCAGTGGCGGCAGAGGGTGCTGTGAAAGCACTGGTCGCCAAGGGCATCCA
AGCGCAGATGGCGAAAGTGGGTATCTGGTTGCTCCCTAGACTGCCCAGTGTACGTTGG

TTGTGCATGCAA

>novel_circ_000513
ATCGTTTATTGATCAAAAATAAGGGATGATAATAATTAAGAATTTGATTCTTTCACTTTG
AATATACCAAACCATACTTGTAAGTAACTCTAAAAACTGTTCGGTGCACGCTCTACATA
CAAAGTGTAAGAAGTGTGTGACATTTAGAGCGTGCACACGCAGTGTGCAGTTTACAGT
AACAGTGTATTTCTGCAGTCTGTGTCGTGTCATAGGCAGATGGTTTTTTAAAGTACGC
ATTATAAATTATGCATACAACCTACACACCACACACTCGTTTCGCACTTTCTCTCTCTCT
CTCTCTCTCTCGCTCTCGCTCTCTCTCTCTCGCGCACATTCACGTTTTTTTGTGTCGCT
ACTTATATCTCACGTTCTCTCTCGCGTTCTCTGTCTTTCTACCTTTTTGTTTTCTGCTTAC
ATTTTTTCGTTTTTCTCTTCTTTTTTTCATGTTTTTTTTTTCTTTTTTAGAAGACCCTATCT
ATAACCCCCACATTATCTCACACAAAGACTAGCGTTTTAAACGATCCAAAATAAACTA
AGGGCACTTTTAGTGGCGTATGCCCTGTGTTCTTATTTGAGTTTCATGAAAAATCTAATT
CTGACCGTTGTTAAACTTTGTTGTTGTTGTTGTTATTTCTTTTTTATCATCATTTGTATTAC
AGCACCTATCGACTTCTAAGAGTCCCTATTTCTTAACTAACTATGACACACTTGCCTTAA
TCTAAGAATTTTATTACGTGCTATGCCTCGCCATCTTAAAAGTATCCGAGAACCTAATGT
GTGTTCATTTTTTATTTTTTTTTATTTTTTCCCCTCCCTTCTTAACTCTTGTTTATAGATGC
GAACTTAATGCCTCAACATCATTTTGTTTTTTTTCTTTCTCTCCCTCTATATCCTTTTCGTT
TCCTCTTTTTTTTTCCTGAAATTTACGAAATTTTTATTTCATTATTTTTATTGGTCGCACAC
CGTATAACAATTATATTCTGGAGAGAAGAAATAAACAAAATTAAACAGATGTTTTTTTT
TATAATTACATATGTTATTTACTCGTTCTGTCTTATGTCACAAAAAGGAATTCATGTGCAA
CGAAGAATAAAACGATAGGAGCTAAAATAAAAACGAGGAGTGGAAAGGATATAGTAA
ACAAATCAAAACAAATATCAGGAAAGTCGAAGTTAAAAAAAAAAAAAATGGAGCACAG
GGATAAATGGAAAAATGTTCGTCTCATTATAGCTAAAAAAACTATCTCGCTTAACAATT
GTAAAACAATGACTTAAAACCTCGATAAATCTAAGGACTGTACAAGTCGAAGTACGAT
GACGCACATCACATTATCAGGCATCATCAAAATTAACTCAGACTCGCTGTTCTTATTTTT
TTTTTTTTTTCGCTTAAGCCGACGTTCGACTAATATTACATTTTTTTTTTTTTTTTTGTTT
CGTTACTTAAAAACACCCTACGTTTTTTCCTTTTTATTCTCATCTTTCTACATCAGCCTA
CGACTACTAGTTGTGAATCTAATCTAAAAAGAGGCTACGCTAAAAATTCTAGAGAAAG
CTTACGGAAAGCAGCGTTGCTTGGAAACTTATGTTCCAAAAAGAAATTGGTTTCTTTT
CGTAAATCACGCCCGGCCAGCCCAAGCACCCGACCCGCGGTGCGCAGCAAGCAGTCC
TTACTATTTATATTACAGTAGATAATATGTTGTAGAATAATATATATTTATTATTAATATGAA
TAGCCAGTCAGTCTTGACGGGTGCGAGGTGCATAGGCTGAACGGTGCCTCACCTGATG
CATTTTCCTTTTTTTTTTTTTTTTTTTTTTTTTCTTTACTTTTTTCAACAAGGAGAGGCAGT
TGTATATATAACCTAACTATCAACTAACTATCTATTTAAACTTAATTACTTATCGATTACTT
CCTATCCAAAATGGTACCTAACCTATATTCATAATCAACACTAACTACACATCTCGCAGT
GTCCTCTTTTTTCGCATTTATATTCTATACAATAGGTTACGATACATTCATTCTTGTTTTTT
CTCACTTTCACTCATTTTACTTTCTCTCTTATCTCACGTTCTGTTTTTTTCTCTCATCATTC
AACTTTTTTTTTCCTTTTCACACATTTTCTTTTTATCCTTCGTTCACGCACTTTCATTTATC
CTTTCAAGTTTCTTTCACTCTGCCTCTTACATCCATGACTCTTCACTCTCTTTTTCTCCTC
TCTGCCCGCTCTCCAGTGCGGCTCCGTATCTGCGGGAGCTGGCATTTTTTTGTCGGCCG
AACCATCGTTCTTCCGCTCGGTCAGCACTATAAAAATGCTGCTCTTCTTTGTTTTGGTCG
TCACAATAATAATTAGTTATTTAGTACAAGTCTATTAGTAAAATTCTTCAAAATTCACTAT
TTAATCACATGTGATTTTTGTTGTCAGTCGTGGTGACAATCGCTTGTGCAAACAATTAC

ACACTCGCAAATAGGTTTTCCCTTTCGCTTTTCTTTCTTTCGTTCTCTCGCTCTCTTATTC
TTTTTTTCTCTCTTAATGCATGGACTCAAATCGAGTAATAATCGGTCTCTTAAAAAATAG
CTCATACCATTTTTTTCATCTTTCATCTCTTGTCATCAAATCGTGATGTACCAAAATAAAC
TAAATATATTATTTACTTCAAGTCTCAACAGTGTAAAAATCTTTCATACTTTAGATGACGT
CAATAATAGTAATCATTAATTATAATAATACAGCATATTATTGTAATATTATAATAATAGCTA
CGATCGCCTCTAACTCTATCTAACTACCTGCCCGCCACTTCGAGCCCAAAGCTCGCAAT
ATTTGGTAGAAAGCTTTCTGCTAATCGTTCCTCGACTCGATCTAAGTCAGACAACGTGA
AAACTGTACCGGATGTAAAAACGGGCTTAGAGGAACGCAGGAGAGAAGGGTGTTTTT
TCTTTTTCTAATGTTTCTCTCTCTTTTTCGAGATATCACATAGATAGAAAATTCTCGATTT
ATATCTTTATTTCGTTTCTTTTTGTTTCCTCGCGTTCAATCCTCTCGCCTATCGTTCAGCT
CCAATCCCATTATCTTTTTCGTTTTCATTTGTAACACGATTTTATTCGTTCGTTCGATATAA
TAAATTAATTTATACTCTACCTATCATATTCCGTTGTAACAAGCCTACTCTGTTTCTTTCT
CTTATTTTCTTTCAGGCGGTGGAAATTATTCTTGGTTTACAATGATCCGCCATTTGATTTT
ATTTTTTTTTCTTCCTAGACTTCATACAGACCGTACAGATGTTTCCGGCCCATCAATCCC
ATTAGTGGCAACTATACACATACAAAATCTATTCGAATTTGTAACCAAAAAACAAACGT
AGTGATATTTAACTGGCTACAACGTAAAAACAATTATATTCTCTTAACTAGAAGATCAAA
ATTTGGCTCAAGTTTCGCATTTACATATCGACGTAAAAATATATTATAATATTTTTCAGCA
TATCCCTGTCTAGCGGATTTGTCTGAAAAGGCACAATTCTAAAAAAAATATCTGTATTCA
CTGTACAATTAAAACTGATATCGATTCGTAGTAACTGTACTCATAAATATAAATCTTCTAA
AAAGTACTATGTTCCTTTCCTTCTTGTTACAATAATTACATATATCCTGAAGTTTTTTTTT
TCTTTAGAAATAAAGCTTTATTTTTTTCTTTTTCTCTCTTAAAATCTTCATTTCTTTTTCGT
CCATAAAATATATTCATTCGTGATTAAAAGAGAACGCGAAAACCGTACTTGTAAAAGTA
GATAAAAATCTCCGCGTGGCAATACATACATACAATATGTGCCTGGCTTGGAATGTCTG
AACGAAATAGAAGAAAACAAAAAGAAATAATCTGATGAGGGTGCAATTAGTGGATGG
GGTGGTGTGTGCTGTTAAGAAAGAGCTCTTTTTTTTATTATTCGAAAAAGAGATACTGA
TCCATAGCTTCTTCTCTTCGCAGCGCAGCCACCGGCCAGCCAGCTGGCACGAAAGTCC
AGTAGGTACTGATTATAAGTAACTGAATCTGAATTTTATTTTTCTTTATCAACTCTAGCAT
TTGGAAGTGCTTTTTCTCTGTATATTATTATTATTATTATTTCTTGGGAAGAATTCTCCTTT
GTATTTCTGAGCAGCGTTTTTTTTATAGTTTACAGTTTTTGTTAAAATATTTTATTCACAC
TCTCAAGCGTTCAGCTTCAACTTATCATCTCCTTAATAAATGTAATACCTAAAGTTAATC
CTGGAAGCCTATTAGCTTGACACTAAACTGTTCGTCGCAGAATATCAGTTTTGTCACTG
TCGTAATGATGTGTTGGCATTACAGCGTATGGAGCCGTGCTGGTGCCGAACAATGTAGA
TGACACGAGCACTCGTACCCCTTTCTTATGACAACATTCATTCTCTAATCGTTGGAAAAT
ATTAACTACTTTCACCTATTTATCTGATGGATAAAATGTATAAAAGATGGCTGGCAAGTC
AATTATCATGAGGAAGAATCTTATCTGACTTTATGTAAT

>novel_circ_000515
ATCTTCCAAACGTTTAAAAATGGAAGCAGGTTACAATTCTCATCCAAATCGTCTCGGTA
CGGAATCCCACTATGGAGGAGATAATTTGTTCAACCAAAGCAGACCTTATATGGTCTCA
TCACCATCATATTATTACACTAGTCCTCCACCTGTTACACCACGACCCAAGGGAGATACT
AGACCTTATATGGTCTCATCACCATCATACTTTTATGTTAGTCCACCACCTGTTACACCA
CGGCCCAAGGGAGATACTAGCTCTCATATGTCAGAATTATACACGGAAGATAGTCTTGT
TCGAAGATTAAGTTTACTCACAAATGCATTAAATCAAGGAAAACCAATTCGTGTTGAAG
ATCTTATGTCGCTTCGATCAGATAATGAGAGTGATCCAAAAGTAGTACAATTATTAGGAG

AACTACATGCTGAACTTCGACTTGCTGCAAATTCAAGATCAAGTGGACTAATAGAATCT
CAGCTTCCTGTATTAAATCCACCACTTTTAGATACAAATGCAGCACAGTCAGGAAACTT
TGATGCACATGCAGTTTATGCTGGTGATGATGCAATGAGTTCTGGAAGATGGTCACAAG
TGGCAACAGCTGGTGTTCGCAGTGCATTATTAGCGCTCATGTCGCGCTATGGTTTGGAA
ACTTGCAATCCTTCCCCTGTTATCACCCGACCCCCAG

>novel_circ_000516
TGAGAGGGATCTATCTGTGGACGGAGACGAGGCGACACCCGAATCTCGAACAGGCGG
ACGCCACAGTGGACGGGCGTGTCGTCTAGTGGCGCGTCGTCGAGGGGTCGCGAGGCG
TTGGCGGCAAGACGGCCGATCGTGCGACGGTGCTGAGTACTATTCCGCGGCTGCGAGG
ACAAGCAACAGCCCGAAGATTCTCCCAGCAAAGGGCAAGATGCACATCGAGGTGCAG
GTGGCGCTCAACTTCGTGATATCGTACCTCTACAACAAACTGCCGCGAAGGCGGGTGA
ACATCTTCGGCGAGGAGCTCGAGAAGGCGCTCAAGGACAAGTTCAAGGGGCACTGGT
ACCCGGAGAAACCGTTCAAGGGGTCGGCGTTCAGATGCTTGAAGACGGGCGACCCGG
TCGACCCGGTGCTGGAGAAGGCGGCGAAGGAGAGCGGCGTGCCGATCCAGGACATCC
TGGAGAACCTTCCGGCCGAGCTGGCCGTGTGGGTGGACCCCGGTGAGGTGAGCTACC
GCATCGGCGAGATGAACGCGGTGAAGATCCTCTACTCGGAGACGGGGGACCCGCACG
ACGAGAGCTCGGCCGACCGGGAGGTGACCAAGACGTTCAACCCCGAGGCCCAGTGCT
TCAGGCCGATCGAGGCCGTGGGCACGTCGCTGGGCGGGCTCAGCCTTAGCCCCAAGT
CCACGTCCCCGTTCCCGAGCTCGTTGGGCAGCAACGCGAGCAACGGGTCGTCCAACA
ACCAGCAGAGCGGCCACGGGTCCGGTTCCTCGTCGGCGCCCTCGCCGACGCCCATCA
CCAGCTCGTTCAAGGGCTCGCCCAGCCCCGTTCCGGCCTTCATCCCGCGCACCACGGC
GCCGCTCACCTTCACCACGGCCACCTTCGCGCAGACCAAGTTCGGAAGCACCAAGTT
GAAGACGAGCAGCAAACGAGCGAACAGAATGTCGCCGACCGAGTTCTCGAACTACAT
CAAGCAGCGGGCGATGCAGCAACAGATCCACCACCACCACCACCACCAGCACCAGCA
GCAGCAGCAGCAGCAGCAACAGCAACAGCAGCAACAGCAGCAGCAGCAGCAGCAAG
CGGCAGCTGCGGGCGGGCTGCCCGTTTCGCAGACCTCTCCCCGAAGCCGCAGCCTCTC
GCCCGGGAGCATAGTGGCGGGTGGCGGGCAACAGCACACCACGGATCCGAGCGCGTA
CTTCTTCCAGCACGGGCCGGCCACGTATCACCCCCAATTCCCCCATCGCAACATCTTCG
ACTCGTCGAGCCACGGCGGCTACCTGTCAGCCGACCTGTACACAGGCGTCAACTTCCC
GTCGTCGTACCTCGACCCGACCACGATCGCCGGCCACCAGTTCTACGGAGGCAGCGTG
AACGGGACGAGCAACGGGGCGGGAAGCAACGGAAACACGCAGAGCGGGGGGAATCT
GGGCCCGGTCGGGTCAGCTGCGACGAACAGCAACGTGAACGGGTCCGGCCAGCAGC
AGGACAAGACGGCGCTCGTCGAGGGGTTGAACAACTTTGGCCTTGGATCGGTGGCGC
CGTACCCGGCCAGCCAGTACCAGCACCTCCTCGTAGCCAACTAATACCCCGCGCGAGT
GGAGACCGAGCGGCTGGCGCGGCTCGAGTCGCGTCTGATCCTCTGCTGCTTCACAGG
ACAGGAAGGGGAGTCGGGGAGGATAGTATGAGGGGGCCGGGCCGTGCGAGCGTGAG
CGAACGGGGCCACGTTCCAGCCTCTATCTCGTGCGTCGAGACGTCTTCGTCGTCCAGA
GTCTAGGTTCCGTACCCTGACACACGTAATACACACACACACACATGTAATACACGCAT
ACATACAAATACATGAACACACACGTACAACCAGATTTTCGCTTGCACCATCCGATCGG
AGCTCACCTTCCTGCTCACCTACCCACGCTAAATACGTATAGAGTTCCCCACTCTGTCT
CTCTCCTGGCGAAACGAGCGTCCAATCCAGCAGAACAACAACAACAACAAGCGGCAA
GGTCGGTAGCTCTGCATTGCTAGTGCAACGATACCCTCCCCTCCTCCCCTCCCACCCGC
CCCTCTTCGAAGATCATCGCGACGACGCCCGAAGCTCATCGACTCCTGTCTTCTCCGAT

CTTTCGGGAGGAAGAGAGGGAAAGAGAGAGAGAGAGAGAGAGAGAGAGAGAGA
ATCGGGGAGAGAGCGTGCACTGCTTCTGCTCTCTTTGCCTGTCTGTCTCTTTCTTTCTCT
ATCTTTCTCTCTCTCTCTTTCGAATATGGCGATCCGCTCGATCTTACGTGTGTGAACAAT
TTACGTGCGCGAAACAGGGAGACAGAGAGAGAGAGAGAGAGGAAGAGGACGGACG
CGATGAAAGCACCGTGGAAGAGACAGCACCGAGGTCGACGCGCGCGAGCATCAAGA
CGACGTGACATGGACGTGGAGAAAGTGAGAGAGAGAGAGCGTGTGAGAGAGAGAAC
GAAAGGGAACGAAAGAGGAAGAGAACGAGAGTTAGAGCGAGAATGAGATTATATGGA
GCGTGTATAGAGGGATTCGTGTGCTAGAAGCGTGCGTGGAACACGTTCGGGAGGAGTG
AAGAGGCGGAGGAACGAAAGAAGAGGAGGTGGGATTGTTCGATGCGACGGTACTCGC
GAACGCGAACTTTGCGACCGACTAACGTATATATTATATATATATAGAAAAGCCGCGTGT
ATCTTGAACATTCTGTAACCGAAAAAAAACAACAACAACAACAACAACAACACGGAT
ACCCGATCAGCGCGATGACGCTTGTCGTTCGACGAATGGCTGCCACTTGTGTTTCGGA
CTAATTTCGGTCGGCAGCCATCTTGAGGCGAATAAATCTTGAGATACCGTACGGTCGCG
TAAAAATGGAATTGCGTGTATACAAGAGACGCGTACTCTGTATAGAGAGACCACAGAG
AGACCAATTCGTGATATGCTCGAGGAAGGATGGAAGAAGAGAATGAGAGAGCGAGAT
GAAAAGGTCGAACGACGCGCCATGCGCCCACACCTGGGACAGCAAAAGAGCCAAGA
GACACACATACAAATTCGTACGCGACGCGTACGTAAAATTCCACACGGGCCAGATTCAT
ACGCAACGGACACACTCACTCCCAACCATTTACCTGGCGACCTATATACATATACATATG
CATATATATATGTATATATATATATATTACATTGTATCTACATTTGCCATGTGAACACACGGA
AATGCCCCGCCTGATGGTGGCACGATCACGAGTATATGATACAAAGACACGGAAGATAT
GCATATATATATATATATATATATATATATATATATATATATATATATATATATATATACATAT
ATACACACACACGGAGGCATCACGCGTCCGTGGTAAAGACACATTGAGGGCGGGGTG
TGCGGACACTTAATATCGCGCACGACTTCACCAACACATACACACAGATATTACCAACG
TGTAATAATTTATTTTTTCACTGGTATAGTCGGAGAATTGATATTGAATATATCTGTAGAG
CCTATGTATTATATTCAAAATATGCACTCACTTCGCGGGTTGTCGCGAAACGGTGTAGGG
ACTACCAGTATCCGGTCTGTCGAAGCGTCGACGAACGAACACCCGGGAAGGCTGTTTA
GTCGTAGCACATCTTCTGCTAGATTATTCCTCTTACGCACATTTGTTCGATTCGGCGGGG
GGGAGGGGGAGGGAAACCGACGACCGAATCGAGGAGGAAGGATGGAGGACGATCTT
TGATGAATCTTGAGAAGACAAACGTGGCGGTATTGGAAAAGCGAAGAGGACGATGATA
TATACGTAACACGGCTGCCGGTGGGATTCTTTCTCGATCGAGTCGCAGATGGTGCTCGC
GGTTCCATACCGTTCGGAAAACTCGTCCTCG

>novel_circ_000517
TGAGAGGGATCTATCTGTGGACGGAGACGAGGCGACACCCGAATCTCGAACAGGCGG
ACGCCACAGTGGACGGGCGTGTCGTCTAGTGGCGCGTCGTCGAGGGGTCGCGAGGCG
TTGGCGGCAAGACGGCCGATCGTGCGACGGTGCTGAGTACTATTCCGCGGCTGCGAGG
ACAAGCAACAGCCCGAAGATTCTCCCAGCAAAGGGCAAGATGCACATCGAGGTGCAG
GTGGCGCTCAACTTCGTGATATCGTACCTCTACAACAAACTGCCGCGAAGGCGGGTGA
ACATCTTCGGCGAGGAGCTCGAGAAGGCGCTCAAGGACAAGTTCAAGGGGCACTGGT
ACCCGGAGAAACCGTTCAAGGGGTCGGCGTTCAGATGCTTGAAGACGGGCGACCCGG
TCGACCCGGTGCTGGAGAAGGCGGCGAAGGAGAGCGGCGTGCCGATCCAGGACATCC
TGGAGAACCTTCCGGCCGAGCTGGCCGTGTGGGTGGACCCCGGTGAGGTGAGCTACC
GCATCGGCGAGATGAACGCGGTGAAGATCCTCTACTCGGAGACGGGGGACCCGCACG
ACGAGAGCTCGGCCGACCGGGAGGTGACCAAGACGTTCAACCCCGAGGCCCAGTGCT

TCAGGCCGATCGAGGCCGTGGGCACGTCGCTGGGCGGGCTCAGCCTTAGCCCCAAGT
CCACGTCCCCGTTCCCGAGCTCGTTGGGCAGCAACGCGAGCAACGGGTCGTCCAACA
ACCAGCAGAGCGGCCACGGGTCCGGTTCCTCGTCGGCGCCCTCGCCGACGCCCATCA
CCAGCTCGTTCAAGGGCTCGCCCAGCCCCGTTCCGGCCTTCATCCCGCGCACCACGGC
GCCGCTCACCTTCACCACGGCCACCTTCGCGCAGACCAAGTTCGGAAGCACCAAGTT
GAAGACGAGCAGCAAACGAGCGAACAGAATGTCGCCGACCGAGTTCTCGAACTACAT
CAAGCAGCGGGCGATGCAGCAACAGATCCACCACCACCACCACCAGCACCAGCA
GCAGCAGCAGCAGCAGCAACAGCAACAGCAGCAACAGCAGCAGCAGCAGCAGCAAG
CGGCAGCTGCGGGCGGGCTGCCCGTTTCGCAGACCTCTCCCCGAAGCCGCAGCCTCTC
GCCCGGGAGCATAGTGGCGGGTGGCGGGCAACAGCACACCACGGATCCGAGCGCGTA
CTTCTTCCAGCACGGGCCGGCCACGTATCACCCCCAATTCCCCCATCGCAACATCTTCG
ACTCGTCGAGCCACGGCGGCTACCTGTCAGCCGACCTGTACACAGGCGTCAACTTCCC
GTCGTCGTACCTCGACCCGACCACGATCGCCGGCCACCAGTTCTACGGAGGCAGCGTG
AACGGGACGAGCAACGGGGCGGGAAGCAACGGAAACACGCAGAGCGGGGGGAATCT
GGGCCCGGTCGGGTCAGCTGCGACGAACAGCAACGTGAACGGGTCCGGCCAGCAGC
AGGACAAGACGGCGCTCGTCGAGGGGTTGAACAACTTTGGCCTTGGATCGGTGGCGC
CGTACCCGGCCAGCCAGTACCAGCACCTCCTCGTAGCCAACTAATACCCCGCGCGAGT
GGAGACCGAGCGGCTGGCGCGGCTCGAGTCGCGTCTGATCCTCTGCTGCTTCACAGG
ACAGGAAGGGGAGTCGGGGAGGATAGTATGAGGGGGCCGGGCCGTGCGAGCGTGAG
CGAACGGGGCCACGTTCCAGCCTCTATCTCGTGCGTCGAGACGTCTTCGTCGTCCAGA
GTCTAGGTTCCGTACCCTGACACACGTAATACACACACACACACATGTAATACACGCAT
ACATACAAATACATGAACACACACGTACAACCAGATTTTCGCTTGCACCATCCGATCGG
AGCTCACCTTCCTGCTCACCTACCCACGCTAAATACGTATAGAGTTCCCCACTCTGTCT
CTCTCCTGGCGAAACGAGCGTCCAATCCAGCAGAACAACAACAACAACAAGCGGCAA
G

>novel_circ_000518
TGAGAGGGATCTATCTGTGGACGGAGACGAGGCGACACCCGAATCTCGAACAGGCGG
ACGCCACAGTGGACGGGCGTGTCGTCTAGTGGCGCGTCGTCGAGGGGTCGCGAGGCG
TTGGCGGCAAGACGGCCGATCGTGCGACGGTGCTGAGTACTATTCCGCGGCTGCGAGG
ACAAGCAACAGCCCGAAGATTCTCCCAGCAAAGGGCAAGATGCACATCGAGGTGCAG
GTGGCGCTCAACTTCGTGATATCGTACCTCTACAACAAACTGCCGCGAAGGCGGGTGA
ACATCTTCGGCGAGGAGCTCGAGAAGGCGCTCAAGGACAAGTTCAAGGGGCACTGGT
ACCCGGAGAAACCGTTCAAGGGGTCGGCGTTCAGATGCTTGAAGACGGGCGACCCGG
TCGACCCGGTGCTGGAGAAGGCGGCGAAGGAGAGCGGCGTGCCGATCCAGGACATCC
TGGAGAACCTTCCGGCCGAGCTGGCCGTGTGGGTGGACCCCGGTGAGGTGAGCTACC
GCATCGGCGAGATGAACGCGGTGAAGATCCTCTACTCGGAGACGGGGGACCCGCACG
ACGAGAGCTCGGCCGACCGGGAGGTGACCAAGACGTTCAACCCCGAGGCCCAGTGCT
TCAGGCCGATCGAGGCCGTGGGCACGTCGCTGGGCGGGCTCAGCCTTAGCCCCAAGT
CCACGTCCCCGTTCCCGAGCTCGTTGGGCAGCAACGCGAGCAACGGGTCGTCCAACA
ACCAGCAGAGCGGCCACGGGTCCGGTTCCTCGTCGGCGCCCTCGCCGACGCCCATCA
CCAGCTCGTTCAAGGGCTCGCCCAGCCCCGTTCCGGCCTTCATCCCGCGCACCACGGC
GCCGCTCACCTTCACCACGGCCACCTTCGCGCAGACCAAGTTCGGAAGCACCAAGTT
GAAGACGAGCAGCAAACGAGCGAACAGAATGTCGCCGACCGAGTTCTCGAACTACAT

CAAGCAGCGGGCGATGCAGCAACAGATCCACCACCACCACCACCACCAGCACCAGCA
GCAGCAGCAGCAGCAGCAACAGCAACAGCAGCAACAGCAGCAGCAGCAGCAGCAAG
CGGCAGCTGCGGGCGGGCTGCCCGTTTCGCAGACCTCTCCCCGAAGCCGCAGCCTCTC
GCCCGGGAGCATAGTGGCGGGTGGCGGGCAACAGCACACCACGGATCCGAGCGCGTA
CTTCTTCCAGCACGGGCCGGCCACGTATCACCCCCAATTCCCCCATCGCAACATCTTCG
ACTCGTCGAGCCACGGCGGCTACCTGTCAGCCGACCTGTACACAGGCGTCAACTTCCC
GTCGTCGTACCTCGACCCGACCACGATCGCCGGCCACCAGTTCTACGGAGGCAGCGTG
AACGGGACGAGCAACGGGGCGGGAAGCAACGGAAACACGCAGAGCGGGGGGAATCT
GGGCCCGGTCGGGTCAGCTGCGACGAACAGCAACGTGAACGGGTCCGGCCAGCAGC
AGGACAAGACGGCGCTCGTCGAGGGGTTGAACAACTTTGGCCTTGGATCGGTGGCGC
CGTACCCGGCCAGCCAGTACCAGCACCTCCTCGTAGCCAACTAATACCCCGCGCGAGT
GGAGACCGAGCGGCTGGCGCGGCTCGAGTCGCGTCTGATCCTCTGCTGCTTCACAGG
ACAGGAAGGGGAGTCGGGGAGGATAGTATGAGGGGGCCGGGCCGTGCGAGCGTGAG
CGAACGGGGCCACGTTCCAGCCTCTATCTCGTGCGTCGAGACGTCTTCGTCGTCCAGA
GTCTAG

>novel_circ_000519
TGAGAGGGATCTATCTGTGGACGGAGACGAGGCGACACCCGAATCTCGAACAGGCGG
ACGCCACAGTGGACGGGCGTGTCGTCTAGTGGCGCGTCGTCGAGGGGTCGCGAGGCG
TTGGCGGCAAGACGGCCGATCGTGCGACGGTGCTGAGTACTATTCCGCGGCTGCGAGG
ACAAGCAACAGCCCGAAGATTCTCCCAGCAAAGGGCAAGATGCACATCGAGGTGCAG
GTGGCGCTCAACTTCGTGATATCGTACCTCTACAACAAACTGCCGCGAAGGCGGGTGA
ACATCTTCGGCGAGGAGCTCGAGAAGGCGCTCAAGGACAAGTTCAAGGGGCACTGGT
ACCCGGAGAAACCGTTCAAGGGGTCGGCGTTCAGATGCTTGAAGACGGGCGACCCGG
TCGACCCGGTGCTGGAGAAGGCGGCGAAGGAGAGCGGCGTGCCGATCCAGGACATCC
TGGAGAACCTTCCGGCCGAGCTGGCCGTGTGGGTGGACCCCGGTGAGGTGAGCTACC
GCATCGGCGAGATGAACGCGGTGAAGATCCTCTACTCGGAGACGGGGGACCCGCACG
ACGAGAGCTCGGCCGACCGGGAGGTGACCAAGACGTTCAACCCCGAGGCCCAGTGCT
TCAGGCCGATCGAGGCCGTGGGCACGTCGCTGGGCGGGCTCAGCCTTAGCCCCAAGT
CCACGTCCCCGTTCCCGAGCTCGTTGGGCAGCAACGCGAGCAACGGGTCGTCCAACA
ACCAGCAGAGCGGCCACGGGTCCGGTTCCTCGTCGGCGCCCTCGCCGACGCCCATCA
CCAGCTCGTTCAAGGGCTCGCCCAGCCCCGTTCCGGCCTTCATCCCGCGCACCACGGC
GCCGCTCACCTTCACCACGGCCACCTTCGCGCAGACCAAGTTCGGAAGCACCAAGTT
GAAGACGAGCAGCAAACGAGCGAACAG

>novel_circ_000520
GACGATTTCGCGGAGCGTCGACCGAGCCACGGAGTGTCTCGTGGCCATCGCGAGAGA
AAAAAAAAAGCGCGGAAGTCTCGCGTCCTCTTCCTTCCGTTATAAATAAAGACCATCG
AACCGACTTTCAAGAGTCGCCCTCTTCGTGCCGGCCATTTTTCATACACACACACAGA
GTGCCATAATCGTCCAAAATAAGAGGAATTTTTGCCAAATCATCGACACACCATTGAAA
TTTTCAAAACGATCAAAGTGATACGGAAATCCTGCCATCTTGAATATCTACTTATACCTT
TTTTATCTTAGCAATAATTAAGGATAAAGGGGAAAGAAGGAAATTTTTGGGAAAAAAA
GTGCAAGTGACGACAATCAAGATCGATTAATTAAATCTCTATCGTTTATCCGTCTATCTA
AATCTATTGAAATCGCGTAAAATAAACGGATAATCGGGTGCAGCGAGCACGGTAGCCG

CTGCCAGAAGCGCATAGTGGATCCCATAAATTTTCCTCGGCGATCGTGAACGCACTAAA
CGCCGAGCCGTGGAGCTGCCTCGAAGTAACAAGCGACGCGAGCCGACCTCTCGACCG
GGCAGTCGTCAGGATGTTTCAGCTGGCCAGCAGCCGGTAGCTTCGTTGCCGGGCCTGG
TTGCGATCCTCAGTCCTTGGAGGCGGAAAAAGCGGGGCCGTTTCAAGGATCGGCCGTC
GACGATGTCCATACGCGTGAGCGGCGTCTACCTGGTGCCAAGCCCGGCGGCCAACAAC
GAGCCGGCCAATGAAAAACCGAGCAACCTGTCGGTGGAGAACAATCTTCACCAACAG
CATCATCATCATCATCATCATCACGCGGCGAAGACCGTGACGATCGTCGCCGGACCACA
GAGGAGCAACAGCCTGGATTATTTAAATTTCGAGGAGAAAAGGCAGATCATCGCGTCG
TCGTTATCGTTGAGCGATTTTTTGGCTCATGGGCCGGCAGCTGCCGCAGCCGCTGCGAA
AGAGGTCGCTGCCAACACCGTTATCGTGACAACGACTTGGAATGGATAAAGCGAAAA
AACAAAATGGGGCGGCATTGCGAACGAACAGTTTGGGCTCTGGGGCGCGGACACCAC
CCCTCGAGAGGAAAAGCAAATTTTCAGCCCTCGGTAGACTCTTCAAACCCTGGAAGTG
GAAACGGAAAAAGAAATCGGATAAATTCGAGGCTGCCTCATTGTCGCTCGAGAGGAA
AATCTCTGTACGAGCAAGCAGAGACGAGCTGGTACAAAAAGGAATCTTGTTGCCTGTC
ATAAGGTCCACGTCGTTTCCCGAAAATGAGACGGGTGGCGACTCGCCGGACAGCCAG
AAGCCGCCGACGCCGCAACAAACGCCCCAACAGCAGCAGCAGCAGCAACAGCAACA
GCAACAGCACTCGCACCAGCAGCAGCAGCAGCAACAACAAGGGAACATCGGTGGTG
GAGGCACCCCTGCAGCAACACCCACGTCGGCCGTGGGCAGCGTGCAGCAATCCCAAC
AATCCCAGCAAGTAGCATCGGCCACGCCGACCCCCACGACTGCCAATCCGCATTCTAC
AGGACCGCCCAGCAATCAACCCTCGCCTCATCCCTCGCCCCTTTATCCGGGGATACCGC
AGCCTGGCCAGCCCAACGGCAACACGCAAG

>novel_circ_000522
CGAAAAAACAAAATGGGGCGGCATTGCGAACGAACAGTTTGGGCTCTGGGGCGCGGA
CACCACCCCTCGAGAGGAAAAGCAAATTTTCAGCCCTCGGTAGACTCTTCAAACCCTG
GAAGTGGAAACGGAAAAAGAAATCGGATAAATTCGAGGCTGCCTCATTGTCGCTCGA
GAGGAAAATCTCTGTACGAGCAAGCAGAGACGAGCTGGTACAAAAAGGAATCTTGTT
GCCTGTCATAAGGTCCACGTCGTTTCCCGAAAATGAGACGGGTGGCGACTCGCCGGAC
AGCCAGAAGCCGCCGACGCCGCAACAAACGCCCCAACAGCAGCAGCAGCAGCAACA
GCAACAGCAACAGCACTCGCACCAGCAGCAGCAGCAGCAACAACAAGGGAACATCG
GTGGTGGAGGCACCCCTGCAGCAACACCCACGTCGGCCGTGGGCAGCGTGCAGCAAT
CCCAACAATCCCAGCAAGTAGCATCGGCCACGCCGACCCCCACGACTGCCAATCCGCA
TTCTACAGGACCGCCCAGCAATCAACCCTCGCCTCATCCCTCGCCCCTTTATCCGGGGA
TACCGCAGCCTGGCCAGCCCAACGGCAACACGCAAG

>novel_circ_000523
AGCCGAAGAAGGAAAAGACTGAACAGAGTGCAAATGGCGCGGTATCGCCGAGCGGT
GAGCCGCAACCGAATCAGGGCACGGCTACGACCGGTAGCTTGCCGATCTCGGCCGCG
CCCCCGAATTCAGGGGACCAGCAAAAGCCGAGCAGGCCGAACACCCTGGAAGCCAG
GGTGGTGGCCAGGAGACTGATCTTGTTCGACATGGAGAAGGGGGTCCTAGACCAAAG
CAGCGAGAACCAGCAGGTGATCGAGAGGTGTTACCCTCTTCCGCCCCAAAACACGTT
GATGCTGTCGGAACTTCCGGAACCGCCGATTCCGCTCAGCGAGATCGGGCCGATCCCA
CCCCCTCCGATGTTCAGCTCCTCGAGTCCAACCCTCTTGTTGGCCAAGCAACGGCAAA
TAAGAAACCCTTTGTCGTCGGACTACGAGGACGAAGAAGACGAAGAGGACTTCGACG

TGGAGGAGGAGGACAACATGTACGTGCCGAGGATGTCGCAGCCTGATCCGACCATCG
ACACGTCCAGGGTGGAGGAGATCCCCGCGAAGGAGCCTAAGTTTCACGCGGTCCCTTT
GAAGAGCGTTCTGAAGAAGAGGGGTTCCGGAAGTGGGCCTGGCACGCCCCAGAACA
CGCCCACGCAGGAGAACAGGCCGTTGACCCTTCGCCAGGAGCTGCACGCCTCCTTCA
AAAGGCCACCGTTGAGGCCTAGGATGAGAATATGCAGTCGACCGGTTAGATTCGGCCT
TGCTCTTCCATGCACCCTCGAGAACAAGGAGAACGCGAGGCCGTATGTGATAAGGGAG
GATGCGGATGGTGATTCAGGGGATGGACAGGTGCTCTATAGGGACGAGTACGACGATG
AGAAAAGCCGGTTGGCAGCAAAGATTGCGCGTAAGGAGTCGCTGTCCCTGAAACTCG
CCCTGAGGCCGGACAGGCAGGAGCTCATCAACAGGAACATCCTTCAGCTGCAGACGG
ACAATGAGAGGCAGGAAACGAAGGAGGCAATTGGTGCCAAGCTCATCAG

>novel_circ_000524
TGGAGAAGGGGGTCCTAGACCAAAGCAGCGAGAACCAGCAGGTGATCGAGAGGTGTT
ACCCTCTTCCGCCCCAAAACACGTTGATGCTGTCGGAACTTCCGGAACCGCCGATTCC
GCTCAGCGAGATCGGGCCGATCCCACCCCCTCCGATGTTCAGCTCCTCGAGTCCAACC
CTCTTGTTGGCCAAGCAACGGCAAATAAGAAACCCTTTGTCGTCGGACTACGAGGACG
AAGAAGACGAAGAGGACTTCGACGTGGAGGAGGAGGACAACATGTACGTGCCGAGG
ATGTCGCAGCCTGATCCGACCATCGACACGTCCAGGGTGGAGGAGATCCCCGCGAAG
GAGCCTAAGTTTCACGCGGTCCCTTTGAAGAGCGTTCTGAAGAAGAGGGGTTCCGGA
AGTGGGCCTGGCACGCCCCAGAACACGCCCACGCAGGAGAACAGGCCGTTGACCCTT
CGCCAGGAGCTGCACGCCTCCTTCAA

>novel_circ_000525
TCGACCGGTTAGATTCGGCCTTGCTCTTCCATGCACCCTCGAGAACAAGGAGAACGCG
AGGCCGTATGTGATAAGGGAGGATGCGGATGGTGATTCAGGGGATGGACAGGTGCTCT
ATAGGGACGAGTACGACGATGAGAAAAGCCGGTTGGCAGCAAAGATTGCGCGTAAGG
AGTCGCTGTCCCTGAAACTCGCCCTGAGGCCGGACAGGCAGGAGCTCATCAACAGGA
ACATCCTTCAGCTGCAGACGGACAATGAGAGGCAGGAAACGAAGGAGGCAATTGGTG
CCAAGCTCATCAG

>novel_circ_000526
AAAAAAAAAAAGAAAAGACGCTGCGTTTTTCGATCCAGATTCGTTTTGTTTTTTGTTGT
TAGTTTGTACGCACTACGATTTATCGCAATTTATTGTATTATCGATGAAATACTGTAACAA
AAATACTGTTAACTATTCTTAAAAAAATTAAAACTTTTGTAAACTGAGA

>novel_circ_000527
ATGTTTCAGATGCATCCGCCATCGCGGTATCGCCGCGTATCGAGGCGAATCAAAGGTAC
GACACTTGAGCATCTGCGACGATCGAGTTCCATCCACGGGGGCCAATCATCTTCGAAG
AGGATCGAACCACGCGGGATCCAAGAGGAATGGTCCCTTGGGCCTTTGGCGTCCCTCT
CGTCCTCGTTTCTGCTCTGGGTCTTTTCCTAAACGGTTACGTGCTCCTCGTGGTTCTCG
GGCTGGGTAAACAGACGCAGCAGCAACAAACCGCCAATACTTTGTTGTTGATACACTT
GGGCGCGGTGGAGGCGGCTGTGTGCCTCATATTACTGATCTTTACCACTGGTTCCTGGC
CAATCGCCGGCACCTGGTGCGTCCTACACGGTTTCCTTTTGGCTCTCTTACACCCGGTC
GCCCTCTGGACTGTCACTGGATTGAATTGCGACAG

>novel_circ_000528
AACTTGAATTGGTAAAACTGCCGGAAGATATGACTTTGGCAGGATTCACTCCGTTGATG
TCAAATCCTCAAGATCCTTGTTATGCCGAGAAGAACGAAGATATGGAAGTCGCTCAAG
TGTGTCTCAGGATAAACAAGATTCTGTTCTTCGGCCAAGTGTTCCTGTGCGGCCTCGAG
ACACCGGTATTGAAGTTGCAAAAGAGCGAAACTGGCGTAAGCGAGTACGTTTCTGTGG
TAGAGGCTTCGAGCACAAGTACGCCGAGTTCACCACCGGAACAGAGCGACTCCGAGC
TTCTGGTAGAAAGTTACAGCGAGGGTGAGGATGAGCCTGTATCCACCATGAGGAGATT
ACCCTTGTGCACAGACGTACCTGACGATAGTACCGCCCCCGTGGCTGCGGTCGAGATA
AGGTCGCTGATCGAGAGAAAAGAGGAACTTGAGAGGCGGCAACGGAAACAGGATCG
TCACAGGCAACGAGTCCAG

>novel_circ_000529
AACTTGAATTGGTAAAACTGCCGGAAGATATGACTTTGGCAGGATTCACTCCGTTGATG
TCAAATCCTCAAGATCCTTGTTATGCCGAGAAGAACGAAGATATGGAAGTCGCTCAAG
TGTGTCTCAGGATAAACAAGATTCTGTTCTTCGGCCAAGTGTTCCTGTGCGGCCTCGAG
ACACCGGTATTGAAGTTGCAAAAGAGCGAAACTGGCGTAAGCGAGTACGTTTCTGTGG
TAGAGGCTTCGAGCACAAGTACGCCGAGTTCACCACCGGAACAG

>novel_circ_000530
ATCCAGAAAGCGGCGACCCAGCTCGAGCAACGATTCTGAACGATCGTTCAGCGGTTG
GAGCGAGCGAATACCCCACGGATGTGGTCGCGAGGGGTGTCGCACAAACGAGCGCGG
CGACTGCATGGACGAATGCACAGCCGCGCTAGTTCTCATGTCGCTCTCGTGCTCGCCA
CACAGCCCTCACAATCCTTTACCGTTACACTGCCAACATAATTACGGTTCCTGGGGAGG
TCGAGGAGGCGGAGGTGGCGGCATGGTGGTCGGCTCGCCGGGAGTCGGCGGCACGTC
CAACAGCAGCAGCAGCAGCGGGGCTTCGTGGCGTAGCGGCACCCCGAGCCCCCCCT
CAGCGACGAGGGGGCGCCCAGTTCCCTGTGGCCGACCTCGGCCCACTCCTCGCACAA
TCAACAGCACTACCCTTACAACGTGTCCGGATCGTCGTCGAGCAGCACCCCAGGCTCG
ACCACCACTCTGGACGAGGGCATAGTGCCCGACTACCTCGAGGAGCAACACCCCCGA
AAGAAGAAG

>novel_circ_000531
GCTAATGCAGCGGTAGTATTTAAATGTACTTGGCCAGGTTGTTTAGAAATAAAGGCGAC
CGTGCCGCTTATCGAGGAGCACGTGCGCCAATCACACTTAGGACCTAAAAAGACTAAC
AGTTACGATAACGAAGAGGACGATGACATTTCCGATCACGAGGAAGAATTCTACTATC
AGGAGGTGGCGGTGGATCATATGAGCTCTCCGCCGACTATGTCGCACAGGGACATGGC
CAGACCTCCTCACGAGGATCCAGAGTACCAGAAACAGCTTCGTTTGGAAACCACTTCT
GCCACCAATAACGCAGAAAGCATGATGGTGAGCATCAGAGAACGAATCGCATTCGCCA
CTTCGCAATCACCGGGTACACCGATCAAGCATATAAAGTTGTCACCCAGGCCATTCCAG
GCGTACCAACAGAGCATGGGCCTGCTGACGGTGTCCACGGGAAAGATGCCATCGTCGC
CCCGACGGATTCGCGGCGAGACGAAGAAGTGCCGCAAGGTCTACG

>novel_circ_000532
ATATTAAAGAAATCGAGGTGAACAATCAACTCGTGGAAAAAGAAAAAAAGAAAGAAA

GAAAAGAAAAGAAAACGTGAGTGTGCGAAGAAGAAACGATCTTTGATGAAAAGAATA
ATAATCTTTAATTGCCCGGAGTCTTTAACAAACAGTAAAAAATTATTAGAGAGAAAAAA
GATTGTTTGACCATCCCCAACGATGGACATTGTTTGCCAAAAGAAAGATAGACTATAAC
CTGGATACACGAACCATGTTTATTCCTTTCTTCCTAATATTAATTCGAAAAATATTGCGGT
CAGAAGAGTATTGATCTCTCTACAGACGTGAGAGAACCCCTTTCAATATTTTTTTTAGC
GTTGGAACGTCGCGTTAACGTGCTGAAGAAAAAGAAAATTAAGTATACACACAGAGA
GAAATAACAGGAACCTGAAAGAATATTATACCAGAAAAAACAAATGAGAATACAATCA
AAGAGGAATAAACGATTCCCCAAAAAAGAAGGAAAAGAAGAAGGAAATTGTAATTGG
AAATGGTGCAAGCGTGACGAGACAGGTGAAACAAACCATAATATTTACATACCAATCTT
ATGAAGAATAAAGAATGAGTTGAACACGATGCATAGACATACAAGTGGAACGTGATAC
CTTTCAAGCTCAACTTCATTAAGACCAACCTAACAAAAACAAAACAAAACAAAAACA
CAAATAAAAAGAAAACAAAAAAATGATGTAAACGAATTTACAGAGGGAGAGAAAACT
AGGAAAAAATCAACGAGACACTGGCCACCTATACAACCAATCAACAAATACACTCAAT
TCTCCCTAAAAAACAAAAGGATCACAAAAAAAAAGACAGGAGAAACAAAACGTAGT
ACATACCAAACTTTTTGTAAGGAAACAAAAAGTGTGAATCGAATAGAGACAAAAGAA
ACAGCGATGTGACCCCAATCACATCCTCTAACCTCCACCTTAAACCAAGGTCGAATTCA
TTAAAACTAAACCAAAAGCAAAAAGAAAAGAAAGCCAACAAACTTACTTACTAAAGA
AAAAGAGAAGAAAACCGATTGATACGCACAGACAAAAAGTATATACCCCCAGAAAATT
CCCCTTCCTCCTGTTCCAAGAAAGAAAAGAGAAAAAGAATCAAACTCGAAGGAGCGA
GGAGTGACACGAGGCAGGTGGAAAGCAACTCTCTTATGGAGAGGCAGAGTAGGGAGC
GGAGATAAAACAGAGAGACAAGAGGAGGAGAGATATATAATGGTGGCGCATGCTCCCC
CCAGTTGTGCACGTATTGTAGAGCAGGCAGTTGCCCCCGTCGGCTCCAGGTACACGCG
AAAATCCATATCGACGCGGTGTACCGTCTCCTACGGCCGTTGACAGAGGCACACGGCC
GGCCGCGCGCCTTCGGTTTTCACCCGTCGTCGCGACGCAGCTTCCTCGCCGACTTACG
CGCCGATCCTGCACACCGCGCCGAGGTGTTGCAAGGCTGCTTACGCCGCTTCGAGCCA
CGTGCCGCTGCCGCAGTCGCGTGTCACTGCTCGAGCGGACCTCTCGAGCAGAGGGAA
GGGAAAGGGAAAGGGTTTCGTCCCCCCAAATCGAGACGATGTGACACTTCTCACGAA
TGTAATAATCATCTAATCGTCCACTTCGAAGATAAATTCAAGGGAAGTTTTTTTCTTTTT
CTTTTTTTTTTTTTTCCCCTCCCCAAAAGAAGGAAAGAAGGTTGTTCCAAACGAGAATT
TTATCAAGAGAATTTTATCGCTCGCCGCTATGAAGAGGGATTTGGATGCAAATGGAAAA
GGGACACGTGTTTTCAACGTCGTACGTCCAAGGAAGAGATGTTTAGGAACAATTCGAA
ACGAGTGAATGGACATATTTTGTACGTCTATCATGTTTTCTTATGATATATAAATAGTACA
CGAGTTATTTTGAAAGGGAAAGGAAAGGGAGAGAGAAAGAAGAAAGGAGGATATTAT
CGAAGAAGATTAATCGAAGAAAGTTATACACGTTCGTGATAAATACGAGTTTTAATTAA
GAGGATATTATTTATTATAATGTTGTTTATAATTATTCTCGAAAAAGAGATAATTGGATAA
ATTTTTCATGAAAGTAGAAGACGATGAACATTTTATTCTAAAGAAGAATTCCTTTGTTTC
TGAAGGAAAAAATAATTAGAAACGATCGAAATAACTTCAAACTCCAACGTTATCGATAT
GAAACCTCTATGAAAAATAAAATTTCAAAGGATTACGTTACGTTAAAAACGGAATTGG
AAAGAAAAGGCTCGACGTCTCCAACACACTTACCAGATAGAACCGTAAATAAATAAAT
AACGAAGTTTTCAAACCTCATCCATTGAAAGTTCACCAAGTGATGGAGGAATAGCATT
TTAAAGAAGACGGGGCGTACATCGACTTGTTCCATCGGGGCCGATAAACGTAGAAACT
TCCCCGTCTCGAAGAAGAAAAGATTCGAGAGAAATCCTCGGGAGAATTTCAAAAGTT
CCCCTTTAGTGGAAAAACAAACGAAAGTAGCCGCACAAATTGTGTAAATTTGCACAAA
TTTCTCAATCATCCCCGGAGAGATCATTTTCGTCCATCGATATTTCATTCATATTATCGATA

TTTCGAAAGGCAGAGGCGGAATGAACGTTTTAAAACTCGTTTACGAACCTCTATCGAC
TATTGATTACAAGGGACTCTGAAGGGAGAGATTCCTCCTTGCGTCGTCGAGTTGAAATC
AGGGGGCGGTTGCAACGTCCTCTTTTTTCCCCCTTTTCACAAGGTGTAGCAGTCTCTCG
ATCGAAACCCCATCGTATCGGTTCCCGAAACTCGACGCACGCCCTTTTGTTCGTGGCAA
GAACAATAGAGGGATGAAGATCACGCTCGATGACAGACGTCGTTCGATAATTGGATCT
CGACCAATTCCGAAACATTTCTTCTTATTCATTTCTATCTAGGAAAACGTCTATTAGAAT
AAATCGAAGAAATCTCTCCAAGAGATATCGTTAACGATAATCCTTTGAATAAAGGGAGA
GGATTATTTTCTTAAAGTGTAATCATCAAGTGAACTAAAAAAAAGAAACTGTAATCCAG
TGGAACAACAATTCAGACGTTTAGAAATTGTCGAGTTTCTCTCTAAAGTTTCCCTTTGT
GGCGTAAGGATATATTTCGAAGGGATATCTCCTTCTCTCCTTCGCTTTGACGAAGGAATT
CAATGAACTCGGAATCCGACGAAATCGATCTCCGATTTCCAACCATTTGTAAAATAATC
CCGCGAAAACTTTGCCCCAATCGAGACAGAAGAGATGTGTATATATATATAGTAATAATG
TATAATAATGTTGTAAAATCACGTTTCGATATGAATCGCGCGTGTAAAGATCAAACATCG
TTCTTCAAAGATTAATTTTCCCTCTCCTCTCCCCCTCTTGTCTCCACAGTTTGCGGAAAG
GAATCCTCGAGGATTAACCCTCCTTTAATGGCCCAAGGATCTCGGAGGAGGATCAAGC
AGGCATTAGTCGTGGAATCGATGAAAATATTCTTATATCCCGAGTGATACAATTGATCAG
TGGAAAAAAAATAGAGTGATCGAGGAGAAAGGATTAAGTTTCCTCTTAATCGAACTAT
CACAGATCGACATTGCATTTGGGAATGGAAAAATCGTGAGTTCGATCTTTCTTTTCTTT
TTAACATTCTCGAATTCGATTCACGAAGAGTGTGTTAAGATTGGAAAGAAGAGATGTTA
ACGAATCATATATACGTGGATCCTTGAAAATTTTCCTCGAATTAATATCGAAGATAAATTT
TCTTTAAGGAGATATCTTGGAAAATTGGTTTTAAAGGAGCCGTGTAAACGATAAACATA
AATTCTTCTTCGATCTCTTTCCGTCTTTCGAAACGAACCCGATCGGAGAAGCGAAACGT
TTCGGTTAAGAAGGAAGGAAGCATGAGCTGGCAATCGAGCAACGAGACTGTTCTCGA
CAACGGTAATTACGTGCTGAACACGTTGCATTATATCAACAAATTGAACGAGTCTGTGA
ACCCAGAGGCGATCGAGTGTCTGGAACGTAAGCATAAGGAGGAGTTGGAAAGGTTGT
CGTTGGGAGGGATACTGGAGGAGGATCAACAGGGGTGCGAGATCAGTTGGGACTCTTT
ATTGTGCTGGCCTAGGACACCTCCTGGAACACTGGTCACGCTACCATGTTTCGATGAAT
TGAATGGGATCCGCTACGACAGCACCC

>novel_circ_000535
AAGATTAGCTGATGTGAATTGAACACACCTTGAAACACAGAAAATTGAAAGAGAAAA
ACACTGAGAAAAAGAAAACATATATAATATGCAGCGGATGTGGTTTATAGAGAACAAA
GAGGACGGGGAATACAAGCAGCAAGAGGAGCCACGTGAGACTAACTTCATCGAGGTG
GCAACTGAACATACATCAGACATGAGGGGATCTAAAAGAGATTGGATTTTTAGAGTCC
AAGTACATAAAGTAGAAAAAAATGAAGTTTTGTATGTAGTGGGTAATTTGCCTGAACTG
GGTGCTTGGAATCACAATCAGGCAATTCAGTTGTCACAGGAACATGGCAGTCGGGATT
CTCCTATACTTTTTGACAGTAATAGTAGTGGAGAATTTTATAGTGACGATGGACATGACA
ATGCTGGAGATAATTCATTTGGTGATATAGATGATGAAGGTGGAAGAATATTTTCACAA
AAAGTTGCACTTCCCATTGATGCTGATGTAGAATTCCGATATTTTGTTGCTGTCATCTGT
CATTCTAATGGTACTAAAAATTCGGCCAAGACTCTTATTATTCGTAGATGGGAGACTCAT
ATGACACCACGCTTTATTAAGAAAAATGCACCTAGCAATTTTAGTAGTGACACATTGCC
TGATCCAGACAAATTTGGTTATTATAATGGCTACTGTAAAATTGAACGAGGGTGGTTGA
CCGATGAAACTGTGATACAATTTAAGCTTTTTAATAATCCAATTAAGTTATGGAAAAATC
GATTACAAAATAGAAAAGTTCACATTAAAATGACACCTGTAAATTTAATTAGACACAAT

TCTTTAGAATTGCAACAATTTGGTGGTGATTGTGTAGATGATAGTCTTTCTATGGATACG
CAAGATATTATTGATCAACCTGCCTTTAGTATTACAGAAGTTGCGGTAATGAATGATGAA
GAAGCTCGCTTCAAGATACAAGAACAATTTGGTCGAGCTTACAATGAAGACGAATTTA
TTATCTTCAATGTTGCAGTTCGATATCCTGAAACTATTGCTTATTTGGTGGACTACTACGT
ATACAGCTCAAGATGTTTTCCAGGAGAACCACCAAGTCATATCGGTTTCAGCTATATTTT
ACCTAGTACATTACAATCTAGCGTTGGGCTTCTAACAGTACCAATTACAAGCACAAAGC
ATAGACCAATTGGACAACTTACATTAGAATATGTTGTGATAAAATCAATTCCAGATTATC
CATGGGATATGAGTATTTCATATGCTAAACATTGGGAACAACGGTGGTCTGGCCTAGAT
GTAGGACATAGGGGATTAGGTACATCTTTTCAAACAAAAAACTGTGCTAATGTGCGTGA
AAATACAATAGCTTCTTTAAAGACTGCATCATATCATGGAGCTGATATGGTTGAATTTGA
TGTTCAATTATCTAAAGATCACATACCGGTTATTTACCACGATTTTTATGTGTCTATTTCA
TTAAAACGCAAAAAACAAATAGAAGCTATGGATATGCTAGAAATACCTGTTAAAGACCT
TACATTAGAACAACTACATCTATTGAAG

>novel_circ_000536
AGAACAAAGAGGACGGGGAATACAAGCAGCAAGAGGAGCCACGTGAGACTAACTTC
ATCGAGGTGGCAACTGAACATACATCAGACATGAGGGGATCTAAAAGAGATTGGATTT
TTAGAGTCCAAGTACATAAAGTAGAAAAAAATGAAGTTTTGTATGTAGTGGGTAATTTG
CCTGAACTGGGTGCTTGGAATCACAATCAGGCAATTCAGTTGTCACAGGAACATGGCA
GTCGGGATTCTCCTATACTTTTTGACAGTAATAGTAGTGGAGAATTTTATAGTGACGATG
GACATGACAATGCTGGAGATAATTCATTTGGTGATATAGATGATGAAGGTGGAAGAATA
TTTTCACAAAAAGTTGCACTTCCCATTGATGCTGATGTAGAATTCCGATATTTTGTTGCT
GTCATCTGTCATTCTAATGGTACTAAAAATTCGGCCAAGACTCTTATTATTCGTAGATGG
GAGACTCATATGACACCACGCTTTATTAAGAAAAATGCACCTAGCAATTTTAGTAGTGA
CACATTGCCTGATCCAGACAAATTTGGTTATTATAATGGCTACTGTAAAATTGAACGAG
GGTGGTTGACCGATGAAACTGTGATACAATTTAAGCTTTTTAATAATCCAATTAAGTTAT
GGAAAAATCGATTACAAAATAGAAAAGTTCACATTAAAATGACACCTGTAAATTTAATT
AGACACAATTCTTTAGAATTGCAACAATTTGGTGGTGATTGTGTAGATGATAGTCTTTCT
ATGGATACGCAAGATATTATTGATCAACCTGCCTTTAGTATTACAGAAGTTGCGGTAATG
AATGATGAAGAAGCTCGCTTCAAGATACAAGAACAATTTGGTCGAGCTTACAATGAAG
ACGAATTTATTATCTTCAATGTTGCAGTTCGATATCCTGAAACTATTGCTTATTTGGTGGA
CTACTACGTATACAGCTCAAGATGTTTTCCAGGAGAACCACCAAGTCATATCGGTTTCA
GCTATATTTTACCTAGTACATTACAATCTAGCGTTGGGCTTCTAACAGTACCAATTACAA
GCACAAAGCATAGACCAATTGGACAACTTACATTAGAATATGTTGTGATAAAATCAATT
CCAGATTATCCATGGGATATGAGTATTTCATATGCTAAACATTGGGAACAACGGTGGTCT
GGCCTAGATGTAGGACATAGGGGATTAGGTACATCTTTTCAAACAAAAAACTGTGCTAA
TGTGCGTGAAAATACAATAGCTTCTTTAAAGACTGCATCATATCATGGAGCTGATATGGT
TGAATTTGATGTTCAATTATCTAAAGATCACATACCGGTTATTTACCACGATTTTTATGTG
TCTATTTCATTAAAACGCAAAAAACAAATAGAAGCTATGGATATGCTAGAAATACCTGTT
AAAGACCTTACATTAGAACAACTACATCTATTGAAG

>novel_circ_000537
ATATGACTCTTTGTCCTTATAATATAATTGCTCACAAAACAATCCAATAAGCATGGATGAT
GAGTTTTATTTTATCAAACAGCAGTGAGGCGAATATACTGTAATGGGGTGGGTTTTGTC

CACCATAATATGGATTACTATATGGAGTTGCCTCTTGCGGGGCTTCGAATTAGCTCCACA
GATATCTCCAAAACAAATTCCTTGTGATAAAACAAGAAAAGTTTTTACTGATTCTTGGG
GAATTATTAGTGATGGACCATTGGGCTCAAATTATACTCAAGATTCTCATTGTGAATGGC
TTATAAAAGCAAATAATAGTCGACAATTTATTACTTTAAGTTTTCGTACAATGGGAACAG
AATGTAGCTATGATTATGTTTTTGTTTATGATGGAGATTCTTTTCGATCTCCTCTTTTAGG
CAGTTTTAGTGGTAAAACTGAACCACAACAAGTAACATCATCATCTGGTTATATGTTAAT
ACTATTGTATAGTGATACAAACTATGTTTTGGATGGTTTTCATGCGGAATTTTCAGTTAC
AAATTGTTCAAACAATTGTACAAATCATGGCAAATGTATTGATAATACATGCTTTTGTGA
GGATGATTGGGGTGGACGAGATTGTTCTAGAGCTCTTTGTCCAAATAATTGTAGTTATA
GTGGAAAATGTGATTTAAAACGATGTCACTGTCACAATGGATATTCAGGACAATCTTGT
TCATTACATAAAATGCATCCTGAAAGCAACAAATGGCACTGGCTTTCTCATTCAGAAGG
TGGATTAAGGCCACGGGCAGCACATACTGCAGTTTATATAAAAGAAACTGATTCTCTTT
ATGTTTTTGGAGGCTATGATCTCAATTATATACTTGATAATTTGGAAGTGTATCGATTTAA
TATAAGTCAATGGGAAGACGAATATGGTAATGAATTAGAAGGCATCTCATCTGCAGAAC
ATTTAGATCCTATACTCCTTGCCCTTGAATTAAAAGGAACAAATACAGATGAAAAAGAA
TTATATGGTCTTCGTACTTCGTCTTTCATATGGAAATTGCTTTCTGGTATTAAAGATAATA
ACAATACCTTTAATTCACGTAATCTTGCTAATGTAGAAAAAAGATATGGCTGGTCAGAA
TTTAGGATTGGAGATCGTGAAAAAAAAATTCATGAAACTAAAGATAATGAAACTAGTA
GAAGTACAGTTAGAATAGAAAGAAATGATGATCTTAAAAGAAAACATTCTAGACATCCT
AGACCTCGATATGCACAAACTGCAAGACACCGTAGAAATCTTGAAAACCTAGATTTTTA
TAAAAAATTTCATACTTCAAATATAAAAAGTGATATATTAAATTGGGAAGAGGAAATTGC
AGAAGATTCTATTGGAGATAATATTTACGTTCAAGAACCTAAAATAAGAGACGAATTATT
TAAAGATGAATCTATTAAATCATCAATTGATAAACTACCTAAACCAAGTCCGCGATATGG
CCATGCTGCTTGTAGATATGATGGTGGTTTTGTTATATATGGTGGAAAAGTACAAGATGG
TTCTTTATCTAATGAATTATGGTATTATAATGTTAATACACGTTCCTGGACATTAAGAGCA
AAAAATTCTCCATTTTATCCTCCACAATTAACAAGACATACACTTACTTTAGCAAATAAT
TATATTTATCTTTTTGGTGGTAGTACAGTTGATGGTGAATTTTCATCAAATTTATATAAGA
TTAAATTAAATTTATCTGACCCTACAGCAACTACAGAAAAATGGAAAGAAGTACATCCT
CGAGGAGGTAAAGAGTTAGATGTACGTGTAGTTGCACATTCAACTGTATATCATCAAGC
TACTAATTCTTTATTAATTTATGGAGGAGTAGTAGCCAGTGTAGCACGATTTAGTAAATTA
TCAGATAGGATGTTTGTATTTCAACTTGACAAAAAAGTTTGGTCGGAAATTCATTATCC
AAGAGCACATTTAAGAGATACTTATGTTCCAAGAGAACGAGCATTTCATACCTGTAATA
TTATAGGAAATTACTTAGTTGTATTTGGCGGATATTCTCATAGACATAATAAAGAAGAAA
TATGTTATGATAATCAGATGTATCTTTATCATTTAGGCTGTCACGCTTGGGTAAGCCATGA
TGTATTAGGTTTAAATGATAAAGACTCTCGTTATCCAAAACAACAGGGAGTATTTGCAC
ATGCAGCAGATGTACGGAATGGAAATACATTGTTATTAGTTGGTGGTTATCATGGAAATG
TAAATGCTGATTTACTTGCATATACATTACCACCAATGCTTGCTCCAGGAGATGAAGATA
ATATAGAACCTGAGCAAATTTGTTCAAGACATAAAAGTTTGATGGAATGTACAGCTAAT
CCAGAATGTGGTTGGTGTTCGGCAGATGAGATATGTTACGGTAGGACTATTGGTAGTAA
TTGTACAACAAATCTTCAAACGACGCGTTGTTTAGGTGTTTGTCCTGCGCTTGGAGATT
GTCATTCTTGTTTAATACATGGTCAACCTGGTGGAGGGTGGGGTACAACTGCACGTGG
AAAAAAGTCTGTGTCTAATAAGTTAAATCGTGGAACTTGTACTTGGTGTGTTCAAAATG
CACGTTGTCATCATAAAGATGATAATTATGGTGTTTGTGGACTTCGAGATGATACATTAT
CTCAAATACCGGGATGGTGGGGATCGAAAGGTACAGAAATTATAAAAGTTGAAGAATG

TCGGGAAATGGATAAAAGACCAGGATTAACATTTTTAAAATATAAACCTCCAGTAAATT
TTACTCAACCTGATTCAGTTACGATAGTTAATGCTACTACAGTAGATTTTAGTGTTCCAT
CTATGCAAGGAGCAAAAACAGAATCAGCACTTGGTGGAGAAATGATTGCTAGATTAAT
TGGTTTTCTTAGACCACCTCAAGATTTTTGGGATAGTACACTAGAACATTTGAAAATTT
GTGTTAGCTACAATAGTGCAACATTACATGTATCAAGAAGTGATGATCCTCAAGAATTG
GAATTAGTTGCAAATTTAACTGCCGATACATCGCAATGTATTCCAACAAAATGGCCAGA
TGGATATCAAATGATGTTATTACCAGGTCGATATTTATTAGATTTTGAATCAAAAAGAAT
GGTTACTGCAAGTTATGCATATGCTAGTAAAATGGAAATTATTCATAACAAAAAAACCG
AGAATCCAAAAGTATTTACATTTGAATATCTCGAACCATTTCAAAACGGTTCTTGTCAG
CAATATAAAAACTGCCTTCATTGCTTAACTGATTCTTCATGCG

>novel_circ_000538
ATGAAAATAGAAACATCACAGGACATGACATAAATATTAATATAAAAACATACGGGAAC
AAACGGCATCATGAAGAATCTCGCGCCGTAGTCGCCACTTGCAATGTTAGTCACACAG
ACACCCTCCTCGACGGACAATCTGCCGAATATGCTGCCGGTACTCGAGGAGGAGGCGG
ATGTCGATGTTGACGATGCTTTCCCGCCACACGTTGACGCGACGAACATCATCATACAA
CCGGAGTCGCCCACCAGCAAGAAGCAGGCACTGAAAACCTTCAACGAGGTCAACGC
GCTCCTCCAGGCCGGCAGGAAAAGGGAGGCTAAGTTGATCATAAGGGAAAATGCATG
GCCATTGAACAACGGGATCAGGACGCAGTTGTGGCCAGCCCTTTGCCAGCAACACGC
ACATGGGAAGAATATGCTTGATGGATTCTATTGGGACATGGTCAATCAAGTGTTTGGAA
CTACAGAACTGCCGGAAAAATCAATCATGTTACCACCATTCGTGGACAGTACTCATTGC
CTGTCGTACCACCTGACGAGAAAAGGCAGGTGCGTTGCGGACAGGGTTGTATCTGTGC
TGGGATACGCTTGTCCCGATATTACCTACAGTCCTTCTCTTTATCCCATCACAGCTCTTCT
TCTTCACTTTATGCCAGAGGAGGAGTGTTATCATTGCATGGCCAGTTTGGTTGCCGCTA
AGGATAAGATGTTCATCACACAAACGAAGCTCCTTTACGAAGTTACCTGGAAAACTGT
GGAACAAATTACGAAAAAGCACGTGAAATCGGCAGCGGTACATTTAGCGAGGCATTGT
TCTGGATCAAGAGCAGAAAGAATTTATATGGATTGGATGTGGTGGATTCTTCAACTTCT
TCCTTTCCAACATCTAGTCAGAGTTATGGATTGTTTTCTTCATGAAGGCATTAAGGTATT
CTATCGAGTAGCAATGGCAATAGTTTTATTATTCTATAAACATTCATCGTTGCAAAATTCT
GAATGGATGAACGAAATTTCGAAGAATGGAATTGATGCTGCACTTTCAAAATTTTGCA
GGCAAATACCGGTAACGTCAGCTAAATTTTTACGTACCGCATTTGGAATACGTGGACTT
AGTTCAGCATACATATCAAGAGTATTTTTGCGCACAGAAATGGCTCTAAAAAGTAAAAG
TGTTTTAACGGGATCCAGATCATTGGCAAGATCACGATCCACAGACAATCTGCCGACG
AGCCAATCTCAAGTCAACATTCAGATGATGTCACACACGCTCACGATACGAGAA

>novel_circ_000539
CGAACTCAAACTCGGAGGAGGGATCCATCGAAACATCGGTCGTCGAGCAGTGTCGCC
CCCTCGAATCCTCGAAGGGGATTTCGGAGGTTGAGAAATTCGCGCGAGATAATACGAA
ACGAAGTCGGGCGGCGGTCGAGCGGAACAAGTGGTTGGTTAAACTGTGATGACCGGG
CACCGGTCGGGCAAAGTACTGGAGTGCAAAGCCTCGATCCCATCGTGCACCGTCCGGA
TACCGGTGCACAGGCCAACGATCGAGAGAAGAGGAAAGAAACGAGCCCCGACGACG
ATCAACGGACAAATATTATTCTTATTATCGTTGTTATTACCATCGTCGAGCGTCGGGCCG
AAGCGTTGAGGAGACGGGGTTATCACGACGAGGCAGGAGGCTGGCAGGACGGTGGA
ACGATGCCCACCACCGGCGTGGATTCGCAGCAGCGAACGCGCAGTTTTTCGTAGGAAA

CGGGAGCACGGCCGATCACGAAAGCGGCGACACGAGGGCCGACGACGAAAGGAAGA
CGAGGCGAGGAGGGACGCGGCAAGGTTGGAACGCGGCGGCGGAGTCGGCCGGCTCG
AAGCGGAGGGAAAGCGAGCAGACGGAGGAAAGGACAGGAAGAGGCGAGCAGCCGG
AGAGTGAGCGGTCTATGACGTTACCGGTGGCCGTCTACGTCACGTTAACG

>novel_circ_000540
GTAAGCTCCCATCATATATATTTTCGTTCAACCAGCTTATCCAATTAATCGAAATAACTAA
GTAAGTATTTATCACCTCATTCTCTCTCTCTCTCTCTCTTTCTCTCTCTCAAAACTTAAGA
TTACAAAATGCAAAATTTTTGAAATTCACTCCATCCGCTCCTTTCTCCCTCCCCTTCTTT
CATCCTCTTTCTTAATACGCGAGGTCAAGTACAGAGCGTGGATGTTGCACGGAAGGCA
AGAGAAAAAGAGAGCGCATCATAGAAAGAAGGAAGAAAGGAAGGAAGGAAGGACA
GGACAGGAGAAGATAGAACGTGGATAGAATCGGAATCGAGTGTGTGTGAACGAAGGG
TGAACGACCGAAAG

>novel_circ_000541
GTAAGCTCCCATCATATATATTTTCGTTCAACCAGCTTATCCAATTAATCGAAATAACTAA
GTAAGTATTTATCACCTCATTCTCTCTCTCTCTCTCTCTTTCTCTCTCTCAAAACTTAAGA
TTACAAAATGCAAAATTTTTGAAATTCACTCCATCCGCTCCTTTCTCCCTCCCCTTCTTT
CATCCTCTTTCTTAATACGCGAGGTCAAGTACAGAGCGTGGATGTTGCACGGAAGGCA
AGAGAAAAAGAGAGCGCATCATAGAAAGAAGGAAGAAAGGAAGGAAGGAAGGACA
GGACAGGAGAAGATAGAACGTGGATAGAATCGGAATCGAGTGTGTGTGAACGAAGGG
TGAACGACCGAAAGGTAGGGGCACTAGGCTGCTACCCTCCGCGACGAGAGAGAGAGA
GAGAGAGAAAGACGTTGGAGGGTTGCGGAGAAGGTGGCGGTCAGAGAGAGAGAGA
GAAGGAAGGTTTCGAACGGATCGGCTCTGACCTCCTCTCTCGCTCCTATCCCCTCCCTC
CTTCGCTCGCCTCTCCTTCATCGTGGCCAGCGAAGAGGATCAATACGGACCACGTG

>novel_circ_000543
ACTCCCCGGTATCCCTTGGATCAGCAGGCTCGCCGCTCGGGATCCAGCCGGCGGACGC
ACCTTCACCCATAAACTTTCCTGCTCCAGGGGAGAGCATGGCCGACACCTCGACCCTG
TCGCCGGAGGCCAACATGCCGGGCTCGCCGGGCTGCCCCGTGGTCAAAGTGAAGAGC
GAGTACCTGCTCGGGATACCGCCGAGCGATCCTCTCCGCGGGACGGCGAGCGTCGGCC
TGGAGCATTCCCGCGCGGACGCGGACCGCGCCTCGCCCGACTCCGTCTTCCCCGACGC
GGCCTCCATTGTCGGCTACGCCAGGGTCGTCGAGGACCAACAACAACAGCAACAGCA
ACAGCAACAACAACACCATCAACCTCATCAGCAACAGCAACGCTCAGCCACGCCGAC
CGTAGCCACCAGGCTGTCGCCGTACTCGCCCATGCAAGCCGTGTCCACGACCCCGATG
CATGGACAGCAGGAGCAGCCGAGCTCGAGGAGCGACAGCCCCGAGAAAGCGGACCT
GTCCTCCGCCCAGACCAAGGAGGAGTCCGACAACTCGGTGTTCGACGGCGGAGGCGG
CCGCTCCGAGGCCTCCAGTCAACCTAGTCACCGCAGCAGCCCGTCCTCCCAATACAGC
CCCAGCCAGAGCGCGAGCCCCGTTCAACAGCAGCAGCAAACGGGGGGCCTGAGAGG
TAGTAGGGTGCCCCCGCACCTGTTGGCCCACCACCAGTTCTGGTCGTCGCAGAACAAC
GGGTTCGCCACGCAGAGGCTGTTGAACGGGGTGATCAGCAGCGCGGTGAGCTACGCC
GGCCAGACGGTGGCGGTCGCGTCCACCAGCAGCGGCGGGGCGGCGAGCAGCGGGGG
GAGCGGGAACGGGGCGAGCTCGGCGGGGCAAGCGAGCACCGGACCCACCACGGCCG
CCCCATCCTCCT

>novel_circ_000544
ACTCCCCGGTATCCCTTGGATCAGCAGGCTCGCCGCTCGGGATCCAGCCGGCGGACGC
ACCTTCACCCATAAACTTTCCTGCTCCAGGGGAGAGCATGGCCGACACCTCGACCCTG
TCGCCGGAGGCCAACATGCCGGGCTCGCCGGGCTGCCCCGTGGTCAAAGTGAAGAGC
GAGTACCTGCTCGGGATACCGCCGAGCGATCCTCTCCGCGGGACGGCGAGCGTCGGCC
TGGAGCATTCCCGCGCGGACGCGGACCGCGCCTCGCCCGACTCCGTCTTCCCCGACGC
GGCCTCCATTGTCGGCTACGCCAGGGTCGTCGAGGACCAACAACAACAGCAACAGCA
ACAGCAACAACAACACCATCAACCTCATCAGCAACAGCAACGCTCAGCCACGCCGAC
CGTAGCCACCAGGCTGTCGCCGTACTCGCCCATGCAAGCCGTGTCCACGACCCCGATG
CATGGACAGCAGGAGCAGCCGAGCTCGAGGAGCGACAGCCCCGAGAAAGCGGACCT
GTCCTCCGCCCAGACCAAGGAGGAGTCCGACAACTCGGTGTTCGACGGCGGAGGCGG
CCGCTCCGAGGCCTCCAGTCAACCTAGTCACCGCAGCAGCCCGTCCTCCCAATACAGC
CCCAGCCAGAGCGCGAGCCCCGTTCAACAGCAGCAGCAAACGGGGGGCCTGAGAGG
TAGTAGGGTGCCCCCGCACCTGTTGGCCCACCACCAGTTCTGGTCGTCGCAGAACAAC
GGGTTCGCCACGCAGAGGCTGTTGAACGGGGTGATCAGCAGCGCGGTGAGCTACGCC
GGCCAGACGGTGGCGGTCGCGTCCACCAGCAGCGGCGGGGCGGCGAGCAGCGGGGG
GAGCGGGAACGGGGCGAGCTCGGCGGGGCAAGCGAGCACCGGACCCACCACGGCCG
CCCCATCCTCCTGCGAGACGATGAAGCCGCCGGCGCAGAGGGCAGCGCCGGCACCCC
CGAGGCCGCCACCCACGGTGATAATGGGCGAGATCGGCGGGGTGCGGACGATGATCTG
GTCAGCGCCACCCCTGGACCCGGTGCCGCCCCCGGCGGCCTCGTGGACGACGGCGAC
GGCGGCGGCTGCCGCGGCCGCAGCGGCCGCTGCGGCCGCGGCGGCCTCCTGCTCCTC
GTCGGAGGAGTCGGCCGCCCAGCTGTTGCTGAACCTCGGGCAGGAGTCGAGGGGGGG
GAAGCAGGCGTCGTCGTCGGCGGCGGCGGGGCCGTACTCGTCGGCGGCGGGGCCCCC
GCTCAACATGGAGAGGCTGTGGGCCGGGGACATGACGCAGCTGCCGGCCGCGCAGCA
GATGCAGGCGTTGAACCTGACCGCGTCGGGGTCCGGGCAGGCGTGGGAGAGGAACG
GGGGGGTGGTCAAGTCCGAGGGGGCGGCCGCGTCGCAGCCGATTTCGGTCGCGCCGC
CCGCGCCCCCGATGCCCCCGCAGGAGCACGAGGAGGACGAGACGCCTATGATATGCAT
GATATGCGAGGACAAGGCAACCGGGCTGCACTACGGGATCATCACGTGCGAGGG

>novel_circ_000545
GGGAGAGCATGGCCGACACCTCGACCCTGTCGCCGGAGGCCAACATGCCGGGCTCGC
CGGGCTGCCCCGTGGTCAAAGTGAAGAGCGAGTACCTGCTCGGGATACCGCCGAGCG
ATCCTCTCCGCGGGACGGCGAGCGTCGGCCTGGAGCATTCCCGCGCGGACGCGGACC
GCGCCTCGCCCGACTCCGTCTTCCCCGACGCGGCCTCCATTGTCGGCTACGCCAGGGT
CGTCGAGGACCAACAACAACAGCAACAGCAACAGCAACAACAACACCATCAACCTCA
TCAGCAACAGCAACGCTCAGCCACGCCGACCGTAGCCACCAGGCTGTCGCCGTACTC
GCCCATGCAAGCCGTGTCCACGACCCCGATGCATGGACAGCAGGAGCAGCCGAGCTC
GAGGAGCGACAGCCCCGAGAAAGCGGACCTGTCCTCCGCCCAGACCAAGGAGGAGT
CCGACAACTCGGTGTTCGACGGCGGAGGCGGCCGCTCCGAGGCCTCCAGTCAACCTA
GTCACCGCAGCAGCCCGTCCTCCCAATACAGCCCCAGCCAGAGCGCGAGCCCCGTTCA
ACAGCAGCAGCAAACGGGGGGCCTGAGAGGTAGTAGGGTGCCCCCGCACCTGTTGGC
CCACCACCAGTTCTGGTCGTCGCAGAACAACGGGTTCGCCACGCAGAGGCTGTTGAA
CGGGGTGATCAGCAGCGCGGTGAGCTACGCCGGCCAGACGGTGGCGGTCGCGTCCAC

CAGCAGCGGCGGGGCGGCGAGCAGCGGGGGGAGCGGGAACGGGGCGAGCTCGGCG
GGGCAAGCGAGCACCGGACCCACCACGGCCGCCCCATCCTCCTGCGAGACGATGAAG
CCGCCGGCGCAGAGGGCAGCGCCGGCACCCCCGAGGCCGCCACCCACGGTGATAATG
GGCGAGATCGGCGGGGTGCGGACGATGATCTGGTCAGCGCCACCCCTGGACCCGGTG
CCGCCCCCGGCGGCCTCGTGGACGACGGCGACGGCGGCGGCTGCCGCGGCCGCAGCG
GCCGCTGCGGCCGCGGCGGCCTCCTGCTCCTCGTCGGAGGAGTCGGCCGCCCAGCTG
TTGCTGAACCTCGGGCAGGAGTCGAGGGGGGGGAAGCAGGCGTCGTCGTCGGCGGC
GGCGGGGCCGTACTCGTCGGCGGCGGGGCCCCCGCTCAACATGGAGAGGCTGTGGGC
CGGGGACATGACGCAGCTGCCGGCCGCGCAGCAGATGCAGGCGTTGAACCTGACCGC
GTCGGGGTCCGGGCAGGCGTGGGAGAGGAACGGGGGGGTGGTCAAGTCCGAGGGGG
CGGCCGCGTCGCAGCCGATTTCGGTCGCGCCGCCCGCGCCCCCGATGCCCCCGCAGGA
GCACGAGGAGGACGAGACGCCTATGATATGCATGATATGCGAGGACAAGGCAACCGGG
CTGCACTACGGGATCATCACGTGCGAGGG

>novel_circ_000546
GTGGTACACTTGAGGCAGAGGCTAGACAACACGGTAAGCAGTTCGAGGGATCGATCTT
TCCCTTTCGACGCGACTGTCGCCATGATCCAGTCCCTCATAGACTGCGACGAGTTCCAA
GATATTGCCACGTTGAGGAACTTGGACGAGCTGCTGGACCATAAATCAAACTTAAGCG
ACAAGCTGTGTCAGATAGGGGACAGTATAGTGTACAAGTTGGTGCAGTGGACCAAAA
GGTTACCGTTTTACCTTGAGCTACCGGTCGAAGTTCACACGACATTGCTCACCCACAA
GTGGCACGAGATCCTCGTGCTGACCACCTCCGCCTATCAGGCGATACACGGCCAGCAC
AAGTTCTCCAACGTCGGCAGCGACGCGATGGGAGCGGATTTTATGCAAGAA

>novel_circ_000547
GTTTCGAACAACATGTATACGTTGCAAACGTGCCTAACGAACATGATGGGCCGGCCGAT
AACCATGGACCAATTGCAGCAAGACGTAGGCCTGATGGTGGAAAAGATTACGTATGTC
ACGTTAATGTTCAGGAGGGTTAGATTACGGATGGAGGAGTACGTCTGTCTGAAAGTAAT
CACCATGCTCTCGCAAGCACGTGGAGGTACGATGGAATTAGAACATCTACAAGAACGG
TACATGAGCTGTCTGCGAAGTTTTGTCGAGCACAGCGCCCCACAACAGCCGAATCGGT
TTCACGATCTTCTCGTCAGGTTGCCCGAA

>novel_circ_000548
TGCACAACAATCTTCAGAAAATGCTGTTAAATCTGGTCGTCATACATTAGGCAATCAAG
TAATACGTAAAGGATACATGTGTATCCATAATCTTGGTATAATGAAAGGTGGCTCAAGAG
ATTATTGGTTCGTTTTAACGTCAGAGAGTATTTCATGGTATAAAGATGAAGAAGAACGT
GAGAAGAAGTATATGCTACCTTTAGATGGATTGAAATTACGCGATTTGGAACAAGGATT
TATGTCTCGGCGGCATTTGTTTGCTTTATTCAATCCAGAAGGCAGAAATGTTTATAAAGA
TTACAAACAGCTAGAATTGAGTTGTGAGACTCAAGATGACGTTGATTCATGGAAAGCT
TCTTTTCTCAGAGCTGGTGTATATCCTGAAAAATCAACAGAGCAAGCAAATGGTGAAG
GAGAGGATAAACCAAAACAACGGTACGAGAAAAATGGTACAGTTAGTACGAAAAATG
AGGGATATGAGGGTGGATCAGAAGCTCAATCATCCATGGATCCTCAACTTGAACGTCA
AGTGGAGACTATTAGAAATTTGGTAGATTCATATATGAAAATTGTTACAAAAACAACTC
GTGATTTGGTTCCAAAGACAATTATGCTTTTGATTATTAACAATGCAAAAGATTTCATCA
ATGCAGAGCTATTGGCACATCTATATGCAAGTGGTGACCAGGCTTCCATGATGGAAGAA

TCGCCCGAAGAAGCACAAAAACGAGAAGAAATGTTACGCATGTATCATGCGTGTAAAG
AAGCACTTCGTATTATCGGAGATGTTTCTATGGCGACAGTGTCCACTCCGGTCCCACCA
CCTGTGAAAAATGATTGGTTAGCATCCGGCGAAAATCCAAGTCTTGGGTTATCACCACC
ATCTCCTGGTGGACCAAGACGTGGAGTGACACAGCCACCCCCTCTTTCTAGTTCTCGA
GCTCCACCTCCAGTTCCTACGGGTGGCCGACCAGCGCCGGCTATTCCTAACCGACCTG
GACCTGGTGGACCTCCGCCAGCCCGTGCTACACCTGGCCTACCTCCTCCTCTTATACCA
TC

>novel_circ_000549
GGTGGATCAGAAGCTCAATCATCCATGGATCCTCAACTTGAACGTCAAGTGGAGACTA
TTAGAAATTTGGTAGATTCATATATGAAAATTGTTACAAAAACAACTCGTGATTTGGTTC
CAAAGACAATTATGCTTTTGATTATTAACAATGCAAAAGATTTCATCAATGCAGAGCTAT
TGGCACATCTATATGCAAGTGGTGACCAGGCTTCCATGATGGAAGAATCGCCCGAAGA
AGCACAAAAACGAGAAGAAATGTTACGCATGTATCATGCGTGTAAAGAAGCACTTCGT
ATTATCGGAGATGTTTCTATGGCGACAGTGTCCACTCCGGTCCCACCACCTGTGAAAAA
TGATTGGTTAGCATCCGGCGAAAATCCAAGTCTTGGGTTATCACCACCATCTCCTGGTG
GACCAAGACGTGGAGTGACACAGCCACCCCCTCTTTCTAGTTCTCGAGCTCCACCTCC
AGTTCCTACGGGTGGCCGACCAGCGCCGGCTATTCCTAACCGACCTGGACCTGGTGGA
CCTCCGCCAGCCCGTGCTACACCTGGCCTACCTCCTCCTCTTATACCATC

>novel_circ_000550
TTTTAACTCATCAAAACAACATGTCTGTGACTACAACTGTGCAGCCAGCTACTGAGAG
TCTTTCTCGCCATGGAAGCAACATTTCTTTGTCCTGCCACAGTTCGTGCGGAAGCGTTG
ACGATGGCCTCCAAGGATCTCATCCTCAGTATCAGGATACAATCCTTACTATTGAGGAA
CTTCGTGCTCAATTGAATTCTTGTTTCACATGTGGAGTTTCGTGGAGCGAAGAACACGT
TTCTCTCGATTGCAGTGAATGTGGTGGTTATTCTCTTCAACGACCGTGCCCACTTTGTG
ACGGACGTTGCCGCACCTCGTGGAAACGCGACCTTACTATG

>novel_circ_000551
AGAACGGAGACTCGGTAACCGGAAGGGTTGGAGGGCAGAGTATGAGGGTCGGCGAG
GAAACGCGGGAAGAAGATTCACAGCTCGGCGAAGCGGTTAATGACTGGCGGTCACCG
AACACAGCTGGACAGGTCTCCACCGGTTGGAGATGAGCACGAAGGAGGCGATGGCGA
CCACCGTTTCTTCGACGGGGGCGACGCTCGCTTCGTCGCCGAAGCTCGACGTCGAGGC
AGAGGACGACGAGGAGGAGCCATCGACCACGTTCGACGCGTTGGATATGGATCTGGT
GAACGGACCGAATCCGTGGCTTCCTGCCTATGGCTTCCAGCTGCAGCATCGTTCCCATT
TTCAACCCACGCACGAGGATCTACGGACTAATGGTTCGTTACTCTGTTGTCTCTTGTAT
GCGCATGCGCATGCGCGCACGTGTACGCGTGTTAGTTATCTATCGTTTCACTTTCCATCA
GTCGGTTAAACGATGCGCACAGACGAAACTGCGACCACTGCGTCGCGTTTAATTTAAA
AAAAAACTCCGTGGTTCTCTAACCTTAGGAGTCTTACCCGCGAGTTAATTTTCTATTGC
TTTTTTTCTCTCTCTCTCTCTCTCTCGTGTATTGAGGCGCACATACAATGATTGAAATAC
GTACGATATGATTGAATACTTTCTACTCGTTAAACGCGATTTACACGCAGTAATAAGACG
ATGATTTTTCTTTTTCTTTTTTTCTTTTTTTTCTCTCTCTCTCTCGCGTCTTAATATCAAAG
GTGATCGGTAAGATGGAAAAATAAGAATGGAAACGAGACGAGACGAATATTTCGAAA
GAATTAAATTAGGATGGAATGGGAAAACGATGTTGCTGAATTGTATCACGCTTTCATTC

GTTCCTACAATCTCACGCGAAGGAAGTGACCGGTTTGCTAATTACGCGTAGATCTCTCG
GTTTCTGTTTTACATTAATCCGTTGGAGAACGAGTCGTGAGTCACGAAAATATTTTTCTT
TCTCATATATATATATACATTGAAAAAAAAGATAACCTCGGTAAAGCGTAAATTTATTCA
GATATATCGAGAGTTGTTGACGAAAATAAAAAGGAAGAAAATAAAATAAAATAAAACT
TTCCAAGGCAATTCGTTCTAATATATACTCGGCCAGATCTGTCCGTGATCTGTCACGAAC
TGTTACATCGAACAGGAAGCCTCGTTATGTACGATGAATTCTTGCTGCTACCCCTCTCTC
CTCTACGCTCTATATATTAATAGATACTTGTGTATCGTTGCCTTAATTTCTATCGGAAAAA
GAGAAAAGTATCGCGCGGGTTTGAATACAAATCGTCTGTTAATGGTAAAAACGCTTGAT
ACGGACTACTTTCTACAATCATAGATCGTAGAACCGAAAGAAGGAAAGAGAAAAAAG
AATTCTGAGCGCAACCGAGATGACGATCTCGATTCGGGGAAGGAAGAAGCCTTCTTCC
ACTCTAGAGAATCCATTAAAATCCACGCGTTACAACAAAAAAAAAACAATTACCCGGG
CGAAACACACGCCAGTTTCTTTTATCGGGTGATCACGGGTTACGCAAAAATGTGCAAC
ACTGACGATGCGGATTCATTCATTCGTTCGTTGGTCGTTTCCTCGTGACCGATTCGAGC
GCGATTCGAATGACGCGTACCGCGCTGCAAAACCAGGCAGATCACCGATTATTTTTGTG
TAAGGCGACTGATTATTCACGTCCGTTGCCCGGTTGCGGAACGAACGGGCTCGCCGCA
ACAGGTGGTTCTCTTTCCTCCGTCATATTGTAGAACGAGTGTACAACTCGTATATATACA
GGATGCCGATATATTCGTGGAACAAAATTTGGAACAAAAGTTGATATATATATATATTTCG
GTTAAATTTATGCAAAGGATTTTGGTGAGATATATACAGGGTGTCGATATATTCGTGAAA
CAAGTTTGGAACAAAAGTTGATATATACATATCTCGGTTAAATTTATTTTATACAAAGGA
TTTTGGTGAGATATATACAGGGTGTCGATATATATATATATATATGGAACAAGATATAGAA
CAAAAATTGATATATATATTGGTTACATTTATTTTATGCAAAGGATTGTAATTAAGGATTTT
GGTGAGATATATACAGGGTGTCGATATATTCATGGAACAAGATTTGGAACAAAAATTGA
TATATATATTTCGATTAAATTTATTTCATGCAAAAGATTTTGGTTAGATATATATGGGTGTC
GATATATTCATGGAACAAGGTTTGGAACAAAAATTGATATATATATTTCAGTTAAATTTAT
TTTACGTAAAGGATTGTAATTAAAGGATTTTGAGATATATATATATATATATATATATAGG
GTGTCGATATATTCATGGAACAAAGTTTGGAACAAAAGTTGATATATATATATTTCAGTT
AAATTTATTTTATGTAAAGAATTATAATTAAAGGATTTTAATGAGATATATACAGGGTGTC
GATATGTATATGAAACAAAGTTTAGAATAAAAATTGATATATATATATCTCGGTTAAATTTA
TTTTATGTAAAGGATTGTAATTAAAGAAATTTTATGAGATATATACAGGGTGTTGATATAT
TCATAGAACAAGGTTTGGAACCAAAGTTTGGAACAAAAGTTGATATATATATTTCGGTT
AAATTTATTTTATGCAAAGGATCGTAATTAAAGGATTTTGGTGAGATATATATAGGGTGT
CGATATATTCATGGAACAAAGTTTGGAACAAAAGTTGATATATATATTTCGGTTAAATTTA
TTTTATGTAAAGAATTATAATTAAAGAAATTTTATGAGATATATACAGGGTGTTGATATAT
TTATGGAAAGTTTGGAACCAAAGTTTGGAACAAAAGTTGATATATATATCTCGGTTAAA
TTTATTTTATGCAAAGGATTGTAATTAAATAATTTTGGTGAGATATATACAGGGTGTCGAT
ATATTAATGGAACAAAGTTTAGAACAAAAGTTGATATATATATATTTCGGTTAAATTTATT
TTATGCAAAGGATTGTAATTAAATAATTTTGATGAGATATATACAGGGTGTCGATATATTC
GTGGAACAAAGTTTGGAGAAAATGGAGAAAGGAAGGAAAAAGCAAAAGTCGGTACA
TAGAAATCTCGGTTAAATTTATTTTATATAGAGAATTGTAATTAAAGAGTTGTGATGGGG
ATAAATCACGATATATTAATTTTGTCGGAAAAAAAAAAAAAAATATGGATTTCACATTCGG
AATGGAATTGACAGTTAACTTGTGCTCGATGAAGTTAAGGTTAATTGAAAAAGTGTCTT
ATTAATTAAAATTAATAAATATTTGAATATCTATAGCTATTATAATAACTATTTATCGAAGT
AGTAATTTCAGTATCGATGGTTAAAATTTTATTCATCTCCGACGAGGTGAAATGTATTTAT
AACATAATAAAATAAACCACGATATTTTTGCATTACCAACTTTTCGAAGAATCGATTTTC

AAAAAACGTTAACGCTTTTCACTTCTTGCAACTTTTGGTCGTTAAAATTTTGTAATTCGT
TCCTTTCTTTAATTGCGAATATACCTTCTGAAATGGAATAATTATATCATATACGATATAAT
TATTTTCGATATATAAAAAAAAAAAAAAAAGAAAGAAAAAAAAAATCAGCGGAAAGAAA
ATGCAAAAATTTATCATCACGCCGCCCGTTTAATTGTAATATACACTTGCCCATTGTCGA
ACAAATCTTTGCGCCGTCGTATTATTTTTATGATTCAGCAAACGAGTGGAGAAGGACAG
GTTTATATGCGCGTGCACGATAAAATAGCGGTGAATCCATAAGAATTCGAATCGAAGCG
TCGAATGCAAAATCTCCAATGACATAACGTATATGCGTCATCACACTCGAGCTATTTAAC
GATTATCGATCGTTTCTTTGGAAAAGAAAAAAAAAAAAAAGAAAGAAAGGAAAAATC
GAACGAGCGATTTATAAAATATGAATAAATTCGTAATTATATTATGTGGATTATTTTCTCT
CCTTTTATTTATGATTAATTTTCGTTTATATCGATATATGTATTTTTTATCTCTCTTCCATATA
TCGTCCGTGAGATATCTTCGTTCGTTTTACATTCCGCTAAAAATATGAAACATTCAATGT
TCGATAAGTTTATCATTTGACCTGACTTTCGAGTCAAGTTACAGAATTTCAAACATTTCG
GATTACATGTGTTAGTTGGAAAAAAAGAGTTTTGCACTCTGTATTATTTTTTTCACCGTG
GATCGAAACGTTTAATTCAATTATGTAAACATGGAGATTGAGAAATAATCGTGTTTCATT
ATGGGTTATTATATCCCATTACGAAGGTATTATATTAATGCCAAGTTTCTTGCTGTGAAAT
GAAATGTTTCTTATTAGAAATTTTCACGGAAAATTCTTCTTCGTCATAAAGTTGTTGTTT
ATACGAGAAACGTTTCATTTATCCTCGATTAATTTTCTCCTGTATAAAGCGTTTGATACGA
TTAAGATGTTTCTGTATAAATTTTTAACTATAACATTGTATTTTACTTTTCTCGTTTCGAG
AATCGAGCAATTTTAATTTACCGCCAGAAACGCCTGTACGCAGGCAACCAGTGAAGCG
TCGGTGTCCGATCGGTAAGGCAAGGAACGTACAGTAATCTGTCATTGTATAGTGTGTGC
TTAATTCCTTGGCGAAAAATTTTCTAAAATTAATTTAGAAACGAGTGGAAGATAATTGT
ACTTTATATATTTTAAACGTGTTAAATATTTATATTTTCTACTTATTACATTTATCTTAAAAT
ATCATCAATGAGTAATGTAACGTATAAGTTACAGTAGTTATTTTTATGTGTGAATAACTTT
TCGATAAGAACACTATGCGTTAGCATAGATCTCACTGCTCGGTGTTTATCATACTGTAGC
ACTCGTTCGTAGCCAGCTAGCTAGCTTATTTGTAAACACTTTTAACCGAAATATCGCGTT
TGAAGTATCGTGAGTGAAAATTCCAGAAAACGAAACTGAAAATAGGCGAGTGAAAATT
AAATACAGGGGAGAAATATTTAAAGCAAGATTAGATAAGGAGATTAACGATAAGTAGTA
ATGGTTTTATAAATTTAATAATTTCACTGTTTTGTTATTATTGTTTGCAGATTCATATCGTG
GGAGAAATATATACACACAATAGTTTTTTAATTAATAATTAATGGAAAATAAAATATGTGT
ACAAAATTCATGGAATTCCACGAAACGTACCAATAACACACGAACGTTTGAAACGTTA
ATGGCATTGCTCGTTCCTTACTCGTGTGTATCATATTACATAAGAAAACATGGCGCTGTT
ATTCTCGATGTGAAGCGATAACCTTGTTCCTACGAAAAGGACAACTTCGTTGGGAGTTT
GTGTACACAATTCGTTCGTCTCGAAACTATTCGAGATAAAAATTTTCGTGGCTCGATCG
AAATATAATATATAAATAAATTTTTTTTTTTATATATAAATATTATAAATATTCGTTAAAAAT
CATCGCTCTTCGTTCACAGATACGGTGCTGGTGCAACAAGTGGATTTTCTGGAGACCAT
TTTAGAAGAAACGTCCGACGACCTGCAAAGCGATTCCGATCGAAGTGGAACCACTTAC
TGGGTCGGCTCGGACTCCGAAACTGAAAGCGTGATACACATACGAGCGAATAGATTAG
CCGGTAAGGAATATACACGTGATATAAAGTTACGAAGGCATTTTTCTTGGATTTAAAATT
CTTTCGATTGAAGAAAAAAATTCTTCGATCGCTATCGATTTATAATTAAAGAAAAATTAT
CCAACAATAAGTCTAAAAATAAAACGAGAGAATTTTTGGACCAGGATGTCGAATATCA
ATTTGGCTAGATCGAACATGGTGGCCGTGTGCCATTTGTTTCATTATATCTTCTAATTAAA
ACGATCTCGCGCTTGAAACGAATTTTGTAACAACAGGTGTAACATTCTTTTTTAAAATT
TTAAAATATATTAAAATTATATATATATATATATATAGATATGTTAAATTTTTAAATATTGCGT
TTGTCCTAAATTCTAGTTTTTTGTTAGTTTTGGTTAGATAATAATTATTATGATACAACTTC

CACTCTCTATATATAAGATTGCCTCGAAAACGGTTCCAACAACGATATTGAAGAACAAG
TGAACGGTATTTCGTTTCGGAATACACGTTGCGAAACCTCATTCTCGCGTCTCGTCGGT
ATTCGCGATGGTGAACATTGTTCACCCGAGGTCCACTTTCTCTTTTCAACTCCCGATCC
CCCGATTCATTGACTCCCGAAGAATGAAATCGTATCTGTATATCTTCATTCAAACCAACA
TTCCGTAACACCAGTTTTCCTCTTGTATAACAAAAAACGAAAACATTTATTACAAACTC
ATTTATGAAACTCGAGCGTATCTATAAATAGATTCGTCGAATCGATTCTTCGATTTCGAAT
TATGATGGGATAAAAAAATTTTCGAAATCTTGTTCGATTAGGAAAATATTATTTTTTATTT
TCGATAATTATATTAAAAAAAAAAATAATATTAGTGAGATGTAAATATTTTTATTCGGTTG
TTTCGAATAATACGTTTAATAAGGACAGTTTCGTTTACTCTTTGTTCTTCGAATTTATCGC
AACGAGATTCGTATCCACGATGGTTGTGCGACCGTTGTAACGTGTCGGTATGTCCGGTT
GCTATGCTTCAGGCGTCACGGGCGCTGCATCACGCGCGCTCTCTTTTTGCGGGAGCGG
CCGTTTGTCGAAACGATTTTTCATCGGCGTGGAACAACCGTTGATCCATTCTCGTTCCC
TCGATAAATAGATCGGTAACGTGGACGCACGTGGAAAACTCGCGTTTGAATTTGATCA
ACTCGGTTTAACTCGGAAGTGGACTCGAACCACTTGTTACTCTAATTGAACGGTAATTG
CATCCCAGACAATTAATCTCTTTTGTTTACGTCTTTACCGCTATCCTTCTCGCACCGAGA
AGAGTACCGTTAAGCGTTTTATTAAAAGCTGTTGGCTCGCTGAAAGGTGGTTCGTTCTG
AACCTGTTAATCACTCTTGCTAATTACGTGTTGTAGTTTCGTAACAAGTGAACGTGGAT
TTATTACATTTTGAACTTTAACGGATTTAAAGAGAAATAATATATATGTATATTATACATGT
TGTATATATTGTATATATTCATTTTCCATCGTTAGATTTTTCCAAAAGAATTTAATAAAATT
ATTAATTTAATTATAAATTTATATCAGTATATTAATTATATTTTATAATTATATTTTATATTAAT
TATAAATTATATAAAATTATAAATTTAATAAAATTTCGCGCGCAAGTAAAATTTACTCGAA
TTAAGTATTTTTTTTTTTTTTTCAATTTTTTTTCAGTTTTTGTTAAAAACGTTAAAAGAAA
GATAACGTATCTCGCGTAACTCAATACCGACATCTGACGGAAATACCAGGAACTTGCAT
TATAGAACAGTATAACAGAAGCGATAAACATTTATCTTGGCCGTTCACCAACTATTCTCT
TTCCTTTTTTTTCACAAGAAAAGCGATTATTCTGAAGCGATTTATTCTGCTAATTATTAAT
CGATTCGCGTATGAATTAAAACGTGTAACTAGCATTTTCGAAGCAATATCTAGAATATAT
TATTCGATTATCGCACGAATAGAGAGTTCCGTTCCCTTTTCTTTTTCTTTTTTTTTTTTTT
TTGTTTTAATTCAGTGTTAAGGTCAATCGTGTCTACGATTCTACGCACGATCCTCTTCCG
TCGCATCTAATATTAGATCACCTTCAACGTGATTCATATAGGATTACCATGGACTTAGTCG
TTGAATTCCACGTGCATGCGATCATGCGAAAATAAACCGAGACGAGAACAATGCATCG
CGGAGCAAATTCAATTGTCGACCTGAAAAGAATTTCGAGTCGAGAGACGAGATTCGAT
CGGTAAGGAGGGGGGAACGAAAACGTTTCGCGCGTTTATTTCATTCGTGTTCCCCAA
AAAATCTCCTTCACCCCCACCTTACGGAATCGGTTTCCATCGATCGGTACGTGCAAGAA
TCGTAACCAACGGTTGTATTAATAGCGAATTTTGCAAACGAGTCTCGCGCGATTGTCGA
TAACGATACCCCGTCGATACTAAAAAGTACGCTCGACTCGATGAAAATTTTTTCCTGGA
AAAAAGAAAAGACGAGAAAAAAAATATCGAAAATTGAAACCAAACGTGGAACGTAAG
CGCGAATTAACACGAATGAGAATCGATCTCCTTCCCGTGTAATCGATGTCGAATTCCCT
TTACGTTTCTTTCACCGCTTTACTTACCCGACGGAAGTGGGGCGAGGAGTGGGGGCGG
CCTGATTGGCGGTGGCCGTGTCGCGGCCAGGAAGCCGTGGTTCGTTCAACCGGAAGC
GACGCTTACACCGGTTGTCGGCTTCGTGACCGTCGACGCGACACGTGACCGGCGACA
CGAGGCCCCGATAACGGGTCGTTAATCGGCAGACGACACGTTTCGCGCCGGCCGACGC
CTATCGTCGCCCACCCTTCGCGACCCTTCGCGCGCCCACAGTGGCTCTCAACCACCGC
TCGGCTCGATGCGATAACTCGAGAGGCGACTCTCCCCCGCGCAACCACTCGGCGGCAA
TTCGAATCTCGGCGGCAATATTTATTTATCTTTAACGTTCGATTTTTTTACGCACGTAATA

TAAAGTGTACGAAAATAATGTATCGTCGAACGATTAGATTTTTTTTTTTTATCTCAAATTA
GACCGCGATCGGATTACATGTTCGGCGTTGTTGGTTCGAGTTTTATGGAAATAGATATAT
TATTTTCGAAGTTTTTTTGTAATACGATAATGAAATTAGTTCGAGATCTTTTATTTTTTGT
TTTTTATTATTATTTTTTTTATTATTGTTTTTATTATTGAATAACGAAATTACTTATTCGTTA
AAGGATTAGATAAGAGGGAAGGTGAGAAATAAGAATAATGGAATATAGGAGAGCACGT
TTATTGGAATTGTAAGGTTTTTTCTCAACGATCTCAAATTTTCTTTCTTTTTTTTGTGAAA
AGATATGTAAAGTATTAAGATATAAATAAATCACGTGAAATTTTATTTTAAGTAATTCATC
AGGGAAATGGTGTCGAGTTGTTGAGTTCGGAAGAAGGTCGAAATTAACAGTCTAAGAT
TGTGATATGATGAAAAGTAGTATTGAAGTATTAGAAATATCGAGAGAAAGAACAAGAG
GAGCGCAAACTTTTCGAATCATTTTTACTTACCCCTTGCCTCTCGCCAATCTTCGCCTTC
ACTTTAACAAGGTTGGCTCGAAAGTAGGTTGGGTTTGGCAGGTTTCGTCGCGCTTTTA
CTCTCACGGCACGCAAACTTCTCTCCCCCTTCGTTCGAGGCAATTTGTATTTAATAAAA
ACAAAGTAGTAGAAGAAGTAAATAAATTGTGAACTAAAAATCTAATTAATTAAATTATT
AAAAAAATTATTAAAAACAAATGAAAAAGTAAATAAATTATAAAATAAAAAATTAATAA
ATTAAAGGCAGTTATTAAAAAAAATTATAAACTAATTAAAGGCAGTTACTAAAAAAAAT
TAATAAATTAAGAAATTAATAAATTATAATAATTATTACAATAATTGTAAATTAAATTAATA
ATTAATAATTAAATTTAAAATTGTAAATTAAAGACAGTTATTAAAAAAAATTGTAAATTA
ATAAATTAAAAATAGTTAGTAAAAAAAATTATAAACTAATAAATTAAAGGCAATTATTAA
AAAAAATTGTAAATTAATAAATTAAAGGCAGTTATTAAAAAAAATTTTTAAAAATTAATA
AATTAATAATAATTATTAAAAAAAATTATAAACTAATAAATTAAAAACAGTTATTAAAAA
AAAAAATTATAAACTAAAAAACAAGAAACTAATAAATTAAAGGCAGTTACTAAAAAAA
AATTATAAACTAAAAAACAAAAAACTAAAAAATTAAAGGCAATTACTAAAAAAAATTAT
AAATTAATAAATTAAAGATAGTTACTAAAAAAAAAAATTATAAACTAAGAAACAAGAAA
TAATAATAATAATAATAAATAAATAAATAATAAATTAAAAACAGTTACTAAAAAAAA
TTATAAACTAATAAATTAAAGGCAATTGCCAAAAAAAATTATAAATTAAAAAACAAAAA
GTAATTATTTAAAGGCAATTACTAAAAAAAATTAAATTAAGAAACAAGAAATAATAATA
GTAATAATAAATAAATAAATAATAAATTAAAAGCAGTTACTAAAAAAAATTATAAACTGA
TAAATTAAAGGCAATTACCAAAAAAAGATTATAAACTAAAAAACAAAAAAACTAATAA
ATTAAATTATTACAATTATTAATAAAAAAAATTATAAACTAAGAAACAAGAAATAATAAT
AAATTAAATAATAATAATAATAATAAATAAATAATAAATTAAAAGCAGTTACTAAAAAAA
ATTATAAACTAATAAATTAAAGGCAGTTACTAAAAAAAATTATAAACTAAAAAGACTAA
GGAACTAATAGATTAAATTACATTCACGTTTTCTGTGGAAAGATGAAAGGCTGGACGG
GAGCGGGAGCGAGTGCAATTCGGGGGCGGCGAGGAAGAAGAGGCGAAGGGGCGGCA
AGTACAATGGCGGGAACGAGCGGGAGCATCAGCGGGATCATCAGGCGGTGTTCGACG
ATTATCCGGCTTCGCCGAGATCGTCGAGATCGTCCGCCTCGTCGAGATCCAGCTCCCTT
CTTCAGTTCGAGTCTCTGGAGAGGACCTGCGCCACCCTCTCCCCGTCGAGCTACAGCT
TCGACTCGTTGGAGTATCCGAACCGGTCGAACGCCTCCCATCCCGGGAACACGTCTCC
GGATAGCCTCGAACAGGACTACGACAAGGCTCTGCCAAATGGTTTTGGAAACGTGGA
CCACTTTTCCAGAATCAGGCCTTACCGTAGCTTCGAGAGTCTGGACACGTGTCAGAAG
GAGGAGGAGGAGTTCGGCCACGGTTGCCTCGCCAATGGATTCACGCCTTTGTACCTGA
AACGAAACGCGGAGCTGTCCAGCAGGACGCGTAGCAACGGCTACCACCGGCCCTCGA
G

>novel_circ_000552

CGCGAACGGGAGGGCGCCAGTGTCCAGCAAGCCGCCCCTGCCGCCTCAACCGCCCCG
CTTCGGGGCGCTGGGCTCGAGAGCGTCGACGGGCGCGTCGGCGGCGACGGCGGCGGC
GTGTTCGTCGCGCCGACGCGCGGCCACGGACCTTGGAAATCCGGCGGCCATCGAGATG
GCGAAAGCCCGCACGATGCAGGCCGCCCAGGAGCGCCCGGTCGGCCTGCTCGAGACC
GACCTCGACGAGGCCGTCGTGCCGTCGACCACGACCCCGAACGCCGCCTCCACCGGC
AAAAAGACCAGGAGCCTGCTCAATCTGAATCACACCTCGACCGCCCCGCGGCCCAGG
CCCGAGCACGCCCTCCACGTACCCCAGTCGCCTGTCGCCGCTTCGCACAGGAGCCCCC
GCGACAAAGACGCCGCCTCCACCATCAACCAACGGCCACACAAATCCATGGAGTTCCT
CCTTGACAAGGAGAACCTGCATTTCGTCAAG

>novel_circ_000553
GGAGCGTGAAGCTGTGTTGGATGGAGAGCTCGTTCGTTGGCACGAGACCCGTGGATTC
ACCAGAGACGCCGATGAGTTTGGCCACGGAGACCGACTGCTGCCCGGGATGCAACGC
GACCGCTACCGAGACGTGCAGCTGCGTCGATTCCGCGACATCCGGCCTCTTCTTGGATT
TGACACCGACGCACGACGATGCCCAACACCACCAACAACAACAACAGCAAATAGGTG
GCGGCGGTAGCCTATGCCCATCACCGTCCTCGTCACTCAATTATCACCACCACCATCAT
CATCATCATCACCGTCAGCTTCACCATCAACAACAATCTCAGCAGCAACAGCGCCATC
ACCGCG

>novel_circ_000554
GCGGCGGTGGAGGCGGCGGCAGCGGCGGCAGCGGTGCGCTGCGACAGCACAAAAAA
CAGCGCAGAAAATCGCGAAGCGGAAGCAGCTCGCCGAGCGGCAGTAGCCGTTCAGGG
AGCAGCAGCAGCAGCCGCAGCGGTAGTTCCGCCGCCAGCACTTCCGGGGCAAGCACG
TCGCCCGGCAGCTCGCCCCACCGAGGCGGAAATGCGGCCGTCACCGAGGACCGCAGG
CCGCTCGCCATTTGCGTGCGTAATTTGCCGGCACGCAGCAGCGACACATCCCTCAAGG
ACGGCCTGTTCCACGAGTACAAAAAGCACGGGAAAGTGACGTGGGTGAAGGTGGTGG
GAGCAGCGGGGGATCGTTACGCGTTGGTCTGCTTCAAGAAGCCCGAGGACGTGGAGA
AGGCGCTCGAGGTTTCCCACGACAAGTTGTTCTTCGGATGCAAGATCGAGGTTGCCCC
ATACCAGGGTTACGATGTCGAGGACAACGAGTTCAGGCCTTACGAGGCCGAGGTCGAC
GAGTATCATCCAAAGGCGACCAGGACCCTGTTCATCGGCAACCTTGAGAAGGACGTCA
CCGCGTCCGAGCTTAGGAAACATTTCGAGCCTTTTGGCGAGATAATAGAAATAGATATT
AAGAAACAAGGCGCGGTATCGAGCTATGCCTTCTGCCAATACTCCGACATAGGCAGTG
TCGTCAAAGCTATGCGATCGATGGACGGCGAACACCTGGGTGCGAATCGAATAAAACT
CGGATTTGGAAAGTCGATGCCCACGTCCTGTGTATGGGTTGACGGTATCGGAG

>novel_circ_000555
ATATGGACGCGTGCAGAGCGTCAAGTTGCTGCCGCGGGGCGAGGAATGCCCCGTGGAT
GGAGGTTCCGGTGGTTCCCTCGGAGAAGGTAACGAGGGTGGTTCCGGTTCCAATTCTG
GCAACGGTGGTGGTGGCGGGGGCAGCGGTTGTTCCTCAGGCAGCACAGGGGGTGCCT
CCGCGACGGTGGCGTTCATGGATATCAAGTCCGCAGCGAAGGCCCACGCGACTGAACA
TACCTTGGACGAACGAGCCCTTACCACACAATACTACGAACCACAGCATCTTCAGCAC
AGGTTTCCCTCGCACGG

>novel_circ_000558

ATACGTGGGCGGTTCGAGGCGAGAGTTGTCAGGGGTGAAGGAACTGGTGACCTCGAG
GAGGGTGCAGAAGAGGCCCTCGGCGAACGTCGTAAGGTCGGATGGAGGTTGCATCGG
GAGCAGTCCAGGGAACGGCGGTGTGATCGCGGCCGGCTTGCTGGGCGGGTGCAGCGG
CGGCAACGACGCCTTAGCCGAGTTAACCATCGAGGACCTGGCATCTATTCCCGCCGGA
AATCACACCTCCGCCAATCACACAGGCCATTCCTCTCATCACAAG

>novel_circ_000559
GTGGTTTGAAGCACGGAAACGGCGGCATAGGCAGCGCGAGCAGTTCCAGAAGCACGA
GTCCGAGTCAACAACAACAATCGCCCCAAACCGGACTCTCCTTCATTCCCCAACCGAG
AAATCAAAATTCGACCAACAGACAACCCTCCACCACCACCCCGAGCCCCCGAGCCTC
GACGGTGGGCAAGCCCGGGGGTGTGAGGGGCGGCCAATCCTACGCGCCCCCGCAGCA
GAACAAGAACTCGGTGCTGGACAAGTTCAAGCTCTTCAACAACAAGGAGAAGAACC
AGGACCGGGGCAAGGCCTCGTCCGGCGTCTCGAAGCGCACCTCGAGCTCGTCCGGCT
TCTCCTCGGCCAGGTCCGAGCACTCGGACAGCTCGACGTCCCTCTGCGACCAGAGCA
AGCTTCAGGTCGAGTCGCCCAAGAGCCGCGCGCTCAAGTCCAAGTTGCAGTCGGCGA
GCAAGGGGGGCAAACAGGCGAGCCCGAAGACCGGGAGGAAGGAGCAGGGCGGCAA
GGGGCAGAGCAAGATAGCGCGGACGAAGGGGGGGGCGGCCGATAAGTTGCTCGTGTA
CGCGGCCCCGCCCCTCGAGGATGTCAAGCCGAGCGGCAAGATCGCCGCCTCGGGGAC
CAAGATGCCCCCCTCCGTCGAGAGCAAGAAATCCTCGAATCTGCACGATTTGCTGAAA
CAACAACAGCAACAACAACAACAACAGCAGCAGCAGCAGCAACAACAACAACAGCA
GTCGAGCGGTGTCGGCCAAGCTTTGCAAAAGGCGAATCCGTCCGTGTTGTTGCCTAAA
CAGGTCGCAGGAGGGCAGCAGCAGCAAGAGGCCGGTGGGAAGAATCAGTCGGGCGC
GAGGAACGAAAATCTGGCAACGTCGAGGCAGCAACCGATCGCCAGGCCTAGGATGGA
GCTGCCTGCCAAGTTGGACGCGGCGAAGATCATCCATCCTCAAAACGCGAAGGTGAAT
CCGCCGCCGAACGGGTTGAACAAAGACATCCTCGCCCAAAAAGGTTTACTGTTGAGC
AACGGGATGATGAAGCAGCAGGAACAGGAGGAGGAGGGGGGGCTCACCGAGGAGAA
TAATTCGACGATCGAGCGCACTTTGAACCAGGCTAACCTGTGCGAGCAGGAGAGGAA
ACGGAAGGAGATCGGCGCGGATCGCGACGATCAAACGTTGGACGGGAAGCAAGTGGC
GTGTTTGAAGGGGGGGCGGGTGGAGGTGCACGAGCTCGAGGGGAGCGGAAGGCAAA
CGCCGTCCTCCTTCCTCCCTCGCAACCCGACGGACGCGAACGACGCGAAGATGACGA
GCGTTCAAGAAGCGACCACGATTGGCCCGATCGCTGCCCCTCCTTGCCACGTCGAGCA
ACAGCAGCAACATCACGCGGTGAATTTTGGCAAACCGCACTCGATGGCGGGGAGCGG
GCCGTTGCAAGGCTCGAGCTTGATCGGTTCCGGGAGTCCGGGTTCCAGCATTCCGAAG
CCCACGGCCTTGGTGAAAGGCACCTCGAAACCGCCGAAGGAAAACAGCGTGCCGGG
CGTACCGACGCCCACGAAATTAAAAGCAGACGCTTTGCACCGCAAGCTCGACCCGAAT
ACGGTCGCGATGGTCTCCCCGATGCCGAGCATATCCGATCTAATGTCGGAGAGTTCGCA
CAGCAATTCGAACAGCACGGGCCAGAGCAATTCAAGCGACAGCAGCGTTATTTACAG
GCCGAGCAGCGAGAGCGGGAGCGAGATAAAAACTATACCGAACAGAAAAATCGATAC
CACGTTCGAGCAAATGGAGAAGGTTTTGTCCGAGAGTTCAACGTGCGGAGGAGGAGG
AGGAGGGGGCGACGACGAAGCGGAGATGACGGTGAAGCCCATGCAACCTCTGCTCCG
CGGTTACACGCCTGGCGCGAGGGCCCTTCAGACCCTGCCCAGCAGAACGAACGCGCG
ACAGTACACGATCCTTCATTCCTCGTCCCATCATCATGTCGGTCACGATTACGCGGACAT
CGACGTCGCCTCCGGTTATCTGAGCGACGGGGAGGTTCTTCGCGGTGGCATGACCGTC
GGTAGCAGGACTCTGTCCGACTTGTGCGACGGTTACATGTCCGAGGGCGGTGCTTCGT

TGTACGCGCGTAGAATCAATCCGTCTTACGGTCATGATCACGAGAG

>novel_circ_000561
TACTTACAAGTTTATTAGTAGTGACCGACGAAGCGCCCCAAGATATCAGACACAAGAAT
CACGATGAACGGCCCAGAGAAGCTTATTTCAATGGATCGGCATTCCTGAAATTGAGCTC
GTTCGTATCTGTGCACAGGCACAGCGGCTTGAGCTTTCGAACTTGCGAGGGCGGCCGG
CTCTTCACCCAAAAGTACGACGAGGACTCGATCAGTCTGGAAGTGACCCCGGACGGA
CTTTTGTTCGCGGCGATTGTCGAGCAGCAGAGATACAAAGCGAGATTGAACGCCAGGC
TGTTGAACAACGCCTGGCACAATGTCAATCTATTCTTTAGACTCGGAAATCTCACCTTG
AACGCAGCGGGCCACACTCAGGTGATCGCCAACGCGACTTACAACGCTGCCATTCTCA
CGTTGCCCGATTACGATATGAACGGGTCTCTCGTCGTTGGAGAAGGGTTCAAAGGATGT
ATCCTGCAGGGTCCGGGGATTCTTTTTAACGACACGATGAATAACGGGGCAGTTTTTGG
CCCCTGCCCCGCGGAAAATAAAGGAT

>novel_circ_000563
GTCCGCCATGCTTGAGTGGGCCATGCCGGAACGGTGGTACGTGCAACGAGGATATAAA
AGGGGATTTCAGCTGCGCCTGCAAGCCGGGCTTCACCGGTGTTCACTGCGAGTCGCAG
CTCGGCGTAAGGCTGTGCGAGCAAAGTCCGTGCAGGAACGACGGTGTTTGTTTAGCG
GTGACCGAGACCGAATATAAATGCGATTGCCAGCCGGGATGGAGTGGAAAAAATTGCG
AAACGAATATCAACGAGTGCTCCCCGAATCCTTGCAGGCACGGCGGTGTTTGTATCGAT
GGTATTAATAATTATACGTGCATATGCGACAGGACTGGATACGAAGGTGCGAATTGCGA
GGTGGACATAGACGAATGCTTGGCTAATCCTTGCTTGAACAACGGCGTGTGCTACGAC
AATTACGGCGGTTACATATGCCACTGCCCGAATGGTTTCGAGGGGCAGAACTGCGAGT
TGAACTTGAACGAGTGCTTGTCGAATCCTTGCAAACATGGCGGCGATTGCGTGGACGA
CGTCGGCTCGTATCACTGCAATTGCCCGTCCGGATACGCCGGCCGGCACTGCGAGCTTA
GAAGTTTGTGCGAGAACGCGGCTTGCCCCTCGAACAGCATCTGCGTGGAGGACGCGC
ACGGGCCGCAATGCGTGTGTAATCCCGGATACATGGGCAATCCCCCGAATTGCACCATC
AATTATTGCGCCAATAATCCGTGCAGCAACGGGGGCACTTGCACGAGCAACAAAGACG
GATTCAATTGCACTTGCCCACCGGAATGGAAAG

>novel_circ_000564
GTCCGCCATGCTTGAGTGGGCCATGCCGGAACGGTGGTACGTGCAACGAGGATATAAA
AGGGGATTTCAGCTGCGCCTGCAAGCCGGGCTTCACCGGTGTTCACTGCGAGTCGCAG
CTCGGCGTAAGGCTGTGCGAGCAAAGTCCGTGCAGGAACGACGGTGTTTGTTTAGCG
GTGACCGAGACCGAATATAAATGCGATTGCCAGCCGGGATGGAGTGGAAAAAATTGCG
AAACGAATATCAACGAGTGCTCCCCGAATCCTTGCAGGCACGGCGGTGTTTGTATCGAT
GGTATTAATAATTATACGTGCATATGCGACAGGACTGGATACGAAGGTGCGAATTGCGA
GGTGGACATAGACGAATGCTTGGCTAATCCTTGCTTGAACAACGGCGTGTGCTACGAC
AATTACGGCGGTTACATATGCCACTGCCCGAATGGTTTCGAGGGGCAGAACTGCGAGT
TGAACTTGAACGAGTGCTTGTCGAATCCTTGCAAACATGGCGGCGATTGCGTGGACGA
CGTCGGCTCGTATCACTGCAATTGCCCGTCCGGATACGCCGGCCGGCACTGCGAGCTTA
GAAGTTTGTGCGAGAACGCGGCTTGCCCCTCGAACAGCATCTGCGTGGAGGACGCGC
ACGGGCCGCAATGCGTGTGTAATCCCGGATACATGGGCAATCCCCCGAATTGCACCATC
AATTATTGCGCCAATAATCCGTGCAGCAACGGGGGCACTTGCACGAGCAACAAAGACG

GATTCAATTGCACTTGCCCACCGGAATGGAAAGGAACGACATGCCTGTCATCCGCCTC
GGATTGGTGTTCGGCTTGTTACAATGGAGGCAGTTGTCTCGAGACGCGTTTCGGTATTA
TGTGTCAGTGCCCAAGATTTTGGACGGGACCGCAATGCAAGGAACCGATCACCTGTCG
CGATCTACCTTGCAAACAAGCTTCCGCTTGTCACGATTAT

>novel_circ_000565
ATTTACAATTAGCGGCAGAACTTGGAAAAACGCTTTTGGAACGGAACAAGGAATTAGA
AAATATTATCAAAGCGCACCAATCGACCGTCGAGGAACAAGCACAAGAGATAGAGTAT
ATGAAAAAACAAACCGCTGCCCTTAGAGAAGTGAACGACTCGCGATTGAAAATTTACG
AGCAACTGGAAGTCAGTATTCAAGATTTGGAACGCGCGAATCATCGTCTAGTAATCGA
GAACACCAGCGACAAAAAATTGATCAAGAGGTATGAGGATCGATCATCTCGTCAATGT
TTGACGATCGAGAGCCTAGAGGCGAGGTGCGAGGAACTTCAGAAGAAAATCGACGAG
ATAAACGAGCAACATGAAAGTCTGCTCCGGCAACAATCTACGAGTCAATCGGCTAACA
ATATCACACAAACACACACCATTCATTGGAAAGTATCGGTCACGCAGGGTAGCAGCAC
ACAACAGAACGCTGCTCCGTCTGGTCAGAAATCTTTCCAAGAGGCAAATGCGGACTC
GAGCGTGCGACAATTACCGACGATTCCGACGAACGACGAAGAAGTTACCGAATTGTTG
AGACAGTTGCAAGAGGCGCGAAGTCAGAGGGCGAGGGAGCAGCAAAAAGTTACCGA
GCTTCGGCAACAATTGGCAACATTGTTGCAGGAAAATAACGCGTTGGAAGAACAGTTG
AACGTTTGGCGTAATAAAGCACAAGACGTGAAAAATCTTCAAGAGGAAATAAATACTC
TGGAAGAAGTAAGGCGGGGTCAATTATGCGGGCGATGTTTACGCGGATTGGACTCTAA
GACGCACGACGAACTCTCTGTAATGTTGAATCAGGAGGAATACGATGATATTAGCATGG
CTGAATCGCTAATGAACGATAGTCAACGTGACACTGAGTCTCTCACTCAG

>novel_circ_000566
CCCCTCCTCCCCTAGAATTCGATCGTTTTAACGGACTCCTCGTGACGTACAGATACAGT
TTCTAGGGGGAAATTGTGCGACGCAACGCAACGTGGGAAAAAAAATCTCGTCGTCTTG
AAACTTTTGTTTCTTGGATTTTTTTTTTACGGCAAATATTTCACGAGAAGAGGTATCAT
ATATCTTTGGTTCATCCATGATTCGGATGAATTGGTCGACGCGTTACACTGTCGACGGA
GAGATGCAAGTATGTTTCGTCCGCAAATTCTTGGGAGAAGTGGCCCCGCAATAACGTG
AGTGTGGTGTGGAATGAAAGAAAAAGAATGCAGCACGGTAGAGGAGGCTAGATGAGC
ACCAATTTTCAGAGAGGTGCATCGCACGCGAAACGTCCAGCGATTGTAGGGAAAAAA
CAAGACGGCGGCGCGTCTCTGAAAGGAAAGAAAAGCGATAAACACGTGTGCGAAAG
AATACGCGGTCCATACCTGGCCCGCCATTTTGGCGGCATGGTCCTTTGGGCCACTATACT
ATTCCTACAAGGAGCTGGTTTCGTAGGCTCGGTAACAGGTATTCCTGGCGTTCCGGAA
AATATAACGGTGATGTTCCTGAATCCAACGTCCGTGCGCGTATCTTGGAGCACGAGTCA
GGTCGAACAAGTCGAGAAATACGACGTCACGTACAAGCCAACGGATGCCAGGAAATA
CAATGACAGTTACAGGGTGGTGGCGGTGGTCGCGGGGAATAGCGAGGCGGTCACGTT
GAGCGGTCTGAGGGCTGACACGCAATACCAGCTAGTCGTCACCGCCGTCAGAGGTGG
GAAAAAATTTCGAAGCCGGCCGATCGTCTTCCGCACGCTTGAGCCGCCGCGAACGTCC
CCGCAACAGGATGCAGCGGTAACCGGCGGCCCTCTTCCACCCCCTCCACCTTCTTCTC
AACAGCCGCAAGCTTACATACAGGTCCGCGGCGTTGAAGTGGGCATTGTCGTGTTAGT
GTTGATCTTTTGGGCGGGCGCGATAGCGTTGTTCTTCAATCGGTGGGGCAAGATTCGTA
TGTTGTTGCCGTATCAGCCCGATTACAAGGAACAATTGAAAGTTCCTGGGACAGGGGT
ATGCGCAGCGGCGAACGCCGCGTACACGCAACATCCCACTCAGCACACTTGCTCTCAG

CATCTGCACTGGTCGAGCCATCACGTGGACTCGTTGGACAGCGGAGGCGGTTTCGGAT
GGGCGAGAACTTCGAGGCCGCGGGTGAACAGCGCCATCGACGTGGCCGGCTTCCTAT
CCCAG

>novel_circ_000567
ATTCGGATGAATTGGTCGACGCGTTACACTGTCGACGGAGAGATGCAAGTATGTTTCGT
CCGCAAATTCTTGGGAGAAGTGGCCCCGCAATAACGTGAGTGTGGTGTGGAATGAAA
GAAAAAGAATGCAGCACGGTAGAGGAGGCTAGATGAGCACCAATTTTCAGAGAGGTG
CATCGCACGCGAAACGTCCAGCGATTGTAGGGAAAAAACAAGACGGCGGCGCGTCTC
TGAAAGGAAAGAAAAGCGATAAACACGTGTGCGAAAGAATACGCGGTCCATACCTGG
CCCGCCATTTTGGCGGCATGGTCCTTTGGGCCACTATACTATTCCTACAAGGAGCTGGT
TTCGTAGGCTCGGTAACAGGTATTCCTGGCGTTCCGGAAAATATAACGGTGATGTTCCT
GAATCCAACGTCCGTGCGCGTATCTTGGAGCACGAGTCAGGTCGAACAAGTCGAGAA
ATACGACGTCACGTACAAGCCAACGGATGCCAG

>novel_circ_000568
ATGCCGACGCAACGAGGAAATTAACGTAGGTTGAAATGTTGGTGTTGACATCGCTATG
AACGAGAGCCGGTGCTACGTCGATTCGGCCGTGCTCGCTGAACATCAACCGTTCAACC
GGAATGGGAAGGCACTCAGGTGGGATCGAAGAAGAGCGTGCTGTTCTACACGACCGA
TTGCGGGGACGGGAAAGAAGATCTCGGACGCAAACTTCAGCAAAGATCCGTCTCTCT
GACTGCTCTGCACACGGGAAATGGGACGCAACAGCAATTTCTAGAACCAAGAATGGC
GCAATTGGAGACATTGGAGGCGAAG

>novel_circ_000569
GATGTTGCGTGGGTGTTTCGCGTATACCTCGATGAATTATGCGTGTACATAGAGGATAGC
GAGAGGGGGATAGGCGAGAAGAGTGGAGATCGTGTGTGTGTCGTCGTGCTGGAGACT
ACCAACTCGGCCGTGCGTGTGTGTGTGTCGTGAAAAAAAGAAGAAAGGTGGGTGTTT
GTTTCTGAAAAGAAAGAAAATTATATATATTATCCTTATCCTTATCCCTTTCGGTGCTCTT
TCTATCGTTTCGTCGTCGTCTGTTGTCTCCGCCCGAGGTCCACGTACCGATTCAAGTCT
GGGAAGAAGCCTGGAATTCCCACCGCAAAAAAACTGCAAGCGAACGCCCTAAAACAT
TCCACGTAGAATCCGTAGCCGGTAAGATGATTCTTTCACGCGTCAAATTCCTCCACCGA
AATAGATCGCCCACGGTTTTATGCCTCTTATCCACGCTTAACCGCTTTTACGAATCCTTT
TGTTTCGCAATTTTTATATATATATAGATCGCCGGCTATTCTTTCGGTCGATAAAATTTTCG
TCTGAAATCTGTTCTATGAATTTTTTTATCTCGAACATCTTTCCAACGATCTTTATTGTTT
TGCTATTACGGATAATACATTTTCATTTGTAAGATTATAGATCGATATCGCTTCGATCTCTC
GGGAGGAAATTTTTCAATTTCTATGGTTTTACTTTCGATCGAATTTGAAGGAGGAAAAG
AAAAAATATTCTCGTCTTTTTCTTCTTCTTCTTCTCGATGAAATAATTATTGTTTGCG
CAATTTTTATCCTTGACATTCCACTTATTCCTTCCAAGCTATGAACTTTCGATACAGATCG
CTATTTCGTCGCAATTCGATCTTGAATAATTCATCGCGGTGGTGAAGGCAGAAACCGAA
TATCTCTGAAATTACTACTATCGTTGGAGGTATGCTTGAAAAAATAACGTATACTCGAGC
GAATGAATTTTACATCGTTTCGTTTCACTTTTTTTTTTTGCGAAAATTTATCATATAAATC
GATATCTTAAAAAAAAAGTAAAACAAATACGAGGAGGAATAGAAAATATACCTAGAAA
TAAATTCTTCTTCTTTTACACACTCTCTTTCTTTCTGTTCGATTATCGAAATATTTATCTTT
TTCTTTCGAAAGAATAATTCTTCGATAAATCCATAATGGAACGTTTTTCAACCTTGAATT

ATCGTTTAGAGGTTACCAATTCGTAAACTCGTTAAAAAGCAAATAAATCTTTCCACGCA
CCCTTTCTCGCCAATCTTCTCGATGATTAACGATGAATCGATCTCTCGATACCAAATAAA
TCGAGATCTAGATCTGCTTTGTCCAAGAAAAAATATCTAACCGTATCGATCGTGTGAAA
TTTCGTAGCGTTTCGTAAAACTATTTATTATTTTTTTATAAGATTTTCCGCATAATTTGCT
CGTAATTTGATCGTAGATAGACATAAGTACATAGACAGAGAGAGAGAAAATGATTACGC
AACGCATCTTCCAGAAACGGCAGGAGCAGTTTTACTCCTTGTTCGTCCGAAGTTACGT
GTACGTGAAATGCCATGTTTTTTAGCTCGCGTTCCAAATCACCTTTAACGTTGCTTGTAC
ACACGTTCGTCCGATCAACAACCTTGAACGACACCTGAACGCGCCGTTTCGAATCCAA
GTGTTTACCTCGGGTCGAGTAACAAAGACGACGCGTGAGAATAATAGGAGAGGGGGG
AAATGACGGGCCAACACGTGGCAGCGTGCAAATAATTCCTCGATATTTACGCTCACCGC
CGTTTAACAAGCGATAGAAATGTATATTTTCGCTGTGGCGAAAAAATCTTTTTAATATTAT
CCGAATGCACGAGTTCTAAAGTTAGACCAGCGTTTTCGAAGGCGAGCCTTCGAAGTTG
CTTTCTCAAAAATAGGTCGAATGTAAGGGAGGATAATAGCACGGCGGCCACGGCCATA
CTCGCCGTCGTGAAAAGAGCGCGGCGTCGTGTCACGATTCCTCCTCTTCCAACCGTTT
CATGCATCAAAATTTATTATACGAGTTGTTCGATCTGAAAACGATTTGCTCTATTTAACG
ATATATTATCTTTCTGCAATATCGGTTTCTACGTTCTTGTACTATTCTTTTATTCCATTAATT
CGAACATGGAAGAGATACATTTTTATAGATTGTATTTCAAAGATCGTATATCGCATATACT
TTTAATTGCTCCTCGTCACCAAGAAGATACGATCGGCACGTACATACTTGGACGAACAA
TGTTTAATTTATATATACGTATATCGTAATTATATCATAGAATACGACGGTTTCATCATCGA
CTCGACCATGTTTATTCGAGGAACGGACGATAGAACGATACGTAGCGTACATATTTAATA
AACGAGCCTTGACATCGCGTCATATACGTGCATAGGGTAACGGTCTATAGAAAGTAGAA
AGCCGTTACGCAAAGTATCAACAGGTGTATTATTACTCGATTCATCCTTAAATACCCTAT
TTTTTTTAATTGCTAAAATATCAATCAATTATCGCTCAACGCACTCGCGTAATAAAATCG
TCCGATTTTTCGATTCGAGCTCGAAGAATTTCATATTCACATTCGTGAATATCGATCGTA
AAAGAAAAATTCGATTTTTAGAAGATTTTTTATTCCTCGAAACTCGTTATCCAAACCGT
GTTGCATCGTTCAAAATCGGAAAAAAAAAAAATTCCATCCAAACATTTTGAGTAATCTCG
CGCGGTAAATAAAGTTGGACACGTCCTATTTTTCCATCTCGTGTATCGATCTCGTGGCA
ACGTATCGATGGCAAACTAACGAGTTTATTTTTAACGCGGTCGAAGAACAAGTTGGCG
AGTGACCGTACCGATAAATTACGCCTCTGATTCCGATCGCTCTTAATTGGTCGAAACGG
ATCGGTTTTGCGGTCGATCGAATCGAAACTTCCTTTCGATCCCGTCCTCCTCGCTTTTCC
GATCGAGTCGCGCCGTCTTTCTTCCCTCCCTCTATCTCTCTCTCTCTGTCTCCTTTTTAC
AGGTCGTTTAGCATCCAGAAAATGGCGAATTTCTCTGCTTCTCTTTTCAAATATCTCTCT
CTCTCTCTCTCTCGTCCGTTCTTATTGTATCTGTCAAACTTTTCGTTCGATCGATTTCGAG
AAGAATTTTCTATAAAACTTATACAAGATATATGTTTAAAAATAAGAATAATCGTCGAG
TAATCTTTCTCGTTGAATTTGTAATCGTATCACGCGGCAAAAAGTGACACAAGCTCGTC
GAGAGTCACGCGTGGTATGTCGAAAGGGTCGAGAGTCGAGAAAGTTATCCGTTCGTTT
CGCCCGTACGTTATTTCCGTTATTAAACGCGATACTTACTTTTTCCAATTATTCTCACACA
GTCTCTCGTGTCTCCCCATTTTCTATTCTCTCTATTTTTAGAACGATATCTTTGCACGAGA
GGGAGTGAAGGAAGTTGGCGAAATGAGAATTCTTGAAATTTATTCGGATTAACACGGT
CGGTGGGTTCTCGCGATTGAAAACTCGACCGAGAGGCGTCGCGTCGGAAGCGAGGCG
AGAAACGATGGAAAAACAAAGGGATATCCGTTCGACGAGAAACCGTGATGAAATCCG
TTCGTTTTCTTCCTCCTCGAGCGCGAGGAAAACTGTGTCACTTTCTCTCCGCATGCTTC
CACGCAGGAACAATACACGCTCCGCGTTATAAACCCAATAATACAGCGTTAAACGTGA
ATGTAATTGTCGATACGGTGACGCGCTCATTGTCGACGACGCCGTGATCCTTTCTTATCG

ACCGTCGATACGATCCTTCGTATTAAACTAAACTCGAACAATGCTTCTTTGTCGTCTTTT
CAAACAAACTCCATTTACACGCTTTTCTCTACTCCGTGGATCGAGAATCCTTTTATATCT
TCTTTCCCTTCAAACTATTTAAATTAATATTCGAAAATGTATCATCAAAGTGTGTAATTTT
ATCGTATCTCGTATTTTACACGATATGAAAAATTGATGTATAATGTCTCGTCTTTCTCGAT
GCGCGCTCTCTGTGCGTTATCGAATCTTGCTCACCAGATACTTACGTACACACCGTAATA
GATTCGTGCAGTTGTCCAGATAGTCTCTTTTCAATGATTCGATCATATCGACCGTTCGAT
TTTAACATCGTTTTCCAGAATAATAGGAGCTAATCGTCAAACGAGTCACGAGCTCCACT
TCCTTCACCACCTTTCTCCAAACTTTTTATTCGAATCGTGTATCGTTATTTCTTCGACTAT
TACAGTTCGTATTCGAGCTTCGTGAAAACTTTTTGAAATTCGAAAACGAAGTTTAAACG
GACGGCCGCGTTATAGGAAAGAGAAGAAAAGGAAAATGGGACAATTAGAAAAATGAG
ACTGCCGAGTGGGACGGATAAGAGTCCGACCCCTCGAGCACAAAGAGTTAACACGTT
CGCCGCTACCAGAAAATGTCATGCACATTCTCATCCACTTAGCGTAAATAACGTGTAAC
ATAACCCACGTATCGCGCGTATATCTTCTTATCGCCCCCTCGCTTTTTTTTCTCTCTCTCT
CTCTTCATTTTTCCCCCTCTCTTTCCATTATATCGTTATTATTGTTGTTGTTATTATTATTTA
ATTATCTATACCGTACGATAATCACACGGAGCAACGGGTTACTCCCTTTCCGTTGCAGAT
CGCAAAATGGGCTGTTTCGGGAGCAAAGATAAGCTGAGCAAAGAGGATATGGATTTTC
TGAAATCGCACACGAGATACGACGAGGCGACTATAAAGGAATGGTACAAAGGATTTAA
AGTAAGTTGTTGCTCGAAAATTTGTTTTTCAAATCGGGATAATAATAATATTATTGGCAA
GTTTCCTCGATGTTTTTTCAAAGTTGAGAGGAAATTTGATTCTTGGAGATATTTGCTTAG
AAATATTCTTCTTCTTGTGTTACAGCAAGATTGCCCGAACGGCAGACTAACGCCGGCAA
AATTTGTCGACATGTATAAAATGTTCTTCCCGTCGGGCAACGCGGAGGAATTTTGCGAT
CACGTTTTCCGCACATTCGACATGGACAAGAATGGCTACATCGATTTCAAGGTAAGAAA
TCGATTTTTCTACACAACTACTCGACTCTTTTCAACGCGTACAATAGAAATTAAAGCTTT
AAATCGAATTTCGAAACGAATTACGTCCGTCATTCTTCCATCGAGAAAGAACGAATAA
AACTTTTTAAATCGAAGCACCATTAATGATAAATAGCGTTGGTGGAAATCGTTCTCTCG
AAAAAAGAGGAGAAGAAGAGGAGGAGGAGGAGGAAAAAGGTGAGTACAATACGTG
GAAAGGCGATAGAAGGGTGGGGCGATGATATAGGAATCTAGCCGATGGCAGAAATTGG
CTAGAGGGGCCAGCACGTCGATGGGGTCAATACGAGAAGTGGAGACACTTCGGAGGT
CATGAAGAGCAAAGAGCCGGCTCGCCCTCGACTTCGTTCTTTAAAACCCTTGCCGTTT
GTCTACCTCAGGCGTAGGATGACCCAGTTCAACCTCTTGCCGTATCTACATCAACGCGA
ATACTCTCAATTCTTTAACTCGAAACGATACGTTCCGTTAATATTGATCGAATCGATCATT
TTACGTTCCAATAAATCTTTGAAAGTTCGTGGAAAATGTAAATTTCTACCCGATTTTCCT
TTTTTTCAATTGCAACGATGAAGCAAGAGTGAACTGAGAAGCAAGAGCGTTCAATTTT
GTTCGTTGCAGGAATTTTTATTGACTCGAAACGATTCGTTCCGTTAATATCGATCGAATC
GATCATTTTACATTCCAATAAATCTTTGAAACTTTGAAACGTGGAAAATGTAAATTTCTA
TCCGATTTTTCTTTTTTTCAATTGCAACGATGAACACTGAGAAGCAAGAGCGTTCAATT
TTGTTCGTTGCAGGAATTTTTGTTGGCTCGAAACGATACGTTCCGTTAATATCGATCGAA
TCGATCATCTTAATACGTTCCAATAAATCTTTGAAACTTCGTGGAAAATGTAAATTTCTA
CCCGATTTTTATTTTTTTTCAATTGCAACGATGAACAGTGAGAAGCAAGAGCGTTCAAT
TTTGTTCGTTGCAGGAATTTTTGTTGGCTCGAAACGATACGTTCCGTTAATATCGATCGA
ATTGATCATTTTACGTTCCAATAAATCTTTGAAACTTCGAAACGTGGAAAATGTAAATTT
CTACCTGATTTTTCTTTTCTTTTTTTTCAATTGCAACGATGAACACTGAGAAGCAAGAG
CGTTCAATTTTGTTCGTTGCAGGAATTTTTGTTGGCTCGAAACGATATGTTCCGTTAATA
TCGATCGAATCGATCATTTTACGTTGCAATAAATCTTTGAAACTTCGAAACGTGGAAAA

TGTAAATTTCTATCCGATTTTTCTTTTTATCAATTGCAACGATGAACACTGAGAAGCAAG
AGCGTTCAATTTTGTTCGTTGCAGGAATTTTTGTTGGCTCGAACGATACGTTAATATCGA
TCGAATTGATCATTTTACGTTCCAATAAATCTTTGAAACTTCGTGGAAAACGTAAATTTC
TATCCGATTTTTCTTTTCTTTTTTCAATTGCAACGATGAACAGTGAGAAGCAAGAGCGT
TCAATTTTGTTCGTTGCAGGAATTTTTGTTGGCTCGAAACGATCATTTTAATACGTTCCA
ATAAATCTTTGAAACTTCGTGGAAAATGTAAATTTCTATCCGATTTTTATTTTTTTTCAAT
TGCAACGATGAACACTGAGAAGCAAGAGCGTTCAATTTTGTTCGAAACGATTCGTTCC
GTTAATATCGATCGAATCGATCATTTTACGTTCCAATAAATCTTTGAAAGTTCGTGGAAA
ATGTAAAACCCGATTTTTCTTTTTTTTCAATTGCAACGATGAACACTGAGAAGCAAGAG
CGTTCAATTTTGTTCGTTGCAGGAATTTTTGTTGGCTCGAAACGATCATTTTAATACGTT
CCAATAAATCTTTGAAACTTCGTGGAAAATGTAAATTTCTATCCGATTTTTATTTTTTTTC
AATTGCAACGATGAACACTGAGAAGCAAGAGCGTTCAATTTTGTTCGAAACGATTCGT
TCCGTTAATATCGATCGAATCGATCATTTTACGTTCCAATAAATCTTTGAAAGTTCGTGG
AAAATGTAAAACCCGATTTTTCTTTTTTTTCAATTGCAACGATGAACACTGAGAAGCAA
GAGCGTTCAATTTTGTTCGTTGCAGGAATTTTTGTTGGCTATCGATGTAACGTCTAGCG
GGACGCCAGAGGAAAAACTGAAATGGGCTTTCCGTATGTACGATGTCGATGGAAACGG
TGTCATTGACATTCAAGAAATGACCAAGATCGTTCAGGTATTATATTTCGTAATTAACAC
GCACACTCTCTTTTTCATACAAATTTCACATCCTTCTTTCATCACGATTAGAAGATGATTT
ATTATAAGAATTGTTTGAAAAGAGAAAGAATCGTCGATTTCATCCCTCCTCTCGTTCCTT
CTCGCAACGAATCGAAGACTTTTTTTTAATATAAAACGAATGTATTGGGTTGGCAACTA
AGTAATTGCGGATTTCAGTTGAAGTTGATGACGACCTAATCAAAGCGATAATCGATTCG
GATCGTCACAGTACAACTCGTGAGATTGCAGAAAAGCTTCATGTATCGCATACATGCAT
TGAAAACCACTTAAAACAACTTGGCTATGTTCGAAAACTCGATACATGAGTTCCTTAGT
GAACTGAAAGAAAAGCATTTAACGCGACGCGTTAACAGCTGCGATTTGCTAAAGAAA
CGTAATGAAAATGATCCATTTTTAAAACGACCGATAATTGGCGATGGAAAATGGGTTGT
TTACAACGATATCGAGCGGAAAAGATGGTGGAGCAGGCCACGTGAACCAGCTCGAAC
AACGTCAAAAGCTGGTATTCGTCGAAAGAAGGTTTTGTTATCAGTTTGGTAGGATTACA
AAGGAATTGTCTATTTTGAACTCTTACTATCCAACCGAACGATCAATTCTGTCGTCTACA
TCGAACAACTAACGAAATTAAACAATGCGGTTGAAGAAAAGCGGCCCGAATTGACAA
ATCGAAAAGGTGTTGTATTCCATCGTGACGATGCAAGGCCACACGCATCTTTGGTCACT
CGGCAAAAATTATTGGAGCTTGGTTGGGATGTTTTGCCACATCCACCATATAGTCCTGA
CCTTGCACCATCCGATTACTTTTTCTTTCGATCTTTACAAAACTCCTCGAATGGTAAAAA
TTTCAATAACGATGATGATATCAAATCGTACCTGATCCAGTTTTTTGCTAATAAAAACCA
CAAGTTTTACGAACGTGGGATTACGATGCTGTCTGAAAGATGGCAAAAGGTCATTGAT
CGAAATGGGCAACGCATTACGGAATAAAGTTATTTAGTTCCACGAAAAAATTGTCTTTG
ATTTTCTAAAAAAAATCCGCAATTACTTAGTTGCCAACCCAATATATAGATTCGAAGATT
CTCGATATCGAGAATGGTATTTTAGATTGGAATTTTATTTAATTGGAAGATAAACCGATT
CGAGCAAAATTTCTCGTCTCTTCCTCCAATTTGAATATCATTTTTCGAAACTCGATTTCC
TTCTAAAAGAACGGCCCCCTAATTGGCTCACAATTAACGAATTATCCGCACGCATCCAG
ACGTCTAGACGTCTGGATTCCCCGAGGATTCGATCGATCGTTACGTAAGATACCAAGTT
CCGAGCATAAAAGTTGGAGGAATAATCGAAAACGATGAATCGGATCGAAATTGTACAT
CGACGAAAATATTCGAAGCGTTCGTCGAACAAAATATTATTATAATAAAATATAAAAACT
AGATATCGACAGAAAGAATGAGACGAGGAGAGTTATTGAAAAATTAAATATTTTTTTAA
ATATATATTACGAATAAAAGGAGAGAAAGAACAGAGATGATTCTGATCTTCGAAACGAG

GATGACGAGGAAAGAAATATTTCTATGAAATGGTTCGTGAAAGGCTGGCTCGGATGGA
TGACTTGGAAAAAGAAGGTTTCATCCACTTTACTACAATTAACCTTCGATATAACTCGA
TCGATCCAGAAACAGGAGCAAATACTCGATTGAATGGAAAGTAAGCGGAGAGCACTC
GGAATGCGATTACAACGCGATGACGGAATCCTGAGAGAAAAATGGATGGATGGAAGA
AACGAAGGAAGGGAAGAAGTCGTTTCGCTCCACTTCTTCTATATACGATTCGAATATCG
TACGAATTTTAATATTTCTTCCAGCGAACAAATCCAACTTTTATCTTTCCATAAAAAAAA
GTCATCTTGGTTTCCAACTGTTTTAAAAATTCATTTCGTCGCCGAGAAAAATGGCCATC
ATTTTTTTCCACCATTTAAAAAACTTTATCGCCAAAGTTCGAAGAACGAAAAATTTTGC
TCGAACGATACCAGATACAGATTCGAAATTAATCAAGTTTTCTTTGAAAAATTCTACAA
ATACTTACAATTAACTAACGCGTTTCAATGTTAAAAAATATATAAATATACGTAAATCAA
CGTTAAAGGGAATAAAGTTTCGATGGAAAAATTCGAATCGAGTGAAAACGAAATCTGT
AATACGATATCGTTTCTCGATTATTTCATCTTTTCTCGAATCGGATACAATAGGTTAAACG
AAACGGAGAAAGAATAAATAAAAGAAGAATAATTTGTATTTAAAATTCGATTCAAATAA
ATCGTGAAAACTCGTATAGAAACAGAATAGAATGGAATTCTAATATTTTTCATTTCGAGC
GGAAATCGAAAGGATAAAATCGAGTAAAAGCGAGAAAAGTTGCAAAATCGATCGATG
GACGAAAGTGGAACAACGAAGATTAGAAAAGAAAAGAAAAGAAAAGGATGATCGAT
CGTTGCGTGAGAAACATCTTGTCAAATAATACGAAATGTTTTCTGTTTGTAGGCAATATA
CGACATGCTCGGTGCATGCTCGAGCAACAGACCGGCAGATAGCGCGGAAGAAAGAGC
GAAGAACATCTTCGCGAGAATGGACGAAAATAATGACGGTCAACTTACCGAGGAGGA
ATTTTTGAAAG

>novel_circ_000570
ATCGCAAAATGGGCTGTTTCGGGAGCAAAGATAAGCTGAGCAAAGAGGATATGGATTT
TCTGAAATCGCACACGAGATACGACGAGGCGACTATAAAGGAATGGTACAAAGGATTT
AAAGTAAGTTGTTGCTCGAAAATTTGTTTTTCAAATCGGGATAATAATAATATTATTGGC
AAGTTTCCTCGATGTTTTTTCAAAGTTGAGAGGAAATTTGATTCTTGGAGATATTTGCTT
AGAAATATTCTTCTTCTTGTGTTACAGCAAGATTGCCCGAACGGCAGACTAACGCCGGC
AAAATTTGTCGACATGTATAAAATGTTCTTCCCGTCGGGCAACGCGGAGGAATTTTGCG
ATCACGTTTTCCGCACATTCGACATGGACAAGAATGGCTACATCGATTTCAAG

>novel_circ_000571
ATCGCAAAATGGGCTGTTTCGGGAGCAAAGATAAGCTGAGCAAAGAGGATATGGATTT
TCTGAAATCGCACACGAGATACGACGAGGCGACTATAAAGGAATGGTACAAAGGATTT
AAAGTAAGTTGTTGCTCGAAAATTTGTTTTTCAAATCGGGATAATAATAATATTATTGGC
AAGTTTCCTCGATGTTTTTTCAAAGTTGAGAGGAAATTTGATTCTTGGAGATATTTGCTT
AGAAATATTCTTCTTCTTGTGTTACAGCAAGATTGCCCGAACGGCAGACTAACGCCGGC
AAAATTTGTCGACATGTATAAAATGTTCTTCCCGTCGGGCAACGCGGAGGAATTTTGCG
ATCACGTTTTCCGCACATTCGACATGGACAAGAATGGCTACATCGATTTCAAGGTAAGA
AATCGATTTTTCTACACAACTACTCGACTCTTTTCAACGCGTACAATAGAAATTAAAGC
TTTAAATCGAATTTCGAAACGAATTACGTCCGTCATTCTTCCATCGAGAAAGAACGAAT
AAAACTTTTTAAATCGAAGCACCATTAATGATAAATAGCGTTGGTGGAAATCGTTCTCT
CGAAAAAAGAGGAGAAGAAGAGGAGGAGGAGGAGGAAAAAGGTGAGTACAATACG
TGGAAAGGCGATAGAAGGGTGGGGCGATGATATAGGAATCTAGCCGATGGCAGAAATT
GGCTAGAGGGGCCAGCACGTCGATGGGGTCAATACGAGAAGTGGAGACACTTCGGAG

GTCATGAAGAGCAAAGAGCCGGCTCGCCCTCGACTTCGTTCTTTAAAACCCTTGCCGT
TTGTCTACCTCAGGCGTAGGATGACCCAGTTCAACCTCTTGCCGTATCTACATCAACGC
GAATACTCTCAATTCTTTAACTCGAAACGATACGTTCCGTTAATATTGATCGAATCGATC
ATTTTACGTTCCAATAAATCTTTGAAAGTTCGTGGAAAATGTAAATTTCTACCCGATTTT
CCTTTTTTTCAATTGCAACGATGAAGCAAGAGTGAACTGAGAAGCAAGAGCGTTCAAT
TTTGTTCGTTGCAGGAATTTTTATTGACTCGAAACGATTCGTTCCGTTAATATCGATCGA
ATCGATCATTTTACATTCCAATAAATCTTTGAAACTTTGAAACGTGGAAAATGTAAATTT
CTATCCGATTTTTCTTTTTTTCAATTGCAACGATGAACACTGAGAAGCAAGAGCGTTCA
ATTTTGTTCGTTGCAGGAATTTTTGTTGGCTCGAAACGATACGTTCCGTTAATATCGATC
GAATCGATCATCTTAATACGTTCCAATAAATCTTTGAAACTTCGTGGAAAATGTAAATTT
CTACCCGATTTTTATTTTTTTCAATTGCAACGATGAACAGTGAGAAGCAAGAGCGTTC
AATTTTGTTCGTTGCAGGAATTTTTGTTGGCTCGAAACGATACGTTCCGTTAATATCGAT
CGAATTGATCATTTTACGTTCCAATAAATCTTTGAAACTTCGAAACGTGGAAAATGTAA
ATTTCTACCTGATTTTTCTTTTCTTTTTTTTCAATTGCAACGATGAACACTGAGAAGCAA
GAGCGTTCAATTTTGTTCGTTGCAGGAATTTTTGTTGGCTCGAAACGATATGTTCCGTT
AATATCGATCGAATCGATCATTTTACGTTGCAATAAATCTTTGAAACTTCGAAACGTGGA
AAATGTAAATTTCTATCCGATTTTTCTTTTTATCAATTGCAACGATGAACACTGAGAAGC
AAGAGCGTTCAATTTTGTTCGTTGCAGGAATTTTTGTTGGCTCGAACGATACGTTAATAT
CGATCGAATTGATCATTTTACGTTCCAATAAATCTTTGAAACTTCGTGGAAAACGTAAAT
TTCTATCCGATTTTTCTTTTCTTTTTTCAATTGCAACGATGAACAGTGAGAAGCAAGAG
CGTTCAATTTTGTTCGTTGCAGGAATTTTTGTTGGCTCGAAACGATCATTTTAATACGTT
CCAATAAATCTTTGAAACTTCGTGGAAAATGTAAATTTCTATCCGATTTTTATTTTTTTC
AATTGCAACGATGAACACTGAGAAGCAAGAGCGTTCAATTTTGTTCGAAACGATTCGT
TCCGTTAATATCGATCGAATCGATCATTTTACGTTCCAATAAATCTTTGAAAGTTCGTGG
AAAATGTAAAACCCGATTTTTCTTTTTTTTCAATTGCAACGATGAACACTGAGAAGCAA
GAGCGTTCAATTTTGTTCGTTGCAGGAATTTTTGTTGGCTCGAAACGATCATTTTAATAC
GTTCCAATAAATCTTTGAAACTTCGTGGAAAATGTAAATTTCTATCCGATTTTTATTTTTT
TTCAATTGCAACGATGAACACTGAGAAGCAAGAGCGTTCAATTTTGTTCGAAACGATT
CGTTCCGTTAATATCGATCGAATCGATCATTTTACGTTCCAATAAATCTTTGAAAGTTCG
TGGAAAATGTAAAACCCGATTTTTCTTTTTTTTCAATTGCAACGATGAACACTGAGAAG
CAAGAGCGTTCAATTTTGTTCGTTGCAGGAATTTTTGTTGGCTATCGATGTAACGTCTA
GCGGGACGCCAGAGGAAAAACTGAAATGGGCTTTCCGTATGTACGATGTCGATGGAAA
CGGTGTCATTGACATTCAAGAAATGACCAAGATCGTTCAG

>novel_circ_000573
GATCAAGACAGCGAGATGTCCAGCGAGATGGCACACTGTCCAGATTGGTCAGCGACG
GATCAATACGATTCGCAGAGCGAAGGTTTAGCGGCGAAACTCGAAGACGTGAATCAGC
CGAGTGCGAAGTCGGGCTACAAGAGAAAAACGACGGAGGACAGCTCGGAGGCGGAC
TCGGAGGACGAGGAGGATGGTGCACAGATGCACAACGATAATGGAAGGAGAGTTAGA
GCTAGATCGTTGATAGACGACGAGCAATTGGCTGTTCTCAAGGGTTACTATGCTATTAAT
CCCCGGCCTAAGAAAGAGGAGATCAGCATGATCGCCAACTACATCAATTTCCCTACTCG
TGTCGTGCAGGTTTGGTTCCAAAATTCCCGGGCGAGGGATCGACGAGAATCGAAAATA
CCACCTCTAGTACCGTTGGCTAACCCGGCCAGTCAGACGACATACGAGCAACCATTGG
ACTTGTCGAAGAAGGAAGGCCTCGCAGAGTCGGTGCGCAAAGACAGTAACGCTGCCT

GTGTAAAGTTCGCATCTTCGTCTCCTGTCGAACAGAGGAACGATAATGCATCGTCGCAT
AATCAAGACGCGGATGATTTGGAGGATTCGCCGCTCGTCATTGACGAAGAAACCACCG
ATTCTGTGGAAACGAAACGTACGGTCCCAACGGGAGAAATTGTTCCAAAG

>novel_circ_000574
GAGCACCTTCGCATTCACAGCGGTGAAAAGCCATTCCAATGCAACAACTGCGGCAAA
CGTTTTTCCCACTCGGGCTCGTATTCGAGCCACATGACCTCGAAGAAGTGCCTGATAGT
GAACTTGAAGAAATCCAGGCACGCGAACGTGAGCAACGTGGACCGCGGCTCGAAAA
AGGCGCAACAATCCCTGCCGCCGGGGCGGCGCGATGTCGATCTCCTCGCGGCGAATAA
TAACACTTTCCTCCCTATTCTGCCCAAGCTTTCACCCTCTGATTATCAGGAGATACAACG
GGAAGGAGCGGGCATATACGACATGCCCACCCTTCTGCCGCAAATGATGGGTTTCGGC
AGTTATTTTCTGCAAACGTCCTTGGGCAAAATCTTAAATCAGCTACATTCCAAGAGATT
GGACGAGGTAGCCGAGCAATTCGAGTCTCATCGGGAACTTATGAGCCCGTCCACGGCG
GATTCCGAGGGCACGGAGGGCACGGAGGGCAAAGAATCCCCGGCGCCCGCCGACCCC
CAAGGCTTGGACGCTGTTCGCCGTATTCTCGAAACGGTGAACACCTCTGTAACGAAAC
AACTGCTCGAGGCGAACGTGCGAAAATTATCCACATCGCCTGCCACGATGAAACGGGA
GTTTGAGGAGGATTATCAA

>novel_circ_000575
GTTGCAGCGAGGACGAGGCAGCAGGTGTCGCGAACGCGGAGGTGGGCGGAGGTTGC
GCGGCCGGGGCGCCAACCACGGGAAGCGGGGGTGGGGGTGGCGGCGGGACAGGTAG
CGAGGCTGGCGGAGGAGGAGGAGGAGGCGGCGGCGGCGAGGGCGGATCGACGAAAC
AGGGTGGCGAGAGGTGTCCCCAGTGCGGCTTCCTCTGCCGGGACGTCCACGTGCTTC
AGCTCCATCTCGAGGACACGCACAAGACCACGTACGCCATCGACAAGAACGATCTCA
ACGCTCAATTCCCCCAAGTGTCGTGCAAGGTGTGCAGCAAAACGTTCGCCAACGTCTA
CCGACTCCAGAGACACATGATCAGCCATGATGAGAGTGCCGTGCTTCGTAAGTTTAAG
TGTCCCCATTGCGAGAAAGCCTTCAAGTTTAAGCATCACCTCAAGGAGCACCTTCGCA
TTCACAGCGGTGAAAAGCCATTCCAATGCAACAACTGCGGCAAACGTTTTTCCCACTC
GGGCTCGTATTCGAGCCACATGACCTCGAAGAAGTGCCTGATAGTGAACTTGAAGAAA
TCCAGGCACGCGAACGTGAGCAACGTGGACCGCGGCTCGAAAAAGGCGCAACAATCC
CTGCCGCCGGGGCGGCGCGATGTCGATCTCCTCGCGGCGAATAATAACACTTTCCTCCC
TATTCTGCCCAAGCTTTCACCCTCTGATTATCAGGAGATACAACGGGAAGGAGCGGGCA
TATACGACATGCCCACCCTTCTGCCGCAAATGATGGGTTTCGGCAGTTATTTTCTGCAA
ACGTCCTTGGGCAAAATCTTAAATCAGCTACATTCCAAGAGATTGGACGAGGTAGCCG
AGCAATTCGAGTCTCATCGGGAACTTATGAGCCCGTCCACGGCGGATTCCGAGGGCAC
GGAGGGCACGGAGGGCAAAGAATCCCCGGCGCCCGCCGACCCCCAAGGCTTGGACG
CTGTTCGCCGTATTCTCGAAACGGTGAACACCTCTGTAACGAAACAACTGCTCGAGGC
GAACGTGCGAAAATTATCCACATCGCCTGCCACGATGAAACGGGAGTTTGAGGAGGAT
TATCAA

>novel_circ_000577
GAATAATGCACATAAAGAGGGAAGAGACCAATGGCGTTGAAGAGCGGCCTCGAAGGA
AGGGTTCTCACAGGGCCACGTCGCAGGTCAGGTCTTTTGCGCTCCCTCCTACAGTAAT
CCATCACATGGCGTGCAAATATGGCTAGTGAAGTAGCCATTGTTATGATACATTTGTTTA

AATTATTTGGAAAACGTAAGAGAAATCATGTTTGCTCATAAATTATTGTCAGGCTATTGA
CGCTACTCGAATTATTGAAAACACAGACCAGGTTACCCGACCGATTCATAAGCCTGTCC
TTAACATTTGCATTAACAATATATTTAATTAATACCAGGTTTATAATTATTTTTTCATTCGC
TATTCAATAAAATGAAAAATATGCATAATACTAATTTTTATTCTAAAAATGTAATATTATAA
TTCGTTACTTTGTATGCACAAATTCCGTCCTGTAAATTTCAATCATGTCATTTTTAAAATT
ATAAATATACATATATAATTGTTAAGTGTGAAAATTGTGTGAAAATATTTTAATATCTGAA
AATGTTTATTACAAATTTAAAAATAAATTTATTATAAATTAATGAACTATAATTGTAAATAT
GTTGAAAGTCAATTCTTAACATGTTAAAAATAAATGTAAATAATTTTTATATGAAATAAC
ATATAAAAAATTAAAAATAAAATGAAAAATTATTTCATACAATAAAATATAATTATAACA
AAATATTGAAAGAATATTATTATGTACGAATGCATGAATGAGTAAAGAATGAGAGGTGC
AATTATTCTATCTGTTTGTAGAGAAACAAAATAAAAAAAAAGCTAGAAATCAATTGAAGA
AATAACTTTGTAATGATAAGGCGAATTTTGTATATTCTTCTTTATATTTTTTATTAAATTTT
ATTTCATCCAAATTTTGATTATTAGAATTATTCCGTATTATTATTGTGTAAGTTAATTTTTT
TTTCAAAATAAAATTATTATCAAAAATAATTTGTGCGGAAAATAATTTTTGAATTATTATT
ATTATTATTCTTGAATAATAATAATTTATAATAAAGTGAGACAGATATGTAAATATTTTTTT
TAAATACATTGTGTAAATTTTATAAGATGCGAAAGAAATCGTAGAAATAGTGTTTATAAC
ATTTAAATAATTGAATGCTATTAAATAATTAGATTTCAATAATTTCTTTTGCATGTGTTAA
AAATAATAAATAATACAAAAATCAAATGTGAATAATATAAAAAGAAAAATTTATTAATTC
AATTTAATTTTTATAAATAAAAGATAGAAATGTGTTAAGTAGAAGTTAAAAAATAATATAT
AAATAATAGTATTATTGAATTAAGAGATAGAAAGATTTTATCTTTAGTTTTGACTTATTTT
TTAAATTTCAAATTTTTGGTTTATATAAAAAAATTATTTTTTATTAATAATAAATGATTTAA
TATTAGTTTATTATTATTTTAATTGTAATTTAGTTTATTATTGTTTGGTAAAAAAGAATTGT
TTATTATTATCTTTCCCAAAGAAAGGATAAGCTTGAATATGCATTTTTATATAAAATCAAA
ATGTATACTAAATCATTTATTTTTAATTGATATTTAAACTTACATAGTATACACAACATGGT
GTAAAATTTTGTTAAAATACGTGATTTAAAAAATTATAAAAAATGATTTTTAAAAATAAT
CTTTAATTGATAATAAAATTACATAGTTTTTTTATATAATTCAAAACTATATTAGCAAAATA
TTATTTAAAAATGTTATTGAAAATTTTTTATCAATTATTATTATTTTTTCGTATTATTATTTA
TGATCAATATATTATCACTCATTTGTTATTATTATAATTTATTTTTATGTAAAAAATAACGA
ACATAATTTTGAACAAAAAATTTTTTTTTATTCTTCTTACTTTATATTTTTACTTTCTTTTC
ATTTCTCTTTATTGTTGTCTTAAGTTATCAATTTTTAAATATAATTTTTTATATATATTCTCA
TAATCTTTTTGATATATAATTATATAATAAAATGTATGTTATGTAATTTTATGATCAATTAAA
TAATTTTGTGTTTATCAAAAAGTATTTAATTTAATATTTATTAAAAAATTAGATGTTGATTT
AATATTTAGATGTTTCGGATTTGTAGTTTGTTACATACATTGAGATTTTGTATAAGATATAT
TGCATTAAGATATAATACATTATCTATATACATATTTATATATATATATATATATATATATATA
TATATATATATATATATATATATATATATATATATATGAGCAGTAAATATTATGTAATGCAGAAT
TTAATTTTATTTTAATTAATTAAGTGTAAGAGTCTAAAATATTGATTTTGATAAGTATATAA
TAATAAATAGATTTACTAATTTAATAATATTAAATGAATAATTCTTTCAAATAATATCATTT
ATGGAAATATTTCAGTTTTCTAATATTTATTCATATAATATTTCTTGAATTCTCGTCTACAA
TAAAATGTTAGACAATGAAATGAATGAACTTTCACAAATAAATTTTATAAATTTCAAATT
ATCTATTTATTTTATTTAACATTACCTAAATTATAATACTTTTTAAAATGATAGTTGATAGT
AACATTTATAAGATTTTTATAAGATTTTATAAGATTTTTTTTTGTAGATTTTAATAAGATTT
ATAATTAAATAATTATAAATATATTTATAGTAATTTATACCATTTTTTACAAAAATCATAAG
ATTTAAAATTAAATAATTGATGAAAGGAATATAGTTATCAACGAAATAATATATTGATAAT
TTTATTTTAAATATCTGAGACAAAAAGTCATAAGATTTTTTTTAAACATTATTTATTTTGC

AAAAAATATAGAAATAGTCGTTGAATTCTACCAATACGGAAGATGTAGATTTCTATCAA
AATCTATGGAAATAATTTCTTTATGAATTAATATATCATATTTGACTCTAGAGAAATAAAA
ATAATATTTATCGCATTTCGAGATCATGAAAAAGAAAAAAAAATTATGTTTCCTAGTTTA
TAAGAATTTTCCTACCGATTTTTAATTAATTCTAAGGAATTTAATAGAGTTAAAAAATTTA
AGTTGGGAAATATAGAGCATTTCAGTGAAATGATATAATAATTTGATATGATATAATATGA
TTATATGATAATTTATCAAAATTCCATTTATGATGTGATATATTTTCTTAACAATAATAAATT
ATATTTCAAGTATGTCATTTATACGAGACTTAGATTTCTCATATTCAATAATCAGAGTAGA
TGCGTAATTACAAAGAAAACTATTTATTTATCATATTCCATCAGAATACAATATGTTCACA
ACAAAAATTAAATAGAGTTAAAAATTAACTCTGCAGATTCATTTTTTGATGAGACTCTC
AGAAATTCCTTCCATAATTGGCATCCTAACTTGCAGTATTTTCAAGTAAGAGAGAAATT
CCTTAAATTAAGCATAAAAAATATGATGATAACCAGAGATTGATCAGCATATATTTATTAT
AAATTTCTCCGAATATAGTTAATTGCAGTTGAATCAATCATATTTAGAGAATATCATCCTT
AAGATCGTGTTTCTAGCCAAGCTAAAATATGTTAAATATATAGAATTGTGTTACCTAAAT
CTTCCTTTAAAGTACCAATCTTCTCAGATAACATCATTCTTTATTTAACTTAGTAAGAGA
ATAGCAAATAACAGTACATGTAATGAAAAAAGTTTTATGTGAGGAAGAAATAAGAAGA
ATACTTATTGCTTACGAATGAATATATATATTATTACTAATAAATAAATAAATAAATAAATAT
ATATATATATATATATATATATTACTTAATAACTCTTGATTAATACATCTTAATAATTTTCGTT
TGATTTTTTTTTTTATCAGATTCATCATAGTGAATCAAAGATAAAAATATTTTTCTAATTTA
TATAATTTAAAACAAAAATAAAAGTGATAACACGCATCAAATTTTGTTTATTATCGAATT
AACATATTTACAACTTTTCAACAAACAACAAAATTGTACCGTATCCATTTCAAATATTAT
TTCACTGTCTCTCAATAAAATAATTTCAAATATATAGTTTGTTAGAAAGTGATAGTAGATT
TTTTTAAACTCCAATGTTCTTTTGTTGAGAATATTAATGAAATACGTTTGCAGTTGATTAT
TAATAAAGATTTGAATAAGATATTTCAAATAACTTTTCTATTGTGTATTGTTTCAGTTATT
TTTGAAATATCAAAAACTTTTTTTTAGAAAAAAACTTTAAAAAAAAATACTATAATAATATT
AGAATTCTATAATAATACTATAATAATAAGAATACTATTAAAAAAAAGTAATTCTTTTTTT
AATATTTTTATCAATTACTTATGAATTTGTTTGTTTTTTTTTTCAAATCAAAAACTAATAA
AGTGAAAAAGTGTACTTATATTGTATTTGAATATTTAAGCACAATATTATACAATAAATTC
TTATTCATATATGTTAAAAATGGAATAATTTTAAAATAGTATATTGCCATAAAAAAATGTT
ATAGTATTAAGATTTAATTTTATTAGAAAAAATAATATTATATTATATACTTAGAAAAAAAT
TAATCTATCAAAAATATTTTTCATTTATTTATCTTTTAAATTTAATACAATATAACTTATGAT
TAGATAAGGCAACGAAATAAATATCTATTTTATTCTACCTGTGTCATTATTTTATAACGTC
CATTTTTTCCATTGCTCGCGATGTTTATAAACAGCTTATTCAAAGATTTCTCATAAAAATC
TTTTCCTGTTATAAACGCAGACTTCTATTAAGTCTAAAAAACACTCACAATCTAAAACA
CAAAGGATGATATTATTTGAGAAAACTGTCGTTTTTGAGACTTTAGTCGACAATTGTATA
TTTTTGATACACTTCAGCCTGATTGGAGCACAGCCCTATCCTAAGAGTGACATATTCCA
AATTGATTTCCAGCTGATTCAACAGCAATCAATATCATTCTAGTTCGAGGAATTTTTCTC
AAATTTGGGGAGTCACTGATCGCAATGTTATTGTTTATTACCGTGCTCTACCGAAAAAC
TGAAATCTTTCGAATTAAACTAGGAAGGAATTTTTTGAGGACACTTATAATTTTTTGAGT
GTTACTTATAAATAGAAGTTCAGGATGAACACGCTAGATATAATAATTAGTTTGTGAATA
TATCATGCTCTGATCGTCGAATTATTTATTGTTATTTGTAAAAAAAAAAATCTCATTGTGA
ATAGATAGTAGTCTGATCAATACTTACATTAATTACATAGTTTAGTTCTAATATCATATCGT
GGTCTGATACGATTATCTGCTGCGACATTGTGAGAGATCCTGTTTTTTTATATAGATACG
CTATAGATGCGCCATGTAACCATTTTTACAGTTCATTCATCTTTTAGAAATATATCAATTAT
TATGACATGTGTCAAAAAACATTTTACAAACACTCAGGTATTAGTTTATATCCATCATCA

TTACACAATAATATTGTTACGGGTGTGAACATGTATAAAAAGAAATTTAAAAAAATATTG
AAAGTATTGAAAAAGTAATAATTTTTTAAAATAATGTTTTTTGAAATTGATATGAGACTA
AAATATGTAGCTATGAATAAAAAGCACATGAATCAATTGTCAAAAGAAAATACGAAATT
GTTGTATAGAAAGCAAGTGAAATTTATAGTGAAAAAGAAAAAGACAGAAATTCTAGAC
AAATTTCACGTAATGAGATTTTTATTATAATTTCCCATTTTTTTGTTCTAATATATAAGATT
TAATATAAGATTTAAGATTATAAAATTTAATATAAAATTTTATGAAAAATTTTTTATTGCCT
GTAATAGAAAATTATTGTAATGAATTATTGGAACTTCTAAATATGCAATATATTTAATAAA
ATAAAAAATTTTAATAAAAATTAACAGTAAATATTTTTCTATATTACGAAAAAATGGATAT
TTTATATAGATATATATATAAAATTTAGCAATATTGCATTATATTTAAGAAACATTGACAAT
CAAGGTATATATTTTATTAGTGTAGCATTTTGTAGCGGATTATATATGTATGTATTGCGATG
GTAATTTATTTACACATATTTAAATGTATATTTATAATTAATATTAATATATATATATATTAATA
TTATATAATATATAAATTATTATATAATATATTTCTGTTAATATTGGCTAGTTGATAATAATAG
ATCAAATGAACAATGTTGTTTTTCATTTGTTGTTTTATTATTTTATATATAAATAATATAATT
GTTTTTTAAATTATTCGATATAAGTTATAATATATTTGAAAATTTAAAACAGTTATAATACT
AATATATGATAATCTACGAAGCTTCTTTTTTTTTTTTCATTGTAATATTAACTGATAGATAT
GCATTTTCTTAAAATGTATTGAATATGATTTATCATTATGACTCTTAATATAATATTAATTAA
TTTCTTAGTATTAGTATATTAAATTATGTAACCAAGAAAGATCTGTCATAAAAAACAATT
GAAGATTAGATACCAAAAATCTTTTAAATACATTAATATACATTAATAGAAAGTTTATGTA
ATATTATAGACTTCTTTCAAGAATGTAGAATATAAAATTCTTCTTTTATTAATGAATAAAA
AATTACATTATTAAAATATTAAAGGATAGTAGAAATAAAAAAATTTAATTAATTCATTATA
CATAGAATGTTAATTCTACTATATAATTATCTGCAGTATTAATATTTCGTAGAAATATTTGA
AAAGAATAAAAACAAAGAGTTTTATTTTATATTCAAAAATAATAATCATTAAATTATTAC
TTATAATATTATTCTTTTTATTAATTATTATTTTTCATTGAAAGCATGAGCATTAAGAAAAA
TTATTTTAAATAAGATATTTTCTTTTATATTTCTTCAATTTTTCTTTTATCATATTATATAAAT
AAATGTAATAATATATATTATATAAATATAATAAAATTTTTTTAATAATTTATAAAATTATTA
AAATTTATTATATTATCTATTATACAATTATTAATTTATTTATTATATATTTATTATATAAAATT
AATTAAAAAATTAAAATTTATTAATATTTATAAATATGTATATTTAATATTAGAAATAAAAA
AGAGGAAAAATATAAAATATATAAAAAGAAAAAATTTTGATTAACGAAATATAATTGCA
CGATCGAACAAAGAAAAAAATTAATCTTTAATTAAGAGCAAAAATTTTTCAGGGATATA
ATAGCGGTTTTTCCAATATGATTGCACATGAGAAAATAACCTTTTGCATGTTTTTAACGG
TTTCATTAAAAATCTATGCAAATATGAATCCTATAAAAAAAGTTCTTAATTCCCCTCTAC
GTACATGAAAAACTCGATCAACAGTTTATGTGTTTATTTTTTTTATCTTATTTTTTGTTGT
TGTTGTTATTTCTGCAATTAAAGAAGTTCCAACAAATAATTGGCATAATTCTTCATAAAT
GAAGAATTTTTAATTTTTTAATATCTCTTTATACTGACTTATGTTAATCATGTAAAATTTGA
TATTGGTGAAATAATAAAATTTTATTATTACAATACATAAATAAATTTTGTATATCGTATGA
TTATATATCAATATTTTGCATCAACTGATTTAATAAAATAATTAATATTAGAAATATAATTA
AATATTAAAAAATTTTATAAGCTTTAATCAATTCTTAATATCAAACATTTTTCATACGAAT
TTATTGAATTTTCTTCAATATATAATGAATTTCAACTATTTCAATACTAATCATATTTCAAT
AAAATGCAAGAAATATATAATGATAGGAATACAAATTTTATCCTAATAATATATTGTTAAA
ATTATCATTTCTAAATTATTAGATATCCAAAATTATATCCTTAATTACCCTTAATTTTAATG
AAGAATTAATAAAATATTAATATTTAATTAATATTTACTTTGGAATTTAATATGCAATATTT
ATTTACATTATTTACATTACATTTGTATATATAGTTTTAAATTTATATAGTTATTTATTATACG
AAAGAAAGAAATACAATATTCTTTAATTTTTATCGAAATAATATAAAAATACTTTAAAAA
TAAATGTTTTTTTTTAATTTTTTATTCTACAAATAAATACAAAATTAATATATATATCAAAG

ATTATTTTAACATTTAGTTAATGAAAATAAATAAAATAAATTTATTCTAAAATTTTATACGT
TTTGTTGTTTTTAATACAAATTTATCTATTATTTTCACAAATAATTTTTATATAAAATTTTTT
ATATAAGAAAAGTATAAATATAAATATTATCGAACTGATAGTAGAGAATAAATTTGCATA
AAAAAACAAGTTGAAAATGTAAAATATTTTTTTGTACAAAGTTTCATTTCTTATAAATAT
ATCAAGTATTTATAGATTTATATTTATAATATAATAATCTTAATAATAAGCAACATAATATTT
TTAAACGATTTATATTTATAATTTTATGTACATTTCATTGTATCATGATTAAAAAAAAAAAT
TTAGTAATTTAACTTACGTTAACGAAATGAATTAAAAACCTAATTTTCATAATAGGATAA
CTATATAATAAAATCTTGAATTTTTATTTATTCCTTTTATTATATTATTCTTTGTCAATTCTT
TTATTATTTATTATTATTTATGTAAGTATTCTTAAATAAAAAAGAATATTGATTATTGAAAA
TAATTATTAAATAATGTAATTATTGTTAAAAATTTAATAAAGTATGATTTTAGATTTCATAA
AAAGATTTAGATTTCTTAATTGAACATAAGGATTGAAAATTATATTTATATTATAAACTTT
GAATGAATTTAATGGTGATTAATTCTATCTATTTTTGTAGCTGATTAATGATTGATTGAAT
TCCATGAATTAATTGTTTTTGACTCCTGCGGCGATTAATTGCTATTTGATTAATTTCCACG
TTTCTAAATGATATCTAATAAATTCTGATTTTCTGGAAAACTGTTATGAATTTTTAATTATT
TTTCCAGTAATTATTTTCGATGAAAATATAATTATATAAACTATATAAACTATATAAACTAT
ATATATAAAACTATATATATAAGAAAAGATAAAAAAGAAAACCAATAAGGGAAATTTTTA
AAAATAAATCTTTGAGATCTCAATGTATAAGAATAAAGATTTTTGCAACTTTTGCAGGA
CTTTTATTTTTTGAGTAATTGGACAAAATATTTGGGATGCAAATATGATTTATGAAATAGC
TATCGCTAATGTATCGAATAATTGTTATTGAGAATTTAGAACAATGTAACGAAATTGTAC
TTACTCATAAATTACTGTTAAAATCTAAACTGCTAAAAATCTAAACTACTGAGAATGTTG
AAATCGCTTCATAATTTTCAGTCTTGGTTTTTTAATAGGAAAAAAGATCCTTAATAAAAA
ATAATCGAAAAGCTTGCTAATTAACCGAGATAAAAATATTATTATATCGTGAATTATATTC
AGTAATAAAATAATTACATGAAAGAAAATTGCGCAATAAAAAAATTTATTTGAAATTGTT
ATATAGCCTTAGTTCAAATCCAATATGTAATTCATTCATTGAAATTTATAATATTCGAATTA
AACATCAAATTTATTTTTTACAAAAATTCTATTTAATATAAAATCTTAAAAAAAATAAATTC
AAATCTATTTTACTAATTTTGAATATTAATTTTTAATATTCTTTTCAAATATTTATATACTTG
TATCCTTTGATCTATAACATAATTGACGACTATATTTACTTTAAATTTCAATGAAAAATAG
AAAAATAATGAAATAAATTAATTTAATTTAATTTTCATTTTAAAATATTTTCATAAATATAT
ATGTATACACATGTACATATATAAAATAAATAATATTTATATATAATTTTTTCATACGAATAA
TGTTTAGCATTATAATTATATATTTCAATTAATATATTTATTATTATATTTCAATCGAATATTA
TTTGACGAAGAAAGCAACATGATCTTTTTTTAATTGTTTTCTACTGATAAATCGAATTTG
AAAAGTCGAATATTTTCCAGTTTTTCTTCTAATTATCTAAAAAAAAAGAATTGGAAATTG
GGAAAAAGTTTATATGATCTAATTTTATTTTAAATAGCATAAATTTTACTTTGTTGTTTCTA
TTTTTAAACTTTTCTCAACAGAATACAAGGATTCTCTGTGCACGTTCGTAAAAACTTAC
GAATATTACATAATTTGTTAATTCACTATCTGTTCATTAATCGTCATCGCGTGCAAAAAA
GTTTTTTAAGTATGCATTTTTTCATCTGAATTTCTCTTTTATTTAAATTTATTTTTTCTTTCA
TTATTAATACTGTATTTTTTTTAAGAGAGAAAATGAAATGAAAAATTATAACATATTTAAA
GTAATATTAATAAAGAAATATAATAAGGAAATTAAAGATATATGTATATGTGATCATTCAA
AAAAATATAAATAGTTTACATTTTTCATTTCTAGAAATAAAAATCAAATTTGAAAATTTG
TATAAAATAATGTAATAAACTATAATATATATATTATATATATGTTATTTAAAATTATTTATTT
TAATTTAATAATAATTTTAAATTAATAATTATTTATTTTTATTTTTTTCAAATCTATGAAATA
TTTTAAAAAATTAAAAATTAAATTTTTAAATTTTCCTTTAATTCTATTATGATCTTTGAATTT
TTATATCTTTTTGTGTTTGATTTATATTATTTTTAAACTTAGTTTTAAAAAATTTATTAACG
ATAAAAAAATCATAATTGATAAAGCTAAAATATTTCATAAATTAAAAGTCAATAAAATTAA

CTGTAAATCACTATTTAAAAATAATGTATAAAAAAATAAAATTAAATAACATTTTAAAAA
AATTTCTCAAAAAACAAAAATACTTTTTTTTTCTCCTTTTTTCACTGTTAAACAAAGAA
ATATTTAACAATCTTGCGATAATTAAAAAAACGCAATAAATCAATAAATTATGTATATTTT
TTATTATTTATATATAATATATATTTGTATACAACTTATACAAGTTTGTGAATTAAAATTACA
TTAACTGATCAAACACGTCGAATAATGCTCGAATAATTTTGTTTAAAAAAAACGTTATAA
TAGTAAATAAATTTGATTTTTCCATAGATAAAAAAAATTTTGATTTTTATGTTCTTGAGCT
TTAGTTAGGAAGTTTACAGATTTAATTATTTATATATAGCGAAACAAAAATTTTAGAATAT
TAATACGTAGTGTTTCATATATTGAATTACTTTTGTAGATCATTATTATTAAAATTATAAAT
TAATTATAATTATTAATTTGTTTTTTATTAAATTCGGATTTCGTTAGTTTGGAAAAATTTTG
AATTGTTAATCGCTTTTTCATTATAATAAAATATTGCTTTTTAACTGATGTATCTTACAATT
ATATAAAAATTTTACTACTAACATAAATATTATTATATATATTCATATTATATATAAGAATATT
TATAATAATAATTATTATAAATAATATTAAGAAAAGCATCTTATGAACAAATTGATTATTGT
AACAATGAAAGATATTATATTTAATATATATCTAATATCATAAATATTTGTTTACTATTAAAA
TTTAAGAAATAAAATAAATACTTAAGAAATAAAATAAAATTTAATAAAAATCTAACTACA
TATTAACAATAACTATATTATTAACAATATATTGTTAATTTTAAAAAGATTTACAAGTAATC
TAATAAATTGAGAAATTTAATTGAATATAATTAAGTTACAGAATGATTGCTAAATTGAATT
TATGATGAATTTTGCTTTTTTTTTATTTATTTTGGAAGCAAAAAACATTGTCTATTTGATAT
TTGTTCAATAAAATTAGTTGTTTGTTAAATAACAAATGAGAATGAGATTTAATATGTAAT
CTTCCAAATAAAAACGACAATTAGAATATTCTTTTGTAATAAAAGAAAGTTAAAGATTG
AAAAATTTTTATTCGCTCAAGATATAGTATTTAATAAATAATCTTCAAACAAATTGTCTTT
AAATTATTATGAAAAATCTATTGTTTTTAATTATTGTATATATAAAAATTTAATAAAACCAA
ATAATATCCAATAATTGAACATTTTAAAACAAAAATTTTTTTATCGAGAAATATCGTAATT
AATGTAATTAATTTTTTTGATATAAAATCTTTTTTTTTAAATACTATGAACTATGTATTTAA
AAAAAAGTGAATCGAATAGTGTTTGATATTCATTTCACTATTGATTATTTTATTTAACATA
ACATACGAAAAAAATTATCTTTGTATTAGAGAATCGTAATGATCTGTACAAATAAATAAG
TGGCAAGGATTTAATCTGATAATTAAATTTTTTTTTAATCAGAGTCGTTATAATCGTAGAA
TTGAAAATGAATGAATATGAATTAATTTCAATAATTGAATTGGTGTTAATTCCATATAAGT
ATGAAAATATACGATATAACTTTTATAGATATAATTTTTATCTACAAATGAGTTTAATAATA
AAAATATCTAATTAACAATTCATTTTTATATAATAAATAATAATAAATAAGTAATCATATCA
TAATTATTTTTCTTCAACAGTTTAATAATATTTTCATTATTTTAGTATGAAAGTAATTAATT
AATATAAAATTAATAATATTTCAATTGTTTTAATTTTTAATTAGATAAATAATTATATTTTAA
CAATTTCAAATTATTAAATATCTATATTTTATTTTATAATATTTACTAATATTTACTAATATTT
AATATTTCAAATAATTATATTTTTAGCAATTTAATTAGTTTATATTTTAATTAGTTTAAAATA
ATTAAAATAATATAAATAATATAAATATTAATATAAATATAAATAAAATAAATATTATTTTAA
AATATTATTTTAATTTTTAAATATTATATATTACATTAATAATATATAAAATAATATTAATATT
AAAATAATATTAAAATTAATATTATTATACACAATAAATATTACATTAATAATTTTTTAATTA
TTTTAAATTAATTTATATTTTTATTTTATTTTATTTTATTTACTATTTAGTATTTATACTATTAT
TAATTATTATTATTATTTATTATTTTATTATTTAAGCTTTATTTTGTAATATTTTAATTATATTA
AGATTTGATTTAAGATGTTGTAAAAAATAAAATAATAGAATAAGATCTAATATAATACAAT
ATAATATAATACAATACAATATAATATAATATAAAGAAATATTGAAAATTATATTTTTAAAA
ATAAAATTAAGATATAATAAAAAAGATAAATGTAAATGGAAGAAATTAAATTTAATTCTA
AAAACATCAAATGCAGACATAAGAAAGTAAAACTATACTATTTATATGTGTAAAAATAA
ATTATATTACAGAAAAATCATTTTCAATTTTCATGAATTGGAAAATTTATTTTTTAAAAAT
TTATATAATATCTATATTTTATGAAAGCAGGTCAAACAGAAAAATATCTTTTTGTCACAAA

TTAATCATTTTCCTTGTAGTATTGCAAATTGTTCACATTATACTATAAACAATTTTATTTTC
TTTTTCACTCTCTTTTGTGCTAAAGTAAATTTAAATTGCTTACAAATAAATCTTAATCGA
CGTTTTTACATACCACTAATATTTTTTCTTAATCTTTATCATTGATATCTCTGAATATATTAA
CACTCTATACTCTTAAAATATATTAAAATTTGATAAAGTCAATTAATGAAGTTCATAACTA
AACTTTATCAATATATTATTTTATGAATAAAGAAATAAACTTATATAAAATATTATTAATATT
TTATTTATTTATTTTTATATTAATGTTATTATAATAATTATAATAAAAATTTTAGTCATTTTTT
AATATTCATTTTATGTTATTTATATTATTATTTATTATTATTATACTTTACATTACGTGCATAT
TTTATAAGATATAATTTTATAAATTATTCATTAATAACTTTTACTAAATTCTCTAATATTTAT
TATAAATTTATTTTCTTTTTATTTTTTGATATAATTAAAAATATATTTAAAACTTTAAATTCT
TAAATGATTAACTGAAAATTTTAAAATATATATATATATATGCTATATCATACATTTAATCAA
TTTATAAATTTTTACAAGTATGCAATATTGGTTATTTATATTATGTTTAAATGAAGCAATTA
GGAAATTTGTTTTTTTTTAATTTTTTGTTATTTAGATTAAAAAATATTTTTAAAGGAAATT
AAAATTTTATTATTCGTCTTAATTAAAAAAGAAATATATTTTCATTTGAAAGAAAAGAAA
AAAAATATTTTCAAATCATTGAAGAAAACTGAGTAGAATTAATTTTTATAGAAAAAATTT
AAATACTAAATTGAATCTGAATTTGCTACTTTATAAGTTTTACAAAAAATTGAAAATCAA
AAAATAATTTATTTAATTCACAATTTTATTTTACAAATTTTTTTTCAGGAAATTTATCAATA
ATAATAGTCATATTTCTACTTTCGTTTCTATGTTTTAATATAAGATTGATTTTATATATAATA
AAATTGTTAAGGACAAAGCTTGTGAAATCCGGTAATCTGGCTTTCATTTTCAACAGCTA
GAATATCTCCAAAAATCTAAACAATAATTTATGAGGATTATATATATATTATAGTTTCTTAA
TATTTTCTAAATTTTCAATTTAATAAAAAATATTACAGCAATGGTCAACATTTCATCACAC
AAACTGTACTTGTACGCATTGCGATGGATCACCATGAAAGCGAGCATAAAAGTCCTGAT
TCCCATGTGCTGTGACGCGAAGGCCCATCCTTCGTCCTCCAACGCAATGATTGGACCTT
CTCAGTATGCATTTTCACAAAAAGCAAGGATTGGAAAAAAAACTAAAGCGACAGTGTT
ACAATTCA

>novel_circ_000578
GAATAATGCACATAAAGAGGGAAGAGACCAATGGCGTTGAAGAGCGGCCTCGAAGGA
AGGGTTCTCACAGGGCCACGTCGCAGGTCAGGTCTTTTGCGCTCCCTCCTACAGTAAT
CCATCACATGGCGTGCAAATATGGCTAGTGAAGTAGCCATTGTTATGATACATTTGTTTA
AATTATTTGGAAAACGTAAGAGAAATCATGTTTGCTCATAAATTATTGTCAGGCTATTGA
CGCTACTCGAATTATTGAAAACACAGACCAGGTTACCCGACCGATTCATAAGCCTGTCC
TTAACATTTGCATTAACAATATATTTAATTAATACCAGGTTTATAATTATTTTTTCATTCGC
TATTCAATAAAATGAAAAATATGCATAATACTAATTTTTATTCTAAAAATGTAATATTATAA
TTCGTTACTTTGTATGCACAAATTCCGTCCTGTAAATTTCAATCATGTCATTTTTAAAATT
ATAAATATACATATATAATTGTTAAGTGTGAAAATTGTGTGAAAATATTTAATATCTGAA
AATGTTTATTACAAATTTAAAAATAAATTTATTATAAATTAATGAACTATAATTGTAAATAT
GTTGAAAGTCAATTCTTAACATGTTAAAAATAAATGTAAATAATTTTTATATGAAATAAC
ATATAAAAAATTAAAAATAAAATGAAAAATTATTTCATACAATAAAATATAATTATAACA
AAATATTGAAAGAATATTATTATGTACGAATGCATGAATGAGTAAAGAATGAGAGGTGC
AATTATTCTATCTGTTTGTAGAGAAACAAAATAAAAAAAAGCTAGAAATCAATTGAAGA
AATAACTTTGTAATGATAAGGCGAATTTTGTATATTCTTCTTTATATTTTTTATTAAATTTT
ATTTCATCCAAATTTTGATTATTAGAATTATTCCGTATTATTATTGTGTAAGTTAATTTTTT
TTTCAAAATAAAATTATTATCAAAAATAATTTGTGCGGAAAATAATTTTTGAATTATTATT
ATTATTATTCTTGAATAATAATAATTTATAATAAAGTGAGACAGATATGTAAATATTTTTTT

TAAATACATTGTGTAAATTTTATAAGATGCGAAAGAAATCGTAGAAATAGTGTTTATAAC
ATTTAAATAATTGAATGCTATTAAATAATTAGATTTCAATAATTTCTTTTGCATGTGTTAA
AAATAATAAATAATACAAAAATCAAATGTGAATAATATAAAAAGAAAAATTTATTAATTC
AATTTAATTTTTATAAATAAAAGATAGAAATGTGTTAAGTAGAAGTTAAAAAATAATATAT
AAATAATAGTATTATTGAATTAAGAGATAGAAAGATTTTATCTTTAGTTTTGACTTATTTT
TTAAATTTCAAATTTTTGGTTTATATAAAAAAATTATTTTTTATTAATAATAAATGATTTAA
TATTAGTTTATTATTATTTTAATTGTAATTTAGTTTATTATTGTTTGGTAAAAAAGAATTGT
TTATTATTATCTTTCCCAAAGAAAGGATAAGCTTGAATATGCATTTTTATATAAAATCAAA
ATGTATACTAAATCATTTATTTTTAATTGATATTTAAACTTACATAGTATACACAACATGGT
GTAAAATTTTGTTAAAATACGTGATTTAAAAAATTATAAAAAATGATTTTTAAAAATAAT
CTTTAATTGATAATAAAATTACATAGTTTTTTTATATAATTCAAAACTATATTAGCAAAATA
TTATTTAAAAATGTTATTGAAAATTTTTTATCAATTATTATTATTTTTTCGTATTATTATTTA
TGATCAATATATTATCACTCATTTGTTATTATTATAATTTATTTTTATGTAAAAAATAACGA
ACATAATTTTGAACAAAAAATTTTTTTTTATTCTTCTTACTTTATATTTTTACTTTCTTTTC
ATTTCTCTTTATTGTTGTCTTAAGTTATCAATTTTTAAATATAATTTTTTATATATATTCTCA
TAATCTTTTTGATATATAATTATATAATAAAATGTATGTTATGTAATTTTATGATCAATTAAA
TAATTTTGTGTTTATCAAAAAGTATTTAATTTAATATTTATTAAAAAATTAGATGTTGATTT
AATATTTAGATGTTTCGGATTTGTAGTTTGTTACATACATTGAGATTTTGTATAAGATATAT
TGCATTAAGATATAATACATTATCTATATACATATTTATATATATATATATATATATATATATA
TATATATATATATATATATATATATATATATATATATGAGCAGTAAATATTATGTAATGCAGAAT
TTAATTTTATTTTAATTAATTAAGTGTAAGAGTCTAAAATATTGATTTTGATAAGTATATAA
TAATAAATAGATTTACTAATTTAATAATATTAAATGAATAATTCTTTCAAATAATATCATTT
ATGGAAATATTTCAGTTTTCTAATATTTATTCATATAATATTTCTTGAATTCTCGTCTACAA
TAAAATGTTAGACAATGAAATGAATGAACTTTCACAAATAAATTTTATAAATTTCAAATT
ATCTATTTATTTTATTTAACATTACCTAAATTATAATACTTTTTAAAATGATAGTTGATAGT
AACATTTATAAGATTTTTATAAGATTTTATAAGATTTTTTTTTGTAGATTTTAATAAGATTT
ATAATTAAATAATTATAAATATATTTATAGTAATTTATACCATTTTTTACAAAAATCATAAG
ATTTAAAATTAAATAATTGATGAAAGGAATATAGTTATCAACGAAATAATATATTGATAAT
TTTATTTTAAATATCTGAGACAAAAAGTCATAAGATTTTTTTTAAACATTATTTATTTTGC
AAAAAATATAGAAATAGTCGTTGAATTCTACCAATACGGAAGATGTAGATTTCTATCAA
AATCTATGGAAATAATTTCTTTATGAATTAATATATCATATTTGACTCTAGAGAAATAAAA
ATAATATTTATCGCATTTCGAGATCATGAAAAAGAAAAAAAAAATTATGTTTCCTAGTTTA
TAAGAATTTTCCTACCGATTTTTAATTAATTCTAAGGAATTTAATAGAGTTAAAAAATTTA
AGTTGGGAAATATAGAGCATTTCAGTGAAATGATATAATAATTTGATATGATATAATATGA
TTATATGATAATTTATCAAAATTCCATTTATGATGTGATATATTTTCTTAACAATAATAAATT
ATATTTCAAGTATGTCATTTATACGAGACTTAGATTTCTCATATTCAATAATCAGAGTAGA
TGCGTAATTACAAAGAAAACTATTTATTTATCATATTCCATCAGAATACAATATGTTCACA
ACAAAAATTAAATAGAGTTAAAAATTAACTCTGCAGATTCATTTTTTGATGAGACTCTC
AGAAATTCCTTCCATAATTGGCATCCTAACTTGCAGTATTTTCAAGTAAGAGAGAAATT
CCTTAAATTAAGCATAAAAAAATATGATGATAACCAGAGATTGATCAGCATATATTTATTAT
AAATTTCTCCGAATATAGTTAATTGCAGTTGAATCAATCATATTTAGAGAATATCATCCTT
AAGATCGTGTTTCTAGCCAAGCTAAAATATGTTAAATATATAGAATTGTGTTACCTAAAT
CTTCCTTTAAAGTACCAATCTTCTCAGATAACATCATTCTTTATTTAACTTAGTAAGAGA
ATAGCAAATAACAGTACATGTAATGAAAAAAGTTTTATGTGAGGAAGAAATAAGAAGA

ATACTTATTGCTTACGAATGAATATATATATTATTACTAATAAATAAATAAATAAATAAATAT
ATATATATATATATATATATATTACTTAATAACTCTTGATTAATACATCTTAATAATTTTCGTT
TGATTTTTTTTTTTATCAGATTCATCATAGTGAATCAAAGATAAAAATATTTTTCTAATTTA
TATAATTTAAAACAAAAATAAAAGTGATAACACGCATCAAATTTTGTTTATTATCGAATT
AACATATTTACAACTTTTCAACAAACAACAAAATTGTACCGTATCCATTTCAAATATTAT
TTCACTGTCTCTCAATAAAATAATTTCAAATATATAGTTTGTTAGAAAGTGATAGTAGATT
TTTTTAAACTCCAATGTTCTTTTGTTGAGAATATTAATGAAATACGTTTGCAGTTGATTAT
TAATAAAGATTTGAATAAGATATTTCAAATAACTTTTCTATTGTGTATTGTTTCAGTTATT
TTTGAAATATCAAAAACTTTTTTTTAGAAAAAACTTTAAAAAAAAATACTATAATAATATT
AGAATTCTATAATAATACTATAATAATAAGAATACTATTAAAAAAAAAGTAATTCTTTTTTT
AATATTTTTATCAATTACTTATGAATTTGTTTGTTTTTTTTTTCAAATCAAAAACTAATAA
AGTGAAAAAGTGTACTTATATTGTATTTGAATATTTAAGCACAATATTATACAATAAATTC
TTATTCATATATGTTAAAAATGGAATAATTTTAAAATAGTATATTGCCATAAAAAAATGTT
ATAGTATTAAGATTTAATTTTATTAGAAAAAATAATATTATATTATATACTTAGAAAAAAAT
TAATCTATCAAAAATATTTTTCATTTATTTATCTTTTAAATTTAATACAATATAACTTATGAT
TAGATAAGGCAACGAAATAAATATCTATTTTATTCTACCTGTGTCATTATTTTATAACGTC
CATTTTTTCCATTGCTCGCGATGTTTATAAACAGCTTATTCAAAGATTTCTCATAAAAATC
TTTTCCTGTTATAAACGCAGACTTCTATTAAGTCTAAAAAACACTCACAATCTAAAACA
CAAAGGATGATATTATTTGAGAAAACTGTCGTTTTTGAGACTTTAGTCGACAATTGTATA
TTTTTGATACACTTCAGCCTGATTGGAGCACAGCCCTATCCTAAGAGTGACATATTCCA
AATTGATTTCCAGCTGATTCAACAGCAATCAATATCATTCTAGTTCGAGGAATTTTTCTC
AAATTTGGGGAGTCACTGATCGCAATGTTATTGTTTATTACCGTGCTCTACCGAAAAAC
TGAAATCTTTCGAATTAAACTAGGAAGGAATTTTTTGAGGACACTTATAATTTTTTGAGT
GTTACTTATAAATAGAAGTTCAGGATGAACACGCTAGATATAATAATTAGTTTGTGAATA
TATCATGCTCTGATCGTCGAATTATTTATTGTTATTTGTAAAAAAAAAAATCTCATTGTGA
ATAGATAGTAGTCTGATCAATACTTACATTAATTACATAGTTTAGTTCTAATATCATATCGT
GGTCTGATACGATTATCTGCTGCGACATTGTGAGAGATCCTGTTTTTTTATATAGATACG
CTATAGATGCGCCATGTAACCATTTTTACAGTTCATTCATCTTTTAGAAATATATCAATTAT
TATGACATGTGTCAAAAAACATTTTACAAACACTCAGGTATTAGTTTATATCCATCATCA
TTACACAATAATATTGTTACGGGTGTGAACATGTATAAAAAGAAATTTAAAAAAATATTG
AAAGTATTGAAAAAGTAATAATTTTTTAAAATAATGTTTTTTGAAATTGATATGAGACTA
AAATATGTAGCTATGAATAAAAAGCACATGAATCAATTGTCAAAAGAAAATACGAAATT
GTTGTATAGAAAGCAAGTGAAATTTATAGTGAAAAAGAAAAAGACAGAAATTCTAGAC
AAATTTCACGTAATGAGATTTTTATTATAATTTCCCATTTTTTTGTTCTAATATATAAGATT
TAATATAAGATTTAAGATTATAAAATTTAATATAAAATTTTATGAAAAATTTTTTATTGCCT
GTAATAGAAAATTATTGTAATGAATTATTGGAACTTCTAAATATGCAATATATTTAATAAA
ATAAAAAATTTTAATAAAAATTAACAGTAAATATTTTTCTATATTACGAAAAAATGGATAT
TTTATATAGATATATATATAAAATTTAGCAATATTGCATTATATTTAAGAAACATTGACAAT
CAAGGTATATATTTTATTAGTGTAGCATTTTGTAGCGGATTATATATGTATGTATTGCGATG
GTAATTTATTTACACATATTTAAATGTATATTTATAATTAATATTAATATATATATATATTAATA
TTATATAATATATAAATTATTATATAATATATTTCTGTTAATATTGGCTAGTTGATAATAATAG
ATCAAATGAACAATGTTGTTTTTCATTTGTTGTTTTATTATTTTATATATAAATAATATAATT
GTTTTTTAAATTATTCGATATAAGTTATAATATATTTGAAAATTTAAAACAGTTATAATACT
AATATATGATAATCTACGAAGCTTCTTTTTTTTTTTTTCATTGTAATATTAACTGATAGATAT

GCATTTTCTTAAAATGTATTGAATATGATTTATCATTATGACTCTTAATATAATATTAATTAA
TTTCTTAGTATTAGTATATTAAATTATGTAACCAAGAAAGATCTGTCATAAAAAACAATT
GAAGATTAGATACCAAAAATCTTTTAAATACATTAATATACATTAATAGAAAGTTTATGTA
ATATTATAGACTTCTTTCAAGAATGTAGAATATAAAATTCTTCTTTTATTAATGAATAAAA
AATTACATTATTAAAATATTAAAGGATAGTAGAAATAAAAAAATTTAATTAATTCATTATA
CATAGAATGTTAATTCTACTATATAATTATCTGCAGTATTAATATTTCGTAGAAATATTTGA
AAAGAATAAAAACAAAGAGTTTTATTTTATATTCAAAAATAATAATCATTAAATTATTAC
TTATAATATTATTCTTTTTATTAATTATTATTTTTCATTGAAAGCATGAGCATTAAGAAAAA
TTATTTTAAATAAGATATTTTCTTTTATATTTCTTCAATTTTTCTTTTATCATATTATATAAAT
AAATGTAATAATATATATTATATAAATATAATAAAATTTTTTTAATAATTTATAAAATTATTA
AAATTTATTATATTATCTATTATACAATTATTAATTTATTTATTATATATTTATTATATAAAATT
AATTAAAAAATTAAAATTTATTAATATTTATAAATATGTATATTTAATATTAGAAATAAAAA
AGAGGAAAAATATAAAATATATAAAAAGAAAAAAATTTTGATTAACGAAATATAATTGCA
CGATCGAACAAAGAAAAAAATTAATCTTTAATTAAGAGCAAAAATTTTTCAGGGATATA
ATAGCGGTTTTTCCAATATGATTGCACATGAGAAAATAACCTTTTGCATGTTTTTAACGG
TTTCATTAAAAATCTATGCAAATATGAATCCTATAAAAAAAGTTCTTAATTCCCCTCTAC
GTACATGAAAAACTCGATCAACAGTTTATGTGTTTATTTTTTTTATCTTATTTTTTGTTGT
TGTTGTTATTTCTGCAATTAAAGAAGTTCCAACAAATAATTGGCATAATTCTTCATAAAT
GAAGAATTTTTAATTTTTTAATATCTCTTTATACTGACTTATGTTAATCATGTAAAATTTGA
TATTGGTGAAATAATAAAATTTTATTATTACAATACATAAATAAATTTTGTATATCGTATGA
TTATATATCAATATTTTGCATCAACTGATTTAATAAAATAATTAATATTAGAAATATAATTA
AATATTAAAAAATTTTATAAGCTTTAATCAATTCTTAATATCAAACATTTTTCATACGAAT
TTATTGAATTTTCTTCAATATATAATGAATTTCAACTATTTCAATACTAATCATATTTCAAT
AAAATGCAAGAAATATATAATGATAGGAATACAAATTTTATCCTAATAATATATTGTTAAA
ATTATCATTTCTAAATTATTAGATATCCAAAATTATATCCTTAATTACCCTTAATTTTAATG
AAGAATTAATAAAATATTAATATTTAATTAATATTTACTTTGGAATTTAATATGCAATATTT
ATTTACATTATTTACATTACATTTGTATATATAGTTTTAAATTTATATAGTTATTTATTATACG
AAAGAAAGAAATACAATATTCTTTAATTTTTATCGAAATAATATAAAAATACTTTAAAAA
TAAATGTTTTTTTTTAATTTTTTATTCTACAAATAAATACAAAATTAATATATATATCAAAG
ATTATTTTAACATTTAGTTAATGAAAATAAATAAAATAAATTTATTCTAAAATTTTATACGT
TTTGTTGTTTTTAATACAAATTTATCTATTATTTTCACAAATAATTTTTATATAAAATTTTTT
ATATAAGAAAAGTATAAATATAAATATTATCGAACTGATAGTAGAGAATAAATTTGCATA
AAAAAACAAGTTGAAAATGTAAAATATTTTTTTGTACAAAGTTTCATTTCTTATAAATAT
ATCAAGTATTTATAGATTTATATTTATAATATAATAATCTTAATAATAAGCAACATAATATTT
TTAAACGATTTATATTTATAATTTTATGTACATTTCATTGTATCATGATTAAAAAAAAAAAT
TTAGTAATTTAACTTACGTTAACGAAATGAATTAAAAACCTAATTTTCATAATAGGATAA
CTATATAATAAAATCTTGAATTTTTATTTATTCCTTTTATTATATTATTCTTTGTCAATTCTT
TTATTATTTATTATTATTTATGTAAGTATTCTTAAATAAAAAAGAATATTGATTATTGAAAA
TAATTATTAAATAATGTAATTATTGTTAAAAATTTAATAAAGTATGATTTTAGATTTCATAA
AAAGATTTAGATTTCTTAATTGAACATAAGGATTGAAAATTATATTTATATTATAAACTTT
GAATGAATTTAATGGTGATTAATTCTATCTATTTTTGTAGCTGATTAATGATTGATTGAAT
TCCATGAATTAATTGTTTTTGACTCCTGCGGCGATTAATTGCTATTTGATTAATTTCCACG
TTTCTAAATGATATCTAATAAATTCTGATTTTCTGGAAAACTGTTATGAATTTTTAATTATT
TTTCCAGTAATTATTTTCGATGAAAATATAATTATATAAACTATATAAACTATATAAACTAT

ATATATAAAACTATATATATAAGAAAAGATAAAAAAGAAAACCAATAAGGGAAATTTTTA
AAAATAAATCTTTGAGATCTCAATGTATAAGAATAAAGATTTTTGCAACTTTTGCAGGA
CTTTTATTTTTTGAGTAATTGGACAAAATATTTGGGATGCAAATATGATTTATGAAATAGC
TATCGCTAATGTATCGAATAATTGTTATTGAGAATTTAGAACAATGTAACGAAATTGTAC
TTACTCATAAATTACTGTTAAAATCTAAACTGCTAAAAATCTAAACTACTGAGAATGTTG
AAATCGCTTCATAATTTTCAGTCTTGGTTTTTTAATAGGAAAAAAGATCCTTAATAAAAA
ATAATCGAAAAGCTTGCTAATTAACCGAGATAAAAATATTATTATATCGTGAATTATATTC
AGTAATAAAATAATTACATGAAAGAAAATTGCGCAATAAAAAAAATTTATTTGAAATTGTT
ATATAGCCTTAGTTCAAATCCAATATGTAATTCATTCATTGAAATTTATAATATTCGAATTA
AACATCAAATTTATTTTTTACAAAAATTCTATTTAATATAAAATCTTAAAAAAATAAATTC
AAATCTATTTTACTAATTTTGAATATTAATTTTTAATATTCTTTTCAAATATTTATATACTTG
TATCCTTTGATCTATAACATAATTGACGACTATATTTACTTTAAATTTCAATGAAAAATAG
AAAAATAATGAAATAAATTAATTTAATTTAATTTTCATTTTAAAATATTTTCATAAATATAT
ATGTATACACATGTACATATATAAAATAAATAATATTTATATATAATTTTTTCATACGAATAA
TGTTTAGCATTATAATTATATATTTCAATTAATATATTTATTATTATATTTCAATCGAATATTA
TTTGACGAAGAAAGCAACATGATCTTTTTTTAATTGTTTTCTACTGATAAATCGAATTTG
AAAAGTCGAATATTTTCCAGTTTTTCTTCTAATTATCTAAAAAAAAAGAATTGGAAATTG
GGAAAAAGTTTATATGATCTAATTTTATTTTAAATAGCATAAATTTTACTTTGTTGTTTCTA
TTTTTAAACTTTTCTCAACAGAATACAAGGATTCTCTGTGCACGTTCGTAAAAACTTAC
GAATATTACATAATTTGTTAATTCACTATCTGTTCATTAATCGTCATCGCGTGCAAAAAA
GTTTTTTAAGTATGCATTTTTTCATCTGAATTTCTCTTTTATTTAAATTTATTTTTTCTTTCA
TTATTAATACTGTATTTTTTTTAAGAGAGAAAATGAAATGAAAAATTATAACATATTTAAA
GTAATATTAATAAAGAAATATAATAAGGAAATTAAAGATATATGTATATGTGATCATTCAA
AAAAATATAAATAGTTTACATTTTTCATTTCTAGAAATAAAAATCAAATTTGAAAATTTG
TATAAAATAATGTAATAAACTATAATATATATATTATATATATGTTATTTAAAATTATTTATTT
TAATTTAATAATAATTTTAAATTAATAATTATTTATTTTTATTTTTTTCAAATCTATGAAATA
TTTTAAAAATTAAAAATTAAATTTTTAAATTTTCCTTTAATTCTATTATGATCTTTGAATTT
TTATATCTTTTTGTGTTTGATTTATATTATTTTTAAACTTAGTTTTAAAAAATTTATTAACG
ATAAAAAAATCATAATTGATAAAGCTAAAATATTTCATAAATTAAAAGTCAATAAAATTAA
CTGTAAATCACTATTTAAAAATAATGTATAAAAAAATAAAATTAAATAACATTTTAAAAA
AATTTCTCAAAAAACAAAAATACTTTTTTTTTCTCCTTTTTTCACTGTTAAACAAAGAA
ATATTTAACAATCTTGCGATAATTAAAAAAACGCAATAAATCAATAAATTATGTATATTTT
TTATTATTTATATATAATATATATTTGTATACAACTTATACAAGTTTGTGAATTAAAATTACA
TTAACTGATCAAACACGTCGAATAATGCTCGAATAATTTTGTTTAAAAAAAACGTTATAA
TAGTAAATAAATTTGATTTTTCCATAGATAAAAAAAATTTTGATTTTTATGTTCTTGAGCT
TTAGTTAGGAAGTTTACAGATTTAATTATTTATATATAGCGAAACAAAAATTTTAGAATAT
TAATACGTAGTGTTTCATATATTGAATTACTTTTGTAGATCATTATTATTAAAATTATAAAT
TAATTATAATTATTAATTTGTTTTTTATTAAATTCGGATTTCGTTAGTTTGGAAAAATTTTG
AATTGTTAATCGCTTTTTCATTATAATAAAATATTGCTTTTTAACTGATGTATCTTACAATT
ATATAAAAAATTTTACTACTAACATAAATATTATTATATATATTCATATTATATATAAGAATATT
TATAATAATAATTATTATAAATAATATTAAGAAAAGCATCTTATGAACAAATTGATTATTGT
AACAATGAAAGATATTATATTTAATATATATCTAATATCATAAATATTTGTTTACTATTAAAA
TTTAAGAAATAAAATAAATACTTAAGAAATAAAATAAAATTTAATAAAAAATCTAACTACA
TATTAACAATAACTATATTATTAACAATATATTGTTAATTTTAAAAAGATTTACAAGTAATC

TAATAAATTGAGAAATTTAATTGAATATAATTAAGTTACAGAATGATTGCTAAATTGAATT
TATGATGAATTTTGCTTTTTTTTATTTATTTTGGAAGCAAAAAACATTGTCTATTTGATAT
TTGTTCAATAAAATTAGTTGTTTGTTAAATAACAAATGAGAATGAGATTTAATATGTAAT
CTTCCAAATAAAAACGACAATTAGAATATTCTTTTGTAATAAAAGAAAGTTAAAGATTG
AAAAATTTTTATTCGCTCAAGATATAGTATTTAATAAATAATCTTCAAACAAATTGTCTTT
AAATTATTATGAAAAATCTATTGTTTTTAATTATTGTATATATAAAAATTTAATAAAACCAA
ATAATATCCAATAATTGAACATTTTAAAACAAAAATTTTTTTATCGAGAAATATCGTAATT
AATGTAATTAATTTTTTTGATATAAAATCTTTTTTTTTAAATACTATGAACTATGTATTTAA
AAAAAAGTGAATCGAATAGTGTTTGATATTCATTTCACTATTGATTATTTTATTTAACATA
ACATACGAAAAAAATTATCTTTGTATTAGAGAATCGTAATGATCTGTACAAATAAATAAG
TGGCAAGGATTTAATCTGATAATTAAATTTTTTTTTAATCAGAGTCGTTATAATCGTAGAA
TTGAAAATGAATGAATATGAATTAATTTCAATAATTGAATTGGTGTTAATTCCATATAAGT
ATGAAAATATACGATATAACTTTTATAGATATAATTTTTTATCTACAAATGAGTTTAATAATA
AAAATATCTAATTAACAATTCATTTTTATATAATAAATAATAATAAATAAGTAATCATATCA
TAATTATTTTTCTTCAACAGTTTAATAATATTTTCATTATTTTAGTATGAAAGTAATTAATT
AATATAAAATTAATAATATTTCAATTGTTTTAATTTTTAATTAGATAAATAATTATATTTTAA
CAATTTCAAATTATTAAATATCTATATTTTATTTTATAATATTTACTAATATTTACTAATATTT
AATATTTCAAATAATTATATTTTTAGCAATTTAATTAGTTTATATTTTAATTAGTTTAAAATA
ATTAAAATAATATAAATAATATAAATATTAATATAAATATAAATAAAATAAATATTATTTTAA
AATATTATTTTAATTTTTAAATATTATATATTACATTAATAATATATAAAATAATATTAATATT
AAAATAATATTAAAATTAATATTATTATACACAATAAATATTACATTAATAATTTTTTAATTA
TTTTAAATTAATTTATATTTTTATTTTATTTTATTTTATTTACTATTTAGTATTTATACTATTAT
TAATTATTATTATTATTTATTATTTTATTATTTAAGCTTTATTTTGTAATATTTTAATTATATTA
AGATTTGATTTAAGATGTTGTAAAAAATAAAATAATAGAATAAGATCTAATATAATACAAT
ATAATATAATACAATACAATATAATATAATATAAAGAAATATTGAAAATTATATTTTTAAAA
ATAAAATTAAGATATAATAAAAAAGATAAATGTAAATGGAAGAAATTAAATTTAATTCTA
AAAACATCAAATGCAGACATAAGAAAGTAAAACTATACTATTTATATGTGTAAAAATAA
ATTATATTACAGAAAAATCATTTTCAATTTTCATGAATTGGAAAATTTATTTTTTAAAAAT
TTATATAATATCTATATTTTATGAAAGCAGGTCAAACAGAAAAATATCTTTTTGTCACAAA
TTAATCATTTTCCTTGTAGTATTGCAAATTGTTCACATTATACTATAAACAATTTTATTTTC
TTTTTCACTCTCTTTTGTGCTAAAGTAAATTTAAATTGCTTACAAATAAATCTTAATCGA
CGTTTTTACATACCACTAATATTTTTTCTTAATCTTTATCATTGATATCTCTGAATATATTAA
CACTCTATACTCTTAAAATATATTAAAATTTGATAAAGTCAATTAATGAAGTTCATAACTA
AACTTTATCAATATATTATTTTATGAATAAAGAAATAAACTTATATAAAATATTATTAATATT
TTATTTATTTATTTTTATATTAATGTTATTATAATAATTATAATAAAAATTTAGTCATTTTTT
AATATTCATTTTATGTTATTTATATTATTATTTATTATTATTATACTTTACATTACGTGCATAT
TTTATAAGATATAATTTTATAAATTATTCATTAATAACTTTTACTAAATTCTCTAATATTTAT
TATAAATTTATTTTCTTTTTATTTTTTGATATAATTAAAAATATATTTAAAACTTTAAATTCT
TAAATGATTAACTGAAAATTTTAAAATATATATATATATATGCTATATCATACATTTAATCAA
TTTATAAATTTTTACAAGTATGCAATATTGGTTATTTATATTATGTTTAAATGAAGCAATTA
GGAAATTTGTTTTTTTTTAATTTTTTGTTATTTAGATTAAAAAATATTTTTAAAGGAAATT
AAAATTTTATTATTCGTCTTAATTAAAAAAGAAATATATTTTCATTTGAAAGAAAAGAAA
AAAAATATTTTCAAATCATTGAAGAAAACTGAGTAGAATTAATTTTTATAGAAAAAATTT
AAATACTAAATTGAATCTGAATTTGCTACTTTATAAGTTTTACAAAAAATTGAAAATCAA

AAAATAATTTATTTAATTCACAATTTTATTTTACAAATTTTTTTCAGGAAATTTATCAATA
ATAATAGTCATATTTCTACTTTCGTTTCTATGTTTTAATATAAGATTGATTTTATATATAATA
AAATTGTTAAGGACAAAGCTTGTGAAATCCGGTAATCTGGCTTTCATTTTCAACAGCTA
GAATATCTCCAAAAATCTAAACAATAATTTATGAGGATTATATATATATTATAGTTTCTTAA
TATTTTCTAAATTTTCAATTTAATAAAAAATATTACAGCAATGGTCAACATTTCATCACAC
AAACTGTACTTGTACGCATTGCGATGGATCACCATGAAAGCGAGCATAAAAGTCCTGAT
TCCCATGTGCTGTGACGCGAAGGCCCATCCTTCGTCCTCCAACGCAATGATTGGACCTT
CTCA

>novel_circ_000579
AGATCAAGATAATGACAGTCATGGTGGAGAATGTGAAAGTTGAAGAGGGCGAACGTC
CGAAGATCGAAACCTTTGATGATATCCTACCTTACGTTGGCGAAGCAGGCCGTTATCAA
TGGTTTCTCTTTATTGTCCTTTTGCCTTTCACCTTTGTGTACGCGTTTCTTTATTTCACGC
AATTCTTCATCACATTACTACCAAACGAACATTGGTGCACCGTGCCAGAACTCGAACGA
TGGAACCTCACGGACGAAGAAAAAATTGCAATATCGATACCCACAGCATCGACAGAAG
AGTTAAAGCAGGAAGGCGCAACTTCCTTTTCGCGATGCAATATGTATAATGTGAATTAT
ACTGAAATAATAGAAAAAGGTATTAGAAAAGCAGATCCATCTTGGCCAATCAAATCTTG
TCAAAATGGTTGGACGTTCAATCATACGATGATTCCATATAGATCTATTGCTGTTGAGCT
GGAATGGGTCTGTGACCACGCATTCCTTAGCTCGGCGGCACAATCGGCCTTCTTCGTG
GGAAGCATGCTTGGAGGTTTAATATTTGGTTACATAGCTGACCATTATGGCAGAATTCCG
GCGTTGGTCGCTTGCAACGCGGTCGGTTTCATCGCCTCTGTTGGCACTGCGTTTTGTGA
CAGTTTCTGGAGCTTCTGCCTCGCAAGATTAGTCGTTGGATCATCATTTGATAATTGCTT
CAATGTACTTTTTATTATCGTGATAGAGTATGTCGGCCCGAAATATCGAACTCTTGTGGC
AAATATGTCTTTTGGGCTTTATTTCGCGGCAGCTGCGAGTCTTTTACCTTGGATCGCCTA
TTGGATCTCAAATTGGAGAATTTTAAGTATGGTCACCGCGTGTCCCATGGTGGTCGCAT
TTATAGGACCATGGATTGTTCCTGAAAGTGCACGGTGGTATATAACCAGTGGAAGAATT
GACAAAGCGATCGAGATGCTAAAGAAATTTGCGAAAGTGAATGGCAAGGAAGTAAAA
CAAGAAATATTCGATGAATTCGAAAAAAGTTGCAGAATGTCGAACGAAAAGGATCAAT
CGCATAATCAATATACCGTCCTGCATCTCTTTAAACTGCCGCGACTCAGTCGTATTACTA
TCATGCTCATTGTTTATTGGCTGCTAATGGTGTGGGTGTTCGATGGTCACGTTTGGAACA
TGAAACTTCTAGATCCTGACGTTTTCATGTCCTTCTCTTTGGCTTCCCTCACGGAACTTC
CGGCGGCTGTACTACTTGCCCTTTTTCTCGATAGATGGGGCAGACGATGGATGGGTTTT
GCATCAATGTTCCTCTGTGGCGTTTTCTCCTTTGTTGCCCTTTCTACCCCTGCTGGCTCA
CCAACGGTCGCTATGGCAATCATAGCACGTCTTGGAGTGAATGTCGCGGCAAATATTGG
TTTCCAATATGCAGCTGAAATGCTGCCTACTGTAGTGCGAGCCCAGGGTGTTTCTTTGA
TTCATATTATCGGTTATGTCGCTCATATTTTAGGACCGTATATTATTTATCTGGCTGACATC
GATGCTGCTTTACCCCTCATTGCTCTTGGTTTGCTATCCTTTATACACTCATTTCTGACCC
TTGGCTTGCCGGAAACTCTGAATCAGGAACTTCCAGAAACCCTTCAGGAAGGTAACGA
TTTTGGAAAAGAACAAAGCTTCTGGTGGATTCCCTGTATATCCTC

>novel_circ_000582
ATTATTTACAATAGCGCCAAACGCGATATATCGATGATATTGAATAAGAAATTATTTCAAC
AAAATGTTGATCAAGAAACATCGAAGACGGAAAAAGAAAATAAGCAAAAAAAATTAA
TCCAGGAATTATCGAATTCCGAGCTGAAATATAAATGGAATATCGTTTGGAAAAACGTG

ATTGTATTTATCTACCTTCATCTTGGAGGTATATACGGATTTTATCTTTGGATTAACGGAA
CGAAGGCATATACAATTTTGTGGTCAATTTTCGTAGAATTTTTTGCCATTATGGGCATTA
CAGCTGGAGCTCACAGATTATGGTCACATAGAAGTTATAAAGCGAAATGGCCCTTACGC
TTCGTGTTGATGCTTCTTCAAACAACCTCTTACCAGAATCACATTTATGAATGGGTAAG
AGATCATAGAGCTCATCACAAATTCACTGATACTGATGCGGATCCTCATAATTCCCGACG
AGGATTTTTTTCTCTCACATAGGCTGGTTAATGGTCCGAAAGCATCCGGATATATTTAC
AAAAGGTGCCACTATTGACTTAAGTGATTTGGAAAAAGATCCCATTGTCGTTTGGCAA
AGAAGATTGTATATCGTGCTGATGCCTCTTTTGTGCTTTGTAATTCCTACATGGGTACCC
TATTATTTTTGGAATGAAAAATTTATATATTCCTGGTACAGCAATTTAGCAAGATATGTTT
TTTCCTTAAACGTAACATGGATGGTAAACTCTGCTGCTCATATATGGGGCATGAAACCAT
ATGACATGAACATTTGTCCAGTAGAAAACAAAATTGTATCTATATTTGCATACGGCGAG
GGATGGCATAATTATCATCATGTATTTCCGTGGGACTATAAAACTAGTGAATTTGGTACC
TATAATACCAATTTTACTACCGCATTCATTGATTTCTGTGCTCGATTAGGATTGGCATACG
ATTTGAAGACTGTTTCCAATGAAATAATTAGAAAACGAGCAGCTAGAACTGGAGACGG
ATCGAG

>novel_circ_000583
ATTATTTACAATAGCGCCAAACGCGATATATCGATGATATTGAATAAGAAATTATTTCAAC
AAAATGTTGATCAAGAAACATCGAAGACGGAAAAAGAAAATAAGCAAAAAAAAATTAA
TCCAGGAATTATCGAATTCCGAGCTGAAATATAAATGGAATATCGTTTGGAAAAACGTG
ATTGTATTTATCTACCTTCATCTTGGAGGTATATACGGATTTTATCTTTGGATTAACGGAA
CGAAGGCATATACAATTTTGTGGTCAATTTTCGTAGAATTTTTTGCCATTATGGGCATTA
CAGCTGGAGCTCACAGATTATGGTCACATAGAAGTTATAAAGCGAAATGGCCCTTACGC
TTCGTGTTGATGCTTCTTCAAACAACCTCTTACCAGAATCACATTTATGAATGGGTAAG
AGATCATAGAGCTCATCACAAATTCACTGATACTGATGCGGATCCTCATAATTCCCGACG
AGGATTTTTTTCTCTCACATAGGCTGGTTAATGGTCCGAAAGCATCCGGATATATTTAC
AAAAGGTGCCACTATTGACTTAAGTGATTTGGAAAAAGATCCCATTGTCGTTTGGCAA
AGAAGATTGTATATCGTGCTGATGCCTCTTTTGTGCTTTGTAATTCCTACATGGGTACCC
TATTATTTTTGGAATGAAAAATTTATATATTCCTGGTACAGCAATTTAGCAAGATATGTTT
TTTCCTTAAACGTAACATGGATGGTAAACTCTGCTGCTCATATATGGGGCATGAAACCAT
ATGACAT

>novel_circ_000584
GTCAAATTTGACTTTTAATTGAATTATCGATAGCGCATGCGCTCTGAATGGCGGCAAAG
GCATCAAAACGTACTTCTGGGGGCGCTGGATATTGTGTAGCGTCGTCTTCAACGAAAA
CGCAGCTACAACGTGGAAGTGGTCTTCTTTGTTTCACCTGCAGTTTATGCTTTATTTTAA
GTTTAATCTGCACATGCTTAGCTACGTTATTGGTTCTTTTCGATCAAAAAGTTGAAGCCG
CCAGCTATCAATATT

>novel_circ_000585
ATTGTCCAGCAGCACGACGGCTATCGGTACTGCAGCCACCGGTGGCGGTGGCGGAGC
GAGTAGCGGTGCGAGTGGCGGAGGTGGCGGTGGCAGTGGGGGCGGAGGTGGCGGGG
CAGCCGGCAACGGGACTTCCGGCGGTGATTTCCTACGCAGAAGTCACCCCCTCTCGGA
GCATACGGCCCTGCATCCCGCCTACCGGCTCAACTACATGGACCACCTTTACCACCAGC

TTCAGGCCTCCACGCACAGTCCCAACGCCTCGTTACACGGACTGGGTGGTTTGGGGCC
CGAGTACCTTTTGCACGCCGCAGGACCAGCGAGCACCCTCGCTTCTTCAGAATTCCCC
TTTTCCATTGACG

>novel_circ_000586
CTGAATGAGGCTCATGGAACCCTTGGGTGTTGCCCCGGATGTGGTGGAGACGGGCGCA
TTGAAAAAATGGTCCGACTTTCCCGTCCAACACCGATTCACAGACTACTCCAATTCTAT
TGGAGAACCAACTCATCATACTATAAGCCCTTTTCCATGTCAACCAACGCTTAACAACA
AACTTGCGTTATGGACTGGCGATATTTCTATTTTACAAGTGGATGCAGTAGTAAATTCCA
CAAATGAAACAATGGATGATAATAGTCCTATGTGCCAAAGAATATTTGTTCGGGCTGGA
TCAGCACTTAAAATGGAAATATTTAATGAAATCAAAGAATGCAAAACTGGTGAAGTAA
GAGTAACACAAGCTCATGGTTTACCAGCACGCTTTATTATTCATACTGTAGGACCAGTAT
ACAATGTAAAGTACCAAACAGCAGCTCAAAATACATTACATTGCTGCTATAGAAATGTT
TTACAAAAAGCACGAGAGCTTGGTTTACGCACGATTGCGCTGCCAGTAATTAATTCTGT
ACGAAGAAATTATCCTCCCGATGCGGGAGCACATATTGCACTGAGAACAATGAGAAGA
TTTCTGGAGCAATATGGCGATTCTGTTACATGTATTGTACTTGTATTGGAACCGTGTGAT
CTTGGAATTTATGAAGTTCTTCTTCCACTCTATTTCCCAAGAAATTTAGCAGAACAAGAT
AACGCGTGTTGGCAACTACCGAACGATATAGGTGGAACGGATGGAGAACCTTTACTTC
CTGATCGACAAATTAGAATTATCGATAATCCTCAACATGCTTTACATGGTGATGAAAGTG
TAGAACTATCTGCGCAATTAGAAACATCTGTGAATATTGGTGAACATGCGTTTGCACAA
ATGCAGGGTGATTTAGATCGACAAAGACTTCTTGGAGAAAGGCCACCTGCTGATCCAC
TTGCAGATATCATGTTGAAACAAATGCAACAGAAAGAAAG

>novel_circ_000587
CTACCTCAGAAGAACTACTTTGATGTCACGATAAGAGGAGAAAAACATAAGAAAGAC
AATGAAGATCCTTGGCGTGGAATTCGCACCGTTGAACGTACCATTGAAACGAAGATTA
GAAACGCTTTCAGCTGCGTTATGGATCATTCTTCTAAGCGGAGGAGATTTAATGGGTTA
CCTTCTTTTTGCTTATCTTCTATTGTACTCGAAAATCACACGTTATTTGTGCTGCTGTAC
ACACTCTGGATGTACTATGATTGGGATACATGTAATAAAGGCGGCAGAAGCGAACGATG
GACAAGATTGATGAGAGGCAACGCGTGGCAACGTTACTTTTGCAACTATTTCCCTATAA
AATTAGTGAAAACTACCGACCTGGATCCGACTAAGTCATATTTATTCTGCAGCTTTCCAC
ATGGAATTCTGTCCACAGGAGTATTCGGCGCATTCGGAACGGAGCATCTCAACTGTAA
AAAAGTGTTTCCGGGTTTGGATTTCCGGGTCGTGATACTAGAACAACACTTTAAAATCC
CGTTCTTCCGTGAATATGCCTATATGGGAGCCGGTATCAGCAGTTCCCAAAGAAGTTTA
ATCCATCAGCTATCGACGAAGCCCGAGGCACCGTTCACCGGAAGAGCAACCATTCTTG
TTATCGGTGGAGCATGGGAATCATTGGAATGTAAGCCGGGCAAATATCGTATCCTGCTA
AAAAGGAGAAAAGGCTTCGTTAAGATTGCGCTGAAGCATGG

>novel_circ_000588
GCTTGACAAGAGCGCCGATGTATCCCTTCTCCACGGGTCAATATCCGTACCCCATGCTT
AGCCCGGAAATGACGCAAGTCGCCGCTTCCTGGCACACACCGAGCATGTACCCCATTT
CCCCGGCAAGCACTGGTTTCCGAAGTCCGTATCCCACGAGTTTGCCCATCACAAGTTCT
AGTTTACCAAGTGATCTCTACCGGTTTTCGCCGACGGGCCTGATGCCTCCGCATCCTGG
ATTAAGTCCGCACGCACACGCGTTGGCGTCCCACGCATTGGTGTCCTCGGCGCCGAAG

ACGGATCATTCCACCCTAGATCACAATCACAG

>novel_circ_000589
TAGCCGACTGAGTTCGCGAACTTCGATCCCTCTTCTGCAAAATCTTCCAAGCAGTGTAA
GGACAGCAAAAGCAGAACGCCGCTTGAAAGAAAGACTAATACAGGAGGGTATAATTT
CGGATGAAGATGAAGAAAGTCATTTAAGTCCTGCCGAACAGAGAGCGTTAAGAGCAG
AAAAACGTGCGGCGTGGAGACAAGCACGTTTGAAATCTCTGGAACAAGATGCTCTTC
AAGCACAGATAGTAATTAAAAAAATGAGTGAAATGATGGACACGAATAAAACGAGTTC
AATGCAGGATTCTACAGATATTCCTATTATTCCTGTTTCTGAATCAGAAAAATCAACTGA
TTTCACTACGTTACGGCCGTCTAGTGCAGATTTTCCCAAACTTGCTGTACGTAGCAAAG
TGGGTCCCCCTAAAGAGATACGCGAATCCGAGAAAGTAGTGGACGAGAAGGTCACTC
GACGTACTGAGGAATATGTGGATGAGGTGACGGGTGAACGACGTGTGCGAACAGTCG
AATACGTCGAGAAACTCATTGAACGACAG

>novel_circ_000590
CTTTCTCCAACAACATCACCAACACCACCTCTGGTAAAGATGTCAGTTAGTGATAAAA
AAAAATTGTTTGAAAGTGCCATGGAGGAACATTTAAAACCTTCCCCTAAACCAGAAAA
AGTATTCAGTTTTCTAAGTCAGGATGAAGTTGAAAAAATGAAACAGGAAGAAGAAAA
GAAGATTGCTACTCTAACGCGAGATGAATTAAAATCGTGGGCACAACTTGATGAAAAT
GAAGGTTTAGAAGATTTAGAAGAAACATTAGAAGATCAGGATAATCGGCGACCTAATG
TGCATGCTCGTTACCTTGCAGTAGCCGACTGAGTTCGCGAACTTCGATCCCTCTTCTGC
AAAATCTTCCAAGCAGTGTAAGGACAGCAAAAGCAGAACGCCGCTTGAAAGAAAGAC
TAATACAGGAGGGTATAATTTCGGATGAAGATGAAGAAAGTCATTTAAGTCCTGCCGAA
CAGAGAGCGTTAAGAGCAGAAAAACGTGCGGCGTGGAGACAAGCACGTTTGAAATCT
CTGGAACAAGATGCTCTTCAAGCACAGATAGTAATTAAAAAAATGAGTGAAATGATGG
ACACGAATAAAACGAGTTCAATGCAGGATTCTACAGATATTCCTATTATTCCTGTTTCTG
AATCAGAAAAATCAACTGATTTCACTACGTTACGGCCGTCTAGTGCAGATTTTCCCAAA
CTTGCTGTACGTAGCAAAGTGGGTCCCCCTAAAGAGATACGCGAATCCGAGAAAGTAG
TGGACGAGAAGGTCACTCGACGTACTGAGGAATATGTGGATGAGGTGACGGGTGAAC
GACGTGTGCGAACAGTCGAATACGTCGAGAAACTCATTGAACGACAG

>novel_circ_000591
CTTTCTCCAACAACATCACCAACACCACCTCTGGTAAAGATGTCAGTTAGTGATAAAA
AAAAATTGTTTGAAAGTGCCATGGAGGAACATTTAAAACCTTCCCCTAAACCAGAAAA
AGTATTCAGTTTTCTAAGTCAGGATGAAGTTGAAAAAATGAAACAGGAAGAAGAAAA
GAAGATTGCTACTCTAACGCGAGATGAATTAAAATCGTGGGCACAACTTGATGAAAAT
GAAGGTTTAGAAGATTTAGAAGAAACATTAGAAGATCAGGATAATCGGCGACCTAATG
TGCATGCTCGTTACCTTGCAGTAGCCGACTGAGTTCGCGAACTTCGATCCCTCTTCTGC
AAAATCTTCCAAGCAGTGTAAGGACAGCAAAAGCAGAACGCCGCTTGAAAGAAAGAC
TAATACAGGAGGGTATAATTTCGGATGAAGATGAAGAAAGTCATTTAAGTCCTGCCGAA
CAGAGAGCGTTAAGAGCAGAAAAACGTGCGGCGTGGAGACAAGCACGTTTGAAATCT
CTGGAACAAGATGCTCTTCAAGCACAGATAGTAATTAAAAAAATGAGTGAAATGATGG
ACACGAATAAAACGAGTTCAATGCAGGATTCTACAGATATTCCTATTATTCCTGTTTCTG
AATCAGAAAAATCAACTGAT

>novel_circ_000592
GTGGCCTCACGAGGGAGGGAACACGGCCCTGCCTGAAATAGGCTCCAGCTGTTGAAG
ACAAGACAGTTGTGATTATTTTATCAACAAATTGCCACATTTAAACGGCACCTCATCTCT
GCCCTACCAGAGAACAAATAGTTTTCAACGTTTGCTCCTAATGTTTACGCGTATTTTCG
AGGGAGAACGAGAAGGGCGCTCTTTTTCTTTTTCTGATGATGTACAAATTGACGAAGA
GGGATGAAAGAGGATTTGATCGAAATTTTAAAAATGGTTTTTCTTTAAATCGTTAACCG
TTAAATTTATTTCATTCGACACGTTATTGAATGTAATAAGTTGGATCGATATCTTATCACG
AATTTTACAATATTTTATTTGTAAAGAGTCACTTTACTTCAAGGAAGTAAAACCAGATTT
TCATGGAATATTTTTTTTTTTTTTTAGAGAACGTTAAGGTTACATACACGTACATTCTCG
TGATGTATCTAATAAAAGTCCAAATAGAGGAAAAAGAACGTCGAGCAGGGACAAATAA
GTGTTTGCGCTTGACCCATATTCCTTTAAATTTGTAAAGTGAAACGTTTGAGAAAGACA
AAAAACTAATCAATAATTTAAAAACCTAATATCCATTAGAAGGAAATAAATTCTCAACC
AATATTGTGAATTTCAAGATACTTTTTTTTCATTTGTACGAATAAAATGAATATGAAGAA
CACTTCATAATTCCTCGATCCTTTTTTTTTCAAATCAAAAATCACACTCGAATAATAACA
AACGCTCGAATAATTCGTATGATCGATCGAGATTTGAACGAAAAATCTTAAAATCAGTG
AAAAATGTCAAAAAATATAACACGATATTAAAAATTAAGAAAAAGAAATTACCCAAATT
AAATGACAACAACAAAATGCATCGATGCATTTTCTCCAAGAATCTCGAAGAATCCTTTC
GTGGATCTAAGAACGCGCTAAAAGGCTAACCTTGACCCGAAAAAGTGTCGAGGAGCG
GAACCGGAAGCGCGGACGTTGCGCGTGGTTGTCGATCGTCGTAGAAACGTCGATTCGC
ACCGGCAACGTTTCACGTCGTGTCACGTCGTGGCTAGGTGGATGTACCCTCGAACAAC
TTGTACTCTCCGACAGATGGAGGGAACACAGAGGTGGTGGAGGAACAGGCAGAGCCA
GGAGGTGGCCGAGAGGAAGAAGAAGGGAAAGAGGTAAACGCGTATCGTAATGCACAA
TGGTGCATTAGCCAGGAGTAGACGGTCAGACGGTCTCTCCCGCGGACCGATTCCGAAA
TTCGGTCTATCGAGGAGCGGAGAGAAACATACATTTTCCGGAAGAAAAATTTCGAAAA
CCCGGTGTGGGAAAAATCGGTCCAGAGGGAGGCGGGCCAACGAAGGAGGGATTCATG
TCACACGCGCGTATGATTCGATCAATTTCCAAAGGATCCGTCCTTCGGTCTTTCGAGAA
CAATTGAACACCCGGTATCGAAGCCAGGAGGAGTCAGGTATCCGCATGATTCTCATAA
ACGCTTGCAAGTTTTTCTCACTTGACATCGAAGGACAACGATTGAAGGAGTTAAGTTG
ATCCGGAGGAAGCTCGAGTATATTGTACATGATACAGAACTGGTGCGAGAGGAAAAGA
AAGAAAAAGAAGAGGAAAAAAAATAGAGGGAACGATTTTTATTTCATCGATTGAAAC
GCCAACTTCTTCTCGAATAAAATCGGCGTTTTGTCGAAGAAGAGATGGCCGACAATACT
ACCAATTCGTCGACGACGGAAAAGAGACGGGGAGAGACGACCGGTAACCAGGTGATA
TCCGAGAAGGCGGTCGTCTGGAAATCCATGACGTTGCGCCAGAAATGGTCCTACTTCA
CAAGAAACATCACCGTGGAGCCTATGATAGCCTGCTACGTGATCCCTTGCATGCTCGCC
TCCCTCGCTACGCAGAATCTCAGCTTGGAAAAAGCGTGCAGGGTGAACTTGGCCTATC
CCGACGACGTTTGCACGGCGTTGGCAAGGAGGAACACGACCGGTTACGAGAAGGAG
GAAATCGCCGTTCAACAATTGGTCGCCGGTATGCAGACGTGGAAAACAGCCCTGTCCA
GCGGATTGCCGACCATCTTGATCCTGTTCATGGGCTCGTGGAGCGATCGCACGGGATTG
AGGAAACCGTGCATGCTGTTGCCCATCGTTGGCGAGTTTCTCAGCAGCGTCAGCATGT
TGCTGTGCACTTACTTCTTCCACGAGGTGCCCATGGAAGCGACGGGCGTGCTCGAGGC
ACTTTGGCCAGCGTTGACAGGGGGTTGGTTCACAATGTTCATGGGTGTTTTCAGTTACA
TCGCGGATATCACGTCCGTCGAGTCGAGGACGCTTAGGATCGGCGCCGCTAACGTTTTC
CTGTCCCTCGGTGTCCCGATCGGGATGGCATTGTCCGGGATATTGTATCTGAAGCTTGG

CTTCTACGGTGTATTCAGCATCTCGACCGTGTGCTACGTGTTGAGTTTCATTTACGGTTT
AGTGGTGATCAAGGAACCGCCTAAGCCGCATCTGAGGAAAATGGAGAAGGCGGATAA
CGAGAGGATGTCTGTGTGCGCCTCGATCTTGGACTTTTTCGCGTTCAAACATATCGAGG
AGACCTTCCGCGTCGCGTTCAAACAGGGGAGGAATAACAGGCAGAAGAGGGTGCTGG
TGCTCATGGTTATCGTTATGGTTGTGATCGGTCCGCTTTACG

>novel_circ_000593
AACGAAAACAGAGCCCTGCGACTGAAGGAGCCTGCATCGTTGGCTCTCTCCGCCGGA
AACTCCGGCAACTACGTGGACCCAGATGGCACCGCCATTGTGATGTCATCCGAGCTGC
TACCTGCACCCACCCCGGATCCAAGGGTGACGTGGAACTACTTCGATGAGCAAGGATA
CGTTTCGAGAGGAGGATTACGAGCAGGGGAGGATCCATATGCTAGGAACAAATTTAAT
CAGGAAGCATCCGACGGGCTTCCCAGCAATCGTGATATCCCTGATACGCGCAGTGCGA
T

>novel_circ_000594
CTCTAGAAACGTGGTCCGACCAAGCGGAATCCGCCAGGAGCGTTATTGTGCAACGGTT
AATCGACTTGCTGACGCGAGGCTCGCGAGTTGCTCGTCCACAGTGAAAGGATCGGTCT
AACGCATCGGTCGATTCGGCCGAAAGCTCGGCCACAATGTAACCGAGGATCTCCGGTG
AAAGGTAATAACACGAGTTCGTTGCCCATTTTTTTCCCTTTTCTTTTCTTTCCTTTTTTTT
TTTTTCCCCCGCTATGCCTTCGCTCGGATCGATCCCACATGGTTAGACGATAATTTGGAA
CCGCGCACGGAGAAAGTAACCTCGCATTTCTCTCGTTCGTTTCACGGAAAATCATCCTT
CCAACATCCAGTAGAGTTAGTGACGTTTCGATAAGGAAGCGAGTAAATCGATTTCGTG
AATGAGTTCGTGGATTGAAGACGGTTTCACATCCACGTCTTTAACATTCTATTGAATTTT
TGATTATAGAATTTATGATAAGGATAATTAAATGACTTTTAGAGCGATGATTGAATGTCTG
GCTAATAATTCGATAGATATGATAATCCAGCATGCTTCATTATCAGGATTATGGATACATT
TATATTTATGTATTTCACTCTTCATTGTATTAAGTCATTTTTTTTGTTCATATTATTAGAGAT
ATAATTCAATAATTCTTATTTTTTGTTGCTTAGAAAATAGAAGAATTAAAGTTCAGAGTG
GAGAAGTCTCGTTTTTAACATAAATTAATTATTATTGTTGTTATCGAGAAGCAGCTCTCT
TCGTGATAAATTAAATAAACGAAGTAAAACGCAAAAAATGTACTCGATCGATGGAAATT
GGAAAGTGTTTACCTTTTCCTTTGATCGATGTTCAATGGCGGCCAGCCTGACCTACATG
ATCTTGCTTAAATTTGCGCATCCGATAAAAATCGAGAGAAAGAATCGAAGTTACGAGGT
CGGGTGCAAGCGGAACAGGTATTGGCCGCCTGGTCGGTGAAAGTTTCTAGAGAAAAA
GTGATTGCAATTTATCCGTAGAATGCAAGGCGAGAATGTCGGGCGTGCGTTCGTTGCTT
CGTTTCCTCTTGCAATCGAATTGCTCACATTTCCCTCGGATCGGGAAGAAAGTGAAACT
TGCGGATAGCGCTGTTCGCTCGTTCATCAGAAACAGAGAAGAAGATTCTTCTGAATCA
CTGTCCTCTCGATGATACGTTTCAAGAAAAATAATAGTCTCCTAGTTAATCGAGTTTTTA
TTATGATACTCAATCGATAAATAAAACTGAATCGAGTATCAAGTTTGAATATCTCGTTAC
GTGTATAGTGACTAATCGGTTTCAGATTTCCAATGAGTGTCAAGACCAAACAATGTTGG
CGTGTCTGAACTTTGTCTGAACTTTGCAACGTTGCAATTTATCCTGTAATGGATCCTAAT
TGTTTAGTTATTTAGATCGATCCGGATTCCAAACTATAATGGATATAAAATTAGGCTAAG
CGTACGTGAAAATATCCAGTAAATTAATTCGGCATTTTAATAAAGACTAAGCAATTAATT
AGTCACGGTAATATTAAATTAATTAAGTCGAAATATCCTGTAAATTTATCTACGTTGGAA
AAGAAAAATTTCTACAAAATATATTCATAATTTATAAAAAAAAAAAGAAAAGGAAGAA
ACCCAAAGAATCATCGAATACAAAGGAAACATTTCAAAAATCCTTTCATTCCAATTTTC

ATGAATAAAACGACAACAAGTTCAATTAATTTCTAAAATCCTGAAAAAGAAATAACAA
CTTTCAATTCTTCTTTAGAATCAACAACGGATAAAGTCACGCAAGAGTATTATTTTCCA
AAATTCTTCTCGAATTTTCAAAAAAAAAAATATATATATACCGTCTTGAAGATTTAACTT
CATGAGATGGCGCGATGGCAAGAATAACGAGGAGAACGAAGAATCGTTCCCGTTCTTC
TGCGAAAGTTAAATGGACCGCGCGGTTAATCCGCCACAATGGCCGCGATTGTAGTGAA
ATATGTGTGCGTTTCCCGGAAATACAGATGGCGCGCTCGCTCGAAAATTGAAACGCGG
CGAATCGCACGAATTCGGATCGATCGGATTTATTAAAACGATCCACATCGGCTGGATCTA
TCCCCTCCCCCTCCCCATTCCCGTTTGCAACCCGTACGTGTTATTTTATTAACGCGCGTG
CTGTCACGTGTTGTACTGCATAATCGCAGTTTCGCCCGTGGCCAGCGAATTTAATGACA
CGATCGAACAGTATCGTCGGATGGGAACAACGGTCATTGAGAGATTTCAAGCGCGAAT
GGTTATCAATAGCGCTTCGTGAGATACGATTCCTTTTTTTATTCGAATACATTCGAAATGA
TTAACAATTCATTCCGATATCGAACTTGGAAATAAAGGAGGAGGGGTCGATCGAGGTC
GAAAAGTCGAGGGAAAACTGATGGAGATTCCCTCTGTAATCAAATGGAACTTGCACCT
TTCCTCGTTAAACTATCCACGTAATCAAGGGAATGCTTTTTCTCCACGATGCATCACGAT
ACTTGTTGCGGCAAAAGGCAACAATTACGCTCTTTATGTTAATTTTACGAGCACTTGGA
TCGTGTATTGCGATCTGCAACACTCGATGACGATGGATTTGAAACAAGGCCACGAGTA
ATGATGATTATTAAACAATTGGGAATCGAGAAAATGTTCCTGCGGAAGATTTCTCGATAT
TTTTCTCCTCGTGAAGAAGGAACTGTTTCGAGAAAATTTATATACGTAAGCTCGATTTAT
ATGTGCAGTGATTTTTATAGCGCAATATAATTGAATTAAGAGAAAGTTTTTAACATTATA
AAGATTAGATAAGAGATTTTAATTGAATTTTAAAAAGATTATAAAATCGAAGTTTATAGG
TTGAACATTCAAACAGAGATTTATTAATATTTTAGAGAAGAAAAAATTACAATTACAATT
TTATAACAAAATCAATTAGTAATACCTGTTGTTTAATTTTATGACTCGTAAATAACTGCAT
GGTTTTCGTCGGAAATCAATTAATAGAATTATGCAGTTTCTTTAACTAATTATCACAGAA
TCGACAGATTTTTAAAAATGGCCAGCCATCGTAAACAATTTATCGGTCCATTAAACGGC
TATTAATAATGCATGGCGCAACGCGGATCGGGTTAATTGGCCGGATAATCGTAATTAAAG
GGAGTTCGATTCTTCCTTTTCCCGCGCCGTATAAAAACTCATCCCAATGGGAATATCGGT
TGAAACTAACTGACTCGAACGTTCGCCGATATCTTCATTGATTTTACACGATAAATTAAT
CTAGAACGAAAATATGTTTCGAGTAAAAATGATTATTGATTATTATATTTTTTTTCTTAGA
ATTACAAAATTTACTTTATCTTTTTAAAGATAAATAATACACTTTTCACATGCAATTAACT
TTCTATTGCAGAGTGATATAGATTAAAAATCACACGAAGAAACAGATATATAATGTATCT
ATATCTCACAAGCATTCCAAGATCTTTTCCTTTGGCGGCAATCTAAAATAAATAAATGCT
AGAAGAACGAACCTACAATTGATTTATCCAAACGTCAATTCAACTTGAACTGTATTTATT
ACAAAAAATTATAAATCACGATTCAAGACAACAACAATAATAATTATTCTTAATTGAAA
AATCTTCCACAAGCGTGAAAGAAAATACGATTCATCCACTTCTCAACGAAACAATATGC
AAATCGCGTCTTCGCTTCTTCAGTTCTGAGACATCTTTCTCGCGTTGTTTCGCGGGAAG
AATTATTGCATCTCTCGGTTAAAACGTGTCCAAACAAACGTGTTAAAACAATCCAAGGA
TAAGGGATCTTCTTACGAACTGTATCGTTGGGAAAGAGAGAATTTATCGTGGGTTTCGA
TGACGATAACTTTTTTCTTTTCACAAAAAGAGTTGATTAATCCACGATTTTGAATTTCCT
TCCGTTTGTTATAGAATCTATCCTTCGTTTCCAGGGGAAACTGTGGAAGAACTTGCAAC
GAGAGAGGGTAAAGTTAACGTATAGAGGGAATGATAAGGATCAGAGATGAAACGATAG
GCTATTTTTAACAGTGGTTACTTTTTGTTGTGACCCTTGGTGGAGCATGATCTGCAATTA
TAATTGTCATGCGTGGATAAGCAACGTTGCTTGAAAATATCACACGGCCCCGTTCTATA
AAATATAATAGCTTACGGTAATTTTAATATATTATTATTCAGAATTAGGTTATCGTTATTTC
TTCATCGAAATTTTTGCTCTATAGGGTGTCTTAGTTAGAAAGAATCGAATTAATTCCGGA

TGTTTCATTTGCTTGTTTTTTGTTTGTCATCAAAATCAAGTAAGATGTTCATCTTGTAAT
TCAAATATTTTTTTGTAGTCAATCAGCATTCATTATCATCGGCCAAAATATATACCAATTAT
ATCTCTTACTTCAAAATCTATATTCAACTTTGACAAAATTTCATAATAAATTAAAAAAGG
AAGAAAGGAAAAGAAAGAGTTAAATCCATGAAGAATTGACTGTCTAATTCTATCATTCA
TATCACGATTCGTTCATATCAAAAAATAAATCAGAGGAGACAAACAAAATTCACTTTAA
CTTCGATTTCAACTTTTGTCCGCGAAACGGATTCACGGATAAGTAGGAACATCGGCTAA
CACATTTACGCGGAGAAACAAGGCGAAACAAAATCCTCGTTGGCATTAAAGTTCTAAA
GTACAAAGTACCGGGATTACACCGGTGGAAACGAATCGCCACGAGTTGCACGCTCCAA
ACTCCATCACGCATAATTATCGTGGACCTCTTGTCGATTCGAAAGAATCTTATCGATCGA
AGAAGAGATTTTCCATTTTAAGAATCGGCCTATGATTGAAATAGCGATCAACGATCATG
AAACACGAGCATTACGTTGTACAATACTGGCAATCGAGGGTGTAACTAGTTTGTTGTGA
GGCTAGATTGATAATTGTTCGAAATTTGGATTTTACACGGGGAAAATTTTATTAGCAGGC
GGAAATACGTGATAGAGCGTCAATTATCCGAATTGGATTGTATTTTCGGGAAATCGAATT
GTTCGGATAATGAAGCATGCACGTGTTTACGTGAATTTATAATTGTAAGAATTATAGTTA
AAATTATCAAAATGCGAGAGAAATGTTGTTATTACAATTCGAGATGTAATTGCTAGAAG
CGATTTATTAACTACATCGAGGTTAATTATTTGATATTTATTAAATGACGTTTACGTTTGTA
CGTTTTAACGTTAAAGTAAGTAAAACTTTTTTAAAATGTTGAGCCGACGAGTTTGACTT
TTTTCTGCGTATCCAATTACTTTGTTTTTGCGGAGTTAAATGCGAACAAATAAAAAAATT
GTGTTAATAAAAAAAATACGGTTAATCACCCTGGCTTTTATATATTTTGTAACAACGAAA
AACTTTTTATATAGAGAAAATTTAACGTTTCCTGAAAAACGGGTTGCCAGCTTCCTGCG
TTACACGATATTTCTATTATTTGCCGGTAACAAACGTTGCCTGTTTCCGGTAGATACAGG
AGAAAAACAAGGAACGCCAAATACAAAGAGACAAATATCCCCTTGACACGTTATTTAA
TAATTATTAAACCAATCTAGAATTGAACAATGTCCAAAACTTTAAAAATATTCGATCGAT
CCAAATTATTCCCTCTCTCTCTTTCTCTGTCCAATCTTATCTCTAGGGTGCAAAAAGAAG
GTCATGGAATAATCAAGGATAATCTCGATAATCCCCATCTCTCCGTTCTTTTCTAATTTCT
AATTACCCCCGCGTTTCGTTATCGCGATTTCCATACAGATGATCGAAAGAATCGATCATC
ACACATTTTTCCCGACCGGACAAAACCGCGGAAACGTGCTCGATCGCTCGTGAAAATT
TCCATTTCTGCTCCAACGATCGCCGATTTTTTTTCCCAACCGATCGAGAAAAAAAAAA
GGGAGGGAGAAACCACCGATGCAACGTGGACAAATTGCGTTCTCGAACGAATCGCAA
TCGTTAAAAATGTTGAAAAATTGTCTGGTCGTTCAAATACGAATTCTAACGTTTGTTAAT
CCACGAAAGGACTTTAGAAGCGCTATTTTTAATCGCTTAATCGATTAAAGAGACGCATC
CCCGTTGACGAAACTCGACTGCGCATTACAGTTTGAACTGTAAACCTACGCAGAGAAG
GTATTTTTTACACAGCCATTAACGTCCTATAATTCATATACAATTTTCCACCATGTTATGC
GATCGAGCACGAAAACGCGTGCCCGTTCGAAATTACGCCTCTCGATTTGCGCGACGAG
GGTTGAAAGTGCAACTGTACGAAGGTGACCGGGCGAAGGTGATGATTATTAATCCTGG
GCCAGGAATAATCGGAAGGTTAAACGACGCATTGTTCCATAAAATATTTCCACGCGACG
TGGCGCACGCGAGACCACGATCACCGATAACTAACGGCTGCGTGAGCTTTCCAAGGGA
TCCCGAGGCCTCGAACAACGTCGCTGCACTGCGGATATCCCAGCCAACGGCCTCCCCC
AATGCCGGCCAAGGCATTTCTCGGTTTCAGGAAACGCGTCCATTCGATTCTAACTCGAT
GTTGAAAATTATTGCAAATGGATGTTTATTCTACAACGCGTGATATGCATTGCGTATTTCT
ATTTTTCATTGTTTCCGGAATGAAAGTTTTTTACGGTTGATTTGATTTCCAACTTCGAAA
AATCGAGTTATAAATTATAGTAAGATTGATGAACGAATTTGTACGACTAATTAATATTATA
GAGATGGAGTAAATTGTTTTCTTTTTACAAGAGACTACATTTGCACGATGAATATTTGCA
AAAGGAATCAGACGAAAAGGAAGAAAAAGTTTCTTTGGATCAATAATAGTTCTTATAA

ATGGATTCTTTATCTTTGGAATATTCAGAGAAGAATAAGAAAACAACTCCTTTTCCCTTC
CTCGTTGAACATCGATCTGTTGAAAAGTTGACGCAAAATCAAGAATCGAACGAATAAT
CTTCGAACCGTGCTCCATCCATCACATTCAACTTTCGAACCTTCTGTATATATACCATCTT
TCCAAAGCGAACGATAGAAAGTCAAGATGGAAATTTCCATTATCCAAACGTCCAAGCA
TCTCTCGAATCTTTTCTTTTCTTTTTTTTTATTTAAACAAATCTTGCGAAAAGATCAGAG
GGAAAGTTTTCACTTATCCCTTGATCGTCCTCGAGAATCCAGAGCGCATAAAGAATTGT
GGGAATCGGTTATACTGGCGTAAATGATTTTACACGGAGGCACGATTAAGTCGAACGCG
AAGGAGGGAGCTCTGTTCAGCTCTGATTCCTGGCGTGATTCGTGGCCCGGAAAAGCGG
TTTCCTTCGATTCATCCATTTCGAAAGGAGAGGGGCAACGCGAAATAATAGATCCAAAT
CAGGCCGTTTAAATTCTAAACCCGAGGAGGAGCCGAGGTTGGATTGCATTAGATTCAC
AAATTAAGAGGCCACCCGGTGTGCCAGGCCGGCTAACAAAATTCTCGCGGAACGTTTA
ATGAATTCTTACGCGAGCGGGCGTAGGCGGCAGTGGATTAAAATTAAACTACGTGTCCC
GATTGTTATAATTTTCATCGGTGCTTGATACTTCTTCGATGGCGAATAAAATTTTATTCTT
AGAGCCAGAAGAAAAAGAAAGGAATTATCGAGAAAACGATATTTGGATCAGTAGCAA
GGGATGTTTATTCAATTGGAAATAGATGAAATTGGATTATTTGTGCACTGTACGATTTTT
CTGAATCGTGGACGTGGCTTATTCGAAGGCTAAGACTCTGCTCTAAATTGATTAAGCTT
TCTCCAATTATCGCGATTCGTAACGCCTCGTAACAAAGAACATACCGAGCATACAGACA
GAGATAATTATGCTTGAAACGATAATTGATCGCAATTGTAGGAAGACAAGTGTCTATCC
ACTCATCTCGGGATCAGAAACGCGTTGAAAGCTGGAAATTTTAAACTTTGGAGTTCGC
CCGAACGACTCGAGTTCGAACTCGTCCGTTTCTTTAATTAAATCCGTTTAATGATTAAA
CGTTTGTCTTGGCAAGAGGAAGATCCGAATTTAAATTTGAATTTAAGTAAGAATAAAGA
TGGAATAAAGAAAAAAAAGAATTTAATTACCTTCTCGAAGATGTTGGAAAATATTCAAT
GGAATTATTTCAGGGTGGATAACGCGTGCCACGGGAGCGGATAACGATCACCGTGGAA
ATAATCACATTCGCCGCCAGTTAAATCTTCTTTGTGAACAATCCTATTTACGGGTCATTA
GCCACAGGCGAGGGGAAATAAGGTGGATGAAATTGACGCAGTCTTGGCGGAATGGAC
GCCGTCGATCTCGGCGTTTAATCAGTCTCCTCCAAGCTATGGATGCTGCGATATGAAAT
GATCTGCCCACACAGATCCTGCTCAATCGATTGCAAAAGGATTACGCAAAATTTCCAGG
CCTTCGTTCGTTATATAACTTTATTATTCTCGAAAAACTTTGCAAGAGACGGATATAAAT
CTGTACAAAGATCTATTAATTGCAAAATTTACTGACTATTTTTTTTTAAATATTGGAAGA
GTAGAGAACGAAGATTGGTCAATCATCATTTATCTGTCATTCTCGTGTTTGTTAAAAAA
AAAAAATCGTGATATTCGAAGAGAATTGTTTCATGACAATTATGGGGACATCAATAACC
AGAATTTCGACCAGAGATATCTACCAGATAACCTACGCCTGGTTTGCCCACTCGATCCC
GTTAATGCTTCACGAAGTGTTGAAGCAGCTGAATCAACTCCTCCATGCTCGAATCAATT
AAATCAGCCGAATTAATTCGATCTCTTAGTCCTTAATCGAGATTGCTCACGCGATTGACT
GATCCTCGAATATCTATCCCTGAAAAAATTGTCGCGGATATTACGCAGCTACACTTTTTT
TTGTTCCAACTGTTCATTAATCCATTCGAAGTTCATTGATATTCATCGATTTCTCTCTCTC
TCTCTTTAAAAAGAAAAAAAAAAAGAAAAAAAAAGAACCAATCTCCACGAGGGAATTAT
CGTGGATTATTAAATTCGTCTTCGTCAAATTTCATCGAGGGCTTAATACGATCCGATTTT
CGTGAACAAACGTCAATGTGAATTTTATTTTAACAGGTGTTCAGAAAACGATTCGTCAC
GCGAATTAAATTGCACCGATTAATGCCTGAATATCGAAGACGATGGGGAATAAAAATGT
TTAAGACTCGTGATCGAGAGAATTAGTTAATTGTTCCCATTACTTTGACTTTGATTATCAT
AGTTTTATTTCTCGTCTCTTGACAGAACTCTCGAAAGAAAATGTAGGGCGAACGATCC
GCTCGTTGGACTGGATTAATTTTTTTATTATTGAAATATCCTCTCTTTCTTCTATTATCACA
CGAAAAACACAATTTTTATAATTCTTATTTCACAATTGTTACGTATAGAATTATTGCGTTA

AAAGAATAATAATTTTTCAACTATTTTTCTATTTTTAAGAATTTCCTCGATCTCTAAAATT
CTTCCACTCAATCTCAATGTTCATATTTCACAAATCCTAGCATACGTTTATTATATTAAAG
AATAATATCGTATTAACTATTTCAGAAACAGATATTAAACCAACTTAAAGAAAAAAAGA
GCCATTTCGAAGAATTATCACTTTTAAGTTCGCTCAACGACTCGCAAATTCCAAGTTGC
CGCTAATTAATGCGATTTTATCCTCAGCTCGCCAACTTCTTCGACCCACCTACATCAATC
AAATTGAATGATACGAAAGGATGGACGAGCGATAAATAATAATTATAATTTATCCGAAA
GTTCGCTAATTAGTTGGAACACTTATACGCGAACTTTGGCGTATTAGTCTATTTCTCGCG
ATTATTCGTGTCACGCGTTCGTCTGCAAGTTAAATACCCGGCCTCAAACTGTTTCAATC
CTCGTACCGGTTTGTCATTGATATATACCGTGAGTGTGGTTTCCGCCACGATCCGGAAG
TTCATTGGTACCGCGGCAAAGATTCCAGAGTTCGAAGCGAACAGAGAAATTTGTGCAG
GTGGTATGAAGGTGTTTGAAAGTGTTTGCCCGATTCTAATTCATAGACTTATGCACGTCT
AAAGTAGATATTTAAATAATCGTGTGATATGCAGATGAATATATATTAGTTAGCTATTCTA
GTTTCACTATTTCATTATTCTATCGAAATAAAATTCTCTAAAAATGTGGTATATTATCAAA
CATTGTTTGCAAAATAATTAAAAAAAAAGAATTTGCATTCTATGCTTAATCGAATCTTTAC
AAAATGCACGATTGGTGAAAAAATGAATCGACAATTAATCGTAACATAGCCTCAATTTA
AATATTAGAGATGCAATAATTTTAGATAGTAAGAAAATCTAAAAACTCGTGCATCTCGTA
ATACTATATGATACTATAAGAAAAGATGAAGGATACTTTCTCGATCGTAAATAAAATTTT
GAAAGATCGGAGAGCAAGTTATCTTTGAGGAAAAAATTGATTCGAAAGGGAATGGTG
AGCATCTAGCGCGACAGTTTTCGAAAAGGGACGGAGCCAGGGTTAATTTCGTACGGAA
GGAGGAATTCTGTTTACGCAGCCGTGCACAAAGAGACTAATTCGAAGCGAAACTACGG
GGCGGAGAGGCAAAAAGGTCTTTTCGCCTCCCTTGGAAACGGTGAATTGAATTCCGTG
AGCGGAACCATCGAATCCTCCTTCCCCCCCCTCCCCTCCCCGGGGTGCAGATTTATACA
TTTAACTCGTCGTGGAAAAAGAATCGACGATCGGATTGCCCGGATTCGATCTCTAGCTT
TCTGCGCGGCTGCTTTAAAGCAATTAAACTAGCTAGACACGGGGTGGAAAATTTCTTTC
CTCTCTCTAAATCTCGAGACTCGTTTGTTGAAGGTCGATTCTCTCCATTCGTTGTCGTTC
TCGCAATGCTTCACCCCTCCCCCTCCCCTTTGGAAACTTGAAGAAATCTTGCGAAAAA
GAAGAAACAATAAAAAAGAAAGGCTATTTCTCGAAGGTCGATTCCACGATTAAAGACT
CGTGAAGTTTTTCTCTGGATGGATAAGACGTTGGAAAGTTCACGTCGTATGAGATACAA
AGTGGTGGATTATATGGTGGCAAGTTAGTCCGGGGCTATGTGAAATTCAGGAGGTTTTC
TTGGATGTGAAACTTTGAAATTTCGGTAAGCGATCTCATCTTCATTGAGATATCACTTTC
CACATAGTTATTGGATAACTACATCGAAATAAAGCAATAAAATATGTTTCTTTCTTAAAA
AAAATCGACCAACGATCGATCGATGATTTAATTATTGCTTAAAATATATTTAAATATATTAT
AATATTAAAAAAGAAATTACTGGTAGCCTCAATGATAATAATTTCCAATTATTTAATCCA
GCAATTCAATTTAATGAATTAGCAACTTAAACTCTTTCAAAGAGATGGGCCAAGCCAAG
GCGATAGCCTTGTAGCGACATTTCATCGCTATTTCTATGCTATCTTTTCTTTTCCCTTTGT
TTTTTCAGACTGCTTATCCGCCTGTGCTGTTTTCGTTCCCTGTCTATAGCCTGCAGATAG
AATTCGTTTCATTCTCGATAGTAGCGATCGAGGGTTTTCGGGGGATTATTGGTTCATGAA
CAACCCCTCTTCGCCCGAATCGGTCGAGACGGTAGCTTTCTATCGGAAAAAGTCGCAT
CCACCGGATGGTCTGGCCGAGATGGAACCTAATCAGTAGGCATTACGTTGCCTCGTCGT
CTTAACAATTTTTCGATAAGATTTTCTACGATTTTAATGGAACCACGTTGAGTCAAGGAT
GAAAATTTGTACAATTTTGAGTTTCAGATTGAAGAAATTAAATCATCTCGAATTGAAAA
GTAAATTGTTCCTATCTCTTTCTTTCCAAGAAGAAATTTTTTTCTTGGAATAACAATAAT
GATGATAACACGATGATAAATTGGTTAAAATATATATGGAAACATGTAAGGCGTATACAA
TTTTTCTATACAATTATTTCAAAGTATTCTCATAACAATGTATATGCCTGTTGTCCAATTTT

CTATCAAGAAATCGAGATTCGCTTCAAACTTCAATACCAATCGAAGTACACGAATTATA
AAATAAATTACGTCATTTACAAGCCATCCATCCATCCAGGAAATAAACAATCTTCACGA
GGTGCACACTGTAATCCCCGAGCATCTCGTAAAATTATCATAGCGAAAAGCCAATGGCT
TATCACTCCATCAGCCTTATCAGGCATCGAGCGCAAAACGCTTCATCTACTCGAATCTC
CATCAATCTATTTCCGCGAGAACAACGGTGTTGGATAACGATCCAACGAGAGATCCGA
CCAAGAGAGGATATTAATTATTACGAGTGTACTCGATATTTCACCGTAACACAGCTCCTT
GCGGATGAATTATCACCCTTCACGGCTATTACGGCTGCCACGGCTAGATTACCCGTGATT
TTCCGGTTTTGAAGAGGTTCTGGACGTGGCATAGGTCGTTATTGGCCGCGATATCTGCT
CGAAAGTGAAACGCGATTCGTTCACTTTCGCGCATATAATAATGCAGCCTGGATGCGAT
GCTTATGTAAACCATTTCCTCTCGCCTTTTCCCAGCCAATCAAGTCGAGAGTTGGCGAG
TACTTGGAGAACGGTTAAAGCGGGATAATTTGAGATCTCTGGGGCAATGATCGCAGGA
TGATTCAGATTTAGAAATTTAGACAATTCATCAGATCATTGTCTTGATCAGAAGAATTTT
TACACGCAATATCGTGTATCATCAACAGAATATACATCTATTCTTATGAATTCAATTTATC
AAAATTAACCAGAAAGAAAGAAAATCAATGATGGAAAGATTATTAGATATCACAGTCT
GATATATATATACCTTTTCTTCTTTTTCTCTCTCTCAATTTTTCCTAATTTCACGGGTTATTA
TTGAAATTCTAGCTATCGAACTTGATTTAATCATTATGAATTTTACAAAGGGACAGGAAT
TTTATCGAGAAATTTCGAGGCAGACGAAACAAAACCGGTCCGCGCGTAATTGCCTGGC
CTGGAACAAAAGGGTGGTCGCGCAGGTGAAAAATACACGGAGGGGGGAGGGAAAAA
CTGGCGCGTTTCCAATTCGTGGCCCCGATACGGAACTCCCTTCTGTATGCACGTTTGTG
TAACGAGATGAGCCCCGTCCTCGAGCAAAGCCGATGTTCCTTTGTGCTACTTTCCTTCT
CGAGGGAAGGTTGGAAACGCTTCTCTGTTGACGCGCCAAGACGTCGATCCGAATTACG
TTTGCACAAGAATCGTTTCCCCTGAATACACCTGTGTGCGGAGGCATCCCAGGATTTCT
GGATCAAAGAGTTCATCGGAACATGAATATTCTTCTGTCCTCTTTCTCTCAACCAGAAA
ACGTTCTCTCAAATCTTTCAGACCCTTCAATTTTCAAATGTTTAGAGCAAATATTCTCTA
TGCTGTTCAAGCTTTATTTTATTAAGAAATTTAATTTATATAATCAGAGTTAATAGTGATTT
ATCTTACATTCGGTATTATATGCGTCAAATTTATCTTTCCCTATAAATAGTGCAGTGCTTTT
TTATAAAACTATAAACGGATTCGATAGATTTCTCACTCTTTATTAAAAGAATCTTAATATA
TTTTTTTATACTTAGCTCCAAATATGGAAAGTTTCTGTGCAAATGTAGTTAGCCGCAATT
CTCTGAATATCTCAGAATCTACTAGGTATTCTGCAGTTCGAAAATCTTCGCCCTTGGGCT
ATTGGGAGGACAGAGGAGGCGGTGAGTTGAACAAAAGGGGTATCCCATGGACAATGA
CGTATCTGTTCGCGCGAAAGTCGCGACAACAAAGCGGGTAAGCTCGGCTAATTGTCGC
GCAACAAAGCCAGAAGGAAAGAATATTGGCGCGTTTCCAACGTCGATGTTTCCATAGT
GGCGATAAGACCCTTGAGGGCATGCGTAATTATACCTTCGGTAAACAAGCAGTACTTTT
CTTCTTTTTTCTTAGTTTTCTTTGTTAACTTTCAATCTCGTGTTAATAACCTCTTCTTGGA
TCAATTTTTATAACCGATTCGAAATCCGATATCGACAATTTCGTATCTCAATAGAAAATTT
AATTTTCATGCAAGTATTATGAATCTAATTCTTTATTCCATGTAGTATAAATTTTTATTCTA
ACATCATTAATATCGATATATAAAAATAAATTACTATATCTTATTATTATGTATGCAATTTGT
TATTCGAAACGGATGTTTTTTCGATCATTGGAAAATAAACATATGCAAAATTTGATTCAA
AAATTTTACCCTCCAGCCTTGCTATATTATCATGATAATTACGATAAATATTATTATGATAA
TTTGCTTTACAAGTGAATTAATTAATTCGTATTTAAAAGATGTACAAGAAATTGCAAAGT
ACATGTAAACATTGTCTTTAATTAAAATTATCGAATCGGTGAAGAAAAAGAACCGTGTA
TAAATTTTTCATAAAGAGAGAAAATTAAAATTTCTTTTCTTTCAATGAAAAGTGTCAATC
CTTTAAAGGTTAGCAGGCGAAACGAATCGCCATTAAAATGTGCAAATAAAATCGGGTG
TAGGTAAATGGGCACGATTGCCAGTCGACGATTGATAAATTATCCCGGGAATATATATGT

GGAGTCACTTACGGTTACGTTGATCGACCGGGAAATTTTGTTTGACACAGCTTTAATGG
GCTCACCTGAAAATGATCGAGTTAATTTTAATATCGATTGAAACTTGAATGGTGCAGGA
ATGTTGGATTTTAATAAGCGGGGTCATGGTATTTGGAGAAATAATAATGCGTTAAATATG
GATACCAACGTTAAAACAATTAATAATAATAAGCTTTTTAATAAAATCATTAATTCATCGA
TCTCTTTATCGCGCAAAAGTATCGAGTTAAAATAAATCCACCTGTTATTTAATTTTTAGA
AACGAGAGTACAATACAACTATATCCCGAAGTTAAAAATTAAAAAAGCAGAGCTGTGT
ATTCAATAGTTTTTAAAAAAATAATTTGCTCTTGTATTGTATTCTTCTCTTAAAATAAATTC
AAAAATTTAATTGCACATCTCTGAGTTCAAACAAACTCTTGTCTCTGATATTCTGGATCA
AGAATCGTACGCAACATCCACTGTTCTATCGCTTCGATATTAATTTTGTTAAATCTCCCC
CACCCCCTCTTAAAAATAGATGGAATCCCTTGACGGGGGAATGAAAAAATGAAAAAAA
TCATTTTGATTTGATATTAAATACAGAGTGAAGACATCTGTTTCGAAATAAAAACTTGCA
AATCTCGAGATCAAATTTTGCAACGCTCAATACCGGGTTCCCGTCTTGCTCCATCATAA
AACCAAGATCCGTCCCATGACGTGAACGTAATCCAGCTCAGACTGCAGTCACGTCGAA
AAGACGTAGAATCGAAACAGGGACGGGAGGAAGAATGACAGGGGTTCCAAAGCCAC
GGGGGATAAGACGGCCGAGGGTGGCGTGTAAATTCGTGACGAGGAGCCAGCCCCACG
CCATCTCTCGATTAATCGTCGCTTCCATTCGAACGGCCTTTCTCGGGAACGATGACAGA
TCTCCCCTCGACGCGTGTGCGATTGAGATGTCCCCATCCTCGCTAAATCGCACGTTAAA
CCGACCTAATCTACCTTCTTCTCTGAGGGGTCGATGTACAGGGTGTTCGAACGAAGAA
GCGATTCCAGAGAGTAAAACAAACGAGTTACAATAATGGCGAAGTATAATGGTTGCAA
TGTATTTGCTACGCAACAGAGTAATTCGATAAAATAATATTTAATGGCGATACATTGTTG
CAGATATTTTTTTGGATTTGGATTAGATCGATCGTTTCATCGATATTCTTTTTCTTTTTGTT
TTGAAGAATCATCCCTTCTAATTTGTCCGAACACCCTGTATATACGGAGCGCAATTCTCC
CCACAGAGAAATCTGTAGGCCGTTCCACGAGAGCCAATTCCATGAACGTGCCGTGACT
TTATTCAAAAAATCCTTCACCCCCTGCGGATTTATTTGTCGGGAGTGCTGGGGGTTGGA
TGGTTTAACTTGGCTGACCGATACAGTGAAGTGTTTCTCGTACTTTTCGGGGAAAAGAA
TTGTAACAATCGATTTTGTTTGAGGGTTGAAAATCTAGTATGGGGAAATAGTGAATTTAA
TCTTAAATAAAGTACTTTATTATATTTTCTCTTTAGTTTTAAAAAAAAATTTATCCTCGAAA
AGATACTTTGTCACAGAAATATAATTGTAATCATACACGCTATTGATCGAAAATTTTGAA
ATTCTATCTAGAGATATCAATAATTTTGCTTATTTTAACATATTAATAATTCGTTATAACGC
GATAACAAAATATTAATTAAAAACTCCGTCCAATAGATGAATAAAATTAATCAAACTAAT
TTCCCTAGCTACCAATTTATTATAGAAATTTTATAATTATAAAATATCCAACTTCTCCTTTT
CACAGCTCTCATTATCGTCAAATGATCAAGAGAATTTTAAAAAAATTCTACCCTTATTTA
CAAGTACTTTCTATCGCGGAATTTTTATTTGGAAATTGGCCGAGAAAAAAGTTTCACTT
CCAATTCTCAAGATTGTCATTTAATCGATCCCATTCCTGTCGAACTTGTATCCTTGGATG
ATTCTAAGACGCAGAAGAAAAGCTGCAGGAATGGAAGACGATCCAATTTCCGGCCAC
GGATTAAACACGGATTCTGCGCGTGAAGGGGCATCACGTTTTCGTCCCAAAATCGTCA
CAATTCGTATTCATATCACATTAATGAAGACAATACGAGAAAAAGTGGAGTTTAAAATT
CAACAGCAAATTGTTTCTAGCTCGTGTAACTCGGGGAACGTCGGACAATAGGTTAGAA
GAGAAAATTTGAGGTGCTTTTCCCGTGTGTTAAGCCGCAAGAAAATTCGCTGAATTTTA
CATTCAAACCGCACAGTGAACTTCCGTCACGAACTTGACTCCGGCTAAAATATTCCGCA
TTTGCGAAACCTTCTATTCACGAATTCCTTTTATACGCGATTCTACCATGTACGAAACAA
TGTTTATGTATACAAAAAAATCAGCATGAAGAAGTCAGCATCGAAGTGATGGATTAATA
AAACGTGGAACGTTTAACAATCGATCGATGAGATTCGATTCGTTTGTTTGTTTAACATTT
CTTTCGAGAAAAATCGTACTTTCGAATTCTTCAGAGATGGGAAAAAGTATTTCTGATAG

CATTGAACGTTGGTCGAAGAGGTTTAAAAGGCTTCGCGAGAAAGGAAATGGATTTTAA
ATAAAAGAGGGCTGTCAAAAGGGGGATGAAAAAACGAGGCGAGCTCGTTCCAAGAA
GACTAATCTCCGGAAAATACGACCATAATCGATGGGAGGAGGGGATAAAACGAATCAG
AGCATTACCCCCATTCCTGTCCTTCTGTTTCTCGCACGAGAGAGAGAGAGAAAAAAAG
AGGGGGGAAAGAAAGAGAAGAGGAGAGCATCTCTCCTCGAGGAAATCCGTCTTGGCG
CGGAGTAAACGAAAGAGAACGAGAACATTCTTTGCGTCTCATGACTCGATCGAGGCA
AAGGGAAGAAACATCGGACCGGTTAAGCCTCGGAAAATTGAGTGCACGCCTCGAAAG
AGTCGCGTCGCACCCTCCTTTGCCCGCTTCCCCTCTCCCAGTTTTCCAACCGATCCTTT
CCAACGCGAAAAATCTTCGAAAAGTTTCGTTGTTGGAATCTCTGCATCCGCGAATTAG
AAATGGACCACGATGAGTTCAAACGCAGACGTCGATCAAGAAATTATTTGTAATGTATA
CTAATAATTGATTAGAAAATTAACCTTCTAATTCTTGATTTCTTGTTTTATCCCCGATAGA
ATGATTTTTAATTTTGAAACGACAATTATTGGAATTACAATGCTATTACAATAAAATCTTT
AAGAATAATTAAAAAAAAAAATAGATTCGATAAATAATTAAACATTAAATTAATAATTTAT
AAAATTTTAATATATAGAAAGATTGGAAGATCTTTAATAATGAATCGTTTTGGAAATTTTT
AAATTAAACATTGTCGAAATTTATGTAACATCTGTTTGGTTTTTATCTCCAAATTTTCACC
AAAGATTACGCTTTTAATCGCATCTCTATGTAAGAAAATATTCCCAGCAGCTTTGGATAA
ACAGTGGGATAAAGGATGCGGGGACGTTGATGCATCTCGACGCCGGATTTTAAATTGAT
GCATGCTAATGTATTCACCGCGAGAGCCGCGCTAATTATTCGCCATCCAACGCGTTCGT
GCTGACGCACAGAGGCGTCCATATCAACGAGGATGACGAGGGGAGAAGAAAAAAAAT
TAAGTTTCGACGTTACATTTTTGTCCTCTCTCCTCTCTTCTCCTTCGTTTTCCATCTATCC
CCAATGAATTTGAGAAATCTGCGAGGATAAAAAAAAAATGAGGATGAAGAGAAAGAT
CATTGAATATCTGCTGACGATTATAATTAAGGGAGAAATATTCGATATCCTGATGCGACC
GCCGTTATAGCAATTTCGAGGATCCTTTATCGAGACGGCTCGATACTGATGATGTAAGAT
GTTCACTTTGCACGCGAGTTGCAATCATGTATCTTCATTAACCACGCATCTAAATTAGTA
GGGTAAATGAAATTGCGGATACCGACATACGACGCATCTCTATCTAGTAGTAACGTTCAT
CGTGTTGAGAAATTTTACGTATATGAGTAGATCTATTTCCACTAATTTTGGTATACAGATT
AATTATCTATAATTCGTATCGAAAGACGATTTATTCGAGTTATTAATAAAATAAGAGAAA
AATAAGAGAGTTTCTTTAGATTTTTATAGATGTCTTTTTATTATTCGTAACAGAAGCAAA
GACTATTTCTTAAACTTCCTCTTCTTCGAATTAATTTCGACTTTTTCTTTGAACTACGAAT
CACAAACTATTTTCTAATTTAATCTTAATCTTATAAATCTCTATACGGAAAGATATATATAT
ATATTCACAAATCAATTCGTTTTGTTACGAGCACACCATCGACCATCGACGTCCAAATC
CACGTTCAGTAACTTCGATTCTGGAATGACTCAAACTTCGCCAATTGCTCCCGTATCTC
CTCCCCCTCTCCAAGTTACTCCCCCATTAATTGTCTCTGCCGTTTGCTGGCTGGTCGATT
CCAGCCAACGAGATCCATCACTGCGAATCGTTTGATCTTTACCCGATCAATCTTCCCCA
GGATCTTCCACAAAACGCGGTACCCGTCTGTACAAACATCCATTACAGCTGTTTCACCG
TGATCCCGGGGTAAAAGATCCTCCCTCGAAAGGACTGGAGGTGCACTCGTTAGACGAG
AAATTCCTCTGTACACAAGATCGAGAGGCGTACGAATAACTTCTCCTCCATCTGCAAAG
CCACTCTTGCGGGGAGGCGTTGCGGGGGTGAATCTTGCCTCGAGAATCGAAACTTACG
ATGTGATCCACGAGATCAGACGTGTGATCGACATCAGTCTCTCTTCGAGCTCTTTCGAT
GAAATTAGTTGTATTGTCGTAGCTTTCATCCTTTTTTTGATCTATTTGAAAATCCTCGAGT
ATGTTCTACGTTTCATTCATCGATTTTAAAGATCTTATTCCAGAGTGATTGGTGTAAAGA
ATTCTATTCTAATCGTTTTATATTAATGTTTCGTTTAGATAATAAGAGGAGAGCCAAAGTA
TCGTGTAAATCTTTGATAGATTGTTTTTTAATTTTATCAATATTTGATAGCTATTTTTTTTAT
TTTTTATTTCTTTTTAGATTACAATAATAAACAGATATGCTTTGTGAATTAACAGATCTTA

TCCAGATAAATATATCTGAATTTTGTTAAGACACATGACATATCAGATTCCCTAGATATAT
ACACGCGTTTAAATTTTGATGTATCAAGCTTTTCCATCTGACCATGGAATTCAGAATTCG
ATTGGAAATTAATTATAATTCCACCATGAAAAATTTCTGAAGAAGGCTTGTATATATTATA
TGCAAATACTCTTGGTCTTAACAATAAAAGTACGAGTCATAAGAGCAATTGAAAGAGA
AGGAAATTCACAAATGGGTTCTGAGCAATTCGATCTTCGACGAGAGACTCGATTTCAG
ACAATTGAAAGGATAAGTAGCATCGCGTAGCATCTCAGCACAACAAGATCGTTTAACC
TTTTTAGAAAGTTAATGGCACGCGAAGAAAAGGAATTCTCCAGCGTTAAGATTGTCAT
GCGATCGGCCCACTTTCGATCTCGATCCGTTCTCCGGGAGAAATCGGTTTCGAGCGCAT
AGAAACCGCATCAGGATGGACACGACAGCTTATGGTTATGTCTAAAGTTAGATTACTTT
CGCGTGACGTATGACTAAAAATTATAGCAACGATCGAGAAGGCAGCATGTTTCTTTCTT
TGGGATATTTATCGAAATCACGATGGAATCTTTAACAATCCTTTAACTTTTTATCCCGGA
AATCGAGGAAATTATTATTTTTATTAATCTCTAATTAATCATTAAATTTCTTTGTATTTCTAT
AATAACAAAAATGGGTAAATTTAATGAAATAATATTTTGCAAAAAGATTATATGATACAG
ATAAATAGTTTAATTTAAGTTTTGCCTTATAGAATATAATAATTTAATTATTTCCTGTCTTT
TTCCTGCTTTATAAAAAATTAATTGATCGATCTTGCAACTGGATGAAATCATGATAACGA
GACCGTGTATCTAATACTTATTTCATTACATCTCTGGGGAATTCATCAGTATAAGATCTCC
TTCGATTTTAACGATCAAATAGAGAGAAGTGCAACCGTGGTAATTGAACCAGCTCGTG
GCTAGCTTCCGGTTACCGTAACTACCGCTTATTTGAAACTCTATATATAAATTTACAACC
AATCCTCTTAAATAAAATTGAAAATAATTATAGAACGTAAAATTGTAAAGATATTTATACA
TATTATATTCTATACAAATTCTAATGAAAAAGAAAAAGACATATTATAAATAAACAATTTT
AAAATTGTTAACAATTCATTTAAACCTCGTCATCTGTTCGCAATAAATATTAATTAAAGA
ATTAAAATAAATTTAATAATAAATTTACACGATCGTATAGCACAAGAATGTTTCCATTAC
GCTTTGTTGACATTTCTCCGATTTAATATTTAATCCTCTTAAATTTCCATACAATACAGAC
AGTCGATTATTTGTTTACTTTTTCTTTTACCGCGCGACACGCGACGAGGCAAATGAACG
TGTATATATATATATATAAATATACAGAAGCTCGTAAATTTTCATAACCCAAGGAATAAAG
ACGGTTTCAGTGAGCAGGTAGCTACGAACGGAGACGGTCGCTCCCCAGTTTTCGCTTT
GAAGCGTAATCGCGTCACTTAAACGTGTTTCAGACTTTTTAAATAATTATTCCTCGTGGA
TCCTCGTCGCGTCCGGACTTCTTTCCACCTCTTCTTCCTTTCTCTCTCTCTCTCTCCCTT
TTCCTCTCTCACGACTTGGATACGTTAATTTAACTTCTAATTTTCTAACTTTTCTTTTTTT
TTTTTTTACCTCTAATTTAGCCGTCGTTCCTCCCGACTCGGTTGAAAAATTTCCATCTTT
CCCGTTCCGGTTGGTTACACGTGTACGAAAGTGTGAACGAAGCTTTCCGGAAACGGAC
GAGAAATGGACACCCGAGTGACTTCCGAGTTTCCGAGCAGGGTGAAAAGAAAAACA
ACGGGAGGAGAGTTTAAAGAG

>novel_circ_000595
CAAGAGAAAGAGCAGCGCATCCGACGAGAAGAATGTGGAAAATTATTCCGAGGATTT
GGTCGAAACGAACGAGGAATCGGGGAAAGGTCACAAGTCGAGGAACAAGAAATTGA
AGGAGAAGGAGAATATCGACATTAAGGGCAAGTCTAGCTACGAATCCCCGAAGAGGA
AGTC

>novel_circ_000596
GTATTACGTTCGTGAAACTACGCATTCTACGCAGAGCTATTGAATGTTTACGATGTCTTA
CGGTTTACGACGAAACGCGAGAAACACAAGCATGGTGAAATGTTAAATTCCAGAAGTT
CGTTTCCACCATCCCCTCTTCGCTTTCACCATCTCTTCATCTTCAACATCTTCCTCTGTAT

TCCTTTTCTCGATGACGCGAAGTCGTTCGACGTTTGAAGGCACATGGAAAAAGTGGAA
AGGGACGAAAGGACGAGTTGGGAATTATTAGAACGAATTATTCAAACGGCGGCGTGG
AACAGGTGGAGCTTATTGCCGGAACACGGCCACATGGCGTCTCGTTACGATGCGTTTG
TGGGATCGACGGCCTCACCTGGAGCCACGCACAGGAGGAGAGAACAAGCGTGGGCTA
ACGAGGGATGGAAAATCTGCGGGGGGTGCGGTGGATGTAACAACGAAAAGAAAAATT
ACATGGCGTAATTTTCTCGCACTGCTACACTGGAAATTGAAATGACGCCGATTCACTCG
GGCGAAAAAGTTTTCTAAGTGATTCTGGTCCTCGGTTCATGCGCGTCGACAATTCGGTA
TTCATTCGAGCGAATGTTTAAAGTGGACGGAATCAAGAAAACCACTCGATGTAAATTG
TGATGCGCATGAACGCTATTCTAGATACTCAAAGTGACTTATATATATATATACATATTTCA
TTGTTGAATATTTTTTATTATTATATCAACAATGATTTTTTATAAGTTGCCAAGAGAATGA
ATTGATACAAGAATGGAACATTTTATTTCTATTATCTCTATTATAGGTAATGCATTAATAAT
TTTTTGTTACGAGTCGATATATAAGAATCAAAGGACTTCAATGTGTAATCAAAAATATCT
GCGACACGATGAAAATAAATCCGTTTCGTGGATTAAAAAATTGGTGAGAAGGAAACCT
CATCGAAATCCTACAATAGATGGTACCAGCTTTCGCAATAGAGCAACTCTTTTTCCAGA
AACTCGAAAATCCAACGTGAAACCAATCAAACGTTACGTGACCAGGAACCCGCGAAA
CGCACGACTCTCGAACATGGTCTCGAGTGGCACTGTCTCGTCACGAGTTTCGAGGAAG
CAAGCGGGGATAAGGAGGGGGCGAGACATTTTGAGCGTACTCCCCTAGGAGCCTCGA
GTCGTTTCATCCGGAATACCATTCTCTCTCTCTCGTTGAAAGAGAAGACGAGACGAGA
GATAATTAATTGTTTCGAGGCAGAAAGTAAAATTCCTTAAATCATCGAGCCAAAAAAAT
TATCCATTATTCCTTTAAGTCCTTTGTGATAGATCTAGCTCTGTGTCTTGCTTCCTCCGTT
GATTTCGATTCTCGTGTCCCAACACTCTCTGCCATCTCGACTTATCCCTCTACGAACTTT
CGAGAGGAACGTGACGAACCTGTCATTTTCGACGTTGAACTCTTCACGGGGAAACGT
GTAAGATGATCCTCGACTCGACGAGGGGGAACAACGAATATATATATACGGAAAAACG
AAGCGTTGTTAATTGGAATACGATACCGTCGAAAGGAAATCGAACGGGGGAGATGAGA
AATGAAAGGAATCGATCAAGGTTAACAGGATTGGAGAATGGCGGACAAGGAGAGCGA
ACAGCGCGATCTAATCTTGGGATAA

>novel_circ_000597
GTCAGCCAAGGGGCCCCGTGTCCCAAGGGTGGGTGTGCCGGATGCTGACCTTCTTGCA
CAATGGCTGAGGCTGCACCATCAGCGGATATGATTTGAAGGGATCAATCGGAGGCGCG
GTCGGGCATCCTAGAACGGTATATAAAGTCGGATCAAATGGTTCGTGGGCACACTCAG
CAATCGGTCGATGAGGAAGATGAAGGCCCTCGTGATCCTGCTAGCTTTTCTCGTCTTGG
TCGCAGCTGCGCCCCGTCAGCGTCGCGATGCTATTTGGAGTGGATATCACGGTGGCTAC
GGTGGTCACCTTGGATACGCTCACGGCGTGGCGGTCGCTGGTCCAGCCTTGGGACCGG
CCAGCGTAGCCGGTCCTCACATCGGATCCACCATGGTGGCAGCGCCGTCCATAGGACC
GGCTAAGCTGTCCGGATCAGTTGCTGGGCCGGTGCACGTTTCCGGTGCTGTGGCCGGC
TCTGCCGTTGTCACTGCTTCCGTTGCCGGACCCGCTCACGTTGAGGGCTACGATGCTG
GTCCCTACGATGGAGGAGTCGGTGTCGGATACGCGGGCCCGGCCTACAGTTACGCGGG
ATACCCGACATTCGCGGGAGTTGGCAGCCACGGAGCGGTGATAGCGGGTCCAGCGTCT
CACGGTGCGATTCTCGCGGGGCCGGCGTCCCATGGTGCCGTGCTCTCCGGACCTCATT
CGGGAACCGCGGCGGTATCCGGTCCCCACGCTGGATCGGTGGTGATCGCCGGCCCCTC
CGGCAAAATCACTGCCCACGGGACAGGTTACGGAGCGATCCACTCTGGCCACTCTGGC
CATTGGTAACGAGTCCTGTTGGATGAGACAGAAGGAAAGGAAGAAAATCAGGAGAAT
AAAATCTCGTCGATCGAAGCGTCCAGTCAAGGTATCGATGTGTTGTCCGATCCGTTGTT

TAGATTTGTTTCGCGACGATCCCTCTTTTAACGAATATCACGATTTTTGTATGCATGGTC
GGTTTTTATACGGTTATATTTTATTATTATTGTTATTATTATTTTCTAAAAAAAGGAATAGA
TAATAAATAGTTTGTAACGTACATTTTCATAGATATAATATACATATATATATTCGTATAGCG
TAACTTTAATTCTGATCTCGTATAATAATACACGCGATTTAGTTACACACGTTTAATCGCG
TTGTTAACTCTTCCACGCTTCTATAGTTTAATCGAGTTTTATATATATACACGGCTTAAAC
CTTTAGTTCGCTTTAAATTCGTTACTCGTCCCGTTTTAACGAGCTGTAAAATAATTGATG
CACGTTTTAAGAGTAAAGAGAGGAGAAAGATCGTATCCCGTGAATTTTGAATCTATTTC
TATTTTGAATATCGATTACGGGTATTTTTTCATCGACTAAAAAGGAAAGGGAAAAATTA
AAAACTTTCTCGAATAGGAGATTTAATATATATATATAGATCATGCTACTATACAGGCAGA
TCACGAAAACGTTTTACGTTTTATCGGTTCTCGATCCTTATCGCCGTGTCGGTTCCGCTG
TGTTTCACCGTGGAAATTTGACACTTACAAGAAGCGACGATTAAGGGTAGATTGAGAA
AGAGAGAGATAATACAAAGTGCTGAAGATCTCGAAGTGTCAGCAAACGATACGAACG
ATAGTGGATACAAATAAAAATCCAAACTACTCTTCAGCCTCGAATTCTTCTTTTTAACTC
AACGAGTATATCCTCTTTATATTGTCAAAATTTTCGTATATACAGAGGATTGTTTTCTCAT
TATTGTCAACTTAAATTCTGATTAATTCTGATCAAGAACGTTGTCGAATTAAATTTCAAA
GTTCTACCTGTATAAATCGCTGGTGCAACGAGCCGGGTTCACTCAGCCTCTTAGCGTAT
TGCGCGTAACATATCATTGGCCGTAGTAAAGGGTAAGGTCGCTAGACGTGTGGCTCGC
GACACGGTCTGGCAATCGTGAAACAAACAACGAGTCGGATTTTCGCGACATTGTCCGG
GGAAAAATCGATCTGTAGATCGATCCGCGGGCACACAGGTGTGCGGGGGCGCGGTCGA
TTTGAATAAGGTCGAGCTAACGAACCGCGAGACCATAATTCACAGAAGGTCGTACACG
AGAATTGCGTTATCTACGGCGGTTGTTCGACTGCGCTGGCTCTATCGCGTTGAATCTATA
CACGACAATTACCGTATGCTTTTTTCTTTTCCTTTCTATTTTTTCCCCCTTTTTTTTTTTTT
TTTTTTCAAAGGCCGATCTTCGCCACAGATCGCGCGACCATGGGAGGGAATATTTTTCG
CCTAGCCAAGCCGTTTCATTACGTTTTTACGACGAAACAATGGCCATTCAAGTTGTTTG
CTTCGAGGCTACCAGGCTCTGTGGAACGCACGGAGGCATTTACGTTATATAAACGATCG
TTTGAAATCCCGGTGAAATTCACCCGATGGATCCATTTGATAATTCGTGTTTGAAAGTTT
AATCCACGAAGAGGGAGGAAAGATTAGGAGTAACGTTTGATCGCAATTTACATTTCTA
CATTTTAAGAGGGAAGGATACGATGATAAAATATTACGAGTCGATGATTTTGATTAAATT
GATCGATCAAAATCACGATAGATTAGAAGGAACAGAATACAGAAGCACGCCATCGTGA
TTCTTAACGTAAAGAAACTCGATGTAAACCAGATCTGACGACGGATGGAAGATTTATTC
GCGAGAGTAGATTCACGAGAGAACAATCGTGAGAACGAGACCGATTTAACTACTAGCC
AGCCTTCATCTTCTATTCCTTTGAGAAGATTTATTATCGGAATCTCGAGGCATTTCGCGA
TGGCTAGGACGAATATCGAACGACTTGGCCTTCTTTATGGGAAAATCGTTGTGCCGCGT
CACAATCAATTTCGTCATCGATCAACAGTCGCATCGAATTCTCGAAAAGGTCATTTCGA
TTTCAATTTAACCAGGTTAAATGGTTAGCACGCAACAACGATTTACAATATTCAACCGA
TTTGTTTTTTTTTTTTTTTAATGGATCAAATAAAAATGAAGCTCTCGGCTATCCATCACGT
TGATTCACGATCGAGTAGTCGTTGCTCCTCGGCTCATGGAAAAATAGATCCAGGTCGTG
AATCGTGGAATTGTAATCTTTGAAAGAGAGAGAGAGAGAGAGGATTTTACGAATCGAA
CGTGAATTATTTTCCACCCCCTCCACTCGGTTTCTTTTTCCCTCCTTTTAATTTTTAACGA
TGGTAAATACGAATGAAACGAATGAAAGTGCGGCGGGGTTGATATTAAATATTTTATTTT
CTCGCGGTTCTCGTTTAACTATTGTACGCGATGAATTTCCCCCTCCGAGATGTGTAAATT
GAAAATGATGCTCACAAAGAGAATAATTTTTTTTTTTCTTCTTCCTTTTCATTTTGCACC
GGGATCACTTCATCGCATCAAAACGCGTGTATGTATGTGAGTGTGTGTTTAAATTACGC
AGGAAAATGGATTATTTATCGCAAATTTCACATTTCTCTCTCGCACAATGCCAGTGGAAT

TAAAGAAGTGGGTAAGCGATGTGCCCTAATTTTAAACTTCGATACGAGCAACGTTAGC
AGTTATTCATTGCCCCCCCATACTTCACGTTAAAATCGTAAAACGTTGAATTACGAATGG
GAGAAATATGGGCGAAGACGATGTTCATTTTACGACTTCAATAAATTATTATTGCGCTTG
AACGTAATCTATTTTAAAACGTCGTGCCTTGCATTTCTCGCCGTATAAAATCCGCGCCGT
CGAATTCTTGGGGGAGGAGGGGCAAAGCAAAATTTCTAACTTCGTTTCTTTTTTTGCTC
GATCGAAATAATAAATATATCGCTTTAAAGGAGTGGATAGTTTGTTACGATAAACTCGGT
ATTCGTTCAGTTTTTGAAATTATATTTATCTAGAAAAGATCCTAATAAAGGGAATGGAAC
AACGACCCACAATATCCCCAATTTCTTTCTATATTTGTAGAATCAAATGCCGCAATACG
AAACGTCACGTCGTCTCATAAATAATTCTGGTCAGATATCTAAGAAGGTTTCAACGGG
AATTGAATCTAAAACAATAGGGCACGTAACGATGGGGATTTTATGGAGGAGAGAAATT
GAACACCGAACATGAAAACGGGAATAAGTGCCATGATAAATCTAAGAATAGACTTCCC
CCTCCCTCTCCCTCTTTCCTCTTCTTATTTTTATTATTAGATCGATCGGAAAAGTTTTCCC
TACTAACGAGAATTTTCGCCATAATGGAACAATATAAAAATCTTCTCCCTTCTTCGATGA
TGAAAGAATCATTTATTATAAAAAAACTTTATACATACAACGATCCTCTCTATATGATAAA
ATATACATTATTGCTTAATGACTGAATGAATAGAAAATTTCACTTTACATGATAATTTTTA
ACCAATAACGTTATATTATAAATAAAATTAAAACAAATTTTTAAAATCCTTTCCCAATCCC
ACTCTCTCTCTCTCTCTCTTTCTTTCTCGTCATTCCTCAACGATCGGCTAGATGGCGATA
AATCATATACTTTGTCACGAATTGCAAGCCGGCCACATATCTATGATCCATGCTACGAGG
TAACCGAACCTTAACGAGAGATCCGGATTGTTTTCGCGAGGTGTCGCGTCGTCAAGGG
ACGAAGAGAAAGAAGGTGTGGCCGATAACGAGGTAGATACGGTTGTCTGTTTATAACG
GGTTGGCGGAGATCGAGGAAGGCCGATCGATGAATCCTTCGACGCGCTGCTCCTTCCA
CATCCATTGAATCGCCTATGGAATACAAAAATCATAGCAAGTGGCCAAGGAGAGGAGA
GAAGAAATGTTCGTTAGAGGAAAGAAAAGAAAGAAAGAAAGAAAGAAATCGACGTC
TTTGTATGAACGACGCGTAGACACGCAGGGATCACGACGTGATCGTAGATCCTTGTTTA
AACATCTAGCTCGAGACACGATCTCGCGGTGGATCGTTGGTCGAGAGGGCGGGGAAAT
AACCGGTTATTTTTCAACGAAGCCTTATTTATAAGGTTTTGGTAACGTAACGCCGCGTTC
GCAGATCAGAAAATCGGTCCAGGAACGGGAGGATAAATCTTGCGCGCATGCCGCGTCC
TCTCTCTCTCTATCTCTCTCTCTCCTCTTGCCTCGATCGTTATTCACACCCCGATCCGCTC
GACCGATGCACCACCGTCGTGGAAATTGAGAAACGTGGCCGTTTTGTACGCGTCACAG
TCTAATTCTTTCGTTTGAAACGAATGGAAGGAGAGAGGGAGGAAAGAAGGAAGAGAC
ATTCTCGGATCTCTCCGACAACTTGTTCCAAAGTTGCGCATGATAAATATTAGGGAATTG
TGGGGGAGAAAAACTTGGAAAATTCCGGATAATATTTAGGATATTATACGTAAGAATTT
GGAAAATCTTGGGATCTTCAATTTTATCAAAAACTCACTCGTTCTCAACTCGCTATAAAT
ATTCGACAAAAGTTGGGGAATTCGCGATTCGAGTTGTTGAAAATCTTGGAAATCCCATT
TATATTTCCCCTCCCCGGATTTAACAAACGATTTCTGCCTCTAGCGAACGTTAAACTCGT
TGGCCGACTTACCTGAAAGTTGGACGAGGCGGGCTGGTGTCGACGCTATTGGAACGA
AGGATCGTGGCTCGTCCGTTTCGGGGTTGGTCCGGCGGCCTCCTCGGGGGATAGGAAG
GCCAATTTTTTTCGAGAATCTCGACGATCCATCGATCACGCCAGTTTCTCGGCTTCTCTA
TCCGGTGGAGATAGGTGGACAACGTAGGTGACACGTCCCCCTGTGGACGAGTGAGGC
CGACGAGATTCCGAGCAGCGACTTTCCATCGGCCATCACGGAGGAACATTGGCGTGCA
AGTTGATG

>novel_circ_000598
CGTGTCAACAGGTCAAATACGGCGTGCAGGCGGTATTCGGGCCATCGGATCCCATTCT

GGGGCAACACATCCACAGCATCTGCGACGCACTGGATATTCCACATTTGGAAGCCAGG
CTGGACCTGGACACGGAGGCGAAAGAGTTCAGCATCAACCTCTATCCCGCACAAAGTT
TGCTCAACGCCGCTTATCAGGATATCATGGAGTTTCTCAACTGGACCAAGGTCGCCATT
ATCTACGAGGACGATTATGGATTGGTGAAGCTGCGAGAACTGGTCAGGTCGCCGAAAT
CTCAGGAGATGGAGGTTAACCTCAGGCAGGCAGATCCAGATTCCTACAGGCAAGTTCT
ATCCGAGATGAAAAGCAAGGAAATACGGAATCTCATCGTGGATACGAGGCCAGAGCAT
ATGCATCATTTTCTACGAATGATACTGCAACTGCAAATGAACGACTACAAGTACCACTA
TCTCTTCACAACTTTCGATATCGAGACGTTCGACCTCGAGGACTTCAAGTACAATTTCG
TCAATATCACTGCCTTCCGTCTGGTCGACGCGGAGGACGTCGGTGTGCGGGGTATCCT
GAGGGACATGGAGCGATATCAGCCGTCCGGGAATACGATTCTCAACAAATCGAGAGTT
ATACAG

>novel_circ_000599
GATTGGTGAAGCTGCGAGAACTGGTCAGGTCGCCGAAATCTCAGGAGATGGAGGTTA
ACCTCAGGCAGGCAGATCCAGATTCCTACAGGCAAGTTCTATCCGAGATGAAAAGCAA
GGAAATACGGAATCTCATCGTGGATACGAGGCCAGAGCATATGCATCATTTTCTACGAA
TGATACTGCAACTGCAAATGAACGACTACAAGTACCACTATCTCTTCACAACTTTCGAT
ATCGAGACGTTCGACCTCGAGGACTTCAAGTACAATTTCGTCAATATCACTGCCTTCCG
TCTGGTCGACGCGGAGGACGTCGGTGTGCGGGGTATCCTGAGGGACATGGAGCGATAT
CAGCCGTCCGGGAATACGATTCTCAACAAATCGAGAGTTATACAG

>novel_circ_000601
GCTGCTGGTAGACATCAATGCAGCTATTTAATAAACCTGCAGAAAGGAGAGTTCATCCT
CCAAGGCGGTGATCCATCTTGGCTTCGTGGTTTGAAGTCGATCCCTGGAAAGCTACAA
GATCTCTATGAGATTAACAAGATTTTAGCTCATAGGCCGTGGTTGCTGAACAAAACGCA
CATAGAGAAACTGACCAAAGGCGCGGACAGTTGGTCCTTGGCCGAGGTCGTTCACGC
GATAGTTCTTCTGGCCCACTTCCACTCCCTTTCGTCTTTCGTCTTTTCCTGCGGTATTAA
CGAAGAGCTGGACAACGTAGCCGGTCACCATTACAAAGAGAACGTCCAGGATAATAG
CAATAACGTACCAACTGCCAAAGACAAACTTTCCAGTAGTCCCAAGAAGATCCCCAAC
GGCGACGGCAAGACTGAAAACATGCCATCGCCGCCATCCTCGCCGAGCATAGTAGGTG
AACAGGAGGTCGGCGTGGAGGCGCTCATGGAACGTATGAAGCGGCTTTCCGAGAAGT
CGGAGTCGTACCAGATCACGCAAGAGGAATTGTCCAAGAGATTCGAGACAGTGGAGA
CCCAGTCCGCCGAGTTGGCAGCCGCGCCCCAGAGGAGTAGTTCGGTCCTCGACTCTGA
CATCGGTCATTTCATCGAAGATCCTACGTTCATCTATCAGGATTTCGCCAAG

>novel_circ_000602
GCTGCTGGTAGACATCAATGCAGCTATTTAATAAACCTGCAGAAAGGAGAGTTCATCCT
CCAAGGCGGTGATCCATCTTGGCTTCGTGGTTTGAAGTCGATCCCTGGAAAGCTACAA
GATCTCTATGAGATTAACAAGATTTTAGCTCATAGGCCGTGGTTGCTGAACAAAACGCA
CATAGAGAAACTGACCAAAGGCGCGGACAGTTGGTCCTTGGCCGAGGTCGTTCACGC
GATAGTTCTTCTGGCCCACTTCCACTCCCTTTCGTCTTTCGTCTTTTCCTGCGGTATTAA
CGAAGAGCTGGACAACGTAGCCGGTCACCATTACAAAGAGAACGTCCAGGATAATAG
CAATAACGTACCAACTGCCAAAGACAAACTTTCCAGTAGTCCCAAGAAGATCCCCAAC
GGCGACGGCAAGACTG

>novel_circ_000604
ACTCATATCCTGTTGATGGATGCGTTCCTCCAGAACAACAGGCTGGATCATGTCTCCAG
GGTGATGGCCTCGCATCCCTCGTACTTGGAGCATTTTCTCCGCACCCAACATTTCATCCT
GAGGGGTGACGGACCACTTCCTTACGACTACAGGCATCTCATCGCCATTATGGTGAGTA
GAACGCTGCTTTTTCTCTTCTCCTCACCTAATTCGACGTAATGGTGGCAACTTTTTTTCT
TCTTCTTTTCTTCTAATCCGGCGATACGATAACACCATCTGGGAGAGACTGGTGGAAAA
GCATTTCTCATACCGACTTGAAGATTTTATCCTGTTTTCTTTTTTTTCTTCCAAAAGGA
AGGAAGG

>novel_circ_000605
GTGCAGCAGCCAATCTACGCAAGGAGGTTATCAGAAACAAGATCAGAGCCATTGGCA
AAATGGCGCGCGTCTTCTCCGTCCTGAGAGAGGAAAGCGAGAGTGTGTTACAATTGAA
GGGCCTCACGCCCACTGGAGCTTTGCCTCTTGGTGCGTTATCGGGTGGCAAAACGTCG
CTAAAAAACGCTCTCCAAGGTTTCTCGCCGAACCACAAAATCACTTCGTTCGCCGAGG
CGAAGGGTCTGGACGCTATAAACGAAAGGATGCCGCCAAGAAAGGACGCCCCACCCA
CGCCAGTGACCGAAGAGAAGCCTGTGATAAAGCCGCCGGCTTTGGCAGAGAAGCGAG
AGCCATGCACACCGCAACCGCAATCGTGAGCCTCACACTCGTCCAATCAAG

>novel_circ_000606
TTACGCGGCGTGCTGCGATTTCCTGCAGAACAACAACTTGCTGAGCATCATTAGGGCG
CACGAGGCACAGGACGCGGGTTATCGCATGTACCGGAAATCTCAGACGACGGGCTTCC
CATCGCTAATCACCATCTTCAGTGCGCCCAACTACCTCGACGTTTATAACAACAAGGCG
GCAGTGCTGAAGTACGAGAACAACGTGATGAACATCAGGCAATTCAACTGTTCACCTC
ATCCTTATTGGTTGCCCAACTTCATGGACGTGTTCACGTGGTCCCTGCCGTTTGTAGGT
GAAAAAGTGACAGAGATGCTGGTGAACGTGCTCAACATATGCTCGGACGACGAGCTG
ATGAGCGACGGAGACGACGGGCTCGAAGAAG

>novel_circ_000607
TGCGTATTGTACCTGTGGGCACTTAAGCTGTGCCATCCAACCACACTGTTCCTCCTCAG
AGGCAATCACGAGTGCCGTCATCTCACGGAATACTTCACATTTAAGCAAGAATGTAAAA
TAAAATACTCGGAGAGGGTGTACGACGCTTGCATGGACGCGTTCGACTGTCTGCCGCT
GGCGGCTCTCATGAACCAACAGTTCCTCTGCGTGCACGGTGGACTCTCGCCGGAAATT
CACAATCTAGAAGACATACGCAAA

>novel_circ_000608
TGTGCGGCGACATTCACGGACAGTTCTACGACCTGATGAAGTTATTCGAAGTGGGCGG
ATCACCCTCCACGACAAAGTATCTCTTCCTCGGCGATTATGTAGACAGGGGTTATTTCA
GTATCGAGTGCGTATTGTACCTGTGGGCACTTAAGCTGTGCCATCCAACCACACTGTTC
CTCCTCAGAGGCAATCACGAGTGCCGTCATCTCACGGAATACTTCACATTTAAGCAAG
AATGTAAAATAAAATACTCGGAGAGGGTGTACGACGCTTGCATGGACGCGTTCGACTG
TCTGCCGCTGGCGGCTCTCATGAACCAACAGTTCCTCTGCGTGCACGGTGGACTCTCG
CCGGAAATTCACAATCTAGAAGACATACGCAAA

>novel_circ_000609
GGTGTTGAAACCCCCTGGTGGCGGAAGCAGCGACATTTTCGGCGCGGCGCCCGAAGA
GACTAGCCCCCGCCGCGTGAAGAACCACAACCAATCGCAGCTGGGCTCCGCTTTGTTC
GGCGATACAGCACCGAACAACAGCAACAGCAACGAAACGCCGCGCAACAAGCCCGG
TAATGATTCATACAATCGCCTGTTTGGCCCTCCCGATGCCCCACCCACCACCCCAAACG
CGAAAAATCACATGCGCAGCAATATCTCCCTCAGCGGGGAGTCGATGTCCTCGTCGGT
GTCGTCGGGCGCCGCAACGTCGCCCGCGAAGAGCACCGGAAGCTCGGACTCGATCGC
GGGCGTTCTCAACGGATACGCGGCCAGCAATGGCAACAATATGACCAACGATGTCACA
G

>novel_circ_000611
GTGGTGTAGCTCTGTGTTTAAGTCCTATGGGAAGACGTAACGGAGAAGCATTAGTACG
GTTTGTTAATAAAGAACACAGAGACATGGCATTAAAAAGGCATAAACATCATATGGGTG
GAAGATATATAGAAGTATATAAGGCATCTGGAGAGGATTTTGTTGGTGTAGCTGGTGGA
ACTA

>novel_circ_000612
AAATTGCAAAAGTACGAGCAAGTCTTTTTCAGATCAGTGGCGTCAAAAAAGAACCACT
GGTCTTGCCAGAACCAGAGGGTGAAGTCACAACACTTATGGAAAAAGTTTATGTACCT
GTCAAAGAACATCCAGATTTTAATTTTGTTGGAAGAATCCTTGGACCACGAGGAATGA
CAGCCAAACAATTGGAACAAGAAACAGGATGTAAAATTATGGTTCGGGGAAAAGGGT
CTATGAGGGATAAAAAGAAGGAAGAGTTAAACCGAGGTAAACCAAACTGGGAACATC
TGACAGATGAATTGCATGTTTTATTGACAGTCGAGGATACTGAAAATCGTGCCACTTTG
AAGCTTGCCAGAGCTGTAGAAGAAGTCAAGAAACTCCTTGTTCCCGTGCAGGCTGATG
GAGAGGATGAGCTAAAGAAACGGCAATTAATGGAACTTGCTATTATCAACGGTACTTAC
CGAGATTCTAATACAAAAGTTGCAGCAGCTACAGCTTGCGATGAAGAATGGAGACGTG
TTGCAGCGGCTGCGGCAGAAACGCAGCGACTTCTTCCAGGCTTAGCCACACCTATGAG
GACGCCCAGTGCTCCATTGGGAGCACCGTTAATACTCTCGCCACGGATATCTGTTCCAA
CAACCGCAGCGTCTCTTTTAAACGGTTCCGGCCCACCAGGATCGCTTCTTTCTGCCGGT
GATCCGCATGGTTTAATTTACACACCATATGCTGATTACGCCAACTACGCAGCCTTAGCA
GCATCGCCTCTTCTCACGGATTACACAGCAGCTGATCATTCTGGTGGGTTGTTTGCACG
CTGATCTTAGTTGCAACAACGTTTTTCATGAATATTTTCTTCGATTGTTGTTGTTATTATT
ATTATTATTATTATTATTATTATTATTATTATCATCATTATTATTATTGTGCAGCAGCCGCTGC
GAAACAACGTAGGCACTTGGGTCAGATTCGCGAGCACCCTTATCAAAGGGCTGGTGCT
TTGTCGTGAATCCGGAGAGCGCAATCGCAATACTGTACGCGCCTCCGCGAAAAG

>novel_circ_000613
AAATTGCAAAAGTACGAGCAAGTCTTTTTCAGATCAGTGGCGTCAAAAAAGAACCACT
GGTCTTGCCAGAACCAGAGGGTGAAGTCACAACACTTATGGAAAAAGTTTATGTACCT
GTCAAAGAACATCCAGATTTTAATTTTGTTGGAAGAATCCTTGGACCACGAGGAATGA
CAGCCAAACAATTGGAACAAGAAACAGGATGTAAAATTATGGTTCGGGGAAAAGGGT
CTATGAGGGATAAAAAGAAGGAAGAGTTAAACCGAGGTAAACCAAACTGGGAACATC
TGACAGATGAATTGCATGTTTTATTGACAGTCGAGGATACTGAAAATCGTGCCACTTTG
AAGCTTGCCAGAGCTGTAGAAGAAGTCAAGAAACTCCTTGTTCCCGTGCAGGCTGATG

GAGAGGATGAGCTAAAGAAACGGCAATTAATGGAACTTGCTATTATCAACGGTACTTAC
CGAGATTCTAATACAAAAGTTGCAGCAGCTACAGCTTGCGATGAAGAATGGAGACGTG
TTGCAGCGGCTGCGGCAGAAACGCAGCGACTTCTTCCAGGCTTAGCCACACCTATGAG
GACGCCCAGTGCTCCATTGGGAGCACCGTTAATACTCTCGCCACGGATATCTGTTCCAA
CAACCGCAGCGTCTCTTTTAAACGGTTCCGGCCCACCAGGATCGCTTCTTTCTGCCGGT
GATCCGCATGGTTTAATTTACACACCATATGCTGATTACGCCAACTACGCAGCCTTAGCA
GCATCGCCTCTTCTCACGGATTACACAGCAGCTGATCATTCTG

>novel_circ_000614
CAAGAAAAACAAAAATGAAAAAAAAATCAGAGGAAGTGAAAAAGGAGGAATAGGTA
AAAGAAAAGAAGGAAAGTTAACGTTTCTCTTATGAATATAAAATTACTTACGCGATTTG
ACTGTGCACATCGGTGTGATTTCTTTTCTCTTTTTATGTGAAAATAACCGTTAACTTTGA
CCTGACAACTGCGATTATGTTTTACTCACCGGT

>novel_circ_000615
ATGTTTTTCAGATCAGGTACCCTGGAGCGACTCGAGGCCCAGAGAGACGAGAAGCTA
ACTGGACACATTATTCTTCTTCAAGCAAGGTGTAGAGGTTATCTGGCACGTCGTAAACT
CAACACTTTGAAACTGCAAGATCTTGCAGTTAGATGCATACAAAGGAATGTGAGGAAG
TGGATGTCCGTAAGAGAATGGCCCTGGTGGAGATTATACGTGAAAGTTGCACCTTTATT
GAACGTCCATCGAACAGAGGATCAATTGAAGGCAAAGACGGAGGAACTTGAGATTCT
CAAAACGAAAGTGGAACGATTGGAGCAGGAACGGAATCATCTGAAACACGACAATGA
TAGATTGGAAGCTAAG

>novel_circ_000616
GACGCAAGATTAGAAATCGAATTAAAGGAAGAAAGACTACGGACATTAAGTCAGGAA
CTTGAAGAATTAACATTTGGCGGGAAAACGGAAGAGGAAGTAGCACAGTTGAAAAAA
GCGAAGCACGAATTAGAGAAAAGAGTAAAGGATCAAGAAGAAGAGTTGGATGATCTC
GCTGGTCAAGTGCAGCTTCTTGAGCAAGCGAAACTGAGGCTAGAGATGAGTATCGAG
CAACAGCGTAAAGAAATACGCAAAGAGATGCAACAAAGAGACGAGGAACTGGAAGA
TGTGCGAGGTAACGCGTTGAAAAAAGTGAAAGCTTTGGAATCACAGCTGGAAAACGA
ACATGAGGAGAGAACGATATTACTTCGAGAGAAACACGAGTTGGAACGTCGTTTGGTT
GCTATTGAGGAACAGGATCGTGCTGAACGTGCCGCAGAAGCTGAAACTATGCACAGAT
TGAAGAGAGACTTGAAAAGAACCAAAGCTTTGCTAAGAGATGCTCAAACAATGCTGG
AGAGATCAAAAGGCGATTCGACTGGTAAGGCAGCTCTACGACAATTGAAAAATCAATT
GGAGGATGCAGAATGTGCTAGAGCAACTGCTGTCAAAGCGAAACAAGCATTGGAACA
AGAACTAAATGAGACACAGGCTTCGCTAGAAGAAGCTCAACGACAACGCTCTGAAGC
GGAAGAACGGGCAAATATAGCTAGTCGTGAACGAACTGAGCTATTATCACAATTGGAA
GAAAACGAAGAAGAGTTAGCTGAAGTTTTAAAGAAATATCGGGCAGCAGTGCAGCAA
GTATCGGCAGAACAGGGTCAATTGCAAGAAGCACAAGTACAGATTGCTGCATTAGAAG
CGGAAAAGTCTGCGTTAAAGGATCAATTATCAGAATTGACACAACGACTCGAATCCGT
TGAACAACTCGGTGATCCAACTGCCAACAGCCTTGCTACACGGCGACTCGAGTTCCGT
GCCAAGGAACTCGAAAGTAAGCTAGAATTGGAACAAACCACGAGAGCACGTTTAGAA
ACTCAAATAGCGAGATTGAAAGAAAGTGTGGAGAAATTACAAACTGAATGTGCACTAC
TTCGTACGAAAGAACAGAGTGCCCAAGATACTTCCAGAAGATTGCAAAGATCATTACG

GGAAGCAAGAGAAGAGGCCAGTTCAGCTCTAGCGCGTGAACAAGAATCTACCCGTGC
TCGTCGCGAATTGGAAAAATCTCTTGAGGCTGCTGAAGCAGAAACTAAAGTCGCCAG
AGACGATCTTAGATTGGCGCTTCAAAGAATCGATGATCTGCAAAGCGCTATTCAAGGTG
AACTCGATTTGGATTGCAGCGAGGAAGGAACAACGGAAAATAGTGATAG

>novel_circ_000617
GCGAGCAAGAATTACGGGTGGGCGAGCAGCGGGTGCAGCATCCTGTCCGAAATTGGC
ACGATGCACCTCGAATTCACCTATTTAAGCGACATCACCGGGAATCCGGTTTTCAAGAG
CAAGGTCGAGAACGTGAGGAAGGTGCTGAAGAATCTGGAGAAACCGAAGGGGCTGTA
TCCAAACTATATTCACCCGAAGACGGGGAAATGGGGCCAACATCACATGTCGCTGGGA
GGGCTTGGCGACAGTTTCTACGAGTATCTGCTGAAAGCGTGGATCCAATCCGGCAAGG
AGGACGTCGAGGCCCGGCAAATGTACGACGAAGCGATCGTGGCCATAGATCAACACAT
GATCAAGACTTCGCAGGGCAAGCTCCTCTACGTGTCAGACTTGAAATACGACAGGCTG
GAGCACAAAATGGGTCATCTGGCGTGTTTCGCCGGTGGTATGTTCGCGTTAGGCGCGA
AAACATTGCAAAACGAATTGTCCGATAGATACATGACGATAGCCGCCGGTCTCACCAA
CACGTGCCACGAGTCGTACGATCGAAGTTACACGAAACTCGGCCCCGAAGCCTTCCAC
TTCATCGAGGGGAACGAAGCGAAGAGCTTGAAGAACGGCGAGAAATACTACATCCTT
CGGCCGGAGACCTTCGAGTCGTACTTCGTGATGTGGCGGCTGACAAAGGATCCCAAGT
ACCGTGAATGGGGATGGGAGGCCGTTCAAGCTCTCGAGAAATACTGCCGTGTTCCAGG
GGGGTTCACGGGGCTCCACAATGTTTACCTGGTCGATCCGCCACAGGACGACGTCCAG
CAGAGCTACTTTTTCGCCGAGACGCTCAAG

>novel_circ_000619
AACTCGGAGATATCCCTCTTCGAGACGAACATTCGTTTCATGGGGAGCTTGTTGGCCTG
TTACGCTCTCACGGGGGACGTGATGTTCCGGGATAAGGCGGCTCAGCTCGGCGAGCGG
ATGTTGCCCGCTTTCCAGACGGAAACCGGAATTCCTCATTCCCTCATCAATCTGCACAC
CGGGGCGAGCAAGAATTACGGGTGGGCGAGCAGCGGGTGCAGCATCCTGTCCGAAAT
TGGCACGATGCACCTCGAATTCACCTATTTAAGCGACATCACCGGGAATCCGGTTTTCA
AGAGCAAGGTCGAGAACGTGAGGAAGGTGCTGAAGAATCTGGAGAAACCGAAGGGG
CTGTATCCAAACTATATTCACCCGAAGACGGGGAAATGGGGCCAACATCACATGTCGCT
GGGAGGGCTTGGCGACAGTTTCTACGAGTATCTGCTGAAAGCGTGGATCCAATCCGGC
AAGGAGGACGTCGAGGCCCGGCAAATGTACGACGAAGCGATCGTGGCCATAGATCAA
CACATGATCAAGACTTCGCAGGGCAAGCTCCTCTACGTGTCAGACTTGAAATACGACA
GGCTGGAGCACAAAATGGGTCATCTGGCGTGTTTCGCCG

>novel_circ_000620
AACTCGGAGATATCCCTCTTCGAGACGAACATTCGTTTCATGGGGAGCTTGTTGGCCTG
TTACGCTCTCACGGGGGACGTGATGTTCCGGGATAAGGCGGCTCAGCTCGGCGAGCGG
ATGTTGCCCGCTTTCCAGACGGAAACCGGAATTCCTCATTCCCTCATCAATCTGCACAC
CGGGGCGAGCAAGAATTACGGGTGGGCGAGCAGCGGGTGCAGCATCCTGTCCGAAAT
TGGCACGATGCACCTCGAATTCACCTATTTAAGCGACATCACCGGGAATCCGGTTTTCA
AGAGCAAGGTCGAGAACGTGAGGAAGGTGCTGAAGAATCTGGAGAAACCGAAGGGG
CTGTATCCAAACTATATTCACCCGAAGACGGGGAAATGGGGCCAAC

>novel_circ_000621
CTGACGAGAATTCCGAGTCGATGAGATCGAAGCAAAGGACGCGAGCTGAAAACGCG
CGGCCGCCGTCGTTGAAGGCTACTTAAAACGACCATTGAGCACTCGTCGGGGTGCTCG
AATTACCTACAACAGGTGGCCCGCAACTCCGCCAATACGAGCGAAACATCTGCAACTC
CGGGCGGCTTGTGGGAGTGCAGCAAGTGCCTGGACGAGATGCGGCTGATGCAGAGGT
TGGCGATCAGCGGACTCCTGTCCGTGATACTGATCGTCCTTCTCACCGGCACGTTCGTC
ACGCGTCGTGATCTCGCGAACGTGGTGGAGCGTGGAGGGGCGCCCGAGGAGCGCAGC
AGCGAGCAGCAAGCGAACCCCGCATGGGTGCAGAGTAACGCGATCCCGGGCCAGGAC
GAGGCGAAACCCCTCGAGGATAAGGATCGATGGGTGGACCCGAGGCAGAGACAGTCG
GAGCCGTCCGCGGTCGGACCACCGCCGGTCCCCGTCCCCGGGCCCTACGTCATTAAAC
CGCCTAATCCACCCCTGGATGACGTGACTAATCAGCGCCGCGAGAAGATCAAAGAGAT
GATGAAGCATGGCTGGGACAACTACGTGAGATACGCCTGGGGGAAGAACGAGCTGAG
ACCGATCTCGAAGAGGGGTCACAGCGCGAGCATCTTCGGCGCGTCGAACATGGGGGC
GACCATCGTAGATGGCCTCGACACTCTGTACATCATGGGCCTTCACGACGAATTCAAGC
AAGGCAGGGACTGGATAGCCGAGAACCTCGACTTCGATATC

>novel_circ_000622
CTGACGAGAATTCCGAGTCGATGAGATCGAAGCAAAGGACGCGAGCTGAAAACGCG
CGGCCGCCGTCGTTGAAGGCTACTTAAAACGACCATTGAGCACTCGTCGGGGTGCTCG
AATTACCTACAACAGGTGGCCCGCAACTCCGCCAATACGAGCGAAACATCTGCAACTC
CGGGCGGCTTGTGGGAGTGCAGCAAGTGCCTGGACGAGATGCGGCTGATGCAGAGGT
TGGCGATCAGCGGACTCCTGTCCGTGATACTGATCGTCCTTCTCACCGGCACGTTCGTC
ACGCGTCGTGATCTCGCGAACGTGGTGGAGCGTGGAGGGGCGCCCGAGGAGCGCAGC
AGCGAGCAGCAAGCGAACCCCGCATGGGTGCAGAGTAACGCGATCCCGGGCCAGGAC
GAGGCGAAACCCCTCGAGGATAAGGATCGATGGGTGGACCCGAGGCAGAGACAGTCG
GAGCCGTCCGCGGTCGGACCACCGCCGGTCCCCGTCCCCGGGCCCTACGTCATTAAAC
CGCCTAATCCACCCCTGGATGACGTGACTAATCAGCGCCGCGAGAAGATCAAAGAG

>novel_circ_000624
GTGAGTCTTTGTGACCGAAGTTCAAAGCTGTTCGTGCGCCACGATTGCCGGATTGTAA
ATCGATTAAAAAATTGCGATGAACGATCGTTATAATCGATTGAAGTTAAAAGAAAAGAA
AAAAAAAAGAAAGAAAGAAAAAAGAAAAAAAGACGAAGCAGAGGGTCTTGTTACAT
ATAATTGCGAGGATGTAAAG

>novel_circ_000625
CTTACAAGAGAAGATAAGTTACCTAAGAAGATATGCGATGATTGTGTGTATAAAGTAGA
ATTATTTTATCAATTTTGGAATACAACAGCAAACGCAGAAAAACAACTTTTGCAATGGC
TAGGAGAAGTCAGTTTAGAAGACAAACAGGGTTATGTTACCAATGTTCTCAATCCGAG
TGTAATGAAACAAGAACAGAGTACAGAGAACAGGTTAGATGGCAATGTGATGCAGCA
AGTAGAGGAGCATCAAAACAATATGGGGATGGGTATGATGGATAACATGGGTTTGGGTA
TTCCCATGATAATATCCAGTGCTAATCAACAACAAATCACCTCAGTTCCTATGGACACC
AGCAGCAATTCTGTACAAACTATTCAGGCAGTACCTGGTCCAAGTTCACAAACTACAC
ATAACCAAATACCACAGAATCAAACAAGCTCTACGCAACAAGAAGATGAAGAGGAAA
GTAGTGAAGATGAAGAAAACTCTGACGAAGAATGTGATGGAGATGAAGGCCTACCTGT

AAAAGAAGAAAGTGAAGAAGATCCCAGCAATAGAACCATAGAGCCTACTACTTTTGTA
AATGTTTCCTTGGCATGTGACGAAGCAGGTCCTTCAGGACTGCAGCAACAGAAGATCT
CAGATATGCCTGAAATGCCAATTCCACAACCAACTGATGGGGATCCCAAATCTGG

>novel_circ_000626
GTACAATGCGCTAGACCCACTCGCTCCAACTTTTCTGATAGTAAAGCTAGAAGATAATT
ACTTTCGGATACCAACGTCGCTGAGCGTGTTCATGATGAAGGAACCGAATCCGAACGG
AGAGGCGAACGCCCAATCGTCGTCGAAAGGCGAAATCGTGGAATCGGGGAAAACGAA
GAACAACAACAACCAATCGGAAGAGAAAACGAAGGATAACGCGAGCACGGACGACC
CGTTCGACGATCTCGACGACGCGCCGTTGGACTTTCGAAAGAGGCAGGAACAGGAGA
AGAGCAAATCGAGCAAACAAGTTCCAAAGAAGAAGGTGCACACATGCGAGATCTGTT
ACGCAACGTTCGACCGTAAGAGCAAGCACACCAGGCACATGTTCAAGCACAGCAACT
CGAGGCCGCACAAGTGCGCCGTTTGCTCGAAAGGGTTCAAAACGACGGCTCACCTGT
CCAGGCACATGGAGGTACACGACGAGCCTGTCAATTTGCACGCGTGCAGCCTGTGCGA
CTTCAAAGCGCGCACCAAACCCTATCTCAAGATCCATTACATCAGAAAGCACACGGAG
GATTACAATTACAAGTGCGAGCAATGCGGGAAGATGTTCAAGGTGCAGTCGGATTACA
CGACCCACGTGAAGGACCACGACACGGAGTCTTGCGTGTGCGACATATGCGGCTCCTC
TTATCCGAGCAAAAGCTCCCTCTACTTCCACAAACATTACAAGCACAAGACGAAGGTG
AAGAAGTTTCAATGCCAGACCTGCAAGAAGAAGTTCAAGACGCAGAAGAATCTCGAC
AGTCACATGGAACTGCACAAGATCAAGTACGTTTGCGAGCAATGCGGGATGGAGTTCA
AGAGCAAATACGGCCTCACCAAGCATCTGAGGACTCATTCAGGGGAGAAATCTTATTT
GTGCGCGATTTGCGGCAAGACATTCGGGTGTCTGAGCTCGCAGAAGATCCATCTGTTG
ACGCACGTCGGCGAACGACCCTACGTTTGCGACATATGCGGGCAGAGCTTCACGCAGA
GGTCGCCCATGATGCTGCATCGCAGGAAGCATCCGGGGGTGCATCCGCCTCCCCCACC
GATCAAAATAACGAACCTGCTTCACGGTGTACAGGACAAAATAATCGTGAACAAGAGC
GGCAA

>novel_circ_000627
AATTCAACAGGAAAATCATGGAAGATATTGCAAAGCGGGGAAATGTTCTCGATTAATAA
ATCGTCTGTGAACGGGAATCGTAGTTGTGTTTTTAAATTTAATTGTTGAAAATAGTGGG
GAGAGAGAGAGAAGAAAAAAATGAGAATAAAAGAATGAACAATTTTGCGAAGACTCT
GACGAGGGATTCGGCCTTTTAAATGGCCGAGGAGAGATCGTACGGCCTTTGAGGAGGA
TCGTAGGACCATTTTTCGGTGGCGATGTTCAGGAAGCGGTCCTTCAACTGGACACAGG
AGAAAAGGATCGCAGAGTAGTCTACGGATAGGCTACTATGACGGACTATTTGGATCCAC
ATTTCGTCAGGGCTCTCTGCCGCGATCCAGAACGACGAACGCTTCAAGATCTACAGAT
CATATATTATGGTCTGCTTGGTCTGGAAGCGTTACGTCCGTGCAGAGATTCCATTCTCCG
GGGGCTTTGCAAAATTGTGCGATATGAAAGGCATCATGCGAATCATGTTCTTTACTACA
CCGGTGAATTGGCCACAAGCTGGTACATATTACTCTCCGGCTCAGTGTTCATCGATGGA
TCGATGTTTCTTCCTCGTTCAAG

>novel_circ_000628
AGCTTGCCACAAACACCAGCTGGCGGTGTGGGTGGAAAAGGAAGCGGAAGTGCTGG
CACCGGATCTGGTATGGTCAATGGTTCCGGTACCGATCATCAAGATATTCGTGACGGTG
CTGCTATAAACACAGAACATGCCTTGGTATATCGGGACGGGAATCTGGTGTCGGGCTCG

TTGGAAGCCCTGGTGCAGCATATGGTGCCCACCGAGGAATATTATCCCGATCGAGCTTA
CCTCTTCGCCTTTTTGCTGAGCGCCAGGCTCTTCATCAAGCCGCACGAGCTTCTGGGC
GAGGTCTGCGCCCTCTGCGAGCATCAGCAAAACCTGAATGGAGAGGGTGGCAAGGAA
CGTCTGCAACGTTTCGTGCCGCGACTGGTGCAACTGCTCGCCGAATGGACGGAAACAT
TCCCGTACGATTTCCGGGACGAGAGGGTGATGAGCCACGTCAGGTCCATCACGCAGAA
GGTCGCTGCGGTCGACGCTGCGGCCAGGCAGGAAGTTTCGGCATTGCTGCAAAACTT
GCTCCTCAGACTGACGGCCCTCGAGAGGTACGAGGAGGGTCTGGCCAGGCTGGCGAC
CGAGGCAACGACGGAGCAACTCGCCCAG

>novel_circ_000629
GTGGACATCACCGAGCTGTGCCCATCGGCCACGGTCCTCGCCCAGCAATTGACCCACG
TGGAACTCGAGAGGCTCTCGTACATCGGCCCCGAAGAATTCGTCCAAGCTTTCGCCAA
GGAATCCCCTCATCTGGAAACATCGTTTAAGGACATGAAGAAAACGCGCAATCTCGAA
TCGTACATCCAATGGTTTAACAGACTGAGCTATTTCGTCGCGACTGAAGTATGCAAGCA
TCCAAAAAAGAAACAACGAGTCCGGGTGATTGAATATTGGATCGAAACAGCCCGTGA
GTGCTTCAACATCGGCAATTTCAACTCCCTGATGGCGATCATCGCTGGTTTGAACATAT
CACCGATATCTCGTCTGAAGAAAACG

>novel_circ_000630
ATCCTGTTAACAAGGATGACAAATTCTATGAAGGAAAATTTAGACCTAATATTTATCAAA
GTACAGCTGATAGACAAGCAGATTCTAAGTTAGATACGTGGGCATCGAAAACGAACAT
TTTTCAGCATTCTAAAGGCCAATTTGGATCAGGAGTAAAAAATACAGCATTTAATTCAA
CGAAGATTAATCCAAATGGTATAAACGCGAATATAAATGGTACAAGAGGTCATTTCATC
ACAAAAGAAATCGAAGTGAATCAAGGCCACTCGAAAATGAGAACTTACAGACCAAAC
GATCTCCCAGGTCTGAATGAATTCAACAAAAGTGATAGAAATATCTATGTCGATTCCCG
AACAAACGTCGATAACATACCACTTACGGTTCTTCAACCACCACCCATTACTTCTGATA
ATCATCCCTCTGGATCGAACAATATCCTACGCAATAATTCTTTTGGACATCGAACTCACA
GGTATCATTGGACATACAACTCTTCCGCGAATTCTGGAAATTTGTCACCCTCTGGTAATT
ATAACAATCGTGTACTCGTGCCGTCACGGGAACTTCAACCACCATATCCATCCCTTAAC
AAGTCTCTGGTCCCTCCATTTCTATCAAATTTCTTAAAGTATTCTCCAATACCATCCCCC
GATTTGCAACCACCAGATGACCACCAGTCTACGAATAAATTTTCTATCGGTTGGCAATT
ACCAAATGACCCAATCGAACGCGAGTTGCCAACTTACGAGAATCGAAACACTTTTCAA
CCATCGAGTCAATTGGTTCCTCCGATTTTCGAATTGAACGTAAGTGGCCGATATCCAAA
CGTTCCATCGACAAATCTCGAACCACCTGACCAAGAATTCGATAGTTTCTTCAACGATT
CCGTCGTCAATAACGCGACACGAGAGATTCCGTCTTTGACAATTCCACCAAGGAAAAG
ATTCGCCAACACCACCGAGAGTTCCGAAAACAGTGAAATTTCTACGACCGATCCAAAC
GTGCCCACCGTGTGGATAGATTACGAAAACTCTTCGACGATCGAGCATCCGGATATTGT
CGAGCCTGCTTCCGAGGAATCTGAAGCCGTAGAATCCGTGGACGTGTTGAGCGACAA
GAAAGAGTGGAAGCCTGTACTGGTTTTTAAAAATGTGACTGAAACGACCACGAAGGC
TTCTGTTATCATGAAGATCGATAAGAAGAACGTGGATGTAGATCTTTTCAATGTAGACG
CTGCCCCTTTCGCGAAAG

>novel_circ_000631
GCGAGGAAGTCGTAACCGTGGCTCCGGCGAGCGGAGGTCAGGACAGTTGGAAAGTGC

AGAGGCTCTCGTTGGGAGGCGGGGAATCGGCGAGCAATCGTCGTAAAGGCTGCCGTC
CCCAATTACAGGCGGGGAGCAAGCGAGCAAGGCCGCCACCGCCGATACCCGGCTTGT
CGTGCAAGGTTAATGCCAAGGACGTGGACGGGTGGTACCCGTTCGACCACTGGCCGA
ACAAGGAGAACACGCCCGGAAATGGCGTGAACAACGGGGAGGGAACGAACGACGAC
GAGGCGAAGCTCGAGAAGAAACTCGGCTCGGACATGGGCTCGAATCTGAGCCGGCAC
AACGGCAAGGGCCTTACGGGGCGGAGGACCCAGTCGTCCGGCAATTTGTGCGACCCG
AAAGGGAAGTTGAACAGCATGGGGAAGATATTGGTCCCGTGTTCCAGCGCGCAGAGG
GAGAG

>novel_circ_000632
GCGAGGAAGTCGTAACCGTGGCTCCGGCGAGCGGAGGTCAGGACAGTTGGAAAGTGC
AGAGGCTCTCGTTGGGAGGCGGGGAATCGGCGAGCAATCGTCGTAAAGGCTGCCGTC
CCCAATTACAGGCGGGGAGCAAGCGAGCAAGGCCGCCACCGCCGATACCCGGCTTGT
CGTGCAAGGTTAATGCCAAGGACGTGGACGGGTGGTACCCGTTCGACCACTGGCCGA
ACAAGGAGAACACGCCCGGAAATGGCGTGAACAACGGGGAGGGAACGAACGACGAC
GAGGCGAAGCTCGAGAAGAAACTCGGCTCGGACATGGGCTCGAATCTGAGCCGGCAC
AACGGCAAGGGCCTTACGGGGCGGAGGACCCAGTCGTCCGGCAATTTGTGCGACCCG
AAAGGGAAGTTGAACAGCATGGGGAAGATATTGGTCCCGTGTTCCAGCGCGCAGAGG
GAGAGATACAAATTCTGTGGCTCGTTGCCGAATCATCTGGACGACAAGGACGCGTTGG
ACGACGATCCGAAGGAAAATAATAACGTAATGACGGATACGATCGGTGGGAATCTTGG
TGGGACATTGCCGCACAAGAAACGATCGTTGGGCGTGCATTTCTCGGACGGAGGACC
AACCGGGACTCTGGACCTCGTGAAACATCGATCCCAAGATTCTTTGGACGAGAACGAT
CCTTGGAGGTATCAGCAGAGCGATCTCGATCTGGTGCGATGCCGTCACGGTAATTTCGA
CTCGGTGAGAAGAAATCGGAACGCCGAGACGTTGTCTGGCGATTCGGCGAGAAGGTA
TACACGAGGCACGTCCCTTGAGGACAGATGTCACCGAAATTCGGAACTGGATCTGGTG
GGCACGCTGCCGAAACGGAAGCAGGACAGCAGGAACGGCGGCGGGAAGAGCGGGG
CGAGCTTGGAAACGAACTCTTCGTCGTCCCAACCGTGCGTCCCAGACGCGGCCGACA
ACGATCTTCTGATGCAGATCGTGCACAAGCCCGATTGCGAGTTGGTCAGGCATCGGCA
ACAGCTCGGCAAGTGCATCGATTTGAAGTTGAGCAAACTGGCTGGCGGGGAACCGCA
GGATCAAGGTTACGCCTCGGAACGGTCCCCGGAGGACGAGCATCCTCCCTCTTTGCCG
GGCCAACCCTTCCCGAATATCACGGCCG

>novel_circ_000633
ATACAAATTCTGTGGCTCGTTGCCGAATCATCTGGACGACAAGGACGCGTTGGACGAC
GATCCGAAGGAAAATAATAACGTAATGACGGATACGATCGGTGGGAATCTTGGTGGGA
CATTGCCGCACAAGAAACGATCGTTGGGCGTGCATTTCTCGGACGGAGGACCAACCG
GGACTCTGGACCTCGTGAAACATCGATCCCAAGATTCTTTGGACGAGAACGATCCTTG
GAGGTATCAGCAGAGCGATCTCGATCTGGTGCGATGCCGTCACGGTAATTTCGACTCG
GTGAGAAGAAATCGGAACGCCGAGACGTTGTCTGGCGATTCGGCGAGAAGGTATACA
CGAGGCACGTCCCTTGAGGACAGATGTCACCGAAATTCGGAACTGGATCTGGTGGGC
ACGCTGCCGAAACGGAAGCAGGACAGCAGGAACGGCGGCGGGAAGAGCGGGGCGA
GCTTGGAAACGAACTCTTCGTCGTCCCAACCGTGCGTCCCAGACGCGGCCGACAACG
ATCTTCTGATGCAGATCGTGCACAAGCCCGATTGCGAGTTGGTCAGGCATCGGCAACA
GCTCGGCAAGTGCATCGATTTGAAGTTGAGCAAACTGGCTGGCGGGGAACCGCAGGA

TCAAGGTTACGCCTCGGAACGGTCCCCGGAGGACGAGCATCCTCCCTCTTTGCCGGGC
CAACCCTTCCCGAATATCACGGCCG

>novel_circ_000634
TATGGGCACAGGGCGACTTCCGGGACGAGGTGACCGGGGGTTTGGCAAGGCTCGATT
TGACGGGTCACGGAGCTCACACTCCCGGTGGAAGCAACCCCAGCACACCGGGCGCGA
CACCCACCACACCCTCCGGTGGATTTTCTACCGCACAACCTAGGTCCCGCACGAACAC
GACACACAGGCCGGATATCCGAAAAGATGTTGCGACACCGACAGAGGTGAAACACGA
ATTGGGAGCCGGTGGAGGGGTTGTCTCCCCTGACGGTGTGGTTGGGGTAAATAACGCG
GACGATAAGGATGGAAAGAAGAACAAACGCCAGAGGCGGCAGAGGACGCATTTCAC
GTCCCAACAACTTCAAGAGCTCGAGGCTTTGTTCATGAGGAATCGGTATCCAGATATGT
CGACCAGGGAAGAAATAGGTCTGTTCACCAAACTTCCAGAACCTCGTGTCAGG

>novel_circ_000636
ATACACCATGGAGAAAAATGAAAAGGAGGAACAAGGCAATCCTATGAAAGAGTTCAAT
AGCAGAACGAAAATAGCCACCATTGAAATTGAAGGATACAACGAGAAGGACGATTTAT
ACAATCCCTTCGAAAATCGTGACAAAAAAAATTCCAATTCAGATTTTGGCGCCCTGGC
ACATCTATTGAAATCATCCCTCGGTACGGGAATCCTCGCCATGCCGAATGCGATCAAAA
ATGGCGGGGTGATATTCGGCGGGATAGGGACGATAATAATCGGCTTGATATGCGCGCAT
TGCGTCCACATATTGGTTCGCTCGTCCCACATTCTGTGCAAACGTACGAAAACACCCCA
GATGACGTATGCCGAGACGGCGGAAGCTGCCTTCCTTTGCGGTCCAAAAACCGTCAGA
CCTTTCGCTAATTTCAGTCGAATGTTCGTAAATGCGGCATTGTGCGCCACGTACATAGG
CGGTGCTTGCGTATACGTGGTTTTCGTATCGACCTCGATTAAACAGCTGGTGGACTTCC
ACACCGGCATGACTATACCGATGCGTTTGTACATACTCACGCTGATACCCGCTGTATTAT
TGTTGGGGCAAGTGCGAAATTTGAAGTTCATGGTGCCGTTCTCCATCGTGGCCAACCT
TTCCATGATGACCGGTTTCGCCTTGACTCTCTATTATATCTTCAATGATATCAAAATACCG
TCCCACGTGAAGCCAATCGCCTCGATCGAACAATTGCCTTCCTTCTTCGCCACCGTATT
ATTTGCCATCGAGGGTATCGGTGTTGTGATGCCCGTCGAGAACAGTATGAAGAATCCTC
ATCACTTCCTCGGTTGTCCAAGCGTTCTTAACATAACAATGACTATAGTGGTGTCTTTGT
ACACCGTGTTAGGCGTGTTCGGATATTTGAAGTACACGGAAGATATTAAGGGTAGTATC
ACGCTAAATATACCAACAGAGGACATATTGGGACAGGCGGTAAAATTGCTCATCGCCCT
GGCAGTTTTATTCACATACGGATTACAACTTTTCGTTCCGATGGATATCATGTGGAGAGC
TGTGAAAGAGAAATGCAGTCACAAGTATCAAGGCTTGTGCCACACAGTAATGAGAATA
TGCATAAGTATATTTACAA

>novel_circ_000637
CTGAATGGACACGGTGGCAAGGTACGACGCATGGATAGATAAAAATTTCTGACAACCA
GGAAACGTGCGGCATGGGTCTGCCGCGTCTCTCCCTGAAGGGATATTTTTTCTACGTGC
TATGCTCATCTGGCCTGTTGTTGGTTATATTGAATCTAGTGCAGGACGAGATATGGCACC
GTATGCGACCTCATCCTGGACGAACGCCACCCGTTCGCGTTTCCGACAGCGCAAAG

>novel_circ_000638
GTATTCAACTTAACTGGCACCACGACCTTATCACGAAACAGTTTAGAATCCTTGGATCC
ATCAAAGAGACCTTGGTACATGAAAGGAGGGAGCAGAAGGCCTTATCCTGCCATAAAA

TCTCATCGTACTGGTCGAAGATTGGCGCGTTTGTGGCCCGACGAAGACGCTTATGACG
ATCGTGTTACCAATCAGTTAATGTACGTGCCCACTGATTATAACAAAACCAGTGGCGAT
CGCCCGTTAAAGAAAATAATGGTTCCACATGGTATGCCAGAGGCAAAAGTTGGCCCCG
ATATATTTCTCCAGCATCGATGCCCAGTCAATACGTGTACAATCATCAGGGATAACGCCG
AGGAGGCCGATTTAATTTTATTTCAAGACTATATAACCCATGTGGGAAGAAGATCTTCG
AATCAACAGGTGTGGATGCTGTATTTTTTAGAGTGCCCTTATCACACGCAGAGCGTGAA
GAACGCTATCATCAATTGGACGGCAACTTATCGTCGTGACAGCGACATAGTCGCGCCCT
ATGAAAGGTGGCAATATTACGACCCTAGTATTACACAAATACCGCAGACTTTTAATTATG
CAGCCAACAAAACTAAAAAGGTAGCCTGGTTCGTTTCCAATTGCCATCCTCGGAATCA
ACGTATGCATTACGCTAGAGAATTGTCGAAATACATCCAAGTTGACATTTATGGGACCT
GCGGCACTTTAAGATGTCCACGTTCGCAGTCTCAAGCGTGTTTCGATATGCTCGACGAG
GATTACAAGTTTTATCTCGCGTTCGAGAATTCGAATTGCAAAGACTATATCACGGAAAA
GTTCTTCGTCAATGGTCTTGG

>novel_circ_000639
GTATTCAACTTAACTGGCACCACGACCTTATCACGAAACAGTTTAGAATCCTTGGATCC
ATCAAAGAGACCTTGGTACATGAAAGGAGGGAGCAGAAGGCCTTATCCTGCCATAAAA
TCTCATCGTACTGGTCGAAGATTGGCGCGTTTGTGGCCCGACGAAGACGCTTATGACG
ATCGTGTTACCAATCAGTTAATGTACGTGCCCACTGATTATAACAAAACCAGTGGCGAT
CGCCCGTTAAAGAAAATAATGGTTCCACATGGTATGCCAGAGGCAAAAGTTGGCCCCG
ATATATTTCTCCAGCATCGATGCCCAGTCAATACGTGTACAATCATCAGGGATAACGCCG
AGGAGGCCGATTTAATTTTATTTCAAGACTATATAACCCATGTGGGAAGAAGATCTTCG
AATCAACAGGTGTGGATGCTGTATTTTTTAGAGTGCCCTTATCACACGCAGAGCGTGAA
GAACGCTATCATCAATTGGACGGCAACTTATCGTCGTGACAGCGACATAGTCGCGCCCT
ATGAAAGGTGGCAATATTACGACCCTAGTATTACACAAATACCGCAGACTTTTAATTATG
CAGCCAACAAAACTAAAAAGGTAGCCTGGTTCGTTTCCAATTGCCATCCTCGGAATCA
ACGTATGCATTACGCTAGAGAATTGTCGAAATACATCCAAGTTGACATTTATGGGACCT
GCGGCACTTTAAGATGTCCACGTTCGCAGTCTCAAGCGTGTTTCGATATGCTCGACGAG
GATTACAAGTTTTATCTCGCGTTCGAGAATTCGAATTGCAAAGACTATATCACGGAAAA
GTTCTTCGTCAATGGTCTTGGACACAACGTTCTGCCAATTGTAATGGGTGCCCATCCAA
CGGACTATGCTCGAAGCGCGCCATACCGTTCTTACATCCACGTGGACGAGTTCGAATCG
CCGAAAGAGTTGGCCGAGTATCTTCATCGTTTGGATCGAGACGACGAGCTGTATAATTC
GTACTTTCGCTGGAAAG

>novel_circ_000640
GCTGTACGAAGCGAAGTGCAAAAAAGGCTTCCAAAGGAATGGTCCGAAGAAGAGATC
GCTCAGCTCACCGAACTTTGGGAACAATTAAAAAATGACGATGATCCCGTGGACCTGA
TCTTCAATGGGCTGACAATAAAGCGACCGAAGCCAAAGATCAAGGAGAAGCTTTTGG
AATTAGGATTGGCCACGGATCGCAAGGATCTTCGCAAAAAGCGATCGAGAAAAAGCA
ACCACG

>novel_circ_000642
ATCTATACGTCAGAGACGTTGAAGCAAGCCGAAGGTGTGGTGAGCACAGTGAGGTGC
GAGGGCAGGGAACATGCCCTCGGCCGCGGAATCAAGCGAAAGCTGGACTCCATCCAT

TCCATGCATTCTACCCTGCATGAAGACCAAGATGTAGCCGAGGCAAAGTCGGAGGAGA
AGAGCCAGAGGAAACTGGAGGTGGGTGAGCTAGTATGGGGCGCCGCAAGAGGAAGT
CCGGCGTGGCCGGGCAAGGTCGAGTCTTTGGGCCCACCGGGCACCATGACGGTGTGG
GTCCGTTGGTACGGGGGCGGGGGCGGTCGGAGCCAGGTCGAGGTCAAGGCTCTCAAG
TCCCTCTCCGAAGGCCTCGAGGCGCACCACCGTGCGCGAAAAAAGTTTAGGAA

>novel_circ_000643
GTGAAGATCCGATGCTCAGCGACCAACAGCAATATCGATACATGACTGTTGACGACAT
GAAATCTATGGGGGATGACTCGATGCGTGTCAACGTGACGGACGACGAGAGGGATAAC
CGGCATTCCGGAGCGCCCACGATAACCGAGATGATCACGAAGGAAACTTGTCAGCGTA
CAAATGCCGCCAATCTGAAACCGGACAATGTCGATATCAATAGGCACCCAGTACACGT
CGG

>novel_circ_000644
GTGAAGATCCGATGCTCAGCGACCAACAGCAATATCGATACATGACTGTTGACGACAT
GAAATCTATGGGGGATGACTCGATGCGTGTCAACGTGACGGACGACGAGAGGGATAAC
CGGCATTCCGGAGCGCCCACGATAACCGAGATGATCACGAAGGAAACTTGTCAGCGTA
CAAATGCCGCCAATCTGAAACCGGACAATGTCGATATCAATAGGCACCCAGTACACGT
CGGATTTTGTTCTGAGATCAGACCGTCCCTAGAGTCCTTAAACACATCGCTGGCAGCTC
TTGGTACCATGTCGAAAGGACAAGGAATGTGGAGGGACAGCTTCCTGGTTTCGTTTCT
GGTGGATGCACGAGGCGGTGCGATGCGCGGCTGCAGGCACAGTGGCGTGCGCGTGAT
CGTTCCTCCTAGGAAAGCAGCGATGCCTATGCGCGTCACGTGCAGATACCTGAGAAGG
GACAAATTGACGAACCCGCCGCCTTTGATGGAGGGGGAGGCTCTTGCGAGTCGTATCC
TGGAGTTGGGACCCGTGGGCGCCAAATTTTTGGGACCCGTGATCATAGAAGTACCACA
CTTTGCTTCGTTGCGAGGAAAAGAACGAGAGATAGTTATACTCCGATCAGATAATGGA
GAGACTTGGCGCGAGCACACCTTGGAAGCCAGCGAGGAGGCCGTCCAAGATGTGCTT
AATGAGAGCTTTGAAGGAGAAGAATTAAGCCAGCTCGAGGATCTCCAAACTTCTCGGA
TCGTGAGGATACTTACGGCAGACTTCCCACATTACTTCGCAGTAGTGTCCCGTATCAGG
CAAGAGGTTCACGCAGTCGGTCCAGAAGGTGGCACCGTTTCTTCGTCGGCTGTACCTC
AAGTCCAGGCCGTATTCCCGCCAGCGGCACTTACTAAAAGATCAGAGTTGGGCTCCA
GGCGCATCCAATACCGGCAGATCTTGTCGCCAAGCTGCTTGGTAACAGAGTGGCTGTAT
CGCCAATCGTCACCGTCGAGCCGAGACGAAGAAAATTCCACAAACCGATAACCTTAAC
CATACCCGTGCCTCAAGCGGCCAATAAAGGCATGATCAATCAATATTCAGGAGATGCGC
CGACGCTGAGACTTCTGTGCAGCATAACTG

>novel_circ_000645
GTGAAGATCCGATGCTCAGCGACCAACAGCAATATCGATACATGACTGTTGACGACAT
GAAATCTATGGGGGATGACTCGATGCGTGTCAACGTGACGGACGACGAGAGGGATAAC
CGGCATTCCGGAGCGCCCACGATAACCGAGATGATCACGAAGGAAACTTGTCAGCGTA
CAAATGCCGCCAATCTGAAACCGGACAATGTCGATATCAATAGGCACCCAGTACACGT
CGGATTTTGTTCTGAGATCAGACCGTCCCTAGAGTCCTTAAACACATCGCTGGCAGCTC
TTGGTACCATGTCGAAAGGACAAGGAATGTGGAGGGACAGCTTCCTGGTTTCGTTTCT
GGTGGATGCACGAGGCGGTGCGATGCGCGGCTGCAGGCACAGTGGCGTGCGCGTGAT
CGTTCCTCCTAGGAAAGCAGCGATGCCTATGCGCGTCACGTGCAGATACCTGAGAAGG

GACAAATTGACGAACCCGCCGCCTTTGATGGAGGGGGAGGCTCTTGCGAGTCGTATCC
TGGAGTTGGGACCCGTGGGCGCCAAATTTTTGGGACCCGTGATCATAGAAGTACCACA
CTTTGCTTCGTTGCGAGGAAAAGAACGAGAGATAGTTATACTCCGATCAGATAATGGA
GAGACTTGGCGCGAGCACACCTTGGAAGCCAGCGAGGAGGCCGTCCAAGATGTGCTT
AATGAGAGCTTTGAAGGAGAAGAATTAAGCCAGCTCGAGGATCTCCAAACTTCTCGGA
TCGTGAGGATACTTACGGCAGACTTCCCACATTACTTCGCAGTAGTGTCCCGTATCAGG
CAAGAGGTTCACGCAGTCGGTCCAGAAGGTGGCACCGTTTCTTCGTCGGCTGTACCTC
AAGTCCAGGCCGTATTCCCGCCAGCGGCACTTACTAAAAAGATCAGAGTTGGGCTCCA
GGCGCATCCAATACCGGCAGATCTTGTCGCCAAGCTGCTTGGTAACAGAGTGGCTGTAT
CGCCAATCGTCACCGTCGAGCCGAGACGAAGAAAATTCCACAAACCGATAACCTTAAC
CATACCCGTGCCTCAAGCGGCCAATAAAGGCATGATCAATCAATATTCAGGAGATGCGC
CGACGCTGAGACTTCTGTGCAGCATAACTGGATTGAGTCATTTAGCACATTCGTTTGAC
TGGTAACCGCTTCGATATTAGGCTTGTAATTTATTAACTTCTATATAAAAAATTGTGTTTA
TCAGAATATATGTACTTTTAGATTTAGGGGTCAGTCTCGGGCAGTCTGGGAGGATGTCA
CCGGTTCGACGCCATTGACGTTTGTGAAAGATTGCGTTTCCTTTACTACCACGGTATCC
GCTCGCTTCTGGTTAATGGATTGTCGGAATATATCCGAAGCCACGAAAATGGCTACGGA
GCTATACACACACGCGACACACGTACCTTTTATGGCTAAATTTGTAGTATTTGCAAAGC
GAGTAGATCCATTGGAGGCGAGGTTGCGCGTGTTTTGTATGACCGACGATAAAGAGGA
CAAAACCTTGGAGAATCAAGAGCACTTCACGGAAGTGGCGAAGAGCCGGGACGTGG
AGGTGTGTTGGAGGGGAAAACGCAATACATGGAATTTTCGGGTAACCTCGTGCCGGTA
TTGAAGAGCGGGGAACAGCTGCAATTGCCTTTCAGAGCTTTCAAGGAGAACCGAGTG
CCGTTCACGGCGAGAATAAAGGATCCGGACGCGGCCGACATGATGGGCCGCATAATGT
TCATGAGCGAGCCAAAGGTTCCCAAAGGCGAATTACCTCAGACCCCTATATGCACGTT
GAACATTTGCTGCCGGAGAAAATCTCGCCGGAGTCTGCCGTCTCCGAGATCGATTTG
TTAGAACTCTCCAAGAATTATAGTTTTCTGCGCGACGGCGGGATAAGTCGTCCGGACGC
TATTCACAGAGCCACCATCCGATTGACCGACATTGCAAATTTGTTGGACAAAGATTGGG
AGAAACTCGCGGAGGAATTAAACGTGCCGCCGAACGAAGTGTCGACGATCAAGCAAG
AGTACGCCAACAAGCCGGCCCAACAAGCTGCGGCGATGCTCAAAGTTTGGCAGAGCA
ATGGAAATAAAGCAACAGGAAACACTTTGGAGAAAGCGTTGAACAAAATTGGCCGCG
ATGACATCGTGAAAAAATGTATATTCAACGTTGAATTGATCACCGATGACGTGGAGAAA
GCTGTGGCGAGAGTTCGATTGGATCAACCTGGATTTGATTCTTTGAAAGAAGAATTGG
GTCCTTCAAGGGACACCTCTCTTCGCCGTGATGCGACCATGGATCCAAAGATGAATCCT
GATTACGATGACTTTAATAAGAGCAAGGAGTCGGGATCTATGGAAGAACTCGATATAAG
CGGCGCTAAACGAGATAAATCGAAACAGCATGTCACGATCACAAACGGCACTGTATTC
AACGGAACCTCGACCCCTATCAAAGATAAATACGCGAGCGAGGAGAAAGAACTGGGG
GATGTAATGGCCGATTTTCTCGAGCACAAATGTCACGTTAGCGATACGATGAGCAAAAA
GTTGCGCGATGAACTTGACGACAAAGATAAAAAACGGCAGAGGGTGATCGCAGACGC
CAACAAAATAGTATCCG

>novel_circ_000646
CTTCCTGGTTTCGTTTCTGGTGGATGCACGAGGCGGTGCGATGCGCGGCTGCAGGCAC
AGTGGCGTGCGCGTGATCGTTCCTCCTAGGAAAGCAGCGATGCCTATGCGCGTCACGT
GCAGATACCTGAGAAGGGACAAATTGACGAACCCGCCGCCTTTGATGGAGGGGGAGG
CTCTTGCGAGTCGTATCCTGGAGTTGGGACCCGTGGGCGCCAAATTTTTGGGACCCGT

GATCATAGAAGTACCACACTTTGCTTCGTTGCGAGGAAAAGAACGAGAGATAGTTATA
CTCCGATCAGATAATGGAGAGACTTGGCGCGAGCACACCTTGGAAGCCAGCGAGGAG
GCCGTCCAAGATGTGCTTAATGAGAGCTTTGAAGGAGAAGAATTAAGCCAGCTCGAGG
ATCTCCAAACTTCTCGGATCGTGAGGATACTTACGGCAGACTTCCCACATTACTTCGCA
GTAGTGTCCCGTATCAGGCAAGAGGTTCACGCAGTCGGTCCAGAAGGTGGCACCGTTT
CTTCGTCGGCTGTACCTCAAGTCCAGGCCGTATTCCCGCCAGCGGCACTTACTAAAAA
GATCAGAGTTGGGCTCCAGGCGCATCCAATACCGGCAGATCTTGTCGCCAAGCTGCTT
GGTAACAGAGTGGCTGTATCGCCAATCGTCACCGTCGAGCCGAGACGAAGAAAATTCC
ACAAACCGATAACCTTAACCATACCCGTGCCTCAAGCGGCCAATAAAGGCATGATCAAT
CAATATTCAGGAGATGCGCCGACGCTGAGACTTCTGTGCAGCATAACTG

>novel_circ_000647
CTTCCTGGTTTCGTTTCTGGTGGATGCACGAGGCGGTGCGATGCGCGGCTGCAGGCAC
AGTGGCGTGCGCGTGATCGTTCCTCCTAGGAAAGCAGCGATGCCTATGCGCGTCACGT
GCAGATACCTGAGAAGGGACAAATTGACGAACCCGCCGCCTTTGATGGAGGGGGAGG
CTCTTGCGAGTCGTATCCTGGAGTTGGGACCCGTGGGCGCCAAATTTTTGGGACCCGT
GATCATAGAAGTACCACACTTTGCTTCGTTGCGAGGAAAAGAACGAGAGATAGTTATA
CTCCGATCAGATAATGGAGAGACTTGGCGCGAGCACACCTTGGAAGCCAGCGAGGAG
GCCGTCCAAGATGTGCTTAATGAGAGCTTTGAAGGAGAAGAATTAAGCCAGCTCGAGG
ATCTCCAAACTTCTCGGATCGTGAGGATACTTACGGCAGACTTCCCACATTACTTCGCA
GTAGTGTCCCGTATCAGGCAAGAGGTTCACGCAGTCGGTCCAGAAGGTGGCACCGTTT
CTTCGTCGGCTGTACCTCAAGTCCAGGCCGTATTCCCGCCAGCGGCACTTACTAAAAA
GATCAGAGTTGGGCTCCAGGCGCATCCAATACCGGCAGATCTTGTCGCCAAGCTGCTT
GGTAACAGAGTGGCTGTATCGCCAATCGTCACCGTCGAGCCGAGACGAAGAAAATTCC
ACAAACCGATAACCTTAACCATACCCGTGCCTCAAGCGGCCAATAAAGGCATGATCAAT
CAATATTCAGGAGATGCGCCGACGCTGAGACTTCTGTGCAGCATAACTGGATTGAGTCA
TTTAGCACATTCGTTTGACTGGTAACCGCTTCGATATTAGGCTTGTAATTTATTAACTTCT
ATATAAAAAATTGTGTTTATCAGAATATATGTACTTTTAGATTTAGGGGTCAGTCTCGGG
CAGTCTGGGAGGATGTCACCGGTTCGACGCCATTGACGTTTGTGAAAGATTGCGTTTC
CTTTACTACCACGGTATCCGCTCGCTTCTGGTTAATGGATTGTCGGAATATATCCGAAGC
CACGAAAATGGCTACGGAGCTATACACACACGCGACACACGTACCTTTTATGGCTAA

>novel_circ_000648
CTTCCTGGTTTCGTTTCTGGTGGATGCACGAGGCGGTGCGATGCGCGGCTGCAGGCAC
AGTGGCGTGCGCGTGATCGTTCCTCCTAGGAAAGCAGCGATGCCTATGCGCGTCACGT
GCAGATACCTGAGAAGGGACAAATTGACGAACCCGCCGCCTTTGATGGAGGGGGAGG
CTCTTGCGAGTCGTATCCTGGAGTTGGGACCCGTGGGCGCCAAATTTTTGGGACCCGT
GATCATAGAAGTACCACACTTTGCTTCGTTGCGAGGAAAAGAACGAGAGATAGTTATA
CTCCGATCAGATAATGGAGAGACTTGGCGCGAGCACACCTTGGAAGCCAGCGAGGAG
GCCGTCCAAGATGTGCTTAATGAGAGCTTTGAAGGAGAAGAATTAAGCCAGCTCGAGG
ATCTCCAAACTTCTCGGATCGTGAGGATACTTACGGCAGACTTCCCACATTACTTCGCA
GTAGTGTCCCGTATCAGGCAAGAGGTTCACGCAGTCGGTCCAGAAGGTGGCACCGTTT
CTTCGTCGGCTGTACCTCAAGTCCAGGCCGTATTCCCGCCAGCGGCACTTACTAAAAA
GATCAGAGTTGGGCTCCAGGCGCATCCAATACCGGCAGATCTTGTCGCCAAGCTGCTT

GGTAACAGAGTGGCTGTATCGCCAATCGTCACCGTCGAGCCGAGACGAAGAAAATTCC
ACAAACCGATAACCTTAACCATACCCGTGCCTCAAGCGGCCAATAAAGGCATGATCAAT
CAATATTCAGGAGATGCGCCGACGCTGAGACTTCTGTGCAGCATAACTGGATTGAGTCA
TTTAGCACATTCGTTTGACTGGTAACCGCTTCGATATTAGGCTTGTAATTTATTAACTTCT
ATATAAAAAATTGTGTTTATCAGAATATATGTACTTTTAGATTTAGGGGTCAGTCTCGGG
CAGTCTGGGAGGATGTCACCGGTTCGACGCCATTGACGTTTGTGAAAGATTGCGTTTC
CTTTACTACCACGGTATCCGCTCGCTTCTGGTTAATGGATTGTCGGAATATATCCGAAGC
CACGAAAATGGCTACGGAGCTATACACACACGCGACACACGTACCTTTTATGGCTAAAT
TTGTAGTATTTGCAAAGCGAGTAGATCCATTGGAGGCGAGGTTGCGCGTGTTTTGTATG
ACCGACGATAAAGAGGACAAAACCTTGGAGAATCAAGAGCACTTCACGGAAGTGGCG
AAGAGCCGGGACGTGGAGGTGTGTTGGAGGGGAAAACGCAATACATGGAATTTTCGG
GTAACCTCGTGCCGGTATTGAAGAGCGGGGAACAGCTGCAATTGCCTTTCAGAGCTTT
CAAGGAGAACCGAGTGCCGTTCACGGCGAGAATAAAGGATCCGGACGCGGCCGACAT
GATGGGCCGCATAATGTTCATGAGCGAGCCAAAGGTTCCCAAAGGCGAATTACCTCAG
ACCCCTATATGCACGTTGAACATTTTGCTGCCGGAGAAAATCTCGCCGGAGTCTGCCGT
CTCCGAGATCGATTTGTTAGAACTCTCCAAGAATTATAGTTTTCTGCGCGACGGCGGGA
TAAGTCGTCCGGACGCTATTCACAGAGCCACCATCCGATTGACCGACATTGCAAATTTG
TTGGACAAAGATTGGGAGAAACTCGCGGAGGAATTAAACGTGCCGCCGAACGAAGTG
TCGACGATCAAGCAAGAGTACGCCAACAAGCCGGCCCAACAAGCTGCGGCGATGCTC
AAAGTTTGGCAGAGCAATGGAAATAAAGCAACAGGAAACACTTTGGAGAAAGCGTTG
AACAAAATTGGCCGCGATGACATCGTGAAAAAATGTATATTCAACGTTGAATTGATCAC
CGATGACGTGGAGAAAGCTGTGGCGAGAGTTCGATTGGATCAACCTGGATTTGATTCT
TTGAAAGAAGAATTGGGTCCTTCAAGGGACACCTCTCTTCGCCGTGATGCGACCATGG
ATCCAAAGATGAATCCTGATTACGATGACTTTAATAAGAGCAAGGAGTCGGGATCTATG
GAAGAACTCGATATAAGCGGCGCTAAACGAGATAAATCGAAACAGCATGTCACGATCA
CAAACGGCACTGTATTCAACGGAACCTCGACCCCTATCAAAGATAAATACGCGAGCGA
GGAGAAAGAACTGGGGGATGTAATGGCCGATTTTCTCGAGCACAAATGTCACGTTAGC
GATACGATGAGCAAAAAGTTGCGCGATGAACTTGACGACAAAGATAAAAAACGGCAG
AGGGTGATCGCAGACGCCAACAAAATAGTATCCG

>novel_circ_000649
CTTCCTGGTTTCGTTTCTGGTGGATGCACGAGGCGGTGCGATGCGCGGCTGCAGGCAC
AGTGGCGTGCGCGTGATCGTTCCTCCTAGGAAAGCAGCGATGCCTATGCGCGTCACGT
GCAGATACCTGAGAAGGGACAAATTGACGAACCCGCCGCCTTTGATGGAGGGGGAGG
CTCTTGCGAGTCGTATCCTGGAGTTGGGACCCGTGGGCGCCAAATTTTTGGGACCCGT
GATCATAGAAGTACCACACTTTGCTTCGTTGCGAGGAAAAGAACGAGAGATAGTTATA
CTCCGATCAGATAATGGAGAGACTTGGCGCGAGCACACCTTGGAAGCCAGCGAGGAG
GCCGTCCAAGATGTGCTTAATGAGAGCTTTGAAGGAGAAGAATTAAGCCAGCTCGAGG
ATCTCCAAACTTCTCGGATCGTGAGGATACTTACGGCAGACTTCCCACATTACTTCGCA
GTAGTGTCCCGTATCAGGCAAGAGGTTCACGCAGTCGGTCCAGAAGGTGGCACCGTTT
CTTCGTCGGCTGTACCTCAAGTCCAGGCCGTATTCCCGCCAGCGGCACTTACTAAAAA
GATCAGAGTTGGGCTCCAGGCGCATCCAATACCGGCAGATCTTGTCGCCAAGCTGCTT
GGTAACAGAGTGGCTGTATCGCCAATCGTCACCGTCGAGCCGAGACGAAGAAAATTCC
ACAAACCGATAACCTTAACCATACCCGTGCCTCAAGCGGCCAATAAAGGCATGATCAAT

CAATATTCAGGAGATGCGCCGACGCTGAGACTTCTGTGCAGCATAACTGGATTGAGTCA
TTTAGCACATTCGTTTGACTGGTAACCGCTTCGATATTAGGCTTGTAATTTATTAACTTCT
ATATAAAAAATTGTGTTTATCAGAATATATGTACTTTTAGATTTAGGGGTCAGTCTCGGG
CAGTCTGGGAGGATGTCACCGGTTCGACGCCATTGACGTTTGTGAAAGATTGCGTTTC
CTTTACTACCACGGTATCCGCTCGCTTCTGGTTAATGGATTGTCGGAATATATCCGAAGC
CACGAAAATGGCTACGGAGCTATACACACACGCGACACACGTACCTTTTATGGCTAAAT
TTGTAGTATTTGCAAAGCGAGTAGATCCATTGGAGGCGAGGTTGCGCGTGTTTTGTATG
ACCGACGATAAAGAGGACAAAACCTTGGAGAATCAAGAGCACTTCACGGAAGTGGCG
AAGAGCCGGGACGTGGAGGTGTGTTGGAGGGGAAAACGCAATACATGGAATTTTCGG
GTAACCTCGTGCCGGTATTGAAGAGCGGGGAACAGCTGCAATTGCCTTTCAGAGCTTT
CAAGGAGAACCGAGTGCCGTTCACGGCGAGAATAAAGGATCCGGACGCGGCCGACAT
GATGGGCCGCATAATGTTCATGAGCGAGCCAAAGGTTCCCAAAGGCGAATTACCTCAG
ACCCCTATATGCACGTTGAACATTTTGCTGCCGGAGAAAATCTCGCCGGAGTCTGCCGT
CTCCGAGATCGATTTGTTAGAACTCTCCAAGAATTATAGTTTTCTGCGCGACGGCGGGA
TAAGTCGTCCGGACGCTATTCACAGAGCCACCATCCGATTGACCGACATTGCAAATTTG
TTGGACAAAGATTGGGAGAAACTCGCGGAGGAATTAAACGTGCCGCCGAACGAAGTG
TCGACGATCAAGCAAGAGTACGCCAACAAGCCGGCCCAACAAGCTGCGGCGATGCTC
AAAGTTTGGCAGAGCAATGGAAATAAAGCAACAGGAAACACTTTGGAGAAAGCGTTG
AACAAAATTGGCCGCGATGACATCGTGAAAAAATGTATATTCAACGTTGAATTGATCAC
CGATGACGTGGAGAAAGCTGTGGCGAGAGTTCGATTGGATCAACCTGGATTTGATTCT
TTGAAAGAAGAATTGGGTCCTTCAAGGGACACCTCTCTTCGCCGTGATGCGACCATGG
ATCCAAAGATGAATCCTGATTACGATGACTTTAATAAGAGCAAGGAGTCGGGATCTATG
GAAGAACTCGATATAAGCGGCGCTAAACGAGATAAATCGAAACAGCATGTCACGATCA
CAAACGGCACTGTATTCAACGGAACCTCGACCCCTATCAAAGATAAATACGCGAGCGA
GGAGAAAGAACTGGGGGATGTAATGGCCGATTTTCTCGAGCACAAATGTCACGTTAGC
GATACGATGAGCAAAAAGTTGCGCGATGAACTTGACGACAAAGATAAAAAACGGCAG
AGGGTGATCGCAGACGCCAACAAAATAGTATCCGGTGTAATAGCAGACGCAGAGAAA
GCGAAGGGGAAGTTAGCTAAAGAAGGGTGGTCGGTGGTTTATGATGATGATGATGATG
ATGATGATGATGATGATGATGATGATGATGATGATACGCGAGACAATAAAGAGGCAAGA
GGTGCTATAGTCGAAAGTTTTGTTAGGGAACAGTTGGTTAAGAGTAAGCCCAAGGACA
AGGTCCTTGCAGCCGAGGAAACGATTACGATCGATCGAACGCCAATCGTCGAGCCGAC
GATAGTTAAGCAGCGTGTCGTTCGCGATGGGAAGGGAGACTCTAATGCGAGGATAGTC
ACGTCGAAAACTATAGTTTCGGTGATCGAGAGCGATAATAAGGGTGATAATCGGGAAG
GAGAAAGGATGAAAGAAACGGATCGAACGTCGGCGGCCGCCACGGCGGACGCGGCG
ACGATCCCCAATGGTAAACAGAGAGTGATAAGCAAGAAGGAGATGATGTCGAGGGAA
GGTAAGGAAATCGACGAGAAATCGGTACCCGCTGGTATCGTAACGCCGAAGAAAGAA
GAGGAAAAGGAGAAGGAAGTCGAAGAATCGAAAGAGACGGTTGCCGGCGTTAACAT
TCCAAAGGAGGGGAGAGCGACGGGTGACAAGCAGGTGTCATCCGCTTACGAGAGCGT
CTACAGCGTGCAATATCCGTCGACGGACGAGAGGCAAGAGGACGAGTCGAAGAAGTC
GAAGGACAAGAGCGGGGGTATATTGTCGGGGATCTTCAAGGGGTCTAAACTGAAGAA
GAGCAAGTCGAAAGGATCGAAGGAAACGTCTTTCGACAGCGGCGACGAGGAATCGA
GAACAGTGGGTAATGGCAAAGAGGGGCCGAAACCTACGTCTACTTTCGTCTCGTACGA
CGCGTACGCGGACATGAAACCTGCCGAGCAAACCGCAACCGACATTAAATTGGCCAC
GATGCAATTCTTGGACGAATCGAGACGCCTCGCTTCCGAGGTGCACGCGCTCGACAGC

GGGTTGATCGACTCGGCTCGAGTCGAGGATAGGGAGGAGGCGGTCAAGGACGGGGAG
GACACCAGCGATCAGGAGAGGGAGAAAGGGGGCGGTTTCTTCGGCATGTTCAAGAGT
CCAAAGTCGAAGGAGAAGAGAGCGAAGAAGAGCAAGGAGTCGTCGTTGGACAGTGG
CGACGAGGAGGGTATTCGAATCGCCACGGAGAGATTGTTGGACGATACGCGAAAGGTT
GCCGAAACAACGATCGTTGTTCCCGTGATCGACGGGAAACAGGATCTGTACACGTTCA
CCTCGCAGGTTCGTTACAACGAGGACACGCCGAAGGATAAATTGAACGGCCATTCGTA
CGATATTTCGGAGATTCGTAGGAAAGAGGAGGGGCGCAAGGAAGCTAATGGAAAATC
GGACGCGGGGAGGAAGCGGAAGGAGGTTGGAAGTTTGGAAAGGCAAGGTGGCGAGA
AAAAGTCAGACTCCCCGGAGAAAAGGGTGGAGAAGGAGGAGGAGGAAGAGGGTGGT
AAGGAGAAGGGTGGCGGATTCTTCGGCATGTTCAAGAGCCCGAAGCTGAAGAGCAAG
AAGAGGAGTTCGTCGAGGGAGAAGAGCTCTTCGAAGGAAGCGTCGTTCGACAGCGG
GGACGAGGAGACGCGGCAGTTGACGGCAGAATTTTTGGAGGATACGAGGAAAGTGGC
GGAGAGCATGCACCGCGAAGGGTCGAGGCAGATCGGCGAAAGGATGACGCCGGCCG
ACGGGGCACCGGCCATCATCGAGGTGACCACGGAGCCCGTGGACAAGGACAGAGGA
GGAGGAGGGATCTTCGGGAAGTTCAAGAGTCCAAAATTGACGAGGGCGTCGCGAAGC
AGGTCCCGGGAAAAGAGCGGGCCGCCGCAAGAGGCGACGAGTCCCTCCTCCGAGATC
TTGGAGGATGCGACGAGGAGCGCGGCGTTGGACGCGAGGGAAAGAGTAGAGGACGC
GACGACGGAAGCGACGGATGCGAGTGACAAAGGCTCGAAGGAAGGTAGAAAGGTTA
AAGAGAAGAGCGGCGGCTTCCTGTCCGGATTGTTCAAGGGGGCGAAGCACGCCGCGG
ACGAGGCTTCGGAGGAGGTGAAGGAGGCGGTCGGCGAGGGCAGAGATCGCGCGAGA
GAAGCGGTGGAAGGGAAATCGAAAGACGCGGACGCGGCGAGGGACAAGGCTGCGTC
GGAAGCGAGGGATGGAAAGGAGAAGGCGATCGCTATTCTCAAGGACAGCAAAGACGC
GATCGGTGGAAAGGCGTCGGATGCCGGTAAAATTGCCGGAGAGAAAGTGGCGGATGC
TGCGAAACGGGTCGAGGAGAAGGTGGTCGACGGCGTGAAAATTGGAAAGGACGAAA
TAAAGAAGATCGAGATAGACGTTAAAGGTGTCGCGGTGACAGGCGTAAACGGAATCG
AGGATAAAATTGGAGAGAAGCTGGGCGAGGGTGGGCGGAAGGTGGCCGATACAGGA
AGAGCAGTCGCGGACAAAGTAACCGAGGGGATAAAGGACGCGGAGTCGGGCGGGCG
AAGCGTGACCGAGGGCGCGAAAGCGACCAAGGACAAGATGGTTAAAGAGGTTAAGG
AGGACATCGAACTGGCGAGGCAGAAGACACCTTGGAGTGGAGTAGACTCGGCAGAA
GGCGCGAAAGAGAGGGCCGCGAAGGAAGGCAAAAAGGGGAAGGAGAAGAGCGGCG
GATTCCTCTCGGGACTGTTCAGAAGCGCGAAACACGCGGCGGACGAAGTTTCCCAGG
AGGTGAGGGAATTCGTGGAGGAGACGAGGAAGGAGGCTGGCGAGGAGGAGGCTCGC
GCTCGCGCGGCAGCGGCGGAGAAGTCGAAGGAATTGGACGAAGCTAAAGAGAAACT
GGTGAAAGACGGGAAGGAATGGGGCACGGCGAAAGTGTCGGACGTCGAGAAAACCG
TTGGCGAGAGGGTAAGCGACGTCGAGAAAAAGGTGGACGCTGCGATTCGTGATGCGG
CCGAGGCCAAGGACGAAGCGATCGCGAAGCTCGCAACGAGCGCAAAGGACGTCGGC
GATAAAGTCGCGGAGGGGGCGAAGGACGCCGAGGCGAGAGTGGAAGCAGCGAGCGA
CGGCGTTAAGGCGGCCAAGGATAAAGTGTCCAAGGAGATGAGGGAAGAGGGCGAAG
CGATGAGGCAGAGGATAGCGTCTGGGATAGACTCGGGCGTGGATGGCGCGGCAGCGG
CGAGAGACAGGGCTGCGAAGGAAGCGAGGAAAGCTAAGGAAAAGGCCGGCGGTTTC
TTGTCCGGATTGTTCAAGGGTGCGAAGCACGGCGCGGATGAAATTTCCGAAGACGCG
AAAGAGTTGGTGGACGAAACGAGAAAGGAAGCGGTGCAAGAGGAGGGTCGGATTCG
TGACCAGGTAACGGAAAAGTGGAAGGAAATCGATGCGACGAGAGACAAGGTTAAAG
CCGTTGCGATCGACGTCGAGGACAAGGTCGGCGAGAAGGTATCGGGTATCGCGGAAA

AAGTTGGAGACGCGGGTAAAGCTGTGGTCGAGAAAACGATACCGGACGATGCCGTGG
AACGAGGCGCGACGATCGACGGCACGAGGATAATCGACGCAGCCAAGGATAAGATAG
CGGATGTAAAAGCGGTCGAAAACGAAGCCGCATCCGTTGCGAAGGACAAGATCTCCG
ACGGTGCTCGGATGACGGCGGACGTTGGAAAAGCGGTTGGAGAGAGGATCGCGGACG
GTGCGGAGAGGACGAGAGACGCGGCGCAAGCGGGCGTGCGTGAAGCGGTGGACGCG
GCGAAGGCGGCAAAGGAGAAAGTGGCGAAGGAGATTCGAGACGACGCGGATGCCGC
GAAACGGAAAATCTCGTCCGGCGTAGGGGCAGTGGTGGACGGGGCGGGCGGAGCGA
AGGACAAGGCGGCGAGAGAGGCCAAGAGAGCTAAGGAGAAGGCGGGCGGATTCCTC
TCGGGACTGTTCAAGGGCGCGAAGAACGCCGCGGACGAAGTTTCGGAGGACGTGAA
AGAATTCCTCGAGGAGACGAGGAGGGAGGCGTCGGAAGAGAAGAGTCGCGTACGAG
ATGCGGTCGACGAGAAGTGGAAGGAGGGCAAGGAGGAGGCTGAGGACACTGTCAAA
GGCGCTGAAGACGCGTTGCTCGCGAAAGTATCCGACGCCGAGGGCAAGGTTGTCGAG
AAGACGGCCGACGGAGTGAAGCGGATCGAGGGAAAAGCGGAGAGTATCGCTCGAAC
GATCGACGGTGCGAAGGACGAGGCGGAAAAAGTTGCAAAAGAGACGAAGGACAAGG
TGGCCGACGGCGTGGACGCGGTCGTGGACGCGGCTGCTAAGGACAAGATCTCGAAAG
AGGCCAGGAAAGGTAAAGAGAAGACGGGTGGTTTCCTCTCGGGGCTTTTCAAGAGCG
CGAAGCACGCGGCGGACGAAGCCTCGGAGGAGGCGAGAGGTTTGGTGGACGAGACG
TGGAAGGAAGCGGGTGCAGCGAAGGAAAGAGCAGCGGCGGACGCGAGGGACGCGA
AGGAGAAAGTTGCGAGCGCCGCGAGAGACGCGAGAGACAGAGTTGGGGAAGCGGCG
GCGGATGGCGGGCGGAAGGTGTCCGAGGTTGGGAAAACTGCGATCGAGAAAGCGGA
GGAGGGAGCGAAACGGGCTGAGCGAAGAGTGGACGCCGTCGCGAAAGACGCGAGCG
AGGCTGCGAAAGCGGCGAAGGACAAAGTGGTCGCCGACACTTTGGCCGCCAAGGATA
GGATGGCGGCGACGGCGAAGGAAACGAGGGACGAGATCGGCGAGAAGGTGGCTGAA
AGCGTCGGAAAGGTATCGGACGCTGGAAGAGCCGTGGGGGAAGAGGTTACGGATGGC
GCGAAGAGAGCCGTGGACAAAGCTGGGGAGATCGTCCGCGACGCGAGCAGCGGTGT
CGAGGCGACGAAGGAGAAAGTGGCGCGGGAGATTAGGGAGGATGCCGAGGCTGCGA
AACGGAAGGTGTCGTCCGGCGTGGACGCCGTTGTGGACGGCGGCGCTGCGGCGAAGG
ACAAGATCGAGAAGGAGGCCAAGCGGGCGAAGGAGAAGGGTAGCGGCTTCCTTTCCG
GTTTGTTCAAGGGAACGAAGCACGCGGCGGACGAGGTCGCGGACGACGTGAAGGAA
TTTCTGGAAGAAGCGAAGAGGGAATCGTCGGAGGAGAAGACGCGCGTTTACGAGGCG
ATGGAGAAGAGGATAGAGGACACGGCCGCGGCGAAGGACAGAGCGATTATCGAAGCG
AAGGAAACGAAGGATAAAATTGCGAGTTCCGTGAGGGACGGCTTGGAGAAAGTGTCG
GATGTTGGCCGAGTCGTCGGTGAAAAGTTGGAGGATGGCGCGAAACGGGGCGGAGAA
AGAATAGAATCTACGGCTCGAGAAGCGGTTACGGGCGCGAGGGATGCCAAGGAGAAA
CTCGGCGAGAAAATGGCGGAAGTCGGCAAGAAGGTGGACGAAGGGATCGCGAGCGG
CGCGAAGGCGATCGGGGACGAAACGAGAGCGGCGAAAGACAGGGCGGAGAAAGAG
GCGAAGGAGGATATGGCCGCCGCGAGGCAAAAGATCTCGTCGGGCGTGGACGCGGCG
ATGGATAGCGCGGACGCGGCAAAGGAGAAGATTGCGAGGGAAGCGAAGAAGGGTAA
AGAGAAGGGCGGAGGTTTCCTCTCGGGCCTGTTCAGAGGCGCGAAACACGCCGCGGA
AGAGGTTTCGGAAGGGGTGGAAGATTTGGTGGACGAAACGAAGAGGGTAGCGGGCG
AAGAGGAGGCTCGTGCTCGCCAGGCGGTGGAAAAAGCTGGTAAAGACGTCGAGGCG
GCGAGGGAGAAAGCGGTTGCGGAGGCGAAAGACGCGAGAGATAAGACGGCGATCTC
GATGAAACGCGGCAAGGACAAGGTGGGCGAGAAAGCGTCCGAGAGCATGAAAACCG
TAGCGGCGGTTGGCAAGATGGTCGTCGATGAAGCGACGAAACGAGCCGAAGCTGTTG

TTGGAGAAGCGGCGGACGGCGTCGAAGCAACGAAGGAGAAATTAGCCGCGGATGCG
AAGGACGCGAAGGACAAACTCGCGGATAAAGCGGCGGACACTGTGCGAGCGATTGGG
GAGAAAGTTAAAGCGGAGGAGGTTGGTCGAGGATCGGGCGATGGAGCGAAAGAGAG
GGCGAGCGAAGAGGACGTCGATGGCGCGATATCGGCGAAGGAGAGAGCCGGCAAGG
AGGGCAAAAGGGCTAAGGAGAAAGGTGGCGGCTTCCTTTCGGGATTGTTCAAAGCCG
CGAAACAGTCGACGGAGGAGATTTCGGACGATGCGAAGGACTTCCTCGAAGAGGCGA
GGAGGGAGGCGTCGGAGGAGAGCGGTCGTCTGCGCGACGCGGCCGACGAGAAGTGG
AAGGGCGCGAAGGCGGCGAAGGACGAGGCGATCGAGGGCGCGAAAGACAAAGTCGT
CGCCTCGGTGAAACGCAGCGAGGACGCGATCGGTGGAAAGGTGTCGGACGTCGCGCG
GCAAGCGACGGACGTTGGAAAGAGCGTCGGCGACGAAGCGAGACGCGCGGCGGAAA
TAACGGAAACGAGGATCCGAGAGATGGGCGACGGTGCGAAAGCGACCAAGGACAAA
GTGGCCGCGGACCTCGAGCGCGTTAAGGCTGGAGCGGCGACAACCGCCGAGGACGCG
ATGGCAACGATCGGCGAGAAAGCGCGCGAAGGCGGCAAAGCCGCGATGGAAGGTGC
GAGGAGGGCCGAGGATAAAGCGGCGAGGGAAATGGAAGCTGGAAAAGAGAGAATTT
CTTCGGAGATGGATAAAGCTGCGGAGAGCGTAGCGGCGGCGAAGGACAAACTGGCGA
AGGAGGGTAAGAAAGGAAAGGAAAAGGGCAGCGGATTCCTTTCCGGACTGTTGAAG
AGCGTGAAGCAGGCGGCGGACGAGGTATCGGAGGACGTGAGAGAATTCGTGGGCGA
GAAGAAGAGAGAGGCCGTTGAGGAAGAGGCGCGTGCTCGCGAAGCAGCGTCCGGAA
AACTTAAAGATCTCGGTGCGGCGAAGGATAAAGCGGTCGGTGAGGCGAAAGAGGCGA
GGGATAAAGTTAAGGACGCGGTGAAGGATGGGAAGGAGAAAGTGGAGGAGAAGGCG
TCCGACATCGCGCGACGAGTGACGGATGCTGGCGAAGCGTTGGGCGGCAAAGTTTCG
AGCGGCGCGAAGGACGCCGAGGCGAAAGCGGAGGCGGCCGTGAAAGATTTGGCGGA
GGCGAGGGACGAGGCAACAGCCGGCGCGAAAGACGCGGTGGATAGAGGCGCGTCTC
TGGTGAAGGGCGCCGAAGAGAGGATCGGCGAGGCCGTGTCCGATACCGCGCGGAAGG
TGACGGATGGCGGGAAAGCGGTTGGAGAAAGGATCGCGGACGGCGCGAGGAAGGCG
GAGGAAAAGATATCCGGTGGCGCCAAAGCGGCCAAGGACGCGGTGGTGAAAGAAGT
GAAGGAGGACGCCGAAGCGGCGAAGGAGAAAGCGGCGGCGGCGATAGACGCGGTTA
CGGACGGGGCAACGTCCGCCAAGGACAAGGCTGGGAGAGAAGCTAAGAAAGCGAAA
GAGAAGAGCGTCGGCTTCCTTTCGGGTCTGTTCAAGGGAGCGAAGCACGCTGCTGAC
GAGGCGTCGGGAGACGCGAAGGAATTCCTCGAGGAAACGAGGAGGGAAGCGACGGA
GGAGAAGAGCCGGTTGCGCGAAGCGGTGGAAGGGAAGGTGAAGGACGCGGAAGCG
GCCAAGGACGGAGCCGTGGCGGACGCGAAGGACGCGACGCGAAAAATTGTGGGAAT
CGCGAAGGACGCCGGAGACAAAGCGGCTGGAAAAATATCCGAGGGTGTGGAGAGAA
CGACGGACGCCGGCAGAGCTGCTGGCGAAAAAATCGTGGACGGCGCGAAGCTCGTGC
AGGCGAAGACGAAAGAGGCAGCAGATGCCGCAAAGGACAAGCTGGTTTCGGATACG
AGAGAAATTGGTGAGAGGATCGAGTCTGCGGCGAGAGACGGCAAAGATCGAGTCGGC
AAAAAAGTGAAAATGGCCGAGGACGAGACGGTGGCCGTCGCTCGGGATATAGCCGAC
GTTGCCAAAACGACAAAGGACAAAGTCGCGAAGGGGGCGAAGGAGGGCGTCGACGT
CGCGAAGGAGAAAGTGTCGTCCGGCGTCGAATCGGTGGTGGACGGTGCGTCGAGTAC
GAAGGAAACGATCTCGAAGGAAGCGAAGAAGGCTAAGGAGAAGGGCGGCGGTTTTC
TTTCCGGCCTGTTCAAAGGCGTGAAACACGCGGCGGACGAAATTTCGGAAGACGGGA
AACACTTGGTCGACGAAGCGAGGAAAGAAACCGCGGAAGCGGGCGAGAAATTAGGA
AAGGGCGTCGACGCGACGCGGGACAAAGGAATCGCAATCGCGAAAGACGCGAAGGA
TAAAGTCAGCAACAAAGTGTCGGAAGGTGTGCAAAAAGTATCCGACGTCGGCAAAGC

TGTTGGCGAAAGAGCGACGGATGGCGCGAAAGAAATCGGCACGGGGATGGAAGCCG
GTGCGAGACGAATGGGAGACGCTGCGAAAGCGACGAAAGATAAAGTGGCGGCTGGT
GTGAAGGATTGCAAGGACGAAGCGATCGCCTCTGCGAGGGATGCCAGGGATAAACTC
GCGGAGAGAGTAGCGGAGGGCGGCGAAGCGATTGGGGAAAAAGTGGCGGCTGGCGC
GGAGAGAGTGGAAGCGGCCGTTCGAAAAGCGGCGGACGAGGCGAAAGAGACGAAG
GAAAAAGTACGCAAAGAAATTGGAGACGATGTCGAAAGCGTGAAGCGGGAAGTTTCC
TCGGGGGCTGGTGCACTGGTCGAGGGTGCAACTTCTGCGAAGGACAAAGCGACGAAG
GAGGCTAAGAAGGCTAAGGAGAAGGGTAGCGGCTTCCTTACAGGCTTGTTCAAGGGC
GCGAAACAGGCGGCCGACGACGTTTCCGACGAGGTGAAAGAATTCCTGGACGTGACT
AGGAGAGAGGCAGCCGAGGAAGAAGCTCGAGCCCGCGAGGCGAGGGACAAGGCAG
CTGCGGACATCGAAAAAGCAGTAGGCGAAAAGGCGTCGGAGGTTGGCAAAGCGGTTG
GCGAGGGAGTGAAACGAGTCGAGGAGCGGACAGACGCTGCGATTCGTGACACGGTT
GATATCGCGAAAGCGACGAAAGATAAATTGATAACGACAGACGCCAAGGATAAAGCTG
CGTCTGTGGTGAGAGACGCGAAGGATAAGGTTAAGGAGAAGGGAAGCGAAGGTGGA
GAGAAAATATCAGAAGCGGTTAAACGAGCGGGAGAGAAAGTTGAGGACGAAAATGC
AAAAGCGGCGAAGGAGGCAGAGAAAGTGGGAACGGAGATTAAGAAAGATGTGGACG
CTGCCTCATCGGCGAAAGATAAAGCGGCGAGGGACGCTAAAAAAGGGAAGGAAAAG
GGCAGCGGTTTCTTTTCCGGGTTATTAAAGAGCGCGAAGCATGCCGTGGACGAAGTGT
CGGAGGAGGTGAAGGAGTTCGTCGACGAAACGAAGAAAGAGGCCACGAAGGAGGA
GGCGCGGGCGCGCGAAGCGTTGGCGAGGAAAGCGGATGCGGCGAAGGATAAGGCCG
GTGCTGAGTGGAACGATGCGAAAGACAAGGCGAGGACAAGCGTGAGAGACGCGACG
GACGCAGTCGGAGGAAAGATAACCGAGGGGGTGCAAAAGGTGTCAGACACTGGGAA
AATCGTGAGCGGAAAAATCGTGGAAGGAGCGAAAGGAGTGGAGGCGAGAACCGAAG
CGGTTGCTCGAGATGCCGTTGAAAGTGGTAAAGCTGCCAAGGACAAAGCAGTCTTCG
AAGCGAAAGTTGTTAAGGACGCGCTCGCGACATCCGCCAAAGATGCTAAAGAGGGTAT
CGGTGAGAAAATATCGGAAGGCGTGGAGAAAGTGTCGGACGCTGGTTCACTTGTATCC
GCCAAATTGGCGGAGGGAGCGAAGCGAATCGGCGGTGGAGAAACGGAATCGGTCGC
GAGAACGACGAAGGACCCTCTATCGGAGGGAATCAAAGGCGACGCTGCGAGGGAGA
AAGCGATGGACGATGGCGGCGAGGCGAGAGACAAAGCCTCGAAGGAAGGTAAAAAG
GGGAAGGAGAAGAGTGGCGGTTTTCTGTCGGGGTTGATCAAAGGCGTGAAACACGCG
GCGGACGAGGTATCGGGCGAGGTTAAGGAATTCTTCGACGAGACGAAGAAGGAAGCT
GTGGAGGAGGAGGCGCGCGCTCGCCGAGCATTGGTCGCTAAAGCGAAAGAGGCGGA
GGCGGTTAAGGAGGAGATCGCGACTGGCGCGAAAGACGCCAAGGAGAAGATCGGTG
AGAGAGTAACCGGTGGCGTGCAGAAGGTGTCGGATAAACTCGTGGATGGAGCGAAGA
TGGTGGAGATAAAAACGGAAACTCGAGCGCGAGAAATCGCCGAGTGCGTTGAAGCAA
CGAGGGACAAAGTGATCGCGGATGCGAAAGATGGCGGCGAGAAAGTTGCAACTGCGG
CGAAGGATACGAAGGAGAAGATCGGTGAGAAGGTGTCGGGTACCGCGCACGCGGTGT
CGGAAGTTGGAAAAGCTGTAGGCGCTAAAATTACCGACGGGATAAAGAAAACGGAGG
CGAAAGTGGGAACAATCGGTCAGGAAGCGAGCGATGGAGCGAAAGCGACGAGGGAT
AAATTGGTGAAGGAAGTGAAAGAAGACAGCTCGATCGTGAAAGAGAAAATCTCGTCT
GGGGTGGATACCGCGATGGATGGCGCGAAAGCAGCGAAGGATAAAGCTTGGAAGGAG
GGTAAGAAGGCGAAGGAGAAAAGCGGTGGTTTCTTGTCCGGTTTGATCAAAGGCGCG
AAACACGCGGCCGATGAAATATCCGAGGATGTCAAAGATTTCGTGGACGAGACGAAG
AAAGAGGCAATGGAGGAAGAGGCTCGCGGCCGCGTGGCGGCGTCCGAAAAATCGAA

ACAAGTCGAGATCGCCGCGGAAAGTGGGAAAAGCGGGAAAAGCGGGAAAGGCAAGG
TTAAAAGTGCGCAGAGAGCGTCGGATTCGGGTAAGGCTGGGAGCGAGAGAGACGCCG
GAAGAATTTTTGGAGAGAAATTGTCGAATCAAGCAGGGCAGAGATTCATCGCGGTTAG
CAGCGACCCTAAAAGCGCCGTGGCCGAGTTTCTGGAAGAAATGAAACGCGACTCCGC
GTGGGACAAGGTGCACTTCCAACACGAGCCGGACGTCCACTTGTACGAAAACGTGGA
CACGATGTTCGTGAAAGAGAGGAAAGAGGAGGGTGAATCGAGAGAGGGTGGAGCGG
GGAAACACGAGAAAGAGTCAAAAAAATCGGCGACGAAAGTTGCTTATTCGGTTTTCG
ATCCACTTTCTGAGCTAGATTCCGAGGCACGGGTCAGGCTGTCCGAAAGAGAGATCGA
GGACAGCGATGGTGGCGGGGAATCGATGTTCAAGAAAGTATCTCGCATTCCACAAAGA
GTTAGTTCCTCGACGGAACGGGTTAGCCCTAGTAGTTCGTCGGATAGTAGTAGTTCGAC
AGTTAGGATATTAAATGTATTTAGAAACGTAGAGCACCTCGAGCCTCGCACACGACAA
ACCGTCACCACAGTCGTAACAAAGTCTGTGCGGACGGCAACGACCCCACCACCCAGT
CCCGCCGAGTACACTGACAGTAGAATTGAAGACGTAACCGAAGAGGCGGCAAGACGA
GCCCAGGATCTAGCCGATCAAGCACAATCATTGGCGAAATCAG

>novel_circ_000650
ATGGTTTCCGGAAAAACGATCTCTCATGCATTATATGCTCAAACCCAACTAGATCATGTG
TGTGCTCTGGATATCGATGCTCCTATAGGAGCTGTCCGTCTTTCAGGGATTATTTGTACA
ATTGGACCAGCTTCAAGATCTATTGATATGCTTGAAAAAATGATCGATACGGGTATGAA
CATTGCTAGATTAAATTTCTCGCACGGAACTCATGAATATCATGCTGAGACTATTGCTAA
TGTGCGACAAGCACAGAAAAACGTTTCCTCTAAAGCTGGATTAAGCATTCCTGTAGGA
ATTGCTCTTGATACTAAAGGTCCTGAAATCCGTACTGGACTTTTAGAAGGTGGTGGAGC
TGCTGAAGTCGAATTAAAAAAAGATCAACTTTTTAAATTGTCTACTGATAAAGCATATG
CAGAAAAAGGCACTTCAAGTATTGTTTATGTTGATTATGAGAATATTACTAAAGTTTTGA
AGCCTAATAGTCGCGTTTTTGTCGATGATGGTTTAATTTCCCTTAAAGTTACTGCCATCC
ATCCTGAATTAGTTGTAACTGTTGTTGAAAATGGTGGGATGTTGGGTTCTCGTAAAGGT
GTCAATCTACCAGGTACTCCTGTAGATTTACCTGCTGTTTCTGAAAAAGATAAGTCTGA
TTTAATATTCGGTGTTGAACATGAAGTTGATATGATTTTTGCCTCGTTTATACGAGATGC
ACACGCTTTGACAGAAATTCGAACAATTCTTGGTGAAAAAGGAAAAAATATTAAAGTA
ATATCTAAAATTGAAAATCATCAGGGCGTAGTAAATATTGATGAAATTATTGATGCTTCT
GATGGTATTATGGTAGCACGTGGTGATCTTGGTATTGAAATACCACCAGAGAAAGTATT
CTTAGCTCAAAAATCTGCTATTAGCAAATGTAATAAAGTTGGAAAACCAATAATTTGTG
CTACACAAATGCTTGAATCTATGGTGAAAAAACCACGTGCAACCAGAGCTGAATCATC
TGATGTAGCTAATGCTATTCTTGATGGAGCAGATTGTGTTATGTTATCAGGAGAAACTGC
TAAAGGAGATTATCCATTGGAATGTGTACTCACAATGGCTAATATTTGTAAAGAAGCAG
AGTCTGCAATCTGGCAAACACAATTATTTCACGAGCTTTCAATGAAAGCAATACCTCCA
ATTGATGCTACACATGCCGTAGCGATTGCTGTTGTAGAAGCATCTGTGAAATGTTTAGCT
AGTGCTATTATAGTAATTACAACTACCGGCCATTCCGCGTATTTAATTTCTAAATACAGAC
CACGTTGCCCAATTATTACAGTTACTAGACACTCCCAGACTGCACGACAATCACATCTT
TATCGTGGGATTTTGCCACTTCACTTTGAAGAACCACGATTAGCTGATTGGGTAAAAGA
TGTTGATGTACGTGTTCAGTTTGGTATGAAATTTGGCAAAAATCAAGGTTTCATTAAAC
CTGGAGATGCAGTTATAGTAGTCACTGGATGGAGAAAAGGCGCCGGATTTACGAACTC
ACTTCGTATTGT

>novel_circ_000651
GTGTTCCATTTTGCTATGTGTCGGACGACTTATTTGTCAACGTCGTTTACCTGAATAAAG
TCTGTTTCTTATTTGTCTCCTGAAAGAAAATGGTCAACCCTGGGAATACTTTACGGAAT
TCGTCAGACGAATGAATCGTGCAACGAGATAAATGTATCCTTCCACCGACGCACGAAA
GGTTTAATACTATTAAAGGAGAAAGAGAAAAAAAAAAAGACATGGGAAGAAGCGGAA
AAAATTCTTTTGATGGAATTGAATAACGCGGTTGAAGAAGAACGAAAATTGATCCCTC
GACACCTAATCGTCTAATCCTTCTATTATTATTACGAGGCGAGAATTTCTTCATTTTTGCA
CCTTTTGAAAAAAAATGTTGAAATCTTGGAAAGTATTTATAAAATATATCAATTATATATT
TATAAAAATATATCAATTATATAATGATAAAAGTATAATATTAATATTTAATATTATTGAATA
AATGTATTCGAATTGCATTACATGAACGGCGGATGAAGAAGAGGAAGCACTCGTCGATA
TTACCCCTGGATCTCGAGGCGTATATATACGGGCGTATTTACGCTGTCGGAACATGCAAT
TTTGGCGTACGACGTGTAAATACAGACGTGTCATTGGACAACAATATACCCGTGGGATT
CAAAGCCTGATCTCTCGGTGTCAACGACCACGGTATTCTGAATTTTTCGATGGTATTCC
GTGACGACGACGAGTAAATTATCGCTAAACAGTGTACCGGCGGATGTCAAGATGCGGG
CCTCGTTCGGGTGGTCGTGGGATCTTTCATCGACGACCCTTTCGAAATACTAGAATATG
GAACAGAATCTTTCTTAATCATCGTCGAACAAAACAAACGGTAGAATTTTTTTGGGGA
GAATTACGAAGAACGAAAGAGGGTAGGATTAAGTAAATTTAAATTTAATACGATAAATT
TTAATAAAAGATATCACAACGCGTTGAAATATCACTAGATGTCACTAACTAATTTTCAGA
AATACAATTTCAATATTTTCTAGCCAATAACAAATGAAAACATTGAAAATCTTCATCAAA
GGAGTTTGATTGAAATTTATGGAGGCTGTTGCGAGTAATAAAAATCAAGCATGGACTGG
TAGATATCATCTAG

>novel_circ_000652
ACATAATAATACGACACGGGACGTTTTGAGCAACACGTATAGTTCATATAGAGTCGCGT
CTGATTCAAAGGATGCAGAGTATCAGTACAGCGTAAATTATAGCAATATACAAGAGAAA
AATTATCTGCAACGAAGTGTTTCTGCGGACAATGTTGTAATACGTTCCCGCGGTTTTAA
ACCCTCTGATAGGCACGATCCCGTGAAGAGGAACTTCCTTGAGAATCTTACTTCAAAA
ATATTGCATTTCGATATGGAGAGTAATGAAAGTGCGCCAGTCAACTTACAAAAATTACT
CACTCCTGCTGCAGATTCGCAGGGAATTATGCAATCTAAAAATAGAAAAATGTTTGCAT
CTTCATATTTTTACGCGCCAACACATCCAACCGTTGAGGATCAAGTTGAACTCGCTCGG
CGAATTTCGCATTCGTTAAGCGATGTAAAAAATATGAAAAGTAAAGGTCAATCTATGTA
TGTGAACAGAAAAAAAAGATCGGTTAAATGGATTCATGATGGCAATGGCGTAGAAGAC
GCAGAAGAACCTTTAACACCTGTTCACAGAGATAAAATTCCATTAAAATGCATGATGAA
CCCGCATGGAAAAGTATTGGATATACATGGTATTCAAGCTTTAGGGGAAGAAGTAAATA
TAGAACCAATGCCAAAAAATCCCGAAAAACTTTTTGATATCGTTCGTGATTTAAACAAT
CAAAGAGGCCGTGGTGCTGAAATATTCGCAAAACGCAGAAAAAGATCTGAGAAATGG
GTTGTAGACAAAGATCAACCACAAACGCCAAACACACTTGGTATGCCAAAAATGCCAA
TATATATGGGAAAATCGATACAGGAAATGAATGGCAGTTCAAAATATTCAAGTCTTCCTT
CGCCTATTGTACCTGAACCAACCAATGAGAAATCATTTTATAATCCTTTTACCGTGGACT
TGTCTTTAGATAATATAAATCAACCGACGACAG

>novel_circ_000653
GTGACCGACTCGAAGGATCTCCAAGTACCGTTCAGCACCGCGGCATCTCTCGGCTACG
GCCTCCATCATATGCTCCACTTGCCACCCCAATTTCTCCACCCTTTGGATCACCGTCTGC

CGTTCGGCGGGTTCAGGCCCCTCGGCCCCTCTGCCTTCGCCCCGCCTAGCAAGTGTTT
GAAAGTAGAGACCGGGAACGGGCCCGTGACCGGGTCCGGTTTGCCGAGCATCGGCTC
GCTGTCGAGCATGTCGAACATGTTCTCGCCCTCGTCCCTGTCCTCCGCGAGCGGAGGC
GCCGTGGTCTCCGTTTCCGGGGCGTCGGCGAGCATCGGGGGCGTCGGAGGCGTCGGG
GGAGGCGCGGGCGGCGCGTCGGGGCAGGAAGTGGCGCCCCAGAGCCCGGCCGGGTC
CGCGAGCCGATCGCCCACCGGGACGGGGACCGGTTCCGTCCCCAATCGAGGTGCCAC
GCCGGACGACGAGGATCGGGACGCCAATGCCACGCCTGGATCCGAGAACACCGAGCG
CTCCACGCCCGAGGAGGGACGCCCTTACAGAC

>novel_circ_000655
GTAAAAAGAAATTTCCATCTGATCCATCCTGCTGTCCGGCATGCGGAGTGACCGTAAG
GCCGCAGGAGCTCGAGCAACATTTCGCCCAGGAACTCGACAGACTTTACAAGATATCC
TCGGCTTCCTCTCGGGCGCGAGCCTCAAGATCCAGCTTGCCACCCGGCCATCCCCAGG
ATCATCCTCACGGGCCCATGCTCCATGCACCCTCGACCGCCGATGGCACGCCTCATGGA
AGATGGGAGACTTACAAAAGGATCAAGGCCAATAGACAGGCTAGAATCCGCGTGAAG
AACCGGAAACGGAAAGCGGACGAGCCATCGTGCCCCGTGTGCAGCGAGAGGCTATCC
GGCACGCCCGAGGAATTGAACCAGCACGTGGAGAGATGCTTAAACAAACACAATAAC
GGAAATCCTGCCGGCCAAAACAATTTGGACGAGGAGGAGGTCGACGTGGAGGGCGAT
GCGGAAACTTTCGAGGAATACGAGTGGGCCGGGCAGAGGAGAGTGCGAGCGTCGTCC
ATGCTCGTAGGAGGATTCTCCGCCGCAGGTTTGGCGACGTCGTCGAGCAATCGTTCGT
CGGCCGGTGGCAACAGCGGATCGAACCACCAGGAGGAGGAGGACGTGGATCTGGTG
GTGGACGGGGACGACGCGGCCGAGTTCGGGCCGGCACAGTATTCCGAGGCCGACGTG
GTCGCGCCTCGCATGGATGGGACGCCGAGGGAGCAGAAGGAGAGAGACGCCCTCCGG
GAAGCGGTGATATCGCCGAACGCGCCCAACACGCCCCACACGCCCCACACGCCCGAG
CATCAAATCGGCCAAGGTTTGGTGGAGGTGAAGCCTGAGCCGGGGACGACGACCCCT
CTCGGCCAGAGCGAGCAGGACGAGGGCGTCTCGACGTCGCCCAGAAGGGATGGCGA
CACGCCGGTGATCGAGGCGCTGAGAGGAAGGATTCGGGAACTCGAGGCGGAAATGCG
CGGCCAACCGTTCAAATGTCTCATTTGTATG

>novel_circ_000656
GTAAAAAGAAATTTCCATCTGATCCATCCTGCTGTCCGGCATGCGGAGTGACCGTAAG
GCCGCAGGAGCTCGAGCAACATTTCGCCCAGGAACTCGACAGACTTTACAAGATATCC
TCGGCTTCCTCTCGGGCGCGAGCCTCAAGATCCAGCTTGCCACCCGGCCATCCCCAGG
ATCATCCTCACGGGCCCATGCTCCATGCACCCTCGACCGCCGATGGCACGCCTCATGGA
AGATGGGAGACTTACAAAAGGATCAAGGCCAATAGACAGGCTAGAATCCGCGTGAAG
AACCGGAAACGGAAAGCGGACGAGCCATCGTGCCCCGTGTGCAGCGAGAGGCTATCC
GGCACGCCCGAGGAATTGAACCAGCACGTGGAGAGATGCTTAAACAAACACAATAAC
GGAAATCCTGCCGGCCAAAACAATTTGGACGAGGAGGAGGTCGACGTGGAGGGCGAT
GCGGAAACTTTCGAGGAATACGAGTGGGCCGGGCAGAGGAGAGTGCGAGCGTCGTCC
ATGCTCGTAGGAGGATTCTCCGCCGCAGGTTTGGCGACGTCGTCGAGCAATCGTTCGT
CGGCCGGTGGCAACAGCGGATCGAACCACCAGGAGGAGGAGGACGTGGATCTGGTG
GTGGACGGGGACGACGCGGCCGAGTTCGGGCCGGCACAGTATTCCGAGGCCGACGTG
GTCGCGCCTCGCATGGATGGGACGCCGAGGGAGCAGAAGGAGAGAGACGCCCTCCGG
GAAGCGGTGATATCGCCGAACGCGCCCAACACGCCCCACACGCCCCACACGCCCGAG

CATCAAATCGGCCAAGGTTTGGTGGAGGTGAAGCCTGAGCCGGGGACGACGACCCCT
CTCGGCCAGAGCGAGCAGGACGAGGGCGTCTCGACGTCGCCCAGAAGGGATGGCGA
CACGCCGGTGATCGAGGCGCTGAGAGGAAGGATTCGGGAACTCGAGGCGGAAATGCG
CGGCCAACCGTTCAAATGTCTCATTTGTATGGAGCAATATAAGAAACCGGTGACTTCCG
TCTGTTGCTGGCACGTGCATTGCGAGCAATGTTGGCTACACACGTTG

>novel_circ_000657
TTTTCACAGAAGAAGCAACGAACCGGAAAGAGGTTCGAGCGTGGTACCTCTGCTCAG
GGACGATGTACAGCTCTCCGTCGACGAGTACAGAAATAAGCTTTATTCGATGATGGACG
AGAAGCATCGCAAGCATAAGAATATGTCTAAGTCCAGAATCAGGCGATTATCGGAGCA
CATCAGGAAGGAAACAGTTAGTAGTAATAGTAATCCAGTTATCGGTCAAGGCGACGCG
ATTGTTGGTAAAAATCTTACACACTCATATCAAGATTTACGTAAGGAGAGAGGTAGAAG
CGACGCATACGAGCCCTCTTACGTAGAAG

>novel_circ_000660
AGGCGGGAGTGCCACCAGAGCCGGAGGATTGCGTGAGGGAGGTGAGCGGCGTGCAG
GCGATCATTAGCAGCGAGGATGTGATAGACAGGAAGACGCTATCCGAAAAGGAAGGCA
TCCCTCTCGATTGCTTGTGGAACATCACGGTCAAAGAAGGATGGAAGATTCAGCTGAC
GTTCTCGGATCCCTTCAAACTGCAACGACCGAACGAGTGCGACGCCAACTTCGTGGAC
GTGTTCAAAGAACGCACTGACATGTCCTCGAGAGAGAAGAATTTCTGCGGAAGTATCG
CCGACACCGTCCTCATCGGGACCAATACCGCCTTTGTCAGGTTTTACACGGAGCCTAA
AGCGCTGAACAGCAGCTTCGAGGCCGTGATGACAGCGCTCAGGGACAGAGATCCCGG
AGACAAAC

>novel_circ_000661
ATAAACATGGAGGCCGTCACGTCCACCGACTACGACTCGTCCAATCTGAGCAGAAATT
TCAATCAGGTGGGGTTCAACACGTACAACTACTCCCAGTTCGAGGAGAAGTACAAGCC
GCGGCAAGTGTTCAAGTACGGGGACGAGTATCTGGAGTACGGGCGGAACTCGGTCAG
GTTGGGGCCGAGCGAGAGGCTGGCGAGATTGTCGTCCGCGAGGCATCAAGAGGTCGC
GGCTGGGAAGATCGATCAGAGGTACGCGAGGAGTCACTCGCAGCCCGAGCGACAACA
CCATTCGAGCCACGAGACGCGGTCCAAGTCGCAGGATTCCCGCGAGTTGACCTTCTAT
CAGATCGATCCGCCAGTTCCCAGACCGGAGAACGTCCACTCGAAGACGCATCGCGGT
GTCGCGGTGGAGAAGGGTCAGGGCAAAAAGGAGTTGACCTTCTACGAGATCGATGGG
CCCGCGTCGCAAGAAAAGGCGGCGAAAGACGGCAAGTCGGAGGGAGGAGGAGGAG
GAGGAGGAGGGAAGGACAAAATGGAGAAAGGGGCACAGAGGGACAAGGCGCCGCA
CGCCGCCGGTGTTCTCCAGGAGAAGGCGCCGTGCCCTTCCAAGCACCCGAAGCAGGA
GCAGAAGGCGATCGCGCCGCCCAATTACCAGCTGAACAGATCCCAGTCCGATCTGACC
GATTGCCAACTGCCCAATTATTACAGGCATCGGAACAATCCTTACATCGATCCGCCCAA
GTACAGGCAATTCGACAGGCCGCCGAGGTATCAGGAAACGCCACCGAGATCCTCCGA
GCCACCGCCGAAGTACTCGGAAGTGGCCAGACGTCAGGACGACTACAAACGCGTCCA
GGAGGAGAGGGGCAAACGGCAAGGTGGTGGAGGAGGAGGAGGAGGAGGAGGGAGC
ATGGTAGTAGGCATCGGTAGCGGTGGTGGCAACGGCGGCGGCGGCGGCGGCGGCGGT
GGTGGTAGTAACTCCAGCAGGGGAACTCATGGCGCCGAGACGCCCTCTAATCTGGCCA
GTATCACGCCAACCGCGCGAGAAGCAACACGCGCTCCTTCGTCGCGGTCGCGGCTCCC

TTACAAGTCGTGCGACACGCGTTGCCCCAACAACAACAATAACAACAACAACAACAA
CACGACGACCACCACCACCAACAACAATAACAACAACAACAACAATAACAGCAGCAG
CGGCGGCAACGGTGGCAGCAACTCGAACAAAGAGGAGGCGTATCAGGATGCGAGCG
AAGAGGGCGAGGTGATATCCGGCGGTTTCAGGTGTCACGGGGATTTTTCGAAAGCGAC
GTCGTTCCATCATCACCATCACCATCACCACCATCATCATCATCAGAATCGGTGTTCGAA
CATCGGAGGCGGTTGCGGTGGCGGGTCGGTTGCCGGTAATGCGTGCCAAAGTGGTTCC
GGTGTGCCGTGCGACCCGTCGACGTGCGACAGGTACAAGGGTGCCGTGGATTGTCTGC
TGAAGGCGAGCGAGGCGGTGCAAGGTCGCCTGTGCGACAGATCGATGCTGAAGATCG
AGAAAGGGTCGAGCAAAGCGGCGGTCCACGGTGTCGAGGGGCTGGTGGTGGTGGGG
AAGTGCGGCGGGGATAGGATGAAATCGGGGCAGGAACTCGGTTACGGGAAGCACGGG
GAGCAAGTGGGCGGCAAGTCGAAGGGGCACGATGTCGGTCACGGGTTCAAGGTGGA
GAACATGAAATACTACGGCGAGTTGAAGGGTTACGACTTGAAATCTTACGGCACTATC
GACAAGCCGGGCGTCGTCGTCTCTGCCCACGAGAAGTCTCACTCGCACGGGGCGGAG
AAGAACGGTCACGACAAGTGTCACTCGAAAGTGTCCTGCTCCTCCTCCTCCTCGTGCT
CGTCCGCGATGCAGAGTTACGGCCTGTCCAAGGTCGGCGGCGGTGAACGTCCAGCCG
AGAAGAGCGTGTACGCCGATCACTATTATCACCGATCGTCGCACTTGAAGCCGCAGAC
CGCGTATCAAGTGTACCAGGAGACCAAGTACTCGTACAACGTGAGCGGGGTACCGGG
GACGCCGGCGCAGGCGAGCGCAGCCGCAGCTTTCTTCGCAAG

>novel_circ_000662
GTATCTCATCCCTTGCGAGTTGTGGCGAGGTGGACGCGTTGCCGGAATTACCAGCCCA
GGACGAGGAACGATTGCAACGCGCCGCACGTTTGTTGCAACAAAGGCTCGTTTTGCG
CCAATGGCTCACTGACCATGGCTTGCACAGCCATTATCAAAAATTAATGCAGATGGAAG
TGATGTCTCTGGAGGATGTGTATTGGGTGGAGGATAATGCGGCACGGGCAGCCCTTGG
CAAGGATTTACCGCGATGGACCCAGGCCAGACAGACGTTGCCTACTTCAAAGGAAGAT
TTAGAAACCCTCAAGGCTGATTTGTGGAGCGCCGTGGTGAAAAATAGCCAGCATCAAG
ACGCTTGGACATGGG

>novel_circ_000663
GTTACTTATTGACCAGAGTGGAGAAAAAGATTGGCTCCCCCGAAAAACCTCTGTCGGA
TCTGGGCTTGATCTCGTACCGCAGCTACTGGAAGGACGTTTTGCTGCAGTACCTCTGCA
ACTTCGGGGGCAAGGAACTCTCCGTCAAAGATATCAGCAAGGAGATGGCCATCGATTC
GTACGATATCGTGAGCACTCTACAGGCCCTCGGTATGATGAAGTATTGGAAGGGGAAG
CACATCATTCTAAAGAAACAG

>novel_circ_000664
TATGTTCGACGTTGGTGGTCAACGTGATGAGAGGAGAAAGTGGATACAGTGTTTTAAC
GATGTAACGGCAATCATATTTGTAACAGCATGTAGTAGTTATAACATGGTACTCAGGGA
GGATCCGACAAAATTGAGACTCAGAGAAAGTTTGGATCTCTTCAAAAGCATTTGGAAC
AATCGGTGGCTTCGCACAATTTCCGTAATTTTATTCTTAAACAAACAAGATCTATTAGCG
GAAAAGATAAAAGCAGGAAAGCACAAATTAGAAGATTATTTTCCAGATTTTGCGAGAT
ATCAGACACCGGTCGATGCTGGGGTGGTGGTGGATCCTTCGGAACCTCCTGACGTCAT
GAGGGCCAAGTACTTCATTAGAGACGAATTTCTTCGTATAAGCACAGCGAGCGGCGAC
GGCAAGCACTATTGTTATCCGCACTTTACATGTGCCGTCGATACGGAGAACATCAAGAG

AGTGTTTAACGATTGTCGGGACATCATTCAACGGATGCACTTGCGCCAGTACGAGCTCT
TGTGACTTCGTTCCTGCCAACGTGTCCTGTCATCTATTCGTATAAAAATTACG

>novel_circ_000665
TTTTCTAGATAAGGTAGCCATTGTTAAACAACCGGATTACACCCCCACAGAACAGGACA
TCCTCAGGTGTCGAGTCCTCACGTCCGGTATCTTCGAAACACGGTTTCAAGTCGACAA
AGTAAACTTTCATATGTTCGACGTTGGTGGTCAACGTGATGAGAGGAGAAAGTGGATA
CAGTGTTTTAACGATGTAACGGCAATCATATTTGTAACAGCATGTAGTAGTTATAACATG
GTACTCAGGGAGGATCCGACAAAATTGAGACTCAGAGAAAGTTTGGATCTCTTCAAAA
GCATTTGGAACAATCGGTGGCTTCGCACAATTTCCGTAATTTTATTCTTAAACAAACAA
GATCTATTAGCGGAAAAGATAAAAGCAGGAAAGCACAAATTAGAAGATTATTTTCCAG
ATTTTGCGAGATATCAGACACCGGTCGATGCTGGGGTGGTGGTGGATCCTTCGGAACCT
CCTGACGTCATGAGGGCCAAGTACTTCATTAGAGACGAATTTCTTCGTATAAGCACAGC
GAGCGGCGACGGCAAGCACTATTGTTATCCGCACTTTACATGTGCCGTCGATACGGAG
AACATCAAGAGAGTGTTTAACGATTGTCGGGACATCATTCAACGGATGCACTTGCGCC
AGTACGAGCTCTTGTGACTTCGTTCCTGCCAACGTGTCCTGTCATCTATTCGTATAAAAA
TTACG

>novel_circ_000666
GAGCGGGAGAGTCTGGGAAGAGTACCATCGTCAAACAGATGAGGATATTGCACGTGG
ATGGTTTCAGCGAAGCGGAAAAGCGGCAAAAAATCGAGGACATCAAAAAGAACATCA
GAGACGCGATACTGACGATAACCGGCGCGATGAGCACGTTAACGCCACCCGTTGCTTT
GGAGGATCCGGCGAATCAGAGCAAAGTCGACTATATCCAGGAAGTCTCAAACTCTCCG
GACTTCGATTATCCCCCG

>novel_circ_000667
ATGTGCCGAGGATGGAGAAGTCGCCGCTGCTTGCGAACGGGTACAACCATCCGCCCAC
GCACCTCAGTCACATGCAATTCATGCAACTGGGCGGACATCCGGGCGCAGGACACACG
GCCATCCTGTCGCCGGCCAGTCTGCCGCATCATCTGCAGGCGCAGGCACAGGCGCGGG
CCGAGCAGGGGCTCAAGGTGAACCCGAACATGTCCAACATGGAGGCGCTGGCGAGGT
CCGGGACGGTTTGGGAAAACTGCCGTGCTGCCTACGAAGATATTGTCAAACATCTCGA
AAG

>novel_circ_000669
ATCATCCGAACGTCAAGTGTTACTTCGGCCACGGTGGCCTTCTCGGGTTAACGGAAGG
GGTTCAGAGCGGGGTCCCGATGATACTGATGCCCATCTTCGGTGATCAATATTCGAACG
CGGCCGCGGCTCAATACAGAGGCGTCGCTATTATCCTGGAATACAACGATTTCACCGAG
GAAAAGCTGAGAAGCGCCATGGACCAAATATTCAATGACACGAGGTACGTCTCCTTTC
TCCGTCTGTTCCTATTTTTGTATCACGTGTGTAAACGAGAGAGAGAGAGAGAAAGGGT
ATAAACGTAGCGATCGCGAATTTAGAATTCCTTCCGTTGGTTATTTAAAGCGTAAATTCT
TCACGCGTCACGTGTTCGTCCACCGTGATCGTTGTGCCAATTACGTCCACCGTTCTCGA
TTATCACGCCGTTGATATGTCGATGCATCGGATCGAGATGCACAGAGAGAGAGAAATAA
AATAGAGAGACGGGGAGAAAGTGAAGGGAAATAAATCGAGACGAAAGAGAGGAAAA
AATAGGGGGAGGAAGGGACACGAAGCGACAAAAAAGGAAAATCGTATAATCTGGCCG

GATATTCGGTCCGTTGAATATCTCACGAGCTGTCCGGACGCTTGGATATCCGGCGAGTG
CATCGTCGTTGCAACACCCCCGTTAATCCGTAAACTGGCGTTTTTCCACCTACCCTCTG
CTACCCCTCTCCCTCTCCTTCCTCCCTCCGACAATCCAACGGTCGGACATTCGCCCTTT
CTCTCTCTTTTTTTTCCCTCCCTTCCTCTTCCCTCCCTCGTCTTCGAGTCTCCTAAATTCG
AGGCACGGAAAGAGCGCACGTGTCTCTCTCCTCGGTCCATCGACAAATACCAGGAGG
CGAGGCGAGGCGAGGTGCGCGGCGTGGCGGCGGGTTTCTCTCTCGTAGCACGGGGAG
GTCGCATGGGAAATTTCACGGCCGCTAAATGTTTCACGGCGCGAATCAACGCCGGTGG
CTCGATGACGACGACGGGGGAAACCGGGACGAAATTAAATTCTACATTATTCAATGGC
CGGCCGGGCCACGCGGATAACTTGTTAACCGCACACGAGCCGTGACGCGGCGATTGC
GTTTACGCGCGTCAGGAGCACGCGACGCCGAATAATTGCTCGTCGCCCTCGTTGAATC
TTTGAAAATCGTGCACACTCCTCCTTCTCGATTTTTTCTCTCAGAAAATGAATGGAAAC
GAGATAACTCTTGTGTTTCGTCCTCGTATTCTCGGTATTCTTTCCTCCTCCCTTTTTGGAA
TTTGATGGAATAGTAAATCGGCCCGTATTCACGTAAACGCGATATTACCCGCGCATTAGA
AGATGGGAGAAGGAGAAGTGGAAAATTCAAGAGGGAGGGGAGAATCGAAACGCCAA
TTTGTGGAGCGGGGCGAACCACGATTTAATTACGATGGCCCGGCACGCGAGCCTCCAA
GGATGAAAGCGGCTTAATAAATCAGCACCGGATATGAAAGCGTTGGCAGCATCGCGAT
CAACGGGCCTCGTACCACTGTGTCCTGGTAATTACGACAGGGGTGGATTTACGAGCTG
GCCGTCTTGAGGGGGATGGGGGAGGATGCTCGTTGTCCGTTGACAATGCACGCGCAAT
TGTATCCTCCGTTTTACTCCACGCCTTGAATATTAATTAATAAATAGAGCCGTGTAATTGG
ATGATCGCACGATTTGCACGATTGTCGTCCGCGTTACGTGATCGAAATACAGCGGGGGC
CGCGTAATCGCCCGTCTCGATCGCGACAATCGATTCCTCCTCCTCCTCCTTGTCGTC
GTTGGTTTGTGCCACCGAGAATTTCAACACGCCTCCTGTCACGAGGCCTTGCTCCCCC
CTCCCGCGATATTCTCCGTGATTTCGCGCGATTCTCACCGATCTCTGTTCGATCGTGGCC
GTTTCTGTGTTCGTGGATCGAGCATACTCGTCGCGCAAGGGCAGAGGTAACGGCACGG
TGAAATGCGAGATCCGGGGAGAAGCGGTCGGTTCGCGTGTCGCCGCGCAATCGTCGTC
GCCGCGCAACGTCCCATGCATGCGAGTGATATAAAATTAACCAACGATTTGGAAACAG
TGTTGGCCGGGATATGTGTGTGTGTGCGCTCGTTGTCGTGGCCGGTGCTGCCGCGTAAT
CACCACTTTTGCTTTGATTTATGGGCCCTGGTTTCTCTTCCCTCGCCCCTATGGCCAACT
ATGTAAGTTCCAAGTATAAGAAAGTCGTCGTCGGGATAAACGCCGTAATGGTCGGACG
ATAGCCTTCGATGGAAAGAGATACGTTATCGTGGGGGAAGGGAGAGGATCCGTGGCTC
GGTTAAACGTCCATCGAGCCAATAATGGCCCGATCGGGATCGCGAAATTCCAGGGTCG
ACAGTGTCAGAGTGGCCTCCATATAGATTCCCGTTCCTCGTCCGCAAACCGTCATCCTT
TGGTACTCTGGTTGATCGATGCGATCTTAAAACGTTATAACCAAAGTTATATTATCGTTC
GTAGCGATTTCTCGAAGAGGGAAGAAGACGGAAAGGAAGACGGAGAGAGAGAGAGA
GAGTTCGTGACCCGCGCCACGAACGACGTGCCGCTTGCTCGATCTCCAGTCTCTCCTT
TCGAGGATCCCACGAGGCGACGCCAAGTGGTCATTCAATCCCCAAACTTGCCCGCGCA
TGATTTCGCTTGTAAGCCAACTTCGAGTTACAGCCATTCCGCTAGAGATCAAACGGCAT
ACACGGCTACTACCCGTCGCCTCTCCTCCCCCTCCGCCTTAAAGACGATCATATTCAGC
TTGTACTTGCACGGCACTAACTGTACGATCGTAACGATTCCATGCTCCCGTGGTCGGAC
AATAAACGTCGTGGAAATTTCGTCCAATTTATCGAGCCCTGGAGACATTCCAACGGCTC
TTCCAGCTGACTCGACCAATTATTATTATTATTATTATCATATACCTTCTTCGCATCTT
GTCCAACGTATCGAAGGGAGCATTTCTTTTTCCTTTTTCTTTTTTCCTTCGGTATAATTAG
CGTCATAATTAATTTTCGAGCAAGAAAGTCATCAGCTGCGCCATATACCACCTTCCTCC
GGTTTCTTCCGTTGATGAACCAAGTTCGGGGGATGATATTAAAACGGCGCTATCGAATT

CGTCTCCCGGCCAGGAGAATTTCTCCCAGACATGATTCGTGCGGCTGCAGAGAGGAGG
AAACGTGTGCGTGCGAACGAGTTGCCACAAGCCGAAGCCAAGTAGTCGGGAGGATGG
CGGCGGAGGGTGGTAGTCAAAGTTTCGACAGTTTGTAAGGAGGTACGAGGCACGCCC
CTGTTCCCCTCTTCCTCCTCCCGCTTCTTCTTCGGTTACGGCTGAGGGATAACATAGTGC
AGCTCCGTCGTCGAGTTAGCTCTTTCCCTCTCCTCTTTTCCTTTCTTCCCTTCTCTCTTTC
TCCTTTATTGCTGTCCTCCTTCCTTTATTCTCGCAACCCTTTCGCTTCAGTTTCACGAAA
ATTCCACTCGCCCCCGTGCGCTCGTTTGCCGGGACATCCCCTAGCTAGGATTTATTTCG
AGAGGCGGAGGTATTCTTATTCCTCGCGCGGAATAAGGAGAGGAAGGTGGTTACCTTG
CTTTCTGCGGCTTCGACGACAGTGCTTGGCCGCACCTGTTCGCTCGTAAGGGACGCGT
AATGGTCGAAGAGGGAAGAGGGATGGAAGAGATATATATATATATATAGGGGAGGAGA
AGAGGGTGGAGAAAGGGTGGAAATGAACGCAAGGTCGGAGGAGACGCTGCGTAATG
ACGCGTGGAACGTCCCCGCGTGGGGACGAGAAAGTAATTGGACGGTTGCCGGGGATA
ACTCGGTTCCGCCACCCTCTTTGATGAATGGCGAGCGTTCGAGTAGCGGTTCTCTTGCG
AAACTCTGTTTCTTAAAACCGTTCACTTCAAGATTCCTCCAAGATTCGTGCGTTTCCAA
ATGCGTGTCGAAGAGGGATGATTTTCGAGGATCGTGAGAATTATAATCTCGTATTCTGG
AAACGAGGAAGATTTTCAAACGCACGAGAGAGATACTTGTCGACTCGATTTAGGCGCG
AACTTCGATAGTGTCGTTTCCCCTCCCCACGGATGACCACGCGTCGGATTAAAAAGTTG
CTTATTTGCAAATGGAATTTTCGCGTCTGCCGGTATTACATTCACCGGGAATATATTCCG
TTGTGGAGTTATATTCGCGGGGCAAAAAGCCGGCTTTCACGGATCCGCTGAATTTCATT
ATTTAAGTTACGTGTACGGGTACAAGTGTTTGAATAATGCATTCGGCTCGATGTACATGA
AAGCCGCATTTAGAAACCGCCCTAATACAGACGTTCACGACCTAACATCTTTATACTTG
CTCTCTCTCTCTCTCTCTATTTTTCTCTCTTTCTTTAGATCGAACGTCGTTTCGATAATAAT
AATTGATAATTAGGATGGAACGTGACTCGCTTTGAGAGCTAGATTCGAGATTTTCTCGC
TGCTATTATTTAATGCATTGCAGATCCATCTATAAAACGTGGGGAATGGAAATCTGTCAT
TAAGAGAAGGAAGAGGGAGGGGAGGGGTGAAGCTTTCTCGTCGACTTGAAACGTATC
GAAAATCTCGAAGTGGATACTCGAAAAGACTCC

>novel_circ_000670
AGGCTTACGTGAATCTGGGCGCGGCGCTGGCCTCGGTCGGACGTGGAACGGAGGCGG
CTGCGGTTTTGCGGGCCGGCGCATCCTTGGATGGTTCCGGGCTCAAGGACAAGAGGGC
TCACGAGGCGGCGAGGGTGCAGGCTCTCCTTCAATTAGGCGCCCTCTACGCCGATCAG
GGCAGATTACAAAGGGCCCTGTCCGCGTACAGAGAGGCACTTCACGCCCTCCCGGATC
ACTATCCACCTCAG

>novel_circ_000672
AATCGGAACCGGCGATCCATCCGACATGCTCAACACCCGCACCAAAGGCTCGAAACC
GATCATCCCCGAGGCGGCCAGGTTCATCGAGGTGGCGACGAACAGCATCACTCTGCAC
TTGAACGCTTGGTCGGACGGCGGCTGCCCCATGATCTACTTCGTGGTCGAGCACAAGA
AGAA

>novel_circ_000673
ATGGTACAAGTTCATCGAGGGCTCGTCCCGCCGCCAACCGGTCCAATTGAACGAGCGC
GTCCGCCAGGTTAGCGGGACACTGATCATCCGGGAGGCGCGTGTCGAGGATTCGGGA
AAGTATCTTTGCATCGTGAACAACTCGGTGGGCGGGGAGAGCGTGGAGACGGTGTTG

ACGGTGACGGCGCCGTTGGGTGCGGAGATCGAGCCTAGCACGCAGACCATCGACTTC
GGCAGACCTGCTACCTTCACTTGCAACGTGAGAGGGAATCCGATCAAGACCGTCTCGT
GGCTCAAGGATGGGAAACCGCTTGGACTGGAGGAGGCTGTGCTGAGGATCGAGAGCG
TGAAGAAGGAGGACAAGGGGATGTACCAGTGCTTCGTTAGAAACGATCAGGAGAGCG
CGCAGGCCACGGCGGAACTCAAACTCGGTGGACGATTCGAACCCCCGCAGATTCGCC
AGGCATTCGCCGAGGAGACGCTTCAACCGGGACCTAGCATGTTCCTGAAATGCGTGGC
TAGCGGTAATCCGACCCCCGAGATCACCTGGGAACTCGACGGCAAACGTTTGTCCAAC
ACGGAACGGCTTCAGGTTGGCCAATACGTGACGGTGAACGGCGACGTGGTCTCCCATC
TCAACATATCCAGCACTCACACGAACGACGGTGGATTGTACAAATGTATCGCAGCTTCC
AAGGTCGGATCGGCGGAACACTCGGCGCGTCTCAACGTCTACGGTCTCCCATTCATTC
GCCACATGGACAAGAAGGCTATAGTTGCCGGTGAAACTCTTCGCGTCACTTGCCCGGT
TGCCGGATACCCGATCGAGAGCATCGTGTGGGAGAGGGACACGAGGGTGTTGCCGATC
AACAGGAAACAAAAAGTCTTCCCGAACGGTACCCTCATCATCGAGAACGTGGAGAGG
ATGAGCGACCAGGCTACCTACACGTGCGTCGCGAGAAACGCTCAAGGTTACAGCGCTA
GGGGAACGTTGGAGGTTCAAGTGATGG

>novel_circ_000674
CGCCAACTTGGAACCCCTCCCCCGTGGCGCACGCAACCCCCCACAGAGAATAGGTGA
CGGACGCTAGGCCGCGCGACTCTGAAAAGGATGCGGGCCCTCTTCTCCTTCGGTAACC
TTCGGTGCCTGAGAGGGTGTCACCCCGATCAAACAGGGGCCAACAACAATGTTGCCCT
GCACCTGCACACCTCCAATAAGATCCCACGATATCTGGCTGGTAAGTCCTCGCCACCTT
CGTGCCTCCGTTCATCCGTTCGTGTTTTTCACAACCACCACCACCACCACCACCACCA
CCACCCTCTCTTTCTCTCGCTTTGCCACAGGATTAATTTCGTCTTTCGTGATCTTGTCCC
TCCCCCTCGTAATTGCAATGTCGCGCGATCTGTCTGTTTGCACAATTTTTTACATTTTCG
TGTCGCTCGATTGCCTTCCTATCCCATCCCTTCTCTCTCTCTCTCTCTCTCTCTTCCCGGA
TCCGAGCTCCCCCATCTATGTGTATCTTTTTCTTTTCATTTTCAACGTGTCAAGCGTGTG
GCAGGCAGCTTATTCCGATTTCGTTTCATCACGATCGAGAGAGAGCAGTTGCCACTTTT
CTTTTTCTTTTTTTTTTTTCGAATCTTCTCGAGCTCTCTTCTCCGAATCCGTCGTCCCTCT
TAGTCGGGAATTATATTTGCATTCGCTCACCACGTGGCGAGGATCGAGACTTGACCTCT
TTTTAATCCCTTTAAATCGTCGCCGACGACGATCGCCTCTTTCTCTTCCCCCTTCGAGAA
CCGATCTCTCGCTCTCCGACATGCGAGTGTATATGAGAGAGAGAGAGAGAGAGAAACC
TACGCTTCTTCTCTTTGCGCTTCTCTTTCCTTCCTTTTTCTTTTTTTTTTTTCCCTCCCCA
CCCCTCCAGTAACCAAGTTGTCGCTGTTGTGTTGTTGTTCGCCAGCCTTCGTAATAAAT
CCCTCCACCCCCACCCCGTATCCCCTCCGACCTGCTCTGCTTCACGACCGCTATTTACG
CTCCAGCTGTTGTCTCGAGCTCTCTTTTTCATTAAGTTTGCGTACTTGTGTCGTAGCGAG
ACACGCGCTAATGAGCCGGCAGTAGAGGCAAAAAGCCCGCGCCCAGAGACGCCCAAA
GCCCTGCCTATATATATATATGTATGTATATACACACACACATATATATATATATACATATATA
TACTCGGAAGATGGGAAAGAAACTCGAAGCGAATAGAAACGCACCGATCGTGCGTGG
CGACCTCTGAATACACACACACACACACACACACACACACACACACACACACACACA
CACACACACACACACACACACACACACACACACACACACACACACACACACACACA
CACACACAGAGAGAGAGAGAGAGAGAGAGAGAGAGAGAGAGAGAGAGAGAGAGAGAG
AGAGAGAGAGAGACATACATACATACACATGTATATAACTCGATACACTCGGTTCATTT
GAATTCATCTTCGTGTCGAACGTCTGTTTCTATTCGCTCGGTTCGTTTCCATCCGAAGGA
AGGAAGGAAGGAAGGAAAGGATTTCTTTCTTTCGAGAAATTCGAGAAAAAAAAAATG

CGGCCGACCGAATCGCAAATGACGAGAACGCCGCGCGCCATGTCTCGTTTCAGATGGT
ACAAGTTCATCGAGGGCTCGTCCCGCCGCCAACCGGTCCAATTGAACGAGCGCGTCCG
CCAGGTTAGCGGGACACTGATCATCCGGGAGGCGCGTGTCGAGGATTCGGGAAAGTAT
CTTTGCATCGTGAACAACTCGGTGGGCGGGGAGAGCGTGGAGACGGTGTTGACGGTG
ACGGCGCCGTTGGGTGCGGAGATCGAGCCTAGCACGCAGACCATCGACTTCGGCAGA
CCTGCTACCTTCACTTGCAACGTGAGAGGGAATCCGATCAAGACCGTCTCGTGGCTCA
AGGATGGGAAACCGCTTGGACTGGAGGAGGCTGTGCTGAGGATCGAGAGCGTGAAGA
AGGAGGACAAGGGGATGTACCAGTGCTTCGTTAGAAACGATCAGGAGAGCGCGCAGG
CCACGGCGGAACTCAAACTCGGTGGACGATTCGAACCCCCGCAGATTCGCCAGGCATT
CGCCGAGGAGACGCTTCAACCGGGACCTAGCATGTTCCTGAAATGCGTGGCTAGCGGT
AATCCGACCCCCGAGATCACCTGGGAACTCGACGGCAAACGTTTGTCCAACACGGAA
CGGCTTCAGGTTGGCCAATACGTGACGGTGAACGGCGACGTGGTCTCCCATCTCAACA
TATCCAGCACTCACACGAACGACGGTGGATTGTACAAATGTATCGCAGCTTCCAAGGT
CGGATCGGCGGAACACTCGGCGCGTCTCAACGTCTACGGTCTCCCATTCATTCGCCACA
TGGACAAGAAGGCTATAGTTGCCGGTGAAACTCTTCGCGTCACTTGCCCGGTTGCCGG
ATACCCGATCGAGAGCATCGTGTGGGAGAGGGACACGAGGGTGTTGCCGATCAACAG
GAAACAAAAAGTCTTCCCGAACGGTACCCTCATCATCGAGAACGTGGAGAGGATGAG
CGACCAGGCTACCTACACGTGCGTCGCGAGAAACGCTCAAGGTTACAGCGCTAGGGG
AACGTTGGAGGTTCAAGTGATGG

>novel_circ_000676
TGGCTTGCGCAGAGGATGAATCGATGGGACCGGTATTCGTCAAGGAACCACCAAATCG
AGTCGACTTCTCGAACGGAACCGGAGCTGTCGTCGAATGCCAGGCAAGAGGAAACCC
CCAACCGGATATCATTTGGGTTCGTGCTGACGGAAGCGCTGTTGGGGATGTTCCTGGA
CTGAGACAGGTCTTGCCGAACGGAAATTTGGTCTTCCCCCCTTTCCGCGCCGAGGATTA
TCGTCAAGAGGTTCATGCTCAGGTTTACAGCTGTCTGGCGCGCTCCCCAGCAGGTTCA
GTTCACAGCCGAGATGTTAACGTGAGAGCCG

>novel_circ_000677
CAAAGGACATCGTTATTCGCAAACTATTGGTGGTTCTCGTCGTGCAGCATGGCTGAGA
AGAAACTCTTTATCGCTTCGCAGAAAGCGATAAAGTTTTAAATATTTCTAATGATAAGT
ACATGATACATCATTGCATTGATATGTTACAGATATTTGTATCAATATTATGGTATATCCTG
TACAAAATAAACAC

>novel_circ_000678
GGATTCGCAATGGGTGCATATAAATATATGCAAGAACTTTATCGCAAGAAGCAAAGCGA
TGTACTCCGCTTCTTGCTTCGTGTTAGATGTTGGCAATATCGTCAATTGACTAAAATGCA
TCGTGCTCCTAGACCGTCTAGGCCTGATAAAGCACGCCGTTTAGGTTATAAAGCTAAGC
AAG

>novel_circ_000679
GCAAGAAGGTGGAGGGTGGAATGGCGACCAGCAGCAGCGGACCAGTCGTGCAGAAA
GTGAAGGTGAAGGAAGAGGCGAAGGATCACTGTACGATGACGACCGGGACCACGTGC
ACCACGACCACCACCGCCACGACCACCACCACGACCACGACCACGAGCACGACCACC

AGTAGCACGGGCACGACCGTGACCAGCACGAGCGCGAGCACGAGCACGGTGACCAG
TTTGGGCGAGAGCATCGCCGCCCTCCATCAACAACCCTCCGCCGCTGTTTGTCACCTAT
CGGCGCCAGCGGCTTCCGTCCTCTCGGCCAACGCCGACCCCTTTCCCGTCGATCTCGA
CCTCAAGACCAAGATCAAGG

>novel_circ_000680
CGACGCAAAGGCCTCACCCACCACCAGTGCCGCCACGACCCAGTCGGCAGGTGGTCG
CAGAAGCCCTTAAAAGATCACCAAGGCCGCCCTGCCCGACCAGGCAGGCCCCACCAC
CGCCCAACACCAAACCGTGGAGATCCGATCGAGATCGGGCGAACAATCGGCAACAGG
TGTATCCCGCTCCTGTTGGGCGCACGCTTGTTTACGAATCTTCGATCAAAGAGTCGTCG
CTTCCCAAAGAAACGAGCAACGAAAGCGGCGGCGATTGCGATTCGCCGCTCGACAAG
AATCGCGAACCCGTTTCTCGAGCCGCGAGTGAAAGGCGCGCGGACGAGGACGACTGG
ACGCGTGGAAATCCGCGGGAAAGGCGGCACCGCGTACCCGACCAGGAACATCGGCCA
AATTCCACTACTGAATCGAGCCCCAACACCGACGCCACCGCCGCCGCCGCTTCTTCCT
CGTCTCCTCTACCGCGAGAGAAAGAGGATCGCTCGAGCCCGTGTCACTCTGTGTCTCT
GCTCGAACATGCGCCCTCTAAAGAACGCTCCTTCAATGGAGCAACCACGTCTGGTGAG
AGATCGCCGGTAAGCGAGAAATCGATAAGCAACGTCGACAGAACGTCGACTGGGACG
AGCGAGAAAGCGAGAATCGAGTCAGAATCGATAATTTCTCGCGCGAAGGAAACGACC
GTTTGCGACAACGATGCCAAAATTACTGGGCTCGAGTCGCGCGTATCTCTGGACGAGG
TACAGTGTTGTCACATTTCGCCCGATGTTTCGGAATCGAAGAAAAGCGGGGAAAGGGG
AGGCCCAAGTTTTTCAGAGAGAAACCAATGCTCGGAGAGAAGCAAAGCTGATGGCTC
GACGCGATTGCCAACGCCAAGCCAGAGATCGTGTCTCGTGAAATATCGAGACTCGGCG
AGGAAAAACGCGGAAAGTTCCACCGCGATCCAATCTTCGGTTTCGAACGTTGACCGTG
CCACAGTGGTTGTTATCGACGAATCGGACCGTAAAGCCGCATCCAACGATCATCCTGAT
CACTGTGACGATCGCAATCACCACGTTAACCAACATAACAACAACAACAACAAGGAG
AATGCGGAGAATGACAACGACAATGATAACATTCATCGACAAGACTGGTTGGAAGCCG
GTGTTCATTATTCGTCCACGCAGATCAGATTGTCCGGAGAAGATGGCGACGTTATCGAT
GGAAGTCGAGTCAATGGATTTGATCGTTGTGAAAACGAGAAATTCGGGGACTTTAATG
TTCCAAGCATACAAGAAAGGATCGCTATGTCTTCCCTGCAAGGTCTACCACCGTTACCG
AGGAGTCTTAGTGGGTTCAATTTGAGTGGTGGACGAAGCGAGGGTTGCGAACCACCT
CCGCCGCCCACTAGATCTTCCAGTAAAACGCAAAGAGGCGGTAAAACCCCGATCCAAT
CATCGTCCAGGCCATCACCTCCTGCTAGACAGCTTACCACATTGGATACACAACTGGCT
ATACTGAGGAGAGAAATG

>novel_circ_000681
ACGAACGAGACGCGATACAAAAGAAAACATTTACCAAATGGGTGAACAAGCATCTGA
AAAAGCACTGGAAATACGTCAAGACTTACACGTGCCTGCACGTGTGCGTCCTTGTCAA
CAATCAACCATGCTGTTCCCCCACTGCAAGCAGACATGTCGGAGATTTGTTCGAAGAC
CTGCGGGACGGGTACAACCTCATTTCCTTGCTGGAGGTTCTCTCGGGCGAGCATCTCC
CGCGAGAACGAGGTCGGATGCGTTTCCACATGCTGCAAAACGTGCAGATGGCTCTTGA
CTTTTTACGTTATAAGAAAATCAAACTCGTCAATATACGCGCCGAGGATATCGTGGACG
GAAACCCGAAGCTGACCCTAGGTTTGATATGGACCATCATACTGCACTTCCAGAGCTG
GCGACGCAAGCATTCCCTCTCCCCTTGGCCAACAATTTCCAACATCTCTCCTCCGTCAT
CTCATCTCTTAATAATCGTCCACCTCTCCGCCTTCCCTTCTCCCTCAACATCGCCTTCTG

GTCCAGTGAACATCACGATCCTCTTTCCAGTCGACGATCTACACACGACGAGCTACAC
ATTCTCGCGCTATCGTCGATCGCTCGTTCCTCCCATTCGTACGTTCGATAAGAATCGCGT
TCGGTGAAACGATAATTCGTTTTTTTCGAGTCTCGGCGCGGGATCTCCGTCCCGTTGGA
GACGTCGATTCTCGATAATCGGCTTTCACGAGCGCGGCCGCGCGTGCCGTGAGTAATC
GAGGAGGGAATCGAGACGCCGGATAAGGCCACGAATGAGTGGCCGTACGTGCCGAAC
CTCTTTTCCCGGAGGTCGATGTTAGCTCGTTGCACGGAATCGGAATCCTCCCCGCGCGG
AAAAGACACGAGAAGGGGGAGAAGAAAGGTTGTTGTTTCGTTGTTGTTGACAACAAC
ACACCATCATGTCGATATATCGGTTCAGCAAGGGCGGCCGGCTATTCGTGCAAGGCACC
AGGGAGGACGAGCCCCGCGAGAACGTCGTTTCGGCTGCCTCGACCGCCGCTACACCC
GCCAACGACGATCTCAGAGCTATTTACACGAAGCGGAGGCTCTCGACCGAGGTTCTAG
GATCGTCGATCGAGTCAACGAAGACGTCGAGGCGCGGCGAGAACGGGACCAAGCGTA
TCGTGACACGGATCGTTCGTAAAACGACCACCTTGACACGGGGCGAGGAGAGATGCG
TGGCCGAGGATCTGACGAAACGCGCCGCATCCGGCTATTTACAGGACACCGCCGCCTC
CTCCTCCTCCTTCTCTTTAAGCCGCCAGGCCTCGCCAAAACCGAAAACCGTCCGGATAT
CCGATATCGTGGTTGGTCAGGAGCCTAACGTGACCGCCCGGGAGGCGCTTCTAAGATG
GGCCAGACGATCGACGGCGCGCTACCCGGGTGTACGCGTCACGGACTTCACCGGCTC
GTGGAGGGATGGATTGGCGTTCAGCGCGTTGATCCATCGAAACAGACCGGACCTGGTC
GACTGGAGGAACGCCCGTGCCAGTCAACCACGAGAGCGGCTCGATCGTGTGTTCAAC
GTCGCGGAGCGCGAGTATGGCGTTACGAGGCTTCTCGATCCTGAAGACGTGGACACCC
CGGAACCGGACGAGAAGTCCTTGATAACGTATATCTCTTCGCTCTACGACGTGTTCCCG
GAGCCGCCGCCTATTCATCCGTTGTACGACGCCGAGGCCCAGAGACGGTCCGAGGAGT
ACAGGGAGTTAGCGAGCTCCCTCCATCTGTGGATCCGCGAGAAAATGTGCCTGATGCA
GGAGCGGGCCTTCCCCCCCACTCTGATCGAGATGAAGAAGCTCGCCGCGGACAGCAC
GAAATTCAAGAACGAGGAGGTGCCGCCACGTTACAGGGACAAGCAGCGCCTCTCCTA
TCTGTTCAGGGATCTTCAGAAGTATTTCGAGGCGGTGGGCGAGGTGGACATCGAGGCC
GAGCTTCACATCGAGGTGATCGAGAAGAATTGGAACCGATTGATGATGCTTCACCAGG
AGAGGGAACAGGCTATAGTCGATGAGATAAAACGGCTCGAACGGCTGCAACGATTGG
CGGAGAAAGTGCACAGAGAGATGAAATCGACCGACAACCGACTGGAGGAGCTGGAG
AGAAGGGTGGAGGACGAGGCGAGACGCCTCGACCGACTTCATCCTTTGGAGGCTAAA
CACGCCGTCGATCTGCTCGAGCAGGACATACGCAACACCGAGATACAGATTCAAAATA
TTTTTACCGACGTGCACACTCTTACCGAGGGACGGTACAGTCAGGCGGCCGAATTGCA
CAAAAGGGTTCAAAAGCTACATCAACGGTGGGTCGCGCTGCGATCTCTTCTTCACAAA
CGTTTGGTACAGCCGCTTTCGGCCGTCTCGTTCCCGGTCGAGGAACGCGTGGTGACGA
AACACCGGACCACCGTGCACGAGACCCGATTGGTCGACACGAACCCGCACTTCCGCT
CGTTGCACGACTGCATCGACTGGTGCAAGGCGAAGATCAAGCAGCTCCAGGACGCGG
ATTACGGTTCCGACTTGCCCAGCGTGCAGAACGAGTTGGACGTCCACCAGAAGGAGC
ACAAGAACATAGAACAGTTCCATCCAAAGGTGGACAGATGCGTGCAGGCGAAGAGCC
ATTTCCACGGCGAGGAGTCCACCCTGTACAGCCAACATTTGGCCGTGCTTCAGAAACT
CTACACCGAGCTGTTGGCTGCCTCGAACAAGAGGCTCTCGGATCTTGACACACTGCTA
GATTTCATACAATCGGCCACGGGCGAGCTGGTCTGGCTGAGCTCCAAGGAGGAGACCG
AAGTGACCCGCGACTGGAGCGACAAGAATTTGAACGTGCAGAGCATAGAACAGTATT
ACGAGCGCACGTTTGGATCCGGGATAGAGTCCCTGATGAGCGACCTGGAGAAGCGAG
AGATCCAATTCTCAGCGGTGCAGGATCGGGGCGAGGCCCTGGTCCTCCAACACCACCC
CGCGGCGAAGACCATCGAGGCGTACATGTCCGCCATGCAGAGCCAGTGGGCCTGGCTC

CTCCAGCTCACCCTCTGCCTCGAGGTGCACCTCAAGCACGCCGCCCAGAGCCAGCAAT
TCTTCCGCGACGTCCAGCAAGCGGAGCAGTGGATCTCGAAGAGGGACGAGACGCTCA
ACACAATCTACTCCCAATCCGACTTCTCGTTGGACGAGGGGGAAAGGTTGTTGAAAGG
TATGCAAGAGCTGCGCGAGGAGCTGAACGGTTACGGGGACCACGTGCAGAAGCTGGT
CGACCAGGCCAAGGACGTGGTCCCCATGAAGCAACGGCGCCAGCCCGTCACGCGACC
CATGCAAGTCACCTGCGTCTGCAGTTACAAACAGGTTAACATGTCGATCGAGAAGGGC
GAGCAGTGCACGTTGTACGACAACTCTGGAAGGATAAAGTGGCGCGTGAAGAACGAA
GAGGGGATCGAGACGCCCGTGCCAGGCGTTTGCTTCGCCCTCCAACCGCCCGACAAG
GACGCCCTCGAGGCCGCTGAAAGGCTGCGACGACAGTACGACAGGAGCATCGCGTTG
TGGCAACGGAAGCAGCTTCGACTGCGCCAGAACATGATATTCGCCACGATCAAAGTGG
TGAAGGGGTGGGATCTGCCGCAATTCTTGGCGATGGGCCAGGATCAGAGGACGGCTAT
CAGGAAGGCGTTGAACGAGGACGCGGAGAAATTGTTATCCGAGGGAGACCCGGCCGA
TCCCCAATTGAGGCGGTTGAAGAGGGAGATGGCGGAGGTGAACAAGTTGTTCGACGA
CCTGGAGAAGCGCGCGAGGGCCGAGGAGGAGTCGAAGAACGCTGGGCGTATCTTCAA
CGAGCAGATCTCCACGCTTCAGCAGTCCCTGGACGAGGCTGAGAGGGCGTTGAACTC
CAGGATAACCGCTCCCCTGCCCAGGGACATGGACACCCTGGAACACCTGGTGTTGCAG
CACAAAGACTTCGAGCAAAGCCTGAGACGGTTGGCCCCCGATCTGGACAGGGTGCAG
CAGACGTTCCGTGGAATCACGTTGAAGACGCCCGCGATGAGGAACAAGCTGGACGCG
GTGACCACCAAGTGGACCCAGATCTGGAACTCGAGCAATCTGTACATCGAGCGGCTGA
AGTGCGTGGAGATAGTGTTGTCGAGGCTCGAGGAGAACACCACGGCGATATCCGAGAT
CGAGGTGAAGCTGGCCTCGTTCGACGAGCTCCCCTCCGATATAAAGGGGTTGCAAAAC
GTGTTGGAGGATCTGATGCTGCTTCAGAACGCCATCGCCCAACAGCAGACCGCGATGG
ACCAGTTGAACGAGGACGCGCACAACGCAAGGAGGCTGGTCGAAAAGTCGAGATCC
GGCCATCGTGGCCCACACTCGGACATGGACCGTCTGGACGACGAGGTGAACAGGTTG
AATTCCAGGTGGACGAACATCTGCGGCCAACTGGTGGACAGGCTTCGTAGCGCGGAA
ACGGCTTACGCGCTCGCGCAACAATTGGAACACGCTTACAGAAACGAGGTCGACTTC
GTGGACGAGTCGTACAACAGACTCGAGATGGAGAATGCCAAGAACCTGTTGAACAGA
GTGGTGGAACGAGCGCCTGCGATCGAAGCGGTAAATGTGACGGGCGGTCGATTGATTC
GCGAAGGAAAG

>novel_circ_000683
CGCGCTCTCCAGGAGCAGAGGCCGCTGAGGACGTTCCGCACCGAGTTCAACATCTAC
GAGAACAGCAGCTGCGAGGAGCGTTACACCACCACGACCACCCACTACACGCAATCG
CAGTACAGTTTGGAACGGCACGAGCGGACAGAGCGCACGGAGAGGAGGGAGACGAG
CGAGACCATAGTCTCGTCCAGCGACACGATACGTTCCCGGGAGAGCGGTCGTCTAGAA
ACTCGAAGCGGATCCGTCGGGCGGGATGCGGTCTCGAGAGAGATCGAAGAGAAAGCC
TCCCGTAAACAGGAGGAGGAGGAGGAGGAGAAGAGGATCAGCGACGCCACTAGTAG
CGAGCGTTTTAGCGAGATTAGGAGTTTAAAGAGGAGTTTAGAGAGGGCGGAGGAGGG
CAGCGTAGGTAGAGAGAGCAGCAGCGTGATCGACGCTAGGGGTATCGTGGATCCACGT
AGCGGGCAACTGTTGACCGTGGGCGAGGCGATAAACCTTAGGATCTTGGACGTTAGGA
GGGGTAGGATAACGTGCTCGAGGGACGGGCAGAGAAAGAGCGTGGCTATCGAGGAGG
CGGCACGCGAGAAGATGATCGAGACGCGTCTCGCCGACCGTCTGCTGGGTCCGTGCG
GCTACGACGAGGAGGGGCGGCCGGTGTCCCTGCTCGGCGCGATCCAGCGGGAGCTGA
GCGACGCCGAGAGAGCGGACGGTGCCGCGGCAGACCGAGCGAAG

>novel_circ_000684
CAAAGGCGCTGAACCCGAGTTCCGTAGCCCGAGAGTGAAACTTTTGTGGGATAGATGG
AGACACGTTTGGATGTTGGCCTGGGAACGTCAACGCCGTCTACAGGATAAATATAATTA
CATACAGGAGTTGGACCGAGTCGCGAACTTTAGTTGGGAAGATTGGCGCAAGAGGTTC
CTGAAGTTCATGAATCACAAGAAATCAAGATTAACGGATCTCTTCAGAAAGATGGATA
AGAACAACGACGGTCTGATCCCACGCGAGGATTTCATCCAAGGAATCATGAACACCAA
ATTTGAGACTTCGAGGTTAGAAATGGGAGCAGTCGCGGACCTGTTTGACCAACACGGC
GAAGGATTAATAGATTGGAAGGAGTTCATCGCAGCTTTGAGACCAGATTGGGAAGAAC
GTAGGACGTACAATGATACGGACAAGATCCACGATGAAGTGAAACGATTGGTGATGCT
CTGTACTTGCCGACAGAAATTCCGCGTGTTCCAAGTTGGCGAAGGAAAATATAGGTTT
GGAGACAGTCAAAAGTTGCGATTGGTACGAATTCTACGATCGACCGTAATGGTACGAG
TCGGTGGTGGTTGGGTAGCTTTGGACGAATTTTTATTAAAAAATGATCCTTGCCGCG

>novel_circ_000685
CCAAGGGAAGAACGAATATCGAGCTGCGAGAGCAATTCATATTGGCGGACGGGGTCA
GCCAGACAATGACGGCGTTCAGATCGAAGCCGAGCCCTACCTCCACGTTGCAGCGTAC
CCATATCTCATCCGCGAGTGCCGGACCCATCACCAAGCGGAGGAGTTTATGAGCCAGC
TGACGCCGATTTTTGAGAGTTTGAGGCAAAAGGAGGACCTCCCGTGCTCGTATCCCCT
CCACATGCACGCCCCCTCTTATCATTGGTGGCGTTGATCGGAGATACTTCGAATCATCAT
AAGCTACTATATCTGTCCCAACTTCCTCTGGTGAGGGAGCGAAGCGCGCGAAGCGTTC
CAATGGGACAATCGCGGGCATCCCGCTCCTCGCTCAGCGCCGGCACGCCGGACAGCCT
GAGCGACAACGAGAGTTCCTTCAAGATTGCCTCTGCAAGGAAATCGAGCACACCGTA
CAGAAGCACAATGACACCTGGAGGTAGCCGGCCATCCTCGAGACCGGCTTCGAGACC
AGCGTCCAGACCGACCAGCAGGCCCGGAAGTAGGCCGGCGTCCCGACAGGGGAGCA
AACCACCGAGTCGATATGGATCCACTCAATCGTTGGACAGCACCGACGAATCGACGAA
CGTGAGCCGTATTCCGCGGAGAACGGCGGTGAGCACGACGGGAAACACTCCTACATC
GAGCAGGCACAACAGCGTTTCAGGGAAACGGCTTGGAACGCCTGTGAACGGGTCGA
GTTCGCGGCCCCGAACACCCACAGGGCTTGTTAGCCCCGCGAGTGGTGTCCCGTCCAG
GTTTGGCACGATCCATAGAGCTTCGAGCATTCCAACCCTGACTGGTGTCGGCACACCG
ATCAG

>novel_circ_000686
CAGCGGCACAGGGTCGCGTGGAGGAGACGCAGATGGACACGCACGAGGGGTCGACG
GTACAGCTGCAGTGCCGTTTCTCCCCGCCCAGGGAGAACGTGACGTGCTTCTGGTTGA
CGCACACGAACGATAATCACGACAACGCAGCGATCGACAATCTGTCGTTATCGCCCCA
GTACAAGGTGTTCATGAATCTGGAGGAGGGTCGGTACGACCTTCAGATAAGGAACGTG
TCGTACGAGAGGGACAACGGGAAGTACGAGTGCCGGGTGAAGGCGAGCGGGACGGG
GCACGACCTGCACCGAAAGTTCATCGCTCTGACGGTGCTGAGGGCGCCCGGGCCGCC
CACCATATCCCCGACCTCGGCCTCGGCGACGGAGGGGCAGAGGCTCGAGTTGCAGTG
CAACACGAACGGGGGCAGCCCCGAGCCCGAGGTGAGGTGGTACCGCGGCAACGAGA
CGGCCGTGCTCCACTCGGGCAGGACGCTGACGGTGGAGCCGAAGAAGGAGGACGAC
GGGGCGACGTTCCGCTGCGTGGTGCGGAACCGGGCGATGCGCGAGGGCGAGACGCTG
AACGCCACCGTCACCCTCGACGTCAACTACTTCCCCCGGGTGGCGGTCGGGCCCGAG

AACCCCCTCAAGGTGGAGGTGAACGGGACGGCGAACCTCGAGTGCCGCGTCGACTCG
AAGCCGGCGGTGGGCATGGTGAGGTGGTGGAGGGACGGCAGCTTCGTGGCGACCAGC
TTCCAGCACGCGATTCGAAGGGTGGCGGTGCAGGACGCGGGCAAGTACACCTGCCAG
GCGGACAACGGGCTCGGCAAGAGGGGGGAGAGCTCCCTGCTGCTCGACGTGCTGTAC
CCGCCCACCGTGTCGATAGAGGGCGAGCCGCTGAGGATCGCCGAGGTCGAGGACACG
GTGACCGTGCACTGCAACGTGACGGCGAACCCGCCGCCCTCGGTGGTCGAGTGGCTG
CGCGACGGCCGCCCCGACTTCCGCCAGCTCGGCTCCATCCTGCGGCTCAGCCGCGTCA
CCGCCGACCACGCGGCCAACTACACCTGCCGCGCCGTCAACGCCATCCACCCGTCGGG
CGGCGAGCGCAGGAACTACTCGGCCACCGCTCGGCTCACCGTGCGCGTGCGGCACAA
GCCGGGCCCGGCGCGGGTCACCCCCGACTCGCCCGTCGCCGTCGAGGGTTCCAAGGT
GATTCTCACGTGCATGGCGAGCCCGGCCGGCTACCCGGAGCCCAGGTACAAGTGGTGG
AAGGAGGGCGAGTCCGGGACGATGCCCTCGGTCAACGTGGGCCCCAAGTACGAGATC
GACTCGGTCCACTTGGGCAGCGAGGGGACGTACAAGTGCCACGCGACCAACGAGATA
GGGAACGGGGAGGCGGCGTCCGTCAACCTCACCGTCCACCAACCGCCCAAGATACTC
ACCAAGCTGCAACCGCACGTCACCAGGAA

>novel_circ_000687
GATCGAAACACAGAACGAGTCGGAAGAGAAAGAAGGAGACTCGAGAATTGTTGCTC
GCCTCCTTTCCGAGGCGTCGGTCGTTGTTCATCCTTTCGTGACGTTTCTGGCTTCCGGC
TAGAGAAGACGGGAGAATGGAGGGTGCGGAGAGTAATATAGGAATAAGAATAAGGTT
CTTGGCGGAAGGATATTTCGTCCAGGTCGTAAGATGACACGCGCGTGTACACGCGGGA
TCGAGGTGTACGAGGGATCGATATTCTCATTCCATCCACGACGAGATCGACGAAAGAA
AGGAAGGAAGGAAGAAAGGGAGGCACGAATCACGCGTCCGCAAAAATGGGCAACGA
TGAATTCTTAGCCGGC

>novel_circ_000688
GTCTTCAGCCTGCTAGCACCAAAGCGCAACCTACCACGATCAGCAAATCTCATAGACC
CAGTTCTGGCGTGTTACCTCAGGGTCATACCCCTGGTCCTGGTCCAGGTGGCCAACAA
AATAGCAGAAGTTTTGCCGCCGCTCTGAGAAACCTGGCTAAACAGGCAGGGCCGGCA
CCTCAGGAAGAAGAACCTCGCGCGAGTCCCAAAAATCGAGCCCCACCTCCTTTGGTTA
GAGGTCCTTCCCCTGCCAAGGAACGATCCACGCACGAGAGAAGACCAGAAGAAATTC
CCTCGTTGTACACGACTGCGAGACCCGCGGATACCAGCAAACATAGCGTGACTGCCGC
CGCTTCCGAATTGCTGGCCCGATCCGGTTTTCAACCGTACCGGCCTGAGCATCATCCGG
CCCACGCTCCACCGGCTTTCGCCCTCGATCCCGCGGCCTACAATCCTTATCACCACGGT
CTCTACCCACCGCCACACCTTCAACACGCTTATAGATTAGAGGAACAGTTGTATTTGGA
ACGGTGTGGAATGCTGAGACCGCCATTATTTCCTGGGTTACCGTCGTATCCTCTGTACG
GACTAAGATATAGTCCGGACATGTTACCGCCCGCGTCCCTCGGACTCATGTCTCCCGTA
ATGCACGAGAGATTGAAACTGGAGGAGGAGCATCGGCTGAGGCAAGCACGGGAACA
AGCCGCGCTGCGCGAGGAGGAGGAGAGGAGAAGAGCGGCACGAAATTCCGCTCCTG
CCGCCCCGGTACCCGCGCCTGCACCTGCATCCGCCGATACTGCAG

>novel_circ_000689
AAAAAAGTCTATGTTATTCGCGTGATGCACACGCGCTCGTTGGAGGGACCATGAGTCT
TGAATGGGCCTAAGGCTCCTGGCGAGTTTGTAATAATTGGCTATCAATGTTGCATTTCGA

TAAAATACGTAGTACCAACGGTGGTCTCTTTTCTGTTTTATTATCAATGATTTACAATTTC
ACGCTATTTTTGCGTATTCTAATTTATTAAAAATGCCTAAACTGTCTATGCGTACCATTTA
TCTGTGTTTTTTTATTGTTAGTCTCGTTCAATGTCATATGATGCATTTGGATGTTATACCTA
TATTTATATACAAGAATGAGAGAATTAAAAATAATAAGAAGATAATGAAATCGTATAGCG
AATTATCTTCAGAAGAATTAAAAACGACCGTTTAATGCCGATCGAGAGACTTATTGCAA
GATATCAATATTCGTATAACATAAAATAATCAATAAATCACATATTCTCAATATCTTATTCG
AGTTGATTTTATAGTCGCTTTAACATTTCAAATTCAACATTTTGCATTAATGTATAAATTAT
ATAAATTGAACTTGAGGATACAACGAAAACAAAATCTTCATAAAATATAAATATTTGTTT
AACTTTCCTTGTATTTAAATTATATTAATTGCAATTTATTATATTTAAAGCACATAATTAAG
AGTGCAGATTTTTCAAACTTCGATATTTTTAAAAAAATTCAAAGTAATAAAGGATTTTAT
AATTGTAATACAGTTTCTAAAGATGATTTTATGATTTTGTCATTATATAATTAAAACACAA
GCAAATCTATGAGATGAGCTCAAATAAGTATATAAAATCTTGGTACTTAAAAACAAATA
AAAATTAAAATATTTTGTCATTTTACATGCATATATGTTCTATCAATTTTACTGAAATGTG
CAAATTTTTTTATTTTCAATGAAAAAAAAAAAAAATAAAAATTTAGAATCATAACCAGCA
ATTTCCAAAGATTTTAATATTTGATTGAGATGAAACGAATGTTCGCAATGATCTATTGATT
ATCGATTATATTATCGCCACGTTTTAACGAAGAGATCGTAACTTCAGTCTCAGTACAAAG
TAGGCGATCACTCTCAAGGTAAGCAAAAAACCTGTCAATGCAACCGCGTCCTGCCAAA
AGACGGCGTTTTGCATCGACATTTCTTCGAGGAACTTCGTCGGGCTCTTGAAATGGCA
GTACGCATCGGAACACTTGAGCTTAGCACGATTATAACCATACACAGAAATCATGGTGC
CTTCAAAACTATATCTAACATAACTTACGTACGAAAGAAAT

>novel_circ_000690
AGTGTGATCAAAAGTTTATAAGCACACCTGATGGACCACCGAATGGAACATTTCACGC
GCCAACGTTGATTAATTACGACGGAGATTCTCGACAATGCGTATACACGTTTTTTGCTG
GGCCACGACAACGAGTCGAACTGATATTTAATTCGTTTGGCCTTCGGGGAACACCTCC
AGATGGATCCGCTGTTGGAGAATTACCCGCATGAACTAGGAAAAACTTGGTTTTCAGT
TGCGCCCACGAATATATGGATGTGTATGCGGAAATACGATCGGAGAATACCAGCAAATT
GGTAGAAACGCCTTTCGGTGGTCGTTTCTGTGGCCCGATCCCACCTCGTAGGCGTGTTT
CTCTTTACCAGGGAATTGCTCTAAGTTTTTACACTGATAAAAATATCACGTTACCTACCC
TGTTCAGCGGTACTTATGCCTTTATCAATTCATCTGAATACGAGGTGGGAACACCAGCT
CCCAGCACACCATGCTCTTTCACGGTAGATTCGGAACATAAACGCAATGGTAATATATTA
TCTCCTACGTATCCTGGCACATATCCAAAGGGTATTGTATGCAGTTATCAATTTATCGGG
AATAAATCGCAACGAGTGAGACTCGAGTTCCGGGACTTTGATTTGTTCTTCGGAGGTC
AACACTGTCCTTTGGATTACGTAAAAGTGTACGACGGTTTAAACGATACGTCGGCCGTT
ATAGGAACTTACTGCGGTCAACAGCGAAATTTGGTATTGTATTCGTCGGAATCTAGTTT
GTACGTGTTGTTTGTTACCCTTCAACGTACGGCCAATACGCAAAATCGTGGATTCAAGG
GCATCTTTGAATTCTCAGAAAGTTTTGTTAAATTAGACTTCATAACCGCTAACAAGGGT
GAGCATATACGAGGATCGGAATGTGATCAGAAGATTTTAAGTAAAAAGGAATCGTCTG
GACAAGTTGTTAGCCCAAACTTTCCATATCCGTACATACCGAAGGTCGTGTGTCGGTAT
TTCATTTATGGAATGCAAGATTCGCAGCATCTCGAACGTGTTCGATTAGAGTTTTCATCA
TTTAATATATCAAAGAATAAGACGATAGGAGATTCGTCTTGTACCGATGGATATTTAAAG
TTATACTTAAAAGGGCAAGAAGCGACGGATTCTTACGACAAATTTGATTACGAATTGTG
CGGCGTGAACAGCAATCCGAACCACGTAGTTAGCGATGGGCCGAGACTTGTGATGGTG
TTTAGCAGCGGAGAATCTCAAGGGCAAGGTTTTAAAGCGCGGTACACCTTCGAAACGG

AATATAAAATACCGGGAACCGCTGCACCCGACGGCAGCTGTACGTTTACATACCGCAGT
TCCTCTCGCAAAAGAGGAGATTTAATTCTCCTCGGCATCCTAGTAATTATCCAAGCGAT
ACAAATTGCACATATTTATTCTTAGCGACGCCAAACGAGCAAGTTACCTTAATATTTGAT
TATTTTAAAGTTCGAACGAAAAATATGAACGTGACGGATGGACATTATGGAATGGAAAT
TTGTCAAGATGATTGGTTGGAAATATATAACATGTATCGGGACAATACGGAAAAATTGAT
AGGTAGATACTGCGGTAACACGGCGCCAGGACCCGTCGAGTCGAATCTCGGTGCTCTC
GGTCTAAGAGTAATTCTCCATTCGGACTCGGAACTGGTTTATAGCGGTTTTAAAGCCCG
TTACACGTTCGAAGTGGCAAAACCTATATTTGGAGATTGCGGATCGAATATAAGTAGCG
TGAATTATGGTATTATCGCCAGTCCGAATTTTCCAAACAAGTACGATGGACCCGCGAGA
AATATGGCAAGTAAAACTTGCAATTGGTTTATAAAAGTTCGACCGAATCAGCGAATATT
GTTAAATTTTGAATCGTTTTCGGTAGAGGGTGACCAATCAGTTCGTGGTTGTCCAGCTG
CTGTACTGCGCATGTGGTATTCCATATCGTCCACGCCTATCGAGTTATGCGGAGTGAAA
ACGTCGGATAAATGGACTTATCTTTCCGAGGATAACAACATGCGACTTAGCTTTATATTG
GCCGACAAAGCGGTTGGACAACTGGGATTTAAAGCAATATGGACGGAAGTAAGCACA
AATACCGACTGCCAGAATCAATTTTTCTGCAGCAAAAATAAATACTGCATTGCAGAGTC
GCTTCGATGTAATGAGGTTTATAATTGTGGCCCAGCTGATAATTCGGATGAAGAAAATT

>novel_circ_000691
CAGGTAAACGAAAATTTGACGGGGGTCACCAGAACCAGGGGGAATCTAAGCGTCGCT
TCCAGAGCAACTGGGGAAACCAACCCCTCGCCCAACAACCACTGGGCAATGCGTACG
GATTGGCGAGTGTGAATGGTGGCAGTGGTGGTGGTGGCGGCGGTGACATAGGATTCGG
AGGTAGGACCGCCACTCTTGGCGGTGTTTCCAGCGATGATCACGAATGGTACCAAGAC
TCCTACCAGTCATGGAGCTAACTTCTGCAAGTCTGTGAGTGAATACGCACGCACGCAC
TCATTTAAGTACGCACGCAATCACGCATGACGAACGAACGAACGAACGAACGAACGA
ACGATCGATCGATCGAGAGTTAAACAATAAAAACAATGAACAACCAATTTCGAGTTGA
CCTTTATGCACAAGGAGGGAGAAGTGAAGTGAAAAAAAAAAAAAAAAGCGAACTGGA
AAGAAAGGAAAAAAAAACGAAAACAAAAACGGGAAGGAAGAGGATAATTGCAAAA
GGAACCCGAAAAAAAAAAAATTGAAAAGGAAAACGAAGAGCGAAATGGAAAAAAGT
TTTTTTATATATAAAATACAGATACAAACGGAGAAACGAAGAATCGAACGAGGTTACGT
TAATCTATATAATAATGCTATTTACTTTCAATCATCAATTTGGCAATTTGCAAATGTTTTTA
ACGCCTACTTTCAATCATCAAATGAACGAACAAATATGAGAAAGCGCGTGAAGCGTCG
TACTAGGCCTCACCACCATCGCCCGCTGTTGTCGTCGTGACTATATATATATATATATATAT
ATATATATACATATATATACACAATATATATATATACATATATATATATACACATCATATATATAT
AGAAAAAAAATATATATATATTTATATATATATATATATATATATACAGTGATGTGCCACT
GGGACTAATTACTACATCGGAGTGACCACCCAACAACCCACCCCGTACTATTATATTATA
TTATAATATACATATATATATATATACATACATGTATATACATATGCATATGTACATATACACA
TATATATCTGTATGTATTATGTATATATGTATGTATGGATATGTATACGTATACGTATATGTAT
ATATTATATAGTGTGTGTGTGTATATATATATATATATGTATATGTATATGTATACACTAATAA
TCTAAGTACTACTACATTGTCACATTGTTTACTGCCAAGTGATACAAAGATCAGCGATGT
TGACATTGGGATTGTGATGGCTTGTATTAATTAAGAAAAATCACGGAAGCCTGCGATTT
TATTATGATGCGATTCTCGACTTTTTGTGTGTATGTGCTAGAACGCGTGTACGGTAAAAT
GGATAGAGAAGATCGTGAAAAAAGCATATGCGTATGTGAAAGATAAAGAAAAAAAAA
AAGAAACAATAGAAGTAATAGCTAAAAAAAAAAAGTGAAGAAGGTAATGATGAATATG
GTGGTGGGAGATACGAGAAGAATTTAGAGAGGGAAGAACCCGTTATATAATAGGACGA

AGTTGAAGAGAAGAGAAGAGAAAAATGAGAAGAGAGAAATGGGAAGAGAGCATAAG
GAGAAAGAAG

>novel_circ_000692
GCTGGACGTGGCCGTGGAGTAGTGCCGCGTGGCCGAGTCGGTGGCCCCCAAGTCCGT
GGGCCAGTCAGGGGGGGACGCAACCCCGCAACTTCAGGGGCTCGTGGGGTCCAGCG
GCTGTCCGCTCGTGGGGGGGTCCGCGCCAAGGGAAGTTTACCAGGTGAGGATGCAGG
TAAACGAAAATTTGACGGGGGTCACCAGAACCAGGGGGAATCTAAGCGTCGCTTCCA
GAGCAACTGGGGAAACCAACCCCTCGCCCAACAACCACTGGGCAATGCGTACGGATT
GGCGAGTGTGAATGGTGGCAGTGGTGGTGGTGGCGGCGGTGACATAGGATTCGGAGG
TAGGACCGCCACTCTTGGCGGTGTTTCCAGCGATGATCACGAATGGTACCAAGACTCCT
ACCAGTCATGGAGCTAACTTCTGCAAGTCTGTGAGTGAATACGCACGCACGCACTCAT
TTAAGTACGCACGCAATCACGCATGACGAACGAACGAACGAACGAACGAACGAACGA
TCGATCGATCGAGAGTTAAACAATAAAAACAATGAACAACCAATTTCGAGTTGACCTTT
ATGCACAAGGAGGGAGAAGTGAAGTGAAAAAAAAAAAAAAAAGCGAACTGGAAAGA
AAGGAAAAAAAAACGAAAACAAAAACGGGAAGGAAGAGGATAATTGCAAAAGGAAC
CCGAAAAAAAAAAAATTGAAAAGGAAAACGAAGAGCGAAATGGAAAAAAGTTTTTTT
ATATATAAAATACAGATACAAACGGAGAAACGAAGAATCGAACGAGGTTACGTTAATCT
ATATAATAATGCTATTTACTTTCAATCATCAATTTGGCAATTTGCAAATGTTTTTAACGCC
TACTTTCAATCATCAAATGAACGAACAAATATGAGAAAGCGCGTGAAGCGTCGTACTA
GGCCTCACCACCATCGCCCGCTGTTGTCGTCGTGACTATATATATATATATATATATATATAT
ATACATATATATACACAATATATATATATACATATATATATATACACATCATATATATATAGAA
AAAAAATATATATATATTTATATATATATATATATATATATATACAGTGATGTGCCACTGGGA
CTAATTACTACATCGGAGTGACCACCCAACAACCCACCCCGTACTATTATATTATATTATA
ATATACATATATATATATATACATACATGTATATACATATGCATATGTACATATACACATATAT
ATCTGTATGTATTATGTATATATGTATGTATGGATATGTATACGTATACGTATATGTATATATT
ATATAGTGTGTGTGTGTATATATATATATATATGTATATGTATATGTATACACTAATAATCTAA
GTACTACTACATTGTCACATTGTTTACTGCCAAGTGATACAAAGATCAGCGATGTTGAC
ATTGGGATTGTGATGGCTTGTATTAATTAAGAAAAATCACGGAAGCCTGCGATTTTATTA
TGATGCGATTCTCGACTTTTTGTGTGTATGTGCTAGAACGCGTGTACGGTAAAATGGAT
AGAGAAGATCGTGAAAAAAGCATATGCGTATGTGAAAGATAAAGAAAAAAAAAAAAGA
AACAATAGAAGTAATAGCTAAAAAAAAAAAGTGAAGAAGGTAATGATGAATATGGTGG
TGGGAGATACGAGAAGAATTTAGAGAGGGAAGAACCCGTTATATAATAGGACGAAGTT
GAAGAGAAGAGAAGAGAAAAATGAGAAGAGAGAAATGGGAAGAGAGCATAAGGAG
AAAGAAG

>novel_circ_000693
ATTATGATTACGACTATTACGGTTATGGGGATTATCGAGGTGGCTACAGTGATCCATATTA
CGATGACTATTATCGATACGAAGATTACTATTTCGATTACGCGCCGCCTCCGCCACCTGC
CAGAGGGAGAGGCAGGCAGCCTCAACCGCTGACAGTGCAGTATGTTGTGTCGCTTAA
AGTTCCTTCGCCATCCCAATACAATAAGCTTTCCGCTTTACTGCATTGGATAATTCTGCT
AATACTACTGCTGTCTGTAAAAAAATGTGGCTGGACGTGGCCGTGGAGTAGTGCCGCG
TGGCCGAGTCGGTGGCCCCCAAGTCCGTGGGCCAGTCAGGGGGGGACGCAACCCCGC
AACTTCAGGGGCTCGTGGGGTCCAGCGGCTGTCCGCTCGTGGGGGGGTCCGCGCCAA

GGGAAGTTTACCAGGTGAGGATGCAGGTAAACGAAAATTTGACGGGGGTCACCAGAA
CCAGGGGGAATCTAAGCGTCGCTTCCAGAGCAACTGGGGAAACCAACCCCTCGCCCA
ACAACCACTGGGCAATGCGTACGGATTGGCGAGTGTGAATGGTGGCAGTGGTGGTGGT
GGCGGCGGTGACATAGGATTCGGAGGTAGGACCGCCACTCTTGGCGGTGTTTCCAGCG
ATGATCACGAATGGTACCAAGACTCCTACCAGTCATGGAGCTAACTTCTGCAAGTCTGT
GAGTGAATACGCACGCACGCACTCATTTAAGTACGCACGCAATCACGCATGACGAACG
AACGAACGAACGAACGAACGAACGATCGATCGATCGAGAGTTAAACAATAAAAACAA
TGAACAACCAATTTCGAGTTGACCTTTATGCACAAGGAGGGAGAAGTGAAGTGAAAA
AAAAAAAAAAAAGCGAACTGGAAAGAAAGGAAAAAAAAACGAAAACAAAAACGGGA
AGGAAGAGGATAATTGCAAAAGGAACCCGAAAAAAAAAAAATTGAAAAGGAAAACG
AAGAGCGAAATGGAAAAAAGTTTTTTATATATAAAATACAGATACAAACGGAGAAAC
GAAGAATCGAACGAGGTTACGTTAATCTATATAATAATGCTATTTACTTTCAATCATCAAT
TTGGCAATTTGCAAATGTTTTTAACGCCTACTTTCAATCATCAAATGAACGAACAAATAT
GAGAAAGCGCGTGAAGCGTCGTACTAGGCCTCACCACCATCGCCCGCTGTTGTCGTCG
TGACTATATATATATATATATATATATATATATACATATATATACACAATATATATATATACATAT
ATATATATACACATCATATATATATAGAAAAAAAATATATATATATTTATATATATATATATATA
TATATATACAGTGATGTGCCACTGGGACTAATTACTACATCGGAGTGACCACCCAACAA
CCCACCCCGTACTATTATATTATATTATAATATACATATATATATATATACATACATGTATATA
CATATGCATATGTACATATACACATATATATCTGTATGTATTATGTATATATGTATGTATGGAT
ATGTATACGTATACGTATATGTATATATTATATAGTGTGTGTGTATATATATATATATATATGT
ATATGTATATGTATACACTAATAATCTAAGTACTACTACATTGTCACATTGTTTACTGCCA
AGTGATACAAAGATCAGCGATGTTGACATTGGGATTGTGATGGCTTGTATTAATTAAGA
AAAATCACGGAAGCCTGCGATTTTATTATGATGCGATTCTCGACTTTTTGTGTGTATGTG
CTAGAACGCGTGTACGGTAAAATGGATAGAGAAGATCGTGAAAAAAGCATATGCGTAT
GTGAAAGATAAAGAAAAAAAAAAAAGAAACAATAGAAGTAATAGCTAAAAAAAAAAA
GTGAAGAAGGTAATGATGAATATGGTGGTGGGAGATACGAGAAGAATTTAGAGAGGGA
AGAACCCGTTATATAATAGGACGAAGTTGAAGAGAAGAGAAGAGAAAAATGAGAAGA
GAGAAATGGGAAGAGAGCATAAGGAGAAAGAAG

>novel_circ_000694
CTGGTCTGACTGAGGTGATTATCTACAGTTCTCCGGATGACAAGAAGAAAAATAGAGG
TTTTTGCTTCTTAGAATACGAATCTCACAAAGCTGCTTCCTTAGCCAAACGAAGATTAA
GTACCGGTCGCATCAAAGTATGGGGCTGTGATATCATAGTTGATTGGGCTGATCCTCAG
GAGGAGCCCGACGAACAAACTATGTCTAAAGTGCGTGTATTGTATGTGAAAAATTTGA
CTCAAGATTGTTCTGAAGAGAAATTGAAAGAAAGTTTTGAACAATATGGTAATATTGAA
AGAGTGAAGAAGATCAAGGATTATGCTTTTGTCCATTTTGAAGAAAGAGACAATGCTG
TCAAGGCGATGAATGAATTAAACGGAAAAGAGATTGGTGGTTCTCACATTGAAGTATC
TCTCGCGAAACCTCCATCTGATAAAAAGAAAAAGGAAGAGATGCTTCGTGCTAGAGA
GCGAAGAATGATGCAAATGTTTCAGGGCAGAAGCGGGGGTTCTCCATCGCATCCAAGT
ATGATGGGAGGACCAATGCCAGTTCGAGGACCAGGTCAAGGTCCTCGAGGTACCGGC
GCAGGTATGCGAGGACAGATGGGAAGAGGCGATTATGCTCTCATGGAAATAGATTATGA
TTACGACTATTACGGTTATGGGGATTATCGAGGTGGCTACAGTGATCCATATTACGATGA
CTATTATCGATACGAAGATTACTATTTCGATTACGCGCCGCCTCCGCCACCTGCCAGAGG
GAGAGGCAGGCAGCCTCAACCGCTGACAGTGCAGTATGTTGTGTCGCTTAAAGTTCCT

TCGCCATCCCAATACAATAAGCTTTCCGCTTTACTGCATTGGATAATTCTGCTAATACTAC
TGCTGTCTGTAAAAAAATGTGGCTGGACGTGGCCGTGGAGTAGTGCCGCGTGGCCGA
GTCGGTGGCCCCCAAGTCCGTGGGCCAGTCAGGGGGGGACGCAACCCCGCAACTTCA
GGGGCTCGTGGGGTCCAGCGGCTGTCCGCTCGTGGGGGGGTCCGCGCCAAGGGAAGT
TTACCAGGTGAGGATGCAGGTAAACGAAAATTTGACGGGGGTCACCAGAACCAGGGG
GAATCTAAGCGTCGCTTCCAGAGCAACTGGGGAAACCAACCCCTCGCCCAACAACCA
CTGGGCAATGCGTACGGATTGGCGAGTGTGAATGGTGGCAGTGGTGGTGGTGGCGGC
GGTGACATAGGATTCGGAGGTAGGACCGCCACTCTTGGCGGTGTTTCCAGCGATGATC
ACGAATGGTACCAAGACTCCTACCAGTCATGGAGCTAACTTCTGCAAGTCT

>novel_circ_000695
CTGGTCTGACTGAGGTGATTATCTACAGTTCTCCGGATGACAAGAAGAAAAATAGAGG
TTTTTGCTTCTTAGAATACGAATCTCACAAAGCTGCTTCCTTAGCCAAACGAAGATTAA
GTACCGGTCGCATCAAAGTATGGGGCTGTGATATCATAGTTGATTGGGCTGATCCTCAG
GAGGAGCCCGACGAACAAACTATGTCTAAAGTGCGTGTATTGTATGTGAAAAATTTGA
CTCAAGATTGTTCTGAAGAGAAATTGAAAGAAAGTTTTGAACAATATGGTAATATTGAA
AGAGTGAAGAAGATCAAGGATTATGCTTTTGTCCATTTTGAAGAAAGAGACAATGCTG
TCAAGGCGATGAATGAATTAAACGGAAAAGAGATTGGTGGTTCTCACATTGAAGTATC
TCTCGCGAAACCTCCATCTGATAAAAAGAAAAAGGAAGAGATGCTTCGTGCTAGAGA
GCGAAGAATGATGCAAATGTTTCAGGGCAGAAGCGGGGGTTCTCCATCGCATCCAAGT
ATGATGGGAGGACCAATGCCAGTTCGAGGACCAGGTCAAGGTCCTCGAGGTACCGGC
GCAGGTATGCGAGGACAGATGGGAAGAGGCGATTATGCTCTCATGGAAATAGATTATGA
TTACGACTATTACGGTTATGGGGATTATCGAGGTGGCTACAGTGATCCATATTACGATGA
CTATTATCGATACGAAGATTACTATTTCGATTACGCGCCGCCTCCGCCACCTGCCAGAGG
GAGAGGCAGGCAGCCTCAACCGCTGACAGTGCAGTATGTTGTGTCGCTTAAAGTTCCT
TCGCCATCCCAATACAATAAGCTTTCCGCTTTACTGCATTGGATAATTCTGCTAATACTAC
TGCTGTCTGTAAAAAAATGTGGCTGGACGTGGCCGTGGAGTAGTGCCGCGTGGCCGA
GTCGGTGGCCCCCAAGTCCGTGGGCCAGTCAGGGGGGGACGCAACCCCGCAACTTCA
GGGGCTCGTGGGGTCCAGCGGCTGTCCGCTCGTGGGGGGGTCCGCGCCAAGGGAAGT
TTACCAGGTGAGGATG

>novel_circ_000696
CTGGTCTGACTGAGGTGATTATCTACAGTTCTCCGGATGACAAGAAGAAAAATAGAGG
TTTTTGCTTCTTAGAATACGAATCTCACAAAGCTGCTTCCTTAGCCAAACGAAGATTAA
GTACCGGTCGCATCAAAGTATGGGGCTGTGATATCATAGTTGATTGGGCTGATCCTCAG
GAGGAGCCCGACGAACAAACTATGTCTAAAGTGCGTGTATTGTATGTGAAAAATTTGA
CTCAAGATTGTTCTGAAGAGAAATTGAAAGAAAGTTTTGAACAATATGGTAATATTGAA
AGAGTGAAGAAGATCAAGGATTATGCTTTTGTCCATTTTGAAGAAAGAGACAATGCTG
TCAAGGCGATGAATGAATTAAACGGAAAAGAGATTGGTGGTTCTCACATTGAAGTATC
TCTCGCGAAACCTCCATCTGATAAAAAGAAAAAGGAAGAGATGCTTCGTGCTAGAGA
GCGAAGAATGATGCAAATGTTTCAGGGCAGAAGCGGGGGTTCTCCATCGCATCCAAGT
ATGATGGGAGGACCAATGCCAGTTCGAGGACCAGGTCAAGGTCCTCGAGGTACCGGC
GCAGGTATGCGAGGACAGATGGGAAGAGGCGATTATGCTCTCATGGAAATAGATTATGA
TTACGACTATTACGGTTATGGGGATTATCGAGGTGGCTACAGTGATCCATATTACGATGA

CTATTATCGATACGAAGATTACTATTTCGATTACGCGCCGCCTCCGCCACCTGCCAGAGG
GAGAGGCAGGCAGCCTCAACCGCTGACAGTGCAGTATGTTGTGTCGCTTAAAGTTCCT
TCGCCATCCCAATACAATAAGCTTTCCGCTTTACTGCATTGGATAATTCTGCTAATACTAC
TGCTGTCTGTAAAAAAATGTGGCTGGACGTGGCCGTGGAGTAGTGCCGCGTGGCCGA
GTCGGTGGCCCCCAAGTCCGTGGGCCAGTCAGGGGGGGACGCAACCCCGCAACTTCA
GGGGCTCGTGGGGTCCAGCGGCTGTCCGCTCGTGGGGGGGTCCGCGCCAAGGGAAGT
TTACCAG

>novel_circ_000697
CTGGTCTGACTGAGGTGATTATCTACAGTTCTCCGGATGACAAGAAGAAAAATAGAGG
TTTTTGCTTCTTAGAATACGAATCTCACAAAGCTGCTTCCTTAGCCAAACGAAGATTAA
GTACCGGTCGCATCAAAGTATGGGGCTGTGATATCATAGTTGATTGGGCTGATCCTCAG
GAGGAGCCCGACGAACAAACTATGTCTAAAGTGCGTGTATTGTATGTGAAAAATTTGA
CTCAAGATTGTTCTGAAGAGAAATTGAAAGAAAGTTTTGAACAATATGGTAATATTGAA
AGAGTGAAGAAGATCAAGGATTATGCTTTTGTCCATTTTGAAGAAAGAGACAATGCTG
TCAAGGCGATGAATGAATTAAACGGAAAAGAGATTGGTGGTTCTCACATTGAAGTATC
TCTCGCGAAACCTCCATCTGATAAAAAGAAAAAGGAAGAGATGCTTCGTGCTAGAGA
GCGAAGAATGATGCAAATGTTTCAGGGCAGAAGCGGGGGTTCTCCATCGCATCCAAGT
ATGATGGGAGGACCAATGCCAGTTCGAGGACCAGGTCAAGGTCCTCGAGGTACCGGC
GCAGGTATGCGAGGACAGATGGGAAGAGGCGATTATGCTCTCATGGAAATAGATTATGA
TTACGACTATTACGGTTATGGGGATTATCGAGGTGGCTACAGTGATCCATATTACGATGA
CTATTATCGATACGAAGATTACTATTTCGATTACGCGCCGCCTCCGCCACCTGCCAGAGG
GAGAGGCAGGCAGCCTCAACCG

>novel_circ_000698
CTGGTCTGACTGAGGTGATTATCTACAGTTCTCCGGATGACAAGAAGAAAAATAGAGG
TTTTTGCTTCTTAGAATACGAATCTCACAAAGCTGCTTCCTTAGCCAAACGAAGATTAA
GTACCGGTCGCATCAAAGTATGGGGCTGTGATATCATAGTTGATTGGGCTGATCCTCAG
GAGGAGCCCGACGAACAAACTATGTCTAAAGTGCGTGTATTGTATGTGAAAAATTTGA
CTCAAGATTGTTCTGAAGAGAAATTGAAAGAAAGTTTTGAACAATATGGTAATATTGAA
AGAGTGAAGAAGATCAAGGATTATGCTTTTGTCCATTTTGAAGAAAGAGACAATGCTG
TCAAGGCGATGAATGAATTAAACGGAAAAGAGATTGGTGGTTCTCACATTGAAGTATC
TCTCGCGAAACCTCCATCTGATAAAAAGAAAAAGGAAGAGATGCTTCGTGCTAGAGA
GCGAAGAATGATGCAAATGTTTCAGGGCAGAAGCGGGGGTTCTCCATCGCATCCAAGT
ATGATGGGAGGACCAATGCCAGTTCGAGGACCAGGTCAAGGTCCTCGAGGTACCGGC
GCAGGTATGCGAGGACAGATGGGAAGAGGCGATTATG

>novel_circ_000699
CTATGGCAGAAGGAAATGGGGAAATAAAAATGGAAGAGAAACAGTCTAAGGAGGAAA
TGGAATATGTAGAAAGAACAGAAGATTTTTCAAAGTTGATTCAATATGGATTAGATGAA
AAAGTAGCTGCAAAATTAGATGAAATTTATAAAACAGGAAAATTAGCTCATGTGGATTT
GGATGAAAGAGCATTAGATGCTTTAAAAGAATTTCCTGTTGATGGTGCATTAAATGTAC
TCACACAATTTCTTGAATCTAATTTAGAACACGTATCAAATAAATCTGCATATCTTTGTG
GTGTAATGAAAACCTATAGACAGAAAAGTCGAGCTGGACAAGGTACTGGTACCAGTAC

TACTCCAAAAGCACCAGATGAAGACAAAATAAAGATGATTCTTGAACGAACTGGTTAT
CCTTTGGATGTAACAACAGGGCAGAGAAAATATGGAGGTCCTCCTCCAAATTGGGAAG
GTCCTACACCTGGAACTGGTTGTGAGGTATTTTGTGGAAAAATTCCAAAAGATATGTAT
GAAGATGAATTAATTCCTTTGTTTGAAAAATGTGGAAAAATTTGGGATTTAAGATTAAT
GATGGATCCGATGGCAGGATGTAACAGAGGATATGCTTTTATCACTTTTACTAATAGGGA
AGCAGCACAACAAGCTGTTAGAGAGGTATGTAGATATTATTTGAATTATTTTTACTATCA
TGTAGGTACATGTTTGTGAAGCGTACAAATTCTGTTATCCTAGTTTACAACATCTTGTTA
TGGATATATCAATTCCCTTCACTTTTGTTTAGAAAAACTTTAGAAATGTTACAAAATTGT
TATTGCTACCAAAATAAGTAAATTTTCGAAATGTTTAAATACATTATTGCACATGTTTTAT
TTCTTTGCTTTCTTCCTGTTTCTATAACTTGTTTTATTGAATTATTTTTATTTATGTTTATGG
TATTTGTTTCAAAATGAACGTTAATCGACTCATCAAATTGAAGATGGTATAAGAACATCT
TTTTCCTTTTCTTCTTTTTTCTTCTTACATTAAACATTTCGAATACATTTTTTTCATATTCC
CATGTGGCTTGTTCTATATGTTGTGACTTGCGTTTTCTTTCTTTATTTTTTTTTTTTTTTC
TTTTTTTTCTTCTCTCTTTTTTATTTATTTATTTTTAAAATTGTAGTTTTAACACAAATGTT
TTAGCATTATTTTACTAATGGTAAAAAAATAATAAAATTTGACATTCAATGTCTTTAGTTA
GGAACAATCCACATATTTGTTTCATACATATACATATATGTCCCTGACCACTAATTTATATT
TAAATATATAATGATGCCCAACACATGCCAAATACTTAACAGCAGCAAAACAGAGAGA
GAAAATAAATGCACATATGTATTACAGTATAAGCACACGCGCGCGCGCACGCGAATACA
TGCAGAGATATACATATTTTTCTGTACATATATTATACATATACACATACATTTGAACAGGT
ACGCATTTACATATACACATACGCGCACACACATCATAAGCGCGCGCGCGCATAGGTGT
ATGCGTACGTACGTACGTACATATGTACGTATGTATGTATATATGTATGTATGTATGTATGT
ATGATGTATGTATGTATGATGTATGTATGTACGTATGTATGTATGCATGTATGTATATATATA
TACATACATGTATATAATAATGTCCTATGGATGAGTTTCTTTTTCCAGCTCTCCTCTTTGC
TCAGTGGGCAAAATTGGGAGTGGAGAACATTGACGATGCGAAAAAAAGAAGTTCGTG
TGGTTTTAATTGGTTAGGGGTATATTTACTTAATTGGTTGACTTTCGTTGGACAGTGACA
ATTTTTGTAACAAAATTTTGAAAATTG

>novel_circ_000700
CTATGGCAGAAGGAAATGGGGAAATAAAAATGGAAGAGAAACAGTCTAAGGAGGAAA
TGGAATATGTAGAAAGAACAGAAGATTTTTCAAAGTTGATTCAATATGGATTAGATGAA
AAAGTAGCTGCAAAATTAGATGAAATTTATAAAACAGGAAAATTAGCTCATGTGGATTT
GGATGAAAGAGCATTAGATGCTTTAAAAGAATTTCCTGTTGATGGTGCATTAAATGTAC
TCACACAATTTCTTGAATCTAATTTAGAACACGTATCAAATAAATCTGCATATCTTTGTG
GTGTAATGAAAACCTATAGACAGAAAAGTCGAGCTGGACAAGGTACTGGTACCAGTAC
TACTCCAAAAGCACCAGATGAAGACAAAATAAAGATGATTCTTGAACGAACTGGTTAT
CCTTTGGATGTAACAACAGGGCAGAGAAAATATGGAGGTCCTCCTCCAAATTGGGAAG
GTCCTACACCTGGAACTGGTTGTGAGGTATTTTGTGGAAAAATTCCAAAAGATATGTAT
GAAGATGAATTAATTCCTTTGTTTGAAAAATGTGGAAAAATTTGGGATTTAAGATTAAT
GATGGATCCGATGGCAGGATGTAACAGAGGATATGCTTTTATCACTTTTACTAATAGGGA
AGCAGCACAACAAGCTGTTAGAGAG

>novel_circ_000701
ACCGGCTTAACACCATTGATGGAAGCAGCTAGTGGAGGATATGTAGAAGTTGGACGAG
TTTTATTAACTAAAGGAGCTGATGTTAATGCTACTCCTGTTCCATCATCTCGCGATACTG

CTCTCACCATTGCTGCTGATAAAGGACACTGCCGTTTGTAGAATTATTATTGTCAAGA
GGAACGCAAGTCGAAGTAAAAAATAAGAAGGGAAATAGTCCATTATGGTTAGCGGCAA
ATGGTGGGCATTTAAATGTTGTTGATTTATTGTACCATGCTGGAGCAGATATCGATTCAC
AAGACAATCGAAAG

>novel_circ_000705
ACGCCACGCATCGTGCTACACGCCCGCTACCGTCACTGCCGCTGCCGCCGCCACCGCG
CCACCTTCTTTCGGAATAATATATCCAGTGTGTATTTTGTGTGGAGCAGCGACGAGAGA
CACTCCTCTACAGGAAAATTTTAACTTCGTCGTGCCATTTACGACGCCCTCAACTGTCG
AGGTCAGTTTTATTGGACGCACGACACCTGATTACAGCATCCGGAGAGCGCGAGCAAG
AGGGACAGGCGAGCAGAAGGGGCTAGAGAGGCAAAAAGAGGAAAGATGGGAGCGA
GACAGAGCAGAAGGTCCGTGGATATAACAACGACTCCGAAGAAGGAGGGGTTGCCCG
CCGACGGCGGTGTCGGGGATGCTGCCGCACCTGGCGATGGCAAGCTGGAAAGGATCG
AGGAGACCGACACCAAGCCTACCACCAATGGTATAGCGCCGCACACGGACGTTCCCG
AGGATAAGGACAAAGATAAAGACGACGCCACGGAAAAAGAGAAAGACAAAGAGCAG

>novel_circ_000706
ACGCCACGCATCGTGCTACACGCCCGCTACCGTCACTGCCGCTGCCGCCGCCACCGCG
CCACCTTCTTTCGGAATAATATATCCAGTGTGTATTTTGTGTGGAGCAGCGACGAGAGA
CACTCCTCTACAGGAAAATTTTAACTTCGTCGTGCCATTTACGACGCCCTCAACTGTCG
AGGTCAGTTTTATTGGACGCACGACACCTGATTACAGCATCCGGAGAGCGCGAGCAAG
AGGGACAGGCGAGCAGAAGGGGCTAGAGAGGCAAAAAGAGGAAAGATGGGAGCGA
GACAGAGCAGAAGGTCCGTGGATATAACAACGACTCCGAAGAAGGAGGGGTTGCCCG
CCGACGGCGGTGTCGGGGATGCTGCCGCACCTGGCGATGGCAAGCTGGAAAGGATCG
AGGAGACCGACACCAAGCCTACCACCAATGGTATAGCGCCGCACACGGACGTTCCCG
AGGATAAGGACAAAGATAAAGACGACGCCACGGAAAAAGAGAAAGACAAAGAGCAG
CAAGAAGAGGTAAAGGAAACGAAGCAAGAGTCGGGCGGAGAATCTCCCGCGGAAAC
GGCAGAGGTCACAACGCCGACGGAAGCGTCCACTCCAAACACTGCCACTTCTCCAGA
CAATAAGGAAACGAAGAAGAAGGAAAAG

>novel_circ_000707
ACGCCACGCATCGTGCTACACGCCCGCTACCGTCACTGCCGCTGCCGCCGCCACCGCG
CCACCTTCTTTCGGAATAATATATCCAGTGTGTATTTTGTGTGGAGCAGCGACGAGAGA
CACTCCTCTACAGGAAAATTTTAACTTCGTCGTGCCATTTACGACGCCCTCAACTGTCG
AGGTCAGTTTTATTGGACGCACGACACCTGATTACAGCATCCGGAGAGCGCGAGCAAG
AGGGACAGGCGAGCAGAAGGGGCTAGAGAGGCAAAAAGAGGAAAGATGGGAGCGA
GACAGAGCAGAAGGTCCGTGGATATAACAACGACTCCGAAGAAGGAGGGGTTGCCCG
CCGACGGCGGTGTCGGGGATGCTGCCGCACCTGGCGATGGCAAGCTGGAAAGGATCG
AGGAGACCGACACCAAGCCTACCACCAATGGTATAGCGCCGCACACGGACGTTCCCG
AGGATAAGGACAAAGATAAAGACGACGCCACGGAAAAAGAGAAAGACAAAGAGCAG
CAAGAAGAGGTAAAGGAAACGAAGCAAGAGTCGGGCGGAGAATCTCCCGCGGAAAC
GGCAGAGGTCACAACGCCGACGGAAGCGTCCACTCCAAACACTGCCACTTCTCCAGA
CAATAAGGAAACGAAGAAGAAGGAAAAGATGAAGAAAAAGTGGTCCTTCAGGTCGAT
CAGCTTCAGCAAGAAGGATAAGAACAAGCCTGCCCGCGAAGAAGCGCCCAAGAATGG

AGACGTCACCAAGGAGGAACCTCTCGCGGAG

>novel_circ_000708
GTCAGTTTTATTGGACGCACGACACCTGATTACAGCATCCGGAGAGCGCGAGCAAGAG
GGACAGGCGAGCAGAAGGGGCTAGAGAGGCAAAAAGAGGAAAGATGGGAGCGAGA
CAGAGCAGAAGGTCCGTGGATATAACAACGACTCCGAAGAAGGAGGGGTTGCCCGCC
GACGGCGGTGTCGGGGATGCTGCCGCACCTGGCGATGGCAAGCTGGAAAGGATCGAG
GAGACCGACACCAAGCCTACCACCAATGGTATAGCGCCGCACACGGACGTTCCCGAG
GATAAGGACAAAGATAAAGACGACGCCACGGAAAAAGAGAAAGACAAAGAGCAG

>novel_circ_000709
GTCAGTTTTATTGGACGCACGACACCTGATTACAGCATCCGGAGAGCGCGAGCAAGAG
GGACAGGCGAGCAGAAGGGGCTAGAGAGGCAAAAAGAGGAAAGATGGGAGCGAGA
CAGAGCAGAAGGTCCGTGGATATAACAACGACTCCGAAGAAGGAGGGGTTGCCCGCC
GACGGCGGTGTCGGGGATGCTGCCGCACCTGGCGATGGCAAGCTGGAAAGGATCGAG
GAGACCGACACCAAGCCTACCACCAATGGTATAGCGCCGCACACGGACGTTCCCGAG
GATAAGGACAAAGATAAAGACGACGCCACGGAAAAAGAGAAAGACAAAGAGCAGCA
AGAAGAGGTAAAGGAAACGAAGCAAGAGTCGGGCGGAGAATCTCCCGCGGAAACGG
CAGAGGTCACAACGCCGACGGAAGCGTCCACTCCAAACACTGCCACTTCTCCAGACA
ATAAGGAAACGAAGAAGAAGGAAAAG

>novel_circ_000710
CAAGAAGAGGTAAAGGAAACGAAGCAAGAGTCGGGCGGAGAATCTCCCGCGGAAAC
GGCAGAGGTCACAACGCCGACGGAAGCGTCCACTCCAAACACTGCCACTTCTCCAGA
CAATAAGGAAACGAAGAAGAAGGAAAAGATGAAGAAAAAGTGGTCCTTCAGGTCGAT
CAGCTTCAGCAAGAAGGATAAGAACAAGCCTGCCCGCGAAGAAGCGCCCAAGAATGG
AGACGTCACCAAGGAGGAACCTCTCGCGGAG

>novel_circ_000711
GACTACGCCGAGGACGAGGACGAGAACGAAGACGAGACCTCAGCCTCGACCACCCT
GTCGAAAATGATGAGCAGCAGCTCGACGACCACCACAACGACCAGCACCACCACCAC
CACCACCACGGCCAGGCCTGTGGTTACTCAGAATTACGATTATCGGGACCCGAGAGCG
TACGAGGGTGATCGTGGTGGGACAGGAGCGCAGTACAACGGGTACCAATCGAGGAAC
GACTATCCCCCGATGTCGGCGCACAGCGTTCACAAGTGGCAGACTCTGGGCACTCGAG
AGAGCGTCAAAGAAACCAGGGGCAACATGCAGCAATATAATAAGAACGGGAAGA

>novel_circ_000712
AAAAAAAAAAGAGAGAGAAAAGAAAAGAACAAAACGGTATTAAACTTTATCGAAGCT
GTAACGCTGTATGTCTGCAAGCCAAAGGATTGTGAATGCGATTGTGAATACGGTGCACC
CGCGCACCGCGTTTGATAACACGAAACAATAAAATTATGCGCAAAATTGCAA

>novel_circ_000714
ACAAAAACAAAAAAAACAAAAAAAAAAGAGAGAGAAAAGAAAAGAACAAAACGGT
ATTAAACTTTATCGAAGCTGTAACGCTGTATGTCTGCAAGCCAAAGGATTGTGAATGCG

ATTGTGAATACGGTGCACCCGCGCACCGCGTTTGATAACACGAAACAATAAAATTATGC
GCAAAATT

>novel_circ_000715
ATTTCGTGGTGTGAGAGAGGAAGCGAGAGGAGAGCGACGCCGGCGCCAGATCCGATC
GACGTAACACACCGCCTAGGAACGACTTTCCACGTGCACGAGCTTCGCTCACCATGAG
GCCTTGGACCAACAAGTCCCTCGGCCTTGGTTTTCTCGGCTTACTTCTCATCGTGTCGG
GCCTTGTTTTCTATTTCACTTCGCCCGCCATTTTTCATTACATTCTTCAAAAGGAGATAC
CGTTAACCACAACATCGAAAGCGTTCGAAGTGTGGAACGACACGAGCGCCCTCCCGC
CCATGTATTTTAAAATTCGTTTCTTCAATTGGACTAATCCAGAGGAGCTGCGCATGCCTG
GCAAGAAGCCCAATTTCGTCGAGCTCGGCCCTTACGTGTTTCG

>novel_circ_000716
GAGATACCGATATACATATGGTACGACTCGTATCCGACCCACAGTGGCGAGGGTTACGA
GGGCCGGGTCTCGAGGGTGAGCCCGAACTCACCTTACGGCGGGGCGAGCCTCAACCT
GACCGACATCCGCGAGAGCGATCAAGGATGGTACGAGTGCAAGGTCGTTTTCCTCAAC
AGGTCGCCCAACAAGAACGGCACCTGGTTCCATCTGGACGTCCACGTTCCAAGAGTG
TCGAGGGAGGATAAAAACGATTGGTTGGTTTTCGTAGCGCCGCCAAAATTCAGCATCA
CGCCGGAGGAGATGATCTACGTGAACGTGGGCGACGCGATAATATTGAACTGCCAGGC
GGAGGGCACGCCGACACCCGAGATCCTTTGGTACAAGGACGCGAGCCCGGTCGAGCC
CTCGTCGACCATAGGGATATTCAACGACGGCACCGAGCTCAGGATCTCGACCATCAAG
AGCGAGGACATCGGGGATTACACGTGCATCGCTCGTAACGGAGAGGGTCAGATCAGTC
ACACGGCCCGCGTCATAATCGCTG

>novel_circ_000717
GTACTATGGGAGCGGGGGTATGGGGGGCGGAGGAGGGCTGCAAGCCACGGACCTCCG
GCGAAGGCTCGAGTCCACGAGGCGTTCGGGAACCACGACTCGTCGTCCCCAGAGTCC
TCCTCGTTCCCTAAGGCGCGATGCTTCCCGACCGTGCCACGACGCCACCGATGACCAG
GAAGAAAGAGTCGAGTTGCAAGCGACTCTGGCCTCCGGTCAGCCCACGTGCACAGGT
TGGCGGGCGTGCAGACGGCAGGACGGGAGGAAGGAGGATGGAGAGGAGGGGGAAG
GAGGGTGGAGGAGGTGGTGGAGCGGCGGATCAGGCGTAATCGGTTCGTCGGCGCGCG
GGGCGAGGATCTTTGGCGCGGATCAGGACGGGAGGAAGGAAGGAAGGAGCGGGAAG
AGGCATCGGCTTCGATGGCGTCGAGGGGATTTACGGTGGATCGCGGACGTGGCAACGA
GGACCCTCCTCCTCGGCATCGTGCTTTTCGTTACGCCAGGTCTGTGCAACCAGGACGC
GGTGCACATTACGGCGATCCTTGGGGAGAGCGTTGTCTTCAACTGTCACGTAGAGTTC
CCCGGTGAGCACCCAGTGCCCTACGTTCTCCAATGGGAGAAGAAGGTAGGAGATGCG
GTACGATATCGGAAGATACCCTCTGCTCTCTATATCC

>novel_circ_000718
GTGATGACAGTGGCACGCCGCGGAGGCTAAGGTGGTGGGGGTGTCGCGGCGGGTGAA
GAAGTGGCGAGGCTCGTGAAAATTGGGGCCGGGAACGGACGATCCGGGCAGCGGTAG
TGGTGGCGGAACGGCAGGGGTACTATGGGAGCGGGGGTATGGGGGGCGGAGGAGGGC
TGCAAGCCACGGACCTCCGGCGAAGGCTCGAGTCCACGAGGCGTTCGGGAACCACGA
CTCGTCGTCCCCAGAGTCCTCCTCGTTCCCTAAGGCGCGATGCTTCCCGACCGTGCCAC

GACGCCACCGATGACCAGGAAGAAAGAGTCGAGTTGCAAGCGACTCTGGCCTCCGGT
CAGCCCACGTGCACAGGTTGGCGGGCGTGCAGACGGCAGGACGGGAGGAAGGAGGA
TGGAGAGGAGGGGGAAGGAGGGTGGAGGAGGTGGTGGAGCGGCGGATCAGGCGTAA
TCGGTTCGTCGGCGCGCGGGGCGAGGATCTTTGGCGCGGATCAGGACGGGAGGAAGG
AAGGAAGGAGCGGGAAGAGGCATCGGCTTCGATGGCGTCGAGGGGATTTACGGTGGA
TCGCGGACGTGGCAACGAGGACCCTCCTCCTCGGCATCGTGCTTTTCGTTACGCCAGG
TCTGTGCAACCAGGACGCGGTGCACATTACGGCGATCCTTGGGGAGAGCGTTGTCTTC
AACTGTCACGTAGAGTTCCCCGGTGAGCACCCAGTGCCCTACGTTCTCCAATGGGAGA
AGAAG

>novel_circ_000719
GTACTATGGGAGCGGGGGTATGGGGGGCGGAGGAGGGCTGCAAGCCACGGACCTCCG
GCGAAGGCTCGAGTCCACGAGGCGTTCGGGAACCACGACTCGTCGTCCCCAGAGTCC
TCCTCGTTCCCTAAGGCGCGATGCTTCCCGACCGTGCCACGACGCCACCGATGACCAG
GAAGAAAGAGTCGAGTTGCAAGCGACTCTGGCCTCCGGTCAGCCCACGTGCACAGGT
TGGCGGGCGTGCAGACGGCAGGACGGGAGGAAGGAGGATGGAGAGGAGGGGGAAG
GAGGGTGGAGGAGGTGGTGGAGCGGCGGATCAGGCGTAATCGGTTCGTCGGCGCGCG
GGGCGAGGATCTTTGGCGCGGATCAGGACGGGAGGAAGGAAGGAAGGAGCGGGAAG
AGGCATCGGCTTCGATGGCGTCGAGGGGATTTACGGTGGATCGCGGACGTGGCAACGA
GGACCCTCCTCCTCGGCATCGTGCTTTTCGTTACGCCAG

>novel_circ_000720
GTGATGACAGTGGCACGCCGCGGAGGCTAAGGTGGTGGGGGTGTCGCGGCGGGTGAA
GAAGTGGCGAGGCTCGTGAAAATTGGGGCCGGGAACGGACGATCCGGGCAGCGGTAG
TGGTGGCGGAACGGCAGGGGTACTATGGGAGCGGGGGTATGGGGGGCGGAGGAGGGC
TGCAAGCCACGGACCTCCGGCGAAGGCTCGAGTCCACGAGGCGTTCGGGAACCACGA
CTCGTCGTCCCCAGAGTCCTCCTCGTTCCCTAAGGCGCGATGCTTCCCGACCGTGCCAC
GACGCCACCGATGACCAGGAAGAAAGAGTCGAGTTGCAAGCGACTCTGGCCTCCGGT
CAGCCCACGTGCACAGGTTGGCGGGCGTGCAGACGGCAGGACGGGAGGAAGGAGGA
TGGAGAGGAGGGGGAAGGAGGGTGGAGGAGGTGGTGGAGCGGCGGATCAGGCGTAA
TCGGTTCGTCGGCGCGCGGGGCGAGGATCTTTGGCGCGGATCAGGACGGGAGGAAGG
AAGGAAGGAGCGGGAAGAGGCATCGGCTTCGATGGCGTCGAGGGGATTTACGGTGGA
TCGCGGACGTGGCAACGAGGACCCTCCTCCTCGGCATCGTGCTTTTCGTTACGCCAG

>novel_circ_000721
TTCCAGCGATGATTCAAGTTCAGACGAGGATGAAAATCATCAACATAATAATGATTCTC
AGTTCATTATTTCGAAGGAAAATTCGGAATTTACATCTTTTGAATCATTATTTCCATCTAT
GAAAGAAGTATATGGTACTCAACCTGAATATCCTAAAAGAAACGATGTGATATTGTATA
AAAGATTTGCATTGATCGGGCGTAATGCAACGTATCCCTCAGATTACATGAAAGTACGA
TTAAAATTAACTCAACAGGTATCATTTCCTGAAGCAAAAATTATTGTAATCCAATTACCG
ACGGTAAAGAAATCTAATATAAATATATACGAACAATACGTTAAAAGAGCTAAG

>novel_circ_000726
ATGATACCTGTCGATCTGATGAATTTACTTGTGCAAACAAGCATTGCATTCAGAAAACG

TGGGTATGTGACAGTGATGATGATTGTGGGGATGGCAGCGATGAAAAAGATTGCAAAC
CAGTTACCTGTAGTCCATCGGAATTCGCCTGTTCAGATAATTATTGTATAACATCTCAAT
GGAGATGTGATGGTGACTTTGATTGTCCTGATAGACGTGACGAAATTGGATGTAAGTAT
GCAAATGGAGAACGCCATGTTCATTGCAATAAAAAAGGAGAGTTTGATTGTGGCGATG
ATTTCTCGTGCATACACGAAAGCTGGGTATGCGACAGGGAAAAGGATTGCCCCAATGG
AGCTGATGAATTGCCTAAAGTATGCCACAATTTTACATGTAGACCAGATCAATTTCAATG
TCAGCATATTCGTAATTGTATATCAGGTCATTTATATTGCAATGGTAAAATAGAATGTGCA
GATGGTAGCGATGAAAAAAATTGCACAACCAAAACTATTGGTTGCGATCCATCGACAC
AATTCGAATGTAGCGAAGGATCATGTATTTCTCTTTCCAGTGTATGTAATAAGAATCGCG
ATTGTATCGGTTGGGAAGACGAAAACGAGAAGCTTTGTGGTGTAAATGAATGTTTAAA
AAACAATGGCGGATGTTCGCAAATTTGCATCGACTTGCCTATTGGTTTTCGATGCGATT
GCAGTGTAGGATATAGATTAATTGATAATCGAACTTGTGATGACATAGATGAATGTTTAG
AACCAGGTACTTGTTCTCAATTCTGTTTGAACGAAAAAGGCAGTTTTAAGTGTAGTTGT
GCCGCGGGTTATCTTAAGGATCCTAGCAATCATACACGTTGTAAGGCAGCAGAAGGTCA
TGCTAGTCTTCTTTTTACTAGAAGACATGATATAAGAAAAGTAGCATTGGATCGTCTGG
AGATGACAAGTATAGTGAAAGATACGAAAATGGCAGCAGCCTTGGATTTCGTTTTTCGT
ACGGGTATGATTTTCTGGAGCGATAGTAGTGAAAAAAAAATCTATAAAGCTCCAATAGA
TGAAGGAAATGAACGTACAGTTGTAATTGATGATGGTTTGACAACATCAGATGGATTAG
CTGTTGATTGGATATATAGTCATATCTATTGGACTGATTCTAAAAAAAACACTATAGAATT
AGCTAATTTCGAAGGCAATATGAGAAAAACTTTGATTCAAGATCACATACAAGAACCA
AGAGCAATAGCTTTGAATCCATTGGAGGGTTGGATGTTTTGGACTGATTGGAGCGACG
AAGCTCGTATCGAAAAAGCAGGCATGGATGGATCTCATCGAACGGTGATAGTTAATAAT
GATGTACAGTGGCCTAATGGGTTGACTCTGGACTTAATTGGCAAAAAAATATATTGGGT
AGATGCGAAATTGTATATAATTGGATCGTGTAATTATGATGGTACAGGTATACGAACAAT
TCTGTATTCTCCGGAAGTATTGAGACATCCCTTTAGTATTACAACATTTGAGGATTATGT
TTATTGGACTGACTGGGACAAGGAAGCAATATTTAAAGCAAATAAATTCACTGGTCAAT
CAGTTGAAGCTGTTATGTCGCTTCGAGCTCTTAAATATCCAATGGTCGTTCATGTATATC
ATCCATATAGACAACCAGATGGAATGAATCAATGTCAGGCAGTAAATGGCCATTGTAGT
CATTTATGCTTACCAGCACCAAGAATTAATTCTAAATCACCTCTTCTTAGTTGTGCTTGT
CCGGATGGTTTAAAATTATTATCTGATGGTTTAATGTGCGTGGAAAAAGTAAGTACAACT
ATAGTACCTACAACACAAGAAATTAATAAACCATTCAAGAGACTTGAACTTTTCAACAT
TACAACTACAACTATTCATTCGATACATTCAATAGATGGAGACGAGGAAAATGGTTTGA
CATCTGAATCAACAGATCCTGGTTTAGTTGCTGGCATAGTGATAGGTGTGACATCCTTA
GGATTATTACTTCTCGCATTAGTAGCAGTATTATGTTATAGGCACTATCTTCATCGTAATG
TAACGAGTATGAATTTTGATAATCCTGTGTATAGAAAAACTACAGAAGATCAATTTAGTC
TTGAAAAAAATAGATTTCCACTCCCTACTGCTACAGTTGGAGAAGAGGCTCAAGAACC
TTTAACGAGTCCTGGAACTAATGATTACGTTTAAGAATGAATTTGTCAATGAAATGGGC
TAAGCAGGCTATTGAAATAATTGAATATTGCTATTGAATATATTGACCAAATAACATAAA
ATGATATATTAAAAAAATAAGAAACACATGTCGAGCAAACCTGTTCAATAATATGCCTGT
ATTTGCCCATCTGCCTGCCAACTCAGCT

>novel_circ_000727

ATGATACCTGTCGATCTGATGAATTTACTTGTGCAAACAAGCATTGCATTCAGAAAACG
TGGGTATGTGACAGTGATGATGATTGTGGGGATGGCAGCGATGAAAAAGATTGCAAAC

CAGTTACCTGTAGTCCATCGGAATTCGCCTGTTCAGATAATTATTGTATAACATCTCAAT
GGAGATGTGATGGTGACTTTGATTGTCCTGATAGACGTGACGAAATTGGATGTAAGTAT
GCAAATGGAGAACGCCATGTTCATTGCAATAAAAAAGGAGAGTTTGATTGTGGCGATG
ATTTCTCGTGCATACACGAAAGCTGGGTATGCGACAGGGAAAAGGATTGCCCCAATGG
AGCTGATGAATTGCCTAAAGTATGCCACAATTTTACATGTAGACCAGATCAATTTCAATG
TCAGCATATTCGTAATTGTATATCAGGTCATTTATATTGCAATGGTAAAATAGAATGTGCA
GATGGTAGCGATGAAAAAAATTGCACAACCAAAACTATTGGTTGCGATCCATCGACAC
AATTCGAATGTAGCGAAGGATCATGTATTTCTCTTTCCAGTGTATGTAATAAGAATCGCG
ATTGTATCGGTTGGGAAGACGAAAACGAGAAGCTTTGTGGTGTAAATGAATGTTTAAA
AAACAATGGCGGATGTTCGCAAATTTGCATCGACTTGCCTATTGGTTTTCGATGCGATT
GCAGTGTAGGATATAGATTAATTGATAATCGAACTTGTGATGACATAGATGAATGTTTAG
AACCAGGTACTTGTTCTCAATTCTGTTTGAACGAAAAAGGCAGTTTTAAGTGTAGTTGT
GCCGCGGGTTATCTTAAGGATCCTAGCAATCATACACGTTGTAAGGCAGCAGAAGGTCA
TGCTAGTCTTCTTTTTACTAGAAGACATGATATAAGAAAAGTAGCATTGGATCGTCTGG
AGATGACAAGTATAGTGAAAGATACGAAAATGGCAGCAGCCTTGGATTTCGTTTTTCGT
ACGGGTATGATTTTCTGGAGCGATAGTAGTGAAAAAAAAATCTATAAAGCTCCAATAGA
TGAAGGAAATGAACGTACAGTTGTAATTGATGATGGTTTGACAACATCAGATGGATTAG
CTGTTGATTGGATATATAGTCATATCTATTGGACTGATTCTAAAAAAAACACTATAGAATT
AGCTAATTTCGAAGGCAATATGAGAAAAACTTTGATTCAAGATCACATACAAGAACCA
AGAGCAATAGCTTTGAATCCATTGGAGGGTTGGATGTTTTGGACTGATTGGAGCGACG
AAGCTCGTATCGAAAAAGCAGGCATGGATGGATCTCATCGAACGGTGATAGTTAATAAT
GATGTACAGTGGCCTAATGGGTTGACTCTGGACTTAATTGGCAAAAAAATATATTGGGT
AGATGCGAAATTGTATATAATTGGATCGTGTAATTATGATGGTACAGGTATACGAACAAT
TCTGTATTCTCCGGAAGTATTGAGACATCCCTTTAGTATTACAACATTTGAGGATTATGT
TTATTGGACTGACTGGGACAAGGAAGCAATATTTAAAGCAAATAAATTCACTGGTCAAT
CAGTTGAAGCTGTTATGTCGCTTCGAGCTCTTAAATATCCAATGGTCGTTCATGTATATC
ATCCATATAGACAACCAGATGGAATGAATCAATGTCAGGCAGTAAATGGCCATTGTAGT
CATTTATGCTTACCAGCACCAAGAATTAATTCTAAATCACCTCTTCTTAGTTGTGCTTGT
CCGGATGGTTTAAAATTATTATCTGATGGTTTAATGTGCGTGGAAAAAGTAAGTACAACT
ATAGTACCTACAACACAAGAAATTAATAAACCATTCAAGAGACTTGAACTTTTCAACAT
TACAACTACAACTATTCATTCGATACATTCAATAGATGGAGACGAGGAAAATGGTTTGA
CATCTGAATCAACAGATCCTGGTTTAGTTGCTGGCATAGTGATAGGTGTGACATCCTTA
GGATTATTACTTCTCGCATTAGTAGCAGTATTATGTTATAGGCACTATCTTCATCGTAATG
TAACGAGTATGAATTTTGATAATCCTGTGTATAGAAAAACTACAGAAGATCAATTTAGTC
TTGAAAAAAATAGATTTCCACTCCCTACTGCTACAGTTGGAGAAGAG

>novel_circ_000728
ATGATACCTGTCGATCTGATGAATTTACTTGTGCAAACAAGCATTGCATTCAGAAAACG
TGGGTATGTGACAGTGATGATGATTGTGGGGATGGCAGCGATGAAAAAGATTGCAAAC
CAGTTACCTGTAGTCCATCGGAATTCGCCTGTTCAGATAATTATTGTATAACATCTCAAT
GGAGATGTGATGGTGACTTTGATTGTCCTGATAGACGTGACGAAATTGGATGTAAGTAT
GCAAATGGAGAACGCCATGTTCATTGCAATAAAAAAGGAGAGTTTGATTGTGGCGATG
ATTTCTCGTGCATACACGAAAGCTGGGTATGCGACAGGGAAAAGGATTGCCCCAATGG
AGCTGATGAATTGCCTAAAGTATGCCACAATTTTACATGTAGACCAGATCAATTTCAATG

TCAGCATATTCGTAATTGTATATCAG

>novel_circ_000729
ATGAATCTTGTTCTTTAAGACAATTTCAATGTGCAAATGGTAAATGTATACCACTTCCGT
GGATTTGTGATGGAACAGATGATTGCGGAGATAAATCCGATGAAACTATCAAGAAATGT
GAAGTACAACATCTGTAGTCGGCAGCGTGAGTGCATCGACATCGCATCGTTGTGAATC
CGATCTTCGGGATAATTATATAGATTATCATATTCAGTTGCAATTTTTATAGAAAAGCATT
TCAATTCTGTTCGCGTTATATTTTACAACAATCTGATCGTATATTTATCACATTAACTCGA
AGCTTTCAAAGATTAAAAAAAGTTTGGTTAAATTTTTTCTTTCATCTTTTTTTGCTTTTAT
CTTACGTTTCATCCATTTAGTGATTTCAATGAAATAGAAAAAAGAAAAAGTATCGAAAA
CAATCTTGAAAAGAGAAATTATTGACAGGAGATTTGATTATTGGAAAACTATTTTATGG
ACAATTGTTTGTTTTCAAATCTGAAAAGTGATCTCTTAAAATAAATTGGTGATTAAGTCA
AGAGAGAAAAGAGGAAAGAAGAAAGAATAAGAATATTGTGAATTGGAAAGAAAAATT
TCTACGAAACAGATAGATTTTTTCATCGAATAAGGATTTCATTCGTTTCAAGGATACGAA
GTTCGAAAGATGCTCTCAATGGTTTCGATATTTCGGTCCTCAAAAATGTTCCGATACGG
AGTTCAAATGTATGAACGGCAAATGCATACCTGGAACCTGGCATTGCGATGGCGACGA
TGATTGTCGAGATGGTTCGGATGAAGATCCAGCAGTTTGCAGATCGGAATCATGCAGT
GAAACGGAGTTCGAGTGCAGCCTAGGAGACTGCATACCGAAAAATTGGCTCTGTGATT
TTCATCGTGACTGCACAGATGGGATGGACGAGAAAAATTGTGGCAGGATGAAGAATTG
CACGGCGGATCAGTTCACGTGTCACTCTGGGAATGGCGAGTGTGTGGCACTCGCATGG
ATGTGCGACGGCCATCGAGACTGTTCGGACGATTCAGACGAGGCTGAGTGTA

>novel_circ_000730
GTAATTCAACCAACTCCAGAACTTTCTCCATGCACAAGCGAAGCAGGTCTTTATGCAA
AAAACGTGATGATCACAGGGATTAACGGTAAAAGAGCACCATTGGCTTCTATGGGCAG
TGTTGATCCTCCAGAAGCAGATTCGAGTTTCAATTATTCAAGGTCTCTTGGTTCCGACT
CGGTCTTCCTAAGAAACGATGAACAAGAAGAGTGTCTCGACACAGAGGATGAAGTGT
CTGGATTTTCGACCGATTCTGAAACAGCCGGGCCAAGTTGTCGACGGTTTCTTCGAGT
ACCCTCGAAAAAGAAGAAAACAATTGAAAAATGGGATGGTTGTGCTGGATGTGTTCCT
AGACGGCGATCTGGAGGCAAACTTTGTCAAGCTTGTCATAGTATTAGTGCAGCTGTTG
AAACCTCTAG

>novel_circ_000731
ATATGGGGAGTGTCGTAGGCAGTAACGTAAACATAAGCAACGTAACAAAGAGGATGGA
TTCAACAACGTTCCGACCAGAAGCGACGTCAACCCTCAGTGAGCGGGAGCAACTTAA
AATTGAATTTTACAAAACTTATGATGTTATGACCGGAGTCAGAATCGCAGCGACTCTCG
GTGGTTTTTTCGGTCTTATGATCCTTCTGCTTGTTTATAAAAGCAGATGTAAATCGAGCA
AACAATTGGAGGATCCAAGATTGACTGCAGCTGCAGCCGCAGCTGTAGCTGAAGCAG
AAGCTGAAGAAAGAGCGCTTGCAGCTGCTCTCGAAGCCATCGCTAGATTACCACCTAG
ACCAGATCGGGGTCCTAGAAGATCTCTCTGTGTCGAGGTAACTCAATCTCATTTGCCAA
GAATTGGTCCGCGTTTTGCATCGATTGGAGGTGGCTATGAAGCTCTATTAACACCACCA
ACAAAGCAATCGAATTTGATACTTCCTGAAGAACAAAGAAGATGCAGTTCGGTAACAT
GTAGCAGCACTGGGAGTAGTTATCTCGAGAGAAGGGGATCTGTGATGCCAGTACCATG
TTTGCCGCTTCATTCTACGTTTTCAACTAAATACACTATCCATGATGAACCCTGGGATCT

TTATTATCCCATTGATATTCAAGTAATTCAACCAACTCCAGAACTTTCTCCATGCACAAG
CGAAGCAGGTCTTTATGCAAAAAACGTGATGATCACAGGGATTAACGGTAAAAGAGCA
CCATTGGCTTCTATGGGCAGTGTTGATCCTCCAGAAGCAGATTCGAGTTTCAATTATTC
AAGGTCTCTTGGTTCCGACTCGGTCTTCCTAAGAAACGATGAACAAGAAGAGTGTCTC
GACACAGAGGATGAAGTGTCTGGATTTTCGACCGATTCTGAAACAGCCGGGCCAAGTT
GTCGACGGTTTCTTCGAGTACCCTCGAAAAAGAAGAAAACAATTGAAAAATGGGATG
GTTGTGCTGGATGTGTTCCTAGACGGCGATCTGGAGGCAAACTTTGTCAAGCTTGTCAT
AGTATTAGTGCAGCTGTTGAAACCTCTAG

>novel_circ_000732
ATATGGGGAGTGTCGTAGGCAGTAACGTAAACATAAGCAACGTAACAAAGAGGATGGA
TTCAACAACGTTCCGACCAGAAGCGACGTCAACCCTCAGTGAGCGGGAGCAACTTAA
AATTGAATTTTACAAAACTTATGATGTTATGACCGGAGTCAGAATCGCAGCGACTCTCG
GTGGTTTTTTCGGTCTTATGATCCTTCTGCTTGTTTATAAAAGCAGATGTAAATCGAGCA
AACAATTGGAGGATCCAAGATTGACTGCAGCTGCAGCCGCAGCTGTAGCTGAAGCAG
AAGCTGAAGAAAGAGCGCTTGCAGCTGCTCTCGAAGCCATCGCTAGATTACCACCTAG
ACCAGATCGGGGTCCTAGAAGATCTCTCTGTGTCGAGGTAACTCAATCTCATTTGCCAA
GAATTGGTCCGCGTTTTGCATCGATTGGAGGTGGCTATGAAGCTCTATTAACACCACCA
ACAAAGCAATCGAATTTGATACTTCCTGAAGAACAAAGAAGATGCAGTTCGGTAACAT
GTAGCAGCACTGGGAGTAGTTATCTCGAGAGAAGGGGATCTGTGATGCCAGTACCATG
TTTGCCGCTTCATTCTACGTTTTCAACTAAATACACTATCCATGATGAACCCTGGGATCT
TTATTATCCCATTGATATTCAA

>novel_circ_000733
ATATGGGGAGTGTCGTAGGCAGTAACGTAAACATAAGCAACGTAACAAAGAGGATGGA
TTCAACAACGTTCCGACCAGAAGCGACGTCAACCCTCAGTGAGCGGGAGCAACTTAA
AATTGAATTTTACAAAACTTATGATGTTATGACCGGAGTCAGAATCGCAGCGACTCTCG
GTGGTTTTTTCGGTCTTATGATCCTTCTGCTTGTTTATAAAAGCAGATGTAAATCGAGCA
AACAATTGGAGGATCCAAGATTGACTGCAGCTGCAGCCGCAGCTGTAGCTGAAGCAG
AAGCTGAAGAAAGAGCGCTTGCAGCTGCTCTCGAAGCCATCGCTAGATTACCACCTAG
ACCAGATCGGGGTCCTAGAAGATCTCTCTGTGTCGAG

>novel_circ_000736
TGTCACTGCGGTCCCACTCCCGGGCGCGATCCCACTACTGCCCGCCACGGAGACTTTT
GGCTGCTGGGTGTCCCCCTCTGGCCCGGTTCGCCTCTCCCTAGCCAACCTGGCCATCCA
GGATTCCTCCAGGATGCCCATATTGATGGCGAGCTTAGCGCGGACACCCAGTCGACGT
GAGTCCCGCA

>novel_circ_000738
CTCACGTCGACTGGGTGTCCGCGCTAAGCTCGCCATCAATATGGGCATCCTGGAGGAAT
CCTGGATGGCCAGGTTGGCTAGGGAGAGGCGAACCGGGCCAGAGGGGGACACCCAG
CAGCCAAAAGTCTCCGTGGCGGGCAGTAGTGGGATCGCGCCCGGGA

>novel_circ_000739

CGGGGGTTCCTGTTGGTGCGGGACTCACGTCGACTGGGTGTCCGCGCTAAGCTCGCCA
TCAATATGGGCATCCTGGAGGAATCCTGGATGGCCAGGTTGGCTAGGGAGAGGCGAAC
CGGGCCAGAGGGGGACACCCAGCAGCCAAAAGTCTCCGTGGCGGGCAGTA

>novel_circ_000740
ATCAAACAAAGAAACAAAATGGGATGAAATGAATATAATTGCAACATTGCATCCACCA
GGAAAAGATTATGGCCATATCAAGATTGATGAACCAAAAACTCCATACAGCTATGAAGG
CTTGCCCGGTGAATTTGAATTTGATGAATTAGATTCTGCTACAATTGCTGCTAAATTAGC
AGAGAGCAGTAAACCAAAAATATTTGAAGAATCAAGTGAAGATGAAGAAGAAACTGA
AACTCTTGAAGAAAGAG

>novel_circ_000741
ATCAATCGGGTTGGCAAGAAAGAATAGAACGTCTCTCGATGAATAGTCAGGATAATAAT
ACCTCGGACTTAGGACACCCTGTACAAATTGTGCTTGCTCATCCAGATCACAGTTTTGA
ATTAAACGAAGAAGCTTTATCAAAAATTCTCCTTGAAGATGATATTAAAGATAGGAGTG
TAGTTGTTGTGTCGGTAGCGGGTGCTTTCAGAAAAGGCAAAAGCTTCCTGCTTGACTT
TTTCTTGCGATATATGAATAGTAAGTATAATAACAACAATCAAACGGATTCTTGGTTGGG
TAAAGAAGATGAACCATTACGTGGATTTTCGTGGAAAGGAGGATCTGAAAGAGACAC
GACAGGCATATTAATGTGGTCAAAAGTTTTTCGTGGTACTTTACCAGATGGTGAAAATG
TTGCTGTGATTTTAATGGATACACAAGGTGCTTTCGATAGTCAATCCACTGTGAAGGAT
TGTGCGACTGTGTTTGCTCTAAGTACAATGTTGTCATCTTTACAAATTTATAATCTATCAC
AAAATATCCAAGAGGATGATCTTCAGCACTTGCAACTTTTTACGGAATATGGTAGGCTG
GCGTTACAAAACTCTGGACGCAAACCATTCCAAAAGTTACAGTTCTTAGTAAGAGATT
GGAGTTATCCTTACGAAGCGAAATATGGTGCAGAAGGTGGAAAAGAGATATTGAATAG
AAGATTAGAAATTTCTGATAAACAACATCCAGAATTGCAAAGTCTAAGAAAGCACATT
AAATCATGTTTCTCGGATATATCTTGTTTTCTCATGCCACATCCAGGTTTAAATATTGCCA
CTAATCCTCATTTCGATGGTAGATTAGCAGAAATTCAACCAGAATTTAAGGAACAACTC
AAGGTATTAATACCGATGTTGTTAGCTCCAGAAAATTTAGTTACAAAGAAAATTGATGG
ACAAATTGTAAAAGCGAGGGATTTATTGGAATATTTCAAAAGCTATATGAAAATTTACA
AAGGAAACGAGCTTCCTGAACCAAAAAGTATGTTAGTGGCAACAGCAGAAGCAAATA
ATTTAGCTGCAGTTACAGAAGCTAGAGAATTTTATATGCGATTAATGGAAGATATTTGTG
GAACAAAAAAACCATATTTAACAACACAACGTTTAGAGGATGAACATGCACGTTGTAG
GGATAAAGCTATATACAAATTTCAGAATAAAAGAAAAATGGGAGGAGAATCATTTAGTC
AGACTTACACAAAAAAATTATGCCAGGATATGGATAAAGCTTTTGTTCACTTTAAAGCA
CAAAATGAAAGTAAAAATGTTTTTAAATCGACGCGAACTGCAGGAGTATATTGCGCAAT
TGTTGCCATTATGTACTTCTTATCCTCTATATTTGGTTTTACTGGATTATATCTATTAGCAA
ATATTTGTAACTTCATCATGTGCATCTGTATATTAACCTTAATGTTATTGGCATACATTAGA
TACAGCGGTAATTATGACACAATTGGAATAGCAATAGATGAAGTGGCTAATGTTTTATGG
AATAATTTAATTAAGCCAGTCTATCAACAAGTTGTGGAGAAGTCAGTGTCCGTAGCAGT
GGCTCAAGCTGCTGAAATGGCTACAAATACAACAATGAATGCCACTGTCACTGCCAAT
GGCAAGCCTAAACTAACATGATGTTCATTCCCTTTGTGTCCCAATTTCTTAAGTGCATTA
GAAATATACACCAATGTATTAAAACCA

>novel_circ_000742

GCGGTATATGCTGGTCTTCCATTAGCAACGGTCGTTGCAAGGAATTATTGTCACAAGGA
GTGACAAAGGAAGATTGTTGCGCGTCGAACGCTGCAGCAGCGACCGCGTACTCGGAC
GAAGATCTCGATAGCGGAAGTCTCTTTTTTGGAGGGTGCTTGGTGGAGGTGTGCAGT
GTCGTCCTTGCAGAGAAAGCTGCACGGAAGTAAGGTGTGAGGAGGGAAAGAAGTGC
GTGGTGCGTAGGGGAAGACCGAGGTGCGTGTGCAGCCCTGAGTGCAAGGCACCAAGA
GGTGGTGGTCCAGTCTGCGGGACGGACGGCAAAAGCTACAAAAATTTGTGCAGACTG
AAGAAACGCGCTTGCAAGAAAGGAAGCCACGAACTAGCGGTTGCCTATAACGGCCAC
TGCCAAAGTTCGTGCGCACGGGTCCGGTGCGGCCAAGGCCGGAGCTGCCTGCTGGAC
CAGAACCTGAGCCCGCACTGCGTGAGGTGCGCCCGCAGGTGCCCCCAGCCGCCCCCG
CAACAACGAGCCGCCGCACGCCCCGTTTGCGGGGCCGACGGAAACACATACAAGAGT
GCCTGCCACCTGCGACTTGCCGCCTGCCGCGCAGGTCGCGCCATTCCCGTCGCCTATA
AGGGCCACTGCAAAC

>novel_circ_000743
AGTTGTGCAATGATGGGTCTGCAGGCCACGGCAGGGAAACGTAAACTGGAGGGAGGT
GTGGGCTGCATGGAGTTCGACATGGATATGGGCGAAAGCCCATCGAAGTTAGGCCGAG
TGGAGGGTAGCTGGTGGGCGATGGAGGATAGTTACACGCCTCCGCTTAGCCAAACACC
GGCTTCTTATTACGATATGGAAGTGAGTCCGCCTTGCCTGCAGACAAATCCTTGTCAAC
CAACATCGACGAGTCCTCAACAACAACAACAACAACAGCAACATCAAACGGCACAGC
AACAACAGCAACAATCATCGACGCCAACGTCAGCGCCTCCACCGTCTACGGTGGCGC
ACCTACACCATCATCCGCAGCACTACTATCACCATCAACGCGGACCTACCAGTCCGGTC
GGTAATAATGGTTACAGTCAACTGTCGAGGCCTCAGCCGCAATGGGGAGCGCCTCATC
ATTACGAACCTCCAACGATACGGCGAGAAGAAAACGGGAAAAGTTACTTGGAGCTTG
GATCTAGTTATCGAGCAAACGATAGATGCTGCGAGGGTTCGAGGTCGAGTTGGTGTCG
ACGTGGCAGAGCGTGTTATAGACAAAGGCGGCTAGCCGTGTTAAATATTTCAATGTGCA
AGCTTGCGAGGTATCGCCAATTTCCGGATCCGAGCCTTCATCGATCGGTTCTCATCTGC
AATACATTGAGGCATCTAGAACGCGAAATGGAGAGGGACAGAAGTCCACCGCCCATG
GAGCCAGTTATACCAGCGCCTATTGCTCAACTTCAACCTCCTGAGCAGGGCAGGTTAA
CGCCATTTCCAATGCCACCAACGTCTTCAAACGAGACAGACGTCGATTCTGGTATCGG
CGATAGCGATGATAGTCGTTCGATTAACTGGGGCAGCGTGCTATCTCTTTCGAGTCAGT
CGCCCTTAGATCCATTGAACAACAACGAGTTACTGGATGTCGATATTGGTCCGGATCTA
GATTTAGACTTCATGCCCGGATGGAAATTGACACCTCTGTCTGCGGACGATATTCTTAG
GAGTACAACGGCGCAAGAGCAGCAACACCAGCAACACCATCATCATCAGCAACAGCA
GCAACAGCATCTGGTATCAACCAGCGGTAGCTGTGCCAATAGCAACGTGGCCAGTGCC
TGCGTTAATACTGGAAGTAGCAGTAGTACGCACGAATCGTTAATGTGCGTTGGATCATA
GCCCCCAATTTTGACATCGTTGAGTCATCTCATCGATTTTTACCCACTGATGCCGCAGA
GCAAATAAGTATGCGGCATTTACAAAGAACCACAAATCAAGATGTAGAGACGACGCAA
TCGCCCGCCTCACGAATTATTTGCAACGCCTGGTCGGCGATTCTCCAGGAGGTCTTCTT
TTTCTTATTTATTACCATTCGTGTCAATTACGCACCGATCGATTATCTCGGTTACGTTCTA
CTTCGTATTACATCGGTAACCAGCATGGGTGGTCTACTCCTTATGGGCGAGGAGGTATG
AGACGTGATACCTCCTGTGTTCGCAGGTGCAACTGGTGCAGAATTCATGGCCCATTGG
AAATATTTAAAATTCAAAGGAAATAGAG

>novel_circ_000744

AAGGCGGAAGGTTGCAAAAAGAGCTGAGCTACGCTTCGGAAGCATTGGGTGAAGCAG
CTGCCTCGACGCATCAACTCGTGATTCAGACACCCAAGGATCCTGGAGCGAATATTTTG
CACCCTGCCGCACTCAAGGAACATTTAGCGATCCTGAAGGCGGCGACGCAGGTCACC
GTACGCTTATTCGATACCACGTGGAAGCTGAAAGACATGTGTTACGCGCTCAACATACC
CAACTTCGACATGCACTACATCGACCAGATCTTCGATAGTATTATGCCGTGCGCAATCAT
TACACCCCTCGATTGTTTCTGGGAAGGTAGTAAGCTGCTGGGACCAGAATACCCTGTG
CACATACCCCGGACGCAGACGCACAAGCCTGTCCAATGGACGAATCTCAATCCATCGG
GGATGCTCGACGAGATGAAGAAACTCCAGTTCATGTTTCCCTTCAAAACTTTGGAGGA
CTACATGAAGAGGGCCGGGATAACGAACGGCTACCAAAGCAAGCCTTGCCTCGACCC
AACGGACCCGGAGTGTCCGGAAACCGCGCCCAACAAAAAATCCCAGCAG

>novel_circ_000745
ATCTTCGATAGTATTATGCCGTGCGCAATCATTACACCCCTCGATTGTTTCTGGGAAGGT
AGTAAGCTGCTGGGACCAGAATACCCTGTGCACATACCCCGGACGCAGACGCACAAG
CCTGTCCAATGGACGAATCTCAATCCATCGGGGATGCTCGACGAGATGAAGAAACTCC
AGTTCATGTTTCCCTTCAAAACTTTGGAGGACTACATGAAGAGGGCCGGGATAACGAA
CGGCTACCAAAGCAAGCCTTGCCTCGACCCAACGGACCCGGAGTGTCCGGAAACCGC
GCCCAACAAAAAATCCCAGCAG

>novel_circ_000746
GCTGTGCTCCTTGACCTTGACCCGTCTGGTGATTTTACGGCCATTAGGCTCTGATTTAAC
CTCGATCAGCATCGCGGTGAAGATGCAGAGCTCGAAGAGAACTTTACGTTCAAATGAG
ATGGCCATCCCCAGCGGTGGCATGCTGGACACTGAACTCGAGCTCCAATTCGCTCTTC
AGTATCCACATTTCCTCAAGAGGGACGGCAACAAACTGCTGATACAGCTTCAGAGGAG
AAAACGCTATAAAAATCGCACCATGCTCGGATACAAGACTCTAGCTGAGGGAGTGATC
AACATGGCTCAGGTTCTCCAGAAGCAGATGGACCTGGAGTTGGAGCTGGTGTCGGAC
AAGGCTGAGAAATATGGCGGACATTCGGTCGCGCTGGCTCGCGTGAGCGTCGTCGCGC
TCAGCTCTCAGCCGGTCGACCAGGACAAAAGACTGAACGACCCGAATGAGAGGCTCT
GTCCCGAATATAGCGACGAGGAGGAAGAGTTCAGCTCAGAAGGTGAGGCGGAAGGCA
GCGACAGCGAGCCGACTCTTGAGATGCACAGGCGGAAGAGTCGGGGAAAGATTCCGA
CGAATGCCAGGCAAAGAAATTTAAAGCAAAAATTCATAGCTCTGCTGAAAAGACGGTT
CAGGGTATCCGAGGATTTAGATCAAGATCAAGAGGAAATTGGGCAGAAGCTTTCAGAG
TCGTTAACTGGATTTGTTAAAGGCGCAGAGATGGAAATCGAAGAACTGTTCGATGAAC
TGGAAGATTTGTCCGACAGTGGTCCAGAATTGGACACCATGTCGGTCAGTAGCACACC
CAAGCCATCCCTTCGACCTTTCTTCAGTTCTAGTCGATCTCTCCTCGCTCCTCCTCATTC
CG

>novel_circ_000747
AACGAGGAATCGGGGGAAAGAAAACTGGTGTCGAAAATCGAATCTACGCCTCGACTA
CGTTTCGAAACACGTGTTCTCGTTTTTTACAAGCGCGTGATAAAAGGATTAGAGAGAG
AGAGAGAAAGGACAACGATAGAGGGACAAACAACCGTTCAAACATTTCATTGAGATT
GTTCTTTGTAATTATGAAAAGGCTGTGAATCGAGGTTACCTATGTATCGCGAAGAGAAC
GAGCAAAACAGAGCCGCGGACTTGGCTCCCCAACAACCGAGTGGTGCAAACACGTTC
GAGCGTTTGGAACATTCTCAGGATAGCAAAAATGGGGACGATGGTCCCAAGAAGGTG

CAAACAGACGCTTCCTCTTCGACTAATACTCCAAAGCCGCGTGCACGGAATTGTGCAC
GATGTCTGAATCATCGGCTGGAGATCACCTTAAAATCGCACAAGAGGTACTGCAAGTA
CCGTACTTGTACCTGCGAGAAGTGTAAGATCACTGCCAATCGGCAGCAAGTGATGCGG
CAGAATATGAAGCTGAAAAGACACCTGGCACAGGATAAAGTCAAAGTAAGAGTAGCG
GAAGAGGTTAGTCTTTTCCTTGTTTCATAATGGTTTCTTGGCGAAAGGCTCTCTTGTCG
TGGCTTGAGTCAGCGAGGTATTTGTATTTTAACGATTTCCTAACATGTTGCCGCTGATA
TAGTTTTCTGTCGTTTTGAAATTTGTTTTTTTTTTTTTTTTCTCTTAATAATTGATCGTA
AAGCTGTAAAAGCTGATGTAAATAGTAAGTTCATAGGATACCGTGGATCCGCTCCCATT
TGGCGTAGAAAATACAATTTCTTCGGTCCCTCAACCACCTAGAAGTCTCGAGGGTAGTT
ACGATAGTAGCAGTGGCGATTCACCAGTGAGCAGCCACAGTAGCAATGGTATACACAC
CGGATTCGGTGGTAGTATCATCACTATACCTCCTACAAGAAAATTGCCGCCGTTGCATCC
TCACACTGCGATGGTCACCCATTTGCCACAGACGTTAACCA

>novel_circ_000750
GTATCTCGAACTTTTTAATAGAGCGACTCAACGAGGAAAGCGAGACCTACGGGATGGT
ACAACGACCCTACCCTCTCCTATCCACTTCTCACCCTTACCTTCCTCCTTCTTACTCGCT
TCCTCGGGCCTTTTCCATTTTTTTTTTTTTTTCCTTCATCGTTAGAACTTTCTCTCCTTTT
TTCTGCCTTGTTTCACCCACTTGTCGGTGCGGCTCTTTCCTTCTCACGACCAGGTCTTC
CACCAGATTTTTCCTGCATAAACAGCAGGTCATTAACCGCCGCTATAGCGGAAGATCGT
ATCTACCTTATCTCGTCGAGACTCTACTATTCTCGTCAATGCGGGTACTCGAGCGGGATG
ATTCCTATCCCCGGCTTCCTCCTCCTCGGATAACGCTCTCGAATGTGTTATTAGTTTGTT
GCGCGATATCGTGGCCGACTATGATTGGCGATAATACCGTTTTCCACGGTGGATTTTCCG
CGAGATTACGATTTACCTCGAAGCGAAAGTAAAAAGGTGAAAACGAATATTGAATATG
GGTGTGTCGTAAGGAATGTGTTTTCTCGGGGACTCAGTGAAACGATGGAAAAAGAATT
GTATAATATAATTGTTTGAAAAGTGATTCTAAGAAAGAGAGATTGAAAGGAAAACGAA
GAATTGGTGTTTAAAAATAAATGTCTCTCTTTGTTCTCTCCGCTGCAAAATTAAGTTGA
AAAACAAGTTATAAGCAAGCTTTGATGGATATTTTTTTGCACGCCATTTCGAAGAATCG
CCTCTCAAGTTTTTTACCTACTTTGCTCGAGCATCCTGTATAAGAGAAAAGGATTGGCT
CTTACTAAAAGCTATAAAAATGAAAAATATGAAAAATATGTTTGAAGATGTTTCAAATT
CAATTCAACGTATTGCTATCTTTAAATTTTTATCAATCTATCAAACTCGATTTTAGATCCT
GAATAATAAACGAAATATTAAATGTTTTTATCGGTTTTTATCATTCTATCGCTCGTTGCG
AATGCTCACAGCTTCGATGCTTTTAATGACTTGGCAATTTGGCCACCGATTTTCGTCGTT
TTTACCTCGCACGCACGACCAGGCGAAATTCACCGGAAATTCGCCGTTGATTGCATACT
CAAAAGTGGGAAGCCACACGCCAAGGCAACGACGATAAATCTAGCATCGAGATCCCC
CCCATGATCCACCCATGGTCGTCATTAAAATTCTGACAAAATATTCGTCCAATTAAAGTT
TATTATTGATTTATAAAACAACGAGGGGGGAAAAAAATCTCGTTCGTTTCCAACTACCA
TATCTATGGCTTACGTTTAAATTTTATATATTCACTTTTAGTGATTTTAAAAAATAGATTTA
AATATTCAATGGTGTTGTACAAAATATAATTTTAACAAATTTATGTAAAAGTGGATGCAA
ATATAACTTTTACACAGCATAAAAAAATAGTGAAGAAAATAAAATTTTCTAAAGATTAA
GATTCTGTCATGGATGAGATTAAATGTTGCTGAAATAACAATAAGCAATTTTAAATAAAA
AAATTGCTTATTTTGTTTATAAATATTTTCCAAAATTTAAAATTTGTTCACAAAGGAGTAT
ATATATTAAGAAAACATTTATATATATGAAAGAATTTCGTATGAATTTAAAAAGTCCAATA
ACTTAATTTTGTGGAGATTTTGCGAATGTGCAAAAGCTATGCAATTCTTTATTTCATTCT
TTTTTGATACCTCTCACCATTCTGGACATACTCCACGAAAAAGAAAATTACGGATTTTTT

TTCAAGGGATGATTTCACCTCCTGAAAATGCTTTTTGGCACGAAAGAAAATTGGTCTGT
GTTACGAATAATAACTGTGCTACTCTTTACATGTATGCAAAGTTTTATAATTTTTTTGAAT
TTCCACGTTTTCTGTCCTCCTCCCTTCTGAATAATACATTCAAAATATTTTTACAAATTGT
GTTTCACTAGTCTCGTGTCGTTGAATAGCTAATTAAATCAAATACATGCACTATAATTATA
TACTTTAACACATTCGAATCGAATATATCAAAAAAATTATCATTTAATAACTCGAGAAAA
CAGTGTACCATTACAGAATCATGATTTAAATATTTCAACATATCGTATCTTCCTAACAAA
AACACGTGGCAAAAACGAGCATTAATAATCAACAAGGAAGAACTTTTTCCTCTCGTTT
AAAAAAAAATGTGCTCTCCAAAGAAATAAAAACACTCAATTATATCGGTGTAACGAGC
AGAAGAATAGCATCACACGTTTCGTAGAAAAATATTCAAGCCTCCCCCCAATTAAAATC
GAATCCTCCTATGGTCCCGCGACCATACCGCGTTTCCTACAGGAACGTTTCAATGAAAT
TACGGGGTGCATTACCATAAATATTGTTTACAGAATTAGGTATGCATAGCACGTAAAGGA
TTAGGCTCACGGGCCTCCTGTATGCAAATCGCAGGCTCATCGCCTGATGTACAAATTTT
CGTGGAACACGCGATGGTTTTCGAGCGAACGATCTAAATCACCGGCTGCCAATACAGA
GCGCAGCCCTTTGTCCCACGTCAGAGAGCGCCGACAAAGTAGAAGTTGCCCTACTGG
AATACCGGTATAGATTTTATGGAAGTAGCTGTACAGTACGTAAGGGAACTTCGTCCCCT
CCCCTCCTTCCCTTTGTTCCGCGAGGAAAATGGGCCGCGAGAATTTTTCACGCGGTTCC
ACCTCACGTAGTCCGCGAAAAGTTCGCCGAAACGAAAGTTATCGAGGTCGACCCCCC
AGCTTCTTGGCTGGCGTCGAGTCCCTCCACACTTCTCCGATTCCGACGGAAAATTAATA
GCCCTCCCACTCGGTAAATTTCGAAACAAACGCGAACCTTTCCTCATCGCGTAAACAC
GCGTTTATATCGTGAATATTGTTATTCGTGATCAATAATATTACAGCTGTGGCGATTCTGA
AGGAAACTTTATCTTTTCCTGGCTCTTTCTCAAATAATTCTTCAAACTTTTTAATAATTCT
TTTATGATTTGGAAATTTAATCCGTTTTAGGGATTGATAGGTTGAGTTATAGATATTAATG
GAATTTTGAATATTGGAAGGATTGAAATTCAAGAAATAATTCGCCAATTATTTAAATACA
TTAAGAAACGTTATGAAACGTTAATTGAAAGTGAATATATTAATTGAAACATATTTGAAG
CTGTTAAATAATTTAATTCGATCAGCTTCGAATACGATTCACTCCACGTATTCCTCTCCTT
ATTTCCTTATAAAGTTAATGGGCCGTCTAATCGATGGATTCAAGTTTGACGGGTCCACAT
ACTCGATCAAGTATGGGATAAAAGAGGAATGTTGCGAATGGAGCAGCTCGATCGGGAG
GGGGAACAGTAATTATACTGTGCGATCTAGACCAATAATAGTGGGCGAGTGGTTTTCAA
TGGTTACAACTTCGGAACGGTTTAGCTTCAATTGAACTTGGACCAACTTGGCGCACTC
AAGGAAGATGAAACGAATGGAAGAGCGAGAGGAAGGCCGCGCGTAACTCGTAACTCT
GAAATCTTGGTAAGGCCAATTCGATCGCGCACTGATGACATCCTTTTATGGTCGGATGC
CAGTCGTTGATCATCGAATTATTTCATTCTATTGTAGTTGGACATTTTCTAATTTATTCTTT
CAATATTGCGATCCTGTACACGATCGTTCGAAATATTTTCTTAAATTTGACAAGCTAGCA
ATTTTATCATGAAGATAATTTGAAATTTAAAAAAATTACATAGGAGAATTTTATTTTAAA
GTGATAGTGATTAATTATATATTTTTTATTATTATGGCGTACATACAAAATTTTATAATTGA
AATTTGTTTTATTTATGATATACAAAATCTTAAAATATGTATACTAGTACAACTCTTAAAA
ATCATAGAATTAACAATTGATTCTTTCGTTCTTAAAAATACAATATATTTATTAAATAAAG
TGAAAAAAATCTTCGCTATAAATCTCATCAAAATCAAGATAAACGATGCAAGACAAATA
TTGTTGTTTACACACTTCAGAATAGTTAAACCATATCTGATAAAGTTACAGAAGAGGAG
ACATAAAATTTTACAATACCAACAATTTTGAAGATACAAATTGAATAAAATAAAAAATG
AATCAAGATGAAACTACGACAACGAAACAGAAGAAGTGAAATACCTAGAGAAATTATC
CAGAGATAGAATCGAGTAAAATACTTCGTCACTACATTTTTCTTCTTCG

>novel_circ_000751

GTGACGGTGGCGGGAACGAGCAACGTGGGACCGCACGGTGGAAGAGGAGGCGGGAA
GGGGGATCCCCTCGCAGCAGGGTTGTCGAGGAGGTGGTGCGTGGTGCGGAGGAGGCG
GGACAGAGGGTGGCGGGAAAGTCGCAGGAAGCGAGAAGGGAGGAGGAGGAGGAGG
AGGAGGAGGTTTGGAGAGAGAACACAAGGCCGCAAGGCTGTGTGTGTGTTTGTTGTG
CTGAAGGAGAAAACGGGAAGAACGTCGTCGAAAAGAAGAGAAACGGGGGAGAAAG
TGTGCGGGAGAAAGAATAGAGAAAGGTAGGGATAACGTGTGCCGTTGACGTGATTAC
GAGTGACGTGACGTGACGTGACGTGACGTCGAGGCGTCGAGATGCTGCTGGATGAGA
TGGCCGCCTCCGTTCACACGCTGCTGTCACTACTTCTCGTCCTCGGTGGTTGTACGATG
TGCACCGGCAGGATCACGAATCAGGAGATAG

>novel_circ_000755
TAATGAAGGGGACGATTCTAATTTTTCTAAGCGTTCTCTCAATGAGAACGATGGGTTTG
CCGAACGGGGCACCGGTAGATTCATGCCAAGATCTGTTGCCTCGTCATCCTGGAGTTAC
GAAGCAGGAAACTCAGCCTCCTCCTTACCAAGTTTTACCAGCTGCAGGACAAGGTAGA
GTCCGCTTAATTCTAGGAAGCCCTCACGGTCTTGCCTACGAAGGTTTCATGATAGTTGC
ACGTGACTCTGAGACCGGTGAATTCGTTGGAGAGTTCGCCAACTTGCCCGATTCAGCA
AAAATCGTCGAGTGCACACCAGGCGTAAAGAACGCGGTGACTCATACGAATACCAGC
AAGAAACACAATCTTGAGTTTGATTGGGAGGCACCTATGGATTACGAGGGAACTATTGT
ATTCAAGTCCACGATCGCGCAGGATTACAGTACCTATTGGGTCGAAGTAGAATCGCCTC
AAGTTAACGTTCGCAAGCGATCCATCGACGTATTGACCTCATCTACCTCGATCACCACG
TTGAGAACAACCACACCGCCCTATTACAGCCCGACCGTCGATTACGAACCGACGAAGA
CTGAGGATCCCTTTTATACCGGATGTGGTTCGACGAAAAATTGTTTCGGTACTCCGTCA
GGATGCATAGAAGAAAAGAATTGTATCGCGGCAGTGACTGTACTCGTTCACGGGGAGC
GATATCTGTTTGAATTGCAAGCACGTGACAGTAAATACGTCGCTGTCGGTTTATCCGAT
GATAGTAAAATGGGCGATGACAGTGTGGTTGAGTGCATGAACGAAGAAGGAGAAATT
GGATTATACATGTCTTGGAACTCTGGAAAATCGAATATACGTCAGCCTATGCAAGAAGG
AGCCGTTGGATTGGAGGCAAGTTCGATCAAAGATGACATAATCTCGTGCAAATTTTGG
AGAGAGAAGATGACTGTCGTTCAAGGCCGCGAATATGATCTCGTCAATACACCGTACA
ATCTTCTTGTGGCCTCTGGTAAAAGTTTAAAAAGTAATGGTATCGGATATCACGATACG
GCACGCGACGCTACGGGTGGCGCAAAGTTATTATCCGACGTTGGCGATTTTACTACGGC
GAGTAACATCTTGATTCGTGTTCACGGTGCTCTAATGCTAGCATCTTGGATCGGTACAG
CGTCCATCGGCATGCTTCTTGCTAGATACTACAGGCAGACATGGGTCAGCTCGCAGTTG
TGTGGAAAGGATCATTGGTTCGCT

>novel_circ_000756
TAATGAAGGGGACGATTCTAATTTTTCTAAGCGTTCTCTCAATGAGAACGATGGGTTTG
CCGAACGGGGCACCGGTAGATTCATGCCAAGATCTGTTGCCTCGTCATCCTGGAGTTAC
GAAGCAGGAAACTCAGCCTCCTCCTTACCAAGTTTTACCAGCTGCAGGACAAGGTAGA
GTCCGCTTAATTCTAGGAAGCCCTCACGGTCTTGCCTACGAAGGTTTCATGATAGTTGC
ACGTGACTCTGAGACCGGTGAATTCGTTGGAGAGTTCGCCAACTTGCCCGATTCAGCA
AAAATCGTCGAGTGCACACCAGGCGTAAAGAACGCGGTGACTCATACGAATACCAGC
AAGAAACACAATCTTGAGTTTGATTGGGAGGCACCTATGGATTACGAGGGAACTATTGT
ATTCAA

>novel_circ_000757
CTACTAACGAGATCGACGTAGATTCCGGAAAGGATATAAGGATCCTTGCACCGAAGC
ACGCGAAGAATGTCAAACCATCCGTTGCCCGTATGGCAAAGAGGCGTTCGTGGACTCT
CAGGATTGCGAACGATGTCGTTGTGTTGATCCTTGTAGAACACAAATTTGTCCCGATAA
CACAAAATGTGCTATTACCTTAGTTGCTACCAAAGATGGTACAGAATACAAAGGAGTAT
GTAGATCAGTCATGAAACCAGGCCGTTGTCCAAATGTATCGAATAGTACTAGATGCGAA
CAAGAGTGTCTCACAGATGCAGATTGTTCTGGAGATATGAAATGCTGTAATAATGGCTG
TGGTGCTTCTTGTATCGAACCAGCCACCGAAGAAATTATATCGACTTCTCCAAGATCGT
TCGTTACTCCACCTGTAGTTGGTGCTGAACCAGCTTCCATCAAGCAACCAGAGGAGCC
GAAAGTCAGTGCTCAGGAAGGTAGTTACGTAACATTGAAGTGCATTACTTTAGGAAAT
CCAAGACCAATGATCACATGGAGAAAAAGCACGACGTTGATTGCACCTTCAGAAGCC
AGACGTCGCATATTACCCGATGGCTCTCTTCAAATTATTAATTTAGATCGATACGATGGT
GGAACTTATGTCTGCACAGCTGACAATGGCTTAGGCCCACCGGTAAGAGCAGAATACC
AATTGACTGTCACAGAACCGAAAGAACTAGCTGCGGCCATCCTCGGGGAGCCTGATGC
ACGTGTGACAGTTGCCATAAACTCTCCCATATCTCTGCACTGCTACGCATGGGGTTGGC
CCAGACCTTTCGTCACTTGGTGGCGAGGCGAGCGCATGTTGCCACTCGCTTCGGAGAT
TTACGAACAGGATTCTGAATACACCCTTCTGATTAGAACAGTCACTCTACAGACTCTAG
GCGTTTACACCTGTCAAGCATTCAACGCAATTGGTAGAGCTGCATCTTGGTCGATCACA
TTGCAAGCTATCGGTCCAGTGTACAATATCAAGCCCGAGTTCGAACAATATATGAAATA
CTTGATTGAGGCTCCTAAAAAAGCTGAGAAACCTAGATATCCTTATAGACCCGATAGAA
CCCAAACTCCAGACTATCGTCAGATTTATGAAGCTAGTAATACGACCAGACAGAATATT
CAAATTTCTACGGTTAGTCCTATTGGATGGACTACTTCGGAATACGGTAGGAAATTTAG
AG

>novel_circ_000758
AGCAAGATACCTGTCTCTTGCCCGCACTTTTGGGAGAATGCCACAACTATACTCAACGA
TGGTACTACGATTCGTACGAACAGCAATGTAGACAATTCTATTATGGTGGTTGCGGTGG
AAACGAAAATAACTTTGTCACCGAACAAGACTGCATTAACAGATGTCAGACTACGATT
ACTACCCCTGCGCCTGTAAGAGAAATCGAATTTAAACCAGAATTCTGTTTCCTTCCGGA
TTCTTATGGTCCATGTTCTAATGAACAAATCAAATGGTTTTATGACAGTAGAGAAGGTAT
TTGCAAGCAGTTCAGGTATGGGGGCTGTCAAAGCAATGGAAATAACTTCAATAATCGC
GAAGAATGCGAATATCGATGTGGAGATGTTCAAGATGCTTGCACTCTGCCCAAAGTTGT
TGGTCCCTGTAGCGGTTTTGTCAAACAATACTATTATGATTATCGAGCAGATTCCTGTTA
CGAATTTGAATACAGTGGTTGTCAGGGTAACAAGAACCGCTTCCAGGATAAGGAATCC
TGCGAAAAAAGATGTCAGAAACAAGTTGTTCAAACGGAAACAACGCCAAACATTACT
GTCACACTTGCTCCATCACTTGAAACTGTTTCGAAGAGCCCGATTTGTTATACTCCTGT
TGATCCTGGTTCTTGCAATAGTGATATCACAGCTTTTTATTACGATTCGCACAATCAGAT
GTGTCAAGCATTTCTCTATGGCGGTTGTGAAGGAAACGCGAATAGATTCCAAACAGAA
GAACAATGTGAACGTCTTTGCGGAAAGTTTTATGGACAAGATACATGTAACTTACCGCC
GGATTCTGGTGAATGCCGAGGTTACTTCCAAAAATATTACTATGATTCAGTCAACCGCG
TATGTCGCGAATTTTCATACAGTGGATGCGAAGGTAATGCGAACAGGTTTAGCTCAATG
GCCGAGTGTGAATCTGTTTGCATTCATCACGAGGAACCGGTGCCTCCTGGAAACGATA
CCAATCTTTTAAGCTTGTCAAGTTGTAAAGAACAAGTTGACAGTGGGTCTTGCACTTCT
GGTGCTACCAAACGATTCTTTTTCGACGTTGAAGAACAAACCTGTCGAACGTTCATTA

CACTGGATGCGGTGGAAATCGTAACAGATTCAAGACCTTTGAGTCTTGTATTAGTACTT
GCCTTAGCACTACTAACGAGATCGACGTAGATTCCGGAAAGGATATAAAGGATCCTTGC
ACCGAAGCACGCGAAGAATGTCAAACCATCCGTTGCCCGTATGGCAAAGAGGCGTTC
GTGGACTCTCAGGATTGCGAACGATGTCGTTGTGTTGATCCTTGTAGAACACAAATTTG
TCCCGATAACACAAAATGTGCTATTACCTTAGTTGCTACCAAAGATGGTACAGAATACA
AAGGAGTATGTAGATCAGTCATGAAACCAGGCCGTTGTCCAAATGTATCGAATAGTACT
AGATGCGAACAAGAGTGTCTCACAGATGCAGATTGTTCTGGAGATATGAAATGCTGTA
ATAATGGCTGTGGTGCTTCTTGTATCGAACCAGCCACCGAAGAAATTATATCGACTTCT
CCAAGATCGTTCGTTACTCCACCTGTAGTTGGTGCTGAACCAGCTTCCATCAAGCAACC
AGAGGAGCCGAAAGTCAGTGCTCAGGAAGGTAGTTACGTAACATTGAAGTGCATTACT
TTAGGAAATCCAAGACCAATGATCACATGGAGAAAAAGCACGACGTTGATTGCACCTT
CAGAAGCCAGACGTCGCATATTACCCGATGGCTCTCTTCAAATTATTAATTTAGATCGAT
ACGATGGTGGAACTTATGTCTGCACAGCTGACAATGGCTTAGGCCCACCGGTAAGAGC
AGAATACCAATTGACTGTCACAG

>novel_circ_000759
ATGAAGTGATACCTGTGCAACCAGATAAGATCAAAGATCTTACCGAGGAGGATGATGA
ATGTGAAGTATATGAGGACGAAGACTTTGTGACTGTCTCATCTAGTCTTACGCTTAGCG
ATAAATCCAGCAAAATGTCAGACCTTACTGAGGCAACTCCCCTAAGTTCACCAAGCAC
AGACAATTCTACTGGAAACGACATGGACGATTTAATGCTCAATGATGATGGTGCCACTC
GAGGCGACATATCTCCTAGAGAAATGGAAAATGCTGAAGGAAGCGGAACAGATTTTAC
CGATACGTTTTTCTATACTTATACATCGGAATTTGAATCAACATTCGAAGGAAGTGGAAC
CAGTGAAATCACAGAAGAAAACGAATTGTTTACAACAGAGTCTGGAATGATGGAAAC
TGATGTTACCGAGGAGACTGTCGAAGGATCCACCATGGATTCCACCGATGAATCGGTG
ACGCAAGAAACTCTAGCATCTGGAGAAACAGATATAACAACCGAATCAGGCGCAACTG
AAGCAACGACAGTTTCTTCTGAGAGCGAAGCAACTGAAGCCACATCGATGACAGAAT
CTATGGAGACTGAAGATACTGGGCCGTCAGAATCCACTGAATCAGGTCCAACAACCTT
TTCATCGGAAAGTGAAAGCACAGTTACGGAACAAGAGATGACTGAAGAATCTGTATCA
ACAGATATAAAACCTGATGGCACAGATAAGACTACAGAAACAAGTGTTACTGAAGAAA
CTACTGAATCAGGATCTATAGAACCAACAATGTCGACAGAATCAGGAATGACAGAAGA
AAGTACAGAATCTGGAGCCACAACAGAATCTGGAGCCACAACAGAATCTGGAGCTAC
AACAGAATCTGGAGCCACAACAGAATCTGGAGCCATAACAGAATCTGGAGCCACAAC
AGAATCTGGAGCCACAACAGAATCTGAAGCCTCAACAGAATCTGAAGCCCCAATAGAA
TCTGGAGCCACAACAGAATCTGAAGCCCCAACAGAATCTGGAGCCACAACAGAATCT
GGAGTTACAACAGAATCTGGAGCTACAACAGAATCTATGCCAACTGAAATTACAGAAT
CAACAGAATCTGGAGCTACAACAGAATCTGAAGGTACAACGGAATCTGGAGCCACAA
CAGAATTTGAAGGTACAACAGAATCTGGAGTCACTACAGAATCTGGAGCAACAACTG
AATCAGTATCAACAGAAGCTACTGAATCAACGGAATCTGGAGCGACAACCATAGAATC
TGGCCCGACAGAAACTACTGTATCTGGTGAAACTACAGAATCTGGTGCAACAACAACA
GAAGAAACGACGATTTCCGGCGAGACTACCGAATCGAGTGAAACTACAGAATCTGGTA
CAACTGAGAGTGGTGAAACAACTGTTTCTGGAGTGACAGAGTCAACTGTTTCCGGATT
GACACCGGTTACTGATGAAGAAGAGACCGAAGTAACTGAGAAAACACACGTAACTGA
ATTCTGGACAACACTCGCTCCGGAAGAGGTCACACGCAAACATCGCGTCTGCAAGGT
GCCTAAGAAGAAAAAGACCTGCAAAGCCATGTCGTTTGGTTGCTGTTACGATGGAATC

ACTGCAGCGCTAGGACCATTTAGCGAAGGATGTCCCACGGCCCAGACTTGCAATGAGA
CCAAACACGGTTGCTGTCCTGATGGAGTATCACCAGCCACTGGCCCAGAGAACAAAG
GCTGTCCCGAATCTCTCTGCGATGAAACATTGTTCGGTTGTTGTCCAGATGGAGTTACC
ATTGCTGAAGGTAATGATTTCGAAGGATGCAAGAAACCTTGCAACAAGACAGAATTTG
GATGCTGTCCGGATAACGAGACACCGGCTAGTGGAGAGAATAACATGGGATGCTGCAA
TGGCACTAAATTTGGTTGTTGTCCCGATGGAATCAAGCCTGCTTCTGGACCAGATGATG
AAGGTTGCGAGGAGGAGATCACTTCTATCACTCCACTTACTGAAGAATATGAAACGAC
CACTGTGCAGGAAGATTGTTCGAATTCCACCTATGGATGTTGTCCGAATGGTATTTCCAT
TGCAACTGGCGCCAATTTCGAGGGATGTGGTATTATCAATGCGGAGAACTGTACTATAT
CGTACTTTGGATGTTGTCCTGATGGAGTATCACCAGCTCTTGGGCCTAATAATTACGGAT
GCCACATGCTCTGTGAAAATAGCACCTATGGTTGTTGCGAAGATGGTATTACTCCTGCA
CATGGTCCCAACAGAGAAGGTTGTTGTCTATCGACACAATACAAATGTTGTCCGGATAA
CATACTTCCTGCTCATGGTCCCGATTTCTACGGCTGTGGATGTCAATACACGAGATTCGG
ATGTTGCCCCGATAATAGTACCACTGCTCGTGGACCGAACAACGAGGGTTGCGGATGC
AAATACACGCCTCATGGTTGCTGTCCAAATCGTTTTACGCCGGCCACAGGACCGAATTA
CGATGGTTGTCCGTGTTATACGTATCAGTTTGGTTGTTGTTCTGATGGTGTCACCATTGC
GAAAGGACCACATGGACAAG

>novel_circ_000761
ACGGAAAGTGAATATTCTGTAGAAGAGCATGCAAAATTAACTGCTACTGATGGAGCTA
GTGATGAAGCATCTCCAGAAGATGAAGGAGGATCATTATGTGAATACCATGACATACCA
CCCCCACCAGATGGTGGATATGGGTGGGTGGTGGTATTTGCATCATTCATGTGTAATATG
ATAGTTGATGGTATCGCTTATACATTTGGTGTTTTTTTGGGAGAATTTGTTACATATTTTG
GAGAAGGAAAAGGCAAGACTGCATGGGTTGGTTCATTGTTATCTGGCATGTACCTCAG
TGCTGGACCTATTGTCAGTGCTTTAACAAATAAGTATGGATGTAGAGCAGTATGTATGGC
AGGAAGTTTTTTAGGTGCAGCAGCATTTGTACTTTCAACATTTTCAACCAGTGTAAATA
TGCTTATGATAACTTATGGTGTTATGGGAGGAATTGGATTTGGTCTAATATATTTGCCAGC
AGTAGTTTGTGTAGGTTATTATTTTGAAACCAAAAGATCATTAGCTACAGGTATTGCTGT
ATGTGGTTCAGGATTTGGCACATTTGCATTTGCACCTCTTGCAACAATGTTATTAGAAG
CATATAATTGGAAAGGAGCAAATTTAATTCTTGCAGGTCTTATTTTAAATTGTGCTGTAT
TTGGTGCAATGATGAGGCCATTGGAATATCCAAAAGCTTCTTCTGTTAAACCATTGTTA
CAAAGAATGGCAGAGGAAAAAAGATTTCAAATGGAACGTGGGAGTATTGGGGGTTCT
TATTTCATGGTACAACTGCCTGATGGATCTATGGAAAAGAGAATGAAAATGCCTATTAAT
ATTGATCCTGGTGTTCATTCCAGTTTCAATTTAGACCAATTAGTGCCTGGAACTCCTTTA
ACACCAGTTCCAACGGTACCAACTCTTCCCACTATATCGGAAGTGAAAGTACAAGAAC
ACTCTTCTAGTGGAGCAACTAGTAATAGTGGCAGTATGGACTTAAAAAACATTTCCACT
AAATCAAAGAGTAGAAAAAATATTGATGATACCAAAGATATAACAGAGAAGTCTGAAA
GCGAATTCAAACCAATAATTCCTAGAAATGCTTCACAGCCTGCTTTCACAACTCATGTA
CAAGGTTTACCTAAAAATGGTTCTGTACCCTTTTTTGATAGAATTCGCAAAACAAGTAC
TGGTGAAAGGTATAAACCAAGTCTTAGTGCCATTAAGAATTCTAGAACAACATTGAATT
CTAATGGTGATATTAGAAAAAGTTTGCATTTGAGACTTTCGACAAGCAGTGTTATGGGT
TCTCGAAATAATAATGCGGAAATAGATGATGGCGAAAGTATTACTTTTACCACCAGTAA
AACTAGTATTCCAAAAGAAAAACCACAGATAATTCGACCACTATCAAGAAAGGATATTT
TTTATAGTGGTAGTGTTGTTAACTTACCAGAATATCAAAGTCAAAAGTCACTTGCAAATT

ATCGTCAAAGTGTTATTTCTTTATCAAAATCCGTTCGTGGAGATATTAAAGACACCGATA
TTGAAAAGGCGCGCGAACAACCTCTATGTCCTTGTTTGGTATTACCTGATTCGTTCAAA
GAAGCTCTGGCAACTATGATGGATGTATCTTTGCTAAAAGATCCAGTATTTCTTTTAATT
GGTATTAGTAACGTATTTGGAATGGCTGGTTTATATGTCCCTTTTGTATATTTATTAGATGC
TGCAGTCTTAGATAATATTGACAAAACTCTTGCATCATATTTAGTATCTATTATTGGAATT
ACTAATACTCTGGGTCGTGTAGCTTGTGGATACATTGCGGATTTTCCACAAGTAGACTC
ACTATTGTTGAATAATATCTGCTTAATTATATCAACAGTTGCTGTGGCTGCAATTCCATTT
TGTCATTCATATCCTGCTTATATTATTATGAGCATTCTTTTTGGAATAGCTATATCTGGATAT
ATTTCTTTGACGTCAATTATTTTGGTAGACCTTTTGGGATTGGACAAATTGACCAATGCA
TTTGGCCTCTTAATCTTATTTAGAGGAGCAGCAGCTATCATTGGTTCACCTTTAGCAGGT
GCTGTTTATGATGCAACGCAAAGCTATAGCATCCCATTTTTTATGGCAGGATTTTTCTTC
CTTGTAAGTACAGTTACTAGTTTCATGGCTCCAGCAATGAAACGCTGTACAACGCCACA
GACTCAGCCTGTGATATTAGATACATTGACTCCAATTGATGAAGATATCGAAGAAGAAA
ATGAAGAGGATATTCCTGAAATTGTAGAAACTGCGCCATCTCCGCAAGAACCTCCTGA
AAAAGAAATTAAACAAATAGAATCTGTTTTATAAAATCTGATCTGTATAAGGAGAAAAA
AAAAAGCCTTCATCTCCGAGCCATACTGTGATAGGGCTGCTGCTTCAAAAAG

>novel_circ_000762
CCAACCGTGGACGACGATGGCGTCGCTGGGGAATAAAGTGGATGAGCAAGTCGCCAA
GGTACCGCAGCAGAATGGACGTAGGAACGAGGAACAACTGCAGATGGAAGATATTCA
CGACAATAACAACGAGAGCATCGAGGTCGAGATGGTGGTGCCGCCGGACGGTGGATG
GGGTTGGGTGATCGTCGCAGCCTCCTTCATGTGCAATCTGTTCGTGGACGGTATCATCT
TTAGCTTCGGTGTCTTTTTAAACGACATATCGGACGCATTCGCGGTGTCCAAGGCGAGA
GTGGCGCTAGTTGGCTCCCTGCAATCTGGATTTTATCTTATGGCTGGTCCATTCGTGTCA
GCGTTGGCCAACAGGTACGGTTTCCGGTTGGTCGCGATCCTCGGAAGTGTGATAAGTT
GCAGCGCTTTCGTTCTCTCGTACTTCAGCACTTCCATCGAGTTCCTTTACATCTCTTACG
GCGTGCTCG

>novel_circ_000763
GAATGCTGTTGAATTGCTCCATTTTCGGCGCCATGTTCCGTCCACTGAAACCAACAAGG
ATTAAGGTGAAATCCACCCCGGAAAACGCTGGTTTAGAGGTAAAGAATAGCTTGATAG
GGAGGGGAGTGTCCATGGCATCATTGCACTGTGTCCAACCAGAAAGAAGTGGTTTCTT
TGGGACAAACAACAATACAGATTATCCCACTGCCGCAGAATTACTTGGAAGCAACCCC
AACATAGTAAACGCTTCCAAGTCCCTACATTCTCTTCACAAGATTCACGTGGAGCTACG
AACCCTAGAGAGAAAGTTGAGCAACTCTGAGAAACGACTGTCAGTCCCGATATATCCA
GAGTTGGACATGAATATAGATGAAAAAATCGTCGAGGAGGAGAATAATCTCCTCGGTG
GAGATGTTGAAAGACTGAATGGCAAAGTGCCCACG

>novel_circ_000764
CGCTTCCAAGTCCCTACATTCTCTTCACAAGATTCACGTGGAGCTACGAACCCTAGAGA
GAAAGTTGAGCAACTCTGAGAAACGACTGTCAGTCCCGATATATCCAGAGTTGGACAT
GAATATAGATGAAAAAATCGTCGAGGAGGAGAATAATCTCCTCGGTGGAGATGTTGAA
AGACTGAATGGCAAAGTGCCCACGATTCGCAGGCACACGATCAGCGGTAGACGACAA
CGCGCGGACTCGGATTGTAGCCAGAAATCGTTGAAGATGGGCAACAGGCGTAACCCAT

TTAGCAAGGATCCCCAGAGACCGTTCTACAGGGATGATATCTTTTATGGGGGATCCCTA
AACAGGTTGCCGCATTACAAATCTCAGCAATCGTCCGTTGGCTATCACATGTCCGTCAC
GCGTTTGCCGACTGCGACGGATGTCGCCGAGCAGGAAAGCGGAAGTTGCTACATTTGT
CCAGAGAGCGTACGTCGAATTCTTACTACCATGCTCGATATAAGCCTTCTGAAGAGCCC
TTCTTTCTTGATCCTGGCCATCTCTGGTGGGCTCACGATGATGGGTTTCTATACACCCTT
CATGTACGTTCCAG

>novel_circ_000765
AAAATTTGTACGCGAACAAAAATTTTGAGTAACAGCTATAGTTTCATCAAGATTTAAAA
CAACATGCCACCAACCACCAGGAACAAAAACAGTTTCACCAGGATTTTGTAAAATCTC
TATTGGTAAACAATCTTGTGGCCATGTAGGCAATTTTGTACGTGGATAAATAATGGAAA
ACCATGTAATAGCTTCATCTCTTTGTTTGCCACCTTCAGCTGCTGATACTT

>novel_circ_000767
AGCGCATTTTCGATGCATCCGCAAAACGCCAAAGAGAATTTCAACGCTGATTCCACGG
TGATCCTTAGCGGGCCAGGGAACGTGAAACATCGGAAAGAGAAACATCTCACGGCAG
TTCAATGGCAGGTCGGTTGTGACGGTCATTTATGGAGACACCAGACAGATTTGGTGAA
ACATCTGCGAGAACAAGCGAAGGATGGCACTCTGGCGGAAGTAATGCAACTTCCGGT
CCTATTATGGCGCGTTAAAACGGAATCGAGCCTGTTGCTAAGAAGCAGAAGAGAGAGC
GGTTCCGGGCATTATAGTAATCCGGATTACGACAGTTACGACGATTACGATATTGAGTAC
GATGGTGAGGATGGCGAGGATGGCGAGGACGGCGAGGATGGGGAGGACGGCGAGGA
TGGGGATGACTCGCAAGTACAGCCAACGTTCATGCCCGAAACTAATCTTGCCCCGGAG
CATCCTCACAGGCATCACCATGGGGAGGAACCTATCGATTGGAACACCGAAGATGATA
ACGATGATCTTATAATACCCGACACGCATCCAGACATAATAGAGAGCGTATTGAACAGA
ACTACTACGGATGCCATGTCAACT

>novel_circ_000768
GACCGAGCACATGGGTGTCGAAGTTCCCAATGGCGGCTGGATCGAGGCAGAGTCCTG
TCAACATCGAAACCGACAAAGTGGAATCCGATCACGAGGCTTTAAGTAGCAAACCTTT
GCGCTGGAAATATCCAGCAACCGCATCCCGGAAACTCGTCAATCCAGGTTACTGCTGG
CGGATGGACACCGATGGCGAAGGCACATTTTTAAGCGGTGGACCTTTAATGGACGACG
TGTATAAACTGGAACAATATCACTGTCATTGGGGATGCAGCGACTCGAGAGGATCCGA
GCACACTGTGAACGGACAAGCCTTCGCGGGAGAGTTGCATCTGGTGCATTGGAACAC
AAGTAAATACAACACCTTTGCCGAAGCTGCAAAAGCGTCCGACGGTCTTGCTGTTCTT
GGCGTATTCTTGAAG

>novel_circ_000769
GAAATCCGGGGACTCGAAGGAGGAGGACAGGAATGAAATTCCTGTGGGCGAGAATCG
TTGCGCCTGTTGCCTGAGGCCACTTTATCTGGGCCGCGGGGTTCGTTGCAAGGATTGC
GGTGAAAAATCGTGCAGAAAAGGCTGCTCCCGTTTCGATCCCTCGGACAACGCTTGGC
GTTGTATCTTTTGCCGTCAACAAAGGAATTGGTTGGAGAGGCACGGACTCGAGGCGTT
CGGTGGATCGATCAGTCAGGAAGATCTACGTTTCTACTTCAATACGGCAAAATCAAGG
GTTTACGTGGAAG

>novel_circ_000770
GAAATCCGGGGACTCGAAGGAGGAGGACAGGAATGAAATTCCTGTGGGCGAGAATCG
TTGCGCCTGTTGCCTGAGGCCACTTTATCTGGGCCGCGGGGTTCGTTGCAAGGATTGC
GGTGAAAAATCGTGCAGAAAAGGCTGCTCCCGTTTCGATCCCTCGGACAACGCTTGGC
GTTGTATCTTTTGCCGTCAACAAAGGAATTGGTTGGAGAGGCACGGACTCGAGGCGTT
CGGTGGATCGATCAGTCAGGAAGATCTACGTTTCTACTTCAATACGGCAAAATCAAGG
GTTTACGTGGAAGCTTGGAAGTAACATCGCGCACTCCCGTAATCCTTTCCTTACCTCTT
TCTCTCGCCGAGCCATCCCCGCGAAACAGATCCCCGTGAAACGAGTCGCGAACTCTTC
TTAATCGAACTTCGTCTCCCTTCTCTCCCTTAATTCTCTCCGTGCTCTTATTTTCGGTATT
TAAAAAAAAGAAAAAGATTTGCTCGTTCGGGCGTTGTGTAACCGCAAGGTGATTGTCA
GGGTCCAAGGCGAGGTGGAGGATCGTGTATATATGTATGTATCGATAATTGAAACGTGC
ACGCGAAGGACGGAAGTCGGACGGACGGACGCGCGTGAAAGCAGGTCTCGTTGGTG
AAAATCGACGAGGGGGGAAGACGTGTTTTCTTTCAGTTCTTTTTTTTTTCATCTTCTTCT
TCTTCATCTTCGAGTGGAAAAAACGAGGAGGAGGAGGAGGAGGAGGAGGAGGA
GGGCCGGAGAAAAAGAAAGGTGGACGGTCTCAGTCGTGGATGAAGAGAGGTTCGAG
ATATAACCGGTTTTGCGGCGTTTAGAAATGGAAAATACCCGACAAGGACGGCAGAGGC
GCGCGAAAGTCGCTAATACCTAATCAAGAATGAGGATGGATCGAAGGGATTTGGAAAC
GTTTGGGGGAACGCGCGTTTGAAGGAGGAAGGGAAGGGGAAAAGTTGATTGGAAAA
GAGATTGTGAGAATTTTTTCTCGCTTACTTTTCTAAACATTTTCTCTCGTCCGTTCAACG
AACGTGCTGTTATTTGCTTAGCTATAGCGGGAGGGGTCTACGAGAGGAGACTCTTTCTC
GAGGAATCTTCGTCGAGTATTTAAAAAAAAATTGCAAGTGGAACTTTGAAAATTTTCTC
AATTTTCAAATCCTCACCGTAACGAAAAGGAAAAGCAAACTAAGATAGACTTAAAAGA
AAAAAAGAAAAAACTTGAATAAAACTTGTTTGGAGAAATTTAAAAAGAATCGTTTAAC
AGAATAATAATAATAATTAAAAATTGCATAATACGAAAGAATGCTCATCAACGCTCGAA
CGAATAAAAACTTTTCCTAGAAACGCACAGATTTCCCCGGGAGAAAAATTATCCTATCT
GGCGACAATGATCGATGGATAGGAAGGGGAAAAGAAAAAAGGAAGAAAAAAGGAAA
TAAAAAAAAAAAAAAAAAAAGGGAGCATAGAGGATCATCGAGAAGGTTGGAGAAGG
TTGTATCTTAAATCCGCCGGAAGGCGAGAGGTACGACGCGCGCACGGTGGGAGGGGC
GATCACGAGGAGGAGGAGGAGAGGATATATCCTCGAGCAAAGATCGATGTCTGTTCGC
GCTCGAGCGGCCTCGAAGCCGCCGTGTGATAACTGATAAATATACCGGTCTCTCTTGCG
GTACCCGGTTAAGAGAATGCGGTTACAAACGCGAAGGGGGAAAAAAAGGACCACGGT
GGTAGATCTTTAATGGTCGTCGATACTTTCGTTTAGAAAGCGCGGCCGGCCGTGTCAGT
TGGACGGGGATTCGCGATCGTGCCGCGTCGTTCCCTTCGAATCGATACCTCGAATCGTC
GCGCGCCGATTTCGACCACCGGTCGGCCATTGTAAACAAACCGTCCGTTCTTTCTTTCT
TTCTTTTTTTTTTCGAAAAATATGCGCGCCGACACGCGCTCCGATGAATATTCGTTTCGA
CGAAGGGAGAAAGTTTCTGCCGCGTGAATGAGTGGCGTGATTAATTAAAGGAGTGGA
AAATGCAGCTACCAGTTCGGTACAAGATTTGGCCGCGGAGAAAGAAGAGGCGAACAC
GATGGAAACCTTTCGAGATTTTGTCGAGAAGATAGTGGTTGGTTTGATCGGCAACATG
GACGTGCCGATCAATCGTGTGGTATATCATCATTCGCCGT

>novel_circ_000771
GAGTGGAAAATGCAGCTACCAGTTCGGTACAAGATTTGGCCGCGGAGAAAGAAGAGG
CGAACACGATGGAAACCTTTCGAGATTTTGTCGAGAAGATAGTGGTTGGTTTGATCGG
CAACATGGACGTGCCGATCAATCGTGTGGTATATCATCATTCGCCGTGTAAGTTGAAAC

ACGTAGGACATGTTGTACAAGCACATGGCAAGCTTTTGGAAAAACACCTTGTACCCTT
GATCGATGCACTCGCGAGTTTGGCTATAGTACTGCAATCAGCTTTAGAGA

>novel_circ_000772
AATTGTGATCCAAATGGGCCACTCATGATGTATGTTTCAAAAATGGTACCAACTTCTGAT
AAAGGACGTTTCTATGCTTTTGGTCGTGTATTCTCTGGAAAAGTAAGCACTGGAATGAA
AGCACGTATTATGGGGCCTAACTTCCAACCGGGTAAAAAAGAAGATCTTTATGAGAAA
GCTATCCAGCGTACAATTTTGATGATGGGTCGTTACGTTGAAGCGATTGAAGATGTGCC
TTCTGGTAATATTTGTGGACTTGTTGGTGTTGATCAATTCTTAGTAAAAACTGGTACTAT
AACCACTTTTAAAGATGCACATAATATGAAAGTTATGAAATTTTCGGTTTCACCTGTCGT
CCGTGTTGCTGTCGAACCCAAAAATCCAGCCGATTTACCCAAATTAGTCGAAG

>novel_circ_000773
GACGTTTCTATGCTTTTGGTCGTGTATTCTCTGGAAAAGTAAGCACTGGAATGAAAGCA
CGTATTATGGGGCCTAACTTCCAACCGGGTAAAAAAGAAGATCTTTATGAGAAAGCTAT
CCAGCGTACAATTTTGATGATGGGTCGTTACGTTGAAGCGATTGAAGATGTGCCTTCTG
GTAATATTTGTGGACTTGTTGGTGTTGATCAATTCTTAGTAAAAACTGGTACTATAACCA
CTTTTAAAGATGCACATAATATGAAAGTTATGAAATTTTCGGTTTCACCTGTCGTCCGTG
TTGCTGTCGAACCCAAAAATCCAGCCGATTTACCCAAATTAGTCGAAGGTCTTAAACGT
TTGGCAAAATCAGATCCTATGGTACAATGTATTATTGAAGAATCAGGAGAGCACATTATT
GCTGGAGCTGGAGAACTTCATCTTGAAATTTGTTTGAAAGATTTAGAAGAAGATCATG
CTTGTATACCTATTAAGAAATCGGATCCCGTCGTTTCTTATAGAGAAACAATTTCAGAAC
AATCAAATCAAATGTGTCTTTCGAAATCACCTAACAAGCATAATCGTTTATTCATGATGG
CATGTCCAATGCCTGATGGTCTTGCTGAAGATATCGATAGTGGTGAAGTTAATCCAAGG
GATGATTTCAAAGTACGTGCTCGTTATTTAAATGAAAAGTATGACTACGATGTGACTGA
AGCTAGAAAGATTTGGTGTTTTGGACCTGATGGAACTG

>novel_circ_000774
ATCCCAGACTGCTCCTCTTCCCACACGTTTCAATCAAGTGCCATACAACGCTCAGCAGA
AGACTGTGATACGCATTACCTATGCCTAGAATCCGTTCGTTCATCACGTAGACGATATTA
GGACGATATTAGTCGAGCAAGATCGAAGAAGAGTGGGAGGCTCGGTGGGGTACGCCA
CGTTGATAAGAAACGCCACGATTAGACGAACTGGAAAGAGAAGTGGAAGACTGCGAG
GGAGAATGTCGACTTGCACCGACGAGGCCGACAACGCACCCCTGCACATGCAGGAGG
CTCTATCCCCG

>novel_circ_000775
ATCCCAGACTGCTCCTCTTCCCACACGTTTCAATCAAGTGCCATACAACGCTCAGCAGA
AGACTGTGATACGCATTACCTATGCCTAGAATCCGTTCGTTCATCACGTAGACGATATTA
GGACGATATTAGTCGAGCAAGATCGAAGAAGAGTGGGAGGCTCGGTGGGGTACGCCA
CGTTGATAAGAAACGCCACGATTAGACGAACTGGAAAGAGAAGTGGAAGACTGCGAG
GGAGAATGTCGACTTGCACCGACGAGGCCGACAACGCACCCCTGCACATGCAGGAGG
CTCTATCCCCGGTGCAGGAGGGGCCAGTGTCTCCGGCATCCTCCTCGCTGAGCAACCA
GAATCAAAACGAGAGCGAGCAGCAGGTATTACTCGCGACGATGCAGCCTGTCAACGTT
CTAGACCTGCACGAGCCAGCCACAGTTCACTCGCTCCATCCCAAGTACCATCATCACC

ACCACCGCCATCACCACCATCACCACCACCACCACCATCAACAGCAACAGCACATCCA
GCCGGATCCGGACGACGTCCAACAGAGGGGCGACGATCCCGACGGTCGAGTGACGCC
TGGCGCCGACCCAGCCGCCGCCAACACGACGCTGGGCGTCGGCGAGCACAAGATGAT
CGATCTGATCTACAACGACGGCCAGAAGACCGTCATGTACACCCACGACAAGGAGATC
ATCTACGAAAACGAGGCGGATCGCGTCCAGGTGGTCGAGTACCCACCCCCGCCACCCT
CGCCGCCATCCCCCTCGCCACCCTCCGCGGTCACCAGAGCCGGACTACTGCCGCCCGC
CTGCGTCACACCCAGATCCGGCTCCGCGACGGCGTTGCACCTTCATCATTATCCCCAAC
TGCAGTTGCAACACGAGGACGACCTTCCACCGGGTGTCCGCCACGCGGTCAACGCCA
CCGTGCCTAATTGTGGCGCGGCTACGACGACCACCACCGTGCTGGTGCTCTCGGAATT
GGTCGCTGCTGACCCCGCCGCTGGTGCTGCCGCTGCCCAACAACTCACCCTCACCGCC
GCCGCCGCCGCTGCCTCCCTTCGTGCTGG

>novel_circ_000776
ACGGCGAAGCCGCGGGGAGGAGGACACGAGCGGCGTTGAGGAGAACTCGCAGCAGC
AGCAGCAGGCGAGCTACGTGGGGGGAGGTCAGCGCGACACGGAGGAAGAGGAAGAG
CTGGGGGCAGAGGAGGCGGCGGCGCAGGCGGCGCAGTCCCTGCAGAGCGGCGCCGA
GGGCGCCGATCCGGAGGTACACAAGAGGGTTGCTGCGGTGCAGGTGCAGGTGCAGGG
TATGGAGGGCGACTGGCGGACCTACTGCGAGCACCCGCTCTCGGTGGCGACCGCGGC
CATGCTCAATCTTCAACAGCAGCACCAGGTCTCGGCCGTGGCGAGCCACGGCGACGA
CCCAGCCCCCTACGTCTACGAATATTACAAACTGCCCGACAAGAACGCCGACGTCAAG
TTGCCGCCTACCCACGAACTTTGGTCCAC

>novel_circ_000777
GGGTGTGATGGGCCAGAAGCTGCTGGTGCAGGAGGCGCCTGGCTCCGGTTCCGGTTC
GGCGAATCGGATGGAAGGCGGCGAAGGCGCCGGTGGGGGGGAGCTGCACCACTTCCT
CCACCAGTACAACCAACAGTCCCACAGTCCCGGGCAGAGCCTGCAGGGTGGCCTTCC
GCTTCCGCTCAGCCCGCAGTCCCTCAGACACGACGGCTCCTCGAGCACCGGGATCGCA
GGAGTGAAAAGGGAGCCGGAGGATTTGAGCTCGTCGAGAGGGGCGCAACAGTCCAG
CAAAAGGCACAAGCAGGCGCAGCCGGACAGTCCCACGCCGCCGGGGATGTACCATCA
TCATCAGCATCAACTGCAGGTTCAACAATACGGCAGCCCCTACGACCCTTACAGCTCGT
GCAGCCCCCGGCTCCAGTCAACGACGTACACGTCGACCTCGGCGACGGGCGCAGCCA
ACACCGGGAATCCGAGCGGGTTGCACCAGGAGGCGACCGCGGTCTATGTGACGGGGG
ACGCCCTGCCACCCTTGGCATCCTCGAGCTCGTCCTCGTTATCGACCACCACCGCTTCG
TACACGAGGTACGAGGTTGTGCCAAGCTCTTACGCGACGACACACGCGATACGCTCCT
CGTCGAGCAGCAGCAAAGTCCTCACCGTTGATCTTCCCAGCCCCGACTCCGGGATCGG
CGCGGACGCGGTCACGCCCAGGCAAGATCATCATCCGCCCACGGCGCTGCATCAG

>novel_circ_000778
ACAAAAACAATAGATTTTGATATAAAAGGAATTAAGGGAGAAGCAATAAAAACTCATA
CCAAATATCAACAAAATAATAATTTTTTGACAATTGTTCCTGCTTTACCCATTTTACCTG
GAGCTCTTGTACCATCAAAGTTATTTAAGCGTCGTTATAATTCATTCAGTCCAACAGATT
CTCCATTTAATATAGAATATGACAAAGTTAATGGTTCTAATTCAGTTAATTTACAATGGAC
AAGCGAAGGAAAAACTGTCATGGCAACAAGAACAGCTTCAGAAAAAGAGAAGAATC
CTGACAGAATATGTCTTGATAGAAGGGGACTTACTTCTTTTCCAAACATAATTGGAGAA

CCACGTTTGCGTTTATTGTCTCTTCAACATAATCTTCTTACAAAGATTGAAAATTGTAAT
TTTTTACAATTAACAAAATTAGTATTTCTTGATTTATATGATAATCAAATTGAAAGGATAT
GTAATTTTGAAATATTAGAAAATTTAAGAGTATTATTGATTGGAAAAAATAGAATAAAAA
GAATTGAAGGATTAAATCATCTTTCTAAATTGGAAGTTTTAGATCTTCATGGAAATCAAA
TTGTACAAATTTCAGATTTAAATAATCTTATATCTTTAAAAGTGTTAAATTTAGCTGGAAA
TAATATTAAAATAATAGGACATAATGATTTTCAAGGTTTAACATCATTAAAAGAATTAAA
CCTTAGACGTAATAAAATTAAGAAGTTATTAGGTTTTGATGAAACACGACAACTACAAA
AATTATATTTAAGTAATAATGATATACATAAAATTGAAGATATAGGAAATCTTGCTAAAGC
ATTACAGCTTAGAGAAATAACAATTGATGGAAATCCCATAACATTAAATGGAGATTATGT
TTCTTTTCTTGTATCATATTTACCAAATTTACAATCTTTATCAACAATGCAAATTACTGAA
CAAATTCGAAGAACTGCTGTGGCATGGAGAACAGCAAAAGAACAAAATAATTCTACTT
TTTTAAATCTTAGTGCACAAGTTTGTATGAATGCTCGACGAGAAGAAATTATATCGAATG
CTAAAATAAATTGGGAACTTCTCAGATCTCATTCTAAATCGTCGATAGATGATCACAATA
AAAATAATAGTCGAAGCAATAATATTAATGCAGTACAAATTCAAAAATCGGATAAATTC
AATATGTTAAAACAAAATTTAGCGAAAATAAAATCGAAAGGTTTTGGGAGTTTAACATC
AATAAATGAAAATGTAGAAGCAACAAAAATAAATATAAAAAAAAGAAGTAACTCTAAT
GATAATTTATTTAGATTAAAAGATACAACTAAAGCATATCAATTAGAATTTAAACTTCCA
CCAATTTTAGATTCTATTATAAATAGTTTAATAAATAATAAATTTGAAAAAATAAAAAAA
AATCTAACAGAAACAAAAACTAAAAATAACATTGAAAGTCTAACAAATAGTGATTCAG
AAATTTTAGAAAGTTCTGAAAATATACAAAGTTATTTAAATTCTCATTTAAAATTAAATT
CATTAAACTCTAAAAATTACATTTCTCATAATATTAATAAAATTACAAATAAAACATTTGA
ATCAATGAATGTTTTTCCATCAAATATTGTTGATGAGCAAGAGAATAACTATTATGATATA
TTTAATTCTTCTAAGTTATCAACAAATAGCAAACAAAATTTAACAAAAATATCTCAAAAT
ATAAAGATTCATGATCAACATGGATTAAATGATTATCAATTAAAATCAAGTATTGATTCTT
CTGGATATGGTTCTTTAAGTTCTAAAACTAATAGTATTGATAGTTGCAAATCAGTCCTAA
GTGATTGTAGCACATCATCCATTAGTAAAAATATAAATATAGAAAAAAGTAAAAACAAA
GATAAAAGAACTGCACAAGCAAAAAAAGTAGTATGTTATAAGAATAATAAAGTAGCAA
TTGCTAGAGCTAAAAAAAAAATTGAAACACCACCAACTCCATTACAAAATTTATCAAA
AGAAAGAGAACAAG

>novel_circ_000779
GTGGGCGATAGAGACACGCTGACGTCGGTGGCCGCGAGATTCGACACGACGCCATCC
GAGCTGAGCAAATTTAACAGGCTCGGAAGCACTTTCATTTACCCTGGCCAACAGTTGT
GGATACCCGTGGAAGGAGAAGGTCAAACAAAAGGCGGAACTGCCGACGCACCTAGTC
CGGATCCTTATCACGATCATGAGTCCCAGGATGACCTACCTCCAGAGGAAAAGGGTAAT
TTTTATTTATTGAACACCGCAATAAAAGAATTTCACTCCTTAAAAGAATCGTGAAGTGA
TTTCAAAATTGATCAATAATAGTATGTGTGAAATTTTATTCAACCTATTAATGATTAATAA
AAAGTAAAACGATATGAGAAAAGACAGGTATATTAAAATGATTGATTTTTATAAGTATTA
ATATATTCTATCGATTTTCAATGTTAATCATCAATGCTGATGACAATTAGATCAATGTTGC
GTGAGATTAAAGATTTTTTTTTTTTTAAGTTATATTCGCAATATATAATCTTATCTTTTGCA
AGTATAATATGTAATGCAATTTGTTTTTAATTGGTTAATGAATAAAGGATTTTAAATCGTT
TATTAATAATTAAATTGGATAGAATTATAAATAAGACATTCGATTCCATATATTATTTCCAT
ACAGAATTATATACATCATAATGTGTAAACATTCTATTCGATTATATTCTATTCCATATTCTA
TTACTAATTTATTTTTTATTTCATTTTGTATTTGACTTTATAATATTATATTATTTTTATTTCT

GTCCTGTAACGCATTTTAATATCGTACTTTTTTTTTTCGTTTTTGAACATCTTATATTATTA
GTTCGATATGTAACTAGATGAAAGCGAAAGAAAAAAAATACTATATGGTTAATAAAATT
TGCTGTACCTGTAAAATTGGCGAAATTTGCTTTCATTTCGATGCAAAAACTAATATTAAG
AGAACGTTACAGTATATGTCAGAACTGTACATTTAGTTTAGCAAGCACAGGCGCCATGT
CTTATCTATTCGATATATGCAATATGCTAGTATGAACATTCTACGTGACACTTTGTATAATC
AGATATTATCGAGACCCAATTCATCTTGATATTTGGCTAGGTGAGTCAATGCGACTTACC
TGAATGCATTCAGTTTTCTATTCCATTATTCATAAAGGCATTCGCGCCAATTTTGTTTTCG
ATTTACATTTGTTGTTGCATGGTGAAGCGTTTTGATATTTTATTGTGACTTGTAACAGCT
GTTAGAAACATCGATCATGATAGATTATTATTTATATAGTGAAATAGTAATCAAATATATAA
TAAGAGTAAAAATAGTATACCTGGTGCAAATAAATAAATAATAAAACATTTTGATGATCG
TTGTTGAAGTTGAAAAATATATAAATACATATTGGAGAAAAAGATTCACAGTAAATTGA
TTATTCGATGGCGCCACTTTACACTGTCATGTCAGACAGTTAATGTTCGTAAAATCAAA
AGAATGGACGGCTAAATATAACATCAAAAACATAAAATACGTTCTGTAAAAGAAGATAT
ATAGTTTTAGTTGATATATATGTGTATATAAAAGAAAAAAATTGACAAATTGATTTTTAAA
GTGTCACGTGTTCTTTGTTGTCTTTATTTCCCGCGCATTCTGTTTATTTTTCTTCATGATC
TAATTATATCAGGTATGCCAGACATTTTAATCGATAATTAAAACTTAATCTAATTAAATTG
TTTGTGTCTTTCTATGATTTATTGGAAAAATACATAATAATGCGTAATTCATTAAATATATT
TAATGAATTCGACTTAACTGTGCATTTGCAAATAATAATTAATTTCAATAATTTTAAGTTA
TTTGAAACAAAGAAAAATTATCTTATTTCTATGTATAAAATAGTTTTTGAAAAGTCAATG
CATCTTTATTTTAATTTTTTTCCATCACTGAATGTATATATCATATAATACATCAGCTGATC
AACGTCATGATGTAAACTATGAAAAGCATATGGCGTTATTATGTGCATCAAACATAACCT
ATAATATATTGAAAAATAAATGGAAAATGAATGTTCATTGGTTTTATTTTATATAAAATAC
AAATATTATAATCATTATGTCATTAAAAATAAAAATAGTATTTGTTATTCTTATTAAATTCA
ATATTACTACTTAAATGTTGCATCATGTAAATTAAAATTTAAAACGCTTCGAATTAAATAT
GATAAGACAACTACTATATTTTTTTACAACATTAAAATTTTTTATATTTTATATTATATGTAA
TTTTATTTTCAATATATAATCTCTATTCTATAAAAAATATTTCAAAATAATTAATATTATCAA
AAGTCTCTATATAAGTCTGTTATATTATTCTTTATAGAACTTCTGGATAACCTAAGGCCTG
TGTCACCCAAATCAAGCCATATGGAAAGAGTAAAAACACCTCTTGGTGTTACAAATAC
AGTTATAGAAGAGGAAGAACAGCCGTTCAAAGAAAGATTTTTAAAAATTACTGTTCGT
CATATCACAGATGGTCAGGTAAAGTTTATATCTCAATTTATTCATATAAAGCTTCTTTGTC
TTTTCTTATTAGTTCTTTTATCTTATTAAAATTATTTTTATTTACAGGGTGTTGTTGGTGGA
GTATTGCTAGTTACACCGAATGCGGTAATGTTTGATCCTAATGTCTCTGATCCTCTTGTA
ATTGAACATGGAGCTGAATCTTATGGTGTTATTGCACCAATGGAATTTGTAGTGAATGCT
GCAATTTATTACGATATCGCGCATATGCGTGTTGGACATACGGAATCTGGAAATTTAGAT
AAGAAGCCAGAAATTTATTATATGAAGAAACCTTCACAAACTGAGAATGTGAAATGTC
AATCGCCAGAAAAGGAGGAAAATTTTCCGGAATTAACAGCGGATGATGAAAATGTATG
TAGTTATACCGAAAGAGATGGAGACGCTTTTCCAAAGGCCTTTGAGAGAGATCTCGTT
ACTCCTACCAATCTCCAAAATGTACATTAATATTTATATTTAATAAATTAAATTTTTATCAA
AGCAATTATCCAATAATAATTCCAATTTTTTTATTAAAATCATAGGAAGAAGATACAACG
ACAACCAAAGAAAAAGAGAAGGAGAAGGAAAAAGAGAAAGAACAGGAGAAAGAAG
AGATTAAAGAGGTGAGCGGTGGTGGTCAAGCAAGTAGAACGTTAGAGGAACGTAGAC
GTTCTATGCTTGACCATCATTGGCCAGTTCCTAGTAAAGATCAATATCCATGGCCAGTTG
AAGATGAACAACAAGATTCTTTAAATTCTGACAAAGTTAGTTGTTCATTATTAGTGGAA
TACAATATAAAATTTTTTATCGAAGTTCAAATTTTAATAAAGATCAATATTTACAGACGGA

TTCTGCAAAATCGAAGGTGATTCCCGAAGGTGAAGAAGGATCTCTAGTCAAACTGTCA
TGCCACGACTCTGGTATTGACATACGTGACCCTAATCCCCCTTTCCCAGTAGTTCAGCCT
AACCCCACAAAGAAAGTATATTCAGACGCAGATATTGTCTTATCTTCAGATTGGGTCCC
TCCTATTACTATTGCTCCGACGAACGTAACAACTGATTCGCTAGATAGTGGTTCTGCTAT
TAACGGCAGGAAAAAGGCGAGTTCAGTGTCTTTTAGCTTAGACAGTAATGCAGAGGA
ACCTGAGAAGGATGAAGAACACAAAGATGACGATAAACAAGAGACTCGTAAAAATAA
GGTAATATTAAAATTTATATACTTGATATCACATACTTGATATACATACTTGATATACATTTA
ATCTAATTCTTATTTATTTAACAATTATTATATTCAGATGTTAAAACGGTTATCATATCCT
TTGTCATGGATGGAAGGATTAACTGGCGAAAAAGAAGATGATAAATCTGGATCTGATAA
AGTTTCGAGCCTTCCTAATAGCGCGGATTCACATCATTCATCAGTTTTCAGCAAAGTTTT
CTCAAGGTGATAATAATTATCCTTGTCTATAAGTCCATAAAATTTTTATAAAAATTTTCTT
GTATCTTATATGGTACAATAATTTTGTTCAAAAATTAATCATATATGCTTTTATGTTTTACT
TTATCATTCGTGTATGTTAGAAATGAGTAATTCAATGAAAAAACAATTTTGCATACTTTT
TATATACATAAGGAAGGTTTTCAATGGTCTGGCCTCCTTTATATGATGTAACGTTTGTTTT
TCGTGTGATTGCTTTCTGCTGAAATTTTTTTAGAACTAAAAGTAATATTTATAGCGTACTT
GAGGCTATTCATAATGAAACGAATTGCTTATATCACGCACACATGTAATAAAAACGTTTT
ATAGTTCTTAGAAACGAGTTTTATTATGAATATCCTCATTTATTTTAATATAGAATATTTAT
TAGAAATTTGCGCGTAGAAGCAAGCGGCTTATTGCATTTGCGAAACGCGCTTTGGGATA
TATCTTTTTTCAATTTCTAAATGGAGTAAAGGAATCAAAGGAATGGGCACGTGTGGCAT
TTCCAATTACGTCCTTGTCTTCAATAATCTTGTACCATCATAGTCCGTTTTTTCAATTTTG
CTTTCCCATTTTTCTTCTTTTTCAAACTTTTTTTATTAATTAGCATTGTAGATATATTCTATA
TCCAAAATAAATTAGTCATTCAAAGCTAGCTATATTTATCAAAATCTTGATGATTAAACTT
TATAAGAGAACTTTCATTGAAATGATATTTCAATGAATTAATTTGATTCAGTAAGCTGTAT
AAGTAAAGTAAATTTTGACTTCTAAAAATTTATAGGGAATACTGATTATTTTTATTACCAT
TTTGAGACCAGTATCAATCAGACTGTGATGGTAAATCTGTGTTAAAAATAAAGTTTAAC
TATCGTTTCCAATGTGACTAACAAAGAATATGTAATGGTGGTGGATATAAGCTCGCCGAT
CAACCTGGTGAGTGACTTTAGCTCGGGCCTGTTTGCTAAAACTCCCAGCGAAGAGAGC
GGTGGGGGCGGTAGAGCTGGGGGAACACCTCCGCTTACCGCCTCCTCTCTCTCCCAGG
ACTCCAGCAATCCTCAGATTTCACCTGGTCCAGGAGGGCTTATAGGGTGAGTTTAGGCC
CAGTCATGAACCAGCAAGTTTATACTGTATCCGTGTTAATCCACTTTGTCCTCATATTTTT
TTTCACTATTGTTCCACTCTCTTTTGCTCTTTCTCTACTTATCCATACTCTATCTCTTCTTA
TCTCTGATTGGCTGTTAACTTTTATATTATTTTTATTAAACATTTATCTTCTTAGATATCTA
GTTTGTTTCTTGCTATTTTCCCATAAATTACATCATCTAAAATCATTCATTCTGTAACATC
CTTTCTTATTAAGTTTTTTAATGAACATATATAAGTATAAATGTAAAATTGTTCTAGTTTTT
TCATAAATTTCACTGTTTCGAGCTTCTCAGGAACAACACTCTTCATCTATCCTATCCTTC
AATACGTTTTATTTATTTATTTATTTATTTATTTTAATCTTTCTCAAAGAGAAAGAACATTC
TTCCTCTGTTTCCCCGCTTTTCAGGTAAACTTACCATCATTTTTTTTTCAGAAAAACTAC
ACCAAGACCGAATAATGTGCTTACAAACAAACACACAATCAATCAGAAACACAGCGTT
TTTAATATTCCCGAGCAGTCATTGAACTTTATTTCACGACGCATACACGTTGGATACAAT
TTTAACATTTATCTTGAATGTTGTTGTTGTTTCCCTTAAGACTTAATTGCTCTTTATCACA
ATTGTATATTGATCAGTGTATATATTGCTTCGAAATTACAGGGAACACGAAAAAATCAGA
GAAATCTCGTGTACTCGTGATTTTGAAACGCAATTTTGAGAACATCTTTCATGCCTATTC
TCTAACATTAACACTTTCTTTCAGGCGGTCTTCCGTGGGCACGTTCATCAGGCAGCAAC
CATTGACGTCGTCAGGTAGCAGCAGCGTGGACAGTAGTCGAGGTAGCAAAACTTCGA

CAACGGCCCCGCGATTGGACTATCGTAGTATGGTCTCTGTCGAAGATATGCCCGAACTG
TTTGTCAGTTTCGACAGTAAGTGTCCAGAGGAATTGAGTCAGTCTTTCTCCCCTTGAAT
TATGGAACGCGAATATATTTCAATCTGTATATTTTTTCGGAATGATTAAATCCAATGAAAG
ATATCATACAATGTAACAGATTTCTCACACTTGAAATATCTTTTCAACATTTTTTATAGAG
TGTTTCCATTTTGTTGGGTGATTCTTTTTCATGGAATGTTTATCCGTAGAATAGTTTATTT
TTCTCCAAGGTGTTTAGATAATTTTTCGTTAGAAAGATCATTATGCGCGCACAAGAGCCT
ACAATGTCAACAATGAAACCTCTACCCTTCGAAGGGATCGATAAAAAAAAAAAGAAACT
CTATGATTAATTACACGTAGCCGCGAAATACAAACAGAAAGAAACAAACAAAATGATG
GCAAACGGAATTACATACCACGCACTGCACTCGCTCGTATGTATAGACGGCTTTTTAAA
CGATTGTTATTTTTGCTAGTGCATTTATTATTGTCCCTTTATTGAAGAATTTTTGAGCGCC
TTTGGATTCGATCTCGACTAATTAGACAGTTTTTCTTTTCACACTATTTATCGCCAAAAC
AAAGAAAAATAAAATTCAATTTAATTTAATCTAACCAAATGCGCTTACGTTACATTGTTA
CACATTGTTTTAATTACATTACAATTCTCGTACATTGTTCTATATGGTTTTTAAATTCTACT
CTAGTTTGTAATTTCCAGTTCTCTACATCTTGTATTATTCGGTGTGCCACTACCCGTGAA
CATTGTTCCACGAATTCGTATCATTATGAATAAAGAATCGCGAGAGAATTTGATATCGTT
CCAAATATTTTCTAAATGCCTGACACAAATTCGAGGAACAATAATTTTCTATTCACGATC
GTATAATTTTTGGATTCTGTCATAGTTTTCGCTTCTCGTCACAGATTCCAAACTCGAGTC
TCTTCATTGTTAAAAATCTTTATCGTTAATTTTAAACGAAAAGAAATTTATTATGTCTTCA
CGATTTTGAAACTCTTTGTGCTATAATCCTTGAAGATTAATTACATATTACAATTCATATAT
TATTTGTGAACTATATATGAAGGCTTTAATTGTTAAAGAAACGTTTTAAAAAAAAATGAA
AAATTGGAAAAATTGGATTTCTGTAAAATCAAGGAGAAAGAGTTTCACGATCAAGTGC
CAATGAATTCATACTAATGCAGATATATCATATGTTTTGTTGTCCCTCTAGTCCAAGCACC
TAATTTTCCCGAACTCGAAGGCCTCGGTAGGTTCATACTGTATTAGCTTGCTTTATCCCT
TTTGTATCAATTCACCATGTGTCCAAAGAACGTTACTTTTTTCACATTGTTAATTTCACC
TGTATCTATTTTTTCCCCCTTTAGTTTTCTTTTTTTTTTTTTTCAAGTCTCTTGTGCAACTT
CTTCTTCTTCTTCTTCTTCTTCTTCTTATGTGTATCATCGTACAATTTTTAAATTATAT
GTTTTTTAAAAAATCTTTTTTACTCACAACCTCCATTCGATTTTGCCCACTGCACTTGCA
CACACGAATGCATTTTATTCTCTTTCTCTTTGTGTATAATTTTTAGAAATAATTTCGTGTA
ATTTTTACGGCAACGATATTGTGCAATTTTTTACTCCGAATCTTGGTCACCTGTCACCGA
TACAATATGGATTTTTCATGGCTGTTTCTCACTATATTTTTGCTCATCTGTATTGTTGTGCC
CGTGTTTCTCGTGTTATTCTAATCTATAATGTTTCTAACATACGTTTGTCGATGCTCATGT
CGCGATCTCATTTGTAATTGTAATTTAAAAACAATTCTTAATTTCGATCAAGAGAAAATA
TCGTCTGCAATAATAATTTAATGCGATGCTTTTTAATTTGTTGAAATATTTTCTAGAAATG
AGCTTTTTGCATATTGTCCCTTATTTTTTTTAATTTTTTTTCTTCTTCTTCTTCTTCTTCTTT
TCTCTTTCTATATCTATAAACGAAATGTATCTAAATATCTAAATATTGTGTGTCTTGTCGG
AAGATTGATTAGAAAAATTGTAAATTTTTGATTAGAGTATCGCATAATTGAGCGAATATT
AAATATCAAAATTAATAAGATTTAATTAATAAGATTTAATTAATTATTCGAGATAAGAAGA
TCTCGTGTACGCATTAATGAATCATTCTGTTGCATTATTTAGAACTTATACCACGACCAG
CCCATTCCTGCGAGGATCCTCCATTGTACCTGAGACTGCGAACCGGAAGACCTAAGGA
TAAGAAAATTCCACGCTCAACGCCTATTATGAGCTACGGGAAAAAGAAATTACGGCCT
GAATACTGGTTCAGCATACCCCGTAACAGGTAAATAAAAACATTGTTTCCTTTGTGACA
CATTTCATTTTCTCTCCCCTTTTTGATAATTTCATTCAATATTGTAAAAATGTTGTTATTGA
TACACAGCAGTCTCATTAGATATCAAAATAAGATATTTTAAAATACACTCTTCTTTTGATA
AAATATTATCAAAATTCTATCATTAATAATTTTAATAATTTTCTAGAATTTAAATAATTACT

CTATTAATTCAAATTATTGTTAGAAGACGACAGATAAATTTAGAAAATTCATCTAATATTA
TAATATTTTAAAACTTTCATTGTAGTATTCTCAATTGTCTCCTCGATTTCCTTTGATTTAAT
TTTTTTCAATTATTTCTGAATTATTGCAAAATCTGTGAAAGATTTTCAAGAGAAAACTCA
CTATCTACGAATATCATTTCCCGGATACGTGTTTCTTTACCTCTATTTATATGTGCGATTTC
CGCGGAATCCTAATTTAGGAACCGGAAACGGCAATGAATCAATCGTTTTTTTACGGCTA
ACTGATATAAAATATCCTCGTACAACGTTTCCAAGTGAGTAATTCTTGCAAGATCGTGC
GTTATTTTTGTTAAATTCACACCTCGTATCACGTTCGCAAGACTAGACCGGCCAAAAAA
GAGCGAAACACGTGATTCCTCTGTAAGAGCGGCAAAATAGGTCTCGAGAATCGGCATC
GTGCTTCTTGCTATTTATACCGATTCTTTGCCTTGCGTTTTATGTTCGTCAACTTTCAACG
AAACACGTTCCACACACGTTTCTTCGTTCTATTTTATCGATTCGAGATGTTTGGACAAA
ATAAATTCGATATAAACTAATAATAATATTCCAAGATAAATGATAATGGCCGCGTTACGAC
TTGTAATCTTTGCTCGACAATTGTCGAAGGTCATGTGTGGTGTGAAGGTATATTCTGTTC
AGCAATCGATTTTAATACGCGGACAATAATCTATATCAATGGCAATTCCTCTTTTATCAAT
TTTTTTATTTTCCTATCAATGTTGTAAATTCAAATTGAATTTTAATTTACATTTACTTTGTA
AATCTAATCTATGATAATTTTATGATCTACTATTTAAATTTGTATTTGGGTCAAGTCTAAA
AATATTAGATGTATCTTATCTTTCTTCCTCTGTGATTGATTCAAATTATTATTATTAATATTC
AATAATTATATTTTTACTTATCCTGATAACTTGTGATTGTGATGAATTATACAAATTCATTA
ACTTTAGAAAATTTTCTAGAAACAAATCTTGAACAAATTGAATAGAATAATTGAATCTAT
TTCAAGTATTTAATCACAATTTTGTTGGATTGAAATAATTACACACTATAATGCTCCCCTT
ACCAGCAATCAATGGGGAGAAATATCGAGTTGAATGTTCACGGTAGTCAAACACGTTT
CAACTTAACTATCCCCATTCATTATGTGAACGTCTCTCGAGTTAATCGAGCCGATCTTTA
ATTAATATCCAAGTTGAACACGTTACACGTGAGAAGTAAGCGCGAGAAATCACGTGAC
ATACTTGGCGTATCATCGAACGTGCCTCCATAACTTTTCCGCTCGATGCTCGTGATACTA
GTCTGTTCGCGCGAAGAAAACGCATCGGAGACGCGCCACGTGTCGGTTGGAAAAAAA
AAAAAAATAATAATAATCGTTTCTTCTTCGTGAATTTTTTCTTCTTTCTTTCTTTCTTCTT
TTATTTATTTATCAATGATACATTATCGGTTTGATCGATAATTAATTGAGGAGCTTTTTGG
GGAAAAGGGATCATGAGAAAAATTTGAATTAAGTAAGTTTAATTTTATGTGTATATATAT
ATATTTATATATTTTGGATATAGATTATTTTTTTTCAGAAAGCTCCTTATTATGTGTTTTATT
TATTGGTAAAACTTAGGTTGAACGAGCTGAATTATTATTTTATCGAAATGTCACGTGACG
ATTGGATTATACATTTATCGAAATTATCTGTTGTAACTGGTGAACAGCTTCGTTGAATTTT
TATTATATAAATTGTTTATCGCTCGACAATTTTAGATAAATGAAATTCTATTGCGTGACCG
TTTTAGCATCGTTCTTAGTGTTGTCATTGGCAATTAAAAAGGATAGAATATATGAGTTGA
TTAGGAAAAAATGATATTTGGTAAAAAAAAAAAAAAAAGCAACTTTGAGTTTATCGC
ACAATAATTTATTAATTTAACTTGATAATGATAATTAAATATTTTGCAATATTTTCCACAGA
AATTGGATAATTTTGCGATGAATTCTTATGTTGGCATCTTATGATCATTGGGATGGACAC
GTGTATAATAGCTTGTTTTTAATTGACAATAATTGAATTATCATTACGTAATAAATAATAAT
ACGTTATTTTAATTTCCTCCTCCAAGAGAAGAGGGATAATGAATTTCAAGATACATGTGT
AGTATAAAGAAGTGATGAGTCTTCTCAGAATGTACATAATATAGAATTTAGATAACAAAT
CTTTAAAAATGTAGATAATTTTTTCCAGAATTATAATTATTCTCTTGCCCAAGAACAATTA
TACTCGTTCAAAGGTAGATTTTCAGAAATAAAAATAAGAAATCTTTGAATAGCATCATG
AGAATCATTTTATAGAATTGTTTCATACAAAAGTTAAAAACTCTGAACGATTCTCTACAA
TTTCAATAACCCATTTAAAAGAATATTCATTGCTTAAAATTTTAATTAAATATTCCAAAAC
TTTAACTTTCTAAAATTTCAATAATAACATTTATTGTTATTTTCAAAGAAATTCTCAATAA
TCCCAATCAAAGAGTTTAAGATTAATCTTTAAAATTACCACTTCCCAAACCCTTCAAATT

CAGAAGAAAAAAAATTTGCATAGAACTTTAATCTTTACTACCTCAACGACACGAATGAT
CGTCGACGATCTTGCAATCGCAAATCTGAAATCCGTCACACGCATATCCGATCCAGTGA
ATACGTTCACATAACAATAAAGAGTGTTGCACAGCGGGAGAAATCGGGAGAGAACGG
TCTGTGTTGGTCATCATCACGCAGTCGGAGATTAGGTAACGCGTCGAAGAAGCGTCTA
ACCTAAGCGATTCACGTTCTCTCTTTTGCATACCGAACTGCCGTTTTAGAATAACATCGT
GTTGCGCTGAATGGTCATTGGAAATTGGAAAATGTTTCTTCACTACGAAACATGTGTAC
GGAGCAACATAGTGACGTACGAGTTGACGCGATAAGAAGAGGTGGAGGATAACGAAT
GCGAGGTGTTGATTAGATAATTCGTTTAGATTCGTTTCGAAAGACAGTAGAGAAGGAA
GGCATTACGTGAGTTATAATCGGAAATGAACCGAATAATTTGTAAACTCGTGCGATTCT
CTTTTAAATAGAAGATCGAAGAAGAAATTAAGAGTTCTAAGATTTCTTTTTTAATGGA
ATAGTTTGTTTTTTAATTAGAAGGAGGAACGTCTGAATTTCATAGTTTTTCGTGTAGAAT
AAAATTTGTAAAAAGGAAGTGTTTATTTTTTTTTTTTTTTGGTGAATTTGTTAAAGGAA
TAATAAGTAATAATTAATTTTGTATTGGAATAATAGTAATTTGATAATATTGTGTTGGAAA
ATATATATAAAAAGAGAAACTATGAGTTTTAAAATAAAATTTTTCCATAATCGCTATCAAT
TTTTTTTTTTTTTTTATAACAAGTACACTTTGATAATAAGCTATTTATTTTTTAATACAATGT
TTAATTTCAAATATCTATTACTATTTTGTTTATTATTTATTGCTATTTTTATTAGTCTTTGTC
ATCTGTCTAAATGATTATTATTATAATAAAGTTGTTATGTGCCATTAAAAAAAGAACGATA
ACAGGTGACAATGATAGGAATTATCGTGAGGCGAACAATCGAGTCGAGTAGATAATGTA
TTTTCGTGCTCGAAGTTAAATTTAAACTAATCAAAAAAAGTTGTGTTCTATTTCGATTTT
TATATTTTGCAAGAAATATCTTAATTTTTTATAATCTATAATATTCTATAATATTAAATATTG
CAAACATTTGACTCTCGTAAAAAAAAAAAAAAAAAAAAAGAAAAGAAAAAAAAAATTA
AAATTTCACAGTAGTTTGCACAGTAGTTTACGTCATCACAGGTAATTATAATGAATATAC
ATAATTCCCGAATGATTCACTCGTCTAATTCTTATGCAGTCTACCAACACAATTTTAACA
AGTGTCTCTCAAGGGTCCTCATCGGTTGCATCACGTTTCGTATTGTTTCGTAGCCTGTTG
AATTTAAAATAAATTCTAAAGAATTTTTATTCGTGATACACAAAATATTTAAAAATCGTG
CGAATAGAAATATTAACTTGTAAAGTTTAAACATCGTTTCGTAGTCAATCGACTTTGAC
AAGTGTTTCCACGGGGTCAACATCAGTTGCATCACGGACGCTTTGTATTCGATTCTAGT
AATAAACTACTTGATCTTAAAAAATAAATTCGAAAGGAGAATAAAATTTTCAGCGTGTT
TTTCAAATTTATGTTGAAAATTCGCGTGACACGAATATTATAATAATCAGAATATAAAGT
TTTAAACAAATTCGAGTGTGTTTCTACGGGGTCATCGTTACGTAAATCACGGACTCTTC
TTTTTTCCGATTCTAAAATAAATTCGAAACGATAATAGTTGAGTGCTTCAAAGTAAGTG
AAAAATATTAATCGAGGTAAACAAAGATTGCGTAATATTTTGCAAGATTCAGAAAGGGG
AACGAAGTGTTGTTAGAGAGGAAATAGGAAACGACCTTGCATGGTCGTCGACGTTTGA
AGCGCAAGTGGTATATCGGTTAGAGAAAGTCAATTCGATCTTGATAGAGGTGCACGAA
CGTGGTGGTGAACGTGTTACGATACCTGACGAGGCGGCAAAATGAATTGGACATTGAG
CAGTGTCGGCAACGTGATGAATTTCGTGTACAACCAGAGTGTGACGAACGCTGTGCAG
AACGTTATACCGTTCGTCAATCGAAGGGTGGACGACCTCTACAGGTTCATCCATGCGTG
GGTGCCCTCGCTTTATGGAGAGCTCGACGAGAATTATTGGCGGGAACGTGGGTACATC
CTGTTGGACACCGACACGGATCTGTCGCCCGAATTGTCGGGCGTGCACGAGGCTGGG
AAAGCGGGGGAAGGAGCCACGGAGGAAGGGAAATCTCGCCAAGACAGCGACGAGAT
ATCCGAGCTGACGAGAGAATCATGGGAG

>novel_circ_000780
AAACATGTGCATAAAAGATCGGAGGAAAATATGAGGAGAGGTTCGATCGAGAGTGAC

GATTCGATCATGTCCTGCTCGTCCTGGGAATTATTGGACTACGAGCCGAGCAAAATTA
CATCTACGAGAAGAAATATCGCGATTATGTCGTGTACTTCTCCATGACAGGCAGCTCTC
CCTGCCCGAACTACAGAAACTTCGAGCACAGCTCTCTTGGAAGCACAGGTACATTTGT
AAGAAACATTCGTAAGACATTTAAGTAAGAAACGAGCAATGTGAATCACGTGCGCAAT
GATCGATGTTTTACACTCCGTGTATATATAGATTCGAAACAGGCGTGAAATTAAGTTTAT
TGATAACAAGCTTAAGAATTGTTGGTCGGAAAATTAAAAATTTGCACAGAGGATATTTT
ATCGAATCGACGATTAATCATTCGGAGATCAAACGAATCAATTCCTTTTTTTTTTTTTTT
TTTTTTTTTTTTTGAAACTTTTAATTTTAACCTTATAAATTAATCGATTGGAATCGAGAAT
TTTGAATTTTTTTTTTTTTTAATAATTTCCAAAACTTTCAAAACAAGGGATCAATCTTTAA
TTATTTGGGGATCGAATAATTGGTTACAATTATTAATACAGTTGATCAATTTGTTTAGCTT
AATTAATCGAATAATCTGAGTTAAAAATTAATTTGAAATAAAATTGATTTAAATCTCCAT
CCCTCTTCTTTTATCAGGAATTTAACTGGCTGAATCGAATACACGAATTAGTCAAAGAA
AATTATTAAGCAATGTATTATAATATCTTGAATAATAGACTAAGAGTTAACATTCCAAATT
CATTTTCGAATTTCGAAGAATCATCAATCAAAATATTGATGATTACAAAATTGAAAGAG
ACTCGTTCTCACGATAGATTATCTTCTCTTATTAAATAAATTTAATAATATCTAATCTATCT
CGTGTGCTATCTTCAAGTTACATTTCACTGACCTTTCATCAAAAGGTGTGCAACCATTT
CTCATGTGGATCTCCTAACAGTGCAATAATGTCAAACTTAATCAATCGATTCTACAAAC
GCATTCAACATTGATTATTAAAAATTCACTTCTAATCATTCACTCCTAATCAAACGATATT
TAATTTAAACAAAAAATTAATCATTAATCATATCTTCATTGCAAAAAAAAGAAAACAGA
AGATTTTTCCAAAGAACAAAATCATCAAATTCTCTTCTAAATACTTGATTATAATATTAC
AGCTAATTCCATCACGAGCAACAGCTATTTTATGCGAGCATTAAAATCACCGTGAAACT
TCCAGTCACGTACACACACACACACACATACACACATACACTTATCGTTGTATGTAAAT
TCGAAGAGAACTGTCCAGCTTGATGAATTTGAAAACTTGCGGTCGATTCAAGGAACAA
CAATACGAGTTCGATGGTATCATCGTGTTCTGCGTACTTCGAATGCGATCGTGATGGCA
AACGACATAGGGACGGGCGCGTGATGATTATGAGGAAACGGGGGATTGCGTTTCGGGA
ACCGTGGCTCGTTCGATTTAGAATCTTGGAGCACGTAGGCTCGTGGAAATCGAGAAGG
AGGAAGTTGGGGGGAGACGTTCGTTGGAAGAAAATTAATGAACCAACGAGAGATAAT
CGGGAGAAAACGGGGAAATCATATTGATTCGCTCCGCCTGCATTTTCGCGCCTCTTCGT
TTCCAAGATCGGGCAGCGAGAAAAGTAAAAAAAGAAAATTATAATACGCGATGATGGA
TTTCCCAGTAATTCGCTGTCGTGATTAGTTTGTGAATAGAAACGTACGTGCATTAATTGC
ACGTAAATAATTGCATTTTTGTCAATACAATCCTTAGCTATCTAATAGAGATTGTAATTCG
TGGAAATAAGATTTTTCTTAAATAACGTAGATTCATAATTTTATAAAAATAAAGCAAATC
GAGTAAATTTGGATAGGAATAAATAATAGAATAAATTGAAGAAAGAGTTGTTTGTTATT
CGCAATATATAATTAATAAATTTTAACAATTCAAGCAATGTAACTAAATCAAGTTTGTATT
CAGGGTTCTATATTTTTCAGATACAGATAGCGAAAGCGAGGATGGCTCGAGAACGAGG
TTAAACTTCAACATCATCCCCTATACGGTGAGTATATTTCGTTTCTTCTTCTTCTTCAAGA
CGAATAGGATTGAAAGTCAAGGAGAACGAGCGTAACCCGAGCTCGGGGAATGCATAA
ATATCTGTTATCGATTGAATCTGCTACGATGCATTTGATTCATGCGACAAGTTGAGAATA
TCTCCTCGTTCGAATCTATTCCACACGCCGGTTATTTGCATACCGCGTTATTCAGTCATT
CCGTTCATTCCAAATCTTAACCTTATCTAGTTATCCTTTTCTATCTACCTGAAAAAAGAA
AAAAATATGATTTTCCTTCGATCGATCGAACACTGATCAAATAAAAAAAATATGATTGTT
CGAATTATTTCCTCGGAATAGATAAGTTCATTTAAATACTAAAATAATAATCAATGATAAA
ATTTTCTGAAAGAACAAGTTTGGGATCAATTAAAAAGCGCGTTTAAATTTACCTCATTT
GTTGGAAAAGATAATTTTAACGGTGTCACGATAAATTTTCTCTTATTCATTCACGTAGCA

TTTTTTCTTTCCTTTTTTTCTTAATTGCCAAAGTTAATTACACCAAACGCTATAAAATAAA
TTGTAATTTTGAATGGTATTCTTTTTATAATCTCCATATATATATATATATTTATAATCCAATT
AAATTAATTCATCGCTTAGCTTGTCAATAACATTACACATTCCTTTATTATTATTGTCTTTA
GACTTTGACTCATTGCTCTATTTATATTCAGTGCACACAATTATCGCGATATTCGTAAATC
ATAGTGATTTGTTTACGATTAATTTTTCTTTTTCTCTTAACCTCGCAAAAGGAATTTCCTA
AGAATTAAGAAATAACAATTTATAGATCAAAGTGGAAGAAAAAAAAAAAAAAAAAAA
AAAAACGGCACGATCCATCGATCAATCGATTTATTAAACTCACGAGACTCGGTAAGTT
GCTAGGCATGATTCATCGTCGAGTATGTACTCTACCAGTCAGTGTCAGGAAACTCTTATT
CATTTTCAATTATCATAATGCTCTAGACCGTTATTGCTTGCTTATTTTCCTCTTAACTTATA
AAATGAAGTAGCATCTATATATATTTTTTTCTTTTTTTTTTTTTATCTCTAAACTTTGTACA
AGATATTTATTTATTACAGAGATGGAAACACCTCTTAATGTCTAATTCCCTCGCTTATCAC
TAATCAATCGAATTAATGAATGATACCAGATTATCTAATTGTGTAATATCCATCATGATTA
CCAATCTTTCGTATTTATATTTTTGAAATGGAATGAAAAATCTATTTAACCTCTCTAATTG
TATTATTCTTTTTTTTTTTATTTTCATTATCATCGATTTCAATGTTTATATTCGTTTATATTATA
TTTATTCAATCGTTTAAATATATTTCGTATCTCGTTCACTTTAAATCATTTTATTACACGTC
GAGAGAGCGAACTGAAAAAAATTTCTTTCAATCTTCTAATTTTTCCTAACTTTTCGCATA
TATCGAAATCCATTTGTGGCACAAAGGAACCATCGAAGAAAAAAAATTAAACGATAAG
TTTCGTGAAAAATATAAATTGTATCATCCAGTTTATCGTGGCACCGCGAAGAATTAATAC
GAACCAATAATACTTTATTAACGGACGTGCCGTGTCACGTCAGAGGTGACATTGTCACG
TATAGGATCTGTGATAATATTTCTTCTTCTTTTTTTTTTTTTTTACGTCACACATGCGATCC
TGAACAGGAAGGATCATCATACTGTAAAAGCAACTACGACTTTTATAAGAATTAAAATC
GTGAAATATAAAATTTTAGAAAAAGCGAAAAAAAAAATTAATAATAATTGACCAGTTAT
ATAATTTGTAATAACGATCCGTCTTATCCTATATCTCGCATATGCCTATAATTAAGAGTTAT
ATATTTCACGTTTTCAGAAAAAAAAATAAGCGAATAATAATTAACGATCGTTGGGGGGAA
AAAAAAAAGGAAATATTTAAAAAAGGAAAAGTCGGTGGAAAATTAATTGCGGAAGTT
CTCACTATCTGTTTGGCGCGAATATATTTATGGTGAAACGAGCTACGGGTTTAAATGCAT
GTGTACATACACACATACATATATACATATATATTACACAAAATTACCCAAGCACCTTGGC
GTCTGGTGGTTGCGCAACGCCACGCTTGTGCTTTCTTGAGCGCATACGTCTCTTTCGCA
AATGGACGCTCACTTTCTCGACGGGCCGGCCGTACATACACGCAGTAATCGATACAATA
TTTCACGAGGAGAAAGAGGCAATGCGAAGTAATCTGTGAAGCTTCTTATGAAAGTCGC
CGACCTCTGACGAAAGAATTGGACGTTAGATCAAACGATCGTACTTTCGTCGCTTCGA
AGATGCATTCAACACCTCGTAGCAATTGAGTTGTTATTATTAAAGCTCCAACCATCCACT
CTTGATGAGTTCTTTTTTTTTTATTATTGATCACTTTAATTATCCATCGAAGATAATTGATC
TTTCTCGATACGGTTAAAGTTTCATTGATTGTTAGATGGATTTGATATCGAACGATATCA
TCTTTTTTTTTTTGAGTAAATTAAATTGTTTTTCTAAAGTGAGTTCGTCTACGTTTATCCA
ATCTTTGGGAGATAAAGATGCGTGGGATCTCGAAGATTGCATTACATTGTGGGAGACAG
AATATTATTTACAATTATTTTTAAAGCCTACGTTCACGCGGCTCGAACAAATCGATAATC
GATTTCGTTGTAACGAAGAAAAATTACGAAATCATTGTACATTTAACTCGATTGCTTTAC
ACATTTTCTTTCGTTACAGAGATACTTAAGAAACGTGGGTATTTCAGGAATAATTTCGAC
ACATCGTAATTAGCACTTCTCTCACAATATATTTCGAGTCAAACTCTTTCAATAATTATCA
CTTAATAACTCGAAATCCGAAATATATTTCGTAATCTTCGTTGCGAATATATTTTTGTGTA
TTTTTGTAAACCTATGAAAGAAACAAATGCCACGTTGTTACAACGCGAGATCGTGATTC
CACGTACTACGTGGATCCTTTCAAACAACTCTCCGGAGAAATTTCTCGAGCAAATATAC
TTTTCGTCCTCCCCCTCCCCCGCGTCTGAATATTTTTAAGCCGCAGCCGAGAGGCCAAA

GAGGCAGGAACCGGAAACCGCGCTCCAGCGACTAGAAAATCAACTACCATTTTGTCC
AACAAGCAATAGATTCGATTCATGAATCGTTGCAATTATCGATCTCGATTTTAATCCGAG
TCTCGATTATTTCACACGATGAGAAGAGAATAATTTTCCAATATAAATATTCAATTCCAG
ACGCTTGCAAAGTATTTACAGTTTTAAATTTCACATTATCACGATTTTTAATCTCTTTTGA
GTAAGGGGAGGGGGTTGTCTCATTCAAATTAAAAATTAAAATTCCATTCAAATTAAAAT
CCATCGGCATCCGGTGAAAGAGCAACTTTCATTCGCTTAGTATACACAACAACAGCGC
GAATGGCGAGGCAATGATCGCTTCTCGGTATAGTTTCCCTTCTGTCGCCGATTATCCTAT
ATACAGAAGGTAGAGGTCTCGATCCACATAGATCTTTCCGTCGATCTTTCTTCGAGGGA
AAAAAAAAACTCCTCTAGATTTCTACCAATCTGTCAACGATTCGTTGCGCGGTCACTGC
TCTATTCTCCAACTCTGTCCTCCACTCGCGTATTATTACCCTCCTCTCTCTGGAAAAAAG
AGAAAACGAACGATGCATATGCTGTCGATCAAAAATTTGGAATTATCTTATCTATTTATAT
ATATCTTTTCCTTCTTCTATTAATATTTTATAACGAATAATCTTTAACGATTAAAAATAAAA
TTTAAAAATCTTCTTTTAAATACTCGCGTATTCTTCTCTCTGGAAAAAAGAGAAAACGA
TGCATATGCTGTCGATCAAAAATTTGGAATTATTTTATCTATTTATATATGTTTCTTTTCCA
ATTAATATTTTATAACGAGTAATCTTTTACGATTAAAAATAAAATTTAAAAAACTTCTTTT
AAATACTCATGTATTCTTCTCTCTGGAAAAAGGAGAAAACGATGCATATGCTGTCGATC
AAAAATTTGGAATTATCTTATCTATTTATATATATTTCTTTTCCAATTAATTCTTTATAACGA
ATAATCTTTTATGATTAAAAGTAAAAATCTTCTTTTAAATATGATCGAAACGATAATATGA
AAAATGAAAAAGTCTTTATAACGAATAATCTTTTACGATTAAAAATAAAATTTAAAAATC
TCCTTTTAAATGTGATCGAAACGATAATATGAAAAATGAAGAAGTCAATGAACAAAGTT
AGACCGTTTATGATTAACAAGATTTCTTCCAAGAGTCTCTCTCCGTTCTTTCGTGATACG
AAACGTCTGAATTACCGTTTCGTCGAAATCTTCCAAGGTATCAATTTAATATCGACTGTG
CGGGTCTGGACGCTAGCGTGGTTACATAAACACGTAGATACTTGGAGCCGGAGTGGCT
CGAACCGTTATAGAAAGTTCGACCGAGCTCTCTCGCGAGTTCGATCCCCCGGATCTCTC
TCCATTATCCAACGACCATGATTGCCCTATAATCCGCGCATCCTGCATCTTCCTCCACCC
GTTCCAACCGATTAGAACAGCTCTCTGCCCTTACGCATCGATTAATGCCCGCGATGTTTT
ATCCGTCGTTTGGGACGCGTGTCTTAAGGATTGATCGCGTGAATTTCGATGGAATATGC
GAATGGAAAATGTGATCGAATTGGAGGGAATAACTTTTTGTTTCTGTATGATCGAGTTAT
TAAAAATTCAAGTTCCAATTGCAGGGATTCATCGACGGTGTATTCGTATTTTGTTGGATA
TGTCGAACACTACTGATGAATGATATAAATATGCTGATAATGGTGGATATACTGTTCATCC
AGTTCCTGATCTTGGATAAAATAAATTTCTTAGTAAAAGTGTAATTAACGAAGATGGAA
GAAGTCAAAATCTAATATTATTTCATTCGAGGAAATGCTATGTCAGGAGAACCTAATATT
AAAGGAATTAATTAATTTTCAAATCCTCCAATTAAGAATGGTATTACTATAAAAAAAATT
ATTAAAAATGTCTAGTTACAATTGTATTGTAAATTTGATCATTATTAATTCATGATCCTGG
GGATCTTAATTCTATATGAATAATTAGTTTTATTGAGGAAGCAAGTATTCCAGACCATAA
GGCTAGAATAATATACAAAATTCCAATGTTTTTATAATTGGTTGATATAAATCACTTTTTA
TTTTTAATTTTGAAGATTAATAGTTTTTGTTAACTTAAAATCTTTTAAATTTATGAAATTT
AAATTGTAACTAAAATTTAAGATTTAAATTAATTATTATTTTATTTAATTTAAATTTTAATT
AAATAAATTATTTAGTGTTAAGAGTGTATATAAAACTTAGGAGAGTTTCTGAGAAATTCG
CAATATCGAAATTCTGATAAAGGCGGAAAATTATATTTCTTCGAAACAAGTCTTCCCTAA
GATCTTTACACGGTGAACAGTTTGCAGATCGTTTTTCGCCGTGTCTTCCAATTAAAGAA
GACCACCTTTGACCATCAATTCCCTCGGAATATATATTTTCCAACGCCGTCTCGAGCGAT
TCAGTTATTCAGAGAGGAGCGTGGTTTAAAAAGGCCGCTCGTTTCAAGCATCCCCGAC
TACGAAATTTTTCCTGAAAGGGCGCGCCTTTGAGGAAGCTCGAAAAGACGATTCGCTT

TTCGTCGCGAATCCTCTCGCTATCTTCTTCTTTTTTTTTTCTTTCCTTCCCTGCGCTGGAA
ATATTCGAGAACCGAGTTAACGAGTGACTCGGCGTGATGACTCGGCGAAGAATTCGAG
GGGAAAAGCGATTTTTGACGGAAAGCGGACTTTGAGAAGAGCCGTGTAAAAGTCGAG
GCGAAACGATATCGGAAAGAAGCAAAAGCGCAGAGTTTTTCATTTGAAAAGGTCCTCC
GGAAACCTCGAAGGATTTGTTCACGCTCGAACAAACTCGAAGAAACTCTGGTTGATG
GAAAAATCTGACCTTTCGTCGATGTAGACATTTATATATATAGGATATTCTTTCTAAATTA
AAATTGTTGATACCTTCCCCTTTCCACAATCATTCCAGTTGTATTATCAATCTAGTTCAAC
GTAAAATCTATCTAAAAAAACTTATGAAATATTTCAGAAAATCAATATCATACCAGTTAT
TTTATTATCATTATATCAAATTCGTATCATCTCGGTTAAAGATCCCTTGAATTTACGAAAC
GTTTTCTTCAGATTCGACAGGAATTAAAAAGATCCAGACAGATTACGATCAAAGACGA
TTCTATCCTTTCTTGATACGACGACGTTCGCCAACTCGGTTGGCCAAGAGCCAGGTGAA
TAAAATTCGTTTGCCATCGAAAGGTTCGGCTGATTAATTAATTGATCGATCGATAAGGGA
TGAAAGTTGTCGCGAAGCAAAGCCTTCCCTTGTCGAGGATGCAGGGATGATTAAATTC
CATCCTCGCCTCGTTACCCTCGTCACGTCTTATCGCCGGATAACTCCGGATCCAAAGGT
TCGCCCGGCGCGAGAGATAAGGGGGATAATTGCGAGGGGGTGGGGGGTGAGTGTTGC
AGCTAGGTATTGAGATGTCGAGGCGCGGAAGAGTTATTATCGGATAACCGCGAAAACG
CGACGACCCTGAATAAAAAAAGTTCTTCTCTCTCTCCTTCCACCCCTCGAAGGGGGTG
GAGGAGGAGGAAGGGTGAGGACGAGGGTAACCGTGGAGAAAGAGGGGAGGAAGAA
AGAATCGGGACAACGGGTGCTTTCTTGCGCGATCTTTTGTGAATGGGATCGAGGCTCT
CCCTCTCGAAGCCCAAGCACCTCTAAAGAGGTGCTCATTCTCGAAACGAATCTCTTGTT
AAATTTGGAGATGAAATGGTGGTGATTGGAAATCGGTTCCTTTATTAAATTTGTATCTTT
GTCGTCTCTTACAAAGCCATTTTATTTATGGATTTCAGGAGAAATGATCTTGGTATGTTG
TAAGAAATTAAAAGAAATATATGTTTTTATTTAATAATAAATTCTTCTTATTGGGATTAAC
GTTTTCGATTCGGTGAACTGAGTCTGATCTTTTTAATTTATTCGAGTGAGGCAATATTTT
GTCGTGTACAAAAGCTACAAGTTGATACGAAGAAGGAACGTTTTAAAAAGCGTTTCAT
TATTATTATTATTCCCTGACAGAAATAATGTTGTTTTTACGATATAAATCTTAAAAGTCGT
AAAAAAATACATTAACTTGGGTAAACAAAGTCAATTAAAATAATGAATGAAAGTCCAG
AGTGGAGAGGTGAATTACGAAGGTGAATTCTCCTCGGATAATAAATATTTGTTATTTTGT
TCAAAATGATGTTTCATTTTGAGAGCCGCTGCGAAGCTAAAGACGCAGAAACTGAGAC
AATGCCGTGTCACCCTTCTCGCTCTGTACCGTTTTACAAAACATCATCCCTCTTCTTTCC
TTGCCCCCACCTTTCTTTCTTTTTCTTCGGCTTAAAAGCTTCATCACCCCGATCCGTGCT
TTTTTTTTTGTCTTCCTCGGTCGATCCTACTTCTCAAGTTGGACCGCTTTTTCTTCTGGA
TGATCTTTCTTAGAGGACCTCTATGACTATTACTTCTTCTACTCGTTTCTTTTTATTCCTA
CCTATAATGGAATGCAGAAAACAAAGGATTCCATTAATCTCTTTGGTGAAGAAAAAAA
ATTACCAAATGGAACTATACTACTCAGTCTTTAAATTTAAAGAAAAAAGTATAATAAATT
AAAAATTATCTTCAATCAAATATAGAGTAGACTTTTTGTAATTGTGAGCTTATTTAACGT
CGCTTCCAAATTTAAGACAATTAAAAATCTTTTTTACATTCTCTCTATTTTATTCAATTTAT
ATTCCTTCTTTTTGATTTCAATTCTTCACTACGAAAACTCGCTACAGCAGCGTCAATTGT
CACACGCGGAAGTAGATCGAGTCGTGTTATAATCAGACGTTCGTCTGTTCATCGTCGAT
TGAAACGCCTGTCACGTCTAAATTCTACCACGCAGTAGATCGCGTGTCGCGTCACCCAT
TTGCTATGCTGATTCTCTCACTTGTGCTATCAACGCGATGCTCAACCGTAAACTTGTTAT
TAACATCCGAAGCCTGATGCTCGTTGATTAAACATTTGTCCAACCGACAAACATATCGT
TAGAAAATCGTGGAAAATCGATGTCTGTTCAATGATGCGGCGTCATTCATTGAACACGA
ATTTATACAGAGAGTAACTGGTCTCTCTTTCAATTTCAATTTTCTGTCACTCGATATCTCT

CCTTGCACGCCATTTCCTTGCCGGATTATGCAAATGTCCTAATGGTCCTTTTGGTTATCG
CGTGAATGTACTTATAAAAGGAGATATTTTAACCTGAGACGAATTTTAATGTGTAAATTA
TAATCCGTTCAAATCAATGAAAAGAAAAAAAAAAATAGAACAATCCTTTCAGGATTAAA
TTTTTACTTCTTCACTTTGCTTGTATATAAAATGATTAATAATTGCAAAATATTTTTGTTTG
TGAAACGTGATACCTTAAAAATTAAGATCGGTAAAAAAAGATTAAAGGATTAAATTTTT
AATAAAAGAATTGTTTCATTTTAATGAAAAAAAAAGTGATTGTATTTTTCCTAGTAGAC
AAAGAAAGAACTTGACGGGATTAAATTGAAACGAAAGCGTGAATTCAATTTTCTACAC
GTTCATCTTGGTTTGCGGCAACCGAGGAGGGAAAGAAGAAATGAAGATGAAACGAAA
CGTTTCTTAAACGTAGCTGAAACGTAGGGAGGACAAGTAACGTTTCAGAGAACGTTGT
ACCGTAACGTCGTTGCAGGGAACCTTGCAAGACGATACACCGGTGCCACTGCACAGG
CATTACAGCGAACCGTTACACGAACATTGTGGGTATTGTGTTTCAGGAACTGCTGTGCA
CGTTATAACGTTATAACGTTTCAAAATGTATCGTGACGTTGGATGTTGCATAAACGTTGC
TCTACGATATTTCATAATGCTTGGAAACATTTCAAAATGACAAATGTTTCTTATCTTGGT
GAAACTATTTCCATTTCTTATCCTATCTCCAACTTCATTGTCTCGAATCTAGTATAGTAGA
AACTCACTAGGGCTATCTATCTGTATCGTCTAGTGACAATCTACAAGTTTAACGGAGTA
GTAAACAAGGTAATTAAGATAGATTGAGTTAGATAATTTATTTTAGATATGTTCTTCTAGA
TATAATAGAGAACAATTATATTTTGTATTTATTAAAAAAAAGTTTACAAAGTCGAATTTAA
TCCATATTTTCACGTGTAATTAAATTTCTTAACGAATCAAACGATAATCGAGTAAATACG
TTATTTCGAGATGAAGTTGTTACTCGCGAAATAATTCGATGGAGGAGATATGATCGTCA
GACGTTCGCAAATTGAAGGATACGGTTTATGACGTTGCGTCAGCACTGGTCCATGAAAT
TGCGTTTCACCGATATTCGCCTGGGTTTCTAAACCGCCTGGTCTGTGGTGGCGCGTCAA
CGACGCGTCCAGATGTAGGATCGCCATCGATGCATGCTACTTCTCGCGATCGATAATCTA
CCTGATTGGCTTTCGAGCCATTCTCACATCGATTCAACCGCATCACGCACATATATTCCC
ACGCAATCACCAACTGTAGCGATTTGCATTTAACTAACGCGGTCATCGATGTGAAAATC
TCTGAATTCCCAATCCATCTGCAAATTTTGCATTATCGATCGCATTATCGTTTTCAAAAA
ATAATTTTTAATCTTAATTAATTCCGGTCAACGAAGATCGAACAGTTTTCTATCCGCGTT
TCTAAGTGAAATCTAATCTCTCGAGTGAAGTTTCATGCGATACAATAGGCATTATCTTTC
CCCATTACCGTTAATCTATTTTTGTCAAGTGATGCGAAACAAACTATCAAAACGACCCC
GCCCACACTCGTGGAACAATATGTTCGTTTCCTTACAAGACTTGTGTCTGTCGATCAAT
TAATTCTTCTTGTCTTGTTTGGCTCAGGTGGGCGATAGAGACACGCTGACGTCGGTGGC
CGCGAGATTCGACACGACGCCATCCGAGCTGAGCAAATTTAACAGGCTCGGAAGCACT
TTCATTTACCCTGGCCAACAGTTGTGGATACCCGTGGAAGGAGAAGGTCAAACAAAAG
GCGGAACTGCCGACGCACCTAGTCCGGATCCTTATCACGATCATGAGTCCCAGGATGA
CCTACCTCCAGAGGAAAAGGGTAATTTTTATTTATTGAACACCGCAATAAAAGAATTTC
ACTCCTTAAAAGAATCGTGAAGTGATTTCAAAATTGATCAATAATAGTATGTGTGAAAT
TTTATTCAACCTATTAATGATTAATAAAAAGTAAAACGATATGAGAAAAGACAGGTATAT
TAAAATGATTGATTTTTATAAGTATTAATATATTCTATCGATTTTCAATGTTAATCATCAAT
GCTGATGACAATTAGATCAATGTTGCGTGAGATTAAAGATTTTTTTTTTTTTAAGTTATAT
TCGCAATATATAATCTTATCTTTTGCAAGTATAATATGTAATGCAATTTGTTTTTAATTGGT
TAATGAATAAAGGATTTTAAATCGTTTATTAATAATTAAATTGGATAGAATTATAAATAAG
ACATTCGATTCCATATATTATTTCCATACAGAATTATATACATCATAATGTGTAAACATTCT
ATTCGATTATATTCTATTCCATATTCTATTACTAATTTATTTTTTATTTCATTTTGTATTTGAC
TTTATAATATTATATTATTTTTATTTCTGTCCTGTAACGCATTTTAATATCGTACTTTTTTTT
TTCGTTTTTGAACATCTTATATTATTAGTTCGATATGTAACTAGATGAAAGCGAAAGAAA

AAAAATACTATATGGTTAATAAAATTTGCTGTACCTGTAAAATTGGCGAAATTTGCTTTC
ATTTCGATGCAAAAACTAATATTAAGAGAACGTTACAGTATATGTCAGAACTGTACATTT
AGTTTAGCAAGCACAGGCGCCATGTCTTATCTATTCGATATATGCAATATGCTAGTATGA
ACATTCTACGTGACACTTTGTATAATCAGATATTATCGAGACCCAATTCATCTTGATATTT
GGCTAGGTGAGTCAATGCGACTTACCTGAATGCATTCAGTTTTCTATTCCATTATTCATA
AAGGCATTCGCGCCAATTTTGTTTTCGATTTACATTTGTTGTTGCATGGTGAAGCGTTTT
GATATTTTATTGTGACTTGTAACAGCTGTTAGAAACATCGATCATGATAGATTATTATTTA
TATAGTGAAATAGTAATCAAATATATAATAAGAGTAAAAATAGTATACCTGGTGCAAATA
AATAAATAATAAAACATTTTGATGATCGTTGTTGAAGTTGAAAAATATATAAATACATATT
GGAGAAAAAGATTCACAGTAAATTGATTATTCGATGGCGCCACTTTACACTGTCATGTC
AGACAGTTAATGTTCGTAAAATCAAAAGAATGGACGGCTAAATATAACATCAAAAACAT
AAAATACGTTCTGTAAAAGAAGATATATAGTTTTAGTTGATATATATGTGTATATAAAAGA
AAAAAATTGACAAATTGATTTTTAAAGTGTCACGTGTTCTTTGTTGTCTTTATTTCCCGC
GCATTCTGTTTATTTTTCTTCATGATCTAATTATATCAGGTATGCCAGACATTTTAATCGAT
AATTAAAACTTAATCTAATTAAATTGTTTGTGTCTTTCTATGATTTATTGGAAAAATACAT
AATAATGCGTAATTCATTAAATATATTTAATGAATTCGACTTAACTGTGCATTTGCAAATA
ATAATTAATTTCAATAATTTTAAGTTATTTGAAACAAAGAAAAATTATCTTATTTCTATGT
ATAAAATAGTTTTTGAAAAGTCAATGCATCTTTATTTTAATTTTTTTCCATCACTGAATGT
ATATATCATATAATACATCAGCTGATCAACGTCATGATGTAAACTATGAAAAGCATATGGC
GTTATTATGTGCATCAAACATAACCTATAATATATTGAAAAATAAATGGAAAATGAATGT
TCATTGGTTTTATTTTATATAAAATACAAATATTATAATCATTATGTCATTAAAAATAAAAA
TAGTATTTGTTATTCTTATTAAATTCAATATTACTACTTAAATGTTGCATCATGTAAATTAA
AATTTAAAACGCTTCGAATTAAATATGATAAGACAACTACTATATTTTTTTACAACATTA
AAATTTTTTATATTTTATATTATATGTAATTTTATTTTCAATATATAATCTCTATTCTATAAAA
AATATTTCAAAATAATTAATATTATCAAAAGTCTCTATATAAGTCTGTTATATTATTCTTTAT
AGAACTTCTGGATAACCTAAGGCCTGTGTCACCCAAATCAAGCCATATGGAAAGAGTA
AAAACACCTCTTGGTGTTACAAATACAGTTATAGAAGAGGAAGAACAGCCGTTCAAAG
AAAGATTTTTAAAAATTACTGTTCGTCATATCACAGATGGTCAGGTAAAGTTTATATCTC
AATTTATTCATATAAAGCTTCTTTGTCTTTTCTTATTAGTTCTTTTATCTTATTAAAATTATT
TTTATTTACAGGGTGTTGTTGGTGGAGTATTGCTAGTTACACCGAATGCGGTAATGTTTG
ATCCTAATGTCTCTGATCCTCTTGTAATTGAACATGGAGCTGAATCTTATGGTGTTATTG
CACCAATGGAATTTGTAGTGAATGCTGCAATTTATTACGATATCGCGCATATGCGTGTTG
GACATACGGAATCTGGAAATTTAGATAAGAAGCCAGAAATTTATTATATGAAGAAACCT
TCACAAACTGAGAATGTGAAATGTCAATCGCCAGAAAAGGAGGAAAATTTTCCGGAAT
TAACAGCGGATGATGAAAATGTATGTAGTTATACCGAAAGAGATGGAGACGCTTTTCCA
AAGGCCTTTGAGAGAGATCTCGTTACTCCTACCAATCTCCAAAATGTACATTAATATTTA
TATTTAATAAATTAAATTTTTATCAAAGCAATTATCCAATAATAATTCCAATTTTTTTATTA
AAATCATAGGAAGAAGATACAACGACAACCAAAGAAAAAGAGAAGGAGAAGGAAAA
AGAGAAAGAACAGGAGAAAGAAGAGATTAAAGAGGTGAGCGGTGGTGGTCAAGCAA
GTAGAACGTTAGAGGAACGTAGACGTTCTATGCTTGACCATCATTGGCCAGTTCCTAGT
AAAGATCAATATCCATGGCCAGTTGAAGATGAACAACAAGATTCTTTAAATTCTGACAA
AGTTAGTTGTTCATTATTAGTGGAATACAATATAAAATTTTTTATCGAAGTTCAAATTTTA
ATAAAGATCAATATTTACAGACGGATTCTGCAAAATCGAAGGTGATTCCCGAAGGTGAA
GAAGGATCTCTAGTCAAACTGTCATGCCACGACTCTGGTATTGACATACGTGACCCTAA

TCCCCCTTTCCCAGTAGTTCAGCCTAACCCCACAAAGAAAGTATATTCAGACGCAGATA
TTGTCTTATCTTCAGATTGGGTCCCTCCTATTACTATTGCTCCGACGAACGTAACAACTG
ATTCGCTAGATAGTGGTTCTGCTATTAACGGCAGGAAAAAGGCGAGTTCAGTGTCTTTT
AGCTTAGACAGTAATGCAGAGGAACCTGAGAAGGATGAAGAACACAAAGATGACGAT
AAACAAGAGACTCGTAAAAATAAGGTAATATTAAAATTTATATACTTGATATCACATACT
TGATATACATACTTGATATACATTTAATCTAATTCTTATTTTATTTAACAATTATTATATTCA
GATGTTAAAACGGTTATCATATCCTTTGTCATGGATGGAAGGATTAACTGGCGAAAAAG
AAGATGATAAATCTGGATCTGATAAAGTTTCGAGCCTTCCTAATAGCGCGGATTCACAT
CATTCATCAGTTTTCAGCAAAGTTTTCTCAAGGTGATAATAATTATCCTTGTCTATAAGT
CCATAAAATTTTTATAAAAATTTTCTTGTATCTTATATGGTACAATAATTTTGTTCAAAAA
TTAATCATATATGCTTTTATGTTTTACTTTATCATTCGTGTATGTTAGAAATGAGTAATTCA
ATGAAAAAACAATTTTGCATACTTTTTATATACATAAGGAAGGTTTTCAATGGTCTGGCC
TCCTTTATATGATGTAACGTTTGTTTTTCGTGTGATTGCTTTCTGCTGAAATTTTTTTAGA
ACTAAAAGTAATATTTATAGCGTACTTGAGGCTATTCATAATGAAACGAATTGCTTATATC
ACGCACACATGTAATAAAAACGTTTTATAGTTCTTAGAAACGAGTTTTATTATGAATATC
CTCATTTATTTTAATATAGAATATTTATTAGAAATTTGCGCGTAGAAGCAAGCGGCTTATT
GCATTTGCGAAACGCGCTTTGGGATATATCTTTTTTCAATTTCTAAATGGAGTAAAGGAA
TCAAAGGAATGGGCACGTGTGGCATTTCCAATTACGTCCTTGTCTTCAATAATCTTGTA
CCATCATAGTCCGTTTTTTCAATTTTGCTTTCCCATTTTTCTTCTTTTTCAAACTTTTTTTA
TTAATTAGCATTGTAGATATATTCTATATCCAAAATAAATTAGTCATTCAAAGCTAGCTATA
TTTATCAAAATCTTGATGATTAAACTTTATAAGAGAACTTTCATTGAAATGATATTTCAAT
GAATTAATTTGATTCAGTAAGCTGTATAAGTAAAGTAAATTTTGACTTCTAAAAATTTAT
AGGGAATACTGATTATTTTTATTACCATTTTGAGACCAGTATCAATCAGACTGTGATGGT
AAATCTGTGTTAAAAATAAAGTTTAACTATCGTTTCCAATGTGACTAACAAAGAATATGT
AATGGTGGTGGATATAAGCTCGCCGATCAACCTGGTGAGTGACTTTAGCTCGGGCCTGT
TTGCTAAAACTCCCAGCGAAGAGAGCGGTGGGGGCGGTAGAGCTGGGGGAACACCTC
CGCTTACCGCCTCCTCTCTCTCCCAGGACTCCAGCAATCCTCAGATTTCACCTGGTCCA
GGAGGGCTTATAGGGTGAGTTTAGGCCCAGTCATGAACCAGCAAGTTTATACTGTATCC
GTGTTAATCCACTTTGTCCTCATATTTTTTTTCACTATTGTTCCACTCTCTTTTGCTCTTTC
TCTACTTATCCATACTCTATCTCTTCTTATCTCTGATTGGCTGTTAACTTTTATATTATTTTT
ATTAAACATTTATCTTCTTAGATATCTAGTTTGTTTCTTGCTATTTTCCCATAAATTACATC
ATCTAAAATCATTCATTCTGTAACATCCTTTCTTATTAAGTTTTTTAATGAACATATATAAG
TATAAATGTAAAATTGTTCTAGTTTTTTCATAAATTTCACTGTTTCGAGCTTCTCAGGAA
CAACACTCTTCATCTATCCTATCCTTCAATACGTTTTATTTATTTATTTATTTATTTATTTTA
ATCTTTCTCAAAGAGAAAGAACATTCTTCCTCTGTTTCCCCGCTTTTCAGGTAAACTTA
CCATCATTTTTTTTTCAGAAAAACTACACCAAGACCGAATAATGTGCTTACAAACAAAC
ACACAATCAATCAGAAACACAGCGTTTTTAATATTCCCGAGCAGTCATTGAACTTTATT
TCACGACGCATACACGTTGGATACAATTTTAACATTTATCTTGAATGTTGTTGTTGTTTC
CCTTAAGACTTAATTGCTCTTTATCACAATTGTATATTGATCAGTGTATATATTGCTTCGA
AATTACAGGGAACACGAAAAAATCAGAGAAATCTCGTGTACTCGTGATTTTGAAACGC
AATTTTGAGAACATCTTTCATGCCTATTCTCTAACATTAACACTTTCTTTCAGGCGGTCT
TCCGTGGGCACGTTCATCAGGCAGCAACCATTGACGTCGTCAGGTAGCAGCAGCGTGG
ACAGTAGTCGAGGTAGCAAAACTTCGACAACGGCCCCGCGATTGGACTATCGTAGTAT
GGTCTCTGTCGAAGATATGCCCGAACTGTTTGTCAGTTTCGACAGTAAGTGTCCAGAG

GAATTGAGTCAGTCTTTCTCCCCTTGAATTATGGAACGCGAATATATTTCAATCTGTATAT
TTTTTCGGAATGATTAAATCCAATGAAAGATATCATACAATGTAACAGATTTCTCACACT
TGAAATATCTTTTCAACATTTTTTATAGAGTGTTTCCATTTTGTTGGGTGATTCTTTTTCA
TGGAATGTTTATCCGTAGAATAGTTTATTTTTCTCCAAGGTGTTTAGATAATTTTTCGTTA
GAAAGATCATTATGCGCGCACAAGAGCCTACAATGTCAACAATGAAACCTCTACCCTTC
GAAGGGATCGATAAAAAAAAAAGAAACTCTATGATTAATTACACGTAGCCGCGAAATA
CAAACAGAAAGAAACAAACAAAATGATGGCAAACGGAATTACATACCACGCACTGCA
CTCGCTCGTATGTATAGACGGCTTTTTAAACGATTGTTATTTTTGCTAGTGCATTTATTAT
TGTCCCTTTATTGAAGAATTTTTGAGCGCCTTTGGATTCGATCTCGACTAATTAGACAGT
TTTTCTTTTCACACTATTTATCGCCAAAACAAAGAAAAATAAAATTCAATTTAATTTAAT
CTAACCAAATGCGCTTACGTTACATTGTTACACATTGTTTTAATTACATTACAATTCTCGT
ACATTGTTCTATATGGTTTTTAAATTCTACTCTAGTTTGTAATTTCCAGTTCTCTACATCTT
GTATTATTCGGTGTGCCACTACCCGTGAACATTGTTCCACGAATTCGTATCATTATGAAT
AAAGAATCGCGAGAGAATTTGATATCGTTCCAAATATTTTCTAAATGCCTGACACAAAT
TCGAGGAACAATAATTTTCTATTCACGATCGTATAATTTTTGGATTCTGTCATAGTTTTCG
CTTCTCGTCACAGATTCCAAACTCGAGTCTCTTCATTGTTAAAAATCTTTATCGTTAATT
TTAAACGAAAAGAAATTTATTATGTCTTCACGATTTTGAAACTCTTTGTGCTATAATCCT
TGAAGATTAATTACATATTACAATTCATATATTATTTGTGAACTATATATGAAGGCTTTAAT
TGTTAAAGAAACGTTTTAAAAAAAAATGAAAAATTGGAAAAATTGGATTTCTGTAAAAT
CAAGGAGAAAGAGTTTCACGATCAAGTGCCAATGAATTCATACTAATGCAGATATATCA
TATGTTTTGTTGTCCCTCTAGTCCAAGCACCTAATTTTCCCGAACTCGAAGGCCTCGGT
AGGTTCATACTGTATTAGCTTGCTTTATCCCTTTTGTATCAATTCACCATGTGTCCAAAG
AACGTTACTTTTTTCACATTGTTAATTTCACCTGTATCTATTTTTTCCCCCTTTAGTTTTCT
TTTTTTTTTTTTTCAAGTCTCTTGTGCAACTTCTTCTTCTTCTTCTTCTTCTTCTTCTTCTT
ATGTGTATCATCGTACAATTTTTAAATTATATGTTTTTTAAAAAATCTTTTTTACTCACAA
CCTCCATTCGATTTTGCCCACTGCACTTGCACACACGAATGCATTTTATTCTCTTTCTCT
TTGTGTATAATTTTTAGAAATAATTTCGTGTAATTTTTACGGCAACGATATTGTGCAATTT
TTTACTCCGAATCTTGGTCACCTGTCACCGATACAATATGGATTTTTCATGGCTGTTTCT
CACTATATTTTTGCTCATCTGTATTGTTGTGCCCGTGTTTCTCGTGTTATTCTAATCTATAA
TGTTTCTAACATACGTTTGTCGATGCTCATGTCGCGATCTCATTTGTAATTGTAATTTAAA
AACAATTCTTAATTTCGATCAAGAGAAAATATCGTCTGCAATAATAATTTAATGCGATGC
TTTTTAATTTGTTGAAATATTTTCTAGAAATGAGCTTTTTGCATATTGTCCCTTATTTTTTT
TAATTTTTTTTCTTCTTCTTCTTCTTCTTCTTTTCTCTTTCTATATCTATAAACGAAATGTA
TCTAAATATCTAAATATTGTGTGTCTTGTCGGAAGATTGATTAGAAAAATTGTAAATTTT
TGATTAGAGTATCGCATAATTGAGCGAATATTAAATATCAAAATTAATAAGATTTAATTAA
TAAGATTTAATTAATTATTCGAGATAAGAAGATCTCGTGTACGCATTAATGAATCATTCTG
TTGCATTATTTAGAACTTATACCACGACCAGCCCATTCCTGCGAGGATCCTCCATTGTAC
CTGAGACTGCGAACCGGAAGACCTAAGGATAAGAAAATTCCACGCTCAACGCCTATTA
TGAGCTACGGGAAAAAGAAATTACGGCCTGAATACTGGTTCAGCATACCCCGTAACAG
GTAAATAAAAACATTGTTTCCTTTGTGACACATTTCATTTTCTCTCCCCTTTTTGATAATT
TCATTCAATATTGTAAAAATGTTGTTATTGATACACAGCAGTCTCATTAGATATCAAAATA
AGATATTTTAAAATACACTCTTCTTTTGATAAAATATTATCAAAATTCTATCATTAATAATT
TTAATAATTTTCTAGAATTTAAATAATTACTCTATTAATTCAAATTATTGTTAGAAGACGA
CAGATAAATTTAGAAAATTCATCTAATATTATAATATTTTAAAACTTTCATTGTAGTATTCT

CAATTGTCTCCTCGATTTCCTTTGATTTAATTTTTTTCAATTATTTCTGAATTATTGCAAAA
TCTGTGAAAGATTTTCAAGAGAAAACTCACTATCTACGAATATCATTTCCCGGATACGT
GTTTCTTTACCTCTATTTATATGTGCGATTTCCGCGGAATCCTAATTTAGGAACCGGAAA
CGGCAATGAATCAATCGTTTTTTTACGGCTAACTGATATAAAATATCCTCGTACAACGTT
TCCAAGTGAGTAATTCTTGCAAGATCGTGCGTTATTTTTGTTAAATTCACACCTCGTATC
ACGTTCGCAAGACTAGACCGGCCAAAAAAGAGCGAAACACGTGATTCCTCTGTAAGA
GCGGCAAAATAGGTCTCGAGAATCGGCATCGTGCTTCTTGCTATTTATACCGATTCTTTG
CCTTGCGTTTTATGTTCGTCAACTTTCAACGAAACACGTTCCACACACGTTTCTTCGTT
CTATTTTATCGATTCGAGATGTTTGGACAAAATAAATTCGATATAAACTAATAATAATATT
CCAAGATAAATGATAATGGCCGCGTTACGACTTGTAATCTTTGCTCGACAATTGTCGAA
GGTCATGTGTGGTGTGAAGGTATATTCTGTTCAGCAATCGATTTTAATACGCGGACAATA
ATCTATATCAATGGCAATTCCTCTTTTATCAATTTTTTTATTTTCCTATCAATGTTGTAAAT
TCAAATTGAATTTTAATTTACATTTACTTTGTAAATCTAATCTATGATAATTTTATGATCTA
CTATTTAAATTTGTATTTGGGTCAAGTCTAAAAATATTAGATGTATCTTATCTTTCTTCCTC
TGTGATTGATTCAAATTATTATTATTAATATTCAATAATTATATTTTTACTTATCCTGATAAC
TTGTGATTGTGATGAATTATACAAATTCATTAACTTTAGAAAATTTTCTAGAAACAAATC
TTGAACAAATTGAATAGAATAATTGAATCTATTTCAAGTATTTAATCACAATTTTGTTGG
ATTGAAATAATTACACACTATAATGCTCCCCTTACCAGCAATCAATGGGGAGAAATATCG
AGTTGAATGTTCACGGTAGTCAAACACGTTTCAACTTAACTATCCCCATTCATTATGTGA
ACGTCTCTCGAGTTAATCGAGCCGATCTTTAATTAATATCCAAGTTGAACACGTTACAC
GTGAGAAGTAAGCGCGAGAAATCACGTGACATACTTGGCGTATCATCGAACGTGCCTC
CATAACTTTTCCGCTCGATGCTCGTGATACTAGTCTGTTCGCGCGAAGAAAACGCATCG
GAGACGCGCCACGTGTCGGTTGGAAAAAAAAAAAAAAATAATAATAATCGTTTCTTCTT
CGTGAATTTTTTCTTCTTTCTTTCTTTCTTCTTTTATTTATTTATCAATGATACATTATCGGT
TTGATCGATAATTAATTGAGGAGCTTTTTGGGGAAAAGGGATCATGAGAAAAATTTGAA
TTAAGTAAGTTTAATTTTATGTGTATATATATATATTTATATATTTTGGATATAGATTATTTTT
TTTCAGAAAGCTCCTTATTATGTGTTTTATTTATTGGTAAAACTTAGGTTGAACGAGCTG
AATTATTATTTTATCGAAATGTCACGTGACGATTGGATTATACATTTATCGAAATTATCTG
TTGTAACTGGTGAACAGCTTCGTTGAATTTTTATTATATAAATTGTTTATCGCTCGACAAT
TTTAGATAAATGAAATTCTATTGCGTGACCGTTTTAGCATCGTTCTTAGTGTTGTCATTG
GCAATTAAAAAGGATAGAATATATGAGTTGATTAGGAAAAAATGATATTTTGGTAAAAA
AAAAAAAAAAAGCAACTTTGAGTTTATCGCACAATAATTTATTAATTTAACTTGATAATG
ATAATTAAATATTTTGCAATATTTTCCACAGAAATTGGATAATTTTGCGATGAATTCTTAT
GTTGGCATCTTATGATCATTGGGATGGACACGTGTATAATAGCTTGTTTTTAATTGACAAT
AATTGAATTATCATTACGTAATAAATAATAATACGTTATTTTAATTTCCTCCTCCAAGAGA
AGAGGGATAATGAATTTCAAGATACATGTGTAGTATAAAGAAGTGATGAGTCTTCTCAG
AATGTACATAATATAGAATTTAGATAACAAATCTTTAAAAATGTAGATAATTTTTTCCAGA
ATTATAATTATTCTCTTGCCCAAGAACAATTATACTCGTTCAAAGGTAGATTTTCAGAAA
TAAAAATAAGAAATCTTTGAATAGCATCATGAGAATCATTTTATAGAATTGTTTCATACA
AAAGTTAAAAACTCTGAACGATTCTCTACAATTTCAATAACCCATTTAAAAGAATATTCA
TTGCTTAAAATTTTAATTAAATATTCCAAAACTTTAACTTTCTAAAATTTCAATAATAACA
TTTATTGTTATTTTCAAAGAAATTCTCAATAATCCCAATCAAAGAGTTTAAGATTAATCTT
TAAAATTACCACTTCCCAAACCCTTCAAATTCAGAAGAAAAAAAAATTTGCATAGAACTT
TAATCTTTACTACCTCAACGACACGAATGATCGTCGACGATCTTGCAATCGCAAATCTG

AAATCCGTCACACGCATATCCGATCCAGTGAATACGTTCACATAACAATAAAGAGTGTT
GCACAGCGGGAGAAATCGGGAGAGAACGGTCTGTGTTGGTCATCATCACGCAGTCGG
AGATTAGGTAACGCGTCGAAGAAGCGTCTAACCTAAGCGATTCACGTTCTCTCTTTTGC
ATACCGAACTGCCGTTTTAGAATAACATCGTGTTGCGCTGAATGGTCATTGGAAATTGG
AAAATGTTTCTTCACTACGAAACATGTGTACGGAGCAACATAGTGACGTACGAGTTGA
CGCGATAAGAAGAGGTGGAGGATAACGAATGCGAGGTGTTGATTAGATAATTCGTTTA
GATTCGTTTCGAAAGACAGTAGAGAAGGAAGGCATTACGTGAGTTATAATCGGAAATG
AACCGAATAATTTGTAAACTCGTGCGATTCTCTTTTAAATAGAAGATCGAAGAAGAAAT
TAAGAGTTCTAAGATTTTCTTTTTAATGGAATAGTTTGTTTTTAATTAGAAGGAGGAA
CGTCTGAATTTCATAGTTTTTCGTGTAGAATAAAATTTGTAAAAAGGAAGTGTTTATTTT
TTTTTTTTTTTTGGTGAATTTGTTAAAGGAATAATAAGTAATAATTAATTTTGTATTGGAA
TAATAGTAATTTGATAATATTGTGTTGGAAAATATATATAAAAAGAGAAACTATGAGTTTT
AAAATAAAATTTTTCCATAATCGCTATCAATTTTTTTTTTTTTTTATAACAAGTACACTTT
GATAATAAGCTATTTATTTTTTAATACAATGTTTAATTTCAAATATCTATTACTATTTTGTTT
ATTATTTATTGCTATTTTTATTAGTCTTTGTCATCTGTCTAAATGATTATTATTATAATAAAG
TTGTTATGTGCCATTAAAAAAAGAACGATAACAGGTGACAATGATAGGAATTATCGTGA
GGCGAACAATCGAGTCGAGTAGATAATGTATTTTCGTGCTCGAAGTTAAATTTAAACTA
ATCAAAAAAAGTTGTGTTCTATTTCGATTTTTATATTTTGCAAGAAATATCTTAATTTTTT
ATAATCTATAATATTCTATAATATTAAATATTGCAAACATTTGACTCTCGTAAAAAAAAAA
AAAAAAAAAAGAAAAGAAAAAAAAATTAAAATTTCACAGTAGTTTGCACAGTAGTTT
ACGTCATCACAGGTAATTATAATGAATATACATAATTCCCGAATGATTCACTCGTCTAATT
CTTATGCAGTCTACCAACACAATTTTAACAAGTGTCTCTCAAGGGTCCTCATCGGTTGC
ATCACGTTTCGTATTGTTTCGTAGCCTGTTGAATTTAAAATAAATTCTAAAGAATTTTTAT
TCGTGATACACAAAATATTTAAAAATCGTGCGAATAGAAATATTAACTTGTAAAGTTTAA
ACATCGTTTCGTAGTCAATCGACTTTGACAAGTGTTTCCACGGGGTCAACATCAGTTGC
ATCACGGACGCTTTGTATTCGATTCTAGTAATAAACTACTTGATCTTAAAAAATAAATTC
GAAAGGAGAATAAAATTTTCAGCGTGTTTTTCAAATTTATGTTGAAAATTCGCGTGACA
CGAATATTATAATAATCAGAATATAAAGTTTAAACAAATTCGAGTGTGTTTCTACGGGG
TCATCGTTACGTAAATCACGGACTCTTCTTTTTTCCGATTCTAAAATAAATTCGAAACGA
TAATAGTTGAGTGCTTCAAAGTAAGTGAAAAATATTAATCGAGGTAAACAAAGATTGCG
TAATATTTTGCAAGATTCAGAAAGGGGAACGAAGTGTTGTTAGAGAGGAAATAGGAAA
CGACCTTGCATGGTCGTCGACGTTTGAAGCGCAAGTGGTATATCGGTTAGAGAAAGTC
AATTCGATCTTGATAGAGGTGCACGAACGTGGTGGTGAACGTGTTACGATACCTGACG
AGGCGGCAAAATGAATTGGACATTGAGCAGTGTCGGCAACGTGATGAATTTCGTGTAC
AACCAGAGTGTGACGAACGCTGTGCAGAACGTTATACCGTTCGTCAATCGAAGGGTGG
ACGACCTCTACAGGTTCATCCATGCGTGGGTGCCCTCGCTTTATGGAGAGCTCGACGA
GAATTATTGGCGGGAACGTGGGTACATCCTGTTGGACACCGACACGGATCTGTCGCCC
GAATTGTCGGGCGTGCACGAGGCTGGGAAAGCGGGGGAAGGAGCCACGGAGGAAGG
GAAATCTCGCCAAGACAGCGACGAGATATCCGAGCTGACGAGAGAATCATGGGAG

>novel_circ_000782
GTCCAGGCGAATGTCCCAGGATATCACCCATTCGAGATCCACCGAGAATCTCACAGTAC
CGTATTTCAGGTACGTAAAAAAAATTCGAAATTCTAACAAATTTCGAGTGGCTGTGCGA
CGAATTGCAAGCATCACGTACATATACATATATATATGTATTTTTAATCTCCATTTTTATATA

TATATTTTTTTAATGAACAAACATTTTTTTCCTTCTTTATTGTTTTTTTTAATATATTATTCC
AGACTGTTAAAAAATTCTGGACTACTTGTTCAAGTGATCAGCAAAAATTTCTTTTTCCC
TTTTAATAATTATTTTTTTTTTAAATACTTCGTCCCGTGATAACGAATCTTTGAATATATTA
TTGCAATTTAAAAAAATATATATATATATAGTTAATTAATTTCTTCTTTTTTCCAAAAAAC
AAAGTTTCATTTATCGGATTAAGGATTAAAAATCCACGAACACGACGCAAGTTGAAGT
AGCACGGCTTAACAGATTTTACTTGTTGCGACATCGGATAACCCGTTCACGCCCCGTGC
TCCTCCATTCGTGAAATTTGTTCTTTTCTTTTCTTTTTTTCTTTTTTTTTCTTCCTCCTCAT
GACCAGTAATTTCATTCACGAAAACCCCATTGTTGCTTGCTATCTTCGCCGGCCGTTCG
TCTCGTGGATTCATCCATCTTGTAGGATCCGTTTATACGACGCATGGTTCGTTGCTTCGT
GAAATTGCATTGTGCCCCGCTTCACTGGCTCTGCTTTTGCAAATAAAACAAAGAGAAA
GGGGAGGGGGTGGGGAGGGAAGGGGATTGGAGCAACGAATAATCGAGATTTTTACTT
GGGAAAGAGACACGCTTACGCAAAAATGTAATGAAATGATTGAAGAAGAAGAGACAA
AAGTTAAAATCATAATTGCGTTGGCTTAGAAAAAAGAGAAGAGGAAAAGTAAAATTAT
TATTAATTTCATTTATGATTATTTTTGTTCTTCCTATAAGAAGAATAAAAATAATTTGTCTC
CCTAAAAGAGATTTTAATTTTTTTCGATGTAGATATATATACTTTTCAAAATTTGATTAAG
GGAATTTGATTGTGCAATGAAGTAAAGACTTAAATAAATATTTTATTAGATCTTCTTGAA
TAATAAAAATAATAGTAATAATAATAATAATAATCATGGACACGATCCAGATTAATTTAAC
CTCACGAAGGATTAAATAATGTGAAAATTAGCAAAGAAAGTGCAAATTTATCGTTCTAA
TCTTGATTTATTGTTTCGTAGGAGCAAGAGTCGTAGCGTGGATCATGGATTCGCGGCGC
CTTTCGACTGGGATTCGTCGAAGAACAAAGTCGATCAAGGACGATTCGAGTCGGTCGA
CGAATTCGCAAAGG

>novel_circ_000783
TTTACGTATTTGCGGTGCTCTTCATCGCCAACTCGATGCAAGCTTATGTGCCTTGCGATG
GTAAACCAGCCCCAACGAACGTGAAGATTTTGGGTTGCGATACTCTGCCTTGCAATTT
GGTTAGAGGAACTAATGTTGAGGCTAACGTTGACTTTAAGGCTG

>novel_circ_000784
TTTACGTATTTGCGGTGCTCTTCATCGCCAACTCGATGCAAGCTTATGTGCCTTGCGATG
GTAAACCAGCCCCAACGAACGTGAAGATTTTGGGTTGCGATACTCTGCCTTGCAATTT
GGTTAGAGGAACTAATGTTGAGGCTAACGTTGACTTTAAGGCTGTTGCAAACACGAAA
ACTCTGAGACCCGTAGTAGAT

>novel_circ_000785
ACCCGTAGTAGATGTTGACCTAGGAAACAGCCACATGCAATACCCACTCCCAGAACAA
AATGCTTGCAAGAATCTGGTGAACGGACAGTGCCCATTGCAAAGTGGACAAGCAGCT
ACTTATTACCTTAAGATGCCAGTTCTGAAAGCCTATCCAAAAGTTGCTCTGACCATCCA
ATTATCTCTTGTTGACGAAAATAACAATTCTGAA

>novel_circ_000786
AATTGTTATTTTCGTCAACAAGAGATAATTGGATGGTCAGAGCAACTTTTGGATAGGCT
TTCAGAACTGGCATCTTAAGGTAATAAGTAGCTGCTTGTCCACTTTGCAATGGGCACTG
TCCGTTCACCAGATTCTTGCAAGCATTTTGTTCTGGGAGTGGGTATTGCATGTGGCTGT
TTCCTAGGTCAACATCTACTACGG

>novel_circ_000787
TGAGCAGGCTCCATTGCGAATCCGAGTCCTTCAAAATGGACTTAATCCTAGACATTAAC
AGCTGGCTGTATCCTATGGAACTTGGTGACAAATTTCGTCTGGTTCTGGCTACGACTTT
GAGAGAGGACGGTTATCCTGATGGAGGGGAGTGGAACGCGACTGAACAGGAAGGCG
GTTCCAGAGCAGACAGTTTTGAATATGTGATGTCCGGTATGGTTTATCGAATAGAAGGT
GATGAAGCAAGCAATGAACCCAGCAGTAGACT

>novel_circ_000788
ATGAAGGCGGCCTATAGAGCTTTCGATGCATTCTGTGAACTTTCAGAAGATGTTCGAAC
TAAATATGAACGTAATCCTATGGGCAATTATGGATACGTGAAGCCAGGACCGTCAAAAT
ATACTGATGAAAAAGAAGCACGACATGTTTTCAATGTGATTGGATGCGGAGGAACTCT
TCCGGATGAAGAAGTGCCTGGTTTCAGATTAGCAGTCAACGAACTTGCTCGAGATTTT
AAACAACTGTCTGCTATGCTTTTAACT

>novel_circ_000789
TATTACTTCGATCATTTCTTTCATTATCTTCTTCTTTAAAAAACAATTCAAGAAGATCACT
AAATGCTACTCCAGCAAGCATACAAACAACTGGAGTTAAAGTGAGCATAAGTCGCACC
ATTACTCCAGCAAAATAAACAGCGCTTATAGCATATAATATAACTGTAAAATAAATAATT
AATAATTTTTATAACAAAATAGTACTATTTTGAATCTTACCGAAAACGCGTTCATCGTTA
ATACGTTTAATGCAGTACCAGAGGCCTACAGGAAATGTTGTAACAAGAATATGTAAATC
AAAGAAAAAACTAAACCATGTAGTAGGTTGATGTTCAGAAACAGATGCTATTATTGGAA
TGTGTATTTTTGCATAGCCAGTATCCCAGAGTGAATAAAATCTTCCACTCCAAGGTGCA
ACAACACCAGCATAAGTTAAACAAATTAAAGCAAATAAAAGAATACCAGCTGTAACTG
CAACTATTCCTCCAAAATATTTC

>novel_circ_000790
TGTTGATCAAAGAAAAAGCACAAGGAAAGAAACTGAAAATGATATTTGAAAAATTGTT
ATTGATCATTTTCCTAATCGGGATTTATTTTTCAAAAATCCAAGGACGAGGATTAATGCT
CGATCCTATTAGTAGGAGCAGTGCTTGGCGAAAAGGATTCCCTGTTGAGCCGAATTACA
ACGATCATGAACTCTTCTGCGGAGGATTAAATATCCAATATGAACAGAATGAGGGAAGA
TGTGGCGAATGCGGTGACGATTACGCTATTCGTCGTCCAAGACCAAACGAGAACGGTG
GTCTTTATGGAACCGGTGTAATTGTCAAAAG

>novel_circ_000791
GATAAGGAAGAAGAAACGTTTGAACGAGGATACGATTAAGCGAAGATGCGAAGACAC
GCAGGATCCAGAAAATGAACGATGAAATAATCGCGAAGGGTTTGACCGGTGGCGGGA
ACATTCCGTTCGCGGACGAGGATGGAGCGGTGGCGGGGCTGGACGATCGCGGCGCGC
GAGAGGTGACGCGGAAGGAAGCGCACGCTTTCGGGACGTTCGGTGGGCCGTCGAGG
AGGATCGATCAAGAGGGAAGGAGCTGCTCGTCGACGGTGCTCGAACGGAATGCGAGC
ATGTCGGTCGGGCAGCAGCAGCAGCAGAGGCAGTACAAGCGATCGCCCGGCCCAAGG
AGGATCGGCTCGAGGAACGAGGGGATCGAGGGGAGCGGGGAGAATCCTCTGCAACG
GTACGAGAACGCCGTGTTGAAGTATCAGAGGGCGAGCAGGCAAGGGAGCCGGGACA
GGATGCAGAATCACGGTTGCTCGAGCAGCGACGAGAGCGTGGCGAGGAACAAGTCGA

AGAGCATCAGCTCGATCGACACGCAATCGGTGAACAACATATTGGACGAGTACGAGGA
CCTCGATTACGGGAGATCGTCGGATCTGAGCGACGTCGGGAGCATAAGCGTGTCCAAC
TTCGAGGCCGTGATGATGGACCAGCGGAAGAAGCAGCAGCAGCAGGAGAGGTCGAG
AGCGTTGGCCGCGTCCAGGATCCAGCCCAAGTGCCTCGCTGCGCGGGAGAAGTCGCA
GCTGGTGGCGAGAGCGAGCCAACCGGCCCAGCAGCCGAGACAGAAGGAGTCGTCGA
GGCACGCGGTCCCGCAGTCGCCGATCGAGGACGGGCCTCGGCCCTCGTCTTGCGTGCC
CGTGACCGTGGACCCCGGCCGGGCGAGAGGTGTCCCGGAGGTGTTGCTTCGCAAGGG
GGAGGTGCAGAAGAGGGTGGACGAGTGGTTGAACCAGACGCAGAGCCAGAATTTCA
CGGTCGGGGCGCGGGAGAAAGCGTTGACCAGGTCGAACAGCAGCGCGGACCAGAAG
AGTCAGAGGAGGTACAGGCAAGACTCGAGGTCGAGATCGATCGACGAGGGCAGGGA
CAAGTCGAACGGGACCTCGTCGAGTTACGACGATCTCAGCCGGGCGGAGAAGAAGGC
GGAGCGCAAGGCGAGCGCCGACAAGGTGAACGTTGGCGTGAACACGAGCAGGGGGA
CGTACAAGGAGTATCTCGCTTCGAAGAACGGAAGGAGCGGCGGCAAGCAGCAGGATT
ACGGCTTCAGCGCGTCGGGCTGCGGCGCGATCGGTAGAACGAGGGACATGAGCCCTT
CGACCGCCGCCTCGAGGATTCCGCAGAGGGGCCCGTTGGGCTCGAGCTTCAGGTCGA
GGCGAGGACTGGACTCGTCGGGCAACGGCGAGGCGAGCCAAGCGGATAACAGCGGG
GGGGAGCAGGCGCAATTTTGCCAGGGCGAAAAGAACTGTCGCGCGAGAAACGCGGC
CGACGCCAGGCCGACGACGACGACGAATTTGGTAAAGACGAGAAACGAGTCCGATAT
CCACGGGAGGATATCTGGGGTGGTGGGAAGCGGCGTTACCGGTGGTTCGACAACATCG
GCGTCCACGTCGTCGTGCTCGGTTATCCCGTGCAGGAAAGCGTCGTTCAAGCGGTCGC
AGAGCCACGATCAAAGCGCGGCGCGGCCGCTCGTTAAGGAAGAGACAGGCCAGAGA
GGGAGCAGAGGAGGGAGCGGCATCTCCAAGGGCCCGGCCAACCTTGGTGACCAAGG
CGAGCCGATCTCGCCAAATAAGGACGGAGAAAGTAGGCGGCCGGCTCTCACGACCGA
TTCATCGCGAACGGCTGGTGATAAACCAGCCGATGTTGCCTGTAAATCGATCGAGGCT
GCGAGGAGCGGCGCTGATCGGCCCACGCGGGATATTATCCACAGGGTTACGGGTGGTG
GTGGCGAGTCGGGCGAGACTAGGAGGTGCTATCCCGTGCAGGACAAGAGAATGGATC
AACAGACGAGGCAGGAGGCCGCTACCACGGTGGTTTACGGCGCGGTTGGCGCCCGCG
TGCGGACCCTCCAAGGTCACCAGGAATCCCGCGTCACGAAGCCGAGGAATCTCCAAG
CCGGCCTCGCCCCGCCGGAAGCTAGAAAGCCGCCGGTTCTCCCGAGGAGGGATCAGA
TCCCTTGCTGCCAGGAGGGGAAGGACGACCGGGCTGCGAAACGGTCGCCGATCGGCT
CCCCTCGATCCACGTTCAAACCGAACAGTCGGGTTTACGCGGCGTTGCAACAATTGAA
CGAGCAGAACTCGGCCAAGTCGAGGTCGGCGGGCAACGGTCCCGCTGACAAGTCGCC
AGCGAGATCCCACTACTCCTCGCCGAATCACGCGGTGGAGAAGATTTACGAGCGCAGT
CTTCAGCCCCAGGTGATTCACGTGGATGACCTCCAATCGATCCTTCGACCTTCTTTGCA
GGTTTCTGCTTACGGCGAAGGAGGGACGGCCGGTCGATCCGATCGCAAGTCGCCTCCC
GGAGAAGTGGTTGTGCTCGCCCAACGAAACGAAAAGGAGGACGAAGAGGAAGAGGA
AGAAGAGGAGGAAGAAGAGGAGGAAGAAGAAGAAGAGGAGGAGGAAGAAGA
AGAAGAAGAGGAGGAAGCGGAGGTTTACGAACGGGTCGGTGAGTTGCGGTACG
AACACCTGGAGACTATCGAGGAAGACGATCAGAAGAGCGAAGCGTCCGTTTGCGGAT
ACCCAGAGATCGGGTTGAGCCGATGCTCGAAGATCGAGCGAGGGCTGGCGAACGGTA
GGTCGCAGGAGAGGAGGAAAAAGAGGAAGAAGAAGGAAGGTTTGACGACGCAGCG
TTCGAAGCCGGATCAAGGGAACGAAGCTGGCAGGACGTTCGTCACGTTTCGCTCATCC
AGTTCCCCCCAGGACATTAAGGCGAGCCTGCAATCTCGACCCGCCGCGACTGGTTTGT
CGGAGTGCAACGAACAGCAGGAAAAACGGGAGATCGCCGCTGCCGAGGAGCCGCCG

CCGAGGATCGTCCACGACGCGGAGATTCTCCCTTACGATTCCGGGAACCAGGCCGACA
GAGGCGAACCGTGTCACACGCGGGAGAACAGCGATCTATCCACCGCCACTATCCCCGT
GGACGAGCTTGAGAGTGAAAGGCAGGCGGCAAAGTTCAAGGTCGAAGACGCGTGTC
TCGGATCGAACGAGATGCCCGATCATCGAACAATGGAGGCAAGATTTCAAAAGGAGGA
TGCCAAGGAGGTCGGTTGCCAGAAGGTCGATAGATGGGAGAGCGAGAAGGAGAGGG
CTAGCTCGAGGGAAATGGAGGAGCGCGGCTCCGTGGTGAACGAAGTGTTGGTGAAGA
ATCTTCAGTTCGAGCAAGATTTGCCCAAGGATTCGGTGGAGCACGTTGGTGGTGGTGG
TAGTGTCGGTGTTGGTGTTGGTGTTGGTGTTGGTTGTAGTGACGCGTTTGGAAAGATCA
TCGAGCATGTCGGTCAGCAGAGAGCGATGGGGTACCACAACGTTTTGTGCGACAATTA
CGAGAACGTGCAGAACGCGAGGTATAAGGAGAGCAAGGTGTACGTGACGAAAGAGG
AGATGGAGCGGCAGAGGCTGATGGAGCTGCTCAGGAAGGGAGATTACGAGTTAGCGA
AAGCG

>novel_circ_000792
GAATTGGAGAGAAGTTATACCCTCTCGACCCCGGGGGGTAGCAGCCCCGTTTGTTGCC
CGCCCTGCAGCCCGCCCCCTGCGCCGGCCGCATACATATCGAGCGCACGTGCGTCCTC
GCGGAGACTCGGCGCCGGAAGCGGAGGCCCGCCGTCACCGGAAAGGGACCCCTTGG
GAAAACTGTTAAGATTCCTCAAGTCCTTCTACAGGGAGCTGGGCACTCGCGATCCACC
CCATTTGCCTGGGTGGGCGTCGCCACTTCCACTCGGCACCCTTTGTCCGGCGCATTTCC
AGCCGCACCTGCAGCTGCTCCACAAAGGCGAGCAGGAGCACAGGCTCGAGAACATCA
AGCCCGACCAGATCTTCGCCCCCGTCAGG

>novel_circ_000793
GAATTGGAGAGAAGTTATACCCTCTCGACCCCGGGGGGTAGCAGCCCCGTTTGTTGCC
CGCCCTGCAGCCCGCCCCCTGCGCCGGCCGCATACATATCGAGCGCACGTGCGTCCTC
GCGGAGACTCGGCGCCGGAAGCGGAGGCCCGCCGTCACCGGAAAGGGACCCCTTGG
GAAAACTGTTAAGATTCCTCAAGTCCTTCTACAGGGAGCTGGGCACTCGCGATCCACC
CCATTTGCCTGGGTGGGCGTCGCCACTTCCACTCGGCACCCTTTGTCCGGCGCATTTCC
AGCCGCACCTGCAGCTGCTCCACAAAGGCGAGCAGGAGCACAGGCTCGAGAACATCA
AGCCCGACCAGATCTTCGCCCCCGTCAGGTTGAGCGACGGCACGGTCTCGGAGGCGC
CTCGCCGCGTGGCCATCGAGGGCGGGCCAGGAAGCGGAAGGACGACCCTGTGCCTCC
GCCTGCTTCACCAGTGGGCGATCCAAGGCGACGGCCCGGCTCTGGCCTTCATCGTCCC
GTTGCGCGAGCTCCGCGGCAGTCCCGTGCTCAATTACCTGGCGCGGGAATTATTCCCA
AGGACGGCAGCCATCGGCGACGCCATCGCCCAGGTGTGGCGCACGCTCCACCTGATG
GAGGATCGCGTGCTGTTCATCCTGGACGGGTACGACGAGTGCGTGGGCGGCAGAGCG
TCCCTGGCCGACGCGGTAGACCTTCTGGAGGGGCGCTTGTTCCCGGACGCGAGGATCC
TGGTCACGTGCTCGCCCGGCAACTCGACCATGCTCTCGCCCCTCGTCCAAAGAAGGAT
CCATTTGGCCGGGTTGGAGTGGCCCCACGTCGAGAGGCTATGTGTCGCGTATTTCATCC
ACAACGACATCGCGGAGAAGGCTTGCGAGTTCCTCGAAGCGTTAAACGTGCAACCCC
AGACCGTGAAACAGCTTGGTCAGCACCCTCTCGGTTGGATCATGCTCTGCTGCCTCTA
CCAGGACTCTGGGAGTTTGCCAACGGAGACCAGCGCGCTGGTTCAAGCAGCGGTCAA
GTGCATCGTGAAGCGGAGTCTGGATCCACCCGTCCCTTACAACGAGGAGATCCCTGGC
CATTGCAGGAAACGATTGGAAGACTTCGGTAAGGTGTCGTTGGCCGCGCTCAGGGAA
GGCAGGTGTTGCTACACGGAGGCCGAGCTCCGCGCACGGGGTGGAGGGATCGAGGTG

ACCAGGCTTGGCTTCCTCACCAAAGGATTGACGTTCGGCCAACGGCGAAAACCTGAC
CTGTACACGCCCATCCACATGGCCGTGGCCGAATTCCTGGCGGCCTACTACTTGACCTC
CGTCGCCCAATACGCCAACATACTTCGAAGAGAGTTGGAGGGCCTCCCGTCGGGGATC
ATCAGCCACTTGGCCGGCCTGTTGGGGCCCAAGACGCATCTGATATTGAACCAATTGTG
CCCGTTGGAGGTGCCGCCAAGGGCTGTGTTCTCCCTGTTGAAGGCGGCAGGCGCCTC
GGACGGAAATATCTCCGCCGTGTGCAGATTGGTCGGCGCCGGGCCCGGCTTCGGCCCC
GCGCCCAACGAGAGGCCGCCCGCTCCTCTCGTCCACACTTCCCCCCTCGAGCTGGAGG
GGTGGGCGAGAATCCTCGAGAGCGCGGCGTGCACCCTCGAGGCTCTCGAGGTTGTGT
TCCAGGTGGAGAGGGGCTCCGATCCGAGATATCTGGACGACTTTTTCGAGGCGTTGGC
CGGGAACGAGAGCGTGAAGCTCGTCAGGATCACGTCCCTTCTTGGACAAGAGTTCCC
CGCGGACGAGGCGCAAAAACTGGCCGGCCATTTGAAGAGCGTCCTCGGCAAGAAGA
AACTGAACGACTTTGAACTGGTTATAACTTGTCTCGAGGAAGCTGCCCACGACAG

>novel_circ_000794
TTGAGCGACGGCACGGTCTCGGAGGCGCCTCGCCGCGTGGCCATCGAGGGCGGGCCA
GGAAGCGGAAGGACGACCCTGTGCCTCCGCCTGCTTCACCAGTGGGCGATCCAAGGC
GACGGCCCGGCTCTGGCCTTCATCGTCCCGTTGCGCGAGCTCCGCGGCAGTCCCGTGC
TCAATTACCTGGCGCGGGAATTATTCCCAAGGACGGCAGCCATCGGCGACGCCATCGC
CCAGGTGTGGCGCACGCTCCACCTGATGGAGGATCGCGTGCTGTTCATCCTGGACGGG
TACGACGAGTGCGTGGGCGGCAGAGCGTCCCTGGCCGACGCGGTAGACCTTCTGGAG
GGGCGCTTGTTCCCGGACGCGAGGATCCTGGTCACGTGCTCGCCCGGCAACTCGACCA
TGCTCTCGCCCCTCGTCCAAAGAAGGATCCATTTGGCCGGGTTGGAGTGGCCCCACGT
CGAGAGGCTATGTGTCGCGTATTTCATCCACAACGACATCGCGGAGAAGGCTTGCGAG
TTCCTCGAAGCGTTAAACGTGCAACCCCAGACCGTGAAACAGCTTGGTCAGCACCCTC
TCGGTTGGATCATGCTCTGCTGCCTCTACCAGGACTCTGGGAGTTTGCCAACGGAGAC
CAGCGCGCTGGTTCAAGCAGCGGTCAAGTGCATCGTGAAGCGGAGTCTGGATCCACC
CGTCCCTTACAACGAGGAGATCCCTGGCCATTGCAGGAAACGATTGGAAGACTTCGGT
AAGGTGTCGTTGGCCGCGCTCAGGGAAGGCAGGTGTTGCTACACGGAGGCCGAGCTC
CGCGCACGGGGTGGAGGGATCGAGGTGACCAGGCTTGGCTTCCTCACCAAAGGATTG
ACGTTCGGCCAACGGCGAAAACCTGACCTGTACACGCCCATCCACATGGCCGTGGCCG
AATTCCTGGCGGCCTACTACTTGACCTCCGTCGCCCAATACGCCAACATACTTCGAAGA
GAGTTGGAGGGCCTCCCGTCGGGGATCATCAGCCACTTGGCCGGCCTGTTGGGGCCCA
AGACGCATCTGATATTGAACCAATTGTGCCCGTTGGAGGTGCCGCCAAGGGCTGTGTT
CTCCCTGTTGAAGGCGGCAGGCGCCTCGGACGGAAATATCTCCGCCGTGTGCAGATTG
GTCGGCGCCGGGCCCGGCTTCGGCCCCGCGCCCAACGAGAGGCCGCCCGCTCCTCTC
GTCCACACTTCCCCCCTCGAGCTGGAGGGGTGGGCGAGAATCCTCGAGAGCGCGGCG
TGCACCCTCGAGGCTCTCGAGGTTGTGTTCCAGGTGGAGAGGGGCTCCGATCCGAGAT
ATCTGGACGACTTTTTCGAGGCGTTGGCCGGGAACGAGAGCGTGAAGCTCGTCAGGA
TCACGTCCCTTCTTGGACAAGAGTTCCCCGCGGACGAGGCGCAAAAACTGGCCGGCC
ATTTGAAGAGCGTCCTCGGCAAGAAGAAACTGAACGACTTTGAACTGGTTATAACTTG
TCTCGAGGAAGCTGCCCACGACAG

>novel_circ_000796
ACATCGCCATGGACTGGTCGGATCACGCGTTATGGTGGCCGGAGAGGAATCACTGGTT

GACGAGAACGAGAAGCACTCTGGACCAGTACGGGGTCGCAGCCGATGCCCTCCTTCA
CTTCACACCGATGCACAAAACTCTGAGAGTCCAACTGCCCGATATGCGCTGCCTCGATT
GCAAGGTCGACTTCTCTGTGAAAACGTTCAACGCCGTGATCAATTTATGCAAGGAATT
AGGTATCAGACACCCGGAGGAGTTGTCGTTCTGCAAACCGCTCGAGCCGAATCATCTG
AAATACAATTTGAAAGATCTGCCGGCTAAGAAGAAGATCGAGAACCAGAAGAACGGG
CATTGGAACGTTCCAGCGGATACGAACACGTTCATCCCGGTGAGCCAAAGTCCCAGGG
GATCGACGGGCAGCCTGGATCAGAGCAGCCCTTTCATGTGCGCGCCGGTCACACCCAA
CAACAGAAACCACAGCACGCCGATCAGCTCGCCCGTCTCT

>novel_circ_000798
GTTGCTCCATCATCAGTATTTCTTTCCTCAAATCTTCTCCTATATTTTATATATTTAATTATT
TCGTTCACTTTTAATGCTACGAAACAAAATATAAATCAAGACTCGAATCGATTAAAAATT
ACAAATGGGAATCAAGCTTATATCCCATTGAATAATACGTTGAATTGTTCGTTCTCCAAT
CGCCGCCATCTTGGCCGGCCAATTGGAAGAACTGAAAGGAAAAAG

>novel_circ_000799
GTTTGACGCATCACGCGGTGCGCACCCTGGGCTCGTTGATGTCCTGCGTGGAGGAGGG
GACGAGGGCACGGAAGACAGCGTCCACGTTGCAGAACCCGAGCAGCAGCCGAAGCC
ACGCTCTGCTCACGATCGTGGTCGGCGAGGGAAGCGAAGAAGCGTCGTCCTCGAGGA
GGAGGAGGCCGTCCCGCGGCGGCAGCAAGCTGCGTCTCGTCGATCTGGCGGGGAGCG
AGAGCGCGGCTACCTGCGGTGGCGTTCATCGGCTGAAGGAGGGCGCGAATATAAACA
AGAGTCTAGTGGCGCTGGGGAACGTAATATCGGCCCTTGCGGAGAGGCCAGGAAGTG
GCCCAGGAAGGAGGTTCATCCCTTACAGAGATTCTTCGCTTACTTGGCTGTTGAAGGA
CGCACTTGGCGGAAATGCCACTACCATTATGCTCGCCA

>novel_circ_000801
ACAAAAAGAAAAAGGGGAAAAAATATGACATAACTCTCCATTATCAATTTGCTTGAAC
GATAAAAAGGACCAAACTAAATATATTGGGAATTGTTTCTCAATAAAGGGGAGAAAGA
GAGAATTGTGTCGAACATAGATCACGTGAGAACGTGGAAATGTCGACCGAAAATCAAA
ACGGTTCCGCTGGTACACAAAGCAACGGAATAAAAACCACAATTGGTTGGTCGTCGTC
CACAAGGACTGTCTCGAACAATTCATCGTTTTTACATACTAGCAACTCGATGTTGAACC
GTGACAAGCCGCGTCAAGTGCCACCACCAACTTTACCGAAATACACGTCGAGTTTTAA
TGCCGGTTGGAATGGCAGCGGCACAACCGAACGATTAAGCAGAGATCGCGAAGTCGG
AGGTAGTTACAGGCTGGCAAGCCTTGACAGACTTGCCCTCAGGCAGAGGATATTGGAC
GGGGAAAAAACGAATGGTGACACCACGTCGATACAGGCGAAGCGCGAGCTGTTCTTC
AAAGGCGATAACGCCGGAATAATATCGTCTCCATCTGTGCCATCGGTCCCGCCTCCACA
ACCTCCGTCGCAGGCCCCGACGTCGATGAGCACATCGTCGACGAGCACGTCGTCGAC
GACGACCACGACAACCACAACACTTGCTTCGCCTAACACAGCACCGCCAACCTCTTCG
ACCGTGTACACAAGCGTCACATCGTCGGCGCCTACCTCGAAGCCACGATTACCTCCGT
CCACGGTGATCTTAAATCAAAACGATGCATCAGAGGACAGTAATAAACGGGAGTCGAA
TCGAAATAGCGAGAACAAGGAGGCTGCTGTCCTTCATCATCCGCGACCAACGCCGCCG
ACCAAGCCCAAAGAACTCGATGGTTATGTGGGATTCGCAAATCTACCCAACCAAGTTTA
TAGAAAGGCTGTTAAGAAAGGATTCGATTTTACTCTGATGGTCGTCGGTGAATCAGGA
CTAGGAAAATCAACCTTGATCAATTCAATGTTCCTCGCCGATATATATAGTGCCGAATAT

CCTGGCCCTAGCCTTAGAGTTAAAAAAACTGTAGCCGTAGAGACTAGTAAAGTATTGTT
AAAAGAGAATGGTGTGAATCTTACTCTGACTGTTGTTGATACACCTGGATTTGGTGATG
CAGTCGATAACAGCAATTGTTGGGTACCAGTAATCGAATATATTGAATCCAAATATGAG
GAGTTCCTGAATGCCGAGTCTCGTGTTGTGAGACGACAGATTCCAGACAGTCGAGTAC
ACTGTTGTCTCTACTTCGTCGCTCCTTCAGGGCATGGCTTAAAGCCGTTGGATGTAGAG
TTCATGCAACGTCTTCACGACAAAGTTAACATTATACCTGTCATCGCGAAGGCGGATAC
CATGACGCCAGATGAATGTGCTCATTTTAAGAAACAGATTTTGAACGAAATAGCACAA
CATAAGATAAAAATCTACGAATTCCCAGAGGTAGAAGAAGAAGAGGAAAGTAAATTGC
ATAAAGTTCTTAGAGAAAGAGTACCGTTTGCGGTAGTGGGTGCAAATACCGTAGTCGA
ACATGACGGTAGGAAAGTTCGTGGAAGGAAGTATCCTTGGGGAATCGCAGAAGTGGA
AAATTTGGAGCATTGTGACTTCATAGCATTACGAAATATGGTTATTAGAACACACTTACA
AGATCTAAAAGATGTTACAAATAACGTACATTATGAAAATTTTCGATGTCGCACGTTAG
CAGGTGTTGGAGTGGATGGGAAACCAACAAAAGTTTCGAATAATTTGTGCCCTCCAGG
AGTGATGAACAGTTTCATGACAGTATGGAATCCACTAGCTCAATTGGAAGAAGAAAAA
AGAGAACATGATAATAAAATGAAAAAAATGGAAATTGATATGGAACAAGTATTTGAAAT
GAAAGTTAGAGAAAAGAAGCAAAAACTTAAAGATTCAGAAGCAGATTTACAAAGAAG
GCACGAACAAATGCGACGTTCTTTGGAGCAACAAGTACGTGAATTAGAAGAAAAGAG
ACGAGCATTTGAGGCTGAAAAGTTAGCATGGGAACAACAAACTGGTCACAGTATTGA
AGAATTGCGTAGGCGCAGCCTAGAAGCAAATTCAAAAGA

>novel_circ_000802
GTACACGAGATCCATTTCCACCAGCTAGAGCAGCGAGTATTTTAGCATTAGCTGCTACA
CAGCAATATTTTTTATTACAAGAAGTTGCAAACCGTATTTTACCAGCATTATGTCCACTT
ACTACAGATGTAGATAAAGGAGTTAGAGATAATGCATTTCGAACTATCCGTGGATTTTTA
TCCAAGCTTGAAAGA

>novel_circ_000803
GAATTAGCCTACAAACAACGTGAAAAAGATAAAAAAAGAAGATTATTGGGAATTAGTA
ATAATAAACCAAAATGGATTCCCACGGGACAATTTGCACCGACGCGTATTGGTGAACAT
ATCAAACCAGTACAAAATGCACGTTATGAAGCTTACCAAATGCGTATTCATGGTCA

>novel_circ_000804
GAAGAGATGGAAGTTCGTCCTCGAATTTCCACGCTGCTCAACAGCCTGTCGGACTACA
GCAACACGATTCCAGCTGCGACCGACCCGGATGCGAAACCTCCACCCGTGCAAGGTG
GTGCACGAATGGGCACTTTGATCGGTGTCTTCCTACCTTGCATCCAAAACATCTTTGGC
GTGATTCTATTCATTCGTTTGACCTGGGTCGTTGGCACCGCTGGAGCTTTTCAGGGATT
CTTCATCGTGTTATGTTGTTGTTGCGTGACGATGCTGACAGCTATCAGTATGAGTGCCAT
CGCGACTAATGGCGTTGTACCAGCTGGAGGGTCTTACTTCATGATATCCAGAAGTTTGG
GTCCAGAATTCGGTGGTGCCGTAGGAATGCTCTTCTATACGGGTACTACACTAGCTGCT
GCTATGTATATCATCGGTGCTGTTGAAATTGTTCTGACTTACATGGCGCCATCGCTTAGT
ATATTTGGAGACTTTACAAAAGACCCTAACATCATGTATAATAACTTTCGCGTGTATGGT
ACTGGATTGCTTATGGTAATGGGCACCATCGTATTCATTGGCGTGAAATTCGTCAACAA
ATTTGCGACGGTCGCTCTAGCTTGTGTCATCCTTTCTATCGTGGCTGTCTACGTAGGGTT
ATTCTATAACTTTAATGGCAACGAATCTCTCAAGATGTGTGTTCTTGGAAGGAGATTGTT

AAAAGATATCAACGTACTTACCGAATGTAATAAGAATACTAGTGGAATTCTTCATCAACT
TTACTGTGGAAACACAACCACTAGCAAATGTGATCCTTATTATATGGAAAACGATGTGA
CTATTATAAACGGAATTCGTGGATTGGCTAGTGGTGTATTTTTAGACAATATATGGGACA
GCTTTCAAGAAGAAGGCCAATTGATTGCCTATGGAAGAGATCCCAAGGATATTGACAT
GATGTCCAATTCTTCCTTCAATCAAATTCAAGTCGATCTTACCACTACCTTCACCATTCT
TATTGGTATTTTCTTCCCTTCAGTCACAGGAATCATGGCTGGCTCCAATAGATCAGGTGA
CTTAGCAGATGCCCAAAAATCTATTCCTATTGGTACTATTTGTGCAATTTTGACAACCTC
AACAGTATATCTCTCCAGTGTTTTACTCTTTGCTGGTACAGTGGACAATCTTCTGCTTCG
AGATAAGTTTGGCCAAAGTATTGGTGGCAAACTGGTAGTAGCGAATATGGCTTGGCCTA
ATCAATGGGTCATCTTGATTGGATCTTTCCTGTCCACTCTTGGTGCTGGACTTCAATCTT
TAACAGGCGCTCCACGATTACTCCAAGCTATCGCTAAGGACAGTATCATTCCTTTCTTA
ACTCCATTCGCCACCAGTTCGAGTCGAGGAGAACCTACAAGGGCTCTAGTATTGACAG
TAATCATCTGCCAGTGTGGTATTCTTCTTGGCAATGTTGATTATCTGGCTCCCTTGTTATC
AATGTTCTTTCTCATGTGTTACGGCTTTGTAAACCTGGCTTGTGCTCTGCAGACTCTTCT
CAGAACACCCAACTGGCGACCAAGATTCAAATACTATCATTGGAGCCTTTCTTTTCTCG
GTCTGTCCCTCTGCATCGCCATCATGTTCATGACAAGCTGGTATTACGCTTTATTGGCCA
TGGGAATGGCTGGCTGTATTTACAAATACATTGAATACCGTGGTGCTGAGAAAGAATGG
GGTGATGGTATTCGTGGTCTAGCTCTGTCAGCCGCTCGTTACTCTCTTTTGAGACTGGA
AGAAGGACCACCTCACACAAAGAACTGGCGACCACAAATTCTCATCTTGGCTAAACTG
ACAGACGATCTGGTACCCAAATATCGCAAATTATTCGCATTTGCTAGTCAATTGAAGGC
TGGAAAAGGTTTGACAATCTGTGTCAGCTGCATCGGTGGTGATTACATTCAGAACACT
GGCAAGACTTTGGCCGCAAAAGTGAATTTGCGCAAGACAATCGCAGAGGAAAAAGTA
AAAGGATTCGTAGATGTCTTAGTGGCTAAGGATGTCGTCGATGGCCTCTGTTCCTTGAT
TCAAACAACCGGATTAGGTGGCATGAAACCTAACACTGTGATTCTTGGCTGGCCCTATA
GCTGGAAACAATCACAAGAGGAAAGAAATTGGAGGGGATTCTTACAGACTGTCAGAG
CAGTTGCCGCAGCCAGGATGGCTCTTTTGGTACCTAAAGGAATCAACTTCTTCCCTGAT
TCAACTGAAAAAGTTGTGGGAAATATCGATGTCTGGTGGATTGTACACGATGGCGGTC
TTCTGATGCTGTTGCCATTCTTGCTGAAGCAACATCGCACATGGAAGAATTGCAAAATG
AGGATCTTCACAGTTGCCCAGATGGAGGATAATTCTATTCAGATGAAGAAGGATTTGAA
GAAATTCCTATACGATCTGAGAATCGAGGCTGAAGTGGAGATTGTGGAAATGATGGATT
CAGACATATCCGCGTACACTTATGAGAGAACTTTGATGATGGAGCAGAGGAACCAGAT
GTTGAGGGAGTTGCGACTAAACAAGAAGGAATCCCTAGGAGTG

>novel_circ_000805
GTGGACACAAGGAGTTCCACAGGCGGCACAGGGACCTCTGCGAGGATACGCTTCTCC
CAACAGTGGGAGCGGGTCCGTCCGCTGTCGGCAGTTGTTCCAGTCCAGGACCAGGGT
GTCCAGTATTAAGTCTGTCCGGACCACCGACCCCGCAACCCGCGGACATCGATAGCCA
CCCAGCCTCGAGAGTTCTACCGTTCCTCTACCTTGGAAATGGAAGAGACGCGGCCGAC
TTGCAACTTCTTCGCGCGTTGGGCGCGACCAGAGTCCTCAATGTCACTTCACAGCTGC
CCGGATATCACGAGGAGAGGGGTATCACGTACAGACAGATCCCTGCCTCGGATTCTGG
CCACCAAAACCTTAAACAGTACTTCGAGGAGGCGTTCGACTTCATCG

>novel_circ_000806
CGTCCGGGCCGCGTCTGGACGCGACGACGACGACGACGACGACGGGCGAAGCTAGG

GGCGGCCCGGCGTGCACGGGCCGGCAACACCACCACCACCACCACCACCATCATCAC
CAGATCGAGCACGAGCTTCAGCCATCGTCGGCGATGTCCACCCTGGTCTGCTGCGAGC
CGGTCTCGGCCGCGGCCCGTTCCCGCGACAACCTGAAACTCGTCCTGAATCGGAGCCT
GAGCGAGCCCGGCCCCAGCGCGGCGGCGGCCGGCGCGTCCGCCGCCTTCCTGTCCGC
CGCCCCCTCGCCCCCCCCGAGCGCCGGGCTGCCCCCGGCGGCGGGGGACGCCTGCCC
CGCGCCCGACGAGTGCGCCGACTGCGCCTCCGCCGACCCCCTGCTCCACCACCGCCAT
TGCCACCACCACCTCCACCACCACCACCACCTCCACCACCAACACCTCCTCCACCACC
AGTCGTCGAAACGCTGCAAGCTGGAGACGGTCAGCCTGCCGACCAGCCCCTCGTCCG
AGACCAACACCCTCCAACGCCAGCTGGTCCTCCAGAGGACCAACGCGATAACCGCCG
ATCAGTTGGCCGACCGGCTTCGATCCTCCTCACGCGAGGGGAACGGGGGTGCGTCGA
GCGTGGTGCTCGACTGCCGCCCCTTCATACTGTACAACGTGAACCACGTGAGGGGGGC
GATCAACGTGAACTGCTCAGATCGGTTCAACAGGAGGAGGCTTCAACTCGGGAAGGC
GGCCCTCGCCGATCTCGCCAACACCAGGGAAGGGAAGGAGTTGCTGCGCAGGAGGCA
TTACAGGGAGGTGGTCGTCTACGACGATTGCACCGACGACGTCGACCGCCTCCCCGTC
CAGCATCCCCTCTTCCTCGTGCTCGTCGCGTTGCTGGACGACAACAGGGAGCCTGCTC
TTCTCTTGG

>novel_circ_000808
ATGGAGCATAGAGGCGCGGCGTCGGATCGGCAGTAGCGGCACCCTATCGGCCGGAGGC
GGAGGGATCGGACGCGAGAGAGGCGGCGTTGTAGCGCGGCGATGGCCGCGACCGACG
GTCAGATCGTGGTTGCGGCGGCGCGGTGGGCCGAGGAGGCGACCTACCTCCGCGAGT
TCGTCTCCAAGTATCGTCTGCCCGCGGTGATCAAGATCACGAAGGGCCAGTACGGGGG
GCTGGGGGTGCCCACCCTGCCGGCGCCCAGCCTGCAGAGCACTGCCCTCCTCGTGTCG
GCCGGCAGGCGGCGCAAGATCGTCGCCCAGGCGGTGAAGATCAAGGAGGGGAGGAG
GGTGGTCGGGGTGGGGCCGAGGCTCGCCATCCCCGACTCGTACGCCGGCTACTTCGAG
ATACTGAGCGAGGAGGGGAGGGCGGTGCGCGGGATAGAGTCGGTGAACGAGCTGTCC
CGCCGTTGCCCCGAGGAGGGCGCCCTGGTGCGGGAGACGGTGCGCGGCATAGCGTGC
AGGGTGGACGACGAGTCGGGGCTGGTGGTCCCGGAGGGGACCAGGACCCTGGCCGC
GGGCGAGACGATCGTCACCGCGGGCGAGGTGACTCTGCCCGGCCGCGGCAGATTCCT
CCGTTGCGTGGACTGTCGGGGGGAGAGCGTGCTGCTCGGGATGGATCAGCGGGGCCG
TTTCAGCGCGGTGGCGCGCGAGGACAACATAAGCGGCGTGCACACGGCCCGCGCGTT
GCTCAGCAAGCGTCTGCCGCTGACGGTGCGGCTGGTGCACGGGCAGCCGCCGAGGGG
GTTGAAGTCGTCGTCGCAGTTCGTGCCCGAGCTCAGGCTGCTGTCCACGTTCGAGGAG
GAGCACGTGTTCGCGTTGCCGTTGCAGCGAGAGGGGGCGGCGGTGGCGCTGCCGCTG
GCCGCGCCGCTCAAGCTGGTCAAGGCGAGGAACGAGGAGGCGCTGAGGTCGATGCA
GGAGTTCGGCAGGCTGGTGGAGCGGGCGTCGCGGCTGGTGGCCGACGTGGCGGACCG
GGCCCACGTGCTGGACGGGCGGCTGGGCGAGAGCAAGCAGGCGGCCAGGCAGACGA
GAGGCGGGTCGGGTTTCCTGAGGCGGTCCTCGGCCGCCACCTCGGACAACAACGGGC
CCCTTCCTCACCACAGGAGCAACGCGGCCACCCACCACCACCACCACCACCACCACTA
TCACAGCAACGGCCAGCAGCAGCAGCAGCAACAGCAGCAGCAACAGCAACAGCAGC
AGCAGCAGCCGCCCTCGTCCGGCTACGCGAGGGACGAGAATCGCGTGCCGCCCTCGG
CGTGCACCGAGGAGTACGACGAGATCGATCAGATTTACGACTACGTGCGGGGTTTCGC
CCCGCTGCCGAAGAGCGTGAGGTCCCCTTACGAGAGCCCGCTGCCGCAGGCGGGGTC
GTCCAGCCCGCCCCTCACCCCCGTCACCGTCACCGTCGCGCCACTGCTCGACGACAGA

CCGGAACCGCCGCCCATCGAGACGATACCGACCAAGAAGATCCAGGCCGAGAAGAGG
ACGAGGCGCGCCGTGAAGGAGGCGCCGCAGCCGAGGGCCGAGAAGCCACCCCTGGC
CAAGCTTTACGTGAAGAACAGCGGCACCCAGAGGGGGCGCCCCCTCATGAGGCAGAA
GAGCGCGTCCCCTCTGAAGGAGACCCCGCCCGGGTACAAGGGGGGCTCCCCCCTTTTC
AACATCAGATACAAGAGCTTGACGAACCTGCAACAGGCCATGGAGCTCGACGGGACC
CTCGACTCGAGCCACTCGGGGGGCAGAACCTCGGGCGACTCTGGTGCTGGTGCTAAG
CTGCCCGAAAAGAGATCGAGGCGTCTGAGCAGACCGCGCTCGTTGACGAATTTAGTGT
GGGAGCTGCGCGGCGGAAGCGGCCTCGGCTGCGCGAGGCCGGAAACCCCGCCGCCG
GCCACCGCCGCACCGCTGCCCCCCACCAAATGCGGACCACGTCTGGCCGTCACCGTG
GTGGCACCCCGACGCGTTAGCACGCTCTACCTCTAACTC

>novel_circ_000809
GATCAAGCGAGAGCGGTATCCACGTAAAAGTCGAGGATGTGGAAAGAAGAGGGCTTA
GCATGAACCAACACGCGGCTACGTTCAGAGTGTTGATACTGATCCTGTTGATCCTCGGT
GATCTCGAATCGGTCACGGTGGTGAACCATCATCCAGACGAGGAATATTACCTGGAGC
ATGAAGTCCTCCACGAGGAGGCGATTAACGAGGCGAAAAAGTTGCACCTCTATCCCGG
ACCAATCCCAGGATGCAAGGCTTGTACGAATACAGAGATGACATACTGCAAAGATGGA
AGTGTGATAAACGATCATTGTTGTTGCGATGGCAGCGTTAACG

>novel_circ_000810
GAAGTGAGAGTCCAGTTTTTGGTCTGGAAGGTGGAACAGGTACAGGTGGTGGGACTT
CCTTGAGACTCGCATCGCATGAATTGCCTAATGAAATTTCTGCTCCATACGAAGTTCCA
CAATTTCCAATTGAACAAATTGAGAAAAAGCTTTTGATACAAAGACAATTAACCGTCA
AATAGTATCGAAGTAAATGAAACGGAAGGTTACGTTGACGGAAATTAAACATATAGATT
ACATTGCTGTACACATATATGTATACATAGTATATAAGTAGCCAGAAACAATGTGGTTGC
GACGTGACAATAGTGCTACGCAGCATGGTAGTGGCGCGGTAGTGATAGGCGAGACGGC
GAAGGGGTGGCTCTCACGGTCGGCCGTTCAGTAGTCGTGCAACCATCGAGGAGGGAG
GAAGTTTTCGCCGGTATGCACTGCGTGCAATGTAGATCGTTTCCGGTCCACCACGAAG
AAATATCACACTCGTGTTGGACGACCGCATGATCCCCTGGCTTGGCTTTACGATAGCCT
TTCATGACCTCGCCACGAGCATTTCCGAGGCCAGGCTATCGTGCATGCAACAGACGTA
AAATACAAGACAAGGAACGTGTAACATGTTCGCCGGTGCGAACGAGTCGAAACGATG
GCTCAGGGATAATAGGATACTCTTGAGAGCTGCCAAGGATTTAGAAGAACGTCGTAGC
CATTATGAACCATCACTCGGACCTGGTATACCAGATGATGTCGATCATCACTTTAATCTC
GAAGAAAATGATTTTGTCCCACACTTTCAGAGGGTATCCATATCTGGTGAAGATACTTC
CGGG

>novel_circ_000811
AAAAAAGTAAAAAAAAAACTGGCCGAGCTAAGAGACGTATTCAGTATAATCGAAGATT
TGTTAATGTTGTTCAAACTTATAGTCGTCGTCGAGGACCGAATGCTAACTCAAATTCATA
ATTGTATCTATATGTATACATTTCATGGTTATGAAAATAAAATAAAAAATTAAA

>novel_circ_000812
AAAAAAGTAAAAAAAAAACTGGCCGAGCTAAGAGACGTATTCAGTATAATCGAAGATT
TGTTAATGTTGTTCAAACTTATAGTCGTCGTCGAGGACCGAATGCTAACTCAAATTCATA

ATTGTATCTATATGTATACATTTCATGGTTATGAAAATAAAATAAAAAATTAAATATAAA

>novel_circ_000813
AAAAAAGTAAAAAAAAAACTGGCCGAGCTAAGAGACGTATTCAGTATAATCGAAGATT
TGTTAATGTTGTTCAAACTTATAGTCGTCGTCGAGGACCGAATGCTAACTCAAATTCATA
ATTGTATCTATATGTATACATTTCATGGTTATGAAAATAAAATAAAAAATTAAATATAAAG
AA

>novel_circ_000814
TAAAAAAAAAACTGGCCGAGCTAAGAGACGTATTCAGTATAATCGAAGATTTGTTAATG
TTGTTCAAACTTATAGTCGTCGTCGAGGACCGAATGCTAACTCAAATTCATAATTGTATC
TATATGTATACATTTCATGGTTATGAAAATAAAATAAAAAATTAAATATAAAGAA

>novel_circ_000815
AAAAAAAAAACTGGCCGAGCTAAGAGACGTATTCAGTATAATCGAAGATTTGTTAATG
TTGTTCAAACTTATAGTCGTCGTCGAGGACCGAATGCTAACTCAAATTCATAATTGTATC
TATATGTATACATTTCATGGTTATGAAAATAAAATAAAAAATTAAA

>novel_circ_000817
GAGCAACGAGAAAAGGAAGGAGAAGTCTCGCGACGCTGCTAGATACCGTCGAAGCA
AGGAGACGGACATTTTCACGGATCTGGCCGCCGCTCTTCCCGTAACACCGGAACAGGC
AGCTCATTTAGATAAGGCGAGCGTTATGAGGCTTGCCATCGCCTATCTTAAAGTGCGCT
CTGTGGTCGACTGTATTCCAGGCCCGATGACCAAGTCCGAGACGTTGAATCAAATGGA
CGAATTATTTTCCAAAGCTCTAAATGGATTCATGCTGGTGCTTTCCAGCGATGGAAATAT
GATCTACCTGTCGGAGAATGTGAGCGATTATCTCGGCATTTCCCAGATGGATATGATGG
GCCAAAGCGTGTACGAGTACAGCCATCCGTGCGATCACGAGGAATTGCGGGAATGCCT
GTCCTCGAAACCGCTCGAGAACAGCGAGAAACGTGCTTGCAGTTTTTTCTTACGGCTT
AAGTGTACGTTGACCAGCAAAGGAAGGAAGGTCAATCTAAAAAGCGCTTCCTACAAG
GTGATTCATTGTACCGGTAGATTAACGTATATCCGCGATCCGGTATCGAATTCGTCGGAC
AACGACGAAACGAGAAATAAAAAGGACGAGGAGGGGAATGAACGGGACACCGGTGC
CTCGCTAGTGCTTTTGGGATGTCCCATACCGCATCCTAGCAACATTGAGATACCGTTGG
GACGTCACACTTTCCTCTCCAAGCATAGCCTCAGCATGAAGTTCACGTACGCCGACGA
AAAGTTGGCCGAGTATCTGGGTTGGAACAGCGAAGAGTTAGTGGGACAATCCGTTTTC
GAGTTCTATCACGCCCTCGACAATTTGGCTCTGGATAAATCTTTCAAATCAC

>novel_circ_000818
GAGCAACGAGAAAAGGAAGGAGAAGTCTCGCGACGCTGCTAGATACCGTCGAAGCA
AGGAGACGGACATTTTCACGGATCTGGCCGCCGCTCTTCCCGTAACACCGGAACAGGC
AGCTCATTTAGATAAGGCGAGCGTTATGAGGCTTGCCATCGCCTATCTTAAAGTGCGCT
CTGTGGTCGACTGTATTCCAGGCCCGATGACCAAGTCCGAGACGTTGAATCAAATGGA
CGAATTATTTTCCAAAGCTCTAAATGGATTCATGCTGGTGCTTTCCAGCGATGGAAATAT
GATCTACCTGTCGGAGAATGTGAGCGATTATCTCGGCATTTCCCAGATGGATATGATGG
GCCAAAGCGTGTACGAGTACAGCCATCCGTGCGATCACGAGGAATTGCGGGAATGCCT
GTCCTCGAAACCGCTCGAGAACAGCGAGAAACGTGCTTGCAGTTTTTTCTTACGGCTT

AAGTGTACGTTGACCAGCAAAGGAAGGAAGGTCAATCTAAAAAGCGCTTCCTACAAG
GTGATTCATTGTACCGGTAGATTAACGTATATCCGCGATCCGGTATCGAATTCGTCGGAC
AACGACGAAACGAGAAATAAAAAGGACGAGGAGGGGAATGAACGGGACACCGGTGC
CTCGCTAGTGCTTTTGGGATGTCCCATACCGCATCCTAGCAACATTGAGATACCGTTGG
GACGTCACACTTTCCTCTCCAAGCATAGCCTCAGCATGAAGTTCACGTACGCCGACGA
AAAGTTGGCCGAGTATCTGGGTTGGAACAGCGAAGAGTTAGTGGGACAATCCGTTTTC
GAGTTCTATCACGCCCTCGACAATTTGGCTCTGGATAAATCTTTCAAATCACTGTTCAG
CAAGGGTCAGTGCGAGACGGTGGCCTACAGATTTCTGGGCAAACGTGGAGGATACGC
TTGGGTCGTGACGCAAGCCACTCTAATTCACTGTTCCAAGCAGCAGAAACCGTTGTCC
GTCGTCTGTGTGAATTACATTCTAAG

>novel_circ_000819
CGGTGTGGAACGCGAGGATGAGGTGTACAGCGCGCGTCAGCTCGCAGCACGTGACAC
CGAGAAGGCCGTGAAGCCGGAGCAGCCGGCCGATCCCATCCTCGTCGAGCCTCTGCT
CACGACCAAGCTTCGTCTCCGCGTCCAAGTGAACAATAATAACAATAACAATAATAATA
ACAACAAGAGTAACAAGAGTAACCTGGAGGAAGCTACGTTGGACGGAAACGAGGAA
CGGTTCTCCCTGCAACCGGAAGTCGATTCGTTGGATCGCGCGAAACCGTTGACGGTGA
CGAGCTCGACTTTCCGCGCGGCGGACAACAAGCCGAGGAGGGCGCAGGACGGCTCG
CGGAAGGATAACACCTTCCCGAGACCGGTCCAATCTACGTCGGAGAAATCGTTCGCCG
GTGTCCAAGCGTTGAAACAATCCCTCGAGGAGTCGATCAAGGAGAACAACGAGCCGA
GAAAGAAGCAGCAGTGTCGCCGTGCTCCGTTCGAGTTCCAGGGTAAACCAGCGCTGG
AATACGATCCGGAGACCGCTGTTCGCCAGGACAGACCGCACGTGGTTACCAGACACTT
GTTCGCATCCATTGCCACTCAGCAGCAGCAGCAGCAGCAGGAACAGCAGATCACTTGC
AGGCCACCGCCGCAAACAGCGACTGCGAGTATCTTCGCACCTCGCACCGAGGACATG
AACAAAGGCTTCTTGACTTTCAGCGAGGATCATCCAGGCCTTACCATGCTGAAGGACG
AGCCGGAGGATCTGACGCATCTGGCGCCAACACCTGGCGACGTGTGCGTCCCCCTCGA
GGACACGCCTTTCCTCTCGGAGATGCTCGACGAGTTTATCCTGAGCAGCGACAACTATT
GCCCCCTGCTCAGCCCGGGCGGGCCGTTGGCCCCCGAGTTGCGGTCCACGGATTTCGG
CGACCCCCTCAAGGACGCCGAGTTGGCCGACACCCCGAGAGGCAAGGATCTCGGGGA
GTCGTTGGCCGACAGCGACCCGTTCATGTACGGCGACTCGCCGAGCAGCCCTTGCAGC
ATCGATCCGAGCGCCGTCTCGCCGCAGCTCGCCAACAAGTATCGTCGAAGCCCGGAGA
GAAGCATAGACTCGTTGGGCAGCCCGACGGGCGGAAGCGGTGCCGACGGTTTGTCCG
AGGACGAGATGTTGATGCTTGGGCTGAACGACAGCATGGCCGACGACGAGCTGGAGT
TGAGGGCCCCGTACATACCGATGTCGGATCAGGACGAGGCGTTGGATCTGCTCATCAG
CAACGATATGGTGATGTGGAGCCCGCCACAGACCACGGATCAGAAGAACGGTTTGAAA
TGGATGTTGGCGGAGAAGGAGCAACGAACGTCGGATTCCAGTTTGGCGCAACTGTTG
AAAACGGATCAGGTCGTCTCGAGAAAGTACAACGACCACGGTGGGGGTTTGGTGAAT
CCGGTCCAAGTTCTCGGCCAGATCCCTAGAAAAAATATAAATCTAGATAACTGCCATTG
GTCCTCCAAAGTGGACAGACCAACGAAGCGTATTCACACTGCCTCGATAGACGTAGGG
AACGACAATAAACGTATCAAGTGCGACGAGTCGACGAAACGCAGCTGCCATCTGGAG
GATCATCTGTTGACCAAACAACAGCAACCGTCTTCGAGGAAATGCTCGTCGAACGGCA
ACAACAGTCAACTGCTGCGCCGCCTGGTGTCGCATCAAAACTCTCCGCGAAATAATAA
CAATTTCGCGAACGAGTCGTTCGAGGGGACTGCGAACGGTCGGCAGAGCGCGAGTAG
GATAGACTCGGAGCTGGACGCGGCGCAGGACGGGGAAGGAGACGGGGGCGGCGGCG

ACGGGGGCGACGGCGGCGGAAGGATGGAGGAGGGGAGGAAGGAGAGCGGAGGAAG
GTGTGGCCGGAACGGCAGCAGCGCCGCGGAGAGCGGCGCAGATACGATCGGTCAGAT
GCCGCAGCGGAATCCAGCGTGCAACAGCGTGCTAATGAATCTCCTGGTATCCGGCTGC
GAT

>novel_circ_000820
AACAACTGGACAGAATCGAGGAGGGTATGGACCAAATCAACGCCGACATGCGCGAGG
CCGAGAAAAATCTCACGGGAATGGAAAAGTGCTGCGGCCTCTGCGTTCTTCCATGCAA
CAAAGGCGCGAGCTTCAAGGAGGACGAAGGGACGTGGAAAGGAAACGACGACGGTA
AGGTGGTGAACAACCAACCCCAACGGGTGATGGACGATCGCAACGGGCTCGGCCCGC
AGGGCGGTTATATCGCCAAGATCACGAACGATGCCCGTGAGACGGAGATGGAGGAGA
ATATGGGCCAGGTGAACACGATGATCGGTAATCTGAGAAACATGGCGATCGATATGGGT
AGCGAGCTGGAGAATCAGAATCGGCAAATCGACAGGATCAATCGCAAG

>novel_circ_000822
GTTTGTATGATGCCCGGTGGCGGCACCGAAAATATGATTGGAAATAATAGGACTATGGC
GAACATTAAACAAGAGATCGAAAATCCTACGACGCCCACGCAAAATTATCAAGTTTGT
TCACCGACCACCACGCTTCAGCATCAAGAGGTGATTTGCAGTAAGATAGAAGTTCCGC
CCGATTATGGCGGAGGCGAAGGTAGCCCTGGAAGTCCGGAAATGCACCATTGTTCCTC
AACCACTCAGCCTTTGGGGACATCAGAGGAAGGTGTCAAAGAGGAAGACATGATACC
CAGAAGGCTTTGCCTAGTCTGCGGTGATGTTGCAAGTGGATTTCATTACGGGGTTGCAT
CTTGCGAGGCGTGTAAAGCTTTCTTCAAAAGAACCATACAAGCGAGTAATTTTACAGG
TAATATCGAGTACACGTGTCCCGCGAATGGGGAGTGTGAGATAAATAAACGGAGGAGG
AAAGCGTGTCAGGCATGTAGGTTTCAAAAGTGTCTTAGACAAGGCATGTTAAAGGAGG
GGGTCCGATTGGATCGTGTCCGAGGTGGGAGGCAAAAATACAGAAGGTCCACCGATCC
CTATACACCTGTGAAACCAGCTCCCCTGGAAG

>novel_circ_000824
GATACACAGCTAGAGTAGAATTTGCCTTAAAACTTGGTTATACAGAACGTTTAGTTCAA
ACTGCACTGGAAAAATTAGGTCCAGATCCAGAACAAAATGAACTTTTAGCTGAATTGA
TAAAATTGGGTGCTAGCTGTTCTCAAAAATCAGTTGATACTTCAGAAGAATCTGATAGT
GTTGTAGATTCTGATTTAGCAACTACTGAAAATAGTGGATGTAGTCTCAGATCCGTTGT
GATCGATGGAAGTAATGTGGCAATGAGCCATGGAAATAAGGAAATATTTTCTTGTAGAG
GAATCAAAATTTGTGTAGATTGGTTTAAATCAAGGGGACATAAAGAAATTACTGTATTT
GTGCCTAAATGGAGAAAGGAAACTTCTAGGATTGATAATCCAATCGCCGATCAAGAAA
TTTTAGGAGAATTAGAAAGGGATCGTTTATTGGTTTTTACTCCATCCAGATTAGTTGGTG
GCAAAAGAATGGTTTGTTATGATGATCGTTATATTTTGAAACTTGCAGCAGAAATTGAT
GGCATTGTTGTTAGCAATGATAATTATAGAGATTTAGCACAAGAAAATCCAGAATTTCG
AAAAGTTGTAGAAGAAAGAATTTTAATGTATACTTTTGTAAATGATCGTTTTATGCCACC
AGATGATCCATTGGGTAGAAGTGGTCCAACTTTAGATAATTTTTTGAGAATTTTTCCTAA
AAAGTCTGATCCAGCACCACCTTGTCCATATGCAAAAAAATGTACATATGGAAATAAAT
GTAAGTTTCGCCATCCAGAAAGAGGTCCTCATCCTCAAAAGTCAGTCACAGAAAAACT
AGTAGAACATGTACAAAAACAACTTCAAGTAAGAGATTCAAATTTAAATATTTTTTTTA
TCAATTTTTTAATACATTAACAAATGTATTTCAGCATCAACCACTTTGTAAAACCAAATC

TACAGTAACTGCTAGTACTCAGTCATTATCTCCAGTTATAAAAAGTAAATCTGCAGAAA
ATGTCACACTTGATCAACCAATAAATTCCTCAATGCAAGGATTACAAGTATCTTCTACTA
TGAGTTCTCAAG

>novel_circ_000825
TCGTCCTCGTCGTCTTCGTCGTCGCGAGAGAGTAGCTCGAGAGGCGAAGGAGGAGGA
GGAGGAGGAGGGCGTCGAAGGGGGGAAGAATGGAGGACGGGAGCCGCGAACGGCA
ACAGCCCCTGCTCCGTTCGATCAACAACGTGCACAGATCGTTCGTTAAGTTCGTGAAC
AGGACGCTGCACAACGTGGTCCTGTACTGGATCGATTACCAGGGCCAGGCGGTGAGCT
ACGGCGCCCTGTCGCCGGGCGATTGCCTCGATATCGACACGTTCGTCACCCATCCGTGG
ATCTTCGTCGACCAGGAGACCAGGGACAG

>novel_circ_000826
TCGTCCTCGTCGTCTTCGTCGTCGCGAGAGAGTAGCTCGAGAGGCGAAGGAGGAGGA
GGAGGAGGAGGGCGTCGAAGGGGGGAAGAATGGAGGACGGGAGCCGCGAACGGCA
ACAGCCCCTGCTCCGTTCGATCAACAACGTGCACAGATCGTTCGTTAAGTTCGTGAAC
AGGACGCTGCACAACGTGGTCCTGTACTGGATCGATTACCAGGGCCAGGCGGTGAGCT
ACGGCGCCCTGTCGCCGGGCGATTGCCTCGATATCGACACGTTCGTCACCCATCCGTGG
ATCTTCGTCGACCAGGAGACCAGGGACAGGTAGGCGGGGAGGCGAGAGACCCTCCAA
AAATTCCAAATTCTTCAAACGATCGTTGTCGTTGTCGGGGGTTGCCTGGTTCCTCGATT
TCTTCTTCTTAAATTGTGGGGCGAAGGGATCGTTTCGTAGGATTAATCAAACATTGTGC
CCCCCTCCCTTTTCTTCGTTCGAGGACGAAGAAAAAGAAAGTTTCGAACCCTTTGCGC
TCCAATCTCGATTCGACGCGGCGATATTTATCTATTTACGTTCGATTCTTTTGAAATAAAG
CTTCGTGTAGATTGCTAAGCCTTTTACGTGCAATCTCACGAAGCATGGAATATAATATAC
TTATCTACATTTTTGGCGTTGGGCCAAGTTTGACGGTCAGTGGGAATAGAAATATTATTT
TTATTTTCGAGTTTTATAAACATGGTAATGAAATTAATTCGAGCGAGGATAAGCTTCTTC
TTCTTTTATTGAATAACGAAATTATTTTTATTCGTTAAGAAATCAATGATAGAGAGAAGA
AAAATAAAGATGTAATATAGAATGAATAAAAGATTATAAGAATATAAACTTTAAAATTCG
ATAAATTTTCTTTCATTACGCGCTTTAAAAATACACAAAATATTAGAATACAAAAGTTTC
GTACGTTGATTCGCGCGTGAAATCATTTTAAGAGCGAACGGGTGGGGGAAAGTCACGT
GGTGTTGTTGGTTCGGTCGAGCGAGAAAGGCCTGGAGCGCAAAGGTTTAACGTATTCG
CGGAGAATCGTTGGTAAAAATATCGGTTTACATTCTCTCTCTCTCTCCTTCTCTCCCAAT
CCTGCTCGCTTAAATATCAAAGTGACCCTTTGTACTCGAGAAGTGGCTCTCTGCCAACT
CGAACAATCTTTGCCATTGGTCCCAGTTTATAAGCGGCGATAGAAATATTATTTTCGATA
TTTTTCAGTTTCGTAAGCGTAGAACATGGCGATGATTGGTGTTCGAGCGATAGAGGGAA
GGAAAGCGTGAAGAAAAAGACGAGAAATAAGAAATAATTGAACAATGAATGAGAGAG
CATGTTGTGTTGACGTTTTACGTTAAACGCATTGCGTACGGCACTCTGTTCGCTTTCGA
GCGCTCTAAAAAAAGTATACCATGCGTATATGCACTTTTTAATACCCACGTTTATAAAGT
GTTCTTAAATATTATTATATATATTTTTTATACATTTATTTATCACGTAAGCTTTTTTACATT
TTAACTATTATTTATTATACAAATAAAATTGAACTGATCGATTATTATTTAGTTTACAAACT
CGAATAACGAGTGCGATATCTTTTGCTCTTTTTTCCATTCGACGAACAGACTTTACATTT
TATCGCTTCGAGTGATTTTAATTTCTCGACTCGTCGATCGATTTTAAAAAACGTTAAAAC
GAGATATCACGTGACTCGTACGCAATACGTTAAAAAGCATTATTATAAGGTTAATGATTG
ATCGAGGACGCTTCATATAATAAAATATCACGCGAAACAACGATATATACATAAAGTTGA

ACAATATTCGACGAATTTTTCATCTCTGTAACGTAAAGATACGCACGTCTCGAGGGCAA
AGGGATTGATTTGTCCTTCTACCGATTTTCGAAAACTAACATTTACAATAAATAAAACAT
AACACACACACACACACACACACATACACATATATAAAACCGATATTTTCACGCGATTC
GAATCAAAATCTCCCCCTCCCCGACTCGTTGTTCGTCCGATCGAATCGAATCGAATCGA
ATGGAATGGAATAGAGAACGAAAAGAGGGTTGGACGCCGGTTTTTCCAGGTACACGG
TGAACCAAAAGGACGTGTTCTTCCCGGAGCCTTGGTTCGCCAGGTATCGCGATCTGCA
GCGAAGCGAGCTGCCCCAAAGGCACGAGAGGACCAGCGTGTACATCACGCTGCCCGT
CTACACTCTGCGCGAGCTGTCTCTCAGAGCCATCAAGCGACGCCTGACCCACGATCGA
CAGGCGTTCCAGCTCGATATACCGAGGAGCCTTCAGTACGAGCTCGCTACCATGCTTCC
ACGGAACGAGGACGAGGAGGAGCAGGAGCAGGAGGGGGAGCAGGAGGAG

>novel_circ_000827
ATTCACGGGAAACGCAGATGGAAATTGGAAGGGTATCGAGGTTGGCCGGCGTCGTTCT
GGCCCTTATCTCGATCACGACCACGTGGTCCCGGCATGGTTGCTGCGTCGCGGAACTTT
ACACCGCGTTAGCGGACATGGAGGAGCTGCTGGAGACGGAGGCGGTGCTCATCGACA
CTCTGAACGGGTACATCAAGGCGCAGGAGGAACGTCTCGCCACTCTGAGAAA

>novel_circ_000828
GTGCACCCGGTGGTCCAAGAGTACAGCCTGATGAAGAAGAACGGCACGTTCCGGAAC
CCGACCGACGAGGATCTGAGAGATTGCGGCCTCATAGTGACCACCCTGGCAACGTCCA
GCTGCCTCACCAGCTTGAACCTGTCGTTCACCCACATAGTGATCGACGAGGCCGCCCA
GGCGTTGGAGTGCGAGGTTCTCATACCGTTGTCCCTCGTCACCCCCCAGACCCGCCTC
GTCCTAGCCGGCGACCAGATGCAACTTGCGCCCGAGATATACAGCGACCTGGCCAGCG
AGCGGGGCCTAGGCATAAGCCTGTTGGAGAGGATCTACGGGATGTACCCCCAGACCCA
CCCGTGCAGGATCCACCTGCATCAGAATTATCGGGCCCACGAGGACATCATCAGATTCA
CCTCGGAGATGTTCTACGAGGGGGTCGTGAAGCCTGCCAACGACATGTTGATACAACA
CCCGGTGTTGAAACCGTTGACGTTCTACGCCGTTCAAGGCGTGGAAGTGCAG

>novel_circ_000830
GTCGCACTCGCACTCGGAGAACGACAAGAGGCGGCCGGGGAGGAGTAGGGCGGCGT
TGAAGTCGGCCAGCGAGCTTTTGTCTAACGATATGGTGTATTGCGGGTTGGCGAGGGAT
AATCACGGGGACGAGAACGAGGAGGATGAGAGAGAGGGGGAGGAGGAAGGAGAAG
GGGAGGAAGATCGGGAGTCGAACGAGGTGTTCGTAGGCGTGGACGATGCCAGGTATT
ACAATCTCTCGTCCAGTATACCGGGAACGGGTCGAAGAAGACACTCGATCAGCACTTT
CTTGGGCAAGGAGAGAGCAAGTTCTTGTTCGAGCAAGCTCGACAACGTGTCGACGAG
GAAACAGCAGAACTCGGCCGGTTTGGCGGAGCCTGACGCGATGGCGCCACCTGTGCC
GACGGTCGACAGCGTGGACGGGGAGGAGCGGAGGCCGAGGTCCTGCAGCAGATCCA
GGCGCAGGAGGCATCGAAGCGCTTCGAGCAAAG

>novel_circ_000831
GAAGGAAAATGTCTTATTGAAGCGCTTGATGCTATTCTTCCACCTACTAGACCTACAGA
CAAGGCTCTCCGTCTTCCTCTTCAGGACGTATATAAAATCGGTGGTATTGGAACAGTAC
CAGTTGGTCGTGTCGAAACTGGTGTGTTGAAACCAGGTATGGTTGTCACATTCGCTCCT
GCTGGTTTGACTACTGAAGTCAAATCTGTTGAAATGCATCACGAAGCTTTGCAAGAGG

>novel_circ_000833
GTACCACCGCGCTACACACTACTTCGGTGGAAGTTAAATCGTGATCGCTTTGGCGAGA
GGGCGAAGGACAAAGAGGAGCATTGAGTGCTTCGCCGTAGAGCTCGTCGAAGAAGGA
GTACAAGCAAGCACGTGACATGAGTACCGTGACCCGAGACACTTGTTGCACGACTGCT
GTCCCTTAAAGAGTCTCGTATACACGGTGTGTTCCTTCAGTGCGACGAGATTAGCCAAC
GTTCGAAATAAAACGCGAGAGAAAGAAGAGAAAAGGAAGAGATAGATACATGCGCGT
GTCGTGAATTCTGTTCAATCTCGCGTTTTCTCGATACGTACTCGTGGCATGTCGGTGATA
TATCGTTTTAATCAGCCTCGTAAGAAACCGCGTTTCGTGCTCGAGGATCATCCGTTGAC
GAGCGAATCGCGAGTGGTAATCGTGTGATGCCGTTAAGACGAATTTCTTTTGTTTCTCG
ACTGTTGAGTGCACGCGTGTTTCGCGGCTCTGTCTAAGATCGGTGAATCGCGGTTTCGC
GAGTTTGATCGCGCGAAAGGGAGGAGGTCGATCGTAGGAGGAAGAAAGAAAGAACAT
GAACGTGATCGTGCGCTCAGGCCGGTGACACCGGTCGAGATTGTGTGTGCGTGTGTCT
ATCTTCGCTGATAACTCCCAGTTGGTGGACTCTCACTGAGACGACGGATATCTTTGTTC
GGTGGATGTCGTCGTCGGGGTTGTAGAAGGGTGGTGTATTCGGCGAAGGCAGGAAGC
AGCGAAGAACCGGAAGGCTGGATCACCTCCGTGGAATGTCTTCGGGCGCCAAGCGGC
TGGTGCAGTCGCTTCGGGGCCGCAGCGGTGGCGGGAGGGGCGGAGGCGGCGGCGGG
CGGGGGGAGGGCGGGGGCGGGGAGGGAAGCGGGGGCGGCGGAGGTGGCGCGAGCA
CAAGCGCGACCAGATTGTGCCACTACGACAGCGGTCACGAGCTCGACGAGATCTCGG
TGGTTGGCGCCGCCATCAGGGGCAGCGAGGGCCTTCCCACCCCCACCACACCCTGCC
ACTGTGGCGGCCTCAAGTACGGGAGCGGGCAAACGTTGTACAGCATAGCCGGTCTGGT
GCCCCCTGCGCCCGCCGCGCCTGCGCACGGAGCACAATCAG

>novel_circ_000834
GTTTGCATCTCGTACAAGGATGAAAGTCGGTAGAGCGTTTAGTTCTAACAAAACTCCA
AGCAAATTAAAGAGAGCAATGTCAACAATGATGTCACCGTTTGGATCAACCAATAGTC
TTACCCCGGCAAGTCAACAACTTTCACAGATGCGACTCGCTAGTTGTAACAACATTAA
CGAGCTGGGTAATGGTGGTTCAGGCTCGCCATCTAGAGATGATGTTCTAGTAGCACCTA
TGTCGGTTCAACCGACACGAAAGGCCAAATGCAGTTCCCTCAGTATGGCTTCATTAAG
AAG

>novel_circ_000835
GTGAGGAAGGTGTGCGGCCGCTCGGAGCTGGAGCCATCGCCGTCGAGCGAGAGGAGC
GTCGCGGACGCAGCCGGTGGAACGGGGAAATCGTCGTCCTCGTCCTCGTCGTCGGGC
GGGATCGAGATGCACGCAGCTGCCGCTGCGCCGCCCAGCCGGACCTTGCCGTCCCAGT
TGCACGTGCAGCTCGGCAATTTCCTGGCAAGCGTGGTCAGGTCTCGCACCTTCTACGG
CACCCTGGCCGACACGATCTGCGAGGAGTATCCGGACAAACGGTGCTGGAACGGCGA
ACGAGTCGGGGA

>novel_circ_000836
GTAACGCAGAGGACAAAACGAAACGCGACAGCAGATGCGGAACTCGTTCGAAACGC
GCCGATTGGTGGAGAGGTGGATAAATAGACGTTGGCGACGATCACGTTTCAGGTGAGG
AAGGTGTGCGGCCGCTCGGAGCTGGAGCCATCGCCGTCGAGCGAGAGGAGCGTCGCG
GACGCAGCCGGTGGAACGGGGAAATCGTCGTCCTCGTCCTCGTCGTCGGGCGGGATC

GAGATGCACGCAGCTGCCGCTGCGCCGCCCAGCCGGACCTTGCCGTCCCAGTTGCAC
GTGCAGCTCGGCAATTTCCTGGCAAGCGTGGTCAGGTCTCGCACCTTCTACGGCACCC
TGGCCGACACGATCTGCGAGGAGTATCCGGACAAACGGTGCTGGAACGGCGAACGAG
TCGGGGA

>novel_circ_000837
AAACGGTGACGATGCTCGCGAGACAGTCGGAGAACAAAACTTTAACACTGTTCGATC
AAGTGTATCGGACGATGGCGGTATCGAGCAGGCCCTCGATAAAAGCCCTGTACCAGGC
GATGGTCGATTACGTGTCACCGAGCAACACGCCGGACAGTTTGCAGCAACCGTTGACG
CGGGACATGCTTCAAGAAAGGTTCATCGAGTTCTTCGCCAAATTGTTCCCGATCGCGTA
TCACGGCGCCGTGAATCCCCGCCAATACGAGCAAGATTTCACGGAGAAGTTCAAGACG
TGCCTGTACGAGACGATGGAACAGATCCAACCGTTCGGCGACATACCGGTCCAGGTCG
CGAAATCGGTCTCGAAAAGCCTGGAGGCGACCCGGGTCCTGGTCCAGGCGTTGACAC
TCGGCAAAACGGTGCTGGACAGGACCGACAGCGTGCTGTTCTCCGGCACAAGCCCCC
AGCAGGAGGCCTGCTACGCCGCCCTCCTCAGGATGACCTATTGTCCAAGGTGCAAGGG
CATCGGTCCCTCCGTCAGGCCCTGCAGTGGATTCTGCACCAATGTCATGAGAGGTTGC
CTGACGCAGCCAGCATCCGAGCTGGACCTGGCTTGGTCCGGTTACGTGGAAACAGTG
GAGAGGCTCGTGGTGGCGGTCGACGGTCGTAACGATCCGTTGGGCCTGAATGCCGAG
AGGGCTGTCAGACAATTGGACACCCGCATTTCGGACGCCATTATGCACGCAATGACGG
ACGGACCGGCGCTCGAGGAAAAG

>novel_circ_000838
GTATCCTCACCCTGAAATATCCTATCGAGCACGGTATCATCACCAACTGGGACGACATG
GAGAAGATCTGGCACCACACCTTCTACAACGAGCTCCGCGTTGCCCCCGAGGAGCAC
CCCGTCCTACTCACCGAGGCCCCATTGAATCCGAAAGCTAACCGCGAAAAGATGACCC
AGATCATGTTCGAAACCTTCAACAGTCCGGCCATGTACGTGGCCATCCAGGCCGTGCT
GTCCCTGTACGCTTCAGGTCGTACCACCGGCATCGTCCTGGACTCCGGGGACGGCGTC
TCCCACACCGTGCCCATCTACGAAGGTTACGCCCTTCCTCACGCCATCCTCCGTCTCGA
CCTGGCCGGCCGCGACTTGACCGACTACCTCATGAAGATCCTCACCGAGAGAGGCTAC
AGCTTCACCACCACGGCCGAGCGAGAAATCGTGCGCGACATCAAAGAGAAACTATGC
TACGTCGCCCTGGACTTCGAGCAGGAGATGGCCACCGCGGCCGCCAGCACCTCCCTCG
AGAAGAGCTACGAGCTGCCCGACGGACAGGTCATCACCATCGGTAACGAGAGGTTCC
GCTGCCCCGAGGCTCTGTTCCAACCGTCCTTCCTGGGCATGGAGTCGTGCGGCATCCA
CGAGACCGTGTACAACTCCATCATGAAGTGCGACGTCGACATCCGCAAGGATCTGTAC
GCCAACAACGTGCTGTCCGGCGGCACCACCATGTATCCGGGTATAGCCGATCGTATGCA
GAAGGAGATCACCGCCCTGGCCCCGTCCACCATCAAGATCAAGATCATCGCTCCCCCG
GAGAGGAAGTACTCGGTGTGGATCGGTGGATCCATCCTGGCTTCCCTGTCCACGTTCC
AACAGATGTGGATCTCGAAACAGGAGTACGATGAGTCCGGCCCAGGCATCGTTCACCG
TAAATGCTTCTAAACGTATCGGCGATACAGCAAAAACGTCGCATAAATATCGAAGAATT
ATCAACCGACTTGGGGGAAACCGAATATACGAGCTGGCATCGGTTTCCTTTGAGAAATT
ATCATGCTGCGATAAAGCAACTGCGTTAATGAAATATCACATTAATACACGGATCATCGT
TAGATCTCGTCGAATATTCATCGTTGTGGTCATCCGTCGCTAGATACGACAACCCGACA
CACTGTTGACTTCTTGATGTCATTTGGACGGAGTCGTACTTCATCTATCGCTTCATTTGG
ACGAGGAAGCTGGCAAAGGTGTTGGTTCTCGATACAGAGCTCGCCTCGCGAGTGGCC

AATCCTTGTTCGAAACAAAATTCGTAAAACGATGGTAGAATCGGTTCATAACCTCACTT
ACCAAATGCATCTTTCGACCATGAAAGGGACATTCTGGACGCGCAAAGGAAGAAAAC
GGAATTTTCAGGAGGAATTGGACCAACTTCGTGGGAAGCTGCGCTTTTTTCACTGTTC
ACGCGAGATTCCTGCACTTCTAACCTCAAATATGCGTTAAATCTATTCTTAGTTCTACCT
AATCGAATTCACCATCGATATTAATTTATTTTGTAAATATTATAAAGTATATATATTGTTATA
TACGATATATCAAAAAGTGAAATGAAATATACATTTTCTCATACCATTATTTATATCCTTTA
GTTCCGACGCGTTGGTACATAACCGACGTCTTTCATTCCCTCTGTTCCACCGTTTCTTGT
TCAAGATTATATCGTCACGTTCGATTCCATCTCTCTCGTTATATTCTTAAGCGTATCGGTG
AAATATCTGTAACGTAAATCGTTGCATAAAAATATATACATACTTGGGATATTTAGAAATT
GAACGTTGAGAAAAATCACGTATAGATTTCGTTGTTCTTTTGCACCATGTCGAATCAAG
TATTTCGCGTCATCGTTATTGAATTTTTACTTACACTTATAAATAAGTTTTAGAAGATAAG
CGATCAAGAACCGAAATATCTTTCGAGAGCAAGAAATAACACGACTCTCGTATTCATTA
GAATTCTATATATAATTAATTATTATCAAATATTTTTATTCCCTATATATTTTACGCATACTGT
TTACGTAAGAAAAAATATTGCTGCTTTCTTCGCGATATTCAAGACCGAATATGTTTGTGA
GAGCGGAGAAAAGGTGGCATTCAAGTAGTTTTAAAATAACTATTATTATGATGTGGAAC
AAACCGTGGATGACCTTGGCCTATTGTGACTAATCTACTCTTGAACAGTTCCAATATATA
TATATATATATATTATTATATATATATTTTTCCAACTTTATGGATATACTTTTTAGAAAAAATCA
AGCTTCATTTTTTTTTTGGGAGAGGGAAGAATTCGATCGATTTTTAATCTTAAAAATTCG
AATCTTGAAAATCGAGAATTTTCGCCGAAGAAGAATCTCTACATTCCTCGAAAGCTTTT
TCCGTCGATAAACGTATAGTTGACCGATAAAAGAGCTACAGATTTATTTTAGTCCAGATG
CGTAATCTCAAAATATTCCTCCTACCTCTGATATCTGGGGGAAAGAAGAGAACAAGTAT
CGCATTGATGGATTAAATGAACGTCGAAGAAAATTTTTATAGCGATAGAAAAGAAAATC
TTAGATCAAAGATTACGAGGCTTTCGAAATAAGATAATATTGATAAGTATGATAAATCTA
CAAAAAATTGTAATTCATAGACATAAAATATAAGCAAAAGTTTACAGAAATATAAATAA
CGAGATAAAGCAATAATCATCGAAAAAATAACAAATCGCTTGCAAAGATAATTTTTTAA
TTAAAGAATTCAAATTTATGGATAAAAAATAATAAATGCTTAAATGTAGAATACAAATGA
AATTTATGATAAAATAGTTTATGTCATATCGAAAATTTTTGATACGTTCACTGTATTCTAA
ATTGGGATCACAATTTTGCTAAAATTACTTAATGTAATTAAGAATGAATTACATCTTATTT
TTATCCATTGAAAAGACATTTATCACGAGAACAGGGAATTTCGAAATATTATCAGAATTT
GAAGAATATATCCAAGCTCACTGAAAATTTACTCAGAAATCTATTTGCACAGAAGATAA
ATCAGTACTGTCGCTTTCCATTCCCATCATTTTCCACGCAAACCTCTTACGTTATATACG
ACACTTTGGTCTCAAACAAAACGAGAGAAAATCCACTTCCGTAACGAAACAAGATCA
AAACTCGCTTCGCAGTATCGGTAGTTTCGAGAACTAACCTAACATATACTCTCAACTTA
ACTATCAAACAGAATGTGTTAAAGCAAAAGGGGCAATTAAGAGCAACACGAATTCTTC
GATCTCGCGAATAATTCGATCTACGATCACTGATTTATCATCTCTTTCCTAAATAGGAGA
GGAGAGAACGGTGAACTAAACTTTTACAGAAACGAAAACTCGCGAAAAGGCTACCCT
CTGGTGACGAAAGGAATAATTAAGAAAAGAAAGAAATTTAAATGACGGAAGAAGTAC
CTACAAGAAGGAACGAAGTTATCGAAGATCTTCAGAACGGCGCATTTTCCTCGGTCCG
CTTAGGGCCTGGGGCCGCCTAGTTGGGCGAGCGTCGCGACGCCACGCGAGGCAAGCC
TTTTTCGTGCCTGTCGGGCAACCACCATCCCACGAGAATGCGGGGGCGTCGTGACGAG
AGCATATATTCGACGTCGGGACGCTCCGGAGGCACAGACGCCACTCCAACTCTGTCCC
GTGCACGAGCATTGAAATCGAAGAAGAAAGAAAATGTGTGACGACGAAGTAGCAGCC
CTTGTTGTCGACAATGGATCCGGTATGTGCAAGGCCGGTTTCGCCGGAGACGACGCTC
CCCGCGCCGTCTTCCCATCGATCGTCGGTCGGCCCAGACACCAGGGTGTGATGGTCGG

TATGGGTCAAAAGGACAGTTACGTCGGCGACGAGGCTCAGAGCAAGAGAGGTATCCT
CACCTTGAAATATCCTATCGAGCACG

>novel_circ_000839
AAAAAAAAAAAAAACACACCGTTCACTGCCGTACCGCTTCGTCTAGTTGCGTTTCCGT
TAGTTCGATTTCTATCGGCTTTGTTCTCTACGTGGCGGTGAGATTTTTCCGACGAACAT
TGTCTGAATATATACGTCATCTTTTGTATATAAGTTTACATCTACGGAATAAATCAGTCTT
ATATTCTTACAACTTA

>novel_circ_000840
GCAAGATTTTGGGTACAAGTTATACAAGAATTAAGAAAAGGTGTGAAGCTAAAAAAGG
TAGAAGCCAATTTGGGTTCAAAAAGCACAACCCGTGGGCGTGGAAAGGGAGCAGAAT
TTGAATTAACACCTTATGAAATTTTGATGGATGATATTCGGAAAAGAAGATATCATCTGA
GGAAGACTCCATCACCAACTCCGAATTTGAAGAAAGATGCTCATGCTGTTATTCTTGAA
TTTATTCGGAGTAGACCACCTTTAAAGAAGGCATCAGAAAGACAGCTTCCACCCCTTC
AAAAGGAATGGACTCTTCGAGAATTACTTATGGAAAGTATAAAGAAACCACCAAATTT
AAGGTCTTCACGCAATAGATACATTCCTAAGATTGAAAGAACATCTCCTCAAAATG

>novel_circ_000841
TGGACTAAATTTCCAAGATTTGATGGCGAGGCAGGGTGCTATTGATTCCCCACCAAAA
ACTCCTTTCATTATGGGATCCGAGTGCGCAGGTGATATCGAGCAAGTCGGCGAGGGCG
TGGAGAACTTTAAAGTGGGCGATCGAGTGGTCGCGCTTCCTGATCACAAGGCGTGGGC
AGAGCTTGTCGCTGTACCAGCCACGTCCGTTTTCGCGCTACCAGCCGGAATGAGCTAC
CTCGATGCAGCGGCGATCACGATGAACTATACTGTTGCTTACATTCTGTTGTTCGAACT
GGCTAATTTAACGCCCGGAAAGAATCTGTTACTCCACAGCGCCGGCGGGGGTGTG

>novel_circ_000842
GAGGGACATCGACGAGGGCATCGCCGGTGAACATCGTTGCGACGGCTACCGTGGTGA
CGGACGAGGCGTTACTCGGCGACGACGAGGTGGACAACTGCGAGGGGGAGAGCGAG
GGTACGGAGAAATCGACCCAGGAGGAGGACGAGGAGGTGGACGTGGACGTGGAGGA
GTGCAGCAGCGTGGACGAGGGGCCGATCCACGAGTCGGTTTCGGCCAGCGCTGCGGC
GGCGGCGGCGGCGGCGGCCGCCAAGTGCCAACAGGACGGCAACTCGGACGAGAGGA
GGACGGCGCATCGGTTGAGGACCAGTTGCAACTCGGACGAGCTTCGGGACGTCGAGT
GCCACCTCGAGACCAAGGAGCTGTGGGACAAGTTCAACGATCTCGGCACCGAGATGA
TCATCACGAAAACCGGCAG

>novel_circ_000843
ATCGTTCTCAACTCGATGCACAGATATCAACCGAGGATACATTTAGTACGGTGTCGACA
AACGGACGACAACAATCTTCGCATAACCGAGCTTCAGAAGGAGGAGCACAAGACGTT
CGTATTCCCCGAGGCGATTTTCACCGCTGTGACTGCCTACCAGAATCAATTGATAACCA
AGTTGAAGATCGACAGTAACCCGTTCGCCAAAGGTTTTCGAGACTCGTCCAGGCTGAC
GGATTTCGATCG

>novel_circ_000844

ACGTACACCGGTTCGATTCTAGTCGCTGTGAATCCATATCAAATATTGCCTATTTATACCG
CGGAACAAATCAAATTGTACAAGGATCGTAAAATCGGGGAGCTGCCTCCGCATATATTC
GCAATTGGAGATAATAGTTATGCACATATGAATCGATACGGCCAAGATCAATGTATAGTG
ATTAGAAATGGTTTGTCAAATCCACGAATGTTTGTGTCTTAGCGGCGAAAGCGGTGCC
GGCAAAACGGAAAGTACGAAACTAATTTTGCAATATTTGGCAGCGATTAGCGGCAAGC
ATTCTTGGATCGAGCAACAAATTTTGGAAGCTAATCCCATCCTAGAAG

>novel_circ_000845
GACATGTCCGATGTGTGCGTGGGTACCAGCGTGGGGACCATCACCGAGCCGGAATGCT
TGGGGCCTTGCGAGCCCGGGACCTCGGTCACTTTGGAGGGGATCGTTTGGCACGAGA
CCGAAGGAGGTGTGCTGGTGGTGAACGTGACATGGCGCGGTAAGACATACGTGGGCA
CGCTGCTCGACTGCACCCGCCACGATTGGGCGCCGCCGAGGTTCTGCGACTCGCCGAC
CAGCGACCTCGACGCGCGCACGCCCAAGGGGCGGGGGAAGCGCGGCAGAGCCGCCG
CCAACGCCACCCCCGGCAACGATCTGAGCAACTTCACCGAGACGAGGAGCTCGGTGC
ACAGCAAGCTGCGCAACGGCGGGGCCAAGGGCCGGCGGGGCGCGCAGGGCTCCCCG
GCGGCGAGCCCGAGCCCGGCCGCGTTCGTCCCCCCGCGGCCGGACCCCGCCGCCAAG
CGCAAGTCGCGCGGCCCCGAGGAGGAGGACAAGAACAAGAGGAGGGCGCCCCCGCC
CACGCCCCCGAGCGGCTCGCCCCCCGCCAGCCCGGTGCTGCTCGAGTGCCCGGAGCC
CAACTGCAACAAAAAGTACAAACACATCAACGGGCTCAAGTATCACCAGAGCCACGC
CCACGGGTCCGCGGACGACGACGACACGAAGGAGGGGGTCACGAGCATGTCCGAGA
ACGACGAGAGCAACATCGAGGCGCCCAGCCCGGCGACGCCCGTCAAGTCGCCGGCCG
ACAAGCCCGAGGGGACGCCGCTTCGCGCGGCGAGCCCCCCTCCGACCGCCCTGCCCC
CGGAGACCCCGCCTCCCCCGCCGCGCGTCGCCTCGCCGAGCATAGCCGAGCCACCCGC
GTCTGTGCTCGCCGCGGAGACAACGGCCACCGCCGCCAAGGGCGGCGGCGGCGGCAT
CGTGAAACCGGGTGTGCTCAGATTCGGCCAGGAGTCCGACTTGCCCGCCCCCCCATCC
GCCGCTATGTCGAGTTCCAACAACGCGAGGCAACAGTCGCAGCAACAGCAACAGCAA
CAACAGCAGCAGCAGCAGCAACAACAGTCGAGTCAGTCGAACTCGCCTCAAAGCCCC
GGGCCACGAGCCAGCCAATCGCCGAGATCCTCCGTGTCCTCGCCGCATCCGGCAGCCG
CCAACATTCCGCCTCCGTTGAATATTCCTCAACCGCCTCAACCGTCCCAATTGTCGTCC
CACCATCAGCAACAAACCTCCATCCTGCAGGCGAGCCAGCAAGCGTTGCAAGCCGGC
CAGACCGTCGCCCCTCCGCCCCCGCCTCCACCGCAGCAGCAGCTCCCACCCGGCCAG
CAAATGCAACAGCAACAACAACAAGCGTCCATCCAACAGCAGCAGCAACAACAGCA
GCAGCAACAGTTGGCGGCCGCCCACCAATTGTCAGTGCAGCACACGTTATCCGCGCAG
ATAGGCCAGCCGTCTCAGGCCCACGTGGTGCAACAAGCCGCCGGATTGCAGCAGCAA
CAGCAACAGCAACAGCAGCAACAGCAGCAGCAGCAGGCGCAAGCGACGGGGCTGCA
GAGCATAGCGAGCCAACACCCGGCCCTGCAGGGCCTGGCCAACCAGATATCGCAGCA
GGCGGGCGGCCTCTCGCAGCCAGGCCACCCAGGCCAAGGCGTGCAGGCGCACTTGCA
GCCGTATCAGAGCGTGTCGCTGCACGCGAAGATGCCGCAGTTCAAGGTTAAGCCGACC
GCCGCCCTGATGCCCGACCAGGACAAGAGCAAGGATCCGCGCGCCGCGTCCAAGGCG
CAGGGGTACAAGAAGAAGTCGAGGAAGTCGCCGGGCGGCAGCCCGCACCCGTCGCC
GCTCGAGGCGCCGATCGTGGACGCGACGCGGGACGACGTGCAGAGCCCCGCCTACTC
GGACATCTCGGACGACGCGGCGCCGGTGCTCGAGGCGGAGGCCGCGGACAAGTCGA
AGCAGGGCCAGCAGGACAAGGACGGGAAGGTGGCCGCGAGCGCCCACCTCCCCTCC
CACTTCAACATGTACCCGTACTACGGCCAGCCCCCCTACCTGGTCCCGAGCGTGGCCG

AGAGCAAGCCGATCGACCAGAAGCCGAGCGAGCTGGTCGCGCCGAAGCAGGAGATG
AAGCCGCTCAACATACCGCAGATGCCGACGCTGGCCAGCCTGCAGAGCGTGGTCGCC
GAGAAGGAGAAGAAGGAGCTGAGCCAGTCGGTCCCCCAGCCGCAACAGCAGCCCCA
CTACTACGGGGGTTACGGGGGTTACATGCCGCCCGGCTACCCGTACCAACCGCCGCAA
CCCGTCTCGCAACAGCAGCAGCAGCAGCAGCAGCAACAGCAACAGCAGCAGCAACA
GCAGCAGCAGCAGCAGGAGCAGGAGATGCACGAGCAGAAGGCGAAGATAAAGCAGG
AGCCGCAGTCGCAGCAACCGCAGCAGCAGCAACAGGCAAGCCTGAAGGAGAAGCAG
CACGAGAATCATCAGATATTGAAGGAGAGTATAGAGATGAAGAGCCAGATGAGCCCGT
ACCACGTGTATCACAGCGGCCAGAGTCAGAGGTCGGCGCCGCCGCCGCCGCCTCCTG
GCCCGTTGCAGGACGACCGGAGGTATTACCTGTACCCGGGTGACCAGAGGCGAAAGG
AGGAGGTGAGCAAGCAGAGCCAACAGGCGAAGCCGCCGCCCCCGAGTCCGAAGCAT
CAACCGAAGCAGGAGAAGCTTCAAGACTTGGGAAAGAACGAGGACGCGAAGAGCAA
GCAGGAGGGCGTGAAGCCGACGATGGAAACGCAGGGACCACCGCCGCCCCCCACCTC
GCAGTACGCGTACATCCATCCGGGCTACATGCAGCCGCAACACTACGGCGCGTTGCCG
TTCGATCCGGGTCACCCGGTGTATCGAGGCCTCAGCCCAATGCTGGTCCCAGGCCCTTA
TTCCGGTAACCCGTATCTACACCAATTGCCGAGGTATCACGCGCCCGAGGATCTGAGCA
GACCGCCCGCGGGCAAAGCTCTCGACCTGCTCCAGCACCATGCCAATCAGTATTATTC
GGCGCACAAGATACACGAGCTTCAGGAGCGTGCGCTCAAATCGCCCACCCCGAAAAC
GTCCGCGGCCTCGGCCAGCCCATCGTCCGCTGGTCCGACACCGTCACGCCCCCCGAGC
GGACCGCCCAGTTCCGTGGCCGGCCCCGGCCCGCCCCAATCCCAGAGTCAACAATCCC
AGGCGAAGCAGCAAGTTCAGTCGGGAGGACAGCAATCGCAACAGCAGCAACCGACG
CCCGTCGACGCCGGCACCGGGACCTTGACCAAGGATAACAGATCACCCCCGCCCCAA
CGACACGTGCACACGCATCATCACACCCACGTTGGCCTAGGTTATCCGTTATTGACCGG
ACAATATCCCGCCCCGTACGGAG

>novel_circ_000846
ATCGAAATTTTGGCAAACGATGATGGAAGTACGAACACCGTTTTAGCGGCGAGTTGGA
AAGAGAAATCACGTTCGAGATTCACGTTCCGTAAAAGGATCAAAGGAAGGAGAGCCT
AATCGAGAGCGAAAACTGTTTTTGCGAGGAAAACAACGTGGAAATATATAAGGGAAG
AGCGATCGTTTAACGCGAAATGCGAGATAAAGGGACGTAAGAGCGCCGGTCGACGAC
GACGACGACAACGACGAGCAACGGACAAGAAGAGACGATACGTCGGATAAGGGCGA
CGCAAATGCACGCTGCCGAGAACCCGTGTTGTTAAAAAAAAAATTAAAAAAAAAAA
AAATAAAAAAAAATAAAAAAAAGAAGAAAAATACGGTGGAGAAGTAAAAGAAGGAAA
AGGAAGAAGCGGAAAGGATTCGAAAGCCGAGGAAAAATTTCGACTAACATCTGTCGA
AGAAAGAAAGAAAGAGAGAGAGAGCGAGAGAGCGAGATGTGTTTGCCGCGTTGAAA
TGAGAAGGAGAGTAGAGAATATCGATCGAAAGCATCGAGCGCGGTAGGCTAGATAGAT
AAGGAGAGAGCGAGCGAGAGCGAGAGCGAAAGAGCGAGAGAGCGAGAGAGAGAGC
GCGCGCGCTAGAGCGAGAGCGAGAGAGAGAGAAAGCGGTGAAAGAAGAAGAAGAA
GAAGAAGAAGAAGAAGAAGAAGAAGAAGAGGACGCGAGGAAGAAGAAGAGGAAA
AGAAAGAGAAGGAAAGAGATCGTTTCGAGAGAGAGAGATAAAACGAGAGAGATATAT
CGGCGAGCTTTCTTCCTCCGAGGCCGAGGTATAAGTGATGTGTTCGACGAACGTGGCG
CGTCTCGCGGGTGGTGGCCGGGGTACAACTGACGCGTGTCGCGCGTGGTGCGATTGCT
GCTTGGGATCGGTGAGACCAGGAGGGCAGTTGGTGATCAGGCTGGCGGGCACCGGGG
AAACGTTTGAAAGGGCATGGCGACCACGCCACTAGGCGGTCGCCTCCCTAACGTAAAC

CGCAAAGGGCCATCTCCGTTCGCCGGTGGTGGGAATAGCGTGAATTACGGTAACGGTA
ACGGCAACGCCGCTTCTGGCAATAATAATCACGCTGTCAAGAACGGCTCGTATCATCGC
AACGCCAACAACACCGGCGGTAACAATAACAACCACAACCACCACAACAACAACAAC
AACAACAACGGGAATTATCTCGATCATCGTTGCCACGGCGGTGAGAAAACCGGCGACA
ACCACGTGGAAAACCACGGTGGGAAAACCGGTTGTACGATCGAGTGCGGTAACAACA
ACAACGGGTACCGCAACGGTGGCAACGGCGTTACCAGCATCGGTGGCGGCAAACACA
ACGGCGGACGTATCGTTGGTAGCAACGTCGGAGGAGGGGGGGAGTCGGAGGAGGA
GGAGGAGGAGGAGGAGGAGGAGGAGGACGAGGAGGAGGACAACAACAACGTTCGT
CGAGGAAAGAGGAACGCGGGTTAACGGGAAAGGATCAACACCGGCGTCATCGTCATC
ATCATCATTACCATCGTTATCATCACCGTAACAATAATAGTAACAACAACAGCAGTAGTG
ATCATCAGCAACAACAACAGAATCAGAATCAGAATCAGAATTCTCATCATCAAGGTCG
AGAAAGATCATCGTCATCGTCGTCGTCGTCGTCGTCGTCGTCGTCATCCTCGTCGTCGT
CGTCGTCGTCGTCGTCGTCGTCCTCGTCGTCGTCGTCCTCGTCGTCGTCCTCGTCGTCG
TCGTCGTCGTCATCGGGAAACCAGCGCGAGTTTAAAACAAGCACGGTGGCTGCAAAC
ACCGATGAGCTGGATCCGGCCGCAACGAACGCGACCAATAACTTCGAATACGACGACA
ACGAATGGGACATTGGGATAGGAGATTTGATAATCGACCTTGATGCGGACATCGAGAA
GACGAGGGACGAAAAATTGAGCTCGGGGACAGGCATGGCTTCAACGCCTGCCTCGGC
AGCGACAGCATCGAATACCACATCGAATCATCATCCGCATCATCATCATCCGCATCCTCA
TCACCATCATCATCACCATCATCATCATCATCATCATCATCATCAACAACTGCAAAATTC
ATCGACTACCAACGGGTTAAACTCGTCCAACTTGATCGCGGTCACGACAAGCCCGAAC
GGTTGCGGTCAATCGTCGAACTCGTCGTCGTCATCGTCGTCGTCATCGTCGTCGTCGTC
GTCGTCGTCGTCATCGTCCTCCTCGTCGTCGTGTTCTTCTTCTACTTCTTCTGTCTCTTC
TTCGTCGTTATCCTCGTCATTATCGTCGTCGTCGTCGTCGTCGTCGTCGTCGTCGTCGTC
GTCGTCGTCGTCGTCCTCGTCGTCGTCATCGTCGTCGTCGTCGTCGTCGTCGTCATCCT
TGTGTTCGTCTTCGTCGATTCCGTCGTCTTGCGTGTTGAACACTTGCGGACGATCGAAC
AGTCCCGGCAACGGGAACGGTGGAAGCATCGTGAATAACGCGATCGTTAGTAACTGTA
GCGGGAACGTGAACGGCGGTTCCACGGCGATCATTATTGCCGGGGCAGACCGTGGTG
GTAAACAGAGTAACGCGTTGTTAAGTAACGTGGCGGTGCACGGGAATAACACAGGCA
AAATGGCCGTTGAACATTCGGCAACGGTCGACAAAGGACTGAAAATGAAGATCAAGC
GCACTAAGCCCGGGACAAAGAGCAGCGAGGCGAAACACGAGATCGTGAAATCGAAC
GAGATCAACGGGACGTTGGCGGCCCCCCAAGAGAGCGGGAACGGGGGCTGCGGGGT
CTCGTTGTCGACGAGCGGCTCCGCAGCGATTTCGGGCGGGGCCGGTGGCGGCCCGTC
GTCGGCCACCTCGATCGCCGCTTCCGTCGTGTCGGTGGTCGGCCAGAACGTGCAGAGC
TCGGATTGTGGTGGTGCGGCCGGTGGAGCCGCGGTCGTGTCCGGCGGCGGTGCGAGT
GTGGTCGGTATCGGTGTCGGTGTCGGTGTCGGTATCGGTGTCGGTATCGGTGTCGGTGG
AAGTAGCAGTGGTTGTGGTGGTGGTGGTGGTGGTGGTGGTGTCGGTGTCGGTGTCGGT
GGTGGTGTCGGTGGTGGTGGTGGTGGTGGTGGTGGTAGTGGTAGTGGTGGATGCGGC
GGAGGTGGCGGGAGCGGCACCGGGGGCGGCGGTAGTGGCGGCGGATGCGGAGTCGG
TGGAGCCGGTGGCGGCGTGGCCGGGTCCGGGTCGGCCGGCGGCGGTGGAGGAGTCG
GTAGCGGCGCCAGTGGCGGTGGTGGTTGTGCCGGCCAATCGAACGCGTCGTCGTTGTC
CTCGTCCTCGTCGTCGTCGTCGTCGTCATCCTCGTCGTCGTCGTCGTCCTCGTCCTCGT
CGTCGTCGTCGTCCTCGTCGGCGTCGTCAACGTCCGTTTCGTCCTCTTCGTCGTCCTCG
TCTTCGTCGGCGAAATCGAAGCCGAGCCATTCCGGGAACTCGGCGGGCGGCGGTGGA
GGCGGGAACGCGGGTTCCTCCTCGAGCACCGGGTCCAAGAGAGGGTCCAGTGGCCAT

CGGCGGGACAAGGCGAGGGAGAAACACGAGAAGCCGCAGAGCAATCACGCGATCAG
TCAGCAGAGCAAATCGGAGATGAACGGGACAGCTAGGCCGAGCGGCGGTGGAGGAG
GAGGAGGAGGAGGAGGAGGAGGAGGGAGCAGCGGTGCGGGAGGATCCGGGACGGG
AGGTCCGAACGGCGGTGGTGGCGGGGGACAAGGTCAGACGTCGAACGGGCCGGGAC
CGGGCGCGGGGCCGGGGCCTGGGTTAGGTGGCGGCGGTATCGGGGTCGGAGCCGGAT
CCGGGGCTGGGGCGGTGGTGCAATCTCAGGGACCGGCAGCGGCCGCGACCGCTCCTC
AGCAACAAAACCAAGGGCCGCCGCCGAGCTCGCGGACGGGATTCTCGGTCTCGCAGG
GGCCCGGTCCACCGTCGTCCGGCTCCGCCGCGGTACCGTGTCCACCCCCGAGTCCGGC
CAGCGCGACCGCCGCTGGCCCGGCTGCCAAACCCGAGCAGACCAAGCTCGCCACGGT
ACCCGGCACTCCGATCACAACGACGCCCGACGACGCCATGGATACCGCGGCCAGTCCT
CCACCTCCGAAGAAACTCAAGACTTCCGCTTCCGAACCAAAG

>novel_circ_000848
GTGCGTGGGAACGAGGTCGTGAGGTGAATTTACTACGCGTCATGAGACACTCGACAAT
ACTCGGAAAATTGGTCATCTTTCTTGGAATTGGATTACCTTCTGGAATACACCATCCTCG
ATGAATCCACATGAACAATAACGGGAGCATCGACGGAAGGAAAAAGAATCTCGAGTCT
GGCAAGAAAGGATGGATTTTTGAAACGATCAACACGCAAACCTATCCAGCCTTCGATA
AAAGCGCACGATG

>novel_circ_000849
CGGGTTTCAACAGACAATCGTCGCGACAAACGGGTCGGCCAACCAACATAATGCCGCC
TGGCAGCACCGTGAACGGCCACGGTGACTCTGTGGCTGGATTGACAGGTAGAAGGGA
GGCTGAGAACGCGCCAACACCAACCACGGCCACGGCCGCTTCCTTCAACGTCACCCT
CTCGGCCCTGGCCAAACAGAAGCAGCAGCAGCAGCAACAGCAGCAACAACAACAAC
AGCAGCAACAGCAGCAGCAACAGCAGGCGTCACAACAGGTGGAGAACGGGAGGGGG
AGGCGAACGAACGTGGTGAAGGAGGTGGAGAGGTTGAAGAAGAACAGGGAGGAAA
GAAGGCAGAGGCAAGCCGAATTGAAGGAAGAGAAGGAGGCTCTGATGAATTTAGATC
CTGGTAATCCAAATTGGGAGTTTCTCGCTATGATAAGGGAGTACCAGAACAGCATCGA
GTTCAGGCCGCTTCGCGAGTCGGACGTGGTCGAGGATCATCAGATAACCGTGTGCGTG
AGAAAGCGCCCCCTGAACAAGAAGGAGATAGCGCGGAAGGAGGTGGACGTGATCAG
CGTGCCCAGCAAAGACCAGATGGTGGTGCACGAGCCGAAGGCCAAGGTGGATCTCAC
CAAATATTTGGAGAATCAAATTTTCCGTTTCGACTACGCGTTCGACGAGACTTGCAACA
ACGAGATCGTGTACAAGTACACGGCGAAACCGCTGGTCCAGACCATCTTCGAGGGCG
GGATGGCCACCTGTTTCGCTTACGGTCAGACGGGAAGCGGGAAAACCCACACGATGG
GCGGCGATTTTAACGGGAAAACTCAGGATTGCAAGAAAGGTATATACGCGATGGTCGC
CAACGACGTGTTCAAATGCCTCAAATTGTCCAAATATCGCTCCCTCAATCTCATCATTTC
CGCGAGTTTCTTCGAAATTTATTCCGGGAAAGTGTTCGATTTATTGGCGGACAAGGAAA
AACTACGCGTCCTCGAGGATGGGAAACAACAGGTGCAGATCGTCGGATTGACGGAGA
AGGTGGTGGAGTCGTGCGACGAGGTATTGAAATTGATACAGCACGGGAACAGCGCGA
GGACCAGCGGCCAGACGAGCGCCAATTCCAACTCGTCTCGATCGCACGCCGTGTTTCA
AATCATAGCCCGTACACCGGCCACCCACAAAGTTCATGGAAAATTCTCGTTGATCGATC
TCGCGGGGAACGAGAGAGGAGCAGACACGTCTTCCGCCAACAGACAAACTCGTATGG
AAGGTGCGGAGATCAACAAGTCACTGTTGGCCTTGAAAGAATGCATTCGAGCGTTGG
GACGCAAAGGAACGCATTTACCGTTCAGGGCTAGCAAGTTGACGCAGGTGTTGAGGG

ACAGCTTCATCGGTGAAAAATCGAAGACATGCATGATCGCCATGATCAGTCCGGGAAT
GAGCTCCTGCGAACATTCGTTGAACACTTTGAGATACGCGGATCGAGTGAAGGAATTG
GCGGCCACCGATCCAACCGAAGTGAAGATTTCCTCGACGGACGACGAACGGGAATTA
AAGATCGAGGCACAGTCGAACAACGTGCTGTCTGACAGCGACTTGGCGCAATTACGAT
CCCTGAACGAGAGCGAAATATCCCAAGATCTTTACACGTTTCACGAGGCGGTATCAGC
GTTGCAAATGCTTGAGGAGGAAGTCTTGGACACGCACAAAATGGTTATGGATCAAACG
ACCAGATTTCTCAATGATGCACACAGTGTGTTCAGTGTGACTCACGAAGTAGACTATG
ACCAAGAAG

>novel_circ_000850
CAAGAGAACGTTTTCCTTCGTCGAGCAGTACTGATGATGATCAAAGCGGTTCTGATAC
GGAACATGACTCGAACGCATTGCGCTCTAAGGTCCACTCTGGAATAGTTCATCATTCCG
GTACTCATTCGGTGCGAAAGCGTCGGGGAAATTTACCAAAGCATTCGGTGAAGATTCT
AAAACGATGGCTTTACGAGCACAGATACAACGCGTATCCAAGCGACAGCGAGAAGCTT
ACATTGAGTCAAGAAGCAAATTTGACTGTACTCCAGGTTTGCAATTGGTTCATAAATGC
CAGACGACGAATTTTACCAGAAATGATTCGAAGAGAAGGTCATGATCCTTTGCAGTATA
CAATATCTCGTCGTGGTAAAAAGATGCCTGCAGGAAATCATCAACAATCATCCAGTCTT
AATTCAAGATCTAATCAAAATTGGGATTCGTTAGCTGGCATGAGCGCTCCAAAACGTAG
CAGAGATCATGATTATGAAGATCATGCTGGATTAATGTATAG

>novel_circ_000851
GAGGAATATAGTCAACGTAGACGTCTATCTACTCCAAGCGACAGTTCTAGTCCTCCTAG
AACAATGAGACTTTTTAAATCTACAGGTACATTGACTAGAGAAGAGGAATCCTTTTGCG
AATCAAATAACACTCTCACTAAAAAAAATCAAGGACAGGAAGTTGAGAGAAAGGGTT
GGTTTAAATCTCTATCTAGGAAGAATAAATCGAATATCAAAATAATGGATAAGAAACCTA
GAATACCAGGAAGTGAAATTTTAACTACTGGAACTGATGAAGAAGCTTCGTCCGTACC
TGAACGAGTTTTTGTACAAAAGAATTTACGTTTTTTTGGGGACACCGATCAGGAATCA
GTTTCATCTAATAGAGATCGCAGAAATAAACGAGATGATCATACCTGTTCAGGACATGA
TGTTATGAATTCCAGCCGTTACAATTTGGGTATTCTATCAACATCTCGGAAACCATCGAG
ACTTACTGAAAGTGCATTGTCTTCCGGTGAAAGTACAACAGGAGATAGCAGTCAACAA
AGTCAGAATTCTCAGAGATCACAGAGATCTGTTGTATATCTTCATGCTGCTACGG

>novel_circ_000852
GACGTGAAGCATCCCGCGTGACCGCGGCTCTGATAGGAGATGGCGAAGGCAGGTTGAT
TGCCACTCCGATCCATAAGGAGAGCATAGCTTTGAAGAGCAAAGAGGATCTAGCTCAG
GAGGCGGCGCTAGTACTTCAACAAGCGGGCAGCCTTCATTGCGTTGCTCATGACAACA
GACGATTGCCAGGTCGTTTCAGCACTTTGCCAGTGCGTCGTAACAAAAAAAGGCCAG
AACTACCGCTTGATGTAAGACGAAAAAGTGAAGAAATAGTCACGAACAACACAGATC
TTATCGACACACCGAAAATAAAGAAAAAAGCATTAGAAAGAAGTATCAGCGATGCAAA
CATAAAAAAGTTAAAAAGTCCATTGGCCAAATTCTTTTCTCCAAAGTTAGAAAGGAAG
CATAAGCTGGTTAGTCGTAGCATCAGTGACGCGGCTAATTTGTTACGGTCCCATCGCAA
AAGGAGCGGTAGCGAAAGTGAAGGATCGCATTTGGAGAGTAATCGAGTGAAGAAGCA
ATTATCCCCTATTATCGAAGCGTCGCCTCACATTGATCAACAACCTTTTCATTTCGGTTT
GAAGATCGAAAACGGTGAAAATAGGCCGGAACCAAAGAAGTGGAAAAAAGAGGAAA

TCATCGATGGCGAAAAGGTGACGGAACATACAGAAACGGTGAAAGAAAAGAAAGAA
AAGATCGAAGAGAAGGACGTGAACATCGGAAAACCACTAATATCGTCGAGAAGAGAA
AACAAGGGAATCGTAAAACATATACCTATTCAACTGGAGGTAGACGATAAGAAAAATA
AAGAGGAGAAGAGAGAACAGAAGAAAGAAGAAAAGACAACAGGCTCGATTCCTTTA
CCAATCATAAAAAATACTGGATTCGACGAAGTAGATAAAATCGATACGAATAGCATGCT
GCACACTAGCCGATACGATCTCCAACAACGATCTATTGAAGAATCAACGATACATAAGA
TGATTCATCAATTATCCAGCGATCAGTCACCACCACCGCAATTGACCAAAACAATGGTC
TCATCTGGTCCAGGATTCAGTCATAACAACAATCGACCATTCTCTTACACTAGACCGAA
TGAATGTTGTGGTATGCCATCCCCGCAAACTGTGATTTACGCTCAAGTGCAGACCGAG
AAGAAAAAAGCGCAAAGAAGTCATTCGGATAGCGACGAGGGGCTTGGCCTTGAAAGA
AAAGATTCGTCGCGAGAAAATAGTCCAGCGGAGAAGGAATATCATAGAATGGATTTCT
CTTATAACAATGAATTACGACACAATAATGAAATCAATATGTTCAAATCTAGATTCGAAG
AGGATAGAAAGAACGATGATATCCATTTCACCAATCGTTATGTTGAAACAGAAGATTAC
GCGAGTAGAGACGTAGATACGAAACGACATCATAAATTAAACGATATTGACAGACGTCT
ATATGATAAACCTGAAAAGTATACATCCACAGTATTCGTAGATACTTTCGATCGTGGTAG
AGCCGATGGTACAGATGCTAGACAACGAGATAATAACGAATTTTCAAATTCTAGACAAA
TTCCAAGCGGAATATCGAATAAGACATCCGAATTAAGCGCGAGACGTGATCTTCTTGAA
TCTAGAATCAAGTCGAGGCTGCTCGCAGATGAAATTTTCGCCGCGAAGAACAGTATGA
ATACACCGAAGAAATCTGAGCACGAGATTATCGACAAACCAATCGTTCCATCTCCAATT
ATTCCTAATCGCGTTGCCTCGCCTAAACGTTACATGGATACATACATCACGGAAACACG
CACCAACAGCAATGGAGAGAAGTATATTTTTGAAAAGGAGATCCACGAAGACAATGGT
AAAGTGTACGGATATGAAAAAAAGATTACTAATAAAATTCCATCGGATGGATACATCGA
TACCAAACCGCGAGATGGATATACAATCGAAACGAGAACGGACAAATATGGAGACAGA
TATATTGTTGAGACGCGAATGCACGAAGGTTATGCGGACGATTTCAAACCATTTCTTAG
TGACAGGAATAGAATCAGTCCAGAAGATCGGTATCAATCACCTAGAAACTTAATTAGTT
CTTATAAGTCATCTGATTTGCGACCCGAAGAAAACACCGTTACTCATCACTTTCGAACT
GAATCCAAGGAAAACAATTTTCTTTCTTCACCTATAATCGCAAAGAAATTCACCATGAC
GGAAAGAAAATTCTCCAAAAGTGATGATTTTTTGGATAGAGATATTCGACCGAGAGAC
GAATACTTCCCTAAGAAGAAGAATTTTGAATCAATCCGTGGTATGAGCAAATCCACGCA
ACGTTTGGATGAAGTAGGTGAAAATTCGCGAATCAAAGCTCTAAAAAGTGAATATACG
ACTAGGTCGCACGAGAACTTAAGCAGCCACGCAGATTACATAAACACTAGAGATGTGC
GTCGACCTCTTTTAGATAGTCCCATTATAAACGGGCGGAGGGAACAGTCACCGTTTACG
AAAAGACATTTGGATAGCTTACGCGAGGGGCCGAACGACGATTACGATACTGATATATC
TCAGATGACAAGTAGCCAACTAGAATCAAAATACTACAAAGAAACGCGCACCACTCAA
CGATATATGAACAGTAAGCACCCGATTCATGATGATTACGATAGTTCTCCATCGAGGATA
CGAACCGATCGTGGAGGTTACAATTCAAGCAGATACGATTCTGATACTACAGCCAGAG
ATTCGATTCATTGTGAAACTCGATACGACGAAACCTCACGAACATTGAGAAAAGAACA
TCGAAAATTTAATGAAGATTCAAAAAAGCAGAGATCCCGCAATCAATTAGATTATTTCG
ATGATTCGGACGCCAGAGAAAATCTACGCGTGAGTATTGGACGATTAGAACCATTCGAT
GAAAGTCCAACACGATCTCCGAGAACTTATCACGACAATGGCAAGTCGAAACACATCA
AGCAAGATATCAGAACGCATACTGAACGCAGACATCGCGAGACTCGGCTCAATGACTC
TGATCGGAAGGATCGTTTGGCCGATTCCGGAATCGAAAATGATTACAGGCGTGACTCG
CAAGAAGTTGATCGAAGGGAACAAATTGAATCGGAAGACGAAGATTTTGTTACGAGG
CAATTTATCAAACATGAGAAACGGCATACTGATCGTAACATAAATTTAACACCCTCCGA

GCAGCGAAAATACGATAAATATTCGAACGATATGTTAAAATCATCGCCGACCACCACAA
AACCACCGTCAGGTACAGATAAGAAATACGAGAAAAAGGCAAAGAAGAGCGGTACGA
TGAGCAAAGTAAAGCAATTGTTTAGTAAAAAGGAAAAAAAAACAAAGGAAGAGAAA
AACAAGAAAAATGCAAAAAGCAGCAGCAGTGGAGCTCTTACAGACGACGAAGTGATG
ATCAGATATAAAGAATATCGAGGTGGTCAATTGAGAAGCGCGAAAAGTACGCGAGACT
TGGGCAGTGACATCAATCAG

>novel_circ_000853
GTCGGTGATCGGGTCTAAGAGGGAAGGAGAGGGGAGATAGAGAGTATAGAAAGAAAG
GAAGGGTGACATCATTTAGAGGCCAGGGTATTAAAGCATATATGTGTGCAGGGGTTTCG
AAGTAAGAAAGAAAGAAAGAAAAAATACGACGAAAAAGAGAGAAAGAGAAAGAAA
AAAGAAAAACTGGTGTCCGACTCTATATGTTATGATTGGGAGTGAAAAGACGGGAAGA
TGCTCGAGGTTCAGCAAACGGAGTTTTTAAGAGAAGGAGAAGCCTGCAACCCTTCTAT
CGACATACTACATCCTGATTTGACCGAAACTTCCGTTTCCGAACGGAAGTCCGCTGAA
AGAAGCGTCTCCTTCAATCGAGACGTCCACGTCAAGAGGATCGGACGTGAAGCATCCC
GCGTGACCGCGGCTCTGATAGGAGATGGCGAAGGCAGGTTGATTGCCACTCCGATCCA
TAAGGAGAGCATAGCTTTGAAGAGCAAAGAGGATCTAGCTCAGGAGGCGGCGCTAGT
ACTTCAACAAGCGGGCAGCCTTCATTGCGTTGCTCATGACAACAGACGATTGCCAGGT
CGTTTCAGCACTTTGCCAGTGCGTCGTAACAAAAAAAGGCCAGAACTACCGCTTGATG
TAAGACGAAAAAGTGAAGAAATAGTCACGAACAACACAGATCTTATCGACACACCGA
AAATAAAGAAAAAAGCATTAGAAAGAAGTATCAGCGATGCAAACATAAAAAAGTTAA
AAAGTCCATTGGCCAAATTCTTTTCTCCAAAGTTAGAAAGGAAGCATAAGCTGGTTAG
TCGTAGCATCAGTGACGCGGCTAATTTGTTACGGTCCCATCGCAAAAGGAGCGGTAGC
GAAAGTGAAGGATCGCATTTGGAGAGTAATCGAGTGAAGAAGCAATTATCCCCTATTAT
CGAAGCGTCGCCTCACATTGATCAACAACCTTTTCATTTCGGTTTGAAGATCGAAAAC
GGTGAAAATAGGCCGGAACCAAAGAAGTGGAAAAAAGAGGAAATCATCGATGGCGA
AAAGGTGACGGAACATACAGAAACGGTGAAAGAAAAGAAAGAAAAGATCGAAGAGA
AGGACGTGAACATCGGAAAACCACTAATATCGTCGAGAAGAGAAAACAAGGGAATCG
TAAAACATATACCTATTCAACTGGAGGTAGACGATAAGAAAAATAAAGAGGAGAAGAG
AGAACAGAAGAAAGAAGAAAAGACAACAGGCTCGATTCCTTTACCAATCATAAAAAA
TACTGGATTCGACGAAGTAGATAAAATCGATACGAATAGCATGCTGCACACTAGCCGAT
ACGATCTCCAACAACGATCTATTGAAGAATCAACGATACATAAGATGATTCATCAATTAT
CCAGCGATCAGTCACCACCACCGCAATTGACCAAAACAATGGTCTCATCTGGTCCAGG
ATTCAGTCATAACAACAATCGACCATTCTCTTACACTAGACCGAATGAATGTTGTGGTAT
GCCATCCCCGCAAACTGTGATTTACGCTCAAGTGCAGACCGAGAAGAAAAAAGCGCA
AAGAAGTCATTCGGATAGCGACGAGGGGCTTGGCCTTGAAAGAAAAGATTCGTCGCG
AGAAAATAGTCCAGCGGAGAAGGAATATCATAGAATGGATTTCTCTTATAACAATGAAT
TACGACACAATAATGAAATCAATATGTTCAAATCTAGATTCGAAGAGGATAGAAAGAAC
GATGATATCCATTTCACCAATCGTTATGTTGAAACAGAAGATTACGCGAGTAGAGACGT
AGATACGAAACGACATCATAAATTAAACGATATTGACAGACGTCTATATGATAAACCTG
AAAAGTATACATCCACAGTATTCGTAGATACTTTCGATCGTGGTAGAGCCGATGGTACA
GATGCTAGACAACGAGATAATAACGAATTTTCAAATTCTAGACAAATTCCAAGCGGAAT
ATCGAATAAGACATCCGAATTAAGCGCGAGACGTGATCTTCTTGAATCTAGAATCAAGT
CGAGGCTGCTCGCAGATGAAATTTTCGCCGCGAAGAACAGTATGAATACACCGAAGAA

ATCTGAGCACGAGATTATCGACAAACCAATCGTTCCATCTCCAATTATTCCTAATCGCGT
TGCCTCGCCTAAACGTTACATGGATACATACATCACGGAAACACGCACCAACAGCAAT
GGAGAGAAGTATATTTTTGAAAAGGAGATCCACGAAGACAATGGTAAAGTGTACGGAT
ATGAAAAAAAGATTACTAATAAAATTCCATCGGATGGATACATCGATACCAAACCGCGA
GATGGATATACAATCGAAACGAGAACGGACAAATATGGAGACAGATATATTGTTGAGAC
GCGAATGCACGAAGGTTATGCGGACGATTTCAAACCATTTCTTAGTGACAGGAATAGA
ATCAGTCCAGAAGATCGGTATCAATCACCTAGAAACTTAATTAGTTCTTATAAGTCATCT
GATTTGCGACCCGAAGAAAACACCGTTACTCATCACTTTCGAACTGAATCCAAGGAAA
ACAATTTTCTTTCTTCACCTATAATCGCAAAGAAATTCACCATGACGGAAAGAAAATTC
TCCAAAAGTGATGATTTTTTGGATAGAGATATTCGACCGAGAGACGAATACTTCCCTAA
GAAGAAGAATTTTGAATCAATCCGTGGTATGAGCAAATCCACGCAACGTTTGGATGAA
GTAGGTGAAAATTCGCGAATCAAAGCTCTAAAAAGTGAATATACGACTAGGTCGCACG
AGAACTTAAGCAGCCACGCAGATTACATAAACACTAGAGATGTGCGTCGACCTCTTTTA
GATAGTCCCATTATAAACGGGCGGAGGGAACAGTCACCGTTTACGAAAAGACATTTGG
ATAGCTTACGCGAGGGGCCGAACGACGATTACGATACTGATATATCTCAGATGACAAGT
AGCCAACTAGAATCAAAATACTACAAAGAAACGCGCACCACTCAACGATATATGAACA
GTAAGCACCCGATTCATGATGATTACGATAGTTCTCCATCGAGGATACGAACCGATCGT
GGAGGTTACAATTCAAGCAGATACGATTCTGATACTACAGCCAGAGATTCGATTCATTG
TGAAACTCGATACGACGAAACCTCACGAACATTGAGAAAAGAACATCGAAAATTTAAT
GAAGATTCAAAAAAGCAGAGATCCCGCAATCAATTAGATTATTTCGATGATTCGGACGC
CAGAGAAAATCTACGCGTGAGTATTGGACGATTAGAACCATTCGATGAAAGTCCAACA
CGATCTCCGAGAACTTATCACGACAATGGCAAGTCGAAACACATCAAGCAAGATATCA
GAACGCATACTGAACGCAGACATCGCGAGACTCGGCTCAATGACTCTGATCGGAAGGA
TCGTTTGGCCGATTCCGGAATCGAAAATGATTACAGGCGTGACTCGCAAGAAGTTGAT
CGAAGGGAACAAATTGAATCGGAAGACGAAGATTTTGTTACGAGGCAATTTATCAAAC
ATGAGAAACGGCATACTGATCGTAACATAAATTTAACACCCTCCGAGCAGCGAAAATAC
GATAAATATTCGAACGATATGTTAAAATCATCGCCGACCACCACAAAACCACCGTCAGG
TACAGATAAGAAATACGAGAAAAAGGCAAAGAAGAGCGGTACGATGAGCAAAGTAAA
GCAATTGTTTAGTAAAAAGGAAAAAAAAACAAAGGAAGAGAAAAACAAGAAAAATG
CAAAAAGCAGCAGCAGTGGAGCTCTTACAGACGACGAAGTGATGATCAGATATAAAG
AATATCGAGGTGGTCAATTGAGAAGCGCGAAAAGTACGCGAGACTTGGGCAGTGACA
TCAATCAG

>novel_circ_000854
AGGGACGACACGAGTTTACTCGCGCAATTTTTCTACGCCGACGAATCCTTGAACGCCG
TTGCCTGCGAGTTGGACTCGTTCGACGGCCGCCAGGAGCCGGAGAGATGCACCACGC
TGGTCAATCAACTGCGCCATTGCCAGGACAAGGTGTTGACGATATGCAGCCAGATAAT
GGACGAGCTGATACCGGAGAGCAGGGCGAACCGGGACTTTAGGGTGAAATTCCCCGA
TGACGTGATGCAGGAGAATCTTGCGGGCCAGCTGTGGTTCGGGGCGGAATGCTTGGCG
GCCGGCTCCTCGATCATGAACAGGGAGGCTGAAAGTTCGGCCATGAGGCCGTTGGCG
AGGGCTTTGACGAAGTCTTTGGACATAGTCAGGAATTTGCTCAGAGAGCACGCGCTGA
AAGGTCACATGAACCTGAAGTATCTTTCTCAAACGCAAAATCCATTGGACCCGTCCCT
GGAGAAGCTGATCGAGTCGTTGAAAATCTTCGATCGGTTGTTCGCCGATTTCGAGCTG
TGTTACGTAGGCGTGATGGTGCCGGTGAAATCGACGAAGGAGTACGAGCAGCAGGAG

CTCGTGTGCGTTTATTTTCGGAGACGCTGCAAAGAGCTCTCGAACGTGGATTGCTCA
GCCAAGCCGACGTGGATAATTACGAGCCAGCTCTGATGTTTACTATTCCACGTTTGGCG
ATTGTTTCTGGCCTCTTGGCTCCGCCTGGAGGCCCTTTGTGCCTCAATTCCCCTGACAA
CATCAGCGAAGTGTTTAGACCTTTCAGG

>novel_circ_000855
GCAAGAAACTGAAGTGCCCGGACTGCGAGAAGCTGTACGGCTACGAGACAAACCTGC
GCGCCCACATACGGCAACGCCACCAAGGTATCAGGGTACCGTGCCCGTTCTGCCAACG
GACGTTCACGCGCAACAACACCGTGAGACGGCACATAGCGCGCGAGCACAAGGCCGA
GCTGAACCTGAAAGCGTTCCAACAGTCGAACCACTCGATGTCGGACACGGTGCCGCA
GTAACGGCGTCCTCGCCACCGCTGTCGTTTTAGCCGCCGTTCCCACCACCGTTCCGGC
CGTCGCCATCGTGTCGCGGCCGTCGCGACGCCACGGGAGACGGCCAGAGCGCGCCCA
GCAAAGAACCGCGAGGCGCTGGAACGAGGAAGGCCGGCGAAAGCAAGCTCGCGCCC
GCCTTCTTGAATCCCGGTCAACGCCATCCCCGTCCTCATGATGAAACATCCGAGCGGCC
CTCCGTGAACTGCGATTGACAGCGACGTAATACATATATACAGAGCCCACACAGCCTTT
GAAAGGAAAACGACAAAGGAAACGATCCAATGCGACCCGCTGTGCCTCGTCTCGGTG
TGATCTTGATAGTAGTTGTCAATTACACACTGCGACCCCGTCTGCAAATCATCGTGTTCC
TTTCGTGTTAATTTCACGGGGATGCGGATTCGGGATGAGGATACTGAAATTAGAGGGAG
GGGGGATGTAGGAAAAATTTGCACGCGATGATCCGAGCCGCTCGGAGATTCTTTTTTTT
CGTTCTCTTGTAATTAGATTTAAGGTGTTGCTCGATCGAGAGTCGCGTGTGCTTCTTTCG
GGGGAAAATTTGAAAAGTCTTCGCGGAGGATTCGGATGCGGCAACGACAGAGGACGG
TTGACCGCGAAATTTAGAAGAATTTAGTCCCGGTGATCGGATCACGAGCGAGGAAGGA
GATTAAACGAGAAGTGAAATGATGGAGGAGGGAATGGAGGAAGGGAAAGGGGTTTG
GAAAAATGTCGACGTCCGATTACTAAGCGTATGGAAGATTGCATTAACAAGAGAGAAA
TGTCGTTTGGGATCAGGAGGATGTGAAACTCGTCGCCTCGGACATGCTACGCTACTACT
ACTACTACGAATAACCATACAAAACTAGTCAATGAAAGTGCAACGTCGAGTAAGAGTT
ATTATTACACTGTAAAAAAAAAAAAAAAAAAAAAAGAAAAGAAGAAGAGGAGCGCGAT
AAAAATAAGGGAAGGTTAGAGACGAAGAAGAAGATCGAAAGATATTCACGACGCCTC
AGAAGAGGGAATATAATAAGCGAGTTTACAAAAAAGACGCATATCTCAG

>novel_circ_000856
GCAAGAAACTGAAGTGCCCGGACTGCGAGAAGCTGTACGGCTACGAGACAAACCTGC
GCGCCCACATACGGCAACGCCACCAAGGTATCAGGGTACCGTGCCCGTTCTGCCAACG
GACGTTCACGCGCAACAACACCGTGAGACGGCACATAGCGCGCGAGCACAAGGCCGA
GCTGAACCTGAAAGCGTTCCAACAGTCGAACCACTCGATGTCGGACACGGTGCCGCA
GTAACGGCGTCCTCGCCACCGCTGTCGTTTTAGCCGCCGTTCCCACCACCGTTCCGGC
CGTCGCCATCGTGTCGCGGCCGTCGCGACGCCACGGGAGACGGCCAGAGCGCGCCCA
GCAAAGAACCGCGAGGCGCTGGAACGAGGAAGGCCGGCGAAAGCAAGCTCGCGCCC
GCCTTCTTGAATCCCGGTCAACGCCATCCCCGTCCTCATGATGAAACATCCGAGCGGCC
CTCCGTGAACTGCGATTGACAGCGACGTAATACATATATACAGAGCCCACACAGCCTTT
GAAAGGAAAACGACAAAGGAAACGATCCAATGCGACCCGCTGTGCCTCGTCTCGGTG
TGATCTTGATAGTAGTTGTCAATTACACACTGCGACCCCGTCTGCAAATCATCGTGTTCC
TTTCGTGTTAATTTCACGGGGATGCGGATTCGGGATGAGGATACTGAAATTAGAGGGAG
GGGGGATGTAGGAAAAATTTGCACGCGATGATCCGAGCCGCTCGGAGATTCTTTTTTTT

CGTTCTCTTGTAATTAGATTTAAGGTGTTGCTCGATCGAGAGTCGCGTGTGCTTCTTTCG
GGGGAAAATTTGAAAAGTCTTCGCGGAGGATTCGGATGCGGCAACGACAGAGGACGG
TTGACCGCGAAATTTAGAAGAATTTAGTCCCGGTGATCGGATCACGAGCGAGGAAGGA
GATTAAACGAGAAGTGAAATGATGGAGGAGGGAATGGAGGAAGGGAAAGGGGTTTG
GAAAAATGTCGACGTCCGATTACTAAGCGTATGGAAGATTGCATTAACAAGAGAGAAA
TGTCGTTTGGGATCAGGAGGATGTGAAACTCGTCGCCTCGGACATGCTACGCTACTACT
ACTACTACGAATAACCATACAAAACTAGTCAATGAAAGTGCAACGTCGA

>novel_circ_000857
GAGTAACGTTCAAGGCGCACAGGTTAATTCTCGCAGCATGCAGCAAACACTTTCAAGA
GCTGTTCGAAGGAATGCCTCCTTCTCCCGCGGGACTGATCGTTATTCTCGACGGAACG
AGTGCCCACAATATGGCGTCCCTTCTCGAATTCATGTACCGTGGAGAGGTACACGTGTC
CCAGGAATCCCTCAGCTCTTTCCTCAAAGCAGCCGAATGCCTTCAAGTAAAGGGTCTG
TCCATCGAGCACGAAAAGCTGGCAGTAGCGCAGAGGCACGCCGCTGAGAACAATAAC
TCGTCCACGGACACCGGGGATGGTGGTGGCGGAAGCGGTGGTGCGAGCAGCGGCAGC
GGTGACAACGGAAGCGGGAACGTGAGCGGAAACGTCAGTGGTACCGTGAGCGGTAG
CGTGGGTGGCGGAGTTGTCGATGACGTTGGCGGAGGAGTCGGCGAGGTGAGCGACAT
GGGTGAAGCGGGTGAAGCGACCATGGTGAGAAACACCATAAAGAGGGCCCGTACGCC
AGCGCCGTCTCCTGCCCCCGCTTACCCCGTGCCCAACCTCTACTCAGCTACCTCTCACT
ATTACGAAACCTTGCCGAAAAGGCTGATGAGATCGCCCAGCGACGTGGATACCCTGGG
AAGGGCGAGTGTGTTGCGCGACGGCACTGCCTTCCGGCCCGCATCAGCGCCGCATCCG
CTTCTTCTGGAGAACCACTTTTACCAGCCGTTCATCCACCCTACAGAAACACCCGCGCA
TCCCCACACCGCGCCACACACACCCACGGAATTGGAACAGGAATTGGAACGAGCGAG
GTCCGAGGAACGCAGCAATTGCGATCTCAAGGAAAGCGTAGCCGAAGATCTACGAATA
AAGCAAGAACCAGTGGCAATGACCGACCGGGAAAGGGCGGAAAAACTGGCAGAAAG
ATTGTCAGGCGAAGTATATACCGGGGGAAGGGGGTTCGATAGTGGGCATTACGGTTCG
AATTCGGATCACATGCAGCCCGGAGGACAAATTGGACCCCCCATGGTGCGTTTGCCGC
TTGCTCATCCACCGACTCCTGATCTCAGCCCTGCACCCACTAATCCCGCTATGTGGAAT
ACGAAAATGAAGAAGGCTGGCACTGTCTCCACACCCCACG

>novel_circ_000860
TTTGCATTGGAACCAATGGCCGCCTCTCTGTTCCGTCCAACAAACAACACCACTACCG
GAATCTTCGAGACCGGTACACCAACTGTACTTACGTTGACGGCAATCTGGAGATCACAT
GGCTCCAGAACGAGACATTCGACCTCAGTTTCCTCCAGTATATACGGGAGGTCACAGG
TTACGTGCTCATCAGCCACGTGGACGTCAAGAAGATCGTTTTGCCGCGATTGCAGATCA
TACGAGGAAGAACACTGTTCAAGCTCACCATTCATGACATCGAGTTCGCCCTATTCGTC
ACGATGTGTCAGATGCAGAACTTGGAGATGCCAGCCCTCAGAGATATCCTCAACGGCA
GCGTGGGCATGTACAACAACTACAACCTGTGCCACATCCGCACGATCAACTGGGAGGA
AATAATCACAGGGCCGAGTGCAATGTACTCCTACGTGTACAATTTTACGTCTCCGGAAC
GCGCCTGTACACCCTGCGATAAGAGCTGCGAGCAAGGCTGCTGGGGAGAGGGGCCTG
AGAACTGTCAGAAGTATTCGAAGACAAACTGTTCGCCTCAGTGCTGGCAGGGCAGGT
GCTTCGGGCCCAATCCGCGGGAGTGTTGCCATCTTTTTTGCGCCGGTGGCTGCACAGG
CCCGAAACAGAGCGACTGCATCGCCTGCAAGAACTTCTTCGATGACGGTGTGTGCACG
CAGGAATGCCCGCCCATGCAAAAATACAATCCTACGACGTATTCGTGGGAGCCTAATCC

TGACGGCAAGTACGCGTATGGCGCAACCTGCGTGAGAAGATGTCCGGAACACCTTCTG
AAAGACAACGGGGCTTGCGTAAGATCTTGCCCGCCCAAAAAGAAGGCGTTGAACGGT
GAATGCGTGCCCTGCGACGGACCCTGCCCGAAAACCTGCAAAGGTGTCGAGAAAGTG
CATTCCGGGAATATCGACAGCTTCAAGGATTGCACCATCATCGAGGGATCCATCACGAT
TCTAGACCAAAGTTTCCAAGGATTCCAACACGTGTACAGGAATTTCAGTTTCGGCAAA
CGTTACGAGAAAATGCATCCGGACAAGTTGGAGGTGTTCAGCACCTTGAAAGAGATCA
CAGGGTTTTTAAATATTCAAGGTGATCACAAGGATTTCAAGAATTTATCGTATTTTCGGA
ATTTGGAGGTGATAGGCGGGAGAACTTTGACCGAGTACTTTGCATCGTTGTACGTCGTG
AAAACGTCGTTGGTCTCGTTCGGGCTTAGCTCGCTCAAGAAAATTTATTCCGGCTCGAT
AGCCATTCTGGAGAACAAGAATCTATGCTACGCGCAAAGTATCAATTGGACCAGGATC
AAGAAATCGTCGGAACACGAGAGTTTGTTGTCCAATAATCGGAACGAGAGCGAATGCA
TAAAAGATGGACTAGTGTGCGACGAGCAATGCTCCGACGAAGGTTGTTGGGGGCCCG
GCCCGGCACAGTGTTTGTCCTGCAAGAACTTTATTTTAGGAAACGATTGCCTTCAAGAT
TGTACCGCGCCAGGAATCTACCAGGCGGATGAGAAGACGTGCAAAGTGTGCCACGAG
GAGTGCGACGGTTCTTGCATAGGGCCGAACACGGATCACTGTAAGAAATGCAAACAC
GCGCGAGACGGTCCATTTTGCGTGCCGGAGTGCCCGGCCTCGAAATATAATGACAACG
GTGTGTGCAAAAGCTGTCACGGGAACTGCGTGGGTGGTTGCGAGGGGCCTGAAAACA
ACATAGGCCCTAATGGATGCCACAGTTGCGATAAGGCGATCCTCAACGACCATGTACCT
GAAGGATGTCTGCAAAAAAAGGAGTCCTGCCCTGACG

>novel_circ_000861
ACGTGAAACGATGCTGTGAGACGAGGCATGCAAGTAAGGCGGTGAAGAGACAGCGAT
GGCAGCAGCGGAAACGCTACCTCGAGGCTCTTCCGATCTTCTCGTTGCTCGTTACTATC
GAAGAGAGGACGAAGAAGTGGGAATCATTGTCGAGATCACCTTGGCAAGGTCCATGG
GTTCGTAGAGGGCATGAGAGCTCATCATGTTCGAACACCGACACAGGCCGCGAACGCT
GTCGACCTGCAACCTCCTCGGCGTGACATTTCTTCTATTGATCGTCGCCGGTAGCTACG
ATGTGTCAGCGGATCTCAACTCCGAATTTGTCAAAGGAAAAA

>novel_circ_000862
ATCGACCAGAGAGAGAGAGATGAGATTGATCGTGACAGTGAGATATTTATTTCACGTATT
ATTATTATTAAAATCGTTACGAAAAATTTTATATCTTAACTTTTTTTTTGATAAAAATTTC
TTCGAGCGAATCATAATATAAAAATTATCACTTTATCGTTGATCATACTTTTTTCAAATA
TTGCTTTTCAATAAATTTTTCGAACAATATAAAAATCATCACTACGTGAAGAAACACTTT
CACGATGTTTCATTGTTTTTGTCCCAACGAAAGAAATTTTAAATTAAAAATAATAATAAT
TCTGGAAAAATTGGAATTACGCGTGATGTAACTCGGTCCCTCTGGGCCCGTTTGTCCTC
GATCAGCCAAACAAATTACACGTTAAACGACGTGCCCTGTCACGCACATTCGTGGCGG
CTCGAGTCTAATGTCACCCCGGGGGGAGCAATTTTCAGGCATTTTACTCGCGACACAC
GTTCCACGCCTCTGGCCCTATTGTCTATCCGTACGAGACGAGCGCGCAATTTATCCTCG
AGGACACTGTCACGATCAAAAAAAAAAAGAAAAAGAAAGGAAATCCAGCCTTCCTTT
TCTCTCGTGCACAGCCTGAGAAATTAAATTTCACTTCAATCCGGGAAACGAATTGGAG
AGGAGAAAAGATTTGGAAGGAAACGAAGATGGATGAGGTGGCGACTACCGGGTTGGC
CGGAACGGTGACGATAAACTTTCGGATTTTATGTGGACGAAGGAATCCGTTTCCCCGCT
CGGTCGTCCCGATCTCGCCAACAGGAACGAGAACGAGGGGGAGGAGGGGAGGGGAG
GGGAGGGACGGAGTTCGTGGAAGAAAACGATGCCGCAGAACGGCGCAGAAATGCGT

CGTCAACGTGTTTGACAGTGATAACGCGTGCGCGCGTGCACCTCTTATCAGCTGCGTA
AACTCGATCTCGCGTGCCTGCTCGATCAGTTGATCTCATGGACGCGGCTGTCTGGAGA
GACGCCTCACGCTTCTATACCTCCTATACGCGTGTCGGAACGTCTCGCGTGACGTTGAG
TCGATAATCGTTGTCGGGGCACGTTTCGATCTCCGCCTCGAATCGGCCCGAAATCGTTT
TCCGATCGCAAACGGTGGATCCACGTGGACGACGCGCGAGGTTTTCCTTTCCCCCGTA
CGCGCGAAGAAAGTGCAAAACACGTGACGGAACGAATTAGCTTCGGATGTTGAGTGG
TGTTTTCACCGGTACATATGGTCGACGAAAACTTGGAAGACACGGGTTTACACGCTTT
GAAAAGTGAACGTTCGTTTCGAAACGTTTTCGAATGTTCCTCGCCTTGGATTTTATTTC
GTCAAATTGACAAGTAGACGACGTGAAACGATGCTGTGAGACGAGGCATGCAAGTAA
GGCGGTGAAGAGACAGCGATGGCAGCAGCGGAAACGCTACCTCGAGGCTCTTCCGAT
CTTCTCGTTGCTCGTTACTATCGAAGAGAGGACGAAGAAGTGGGAATCATTGTCGAGA
TCACCTTGGCAAGGTCCATGGGTTCGTAGAGGGCATGAGAGCTCATCATGTTCGAACA
CCGACACAGGCCGCGAACGCTGTCGACCTGCAACCTCCTCGGCGTGACATTTCTTCTA
TTGATCGTCGCCGGTAGCTACGATGTGTCAGCGGATCTCAACTCCGAATTTGTCAAAGG
AAAAA

>novel_circ_000864
GTAAGTTCACGCGTCCAACTCCGGAGCGGAGAGACTGACCTCAAGGAAGGAGGAGG
ACGGTAATGCACAAGGCTCGAGGGGCCCGCGAGATAAGGATCGGGTGTTCGTTAAGCC
CGGGCCCCAAAAATCCGGCGCTTAAAAACAAAACGGGGCCCCGTGGTGGTCGTCGCG
CCGTTTTCGTGGAACGGCCGCGTATGTATAAATGGTTGGAACGAGTCTACTCTCGGGGC
AGGAGAGGAGGCTTGGTCCGTTTGCCTGTGTAATAGGGAGGGGGGGGGAAGGCTTAG
ATCGGGAGGGTAGATTAACAGGGGGATACACGTGTCAGGCTCAGATTGACGCGATTCC
ATTTTAACGAATTATCTTGACCCTGTCGGGGTAGGCAGGGTGGAGGGGTGTCGTTTTTC
TACTCTTTCGTGGAGTTGTATATGTTTGATAGGCGCTTCTCTCCACTTTGGGAAAGAGAT
AGATGTATATGTTCCGGATTTTTGGACGAAGATCGGGAGGGATGATAATTTGTCCAGTG
TTTCGTTTCGGTGAAACGGAGACTTTTTTCTTTCTTCGAGTTTGTTTCAAGTCGTAGAA
TCAAATTATCATAGTCTTTTTTTTCAAAATTGTAGATTCTTAAAGAAACTAGAGGATATT
AATACAGATGTACATTTTAATTATTTTTCGAATAATTTATCGAAAGGTTGCGACACCCAA
TGAGGATAAAAAATTTTATATTTTATTTAGAGATGTAATAGACATTTTTATAAATAGACAG
TTGCTACGAAAGGGGATAAAATATATAAATATTGTCGTATTTGATTCAACTTAATTTTCCA
CGGTATTACCTACACAAAACGTTCCAAGTCTTATCGCGGACAGTTAGAATTCATAGATC
GAGAGACTTTAAAGGTTACAATGGTATCTAAATCAAAAGTAACGGGTGATTCGAGCGA
GCTTTAGCGCTGGCATCGACTTAGCAACGGTTGAAAATCAACGCTCTTCCACGAAAGA
CTTGGAGACTCTGTAAATCAATTTGCATGCCCGGTTTATAGAGCGCGTTAAAATAAAAC
GCGGCCCGCTTCGCATTTATGTAAATGGAAAACTGTCCACCGTCAGGCGCGACATGCG
TCTGCACTTAATTCATTTTTCTGCCGGCAATTTTATACCGCGTTGTTAAACGGCCCGATT
CTTAAAGTGAATACCGCTCGAAGCGAGTATGATTGCCCGCGGCCGTACAGGGACCCCC
ATGAAAAATGAGTTTGTATAATTTTAATAGCACGTCCCTGGCCTCGGCCGATGGTTACA
AGCGCGAAGATTGATCGATACATTGGACGCGGATAGATAATACGTAGCAACTTCGAACT
CGAGAGACTCTTCTCTACTTTTTTAATTGTACGAAATTGCCCCCTCGGTCTTTAAAAGG
TTTTTGAACTCTGTCTATTCTTGGTGGAATTTTAGAAAGTTTCACGAGGCCAAACTTTT
GTTAAATAATTTCTCTTCGGTATTCTTTTCGTAATAACGTTCTGATATGCGAGAACGCAA
GAGTATAAAGAAATTTGTGTAAACTGAGAACGTTTATTTCAGGTATATGGGGATATAATT

AACGATTTTTGGAAAAAAATAAATGATAAATTCGAAACAATTTTATGGCATATCTTCGA
GACGGGGATAATTCATGCGAATCCTCTTTGTTTGCACGTAATTTACGCGCAGCCTCGTG
TCATAATTCGAATTGATGGCGCGATTTGTCGTGTTTTTCACCGACTCCTCTATAGATTTG
CCGTGTAGATAGGGTCAGAACCGGAGCTGACAGAACGCATCTCGGGCGCACGTTTTAC
AAGTTGTTTTACAACCCGGGGATACAACGCCACACGCTGAGAACTCGAGGACCTCTCC
CTCTCCTCGCGATAGCCGCCGCTCTCCTTTGTCCTGGACACGGATTCGACTTTTCGACC
AGGTTCCTTCTGCTTTGAAACCTTCCTTCTGTCGACACCTTTCCCCGACGTGGTTCGCG
CCTGATGGACAGTTTTTTTGCACAACGATTTTCCTTTCTGTTTTCCACCTTTGTTTCGAG
AAATTTGAGAAATTATACATCCATCTGTCGATGATGAAGCTTCTTGTGAATGGAAAAAA
AGAGAGGATAACTAAGAGAGAAATTTTACAGTTTTTGGAAAGAGAGTTTTTTTTTGATA
CAGCATTCCTTTTATCTTGCTCCATTTTATTTTCGTTTAATTTTCCTTATACGATTCAACTG
TGTATTTAAATTAATTTTTCTATTCAATGGAATTTTTTATTATAAAGTATTTTATATTAATTC
ACTAGATGTTATCAATTGGCTTGATTTGTGAATGGAAAAGAAGAGAGGATAATTTCAAG
AGAGAAATTTTACAGTTTTTGGAAAGAGAGTTTTTTTTTGATACAGCATTCCTTTTACCT
TGCTCCATTTTATTTTCGTTTAATTTTCCTTATACGATTCAACCGTGTATTTAAATTAATTT
TTCTATTCAATGGAATTTTTTATTATAAAGTATTTTATATTAATTCACTAGATGTTATCAATT
GGCTTGATTTGTGAATGGAAAAGAAGAGAGGATAATTTCAAGAGAGAAATTTTACAGT
TTTTGGAAAGAGAATTTTTTTTTGATACAGCATTCCTTTTACCTTGCTCCATTTTATTTTC
GTTTAATTTTCCTTATACGATTCAACCGTGTATTTAAATTAATTTTTCTATTCAATGGAATT
TTTTATTATAAAGTATTTTATATTGATTCACTAGATGTTATCAACTGGCTTGATTTCTACTA
ATCTAAATATATTGAATGTTGAATTTTCCAATAATAATTATGAATAAAGGAAAACAATAA
CTTCTTTAATTAATTTCTATAATCAGCTTCATTTTCCTTTACTATATAATTACGATATTATAT
AAACCCATCGCATTCTCTTATCAGAGATTTAACTCTCGAATCCTATCCTCAATTATCTTTT
GATTTTTCCTTCCATCGATTATTTTTCAAAATCAAGAATCATTAAAATTGAAAACAAATA
TTATCAAGACAATTATCGAGAAAATATAAATTTTCCTTTCTTTTATCGCAACTGGAACTC
GCTTTGCTCGTTAAACTTTGGATATTAGAAGTAATTAAGCGATCAAAAAGTTTATATATAT
ATATAATCTACCACGCTCAGAATTTATAATCGATCGAACGGCGCCAGTGGTTGATACTGG
AAATACTGGATATTCCGGAATAAGAGCACTCTCACTTTGCATGTCGACACTCTTTCCGT
AGTAGGCTAACGTTCCTCGCCGATAAAAGTGCTCACCGGTTATCGGCCTTAGAAGCATC
GCGATCGTTCGACCAGCAAATTTTAAGAAACAATCTCGATCCTGTTATGGATATCGTTCT
ATCTTGAGGTCCTTTCAATGTCCTTCCTTCTCTCTTCTTCTCTTTTTTTACAGGAATATTA
TTAAGAATAATATAAGCGCTTGTATTTTATAAATTTTTCGATAATCCTTTAAAAGAAATCG
AGATCATCGTGCAGATTTCAATTTTTAATGACAAACGATAGATCAACAAATTATTTTATTT
AATCATGGCTATTGGATCTAATATTCTGTCGAAATTTGCGTAGATGATTTCAATGCAACT
GGTCCAAACTGACGATGCATTTAAATCATTTTCGATCGATGCATACGTAAATACGTTTTA
CGAGGAACGTACAATCAACATTACCCCTGCTTCTAGGAATAGGAGGAATTACGACGCG
GCGTGTACACGTGTACACTGAGTAATGGAAGGAAGAGCCGCCTCAATTATCAAGCTCG
TTTGTATCAACGTTGTTTACAGCGTCTGGCCGCCCCTGCCAAGCGTTTATATATCCAGGA
CGCGATTTTACAGCCCATCCAGCGCATCGCGTAGTATAAAAGCACGCGCGTGCCCCTCT
CGAAGGAATAATTCTTCATTAATTGCGATCGCATGCGCGGTTCACATCCCACCCTTCGA
GGATGCGCCTTGGATGCATTTCCCAGGCTAATATTTCGTCAAATAATACATAATATAATTT
CTGCTCTCTTACAGATGATGATCTCGTCGTTACATCAACCTGGTCGAGTGTTATAAATAT
AAACTCCATAACGTATTATGGAGGAATAATAAGGGGAGATTGAGCGCGAGCCATAAAA
GTCGTGGTCGGGTTCGTGGTCGCCATCATAGAGAGGGCTATTCTATTGTCTCATATCTCT

GGAACCGTATCGAGGGAGGGGGGTGAAAAAAAAGCGCGTCTGCCGAACGTTCTAGAC
TGTGAGGATCCTTTGGACCTGTCGTCCGCGGGGAAACGCTGTCCTGTCCTCGAAATTC
TCTCCCTCCTCTCTCCCTATCCTTTCCCTACGCTTGGATTCTTTTGTTTCCCGCCGCGGA
ACCCCAGTCGAGATCCTATCTGACGTAAATAATTATCCTGGAGAGGCTGCAGATGCCCG
CGTTCGAGTGGTACCCCTCTCGGGGATTTTCATTTAATCGTGGATCTCGGGACCGCGG
TTCGAAATATTCTGGATATTTGGGGAGGAGGAAACTGGAGTTTTGGAAAGGATCAAAG
TGATCAAGCGGCGATGTGTGTGTCCATCCCTTTTATTTAATCAAGGATCTTGGCAACG
ACGGAAATATTCTTAGTATATCTTTCGATTGTTTGTATTTGTATTTATTTGAAAATGAATAT
TTAGATCAATAAAGTTAAAATTCTCGAAATTTCGAGCCAAGGATCTTGATTATTGGGAG
AGTGAGGCAAAGATGTGCCTCGCTTTCTCTCATTTCTTTTATTGTATTTCATCATCAAAA
TATCGACGAATTTCATAATTTAACGTGGACGATTATCTCCTCAATATATTATTTATTTTTCC
AATGTTTACATCTGTTGGTGCGTTTTTATTTCGACCAATCGTGACGTAGAAGGAAAGTG
GGAATTTTCAGAAATAATCTTTTAACGGTGCAATCTCAGCGGCGGAATCAAGTGATTGC
GGGGTATTGTTGTGAACACGGTTTCCTTTTTTAGCGTGAACGAACCCGATAAGGAACG
GTTTTATGGCTCACGCGTAATTGAACTGCGAGCAGCTTGATTTCGTTTTAAGGATACGG
GTCGTGAGAAGCGATCTGTTTTGGAACGTACTATACTCGAAGCTTGATTTCAGATCGAC
CAGAGAGAGAGATGAGATTGATCGTGACAGTGAGATATTTTATTTCACGTATTATTATTA
TTAAAATCGTTACGAAAAATTTTATATCTTAACTTTTTTTTTTGATAAAAATTTCTTCGAG
CGAATCATAATATAAAAATTATCACTTTATCGTTGATCATACTTTTTTTCAAATATTGCTTT
TCAATAAATTTTTCGAACAATATAAAAATCATCACTACGTGAAGAAACACTTTCACGAT
GTTTCATTGTTTTTGTCCCAACGAAAGAAATTTTAAATTAAAAATAATAATAATTCTGGA
AAAATTGGAATTACGCGTGATGTAACTCGGTCCCTCTGGGCCCGTTTGTCCTCGATCAG
CCAAACAAATTACACGTTAAACGACGTGCCCTGTCACGCACATTCGTGGCGGCTCGAG
TCTAATGTCACCCCGGGGGGAGCAATTTTCAGGCATTTTACTCGCGACACACGTTCCAC
GCCTCTGGCCCTATTGTCTATCCGTACGAGACGAGCGCGCAATTTATCCTCGAGGACAC
TGTCACGATCAAAAAAAAAAAGAAAAAGAAAGGAAATCCAGCCTTCCTTTTCTCTCG
TGCACAGCCTGAGAAATTAAATTTCACTTCAATCCGGGAAACGAATTGGAGAGGAGAA
AAGATTTGGAAGGAAACGAAGATGGATGAGGTGGCGACTACCG

>novel_circ_000865
CACGATGCCCTCAGATAGTATGGATGACATCGAACTGAAAAATATTCAACTGCAATTTC
CACCGCTGAGATGTCTATCGAGAGATGACAGTTCCGTGCGAGAGGAGAGGGTTGAAA
CCGACAAAGGAAGTATTTTGGTGGCTGTGCAAGGGAATCGAGCGAAACCTGCCATTCT
CACTTATCACGACTTAGGCCTTAACTATATATCGAGCTTCCAGGCGTTCTTCAATTATATC
GACATGAGAGTTTTACTGGAGAATTTCTGCGTTTATCACGTGAATGCACCTGGCCAAGA
AGAAGGTGCACCCACGCTGCCTGAGGACTACATCTATCCGTCCATGGACGAGCTGGCT
GAACACCTGCTCTTCGTATTGAGCCATTTTGGGCTGAAATCCGTAATCGGTTTTGGAGT
CGGTGCAGGGGCCAACATACTAGCGAGATTCGCACTCGCTCATCCCGAGAAGGTCAAT
GCTCTTTGCTTGATAAATTGCGTGTCGACACAAGCTGGCTGGATCGAATGGGGATATCA
GAAACTGAACGTTCGCCACTTGAGATCGCAGGGGATGACTCAAGGTGTATTGGATTAT
CTGATGTGGCATCACTTTGGCAGGGGCACGGAGGAAAGGAATCACGATCTTGTACAAG
TGTACAAAAATTATTTCGAGCGTCGCGTGAATCCGACGAACCTGGCATTATTAATCGAC
AGCTACGTTCGCCGTACAGATCTGAACATCACGAGAGAATTAGATCCTACGCGTAAGA
AGGAAGGCCTCACGCTTGGTGTACCTGTGATGAATATCACAGGAGCTTTGAGCCCACA

TGTCGACGATACCGTCACGTTAAATGGACGATTAGATCCGATGAATAGCTCTTGGATGA
AGATTTCTGATTGCGGAATGGTACTGGAGGAGCAACCGGGCAAAGTAAGCGAGGCCTT
TCGACTATTTCTGCAGGGGGAAGGATATG

>novel_circ_000868
GTGGCGTGATGTTGCTGATGCGATGTAATTTCCACACGTCGTCGAGTGGTTATGCGATG
CATGAAGTTGTGTTTCGTCGTGTGAATTTCGATGATTGGGTAGTTGATTGGGAATTGGA
TCATTTTAAAATTTTGTAGTCGTAGTGAGTAGTAATTATTATCACATTATTTAAGTTTTATT
TTATAAATGTATTGTTGTAATTGTCTGAAATGTGCAATGATGTTCAAGTATCTTATCTATAT
TCATGTTACAGATTGAAGTGTTTAAATTATTGCATAAAAGTGATTATTCGAGTAAAAAGA
TAAGTAAATTGAATTTGGTATTTATATAATTTAAATATCGTCAAACATAAATGAAAATTTA
TGTTTCAATCTATATTTAATAAATATTTTGCTACGTAAACATCATGTATTTATATTTTATATC
ATTCGTATGAAATATAATTAATTTAACGTTAATTAGATTTAATTCAACGAGATATATTACTA
ACAATATATCCTCTTTTTTGTTTCAGCTTGATGATACGGCGCTCCAGCTCTACAAAGATG
GCGACTATGGTGCCTATTTGGACCTCGAAGCTTCCATCAGCGAGCAACAAGAAGAATT
CGAAGGGTTCCAAACCAA

>novel_circ_000872
GTATTCCTCCTGACCAACAAAGATTGATCTTTGCTGGAAAACAATTAGAAGATGGTCGT
ACTCTTTCCGATTACAATATTCAAAAAGAATCTACACTTCATCTCGTTCTACGACTTCGT
GGTGGTATGCAAATTTTCGTTAAGACTCTTACCGGAAAAACCATCACTTTGGAGGTTGA
AGCATCCGATACTATCGAAAATGTTAAAACTAAAATTCAAGATAAGGAGG

>novel_circ_000873
GTATTCCTCCTGACCAACAGAGATTGATCTTTGCTGGAAAACAATTAGAAGATGGTCGT
ACTCTTTCCGATTACAATATTCAAAAAGAGTCTACACTTCATCTTGTTTTGCGACTTCGT
GGTGGTATGCAAATTTTCGTCAAAACCCTTACAGGAAAGACTATTACTCTCGAAGTTGA
AGCTTCAGATACCATTGAAAATGTTAAAGCCAAAATTCAGGATAAAGAAGGTATTCCTC
CAGATCAACAGAGATTGATCTTTGCCGGTAAACAACTGGAAGATGGTCGTACTCTTTCC
GATTATAATATTCAAAAAGAATCGACCCTTCATCTCGTTCTACGACTTCGCGGTGGTATG
CAAATTTTCGTCAAAACCCTTACAGGAAAGACTATTACTCTTGAAGTAGAAGCTTCTGA
TACTATTGAAAATGTAAAAGCTAAGATTCAAGACAAAGAAGGTATTCCTCCTGACCAA
CAAAGATTGATCTTTGCTGGAAAACAATTAGAAGATGGTCGTACTCTTTCCGATTACAA
TATTCAAAAAGAATCTACACTTCATCTCGTTCTACGACTTCGTGGTGGTATGCAAATTTT
CGTTAAGACTCTTACCGGAAAAACCATCACTTTGGAGGTTGAAGCATCCGATACTATCG
AAAATGTTAAAACTAAAATTCAAGATAAGGAGG

>novel_circ_000874
GTGGAATGCAAATTTTTGTCAAAACACTCACTGGAAAAACTATTACTTTAGAAGTTGA
AGCTTCAGATACTATTGAAAATGTTAAAGCTAAAATTCAGGATAAAGAAGGTATTCCTC
CTGATCAACAGAGATTGATCTTTGCCGGTAAACAGCTGGAAGATGGTCGTACTCTTTCC
GATTACAATATTCAAAAAGAATCTACACTTCATCTTGTTTTGCGACTTCGTGGTGGAATG
CAAATTTTTGTCAAAACACTCACTGGAAAAACTATTACTTTAGAAGTTGAAGCTTCAGA
TACTATTGAAAATGTTAAAGCCAAAATTCAGGATAAAGAAGGTATTCCTCCTGATCAAC

AGAGATTAATCTTTGCCGGTAAACAACTGGAAGATGGTCGTACTCTTTCCGATTACAAT
ATTCAAAAAGAATCTACACTTCATCTTGTTTTGCGACTTCGCGGTGGTATGCAAATTTTC
GTCAAAACCCTTACAGGAAAAACTATTACTCTTGAAGTAGAAGCTTCTGATACCATTGA
AAATGTAAAAGCTAAGATTCAAGACAAAGAAGGTATTCCTCCTGACCAACAGAGATTG
ATCTTTGCTGGAAAACAATTAGAAGATGGTCGTACTCTTTCCGATTACAATATTCAAAA
AGAGTCTACACTTCATCTTGTTTTGCGACTTCGTGGTGGTATGCAAATTTTCGTCAAAA
CCCTTACAGGAAAGACTATTACTCTCGAAGTTGAAGCTTCAGATACCATTGAAAATGTT
AAAGCCAAAATTCAGGATAAAGAAGGTATTCCTCCAGATCAACAGAGATTGATCTTTG
CCGGTAAACAACTGGAAGATGGTCGTACTCTTTCCGATTATAATATTCAAAAAGAATCG
ACCCTTCATCTCGTTCTACGACTTCGCG

>novel_circ_000875
GTTGAGGCCTCAGATACCATTGAAAATGTAAAAGCGAAAATTCAAGACAAGGAAGGA
ATTCCACCAGATCAGCAAAGATTGATCTTTGCTGGTAAACAATTAGAAGATGGGCGTAC
ATTATCTGATTATAATATTCAAAAAGAGTCAACACTACATTTAGTACTACGTTTAAGAGG
TGGAATGCAAATTTTTGTCAAAACACTCACTGGAAAAACTATTACTTTAGAA

>novel_circ_000876
ACAGCGAATTAGAGCCACCGTTTCGACGAGCTATTTCTCTTAGGCTACCCAAATCATCG
AATTCAAATACTATTGATCGCAATCGATTATATGTATCGACATATCATGGAAAATCATCGA
TGTCCATGACATGGACACCAGACAAAACCATACCAGTTTGTCCCGCCAGGCATTCCAC
ACCTACGATTGACGCACCTGCGCCGGAATATCTTGCCCGAAATCTTCTTCAACTTGGTT
GTCCTGTTGTGTCAATGCCCACACCTCGTGGAGGAGTAGGGTCTGAACCTCCGCAGAG
TAGCGATAGCGATGAATACTCGACGACGAATACTCAACAGCATAGTCATGGACATGGAT
ATGGTCAAGGACAGGTGGATATCTACAACGTGCCCAAAGTCCAGGTGTCGGGACCATC
GAACAATGATGAATCCTCTTCCGTCATCAATGG

>novel_circ_000877
ATCCATCCGCTTCCGGTTCTTCCTGCGAGGATGACGAGGAGAGTCGGCAGGTGGACAT
CAGGAAACGCAAGAACAGTTTGTACGCGTCTACCTCGACGCGCGCTCCATCAATAGAC
AGGGAGAAAGAGTTGAGCTCCGCCGAGGGGGCGTTGGTCGACTGCGTGGCCCTGGTT
CATCTCACGGATCAATCGCCAACCGAGTCCAATCAACCGGTCCGTCATCCATCGCCCTA
TTACTACGGCGACTTGTTCAAGGTACCGGAGCAAATAAGCAAATCACAGCCGAACAGG
TATAGGAAGAGCGTGAGCCTCGACGTACCGGGTGAACGGTCTCGAACACCCGGTATAC
CGAAGAGAAACTCGGTGGCGGGTGATGGGGGTTCCTATTCGTCCCAGCAACAGCAAC
CGCAACAGCAGCAGCAATCACAGCAGCCGCTACATTATCAGCAGCAGCAGCAGCTACA
GCTACAGCAACATCAATTACAGCAACATTATCAAAGTCAGGATCTAGTGAGCCCCACGT
CGGGGAGCGAGCATGCTGTCCCAGCCAGAAGCGAGATCCTCTCGTCGTGCATCATCAC
CGAGTGGGATCACACCCTCATGCCTCCTCATAGATTGTTAAGCCATGACCTACCGACCA
CCGTTTGTACCTGCGTCGCATCGGCTGAGGAAGAGGAGGATGAGCTAGATCAACGGCA
GCCGCAGTTAACGCGGCACCAAGTGCGCAAGTTACGCCGTACACGGAGGATAGACCC
GCAGCCGATACTCGTCGAGGACGAGGACGACGTGGAGGGAGAGGACGAGGGCGAGG
AAGAGGAGGAGGAGGACGTGGAGGTGGAGGAAGACGAGGAAGAGATGGCTAGGCA
ACGTCACCTCTACGAGACGGCCTTCGACTGCAAGGTCAATCGCTCCGACGATGACCTC

GATGACCTCGATCGGGTCACCAATCATCCCGTGTTGCATTCCCAGATAAGAAGCAGCA
GCGCGATACCAGTGAGCAGAACGAGTTCATCGACGGACACGTCGAAGCAACAGCAAC
AGCAGCAAGCTCCGTACGCTGGTCAATCCCACAGCAACAAAGATCGACGCAAAGTCG
TACTTCTCCGCCCAAAACCTATTCCGAGCCGTCTTCAGCAGCAGCAGCAGCAGCAACA
GCAGCAATCAGACAATATTTCTACTCTGTCCCAGGACATCGAATCCCTCCAGATCAATT
CGAGCGACGAGAAGACCGGTTCGCCGAGATTACCGGCCCGTGGTTACACACCGTCGC
CGCCATCCACGGCACCGTTGCCCGCCAAATTCCACGGCAATCGTGACCACTTGTTGTTA
AACATACGAAGCACGCCCAATCTACCCTCTCAACCGGACCATCCGCGCCTCAAAGATC
TCCGATTGCCTGTGAAATCCTCGTTGAGGGCGAAGGACTCGCCGCAGAGCAGCGAGG
GTAGTATCCTCGAGATCAAGAGTAGACCGAAGAGTTTTGCTCAAGAAACCGTGGAGTC
GTCGCAAGGGCGACGTTTCGACAAGGAGTTGATCCTAGAGTTCAAGGGGAGGCCGAG
GGAGGACAGGCAACGACCGAGGAGCCTGATCCGCGAGAGCAAGCCCATCATGGAGTT
CAAGGGGAGGCCCCACGAGGCCGAGAGCGATCTGTTACGGCCCCGAAGGGACGTCGG
CCTGTTGGAATTCAAACTGCGCACCAGCAGACGCAGACCAGGCTACTCGAGCACCGA
GAGCATGGCCACGAGTAGCAGCGGCGGCAGCATGGAGTCGCTGCGAAGCAGCACGAG
CGAGGGGAACAGATCGACTAGCAGCTCGGAGAGCCGTCACAGCACCAGCCTGAGCTC
CCACAGCTCGGACAGCGGCGGCACGAATTGCTACCACCACCACCAGCAACCCTCGAT
GACCGGTTTCTTGACCCACCACGCGAACAAGCTGCACATACTGTCCCCCATCTCCGAC
AAGTCGTCCCAAGAGCCCGCGTCCGAGACGTCGGACAACAATCGGAACAACAACTCG
CAGAAAGCCTCGCCCGAGGAGAACGTGACCACCGAGAACAACGTGACCGCCACGAA
CAACGTGACCAACACGAACACGACCGGGAACACGAATTCGGTTACCTCGCCGATGGA
CACGTCGTTCAAGAAGAGGCGGACGCCGCAGAACAAGAATCTGATCAATTTGGCGTT
GCAATCCTCGTCGTCGGGCGATGCGGAGATCCAGGGGTCGGACAGCGGGATCTCGATC
GAGTCGCGGGCCGGGATCAAGTGCAAACCGTTCGGTTTCCACTTGTTGAAACCGCAAT
TGGACAATATCAATCTGGTGGACAACGAGCCCGAGCTGTCCGATCTTCCTTTCGACATG
CCGAAACTGCGTAGAAGGCGATTGCTTATGCAACAGGACGCGACCACATCTGGGAGC
GCGACGAGCGTGGATTTGAGGGACTTGCCGTTCGACATGCCGAAACTGAAAAGAAGG
CTCAGATGCATGCAGAGCACGGAATCCAGTGGATCCCAAGCATCCTCCAGCCTTTCCG
TGCGTGACGTCGAGCCGCCTGCGTTGTTCG

>novel_circ_000878
TGAGGACGCTGGGCGTAGCGGCGACGAAATGAAGCATGCAGCATGCGTGGTAGCGGT
GACGGCTGCGCGGTCCTGCCTTCAACCCCTGGTGTCCGCCAGCCGCTACCTCGCCGTT
CAAGCCCTTCCTGGTGATCCTCTTTACTTCGTCGTTGAGGCGAAGTCGAGAGTGAAGG
AGGTGTACGCGCAGACATGCATGCTTCTCGGCCAGCAGGGCATGCGAGACTGCGAACT
GTTCGGCCTGGCAATATTGTCCGACGGCGAGTACCTGTTCGTCGATCCCGAGAATAAAT
TAAGCAAATACGCGCCGAAAAACTGGCGGAGCTCTCATACATAC

>novel_circ_000879
ATTTCGTACGAACCGTGGTTGCACGGTACGAAAGGCACCGAGAGCGACGAGGATGAT
GTAGCCGTCCGGCGGCTGCCGATTCACGAGAATAAGCATCGAGAGGATGTACACGAAC
GAACAACGTGCACGAACAAACGAGAACGAGTTGTTTGTCGCGATTCCTCGAGCCGAT
TCCATTCGCGTAACGCGTTAGAGAATAGAAAAATCGTGACCAAGAAAGAATCATAACT
GCCAGATATATAATTAAAAAAAAAAATCCTTCCGTTCCATCCTGAGAAAAAAAAGAAA

AAGAGCAGAAAGAAGAAGGAAGGAAAAAAGTATCTCTCTCCGTTCTCACCCTCTTTC
GAGATCAAAAAGACTGAATATCAAAGAGAGGATCAAGATGTTGGGAGTGAGTCAACC
GGGAAACCCACCCTCGAGCCTGCGACACTCGGCGAGTTTCTCGTGTCTTCAACGGGG
CACGGCGAGCGAGGGACACAGGGCGAGGACCTCGACTCTGCTCCAAACACAGCCGA
GCCTCCAGCTGAGCAACAGCCATCACCAGGCGTACGCGCCCGGCCAGGCGACCTCGT
CCCTCCTCCAACAGTCCTCCTCGGCCGTGGGGACCGTTCAACACCGCATCGAGGCGTT
GGAAGCCAGCACCGTGCAAGACACCGGCAACGACTACGGTTTCGTCAAGCTACGCCC
ACGCCTGCGTAGCACCAACGCCACCCTCCACTACGAGGAGGAGCTGGCCGCTCAGAG
GGGCGCTATATGGGACCGGGACGCGTCGGCGTTCAGCGACCGCGAGTGCGACGGCAA
TTACAGGGGGGACCCGCCCTCCCTCAAGCTCAAGGACAACAGGGAGCACTCGCCCAA
GGGCGGGAAGAATCAGAACCCGCTCAGGGGCGCCCTCGTACTGTTCACCTCGACCTC
GTCGTGGTGGAAGGCAAGGCAACGGGAGGATCAGCTGAACGGCGGCGTGGCGGCGG
CGTTCGCCTCCTCGTCCGAGCGAAAGTGGTCCAGCACCTCGATGGCGTCCCCCTCGAA
CGAGAGAAAGTGGCCCTCGCTCGTCACCTCGCCCACCAACGATCGCAAATGGTCCCTT
GCGGCCAACAACAACAGTAATAACAACAACAACAACAGCAACAACGCTTTGACGTCG
CCGGGTAACGAGAGGAAGTGGACGAGATCGTCGGTCACGTCGAACTCGGGCGGGCAG
CAGCAGGACAGGAAATGGCGGTCGCTCGGCGCCCTGTTGAGGGCCCCGACCGGGGCA
GCGACGCCCGCCTCGCACGCCGTCCAACACTCCTTGTCCCACAACATGAGCGTCTCGA
TGATCGAGGACCACCACTTCAGGGAGGACGCCCGGGGCTCGGCATCGTGCAAGTCGA
CGCGTTACCAACAACCGGCGGCCACCTCGTACTCGGCCCGGGTGTCGCGCGTCACCCG
GGACGTTTACAGGGAGAACGGGTCGGAGTTCGGGCAGGCGGGGGGCAGGTCGAAGG
TGAGCCGTAGGCTGTATCAGGAGGATTGCTTGGACAGGGACGGGGCGGAGGAGAGAT
CTCACCAGGCGCCGAGGCAGCAACACCAGTTCGACGAGGAGTGCAGGGGAGGGCGC
GCGAATCGGGAGAGTGCAATCTGCAAAACGGAGCAGCAGACGATGAACGAGACTGCG
GGTGGCACGCGCGCTTCCACGACGAGATCGAGCCGGGCGCAAAGCTTCTATCTCCTCG
ACGATTTCCTGCGGCCCCAGCCCCAACAGAACAACGCCACTAATAATAACAATAACAA
CAACAACAACAAGTGCATGCTGAACGTTTACCTGTCGCCGTCGCACGCCGCGTCCAAG
TCCACGTGTGGCGGCAACGATCTGCTGCACTCGGGCAGATCGGGCGACCAGGCGAAG
CACCAGGTCCCCCCGCAGGCAGGGAGCGCCAACGTGACGAACATCACTCCGCCGCGG
CGCCACAACTCCAGCTCGGACAGCCTCGAGGTCGCGACCAGTCCGCTTCCGCCGCCT
GTCCCGCCCCCGCCACCTCAGGTCGCCGCGACCCGCGACAGGGAGAGGGCGGAGAAT
TTGAAAGAGAAGGACGTGTGCCAGTGCAAAGTGTGCTGGACCAATATGCAGCAGCAA
AGATGTTTCGTAGAAGAG

>novel_circ_000880
CTGGGAGATTGCCAATGACAGCTCCAGAAGCACTCAAGTATTATGGCTCGAGGCTCAC
CGAATTTGAGCGCAACGAAATCGAGAAATACTCGGAGATTTGGTACCTCGGCCTGTCG
GCCACGAAAATTCACGGCGAGGAGGGCTCATCCCAAAATGGTGGATACGACGACGAA
AACGGTAGTTACAACAAGGTCTTCCACGATCATATCTCGTACAGATACGAGATATTGGA
GGTGATCGGGAAGGGAAGCTTCGGACAAGTGATCAGAGCGTTGGATCACAAGACGGG
ACAGTACATTGCCATCAAGATCATCAGAAATAAAAAGAGATTCCATCATCAGGCGTTGG
TCGAGGTGGAGATTTTGGAACATCTCAGGAAGAAAGATTTAGAAGCGAATGCGTCGCA
CAATGTGATACACATGCTAGAATACTTCTACTTCAGGAACCATTTGTGCATCACCTTTGA
GTTAATGAGTTTGAATTTATACGAGCTAATCAAAAAGAATAACTATAAGGGATTTAGTCT

GAGCCTAATACGACGATTCGCCAATTCTTAATTAGCTGCCTGAGACTGCTGTACCGAG
AGAAGATAATTCATTGCGATTTGAAACCTGAAAACGTGCTTCTGAAACAGCGGGGTAG
CAGCTCGATTAAGGTGATAGACTTCGGATCCTCGTGTTACAGCCATCAGCGTGTATATAC
ATACCTTCAGTCCAGATTTTATCGTAGCCCGGAAGTGATTCTGGGCCTTCCATACGGTAC
ACCGATCGACATGTGGAGTTTGGGCTGTATCCTGGCCGAACTCTACACAGGTTGCCCTT
TGTTCCCGGGCGAGGATGAAATCGAACAACTTGCCTGCATTATGGAAGTTCTTGGCCT
GCCACCGGAACATATTATTAATCACGCATCCCGTCGCAGGCTTTTCTTCGATCCGAAGG
GAAGTCCTCGATGCGTGACGAACAGTAAGGGCAAGAAGAGATGGGCTGGTAGCAGGA
ATCTTGCCATAGCTCTTCGTTGCACCGATACACTTTTCGTCGACTTTGTTCGCAGATGCC
TCGAGTGGGATCCAAAGAAACGCATGACCCCCGACGAGGCGATGCGTCACGAGTGGC
TGAACTCGTCCTCGTTTCACGTGAGCTCCTCGACGTCCACGATAGCCGCGTCGACGGT
GAACGCGAACACGAACGCGAACGCGAACGCAACTAGCATGGAAACGTCGTCGCAATC
CGCCCACGCTCAAACGGTGAGCGTGACGGTGAGCGCGCCGAGGCAACGGGCGACCA
CCGTCGAGGATCCCCCGTACACGATGTACCGTTTGTGCAAAGGTCGTAAGTACGTGCA
GAGGATCAGCACGACCGAGAACACGGACAACGGGGGCCTCGTAGTGAAGAGCAAGC
TGAACGGAAGCGCGAGCAGCCACGCGCTCGCGAGTAGCACGCAGACTACTACCTCGA
GGCACGCCTCGACCGGCGATATCGTCGCGAGTCTGGACCCAAATCTCGACGATTCCG

>novel_circ_000881
CTCTAGTATGAGTTCTCGAGGCCGTGGATTTAATTCTCGGGGAGGCAGTTCTTATAGAG
GAAGAGGTGGAGGTAGTGGAAACGAATGGAATAGTGGATCAACAGGAGGAAACATGT
CTAGCAGAGGAGGTTATTCCTCATCTAGAGGAGGTCGATTTAAATATTCAACTACTAGTT
ATGATTCGCGTTCAAAATATAATTCTGGAGGTTCAGAAAGATATTCTAGTAGAGGAGGA
AGAGGTGAACATTCAAATAGTTACAAAAGACCAAGGGATTCTTATTCTGGAAGAGATG
AACATCGTTCATCAAGTGATTCAACTCGTAAAAGGATGAGAAGTGATTCATATCAG

>novel_circ_000882
CACAAGAGTTATCAAGCCAAACGGGCCGGACATTGGTCGTAAGGTGAAGGTAAAATC
GACTGGAAAACCCATACCATCATCATCAAATTCAAGACATAGGGAATCAACGAGCATTC
CAACTTCTGGAAGTCAGGCTAGACTGTCGTCTTCCTCTTACAAACCGGCTAATCCAGC
AGCGGCTCGAAGCCAACCGGAGAAAAAAATCCCTGACATTATGCGCAGACCATTAAA
AGAAAGACTTATTCATCTTCTTGCTTTAAGGCCATATAAAAAACCTGAACTGTATGATCG
TATAAATAGAGAGGGTTTAAAAGAACGAGAGCGTAACATCATGACGACAATCTTAAAG
CAAGTAGCTTTTATGAGGGATAATACGTATCATTTGCACAGATATGTTTGGAATGATGTG
CAAGAAGACTGGCCTTATTATACCGAACAGGAAAAGGCAATGTTAAAAAGAAGAAAA
CCTCAAAATCTTACACCTCCTGGTTCCAGTGATGGTGGATCAAGTGGCAGCGGACAAT
CGCCGAATTCCACTCATCCAGGTTCACCGCCTGCAATCACAGCACCACCTCCATCTTTG
TTGAACAATAAGCGACCAGGGTATTATCAGGGAAATGATGGGCTGCCGACGAAGAGAC
CACGGATATCCCATTATAAGAAGTCAGAACAAAATTCTGGATCGTCGATCACGGGAGA
GAACGGAAGGACGGCTGGTAGTGGTAATAGTAATAACAGTGTTAGTGTTGTTTCTGGA
GCTGGTAGTACTAGTGCTAACGGTGGCAATAGTAGTAACAGCGGTGGTGTGGTTGATG
GTTGGGATCAGAGACAGCACCAACGTGATCGTCGTGGCGATTATCGGCCCGAAAGAAC
AGCGAACAGTGACGTGCTACGAAGCAGCAGCTACAGCACTGGGAAGCTACCGTGCTT
GACACCAACCAGTGACAGTGAAGAGATCAACCAAACTGTTGGTCATCGGGACGGAAT

CATAACGAGCACCGGTTCATCCGGAAACAACGCAGCTAGCAATCCGGCCACTGGCCAT
AATTCTCATAGTATAGCCCATAGTAATTCATTGAGCGGCAATCGTCATCATACAGGCAAG
ATCGGTACCGATACCGGCAATTCTGGGACGGATTATGTGACGCGTCTTATGCCAAGTGT
CGTGAGTGACGGTGGTAGTAGTGCAAGTAGTTTTAGTGGTAGTTCGAATGGAATACTC
GCTGATAGGAGAGACAGAAGCGACAGGAATGACAGGAGCCGGGCAAGCGAAAGGGA
TCGTGACTCAAGAAAGAAAAGCAACGGGACAGGTGGTAACTCCTACAATGATTTAACC
GCCCCTGATTACACAGCCACCGAGGATAACACTGTCAGTACATCGTCTTGTGCTCTTGA
ATTACCTGAAAGTCCTAAATCGTCGGAATATCCGGACTACCTCACGTATTACACGACAAT
AAGTAGTTCAGAACAGAGAAGACGTTACAAAGCAGAATTTAATGCTGATTATGAAGAG
TATCGACGGTTGCATGCTCAAGTAGCAAATGTGTCTAAACGGTTCACGCAGCTTCAGG
AACGTTTGAAACAAGAAGAAGCCTCTGGGAACTGGGAAGAGTACGAGGAAGTAAGA
CGGCAAATTTTGCACGACTATAACGAAACTAAACGGGACCCTGTACATAAAGAGATAA
AACGTCGATTTCATTATCTGCACGAGAAGCTAAGTCACATTAAACGGCTAGTATTGGAG
TACGACACGCAAAACTGTGGCGGTGGGATGACAAATAGCAGTAATGCTAATGGTACCA
ACGAAATTAAGGAGATGGACAGTTTGCACTACTGACACAAACTGAAAAGGCCGCCTC
TTATATTCAATCTTTTCTCAATTGTCG

>novel_circ_000883
CAATTGTCCTTCCCATCTACGCAATCCAGCCATGGATCAGCAGGCTTTACGTTTAGCCTT
TCTGGCAATCAAGACATTGAGGGTCCTCAGGGTGGTTTCGAATGCATCCAGCAAACAG
GCCCTAACGTTCCATTTTTCCTGTGCAGAAGCCTGGAGAGTCTTGGCGCATTGCCGTGT
AAAATGCGGATACAGGCGAACGACGATGTGTACGAGACAACCAGGCATCGCATGGCT
GTCGCCGAGGAGAATAATAAGAACAAATGCACAAGAGTTATCAAGCCAAACGGGCCG
GACATTGGTCGTAAGGTGAAGGTAAAATCGACTGGAAAACCCATACCATCATCATCAA
ATTCAAGACATAGGGAATCAACGAGCATTCCAACTTCTGGAAGTCAGGCTAGACTGTC
GTCTTCCTCTTACAAACCGGCTAATCCAGCAGCGGCTCGAAGCCAACCGGAGAAAAA
AATCCCTGACATTATGCGCAGACCATTAAAAGAAAGACTTATTCATCTTCTTGCTTTAAG
GCCATATAAAAAACCTGAACTGTATGATCGTATAAATAGAGAGGGTTTAAAAGAACGAG
AGCGTAACATCATGACGACAATCTTAAAGCAAGTAGCTTTTATGAGGGATAATACGTAT
CATTTGCACAGATATGTTTGGAATGATGTGCAAGAAGACTGGCCTTATTATACCGAACA
GGAAAAGGCAATGTTAAAAAGAAGAAAACCTCAAAATCTTACACCTCCTGGTTCCAGT
GATGGTGGATCAAGTGGCAGCGGACAATCGCCGAATTCCACTCATCCAGGTTCACCGC
CTGCAATCACAGCACCACCTCCATCTTTGTTGAACAATAAGCGACCAGGGTATTATCAG
GGAAATGATGGGCTGCCGACGAAGAGACCACGGATATCCCATTATAAGAAGTCAGAAC
AAAATTCTGGATCGTCGATCACGGGAGAGAACGGAAGGACGGCTGGTAGTGGTAATAG
TAATAACAGTGTTAGTGTTGTTTCTGGAGCTGGTAGTACTAGTGCTAACGGTGGCAATA
GTAGTAACAGCGGTGGTGTGGTTGATGGTTGGGATCAGAGACAGCACCAACGTGATCG
TCGTGGCGATTATCGGCCCGAAAGAACAGCGAACAGTGACGTGCTACGAAGCAGCAG
CTACAGCACTGGGAAGCTACCGTGCTTGACACCAACCAGTGACAGTGAAGAGATCAA
CCAAACTGTTGGTCATCGGGACGGAATCATAACGAGCACCGGTTCATCCGGAAACAAC
GCAGCTAGCAATCCGGCCACTGGCCATAATTCTCATAGTATAGCCCATAGTAATTCATTG
AGCGGCAATCGTCATCATACAGGCAAGATCGGTACCGATACCGGCAATTCTGGGACGG
ATTATGTGACGCGTCTTATGCCAAGTGTCGTGAGTGACGGTGGTAGTAGTGCAAGTAGT
TTTAGTGGTAGTTCGAATGGAATACTCGCTGATAGGAGAGACAGAAGCGACAGGAATG

ACAGGAGCCGGGCAAGCGAAAGGGATCGTGACTCAAGAAAGAAAAGCAACGGGACA
GGTGGTAACTCCTACAATGATTTAACCGCCCCTGATTACACAGCCACCGAGGATAACAC
TGTCAGTACATCGTCTTGTGCTCTTGAATTACCTGAAAGTCCTAAATCGTCGGAATATCC
GGACTACCTCACGTATTACACGACAATAAGTAGTTCAGAACAGAGAAGACGTTACAAA
GCAGAATTTAATGCTGATTATGAAGAGTATCGACGGTTGCATGCTCAAGTAGCAAATGT
GTCTAAACGGTTCACGCAGCTTCAGGAACGTTTGAAACAAGAAGAAGCCTCTGGGAA
CTGGGAAGAGTACGAGGAAGTAAGACGGCAAATTTTGCACGACTATAACGAAACTAA
ACGGGACCCTGTACATAAAGAGATAAAACGTCGATTTCATTATCTGCACGAGAAGCTAA
GTCACATTAAACGGCTAGTATTGGAGTACGACACGCAAAACTGTGGCGGTGGGATGAC
AAATAGCAGTAATGCTAATGGTACCAACGAAATTAAGGAGATGGACAGTTTGCACTACT
GACACAAACTGAAAAGGCCGCCTCTTATATTCAATCTTTTCTCAATTGTCG

>novel_circ_000884
CAATTGTCCTTCCCATCTACGCAATCCAGCCATGGATCAGCAGGCTTTACGTTTAGCCTT
TCTGGCAATCAAGACATTGAGGGTCCTCAGGGTGGTTTCGAATGCATCCAGCAAACAG
GCCCTAACGTTCCATTTTTCCTGTGCAGAAGCCTGGAGAGTCTTGGCGCATTGCCGTGT
AAAATGCGGATACAGGCGAACGACGATGTGTACGAGACAACCAGGCATCGCATGGCT
GTCGCCGAGGAGAATAATAAGAACAAATG

>novel_circ_000885
AGAAGTTCGATATCACCCGCGGAACGCGAAAAGTATAGCAGGCCGCGATCAACCGACC
CCCAACACCACGATCACCTCCCACTCAAGAAGATGAAGAAGGAGCAGGACAAGGATA
TAGGCCATCAAAGCGATGGTGAAAAGAGCGACCAGGATTTGGTGGTTGACGACGCGA
GCGAGGGGCCGACGAGCCCCACGGCGAATGGCACAGCGTCGCCAAGGGAGAACGGG
CTCGACAAACTCGGCCCGTCCTCGGTGCCTTTGAGCAGCCAGGTGAAGAAAGAAGTC
CCGCCACCGTCGCCGAGAAGCGGCACGAGCAGCAACGCGAGCACACCCTCGGCTAAG
AAGATGGAAGAAAGAGAAAAACCGACTACGCCTATCTCGAAACCCGTCACGCCCACG
TCAGCTGGTGGTAGCAGCTCCGGGTCCGGTGGTCTGAAACCATCGAGTTCCAGCAAAC
CACTGGTCTCGGCTTCGGCGGTCGGCCCTTATCCGCCGCACTATCCGCCGCCACATCAT
CCACCCGCAGGACCTCATGTGGACGTGCTGGGTTATCCCCCAGCCCTGAATGGATATCC
CGCAAGACCGCCTCTTCAGCAACTCTACGATCCTCACGGAAGCATGAGGGCTCCTCTT
GGACCTCTCGGTGTACCCGGTGGAAAACCCGCGTACAGTTTCCACGTGTCGAGCGAG
GGCCAGATGCAGCCTGTTCCATTCCCTCACGACGCTCTAATTGGACAAGGAATACCGC
GTCATGCACGCCAGATAAATACCCTGACGCACGGGGAGGTGGTGTGCGCGGTGACGAT
CTCGAATCCGACTAAATACGTTTACACGGGTGGCAAAGGATGCGTCAAGGTCTGGGAC
ATCAATCAGGGCGGGAGCGGTAGCGCGAAACACGTTTCGCAGCTTGATTGCTTGCAAC
GAGACAACTACATCAG

>novel_circ_000886
AGAAGTTCGATATCACCCGCGGAACGCGAAAAGTATAGCAGGCCGCGATCAACCGACC
CCCAACACCACGATCACCTCCCACTCAAGAAGATGAAGAAGGAGCAGGACAAGGATA
TAGGCCATCAAAGCGATGGTGAAAAGAGCGACCAGGATTTGGTGGTTGACGACGCGA
GCGAGGGGCCGACGAGCCCCACGGCGAATGGCACAGCGTCGCCAAGGGAGAACGGG
CTCGACAAACTCGGCCCGTCCTCGGTGCCTTTGAGCAGCCAGGTGAAGAAAGAAGTC

CCGCCACCGTCGCCGAGAAGCGGCACGAGCAGCAACGCGAGCACACCCTCGGCTAAG
AAGATGGAAGAAAGAGAAAAACCGACTACGCCTATCTCGAAACCCGTCACGCCCACG
TCAGCTG

>novel_circ_000887
ACAGAGATAGCGAAGAGGTTAAACGCCATAATTGCTCAGATCCTGCCATTTCTCTCGCA
GGAACACCAACAACAAGTCGCTACTGCTGTTGACAGAGCCAAACAAGTGACCATGAC
CGAGCTCAACGCCATTATCGGGCAACAAAGGCCAGACCTGCCAAGGCTTCTTCAGCAG
GTGCACGCCCAACAACTACCACCTGGCGCCGCGATGGGCCCGCATCCAGGCATACCTG
GCCTTCCTGGGCTCGGCCCTGGCGCTGGTCTTCCCGTTCCTACGTCAGCCTCCGCAGC
ACTTCTCGGATTAGGTATTCCACCGGGTGCCGCAGCAACCGCTGGTGGCACAGCTGCT
CATCCCCTTTCGATGTTGACCAAGCCGGACATCCATCGTCAAGCGGACGATTTAAAGCA
CAACGGAG

>novel_circ_000888
TTCCAGACTGTTCCTTCGTTGCTCCCCGAACGGCCCTTACACGAAGTACAAAAAGTAT
GGTACCGCGACCGGTGAACAGCACCTTACTAACGTCAACGTTGCCTCCGTTCCGTGAA
TCGGCCGTTCCCGGCTAACGTCCTTGGGCTTCTAATATTTACACGCTCGCAGCGCGAGC
CACCGCCACGATATTCCCAACGTTGACGATAAAGCCAACGGCGTAAAGAACTGAATTG
AATTTTGCGCGCAAACAGGATCAGTCGCCTTAGAGTGTAAATAATGGCGGGGCAGAGC
GTTGTGACGCGAGCTTTCCCCGAGATACTGCCCCTTTTCATGGAACCAGTAATTCGACG
TTATCTGCGGATGTCCCTTTATGAGCGACGTCGCGCGTTGCGGTTTAACTTCCATTCATG
TAATTCGATCGTTTCGAGAAGCCCGAGTGTATATAATATAGCCTGTGCATCGACGTGGAT
AAAAATAAAAAAAAGAAAAAAGAAAAAAAAGAAGGAAAAAGGAAGAGAGAAGAAA
GGGAAAAAAAGAAAGAAAGAAAGAGAGAAGTACAAGAAGCGTTTGGAGAAGGCTTT
GACGACACGGAGTAAAAAAGGGCGTAAAAACTTACGAGCACGATAGTTGTTTTTCACC
TATACGACTGAGGCAAAAGAAAAAGAAAAAAAAAGGATACGGTGTAAGAGGTCGGAA
TAAAAGACGCGTGAAAGAAACAAGACACGCAAGTGCTGCAAATCGAGAGAAAGAAA
CGTAGTGTAAAAAAAAAAATGTTGCGGTGCTGCTCGCCGCCGGTTAGCCCCTGGGCTT
GAAAGTCTATTGTCCTCGGTTCATTACACATTTTCAGAAGTGTCCTGGCGAAGGACGGT
GGGTGGAGGAGGGACCAGTCGCTGACCAGATGCATGACAACGAAACTGAAATATCAG
ATTTCGCGCTAACGTTGCGTCTTTTTACTTACCGTAGTGCGTTTTTCGCGAGTAAGGGC
CACCAAAGGCCCTTATGGTGGAAAAAAGAACAGCGGGGCGAAAAAAAAAAAAGGGG
GAAAACATTTTACAAAACGTTGTTCTCCGTTACCAGGAAAGCAGGCAGAGGGGGAGA
GATTGGGATTTTCAGCTCCGATAATAGTCACTTTTGGGGTAACCGAGCGAATCCATTGA
ATCACCGATCGCCAA

>novel_circ_000889
TTTCTCGTAGTCATCGATGACGCGTTTAGAGGAGTCATAAGGAAGAGCTAGACAACTT
GTAACAATACCAGCCTCCAAAATAATGTGCTCGAAGACGCAAACACGTCAATTAAGTT
GATTTCACAGTTACGAAACGAGACATGGGAAAAAGGACGAGAAGAAGAAACATTTTT
AACGATAAGCTTTGCCACCGCCTGCCACTATACTTAGGTCGGGGTCCTCCTCCGCCTGG
GAAACTTTTAAAGAGGCCGAAGAAGAATATTCGCAAATGGAGTACTCAAGTTGCAGTT
TGCGGTCAGTACGCTCATATTATGCAGCCGTGATCTTTGTAGATTACAAACGAAAGTCT

TTTCTCTTAGTGGCGTTCCGGTGATGTTAAACTTTGTGGGCCAGCGGCCGTCCATTAC
GGTGATAACATTGCAGCCATCAAGCTTACCACACTGCGCTTACTCCTCCCCCATCTTT
CGAACAAGAAGAAGAAGAAGAAGAAACTGCTTTTAATCTTCAAAGAGGATTCTTTTA
ACCCTTTGTACATAAGTAGATTTCTCCCGTTCCTTGCGAAACGAAATTTATCATAATCCC
TTGGCGTTTCTTAAACTTAAGTAAATACGAATCTTTACGAATATTGTATATACGAATATCC
ATAATTCCACGAAATGAATGAAGGGTATGATAATTTGGCTTTGGAAAGAAGACACGATA
ATTCTACGAAACGAATAAAGAATTATCGAAAGAGTAGAAG

>novel_circ_000890
GTCGGTGAAGCTGTTGCCGGACGGCAGAACGCTGATAGTCGGGGGCGAGGCCAGCCG
ACTTAGTATCTGGGATCTGGCGAGTCCAACGCCGAGGATCAAGGCCGAATTAATCTCG
GCCGCCCCCGCCTGTTACGCCCTGGCCATCTCCCCGGACTCGAAGGTTTGCTTCAGCT
GCTGCAGCGATGGCAGCATCGCCGTCTGGGATCTTCAGAATCAGTCCCTGGTGAGACA
GTTCCAAGGCCACACGGACGGCGCCTCGTGCATCGACATATCGGCGGACGGGTCGAAA
TTGTGGACCGGCGGATTGGACAACACGGTCCGCTCGTGGGATCTGAGGGAGGGTAGA
CAGCTGAAACAGCACGACTTCACGTCCCAGATATTCTCGCTCGGCTACTGTCCAACGG
GGGAATGGTTGGCCGTGGGCATGGAGAACTCGAACGTCGAGGTGTTGCACGCCTCGA
ACCCGGACAAGTATCAGCTACACCTGCACGAGTCTTGCGTGTTATCGTTGAGATTCGCC
TCCTGCGGCAAGTGGTTCGTCTCGACCGGCAAGGATAATTTGCTGAACGCGTGGCGCA
CGCCTTACGGGGCCTCGATATTCCAGTCCAAGGAATCGTCATCCGTGCTCAGCTGCGAT
ATATCAGCCGACGACAGGTACATCGTTACCGGTTCAGGTGACAAGAAAGCGACCGTCT
ACGAGGTCATGTATTGAAACCGCAACGAAACCGAACCGTACCAACCAGCCAAACGTAT
TCATCAGACCCCGAGCAACTCTCCACAGC

>novel_circ_000891
AGGAGCTCGATATCGCCCGGTGATAAATATAGTCGCCCGCGTACACCGCAGGAGACGC
ATCATTCCCATCATTCTCAGAATCACCATGACCATCCCATGCACATCAAGAAGATGAAG
AAAGAGCAGGACAAGGACATCGGTCATAGTGACGGGGAGAAGAGCGACCAGGACCT
CGTTGTGGACGACGCGAGCGAGGGTCCGACGAGTCCCGTGGTGAACGGCACAACGTC
GCCAAGAGAGAACGGGCTGGACAAGGTGGCGCCGATAGGCGCCGGGGTGCAGACGA
AGAAGGACGTGCCGCCCCATTCGCCGCGAAGCGGGACGAGCAGCAACGCGAGCACA
CCCTCGGCCAAGAAGATGGAGGAACGGGAGAAACCGACCACGCCGATCTCGAAACCC
CTGACCCCGACCTCCGGCTCCGCGATCGGTGGCGGTTTGAAGCCGTCCGGCTCGGCCA
AGCTGCTCCAGGGACCACCGCCCCCCGGCGTGCCCAGCGCTTATCCGCCGCACTACGC
CCCGGGTCCACCCCCTCACCATCTGGCACCAGCTGGCCAGCATCCTCACGTCGACATG
ATGGGTTACAACGGCTACGTGGCGCCCAGGGCGCCACCTTCCCTCCAACAGCTCTACG
ACCCCCACGTGGCCATGAGGGCCCCCATGGGATCCGTAGGAGTGCCCGGAGGCAAAC
CCGCGTACAGTTTCCACGTGTCGAGCGAGGGCCAGATGCAGCCGGTGCCCTTCCCACC
GGATGCGCTCAGCGGACAAGGGATACCGCGCCATGCGCGGCAAATTAATACCCTCAGC
CACGGGGAGGTCGTGTGCGCTGTCACGATCTCGAATCCGACCAAATATGTGTACACCG
GCGGCAAGGGATGCGTCAAGGTTTGGGATATAGGGCAAGGTGGCACGGGCAGCACCA
AGTCCGTGTCCCAGCTCGACTGCCTCCAACCTGACAATTACATCAGGTCGGTGAAGCT
GTTGCCGGACGGCAGAACGCTGATAGTCGGGGGCGAGGCCAGCCGACTTAGTATCTGG
GATCTGGCGAGTCCAACGCCGAGGATCAAGGCCGAATTAATCTCGGCCGCCCCCGCCT

GTTACGCCCTGGCCATCTCCCCGGACTCGAAGGTTTGCTTCAGCTGCTGCAGCGATGG
CAGCATCGCCGTCTGGGATCTTCAGAATCAGTCCCTGGTGAGACAGTTCCAAGGCCAC
ACGGACGGCGCCTCGTGCATCGACATATCGGCGGACGGGTCGAAATTGTGGACCGGCG
GATTGGACAACACGGTCCGCTCGTGGGATCTGAGGGAGGGTAGACAGCTGAAACAGC
ACGACTTCACGTCCCAGATATTCTCGCTCGGCTACTGTCCAACGGGGGAATGGTTGGC
CGTGGGCATGGAGAACTCGAACGTCGAGGTGTTGCACGCCTCGAACCCGGACAAGTA
TCAGCTACACCTGCACGAGTCTTGCGTGTTATCGTTGAGATTCGCCTCCTGCGGCAAGT
GGTTCGTCTCGACCGGCAAGGATAATTTGCTGAACGCGTGGCGCACGCCTTACGGGGC
CTCGATATTCCAGTCCAAGGAATCGTCATCCGTGCTCAGCTGCGATATATCAGCCGACG
ACAGGTACATCGTTACCGGTTCAGGTGACAAGAAAGCGACCGTCTACGAGGTCATGTA
TTGAAACCGCAACGAAACCGAACCGTACCAACCAGCCAAACGTATTCATCAGACCCC
GAGCAACTCTCCACAGC

>novel_circ_000892
AGGAGCTCGATATCGCCCGGTGATAAATATAGTCGCCCGCGTACACCGCAGGAGACGC
ATCATTCCCATCATTCTCAGAATCACCATGACCATCCCATGCACATCAAGAAGATGAAG
AAAGAGCAGGACAAGGACATCGGTCATAGTGACGGGGAGAAGAGCGACCAGGACCT
CGTTGTGGACGACGCGAGCGAGGGTCCGACGAGTCCCGTGGTGAACGGCACAACGTC
GCCAAGAGAGAACGGGCTGGACAAGGTGGCGCCGATAGGCGCCGGGGTGCAGACGA
AGAAGGACGTGCCGCCCCATTCGCCGCGAAGCGGGACGAGCAGCAACGCGAGCACA
CCCTCGGCCAAGAAGATGGAGGAACGGGAGAAACCGACCACGCCGATCTCGAAACCC
CTGACCCCGACCTCCGGCTCCGCGATCGGTGGCGGTTTGAAGCCGTCCGGCTCGGCCA
AGCTGCTCCAGGGACCACCGCCCCCCGGCGTGCCCAGCGCTTATCCGCCGCACTACGC
CCCGGGTCCACCCCCTCACCATCTGGCACCAGCTGGCCAGCATCCTCACGTCGACATG
ATGGGTTACAACGGCTACGTGGCGCCCAGGGCGCCACCTTCCCTCCAACAGCTCTACG
ACCCCCACGTGGCCATGAGGGCCCCCATGGGATCCGTAGGAGTGCCCGGAGGCAAAC
CCGCGTACAGTTTCCACGTGTCGAGCGAGGGCCAGATGCAGCCGGTGCCCTTCCCACC
GGATGCGCTCAGCGGACAAGGGATACCGCGCCATGCGCGGCAAATTAATACCCTCAGC
CACGGGGAGGTCGTGTGCGCTGTCACGATCTCGAATCCGACCAAATATGTGTACACCG
GCGGCAAGGGATGCGTCAAGGTTTGGGATATAGGGCAAGGTGGCACGGGCAGCACCA
AGTCCGTGTCCCAGCTCGACTGCCTCCAACCTGACAATTACATCAG

>novel_circ_000893
AGGAGCTCGATATCGCCCGGTGATAAATATAGTCGCCCGCGTACACCGCAGGAGACGC
ATCATTCCCATCATTCTCAGAATCACCATGACCATCCCATGCACATCAAGAAGATGAAG
AAAGAGCAGGACAAGGACATCGGTCATAGTGACGGGGAGAAGAGCGACCAGGACCT
CGTTGTGGACGACGCGAGCGAGGGTCCGACGAGTCCCGTGGTGAACGGCACAACGTC
GCCAAGAGAGAACGGGCTGGACAAGGTGGCGCCGATAGGCGCCGGGGTGCAGACGA
AGAAGGACGTGCCGCCCCATTCGCCGCGAAGCGGGACGAGCAGCAACGCGAGCACA
CCCTCGGCCAAGAAGATGGAGGAACGGGAGAAACCGACCACGCCGATCTCGAAACCC
CTGACCCCGACCTCCGGCTCCGCGATCGGTGGCGGTTTGAAGCCGTCCGGCTCGGCCA
AGCTGCTCCAGGGACCACCGCCCCCCGGCGTGCCCAGCGCTTATCCGCCGCACTACGC
CCCGGGTCCACCCCCTCACCATCTGGCACCAGCTGGCCAGCATCCTCACGTCGACATG
ATGGGTTACAACGGCTACGTGGCGCCCAGGGCGCCACCTTCCCTCCAACAGCTCTACG

ACCCCCACGTGGCCATGAGGGCCCCCATGGGATCCGTAGGAGTGCCCGGAGGCAAAC
C

>novel_circ_000894
CAACAGAGACCCGATATATCAAGATTACTGCAACAAATGCATGCTCAACAACTGCCAC
CGGGGACGGCGCTAGGCGCTCATCCGGGGCTACCCGGACTTCCGGGACTAGGACCAG
CTGCTGGACTTCCGGTGCCAACTTCCGCCTCCGCCGCGCTTCTTAGCCTTGGCTTGACG
CCGGGGGCCGCCACTGCACCCGGCACAACAGCGGCTCACCCGCTATCGATGTTGAGTA
AACCGGAATTGCATCGTCAGCCCGACGATTTAAAGAGCAACGGTGGTCTCAGCTCGAC
CGAGGAGAGACACAGGAGCTCGATATCGCCCGGTGATAAATATAGTCGCCCGCGTACA
CCGCAGGAGACGCATCATTCCCATCATTCTCAGAATCACCATGACCATCCCATGCACAT
CAAGAAGATGAAGAAAGAGCAGGACAAGGACATCGGTCATAGTGACGGGGAGAAGA
GCGACCAGGACCTCGTTGTGGACGACGCGAGCGAGGGTCCGACGAGTCCCGTGGTGA
ACGGCACAACGTCGCCAAGAGAGAACGGGCTGGACAAGGTGGCGCCGATAGGCGCC
GGGGTGCAGACGAAGAAGGACGTGCCGCCCCATTCGCCGCGAAGCGGGACGAGCAG
CAACGCGAGCACACCCTCGGCCAAGAAGATGGAGGAACGGGAGAAACCGACCACGC
CGATCTCGAAACCCCTGACCCCGACCTCCGGCTCCGCGATCGGTGGCGGTTTGAAGCC
GTCCGGCTCGGCCAAGCTGCTCCAGGGACCACCGCCCCCCGGCGTGCCCAGCGCTTAT
CCGCCGCACTACGCCCCGGGTCCACCCCCTCACCATCTGGCACCAGCTGGCCAGCATC
CTCACGTCGACATGATGGGTTACAACGGCTACGTGGCGCCCAGGGCGCCACCTTCCCT
CCAACAGCTCTACGACCCCCACGTGGCCATGAGGGCCCCCATGGGATCCGTAGGAGTG
CCCGGAGGCAAACC

>novel_circ_000897
GTTTGTTGAAAGGGTCTGTTACGGCTGAGCGACCGCGCACCCTGCGCCGGCTGCGTAA
CGTTTACGGGCCATCCGCATGCGGAATGGGAACCGGAGGTGACGGGGGCGGAGGCGG
GGGCGAACGCCTCGAGGACGGAGAGGTGTCGTTCAGATTGCCGTCGGAACGGGATCG
CGAAAGGGACGAGAAGTCCCTCAGCATAATGAGCCCTTTCGACGAGCAGGAGGAATG
GGCCAAGATTTCAGAGATCATGGCGTCGATCGGCACCGGCCTCGTCAGGGAGTCGGTC
TTCGTCACAGAATTGGAGAAAGAGTTCCAGACGAGACTAGGTTTGAGCTCGGACACT
AGTAGCAGTCCTATCGTCTCCACTTCCGTTGGCCAATGGCTGTCGACGCTGGGCCTGGC
CGATTACGAGACCCTGTTCATCAACTACGGATTCGACGACCTGGATTTCATAAACGGGG
TTTTGGACGAGACCGATCTGAAGGATATGGAGATCACTAGCGATCAAGAGCGGGCGGT
GATAATGGACGCGGTCGGTTTGTTGAACAAACGGTTGGACAAACGGTCCAACGTTCAA
TCGGTGGACGAGTGGTTGAAAAGTATCCATTTGGAAAATTACGCGGAAACGTTCAAAA
AGCACTTGTACACGGATATGGAGCGCGTGAGGTGCGTGTGGGAGGTGGAATTGGCGA
CCGTGCTCGAGATACAGAAGCCGGCGCACCGTAAGAGGATCCTCGCGTCCGTCAGTGG
ATCGAGCGCGCGTGCGCAGAATCGTAACTGCGCCGGGCCCAACTTGGAGGATCTCAAT
AAGGATTTGAACACCTTG

>novel_circ_000898
GGCACAGATCTTCCTCCGGCGTGGAGAGCGATGCCGACAGCGAGAACTTGCCACCGA
TTCCGGTCCAAGGGAACGACTCATTGGGCAGCCCTTACGAGAACGTTCGCCCAGGGGA
TGATCTCTCCCCGGCGGAAGGGACATCGCCCACTCAATGGCAATTCTATCACAAGCCTC

GGGACGATGATCGCCGTGTTTCCGGCACTTCCGTTATGTCTTTGTGAGTATCGTGTCGC
CCCTTCCTCCTTTTAACACCGCCGTTGCTTTCGATGTACATTCCTCCTTTCCCCTCTCTTT
TTTTTGCCGCCATACATCTCGTAATAATAAAATGCCGTTATTGCGCTCTGTCTGCGATTGT
CAATCTGCAAGGAATAAAATAATTCGATGTAAAAAACTTTACTTAAAATTTAAAAATTTT
TTAGATCGTAAATCTCTTTCTCTCTCTCTCCTTTTGATAAAAAAGTGGTCCCGTTCTTAA
TTCGTTTAAATCACAGACGAAATAAGAAATTAAAACAATCGAGACGTATTTAAATCACG
ACGATCGAAATGAACAGAGATCCGGAGAAAAAGTATCTCCCTACGTAAAAAGCAAATG
CAACAATGCAACAATAACGGTGTTCCATCGATGCAAAAAACGGCCAGGTCTGGTTTCG
ATCATCCTTCCAACAAGAACGAACGCATCGCTACACGCCATTCGCATTTTGCATTTCCG
TGTAACGATTGCGTGAACTTCGAGAAGAGGGGGGCGAAAAAAAAACACACGGAAGA
GAAAAGAAAGAGAACGGAAAAAAAACGAGAAGAAAAAAATAAAACAAGAAGGAAA
AAAAAAAGAAACAATTGCACCGAGCTGTTACTTGTCTCTCCCTCTCTCTCTCCCTATCC
CTCCCCCTGCATTTGTGCTCATCCGGCGTCATCTTGGGGCGGAGTGAGAGAAAAGAAT
ATCGCGTCGGTGCATCGGATAAGAGCGGAGTCAGAGTGGAGTAGTAGAGATCGGTATA
ATATTTTCTAGAAAAGAGCGGATACGCGGGGCGCATAACCGTGGCGGCTTTAATATAGG
AGATGTCGGGTAAACAAAAGCATAGACGTTGGACGTGGGGCACACGGAGTGAACGAA
CGTGGCTCGGATGATGGTTAGTCGTCGTCGTCGAGTTGGTACCGTTTTAGCAGCACCGA
TGCTCTGCACCGCACAGCCACAGCCGAAGTTCTGGGTGGGAGAG

>novel_circ_000899
AGCCGTGGTGGAGGTGTTGCTGGCGGCGGGCGTCGACGTGAACACCAGGACGTCCGC
GGGGACCGCGATGCACGAGGCGGCGCTCTGCGGGAAGATGGAGGTGGTGCGGGCACT
CCTGGACAGAGGGGTCGACCTGGGCATCCGGGACTCGCGACAGAACACGGTGCTCGA
TCTTCTGGGACAGTTCCCGCCGCACGTCACTCAGGACATCACCGCGGTTATAAAAAGG
CACAGATCTTCCTCCGGCGTGGAGAGCGATGCCGACAGCGAGAACTTGCCACCGATTC
CGGTCCAAGGGAACGACTCATTGGGCAGCCCTTACGAGAACGTTCGCCCAGGGGATG
ATCTCTCCCCGGCGGAAGGGACATCGCCCACTCAATGGCAATTCTATCACAAGCCTCG
GGACGATGATCGCCGTGTTTCCGGCACTTCCGTTATGTCTTT

>novel_circ_000900
GGGAGATCACAAGTAATGTATTACGCGTTGCACGTGAAGCACGTCAACGAACTCTTGG
TCCATTTAGTCCATCGTTTAATGTACAAGAAATATTGTTAGAAAGTTTACAAAAGTTTCT
TCCTAATGATGCTCATATTCGTGTGAGTGGTAAACTTCATGTTTCCTTAACAAGAGTATA
CGATGGAAAAAATGTAATTGTTTCACAATTCTCATCTAGAGAAGACTTATTACAAGCATT
ATTGGCCAGTGCATTTGTTCCCATTTTTTCTGGTCTTTTACCACCAAGATTTCATGGTATT
AGATACATGGATGGTGGTTTTAGTGATAATCTTCCAACACTTGATGAAAATACTATTACT
ATTAGTCCTTTTTGTGGAGAAAGTGATATTTGCCCTAGAGATGTTTCTTCTCAACTTTTC
CATGTCAATCTTGCAAATACAAGTATAGAACTTTCAAGACAAAATATATATAGGTTTGCA
AGAATACTTTTTCCACCAAATACAGAGATACTTTCAAATATGTGCAAACAGGGATTTGA
TGATGCATTAAGATTTCTTCATCGAAATAATTTACTTAATTGTACTCGATGTTTAGCAGTA
CAATCCACATTTGTTGTTTCAGAAACTCTTGATGATAATATGGATTATGATCCTGAATGTT
TAGAATGTAAAATGCATAGACAGGAAGCATTAGTATCTAATTTACCTGAAACAGTTATG
ACCATATTTCAAGATGCAATTGATTCAGCTAATAAAGGTTTAATTAATTGGTTATTTAAAC
ATCGAAGTGTAAAACTTTTGTCATTACTTAGTTTACCATGTACATTGCCAGCAGATGTTG

TGTATGCAACTTTTACAAA

>novel_circ_000901
GCATTATTGGCCAGTGCATTTGTTCCCATTTTTTCTGGTCTTTTACCACCAAGATTTCAT
GGTATTAGATACATGGATGGTGGTTTTAGTGATAATCTTCCAACACTTGATGAAAATACT
ATTACTATTAGTCCTTTTTGTGGAGAAAGTGATATTTGCCCTAGAGATGTTTCTTCTCAA
CTTTTCCATGTCAATCTTGCAAATACAAGTATAGAACTTTCAAGACAAAATATATATAGG
TTTGCAAGAATACTTTTTCCACCAAATACAGAGATACTTTCAAATATGTGCAAACAGGG
ATTTGATGATGCATTAAGATTTCTTCATCGAAATAATTTACTTAATTGTACTCGATGTTTA
GCAGTACAATCCACATTTGTTGTTTCAGAAACTCTTGATGATAATATGGATTATGATCCT
GAATGTTTAGAATGTAAAATGCATAGACAG

>novel_circ_000902
ATTGAGGATGTCCTCAAAGAGGAAGTCACCACCAACGAAGCTATCGGAGGGCGGGGG
CGGCACGGGTACAGTGGCAGGCGCAAACGAGGAGGAGGAGGACACGACCTCGTCGG
TGACCGGTGGTCCCGGTGGCGAGGTAGCCGTCGGTGAAGAGGGTCCCACCACCCCCG
TCGACATCGAGGACTGTTACCCCCACCAGAGGGGAGGTGACTGCGAGTCGTCCGGCT
GCTCGTCGCCGGCGACAACCAGCGAGCCCGATCTCAGGGACTCGCCCTCGCCATCGTC
GAATTCCTTGCGGACCAGCAAGCGGCAGAGGATCGTGTTGACGGGGCAGGAGGCACT
TGCCTACCATTATCCCCCCCACCACCACCACCACCATCATCACCATCACCACCACCATC
ACCACACGAACACATCCTCGTCGTCGGGCGGTGTCGTGGAACCCGCCAGCTCCTCCGA
GTGCGGCTCGCCCCCCACACCCTTAACCGTCGACCCCTATTACCCCACCCATCCTCATC
ACAACAACAACAACACCGTCACCCAGAATAATAACAACAACAACAACAACAACAACA
ACGGCGGGAACGCCACCACAGCGACTACCAAGAGATCCATGGACGACGTGCTGAAGA
GGTTAACCTTCAAGATGAACAGCGAGTCGTTGTCGCTGCAGGACGACACGAGTAATAA
CAGGCGGAACAGCCCGCCGCCCACCTCCACGCCGACCGGGCACAACGCGCATTG

>novel_circ_000903
TCGTATCCTGCGCACATGACCGTCGACTCGCAGAAGCAAATCGAGATGCAGCGGCTGC
AAACGGAGCACTTGAAGCGGCAGCAAGAGCACATCATGCAGCACAACATCCAGGAGC
TACAGGCTCAGATGACAAAGAACCAGCTGAGCATGTCGGGGCCGCAATCCTTGATGTT
CCTACCGTTTCTCGAACAGCTCAGAGGGTTGCCGGTGCAATCGCCTATGCCGCCGCCG
CCTGTTACGTCGACGTCAACCACCACCAACAAACATATCAATTCGATCGCTAACATGAT
CAGCAGCCACAGGGAAGGGCCAAGCTGGGCGACGGCGCATCTCGCGCAGATGACCAC
GCAGATGGAGAAGGAAGCGTCGCCGACGCCGATCTCTTCCGCGGTGGCGCCGACCGC
TGCCCCCTTGCAGGACCTCGACGCGCCCCTCAACCTCACCAAGCCCAAGTCCTCGTCG
TCCGGGGCCACGGCGTCCTCCTCGTCGCCCGGAAGCGACTCCCACTCGACCGGGGCC
GGAAGCAGCGGCCAGCAGGAGCAACCGCTCGCGGCCACCGCGCCCAAGCTCTTCCCA
CCCGGGCTACCGATGCCTCGAAATTATCTCACCACCCTTCCCTACGCGGGCCTCCCGCC
TCACCTCAGCTCCATCTCCTCCCCGATGGGCAAGGTAATGGCCAAAGACGAGGCGGCT
GGACCTGGGGGTGCGGTGGCGGCAGCCGCGGTCGCCGCCATGGAGAAGCACATCGCG
ATGCACAGTATTTACGCGATACCGCCGAGCGCTGGCGCGATGCCCCCGTCGCCGCAGA
CCCAACGACCCGGGGGTCTCAAGCACTCCAGTTCTCGGGAAGAGGCTGCACAGGAGG
AACAGGACTACCTGTCGACACCTCACA

>novel_circ_000904
AAAATTTAGCGCCATCGGAATTAAAAAAATTGAGAAACAAGCAGAGGAAGCAACGTA
GAAAGGCTGAATTGGAAAGACAACAGGCTGCTCAAGCTCAAGAAAAGAGAGAACAG
CACAATAAATCACGGCAACAGACCGATCCTGATTTAGAACAACCTACGCTAGATGAAC
TTATACCGGAGAAACTGGAGAGGATCGAGGACCCGTTAGAGCAGGCAATCAAATTTTT
ACAACCATTACAAGATTTGGCATCGAATAGAATAGAGACGCATCTTATGGCATTTGAAA
TATACATTCGTAAAGGTCGTACGTTGTTAATGTTAAGGTCGATAAAACGTGCACACGGT
TTAGATCCTAATAATCCAGATCTTCATACATGTTTGGTACGTTTCATGTTATACATTAACC
GTTCACCGTTGGAAGGACCCGTAGGCGAAGTAGTGAAACGGCAAACATCGGGAATTTA
TTCAGCTTCAACAGCCACCCAATTGAATGCTGAATTCCTAAAAAAGAATCGAAACTCA
CTGCCGCATCTACTTCAGGGCGCGAGGATGTTATATGTCCTGGATCCCTCTGCTCAAAC
AAAAGCTCTCTCACTTATCACAAACATTGATGGTCTCGAAAGTGTGACACTCCAGAATT
GTACAAAAGTATTGGAGTCATTATGCAACGGCGACTTTGGTCATTGCGATTCAACCATC
GCTGATTATATGGTGAAATGTCATAAATACTTCCCATACGCGACCGCTTTTCGACCGCCA
GAAACGAAAGTGACGGTAACGATCAAACATCAAGAGAAAGAGAATTCCATCAAGAAC
TGATCCAGGCACGCGCTGTCCAGCGTTACTGAATCGTTATCGTAGTTAGACGACGAGG
CAAGCGAG

>novel_circ_000905
GATTTTGCTTTTCAGGCTCGCGTAATCGACAGACGTGCCGGATCGCGCGGCGTCGCCG
GGGCTCTTCAGTGGTAGTCGAGAGGAATCTGCGGCGCAAAAAGAGAAAGAGGGAAA
AAAAAGGGAAAAAAGGAGGGAGGGGGGGCGAACACATCGCTAAGGAAGGCTAGGAA
GGAGATAAAGGGAGAATCTGGTGGAGAGGATCGTCGAGGAGAGGAAGTGGGGCGCC
GGTGCTGGCCACCGGGACCAATCATCCTTCCCTTCCTCAGGATGTCGTACGCCAATCGA
TTCCCGTCTTCGCAGCACACCCGTTATCGAAGCAGCTGGGGACCCTCCTCGGTCACCG
TGTTCAATCGGGCCGATGTGCCCAGGCCCTCGCCGGAAGAGGAAGAGTACGCCGAGT
TCTATCACGAGCGTCTCCACTCGGCACAGAGCAGACAGTTGCTGCCGTTGCAGGAGGA
CCTGGCCGATTGGATCAATAAAACGTTGA

>novel_circ_000906
GCTCGCGTAATCGACAGACGTGCCGGATCGCGCGGCGTCGCCGGGGCTCTTCAGTGGT
AGTCGAGAGGAATCTGCGGCGCAAAAAGAGAAAGAGGGAAAAAAAAAGGGAAAAAA
GGAGGGAGGGGGGGCGAACACATCGCTAAGGAAGGCTAGGAAGGAGATAAAGGGAG
AATCTGGTGGAGAGGATCGTCGAGGAGAGGAAGTGGGGCGCCGGTGCTGGCCACCGG
GACCAATCATCCTTCCCTTCCTCAGGATGTCGTACGCCAATCGATTCCCGTCTTCGCAG
CACACCCGTTATCGAAGCAGCTGGGGACCCTCCTCGGTCACCGTGTTCAATCGGGCCG
ATGTGCCCAGGCCCTCGCCGGAAGAGGAAGAGTACGCCGAGTTCTATCACGAGCGTCT
CCACTCGGCACAGAGCAGACAGTTGCTGCCGTTGCAGGAGGACCTGGCCGATTGGAT
CAATAAAACGTTGA

>novel_circ_000908
GCCGAAATATGGAGCGTGTTCATCGCCATTTTGAGGAAGAGCGTGCGGAATCTGCAAG
CGTGTACAGACGTAAGCCTCATCGAACACGTGCTGCTTCGATTGTCCCGCGCGGAAAC

TGTCGTCGCAGATCTATTAATCGACATGCTTGGAGTGTTGGCCAGCTACAGCATAACGG
TGAAGGAGCTGAAACTTTTATTCGGCGCTATGAAAGCTGTGAAAGGGAAATGGCCACG
ACACTCGGCGAAACTGTTAAACGTGCTCCGTCAGATGCCGCAACGGAATGGACCCGA
CGTGTTTTTTAGTTTCCCCGGTAGAAAGGGATCGGCAATCGTTCTGCCGCCTCTCGCAA
AATGGCCACACGAGAATGGTTTCACGTTTACCACATGGTTCCGCCTGGACCCCATCAA
CTCCGTTAATATCGAACGAGAAAAGCCGTACCTCTACTG

>novel_circ_000910
TTTTAAAACTAGCAAGGGAGTGGGATACTCCGCGCATTTCGTAGGGAACTGCTTGGTC
CTCACCTCTATGAAAGTGAAGGGCAAGGGCTTCCAGCATTGTGTCAAATATGAATTTCA
ACCACGAAAGTGGTATATGATCGCCGTAGTGTACATATATAATAGGTGGACGAAGAGCG
AAATTAAATGTTTGGTCAACGGTCAATTGGCTTCGAGTACTGAAATGGCGTGGTTCGTT
TCAACAAACGATCCGTTTGACAAATGTTACATCGGGGCTACACCGGAACTGGATGAAG
AGCGCGTGTTTTGCGGACAAATGAGCGCTATTTATCTGTTCAGCGAAGCTCTGACTACG
CATCAGATATGCGCTATGCATAGACTGGGGCCTGGATATAAGAGTCAGTTTCGTTTTGAC
AACGAGTGTTACCTGAATCTGCCTGACAATCACAAAAGGGTGAGTGATGAGCCGGAG
CTCACGAGCATGGTGGACCAAAGTATGCAAACTGTGCTTTACGATGGAAAGCTATCGA
ATGCGATTGTGTTCATGTACAACCCGGTAGCAACGGATTCTCAACTTTGCTTGCAAAGC
GCACCAAAAGGAAACGTCTCCTATTTTGTACATACACCACACGCCCTTATGCTACAGGA
TGTTAAAGCTGTCATCACGCATTCCATTCATAGCACGTTAAATTCCATTGGTGGCATTCA
AGTGTTGTTCCCTTTGTTCTCGCAACTAGATATGCCTTACGATTGTATAGCTCCCAACGA
TGTTAAACGAGATCCAACGTTATGCTCGAAACTGTTGGGGTTTATTTGCGATCTTGTCG
AGAGTTCGCAAACGGTGCAGCAACACATGGTGCAAAACCGTGGGTTTTTGGTGATCA
GTTACATGCTGCAGAGAGCGAGCAGAGACCATCTTACGACGGAAGTCCTCGCGTCCTT
TTTGGAGCTGACGAAACATCTGGTCACTTGTCTTAGCGCGAACAGCGACTTGTTATTG
AAACAG

>novel_circ_000911
TTTTAAAACTAGCAAGGGAGTGGGATACTCCGCGCATTTCGTAGGGAACTGCTTGGTC
CTCACCTCTATGAAAGTGAAGGGCAAGGGCTTCCAGCATTGTGTCAAATATGAATTTCA
ACCACGAAAGTGGTATATGATCGCCGTAGTGTACATATATAATAGGTGGACGAAGAGCG
AAATTAAATGTTTGGTCAACGGTCAATTGGCTTCGAGTACTGAAATGGCGTGGTTCGTT
TCAACAAACGATCCGTTTGACAAATGTTACATCGGGGCTACACCGGAACTGGATGAAG
AGCGCGTGTTTTGCGGACAAATGAGCGCTATTTATCTGTTCAGCGAAGCTCTGACTACG
CATCAGATATGCGCTATGCATAGACTGGGGCCTGGATATAAGAGTCAGTTTCGTTTTGAC
AACGAGTGTTACCTGAATCTGCCTGACAATCACAAAAGGGTGAGTGATGAGCCGGAG
CTCACGAGCATGGTGGACCAAAGTATGCAAACTGTGCTTTACGATGGAAAGCTATCGA
ATGCGATTGTGTTCATGTACAACCCGGTAGCAACGGATTCTCAACTTTGCTTGCAAAGC
GCACCAAAAGGAAACGTCTCCTATTTTGTACATACACCACACGCCCTTATGCTACAGGA
TGTTAAAGCTGTCATCACGCATTCCATTCATAGCACGTTAAATTCCATTGGTGGCATTCA
AGTGTTGTTCCCTTTGTTCTCGCAACTAGATATGCCTTACGATTGTATAGCTCCCAACGA
TGTTAAACGAGATCCAACGTTATGCTCGAAACTGTTGGGGTTTATTTGCGATCTTGTCG
AGAGTTCGCAAACGGTGCAGCAACACATGGTGCAAAACCGTGGGTTTTTGGTGATCA
GTTACATGCTGCAGAGAGCGAGCAGAGACCATCTTACGACGGAAGTCCTCGCGTCCTT

TTTGGAGCTGACGAAACATCTGGTCACTTGTCTTAGCGCGAACAGCGACTTGTTATTG
AAACAGTTGTTTTACTTTTCATTCCTAACATGGCAGCTATTGGACCACGTCCTTTTTAAT
CCCGCTCTGTGGATATATACGCCGGCTCCGGTGCAAACCCGGCTTTATTCGTACTTGGC
CACAGAGTTCCTCAGCGACACGCAAATATATTCTAACGTGAGGAGAGTGTCGACGGTA
TTGCAAACGGTGCACACGCTTAAGTACTATTACTGGGTCGCCAATCCTCGGGCAAAGA
GTGGAATCACTCCCAAAGGACTTGATGGACCGAGGCCACAGCAAAAGGATATTTTGAC
AATACGTTCTTACATTTTGTTATTTCTCAAGCAATTGATCATGATAGGGAACGGGGTGAA
GGACGACGAGTTGCAGAGTATATTGAATTATCTGACGACAATACACGAGGATGAAAATT
TACACGACGTTTTGCAAATGCTTATATCTTTAATGTCGGAACATCCCTCGTCCATGGTGC
CTGCTTTCGACGCGAAACAAGGCGTCAGGACGATTTTTAAGTTGTTAGCAGCGGAGAG
CCAATTAATACGTTTACAAGCCTTGAAATTGTTAGGATTCTTTTTAAGCCGCAGTACACA
CAAAAGGAAATATGACGTAATGAGCCCCCACAATCTCTACACGTTATTGGCCGAAAGAT
TATTGTTGAACGAGGAAACATTGTCATTACCTACCTACAACGTTTTATACGAGATCATGA
CGGAGCACATCAGCCAACAGATCCTCTACGCGAGGCATCCTGAACCAGAATCCCATTA
TCGTTTAGAAAATCCAATGATCCTCAAGGTAGTGGCGACTCTGATTCGACAATCGAAAC
AAACGGAACAATTGCTCGAAGTGAAGAAGCTGTTCCTCTCGGATATGACATTGTTGTG
CAACAACAATCGAGAGAATCGTCGAACTGTTCTACAGATGTCTGTATGGCAGGAGTGG
CTTATAGCCATGGCGTACATTCATCCGAAGAACACGGAAGAGCAAAAAATCTCGGACA
TGGTGTACTCGTTGTTTCGCATGCTTCTCCATCATGCTATCAAACACGAGTACGGTGGAT
GGCGCGTATGGGTGGACACTTTGGCCATCGTGCACTCCAAGGTATCCTACGAGGAATTT
AAGCTTCAATTTGCTCAGATGTACGAGCATTACGAGAGGCAAAGATCGGATAACATCA
CGGATCCTGAGCTGAGACAGCAAAGGCCGATCAGCACTATCTCTGGCTGGGATCAACA
GCATTCGGGGAATAATGGTTACAACAAACCGTCACCCTGGAGCAATCAACAGACTCAC
GATGTGCAACACGTGGAGAGGAATTACAAGGAGTTCGACACAACGGAGGGTAATGAC
AGGCTGGAGGAGTCGTGCAGTTGCGACTTGAATTCGATAGACAACACGGAGAGCGAC
GGGAGCCCTGCGATCGTCAAGTCTCGTAGCGAAACGTTGGACACGGCGCAATCGAGC
GAGGCGATATCCCTGGACTCGAGGCTGAGCGACAAGCCAGAGACGCCGACTACGGCG
CGAGAAATCCACACGGATACGCCTACGATCCTCGAGGAAGGGAACAGCGAGGCTGCC
TCCATTCCGACCACCCAAGAAACCAGCGAGGCGATATCGATAGAGAGCTCGAGCGATT
TCTACAGCGAGGTACACAAGATCGAGGGTTCCAGCCCGGATTCCATGGTGGTGCAAGA
AGTTCTGAAGAAAGACGACGATCCTTCCGAGGCGAAATCGCAGGATGAAGCTGTCTCT
CCGTCCGAAAAGAAAGAGGAGAGCGTGTCCCAGGAAGACGTGGAGCAGCCGAAGGA
AACAACGGACGAGTGTGACGTTTCCCACGTGCAAAAGGGCGACGAAACCGAGGGTG
AAACCGAGGAAATGGATGGTCAAACGGAAGTGGAGGAGGCTGGAGGAGAGGTGAAA
GTTGAAGCTGGGATCGTCGAAGCCTGCGTCGAAGCTGGAACCGAGGTGGAAACCGAG
GGAGAGACTGAGGTCGAGGCTCCATCGAGGTTGGAAGAGGCGAGGAACGAAGAGATA
TCGAGTTGTTCGATGGACAACGTTGCTTCCAACGAGGAGACTTCCCCTGATATTGTTCT
CGATAAGGGGGAAGAGGGAAGGGAGAAAAGTGCGGAGGAGGACGTTGCCGGCTCGG
AGCAGCAAGCCGATACGGAGCACGCGCCGTCGTCCGTTCCCTCGAGCGAGACGCCAG
AGCCGTTGATCATCAAGGATATCGAGAAGCTGCAATTGGAGAACGATCGCGAGGTGAT
CGACAGCGATACCTCGGAGGCTTACTTGACACCGACCGAGAATCAAGATAATCTGGCC
GACATGAAGCCGAAGGAAGCGATGGAGAAGATGGAGGACAGCGAGAAGGGGGTGGA
GAAGGGTGTCGACGTTGAGAAGAGCGAGAACGACGACGGGGAGGCTGGGAATAATTC
CAATTGCTCAACTAGCGTAATAGAGAGTAACGAAGCGGGCGACGAGGAGGAGAAGAA

GGAGGACGTAGACGTAGACGTAGAGGGGAAAGTGGATACATTAGTAGTGACTAGTGA
GTTGTTAAGTAGTGAGAAATGCGCCGTGGACTCTGTGAAAGAGGAGAAAAGTGCCGT
TGTTAACGTTGATAACGGGGTGAAGGTTACTTACGACAAACAACATTCAAGGGAGCCG
TCCACTATTTCTACTAACGTAAGTGATAATCAGTCAGACATTCCAGTGAGAACCGAGAA
CGAGGTAGAAGTAACAGATAGTGTTTGTAGTAATAGCGATATTCAAAGTTCTGTAGATA
ATGTGACGCAAGTGCCAAATCCTAAACAGCAAATTCCAACAATATCTACGGTCTGTGAT
GCAAATAAAAATATGCCCGACGGTGTGCCTCAAATCGTTAGTTCTACAGTGGTTGCGAG
GGATAATTCTTTGTTGATGAATGAGTTAACTTCGGATCACGTAGATGCTCATCGCAGATC
CAGCTTGCCGGCTGTGTCCTCCGAGACGACCACCGACGATAATAACGTAAACGAACCT
CCCTCCTTACCTGTCCCCTTGCGGAAAACATCGTCACCTCAGAAACGACCAAGGAGTG
CCTCAACGTCCACGCAAGTGGATCCGAATCATTTCG

>novel_circ_000913
GTGCTCAAGTACTGCGACCATCTGCACGGAAAATGGTACTTCTCGGAGGTGCGCGCGA
TATTCTCGCGGCGATATCTGCTCCAAAACGTCGCCATCGAGATATTCCTGGCCAGCCGA
ACCTCGATCCTGTTCGCGTTCCCGGACCAGGCGACGGTGAAAAAGGTGATCAAGGCG
CTACCGCGGGTCGGCGTTGGCATCAAATACGGGATTCCGCAGACTAG

>novel_circ_000914
GTGCTCAAGTACTGCGACCATCTGCACGGAAAATGGTACTTCTCGGAGGTGCGCGCGA
TATTCTCGCGGCGATATCTGCTCCAAAACGTCGCCATCGAGATATTCCTGGCCAGCCGA
ACCTCGATCCTGTTCGCGTTCCCGGACCAGGCGACGGTGAAAAAGGTGATCAAGGCG
CTACCGCGGGTCGGCGTTGGCATCAAATACGGGATTCCGCAGACTAGGAGAGCATCGA
TGATGTCGCCCAGACAATTAATGAGGAGCTCCAATATGACGCAGAAGTGGCAGAGAAG
AGAAATATCAAATTTCGAGTATCTCATGTTCTTGAATACCATTGCTG

>novel_circ_000916
TGCGTGGAAACACCACATGTAATATCTGCTACAAGACATTTGCGTGCAACTCCGCGCTG
GAGATCCATTACCGGAGCCATACGAAGGAACGACCTTTCAAATGCACCATATGCGACA
GAGGATTTTCGACCAAGAACGATGGTTCCGGGCAGCAAGCATGCGTCTGTGCGTCTCA
ACCGTTGCCCGCTAACAGTTCGATCACTTCACCCGATCCTCAATTCGGATCACCGTGCG
CCAGTAGTCGAGAAAAAGCGAAACGCAGGCGGCGGCGGCGACCTGAGGATCGGAAG
AACCGGGGGCGGAAAGGAGCCGGAGGGGGGCGGGGGCTTACGCCTGTCTATCCTGCC
ATCCGCCTACCCCCGCTGATGTCGCCCGCCCTCGGACTTCTACCCTCGGCGCACAGCCT
CCTGGGAAACATGAAGCAGCATATGCTGACCCACAAGATCCGGGACATGCCGCCCCAT
CTGTTCAGCGATTCGAAGCAACAACAGCAGCAGCAGCAGCAGCAGCAGCAACAACA
ACAACAACAGCAACACCAGCAACAGTCTCAGGAGCACGAGACCTCGAGAAGTCCTAT
GTGCGCCGAGGACTCGAGCTTACCGCCGCCTCCGCCGCCTCCGCCGCCTCCTCCCCCG
ATGCCCCCCACCTCGATAGAGCAGAGCATCGCCGTGAAGAGATCTCCGCCCGAGGGAG
AGCTGCCAGCGCCAAAGAGACCAGCCAGCGTGCCGAGCAAACACTTGTGCCAGATTT
GCAATAAGAATTTCTCCTCATCTTCGGCGCTCCAGATCCATATGAGAACTCACACGGGC
GACAAACCGTTCCGATGCACCGTCTGCCAGAAGGCCTTCACCACCAAGGGCAATCTCA
AG

>novel_circ_000917
GTCCGGCCACTTGGACGGGAAGGACGGGAAGGACGAGAAGCCACGTAGCCACGGTG
GTTCCCTCGCCCGATCGGGGAAACGACGGATCTCACTCGCGGAAATCGGTGACGATGA
GCTAGCGACACGACGATGAGGAGGATGAAGCAGCGAGCGCTGCTCGCCTGGCCATGG
TTGTGGCTGTGGCTGCTCGCGGCCACACCGGGTTCCCCGTCTTCCGACGCTTCCCCGT
CGACCACGCTCTCGTCATCGTCGTCGTCATCGTCGTCGTCGGCGGCGGTGGCGAGCAC
AGTGACGAGGTCAGTCTCGCCCACCATCATCCCCACCTCCACCTCCTCCTCGTACGAG
GGGACGAACAACCACGACGACCAACGACCAACGTATCCCCAACAACCTGACAACGAG
TTCCGTTTCACCCTTCCGCTCTACAACGTGTCGATACCCGAGAACTCGATCGGCAAGAC
GTACGTGGTCCCGTCCGTGGACGAGAGGATGGGTATAAAGCTCCCGTCACCGGACGCC
AACCTCGACATCCGTTTCCGCATAATGAGCGGTGACCGCGACCGATTCTTCAAGGCCG
AGGAGCGGACAGTCGGTGATTTTTGCTTCCTATTGATTCGGACTCGCACGGGGAACGT
GGACGTGTTGAACAGGGAGAGGAAGGATCATTACGTGCTCGAGGTGCGCGCCACATT
GAACAAGAACGACGGGAGAAATCTGGTTACGATACTCGAGGCGGACACCACCGTGGT
TGTCACGATCTTGGACACGAACGACCTGAACCCCCTCTTCTACCCGACCAAGTACGAG
GCCACCGTCGCGGAGGACACGCCTATGCATCGGAGCATACTCAGGGTAATCGCCGAGG
ACGCAGATCTCGGCAGGAACGGGGAGATTTATTACAGCTTCGCCGAGGAAACCGATCA
GTTCGCCGTTCACCCGGTCAGCGGGATAATAACGCTCACCAGGCCGCTCAGATACACG
GAGCGAGCGATCCACGAACTCGTGGTGCTCGCAAAGGACCGGGGTGCGTTGTTCCGG
GGAAGCAGCAGGGCCAGCACGGCGAAGGTGACGATCAAGGTGCGCCAGGTGAACCT
CCACTCGCCCGAGATCTACGTGCACCACCTGCCCGACATCGTGGAGCACTCGAACGCG
GACATCTACGCGATCGTGCGGGTGATCGACAACGACGAGGGGGTACACGGGCAGATC
GCGAGCCTGGACATCGTGGGCGGGGACCCGGCCGGCCACTTCAGAGTTCGTCCAGCC
GGCGGGCCGCACTCGGGCGAGTACAACATCGAGGTGTTGCACCTGTTGGACAGGGAG
ACGGCGGTGCAGGGTTACAACTTGACGCTGCGCGCGACGGATCGGGGCGTCCCGCAA
CGGTTCAGCTACAAATTCGTGCCCGTCCATCTGGCCGACCTGAACGACAACGCCCCGG
TGTTCAGCCGGGAGATATACGAGGTGAGGGTGGCCGAGACGGCGCCCGTCAACACGC
CGGTGATCCAGCTGAAGGTGACGGACGCGGACGAGGGGAGGAACGCGTTGGTGTTCC
TGGAGATCGTGGGCGGGAACGAGGGGGGCGAGTTCTACGTGAACGCGGAGACGGGG
ATGTTGTACACGGCCGTGAGCCTGGACGCCGAGAAGAAAGCGTTCTACACGTTGACG
GTGTCGGCGGTCGATCAAGGGAACGCGGGGACCAGGAAGCAATCGTCGGCCAAGGTG
AAGATAAACGTGGTGGACACGAACGACAACGATCCGACGTTCGAGCAATCGGAGATG
GAGGTGTGGATCGACGAGAACGAGCCGGCCGGCACGTCGGTCGTCAAGATAACCGCC
AAGGATCGTGACTCGGGCGAGAACGCGTACATCTCGTACAGCATCGACAACATCCAGA
AGGTGCCGTTCGAGATCGACCACTTCTCGGGTATAGTGAAGACGAAACAGGTGCTCGA
CTACGAGACCATGAAGCGGGAGTACTTGCTGCACGTGCGGGTGAGCGATTGGGGGAT
GCCGTTCAGGCGGCAGGCCGAGATGCGGCTGCACGTGAAGGTGCGCGACGTGAACGA
CAACAGGCCCCAGTTCGAGAGGATCGATTGCTCGGGCCACGTGCCGAGGTACGTGTCG
ATCGGCTCCGAGATCATCACCGTCTCGGCCATCGACTTCGACGCGGGCAACATCATCA
GTTACCGCATCGTCTCCGGGAACGAGGACGGCTGCTTCGCCCTCGACTCGGCGAGCGG
CGTGCTGTCCGTCGCCTGCGACCTGTCCGACGTCAAGGTGGCCGAGCGGGTGGTCAA
CGTCACCGCGACGGACGGCTCGCATTTCGCGGACGTGAACCCCGTCCAGATGCACCTG
GTGAACGCGAAGCGGAACTTGGGCTCGCAGGGGAGGATACTGTCGGATCAGAGCGGG
GCGTTCGAGTGCCGGGACACGGGCGTCGCCCGCCGTTTGACCGAGGCAATCGCTGCC

GCCGAGAGGAACAACATGCCCTCGAAGGACGACGAGTACGCGCTGACGCCCAGCCGA
TACGGCGAGAACGTGCACGCCCCCGAGTTCGTCGACTTCCCCAGCGAGATAAAGGCG
AACGAGTCGGTGGGGATAGGCACGACCCTGGTCAGGATCCGCGCCAGGGACAAGGAC
CTGGACTACAACGGGAAACTGGTGTTCTGCGTCTCGGCCGGGGACCGGGACTCCGTG
TTCAAGATCGACCCGGACACGGGCGATCTGAACGTGATCGGTCACCTGGACAGGGAG
CGGGAGAGCGAGTACTTCCTCAACATCAGCGTGTACGACTTGGGCAAGCCGCAGAAG
TCTGCCTCGAGGATGCTCCCCGTCACGATCCTCGACGTGAACGACAACGCGCCCAGGT
TCGACAAGTCTTTGGCGAGCTTTCGCGTCTCCGAGACGGCGTTGAACGGGACGAACG
TGTGGCGGGCGAACGCGACCGACGCCGACCTCGGCGAGAACGCCCGTGTCACGTACT
CCCTGGTGACCGAGACGAACGACTTCCGGGTGGATCCCGTCACAGGCGTGCTCACCG
TGTTCGGCAGGTTGGACAGGGAACGGCAGGAGGTGTACGAGCTGAGGATACGGGCGC
AGGACAACGGGGGCAGGGGGACGGACGCGCCCCCCCTGTACTCGGACGCTTTGGTCA
GGGTGATGGTGGACGACGTGAACGACAACGCGCCGAGCTTCGCGTTGCCCAGTTACA
CGGTGAAGATTCGCGAGGACGTGCCCGTGTGGACGGTGGTCGCCGTCGTTGATGCCAC
GGACCCGGACGAGGGGGGCGGGGGCGACGTCGAGTACTTCCTGTCCGACGCCATGGA
GAGCGAGGGCTACTTCAAGGTCGACAAGGTGTCGGGCACCATCAGGATCACCCAGAG
CTTGGACTTCGAGGAACGGCAGATGCACACGTTGACGATAGTTGCTAGGGACAGGGG
CGAACCGTCCTTGTCCTCCGAGACCATGGTTATCATAGACGTGGTGGACGTGAACGAG
AACGTCCATCCCCCATTGTTCGACGACTTTGTCGTTTCCGCGAGCGTGTTCGAGAATCA
AGCTGTAGGCACCCTGGTAACCACCGTCCGCGCCAAGGACGCCGATCCCCTTGGCGGG
GACTCGAGGATCGGATACACCATACGTGGAGGGGACGGGATCGGGATTTTCTCCATCG
ACAACGAAG

>novel_circ_000918
GTAACATCAAGACAAAGGCTGTGCTCGACGTGGAATCGAAGAGAGGATACTGGTTAAC
GGTATACGCGCAGGATCACGGTGTAGTTCCTTTGAGCTCCAGTTTACAGGTGTACGTGG
AGGTGCTCGACAAGAACGACAACACACCATTGACCGAGGTTCCGGTCTATTATCCATC
GGTCCTCGAGAACTCGCCGGCAGGCGTGAGCGTTCTCCAGATACGGGCGTTCGATCGC
GACGTCTCGCCTCAACGATTCACCTTCTCCATCACGAGCGGCAATCCGGAGGGTTATTT
TCTCATCAATTCCACCACGG

>novel_circ_000919
GTACTCGCGAAGGATAAAGACATCGGAGATAATGGAAAAATTCAGTACAGTATCAAGG
GTGGGCGAGGCAAGGGCAAGTTCAAAATTCATCCCACAACTGGAATGGTCTATTCTCA
ACGAGGATTCGAAGCTGGACAGGAGTACGAGATGATGATAAGGGCGGCAGACTGCGG
GGAACCGCAGCGCAGCCATCAAACACGGGTGTCCGTGCAAGTTGTCGAGGTACCAAA
GGAATCCGAGAACCCGCCAGTCTTCAAAACGAACAATCAGAGTGTCGAGGTTACGGA
GAGCGACAAGGTCGGGTTTCTCGTGGCACTGGTCCAGGCCACCGACAAAGATGGCGA
CTATTTGTGGTACGATATCATTGACGGCGACAAAAGGGACGAGTTCTTCATCGGCCGGG
ACAACGGGAACGTGCTGCTCGCGAAGAAACTCGATTGGGAAACGCAAAACTTTTACA
ACCTGACGATCCGCGTGACGGATAGCGTGGAATCTGCCGTGACTCAACTTCTGGTCAA
CGTGATCGACATCAACGATCACAGGCCTGAATTCACGGAGAGCGTTTATCACGTGGAC
ATCTCGGAGAACGTGGAAAAAGGGGAGAGGATACTTGAGCTTCACGCGAGCGACGAG
GACGAGGACAAGAAAGTGTTCTACAGCCTTCACGCGGCCCAGAATCAAGCGTCGTTG

GAGATATTCCACGTGGATTCGGTGACGGGTGCCGTTACGTTGAACGAGGTATTGGATCG
GGAGACGATCGAGGAGCACGTGCTCACCGTGATGGTTAAAGATCAAGGGACACCGGC
GAAACGGAACTACGCAAGGGTTGTAGTAACGGTGCACGATCACAATGATCATGCCCCA
GAGTTCATCAGCGAGATAATCCAAGGAAAAGTGTACGAGACATCTCCCATCGGCGCTG
CCGTGGTCCAAGTGTACGCCATAGACAGGGACAAAGGGGACAATGCCAAGATCACTTA
CTCGATTACTTCTGGGAACGTGGGTAACATGTTCACGATAGATCCGAATCTAGGCGTGA
TCCGAGTTGCACGAGAATTAGATCTGAGCGCCTCCTCGGAATACATACTGTTGGTAAAG
GCGACGGATCACGGCTCCCCGGCGTTGGCCAATACGGTGCCCGTACACGTGATGGTAA
CCATGGCCGACAACGCGCCACCTAGATTCATCCAGAAGGAATTATCGGCCGAGATTTAC
GAGAATCAACAGGTGGGCACTTACGTGAAACACGTGGAGGCAAGAAGCACGTCCTCT
CTGCAATTCGAGATCGTTCGAGGGAACAAGGACGACACGTTCTTCGTGAATCCGAGCA
CGGGGATCATAGTGATCAAGAACGAGCTGGATTACGAGAAGACGAAATTTTACAACTT
GACGATAGCCGCGACAAACATGGCAAGCGCGAAGGCGCATTGCAACGTGATCGTCCA
CGTTCTCGATCGAAACGACAACGCGCCGAGATTCCTGCAGGCCGTGTACAGCGGGGA
GATAAGCGAGGGCGCCACTATAGGCTCCTTGGTACTCACCAATACGAGCACGCCATTG
GTCATAAAGGCGGAGGACGCCGACTCCGAGCTGAACGCCCTCTTGAATTACGACATAG
TGGAGGACTTGCCGCGAAAATACTTCCACATTGACTCGAGCACGGGCGCTATCAGGAC
GGTAATGGTTCTGGATCACGAGACCGTTCCTAAGTTCACGTTCCACGTGAAGGTCTCC
GATCTAGGGAAACCGAAACTGTCCTCAGAGACCACCGCAAAAGTGATGATCCTCGTCA
CGGACGTGAACGATTGTCCGCCCAAGTTTCTCGAAAACGACTATAACACCACAGTGCT
GCTACCTACGTACAAGAATGTAGCCGTGGTCCGAGTCAGCGCCGTGGATCCAGACAGT
TCGGAAGGCGTCCCGCTCAGATACGACATCATCGATGGAAACAAGGCGCACACGTTCG
ACATAGACTCCCAAACCGGTGTGATCACCGTGTATAATCCGGAACGGATGAAGAAAAG
TTATCTGTTACGGGTGCGCGTCTCGGACGGGAAGTACTCGAGCGTGTGCCAGGTGAAC
GTGATCGTGGAGAAGTCGGAGAACTCTGGGCTGATGTTCCAGAAGGACGTCTACGAG
GGCACAATACCGGAGAACTCGACTCGGATCACCACTGTCGCGGTCGTGAACGTTTTGG
GGAGCGCGTTGAACGAGCACATTGTCTTCAGCATTCTGAACCCGACCGACATGTTTGT
AATTGGATCCACGTCCGGCGCGATCCGAACCACCGGGATTAGATTCGACCGGGAGGTT
TGCGACCATTACGAATTGATAGTGGAGGCGAGGAGTCAGATGCCGGGCAGGGAGAGG
CCGAGGGTGGCGCACGTGATCGTGAACGTCACTATATTGGACATCAACGACAACTGTC
CAATGTTTGTTAACTTGCCCTATTACGCCGTCGTGTCCGTGGAGGCGCAGAAGGGCGA
CATTATCACGAAGGTGCATGCAATAGACATGGACAGCGGCGACAACGGAGAAGTTAGA
TACGAGTTGAAGAAGGGCCACGGAGAGTTATTCAAGGTGTGCAGAAAAACGGGCGAG
ATATCTCTGAAACAGAATCTGGAGGGTCACAATCGCGAATATCAGCTCACCATTGCCGC
ATACGATGGAGGCATTACACCGTGCTCTATCGAGGTGCCGGTGAACGTAAAGGTGATA
GATCGATCGATGCCAGTGTTCGACAAGCAATTTTACACGGACAGCGTGCTGGAAAGTA
TCGAGATACATTCGCCCCTAGCTTTGTCCATACAGGCCGAGTCGCCGTTGAATCGCAAG
TTGATATACAGTATAACGAAAGGGAACGATTTCGAGGAATTCGCTCTGGATTTCAACAC
AG

>novel_circ_000920
TAATGATGACTCGGATAATTGAAGGAGTTGCTCGGTTTCGCGGACACCAAGAAGAGAC
ACTGAATGCAAGTCACGTGGTGAACACGTTTATACAAGACCGTTGGATACCGTGAATG
AATTTGCGGATGCTACCAGAAATGTCTGCACCTTTGAACATGTTGTGCAACTTAACAGG

TTGACTCCATTCATTTGATTGATTTTTCTCTTTGGAATATTATGGAGAAAAATTACTGTAA
TCGTGCATAGTCATTGACAAGCGGTTAGTTGTCGTGAAAAATGACGGACATCGTGCAC
GAGCCGTGCAATTATGGAAGCTGTGTATGGCAGACGAAAGTCTCGGACCGCCCAGGCG
GCCATTTCGTACGATATTCTGATTTTCCGATGTTTAACTGGATGTGGATTTATCGCGCGA
GAATCAACTCAAAAAGAATCAACCGAAGGAAACTTATAGTTACTGTTCCTTTTCGTGCA
TGAAAATCATCATAGAAAGAAGAGAGAGAGAGAAGAGAAAGAAAGAGAGAAAGAGA
GCAGTGCAGTAGCGTGATACAGTGCAACACAGCAGACCGATAGAACTAGTATTTTTTAT
ACTCACAAGGACCATTGTTACTGTCGGGGGCTGCGAAACAGGGAGATACAACAACAC
TTTAATTTCTAGTTTAATCAGATAAGCATTCGATAAAGAAATAAATTAAATAAGGGAGAG
ATTGGTAAAGAGAGGAGAATTAATTAATTAAAATAAATAAATAAATAAATAAATGAATG
AAATAAACTACTATAAATAAAAATGGTAATAATAAATTGGAACGTAAATTTGAATTATCG
TGTGGGGGGGGTGTGAATTAAATCGAGATATAAATATAATTATTTACGATTAATTTTCAC
GCTTACGAACAAATTAACCGTATAATTGTATAACTGCAGCTTGTGTTACGTGCGATACAT
TCCAACATCCCCAATTTGCTACTGTTGTCTCACCACTACTACACCGTGACTTGATTAGAA
TTTAGTCACCGAATACCATGTATACAG

>novel_circ_000922
CAGCAAAAATTCGTGAAGAGGACGGCCGAGGAGTTGGAGCCAGCCGGCGGCGGTAG
CGGAGACGGTGGCTTCGGCGAGCCCCCCGTGAAGCTGCAATGCACGCAGGCGCAGCA
TCAGCACCAGCAGGGGCCGGGGGCCGGCGGCGGGGGCGGGCCCGGGCACCACGGGC
ACGGCCAGCACCCGGCCGGGAACGGGGCGAGCGCGACGGGGCCCGCGCACGGCGCG
GGCGGGGGCGGCGGGGGCGGCGCCGGTAACGAGGGATTGACCAAGTTCTCGGTGGAG
ATCGTGCAGCAGCTCGAGTTCACCACGTCGGCGGCCAACTCGCAGGCCCAGCAGATCT
CCACGAACGTGACGGTGAAGGCGCTGACGAACGCCTCGGTGAAGAGCGACCTGCTCA
ACGCGTCCTCGTCCCCCAAGTCGGGGCCGGACACCGGCCCCGCCCCGCCCGGCCCGC
GCCCCGGCGGCGGCCCGCCCGGCAACCCAGGCGCCGGGACGGGGCCCGGCCCGGGC
GCCGCCGGCGGCGGCCCCGCCGCCCCCGGCGGCATCGGCTGCGTCGACATAGGCAAC
CTGGTGGAGTGCAAGCAGGAGCCCGACAACGAGTTCGTCGATCTGGAGCAGTGCGCC
GCCGCCCTGGAGAAGGACGCCGCGGCGAACGGAGCGGGTTTCCCCGGCTTCTCCGAC
TTCATGGGCGACGACACCGGCGACGAGATCATCACCTCGGACGCGTTCAAGGACCTCA
TCTCCGAGATATCCGACCTTCACCCCGAGTTCATGAAGGACTTCGACTTCGAGGAGAA
GATCCCCGTGGAGGCGTTGGCGGCGAGCAACGCGGCCGCGGCGGCGGCCGCGGCGGC
CGCGGCCGCCGTCGCCAACACGAACGGCAGCAACGGAGGGGGCGGCGCGAACAACG
GGCTAGGGACGAACAACGGGCAGATCAAGATCGAGGACGACAAGGACGGTGGATTGC
ACCAGCAGCAACAGCAGCAGCAAGCTCAGCAGCAGCAGCAGCAAGCTCACGGGAGC
GTGAACAACGGGAACGGCGTGGTGAACAACGGGCAGACGGGGGCCACGAACAACGG
GACGGGGAGCGTGGCCGCGAATTCGAGCCCGGGCGCCCTCGCCTCCGCCCAGTACTC
GCCCGCCAGGCTGCCGTACTCCGGCCTCGACTTCAAGTCGGAGATGAGCCCCGCCGCG
CAGACGTTGAAGCAGATGGCCGAGCAGCACCAGCACAAGAGCCAGCAGCTGGGCCT
GGGCGGCTTCAACCCGGGGGCGGCGGCCGCGGCCGCAGCGGCCAGGGGGCCCGCCG
CGCGCTCCCCTTACGCCGAGTTCCCCCAATTCGGCGGCACGTCGGACTACCTGGGCAG
CCCTGGGAGCGGCGCCGGGCCCGGGGCGGGCGCGCAGGGCCAGGGGGCGGCCGGGG
CCGGGTCGTATCACAAGAACAACGGGGCGGCCGGGTTCCAGGGGCAGCAGGCGGGCG
ACATGTTCGTGGGATCCCAGACCCAGTTCGCCGCCGGTCTTGCGGACATGAAGAGAGC

GCAGCCGAGCAGCGGCGGGAAGCCGAGCATGCTCGGGCCGAGCGCTGGTTACAAGCA
GCAGTATTCGCCGTACGGCAGCCCGGGATCGATGCCGAATCACGGCAGCCCTGGCTAC
CCGCTTCCGCCCAGGGGATCCCAGGCCGGCGGACCCAATCAGACGGGATCCCAGGGA
TCCTTCACCAGCTCCACGCCGCCGAGGCCTCCCTCCGGACCCGGCACCTCCACTTTGC
AGATCAACCAGGCGCAGCAGTTACACATTAACAATCCCGGCCATCAGATTCAG

>novel_circ_000923
CAAAAATTCGTGAAGAGGACGGCCGAGGAGTTGGAGCCAGCCGGCGGCGGTAGCGG
AGACGGTGGCTTCGGCGAGCCCCCCGTGAAGCTGCAATGCACGCAGGCGCAGCATCA
GCACCAGCAGGGGCCGGGGGCCGGCGGCGGGGGCGGGCCCGGGCACCACGGGCACG
GCCAGCACCCGGCCGGGAACGGGGCGAGCGCGACGGGGCCCGCGCACGGCGCGGGC
GGGGGCGGCGGGGGCGGCGCCGGTAACGAGGGATTGACCAAGTTCTCGGTGGAGATC
GTGCAGCAGCTCGAGTTCACCACGTCGGCGGCCAACTCGCAGGCCCAGCAGATCTCC
ACGAACGTGACGGTGAAGGCGCTGACGAACGCCTCGGTGAAGAGCGACCTGCTCAAC
GCGTCCTCGTCCCCCAAGTCGGGGCCGGACACCGGCCCCGCCCCGCCCGGCCCGCGC
CCCGGCGGCGGCCCGCCCGGCAACCCAGGCGCCGGGACGGGGCCCGGCCCGGGCGC
CGCCGGCGGCGGCCCCGCCGCCCCCGGCGGCATCGGCTGCGTCGACATAGGCAACCT
GGTGGAGTGCAAGCAGGAGCCCGACAACGAGTTCGTCGATCTGGAGCAGTGCGCCGC
CGCCCTGGAGAAGGACGCCGCGGCGAACGGAGCGGGTTTCCCCGGCTTCTCCGACTT
CATGGGCGACGACACCGGCGACGAGATCATCACCTCGGACGCGTTCAAGGACCTCATC
TCCGAGATATCCGACCTTCACCCCGAGTTCATGAAGGACTTCGACTTCGAGGAGAAGA
TCCCCGTGGAGGCGTTGGCGGCGAGCAACGCGGCCGCGGCGGCGGCCGCGGCGGCC
GCGGCCGCCGTCGCCAACACGAACGGCAGCAACGGAGGGGGCGGCGCGAACAACGG
GCTAGGGACGAACAACGGGCAGATCAAGATCGAGGACGACAAGGACGGTGGATTGCA
CCAGCAGCAACAGCAGCAGCAAGCTCAGCAGCAGCAGCAGCAAGCTCACGGGAGCG
TGAACAACGGGAACGGCGTGGTGAACAACGGGCAGACGGGGGCCACGAACAACGGG
ACGGGGAGCGTGGCCGCGAATTCGAGCCCGGGCGCCCTCGCCTCCGCCCAGTACTCG
CCCGCCAGGCTGCCGTACTCCGGCCTCGACTTCAAGTCGGAGATGAGCCCCGCCGCGC
AGACGTTGAAGCAGATGGCCGAGCAGCACCAGCACAAGAGCCAGCAGCTGGGCCTG
GGCGGCTTCAACCCGGGGGCGGCGGCCGCGGCCGCAGCGGCCAGGGGGCCCGCCGC
GCGCTCCCCTTACGCCGAGTTCCCCCAATTCGGCGGCACGTCGGACTACCTGGGCAGC
CCTGGGAGCGGCGCCGGGCCCGGGGCGGGCGCGCAGGGCCAGGGGGCGGCCGGGGC
CGGGTCGTATCACAAGAACAACGGGGCGGCCGGGTTCCAGGGGCAGCAGGCGGGCG
ACATGTTCGTGGGATCCCAGACCCAGTTCGCCGCCGGTCTTGCGGACATGAAGAGAGC
GCAGCCGAGCAGCGGCGGGAAGCCGAGCATGCTCGGGCCGAGCGCTGGTTACAAGCA
GCAGTATTCGCCGTACGGCAGCCCGGGATCGATGCCGAATCACGGCAGCCCTGGCTAC
CCGCTTCCGCCCAGGGGATCCCAGGCCGGCGGACCCAATCAGACGGGATCCCAGGGA
TCCTTCACCAGCTCCACGCCGCCGAGGCCTCCCTCCGGACCCGGCACCTCCACTTTGC
AGATCAACCAGGCGCAGCAGTTACACATTAACAATCCCGGCCATCAGATTCAG

>novel_circ_000925
GTGTCCGCGGGTCAGCACATGCAGCTAACAGGCGACCTGAAACCGGGGGTATCGGTG
GCGGCCCAGCAAGGCATGTACTTCAGCCAGCAGCAAACGCAGAATCAGTCGGCCGGT
CAAACGGACACGTACTGCTCGGTATCGCAGTCCCAGACCATCAACTTCACCCAGCAGA

GCCTGAGACAGAGGGCCGCGGCCGCGGCAGCGGGCGGCGTCCCCCAACCCGCCGGC
GCTGCGCCGGCACGCAGCCATCCCCAGCAAGTGCAGCCGAACCAGGTGGATCAACAT
CAACACGCGAAAATTCTTCAACAACAGCAGCTGATGCGCGCCCAGCAG

>novel_circ_000926
GTGTCCGCGGGTCAGCACATGCAGCTAACAGGCGACCTGAAACCGGGGGTATCGGTG
GCGGCCCAGCAAGGCATGTACTTCAGCCAGCAGCAAACGCAGAATCAGTCGGCCGGT
CAAACGGACACGTACTGCTCGGTATCGCAGTCCCAGACCATCAACTTCACCCAGCAGA
GCCTGAGACAGAGGGCCGCGGCCGCGGCAGCGGGCGGCGTCCCCCAACCCGCCGGC
GCTGCGCCGGCACGCAGCCATCCCCAGCAAGTGCAGCCGAACCAGGTGGATCAACAT
CAACACGCGAAAATTCTTCAACAACAGCAGCTGATGCGCGCCCAGCAGGTGATGCAA
CAACAACAACACATGGCCGGAGGAATGGGTGGCGTGAGACCGCCACCACCCGAATAC
AAAGCGGCTCAGGCCCAAATGATGCACGCTGGTATCGGTATGGCTCAACAGGCCAGGT
TTCCCAATACCGGGCCTATGCGCAGGGTTACGCAACAACCCATGCCACCTTCAGGTCC
GATGATGAGGCCGCAAATGGCGCAGCAACAACAACAGGCGTTGCACGCCGCCGGTGG
CAATATGTACATGGGCGGTGGTAGTATGGCCGCGATCGGCGGCATGCATCAAATGCATC
AGCGTCTTGGATATCCTAGGACCAACAATCAACGGCCGCCTAACGTCAGCGTTGGCCC
GCCAGATGGCCTTGGTAATAGCATCGCTGGCCGTGGCGCGCAGCAGGAGTGGCGACAC
GTTCTCATGCAGCAGCAGGGCTTTCAGGCGCAGATGCGATCGCAATTTAATCAGCAAG
GTCACCAAGGTGGATTCGGTATGGGTGGCACGGGTGGCATGCAGATGAACGCCGCACA
AATGCAGCATCAACAGTTGATCCGCTCGCAGAGCGGGGGTATAGCGGGCTCCGGTATG
GGCAACAGCACTCAGATGCAGCAGCTACTGTCGCAGCAACACCAGCAGCAGACATTG
GCCATGCAGCAGAGTAATAATCAGATGTCACTGCAGATGCAAATGTCGCAGTCCTCGTC
GGTCAACTCTGTCAGCAGTACTGGAACTGGTTCCCCCCTACACCCGCATCAACAATAC
GGCGCTGGCAGTCCAGGAGTGCGCAGTTTGCCACAACAGCAACAGCAGCACGCGCAA
CCGCCAGTCACCACCACCACGGATCCATCCGTAACCGCGGCGGACTTTAGCCTCGAAT
TTCTAGAGAATCTTCCGGCCGGAGACACGTCGAACTTCAGCGCTCAGGAACTGCTGAA
TTCCCTTGACTCCACGGGGGGCTTCAACCTCGATATTCT

>novel_circ_000927
ACGGACCTCTGCGGTATCAAATGGAAGAAGCTCGTGTGGGGCGAAGTAGCCGGCGATT
TCGGGGGTACTCCCCTCGAGGACCCGGTGTTGTCGAGCTTCTCGCGGTGCCTCGCGGG
TGACATTCTCTGCGTGTGGCGGCGGGTCGCCGCTACGCCGGCCACCTCCGGCCCGGCA
ACCGCCTCGGCAACCGGAGCTGGCATCTTCGACCTGGGCATCGCACCCTCGCCCGCAC
CACCACCCCTCTCCCTTACCGCCGCCAAGGAACTCTGGATTTTCTGGTATGGCGAGGA
ACCTGATCTCTCTGGTCTCGTGTCACCGGAACTCATCGCGTGTGAAAGTGAGCAAGGT
TCTTGGGAAAGCGGATTATCATACGAGTGCCGGTCACTTCTTTTCAAAGCTTTGCATAA
CTTGATTGAACGGTGCTTACTATCTCGTGATTTTGTTCGCCTAGGAAAGTGGTTTGTAC
AACCTTATGATGGATTCGAAAAACACCGTTGCAGTAGTAGTCACTTGTCATTCTCATTT
GCATTCTTTGTGCATGGAGAAAGCACTGTATGTGCAAGTGTGGATGTCAGGCAGCATC
CTGCTGTGCGACATCTTACAAGGACTTGCTTACAACGTACCCAAACTTCCCAATCTGGT
GTTAAAGTGATTCTAGCTCCTTATGGACTAGCAGGCACATTAATTGGTCCAGTGGGACG
TATAGATAGTCAACTTCTTGAAGAGTGGAAACACTTTTATCCAATCAATAGCAGTAACAT
AGAAGCAGGACTTCCACCTCTAGTAGAAGTACTTGTTGGTGGTGTCCGCATGCGTTATC

CTTCTTGTTATGTCCTGGTCACAGATATGGATGATAGTCCACCTGATACTCCTCTTTCTC
CACCAAGTAGTCCTGCTAGTTGTGAACATCCTCTCTTGAGTCAGCAGGAATTACAAGC
AGCTACAGAATTACCAGAACGTGTATGGGCAGAATGTACTTTGAGCTCACCAATTTCTG
CTTCCAAGACAGAATCTTCCACAGAACTTGGAACCTGGACCTTTATTGATCCAACACA
AAAGTCTTCCTGTATCTGTTCAAA

>novel_circ_000928
GTGATCATGGCTAATCGGGGTACAGCCCAGAGGCCGAATGGTTCGACACAAGGAAAGA
TTTGTCAATTTAAGCTGGTATTACTTGGCGAATCTGCTGTGGGAAAGTCAAGTCTTGTT
TTGAGGTTTGTTAAAGGACAGTTTCATGAATATCAAGAAAGTACTATTGGAGCTGCATT
TTTAACACAAACTGTATGTTTGGATGATACAACTGTAAAATTTGAGATTTGGGATACAG
CAGGGCAGGAACGTTATCATAGTCTTGCACCAATGTATTATCGTGGTGCACAAGCAGCT
ATTGTTGTATATGATATAACAAATCAAGACACATTTGTACGTGCCCAAACGTGGGTGAA
GGAATTGCAACGACAAGCCAGTCCAAGTATAGTTATAGCATTAGCTGGAAATAAAGCT
GATCTTGCAAATAAAAGAATTGTAGAATTTGATGAAGCTCAAACTTATGCTGATGAAAA
TGGCCTTCTTTTCATGGAAACTTCAGCTAAAACAGCAATGAATGTTAATGATATATTTTT
GGCAATTGCTAAAAAACTTCCAAAGAATGAACAATCAGGAAGTGCAAGTACAAGTGG
TCAAGGCCGTCGATTGGTTGAAACAGAAGGACAAAAGGCAGCAACTGGCAATTGTTG
CAAGTGATTATCCAAATCT

>novel_circ_000931
GTAATTTTTTTTTCGTGTATTATTAGTATCAACATTGAAATTAAATTTTCGCGTTTTTTAA
TCCACAGAAAAAACACACTTCCACTTTTACAAGTTATTCAAGATCTTAAATTGAACTGT
ACTTTTTTACCTAGAAATGTAAAGAATACAATGAATTTATAACAAAGGTGTAATAGGATG
TCGTTCTTACCAATTAAGTAAATTAGAATATTTGCCCGAATCGTCGATGTATTAAAATTAC
GACACAAACTGTGGATCTATTTCAAACAAGTTATTACTATTAGTAATAA

>novel_circ_000932AAGAAAACAGTCGTGGCAGTACAAAAGCAAAATGGTATAATTGAA
CAAAATTATAGTAAAGAAAGTAGTGGTAGTGCTGTTGAAAGTAGGGGAAAGGAAAATA
AGGAACAAAACCATTGTCAAGATGATAATGAAGAACATATAGATGGTAGTGAAAAAAA
AAAGAAAGTAAATAAACAAAAATGGGTACCATTAGAAATAGATATAACAAAAAACCGA
AGTAAAAGAGATCGTTCTCCAAAGTATCACAATCAAAGAGAAAAGAATGGAGAAGAA
ACAGATTCGTATAGAAGTAGGGAACATGATAGACCTGCATATATCACAAGAGGTGGACG
AGGTGGACGAAGTTACAGAGGAAGAGGAGGACGAGGTGGACGAGGCGCCTACCGTG
GTAGTTTTAGACAGAGGCATGATCAAGTCTACATGCAGATGCACAAATTTGGGCTTCTA
GATCCTAATTACATGATGGCTTACATGGGAACGTTTTATTTTAATAATACAAATTTCATTA
ATATAAATACAACAACACTCAAAGAATTTTTAAGAAATCAAATAGAATATTACTTTAGCG
AGGAAAATCTTCTAAGAGATTTTTTTTTGCGTCGTAAAATGGATGCACAAGGCTTTTTA
CCTATTACTTTAATTGCATCTTTCCATCGTGTACAAACTTTGACGACAGATGTTGGACTT
GTTATTGAAGCAATTATGGAATCTGATAAATTAGAATTAGTTGATGGATTTAAG

>novel_circ_000933
GATGAATTTGCTGGTGATGGTGTTGATTTATATGATGACGTGATAGCAGCTCCTGCTGGT
GGTAATGGCGGTGTTTCTACAGGAAATAGTGGAGATGGCGGAGGTGATACTACTTCAC

CAAATGAAGAAACAAATGGTAGTGCACCTTACCATCAACTTGGAAATAACATCCAGCC
AAATCAAATTGGAAGACGTCATCAATTATATGTTGGAAATTTGACTTGGTGGACAAGTG
ATCAAGATATAACTGATGCAGTACAAAGTATTGGTGTATCAGATTTTGTTGAAGTAAAG
TTCTTTGAAAATCGAGCCAATGGACAATCGAAGGGTTTCTGTGTAATATCCCTGGGTTC
AGAACAAAGCATGAGAATATGTATGGAAAGATTACCTAAAAAAGAATTACATGGACAG
AATCCAGTAGTAACATTTCCTACTAAACAAGCATTGAATCAA

>novel_circ_000934
TGGCCAACGTGGTTCTACAGGCCGGATTTCCCCCTCGGCACTCAACTGCCCACCGGCC
ACGTGTCGAATCCAAGCAACGTTCCTACTTCGTCCCCTCTGTGCCCCAGGACGACTCA
CTTGCAATCAGAGGCGTCCAAGGTTCCCGAGTTTCTCGATAGCAGCAAAATCCATGGA
CAGACGCCGACGATATGCACG

>novel_circ_000935
AAAAGCCCGACGAAAGGAGAAGGATGCGGGAACAACAGAGGATCCGAAATTGTATCT
CGAGGAGGCGGCTCTTGAAAGGCAACTGCCAGAGCTGGACTTGAGGATGTTGGTCGA
TTTGCCACCTGGTTTGGATTACAACGAATGGTTGGCCTCTCATACTCTTGCACTATTTGA
TCACATCAATCTTGTTTACGGAACGATATCCGAGTTCTGTACGATGACGGGTTGTCCTG
ATATGACCGGTCCTGGCTTAAGAACTTATCTGTGGTTTGATGAGAAAGGAAAGAAAAC
GAGAGTGGCAGCGCCACAATATATAGATTATGTTATGACTTTTACACAACGCACCGTTA
GTGATGAAACTATATTTCCTACGAAATATGCAAATGAATTTCCTAGCTCGTTCGAATCGA
TAGTGCGTAAGATCCTGCGGCTACTGTACCATGTGGTGGCACACATTTACCACTGCCAC
TTCAGAGAGGTAGCACTCTTGGGGTTGCATGCTCACCTTAATTGTGTATTTGCCCACCT
CACGCTCCTTAATCAACGCTTCAACCTTATCGATCCCAAGGAAACGGAGATCTTAGGAG
ATTTAGAGGCTGCCCTCTTGGGTGACTCAACGTCTTCATCAGCGCCTTCTTCACAAACC
TTAGCCATTCAGGAGACTCCGACCACAGCCACCTAGATCGCGATGCACAGCAAGCAAA
AGGTTGCAGTTCCTGAGACTAG

>novel_circ_000937
GTTGTCCAGGCGGCACCGGTCCCCGTACCTGTCCAGGTACGTAGCAAGAACGATGGCT
CGGGAGTGGTGGCAGCGTCCGTGTCGGGGGTACGGAGACGAAGCGGCTCCCAGGGTT
TACGATCGCCCGCCGCCAAGAGGCCACTCTCCGCCCCCGTGGCTCTTCAGGGATGGCT
ACACAAACAGGGATCCGAGGGGCTGATGCTGTGGAAGAAACGGTGGTTCGTCCTCTC
CGAGTACTGCCTCTTTTATTACAAAGGTCCCGAAGAGGAGAAATTATTGGGTTCGATAT
TGTTGCCATCGTACAGAGTTACCGTGTGCAAACCGGAGGACAAGGTTAACAGGAAGTT
CGCGTTCAAAGCTGAACACGCGAACATGAGGACGTATCACTTCGCGGCCGACAGCCG
TGAGAGCATGAACCAATGGGTGAACGCGTTGACTTTGGCGACCCTGCTTCAGGATCCA
AGCCC

>novel_circ_000939
CGGAGGGGGCGAGGCGGCAGTGGTGATCGAGATAGGAGGGGGGCAGCAGGAGGGCG
AACGAAGCGCGAGGCCCAGCGTCTCCTCCATATCCTCGATCCTGAACCAGAGCGCGGA
CGACAGCGATTCGGGTTTCCACGGTTTCCAATCGCGGGACGATCCCAGCCACGCGTCC
AACAACAACTCGAGCCCGAACAGCGTGAACACCGCCTCGTTTCCGAACCTGAGCGGC

AGCAACAACGGGAACAGCAACAATGGGAATAGCAGCGGCAACAACGCCAACAATAAC
GCCACCATCACCGTCACCAACAACAACAACAACAACATCAGCCTCGCCGGGAACAAC
AACAACAACAACACCACCAACGACGTTCAACCGATGGTGAACGGTTGGATCCAACAA
CAGCAATCGCCTTACGGCCAACCGAGCACTTCTCAACAACAACAACAATACGGCCAAT
TGATATCATCCGTCGTGATGGCCGGCCAACCGTCCCTGCAACAAACCCACCACCACCA
CCACCACCAAGCGTTGCTCCACCAAACGCACCTTCCCCACCCAACTGCTACCCAGATC
CTTCAACACAACAAGCAACAACAACAATCTCAACAACAGTTGCCCAACGTGCAAACT
GTCCAACCTATGCCGAGGAAATTCGGCCAACCGATCTACGCGAACGCGCCGCCTAAAC
CCAGGCGGATAACCGACGGCTCGACCGAGTACTCCACCCCCTCCCCGGACCCCGACTA
CAGAAAATCGCCCGTCTCGCCGGACGTCGCAAGCATGGCAGGCGGCAAGAGTCCAAT
GCCCGACTACGAAAGGAGTGGCGCGATCTATGGTGGGGCGGGTAGAACTGTTGCCGG
GCAACAGAGCCTCAGGACTGATAAGTCCGGCCTGAATTACGGTTACGGGGGGGGGCA
GGTGCAGGCGCAGACCGAGCGGCGCACGCCCGACACGTACGGTCGCAGCGCGGCTAA
ACCGCGGCCGGGGAGAGGGAACGGGGACTACGAGGAGGTTTACGGGGCGCCCCAGT
TGTACCAGAGGCCGGCAGGCCCCGTCGGCTACACGAAGGGGGCCTCGCCCGCCCCGA
TCCCGATCCCCCTTTACGCGCAACAACAGCAACACCATCATCAGTACCAGCACCAGAT
ACATATCGCGACCACGAACGTGCCGATAATGAGACAGCCGAGGGCGCAGCCACCGCC
ACGGCCGCACAGCGCTGACTTCTTGGAGTACGAGGCGATCAGGAAGCCTCAACAGCA
GATGCCGCCGCAGTGCGAGGAATCGGTGAACCAACAGAGGCGTCCACAGAGGCCTAA
ATCGAGTTTAGACATCGTCACTCCGTCGGACGCAGCGAACGATGGATACTTTTATTCCG
AGGAAAGATACGCGGCACAGATGCGGCAATCCGCGGTTTATCTCCACCAGACGCCCCA
GCATCACCAGCAACAAAGGAACCAATCGCTTTCCCGGACGACAATGCCCTCGAAAGC
GACCGGGATCCGCGACAGAAGCGTAGACGAAAGTAGGATAGCTACGCTACCGGAAGT
TTCCTGCTCGGGCCTGAGGCGGGGACAACACGTTCATCAGCAACACGTGATACCTCAC
CAATCGCTCCAACGGCATACCGA

>novel_circ_000940
TGCCGATAATGAGACAGCCGAGGGCGCAGCCACCGCCACGGCCGCACAGCGCTGACT
TCTTGGAGTACGAGGCGATCAGGAAGCCTCAACAGCAGATGCCGCCGCAGTGCGAGG
AATCGGTGAACCAACAGAGGCGTCCACAGAGGCCTAAATCGAGTTTAGACATCGTCAC
TCCGTCGGACGCAGCGAACGATGGATACTTTTATTCCGAGGAAAGATACGCGGCACAG
ATGCGGCAATCCGCGGTTTATCTCCACCAGACGCCCCAGCATCACCAGCAACAAAGGA
ACCAATCGCTTTCCCGGACGACAATGCCCTCGAAAGCGACCGGGATCCGCGACAGAA
GCGTAGACGAAAGTAGGATAGCTACGCTACCGGAAGTTTCCTGCTCGGGCCTGAGGCG
GGGACAACACGTTCATCAGCAACACGTGATACCTCACCAATCGCTCCAACGGCATACC
GAAATGAGCAGCAACGAGGCGAAGCTGAGGCGGTCGTTGCGGGAGAAGAGTTACGA
GGCGAGCAGCCGAGAAATGCTGAGTCACATGGCGGAGGAAGGGACGGTGCCGCGAA
GAATTCCACGCGAGTACGAGTGTTCGATGGGGTGGGAAACAGGGGGACGAGTCAGG
GTAGCGCTCACGTGGGCCAACCGTGTCACGCTCCTCACGCCGCCGCCAGACGATGGA
ACGAGCAGCAACAGTTCTGCAGGTCGGCGTCCGCGCGTCTACCGAGGACCAGGCACC
CGGCCACCTTGGACCCCGACGACGACGATTACGAACGATCGTCCGAGCAGGACTCGC
GGGATGGTGAACGAAAGATACAACAG

>novel_circ_000941

CGTCCGGATCCGGATCGGTGATAGGAAGGGATCCGCTTCCGCCGAGGGCTGTCAGCAC
TTCGCAACTCGCTCAAAGGACTCAAACTCAACCCCCGGCTACGGTGTCCACCCTTCCG
TTGCCGAGGAGCAATGCCACGCACAGGCAGCCCCTGCCCCTCCACCAGTCCACCCCTC
AATCGGTCAGACAATCGGAAAACGACGCGAGCAATTCGCAGCTATTACGAGTCGCCAG
CAATGGCGACATCGGGACAGGATCTGCAATGTTGAACAACAACAACAACGATGGAAA
GGAAATAAGGTTAACTTCTCCGGGCGGGCACGCAACTCAACGATTGATGAGTCCGGCC
GGAGCGTTAAGGGTCATTTCCATGAACGACCACCGTGTCTCTAGCCCTAGTGACAGCG
ACATACGAGTTCACAGCCCAA

>novel_circ_000942
CGTCCGGATCCGGATCGGTGATAGGAAGGGATCCGCTTCCGCCGAGGGCTGTCAGCAC
TTCGCAACTCGCTCAAAGGACTCAAACTCAACCCCCGGCTACGGTGTCCACCCTTCCG
TTGCCGAGGAGCAATGCCACGCACAGGCAGCCCCTGCCCCTCCACCAGTCCACCCCTC
AATCGGTCAGACAATCGGAAAACGACGCGAGCAATTCGCAGCTATTACGAGTCGCCAG
CAATGGCGACATCGGGACAGGATCTGCAATGTTGAACAACAACAACAACGATGGAAA
GGAAATAAGGTTAACTTCTCCGGGCGGGCACGCAACTCAACGATTGATGAGTCCGGCC
GGAGCGTTAAGGGTCATTTCCATGAACGACCACCGTGTCTCTAGCCCTAGTGACAGCG
ACATACGAGTTCACAGCCCAATTGAAAGGAAAATGGTGAGCCCAATCGGCAGAGAAG
TGGATCGTGGTGGAGGCACGGCTATTAGACAGAATCAACGTGTATGGGAGTACAACGA
GTTGCTGCACAAAAGG

>novel_circ_000943
AGTATATGGTCTTGCTGTAAGACGATTATTGGAGGAATATGGATTGAAATCAGAAGAAA
TTAAAGGTACTGGTCGACCTAATCGATTATTAAAAAGTGATGTTTTAACTTATATTCAAA
CAAAGAATATAAAAAAAGTTGCACCAAAAACAGCACCTCCACCTAAAGATCAAAAGC
AACCTGATATTCCTTTAAAGAAACATGTACCCAGTGGTGGTCCCTCCACTTATCAAGATA
TTCCAGTTTCTAATATACGCAGTATCATTGCTAAAAGACTTGGAGAATCAAAAATAACTA
TCCCGCATTCTTATGCGACTATCGATATTAAAATTGATAAAATAAATGAAATTCGTAAGG
AACTCAAGGCTGATGGTATTAATATTTCGATAAATGATTTCATTACAAAAGCAACTGCAC
ATGCACTAGTTGAATGTCCATTTATAAATACATTATATAAAAATGATCAAATAATTCAAAT
GCCAAGAGTTGATATTTCTATCGCAGTTGCTATTGAATCAGGACTTATTACGCCAATTGT
TTTTGATGCTACAGCTAAAAGTATATTGGATATTTCGAAAAATATAAAGGAATTAGCTGA
AAAAGCTAAAACTGGTCAATTAAAACCGGAAGAATTCCAAGGAGGAACATTCACAATA
TCCAATTTGGGAATGTTTGGTATTAAGCATTTCAGAGCCATTATAAATCTTCCTCAAACA
GCAATATTAGCAGTTGGAAGTGGTCGAGAAGAACTAAATGCTGCATTACAAAAAGTAA
CAAAAATGTCAACATCTTTATCGTATGATAGACGAGCAATTGATGAAGATCAAGCAGCT
GACTTTTTAGCTGTTTTAAAAGCTATGTTGGAAGACCCATCTTTTCTTATTGCAGGAAGA
TTACGAGCACTAAG

>novel_circ_000944
GTGGTATAGCGTCATGTATAAGAAATTTTCGAACCGACTTTCTGGAGGAGTGGCCAACT
CCCGGCACAGGGCCCCATTTCGACACCTCGATGCAGTCCAACTTCACCGGATTGGTGG
GGAAAACCGTGCATCTGGTGTGCAAAGTGAAAAATCTCGGCAATCGTACGGTTTCCTG
GGTGAGGCATCGAGACATACATCTGTTGACGGTCGGCCGTTACACGTACACGAGCGAT

CAACGGTTCGAGGCGTTGCACCTTCCCCACGCCGAGGAATGGACGTTGAGGATACGAT
ATCCTCAGAAGAAGGATTCCGGTATTTACGAGTGCCAAATATCAACGACACCGCCTATC
GGCCATCCCGTTTACCTGACTATAGTCGAACCGATCACCATCATCATAGGAGCGCCCGA
TCTGTTCGTGAACAAGGGTAGCACGATCAACCTAACCTGCGTGGTGAAGTACGCCCCG
GAACCACCGCCCATGATGATCTGGAGTCACAACAGCGAG

>novel_circ_000946
GTTAGTATCGTTTCGTGTAAACTCTCCGGGGAAGGGGGGGGAAAAATGGACGGTAAAT
CACAGAGTTTAGAAAATCTACCGATATCGCTCTCCGATTACACGGTAAGAGAAATCGTT
GAAGGAATGCGCAACCGCTGCGTTATATTGTCTTCATAATATTCCCGAATGAATTCATCG
ACGAAAGTTGAAAAAAAAAAAAAAAGAAAAGAAACGATAATTAAGCATTTTTCTGACG
GATTCAATTTCGAATCTTCGATACGATCTTATTACAAAAATTTTTATGAAGTTGGAATAG
ATATGAAATTGTAGATAAATTTTATTTTTTATTTTCTACGAATTAATAAAAAAAAAAAAAT
TGATAATTCACGTTGGAAAAGAAAACTAGATATTCGAATCAGTATGGAAATAACTCGAT
ATTAGTAACGTTAATTACGACATTAATAACGATTTAGATCGTTTCTTATTAAGATTAATTG
ATACGCGAGGTTATAATAATTTCCTACTTTTTTTTTTTTTTTACACAACCTGTTTTTTTA
GACACGCAGAATTTGCAGAATCGATCGCAGAAAATGATTCATGCTCGACCTCGACGGT
CCAAGTCTCGACACGTGTTAATACACGTTAATTTTTTCTTTTTCTTTTTTTCGAAAATTC
GATTCTTGTCGAAAAAAATCCGAATCGAACGTGACGATCTTTCCAACTTCCTCTCCTTT
CCCTTCTTTATTTAATAATCTCGAAATGAAAATTCATTATTCGATTTATAAATCGATTTAA
AGGGATCAACAAACACACGTTTCTCGATATTTCGTAATAGCCGATAATTGGTCGAGATA
CGCCTCACGGTCGTGAAAAATTGTTAATATATTGTAGCAAGGTGGGAAACACGTGATCG
GGCTTGCTGCGCGAATTATAACAAGTACAAAGCGGTGGAACAAGTGCGCGAACGAGA
TGGGAGGGGTGGGGGTAACGGAGGACAAACGGTGGTGGACGAATAGCCAAGCAAAG
TCGAGCACAACCGAGCGAGCAGCTGACGCGGCGAAATCAATATTCCCACGTGAAGAG

>novel_circ_000947
GTTAGTATCGTTTCGTGTAAACTCTCCGGGGAAGGGGGGGGAAAAATGGACGGTAAAT
CACAGAGTTTAGAAAATCTACCGATATCGCTCTCCGATTACACGGTAAGAGAAATCGTT
GAAGGAATGCGCAACCGCTGCGTTATATTGTCTTCATAATATTCCCGAATGAATTCATCG
ACGAAAGTTGAAAAAAAAAAAAAAAGAAAAGAAACGATAATTAAGCATTTTTCTGACG
GATTCAATTTCGAATCTTCGATACGATCTTATTACAAAAATTTTTATGAAGTTGGAATAG
ATATGAAATTGTAGATAAATTTTATTTTTTATTTTCTACGAATTAATAAAAAAAAAAAAAT
TGATAATTCACGTTGGAAAAGAAAACTAGATATTCGAATCAGTATGGAAATAACTCGAT
ATTAGTAACGTTAATTACGACATTAATAACGATTTAGATCGTTTCTTATTAAGATTAATTG
ATACGCGAGGTTATAATAATTTCCTACTTTTTTTTTTTTTTTACACAACCTGTTTTTTTA
GACACGCAGAATTTGCAGAATCGATCGCAGAAAATGATTCATGCTCGACCTCGACGGT
CCAAGTCTCGACACGTGTTAATACACGTTAATTTTTTCTTTTTCTTTTTTTCGAAAATTC
GATTCTTGTCGAAAAAAATCCGAATCGAACGTGACGATCTTTCCAACTTCCTCTCCTTT
CCCTTCTTTATTTAATAATCTCGAAATGAAAATTCATTATTCGATTTATAAATCGATTTAA
AGGGATCAACAAACACACGTTTCTCGATATTTCGTAATAGCCGATAATTGGTCGAGATA
CGCCTCACGGTCGTGAAAAATTGTTAATATATTGTAGCAAGGTGGGAAACACGTGATCG
GGCTTGCTGCGCGAATTATAACAAGTACAAAGCGGTGGAACAAGTGCGCGAACGAGA
TGGGAGGGGTGGGGGTAACGGAGGACAAACGGTGGTGGACGAATAGCCAAGCAAAG

TCGAGCACAACCGAGCGAGCAGCTGACGCGGCGAAATCAATATTCCCACGTGAAGAG
GTGAACGATGTAGATATATAGGAGAGAGAGAGAGAGAGGGGCAACGTGAGCGCCGCA
CCGCGTCGAACCGCGCCGTCTTCTTTCCTACCACATTTTTCTTCGTCTCGCGTTATTCGT
TTATTTTCTTATCTCTCTCGGTTCCCGGTAATTCACTCGCCTTCGATACGATCTCTCGCTG
CTACAAATTAACAAACTAACTCTTGGCCAGAGCGTGTTAATGCGTTGCTTTGGAAAGAT
TTGAAGCGTAATATACATTGCAACCGTTTGCAATCGTTCATTAAACGGTTTTTCGAAAA
ATTTTTCGCAAAGAAAGGATTCGAACACGGATTCTACAGATATAGATATATGTAGGAAA
AAAGAAATCGTTTCTCCAGATGATTATCTTTTATAGATATTTTAGAAATTAATTTCGAACA
GGTTTATATAGCTTTTCAAAATTGATAGAAAAAAAAAATGAAAAGAATATTTAATTATTG
GAGTATATCGTAGATCGGTAAAAAAAACCGATTGGCGCGTTCTTGCAGAAAATGTCAC
GTGACCGAAAACCTCGAGATTAATTCGTCTGGAGCAGGGGGTGATAAACACGTTAGAA
GCACGATGGAGGCATATCGGCATCCGTTAACCATGAGATTGAGTCGAAGAGATTGAGG
GGATTATATATATATATACATGTATATAGGGGCAGAAATAAAGAATGAGCATATGAGCGGG
GGTTGGAGAGGGGGGAAAAACGAAACGGCAGAAGCGCAGGCGGGCGGCCGCAATCA
GGTGACAAACCCTACGTCGCGGCATCGTCGCCGTTGCCCGCGTGGTCGTAGTGTGGTC
TCGGTAGTCGCGCGCACTTTCGGTCTTCTCCTACTCCTGTCCCCCTATCCCCCTCCGCCC
CCCTCCCTCCCCTCCGCCTGTCGATCTCGACCTGTGCGCCAGTGTTTCTGGACGAGCTT
CCTCTCGGTCTGTATAGGATCTCGCCGCGCAGCAAACATAGAGAGAGGAGATGAGAGA
AAGAGATCGCCGGGAGTCGCCGGCTGGCACTCGTGTGACGCGTCTCGTTGCGTTTGTT
TCGCACGGTTCGAACCGTTGCACTCTCTCTCTCTTCTCTGTCGCGTTACACGGGAGAAC
GACGAGAAAATCGCGCGCGCCACGATTGCAAGTGGATAGCCGTTATTCGTTGGTTGGT
TCGTAGTTTCATCGATCTTTCGAGGATCGTATAGAATAGAATAAGAGAGTGGCGTGCAG
TTTTTTTCGACCGCGTCTTTCGACCGTCTGCCTGTGTGCTCTACAGTGTTGACCAAACC
CGCTAAAATTGGCTACTATTTCCACCGCACGATGTCCCTAGTTGACCGACACCATATTCG
AACGATGCGCAGTCGCGCAAAATCGCTTCGAAAACGATAACGAGAAAGAGGTATATAT
GCTTACTTTGTTTCAACTTGTTTCGTAGCTGTCACGTTCGTACAGTTTTCTTCGCGTCGC
GACACGCGTTGAACGTTTTTCCTTTACGTAACGTACGTAAGAAACCACTTTTCCTACGG
TAAGTCGTAGAGAATCGTATCGAGAGAATCTGCCGACTGACATTCTTTAGAAAACGTG
CCAAGAACTTTCGATGGACAACTTCGTCTCGCGAAGTGTGACGCTCCTCAAGAGGAA
AAAACTTTCCGCGAAATTGAATAAAAGTATTGCGTAATTATCGGAAAGAGGCAAGCGA
AATATTTCAACCGATATTTCCAGATACTTCGAGAAACCGTGTACTTAGCCTTGTTATCGC
TATTATTAAATTCCACGATCGCCGTTTAATACGCTGTATGCGCGAACTTTTATCTCGTTTA
TTTTTATCCCTTTCTCTTACTTTTCGAGCGGTTTGTACGGTAACCACCACTCTCGAACGT
GCGGTACTCGAATTAATGTTTGCAAAACGACGATTTATTTTACGTGTAAGTACAAAGTG
GACGCTTTTATTGCGACTAACACCGTGCATATCCAAAATAGCGACGTGTCCTCGTAAAA
CGTGTTCATTCCTTTATGAAATTATAAACAAACATTCGCCGAGACTGGAAACTGAGATA
GTCGTTTTTCCAAACCTAGTTTTTGTTCCCTTTTTGCTTCGGTTCGTTATAACTCGCGGC
CGCAGGAAAGATCGATCGCACGTTTTCCATGTGCGAGGCAGCGTGGCCGGTGTGAAA
CGATGGTGCGTTCGGCAAAGAGAACGGTAAAGCAACCGTTAGTAGGTGAAAGAGCAC
GGGACCGGGGCACGATACGAAACACGATGAGCAACGAACTCTCGAGCGAGCGAACG
GGAGCGAATGACTCAGACGACTTGGGTATCCGGCGTGACATAGATCTACCTATGTAAAT
GCTCCGCGACTATATTATTGTTCTACTCTTTCCACCACTCATTACGAGTAATAATTTATCC
TCTACGATGCATGAAAATAATAAATAACGTTAAACACAATTTTTTTTTCAGAATTAATAT
AATAAATTTTTAATAATTGGAGAATTAGAAATTTGAAAAGAAATTTGTTTGTGAGATATA

TATATGTAAGATTCTTCAAAGAATTCTTTTAAAATATCGATCATGAGCGGCGCATTATTT
CGATCGGAGAGGACAACGACTGTCTAAAAATTGTAATTTCATAGCTCGGTAGGAAGGA
AAAATTCTTTCCCCGGGGTGTACGCCTTTACCGGCGACGTACGAACGTTTTGACCTGAA
TAAAGTGACCGGATCCCGATCGATCGCGCGAAGTATTTACGTGGCAGATACAATGATCG
TGCACACACGTTTATTGATTTTTCACGCAAGTTAAAAATTAGAAAATAAATTATGCATCG
TGAATGCGTGCATCAACCTAATTATTATATTTTCGAGAAATCGAGAATAATCTTTTCCAAT
TAAATATATTTATTTATTATCGATAATGATAATTGATTCGAAAGGAAAGAATCGAGCAAA
AGTGGATCGAATTTTTTTTTCTCTTTCCGCCATTAATGTGTAACAAAAATAAATAAACGA
CGCTTCTAATAGATCAAAAAAAAAAAAAAAAAGAAAAAAAAAAATTCGAAAAACGGG
GCACGGAAAGGGCCCCTTCTCAGAAAATCGTATGCCCGTCCGGCTTTGTAGGTGAATG
TACCGTTTGTTTAATTCCGCTATACCACGCGATGGATTCTCCTTTAGCGTTACTCGGAGA
AAATCAATACACGGACCATAAAAAATTAATCACCGCGATTATTTGCGAACACGCGCCAT
CCGAGTGTTAATTGACGGGAATTTATCGTTTCTCTATATTTTTTCCCAATTATTCATTCAA
TTTTGTTCGGTAATTTTAAGAATAATTGTTAACGGTTAGAAAGTTTTATTTATTTTTTTTT
GTTAAAATATATCAAGATTTATCGTACGTGTATATATATATATATATTATATCGCGTATGCGA
CACGCACGCGATAAGAGACAAGCGTGCGTTTCACGAACTAAGTGTAAGTTTCGTAAAG
AAGTACTTTCGTATCGATTCGAAGAGGTAAGCCGTCGTACTTGACCGTGAGACATCATG
GCCGAATGAAAAGTATGTAATGAAAACGTGTACCTTTCTTGAACACTCTTAAAATTCTC
TTATAAAATTGTATTCGATTCGTTGTTACGATTTAAAAAGGAAGAATTTTCTAAAACGAG
GATACGAAGAGATATCGAATCAAAATTCGTGTATGTATTTGTTAATTCATCCGAATAAAT
TGGTTTTGTCACTCGTCGAAGAAGCCATTCTTGAATTTCGTGATGGCGAGATATCGATC
GATGGCAGCACTCACCGGCGTGTATCCTCGAGATAAATTTAGATGCTGGTTTTTAGATC
CGTGGCAACTTTGGCATTGGTTGCGAAAATTTCTCAGTCTTTCGAAGTAATAGCATTCA
TTCGGATTATAAACGCGCATGCAGTAAAGAAAAGAACTTAACCTAACCTAACAAACATT
ACGTACACATATATATTATATATATATATGTACAAATACATATTTGGAATATGTAAATTGCA
AAGATCGATAAGGGTATTTCAAAAGAGAATGAAACAAAGAAGAAGAAAGGAAAGGTT
CTTTTCTGGAAAGAAGTTGAAAAGTCTGGATGAAAACCGAGTATTTTACGCGTCATCGT
AATCGTATAAATTCGCGCACGTCGACGATTCGACATAGTTTTTAGTATATAATATCCATTT
TGAAGACAGGAATGATCCATCGGGAATTGCAAATATTTCGAAGAATGTCTACGCGGAAT
TCTTATGGCGGTCATTACCCGTTCATTCACCATCGCGAAAACGTGACGCGTTTGAAAAT
GTGTCGTCGGTTGAAACTATCGTAATCGTCGATCACGTAGGAATTGGTTTTCCCGCAAT
TACTTAGTAACGTATGTGTCTCTCTCTCTCTCTCCGCGCCATACAGAAAGCCTTCATTAC
ACGGTCGCCGAATGTAAATAATTTCTAAAAGCGCTCATGCCGATAAGCGTATTACCATCT
ACGCCTACGCATAGGGTGACTCATGCTCTCGATAGTCGGTACGCTCATATAACGCACGA
TACGTATAAGTACATATGTGCATACGTGTAATCTCCACCCTTAGCTGGTTATATACTTCTG
TCCATAAGTATACGTGAACGGCTTTCTTTATTAAGACGTTTATCTGAAAGAAAGAAACG
TGAAATCGATAATGAATTCCGCTAAATTTTATACATATTTTATTTTTTTATCGCGTTCGAG
AATATATATTTTAATAACCGCGAGCAATTTATTTACTTACTTATCGATTATTGTTTGACACT
TTGACACTACGTATAAAACTTATCGTTTGAATTATTAATTTAAATCTTGTATACGTTTCGT
TATCGAATGGTAAAAAGTTAAATTTCTCACTGTTCCTTCCATTGCATCTGTGCTGCCCTC
TTTGGTAAAAATATCCTAGTATTCTTTCACTTTCTAAGATACGTTGAAATTTGAAGTTCC
ACATGTCTTCTTGGATTTTTTTGGATAAAATTAAAAAGTACCAAGTATTTTTGTAAAAGT
TTAGAAACAGTGTAATTTATTTGTCCTGTAACGGATGACGCAAACAGAAGCGATAAAC
GAACGGTTTTTACAGATGTAACGAATAGGACATAACCTAAAAGAAGAGCAAGAGTTCA

AACGAATGCAAATTTTGGGGTGTTGGAGATGGCGGCCACAGAGAGCACGATCCGTTT
CAGCGGGCACACATTGGACAAGGCTACAAAGGCCAAG

>novel_circ_000948
GGGTGTTGGAGATGGCGGCCACAGAGAGCACGATCCGTTTCAGCGGGCACACATTGG
ACAAGGCTACAAAGGCCAAGGTAACCTTGGAGAATTACTACAGCAATTTGATAGCTCA
GCATATCGAGCGAAAGCAGAGGCTTGCGAAATTGGAGGAATCGTTGAAAGACGAAGG
GCTGTCGGAGCAACAGAAGCAGGAGAAACGGTTGCAACATGCCCAGAAGGAGACGG
AATTTCTTCGCTTGAAACGGTCCCGGCTCGGCGTCGAAGACTTCGAACCTCTCAAAGT
TATCGGCAGAGGTGCTTTCGGGGAG

>novel_circ_000950
TAAAAAAAAAAAATAAAAGTGAATAGTGAATAAAGTCGAGCAACAAAGAGGGAAACG
TTGATCATCAAGAAGAGATAATCGATGTAATAATCTAGTGGTTGATGTATATATCTTTTAT
AGTAATTTTACATTAATTAAAAATAGAAATCTATAATTA

>novel_circ_000951
AAAAAAAAAAAATAAAAGTGAATAGTGAATAAAGTCGAGCAACAAAGAGGGAAACG
TTGATCATCAAGAAGAGATAATCGATGTAATAATCTAGTGGTTGATGTATATATCTTTTAT
AGTAATTTTACATTAATTAAAAATAGAAATCTATAATTA

>novel_circ_000952
CGGTGCAGAGGGGACGAGTGCCGCCGTCGCAGCCGTCGCTACCAGGTCTGCCGGGCC
AGTTCGCCCTGACGAACGGGGACGCGGTGGCGTGCGCGAGCCTGAACGGCCACTCAT
ACCTCTCCTCGTACATAAGCCTGCTGCTCAGGGCGGAACCTTACCCGACCTCGCGCTAC
GGCCAGTGCATGCAGCCCAACAACATCATGGGGATCGACAACATATGCGAGCTGGCCG
CCCGGCTCCTATTCTCGGCGGTCGAGTGGGCAAGGAACATCCCGTTCTTCCCCGACCT
CCAGGTCACCGACCAGGTAGCCCTTCTCAGGCTAGTGTGGAGCGAGCTGTTCGTCCTG
AACGCGAGCCAGTGCTCGATGCCGCTCCACGTGGCGCCCCTCCTCGCCGCGGCCGGCC
TCCACGCGTCCCCCATGGCCGCGGACCGCGTGGTCGCCTTCATGGACCACATCAGGAT
CTTCCAGGAGCAAGTGGAGAAGCTCAAGGCGCTGCACGTAGACTCGGCCGAGTACAG
CTGCCTCAAGGCCATCGTCCTCTTCACCACAG

>novel_circ_000953
AGGTGCTCATTCAAATGGGCTGCGTTGGAATTTGCGGATCCGACGTTCATTATTTAGTG
AACGGTAGAATCGGTGATTTTGTAGTGCGCGAACCTATGATAATGGGCCACGAGTCTTC
CGGAGTGGTGGTCAAACTTGGAAAGGATGTGAAAAATTTGAAGGTCGGCGACAGAGT
GGCCATCGAACCTGGAGTATCCTGTAGATATTGCAAGTTTTGTAAGGAAGGACGTTACA
ACTTGTGCAAAGAAATGGTATTTTGCGCTACTCCTCCAGTGCATGGTAGCCTGAGACGT
TTCTACAAACATGCTGCTGATTTTTGCTTCAA

>novel_circ_000954
CGCCAAACAGCCCACTCCATCTTAGCAACAGGGCTCACGTTTTGCTCCTGCTGAATAG
ACCTCAAGCCTCCCTCACGGATGCTGATCATGCTGTTAGACTACGACCGGACTGGGGC

AAGGGTCATTACAGACGAGGGGTGGCTTTGTCCGCGCTAGGAAGACACGAGGAGGCA
CTGTTTGCTCTTTGCATTAGCGTCGCTATTGACAAGAATCCGCAAGCTGTGCGTCACGA
ATTAATCAAG

>novel_circ_000955
CCCAGAAAATACTCGACCACACGAAGATTCGACGATTTTCCTGCCTTTCCGGATAATCG
AATTGCAACGACGTTTGCACGTTTGAAGGATCGTGGATTTTTCTATTTCGCGAAATTA
AAATTTGTGAGAAATTATTTCGTGAATTAAAAAAAAAAAAAAAAAAAAAAAAATAGAAA
AGAAAAGTGAACAAATCGTGAAGGAACAATTGAAAAAAAATGTGTTTCATACGTTGCC
TCTTGATCTCGATGTTCAACTATTTTCCGATCATCACGTGAAAAATATCGATCTTCGAAG
AACGATCATGTTTCGAGCATGATGCGCCGCCGCTTCGCTACGGAAGATGTTCGATCGAA
CGTTAGAATTTCTCCCGTCGAGAATTTGAACAATCATGAAAATTGACGGAACTATTATA
AAATTCGTCGAATAATTACGCGTTTTATCAAGCGGATCGAGGATCGAGAGGGGAAAAA
AAAAAAATATCTCACGAGGATTTTTTTAAATAGAACGAAAGTTGAAAGTATTTTTTTT
GGAAAAGTCCTTCGAGTGTATTTCTTTATTTTTTTTTCTTCTTCCTCTTCTAACTAAACG
AGATCTAATCTAAGAGGATAATCGAGTGAGTTGTACTCGGTGTAAAAAAAAAAAAAAA
AAAAAAAATGGTGTTTCGTCGGCAAACCAGCGAACCGACCGGAAGTGTGACGAAGA
GCCCCAGCATAGCGCATAGCACCAGCTCGTCCGGGATCTTGTCGAATTGGTACACGAA
AATCACTACGAACGTGAAGAAGAGGCAACCGTTTAGCCCGAAATCTTTATTGAAGAAG
GCGTTG

>novel_circ_000956
GAACTTGGATCGCAACCATTACCGACGATCGTAACCGAAGATGCAGATACTTCGGGTC
AAGCGTCTCAGGATCAAGGTCATAGGCCACGAGCAGGGACCTGGGGTTCGCATTCTAG
AACTATTAGGAAGCCCACCTCGGGCAGTGCTCCTGGTTCTGGAAAATCAACTCCTCTC
CCGCAGCAAGTGGAGCCTTCAAGTTCATCAAGTAAAAGCAAGAAAGTTTCTGGCACG
CGATCTGGCTCCACCAGTCGTAGTAATAGCCGTAGTAATAGCGCAGAGCGCAGAAGAA
GTGGTGGAACTGTTGATGACACAGCCAGTCCTACCAAAAAACCAGCACTCTTTGATGC
GTTTAGACCAAGGAGTAAATCTGATGCTAGCAAACGAAAGCCCAGTATTATAGCGAATA
TGAAGAGCGCTATGCAGCATTCTTTGCACAGAGGCTCACACGGAAGTTCAACGACAGA
TATACGTACAGATAGGGATCATCATAGAGATGGCAAGGATCATAAGGAAGGTAAAGAAC
CTATGGGACGTCCTCGAGCAGGTTCAGAAAGTAGCAGAAATCCTGTTAGTAAAGTTAT
GGATCTTATTAGACACAGAAGTCACAGTACCCTTAGCGCAGATGACAAACGAAAAGCG

>novel_circ_000958
CAAAAAACGCGGACTCGTCGAGCGCCGAGGATGCCGTAAAGAGCAACAAGGACGAC
GCGACGAAGACGGAAGAATCCGAGGACAGAAACGAAAGCGCCTCCTCGAAAGCATCT
AGTCCATCTTCCTCCGAGAAATCGAGCTCGGAAAGGATCGATCAAAAATCGAATCCTA
GCGGGTCGAGCGTCTCGGAGTCGAAGAGTCAAACCGCTACCTCCACGGTCGCCTCCG
CGTCGTCGACAACGTCGACGGCACCGTTGACAACGTCGACGACCGCGTCGTCGACGG
CTACCACGCTGACGACGACGTCGACGACGTCGACGACGGCTGCCACGCCGACGACCA
CGATGACGTCGACGACGTCGACGACTACGGCGGCGACGACCACTACCACTACGACGG
CCACGACGATCCCCGCGACCACCGCGTCGTCCAGGTTCACGACGTCGACGGTCACCTC
GCCAACGTCGAGCACCAAGCCCCCGTTGAGGAGGAGGCACACGACCGGGCCGGGGA

TGACTTTCCCCGCAACCGATCCGCCGACGTACTCCAACAGCATGACCTTCTCCAGGAC
GAGTCCCCTCCCCAGCCCCCATTTCGACAAAAGGTTCTTCGACAGCAGTTTGATAGAG
ATGAAGAGTCAGGCGAGCAGCTCCAGCACCCTCGACTACGACTCGACCGAAGAGGTG
TGGGTTCGCAGAGTGGACTTCGTCCAGGAGAGGAAGAGGAAG

>novel_circ_000959
ATACCTCGATGGAGAGTTCCGGTGGCGACAAGCAGAACGAATCGGACGAGTGCGATG
TGACTGCCAATAATCAGCATCGTAAGGATAAAATGACGGGCCAGGTGGACAGTGAGGT
GAAAATAAGTAAGGATTTGGGGGAGCAGGAGGCGTTGAAAAATTTGAAGAACTTGGA
GAATATCAGTCAGAATGACGAGGCTTCGAATAAATTATCAAAGGCCGTGTCCCAAGAG
GACGAGGATGACGAGGACGAGTGTAAAGGGTCCGACGAGGACGAGCTCAAAGACGA
GAAAACGGATGGGAAGAAGGACGAGGATACGTCGAGCAGCGCGACGTCGACGAGCA
CCTCCGACGACGAGTCCGAGACGAGCTCGGACGATAAGACGGAAGCCTCCAAGGCCG
AGCACGCGAAGGCGGCCGCGAGCGAGGCGAAGAAAGAGGATCAAGCGTCTAAGAAC
GACGACACAACGGACGACAGTGACATCGAGGACGATTTAATTTCGGCCATCAATTATA
ACACCATCACCTCGTTGGCCGCCCTCAAGGATATGCTTCAATCCTCCGGCGAGGATTCG
GACGGCGTGGACAGTTTCTTTCATCATTACTTCCTATCCCACGTGAACAGGTTACCGTC
GAGGAATCAAACCCATAGCCCTTATCCGTGGGGCAGAAGATTGTCCGAGTGCAAGGAA
GAGGACGAATACGAGAACGAAGATGCAAAAAACGCGGACTCGTCGAGCGCCGAGGAT
GCCGTAAAGAGCAACAAGGACGACGCGACGAAGACGGAAGAATCCGAGGACAGAAA
CGAAAGCGCCTCCTCGAAAGCATCTAGTCCATCTTCCTCCGAGAAATCGAGCTCGGAA
AGGATCGATCAAAAATCGAATCCTAGCGGGTCGAGCGTCTCGGAGTCGAAGAGTCAA
ACCGCTACCTCCACGGTCGCCTCCGCGTCGTCGACAACGTCGACGGCACCGTTGACAA
CGTCGACGACCGCGTCGTCGACGGCTACCACGCTGACGACGACGTCGACGACGTCGA
CGACGGCTGCCACGCCGACGACCACGATGACGTCGACGACGTCGACGACTACGGCGG
CGACGACCACTACCACTACGACGGCCACGACGATCCCCGCGACCACCGCGTCGTCCA
GGTTCACGACGTCGACGGTCACCTCGCCAACGTCGAGCACCAAGCCCCCGTTGAGGA
GGAGGCACACGACCGGGCCGGGGATGACTTTCCCCGCAACCGATCCGCCGACGTACT
CCAACAGCATGACCTTCTCCAGGACGAGTCCCCTCCCCAGCCCCCATTTCGACAAAAG
GTTCTTCGACAGCAGTTTGATAGAGATGAAGAGTCAGGCGAGCAGCTCCAGCACCCTC
GACTACGACTCGACCGAAGAGGTGTGGGTTCGCAGAGTGGACTTCGTCCAGGAGAGG
AAGAGGAAG

>novel_circ_000960
ATACCTCGATGGAGAGTTCCGGTGGCGACAAGCAGAACGAATCGGACGAGTGCGATG
TGACTGCCAATAATCAGCATCGTAAGGATAAAATGACGGGCCAGGTGGACAGTGAGGT
GAAAATAAGTAAGGATTTGGGGGAGCAGGAGGCGTTGAAAAATTTGAAGAACTTGGA
GAATATCAGTCAGAATGACGAGGCTTCGAATAAATTATCAAAGGCCGTGTCCCAAGAG
GACGAGGATGACGAGGACGAGTGTAAAGGGTCCGACGAGGACGAGCTCAAAGACGA
GAAAACGGATGGGAAGAAGGACGAGGATACGTCGAGCAGCGCGACGTCGACGAGCA
CCTCCGACGACGAGTCCGAGACGAGCTCGGACGATAAGACGGAAGCCTCCAAGGCCG
AGCACGCGAAGGCGGCCGCGAGCGAGGCGAAGAAAGAGGATCAAGCGTCTAAGAAC
GACGACACAACGGACGACAGTGACATCGAGGACGATTTAATTTCGGCCATCAATTATA
ACACCATCACCTCGTTGGCCGCCCTCAAGGATATGCTTCAATCCTCCGGCGAGGATTCG

GACGGCGTGGACAGTTTCTTTCATCATTACTTCCTATCCCACGTGAACAGGTTACCGTC
GAGGAATCAAACCCATAGCCCTTATCCGTGGGGCAGAAGATTGTCCGAGTGCAAGGAA
GAGGACGAATACGAGAACGAAGATG

>novel_circ_000961
GATCAGATTTTCATGTGTCTGGACAAAATTACGGCACCCAACCAGGGGGCCAGGTGGG
CGGGGGAGGGGGTAGTGTAGGAGTACCTGGGGGCAGAGGACCTCCACCACTCGGCGG
CTTACAATCTATTGGACAGTCAGCAGCAGGAATTCCACCAGGAGGTCCTGCTGGAGGA
CAAGCACCACCACAAACCCCGGCTCCCGGGGTACAGTCACAACCGCCGCAAGGTCCG
GCTCCCACCACTACTCCCCCGGTACACACACCGTCACCTCAGGAAATGGGAAAACAG
GCCCATTTACAACCTCAACCACAACCTCTTTCACAAGTATATGGGCCAACGCAAACAA
GGCCGACGTCACAAGGTTACTATCAAGGACCTAGACCACAACAACCTCGAGGCATTAG
TCACAGAGGGGGACAAGGAGGTACAGGTGCACAAGTTGTAGGAATGTCAGGAGTTGG
TGGAGGTGGAGGCCAACCAACTGCGATTTATCACCCAGGTGGTTTGCCAGTTCAAACT
GGAGCAATATATCCAGTTCCTAATCTTCATCCTGGCCCACATCAACAATCTTTATACGCA
ATGAACAATCAAATGCCCCTTCAGGTAGTATTCGGTGGTCCTCCTCAAAGGCATCAAAC
GCATCAAAATCAGTCTTATTTCCCACCTTTTCAACATTCGGCTATTCTAACGCAAAATAT
GTTTGGATATGGTGCACCTGCACCAACTCCTCAACCTTATTATTGGCCTACTGGTCAAC
CACCGAATACACCACTAATAAGTAGGGCAGGTGCAAGCGCTGTTACTGGTGGGGCACA
ACATGTTGCAGGCCCGCTGGCCGGTGCCCAAGGAGCCCCAGTAGTACCACAAGGTAC
ACTTCAACAGCCTACACAACAAGCGCAACCTTTACCTCCAATGGGTATATCTCTTTCTC
AGACTG

>novel_circ_000962
AAATCTTCCAAAAGCGATTTGCATATCGTTACCATTGGTAACTATAATTTATGTTCTGGC
GAATATTGCTTACTTTGTGGTGTTAACGCAAGATGAAATTCTGGCATCGAATGCTGTCG
CTGTCACTTTCAGCGATAAGCTGTTAGGCGTGATGTCTTGGATAATGCCAGTATTCGTG
GCTTGTTCAACCTTCGGGGCGTTGAACGGAGCAATTTTCGCATCATCACGATTGTTTTT
CGTAGGAGCTCGTAATGGACATTTGCCCGCCGCCATTGCCCTTATTAATGTTCAAAACTT
AACACCCACGCCTTCGCTTATCTTTCTT

>novel_circ_000964
ATATACGATTGCAGCTGAACGAACAGCTGAGATGTCTCGACATCCGGATGGAGGCCCA
AGTCGCCATCGTGGCGGAGCTTCAGGATTTTTTTCGAAAGAGAGCCGAGCTCGAGCTC
GATTACAGCAAGTCTCTTGACAAGTTGGCCAGAAGCATACAGCTCAGACACAAGGAG
CAAAAGCAAAAGCGAGAACAGTGGCCCCTATTCTCGAGCTACGCTTGCTGGCAACAA
CTCATCAACGAGACTAAATCCTTAAGCAGAGATCATGCAGCTTTGTCAGAAGTATATAG
CACACACCTCGTTGGTCGTCTTAATCAAGTGATGGAAGATGTTCAACGGATATACAAAC
GTTGCCGTGAAATAGGTTATGAGACGCACGAGGAAATTCTTCGAGTATTGGATGAGCT
GCATACCACGATGAAAACGTATCAGACATACCAGACCGGGTCACGGCAAGCGGAAAC
GAAACTTCGCGTCGCGGAACAGCAGCGTAGCAAGCTCGAGGTAGCGAATGCTCCTCC
GGAGAAACTCGCGCGCAGCAAGAAATACAAACTCATGGAAAAGGAAGTGAACAAGA
GGAAAGTTAAATATCAAGAGGCGAAGCTGAAAGCGTTGAAAGCAAGAAACGAGTACA
TTTTATGTCTTGAAGCATCCAATACGACAATACATAAATATTTTGTGGACGATCTTTCGG

ATCTTATTGATTGTATGGATTTCGGTTTCCATAATTGTATCGCCAGAGCTTTGCTGATGCA
TTGTAGCGCAGAGGAAGGTAGACAACGTTCACTACAATCTGGTGCTGAACAATTGGCT
GCTTGTGTTGGTGCTCTAGACTCGAGAGCGGATAAACAAAGATTTCTGGAATCTCATCA
TGCTGCTTTCATGATTCCTAAGAAGTTCGAATTCCAAGGACAACGAGGGGATGAGGTA
CTCGAAACTCCCGAACCAGAACTTCAAAAATTGTTGCATGCTGAAATGGAGCAACGAT
TGGCTCAATTACAACAAAGATTAACTTCTTTGAGAACAGAATCTGAAGAAGTTTGGAA
AACTTTGGAAACAGCAGAAGCTAGTCTATTGGAAATGTTAACTGCAAAAGACTATGAT
TGTTCAAGATATTTTGGAGAGAATGCTGTTCCCACTTCCAGACCACCAGAAACCGTGC
AAATCAAGCTTAGAGCCGACAGACAGGAAACTGAAGAATTCTACCTCACAAAATTCA
GGGAATATCTTCTTGGAACCTCAAGAATAGCTAGGTTAGATGCAAAGCAAGAATACATT
CGGCAGAGCCTATTGGATGGTTCTACTGCTAGCCCAAATCCATCAATATCAACAACGAA
ACAAAAACAAGCTCGGAGAAAACGAATTGGTAGATTGCAAATGAATGGTCAACCAAA
ATTATTTGGTGGCTCATTAGAAGAATATTTAGAAAGCACAAATCAAGAAATACCTCTAAT
CATGAAAAGCTGTATCAGAGTGATCAATTTGTATGGTCTTCATCATCAAGGTATTTTCCG
AGTCTCTGGCTCCCAAGTAGAAATTAATAATTTCCGAGAATGGTTTGAGAGGGGAGAA
GATCCATTGGCTGATGTCACAGACGCATCAGATATAAACAGTGTTGCTGGTGTATTGAA
GCTCTATTTGAGAGAATTGAGAGAACCACTATTTCCTATTATTTATTTTGAACATTTAATG
GAACTAGCGCAATTAGAATCAAAGCAAGAATTCGTTAACAAGATGAAAGAATTGATTT
CCAGTCTTCCAAGACCAGTTGTCATAGTAATGCGATACCTGTTTGCTTTTCTCAATCATC
TCTCAGAATTTTCGGATGAAAATATGATGGATCCATATAATTTGGCAATTTGCTTTGGTC
CCACATTAGTACCGGTTCCAGAAGATAAGGATCAAGTACAATATCAAAATCAAGTAAAT
GAACTTATCAAGAACATTATCACATTCTGCGAAGAAATATTTCCAGAAGACATTGGAGG
TACTCAATATGAAAAATACATCAGTAGAGAACCTGATGACGTAGATGTTGGAGACTCGC
CGACTGATCAAGCCCAAGAAGATATGGATTCTGAAGTGTATCCATCTGAGGATGAATCA
GAAAACCTTGAAGCCACAGCGCAATTCGATTTCAATGCAAGATCCGAAAGAGAGTTAA
GCTTCAAAAAGGGAGATACCTTGACTCTATACACACAAGTCAGTAATGACTGGTGGCG
AGGTGCCTTGGCCGGTAGAGAGGGGCTTATTCCCGATAAATATATAATGATCAAGATAA
AGGACGAAGAACGTGAAAAGGAACTCCTGAAATCGTCCAGCGAAGAATCAATGCGAA
GAAGGACTTCTAGCTCTGCCGATAGTGTTCTTTCAAGTAACAATTCACCACTGATGGGA
CCATCCGGAAATCCAAATACTTGGCCTCCTGGTACTACATCTGATATGCAGTCTACCACG
ATCACAACAGAGAATAGCAGTAACAGCGGTGTTATCCCAGCAGTCGTGACAAATGTCC
CTTGCATCTCATCAACACAACCAATCATCAGTCGAGAG

>novel_circ_000966
GTGGGCTATCGTCACCCCTGCAGCGCGCCTCTACCGCCTCGCCCCACGGGGGTGTCGG
GGGTGTGCCGCCTTCGTCCCACGTGTCCTCCCAGCAACCACCGCCTCCACCCCCGGCA
GCAGTCTCCCTCCCCCCGCGCTCCACACCCACGCCCTCCTCGGCCACCTCGACCACGT
CCAGCCTCCTCTTCCCCCCTCCCCTGCAAAACGTCGAGACGTTCGGCTCGTTCCTCGG
CGAAGAGATCGTCGCCAACTGGAGGCGGACGTTGGAGAGGTCCATCATGAATCAACA
ACAGCAACAACAGCAACAGCAACAACAATCGAATCCGTTGAGTCAATTGTCGCAACA
ACAGCAACAACAGCCGCTGATCGGCCTACACCCGCCCACCACCACCACCACAGCCGC
GAGCAGCGGCCTCAATCATCTTCCGTCGCCCACCACCGACCCCTCGTCCAACAGCTCC
AACACCCCCGCCAAATCGAACACCCCCCTGGGGACGACCCCGCTCAGTTGCCTGGAC
GCGGAGAAGGCGCCGACCCCGGTCTCGGGCCACGAGACCCCGGCGCCCCCCACGCCA

TCCTCAACGACGGCGTTAACGTCCCCGCCGAGCTTTCAACCGCCGACCTCCGTCGTCG
AGACCGCCGTCAACGGCGCAACCCTCACGCCCGCCGCCCTTGCCCCGGCGCCGCCCC
CCAAGAGCCCGCCCGACCAGGAATCTTGCCACAACAATAATAACAACAACAACAACA
GTCATCATCTGGCCAACAATAACAACAACAACAACAACAACAACAACAACAATAATAA
TAATAGTATAGGGGGGTTGAGCGTGCTGGACACGCTGAAGAGCCCGTTCCGGTTCGAC
GAGAGGACGCATCCGTTTCGATTCAGCGACGAGTTCGGGGCGACGGGTATGCCGGTGA
GATTGGGCGAGTCCCTGATCCCGAAGGGGGACCCGATGGAGGCGAGGCTGCAGGACA
TGCTGCGTTACAACATGGACAAGTACGCGTCCCAGAACCTGGACACGTTGCACATAGC
GAGGAAGGTGAGGGAGCTGTTGTCGATACACAACATCGGGCAGAGGTTGTTCGCCAA
GTATATACTGGGGCTGAGCCAGGGGACGGTGAGCGAGTTGCTGAGCAAACCGAAGCC
GTGGGACAAATTGACGGAGAAGGGGCGAGACAGCTACAGGAAGATGCACGGGTGGG
CGTGCGACGAAAACGCTGTAATGCTGCTTAAATCTCTGATACCGAAGAAAG

>novel_circ_000967
ATCGGTCAACTGGTGGAGGAGGTGTCCAGGTTACAGCAGAGCCTGCAGCGTCTCCAA
GAGACGAGCGCACAGGCGACCGCTCGGCTCGAGGAGGAACTGGAGGCGCGCAGACA
GCACATCAACAGACTGGAGAGCAAACTGGAGCGGCAGAGGGACTACGACGACCTGA
AGCGCGAGATAAACGCGCTAAGGTCGGTCGATCTCTCCCAGATACCAACCGGCGAACC
TGGCAAGAGCTTGGAGCACCTTCTAATGGAACGCTCGAAGGCGCTTCAACAGACCGA
GAGCTTGAAGCCATCCAACACCCCGGACGCGCTCGGTGGGCTATCGTCACCCCTGCAG
CGCGCCTCTACCGCCTCGCCCCACGGGGGTGTCGGGGGTGTGCCGCCTTCGTCCCACG
TGTCCTCCCAGCAACCACCGCCTCCACCCCCGGCAGCAGTCTCCCTCCCCCCGCGCTC
CACACCCACGCCCTCCTCGGCCACCTCGACCACGTCCAGCCTCCTCTTCCCCCCTCCC
CTGCAAAACGTCGAGACGTTCGGCTCGTTCCTCGGCGAAGAGATCGTCGCCAACTGG
AGGCGGACGTTGGAGAGGTCCATCATGAATCAACAACAGCAACAACAGCAACAGCAA
CAACAATCGAATCCGTTGAGTCAATTGTCGCAACAACAGCAACAACAGCCGCTGATCG
GCCTACACCCGCCCACCACCACCACCACAGCCGCGAGCAGCGGCCTCAATCATCTTCC
GTCGCCCACCACCGACCCCTCGTCCAACAGCTCCAACACCCCCGCCAAATCGAACACC
CCCCTGGGGACGACCCCGCTCAGTTGCCTGGACGCGGAGAAGGCGCCGACCCCGGTC
TCGGGCCACGAGACCCCGGCGCCCCCCACGCCATCCTCAACGACGGCGTTAACGTCCC
CGCCGAGCTTTCAACCGCCGACCTCCGTCGTCGAGACCGCCGTCAACGGCGCAACCC
TCACGCCCGCCGCCCTTGCCCCGGCGCCGCCCCCCAAGAGCCCGCCCGACCAGGAAT
CTTGCCACAACAATAATAACAACAACAACAACAGTCATCATCTGGCCAACAATAACAA
CAACAACAACAACAACAACAACAACAATAATAATAATAGTATAGGGGGGTTGAGCGTG
CTGGACACGCTGAAGAGCCCGTTCCGGTTCGACGAGAGGACGCATCCGTTTCGATTCA
GCGACGAGTTCGGGGCGACGGGTATGCCGGTGAGATTGGGCGAGTCCCTGATCCCGA
AGGGGGACCCGATGGAGGCGAGGCTGCAGGACATGCTGCGTTACAACATGGACAAGT
ACGCGTCCCAGAACCTGGACACGTTGCACATAGCGAGGAAGGTGAGGGAGCTGTTGT
CGATACACAACATCGGGCAGAGGTTGTTCGCCAAGTATATACTGGGGCTGAGCCAGGG
GACGGTGAGCGAGTTGCTGAGCAAACCGAAGCCGTGGGACAAATTGACGGAGAAGG
GGCGAGACAGCTACAGGAAGATGCACGGGTGGGCGTGCGACGAAAACGCTGTAATGC
TGCTTAAATCTCTGATACCGAAGAAAG

>novel_circ_000968

ATCGGTCAACTGGTGGAGGAGGTGTCCAGGTTACAGCAGAGCCTGCAGCGTCTCCAA
GAGACGAGCGCACAGGCGACCGCTCGGCTCGAGGAGGAACTGGAGGCGCGCAGACA
GCACATCAACAGACTGGAGAGCAAACTGGAGCGGCAGAGGGACTACGACGACCTGA
AGCGCGAGATAAACGCGCTAAGGTCGGTCGATCTCTCCCAGATACCAACCGGCGAACC
TGGCAAGAGCTTGGAGCACCTTCTAATGGAACGCTCGAAGGCGCTTCAACAGACCGA
GAGCTTGAAGCCATCCAACACCCCGGACGCGCTCG

>novel_circ_000969
GTGAGTGTACGATGCACCGATTCCGGGACGGGAAATTATCGAATTAATTTACGAGGAG
GAGGAGGGATTAATCCCGCAATTATCTCTCTCTCTCTCAACGTGGAGGAGGAAAGA
AAGGGAAAGAGAGAGGCGAATTCGAGGAGGGGGTTCGAGTTAACGCACTAATCAACC
GTTCGAAGGCGCTCTGGATGTATCCACGATGGAGGAGGAGGTTTCGGTTATGATCCGC
GGATGGAGGAGGTGGACACGGGGTGCGAGAACAGGGAAGGAAGAAGGCGAAAAGG
GAACGAGGAGGGGGAGAGGAGCGATTCTCGACGTTGGCGGAGAATCGAATGACGAC
GTTATCGGTCAACTGGTGGAGGAGGTGTCCAGGTTACAGCAGAGCCTGCAGCGTCTCC
AAGAGACGAGCGCACAGGCGACCGCTCGGCTCGAGGAGGAACTGGAGGCGCGCAGA
CAGCACATCAACAGACTGGAGAGCAAACTGGAGCGGCAGAGGGACTACGACGACCT
GAAGCGCGAGATAAACGCGCTAAGGTCGGTCGATCTCTCCCAGATACCAACCGGCGAA
CCTGGCAAGAGCTTGGAGCACCTTCTAATGGAACGCTCGAAGGCGCTTCAACAGACC
GAGAGCTTGAAGCCATCCAACACCCCGGACGCGCTCG

>novel_circ_000970
GCCAGCAAGGTCAGCAAGGCGAACAGGAGTTGGCAACGAAGATGTTGCAGATCCAAA
GTAAAAGATTCTATCTTGATGTAAAACAGAACAGACGTGGGAGATTTATCAAAGTAGCC
GAGATCGGCGCCGATGGAAGGAGAAGCCAAATCTACCTGGCACTCAGCACAGCATCC
GAGTTTCGGAATTACCTTTCAACGTTTAGTGACTTTTATGCATCTTTAGGTCCACCAAAC
TCGGAGAACGTGCCAGATGATGGGAAATTAAAATCAGAAGTGATGACAAAGGATAAC
AGGCGGTATTACTTGGACCTCAAGGAAAATACTCGCGGCCGTTTCCTGCGGGTGAGTC
ACCCTGTGTCGCAGACGATAACACGAGGAGGACCCAGGACGCAGATCGCGATACCAG
CACAGGGTATGATCGAGTTCCGTGACGCGTTGACAGACCTCCTCGAAGAATACGGTAC
GGACGATGGTGGTTTCAAGGGTGATTTACCAGAGGGACGATACATGCGCGTGGATAGC
AAGAATTTCTATTTTGATATCGGCCAGAATAACCGTGGTATCTACATGAGAATTTCTGAG

>novel_circ_000971
CTCCACCCGGCGAAGAGGACGAAGAAGAAGGATTACGGAACGGATTCTTGAAAGGGC
AGGAGAATGACACGAACACGCACAGCTTTACTCGGCAGAGCGGATACAAGCCGCGTA
GAACCGGAAGTAGCGATGGTGGCGCGTACGCCGGCGCTACGACACGGCACAAGAACG
ACACAACCGGTTCACCGTCCCAGCCCAAGATTATCTTTAACGAGGACGAATACACGAG
GATCACGACACCCCGCCAGGATATGCTGTTCAAAAAGGGTTATCTGTCGAGGAAGAAA
CCCTGGAGCGGGAACGCCAACACCAGCGCGACTTCCTCGACCACCGAGAGCCAATCG
GCGTCTCATTCCACCGCAGGTAGAGGTATAGATGGTAGCGAAACGACTGAGGATCAAC
AGTTGTTGGACAGGGACTGCGGTACCGGCGAATATCCACCAGTGATGGATTCCAACGC
CCAACTGGGCTATGGGACGTTTTATGACCACATGGGTGGTTACTATTACGAATATCCTGT
GATGTTGGTTGGTCCAGCGCCGATGCAATCTCAAGTTGCACCGAGTGTTTTGGCGGCC

GTGCCTTGTGCACCTGTGCCTCTTAGGCCCATAGAATGGATTAATCCAACTTACGTGCC
CAAACTGCCTAATCAACCATACTGTATAATGAATTACGAGAACAATCAAAGTATGGAAA
ATACGGTGGTAATGGAAGAGCAAGAAAATACGGTTCTGCCGACGGAGAATTCAAATGG
AATCAGTAACGAGAGCGGTACAGGTAGCACCAGTTGCAGCGGTAGCATGGCCGGGGA
GACGGAAGAGCAGCCCATAGAGTTCACCAACGCGAAGGAGGAACAGCAAGCGGACG
AGCAGATGGAGGAACAAATGGAAGAGCAAATGGAGGAACAATACGGGGATGAGCAA
CAAGCGGAGGAGCAACCCTTGGAGAACGGTGGCCCCTATTTTGAACCAATGTTGATGC
AGCAACCGGTACACGTATCTCACGTGATACCAGCGATTCCTCAGCCTTACATGTATCCC
GGCCATTACATGTTTGGTCCTCCTTTGATCAACGTGAATG

>novel_circ_000973
GAGACGGTATTCAACAAGTCGTGATGCATTGGAGGTTCGGCGGTCCTTATCCAAAAAC
CGACCAGAAATCAGTGTTACAAAAGCAATTTGTGTGACAAAGCATAAAAGTGCAAAAT
TATATTTTAAGACATATTCAGAAGCTTTCTTATCAGTGGACTATATATCCTTGGAAGAAA
GTAGACATTCTTAGTGACACTGAAAGTACAAGTGAAAAATATAAAAATTGGGGGGGCT
GTTTGACCGATGGACAAACGGAAGATGATGCCCAAACGCCCTCGAATGGATAACCTGA
GGGGTCCTATTGCAAATGGACCCATTCAGACACGACCATTAGTAGCTTTGTTAGATGGT
CGAGATTGCTCTATAGAAATGCCTATTCTTAAAGATGTTGCAACAGTAGCCTTCTGTGAT
GCACAATCTACATCAGAAATTCATGAAAAGGTATTAAATGAAGCAGTAGGTGCATTGAT
GTGGCACACCATAATTTTGACAAAGGAAGATCTCGAAAAATTTAAAACATTACGGATTA
TTGTGAGAATTGGATCTGGAGTAGATAATATAGATGTCAAAGCAGCAGGTGAACTCGG
CATCGCTGTGTGCAATGTGCCTGGATATGGTGTTGAAGAAGTAGCTGATACTACTCTTT
GTCTCATTCTAAATTTATATCGACGTACATATTGGTTGGCAAACATGGTGCGTGAAGGGA
AAAAATTCACAGGTCCAGAACAGGTTAGAGAAGCTGCAACAGGATGTGCAAGGATAC
GGGGTGACACACTTGGAATAGTTGGATTAGGTAGGATTGGTTCTGCAGTTGCCCTGCGT
GCCAAAGCTTTTGGTTTCACCGTGATCTTCTATGATCCTTATTTACCAGATGGAATTGAA
AAATCTCTTGGTCTTAACAGGGTTTACACATTACAGGATTTGCTATTCCAGTCAGATTGT
GTATCCTTGCATTGTACCTTGAACGAGCACAATCATCATTTAATTAATGAATTCACTATC
AAACAGATGAGACCTGGCGCCTTTTTAGTTAATACAGCAAGAGGTGGTCTGGTTGATG
ATGATGCCTTGGCTGCGGCATTGAAACAAGGCAGAATTCGTGCAGCAGCACTAGATGT
ACATGAAAATGAGCCATACAATGTTTTTCAGGGTCAGTGCTCACAGTGTCCATTAAAAG
ACGCACCCAATCTATTGTGTACACCACACGCAGCATTTTACAGCGACGCAAGCTGCAC
CGAACTTCGTGAGATGGCAGCCAGTGAAATACGAAGAGCTATCGTTGGTCGCATACCT
GACTGCTTACGAAACTGTGTGAACAAGGAATACTTCCTTTCTTCGACCGG

>novel_circ_000975
AACGAATGACGTCGATATATAGAAATACAATCAAGAGAAAAGCCATAGGGAGCCTGCA
AGACGTGGATCTTATTCTAATATAATTTAAATTAAAAATGTCTTGCGTGGGGATGTCCTG
TTACGAAGATGGCTTCCGGTTGGCTGGTCCCGCATCGGAATGCTCTCTCAGGAGCAAA
GCTCGCATCGATGCCCTTTTCGAGGACTCGAGATCGATTTACGGATCCAACATTGGCGG
ATGGTACGGTGGGGTATCGACTATGAACTCGAATTACGTGGAGAACGTGGGGGCGGAA
GAGCAAAGGCGGGTGAACAGCGCCGTGCAAGCGCGGATCGAAGCGATGTTCGCCTCG
GTGGAGGCCGAAAGCGGAACACCCGAAGAATGCGTCGCTGCAATTTTACCG

>novel_circ_000976
GTACGTTATCACAACGTTGTGCGGGGAATCCTGTCCGTGCCCCGACGTTGTGATCGCCC
TGCTTTTCTGGATCGGGTACACGAATTCGGCGCTGAACCCGTTGATCTACGCGTACTTC
AACCGCGACTTCCGAGAAGCTTTCAAGAATACTCTGCAGTGCGCGTTCTGCTCGCTCT
GCAGACGAGAGCCGTCCGATCTCGAGGCGCTCGATTTCCGTCGGCCGTCCCTAAGGTA
CGATCTGGTCTGACACGTCGAG

>novel_circ_000978
CTGGTGGCCTAATGGCAGGTTCCGTGGCTACCTTGGCGATCCTATGGGTCTCTCTACAA
ACCATGGGATTCCTTGGAAATACGCATACGATCGGTGCCTATAAATTGCCAATTTCCTTG
CAGAACTATCTTGCCAGTTATACAGGTATCCTTTCAGGTTTGCCGATGACAAACTACCC
TTACGAGGGCGGCGTCGTAGACACGGGTCGTACCCATAATCGGCGTTACCCTCACATTG
ACGAGGCGGTCTTGAACAACTCGATCAGTTTCGCGCAAAGCTTGGTCGATCGTATGAG
CACCCTCGAAAGGAATATCGCGAATGCTGGCGTCGAATTGATGGCTCACACGCCGGCT
GGAAAACAATTCCTTGATTCATATCCTTCCGAGGATGCTTTCGAGAGAGGCCTCGACGC
CATAGTGGCGATGAAAGCTTCGTCCTATCTCCTTCATCAGAGCTGTCACAGATTCGGGC
TGGACAAAGACGATTGCGCTCGTTTCATATCGACCTTGTCGCTGGAGGGGACTCCGTT
GAACTCGGCCTGTCCGTCATCGCGGCACAAGGGGTGCAATTCTCTGGCCAAGTACCGA
AATATCGACGGCAGTTGCAATAATGTCCAAAATCCAAGCTGGGGTAGTGCGATGACCG
CGTACACCAGAATTCTCTTCCCCCAGTATTTCGACG

>novel_circ_000979
CTGGTGGCCTAATGGCAGGTTCCGTGGCTACCTTGGCGATCCTATGGGTCTCTCTACAA
ACCATGGGATTCCTTGGAAATACGCATACGATCGGTGCCTATAAATTGCCAATTTCCTTG
CAGAACTATCTTGCCAGTTATACAGGTATCCTTTCAGGTTTGCCGATGACAAACTACCC
TTACGAGGGCGGCGTCGTAGACACGGGTCGTACCCATAATCGGCGTTACCCTCACATTG
ACGAGGCGGTCTTGAACAACTCGATCAGTTTCGCGCAAAGCTTGGTCGATCGTATGAG
CACCCTCGAAAGGAATATCGCGAATGCTGGCGTCGAATTGATGGCTCACACGCCGGCT
GGAAAACAATTCCTTGATTCATATCCTTCCGAGGATGCTTTCGAGAGAGGCCTCGACGC
CATAGTGGCGATGAAAGCTTCGTCCTATCTCCTTCATCAGAGCTGTCACAG

>novel_circ_000981
AGGCAGTGATAAGAAGATTACTGTGACCACAGTAATCAACTAAATGTTCAGACTTCCA
AAGGAATAAAAATGCTGGAATTGCTCCAATGGTCATAGCTACACCAATGGGAAGTTCAT
CACCTCCCAACTTCTGTAAATGTCTAAATATAAAAATACGAACTAATTTTTATTGTATAAA
TATATTTTCTTATTATAGCTTAAACAACAACACTTAACTGATTTTAAAATTAATTCTATCAT
TAATCCAATAATTTCAAGTCAATTTTATTGTTCTCATGATAACGTAAAATAATTAATAAAA
AATCTTACAAAGGCAAATAACTATCCATGGCGCTCCAAAAAGTTCCCATAACGATGAGT
ACAATTACAAGAGCGGCGGTTTCACTACCATATCTTTTGATAGCGCTCATGGGAAGTGC
CAGCATGCCGCTTCTGGTATGCCACCACCATTCCGGTGGACTAAGTGGCATGCCGTTGG
CACAAAAAGCTACAAGAGCTGCCAAAAGCATCATTACAGCATGTATACCAATTGGCAG
ATAGTAGAAAGAATTCAAATTTT

>novel_circ_000982

TAGCTTTTCCTGACGTCTTTTATTACATCTTATCACAAAGGCTTTAATTAAGATGGTATAT
TTTTTTTATCTCCTTTGTACTTACATTCTTTACATACTGCACAAATAAAAACTCCTTCTAG
AATATCTTTTCATAAGTCGACTGGTTAAGTAGTCAAGTCCAAAATGAATCGTGTATGTGT
CACGATTTATTATTGTAAATATGTTATTTACACATATATCTAAGCTTCAAAAGCTTTATATT
TTTCTTTTTTCGCATTTATGGAGAGGGTTTGGATTTTTTGGCTATGGTATGTCTTCATTTT
ACATGTAACAAATACAATAGATAACTAATGTAGCAGGTCATAATTAAGAACATGATGATA
GACATATAACGTAACTATGTAAGACTGGATTCATCCTATCTGCCCTCTGAACGTTTTTTC
AACCGTTCTTTTAATCTCGTTATACAATATATATGTAAAGCTGTATATCTGTGTTGTGTGT
CTATAATATTTACTTAAATGTTAACTTCTTACAGACCGGTTACATCTTTGAAAGGAAAAA
GATCTATATATATTTCTATATAAGCCAAACTTAGCATATCTAAGTAGATATTTTTAATATCA
G

>novel_circ_000983
AGAAACGAACGGTTCGAAAGAAGATCTTTTGGTCGTGATTATAGTGATAGAGAAAGGG
AACGAGATCGAGATAGAGCAACTGAACGGAATGATCGTAATCATCAATCGGAACGTGA
TAAAGATCGCGGTCGTGAACGAAGATTTAGTAATGATC

>novel_circ_000984
CAGGTCTGGTGTAACTGAAAGCAAAGAGGAGCAAAACGTGGCGGATTCGGTGACCCC
TCGGCTGTGACCAGAGGCGCACAGAGGAATTAGGGCAAGGAAATGCGTGAATATAAA
ATAGTAGTGTTGGGCAGTGGAGGTGTAGGCAAGTCCGCCCTCACTGTCCAGTTCGTGC
AAGGAATCTTCGTGGAGAAGTACGATCCGACGATCGAGGACAGTTACCGCAAACAGG
TCGAGGTCGACGGTCAACAATGTATGTTAGAAATCCTAGACACAGCCGGAACGGAACA
ATTCACAGCTATGAGGGACCTTTACATGAAAAATGGGCAGGGATTTGTGTTAGTATATT
CGATAACGGCGCAATCGACGTTCAACGACTTGCAAGATCTCAGGGAGCAGATTCTACG
GGTGAAGGACACAGATGACGTGCCTATGGTGCTGGTGGGCAACAAGTGTGACTTGGA
AGATGAGAGGGTAGTGGGCAAAGACCAGGGCGTCAATCTTGCCCGGCAATTTAATTGC
GCATTCATGGAGACTTCTGCCAAAGCCAAAATTAATGTTAACGATATTTTCTATGACCTG
GTACGACAAATAAACAAAAAATCGCCAGAGAAGAAGATGAAGCAGAAAAAGAAATC
GCTGTGCCTACTTCTGTAAG

>novel_circ_000985
GTCTGGTGTAACTGAAAGCAAAGAGGAGCAAAACGTGGCGGATTCGGTGACCCCTCG
GCTGTGACCAGAGGCGCACAGAGGAATTAGGGCAAGGAAATGCGTGAATATAAAATA
GTAGTGTTGGGCAGTGGAGGTGTAGGCAAGTCCGCCCTCACTGTCCAGTTCGTGCAAG
GAATCTTCGTGGAGAAGTACGATCCGACGATCGAGGACAGTTACCGCAAACAGGTCG
AGGTCGACGGTCAACAATGTATGTTAGAAATCCTAGACACAGCCGGAACGGAACAATT
CACAGCTATGAGGGACCTTTACATGAAAAATGGGCAGGGATTTGTGTTAGTATATTCGA
TAACGGCGCAATCGACGTTCAACGACTTGCAAGATCTCAGGGAGCAGATTCTACGGGT
GAAGGACACAGATGACGTGCCTATGGTGCTGGTGGGCAACAAGTGTGACTTGGAAGA
TGAGAGGGTAGTGGGCAAAGACCAGGGCGTCAATCTTGCCCGGCAATTTAATTGCGCA
TTCATGGAGACTTCTGCCAAAGCCAAAATTAATGTTAACGATATTTTCTATGACCTGGTA
CGACAAATAAACAAAAAATCGCCAGAGAAGAAGATGAAGCAGAAAAAGAAATCGCT
GTGCCTACTTCTGTAAG

>novel_circ_000986
GGGCGCAGTATGTGCCCGGCGTTGGGCTTGGCGCTGCACCCCAGAACAGGAGCTTCAA
TTTCGAATATATACCGAATTATCCACCGACCAAAATCGACAAATACAGGGGTGGTTCTG
GTATACAAAAGAGAATGACTCAAAGGGAAGATACGTTGAGATTCGAGCAGGGCCGTAT
TAAGGCCTTGCAGGAAGAACGTTTGCATATACAGAAGAAAACATTTACTAAATGGATTA
ATTCATTTTTATTGAAGGCACGTATGGAAGTAGATGATTTATTCACGGATCTAGCGGACG
GCAAGAAGTTACTGAAATTGCTGGAGATCATCTCTGGTGAACGATTGGCCAAACCTAA
CAATGGTCGTATGCGAGTACACAAAATCGAGAACGTGAACAAATCTCTAGCATTTCTTC
ATACAAAG

>novel_circ_000987
TATATCGAAGAATTTGGGACTGATCATGTTTACAACTGCGACACTTTCAATGAAAACGA
ACCCTACACCAGTGAACTCAAATTTCTGAGGAACATAGGGCATTCAATTTTCGAAGCTA
TGAACAGCGTTGACTCAAAAGCGATATGGTTAATGCAAGGATGGTTGTTTTATCATGAT
TCAGTTTTCTGGACAGAGCCGAGAACTCGAACATTTTTAACTTCAGTCCCATTGGGAA
GAATGATAGTACTGGATCTTCAATCAGAACAATTTCCACAATACAAGAGATTAAATTCTT
ATTATGGTCAGCCATTTATCTGGTGCATGTTGCACAACTTTGGTGGCACCCTTGGAATGT
TTGGTTCCGCTGAAATTATTAATCATCGAGTTTTCGAAGCTAGAAATATGAATGGCAGTA
CAATGGTCGGGACAGGTCTAACACCAGAAGGTATAAACCAAAATTACGTCATCTACGA
GTTGATGAACGAGATGGCTTATCGTAAGAAACCCGTCAACTTGGATAAATGGTTCGAA
AATTTTGCCAATCGAAGATACGGCGATATCAAAGGGAACGAGCACACTGTTACAGCAT
GGAAAGGTTTTAAGAACACGGTGTATAATTTCTCGGACACCAGGAGAATTAGGGGGAA
ATACGTGATCACCATTCGACCAAACTTAAATTTCTTTCCATGG

>novel_circ_000988
AAAAAAGGAAACACCACCGAATGAAAAACCAGAAATTGTTAAAACTCATTTACGTAAT
ATGATTATCGTACCAGAAATGGTAGGCTCAATTGTTGGTGTTTATAATGGTAAAACATTT
AATCAAGTTGAAATTAAACCCGAAATGATCGGTCATTATTTGGGAGAATTTTCTGTTACT
TATAAACCTGTAAAGCATGGTAGACCAGGTATTGGTGCTACTCATTCTTCAAGATTTATT
CCACTAAAGTAATTTATATTTGTAATGTGAATAAACTGTACAAAAAATAA

>novel_circ_000989
GAAACACCACCGAATGAAAAACCAGAAATTGTTAAAACTCATTTACGTAATATGATTAT
CGTACCAGAAATGGTAGGCTCAATTGTTGGTGTTTATAATGGTAAAACATTTAATCAAG
TTGAAATTAAACCCGAAATGATCGGTCATTATTTGGGAGAATTTTCTGTTACTTATAAAC
CTGTAAAGCATGGTAGACCAGGTATTGGTGCTACTCATTCTTCAAGATTTATTCCACTAA
AGTAATTTATATTTGTAATGTGAATAAACTGTACAAAAAATAAT

>novel_circ_000990
AAATCCCGCGGAGATGTCGCACACGTGCCGCGGCACAATATCCTTGCACGGGGCCTTA
ATACACACCGTGGACGCTTGCACATTCGTTGTGAGCAACGGTGGTACGCAAACCTTCC
ATATCAAGGCAGCGACAGAGGTAGAACGTCAGCAATGGGTCACGGCCCTTGAGTTGG
CGAAGGCCAAGGCTATCCAGGCCATGGAATCTGAAGAAGAGGAGGAGGAATTTCAAG

ATAATGACAGTCAAAAACCAGAACAAGTCTCTGTAAAGGATCTATCACAGCGTTTAGA
AAATTTACAAGCCTGTAATGACTTATTAGTAAAAAAAGGGACAATGCTTCAGCGGATCT
TAAACGAGCTTGAAGCGTTGGAGCCTCCATCGGCCGAATTAGGGACAAAAATAAAGAC
AGTCAGTGAACGAGCCACACTCTTTCGCATCGCAGCCAGTGCTATGGTTAATGCAAGC
AATGAATATTTGCAATTAGCGCAGCAACAAGAACCAAAATGGAAAAAGATGTTGCAAC
ATGAGCGCGATCAGAAAGTACGAATAGAAAAAATGGTAGAACAATTGGCTAGGCAACA
TTCTCATTTGGAAGAGGCTGCACAACATGCCTTACCTTCGGCAGTTCCAACAGGAGGA
CACAGAGCTTCGC

>novel_circ_000991
AAATGGTCGAGGCGATGAAGAAGGTCGCCTCGTTGGATGTGGAGTTAACCGTCGAAG
AGAGGAATCTTCTTTCTGTCGCTTACAAAAATGTGATTGGCGCGAGAAGGGCATCCTG
GAGAATAATCTCCAGCATCGAACAAAAGGAAGAGAACAAAGGTGCCGAGCGGAAACT
AGAGATGATCCGCCAGTACCGATCTCAGGTTGAAAAAGAGCTGAAAGACATCTGCGCC
GATATCCTCGGAGTTTTAGACAAACATTTGCTCCCATGTGCATCTACTGGCGAATCGAA
AGTCTTCTATTATAAAATGAAGGGTGATTATCACCGTTACCTTGCTGAATTCGCCGTTGG
CAACGATAGGAAGGAAGCCGCAGAGAATTCTTTGGTCGCTTATAAAGCAGCAAGTGAC
ACCGCCATGACGGATCTACCACCAACTCATCCCATCCGCTTAGGATTGGCGCTCAACTT
TTCCGTGTTCTATTATGAGATTCTCAATAGCCCTGATAGAGCATGTCGTCTTGCGAAGGC
AGCCTTTGACGACGCGATCGCGGAACTAGACACCTTATCCGAGGAGAGTTACAAAGAT
TCTACCCTCATTATGCAGCTTCTTAGGGACAATCTTACTCTATGGACGTCAGACATGCAA
GGAGACGGGGAGGGCGAACAAAAGGAGCAGTTGCCAGACGTGGAAGATCAGGACGT
ATCGTAACTCTAACTGTA

>novel_circ_000992
ATCTTATACAGAAAACAGAGATGAAGGTGCATGGTTCTATCTTAACTGTTTTTCCAACT
GTTTTTTCAGTTAATCGATCAACCATGTAACTCCAGCTCTGGGGAGGAGACGAGTTTAT
CCCTTAGATAAACGAATGATTTACACTCGATTTACACGCATACATTAATAATATTCGTTAT
TTATCATAGTATGCGAGAATAATGATGCGCGCAGTAAAAAAGCGGGGAAGAGGACGAG
GAGGAGGAGATTGGGGGTGGGGTGAAAACAAACGAACACAATATGCATTTCGATCGA
CGTCGAGCCATTAATTCGTTATTGATAAAACATTGCGGAGAGGATAATGCTTATTCCTTA
TTTCTCACTCGCTGGAGCAAACAAAAATGAGAGTTTATTCCGACTGGATTAGCGCATAT
TCCTTTTCTTTTTTTTTTCTTTTTTTTTTTTATTTCATTTCTGATGCTTTTCTGGTTTCATTG
CTATTACGATCGAATATATGACTACGCGCTGCAACAGTCGGTGCCAACTCAACTGGTTC
TCGACATCGTACACTGAAAGAGACAGCATCTTTTTTCTCTTCTCGATACGCAAAGAAG
GCAAAGAATAATGCCTCTCTACACACGCTCTTGCAGCTTTTCTATTTAACCGCGTTTAAT
GAGTATGTCCTTTTATTTTTTATAACTTTTCTGCGCGCTTCGTAATTTAGTGTGTATTATTT
CGAATGATATTTTTTTTTTTTTTCATTTTTGTATTTACTTTTTTTTTTTTTTTGGAATAACC
GTACGTGCGTGCTTAGAACACGCAACCGTGTTAAAGATCTCCCCTTCCCACCCCGAAC
TGCCACCTGCTGAATTGTTTTCTTACAAGTTTGCTTACAAGTTATTAAGAGTTTTTTGCT
TTCTTGTGTTTTTTCAGTTTTATATACGCATAATAACACTCACTACAGTTAGAGTTACGAT
ACGTCCTGATCTTCCACGTCTGGCAACTGCTCCTTTTGTTCGCCCTCCCCTACGTAACA
CAAAAAGAAAATTTGTTAATAAACTGTACTCGATGATTATACAGTTACATAAAAATGTC
AATTATGTAACAAACTAAGATAGGGTGGGGTCGATTCAACTCATAATTAAAAGTTGAGT

TCATTTCAACCCTACCCTGCTAATTGAAGGATCTACTAAATGGTTAAGAAGGAAGGAAG
GAAGGAAGGAGAAGGATCGATCGAGTTGCTCACCAGGTGACTTTATAATGCAAGGGC
GTATGGAAAGGATGCCTGAGTTCAAGCATTGGTGGAAACCGCGGCAGCCTCGTTTTCG
CGGTCAGCCTCGAACTCTAAATCACAGACCAATAATTCTCTACCCATATATAAATATATAT
GTATATAATTTCAATAATCATTATACAGAATTATATGTGAATAATAATGCAACTAAAAAAT
GTCTTACCTAATAGAAAACTTTACTTTCTTTTTTGGTTTCAATTCAAAAATTTTTTTATAT
CATGATACACAAATTTCATCAAATAATAAAAAAAAGGATCTCATACGTTCTCTGCACAA
TTTCGTGAAAAAAATCTGATATATAAGAGAGAAGATTTTATCGCGCTGCTGCAATACATA
TTATTGATCGTACGACACAACTTCTACCGTTTAGTAGTGGTAGTTTGTGAACGTTTTAA
ACGAGCAATTTTTTTTTTTTTTTTTGAACAAAAAAGATATATACTAACCGTCTCCTTGCA
TGTCTGACGTCCATAGAGTAAGATTGTCCCTAAGAAGCTGCATAATGAGGGTAGAATCT
TTGTAACTCTCCTCGGATAAGGTGTCTAGTTCCGCGATCGCGTCGTCAAAGGCTGCCTT
CGCAAGACGACATGCTCTATCAGGGCTATTGAGAATCTCATAATAGAACACGGAAAAG
TTGAGCGCCAATCCTAAGCGGATGGGATGAGTTGGTGGTAGATCCGTCATGGCGGTGT
CACTTGCTGCTTTATAAGCGACCAAAGAATTCTCTGCGGCTTCCTTCCTATCGTTGCCA
ACGGCGAATTCAGCAAGGTAACGGTGATAATCACCCTTC

>novel_circ_000993
AAAAAAGAAAAAAAAGAAGAAACGCAATAACATCTTTGTCTTTATGTCTTAAGTCTGG
CTTTCGCAGTATCAAATTTTATGTTAAGTCGAAAACAAATTCTTTTTAATTTTGTGAGTA
AGAGAAAAATATATATATTCGCTATTCTCGTCGTAAA

>novel_circ_000995
GACCTGATGACGAAGAACAAGCAACGGAATTGGCGACGATTCCTGGCCGGCTGCTTG
GTCGCCGTGGTCGTGCTCGGCCTGGTCATCGCGGCGACCGTTCTTTTGACCGGGAGCC
CGGATTCCTCGGCCTCGAGGACACCCCTCGCCTCCGGCATTTCTCTCGAGGAGTGGCT
CGCCGGCTCCTTATCCCCGAAAAGCTTCAACGGGACATGGATCACCGGAAACGAGATA
CTGTATCGCGATGAAATTGGGAATCTCTTGATCTATAACGTCACATCGAGAAAGCCGAG
GAAAATCCTGGACTCGACCAATAACGTGCTGATATTGAGTTTCGATTACCAACTGTCCG
CCGACCGGAAATATCTTCTGCTGGCCATCAACTACCAGAAG

>novel_circ_000996
AGTGGCACGACCAATCCTACGGTGAAGCTGTTCTACGTCGACTTAGAGAAAGTGATCC
AAGGTAACACGAACTTAACTGAAATCGAACATCCGAACAAGTTATCCACCGAGGAACG
GATTTTATCCGCTGTTGCTTTTCCTACCGATAATCTCGTGTACGCGACCTGGATGAACAG
GGTGCAAAACAGGGCGTACTTCCATTTCTGTTACGTCCACGGCCATCTACCCAATTGCA
CCACCGCGCTGACGCACATAGAGAGCCACGGATGGGTGGAACAGTTCGAGCCACCGA
TGTTCAGCGATGACGGGGACACGTTTCTTACGATCTTGCCCAAAAAGCAACACGATGG
AAGTTACTGGAGGCATGCAGTCGCAATTACCAACGTTTCTTCCACCACGCCGAGGAAA
ACCGCCCTCACTTCCGGCCGCTTTGTCGTCACGGAGATAGTTTCTTGGGACCAGAAGA
AATCGTATCT

>novel_circ_000997
AATTCCACGAAGATGCTGACGTGGGAGGCGAACGAAGCAGTCTCCGAGATTATCGCGG

AGAAATCGCAACCGGTGGTGCGCCGTTTCAAGGTGCCGGTTCCCGGAGGATTCGAAG
CCCGTGTCAGACTTCTGATTCCGCCGAATGCTGATTTGTCGGGCGCCACCAAATATCCC
ATGCTGATCTTCGTATACGGTGGGCCAGATTCGTATCAGGTGACGGAGAAATTCGATGT
CGATTGGGGCACGTACCTTGTAACGAATAAAAGCATCATCTACGCGACGATCGACGGC
CGTGGATCCGGCTTAATGGACAACGGCATGCTATTCGCCGGATATCGAAACTTGGGAAC
CGTGGAGATCGCGGACCAGATCAACGTTACCAG

>novel_circ_000998
GGTGGTGGCGGTTTGGTTCAGCATTTGAAGAAGAGCTACAGCCTGAGCGACCTGACC
GGCGAGAAAGAGGACGAGAATAGAAACACGCCGCACATGCACGACGAGACGGACAT
GGAAGTGGAACCTCGTCGAAGAGAGCACGTGGGTAGAAGGGGGTACAGCCCTCGTCG
GACACAGTCGAGCAGCAATCGCGTGGAGATGTACTTTTCCGAGGTGGACGTGGACGT
GAGGCAACCGCGGTCCAGAGAGCCGACAACCAGCTTAACCAACATAAGGTCCTCGGA
CGACATATCGAGCGGGTACAGCAGCGGGGAAGCGCTGCAATCTAGCCGCACGTCTCAA
GGCGATTCGTTGGTGCGGACATCGAGCGTCGGCGCAAGAACACGGGCAAAACCTCGC
TCAGCGACCAAGAAGACCCCAGAG

>novel_circ_000999
ATTAGTGTTCGGCAATTTGTACCCGGCCTACGCTTCTTACAAGGCGGTGAGGACGAAG
AACGTGAAAGAATACGTGAAATGGATGATGTACTGGATCGTGTTCGCGCTGTTCACGT
GCGCGGAGACCTTCACCGACGTCTTCTTCAGCTTTTGGTTTCCATTCTACTACGAGATC
AAGACTATCCTGGTGATCTGGCTACTGAGTCCGGCCACCAAAGGGTCGAGCATCCTTT
ACAGACGTTTCGTCCATCCCGCGTTGATCCAACGAGAGGCTGAGATCGACGAGGCTCT
GGCCCGCGCCACGGAGCAGGGTTACACGGCCGTGTTGCATCTGGGGTCCAAAGGTGT
GAATTACGCCACCACCGTTCTCATGCAAACGGCCATCAAG

>novel_circ_001000
TTTGATGCGTCGGATCGGGGCGACGGGGATCGAAAAGAATTCGGAGAGGGGACGGTC
GCGGGTATAGCGAGGAGAATCCAGGCGGGACGAAGGGGGAGGTGAGGGAGAAATTC
GGCCCGCGCGTGGAATGGAAATGAAAAGGGAGGAGCCTCGACCGGATGAAATCGAGG
AGAATCCGTTTCTCTGGATCGTGTGCAAAACGGGGGCCCCCCGTTTAATAATAACGAGC
TTTCACGCTGTCGCACGTTACTGCGCCGATCAGATGCCAAAGAAAATGAGCCTGGGCT
TCCTTCGAACGCTTGCCGCCCTCCTGGTAATAGTGATGCCCCGATCGCTCACTTACGTT
CTCCTGTATCCGATTTTCAGATTAGTGTTCGGCAATTTGTACCCGGCCTACGCTTCTTAC
AAGGCGGTGAGGACGAAGAACGTGAAAGAATACGTGAAATGGATGATGTACTGGATC
GTGTTCGCGCTGTTCACGTGCGCGGAGACCTTCACCGACGTCTTCTTCAGCTTTTGGTT
TCCATTCTACTACGAGATCAAGACTATCCTGGTGATCTGGCTACTGAGTCCGGCCACCA
AAGGGTCGAGCATCCTTTACAGACGTTTCGTCCATCCCGCGTTGATCCAACGAGAGGC
TGAGATCGACGAGGCTCTGGCCCGCGCCACGGAGCAGGGTTACACGGCCGTGTTGCAT
CTGGGGTCCAAAGGTGTGAATTACGCCACCACCGTTCTCATGCAAACGGCCATCAAG

>novel_circ_001001
ATCCTCGTAGAATACGACGACGTGGAGTGGCAAAGGAGGGAATGGCTATCGCCGCACA
GGGACTCGGTGTTCTCGTTCTTCCTGGTGGAGAAGGGTCTGTGCTGGGCCGAGCGGCC

TGATCCAAGGCATGCCAGCCTCATCGCCATCGATCACCACAATCACGCGAATAATAACC
ACCACCATCACCGTATCAACGGGAAGCTGCTGAGGGGCGCCACAGCTGTCGCCAACA
CGGTCGCCTGGCCGGCTCTCACGTTTTATCCGCTGGTGGCGCGTGCAGAGTTGCCGGA
AGACGCGATGCCTCTCGAATTCATGCAAGATCGGAGGCTGGACTTTGTCGATTACTCGA
AGCTGAAGCCGTTTACC

>novel_circ_001002
ATCCTCGTAGAATACGACGACGTGGAGTGGCAAAGGAGGGAATGGCTATCGCCGCACA
GGGACTCGGTGTTCTCGTTCTTCCTGGTGGAGAAGGGTCTGTGCTGGGCCGAGCGGCC
TGATCCAAGGCATGCCAGCCTCATCGCCATCGATCACCACAATCACGCGAATAATAACC
ACCACCATCACCGTATCAACGGGAAGCTGCTGAGGGGCGCCACAGCTGTCGCCAACA
CGGTCGCCTGGCCGGCTCTCACGTTTTATCCGCTGGTGGCGCGTGCAGAGTTGCCGGA
AGACGCGATGCCTCTCGAATTCATGCAAGATCGGAGGCTGGACTTTGTCGATTACTCGA
AGCTGAAGCCGTTTACCCAGGACTGGGAGCTTACGAAAGGCTCGGTGCCTTGGGCGA
GCGCCGTCAGGCGATGGGCAGAGATGCAGGACGGGCAAAGGATCCTGCTCACCACGC
CGAGCGTTCTGGTCGGCTTCAGGGTCGAAGTTTATCGGGCCGAGGGCACCACGCAATG
GTACACTGCCGTCATCGTTGGTTACAACGAGTCGACCAAGGATTTGACCGTCACCGAT
GACACAGTTCTCGAGGACCACAACGAGGACCCCAGCCTAGTACAAATGCGGTTGATC
GGCGACGGAGTCGTGGAGAGCATCATGAGAGGCGAGGTCGTCGGCATGACACCGAGG
AGGTCGAGGTCATCGACTGCTCTGACGCACGCGCTTGTTGTG

>novel_circ_001003
ATCCTCGTAGAATACGACGACGTGGAGTGGCAAAGGAGGGAATGGCTATCGCCGCACA
GGGACTCGGTGTTCTCGTTCTTCCTGGTGGAGAAGGGTCTGTGCTGGGCCGAGCGGCC
TGATCCAAGGCATGCCAGCCTCATCGCCATCGATCACCACAATCACGCGAATAATAACC
ACCACCATCACCGTATCAACGGGAAGCTGCTGAGGGGCGCCACAGCTGTCGCCAACA
CGGTCGCCTGGCCGGCTCTCACGTTTTATCCGCTGGTGGCGCGTGCAGAGTTGCCGGA
AGACGCGATGCCTCTCGAATTCATGCAAGATCGGAGGCTGGACTTTGTCGATTACTCGA
AGCTGAAGCCGTTTACCCAGGACTGGGAGCTTACGAAAGGCTCGGTGCCTTGGGCGA
GCGCCGTCAGGCGATGGGCAGAGATGCAGGACGGGCAAAGGATCCTGCTCACCACGC
CGAGCGTTCTGGTCGGCTTCAGGGTCGAAGTTTATCGGGCCGAGGGCACCACGCAATG
GTACACTGCCGTCATCGTTGGTTACAACGAGTCGACCAAGGATTTGACCGTCACCGAT
GACACAGTTCTCGAGGACCACAACGAGGACCCCAGCCTAGTACAAATGCGGTTGATC
GGCGACGGAGTCGTGGAGAGCATCATGAGAGGCGAGGTCGTCGGCATGACACCGAGG
AGGTCGAGGTCATCGACTGCTCTGACGCACGCGCTTGTTGTGCCGAGACCTGGAAGG
AGGCCCCGAGGACGTCCTGGGAACGCCACACAGTTGCACACAGCGCCTAGGATACAA
AGCCCTATTTCTCAGCATCTCGAGAAAG

>novel_circ_001004
CAGGACTGGGAGCTTACGAAAGGCTCGGTGCCTTGGGCGAGCGCCGTCAGGCGATGG
GCAGAGATGCAGGACGGGCAAAGGATCCTGCTCACCACGCCGAGCGTTCTGGTCGGC
TTCAGGGTCGAAGTTTATCGGGCCGAGGGCACCACGCAATGGTACACTGCCGTCATCG
TTGGTTACAACGAGTCGACCAAGGATTTGACCGTCACCGATGACACAGTTCTCGAGGA
CCACAACGAGGACCCCAGCCTAGTACAAATGCGGTTGATCGGCGACGGAGTCGTGGA

GAGCATCATGAGAGGCGAGGTCGTCGGCATGACACCGAGGAGGTCGAGGTCATCGAC
TGCTCTGACGCACGCGCTTGTTGTG

>novel_circ_001005
CAGGACTGGGAGCTTACGAAAGGCTCGGTGCCTTGGGCGAGCGCCGTCAGGCGATGG
GCAGAGATGCAGGACGGGCAAAGGATCCTGCTCACCACGCCGAGCGTTCTGGTCGGC
TTCAGGGTCGAAGTTTATCGGGCCGAGGGCACCACGCAATGGTACACTGCCGTCATCG
TTGGTTACAACGAGTCGACCAAGGATTTGACCGTCACCGATGACACAGTTCTCGAGGA
CCACAACGAGGACCCCAGCCTAGTACAAATGCGGTTGATCGGCGACGGAGTCGTGGA
GAGCATCATGAGAGGCGAGGTCGTCGGCATGACACCGAGGAGGTCGAGGTCATCGAC
TGCTCTGACGCACGCGCTTGTTGTGCCGAGACCTGGAAGGAGGCCCCGAGGACGTCC
TGGGAACGCCACACAGTTGCACACAGCGCCTAGGATACAAAGCCCTATTTCTCAGCAT
CTCGAGAAAG

>novel_circ_001006
GACTGGGAGCTTACGAAAGGCTCGGTGCCTTGGGCGAGCGCCGTCAGGCGATGGGCA
GAGATGCAGGACGGGCAAAGGATCCTGCTCACCACGCCGAGCGTTCTGGTCGGCTTC
AGGGTCGAAGTTTATCGGGCCGAGGGCACCACGCAATGGTACACTGCCGTCATCGTTG
GTTACAACGAGTCGACCAAGGATTTGACCGTCACCGATGACACAGTTCTCGAGGACCA
CAACGAGGACCCCAGCCTAGTACAAATGCGGTTGATCGGCGACGGAGTCGTGGAGAG
CATCATGAGAGGCGAGGTCGTCGGCATGACACCGAGGAGGTCGAGGTCATCGACTGC
TCTGACGCACGCGCTTGTTGTG

>novel_circ_001007
AGAAGAACTCTGCGCGCAACAGCCGGCGGCGACGGGTGAGCGAGGGTGGTGGAGGC
GGGAGCCGCGAGAAGGAGGCAGCGGTAGCAGCGGGCGAGGGTGGGAACGTATCGTC
GTGCCGGCAGTCGGAGGACAGGGAGAATAAGGGCCCCGCGTCGTCGGGCAGGCCCGG
CAATCGCGTGCCACGGCCCGTCTTGGTTGCGGAGGAGGCGAACAACACCGCCTCCAA
GCTCCGGAAACGCGGCACCGCGGTCAAGAGCCCGATCGAGAATCAAAGACCCGTGAG
ACGGAGGCCGAGGCGACTCTCCTCCGGTTGCCAGGAGACGAGGACAGAGGCGGCCG
CCGCCGTCGTCGTTGCTGCTGCTGCCGCCGCCGCTGCTGCTGCTGCTGCTACTGA
ACAGGAGGAGGAGGAGGAGGACGACGAAGAGGAGGAGGACGAAGAAGAAGAAGAG
GAAGAAGACGAGGAAGAGGAAGAGGATATCGAGCAACAACAGCAGCGCAACGTAAA
ATCGTCGTCCGAGAAGGAGGAGGACGAGGACGAAGAGGAGGAGGAACGATTGGGCG
GCAGATCCGAGGCGGAGAACCGTGGCGAGCAGGAGAAGGAGGACGGGGGAGGAGG
AGGAGGAGGAGGAGAGCGTTGCTGCGATCCTCTGACCAAGAGGCTCAGGACAAGTCC
TGTCGCTGCTGCTAGAAAGCAGCCCAGGCCGGAGAGGAGGAAAGGAGGGAAAGGTT
GTTGCGACGAGGAGGCAGCGTCCGATCTCGAGGAAACGGAAGAGGAGGAAAGGAAG
GCAGAGGACGAGGAGGAGGAGAAGGAGAGAGGGGGGAGGGTCGAGAAGAAGCTAG
AGGAGGACGAGGAGAGCCAGTCGAGGGAGAGGGACAGGGAGCCGGAGCCACCGCCT
CCGAACAAAGTAGATCCGGGGGGCGACACCGTTCGATTTAACGCCGCCGGTCGGAAG
GAGGAGGAGGAGGGGGAGAGGCGGCAGCAGCAGCAGCAGCAGAATCGGAGGCAGC
AGGTGTTGGTGGTGAATGGATCGTTGCTCGACTCGTCGTCGTCGTCGTCGTCGTCGTC
GGCCCTCGTCGTCGTCGAGGAGAGCGGCGCCTCCTCGCCCCTCGATCCCGACGCCTCG

GCCCCTCGGCCGTTGAAATCGGCTTGTTGCACCGTCGAGTCGAGGGCGAGCGCCAAG
CCAGGGGGAGGCTGCGTCCTCGAAGGCCAGGCGGAGGCGGCGGAGGAGGAGGAGGA
GGAGGAGGAGGAGGAGGAGGAGGGGAGAGCGAGGACGGGGAGGACGGGGAGGA
GGGTTCGGCGGAGGAGCGCGACCGGGGGAGGCGGCGGAGGCGGAGGAGGAGGAGG
AGGAGGAAGGGACAGGGGGAGAAGGAGGAGGAGGAGGATCGGGAGCAGGAAGCGC
GGGAAGAGCAGCGGCAGCAGCAGGAGGAGGAGGAGGAAGAGTCGCGGAGAGCCGA
GGAGGTGACTACCGAGGTAGGCACGGAGGATAGCGTGGGTAGCGCGAGCGGCGCGAG
GGCGGCGAGCGTGGAATCGGTGGTCGAGCTGCTGGAGTCGAGCAGTCAGGACTCAGG
CTCCGTGCTGGAGAGGCTGAGTCCGCTTAGTGGCAGCGGCGGCGGCGGCGGCGGCGG
CGGCGGCGGCGGCTCTGGCAGGCAGAGCCTGCTCCTGGAGCAGCAACGGGAGCAGG
AACGGAATCGGCACAACGAGAGCCCTGTCATTCTAGCCGAGAGGCTGAACAAGCCGC
CACCGCCGGTACCCGGTCACCATCACCATCACCTTCAACAGTACCATCAGCACCATCAT
CACACGCCCCCTCACCACCACCACCAACAGCAACGTTACTCGACCGGTAGTCCCGTGA
TACATCACCATTCTCAGCAGCAGCAACAGCAACAGCAACAACAGCAGCAACAGCAGC
AACAGTTGCACCAGGTAGCGAACAGCGGGCACCATCAAGCGCAGCATCAGCAACAGT
CGCAGCATCATCACCAGCTGACCCCCTCGAGCCTGCTCGAGGTGCAACAGCAGTCGCA
CGCATCCAGGGGCGGCGATGAAGCCGGCCTCATGGAAGTGGAGGCCGGGGCCGGTAT
ACCTGGAAGCGGCGTGCTCAGCGGCGTGCCAGCGGCGGTCGGGATTCGGGCGGCGGT
CGGAGGTGTGGCCGGCGGCGGAGCGGCGAGCGGCGCGTATGGGGACAGCGGGTCGG
ACAGCGGGGTGAGCTCGTTGAGGAGCGCCGGGTCCGGGGACGAGAGGAGCGGGAGC
AGGAGTTCGGCGCTCAGCGTGGACGAGGCGACCAACTCGTCGACGCCCGCCGCGGCC
ACCACGCCTGCAAGGGTCTGGCACGTGCAGAGCGTTCAGCACACCTCGCTGCTCATG
GCCCATCCTCAGCCGCCCCCCACCGCCGGCCCGAGCTCGGCGGCCGCGGCGGCCGCG
ACCGCTCCGCCGGTCGGCTACCAGAGCCCGGCACCTCCGCCCGGTCACCACCATCCCG
CCGTCGCGTCCGAGATGCTGTGGCGGTCGCAGAGGTACCCGCCGTTGCCCCACTCGTT
GCTCGGACCCGCCCAGCCCACCACCGAGGAGATCGTCGAGAGGGAGAGGATACTTCG

>novel_circ_001008
AAATGTGTTTCAGTAAAACTTGGAAGAGAACAATCGAAAGTATCAACAATGGGCGCAA
TTGTTGATTCGAAGCAATCTTCTAAACGAGCAAAAGCGACATTTGGCAGTAGCCGCGT
CGTGGATAATAATACTTGATAATGATAATAATAGATGGGCGATATCGAAGCCAATTTCTC
CCGACTACGCTTTTCTTCCTGGCTGATGGCTCGAGTAAACATTGCCCAATAAATTTTTAA
TTCTTTCCCCCTCCTCTCCTGTTCAATAAGCGATTCTTATATACCGGGTGTTTGCAAGAA
TAAGAAATTCTTATCACTTTCAAAATATATTAAAATTTTCATTATACAATTTCAGTTAATT
GGATGATGCGAAAAAGAAAAATAGAAAATCGATTGTTGAAAATCGTTAATTTGGAAAA
AAGAAAAAGAAAGAAACGTTTTTTTATTAAGCGAGAAAAAGAACTTGTGTGTGTAAC
CTTGAAACTTTTGTACGTGTTCCAGGGAGCGGCGAGAAGCGGAAGCTCGGCTGGAGA
AGCGTGAACGAGAAAGAGAGGCCAAGATGGAAAGGGAGAGGCTGGAGAAGCAAAG
GGCCGCCGAGCAGGCAGTTCACAAACACTTCGAGGAGTCCCTAAGGCTGGCGCAACA
AAAG

>novel_circ_001010
GTAAAACGAAAGAAGGGCGCAGCACGGAGGCAGCAAACGACCGCGGAAGATCAGCG
GAGGCGAAAGGAAGATAAACAAGGAGGAGGGGCTGGTGTGGGCAGTGAATCTAGTAA

CGAGAGCGAGGCTGGTTCTGCCAGCGATACGAGTGAACAGTTGGGTTCCATTCAACCT
ACATCGAGGGTGCGGCATACCACGGCCAATTCCAACAATTCCTCGGGAAACGGTTAGT
TGACGTGTAGTAGCTTCTTGTAAATGGACTTCCCATCAGGTGAACTAACTATTCACTTG
CAGG

>novel_circ_001012
GCCACGAAAGAATTTATAGAAGCAGTTAATAAATGCCGGGCTGGAAGGAGAGCTGGTC
CAGTATCATGGAGCACGGCTGTAAAATTTTTGACAGCAAGGAAATTCGAAGTAGCGAG
AGCACTAGCATTGTATGAACAGCACGAAGCCACCAGAAGAAGAGAGGGTCTTGCACT
TTTATATCCTACTCAGGAACCTTTGCTCACTGAATTACGCACAGGAAAATTTACAGTTTT
ACCTTCACGGGATGCTACAGGGGCAGCCATAGCTATTTTTACAGCACATTTACACCTTC
CACAAAATACCACTCATCAAACAACATTGCAAGGCGTTGTATATCAATTAGATGCAGCT
CTAGAAAGTGTAGAAACTCAAAAACACGGTCTGGTTTTTATTTATGACATGTCGGATAG
CAAATATCAGAATTTCGATTATGATCTATCCCAAAAGATTCTCACCCTTCTAAAGGGTGG
TTATCCAGCTAAATTAAAAAAAGTCTTAATAGTGACGGCACCGTTGTGGTTCAAAGCCC
CCTTCAAGATCCTTCGATTATTCGTACGGGAAAAATTAAGGGATAGAGTATTCACCGTAT
CCATTCCCCAATTAACATTACACATCCCACGGGAATCATTACCGCATCGTTTGGGCGGC
ACGTTGGAGATACAGCATGAGGCATGGCTTCTTCACTGTCTTAAATCCATGACAAACAG
GGGCGGGGGTGAACTTTGCGAGGTTACCCCCAGAGTGGGTACTCCTCTTTCACCCACA
ACGAAAAACCAAACAACAGTTCACCAGAATCCCAATGGAACTACGGTCAGTAGCTCA
ACTAGTCCTATAATTGATAAGAACAAGGTGAAAAATGGTATCTCGACTATTGGGGATATC
GAGATCACGAACGGTGATGTCTGGATGGGTACCGATGAGGCGCCATCCCCTGTTCAGC
CTCCGTCCTCGGCTAGTTCAGGTTTCAGTGACGACGACAGCCTTCACGGGGATCTTGG
CCTTCAAGCCGTTACCATGGAACAGCTCATCGAAGAGATACACTCGAGGGGGCGCGCT
GGGCTCATAGCAGAATACGCGGAAATTAGGCAAAGGCCGCCCGAGGGTTCCTTCAATA
ATGCCAAATTGAGATCGAATCAATCGAAAAATCGATACACGGATGTGCTTTGCTACGAT
CACAGTAGAGTGTGCCTGTCGCAGATAGACGGAGACGCTACGTCGGATTACATAAATG
CTAATTTCGTCGACGGTTACAAGCAAAAGAATGCGTTTATCAGCACCCAAGGCCCGCT
GCCGAAAACTTGCGGTGATTTTTGGAGAATGGTCTGGGAACAACAAACATTGGTTGTT
GTTATGACCACAAGAGTGGTGGAAAGGGGACGCACGAAATGTGCACAATATTGGGGT
CCTGAGCCGGGCGACGAAGTACAAGCAGGTGGTTTTACAGTAACCACTCTCGAAGTTG
ATACAAATCCAGATTACACGATATCGATGCTTCTTCTTACAAACAAAAAGACTGACGAG
GCAAGAGAAGTTTGCCATATGCTATACACGGCATGGCCGGATTATGGAGTTCCACAATC
GGCCAGAGCTTTATTACAATTTTTAGCCTTAGTAAGACAGCAACAAACTAAGTTGCTTG
CTAGTAGAGGAGATACATGGGCCGGGCATCCGCGAGGACCTCCTATAGTTGTTCATTGC
AGTGCAGGGATAGGAAGAACAGGAACATTTTGCACATTAGATATTTGCATATCGCGACT
AGAGGATACCGGAACTGTAGACATCCGTGGAACTGTTGAAAAAATCCGAGCACAGAG
GGCCTACAGTATTCAAATGCCAGACCAATATGTATTCTGTCATCGTGCTCTAGCAGAGTA
TGCGCTTTCCAGGGGAATGCTTAGTTCACAACATCTTGCTATGTTACCTCCAACCATAG
AAGAAGATTCTGATTAGATCTCTGTTATCATGTACCAGATTGATGTTCTTCCCGAGTATA
CATAACAATCTCTCTTACATAGCCTATTTCTAGCTTAAATAACTAAAGGTTATTTTTTGCT
ATCATCTCCATCGATGTAATATTTCAAATTTCTATTCTTACGTTAGGCTATACGTTATAGA
GGATATCTAAGGAAAGACTAGTTTTTTCAAGGCTAAATGTAAGAACAGAAATTTTTTTT
ATTTCTGTTTCTTGGTATTTTGCTCTGTGAATAAACGATTCAATG

>novel_circ_001013
GCAAATCACCGTTCTTCCCGAGTTTGGAGGATCAGAGCGGCACGCTGCTAGAGGATCC
TAACGCGCCGAGCCAATTGGACGGCGTCGGTGGGGTCGGGGTCGGGGTCGGCGTCGG
TGTTGGAGTCGGGGTCGGAGTTGGTGTGGGTGTCGGCGTGGGCGTCGGTGTTGGGGT
CGGCGTCGGTAGCGGAATAACTGGATCTCAGACGGCACCGCTTTCCTCCCCCAGTCGG
AGCAGTCCTCACAGTCAAGGCAGTGAAACCGGAGAACGCTTCTCCAGGAAAGTGTTC
GTCGGCGGCCTGCCACCGGACATCGACGAGGAGGAGATCAAAGCGAGTTTCCGCCGT
TTCGGATCGTTGGTGGTCGATTGGCCTCACAAAGCTGAAAGTAAATCTTACTTTCCACC
GAAGGGCTACGCTTTCTTGCTTTTCCAGGACGAAGCGTCCGTGCAACAGTTGATCGAC
GCCTGCATCCAAGACGAAGAGAAACTGTATCTGTGCGTCAGTTCGCCGACAACGAAA
GACAAACCGGTCCAAATACGGCCGTGGCGATTGAGCGACGCGGATTTCGTGCTGGACG
CCTCGATGCCTTTGGATCCACGGAAAACGGTATTCGTCGGTGGTGTCCCGAGACCGTT
GAAAGCTGTCGAGCTGGCGATGATCATGGACCGACTTTATGGGGGCGTGTGCTACGCC
GGTATCGACACCGATCCCGAGTTGAAGTATCCGAAGGGAGCTGGTAGGGTCGCTTTCA
GCAATCAGCAAAGCTATATAGCTGCCATATCGGCCAGATTCGTTCAGTTACAGCACGGC
GACATAGACAAGAGAGTCGAGGTCAAACCGTACGTTCTGGACGATCAAATGTGCGAC
GAGTGTCAGGGACAACGTTGCGGTGGCAAGTTCGCGCCGTTCTTTTGCGCGAACGTTA
CCTGCCTACAGTACTATTGCGAGCACTGTTGGGCGACGATTCATTCACGGCCGGGTCGA
GAATTTCATAAGCCGTTGGTGAAAGAGGGTGCAGATCGACCTCGAACCGTGCCGTACC
GCTGGTGTTAACCCCAGCTGCGTTGTCCCTAATTATCTCGCAACCCTAGCTAATTAACTA
CCACTTCTACGTACCAACTCCTCCTCCTCCTCCTCCTCCTCCTCATCCTCCCATCTA
CTCTCCTACCGCTGTCACCCACCCTGCCATTCAATCGAGAACCCCCCCACCCCCCACTC
CCTGGCCCGTCCATCACCATCCCATGCTCGCATACCTACCTGCCTTCGGGCACAGGCAC
AGCCCGAGAAACCGTTATTACCGCTACTACCCACTACTGATCGTCGGTACTATCGCTATT
CTACTACTGTCGCTATTATCGCTCTATTTACCATTACTATTACCCACACACGCTCTATTATC
GAAACCATCACGATTTATTATTATTATTATTCCACTATGAGTTTATTTTTACTCTCCGATCC
ATTGCTACTTGGACGTGTTTACACGGCGTGCGCTATAACCCAATCAGACATCGCCGAGA
CCACGGCCGAGAAATATATCATCGTCGTACCCCACCGCAGCCGCAGCCACAGCCATCG
TCGCGCCACCGTGCTCCAACATATACCGACCGGAATATACCGTCTCGACGAAACGCATC
GCACCGGACTCTTCCTCACCGACGACAACTTTCACCGACTTTCGTCTCCACCACCAAG
TTTGTTGTATCGTGCCGCGTGTACAAGTGCGTGGTAACACAGCATACATACATATATACA
TATACACACACCGAACCGCCCACGAGAGGAGAGCAGCGGTTTAGCAGCGGAGGGAGG
GTTGCTGATCGACGACCGATACAGCAGCAGCAGCAGCAGCTCGTCGTCGATGGGACG
GACGGGGCAACGGCGTGAGCGAGCTGATTCCTCGAGCGGGAGAGAAACAATGGAAC
GAGGTCGAGAAGAGACGAAGAGAGAGAGAGAGAGAGAGAGAAAGGGAGCGACGTC
GAGGACAGAAGAAGAAGAAGAAGAAGAAGAGGAAGAAGAAAGAACAACGCGGAGC
ATCGCCCTTCCCGACCGACCGAGATGGAGCATGCGTTAGTTACCGACATCTATATCGAT
CCACGCGTGTACACAGATGCGTATTTATGTAT

>novel_circ_001014
GCAAATCACCGTTCTTCCCGAGTTTGGAGGATCAGAGCGGCACGCTGCTAGAGGATCC
TAACGCGCCGAGCCAATTGGACGGCGTCGGTGGGGTCGGGGTCGGGGTCGGCGTCGG
TGTTGGAGTCGGGGTCGGAGTTGGTGTGGGTGTCGGCGTGGGCGTCGGTGTTGGGGT

CGGCGTCGGTAGCGGAATAACTGGATCTCAGACGGCACCGCTTTCCTCCCCCAGTCGG
AGCAGTCCTCACAGTCAAGGCAGTGAAACCGGAGAACGCTTCTCCAGGAAAGTGTTC
GTCGGCGGCCTGCCACCGGACATCGACGAGGAGGAGATCAAAGCGAGTTTCCGCCGT
TTCGGATCGTTGGTGGTCGATTGGCCTCACAAAGCTGAAAGTAAATCTTACTTTCCACC
GAAGGGCTACGCTTTCTTGCTTTTCCAGGACGAAGCGTCCGTGCAACAGTTGATCGAC
GCCTGCATCCAAGACGAAGAGAAACTGTATCTGTGCGTCAGTTCGCCGACAACGAAA
GACAAACCGGTCCAAATACGGCCGTGGCGATTGAGCGACGCGGATTTCGTGCTGGACG
CCTCGATGCCTTTGGATCCACGGAAAACGGTATTCGTCGGTGGTGTCCCGAGACCGTT
GAAAGCTGTCGAGCTGGCGATGATCATGGACCGACTTTATGGGGGCGTGTGCTACGCC
GGTATCGACACCGATCCCGAGTTGAAGTATCCGAAGGGAGCTGGTAGGGTCGCTTTCA
GCAATCAGCAAAGCTATATAGCTGCCATATCGGCCAGATTCGTTCAGTTACAGCACGGC
GACATAGACAAGAGAGTCGAGGTCAAACCGTACGTTCTGGACGATCAAATGTGCGAC
GAGTGTCAGGGACAACGTTGCGGTGGCAAGTTCGCGCCGTTCTTTTGCGCGAACGTTA
CCTGCCTACAGTACTATTGCGAGCACTGTTGGGCGACGATTCATTCACGGCCGGGTCGA
GAATTTCATAAGCCGTTGGTGAAAGAGGGTGCAGATCGACCTCGAACCGTGCCGTACC
GCTGGTGTTAACCCCAGCTGCGTTGTCCCTAATTATCTCGCAACCCTAGCTAATTAACTA
CCACTTCTACGTACCAACTCCTCCTCCTCCTCCTCCTCCTCCTCATCCTCCCATCTA
CTCTCCTACCGCTGTCACCCACCCTGCCATTCAATCGAGAACCCCCCCACCCCCCACTC
CCTGGCCCGTCCATCACCATCCCATGCTCGCATACCTACCTGCCTTCGGGCACAGGCAC
AGCCCGAGAAACCGTTATTACCGCTACTACCCACTACTGATCGTCGGTACTATCGCTATT
CTACTACTGTCGCTATTATCGCTCTATTTACCATTACTATTACCCACACACGCTCTATTATC
GAAACCATCACGATTTATTATTATTATTATTCCACTATGAGTTTATTTTTACTCTCCGATCC
ATTGCTACTTGGACGTGTTTACACGGCGTGCGCTATAACCCAATCAGACATCGCCGAGA
CCACGGCCGAGAAATATATCATCGTCGTACCCCACCGCAGCCGCAGCCACAGCCATCG
TCGCGCCACCGTGCTCCAACATATACCGACCGGAATATACCGTCTCGACGAAACGCATC
GCACCGGACTCTTCCTCACCGACGACAACTTTCACCGACTTTCGTCTCCACCACCAAG
TTTGTTGTATCGTGCCGCGTGTACAAGTGCGTGGTAACACAGCATACATACATATATACA
TATACACACACCGAACCGCCCACGAGAGGAGAGCAGCGGTTTAGCAGCGGAGGGAGG
GTTGCTGATCGACGACCGATACAGCAGCAGCAGCAGCAGCTCGTCGTCGATGGGACG
GACGGGGCAACGGCGTGAGCGAGCTGATTCCTCGAGCGGGAGAGAAACAATGGAAC
GAGGTCGAGAAGAGACGAAGAGAGAGAGAGAGAGAGAGAGAGAAAGGGAGCGACGTC
GAGGACAGAAGAAGAAGAAGAAGAAGAAGAGGAAGAAGAAAGAACAACGCGGAGC
ATCGCCCTTCCCGACCGACCGAGATGGAGCATGC

>novel_circ_001015
GCAAATCACCGTTCTTCCCGAGTTTGGAGGATCAGAGCGGCACGCTGCTAGAGGATCC
TAACGCGCCGAGCCAATTGGACGGCGTCGGTGGGGTCGGGGTCGGGGTCGGCGTCGG
TGTTGGAGTCGGGGTCGGAGTTGGTGTGGGTGTCGGCGTGGGCGTCGGTGTTGGGGT
CGGCGTCGGTAGCGGAATAACTGGATCTCAGACGGCACCGCTTTCCTCCCCCAGTCGG
AGCAGTCCTCACAGTCAAGGCAGTGAAACCGGAGAACGCTTCTCCAGGAAAGTGTTC
GTCGGCGGCCTGCCACCGGACATCGACGAGGAGGAGATCAAAGCGAGTTTCCGCCGT
TTCGGATCGTTGGTGGTCGATTGGCCTCACAAAGCTGAAAGTAAATCTTACTTTCCACC
GAAGGGCTACGCTTTCTTGCTTTTCCAGGACGAAGCGTCCGTGCAACAGTTGATCGAC
GCCTGCATCCAAGACGAAGAGAAACTGTATCTGTGCGTCAGTTCGCCGACAACGAAA

GACAAACCGGTCCAAATACGGCCGTGGCGATTGAGCGACGCGGATTTCGTGCTGGACG
CCTCGATGCCTTTGGATCCACGGAAAACGGTATTCGTCGGTGGTGTCCCGAGACCGTT
GAAAGCTGTCGAGCTGGCGATGATCATGGACCGACTTTATGGGGGCGTGTGCTACGCC
GGTATCGACACCGATCCCGAGTTGAAGTATCCGAAGGGAGCTGGTAGGGTCGCTTTCA
GCAATCAGCAAAGCTATATAGCTGCCATATCGGCCAGATTCGTTCAGTTACAGCACGGC
GACATAGACAAGAGAGTCGAGGTCAAACCGTACGTTCTGGACGATCAAATGTGCGAC
GAGTGTCAGGGACAACGTTGCGGTGGCAAGTTCGCGCCGTTCTTTTGCGCGAACGTTA
CCTGCCTACAG

>novel_circ_001016
GGGGATCCAAAAGGAGTTGGAAAGGGATATCCTCGATACGATCGGCAGGATACTCGTG
GATCCTGGAACTACGATTTATGGGGGACTTTAGCGCGGCGAGTCTCTTCTCTTCGAGTA
GTAGTCAATTGTCAACCACGGGCAAATCGTCGATCGGGCAAATCAACGCAACCAGCAA
TACCAACGTGGCCGCGTTACAATCGACGACGTCCACGACGATGCAGGACGATCTGTTG
ATCGAGAAGCAAAGCGCGGGTGGGAAAACGGCGCCCCTGTCCCCTGGGAGCACGGG
CTTGGAAACGGTCGGCGGTGGCCGCGAGGAGAAGGCGGCGAAAGACGGGGTCGAGG
TCGAGGGTATCCCCATCACGCCGACGACGCCCTCCTCGGGCACGGAGAAGGAGATCGC
GTGCAGCACGAGCTCGGCGTTGCAGAGTTTCACGGAGGGCGGCAGGGTGCCGAGCCT
GTGGTCCTCGGCCCCGGACGAGACCGGGCTGCACGCGTTGCCCGTAAACGGGTCCATC
GCCGCCTTCCAGAACTTTCCGACCGGCGGCCCGACCGGCTTGTTCGCCGGCACGGGG
CCGGCCGGCGCGCGCCGCGCTATCACGGCCAGCCACAATTTCCCGCAACAGCACGGG
ACCGCGACCGCCCCCACCGCCAGGCACGGCGGCCTTCAAGTATCGTCCGCTCAACAAC
AGCAGCAACAACAGCAACAGCAGCAACAGCAACAACAGCAGCAACAGTCGCAACAG
CAGCAGCAACAGCAGCAGCATCAACAGCAACCGGCAGCTACCTCGCATCCGGGTGTTT
ACCTTCCTGGGAAGGGGTACGCGGCTTGGTCAGGTGGGCCGCAAGCGCCTCAGCAAT
GGAGCGCTGGGCCGCAAGCTGCAGCGGTGGCGAGCCCCGGCCTCTCGCCCTGGAACC
GTGGCAGATCGGTGCCGAATCTTCCTCCCCTGCACTCGTCCCTGCACGCAGCGGCCGC
GGCAGCGGCCGCGGTCGGATTGCAAGGAAGGAAACCGAGTCCCACGTTTCCCGGCCA
CCCGGGGCCGGCTGCTCATCCCTCCTGCATCAGCCCCGTTAAGTTTCGAAGGAGCACC
TCGTATCCCGGCAAGGGTAACATGTACCCGCCCCAACCCGCACCGACCTTCGAAGTCA
CCACCGCAGAGGAAAGGGATTTGTTACAGCTGCCGCCGTTCCAGCAGGATCGCATGAC
AGCTAATGGAAACTCACCACTGGACAATATGAGAACGTTAGAGCATTATTTGAGCGAC
ATTATGCGGGCTGGTACAGCAGATACTCCGGAACACGTGAAAG

>novel_circ_001017
GCGGCGCTGTCACCTCTAAAGATGTCAGAGCTCCGAATCGATTGTTCCGAACTAACTCA
TTCGAAACATTAAGACGCCTACACCAGAAACAGCAGAATACTCGGACAAGATGTCAAT
GACGGGAAGCTTAATGGGTTACGAGAACAATAACGAGATAAATCAAGCAAAGTGTAAG
AAGCCGTTAAAACCGCTTCCGATAGTGTCTAGCATAAATTCCAGTGGTATTACAACAGC
CAACAAAACGAAAAAACCCCGAAGATCGGCCAGACGCAGCTCTTGTATACGAGGTCA
CGTGAATAGTTTGTGGTCCGTCTGGTACGGTGTTTTGGCGGTCGCTTTTCAAGCTTACA
TCGGGTTAAGATACGCGAAAAGGTTCGCCGCGTATTTATCGTTACCTTGGCCTGCGGAC
GCTCCACCGCCAAAGGTGGAGTTGTACGCGTGCTTGGTGCTGGCCGGTGCTGGAGTCG
TTTTGCTGCCCGTTCTTCTCGGCGCGGCATTTTTAAAGCTCGGCAACCTGGCGAACGAT

GGGGTGAAACTTGGCCGCCATTTGAGCGCGTGTTCGCGCGATCCGCCTTCGTCCCTGC
TCACCAACAATCCCGACCACAGTTTGGCAAACAATTTATGGCGGCACGGGGGTCCCAC
GGCCGCTTTCGTGCACCTTTGTACAGCCATGTGCTTCCTCCTACCCTCGCTTCTCATGG
AGGCTAGGCTGATACACGCTGGATTTTTGCCAAAAG

>novel_circ_001019
GCAGCGTGATATTTGCGAAGAGACAGCACCTCTCGAGGTGCTCGCGGTCAAAGTGGAT
TACATGGAGGAAACGAACATGATGGACTCGGTGCCGGTTTCTGCGTCAGGGTCAAAGA
CCGGGTTGCAACCACAGCAGGGGGTACCATACGATCTTGAGAGTGGGTTCGAGCCTCA
GACCAGAGCACGATCGCACACGTGGCCGCTGCCAAGTCCGGAGGAATATATCGAGGG
AAACAAGAAGCCGAACGTATCGGTTCGAAAAGAGGGCGTCGAAGATGTCGAAAGTGG
CACGCCGCCTCAACATGGTTCTCTCGGCCAAGGTACCGGCTTGCTTCCGGTCAAGAAG
AATTCCTCCCGACGGAATGCCTGGGGAAATCACAGTTATGCAGATCTCATTACTCAAGC
CATTACCAGTGCTCCAGATGAACGGTTGACTTTGTCTCAGATCTACGAATGGATGATGC
AGAATATTCCATATTTCAGGGAGAAGGGAGAAAGCAACAGTAGCGCCGGATGGAAG

>novel_circ_001020
GTGAGTCTGGTTTTTCATTATCATCGACGAATAGATCATAAATCTAACTAATTAAATTATA
TTTACTCTTTAAAAAGATAAAATTAGGTAAGTTACAATTATCTATAATTAAATAAGTATTT
AAGATGAATCTTTTTTGTTTGCAAAGTTCTCGTAAAAAGGTAGAAATTACGGTGAAATA
GTCGAGACAGTAACAAATGAAGTAAAGTTAGCATCGTGATCTGCTTAGTTGATAAGCC
AGAACGACTAGTAATTGCGTTCTTCAATTTAACGATCGTCTGCTACTTGGATAAAAATC
ACACGCGCGTTGTTACAGGTTGCAACACAGACGTGGTTAAGCTGTTAATGGCGTTTCA
TTAAGTATTTAGCATACTAATAGGCTCCTCCTAAAAACACGTGATAAAAGGTTATTTTA
TAAAATTCAAATTCTTATCTTTTTCTTATCATTTATTCAAAATTTTTCGACATTTTTTTCT
TTTAAGTAAGAAGTAAAAATAATGAAGAATGTTATTTCAGTTTGCACATATCAATATATT
CGAAACCTGAAGCCATTTCCTTTCGTCATCTGATAAAGAAAAGTTAATGTATCGAATAA
ATGTATCGTTACATTTTCATTCACGTTTTTCTCAAGTCTTTTTGCTTCGGTATCTGCTGAG
AGACTCAGTTAATCAAACGTTTCGTAGATCATCGATTATTGCAGTGTACCATTGAAACT
CGTCAATTAGAAATGTATCGCACGATTAGAACTTTTTTTTAAATCGAACTTCGCAAGAA
TTTAATAGTCTTTTCAAAAAGAAAAATTGGCATGAAAGTTCTTTCTACAGAATTCTTTTA
ACTTCTCCTAGTTATGGCAATTTATGTGTGGTAGAATTTTGGATATAAGAATCGAGATATT
TACAATAATTGAGGTAATCAATCAACTATATAAATTTCCTTATTATCATATTTCTTATACTA
AAACTACTGAAGTTTTAGAAGTAGAGTCTATAATTTTCTTTCTATGGCTTCTTATCGTGT
TCATATTCATAGTTAATTATAAGTTATCTACTCTACATTAACGATATGCAATGAAAGAGAT
TCGTATGTCAAAATAAGTAAATCAAAAATGTAGCTATCGAAATTTGATTGTAATAGAATA
CTCAATAATTTGTATTATATTCAAATATCTGAATATTTATTAATCTTTAAATGTCGAGATAT
TCTCAAAACCAGTATCTTGAAATTTATATAATAAATTAACAAACCAATTAATACAATGTA
AATTCTAATTAGAAAATCTATTATTCAAATTATAATTATTCAAATTATATTTTATTGTTATAT
ATTTATATTAATTTAAATTTCTACAATAAATAATAAATAAGAAAAAAAGACTTCAATAAC
GATGCTTTTATCTTTTAAAACATTTATAATATTAAAAAAAGACCGAAATAGTTTGAAAAA
TAATGTATTGATAAATTTATGTGATCTAATAAATAAATTAAATTCATAAATAATTAAAATAT
TTTATAATATTTTATATCATATAAAATAAAAATCAATTGATATTGCATTAATCTTTTTGAAA
TGCATGATAATATATGATAATGACGTATGATAAAAAAGGAATATTCCACAATATGAAAAT

TTTTTAACCGAATGAACAATCGTATTTCTCTATTATTTCGAATATTAAAGATTCCACTAT
ATTGATTTAGTATTTGAATAACTCATTCGATTCATATGTCTGATTGAATTATAATTTAAATT
TGCAATAAGACAATAAAAGAATTCTTAAAATTCCTTATAAATTGTTCTTTGAGGTCTGTT
AGTTCATGGCGACGAGGCAAATTTCAAGGACGTGAAACACGCAATAGTTAAAACTTCG
TCGGACTGAGCACGGAAATGAATGGGAATCTTCTGAGAGAGGGTGATTGAAG

>novel_circ_001021
AACTCTATAAGGCACAACTTATCGTTACATAGCAGATTTATGCGAGTGCAGAACGAGGG
TACCGGAAAAAGTTCCTGGTGGATGATCAATCGTGATGCGAAACCAGGGAAATCACGT
AGAAGAGCAATAACCATGGAGACGAGCAAATTTGAGAAACGACGTGGTAGGGTAAGG
AAGAAGATCGAGGCGCTAAAAAACGGTGGGTTACAAGCTGACACGACAACTAGCCCC
AGTAACAGTGTGAATGAGGGGTTGGATCTCTTTCCAGACAGTCCGCTTCAGTCTGGCA
GTGGATTTCAACTTTCACCTGATTTTCG

>novel_circ_001022
AACTCTATAAGGCACAACTTATCGTTACATAGCAGATTTATGCGAGTGCAGAACGAGGG
TACCGGAAAAAGTTCCTGGTGGATGATCAATCGTGATGCGAAACCAGGGAAATCACGT
AGAAGAGCAATAACCATGGAGACGAGCAAATTTGAGAAACGACGTGGTAGGGTAAGG
AAGAAGATCGAGGCGCTAAAAAACGGTGGGTTACAAGCTGACACGACAACTAGCCCC
AGTAACAGTGTGAATGAGGGGTTGGATCTCTTTCCAGACAGTCCGCTTCAGTCTGGCA
GTGGATTTCAACTTTCACCTGATTTTCGTCCTCGAGCTTCGAGCAATGCCTCATCCTGT
GGTCGATTAAGTCCAATCACGGCGATTCCTAGGAAACCGGAATGGACACCAACATACA
CACTCTCATATAGTCCAGAACAATTAGCTGGTAGCCTCGCAGAAACGATGAAATTGGAA
TCCTATCAAATGTATCATACGACACAACCGAGCCACCAGCATCATACAGGACCACCACC
TTCTTATTACGAATCCCAATATCAGAGGAGCAATAGCTTGTCGTCAGGATCGTCCTCCTT
CGCTCTGCAGCCAACTCCTCAATCAGCAAATCAACAAAGATGCCCAATTCACGGTTTG
CAACCGTGTGCCTGTCAAATGAATTTGAGTCCAGTGGCTGGAATGTCGCCATCGTACC
AGCAAAGCGAACCAAGTCCCACAACGTTAGGAAATAATCAACAGCAAACTCTTCAATA
CATGATGCAGACGCAACAATGTCAGCAGCAACAATTGCCACAACGACAACAGCAATC
ACAAACAGGAGTAACAGATTCCAGTTCATCACAAACATCTACCCCATGTGACCCCACA
CCAAGCACTATGATGGGTCAATTGATGGGAGCCTTGAACAATTCAACACTTCTTGATGA
TTTAAATATTAACATCGAATCGCTGCATGGTGGATTTGATTGTAACGTAGAAGAGGTGAT
CAAACACGAGTTGTCGATGGATGGTACATTAGATTTCAACTTCCAACAGAGTGTAATGG
GTACGGCTGCAATCCAAGTAAGCGACAATAATATCGGTCAGAACGGCGCCGTTTCGCA
GAACAACGTGATAGGTACGACGACGACCGGAAACGCGACCGCAGGTGTTTACGTGAG
CACCAATGCGGCCACTCCTGCTGCACCTCCCTCTTGGGTTCATTAGCAGTGGGCCCCAC
TGCCCTCGACATCCTCGTCGTCCTTGTTATCGATGGTACTAGACAGACAAGAAGACTTC
CAGTTTGAGGGACAGTTCATTG

>novel_circ_001023
AAAGCACAAGTTCGCATTGCGTAGGATGTGGTGGTCGAATTCATGATCAATGGATTTTG
CGAGTTGCACCGAATCTGGAATGGCACGCTGCTTGTTTGAAATGTGCAGAATGCCAGC
AATTTTTGGATGAGCACTGCACCTGCTTCGTGCGAGATGGAAAAACTTATTGCAAGCG
AGATTATGTCAGAATTGATTTCAGACTGTTTGGGACAAAATGCGACAAGTGCAGTGAG

TGCTTTAGCAAGGATGACTACGTAATGAGGGCAAGGAGCAAGATTTATCACATAAAGT
GCTTCCGATGTTCAGCCTGCATGAGACAGTTAGTGCCTGGCGATGAATTTGCCTTGAGA
CAGGATGGCCTGTTCTGCAGACATGATCACGACGTTCTCGAAGGCGGAAAGCTCTGCT
CTGGACCTGGTGGTGTTCCTGGAAGTGAAAACAACAACAATGCATCCCTTATGAATAAT
AATCATCATCTGCATCCCAACGACGGCTCAATATCAG

>novel_circ_001026
GTACTGGTGAATCCGGCAAATCCACCTTCATCAAACAGATGAGAATCATCCACGGGTCT
GGTTACTCGGATGATGACAAGAGGGGGTTCATTAAACTCGTGTACCAAAACATTTTCAT
GGCCATGCAGTCCATGATCCGAGCGATGGACCTCCTCAAGATCCAGTACACCGAATCTT
CGAATATAGAAAAAGCAGAACTCGTGCGAAGCGTGGACTTCGAAACTGTTACAACGTT
TGAAAGCCCATACGTAGAGGCAATTAAAGATTTATGGGCAGACGGTGGTATCCAGGAG
TGTTATGACCGCAGGCGAGAATATCAGCTCACAGATTCCGCTAAATATTACCTATTGGA
GATAGATCGAGTCGCAGCACCGGACTACCTCCCAACAGAACAGGACATTCTTCGCGTG
AGAGTACCCACTACTGGTATAATTGAATATCCATTTGACTTGGAAGAAATTCGATTTAGT
TATCTTAGTGACCTAGACCGTATTGAAAAGCCTGACTTCCTTCCTACTGAACAAGACAT
TCTTCGGGCTCGAGCTCCTACTACTGGCATTATAGAATATCCGTTTGATCTGGACTCCAT
CATATTTAGGATGGTAGACGTCGGTGGACAGAGATCAGAAAGAAGGAAGTGGATTCAT
TGTTTCGAAAATGTTACTTCTATTATCTTTTTAGTAGCTTTAAGTGAATATGATCAAATTT
TGTTTGAGTCAGAAAATGAGAATCGAATGGAAGAAAGTAAGGCATTGTTCAAAACAAT
TATTACATATCCTTGGTTCCAACAGTCCTCTGTTATTCTTTTCCTAAACAAGAAAGATTT
GCTAGAAGAGAAGATTATGTATTCTCATCTTGTTGACTATTTCCCGGAATATAATG

>novel_circ_001027
TTATCTTAGTGACCTAGACCGTATTGAAAAGCCTGACTTCCTTCCTACTGAACAAGACA
TTCTTCGGGCTCGAGCTCCTACTACTGGCATTATAGAATATCCGTTTGATCTGGACTCCA
TCATATTTAGGATGGTAGACGTCGGTGGACAGAGATCAGAAAGAAGGAAGTGGATTCA
TTGTTTCGAAAATGTTACTTCTATTATCTTTTTAGTAGCTTTAAGTGAATATGATCAAATT
TTGTTTGAGTCAGAAAATGAGAATCGAATGGAAGAAAGTAAGGCATTGTTCAAAACAA
TTATTACATATCCTTGGTTCCAACAGTCCTCTGTTATTCTTTTCCTAAACAAGAAAGATT
TGCTAGAAGAGAAGATTATGTATTCTCATCTTGTTGACTATTTCCCGGAATATAATGGCC
CTAAACAGCAAGCTGTACCAGCCAGGGAATTCATCTTACAGGTGTATCTTAGTTCGAAT
CCTGATCCTGACCGCATGTGCTACTCTCACTTCACGTGTGCGACAGGCCCACAAAGAG
ATGCCATACCTGCTAGAGAATTCATTTTACAGATGTTTGTCGACTTGAATCCTGATATTG
AGAAGATTATATATTCACATTTTACATGTGCCACAG

>novel_circ_001028
GATGGTAGACGTCGGTGGACAGAGATCAGAAAGAAGGAAGTGGATTCATTGTTTCGA
AAATGTTACTTCTATTATCTTTTTAGTAGCTTTAAGTGAATATGATCAAATTTTGTTTGAG
TCAGAAAATGAGAATCGAATGGAAGAAAGTAAGGCATTGTTCAAAACAATTATTACATA
TCCTTGGTTCCAACAGTCCTCTGTTATTCTTTTCCTAAACAAGAAAGATTTGCTAGAAG
AGAAGATTATGTATTCTCATCTTGTTGACTATTTCCCGGAATATAATGGCCCTAAACAGC
AAGCTGTACCAGCCAGGGAATTCATCTTACAGGTGTATCTTAGTTCGAATCCTGATCCT
GACCGCATGTGCTACTCTCACTTCACGTGTGCGACAGGCCCACAAAGAGATGCCATAC

CTGCTAGAGAATTCATTTTACAGATGTTTGTCGACTTGAATCCTGATATTGAGAAGATTA
TATATTCACATTTTACATGTGCCACAG

>novel_circ_001029
GCCCAGCACCATGGCGGCTGAGGAGGGTGCGCTGAGTGGCGGACTCGGGGCGAAATT
AAAATGTCCGACCCCTATATCACGCTTGAGGGTGGAGAAGATCGAGGGCGGCCTGATG
GTGGCTGGGGTTGTAATAGCTGCGAACTGCCAGATTGCTCTTCCGGCATTCGGCTTCCT
CTTTATGCTCGTCGGCGCTGTACTCACCGGTGATCGAACTCGACGATGGCAATGGAGC
GTTTATGTCACGAAGAGGAAAAAGAAGAAAAAGGAAGAAGAAGAAGAGGAGGAGG
GGGAAGGAGGAGGAGAGAGAGGAAGAAGAGGTGGCGTGGAAGAAGAAGAAACGAG
GGAAGAAAGAAGGTTCACTAACGGGCAACAGCTGGCCGTTTACTCGTTTGGCCACGG
TTTCTTGGATACAGGTCTGGACCGTTGTTACCAACCGATTTATCAAAACCG

>novel_circ_001030
GTGATGAACCAGGAAGTCAAAAAAGAGACTCCGTTGCAATTCAAATTCCGCGCCAAAT
TTTATCCCGAAGATGTTGCCGAGGAATTAATTCAAGATATCACCTTGCGTCTTTTTTATC
TTCAGGTGAAAAATGCTATTCTTACGGATGAAATTTATTGTCCTCCGGAAACATCTGTAT
TATTAGCCTCTTATGCAGTTCAAGCGAAACACGGAGATTTTCAAAAAGGTACGCATACT
GCTGGTTTTTTGATAAATGATCGATTATTGCCACAACGAGTTGTGGATCAACATAAAATG
AGCAAAGAAGAATGGGAAAGTTCGATTACTAATTGGTGGCAAGAACATCGTGGAATGT
TACGTGAAGATGCAATGATGGAGTATTTAAAAATCGCTCAAGATTTGGAAATGTATGGA
GTAAACTATTTTGAAATTCGTAACAAAAAGGGTACAGATCTATGGTTAGGTGTTGATGC
TTTGGGTTTGAATATATACGAGAAAGATGACAAATTGACACCAAAAATCGGTTTTCCAT
GGTCTGAGATAAGAAATATTTCATTTAATGAAAAAAAATTTATAATCAAGCCAATTGATA
AAAAGGCACCAGATTTTGTCTTTTTTGCCACTAGAGTTAAAATAAACAAGCGAATTTTG
GCATTATGTATGGGCAATCATGAACTTTACATGCGTCGACGTAAACCGGATACAATTGA
CGTACAGCAGATGAAAGCGCAAGCAAGAGAAGAGAAAATTGCCAAACAGCAACAAA
GAGAAAAGTTACAATTAGAGATAGCAGCAAGAGAACGTGCGGAGAGGAAACAGCAA
GAATATGAAGAAAGGCTTCGAAATATGGCGGAAGAAATGGATAGGCGGCAAGCTGAGT
TAAATGAAGCTCAAGAAATGATTCGACGTTTAGAAGAACATCTTAAACAATTACAAGC
TGCTAAAGAAGAATTAGAAGATCGCCAGAAAGAACTTACGGCTATGATGGAAAAACTT
GAACTTTCACATGAAATGGAAGCTGCTGAACGTGCTAAACTTGAACAAGAAATACGAG
CCAAACAAGAAGAAATACAACGTATACAGTCTGAAGTAGTAGCAAAAGATGCAGAAG
CAAGGAGATTGGAAGAAGTATTTGAAGCTGCCAAATTAAGACAAGAAGAAGCTGATC
GAGCATTTCAAGCTAATACAACACCCCATCATCATCACGTTGAAGAAAATGAAGAAGG
TGAAGAAGAAGGAGAAGATGAAGTTTCTCATGGTGATGTTACTAAAGATCTTGCAACT
GATGAATCAATAATTGATCCAGTCGAAGAGCGACGTACCTTGGCAGAAAGAAATGAAC
GTCTTCATGATCAACTTAAGGCATTGAAGAAAGATCTTGCACAATCACGTGATGAATCG
AAAGAAACCGTTATGGATAAAATTCATAAGGAAAATGTTAAACAAGGTCGTGATAAATA
CAAAACGCTTCGTGAGATTCGCAAAGGCAATACTAAGCGCCGTGTTGATCAATTTGAG
AACAT

>novel_circ_001031
ATGTTATTGTTGTGCACTATCTCTTTCTCCACAATCAGCTTTACTATGGCATGATTTAGCT

TTGTGCTACTTAATGCAACTACAATATGATCCATCAATTAATCATAAAAATCTTGCTAGTA
AAAGTCTTGCTGCTGCAAAACATGCTATTAAATTAAATCCTTCAATATGGATACATTGGA
ATCTCTTAGGAGTTATTTGTATGTCACCATATATAAAAAACTTTGCATTAGCACAACATG
CTTATATCATGGCAATTGATACAGAAATAAATAATGCCATAGTATGGTGTAATTTGGGAA
CATTGTATCTATATACAGAAAATTTATATAAAGCAAATGAAGCTTATTCTCGAGCTCAGC
GTGCAGATCCAGCATACATAAATAGTTGGATAGGACAGGCTTTAATTGCCGAAATGATG
CATCGAAAAGAAGCAATGGATTTATTTAGACATGCAACACAATTAGGATATCACAGTCA
AGCTGCCATAGGATATACTCATTGGGTACTTGATATGATTTTAAATTCTGATACCAAAAA
GAACAGTTTAAATATATGTGTTACAGAAAATGCTGTATTTGCTGCTACTGATGTTATGAC
ATGGTATATAGAAAATCATCCAAATGATTGTTATGCTCGAAATGCATATGGATTACTATTA
CAAAGACAAAAACTTTATAAATCAGCTGCAGAACAATTTGCAGTAGCTGTATGTAATAG
TATTAATAATAAAAAAGATCTAGTCTGTGTAAATTTAGCACATGTATTAATAAAACTTAA
AAAATATAATGAAGCTATAAAATTATGTCAAACAGTTCAAAATATCAATTATAATTCTCA
ATGTCATTTAGCATTGGCTTTATTCAAAGCTGCAAAATATGAAGAAGCATATACCACATA
TGAAACAACACTGCAATTTTTGGCGAATACAGAAATAGAGAAATCATATGCACTGTGC
GCAATGGGTGCAATAGCATATACTTTTCAAAAAGTAAATGATGCAAAAACTTTACTTTT
TCAATGCATACAAATACAACCGCCTGTTATAACTGGCCTTTTAGCAGCTGCATCATTAGG
AATATTACATGGTGATATCAATTTAACTACATTGGTATTAAATGAATTGCAATTATATGAA
AATCATCTTGAATATGGTTATCATGTTGTGAATTTATCTGCATATTTTTATTTAATTGGAAA
TGATGTTAAAAAAGCTATTACTATTCTTTCTAAAGCAATTTTTACACATCCTAGTAATGTA
AAAAATTGGATTCGATTACTCAGGATATTATTGGAAACTAATGTACATATGTTTAGCAAA
TGTGCTCAGAAAGTATTGTTCCTTAATAAATATATTACTTCTGAAAATCATGCAAATGTT
GCATGTGCTCTATCTTATAGTTATTTTATGCAAAATTTAGTACCAGCCAATATTATAGCTAT
GCAAAAACTTGTTTTCACTTATCCTGGTAATATTGAAAGCTGGGCTATGTTTATTGCAGT
ATTTTTATCAAG

>novel_circ_001032
AATGTCGGAAGCCGAGGATGCCTCCTCGGGCAAAGGCAGTAACCTGACAGCGGTGGA
GGCGATCTCGGCGAGGTTGGCGGAAGTAACTCGTCTCGCCGAGAAGTTGGACGCGAA
ACTTTGCGAGGCTGGCGCCAGGACTCGTCCAGTCCCGATGTCCTCGTCCGCCGTCTCC
TCCACCCCCTCCCCCCTCTCGTCCTCGAGCGCGCCCCCCACTTCCGCCTCGGGTCAAAT
CCACACGATTTCCGCTGTCACAACTTCGTCGATCGGGATCACAGCACCGTTGATCAGCT
CCTCGACGGTGCAGCTTCAGCCCAAATGCGTGTCCACGATACTCTCCTCGGCGACGAG
CAACGAGAACGTTGGCACGACGACCAAAGATTCGGCGCAGAGTGTGAATGGCACGGG
AGTTGGTGATGAAAAGGTGGAGAGCAAGGTGGAGGATGAGGGGGAGGCTGGAACAG
GGGAAGAGAATAGACAGGATGATTTGAGCGGGATGAATGATGAGAAGTTGAAGGGAG
AAGTTGTGGAGGACGTGATCGAAAAGTCATTCGACGATAATCTAGCCGAAGATTCGAA
CATGGACGAATACGTAACTGCGACTGAATGTTCAATCACTCCGACAGTTCGTTCGAGG
AGCGAATCTTTCGTTACGTCTCCAGAATGGGAAGATCTCGCTGCTCCAACATCCGTGAC
ACATACTTGTTGCGATACTGGGTGGAACATCGAAGAAACTAAGCTTAAACGGACGGAA
AGTTTGGAAACGGAGAAAATCGACGCTTCGATGGATATTCCAAAACCGGAAGTCGAG
GGGACGGAGAACGAGGGAAAACAGAGTGAGAAGCTCGTGTCGATGGAAAAAAGAGT
CGAAGAGAAATCCGGGAAGATAGTGAAAGAAGTTACGGAAACGACGACTTTACGAGT
GTCTCACGATACTCGTTTAGATATTGCGTCTTACAAGGTGATATCCAACACCACGCAAG

ATCATCGAGAAAACGTAGATGTTAAGGAAATCAAGGATCTCGAACGCATCGTGTTGCC
GGATGTCCACGAGAAAACGAATGGATCGCACTACGAGATGGATTCCGTTAGCAAATCG
GAGTCGTACAGTCGCAGAGACGAATTGTCCACGAAAACGGACGACATCGAGCGCGTG
GTAAAATTCGCGAAGATCTGCGAAATAAATCAAGAATCGCGCACGATCTCTGTCGATAC
ATCGGCGAAGGATATCGCTCTGTCCTTGAAGGAATACAGGCGACGTCACGAGGACAGT
GGGATCGAGATGTCGCCGACGAAGAAAGACGAAAACCTCGAGGGCCAAGATTTCCTC
GAAGCGGCGAGGAAG

>novel_circ_001034
GAGTACCTTCGCCATTTTATGGAACGTACAGGGACCAAAACATCAACGAGAGGGCTAT
GGAGAGCCCGCCCAGGTACGTGGCTCTCAAAGACCTATTTCTTCCGCAAGAGAACGAC
GTGAAATCGATACCTGCGGATCGTCAACATTCTACGCGACACGATTACTCGGTCCCGTG
GAATTTGAACGCGGATCCCAGTTGCGCAGGTGGCGGCTATCGCGAGAGGCGAATTACG
ACGGAAGAATGCGAGGAGATAACGGATCGAAATACGATAGCGCGGCGACTCGGGTATT
CCGATTTCGAGGCGAACGATTCGTTTCGCGATGATTTCTGCTTCGATCGCGAGTATTGC
CGCTCCGCGAGGCCGAGGACGGAGGACGGAG

>novel_circ_001036
GGCAATCAGATATTTAAAACGGTGTTCGTCGCATTTTTCCAAGGAGAACAGCTGAAGA
GCCGAATAAGAAAAGTGTGCACCGGTTTCCACGCCTCTTTGTATCCATGCCCGCATAGC
CACGCGGAACGTCAAGAAATGGTCAAAGGCGTTCGAACGAGACTGGAAGATTTGAAC
TTGGTTTTGAATCAAACGCACGATCACCGCCAACGCGTTTTACACAACGTGGCCAAGG
AATTGCCGAATTGGGCGATCATGGTTCGAAAGATGAAGGCGATTTATCACACGATGAAT
CTGTTCAACGTTGACGTAACGAAAAAGTGCCTTATAGGGGAGTGTTGGGTACCTGTATC
GGATCTTACCATCGTCAGAGACTGCCTTAACGAAGGATCGCGTCTCTGCGGTAGCTCGA
TACCATCTTTCCTCAATGTCATCTACACGAACGAAAACCCGCCGACGTTTAACAGGACG
AACAAGTTCACCAGAGGTTTTCAAAACTTGATCGACGCGTATGGCGTGGCGTCGTACC
GCGAGGCTAATCCAGCCCTTTATACGATTATCACCTTCCCCTTTCTATTCAGCATCATGTT
CGGCGATTTTGGACATGGTATAATTATGACTCTGTTCGCTCTGTTTATGATCGTGAAGGA
GAAGAAGTTTATGGCTGAAAAAACAACGAACGAAATTTGGAATATATTTTTCGCCGGC
CGTTACATTATTCTTCTCATGGGTTTGTTTTCCATTTACACTGGCATCATCTATAATGACG
TATTCTCAAGATCAATAAACATATTCGGGTCTAGTTGGGACATACGGTTCGACAATAAA
ACAATCATGATTAATGAGCTTATGGAATTGGACCCGGCTAAAAATGATTACAAACAATA
CCCTTATCCGTTGGGTATGGATCCAGTTTGGGTGCTTGCCGAGAACAAGATCATATTCTT
GAATTCGTACAAAATGAAGCTGTCCATCATCTTCGGCGTCGTCCATATGATATTCGGCGT
GTTCATGAGTACCATTAATATTAT

>novel_circ_001037
GGCAATCAGATATTTAAAACGGTGTTCGTCGCATTTTTCCAAGGAGAACAGCTGAAGA
GCCGAATAAGAAAAGTGTGCACCGGTTTCCACGCCTCTTTGTATCCATGCCCGCATAGC
CACGCGGAACGTCAAGAAATGGTCAAAGGCGTTCGAACGAGACTGGAAGATTTGAAC
TTGGTTTTGAATCAAACGCACGATCACCGCCAACGCGTTTTACACAACGTGGCCAAGG
AATTGCCGAATTGGGCGATCATGGTTCGAAAGATGAAGGCGATTTATCACACGATGAAT
CTGTTCAACGTTGACGTAACGAAAAAGTGCCTTATAGGGGAGTGTTGGGTACCTGTATC

GGATCTTACCATCGTCAGAGACTGCCTTAACGAAGGATCGCGTCTCTGCGGTAGCTCGA
TACCATCTTTCCTCAATGTCATCTACACGAACGAAAACCCGCCGACGTTTAACAGGACG
AACAAGTTCACCAGAGGTTTTCAAAACTTGATCGACGCGTATGGCGTGGCGTCGTACC
GCGAGGCTAATCCAGCCCTTTATACGATTATCACCTTCCCCTTTCTATTCAGCATCATGTT
CGGCGATTTTGGACATGGTATAATTATGACTCTGTTCGCTCTGTTTATGATCGTGAAGGA
GAAGAAGTTTATGGCTGAAAAAACAACGAACGAAATTTGGAATATATTTTTCGCCGGC
CGTTACATTATTCTTCTCATGGGTTTGTTTTCCATTTACACTGGCATCATCTATAATGACG
TATTCTCAAGATCAATAAACATATTCGGGTCTAGTTGGGACATACGGTTCGACAATAAA
ACAATCATGATTAATGAGCTTATGGAATTGGACCCGGCTAAAAATGATTACAAACAATA
CCCTTATCCGTTGGGTATGGATCCAGTTTGGGTGCTTGCCGAGAACAAGATCATATTCTT
GAATTCGTACAAAATGAAGCTGTCCATCATCTTCGGCGTCGTCCATATGATATTCGGCGT
GTTCATGAGTACCATTAATATTATACATTTCAAGAAATACTCGAGCCTTTTCTTAGAATTC
TTGCCACAACTGCTTTTCCTTGTTGTATTATTTCTCTACCTGGTAGTCTTAATGTTTGTTA
AATGGGTTTTATATAGCCCCACCTCGCCAGACATGGCGTATACTCCTGGCTGTGCACCAT
CGATATTGATCACGTTTATTAATATGATACTGCGGGGTCACAGCCAAGTGCGCGAAGGT
TGTTCAGAGTACATGTTTCCGGGCCAGACCACTATTCAGCTTGCTTGCGTCATAATCGC
AGCCCTATGCGTGCCCGTCATGCTGTTTGGGAAACCGCTTTTTTTCTTATTGCACAAAA
AGAACGCACAGCCGGGAAAAGTCCTGAGCGATGGGATCGCGTCCCAAGATATTGAATT
GCAAGCTAAAGGGTTGCAAAACAACCCGTCGACCAGCGACGCAACCGATGAACACGA
GGACGAGTCGTTCGGTGAAGTAATGATACACCAGGCGATTCACACTATAGAGTACGTTC
TCTCAACGATATCACACACTGCCTCTTACCTACGTTTGTGGGCTTTGTCGTTGGCCCAC
GGACAACTTTCCGAAGTTTTGTGGTCGATGGTGCTGCGCAAGGGTTTACTCGCTGCCG
AGGGTAATTACGTGTCGGCCATCATGTTATTCTTCGTGTTCGCCGCATGGGCCTCTTTCA
CCGTTGCCATTCTTGTCATGATGGAAGGCCTGTCTGCCTTCCTTCACACACTTCGACTT
CACTG

>novel_circ_001038
CGATACGTGACCTCACTTTTAACGCCGATATACAACAAGCTTGGATTTAAGGATAAATC
GAAAGATGATCATGTCACGTTGTTGTTCAGATCGCACGTTCGCAAATGGGCGTGCAAA
TTCAACGTAACGGATTGCAAGGAGCAAGCTTTATCTCACTTCGATGCTTCAAACAACG
GTGCAACACTTGAAGCTAATATTCGTAGCGTATCTTACTGCACGGTCGCCGAACAGAAT
GACAGGCAATTGTGGAATCGTTTATGGGAACTTTACACACAATCGACATTTTCAGCGGT
AAAATCAGTAATTCTTCAATCTCTACCCTGCGCCACCGAAGATGTCCTCCTCAAAGATC
TGCTCCAAAAGGCGATTACGAAGGATTCGGGAATACGATTCGAAGATCGTTCGAGCGT
GTTCACTTCCGTTATCAATTCGAGCCCGGAAGGGGTTGAAAGCGTGATAGATTTCGTTC
GAGAAAATTATGACAAAATTTACGAGCAAGTCTCCAACGTGAACGGTATAGTGAATGC
AATTGGAAAGAAGGTGTTCACAAACGAGCTTTACGCAAAG

>novel_circ_001039
GTATTCCGTCAAGCCATTTTGGAAATTTTGAAGCGTCAAAATGTGCGACATCATTCGAA
TAACCGCTCCAGCTGTAAAGAATATTACGAATTGTTGACGATTTCTAGTATTTAGTTTTT
AATCAAAGCTTCTGGATGAAGCAATTATTAATTTTGTCGTACACGTTTTTATCGAATTTG
TTTTTGTTTATGAATATATATTAACATTAGAATTGGCAATTTCTAAATATATGCATGAATAT
TTATATACGAGTAGCATATTATTTTTTCCAAAAATATCTGTATCCTTTTTTATAATAGAACG

AAAAATTCGTCATTTCGACTGTTTCACGGTTAAATATCTTTTTTAACCTTTAAAAGAAAA
ATTGATTATTGATCGTTGCACAGAGAATTTGGATTTTTATAAAATATAAAAATCTGAATGA
TAAAAAAATTGTAATAACAAATTATAACTATAAGCATTTTCTTCAACAACTATGGAAGTAC
GGAATTTTTAAAATGCAAGAAATACGCGAAATACATTTTAAATTTTCATTATTGACTCTT
TCTTCGATTTTTTCCTATAAAAATTATTTACTTATCGCAATAACTTAAAGTTCTTTTCCTT
TCTAAATCTTGAAAATTTTACTTTTCAAAGTTTTATTCCAAAGCAAAATCATCGAGACA
GAAATTAGTCTCCTTTTATCAATTATTATCGTGCCTGTAACGTGTTTAAGTAGTCCACTTA
TTACATTGGCATAAAATTCTTTTCATCTTTATCATTTCTTATTAATAATAACGTTATTAAAC
ATTTTACGTAACGCAATAAAATCTACTTTCATAAATCTCGAATTAAGAAAGATTAAATTA
TTATCTTTCGAATCACTTCTAACTAAATTTTTCATTTATCAAACGTTCTTTTAATTTATAAT
TATCGATAGATATTATTTAAGTCGTTCGTCTTTTTCATTCCAAATTATTAAATTATTAAAAA
TATATATTTAAAATGATTAAAAGAGAAAGATTAAAAGAAAAAGAGAAAGACACGATCG
TGATATTTTTTTCTATCGATTCAACGATTCTATCCTCTGTTATTCTTCTAGAATAATAAATA
GATCTATGAAACAATAACTCTCGACTTTTCGTCGTCTGATAATATTTAGTTTCAAGATTA
AATGTATTAACCGACTGATAAGATATGTTACATATTATCTCGTAACGTTGATCAATAAATA
TAAATTATTGGAAATTAATAACGTGTTTGTTCACATATATATTTTTTTCTTCTTTTTTTTAT
CTGATGCTAAAACCAATGACTCGTACCTTTGTCTCAATATCTCTCGTGATTATAACGTATA
TACGTAGAAATATTCTTTTTTTATTTTCGAAAATAAAAAAATCTTTTTCAAAGACCTCGA
CAATTTTCTCCTTCCTTACTTAATGAAAATTTCAAGTATTTTTGAAAACTGTTTTTGCAT
GTGCATCCATTAAAATAAAATGTAACGAAATGATGATGACAAGGATGAAGAATAAAATA
AGAGCAATCAAAAAGTTTGATTTCATTGGTTCGAGATAAAATTCCAATATTGATCTCGAT
CGACTTACGAGCTAAACGCGTAGCAAGTACTTCCCTGCTGATTAATTTTTACCCGTCGA
AAATAAATTTTAATCACTCTTATAAAATTATTACGTATCGTTTCATCACTCACACTTCTGA
TACGCGATATTGTCGAACAATTTGCTGATTTCTGCGGGCGTGGTCGCGTCCTGGTTCAT
GGGTCGTGTGTTTAAACCCGCATCCGTAACGAAAGCGACCCCCTGC

>novel_circ_001042
ACGGAAATGTGAGGCCGACGTAAAGGAGGCGAAGGCGGAGCTGGCGCAACGCAATAT
GTTCCCTAGCGCTCAAATTAAAATGAGTATCTTTGTCAGATTGCTGTCGCCAAGCAGTT
CGCCGGCCACGTCGCCCACAATGTCCAATTCTTCGTCACCGACGCCACCGACTCCGGC
CGCAGCATCCTACAACCAGAAGATGACCGGGATACCATGCGTTGCGGCCGCTTCCAGA
TACACGGCTCCGGTCCACATCGACGTAGGCGGAACGATATACACCTCCTCCCTCGAAA
CTCTGACCAA

>novel_circ_001043
GGTACAAATTAAATATAAGATTTGAAGCCATAAGTGTAGTGTGTGATATTGGTAAACAAT
TTGAAACAATTTTGAACTATATTTATAGTTCAAATATAAAATGTCTTGATATTAATGCTTT
AAATATTTAAAAATTACTAAAAAAATATATGTCAAATGTTATATTGGGATAATACAAAATA
TACCACATATATATAAAGTGACAGAATTGTTTTATATATTCAATTTTTAATGTTATGTCATG
GAAAAATGTAGTTCAATTGTATTTATACATGGATGTAATTTTGCAACAGATGTGCTTTAA
GGATAAGTCAAAATACTTTCAAAATGCAATTGTTCAATTATTCTTTAATGGATAATGTAG
TTCGAAAAAGAACTACCTATTATATACAAGAAATCAATATTCAAACATGGTTAATATCAG
AGTGTGATATTGAATATAAACATCAACATCCCAAATAGATTTATTTCAAAAAGATTAAAA
GATACCTAAATAACAGATGAAATAAGAAGTTACATGCATGACAGGAATAGGAGACGTA

GGTCGCAGACGCGTTGAATGGCGCCGTAGCGCAAATACAATCTCCTATCATCATTAAGT
TATAATTCATGAGTGGAGTATATAGAAGCGAATATAGATAAACTGAAAAGGTGGCTAGA
CGCTGCCCAGAGTAAAGAGGGGCTGCGTGGGCATGCAGCAGCCTCAGTCACGCCCTC
TTCTCGGGGACTCAGTATCCTCTACAACTTCCCCGACTGCTCGGCGTAATAATCCTGTT
GCTGTTTTGACACATCAACAATTACAACAGTTACAACAACTGCAGGCTCAAAATTCGA
GTGGTCAATCATTAACATTTGCTTTACAACAAACATCAAATAATCCACAAGACCAAGGA
AATGGGTCAACGACTGGAAATCACATAGTATATGTAAATCAATTAAACCATTTAAATCA
AGGTACCATAAATCCCTCTGGGACTGGTATGGGCCTACCCCCGGCCACCCCCACTTTCG
GGGTGGGCCTTAATATGGGATTGCCACTATCACTTTTAAATACCAGCCTTACATCCCTTA
CATCTAATGTTATCACTACATCAAATCCTCTGCTCAATTTGGGCCTGCCAAATATAAATA
CAGGACTAACTACAATAAATCCTAGCCTTAATCACTTAAATACCATAAATTCAAATTTAA
CTTCAAATATTGGTTCAAACAGTATTAATAATGGTTTATCTGGTTTGGGACAGGATATAA
ATAATATAAATACTGGAATTAATAGACTCAATACATTAAATTCAACAAATATAAATTCTGG
ATTACCCAGTGTAAATACTGGAATTACCAATGTAAATCAGAATTTGACAAGTTTAAATTC
AAATATAAACCAGAATGTTACTAGTTTAAGCACAAACTTTACATCTCCAGGTTCTGGAG
TATCTGGTTTAACTACTATTAATCCAAATATAAATACAAATCTAACGACTACCAGTATAAA
TCAAGGTCTTAATCGATCTATTAATGCTTTAACAACTGGGTTGACTCAAACTAGTCTGAA
TTCCAGTAGTACACAATTTCCATTCCAAACCATACAAAGTATACAAAGCATTGCACCAC
ATATTGTTGGATCAAATATTGCACATGTACCAAGTTCTGCAATATCGCAACATCCAAATT
CAAACGTTTCTACCATTTCACAAATATCAAATACTGGTACTTTGACAAGTTCCAGTATAT
TTACAAGTACTTCTAGTGAACCAATTCATTCAACTACAACAAATGTGAATCACACTTCA
AACGTGAATCTTACTACAAATATTTCACATCTTGGTACCATGCACTCAAATTTATCTAATA
TATCAACATCTGTGCAGCATATATCTGCATCAAATGAACCGAATATGTCACTGAGTCATA
CTTCTTCTCTAAATTCTTCTCAATCAACTGGATTTCAACCTCAGCGACAGTATTCACAG
GGAATTAACCCTGGTTTAAGTTCATCAGTAACATTAACGGGCTCGTCTCAGCCATCAAA
TGTGGTCAGTACAATAAATACTGTAAAATCTATGCAAAATATTCAAAGTCAAAATATTAG
TTCTCCAAGCAGTCCATTTTCAATACCATTAAAAAGTCCAGCATCTAATATTGCACCACC
TACACCAAGTCCTAGTCCTAACAGACTATTACTTAGAAGTCCAGCATCAAATTCAATAC
AATCTAGAAATAGTCCAAGTCCTGTTGGAACATCAACATCAAACAATAATTTTAATATAC
AAATGCAAAGTCCAATGCAGAGTCCAATGAGTGTTAGTCAAATACAGAGTCCTGTACT
TAGTCCATATCCTCCTGCAAAAAGCCCTCATCTGTTAAGTGGAAATAACTCTCTGAACA
ATAGAAGTCCAGCACCTGGTGGGAGTCCTGGTCCACCTGTGGTTAGACCTAATACACC
AATACTTCAACAAGGTATGCAAGTATTGCAAATAATTCATGGTACACCACAGGGTTATC
AATCAACGCCAACACAATTAGTAACTAGAACGCATTTAATTGGAAATCAACAAATTCAA
ATAGCTGCAACTAAACCTGCAAAACAGCCACCACAAATTTTACCAAAACCTCCAAATC
AACAAACTGCTGGATCACAACAAAAACAGCAACGTGTTACTACTACAATTACAAATCA
AGTAACACAACAAACTCAACCACAATTGGTTCTTGCTGGACCACAACCAAATCCTACT
ACAGCTACAATGATACCAACAGCACAAGGTTTATTATTAAATCAG

>novel_circ_001044
GTGCTAAGTGAAGAAAGAGTTACTGGAAATGTAACAACAGAATTGCATACAAGTGGTG
ATACACAAGGTCGTATTAGTTATGAAAATCGTGGAGAATGTTTTTTTCTGCCATATACAA
AAGATGATGTTGAAGGAAATGTTACTTTGCGTGCTG

>novel_circ_001045
ACGGCATTCCTGACTGTGGCTCTCCTACCATTCAGACTAGCTGCAATTACGGCTCTGGT
GATCATGGCCTGGCTTTTGGCTTGTCTAGGCCTCCTTGGATTGTCCGAGGAAGATCTTC
GACGCGCACCTTTGACGGGGTGGAGACGAGACATGCGAATCGTGATCTGCTGGATGAT
GCGAGCATTGTTCATCTGCGGTGGATTTCATCACCTGAAGGTCAAAGGTCGTAGAGCA
GAATCAAAGGATGCCCCCGTGTTAGCCCTAGCCCCGCATTCAAGCTTCTTCGACGCACT
TCCGGTTGTTTACCTCGGCGGACCGAGCATCGTCGCCAAAGCGGAAATCGGGCGCATT
CCTTTTTTCGGAA

>novel_circ_001046
GCGGATCTGCTTGGCCATTCTCTTTATGCGTTCATTCATCCCAAAGATGAAGAGGAGCT
TGCGAGGAACCTCAACCCGGATGAAATGCAAGGCGTGGTATCCTCGTTGCCACAGATC
ACCGACGGAACGAACGACAATTCCAATTCATCGGAGGATTCAACGTCTTCGAAGAACG
GGAAAACGTTCCAGAATCAGAGGCGGACTTTCGAACTTCGAATGTTGCATCGTACTTC
GTCGAGGCGGGAGCATACACAGTACGAATGGTTCGAGTTCTCTGGAATACTCAGGCTT
GCGGACGCTTGCAAAACTTCTACGTCGAATGTTAATCGATCGAGACATCGAGAAATTAC
TTCAACCAGTAACGACATCGTTTTCGTGGGTGTAGCACAATTATTGATGAAGCAGCCAC
TCACGAAAATTTCGATAATAGATGCTAATAAGAACGAGTATATCACTCGACATTTGGTAG
ATGGCAGGATCATTTATTGTGATCACAGGGTGTCAGTTGTGGCTGGATATTTGTCGGAA
GAAGTATCGGGTATGAGTGCTTTTGGTTTCATGCATAAAGATGATCGTATTTGGGCGATG
GTTGCACTTCGTCAAATGTACGATCGCGCGGAAACTTGTGGATCATCTTGTTATAGATTA
ACGTCAAAAACAGGAGAACCCATTTATCTACGTACTCATGGATATTTAGAAGTAGATAA
GGATACGCAAATTGCAGTATCCCTTGTGTGCATAAATACATTAGTATCGGAAGAGGAAG
GAATACAACTTATGCAACAGATGAAGAAAAGATTCTCGGCTACGATTTCTGAAACCATG
AGAGCCATAATACAAAATGGAGATGATGCATCGATTGATTTGGGCTCAGATTCTCAAAA
TCCAAATTCGAAAAGTAATATGGAGGATCCCGCACAATTAGAGGATGCCATCACATATC
TAGTTAGTGATCTGTCATCACCTCTTCCAGAAGATTGTCTATCACCTCCTACGCAAAATG
AACAGTATGCGAAAGCTGCTATGATATCACAACATTTACCTCCTGCTGAAGCCCAAGCT
CGAAGACTTGGAATTAAGAAAATCGATCGTTATTTAATGGTGCAAGCCAAGGCAACAA
ATAATCAGAAATCAGAATCAAAGACAAATAATAATAAGAATAACTCTAATGAGAGACAA
GAAAACACGAGATCGTCGAGAAAAACTGTACATGAAGTTACAAAACAAACGCGTAAT
AGTACACAAGATGAACAGAATTCATCGCGAATTAATAACATGTCTAATGTAGAGTCATG
CGTAAATACCCAACGAAAACCTGGAATATCTCAACTTGAGCTTCATCAAAATGTGGATA
TTTTGAATCCTAATACTACAGTTAAGAAACCTGTTGCAACAGTAGAAAATAATCCATGT
AACGTTAAAATTGAACGTAACATGGATGTTTATACGCCCGAGACAATGATTGAATACTT
TGAAAAGAATGCAATTGATAATAATTCTGATTTAAAGAGTCATCCTTATCCATTGAAGAG
GATATATAGTGACGAAGATATTAAGACAGGTTGCACTAAAAAAAGACAGTATAATAATG
TTCCTTATGCGTCATCGGATACAAGCGATGAGCAGAATATCTCTTACATTGATTGTAAGC
CTTTCGTCGATGAAGAATTTTCAGAACTTTCAACGGATTTCAACAATATAAATCCTCAAT
CACTAAAGATTGCTGAATCGCCAAATTCAAGTCTAAGTGAAGTCATAACTGATTATCAG
CAAGTGGATTCTAGTATACCACTTATAGCTCCAAATCCTGAATTGGAAGCGGAATATGG
CGATCTTCAGGACAATGCATTGCTGAGCCCAGGCCTGGAAGCAAATCCAGATCTGATA
ATGATGAAAATATTTGATGATCTAAGGCATATACCAATTCTTGAAAATACTTTTAATGAA
ACAGAAGTACAATTAACAGACAATATGGTAATCAATAAAGAAATAAGGAAAAAACATC

TTCAACTTATGAATAATATAGCACGCCAAGAATCACAATTGAATGTTTGGCACGAGAC
TTCAAAAATCCAGCTTTGCAAGCGAAAAGAGGAAGTTTAACACCATTAGAG

>novel_circ_001047
CAATCCAAGGGCGTCACGGAACATGGCGGAGAAACAACGCCGAGACAATCTTAACAC
GAACATCTCGGCGATGGCCGCGCTCGTACCGACCGTCGCCGAAAGCCCGAGGAAGAT
GGACAAGATATCTATCCTGAGATTGGCTGCTAATTTCCTCAGAATACATTACACCGTTGG
ACGTGGCGTGTCCGACTTTCTGCCTCGAGAACTCGGCGATCTGGATTTGGAACAGTAC
ATAGGCGATAATTTGATCAAAAACGGGAGCTTCTTTATCGTTGTCACGACCACCGGGAA
AATAGTATACGTCAGTCGGCAAGTTCAAGAGCATCTTGGTCATACTCAG

>novel_circ_001048
TAATCGGTTCTACTAGCGAAGCATTCAATAATCGAAAAACCTCGGACATCCCTCAGTTC
TTGTTATTCATCGAGCTTCGGCTCCAATCTGGCCGATAACGAAGGATGGTAAGCTCCCG
TTCGCCTGGTGTAAATGTTTCACCAACTAATAGCGTGGCTACATCACGTGTCGTCGTGT
CTGACAGTGACGTAGCTCGTGATGGTGACGCCATCTTACCGAGCACCCGCCAATCATAT
CGTGTGGAGAACGAGGGTGGTCATGTAAACGGAGCTCCTGGTGCTGTCGAATGGAGC
CAAAGTGATCCGCCTTCGCTTTCTATGTCGGTGGACTTGCATAATCTGGTCAATCCGAA
TCTTCAAGACTCTGGGCATTATTCCGCTAGTCCCGAACACGTTGATGCCATCGCGTCAT
CTTGTAACATTAAATCTACGAATAATCACGAAGAAGAGGAAAGCTACGAAGCATTTGG
ATCTGTCGAAGGTGACGCTGATGACGGGGTGGAATCACCGGGTAGCAGTTCTGCACCT
TCACCCACAAGTACTAGTTCTTTCAAAAATCCAAGAAGTCCCAATTCAACTATAGGAA
GACATTCTTGTCTACGGACATCACTTAGGCGAACACCGCCTTGTTCCGGAAGGAAGAG
ACTTAGTTCCAATGCATTAGCAAGTCAACTTTATCGAAGCGGGAGCTTTAATAGTTCTG
GTATAAGCTCCAACTGTGATCCCGCGGACGATATGTACAGCGATGTCTCTCTCGAGGAT
GATGTCATTGATCTCAATCATAAA

>novel_circ_001051
GTATATCAATCTGCAAGTGGATACGTACCTACTACAACTTCTCAATATCAAGCACAATCT
GTAGCCACAAATACTGTATCAAATAGTAATACAGGCTATCAAAGTGGATATCAAGGATC
ATCTTCATTTCAATCAACTCCACAAGCTTACCAACCATCTGCTACTACATTTACTTCACC
TATAACTCAAACATCTGGAGCTTACCAAAGTGCAGCACAATCT

>novel_circ_001053
GTGAAAAAGGAATTCATAGCGATGCTACGTGAACACAAAGACATCGACAGGCATTCGC
ATTGGAGCGATTGTAAGAAAAAATTGGAATCTGATTGGAGATACAGAGTCGTAGAATC
GGCAAGCACGAGAGAAGATTGGTTTAGGGATTACATTCGTATGCTAAAGGAGGAGAGG
AAAAAGGAGAAGGAGAAAGACAAAGACCACCGGCACAGGGAAAAAGATCATCATAA
ATCGGAGAAGAAGGATAGGGACCGTAAGGATGTTGATAAGTATAAGGAAAAATCGTCA
AAGGATCGGGTTGACAAGGACAGTTCAAAGGACAAAAAACGCAGGAGCGAAGTACC
TTCGGAGGAAAATGGTAAAGAAAAGAAGGATGCGGTATCGGAGAAAGAAAGCGGAG
AAATTGAGGATAATGATGAAAAACCGTCGAAAAAAGAAAATGACAAGGAGAATGCGG
AGGATCAATCTGATTCCGAGGAAGATCGTGAGAAACAGAAACGAGAGCGTGAAAGAA
GGGCGGAAGCGAGCCTTCGTGAAAGAGAAAGAGAAGTTCAAAGAACTCTTGCTACAC

ATCTTCGCGACAGAGACAAGGAAAGGCAACATCATCGTCACACAGAGGCTGTACAGC
ATTTCAGTGCTCTTCTTGCTGATTTAGTGAGAAATGGTGATTTAGCTTGGAGAGAAGCT
AAACGACAATTAAGGAAGGATCATAGATGGGAATTAGCAGAAAGCTTGGATCGTGAGG
AGAAAGAAAGATTATTTAATGAACATATAGAACAGCTTAGTCGCAAAAAACGTGATAA
ATTCCGAGAACTTCTCGATGAAGTAGGAGCTTCTACTGAATTAACTGCATCGTGGCGAG
ATATAAAAAAATTACTGAAAGACGATCCTAGATATCTTAAATTTTCATCCAGTGATCGAA
AATGTGAAAAAGAATTTAAAGAGTATATTAAAGATAAACTTGTCGCAGCTAAAGCCGAT
TTTAGAGAACTTCTACAAGAAACAAAACTTATTACTGATAAGACATACAAAAAAGTAC
AAGAAAATAGCGGACATTTAGCAGAAATTGAAGAAATTTTGAGGAAGGATAGAAGATT
TCTTGTATTGGAAGCAGCTGCTGCTGAACGAACACGTTTATTAATGGGATATTTAGAAG
AATTGGCACGTAGGGGTCCACCTCCACCGCCTACTGCCTCCGAACCTTCAAGAAGACC
AACCACAAACTAAACGAGGCCCGCATCAAAGCATGGTATATATATGGTGTCCAAATAAC
AAATCAGTGATTGAATTTATAGCTGTAGTTGAATTGAAGTACTTTACACACGAGATGATT
ATCGCCTGTAATTATAGGATTCAATAAAACTAACGCCAATTCCACACAGCTAAAAAAA
TGTGTGTCATACTGTAAAGATGCCATAGTGTAAGCATATCATTATTTGTTGGATTACATTA
AAATAGATACTAGTTAACGAAAGAATTATTATAGTAATATCATAAAGTGCGAATTTTATAC
ATATTTTATCCAGGTTTGTTACATAAGCACCCCATTATGAATCTTTTGTATGAAAATTACA
TGAGAATTGCAACGAGTGTTTGTCGCACACTCTCAACTAGTTTAAAAGCATGCGCAGT
GAATTTCTATAAACTGATGTTAAAAATATAATAGTCCAAAAAAAATTTTAATCCTTATGTA
TCTAAAACAAACAAAAAAAAAAAAATGTATAACGTACGACAATCATGCACCATAATTTTT
TGACGGAGTATATATAGTTCGAAAATAATTTTCAATTTTCGAATTATTTTTCTCATACAGC
TTTATTGGATCATGAAACGTAAGTATCCTATAAAACATTGTCATAAAAAATTTTGATTCTA
AAGAATTCGTAAAAAAAAAAAAAAAAAAAATTATCACGCATACAAAAAATTCTATTGTTT
ATAGTAACAATTATTTACTAATTTATTCATGTGGCATTTTTACGGTACGAAATTCATAAAA
CGTATATTAGGAAAATATATTTTCTTCAATAAATCATTCATAAAAATTTGTGAAAAAAAA
AACAAATTTTACACATCTCCCAACAATTTATTATTTTCGTTTTTTTATTTCTAATGTTTTAT
AATATTTCATTACAGACTTGCGTAAACAATTAAGTGTAAAGTAGTACAAATAATTGAAG
ATAAATCATAAAAATTATCAAAATATTAATTTGTAAATTTTATTTTATATCGAAGAATATTG
AAATCGTTTATTTTTATCCGAAAAATATTAATTAATTTTAGTACGATTATGTAATATTATAA
TGTTACAATATATTCAATGTATATAAAATAAATACTATAAAATTTATGCGCATTGTAACATT
ATAAAAAAAAAAAAGAAAGAAAGAAAGAAAGAAAAAAAGAAAGAAAACAATTTTGC
TTTCTCAATCATATACTTATTCATTGCATGATTTTGTTTCAAATATTGGAGAACAATAATA
ATTATTTATTACATATCATTTGATCAAATGAATATAAATAACGACTTATATACTATCTCTAAT
AAACAGCTATATATTTACATATTCTTTCATTCTTACAAGCTATGTCAGTCATTGAAATATA
CGCGGTGATTTCAACGCCTGGAACGGAGATTTCAACGCTTGCATCTATCTAAGAAAG

>novel_circ_001054
GTGGTGCCACCACCTGGTGCCGCGGCCAGCTCCCACGGCCGCTCGTTGTCGACACGC
AGACGAACTCTCGAGTGCACCTCCTCCCCCGGGCTGACCGTGCACCCGGCCGCGGGA
AGTGGATCTGGCACTACGTCGCCCCGCAGGGTCGGCTCTTTCCGAAGGAGCACCGGTT
CCGCCGATCGGCCCCCGTTGATGTTCTGCCGAAGGAGGCCCTCCTGGCCGGAAGTCGA
CATACAAGCCACCTCCGG

>novel_circ_001055

GCGAAGGGGCGAAGAAGCGTGGACGGAAACGGGACGAGGACACGCGGCGGCGAGG
AGAGGAGGGACAGGGCGATGACTTAAGTCACTCCCTGTTTGACCGAGGAGGAGGAAG
GAAGGAAGGAAGGAAGCGGCGCCCCGCGAATGGGGGGAGAATCGATACGACCGTGA
CGCGTAGAGGAAAAGAATCGCGAGGAAAGTTCGAGCTGTTTTTCTCTCTCTCTCTCC
TCCCCGTCGCTTCGAGATCGATGCGTGCGAACAAAACCGGGCCTCGTAACAAATCCCA
TCCTCGTCGGGAACGCGAGGCGCCCCCGAAAACGATTGGCCCGATCATCGTTTCAGCG
TGAGAGATAGAGAGAGACATAGACGGATAATCGACGAGATGAGCTTCTTCAACTCGTT
GCAACAGTACGTGACGGACAGCGTGGCTAGCTTGAGCCTGAGCCCGAAGAGGTTCTC
GTTCAGCCGCGAGGATTCCGGGTCAAGCGCGACGGGAAGGAGCGGCTCGACGGGGA
GCGTGACGAGCACCACCAACACCAATTCGACGGCGCAACAGTCCACCCCTCATGGGTA
TCCAAAGGTGGTGCCACCACCTGGTGCCGCGGCCAGCTCCCACGGCCGCTCGTTGTCG
ACACGCAGACGAACTCTCGAGTGCACCTCCTCCCCCGGGCTGACCGTGCACCCGGCC
GCGGGAAGTGGATCTGGCACTACGTCGCCCCGCAGGGTCGGCTCTTTCCGAAGGAGC
ACCGGTTCCGCCGATCGGCCCCCGTTGATGTTCTGCCGAAGGAGGCCCTCCTGGCCGG
AAGTCGACATACAAGCCACCTCCGG

>novel_circ_001056
AGAACAGTTTCGTGGAGGAGAGGTCTCGACTGAACTTGAACGGGAGCCGTCAGCTCT
CGAAAAGCGCCACGGAGCTCCGGAACAACGACAGCGGTTCGCCGTCACGACGCGGT
GAACAAGTCGAGTCGGGTGTCGTCCATCACCATCGCCATCACCGACACGCCACTTCGG
TGGCGCAGCGTACGCACATCTTCTTCGCTACCTTGAAAAGCCGGTGGGGTCGCAACAG
GAGCAAGGAACGGAAAAAGTCGAAGGATGGCGGGAGCGTCTTACCGCATGGCGGGAT
CGAGTCCGATTACGCGGCCGATTACTCGTCCGAGCACAGCAAAAGCTCGTCGGCAACG
CAGAGCCCAGCCAGGCACTGTCTCAATCATCCGGAATCACCGTTGGTATGCGGAGGAA
GGCAGAATGTCGTGACAAAAGCGGAAGACAGTCCTGGAAAATACAGCGAGGCGCACT
CGAAAGGATCGTTGAGCTTCCAGCCGTCGAAAGATTCGAACGAGGAAGCTCGAGGAT
CGATCAGCCAGGAAGATAGCGCATTTCTCCAGGAGGAACTCGCACGTAGAAGGGAATT
GGCGTTGAGGCAGCATGCCTTCTTTCAGCTTCGTTTGCACATCAGAAGAGGGATGAAT
CTCGTCGCTATGGATAGATGCGGCGCCAGTGATCCTTACGTGAAAGTGAAAAGCGGAG
GTCGATTGTTGCACAAGTCGCGGACCGTTCATCGCGACCTAAATCCAGTTTGGGACGA
AAGTGTGACCCTGCCTATAGAGGATCCTTTTCAGCCTCTTACGTTCAAGCTGGCAAAAG
ACCTTGGTCGTTTCGACAGGTCTTCGACTATGACTGGGGTCTGCAGGATGACTTCATGG
GGGCGGCTCAATTCGACCTCGCGCAGCTCGATCTGGGCCAGCCGCAAGATATCGTGTT
GGAGTTGAAGGATCACAATAGGCCGAAACAACACCTAGGCGAAATTTATCTGACCGCC
ACGCTTTGGCCGAAAAATCAACAGGAGAAAGAGCAG

>novel_circ_001057
TCTATTAGTGAACTCGTTAGAAGTTCTTTCTAACAGGCTGATGTATTAGACATCGTGATA
GTGAGAGATGATTAGTGAGAATTATTTGAGAATTGTATTTGGTGTGCATCCCTCTAGTG
GCGAATATAGGAGGATCACCACACATTAATACGCGACCGCACCCGTCTCTATGTGTGGT
GATTCATAGATATAATATTTATCGAATTTTACGCCAAAACTAAATGCAAAATGACAGAGG
TTCACAATTTAGGCGTTGATGCTATTAGTTGTCATGCTTGGAATAAAGATAGAAAAGAA
GTGGCAATTTGTCCAAATAATAATGAAATTCAAGTTTATAAACGAACGTCGAGCGGTTG
GAAATTGTTACAAAACTTGCAAGAACATGATATGCATGTCATGGGTATTGATTGGGCAC

CAAATACCAATCGAATAGTAACATGTTCTGCGGATAAAAATGCATATGTTTGGACACAA
GAAGGAGATGGAAAATGGAATCCGGCATGGGTTCTTTTAAGAATAAATCGAGCAGCAA
CTTGTGTAAAATGGTCTCCATTAGAAAATAAATTTGCCGTTGGATCTGGAGGTAGAGTA
ATAGCTGTTTGTTATTTCGTATCTGAGAATAATTGGTGGCAGTGCAAGCATATAAAACGC
CCATTGAGATCCACCGTAACTACAGTTGATTGGCATCCAGACAATAAAGTTCTAGTTGC
TGGATCCACTGATTATAAAGTTCGTATATTTAGTGCATTCATTAGTGATATGGAAGATGCA
CCTGGTAATGGTCCTTGGGGTCATAGCAATACTTTAGGAACGTTACTCGCTGAATTCCA
AAATACTCCTAATGGAGGAGGTTGGATACACTCTGTTGCATTTAGTCCTTGTGGTAATA
AAATTTGTTGGGTAGCACACAATTCATCGATTTGCATTGCAGATGCTACTAAAGGAAAT
GCAGTTATACGATTGTATACGGAGCATTTACCATTTTTAAGTTGTGTATGGATGGGGTCC
AATTCCATTGTTGCTGTGGGACATAGTTGTATGCCTATGTTATATTCCATAGACGACCATG
GTCAACTATATTTTGTATCAAAACTAGATAATACACAAAAAAAAGAAGCTGCCGGTTTG
TCTGCCATGCGTAAATTTCAATCGTTAGATCGCCAAGCGAGAACTGATACGAATGACAA
TGCCTTGGATAGTATTCATCAAAACACGATTAATTGTGTACGCAAAGTATCAGATAACG
AATTTAGCACGAGTGGTTTAGACGGTCAATTGGTAGTTTGGGATCTTAAGCTTCTGGAA
AATTCCATTGCTGGTCTCAAGATATCATAGATAAAAATCTTTTTTTTATAACGAAGGAA
GACGAAGGAATCAAAGGAAGATATTACATCCGAAGAAAAGATGTATAAAT

>novel_circ_001058
AAGTGGCAATTTGTCCAAATAATAATGAAATTCAAGTTTATAAACGAACGTCGAGCGGT
TGGAAATTGTTACAAAACTTGCAAGAACATGATATGCATGTCATGGGTATTGATTGGGC
ACCAAATACCAATCGAATAGTAACATGTTCTGCGGATAAAAATGCATATGTTTGGACAC
AAGAAGGAGATGGAAAATGGAATCCGGCATGGGTTCTTTTAAGAATAAATCGAGCAGC
AACTTGTGTAAAATGGTCTCCATTAG

>novel_circ_001059
GAGTCAATCGAAGGCCTCTTATCTGGCATATCGGCGAGAACGAGGTGCGCCTGATACC
GAAGGTAGTTACAAGAGAACCATGTCACCGACATCTCGCCTAGAGGATTGGCCACCGC
CACGGGATCACGATGATCCGGTTCTTCTTCGCGTCACGCCTCATCATCCGTTGCAGCAT
CATCACCATCACCATCATAACAATCACCATCACCAACAACAACCGGAGCTTTCCAAATC
GCACAGCGTGGACGCTCTTCATCACCGGTTAGAAGAACGATCGGCGCAACAGCAACA
GCAGCAGCAGCAACAACAACAACAACAACAACAACAGCAACAACAACAACAACAAC
AACAAAATTCGGCTGAGATGATTGGAAAGTTGTCGCACGATTTGAACAAGAAATTGCA
GTTGAATGATGCGAGATCACGAACCAGTGAGAATAATAATAACGATAATTTGGAGGACC
GACATCAGCATCATCACCATCATCATCATCATCATCATCATTATCATCAAGAGAGGAGAC
ACGATTATGAACAACGTAAGGAACGACAGTCCCCTCAGAGTATCGAAGTTCTTAACGA
TAGAAATCGACAGTTGGAACGCGAACGCAGGAAGTTGGCAGCTAGTTGCGAGCCTTTA
TCTCAGAACAGAGAGAAACAGAATCGGAGTATGGAAATACCTTCTTCGGTGGTCGAAG
AAAATTTGTTTCGCGAACGGAGCGCCACCATTGAGATGACCTCACAGAACATCGAACT
TTTGAATCGACGTAACGAAAAGAGAACAAACGTTACATCGACAACGACCAGCACGAT
CACCACTTGTATCACTACATCGTCAACAACTACTGCAATGACGATCACAACTGATAGTT
GTTGGCCACGCAGCCTCGAAGTGCAGAGTTCCATCGAAGCGTCACCTGTTTCGGAAA
AGCCTACACTACCGACCAGTTCTCCTCCAAGATCACCGGATCAAGTCGAAGGAGATAG
TTCAAGAGGAGCAGTACCGAGACGATCCGGAAGCTGTTCCAGTTCTTCCTCCTCCTCT

TCTTCCTCCTCTTCCTCTTCCTCCTCCTCCTCTTCTTCCTCTTCCTCCTCCTCATCCGGTA
AATCCTCGGATCTTCATATTTCACCACGAAACTTGTCCAATAGTTTCTCCAAAAACGAG
AATTCTTTCCTTAACAATAGAAAAGTCTCGAGCCCCACTCAAACGGACAACACTGGTT
CTTCCAATAGATCCAGTTCACCTGTTCGCTCGAATCAAACGAAAGAGATAGAATTGCG
AATGGATAGGTCCTCTTTATCGAGCAAATCTAGATCAGGAGGAAGATCTTCGACTTCTT
CCGTATCGAGCTGCGTATCGAAAGCTTCCTCCTCGTCGTCATCGAGAAATTCTCCGGTC
GAAGAGGACATATCGTTTTCTCCAATGTCGCCATGCGTATCACCTCAACCGGGAATAGA
GGGATTGACACTGTTACAACGTACAGAGGTTGTGCTTCGAGTGAACACGGCTACCAGT
GACGTTGCCTCGCAGACAGATATTCCAGAAACAACAGAGATTGAATCATCGACAGTCA
AGATTCGGGAAATTCTTCTTTGCAGAAAGAAATTACCAGAAGAGATCGAATGCGAGGA
ACTGGGTCGAGACTTGGCAAGTCAGCTCAATCCGAACGATAAGCTGGTACCTCTTTTA
GTTCCAGCTCCAGAGCATAAAAAACCCACAGACTATGTTACCGGTCTGTTCAGGGTGG
AAGCGACTTTGCATCCGCGACCTAAACGTCGATCGTCTCTCGAAGAACCAACGACCCC
TTGCTCGGATAATGGAGACGAGGAAAAAAA

>novel_circ_001060
GACTGAAATGGAGGCAGTGTCTGGGGCCAAAAGCGGCACTTTGAAATTTATACGGACT
TTGACTAATATTTCGAAAGATGCTGGCGGTGTGGCACATATGCGATGTGAGGTTGTTGG
AGATCCTCCACCAACTAAAATCAAATGGTTCAAGAACGAAGCACCTCTCGAAGAAAA
ACGACCGAAAATTACAATTAAGAAGATACATGCACAAGCACATGAACATGCTGCGAAG
AATATTGCTGGAAGTCGTTTAAAGATCATCAACTTAGATGTCTCTGACGTAGGTTTCTAC
ACTTGTCGCGTAACAAATGGAAAAGATCAGATTCAAAGCGAAGGAACTCTTCGAGTCG
ATTCTTCCAAAAACAGACATG

>novel_circ_001061
CACTGAGAATATAACAAACATTGAAGTTTGAAACAAGTTTGGATCTATACTCTGAAGAA
AATTCCACGTGTAAAAGAGAAATTCTTGCTTGCATAAAAACTATAAAGATCATGATACA
ATATAATTTGGCCAAATATAAATAACTATATGAAATAAAAAAGAAACGACACAGTTTGG
AAACAAATCTATCCTCTACAAAATCTATACAGAAAATGAAGAACGAAATCTTTAGCATT
TGAAGAATCTAAAGTTAAAACAAGGAAGATTCTACTATTTCACATAAGAATTAAAAAA
GAACAATGATCTATTACTAAAAACTCATCTCTATTCTTCTAAAAAGTTCTTTCATAAAAA
GGGAAAAAAAAAAAAAAAAGAAAAACTCAATTGACAAAAATTAAAACCAAGAATCC
AATAGGATCCGCCTACGCGAAAATAAGAAAAAATTCCAAAGAAAATGCTCGTCAGAAA
TTTTCACCGTGAATCTCCAACATCGAATCGAAATTTCCTCGACAAAAGTAACGACGAG
GAAGCAAGCAACCCTGTCTCAAAATCCATCTCCAATCTTCCGAAAGATCCTCTCCTCTC
GTAGGGAAGAGAGAGAGGGAGAGAGAAAGAGAAAGAAAGAGAACGATACCACCGA
GAGAATCTCGAGATTCCGAGTATTCCCTGTGAAAACAGATCTTTCGTAGAGGCAAACA
GCGTCGAAATTTCACTGCAACCTATATGGAGAAACACGTTGGACCCTTGGGAGGGGGT
GGGAGAAAAAATTCGGCCGCGATAAATTCGGATCAGCGGCGCGGATAAATCGAAGGC
AGGGAGAAATCGAAGCGAAGGAAGGGAGGAGCCGGAAGAGGAAGATCGCGCGACCG
GATAAAACCTCGCGCGAATAAATTGAAAGAAATAGACAAGGACAAAGGGCAGGTGGA
GGGAGGGGTGGTGGACGGTCGAAAACGCTGATAAACGGTTGAATAATGGGCGGGCAA
ATTGCAGGAGCGGAGGAGGAGCGCGTGATAAACGTACGGACGATGCAAATCCTTTTGG
GCGAGGCGCGGACGTTTCGGAACTGGATGGGAAGCGCGGCGTTTCCGCGCCTTGTGT

GTCCAATTTTTTATCGTGGAAATGATGACTCTGATGATGATTGAATTTTCGATACATAGC
TCTTTTCTTACATCTTTTTTAATCGATCGAGATGAAGGGTGGAAGAATTAAGGATCGGAT
GAAAGAGAGAATTTGGAGAGAGTAAGAGGGAAGAAAAAGAATTTTGAGACAAAGGA
TTTGGAATTGAAAGGAAAATCTGTATTTAATTGGGAGGAAGTAAAAAAAGAGAGGAGC
CAAGACAAATTTAGAAGAAAAATATAAAACTCGCGAAATATCAAGGAGAAGAGAAGG
ATCTCGGGAAGAAGTTTGAAAAAAGAAGAATCGGAAGAGAAGCCCATAAAGGGAAG
GAAAATCTGAAGGTGGTAGTTCAACGCCGCAAATCCAATTTTAAAAGCTTCACGTGAG
ATTAAACGCCACTTGAACGCTTCTCCCATGACTTTAAACTCGCTTCTTAATGGCGCAAT
AAGTTCCAACGTGTCCACGGCTATCAAGCGAAAGTTACAAACTATCCTGTTGGAAAAA
AGTTAATAGCTGATCGTAAAGGGAGTAAACCACGCATAATAAAAATCAGCCTAAACGC
GCAATTTTCTCAAATAGTGTTTCCTTTGTGTTCCTTTAAAGAAAAACTGCATCTAAGAC
AGTGATTCTAATCTCGATTTTGATTTCAAATTTCGATGATAAGATAAGATAAACGTAACA
CGACGTAGACGAATTAAAAGATTTAAAAAATTTATCTGAAGTGATATTTTTCGTTTCTTT
TTCTTTTCTTTTTTTTTTTTTTTGTTGTTGTGATCGTGAGATTTTTGTTGTTGAAATTAAT
GAAAATGATGACGACTACTGCCGGTGTTGGTGGGGCCGGGATGCTGAGGACCAGGCG
CAGGGATTGCAGCGTGAAACCGAACAGGAAACTCGACCGGAAATCCTCGAGGGGGAT
GATTTATGAGAACGAGGAGCTTAGACTGAGGACTATTCACATTAACGCCGAAGTGGAG
CAAG

>novel_circ_001064
GAGAGCGACAATCTTTGGTGGGACGCATTCGCGACTGAATTCTTCGAGGATGACGCAA
CCTTAACCCTCACCTTCTGCCTGGAGGACGGACCCAAGAGATACACGATAGGAAGGAC
GTTAATACCCAGGTACTTCCGTTCAATATTCGAGGGCGGTGTGACGGAACTTTACTACA
ATTTGAAACACCCGAAGGAGTCATTTCACAATACCAGCATAACCCTCGACTGTGATAAC
TGCGTTATGGTTACGCATCACGGAAAACCGATGTTCACGAAGGTATGCACGGAGGGTA
GATTAATATTGGAATTCACGTTTGACGACCTTATGAGGATAAAGTCGTGGCACATGTCG
GTGAGGACGCACAAGGAGCTCGTGCCTAGGACTGTGGTCGGTATGCAACAGGACCCG
ACGATGCTCGAACAGCTTAGCAAGAACATCACGAGGCAGGGCATCACCAACTCCACC
CTCAACTATCTTAGG

>novel_circ_001065
GCTGTAGCCGCCTAAACACGCCATTGGCAGCCACTACCACACTGGAGGATCCGGGAAC
ACCAGCCTCTTGGAAGGGCCCGCCGCCCGGCTCGGAGGCATCGCCCTTTACACCCAAC
TCCGTTGGCCAGGGTGGTGGCCCTGGCTCCGGTCCCTACAACAATACAGGTGGTCCAC
CGAGCAATGCGGCGTTCCAAGGCCCAAGCCCTTTCCCCGGTGGTGCTGGTAGTCCAGC
CGGGGCGCCTCCTTATCAAGGGCCACCCCCTGGTTCCGCGACCCCTCAGTATACTGCCT
CGCCAGCGCCCAGCGGCTCCTCGACACCCGGCCCTGGGCCACCGCCCAACTCGTCGG
GCTTCCCACCGCCACCCAATAATTCCGGGCCCCCGTATAATGGGCCCGGGCCGAGCCCA
TTCGGCTCGCCATCTGGCGGGCCTCCGCAGTTCGTCGGTCGACCCGGCTCCTCGGGGC
CGCCGTTCGTACCTCCGGGAGCCGGGAATCCTCACTTTCCTTCGCATGGTCAACCGTTT
GGCGGGCCCCAATATGGTATGCCACCTGGAAGCCCGTTTGGACCCGGCCACCCGATGA
CAGGACCTATCGGGCCTGGACATCCAGCGATGATGGGACCCGGTGGACCCGTCGACAG
GATGGACCAAGG

>novel_circ_001066
GACGAAGTGCTGACAATATCTGAAAAGGTCGTGGTGCGTGTTCATGATCTCGTTACGT
GGTTATCGCCTGCATTGGAATGGTCATGGGGTCGGGAAGTATCTTACCCACCGACCCCA
ACGTCGTCTCCGGAGAATAGCCCGTTAAGGTATGAGATACCTCAACCGCAGGTGCTCA
CCGATCCTGGCATTGATTTTTCGGACGTCGATAAGCAACAGAAGGAGTACGAAAGCAA
TCACAACACGCGAAAATCGGACTATAGTTCTCAAACGAAAGTGGTGGTGCTCTCGTAT
CCCAGGTATTGCCGATATCGAGCACTCTTACGTAGGCTCGAAGGCGCCGAACCGTCCT
GGCTTTGCTCGTCCATAGCAGCCGCACTGGGTGGATTCACCGCCACACCGGGTACAAG
GATACTCTTCTGTAGAGATACCTTTGACTATCCGGATCTTGAAACTCACGAGCTCCTCT
GCAATCATTTAGCACCGAAATTGAAAGGAAGGCCGAGGAGGAAACGCAAAAAGCGCA
GCGCATCGCCCGGTGAATCGAGTAATGAAAGTGAAGCTTCCGTTGCGTCCACGTCGAA
AAGTGCCCCGGCGAGTTCGAACGTTATGGTACCGAGCGTTGGTAGGCCACCTTCGTCG
GTAGTACGGCGAAGCGAGCGGAAGACCAGCGCGGAGGAAAAGAAATTCATAGCGGAC
GTGCAGAATTTCATGAACTCGCGGGGTACGCCTGTCGGAAAGATGCCACTGCTGGGCT
ACAGGCAGA

>novel_circ_001067
GACGAAGTGCTGACAATATCTGAAAAGGTCGTGGTGCGTGTTCATGATCTCGTTACGT
GGTTATCGCCTGCATTGGAATGGTCATGGGGTCGGGAAGTATCTTACCCACCGACCCCA
ACGTCGTCTCCGGAGAATAGCCCGTTAAGGTATGAGATACCTCAACCGCAGGTGCTCA
CCGATCCTGGCATTGATTTTTCGGACGTCGATAAGCAACAGAAGGAGTACGAAAGCAA
TCACAACACGCGAAAATCGGACTATAGTTCTCAAACGAAAGTGGTGGTGCTCTCGTAT
CCCAGGTATTGCCGATATCGAGCACTCTTACGTAGGCTCGAAGGCGCCGAACCGTCCT
GGCTTTGCTCGTCCATAGCAGCCGCACTGGGTGGATTCACCGCCACACCGGGTACAAG
GATACTCTTCTGTAGAGATACCTTTGACTATCCGGATCTTGAAACTCACGAGCTCCTCT
GCAATCATTTAG

>novel_circ_001068
TTATTGGGCGGTGCTTGCTTCAGCCATGGCCCGTACACGTTCTACAAAGCGGTTAGAAT
AGGAAATGGTCGCGTACTTCGTCTTGGAAGCTTCTTCCTCACGAAACTGTGGAGCGAT
GCCGATCTCGTCAGCATCGGGGAACTTCAGCTGTTGTGGATGGACAGCCGGGGACCGA
ATCAACCTCTGGCATCGTTGCGGTTGTACTTCTTGCCGGAGAACACCCCGGATGGCCG
AAGGGACACGCATGGAGAG

>novel_circ_001069
GGGAAACACGTGAGACACAGCATCGTCAGGGACAATCGTGATCGAGGTGGACGTTCA
AACTCGCGCGTAAATGCTTCGAAGGCCATCGTCTTGGAACCATTCGTCCGGTTGACAG
GGAGCCGGGTTGGTTTCACTTCGAGAACGAGCCCAAAGACTGACTCTGCATCGACCA
ATACCTAGTGGGGGAAGAAGGAAGGCCGAGAGGGATTAAGGTGAGGCGAAGAGGAA
GGAAACGGATCTAAGCGGCGAACGGTTGGTTGAATTGCCAGGTCCGCGCATGCTTCAG
CAACGAGGCAGTTCAGTAACGACACCTCCCGCGACGTCATCCTCCAACATGTTCCCTC
AACAGTATTGCCTACGATGGAAATACCATCACAGCAATCTACAGACCATGTTCTCACAG
CTGCTCGAGCGGCAAGCTTATTGCGACGTCACTCTGGCCTGCGAGGGCAAAACTCTCA
GAGCGCACAAGTATCTATATTATCGATAGTTTCGATACCATCTTATCGATGGTAAAAATAT

CGATGCTTGCTAGTATATCGATATATCGATAGTTGCCGCCATGTTTAGTTATTAGTGGGAC
GAGTGTCGTGTGACATATCAACGATTGTTGTGCCATTTCTAGATATTTTTAATAACACAA
TTTTTAATATCTACTACGTAACTTAATAATACAATACCAATTATTGAAATTAGAATTAAATT
GCCGATATATTCCTGTTTAATTCAACGTCGGTGTTTACTTTGTTGCAAAAACGTTACATA
ACCTCTCACAGTAGGTGGTTCTGTCAGCGTGCAGCACGTACTTCGACACGATTTTGTCC
CAGTACGAGGAGAAGGACCCCATTGTCATAATGCGGGACGTCAAGTTCTCAGACATCA
AAGTGCTGGTCGAGTTCATGTACAAGGGAGAGATTAACATCGACCAT

>novel_circ_001070
GGGAAACACGTGAGACACAGCATCGTCAGGGACAATCGTGATCGAGGTGGACGTTCA
AACTCGCGCGTAAATGCTTCGAAGGCCATCGTCTTGGAACCATTCGTCCGGTTGACAG
GGAGCCGGGTTGGTTTCACTTCGAGAACGAGCCCAAAGACTGACTCTGCATCGACCA
ATACCTAGTGGGGGAAGAAGGAAGGCCGAGAGGGATTAAGGTGAGGCGAAGAGGAA
GGAAACGGATCTAAGCGGCGAACGGTTGGTTGAATTGCCAGGTCCGCGCATGCTTCAG
CAACGAGGCAGTTCAGTAACGACACCTCCCGCGACGTCATCCTCCAACATGTTCCCTC
AACAGTATTGCCTACGATGGAAATACCATCACAGCAATCTACAGACCATGTTCTCACAG
CTGCTCGAGCGGCAAGCTTATTGCGACGTCACTCTGGCCTGCGAGGGCAAAACTCTCA
GAGCGCACAAG

>novel_circ_001071
CATCGTGAAATTCATCGATATCGTTGGAAACAATTTCGACCTACCATTATCTTCGTAATT
CGCATTCGTCGATTTTTACAACGCGAGATCGAGTGTAATCGAATGTCGCCGTTCGATCC
GCGATCGCGTCCGGACCTGTGGTCGCAGATCCGCGAGTAACTATCAAAACATCG

>novel_circ_001072
TTAATTTCCACTAAGCAAATTATTGATAAAAAATGGGAATTAACATCAGAAGGTCATCAT
GTAATGAATTATGGAAGTCATGAAGCAGCAATTTATAATGCAATTCCTGATGATGGAATG
CCACAATCTGAAATTATACAATCAATTCCATTTGCAAAAATTGGATTTTCAAAAGCATTA
CAAGCTGGTTGGATTGTAATTGACAAAAGTAATGGTACACCTATTGTGAAAAAGAAAG
CAACTTCTATTATAGATATTATACAAAATGATTTAAAGGATCTAACAAGTTTAACTGATC
AGCTTAGAAATGATTATAAAAAAAGAAAACTGATTCAAGAAGT

>novel_circ_001073
ATGTTTAAAGGTAATATAGATGGTGATACTATAAAACTTAACAAATTTGAAGTACCAATT
ATTGCACAATGGATTAGAATAAATCCTACTCGATGGCGGGATAGAATATCATTAAGATTA
GAATTATATGGATGTGACTATGTATCAGACATCCTTTCCTTCAATGGTTCATCCCTTTTAC
GATATGATTTATTAAGGGAACCAATAGAAACTGACAGACATTTTATACGTTTTCGATTTA
AAACGAATAATGCAGATGGTATTTTAATGTATTCTCGTGGGACACAAGGAGATTATATAG
CTTTACAATTAAAAGACAATAGAATGATATTAAATATAGATTTGGGATCTGGTATTATGAC
AAGTCTATCTGTTGGAAGTCTTCTTGATGATAATATGTGGCATGATGTTTTGATATCGCGT
AATAGGAAAAATATTTCATTCTCTGTAGATAGAGTATTAATTAAAGGAAGAATAAAAGG
AGAGTTTCATAGACTGGATTTAAATAGAGCACTTTATATAGGTGGTGTACCTAACAAAC
AGGATGGATTAGTAGTCAATCAAAATTTTACTGGATGTATAGAAAATTTTTATCTTAATG
CAACTAGTATTATTCATGATTTAAAGGAAACAGAAATTATTGGTGAAAATCTTAGATATT

ATAAAGTGAATACACTTTATAACTGTCCTGAACCTCCCATAATTCCTGTTACATTTTTAA
CTCATGGTTCATATGCTAGACTTAAAGGATACGAAGGAGTATCTTCTTTGAATGTTTCTC
TTACTTTTCGAACATATGAAGATAAGGGTATTATTTTATATCATCAATTTACTTCTCCGGG
TCATGTAAAGCTATTTTTAGAAGATGGTAAACTGAAAATTGATATTCAAACAAAAGGAA
ATCCTCAAGTAATTTTAGACAATTTTGATGAAAAGTTTAATGATGGCAAATGGCATCAA
GTTATTCTAACAATTTCGAAAAACAATCTTATTCTAAATGTCGATGGAACACCCATGCG
GACCAGACGTATATTAGATATGGTTACTGGTCCTATTTATATGATTGGTGGTATGAAAGG
AATAGAAAGCAGTCGCGGTTTCGTTGGTTGCATGCGTATGATTAGTATCGATGGTAATTA
CAAATTACCAACTGATTGGAAAGAAGAGGAATACTGTTGCAAGAACGAAATTGTTTTC
GATGCTTGTCAGATGATGGACCGTTGTAATCCGAATCCGTGCAAACATTTTGGCGTATG
TCGACAAAATTCTGATGAATTCTTCTGTGATTGTGCCAATACAGGATATACAGGAGCAG
TTTGTCACACTTCTTTAAATCCCCTTTCATGCGAGGCATATAAAAACATAAACTCAGTTA
ATCAACGAGCAGATATTAAAATAGATGTTGATGGAAGTGGTCCTTTGAAACCTTTTCCA
GTTGTTTGTGAATTTTATACAGATGGACGTGTAAGAACGATTTTGCGACATAATAATGAA
CGCATAACACCTGTGGATGGATTTCAAGAACCAGGTAGTTTCGTTCAAGATATTATTTAC
GATGCGGACATGGATCAAATCGAAGCTCTTTTAAATCGATCTACTAATTGTAGGCAGAG
AATTAGTTACGAATGTGTACATTCTAAGTTGTTTAATTCACCAGTACCTCAAGGAGATTA
TTTCAGACCAAATTCATGGTGGGTTAGTAGAAATAATCAAAAAATGGATTATTGGGGAG
GAGCTTTGCCAGGTTCGCGTAAATGCGAATGTGGTATACTCGGAAATTGTGCAGATCCT
ACTAAATGGTGTAATTGTGACTCTGATTTGGATGGTTTATTTGAAGATAGTGGAGATATT
ACAGAGAAAGAATATCTTCCAGTTAAACAATTGCGATTTGGTGATACTGGTACACCAGT
TGATGATAAAGAAGGACGTTATACACTTGGACCACTTATTTGTGAAGGAGATG

>novel_circ_001074
GTAATATTCGAGCAAACGCTAAAGGTGAAGCAGATGTACACATCAAAGATTTCATTATT
TCTTTGACTGGAAAGAATAATATTCTTGGACGTGCAATAGTTGTTCATTCTGATGAAGAT
GATTTAGGAAAAGGGAACAGTAGTCTTTCTACAACCACTGGAAACTCGGGAGATCGTT
GGGCTTGTGGTATTATCGAAGCTAACTAGATTTCACTGAATTTGATTCTTCATTTGATTTT
ATATTTAAAATTTAGAATTTAG

>novel_circ_001075
GAACAATTTAATGGTCAAGGTTCATGGGGAAATGGTGGAGCAATGAGAATTGCACCTA
TTGCACTGTTTTCCTATCAAAATTATGATAAACTTTTAGATACAGTTAGGAAAGTAACTC
AAATTACTCATACACATAAAATCGGAATAGATGGTGCTATTTTACAAGCAATAGCAATTT
ATCAAAGTCTCCATTTAAACCCTAATGAAGAATTAAATGTAATAAATTTTATTGATGATTT
GATTAATAAAATGGACCAAATAGAAAAAGATGAAGAAGG

>novel_circ_001077
GATTTGGCAAGTTTAACCTTAAGGTTATGGCCGTCTGCAGTATGATCTTTATGAACGTGG
CTTTCAGTATAACAAGCATCGGTTTCATATTACCATCAGCGGCATGCGACTTCAGAATG
ACCACAATGGACAAGGGACGTCTGAGTGCCGCCCCCATGTTAGGTATGTTAGCTGGTT
CATATATATGGGGTTGCTACGCGGCTATCAAGGGGGCGAAGATTATCTCTATTGGTCGCAC
TATTTCTACATGGTATCTCCGAATTATTGGCATCGGTAGTGCCATTGTACTGGGTTTTCTT
ATTTTTAAAATTTCTCAGCGGTGCAGCTATGAATGGGCAGTCAGCTGTTGTGTTTCTTTA

TCTGAGTGAATTCCAACCAACCAAACATAGGAGTAGAATGCTCTCATGGATGGAAATG
GCTTGGGTGCTAGGAATGATAATTTTAGCAG

>novel_circ_001078
TGTTTTTAATATTCCATCCAGTGGTTCCGAAGAACTGTTTCATCTTTATAACGCTGTACA
AAAATGGGTCGGAAGAAGATTCAAATTTCGCGCATCACGGACGAACGGAATCGTCAG
GTGACCTTCAACAAAAGGAAGTTTGGGGTAATGAAAAAAGCATATGAGTTGTCGGTAC
TATGCGACTGTGAGATCGCTCTGATTATCTTCAGTTCGAGTAACAAGCTATATCAGTATG
CAAGCACCGACATGGACAAGGTTCTTCTCAAGTACACCGAGTACAACGAACCCCACG
AGTCTCTTACCAACAAGAATATCATCGAG

>novel_circ_001079
ACGTCCAAGTTTAGTCACTGGAATCCATTCTTTACTGTCTTCTTTACCACGTCCACGAC
CACGACCTCGTCCTCTACCTCCACGACCTCTACCTCTTGGACCTCCACGATCACCTCCT
CCACGAGAACCAAATCCTCCACGAAAACCTCCACGCGCGGCTGGAGCA

>novel_circ_001080
GGTAAGGTTAAGTGTACTGATCTGAGGACAAAAGATAAAAATGAGTTGCTCAAGCAAC
TGGAAGAACTCAAAACTGAGTTGACAAATCTTCGAGTGGCAAAAGTTACTGGTGGTG
CTGCATCAAAACTTTCGAAAATCCGTGTGGTAAGGAAAGCAATAGCTAGGGTATATATT
ATTATGCATCAAAAGCAAAAGGAAAATTTGCGTTTGTTGTATAAAAATCATAAATATAA
GCCTTTGGATTTAAGGCCAAAAAAAACTAGAGCTCTTAGAAGAGCATTAACACCTTATC
AAGCCAACAGAAAGACTCTAAAAGAAATACGCAAGAGATCTGCTTTTCCTCCAAGGA
AGTATGCTTTAAAGGTGTAAATTGCAAGGTACTTAATATTCAAGAAATAACAGAAGCTG
TAAAAATTTAAAGACGAATGCCAGGGTTTCTAGAAAGCACAAAGTTCAGAAGTTCATC
ACTATCCTCACAGACAAATGGTTTCGATGATGGCAAGCTACATCATGCCATTTAATTCC
ATCATTGTAAAAGTTATTCAGAATTGATAAACAAGATTCGTCGTTACCCTGCAATATAAA
TTCATGCATTAAAAAACTATTAAAAATAAAATTTGAATAAAGCAAATAGAAAATTCATAT
ATATATATATAAATGATTCCAGTTATAATTATAAGTGAAATTATAAGTGAAATTGAATTAA
GATAAATTTTTTATCAATATTATCTAAAACTAATAAATATTTCAAATTTTCATTGCCATTAA
TCGTTCGAATTTATTTGTTGTGCGTATGTGTTGTATTTATAAAAAAATAATAAATTACCTG
AGCAGCTTCACGATTATCAGGTTGTGCTTGACCATATCCACCAGTGTGACTCCAATCTC
CAGTATTACGCTGTGTAGTTGGACCAATTTTAGCTCCTGAACCAGACCAGAACCATCCA
TTAATGTTCTGCGGTTGAAGATCTGGTCTGTCACAGCCATTGAAATTACATTTGCGTCC
AGATGTCCAAATATATCTCACATTTCCTATAAGAAATAAAAATTAATTTTTTAAAATAAA
AATAAAATTAAAATAATATTCGGTTTAATAATTAACCTCGTGCTATTCGTTGTTTGATAAA
TTCATTTTCTTGCGGTGTTTCAAGAGAAACTGCATCCATACAATGCCTGCGACAAATATT
ACGAGCATCCAGCCAATCTACTTCGAGATTTCTAGTCGGTTGATGTTCCCAACTGAAAA
AATATGAATGTGCAACACCTCTAGCATCCCTATAAGTGGCATGTCGCACCCCTAAAACA
AGATTTATTTTTTTATTAAAAAGATTAAAATAAATCAAACGATTAAAAAACACTTACGAT
TGGCACAACTGCGTGGATCAGGAAGAGCAAGTCGACGTTGAGCATTCACCACAATAAT
G

>novel_circ_001081
CACCCAGCGCATGTGCGAATATCCCCCACCATACTGCCGAGTGAATTACCGGAGCCGTC
TGCCATCTGATTGAACTGGAGGTAAAGAGAGAGAACCAACCGATAAAACGATGCCGC
AGGCACGCTGGTGGAACGCTGTTATCGCGAGCAGTCGGCCTAGGACGTGGGAACAAC
TGACTGCCTCCTCCTCCGCGGGTTCAGGGACATCGACAGGAAGAACATCCATATGTCC
GACGACTGAAGTTGTAATCCACCGGCCGGCTACCGTCAGAACATTACACCGCGGCATG
GCGAGCAGTCAATCTGGAACCAAAATCCTCACCGAGGATAATGTTTTCACGAATCTTC
GGAAAATGGAGTACGCAGTGCGCGGACCACTCCTTTTACGTGCTCTCGAGATCGAAAA
GGAGCTTCAGAAG

>novel_circ_001082
TTAGGTGGAGGTTGTGAATTAGCAATGATGTGTGATATTATTTATGCTGGAGATACAGCA
AAGTTTGGTCAACCTGAAATCACTATTGGTACCATACCTGGTGCAGGTGGTACTCAAAG
ATTAACTAGAATAATTGGCAAGAGTAAAGCAATGGAAATGGT

>novel_circ_001083
ATCCATCCGCAGAGGAGGACTCCTCTGGTCTGACTGGACTTAACTGGCTTCACTTGTA
AATTTTGTGGCAGGTTGACCGAAAAGTAATATGTCTACTAAAAGTCGTTGTCAAGGAA
ACGGATTTGTATACAATATGCTCTGAAATTTGATACGCCTCATGAAGATATTCATCTCTAT
TAAGATACAATAAGTGGTTGCATCAACAAAAATACGAAACGTTGGATTAGTATCTATAAT
AAATGAGAAGTTTAATACCTTATATGGATAAAATAATCAAATTATTAATGTATATCCGATA
AAAGAAAAAGAGATTGAATTTTATAACAAGACGTGAAATAGTTTTGAACAATAATTGG
TTACATAATTTTCAAATATTATTATGTATGATAAGCTACTTGTTATTTAATGATTTGCTTTAT
AGTTTAATTATACAAGTGTTTACAAATACTGGGAACTGGAAAATATTGAATTAGGTATAA
ATAATATTATTTAAATATAATATAAAAATAATAAATATAATTATTTTTAATTTATTGTAATTT
CTATATATAATAATCTTAACTTGTTTTATAATAATATTATATATTATTAATACAAGATAATTC
TAAATAATTTTATTTTATATATTTAAAAGAAATCAATAATTAAAAACAAATAATTAAATTT
AGTTCTATTTGAATATCGCTCCAACCAGTGATCATCAAGAAATTAACCAATAAGAATTCT
GTTAAATTATTATTCCCAATCTTATATCATATCAACAATAGATAAAATGAAGTAATTGCTG
ACGTGAGGTTATGTGCGTATGGTATGGCTAAAGCTGCCACGGCGCGGCGCTTTGTTGGT
TGAGCGTGGTCAGCTCTGATTGGCGATTGCATACTAGCCAATGAGCACTCTCCATCTGT
CTACCTTAGCGGCCACTAACTCACCAACCACATCCACGAATCGAGCATTATCGGCTAGT
TCGTCGTAGAATAAGCATCATCGTCGATTGGACTTTTGTCGTCCCGGCGTTTGAAAATG
CGATCTTTCTGCTCATTTTTGCTTCTGTTGGCGATTACAATCGCCACAGCAGAGATCTAC
TTTGAAGAAAAATTTCTTGATGATTCCTGGGAAAAAAATTGGGTGTATTCTGAACATCC
TGGAAAAGAATTTGGAAAGTTTATTTTAGGGCATGGAAAATTCTGGAATGATCCAGAA
AATGATAAAGGTATCCAAACATCTCAAGATGCAAGATTTTATGCCTTAAGTAGAAAATTT
AAACCATTCAGTAACAAAGACAAAACACTTGTCATTCAATTTACTGTTAAACATGAAC
AAAATATTGATTGTGGTGGTGGATATGTTAAAATATTTGACTGTTCTTTGGATCAAAAAG
ATATGCATGGTGATAGCCCATATCAAATTATGTTTGGACCTGATATTTGTGGACCAGGAA
CAAAGAAAGTTCATGTGATCTTCAGTTATAAGGGAAAGAATCTTCTGATTAATAAGGAT
ATTCGTTGTAAAGATGATATTTATACTCACTTATATACTTTGATAGTTAAACCTGATAATA
CATATAAGGTTCTAATTGACAATGAAGAAGTGGAATCTGGTGAACTAGAAGCAGATTG

GGATTTTCTTCCACCAAAAAAAATTAAAGATCCATCTCAGAGCAAACCTGAAGATTGG
GATGACAAACCTACCATTCCCGATCCTGATGACCAAAAGCCAGAAGATTGGGATAAAC
CTGAGCATATTCCTGATCCTGAAGCTACTAAACCTGAAGATTGGGATGATGAAATGGAT
GGAGAATGGGAAGCTCCAATGATTGATAATCCTGAATATAAAGGTGAATGGAAACCAA
AACAAATTGATAATCCTAACTACAAAGGACCATGGATTCACCCAGAAATTGATAATCCT
GAATACACACCTGATCCAGAATTATATAAAAGAGATGAAATTTGCGCTATTGGTTTTGAT
CTATGGCAAGTGAGATCTGGAACAATTTTTGATAATGTTCTTATCACAGATGATCCTGAA
GTTGCTCGTAAATTTGGCGAAGAAGTTTGGAAACCTACATTAGAAGGTGAAAAAAAGA
TGAAAGAAACTCAAGATGAGGAAGAAAGAAAACAAAGACAAAAAGAAAGCAAAGA
AAATGAAGATAATAAGGATGATGATGATGATGAAGATGCAGATGAAGAGGAAAATAAC
GTCCCTGATACTGAA

>novel_circ_001084
GTTTCCAAACTTCTTCGCCAAATTTACGAGCAACTTCAGGATCATCTGTGATAAGAACA
TTATCAAAAATTGTTCCAGATCTCACTTGCCATAGATCAAAACCAATAGCGCAAATTTCA
TCTCTTTTATATAATTCTGGATCAGGTGTGTATTCAGGATTATCAATTTCTGGGTGAATCC
ATGGTCCTTTGTAGTTAGGATTATCAATTTGTTTTG

>novel_circ_001085
ATAATAAGGATGATGATGATGATGAAGATGCAGATGAAGAGGAAAATAACGTCCCTGAT
ACTGAAGAGCATGATGAATTGTAAATAATGTGTCGTAATCGATACGAACACAGACTGTA
TTCCAGGAGCTGTGTAACCGCGATACGCACTAACTTTCCATGAAAAACACAAAAAATT
ATAATAGAAAAAAAAAAAAAAAGAAAAACTAATCATTCTCAGAAAATCAACGGATTCCT
TTAAATTGAATTAGTCAAAAAAAAAAAAAAAAAAAAAATAGTTATAAAACTTGTAAAAA
ATTTTTTATGTCAGTGAATTTTTCGAAATTTCTTCATCTTTTTTTTTATTTTTCTTTTATATT
TGAAATATGGCCTCATTTTCATAGCCTAAACTTTTTAATCTAATCTTCAATTATTCGATCG
TGTTCCATGAAATTCATTAGTGAAAGGTTACATCATAAATATTAAGTGTCCAAAAAGAA
GTCAAAGTGCTCATTATGTGTTATCAAAATACGTTATGTGAACGTAATTATATACATGGCA
TTATATGCTGTATGGCATATATATATCAGAGTCATCTTGCCCTATGTCTCTCCCATGGCAC
AGCTCCAGAAAAAGACTCTAAGTTGTTCCGTTTTTGTTTGTCAAGTGTTTTTATATACAA
TTGAAAGATAATTGAGATGTAATTTAATGATGCTATATTATATATATATAAATATATATATTA
TATATTATTTCGATTTCCGTACAGTGTACGCGCGTATTAAAAAGCTCGCTCCAGTTAAAA
TGTAAGAATGATCGGATGTGTGCAGATATGACTGAATGAAATATAAATATAAATACGATT
GGTATGATTATGTATTAATAAATACAAAACCAA

>novel_circ_001088
AATTAGAGAGGAGAGGCCATCCAGAATGGTTTTAACTGTGACCGAAGACACACCAGC
AGACATGCTGGCCGGTAGTATGGAGCTTCTGGTCCAGTTACCTCGGGAACATCATCTTC
AAATGCAAAGAGTCACGGTACAGAGAAGTACACCGATGATGGATCTCCTCGTGCAAAT
CGCCACGGCTCACAAATTGACAGCTTCCAACTATACTCTACAAGCGATTGGAGAACGT
GGGATGGTTTTATCTCATCAACCCAATACACCAATTGGTGCGTTGGATGCACTTCAGGT
AAAATTATTACCTAAACAGGGGACTTTGGTACCAAGAAAAGCGAAGCAAGTTAATCAA
CCGTTTGAAACTACTTTCAGGTTACAGGTACATCTACCAAGAAATCAATTGTATGTGTC
GAGAGTCAGTCCCAAGATGAACTTAGGTGAGATCTTGGAAGAAGTATGTCGTGAAAA

AGATCTAGATAAGAACAAATATGAACTACGTCATCCAGCAAGCTTGGAAACATTGGAC
TTATCATTGTCACTGCAGGATTACCACTTACAGGAAGTCACCCTGTATGCTAGACAAGG
CAGAACACTAGGTTCAGCACTTAGCACTCAGGATATAATGGCTCTTCAAAGGCAAGAA
GAGCGACGTAGGCAGCAGGCAAAACAAGGTGTTTTTGGATTTGTATTCAAAAAATCTA
AAGAAGGTTCCTTAAGTACAGATAGTCTTGGTGAACGTAGTGCGTCACCAGCAAGAAG
TGACGAAACTGGAAGAAGTACGAGTCCTCTACAAGCACCTACTAGACCTCAAAGAAA
ACGGAGACCAGCTCCAAAACCACCTATTCAAGCTCAAGCAGAGATTAGTAATAAAAAC
GATATTGAAGAAAATTCTGGAGATTCTAGCAAAGATAAAGTGATGATTAGCCACAGTCG
AAATAGCAGCGATAGCTCCGGTTATCATGAGGCTTCTGTATTAAGTGATAACCCAGATTC
TGCGGGAAGACTTCCAGAAACATTGCCCAGAAGGAACAAAGTTCCTTTGGAAATGTC
TCGGAAATTAGCTCAAACGTCACAGTCCAGTAAAAGTCTAAATAATTTGGCTTCTGTAC
CTGGTACACTTAGTCATGGCATTAGCAATACTTCATTAAGTTCTACAGGATTACGAAAG
AAAAGAGTTGCACCTCCTCCGCCAGTAACTAGGCCTTTATCATCGGCGATTTCTACTCA
AGCATTAGAACGTATAGTTGACTCTGAAGAATCCTTAACATCAGATATGGATCCTTCTAA
ACCTCCTTCTGACATTGGTGTTCCATCTAAAGCGAATTCAGATATAGAAGAACGTCCAA
AAGCCAATTCAGATATTGGAATAACAACTGTTAGCTCAATATTGTCTTCGAATATTGATT
TTATAAAATCTGATACTACAAAAGTGGAATTAGAATCAAAAAGCGGCAAATCTGATCTT
GAATCGTGTCATAGATCTGATTCTATAAATGTTAAGTTACCCAGCACGGAGTCAAAACA
CCCTGCACCAATAGAAGATGCTCCTCTAGACGCTAGAGTACTGCAGACGAGAG

>novel_circ_001089
AATTAGAGAGGAGAGGCCATCCAGAATGGTTTTAACTGTGACCGAAGACACACCAGC
AGACATGCTGGCCGGTAGTATGGAGCTTCTGGTCCAGTTACCTCGGGAACATCATCTTC
AAATGCAAAGAGTCACGGTACAGAGAAGTACACCGATGATGGATCTCCTCGTGCAAAT
CGCCACGGCTCACAAATTGACAGCTTCCAACTATACTCTACAAGCGATTGGAGAACGT
GGGATGGTTTTATCTCATCAACCCAATACACCAATTGGTGCGTTGGATGCACTTCAGGT
AAAATTATTACCTAAACAGGGGACTTTGGTACCAAGAAAAGCGAAGCAAGTTAATCAA
CCGTTTGAAACTACTTTCAGGTTACAGGTACATCTACCAAGAAATCAATTGTATGTGTC
GAGAGTCAGTCCCAAGATGAACTTAGGTGAGATCTTGGAAGAAGTATGTCGTGAAAA
AGATCTAGATAAGAACAAATATGAACTACGTCATCCAG

>novel_circ_001090
ACACACAAGGGGGAATGTCCCGTAACACTGGACCTGGTTTGTATGAATTCCTTATGGA
AGCAGAACTCCAACAGTATTATCCTGGAATTCGAGGGGACTTGAAAGTACAGACAACT
GCTCAATTGAAATATGTAACAGAAGATGATTTGAATGCAATTGGAATGAGCAAACCAG
AAATGCGTCGTTTAAAAAAATATTTTCAAAAACATTTTCCACAAAATTATTTATCTAAAT
TCAAAAAGATGTTACTACCAAAACGGGAAGAACCAACTACAGGTGCTTTAACTATGTT
GCCAGAAGAAAGACAAGATAGACCACCGATTCGTGTTCCTAATAAACATATGATACCA
GCAGATGCAATTATTGTGAACAAAGAATTAGGAACAGGAGAATTTGGAGTTGTTCAAC
AAGGTGTTTGGACAAACGATGGTGAAAGAATACAAGTAGCAATTAAATGTTTATCACG
AGAAAGAATGCAGAATAATCCAATAGAGTTTCTAAAAGAAGCAGCCATAATGTATGCAA
TAGATCATGAGCATATTGTAAGATTGTATGGTGTAGTTCTAGATACAAATTCTTTGATGCT
TGTCACAGAATTAGCACCTCTGAGATCATTATTAGAATGTTTGAAAGAACCGAGTTTAC
GTAGCAGTTTTCCTGTACTTTCATTATGTGACTTTTCTGTACAAATCGCAGATGGAATGC

AATATTTAGAGGCAAAGCGATTAATACACAGAGATCTTGCTGCTCGAAATATTTAGTAT
TTTCTAAAAACAAAGTTAAAATATCTGATTTCGGATTATCTCGTGCTTTAGGAGTTGGAA
AAGATTATTATCAAACGAATTTCAATGTAAATTTGAAATTACCAATAGCATGGTGTGCTC
CAGAATGTATATCATATTTAAAATTTACTTCTGCTAGTGATGTATGGGCCTATGGAGTAAC
TTTATGGGAAATGTTTAGTTATGGTTTTCAACCATGGGCAGCATTAACAGGTCATCAAAT
TTTAGAAGCAATAGATGATCCTAATTTTCAAAGATTAGAACAACCAGAATGTTGCCCAA
AAGAATATTTTTCACTCATGCAACAGTGTTGGCAACATGAACCATCTAAACGTCCCAAA
TTTTCTGAATTAATTAATCTTTTACCTGATTTAAAGCCAGAACAAGTTCAAGCTGTTCAA
GATAGTATGGAAACAAATCAGCTTGTTTACAGACAAGGAGATGTTATTACTGTTCTTGA
TAAAGGAAGTAGTAACACATTATGGAAAGGTGTTTTAAATAATGGAAAAACTGGCTTTT
TTAATCCTGCTCATACAATAGCATATCTTGGATCTAATTTACCAAGTAATAAACCAGGTG
AATTCACACGTGGTGATGGCAAAAATACATTTTCTTCTCAAAGGCGAAAGATTCGAAC
GGATATGATTTCATCTCCACAAGGTGATTTGAAACATACTGGTCATGTTGGATTAGATGG
AGCTTATTTTGGTGATATCGGTTTTCTCGGCGGAAAGTATTCACATTTACCACGACAAGT
GGTTCCACCATATAAACCACAAGAAGATGTAACGGATAATTCTAGTCAAGCTTCGAGCC
AGGAAACAAGAAGCGCGGATACAAATAGAGAATTATTGAGAGATTATAGGAATACACA
ACAGGATGTTCAAAATAAACATGAAAATTTGTGGTCAGATGTAAATGAAGTGTGCCATA
CTGCTAATTCAAGTAAACAGTCTGTTCCATTAAATATGGCTAGCAATAATGATCTTGGTG
CAGATCATGAATATCATGAAATAAGTGATGAAGAAAATCAAGATAGCCCATTAAGATTT
GATAAAACATTAAATTTTGATTTTGGTCCAAGTTTATTAGCTGAAATGGATCAAATGTTT
AGATCACTAGGATCTTCTCCTCCTCCACCACCTCCAATTCATCCTTTATCAACTGAACAC
GAATCAAGTAATGTTCGAAATGAACTGAGAGAAATACAAGCAAAGCAGAGCAATAAA
AAAAAGCAAGCCACCGTGAAGCCTATCTCAGCTGCCGATCAGAAAACTCTCTACTCAG
CCATTGCTATGGCACAGGAATTGACAGCACGTTCCATGACAGATCTTGAACATCCACCG
GAGTCTCCCCGAACACCTGCCAGTCCTTCAAGACGCAGAAAGTTCTCTTTTAAGTTGC
CACATCAACACAGTCCCAAACCAGACAGACGACACTTTTCAGAAGAAGCTGCCAGTAT
ACCTGACATACAG

>novel_circ_001091
GAAGTGGAAGCAAAGATGGAAAAGGTGGGAAGGATAATGCATCACCGTGTGGTCCTT
ATGCAGCGGCTGAGTGCGGGGACGCGCCTAAAAAGAAAGGATTCTTCAAAGGTTTCT
GGAAGAGGTCGCGTCATTATTCATTGGAGAACCAATAGCCCAGGTTAGGCAGTAGGGT
GATGGGCCACCAGCAACGGCAGACATCGCGGCAACAACACCATTATCGACAACACGT
CGCTACAAGCGCTTTGACGCACAGTGCTTGTTGCTGCATCGTCCAATAGCCTCGGTCCC
GTTTCGTGGTAGAACCAGCGACAAGGTTCGCGTTCTTTCGTCCATTTCATCGAGCTTTT
ATTCCGCCCGATCAGTGGTCTCCGAATACAACAACAAGCTACGTCAATCCGTGG

>novel_circ_001094
GAAGTGTGTTAGAGAAAGTTTAAAGGGATAGAAACCTAAAAAAAATGGAAAGGGCGT
GAAAGAGAAAAAAAAATGATGAATGATCGAGTAAGAAAGAGGAAATGTGCGAATGAG
AATAATAAGGGAAAAAGGGAGGTAGCGTGGAAGGATTGATTTTTAAGGATTGAACGGC
AAGAATCGGAAAAACAAGAAGCGGTTGTCTAAAGGTATGTCG

>novel_circ_001095

AGAAAGAGTGAGCGAAATATGAAGAAGGATGAAAGAAAAAGAAAGAGAGTGAACGA
GAAGAAGTGCGAGGAAGTGTGTTAGAGAAAGTTTAAAGGGATAGAAACCTAAAAAAA
ATGGAAAGGGCGTGAAAGAGAAAAAAAAATGATGAATGATCGAGTAAGAAAGAGGA
AATGTGCGAATGAGAATAATAAGGGAAAAAGGGAGGTAGCGTGGAAGGATTGATTTTT
AAGGATTGAACGGCAAGAATCGGAAAAACAAGAAGCGGTTGTCTAAAG

>novel_circ_001096
AAAAGTATAGAGTTTACGCGAGTATATCCTTTGCTGTACTGTTATTGGGCATCGGAGTTC
CTCTTTGGTGGCATACTACTGCAGTTCCGCGTGTACCTCTTCCTTATGATGGAATTGAGC
AACTTTCCAACTTAGAAATCAACATTAAAACAAAACTCATCATACTAGCTCTTTCTCGA
GATCGTGCTGAATTGTTGGTTCACGATATAAAAGAAGCTTTCAAAAATGCCAGTATCTG
TCAAGTAGAGATCATTTATCGTGTAATATCCAATACTTTCGCATTTACGCATCATCAACTG
GAGAAGATAGCGTCCAATTTTGATGTTGACGTAGGGCAACTTTTACTCTTTGAAACAAC
TAACTTGAATAATGCAGTTCTCGTCGGTTCGGAAAGAACTATTTATTTTTCTACCGAAAC
CACCTCTTCCACTTTGATACAGATATTATCGGAATGGATGTTGCACGAAAATTCGTTGGC
TTTAACAAAGAATGCGCTCACCGAACCGACCCTTTATAGCTTAGATGAGAAGAATAGA
AGAAGATTTCCTGCTAGTTCGGCATACGATATATTGATCACTGTAGTTAATCCTGATCCA
GAGAAATTGAAGATTGATTGGAATCTTCGCGAGATGGTCGAAG

>novel_circ_001097
AAAAGTATAGAGTTTACGCGAGTATATCCTTTGCTGTACTGTTATTGGGCATCGGAGTTC
CTCTTTGGTGGCATACTACTGCAGTTCCGCGTGTACCTCTTCCTTATGATGGAATTGAGC
AACTTTCCAACTTAGAAATCAACATTAAAACAAAACTCATCATACTAGCTCTTTCTCGA
GATCGTGCTGAATTGTTGGTTCACGATATAAAAGAAGCTTTCAAAAATGCCAGTATCTG
TCAAGTAGAGATCATTTATCGTGTAATATCCAATACTTTCGCATTTACGCATCATCAACTG
GAGAAGATAGCGTCCAATTTTGATGTTGACGTAGGGCAACTTTTACTCTTTGAAACAAC
TAACTTGAATAATGCAGTTCTCGTCGGTTCGGAAAGAACTATTTATTTTTCTACCGAAAC
CACCTCTTCCACTTTGATACAGATATTATCGGAATGGATGTTGCACGAAAATTCGTTGGC
TTTAACAAAGAATGCGCTCACCGAACCGACCCTTTATAGCTTAGATGAGAAGAATAGA
AGAAGATTTCCTGCTAGTTCGGCATACGATATATTGATCACTGTAGTTAATCCTGATCCA
GAGAAATTGAAGATTGATTGGAATCTTCGCGAGATGGTCGAAGAGTATGTCGAACCCT
TTCTCGATGAGGTTTCAATCTTGAGTAATTTTTCGGTGAAATCGCAGTGGTTGTATTTGT
TACCTTTGGACGTGACTCCGAAACGTGTACCTGATAGTAGTCCGTTAGGTAGACACTTT
GCATTGTCGGAAGACGTTTTGCCACAACTAATCACGCCGTTGGAAAAGAAATTAGCCT
CGCAAGTTAGTCTTCGATCAACTATCAATTTCGTCATCTATGTTGTTCCTTGCGATAATG
CTCCATTGTATATTTACACACGTTCTGGTCATCGATCAAAAATTAACGCTAACGTTGAAG
CATTTCTTTCTCCAAGGTGGGGTGGTGTAATATTAATCAATCCACCCGTGGAAACTTGTA
TGACTGTGAAATCTGATGAATTAGTTACCATTGTTCCTGAACAGACTATAGTTGTTGGA
ACATTTCTTACGCAATTGAAACTTCTCTTAGGTATTCCCGAGCCGAAATTTTTGAATGGT
GTTACCATTGTTCCTTTACCAGGATTGAAACTACATAATTGGGAAGTTGATGCATTGCTA
CGTGTACGCACGATTGAGCAATTAACATCAGCAAAATTGACCTTGCAATCACTTGCTCG
ACTTCTTCAAGAAATTGGTAACATTGTGATCACAGATATTGTAGGAAATAGAATAAAAA
CGGCCTTGAGTTTAGTGGAAAAATCAGCTCAACAATTAACACAGGGCGATCTAGCTAC
AGGTTTCTTATTGAGCAAAGAAGCATTCATTACCGCAGAAGCAGCATTTTCCGATCCAA

CACTTTTTGCTTTGTTGTATTTCCCTGCAGATCAAAAATATGCTGTATATACACCTTTGTT
TTTGCCAACTATGGTACCAGTCTTGCTTTCCTTAAAAA

>novel_circ_001099
CCGAGGCAACTCGCTACGCGAGCGACTGGGTCCCGATTTTCCGGACGCGGCATCATCC
CGACACGAGAACGACTGGACCGTCGCCACTGGACATCGTCGATATACAACGGCCCGGT
CCGACAAGCACACACACAACCCTCGCTCGGTGTGGTATCGGTCGTCTCGTGTATTTCGT
CCCATCTGTGCGCGCACGGCGTACGTGTCTGCGTGTCCGTCTGTCGGCGCGTGTACCC
GCGTACGAACGATAATGCTGGCGGCTGACGGTTTCGGTGATCGACGACGACGCGGCCG
GTGAACTAGTGACAGTGTGAAATCGATCGTCAGCGGGGAAACCGGCTAGCGGTCGGC
ACTAAATATACCCGAGGAATAATGGCCAGCGCGACAATGGGACTCCGTCGCAGAGTGC
CAGTGAACAATTCCCCGACCCCGTCGGGACAATCCGCGTCGGCCACTGGCTCGTCCAG
CAAACGTTCAAGGTCCGAGAACAACAACAGCCCGTCCCACGGTAATCTGAACTCGAT
GAACGCGAGTGCACGGGACGAGCCACCTACCAAGAAGTCACGTGGTACGTCGACGAA
CGCGACTAAAGCGACATCTTCGTCGTCTTCGAACGCGTCCTCCTCGTCGCCCGAGACG
AGTCCCTCGTCGAAAAATCGCGGTGGTTCTGTGTCGAGGTCCGGAAACGCGCAAACG
AAAACGTCCACGGCATCGGTATCCACGACGTCGGATCCCGTGTCCAATAACAGTGTGC
TGTCCAGTAGTTGGCAAACCACGAACATGTCCGATGTGTCTACGTACGCTTCCGTGGCT
GGATTGAGCATGGCCTCGAGTCCAACCGCCGGTCTGTGCGCCGGCAGCCTCAGCATGC
CCACCCCGATACCGTATATGTCTTCGTCTATGGCCACGTTCGTGGGAAACGCGACGAAC
CTGGGCAAGAGCTCGTCCGTCTCGTATCTGGACATTTCGAATATGACCGGTGGTTCGTT
GAGCTCGGCCGCATCCGGATTGAACAATGCCTACGTCGGGAATGGAAACGGTGGTAGC
GCGATGGACTCGAAGAACGGCGTGCCCATGCCGTTCTCTGGAATCGCGACTGGCGTAA
ACGGGGCCGGTTACGGGATGCAAAAGGGACAAGCGGAGAACGCGAGCCTGCCGAAG
AAGTTTCAGAACGATAGTATGTTCAACGCGTATCAACCGTGGGTGATCAAAACGTACG
GCGATTTGGCCAAGACTAAGACGATCACGATTAAAAAGTACGCGCGTATATTGCGAACA
TTGAGAGGTGAGGAAGTGAACAGTGCGGAGAACAGTAAGTTTCGATTCTGGGTGAAG
AGCAAGGGGTTCCACATCGGGCAGCCGGAAGGATACGACGCGAAACCGGCGGACAG
GATTATCGGACGTCACGCGGTGACGAGTCCCGGATTGGATCCACCGTTGTACGTGCCC
ACACAGTTGCCGCATAACAAG

>novel_circ_001100
GAAAACATCTCGACCCCGGAGCCAGCCGACATATCCGAGGAGGATCACTACTCGAGCG
CGCTCATGAAAGACGCGGATAAGCTGAAGCTGATGCTGCTCGCGTGGAATTATAATCTT
CAACAGCAGGCTCAGGCTCAGGCCCAGGCCCAGGCACAGGCCCAGGCCGCCAACGC
GGCCCTCTCGCCAAATAACTCCTCGACAACCACGGCCACCACCGTTGGCACACCTGTA
ACCAGCCAATCAGCCATATCCACGGCAAACAACACCATTAACAACTCCACACTTACAG

>novel_circ_001101
GCGCGCTCATGAAAGACGCGGATAAGCTGAAGCTGATGCTGCTCGCGTGGAATTATAA
TCTTCAACAGCAGGCTCAGGCTCAGGCCCAGGCCCAGGCACAGGCCCAGGCCGCCAA
CGCGGCCCTCTCGCCAAATAACTCCTCGACAACCACGGCCACCACCGTTGGCACACCT
GTAACCAGCCAATCAGCCATATCCACGGCAAACAACACCATTAACAACTCCACACTTA
CAGACTCCCGAAACGGGTCATCAGAGTTGGTGGACATGCCGGCCGGAACCGTTCCTAA

TTTGGGCGCGGTTGCCGGAGTCGGTGGCGAGGACATGGCATCCTTGTGGGCATCCTAC
GCGATGGGATTAAAGAAGACACCGCCGCCGGCGTCCGCGGCGACGCCCTCGCGATCG
GATCGTGATCGCGAGTCCAGCCCCCCGGGGGAGGGGACGGGGAGCGCGCACGACGA
GACGAGCAGCAGCGGCAATAAGGAGGACGAGGACGACGACGACGAGGATGCCGACG
AGAGGTTGGACCCCAGACACCACGACCCGGAGCGCCAGAAGGCTTTCAACATGTTCG
TCAGGCTGTTCGTCGACGAGAACTTGGACCGTATCGTGCCCATCTCGAAGCAACCGAA
GGAGAAGATACAGGCGATCATCGACAGTTGTACCAGGCAATTTCCAGAGTTTGCGGAG
AGGGCAAGGAAAAGGATCAGAACGTACCTGAAGAGTTGTAGGAGGAACAAGCGTGG
CAGGGAGGGCGCACCTTGGGACGCGGCGAGGCCCACTCCGGCGCACTTGACCTCTGT
GCAGGCCGAACAGATTCTGGCAACGGCCTGCGAAAATGAGAGCGACAACGCGAAAC
GCATGAGACTCGGTCTGGAGCCCGTCTCACAACCAATGCCAACTCTTCCGGCTGCGAC
G

>novel_circ_001102
ACTCCCGAAACGGGTCATCAGAGTTGGTGGACATGCCGGCCGGAACCGTTCCTAATTT
GGGCGCGGTTGCCGGAGTCGGTGGCGAGGACATGGCATCCTTGTGGGCATCCTACGCG
ATGGGATTAAAGAAGACACCGCCGCCGGCGTCCGCGGCGACGCCCTCGCGATCGGATC
GTGATCGCGAGTCCAGCCCCCCGGGGGAGGGGACGGGGAGCGCGCACGACGAGACG
AGCAGCAGCGGCAATAAGGAGGACGAGGACGACGACGACGAGGATGCCGACGAGAG
GTTGGACCCCAGACACCACGACCCGGAGCGCCAGAAGGCTTTCAACATGTTCGTCAG
GCTGTTCGTCGACGAGAACTTGGACCGTATCGTGCCCATCTCGAAGCAACCGAAGGAG
AAGATACAGGCGATCATCGACAGTTGTACCAGGCAATTTCCAGAGTTTGCGGAGAGGG
CAAGGAAAAGGATCAGAACGTACCTGAAGAGTTGTAGGAGGAACAAGCGTGGCAGG
GAGGGCGCACCTTGGGACGCG

>novel_circ_001103
ACTCCCGAAACGGGTCATCAGAGTTGGTGGACATGCCGGCCGGAACCGTTCCTAATTT
GGGCGCGGTTGCCGGAGTCGGTGGCGAGGACATGGCATCCTTGTGGGCATCCTACGCG
ATGGGATTAAAGAAGACACCGCCGCCGGCGTCCGCGGCGACGCCCTCGCGATCGGATC
GTGATCGCGAGTCCAGCCCCCCGGGGGAGGGGACGGGGAGCGCGCACGACGAGACG
AGCAGCAGCGGCAATAAGGAGGACGAGGACGACGACGACGAGGATGCCGACGAGAG
GTTGGACCCCAGACACCACGACCCGGAGCGCCAGAAGGCTTTCAACATGTTCGTCAG
GCTGTTCGTCGACGAGAACTTGGACCGTATCGTGCCCATCTCGAAGCAACCGAAGGAG
AAGATACAGGCGATCATCGACAGTTGTACCAGGCAATTTCCAGAGTTTGCGGAGAGGG
CAAGGAAAAGGATCAGAACGTACCTGAAGAGTTGTAGGAGGAACAAGCGTGGCAGG
GAGGGCGCACCTTGGGACGCGGCGAGGCCCACTCCGGCGCACTTGACCTCTGTGCAG
GCCGAACAGATTCTGGCAACGGCCTGCGAAAATGAGAGCGACAACGCGAAACGCATG
AGACTCGGTCTGGAGCCCGTCTCACAACCAATGCCAACTCTTCCGGCTGCGACG

>novel_circ_001104
GTGGATGGAAGGAACTGAGAGCTTCTTCAGAGTTACTTCAGCTCTGAGTCTGCTGTAT
AATCGTCGTACGAGAAGAGAGATATATATAGCGAAGGAAGATAGGATAGACGGTGGTT
CCCTGGTCGTAGCGTACGTCACTGGGAACACGTGGTGTGTTGTGATATGATGTACCATC
GGATTCCACCGTTTGGATACGGTGCGCTCAGTCAGTGACCAGTTTTGTGCGACTTGGC

GATCGTCTGTGTCTTGATGATCACAGACGAAGAAATTACACGAGGTTGTGAATATTTG
TTATTAAACGGATATTTGAAAGGATCGAGAGAAGGGGATTCGGCTCGAGATCTTTCTCG
AGATCTTTTGTTGGTGTATTTTATATATACGGTGTGATTGTGGTGCGCGTGTTTTGAAGG
ATACTGTACACCGATGCGATGCGGTGTGTATCCTCGTCGTAACGATTCGAAACGATCAA
TTACTGGGAAACAGTTGATTAAGTGTATCATTATGTGAATATGTCAGTGGCCGCCGGTG
GGTTTCTCCTCGGAAAGACCGAGCCTATTGTTGTCTATTCAACGTTCGTGACGTTGGTT
TAAAACAATCGATTGCTCGAACACTTTTGATCGACTCGAAGAAGAAAACCAGACTTGT
TAATTGGAATGGTGATAAACGAGGAAAGGAAAGAGGGGGAAACATGAGGAAAAAGA
ATCTAAGAGTGGTGCACAAGCAGGTGTACGATCGCGTGCAGATCCAGCAGGTACACGA
ATTCGATTCACCAGCCACGTGGTTGGTTTCCTTAGTATTTTTTTTTCTTTTTTTCTTTCTT
TTTTTATCAACACAGCGATCGATCGATCTCCGCGCCAAGCCGATTCCCGATCGCCACTG
GCTAAAACTGGGTCAGAATGAATTAAAAATCTGGAACGTGAGAGATAAATTTCTCGCG
CGCGTGCAGTGGAAGGGGAGAGTTTGTCGCGACCAATCGCGACAAACGTTATTACAGT
TCATTTACAGGGTGATCCAAAAGTCGTGCCCCTAAAAAGAAAAGAAAAAAAAGGCTC
GATTCGTATTACGATTTTTTCCCTTCTTTTTTTCTTCGAATTTCTCTTCGAAATATCGGCA
CGATTTTCGTAATTTTTTGCAAAATGTTAATTAACTGCATACTGTTCGTCATAGAAGAAT
TTCTTATTTGGTTTATTCATTTATTTGTTTGTTTATTTTTGTTTCGTTCGATCTTTTTTTAAG
ATTTCTACCCGCAATAAACCGTATGTCAATAAACTGTATGTCACATTGCTAATTATAGTC
GATTGTTCGAGTCACTATTTATTGATACGGATATTTCAGTTGACATGAAATATTGTTAATT
ATAGACGATTACATGAATCGTCTGCGCTGTTTATCGTCATGCGTACTTACTCATGGACGT
GCTTGTTAATAAGACGCACTTAATTCCTTCAAGCAAAATCCGATAATCGTAAATTTCGTG
GAATTTTCATTATTTCAATGATGAAAATCGAAATATAATTTAGCGGTGAAACAAGCGGC
GTGTGTTAATAATTTTATTCGCGCGTAGAAATGAATAATCGGGAGGGGGAAAAAAAAA
AATCGGATAAAATTATTGCTCGTAAAAAATATATATATCCTTACGTCAAATTAATCGAGG
AGGGGGGGGCGGGGAGAAAAAGAAAAGAAAAGAAAAAAGTATAGTTTAAAATATACC
TATCGTTAAATTTGTAGCCATTTATTATTGCCAATTAAAAATTTGCAATAATAAAAAAAA
AAAAGAAAAGTCATCGAATTACTTACTTTTAAACGGCAACAATAATATTAATAATATTAT
TATTGCCACAGAGATGTATTATCCTTCAGCCATCAGCCATATTCCCCAGAAAGTAAATAC
TCTCTATTATTATTCACCTCTTAATATTATCATATTAATTAAGGAATGTTAAAAAATAATTA
ACCGACGTTAATAATAATTATTAGAAGCTCATCTCTCTCTCTCTCACTCTTTGACTTATCT
CCAAAGTACGAAACACCTCAACCGTATTTTCCACCGGTAATCCCGCTTTGGAGAGCTA
AGATCGAGCGGTTGTTACAGCAAACGGATGTCCGGTCAAGTTTGGAGCATTTCTCGAG
TACACCGGTGAAATCACTTCCGGGTAAAAGGGAGGATACACCACCGGCATCGCTAACG
TCCCTGGCGCCGCTAACCAGTTTGGCGAACTTGCCCAGCAGCACTCTCTCCTCCACAC
CCCTGTCCCTTGCACCGGCGTCGAAACTGCTCACCCCGGCGGACAACAAGGGTCTCAT
GAG

>novel_circ_001105
AGCGGATCTTCTATGCCGTTCTCCACCGACTCGAAACGGGGGGAGAGAGAGAAAGAG
GATTCGTGCATCAAGATCGGATCGATTCTCAAGAACGAGAAAGTAATGGCCCATGAAA
ATTCACGAGGTACGGAGCAGGGAGCGATAGGCTGCGTGTGAAGGAATATCCGTGCGTC
GAAAAGGACAATTAGACCCTTTATCCGGCCAGGTGAAATTCGAGGATGGAAAAGAGC
GGTGATGCAGACGTGGAAGGTGCAGTCGCAGTGAAACGACCG

>novel_circ_001106
GATGGTTTTCTGTGATATCTGGGAACAATACAACGGGCAGTGCAAGGGTGTACAGAAG
AGTAGATTCATTGGTACGAGCAGGGGTACCAGCGTTTCTTTTAGCCGCACCTTTAAGGG
CATACATCTTGACCCACTCGAAAATGGAAAATGGCAATCTACGAGCTCATTATACAAAG
ACCACGATCAGAGCTGGAGAGATTCTACGATTAATAGCGGTTTTCCAAGACACGAGGA
AGTGTAGTACGGTTTCCTTCGGAATTTCTGGCAGCTCTTCCGAAAAGGATCAGTACGCA
CAATGTTTGGATCCACATGGTCGTGAGGTATTCGCTTCTTTGTCGGCAAGAGGGGAATT
CTATGCTATTTGCCAGAATGGAAGTATCGATACCGGAAGCGATGCAGTATTGTACAAAG
TGCATCATCTTGCGAAAAGACCATTGCCTCTCAGGGTTCGCTTAATAGCTGGTCCATTA
CCTGTACCATTGCCAAGGGAATATGGCGGCTTAATGCAACTGGAAAGTTCGACTCGAG
GTCCAATTGTTTTAGGATGCATTGTACCGGAAAGACCAGTTCACAATCCTGAAATGCTT
GAGCTGGTTGTGACCGGAAATGGTGCACCAAGAGTGAGAAGAGCACGATTAGGTTAT
CCATCAGAAGCTAGACTTCTGGCATCACCGAAAATGCAACGATTATTATCTGCCTGCAG

>novel_circ_001107
TTCAGAACTTGAAGAATCTGATCTACATAGTGAAACTAATAAAAATTATGGAGAATTTG
TTGAACATCCTTTTTCTTGGGAATTTGAATTACCACCTTCAATGATGCTTAGCTCTAACT
GTGCTTCATATGATCGTGTTTCATGTACTATATGGACACGACGTGATATAAAAAGGCTG
AACTATCAGTGCTGGATATCAGTTCGGCTATTATTGCTAGAGTAGAATCAATGCCTGAAA
GTATGAATTACTTTTATGGAGGTCTAATGCTTAGTGTGGTATTATCTTTAATACCATCTATT
AAACGATTGAGCGATCACGTTGGAATGGACAATAGTAGCAATGTAACTAATTCCTTAAT
ACCAAATGACTTAACTTATGTCAATTTGGAAACATATAGCGATATTTTATCTAAAGTTATA
GATTTGGCATTTGGAACAACTCTTTG

>novel_circ_001108
AGGTGGGAACAGTTGGTCGTATCGAAGCTGAAATGGGAATTATCCGCCGTAACGCCTG
GTGACTTTTTGATGCACATCCTGAGCAGACTGCCAGTACCACGTACTTGGGATCCAGTA
ATGGTCAGAAGGCACGCGCAAACTTTCATCGCTCTTAGTGCAAGAGAGTACAAATTCT
CGATGTACACACCAAGCATGATAGCGGCAGCGAGCGTCGCGGCAGCCCTTCACGGACT
CGATTGGACAGGGAAAAGCGGTTACGGACTGGCCGGCCTCCTCGACGAGCTAACTCG
CATCACGGCTATCGAACAG

>novel_circ_001109
GTATACGCACGATCGTAGAATCATAAAAATCATCGATAAACCGAGCGCTTCGAAAACAC
ACGTTGTATATATTCAGCTTCGCTCGAGGCGGAGCGACAGAGCCTCGTTGCACGCTCGT
TCCAACAACCGACCGTGTTCCGTGTTTTTAAAAAAAGCGCGGGAAAAAAGCATCGGC
GAAATTATCATCTGTCACCGAAGCTCTCGAGGTGTTTCGAGGAATCGGCTCGATCCAGC
TTGCAAGAAAGTCGACGTTCCGACGACAGTTGGCGGTGGGAACTGATTGGTGAAGAA
AAGAAAGGAGGGAGGGGAAAAAGGAAAGAAAGAAAGAAAGAAAGAAAGAAATCTG
GGAGGAGAAGAGGAGGAGATCCTCGTTGAAGCGGATCATGCGGATGATCGTCCGGTT
GACGATCGTCTCCTGATCGTTCATTCTCGTGTCTTGTTCATTCAAACAGTTGTGGCCGC
GTTATGCAGTGATATATCCTCCCTTGAGAAAGCTCCCGCGATTGTGAAAGAGGAAGAAT
CATGTCGAAGAGTGGTGACTCGTCGGCAGGTACCACGAAGAACCCCGAGCACGAGAG
CGGTTACTTCGAGGATGGAAGCGACGTGGAGACGATCGTTGAGGAGCCAATTGTCATT

GAAGAAAGGAACTACGGATCGACTTGTTACACATTATCCAAGGACATTGACGATTTCG
ACATGAGGGATTTAACGAGCCAGCTCCCGATTACGACGAGGACAGAG

>novel_circ_001110
TAAACCTGGCAGACAGCGGAGGCCAGAGGGTTGACATGACGCACTTCGATCTTCTGA
AAGTCCTTGGAACCGGAGCTTACGGAAAGGTGTTCCTGGTGCGCAAAAGAACTGGAA
CCGATGCAGGCAGGCTCTACGCCATGAAAGTCTTGAAGAAAGCGTCCATCGTCCAGAG
AAAGAAGACGACCGAGCACACCAAGACGGAGAGACAAGTTCTGGAGGCCGTTCGGG
ACAGTCCTTTTTTAGTTACATTGCATTATGCCTTTCAAACGGACGCAAAGTTACATCTGA
TTTTAG

>novel_circ_001111
CGCGTTCAGATTGGCGGAAGAGAAAAGCTGAATGGAGGCACCGGCACTCTTGAGGAC
CCTTGCGCTTCTCACGATATGGCTGCTCCAGGTTGCGGTGCCGGGCGCCGTTGCGTCCT
ACACCATCGGCGTGGGCAGAGCGGACACAACCGGACCCGTTGCTGAAATAGTTTTCAT
GGGCTACGCGAAGATCGATCAGAAGGGATCAGGACTCCATCTCCGAACGTTTTCACGG
GCATTCATCATCGACGATGGGGTGGAGAGGTTCGTGTTCGTCAGCGTGGACAGCGCTA
TGATAGGAAACGGGATACGTCAAACGGTGGTGGAGAATCTCCAAAAACAGTATGGCGA
TTTGTACACGGAGAAGAACGTCATGATCAGCGCAACCCACTCGCACTCCACCCCTGGT
GGATTCATGTTGCACATGCTGTTCGATCTCACGACGTTCGGTTTCGTTAGGGAGACGTT
CGATGCCATGGTGAATGGGATAACCAAGAGCATCGAACGTGCCCATAACGCCATGGTG
CCTGGTCGGCTCTTCATCACCCATGGGGAAGTTCACGGCGTGAATATAAACAGGAGCC
CGTTCGCTTATCTGAACAATCCAAAAGTGGAGAGAGACAAGTACAGGGACAATGTGGA
TAAGATTTTGACGCAAATACAATTTTACAAGAACGAGGACAACAAACCGTTGGGTGTA
ATTAATTGGTTCGCCATCCATCCGACTAGTATGAACAACACGAATCATCTGGTGTCCAG
TGATAACATTGGCTACGCATCGGTTCTTTTCGAGAGAATAATGAACAATGATTCACTGAT
TGGCAAGGGCCCATTCGTGGCAGCCTTCGCCTCCAGCAATCTCGGCGACGTTTCACCC
AACACACGTGGCCCGAAATGCGAATTTTCCGGCAATAATTGCTCGAAACAATACACTT
GCCCAGGGAGAAAGGAGATGTGCTTCGCCTCTGGACCAGGGAGAAACATGTTCGAAA
GCACCAGTATCATAGCCAATAGGATGTTCAAGGAATCTTGG

>novel_circ_001113
GTGAGTGTGCCATATTTTACATCAACGTATCTTATCTCCCCACTTACTGGTATGTTAGAAT
AATTCTATTACAATTATTTATCCATTTAAGAAGAAGAAGAAAAAAGACACTCCCACTGA
AGTACGTACAAATTTCCATACTCTATTTAAATCACTCTCACAGATCCGAAAAAGGATCC
CTCTACAGCCAATCAAGATTCCATTTAACAACACTTTTCCACCTTATCCGATCACTCCGC
TCGACCACTCCGTCCATCAGCTAGAAAGGGGACCAGAAAAAAAAGGAAAAAAGAGA
AACTCTCGACCCAACGATATTTTTTTCGTAACGCTCTATTCTCGTCCATCCTATCCGAAC
CAAGATGGTGGGTCGCGGAGCGTAAATACGAACGATTAGCGTGTCATCCATCATCCAA
GTTGTGACGTTCGGTAACCGAAACATTCGTTCGAACAACGAACTCTGTCGCGTTTTCG
TTTCCCCCAATGAACTTGGGGCAGACGCCCCCTTCCGGTCGCTGGGAACGAGCTTTTT
ATCGCTAATCGGATTTTCCGAATTCCTGAAAGTCGCGGAGATAAATAGATTCTTCCACC
GATCGATTCCAGGAGGGAGAAGGATAGATGTAATCGAAGATCAACGTTTGCAATGAGA
TCGGCGATGACGAGGGGAAGGAAACAGAGTGCAATGTCTCTTTTGAAGGCTCGTGCG

ACGAAGGATTGCGTTGTAGACGGCTCTGTTTCTTGTTGAAGATTGTTTCCG

>novel_circ_001116
CAAATTGCAAAGTGGGCGGGAACAAAATCGACATGGGCGACTCGTTCAGCGTAAAGG
TACCGAAGAAAGAGGCTGATGTTATTTTTGTAATCGAACAACAGATACCGAATGATAAA
GTGTACAAGGAAATGATTACACCTTTAATGTCTGAACTTAGGGAGGAATTGAAACAAC
AAGGAGTAACAGACGTGCACATTGGATTAATCGGGTACAGCGAGATGATGAAATGGCC
ACAACACTTTACATTAAATGGCGATACCAACATAGATGGTGAAGTCAAAAATATGAAGT
TTGAGGAAGGAAAACCAATAATTTCTTACCAGGAAGCGAAAGAAGGCAATACGGAAA
AAAAAATTGATTATCTACACCAGAGAATGGATGTCGAATTAGGTACTTTCAAATTGACC
GATGCTTACGAGGCAGCTATTCGATATCCATTCAGACCTGGTGCAGCGAGGGCTGTGGT
CGGAGTGATCGCGAACCCCTGCGAGAAGAGTCCATTCCCCATTTCG

>novel_circ_001118
GAGTATTACGGCCGCGTTGGCGTATTGGCTAGCGGGAGCAAGTACAAACCGATCATGG
AGTACAAAGTGCCGGAGCACATCGAAAAGTTGGCCAGCTCGAAAACTGGTCTTCCTTC
GGGGCAACAGTACTATCTGGACGGGAGCGTGGACGTTACCGACAATCAAGATGGAAA
GAAGTTCAACCTCGAAAAGGTGGCATTCGTGCTCAATGGTCAAAAGTTGATCGTGATC
GACGGCCCCGTAACGTGGACCAGCAACAGCGTCAACGTGGACACGAATATCGGTTAC
GGGGACAAAAATCTGGCCTTCAAACTGGATGGTCAGTGCTCCAAAGACGATCACAGG
CTCGTAGTGTCCGCCATGCCGTCCAGCGATCCCAACATCGGTTTCAACCTGAACTGGC
AATTGAAGAAGACCGACAACAACTTGGAGAACAAATTCTACTTCGTCCACGGTCCTGA
TCCAAACTCTCAGACGAATCGCCTTTCCTTGACGCAGAAAGCTGTTTACAAGCTGAAC
AACAAGGAATTCTTCTTATCCGTATCCAACGAGCTCACCTATCCTATCGTAAATTTGAAA
TTAAAATACGAGGGAGAACTGACCCAAAAATCGGTCAGCAGCGACCTCGAATTCAAGT
ACAAGGAGTTCAATTTCGGTACGGAATTGTCTGCCAAGGTGGGGACGGAGAAGCCCG
GTGATTACGAGGTCGAGTTCGAGGCTCAATTAATGGAGAACGGGATCGAGTTGAAGGC
GAAGCGGAAGATTTTGGACGGGCAGAAGAGTCAATTCTCCAATTCCCTCGAGTTGAAG
CCGAGCGGTAAGTATACCGCCGACGCGGTTGTGCTTTACAGCAT

>novel_circ_001120
ATACAACGTTGCTATCCAAGGTTGAGCCCAATCCCAACGAGTCGATCTTGTACGACAAT
TCGTTGTTGAAGGAGTACTTGCGGAAATCTTCCGGGAATTTGACGCGAGGTTTGATCA
ATCCAAACTGATTCTTGGCGTTCAACTTGTCCGGATCTGTCGACGCTCTGAGATTCCTC
AGATGTGTCTGGATGAACGATCCGACTTGATTCACCGTCTCCTTGTCCAACACCTCTTT
CAACTGGTTGGCAGCGTGCGGACACGGGCACGCGACCAACGACAGATACGTGTTGAT
CCTGATTTCGCTGTCCTCCTCCCGATCGGCCAACACTTTGAACATCACGTTCTTCCA

>novel_circ_001122
GAAAGGGGAGTCGAGAACCTTTGATGTGTCGTTGGGAGGAATATTGGGAGGAAACGC
GAGTCTGAAACGGATGTGCAGACGGATGTGCAAAGTACCGTGTGCTACGAAACGAGT
CGAAAATTGACTCGAGAGGGGGAAAATTGTTGATACTTGGCGGTGTACTCCCCCCCCT
ATTGTTTGCTTTATCACTGTCGTCATCCGCGAGGGAAAACTCTTCCTCTCGAAGACGTG
GCGCTCGATGAAAGAACACGGGCCGCCAAGTGGAAAGGCAAGAGAAGACAGAACGG

AAATAATCGAACGCGTCGTTTGAGAAAACGTCGTTTTCCCATGTAACACGGAAGCCTA
CCACCGGTTATCGATTGATCGCCGGTCATCGCTCATTGTCCGCGTTTATTCTTCCCTCTA
GTCGCGCCCTACAGAGTCGCTTCCTCCCCCGCCCAGTTAACGAAAATTCGGCCTAAAA
CTCAGCCAGTCCCCCCGGTGCCTTTCGACTGGCAAGCAACCGAAGTACGAGCGTAGAA
TCGGGACGCTCGATATATATATCTAAATAGAAAGAGAGAGAAAGAAAGAGAGAGGAAA
TAAAGAGACAAGGAGAAGAATATAGATAGAGAAGATAAAAGTCTCGAAGAAGAAAGG
AAGGAAGGAAGGAAAGAACGGTCGAAGCGAAGGAAAAGTATTACTTCGAAAGCCAG
GCCGGTTAAAACCGGTGGAAAGGCATACATATATGATGGCGGTGTGCATGTTTTGGCTT
CTCACGTACGTGGGACAAGGGCTGGACCTGGCCGTGGGCAATCGTCCGGTGATCATAA
TGCAGGGTGCGCAGGAGCAGCAGGACTACCGGCAGCACGGCAGCGGCATCAGAAAG
AAGAGCGAGTTGAATTCGGTAGGCAATAACCTGTTGCACTTCAGAATATATCCTCCCGA
GGCCTCTCACAGGGACGACCTGTCCAATTGGCTCCTGTTGAACGACGAGCCGCGGATC
TCCTCGTGGACACCGGCCGGCATATTGATCGACGATATGATCAGGGAGCCTAGGGAGCT
CCAGACCGTATTTTTCGCCCTGTCCCCAGCTATGCTGAACACGCTGTACACGGAGTACG
TGAACACCGCCTCCTCGCAACCGGCCGACCACTTTCTCCACGAGGATCAGCAACAAG
CCGTTGGCCAAGGTCCGTTGGTTGGCCAACGCGGCAAGAAGCTTCGTATCGAATGTAG
ACACTCGAGGAACACGGTCAGCCTGCCTATAAGGGTGTGCGACGACGAGAAGACGAC
GGGGCGGAACGGAAGGCTCCAGGAAGCCAGACAATCGAACTACGACGATCTCTTCGA
GCAGAAATTGAACACTGCTGCTAATATGAAGACTGACCCGACGTCACGGGGATCCGAG
AAGCTTCAACGGGATTATCAGAATAACGGCGCAACGTTTGTCCCGAGGCCACCGTTGT
TCGGCCTGGACGCGGGCAAACCTCCCGGAAAGAGGAGCCAAACTCCTGTTGGACGCC
GCTCGATCGACCTCTCAGGAGAGTCGGCCCCGCATCAAGCTCAACCGCCGTCCTCCCT
CGGGAACGGTATCAGCGTCGCTTCTCAACTTATGCTCAGATCCACCAGGGGAAGCATA
CAGTACGACGTACCGCAAATCGAATGTCCTATCTCAGAGGACGGGATGGAAAGGTTTG
CGTGTCCCTCAGCGGACAGAATGGGTAGATACCATTGCATCGACGATCACGCCTTGTGC
GACGGTTTCATAGACTGCCCTACAGGCGAAGACGAGGACAGGCAAGCCTGCATGTTTT
ACAAAACT

>novel_circ_001123
GTCCATTAAATGATGAAGAAATTTTAACTATGTTTACTGAAAATAAGGAACCTACAGTA
AAAGAACTAACAGATGATACTTTTGAACATTTAACTCAAGCAAGTAGTGGAGCTACAA
CTGGAGATTGGTTTGTTATGTTGTAGGTTTTAATTCTTCCTAATACTTTATATACTCTATTT
TAAAGATATTTAATATATACATATAGATATATATATATATATTTTTTTTTTTTTAAAAATAGT
TATAGTACAGATTGTGTGGAATGTGTTAGAATGATTGCAAGATGGGAAGCTGTAGGTGC
AAAATTAAAGCAAAGGGTTAATGTAGCACGTATTGATAAATATACAACTGGAGCATCTA
CAGCAAGAAGATTTAATGTTTATAAAGCTCCTGAATTTATATTGTAAGTATACAGTTTAA
AAAATAAACAATAATAATAATGTAAAAATCTACAATAATAAAAAGGATTAATAATATAAT
GATGTTATAATATTTAACATAATTTATTTTGTACTTGTAGATTTAGACATGGAAAAATGTA
TCGTTATCAGATTACAAAATATGATATTAACTCTTTTGTATCATTTGCAAAAGAATGGTAT
AAAAACGCACGTGCGGAATCTGTTCCTGTACCACAAAGCCCATTGTAAGTTTGAACAC
ATTTATTAATAGATTCAGCCTTTTATTATAATTATTATTAATTATTAAGCAGATATTATAATT
ATTAAATTCTGATTAAAAATTTCTATTTTTAGCGATGATCTCGTACAAATGATCGCAAATT
GCCTTCGTGAAAATCCGTGGATAATGAAACTCGGTAGTATAACAATTGGTGTATTTATTA
TCATTTCTGTAGCTTCTAAATTTAGACGTAAAACTGAAATTCCACAAAAGAAGGACTAA

TTGTTTCATTTACCAAAGATATATATTATGGGTACTTTGTCAACTATTAGTATTTATAATTT
TATATAAAGCACAATACATATATACATACATACATACATACATATATATATATATATATATATA
TATATGTATGTATATAAATTTTTTACTTAAAGACAACTTTTTATTTTTATATTTAACAAATT
ACATAATCTTTTATTTTTCTAATTTTATTATATTTGATTAGTGCCCATAATATAGATAGAAG
ATAAAAATACGTCTATATTTCTAATAATTGCATTTTTTATTTCATATAAAACATATGTAATA
GTCATTGCAAATCTATCATTTTTTAAACAAATGTTTTACAATAAATACTTTTAAATGAAAA
TTATTGCTATTAAATACATGATAAAATATCTATTATATCATGGTATTTTTCTTCCCAGAAAA
AAAATCTGAATTAATATAACAAAATACCTTATATATTATACACAGATCTATTTAAAAATTA
AATAAAATTGAAAGTAACATATTTATTTGGATAAACGTTGTACTTTAACTTATTTTTTTGT
TAAATAAGATTGAATGTAAATTGTTTCACATTCATTAGGTAACAATACATTTCCTTTATTA
GTTTTTTATTATGAGTAAAAAAATAAAGTAAAAATATGATATCAAATGTATTAAAATTCTT
TGTATCTTTCTGTAACTTAATAATTTCAGAATAATTGTTTTGTTTTATGTCTTAATATCATC
ATAGTTCACAATAATGCTATTATTCGCTCACAAGACACTGTTTTACATATTATAGAAGTA
GTTTTGTGCAATAGTATATTTACTGTGATCACGATTTAGTTTTATTAATTAAAAATATTCCA
TATATTTTAAACATTATCAACTGATATATTTATCCTAAAAATCATAGATGTGTCAGCTAGT
GCCATTCTGATACTATTTTCAAACTTTTCTTGTATACATGAAAATTCCATCATATTGCTTG
GTTTTGAGTCAATCCAAAATCCATCAAATTCATAGAAAAGATAACTATAAAATTGATGA
AATGCACGAATTGTTGGAAAACCTTTTGAAGAATTATAAATATGAGTTTTAGCACTTCC
ATCCCTTAGTAATTTTAATGCCATACTTGTTAAATTTATGCCAACTATGGCAAATGCATAA
CCATAACGTGGATGTGTAGAATGCGACAATACATGTGTTGCAGCACTGGGATATTCCTG
AGCAAAATAAACTAAATTTTCTAATCCCAAAATTCCCATTCCACGAAAATCAGTTTTTG
GATCATCACCTTGAAAGCCAATGTGTTGCCATTGTTTAGTAACTCTAGCATCTAGAGGC
TCATATGGCATTAACAAATTCCATAACTTTAATAGTAATAACTCATGATCTGGATTATCAG
CATCATAAGGTGTTCTCCTAAGCTCTTCACATTCCACACAAAGTTGTCTATAGCCCCAA
ATTAATTCTATACATTTACCAAATGATTTAGAAAAATCAGGATGAGCTGCTGGGTTTATT
TTCTTAGTTACCAATACATTTTCAATAGCTTCCTTTACAATTTTTCGTTTTGCTTGGTTTG
TAAATGCACATTGATCAGCAAGATCATTCAAGTAGATTAAAAGAGTTTTGATATTCGCAT
TTTTAGATAATTTAAGAGATTTTTCTACAGCTAGTGTTCTTGGTGCTCCAGGAAGTTCTC
CATAACAAATTCTTTGAAGTTCGCACATTTGCGTTGTATGCCTTAATAACCATTTTATAA
CTGGTCTGAAATACGAGCACACTAGTGACCAGAGGTATATAAACATGTTTTTTGATAAT
AAAAAACCTTGATACGAAGGATATTTGCATAAATCCTTTTATATTTAAGTTTAAATTAAC
CTCTTTCCGTCATTATGTTTCAAAATATAAGTTTCTTTTATTAAATTTAAAAAATTTTGTC
ACTAATTTAAAAAGATTCTAATACAAAAAATCAACACTTTTGTATTTTATATAATAATAAA
TATATTTTCCTTCTTTCACTAGTATCATTAAAATCAATGCACATAACCTATACTAATATAAC
TAACATTTATGACAGAAAGTCTGATTTTATTTAATTGAAAATAATTTCATCTATTAAATTT
ATTTATAATCAACAAATATTTGATTTCTATCGCAAAAAAATTATTGATTTTTTAAAATACA
TGACTTTAATAAAGAAAAATCAATAATAACCTATTAAACTAGCATAGTATCGATACCAAT
GGTAGAAGAGGTCACTCACTACCGTCATCTTATATCAAAAATTTTATGTGTCTAAATAGC
ATACATACCTATTTTTTGAAATTTTAGATAATTTATGATTAGAAACAAACATTATAAATATA
GATAAAAAAATGTACAAAAAGAAAGTGTAAAAATAACTTCAACTTAAATATTTGTATCG
TTCTTATAAAGCAATAGAAAGAATAGAAAATTATTTTCAATATTTTTATTTTTCACCTTAA
ATATCTATCCTTACTAATATACAATGTTTGTAAACACAAATGGAATTTTATTTTTTATTGTA
ACTTAAAAAGTATAATGAAGAAACAATATCATATCATAAAATGAAGTATGTACAGAGAA
ATAACTTCAATCAATTAGATTTTTAAATAATCATCCATCTTTGATTAAAAAGTTTGTCAAA

TCGGTAATCTCAAGAAAGAACAAAGAAATTATCATAATGGAAAACGAACATTTGTCATC
GACAGAAATTTCAACAGATTGTTCGAGCAATTCGGACAGTATACAAAATGTTGATAATC
AATGTAAAGAAATATTGGATGAAGAACAGGGTAAAAAAGACTTGGCAGAAGCTGATTT
AACATTAGATCTTGGAAGTGTGTTACCAAACTATGATCGCGTTTCTGAAAATGATGATT
CGTTAGAGAAACTTTCGGCTGTTCTCGAACAGCCAGAAGATTTGGTGATTTTAGAAGG
TACACAATTAGCAGAGGATGATCAAGATGATAAAGATTTAAAGTATGTAATACTCTAATA
AATTATGTAATACTTCCTTTTTTAATCAAAAAAAGAACAATTATATGTTGTGTTGAATTG
AATGAATATTGTATTGAATAATTTTCATAATTAATAGTTGTATTCTTCGAGAACGCATAGA
GTCTCTTAGTGAAGTGATGCAAAATTTAAAACAAGAATTAAAAAAGGAAATAGAACTT
TGGAAGAAGGAAAGAGAAGAATTGCAACTCTTACGTGAAAAAGACGATGAATTGGCT
TTGGAAGAAGCAACTGCAGCTGCACGTGCAGCTGCAGCAGCTTATGCAGCTGAATCGC
CATTATCTAACAATTTAGGTAATCTTTTTTAGAAGATAAAACAAAATATTTTAAAAATATT
CAAAGAATCAATTAATTTATGATACAGGAGATCTTTTAGATATTACTTCTGAAGATACGT
TTAGAGAATTGACAATACTTGAATATGAAAAAAGGTTAGCTAAATATCAAGATGAATATT
CCTTTAGCCAAGCAGAGAAACGTTACAATGCTCGATGGAAAATGGTTGCAAACGCATA
TAAACAAAAATTGATGGAAGTAGAACGACTTTGTAACGAAGAATTGGAAAAGGTTAA
AAAAAATGTGAATCATTTGCAACCACTTAAAGAAATGATATCTCAATGGTATACTGATG
AAGAAAATCATGGTGATTCAATCAAAAGAACTGATTTTGTTGCAAATACACAAAAAAT
CAATACTTTTAATGAAAATAATACGCGTAATAGTCACATATTTCAAAAGATCGATGCAGA
AGTCAATATGGCGCCAGAAATATTCGTTGCTCGATTCAAATCCGATGATTTTACAAAAAT
GGATAAATTTTATGGATAATTATATTTTTTTCATATCTATTGAAATATTATATTTTCACTAT
ATATTTAATATATATTTACTTATATTATGTATGTGTAACATTTTAGAATTGTAAAAAAAATT
AGCATAAATTTTAAATTTAAATTTTGGATAACATTTTAAGGGAATAAATTATTTATTACTA
GAAAGTATACATACCTTTCGTTGCTTTATGAAAAAAGATTTTATCCTTGAAGAAAAATCT
TGACACAATAACACAGTTATATATAAATTATGTTCATTCTATTTTTTCTTTAATTGAATAA
AAAACTATGAATATGAACCAGAAGATAGTTGTAAAATTTTTGAAAAATTATGCAAACAC
AATGAAAAAAATTTATAAAACTTATAATTTTACAATAACTTTGAAGCAAATTAATTTCACT
GAAATGAAATATAAGCACTAAAATTTAATAATATATTGTTTAATTTACAAAATAACCGTCC
GGAGTACACTATTTGAAAGTATTTCCGCGCGTTCCTTTGTTCACTAACGAATATAGACTA
TTCCATTAAATGACAGTATTACCACTCTTAATACGTTTTGTAAGCAAACTTCTGTCATAA
TTATTTGAAATGAATTTCAAATTTCTAATTATTATAACAAGTTTTTATAATAAATTAATAA
ATAAGAATGAAGAATAATGTTTTTAAATATTTATTGAATTTTTGCTATTCTAATATCATTAA
AAGAAATTATAAAATAATTAAAAGGTTAGAATTTAAAATGAATCGATATTTAAAAGAAAT
GTTGTATTTAAAAGAAATGATTTTAAATAATTATTTATTGAATTTCCAGATTGAATAAAAT
GTAGTATTCGTCAAAATTATTGCATTAAAAATAACAATTTTGTTAATTTTAAATAAACAA
AATTGTTCATTTAAATCATATAAATAACTTCTATTAATATAAAAAATATAAAGAATTGGCA
AAAAAAAAAATACATTAAATTTATCTTTTATGTTGTTAAATATTGTAAACAAATTAAAAT
GTTTAAACGAATAGAAAGTAATAGTGAAAATGAATTTTCTCTACCTGCAAAACGTCCAA
AGTATGATATAAAGAAAGGGAATTTGTCAGATACTTCTATAAACAATAAAGATTCAAGA
AATAAAAAATATGGAAGAGATGATGTTTGGGGAGATGATTTTGCAGAAGAGGATATTGA
AGAAATGGATTTTATAGCTACTCAAGCTTGTTTGCAGGTTTTATATATATATATCTTTATTT
TTCTTTATTTATTTATATATCTTTTTATGGTTTTTTAATATATAAATTTATTTGTTTTTATATAT
ATTATTTTTATATTATATATATTAATTTATATATTTTATATATAAATTTATTTTTCAGGATGATA
ATATTGTAAGTCAATTAGATATAGATAAAAAAACATTTATAGAAAATGCAATACCATCTA

CTAGTAAAATTAATTTAAATAATAATGATCATAAAATATCAAAAACTTCTGCACATGTATT
AAGTAAACAAAGAAGAAAAGAAGAAAACATAAAAGATATAAGTACTGTAGTTTCTGTT
GATTATAATGAGTTTGAAGATAAATTAATAAATAAAAAAATATATAATTCAACATTTAAAT
TAGATGACAATGCTACTGCGGGTAAGTAGGAATTTCTTAAGAAATCATAATATTTTATTAT
AGTTTTGCATATTATTGAAAATAATATCATAATTAGAATATGCAAAGGAATTAGAGAAGT
TAAAATATGAAAATCAGAAGTTATTAAATGACTTCATAACTAAAGAAGGAGAAGCTGTA
TTTTTACGAAACCAATTGCAACAAACTCAGTTAAGAGCAGAAAATGATAGATTGGAAA
AAACACGTTTTATTGAAGAACAAGAAAATCAACATAGATCGGAAATTAATATTATTTGT
AAAGAAAAAGAAAATTTAAAAACACAACTTGAATTGCAAGTTAGTAATTTATTGATAAT
TAATTTTTTTATAATTCTATTTCAAATAATATATTCTGATTTTTTCTTTTTATTATAGACATT
TGAAATAGGAAATTTAATAGAACGTTGTAAATTATTAGAAAGTGGAAATATTAAATTAAC
AGAACCACATACTATAAATTTAAATCTTTCTATGAATAAAAATAAATTTAGTTCACCAAA
GAATCGGTAAATAAATCTTTAAATATTAAAAATATATAATAATAAATATTTTTTTTAAAGAT
AAATATTAAGAAAATATTTAATAATTATTTTCAGATCATCAGTTTCAATCATGAAACCAAT
AAAAGTTACAGACACTTATGTACAAACTAGTAAAATGATTAATAAAAGTAATCATTTTCT
CAAAATATGGAATCCATGTAAGAAGATTATAATTTAATATGAAAAATATTAATTTTTATCA
TTTATATAATTCATAAGAATGAATTTAATAAGAACATGTTGTAGATTTTCCTCTTGCAAAA
ATTCCAAAATCAATATACGATCCACCACAACCAGAGAAATCTATAGTTGATATTCAGATT
ATAGAAAAAATAGGAAGACGAAATTTGCCAATTTTACAAGAAGAAGATACATCAAGAA
TATTCGGTAATATTTCTATTTAAATTATATTTTATTCAGAGCTTAACTACTTTGTACATTTTA
TAGAAAATCCAGAATTAGTAAAACCAATAACGACTATGATTGATGATAAAAAATTATCA
GTGGAATTTATTGTGCCAGAAATAGTTGCTCTTCAACGAAAAACCAGCTTAGAACTTG
AGGCAGAAGAAACTATACCATTAATAAATAAAGTAAATTATTTTCATATATATTATTAAGT
TTATTTTTATATATATTAACTTTATTTTATTAAACAATATACAAATTTATTAATAATTATATTT
TTAGTTAATTTCTACTGCAAGAGAATTAATTTTAAATGTAATAATTATTCTACAAACGATT
TCCCAAGCTATGAAAAATGACGATATTCGAGACATGAATGACATTTATTTTTCTTCTTTAT
ATTCGAGTTCTGACTTCAATGGAAAATGCGTGTGTTCTGCTAATGCATGGCACGAATGT
GAACGTGGTGTTGAAATTAGAAGAGTATTTGGCGTATTATCGTATGTAAGTGTGGAATC
AAGTTATCTTAGCAAATATATTGCAGGAAAAACACGTTTACTCATAGAGAGAGATGAGT
CTTATAAACATTATTCCTATCAAATGGTTCGTTACAATACTTGGTTGAAAGAGAATCAAG
ATTTTGAAATACTTAAAATGATATTGCAGTTCGTTGTTTTAGTTGGGACTACGGTAATAT
ACATTACTTAATGATACATATTTTGATAATTTAAAAGAATATTATTAATATTAAAGTTTTAT
TATTTATTTTTAGAGAAGGTCTCATCAATTTAGTGGTGTTATATGTGCAATTATGATGATT
ATTTACAATGTTCATGAAAAAGTTCAATATTGTTCTGAAGGGTATGCAACTGATATATATA
TATTTAATAAATTAATATATTTTAAATTAAGTTCAAATTTTTTTAACTTAAAATTTTTTATT
CCAGAATGGAATATATTTATCAAATATTTAAAGAAATAATATTTAGTAGACCTTTGCCATA
TTGTTATACATTACTTAATAATTTGATGATGATTTTTGTGAAGTCTACAACTCATTTAAAA
AAACTTTGTATTAATTCACGTATGTAATTTAGTAATATTAATGAACATTTATGATTTATATA
AAAAATATACATTTTGTTAGTTTTATGTATATTAACATTTTTAAATGATAAGTCTAATAAAT
TTTTTTCATATTTTATAAATAATTACTTTATTATTTTTATTATAGAATCAATGGCAGTGAATA
ATTGGAAAGGTTCATTACATTTTACTCCAGGTAAAATAATATTTTTTTTAAATAAATCGTA
ATTCAATTTTAAAAATTATTATCATATCATATCTATTTTAGATGCTTGTCCATTACAAATTC
TTTTAGCACAGGAGGAGAATTATCACTTTGATATATTATCTGCAATTGACATTACGGATAT
ATTATTACAAATTGTACAATATATATTACATACAGATACTATTCCTTTAAGATCTGAAACTT

TGAATTCTTGTAATTGTTGTATGAAATTGTTAAGATTCACTATTAAAACGTTGTGTAAAT
GTTCTGAAGCAAATCTAGCTGCCATTGGAAATTTTAATCCAGATAATTTCCCTGTACAA
AGGAAAAGTTCTTGTAACTTAAAGAACATTTGTACGAAACACTCGCTGTTTAACACAG
AAATAAATAAAATGTGTATAGGTGAAATATACCAGGATAAGTTTTCAGACGAAAATGCT
TGGTCAATTATGAAAAAGAAACAATCAAAAGTATTAAGAGACGGAATAAGATTTTTGT
CGCATTTGGCAATATGTGACCCCGATTTTGTAATTCGATTGACGGATATCGAAGATTCCT
TTCATTTATTTATGAGGAATTTAAATACTTTTGAAAATTTTATTCTTCATGAAAATGAACG
TAAGTATAAATATATTTTGCGTTAGAAAAAATGTAATAAAACTAAATTTTAAACTTCCTC
TTTATTGATATCATTTTTCTATCTTTTTTTATTCTTACCACATGATACGAGTTATTTTAATTT
TATCCCTATTTATCGGTCCACGAGAATTTTTATATTTCCGCTTTGTTGCTTACTCATTTGCT
CTTGGTCATCAGTAAATTTCAATTCTTTTAATATTTTTGTTAAATTAAATTTTTTCGTAGA
ATTTTTTATATATTTTTTCATTTGATTTTATTTTCAGAAAATGAAATATTAATTTATTAATTA
TAATTTTTTTTTTTATAGAAGAGGCTATGAATCGAATAAAGCAAACTTTTATTTTTGATAA
AACGCAACAATCTGAAACAGAACAATTTGTAACAAAGAATTCCACTAAGCAGTTGGAT
TTATTATCAAATTTTGAAAAAATAGTTGTTCAACCAATATCCGAAAGACCTAAAAAGAA
TGAAAATTATAATAAAACTTTACTTGCATTCAAATCATTGTTTAATACTAAAATGGATTTC
TCGTAATTTAAAAATTTTAATCATAAATTATATTTTAATCATAAAAAATTTAATCATATATTA
TAATAATAAGATCAATATCGAAGACTGAATTTATTTGAATTTGAGTACAATTAATTATTAT
TTCATGTATATATTATTTTGTGAATTATCTTCAACTTTGTTCAAAATATCGTTCTATAATAA
AAATGTAATATCTCGCAATATTTTATTATGGACGATTCTTTAATTCAAATTAATTGAGCTC
GACATGTATTTTTAAATAATTTTTATCTCCAAATCATTGATTTATTATAATGTAAATAAAAT
TATTTTATCAAGAAACTATTATTGAGAAATATTTTACAGTGTTAATAAAATATTTTATATTT
TAATTATATAATGTATTTTAATAATTTTAATATCTAACACACATATAATTCCTATTTACATCC
CAACCAATCTTTCTTCCTTTCTAACATCAAAAATTAGAAGAAATTGTTTATAACTATGAC
AAAATTTTTAACTTGAGAAACTATAATAATTAAAATTAAAATTAAATAAAAAAATTTGTA
TGCGTTTACAAACATTAAAATACGTCATTTATTATAATCATAAAGAAACATAAATCCAAA
TACAAAGATATTTAAATTAAAAGACTTTCAAATAAGGATCTTACCTATTATTCTTTTGAA
AAAAATGTCATCTATATATCACATCTCCACTGGACTTTATCGATCAAGCTTCTAAAGATG
CCACAAAAAATAAGTGTCGCTAATGAAAAGATAAAAAAAAATATATATATATATATATATA
TATATACATATAAAACACTTTTTGCACGGGGTTGAAGGCATGTCTGTGGCGTTGGTTTG
GAAAAAAGGAAAAATCCTGGCGCCAAGGCGTGATCTAATCAGAGACGGTCAGTGTTT
CTCTCTCCTGGAATTCATTTATCTGTGGCGCAAATGCAATTTGTAGGAATTCTCCTCAAG
GCCTGGCAATGTTCGGTGTTGATAACGCCAGCCGGAAATTGATAGTACACTATCACGAT
AGCATGTCGACGGAAGTGATTTTCCCGCCTTCCGTTCGGAGCGTAATAATCCCAAAGC
GCGGCATCCCTGTTTCATTCATAGATGTAGAACGATTCGAGAACCGATTTTCGTTTTTAT
CCCTCGTCCAGTGTGTATCGTCTTTTCATTCTTATCCTTTACCTCGGCCGTTGTTCACGT
GATTCGCGATTTGCCGTTTCATCCTTCTGATGATGGATTTTGAGGACATGTGCATAAAAA
TTCAATCATCGATTTAAAAGCGAAATTGCGAAATCGAAAGGGAAGACGCTGTTCTTTTT
TTTTTTTTTTTTGAATTCGATTTATGAGTTACGGTGAATTGTTTCCTTGAGGAATAAAAA
AAAGAATGAGGATCTCTTCAACAACGCTGTTTCTTCTGTTGGCCTTCTTGATTCAGTGT
TCCATTTGCTATGGTGCTGCGACAACTGCGAATAGATGTTCGTCCATAAAGCTGGTGAA
TGGAAGAGTTCGATTGAGGGTCAGGGGAAGGATCATCAAATTTAGTTGTTACGACGGT
TTCACTCTTGTGGGTACCAAGTATGCAACTTGTTTTCGAGGCCAATGGGATACACCTAC
GCCCATATGCGTCAATGCAGAATGTCCTGTACCACAAACACCAGAGCATGCTCTTATAG

TGCCTAAATATAATGGAGCAATATTATTATATTTTTGCGAGCCTGGATACACGTTGTTTGG
TTCACACGAAATCTATTGCAATGGAAAGCAATGGAATGGCACAGTGCCTTATTGTCGAG
ACACAAATGCGGCACCCCCGACGCAATGCGATTTCGAGAAACCGGATCTTTGTTGGTG
GGAGCAAGATCCACAGCACGATTTCGATTGGCGGCGACACAACTTCGAGACACCAAG
CTCCCATATAGGAACTGGTCCAACTCATGATCACACATTGGGACCAGGAAATGAGGGTT
ATTACTTGTACATCGAAGCATCTGGTCGGCTGCAAAATGACTCTGCAAGAATTATTTCT
CCTATTTATAACGCATCGTACACTGACTCTGGCTGTTTCTCATTCTGGTATCACATGTATG
GAGCAACGATAGGCGCTCTAAACATATACTTCAAACAGGAGAACCTCTTACCTCGTTTG
ATGTTCACCAAGGAGGGTGACCAAGGAAACCAATGGCTGCACGGTATTTTCAATCTTC
CCAAGGCTAAGAAAGGTTTTCAGATTATAATCGAAGGAGTACGAGGCAGCAGCTATGT
GAGCGACATCGCTGTGGACGACATAGCCATTTTACAGGGAAACAAATGTCGTATCGAG
AATAAAAACGAAACTGTGACGGTTACCGAGAGCGACGATGATCAAATCGAACTGGTG
AACGCGCAGCAAACTTGCCGTGGAAGATGTAAAAATAGTGTGACATACAATTTTACAA
CTTCGATGCCACCCACGCCGGAAGTCTGTCTCTGTACCCTCGATTGCGAAGAACAGTC
AATGTGCTGCCCGGATTACGCTGAGTATTGTGTTCTAGCCGAGACCGAAGAGATGATCA
CGGGAACGGAGCAGATCCCGACTACGAATCACGAAGGGGGGGTAGTTTTCGTCCCAC
CCGCGAGAAAGATCACGGTGACCGACGTGGCGACGGTGAGGACAGGTTTCTCGATAA
ATCCAAAGGACGATATCGATCCTGTCACGTTGAGGCCGTCCACCAGCACCACGTTGAA
ATCATCGTCCGTCACGCGGAAAGTGGAGAAACCGATGAGAATTACCAAACCACCAACC
ACTTACCACCAAAACACGACAACAACAACGACAACAACAACAACGACGACGACGAC
GACGACGACGAGGGCAAGAACAACGAGAAGAACAACAACCGTCCCAACAACGACGA
AAATTGCACAGACAAAGGAGACGCCGTTCGTTCGACCCACCACCACCACCACCACAA
CCACCACCACCGGAAGATACGGTTCGGTGAACGAGAAACCTGATTCAGATATGAGGAG
GCAAGATCCCGAGATACATCGGGCATCGAAATTCAGTTTGCCGGGGATAATCGGTGTG
GTGGTTGGAATTTTAACGGGAATGGTGATCTCATTGTTGGTCGCCACCATAATCGTTAG
ACGAAGAAAAACGTACAAACGTGGAACTAATGGATCGGCACTCTCGGAGGATAGCGA
CGTTCGATTTCTAACGTCAGACGAGATTTTGGATTTCACGTTAGCCAGACCCAGTGACG
AGAC

>novel_circ_001124
GGATGGACGAGCGATTCGACGATCGAGAGATTACACGGAAGGAGCGTTAATCCTTTAC
CGGTCACGCATATGAGCAAGTTCTTTGACGATCGACCAGAGTCAGGTGTCGCCACGGG
TATGCAACTGGCGAAGAGCTATTCGAGAAATCCTTACAACGAGGAAGTTCAAGTGTAC
GAATTGACCGGGGGCGTGGAATTGGTGCCGCTTTTAACCAGCAGGCGTTACTGCTCGC
GATCGAAGGCCGCTCGTCACATTGCCACCGACGCCACTCGACCTGGATACACAATAGA
CTC

>novel_circ_001125
GAGCGCCGCGTTGGATCAGGAGATGAAGAACGTGTTGACGTTCTGGATGAACAGGGG
GGTGGACGGTTTCCGGATCGACGCCATCAACCACATGTTCGAGGACGCGAGGCTGTTG
GACGAGCCGAGCGCCAACAGGACCGACCTGTCCAAGGACGATTACGAAAGCTTGGTC
CACTTGTACACGAGGGATCAAAGCGAGAC

>novel_circ_001126

GTGGACGTGATCGATGGAAGGGTTCTGAGCGTGAAACGGGAATTGGGTAACGACACG
GTGATAGTTATGATGAATTTCTCCAAAAATCCCGTCACTGTCAACCTCACCAAGCTGCA
TCCACCTGCCGATCTCGTCGTTTACGCTTGCAACGTTGTCGGCTCCGGTCTCAGCCACG
GCAACTGGATCTATCCGGCCTCGATGACTATCCCCGGATCTAACTCAGCCGTATTCACC
AATTACAAATTGTATTGGCGATATTGGCAAG

>novel_circ_001127
GTGGACGTGATCGATGGAAGGGTTCTGAGCGTGAAACGGGAATTGGGTAACGACACG
GTGATAGTTATGATGAATTTCTCCAAAAATCCCGTCACTGTCAACCTCACCAAGCTGCA
TCCACCTGCCGATCTCGTCGTTTACGCTTGCAACGTTGTCGGCTCCGGTCTCAGCCACG
GCAACTGGATCTATCCGGCCTCGATGACTATCCCCGGATCTAACTCAGCCGTATTCACC
AATTACAAATTGTATTGGCGATATTGGCAAGGTGTAGACTG

>novel_circ_001128
GGAAATTGAAGGTATAAGGTTATCACGAGGGCACGCCCACGAGGAAGAATCGATATATA
TTAACGAAGTCTACGAAGTTTCGAATGGACGAGGAATTGGAAATGAGGAATGAAGTG
AAATATTTTACACGGATCAATTTCTTAAATGCTCGAACGAATATTTACGAGTGAATCGTT
TCATTCTTTCTTTATAAATAACAATAAGTATTTTATATGATATATTAAAAAATAGAAAGAA
AATGGAAGAACGATGTAAATGGTTTCATCGTTCTTTGAATGACATTATTTGGAACACTTT
ATTAAAAGAAATGAAAGGACGAAGAAAGGAAAGGTTAATAATGTAAAAAAATTCCTAT
TGAAATATACATCTTCGATGATAACCTGAAAGATAATCAGAAAAATTTGAATCGTACGA
GGTGAAAAAAGAGGAAGAAAAAGAAGAAACGATTCGATTTGTCGAAATATCTTTTCC
GATATTCAATTCTATGGATCGATCTGTAAAATCTCAAATGCAAAGTGGATGTAATCGAAA
GAGAAGGGCGCGAAGAAAACGAAAAAAAGGAAGTTTGTTAATTTCTTTTCCAACTTG
CCGATTCGTCGAACAACAGGGATGATCAGCGAACCAAGGTTGATAAAGATTTAAAGCG
CGTGCCTCGATTTTTTTCCAGTCCCACGACGGTAATTGCCATCCTGCCTGTAGATCGAG
TGGCTACAGCGTGATTTACAGCGCTCTTGGGACACTAAGATGTGGTGTCTCCTCCTCTT
CGTGCTTCTTACCCTCAACGGATCATCCTCTCCTCTTCCAAG

>novel_circ_001129
ATGGGGCCGCGGCCAGCCGGATGATATCAGTGCGATCAATCTAGGAGATGAGAAATTC
GACCCTGACTCGGATAAAAAGCCGCGCGCAGGACAAATTCTATGGATCCGTGGTCTAA
CACGACTACAGACACAGACGAGCAAAAGAACTCTGGTGCAGAATGTATCTGTGACAG
GCAGATCCCCCTCTCAGGATTACTATATG

>novel_circ_001130
ACGAAGAAATAACTGGGAACAGCCATGTGAGCGCAGCCAAGCCCGAACCTGCGGAGA
ACCATCATGCAGTTAGCCACGCACCTGAAGGAAAGAAGGAGAAGAGTGTTTTACAAG
CCAAGCTAACTAAACTCGCCATACAAATCGGTTACGCCGGCTCGACCATAGCAGTGCTT
ACCGTTATCATTCTAGTCATTCAGTTCTGCGTTACCACATTTTACGTGCAGGGGAAAAG
TTGGAAAAATACGTACGCTGGTGATCTGGTGCGGCATTTGATCATTGGTGTAACGGTAC
TTGTAGTTGCCGTTCCCGAAGGTCTTCCTCTAGCTGTTACCCTCTCCCTCGCTTATTCCG
TTAAGAAAATGATGAAAGACAACAACTTGGTGCGTCATTTGGATGCTTGTGAAACGAT
GGGTAACGCGACGGCAATCTGTTCGGACAAGACTGGTACCCTGACAACCAATCGAATG

ACCGTTGTTCAATCGTACATATGCGAAAAGATGAGTAAAATAACCCCGCAGTTTTCGGA
TATCCCGTCGCATGTCGGAAACTTAATGGTTCAAGCTATTTCTATTAATTCGGCGTACAC
ATCCAGAATAATGCCTGCGCAGGAACCTACAGATTTGCCGCTTCAAGTCGGCAATAAA
ACCGAATGTGCCTTACTTGGATTTGTAATAGCCCTGGGCATGAACTATCAGACGATACG
AGACGATCAACCTGAGGAAACTTTCACGCGGGTGTACACGTTCAATAGCGTTAGGAAG
AGCATGTCCACCGCCATCCCGAGGAAAGGTGGTGGATACAGGCTCTTTACCAAGGGCG
CTTCCGAGATCATCATGAAGAAATGTGCCTTTATATATGGTCGTGAAGGTCATTTGGAA
AAATTTACCAAAGAGATGCAAGAGCGTCTGGTAAAGAACGTGATCGAGCCGATGGCGT
GTGACGGTCTCCGTACTATCTGCATCGCGTATCGCGATTTCGTTCCTGGAAAGGCAGAG
ATTAATCAGGTTCATATCGACAACGAGCCGAATTGGGATGACGAGGAAAATGTCGTGA
ACAATCTCACGTGTTTGTGCATCGTGGGTATCGAGGATCCGGTACGGCCCGAGGTGCC
GGAAGCTATCAGGAAGTGTCAAAAAGCAGGCATCACTGTGCGAATGGTGACTGGAGA
CAATATAAATACTGCGCGTTCCATTGCATTGAAATGTGGAATTTTGAAACCGAACGAAG
ATTTCCTGATCCTCGAAGGGAAAGAGTTCAACAGGAGGATTCGCGATAGCAGTGGCGA
GGTACAGCAACATTTGTTGGACAAAGTGTGGCCGAGGTTAAGGGTGTTGGCTAGATCG
TCACCTACAGACAAGTATACACTCGTGAAAGGCATAATCGATAGTAAAGCGAGCGTGA
GTCGCGAAGTTGTTGCTGTAACCGGTGATGGAACGAACGACGGCCCTGCTTTGAAAA
AGGCTGATGTCGGTTTTGCCATGGGTATAGCTGGCACCGACGTCGCCAAGGAAGCTTC
CGATATCATCTTGACGGATGATAATTTTTCGTCGATCGTGAAGGCAGTTATGTGGGGTAG
AAACGTCTACGATAGTATAGCAAAGTTCTTGCAGTTTCAATTGACCGTCAATATCGTCG
CTGTTATAGTTGCTTTTATCGGGGCATGTGCCGTGCAAGATTCCCCTCTTAAAGCGGTG
CAGATGTTGTGGGTGAACTTAATCATGGACACATTAGCATCTCTTGCATTGGCCACCGA
AATGCCTACGCCTGATCTTCTTCTTCGTAAACCCTATGGTCGCACGAAACCGCTCATTTC
CAGGACAATGATGAAGAACATTCTTGGCCAAGCTATCTATCAGTTGACTGTAATTTTTAT
GCTTCTTTTCGTTGGTGATAAGATGCTCGACATCGAAACAGGCCGAGGAGTTGCGCAG
GCTGGTGGCGGTCCAACGCAACACTTTACTATTATCTTTAATACATTCGTCATGATGACA
CTTTTCAACGAATTTAACGCTAGAAAAATCCATGGTCAGCGTAATGTCTTCCAAGGAAT
ATTCACCAATCCCATCTTTTACACTATCTGGATCGTGACATGTCTATCGCAGGTAGTTATC
ATACAATATGGTAAAATGGCGTTCAGCACGAAAGCTCTCACATTAGAACAATGGATGTG
GTGCCTATTCTTCGGAGTCGGTACTCTATTGTGGGGCCAAGTAATTACAACTATTCCTAC
GCGCAAGATTCCTAAAATCCTTTCATGGGGCCGCGGCCAGCCGGATGATATCAGTGCGA
TCAATCTAGGAGATGAGAAATTCGACCCTGACTCGGATAAAAAGCCGCGCGCAGGACA
AATTCTATGGATCCGTGGTCTAACACGACTACAGACACAG

>novel_circ_001132
ACGAAGAAATAACTGGGAACAGCCATGTGAGCGCAGCCAAGCCCGAACCTGCGGAGA
ACCATCATGCAGTTAGCCACGCACCTGAAGGAAAGAAGGAGAAGAGTGTTTTACAAG
CCAAGCTAACTAAACTCGCCATACAAATCGGTTACGCCGGCTCGACCATAGCAGTGCTT
ACCGTTATCATTCTAGTCATTCAGTTCTGCGTTACCACATTTTACGTGCAGGGGAAAAG
TTGGAAAAAATACGTACGCTGGTGATCTGGTGCGGCATTTGATCATTGGTGTAACGGTAC
TTGTAGTTGCCGTTCCCGAAGGTCTTCCTCTAGCTGTTACCCTCTCCCTCGCTTATTCCG
TTAAGAAAATGATGAAAGACAACAACTTGGTGCGTCATTTGGATGCTTGTGAAACGAT
GGGTAACGCGACGGCAATCTGTTCGGACAAGACTGGTACCCTGACAACCAATCGAATG
ACCGTTGTTCAATCGTACATATGCGAAAAGATGAGTAAAATAACCCCGCAGTTTTCGGA

TATCCCGTCGCATGTCGGAAACTTAATGGTTCAAGCTATTTCTATTAATTCGGCGTACAC
ATCCAGAATAATGCCTGCGCAGGAACCTACAGATTTGCCGCTTCAAGTCGGCAATAAA
ACCGAATGTGCCTTACTTGGATTTGTAATAGCCCTGGGCATGAACTATCAGACGATACG
AGACGATCAACCTGAGGAAACTTTCACGCGGGTGTACACGTTCAATAGCGTTAGGAAG
AGCATGTCCACCGCCATCCCGAGGAAAGGTGGTGGATACAGGCTCTTTACCAAGGGCG
CTTCCGAGATCATCATGAAGAAATGTGCCTTTATATATGGTCGTGAAGGTCATTTGGAA
AAATTTACCAAAGAGATGCAAGAGCGTCTGGTAAAGAACGTGATCGAGCCGATGGCGT
GTGACGGTCTCCGTACTATCTGCATCGCGTATCGCGATTTCGTTCCTGGAAAGGCAGAG
ATTAATCAGGTTCATATCGACAACGAGCCGAATTGGGATGACGAGGAAAATGTCGTGA
ACAATCTCACGTGTTTGTGCATCGTGGGTATCGAGGATCCGGTACGGCCCGAGGTGCC
GGAAGCTATCAGGAAGTGTCAAAAAGCAGGCATCACTGTGCGAATGGTGACTGGAGA
CAATATAAATACTGCGCGTTCCATTGCATTGAAATGTGGAATTTTGAAACCGAACGAAG
ATTTCCTGATCCTCGAAGGGAAAGAGTTCAACAGGAGGATTCGCGATAGCAGTGGCGA
GGTACAGCAACATTTGTTGGACAAAGTGTGGCCGAGGTTAAGGGTGTTGGCTAGATCG
TCACCTACAGACAAGTATACACTCGTGAAAGGCATAATCGATAGTAAAGCGAGCGTGA
GTCGCGAAGTTGTTGCTGTAACCGGTGATGGAACGAACGACGGCCCTGCTTTGAAAA
AGGCTGATGTCGGTTTTGCCATGGGTATAGCTGGCACCGACGTCGCCAAGGAAGCTTC
CGATATCATCTTGACGGATGATAATTTTTCGTCGATCGTGAAGGCAGTTATGTGGGGTAG
AAACGTCTACGATAGTATAGCAAAGTTCTTGCAGTTTCAATTGACCGTCAATATCGTCG
CTGTTATAGTTGCTTTTATCGGGGCATGTGCCGTGCAAGATTCCCCTCTTAAAGCGGTG
CAGATGTTGTGGGTGAACTTAATCATGGACACATTAGCATCTCTTGCATTGGCCACCGA
AATGCCTACGCCTGATCTTCTTCTTCGTAAACCCTATGGTCGCACGAAACCGCTCATTTC
CAGGACAATGATGAAGAACATTCTTGGCCAAGCTATCTATCAGTTGACTGTAATTTTTAT
GCTTCTTTTCGTTGGTGATAAGATGCTCGACATCGAAACAGGCCGAGGAGTTGCGCAG
GCTGGTGGCGGTCCAACGCAACACTTTACTATTATCTTTAATACATTCGTCATGATGACA
CTTTTCAACGAATTTAACGCTAGAAAAATCCATGGTCAGCGTAATGTCTTCCAAGGAAT
ATTCACCAATCCCATCTTTTACACTATCTGGATCGTGACATGTCTATCGCAGGTAGTTATC
ATACAATATGGTAAAATGGCGTTCAGCACGAAAGCTCTCACATTAGAACAATGGATGTG
GTGCCTATTCTTCGGAGTCGGTACTCTATTGTGGGGCCAAGTAATTACAACTATTCCTAC
GCGCAAGATTCCTAAAATCCTTTC

>novel_circ_001133
CTATTGGTCCTCCAATTTCCTGGAGGCCAGTGGTGTCAGTGACACCAAAAAATAATACG
AGTTGCTAGCGTGAAGAAGTATAGTAAAGTTGGCTTAAGCCAACATCGGTGGAAAAAA
CAAAATGGCAACAATAGATGGCAGACCGACCCAATATGGTATCAGTCTTAAGCAACTTC
GTGAGCTCATGGAGCTCCGAGGACGTGAAGGTGTCACTAAAGTCAATAGCTATGGTGG
TGTGCAGGAGATTTGTAAAAAATTATACACTTCACCTAGTGAAGGTCTCAGTGGATCAG
CAGCTGATATTCAACATAGACGAGATACATTTGGTTCCAACCTAATACCTCCAAAACCA
CCAAAAACATTTCTACAATTAGTGTGGGAAGCTCTTCAAGATGTTACATTAATTATCTTG
GAAGTAGCAGCATTGGTTTCATTAGGTCTTAGCTTTTATCATCCAGCAGATGATGAAGA
AAAACCTTTAATAGATGAAGATGAAGCAAAATATGGTTGGATAGAAGGAGCTGCTATAT
TTATTTCTGTGATCTTGGTGGTAATAGTAACAGCTTCCAATGATTACTCTAAGGAAAAAC
AATTTAGGGGCCTCCAAAGTCGTATAGAGGGGGAACATAAATTTTCTGTCATTAGACAA
GGAGAAGTCAAACAGATTTCTGTAGCTGACATTGTTGTTGGTGATATATGTCAGATAAA

ATATGGAGACCTGCTGCCAGCAGATGGTATCCTTATACAAAGCAACGATCTCAAAGTGG
ATGAGTCCAGTTTAACCGGAGAGTCAGACCATGTTAAAAAAGGAGAATCGTTCGATCC
CATGGTACTCTCAGGTACACATGTGATGGAGGGTTCTGGAAAAATGTTAGTTACTGCAG
TAGGCGTTAACTCGCAGGCTGGTATTATCTTTACTTTGTTGGGTGCTGCTGTTGATCAAC
AAGAACAAGAAATCAAGAAAATGAAAAAAG

>novel_circ_001135
GCTATGACATGTCACCGTTTATCAGGAGATATGCAAAGTACCTCAATGAAAAGGCTCTT
TCTTATAGGACTGTGGCATTTGATTTTTGCAAGGTGAAGAGGGGAAAGGATGATCGTAC
TTTACGTACAATGAATGCTGAGAAATTGTTGAAAACCTTACCAGTACTACAATCACAAT
TGGATGCACTATTGGAATTCGATTGTACTGCCAATGACCTAACCAATGGCGTTATAAATA
TGGCTTTTATGCTTCTTTTTCGGGATTTAATTAGATTATTTGCATGTTATAATGATGGCATT
ATTAATTTATTAGAAAAATATTTTGATATGAATAAAAAACAATGTCGCGAGGCTTTGGAT
CTATATAAAAAGTTTCTCATACGAATGGATCGGGTTGGTGAATTTTTAAAAGTTGCCGA
AAATGTTGGCATTGATAAAGGAGATATACCTGATCTAACAAAGGCTCCTAGTAGTTTATT
AGATGCATTGGAACAACATTTAGCTTCGTTAGAAGGAAAGAAAGGTTCTGCAGCAAAT
ACTCCCACGCAATCTGCAAGCAATAGAACGAATGTAAAGTCGGGAGTGTCCGCCCTGT
CTTCCACCAGTACTGCGTTTGGAACAGCAGCTAGTAATAACCGCCTTGACCATGCCGG
AAATGGACATATCGATGAAGCGCTTCGACAACAAGCTCTTGCTGAAGAGGAGGCTGCT
ATGAATCAATACAAGGCAAAAGTGCAATCTCCATCCAGTGGCCCCAGTACGAATCCATT
TCTTAGTTCACCGACAAACAATGCTAATCAACCAATTGTAGATTTATTTGGTGCACCATC
AACGATAACGAATACTGAAAGCCAGCCACAAAAAGCATCAGACGATTTACTTCAATTA
GCAGGCAATCCATTTGCGGACATGTTTGGAACACCTCAATCTATAGCTGCACCGACTAC
ACAAGCACAGAATAATATGTGGATGACTAATGGTAACGGTTTTGCTGCAGTGCCTCCAG
CAAATAATAATTTTGTTACAGATAATAGTTTCTCTTCTGTATTTGGTAATCAAGATTCTCA
ATCTGCTGGTGCTCCAGGAACGGCCGGATCCGTACCAAACCCTTTTATGTCCGACTTCC
CTTCCCTCGGTTCCCAGTCAACGCAGCCAAACGCAGCCGCTTTCGGTCTCTTCGAGCA
GGGTGCCGCGACCAATGTTGCTGCTAATACGAACAGTGGTGATAACCAGCAACAAACG
CAGACCGGAGACCTGTTCAGTGCTGGCGGCCAGGCAGATCTCTTTGGGAGCGACTCT
GCAGTGCTCAAGACCGTGGAGGGTGTCGCTGGGGAAGCCGCAGATACGGCGTCCTCT
GTGACTCTAGCCTCCGGTAAGTCCACTGCCACGCCGCCTCCCAGACCGCCGCCTCCCG
CGACAGCAATAAACGGTACGCCTAGACCAGCTTCTCCAACGGTGTCCGGAGTGGCTGT
TGGCAAATTATCTTCTGGAACAACGATCACTACCGCCACCGCTCCAAGTAAGAGCGCG
TTTGACGATCTGAACGATAGCATTCGAATGGCACTGGGCGGGTCTCCGTCAAGGCCGG
CACCCATAGCTCAACAACTGCCGACCGCGCAACAAGCACAGCAACCTCTTCAACAGG
GTTTTGGCATGTTTGATATGGCTACCAATATGGCTGCCACCGGGCAGTCGGTCATGTCA
GGCGGACCGATGGTCGGCTACGGTATCCCCACCCAAGTCCCGGCGGGGTACGGTTCCC
CGGCAAAGCAACCGATTTCAGTAGCAGGACAACAACAGAATGCAGCGTCTACTGGTA
AAGTCCTGACTGGAGATTTGGATAGCAGTCTTGCTAGTCTTGCTCAAAATTTGACCATC
AACAAAAGTGCTCAGCAACAAGTCAAAGGTATGCAATGGAATTCGCCTAAAAATGCTG
CCAAAACTGGTGGCTCAGCTGGAGGATGGACACCGCAACCTATGGCAGCTACAACTG
GCGCTGGATATCGTCCAATG

>novel_circ_001136

ACCTCGTGCGATGTACAAACGAACCCAACGTGTCGATACCTCAGTTGGCGAATCTGTT
AATAGAACGATCACAGAATACGAACTGGACAGTCGTTTTCAAAGCTTTGATTACAGTA
CACCATATGCTTTGTTATGGCAATGAGAGGTTTACACAGTATCTAGCATCCAGCAACAG
CACGTTTCAACTCAGTAATTTTCTTGATAAAAGCGGCGTCCAAGCATTATGCAGCTATG
TGTATCTGGCCTCTGTCTATGATTTACTAACCTGGAACTAATTTTCTCTCTATTTGGGCAC
AACAGGAGCTCGCATCGGCTATGACATGTCACCGTTTATCAGGAGATATGCAAAGTACC
TCAATGAAAAGGCTCTTTCTTATAGGACTGTGGCATTTGATTTTTGCAAGGTGAAGAGG
GGAAAGGATGATCGTACTTTACGTACAATGAATGCTGAGAAATTGTTGAAAACCTTACC
AGTACTACAATCACAATTGGATGCACTATTGGAATTCGATTGTACTGCCAATGACCTAAC
CAATGGCGTTATAAATATGGCTTTTATGCTTCTTTTTCGGGATTTAATTAGATTATTTGCAT
GTTATAATGATGGCATTATTAATTTATTAGAAAAATATTTTGATATGAATAAAAAACAATG
TCGCGAGGCTTTGGATCTATATAAAAAGTTTCTCATACGAATGGATCGGGTTGGTGAAT
TTTTAAAAGTTGCCGAAAATGTTGGCATTGATAAAGGAGATATACCTGATCTAACAAAG
GCTCCTAGTAGTTTATTAGATGCATTGGAACAACATTTAGCTTCGTTAGAAGGAAAGAA
AGGTTCTGCAGCAAATACTCCCACGCAATCTGCAAGCAATAGAACGAATGTAAAGTCG
GGAGTGTCCGCCCTGTCTTCCACCAGTACTGCGTTTGGAACAGCAGCTAGTAATAACC
GCCTTGACCATGCCGGAAATGGACATATCGATGAAGCGCTTCGACAACAAGCTCTTGC
TGAAGAGGAGGCTGCTATGAATCAATACAAGGCAAAAGTGCAATCTCCATCCAGTGGC
CCCAGTACGAATCCATTTCTTAGTTCACCGACAAACAATGCTAATCAACCAATTGTAGA
TTTATTTGGTGCACCATCAACGATAACGAATACTGAAAGCCAGCCACAAAAAGCATCA
GACGATTTACTTCAATTAGCAGGCAATCCATTTGCGGACATGTTTGGAACACCTCAATC
TATAGCTGCACCGACTACACAAGCACAGAATAATATGTGGATGACTAATGGTAACGGTT
TTGCTGCAGTGCCTCCAGCAAATAATAATTTTGTTACAGATAATAGTTTCTCTTCTGTATT
TGGTAATCAAGATTCTCAATCTGCTGGTGCTCCAGGAACGGCCGGATCCGTACCAAAC
CCTTTTATGTCCGACTTCCCTTCCCTCGGTTCCCAGTCAACGCAGCCAAACGCAGCCGC
TTTCGGTCTCTTCGAGCAGGGTGCCGCGACCAATGTTGCTGCTAATACGAACAGTGGT
GATAACCAGCAACAAACGCAGACCGGAGACCTGTTCAGTGCTGGCGGCCAGGCAGAT
CTCTTTGGGAGCGACTCTGCAGTGCTCAAGACCGTGGAGGGTGTCGCTGGGGAAGCC
GCAGATACGGCGTCCTCTGTGACTCTAGCCTCCGGTAAGTCCACTGCCACGCCGCCTC
CCAGACCGCCGCCTCCCGCGACAGCAATAAACGGTACGCCTAGACCAGCTTCTCCAAC
GGTGTCCGGAGTGGCTGTTGGCAAATTATCTTCTGGAACAACGATCACTACCGCCACC
GCTCCAAGTAAGAGCGCGTTTGACGATCTGAACGATAGCATTCGAATGGCACTGGGCG
GGTCTCCGTCAAGGCCGGCACCCATAGCTCAACAACTGCCGACCGCGCAACAAGCAC
AGCAACCTCTTCAACAGGGTTTTGGCATGTTTGATATGGCTACCAATATGGCTGCCACC
GGGCAGTCGGTCATGTCAGGCGGACCGATGGTCGGCTACGGTATCCCCACCCAAGTCC
CGGCGGGGTACGGTTCCCCGGCAAAGCAACCGATTTCAGTAGCAGGACAACAACAGA
ATGCAGCGTCTACTGGTAAAGTCCTGACTGGAGATTTGGATAGCAGTCTTGCTAGTCTT
GCTCAAAATTTGACCATCAACAAAAGTGCTCAGCAACAAGTCAAAGGTATGCAATGGA
ATTCGCCTAAAAATGCTGCCAAAACTGGTGGCTCAGCTGGAGGATGGACACCGCAACC
TATGGCAGCTACAACTGGCGCTGGATATCGTCCAATG

>novel_circ_001137

ACCTCGTGCGATGTACAAACGAACCCAACGTGTCGATACCTCAGTTGGCGAATCTGTT
AATAGAACGATCACAGAATACGAACTGGACAGTCGTTTTCAAAGCTTTGATTACAGTA

CACCATATGCTTTGTTATGGCAATGAGAGGTTTACACAGTATCTAGCATCCAGCAACAG
CACGTTTCAACTCAGTAATTTTCTTGATAAAAGCGGCGTCCAAGCATTATGCAGCTATG
TGTATCTGGCCTCTGTCTATGATTTACTAACCTGGAACTAATTTTCTCTCTATTTGGGCAC
AACAGGAGCTCGCATCGGCTATGACATGTCACCGTTTATCAGGAGATATGCAAAGTACC
TCAATGAAAAGGCTCTTTCTTATAGGACTGTGGCATTTGATTTTTGCAAGGTGAAGAGG
GGAAAGGATGATCGTACTTTACGTACAATGAATGCTGAGAAATTGTTGAAAACCTTACC
AGTACTACAATCACAATTGGATGCACTATTGGAATTCGATTGTACTGCCAATGACCTAAC
CAATGGCGTTATAAATATGGCTTTTATGCTTCTTTTTCGGGATTTAATTAGATTATTTGCAT
GTTATAATGATGGCATTATTAATTTATTAGAAAAATATTTTGATATGAATAAAAAACAATG
TCGCGAGGCTTTGGATCTATATAAAAAGTTTCTCATACGAATGGATCGGGTTGGTGAAT
TTTTAAAAGTTGCCGAAAATGTTGGCATTGATAAAGGAGATATACCTGATCTAACAAAG
GCTCCTAGTAGTTTATTAGATGCATTGGAACAACATTTAGCTTCGTTAGAAGGAAAGAA
AGGTTCTGCAGCAAATACTCCCACGCAATCTGCAAGCAATAGAACGAATGTAAAGTCG
GGAGTGTCCGCCCTGTCTTCCACCAGTACTGCGTTTGGAACAGCAGCTAGTAATAACC
GCCTTGACCATGCCGGAAATGGACATATCGATGAAGCGCTTCGACAACAAGCTCTTGC
TGAAGAGGAGGCTGCTATGAATCAATACAAGGCAAAAGTGCAATCTCCATCCAGTGGC
CCCAGTACGAATCCATTTCTTAGTTCACCGACAAACAATGCTAATCAACCAATTGTAGA
TTTATTTGGTGCACCATCAACGATAACGAATACTGAAAGCCAGCCACAAAAAGCATCA
GACGATTTACTTCAATTAGCAGGCAATCCATTTGCGGACATGTTTGGAACACCTCAATC
TATAGCTGCACCGACTACACAAGCACAGAATAATATGTGGATGACTAATGGTAACGGTT
TTGCTGCAGTGCCTCCAGCAAATAATAATTTTGTTACAGATAATAGTTTCTCTTCTGTATT
TGGTAATCAAGATTCTCAATCTG

>novel_circ_001138
TTGGAGTACGCAGGAGCCAGAGCCGCACAACTCACAGTTGGTAGCGTCAAATCAAGA
ACTGCAGAATCAAACTCCAAATCAGAAAGTAGATCAAGAATTTGCTAAAATGACAGAT
CAACGAGAACAACAACCTCAACCACAACAGCAGCAACAGGCGCAACAGCAACAACA
AACCAAAGGCAATGAAGAACCAAGAACAAATTTGATTATCAATTATCTTCCACAGAGTA
TGACAGAAAAAGATCTTTACAGTCTTTTTGTAACTATTGGGCCTGTGGAATCTTGTCGT
GTTATGAAAGATTATAAGACAGGGTACAGTTATGGTTTTGGATTTGTTAATTATGCAAAA
GCTGAAGATGCAGCCACAGCTATAAGTACTTTAAATGGCCTTCAAGTTCAGAACAAAA
GGCTAAAAGTATCCTTTGCACGACCATCTGGTGAAGAGATTAAAGAAACTAATCTTTAT
GTAACAAATTTGCCGAGAAATATAACTGAAAGTCAGATTGATGATATATTTAGTAAATAT
GGAAATATTGTGCAAAAAAATATTTTAAAAGACAAACTTACTGGATTACCAAGAGGTGT
AGCATTTGTAAGATTCGATAAACGGGAAGAAGCACAAGAAGCGATCGCACGACTACAT
GGAACGATACCAGAAGGTGGATCAGAGCCACTTAGCGTAAAAATCGCGGAGGAACAC
GGAAAACAAAAAGCGGCATACTACGCTGGATGGCAGGCTGGTTACAATCAGAGTAGA
G

>novel_circ_001139
GTGCAGGAAGTATGGATGTTATAAGATTAGTAGAAGAGGTAAAAGACAATTTGGGAGT
AATTTTGCAAAATATCGACGTCTTCATGTCTCCGGTTTTCATTCAATTTGTAAATAAAGT
AGTTTTAACAGCACGAGGTATTTCTACCTCTAAAGAAATCAAATACGATGCTGTGGAAG
TACAAACGAACAATATGACTCTGAAATTCCCTAGACAATTATTTATTAATGGAGAATTTG

TAAATGGCCATGGAAATCCTATAGACACAATTAATCCTCATGACGAAACAATTATTTGTT
CTGTTGAAACCGGCACTGTGGAGGATGTGGACAAGGCAGTTAAGGCAGCCAAGAAAG
CTTTTGAAGAAGGAGAATGGAGCAAAATTAGCGCCAGAGAGCGTGGAGTTCTTCTTTT
CAA

>novel_circ_001140
GTAAAAGACAATTTGGGAGTAATTTTGCAAAATATCGACGTCTTCATGTCTCCGGTTTT
CATTCAATTTGTAAATAAAGTAGTTTTAACAGCACGAGGTATTTCTACCTCTAAAGAAAT
CAAATACGATGCTGTGGAAGTACAAACGAACAATATGACTCTGAAATTCCCTAGACAAT
TATTTATTAATGGAGAATTT

>novel_circ_001141
ATACGGTTGCGTTGAGAATGCTGGAACTGGTGGTACCGCAGCACGTAGTCCGTGGTCA
AAATATCAAGTTAGAATGCAACTTCAATCTAGACGGTGAAACGTTGTACTCGGTGAAAT
GGTACAAAGACGGAAACGAGTTCTATCGATACGTACCACAGGAAAGGCCGCCTGTGCT
CGTTTTTCAGCTGCCAGGTGTCACGGCGAACATTCACAATTCGACCGAAAGATCGGTC
GTTCTCCATTCTGTGAATTTAATGAGTACTGGAAGATACAGATGTGAAGTGTTAGCGGA
GGCGCCATATTTCCAAACGGTTTCCGATCACTCGGACATGCTGGTAGTGGCTCTGCCGG
AAGACGGTCCTATTATCACTGGTAGACCAGGAAGACATCGTTATCAAGTCGGTGATGTG
GTTCGGTTCAATTGCACTTCGGCGAAATCGAAACCAGCTGCCATACTCAGCTGGTTCAT
CAACGGGGAGCCTGTTGACACGCAATACTTGAGAGGGCCTCACATCACCGAGGTGGAT
CGTGAGGGTTTGGAAACGGCTGTGCTAGGTCTGGAGTTTCGTCTGCGTACGAAGCACT
TCAAGAGGGGTGATCTGAAGATCAAATGCCTGGCAACGATAGCGACTGTATATTGGAA
ATCGAACGAGCTCAGCATAGAGGGTGAAAGGCCCCTCAAGATGCCCGTTATGGAGAGC
AGGGAGACAAGGGCGCAAGGCCATACGCATGCGGAACATATCTTAGGAG

>novel_circ_001142
AAACCAGAGACGAGTCGAGAAAAAAAGAACGAAATCCGGGGAGAAAAAGTAGGAAG
GGAACCGAATCGAAGAAATCGAAATCGAATCAAGAAAGCCGATCCTTTCCTCGCTGGA
TGAGTCGCAAGCTGGAAAGGGGCCTCGCCACGGTTCCAACCGTATCCAGTGAATCGCG
ATAGAGAGCACCCTCGACACGTCTGCCCGTTGGACAAAAGGGTTTGGCCACTCGTCGC
CCCTCCAATCCCCTCTCGCCATCACGCTCCTCCAACAGGGAATTCAGGATCGAAGATCG
CCCGTTGATTCGTCACGGGAGAGCGAGAGGATCGTTGAAGGCAAGATCGTGTCGGCG
CCAAAGTATCTCATGAAGTCGACAGGCGACAGCGGCAAAGTCGGGGAGCGATTGTTG
AGCATAACCGACACGGGATCGAGCCAATATCGCGGCACCAGGTGAATCGAAATCGAGG
AACGCCTGCCCCGAAGCGAATCCAACTTTCGTACCATGTACAAGATCCTTCGACGCGG
TTCAAGCCGCGTTTTCGTCGTTTGCGCGGTCCTTCTCACTTTGCTGCTATGTTTGTACTA
CGTGAATCAGGCACAGGCGCCATCCACGGGCCCATCTGCGGGAATTTTGAACGAAGA
GCTCAGATACGACAGAGGTCTTACCTCCATCACGCCGGATTACGTGGAATCACCGGAC
GCTAAGGTCTCGTTGGACACCTGCCCCATCATTGTGCCTAGAAACGTGGATATCGACAC
GCAGCAGGAGTTCGAGAAGTTCGATTTTCAA

>novel_circ_001143
GGTTTAAGAAATTGATGCACGGCGAAGGGGCCTTGAAGAACACCGTGTTGACTGCTCT

GGCGGAGATGATTGGCACTTCCATGTTGGTGTTCCTTGGATGTATGGGATGCGTTGGAA
GCCTTGGTGTCGTTCCTTCTCACTTCCAAATAGCCTTAACCTTCGGCCTGGCTGTGATG
ATTGTGATTCAGTCCCTTGGCCACATCAGTTTAGCTCACATTAACCCTGCCATCACGGT
GGGATCGGTTGTGCTTGGAATGAAGACTATTCCCGAAGGTTTGGTGTATCTCTTGTCTC
AAGTAGTCGGTGGAGTCTTAGGATTTGGAATGTTAAAG

>novel_circ_001144
ATCAACAAGTTGGAAGAATAATTATTTCAACCATTTTTGCTGATGCTGTTCTTGTGTGC
CAAGGTAGCCTCCTCTTAAAGATGTAACATCAACTATTGAAAGGTGTAGCTTGTGATCA
GTGATTGTTAATTGTTGAACAATTTTGTGCCAGTGCAATTTTGAGAAACTTTGAAAAAC
AAATCCCTTTCAAAGAAAGAAAGAAAGAAAGAAAAAAAATTAGAACATTTTGAAAGA
ATCAGTGTTGAACAGTGGAGGGCTTGCCCGCCTATTGGGAGGACTTAGCCGTGCAGCA
TAGTGTAAATGCAGCTGCAACGGCCAGCATTTTTATGAATTACGACCTGGACAAACCG
AGGATGGTCTCGCACGTCATTCCCGTCGCTGGG

>novel_circ_001145
AATGTCCCGATGGTCAGTTACCATGTGACAACAAAGTGTGCATCAACAAAAACTTCTT
CTGCGATAACAACATCGATTGTCACGATGGGAGCGACGAACGCGATTGCAATAATGCC
GTGACTCAACCACCAAGATGTCGCGAGGACGAGTTTACTTGTCGCGATGGAACCTGCA
TTCCGCAATCAGCCCTCTGCGACGAACGACCCGACTGTCCTTACGCGGAGGACGAGGC
CAATTGTCTTCAAG

>novel_circ_001146
TTCGATGTCGGTCAGACGAGTTCGAGTGCTTAGACGGAAGTTGCGTGAGCCAAACCGC
GAGATGCGATGGAAGATCCGATTGCAGGGATCGTTCTGACGAGTACAATTGCACCGTG
ACCACCTGCAGCGGGGATCAATTCAGGTGTCTAGACGGTACCTGTATCAGCATCGACA
AAAGGTGTAATCACAACATAGACTGTCGCAATGGGGAGGACGAGAAGCAGTGTG

>novel_circ_001147
AATGACGATCTAGTATTTGATCAGGATGGCAAGCAATCTTTAGACGAGATACCTTTGATT
GAGACACGCCAACAAGACTCCATACTTCACAGGTTAAGAAGAGGATTGTTCGATTTTT
TTAATCCAACTACATCTACTTCTACTGAACCTTCAGAACCTGGAGAAAATAACGCGGAC
AATGAGTTGGACAACGATCCTCTCGTTCAATCCAACGATGACGAATATTTGGAAAAATC
AGCCCATGATACTGCAGATGAATTCGAAGTGGAAGCTGATGAGGACAATGAAGTTGGA
AATGCGGCTGAAGGTCGAAGAGAATCAGCAAATTTTGGAAACAGCGACGACGAGGAT
CTTGCTGGATCTGGAGAAATGGAGGGCAGCGCTGATGATACGCGATTGCACAGTTTTAT
ACCAATAAGAGAAGAGAAAAAGTATTATAGAATAACCTTGACAGTTGGAGAACCGTTC
AGAAGAGAGTATGTGGATAGGAACAGCAGAGAGTACAAGGAGCTAAGTGGCAATCTA
ACTCAACCTTTGGAAGAGTTGTTGAACCGTTATATTCCTAATGAAAGTCACCATGCCAA
CGTTATTAAAATTTCGCCAACGTCCGACAGCTTCACGTCACAAGTTACCCTAGACATCG
GTTCGACGTTCACGGATGAGATGGAGGTGCGGAACGTCATAGAGCAACAGCTGCACTT
ACATTCGTTGGGCAACGTCCAAGTTCGACCAGAAGAATTCTCGTTTAGAGTATTTCAA
GTTGGAGAAGAGATAGAGGAACCGGAATGCGACCAATCGACCGAGATAAGGTGTAAA
GATGGTACATGCGTGCCGGTCGAATGGCGATGCGATGGGATTTCTAATTGTAAGGATGG

TTCTGACGAAGAAGGTTGTCCTGAAACGACGACACATGTCAGCGAGAATATCACAGAA
TTTTACGAGGAAATAACTAAGGAAATTAAAAAGGAAGAAGACGTTGAAGGAGTAAAA
TTAAGCAAATGTCGCGCCGATGACACTGTTCGTTGCAGCGATGAAAGTCGTTACATATG
CTCTGTACAGAAATGTGACGGCGTCAAGGACTGCGATGATGGAGATGATGAAGTTGGT
TGTCCCCATCCAGGATGCAGTCCTGGAGAATTCGCCTGCGACGTATCTAGGTGTATACT
CGCATCGCATCGATGTAATTTCATAAAAGAATGTGACGATGGAAGTGACGAATATGACT
GCAGCTATCCTG

>novel_circ_001148
ATAAAAGATGAAGGGTTGAAACCTGGTACGTCCGTAGAAGAAACAGTAGTAGAAGAA
CGTTCAAAAAAGACGAAGACTCGTGCGGAGATTGTGGACAATATTAGTGAATGGAGTC
AACAACAACAAAGAGCTCTGGAAGCAGCCCTTATAAAATATCCTAAAGGCACATCTAC
AGATAGATGGGAGAAAATCGCAAATTGTGTTGAAGGAAAAAGCAAG

>novel_circ_001149
AAGTAGCAAGGACGAGACTTAGAGAGGAAGGTACGAAATACCAGACTTTCTGGCAAA
CATTGAGCACAGTCTACGCTGAAGAAGGATTTCATGGTCTATATCGTGGTCTTGGTACT
CATCTAATTAGACAAATTCCAAATACAGCAATTATTATGGCTACGTATGAAGCGGTGGTA
TATTTGCTAAGCAAGCACTTTCACGAACGATCTTTAAGCATAAACAATGAAAGCGAGTC
CAAGTTCTATACAGATCCAAAGGGTAAACGAGAATTGGCCTAGGACTTACTCACGGCC
CCTGAGTAAAGGGCCAATAATTCGCATGCTGCGGCGAGTCTTGAAATTGCATCTGTTTT
GAAAAAAAGAAAATGAACCATAAGAACAACAACGCCGTCCAAGAGAGAGTCCATACG
CCGGGAGAGAACGCATTTCCGAGTTTGGGGCCATGCCGGCCGGCCAGGTCCAATCGA
ACATGTCGAAGGTAGCAAAATAGGTATCACAGCGCGTTGTACGCTGCTGTGTGCTCTAT
AAGTGTAGCAAAAAGTAGTCCTTCCTCCTTTCAGGTAGCGTACCTACGTACGCGAGGA
ACAGCCTAAACTGTCTGTCTGTACCTTCGTCGACACATTCACACACACATACACGGAG
GCGAGGATCGTGAAGCTGGATTAAATGAGTCAATTACATAGACTCAGAATGCTCCAAA
A

>novel_circ_001150
GGTATCCTGGGATTCTACAAAGGCATTATGGCCTCCTACATAGGAATAAGCGAGACTGT
GATACATTTTGTGATCTACGAGGCTGTGAAGGCCTCTTTAGCAACATACAAGGCTCCCA
ATGCAGATCGAAAAACATTGCGCGATTTCTTAGAGTTCATGGCGGCTGGCTCGTTCTCA
AAAACGATTGCCTCCACCATCGCTTACCCCCATGAAGTAGCAAGGACGAGACTTAGAG
AGGAAGGTACGAAATACCAGACTTTCTGGCAAACATTGAGCACAGTCTACGCTGAAGA
AGGATTTCATGGTCTATATCGTGGTCTTGGTACTCATCTAATTAGACAAATTCCAAATAC
AGCAATTATTATGGCTACGTATGAAGCGGTGGTATATTTGCTAAGCAAGCACTTTCACGA
ACGATCTTTAAGCATAAACAATGAAAGCGAGTCCAAGTTCTATACAGATCCAAAGGGT
AAACGAGAATTGGCCTAGGACTTACTCACGGCCCCTGAGTAAAGGGCCAATAATTCGC
ATGCTGCGGCGAGTCTTGAAATTGCATCTGTTTTGAAAAAAAGAAAATGAACCATAAG
AACAACAACGCCGTCCAAGAGAGAGTCCATACGCCGGGAGAGAACGCATTTCCGAGT
TTGGGGCCATGCCGGCCGGCCAGGTCCAATCGAACATGTCGAAGGTAGCAAAATAGGT
ATCACAGCGCGTTGTACGCTGCTGTGTGCTCTATAAGTGTAGCAAAAAGTAGTCCTTCC
TCCTTTCAGGTAGCGTACCTACGTACGCGAGGAACAGCCTAAACTGTCTGTCTGTACCT

TCGTCGACACATTCACACACACATACACGGAGGCGAGGATCGTGAAGCTGGATTAAAT
GAGTCAATTACATAGACTCAGAATGCTCCAAAA

>novel_circ_001151
GTTTCGTCGCATGCACATTGACAAATCCTATTTGGTTCGTGAAGACCAGGCTGCAACTG
GACCATCGAACCAACAAAATCACTGCGATGGAATGTATGCAAAGGATATATCAACAATC
GGGTATCCTGGGATTCTACAAAGGCATTATGGCCTCCTACATAGGAATAAGCGAGACTG
TGATACATTTTGTGATCTACGAGGCTGTGAAGGCCTCTTTAGCAACATACAAGGCTCCC
AATGCAGATCGAAAAACATTGCGCGATTTCTTAGAGTTCATGGCGGCTGGCTCGTTCTC
AAAAACGATTGCCTCCACCATCGCTTACCCCCATGAAGTAGCAAGGACGAGACTTAGA
GAGGAAGGTACGAAATACCAGACTTTCTGGCAAACATTGAGCACAGTCTACGCTGAAG
AAGGATTTCATGGTCTATATCGTGGTCTTGGTACTCATCTAATTAGACAAATTCCAAATA
CAGCAATTATTATGGCTACGTATGAAGCGGTGGTATATTTGCTAAGCAAGCACTTTCACG
AACGATCTTTAAGCATAAACAATGAAAGCGAGTCCAAGTTCTATACAGATCCAAAGGG
TAAACGAGAATTGGCCTAGGACTTACTCACGGCCCCTGAGTAAAGGGCCAATAATTCG
CATGCTGCGGCGAGTCTTGAAATTGCATCTGTTTTGAAAAAAAGAAAATGAACCATAA
GAACAACAACGCCGTCCAAGAGAGAGTCCATACGCCGGGAGAGAACGCATTTCCGAG
TTTGGGGCCATGCCGGCCGGCCAGGTCCAATCGAACATGTCGAAGGTAGCAAAATAGG
TATCACAGCGCGTTGTACGCTGCTGTGTGCTCTATAAGTGTAGCAAAAAGTAGTCCTTC
CTCCTTTCAGGTAGCGTACCTACGTACGCGAGGAACAGCCTAAACTGTCTGTCTGTACC
TTCGTCGACACATTCACACACACATACACGGAGGCGAGGATCGTGAAGCTGGATTAAA
TGAGTCAATTACATAGACTCAGAATGCTCCAAAA

>novel_circ_001152
CATTGTCAAACAGAATGGGATGCGGGTGCATTGGAAAACGGTGAATTAACGCAACTGC
GAGCAGCCTACAACCATCAATTGGAAGAACAAGTTGCGCTAGCTAAATTGGATATTATT
AATGCACTTCAAGAACAAATTCAGGTACTTTTAACGATCGAGTCGGACGTAGAAGACA
ATTGGCCAATAGAATTGCTAGAATTGCGAGATAAACTAACTGGTAATGCAAAAAAAGA
AATGCAACTGCTCAAAAATACACATATTGAAGAGGTGCAACATTTAAAAGAAGAGCAT
TCTCGAACTGTGACTAAGATGATTGATTGTCATCGAGAAGAACTTAATAAAATTAAATC
AGAATATCTCCAAAATTATTGTGGTGATAGAAGTTTGTTAATAGATAATAAGATTTTTGA
AGAAAGAAATAATTTAACTAAAACATGCACCACTCTTAAAACTTTAATTGAAGAATTGA
TAAAATACTTTATTGTTTGTGAAGAAGAAATTAATAACACTCTTTTTACTGAAATTACTA
AAAAGCAATTATCTGATAGTGTTAATAATGAAAAAACCATGCAATTTGATGAAACTGAA
GGATTGAAATGTAATGAATTAAAAATGAGTTCAAAAAAGGATTCATTGAATTTATCTGA
GATGATTGTCCGAAAGGTGCATTTTGCTCCAAAAACTACAGAAATAGTTTCAATAATAA
ACAGCAATGTTGAAACTTTACCAACTATTCTAGAAGAAGATGATAATATAACAGAAAAA
TTAAAACAAGAATTAAACAATTGCATACTTCGTTTGAAGTCTGAGAGTGCTGAAATTCT
TAATACTTCATCAATTGAAGGAAAATTATCTTCGAAAGATACTTTTTGGTTGAATAAAAT
GAATGAAGAATTAAATTTGAAACTTCATCATGCTGAAACTTTAATTATGGGTTACCAAG
AAGAAATTGATCATCAGAAAATGACTATTTTCGATCTCCAAAGAAAGTTGGTTAATGCA
GAGAATAAAAAAGAAACAATCACAGAAGGTTATGGGGAAAATTATGATGTGGGTATTG
ATACTACATTACAAGACTTTTCACAATTACAAGAGAAAGTCAGACATGTATTATCAAAT
GGAGGAGGAGATTGTACAGAATTGTTACAATTGATAGATGAATTGTCTAGACAGAGTGA

TAAATTGATGGAAGAAGCTAAAAAAGAAAAGGAAGACTTACAACAACAGCAGGTGCC
TTTAGAACCTACTCCTACCCCATACATTCACAGGGTTTGCCACCGAAAGATCGAGGCAG
CAGATAAACAATTAAAAGCAACCCGTAAATTTTTGGATGAACAAGCAAGTGAAAGAGA
AGCAGAGAGAGATGAAGCTGCAAAACAAATACATATTCTGCAGGAACAACTTAAAGA
GCGTGAACGAGAGAAAGAAAGAGATCAACGTATCACATCTGAA

>novel_circ_001153
GTATTGGAATAGACGATACGTTTGTGATGCTAGCTGCTTGGAGGCGAACCAGTATAATG
AAACCGGTACCTGAAAGAATGGCTGCGACTCTTAGCGAGGCTGCTGTCTCGATTACTAT
TACTTCCTTGACTGACATGATATCGTTCTTCATCGGTATTCTCTCACCTTTTCCCTCTGTT
CAAATTTTCTGTATTTATTCAGGATTCGCCGTAGTCTTCACCTTCGTGTTTCACCTGACC
TTCTTCACAGGCTGTGTAGCCATCAGTGGCTATTGCGAGCAAAAGAATCTTCATAGTGT
AGTATGTTGCAAGGTGCAACCTCTCTCCAAGTCTT

>novel_circ_001154
TACACGGACTGGGAGGAGGTGTACAACGGGTATATTTACCAAAAAATGGTCGCGGACG
TGGTTGGCGACTACTTCTTCATCTGTCCCTCGATCCATTTCGCCCAATTGTTCGCCGATC
GTGGCATGAAGGTCTACTACTACTTCTTCACGCAAAGGACGAGCACCAATCTATGGGG
CGAGTGGATGGGCGTATTGCACGGTGACGAGGTTGAGTACGTGTTCGGCCATCCCCTG
AACAAGTCTCTCAAGTACAGCGACAAGGAACGAGACCTGTCATTGAGGATGATACTCT
ACTTCTCCGAATTCGCCTATTTGGG

>novel_circ_001155
TACACGGACTGGGAGGAGGTGTACAACGGGTATATTTACCAAAAAATGGTCGCGGACG
TGGTTGGCGACTACTTCTTCATCTGTCCCTCGATCCATTTCGCCCAATTGTTCGCCGATC
GTGGCATGAAGGTCTACTACTACTTCTTCACGCAAAGGACGAGCACCAATCTATGGGG
CGAGTGGATGGGCGTATTGCACGGTGACGAGGTTGAGTACGTGTTCGGCCATCCCCTG
AACAAGTCTCTCAAGTACAGCGACAAGGAACGAGACCTGTCATTGAGGATGATACTCT
ACTTCTCCGAATTCGCCTATTTGGGAAAACCAACGAAAGAAGACTCTGAATGGCCATC
GTATTCGAGAGACGAACCAAAATACTTCATCTTCGATGCGGAGAAGACTGGACTTGGT
AAAGGGCCACGTACCACGTATTGTGCTTTCTGGAACGAATTTCTACCAAAGTTGAAAG
GAATACCAG

>novel_circ_001156
TACACGGACTGGGAGGAGGTGTACAACGGGTATATTTACCAAAAAATGGTCGCGGACG
TGGTTGGCGACTACTTCTTCATCTGTCCCTCGATCCATTTCGCCCAATTGTTCGCCGATC
GTGGCATGAAGGTCTACTACTACTTCTTCACGCAAAGGACGAGCACCAATCTATGGGG
CGAGTGGATGGGCGTATTGCACGGTGACGAGGTTGAGTACGTGTTCGGCCATCCCCTG
AACAAGTCTCTCAAGTACAGCGACAAGGAACGAGACCTGTCATTGAGGATGATACTCT
ACTTCTCCGAATTCGCCTATTTGGGAAAACCAACGAAAGAAGACTCTGAATGGCCATC
GTATTCGAGAGACGAACCAAAATACTTCATCTTCGATGCGGAGAAGACTGGACTTGGT
AAAGGGCCACGTACCACGTATTGTGCTTTCTGGAACGAATTTCTACCAAAGTTGAAAG
GAATACCAGATCCAGCGCCCAATACCTGCAAGGTGATAGCCTCGAGCGTGTCGGCCGG
CGAGGGGGGGCTAGGGAATTCCCTAGCGATCACGGCGCTGTCGTCGATCCTTGTCCTG

TCGAGAGTGATTTAAA

>novel_circ_001157
AGGACGAGCACCAATCTATGGGGCGAGTGGATGGGCGTATTGCACGGTGACGAGGTTG
AGTACGTGTTCGGCCATCCCCTGAACAAGTCTCTCAAGTACAGCGACAAGGAACGAG
ACCTGTCATTGAGGATGATACTCTACTTCTCCGAATTCGCCTATTTGGGAAAACCAACG
AAAGAAGACTCTGAATGGCCATCGTATTCGAGAGACGAACCAAAATACTTCATCTTCG
ATGCGGAGAAGACTGGACTTGGTAAAGGGCCACGTACCACGTATTGTGCTTTCTGGAA
CGAATTTCTACCAAAGTTGAAAGGAATACCAG

>novel_circ_001158
AGGACGAGCACCAATCTATGGGGCGAGTGGATGGGCGTATTGCACGGTGACGAGGTTG
AGTACGTGTTCGGCCATCCCCTGAACAAGTCTCTCAAGTACAGCGACAAGGAACGAG
ACCTGTCATTGAGGATGATACTCTACTTCTCCGAATTCGCCTATTTGGGAAAACCAACG
AAAGAAGACTCTGAATGGCCATCGTATTCGAGAGACGAACCAAAATACTTCATCTTCG
ATGCGGAGAAGACTGGACTTGGTAAAGGGCCACGTACCACGTATTGTGCTTTCTGGAA
CGAATTTCTACCAAAGTTGAAAGGAATACCAGATCCAGCGCCCAATACCTGCAAGGTG
ATAGCCTCGAGCGTGTCGGCCGGCGAGGGGGGGCTAGGGAATTCCCTAGCGATCACGG
CGCTGTCGTCGATCCTTGTCCTGTCGAGAGTGATTTAAA

>novel_circ_001159
TTGATCTACCTTAACTTTCCATCTTGATTGGTAATCGGTTATCAATCAATCAACAGGGAA
ATTTGTTACCGACCGTAAATCTTCTTATCGAATGCAAACCTTTTTATCGGCATTAAATTAA
TTGAATCGAATTCCATATTCCAGTATTCGCAATTTGCAAATTGTGCATGCCATACGCATG
ACATATACATACATATATGTACATTAAATTATGATCATATTTAAATTGATATTTCGGGCCAA
AAAGCAATTTTTTTAGTTGCCCCAACAATATCTTTACATCATATTTTTCAATTTTTTTTTT
TTTTTTTCTTATATACATGATCTTCAGTATTGTATTTGTACGTGATTTCATTAATCACAACG
GGCAAAAAAAATACGTTTCTTTAATCTAAAAGACAGCGCGTGTCTCTTCTCCCTTCGTT
CCATGATTTTTTTGTTGCTTTCTTAAAATATATCAACTACTATTTTGAACCAAATATCGA
CAATTTGTCAGCTCATGAGATAATACTTTGATGCTTTTAATAACTATGACTACTTTTTACT
TTTTATTTTTCTAGGATATTGCTCTCAAAATATGATGAATGATCTTTGAAATTCTTTTTTTA
AGAGAGATATCTAAATTCTGGAATCAAGCCATTGGTTATAATACTACATTAGATACAAGT
ATTAGGTATAAGTACACGTGTGATTGAAATTTTATTAAATAAATTTAGATTAATTTTAAAT
TTCAAAACTTACACGGAATTACTGTGGAGGAAGACATAGGTCCTAATCTGCTGTAAAGT
TGTGATGGAAAAGTAAATACGTTTCACAAAAATATTTGCTTTTATCTACAAATAATTTTG
TAGAATTTTTAAAAAAAATAATTAGTGTACAAAGATACTGTATCAAGATCTGTTTTAACA
AATTTTTTTTTAATTTTGTAGGCAATAACCCGAGATCTTGACCAACAACATGCACACAA
AAAGAAATACGCAATAGCTCTAGGTAGTGAAAAATCCTGGTAAATCGCATTTCGCTGAC
AATTGGTAGCAACAATAAACTTTAATAATTTCTACAATGTTGCTTGAATGAATGCTTGAT
CATAAGTTGATCTTATAAAGAAGCGAATGGCAAATTTATAAAATCTAAAGCATTCTAAA
GTATTTGTTCTCTGAGCACAATGATAAAAAATTATCTTTTGAAAAATATTTTAAAAATATT
ATAATAATATGCTGATATCTTAAAGCTTGGTACAATTATTACTTTCGCGAATCTGTGCCTG
CACACTTCGAAACGTTCGATCGAAAGTATCGAAAACTTTGTCCCAAGCCATTTACTAAT
TTTCTCTACAGCAAGTACGAGCCGACGTGTATTGTTAATAATATATTATCGAACTGCGGG

AGGGAGCGTTTCACGGCCTTTTAGACCTCTCGACGAAAAACCAATACGATATTGGTCG
ATCCAATCGTTCAAAATCATCGAAGATAACATCACCATCGACAAACCAGGATCATCAAG
AAATGTTTATACGCTTGAAAACTTTTTTCTTCATTCTCTTTTACCTTAAGAAATATTGGAC
CATTATCCTCTTCGAATATTTTGAACGATCGTTTTAAAGGATTCACAATGTTTTCTCGATC
CATGCGTGTGATATAAAATAGCTCGAGACTCGTGAACATTAACTAGGGAATGAAGGATC
CAGTCCTCCTTTTTATCATTTTAAAACATTCCTCGAACTCTCGATTAAATTCTTACTTATA
AATAGAACTCGATTATCTTCCATAGATAACGCGTGGGAGGCCACACCGAGTTATTTTAAT
AAGCGGACGAAAATGTTTACGAGAAAAACATCTTTCTAAAACATTCTCTAGTATCATAC
AAGACTCTTAACTCTAGTCAGAGTATTGTCCGAGAGACAGGGCAAAATATAATAACGTT
TTGGATAGTTTTTTTAGAATCTCGTGTGATACAGAATGTTCGAATATTTTCCACTAATTT
TCGATAAAAAGAGAAATAAATGTATATACGTGTGTGTGTGTGTGTGGCATAGCCTCTGA
TCTCGCTGTGCGATAGAATGAACAAAATTCTACATCATTATCATTAAAAGTCAAGTATCA
TCGAGATAATAATCGATATCGATCAAACGAAATTAATAATTATCCGTTGTTATATATCGAA
AAAGAAATAAGAAAACGAATGGCAGTTGCGACTAGAAGAAGAGTAGAAAATATAGGC
GAACATGGCGAACTCTACACCCGTCGATCAGAGTAAATTAGAGATCAATAAGTTGTACA
ACCCTCCCGGGGCTTCATTCAAAAAGCTCAAAAAAGCGATTCTAAAGGGTTTGAGAGA
AGCGGTAAGACAATTTTGAAACCTGTCAACATTCTTAGGAAAACAGAAATCCTCTTATG
AAACCTCTATTTCCGCTCCAATCGAGTAGAACGCTACAAAAATAGTGCGAACAACTGT
TCGATACAAATACGATACATACGAACGAGTATATCTTCGATCCATATATAAAAAGTAGAA
AAAGTAACATGGTGACCACAGCGGCAATATAAAGCAAGGTTTGAAGCACGAACCGGT
TCTCTCTCTTTCTTTCTCTCTTCAAACCAGGTTGGATTAAAAATTATATCTTAATGTATAA
TTTCACCCTTCGCACCATTTCTTTTTTTTTCTTCGTTTATATTTCTTCGACAAATTATCAAT
TCTCTTCAATTCTTTCTCATAGAAAAATTCCCTACGAATGCAAAGTAACAATAAAAGAT
GGATAAGCGTTATTACACTACCACTGTATAGCTTGTATTTTGTTCGCTTGTTTCAAATGG
GTGGGTGCAATGGGTGAAATGTGAAAAATACAAGATATTAGATAGAAAAGAAAAATGT
ACAGAGAAGATAACTCTTCATCTGCTTCTTCGCATCGCCAATCCATTCGAGCTCCACAC
TTGCATAACGTAAGCGTAAAAAGGCTGCACTCGTTTCAATAATGTCTTTAGGCTAAACC
GTCTGTATTTAACTTGTCTAAACCCTGTGTGTGATTTTTCTCACAATTGCTATCTCGATTT
CG

>novel_circ_001160
TACGCTTCACAAGAGAAAAATGGCGAAAGGTTTATGACCTCGTCTGACTTCGTCCGAA
CTTATCTTGGACTCTACACCAATCCTGATTACAATCCTGACTCCGTCAATTTGCTGGCTG
GTATCGTCGATACCAGTAAAGATGGACTTATATCATTCGCCGAGTTTCAAGCGTTCGAA
GGATTGCTCTGCGTACCAGATGCTTTGTATAAGACAGCTTTCCAGCTGTTTGATACTAAT
GGAAATGGAATGGTTGCATTTGATGAGTTCGCTGAGGTGATGCGGAAAACGGAACTCC
ATCGGCGGATGCCCTTCAATATGGACAGCAGTTTCATTAAACTTTACTTTGGAAAGGAT
AAGCAGCGACTGATAAGCTATGCGGAGTTCAGTCAATTTTTGCAC

>novel_circ_001161
TCACGAGTTGCTCGGTTGCGTCATCGGCGGCGCTCGACGATGCATCGTCTCTCCCGAG
GATCTCCACGGGTTCGCCTATCCTCCCCGCCGTCTTCCCACCAGGAGGCGCGGCGCGG
GTGGCGTTCAAGGTCACGGCTTCGTCCACCACGTTTCCCGACAAGTCAGACAACACC
GCCACGTCCTCGGCTTTCGTCGGGTTTGCCAGGTACTGCGCTCCTCTTAACACACGCTT

TGGCGCTTCTACGGCATTATTTCATCCGTATTAATTTAGACGCTTTTGGCAGCCTGTC
CGCGCCCGATGGAAGCGCTTCACCGGCGCTGCTCTCGCTCGCCAACGGGTCAACACTT
GGCGTCACGCTGCTCGCCTCGATCCTCGATGACGGACCGTCGTCGATCGTTTTCTTCTT
CTTTTGCTTCGACGCAACCTTCCCCATCGTCGATTCTTCCAACTTCGTCCCCCCGGCAA
ACCCGACCGTTTTCTTTTGTTTCCCGATTATTTTATCCCCATCAACGGGGATAGATTTCG
CGGGTAAGACACGATTAATCGTATCCCCTTCTCCGGATTTCGTGCTAATTTCGAATTTCT
TCAGGGGGTCGTTCTTCCTGGTCGCCGCCTCCGGAGGTGTCTTCACCGTGAAGTAAAC
GCTGTTTCTCGAAGTGTCCTTCACGTAAGGCTTCTTCACAGGTAACACAACTTCATACT
CCCTTTCTTCCTCTTCTTCTTCTTTCTCCTCTTCGATATGGCGTTTGTCTAATTTCTCCAT
GGGTACTTTTTTAGGTATTGGTATCCTCACGCTTTCCCTGAACTGTTCCACGCCTCCACC
GTTCCAGACGGCTGCAGAGAAATCCGGGACGCTGTTGGATTCAACGTTGAACGTGTTG
GATTTTTCAATTTCCTCCTCGTTGTTCGGGACGTCTGATTGACACAACCTTGAACAATC
CTCGTTCGTTTCAGGAGGGGAAGGTTTCTCGGTGGTTTTCTTCCTCTTCTTCATCTTCTT
CAACGTCTTCCTCATCTTTTGCGCCGCAGCCTCCTCCTCGCTCTGCGACACGGAATCGC
CGTCCACGTTCCTGAAGAAGCCAGGGACAGGTTTCCGCCCCTTCTTGATCAACGTCCC
TCTGCGCACCTCGTTCTCGATCTCAGCTTGCTCCTCGGCCACCAAGCTGTCCAATACCT
TCGCCTGGACCTGAGC

>novel_circ_001162
TCACGAGTTGCTCGGTTGCGTCATCGGCGGCGCTCGACGATGCATCGTCTCTCCCGAG
GATCTCCACGGGTTCGCCTATCCTCCCCGCCGTCTTCCCACCAGGAGGCGCGGCGCGG
GTGGCGTTCAAGGTCACGGCTTCGTCCACCACGTTTCCCGACAAGTCAGACAACACC
GCCACGTCCTCGGCTTTCGTCGGGTTTGCCAGGTACTGCGCTCCTCTTAACACACGCTT
TGGCGCTTCTACGGCATTATTTCATCCGTATTAATTTAGACGCTTTTGGCAGCCTGTC
CGCGCCCGATGGAAGCGCTTCACCGGCGCTGCTCTCGCTCGCCAACGGGTCAACACTT
GGCGTCACGCTGCTCGCCTCGATCCTCGATGACGGACCGTCGTCGATCGTTTTCTTCTT
CTTTTGCTTCGACGCAACCTTCCCCATCGTCGATTCTTCCAACTTCGTCCCCCCGGCAA
ACCCGACCGTTTTCTTTTGTTTCCCGATTATTTTATCCCCATCAACGGGGATAGATTTCG
CGGGTAAGACACGATTAATCGTATCCCCTTCTCCGGATTTCGTGCTAATTTCGAATTTCT
TCAGGGGGTCGTTCTTCCTGGTCGCCGCCTCCGGAGGTGTCTTCACCGTGAAGTAAAC
GCTGTTTCTCGAAGTGTCCTTCACGTAAGGCTTCTTCACAG

>novel_circ_001163
GTCAGTTTCGTCGTCGGCGTACAGCAGTACGGGTAGCGGTAGTGGTACGGGCACGAAT
GTGACGACGAACGGAACCAGCGGCACTAGATTCGGCTTGTCTACATCGTCGTCCTCAT
CGAGCATCTCTGCCGGCGGTTCGTCCAGAGACAGAGGGATCCCCGAATCCTCGTCTAG
GCTAGAAATAACGGCGGACTTGATCAGGTATTCGCCGTCCGGTTACATACCGAATATCC
GTCAAACGAGCAGCACCACTGGCAGCACCAGTGGGATCCACCATCATCATCACCATCA
CCACCAGTGGAAGCACCACTCGACCGGTTCGTCGTTGACGGAGTTGTTCGGTGGTGA
CGATCGTGACGTAGCCGTCGAACGTACCGGCAGAGATCATCGTCCAGAGTCGTCGCTC
TCCTCGTCCTCCTCGTCATCTTCCTGCGCCGTTGGTGGTGCACTCTGGTCCAGGTCGAA
GAGAAGGCCACTGTCCAGCAGCACGGTGCTCAACAGCGGGCATGCACGCGCGGACAG
TGCCGCGTCGCCTGGCGAGGTAACGATTAACGAGCGCGCCACGATCCGCGACCTAGAC
GACGAGGACGACGACGACGACGACGACGATGACGACGACGACGACGATGACAACGA

CGACGACGACGAGGACGAGGACGGGAACAACACCGACGACGATCAACACAACAACA
ACAATAATGGGAATAACAGCAGCGGTGCCGAGGACACGTGCGATTCCGCTCGATATGG
GTCGAACAATAACAACAACAACAACCGTGACCGTGACCGCGACCGCGACGACAAGGA
CGACAAGGACGACGGGGACGACGAAGACAAGAACAAGCTGAACAACCGGCATCATC
GCCAACAGCAGCAGCAACGCGGTGCTGCCCATCGGATCGACGAGGTTTCACCAGCTA
AGCGTAACCTCTTGTCGTCTATACCCGGTGGCGGTGCTGGCGGTGCTGGTTTGATCGGT
GTTAAGCTCGACTTGCTCACTAACCTAGCCACTAATCCGAGTAACGGCCAACTGCTGAT
TAACAACAACAACAACGGTCGGAAGATTATCCAAGAAGGTGTGGAAGAGAAGGAGG
AAGACGTCGAGATCAAAGACGTCGAGGGCGGGAATCGTGGCGAGGTAATACACGGGA
TCAACGAGGCCGCCATCTCTGTCGACGCGATTGCGTTCCTACGTGGTGGTCGTACCGG
GGATCCAGAGTGTCTCGAACACGGTGAGGAGCAACGGACCGCGTCTACCTCGTACGAT
ATCGATCAGACCGGGTTCGTAGGCGGCATTACGCCGCCCATCTTGCAAAACGGCGTGG
CGGGAAGACCGTCCGGTACTTATGGGGACGACCCATCCGTGCCTTCTACATCCAGGAG
ACCGGATGGTCACTTCGCGGGGACAAACACTGCCAGTAATCTGACTTCCTCGTCGCCC
ACCGCACCGTCCACGGCTCACCAAAATCTCTATCGCGGGGTCGTCGATCAGTTT

>novel_circ_001164
GAAGGGGTTTCTATAGAAGAAAGAAAGAGAGAAAAAGAGAGAGATTGTGAGACTGG
TGAGACCCGGGTGCAATGTCGTATCACCGTGGGGGCCAGGCGGGCGCCGTAGCAGTTT
CGTACAGCACCAGTCCAATGGCACCGCATTCTCCTAGCAGACGATGTTCCAGCAATAA
CAGCGGCAACAACAGTTCCGCTATTGGCACCTCCACCTCGAAGCTGAACACCTACAGA
TCTACTGCTTCCTCGTCGATCTTAGATCGACCAACAAATTTCTACTCGACATCGTCGTCG
ACGTCATCGTCGTCGGGCAGCTCGGGCTTTCGTTCGAACTATACGACCGAGTACAGGC
GATCCTACTGCCCATCAGGCAGTTTCTACTCATCAACGGGTACGAGCATCCGAAGGAGT
TACTTATCGGAATACAACCGGTCTCGCTGCTCACCCACAAGGTCAGTTTCGTCGTCGGC
GTACAGCAGTACGGGTAGCGGTAGTGGTACGGGCACGAATGTGACGACGAACGGAAC
CAGCGGCACTAGATTCGGCTTGTCTACATCGTCGTCCTCATCGAGCATCTCTGCCGGCG
GTTCGTCCAGAGACAGAGGGATCCCCGAATCCTCGTCTAGGCTAGAAATAACGGCGGA
CTTGATCAGGTATTCGCCGTCCGGTTACATACCGAATATCCGTCAAACGAGCAGCACCA
CTGGCAGCACCAGTGGGATCCACCATCATCATCACCATCACCACCAGTGGAAGCACCA
CTCGACCGGTTCGTCGTTGACGGAGTTGTTCGGTGGTGACGATCGTGACGTAGCCGTC
GAACGTACCGGCAGAGATCATCGTCCAGAGTCGTCGCTCTCCTCGTCCTCCTCGTCATC
TTCCTGCGCCGTTGGTGGTGCACTCTGGTCCAGGTCGAAGAGAAGGCCACTGTCCAGC
AGCACGGTGCTCAACAGCGGGCATGCACGCGCGGACAGTGCCGCGTCGCCTGGCGAG
GTAACGATTAACGAGCGCGCCACGATCCGCGACCTAGACGACGAGGACGACGACGAC
GACGACGACGATGACGACGACGACGACGATGACAACGACGACGACGACGAGGACGA
GGACGGGAACAACACCGACGACGATCAACACAACAACAACAATAATGGGAATAACAG
CAGCGGTGCCGAGGACACGTGCGATTCCGCTCGATATGGGTCGAACAATAACAACAAC
AACAACCGTGACCGTGACCGCGACCGCGACGACAAGGACGACAAGGACGACGGGGA
CGACGAAGACAAGAACAAGCTGAACAACCGGCATCATCGCCAACAGCAGCAGCAAC
GCGGTGCTGCCCATCGGATCGACGAGGTTTCACCAGCTAAGCGTAACCTCTTGTCGTCT
ATACCCGGTGGCGGTGCTGGCGGTGCTGGTTTGATCGGTGTTAAGCTCGACTTGCTCA
CTAACCTAGCCACTAATCCGAGTAACGGCCAACTGCTGATTAACAACAACAACAACGG
TCGGAAGATTATCCAAGAAGGTGTGGAAGAGAAGGAGGAAGACGTCGAGATCAAAGA

CGTCGAGGGCGGGAATCGTGGCGAGGTAATACACGGGATCAACGAGGCCGCCATCTCT
GTCGACGCGATTGCGTTCCTACGTGGTGGTCGTACCGGGGATCCAGAGTGTCTCGAAC
ACGGTGAGGAGCAACGGACCGCGTCTACCTCGTACGATATCGATCAGACCGGGTTCGT
AGGCGGCATTACGCCGCCCATCTTGCAAAACGGCGTGGCGGGAAGACCGTCCGGTACT
TATGGGGACGACCCATCCGTGCCTTCTACATCCAGGAGACCGGATGGTCACTTCGCGG
GGACAAACACTGCCAGTAATCTGACTTCCTCGTCGCCCACCGCACCGTCCACGGCTCA
CCAAAATCTCTATCGCGGGGTCGTCGATCAGTTT

>novel_circ_001165
GAAGGGGTTTCTATAGAAGAAAGAAAGAGAGAAAAAGAGAGAGATTGTGAGACTGG
TGAGACCCGGGTGCAATGTCGTATCACCGTGGGGGCCAGGCGGGCGCCGTAGCAGTTT
CGTACAGCACCAGTCCAATGGCACCGCATTCTCCTAGCAGACGATGTTCCAGCAATAA
CAGCGGCAACAACAGTTCCGCTATTGGCACCTCCACCTCGAAGCTGAACACCTACAGA
TCTACTGCTTCCTCGTCGATCTTAGATCGACCAACAAATTTCTACTCGACATCGTCGTCG
ACGTCATCGTCGTCGGGCAGCTCGGGCTTTCGTTCGAACTATACGACCGAGTACAGGC
GATCCTACTGCCCATCAGGCAG

>novel_circ_001166
GTATTCGCATGTCTGTACCTCAGAATATTCTGACTCCTGGATGTACAATCAATGATGGTT
TAATGCGCGGAAGAGGCAGTGGAACTATACCTCAACGTGGATTGATGCGTCGTCCTATA
CCATCTCGCGGTGGACAATTAGAAATTGCTGGAGTCGTCGTTGGACAATGGGGTCCAA
ATTATGGACAACCTGGTCGTTTACCTCAACCTCTTCAGGGACATCCTCGTGGGCGGCTT
CCACCTCAAGTAACTTCAATTGGTGGTCTTAAGTATGGTCTCCCTCGTGGTTTCACTCCT
ATGGGTCGTCCCACATTTCAAACGCGAGGAAATAATCGTGCGCAAATCGCAGGACGAG
GAAAAAAGACACAAATGAAAGCAACTGGCAAAAAGAAAACTACTGTTAAAAAGAGT
AAAGAAAGTGAAAATAAGAAAGAAACTAGAGGACGGGGTAGCAATATCGATGGAATA
ACTGATTATAGTGATTCAATTCCAAATATAAATGCAACAAAAGACGCACTGCCAGTACC
AGGCCAATTTGAGGATGCCATAGAAAACGGCAAAGGAATAGAAAAAGGACAAGGAGA
CGCGTCACTGGTTGCTCCAGTCGCCGCAGAAAATTAG

>novel_circ_001168
GTGAATCGGCCGCTCACTATGAAGAAGGAGGGCATACAGACGAGGAACAGGAAACTG
TCGTCGAAGAGCAAGAAGAAGAAGGCTGGCGGTTGTCTGGGGCTTGGTGGCGTGATG
GGTGATATGATCAAGGCCAGCGGCCACCTCGACCTCGACAACAAACCCTTCCACTCGG
GCTTCGGTTCACCCATGAGTACGTCGCAGCATCATCACACGATGCATCCAGCGATGCAG
CACTACATGTATCACACGGGGGTGGGGGTGGCGGGTGGGCTCCACCAGGGATTCGCGC
CCCCAGCGCCGCCACCGATCCACCCCCACTCGCACTCGCACTCCCATTCCCATCACATG
ACGAGTCTTCAGGGTCTCCAGTTGGCAGCCACGACGAACGCGATG

>novel_circ_001169
AGGGCCGCGAATGCGTGAATTGCGGCGCGACATCGACGCCACTGTGGAGACGAGACG
GTACCGGTCACTACCTCTGCAACGCTTGCGGCCTCTACTACAAGATGAACGGGCAGAA
CCGACCTCTCATCAAGCCGAAACGACGGCTGTCGCTGCAGAGTGCGGCGAGGCGGGC
GGGCACCAGCTGTGCCAACTGCAAGACCGCGACCACCACGCTCTGGCGAAGGAATCA

GGCCGGAGAGCCAGTCTGCAACGCGTGCGGCCTCTACTACAAGCTGCACAAC

>novel_circ_001170
AAGGCGGGGTCAAAGACAGACGCGATTATCCTTGGATCGTGAACGAACCGGTGAAGG
GAATCACCTATGATCTCCCCTCGGAGGAGAGGCCTGACCAGGACCACCACCAGGCTTC
CGGCAGAGGGGCACCTCCGTTCCTGCCACCGTTCCTAATCCCTCCCTCCCTCCCTCCCT
CCCTCCCTTCCTCCCTCCCCCTTATTCTCCCCTGTTTCTCGCGGCGCCCCCACCTCCTCC
TCCTCCTCCTCCTCCTCCTCCCGCTCCATCCTCCACCATCCGTCGCCCGTGGACGTCGC
TCCCCGGGACGGAGGAGCAGCGCACGCCGGAGGAGGTGATCGTCGACCCTCCCCCCC
CCGTCGAGCATCCCGCCCCGCGGGACAAAGGACGCGGCAAAGAGTTGAAGAGGAGA
AGAAGAGGATGGACATGAGCAGCGAGTCGAGCGCGGCCAGGTGGTACGAGCCACCTA
GGTCCACCCTGGAGCCGCCCTCGGGGGGTGCAGGAGTCGCGGGAGTCGTTGGAAATC
CGACCGATTACAGGGGTTACTACCCGCACGCGGGGCCGACCGCCCATCACCCCGCAGC
AGCGGCCGCTCACTATGCCCACACAAGGATGTCGAGCAGCGGAGTGGCGAGCGGGCC
CGTGAGCCAGGTGTGCCGGCCGCACTTCCACAGCTCGGTGCTGCACCCGTGGCTGTCG
GGGAGCGGGGCGGACACGTCGAAGACGCCGTGGGGATTCCCGACGACGACCGGCTC
GGGCGGGAGCGGGGTGGCCGACGACAAGCCCCAGAGCCCCCTGGGATCCTCCCTGCC
GCCGAGCACCGGCAGCCTCTCGAGCCACGGCGGGGCGGGGGCCGGCCACCACCTGTT
CTCCTTCCCCCCGACGCCCCCCAAGGACGCCACCCCCGACAGCATCACCGCGAGCACC
ACCTCGGCGGGCAACAACAACAGCGGCAACAGCAACAACAACAACAACAACAACAC
GACCAGCGCCAACAACAACGCCAACAACCCCCTGTCGGGGCTCGGGGGTGGGGCGA
GCAGCGAGTACCAGGCGGCGGTTGCCCACGCGGCGGTGATGGGCGCGTTCATGCACC
ACCAGGACGCGCTGGGCGGCGGGGGAGCGGGCTCGTGCGACGTGAAGCCGAGCGTG
ATGCTGGCCCATCACCACGGCGCGCCGTCGCCGCCCCATCAGCAGCACAACGGGACG
GCGAGCGGCGGGAACGGAGGGACAGGGGCGAGTGGAGGCGGCGGGGCGAGCGGGC
AGGTGAAGCAGCGCGAAGGTAATAATCAGTCGGGCAGCCAACAGGCGAGCACCGCGC
AACAGCAGCAGCAGCAGCAGCAGCAGCAGCAGCAACAGCAGCAGCAGCAGCAGCAG
CAGCAGGAGGCGGCCGCGTATCAGGGGAGCAACGTGGGCGGCGGGGGTGGCGGCGG
CGGCGGCGGGGGGGCGAGTTCGCCCTCGTGCAGGGAGAGTTACGCGGCTGCGGCCGC
CGCCGCCGCCGCCGCCGCGGCCGCGGCCGCGGCTGCCTCCAACACCCACCACGCGAC
CTCCGGATCCCAGTACGACCCGGCAACCAGCGCGTATAACATGTACCAGCACCTGCAG
TATCCTAGTACCACCCACCACCACCACCATCACCATCATCACGCCGCCTCCTCGTCGTC
TTCTTCGTCTTCGTCCCACAATCCGCACAACGGGGCCGCCGCGGTCTCCGGTATTTTCG
GCCACCACGCGGCGGCGGCCGCGGCCGCCGCCGCTGCAGCCGGGGCCGGGAACGCG
GCCGGCGTGGCCGGCGTGAACGTCGACTCGAAGCTGTTGGTCGGCCACGTGCACGCC
AACACGCCGAGCTCGCCTTCCGCGCAGCAGCAGCAGCAGCAAAAGCCCAGGAACAA
GTCGAGAACGTCCGCCGAGGGCCGCGAATGCGTGAATTGCGGCGCGACATCGACGCC
ACTGTGGAGACGAGACGGTACCGGTCACTACCTCTGCAACGCTTGCGGCCTCTACTAC
AAGATGAACGGGCAGAACCGACCTCTCATCAAGCCGAAACGACGGCTGTCGCTGCAG
AGTGCGGCGAGGCGGGCGGGCACCAGCTGTGCCAACTGCAAGACCGCGACCACCAC
GCTCTGGCGAAGGAATCAGGCCGGAGAGCCAGTCTGCAACGCGTGCGGCCTCTACTA
CAAGCTGCACAAC

>novel_circ_001171

CAAGGATGTCGAGCAGCGGAGTGGCGAGCGGGCCCGTGAGCCAGGTGTGCCGGCCGC
ACTTCCACAGCTCGGTGCTGCACCCGTGGCTGTCGGGGAGCGGGGCGGACACGTCGA
AGACGCCGTGGGGATTCCCGACGACGACCGGCTCGGGCGGGAGCGGGGTGGCCGAC
GACAAGCCCCAGAGCCCCCTGGGATCCTCCCTGCCGCCGAGCACCGGCAGCCTCTCG
AGCCACGGCGGGGCGGGGGCCGGCCACCACCTGTTCTCCTTCCCCCCGACGCCCCCC
AAGGACGCCACCCCCGACAGCATCACCGCGAGCACCACCTCGGCGGGCAACAACAAC
AGCGGCAACAGCAACAACAACAACAACAACAACACGACCAGCGCCAACAACAACGC
CAACAACCCCCTGTCGGGGCTCGGGGGTGGGGCGAGCAGCGAGTACCAGGCGGCGGT
TGCCCACGCGGCGGTGATGGGCGCGTTCATGCACCACCAGGACGCGCTGGGCGGCGG
GGGAGCGGGCTCGTGCGACGTGAAGCCGAGCGTGATGCTGGCCCATCACCACGGCGC
GCCGTCGCCGCCCCATCAGCAGCACAACGGGACGGCGAGCGGCGGGAACGGAGGGA
CAGGGGCGAGTGGAGGCGGCGGGGCGAGCGGGCAGGTGAAGCAGCGCGAAGGTAAT
AATCAGTCGGGCAGCCAACAGGCGAGCACCGCGCAACAGCAGCAGCAGCAGCAGCA
GCAGCAGCAGCAACAGCAGCAGCAGCAGCAGCAGCAGCAGGAGGCGGCCGCGTATC
AGGGGAGCAACGTGGGCGGCGGGGGTGGCGGCGGCGGCGGCGGGGGGGCGAGTTCG
CCCTCGTGCAGGGAGAGTTACGCGGCTGCGGCCGCCGCCGCCGCCGCCGCCGCGGCC
GCGGCCGCGGCTGCCTCCAACACCCACCACGCGACCTCCGGATCCCAGTACGACCCG
GCAACCAGCGCGTATAACATGTACCAGCACCTGCAGTATCCTAGTACCACCCACCACCA
CCACCATCACCATCATCACGCCGCCTCCTCGTCGTCTTCTTCGTCTTCGTCCCACAATCC
GCACAACGGGGCCGCCGCGGTCTCCGGTATTTTCGGCCACCACGCGGCGGCGGCCGC
GGCCGCCGCCGCTGCAGCCGGGGCCGGGAACGCGGCCGGCGTGGCCGGCGTGAACG
TCGACTCGAAGCTGTTGGTCGGCCACGTGCACGCCAACACGCCGAGCTCGCCTTCCGC
GCAGCAGCAGCAGCAGCAAAAGCCCAGGAACAAGTCGAGAACGTCCGCCG

>novel_circ_001173
GCGGTGACAAGCGTCGCGCGGGGGAAGGAGAATGGAGGCGGCGGGGGAGGCTGGCG
ATGACAAGAGCCCCTCGAAGACTGAGCCGGATGACCAGGGATCGAGGCAACTTAGCG
TGGACCAACTGCGCACTCTCTCGATCGACAAGGTTACGGTGGAGAAGATCGTGTTGGA
ATCGTCGGCGGGCTCGCGAGAGGTGCTGTTGACGAAAAATGAGAGTTCGAGGCGAGT
TTCGATAAAATCCGAGGTGGAAAAGCCGGCGAGCGGCGACAATTATCGCTCCTCCCTT
CCCGACGACATTAAAGCGTTCGACGGGCGTCGGTCGAAGAAACCGGAGAGGATCAAA
CCGATCAGACTGACCAAGAGCCCGTCCATAGGCAGCTTGGCGGTCGCGACAGGCGTC
CCTGTGGACGAGGCCAAGGGAGAGGACAAAGAGAATCTGTTGATCACCGAGGAGGA
GGACAAAGAGAATCTGTTGATCGCCAAGGAGGAGAAGGTGAACGCGAGAAATCTGTT
GGAGAGGAGCCAGACGAACATCGAGGTGCTGCAGCGGAACCTGAACACCAGGATCG
GCGCGAGCACCGCCCAAAGGTACAAGAAAAAGTTGCTCGAGTCCCGGGAGAATCGGC
GTCCAAAGTTCGCGGGGAGGATCGCGTTCGACGAGGAGTTCCTTCAGAAGCACAATC
TCGACAAGAGATTGAAGATCGAGGAGTACGGGTTGCCGGACCTTCCTCCGCAACCGG
AAGAAGAGGAGGAGGAGGGTAGTTCGAAGGCAGGGTACAGCGAGAAGGTGTTGGAG
GTGGAGAAAAGGTGGTCAGGGGAGTTTCTGAAGAATCAATTGGACCGCCGCTCCTCG
GAGTCGTCCAAGTCCTCTTACGTGGGGGAATACGGGAGCGTTTCCTCGAGGGAGAGCA
TAGCCGAGGACTACGATCCTAGAGCAGCCTCCTCCGCCGTTCTTTTCCAAGCTGGAAA
AGAAAAGTTCGAGGCGGGGCGGCCGATTAGTTTGAACTGCGCGAAAACTCTGGCCGC
GAGCACGTTGAGCACGCTCGACTCCGACTCGTGCCTCGAGGACAGGATCAAGTCGCT

GGAGGAGGACATCGAGGAGGATGGGGACTCGGTCGCTCGTCGACGGATAGGGAGATC
GTCCGATCCTTGGAGGGAGTCCATGCTCCCGTTCGAGTTTTGGAATCGAAAGGAGACT
ACGGAGTCGAGGATCGACGAGACTCGAAATTCGAGAGTCGCTTCATTGGAGTCGACG
AGGGTGGACGGGTACGCGAGCCTGATAAGGAAGTTCACGATGGAGGCGGGCTCGTCG
CGGTCGAGGAAGGAGGAGAAGCAGAAGAGGAAGCTCGGTTTCCGCCGCCTTCTGCCC
GGCTTCTTCTCGCCCAAGGACTCGCGAAAGGATTACAAGAAGAAGGAGGGGAAGGAG
AGGAAGAGGGCGGGCGACGACAGGCACTTCGCCCGCTACCAGCAGAACGGGAACTA
CGCCAGGTCGGCGGACACGATGAACCTGAACGAGGACATCAAGAGGAACGTGAAGCT
GGACAACAGCCTGAACGGGTCCATGATCGAGGAGAGGCTGGACGAGATCAAGCGGGA
GCTGTTCCCGCTCGATCAGGGCCCCATCACGAGCACCCCCGACCACCTGGCGCGCGAG
GACCAAGGAGGTGGCGGCCAGCTGCCCGCCCGCTCCTACACCACCGACTCGAGCCTC
AGCTCGATCGCGCCCGACGAACGGTGGGCGGGCGAGAGGAGCGGCCAGCTCCTCGGC
GTGTCGCCCGACGCGAGGAAGTTCGAGCAGAGGCAGAGGCAACGTTACGGCCAACCG
TTGGAACGGAAGCACAGTTTGCAGGAGCCGAACCACTGCGCCCCCAGGCAGCAGCCG
GCCTTCTTCCAGAGGAACCACGGCCCGTCGGGGAGGATCTCCGCGCCGCCGGCCGAG
AGGTACCTGGCCAGGCCGAGGCCGGTCGACCGCCCCCTCCCAGCCATACCCCGCTCCA
GGCTCGACCTCTTGTCCAACTACGAGAATTACCTGGAGGCGGGCGAGCAGGCGTTGTA
CGAGAACGAGAACCCGCCGCCGGGCTTGATCCTGAAGTCGGTCTCGGCCCAGGTCAA
GATCACCAGGCAGCCGATAGTGAATCAGAACCAGCGCGCCCCGTTGCTGGGCAGGTC
GCCCAAGTACCTGTCCTCGGGCTCGAGCCAGAAGTCGGGGGACTACGGGGGCGAGTC
GAGTTGCACACCGAACTCGAGCCAGAAGAGCGAGTTCTCGCCGACCAGCTCCAAGAG
CGGCGAGTATTACCTGCACTCGCCCCGCGACACCGGCTCCCCGAACGAGGAGGACTCT
CGAAGGGACGAGGGCATCTACGAGAACGAGAAATCACCGGACGAGCGAATCTACGAC
GAGACGCCCTGCGAGGAAGCCGACTCGAACCTCGTCGAGGACAAGAGAGGGTCTCCT
CGTAGGAGTCGCTCGAGAGGCGGCGAACGAGCCGCTTCCCCGGCGAGGGGAAGCGC
GGAGAAGCTGTGCGGGGAGCAGGGAAAAGCGATCTCGCCGCTGGGACCGAGGCGGC
CGCCCTCGGCCAGAAGGGCGCAGCCCAACGAGCAGATCCTTATAGCGTCCCCGAAGA
GGGACGTGCCGTACGAGACGCGAATACCGAGGCCGTGCAACGAGTCGAGGCGCTGCC
TCGTCGAGTCGCCGGTGCACATGACGAACGCGCCTCGAAAACTCGTGGTCGACCCGC
GGGGCCAAGATTCGAGCGACGCCTCGAAGGCTCAGGTTAGCTTGGAGATACCGCGTCC
AGAGCCTGTTTACGCGCGCCAGCGGAACCCTGAACCAGCTTCTTCGCCCGAGAAAAA
GACGAGCAGTCAAGAGAGAAAGGAGGGGGAGGAGAGGGTGGCTTGGGTCCAGGCGT
CGTCCTCGCCGCAAATCGGCGAGACTGGCTCGCAAAAGGTCGACAGGTCGATCGAGG
ATCATCGTCAGAATTCTATCCCAGCTATGGAACCTCAGCCACTTCAACGTAATCCTAATT
TAGAGGAGCAACGGGTCTCCAATCAGCTCGACTCGAGGCAGAGAAGTTTGGAAGATG
GCGCACAGTACAACCAGAGGACCTACGAAGCGACGCAGCGGATGGGTCCGTTGTCGC
CCTCGAAGCAGGAGACTCGACAGCACCTGGAAGCGTTCTACTGGCAGCAGAAGGCGC
TCGAGGCGCACCGGAAGTCGCTCGCGTCCCCGGCGAGCGAGAAGCAGATTGGGCGAA
AGATCGACTTGCCGGAGGTGAGGGAGGCGGTGTACTGGCAGCAGCTCAAGAAGCTGG
ACGAGGAGCAGCAGAGGCGCATCTACGAGCAGAATCCGATGGACGAGTCGTCGTACT
GCAAGGCGAAGAGAGTATCCGCGCCGAGGCAGCAGGGACCTCCTGTTCGAGTGGCGG
CGACCGGTGGTCTTCCTCTGCCTGTCATCGCCCATCATGGAAACCCATCCTCGTGGACC
TGTGGCAACGCGAAGCCGAGCCCCGGCGGGAAACCGCCGCTGATGCAGAGCCAGAA
GGGCCAGAACCAGCCGGTGCTGATCGTCCGCCCGCAGCAGGCGATCCGCGATCCGCG

GGTGGACGCCAAGCCGCTCGACTCGGTGCAGAGATCGAAGAGCGCGTCACCGCATTT
TCACAGGGGAGACGTTCCTCAGGCGCGGAAGCTCGAGCTTTCCTCCTCGATCTACGAG
GAGGAGCCGACCAACGACGTCGAGGGGAAAGCAGTTGCTCACCCTCCTCCTCCTCCT
CCGATATTCAAAAGGGGTAGCTTGATCGGGGGAGAGTCGGTGGAGTACGGAAGCGGG
AGCGGCGCGAAGAGGGTGAGCTTCTCGAATCAGTCGGGCGTGGGCGGGCAGGAGCTG
GCCAGCGGAAGTTGGCCCACGAAACACGGCACTGCCCCGGAACCGCCGACTCGAAG
GCATCGAAGCGAGGACAGCGTGTCCGACACGGACTCGGTGTTCTCGTACCCGGACCG
CCTTCGTGATCCTTCCTGCGAGTACGATGCCGACAGGCCTCTTCCGCCTCTGCCCAAGG
ACGCGACGAGAAGAGGCGTCGCGTACAACGGGGACACGACGCGTTGCAACGTCGGA
GTGGATCCTAGACGCGGCGTCAGCCCGCAGCGAGTACTTCGACAGAAGG

>novel_circ_001174
AGGAAGAGTGGAATTCGGATGGCAAGGGTCGACAGTCCAACGACATCGGTGGGTCCG
GCAGGGGGGAGGGTGAGGCAGGCTCGAACGAGGTTCCAACTGGGAGGAGAGGCGGC
GGTGGCGGCGGCGTGGGTAGCGGAGTCGGCGGGGGCGGGGGCGGAGGAGGAAGCGT
GCTGGGGGTTGGCGGAGGTGGCGGAAGCGGAAGCATCGGGGGCGGCTCGGGGGGCA
GAAGCGGATCAGGGGGAGGCCGATCGAGGGACGAGCCGAGGAGGCACACCCTCGGC
GGCGATCACCAACCGTCCCTACATCATCAGCAGTTCAACGCGGCCCAGCAGCTACACC
CCCTTCATCCCCATCATCTGCCGCCGCCACACGGCCAATACGGTACGCCGCCCACGCGT
CATACCACCATGGATCTCGAGATGGGGACCAAGTCTCGCCAGAGAAAGTCGCCTCTTC
CGCGCGGGTACCCACCTCCCTCTACGACGATGTTGTTCGATGACGACCCGGGCATCATG
TCCGAGGTGGAGACATCGAGCACCGGATTTCGACGGGGTGGCAAGCAAAGAAGCAGC
CTCCCCGTCGTACGGACCCCCAGCAAAACTCTGGAGCGACCACTAGGTCTAGTGTTCC
TGCAGTATAGGAACGAGACGAAGCGGGCGCTCCTACCGAACGAAATCACTAGCATAGA
CACCGTTAAGGCTCTCTTCGTACGAAGCTTTCCCAAGCAACTCACTATGGAATATCTAG
ACAGCCCCCACGTCAAGGTTTACATACACGATAGCAACAAGGATATGTTCTACGAACTC
GAAGATCTCAGATCGCATCTGAGAGACATCCGAGACCGAAGCGTCTTGCGACTATTCG
AGAGCACTGACGGTGTGACAGGGATGCCAGGGCCATTGGGGATACCAGGGACAGGAA
CGGGGCTACCACCTCACTGGGAGGACCAGAGCTACTTCAGCGAGCCGGAATTCGACA
GCGAATACCAACATCAGCATATCCACAAGAGCAAG

>novel_circ_001175
GTGGGTCCGGCAGGGGGGAGGGTGAGGCAGGCTCGAACGAGGTTCCAACTGGGAGG
AGAGGCGGCGGTGGCGGCGGCGTGGGTAGCGGAGTCGGCGGGGGCGGGGGCGGAGG
AGGAAGCGTGCTGGGGGTTGGCGGAGGTGGCGGAAGCGGAAGCATCGGGGGCGGCT
CGGGGGGCAGAAGCGGATCAGGGGGAGGCCGATCGAGGGACGAGCCGAGGAGGCAC
ACCCTCGGCGGCGATCACCAACCGTCCCTACATCATCAGCAGTTCAACGCGGCCCAGC
AGCTACACCCCCTTCATCCCCATCATCTGCCGCCGCCACACGGCCAATACGGTACGCCG
CCCACGCGTCATACCACCATGGATCTCGAGATGGGGACCAAGTCTCGCCAGAGAAAGT
CGCCTCTTCCGCGCGGGTACCCACCTCCCTCTACGACGATGTTGTTCGATGACGACCCG
GGCATCATGTCCGAGGTGGAGACATCGAGCACCGGATTTCGACGGGGTGGCAAGCAA
AGAAGCAGCCTCCCCGTCGTACGGACCCCCAGCAAAACTCTGGAGCGACCACTAGGT
CTAGTGTTCCTGCAGTATAGGAACGAGACGAAGCGGGCGCTCCTACCGAACGAAATCA
CTAGCATAGACACCGTTAAGGCTCTCTTCGTACGAAGCTTTCCCAAGCAACTCACTATG

GAATATCTAGACAGCCCCCACGTCAAGGTTTACATACACGATAGCAACAAGGATATGTT
CTACGAACTCGAAGATCTCAGATCGCATCTGAGAGACATCCGAGACCGAAGCGTCTTG
CGACTATTCGAGAGCACTGACGGTGTGACAGGGATGCCAGGGCCATTGGGGATACCAG
GGACAGGAACGGGGCTACCACCTCACTGGGAGGACCAGAGCTACTTCAGCGAGCCGG
AATTCGACAGCGAATACCAACATCAGCATATCCACAAGAGCAAG

>novel_circ_001176
GTGGGTCCGGCAGGGGGGAGGGTGAGGCAGGCTCGAACGAGGTTCCAACTGGGAGG
AGAGGCGGCGGTGGCGGCGGCGTGGGTAGCGGAGTCGGCGGGGGCGGGGGCGGAGG
AGGAAGCGTGCTGGGGGTTGGCGGAGGTGGCGGAAGCGGAAGCATCGGGGGCGGCT
CGGGGGGCAGAAGCGGATCAGGGGGAGGCCGATCGAGGGACGAGCCGAGGAGGCAC
ACCCTCGGCGGCGATCACCAACCGTCCCTACATCATCAGCAGTTCAACGCGGCCCAGC
AGCTACACCCCCTTCATCCCCATCATCTGCCGCCGCCACACGGCCAATACGGTACGCCG
CCCACGCGTCATACCACCATGGATCTCGAGATGGGGACCAAGTCTCGCCAGAGAAAGT
CGCCTCTTCCGCGCGGGTACCCACCTCCCTCTACGACGATGTTGTTCGATGACGACCCG
GGCATCATGTCCGAGGTGGAGACATCGAGCACCGGATTTCGACGGGGTGGCAAGCAA
AGAAGCAGCCTCCCCGTCGTACGGACCCCCAGCAAAACTCTGGAGCGACCACTAGGT
CTAGTGTTCCTGCAGTATAGGAACGAGACGAAGCGGGCGCTCCTACCGAACGAAATCA
CTAGCATAGACACCGTTAAGGCTCTCTTCGTACGAAGCTTTCCCAAGCAACTCACTATG
GAATATCTAGACAGCCCCCACGTCAAGGTTTACATACACGATAGCAACAAGGATATGTT
CTACGAACTCGAAGATCTCAGATCGCATCTGAGAGACATCCGAGACCGAAGCGTCTTG
CGACTATTCGAGAGCACTGACGGTGTGACAGGGATGCCAGGGCCATTGGGGATACCAG
GGACAGGAACGGGGCTACCACCTCACTGGGAGGACCAGAGCTACTTCAGCGAGCCGG
AATTCGACAGCGAATACCAACATCAGCATATCCACAAGAGCAAGACGGCGAAGAACTC
GACTTCCGGGAATAGCGGATACTATGTCGGAGGAAGCAGCACTCTGCCACGAGGAGG
ACAACTAATGAGGGCGTACAGTCCGGCGGCGTCGTCTGTGGTCGGAGGACCAAACGC
GACGCCGACACAACCGAAACCTCTCGCCACTCCAG

>novel_circ_001177
GTGGGTCCGGCAGGGGGGAGGGTGAGGCAGGCTCGAACGAGGTTCCAACTGGGAGG
AGAGGCGGCGGTGGCGGCGGCGTGGGTAGCGGAGTCGGCGGGGGCGGGGGCGGAGG
AGGAAGCGTGCTGGGGGTTGGCGGAGGTGGCGGAAGCGGAAGCATCGGGGGCGGCT
CGGGGGGCAGAAGCGGATCAGGGGGAGGCCGATCGAGGGACGAGCCGAGGAGGCAC
ACCCTCGGCGGCGATCACCAACCGTCCCTACATCATCAGCAGTTCAACGCGGCCCAGC
AGCTACACCCCCTTCATCCCCATCATCTGCCGCCGCCACACGGCCAATACGGTACGCCG
CCCACGCGTCATACCACCATGGATCTCGAGATGGGGACCAAGTCTCGCCAGAGAAAGT
CGCCTCTTCCGCGCGGGTACCCACCTCCCTCTACGACGATGTTGTTCGATGACGACCCG
GGCATCATGTCCGAGGTGGAGACATCGAGCACCGGATTTCGACGGGGTGGCAAGCAA
AGAAGCAGCCTCCCCGTCGTACGGACCCCCAGCAAAACTCTGGAGCGACCACTAGGT
CTAGTGTTCCTGCAGTATAGGAACGAGACGAAGCGGGCGCTCCTACCGAACGAAATCA
CTAGCATAGACACCGTTAAGGCTCTCTTCGTACGAAGCTTTCCCAAGCAACTCACTATG
GAATATCTAGACAGCCCCCACGTCAAGGTTTACATACACGATAGCAACAAGGATATGTT
CTACGAACTCGAAGATCTCAGATCGCATCTGAGAGACATCCGAGACCGAAGCGTCTTG
CGACTATTCGAGAGCACTGACGGTGTGACAGGGATGCCAGGGCCATTGGGGATACCAG

GGACAGGAACGGGGCTACCACCTCACTGGGAGGACCAGAGCTACTTCAGCGAGCCGG
AATTCGACAGCGAATACCAACATCAGCATATCCACAAGAGCAAGACGGCGAAGAACTC
GACTTCCGGGAATAGCGGATACTATGTCGGAGGAAGCAGCACTCTGCCACGAGGAGG
ACAACTAATGAGGGCGTACAGTCCGGCGGCGTCGTCTGTGGTCGGAGGACCAAACGC
GACGCCGACACAACCGAAACCTCTCGCCACTCCAGATGGTTGGATAGGTGGTGGCGG
GGTGCCGCCGGCCAAGCCGCTTCGCAGCTACCAGTGTGGTAAGAGTCCCCTTGGAAG
CCTGGGGGGCTCGGCTCGCTTCTCTAGAGACAGCTCGGTCTCCCTCTATAGTATAGCAG
ATCGATTGCATGGGGAAAGCGGTTATATGAGCAGTCCCGAAAGAGGTGGAGGAGTTGG
TTCTGGAACGGGAAGATACCCACCCGGCCCCTACAGCGCAGGAAGCAGCTACGAGGA
TCCGTACTATTCGCAATATTCGGGCACTGTGACACCTGTCATTGACGAGGAAGCCAG

>novel_circ_001178
GTGGGTCCGGCAGGGGGGAGGGTGAGGCAGGCTCGAACGAGGTTCCAACTGGGAGG
AGAGGCGGCGGTGGCGGCGGCGTGGGTAGCGGAGTCGGCGGGGGCGGGGGCGGAGG
AGGAAGCGTGCTGGGGGTTGGCGGAGGTGGCGGAAGCGGAAGCATCGGGGGCGGCT
CGGGGGGCAGAAGCGGATCAGGGGGAGGCCGATCGAGGGACGAGCCGAGGAGGCAC
ACCCTCGGCGGCGATCACCAACCGTCCCTACATCATCAGCAGTTCAACGCGGCCCAGC
AGCTACACCCCCTTCATCCCCATCATCTGCCGCCGCCACACGGCCAATACGGTACGCCG
CCCACGCGTCATACCACCATGGATCTCGAGATGGGGACCAAGTCTCGCCAGAGAAAGT
CGCCTCTTCCGCGCGGGTACCCACCTCCCTCTACGACGATGTTGTTCGATGACGACCCG
GGCATCATGTCCGAGGTGGAGACATCGAGCACCGGATTTCGACGGGGTGGCAAGCAA
AGAAGCAGCCTCCCCGTCGTACGGACCCCCAGCAAAACTCTGGAGCGACCACTAGGT
CTAGTGTTCCTGCAGTATAGGAACGAGACGAAGCGGGCGCTCCTACCGAACGAAATCA
CTAGCATAGACACCGTTAAGGCTCTCTTCGTACGAAGCTTTCCCAAGCAACTCACTATG
GAATATCTAGACAGCCCCCACGTCAAGGTTTACATACACGATAGCAACAAGGATATGTT
CTACGAACTCGAAGATCTCAGATCGCATCTGAGAGACATCCGAGACCGAAGCGTCTTG
CGACTATTCGAGAGCACTGACGGTGTGACAGGGATGCCAGGGCCATTGGGGATACCAG
GGACAGGAACGGGGCTACCACCTCACTGGGAGGACCAGAGCTACTTCAGCGAGCCGG
AATTCGACAGCGAATACCAACATCAGCATATCCACAAGAGCAAGACGGCGAAGAACTC
GACTTCCGGGAATAGCGGATACTATGTCGGAGGAAGCAGCACTCTGCCACGAGGAGG
ACAACTAATGAGGGCGTACAGTCCGGCGGCGTCGTCTGTGGTCGGAGGACCAAACGC
GACGCCGACACAACCGAAACCTCTCGCCACTCCAGATGGTTGGATAGGTGGTGGCGG
GGTGCCGCCGGCCAAGCCGCTTCGCAGCTACCAGTGTGGTAAGAGTCCCCTTGGAAG
CCTGGGGGGCTCGGCTCGCTTCTCTAGAGACAGCTCGGTCTCCCTCTATAGTATAGCAG
ATCGATTGCATGGGGAAAGCGGTTATATGAGCAGTCCCGAAAGAGGTGGAGGAGTTGG
TTCTGGAACGGGAAGATACCCACCCGGCCCCTACAGCGCAGGAAGCAGCTACGAGGA
TCCGTACTATTCGCAATATTCGGGCACTGTGACACCTGTCATTGACGAGGAAGCCAGTG
GGTGGTGCAGCGATACGGAGCTGTTGGAGGAATCGTACAGCCTTTACGGTGTGAAACC
GCCAGGCCGTCCACCGTCCGGTCCTCCGCGATCTCCGTTTCCCCCAGGGGCACCACCA
CCTTTGCCACCTGGTAGTCAGAGCTACGACGCTACTCGGATAAGGGTAGAGCACATGG
AGAGACAGTTGGCCAACCTGACAGGACTAGTGCAGAAAGCTCTCACCCATGCACCGC
ACACGAGCCCATCACCACGTGATTACTTGCAAGTTCCCGCGGGACGTGATCCTTACGC
GAGAGGGCCAGGCGCCGGGGACGAGTTCGACAAACATTCTAGCTCCAGCTCTGCCTC
ATTGCCAGTGTCTCAACCTGCCGTTACAGATGACTCGTACTTAAGGACTGACGTAAAA

CCACCAAAGTTAGGCAAAGACAAGTCGGTGTCGTTTGAAAAATCAGTGTCCTTCAGTG
ATGAGCCTCCAGACATGAATTCTCCCAAGCAACATTCCCCGCAGCACGCAGCGGACAC
AAAACCAACGAAACCGGCGATCAAATCCTCCACGTTGCCGAGGATGTCTTCGCAAGA
GAGAGACAGGCATAAACCTACCCCGCCGCCGAAACCGGCCGCGCTGGTTGCCGGCCA
ATACGTATATCGGGACATGGCTTTGACACCGGAAATGTACAATCAGCTGAGAGGCTTGC
AGAAAAAGGCCAAGGATCTGAGGCAAGAAGTTAGGAACTTGAGGCGCATGTCTCAGG
CTCAGGCGCACACCATACGGGAGACCATTTTGGATACGTTCATTACCATCAGGACAATG
TTGCTGTCGTGCGGCGACGCAGCCTGGGACGCGGAGAAGATTCGACTAAGCCGCGAG
GAGGACCTCTACAGGCAGGAGATGTTGAGGCTGGTAAAAGATCTAACCGAGCTGGAA
AACACGGTGGAGGAACTACGTGGCAATGTCATAAATAGAAAGACTAGAGTGAACATGT
CGGATGTGGAGAACATGGCCTTGATAATGAGCAAATCTAGCAAAACGGTAGCGGATCT
AAAGGTGCGATTCCCAAGTTTACAAGAGGGTATGAAAGGTCTGTTGAGTTCTGAAATG
GAGATGGTTGTGCGAGCGGAAAAGTTCCTCAAGGAAGAGCCAGAACGATTGGAGTCG
GCGTTGAAACGTTGCAAAAAGCTCACAAGTACCCTCGTGACGCTCAAACGTTTGGCGT
CGGTGCAAGAGCAGCGATTACCAAACGCAGCAGCGAGCATAGACGCCGAAGAGACGC
CACCAATAACACCAACCTCTGCTCAACATTCCAAG

>novel_circ_001179
TCGCCTCTTCCGCGCGGGTACCCACCTCCCTCTACGACGATGTTGTTCGATGACGACCC
GGGCATCATGTCCGAGGTGGAGACATCGAGCACCGGATTTCGACGGGGTGGCAAGCA
AAGAAGCAGCCTCCCCGTCGTACGGACCCCCAGCAAAACTCTGGAGCGACCACTAGG
TCTAGTGTTCCTGCAGTATAGGAACGAGACGAAGCGGGCGCTCCTACCGAACGAAATC
ACTAGCATAGACACCGTTAAGGCTCTCTTCGTACGAAGCTTTCCCAAGCAACTCACTAT
GGAATATCTAGACAGCCCCCACGTCAAGGTTTACATACACGATAGCAACAAGGATATGT
TCTACGAACTCGAAGATCTCAGATCGCATCTGAGAGACATCCGAGACCGAAGCGTCTT
GCGACTATTCGAGAGCACTGACGGTGTGACAGGGATGCCAGGGCCATTGGGGATACCA
GGGACAGGAACGGGGCTACCACCTCACTGGGAGGACCAGAGCTACTTCAGCGAGCCG
GAATTCGACAGCGAATACCAACATCAGCATATCCACAAGAGCAAGACGGCGAAGAAC
TCGACTTCCGGGAATAGCGGATACTATGTCGGAGGAAGCAGCACTCTGCCACGAGGAG
GACAACTAATGAGGGCGTACAGTCCGGCGGCGTCGTCTGTGGTCGGAGGACCAAACG
CGACGCCGACACAACCGAAACCTCTCGCCACTCCAG

>novel_circ_001180
ATCGATTGCATGGGGAAAGCGGTTATATGAGCAGTCCCGAAAGAGGTGGAGGAGTTGG
TTCTGGAACGGGAAGATACCCACCCGGCCCCTACAGCGCAGGAAGCAGCTACGAGGA
TCCGTACTATTCGCAATATTCGGGCACTGTGACACCTGTCATTGACGAGGAAGCCAGTG
GGTGGTGCAGCGATACGGAGCTGTTGGAGGAATCGTACAGCCTTTACGGTGTGAAACC
GCCAGGCCGTCCACCGTCCGGTCCTCCGCGATCTCCGTTTCCCCCAGGGGCACCACCA
CCTTTGCCACCTGGTAGTCAGAGCTACGACGCTACTCGGATAAGGGTAGAGCACATGG
AGAGACAGTTGGCCAACCTGACAGGACTAGTGCAGAAAGCTCTCACCCATGCACCGC
ACACGAGCCCATCACCACGTGATTACTTGCAAGTTCCCGCGGGACGTGATCCTTACGC
GAGAGGGCCAG

>novel_circ_001181

AGAGAGACAGGCATAAACCTACCCCGCCGCCGAAACCGGCCGCGCTGGTTGCCGGCC
AATACGTATATCGGGACATGGCTTTGACACCGGAAATGTACAATCAGCTGAGAGGCTTG
CAGAAAAAGGCCAAGGATCTGAGGCAAGAAGTTAGGAACTTGAGGCGCATGTCTCAG
GCTCAGGCGCACACCATACGGGAGACCATTTTGGATACGTTCATTACCATCAGGACAAT
GTTGCTGTCGTGCGGCGACGCAGCCTGGGACGCGGAGAAGATTCGACTAAGCCGCGA
GGAGGACCTCTACAGGCAGGAGATGTTGAGGCTGGTAAAAGATCTAACCGAGCTGGA
AAACACGGTGGAGGAACTACGTGGCAATGTCATAAATAGAAAGACTAGAGTGAACAT
GTCGGATGTGGAGAACATGGCCTTGATAATGAGCAAATCTAGCAAAACGGTAGCGGAT
CTAAAGGTGCGATTCCCAAGTTTACAAGAGGGTATGAAAGGTCTGTTGAGTTCTGAAA
TGGAGATGGTTGTGCGAGCGGAAAAGTTCCTCAAGGAAGAGCCAGAACGATTGGAGT
CGGCGTTGAAACGTTGCAAAAAGCTCACAAGTACCCTCGTGACGCTCAAACGTTTGG
CGTCGGTGCAAGAGCAGCGATTACCAAACGCAGCAGCGAGCATAGACGCCGAAGAGA
CGCCACCAATAACACCAACCTCTGCTCAACATTCCAAG

>novel_circ_001184
AGATGAATGGTACCAGACTTGGTAGGGGTCGAGGTGGCCGCCTTGCCGTGTACGTGGG
TTGCGGGATGGTGGTCATACTCCTGGTGTTCCTCTACCGCGCCGCCACCTCAGAAATGG
CCAGGCTTCAAGAGCTCAACGTCCAATGTTCTCATCAACAGGAGGCTCTTGCCGCTCA
ACTTCAAGTGATATTCGAATACAAAGTGAGGTTAGAAAAATCGTTGGCCGAGGAAAAG
AGTTCGAACGCGGCTGTGAAACAGGAGCTTCAGCAACGAGCGTCCAGAGAGAAATCC
CTCAGGGATAAGGACAGTATAGAGGCGATGCAGAGATTCAACTCTTTGCAACAAACGT
ACAAGTTGCTGCAGACAGAGCATCAGGATTTGAAGGAGGAATGCAAGAAACGGGACC
AGCAAGCTTTAGAGGACACTAGTAAATTGGAGACAACATTGAGAGAGCTTCGTAGCCG
GATTAGGCAGGCTAGGGAGGACAAGGAGAAGGCTTTGGAACATTTGAAGACCAAGTT
CCTCGAGTTGGACACGCAGAAAAACGAATTGGAACAAAAGTACAATGACATGTTGAA
AAATAACGAGGACACGGATAGCACGATTGAACATTTGAGGAAGGAGGTGATTCAATTG
AAGCGCGAGCTGGACGAAGCTAAAAAATTATCACGCAAAGTTACTTCTCTTGGATCAA
GCGTATCGAATCCGCAACCAATAGCCTCACAAGCGAGATCTGGGGAGGAAGAGAACG
CAGAAAAATCTGTTGATCGGTCGCAAAAACAGCAG

>novel_circ_001185
TTTGCTCGAGGCTGACCGAATCTGCCGTGGGCCGTTTGAGGCGCGTCGGCGATCCGTC
GAAGAAGTATGGCAGTTACGCGTACCAACCTTACAACGAGAACACAGGGGCGGATGG
AATTTGGGTCGGTTACGAGGATCCCGACACGGCCGGGAACAAGGCTGCCTACGCAAA
GGCGAAAG

>novel_circ_001186
TATCAGAGTTCGCTCAAGAGTTGGGCGCTGCAATGGAGAGCCATTCGGCGAACGTACG
ACGGCTGGTCGACGAATTTCGTAGCCGATCCGGGGAGACCAGAGTCGATTCGGGAGG
AATGCGCCGAGTTTGGGAAAGTTTACTGCGCCAGGTACAATCGGATGCAGAAGCTCAC
TTAGATTTGGCTGCGGTCCTCCAACAGCAGCTGTCCAGACCCACCTTGGAAGCCAGCT
TTCATCGTAAAGTCCAGTCTAAAAAGGTATTTTCTCATAGAGAAGCTTATGAACAGGTG
GTTTCCAAGGCCGAGGAGAAATTGCAGCGTGCCCGAGCGGATTACAAACGCGCTTAC
GCAGCGCTGCTGACGGTAGATGGCGGAAGCGAGCAGGAGCTGAAACGCGCCTACTAC

GAGGCGCATAACGCGTACATTCTTCAATTGAGAGCCACGAATGCCATCACAGAACGGT
ATCAATCGCAATGCCTGCCCGGCCTGCTTGGCGAAATAGCGGAAGTTTACGAGGAGTT
ATGCGGATTGGCTTGCAAATGCATGGTTGGCATATCGGAGGCTGCCGCGGAGCGGGCC
AGGGAGCAGACAAAGCGTTATCAGGCAGTTGCCAAAGAGGCTCAAGTTGTTGTGCCT
ATGAACGATTTGCAAATCTTGGCACGAAACTTATCAGCAACTGCAACCCCGTCCAAGA
AACCCTCGAGGCGGCTTTTCGTGGCACCCGGCCCTCCTGAACAGGTTTCTATGGAAAG
AATCAATCAGATACCTTCCCTGAGGGACGAGTTAGCCCCAACAGGAACAAGCACTCTG
CCACTGATGGAAGATTTACGACGGGAATACGATAGTCTTACTCAAGAGATCACTCGACT
CCAGGACGCTTTGGATGCGCTAATTCGCATGCAACGAAAGAGCGCTGAAAGCAATCTG
TACACGAAAGTGGCCGAGTTGCAAGAGGACATCTCCATGAAGCGTTTTGCACTCGGGG
AGACGCAACTATACCTTGCAGCTGTGCAAGCACAG

>novel_circ_001187
GCCAAGAATCCCAAGCCGTTCCATGAGAGTGCGTCGACCCCCAGCGCGGCACCAGCC
CTCCCCATAACCATCCCCTGGTCGAGGGATCCATCCGTCCATACCGCGGAGTGGGAAG
AGAAGGGAAGAATAGAAGAAGAAGGCGGAGGGTGGCTGGCGTGCCAAGAAGATGCG
ACTGTTCGCGTGGCCTGGCAGCGTGACTCGCGGTGGACTCATAAACGATGAATTGGCG
GGCAGCGATGTTGTTGGCTCGGCCGAGAGACGCGGGAGAAGCAGCGGAGGAGGCAG
CAGCAGCAGCAGCAGCAGCCAGGGCAGCCAGGGAATCGGTAGATCGGGCGCGCGATC
GGGCGAGCACCGCGGGGAAACCATCCGCTTGGCATGTACGTCATCTATCCGGCAGACG
AGTTGCTGCGTATCTATGAGTGCCCGGAACCCCGTGATGAGCCCCCGTGTTCCACTGTT
CGTACGCCACTGTTGACCGTTGACGGTGGGGAACGGCGTAGCGGTTGTCAAGCGGCC
GTGATCGCTGTGAACGCGGCCGAGAGCGCGGGTAAGCAGCGCCACGAGGGCGACGCG
AGGAAGGGGAGGAGGCGCGACGGGCGCACGACGCGAAGGGCGAGAAGGGCGAGCA
AGGGTATCCGCGGCGAGCAACGCGTCACCGCACGGACAATGCAAAACAGCCGCGCTG
CCGCCGCCGAGGATCCCGGCAATCCTCTCAAGGATCTCACCCAGCCGGGGCTCAATGA
GCCGCTCAAGCAGCACAATGCGGCCACCCTTAAGCTGGTGCAGACCG

>novel_circ_001188
CTGTTCAAAATTCGTTCGATATTAAAAAAAAAAAAAGAATGAACGTAGTCGTTAAATTT
TGATATTTAACTTTATACAATGATAGCACAAAGCCGTAAGAAAGTCTTTTGATACATAAA
AGATTCAACAAACATTTCAATGCAATTTTATTCACTATTGTAA

>novel_circ_001189
AAAAAAAAGGTAAAAGTGCTATGAGCGAGGTTGTGACTCGTGAATATACAGTGAATCT
CCACAAACGTCTCCATGGTGTTGGATTTAAAAAGCGTGCTCCACGAGCTATTAAAGAA
ATTCGCAAATTCGCTGAAAAACAAATGGGAACTCCAGATGTGAGAATTGATACACGAC
TCAATAAACAACTTTGGTCTAAAGGAATTAGGAATGTACCATTTAGAGTTCGTGTACGA
TTAAGCAGAAGAAGAAATGATGATGAAGATTCTGCAAACAAATTATATACCCTTGTTAC
TTATATACCAGTTGCTTCCTTTAAGGGTTTACAAACAGAAAATGTTGATGCCAGTCAAG
ATTGATTTATTTTTGATTAATAAATAATTTATCTCAA

>novel_circ_001190
GGAACTCGCGAGAGGATCGAACGCCATTCTGGAACCAAGAGCCATCGCCAAGGCGCC

AGAAGGAGAGGGTTGCCCTCGATGCGGAGGATACGTTTACGCTGCCGAACAGATGCTG
GCCAGAGGAAGGGCTTACCATAAGCAATGCTACAAGTGCAAAACCTGTCACCGGTCTC
TGGACTCGACGCTTCATTGCGATGGTCCTGATCGCGAGATATATTGTCGAG

>novel_circ_001191
ATGGTGGTGGTGGTGGTGGTTGAGCAGCCGGTGGAGGAGGAGGAGGAGGGCGGGTG
CAGGGCGGAGGAGGGCGATGTGGCTAGCAAGTGGGAAAACTCGAGGAGGTGATAAC
GACTTCTGACCTTCCGTGAGTGTGCGTGCCGCGGCGTAGGGGGGCGCCGCCGCCTTCG
ATTGCCAATTCGAGAAGGCTCACCATTCGGAAGAAAACGAGGAGGAAAAGGCGAAAG
AGAAAGGAGGAGAAGGAGAGGAGGAGGAAGAGGAGGAAGAAGACGAAGGAAGAG
ACGAAGGGTGGAGAGAGAGGGGAGAGAGAAACATTATAAATAAAGAATAGGACGAAC
AACACTAAGGAATAAAACTAAATTGGCAACTCAGTGGAGAAGAACTCGAGAGAGATC
GAAGGAGCGGGAAGGGGTTGGAAAAAAAGCGGGAGAGGAACCTCGGACAAGTTTTG
TAGTCCCTTCCCTTCTCCCGGCCAGAATCGGCCGGACGAGCACACACGAGACAACAAT
TCTTCTTCACAGCGGGTCAAGTCGCTGCATTGCTCCCTACATTGGGGAAAGAAAAATT
AATAATAATAATAATAATAATAAGAATAATAATAATAATAATAATTATAATAATAATATTATT
AATAATAATAATAATAATAATAATAACTTCTACCTTGAGAACATATACCTCGTGCGTACAA
GTACCTTCAAACTTAAACCTCTTTTTCGCAGCGTAATAGTGCCTAGGCCCCTCCGAAGA
ACGCGAAATACAAATAATAATAATAATAATAATAATAATAATAATAATAATACAAAATATT
AATAATAATAGCAACATTATATATATATATATATATATCTATACGGAGACAAAAAGAATCAGA
GAAAGAGAGCGTGGGAGAAAATTTTAAATAGAAGAGGAGAGGAAGAGTTTTAACGTT
TTAATCATCAAGTTTGCGTTTGGAAAGTCACGCAGCCCTTTTTACGTACATATACAAATA
CACATTGTGCATAGATAGATAGATACACATATACACGCATAAAAGGTCCCCCGGTGGCC
GTATGTATACGGACGTGCGCGAGAGAGAGAGAGAGAAAGAGAGCCGCGATTGGTCGG
AAGAATAAATAATATCCTCGATTCGAGATCGACGGACGAACGAACGAACGAACGAACG
AACGAAGTTACGTTGCGAAAGAAAACGATTTCCAAGATTTGGTTCAGAAATTCTCTTT
CGTTGTATAACGAAACAAATCTCTCCCCCTTCGAGCCCTTTAATGATCGATTGATAATAC
GAACCAGGACAGCAAAGTACCTGCAGACACAGATATATTTATAAGAATAATATTTATATG
ATATATATATATATATATTATATAAACGAATGAATATATAGTAACACACACGTACACACACA
TACATACACGCATAAGTAATAGAGGGTCGAGCAACAACAAGCTAAAGGGCAAAGAGA
ATAATAATAATAGCGACAACAACAAGTAGAAGCAAGAATAATAAAATAAAATAAAATAA
CGATAGCTGTTAAGAAGAGAGAAGAAAGAAGAGGATCGTACAAGAACAGAGTGTGAA
CGCTTATACATAATTCTAGAAACGCGCTACGGTTAGAGTACTTCCACTTTGCGATTAATT
AATTTACTACGAGCGATTGCGGGGCCTGTTCTACGTGTACACGATCAAG

>novel_circ_001193
AAAAAATTACTTTTGGAATGGTTGTTACGCTGGTACTCGGAAGAACAAGTGGTTGTGG
TTTAGTGATCGGTGGATGTTTGCTTTGAGATGATGATGGATTTTTAAGAAGCTGAGGGG
AAAGATACCTCAACACAGCAACGGGCGACGAAAAACGACTCGATCGGTATGCACCTG
GGACCGCGGTTCGAGGCGCACTTCAAGATGCGGTAGAGGAAGTCCCTCGACCCGAGC
TCACCCGAATCCAGACCGGTGCAACAACCGACAGTTATCCCGCGATATTCCCGGTGAC
ACGTACGAAGCATAATAATCGCATGCATAATAACAACAATCAACGGAGCGAGACTCCTG
GAACGTAGAAAAGAAGGAAAAGAATCCGGCAAAGGATTCCTGAAATACCCATCCATC
CACTCAGTGCGGCCAACCCAAGGCTCTCTCTCAGTTTCTCAGGCCGAACGAGCCCGG

GAGCCTCGGATCGATCGATCGATGAGAACCACTGCCCTCTGAAAGGATCCCTTTAGAG
GAGCGCGCCTAGGCGCGCGCGATCGTCCTCCCCTTCGAGAAAAAACGTCATCGTCGAC
CTTTCCCCCTGCCCCTCCCCGCCGTTAAACCGTTTCCCTCCCCCGTCGAGGGGCACCTT
TTCGAGAGAAGAGAAGTGAAGAAGAAGAAACAGAAGAAACAGAAGAGGAGGA
GGAAGAAGGAGAGACCCGGTCGTCGTCACGGTGGCAAGCTTAGTGAGCGGGAACGTA
GCGGTGCGTTACGATGAGGCGTCAGGAGTGGTTGGCACGGGGGGTGTCGTGTTGGCA
GCCACTCCCAGGTTAGCCGCCGCTACGGACCAGGCCACGTCCGCCACCATGGACACCA
GCGACTCCAGCATGGACACGACTAACGGGGGGGGCTCGAGCGCCGGGGTTGGCGTGG
TCGGTGGGACGATCGCCTCTGTGGTGGCAGGGGCCGCGTCCTTGACCCTGGTCAAGGC
CGAGACGCCCGAACACCTGGCCGGGACGTCGACGACGGCCGCCGCGACGCCGACGC
CCCCGTCGGTCCCCGTCGGATCGGCGGTGGCCGGGACGGCCGGTGGCGCCCTCTTCCC
CGGCATGGCCGCGGCGGGCAAGGGCGCCGCGAGATCCGACGACTGGCTCGCGAACGC
GAACAGCCCGGTCGGCTCCCCGTCCGCCGCGTTGCAGCCGCAGCACGTGGTCTACGG
GAATCCTCAGCAACAACAGCTCGCCGCCGAGACGCAGCAGCAGCAGCAGCAGCCGCC
CCTCGCCCACTCAAGCCCCCTCGCGCATCAGCAGCAACAGCCCAACAGCAACAACGG
CTACGCCAGTCCTATGAGCACCAGCAGCTACGATCCTTACAGTCCCAACAGTAAGATAG

>novel_circ_001194
ATACCTCAACACAGCAACGGGCGACGAAAAACGACTCGATCGGTATGCACCTGGGAC
CGCGGTTCGAGGCGCACTTCAAGATGCGGTAGAGGAAGTCCCTCGACCCGAGCTCAC
CCGAATCCAGACCGGTGCAACAACCGACAGTTATCCCGCGATATTCCCGGTGACACGT
ACGAAGCATAATAATCGCATGCATAATAACAACAATCAACGGAGCGAGACTCCTGGAA
CGTAGAAAAGAAGGAAAAGAATCCGGCAAAGGATTCCTGAAATACCCATCCATCCACT
CAGTGCGGCCAACCCAAGGCTCTCTCTCAGTTTCTCAGGCCGAACGAGCCCGGGAGC
CTCGGATCGATCGATCGATGAGAACCACTGCCCTCTGAAAGGATCCCTTTAGAGGAGC
GCGCCTAGGCGCGCGCGATCGTCCTCCCCTTCGAGAAAAAACGTCATCGTCGACCTTT
CCCCCTGCCCCTCCCCGCCGTTAAACCGTTTCCCTCCCCCGTCGAGGGGCACCTTTTCG
AGAGAAGAGAAGTGAAGAAGAAGAAACAGAAGAAACAGAAGAGGAGGAGGAA
GAAGGAGAGACCCGGTCGTCGTCACGGTGGCAAGCTTAGTGAGCGGGAACGTAGCGG
TGCGTTACGATGAGGCGTCAGGAGTGGTTGGCACGGGGGGTGTCGTGTTGGCAGCCA
CTCCCAGGTTAGCCGCCGCTACGGACCAGGCCACGTCCGCCACCATGGACACCAGCG
ACTCCAGCATGGACACGACTAACGGGGGGGGCTCGAGCGCCGGGGTTGGCGTGGTCG
GTGGGACGATCGCCTCTGTGGTGGCAGGGGCCGCGTCCTTGACCCTGGTCAAGGCCG
AGACGCCCGAACACCTGGCCGGGACGTCGACGACGGCCGCCGCGACGCCGACGCCCC
CGTCGGTCCCCGTCGGATCGGCGGTGGCCGGGACGGCCGGTGGCGCCCTCTTCCCCGG
CATGGCCGCGGCGGGCAAGGGCGCCGCGAGATCCGACGACTGGCTCGCGAACGCGAA
CAGCCCGGTCGGCTCCCCGTCCGCCGCGTTGCAGCCGCAGCACGTGGTCTACGGGAAT
CCTCAGCAACAACAGCTCGCCGCCGAGACGCAGCAGCAGCAGCAGCAGCCGCCCCTC
GCCCACTCAAGCCCCCTCGCGCATCAGCAGCAACAGCCCAACAGCAACAACGGCTAC
GCCAGTCCTATGAGCACCAGCAGCTACGATCCTTACAGTCCCAACAGTAAGATAG

>novel_circ_001197
ATTACATAAAATATTTATTTTACAAGTAAACATCATTTAATCATATAAAATACTTTGTCAAT
TAATTTATCGCTATTAACGTTTTTTCTTTGCTTTCATCTGTATCCTACGACTTTTTCCTAAT

CTCTGCTTTGATTTTCCTTTTTTTTTCTTCGAATCTTTTCCTG

>novel_circ_001198
AAAAAAAAAGGAAAATCAAAGCAGAGATTAGGAAAAAGTCGTAGGATACAGATGAAA
GCAAAGAAAAAACGTTAATAGCGATAAATTAATTGACAAAGTATTTTATATGATTAAATG
ATGTTTACTTGTAAAATAAATATTTTATGTAATCTTGA

>novel_circ_001199
AATGGAAAATTGTTATCGCTGCATCGTCATGCACATGAAGCATCCGAACGTCCTTCAAT
ACAAAGAGACCTGGTGTATCACGGGATCGTCGAAGCCCACGCTCCCCTCGATATGCGG
CGATTTGACGAGCGACGCCATATTAATTTCCCTATTCCGCACGGGGGCAGCACCGATGC
CCTGCCCGTTCAAGGGACCCCTGGAATTCACGTACAGCCACGGGGAAGCGGAGTGTA
AATCTCCTTTGTCCGCAGCGGAGACGTGCACCCAAGAATCGCGATTACTTTTTCGCTAT
CAAGCGTGTGCCAACGTGTTATCCTCCGAGAGCGTCGACGTGGAGTTGGAGTGTCTAG
CCACGTGGAAGGAGAGCAGCACGCACAACCTCGTGGCAAGGTTGCACGCCCCGCGG
AAGACCAGCGATGAGGACAGCTACAGATGCTTCATCTACGAGCAAACTTCGAACAATT
CGTGGAATTTGGCGCAGAGCGAGGACGCCTCTTGCACGGGATTGATCAGCGTTAAGGA
GGCTGCCAAGACGTTCAAGATGAAACAGA

>novel_circ_001200
CCATGGCGAGGACCTCATCGTCACGCCGTTCGCGCAGATCCTTGCCTCTCTGCGCTCG
GTCAGGAACAACTTCCTGTCGCTTACTAATGTGCCCACCAACAAATCACGAAGGAGTA
GCGGTGCACAGGGTAGCAGCACGCCGCAGCCACGGATCTTGGCACCGGGGGAGGAAA
ACTACATGAAATTGGCGGTGGAGACTATGGAGGAGCTGGACTGGTGCCTGGACCAACT
GGAAACCATTCAGACTCATCGATCCGTGTCCGACATGGCCTCTCTGAAG

>novel_circ_001201
AGAGAGAGCGGATGCTTCTCGAAAGGAGCGAGTCCTTCCACTGTCGACAATCAGTTCC
CGTTGCACACTCGCGCGAGAACAACGCCAATCAGCCAAGGATCTTCTCCTTCCCTCCT
GAACCTGAAAGCTGAAATCAAACTTCGGATCATCCGTGCCTCGTCGCTTTCCACCGCA
CGCGCGCGCGCGCACGCACGCACGAAATCTTTCCTTCCGTCTGGCAATTCTCTCGCGC
CGGTCGAACGTGGCAACCACCGAGGCGGACCGCTCTCCTTCCCAAGAAGAATCAATC
CGTGCGATGAACGACCGTTGGAAAAGTTTGAGGTTTCGTGATGGGATCGGGATCCGTC
GGAGGCTGTTCAGTTGAAAAGATTTATCGGGCAGGGAGAGGGGGGAGACCGATATATA
GCCAGCGGGTGAGATCGAGTTTGTGGAAAATCGATGGAGAGAGAGAGAAGGAGAGG
CGATTAAAAGGAGCGGTGAAAAGGATGTAGTAGTTGTAGTCAGCAGTCGTAGTAGCAG
TCGAAGGAAATCGATATAGTTTCCAGCACGTGGCTGGCTTTAAACTGGCGGTTTAGTTT
ACGCCTATATACACTGATAACCTCGCTTAAAAGAAAAATAAAGAAGAAGAAGAAGGAG
GAGGAGGAATAATTCGTGCGAGTGTCGTTTGATCGAGTGAATCTCTCGAAAAATCTTCT
TTTCCCTTTACGACGTTCTTTTTTCTTTTTCTTTTCTTTTTTCTTTTTTCATGTTAACTGGG
AGCACAAGATAGAGAAACATTAGTAAGGAAGTAATATAGCAGAAGGTGAAAATCGTGG
TGTGCCGATCGTGTTCTCTCTCGACAATCCCCAAGCCAGGGATCTCTCCTCTCTCGGGA
AGATCAGTGAAAAAACAGTGTTGAAAATTGTGAAGTGCAAGCTTGTTAATCGGAGAA
AGATCGAGAAAGTGAGAAAGAAGAAGGAAGATCTTTGAAGAATTGTTTCGAGGAGTC

TTAGGAGTCTGGCGCCTTCATCGAGATCCCAAACAGGGATCATTGAGAATTTGTACGC
GAATTGAGAGCAAAGGCCTATATTATATCAAATTTTCATCGTCCATCCTTCATACTTTCTC
GCCTTAATCATCGGTTCGATAGAAAAACCGAATATTTCGAAAGAAGCTTGGTCGTCGAG
GAGCATAGGCGGGATAAGAAATCGATGTGATAAGGGAAGAGGAGGAAGAGTTTGAAA
ATAATTCGATTAACTTCGAATCCGGCAACCCTCTTTATCGAAGTATCGAGTTGAAAGGG
TTGAAAGGGAGGGATTCTCGCGGCTTATCTCGCTCTAATCCGAAAGGAAACTTTTCAA
ATTATCCTAGTCGAATAAACTGGGACTGAGAATACCTGAACGGTATTCAAAAAAACCAT
CATCGTGAAAAGGAAGCAACTGGAACGCTGTAACGTAAATTAAATAATTGTTCGAGAC
AATCCAAAGATATCCTCGAATCGAGGCCAGATCTAAAAACTAAAAATTAAACTAAAATA
GGATCATTTCTATAGACGTATTTAATAGAATAGATGTTGGATCTCGACTTTCAAGGGGCT
CAACAACTTCTAGACTACTTTTTCCAACCAAAAGCCATCCATCCTCGAATTTCTAGCAA
AGCTATTTCAAACGATACACTTTTATAAATCCTAGACAAGGAATTCTCAAAGAATTCGA
CCATCAAGCTGGACCAGGTCGACCAACTCCTCTTGGTAAATTTTTTTAAAGGCCAAATC
CGACTCAGCCTATTAATCGTCACTTGGCAACTCGTGGGATCTTAGTTTCTCAGGCCTCT
GAAACTACTCACTCCATTACTAAAGCTTTAAAAAAAGAAACAGTGAAACAAACAAGC
GGGAAAGAAAATTCTCCACTCGACTTTTCCACTCGACTCTCGTAGATGCTTCTTCGCAG
CAATTCCTTCCTCTGGACACTTTCTGGACAAATGGGAAAGCGATGTAGCCTTGGCGGC
GGGGGTGGGGGTGGTGCAGCTGTCTCGGGGGCCACCCCCGAGGCTAAGAAGGGTTCC
ATCGGCCACAAGTGCCAGCAACAGCAGCAGCAGCAGCAACAGCAGAGCCAACACCT
GCTGCAGCATCATCATCAGCAACAGCAGCAGCAACAGAAGCAACAGGAGAGGGATAA
ACAGCAACAGCAACAGTACCAGCAACAGCACCAGCAACAGCACAAGAGTAAGGGGT
CCGCGGTGAGGGGGGGCGAGTTGGCGCCTCTGAGCAATCTCGACGAGGATCTGGACG
ATCTTGAGGACGAAAAGGGGATCGAGCAGAGGGGGGAGGAGGAGGAGTACGCCAAG
GAGGGCGGGACCCAGCATCGGTTGAGCCCCAGGCTGCACGATATCGAGGAGGAGGAC
GATGAAGACGGCCGAAACACCAAGGAAAGGCCGTACCAGAAGAAATCGCCAACCCC
GAGTGGCGGTGGAGGCAGCAGAACGGGTTGGCGATCGCGCAGAAGCATCACCACGGC
AGGTGCACCGGCTCACCAGCACCACGTGATCGCCAGTGCCGCTGCAACTGCACAGGC
AAAGATGCAGGCCGAACAGGGGAGCATCGGTGAGCTGCGGGGCTACCACAACCTCAG
ATCCAGAAGACACACTTTGGCCAATGTTCG

>novel_circ_001203
TTATAATATTATAAAAATAAACGTGAAAGATACAAATGAAAAGAGAAAATAATTAATAA
GTGATATTCATATCAAATTCATCCTTGTGATAAATAAGAATATCTTTGAACAACATATTAG
GTCCTTCATAATTGAAATCATAGAGAGGTTTATCT

>novel_circ_001205
GAGGAACAGCCACGAATCCGTCTTCGTCGTCCCCGACGAGGTTCCCAGCGACGTGCT
GTACAACAGGCTGGTGGTCAGCGAGGACGGTAGCGAGACGTTCAAGTATTCATCCCAG
CCTTACGGCTTCCCCGAGAGGCTTCTTCTGCCCAAGGGCAAGAAAGAAGGTATGCCGT
ACAATGTGCTCGTGGTCGTCTCGCCATTCGACGACTCGAACGTCGTCCAAATAGACTC
CCCTGTGTGGGGGCGCCACATTTACGACGGACGTGCTATGGGCTTCCCTCTGGACAAG
CCTGTCGATCCTTTGCTCTTAGTACTGTCCAACATCCACGTGAAGGAGGTCCTCGTTCA
TCATAGGGAGATGGAGGAGTTGAACGTTGCCCTGTAAACGTGTAGAATTTAATACGTAG
ATTTAATCTTTTCTTCCTTATTTTTTTCTTTTTTAAATTATTTGAAGTAGAAAAAAAAAAA

GAATTCGAAGTTTAAAGGAAGAGAGATGTAGAGAGAAAAAAAGAGATGTG

>novel_circ_001206
CACATTGTACGGCATACCTTCTTTCTTGCCCTTGGGCAGAAGAAGCCTCTCGGGGAAG
CCGTAAGGCTGGGATGAATACTTGAACGTCTCGCTACCGTCCTCGCTGACCACCAGCC
TGTTGTACAGCACGTCGCTGGGAACCTCGTCGGGGACGACGAAGACGGATTCGTGGC
TGTTCCTCTCGATCGTGTTGCTTCCGCTTTTCACTGCGAAAAAAAATCGAAAGGAATG
AAAAAAAAGTTTCGAGAGATTGATTATTGTCCATTAATAAAGATTTTAGAGCAAAGAAG
TTAAATTTTTTAATTACTTTTTAGCCTTCTAACCTAAGATCTAGAAGATTGAATATTAATAT
ATAAAATAGAGGAATTTTTTTTGTAAAAGTAACACATAATTATTTTCAATTATAAAATTAT
ATAAAAGATTGCCCTTCTCTGTAAATAGAAAGAAAAATTCATTGCAAAAATAAACGTGT
TTTGATCGGATTCATTATCTCTTTCCAACAACAATAGAATATACGTAATAGATATAAGAG
AAACTATTCTTATTTATACAGTTTGTAAACGATAAAAATGATACGTACGGTTAACCACGA
ACTCGTCCATCTGCATGAAGTTCATGTAATTGTGCACGAG

>novel_circ_001210
CAAGGATTGAACGTTCTTGGTAACATCGTTCAAGGTAACGGAGACAGCGTTAACGTAC
AATTATACGGACAATTGGATCTGCTCGTGAGAAAAGTCTTAGGTTTCGGTTACGAATCC
AACGTCAAGTATCAAGTCGTGCCATCCGCTCTTCAAATGTGGTCTACCAGCTTGAGGGA
TCCTGTATTCTTCAGCATCTACAAGACCATCCTTGATTATTATCACAAGTA

>novel_circ_001211
ACTTGAACATATTTCTGCATATGAAGTGGAACCGTAGCACCAGTTTCTCTCTGAGGGAA
GGCAAGTCCATTGCGGAATCTCATAGTGGGATAATATCCAGTCGGTATGGGATGATCTA
GGCTCACATAACTAACCTCACCCATGTCGTTGCTAAGTCTCTCAAGGTAATATC

>novel_circ_001212
TGGGATAATATCCAGTCGGTATGGGATGATCTAGGCTCACATAACTAACCTCACCCATGT
CGTTGCTAAGTCTCTCAAGGTAATATCTGCGAGATGAATTTTAATTCGTAGATGTCGTTG
TATTATATTTCTTACGCGAGAGAATATATGTGTAGAAAAATTGAAACTGTCTATAAAAATA
TTTTTATAGTTTGGTAAAGTAACGTACCTGTTCAATACTTGCTTATGGAGGAAGAAATAG
AATTCACCCCGAATTTGTGGAAAGTTGAGCGAGTTTGAAAGCATGAAAG

>novel_circ_001213
TTTGAAAGCATGAAAGGTGGATAATTGTGATTCAACATGAAGTAGAAATGGTTGAGGC
CAACATCTTCGGTGAAGTAGTTTAATCTCTGCTCGGGCACGTTGTGTTTGGTTAAGTAC
CATCCGGTATAGTTCGCCGCAAGTAAGTAATAATCGATGTT

>novel_circ_001214
GTGGATAATTGTGATTCAACATGAAGTAGAAATGGTTGAGGCCAACATCTTCGGTGAA
GTAGTTTAATCTCTGCTCGGGCACGTTGTGTTTGGTTAAGTACCATCCGGTATAGTTCGC
CGCAAGTAAGTAATAATCGATGTTGTTGTAAGTCTTTTTCATGTCAGCTATTAAAAATGA
AAGAAAATTTTCATGAATAATTTTTTTAATTTCAAAAATTAACCCGTTTCTTCCTTTCGT
AAAGAATTATAATTTTATTTCATATGTGCTATAAATTTTGCATTTTGCGAAAATTCATTCTT

AAATATACGAATAAAAGAAAGAATAGAAGATTGTATATAATAAAATTATAATATTTAACA
GACTCATTATTGGTTGAATAAATTTTCGAAACAGTCTAAATATATTTTATTCGTTGTCTCC
GAGACAATTTGGTTAAGTCATATAGGAATAGATTACGTTCAAAATGAATTGAACAAATT
CAAATCGATAAAAATATTTTTCATCTAAATTTTTCAATTCCCATTTTCTAAAATTGGATAT
ATAAAGATTGGAATTCGAATGAAACTTTCTACCAAAGTTTCAAAATTGAATAAAAATTT
TCATCGATTGAATTTCAATTCTATCACTCTACAAGTGTTCATCGAGTTTCTTTTAATCACT
TGTTCATTATGTATCATGTATTCGTATATAGAAATAAAAATAAAAAATAAAAAATTTCTAC
ATAGCCAACCTGTGTCACCCATGGCAATGTTGTAAGCTTTCTGCATGACCTCATCATTG
AAATATAAATGTGGCATCACCTCGTACATTGGCGGCAATTTCATCAGTTTGGTGTCAGG
ACG

>novel_circ_001215
CAAGTAAGTAATAATCGATGTTGTTGTAAGTCTTTTTCATGTCAGCTATTAAAAATGAAA
GAAAATTTTCATGAATAATTTTTTAATTTCAAAAATTAACCCGTTTCTTCCTTTCGTAA
AGAATTATAATTTTATTTCATATGTGCTATAAATTTTGCATTTTGCGAAAATTCATTCTTAA
ATATACGAATAAAAGAAAGAATAGAAGATTGTATATAATAAAATTATAATATTTAACAGA
CTCATTATTGGTTGAATAAATTTTCGAAACAGTCTAAATATATTTTATTCGTTGTCTCCGA
GACAATTTGGTTAAGTCATATAGGAATAGATTACGTTCAAAATGAATTGAACAAATTCA
AATCGATAAAAATATTTTTCATCTAAATTTTTCAATTCCCATTTTCTAAAATTGGATATATA
AAGATTGGAATTCGAATGAAACTTTCTACCAAAGTTTCAAAATTGAATAAAAATTTTCA
TCGATTGAATTTCAATTCTATCACTCTACAAGTGTTCATCGAGTTTCTTTTAATCACTTGT
TCATTATGTATCATGTATTCGTATATAGAAATAAAAATAAAAAATAAAAAATTTCTACATA
GCCAACCTGTGTCACCCATGGCAATGTTGTAAGCTTTCTGCATGACCTCATCATTGAAA
TATAAATGTGGCATCACCTCGTACATTGGCGGCAATTTCATCAGTTTGGTGTCAGGACG
GTGGATCACGGCTACGCTTAACGCGTA

>novel_circ_001216
GCCGGTTTCCTATTTCTCGCGAGTCTCTGCTTGCTTGTCCAGGCGGTGCCCAACAAGGT
CGCCGACAAGACGTATGTCACCAGGCAGAAGAACATCTACGAACTCTTCTGGCACGTG
GACCAACCGACCGTTTACCATCCGGAACTCTACCAGAAGGCGCGCACTTTCAATCTCG
TTGAAAATCTCGACAACTACAATGACAAAGAAGCGGTGAACGAGTTCATGCAACTCTT
GAAACATGGAATGCTTCCACGTGGCCAAGTGTTCACGATGATGAACAAGGAAATGCGC
CACCAAGCCGTGGTGCTTTTCCGTCTTCTGTACAGCGCCAAAACGTTCGACGTGTTCT
ACAACACAGCTGTCTGGGCCAGATTCAACGTAAACGAACAAATGTACTTATACGCGTT
AAGCGTAGCCGTGATCCACCGTCCTGACACCAAACTGATGAAATTGCCGCCAATGTAC
GAGGTGATGCCACATTTATATTTCAATGATGAGGTCATGCAGAAAGCTTACAACATTGC
CATGGGTGACACAG

>novel_circ_001217
TTTGTTCGTTTACGTTGAATCTGGCCCAGACAGCTGTGTTGTAGAACACGTCGAACGTT
TTGGCGCTGTACAGAAGACGGAAAAGCACCACGGCTTGGTGGCGCATTTCCTTGTTCA
TCATCGTGAACACTTGGCCACGTGGAAGCATTCCATGTTTCAAGAGTTGCATGAACTC
GTTCACCGCTTC

>novel_circ_001218
GCCGGTTTCCTATTTCTCGCGAGTCTCTGCTTGCTTGTCCAGGCGGTGCCCAACAAGGT
CGCCGACAAGACGTATGTCACCAGGCAGAAGAACATCTACGAACTCTTCTGGCACGTG
GACCAACCGACCGTTTACCATCCGGAACTCTACCAGAAGGCGCGCACTTTCAATCTCG
TTGAAAATCTCGACAACTACAATGACAAA

>novel_circ_001219
TAACAGCATCGAACGCCAGAGCTCGGAATCACCGTTCACGACGTCAACTATAATGCCC
AGCGACATATTCTACGACAAACTGAACAAAGCGATCGGCGGAAGCGAACCATTCACTT
ACTCTGAAAAAATGTTGGGCTTCCCCGAACGTCTCATCTTGCCCCGTGGTAAACCGGA
AG

>novel_circ_001220
GTAATCAACGCGTTGGAGATGCGTTTAATGGACGCTATCGATTCCGGATACTTGATAGA
CGAATATGGAAAGAAGATAGACATTTACACACCAGAAGGTCTAAATATGCTTGGCAATG
TAATAGAAGGAAACAGTGACTCTATTAACACGAAATTCTACGGTATGTACGATATTCTTG
CTCGCGATATCCTGGGCTACAACTTCGACTTCCAGAATAAGAACAACTTAATCCCGAGC
GCTCTTCAAAGTTATTCCACCAGCATGAGAGATCCAGCTTTCTATATGCTCTACCAAAA
AATCTTGAGTTATTTCTTAAGATACAAAAAACTGCAGCCTCAGTACAGTCAAAGCGAA
CTGCAGATGCCAGGAGTTAAATTCGAATCTGTGAATATCGACAAATTGTACACGTACTT
CGACAAATGTGACACTCTGATCAACAATGCCGTGGCAGTAGAAAATTTCAAAGGTGGA
ATGTATTTACGTTTGAAGGCGCGTCGTGCTTGTATGAACTACGAGCGATTCACTTACAA
AATAAACATCAACAGCGATAAGGAAACGAAAGGAATGATGAGAATCTTCCTTGGGCCA
GCCTTTGACGAGATCAAACACGATATGGTCTATCTACAGAAATATTTCTATCTGTTCATG
GAAATGGACCGATTTGCCGTAACAC

>novel_circ_001221
GTCTAAATATGCTTGGCAATGTAATAGAAGGAAACAGTGACTCTATTAACACGAAATTC
TACGGTATGTACGATATTCTTGCTCGCGATATCCTGGGCTACAACTTCGACTTCCAGAAT
AAGAACAACTTAATCCCGAGCGCTCTTCAAAGTTATTCCACCAGCATGAGAGATCCAG
CTTTCTATATGCTCTACCAAAAAATCTTGAGTTATTTCTTAAGATACAAAAAACTGCAGC
CTCAGTACAGTCAAAGCGAACTGCAGATGCCAGGAGTTAAATTCGAATCTGTGAATAT
CGACAAATTGTACACGTACTTCGACAAATGTGACACTCTGATCAACAATGCCGTGGCA
GTAGAAAATTTCAAAGGTGGAATGTATTTACGTTTGAAGGCGCGTCGTGCTTGTATGAA
CTACGAGCGATTCACTTACAAAATAAACATCAACAGCGATAAGGAAACGAAAGGAATG
ATGAGAATCTTCCTTGGGCCAGCCTTTGACGAGATCAAACACGATATG

>novel_circ_001222
ACAGAATATGTAATTCGTTTACAATTTGGATAACACGATATTAAGCAATTGCTTCCGTAG
TACTTTGCCGGATGGAGTCTTGGGTAAAGTTTCGACGAATTTAACGCCGCCTCTCAGCC
ACTTCTGCGGCGATAAATTTTCTAAAAGCAAAATTGTCGTTAATAGCGCATTTCACACT
TTGCTTGAACCATTCAAACACACGGACGCAAGACTCACTTTTCACGAAATCCACAATT
TCTTCGGCCGTCACGTTGCTGCCTGGTTGCCTCACCACCACGGCCATCGGCAACTCTC
CGCACACCTCGTCGGGTTTACCCAACACAGCCACGTCCTTGACACTTGAATGCGTTAG

CAACAATGCCTCGATCTCGCTTGGCGCCACTTGGAAACCTTTGTACCTGATGATTTCCT
TTATTCGCCCCGTGATGTACAATCCTCCTTCCTCGGTGAAATATCCGAGATCGCCCGTGT
GAAGCCAATTCTGCTCGTCGATCGTTTCCGCGGTGCTTTTCGGATTCTTGTAGTATCCCA
ACATTACCTGATCACCGGCGAAGCAAATTTCCCCCACTTTCCCCGCGCCAACCGTCTTC
CCCGTTTCCATGCTCACCACCTTGCACTTGAAACCTGGCAACGGGGGGCCGATCGAGG
CGTCTTTGCACGATCTCTCGCTCAAATTCGACACCATCGACAATTCCGTCATTCCGTAA
CCGTTTCGTATGTTTTTCACTTTCGTGCGCC

>novel_circ_001223
ACATACTCAGGACTATTCTGCGTGGTGGTAAATCCATACAAGAGGCTGCCAATTTACAC
GGAAAAGATAATGGAGAGGTATAAGGGTATTAAGAGACACGAAGTTCCACCCCATGTT
TTTGCCATTACGGACACCGCATACCGTTCTATGCTTCAAGATCGTGAGGACCAGTCAAT
TTTATGCACCGGTGAATCCGGCGCTGGTAAAACAGAAAACACGAAGAAAGTAATTCAA
TACTTGGCATATGTTGCTGCCTCGAAACCGAAATCGAATGCGACACCAAGTCCGGCATT
AATCATAGGTTCCGGAAATATTTCGGATAAATTTGCGGTAATTAACGGTGAATTGGAAC
AACAACTTTTACAAGCAAATCCGATTTTAGAAGCTTTTGGGAACGCAAAAACAGTGAA
AAATGACAATTCATCCCGATTTGGTAAATTTATACGAATAAACTTCGATGCTTCCGGTTA
CATTGCTGGTGCAAACATAGAAACGTATCTTCTGGAAAAGTCAAGAGCAATTCGACAA
GCAAAGGATGAAAGAACTTTCCATATATTTTATCAACTTCTAGCTGGTGCCTCACCTGA
ACAAAAGA

>novel_circ_001224
GTCGAAGGGTACGCCGCGGCAGGCCGCGGCTCGGAACGAGAGGTGGAAAGCATGGAT
CCCCGTCCGTGCAAGGTAATTCGCCATCGAGGAGCGAATGGTTACCCTTGGACATGAG
AACTACTCCTCCTGCTGCGATGCCTGCTTTCCGTGGTTTCGAGGAATCCTCCTCCGATG
GCGTGGTGCATCTAGGGGAGGGAATAGCAATTTGCGAGGAACAACTAAGGGCTGTGA
AATGGAGCGATTACAGGAAATTGACACGTGGCCTGGCTGCAATTTTGTTTAGCCCTACC
GAGTTGGCCACTTGCTCGGTAACGGGGCAACGATGGAGTCGAGCTGGGACTGCCACT
GAGAGACCGGTGAAACCTGCCTTGGATAAGGCCAAAGTCCAGGCTATCATATCTTACG
TGACATCGAGATTTCCTACAGTCGATGTGAGCAGTGTGAAGCAAGTGTTGGCTTACAA
ATGCAAGGAGAACTCGACAGCGCTCAAGATGAAATCTATTAGATACATTTG

>novel_circ_001225
ACAAATCCATGCAAGCATCCGATAATATTTATGCGTGATGTGGAATTTGAACATCTACAG
TCACTTTTGGAATTCATGTATGCTGGAGAGGTTAACATATCTCAAGCAGAATTACCTACA
TTTTTAAGAACTGCTGAGTCTTTGCAAATCCGTGGCCTCACTGACTCTCAAAATAATCA
GCACAACAACGAAAAGCATTTGAAGACAAACAATATACATGCATCAAATGGTCGTGGG
TTGATCTCACCAAATTTAGAGGAGGAGCGTAGTAAAACTCCACCAACCTCAAGTCCTC
CACCACTAAAAAGGCTGTGCAAACGAAGTGATTCGCCTCAAATATCTAGTCCAGTACC
AGCTGCGGCGGCCTGTGCATCTGGAACACCACGCACACGGCCATTAATTGAGCCACAG
GTTCAACTTGACTGTTATAAAGATCTCGATATTGTTGAG

>novel_circ_001226
GAAGGTGAGGCGATATCTTTCGTCGCCACAGCGTCCAAGCTGGCCGCCGTGGAGGATG

CCGCTTCGAAGGGGCCCGAGGCTTCGATTCGTTCGAAAGTCTCGCCCAAGGCAAGGG
CCGCCTTGCAGGCCCTTCGGTCGCCGAGAAACAGAATATCAACACCTCTTACCCCTCC
TCCAACGACAACCAAAGACGTCGAGACCATTACAGAAAATGAAATTGAAATTAATTGA
TTGGAATTAAAGATTATGTACCTAGGTAGCAGTGAAAATCGGCCCAATGGCCCTGCAAA
GCTTAAAAAAATTCTCGAAAAAAAGGAAGGCATTCTTAACAAATGGAGAAATGAGGT
CTGCAGTGGAGGTGAATTACCAGAAGTTGAGATCATCAGTTTGCTAGAAGAACAAATA
CCTAGGTACCGTTTGCGAGCTGACACTCTTACTCAATTCCAAGGATACGAAAACGCTGA
TTGGTTCATTCCATCACCGGCCCTAAAATCTGTCGACACTGATTTGAAATTATCGCCGGA
TCAAATCCGGGAGACTCTTAATTACTTTA

>novel_circ_001228
TGTGAGAAGCGTAATGCACAAATATCTGGAAAAGAAGAAGGAAGTTAACTTCGATAAA
ATATTCAATCAAATGTTTGGTTACCTCTTATTCAAAGACTACTGCGAGAATGTGGCGGA
GGAGCCGATTCCGCAACTTAGGTTTTACGAAGAGATCAAGGCGTACGAGAAACTCGAA
TGCCCGGAAGAGAGGAGAAAACTTGCCAAAGAGATCTACGACAATTTCATCATGAAG
GAGCTGCTGGCACACTCCCAT

>novel_circ_001229
CTGACAATGAACGACTTCAGTGTACATCGAATAATAGGAAGAGGCGGATTTGGAGAAG
TTTACGGGTGTCGAAAAGCAGACACGGGGAAAATGTACGCGATGAAATGCCTTGACA
AAAAACGAATAAAAATGAAGCAAGGCGAAACTTTAGCGCTCAACGAAAGGATAATGC
TTTCGGCAGTCAGTACCGGAGTCGATTGTCCATTTATAGTATGCATGACGTACGCCTTCC
AAACGCCGGACAAACTGTGCTTCATTCTGGATCTGATGAACGGTGGCGATCTTCATTAC
CATCTAAGTCAGCACGGAGTGTTCAACGAGCGAGAGATGAAGTTTTACGCGGCAGAA
GTGATATTGGGATTGGAGCACATGCACCGTAGATACATCGTTTACAGAGACCTGAAACC
TGCCAATATCCTTCTGGACGAGCATGGCCATGTCAGAATATCGGATTTGGGGTTGGCTT
GCGATTTCTCCAAAAAGAAACCGCATGCAAGCGTAGGGACGCACGGGTATATGGCACC
GGAAGTGCTTTCGAAAGGTGTAACCTACGACTCCAGCGCCGATTGGTTCTCGTTCGGT
TGTATGCTGCATAAATTGTTAAAAGGTCATAGTCCATTCAGACAGCATAAAACAAAAGA
CAAACATGAAATAGATCACATGACATTAACCAAG

>novel_circ_001231
TATCAACGTGGGAGCGGTTAACCGTTAGATAGAATCAGGAAATGAAAATGCGAGTCGG
ATCGACTCTCCACCTTTTCAATATCTTGCTTCAAATCCACCTCTGGACCACCACGATCCA
CGTCGCTGCTGGCGGCGAGGATAAACTTCGCGTGATCTTCCAATGGAAACAGCTCGAT
TACGAATGGCCGAGCAACGAAACCAAACTTCTCTTCCCAGGATACAAACAGGAAGAC
AACCTTCCCCTCGGCCTCGAGATCACGAGCACCAGGATATTTGTCACTGTGCCGAGAT
GGAGGCGCGGTGTCGTTGCCAGTTTAAATTATTTTTACGTAAATGATACGCGGGAATCT
CCTACTTTGATACCTTATCCTTCTTTCGAGGCGCATCAATACGAAGCTGGAAGCGTGCC
GGAGATCATTTCGCCGTTTCGAATCCGGGTGGATCGATGCGAAAGATTGTGGGTCCTCG
ATACTGGATTTACAGATATTTTGCAAAATCCGGAACAAGAGGCGCCGCCTGCGTTGCTC
ATTTACGATTTAAAAAACGACCGATTGTTACGAAAATTCGTTATCCCCGAGGATCAAAA
AACGCACGATTCTCTTTTCGCGAATATCGCGCTCGAAGATTACTCCTGCGAGGATACCT
TCGCTTATCTGGGTGATCTGGGTGGCCCCGGATTGGTGGTCTACTCGTGGAAGTCGAG

AAAATCATGGCTCGTCAAGCATCGATTCTTTCAACCCGATCCGCAAAGCGAGGAATTC
AACGTGTCGGGAATTTCGTTCCAATGGACCGACGGTCTGTTCGGTATGAGCATCGCGC
CATCTAACGATGGATACAGCGTGATGTATTTCCATCCACTTTCCAGCACCATGGAATATT
CGGTCAGTACCAAGATATTGAGAGATTCCGAGCGCGCCAATTCTCCAGACAACTTCAA
AGAATTTCGTGCTCTCGGATCTCGAGGACACAATGGTCAGAGTAGCGTCAGTTTTTTG
GACCCGGACACCGGAGTTCTTTTCTACGCGTTGACCAATTTAAACGCTATTGCGTGTTG
GAAACCTCGAAACATGTTCACGCTTCACCAACAAGGTCTTATTTATCAAAACAGTATCA
CGATGGTTTTTCCTAACGATTTAAAG

>novel_circ_001233
GCGCTCGAGGAGAAGGCGAGGAAGACCCAGGAGGCGCTCGAGAAGGAGGAGAAGCT
GCGCAAGGAGCTCGAGGAGCAGAACAGTAAACTCGTGACGGAGAGGGATGCCTTGCA
ACGGCAATTGGACGGGGAGAAGGGCTCCCTGTCGGAATATATGGAGAAATCGTTGAAA
TTGGCCGCTCAGAAGGCCGATCTCGAGTCGCAACTTCAGGATCTGAACGACAGATTCA
AAGAAGAGGAGGATACGAGGAACAATCTGTTCCAAAATAAGAAGAAATTGGAACAGG
AAGTGGCGGGCCTGAAGAAAGATATCGAGGATTTGGAACTTAACCTGCAAAAGTCTG
AGCAGGACAAGGCGACCAAGGATCATCAGATCCGTAACTTGAACGACGAGATCGCTCA
TCAGGACGAGTTGATCAACAAATTGAACAAGGAGAAGAAGAATCAGGGCGAGGTTAA
TCAAAAGACCGCCGAAGAGCTTCAAGCTGCCGAGGACAAGGTCAATCACTTGAACAA
AGTGAAGATCAAATTGGAACACACTTTGGACGAATTGGAAGATTCATTGGAACGTGAA
AAGAAATCACGGGCCGACGTAGAGAAGGCGAAGCGAAAGGTGGAAGGTGACTTGAA
ACTTACTCAGGAAGCTGTCGCGGATCTCGAAAGGAACAAGAAGGAACTCGAGCAGAC
CATACAACGCAAGGACAAAGAATTGTCGTCGTTGACGGCGAAACTCGAGGATGAACA
GTCGTTGGTGGGCAAACTGCAGAAACAGATCAAGGAGTTGCAGGCCCGTATCGAGGA
ATTGGAGGAAGAGATCGAAGCTGAACGCGGCTCGCGAGTGAAAGCCGAGAAACAGC
GCAGCGACTTGGCGCGGGAACTCGAGGAACTAGGCGAACGTTTGGAGGAAGCTGGTG
GCGCCACGTCCGCCCAAATCGAGCTCAACAAGAAGAGAGAGGCCGAGCTTAGCAAAC
TTCGCAGAGACCTCGAGGAGGCCAACATCCAACACGAAACAACTCTGGCCAATTTGC
GCAAGAAGCACAACGATGCCGTTGCCGAGATGGGAGAGCAAATCGACACGCTCAACA
AATTGAAAGCTAG

>novel_circ_001234
ATACAAAATTCTGTGCGCCAATGCCATCAAAGAGCCTTGCGACCCGCAAAAGGCGACC
CAACTGATTCTGGACGCGATCAATCTGGAGCCCGAGTTGTATCGTATGGGCAATACCAA
GGTATTCTTCCGTGCCGGAGTGTTGGGTCAGATGGAAGAGTTCCGTGACGAACGATTG
AGCAAAATCGTGTCCTGGATGCAAGCCTACATCAGAGGTTACCTGTCGAGGAAGGATT
ACAAGAAGCTGCAGGAGCAACGTTTGGCTTTGGTCGTGGTGCAAAGAAATTTGAGGA
AGTATCTGCAAATTCGTACTTGGCCGTGGTGGAAGTTGTGGCAGAAAGTTAAACCTCT
CCTCAACGCAACTCGTATCGAGGACGAGCTTGCCGCGCTCGAGGAGAAGGCGAGGAA
GACCCAGGAGGCGCTCGAGAAGGAGGAGAAGCTGCGCAAGGAGCTCGAGGAGCAGA
ACAGTAAACTCGTGACGGAGAGGGATGCCTTGCAACGGCAATTGGACGGGGAGAAGG
GCTCCCTGTCGGAATATATGGAGAAATCGTTGAAATTGGCCGCTCAGAAGGCCGATCTC
GAGTCGCAACTTCAGGATCTGAACGACAGATTCAAAGAAGAGGAGGATACGAGGAAC
AATCTGTTCCAAAATAAGAAGAAATTGGAACAGGAAGTGGCGGGCCTGAAGAAAGAT

ATCGAGGATTTGGAACTTAACCTGCAAAAGTCTGAGCAGGACAAGGCGACCAAGGAT
CATCAGATCCGTAACTTGAACGACGAGATCGCTCATCAGGACGAGTTGATCAACAAAT
TGAACAAGGAGAAGAAGAATCAGGGCGAGGTTAATCAAAAGACCGCCGAAGAGCTTC
AAGCTGCCGAGGACAAGGTCAATCACTTGAACAAAGTGAAGATCAAATTGGAACACA
CTTTGGACGAATTGGAAGATTCATTGGAACGTGAAAAGAAATCACGGGCCGACGTAGA
GAAGGCGAAGCGAAAGGTGGAAGGTGACTTGAAACTTACTCAGGAAGCTGTCGCGGA
TCTCGAAAGGAACAAGAAGGAACTCGAGCAGACCATACAACGCAAGGACAAAGAATT
GTCGTCGTTGACGGCGAAACTCGAGGATGAACAGTCGTTGGTGGGCAAACTGCAGAA
ACAGATCAAGGAGTTGCAGGCCCGTATCGAGGAATTGGAGGAAGAGATCGAAGCTGA
ACGCGGCTCGCGAGTGAAAGCCGAGAAACAGCGCAGCGACTTGGCGCGGGAACTCG
AGGAACTAGGCGAACGTTTGGAGGAAGCTGGTGGCGCCACGTCCGCCCAAATCGAGC
TCAACAAGAAGAGAGAGGCCGAGCTTAGCAAACTTCGCAGAGACCTCGAGGAGGCC
AACATCCAACACGAAACAACTCTGGCCAATTTGCGCAAGAAGCACAACGATGCCGTT
GCCGAGATGGGAGAGCAAATCGACACGCTCAACAAATTGAAAGCTAG

>novel_circ_001237
ACCTACTCTGGCCTCTTCTGTGTAGCTATTAACCCCTACAAAAGATTCCCCGTATACACC
CAAAGATGCGCGAAACTTTATCGAGGCAAAAGGCGTAACGAGGTGCCGCCTCACATTT
TCGCCATTTCCGACGGAGCCTACGTCAACATGCTTACCAACAGCGAGAATCAGTCCAT
GTTGATCACCGGTGAGTCGGGAGCCGGGAAGACCGAGAACACGAAGAAGGTAATCGC
GTACTTCGCCACCGTCGGCGCCTCGACCAAGAAAGCGGACGATCCAACCCAAAAGAA
AGGCTCTCTGGAGGATCAGGTCGTTCAAACAAATCCTGTACTGGAGGCGTTCGGCAAC
GCCAAGACCGTCCGTAACGACAACTCTTCGCGTTTCGGTAAATTCATCCGTATCCACTT
TGGACCGTCTGGAAAATTGGCCGGTGCCGATATCGAGACATATCTGCTGGAGAAGGCT
CGTGTCATCTCGCAACAGGCTCTGGAACGCTCTTATCACATTTTCTACCAAATGATGTC
GGGCTCGGTTCCTGGATTAAAGGAAATGTGCTGCCTCACGAACGACATCCACGACTAC
GTGTTCGTCTCTCAGGGCAAGACAACGATACCAAATGTTGACGACGGCGAGGAGTGTA
CTTTGACAGACAAATGTTGCTGTTGTCCAACAACATCCATGACTACTACTTTGTGTCGC
AAGGCAAGACGACTATCCCTGGCCTCGATGATGGCGAGGAACTTTTAATCACCGATACA
TGTGTCTCTTGTCCAACAACATATACGATTATGTTAACGTATCTCAAGGAAAAATCACGA
TCCCCAACGTCGACGATGGCGAGGAATGTGTACTGACGGACCAAGCCTTCGACGTGTT
GGGTTTCACCCAAGAGGAGAAGAACGACATCTACAAGATCACCGCCGCTGTGATGCA
CATGGGTGGTATGAAGTTCAAGCAAAGGGGTCGAGAGGAGCAGGCCGAGGCCGATGG
CACAGAGGAAGGTGAACGCGTGGCCAAATTGTTGGGTTGCGACTGTGCCGATCTTTAC
AAGAACTTGTTGAAACCAAGGATCAAGGTCGGTAACGAGTTCGTCACCCAGGGTCGT
AACAAGGATCAGGTCGCTTATTCGGTGGGCGCCATGTCGAAAGCAATGTTCGACAGGT
TGTTCAAATGGTTGGTGAAAAAATGTAACGAGACTTTGGACACGAAGCAAAAGAGGC
AACACTTCATCGGTGTACTGGATATTGCCGGTTTTGAGATCTTCGAT

>novel_circ_001238
TTAATTGATGGTCGTGGGACAAAACGTAATGTGTTACGAAGTACAAGTACTGGTTCTTC
AACTACTACACCAACACCAACTCATACACCTATTCCTAAAAATTCTTTAACTGATCCATT
ATTGAGTCATTGTTCCTTTGATGGATCCGATCCTCTTTCACAATTTGCTAGAGAAGAATT
AGATCCATTATCTAAAATGGCTGCAGATGAGTGGGATTACTCTTGTATAACTACTGGTAA

AAAATCTGTGGATACTGCAGAAGAATTAGTAGAACCATGGTCAGCAAGACGTACTACA
ATATTGAATAAATACACAACATCAGAGAAGTTATCAATAGCTACCAGTTTTTACCAGGT
GGTGAAAAAG

>novel_circ_001239
GTGGCCCTTTTGAAGAACAGCTTAACGCGTACAAGAATAAAAGTTTCTGCTGCTATCG
AAAATTTCAACTCATACTACGAAACATACGTGGATTACGATCCAATGTTTGTACAGCCA
CAACCTTCCAATCCATGGATTACCGACGATCAAACATTTTGGCAGCTAAACAGTCCTCT
AGTGGAAATTCCTACGGAAAAACGTGTTAAGCGGTGGGCATTATCCATGGAGGAGTTA
ATGTCCGATCCTACTGGTTTGCAAGAATTTACGCAGTATTTGAGAAAAGAATACAGTCA
TGAAAATATACGATTTTGGTTAGCGGTTAAAGATTTGAGGCATAGTTTTCAAGCAAAAA
TACCCGACAAAGTTAACGAAATCTTCAGAGAATTCTTAGCACCCGGAGCACCTTGCGA
GATAAATATAGATGGGAAGACGATGGAAAAGGTTCATCAAGAGATGAAAAATCCAAAT
CGATTCACGTTCGATTCCGCCGCTGAACACGTGTACACGTTGCTTTTGAAAAAGGATTG
TTATCCTAGATTTATTCGTTCCGATCAGTATCGAAATTTATTAGCCTCTGGTGTGCAACCT
TCGCAAAAAAAATGGTTTTTTTCGGGGGACAAGCGAAAAAGAAAGTTTCTTCGACTT
CGACTTCGATTTCGGCCCCAACTCCGACAACGAGCACGTTGCAACATCATGCTGTAAC
CGCCGGCGGGGGAGGAGGGAGTGGAGGTGGTAGTAAACGAAGGGGAAGCGATCGAA
GCCTTTCCGGATCTGCTCACGAGTTAGCCATTTGTGGTATTCGAGATACAAGTTCGATG
CCTAGAGTACCGCATTCTCACAGTCAGTCGAATCTCACCGATATTCCATATAGGGGAGA
TCTGGCTCGACTCGTAAAGATTCCAACAAGGCCTATCCCGCATAACGTTCAGGATGCTG
GCGCATCGGAAACATCGACGGCTCGCCCTATGGACGACGTTTGCCCGTGGGACGTGGC
TCCGGGGCCGAGTGTAGAGCACGATATAGATGCGGGCGCGCAGCTACGCCATCCAATAT
CATCTGGAGTGACGTCGTCGGTTGAAAGCCAAGCGGAAGAAGGAAACAATAACTTGA
TTAGCCAGTCGCATTCCATCGAAGTTGCGGCACGCCCGTCTCGCAAAAATTCATCGCAA
TTCGACTCCTGTAGTTCCTCATCGGACGTGAGCTTGGCCGTCACGGAAGCGGTATCGG
AACATCTTCGAAAGTCTTGCAGTTTGCAGCAAAGCAGCAGCACAGGTGGGACGAGTA
CCTCGGAACGAATCGGTATGACGACGCGGAATTATTCGATCGGCTCGGTCGGCGGAAG
GGCGAAACTTGGAGAGATCGGGCGACCGTCGGCTTCGTCCTTCAATTATCCGTCGCCG
CAGCATTCGTTCGAATACTCGCACGTGGTGACGGAGATCCGATCCGAGGGGAAGGAGA
TCGCCGAGGTGGTCGAGACCGAGACGAGGTCAAAGGTCACGTTGGTGGAGAAGGAA
CACGCGAAGAAAACATTGAGCAAGGCCCCCCTGATAAGCATTAGCGCGATCGTGGGCG
ATCTGGCGAGCGATAGCTTCGCGGTTCAAGCGGAGGAAAGGAAGGAGGACGATTGCG
GCAAAGGTGTGGCGGCGGTTAAGGAAGGGGAGGTGGAAATTGAGAAAAGGAAAGGG
GAGGTGGAGGGTGATAAAGGCGACGCGATGGTCGCGGAAGAAAATTTGGCGATTTCG
ACTCGAGTGGAAACGGAGACGGAGGAATCGCTGGAGGAGGCGCAAGTCGTTCCTGTT
TGGGAGCCGGACACCCGACAGGACGACCAAATAGCACCCGTTACCCAACCAGCTCCT
CAACAAAAGCGTGACAATAACGTTAACGAAGTGTGCCCGTGGGAAGACGA

>novel_circ_001240
GTGGCCCTTTTGAAGAACAGCTTAACGCGTACAAGAATAAAAGTTTCTGCTGCTATCG
AAAATTTCAACTCATACTACGAAACATACGTGGATTACGATCCAATGTTTGTACAGCCA
CAACCTTCCAATCCATGGATTACCGACGATCAAACATTTTGGCAGCTAAACAGTCCTCT
AGTGGAAATTCCTACGGAAAAACGTGTTAAGCGGTGGGCATTATCCATGGAGGAGTTA

ATGTCCGATCCTACTGGTTTGCAAGAATTTACGCAGTATTTGAGAAAAGAATACAGTCA
TGAAAATATACGATTTTGGTTAGCGGTTAAAGATTTGAGGCATAGTTTTCAAGCAAAAA
TACCCGACAAAGTTAACGAAATCTTCAGAGAATTCTTAGCACCCGGAGCACCTTGCGA
GATAAATATAGATGGGAAGACGATGGAAAAGGTTCATCAAGAGATGAAAAATCCAAAT
CGATTCACGTTCGATTCCGCCGCTGAACACGTGTACACGTTGCTTTGAAAAAGGATTG
TTATCCTAGATTTATTCGTTCCGATCAGTATCGAAATTTATTAGCCTCTGGTGTGCAACCT
TCGCAAAAAAAATGGTTTTTTTTCGGGGGACAAGCGAAAAAGAAAGTTTCTTCGACTT
CGACTTCGATTTCGGCCCCAACTCCGACAACGAGCACGTTGCAACATCATGCTGTAAC
CGCCGGCGGGGGAGGAGGGAGTGGAGGTGGTAGTAAACGAAGGGGAAGCGATCGAA
GCCTTTCCGGATCTGCTCACGAGTTAGCCATTTGTGGTATTCGAGATACAAGTTCGATG
CCTAGAGTACCGCATTCTCACAGTCAGTCGAATCTCACCGATATTCCATATAG

>novel_circ_001243
AATGGTATATGTGGCGATGTTATGAACCATATGGTTGCTTTTATATTGGTTCACCATGGTC
GGGAGAACATAGACCAGTATCAACATTTCCAGATCGTCCGGACTCTATAAATCCTCGTT
ATTTACTTTATATTCGTGAAATCATAGAACAACCGCAAGAATTGAAGATAGATAAAGAC
GAAACTATTCATGATTCAGCTCTTAAAAAACAGAATAATCTTTATCTTATCGTTCATGGA
TTTCTTGATAATGGTGACAAAACGTGGGTTATGAGAACTATGAAAGAATTACTAGAAAA
AGAAGATTGTAACGTGGTAATAGTGAATTGGATTGCTGGTGCAGGTCCGCCATACACTC
AAGCCGTGGCCAATACAAGATTAATCGGTGCAATGACAGCTCGTTTAGTTTATCAATTG
ATTGAAATTGGAGGAATAAATCCTTTGAAAATGCATTGCATTGGACATAGTCTTGGTGC
ACATACTTGTGGCTACATTGGATACACTTTGAGGAAAAGATACAAATATAATCTTGGAA
GAATTACTG

>novel_circ_001244
AATGGTATATGTGGCGATGTTATGAACCATATGGTTGCTTTTATATTGGTTCACCATGGTC
GGGAGAACATAGACCAGTATCAACATTTCCAGATCGTCCGGACTCTATAAATCCTCGTT
ATTTACTTTATATTCGTGAAATCATAGAACAACCGCAAGAATTGAAGATAGATAAAGAC
GAAACTATTCATGATTCAGCTCTTAAAAAACAGAATAATCTTTATCTTATCGTTCATGGA
TTTCTTGATAATGGTGACAAAACGTGGGTTATGAGAACTATGAAAGAATTACTAGAAAA
AGAAGATTGTAACGTGGTAATAGTGAATTGGATTGCTGGTGCAGGTCCGCCATACACTC
AAGCCGTGGCCAATACAAGATTAATCGGTGCAATGACAGCTCGTTTAGTTTATCAATTG
ATTGAAATTGGAGGAATAAATCCTTTGAAAATGCATTGCATTGGACATAGTCTTGGTGC
ACATACTTGTGGCTACATTGGATACACTTTGAGGAAAAGATACAAATATAATCTTGGAA
GAATTACTGGCCTTGATCCTGCTGAACCGCATTTCAGCAATACGTCCACAATGGTTCGA
CTTGATCCAACGGATGCCACTTTTGTTACGGCTATTCACACCGATTGCAATCCTTTTATC
AACTTAGGACTCGGTATTACTCATCCTGTCGCTCATATCGATTTTTTTCCAAATGGAGGT
CGCAATCAGCCTGGTTGCAATGAAGGTGTATTGAATTCTATCACTTTAGAACGTGGAAG
TTTCTTTCGAG

>novel_circ_001245
AATGGTATATGTGGCGATGTTATGAACCATATGGTTGCTTTTATATTGGTTCACCATGGTC
GGGAGAACATAGACCAGTATCAACATTTCCAGATCGTCCGGACTCTATAAATCCTCGTT
ATTTACTTTATATTCGTGAAATCATAGAACAACCGCAAGAATTGAAGATAGATAAAGAC

GAAACTATTCATGATTCAGCTCTTAAAAAACAGAATAATCTTTATCTTATCGTTCATGGA
TTTCTTGATAATGGTGACAAAACGTGGGTTATGAGAACTATGAAAGAATTACTAGAAAA
AGAAGATTGTAACGTGGTAATAGTGAATTGGATTGCTGGTGCAGGTCCGCCATACACTC
AAGCCGTGGCCAATACAAGATTAATCGGTGCAATGACAGCTCGTTTAGTTTATCAATTG
ATTGAAATTGGAGGAATAAATCCTTTGAAAATGCATTGCATTGGACATAGTCTTGGTGC
ACATACTTGTGGCTACATTGGATACACTTTGAGGAAAAGATACAAATATAATCTTGGAA
GAATTACTGGCCTTGATCCTGCTGAACCGCATTTCAGCAATACGTCCACAATGGTTCGA
CTTGATCCAACGGATGCCACTTTTGTTACGGCTATTCACACCGATTGCAATCCTTTTATC
AACTTAGGACTCGGTATTACTCATCCTGTCGCTCATATCGATTTTTTTCCAAATGGAGGT
CGCAATCAGCCTGGTTGCAATGAAGGTGTATTGAATTCTATCACTTTAGAACGTGGAAG
TTTCTTTCGAGGTGAGTTTTTTTTTATTTTTCTTTTTCATATATTTTTATATATTTTTTTTT
TGTTTTATGATTAACTACTTGTTTTAATATTTTATTGTAAATATTTATTGTAAATAAAGTATA
TATGTATGTATAATATTAATAATTATTTTAAAAAAATACATTTTTAATTTGATAATCTTGAA
ATATATTGTCAAAAACAATATTTTAAATATTAATTTTTCCTATATATATAATCATCGTTTATA
ATGTGATATCAGCCTCATATTTAATTTTATTATATCTGTTATATTATTTATTATATTTTTTCAT
TTTTTTATGTTATAAAAAAATTTTAAAGAAGTTCTATTGGAAAATAAAATAGAAATTTTT
CGCTAAATAAAGTCGTTTATATAATCGTCAACTGTTCTAATATTTATATTTTCAGTTAATA
AAATCACAATTGTCATATATAATGTCACGAACGCATCACGTATTAAGCAGAATTCAATAT
AGTAAAAACTATTTGCAAATGATTCGAAAATTAAGATCGATTATTGATTAGGTGTAAAA
AAAAAGTTATATTTTATGGCCATTGAAATCGGCGAACTTCATTGATAAATTGATCTAATT
GATAATTATTATTCAATAATATTATATATATTATTATATATATTGTTATAATAGTATATAATAAT
ATCGGAAAGAATAATTGTCTATACAGTTTTGCAATAATTTAATTTGTTTTTACTTTTTAAA
ATAGGATTAGTGGAATTAGCAAAATTTATGTCCTTTAATTCACCTCTTTTCTTTCTTATTT
TTCTACTTTAATATATTTTCTATAGTGTTAATAATATTGATATTAGAAAAATTAAATATAATA
TTTTTAAAAATATGTATTAACAATAATATCATGTATAATATTGTTAAAGTCTAAACTGAAT
GATTTTGAATATTATACAAAACCTCAAAGATGCTTGAATTGATGATATTACTCGAAAAAT
CATTTAGTACTGTTCTGTATTGTACTATCTGAAGAATCATCTAAAATTGTACAATTATTTG
TATTATAAATTTGAAGTGAACGATAATTTATTTTTAACTATTTATAAGTACTGATTGGCGA
CTAAATGTAATGCTATTCTAATTCTACTCTATTGATATGAAATGAGTTCTATTTGCTGAAT
TCTATTTTATTCTATTGATTGATCTGATCTGAAAGACTGTCGTAGGCCTTGGTGTTTCAAT
CATTATTTTCTGAAAATTCCAAGAGGGATTTAGGGGAATTAATCGCATTAAAGAATAATT
TCAAAAGATGGCCTCTCGCTTCGCAGTGTACAAGAGTTTGCGATCGCTCCATTACAAAA
TACTACGTGCATAAGTGTAATGTGTGATCATTTCCATTTTGATTGTTGTGAAAATCTGGA
AAGCATTAGAGATCTGTTCTCAATTGTAAATTATTGCTAGACTATTGAAAATGTTCAAAC
CATTAAAAATAAAATCCAAGCTGTCTGACTGCTTCAAAAATGTTGATTTTGTGCTTAAC
AGATATAAAAATATTTTTAGATAATCATGAAATTATCTTCATGTATGTACATTATTGTACAT
ACATATTTTATTATCAATTTATATAAAATATGCGATTTTATCAATTATTAATATATATATATAT
ATATATATATATATATATATATATATATATATATATATCGAAATATAATAAATGCTTAATAATAA
ACTTGAAATTAAGAAGCAAATGCCAGATTATATAACTCTTTGCACTCCAAGTCTGTTTC
GCTCGCTCGAACTCCAGACGATTTGAACCCGCGCATTCAGTGTATTCATTGCGTTCGGT
GGAATTTCTTACAGATAATTTATGATTTTATATACAAATTAATGCATAAAATTTTTATATTT
TAATATTTTATATATCTTTACATAAAGGAAAAGAAATTATTGAATATTAAAGTCTATATTGT
TGTTTTGTGCGTTATTGTATGATTATTAAGATGCATCCTCGGTTTGTCAGGGCAAGTAGA
GTAGTACTTTTTCTTCTAATTTTTTTATTGCATAATTATTTATTTTGTGCACTATTTCATTAA

TAAATTCATTAGTAAAAAATAGTTTAAAAAAATCTAACGGTTCATTTATATTCGAAGATA
CTTGCGGTCCTGCAGATTTTTCGTCGGATACAAATTCTATTCTTCGCGGTATATTTTCCGA
TTCTATACATATTATCCATTTGGAATTTTCTGTATCTTCTGCTTATATAATATCTTGACTACA
ACTATTGCTTTCACATTCACTATCTATTATTCTTATTTTTCGCCTTCTTATATCTCTTATCTC
ATTTCCATATTCATGGTTTTCTCTTTCATTAATTTCTTTATGAATAGAAGCAATTTCAATAA
CGAAAAATCAAACTCATCTTCATTTGAACTAATTTTATTAACTCATTTTATGCTTACGAA
ACTCAAAAACTTTAAAAATAATATTTCTATTGTCATGATGTCAAACTCAGATTAATGCCA
AAAATATCGTTTGACTCACAATCACGGTCTAATTTGAGATAAAAAAATCTAACCATTTAA
TAATATATTATTCTACATCTTATAAGACTATAGATAAAGAGCTTAGAATCCTATTATGGAA
CGATATTCTAAAAAAATCAAACATAAATAAATATTGTCGCTGTTCAATGCCGCATCAATC
CAAGTGATGTCGGAGAGTCATCACTGGAGTTACGGCATTAACTCAAGTGGTGACCAAG
AATCACCGTTGAAATGCAAAAGGTTAACAAAATATTATCAATATAATAATAATTTAAATA
ATAAACAAATTTTCAATTTCAATTATTTTTAAACATATTACAAAAATATTTAAAGATCAAT
ATTTAAATTATTTAAACAACTTTACTAAACACATTAAACAACTTTATTAAACATATATACA
CACACACACACACACTCACATAATCGCTCGTATTATTACAATGAATTCTGCCTATACGTA
TGCACTTATGACATAAATTATTTTACAGTGTTATTGGTTATTTTACAGTGACCGGATAAAA
TAGGTCACCCAAATCCAATTCTTATCTTTTGTTTATAGTTTATATAAACTGAGGTTGGATG
AACTTACTATTAATCCAATGAGGCGCAGTAGATATAAACAAGTGCACGTGATCGACGTA
TGGACAAGACAGGACCGATTCACAGATTCCAGATTCCTGCGAATTAGGAAATAAGAAG

>novel_circ_001246
ACACTAAAGCAAAATACAGAACCGGCAAGTAGTAGTGGAATTGATGTCCAGAAATCAA
CCGAGCCAAGAAAAAAAGAATCTGTATCAACACAAACTCAAACTTCTTCAATCAATCG
ATTAACAAGATTTAATCATCAAGAAAATTCCAATTTATCTTCTTCATTGCAAAGTGGTAG
TATAAATAAAACAAAAACATCCAATGTTGGTGTGGATCTTCAAACATCTCCTCCTTCTC
CATCTCCATCTTCAAGACGACATCATCATCATCATCATCATCATCATCATAATAACAAACA
TTATAGGCATCATGATGTGCTTACAACACGACGTCAACATAATCATTCTCAAGATTCTGG
TCGATCTAGTGATAGTTCTGTCTCTCACAGTCGTACATCTTTAGAATCAACTGGATATCG
ATTATTGGATTCTCCACATCGACATCATCATCGTTCTGTCACTCAGACATCGACAAGTCA
AGTACAATCTTCACCCAGGCAACATAGTGGTACATTGGAAACTAGGGGACACAAAAGT
GGTACTTTAGAAAGTATAAAAAATCAGAAATCAGTACCATTGGGTGAACCACCTCCATT
AGGTTTGTCACTTTCAACTAGTACTCCTCTATTTAAAAAAAAGACATTTGTTGATTCAC
AACGGGAGAACAGAATTGGAAATTTAGAATTGGAAAAGAATAAAAATGGTAGAGAAA
GTAGTAGAGGTTCATATGGTCCTGAACCAAGTACTTCTCCATATCGTGATTCATTGATGG
AATCTAGAATATTTAGACATGAAATGCCTCCATATCGTCCACCAGAAGTTCAATTGAGTC
ATAGTTATAAATCGCAAGGTGAAAAATATGGCTCCCTGGTGGAAAGTAGTAATCGTTAT
CATAGAGATAGAGAAAGGGAAATTCACCATAGAGAAAGAGTAAATAGTTTGGACAAAA
CAAATACTTCAGCCTTTTATCCAAGTTCCATACATCCGCGCAATATGAATAAAGATAATA
CTGGTAGTGTAGGAAGAAGGCATCATGGATGTTCAGTATCACTTTCAGAAGCGAATGTT
AGAGATATGTATAGTGCAATGTTAGTTGAAAGAAATGAGCCTTCTAAGAGTCTAGTTAG
AGGTACAAGCATAATGAGCAATACATCAAAGCAGAGAAGTCTCGAAGTTATGGAACGA
GAAAGAGAACGCGAACGTGATAGAGAAAAAGAATATAGAAGAAACACAGCTTGTAAT
AATTCATTGGATT

>novel_circ_001247
AACTAGTGCTTGCGAGAGGGTTAAATTCCAGGGTGGCGGGCTTCGTCAGCAGGAAGC
GGCGAGTGTCGCGGAGCAGTAGCTAGTGAGAAGGAAAGAAACGATAACGAAGAAGA
CGAAGACGACGCCGTCGGGGAATAAGTGTCGAAGGGTGGCGGCGGAGGTGACCCGC
AGGAGGTGGCGGCGGAGGAGGGTGGTGGTGACTTTGGAAAAGACGACTCCTGCGAC
TTGAGTCGCGAACCGTGGTTGGTGGTGGTAGTGGCCACGAAAAGAGTAGAGTAACCG
TTTTCTGGCCGACGAGGGAAAAAACAGAAGCTTGGTGGTGGCGTTTGTGAAAGCAAC
TTGTGGCAAGATGGGCTGCGCCATGTCCGCGGAAGAGCGGGCCGCATTGGCTCGTAGC
AAACAGATCGAGAAAAATCTAAAAGAGGATGGTATTCAAGCCGCCAAGGATATCAAGT
TGCTATTACTTG

>novel_circ_001248
AACTAGTGCTTGCGAGAGGGTTAAATTCCAGGGTGGCGGGCTTCGTCAGCAGGAAGC
GGCGAGTGTCGCGGAGCAGTAGCTAGTGAGAAGGAAAGAAACGATAACGAAGAAGA
CGAAGACGACGCCGTCGGGGAATAAGTGTCGAAGGGTGGCGGCGGAGGTGACCCGC
AGGAGGTGGCGGCGGAGGAGGGTGGTGGTGACTTTGGAAAAGACGACTCCTGCGAC
TTGAGTCGCGAACCGTGGTTGGTGGTGGTAGTGGCCACGAAAAGAGTAGAGTAACCG
TTTTCTGGCCGACGAGGGAAAAAACAGAAGCTTGGTGGTGGCGTTTGTGAAAGCAAC
TTGTGGCAAGATGGGCTGCGCCATGTCCGCGGAAGAGCGGGCCGCATTGGCTCGTAGC
AAACAGATCGAGAAAAATCTAAAAGAGGATGGTATTCAAGCCGCCAAGGATATCAAGT
TGCTATTACTTGGTGCCGGCGAATCTGGAAAAAGTACCATCGTTAAACAAATGAAAATT
ATTCATGAAAGCGGATTTACACCTGAGGACTTTAAGCAGTATAGGCCCGTAGTATATAG
CAACACGATACAATCTTTAGTCGCTATTCTCAGAGCGATGCCAAATTTGGACATCAACT
TTTCGACCAATGAGCGAGAG

>novel_circ_001249
TTTTCTGGATGATTTAGACCGATTAGGAGCAAGGGATTATCAACCTACGGAGCAAGATA
TTTTAAGAACTCGTGTCAAAACAACGGGCATAGTGGAAGTCCACTTTTCCTTCAAGAA
TCTTAATTTCAAATTATTCGATGTAGGCGGTCAAAGAAGTGAACGTAAAAAATGGATCC
ATTGCTTTGAAGATGTCACTGCGATCATATTTGTGTCGCGATGTCTGAATATGACCAAG
TTTTACATGAAGATGAAACGACG

>novel_circ_001250
CTAGGCAGTGAGATCGACTTAAGCACGAAACAAATTGCCAATATAAATGATGTTTGCGA
CAGTATGAAGGAACAACTTCTTGTCTTGGTTCAATGGGCTAAATACATACCAGCTTTTA
GTGAGTTAACCCTTGACGATCAAGTAGCCCTTCTGAGGGCACACGCAGGTGAACATCT
ACTCTTAGGTGTTGCTAGACGCAGTATGCAACTTCAGGATGTTCTTCTTCTTGGAAATA
ACTGTATCATCACCAAAAACTGCCCTG

>novel_circ_001251
CCGTACAAAATGAAAGGGATCGTATAAGTTGTAGAAGGCCAAGTTACGAAGAACAAA
GTAACAATGGAAGTGGACTCTCTGTTGTTTCTCTTCTTCAAGCAGAAATGCTCAGTAGA
CAAGTTGCCTCTCTTGAGCTAGGCAGTGAGATCGACTTAAGCACGAAACAAATTGCCA
ATATAAATGATGTTTGCGACAGTATGAAGGAACAACTTCTTGTCTTGGTTCAATGGGCT

AAATACATACCAGCTTTTAGTGAGTTAACCCTTGACGATCAAGTAGCCCTTCTGAGGGC
ACACGCAGGTGAACATCTACTCTTAGGTGTTGCTAGACGCAGTATGCAACTTCAGGAT
GTTCTTCTTCTTGGAAATAACTGTATCATCACCAAAAACTGCCCTG

>novel_circ_001252
ATTTAGTAGAAATTGTATAATAGACAAAGACAAAAGGAACCAATGTAGATATTGCAGAT
TAAGAAAATGTTTCAAGGCTGGCATGAAAATGGAAGCCGTACAAAATGAAAGGGATC
GTATAAGTTGTAGAAGGCCAAGTTACGAAGAACAAAGTAACAATGGAAGTGGACTCTC
TGTTGTTTCTCTTCTTCAAGCAGAAATGCTCAGTAGACAAGTTGCCTCTCTTGAGCTAG
GCAGTGAGATCGACTTAAGCACGAAACAAATTGCCAATATAAATGATGTTTGCGACAGT
ATGAAGGAACAACTTCTTGTCTTGGTTCAATGGGCTAAATACATACCAGCTTTTAGTGA
GTTAACCCTTGACGATCAAGTAGCCCTTCTGAGGGCACACGCAGGTGAACATCTACTC
TTAGGTGTTGCTAGACGCAGTATGCAACTTCAGGATGTTCTTCTTCTTGGAAATAACTG
TATCATCACCAAAAACTGCCCTG

>novel_circ_001254
TTATCATTTTCACCAACAACACCTGGTTGGAGTCATGCAAGGCTTCTTGGACTTCTAGC
AACTATCCTTGCACTATCTTCCTTCATGTTACTTCTATGCACAGCTGCAGCATTCTTCTTC
GTTTCTTTCAATACTTTTGTCTTCACTGCAGCTGAGTGTATTCTGTTAGGTGTCAGAACA
ATTCATGTTATGATACGATATATCATTCATTTATATGATACTCGAGGAGCTGGAACTTCCT
CACAACGTTCTTGGGATAAAAGGGGTCCTTTAACATATTATACAGATTTAGCAGCAGAA
TTAATAGTGCTAGCTGTAGATTTCCTTCATCATGTTCATATGCTACTTTGGAGTAATATAT
TTCTAAGCATGGCATCCCTTGTAATTTGTATGCAACTTAGATATCTTTTCTATGAGATACA
ACGCAAAATTACAAAACATAGGAATTATCTTGCTGTTTTAAATCATATGGAACAGAACT
ATCCAATGGCAACTCAAGAGGAATTAGCAGAAAACTCGGATAATTGCGCTATTTGCTGG
GAGAAAATGGAAACAGCTCGAAAGTTACCTTGTGCTCATCTTTTTCACAATTCTTGTTT
ACAATCTTGGTTAGAACAGGATACTTCTTGTCCAACTTGTAGACTTGCTTTAAGCATGC
AACCTAACCACCGAGAAAACACACAAGAATTACAAAATGAACCACAAACACCAGCTA
GACGGAATGAAAATCATTTCTTTCATTTCGATGGCTCAAGATATGTATCTTGGTTACCGA
GCTTCTCTGTTGAAGTTTCTCATAACAGATTGCGTGGAAATATTTCTACTATTACACATA
ATAATTCGCAAATGGATGCTATGATTAGACAG

>novel_circ_001255
TGTATTCTGTTAGGTGTCAGAACAATTCATGTTATGATACGATATATCATTCATTTATATGA
TACTCGAGGAGCTGGAACTTCCTCACAACGTTCTTGGGATAAAAGGGGTCCTTTAACA
TATTATACAGATTTAGCAGCAGAATTAATAGTGCTAGCTGTAGATTTCCTTCATCATGTTC
ATATGCTACTTTGGAGTAATATATTTCTAAGCATGGCATCCCTTGTAATTTGTATGCAACT
TAGATATCTTTTCTATGAGATACAACGCAAAATTACAAAACATAGGAATTATCTTGCTGT
TTTAAATCATATGGAACAGAACTATCCAATGGCAACTCAAGAGGAATTAGCAGAAAAC
TCGGATAATTGCGCTATTTGCTGGGAGAAAATGGAAACAGCTCGAAAGTTACCTTGTG
CTCATCTTTTTCACAATTCTTGTTTACAATCTTGGTTAGAACAGGATACTTCTTGTCCAA
CTTGTAGACTTGCTTTAAGCATGCAACCTAACCACCGAGAAAACACACAAGAATTACA
AAATGAACCACAAACACCAGCTAGACGGAATGAAAATCATTTCTTTCATTTCGATGGCT
CAAGATATGTATCTTGGTTACCGAGCTTCTCTGTTGAAGTTTCTCATAACAGATTGCGTG

GAAATATTTCTACTATTACACATAATAATTCGCAAATGGATGCTATGATTAGACAGGTGC
AGCAATTGTTTCCACATTTTCCGCGTAATCTAATTGTAGAAGATCTCAGAATGACCAGAT
CAGTTGAATGGACTGTTGAAAATATATTGGATGGGGTGCTAATATCTCCTCATCGTATAA
TCGAAGAACCACAATCGGAATCCGTCCTTCAAATCCATACCAGTACTACTGAAACTAGT
GTTTCTTTAAATAGCCCTTCAGCACCATTATCTTCAGATCCAAGATTTGATATTCCTCTG
GCTGATAG

>novel_circ_001256
CAGAAGTATGTTCTGATAGACTGCAAGCAAATAAAAAAGCTAGAGTTTCAGGAATTGT
AATAAATACTTGTGGTTGGGTAAAAGGAGCTGGATATAAATTATTGACACATGCTGCTC
AAGCTTTTGAAGTTGATGCAATTCTAGTACTTGATCAAGAAAGACTCTATAATGAACTT
GTTAGAGATATGCCAGACTTTGTTAAA

>novel_circ_001257
TGATGAATATTATGGTGGCCTATGGGCAACATTTAATTTTGATGGTCTACAGAAATGGAT
AGAGGACCTAAAAGTTTCCAAAAATAATACTAAATATGTATCTGAAGTAGATTTAGAAT
CTGAAGAAAAGTTTTTAGAAACTAGTAATGAATATTCGACTGTTGAAAATATTGAGGAA
ACTTGGTATATTTCAAATGATGCTGATCTACCAGTAGTGTCTTCAAAAGATACTCAAACA
GATAGTATTGGAGATGGTAGTAGTAGTGGTGATGAAAAAGCAGATGACGATAAAGTTG
AAAAAAAAGAAAATGTAAAACAATGGAGTATAGATCGTATAAGAAAAGAATACAGGA
AGTTTAATATTGACTTAGCACCAAAGTTGTTATTTGCACGAGGTGAATTAGTTGAGCTTT
TAATCTCCAGTAATATTGCTCGCTATGCGGAATTTCGCGCAGTATCCAGAGTAGCTACAT
TCATGGATGGGAAACTGACACAAGTACCTTGCTCAAGAGCTGATGTATTTGCAAATAA
AACTGTTAGTGTTATTGAGAAAAGAATGTTAATGCAACTGCTCACTTCATGCATGGAAC
AAGGAGCTGATAGTCCAGAATTCGATG

>novel_circ_001258
AGAAAAAAGGGTAGAAAGCATACGCATAGAACGTGTTATGAAATTGTTGCCGTCGATT
CAGCTGCCGCAATTACCGCCGGTTTCGGCCGGGGGTGGAATGACGGGAATGGACTCTC
GGCTACCACCCGGTATATCTTTACCTTTTAGTCCTACTGAGCTACTCTGGCGATACGGTC
CCGCTATGAATTTTCCACCGACTCACCCGCCACCAAGTCCCTTTCTAGATTTCAAGACG
CACCTACCGGCAAGTTTGGCTTCAGACCCACGACTGTGGTCCCGCGAGGATGTGACGA
CGTTTCTTCGATGGGCTGAACGGGAGTTCGATCTACCCCAATTCGATATGGACATGTTT
CAAATGAATG

>novel_circ_001259
GTAAGTAGAACGAACTGTTGCTCGACGAGCCACCCCTCCATCCCGATTTCCAGTACTC
GTGTGAGAAATTATCGGATTTTAATTACCATCCGTACGAATACGAGGATTGAAGTTGGC
AGCACTGAATCCACATCTCTTTCGCGTGTTCTTGCGGTGCGCGAGTCAATATCAACGGT
AAAAATTCTTTGTTAAAACGTTGAAGAAGAATTGTTCAGAATTCATGCAGCGGAACGA
GTATTCCCGGCAGAATCTGAAGTATTCATCGGGATGATAGAATTTTTACTGAGCTTTGAA
ACATCGGGAGTTACATCGGTGGAAGCTTTTAATTTCTGCATTG

>novel_circ_001260

GTAAGTAGAACGAACTGTTGCTCGACGAGCCACCCCTCCATCCCGATTTCCAGTACTC
GTGTGAGAAATTATCGGATTTTAATTACCATCCGTACGAATACGAGGATTGAAGTTGGC
AGCACTGAATCCACATCTCTTTCGCGTGTTCTTGCGGTGCGCGAGTCAATATCAACGGT
AAAAATTCTTTGTTAAAACGTTGAAGAAGAATTGTTCAGAATTCATGCAGCGGAACGA
GTATTCCCGGCAGAATCTGAAGTATTCATCGGGATGATAGAATTTTTACTGAGCTTTGAA
ACATCGGGAGTTACATCGGTGGAAGCTTTTAATTTCTGCATTGGTACGTGGGGATACAT
AGGTAGAATATAATTTTAATACACGTGGTATAATGGCAGATATGTTCGAATATTTTTATTT
CGATATCTTTTCGTCATGGGAATGATATAATTTTAGTGGAAATTTTAGTTTGGAGAGAAA
ATTATTTCTATATTTCGTTGTAAGTAATTAAATGATTTAAGAAATAAAAGTTTAATTGGAT
TATATGATTATGTAAAATAAAATAGATTAATATTGTTTTTCCCTATAATTAATCGTATATTTT
ATAGTGATTCCAAAAATTTTTTATTATATTTCCTTTTGTACAATGTTAATGGAAGTAACAG
TATATAGAAGAAAAATTCTATGATGTTTTGTTTATCAATGTTTTGTTAATTAGTGTAATAA
ATGTAATGAAGAATAATTCAAAGGAAAAAATAGATATGTAAATATAAGAAATATGGAAA
TGTTTTTTTTTTTATTTATTAAAGATAAAATGTGCTGTGTAAACATATATATCGTAATTTTA
AACAAATTTGTGATTTTTTATATCTTTTTTTAAATTGGATAATACGAGAATGACATGAAAT
ATAAAACTTTAAGATAAATAAATTATGAAATTACATTCAAGAATGTTAAATACAAATATG
AATACAATTATAATAATGTAGCCATTAATTAAAATTAACAATTGAAATCCATTTTATCAGT
AAATAAATTTCAAATGTTTCATATTATTCTCCATTTTATTTCTTGTTATTATACCAAAAATA
AAATAATGTATTTCAAATTTCTCTTACAATTTCAAAATTAAAATCATTATATAATAACTTAT
ATATATATTATATATTATTTCTTTCTTTTAATTTTTTTTTTTTTTTGAATAGAAACATACGTT
AAAAACATACGTTCTTGTCACAATTGAAAGAAGAAAATTTTCATCGAGCAAAAGGTAT
TACGAAATCATAATATCATTTTGCTTCGAATTTTAATCAGTTTTTAAAGAATCTCGTTGTA
CATTTCAAAATCTAAATCGTATTTACATTCTATTGACAGATCTATATGGTTAAATTCCCGA
AACAGAGGAGAAATTACAGATCTGTTAAACGTTTACGAATGATTTCAATCGAAACTTCA
CAAAGGTTAGTTCAGTTATTACCGAAATTGTGCCAAGTGCAAACATTATCTCTTGACCC
TCGAAAGCTTCGCGCTAATTGGCAACATCCAGGGGATGAATGCTACCCCCCTTCCCCCT
CCATCTCCCCCTCCCCTTCTCTGATCAGACTCTGCCTACAGTGGGGTTTCGAGGATAAT
AATCGAGAGGCGCACAAACTGCACTGGACAGCTCGTGTACTCGCCGCCCTTTTCTCTA
TGAGGCTCTGTGTTTGCACCAGCCACGTATTTCCCATGTGACCCGTGTGCATTTCGTGT
ATAGGTCACCCTACAAATCGTCGATCGTGTTACTC

>novel_circ_001261
GAAGGTGAGAGGGGATACTTAGAGGGGCTGGCTTCAAGGTATGCAGACCTCTTCAGA
ACGCATTACGGAGACGTCCTGGAACCGCTTGAGGAGCTACGCCGCCGTGAAACAAAT
GCTTCTCCCAGAATGCCCAACACTCAACTCACTACCAGTCATTCACAGCCCATTTACGT
GCCCGGGAAATACTCGCCCTCGAGCTGTCTCAGCGATAAGGAAGAGGACGAAATCTAT
GGCTTTGGATACGGTGTATTCAGCAGGCAAATGCTTCAACAACGACAACAGAAACTTG
CTCTTGCCGCGCAAAATACAACTGCAGTGACACCTGGTGGACATCAACAACCTTATCA
AAG

>novel_circ_001262
ATTGCAATTCCCGATGTAGTAGAAGCTGCTAAAGATGCAGATATTCTAACTTTTGTAGTA
CCTCATCAATTTATAAAGAGAATATGTAGTGCATTATTTGGAAAAATAAAGCCTACAGCT
ATTGGCCTTTCTCTTATAAAGGGTTTTGATAAAAAGCAAGGAGGTGGTATTGAACTTAT

TTCACATATAATTTCAAAACAACTTCACATTCCTGTTTCAGTTTTAATGGGAGCAAATTT
GGCTTCTGAAGTAGCAAACGAAATGTTTTGTGAAACTACTATTGGTTGCAAGGATAAA
AATATGGCTCCTATACTGAAAGATTTAATGGAAACATCATATTTTAAAGTTGTAGTAGTT
GAAGATGTTGATTCAGTTGAATGTTGTGGAGCATTAAAG

>novel_circ_001263
GTCCTATGGGTGGTGGAAATTCTGGAGGTGGTGGTGGTGGTTATGGTAATCAAGGAGG
ATATGGAGGTAGTTCTCTTGGTGGTGGAAACATGGGGGGTGGAGGTGGTGGAGGTGG
AAGAAGATACTAAAATGCATTATCACTGGTTACTTCCCTCATGTGGTGAACCTACTACTT
GTCTTCTAGTCGCCAGTACTTCTGTACTGTTTAGTATTGGAGCAGGCAGGGCTATAAGG
CTAGAGACAAGACCAGGCAGGACAATCAGGATACAGGACAGAGAAGCGTCATCTTAA
TAGATCACAGTGAGGGAGGCTTTTACTTGTATTTCTTTTGGTATTTAGCACCTATTGCATA
TATCTATATTCAACTATTTATGTGAAAATACTGCATCACAATTTTCTAGATTGTGTCTGAA
CTCATTGTTTGGCACATATAATATCCCCTGAAAAAGAAACAATTATTAACGTGTAATTAT
CGTCATTGTTAGTAAAAAAAGATATGGATTAATTTGATTATTCAGTTACAATGGCTAGGG
ATACATCCGTGCCATCCAAATATTAAACTTTGTAACATTTTATTATACTTATTATTGTGTAA
CATCTTTGAAAGTGACTACAGATCTAGGATTTTAGATATTGGAGAGCACGATTGTTTCTC
TAATTTAATGAAATATATCAATATAGCGGTATATGTAAGAATCTAGGTTTTGATATGTTGAT
GATTTGTTTCATAATAAGCAAGGTGGTTCTTATATAGACTAAACATCTCAGGATAAAAGG
GAAATTGCATGAGAAAAATGTTAATACAAAATGAAATTAATGAGTTTGCAAAAGAACC
AGGTACATAAATATCAGTTAGCATTTTTGTAGGTTACCTAGATTAATTAATTATTTAAGTT
GTAATGTTTTAAGAGTTAGTTAATAAAAATGACCAGAACCTTCCACGTCACTTTTTAATT
TACGAAATAAAATGTCCATAGCATAGGATAAGTAAAAAGTACAAGAAGATGTATCAGTT
AAAGGAATAGGGAAGTATAGTTAGATAAGGGTATATTTGATTATTATATATAAATAATAAT
GGAAAATTTCAAATATTATGTATAGAAAGCATTCTTGTATTTTCTGTCTGTGTAGTAGCG
CTCCGTGTCACAGCTACCATTGTCACTCGTCATAGGAG

>novel_circ_001264
CACGGGATCCAGTGATCATCGAGAGGAGTAACCTCGTTAATATCTCGAAATTAATCGTG
AAGGAGCTGATCGAAACGTCTTTGAAGTATGGTCGAATGCTCGACTCTGATCATATGCC
GTTGCAACATTTCTTCATCGTTCTTGAACACGTGCTTAGGCATGGTTTGCGACCAAAGA
AGGGTCTCCTTGGACCCAAGAAGGAGCTTTGGGATATTCTACAGCTCGTTGAGAAATAT
TGCCCCGAAGCGCAGGACATTACGTCCAGTATTCGTGATCTACCCACTGTTAGGACCGC
CATGGGTAGGGCAAGGGCATGGTTGCGCATGGCGTTAATGCAGAAAAAGTTGGCGGAT
TACTTGAAAGTTTTGATCGATCACAAGGAGGACATACTGTCCGAGTACTTCGAGCCAG
ATGCTCTGATGATGAGCGAGGAGGCGATCGTTATAATGGGCCTGTTGGTCGGTTTGAAC
GTGATCGATTGCAATTTCTGCGTGAAGGAGGAAGACCTCGATTGTCAACAAGGGGTGA
TCGATTTTTCATTGTATCTGCGGAACAGCAATCACATACCCGGCGAATCCCCGGACGAC
GAGCTGGAAAACGACAACATGACGACCGTGCTCGATCAGAAAAATTACATCGAGGAG
CTGAACCGACATTTGAACGCTACTGTGACCAACCTTCAAGCTAAAGTGGAATCATTGA
CAACTACGAACGCTCTTATGAAGGAGGATCTCTCCATAGCTAAGAATAATATACTGTCC
CTTCACGAGGAGAATAGACAATTGAAGAAAGAGTTAGGAATCGAGATCAAAGACACG
AACGAGAATGGGAAACCACCGATCAAAATCACTGAAACGACCACGGAAATCGAGGAG
TTGAGAAGTAGATTAGAGGCTGAAAAGAAAATGCGGCAGGATGTAGAGAAGGAGTTA

GAGTTGCAGATGAGCATGAAGTCGGAAATGGAAGTGGCTATGAAACTGTTGGAGAAA
GATATTCACGAGAAACAAGACACGATTATATCGTTGCGACGGCAACTCGATGAGATCA
AATTAATTAACTTGGAAATGTATAAAAAGCTACAG

>novel_circ_001265
GACCGCCATGGGTAGGGCAAGGGCATGGTTGCGCATGGCGTTAATGCAGAAAAAGTTG
GCGGATTACTTGAAAGTTTTGATCGATCACAAGGAGGACATACTGTCCGAGTACTTCGA
GCCAGATGCTCTGATGATGAGCGAGGAGGCGATCGTTATAATGGGCCTGTTGGTCGGTT
TGAACGTGATCGATTGCAATTTCTGCGTGAAGGAGGAAGACCTCGATTGTCAACAAGG
GGTGATCGATTTTTCATTGTATCTGCGGAACAGCAATCACATACCCGGCGAATCCCCGG
ACGACGAGCTGGAAAACGACAACATGACGACCGTGCTCGATCAGAAAAATTACATCG
AGGAGCTGAACCGACATTTGAACGCTACTGTGACCAACCTTCAAGCTAAAGTGGAATC
ATTGACAACTACGAACGCTCTTATGAAGGAGGATCTCTCCATAGCTAAGAATAATATACT
GTCCCTTCACGAGGAGAATAGACAATTGAAGAAAGAGTTAGGAATCGAGATCAAAGA
CACGAACGAGAATGGGAAACCACCGATCAAAATCACTGAAACGACCACGGAAATCGA
GGAGTTGAGAAGTAGATTAGAGGCTGAAAAGAAAATGCGGCAGGATGTAGAGAAGGA
GTTAGAGTTGCAGATGAGCATGAAGTCGGAAATGGAAGTGGCTATGAAACTGTTGGAG
AAAGATATTCACGAGAAACAAGACACGATTATATCGTTGCGACGGCAACTCGATGAGA
TCAAATTAATTAACTTGGAAATGTATAAAAAGCTACAG

>novel_circ_001266
GACCGCCATGGGTAGGGCAAGGGCATGGTTGCGCATGGCGTTAATGCAGAAAAAGTTG
GCGGATTACTTGAAAGTTTTGATCGATCACAAGGAGGACATACTGTCCGAGTACTTCGA
GCCAGATGCTCTGATGATGAGCGAGGAGGCGATCGTTATAATGGGCCTGTTGGTCGGTT
TGAACGTGATCGATTGCAATTTCTGCGTGAAGGAGGAAGACCTCGATTGTCAACAAGG
GGTGATCGATTTTTCATTGTATCTGCGGAACAGCAATCACATACCCGGCGAATCCCCGG
ACGACGAGCTGGAAAACGACAACATGACGACCGTGCTCGATCAGAAAAATTACATCG
AGGAGCTGAACCGACATTTGAACGCTACTGTGACCAACCTTCAAGCTAAAGTGGAATC
ATTGACAACTACGAACGCTCTTATGAAGGAGGATCTCTCCATAGCTAAGAATAATATACT
GTCCCTTCACGAGGAGAATAGACAATTGAAGAAAGAGTTAGGAATCGAGATCAAAGA
CACGAACGAGAATGGGAAACCACCGATCAAAATCACTGAAACGACCACGGAAATCGA
GGAGTTGAGAAGTAGATTAGAGGCTGAAAAGAAAATGCGGCAGGATGTAGAGAAGGA
GTTAGAGTTGCAGATGAGCATGAAGTCGGAAATGGAAGTGGCTATGAAACTGTTGGAG
AAAGATATTCACGAGAAACAAGACACGATTATATCGTTGCGACGGCAACTCGATGAGA
TCAAATTAATTAACTTGGAAATGTATAAAAAGCTACAGGAATGTGAGCACGAACTAAC
GCAGAAAGGGGAAATGGTGAGCCGGCTCCACGCCAAGACGAATCAAATAGGGAAGAT
CCTGAATAATCTCGAGAAGTGCAACCACATGAAAAAGGAGGTGGAGAATATTCGTAGC
CCGACGACACCGAGCAGTGTAACGAAATCTATCCTAAATAAGACCAGTCCCACCTCGC
CCAGGTGTTACGCTGACAATGCGGTTGATTATCAACAACAACAACAACAGCATCCCCA
GTCCAATAATCAGCAACAATCGGCTAACAATCTGCAAAAGTCGACTAATAACCAGCATT
TATTTCAGGAGTGCGAAGGCTCGCTTAAGCATAAAACAGAACTGATCACTAAATTGGA
GGCTAAGACGCTATCGATGACTGAGACCATCCAGAAAATGGATGAGAAGTGCAAGGA
AATCGACGACGTGAAATCAGGAGCGGTGGAGAGGGTGAAGATTTTGGGCGCAGAGGC
TGCCGAGAGGGAGGCGAGGGCGAATGGGGTCGAGAGGGAATTGCGACTCGAACGTG

AATGGCGGACCTCCTTGCAGGAAGCATCGATCTCCAACGCGGAGAAGATCTCTCAATT
ACATCAGGAAATCGATCAGTTGAGGCGAGTGTCCGAGAAATACCTGGCCCTGCAGGAG
GAGCATTACGCGTTGAGGGAGATCTGCACCGAGCAGGAGAGAACTCTGGAGGAACTT
GGGGGGCAATTGAGCGCCGCGAAATTGGCGGCCGTCGAATTAAGGGAGGCCGCTGAC
AACGCTCAACACCAGCACCAACAGCACCACCAGCACCACCAGCACCACCAGCAGGA
GGGAGCGGCGACCTGGGCGAACGATCGGCTGGTCACCCATTGCAAGAGCTGCAACCG
TGAGTTCAACATCACTCGTCGCAAG

>novel_circ_001269
CACACTCTAACGGGCCACTGCGGGAAGGTGATGGCTGCGAAGTTTCTTGGCGAGCCTT
CAAAAGTAGTTACTGGCAGTTACGATCGTACCCTAAAAATTTGGGACCTGCGTAGCAA
AGCGTGTATAGAAACAAAGTTTGCGGGCTCGAGTTGCAACGATCTTGTAACCTCAGAC
GGCGCGGGTTCTACAATAATTAGCGGCCATTTTGACCATAGGATTAGATTTTGGGACAC
AAGAGCCGAATCCAGCTCAAATGACATTTTATTGGAAGGAAAGGTCACTTCGCTGGAT
TTATCTCGTGATGCAAATTACCTTTTGAGCTGTGTCAGAGATGATACATTGAAACTCATC
GATCTGCGGATGAAGAAGATCATTGGATCATTTAGCGCCGATGGGTTTAAAGTCGGTTT
CGACTGGACACGAGCTACTTTTAGTCCAGATGGTCAGTACATAGCGGTTGGTTCTTCAG
ACGGGTCCGTTTTCATTTGGTCTGTCGCCACTAACACGATTGAGACTGTGTTGAAAAA
CCATTC

>novel_circ_001270
ATACTTCTCTCGCACTTCGACGCGTTCAAATTCCCTAGCTCGGAGGTGGTACAGTTCCG
GGCCCTGGTCACGCCTTGCATGCCCACCTGCGAGCCGGTGCAGTGCGATCAAGAGGA
GGCGACCGGCGAGCTTCGGTCCGTCATCTCGTACGGGAAGCGGCGTCGCCGCCGCTCG
GCCGGCTCCCAGACCCGCGAGGACCTGCTTCTCGTCCAGTCGATCCAGATCACGGACA
AGTTCGGGTTCGAGCACGACAGCAAGGCGTCGAACGCGAGCTCGGCCAGCAGGGAC
ACGGTGTTCGTCGAGAGCGAGGACATATCGTCAACCATGGGCATGTGCATCAACCTGG
GCGAGGCGATCGTCGCGGGGACCGTGTTCCTCGTCGCGCAGATCGCCATCATAGCCGC
GTGGACGTTCACCTGGCAGAGACGTAGGCAGATGCTCAAGCATCAAGAGGCGCTCTC
GGTCTCGGTCTCGGTCCCGGGGATGCACTCCGTCCCAGGACGCACCGACAGCCTCT

>novel_circ_001273
GCGCTTTTGCAAGCACATGACGTAGCGGGACATGAAGTTTATGGCGAAGAAGCCACCA
GAGTTACGCCACCTCCTCTTCTCCCCTATCTAAACGGAGGCGACGATCTCGAGGGACA
GAACGGTGATTTGGACCTCGAAAATGTAACGCGCGTCAGGCTGGTCCAATTCCAAAAG
AATACGGATGAGCCAATGGGAATCACTTTAAAAATGAACGAAGATGGAAAATGTGTGG
TAGCACGAATTATGCACGGTGGTATGATACACAGACAAGCGACCCTTCACGTGGGTGA
CGAGATCAGAGAAATTAATGGGATACCGGTGGCCAATCAATCTGTCAATGCCTTGCAA
AAGATACTCCGGGAAGCACGAGGTTCGGTGACGTTCAAAATCGTGCCATCGTACAGGA
GCGCGCCACCCCCCTGTGAG

>novel_circ_001274
GCGCTTTTGCAAGCACATGACGTAGCGGGACATGAAGTTTATGGCGAAGAAGCCACCA
GAGTTACGCCACCTCCTCTTCTCCCCTATCTAAACGGAGGCGACGATCTCGAGGGACA

GAACGGTGATTTGGACCTCGAAAATGTAACGCGCGTCAGGCTGGTCCAATTCCAAAAG
AATACGGATGAGCCAATGGGAATCACTTTAAAAATGAACGAAGATGGAAAATGTGTGG
TAGCACGAATTATGCACGGTGGTATGATACACAGACAAGCGACCCTTCACGTGGGTGA
CGAGATCAGAGAAATTAATGGGATACCGGTGGCCAATCAATCTGTCAATGCCTTGCAA
AAGATACTCCGGGAAGCACGAGGTTCGGTGACGTTCAAAATCGTGCCATCGTACAGGA
GCGCGCCACCCCCCTGTGAGGTACAATTGTTCAGGATTAAGCCTCTGCCAGTGTTAATA
TTCGTTCGAGCACAATTTGACTATGATCCGTTGGAGGACGAGCTGATACCGTGCGCCCA
AGCCGGGATCGCGTTCAAAACCGGCGACATCCTGCAGATCATCAGCAAGGACGATCAT
TATTGGTGGCAAGCGCAGAAGGACAACGCGGCCGGTTCCGCGGGGTTGATACCTTCTC
CTGAACTTCAGGAACGACGTATCGCTTACATGGCAATGGAAAAGAACAAGCAAGAAC
AAG

>novel_circ_001275
ACATCTTCATCAACCAAGACAACCACAATGACAACTGCAGCAAAATTATATGGCAAGG
ATTTGAGTGAATATGATGATGTGGATGTGGATGATTTATTGGCACAACTTACCCCAGAA
GAAATTAATATACTAGCTAAGGAAGTGGATCCAGATGATAGCTTTATGCCACCATCACA
GCGTTGTAGCTATGAATGTGACAAGAGTCCTACAGGACCATTAAATCGTAAGAAACTCA
TTGAACATATCAACAAACAAGCTTTAGAAACACCAGACAGACCTGAATTAAAACCTTA
TGTACCTGGTGTAATAAGAGGAAAAAAGTGGGTTCCACCTCCTCAAGAATCAACAAAA
GAAAAAGAAGCAGAAGAACAAATTGCTATTGATCTTGGAGAAGAATATGAACAAGCAT
TATCTAATGCTACTCAAGAAGAAATTATTGATCTTGCTGCTATTCTTGGATTTCATTCCAT
GATGAATCAAGATCAATATCATGCTTCTCTATTAAATTCTGGCCAACCTGTTGGTTTGGG
TTGGGATGGTATAACAAAAGCTAGTCAACCAAAACCTTACCCAATGGAACCTCCCAAT
GATACAGATGTTGATGCTACTATTAAGCAAGTTAGAGAAGATGATGTTTCATTAATTGAT
TTAAATTGGAATAATATCAAAAATATATCTGATGAAAAATTTATTCAATTATTCGAAGGAT
TAGAGATAAATACACATCTCGAATCTCTAAGTTTAACGAATGTGGGACTCACTGACAAG
ACTGCACAAAGGTTGGCAGATGCCTTAGAAAAAAATTCAACGCTCAGAGTACTCAAC
GTGGAAACAAACTTTATAAGCCCATCTGTGATAGTAAGGCTCATCAGGGCATTGCTTAA
AACAAAATCAATAGAAGAATTTCGATGTTCGAATCAGAGGTCGCAGGTATTGGGAAAT
AAAATTGAAATGGAAATTACACAATTGGTGGAACAAAATCCAACATTGCTCCGACTTG
GTCTTCACTTGGAATTCAATGATGCTAGACATCGCGTAGCTGCACATTTACAACGCAAC
ATTGATAGGAATGGGAGCAATTGTATGGGGGACCATGGAGATCATATGACGAAATGCTT
CTAATTCTTCCGAATCTGCTTCGTAGTCTTTCATAGCGCTTTATTAGCACATCATCATTCA
TCCATTCATCCTGCTAAATTCATTTATTATTTTTCTAGCTAGTGATATAGAGTCTGTCGTG
TGGCACGCGCGAAGCAAGAGGCCGGTGAACCTGCTAAAAGTTTCACTGTCCCAGTAG
GTGGTGTAAGAAAGGAATCGTAGCGCTGATTAGCGCATACGGAAAGACCTAACGCTGC
GACTACAGTTCCGATTCTTTAACATGAATCACAGGAAACCTGCCTTGATTCAATGAAAG
TACGGCAGTCCCGGCTCGGGGTGGCGACATCTTAACGCGAAGTGGCGTTCGCCTAG

>novel_circ_001277
ACATCTTCATCAACCAAGACAACCACAATGACAACTGCAGCAAAATTATATGGCAAGG
ATTTGAGTGAATATGATGATGTGGATGTGGATGATTTATTGGCACAACTTACCCCAGAA
GAAATTAATATACTAGCTAAGGAAGTGGATCCAGATGATAGCTTTATGCCACCATCACA
GCGTTGTAGCTATGAATGTGACAAGAGTCCTACAGGACCATTAAATCGTAAGAAACTCA

TTGAACATATCAACAAACAAGCTTTAGAAACACCAGACAGACCTGAATTAAAACCTTA
TGTACCTGGTGTAATAAGAGGAAAAAAGTGGGTTCCACCTCCTCAAGAATCAACAAAA
GAAAAAGAAGCAGAAGAACAAATTGCTATTGATCTTGGAGAAGAATATGAACAAGCAT
TATCTAATGCTACTCAAGAAGAAATTATTGATCTTGCTGCTATTCTTGGATTTCATTCCAT
GATGAATCAAGATCAATATCATGCTTCTCTATTAAATTCTGGCCAACCTGTTGGTTTGGG
TTGGGATGGTATAACAAAAGCTAGTCAACCAAAACCTTACCCAATGGAACCTCCCAAT
GATACAGATGTTGATGCTACTATTAAGCAAGTTAGAGAAGATGATGTTTCATTAATTGAT
TTAAATTGGAATAATATCAAAAATATATCTGATGAAAAATTTATTCAATTATTCGAAGGAT
TAGAGATAAATACACATCTCGAATCTCTAAGTTTAACGAATGTGGGACTCACTGACAAG
ACTGCACAAAGGTTGGCAGATGCCTTAGAAAAAAATTCAACGCTCAGAGTACTCAAC
GTGGAAACAAACTTTATAAGCCCATCTGTGATAGTAAGGCTCATCAGGGCATTGCTTAA
AACAAAATCAATAGAAGAATTTCGATGTTCGAATCAGAGGTCGCAGGTATTGGGAAAT
AAAATTGAAATGGAAATTACACAATTGGTGGAACAAAATCCAACATTGCTCCGACTTG
GTCTTCACTTGGAATTCAATGATGCTAGACATCGCGTAGCTGCACATTTACAACGCAAC
ATTGATAGGA

>novel_circ_001278
AAATTGAAAAATATATCCTATTCTTCGGTAGGAACGAAAAATTCCGATATTGTGGAGGG
AACGGTCACTCAACATTTCACTTATCTACACGGTATCCTTCAAAATGCGGAGAAACAAA
TTATCGATTCATTGCGGGCGCATAAAAATTCCCGGAATAAAAATATCGACGAAATTTCC
ACGCAGTTAAAAGAACACGAGGAACGACTGCAGTCTGCCATTGTGGTAACTACAGCA
ATTACAGAAAGTCTTGATAAAATAGATCTTCAACAGATTATACAAAAACTAAAGCCCTT
GACCGATATTCCTTGCCATTTGATACAAAATAATGTGCCAGACGACCAAGAGATAAA

>novel_circ_001279
TAATACAGGCAAGAAATATAAATTGTGCTACCACAGAGTACTGCATACATTCTTGCATAC
ATACGCATTGCAACATTTTATTATATTTGATCACCATAGAAGAATATTAAAACTTCTGACT
ACTATCAAATGAATACCTCGATTTTGAGCATTTTATGCTGTAAAATTAAACTTTACTTTTC
ATCGAGTGGAGCACCAGCTCTTATTTTAAAAATCTGTTCTTTAGTATAAGAACTAAACTA
ATGAGGCATTCCAATATTAAAAACTTGTCACTCGATGAGAAAAAAGATATAGCAATGAA
TAGAAATAAATGGCAAATTGAAACAACTGAGCACATCTACAAAATTAACCACCATAGA
GA

>novel_circ_001280
GGTACGTGTACATGAATGGGGACATTGGGAAGCTCCCACCAGGGGCGGTATATCCAGC
CACCCCTGAACCTTTGGGTTCTGTTGGGGGTGCACTTGGAAGTACTGCATCCAATTTGG
AATATAACATCATGAACGGTGTTCCAAGTGGAAATGAATTGATATTGGAGGCGGGTGTT
GGTAATTTAGAACCTTCACTGCAGCATGCTATAGGTATAGGAGGTGCTGCAGGTTATGG
TCCAGTGGATGTTGCTCCTTTAAGTGATGTGCCAAATCAACCTGCTCCAATGAGTGACT
TATCTGCCCCTGGTATACCATTAGAACAACTTAAACAAATGCTCTCTTCACAACTTGAAT
ATTATTTCTCCAGAGAAAACTTAGCTAATGATACATATTTATTATCACAAATGGATAATGA
TCAATATGTACCAATATGGACAGTAGCTAATTTCAATCAAGTAAAAAAATTAACAAAGG
ATATAAAACTTATAACAGAGGTTCTTAGAGAATCTCCAAATGTACAAGTTGATGAAGAG
GGTCAGAAAGTAAGACCTAATCATAAACGATGCATTGTAATTCTTCGTGAAATACCAGA

TAGTACACCATTGGAAGATGTAAAAAATTTATTCTCTGGAGAAGGATGTCCAAGGTTTA
TTTCATGTGAATTTGCCCATAATAGTTCTTGGTATGTAACTTTTGAATCTGATGAAGATG
CTCAAAAGGCCTATAGGTTTTTACGTGAAGAAGTTCGGGAATTTCAGGGAAAACCAAT
AATGGCTAGAATCAAAGCTAAACCAATGAATAGGCTACCAATCCCGGCAGTTGCTAATG
TGGGTGGAATTAAAAATGGTTATAGAACACCACCACCACCACCTGTTTATGATCCAAAT
AGTTATACAGCTGGTCAGCAACGTTTTCTTTATACAAATGGCACTACTATGCCACAAACT
ACTATGCCAGCATATGCCAATCAGGTTCATGTATATCATCAACCATTTTATCCTCCTGGAA
TGATGCCTTGGGGACCTGCAAGTCCTGCTTATTTTGATATGTCAGGTGTTTTTATGAATG
GTATTACACCACATAATACATTTAAACCACAGAATGCAAGATATAATCCTCGTCACAGAA
ATAAGCAAAGAAATGGATCTGAAAGACCAGGCTTGATGGATAGTGGAGGTAACATGGT
GCCAATGGGACGCATTTCACATCCACCTATAAGTGGGGCACCAATATCTTTAGGAGCAA
CAGTTCCGACATTTACTTCAAGCTTGCCTACGGTGAAACCAACTTCTTCCTACCAAACT
GGCACAAAGTTCCATTCCTCACATACAATTGTAGCAACTGGAGAGACCCACTATAATAG
CAGTCATACAAAAGGATCCCAAGAAAATGACACTCAAACTCTAGATTCTGGAGTACAA
TTTCCGCGTCATCGTATGCATCGTAGGACCAAAGACCAAGAGTCATTATCAAAGGCTAA
TAGTAGCCCCTTACCGTCAATCAGAGAATCTACCCCGGCCAGTAATAATAACACTCAAT
GTAATCGAGGGATACAATTCGATTTGGAAGCTTCTGCTTTCCCTCCTCTTCCTGGCCTAG
ATGCTGATCTTGCAAAATCCCATAATGCTTCAGTAGAAACTGTTGGTACAGATTGTACG
CAATCGCAGAATAGGCTTTCAGATGTAGTTAAAGGTACTGCAAAATTGAAAAGCATCA
AAGAAAAAGAACCTACAGGAACTTCACAACAATATCATCAACAAGCGACAAGTAACA
GCAGTAGGTCTGCAAGTCCAGGGTCAAGTGCTGGTGGTCATGCTGGTTCGCTAGAAGC
TCCAACTGGTTCTGGCAGTACATCAACTACTCCTGTTGCATCTACAAATGTCAGCTCTA
ATGCTTCTGCTAGCAACAATGACTCCACAGACATCGCTCTCAGCACCATCACTCTCACT
CCCCCGTCATCTCCTGATAA

>novel_circ_001281
ATGACAACACACCAACTCCAATTGTGGAGAACTCGTCCAAGGGTAAAATTTACTTCGA
TGGCCGACGCCGCAAGCGCAGGTCTTTAACCGAGAAAAAGGAGCTGGAACGTTCCGA
GGAGGACGAGATGTACAAGCAGTTCGTGGGACAAGCCGAGGGGCATCGAAATTTTTT
ACGAAACGATGCCAGATACTACTCCGTGGAGAGAAGACGAAATGATCTGAGCGAGGA
GTATGGCCAGGACTCGGAAATGGAATCGGATCCCTCCGTGACTTGGAGACCACGTAGA
GCCCTGTCAAGTGAATATTTCATCGAGATCATGGTGGCTGCGGACGCGGAGATGGTGA
GGTACCACGGCAAAGGTTTGCTCGGCTACATTTTGGGCCTGATGAGCACG

>novel_circ_001282
TTTTCCTAATGAAGGAAGGGGGAGGAGGGAAGGTGATTTATCGTTCCAGAGTTAATTA
CATCGAGGTCGTTGTGGCAAAAGAAGTGTTCGCTCGCTGGAAAAACGAAAGTTGCAC
GCTGGGTGGACATGTGATACGGTGAGGTGGTAATTAGCCATGTTTTTTTGTCCGAGTAT
GAAGGTCGAAATTTTAACTTTCGTTAAATGGAATGCCCATAATGTTTTATCGTAAAAAA
AAAAAAAAAAAAAAAAGAAAGAAAAAAAAAATATCGAACGTGAAATAGAGAAACGTC
AGAATTTTTCAAAATATTTTTCGAGATGGAATATATTATTATAAATCTAAATATAAATATTC
AACAGCTTACTTTTTCATCGAATTATTATACTTTATATGCAAATCGTTATTTTCATTTTTCA
CTCTCGAAAACTTTTTCTTCGGCAATATGAGTGTAAAAAGAGTTTAATGATTATTCTGTT
TTATTTAAAATAACGAATTGTCTCTCTTTCGTTCATCGATATAAATTTATTTTTATTCAACC

ATTTTTTAAATAATCTTTTTTAAATATCTACGAGCGATATTCGATTGTCGATGTACATATAA
GTCCGATTAATAAAATATTCACAGATAATAAAGCTGATCGCAATATTCAATCGCGAAACA
AGTTCGATATTGGCGTTCGAAATTAATATCTGTGCCAAATGTACTTTCCATACGAAGGAG
CAAAACTTAACACGCATAATACCTTTGAATACTTTCCTGATCGGGCATTGCACACGCCA
CTAAGCATCGTCCTACATGCATTCAAAGAGATTCACTATCCGCGATATCCGTTTAATTTT
GTTCATTGTAATCTCCAATATGAAACTCGACTCGATTCGATGAAAATTAATTATGCATAG
TTATGCTCCAGATGTTCAATCGTCTGCATTAATATTTGGTCGAAAATTATCGAGCTTTTG
CTGATTGTTCGAATTTTCTTTCTTTTTTTTTTTTTTTTTGCTCAGTGATATTAATTTTTA
ATCTCTCGACTGGAATAACTCGCTTAAAATTTTATGATACCTTTTATAATTTAATATTTAA
AAAGAGTAGAATAATGGATAATCTGATGAAAAGTTTAAAAGAAATATTTCCAAAAATAT
TGTTTTCTAATAAATTAACCGATTTAAGACACCCGGTATATTGAAATATTTATTCATAATAT
CACGAATTTTCTTTTCGATTGACTATTTCTTTGTCACTATTTTTTACATAGTCAATTATTAT
ATTACAAATTCTTCTAATCGATTATAAAATAATCTGTCAATTACAATTTTCTTTTTCGAAT
CAAAATTTTCAGATCCGAGTTTCCACTTAAAAATTTTATCTTCCAATAATTTTTATAAATT
CACCACGCGTAGAAATGTATAGAAATCCTATTATTTTTATTTTTCTTCTTCGAATTTATGA
AATACCCTATATACGCTGAACCGTCTAGCCGTATCGCGAATTGTTTCGCGAATCGATCGT
GTACATCAAAGTTTGGCGCCCGCCACCTTACAAGTTTCTTTGGGAACTTTGTACCATGG
AGAGTGAGAAAGAGGAATAGGAAGGGTAGAGCGACATATGGAGAATGGAGATGGGAA
AAAGGGGGGCACCGGTTATGACATAATTGCTTAAGAAAGTTTGGAATGCGAATACACA
GCTAGAATGTAAGTAGTTCACGCGCATTTCGCTTAAGTCGTCCATGAAGGTAGTAGCTG
AATGACTGCCATGCTTATATCCTGCTTATGAATACGTTATTCTATTCTAGATTATTCTTTTA
TCTTTTGTTTCCATTCGGAGAAACGAGATCGCATCATAATTACAGTGATATTTGACCCTT
TACACTTCCATGATCCGTATGAACGTGCTGAAAAGTCTCTCTCTTATTTTCGTGACACAT
ATTTAATCTTGCGATATTATATAGTTGTAGAATATAAAATTATAATAATATTAATATATAATA
TTATTATAATTATATATTTTATATATTATTAATATATAATATTAATAATATATAAAATTGTAGAA
TATCAATACGATATGAAGAGAGAGAGACAAATTTGTAAATTCTTTCGTGAAAAGAATAGGA
TAATTTTGTAAAAGTATCATTCTTAATTATTTCATTCATTAATAATTTTTACTTTGTTCCGA
TTGAATCGAATAACAACGAATAAATAAATTTTTCAAGTATACTATATATTTGTATATATATA
TAATTATTTATTATGTTTATTTGTGAAAATATTAATTAGAATACTTTATTTTTTCAGGAAAT
AATTTATTCGTGGCCATGCTTGTATGTTCCATTTAGTAAAAAAACGATTATTTTACCCGA
ACATAAAGATCGGAATGTTTTCGTGGTTCGTTAATCGAGAAAGGATTTTCAAAAATTTC
AATTTTCGGCGCGGTTTGAGTGGAAAAGGAACCATGCCGCGCCAGCCAATCAGAAAG
CTTGGAAGCGTACCTGCTGCGCGCGACGTCATTCGGCTGCCGAAAATCATCGTGGCAG
GACGTGTGATACCGCTCGATATATATAACCTCAATTACGTAATAGATATTTCTGTTATTATT
TTGTTAATTTACGGTTTAAATTGTTATTAGTTTTGTTTTTGTTCAATGTGTGTTCGTTTCAT
AAGAAATTGTTATGATAGAATTAAGTGATATTGTGAATTTTCTTTGCTTGTTTTCGTTGAT
CGATTATTCGACCGTTGAAGTTGCCGGTATCTCACGTTATTTATTCTTTTAAGGTAGAGT
TCGATTCGATATTATTGTATCATTTTACTATATTTGATTTCAATTTCGGGTATTTAATTTAAA
TCTATAATTTCATTATGTACAGATCATTGATCGATTTGAAGGAATGAGCGTTATGCATTTT
TGACGCTACGATGATTAATGGCGAATGAGATGAGTCTTCATGCAGCATTATGGGTTATGT
TTGATTTATTGCAACAATGAT

>novel_circ_001283
CACAAATTTCGTCGAACGAAGCTTTAGAAGAATTCAAAATAAAAACAGAACAAAGCA

CAGCAACAACGAAAAATTGTAAATCTAACGAATAAATAGTGTAATGACTAGAATAGGA
AGAATTGTACAAAAAGCTCATGGATCAAATGATAGATAATGAAAAAATAAATCAAATAA
TGCGTAAAGATTATTTCTGTACATCAAATTCAAAAGATCGTCCGTATTTTATAACAACTT
TATACTCGATAGAGTATAAATCATTTATAAGAATATATAAAGATATACGGCTGTCTCTTTC
ATAATTGACAAAAAGAAGCTTCACAATGTCAAACAGCGGGCAATCGCTACAAAACAA
GATTGACTTATAAAAAAAAAAAAAAAAAAAGAAATAAAAATTGGATTATTTGACGAATAT
TTAAAATAATTACTATAATTATGGATGAGATAAAAGTGGCGAACTTGGGCCAAGCGGCA
TCAGCCGCATGCTCGATGGCTACAAATTTCTTGGCGCCAGTGAAGACCGAACGGCAGA
TCTACGAGAATTCGATGCTCGAGGACGATCCCAATGCCAATTCGGTGGTATCAACTTCA
CAAGAAGACAGAACCGTACCGAGATGGGGTCCTAATCACAAGGGTGCTCAAGAATTG
GCAAATCTTTATAGCACTG

>novel_circ_001285
AATTTTGGAGAGCTTCAAGATGCCCAGGAGCACAAGTTTTCACATCCATGACATTCTGC
AGCTTGATTCGAAGCCGTCCCAGGAGGAAACGGAAGTGCAAGGTAGTACCACGGTGC
TACCGACCACGAACGATGTTCCGTCGACATACCAACAGTTCTTCGAGCACACGACGGC
CATGTTACAGCCCGCGATATACGCGAATCTGAACAGAGGCACCTTACCACCCCCGCCG
CCACCTTTATTGGCATGGCCGACCGGTCCAACGTTACCGACCACGTTGCCTCAGCCTTT
GGAAGAGGTGAATG

>novel_circ_001286
ATTCTTATAAACTATAAAAATGCCTCGAGTAAGGAGACAAGTGGTCTTAGAAGATCCTG
CTAGGCCTGTCACACCAGATATGGTGGAGACTAAATTACTAAAAGAAGAATTTTTGGAT
GAGGGCTCTTTGGATGAAGTACAAGCACAAAAAGTAGCTGAAGAAACACCAAGTCCT
CATCTTCAAGACCACCAATATGGACAAACACCTTGTTATCAAGTAACAAGGAAACCTA
CACAACCACCACCACGAACTGAACAAATTTCAGTTCAAGCAGTGAAACGAAGACTGA
ATTTGGAAATGGGAACTACAGGCCCTAGCCAATCTGCCTTTAAGGCTCCTAGAGGTAA
ACGTAGAAAATCTGGATCAAGTTCTCTAGCTGGCCATACACCTACAAAAAGTAAAACA
GTAGAGAGAACGCGGTACGATACATCACTGAGCTTACTTACAAAAAAGTTCATACATTT
AGTTGAAAGCAGTCAAGATGGTGTTGTAGATTTAAATGTAGCATCTGAAAAGTTAGAA
GTACAGAAACGTCGTATTTACGATATTACGAATGTATTGGAAGGCATAGGTATTTTGGAA
AAGAAAAGTAAAAACAATATACAATGGAAAGGCGGTCAATTACCAAATGAAAGGAAC
GATATCGCTGATCTCAGAAAGGAAGTGGCAGATTTAGAAGCTAAAGAAAATACTTTGG
ATCGATTAATTCACGGTGCAGACAAAAATCTTAGGGAGCTCTGTGCAGACAGACAATA
CGCTTATGTAACGTATCATGATTTACGTTCTGTCCCAATGTACAAAGACCAAGCTATAAT
GGCGGTGAAAGCTCCACCAGAAGCTACACTTCATGTTCCACAACCTATTAATAATCTTG
GTCAACAAAAGCTTCAAATGCATATGAGGTCATCTCATGGTGAAATAGAGGTATTTCTT
TGTCCTGATGATCCTGCAGTTAAAACGTCTCCCACTCCGGGTTACACGACGACGCAAC
CTGTGCCTTCGTCAAAAGAATCCGAAATACCTTGTCTCCCTCCTGAACTGCTGGCCAAT
GGAGGAAGCGGGGTTCGGGTTGAACCAGTACCTTCTATCGAAAGTTCGTTGAGTACTC
GTTTGTGTACTCCGGTTATATCTACAGTTACCAGTTTAGCAGGCATGAGGGACGCGCTT
TTGTGTGAATCCGATGATTATGGACCAATGGGTGGTGGCAAATTCCAACTCCAAACGG
AGGATCAAATTAGCACTCCAG

>novel_circ_001287
GAGGTACCCTATTTGGAAAACATTTAGTTAGAGATCTAGATCTACAAAGACCATGTTCT
TGTCAAAGAAGACGCAAGCGTTGTTTTTGTTTCCGTCCAAATCGCAATGAAAATTGGC
TTTTTTCAAGATATTCCACTGGTTGGAAATGTGGTCTACATGCTGACTGGACAGAATTA
ACTAGTTGTGTTGATACTGAGTTAAATAAAATTGAGGGGGATGGAATAAAACGTAGATA
CTTTTATATAACTATAATAAGGGATCCTGTTGCACGATATTTGTCTGAATTTAGACATGTA
CAAAGAGGAGCAACCTGGAGAGGAGCTCGTCATTGGTGTGGAGGAACTCAAGCAAAT
ATACCACAATGTTATCCTGGTTCTAGTTGGCAAGGAGTTTCTTTAGAACAGTTCATGGC
GTGTCCATATAATTTGGCAAGCAATCGTCAAACAAGAATGTTGGCTGATCTATCAATTGT
TGGTTGTTACAATTCTACACTCAGCAGTTTGGAGAGAGATCGTCTCATGTTGGCTAGCG
CTAAGCACAATTTGCAATTCATGCCTTTCTTCATGTTGACTGAATATCAAAAA

>novel_circ_001288
AAAATCGGATCGTATCATCGTATCATCGAAACCTGTTCGTCAAAAATCTTCGAAAAATT
CGTCGAGAAACATCGAGAGAGGAAAAGAATATGAAAAAAGAGAGAGAGAAAGAGAG
AAAAGAAAAACAATTAAAAATTAACGAGCCAATCCGAAAATGTAATTAGCTTGAAGGG
ATTTTAATATCTTCTATATGTGTGTACACGTATCTTACTGGGGAGGAAATTGGCATAATAA
AACGAAGGCGTGGGTAAAAGTGAAGCTGACACGATGCTAGTTCTGTAGGACGATTAG
GAGAACAAAGGAGGGGGCGAGGGGGGGCTTATCTGGCGGGCAAAATGTCAATTAACA
CCGAATCAATGGGGGATTTAAAGGATCGATTGACAATGGAAAAGATGGATTCTGTGCC
AGGATGTTTCGAACTCGCACCACGTCCCGCTTTTTTATGGCCGTTCGTTAGCAGAGTTG
CCTGCCTACGCCCGAGTTAAAGCTAG

>novel_circ_001289
GTGATGAGATGTTCTCAGATACTTACAAAATAAAATTGGTTGATGATGTTCTATATGAAG
TATATGGCAAGGTAATTACTCGTAAATCAGGTGACATAGAAATTGCTGGTTTTAACCCAT
CTGCTGAAGAAGCTGATGAAGGAACAGATGAATCTGTAGAATCTGGTGTTGATATAGTT
ATGAATCATAGACTTCAAGAAACTTTTGCATTTG

>novel_circ_001290
GTGATGAGATGTTCTCAGATACTTACAAAATAAAATTGGTTGATGATGTTCTATATGAAG
TATATGGCAAGGTAATTACTCGTAAATCAGGTGACATAGAAATTGCTGGTTTTAACCCAT
CTGCTGAAGAAGCTGATGAAGGAACAGATGAATCTGTAGAATCTGGTGTTGATATAGTT
ATGAATCATAGACTTCAAGAAACTTTTGCATTTGGTGATAAAAAATCTTATACTTTATATT
TAAAAGATTACATGAAAAAATTGGTAGCAAAGTTGGAAGAACAAGCTCCTGATCAAGT
AGAAGTTTTTAAAAAAAACACAAATAAAGTAATGAAAGACATATTAAGTCGTTTTAATG
ACTTACAATTTTTCACTGGTGAATCTATGGACATTGATGGTATTGTTGCGTTATTAGAATA
TCGCGAGATTGACGATGAATCCGTGCCTGTTCTCATGTTATTCAAGCATGGTCTTGAGG
AACAAAAGTTCTAAATAATTTTAGGTATACAACAAATTGTGGAAAAAATATTATAAACT
CAAATCAAGACTGTACACAATGCTGAAAAGCTTGTGAAATTTAGTTAATGGAAAACAC
GATTTTACTTCCAATCTTATTCTATAACGCAGTTAACTTATTTTTTTTAAACAACAATCG
ATGCCAGTCTCAATGGAACTAATAATT

>novel_circ_001291

GGCTAAGTCTGGACAACAGCAACATGTCCATGTCCTCAGTGGGCCCGCAAAGTCCACT
GGACATGAAGCCCGACACAGCAAGCCTCATCAACCCCGGAAACTTCAGTCCTTCCGG
CCCCAACAGCCCGGGATCCTTCACCGCTGGTTGTCACAGCAACCTTCTAAGTACGTCG
CCGAGTGGCCAGAACAAAGCAGTCGCACCCTACCCGCCGAACCACCCTCTCTCCGGA
AGTAAGCACCTCTGCTCGATTTGCGGTGACCGTGCCAGTGGCAAGCATTATGGTGTCTA
TAG

>novel_circ_001292
AGCGATCGAGAGTTGACCGTTCGAACGCGAAACAACCAAAGAGGAGTGCATTGAACG
AAACCAAATCGATACCAAAAGAAGAGGGGAAGTTAGCTCGATTTAGAGAAGGAAGGA
GGAGAAGGAGGAGAAGGAGGGAGAGGTGGAGGAGGAGGTCGAGGAGGAGGAGGAG
GAGGAGGAGGAGGAGGAGGTGATATGGTGGTCGGCTAGTCGTTTTTTTCGTTTCTCTT
CGAGATGACGCGACAAGGATATCGTGCGTGTTTCGCTTGAGAGTAGGATCTTTCGGATC
TTTCGGTGAAGTGTAGAAGAAGGGGGGGGAAGGAAAAGAAAAAAAGAAAAAAAAA
AAGAAAAGAAAAGTAAAAGTGACGAGTGAAAACAGGAAGATCAAGACTGCCAAGAT
GATGAAGAAGGAGAAACCGATGATGTCGGTGACCGCCATCATCCAAGGGACTCAGGC
CCAACATTGGTCCCGTGGCAACACTTGGCTAAGTCTGGACAACAGCAACATGTCCATG
TCCTCAGTGGGCCCGCAAAGTCCACTGGACATGAAGCCCGACACAGCAAGCCTCATC
AACCCCGGAAACTTCAGTCCTTCCGGCCCCAACAGCCCGGGATCCTTCACCGCTGGTT
GTCACAGCAACCTTCTAAGTACGTCGCCGAGTGGCCAGAACAAAGCAGTCGCACCCT
ACCCGCCGAACCACCCTCTCTCCGGAAGTAAGCACCTCTGCTCGATTTGCGGTGACCG
TGCCAGTGGCAAGCATTATGGTGTCTATAG

>novel_circ_001293
AGCGATCGAGAGTTGACCGTTCGAACGCGAAACAACCAAAGAGGAGTGCATTGAACG
AAACCAAATCGATACCAAAAGAAGAGGGGAAGTTAGCTCGATTTAGAGAAGGAAGGA
GGAGAAGGAGGAGAAGGAGGGAGAGGTGGAGGAGGAGGTCGAGGAGGAGGAGGAG
GAGGAGGAGGAGGAGGAGGTGATATGGTGGTCGGCTAGTCGTTTTTTTCGTTTCTCTT
CGAGATGACGCGACAAGGATATCGTGCGTGTTTCGCTTGAGAGTAGGATCTTTCGGATC
TTTCGGTGAAGTGTAGAAGAAGGGGGGGGAAGGAAAAGAAAAAAAGAAAAAAAAA
AAGAAAAGAAAAGTAAAAGTGACGAGTGAAAACAGGAAGATCAAGACTGCCAAGAT
GATGAAGAAGGAGAAACCGATGATGTCGGTGACCGCCATCATCCAAGGGACTCAGGC
CCAACATTGGTCCCGTGGCAACACTT

>novel_circ_001294
GTCGATCTGGACGACACGATGGACAATTTCTTCAACACCCTGTATCAAAAGATGTTCAC
CGTGTTGAACAGTCAGTACAATTTCGACAATAAATACATGGAATGCGTGGGTGAGCAC
ATGAAAGAGATCAGGCCGTTCGGCGACGCCCCGCAGAAACTCGGGGTACAGATCAAG
AGATCGTTCGTTGCGACGAGGGCTTTCAGCCAGGCCCTGACCGTGGCCGCCGACGTCT
TGAAGAACATGCAATCGTTGAAGCCATCGGCGGAGTGCGCAGCTGCTCTCACGAGGAT
GACCGTCTGCCCTTCGTGCAGCGGGATAGCCGGCAACGTGTTGGCGTGCGGCGACATG
TGCGCCAACGTGATGAAGGGTTGCCTTGCCCAGCATGCGGCTCTGGACACTGAATGGA
ATCACTTCGTCGAGGCCGTGGACAAAGTGGCGGACCGATTGTTGGGCCCTTTCAACAT
CGAGATGCTGGTACGGCCGCTCAACTTGAAAATCTCGGAGGCGATCATGAACTTTCAG

GAGAACAGCAACGACGTCTCCCAAAGGGTGTTCACCGGCTGCGGCCGGCCCGTGTTG
GGGAGGCGTAGGCGTCGGGACAATCGGGAATTGGAACTCGAGTCGTTGAATTTCGACC
AGGACACGTTGACGGACGATCGCGTCTCGGCCGCGGCTATCCTCGACAAATTGGTGAA
GGAGACGCGGCAGAGAGTCAGGGATTCGAGGCAATTTTGGGTTTACCTACCTTACAAG
ATTTGCAACGACGGCCTCGTCGTGCCTCCTAGCAACACCAAGGAATGCTGGAACGGCA
CGCACGTCGACAAATATATTTATCCGGTTTCGTCCAATGGCGAGGATCAGAAATTGAAT
CCAGAAGTGCTTAGCACGGGATCAAGGCCGACAATTGTCAGGGACCAGGTGTTCGCG
TTGACCACCATCACGAATAGACTGAAATCCGCTTACAACGGGCAGGACGTTGATTGGA
TAGATACAG

>novel_circ_001295
GTCGATCTGGACGACACGATGGACAATTTCTTCAACACCCTGTATCAAAAGATGTTCAC
CGTGTTGAACAGTCAGTACAATTTCGACAATAAATACATGGAATGCGTGGGTGAGCAC
ATGAAAGAGATCAGGCCGTTCGGCGACGCCCCGCAGAAACTCGGGGTACAGATCAAG
AGATCGTTCGTTGCGACGAGGGCTTTCAGCCAGGCCCTGACCGTGGCCGCCGACGTCT
TGAAGAACATGCAATCGTTGAAGCCATCGGCGGAGTGCGCAGCTGCTCTCACGAGGAT
GACCGTCTGCCCTTCGTGCAGCGGGATAGCCGGCAACGTGTTGGCGTGCGGCGACATG
TGCGCCAACGTGATGAAGGGTTGCCTTGCCCAGCATGCGGCTCTGGACACTGAATGGA
ATCACTTCGTCG

>novel_circ_001296
GTGAGTATCTTATGCGTGCATCCTCGATACGCGCGGAGATAAAGATCAACGATTCAGTT
ATCGTCCACCTTCGAAGAAGAGGAAGAAAAGAAAATGGAGGAGGATGAAGCTATACG
ATGCTAGGAGAAAATTGTCGATCAAAATTGTTGTTATTGTTGTTTTTATTATTATTATTATT
ATTACTTTCTTTTAAAAAAAATTTTCCCTCTTGAAACGATCGGGAAACAGATGGTGTTT
CGTGCGACGAAATAACACGAATGTTAATCAAGTGGAATTTTTGCCGATTAAAATTACAA
TTTTCATTACGAGTGGTCCTCGCGAAACGGGATAAAACGGGCCTCCTCGTGTTCCCTCG
CAGAGAACCAGTTTCGATCGTCGATTCGTCGCTTTGTTAACCGCTTCCCCTTCTTTTTTT
TTTTTTTTTTAATGCTTTTTATCCCTCCTCTCCTCTTCCGTGGATCCACGAGAAGTCCA
AAAGAAGGAGGAAAGCGTTTCACACGATTCGCTGATTGAAAGTGCATATTTGTACAGG
TGCAAATATCAACGCGAACAAATTTGATGCAGATGGAAGATTATTTACGCACCCCGATA
TCGATATCGATGCCTTTGATAAATTCGATCGATTTAATTATTGCCAAACGTCCCCTAATTG
GGCTCGTTTAAGAATTTACGTTTTCAACTAGTTTGATGAAAGTTGAAGAAGAATATTTT
CGTCCCTAGATTTCTAGGGATCACTAGGAACTTTGGGAGTTATATCTGTACTCGAAAAT
GGAATGATTAAAAGAAGAGAGAAATTTCAAATTTAAATTTAAATATTCTCGAATAGTTG
ATAAGAATAATTTCCCTTTTACAGATATTGCGATCGAAGTTTAACTATAATTAACCACGTA
TGGCGATACGGAGGAAAGATATATACGCGAATGCGTGTTTGAAATTGCTCCGTCGAAAC
GAGGCTGAAACCTTTTCTTCGTTGTGAAACCTTGTGCACCTTTTAGTTATTTTCATCTCG
TTATAGACGCATAATCTTAGATATTTTTCTTCCACATTTTTTTTCTTCCTGCAAGTAATTG
CAATCGACCGCAGAAAACCTATCTCCAAAGCAAGTTTCCGACAAGCGAAAGAAGGAG
GAAAAAATATCACGTTGACTAAACCGCAAAAATATGTCGTCTCGGGGAAAAAGGGAA
GGATGGAAAAAAAATGAAGAGATGCCACCGTGAGTTATTTGACGACAGTGTAGACAC
CGGTGGTGGCGCACGCAAGTCGAATGGAAAGAGCGTGGTGTTGGTGCGTGCACGTGA
AACATCGGCCCCTTTGATATGGCAAACGAGACGCGTGGGTTGCGCGCTCGTCGACCGA

AGAATGCACCTTGTTTGCCCAG

>novel_circ_001297
CGAAATCCACGCAAGAATGAAAAAAAGAACAGTTATAAAAAAGAATGGAAATGATTAT
AAAAAAAAAGAAATTTAAAAAAGATAGAAAACAAAACATATAGAGTTTAAAGAAATG
ACGAAAAAGAAATATTTACAAGAAAATGCAGAATAGATAAAATGAACAGCAAAA

>novel_circ_001298
TTTTGCTGTTCATTTTATCTATTCTGCATTTTCTTGTAAATATTTCTTTTTCGTCATTTCTTT
AAACTCTATATGTTTTGTTTTCTATCTTTTTTAAATTTCTTTTTTTTTATAATCATTTCCATT
CTTTTTTATAACTGTTCTTTTTTTCATTCTTGCGTGGATTTCG

>novel_circ_001299
AATGAAAAAAAGAACAGTTATAAAAAAGAATGGAAATGATTATAAAAAAAAAGAAATT
TAAAAAAGATAGAAAACAAAACATATAGAGTTTAAAGAAATGACGAAAAAGAAATATT
TACAAGAAAATGCAGAATAGATAAAATGAACAGCAAAATGAAATGTTATGCCAAT

>novel_circ_001301
GGGTGGACAAGGCGTACGATGCTTAGGGATGCTTGGTCGAACGACGGTGGGAAAGGC
TGGTGGCGATGGTGGTCTAGGTTGGGCGGATTGCTGGCGATGGCGACGGCCATTTCCT
GTCTTGTTGCCCCGTTTCCGGGTGCCTCGGCCAATTCGGAAGCGAAGCGTCTCTACGA
CGACCTGCTGTCCAACTACAACCGCCTCATCCGCCCCGTTGGCAACAACAGCGATCGC
CTCACTGTCAAAATGGGACTGCGCCTCTCCCAGCTCATCGACGTT

>novel_circ_001302
GTTGACCGTTCGAATGGAAGTCGTTTGAGCGGTCGAGAGGATCCTCCCACGAGGAAG
AATCCCGGTTAGAGAGCGAAGAGAGCTGCGAGGAGAGAGAGGGAGAGGGAGAAGAG
GAAGACACAGGGTAGCGGATCGGTAATAATCGAGACACGATGGCGGGCGAGGACACG
CAAATGTTGGCGAACGGAAACGTGGAGGCGCTCACGATAATTCCACCGAAGATGGCG
AACGGGAACGGGCTGATCTGCGGGAAGCAGCAGCACAACAACAATGGGTCGATACCT
AATGGAAAGTTGCACGAAATAACAGCGGCGGAAAACGGGAAATTGATCATGTCCGAC
GAGAAGTCGAGCAGCCTTCACACGGAGTTCGACGATGCCGATGTGTCTTGCGGCTGG
GGCCCGTGCAGGCCACACTGGCTTCAATATTTCGCCACGAAGCAGGCCTTCCTTGTCA
CTTTCTGCATCACCTGGGTTGGTTTGGGTTCTTCAGGGAATGTACTACACGTACTTCGT
CTCGGTGATCACGACGATCGAGAAGCTGTTCCAGATACAGTCGAAGACCACCGGTATT
ATAATGTCAGCGACGGAGATCGGGCAGATCGGTTCCTCTCTCCTGCTTACGTATTACGG
GGGCCAAGGTCACCGGCCTAAGTGGATCGCTTGGGGCATGATCCTGTTCGCGGTGAGC
TCGTTCACCTGCTCGATGCCCCATTTCATCTTCGGTGAACAGCTGATCCATCAGAACGA
GATGTTTCTCAGCGGGGTCGAGGGTGGTGACCCGAACAACACCCATCCCATCCCTGCC
AACCTTTGCAAATTGCAGGAACGATCGGAAAATTCTACGGTCGCCAG

>novel_circ_001303
GTTGACCGTTCGAATGGAAGTCGTTTGAGCGGTCGAGAGGATCCTCCCACGAGGAAG
AATCCCGGTTAGAGAGCGAAGAGAGCTGCGAGGAGAGAGAGGGAGAGGGAGAAGAG

GAAGACACAGGGTAGCGGATCGGTAATAATCGAGACACGATGGCGGGCGAGGACACG
CAAATGTTGGCGAACGGAAACGTGGAGGCGCTCACGATAATTCCACCGAAGATGGCG
AACGGGAACGGGCTGATCTGCGGGAAGCAGCAGCACAACAACAATGGGTCGATACCT
AATGGAAAGTTGCACGAAATAACAGCGGCGGAAAACGGGAAATTGATCATGTCCGAC
GAGAAGTCGAGCAGCCTTCACACGGAGTTCGACGATGCCGATGTGTCTTGCGGCTGG
GGCCCGTGCAGGCCACACTGGCTTCAATATTTCGCCACGAAGCAGGCCTTCCTTGTCA
CTTTCTGCATCACCTGGGTTGGTTTGGGTTCTTCAGGGAATGTACTACACGTACTTCGT
CTCGGTGATCACGACGATCGAGAAGCTGTTCCAGATACAGTCGAAGACCACCGGTATT
ATAATGTCAGCGACGGAGATCGGGCAGATCGGTTCCTCTCTCCTGCTTACGTATTACGG
GGGCCAAGGTCACCGGCCTAAGTGGATCGCTTGGGGCATGATCCTGTTCGCGGTGAGC
TCGTTCACCTGCTCGATGCCCCATTTCATCTTCGGTGAACAGCTGATCCATCAGAACGA
GATGTTTCTCAGCGGGGTCGAGGGTGGTGACCCGAACAACACCCATCCCATCCCTGCC
AACCTTTGCAAATTGCAGGAACGATCGGAAAATTCTACGGTCGCCAGCTGGATCTCGT
CCACGGCTCAAACGACTCACACGATAAACGACTGCAAAGGCGACTCGTTGACGGAGC
AGAGGATACAGAGCAAAATAACCACGGTGGTCCTGGCGATATTTTTCGTCTCCTTGTTG
GGCGTTGGAATGGGTCAGACGGCGGTCTACACCCTCGGTATCCCTTACATCGATGACAA
TGTGGCCAGCAGAGAAAGCCCGCTCTATTTCGCGATCACGATCGGAGTGAGGATCCTC
GGTCCAGCCCTGGGCTTCATTCTGGGCTCGTTGTGCACCATGATTTACGCGGATTTGTC
GGCGAATCCGCAAATCACGCCGACGGATCCGAGATGGGTCGGGGCATGGTGGCTTGGT
CTGGTGCTGATAGCAGCCATGCTGATGTTGGTAAGCATAGGAATGTTCGCGTTCCCGAC
GCGGTTGCCCGCGAGCAGGACGCCGCCGAAACGGGCCGACGCGAAAAAGCCTAGTCT
CCGAG

>novel_circ_001304
CTGGATCTCGTCCACGGCTCAAACGACTCACACGATAAACGACTGCAAAGGCGACTCG
TTGACGGAGCAGAGGATACAGAGCAAAATAACCACGGTGGTCCTGGCGATATTTTTCG
TCTCCTTGTTGGGCGTTGGAATGGGTCAGACGGCGGTCTACACCCTCGGTATCCCTTAC
ATCGATGACAATGTGGCCAGCAGAGAAAGCCCGCTCTATTTCGCGATCACGATCGGAG
TGAGGATCCTCGGTCCAGCCCTGGGCTTCATTCTGGGCTCGTTGTGCACCATGATTTAC
GCGGATTTGTCGGCGAATCCGCAAATCACGCCGACGGATCCGAGATGGGTCGGGGCAT
GGTGGCTTGGTCTGGTGCTGATAGCAGCCATGCTGATGTTGGTAAGCATAGGAATGTTC
GCGTTCCCGACGCGGTTGCCCGCGAGCAGGACGCCGCCGAAACGGGCCGACGCGAA
AAAGCCTAGTCTCCGAG

>novel_circ_001305
CTGGATCTCGTCCACGGCTCAAACGACTCACACGATAAACGACTGCAAAGGCGACTCG
TTGACGGAGCAGAGGATACAGAGCAAAATAACCACGGTGGTCCTGGCGATATTTTTCG
TCTCCTTGTTGGGCGTTGGAATGGGTCAGACGGCGGTCTACACCCTCGGTATCCCTTAC
ATCGATGACAATGTGGCCAGCAGAGAAAGCCCGCTCTATTTCGCGATCACGATCGGAG
TGAGGATCCTCGGTCCAGCCCTGGGCTTCATTCTGGGCTCGTTGTGCACCATGATTTAC
GCGGATTTGTCGGCGAATCCGCAAATCACGCCGACGGATCCGAGATGGGTCGGGGCAT
GGTGGCTTGGTCTGGTGCTGATAGCAGCCATGCTGATGTTGGTAAGCATAGGAATGTTC
GCGTTCCCGACGCGGTTGCCCGCGAGCAGGACGCCGCCGAAACGGGCCGACGCGAA
AAAGCCTAGTCTCCGAGACTTCCCTAAAGCGGTGAAGAGGCTGTTGAAGAACGACAT

TCTTATGTTCCGGACGGCCAGCAGCGTGCTGCACATCCTACCGATCGCCGGCCTCTACA
CCTTCCTGCCAAAGTACCTCGAGAGCCAATTTCGCTTGCCAGCTCATCATGCGAACATG
ATCTCAGGTGTCGGTGGTATTTTAGTAATGGGTTTGGGTATTATAATTAGCGGAGTGTTT
ATCCTGAGGGCAAAGCCTAACGCCAGGTTCGTCGCAGCCTGGATCGCCTTCACAGCTG
TAATATACGCGATCGGCATGGGTGTGCTGATGTTCATCGGTTGCCCTATGGACGATTTCG
CTGGTCTAGTTTCCCATTCCGACGG

>novel_circ_001306
CGATCACGATCGGAGTGAGGATCCTCGGTCCAGCCCTGGGCTTCATTCTGGGCTCGTT
GTGCACCATGATTTACGCGGATTTGTCGGCGAATCCGCAAATCACGCCGACGGATCCG
AGATGGGTCGGGGCATGGTGGCTTGGTCTGGTGCTGATAGCAGCCATGCTGATGTTGGT
AAGCATAGGAATGTTCGCGTTCCCGACGCGGTTGCCCGCGAGCAGGACGCCGCCGAA
ACGGGCCGACGCGAAAAAGCCTAGTCTCCGAG

>novel_circ_001307
GTAACTGGACATGTTGCGCGACTTCATGTACGAATTGATCTCGGGATTGCTCTTCCAAC
AATCGAACGGCACCTCGTCTCCGCCCAGATGAACGTATTGATCCGGGAACACTTGCAC
GATCTCGGCGAATAAGTTGCGGAGGAACTCGTACAGAGCCGGATTCGTCGGATTCATC
GGGCCCAATTTACCATTCGGCTTTCCCTTCACATC

>novel_circ_001308
AAACAAAGTATGATGGTTCGCCAACTGGAGGAGGAGCTGAGGATGCGGATGAGGGGG
CCGAGCGTCGAGATGCAGCAACAAATGGAAGTTTTGTACAACGAGAACGAGCATCTG
ACGCGAGAGATCGCCATACTTCGCGACACGATTAGGGAGCTGGAATTGAGAATAGAGA
CACAGAAGCAGACACTGCAAGCTAGAGATGAAAGCATCAAGAAGCTGCTCGAGATGC
TCCAAAACAAAGGGATGGACAGCTACCGTCCGAAAATCTGGATTCCGAGCAGACATCC
CAAGAGCTTCTCGTTGCTTTCTGCTGTTCCAGGAAAAGAGGAGGAAAGGATCATGTTC
CAGCAGATGCAGTCGATGGCCCAGAAGCAGACACGTTCTTGCCCATCACGACATACCG
CTATCATGGCCATTCGTCGTAATCGATGCACGAGCAGCGGGACGGTCCGAAAGTCGTC
AAGTCGCGTCAAGACGAGCCGATCCCAGGCCCAAGGGAGGACTTTGCGACTGTTTGG
CACCGACCTGATGCGACATGCGATAATACGACGTCGTTCTCCTCGCGAGCTCAAAGCG
ACGAACGAGAGCCTGAAGAGCCTGCAGACGCAGCTGGAGTTGGCGTACGCGTCGACC
GCGTCATCCGGGGCAGCCGTGGCGGGCGGAAGCGCTGGTCTCGGGCTAGGTAGCGTAT
CAGGTGCAGGCGGTACCGTCCTTGGAGGTAGCGTGGCGGTTCCAGGCGGCGTGCCCG
GTAGCACCACCCTGACGGCCATCTTGGAGACAAAAGACGCGCGCATACAAACCCTGG
AGAAGGAGGTGGCTTTGCTCGAGGCCGAGTTGCAACGTATTAAGGAATGCGGCGGTA
GATTAAGGGAGCAGCTGTTGAAAGCGACCGCTGTGCCGAGTAGTAGCGGGGGCGGTA
GTGCGCAGCAGCAGCAAACGATAACGCAACAACAACAGACGCAACAACAGCAACAA
CAGCAACAACAGAATAAGGTTGCCGTGGGAGGTATCGAGTCGCAGATGCGGCAGGGG
TTGGTGACGGCCATGGGTGGTCCGCAGCAGCCCGGCACGTTGGCCTTGGCCAGGGGA
CCGTCCGTGATGACCTCGCTGGTACCGTACGGTCACACGTCGACCTCGGCATTAACCA
GTTCATTGCTTGCCGGTTCGACAGGTGGGACCACGAGCGGCGGAGGCGGTGGGGGCG
GTTCCTCGTGCCTCGGCGGCATCTCGTCGGGCGCTACCACCGGCGTGGTCAATCCCTAT
ACCGATCGTTACGCGGATCATCATCTCCATCAGCTTCATCATCACCCTCATCATCATCAT

CATCATCAGCCTCATCTCCATCAACACCACCACCAGCAGCAGCAGCAGCAGCAGCAGC
AGCAACAACAGCAGCAGCAGCTGCAGTTGCAGCATCAGCAGCAAATGCAGCAACACT
TGAAACAAATTGAGCCTGCGGCGCAGGGCGCACTGGCGCTCCACGCGCACACCTTCA
ACAAGCACGACATGAACTTCAAACGGCAACTGGACGAGCTACGGATGGAAGTGCAGC
GTAGGGATCAGGAGCTGCTCGCCATGTCCGCGAAAATGAAAACCCTCGAGGAGCAGC
ACCAGGATTATCAGCGGCACATCACTGTGCTCAAAGAGTCGCTCGCCGCCAAGGAGGA
ACACTACAACATGCTGCAGGCTGACGTGGAGGAAATGCGGCAGCGGCTCGAGGAGAA
GAACCGGATGATCGAGAAAAAGACGCAGGCCGCGATGCAGGCGCAACAGGAGCGGA
ATCGTATGAACACCGAGCTCACCGAGCTCAAGGATCATATGGACATTAAGGACAGGAA
GATCAACGTGCTTCAGCGCAAG

>novel_circ_001309
GAAAAGAGGAGGAAAGGATCATGTTCCAGCAGATGCAGTCGATGGCCCAGAAGCAGA
CACGTTCTTGCCCATCACGACATACCGCTATCATGGCCATTCGTCGTAATCGATGCACGA
GCAGCGGGACGGTCCGAAAGTCGTCAAGTCGCGTCAAGACGAGCCGATCCCAGGCCC
AAGGGAGGACTTTGCGACTGTTTGGCACCGACCTGATGCGACATGCGATAATACGACG
TCGTTCTCCTCGCGAGCTCAAAGCGACGAACGAGAGCCTGAAGAGCCTGCAGACGCA
GCTGGAGTTGGCGTACGCGTCGACCGCGTCATCCGGGGCAGCCGTGGCGGGCGGAAG
CGCTGGTCTCGGGCTAGGTAGCGTATCAGGTGCAGGCGGTACCGTCCTTGGAGGTAGC
GTGGCGGTTCCAGGCGGCGTGCCCGGTAGCACCACCCTGACGGCCATCTTGGAGACA
AAAGACGCGCGCATACAAACCCTGGAGAAGGAGGTGGCTTTGCTCGAGGCCGAGTTG
CAACGTATTAAGGAATGCGGCGGTAGATTAAGGGAGCAGCTGTTGAAAGCGACCGCTG
TGCCGAGTAGTAGCGGGGGCGGTAGTGCGCAGCAGCAGCAAACGATAACGCAACAAC
AACAGACGCAACAACAGCAACAACAGCAACAACAGAATAAGGTTGCCGTGGGAGGTA
TCGAGTCGCAGATGCGGCAGGGGTTGGTGACGGCCATGGGTGGTCCGCAGCAGCCCG
GCACGTTGGCCTTGGCCAGGGGACCGTCCGTGATGACCTCGCTGGTACCGTACGGTCA
CACGTCGACCTCGGCATTAACCAGTTCATTGCTTGCCGGTTCGACAGGTGGGACCACG
AGCGGCGGAGGCGGTGGGGGCGGTTCCTCGTGCCTCGGCGGCATCTCGTCGGGCGCT
ACCACCGGCGTGGTCAATCCCTATACCGATCGTTACGCGGATCATCATCTCCATCAGCTT
CATCATCACCCTCATCATCATCATCATCATCAGCCTCATCTCCATCAACACCACCACCAG
CAGCAGCAGCAGCAGCAGCAGCAGCAACAACAGCAGCAGCAGCTGCAGTTGCAGCA
TCAGCAGCAAATGCAGCAACACTTGAAACAAATTGAGCCTGCGGCGCAGGGCGCACT
GGCGCTCCACGCGCACACCTTCAACAAGCACGACATGAACTTCAAACGGCAACTGGA
CGAGCTACGGATGGAAGTGCAGCGTAGGGATCAGGAGCTGCTCGCCATGTCCGCGAA
AATGAAAACCCTCGAGGAGCAGCACCAGGATTATCAGCGGCACATCACTGTGCTCAAA
GAGTCGCTCGCCGCCAAGGAGGAACACTACAACATGCTGCAGGCTGACGTGGAGGAA
ATGCGGCAGCGGCTCGAGGAGAAGAACCGGATGATCGAGAAAAAGACGCAGGCCGC
GATGCAGGCGCAACAGGAGCGGAATCGTATGAACACCGAGCTCACCGAGCTCAAGGA
TCATATGGACATTAAGGACAGGAAGATCAACGTGCTTCAGCGCAAG

>novel_circ_001310
CTGGACGAGCTACGGATGGAAGTGCAGCGTAGGGATCAGGAGCTGCTCGCCATGTCCG
CGAAAATGAAAACCCTCGAGGAGCAGCACCAGGATTATCAGCGGCACATCACTGTGCT
CAAAGAGTCGCTCGCCGCCAAGGAGGAACACTACAACATGCTGCAGGCTGACGTGGA

GGAAATGCGGCAGCGGCTCGAGGAGAAGAACCGGATGATCGAGAAAAAGACGCAGG
CCGCGATGCAGGCGCAACAGGAGCGGAATCGTATGAACACCGAGCTCACCGAGCTCA
AGGATCATATGGACATTAAGGACAGGAAGATCAACGTGCTTCAGCGCAAG

>novel_circ_001311
GTGGACTTGCACGAAGCGAACAAGAAACTGCAAGCTGGCGCCGCGAAGGCTGGTATC
GATACTACTCAAGAGAGAAGACAATTGGAGGAGCAGAAACGCCAACTGGAAGAACA
GAGAAGACAGACGGAAGAACGAGCGAAGTCCGTAGATCAGAAAGCTAGAACGATAG
AGGAGAAGGAGAGGACGCTCCAAAGTCTCGACCAGGATTTGAAGAAACGGAAAGCG
AAGATGGATCAGTTGGAGCAGCAGTTACAAAGGAGCGGTGGCTCGCCGGACAAGAGA
TTGGCGGAAATGCAGAAGGCTCTGGAAACTGCCGAGAAGGAATTGGAAAAGGCGAA
AGAGGAATCGACCAGAAGCTCTGCCGAGACCGAAAGGTTGCTGCAGCTGATGCAGAT
GACCCAGGAAGAGCAGAATGCCAAAGAAAGACAAATCAGAGAACTTCAAGATGCACT
CAAAGCCGCACAAGCCAAGTTGAAACAAGCCGCAACTGCACAGCAACAAGAG

>novel_circ_001312
GGTGCTTACCATCAGCTCGGATCTGCCCACCGTACTCCAAGTGTTGAACGAGGTCGTA
CCTAATCTCGAGGAGGTGAGTGTTGCTACCAGAAGAGGATCACACGGGAAAAGAAAT
GACTCAATCAACGGATCTCGACACGGGAGCGACGAGATCGACGTGCGCATGCTGGTG
CATCAGAGCCAAGCCGGATGCATAATCGGCAAGGGAGGTTTAAAGATCAAGGAGCTTC
GCGAG

>novel_circ_001313
AAAACTGGGGCAAGGATCAAGATTTATTCTCACTGCTGTCCCCATAGCACTGACCGGC
TGATCAGCATCTGCGGAAAACCGACCACTTGCATCGAGTGCATTCGCGAGCTGATCGC
CACTATTAAAACCTCGCCTCTGAAGGGAGTGAACAACCCTTACGATCCGCACAACTTC
GACGACTATTACGCGGACGACTACGGCGGCTATGGGACCGGCGACGGCGGCCAAGGG
AAAGGGGGTGGCTTCGGGGGTCCTGGGGGGCGCGGCGGTGGCGGAGGCGGCGGCGG
CGGCGGGGGTATGCCACCCCGACGCGACAACCGCGCTGGGCCGGGCGACATGAACCG
CGGGTTCCCGCCGCCACCGAGAGGTGGGCCGCGCGGGGGAGGCGGCGGAGGCGGTG
GCATGTCTGGCGGGCCGCCTGACCGTGGCTACGGGGGCAACTCTCGCGGAGGAGGCG
GGGGTGGAGGGTACGAAGGCGGACGAGGCGGATACGGCGGCAATAGGGGTGGCCCGC
CCCCGTACGCCGCTGGAAATTACAATG

>novel_circ_001314
TGGGACAGTTCGGAATCTGAATATTCGGAGCCTGAGGAGTCAAATGAGTTAGGGGCCA
TCGTAAGCCCCGAGATCTGCGCGATGGACACTGTGGACAGTGCGAGCAAGCGGCAAG
CTACTCGTGTCTTCAAAAAATCCAGCCTTAATGGAAAGATCACGGTATACCTCGGGAAA
AGAGATTTCGTCGATCACATAACTCACGTCGATCCTATCGACGGGGTCGTGCTGATCGA
CCCGGACTACGCGAAGGATCGCAAGGTTTTCGGCCACGTGCTGGCCGCGTTCAAGTAC
GGGAGGGAGGACCTGGACGTTCTCGGGCTGACCTTTCGAAAGGATCTCTACTTGGCGG
CCGAGCAAATCTATCCCGTGGTGGCTGGCGCCCAGCAGAGGAAGCTGACGCGGTTGC
AAGAGAAGCTGATCAAGAAATTGGGGAGCAACGCGTACCCTTTCTATTTCGAGTTACC
GCCTCATTGCCCTGCGTCGGTCACGCTGCAGCCGGCGCCAGGCGACACCGGGAAACC

GTGCGGCGTCGACTACGAATTAAAAGCGTTCGTAGGTGAGACGCAGGACGACAAACC
GCAGAAACGAAACTCGGTGCGGCTCGCGATACGGAAGATCATGTACGCGCCGTCGAA
GCAAGGCGAGCAGCCTTCCGTCGAGGTGAGCAAAGAATTCGTCATGTCGCCGAACAA
GCTTCACCTGGAGGCGTCGTTGGACAAGGAGCTGTACCATCACGGGGAGAACATTGCT
GTGAACGTGCACATAGCGAACAACAGCAACAGGACGGTGAAAAAGATCAAGGTGTCG
GTGAGGCAATTCGCGGACATTTGCCTCTTCTCGACGGCGCAGTACAAGTGCACGGTGG
CCGAGGCCGAGAGCGA

>novel_circ_001315
TGGGACAGTTCGGAATCTGAATATTCGGAGCCTGAGGAGTCAAATGAGTTAGGGGCCA
TCGTAAGCCCCGAGATCTGCGCGATGGACACTGTGGACAGTGCGAGCAAGCGGCAAG
CTACTCGTGTCTTCAAAAAATCCAGCCTTAATGGAAAGATCACGGTATACCTCGGGAAA
AGAGATTTCGTCGATCACATAACTCACGTCGATCCTATCGACGGGGTCGTGCTGATCGA
CCCGGACTACGCGAAGGATCGCAAGGTTTTCGGCCACGTGCTGGCCGCGTTCAAGTAC
GGGAGGGAGGACCTGGACGTTCTCGGGCTGACCTTTCGAAAGGATCTCTACTTGGCGG
CCGAGCAAATCTATCCCGTGGTGGCTGGCGCCCAGCAGAGGAAGCTGACGCGGTTGC
AAGAGAAGCTGATCAAGAAATTGGGGAGCAACGCGTACCCTTTCTATTTCGAGTTACC
GCCTCATTGCCCTGCGTCGGTCACGCTGCAGCCGGCGCCAGGCGACACCGGGAAACC
GTGCGGCGTCGACTACGAATTAAAAGCGTTCGTAGGTGAGACGCAGGACGACAAACC
GCAGAAACG

>novel_circ_001316
GTTCCATTTACTAACATTGAGAACGGCCCTGCGAGACGATATTTACGATAATTCAACCG
ATAATGTTGCAAAATATCGATTCCACTTCGTACGGGTGTATCGCCTTCCTGTTTAAAGAC
TTCCGCTATGTGCTCGGGTCTACAGCAAGGAACAAGGGTAAATAGGCAAAGATGTAAT
TATAGGCGAGATTATCGAAAATTATTACCTGGGAAAAGGTTACCTGTGAATCATGACAAT
ATCTCCTGAAAGAGGCCCGTTAATCCTCACGATGCAACCGTATTCGTTGAACAAGTATT
TTAAAGGTGTGATATTGCGATTCCACGTTAACCGTCCTGAAAATGGCTCAATTGAAATT
TAATAATTTATCTTCACAAAAAAAACAAAACTATCTCAAACTAAACAAACGAGTTTCAC
GTGATTCTTTTTTTTTCTCTCTTTTTCTTTTTCTTTTCCGATTGTTTCAAAAAATGGAAAA
TCGCGTAATTCGAAATCCGAAGAGCAAATGAGAAATTTTCTTCTTAACTTTTCTTTACA
CGATAGATTCATCGATCATTCTTCAGATGATTTTCTTTTTCCTTTCTTTCTTTCTTTCAAA
CTTTTTCATCGATACTTTTTTTTTTTATTATTATTAGAAAAAAGATCGGGCTCTTCGCATT
TCTACCTATCGATACTTCAACAGATCGTACCCTGGGTGAATCTGTTGATTAGCAGACTG
GAAAGCAGTTGCGTACCGAGGAGCGGCACGTATTTCCAATATTTCTCCCAGATCTTAAG
AATCGCCGGTCCAGGGATCTCGTCGAAGGGCAACGGCGCTTTGTCAAAGATCTGCGTG
TCGACCTTCATCGTGGATACCTCGAACGTGGTCGGTGACACCTCGGTAGTCGCTGTATG
GTCCAACAGGGGAATCGTGTCC

>novel_circ_001317
TTGGATGAGGCTTAGAATGCTGATCGATCCGAATGGAAAAGTTCCGGTGAAAGTGGTC
GCGAGAACTTTCGCATCTGGGAAAACGGAAAAGCTTGTTTATCAGTGCCTCGCCGATT
TAGGTCTATCCAGCGGGAAGAACGATGTGATCGAGCCTGAAGACTTCACCTTCGACGC
TTTCTACGCGTTGTACCATAAAATCTGCCCTCGAAACGACATAGAGGAATTGTTCCAAT

CCATCACCCAGGGCAAAGCGGACACGATCAATCTCGATCAATTGGTCATGTTCCTGAA
CGAGAAGCAACGAGATCCAACGTTGAACGAGATCTTGTACCCGTTGTACGACGAGAA
GAGGGCGTTGGAGATAATAAACGATTACGAGCAGAACGAGATAGCGAGGAATCAAA

>novel_circ_001318
GTGTCAGAATGTTCAAAATCGGAGGGGCCGTCGTTGTCCACCTCGCCGGGATCGGACT
TCTTGACGTCGCCCGCTCATCGTTCGGTCCCTCTCACGTCCCGCCATTTCTCGAGGACG
TCCTCGAGGGCGTCCCAGAACTCGTTGCAAAGCCCGAGCAAGAGTACCGGCTCGTCC
CCGCCGAAAACCGGCAGCTCCAACTCCCTGAACAAGTTTCACAACCGTTTGGTGGAC
AAGCTGAAAAGGTCGTTGAAGAAGGCTGAGGACACGACGGAGGATCAGAGAAATTT
GTCGTGAAACGGTCAGGGGGTTTTGGGCCGGCCACCGATTCCCGGCTCGAAACCGATC
GAGATAAGGCGCACCTCCGCACCCGTTACCGCGTCCGCGACCATCTACGAGGGGGTTG
TACGATCCTGCCCTGATCAATCGTCCTCGGCATGGTAGAGCACGCACGCGGAGAGAGA
GAAACGCGACGACAATATAACGATAACAAAGTAGCTGGCAACAACGAGACAGGATCG
GATCAAAATAATAATCCCCCCTTCGATTCGTTTACGGGTAACAACAACAACACCGATAA
TAACAACAATAACAACGACGAGGACGATCGATATTTACGCGTGAAACCATGCTTGAAA
CGTGACTCGAAGAGAGGAGGAGAAAGTGGCGCGATCTTTCGCGAGAAGAGGAGAAC
TTCGATCGCGGGAGAAGAGAAGGAGGAAGAAGAATCGGAAAGGAGATCGACAAAGA
TGGCGAAAGATTTTCGTGAATGATGTTCCTACCAGCCGAATCCTCCGGTACCTGTACGT
TTCTAACCGCTCTCTTCTGCGTTTCTTTTTGTACATGCCGATTTACAGTAATTGAGAAAC
TGTAAACGAGATTTTTATCTTTTTTTTTTTTACTTTTTCGTTTCTTTTTTTTTAATCTCCCT
TATTTCGGGAAGAAGTCTGGATGAACGAGCCACGAACGAGTCGGGAAAATTTTAACTT
TCTTATTTTCGATTTTCGAAATTGAATTATCGTTCCGAACGAAATGAACGGGATTCTTAT
TGTGGATAATTGTTTAGGATGACGAAGATATAGAGAAAGAGAGAGAAAGAGATAGAGG
AGAAAGAAGGAGAAAGAAGGAGGAGGAGGAGAGAAAGATATGCGGAAGAGAGCGA
GGGGATTAATGAACGAATTAGAAACGAGCCTCCGTCCACTTATACGCGGAAGCTCCTTT
GTAAAAAAAAAAGGAAAGGACGAGGGAAGGACAACGAGGAGGTGGAAAAAACGAG
CGACGAGAGAGAGATCCAACGAAAGAG

>novel_circ_001320
GATTGAGGAGGAGCAGCAGGCTCCGGAGGAGGTACGCGAGGCAAGCGAGATCGTCG
AGCGCCACGAATCACCGTCAACCGTCCACCCTGTGCCCGTGTTGCGAGCGGATGTACA
AGATGAACAGGAGCTTGACCACCCACATGAAGTACGATTGCGGCGAGCAGGCGGCGA
GCTTCGCGTGCCACATTTGCTTGGCCACGTACGGGAAGAACATCAAACTGCGCAGAAT
CGGCATCGCGAATCGCACATCGGGCAACGAGAAACAATCCAAGGGCAAACGCGGAAG
GATTCACGTGCCACCAGTGCGGCAGATCCTACCAATTGAGGCACAATCTGGTCAAGCA
TTTGAGGTTCGAGTGCGGTGGCCAGAAACACTTCACCTGTTACCTGTGCCCGGCCAGA
TACACCCAGAACGGCAAGCTGAGGCAGCACATGCTTAACGCGCACAATATTTTCGTCC
CACCGCGCAGAACGTGGATAAGATCCGCCCACGGTTGAATTACAATAACAATAATAAC
AATAATACGCGGGGAGAAATGGAAGAGAAAAGAACGACTGACTGACGAGGATAATTC
GAAAGGGGATAATCGTGAAATTTCCTGCTCATCGTGTGTAACTAAATTTAGATTTAAAG
AGGCAATAAATCGTCGACGAATGCTGGCTGCGTCCCTTTCATATGCCGTCACATTTCTT
CCGTTTGGATTTTCAGCACCTCTTTTTCTCCATTCTCGAAGAGGGCTCGAAGAGAGTAT
ATTTTTTCCAGATTTTCCCCTCAGAGTATTCAGGATCTGATATATTCGTCGTGGAAGATT

GGATTCTCATCAACGAAGAACGGAACAAACGAAGAAGAATCGGAAAGCAGCGACAG
GAAGATGGACGAACGAAGTCAGCCATCGAAGATATCCCGCGTCTATTCGTCCCCCTTAC
CCTTACTTTCCCCTTCCCTATGTTGAATTGAATAAAGAATTGAATAAAAAAATTTGATC
TACCCTGATAGTTACGTGAGCTTTCGATTCCCCTGCCAGCAATGCGGACGGGGATACAC
GAGGCGGTGCAACTTGAGGAGGCACATGAAATGGGAATGCGGAGGGAAACGCCAGTT
TCCGTGTTACTACTGCAGCTACAGTTTCACTCAGAAGACGAGCCTACACCGTCATCTGA
TCGCTATTCATAAGATAGATATTGTCAATGGAGTAGAGCCTAATCTTATGTTGTAAACGA
TCTCGCGACATTTTTTTTTTCTTCTTCTCCCTTCTCCTCTCCTCTCCTTCTCTCTTATTAAT
TTTCAACGAGAATCCTCCATCCTGTGACAACGATCTATCCCAAGGATTCGTCGGATTCG
CTTGGAAAAGGAAGAATCGTTAACCCTTCTCCGTTTCATTTAACGGATCAGAAAAAAA
AAGAAAGAAAGAAATGTAATCCTGTGCCTGTACCTTCACCTTTCCAAAGTAAACGAGG
CTTCGAATCCTCCTTGCCTGCCTACCTGCCTATCCGCCTGCTTGCCTGCTCCCACTTTGC
GAGTTTTATCCATTTTTCTCCCATTAATATATATATATACATACACACACATATATATATATAT
ATATACACACATATATATATTTTGTTTTCCATCTTTCTCGACACGCAACGACCGGATGCGG
TGAAAGGAAACGGCCGGGGGATGGATTATCCCCCCCCGATTGGAAAAGGGAATCGAA
GATTTGACGATTCCTTGGATTTATTATTGTTGAATCGAGAGAGGAGCGTAAAACGCCTC
TCGCGTTTTTTCGGCCGTTCCTTGTTTCTCGTGATTAACGCGAATTTCCAATTGTTAATG
TTCTTCTAGGTATTGTATAGCGAAGATGACCCCGCCCTTCGACGATTCTCACGAAGATTT
CAATGGCGAGCAACGTCCATTCTCTCTCTCTCTCTCTCTCTCCCTCACACTCTGGCAAG
GAAAAGAGAAGAAGCATCGAGAAGCTGCCACGTCGATCTCGAAGAGAAGAACGGATT
CGTCGAAGGGAGGAAGATGGAGAAGGAGGAATCGATTCTCGAAGAAGAAGAAGAAG
AAGAAGAATCTTTCTCATTCGACGCGCCAAGAATTGTTTTCGCTTCAAAATTTGGGGA
GGGAGAGGAGGAGGAGGAGGAGAGGGGTCATCCTCGGCTTCTTCCTTCCTTGTTGTT
GGTCGACGGATATCCCGTTTCGCTCGAAGATTGAATCGCCCGAATCGAAGATGTGAATC
GTCGGGGCCCGATTGGTGATCAACGATGGCGTCGCGACGAAGAGAAAAAAAAAAGCC
TGCTGCGTACATCTATCGAAGATAAGAGCAAAACGGATTACCAAGTGTATCCAAAGTGT
CCGTCACCTGCGATCGATCATCTGCCGAACAACACGCCGCCCTTTAACCCGGCTATAAA
TTGCTCCGGTTACTCGAGGCTTCTGTTTCAAGATTATCCCGGCGGGATGCCGGACGCGG
ACAGGCCTTACAGATGTTGGAACTGCGGCAAATTGTACACGCACAAATCCACGTTGAA
ACGGCATCGCGAAACGGTCTGCGGCAAGATAAGAAACACGAACGGCAAGTGGAAGTG
TCTGCGCTGCCCGAGGTCCTATCGTTCCGAGGGGAATCTCGAGCGCCATCTACG

>novel_circ_001321
AATCGGCATCGCGAATCGCACATCGGGCAACGAGAAACAATCCAAGGGCAAACGCGG
AAGGATTCACGTGCCACCAGTGCGGCAGATCCTACCAATTGAGGCACAATCTGGTCAA
GCATTTGAGGTTCGAGTGCGGTGGCCAGAAACACTTCACCTGTTACCTGTGCCCGGCC
AGATACACCCAGAACGGCAAGCTGAGGCAGCACATGCTTAACGCGCACAATATTTTCG
TCCCACCGCGCAGAACGTGGATAAGATCCGCCCACGGTTGAATTACAATAACAATAATA
ACAATAATACGCGGGGAGAAATGGAAGAGAAAAGAACGACTGACTGACGAGGATAAT
TCGAAAGGGGATAATCGTGAAATTTCCTGCTCATCGTGTGTAACTAAATTTAGATTTAA
AGAGGCAATAAATCGTCGACGAATGCTGGCTGCGTCCCTTTCATATGCCGTCACATTTC
TTCCGTTTGGATTTTCAGCACCTCTTTTTCTCCATTCTCGAAGAGGGCTCGAAGAGAGT
ATATTTTTTCCAGATTTTCCCCTCAGAGTATTCAGGATCTGATATATTCGTCGTGGAAGAT
TGGATTCTCATCAACGAAGAACGGAACAAACGAAGAAGAATCGGAAAGCAGCGACA

GGAAGATGGACGAACGAAGTCAGCCATCGAAGATATCCCGCGTCTATTCGTCCCCCTTA
CCCTTACTTTCCCCTTCCCTATGTTGAATTGAATAAAGAATTGAATAAAAAAATTTTGAT
CTACCCTGATAGTTACGTGAGCTTTCGATTCCCCTGCCAGCAATGCGGACGGGGATACA
CGAGGCGGTGCAACTTGAGGAGGCACATGAAATGGGAATGCGGAGGGAAACGCCAGT
TTCCGTGTTACTACTGCAGCTACAGTTTCACTCAGAAGACGAGCCTACACCGTCATCTG
ATCGCTATTCATAAGATAGATATTGTCAATGGAGTAGAGCCTAATCTTATGTTGTAAACG
ATCTCGCGACATTTTTTTTTTCTTCTTCTCCCTTCTCCTCTCCTCTCCTTCTCTCTTATTAA
TTTTCAACGAGAATCCTCCATCCTGTGACAACGATCTATCCCAAGGATTCGTCGGATTC
GCTTGGAAAAGGAAGAATCGTTAACCCTTCTCCGTTTCATTTAACGGATCAGAAAAAA
AAAGAAAGAAAGAAATGTAATCCTGTGCCTGTACCTTCACCTTTCCAAAGTAAACGAG
GCTTCGAATCCTCCTTGCCTGCCTACCTGCCTATCCGCCTGCTTGCCTGCTCCCACTTTG
CGAGTTTTATCCATTTTTCTCCCATTAATATATATATATACATACACACACATATATATATAT
ATATATACACACATATATATATTTTGTTTTCCATCTTTCTCGACACGCAACGACCGGATGC
GGTGAAAGGAAACGGCCGGGGGATGGATTATCCCCCCCCGATTGGAAAAGGGAATCG
AAGATTTGACGATTCCTTGGATTTATTATTGTTGAATCGAGAGAGGAGCGTAAAACGCC
TCTCGCGTTTTTTCGGCCGTTCCTTGTTTCTCGTGATTAACGCGAATTTCCAATTGTTAA
TGTTCTTCTAGGTATTGTATAGCGAAGATGACCCCGCCCTTCGACGATTCTCACGAAGA
TTTCAATGGCGAGCAACGTCCATTCTCTCTCTCTCTCTCTCTCTCCCTCACACTCTGGC
AAGGAAAAGAGAAGAAGCATCGAGAAGCTGCCACGTCGATCTCGAAGAGAAGAACG
GATTCGTCGAAGGGAGGAAGATGGAGAAGGAGGAATCGATTCTCGAAGAAGAAGAA
GAAGAAGAAGAATCTTTCTCATTCGACGCGCCAAGAATTGTTTTCGCTTCAAAATTTG
GGGAGGGAGAGGAGGAGGAGGAGGAGAGGGGTCATCCTCGGCTTCTTCCTTCCTTGT
TGTTGGTCGACGGATATCCCGTTTCGCTCGAAGATTGAATCGCCCGAATCGAAGATGTG
AATCGTCGGGGCCCGATTGGTGATCAACGATGGCGTCGCGACGAAGAGAAAAAAAAA
AGCCTGCTGCGTACATCTATCGAAGATATTTTGCACGCCAAAGAGATCGAATCGCGCGA
GATGGACGGTTGTTTTTTTTTCTTTTTGCACAGATTTGTTTTTTTCTTTTTCTTCTTTTTT
CTTCTTCCGTTTTTCATTCCTGTGCGCGTTGATCCGATTTTTAAGCACAAATTATTGTATC
ATTATATCGTAGAATTATCGATCGTTGAATTTTTCGATACACGTTGTAAAAGATTCGAAG
GCTGGACACGTTTAACGTATTTCAACAATTGTCGTTTTCGTTTTATTAAAATAGTATATTA
CACATCGAATATTACAATTTTAAAATCGTCTCTCTAGAACCGTCGATAGTAGATTTGATG
TGCCATCGAGAGTGTCTAGAAGATAGATGATATAAATAATAATAGTAAATAAAAATAATA
ATAATAAATAATAACAATAATAATGATGATGATGATAATAAAAATTTTACCGAAACATCTT
CTACCTGTTCACCACCAAAATGGATGCTCTCACTCAAGAAGATCAATCACATCCAAGAT
AATTTGTATTTTCGTTTCCTTTCTTAAATTTTATAGAATATTTTTTCTTTTTTTTTTCTTCTT
TGAAATGGAATTTCTCCTTTTATATTTCAATTACACAATGCGTACTCTTGATCGACGTCG
TCTAGTATCGAAGATATTCGTCCTGCGTGTCATTTCAGTGTACAA

>novel_circ_001324
TGGATCGTTCGTTTTGCCTGGTATACGGGAATTACGGAGCCGATTCGTATCAGGAGGCG
AGGAAATATCAGGGATCGGGGATCGGGGACGTCTACGGATCGCTTCGACGGAGAAGA
AGCAAGAAAAAAAATTACGTGTGTCCAAAGTGTGGGAACGGTTACACCGTGGTGAAG
AGTTTGAAGCGCCATCTGCGTTACGAGTGCGGCGTAGCGCCGAGGTTCAAATGCCCGT
ACTGCGGCACGCGCAGCAAGCAGAGAGCTCACGTGAACGAGCATATACGTAGGAAGC
ATTCGGGACAACGGATCTACATTATCGACTCGCCTTGATCTGTGACGGGCTGCCGTACA

CAGTAGATATACCCATCGAATAATCGACTCCTCCGTCTTCTCTTTCCACGATATTAGGATT
AGGACCGCATCTCGCCAAG

>novel_circ_001325
GATTCCATATTCTACCGCCCCCGCTCGGACAAAACGTAATCTTGAAGAGCCGATCGGGA
GCAATGGGGAAACGTTGTTTGGAATCGGTCGGGAAAACGGTGAAACGGAGGGGCTTA
AAGATAAACGCCTCCGACAAGAAGAAGCCGTTTCAGTGTCAAAAGTGCGGTCGCGGT
TTCACCTTGAAGAGAAACAAAGATCGCCACGTGAATTACGAGTGCGGCCACGAGCCA
AGATTTCAGTGCCCTTACTGCGGACTGAGGAGCAAGCAAACGTCGCCCGTCTACGCCC
ACATCAGGAAGAAACATCCTGAAGAGGAGGTGTTCATCTTCGACATGAAGCTGTGAA
GATTTTCGAAGCCCGAATTTCATTTCTCGCTCACTTTGCCAACGATCGCGTCCATCCATC
TCTTATAAAGTCTGCAGTTGTTTGCCAGTGTGTGCTCTAAAATACTCTCCTAAATTATTC
TTCTCCGCGATTGTTGTCGCTCTCGACAACAAAATTAAATCAATCCGCTTTTCCGTAGA
GATCTCGTGTCTTATTTCGTGTCAACGTCTCGGATCTCGCCCCATCTGAGAGCCTGCCG
AGAATTCCATTTTTCGGCAAACGAAATTTTCGAGAAAGAAGAGATTGAATCCATCGAG
TTTTATCGAGGATATTTCGTGTAAAAAAATTAAGAATAAAAATAAAACTTTACTTTTTTT
TTCCAAGTAAGATATGCATGCTTGTTGATGCGAAAGAAAAAAAAAAAAAAAAAAAGA
AAAAAAGAAAAAAAAAGAAAATATATAAAACGCAACGATTCTTCGTAGTTTAATCCTC
CTCTTATGCGAATTTCTGCTAGAAAAAAGAAAAGAAAAATGAAAAAAAAAAGAAAAA
AAAAAAGATTGTTCGAGTACCAACCTTTCTTACGCGTTTTTTTTTCGGTGTCTGAAACG
GATAGCCGAACAACAAGGATGTCCTCACCACCTGAAGATAAATGGTACACAACGATCT
TCATTCCGGTAGTTTTTAATAATTATCGTTAAAACGTATAGATTAAGACGTATTTTCTTCT
TATAGATATATATATATATATATATATATATATATATATATATATACATACAAACATACATATATATA
TATATATATATATATATATATATATATATATACTATATATATTAGTTAATTGAAAAAAAAAAAG
TTCATTAATAATCGCGCTTTTGAAATTGAAAAACGAAAAAAATTGCAAAAAAAAAAAA
AAAAGAAAAAAAAAGAGTGAGCTCTCTGGAAATGATCGATATGATGGCTCGACGTTTTC
ATCTAGAAGATCATCCGCAACTTGTTTAAG

>novel_circ_001326
AATCGGCGCGAGGAAGCGGACAAGCGACGAGTAATAGTAATAGCGACACCGGTTGGG
GACGGGGCGGCGAGGAAAACCGACGACACCAGTGTCCCAATTGCTCGCGGAGCTACA
AACATCGTAGCCACATGATACGCCACTTTAAGTACGAGTGCGGGACGCCTCAGAGATT
CGAGTGCCCTTACTGCAAACATCATCTCCGCCAGCGTACCAACGTGTGGACCCATATAC
GAACGTTCCACCCGAGATACGAGCTTTACTGCATCGATATCGCGACCAACGCGAAACT
CTATCACCAGGAGCAGTATCGGACCGATTGATTCGATCGATTCAAATAATAATCGATCGA
ATTAATCTCGTCTCTTCTCTCTTCTCGTCCAGAAACGATTTCCATTAACAATAAGGGGAC
ACCTGTGGAGGAGATGTTCTTCTCGAATCTCTTCGTAATCTATTTTTTTTTCTGAGGATT
ACGAAGGAAGATGCTTCGAAGGAATAAAAAACTAATCGATCATCGATATTCAAAGCTC
CTTAATTGTTTTTTTCTTGACAATGGAGGAATATTGTGATGATATTGTTGATGACCTTCTA
ACGTGTATAAGTGTACGTTCTCCGAGTAAAAGGAATCGACGGTTAATGGAAGAGAGGA
CCGCAGGTTTGTAGAAAGAAAGAAAGATAATAGGGGTGTGAAAGAGAGAACGGAGGT
GGAGCGATTCCACGCCCGTTCCGGCCGCGTGTTCACTATCGTGAGGCGGGACGGGATG
TACGAGTGCCCGAGTTGTCGCAACCTCTACAAGTGGAAGAAGTCGATGCTCTCCCATT
TGAGGAACCAGTGCAAGCAACCGCCCCGGTTCGAATGCCCCCACTGCACGATGAAGA

ACTACCAGAAGTCGCACATGATACGCCACTTGCGGGTCCACCATCCCCAGCTCAGCCA
AACGTTCTGGGATCGGAAGCTGAACGGTTTCTTCCGGCTGTAAAGAGAGAGAGAAAA
GAAGAACTTTGATATGATAATCGAGCGATCTTATTGTCCTTTACCTCCGATCGAAATATT
TCAAATATTTATTGCGCCCAATAATAAAAAAGAAAAAAAG

>novel_circ_001328
GTAATTACCAAGGGGAGCGTTACAGCGTGAAGTGGAACAACATGGGTTATGTCGGTTA
TACGTCGGAGAAAAACTACTTGGACAGGTATAGATATCACCCGAACGAGCGTAAAAGG
TTCGAATGCCTCAACTGCGGATGTCGTTTCACCCAGAAGACAACAATGACCAGGCATT
TGCGCTACTTTTGCGGACAGGGGCATCGTTACCAGTGCCCTTACTGCGAGATGAAAGC
CTCGTGCTCGTCCAATATATACAGACACGTGAGATCGAGACACGCCGGATACAAAGCT
CACGCCATAAAACTCTTCTCCTCGATCAATCCCCGTAGAAATTTAGAAACCATGTAAAA
AAAAAAAACAAAAAAACAAAACAAAACTAAAAAAAAAAGCGGACGATATCTCCGAA
ATTTGGAAATTTCTGTGTGTTTTTTGACGCGAGGAAAAAATCAACGAATGGAAAAGTG
CATTCCAGGATAAATTTACCATTCTCGATAAACCAATCGAAGCATCGAACGATACTAGG
ATAAAAATAGAAAGAGAGAGGCGAATTCTAATGGAAAGATGATATTCAACCTGTCCAC
ATTGCCTTACCTGACCGTTGTGGGTACGGCGGAACGAAGGCGGAGACGGTACAGCGG
CGGTATCCATCAGCCTGTGAACACAGCCTCCCGTGGAAACTTCGAATGTCCAAAGTGT
CAGAAGACCTACAAGTGGTATCGCGGCCTCCACAGGCATTTGAAGTACGAGTGCGGTA
AGGCGCCCCGTTTCAAATGCCCGCACTGCATGTACACAGGGAAACACAGGTCGCACGT
GTACTCGCACATCAAGAGCAATCATAGCAATTGGCCGATATACGCTCTGGACACTCAAC
AGGG

>novel_circ_001329
ATATACGGATATTGGAATGGCCAAACGTGAGGACGCAAGCCGGTGTCTCGAGTTACATC
TGTTCAAAGTGCAACGCGGGATTCCGAAGGATGTGGGACCTGATCAGGCACAAGTGT
GGCCAGATCCCTAGATACGCGTGTCCTTATTGCCATAAGAAGGACAACTCATCCTCGAA
CGTTTACAGGCACATCAGACGATGGCATCCTAAATTGCCAGTCGGTGTAAACAAAATGT
TTTGAACGGTGTCCCGAGATACTTTCGGTGAAGTATTATATAGTCTAGAACCATTAAAA
AAAAAAAAAAGGAGAAAAAAAAAAAAGAAAATAAGAGAAGAAGAAGAAAAAAAAAA
GTTGAATCGAACCGAATCGAATTTAAACTGGCTACTTTTCATCCCTCGTTTTCATCACCC
TTTCAATTTTTTACCTTCTCAATTTAATCGTTCGATTTTTTTTTTTTTTTTTTTTTAATTC
CAAATAATAGCGTCAAGTTGTATCGGGTTGGATGGGTACGAGTACGTAATTTCCGTTAA
CGTGTGAATGCGTGGATGGGAGCGTGTGAGCAAGGAAATCTCGTCTTCCGTCAGGATT
CCACAGGAGGGAAAAAATCTTATCCATCGTTTCCTTGGTGTTTCTTGCCTTGATTTAGG
TGTAACCTGTGTAAACAAACGCTTGTAGATTATATATCTACGCTTCAATCTACGGAAATC
AATATTTTTTTTTCTCTCACTTTCCCGATACGTACACGTGCACGCATACACACACACAT
GTATCGTACACAATGATATCGTACACACGTGCACGTATACGTATGTACACGTAGCGAGG
GACCGCATAGAGATCGAAAGGGGGGACGAAATATCTCCCACTCCCCCCCCCCCCCCTC
CCTTTACCTTGCTAAGTCTCGAAGGTATCGAAGCTCTCGAGAACGTTTGCTCTACATAT
CGTGTTTGTGTTTTTAATTTTCAGAATGGAAATACTCGACGTCCCGTACAAGGACGAAC
GCTATAGAGTGTTCGAAGTGCGGCAAGACGTTCCCCAAGCAAAGAGATTTGACGTTCC
ACGTGAGGCACGCGTGCGAAGAGAAATACAAGATTCGTAGCATGAAAAATTCGGATGC
GTATCTCTACGGCAACGATTTGGACTGCAATCGATGCGGGAAGAGGTTCAAAAGGCGG

AAAGATCTCAATTATCACATACGTCATTTGTGCGGTATACGTGGTATACAGTGTCCTTATT
GCAACAAATGTTACACGTATTCGTCCAACGTCAAGTATCATATAAGCCGTTACCACGAG
GGGAAAGAGGTTTATTACAATAAACTTTTTTGAACTTAATTTCGATCGGATCACCGCGA
ATCATTTTGCCGTCGAGAAAAGAAAAGAACGCGGTGTTTTTTCCCCGAGCCGGTTCCT
TTTCTAATCGTACGCTCGATCCCTCAAGCTTGCCAATATCTTCGTGCTCGTATCGTATCTC
GCCGAACGAGGAATAACGTTCGAATTTTCGAAACGAAACAATTCTTTCGATTTCGTTTC
GAATTATATAGGAAAATTTTTCTCTCCTAATCGTCACGATTCTCTTTCCGTTTCTCTCTTT
CTCTCTTTCTCTGTCTCTCTCTCTCTTCCTCTCTAGTCACTCGTCGCCACAGTCTGCTGC
GAGCATTCGAACTGCGTGTTTCGAAACCCCGGCGACGTCTCGCCGATATTCTCGCGAA
CGAACGAATTGTAAAGGATCTCGTAAGCATAATGTAAACGTATTTGAAAATAAGTAATT
ACCAAGGGGAGCGTTACAGCGTGAAGTGGAACAACATGGGTTATGTCGGTTATACGTC
GGAGAAAAACTACTTGGACAG

>novel_circ_001330
ATATACGGATATTGGAATGGCCAAACGTGAGGACGCAAGCCGGTGTCTCGAGTTACATC
TGTTCAAAGTGCAACGCGGGATTCCGAAGGATGTGGGACCTGATCAGGCACAAGTGT
GGCCAGATCCCTAGATACGCGTGTCCTTATTGCCATAAGAAGGACAACTCATCCTCGAA
CGTTTACAGGCACATCAGACGATGGCATCCTAAATTGCCAGTCGGTGTAAACAAAATGT
TTTGAACGGTGTCCCGAGATACTTTCGGTGAAGTATTATATAGTCTAGAACCATTAAAA
AAAAAAAAAAGGAGAAAAAAAAAAAGAAAATAAGAGAAGAAGAAGAAAAAAAAAA
GTTGAATCGAACCGAATCGAATTTAAACTGGCTACTTTTCATCCCTCGTTTTCATCACCC
TTTCAATTTTTTACCTTCTCAATTTAATCGTTCGATTTTTTTTTTTTTTTTTTTTTAATTC
CAAATAATAGCGTCAAGTTGTATCGGGTTGGATGGGTACGAGTACGTAATTTCCGTTAA
CGTGTGAATGCGTGGATGGGAGCGTGTGAGCAAGGAAATCTCGTCTTCCGTCAGGATT
CCACAGGAGGGAAAAAATCTTATCCATCGTTTCCTTGGTGTTTCTTGCCTTGATTTAGG
TGTAACCTGTGTAAACAAACGCTTGTAGATTATATATCTACGCTTCAATCTACGGAAATC
AATATTTTTTTTTCTCTCACTTTCCCGATACGTACACGTGCACGCATACACACACACAT
GTATCGTACACAATGATATCGTACACACGTGCACGTATACGTATGTACACGTAGCGAGG
GACCGCATAGAGATCGAAAGGGGGGACGAAATATCTCCCACTCCCCCCCCCCCCCCTC
CCTTTACCTTGCTAAGTCTCGAAGGTATCGAAGCTCTCGAGAACGTTTGCTCTACATAT
CGTGTTTGTGTTTTTAATTTTCAGAATGGAAATACTCGACGTCCCGTACAAGGACGAAC
GCTATAGAGTGTTCGAAGTGCGGCAAGACGTTCCCCAAGCAAAGAGATTTGACGTTCC
ACGTGAGGCACGCGTGCGAAGAGAAATACAAGATTCGTAGCATGAAAAATTCGGATGC
GTATCTCTACGGCAACGATTTGGACTGCAATCGATGCGGGAAGAGGTTCAAAAGGCGG
AAAGATCTCAATTATCACATACGTCATTTGTGCGGTATACGTG

>novel_circ_001331
ATATACGGATATTGGAATGGCCAAACGTGAGGACGCAAGCCGGTGTCTCGAGTTACATC
TGTTCAAAGTGCAACGCGGGATTCCGAAGGATGTGGGACCTGATCAGGCACAAGTGT
GGCCAGATCCCTAGATACGCGTGTCCTTATTGCCATAAGAAGGACAACTCATCCTCGAA
CGTTTACAGGCACATCAGACGATGGCATCCTAAATTGCCAGTCGGTGTAAACAAAATGT
TTTGAACGGTGTCCCGAGATACTTTCGGTGAAGTATTATATAGTCTAGAACCATTAAAA
AAAAAAAAAAGGAGAAAAAAAAAAAGAAAATAAGAGAAGAAGAAGAAAAAAAAAA
GTTGAATCGAACCGAATCGAATTTAAACTGGCTACTTTTCATCCCTCGTTTTCATCACCC

TTTCAATTTTTTACCTTCTCAATTTAATCGTTCGATTTTTTTTTTTTTTTTTTTTTTAATTC
CAAATAATAGCGTCAAGTTGTATCGGGTTGGATGGGTACGAGTACGTAATTTCCGTTAA
CGTGTGAATGCGTGGATGGGAGCGTGTGAGCAAGGAAATCTCGTCTTCCGTCAGGATT
CCACAGGAGGGAAAAAATCTTATCCATCGTTTCCTTGGTGTTTCTTGCCTTGATTTAGG
TGTAACCTGTGTAAACAAACGCTTGTAGATTATATATCTACGCTTCAATCTACGGAAATC
AATATTTTTTTTTCTCTCACTTTCCCGATACGTACACGTGCACGCATACACACACACAT
GTATCGTACACAATGATATCGTACACACGTGCACGTATACGTATGTACACGTAGCGAGG
GACCGCATAGAGATCGAAAGGGGGGACGAAATATCTCCCACTCCCCCCCCCCCCCCTC
CCTTTACCTTGCTAAGTCTCGAAGGTATCGAAGCTCTCGAGAACGTTTGCTCTACATAT
CGTGTTTGTGTTTTTAATTTTCAGAATGGAAATACTCGACGTCCCGTACAAGGACGAAC
GCTATAGAGTGTTCGAAGTGCGGCAAGACGTTCCCCAAGCAAAGAGATTTGACGTTCC
ACGTGAGGCACGCGTGCGAAGAGAAATACAAGATTCGTAGCATGAAAAATTCGGATGC
GTATCTCTACGGCAACGATTTGGACTGCAATCGATGCGGGAAGAGGTTCAAAAGGCGG
AAAGATCTCAATTATCACATACGTCATTTGTGCGGTATAC

>novel_circ_001332
ATATACGGATATTGGAATGGCCAAACGTGAGGACGCAAGCCGGTGTCTCGAGTTACATC
TGTTCAAAGTGCAACGCGGGATTCCGAAGGATGTGGGACCTGATCAGGCACAAGTGT
GGCCAGATCCCTAGATACGCGTGTCCTTATTGCCATAAGAAGGACAACTCATCCTCGAA
CGTTTACAGGCACATCAGACGATGGCATCCTAAATTGCCAGTCGGTGTAAACAAAATGT
TTTGAACGGTGTCCCGAGATACTTTCGGTGAAGTATTATATAGTCTAGAACCATTAAAA
AAAAAAAAAAGGAGAAAAAAAAAAAGAAAATAAGAGAAGAAGAAGAAAAAAAAAA
GTTGAATCGAACCGAATCGAATTTAAACTGGCTACTTTTCATCCCTCGTTTTCATCACCC
TTTCAATTTTTTACCTTCTCAATTTAATCGTTCGATTTTTTTTTTTTTTTTTTTTTTAATTC
CAAATAATAGCGTCAAGTTGTATCGGGTTGGATGGGTACGAGTACGTAATTTCCGTTAA
CGTGTGAATGCGTGGATGGGAGCGTGTGAGCAAGGAAATCTCGTCTTCCGTCAGGATT
CCACAGGAGGGAAAAAATCTTATCCATCGTTTCCTTGGTGTTTCTTGCCTTGATTTAGG
TGTAACCTGTGTAAACAAACGCTTGTAGATTATATATCTACGCTTCAATCTACGGAAATC
AATATTTTTTTTTCTCTCACTTTCCCGATACGTACACGTGCACGCATACACACACACAT
GTATCGTACACAATGATATCGTACACACGTGCACGTATACGTATGTACACGTAGCGAGG
GACCGCATAGAGATCGAAAGGGGGGACGAAATATCTCCCACTCCCCCCCCCCCCCCTC
CCTTTACCTTGCTAAGTCTCGAAGGTATCGAAGCTCTCGAGAACGTTTGCTCTACATAT
CGTGTTTGTGTTTTTAATTTTCAGAATGGAAATACTCGACGTCCCGTACAAGGACGAAC
GCTATAGAGTGTTCGAAGTGCGGCAAGACGTTCCCCAAGCAAAGAGATTTGACGTTCC
ACGTGAGGCACGCGTGCGAAGAGAAATACAAGATTCGTAGCATGAAAAATTCGGATGC
GTATCTCTACGGCAACGATTTGGACTGCAATCGATGCGGGAAGAGGTTCAAAAGGCGG
AAAGATCTCAATTATCACATACGTCATTTGTGCG

>novel_circ_001333
ATATACGGATATTGGAATGGCCAAACGTGAGGACGCAAGCCGGTGTCTCGAGTTACATC
TGTTCAAAGTGCAACGCGGGATTCCGAAGGATGTGGGACCTGATCAGGCACAAGTGT
GGCCAGATCCCTAGATACGCGTGTCCTTATTGCCATAAGAAGGACAACTCATCCTCGAA
CGTTTACAGGCACATCAGACGATGGCATCCTAAATTGCCAGTCGGTGTAAACAAAATGT
TTTGAACGGTGTCCCGAGATACTTTCGGTGAAGTATTATATAGTCTAGAACCATTAAAA

AAAAAAAAAAGGAGAAAAAAAAAAAAGAAAATAAGAGAAGAAGAAGAAAAAAAAAA
GTTGAATCGAACCGAATCGAATTTAAACTGGCTACTTTTCATCCCTCGTTTTCATCACCC
TTTCAATTTTTTACCTTCTCAATTTAATCGTTCGATTTTTTTTTTTTTTTTTTTTTTAATTC
CAAATAATAGCGTCAAGTTGTATCGGGTTGGATGGGTACGAGTACGTAATTTCCGTTAA
CGTGTGAATGCGTGGATGGGAGCGTGTGAGCAAGGAAATCTCGTCTTCCGTCAGGATT
CCACAGGAGGGAAAAAATCTTATCCATCGTTTCCTTGGTGTTTCTTGCCTTGATTTAGG
TGTAACCTGTGTAAACAAACGCTTGTAGATTATATATCTACGCTTCAATCTACGGAAATC
AATATTTTTTTTTTCTCTCACTTTCCCGATACGTACACGTGCACGCATACACACACACAT
GTATCGTACACAATGATATCGTACACACGTGCACGTATACGTATGTACACGTAGCGAGG
GACCGCATAGAGATCGAAAGGGGGGACGAAATATCTCCCACTCCCCCCCCCCCCCCTC
CCTTTACCTTGCTAAGTCTCGAAGGTATCGAAGCTCTCGAGAACGTTTGCTCTACATAT
CGTGTTTGTGTTTTTAATTTTCAGAATGGAAATACTCGACGTCCCGTACAAGGACGAAC
GCTATAGAGTGTTCGAAGTGCGGCAAGACGTTCCCCAAGCAAAGAGATTTGACGTTCC
ACGTGAGGCACGCGTGCGAAGAGAAATACAAGATTCGTAGCATGAAAAATTCGGATGC
GTATCTCTACGGCAACGATTTGGACTGCAATCGATGCGGGAAGAG

>novel_circ_001334
GGGCGTTGCCGTTGCCGGCCTACGACGAGTCGAGCGCCACGGACGGCCACGGCCACG
GCCACCACCACCACCACCACCACCACCATCACCACCAGAGGGGGGCGACGGCCGAGT
ACAGGGGAAGGAGGGCCGCGATCAAGAACGAAGACGGCTCGTTCTGGAATTCTGGAA
AGATGTCCTACCATTGCCCCATCTGTAACGCGGGATACACGTACAAGAAAACGTTGAA
GACCCATATGAAGTACGACTGTGGCAAGGAGCCGAGGTTCAAGTGTCCTTATTGCAGC
AAGAGGGACAAGTGTTCGTCCAACATTTACAAGCATATCAGGATGAGACACGACGGGA
TGCCTGTGATCGTGCATAGGAATTAGAAAATTTATTCAATCCGTGGATCGTTGTAATTTT
TCGTTTCCTTCCTTCTTCGTTTCGAATCCCCTCTTCCTCCTCTCCTTCCTCCCATTTGGTA
ATTCAGTGATGATTCGAAGAGAATTGAAAAGAGAAACATCTCTCCGGAGAGAAAGAG
AAGAACGTCGCAGATTTCGTTGGACGAAAAAGCGAGAATGAAGTGAAAACAAAAAA
GTGAAAAAAATGTTCCCTTGTAAAGCTTTAGAAATTATAGAGGCTATACGTCGAGGTAA
TACGTGTGTAATATGTATATTTCGATATTTTTCGCAGACAGATGTAAAAATTTTTATGTTA
GAGAAAGTTGATGAAGAACGAGAAAGAGAGAGAGAGAGAGGGAGAGGGAAAAGAG
AGAGAGAGAAAGAGAAGAGAAGAGAAAGAAGAGAGAAAGAGCAGGACGAGAAAG
AAAGGAAAGAAGAAGGGGAAAAGAGAGAAAAGAGAAAAATTGGAGGAAGAATATTT
CATCGA

>novel_circ_001335
GATACGCGAAGGAGCCGTCGAGGCAGTCGACGTGGTCGAATCAGCCGGATCAAGTTC
AGAGATACTTTTGCCCGAGGTGTTGCAGCTCGTTCAGCAAGAAGGCGAACATGCTGAC
CCATTTCAGATACGAGTGCGGGAAGGAGCCCCGGTTCCAATGCCCTTACTGCGGCAAA
CGGGACAGAAAATCGTCCAACACCTACAGGCATATCAGAACGTATCACCAGGGTAGCA
GGATACAAGCGTACAAACTCTATTAAACTCGTGTTCGTGGCACGCGTCCATCCCAAGA
AGGAAAACAAAACAAAACAAAAAAAAAATAAGAAGAGAAAAGAGAAGAGACTCTTAC
GTGACGTAATATTGTACAATATTGCGATTACATATATATATATATATATACAATTGTAGCTC
GCGTGCCCGTGTTCCTTCGTCGAATGTGCGTGAAAACTGACACTCGTGTGACTTGTGTA
TGTACGTACGTGTGTATGCGCGCGTGTATACGAGATGTTAAAAATAGACAGATCTTCCTT

GTCCTCGTGAGGATCATCGCTGTTCTTCTCTCCTCCCCCTTCTATCTGTTCACCCCTCCC
TACGTTTCATCGCGCGAAAATTGCAAGGAGTGGCAGGAGAGGAGGAGGGGTTTTTTT
CTCTTATAGAGAGGCGTTGTCGTCCCGACACGACACACACACAGGATTCCTCCTTCGTT
TTCGGATTCGTCGCTCGAATCGCCACGATCGCTCTATCTCTGTCCCATCCGTACCGTTTT
CGTTCGTTCGTTCGTTCGTTCGTTCGACCACGTTCGGCTCCCCCGCCCCTAACGCTCTA
ACGGTCGCCTCTGTTTTCTTGTTCGTAGGGGCGTTGCCGTTGCCGGCCTACGACGAGTC
GAGCGCCACGGACGGCCACGGCCACGGCCACCACCACCACCACCACCACCACCATCA
CCACCAGAGGGGGGCGACGGCCGAGTACAGGGGAAGGAGGGCCGCGATCAAGAACG
AAGACGGCTCGTTCTGGAATTCTGGAAAGATGTCCTACCATTGCCCCATCTGTAACGCG
GGATACACGTACAAGAAAACGTTGAAGACCCATATGAAGTACGACTGTGGCAAGGAG
CCGAGGTTCAAGTGTCCTTATTGCAGCAAGAGGGACAAGTGTTCGTCCAACATTTACA
AGCATATCAGGATGAGACACGACGGGATGCCTGTGATCGTGCATAGGAATTAGAAAATT
TATTCAATCCGTGGATCGTTGTAATTTTTCGTTTCCTTCCTTCTTCGTTTCGAATCCCCTC
TTCCTCCTCTCCTTCCTCCCATTTGGTAATTCAGTGATGATTCGAAGAGAATTGAAAAG
AGAAACATCTCTCCGGAGAGAAAGAGAAGAACGTCGCAGATTTCGTTGGACGAAAAA
GCGAGAATGAAGTGAAAACAAAAAAGTGAAAAAAATGTTCCCTTGTAAAGCTTTAGA
AATTATAGAGGCTATACGTCGAGGTAATACGTGTGTAATATGTATATTTCGATATTTTTCG
CAGACAGATGTAAAAATTTTTATGTTAGAGAAAGTTGATGAAGAACGAGAAAGAGAG
AGAGAGAGAGGGAGAGGGAAAAGAGAGAGAGAGAAAGAGAAGAGAAGAGAAAGA
AGAGAGAAAGAGCAGGACGAGAAAGAAAGGAAAGAAGAAGGGGAAAAGAGAGAA
AAGAGAAAAATTGGAGGAAGAATATTTCATCGA

>novel_circ_001336
TAACGAGGGAACGAAGTCCGAAAAAAAATGGAAGACGACCAGCAATTCTGCCTTAGA
TGGAATAATCACCAGAGTACATTAATTCAAAACTTCGACACGCTTCTCGAAAGCGGGA
CGCTTGTCGACTGCACGCTGGCCGCGGAGGGGAAATACTTGAAAGCGCATAAAGTAGT
GCTATCTGCTTGCAGCCCCTACTTCGAGGGATTGCTGAGTGAGCATTACGACAAACACC
CGGTGTTCATTCTAAAAGATGTAAAATTCAAAGAATTAAAAGCAATGATGGACTATATG
TACAGAGGGGAAGTAAATATTTCCCAAGACCAACTGGCAGCACTTCTTAAAGCCGCTG
AATCATTGCAAATTAAGGGTCTATCAGAAAGTAGAACAAGTGGTAGTAGTAAAACGGA
CTTTAGGCAACAAAAAGTAGTTCCACAAACAACATCTCAAATGGACATTCCACAATCG
TCTTCCGGTCTCACGATAGAAAAGAACAAAGTTCCTAGGCAGGGTATGGCACAAGGTT
CCGTAGGGGACCTGCCCGAAGACTCGGCCAGTCCCCAAATACCTAAGGGTCTCTCCTC
GAGGGAAGGTTCTCAAAGTCCCACTTCGAGAAAAAGAAAAAAATTTAGACGAAAAAG
TATAGGCGATGATAATTCTATAGAAAACCACGAAGCGTCGAATTCAAGCGATATGCCTC
AACAAATGAGTGTTCCTGCGCTTGGAATTGCACCTGTAGCAGATGAAAAAGTACACGC
AGACCCTACAGATTCCATTGGCAGGTCAGCTTTAATGACGCAGCTGACGAAACCTGCC
GATGAAATGCTGCAGTTGCCCTTGGAGAAACCTGAGCCAAATGATAATCTCATCGAGC
CAAAGTCTGAATATCTAGAAGATCAAGAAGAAAGCGTTGAAGATCTGACGCTGGATGA
CGATATGAACGATCTGAACGAGATGGAACAGGATAACAATAGAGCCGGACCGTCCCAC
GATCCCTCCCAACATCCTGCAGGTATAGGTGCATGGCACGTTACCGGCGATCGAAGTAA
CGCGGGAGGAGTCGTGGGCTCGGTGGCAGGAGCTCCAGGAACGACAGACGAAGTCT
TTCTTGCAGCCCAGGAGGCCGCCCAGGCCCACCGCGACTCTCAAG

>novel_circ_001337
TAACGAGGGAACGAAGTCCGAAAAAAAATGGAAGACGACCAGCAATTCTGCCTTAGA
TGGAATAATCACCAGAGTACATTAATTCAAAACTTCGACACGCTTCTCGAAAGCGGGA
CGCTTGTCGACTGCACGCTGGCCGCGGAGGGGAAATACTTGAAAGCGCATAAAGTAGT
GCTATCTGCTTGCAGCCCCTACTTCGAGGGATTGCTGAGTGAGCATTACGACAAACACC
CGGTGTTCATTCTAAAAGATGTAAAATTCAAAGAATTAAAAGCAATGATGGACTATATG
TACAGAGGGGAAGTAAATATTTCCCAAGACCAACTGGCAGCACTTCTTAAAGCCGCTG
AATCATTGCAAATTAAGGGTCTATCAGAAAGTAGAACAAGTGGTAGTAGTAAAACGGA
CTTTAGGCAACAAAAAGTAGTTCCACAAACAACATCTCAAATGGACATTCCACAATCG
TCTTCCGGTCTCACGATAGAAAAGAACAAAGTTCCTAGGCAGGGTATGGCACAAGGTT
CCGTAGGGGACCTGCCCGAAGACTCGGCCAGTCCCCAAATACCTAAGGGTCTCTCCTC
GAGGGAAGGTTCTCAAAGTCCCACTTCGAGAAAAAGAAAAAAATTTAGACGAAAAAG
TATAGGCGATGATAATTCTATAGAAAACCACGAAGCGTCGAATTCAAGCGATATGCCTC
AACAAATGAGTGTTCCTGCGCTTGGAATTGCACCTGTAGCAGATGAAAAAGTACACGC
AGACCCTACAGATTCCATTGGCAGGTCAGCTTTAATGACGCAGCTGACGAAACCTGCC
GATGAAATGCTGCAGTTGCCCTTGGAGAAACCTGAGCCAAATGATAATCTCATCGAGC
CAAAGTCTGAATATCTAGAAGATCAAGAAGAAAGCGTTGAAGATCTGACGCTGGATGA
CGATATGAACGATCTGAACGAGATGGAACAGGATAACAATAGAGCCGGACCGTCCCAC
GATCCCTCCCAACATCCTG

>novel_circ_001338
CGGCGGCATGGCCGGGCAGCACTACTGCCTGCGCTGGAATAACTACCAATCAAACATG
ACGTCCGTTTTCCATCAACTGCTTCAAACGGAAGCTTTTGTCGACGTTACACTGGCATG
TAACGAAGCTTCCTTAAAAGCGCACAAAGTGGTATTATCGGCGTGCAGTTCCTACTTTC
AAAAGCTCCTACTTTCAAATCCCTGCAAACATCCGACAATCATTATGCCACAAGATGTA
TGCTTCAATGATCTCAAATTTATAATAGAATTCGTCTATAGAGGGGAGATCGATGTTTCG
CAAGCAGAACTTCAG

>novel_circ_001339
ATCACCACAGTAGAACGTCAAGTCTTCGACTTTCTGGGCTTTATGTGGGCCCCGATACT
GGTCAATTTCTTTAACATTATATTTGTAATCTTAGGATTTTTTGGGGCTTTTCAATATAGA
CCAAAATATATTATATCTTACTGTATATGGAATACACTATGGTTGGGATGGAATATATTTAT
GATATGTTTCTATCTTAATGTAGGTGTATTGGATAAAAATAGTGATATTTTAAATCTTGGT
ACTGGTAGTTTTTCTTGGTGGCATGTCAATGGACCAGGTTGCAAAGCTATTTATGATGTT
ACTGAACCAGAATTATTTCGACCTGCCCGACCTACAAATGTAACAAATTGTGTACTAGA
TTATGAAATAGTTGAAATTTTACACGCATCTACTCAATGCATCTTAGGTTTTATTGCAATA
GTTGGTGGTATTTATCTAAGTAAAGTTTTTTTGGAGGAAGATGATAGCT

>novel_circ_001341
ATAATGAAGCACAGGTACAACGGGTTCGAGAAGGAGATGTACGCGAACGCGTCCCGC
GTCGCAGTGGTGAATCAACTGGCCAGGCAGTTGTTGCACGTCGAGCATCCAAACTCGG
AGCAGATCGTGGCGCGGCAGAACGAGCTGAACCAGAAGTGGGCCGAGCTGAGGGAG
AAGGCGGAGAACAAGAGGGACGAGTTGAACTCGGCCCACGGGGTGCAGAC

>novel_circ_001342
ATAACGTTCATACTGACGGCGATGACGCTGGTCGCCTCGAGCACCGATACCGCGGATCT
ACCGAGGCCGAGCTACGCCAGGACGATGCTGAAGGTGTCGCGAAGCCTTCCGAGCGA
GACCGAAGAGTTGAAGGTGCGCACGAGCGAAGGGAACGTGGCCACGCTGATCGTGA
AACGTAGGGAGAAATCGGCGACGAACGAGACGAGGGCCGGCGTCGCGGAGGATCTC
GAAAATTCCCGCAATTCGGACGTGATAACAGCGACAAAAAACGCGGAGAACGGGACG
ACGAGATCCGAGGCGAGGAGCAAGGAGGAGGTGAAGAGGCTGGAGGACGCGCAGAT
AGAGCAACTGAGGGCGAAACTGTCGGACGGAGGGGCGGAGGGAGGGCGGAGGAGCT
CGTCGAAAGCTGGCAACGAATCGGCGAGGGAGGAGACGGACGAGAACGATCGGCCG
CCTATCGGCGAGAGGAATATCGATTACGGGAACTGGACGCCCCTGGGTATGGACGGAA
GGGCCGTGGTCCGTCTCGAGGAATCGAGCAGCACCGAGGAGTATCAGAATTGGAAAC
CACTTCGAGCAAACTCGAAGACGAGCACCGCCGCAACCACAGCAGCGACCAACGAG
GAGAGATCGAACTACGCGAGGTTCGATCCCGTCTTTCCTGCCGGTGGATTGCTGTTGTC
GCGTCATTTTCAAGACAGGTCTCAGAGGAAGCTGGAGTTGAGCGAGAGGGGAGAGAG
CGAGGAGGGGGCGCGGTACGCGTTCCTGACGCACCAGATACGCTCGTCGAGGACCAA
CATCGGGGCGAACGCGTCCAAGAACAGGGATGGAAAGAACGTGCCGGCGGAAGTTAT
GGTCAGGTCGGAGATAAATGTGAAAACGAACCCGAAGAGGTCGGCGATGACGTTGGA
CGGGGATGGTACACCCGTGATCCACGGGAAAAGGGTCCCCGACGAGCCGATCGACAA
GCTTCAAACATGGCGGAACGCTCGGGTGATCAACAATAAATTGGTCCACGATGGGGAA
GCTAGCGGAGGGGATGGGACGATGGGGGATCCTAGTTTCAACTTTTATCCGACGGAGA
ACGTTTTGGAGAGGCAGAGGTTCGAGAGGTTTTTCCAGAACGTGAATAGAAGGTACG
GCAAAGAGTACGAGGAGGAAGGGAAGAACGTGTTCTTCGAGTGGGATCCGAAGAATT
ACAAGAACGAGGCGTTGAAAGCGGAGGTGTACGAGGCGAGGTCCGACACCTATCGAA
GCAACGGTCGGAAACGGATGCTCCACCCTGACGGGGTGTCCGTCTACCCGGTGTCCCA
ACTGTACACGCCCGAGAGCCAGAAGATAGCGCCGATCGCGTTGAAGCCGGGCGCGAG
GGCGCCCGTCCTCCAATACGCCCATCCGGAACTGGGCGTCCAACCGGCCAAGATCGTG
AAGAACGAGAAGAAACGGCCCGACAATTTCCCCGAGAATCAATACTCGTTCACGGAG
CAAAGGCAGAAGAAGAAATACGTGTTGAACGACAAGAATATCGTGGACACTTACACG
ACCAAGAACTATTATCCGAATCAACATTTCTACGGGTTGAAGAGGCCGAGCGACGCGC
CGTTCTGGGTGAAAATCTCCGAGAACTTGAAGAATCAATTCTCGAACGGCGTGGAGAA
GATGTCGCAATTCACGAAACCGGTGATCGATCCACTGGTGGAGGCGACCCATAAAATT
TCCCAAAATTTGGGCCTCTCGAATCGGGGCAAGGAGGCGCAGGACAAGGTCGGCACG
GTCGCATCAGGCACCACGAGCATCCTTATACCGGCGCTTGGCCTCGTCGCATCGGGCG
CTGCACTCGGGATCGGGGCGGTCGCCGTCGGCAGATACCTGGACGTGGACGTGTTGAA
AAGATCGGGCGACGCGTTGGCCGGGACCGAGGTCGAGTATCGAAGAGCTTTGGACGA
GGCGATGGGGGGCGGGGAGCGAGGGAACGATGATAAGAGCTCGAGCGGAGCGACGT
ACGACGGGGGCGTATACTTGTTCGAGGAGGGGGGAAGGAAGGGCGAGGGGGAGAAG
GTTGGAAACGGAGATCGAAACGGAGTTTTGTTGATCGTCGAGGAAGAGGATGGGGGG
AAGGGGAGGGAGAACGGGGACGAAACGATGGATTCGAAACGGAGTTTAAGAAGGAG
ACGGAGCCTCAATTACTTGGACGTGTTCCAGGCCGAGGGAAATTTGAAAAATTTGATC
AGGAACAAACGTCTTCAACGAAAGGGAGCCGATTCGATCAGCGAGATCGTGGAGATA
GATCTTCCAGCGCGCGAGGGAAGCGTGGACGTGACGGAATTTCTCGTCCCGAGGAGA
AAGATCGAGTCCTCCCCCAACGGGGGGAAGGAGGAGGATTCCGCCATTTTGATAATCG
ACAATGGATCCACGTTGGCTAAATTTCTGTCCAGCAACGATTCGAAAGATTCGGACTCG

AAGAACTCTGTGGAGGCGAGAAATTCGGTGGACGAGAGGGACGAGAGACGGAAGAG
GAGAAGGAGGAGGAGGAGCGTAGAAAGCGATCAGGAATTGGAGGATGCGCTTCAGAA
TTTGGAGAACGCCGAGATAGCGGAGGTGGCCCACATAGATGGAGACTGGACGAATAC
GCCTTGTGCAAAGAGGATATTTTGCGATACCATGATCCAGAGAGGGCCTGACGCTGTTA
TTACGATGGAGAAGAAAATGGCCGCCCTTTTGGGCCT

>novel_circ_001343
GCGTTTCCACTGGCGGAGCTATGAGGGGCGGCAAATTCTTCATCTTCAACAAGAAACT
GCTCTGAGCGTGTCTCTTCTTCGTCGTTCGCCGTCTGCTTAAGATCCGAGGAAGAGGA
AGAAGAAGGAACAGAAGAAAAAGAAGAAGAAGAAAAATAAACCTCCTCCCTCT
CCCTCCCCAAGAAAAAGAAACGTATCAAAAAGGATAGTGGCGTTCCCTCTTTCCTAAA
TTCGTTTAATTCTTCGTCCGATTGAGAAGGAAACGAAGGTCTGAACTAAGATCTTCGAT
AATCCTCCGGTTGAGTAAGAAGATCAAGGCAGGAAGAGGAGAATCCGCCGAGATCGT
GACCGCGGTTTTCTTTCTTTTCTCCGCATAAGAAAAAATCGAGGCGAATTTATATTTCTC
GCTCGACGATTTTCCATTCTTTCTTTCTTTCCCGAAATAATCGTATTTACGGAGGAGAGT
TCGAATATACATATATCACCTTTCTCTCTCTCTCTCTCTCTCTGATCAATCGAATCGAATC
GAATCGAATGATCGATCCTCCTCGGTTGGTCGCGATCGAGGAGGCGTCGGGGCAGGGG
AGGGAAGGGTGTTGGTCGAGGAGGACGTAACGCGCGGCGCGTGGCGATCGATCGATC
GATCGATCGGGCGAAGGTGGAGGGGGAGGACGAAGGGAGAGGCGACAGGAGGAGGC
GGAGGCGGAAGAGAAGGAGGAGGAGGAGGAGGAGGAGGAGGAAGGGCGATTCGGG
GAAGGTTTGACGATCTCGCCCCGTTCCTCGGCCGCCGTGTCGCTGCTGGCCGTGGCGA
GTGGAGGATGGGGGGGACGAGGAGGTGGTGGCGGCGGTGGTCGCCGGTGATGTTGTT
GCCGCTGCTGTTGTGCGCCGCCGTGTCGCTGGCCGCTTGCCAATTCGACGCGACTTGG
AACACGGCCGCTCTCGACGACGATTACGACGAGATCAGGAGAGGATACGTGGCCGATT
ACCGTGGGATCGAGGAGTCGGTGCTCGGCAGTGGCAACCGCCGGGAAGAAAGGGGC
GCTCGGGATCTCGATCCACGGGATCCGTTCTACGAGAGAATCGGCGCCTACAGGCGCA
ACAGGGCGATAGATTCCAGGTCGACGGAAGCGAACACGAGATTGGCGGAGGAAGGTG
GCCAACAATCGAGGCTGGCGAAATCGAGCGCGAAGTCGAGCGACTTGGGCCGCGACA
AGGGGCGGAGATACAAAAATGTGACGATCGGCGACGGGATATGCCAGAGCATCGACAT
ACGGAACACCGCGTCCGCGTTCGGAATTATGAAGGACTGCAGGGTGATAGAGGGCTTC
CTGCAGATCGTCCTCATCGAGAACAACTCGGAGAGGGATTTCCAACCGATCAGTTTCC
CGCTGCTCAGAGAGATCACCGGATACCTTCTACTGTACCGCGTCAACGGATTGAAAAC
ATTGAGCAATCTGTTCCCCAATCTCGAGGTGATCAGGGGAAATATTTTGCTCACCGATT
ACGCGTTCATGGTGTACGAGATGCAGAATCTTCAAGAG

>novel_circ_001347
CAAAAAAATAGAAAATATTGGTAGAATGACGGCCATGACGCAGGAAGAAATTATGGCT
GGTGCCAGAATTGTGGCCCAGGGTCTGGAAGCTCTCCGTGTAGAACATGGAGGACTTC
TTCAAGCTTTGCAGACCCAGGATGCGCCAGTCGCAAGAGACAAAGCTAGTTTATTATC
AAAGAATATCGAGATGATAGAATTAGGCCTTGGAGAGGCACAAGTTATGATGGCTCTTG
CAAATCATTTACAAATGGTAGAGGCTGAGAAACAAAAGCTTAGAACACAAGTAAGAA
GGTTGTGCCAAGAAAATGCCTGGTTAAGAGATGAGTTGGCTGGTACGCAGCAAAAATT
ACAAGCCAGTGAACAAGCACTAGTCCAGTTGGAAGAACAAAAGAAACATTTAGATTTT
ATGGAAAGCATGAAACAGTACGATCCTGATCCTTCCGCGGATGATGAGAATGCTAAAG

ACAGGCCGCCAGATGATCCTGTGGTTGATCTATTCCCCGATGATGATGCGGACGACCGA
AATAGTAAGTCAATATCACCGACACCGCCTTCACAATTTGCACAACAAGTGAACGCTG
GATACGAGATACCTGCACGTTTGCGTACGCTGCACAATTTGGTTATACAGTACGCTAGC
CAAGGCCGTTATGAAGTAGCTGTCCCCTTATGCAAGCAAGCATTGGAGGATTTGGAAA
AGACTTCTGGCCACGATCATCCCGATGTTGCCACAATGTTGAACATCCTCGCTTTAGTG
TATAGAGATCAAAATAAATACAAAGAAGCGGCGAATTTGTTGAACGATGCCTTGGCTAT
TCGTGAAAAGACGCTCGGTGAAAATCACCCCGCAGTCGCTGCCACGTTGAATAATTTG
GCTGTTTTATATGGAAAACGGGGCAAATATAAAGAGGCTGAGCCGTTGTGCAAACGTG
CCCTCGATATTCGAGAGAAGGTCCTCGGTCGTGATCATCCCGATGTCGCTAAGCAATTG
AACAATCTTGCGTTGCTGTGTCAGAATCAGGGTAAATACGAAGAGGTAGAACGCTATT
ACCTGCGAGCGCTAGAAATTTATGAAGGTAAACTGGGACCGGATGATCCTAATGTTGC
AAAGACGAAAAATAATCTGGCATCGTGTTACTTGAAGCAAGGAAAATACAAGGATGCT
GAAGTTTTGTATAAACAAGTATTGACTAGAGCACACGAAAAAGAATTTGGTGCTATTGC
CGGCGATAACAAACCAATTTGGCAGGTTGCGGAAGAAAGAGAAGAAAACAAGCATAG
AAATAAAGAGAATACTCCATATGGGGAATACGGAGGCTGGCACAAAGCTGCTAAAGTG
GATTCTCCTACGGTTACAACTACCCTAAAAAATCTTGGTGCATTGTATCGAAGACAAGG
AAAATACGAGGCTGCAGAAACATTAGAAGATTGTGCTCTTAGGTCACGAAAGGAGCA
GACATTGGAGTTCGTGAAGCAAGGAAAAGTTGCGCAGCTTTTAGGAGAAGAGAAAGG
ATCGACGAGACGCGGCTCGCGATCCAGTTTAGCTAATAGCGAACACGAGCAACATGAC
GAG

>novel_circ_001348
GAGCGATGTCTTCCTGGAACCGTATCTGCAGCAGCACGGAACCGTTCAGGTCGTCAGC
TCGCCGAGCACGCCTCCGCCGAACCCGCTGCAGCATCATCAGCTCACGCAGCTGCATC
ATGTACAGAGCGAGGAGTCGCTTTCCTCGGAAGGGTCGGCCACCTCGGGTGGATCTTC
GAGCTCGACGGAAGATTCTGGCAGCGAGGTCGCGTGCGACATCACCCTGATCGAGAG
GCTTATACGCTCCCATCCGATCTGGTTCCTTCCTGGCATCCAAAGAGCAGGCGCTTTCC
ACTTGTTGCAAGGCAAAGAGGAAGGG

>novel_circ_001349
ATAGAGATGGCAACAAAGTTACAACCGTGGTGGCTACACCTGGTGCTGGTCCGGATCG
TCCCCAAGAGATCTCCTACACAGACACTAAAGTAATCGGTAACGGCAGCTTCGGCGTT
GTATATCTAGCGAAATTATGCGACACGGAAGAGCTGGTCGCCATCAAGAAAGTTCTTCA
AGACAAACGATTTAAGAACAGAGAATTGCAAATCATGCGGCGCCTCGAGCATTGTAAC
ATCGTGAAACTGAAATATTTCTTCTACTCCAGCGGTGATAAGAACATCTTAAACGCAAC
TAATCCAGTTTTTCATGTGGACAAGGACGAGGTTTATTTGAACCTAGTGCTGGAATACA
TACCTGAAACTGTGTATAAAGTTGCTCGGCATTATAACAAGAGCAAGCAGACCATACCG
ATTAATTTCATTAAGCTGTATATGTATCAATTATTCCGTTCTCTGGCGTACATCCACTCGC
TGGGAATCTGCCACAGGGATATCAAACCGCAAAATCTTCTGCTCGACCCGGAATCTGG
CGTTCTCAAGCTCTGTGATTTTGGTTCCGCCAAACATCTGGTGAAAGGGGAGCCCAAT
GTGTCTTACATCTGTAGTCGTTACTATCGCGCGCCCGAATTGATCTTTGGTGCTATTGATT
ATACTACAAAAATCGATGTTTGGAGCGCAGGTTGTGTAGTGGCAGAGTTATTGCTCGGC
CAACCGATATTCCCAGGGGACAGCGGAGTGGATCAATTGGTGGAGATCATCAAGGTGC
TCGGCACCCCGACACGTGACCAGATACGTGAGATGAACCCCAATTACACCGAGTTCAA

ATTCCCACAAATTAAGGCGCATCCATGGCAAAAG

>novel_circ_001350
TGTTACAGATACTCAGCATAATGTATTGGAACCTGCACAAAAGACTTTGGAATTGGCAA
AAACACCAAATTGCACAATGGTGGATGACTTATTATGTCCTCCAGGTCGTCAGAGTAGA
CCACCAAAAATAGCTATAATTTTAAGAGGTCCGCCAGGTAGTGGAAAATCTTTTGTGGC
CAAACTTATTAAGGACAAGGAAGTTGAACAAGGAGGTTCTGCACCAAGAATATTAAGT
CTTGATGATTATTTTTTGGTAGAGAAAGAAATAGAGTCCACAGATGATAATGGGAAAAA
AGTTACAATTAAGGATATGATATATGAATACGAAGAAGTAATGGAACAAAGTTACATTA
CATCTCTTGTTAAAGCTTTTAAAAAAAAATGTTACCGATGGTTTCTTCAATTTTATTATATT
GGATTGTATAAATGAAAAAATATCCGACTATGAAGAGATGTGGAGTTTTGCTAAAACAA
AAGGTTTCAAGGTATACGTATGCGAGATGGAAATGGATTTACAAATTTGTTTAAAAAGA
AATATTCATAATCGCACAGAAGATGAAATAAATAGAATTATAGATTATTTTGAACCAACT
CCAAGTTATCATCAAAAATTAGATGTTAATTCAATGTTGCAGGAACAAGCAATTGAAGA
AGTTCATATGGAAGATAGTGAAGAAACGCAGGAGAAATCATCTCAGCAAAATGAAGAT
AGTCAAGATAGTCAAGATGATATGCAAGATGCTATTGGAGTTAGCAAATGGGAACGTAT
GGAAGCTGAAGATAAATTAGATCGATTGGATGGACTTGCAAAAAAGAAAAATGAAGG
AAAGGTACAAACTATGAAAGATTTTCTTCAAGTTCCTGACTATTACAATATGGAAGATA
CTTCCGGTAAAAAACGAGTGAGATGGGCAGATTTAGAAGAACGTAAAGAACAAGAAA
AAATGCGCGCTGTAGGATTTGTAGTCGGTCACACTAATTGGGATCGTATGATGGATCCT
ACAAAAGGAGGAAGCGCCTTAACACGCACAAA

>novel_circ_001352
GTCAAGATCCCGTAGCCCGCGCAGAAGATCTTTAACCCGTAGTCGCAGCCGAGATCGT
CGTTCTCGTTCGGATTCCCGTGACAGACGTTAGATGCTAAAAGTATTCCAGACAATGGA
TCTACTAAATATGGCAGAAAGAAGATAAAAGAAAAATGATTTTATATGTATATAAAAAA
GATCGTTATTACACGTACATACAGTTTAACAATTATTATTATAATTAAAAAAAATAATATT
TGTAAGAAATGCGAAAGAAATATATACACAATGTTTACTATTTCAATTGTTGAGTTTGTA
TACATATATAACATGAAAATTAATCATATATACACATAAATGCTTTTGAGTAACTATATATA
TATAATATTTTCACAAATATATGACATATTTTTTGTATACAGGTACATTTCACAGACATAA
AATTTTTTATAATTGAGTAATTAAATATCGAGTAATGAATTAGATATCTATTTCTTAGATGT
CTATAAGATTTATTCATTGCTTTAAAGTGAATAACACTTATATTATTTACAAGTAATACATA
AAAATTCGGTTATGAATTTCAATTGAGAGAGTTATAGCGATAAACATAACATTAATTGTT
CAAATCATTTTTGTCAATTTTGTCAATTCAGAATAGAAATGATTTTGATGTGAAGAAATA
AGAAATCTTCATTTTATGTTGTATTAATAATTTTGTACTCTTATAAATTTAAGATTTTCAAA
TAAAAAAGTATATGTTATAATTTTTATTAGTTTTCCTATTGTGAATAATAATTTGATAATTA
CTAATAAATTAACGAAATAATATTGCATTGAAATTATATTTTTTATCCTATTTTAATATTTAA
TTTAAAAAAATTTAATATATACATGGTTAAAATTAATGGTGCAAATGAAATCATAGAGCC
CAAAATAAACAAAAAAAAAAACAATAATAATAAAATTATATTATATATTAGATAGTTAAC
TTCTTTTTTAAAATGCATTTAAAGTTCTTTTACACATATGATTTATTTTGAACTGGATATTA
CTGTTAGTAAGAGAGTATTTTATATTTGTAAATTATAATAATATAATATTATTAAAGAATAA
TAAAACAAAATAAAATTTTATTTAAGATTTTGTTTTTATAAAAATCGAATTTAAAAATTT
GTTAAATTAAGTCTAATTAAGTTATAATTTTTAAAAAAAATTACAAACATTTAGAATTTC
CAGCATTTTTCGTGATAACATTTTACTAAAATTTAATAGTTTAATACAGAGTATTCATAAA

AAATGTAGAACAGAAGGGTGATTTTCGTTATTTAAAATGATGAAAAAATTTCATATATCC
GATAATTGAAGAGTTGTAAATATTTTTAAATTTATATTAGATGAGATGGTGGAAAAGCGA
GAATGTTCAGCTGAGTATATGACTAATATTTGCAATATCGCTTATGTAGCTTTTTAATGAA
AATAAATACATTGATATTCTTTTATACACAAATGATTATTTTTGAATTTCAGTGTAATATAC
AATGTAAATACTATGTAATGTAATTATGCAATATTCTTATATCGTTAAAAAAATTTAGTACT
TTTAATTGTGCAAATTGAAATTTAATTATTATTTTTTATTATTTTATATTTAATTTATTATTTA
ATAGAAATGGACGAATTTCAGAATATTCTACATTGCATGGTAATTACAAAATATTTAATTT
AAAATCATAGATAAATTATTGTATTATATTACATTCAATTTACTTTACTTATGAAATTCATA
GAAGCAAATCAAAAAATATATTATAAGATTTTGATTTAAAAAGCTTGAATGTGTTTTCGT
ATGCATAATTGCTTTTTAAATTATTTTCACATGTACAAGAAAATTTGAAATATTAATGATA
CTTGAATCAATTTGAATTTCAGATTATTAAATATGAATTAATCACTTCACAAATTTTTATT
TTTCTTTCATTTATACTCAATATTTCTTATTATGCAGAGATAAAAATAATAGTCGATCTGA
ATATTATATATTAATAATACGAATTAATATTATAATTACACATTAATATCAAATATCTATAAA
AATATACATATATTATAATTATTTTTCTTATATATACTTCGAATGAATATTACAAATTTACTA
ATTTGTTTTATTTCTTATATTTTTATTTTTGATATGTCAACAAAAGAATTGCGACCAGGAC
AAAGCAATATTATCTTAGCAATAGAAAAAATTGTTGACTTAGACTTTCGTATTTTTTTATT
TGGTACACACAAATAAAAGATTCGATTTCGGATATTATTATCTCATTTTTTCTTTCTTTAT
TTAGTCTCTTTTTCTAACATCCACGATTAGTATTTAATTGTTAGTAATTGTATTTAATAACC
ATATTACCATAGAAAATATATATATTATATATATTAAATATTTTGTATTTTTTGTATATTTAAT
TTATTAAATTTATAAAATTTTGAATAATTATATTTTTTATAATTTGCAATATAATAATGATAA
TGATATGTTTTTAGAATTAAGAAAAATAGAATATAAGAATTGAAATATAATATTAATAGGA
ATAAAAATTATTTATATGAAAAGGTGTAAACAAATAATATTCGATTATGTGCAAGAGCTT
TGATTATTGAGCTTTTTAATTATAAATCGATAATGACTTTATATGTAAGTTTTATTGTTTGG
ATCTTATATATATATATTGTGAATTAATTTATTATTAATTGTGTATAATTAATAAAAATTAGT
TTAATTTTTTGTGTATGTCAGAATAGAAATTCTAAATAATCAAGTCATCCCATTGAAAAT
GCGAAGTGAACGTAATAATAAAGTATTCCAGTATATAATTTTAGTAATATATAAATAATAT
CCAAAAATGTTATCTAATGTTTGTTAGAAGTCCTAAAATAAAATATAATGTAATATAATGT
AATGTTATATATTGTGAAAAAAATATCGTTGAAATGAAAAAACAAATAGATGAAGATAT
GGTAAAACATTCATCCTTTGAATTTGACAGCTAATTTCAATAATTGAAAAAAAAATTTTT
TTTCTATGTATTGATTTTATGTATTGATATTTATTGTTATTTGTAAATAGAGATTCAATGTA
AATATATACTTTGACCGCCGGATATGACAATTAGATAATTTGTGAATACATCGTGCTCCAA
TCGTCAGACACGATTATCATTACTTGTAAAGAATTTTGAACTCTTATTATAATCTGATCGC
CAGATATGACATCATAGATAGTTTGTTCTAACATCACATCGTACTCTGATCATTGGACAT
AATTACCAACTTTAAAATAGTGAAATACCCAACTCTTTTATAGACACGCGTCCTGCACG
TGTAATACAATAATACAGTCTACTTATCTTTAAGAAATATAGCACTTATTACGATATCTCT
CAGAGAAAATTTATAAATACCCAGCTATTAGCTTCCATTCATCAAAATTATTACAATACTT
CTGTTGAAGGCGCAAACAGATTTGTTTCTATAAACTACGAAAAATAAGAATTCATTTATA
TTTAAATAAAATATTATATAATGTTGAATTATTATGAAATTTGTTGTAATTATTCAAAGTAT
GTTTTTATATAATATATATAATTTTTGTTTAAGTTTTTGTGTTTATAATCGTAAAACTGGAT
AAATAATCGAATATTGATAGATGCACGCTACTTTGGACAAAATTAGATAAGAAAAAAAA
ATGAAGTATTTTTCTATTTTGATAATAAACAATAAACTATATTGGATGGATATTAATATATA
ACTATACAAAACCGTTATAATGATTTACAATATCTAATCGTGTTCAATGGCTAAAGTATGA
TGAAATTTTAATAAACTCCAAATTCATACTAAAATGCAAAAAGTTAATATCTATGGAAAA
CCTTATTATATATAGATAATTATCTGTAATATATTTATATTATCTATAATATATTTTAGAAGAG

ATACATCGTAGCCAATAATAATATGAATTAATATTGAATCTGAAAGCTGACTTCAAATGA
TCCCTAGATGATCAAGAAAGAATTCCTTGGAATATATGTTACATAAAAGTACATAATGCT
TAGAGTTTGATTCACATATTTGCTATAAATCTTGAAGAATAGGTTGATGTTTGTTGCCAC
AGAAGCTGATTGAAGTATTATATATTATTCTCAGAATTACATTGCACTCTAATCAGACTA
AAATATTTTTAAATATTTAATTCTAACATTTTATGACATTAAATATTATTCTCACAAATGTT
TTTCTAACATCTTGAATTCTTCATTTTAGATTTGCGTAATATTCGATAATGATAAGAAAAA
ATTTTCATGAAATATCTTTCCGAAATAAAGAAATTATAAATCTTGCGGATTCATTATTCGC
GAACACTTTCAATTTTGATTTTAAACATTTAATATTTCTAAAAAAATTTTAATATTTTATTT
AATAAAAAAATTTTTTAATTTCAAATTCAAAATTATAAATGTGAAATGAAATGTTTACAAG
CAATGATCGCGAGATTTATTTATAGTATATTTATATATTATATATTATAGTCTATATTTATATAT
ATTATAATATATATATTATATTATTATATTATTATATTATTATATTATTATATTATATATATATATAT
ATATAATCTATTTGAAGCACTTTTATCTGATTGAAATATAATCTGAATAATGTTCCGTTGA
ATAATTATTCAACAACTTAGCCGACAACAATATACGCTTGAAATTTATAGTACGAGCTTC
GAAAACTGTTTTTAGGTTTGGAGGAAGCTGATCTGTGCGTTTATTAATTATGATATATTTT
ACGGATTTCCCAAATTCAGCAGAATTGGGAAAGCACTAATATTTTGCATTTTGGAATGG
ATTATGAATTTGAAGTTTATTAAAGATTTCATCATATTCTAATCTCTACAATTATACAATTA
TATTATCATCATTATGAGATACTACCTTGTACAATCATGCATATCAATATGATATATCAATA
CAGTCTATTGGAAAATATTCTTATTTTTTCATCTATCAATCAGAGATTTCTTTCCACATATT
GATATCCTATTATTTGTACCTATTTATGATTATTATCTATTTATGATTACACAAAGACTTAAA
TAAAAATATTTAATGCTAATGTACAACAATAGAAGAAAAATTTTTAAAAATTCAATTCAA
TTCTATTAGATTATAGTTAATCTGTGTTGATGATCCTATAGCGAAGTAAATGATAGATATT
GAATTTGATTTGAAAATATATATTCATATATTATTTTCAATGACGTTTTATCCATAATCAGA
TAATTAATATTATGTACAAATTTATCAAAAATTATTTCAATTTCATTATGCTAAGGAAAAA
TATCAAAATATCATCAATTTTAAGAAAGAATATTCAGAAGATGATTCACTGGCATATTTC
AAAGAATTGAATTTTTAATAAATGGTAAATAATTAATCTTATCTACTTATTTGGCGAATTA
TTTTTTAATTTAATATAGATAGTAATTTATTATTTATTTTTTTATTTTAAATATATATTTATTT
GTTAAAAAATTTTATATTCGTCTTTTTATATCATAATTCAAGATTATTCATTTTAAATAAAT
TCGATTATTTATCCCATACGAATAACAATTAATTAAATATGTTATGAATAAGATGTAATTTA
ATATAATATAATTTAATATAATTTAATATAATTTTTTTTTAAAAATATACTTTTTTCAGTGTT
ATTTGTATTTGTTTATATACAGAATATATTCAATGGAGTTTTTTATGAGAAAATTTAATCAT
TTGCTTTACACATTTAGAAACAATTTGGCACAAATTAGTTGGAATGTCAATATAATCTTG
TTGTCACCTGAGACGACATTTATAGTCATACCAGTTTCAACGGCAATTACCGTCATTTTA
CTTGAGGGAAAATACTAACATGTATGGAACAGCGTTTTTTGAAAAAATAATGGACTTGT
CAGTGGTTCGGACTCATTCTAGGAAGCTATCTTTGGTCCGAGGTGCCGTTTAGGGGATT
GGTCCTTATTGGATACTTGTACACTGAAGATTCGCAAACTCACGAACAACATAGCAAA
GAATACCAGTTTGAAGAGAACTCTTGAAATCTTGCGAGAACTTCAAGAAGAACATTAC
AATATAAGAATTTCAGAACTACAAGAAAATCAAAATTATAAAAACATCAGAACTCTTTA
AACTTTCGAACTTTAATGAAAGTGAAATATCATTGGCATATAGACTTTGTCTTCTTCTTG
TTTAATGAATTGAATTTATCAACAGCGGACTTTGTATAATTAATAATTAATTTTATTACTAT
ATTTCATAGCTCTAGGAACGAATTGGAGCTAACATTTTGTAGTCAGATAAAAATAAATAT
ATGAATTTATAACTTGTGAAGTGTAAAATTTGTGCCATTCAAATCTCGTCGTGCTTGTAG
TGCAACACAAGAATTTTAAGAACAACTTTGAAACTACTAATCACAATCAAGTATATCAT
CAAACACCTGCCAGATTTTATCTGCAAGGAAGCTTGTCTTCTGCCAAACTCCAGATCA
ACGAATTCTGGAATGTCTTCACGCAACATACAAGGGATATACCCCAGCTGGATGATATC

GTCTTGATCATTTTAATCCATTTTTATATAAGTCTCCTCTACTAAGATAATATAGAAGTTG
CATTGAAATCTTTCATTAGAGAACATTCAAAGGATCATAGAAGAACAGATAGAAGATAC
AGAATTTGAAGCAGCTCATGCCGCAGTATGAAATAAATTTGAAAACTCGTATTGCGAGA
TCGATACTATCAAGTACCAAACACTTAATTCTGTACTTACTTTATCTATAATCTCAAACAA
TAGTTTTGACATATATTCTTTTATTTTATTCTTTTATATTCTTTATTCTATTATATTCTATAAT
TTATTCTAGAATAAAATTACCAACAATAGAATTATTGACTTTCGGAAATTGTAATCTTTG
GATAAAGTTATAGAGACATGTTCGAATCATTGATACACAATAACGACGTATTAATAAATA
TTGAAAAATTTCACTATTTGAAGTCATCTTTCAAGAGCGCTGCAGCGAGAGTTAAACA
GTCTCTCGGTATCTCTGGAG

>novel_circ_001354
AAAAAAAATTTGATGATGGTTAAAAGAATTGCTTAACGAAAATCCGATAGACGCAAAA
AGAATTAGCAAAAAGATTGGGAGTCACCAGACAAGCAGTATCCATCCGACTGAAATAA
TTGGTAAGATAAAAAGGGTGATGATCCATATGAA

>novel_circ_001356
GAATACAAAGAGTTCAGAGACGCGGAAAATATAATGCCACTGGCGACTAGCGACTTTC
GTAACCCTATCTGCCAGGAAATGAAGTCGATGCGATCCGGAGTCATGTCAACAGAACC
ATCTATGCATCAGCAGATTTTCGCAACAGAGATGAAGATATCCACGCTGCAACCAATGG
ATAACTCAAGTTATGGAAAAATATACAATGAGCAAGGGAGCATAGACAGCTCCGATAC
GTATGCTAGCTGTCAAACTCACCCATCCCATTCGCAGGTTTCTTCTGCTGGTCATTCGG
CGGATAATAATAAGGGTGATTTGACTGAAGAAGCAGACAGTAATCTCTATGTGAATCCC
CTGGAAGCTGCAGAAAAAATGTGGAAATAGAGTGAAGAAATCTGCTTCCGGTGAAATTG
GACGTAATGTCGACATGTCACCGAGTGTTGAATCGTTAAAGGATCTTCGTCCCTTTAAT
GAGGGTAGCAAGATTACCTTAAATGATATAGTGCCCAAGCACAGAAAAATTCGAATTCA
GGAG

>novel_circ_001357
GAATACAAAGAGTTCAGAGACGCGGAAAATATAATGCCACTGGCGACTAGCGACTTTC
GTAACCCTATCTGCCAGGAAATGAAGTCGATGCGATCCGGAGTCATGTCAACAGAACC
ATCTATGCATCAGCAGATTTTCGCAACAGAGATGAAGATATCCACGCTGCAACCAATGG
ATAACTCAAGTTATGGAAAAATATACAATGAGCAAGGGAGCATAGACAGCTCCGATAC
GTATGCTAGCTGTCAAACTCACCCATCCCATTCGCAGGTTTCTTCTGCTGGTCATTCGG
CGGATAATAATAAGGGTGATTTGACTGAAGAAGCAGACAGTAATCTCTATGTGAATCCC
CTGGAAGCTGCAGAAAAAATGTGGAAATAGAGTGAAGAAATCTGCTTCCGGTGAAATTG
GACGTAATGTCGACATGTCACCGAGTGTTGAATCGTTAAAGGATCTTCGTCCCTTTAAT
GAGGGTAGCAAGATTACCTTAAATGATATAGTGCCCAAGCACAGAAAAATTCGAATTCA
GGAGTTTCACAAATCATCAAGATCGCAAACGGAGCACAGTTGAAAAATTGCTGAGGG
CGATCATACATACTGAATGCCCTGATATTTATAGCGTAAGACCTCGTGCGCAATTTATCA
CCGATCAGGATAGCATCGTGACTCGTAATATCACAAAAAAGGAGACCACAGAGAACAA
TTATTATGGAGGATTACGAGGTGGCAAGCCATTTACGAGCAATAACAGTCTAGCCTCGG
CAACAAGAATAATAAATCATCATTTATTTGGCTCTAACATTGGACCTAGACATTACACGG

>novel_circ_001358ATACAAACTGGAAGTGGCCGGCAAGTCTCTACCCGTGTTGACAAA

CCTGGACAAAGGTCGATACGGTGTCTTAATCTTTGAGAATCTGAACAAGTACCTGCAG
ATGGACAAGTGGAATCGCGAATTGCTAGATAAATATTGCAGAGAGTATTCGGTCGGCAT
CGTGGGATTCGCGCCGTCCGGCGAGGAAAGTCTCGTCGGTGCGCAGCTGAAGGGTTT
TCCTTTGTTCGTGCATACTAATCTCAGGCTGAAGGATGCACAACTGAACGCGGCGTCTC
CTATCCTCCGATTAACCAGATCCGGAGAAACGGCCTGGGGACCACTTCCTGGCGGTGA
CTGGACCATTTTTCAGGCCAATCACAGCACCTACGAGCCTCTGGCATGGGCACATCGG
GACAGCCTCGACTATCCGGCCAATAAAAGTCCTCTGGCGACTGTCATTCAG

>novel_circ_001359
ACTCGTGAAACAAGGGGAACCAACGGCGCTGCACGAGATCGATAAAGGCGATAGGGT
AACGTTCGAAAATCGTTCCGGCCGGGAAGCATCGTAGCTTTCCATCGTCCCTAATTGAC
AGGTAGAAAGAGGAGGAGGAGGAGGAGCAGCCTCTCTCGCGGAGGAGAGAGAAGAA
AAGAAAAGGCGACGCGCACGACACACGTTAACATCGTGGAGAAAAGCTAGACGAGG
ACGAAAGAAAGAAGAAGAAGAAGAGGAGGAGGAGGAGGAGGAGGAAGGGACACG
AAATTTACGAGTGAACCGGGTTTACTTGGTTGTCTAGGAGGCAAACACACCGGGTATAT
CTTGAGCCAGTCGTGTGCCGTAACAAATGACGGCGCCGTAAAATCAGCTAACCGTGGA
GCCGGAAGTGGAGGCCGGATTTTCGCGAAAGTTTCAACGGCGCCGGATAAAAAAAAA
TTTGAGTTGACTTTGGACTTTGGAGTTTGCAAAAAAAAATTCGCAACATCCACGTTGAA
CTGAAAATTCTTCGTCCTTGAAAATTCTTTCTTTCCTTTCACGATACAGTCGCGATATGG
TCTCGAGCAAACAGTACCGGAAAAGATATTCGTCCACATTTATCGATTAAAGGAGAGA
ATGTCGAGGATCCGTCGTGGCACCGATCGCTGAGAAAAGAAGGAGGTGAGATCCATC
GAGGGGGGAAGAGACGTTGGGCCAAATGGGTTAAAAATTCGGATCGCGATGGACGGC
GCGACCCCGTGCTGCTCGCAAAATGGTGATCGTGCTGCCGTCGTGGAAACGCAACAA
GTGGCGATCTTAATTCCAAGTTGTCATCGATGTTTTCGAAAGGATTCGACGATCCACGA
CCCCCCGTTTTCGATCTGAGCCTCTTCGAGTTGACCTGAGCCAACAACCCCCTAACTAG
AGAGACATAAGAAGAGAAATACCGAATTCAAGGATTTTTATTCTTTAGTCATTCGTAGC
AAAAATTCCGTTGTTTTTATCGAGACGCTGGGATTAATTCTCTTTCTCTCTTCTCTCTTT
CTCCCTGCGATAGAAAAATCCCAAGTATCAAAGGAAATTTTCCAATTAATTCCTGTGTG
CTTTCGAGAAAGAATACTCGATTCTGAGATTTCGAGCGGAGAGGGGAGATCGAGTCCT
CCTTAGTTAGATTGGCTCGCTGTTAATCTTCGACGAGAAGGTTTTTCGATCGAGGTTGA
AGAGGAATCTCGAAGGCAGATTCGATATCCTCTCGATATTGTTCAAGGGAAAGAGAGT
GGACGAGTATGAGCAGCGTCGATGCCCGGCGAAGCAAGAGAGGGTATCCTTGTTCCG
GAAGTGAGGGACGATTGTCGCCTCCGGGCGCTCCGACTCTATACGAGGTGATTCGAGG
TACGTTTCGCGTCTCGACGGCGGGGCAGGGAGGAACAAACCGGAGGATAATCTGAGA
GACAACAGGACGGTGCCTCGTCGAATGAGACGATGGTGATGAAGATTTCCGCGGTCG
AGGCTGGCGTTCCTTTACTAAGCGCCGTCGGTCCGCGCTGCGTCGTCAAGCAGGCCAC
GGTGAAACGATGCGTCGCGGCGCTGCTTCTCGTCTCCGTCGCCAGTATATTTTATTACA
CGCATTATATCATCAGCAGCCCGCTGTCCAG

>novel_circ_001360
GAATTTGCTCCGGTTTGCGAGAAGTCCGCATCCAGATACCGACGGCCGTGAAGAAAGG
TGACTCTGCGATACTGAACTGCTGGTATGACACCGAGGGTGATCTTTTATACGCCGTCA
AGTGGTACAAAGGGGGTCGAGAGTTTTATCGCTATGCCCCGAACGAGATCCCGATCTT
CAAGACTTTCCCCATCGGGAACTTGACGGTGAAGAAAAGCGAAAGCAACGCGACCCA

AGTCGCCCTGACCAACCTGGAGTTGGACGCTGCGGGGGTGTACAGTTGCGAAGTCTC
CGCTGATGCTCCCTCCTTCCAGACGGCCTGTGTCCAAGACACCATGAACATCGTAG

>novel_circ_001361
TCTAAGAATATTCTAATCTCTAACCGTTGATGTTCCGTCTCCTTTTGGATTTTCTTCGGTG
TTAGGGGTTCCATTTTATGGGCATGGAAGTTCTCCTGATTATTTTTCCCTTATTCGTGTTT
TTGTTTTTTCCCCATTTTAGAGAGTAGGGCATCTCGAGAGGGTTCTTGAAAATTATTTTT
TTTTTTATTTTCTTTATTTCTTATTTCTTTTATGTGTTTTTCGTTTTTTTTGTATTTTTGTATT
TTTCCACAAAGGGCAGTGGTTTTACCACGAGACAATATTTTTGGTGTTCTCACACCTTT
CACACACAATTCTTCGATCTTTAATTCGTCGTCATTTTCGTACGTAATTTATGGTCTTACG
TATGGACACTACTACTAAAACTTTAATACTCCTCCTTTTCTGTTCTTCGTAATTCATCATA
CTCCAAACACTTCTTCTGTAATCTGTAGTAATCGTGGGTTTATTTTTCGTCGACGCCGTT
TTATTGACCTTAATCGTGTTGTGGAAAGAGGTCCTTCCATCCGTGGATAAGTTTCCAGC
TGCTGGACTTGAAGGAAAAACCAAAGGAGACCAACGAGGATTCGAACCCTAGAACTT
CTT

>novel_circ_001362
ACGAAGAACAGAAAAGGAGGAGTATTAAAGTTTTAGTAGTAGTGTCCATACGTAAGAC
CATAAATTACGTACGAAAATGACGACGAATTAAAGATCGAAGAATTGTGTGTGAAAGG
TGTGAGAACACCAAAAATATTGTCTCGTGGTAAAACCACTGCCCTTTGTGGAAAAATA
CAAAAATACAAAAAAAA

>novel_circ_001363
TACGAAGAACAGAAAAGGAGGAGTATTAAAGTTTTAGTAGTAGTGTCCATACGTAAGA
CCATAAATTACGTACGAAAATGACGACGAATTAAAGATCGAAGAATTGTGTGTGAAAG
GTGTGAGAACACCAAAAATATTGTCTCGTGGTAAAACCACTGCCCTTTGTGGAAAAAT
ACAAAAATACAAAAAAAA

>novel_circ_001364
ACGAAAAATAAACCCACGATTACTACAGATTACAGAAGAAGTGTTTGGAGTATGATGA
ATTACGAAGAACAGAAAAGGAGGAGTATTAAAGTTTTAGTAGTAGTGTCCATACGTAA
GACCATAAATTACGTACGAAAATGACGACGAATTAAAGATCGAAGAATTGTGTGTGAA
AGGTGTGAGAACACCAAAAATATTGTCTCGTGGTAAAACCACTGCCCTTTGTGGAAAA
ATACAAAAATACAAAAAAAA

>novel_circ_001366
TGAACGCGAGTAGCTACATAGTGTCAGTGGTGCTCGTCCTGATAGTCGGAGCACTGGT
GGGTGTCCTTGCGACGGTATCGCATCATCTTCATCATCGTAGGCTGCTACTGCACGAGC
CTGCTTCCGCCTCGATCTTGCACCACCATCGGCATCATCATCACCGCCATCATCATCATC
ATCATCATCGCGAGGAACTTCCGGCGTCGCACCAACGACGCCTCCTGCTGCAGGACCA
CCACCGTCATCCTGGGACGCCG

>novel_circ_001368
TAGCCTGTGGAAATGCAAACGGTGTCAATTTCATGTTAGTGTGGCGCGTCAGAAAAAG

AAAAAAAGCAAAGGTGATGGTCCGAAGATAATAATACCTGGCAACTGTGCTGAATCGT
CTAAACCAATTCTGGAGCCTATGTGTAAAAATGCTATAGTGCTTGGAGGATCTGATGGC
TTTGGTTTTGCAGCGGCTGATCATCTCTTGCTCAAAGGAGCACGTGTTGGTGTTTTCTA
CTGTCGTAATAGCTGATCATGATCCTGAAGAAGGGAGAATAGCAGTCGAAAGACTGTG
CAGTTTTCATGGAAAAAATCGCTCATACTACGCTCATTATAATATAATGAGTGATTGTCAT
AGTCACAGTAGTTTACACGATGCTCTTTGCAAACTCAAAGTGATTCATATTATTTTTAAC
AATGTTGATAAAGAAAAGACATCGAATCCGAAGATCGAAGAAAACACTATACACAAAA
CGATTCGAATAGGCTTGGAACTTTTAGGGAAGAATAATGGTGGTTCTGGCGGAATAATA
ATAAACTGTGCGAGTATTTTCGGGTTCTTGGGATGGCCTCAAGATCCATTCCCAATTTAT
TGCAACAAAGAACCTGCTATAGAAGTCACCCGACATTTCGCGAAAAAGTATAATGTGG
AAACAACTGGAGTTCGTTTAGTGGCACTTTGTCCAACGAATAAACACCTTCGCGACAT
AGGATTGCCAATTTTTCCAGACCCTCTTCCAAATAAAACTATGTGTGAATTGCCAACTT
GTATTCCGCATTCGAAGTATCACATAGGAACAGCTGTAAGTCATATATTAGCGTGGGCA
AAAAACGGAAGTGCGTGGTTGGTCGAACCTGCAATCAGCGTCAATCAAATTCCTCAAT
TACTTCATTTTCCGGAGAAGGAAGGCGAACAAGTTGACCCCAAAGTCTATGAAACACA
A

>novel_circ_001369
ACTCGTTGGAGGCGCCAGGGACGAGCAAGCGGTTTCAGATCCTCTACAGAAACGAGG
AGGTGACACTCGGCACGTCGGTCCTATTCCGTGCTCACGTGCTCGTGCACAGTCACAA
GATCGAGGAGGTGCTTTCCCGAACCCACTTCAATCTCGGCGTCGAGCTATGGTTCAGC
GAGCCAACGCAGCCTGGGAACATGGCCTGCGTGTCCTCTAGGTGAGGCAACGTCTTG
GCCCCCATAGAAACGCGGCGATCGGCCGAGCGTGAGGCTTGGGAAACAGATTGCGGT
TTCTGGAAGGGAGATTACTACAAGCGTTATGGAAGAACCGGCGTTGCAGCTGAACTTC
GCGCCCACGAAGGGACTTCACTATCATTTGCCGGTGCTCTTCGATTACTTTCATCTTGC
CGCAGTCTCCATCACGATCCATGCCTGCCTCGTGGCCCTTCATCAACCCTATATCAAGA
AGAGCATACTTCATTATGTGCAGAGCTGCGCGCCGCGTGGAGGGAAGCCGTGGCTGCA
ATTTAAACAGTCTGCAGCGAACGGAGACAACAATGCCCAGTTAGGAAATATCGAAACG
ACGACGAGATGCGTCGGCTCGGCCACGAGAATCCAACACGCGAAACTGGTTCAGCAG
GAAGTAATCAGGTTACTTTTGGCCGCAAGGGAATCTTTGCTCAACGACTTGGCCGATTT
AGCGCGGTTGCTACCTTCTTGCCAGCAAAGGGCGCTCGAGCTCGCTCAGAACACGCA
CAAAGAAATTACCAAGTTGCAGATGATGGACACGGAGGAAACCGATATCGCTCAACTC
TGCGCACAAAATATCGTTCTCTGGCAACACTTTCTGGAGGTGTTCTCGGGGCGTGAAG
CTGTTCATCAACACCTTGCGAGGATCCATCATCAATTGAGGGTGAAACGTTTCGCGGA
AGGTTTCTTCGTGTTGGAGAATCCCAGGACGTCTGCGTGGGGTTGTTACGACGCGAAT
TATCAGTCGTATCAGGCAGTGAGCGAGGCTGCACGAAGATCGCGATATCTCGCCTCTTT
GCCTCCATTGCCTGTGCATTGTCCGGAATTGGACGGAGATCTTCATTCCCTGCCTTTGAT
CTTCGAGGATCAGTACGTGGATATGAAACAGAGGCACAGAAACAGCG

>novel_circ_001370
GTTGCCTCGGACGTTGAAACGTGGGGACATGATGGGAGGAAGGAGCCGGGAACCTCG
GCCAGACCCTGAACGACAGTGTTCATCCCTCGTGAATCGTCGCGACGTGCGGAGCTGG
TCTTCGTTCGATTAAAGGGGAGGATTGGAAGGACGAAGGGTGGAGTGGGTTGGCGTC
GCGAGCGGCGTGATCGAGATGAGCGATCTACAGGCCACACTCGAGTTCTCCCTCGAGC

TTTGCAAGTTCTACAATGTCGACCTGTTCCAGCGTGGGTATTACCAGATACGAACCGCC
CTCAGAGTGTCGCCGAAACTGCCTGTCAAGGTGGAGGTTAACCAGCTGCGGAACC

>novel_circ_001371
CAACATACCAGGCCATTCAAAGCCGCCGATGTACACCGAATTGCACCAGGATAACAGT
TTGTCTTTGTTCTTCAACTCGCTGCAAGAGCAGCAGGCTGGGAAATACACGTGCAAAG
CCACTTACGCGAATTCGATTCAATTGCAGAAGTCTGTGACGATCGATACTATAGTTGCG
ATCACTTGGGACAATGCACCGCTCAATCAATACCCCATTCTGGGCGAGGACTTCGCTAT
TCAGTGCAAAGTGCGGGCAAGGCCTTCGCCTTCGGTCGACTGGCTGTACAACGGCGA
ATTAATTAAGACGAACGACCATTACATTATAGACACGTATGCACTGAAGATAAAAAATG
TTCAAGAGTCGGATGATGGCGTTTACACTTGCCGCGCTTCCGTATTTTCCACCGGTGAG
CTCAAGGAACGACCTATCCGCGTCGAGGTCCACATACGGCCCACGGTGAGCGAGATGC
CCAACCCCATAAACATGATCGAGGGAAAGGACGCGAGTATAAAGTGCGAGGCCCGAG
GCAAACCCCCCCCAAAATTCACCTGGGTTAAGTCGTTGACCCACCAGAATCTGTCCAA
CGTGGATCGTTTCTCGGTCGATCCGGACACGGGCGTGCTCACGATCACCAATGTGAAC
AGGGACGACGCAGGGGAGTACCAGTGCACGGCCACGAATCTGGCTGGGACGGCGAA
CACCAACATCATGGTGAACGTGATCGTCAAGCCCAAGATCATCGAGTTCTTGAACAGC
ACGGTGGTCGAGGACAAAGAGGCGACGCTGGTGTGCAAGGCGTTCGGCAGGCCGCC
CCCCCAAGTCACTTTCAGGAAGCTAACCGCGGAGAAGGCGTACGTAAAGGGTCCCCA
ATTGGACGACGACAGGATCATACTCGAGAACAGTGGCGATGACAGCGAGACCATCGG
CACGCTGACCATCAGCCAGTCCCTCAGATCGAACGACGGTTTGTACGAGTGTATCGCG
CAGAACGCCGGTGGTGACGCGCGTAGAATCGGCCATCTAACCGTCGAGTTTCCACCAT
CGTTCGCCTCCATGTCGAACACAAGCGTGTGGTCGTGGGACCAGAAACCCGTGAACAT
CAGCTGCATAGCCGAGAGCATACCGAACGCCACCATACGTTGGACCATGTACGGCGAT
CAGAAGATCGACAATGACAAGATGATCACGCAAATCGGTAACGGGCCACGATCTATAC
TCACCATCGTCCCCTTGGACAAGCGGTATTACACCACTTACAAGTGCATCGCGTCCAAC
GCGCACGGGACGCGGCAACGGAACATCGAGTTGAGGGAGGCCACGAAGCCGGGCGA
GATGTTGCAGGCGAAGATGGCCGAGATCACCGCGACCACGATTAGGTTCGATCTTGTA
CCGCCTAGTACGCATCCCGAGCAACCTCTCAGAACGATTAACGTGCAGTATAAGGAGG
ACGGGCAGACGTGGATTCAGGCTAGGAACAAGACGTGGTCTGTCGATTCGTACTACGT
GCTCGAAGGTCTGAAACCGCAGACTATGTACGAGTTCCGGTTCGCGGCCAGAAACGAT
GTCGGTCTTGGTAATTGGGGGGCGTTACACCGCGAGATGACGCCCGGAAGGACGGTTC
CTAACGAGCCGAAGATAGTAATGTCGAGGGAGGAGTACGATATGTCCAGGTACAGCAA
TCAATACGAGTTGACCTGGCTGGCGCCGGCTGATAACGGCGAGCCGATCGACATGTAC
CAGATCAAATATTGCCAAATAAGACGCGTTTCCGGCGAATGGGAGACGTTGGAGGATA
CCTGCCGGACGGAGGACATTAGGACGCAGGGAAGAGTGAGGCATTGGTTGAGAAATC
TCTACTCGGACACGTTCTACAAGGTCGAGTTGAAGGCGCACAACGCGATAGGTTTCAG
TAAGCCTGGATCAGCGAAGTTTAAGACGGCCAGGG

>novel_circ_001372
TTTACGCTGCCGAGCCTTCCTTGGAGATTCTTCCGAGTGGAGAGACACAGACGAAGGC
AATCGGGTCCAGCATCATTCTGACTTGTAATCCAAAGGTCGAAGACACAAAACTGATC
TCTGATATGCAATGGATCGATCCGCAAGATCGTGTGATCGAGCCTTTGAAACTCGTCGT
TTTGCCGATCGAAAGCAACATACCAGGCCATTCAAAGCCGCCGATGTACACCGAATTG

CACCAGGATAACAGTTTGTCTTTGTTCTTCAACTCGCTGCAAGAGCAGCAGGCTGGGA
AATACACGTGCAAAGCCACTTACGCGAATTCGATTCAATTGCAGAAGTCTGTGACGAT
CGATACTATAGTTGCGATCACTTGGGACAATGCACCGCTCAATCAATACCCCATTCTGG
GCGAGGACTTCGCTATTCAGTGCAAAGTGCGGGCAAGGCCTTCGCCTTCGGTCGACTG
GCTGTACAACGGCGAATTAATTAAGACGAACGACCATTACATTATAGACACGTATGCAC
TGAAGATAAAAAATGTTCAAGAGTCGGATGATGGCGTTTACACTTGCCGCGCTTCCGTA
TTTTCCACCGGTGAGCTCAAGGAACGACCTATCCGCGTCGAGGTCCACATACGGCCCA
CGGTGAGCGAGATGCCCAACCCCATAAACATGATCGAGGGAAAGGACGCGAGTATAA
AGTGCGAGGCCCGAGGCAAACCCCCCCCAAAATTCACCTGGGTTAAGTCGTTGACCC
ACCAGAATCTGTCCAACGTGGATCGTTTCTCGGTCGATCCGGACACGGGCGTGCTCAC
GATCACCAATGTGAACAGGGACGACGCAGGGGAGTACCAGTGCACGGCCACGAATCT
GGCTGGGACGGCGAACACCAACATCATGGTGAACGTGATCGTCAAGCCCAAGATCATC
GAGTTCTTGAACAGCACGGTGGTCGAGGACAAAGAGGCGACGCTGGTGTGCAAGGC
GTTCGGCAGGCCGCCCCCCCAAGTCACTTTCAGGAAGCTAACCGCGGAGAAGGCGTA
CGTAAAGGGTCCCCAATTGGACGACGACAGGATCATACTCGAGAACAGTGGCGATGAC
AGCGAGACCATCGGCACGCTGACCATCAGCCAGTCCCTCAGATCGAACGACGGTTTGT
ACGAGTGTATCGCGCAGAACGCCGGTGGTGACGCGCGTAGAATCGGCCATCTAACCGT
CGAGTTTCCACCATCGTTCGCCTCCATGTCGAACACAAGCGTGTGGTCGTGGGACCAG
AAACCCGTGAACATCAGCTGCATAGCCGAGAGCATACCGAACGCCACCATACGTTGGA
CCATGTACGGCGATCAGAAGATCGACAATGACAAGATGATCACGCAAATCGGTAACGG
GCCACGATCTATACTCACCATCGTCCCCTTGGACAAGCGGTATTACACCACTTACAAGT
GCATCGCGTCCAACGCGCACGGGACGCGGCAACGGAACATCGAGTTGAGGGAGGCCA
CGAAGCCGGGCGAGATGTTGCAGGCGAAGATGGCCGAGATCACCGCGACCACGATTA
GGTTCGATCTTGTACCGCCTAGTACGCATCCCGAGCAACCTCTCAGAACGATTAACGTG
CAGTATAAGGAGGACGGGCAGACGTGGATTCAGGCTAGGAACAAGACGTGGTCTGTC
GATTCGTACTACGTGCTCGAAGGTCTGAAACCGCAGACTATGTACGAGTTCCGGTTCG
CGGCCAGAAACGATGTCGGTCTTGGTAATTGGGGGGCGTTACACCGCGAGATGACGCC
CGGAAGGACGGTTCCTAACGAGCCGAAGATAGTAATGTCGAGGGAGGAGTACGATATG
TCCAGGTACAGCAATCAATACGAGTTGACCTGGCTGGCGCCGGCTGATAACGGCGAGC
CGATCGACATGTACCAGATCAAATATTGCCAAATAAGACGCGTTTCCGGCGAATGGGAG
ACGTTGGAGGATACCTGCCGGACGGAGGACATTAGGACGCAGGGAAGAGTGAGGCAT
TGGTTGAGAAATCTCTACTCGGACACGTTCTACAAGGTCGAGTTGAAGGCGCACAACG
CGATAGGTTTCAGTAAGCCTGGATCAGCGAAGTTTAAGACGGCCAGGG

>novel_circ_001374
CGGAAACCTCGCAAGGCACCACATCCCCAGACACCGTCCAATGCGGGGCGTGCAGGG
TGTCCTACCCCCTTGGCGAGATCGTTCGGTTCATCGAGCACAAGGTGAACCACTGTCG
GATCAATCTTCAAGGTTGCCACAGTCCACCAGCAACCGCTGCGCCGGAAGACTCCGAT
CCGGAGGACGCGCTCGCCCTGAAGACCGTCGACCAAGACTCGAAGCTGAACTCGGTG
CCCAGCATCTCGGCGCCCATTAACAAGAGGGGTGGCGGCAGATTGGAGAGCCCTCCG
ACGCCTCAGGGCTCCGGCCCGGAAGCTGGAAGCCCGATCGAGCTGAAGGCGAGCGCG
AGCTCGACACCTAAGAGGCGAACGGAAGCCTCGAACGAGGACAAGGAAGATCTGCC
GAAGAAACAGAAGACGGAGTCTGTCGACGCGGACACCAACACGGTCAATTTTG

>novel_circ_001375
AAGCATGACAGCGATCTGTTTCGTGTGTAATCTACCGATCCTGAGCCACCAGGTCGGCC
TGGTGTGGCAAGGTGGAAATGGATGGGACGATCTTATGCGGGAACAGGTCGAGGAATC
CACCTTGAAACAACGTCTTGGAACTGGAAGGCGCGATTCGGCGACACAGATCACTTC
GATATCCCCGCCAAACGAGCCGCAAGAACCTGCGACCAATCAACGTCGACGACGATCG
AGCTTGGCACAATTGACAGATATTTTGCGCGAATGGAGCGGGGGTGGCGGTGGAAAG
GGCGAGCGGGGGAACAAGCTGTATCGTCGCGAAACATTGGGAGATATAGCGAAATCTT
TTCCGTGGTCCAGGCAAACGACCACGGATGCGAGCCATCTTGAGAGGCTGCGCAAGA
GGAGGGAGAGCTCGGTGGACAGCGGGATCAGAAGCCAGTCGTCTACGAAATCGAGAA
AAGATTCAACGATCACCGAGCTGAAAAATGATTTTGCCAGATTATGGGGAAAGAAGGA
GACGCCTCAGCCGCAACCACCCCCTACGGTTATTTCGCCCACCTATCGAGGGTTCAGA
GAGAGCGATTCGAGAGGATCTCGACGGGGGTCAGGGGAGAGCGGAAGGGCGAAAAA
TCAAGGGGAACGGAAGCATAATTGCCGTCACCACAGGCGCAGGCAATCGCAGCAATC
GCAGCAATCAATGGACAGTGGAAGCGGCGTGATGCCCGTGAAATATTACAGAAGCGAT
CAAAGACCGAGCGCTTCGAGTACGGATAGCGCGGCCAGCAATGCTGTCAGAGAGCAT
TTGGAAGCGCGTAATTCCATGGACAAAGGCGAACGATCGAGTAGTATCGAGGGCAAAA
CATCCGTGACAACAAGTAGCGAATCAAAAACGTCCACCACGACTACCACCACAACTGT
GTCGTCGACGACTACCGCGGTGACCACGTCGACCACAGTGACGATGGAGCCGAAGGC
CGCGACCGTCGTCGACAGTAAAAATTTAACCCTCGAGTCGTCCATCACTACCCCTACCA
TCGTGATGTCATCAGTGACGCCACCAAGCGTCTCTCCAACGGTGGGTAGCAGCCTTCC
ACTTCCAGGAACCTCGACTTCCGGTAGTTCCAGTCCCGTTGGCTCGCCAAACGTCAAC
ACACCGACCCACCCATTGATTAGCACTAGAAGAGACTCGACCACCCAGTGTTATCAGA
AGGGAAAGGAAGTATCACCGAACAAGTTGTCGAGATTGATACGGCAATCGGCTGCCAT
AGATGAGTCGATGCCACCAGCACGACGACGAGGAAGTCAACCGACATTGTCACCGGA
TACTGACGAAGGAGGACGAAGAGCTCGAAAAGACTCGCTGAGCCCCGATTCCGCTTC
GTATCCGAGGCGCCGTGATTCGAAGAGCCATCTGAGTCCAGAGAGAGCAGTCGATAAA
AGAGACATCAGTCCTATCAGGGAGAGATCTCAGCTGACGCGACAGTCAATGTCGATGG
CAGGACGGTCGCCACCAAGATCACCGGATAGTTCTTCCTGTTCCTCAAGGGATCCAAG
TCCGTGTGCTCGTGCTCCAGGAAGTATACCTCATGCTCCTATTGGAAGAAGACAATCGA
CAACGGACGAGATATTTATTGCTCGTGGATTTAGGCGGCAGAGCACCACGGAAGAGAC
AATAAGATGTCGAAACTTTCGAAGACAAAGTTCGCAAAGTGAAAAACTGGTTCAAAG
ATATCAAGGACGAAGAGACAGTTCCACACAAATCACCGATGGAACATTTGCTACCATG
TCTGTCGAGATATCGAACACGTTTTTCGATAGCAGCACTCAGACTGAACCATCACCATT
GTACGACAACAATCACTATCATGAGGAATGTCTAAAATGTAACAGCTGCGGCTTGAATC
TCACAGGACCCAATCAGAAACGGGCTAGACGGTTCAAGAACCAAATACTTTGCGATCT
TCACTTCGCTGACGTGGCGCTGATGGAGTGCTCGGACTTCATGCAGCAGTTGCGCAGC
TTCAAACCACAAAGTTTAGGCTGCGCAGTTGCCCGAAGGAAGTCATCAACCACTTTAA
TCTTCCCCTTACCACCGCAAGCTTGTTCAG

>novel_circ_001376
AAGCATGACAGCGATCTGTTTCGTGTGTAATCTACCGATCCTGAGCCACCAGGTCGGCC
TGGTGTGGCAAGGTGGAAATGGATGGGACGATCTTATGCGGGAACAGGTCGAGGAATC
CACCTTGAAACAACGTCTTGGAACTGGAAGGCGCGATTCGGCGACACAGATCACTTC
GATATCCCCGCCAAACGAGCCGCAAGAACCTGCGACCAATCAACGTCGACGACGATCG

AGCTTGGCACAATTGACAGATATTTTGCGCGAATGGAGCGGGGGTGGCGGTGGAAAG
GGCGAGCGGGGGAACAAGCTGTATCGTCGCGAAACATTGGGAGATATAGCGAAATCTT
TTCCGTGGTCCAGGCAAACGACCACGGATGCGAGCCATCTTGAGAGGCTGCGCAAGA
GGAGGGAGAGCTCGGTGGACAGCGGGATCAGAAGCCAGTCGTCTACGAAATCGAGAA
AAGATTCAACGATCACCGAGCTGAAAAATGATTTGCCAGATTATGGGGAAAGAAGGA
GACGCCTCAGCCGCAACCACCCCCTACGGTTATTTCGCCCACCTATCGAGGGTTCAGA
GAGAGCGATTCGAGAGGATCTCGACGGGGGTCAGGGGAGAGCGGAAGGGCGAAAAA
TCAAGGGGAACGGAAGCATAATTGCCGTCACCACAGGCGCAGGCAATCGCAGCAATC
GCAGCAATCAATGGACAGTGGAAGCGGCGTGATGCCCGTGAAATATTACAGAAGCGAT
CAAAGACCGAGCGCTTCGAGTACGGATAGCGCGGCCAGCAATGCTGTCAGAGAGCAT
TTGGAAGCGCGTAATTCCATGGACAAAGGCGAACGATCGAGTAGTATCGAGGGCAAAA
CATCCGTGACAACAAGTAGCGAATCAAAAACGTCCACCACGACTACCACCACAACTGT
GTCGTCGACGACTACCGCGGTGACCACGTCGACCACAGTGACGATGGAGCCGAAGGC
CGCGACCGTCGTCGACAGTAAAAATTTAACCCTCGAGTCGTCCATCACTACCCCTACCA
TCGTGATGTCATCAGTGACGCCACCAAGCGTCTCTCCAACGGTGGGTAGCAGCCTTCC
ACTTCCAGGAACCTCGACTTCCGGTAGTTCCAGTCCCGTTGGCTCGCCAAACGTCAAC
ACACCGACCCACCCATTGATTAGCACTAGAAGAGACTCGACCACCCAGTGTTATCAGA
AGGGAAAGGAAGTATCACCGAACAAGTTGTCGAGATTGATACGGCAATCGGCTGCCAT
AGATGAGTCGATGCCACCAGCACGACGACGAGGAAGTCAACCGACATTGTCACCGGA
TACTGACGAAGGAGGACGAAGAGCTCGAAAAGACTCGCTGAGCCCCGATTCCGCTTC
GTATCCGAGGCGCCGTGATTCGAAGAGCCATCTGAGTCCAGAGAGAGCAGTCGATAAA
AGAGACATCAGTCCTATCAGGGAGAGATCTCAGCTGACGCGACAGTCAATGTCGATGG
CAGGACGGTCGCCACCAAGATCACCGGATAGTTCTTCCTGTTCCTCAAGGGATCCAAG
TCCGTGTGCTCGTGCTCCAGGAAGTATACCTCATGCTCCTATTGGAAGAAGACAATCGA
CAACGGACGAGATATTTATTGCTCGTGGATTTAGGCGGCAGAGCACCACGGAAGAGAC
AATAAGATGTCGAAACTTTCGAAGACAAAGTTCGCAAAGTGAAAAACTGGTTCAAAG
ATATCAAGGACGAAGAGACAGTTCCACACAAATCACCGATGGAACATTTGCTACCATG
TCTGTCGAGATATCGAACACGTTTTTCGATAGCAGCACTCAGACTGGTAAGTTCATAAA
TTTCATTCATCATTTATCACAGATGAGATATAGACGAGATATTTCAAGGAGGGAATTTTT
TCCAAAGATATCGGTAAATTTATCCTTCCAAAAAATTTTACTTTTTTAAATAAAAAGAAT
AAATTATTAATTACACGCACACTCATATTTTTACGTAAATCTTATCAAAGAATAAAGTAC
TTTGTCTTAAATTCCTTCCAAATTTCTCGTCTCTCATTGTAAGTATAATATTATCTCATTGT
ATATAGTCATAGACAATCATTCCTCCATAGACAAAAGATTTTTTATCACGCATGTCGAT
CCTTGTTCGTCAATTGATCCAAATATCTCTTCGTTCTTCGATACTTTCATCGATTTCAATT
AATCGAAATAACTTCAACCCCATCTATCCAAAATCCACTACAAAAATTATATCCCCTCGA
TTCATCCTTGATCATCTATCTCCCAAAAGCACGATACAACGAACACAGAGATTCGAAAG
ATCGGTTAAATCCCCTCCTCCACAAATTCCAATCGAATTTCGCGGCCAGTGCCAATATCT
CTCGACCATAATCGAATCGAACGACGGCATGCGCCCTGGCAACGGCGTTCGCCGTTGG
TAAGCCGGCTTCGCGACGCCGTGCAGCGTCTCCGTGGACGCAGGCGTGGTGTCAGCTC
CCCTAGTTCCGACAATGTGACACTGGGTGCATGGCAGTCGATAGCCAGCCGAAGAGGA
AAACCAGCCTCGAGGAGCTGCTAACTCTAAACGTGTGCGTGCTCTTTTCAAAACCCCT
TCGTACGTCGTTCCACGTCCATCGTGTATCCTCGAGGAGCGAGACGAGGGGAACATCC
TCCCTCCCCCTCGAACAGAAATATCAACAGCAATGCGACTGTAGAATCGTGGAATCGT
GAAGTGAACTCGGGTGATCGTGGATCGGATTGTTGCCCTGATCGACGATCGAATAACA

CACACACACACACACACACATACACACACACATACACACACACAGACCATCCTGTCTGGA
TGGGACGAAACATCGATTCTCAGGTATCTGACGAATATCAAGAGGATCGACCATGACTT
GAATCTGATCAAAGGAGAAAAACAAAGTGTTTCCATTAGGAGGTGTTGACAAATCAA
AGTCCTGCCGTCGATGATCACCTGTTCGTGTTGTGTTTACTCGAACGACGAGTATTTTT
CAGCCTGTAATTAGGAATAATTTAGGAATCCGATTTGAGAGAGAGAGAGAAGGGCTCG
ATTTTCCACGTTGGAACGATAGGTGGTGCCCTTCGAACGGTGATCGAAGGATCGATCGA
TCCTTGATAAACGTGTATACAAAAATTCGTCCTTAGAAATCGCATCCATTGTCTGACACC
AGGGCACCAACCACTTGTGGCCGTGTGGTGCGGCGGGTATCACGAGTAGTAGTTGAAA
GCCGGCCAGAGACACACTTGTGCACCAGCAGATCTCTTTTGGATATCGTCTGTTCGAG
GGAGGGCGATCTGTGATCTTGGACTGGGCGATCCGATCCAAATCTTCGATGATCAGAGA
TTTTTGTTGTTCGATCGGTGGTGCGCGCTCGAGGATGTTCGTTGGTGGTGTGTTCGTGT
GACATGTGACACGGAGGTGTGCGACGAGTGTGATGGGAGAAAGGACTCGTCGCCACG
ACGAAGGATTCCAGCGACGGGATGACGTTGTTGCCGCCTCTCGACGTGCTCACGCTTT
CTCGGAG

>novel_circ_001377
AAGCATGACAGCGATCTGTTTCGTGTGTAATCTACCGATCCTGAGCCACCAGGTCGGCC
TGGTGTGGCAAGGTGGAAATGGATGGGACGATCTTATGCGGGAACAGGTCGAGGAATC
CACCTTGAAACAACGTCTTGGAACTGGAAGGCGCGATTCGGCGACACAGATCACTTC
GATATCCCCGCCAAACGAGCCGCAAGAACCTGCGACCAATCAACGTCGACGACGATCG
AGCTTGGCACAATTGACAGATATTTTGCGCGAATGGAGCGGGGGTGGCGGTGGAAAG
GGCGAGCGGGGGAACAAGCTGTATCGTCGCGAAACATTGGGAGATATAGCGAAATCTT
TTCCGTGGTCCAGGCAAACGACCACGGATGCGAGCCATCTTGAGAGGCTGCGCAAGA
GGAGGGAGAGCTCGGTGGACAGCGGGATCAGAAGCCAGTCGTCTACGAAATCGAGAA
AAGATTCAACGATCACCGAGCTGAAAAATGATTTTGCCAGATTATGGGGAAAGAAGGA
GACGCCTCAGCCGCAACCACCCCCTACGGTTATTTCGCCCACCTATCGAGGGTTCAGA
GAGAGCGATTCGAGAGGATCTCGACGGGGGTCAGGGGAGAGCGGAAGGGCGAAAAA
TCAAGGGGAACGGAAGCATAATTGCCGTCACCACAGGCGCAGGCAATCGCAGCAATC
GCAGCAATCAATGGACAGTGGAAGCGGCGTGATGCCCGTGAAATATTACAGAAGCGAT
CAAAGACCGAGCGCTTCGAGTACGGATAGCGCGGCCAGCAATGCTGTCAGAGAGCAT
TTGGAAGCGCGTAATTCCATGGACAAAGGCGAACGATCGAGTAGTATCGAGGGCAAAA
CATCCGTGACAACAAGTAGCGAATCAAAAACGTCCACCACGACTACCACCACAACTGT
GTCGTCGACGACTACCGCGGTGACCACGTCGACCACAGTGACGATGGAGCCGAAGGC
CGCGACCGTCGTCGACAGTAAAAATTTAACCCTCGAGTCGTCCATCACTACCCCTACCA
TCGTGATGTCATCAGTGACGCCACCAAGCGTCTCTCCAACGGTGGGTAGCAGCCTTCC
ACTTCCAGGAACCTCGACTTCCGGTAGTTCCAGTCCCGTTGGCTCGCCAAACGTCAAC
ACACCGACCCACCCATTGATTAGCACTAGAAGAGACTCGACCACCCAGTGTTATCAGA
AGGGAAAGGAAGTATCACCGAACAAGTTGTCGAGATTGATACGGCAATCGGCTGCCAT
AGATGAGTCGATGCCACCAGCACGACGACGAGGAAGTCAACCGACATTGTCACCGGA
TACTGACGAAGGAGGACGAAGAGCTCGAAAAGACTCGCTGAGCCCCGATTCCGCTTC
GTATCCGAGGCGCCGTGATTCGAAGAGCCATCTGAGTCCAGAGAGAGCAGTCGATAAA
AGAGACATCAGTCCTATCAGGGAGAGATCTCAGCTGACGCGACAGTCAATGTCGATGG
CAGGACGGTCGCCACCAAGATCACCGGATAGTTCTTCCTGTTCCTCAAGGGATCCAAG
TCCGTGTGCTCGTGCTCCAGGAAGTATACCTCATGCTCCTATTGGAAGAAGACAATCGA

CAACGGACGAGATATTTATTGCTCGTGGATTTAGGCGGCAGAGCACCACGGAAGAGAC
AATAAGATGTCGAAACTTTCGAAGACAAAGTTCGCAAAGTGAAAAACTGGTTCAAAG
ATATCAAGGACGAAGAGACAGTTCCACACAAATCACCGATGGAACATTTGCTACCATG
TCTGTCGAGATATCGAACACGTTTTTCGATAGCAGCACTCAGACTG

>novel_circ_001379
TTTCCTGGTGAGTGCATCCTCACATACATGGAGTTTCAGCTGGATAGTGCAGAATACTG
CCAGGCTCAACACAAGTAGAGAGACACGCCCAAATACCCTAGAATTTGCTAGGTACTA
TTAAAAAAGAAATAAGGAAAAAACATCTTAATCAGGCAAAAAAAATTTTTATAATATTG
GGATCATTGCCTCAAAGTCAGAAAATCATCAGCTAGTCATCACGCAAAACATAGAAGA
ATTAAATAGAATCAATTGTTAATTTACAATATTAAATGCAAGCATTGTAGTTACTGAGCG
CACCTGATCCAGGCGCACCCAGTGCCAAATAGATTATCTTCCGAATCATAGTCAAGGAT
CCCAAGTGGGAATCAATCGCATATCAACAACGCCTAATGTAACGTAGCCCTCACCCCTT
CAGTGAAATTTGCACCTTAACAACCTTTTCGAGGTCTACATGTAAACCCATTGTTCTGT
CCTCTGTAAAAAAATAACAAAGTATTCAATTAGTTAAATGAGAAATAACTTTATAAGAC
TGACATCTATGGGCTAAGTTGTCAAATTCAGCCTTTGTTGCTTGATAGTGTGGCAAAAA
TTGCAAAAATAAAGAGAAACAGTGTCGTACAAAATTATTAAAAACATAAAAGTTAAAA
TAATAAAAAAGTAGGAGTTTTACACCACTTGTTGTCCAAAAGGAAATGAGCAACATGA
GGGTGAAGGATATCAGACCGGAGCCCGCCGAGATCGAATACAAAAACATGAAGTTCCT
CATTACTGATCGGCCCAATGATCAGACCATTCATACTTTTATTCAAGAATTAAAAAAGCA
CAATGTAAAAGAAGTAGTGAGGGTCTGTGAACCAACGTACAAAATTGAAGAACTTAA
AGCAGAAGGGATCAATGTCATAGATTTGGTATTTGACGATGGCACATTTCCACCAAACG
AAGTTATCGATGAATGGTTTGAATTGCTAAAAAATCGATTCCGCGAGTCTCCAGATGCA
TGTGTGGCAGTACATTGTGTCGCAGGACTTGGTAGAGCACCGGTCTTAGTTGCACTTG
CTCTTATTGAGTTGGGATTAAAATACGAAGATGCAGTCGCGTTAATTAGGGA

>novel_circ_001380
GCAAGCACGGGAGCACTCGGCGAAAAATGTGGACGGCGGAGCCGTAATTCAAGGATT
TTGGTAGGAGGGAAGCCGAGGGACGCGAGGAAGCGGTGGAGGAGAAGGAGGAAGA
AATGGCGTCCACGAGCGGGCACAAGAGGAACGGCTCGAGTTCGAGCATGTTCACCCA
CAAACGGACCGAGTCAGGCGGCGGTTTTATCGGGCACAAACGCTCCGAGAGCGGTCA
CAAACGTGCCGAGAGCATATCCAGTGCCTTTGGCCACAAACGATCCGAGAGTGGCCAC
AAGAGGAACGAGAGCAGCGGGGGTGGTTTCGGCCACAAAAGGTCCGAATCTGGTAGC
AATTATCATGCTGGGCATCGAAGGAACGAAAGCATGTATGCTATGACTGGTCTGTACGC
TGAGAGCGCGACCACGGGAACCGACGAGACCACAGACCCACTGCAAGCAACCGGTA
GCAGACTGACTCATGACACCGTCATCAGGTGTCACAGTAGAAATCCCAGTTCTGGCCT
CGTGGACCATAG

>novel_circ_001381
GGATGGTGGTGAACGGTTTCGTGAACGTGGTCATAACCACGATAGAGAGGAGATTCGG
TTTGAGGTCGTCTCAGACGGGGTTGATAGCGGGCGGATACGACATAGCCAGCTTCCTT
ACCCTCGTCCCAGTCAGCTTCCTAGGAGGACGCTCGAAAGCGTCGAAACCGAGGTAC
ATAGGAATAGGTGTTCTAGTCTTAGGCTTAGGAAGTCTGCTGTTCGCATCCCCTCATTAC
ATTGCTGGACCATACAGGGGTGGGCAACAGTCGGAAAATATTTGTCAAAGGGTGACGA

ATACGTCCTCCCGTTCGCTATCTTGTAGCGGATCAGTGGGCCAAATAGACCAAGAACCA
TACAGTGGATTGTACTTAAGTATCTTTCTGATAGCTCAACTTCTACATGGGGCTGGCTCC
GCTCCTTTTTATACGCTTGGCGTTACGTATCTAGATGAGAATGTGTCGAAAAAGATGTCC
TCCGTGTACGTAGGAATTTTCTACACAATGGCCATAATAGGGCCTGCGTTAGGATACGT
GGTTGGAGGTGAATTGTTGAAGCTCTATACAGACTTTATCACCGTGGATCCATCAAA

>novel_circ_001382
TCGGGGGTGCCGGACATGCACGAGACCCTGTCCAGTTTTTCGGAACAGTTCAGGGAC
GGGCCGACGAAGCCGGAAATAAAGCCGGAAGCAGGTAAGGAGACGGAGAGGACGGT
GGAGCCGAAGTGCGGTTGGTTCTGGTTCAGACCCAAATATTTGCAACGATTTCGAACC
GCGAAATGGGCGCTCTTCTGGTTATGCTGGACAGGTGCGATGCAAGGTGGATAGGGTC
GACGGTGACTGACGATAACCGGAAATCGAGTTTTCCGGCCCGGTGGCAGGAGGTTGA
CGCGGTGGAGGGTGGTGCGCGTCGACGAAAGGAGAGCGAGGATCGAGGCGAGAAGG
GTGGCGAGACGAAGGTCGTCGACGTTGGCTAAAAAGGGGTGGAGGGAAAGGTTGGA
ACACGGTAGCCGTATTCGGTTTAAATAAGTTTGTTCGCCCCCCCTCTCCCTCTCCTCTCC
ACTCGGCCGACAAAAGAAATCGTCGTGCGGAGGTTCGTGCCTTCTTCGAGGCGGACG
GGATTGAACAAATTGGTGGACAATGGAGCCGGCAATCGTCTGGATGGTGGTGAACGGT
TTCGTGAACGTGGTCATAACCACGATAGAGAGGAGATTCGGTTTGAGGTCGTCTCAGA
CGGGGTTGATAGCGGGCGGATACGACATAGCCAGCTTCCTTACCCTCGTCCCAGTCAG
CTTCCTAGGAGGACGCTCGAAAGCGTCGAAACCGAGGTACATAGGAATAGGTGTTCTA
GTCTTAGGCTTAGGAAGTCTGCTGTTCGCATCCCCTCATTACATTGCTGGACCATACAG
GGGTGGGCAACAGTCGGAAAATATTTGTCAAAGGGTGACGAATACGTCCTCCCGTTCG
CTATCTTGTAGCGGATCAGTGGGCCAAATAGACCAAGAACCATACAGTGGATTGTACTT
AAGTATCTTTCTGATAGCTCAACTTCTACATGGGGCTGGCTCCGCTCCTTTTTATACGCT
TGGCGTTACGTATCTAGATGAGAATGTGTCGAAAAAGATGTCCTCCGTGTACGTAGGAA
TTTTCTACACAATGGCCATAATAGGGCCTGCGTTAGGATACGTGGTTGGAGGTGAATTG
TTGAAGCTCTATACAGACTTTATCACCGTGGATCCATCAAA

>novel_circ_001385
ATGCAGTTGTATTAATGGGAAAAAATACTATGATGAGGAAAGCAATCCGTGGTCATATT
GAGCGTAATGCAGCACTTGAGAAACTTCTTCCACATATTCGTGGAAATGTTGGTTTTGT
TTTTACCCGTGGAGATTTAATTGAAGTGAGAGACAAACTTTTAGAAAATAAAGTCAGA
GCACCAGCTAGAGCAGGTGCCATTGCACCATTGAGCGTAATTATTCCTGCTCAAAATAC
TGGTCTTGGTCCTGAAAAAACCTCATTCTTTCAAGCTTTGAGTATTCCAACTAAAATTT
CTAAGGGTACTATTGAAATTATT

>novel_circ_001386
GGCCGTGAAAGCATTCCAGCAGGTGCTATGGGTGGAACCAGGATTTCCACGAGCCTGC
GAGGTTCACCTACGGCTCGGCTTGATGCTGAAGGTCCACGCTGACTTTGATGCCGCCT
TGAAGCACCTGACACTCGCTTTGATCGATGCCACGACACCTGCCTCTTTTTCCAAGCTT
GAAATAAAATTTCACATAGCTCATTTATACGAAGTCCAGGGGAAGTATCGTCTTGCTAA
AGAACATTACGAGGCATTGCTCAAGGAAAAAACTCTGCCTTCGCACCTGAAGGCCGA
CATTTGTCGTCAATTAG

>novel_circ_001387
GGGTAATAACAATACACCAAATTGCACGGGTTCACTGGTGGGCCTGGGTGGTGCTAAA
TCAGGTGGTAATACCCCGATGAACCCATCGCTACAACAGCGGATCAACTTCCTCCAAA
GTCATCTGAGCCAAGCACCAATGCCTTCCGTTGCTACCAAGAGGCGGCAACTGCCGTC
TATAGAAGAGGCTTGGAACTTACCCATTAGTGCTGAGATGTCTAGTAGACAGCAACAA
CAGCAACAAACACCCACTGGTCCGGGTTATAAATATGGTTCCACTCCTTCTGGACCACC
ACCTCCTTATCCTCAAGGACAAGGGCAGAATCTAAATACAAAAAGATTTAAGCCGGGA
GAAGAACCAATTTCTCCAGGTTCACAACAGAGACCACCACCATTTTATCTCACGTCTC
AACAACTGCAGATGTTACAGTTTCTTCAACAAAATCATGGAAGTTTAACGCAACAGCA
GCAAGGTTTGCTTGCACAATTACAACAACAATACAGATGTATGCAACAACATCAACAA
CAAATTAGATTACAACAGCAACAAGCTGCTCAAAGAGGTTTAAGGCCAGGACAACCT
GGTTATCCTACAGGTTACAATCATTCACAACTAGGACAATCTGGCGTGATCAAGAATTA
CGGGATACCTCAGCAACCGTTGCAACAAGGTGGAACTGTTGCATTACAAACAGGATTC
TCAGATTCTAATGTCGGTTATAACACGGCAGCAACTGGGAACAGTCAAACACCAGGAA
TGCCTTACAAATCTGCCTCTGATTCAAACTATCCTCAAAGGCAATTGACATCCGGCCAA
TATAACCAATATCAGCCTAACCAATACACTGCCCAGGGTTACACACAAATATCGTCCAC
GACGAGTAATTATCCCGAAGGTTCCGCGGGAAATAAAGACCTGGGAGTAACAGATCAA
GAATTACAGGCTTTGTTATCACAAAAAGATATTGCGACGTCCTTGGCGGAGGATTTATT
GAAACATTTTGGTTCAGAAGATTTAGACGTAAAAGAAGAAGCACCGAGCATCATGAAC
AATGGCACATTAAGTTCAGGTCCGTTCTCACCGTCAAATCTCGAAGAGAGTACAGAAA
AAATCAAAGAGGTAAAACTAGAGAAGGTTGAGGATAGTCAACCTTCGTCTACGTCGAA
ACTCGAAGCATACGAAAAAAGCAATACAAAGACATCGACTTCTGTAGTTGAGACGGTG
AAATGTGAAGCGACTTCGAGTACAAATAAAATCGAATCAGTTGCTCGGATTGAACCGG
TGCTTAAATTGGAAAGCTTGTGTGAATCACAACCTGAACAAGAACTTAGTATAGATATG
GATTCTAAGGGTATTATAGAAGCATGTAAAGGTCAGGGATTAAAAGGTGTACCAAATTG
CTCCATTCTGAGTGATCGTTCACCTCCACCCGCTCCACCTGATCCTCCAAATCAGAGAC
TAACCAAGGAACAACTCTTACCCCCAACTCCTAGCGTTTATTTAGAGAATAAAAAAGAT
GCGTTCAGTCCACAACTCCAAGAGTTTTGTTTAAAGCATCCGATAGCTGTGATTCGCGG
TTTAGCAGCCGCTTTGAAATTAGATCTTGGATTGTTCTCAACGAAAACTTTGGTAGAAG
CAAATCCAGATCATGGTATCGAGGTAAGAACACAAATGCAACAGACCAGCGATGAGAA
TTGGGAAATCTTTTATGAAACTATTTAATTTTTATTTGAAACATTTTTTAATATTTTCAAA
AACGAATGAGCAGATAAATCTATGGGATATTATATTTATCATAATCGAACTTTGTATATAT
AAAATATGAGTAAAGTTAAAGTATTTCAATAGAGCTATTTTTTTGTTATTATATACTCATA
AAATATATATAAAGGTTAGCTTTGTTTTTAAAAAGAAATATATATTTTTTAATATATTGAT
ATATTTCAATTTTTTTTAACTTATAAAAAGATATTGGAACAAGGTATTGTATGAACATTTA
TAAGTTTGGAAGAAAATTGAATATAATTAAATATTTCTTAAATTATAATTTTTTAATTATAA
ATATTTTTTTTATATTAAAGAACATATTGCATCTACTATCATATCAAAATATTTATATATATA
ATTATATTTAAAAAAAAAATGATTTGAATATCATACCTTTATATGTTTACTTACAAAATCAT
GAATTAAATGAAAGTTTCCTCTTTGAAATCATTCAACTTTTATTTCAAATATTTTTCGTTA
CTAAGAAATTAAAATGATACTAAATGAGATGTTAATATTAATTTGATATTTATTAATTAATT
AATTATAAATATTAAATGTGATATTTTAATAATACTCTTTATACATATTAATAACATATTAAT
TGGTTGTCGTGAATTGTTTGTATAAACAATTATATTAACAATGATTTGTGTTTACATGTAA
AGAAAAAAGTTTCGTAAAGTAAGAGATATTTCAAATTAAAATCGATGATAAGGAAGCT
AATCTATAAATTCACTGCATTTTCTTTAATGTATCTATCTTTCTTTAATGATCTAGTGATAG

ATAGAGATCGCTTACGAATTGTTGTTGATTGCAGAAATTCTGTGGAAAAATCTGAAGGA
AATGCCGATACATGGTGTAGTATAGGCGTCTTGTATCAGCAGCAGAATCAGCCTATGGA
TGCTCTGCAAGCGTATATATGTGCTGTACAATTAGACAAATCACACTCAGCTGCGTGGA
CTAATCTGGGCATTTTGTACGAAAGTGTTAGTCAGCCAAAAGACGCTCTAGCTTGTTAC
GTCAACGCATCCAG

>novel_circ_001389
ATTCAGAACTTCTACACATGTTGGAGCATAATGAATCGCTGAAAGCCAAGGTTGAAGA
GGCCGTAGCTGTATTGCAAGCTCATCAGGCCAAACAGGCTGTGGCTTCTAAAAAAGAA
TAAACATTAATTAAGGTATTATTTATTCTTCCTTAATTGTTCGTAACAATGTTCAGTTTT
CTTCCTCATTCTGCGATTATCTTTGGTTTAAATAAATTTCTTCTGAAGCTTTATGGTGGCT
TAAGCGATTATTACATGCAAGAATGACAGGAACAAATTGTTATTCAAAACTAATAATTG
ATCATGAAAGGGTT

>novel_circ_001390
ATCAAGGTGTAAATTTATATGTAAAGAACTTGGATGATAGTATAGATGACGAGCGGTTAC
GCAAAGAATTTGCACCATTTGGAACAATTACCTCTGCTAAAGTTATGATGGAAGAAGG
ACGCAGCAAAGGTTTTGGATTTGTCTGTTTTTCCGCTCCTGAAGAAGCTACTAAAGCT
GTAACAGAGATGAATGGTCGCATAATAGTTACTAAACCATTATATGTTGCTCTGGCTCAG
CGTAAAGAAGATCGCAAAGCACATCTTGCTTCCCAATATATGCAACGTTTAGCAAATAT
GCGAATGCAACAAATGGGTCAAATTTTCCAGCCTGGCGGTGCTGGAAATTATTTTGTTC
CCACTATTCCTCAACCACAAAGATTTTATGGACCTGCACAGATG

>novel_circ_001391
AACGTCAACAAGAACTTAAGAGAAAGTTCGAACAATTAAAATTAGAACGTTTAAATCG
TTATCAAGGTGTAAATTTATATGTAAAGAACTTGGATGATAGTATAGATGACGAGCGGTT
ACGCAAAGAATTTGCACCATTTGGAACAATTACCTCTGCTAAAGTTATGATGGAAGAA
GGACGCAGCAAAGGTTTTGGATTTGTCTGTTTTTCCGCTCCTGAAGAAGCTACTAAAG
CTGTAACAGAGATGAATGGTCGCATAATAGTTACTAAACCATTATATGTTGCTCTGGCTC
AGCGTAAAGAAGATCGCAAAGCACATCTTGCTTCCCAATATATGCAACGTTTAGCAAAT
ATGCGAAT

>novel_circ_001392
ATTCGCATATTTGCTAAACGTTGCATATATTGGGAAGCAAGATGTGCTTTGCGATCTTCT
TTACGCTGAGCCAGAGCAACATATAATGGTTTAGTAACTATTATGCGACCATTCATCTCT
GTTACAGCTTTAGTAGCTTCTTCAGGAGCGGAAAAACAGACAAATCCAAAACCTTTGC
TGCGTCCTTCTTCCATCATAACTTTAGCAGAGGTAATTGTTCCAAATGGTGCAAATTCTT
TGCGTAACCGCTCGTCATCTATACTATCATCCAAGTTCTTTACATATAAATTTACACCTTG
ATAACGATTTAAACGTTCTAATTTTAATTGTTCGAACTTTCTCTTAAGTTCTTGTTGACG
TT

>novel_circ_001393
CTTCTTCAGGAGCGGAAAAACAGACAAATCCAAAACCTTTGCTGCGTCCTTCTTCCAT
CATAACTTTAGCAGAGGTAATTGTTCCAAATGGTGCAAATTCTTTGCGTAACCGCTCGT

CATCTATACTATCATCCAAGTTCTTTACATATAAATTTACACCTTGATAACGATTTAAACG
TTCTAATTTTAATTGTTCGAACTTTCTCTTAAGTTCTTGTTGAC

>novel_circ_001394
AAACATTAGTGAAAAGCTTAGCTTTTTCTCCCAATTCCTTCTCGCGTTCTTTACGAGGTA
TAAATTTGCCAACATATACCTTTTTTCCATTTAGTAACATGCCATTAACTCTATCAATAGA
TTTATTTGCTGCTTCTTCAGTTTCAAAATGAACAAAACCATAACCTTTAGAAACACCAG
ATTCATCTTGAGCTACTTTGCAACTAAGTATGTTACCAAATGCAGAGAAC

>novel_circ_001395
TTTTTATCTAAATTTTTTATAAATACATTTCCAACACCAGATTTTCGAAGAGAAGGATCA
CGTTGAGACCACATGATACGAATAGGCCGCCCCTTTATCATATCAAAGTTCATGGTATCA
AGAGCACGTTCCGCTACAACAAAAAAAAATACAAACAATTATATTATTATATTATTATAA
TTAAAAAAATATATAATTTTCGTAAATATTTCTATTAAATTTAATTATTTTTTTCTTAATTTA
TTTAGTATATATATAATATCATTATTTTAAAAAATATATTCAATATAAATTACAGATTTCAAA
ATATTTCCAATTATCTTATGTATATTCTAAATGAATCATAAATAAAGTTACATTACATTGAA
TAATAATTTGAAAAATATATTAATAATGAGAACATATTGAAGAACTAAAATACGTCGATA
GAATCATGTGGTATTTTCCTTTTTTCTTTTTATTTTTTATTCAAGTTATAATTGTATATGTAT
ACAATGAATATGTTGTATATTAAAATCTTTTACTTTTTAGAATATAATAAATTCATATCAAG
CTGTTCGATATCACCTGGAAAATGAAAAAGAAAGTAATAAAAAAATAAGGCATGACTC
AACCAACCGTCAATTGCTATCTACTTTGTTCGAAATAGGTTTCAATATGGTGGTATCGTA
TGATCCGAAACACGTGTCAAGCACGGACAAATGATGTAACAAACAGTAATTTTTTTTA
ATCTAAAATATTTGATTCAAAAAGAAATAATGAAATATAGAATCATTATACTCACCATCA
GCCGGTTGCTGGAAGTTAACATAAGCATAACCGAGCGAACGTCGGGTTATCATATCACG
ACAGACGCGTATCGAGAGTACAGGCCCAGCTGATGAAAACTTCTCGAAAAGCATCGCC
TCGGTGATATCACTATGCAAGTCACCCACATAAAGTGAGGCCATAGGGTAATTCGGA

>novel_circ_001397
GTCGAAGCCCGAACTCAAGGATGCCGTGGGTGAGGAGACAAATTATCCACCACCACT
GAAACCCGCCTCCCCCCCCTTGACCGCCTTAAAACGAATACTGCCAGCCGCCTCGAGG
CCATCGATATCGAAACGAAGCGTCTCAACCGATCACCAGGACGAAGCACCAAACCTAC
CCCATGGATCTAACCGAGGCATTCGCCCCGGGAGGGGGTGGAGAGGAGCTGCCAAAG
ACCGACTTCTTCGACTTCGTCGTCTCTCCACCTCCCCACTGCGCCTCCGCAGATGGCGT
GTCGGACGAAGAAAGCTTCCTCCATCGCACCACCTGCAGGAGCATCGGCTACCTTCAA
CAAGGTCACGAGGAGCACGAGACCGGTCGGGAACTGCATGGCTCCCCGTTCATCAAG
GAAACGAACAACAACACGCTGACAGCTTTACCACCTGTCTCGACTATCACCGGCTCCC
TCAGCCACCATCATCATCACCACCACGTGAGAACGCATCACACCGCCTGCAACGTTCA
ACAGAGCAACGAACAGACACCGATGGACCAATCCGACTGCGACTATTGGGGCACGGA
CGAGAGCAAGGAGCAAACCTGTAACATACTCCTGGAAGATCTGAACAAGTACTGTTGG
TCGGCTCACGCGAATGCCCAGAGCCACGGGAACGACAACGTGAACGTTCACCAGGGT
TCGAACGAGCATCACCAAGGGGTGGGTCGAGGATGCTCGGCGAACGACAGACAGAAC
ACGGACGGGGCGATATACACTCTCACCGTGTTGAACAACGAGGCGAACTCGATGGAC
GCGTTGGACTGTTGCAAGAGTCCAGCCCCAACGTCAAGCGACTCCTGGTCGCTCAGA
CCTAACCTAGATCTGGACGCTATACTGAGCATGGAGCCGGCGTCCGCCGAGCAGGAAC

ACGACCAATCGAATACCGTGACAGAGGTTTCGCGGACGGTGAACGACAGGTTTCACC
CGGACCGTTTCTCCGTCGCCTCCTCGCAGTACGCGACCGACGACAGCGGTTTCGTCGA
GAGCAAGGAGTTGTGCGGCCGAGGCGCATCAAGCGGCGGCAATTGCACCGGCGGCGG
CGACAACAACAACGATTGGAAGCTGTCGGATCAGAATCTGCAGGAGGCTGCGGCCGC
TGCCGCGGCCGCTGTCGCTGTCGGCGCCGGCGACTCCGCCGAGAGCCTGCTCAGGAG
CGCTCTTCAAGGCAAACTGTACACAGGCCCTGCCCAAGTGGTGTCCTCCTCGTCGCCG
ACCACCCAAGGCTCCGCTATATCGATGCCTGTCACGAGCAACGCCGGTTTGCAGATAGT
CACCGATCAACAGCAGCAGCAGCAGCAGCAGCAGCAGCAGCAACAGCAGCAACAGC
AACAACAGCAGCAACCGCAAACGCAGCAGGACGAGTCCATGCACACGTGCACCGAC
GAGGACTTGTTGCTCTCCCAACTGGACCAAACGACCTATCGCCCGGGCGACTACGAGA
AGCTGAAGAGCATAGCGAACGAAGTGGTGGAGTCTTACTGCAGCCTGGAACCGGTTT
GCAACGTGTCGGCAACGACCACGGTTATGTACACGTTGGACCCAACGAGCGGTAGCCT
CGGCACGATCACGCTTCCCGCAGATCTGGGTCAAGTGAGCACGGTCACGGTGGTGAC
AGCTGCTCAGCAGGAGGTTCTCCAGCAACAGGCCGCGCCTCGAACGGAGGTGGAGCA
GCAACAGTCCGCCAGCCAACTTCAAATAACCCCGTCGCGAGTCGTCACGACGAAAGC
CCCGAAGAAGTACTGCAGGCGCGCGAACAGGAACAGCAGCAACGCGAACGCGGCCA
GCAATGGGAGCAGCGGTTCTGAGACGGGTGGAAGCACAGGCGGGCAGCAACAGGGT
GGTTCGGGCGGCTCGCCTGGCAGCGTGCAACGGAAGGAGCGCTCGTTGCATTACTGC
AGCATCTGTAGCAAAGGTTTCAAGGACAAGTACAGCGTGAACGTTCACATTAGAACGC
ACACGGGCGAGAAACCGTTCGCCTGCTCCCTGTGCGGCAAGAGTTTCCGCCAGAAGG
CGCATTTGGCCAAGCACTACCAGACCCACGTGACGCAGAAGCCCAGCGTTCAGCAAC
AGGCGGGCGGCGGTGGCGCCAGTAACAATGGGAATAGCGGGAATCCAAGCCCGTCTG
GCGAGACGAGCGGCACGACAACTAGCACCGTGACGAACAGGAGTCAATCACACCAG
GTGTCGGTCGATCCCGCGGGGAACGCCTGCAATCCACCTAGTTAGTCGCGTTGGATGG
GCCAGACAACCTACTTCCGACGGTTCCCAATTGGATTGCAGTTAATCCTTGCTACTCCA
TTTACTCTAACTTTCCCGGTGGCACGGTGCGGAAAGTGGATCGGCGATTTTGCACGATG
GTGATGGTCAACGAAAGGATACACC

>novel_circ_001399
AAAAAAAAAAGAAAAATCATCCGTCATTTCCGTTTCGTGTAACGCTTACAGCGATACTT
CCTGTCTTATTTTTATATAATTAATTCTTCGCCACTAAGTGGCTGTTTCTCGATATTGTTTA
TTATCTAAATAATGTGCTCTGTTTCTTAATTATTAAAAAATATATATATAATTTGCTCTGAT

>novel_circ_001401
AAAATATCCTGTACGTCGATCGTCGAAATTCTTCATCGCGTGCCTCTTTTCCTCTTCGAA
TAAAAATATAAAAGTCAGATGGAGAGAAGCTGGTTAATGCGACATCGAAAACTCCAAT
TTTCACAGATTGGTAGTAACAACGAGCCGTCGCACGAAATGTTGGCGTGAAATGCGGA
GACCTTGGACGCGATAAGAAGGAACGGTCGAGGGTCGCACATGTTTTATCTGGTCGCA
GTGCAACATCAACGTCTACTCTTGTTGAAGACGGGAATTCCACGAAGTATGGGTCCAG
CTCGAGGGCCGTCCAGTCCGGAGAACATGCTGACGCCATATTCTCCGCGATCTTATATC
TCGAGGGGCTCGGGAGGAGCTCCGTTCGCCATCTTCAAAATTCTTCTTCTTCTTCTTCT
TCTTCGTTG

>novel_circ_001402

AGAGTTATACACGTCGACATTAACGGTACGAAAAGTATAAACAATTCCTACTACGCGTC
TCCTTGAATAATCTCCGAATATATCAGTCTTAATTCGACTTCGAACACGATAACATCGGG
CGCCGAACCAAAACAAGAGAAACTGCGACGTTCTCGAGACGGAGACCAACCACGGC
GATCCTGTTGGCCGCCGTAGGAGATGAAAATGCGATCTGTCGGTGGAGGGGAGGGTGT
ATGGCCGGCGCATCGACGCGTATCGACGGGTCGGAAAAACCGCGGCAATCTATCCGGG
GATTGCTCGTGGAATCGGCTCGAGAAGGCGGCCCTCTCTGATTTCCTGGTGTTCTTGCG
CGGCGATGTGCCGCTTTGGCCGCTGCCAAGGAACGAAAGATAAACAGGCATAGCAGA
A

>novel_circ_001403
ATATCCGTGAGGCAAGGATACGTCGCGGCCACTACCTGCCAGGTGGATCTGGTTCCTG
GAATTTCTTCCTCTTTGTCCGTTCGTTCGCTCGTTCGCTCGGCGGAAGACACGGTGCGC
GCGCGGACACACGAGGTGATCATCGGCACCTGGTGATTTCAATGCTCGGTACGGGCGC
GACGAGAGGAGGACGACGACGGATACCCGTGGCGTGATATCGTGCGAGTATTATTCTC
CAAGTGTCTTCGATATCCCTACGATAATAGAAAAGTAAATCTGTGGAGAGAGAGAGAG
AGAGATGTTCCTCGAGCTGCATCGGGACGAGTGTCGAGGTTTCTCGTGGACCAAGCTG
GTGGACATGGAAGCATGAGAAC

>novel_circ_001404
CTATATTTCATCAGAAGATTCTGTGGATGATCCAGATTGGATTTTCGAATCCGAAAAAA
AAAATATGAAACAATCTACTTGTATTTCAACAAAAAATCGTCAAAAACCTTATTCTCGT
CCTTCAATTGAAGATAAAAAAGTTCGAAAAAAAGAACAAAATAAAAATGCAGCCACA
CGATATAGACAGAAGAAAAAACAAGAAATAAAAGAAATATTAGGTGAAGAACGTGAA
CTTACTGAACATAATGAAAAACTAAAAAATCAAGTAACAGATTTACAACGAGAAATTG
GATACTTAAAAGGCTTAATGAGAGATTTATTTAAAGCTAAGGGTCTTATTAAATAATCCA
TTTATATATGAACTATGTTAT

>novel_circ_001405
TGTTTGAAGCTTCACGAGCTATGAGAATATAGGACAAAGAAGAGGTGCCCTGGGAAAC
GTGAAGTTAAGCAGTTTCAACGGTTTCCTCGCCGCACACGCGGAGGGGAATAATTTTT
CCTTTTTAACGACGACCTCTCTTTTCGTCTGCATTAGACAAGTTTCTACATGAAAACATT
ATGGGATCCAGGATGGAATCTAAAATGTTGAAAGACGACGAGAAGGACAATATGAGGC
TATTTTGCGCTCAGAACGACGATGAATCGTCGACGATGAACACCAAAGATGGCTCCAG
AGGGGGCAGGAAGCAGGAAGTGGATCGCTTCGGATACTATCAAATGATCATGTTCGCG
ATAATCAGCCTGCCTCTGTTCTTGTCAGCAGGATTCACGCTTGCCTACGTGTTCACAGC
CGGGGAAGTTAAATACAGGTGTACGGTGCCAGGATGCGAGAATTCATCGAACACGCAA
TTCAACGTGCCTTGGATGATTGATTCCGTCCCGGATGTATCCGAGACGTCCAAATGCAT
CCGTTATGTCGTGCAAAATCACACAGGGGCGTGCACAAACGAAACTTTCAGTAACGTT
ACCAAAAGGTGCGATTCGTGGATCTACGATCCATCCGAGAATACGATTCAAAGCGAGT
GGGACATCACCTGTGACACCAATCGATGGAAACTCACTCTTGTGGGCACAGTAAATAA
CATCGGTCAATTCGTCGGGCTGATCTTCGCCGGCTACATATCGGACAGATATGGAAGAC
GCACGATACTGACGCTCACCACATCCCTGAGCGGAATCAGCGGTCTGATACATTCCTTC
TCCGTGAATTATTGGATGTTTCTCGCGTTCGAATTTATCGACGCGACTGTCGCGGCCGGT
ATCTACAGCGCTGGATTTATTTTAGGGATGGAGATGGCAGGGGTAAGAGGCAGAGTATT

AGCCAGCACGATTATCTGCTGCATGTTCGCAGTCGGTGAGATGTTACTCGGATTGATAG
CGATGTGGCTTAGATCATGGCGATTGATCCTTCGCATGGTTTACGGGCCGGCATTGCTG
GCCATCTTATTGCCGCTCCTTATCCCAGAATCAGTCAG

>novel_circ_001406
CCTGAACCTACCAGCACACCGTCCAGCACTGAACCAGATACGACAACGAGCCCTGAA
CCGAGCAGTACACCGTCCAGCACCGAACCAGCTACAACAACGAGCCCTGAACCTACC
AGCACACCGTCCAGCACTGAACCAGATACGACAACGAGCCCTGAACCTACCAGCACA
CCGTCCAGCACTGAACCAGATACGACAACGAGCCCTGAACCTACCAGCACACCGTCC
AGCACCGAACAATCAACGAATACTGAACCAAATACGACAACGAGCCCTGAACCTACTA
GCACACCATCCAGCACCGAACCAGATACGACAACGAGC

>novel_circ_001407
GTTCAGGGCTCGTTGTCGTATTTGGTTCAGTATTCGTTGATTGTTCGGTGCTGGACGGT
GTGCTGGTAGGTTCAGGGCTCGTTGTCGTATCTGGTTCAGTGCTGGACGGTGTGCTGGT
AGGTTCAGGGCTCGTTGTCGTATCTGGTTCAGTGCTGGACGGTGTGCTGGTAGGTTCA
GGGCTCGTTGTTGTAGCTGGTTCGGTGCTGGACGGTGTACTGCTCGGTTCAGGGCTCG
TTGTCGTATCTGGTTCAGTGCTGGACGGTGTGCTGGTAGGTTCAGGACTCGTTGTCGTA
TCTGTTTCAGTGCTGGACGGTGTGCTGGTAGGTTCAGGGCTCGTTGTCGTATCTGGTTC
AGTGCTGGACGGTGTGCTGGTAGGTTCAGGGCTCGTTGTCGTATCTGGCTCGGTGCTG
GACGGTGTGCTGGTAG

>novel_circ_001408
CACACCGTCCAGCACTGAACCAGATACGACAACGAGCCCTGAACCTACCAGCACACC
GTCCAGCACTGAAACAGATACGACAACGAGTCCTGAACCTACCAGCACACCGTCCAG
CACTGAACCAGATACGACAACGAGCCCTGAACCGAGCAG

>novel_circ_001409
CTGCTCGGTTCAGGGCTCGTTGTCGTATCTGGTTCAGTGCTGGACGGTGTGCTGGTAG
GTTCAGGACTCGTTGTCGTATCTGTTTCAGTGCTGGACGGTGTGCTGGTAGGTTCAGG
GCTCGTTGTCGTATCTGGTTCAGTGCTGGACGGTGTG

>novel_circ_001410
GTGGTGCTGTGCTTCGAACGGATCTTCTGGGATCCGACCGCCAATTTGTTTGGCCACGT
GGGGAGCACGACCGCGTCGCGTGGTGAGCTCTTCCTCTTCTGGAATCTTTACAAGGCG
CCGGTGCTGTTGGCCCTGGTCGCTGGAGAGGCTGCCTGTGTCATGGAGAATGTCAGCG
ACGATGTGATCGTTGGACGTTGCATAGCCGTCTTGAAGGGTATCTTCGGAAACCAAGT
GGTTCCGCAGCCGCGCGAGAGTGTAGTGACGAGATGGCGAGCGGATCCATGGGCGAG
AGGCTCGTACAGCTTCGTCGCGGTCGGCAGTTCCGGTAGCGATTACGATCTTTTGGCCG
CCCCTGTCGCGCCCCCTGCCACCCCTGGCGCACCGCCGCCGCAGCCCAGGGTTTTCTT
CGCAG

>novel_circ_001411
TTCGCAACGGCTACTCGTGCGTACCTGTTGCTCTGTCCGAGGGCCTGGACATCCGGCT

GAACACGGCTGCAAGGGCGGTCCGCTACGGGGTGAACGGGGTGGAGGTGTGGGCCG
CACCCTCGCGCAGCCCCCACACAAATCACACGGTGTACAAGGCGGACGCGGTGCTGG
TCACCCTTCCCCTCGGAGTGCTCAAGGCGTCCGCGCCGCCGTCCGCGGTCGCCTTCAA
TCCTCCGCTTCCGGACTGGAAGTCGCAGGCTATTCAGAGGCTCGGTTTCGGCAATTTA
AATAAGGTGGTGCTGTGCTTCGAACGGATCTTCTGGGATCCGACCGCCAATTTGTTTGG
CCACGTGGGGAGCACGACCGCGTCGCGTGGTGAGCTCTTCCTCTTCTGGAATCTTTAC
AAGGCGCCGGTGCTGTTGGCCCTGGTCGCTGGAGAGGCTGCCTGTGTCATGGAGAATG
TCAGCGACGATGTGATCGTTGGACGTTGCATAGCCGTCTTGAAGGGTATCTTCGGAAA
CCAAGTGGTTCCGCAGCCGCGCGAGAGTGTAGTGACGAGATGGCGAGCGGATCCATG
GGCGAGAGGCTCGTACAGCTTCGTCGCGGTCGGCAGTTCCGGTAGCGATTACGATCTT
TTGGCCGCCCCTGTCGCGCCCCCTGCCACCCCTGGCGCACCGCCGCCGCAGCCCAGGG
TTTTCTTCGCAG

>novel_circ_001412
TTCGCAACGGCTACTCGTGCGTACCTGTTGCTCTGTCCGAGGGCCTGGACATCCGGCT
GAACACGGCTGCAAGGGCGGTCCGCTACGGGGTGAACGGGGTGGAGGTGTGGGCCG
CACCCTCGCGCAGCCCCCACACAAATCACACGGTGTACAAGGCGGACGCGGTGCTGG
TCACCCTTCCCCTCGGAGTGCTCAAGGCGTCCGCGCCGCCGTCCGCGGTCGCCTTCAA
TCCTCCGCTTCCGGACTGGAAGTCGCAGGCTATTCAGAGGCTCGGTTTCGGCAATTTA
AATAAG

>novel_circ_001413
GTTACTTCGGCAAGTATCTGAACAAGTACAACGGATCGTACATACCGCCCGGATGGCG
GGAATGGGGAGGTCTCATAATGAATTCACGGTACTACAACTACAGCGTGAACATGAAC
GGGAAGAAGATAAAGCACGGGTTCGAGTACAGCAAGGACTATTACCCGGACCTGATAG
CGAACGACAGCGTGACTTTTCTACGCCAAAGTAAACACAATTTCGCACGGAAGCCGGT
GATGCTGGTCGCGAGCTTCCCGGCGCCCCACGGCCCGGAGGATTCGGCACCCCAATTC
TCGCATCTCTTCTTCAACGTCACCACTCACCA

>novel_circ_001414
ATACAAAACGACGAGCTGTTCAAATTTCTTTGGGAAAGGGAGACGAGTTCCGAGAAA
CTCTATCACTCGGAAAGGAGCCGAAAATTTCATTAAAAAGTCCCGTTGTTAAGGGAAG
GAGGCGAGACACGAGTCTAATTGCGAGAGGATTTCAAAGAGGATTCGCGACGTTAAC
GACTCGTCCACTGACCCGTATATACGCGTCCCGGATAAGGACGAATTTTCGCCCTCGAT
GTGGCCGCAAGCCACCCATCCAACCGAGATTTACCGTCCTTTATATTTAGCATCGAGAA
AGGCGGCCCTTTATTGAGACGTCATCGGTTAACGCAAAACGAGAGTAATGCGATTGGC
CCCTGGTCTCGAGGTGGTCCTCGCCCGCCACGCAAAAAGTTGCCCCGTTCCGTCCAAA
CCGAATCGGCCTCCCTTCCCTTCCCCTCCTCGTGCAACTGTTATAAATAAGCCCCCCCTT
TCCCCCTCGTACGCTGTTCGATAGCGACGGTGGGTACAGGTATGTAAACGCTGCCGCG
GCTACCAACGAAAGGGACGGCGTGGTACTTTTGAATTAATTATTTGATGGTGCCCCGTA
TGAATAATTCAAACGGTGCCCCCGAGATGAATCCGAGAAGACCGATGCGCCTTGCCTG
TGTGCATCGAGGGGTTGGCCCGGAAAATGGGATTGCCTTTTTGATTTATCGCGGGAGG
AAAATTCCCTTGGAAATCACCAAGTTGAGAAACGTGAATCGTGGGAGGGAATTTTCAT
CATTACGTTACGTTTCCTCGAGGCGAACGAGTAATCGGTTCGCGAACATCTTAAAAAGC

GAATTAATCGAAGTGATAGATTCCGCGAAGAGTTAATCGTTGATTAGAGTATTCGAAGG
AGAGGGGGGGGGGGTTGGAAAAACGGGAGCAAAAAGCAAGCCGGGGTAATCGATCT
GTTACATGAGTCAGTTCCTGCGACGAACCGAGCGTGTCCATAGCACATTAATTACTATT
GCGATAGCCGGCCTCTGCTAGAGATATTTCTTTTATTAAGCCCGGTTGAAACGAGCCCT
GCCACGCCTCGTGATCATTACTCACCACGACGGCAACACTCTGATTCGGTGCCTTTTTT
TGCCGAACGCCCTCGATCCTATTGAGACAAACCGAGTACGGGCTTATAAGTACATATAC
GTGTGACAGGGGAAACAAAGAGCGTACTAATCGAAAGAATAGTGTGTGAATAGATTTA
ACGCGTTCAATGCGCCGCGTTAGGGAAAAAAGAGAAAAAAAAATCGTACGGACTCCT
CTCGACGGTGGATATTGACTTTTGATAAATATAAAAAACAGTTATAAAAACGCGAAATT
ATTACTGAATGGGAGAGAATGGAAGAGAAAAGCCAGATACGTAGAAACGCATTTCATA
TTTCTTGGAAAAATTCATGACGCGACTGTATGCATCATTCACCATAAAATATCCATAGAA
ATCTCCATGACGCATACGGCATTCACATCGTTTACATCGAATGTATTAGGATGCAAATAC
ATTTTTTCAGTTTCATAAAAGTTTCCAGAATTTTTATTTCTCATTTTTCTCTTTATTGATCG
ATACGTTCAAGATAAAAATTAACGGATTAAATTATTTCTTGTAAACAATCTTTTAATTATA
AAAATACCACCCGATTACTTCTCGACTCTTCAGAATTTTGCGCGATACCATTATAAAAAT
TAATCGAATCGATGATATTTTATACGATTCTATCGTTCGAATCGTGGAATATCGAGGGGTC
GATTCGAGCTTCCCTAGCCTCGGCTTTCCGCAGCAGAGTGTAATTCACTTTATCACGAA
CACACGTTCCAGGTTACTTCGGCAAGTATCTGAACAAGTACAACGGATCGTACATACCG
CCCGGATGGCGGGAATGGGGAGGTCTCATAATGAATTCACGGTACTACAACTACAGCG
TGAACATGAACGGGAAGAAGATAAAGCACGGGTTCGAGTACAGCAAGGACTATTACC
CGGACCTGATAGCGAACGACAGCGTGACTTTTCTACGCCAAAGTAAACACAATTTCGC
ACGGAAGCCGGTGATGCTGGTCGCGAGCTTCCCGGCGCCCCACGGCCCGGAGGATTC
GGCACCCCAATTCTCGCATCTCTTCTTCAACGTCACCACTCACCA

>novel_circ_001415
CAACAGACCGCCATGATATGACACTGGATTGAGAGGAAGAGCGTGCAACCGACGACC
AACCACCATGAGGAAGTTACTGCAGTTTTTGGCGGTGATATTGTTGGGTGTCAATGCCA
GGCGGACGGACGCCGACGCCCCTGTCGACGACGTTTACGGTGGTCAGCGTCGTGGCT
CCCTTCACAAGTCTAATTACAACAACAACAACAACAATGGGAACAACAATTACAATTC
TCAGCTGAACTCGCCCTATCTCCCGTACCAGCAGCCCAGGGAAAGAAAGCCGAACATA
GTGCTGATTCTTACGGACGATCAGGATGTCGAACTCGGTTCGCTGAACTTCATGCCACG
GACCCTAAGGCGAATAAGGGACGAGGGGGCCGAGCTAAGACACGCATACGTGACCAC
TCCTATGTGTTGTCCAAGCAGGAGCTCGTTGCTGACCGGCCGTTACGTTCACAATCACG
AGGTCTTTACGAACAACGACAACTGCAGCAGCCCCCAGTGGCAACGAGATCACGAGC
CGCACTCGTTCGCCGCTTACCTGAGCAACGCTGGATATCGTACCGGTTACTTCGGCAAG
TATCTGAACAAGTACAACGGATCGTACATACCGCCCGGATGGCGGGAATGGGGAGGTC
TCATAATGAATTCACGGTACTACAACTACAGCGTGAACATGAACGGGAAGAAGATAAA
GCACGGGTTCGAGTACAGCAAGGACTATTACCCGGACCTGATAGCGAACGACAGCGTG
ACTTTTCTACGCCAAAGTAAACACAATTTCGCACGGAAGCCGGTGATGCTGGTCGCGA
GCTTCCCGGCGCCCCACGGCCCGGAGGATTCGGCACCCCAATTCTCGCATCTCTTCTTC
AACGTCACCACTCACCA

>novel_circ_001416
CAACAGACCGCCATGATATGACACTGGATTGAGAGGAAGAGCGTGCAACCGACGACC

AACCACCATGAGGAAGTTACTGCAGTTTTTGGCGGTGATATTGTTGGGTGTCAATGCCA
GGCGGACGGACGCCGACGCCCCTGTCGACGACGTTTACGGTGGTCAGCGTCGTGGCT
CCCTTCACAAGTCTAATTACAACAACAACAACAACAATGGGAACAACAATTACAATTC
TCAGCTGAACTCGCCCTATCTCCCGTACCAGCAGCCCAGGGAAAGAAAGCCGAACATA
GTGCTGATTCTTACGGACGATCAGGATGTCGAACTCGGTTCGCTGAACTTCATGCCACG
GACCCTAAGGCGAATAAGGGACGAGGGGGCCGAGCTAAGACACGCATACGTGACCAC
TCCTATGTGTTGTCCAAGCAGGAGCTCGTTGCTGACCGGCCGTTACGTTCACAATCACG
AGGTCTTTACGAACAACGACAACTGCAGCAGCCCCCAGTGGCAACGAGATCACGAGC
CGCACTCGTTCGCCGCTTACCTGAGCAACGCTGGATATCGTACCG

>novel_circ_001417
CAACAGACCGCCATGATATGACACTGGATTGAGAGGAAGAGCGTGCAACCGACGACC
AACCACCATGAGGAAGTTACTGCAGTTTTTGGCGGTGATATTGTTGGGTGTCAATGCCA
GGCGGACGGACGCCGACGCCCCTGTCGACGACGTTTACGGTGGTCAGCGTCGTGGCT
CCCTTCACAAGTCTAATTACAACAACAACAACAACAATGGGAACAACAATTACAATTC
TCAGCTGAACTCGCCCTATCTCCCGTACCAGCAGCCCAGGGAAAGAAAGCCGAACATA
GTGCTGATTCTTACGGACGATCAGGATGTCGAACTCG

>novel_circ_001418
ATTGGTATCGTCCCCGGCGAGGCAGAACGAGGCAGGTGGCAGCGGAGGCGGCGGCGG
CGGAGCGATGCAACACGCGGCGACTTTCGCGGTGAGGGTGGGCACGATGGACGGTGT
GGTGACGCATCATCTGCGCGCCGAGACGAGGAGGGACCTGGCCGCCTGGGCCAGGGC
GATCGTTCAAGGCTGCCACGCGGCCGCTCATTCGTTACGCGAGTACACCGTTCGATGC
ACGTGGCAAGGGAAGGCATGCCAGCTGGTTGTAAACCACGAAGATGGGTTCGCGTTG
TACGCGGCCGGGGCCAGAGGCGGTGGCAACGGGATGAGTCCAAGCTCTTCCCCGACT
CCTCTTTGGAGAAGATCCTTCGATAAACTGAAAATGTCCGCTGACGATGGGGCGCGTC
TACTGTGGCTCGACTTTGGCGGCGAGGATGGCGAGATT

>novel_circ_001419
AGGGTCGAATCCTGGAACTTCATTCCCCGGATGGAGTGCACGAGTGCTGGTTGAGAGC
CGCGGATAACGCGGAGGCGAACGTGTGGTTCAACGCTCTGCACTCGGCTCTAGCAGCT
CTCACACTGAAGGCGCTGCGACTGGCGTCCGCGCTTCCGGATCCGCCGCAGCTTCAGC
ACATTGGCTGGCTCGCGAGGAGACACTGTCTTCAGGTGAGTGATCTCGCTCGTTTCGT
TGCATGCGTCCCTCGAAAATCGAGAGTGTAAGCCGTGAAGATGGATCCGCGCCATCAT
CGAACAACCATTTCTAATCTCGTATATCCTTTCTCTCGGGTAGAGATGATCGAAATTTGG
ACGTTTATTATCCATGTATATAATTAATGATAAGTAATAAACACACACTGTACATTTATAAT
TGTTTCTTTGATTCCGTGCGAGATAAATTTAATCGAGGAAAGGCATCGATTTTGAAAGA
GAAATGGGAAAGAGGAGACGAGATACGGAGACGAGTTACAAAGTGTGGAATAATTAA
AAGAAAAGGGATGAAAAGGAGGGATTAAATTGGCTGGGGAAGTAGTAAGAATTCATA
GGGATGAAATGCAGCGGAATTAATAGTAGAGTTCCTCAATGGTGGACGGATAAACAGA
GGATAGAAACAGAAATTTCTACAAAGGGTGGAAAGATGAAAAGTGGGATGGTTAATAA
AAAGGACGAGGGGATGTAAGGGAAAACACGACTGGGATCGGCGACTGGAATGATTTG
ACTGATAAAACGGCGTAGCTTGGGAGTTGGCAGCGCTGATTGAACCGGAAGAAATGG
AAAAAGAGCTGCTAAAATATAG

>novel_circ_001420
AACTTCAAATTTGAAAGTTCCAACTTTGGAAAAATGGAGGAATAGAATGAAAGAAAA
CGAGAAGATAGATGGATTCATAGCGCGTGAACTATCATAGAGCTACTTCCAAGTCGATG
GATTCGTAAAGCAGAGCGGAAGTTTAATCAAGTTAAGATCCAAGATATCGACTCGACG
AAAGCCGCGAGCTTCTTTCAATTGCACGGACAATTTTCTCGGCTTCTGGCTCACGTTTG
ACAGTATGCCCCTGCTCCTCCGCTCAACGAAGATTGGCCATGATTAACGGCATACACGG
CCCGAGCACGATCGATTCCTCCGACCAATGTCTTCCTCCATATTCGGTTAATTAAAGTGC
TCGTATCCTTCGAATAAAACATCATTTCCAAACCCTGCTTCTTGCAAATTTCCCAATGCA
AAAAAAAAAAAGAAACTACAATTTTCTTACTGAAAAAGAAAAAAAAAAAAGAACCG
AGATCCATTAGACTTTACAATGTCTATAAAACACGCACTAAATTCTCCACGATCGGGGA
AAAGAAAGAGTAAAAAAAGGGGAGAGAACCTAGCCCGAAAGATATACGTCGTCAAGT
GGCTGTGTTATATCGCGGCAAGTGAATTATTGCCGGTGTGAAAAAAAGGAAGGAGGGG
GGGATCGACGAGATACACGAAAGGAAGAAGGATGCCTCGATATTAACCTCCCTTTATTT
GAACAACCGGCGACCAACATCTCTGTTCAAGAATTTTTTTCCATTCTCTCATTGCCTCT
CTCATTGGGAATAAATTGGACGAACCGAATATCGATCGGTCCTTTCCTTTCGATCAATG
GGGACGAAATGTATGACACACGCAGGACGAGTTCCTGATTCTCACGATCGAGAGAGGC
ACACGACGAGGTCCTCTCCCGCAAAGCTGGTGGTGGAGATTGAATCGGCGAGATGAG
CGCCGCCTTGAAATAGGATCAACGTTTCGAGAGCCCTGTTCTCTGCTGGATTGGATTCG
GGGGGGGATCCTACGCGCGAGATATTACGCGAAGCGTGGAAATTAATTCGGCGGCTTT
TTCGCCAATTGAAATTGTCGTTGAAATTGTATGATAACGTAACGGGACGTAATGTAATCT
CGCCAATTAAAATATCAACGAAAACGTTGAAACCGAATGATTTAATGGGGAAGGGAA
GGGTATGGAATTAATTTTCGCGATACAATAACAATTTCTTCAAACGAATTTCCCCCTGTG
AATTTGCAACGAGGGAAATAATGGAGGAAAAACCGTTTAATTGAGGAAAAAAAAAAA
AAAAGAAAAGAAAAGAAACGAGATATTCGATAAATATTTATTCGCTCATCCGTTCATGA
TTTATTTCGCTTCGAGAAAACTGTCTGGTTACGGTTCGAGGAATGAGCAATGAGCAAA
CGGGAAAAAGCTTAAATATAGAAGTGATGAAATAAGGAAAGGCAAGACCGCGAGCAG
GTTATTTTTCAACCGGCCTTGCATAAGCTGATTTTCCGGCTGTTTAATTGTCAGGAAATA
TAGGATGACTGAGGCAGAGAGGCTAAAGATATTGAATCATTCCGGAATAAAGGGAAAA
ATAGCGAAG

>novel_circ_001421
GTAGGCGCGGACTCGATCCTAATTGACGGCGACGAATCAGCTCGCTAATTGACCAAAG
GATCCTTCCCCTCCAAGGATGACGAGTTTTTCGATAGTTTGGAGGAGTAGTAGTTTCAA
AGAGACTTCTCGTCGGGAATCCTCGAGTCACGGATTGTCTCGGGACTTGTTCCGGGCT
AATAAGAAGTAACCGAGATGTGGCCTAACTCCAACCGTTCCTCGGGATCAACGCGAGG
AGAGAGGGGTTGTCCTCCACTGTCTCTCCGCTAAGGAAAGGAAAAGTTTCTTCTCAGA
GTTTTCTATGATTTATTACTAAAGATAACACAAAGAATTTCGCTTCGACAACTTAATTAC
GCTGCCGTCTACAAGATCGTCGAAAG

>novel_circ_001422
CGTGCAGTTTTGGAGGAAATCGAAATGGTCGAGCTGTGCGGAAGCGGGCAACCGACT
TCAGCGATGGGAGTGATGGGGATCGGTTCGGTTGTGTCCGCGGCTACGTCGTCCTCGT
CCTCGTCCACGACCACCACAGCAGCCTCGTCCTCGGCGTCTGGACGGGCCGGGGTCCT

GGAGACCCAAGTTCGTGGCCAGTGGTATCGCGTGTTCGTCTCGTTGGAGGACGATTAT
CTGAGCATCTCGTTGGACGAGAGCTGCGAGACAGCAACGACGGGCAACAACGCCTTG
AACAACGGTAACATAAATAACAACAACGTGGACAGCCTGAACGATCCGGACGTGCCG
GACTCGGTCGCCAATCAGAAACGTATCGTGCGCGTGGTGAAAAGCGACAACAACGGT
CTCGGGATCTCGATCAAGGGCGGGAAAGAGAACAAAATGCCGATCTTGATCTCGAAGA
TATTCAAGGGGATGGCGGCGGACGCGACCGAGCAGCTGTACGTGGGCGACGCTATATT
GGCCGTGAACGGGGAGGATCTGCGCGAGGCGACCCACGACGAGGCGGTGAAGGCGC
TCAAGAGGGCCGGCAAGGTCGTCGAGCTTGAAG

>novel_circ_001423
ATATACGTTCGAGGATCTCCAAGAAGCGGCACAATATTTGCTGGACCGGATCAAATTGA
AGCCAAAACTTGGAATCATATGCGGTTCTGGAATGAGATCAAATGAGCAATACGAGTT
GTGTCCAGGTTCGCTGGCCGATTGTCTCGAGAATAAGCAGTACATCCCCTATCAAGAAA
TCCCGCATTTTCCTGTCTCCACCGTGAAGGGTCACCGAGGCCAGATGGTTTTCGGTTAT
TTGAATTCCGTACCAATTATGTGCATGCAAGGCAGATTTCACTATTACGAGGGTTATCCA
CTTTGGAAGTGCGCCATGCCAGTAAGAGTTATGAAGCTCGTAGGTGTGACTCATCTGAT
CGCGACGAACGCGGCGGGAGGTCTAAATCCCACATACAAGGTTGGCGACATTATGATA
GTGAAGGATCACCTGAACATGATGGGTTTTGCGGGGAATAACCCTCTTCAAGGGCCAA
ACGACGACAG

>novel_circ_001424
AATATAGTAACACAATTCTTGTTGCTCTATTAGAACACAAGTATTTTTTTCAAACTTTGTA
TATAAAAGTTTATTATAAGTAAATAGTCTTGCTAAAATATATTTTTTTATAGCCTATTTAAT
ATAAAAAGGCGAGGAGTCTTCGAGTTTTCTGTATTTCAAATAATAACACATCATGCTCG
TTTGACGTCTCTATGCAGTCTTTTAAAACGCGAAGGCTATGCCTAGAAAA

>novel_circ_001425
TTTGATTATGTTGTTGATCATCAACACCAAGAACACGAATACTTGGTATAGTAGATTCTT
CACTGGCATCACCAACAGTAGTACCTGTTACTGATATTGGTATTACTGAAACTGGGGTT
TCTTGTTCTTCTGATGGTCCACTGCTTGTCTAAAAGTAAATCATTCATTAAATAATGGAA
GTATAATATTATATAAAAATATTTAATATTTTTTATACCGAAGGTGGTAAATCAGCTGCTG
GTGAAACTTGTAAAGGAGTAGTAGGTACACTACGACCAGTACCACCATCTTCTAATTGA
ACAAGATCTATTCGTGTATCATCCATGCTTTCTTGAATTACTTGTGCTACACCAGAACTA
GATGATCCAAATATCGTTCGAGTTGTTGATCCAGAAGGAGTTGATAACGAAGGAGTTGA
TGAAGCAGTTGTTGGTGCAGATGAATCTCCAAAAGTAAAGCGTCCTTGAGGAACTTGA
GGTGAACTTACAGCTTCTCCAAATCCATCTCCTCGACGTGGTACAAATAAAGTTGGAGT
ACTTGGTACTATGCTATCATCACCACCATCCTCATATCCTTGTTGTGGCTAAAAATCATTT
TATTAAAATCTCTTCATTTCTTTTTACTTTATTATAAAAATAAAAATGATTACTAAAAGAA
GATGTTGTTGTTGTTGTTGCCTAGGCATTGGTGCAATTCCACGTGAAGATCGAGTTGTA
AAAGATGTAGGTGGTTCTCCTGTTGGTCCAGCACTACTAATTGCTTCTGATTGTTGTTGT
TGTTG

>novel_circ_001426
ATACTGGCGCGCCCTACGTATTATACAAGGACAACATGGAGAGACCCCAGGGGCAATG

GGGCCCCGGGCCGGGATCTCTCCAGGACGGGGGGCTGTACCCGCAGCAACAGCAACA
ACAGCAACAGCAGGGCTCCTCCTCGTCCGGCAGCCCACAGCAGGCCTGTGGTCCCCC
TGTCGAGGGAGAAAGCGGTCCCCCGCCTTCGTTGGCCTCTCCCGTGCCCTCACCGTAC
CCCTCGGCACCGCCTGAGCCTCAAGCCTTAACCCCACCCGACGACGATATACAATCGA
ATCAAGCCACCTCTCAACAGCAGCAGCAACAGCAGCAGCAGCAACAACAGCAGCAG
CAGCAGACGCAGCAACAAGTGCAGCAGCAGCAGCAGCAGCAACAGCAGCAGCAACA
ACAGCAGCAGCAGCAGCAGCAAGTTCAACAGCAGCAACAGCAGCAACAGCAGCAAC
AACAGCAGCAGCAGCAACAGACGCAGCAACAGGACCACTCGCCCCAACAACAGTTA
ACCCCTCACGAGGTGGATCGCGCGGATTGTTTCCCAGGGGCGGGAGAGCTGCAGCAG
TATCCGCAGCACTACTTCAAGGATGCCAGGCCGCATCCGTCGCCGTCGGTGCCACCGC
ACATGCTGACCCCGGGTGGTTTCTCCGCGTTGCACTATCTGAAGCAGCCGGGCGTGAT
GCTCACGTCCCTCGGGCAGGGGGACGGTGGACCCAGCCTGGACGCGCACCAATACGC
GAGCCCCGGGGCACCGAACATGGCGACCGGGCTGCCGGACATCGTCCAGCAGAGCGG
CAAGTCTGGGAAAGGGGCGAACAGCGACCTACGTTTGTTCAAGTGTTTGACGTGCGG
GAAAGATTTTAAGCAGAAGTCGACGTTGTTGCAACACGAGCGGATCCATACGGACAGT
CGGCCATACGGGTGTCCAGAGTGCGGGAAGCGGTTCAGGCAACAATCGCATCTAACGC
AGCACCTGCGCATACACGCGAACGAGAAGCCGTATGCTTGCGTGTACTGCGAGCGTAC
GTTCCGGCAGCGTGCCATCCTCAACCAGCATCTGCGCATCCACTCGG

>novel_circ_001427
ATACTGGCGCGCCCTACGTATTATACAAGGACAACATGGAGAGACCCCAGGGGCAATG
GGGCCCCGGGCCGGGATCTCTCCAGGACGGGGGGCTGTACCCGCAGCAACAGCAACA
ACAGCAACAGCAGGGCTCCTCCTCGTCCGGCAGCCCACAGCAGGCCTGTGGTCCCCC
TGTCGAGGGAGAAAGCGGTCCCCCGCCTTCGTTGGCCTCTCCCGTGCCCTCACCGTAC
CCCTCGGCACCGCCTGAGCCTCAAGCCTTAACCCCACCCGACGACGATATACAATCGA
ATCAAGCCACCTCTCAACAGCAGCAGCAACAGCAGCAGCAGCAACAACAGCAGCAG
CAGCAGACGCAGCAACAAGTGCAGCAGCAGCAGCAGCAGCAACAGCAGCAGCAACA
ACAGCAGCAGCAGCAGCAGCAAGTTCAACAGCAGCAACAGCAGCAACAGCAGCAAC
AACAGCAGCAGCAGCAACAGACGCAGCAACAGGACCACTCGCCCCAACAACAGTTA
ACCCCTCACGAGGTGGATCGCGCGGATTGTTTCCCAGGGGCGGGAGAGCTGCAGCAG
TATCCGCAGCACTACTTCAAGGATGCCAGGCCGCATCCGTCGCCGTCGGTGCCACCGC
ACATGCTGACCCCGGGTGGTTTCTCCGCGTTGCACTATCTGAAGCAGCCGGGCGTGAT
GCTCACGTCCCTCGGGCAGGGGGACGGTGGACCCAGCCTGGACGCGCACCAATACGC
GAGCCCCGGGGCACCGAACATGGCGACCGGGCTGCCGGACATCGTCCAGCAGAGCGG
CAAGTCTGGGAAAGGGGCGAACAGCGACCTACGTTTGTTCAAGTGTTTGACGTGCGG
GAAAGATTTTAAGCAGAAGTCGACGTTGTTGCAACACGAGCGGATCCATACGGACAGT
CGGCCATACGGGTGTCCAGAGTGCGGGAAGCGGTTCAGGCAACAATCGCATCTAACGC
AGCACCTGCGCATACACGCGAACGAGAAGCCGTATGCTTGCGTGTACTGCGAGCGTAC
GTTCCGGCAGCGTGCCATCCTCAACCAGCATCTGCGCATCCACTCGGGTGAGAAGCCA
TACCAATGTCCGGAGTGCGGAAAACACTTCCGTCAGAAGGCGATCCTGAATCAGCACG
TCCGCACACACCAAGACGTGAGTCCGCATCTAATCTTCAAGAATGGGATGACGCCGAC
ACTCTGGCCCCAGGACGTTCCGTTTCCGCAGGAGGAGGGGAAGGAGGAGGTGGGATC
GACGTTCGGCGACACGGACACGCAGTCGGGCGCGTTCAGCCCGGCGCCGGACGCCAA
CTCGATCCAGTATCCCGCCTACTTCAAGGACCCGAAGGGCGGGAATCACGCGGTTTTC

GGTGCGGGTGGCACGGGCAGCTTCGGCGCCCTCCAGTACATCAAGCAGCAAGGCGGC
AGCAAGAGCTGTCTGCCGGACGTGATACAGCACGGCCGCTCCGCGGGCATGCCGTTGT
ACGTTCGGTGCCCGATCTGTCAGAAGGAGTTCAAGCAAAAGTCCACGTTGCTGCAGC
ACGGGTGCATACACATCGAGTCGCGGCCGTACCCTTGCCCCGAGTGCGGCAAGAGGTT
CAGGCAACAGTCCCACTTGACCCAACACCTTCGCATCCACACCAACGAGAAGCCGTA
CGGGTGCGTGTACTGCGGCCGTAACTTCCGCCAGCGCACCATCTTGAATCAGCATCTG
CGCATCCACACGGGCGAGAAGCCGTACAAGTGTCAACAGTGCGGCAAGGATTTCCGC
CAGAAGGCGATACTGGACCAGCACACGCGCACCCACCAGGGCGATCGGCCCTTCTGC
TGCCCCATGCCCAACTGTAGGCGACGCTTCGCCACCGAGCCCGAG

>novel_circ_001428
ACGTGAGTCCGCATCTAATCTTCAAGAATGGGATGACGCCGACACTCTGGCCCCAGGA
CGTTCCGTTTCCGCAGGAGGAGGGGAAGGAGGAGGTGGGATCGACGTTCGGCGACAC
GGACACGCAGTCGGGCGCGTTCAGCCCGGCGCCGGACGCCAACTCGATCCAGTATCCC
GCCTACTTCAAGGACCCGAAGGGCGGGAATCACGCGGTTTTCGGTGCGGGTGGCACG
GGCAGCTTCGGCGCCCTCCAGTACATCAAGCAGCAAGGCGGCAGCAAGAGCTGTCTG
CCGGACGTGATACAGCACGGCCGCTCCGCGGGCATGCCGTTGTACGTTCGGTGCCCGA
TCTGTCAGAAGGAGTTCAAGCAAAAGTCCACGTTGCTGCAGCACGGGTGCATACACAT
CGAGTCGCGGCCGTACCCTTGCCCCGAGTGCGGCAAGAGGTTCAGGCAACAGTCCCA
CTTGACCCAACACCTTCGCATCCACACCAACGAGAAGCCGTACGGGTGCGTGTACTGC
GGCCGTAACTTCCGCCAGCGCACCATCTTGAATCAGCATCTGCGCATCCACACGGGCG
AGAAGCCGTACAAGTGTCAACAGTGCGGCAAGGATTTCCGCCAGAAGGCGATACTGG
ACCAGCACACGCGCACCCACCAGGGCGATCGGCCCTTCTGCTGCCCCATGCCCAACTG
TAGGCGACGCTTCGCCACCGAGCCCGAGGTGAAGAAGCACATCGACAACCACATGAA
TCCGCACGCGGCCAAGGTGCGCAGGAACTCGAGCAGCGACTCCAAGCCGCCCGGCAC
GCCGGCCGGTCTTGGCCCCGTCCCCGTCCCCAGGGGGTTGACGCCGACCGTGGTCAA
GCCGGAGCTCTACTTCCCCCAATGTTACGCGCCCGCGTTTAATCACCAACCCCCGGTGT
CGACGGCGCAGTTCCCGGCACAGGCGAACGGGGTGTCCGTCGCCGGCGAGTTCAAAC
CACCGACCGGCCTGCCGCCGCAGTGACTAACCGTCCATCCTGCCGCCTACTAGCAAGC
AGGAATACCGCTGAGGTTTCAGCGATGCTTCGGTCACGCGTTACAGAGCGACGCCGCC
GCCGCCGCCGCCGCAGCAGCAGCAGCAGTGACCGCCGCAACGACGTCGTCCAGTGCC
GCGACGCACCGATGCACCCAGCCGATCCAGCAACACTACCCGTGACATTTATCGTGCA
TTTACTAGCCTCTTTCTTTTCGTTTCAAG

>novel_circ_001429
ATGAAAATTTAAAACAGATACAAATATATATAGATGATGAAATAAAACAAAAATATATAA
AAAAAATTGCAGAAGAAAAAGAACAAATAGGACGGTCTCTATTGACAGCTTTAACTTT
TATACACCTAGTTAAAAATCAATCTTTAGTTAAACAATAATTTAAAATAATTAATTGTACA
TAAAATTT

>novel_circ_001431
CATCCCTGGCAAGTGAGCAGAGACGGCCAGAAGATCATTGGCCACATGGTATTGCGAA
AGCAGTCCGGATCCGGGAGCAGTAGTAGCATACTCGGGTTGAAAGTGGTCGGCGGCA
AACTGTTGGAGGATGGCTCTATGGGCGCCGTCATCGAGAAGGTGAAGAAGGGCAGCA

CGGCCGACATAGAAGGTCAACTCAGACCCGGTGACGAGGTGATCAAGTGGAACGGCA
GATCCCTTCAGGGCAAGAGTTTCGGCGAGGTTTACGACATCATCGCCGAGTCGAGGCA
GGATCCCCAAGTGGAGCTGGTAGTCTCGCGGAATATATCGTCGACCGCGGGTCCCATG
GCTACGGGGGGGCCTATGACCGGGGGGCCGATGGCGGTGAGGAAGACCGCGCAGACC
CAGTGGAGGCAAAAGCACCCGGAGACGATATCCGGCCCGCAGCATCACAAAG

>novel_circ_001432
CATAAGTTAAAGTTCTCACAAAGAGATAAAGTAAAGAAATTCATTACATTTACACATAC
TGGAGAACAAACTGCAATATATTGTTTAGCACAAAATGATTGGAAGCTAGACTTGGCA
AGTGATAATTATTTTCAAAATCCTGAGGCATATTGTAAAGAACCAAAAAATACAGTAGA
TAAAAAGAAACTAGAAATATTATTTAGTCGATATCAAGATCCAAACGAGCCTGATAAGA
TTACAGCAGATGGAATAATGAAATTTTTGGATGATTTGGATTTAAGCCCTGAAAGCAAA
TTAGTATTAATAATTGCATGGAAATTTAGAGCAGAAACACAATGTGAATTTACTAAAGAT
GAATTTATGAATGGAATGACAGATCTAGGTGTTGACAGTATAGATAAATTAAAAGCTTG
TTTAAGTAGTTTAGAAAATGAATTGAGAGATCCTCAAAAATTTAAAGACTTTTATCAGT
TTACATTTAATTATGCAAAAAATCCAGGACAAAAGGGTTTAGATCTTGATATGGCTATTG
CTTATTGGAACATTGTATTAGATGATAAATTTAAGTTCCTGCCTTTATGGTGCCAATTTTT
ACAG

>novel_circ_001433
ATGGAATACGGGAAGGATCTACGATTCAGCGAGCAGTGGGACCGTTTGCAGAAGGAG
TTCACCTGCTGCGGCGTGACCGGCCCTAAGGATTTCGGTAGCCGATGGCCGCTCACCT
GTTGCGGGCCGTCCGGCAACGTTAGCGACAGTTGTCAACCGTACGCGAGGGGATGCG
AGGAGTCGTTGATAAAATGGCTACGGAAGACCGCCGACTTGCTGTTCGTCTTGGGCTT
CTGCGTGATAGCGTTCGCCAAATTGTGTTTCCTCGGTATACTGCGTTACGAGATAAGGG
AGATGATACAGAAGATACGATTGTTAAGGGAGCCACCGCCTCCGGCGACCACCCTCGC
GCAACCACCATTCTCCCAAGTGATACCGGAAGCGACCAACAACGGGAGCATCGTTCGA
CGCACCACTCTGCCTAACGCTGTCACCCCTTCCGGAAACGCATCGTTGTTGATTCAAAC
GGAGGAATGCAACACCGGAAGGCATCCTTTGTTGGCGAACAATTTGCAGGATGGCGG
GGCGGACAGCGACACGAACAGTCATTGCGCGCTGATCCTCGAGGAAACAACGCCTAC
CTCGGCGAACCATAATTGCGGTAACAGAGAGAAAAGCAATGGCAACAACAATTACGA
AATGCGAGAATTCAACAGGAGGCTGTGGTTGTCGAACGGTGGCAGCCCGGGCACGGG
TATAACAGCCGGTGGTCAAAGCAGGCGCACCTGACTATGGAAGTGGTGGCGAAACAC
GTCGAACCGTCTCCTCTACAGGAGCAATCGGTCTGCCGAGATCACCGTGTATAA

>novel_circ_001434
GAGGTTACAACCATTTACGCAGTCCCGTGTGCCAAGACGAGAATCAGAGGGATGAAA
CCTCGCCGCCACCCCACAGCCATAACACTCAAGCGCCCAACTCTCATTCCACTACTCC
GAATAGCAACAATAACAATAACAATAACAACAACGCGAACAACGCGTATATACATCTGC
GGAACTTGAATCAGCTCAAAACGGAGTCGAACTACAGCAATCACCATATAACGGAGCA
CCTCCAAGGGGGTGGGGCAGGTTTGACGAGCGGCAACGATCTCGCGGGCACGGGGAC
CAACGAGAGCGACTTAAGGGTTAGCCAAGCCGCCACGGAGAGCTACATGACCCCGGC
GAATTATTCCGGATACGATGAAGCGTCCGAGTATCACAGCCTGCCGCAGGATCATCAAC
CGCCGCACCCCTTCAATCTCGACGGCTCGCCGGAATTTACAGCACCAGCGGAATTCAT

CTCGAGCCCAAGTATCAGCAGTCGCCTTTCAAAAATTATCCTCGAG

>novel_circ_001435
ATGTCGACGCGGCGAAGGCCTCTCTGGAGAACGTGGCGGGCAACATGTACGAAAGCG
TATGCGTGCCAGATTTAGGTGATTTCTTGGGATACCAGAGCGGCGGCGGCGGCGG
CGGCGGTCATCAGGTCGCGACGCCGGAACACAAGAGTCCAGCGACGCCAGCGAGTTC
CGCAACTTCGAACTCGTCGGGCCCGCAAAACGGCGGAAACCAAGCGGGGGCCCCGTT
GCAACCACCCTCCGCCTCGGTCTCTCTGCCCTCGAGACAGTATCATAACGATG

>novel_circ_001436
ATATACCGGCACAAGTGCCGCCGTTAACACCTGGGACCAACAAGAAGATGACCGAGG
CCCTGGAGGCCTCTTTCGCCTCCTGGGAGAAGGAACGAGTCCGCCTCAACATAACCAA
AGATCCGAGGCAATGGTCGGAAGCAGCTGTCGCTCACTGGTTGCACTGGGCCATCGGC
GAGTTCTCCCTCGCTGGTGTAGCGATACAACCCTGGCAGAACATGACCGGGAAACAGA
TTTGTGCCATGGGGAAGGAATCCTTTTTGGCACGTGCTCCGGCCTTCATGGGCGATATC
CTGTGGGAACACCTCGAGATACTTCAAAAAG

>novel_circ_001437
CGGGATGCTAGAGGCGTGGGCAGTTATCTGGGAGGCGGCAGCATTAGCGACGGTGGC
GGCATGTTGACGGGTATGGCGAAGTTGATGAAACAAGAGCTGGCCTCTGACGCCGAG
GAAGAGGGCCTGGTGCCTGCCCTCTCCGGCAGGTTCACCTCGATGCGGAAAGTGCCCT
CTCTCTCCGACCTATCCGATCCAGAGAGCTCCTTGG

>novel_circ_001438
GCAAAATGGATATGGAATCCCTGTACGACTACGGGGAGGAGGAACAGGATTTCTACGA
GGAGAGTCCTGGTACCAGGAAAGGTTACCCGATCGATCACAGATCCATCAAGGTCAGT
GATACTCTCTGATCGTTCATTCAAGCGAGGCAAAGACGGAAGGAAGAACTATCGTCGC
TAAATTTAATCGCACACGATCCACAGCGTTATTCGTCGAAAGAGATCATCCCGTAGGAA
TCGTAAACGAGTTATCCGAAAAGGAAGGAAGGAAGGAGCGTCGGTAGCAGCGGAAA
GGAAGAGTAGTAATCCTTCGAAAGCGAAAGGGACGTGGAAGAGACGGAAAGTCGCG
GGATGCTAGAGGCGTGGGCAGTTATCTGGGAGGCGGCAGCATTAGCGACGGTGGCGG
CATGTTGACGGGTATGGCGAAGTTGATGAAACAAGAGCTGGCCTCTGACGCCGAGGA
AGAGGGCCTGGTGCCTGCCCTCTCCGGCAGGTTCACCTCGATGCGGAAAGTGCCCTCT
CTCTCCGACCTATCCGATCCAGAGAGCTCCTTGG

>novel_circ_001439
GCAAAATGGATATGGAATCCCTGTACGACTACGGGGAGGAGGAACAGGATTTCTACGA
GGAGAGTCCTGGTACCAGGAAAGGTTACCCGATCGATCACAGATCCATCAAGGTCAGT
GATACTCTCTGATCGTTCATTCAAGCGAGGCAAAGACGGAAGGAAGAACTATCGTCGC
TAAATTTAATCGCACACGATCCACAGCGTTATTCGTCGAAAGAGATCATCCCGTAGGAA
TCGTAAACGAGTTATCCGAAAAGGAAGGAAGGAAGGAGCGTCGGTAGCAGCGGAAA
GGAAGAGTAGTAATCCTTCGAAAGCGAAAGGGACGTGGAAGAGACGGAAAGTCG

>novel_circ_001440

ATCGCGGTGTCAGATGCTCGCGTGGTGTACATCGAGGAACGCGAAGATGTAGCTCGAT
CGACCGTCGCCATCGCGTGATCATCAAGAATCGAATACCACCGCGGTAACAGTGGACA
TACTTATCTATAATTAAACTATATAATGCTGACAGTGTACCCCGAACGTGAACATTTACTC
TCGCGTTGAATACCAGTTGACCAGTTTGAATCTCTGCAAAGAGCAAACAACGATCTAC
CTCGTATTCGACGCCTGCTAAAATAATACTCGGCTTTCGAACGTTGGAAGAAAGGATCG
GTCGTTGAAAAAAAAAAAAGAAAGAAAGAAAGAAAAAAGAAAAAGAGAAAGGAAA
AGAGAAAGAAGAGGAATAATAAGAAGGAATCGGAGGATGGGGGGACACGCGGAGGG
AGCGAGGATCGCGTGGTCAGTGGCACGGCTTCCGTCGTGGCCGTGGAGAACGAGATT
CTTAGGCCAGCTGGCCTGAGATATGCCAGCAGCAACAGACCTCGTAATCACGGAACAA
TCGAGAACGATATCATCCTCGTCATCGTCATCCTCCTCCTCATCGTCATCGAGAACGGA
AACGGCGGTATCGAGGTGTTCTGGGGGTGTCGCTGGCCGACAGCTGGCGGTAGAGATC
GAGACGATCGAGCGCCGGGTCTGCGCGACCCGACGCGACCTCTTCGTGCCCGTGACC
GTCGACCCTCGGCCCAGCCGGGCCGTCCACTGTCCCGATCTAAACGCCTGTTTGGTGA
ACGAGTCCCGATCTGGCCACGACCAGGTCGACTGCCAGGCGATTCACTTCGGTTACGC
GATCCCCGTGAACGTGGTCCCCCTGGGAACCGATCCACGGCTCGCCGAGCTCGTCTTG
GACCACTCGACCGAGTTGAGCAGACGTGCGGCGAGCTTCCTGCTTGAGCACCATCCCC
AGCTGCGCCAGCAGCGGCAGACGCAGCAGGAACGTTGTCCAAGCATGGCGGCAACCG
CGAAGCACGATCTGCGACAGAAATTCCCGACGGACACCTCGACGTCCGGTGACGAGG
ACATCGCGGCGATCGCGAAACAGATCAGCGACCACGCGGAGGCCATCTATCAGACTTG
GAAGAGTCGTGGGCTAGCCCCTACCGAGATATTGAACTGCCACAGCAATGCCACGGCA
GCGGACAAGTTCGGAAGCGCTCTCACGCCGAGCTCCGCGAATTCGAGCAGCGCCAAC
AGTACTGGGAAAAGTTCGCCGACGAGCGGCACACCGGCCACGGCGGCGGTTGATATA
CTGAGCCAAACGCCGAATCTCGATAACGGAAATTTGGAGAAGCTGGTGAACAATTTCG
TGGTGGAGGACAAAGCTAGGTTAGCCGCGTCGAGGCAGAGGTCGCCGTCCAAGACTC
TACCCTCGTCGATACAGTTCGCGCTAGAGAAGTTCGAGAAGAACTCGATGCAGCAACA
GCAGCAACAATCGCAGACCTCTCCGTTGAAGAGCGGTAACGTCGGTGGCAGCGGTCA
GGGGAAGGCGAAGCAAGCGACTATCCTCCTGTCCCCGACTTCCCCGTTCACGAACAA
ACCTTCGCAGATAGTGAAGCCTCAGGTTTCCCCGAAAACGCCGGGCGCGCATCATCGG
GAAGTGTTTTTGGAAACTATAGAGACCACCTTTCCCGCGGATATCACGCCGTCGAAGAT
CGTGGTGCAGCAAAAGAGGCCAACGAGTACGGTCATCACCTCTCCCACCGCTTCCTCC
AACCTCGACGATTCTTCCACGAATTCAGGTTTGACCACTTGGCCCCTGAAGAACAAGT
TGAACGACAATAAAAGAATAACGGATCCTTGTAGATCCTCGCCGCAACAGGAGGGCAA
ATCGTTGACGAGCAAGGAGGAAAAGAGGAACAGCGGTTCCAATTCGAGCGCGTACCT
GGACGAGGTTGCACGCGAGGAGGAAAGACTGATAAACGCCCTGAAGACGGGCTCGGT
GATCACGGAGGAGCCCGTGGAGAGGACGGTGCCCCTCACTCGTCAGAAACCCGCGAT
CAAGAGGGAGAAACCGAAGGTGGAGCCCGTTAAACCGATAATCATCGGCCACAATCAT
CATGGAGGGGGCCTGGTCCCCGCTTCCGCCACCATGGTGAACAACGTGACGTCCATCG
GGACACAGGGAACCAATCCCGGTGCGACGTCAGACGCGAAATCTCTGAGCAAGGATG
ATCTGTCCAACATGTCGGTGGTGGATTACGCGAAAGTGAGATACAGAGCGGCTCAGCA
AAATCCTCCGACTCAGCAGAGGTTGGAGGAGCAGAGAACGAACAACAACAGTTTCAA
TTCCACGGAGCAGTTAGTGTCTACCACGAGGTCTAAATTCGAAACGGAGATACAAAAG
GACAAGGAAGCGGGTAAAGAGGAAAAGGATCCTGTCACGTCTAGATGGGGCCCTAGA
ATCAATTCGCCTAGGCCAAGAATCGAGCAAGTACCTCATCCGGAATTGACCACCCAAC
AGAAGCAGCATATCAGAGCTGCCGCCGCGACTGGAACCGGGAACAATCCTATCAGACC

GTTTTTAACCAGGGGATCGGTCGCCGAACGCGTTTTGATCTTCGAGAAGTGCCCGAGT
GAGCTGTTGCTCGACAAACGAGGGCCACGTCAACCGGCCATCAACACTTGGAGAACG
GGACACGAGGTTCAAAACAAGGCTCAG

>novel_circ_001441
AAATGGACGATCCACCGCGGAATACGTCGTGGAAAGAACCAAGCTGTGCCAGGAAGC
CAAGGCACTGACGCCAAAGCCCCATCCGACTCGGAAAACGCCCCTCAGGTTCAGGGC
TCGTGTAGCAACTGTGGCGTTGCTTGTCATAGTGTTCGTGCGACGCCAAAAGGACACG
CGTGTCACAGCTGCTATCTGCACTGGAG

>novel_circ_001442
CCTCGGACCTGGACTGCATAAGGGCGCTGTTCAACGAGAAGGAGAAGGAGCTCTCGT
TGGCGGTGGCGAAGGTGGAGGAGTTGACGCGACAACTCGAGGAGTTGCGGGGCAGG
CAGAACGCGGCCGGGACCGGAAGCGGAGGTGGAGGCGCCGGAGGCGTCGGCGGCGG
AGGCGTTGGAGGCGGCGGCGGCGGTGGACACTTGTCGACGCCAGCCAGCGCCGAGCT
CGAGAAACTCAGGAGGGAGCTTATTTATCGTAACAAGATGAACGAGCAGCAGAACCA
AATGGTGTCTCAGCAACGGTTGGCCCTAGCGCAACGGCAAGCGGAGATGGCGTCGATT
GACGCGAGGATAGCTCAGCTGCAGGGTCGCCTTCAACGCAAGAGAGCGTTGAACCAA
CGGCTGAGCCAGCAGCTAGGCTCTGGGAATCGCACGAGCGCGAGCACCGGGTTCACA
GAGTCGAAGCTGGACTTCAATGGCGGGGGGAAGTCGAGGCCTGCTGGAAATATAGCC
GCTATCGAACCCTATTCCCATATACCGAACGACAACGATTTCAATTTGAACAAGAACGA
CCCGAAATACCAGACACTGCCTTACAACACCAAATTCACGGTGAATTTCAAGGCTGCC
GAAGACGACGTTAATAAGAATAAGATACAACACTCGGCCAGTGCATCCCAATTAGGTC
AGAGGGCAGCTTTTCAACAGTTTGGTCATCAAATCGCCCACAGCCAATCCCAATCTCAT
ATTCAAGGTCAATCCCAATTACTGGGCACGAATCAACACAATCAGAATCTGCCTAATCA
ATCCGTCAACATGCAACAGACTTGCAACAACAACAACAATAACAACAACAACAATAA
CAATAACAACAGTAATAACAACAACAACAACGCCAATAGCACGAACAACAACCTGATT
AGCACATCCCACAACTTCAGCCCCGAAGGTTTGTCGCAAAATCTTCGGATCCATCATCA
ACAGCAATCGCAGTCGCAGACGCAACAGCAGCAGCAGCAACAACAACAGCAACAGC
AACAGCAACAACAACAACAACACGGAAGCATTCAACAGAAGCAACACAACGTTGGT
TCGTCGACATCGAATCTCGGCAGCACCGTATCCATCACTCAAACACAAAATCACAGAA
ACCTTCAAACGCATCAGAAACCAATATCCAGCGTGGCGCCAAGTTTCTCCGTCAAGTC
TCAAATCTATCAGACTTCCTCCACCAAGATTCATCCCGTGATGCCGCAAACTTTGAGCC
TGATAGGCCGTGGACACAATAACGCGCAAAATTTCGTTGGTCTGAGCACTCAGGCAGG
TGGGAATCAGCAACAATCGAATCAATCCTCTTCTAACAATCAAACGTCCACTCAGGAC
CAGACGTACGCGTCGAGGCACAACTATCCCGGTATTCAACACTCCGCCCAAGCGAATA
CTCACCCTGTGTCCTCATCCTCGGTGTACCAGACGCAGAATTCTCCCAATCCTTCGTCG
ACCATTCACACGTCGTCGAGCGGGACCAGTTTTGGAAACTCGGTTCAAAGATACAATC
AGGCTCAACCGTTAACCGGGCCCGCGTCCAACAACGAACAATCCGATAAGATAAAGTT
TTCGGAAGGGAAGGTTCAAGAAAAATTCGAGCACGCCCTTCCGGCTAAACACGAACA
GCAGAGGTACGAGGCCAATCAGGTGTTCAAGTACGATTCTCATCAGATAAAGTACGAG
CAGCATTCCAAGTACGATCAACACGGGAAGTACGATCAGGCGAATCAACAAACGAAG
TTGTACGAACAATCGAGCGCGAAGCACGAACAAATTGGAAATCAGACGAATCGTTACG
AATCGAAGATGGATCAAGGGAAGTACGAAAGCGGGCAGAACAAGCACGAACAAATTC

ACGGAACTAAGTACGATCCTAATCAGAATACTCAACAGTACGGTAAACACGATCAACA
CGAGGTGAGGCCGTCGATCAAGTACGAGCACAATGCGGGATTTAAGCACGAAACGAG
CTCGAACAAGTACGAGGGCGGGGGCCAGCAATTCAAGCAGGAATCGGTGAAATTCGA
GGGGAACAAATTCGAGCAAAGTTCCGCGACGAAGCACGAGCACAATGGGAAATTTGA
GATCCCCGCGAAAACTGTGGAGAAAAGCTCGTTCGAATTCGATAGGTTGAAAAACGA
GAACGAAAGGAAAAGTATGATAGAGGACAAGACGAAACCGGCGCTACCTCCGAAACC
GAGCAAACCGAATCCCCCTCCGCGGCTGACCCATCACGAGAAGATGGACAATCCGGG
CGACGGGATCGCCGATTGCAAGAACAATTTAGGGATCAATACGAG

>novel_circ_001443
TTACCAGTGGGCGGGGGTGGTGCTGTGCTGGTGCTCAGCGAGCTTGAAAGCATGGCA
GCCAGGCAACAGCGGGAAATTGCCCAGCAAAGGCGCCTCCTGGAGCAGAGAGAGGC
CCGTCTAGCCGTGCTTCGAGGCGCACAGGAGCCAGCACAGCAGGACAAACTCGCCAG
ATTGAGGCACAGGTTGGACCAACAACAAAGCAAACTGAATCGGTTGCGATTACTCAG
GTCGCAAACCGACCAGTCACGTGCCAACAACGCCACGCTGA

>novel_circ_001444
GTCGCAGGGGAGCCGCGAGGATGTGAGCAAGGATGCGCGCAGGAGTGCGTGAGATCA
TCGTGATCGCCGGGAGATGGCCGCGACGAGCGTTTCAACGTGATCCCTGGAGGCAAG
CGGAGGGGAACGCGCCGTCGAGCGATGCACTGAGGCCCCGAGCAGCGAGGAGGACG
ACACAAGGCAGCTAGGACGAGCGCAACGAGGCGAGGAAGGATGGAGGAGGTGCGAG
GCTCCCCGGCGCGACGCCTCTCGCAGATCCTCGACCCGATCGCGCGCCTCGCCACCGA
TTTCGGCTCCAACTCGGCCCCCTGCAGCCCCAGGCTGGGCGCGCGGGGTCACGAGGC
GAGCGGCGGCGGCACGATCCAAACGACGGGGCAGGCCGAGGTGGCGGCGCAGCGGC
GACAGCCGGAGCCGAGTCGAGGCGGCGGGCTGCTGCCAAGCGGCGCGGAGAGTCCT
CGACTGTGGCCGAGGCAGCTCCGCCAAGCGCCGGCTCCGCCGCCCCGCCCGAGGCAC
GCCAGCAACGCGAACAACAACCACGCCGCGGCGATCCAGGGCCCTCCAGGAACTCTT
CCGCCTCCGGTGCACGGCAGGGACTCGCCCTACGTCAACGTGCCGAATTTGCTGTTTC
AAAAGCAGGACAAGAAGGGGTTCGCGGAAGCCGCGAGAAGCGCGGGCTACGTGGAG
CTCAGGGACGCGAAGCCAAAGTGCAGCCCGTACGATTTCACGTCGGAGTCGAGCGGC
CACGTGGAGTACGCGGACAAAGTACGGACGTTCGCCGCGCAGACCGATTTCGAAGCT
TCCTCGGCCTGCTACGACGCCAAGGACCTGGCCGCGTTCGCAAGAGCCAGGTCCAATT
TCGATCCCGGACCTGCGCCGCGCGGGGGACCCTTCGATCGGGCGTACTCGTTCTCGGG
CAGGCAGCCGGACCAAACCGGAAGGATGTTCGCCGAGGGCCGGGTGTACGGGGAGC
AGAGGAACGTCGTGGGGGCCGCAGCTGGTTCCGAGGCGGCCAAGAAGGAGAAGCTC
GAGACCAGACCCGCTTACGGCGCGTCCAAGAGCTTCGATGGATACTCGAGCGCCGTGG
AGGAGCTTAATTGGCAGGAAAGGTGCCTCGAGTTGCAACTCGAGCTGCACAGAAGTA
GAAGCCAGGCGACCAGGGTGCGAGATATGCTTCGAGAGAAG

>novel_circ_001445
GTACGGATGATGGAGGAGCGGCTAGTGAAGGGTGAATATTGCTTGAGCACATCCGGCG
TTGGTTCTCCACCGCATTCGCTGCCGTCTACTTCCGGTACGCAGAATGACAAGATCGAG
GATGTCCATTGTGCTTGTATCTCGAAGCTAGAGACACAGGTCGAGGAGCAGAGGCAGT
TGCGGCTTCAAGACGCGAGGCAGGTCGAGGCGAAAGCCGCGAGAATAAAGGAATGG

GTGACCAATAAGCTGAGGGAATTGGAACAGCAAAACCAGCATCTGAGGGAACAGAAC
AACAAGTGTAACCAACAGCTGGAATTACTGAGAAATCACATAACCAACATCGGCCTGA
AACCGCCTGCCCCCACGAGAGCGTCCCTATCCCTGGAAGTAGACCCGACGTTGCGCAG
ACGATCGGAAAGCTTGGAGGGAACGCCACAAGCTATACCGGAAAGCGTGCAGAGCTC
TGTAGCGGAACCACAAGCCGGCACCAAGCACCGAAGACACCTGTCCGCTTGCGGAAC
TATACAACGAGGAATCACAACCACCAATATGGACTCAAG

>novel_circ_001446
AACATGCTCGCAGAACAGACATAGCGGATGATTCTACCTTCAGCTCCAGACGCCGGTT
CTGAGTGAGCTAACCTTCACAGAGTGAGGGTACCTCTTATCTGTTTGATCATTGTTCAT
CGAGCCTGAAGCAGTGCTACAAATTGACAGAACGATGAAGTGGCTCGGTGCGAGTAG
CGCTGGTGCTGCGGGTGAACCGGCACCTTCTATGCAATTACACCACCCTTCCGCAAAT
GGACATATACCTACGGATGCAGAGCAATGTGGTCAAGTAATGGCTGGTGGTGACAGTAT
GCGTATACAACCTGGTCAAACGCATCAAATAGGTGTGAACAGAACTCAAGATGATGCT
ATGGTGGGTTATGTATTTCAACGACCAACAGAAGCAGAATTTAATGCTCAATCTTCAAC
ATTTCAAACAAAACAAGCACCCCGTGCTTGGGCTCTTGCAGATGATGCTATTATCGATA
ATAATCCAGAAAAATGGAAATATCCCATTACTAAACTGAGTGTACCACAACAGAATCAA
CCTCAACAATTAGGTCTACAAACTATGAACAATGTTCATCTACCTTATGAAATCCATCCA
ATGCAACTTAAAAGTGGAGCACCAGGAACAGAACATTTAGTTTATCTTAATAATCAAAT
GACAGCACAACAACAAGTTGCTCTCTTTCATCATCAACAACAACAACAACAACAACA
GTTCAGGAATGGCCAGATCGCTCCCTCGGCAAAAAAGCTTTGGGGCGTGGACGAGGG
CGGCTCCAAGGACGAAGGGGGTGTAAAAGGTGGTGGGATACTTCACCTGGGCGACCA
CCAGATGTGGCGAGACTCCACCTGGAGCACCTCAG

>novel_circ_001447
CTCCAGACGCCGGTTCTGAGTGAGCTAACCTTCACAGAGTGAGGGTACCTCTTATCTG
TTTGATCATTGTTCATCGAGCCTGAAGCAGTGCTACAAATTGACAGAACGATGAAGTG
GCTCGGTGCGAGTAGCGCTGGTGCTGCGGGTGAACCGGCACCTTCTATGCAATTACAC
CACCCTTCCGCAAATGGACATATACCTACGGATGCAGAGCAATGTGGTCAAGTAATGGC
TGGTGGTGACAGTATGCGTATACAACCTGGTCAAACGCATCAAATAGGTGTGAACAGA
ACTCAAGATGATGCTATGGTGGGTTATGTATTTCAACGACCAACAGAAGCAGAATTTAA
TGCTCAATCTTCAACATTTCAAACAAAACAAGCACCCCGTGCTTGGGCTCTTGCAGAT
GATGCTATTATCGATAATAATCCAGAAAAATGGAAATATCCCATTACTAAACTGAGTGTA
CCACAACAGAATCAACCTCAACAATTAGGTCTACAAACTATGAACAATGTTCATCTACC
TTATGAAATCCATCCAATGCAACTTAAAAGTGGAGCACCAGGAACAGAACATTTAGTTT
ATCTTAATAATCAAATGACAGCACAACAACAAGTTGCTCTCTTTCATCATCAACAACAA
CAACAACAACAACAGTTCAGGAATGGCCAGATCGCTCCCTCGGCAAAAAAGCTTTGG
GGCGTGGACGAGGGCGGCTCCAAGGACGAAGGGGGTGTAAAAGGTGGTGGGATACTT
CACCTGGGCGACCACCAGATGTGGCGAGACTCCACCTGGAGCACCTCAGATCATGCCG
TCTCACAACCAATTTCCATGGGTACAGGTAGACGTGTGGGTGTCGGAACTGGATACCAT
CATCAAACATCAGAAGTTGGAACAGTTCTTAGCCCTAGAAGCAGTGAAACGGGTGGTC
TGGGGGTTAAAATGGTCGAATATGTTCTTGGATCAAGTCCTACGACACCCAAAGATTTG
GAGCCCAGAATGGTTTCCCTGAGATTGAATACTGATGCAGACAAGAAAGAAAAAGAG
AAGGGCTCGGCAAGCCCATTTGACAGTTCTAAAGACGATACAGGTCCACAAAATAACG

GATTAGCATCCCAGAATGGTCTTGATGATGATAAAGGATTTAATCGTACACCTGGGAGT
CGCCAACCTTCTCCAGGCGAGGAGGAATTTCAGAAGAACGCGGCTGCTACACTGGCT
GTGAGCGCCGGTGGTGGCGGGGTGGTACTGTTGAAGCCTGGTGTCGATGTGGTCGGA
AGCGAGGAACATTTCATGGCCGCCTTTGCCCCAGCTCCGCCGCATCATCTTCAACATCA
TCAAGCACCACCGACGCATCATCTCCAACATCCACACTCGCACGCGGCGCAACTCTTT
GGTGCACAGGCTCCGCCACCCCCACAGGGCCCTCCGCCTTCCCAGCAGCAACAACAG
CAACAGGGACCCCCGCAGCCGCAAGACACCACTACTCACTTTGATGTTCAG

>novel_circ_001448
ATCATGCCGTCTCACAACCAATTTCCATGGGTACAGGTAGACGTGTGGGTGTCGGAAC
TGGATACCATCATCAAACATCAGAAGTTGGAACAGTTCTTAGCCCTAGAAGCAGTGAA
ACGGGTGGTCTGGGGGTTAAAATGGTCGAATATGTTCTTGGATCAAGTCCTACGACACC
CAAAGATTTGGAGCCCAGAATGGTTTCCCTGAGATTGAATACTGATGCAGACAAGAAA
GAAAAAGAGAAGGGCTCGGCAAGCCCATTTGACAGTTCTAAAGACGATACAGGTCCA
CAAAATAACGGATTAGCATCCCAGAATGGTCTTGATGATGATAAAGGATTTAATCGTAC
ACCTGGGAGTCGCCAACCTTCTCCAGGCGAGGAGGAATTTCAGAAGAACGCGGCTGC
TACACTGGCTGTGAGCGCCGGTGGTGGCGGGGTGGTACTGTTGAAGCCTGGTGTCGAT
GTGGTCGGAAGCGAGGAACATTTCATGGCCGCCTTTGCCCCAGCTCCGCCGCATCATC
TTCAACATCATCAAGCACCACCGACGCATCATCTCCAACATCCACACTCGCACGCGGC
GCAACTCTTTGGTGCACAGGCTCCGCCACCCCCACAGGGCCCTCCGCCTTCCCAGCAG
CAACAACAGCAACAGGGACCCCCGCAGCCGCAAGACACCACTACTCACTTTGATGTT
CAG

>novel_circ_001449
GTTTAGGCCTTGGCGCTTCTTCAACTGCGCGAAGAGATTCCTTCGATAGAAATACATCT
GCTTTCAGTCCTAGTTTAGAATACAGCCGCGCCAAGTGGCCGCAGAGTTACGGAGCTT
TAGGCACTGTAACAGCATCGCCAAGCCCGTTGGGATTATCCTTAACACCGCCACCGACT
TTGGGAGGATCTTTAGGGGGGTTGGTCGGTGGGGCCAGCCGTGTATCAGCTGCCCCCG
GCGCCGAGGCCAAATTCCGGGCTAGCGCGGTCCCAGCTTTGACAGCTAACGGGGTCTT
CGGCTCTAGCAGCTCTCTTTTCCCGAATTTGGTTGGCAAACCTGGCCGCGGCGGCACG
ACCAGCATCGGAGATAAAAATGCCGGTGGACGATCACGTTTGCTAGAGGACTTCAGGA
ATAATCG

>novel_circ_001450
ATGTCCAGGTACCTCGGTCTGATTCTGCTCGTCGTCACAACGTGCGCCCACGGAGCCA
AAAAATGCGAGGGGAACGATCTGGGCTTGAAATACGTGTGGGGCGATGCGCTCACGG
ATCCAGCCGATTGTATAGGTCCAAATAACGTGCAGTATCCGTCAGCCGCAATATCGAGA
AGTTACAAGAATCTTTGGACGAACAGCCGAATTGCGAGATCTCATCAGCCACGAACGA
TCGATCCCGAATTAACGAGGCAGGCGCTATTGCACAATTACAAAATCACCCATGGAGAT
TATTCGATGGCCGAATCCTCGCGAAATG

>novel_circ_001452
TTGATTTTCAGTGGCGAGTACACGAAGGCGATGAGCAAGGATTGGCACAGCGGCCACT
TCTGTTGCTGGCAGTGCGACGAGTCTTTGACCGGGCAACGCTACGTGCTGAGGGACG

AGCACCCTTACTGCATCAAGTGCTACGAGAGCGTGTTCGCGAATGGGTGCGAGGAGTG
CAACAAAATCATCGGTATCGATTCCAAGGATTTGTCGTACAAGGATAAGCATTGGCACG
AGGCCTGTTTCCTGTGCAACAGGTGCAGAGTGTCCTTGGTGGACAAACAGTTCGGTAG
CAAGGTGGACAAGATCTACTGCGGGAATTGTTACGACGCCCAATTCGCCAGCCGTTGC
GATGGATGCGGCGAGATCTTCCGTGCCG

>novel_circ_001453
GTGTACAAAAACAACGAGCTGTTCTACTACATCGACGGTTATGACGTCCAGAAATCAA
ATTGGATGCGTTACGTAAACCCGGCTTATTCGTCCGAATCGCAGAACCTAATAGCATGC
CAGTACAAGATGAACATATATTTTTACACGATCAAGCCGATACTACCGAATCAAGAGCT
TCTGGTGTGGTATTGCAGGGAGTTCGCGGAAAGGCTCAATTACCCGTTGACGGGTGAA
CTAATGCTTCAAAGAATAC

>novel_circ_001454
AGGTGTGTTCGTGAGAATCAAGGACAAGAATCTTCACGTGGAGTGTTTCAAATGCTCC
ACTTGCGGCACTTCCTTGAAAAACGTCGGCTACTATAATATCAACAACAAATTGTATTG
CGACATACACGCGAAATTGGTCGCCAGGCAAAATGCACCTGCTGGCATGGTTCCAATC
ACTATACCACCGGGCGGTAATAAGGCTCCCGCGAGCACCATTTCCGCCGCTCTCGCGCA
TGCTCCGTTATCCCCACCCTTGAGCAATCACGCATCGTCGCCCCAACCATTCTCCGCTT
GCAATCGTTCGAGTCCCGATTACTTCGGATCCCCGAATTCTCAACTATCGCCCCTCGATA
AGAACGGATACATCAGCAACGGCAACTGCAACTGGGAATCGAAAACGTTTACGAGCA
CGATCACCATTCAGACGGGTTGCACAGAGCCCACCCGCCTTGGCACTACGCCCGTCGA
TCCACCCAGTTTCCGATCAGTCCAGGCTCCAACGTCGAACAATTTAATCGGTCCTAAAC
CGTTTGGATCGTCCAATATATCGTCCCCTCCGCTAGCGAGCACAGGAGGTAGCACTCTG
CCACGACCCCAGAGTCAGACAGTGACCG

>novel_circ_001456
GTGAATACCGGATCACCGGCGGAAGCGGCAGGTCTGAAGGCCGGCGATGCAGTAATC
AGGGTGAACAACACGGAGATGTACAATCTGAGGCACAAGGACGCGCAGGACATTATC
GTGAGAGCCGGGAACAACTTCGAGTTGACCGTACAGAGAGGTGGAGGCACCTGGAA
ACCGCACGTGTCTCCGATAAGCTCTACCCTGCCCTCGCCGTCCCCCACCAGCGGATTG
AGCAACATAGCTCCAGTTACGAAAACATCGTTGGCCGCCAAGAAACAGGATGGGCCG
CTCATAGGGAGCGGTCACAATTTCAGCCCGAAACCATTCCTTAACGGGACGGGGGACG
GGTCGATCAAATCGATCGTTAACAAACAGTACAACAGCCCAGTGGGTATATACAGCGA
GGAGACTATCGCGGAGACTCTTTCCGCCCAAGCGGAAGTTCTAGCCGGCGGTGTGCTC
GGGGTGAATTTCAAGAAGAACGAGAAGAATTACAACGCGGAGAACAGCGAGGTATTC
AAAATGGTTCAGGAAGCTGACAAAGAGCCAAAGACACCGGAACCCGCGGAGCCAAC
TGCGCAGAGCGGCGTGATTACGCCGTGCTCGCCGGCCCTGGCAGGACTGAGGCCAGT
CTCGGCGCCTGAAACGAAGCAGCACTCCCAACCGTCCACGCCGCAATCGAGCCTACC
ACCTGGACAGAACGTTTGTGCCGAATGCGAGAGGCTCATCGT

>novel_circ_001458
TTCATCATGATTGTTCAAATTCGATTACTTTTTTACTTTCCATTTATTCACTGCTAGCTT
TGCGAATTTTCAAGATGTTGCAAACAATAATGAAAGTTCTGGAAAGATATCGGCTATAC

TCAACAAATTGTTCGACACGGCATTACCTTCAATTCAAATGTTTATTTTAAAACATAAAT
TAGATCCTATGAAATTGGATGATCTATCCCAAAATCTGAAAGGAGTAATAATTCACCATA
GAACTCTTAATTTAACAAACGGTTCTCTTCAAGGATTATCAAATGTAAGACGGGCAAAC
GATATTATTTTGTCTTTTGAAAACAAAATTCTAACTCTAGATGCAACTCTAGGATTCGAC
GATCTTAAG

>novel_circ_001459
AAAGAAATGATGGACAATGAAGACAAGATAGGAAAAAAATAAAAGAAATAAAGAAGA
CGATGCTAATTCTCTTTGTGTATGGGAAAACCGAGCGGGGTCCGTTGAACAAACTAGG
AGGCAACCCGCCAGACTTTCGAATAAGCTATTCAACCAGTGCAATATACCGTGCAAATA
TAGCGCATCCCGTCTAGACGACGTTGCTTTTCCATCTGAACGAGAAGTTACCTCCTTAT
TTCGTGATTTTTCCATGGAAAACTTGGCGAAGAAATAGGAGAAGCAATAGCAAAACAG
TTGAAGTCGAATCGATTCGAGATCATCGATCTTGCCCAATCGTTTTCGCTCGTTAAACA
ACGATAGTGTCTTGTTCAATTGATAAACAAGTGAAAGTTTTCTTTTATTATAGTCAATAA
ATATTAATGCCTCTTTTTTTTTTCTTACTTCTATCAATTTAATCTATCAATAATTTAAAAAG
TATATATTTAATAATCTGATTTGATTAAAATTGTATTCAAAACTAGCTATCTAGTTATTTCT
TCAATGTTCAATATATGTAATTCAAAGCTGAACAACCGCGAGAAACTGGTGCAAACTC
AACTCGAATGACTTTATCTCGGCATACGTACACACAATACTCTTTATG

>novel_circ_001461
ATCGCAATGAAGGAGACGTTGACGAAGCAAGAGATCAAACGCGAATGGGAGGAAAA
CAGCGTGGACGAAGGTTCACCCAGGATGGCCATGGTTGGGACAAATCCAGATGAACA
ATCCGTCGAGAGTCCCCGGACGGTGATCACAGCGAGAAGGCACGTGCGCACCATCAC
CACGGCTGGTCATATAACCGAGATGGTGGTTGATCCGGAACCAGACTCACCCGATAATC
TCCAATCGCAGCGGCAACAGCAACATCATCGATCGATGGATGAACAGCAAACTCGATC
GCGCACGTTTCAAGAAGAGCAACAGCAAGATCAAGCATGTAAACAAAATTCGCAGAC
CTACGTGCAAATTTCTCACACCGAAACGGAAATTCAACAACAGGATCGTTCTCGAGAG
CAACGTATAGTTTATTTAACGAGCAATGGGCAAGAAGTGCGAGTCGTAGCAGAAGCGG
TAGATCCATCTACATTGACGGTGAAAGAGACCGTTAGGTATGAGACGTCAGAATCCGAT
GGGACAGAAGCGGATCGCATATACGCTTATCCGGCGGATGGCCAACAACAATTGCAAA
GAGAGAACCACGGGATCGCTGTTCAAGCGCAGGAAAGGCGAGTCGTTCAATCAGCCA
ATCATCAAAGATATAGTCCACGAGAAACTAACCAATCCAGTGGCGGAAATGGGTCAAC
CAGTAGATCCTATCATCAAGGCTCACCAGTCCTAGTTCCAACTAGCGAGGAGTACGAG
GGTGGCGCCCCCATAGTTTCTCATGCGGGGACAACCGTTAGCGTGCGACTCGATTCGC
CTACAGCGCCCTCTTATTCTCCGCCAATGAACGACATCGGTATTCGAGCGAGTGCTGGA
CTTCAACAATCAACTGGTCAGCATCATCATCAACTTGTCGCAGGATACGTGAACGCTGG
AGGAGTAAATATCAAGTACGAAACACCGGAAACGTCAAACGCCGCAGTGGTAGCAGC
GGCTGCTGCTGCGATTCCAGTCGACAACATTAAAATATCGAACACGTACACTACTCTCG
AAACGGTACCGATTCCTCCCACGCAGGCTGTTCAGTATCCTCAGTATATATCCGGTAGT
GAGACGTTTCAGCAAGCTCCAACTTATACGTATACAAAACCAGGGGAACAAGTGATTC
TGACGTATCCGTCACCAGTTCAATTGCCTTCGCGCGTTCCTGGAGTAGAATCAGGGAG
CACATATATGAAAGGTGATCCAACGTTAGCATCTTCGTTGGGAAGTACGCGTGGAGTAG
CATCTCTGCATTACGAACAACCAGGATCTCCAGGATCTCAAATGGCGTACGGAAGCGG
GACTAGTACGTATCCATATGCCAAAGCGACTCCTACAGCCGATTATTGGTCGACCGCAG

GAACTCCATCTCCACCTACGTTCGATTGCGTCCAGGGATACCAAACGGCAATTTCCGTC
GGCGATAGTAACATACTCTATTCTGGCGGTGTATACAGTGTCTCAACTGGAAACACTAC
GTCTTCATGGTCTAACAATCTGTCACTATCCAATACCGAGGAGCATTTCGAGGGTTCGA
TCATGACTGTGGATCCTAAAGAGTGTCCTAATTGCGCAATTTTAACTAATGTATTAAGGA
GGGACGAAACTGGGAATTACGTGTGTCAAAATTGTATCTATGCAGCAAACAAGATAAA
CGGTATAAATAGATCATCCATTAAATGCGGCAAACCGAAGCAAGCTGTCGCCACGGCG
GGTGTTCGAAGAACCGGTGTTCAATGCGCGAATTGCAGGACAAGCAATACGACTCTGT
GGCGACGAAATAACAACGGCGAGCCAGTGTGTAATGCATGCGGTCTCTACTACAAATT
ACACAAC

>novel_circ_001462
TTCGTTATATCGTTCGATAAGCGATGTCAGTAGTATGACGGCGGCCAGCGCGGCAGGAA
CAGGAGGTACTTACGGCCTTCCTTACATGACCGGCAGCCCAACAGAATTGACCGCCTC
GCCCCAGCAATTGTGGAACGCTCAAGGTCTGGGGACAGGACTTCCCGCCATCTCGGA
GGATTACGGCGCTGGCAGCAAGTCGACAGGCACGGTGACGCATCAGGCGTTGCCAGG
ATTTTCGCAACCGTTCTGCGGCAGGGCGAGCTTTCGAGGATACAGCCCGTCTTATCCGA
CGCAGCAAAGCAACGCCGGGGTCGGGGCTGGGACTGTGGAGCCCTCGTCGTGGAATT
ATGGCTCGCCTTCGAACGACACCCTGGCCACCCAATACGCCGCCCCTTCGAGGCGGCA
ACCCGTTAACCCTCCGTCGACACCCGCGCAGCATCAGCTCACAGCCACTGCTAGTCTT
AGCGCGA

>novel_circ_001463
AATTGGCCGAATGCCAAGTGGACGAAATGACGGGCTACGAGAAAGGGACGGAGAGAT
CCGTGAAACTGATCTCTGGCGAGAGCATCGAGGAAACGAACAACGACGGTGGATCGA
TGGGATTGTTGGAGACGGACGGCGGGGACGTGGTACTTTTTCGTCAGGAGACACAAC
AACAGAAGCAACAACAACAACAACAACAACAACAACAACAACAACAACAACAACAG
CAACGGCAACGCCACCAACTTTACCAACTACCTTACGAAAAGCATGGAAGCGAAGAG
GGCGGGGACATGGTGGGCGTGCTGGAGGCGGGACAACAGGAAATTGAAAATGGCGC
GGTCGGCGAGGCGAGTGACGCCGAACCGGTAGAAATCCCCGTCTCACCGGAACAAGG
AAACGACGTAGACGACGCTTACGGCGTGCACTCGCTCGGCTTTAACGCTCAGGACCCT
CTGGTTCGAGAAAGGGGTTATCTCGATCAGGACTCGTACGGGGGCGGCGATTTAGGCG
GCGATTCCTCGAAGCAGGAGGAGCAGCAGTTGCAGCAACAGCAGCAACAGATCTCTC
AACCTCTCCACCACCATCATCACCAACAACAGCAGCGTCAGCAATCGCACCACCACCA
CCATCACCACCACCACCAGCAGCAGCACCACCATCACCACCAACAGCATCAGCAGCA
GCAGCACTACCACCGCCATCAGCACAGCGACCATCACCAACACGACCAACAGCACCA
TCAGTCCGAGTACGTTCAACTGGGGGCGAGAGAAGTATCGGGTGGCGAGGGTGGGGA
TAACGAAGCGCCGAGCGAGGTCCCCGTATTGCCGGAGGAAAATCGGGCCGGCAGTCA
CGCGGCTTGCTCCACGATGCATCGTTACGGTACTGAAGCGCTCTCGCCGGCAGCGGCC
GATCATCACAACAACAACCACCACCACCACCACCACAACCACCACCACCACCACCAC
CACCACCACCACCACAGTAACAACCATCACCACCACCATCAACAGCACCATCACCAGC
ATCTACAGTCGACGGACACGGCCCAACAACACGGCGCGCTCTCCTCGGCGAGCCATCG
CCACTTGGACGAGCAGGAGGCGCAGCCGCAGTCACAGTCGCATCAAAAACCGGACGA
GGACGTCGTGGAGCGAGCGAGTGGAATCGCGGTAGCGATGAGCGGCGCACGCGGCGA
CTTCAACCTCGGCATGCTGTTTCCGAATCTGGCCACCACTGGCGCGGCCGGTTCCCCA

GCAGAGATAGGTGGAAACGATGGCCAGCAGCAGCAACAGCAGCACAATCGCCATCAC
CAGCAGCAGCAGCAGCATCATCATCACAATCATCATCACTTGGAGGAAGTTTTGCCGG
ATACGGCGTATCTTCAGCATCAGATGCGCGGCGGAGGTGGCAACGGAGGAGGTGGCG
GTAATGGCAACAATGGAGGAGGTGGCGGAGGAGGTGGCAGCGGCGCGGAGGGCAGC
GGTGGAAGCGGCAGTGGAGGAGGAGGAGGAGGAGGCGGATGCTCACCGGCGCTTCG
AACCGGTAGCTCACCGGGTTCCAGTCGTAGCCCGCACGAGGATCAAGAGCTCTCGTCT
CCTCATCGCCAGGGGCAGGCAGAGGGAGGATACAGCCCGAGCGAGCAAGGGAGACA
GTCGTACGCCCATTTGACAGCTATGCAGCCACCTTCCTCGGTACAGTCGAACCACGGC
CTTGGCCAGGATACCGACCGAGTCTCCGATCAGATTTACATGGACTCGATATACGCTCA
TCACACTGTCGGCACGCCTCATCATCATCAGGAACACGAGCCAGGATCGTCGACTCCT
CACTCGCCCAGCGGACCGCTTACAAGTTCGTTATATCGTTCGATAAGCGATGTCAGTAG
TATGACGGCGGCCAGCGCGGCAGGAACAGGAGGTACTTACGGCCTTCCTTACATGACC
GGCAGCCCAACAGAATTGACCGCCTCGCCCCAGCAATTGTGGAACGCTCAAGGTCTG
GGGACAGGACTTCCCGCCATCTCGGAGGATTACGGCGCTGGCAGCAAGTCGACAGGC
ACGGTGACGCATCAGGCGTTGCCAGGATTTTCGCAACCGTTCTGCGGCAGGGCGAGCT
TTCGAGGATACAGCCCGTCTTATCCGACGCAGCAAAGCAACGCCGGGGTCGGGGCTG
GGACTGTGGAGCCCTCGTCGTGGAATTATGGCTCGCCTTCGAACGACACCCTGGCCAC
CCAATACGCCGCCCCTTCGAGGCGGCAACCCGTTAACCCTCCGTCGACACCCGCGCAG
CATCAGCTCACAGCCACTGCTAGTCTTAGCGCGA

>novel_circ_001464
AATTGGCCGAATGCCAAGTGGACGAAATGACGGGCTACGAGAAAGGGACGGAGAGAT
CCGTGAAACTGATCTCTGGCGAGAGCATCGAGGAAACGAACAACGACGGTGGATCGA
TGGGATTGTTGGAGACGGACGGCGGGGACGTGGTACTTTTTCGTCAGGAGACACAAC
AACAGAAGCAACAACAACAACAACAACAACAACAACAACAACAACAACAACAACAG
CAACGGCAACGCCACCAACTTTACCAACTACCTTACGAAAAGCATGGAAGCGAAGAG
GGCGGGGACATGGTGGGCGTGCTGGAGGCGGGACAACAGGAAATTGAAAATGGCGC
GGTCGGCGAGGCGAGTGACGCCGAACCGGTAGAAATCCCCGTCTCACCGGAACAAGG
AAACGACGTAGACGACGCTTACGGCGTGCACTCGCTCGGCTTTAACGCTCAGGACCCT
CTGGTTCGAGAAAGGGGTTATCTCGATCAGGACTCGTACGGGGGCGGCGATTTAGGCG
GCGATTCCTCGAAGCAGGAGGAGCAGCAGTTGCAGCAACAGCAGCAACAGATCTCTC
AACCTCTCCACCACCATCATCACCAACAACAGCAGCGTCAGCAATCGCACCACCACCA
CCATCACCACCACCACCAGCAGCAGCACCACCATCACCACCAACAGCATCAGCAGCA
GCAGCACTACCACCGCCATCAGCACAGCGACCATCACCAACACGACCAACAGCACCA
TCAGTCCGAGTACGTTCAACTGGGGGCGAGAGAAGTATCGGGTGGCGAGGGTGGGGA
TAACGAAGCGCCGAGCGAGGTCCCCGTATTGCCGGAGGAAAATCGGGCCGGCAGTCA
CGCGGCTTGCTCCACGATGCATCGTTACGGTACTGAAGCGCTCTCGCCGGCAGCGGCC
GATCATCACAACAACAACCACCACCACCACCACCACAACCACCACCACCACCACCAC
CACCACCACCACCACAGTAACAACCATCACCACCACCATCAACAGCACCATCACCAGC
ATCTACAGTCGACGGACACGGCCCAACAACACGGCGCGCTCTCCTCGGCGAGCCATCG
CCACTTGGACGAGCAGGAGGCGCAGCCGCAGTCACAGTCGCATCAAAAACCGGACGA
GGACGTCGTGGAGCGAGCGAGTGGAATCGCGGTAGCGATGAGCGGCGCACGCGGCGA
CTTCAACCTCGGCATGCTGTTTCCGAATCTGGCCACCACTGGCGCGGCCGGTTCCCCA
GCAGAGATAGGTGGAAACGATGGCCAGCAGCAGCAACAGCAGCACAATCGCCATCAC

CAGCAGCAGCAGCAGCATCATCATCACAATCATCATCACTTGGAGGAAGTTTTGCCGG
ATACGGCGTATCTTCAGCATCAGATGCGCGGCGGAGGTGGCAACGGAGGAGGTGGCG
GTAATGGCAACAATGGAGGAGGTGGCGGAGGAGGTGGCAGCGGCGCGGAGGGCAGC
GGTGGAAGCGGCAGTGGAGGAGGAGGAGGAGGAGGCGGATGCTCACCGGCGCTTCG
AACCGGTAGCTCACCGGGTTCCAGTCGTAGCCCGCACGAGGATCAAGAGCTCTCGTCT
CCTCATCGCCAGGGGCAGGCAGAGGGAGGATACAGCCCGAGCGAGCAAGGGAGACA
GTCGTACGCCCATTTGACAGCTATGCAGCCACCTTCCTCGGTACAGTCGAACCACGGC
CTTGGCCAGGATACCGACCGAGTCTCCGATCAGATTTACATGGACTCGATATACGCTCA
TCACACTGTCGGCACGCCTCATCATCATCAGGAACACGAGCCAGGATCGTCGACTCCT
CACTCGCCCAGCGGACCGCTTACAAG

>novel_circ_001466
CACACTATTGTTACAAAAATAGACAAAAATGGAGTAGAAACAAAAACAGAAGAATTTT
GTGATGTAAATGAATGTGATTCATTTGGAAAAAAAAAATTATCATCAAATGAAAATAGT
AATTTTCTCGATAGTGATTTAAATTTCTTTTCTTGGGATAAATTTTTTAAACCTAATCCAA
AATTATGATAAAATT

>novel_circ_001467
GGCCCAGAGGCCCCGCCAAGGCCAAACAGGCAGCATAAGGCTCCATCATCAACGTCG
AATTCCGGTCAGCCTGCGAGCAGTAATGACCAAAACAATAAACAAATGCCACAGTCTT
CCAGTATACTCGATCAAGCTTTATCCGTTGAAAGCGATAGTGATGATGACCTCGAAGAT
GCAGGAGGAAATAATGCGAGGAATGATGGTACATTACTTGCCAGTGATCCACCAAAAC
CTTTACCCGAATTTTCTCCTTTCAGACCATCCGCGGATCCATCGTCTACGTCGTCGCACA
ATGCTCAGAACTCGCACCTCGAAGGTAAGCCGAAAGGTGGAGCACCGAATCGGCCAC
TTCCACCAACCCCCGACGAAGAAGAATCAGGAGATCGTACGCTAGTCATGAAACGG

>novel_circ_001468
ACCATCCGCGGATCCATCGTCTACGTCGTCGCACAATGCTCAGAACTCGCACCTCGAA
GGTAAGCCGAAAGGTGGAGCACCGAATCGGCCACTTCCACCAACCCCCGACGAAGAA
GAATCAGGAGATCGTACGCTAGTCATGAAACGGAAACTTAGTCAGATGTCAGACGATC
GCACGGCGAACATCAACACCAACACCACTACCACCACCACCACCACCACCACCACCA
CCACTACCAATAATAACAACAACAACCGACGCTCCGAGGCCGAGGAGCAACTCCTGCT
CAAAGAGTGGGATTTCACCCGTTTTTTCCAAGGTTTAACGAACGATTGGATAAAATGA
AACAGCAAGAGTCGTCGCAAGAGTCGTCGTCGAGCGCAGAGGCGTGCAACAAGCCC
CCCTCCTCCTCCTCCTCGGTTAACGAGAATCACCTTTCCTCCTCGTCCACGGAGAAAAC
GTTAAAGAGGCAGGAACAATTGTCGTCACGCAGAAAGTACGATAATCAGCAACAGCA
ACAGCAGCATCATCATCATCAAAAGCATCAGCAACAGCAGTTGAAGGCGGTGCACAG
GCGGCAAGAGAGCGACTCGAAGCTTGGTGGGAACACGTCCAGCGCTTTTGCACGCGC
TTTCCGCCGTGAGAATTCCGACTTTTTCCCGTCCGCGAGACACTCTGCATACTTACAGA
AGTCGGACACCTCGAGATCGAGCATATTCTCGAGCGGGAACAGGCGTGGCAGCGAGAT
AAGCGTTGCAGGTATCGCCGGGAAAAAGGGGAACGCTGTGAGCGCCGGTGAACCGGT
GCTTACAGACTTCTCGTTCGGTCGTGACGGTGTTCAGAGGCCTAGAAGGGAGAAAAC
CGAGAGCGAGATCGTTTTTGGTAGTAGGCACGAGGCAAGAAGGCTCGACTTTGGACG
CGATAAAGATGACACCGCGAGGAGGCGATGTTGTAGGCCATCGGACGCGGTTTCCGCC

GCGGTCGACGAAGCGGGAACGATTAAATCTACGGCCAGTACAACGGCTAGCGAGTAC
AGCCCCATTGTCACCCAGAATCGAGAAAGCGGTGATCGTTCGAGAGGCGGAGGCAGC
GGTGGCGACTTCCAGAGGTCGGATTCGTCTCCCGGATCTCGGCCGAGCTCCGTGTTGC
CAGATCTCCTCACCTCGTCGCCTGGCCAGCGACAGGATAAGACAACAAGCGAAGAG

>novel_circ_001469
AAACTTAGTCAGATGTCAGACGATCGCACGGCGAACATCAACACCAACACCACTACCA
CCACCACCACCACCACCACCACCACCACTACCAATAATAACAACAACAACCGACGCTC
CGAGGCCGAGGAGCAACTCCTGCTCAAAGAGTGGGATTTCACCCGTTTTTCCAAGGT
TTTAACGAACGATTGGATAAAATGAAACAGCAAGAGTCGTCGCAAGAGTCGTCGTCGA
GCGCAGAGGCGTGCAACAAGCCCCCCTCCTCCTCCTCCTCGGTTAACGAGAATCACCT
TTCCTCCTCGTCCACGGAGAAAACGTTAAAGAGGCAGGAACAATTGTCGTCACGCAG
AAAGTACGATAATCAGCAACAGCAACAGCAGCATCATCATCATCAAAAGCATCAGCAA
CAGCAGTTGAAGGCGGTGCACAGGCGGCAAGAGAGCGACTCGAAGCTTGGTGGGAA
CACGTCCAGCGCTTTTGCACGCGCTTTCCGCCGTGAGAATTCCGACTTTTTCCCGTCCG
CGAGACACTCTGCATACTTACAGAAGTCGGACACCTCGAGATCGAGCATATTCTCGAG
CGGGAACAGGCGTGGCAGCGAGATAAGCGTTGCAGGTATCGCCGGGAAAAAGGGGA
ACGCTGTGAGCGCCGGTGAACCGGTGCTTACAGACTTCTCGTTCGGTCGTGACGGTGT
TCAGAGGCCTAGAAGGGAGAAAACCGAGAGCGAGATCGTTTTTGGTAGTAGGCACGA
GGCAAGAAGGCTCGACTTTGGACGCGATAAAGATGACACCGCGAGGAGGCGATGTTG
TAGGCCATCGGACGCGGTTTCCGCCGCGGTCGACGAAGCGGGAACGATTAAATCTACG
GCCAGTACAACGGCTAGCGAGTACAGCCCCATTGTCACCCAGAATCGAGAAAGCGGT
GATCGTTCGAGAGGCGGAGGCAGCGGTGGCGACTTCCAGAGGTCGGATTCGTCTCCC
GGATCTCGGCCGAGCTCCGTGTTGCCAGATCTCCTCACCTCGTCGCCTGGCCAGCGAC
AGGATAAGACAACAAGCGAAGAG

>novel_circ_001470
AATCGAGAAAGCGGTGATCGTTCGAGAGGCGGAGGCAGCGGTGGCGACTTCCAGAGG
TCGGATTCGTCTCCCGGATCTCGGCCGAGCTCCGTGTTGCCAGATCTCCTCACCTCGTC
GCCTGGCCAGCGACAGGATAAGACAACAAGCGAAGAGTACCGGCAAGCAGTTAAATC
ACCACAACTACTTGCACATCAACAGAAACAGAGATCTTTCTTGACCTTTGGATTTGGTG
CGGGACCAGCTAGAAGGGAATCTCATGTAAATGTTAATGTGACACCGACCAGCCATGA
CCTTACATCTGATACACCTGAAATACGCAAATACAAGAAGCGATTCAACAGTGAAATTC
TTTGTGCTGCGCTTTGGGGAGTAAATCTATTAATTGGTACCGAAAACGGCCTTGTGTTAT
TGGATAGAAGTGGTCAAGGAAAAGTATATCAATTAATAAGCAGAAGACGATTTCAACA
AATGGAAGTTCTTGAAGGACAAAATATTTTGGTTACAATTAGTGGAAAAAAGAATAGA
GTACGGGTATATTATCTTTCTTGGCTTAAGAGTAAAATTTTGCGAACGGATGGACATAGC
GATCAAGTTGAACGGCGCAATGGCTGGATTAATGTTGGAGATCTTCAGGGTGCGGTAC
ATTTTAAGATAGTCAAATACGAAAGAATAAAGTTTCTTGTCATTGCATTAAAAGATTCGA
TAGAAATTTATGCTTGGGCTCCGAAACCTTATCACAAATTTATGGCGTTTAAATCGTTCA
GTGAACTTGCACACAGACCATTACTTGTCGATCTTACTATAGAAGAGGGTTCGAGATTG
AAAGTTATTTATGGCAGCGCCGATGGTTTTCACGCTGTTGATTTGGATTCTGCTACGGTT
TATGATATTTATCTGCCAAAACATACTCAAGGCCCCATTTGTCCACATTGTATCGTTGCAT
TACCAAACAGTAATGGCATGCAGTTACTGTTATGTTATGATAATGAGGGTGTATACGTGA

ATACTTATGGTAGATTATCTAAAACCATGGTTCTACAATGGGGTGAAATGCCCACTAGTG
TTGCATATATCGGTACAGGACAAATCATGGGCTGGGGTAACAAAGCTATTGAGATTAGA
AGCGTAGAAAGTGGACACCTTGATGGTGTGTTCATGCACAAGAAAGCTCAACGGCTC
AAGTTCCTTTGCGAGCGTAATGATAAGGTATTTTTCTCGTCAGCTAAAGGCGGAAGTGC
GTGCCAGATTTACTTCATGACATTAAATAAACCTGGCATGGCTAACTGGTGATCCCGAAT
ATGGCCAAATCTGGCCCTCAAATTATCTTTACGAATAAGGACACAAAATCTTTCTCGTAT
ATCGAGATACGCATCATCGCCATTCATGTTAATTCGTTTTTCACTGCCAGGATTTAAGGC
CATGGAAAGCAAGCAAT

>novel_circ_001471
ATAGGAGGTGGTTCAGTGATAGATACTTGTAAGGTAGCAAATCTTTATAGTTCTGATCCA
CAAGCAGACTTCTTAGATTATGTAAATGCACCTATTGGTAAAGGAAAACCAGTTACAGT
ACAGTTAAAACCATTAATAGCTGTACCAACTACATCTGGTACTGGTTCAGAAACAACTG
GTGTTTCTATCTTTGATTATCAACCACTTAAAGCCAAAACTGGAATTGCCAATAGAGCA
TTGAAACCCACTTTAGGTCTTATTGATCCAAACCATACATTGAGTTTGCCAGAAAGAGT
CTGTGCTTATTCTGGCTTTGATGTACTATGTCATGCTCTTGAATCTTTTACTGCATTACCA
TATACTGAAAGAACTTGTCCTTCTAATCCGATACTTAGACCAGCCTACCAAGGAAGCAA
TCCTATTAGTGATGTATGGTCAAGATATGCTCTTCAAATAATGCATAA

>novel_circ_001472
CTACCAGTTTAACTAGACTCAATCTTGCGGGTAATCCTTTGACAACATTACAAGTAACA
CCATTCCTGAAGACTCCCAGTCTTACTAAACTCGACGTAAGCAGATGCGCTTTGGAAA
GAGTATGGAGTGAGGCTAGAGTTCCATTGACTACTTTACGGTAT

>novel_circ_001473
GGAAAAATCTTCCGGTCCACTGCGTGGTCGAGACCATACACAACATCGTCGAGACACA
CGTGAGCAATTCTCGTGAGAATTGGCGTAGGCCTCAGGTGGAGACGGACTCGTACGTC
ATCATTCCTGTAGCTATGCCTTTTCAGGATCTGGTGGGGGAGGCTCTGGTGCGGCTTGG
ATACTCGAGCGACCTGATACCAAGCGCGAGGGGCAGTATCGTGATAAGGAATTGGAAA
CCTCTGCCGATGGAGAAGGTCGCGGACGGCCCGCTGTTGACGGTGGGCGACATATTGG
CCGAGTTGACGTCGGTCGCAACGCTCAAGATCCAAGTGTACAGGTCGAGGCCACCGC
CCCCGAGCCCCGCCGCGGAGGTCAGGAACAAATTGTTGAGACTGTTGCTGCTACACA
GCCATGCGCTCCTCGTCTCTGCCGGTTGTCCCCTCGACGAG

>novel_circ_001474
AGCCAAGCTTTGGCATTGACACACAAGGCCTCGCCACCTTCGAGGCCGAGCAGTGTG
TCCTCGTTACCGAGGAGCCCTCAGTCTCACCAGTCCCAACAGTCTCAGCCGCAGAATG
CTACGCAACTTCCGCCTGTGCATCCTCATCAATCGCAGACACCCCTAAGCACACCCCA
GACACCGTTTTCCGCGCACAATCAACACCCCAAGCAGGAGGCGAACACGAGCCCGAA
ACAGGAGGGGTTCGATCTCAGCAAATCTACCTCGAGCACGGGGAAAAATCTTCCGGTC
CACTGCGTGGTCGAGACCATACACAACATCGTCGAGACACACGTGAGCAATTCTCGTG
AGAATTGGCGTAGGCCTCAGGTGGAGACGGACTCGTACGTCATCATTCCTGTAGCTATG
CCTTTTCAGGATCTGGTGGGGGAGGCTCTGGTGCGGCTTGGATACTCGAGCGACCTGA
TACCAAGCGCGAGGGGCAGTATCGTGATAAGGAATTGGAAACCTCTGCCGATGGAGAA

GGTCGCGGACGGCCCGCTGTTGACGGTGGGCGACATATTGGCCGAGTTGACGTCGGTC
GCAACGCTCAAGATCCAAGTGTACAGGTCGAGGCCACCGCCCCCGAGCCCCGCCGCG
GAGGTCAGGAACAAATTGTTGAGACTGTTGCTGCTACACAGCCATGCGCTCCTCGTCT
CTGCCGGTTGTCCCCTCGACGAG

>novel_circ_001475
AGCCAAGCTTTGGCATTGACACACAAGGCCTCGCCACCTTCGAGGCCGAGCAGTGTG
TCCTCGTTACCGAGGAGCCCTCAGTCTCACCAGTCCCAACAGTCTCAGCCGCAGAATG
CTACGCAACTTCCGCCTGTGCATCCTCATCAATCGCAGACACCCCTAAGCACACCCCA
GACACCGTTTTCCGCGCACAATCAACACCCCAAGCAGGAGGCGAACACGAGCCCGAA
ACAGGAGGGGTTCGATCTCAGCAAATCTACCTCGAGCACGG

>novel_circ_001476
GTAACATCCTACATCAGAGAATCATCATTTTTCTCTAAATGGTGGCAAGCCTGTGACGT
GATTAACCGTTGACCAAAGTACCAGAGAAAGGTGTCAAAATTGAGCAGGTCGTCCTCC
CGTCCGGCGGTGATCTCGCCAAAATTTGGAGGATGTTGATGCCCGGTGCTGCTGGCCT
CAGTGCCGTAGGCGCGGTGGGCGCCGTCGGGGCGCCAGGGCCGGACGAATTGGTCTC
GGGCCTTGGCGCTTTGGCGGGCACCACGCCCCAACAGAAAGACACCACATGGACGAA
ACTTTTCGTCGGCGGTTTACCATACCACACCACCGACAAGAGTCTCAGGGAACATTTC
AACGTGTACGGGGACATCGAGGAGGCGGTCGTCATTACCGACAGGCAGACCGGCAAA
AGTAGAGGTTACGGTTTCGTGATCATGGGAGACAGGCCAGCGGCGGAGAGGGCGTGC
AAAGACCCGAACCCCATAATCGACGGCCGCAAAGCAAACGTCAACTTGGCGATTCTTG
GAGCAAAGCCCAGGGGTAATCTGCAAGCGA

>novel_circ_001477
ACGATGTATACTGACGGTCTCCTTACACTCCACTATGGGAAGCATCCGGCGACCTTGGC
CGCGGAGGTCGGAGCCTGGTACACCGGGGACCGTCACGTGGACGTGACGTTGGCATG
CGACGATGGGTCGGTAGTGAAGGCCCATCGCGTTGTTCTGGCCGCAGCTAGTCCCCTT
TTGGCTAGTCTCCTTCGTAACCCCGCCCTGGACCACGTGGTCCACTTGTCAGGGGTGC
GAAAAACCCAGCTCACCCACCTCCTGGAATTCCTCTACAACGGAGAAGCCCTCATCCC
GTCGACGGAGCTCACGCCGCTCAGGGAATTGTTCGAGCTTCTTCAGATAAAGTCGGAG
CTGTTCGAGCCTAATCAACCGCAGACCTCCGCCAACTCCGACCCCGAAAGGATACCCA
CCCCCCAACCCTCCGAGGGCCAGGAGAGCTCCAGCTACGAATCGCAGTACGACGGCA
GACAATCCAACAATCCCGCGGACTGTTGCTCGGTGCTCATAAAGACGGAGGGCTGCGA
GGAGGAGGCGGAGGTGGACGTGGAGGGCGTGGAGGAGGCGCTGCTCACCGAGGGGA
ACAGGGAGAGCAGCATAGAACCGCCTAGGCGGAGGGACAGCTCCGACCCCGTGAATC
TGAGCTTGAACTCGGGCACGTCGACCACCAGCGAGAGTTCCCACGACATCGTGCCCA
GGCCGGAGAAACAGTTGCTGGAGAGGCGGGAGTCACTGGAGGAGGCGGAGGAGAGG
AGGAGGCAGCTGGCCGCCCGGCTAGCGCTAGGCCTGGAGCCCGGGAAGCGAAAGCCC
GAGGAGATCCCGATCCCGCCCGCCGAGGCGTACATCGTGACCCCTCACAGGAAGCGA
AGGCCAGGCTTCCACAACGCGCCCGCACAGAACCCTGCCTTCGTCCCGTTCAACCCCG
GATTCGAGACCCCGAGGAGATTGCAAGCGCCTCGTCCCCTCAGCGTCTCCACACCCCC
CTACCTG

>novel_circ_001479
CTTCCTATTTCTTCTCTAAAAAAAAATGCTAAATACGTTCTAAATCATAATACCGTGTATA
CATGAATTTATACATTATTACCAGTTACGAAAACGGTGGACATCTGACTATCAGCACAAA
TATATTCTGTCAATTTCTTTTTCTTTCCTTCTTTTTTTTTTCTTTTTCTTTTTTCTTTTTTTA
AATATGAAACATACGACAATAGCTACACATCAGCTTAAATTCGATTATTATTATTATTATTA
TTATTACACGTTACTCGAACACCTTGGTTGGTTCATAATAATATAGAAAAAATGAGAGA
GAAAAAAAAAAAAAAAAAAAAAAAAAAAAAAAAGAAGAAGAGGAGAGAAAAAAAAA
AGAAAAAATACGCATGGAATAATTAATGAATAGCGTGATTAACTTTGACGATCAACACC
ACTGTTTTTTTACGTATAAAAATAGCGTTATAACTATTATGAGAGAGAAACAAGTATGAA
AATACTTTCGTATATAGTTGTTACTTGCATAATCTCTGTCCACCACGTTATTCGTAAATGA
ACATATTTCCAAGTTCACAAGACTAATAACTTACATCGACGCGTCATATGTATTCGTTGC
CTGAGTGAACAATTGGACAATAAAATCTAGAATTTCTCTTTGATTCATATTATTGAAGTA
TAGGTATATAAAAGAAACTTTAATAATGAAATTACATAACAGTATAGTGACTGAATACTG
ACAAAGTCATTTGTATTCCATTGATTGTAAATTTTGCAAAAGAAGAAACAGAAATATAA
TATATATATATATATATTTTATATAATTATAGATATTTATATATAATTATAAATATATATACATAT
ATATAGAAGAGAGAAAGAAAGAGAAAGAAAGAAAAGATATATATATATATATATATGTG
CATACATATATACATACATATATATATATTTGTTTACAAAAAATTCGAGATAATAGTGTCCA
AATGTCACTTATTCTTTTTCTATTTCGCAATAAAATAACGCAACGCAATACGGCAAAGTC
GTTATGCAAAGTTTGCGCATATAGTACCACCTTGTCATAAAAAAAAAAAAAAAAAAAA
AGAATGAATAATAATAATAATAATAGTAGTAAAGTAAGAATATATAAAGCGGTAGTGGTA
CTAATCATAATAATAATAGTAATATTACGGCATGAATCTACACTCTTACATATAGACTAAG
GTCCAACTAAACAACTTGTGCCGTAAAATTGTACACTATGTAACGAGAAAGTGAGGTG
GATCACTTTTCGTTGTGAATATTTCTTCTTGTAATGTTCTTGGCAGGGCCTCCGAGTTGA
CAAACAGACTCGACGATCAAAAATCAATCATATAGAATTAGAATTTGAAA

>novel_circ_001480
GAACGGGCTAAGAGGAGGACATTGTTCGTCGCGCTGTTATCCTCGGCGGGGTGGGGCG
GAAGGAGGAATATTCCGGAAGATATCTCCGGTTTTTCGTGAGAACCACCTCTTCTCCGC
CGTCGTACGTGAGAAATAACTGGCAGAGGTCGGCCTTGAGGTGTGGAAACACAACAC
AACGGGGCCGGTGGTCGGTGGTTGGTGCGCCGCCGTTGTAGCGGTGTAGTGGTGGTAG
TAGTAATCTCGGAGCGGCGCGGGAGGGGAGGGGGGCGGGTGGGTGGGGGGACTCGG
CATCGCGCAGAGAGAGAGAGATTTATTCGAGTTTCACAAGCTCTCTCGATCACCGGCACC
GGTCGTGACCTCGATAGAGCTCATCCACCTGGTTGTGTTGGATAACTCGAAGCAGCAG
TGGCAGCCGCCGCCGCCACAGGACCCTGAAAGGTCGAGAGCTCTCCTTTGCAATCTCC
TTTCGCGCGATATTCGCCTCTTCTTCTCCTTCCTTCTTATTCTTCTTCTTTTTCGGTTGCA
CGAAAAGTAGCGACGATTTTCCTACCCGTTATCCTTGTCGAACGGCGCGCGCTTCCTTT
GTTATGTATCGTTCCACGTGTCTGTGCGCAGAGTAGCAGAGTAGTCGCGCGCCAAGGT
GGAAAGAGATAGGGATATATTCGCGGCTGTTCGGGAGAGAAAAGGATGAAAACGATG
GGCGACGTGGTCGCGGATCCCATCGAGGAGGAGAGCAGCGGGGAGGGCGGCACTGC
GCAGAATCCGAGCGGCGGGAAAGAATTCGGGCAGAGGACGCTCAGATCGTTGAAGAG
GGGGCTCGGCAGGCTTTGGAGGAGGCATCGTGGCAATGCCTCGATCACCGAGTACGAC
CCCTGCTACAAGGTCGCTTATCTTGGGAACGTTCTCACTGGATGGGCCAAAG

>novel_circ_001481

GTGTGAACATACTATGAAAGAATTGGAATACTACAGAGGACAGCATATAGCAGTCATGA
ATCAGTTAGAGGCAACATCACAAGAGAGTTCCGCACTGCGGGGCAAATATGGAGATCT
GGTAAATGATAAGCAACGCCTGGATCGGGAAGTTCAGGCGTTGCAGAAAGAAGTGTC
CGAATTGCGATGCCAAAATCAAGAAGTTCTTGTTTCCGATGCTGGTAATAGCGACACCA
TGAATCAACATTATTTATCTGCGCTTCGAAAATACGAAGCCGTTAAAGATGAGTATGATG
CCCTTCGAAAACGGTACGATGACTTAATATCGTCTCATTCATCGGCTGTTAACAAGTTG
GAATTATCGCAAGAAGAAGCTGCCAGATTGAAGAAACAATACGATGAAATTGTTCAAG
AGCGCAATAGTGCGGTTCGTGAGCGTAATGGTTTGAAGCAACAGTGCACAGCTGCGAT
TAGACAATGGGATATTGCGTTGAGGGAGAGAAACGAATATCGCGAAGCTTTGGCCAAA
GTGCAGCAACAGCACGAGGAGGCGGTGAAAGAAATTAATCATGCAATGGTGCTACGC
ATGAAGGCTAGCAAGGATATGAAACGATTAACAGAGGAGAGGAATGCTGCGTTGCAA
GAATACAGTTTAATTATGGGTGAAAGGGATACGGTGCATAAGGAGATGGAGAAGCTTG
GGGATGATCTTACGCAGGCATATACAAAGATCACCCATATAGAAAATCAGAACAAGCAA
TTTATGGAAGAGAAAAAAGCTTTATCCTATCAGATAGAAACTTTAAGGAGGGAAATTTC
ATCGGCTCTGCAAGATCGGGACGAGGCTTTAAAGCAATGCAACGAACTGCGTCAAAA
GTTCGGCGATTATTCCGAGGGTTCGAGTCGAGATTACAAAAATCGGATGGAATTGCATT
CGTCGTACAACCACGAGCGTGACAATTCGAGCAAAGAGGCCGAGAGAGAGAGCAAC
ACCGCTGATTACACTAAACGCGACAAAGAGCGTATGGATAATTTGGATCAAGCGAATCT
CGAGTTGGATAAACTGAGGAAATCCGTTGACAAGTTGCAGACGGAGCTGGAGGAAGC
TTTGCAAGAAGCTGAGGTGTCGAAGCGGAGACGAGATTGGGCGTTCAGCGAGAGGGA
CAAGATAGTGTTGGAGAGGGAAAGCATCAGAACGTTGTGCGACAGGTTGAGGAAGGA
GCGCGACCGAGCCGTGTCGGAATTGGCCGGGGCTTTGCGCGATTCCGACGACATCAAG
AAGCAACGGAACGAGGCGTCGAAGGAGTTGAAGGATCTCAAGGAGAAGATCGAGTC
CGGCGATCACGCGTTGCGAGCGAATCAATTTGCCCAAACGTTGGGCATCCACGATTCG
ACGCTCGACACCGACGCGAACGATTGGGAAATTATCCCCGTCCACTTGGACCTCGGCC
GACTTTGCCTAGATTCTGATCGTGACCTTGGATTGACGCTGGTCGGTGGCCGTGACAAT
CCTTATTATCCCAACGACACGGGAATTTACGTCGCCCAGGTGATATCCGGAAGCGCGAC
CGACGGCAAATTGAGAGCGAACGATTGCATCGTGCGAGTGAACAACGTGGATTGTACA
TCCGTTTCCACCCGTATCATCATGGAAACTTTACGTACCTGCTCGGGCGGTTCCGCCAC
GTTGACGGTAAGGAGACGACGTTTGACCAGAAGATCGTTGAGGACCACTCAATTGTCC
GTTGGCTCGGTTCCTCATGGTATTTCTTTGGAACTCGGTGTATACATTTCAAAGATCTCG
CCCGGCAGTCTAGCGGCTAAAGATGGCAATCTTGCCGTTGGGGACAGAGTTCTGAACA
TTAATAGTAAACCGATGGAAGGGATCAATTCCAGTCATGAAGCAATGGCAACATTGAA
CGATACGAGCACGGATGTGTTGACTATTACAACATTGAAGGGGATACCATTGCCTTCGG
CTACCAGTTCCGAGACAATGACCATCGACGCCAGTTTCGGCGCAGAGAAACAGAAAA
TGGTGAACAGTTGTTCCCAGACGGAGCAAGGGAGGATATTGTTGAAAATCCCGTCGGA
CGATTACGAGAGAAGGCACGTTGCTTCGAACTTCGGCGACAGAAGTATATACAAAGTA
TCGAAATCGGTGAGCGGCGAGAAGCCGAGCGGGATCAGCAATGCCTGGGACAATATA
AGGGAGAAGATCGACATAGTTCGGGGGCGGAAGCACAGCAAGGATCGGGAGGAGAA
GAAGAAACGGCATCGAAACTCGAGCCCGAACACGTTCGAGCAAGAGCAGGACGCCAT
AGCCGAGTTGGATTCCGTGATAGAGAGTTATCACAAGAAGGCGAACAACGGGGTGTT
GAAACGGAGCAAGCGGCGGGGAACCGAGAAAGCCGTGGAGAAGAACGGAGGTACGT
GGCCGAAGGCCAGGGGCGGACCCCTGATACAGAACGGCACCGGTACCATTTTGCACT
CCCGTAAGACGAAAGAAAGATTGCCCCTAAGCGTGCTCCTCAACCAACAACCGCCCA

AGTACGAGAGCACTTACAATTACAACCGTATCTCCAACCCCATCCCGTTGACCAATTTT
CCCGTCAACGTGAACAATCGGCACACCGTGTACAAATCCGTAGAGAAACCGTTGTCGA
ACTTCCTTAAAACCGCCGGCCCGTTATTCAGTCAGAAATCCTTTACTCCAGTGGTCCAG
TTCAAAGATATACCGATAGATAAGCAGAAATCGGCGGCGGCCGAGTACGAGAGCACGG
AGGTGAACAGGCTTGGCTCGACCCTAACCCCGTCCGAGACCAGCATCGACTTCTCCGT
GAAGTCGGGCAAAGACGTGGATTATTTCACGAGGAAGAGGGCGCAAAAGTATACGGT
TGGGAGCGAGGAGCGTCAACAAGTGGACGCGTCACAGCACAACAGGGCCCAGTCCC
AAGTTTATTCGGGGGCCGGATCGTCAACCTCGTCGACCAGCGGGCCTAGACAACAATT
GGCAACAACCGGTAATTTTTCATTTCCCCCGTACACGCATTCGCATCCGCATCCCCATC
AACAAAACTCTTTGCCGTCGAGGTACCCCTCCCCGCCCTCCTTGCCGTCTGCACAGTC
GGGGGAGTCGATAGGGCTTCCCGATGCACGATCTTATTGTTTCGAACCTTCGTATAGCC
CCGGCCCGCAAACAGGATTCGCAGGGCATTTGCACACACCCTCCGTAGACTTGCATTA
TCACAAAGCTCGCGGACCGCCGATCCACGCCAACGCGTACGACGTAGTGTCGGCTGTC
GCGTCGTCGTACACGCACGGTTACGAGGGCGGCACGTTTCCGAGGAAGAAGGAGAAC
CAACGGTTTCGAATACCGTCCAATCCTAGCGTCACGTCCAAGAGCAGCGTGGGCAAAT
TGTCAACGGGCAGTATAGAGAGAACTTCGGAAAGGGGGAGCCCGATGCCGACGTTCC
ACGTCGAGGTGCTTAGCCCCGGCACGGGAGGGAGTAGCTCCGGTGGAACTATAAGAG
GAAGTGGCGGGAACAAGAGATCCAGCATGCCCGATTATTGCTACTCCCAACCTAGGCC
CGCACCGGGCGAACTGAGAAGGGTTCATATAGACAAATCCGTCGAGCCGTTGGGCATA
CAAATTTCCTGCCTGGAGAGCGGAGGTGTATTCGTTTCCACGGTCAGCGAGCACAGTT
TAGCGTCCCAAGTGGGCCTTCAAATCGGCGATCAATTGCTCGAGGTCTGCGGCATCAAT
ATGAGGAGCGCCACTTATCAACTTGCTGCCAATGTGTTACGCCAGTGCGGTAATTCCAT
TACGATGCTGGTGCAGTACAGTCCAGACA

>novel_circ_001482
GTGTGAACATACTATGAAAGAATTGGAATACTACAGAGGACAGCATATAGCAGTCATGA
ATCAGTTAGAGGCAACATCACAAGAGAGTTCCGCACTGCGGGGCAAATATGGAGATCT
GGTAAATGATAAGCAACGCCTGGATCGGGAAGTTCAGGCGTTGCAGAAAGAAGTGTC
CGAATTGCGATGCCAAAATCAAGAAGTTCTTGTTTCCGATGCTGGTAATAGCGACACCA
TGAATCAACATTATTTATCTGCGCTTCGAAAATACGAAGCCGTTAAAGATGAGTATGATG
CCCTTCGAAAACGGTACGATGACTTAATATCGTCTCATTCATCGGCTGTTAACAAGTTG
GAATTATCGCAAGAAGAAGCTGCCAGATTGAAGAAACAATACGATGAAATTGTTCAAG
AGCGCAATAGTGCGGTTCGTGAGCGTAATGGTTTGAAGCAACAGTGCACAGCTGCGAT
TAGACAATGGGATATTGCGTTGAGGGAGAGAAACGAATATCGCGAAGCTTTGGCCAAA
GTGCAGCAACAGCACGAGGAGGCGGTGAAAGAAATTAATCATGCAATGGTGCTACGC
ATGAAGGCTAGCAAGGATATGAAACGATTAACAGAGGAGAGGAATGCTGCGTTGCAA
GAATACAGTTTAATTATGGGTGAAAGGGATACGGTGCATAAGGAGATGGAGAAGCTTG
GGGATGATCTTACGCAGGCATATACAAAGATCACCCATATAGAAAATCAGAACAAGCAA
TTTATGGAAGAGAAAAAAGCTTTATCCTATCAGATAGAAACTTTAAGGAGGGAAATTTC
ATCGGCTCTGCAAGATCGGGACGAGGCTTTAAAGCAATGCAACGAACTGCGTCAAAA
GTTCGGCGATTATTCCGAGGGTTCGAGTCGAGATTACAAAAATCGGATGGAATTGCATT
CGTCGTACAACCACGAGCGTGACAATTCGAGCAAAGAGGCCGAGAGAGAGAGCAAC
ACCGCTGATTACACTAAACGCGACAAAGAGCGTATGGATAATTTGGATCAAGCGAATCT
CGAGTTGGATAAACTGAGGAAATCCGTTGACAAGTTGCAGACGGAGCTGGAGGAAGC

TTTGCAAGAAGCTGAGGTGTCGAAGCGGAGACGAGATTGGGCGTTCAGCGAGAGGGA
CAAGATAGTGTTGGAGAGGGAAAGCATCAGAACGTTGTGCGACAGGTTGAGGAAGGA
GCGCGACCGAGCCGTGTCGGAATTGGCCGGGGCTTTGCGCGATTCCGACGACATCAAG
AAGCAACGGAACGAGGCGTCGAAGGAGTTGAAGGATCTCAAGGAGAAGATCGAGTC
CGGCGATCACGCGTTGCGAGCGAATCAATTTGCCCAAACGTTGGGCATCCACGATTCG
ACGCTCGACACCGACGCGAACGATTGGGAAATTATCCCCGTCCACTTGGACCTCGGCC
GACTTTGCCTAGATTCTGATCGTGACCTTGGATTGACGCTGGTCGGTGGCCGTGACAAT
CCTTATTATCCCAACGACACGGGAATTTACGTCGCCCAGGTGATATCCGGAAGCGCGAC
CGACGGCAAATTGAGAGCGAACGATTGCATCGTGCGAGTGAACAACGTGGATTGTACA
TCCGTTTCCACCCGTATCATCATGGAAACTTTACGTACCTGCTCGGGCGGTTCCGCCAC
GTTGACGGTAAGGAGACGACGTTTGACCAGAAGATCGTTGAGGACCACTCAATTGTCC
GTTGGCTCGGTTCCTCATGGTATTTCTTTGGAACTCGGTGTATACATTTCAAAGATCTCG
CCCGGCAGTCTAGCGGCTAAAGATGGCAATCTTGCCGTTGGGGACAGAGTTCTGAACA
TTAATAGTAAACCGATGGAAGGGATCAATTCCAGTCATGAAGCAATGGCAACATTGAA
CGATACGAGCACGGATGTGTTGACTATTACAACATTGAAGGGGATACCATTGCCTTCGG
CTACCAGTTCCGAGACAATGACCATCGACGCCAGTTTCGGCGCAGAGAAACAGAAAA
TGGTGAACAGTTGTTCCCAGACGGAGCAAGGGAGGATATTGTTGAAAATCCCGTCGGA
CGATTACGAGAGAAGGCACGTTGCTTCGAACTTCGGCGACAGAAGTATATACAAAGTA
TCGAAATCGGTGAGCGGCGAGAAGCCGAGCGGGATCAGCAATGCCTGGGACAATATA
AGGGAGAAGATCGACATAGTTCGGGGGCGGAAGCACAGCAAGGATCGGGAGGAGAA
GAAGAAACGGCATCGAAACTCGAGCCCGAACACGTTCGAGCAAGAGCAGGACGCCAT
AGCCGAGTTGGATTCCGTGATAGAGAGTTATCACAAGAAGGCGAACAACGGGGTGTT
GAAACGGAGCAAGCGGCGGGGAACCGAGAAAGCCGTGGAGAAGAACGGAGGTACGT
GGCCGAAGGCCAGGGGCGGACCCCTGATACAGAACGGCACCGGTACCATTTTGCACT
CCCGTAAGACGAAAGAAAGATTGCCCCTAAGCGTGCTCCTCAACCAACAACCGCCCA
AGTACGAGAGCACTTACAATTACAACCGTATCTCCAACCCCATCCCGTTGACCAATTTT
CCCGTCAACGTGAACAATCGGCACACCGTGTACAAATCCGTAGAGAAACCGTTGTCGA
ACTTCCTTAAAACCGCCGGCCCGTTATTCAGTCAGAAATCCTTTACTCCAGTGGTCCAG
TTCAAAGATATACCGATAGATAAGCAGAAATCGGCGGCGGCCGAGTACGAGAGCACGG
AGGTGAACAGGCTTGGCTCGACCCTAACCCCGTCCGAGACCAGCATCGACTTCTCCGT
GAAGTCGGGCAAAGACGTGGATTATTTCACGAGGAAGAGGGCGCAAAAGTATACGGT
TGGGAGCGAGGAGCGTCAACAAGTGGACGCGTCACAGCACAACAGGGCCCAGTCCC
AAGTTTATTCGGGGGCCGGATCGTCAACCTCGTCGACCAGCGGGCCTAGACAACAATT
GGCAACAACCG

>novel_circ_001483
AATTATAGAAGTGATATATATCTTAATTGTCGAAACCTACACAACGAATCGCGCCACGTA
TGTATGCTTATATATATATATATATATATATATATATATATATATATATATACGTTG
GTAATCTTAATTTCTTTCTCTTCTTCTTCGTTTCTTAACCACGAACGAAATTGACGAAAG
GGAAATAAACATTGTGGAAATATATAATCG

>novel_circ_001484
ACCCCGAGGGAGGACAGGGCAGCGTCGAGTCCTGGTAATCAGAATAATCACACACCC
ACACCATCCCCGTCACCGGTCGGCGATGCACCCGTTGGCAGGGCTCTTATGGAGGCTG

CGCTTCAGCAAGCCACTTCCGGATCTCAGCCACCTCTGGTGGTGACACCGCCTGGTTA
CTGGGTCGACGGTACCGATCATCGACACGCCCTGGACTCGGCTGGCAGGGCGTTACTT
CCGGCCCAGCCTGCATGGCAACCGAGGATAGACCAAGACGACACGGCCAAGTGTTAC
AGGCGATTTTTCGTCGGCAGG

>novel_circ_001485
ACGGAAGTAAGACAGCCGAAGATAATTAGATAAGTTTCGATGACTAACTACTGGTGGCT
AAGGTGGATATTACAGCGAAGATAAGATCTTTGCGAATCGTGCAACTCTTTAATTGCAG
AAGTAATTACTGCATACCATTCAGTGCAATAACGATGACGACCTTAGACTCACAGACCG
ATAAAACTTTAAGCAATCTTGATAAGCTTTCTAACACGGAGCTAGCTGAAATTCCATCC
GATCATGGACTTCCTGTTGGAATAAGCCCGTTGGAATCTCGGGTAGCCGGACATCCGTC
GTTAGACATCGAACGGCAGACGATTGGCATGTTACGTAGATCAGACGGCCGTGTATATA
AACCTGTTGTTAAACCGTTACTTGGGAAAAGAGAAATCTCGTTTTACGAAAATTTACA
AACATCTCAAGATCCTGTTATGTTACAATTGAAAAATTATGTTCCAAGATATTACGGCAC
GACAGAGTTGCAAATTTTTGGCAGACGAGTAACATTCCTTACGCTGAAAGATATTACCG
ATGGTATGGCGGAACCGTGCGTGATGGATATAAAAATAGGTAGACGAACGTGGGACCC
TTTAGCCACGCCGGAGAAAAAGGCAACCGAAGAATTAAAATACGCCGAGTCTAAACG
CACTTATGGATTCTGTATAACCGGATTTCAAGTATATTGCGTTTCGTCCGGCCGATTAAA
ACAATTTGGCAAACATTACGGCAAGACTCTCGATGCTAAAGGTGTCGTGGAAG

>novel_circ_001486
GTAGCATGGTTGCGTGTGGATACACAAACTATTCTAACGATAGCGAATCATGTGATAAC
AAAGAATCATAGAATCGGAGTAACGCATACCGAACGCAAGACATGGCATTTACATATAC
GCGATGTGACCGAATCCGATCGAGGTGCTTATATGTGCCAGATAAATACCGATCCGATG
AAGAGTCAAACCGGTTATTTGGATGTCGTAGTTCCGCCCGATATTTTAGATTATATGACA
AGCACGGACATGATAATCAGAGAAGGCAGCAATGTGACATTACGTTGTGCTGCTAAAG
GATCTCCAACACCAAGTATTACATGGAGGCGAGAAGGTGGAGAAAGTATATTTCTCGA
GAATGGAGAAGAAG

>novel_circ_001488
TGAAAATTGTCGAGGGTTCGATCTTCAATATCACTAAGATAAATCGATTGCAGATGGGT
GCGTATTTGTGCATAGCTTCGAACGGTATACCACCTACCGTCAGTAAAAGAATAATGCTT
ACGGTACAATTTAGTCCAATGATTTCAATTCAAAATCAATTAGTCGGGGCACAAGAGGG
ACAAAGAATGACTTTGGAATGTAACTCGGAAGCGTTTCCACAATTGATCAATTATTGGA
CAAAGGAAAACAACGAAATTATAGAAAATG

>novel_circ_001489
ATCTTGATTTGAGAAAAATGGCAAACGAAAGTTCTTTCACTTTTCTGTTAGAAGATTCG
TTCAGCGACAATGATAAAAGTATGACCGAAGATTGTTCGTCGAAATTAAAAGATGCTTT
GGAGAGCCTTGTTCAGAAATACGACGGGGAATTATTGCAACGGACACTGGAACAATCT
TTGTCCTCCTGCTCGGATAATTTATTAGATGAGAAAATTTTCAACGAAATTTTTCCTCTC
GCCCATGATCTTCTATCCAAGACGTTGAAGCAGATCGGCGAAATGATAAACAGAAACG
AAGACACGGAGCGAGACACGAAACGAGAGCTGTTCGTTTGCCACGAATTGTTAAAAA
TATGGGAAAAATCTATGGAACGTGTGTCGAAACTTCAGAGAACGTCTGCCACGGATCT

TAAATCCATATTGGAGAACGTACTCGTCACGGTTGAATTAATATTTCAACACTGTAGAG
GAAGTAAAAAATTATACGGTGTTTATTCGATAAAGTATCGGAGGAATTGACGAATCTC
TTTCGTAAAGCAAAAACGATCTTGAATCTATTCTTGGCCACTTTGGACGGTGTAATAGC
CTTCGACACGGACACGGAATCGGAACTGTTGGTAAAAG

>novel_circ_001490
ATGCAGTTTGGGATTACTGTCGATCGTAACTTTTACGGCGCTAGTAGCGATCGCCTCGA
CGACGACAGTCCATCAATTCGATATTTCAGGCGAGTAGAAATTTTTAGTAGCAATATCA
CGAATAACAATGAATGAATATTTATTAAGCTGTTTTAAATATATTGCGCAATATTCTACCG
TGTCGATAATTTGGAAATTTTTCCGAAAAATCTCGACGTGTAAATTTTTGCATTCTTTTA
AAATTATGAATTTTAATTAATCAATAATCAAATTTTACCAAATTTCACTGTAAAGAATTTG
GCCTTCAATTGTGCGCAAAAATTCTCAAAAATTCCCCTTTGAACTTTGCAAAGTTCAAG
GAACATCTCTCTGCCACACGTGTTGTGGCAAAATCAATTTTTTCGATCGATGAAATAAA
TAAAAATAACAAAAAGAAAAAAAGAGAAAAATGTTACGCAACGTGGAAAGAAACGT
AACACGTGTTACAATTTCGAGGGCAACGATCGAAGACAATAGTGAGATTCCTCTCACG
AAAATCGATGCTTCTTGTTGTTATTGTTGTTGTTGATCGCGATGATGTTGCATTTAATTAA
TAAAGCAACGATGTAATCGTCCTTTGTGTCGCGTGCTAACATGCTAATTAATAATCTTTA
CGATCAAGAAAAAAAAAAAAAGAAGAAGAAAGATAGTAAACAATTAAAAAAAATTTCA
AAATAACAAATTTATTTCGCATTAAACGAGTGTGTTTTTTTTCTTCTTCTCCAAAGTGTT
GAGGATATCGTATATTGTAAATTTGTTTTTTTTTTTCCATCCACAGATTATCTGTACGGGA
TAGATCACGATTTACAGGCAAGAGCACGTGCAGGGATCGAGGCCGAAAGATTTACCTA
TCAGACGAAGGCTAG

>novel_circ_001491
ATGGCAAACCTATGTTACGACAAGGAGATACTGGAGATTGGATTGGGACATTTGAAGG
ACATAAAGGAGCAGTGTGGGGTGTTGCATTAAATCCTCAGGCTACGAGAGCTGCTTCA
GGTGCTGCAGATTTTAATGCTAAAGTTTGGGATGCGATAAAAGGAGAAGAAATTCATTC
GTTCCAACATAAACATATTGTTAAATCTGTCAATTTTAGTACAGATTCTAATTATTTGTGC
ACTGGCTCCAATGAGAAACTTGTACGAATTTACGATCTTAACAAACCGGATGCAGCAC
CACAAGTTTTCTCAGGTCATAAAAATGGTATCAGACATGTTACCTTTTTCAATAATAATA
CTGCATTAATTACTTGTGGCGATGATAAAACATTAAGAGTTTGGGATAGGAATAGCGGC
CAAGAAGTCAAAAGGTTAGATTTTCCGGCAATTCCAAATTCCATGGAAGTATCAAGAG
ATGGAAATATCATTACCACAACTCATTCCAACATAGTTACTTTTTGGAATAGTAGAGAAT
TAACCAAATTACGCGAATACACTGCACCAACACAAATGAATAGTGCCAGTTTGCATCCA
GATTGTAGTATTTTTGTATGTGGAGGAGAAGATTTAAAAATGTACAAGTTCGATTACACT
ACGGGCGCGGAGATTGAATCATTTAAAGGACACTTTGGACCCGTGCACTGTGTACGTT
TTTCACCGGATGGTGAATTATATGCTAGTGGTTCAGAGGATGGTACATTGAGATTATGGC
AAACAACTGTTGGCAAAACTTATGGTCTCTGGCGATGTATAGAACAAACACCTGCAAT
ACAAGAAAATACTACTGTGCTTAATAATAAACAAGAAGTTCCTGCCAGTTAAAGATCTG
ATGATTCATAAATAATGTATTTTAATACCTTCCAAAAGAGAAAAAAAGAGAAGAATACT
TCATCGAGAGATGTACGTATTATATATAAATGATAAATCACGAATTTTCATAGCAATTTGT
ATCGTTGAAATTTGTATTTAATCGGCTGATATCTCTCATATGTTTTGTACATATATCAGATT
TCTATAACGTGTCTTGAATCAGCCTAAATAATCGCGATATTCCGGGAAAAGCAATTTCAT
TCGTAGTTTAATAAGCAATGGAAGTCTGGGACGCCGTTTACTATTTATTGTAGAAAAGA

AAAAAAAAAGAAATATTAACATTTATTAACATTATACATGTCACTATTATACTACAAAATT
AGATAGAGGTGTAATGAGAAAAAGAAAGACAATTCAGAATTAAATTTTGTGTTCGCAT
TTGTTATTTTCGCGTTGTAATATTACTATACAAGTATAAAAAGGATTTATTCGGGCCACCA
CTAGGCGCAACTTTTTTCAATATACATATCACTCATTCGCCTATTATTATTATTCACATTTT
TATTCCTTTTGTCTAACAAACGTCACAATACCGTGACAATTTATTAAAGTCCTTTTTCCT
CACACAGCAATTACGTGCTACGAGATCACTGTACAATTTTTCCTTCAAACTGGCAATGA
CAGCTTTATACAAGTAGTATATCATGTACTGACCTCTTTTGACTTTCCTTATTCCAGTACA
CCTTCTTACGTATATATATATATATATACGCATATATATATATATATATATAAAACATAAATT
AAATACTTCTTATAATCTACAAAATAGTACTAAACATAACAAGAAAAAAAAAAAAAGAA
AAAGAGAGAGGTATTCGTTACAAAATGAAACATTTAAAACATTTCCAGTCGAGTATATA
ATACATGAGCGAATCTTGATTACTTCTTGACCTCTCTTCATAAAACGATTTCTATCAAAA
TGCACGAGTTTTGAGACGCGTGCATAACACATTCACCGTGATTCTTGACTCTCTTACAA
AAAAATTGTTCAACCTTTTACGATGAGTGCAGCTCGTTAAAAAAAACTTAACATCACTG
TATGATCTCTCGGAAGGAAGACAGTGATTCTCTTGCGTGTGATAGTTAGTAAGCAACCA
GTTATAGGAGAGAAGTCTTTGTTATAACATTTTTTTTCTTCCACCGCTCAACCTATGAA
TTGCGACAGATCGGGCAACTTGAATCCGTTGAACGAAACTGGCGTTGGCGCGCTTCTC
AAACTATTTCTGTGCGACACGAAACCAGGGAAACTGGTCGGCGTGGTAAACTGTTGA
AGTCTCGAGGAAGCTTCCGTCGCCGCCAAAACGAAACCGCACTGAGCCGTGCACACG
TCGTCGCGTAGAATCGCCTCGCGAGATGCCTCGCGTAGAAGCTGATTGCAGTCGGTAAT
CCTCAGAGAAGCGCATTGAGTCTGAAAACAGAAACGAATTGCAACTTGAATTCATTCC
ATTAACGTGTGTAAACGTCAAAAACAAGTACTTTCCAGAATAGGAAAATAAATATCCAA
TTTCATAATAAGTATTCGATTTAGAATTTAAAAAGAATTAAATACGTACGCCTTCGGCGG
TGCAAACGCAGCTGGTGCAAGGCGTAGGGAAGCCAGACTCTCCGAGAGCAACGTTTC
TTCCTCCGATCTGGCAACCGTGCCTCGTTTCTCTCCACGCAGCGAGATCCATTTGCGGC
ATCGAGCCGCAAGGGACTCGAGGATTGAGAAAATTGCTCGGCAAGTCGAACGCCGCC
CTCTGCATGTCGTTTTGATTGTCCATGTTCTCGCACATAACCTTCGACAACGTCGATTTA
CGAATCTCTGCGAGCTGGTGCTCGGTGAAGCGGATATTCGGGTCGTCGGTCTCGTACC
AGAATCTATCGCACTTCCTCGCCTGCCTGAATTGTATCGCGATTATACAAGCGAACGTG
GGCCCCACCAATCCGCCTTGAAGGGGTCTCTCGCTCATTCCGCCTGGGAACAAGTCGA
TGTCGTCCACGGAGGCGTAGATGCGTTTCATCCGAGCTATCACCTCTGGCGCCATCTCT
CGCGACAAATCTTCGAAAGTGGTCGCTCTCTTCAGATTACACAGAGCCCTGTAATTATT
GTACGACGGTATACCGTGATCCCTGGCACGATGAACGTTGAGAGCGACCAGATCGATG
CCAGAGTACGGTATTCCTCGGATCTCGAACAGGTGATTGGTCACCTCGCCGGTTATAAA
TTGATCCAAAGTCTCCATAGGTGTCGCGACCAATCCGCGCGTCATTTCGTCCATCATGT
TCGACTGATAGAGCACGTCCGGGTCGAAGAAGCCATCTCTGAGCAAGATGGACGGTTC
CATGTTCTGATAGTTGCGATCCATTCTCGGCAGATGGGGCCTCAGTAACGAGTGACCGA
TCCTGTACGCCGCGGACGCGAATTCGGTGAGCACGCTGGGATTGCAAGTGGGGGAATA
TTCCTTGTAATAGCCTTGCGGCAACAGTTTCAATCCGTAAAGCGTGACGGCGTTCCAGC
CGAGTATCCGAGGTAGGAATTCGTTGTACGTGATGTGCTGTAGCATCCCGCTGATTATTC
GTCGAGATTGTTGGAACAGTTTCTCGCCGTCCCAGTGAGGATTGATCTGCCTCAATCCC
TCCACGATCCTGTTATGCTCGCGCACCCACATGGTATGCATCACGGTTAATCCAGGCTG
CTCGGAGGCGCGGCCGTCTCCGCCGATAAAGCAGTAACCTGATTTAGCTTTGCACTCG
GGATGCGTGGGCGATTGAGGCAGGAGATCCTTGGCCCCTCTGTGCGGACTTTGGGTGA
TGTTCATGCGACCGTTGAAGCCACGCAGTATGTTGCAGATGCAAGTGTTCTCTCCATAC

ACTACCGATCCGTCGAGAAAAGCGGTGTTCTGATTGATTTGTTCCCTCGGTCCGAGGTG
TTGTTGGCCAGGCAGCGACCTCATGGACGGGAAACACATTCGCGCGCCAGACGTTACG
TTCACGGTGGGATAGTAATGGTCGCCAGGGGGGACGGGGAAGGGATTACACTCGGGAT
GAACCGTTCTGGGCGAATCGCAAGAGCGACAGCTGGGTATAGACTCGGCGAAACCTTT
GTGAATGGGCGTCATGGTGAGGTCGTGGTCGAGAAATTGAGCGAACTGCATTACCATC
AGAGTGTATCGATTGTGCAAGTTGGAGATGTCGGGATGAATCACCGTGGAGATTACTC
GGGGATTCGGTAGAGGCACTCCCGTCACCGAGGTGCTTCTCGGCTTCGACACTCCGTC
TTCGTAGACGGGAGGCAGCAATCTGGCGAATGTCGTCAACGATTTACCCAAATTGGGA
TTGCGTAAATTATTGCAGTGGCCGGAAAGGGTTCTGTACGGAGAGGTTGGGTCGCAAG
GGCTATCGTCCACGGGGCACTCGACTTCGTCGTGATTCGTAGGCTTCTTCTGGGACAG
AAATCCGGAGATATCGACGTTTTGCAGCAGATCTGAGAAGTCGTTGTTGGGAAAGCCC
AGTATATTGTCCGTGGTGTCGAAGATCTGTCGTTTCTTCCGCTTCAGCCCGTGTATCCCG
TTCAGAATTTCGTTGGTGGCCAACTCGTACATGATGGAGGAGTTTGCCAATAGGAGGG
CATCTCTGTTGGCTTTCGAGAAGCTGGCAGCCGTCCCGGCTGGCGATTTCGGATCCGCT
ATTCTCTGCTGCAGCCACGAATTGTACTCCAGTTGTTTCCTCTCGATCAAATCTGCCTCA
GCTTTCTTCACGGCTGCCGCTATTAAACTCGGGCTGAGTTCCGGGATGAACTCGGGGAT
CACTGATCTGGTTCCAATCGCTACCTCCTTGGCGGGCTCTGGAAGTGGCTCTTCTCGCC
AAGGAGCGATGTCGAGTACGCTGTGCTGGTCGCAATTGATCCTCGCGTTCAGATACGG
GTCCTGCTGTATGAACGCTTTCGGTTGAATCTTGTCGATCTTCGTGTTGCCGCACAGGA
TCCCGGCCAGAGAAACTTTTCTAATTTCGGCCAGTTGAGTCGGATTCAATCCGGACGG
CGGGATGTCGTTCTCGTACCAGAATCGATCGGAGTTTCTCGTCTTGATGAACTGCCTCT
TGAGAAGGCATTCGAACGTCGGACCTACCGTTCCACCCCTTCTAGGAATCTCTAAAATT
CCACCGAGCAGCAAATCTACGTCTTCCACCCGCGAGTATACGGATTCCAACAGCCTCG
CGTGTACCGGGTGCATTAGATGCTCCAAATCCATGAAATTTTGGATCTATGGAAAAAAA
AAACAAAAGCTACGTTTTAGAATATATCGAAGAACGAAATGAATTTCTTAAATACATTAT
GCAGTAGAAAAAAGAATTTTTATACAAATGCAAATTAAACTGCACAAACGTACGGTCA
CATTGCGTCCAGAACAATCGGCTACGAACTCGACGTATCCGGGGATCCCATGATCACG
GCCCACGTGTATAAAGCGCGCGTTCGCGTCGACTCTCGATTCGAAAGCGGATTGGCCG
AGATCGAGGCTCACGCGATTGGCCGAGGCGATCACGTGGTCCTCCAAGCACGTGGCGT
TGTCGATTCTGTGGCTGCGCAGCGACGTCAGAGCGCGCAGAGCCGCCAGCGCGACAG
CATCGTACGTCCCTCCCACGTTCGAGGACGAGTAACCGTTGTAGAACCCGCTCGAAAC
GGGCATCAATTCCGGATCCGTTCGAGCACCCTCCCCGAGAATACTCGGTACGTATTCGT
TCAAAGTGACGTGTTGAATTTGCGCCATCACCAATCGTCGAGCCTCCAAGAACAACTT
AGTGTCGTCCCAATGCTCGTTGGCGTCGGCCAACGCCTCGGCCAATCGATTGTGCTCG
CTCAAAAAGGCGGCGTACACCATTCCAAGCGGCTTGTCCAAGCTGCTGTTGCACACTT
GGCAGGCAGAAACGTCGACCCTTCCTCGATTGTACGTCCTGATTCGGTGCAGCTGTTC
GTCGGTGGATCCGTAAATGTGGGAACCGTCCAAATATGCCGACGCACCGTTCATTTGTT
CCCTGCTTTCGAAATTACACTTGTGCACAGTGAGAGTCGGCACGGACCGCCAATAACC
TATACATCCGTCTTGCTCGTCGCGGCTGTCGTAACATTCTGGATGCCTCGCTTTCCCTAT
GGTGCAACATAGGTCCAGTTTTCGGGATGGATAGACGACGGACGCGATGTCGTTGAGT
ATTAGCTCGGACCATATCCCGGATAAGCTGGTCAGACCCTCGTGTGCCTCCGGAGTGGG
GCGTAGATGGTTGAGCAGTGAGGTCACCACTTTCGTAGGTGTCGGAAGCACGTGATTT
CCGACCGACTGGCGAGGACTAGCGACATCTGGTGACAATGAAACGTAACCGCGTGTTT
AGACGTGAAATTATCGTCTCGACGCGATGGTCGATATAGAAGAGGTTAGTTCGTAAGGG

AAAGAAAAAAAAATTGTAAATCGTTATCCGAGAAGCGTTTAATCGAAATGATTAATTGG
AAATATTATTAATAATTCGTATTTAAGTGATATGGACGTGTAATGATAATTCGTGTAAAAT
TTTTCAAACAACAATCGCGTCAATTACGAGCGAATTTCTCGAGTTACGAAAGAGTACA
ACAGAGATATTATTCTTCAAATTGCGCTTGTATTCGTAAATAATTTTTTTCCTTATTATATA
ATAAAGCTGCGGTTTCTACACTCTTACTTCACGAGGGTGTACGATGTAAATGAGATTAA
ATCGTTGCACGTAGTAAATTGATAATATCTGTGTCAAATTAGACGTTACCTAACACAGTT
TATTATTCTCGGTTACATTACACGGAAAATTGATTTTTACGCTACGCGCACGTGAATCCC
TATAGATTTTAACGCGACAAATTTTATCAAATTAGCGTAATTGTACCTTTGCCTCTGCTTT
TGACAGCGAGAGACGAGAAATTGTACGTAATTATGAAACGAACGTAATCGAAGATTGG
ATGGAGAACAGGTTATAAATATTATAATAAATCGTACTTTCATCTTTTATTATTACAGTGT
ATTATAAAACTTTCTTCGCTAAATACAAGGAAAAATTTTAAAACAAAAATACCAAAAGT
ATAGTAATTGTAAAGTACAAATTTTTTCTTTCTTCAAAAAAAGAATATAATAAATATAATA
TCAACGAGGAAAAGAAAGGATCGAGTAATGGAGATAGGTCAAATTAAATCGATCTCTC
GTTCGATTAAACGTATAATCAATATCTAAATTTTTTTTCGACATCATCCCATAAAATGACT
TTCTACTTCGATGTAATTATATCGCACGTGCATACACCTGTGCTTGATCCTCTGCATCCCA
GTTAGAGATAGTGAAATTTATTACGCACAACTTTCTAGATCGAGGTGGTTATTCATCTTC
CGATTACGTCCAACATTGAAACCGAATAACGTTGGTTAGAAATCGAAAGTCTTCCGTCA
TTATTGTTACCACGGATGCCATAAGCACGTTCTGCGGTTTTACGCATGTTTCTATCAATG
TATCCTCGTTTAAAGTTAAACAAACGAATTAAACGAGCGAATTGTTATTCGATTAAAATT
AAAAAAAGAAACTCGTAAAAATTCGAATTCGAATATTTGTTATATAAAAAGTTACATTT
CATTCGATTTCAATTAAATAATTCGGATTATTTATACCATTTTGAGTTTCGTTTCTTTTTTT
TCCAACAAGACGTCACCGACAAGCGGCGAAAAGAGAACCTAACCTAGTCTCGCGACG
CGAGATTCTGTGTCATGGTTACTCAACCGGTAAAGTAGATTGACATCACGTGGTTTTTA
CGACTCGCGCCTATATTCAGACTGTACGATGGTGTGCATACGGAAGGATTTATGACGAG
GATGGTACGGATATTCGTCCAACAATGAAAAACGAGCGAAAAAATATTTATAATAGGAT
CCGTCCTCGTCCCTAATAAGATTGATTCATGCGCATGTAGATAAGCTTGCAGCATTGGA
GCGGTATTTAAATTAATATTGCCATAAATGTATAATACCTTGTTCCCTCTGACTGTCATTG
GAATTGGAATTGATAATTCTCTTTTTTAAATTTTTTAAAGCTATCGTAATAGTACACATAT
ATATACTCGAATTCAACTAATTGTCACACGATAACAAATTCCATTTCTACTTTTTTCTTGC
AAATATAATGCAATGGATGTAATCAGAGTATAAAGATTCGTAACTATATTTATAGAACAC
TTATTATTATTTTATTTCATTCTTACACTATTTTTGTAGCATTTCGATATTAACAATTCCAA
AACTGGAAGATATTCATTTATGAGTAAAATTGAACTACGATTAAATTATGGACGTGCAA
GGATGTTGTTATATAAATCTAAAGAACGAATCAATGGTATGTTGCGCTCAAACGAGATA
ATATCGAGCTATTTAGAAAAGCGAATTTGACAAGTTCGATTATTATTCCAGTCAACGTCG
ACATTTTAGCCACTCATCAGAGACTTTCAATTGTGTCGACGCGACGAAAATACGACGA
CGATCGAGATAATTTGGGTCAACGTAGTCGAAAGGCAACGTTGGCGCAGGGAGCCACT
GGTAATGGAGGAACACCTGAATCACTTTCATCGCCTCTTTTCACTTTCATATTTCCGTCT
TCATTTTTTTCTTCTTTCTTTTGCACAGGTGTCAATCGCCTTCCGCCTTACCTTTCGCAAT
ACACAGAGCAGGCACGCTTCCTGATAAAGCGGCACAATCTCCAGTTGTTTAATTAACG
CTTAATCCTGATCTTCGTGCTTCATAGATTCCAATTCGACGTGACAGATATTCGCAGCAA
ATATTTCTGAGAGATAATTCTATATATTTGTGGATTAAATTGAAGTTTAAATAAATATTTAT
ATTTTTTTCATTATAAGATCGATCGAAATTATTATTTAAAATTAGAAACGATACACAGAG
GTTTTTATGTAGATAATTAAATTATTATTCTTACTGAACAATATTAATAATATAGTGCATAG
AGTTCTATTTATTTTAGAGATAGTATATTACTATAAAATAAGCAATTGAGAGTAACTGATG

TTATTATACTAAATAAACAATAAGAATTTGAATGTAATTAATTTTTCTATGTAGTTTACATA
TATTTTTTTTATACATTTCAGTTATGTTTTTTATGTATAATCTTCTATAGAATTATAGTTTTC
TAATGTATCTCTAATACAAATCTCGGTACAAATGCAATTGTTGAAAACGTACAGCAAATA
AAGGAAATATGATAAATAATTAGAATATCGCCAATTCCTAAAAGTGGCGTCAAATTTTCA
GAATTTCAGAAGTTCTTAAAAATGACATCAAAATCCTAGATTTTCTGAAAAATGTTCAA
ATTAATTAAATAAAATAAATACTTGCGTATTTTTGACTACAAAAATCACACGACTCATTT
AATTCGACATTGAACAGAAAACAGATCCAGGAAAAGCTATGTAATTCCAACCAATTCT
ACAATCTTTCCTCTTTAAAATGAGCATTGATTGAGCATTTTACGATATTTCTATCGAAAAT
TATGCACTTTCAAAGATTAGTGATTCTCTCCTCAAAAATCCCTCGACTCATTTAATTCGT
CATTGAACAGGAGAGAGTCCAAGGAAAAACTATTGAATTCTACGTTATTCTACAATCCT
TCCACTTTAAAATGAGCTTTGGTTGAGCATTTTACGAAATTCCTATCGAAAGTTATGCAA
TTTTAAAGATTAGTGATTCTCTCCTCAAAAATCCCTCTACTCATTTAATTCGTCATTGAA
CAGGAGACAGTCCCAAGAAAAACTATTGAATTCTACGCTATTCTACAGTGCTTGCCCTT
TAAAATGAGCTTTGGTTGAGCATTCTACGAAACTCTTATAGAAAGTTATGCAATTTCAA
AAATTAGTGATTTTCTCCTCAAAAATCCCTCGAATCATTTAATTCGTCATTGAACAGGAA
ACAGTCCCAAGAAAAACTATTGAATTCTACGCTATTCTACAGTGCTTCCCCTTTAAAAT
GAGCTTTGGTTGAGCATTTTACGAAATTCCTATCGAAAGTTATGCAATTTCAAAAATTA
GTGATTCTCTCCTCAAAAATCCATCGAATCTTTTAATTCGTCATTGAACAGGAGACAGT
CCCCGGAAAAAATTTAGGATTCTACACTATTCTACAATTCTTCCCCTTTAAAATGAGCTT
TGTTTGAGCATTCTACGAAATTTCTATCGAAAATTATGCAATTTCAAAGATTAGTGATTT
TCTCCTCAAAAATCCCTCGAATCATTTAATTCGTCATTGAACAGGAGACAGTCCCCGGA
AAAATATTAGGACTCTACGCTATTCTACAATGCGTCCACTTTAAAATGAGCTTTGGTTGA
ACATTCTACGAAATTCCTAACGAAAGTTATGCAATTTTAAAGAATAGTGATTCTCTCCTC
AAAAATCCCTCTACTCATTTAATTCGTCATTGAACAGGAGACAGTCCCAAGAAAAACTA
TTGAATTCTACGCTATTCTACAGTGCTTGCCCTTTAAAATGAGCTTTGGTTGAGCATTCT
ACGAAACTCTTATAGAAAGTTATGCAATTTCAAAAATTAGTGATTTTCTCCTCAAAAAT
CCCTCGACTCATTTAATTCGTCATTGAACAGGAAACAGTCCCAAGAAAAACTATTGAAT
TCTACGCTATTCTACAGTGCTTCCCCTTTAAAATGAGCTTTGGTTGAGCATTTTACGAAA
TTCCTATCGAAAGTTATGCAATTTCAAAAATTAGTGATTCTCCCCTCAAAAAATCCCTCG
AATCATTTAATGCGTCATTGAATAGGAGACAGTCCCCGGAAAAAATTTAGGATTCTACA
CTATTCTACAATTCTTCCCCTTTAAAATGAGCTTTGTTTGAGCATTCTACGAAATTTCTAT
CGAAAATTATGCACTTTCAAAGATTAGTGATTCTCTCCTCAAAAATCCCTCGACTCATTT
AATTCGTCATTGAACAGGAGAGAGTCCAAGGAAAAACTATTGAATTCTACGCTATTCTA
CAATGCGTCCCCTTTAAAATGAGCTTTGGTTGAACATTCTACGAAATTCCTAACGAAAG
TTATGCAATTTTAAAGATTAGTGATTCTCTCCTCAAAAATCGGTCGAATCATTTAATTCG
TCATTGAACAGGAGACAGTCCCAAGAAAAACTATTGAATTCTACGCTATTCTATAGTGC
TTGCCCTTTAAAATGAGCTTTGGTTGAGCATTCTACGAAACTCTTATAGAAAGTTATGC
AATTTCAAAAATTAGTGATTTTCTCCTCAAAAATCCCTCGAATCATTTAATTCGTCATTG
AACAGGAAACAGTCCCAAGAAAAACTATTGAATTCTACGCTATTCTACAATGCGTCCCC
TTTAAAATGAGCTTTGGTTGAGCATTCTACGAAATTCCTATCGAAAATTATGCACTTTCA
AAGATTAGTGATTCTCTCCTCAAAAATCCCTCTACTCATTTAATTCGTCATTGAACAGGA
GACAGACCCAAGAAAAACTATTGAATTCTACGCTATTCTACAGTGCTTCCCCTTTAAAA
TGAGCTTTGGTTGAGCATTTTACGAAATTCCTATCGAAAGTTATGCAATTTCAAAAATTA
GTGATTCTCTCCTCAAAAATCCATCGAATCATTTAATTCGTCATTGAACACGAGACAGT

CCCCGGAAAAATTTAGGATTCTACGCTATTCTACAGTGCTTGCCCTTTAAAATGAGCTT
TGGTTGAGCATTCTACGAAACTCTTATAGAAAGTTATGCAATTTCAAAAATTAGTGATTT
TCTCCTCAAAAATCCCTCGACTCATTTAATTCGTCATTGAACAGGAAACAGTCCCAAGA
AAAACTATTGAATTCTACGCTATTCTACAGTGCTTGCCCTTTAAAATGAGCTTTGGTTGA
GCATTCTACGAAATTCCTATCGAAAGTTATGTAATTTCAAAGATTAGTGATTCTCCCCTC
AAAAAATCCCTCGAATCATTTAATGCGTCATTGAATAGGAGACAGTCCCCGGAAAAATT
TTAGGATTCTACACTATTCTATAATTCTTCCCCTTTAAAATGAGCTTTGTTTGAGCATTCT
ACGAAATTTCTATCGAAAATTATGCAATTTCAAAAATTAGTGATTTTCTCCTCAAAAATC
CCTCGACTCATTTAATTCGTCATTGAACAGGAGACAGTCCCAAGAAAAACTATTGAATT
CTACGCTATTCTACAATGCGTCCCCTTTAAAATGAGCTTTGGTTGAGCATTCTACGAAAT
TCCTATCGAAAGTTATGCAATTTCAAAAATTAGTGATTCTCTCCTCAAAAATCCATCGAA
TCTTTTAATTCGTCATTGAACAGGAGACAGTCCCCGGAAAAATTTAGGATTCTACACT
ATTCTACAATTCTTCCCCTTTAAAATGAGCTTTGTTTGAGCATTCTACGAAATTTCTATC
GAAAATTATGCAATTTCAAAGATTAGTGATTTTCTCCTCAAAAATCCCTCGAATCATTTA
ATTCGTCATTGAACAGGAGACAGTCCCCGGAAAAATATTAGGACTCTACGCTATTCTAC
AATGCGTCCACTTTAAAATGAGCTTTGGTTGAACATTCTACGAAATTCCTAACGAAAGT
TATGCAATTTTAAAGAATAGTGATTCTCTCCTCAAAAATCCCTCTACTCATTTAATTCGTC
ATTGAACAGGAGACAGTCCCAAGAAAAACTATTGAATTCTACGCTATTCTACAGTGCTT
GCCCTTTAAAATGAGCTTTGGTTGAGCATTCTACGAAACTCTTATAGAAAGTTATGCAA
TTTCAAAAATTAGTGATTTTCTGCTCAAAAATCCCTCGACTCATTTAATTCGTCATTGAA
CAGGAAACAGTCCCAAGAAAAACTATTGAATTCTACGCTATTCTACAGTGTTTTCCCTT
TAAAATGAGCTTTGGTTGAGCATTCTACGAAATTCCTATCGAAGGTTATGCAATTTCAA
AGATTAGTGATTCTCTCCTCAAAAATCCCTCGAATCATTTAATGCGTCATTGAATAGGAG
ACAGTCCCCGGAAAAATTTTAGGATTCTACACTATTCTACAATTCTTCCCCTTTAAAATG
AGCTTTGTTTGAGCATTCTACGAAATTTCTATCGAAAATTATGCAATTTCAAAGATTAGT
GATTTTCTCCTCAAAAATCCCTCGACTCATTTAATTCGTCATTGAACAGGAGACAGTCC
CAAGAAAAACTATTGAATTCTACGCTATTCTACAATGCGTCCCCTTTAAAATGAGCTTTG
GTTGAACATTCTACGAAAATCCTAACGAAAGTTATGCAATTTTAAAGATTAGTGATTCTC
TCCTCAAAAATCCCTCGAATCATTTAATTCGTCATTGAACAGGAGACAGTCCGCGGAA
AAATATTAGGACTCTACGTTATTCTACAATCCTTCCACTTTAAAATGAGCTTTGGTTGAG
CATTCTACGAAATTCCTATCGAAAATTATGCACTTTCAAAGATTAGTGATTCTCTCCTCA
AAAATCCCTCGACTCATTTAATTCGTCATTGAACAGGAGACACTCCCAAGAAAAACTAT
TGAATTCTACGCTATTCTACAATCCTTCCCCTTTAAAATGAGCTTTGGTTGAGCATTCTA
CGAAATTCCTATCGAAAGTTATGCAATTTCAAACATTAGTGATTCTCTTTTCAAAAATCG
GTCGAATCATTTAATTCGTCATTGAACAGGAGACAGTCCCAAGAAAAACTATTGAATTC
TACGCTATTCTACAGTGCTTCCCCTTTAAAATGAGCTTTGGTTGAGCATTTTACGAAATT
CCTATCGAAAGTTATGCAATTTCAAAAATTAGTGATTCTCTCCTCAAAAATCCATCGAAT
CATTTAATTCGTCATTGAACAGGAGACAGTCCCCGGAAAAATTTAGGATTCTACACTA
TTCTACAATTCTTCCCCTTTAAAATGAGCTTTGTTTGAGCATTCTACGAAATTTCTATCG
AAAATTATGCAATTTCAAAGATTAGTGATTTTCTCCTCAAAAATCCCTCGACTCATTTAA
TACGTCATTGAACAGGAAACAGTCCCAAGAAAAACTATTGAATTCTACGCTATTCTACA
GTGCTTGCCCTTTAAAATGAGCTTTGGTTGAGCATTCTACGAAATTCCTATCGAAAGTTA
TGTAATTTCAAAGATTAGTGATTCTCTCCTCAAAAATCGGTCGAATCATTTAATTCGTCA
TTGAACAGGAGACAGTCCCAAGAAAAACTATTGAATTCTACGCTATTCTACAGTGCTTC

CCCTTTAAAATGAGCTCTGTTTGAGCATTCTACGAAATTCCTAACGAAAGTTATGCAAT
TTCAAAGATTAGTGATTCTCTCCTCAAAAATCCCTCGAATCATTTAATTCGTCATTGAAG
AGGAAACAGTCCCAAGAAAAACTATTGAATTCTACTCTATTCTACAGTGCTTCCCCTTT
AAAATGAGCTTTGGTTGAACATTCTACGAAATTCCTAACGAAAGTTATGCAATTTCAAA
GATTAGTGATTCTCTCCTCAAAAATCGGTCGTATCATTTAATTCGTCATTGAACAGGAGA
CAGTCCCAAGAAAAACTATTGAATTCTACGCTATTCTACAATGCGTCCCCTTTAAAATA
AGCTATGGTTGAACATTCTACGAAATTCCTAACGAACGTTATGCAATTTCAAAAATTAG
AGATTCTCTCCTCAAAAATCGGTCGAATCATTTAATTCGTCATTGAACAGGAGACAGTT
CCAAGAAAAACTATTGAATTCTATGCTATTCTACAGTGCTTGCCCTTTAAAATGAGCTTT
AGTTGAGCATTCTACGAAATTCCTATCGAAAATTATGCACTTTCAAAGATTAGTGATTCT
CTCCTCAAAAATCCCTCTACTCATTTAATTCGTCATTGAACAGGAGACACTCCCAAGAA
AAACTATTGAATTCTACGCTATTCTACAATCCTTCCCCTTTAAAATGAGCTTTGGTTGAG
CATTCTACGAAATTCCTATCGAAAGTTATGCAATTTCAAAGATTAGTGATTCTCTCCTCA
AAAATCGGTCGAATCATTTAATTCGTCATTGAACAGGAAACAGTCCCAAGAAAAACTA
TTGAATTCTACGCTATTCTACAATGCGTCCCCTTTAAAATGAGCTTTGGTTGAGCATTCT
ACGAAATTCCTATCGAAGGTTATGCAATTTTAAAGATTAGTGATTCTCTCCTCAAAAATC
CCTCTACTCATTTAATTCGTCATTGAACAGGAGACACTCCCAAGAAAAACTATTGAATT
CTACGCTATTCTACAATCCTTCCCCTTTAAAATGAGCTTTGGTTGAGCATTCTACGAAAT
TCCTATCGAAAATTATGCAATTTCAAAGATTAGTGATTTTCTTCTCAAAAATCCCTCGAC
TCATTTAATTCGTCATTGAACAGGAGACAGTCCTAGGAAAGACTATTGAATTCTACGCT
ATTCTACAATGCTTCCCCTTTAAAATGAGCTTTGGTTGAGCATTCTACAAAATTCGTATC
GAAAGTTATGCAATTTCAAAAATTAGTGATTCTCTCCTCAAAAATCGGTCGAATCATTTA
ATTCGTCATTGAACAGGAGACAGTTCCAAGAAAAACTATTGAATTCTATGCTATTCTAC
AGTGCTTGCCCTTTAAAATGAGCTTTGGTTGAGCATTCTACGAAATTCCTATCGAAAATT
ATGCACTTTCAAAGATTAGTGATTCTCTCCTCAAAAATCCCTCTACTCATTTAATTCGTC
ATTGAACAGGAGACACTCCCAAGAAAAACTATTGAATTCTACGCTATTCTACAATCCTT
CCCCTTTAAAATGAGCTTTGGTTGAGCATTCTACGAAATTCCTATCGAAAGTTATGCAAT
TTCAAAGATTAGTGATTCTCTCCTCAAAAATCGGTCGAATCATTTAATTCGTCATTGAAC
AGGAGACAGTCCCCGGAAAAATTTTAGGATTCTACACTATTCTACAATTCTTCCCCTTTA
AAATGAGCTTTGTTTGAGCATTCTACGAAATTTCTATCGAAAATTATGCAATTTCAAAGA
TTAGTGATTTTCTCCTCAAAAATCCCTCGACTCATTTAATACGTCATTGAACAGGAAAC
AGTCCCAAGAAAAACTATTGAATTCTACGCTATTCTACAGTGCTTGCCCTTTAAAATGA
GCTTTGGTTGAGCATTCTACGAAATTCCTATCGAAAGTTATGTAATTTCAAAGATTAGTG
ATTCTCTCCTCAAAAATCCCTCGAATCATTTAATTCGTCATTGAACAGGAGACAGTCCC
CGGAAAAATATTAGGACTCTACGTTATTCTACAATCCTTCCACTTTAAAATGAGCTTTGG
TTGAGCATTCTACGAAATTCCTATCGAAAATTATGCACTTTCAAAGATTAGTGATTCTCT
CCTCAAAAATCCCTCTACTCATTTAATTCGTCATTGAACAGGAGACACTCCCAAGAAAA
ACTATTGAATTCTACGCTATTCTACAATCCTTCCCCTTTAAAATGAGCTTTGGTTGAGCA
TTCTACGAAATTCCTATCGAAAGTTATGCAATTTCAAAGATTAGTGATTCTCTTTTCAAA
AATCGGTCGAATCATTTAATTCGTCATTGAACAGGAGACAGTCCCAAGAAAAACTATTG
AATTCTACGCTATTCTACAGTGCTTCCCCTTTAAAATGAGCTTTGGTTGAGCATTTTACG
AAATTCCTATCGAAAGTTATGCAATTTCAAAAATTAGTGATTCTCTCCTCAAAAATCCAT
CGAATCATTTAATTCGTCATTGAACAGGAGACAGTCCCCGGAAAAATTTTAGGATTCTA
CACTATTCTACAATTCTTCCCCTTTAAAATGAGCTTTGTTTGAGCATTCTACGAAATTTCT

ATCGAAAATTATGCAATTTCAAAGATTAGTGATTTTCTCCTCAAAAATCCCTCGACTCAT
TTAATACGTCATTGAACAGGAAACAGTCCCAAGAAAAACTATTGAATTCTACGCTATTC
TACAGTGCTTGCCCTTTAAAATGAGCTTTGGTTGAGCATTCTACGAAATTCCTATCGAA
AGTTATGTAATTTCAAAGATTAGTGATTCTCTCCTCAAAAATCGGTCGAATCATTTAATT
CGTCATTGAACAGGAGACAGTCCCAAGAAAAACTATTGAATTCTACGCTATTCTACAGT
GCTTCCCCTTTAAAATGAGCTCTGTTTGAGCATTCTACGAAATTCCTAACGAAAGTTAT
GCAATTTCAAAGATTAGTGATTCTCTCCTCAAAAATCCCTCGAATCATTTAATTCGTCAT
TGAAGAGGAAACAGTCCCAAGAAAAACTATTGAATTGTACTCTATTCTACAGTGCTTCC
CCTTTAAAATGAGCTTTGGTTGAACATTCTACGAAATTCCTAACGAAAGTTATGCAATT
TCAAAGATTAGTGATTCTCTCCTCAAAAATCGGTCGTATCATTTAATTCGTCATTGAACA
GGAGACAGTCCCAAGAAAAACTATTGAATTCTACGCTATTCTACAATGCGTCCCCTTTA
AAATAAGCTATGGTTGAACATTCTACGAAATTCCTAACGAACGTTATGCAATTTCAAAA
ATTAGAGATTCTCTCCTCAAAAATCGGTCGAATCATTTAATTCGTCATTGAACAGGAGA
CAGTCCCAAGGAAAACTATTGAATTCTACGCTATTCTACAGTGCTTCCCCTTTAAAATG
AGCTTTGCTTGAGCATTCTACGAAATTCCTAACGAAAGTTATGCAATTTCAAAGATTAG
TGATTCTCTCCTCAAAAATCCCTCGAATCATTTAATTCGTCATTGAACAGGAAACAGTC
CCAAGAAAAACTATTGAATTCTACGCTATTCTACAATGCGTCCCCTTTAAAATGAGCTTT
GGTTGAGCATTCTACGAAATTCCTATCGAAAGTTATGCAATTTTAAAGATTAGTGATTCT
CTCCTCAAAAATTCCTCTACTCATTTAATTCGTCATTGAACAGGAGACAGTCCCCGGAA
AAATTTTAGGATTCTACACTATTCTACAATGCTTCCCCTTTAAAATGAGCTTTGGTTGAG
CATTCTACAAAATTCGTATCGAAAGTTATGCAATTTCAAAAATTAGTGATTCTCTCCTCA
AAAATCGGTCGAATCATTTAATTCGTCATTGAACAGGAGACAGTCCCCGGAAAAATTTT
AGGATTCTACGCTATTCTACAATGCGTCCCCTTTAAAATGAGCTTTGGTTGAACATTCTA
CGAAATTCCTAACGAAAGTTATGCAATTTTAAAGATTAGTGATTCTCTCCTCAAAAATC
GGTCGAATCATTTAATTCGTCATTGAACAGGAGACAGTCCCAAGAAAAACTATTGAATT
CTACGCTATTCTACAGTGCTTGCCCTTTAAAATGAGCTTTGGTTGAGCATTCTACGAAA
CTCTTATAGAAAGTTATGCAATTTCAAAAATTAGTGATTTTCTCCTCAAAAATCCCTCGA
ATCATTTAATTCGTCATTGAACAGGAAACAGTCCCAAGAAAAACTATTGAATTCTACGC
TATTCTACAATGCGTCCCCTTTAAAATGAGCTTTGGTTGAGCATTCTACGAAATTCCTAT
CGAAAATTATGCACTTTCAAAGATTAGTGATTCTCTCCTCAAAAATCCCTCTACTCATTT
AATTCGTCATTGAACAGGAGACAGTCCCAAGAAAAACTATTGAATTCTACGCTATTCTA
CAGTGCTTCCCCTTTAAAATGAGCTTTGGTTGAGCATTTTACGAAATTCCTATCGAAAG
TTATGCAATTTCAAAAATTAGTGATTCTCTCCTCAAAAATCCATCGAATCATTTAATTCG
TCATTGAACACGAGACAGTCCCCGGAAAAATTTTAGGATTCTACGCTATTCTACAGTGC
TTGCCCTTTAAAATGAGCTTTGGTTGAGCATTCTACGAAACTCTTATAGAAAGTTATGC
AATTTCAAAAATTAGTGATTTTCTCCTCAAAAATCCCTCGACTCATTTAATTCGTCATTG
AACAGGAAACAGTCCCAAGAAAAACTATTGAATTCTACGCTATTCTACAGTGCTTGCC
CTTTAAAATGAGCTTTGGTTGAGCATTCTACGAAATTCCTATCGAAAGTTATGTAATTTC
AAAGATTAGTGATTCTCCCCTCAAAAAATCCCTCGAATCATTTAATGCGTCATTGAATAG
GAGACAGTCCCCGGAAAAATTTTAGGATTCTACACTATTCTATAATTCTTCCCCTTTAAA
ATGAGCTTTGTTTGAGCATTCTACGAAATTTCTATCGAAAATTATGCAATTTCAAAAATT
AGTGATTTTCTCCTCAAAAATCCCTCGACTCATTTAATTCGTCATTGAACAGGAGACAG
TCCCAAGAAAAACTATTGAATTCTACGCTATTCTACAATGCGTCCCCTTTAAAATGAGCT
TTGGTTGAGCATTCTACGAAATTCCTATCGAAAGTTATGCAATTTCAAAATTAGTGATTC

TCTCCTCAAAAATCCATCGAATCTTTTAATTCGTCATTGAACAGGAGACAGTCCCCGGA
AAAATTTTAGGATTCTACACTATTCTACAATTCTTCCCCTTTAAAATGAGCTTTGTTTGA
GCATTCTACGAAATTTCTATCGAAAATTATGCAATTTCAAAGATTAGTGATTTTCTCCTC
AAAAATCCCTCGAATCATTTAATTCGTCATTGAACAGGAGACAGTCCCCGGAAAAATAT
TAGGACTCTACGCTATTCTACAATGCGTCCACTTTAAAATGAGCTTTGGTTGAACATTCT
ACGAAATTCCTAACGAAAGTTATGCAATTTTAAAGAATAGTGATTCTCTCCTCAAAAAT
CCCTCTACTCATTTAATTCGTCATTGAACAGGAGACAGTCCCAAGAAAAACTATTGAAT
TCTACGCTATTCTACAGTGCTTGCCCTTTAAAATGAGCTTTGGTTGAGCATTCTACGAAA
CTCTTATAGAAAGTTATGCAATTTCAAAAATTAGTGATTTTCTGCTCAAAAATCCCTCGA
CTCATTTAATTCGTCATTGAACAGGAAACAGTCCCAAGAAAAACTATTGAATTCTACGC
TATTCTACAGTGTTTTCCCTTTAAAATGAGCTTTGGTTGAGCATTCTACGAAATTCCTAT
CGAAGGTTATGCAATTTCAAAGATTAGTGATTCTCTCCTCAAAAATCCCTCGAATCATTT
AATGCGTCATTGAATAGGAGACAGTCCCCGGAAAAATTTTAGGATTCTACACTATTCTA
CAATTCTTCCCCTTTAAAATGAGCTTTGGTTGTGCATTCTACGAAATTCCTATCGAATGT
TATGCAATTTCAAAGTTCGAAAATAAAGAAATCGGTAATGGAATAGTATTTACAGAATT
AGTATTTACAGAATCCTTGTTGAATGGGAATCAATAAAAATTCAATAATCAGAAGCTAA
AAATCAAAAGCTAAATTTTTGAAAACTTTATTTAGGGGCTACCAACTCGAATTTACTAG
AATTTACTAGANNNNNNNNNNNNNNNNNNNNNNNNNNNNNNNNNNNNNNNNNNNNNNNNN
NNNNNNNNNNNNNNNNNNNNNNNNNNNNNNNNNNNNNNNNNNNNNNNNNNNNNNNNNNNN
NNNNNNNNNNNNNNNNNNNNNNNNNNNNNNNNNNNNNNNNNNNNNNNNNNNNNNNNNNNN
NNNNNNNNNNNNNNNNNNNNNNNNNNNNNNNNNNNNNNNNNNNNNNNNNNNNNNNNNNNN
NNNNNNNNNNNNNNNNNNNNNNNNNNNNNNNNNNNNNNNNNNNNNNNNNNNNNNNNNNNN
NNNNNNNNNNNNNNNNNNNNNNNNNNNNNNNNNNNNNNNNNNNNNNNNNNNNNNNNNNNN
NNNNNNNNNNNNNNNNNNNNNNNNNNNNNNNNNNNNNNNNNNNNNNNNNNNNNNNNNNNN
NNNNNNNNNNNNNNNNNNNNNNNNNNNNNNNNNNNNNNNNNNNNNNNNNNNNNNNNNNNN
NNNNNNNNNNNNNNNNNNNNNNNNNNNNNNNNNNNNNNNNNNNNNNNNNNNNNNNNNNNN
NNNNNNNNNNNNNNNNNNNNNNNNNNNNNNNNNNNNNNNNNNNNNNNNNNNNNNNNNNNN
NNNNNNNNNNNNNNNNNNNNNNNNNNNNNNNNNNNNNNNNNNNNNNNNNNNNNNNNNNNN
NNNNNNNNNNNNNNNNNNNNNNNNNNNNNNNNNNNNNNNNNNNNNNNNNNNNNNNNNNNN
NNNNNNNNNNNNNNNNNNNNNNNNNNNNNNNNNNNNNNNNNNNNNNNNNNNNNNNNNNNN
NNNNNNNNNNNNNNNNNNNNNNNNNNNNNNNNNNNNNNNNNNNNNNNNNNNNNNNNNNNN
NNNNNNNNNNNNNNNNNNNNNNNNNNNNNNNNNNNNNNNNNNNNNNNNNNNNNNNNNNNN
NNNNNNNNNNNNNNNNNNNNNNNNNNNNNNNNNNNNNNNNNNNNNNNNNNNNNNNNNNNN
NNNNNNNNNNNNNNNNNNNNNNNNNNNNNNNNNNNNNNNNNNNNNNNNNNNNNNNNNNNN
NNNNNNNNNNNNNNNNNNNNNNNNNNNNNNNNNNNNNNNNNNNNNNNNNNNNNNNNNNNN
NNNNNNNNNNNNNNNNNNNNNNNNNNNNNNNNNNNNNNNNNNNNNNNNNNNNNNNNNNNN
NNNNNNNNNNNNNNNNNNNNNNNNNNNNNNNNNNNNNNNNNNNNNNNNNNNNNNNNNNNN
NNNNNNNNNNNNNNNNNNNNNNNNNNNNNNNNNNNNNNNNNNNNNNNNNNNNNNNNNNNN
NNNNNNNNNNNNNNNNNNNNNNNNNNNNNNNNNNNNNNNNNNNNNNNNNNNNNNNNNNNN
NNNNNNNNNNNNNNNNNNNNNNNNNNNNNNNNNNNNNNNNNNNNNNNNNNNNNNNNNNNN
NNNNNNNNNNNNNNNNNNNNNNNNNNNNNNNNNNNNNNNNNNNNNNNNNNNNNNNNNNNN
NNNNNNNNNNNNNNNNNNNNNNNNNNNNNNNNNNNNNNNNNNNNNNNNNNNNNNNNNNNN
NNNNNNNNNNNNNNNNNNNNNNNNNNNNNNNNNNNNNNNNNNNNNNNNNNNNNNNNNNNN
NNNNNNNNNNNNNNNNNNNNNNNNNNNNNNNNNNNNNNNNNNNNNNNNNNNNNNNNNNNN

NNNNNNNNNNNNNNNNNNNNNNNNNNNNNNNNNNNNNNNNNNNNNNNNNNNNNNNNNNNN
NNNNNNNNNNNNNNNNNNNNNNNNNNNNNNNNNNNNNNNNNNNNNNNNNNNNNNNNNNNN
NNNNNNNNNNNNNNNNNNNNNNNNNNNNNNNNNNNNNNNNNNNNNNNNNNNNNNNNNNNN
NNNNNNNNNNNNNNNNNNNNNNNNNNNNNNNNNNNNNNNNNNNNNNNNNNNNNNNNNNNN
NNNNNNNNNNNNNNNNNNNNNNNNNNNNNNNNNNNNNNNNNNNNNNNNNNNNNNNNNNNN
NNNNNNNNNNNNNNNNNNNNNNNNNNNNNNNNNNNNNNNNNNNNNNNNNNNNNNNNNNNN
NNNNNNNNNNNNNNNNNNNNNNNNNNNNNNNNNNNNNNNNNNNNNNNNNNNNNNNNNNNN
NNNNNNNNNNNNNNNNNNNNNNNNNNNNNNNNNNNNNNNNNNNNNNNNNNNNNNNNNNNN
NNNNNNNNNNNNNNNNNNNNNNNNNNNNNNNNNNNNNNNNNNNNNNNNNNNNNNNNNNNN
NNNNNNNNNNNNNNNNNNNNNNNNNNNNNNNNNNNNNNNNNNNNNNNNNNNNNNNNNNNN
NNNNNNNNNNNNNNNNNNNNNNNNNNNNNNNNNNNNNNNNNNNNNNNNNNNNNNNNNNNN
NNNNNNNNNNNNNNNNNNNNNNNNNNNNNNNNNNNNNNNNNNNNNNNNNNNNNNNNNNNN
NNNNNNNNNNNNNNNNNNNNNNNNNNNNNNNNNNNNNNNNNNNNNNNNNNNNNNNNNNNN
NNNNNNNNNNNNNNNNNNNNNNNNNNNNNNNNNNNNNNNNNNNNNNNNNNNNNNNNNNNN
NNNNNNNNNNNNNNNNNNNNNNNNNNNNNNNNNNNNNNNNNNNNNNNNNNNNNNNNNNNN
NNNNNNNNNNNNNNNNNNNNNNNNNNNNNNNNNNNNNNNNNNNNNNNNNNNNNNNNNNNN
NNNNNNNNNNNNNNNNNNNNNNNNNNNNNNNNNNNNNNNNNNNNNNNNNNNNNNNNNNNN
NNNNNNNNNNNNNNNNNNNNNNNNNNNNNNNNNNNNNNNNNNNNNNNNNNNNNNNNNNNN
NNNNNNNNNNNNNNNNNNNNNNNNNNNNNNNNNNNNNNNNNNNNNNNNNNNNNNNNNNNN
NNNNNNNNNNNNNNNNNNNNNNNNNNNNNNNNNNNNNNNNNNNNNNNNNNNNNNNNNNNN
NNNNNNNNNNNNNNNNNNNNNNNNNNNNNNNNNNNNNNNNNNNNNNNNNNNNNNNNNNNN
NNNNNNNNNNNNNNNNNNNNNNNNNNNNNNNNNNNNNNNNNNNNNNNNNNNNNNNNNNNN
NNNNNNNNNNNNNNNNNNNNNNNNNNNNNNNNNNNNNNNNNNNNNNNNNNNNNNNNNNNN
NNNNNNNNNNNNNNNNNNNNNNNNNNNNNNNNNNNNNNNNNNNNNNNNNNNNNNNNNNNN
NNNNNNNNNNNNNNNNNNNNNNNNNNNNNNNNNNNNNNNNNNNNNNNNNNNNNNNNNNNN
NNNNNNNNNNNNNNNNNNNNNNNNNNNNNNNNNNNNNNNNNNNNNNNNNNNNNNNNNNNN
NNNNNNNNNNNNNNNNNNNNNNNNNNNNNNNNNNNNNNNNNNNNNNNNNNNNNNNNNNNN
NNNNNNNNNNNNNNNNNNNNNNNNNNNNNNNNNNNNNNNNNNNNNNNNNNNNNNNNNNNN
NNNNNNNNNNNNNNNNNNNNNNNNNNNNNNNNNNNNNNNNNNNNNNNNNNNNNNNNNNNN
NNNNNNNNNNNNNNNNNNNNNNNNNNNNNNNNNNNNNNNNNNNNNNNNNNNNNNNNNNNN
NNNNNNNNNNNNNNNNNNNNNNNNNNNNNNNNNNNNNNNNNNNNNNNNNNNNNNNNNNNN
NNNNNNNNNNNNNNNNNNNNNNNNNNNNNNNNNNNNNNNNNNNNNNNNNNNNNNNNNNNN
NNNNNNNNNNNNNNNNNNNNNNNNNNNNNNNNNNNNNNNNNNNNNNNNNNNNNNNNNNNN
NNNNNNNNNNNNNNNNNNNNNNNNNNNNNNNNNNNNNNNNNNNNNNNNNNNNNNNNNNNN
NNNNNNNNNNNNNNNNNNNNNNNNNNNNNNNNNNNNNNNNNNNNNNNNNNNNNNNNNNNN
NNNNNNNNNNNNNNNNNNNNNNNNNNNNNNNNNNNNNNNNNNNNNNNNNNNNNNNNNNNN
NNNNNNNNNNNNNNNNNNNNNNNNNNNNNNNNNNNNNNNNNNNNNNNNNNNNNNNNNNNN
NNNNNNNNNNNNNNNNNNNNNNNNNNNNNNNNNNNNNNNNNNNNNNNNNNNNNNNNNNNN
NNNNNNNNNNNNNNNNNNNNNNNNNNNNNNNNNNNNNNNNNNNNNNNNNNNNNNNNNNNN
NNNNNNNNNNNNNNNNNNNNNNNNNNNNNNNNNNNNNNNNNNNNNNNNNNNNNNNNNNNN
NNNNNNNNNNNNNNNNNNNNNNNNNNNNNNNNNNNNNNNNNNNNNNNNNNNNNNNNNNNN
NNNNNNNNNNNNNNNNNNNNNNNNNNNNNNNNNNNNNNNNNNNNNNNNNNNNNNNNNNNN
NNNNNNNNNNNNNNNNNNNNNNNNNNNNNNNNNNNNNNNNNNNNNNNNNNNNNNNNNNNN
NNNNNNNNNNNNNNNNNNNNNNNNNNNNNNNNNNNNNNNNNNNNNNNNNNNNNNNNNNNN

NNNNNNNNNNNNNNNNNNNNNNNNNNNNNNNNNNNNNNNNNNNNNNNNNNNNNNNNNNNN
NNNNNNNNNNNNNNNNNNNNNNNNNNNNNNNNNNNNNNNNNNNNNNNNNNNNNNNNNNNN
NNNNNNNNNNNNNNNNNNNNNNNNNNNNNNNNNNNNNNNNNNNNNNNNNNNNNNNNNNNN
NNNNNNNNNNNNNNNNNNNNNNNNNNNNNNNNNNNNNNNNNNNNNNNNNNNNNNNNNNNN
NNNNNNNNNNNNNNNNNNNNNNNNNNNNNNNNNNNNNNNNNNNNNNNNNNNNNNNNNNNN
NNNNNNNNNNNNNNNNNNNNNNNNNNNNNNNNNNNNNNNNNNNNNNNNNNNNNNNNNNNN
NNNNNNNNNNNNNNNNNNNNNNNNNNNNNNNNNNNNNNNNNNNNNNNNNNNNNNNNNNNN
NNNNNNNNNNNNNNNNNNNNNNNNNNNNNNNNNNNNNNNNNNNNNNNNNNNNNNNNNNNN
NNNNNNNNNNNNNNNNNNNNNNNNNNNNNNNNNNNNNNNNNNNNNNNNNNNNNNNNNNNN
NNNNNNNNNNNNNNNNNNNNNNNNNNNNNNNNNNNNNNNNNNNNNNNNNNNNNNNNNNNN
NNNNNNNNNNNNNNNNNNNNNNNNNNNNNNNNNNNNNNNNNNNNNNNNNNNNNNNNNNNN
NNNNNNNNNNNNNNNNNNNNNNNNNNNNNNNNNNNNNNNNNNNNNNNNNNNNNNNNNNNN
NNNNNNNNNNNNNNNNNNNNNNNNNNNNNNNNNNNNNNNNNNNNNNNNNNNNNNNNNNNN
NNNNNNNNNNNNNNNNNNNNNNNNNNNNNNNNNNNNNNNNNNNNNNNNNNNNNNNNNNNN
NNNNNNNNNNNNNNNNNNNNNNNNNNNNNNNNNNNNNNNNNNNNNNNNNNNNNNNNNNNN
NNNNNNNNNNNNNNNNNNNNNNNNNNNNNNNNNNNNNNNNNNNNNNNNNNNNNNNNNNNN
NNNNNNNNNNNNNNNNNNNNNNNNNNNNNNNNNNNNNNNNNNNNNNNNNNNNNNNNNNNN
NNNNNNNNNNNNNNNNNNNNNNNNNNNNNNNNNNNNNNNNNNNNNNNNNNNNNNNNNNNN
NNNNNNNNNNNNNNNNNNNNNNNNNNNNNNNNNNNNNNNNNNNNNNNNNNNNNNNNNNNN
NNNNNNNNNNNNNNNNNNNNNNNNNNNNNNNNNNNNNNNNNNNNNNNNNNNNNNNNNNNN
NNNNNNNNNNNNNNNNNNNNNNNNNNNNNNNNNNNNNNNNNNNNNNNNNNNNNNNNNNNN
NNNNNNNNNNNNNNNNNNNNNNNNNNNNNNNNNNNNNNNNNNNNNNNNNNNNNNNNNNNN
NNNNNNNNNNNNNNNNNNNNNNNNNNNNNNNNNNNNNNNNNNNNNNNNNNNNNNNNNNNN
NNNNNNNNNNNNNNNNNNNNNNNNNNNNNNNNNNNNNNNNNNNNNNNNNNNNNNNNNNNN
NNNNNNNNNNNNNNNNNNNNNNNNNNNNNNNNNNNNNNNNNNNNNNNNNNNNNNNNNNNN
NNNNNNNNNNNNNNNNNNNNNNNNNNNNNNNNNNNNNNNNNNNNNNNNNNNNNNNNNNNN
NNNNNNNNNNNNNNNNNNNNNNNNNNNNNNNNNNNNNNNNNNNNNNNNNNNNNNNNNNNN
NNNNNNNNNNNNNNNNNNNNNNNNNNNNNNNNNNNNNNNNNNNNNNNNNNNNNNNNNNNN
NNNNNNNNNNNNNNNNNNNNNNNNNNNNNNNNNNNNNNNNNNNNNNNNNNNNNNNNNNNN
NNNNNNNNNNNNNNNNNNNNNNNNNNNNNNNNNNNNNNNNNNNNNNNNNNNNNNNNNNNN
NNNNNNNNNNNNNNNNNNNNNNNNNNNNNNNNNNNNNNNNNNNNNNNNNNNNNNNNNNNN
NNNNNNNNNNNNNNNNNNNNNNNNNNNNNNNNNNNNNNNNNNNNNNNNNNNNNNNNNNNN
NNNNNNNNNNNNNNNNNNNNNNNNNNNNNNNNNNNNNNNNNNNNNNNNNNNNNNNNNNNN
NNNNNNNNNNNNNNNNNNNNNNNNNNNNNNNNNNNNNNNNNNNNNNNNNNNNNNNNNNNN
NNNNNNNNNNNNNNNNNNNNNNNNNNNNNNNNNNNNNNNNNNNNNNNNNNNNNNNNNNNN
NNNNNNNNNNNNNNNNNNNNNNNNNNNNNNNNNNNNNNNNNNNNNNNNNNNNNNNNNNNN
NNNNNNNNNNNNNNNNNNNNNNNNNNNNNNNNNNNNNNNNNNNNNNNNNNNNNNNNNNNN
NNNNNNNNNNNNNNNNNNNNNNNNNNNNNNNNNNNNNNNNNNNNNNNNNNNNNNNNNNNN
NNNNNNNNNNNNNNNNNNNNNNNNNNNNNNNNNNNNNNNNNNNNNNNNNNNNNNNNNNNN
NNNNNNNNNNNNNNNNNNNNNNNNNNNNNNNNNNNNNNNNNNNNNNNNNNNNNNNNNNNN
NNNNNNNNNNNNNNNNNNNNNNNNNNNNNNNNNNNNNNNNNNNNNNNNNNNNNNNNNNNN
NNNNNNNNNNNNNNNNNNNNNNNNNNNNNNNNNNNNNNNNNNNNNNNNNNNNNNNNNNNN
NNNNNNNNNNNNNNNNNNNNNNNNNNNNNNNNNNNNNNNNNNNNNNNNNNNNNNNNNNNN
NNNNNNNNNNNNNNNNNNNNNNNNNNNNNNNNNNNNNNNNNNNNNNNNNNNNNNNNNNNN

NNNNNNNNNNNNNNNNNNNNNNNNNNNNNNNNNNNNNNNNNNNNNNNNNNNNNNNNNNNN
NNNNNNNNNNNNNNNNNNNNNNNNNNNNNNNNNNNNNNNNNNNNNNNNNNNNNNNNNNNN
NNNNNNNNNNNNNNNNNNNNNNNNNNNNNNNNNNNNNNNNNNNNNNNNNNNNNNNNNNNN
NNNNNNNNNNNNNNNNNNNNNNNNNNNNNNNNNNNNNNNNNNNNNNNNNNNNNNNNNNNN
NNNNNNNNNNNNNNNNNNNNNNNNNNNNNNNNNNNNNNNNNNNNNNNNNNNNNNNNNNNN
NNNNNNNNNNNNNNNNNNNNNNNNNNNNNNNNNNNNNNNNNNNNNNNNNNNNNNNNNNNN
NNNNNNNNNNNNNNNNNNNNNNNNNNNNNNNNNNNNNNNNNNNNNNNNNNNNNNNNNNNN
NNNNNNNNNNNNNNNNNNNNNNNNNNNNNNNNNNNNNNNNNNNNNNNNNNNNNNNNNNNN
NNNNNNNNNNNNNNNNNNNNNNNNNNNNNNNNNNNNNNNNNNNNNNNNNNNNNNNNNNNN
NNNNNNNNNNNNNNNNNNNNNNNNNNNNNNNNNNNNNNNNNNNNNNNNNNNNNNNNNNNN
NNNNNNNNNNNNNNNNNNNNNNNNNNNNNNNNNNNNNNNNNNNNNNNNNNNNNNNNNNNN
NNNNNNNNNNNNNNNNNNNNNNNNNNNNNNNNNNNNNNNNNNNNNNNNNNNNNNNNNNNN
NNNNNNNNNNNNNNNNNNNNNNNNNNNNNNNNNNNNNNNNNNNNNNNNNNNNNNNNNNNN
NNNNNNNNNNNNNNNNNNNNNNNNNNNNNNNNNNNNNNNNNNNNNNNNNNNNNNNNNNNN
NNNNNNNNNNNNNNNNNNNNNNNNNNNNNNNNNNNNNNNNNNNNNNNNNNNNNNNNNNNN
NNNNNNNNNNNNNNNNNNNNNNNNNNNNNNNNNNNNNNNNNNNNNNNNNNNNNNNNNNNN
NNNNNNNNNNNNNNNNNNNNNNNNNNNNNNNNNNNNNNNNNNNNNNNNNNNNNNNNNNNN
NNNNNNNNNNNNNNNNNNNNNNNNNNNNNNNNNNNNNNNNNNNNNNNNNNNNNNNNNNNN
NNNNNNNNNNNNNNNNNNNNNNNNNNNNNNNNNNNNNNNNNNNNNNNNNNNNNNNNNNNN
NNNNNNNNNNNNNNNNNNNNNNNNNNNNNNNNNNNNNNNNNNNNNNNNNNNNNNNNNNNN
NNNNNNNNNNNNNNNNNNNNNNNNNNNNNNNNNNNNNNNNNNNNNNNNNNNNNNNNNNNN
NNNNNNNNNNNNNNNNNNNNNNNNNNNNNNNNNNNNNNNNNNNNNNNNNNNNNNNNNNNN
NNNNNNNNNNNNNNNNNNNNNNNNNNNNNNNNNNNNNNNNNNNNNNNNNNNNNNNNNNNN
NNNNNNNNNNNNNNNNNNNNNNNNNNNNNNNNNNNNNNNNNNNNNNNNNNNNNNNNNNNN
NNNNNNNNNNNNNNNNNNNNNNNNNNNNNNNNNNNNNNNNNNNNNNNNNNNNNNNNNNNN
NNNNNNNNNNNNNNNNNNNNNNNNNNNNNNNNNNNNNNNNNNNNNNNNNNNNNNNNNNNN
NNNNNNNNNNNNNNNNNNNNNNNNNNNNNNNNNNNNNNNNNNNNNNNNNNNNNNNNNNNN
NNNNNNNNNNNNNNNNNNNNNNNNNNNNNNNNNNNNNNNNNNNNNNNNNNNNNNNNNNNN
NNNNNNNNNNNNNNNNNNNNNNNNNNNNNNNNNNNNNNNNNNNNNNNNNNNNNNNNNNNN
NNNNNNNNNNNNNNNNNNNNNNNNNNNNNNNNNNNNNNNNNNNNNNNNNNNNNNNNNNNN
NNNNNNNNNNNNNNNNNNNNNNNNNNNNNNNNNNNNNNNNNNNNNNNNNNNNNNNNNNNN
NNNNNNNNNNNNNNNNNNNNNNNNNNNNNNNNNNNNNNNNNNNNNNNNNNNNNNNNNNNN
NNNNNNNNNNNNNNNNNNNNNNNNNNNNNNNNNNNNNNNNNNNNNNNNNNNNNNNNNNNN
NNNNNNNNNNNNNNNNNNNNNNNNNNNNNNNNNNNNNNNNNNNNNNNNNNNNNNNNNNNN
NNNNNNNNNNNNNNNNNNNNNNNNNNNNNNNNNNNNNNNNNNNNNNNNNNNNNNNNNNNN
NNNNNNNNNNNNNNNNNNNNNNNNNNNNNNNNNNNNNNNNNNNNNNNNNNNNNNNNNNNN
NNNNNNNNNNNNNNNNNNNNNNNNNNNNNNNNNNNNNNNNNNNNNNNNNNNNNNNNNNNN
NNNNNNNNNNNNNNNNNNNNNNNNNNNNNNNNNNNNNNNNNNNNNNNNNNNNNNNNNNNN
NNNNNNNNNNNNNNNNNNNNNNNNNNNNNNNNNNNNNNNNNNNNNNNNNNNNNNNNNNNN
NNNNNNNNNNNNNNNNNNNNNNNNNNNNNNNNNNNNNNNNNNNNNNNNNNNNNNNNNNNN
NNNNNNNNNNNNNNNNNNNNNNNNNNNNNNNNNNNNNNNNNNNNNNNNNNNNNNNNNNNN
NNNNNNNNNNNNNNNNNNNNNNNNNNNNNNNNNNNNNNNNNNNNNNNNNNNNNNNNNNNN
NNNNNNNNNNNNNNNNNNNNNNNNNNNNNNNNNNNNNNNNNNNNNNNNNNNNNNNNNNNN
NNNNNNNNNNNNNNNNNNNNNNNNNNNNNNNNNNNNNNNNNNNNNNNNNNNNNNNNNNNN
NNNNNNNNNNNNNNNNNNNNNNNNNNNNNNNNNNNNNNNNNNNNNNNNNNNNNNNNNNNN
NNNNNNNNNNNNNNNNNNNNNNNNNNNNNNNNNNNNNNNNNNNNNNNNNNNNNNNNNNNN

NNNNNNNNNNNNNNNNNNNNNNNNNNNNNNNNNNNNNNNNNNNNNNNNNNNNNNNNNNNN
NNNNNNNNNNNNNNNNNNNNNNNNNNNNNNNNNNNNNNNNNNNNNNNNNNNNNNNNNNNN
NNNNNNNNNNNNNNNNNNNNNNNNNNNNNNNNNNNNNNNNNNNNNNNNNNNNNNNNNNNN
NNNNNNNNNNNNNNNNNNNNNNNNNNNNNNNNNNNNNNNNNNNNNNNNNNNNNNNNNNNN
NNNNNNNNNNNNNNNNNNNNNNNNNNNNNNNNNNNNNNNNNNNNNNNNNNNNNNNNNNNN
NNNNNNNNNNNNNNNNNNNNNNNNNNNNNNNNNNNNNNNNNNNNNNNNNNNNNNNNNNNN
NNNNNNNNNNNNNNNNNNNNNNNNNNNNNNNNNNNNNNNNNNNNNNNNNNNNNNNNNNNN
NNNNNNNNNNNNNNNNNNNNNNNNNNNNNNNNNNNNNNNNNNNNNNNNNNNNNNNNNNNN
NNNNNNNNNNNNNNNNNNNNNNNNNNNNNNNNNNNNNNNNNNNNNNNNNNNNNNNNNNNN
NNNNNNNNNNNNNNNNNNNNNNNNNNNNNNNNNNNNNNNNNNNNNNNNNNNNNNNNNNNN
NNNNNNNNNNNNNNNNNNNNNNNNNNNNNNNNNNNNNNNNNNNNNNNNNNNNNNNNNNNN
NNNNNNNNNNNNNNNNNNNNNNNNNNNNNNNNNNNNNNNNNNNNNNNNNNNNNNNNNNNN
NNNNNNNNNNNNNNNNNNNNNNNNNNNNNNNNNNNNNNNNNNNNNNNNNNNNNNNNNNNN
NNNNNNNNNNNNNNNNNNNNNNNNNNNNNNNNNNNNNNNNNNNNNNNNNNNNNNNNNNNN
NNNNNNNNNNNNNNNNNNNNNNNNNNNNNNNNNNNNNNNNNNNNNNNNNNNNNNNNNNNN
NNNNNNNNNNNNNNNNNNNNNNNNNNNNNNNNNNNNNNNNNNNNNNNNNNNNNNNNNNNN
NNNNNNNNNNNNNNNNNNNNNNNNNNNNNNNNNNNNNNNNNNNNNNNNNNNNNNNNNNNN
NNNNNNNNNNNNNNNNNNNNNNNNNNNNNNNNNNNNNNNNNNNNNNNNNNNNNNNNNNNN
NNNNNNNNNNNNNNNNNNNNNNNNNNNNNNNNNNNNNNNNNNNNNNNNNNNNNNNNNNNN
NNNNNNNNNNNNNNNNNNNNNNNNNNNNNNNNNNNNNNNNNNNNNNNNNNNNNNNNNNNN
NNNNNNNNNNNNNNNNNNNNNNNNNNNNNNNNNNNNNNNNNNNNNNNNNNNNNNNNNNNN
NNNNNNNNNNNNNNNNNNNNNNNNNNNNNNNNNNNNNNNNNNNNNNNNNNNNNNNNNNNN
NNNNNNNNNNNNNNNNNNNNNNNNNNNNNNNNNNNNNNNNNNNNNNNNNNNNNNNNNNNN
NNNNNNNNNNNNNNNNNNNNNNNNNNNNNNNNNNNNNNNNNNNNNNNNNNNNNNNNNNNN
NNNNNNNNNNNNNNNNNNNNNNNNNNNNNNNNNNNNNNNNNNNNNNNNNNNNNNNNNNNN
NNNNNNNNNNNNNNNNNNNNNNNNNNNNNNNNNNNNNNNNNNNNNNNNNNNNNNNNNNNN
NNNNNNNNNNNNNNNNNNNNNNNNNNNNNNNNNNNNNNNNNNNNNNNNNNNNNNNNNNNN
NNNNNNNNNNNNNNNNNNNNNNNNNNNNNNNNNNNNNNNNNNNNNNNNNNNNNNNNNNNN
NNNNNNNNNNNNNNNNNNNNNNNNNNNNNNNNNNNNNNNNNNNNNNNNNNNNNNNNNNNN
NNNNNNNNNNNNNNNNNNNNNNNNNNNNNNNNNNNNNNNNNNNNNNNNNNNNNNNNNNNN
NNNNNNNNNNNNNNNNNNNNNNNNNNNNNNNNNNNNNNNNNNNNNNNNNNNNNNNNNNNN
NNNNNNNNNNNNNNNNNNNNNNNNNNNNNNNNNNNNNNNNNNNNNNNNNNNNNNNNNNNN
NNNNNNNNNNNNNNNNNNNNNNNNNNNNNNNNNNNNNNNNNNNNNNNNNNNNNNNNNNNN
NNNNNNNNNNNNNNNNNNNNNNNNNNNNNNNNNNNNNNNNNNNNNNNNNNNNNNNNNNNN
NNNNNNNNNNNNNNNNNNNNNNNNNNNNNNNNNNNNNNNNNNNNNNNNNNNNNNNNNNNN
NNNNNNNNNNNNNNNNNNNNNNNNNNNNNNNNNNNNNNNNNNNNNNNNNNNNNNNNNNNN
NNNNNNNNNNNNNNNNNNNNNNNNNNNNNNNNNNNNNNNNNNNNNNNNNNNNNNNNNNNN
NNNNNNNNNNNNNNNNNNNNNNNNNNNNNNNNNNNNNNNNNNNNNNNNNNNNNNNNNNNN
NNNNNNNNNNNNNNNNNNNNNNNNNNNNNNNNNNNNNNNNNNNNNNNNNNNNNNNNNNNN
NNNNNNNNNNNNNNNNNNNNNNNNNNNNNNNNNNNNNNNNNNNNNNNNNNNNNNNNNNNN
NNNNNNNNNNNNNNNNNNNNNNNNNNNNNNNNNNNNNNNNNNNNNNNNNNNNNNNNNNNN
NNNNNNNNNNNNNNNNNNNNNNNNNNNNNNNNNNNNNNNNNNNNNNNNNNNNNNNNNNNN
NNNNNNNNNNNNNNNNNNNNNNNNNNNNNNNNNNNNNNNNNNNNNNNNNNNNNNNNNNNN
NNNNNNNNNNNNNNNNNNNNNNNNNNNNNNNNNNNNNNNNNNNNNNNNNNNNNNNNNNNN

NNNNNNNNNNNNNNNNNNNNNNNNNNNNNNNNNNNNNNNNNNNNNNNNNNNNNNNNNNNN
NNNNNNNNNNNNNNNNNNNNNNNNNNNNNNNNNNNNNNNNNNNNNNNNNNNNNNNNNNNN
NNNNNNNNNNNNNNNNNNNNNNNNNNNNNNNNNNNNNNNNNNNNNNNNNNNNNNNNNNNN
NNNNNNNNNNNNNNNNNNNNNNNNNNNNNNNNNNNNNNNNNNNNNNNNNNNNNNNNNNNN
NNNNNNNNNNNNNNNNNNNNNNNNNNNNNNNNNNNNNNNNNNNNNNNNNNNNNNNNNNNN
NNNNNNNNNNNNNNNNNNNNNNNNNNNNNNNNNNNNNNNNNNNNNNNNNNNNNNNNNNNN
NNNNNNNNNNNNNNNNNNNNNNNNNNNNNNNNNNNNNNNNNNNNNNNNNNNNNNNNNNNN
NNNNNNNNNNNNNNNNNNNNNNNNNNNNNNNNNNNNNNNNNNNNNNNNNNNNNNNNNNNN
NNNNNNNNNNNNNNNNNNNNNNNNNNNNNNNNNNNNNNNNNNNNNNNNNNNNNNNNNNNN
NNNNNNNNNNNNNNNNNNNNNNNNNNNNNNNNNNNNNNNNNNNNNNNNNNNNNNNNNNNN
NNNNNNNNNNNNNNNNNNNNNNNNNNNNNNNNNNNNNNNNNNNNNNNNNNNNNNNNNNNN
NNNNNNNNNNNNNNNNNNNNNNNNNNNNNNNNNNNNNNNNNNNNNNNNNNNNNNNNNNNN
NNNNNNNNNNNNNNNNNNNNNNNNNNNNNNNNNNNNNNNNNNNNNNNNNNNNNNNNNNNN
NNNNNNNNNNNNNNNNNNNNNNNNNNNNNNNNNNNNNNNNNNNNNNNNNNNNNNNNNNNN
NNNNNNNNNNNNNNNNNNNNNNNNNNNNNNNNNNNNNNNNNNNNNNNNNNNNNNNNNNNN
NNNNNNNNNNNNNNNNNNNNNNNNNNNNNNNNNNNNNNNNNNNNNNNNNNNNNNNNNNNN
NNNNNNNNNNNNNNNNNNNNNNNNNNNNNNNNNNNNNNNNNNNNNNNNNNNNNNNNNNNN
NNNNNNNNNNNNNNNNNNNNNNNNNNNNNNNNNNNNNNNNNNNNNNNNNNNNNNNNNNNN
NNNNNNNNNNNNNNNNNNNNNNNNNNNNNNNNNNNNNNNNNNNNNNNNNNNNNNNNNNNN
NNNNNNNNNNNNNNNNNNNNNNNNNNNNNNNNNNNNNNNNNNNNNNNNNNNNNNNNNNNN
NNNNNNNNNNNNNNNNNNNNNNNNNNNNNNNNNNNNNNNNNNNNNNNNNNNNNNNNNNNN
NNNNNNNNNNNNNNNNNNNNNNNNNNNNNNNNNNNNNNNNNNNNNNNNNNNNNNNNNNNN
NNNNNNNNNNNNNNNNNNNNNNNNNNNNNNNNNNNNNNNNNNNNNNNNNNNNNNNNNNNN
NNNNNNNNNNNNNNNNNNNNNNNNNNNNNNNNNNNNNNNNNNNNNNNNNNNNNNNNNNNN
NNNNNNNNNNNNNNNNNNNNNNNNNNNNNNNNNNNNNNNNNNNNNNNNNNNNNNNNNNNN
NNNNNNNNNNNNNNNNNNNNNNNNNNNNNNNNNNNNNNNNNNNNNNNNNNNNNNNNNNNN
NNNNNNNNNNNNNNNNNNNNNNNNNNNNNNNNNNNNNNNNNNNNNNNNNNNNNNNNNNNN
NNNNNNNNNNNNNNNNNCTTAGTGATTCTCTCCTCAAAATCCCTCGAATCATTTAATTCG
TCATTGAACAGGAGACAGTCCCGAAAAACTATTGAATCAACGCTATTCTACAGTGCTTC
CTTTAAAATGAGCTTTGGTTGAGCATTCTACAAATTCGTATCGAAAGTTATGCAATTTCA
AAAATTAGTGATTCTCTCCTCAAAAATCGGTCGAATCATTTAATTCGTCATTGAACAGG
AGACAGTCCCCGGAAAAAATTTTAGGAATTTTAGAAAATTAAAATTAACAGTTGATCCTT
TGACTACTCGATAAAAAATTTCAATTCTTGTTTCTTCAAATAATTTACTGCCGAAACGTT
CGAAAATAAAGAAATCGGTAATGGAATAGTATTTACAGAATTAGTATTTACAGAATCCTT
GTTGAATGGGAATCAATAAAAATTCAATAATCAGAAGCTAAAAATCAAAAGCTAAATTT
TTGGAAAACTTTATTTAGAGGCTACCAACTCGAATTTACTAGAATTTACTAGAAAATTA
AAATTAACAGTTGATCCTTTGACTACTCGATACAAAATTTCAATTCTTGTTTCTTCAAAT
AATTTACTGCCGAAACGTTCGAAAATAAAGAAATCGGTAATGGAATAGTATTTACAGAA
TTAGTATTTACAGAATCCTTGTTGAATGGGAATCAATAAAAATTCAATAATCAGAAGCTA
AAAATCAAAAGCTAAATTTTTGAAAACTTTATTTAGAGGCTACCAACTCGAATTTACTA
GAATTTACTAGAAAATTAAAATTAACAGTTGATCCTTTGACTACTCGATACAAAATTTCA
ATTCTTGTTTCTTCAAATAATTTATCGCCGAAACGTTCGAAAATAAAGAAATCGGTAATG
GAATACCACGCGCAAACCAGCAGGTACTACCGTTACGGTTTCGATAGTGGCGAGGAAG
TGGTAGGGCCTGCGGTGTGGCGCTTGCCAACGCACGGCCGCGTGGAAAGGGGATAGC

ACCGTTAATGGTCGCGGCGAGAAACCTTCCTCGTGGTTGAAATGCCGCCGCTGCTGCG
CGGAAGGACGGCACGCCGACACTCCTTTTCTCGGCCATGAAAGTAAAAGTTCGCTCGT
GGCGCTCCCAGCGGCGAGAAGAGGAAGGAAAATGGAGCAGGAAGGACGGACCGCTA
TCTCCGCCTCGCCACACGTTCGCTCTCCTCCCGATACTTTTTTATTATTCCCGGCCCGA
CTTCCTCGTCTCTCTCTCCCTCGTTGATTCTTCCTTAAACGTTTGTTTGCCAAACTGATT
TATTTGTTTGTCCAACGAATTGACGAATGAAAGGTATAATAATAAGGTATAGTATAATTG
AAGTTTAAACGTGTTTTGCGTTATGGATTTAAAGACGTCGTCCCTTTGTTACGGTTTCTT
TTAGTATTCGCAGCAGAAGCGAGCGTTCGAGAGAAATTTGCATTTTTCTGGGCATAAAT
AAATTACTCGAGGTAAACGATGGGTTATGTCATTTTTTTTTTTCATCGTAATGCGAAAAA
AAATTTTTTCCTCGTTAATTAACGATTTTAAAATTTTATTGAAACACGGGGAAATAAATT
TCAACGAATTTTCGATAATTTGCAATATGGATATGGATATAGGAAAAGAGAGATAAAGAT
GGATGGTTTTTATCATTCGTGACTATCGATTATATTTATTAATACTTAGAGTCATTAATTAC
AATTTAAATATTCGATAATTCGACGAATACCAAAGTGACGATTCGATTGGTAATGCGAAA
TTTAAAAAGACGTCGAGAAGACGTCTATCCTTTCTTTGTTCGAAACAACATTCCAAAA
AAGATAATCGCTTTCGGATCCTTTCCACACAGAATTATACTCGAAAAAGCATAATAATAA
TAAATAAATTATTCGCCGTTTCCTCCTTCCAACATGCTTCTTCCTCTCCTCTCCTCTCGAA
TAAATCTTTTCGAGGTTTCGTAACGAAGAAGAGAAATAGCTTCTCATAATTCCGATTCA
CTTTCTACGCCACGGTTCAACCACATTCCCAATCGATTCGGCGAAAGGTATCACGCGTT
CGATAATTACGATTGTACGCTGCGGATCAATGATGAGCTTTTTCTTTTCAAAATTTTCTT
TTTTCCTTTGGAAAGAAATAAAAAACATTGAGTTGTGAAAAATAATGCTACACTAACCG
AACTAAATGAATATTATTTTTTTTTCGAATACGTATGATACGATATAATATACGAAACATA
ACCTAACTTTTTCTCTCTTAATAAATTTTATGCGAATCGATGATGAGCTTTTAATCTTTTC
TTCAAAATTTTCTTTTTTCCTTTGGAAAGAAATAAAAAACATTGAATTGTGAAAAATAA
TGCTACGCTAACCGAGCTAAATGAATATTATTTCTTTTTCGAATACGTACAATACGATATA
ATATACGAAACATAACCTAACTTTTTCTCTCTTAATAAATTTCAAGTATTATTCGAGATAC
GCGGATCGATTATGAGCCTTTTCTTCTTTTCAAAATTTTCTTTTTTCTTTTGAAAAGAAA
AAAAGAACATTGAATTGTGAAAAATAATGCTACACTAACCGAGCTAAATGAATATTATT
TTTTTTCGAATACGTACGATACGATATATACGAAACATAACCTAACTTTTTCTCTCTTAAT
AAATTTTACGCGAATCGATGATGAACTTTTAATCTTCTCTTCAAAATTTTCTTTTTTCCTT
TGGAAAGAAATAAAAAACATTGAATTGTGAAAAATAATGCTACACTAACCGAGCTAAA
TGAATATTATTTTTTTTTCGAATACGTACGATATAACCTAACTTTTTCTCTCTTAATAAATTT
TAAGTATTACTCGAGATACGCGGATCGATGATGAGCTTTTAATCTTCTCTTCAAAGTTTT
CTTTTTTCCTTTGGAAAGAAATAAAAAACATTGAATTGTGAAAAATAATGCTACATTAA
CCGAGCTAAATGAATATTATTTTTTTTCGAATACGTACGATACGATATATACGAAACATAA
CCTAACTTTTTCTCTCTTAATAAATTTTACGCGGATCGATGATGAGCTTTTAATCTTCTCT
TCAAAATTTTCTTTTTTCTTTTGAAAAATAAAAAATATTGAATTGTGAAAAATAATGCTA
CACTAATCAAGGATGAATGTTATTTCTTTTTCGAATACGTACGATACGATATATACGAAA
CATAACCTAACTTTTTCTTTCTTTCTAAATTTTAAGTATTACTCGAGATACGAGTTAAGTT
AATTAAAATGGAAACAAAATATTTGTATTATTAACGTAACAACGATTTGTTTTTTTCTTTT
AAATTTAAATAATTATTTTTTTTTCTTTCTAAATTTTAAGTATTACTCGAGATACGAGTTA
AGTTAATTAAAATGGAAACAAATATTTGTATTATTAACGTAACGATACAATTCGTTTTTTT
CCTTTAAATTTAAATAATTATTTTTTTTTTTTTTTCGTCAAAAAGTTGCAGTAACTCAACC
GTGACGAGTTCCATATTATAAATTCAATGAAGCAAGAGCCGGCCTTCTTCCATATCTCCG
GAGAGGAAAAAAAAAAAAAAAAAGAAAAAAAAAGAAGAGAAGAATTCGGCGAGGAG

GAGAAGAGGCGGGGTAAAGGAATTAGGTAATTACATCGAGAAGCTAGCGAAACTGTC
ACGTTACCTTGAATGAAAA

>novel_circ_001492
ATGGCAAACCTATGTTACGACAAGGAGATACTGGAGATTGGATTGGGACATTTGAAGG
ACATAAAGGAGCAGTGTGGGGTGTTGCATTAAATCCTCAGGCTACGAGAGCTGCTTCA
GGTGCTGCAGATTTTAATGCTAAAGTTTGGGATGCGATAAAAGGAGAAGAAATTCATTC
GTTCCAACATAAACATATTGTTAAATCTGTCAATTTTAGTACAGATTCTAATTATTTGTGC
ACTGGCTCCAATGAGAAACTTGTACGAATTTACGATCTTAACAAACCGGATGCAGCAC
CACAAGTTTTCTCAGGTCATAAAAATGGTATCAGACATGTTACCTTTTTCAATAATAATA
CTGCATTAATTACTTGTGGCGATGATAAAACATTAAGAGTTTGGGATAGGAATAGCGGC
CAAGAAGTCAAAAGGTTAGATTTTCCGGCAATTCCAAATTCCATGGAAGTATCAAGAG
ATGGAAATATCATTACCACAACTCATTCCAACATAGTTACTTTTTGGAATAGTAGAGAAT
TAACCAAATTACGCGAATACACTGCACCAACACAAATGAATAGTGCCAGTTTGCATCCA
GATTGTAGTATTTTTGTATGTGGAGGAGAAGATTTAAAAATGTACAAGTTCGATTACACT
ACGGGCGCGGAGATTGAATCATTTAAAGGACACTTTGGACCCGTGCACTGTGTACGTT
TTTCACCGGATGGTGAATTATATGCTAGTGGTTCAGAGGATGGTACATTGAGATTATGGC
AAACAACTGTTGGCAAAACTTATGGTCTCTGGCGATGTATAGAACAAACACCTGCAAT
ACAAGAAAATACTACTGTGCTTAATAATAAACAAGAAGTTCCTGCCAGTTAAAGATCTG
ATGATTCATAAATAATGTATTTTAATACCTTCCAAAAGAGAAAAAAAGAGAAGAATACT
TCATCGAGAGAT

>novel_circ_001493
GAATAAACAACACTCTCGTCCTCGAAACTGGTTAAAGCGACTTGAAAAACATCGTCGA
GGCCGCCATAATATTCTTCCTGCCTGAACTTGGCCTACAGTTTTGTGCGCTTGCTACGTT
CACTACGTTCAGTGTCGCGTCGTACGTAGATGGTGACGGTTCGTTGGGAGAGGATTTTT
AGTTAATGGAAAGTTCACCGGGAATGTGGAAGTATATTTGCGAGTAAATCTTTTCCTCG
CGCTCTACCGAGTGTAGGTGCATCGAAGAAAGGGAAAAGTATCGTCATTCGCAGTTGC
AATCGTACATTATGGCAAATTTAAGACAAACTCCGTTGACGTGTAGTGGGCATACTAGA
CCCGTGGTGCATCTCGCATTCTCCGATATCACTGAATCTGGATATTATCTGATTTCGGCG
TGCAAAGATGGCAAACCTATGTTACGACAAGGAGATACTGGAGATTGGATTGGGACAT
TTGAAGGACATAAAGGAGCAGTGTGGGGTGTTGCATTAAATCCTCAGGCTACGAGAGC
TGCTTCAGGTGCTGCAGATTTTAATGCTAAAGTTTGGGATGCGATAAAAGGAGAAGAA
ATTCATTCGTTCCAACATAAACATATTGTTAAATCTGTCAATTTTAGTACAGATTCTAATT
ATTTGTGCACTGGCTCCAATGAGAAACTTGTACGAATTTACGATCTTAACAAACCGGAT
GCAGCACCACAAGTTTTCTCAGGTCATAAAAATGGTATCAGACATGTTACCTTTTTCAA
TAATAATACTGCATTAATTACTTGTGGCGATGATAAAACATTAAGAGTTTGGGATAGGAA
TAGCGGCCAAGAAGTCAAAAGGTTAGATTTTCCGGCAATTCCAAATTCCATGGAAGTAT
CAAGAGATGGAAATATCATTACCACAACTCATTCCAACATAGTTACTTTTTGGAATAGTA
GAGAATTAACCAAATTACGCGAATACACTGCACCAACACAAATGAATAGTGCCAGTTT
GCATCCAGATTGTAGTATTTTTGTATGTGGAGGAGAAGATTTAAAAATGTACAAGTTCG
ATTACACTACGGGCGCGGAGATTGAATCATTTAAAGGACACTTTGGACCCGTGCACTGT
GTACGTTTTTCACCGGATGGTGAATTATATGCTAGTGGTTCAGAGGATGGTACATTGAGA
TTATGGCAAACAACTGTTGGCAAAACTTATGGTCTCTGGCGATGTATAGAACAAACACC

TGCAATACAAGAAAATACTACTGTGCTTAATAATAAACAAGAAGTTCCTGCCAGTTAAA
GATCTGATGATTCATAAATAATGTATTTAATACCTTCCAAAAGAGAAAAAAAGAGAAG
AATACTTCATCGAGAGAT

>novel_circ_001494
ATCGTTGGGATTCAACGGACTGTGTTAACCGGATCAACGTACTTGAGCGTCTATTGACC
TTTACTTAAAAATTTGAATAAATAAAAAAAAGAAAGAAAGAAAGAAAAGAGAGAGAG
AGAAAAAACAGATATTTCTATCCGCTTAAAAATAAATTTATTATACTAATCAATTCTCAA
GAATTCTCTAATAGAACGATCTATCCTATTCGTTCGGTTAAACACAAATAAATTAAAGTA
AAAAGTCAGAAGAGAAAGGAAAACAGTATGCGTTCAAACGTATCTGAAATTCAAATTA
CATACAAGTTTTGAAAAAAATTTACATAAAATAAAAGAAAGAAGGAAGGAAGAGGAA
AGAGTTAAATAAAAAAAAAAAAAGAAAAGGAACCAGATTGAGAAGAAATCTTCTCTTC
AATGAATGATTTATATATTGCTTTAATAAATAAAAAGGAAAGAAAGAATTCATGAGAGA
GAAAAAAATAAATGATTGAACGAAGAAGCAGGAAGGAACGGGTGTGTACAGTGATGT
ATTAGAACGCCTAAAAGAAAAAAGAAAAGAAAAAAAAAAAAAAAAGTATGGAGAACG
CGGCGATCGCACTCGACTCGACTGTTAGAAGTCAGGATTCGAATAATTCAAAGAATTC
GAAGGACTCGAAGGACTCGAACGCATCCGATTCGAAGCCAGAAAGTTGCCTCGAGTC
CAAACATTCGAAGGAAATCCTTATTTCTGAGAAGCAACAACGGCAACAGCCCCGAACC
GTGACAAGCACGACGGACGATCGACAGAAGCTGAAGAACAAGGAGTCGAAGGATGA
ATCAACTATTCTGATGTTCACTTCTTGTGGCCAATTGCTTTTAGGTGTAGTACTTGTAGT
GTTTGGTATCCTGGTATTGGTCCATGGCGCTGCATTAGGAGGTTCCGGTGCAGGATTGT
GGGCAGGTGCGGGTGCACTTGCCGCAGGAGCGTTCGGTGTCGTAGCAACACTTGCAA
CGTCAACGAGCAAAACGAATTCCAGCTTTTCCACGGCTCACCTTGCAGCGAGCCTCAT
TGCCCTTGCCCTTTCGAATATGGCTGCAATCACGGCCTTGACTGCTATTGTCAGGGACT
CCCAGAGAACACCGGAAGTGACCTTGCTCACTGTTCCG

>novel_circ_001495
GCATCGAGTGCGGTTATCCAGCAAACATAAAACACGGAGGCTACACACTTATCAACAA
TACCGTTAGTTATCTCAGTCAGGTGCTTTACTCCTGCGATGAAGGATTCGAGATGACTG
GTCGTGCAAGATTAACTTGTGACATCGATGAGCGTTGGAATGGACCACCGCCGCGTTG
TGAACCAATCCTGTGCGATCCTCCAACACCTGTGCCGCACAGTTACATTCAAATCGATG
AAATCGACGAAACCGAAACAATGCCATCGAAATCGAGCTTCAATCGAAGTCTTCTCGT
AGGCAGCATCGTCACTTATACTTGTGAAAAGAATTATAGACTCACTGGGCACCGACAA
ATATTATGTTTACCCACTGGATTATACGATCACATCGCACCCACTTGCATAGAGGAACCA
CGCACCACAGTGACCGCCCCACCTCGAATAACTGTACGAACAACGACCGCCAGAACA
CGGCAACCATTCTTACCGCCACGAGTCCGACCCACGACAATCTCGACGACCACAACCA
CGAGCACCACCCCTGTACCGCCTCGAAGAGTGGCCAGCACCGCTGAATTACCCGCGAC
GGTTCGCGAAACCATCGCGAGGAAACCGAATGTCGGTCTTCAGAGGCCAGCACCTCC
TCCTTCGCCACCTTTGAACGACGACAGCAGTGATCATCCTCAGGACAATGAGATTTCG
GGCAGTGGGGTGGATAACGCACAGGTTGGGATTGGAACCGGTGTTCCAGAACCTTCTG
GACCCTACAGGGTGGACAATGCCGACCACACGAGTAACACTCATCAAGCGAAATTAAA
TCTAGGCGCTGTGATCGCCTTGGGTGTCTTCGGCGGTTTCGTATTCCTAGCCGCTGTAAT
CACCACCGTTGTAATACTTATACGCAG

>novel_circ_001496
CGACGGTAGTGATTTATTGGACGACGCGGGAGACGATGCCAAATCAGTTCGCACAAAC
GACGACAGTAAAAAACAAAATCACAGCGAGATCGAGAAAAGGAGGAGGGACAAAAT
GAACACGTACATAACCGAGCTTTCCGCAATGGTTCCCATGTGCCACGCCATGTCCCGCA
AACTCGACAAGCTGACCGTGCTCAGGATGGCTGTCCAACACTTGAAGACCATCCTGGG
CGCCGTCACCTCGTACACGGAGGGCCACTACAAGCCGGCGTTCCTCAGCGACCAAGA
GCTCAAAACGCTCATACTTCAA

>novel_circ_001497
CAATGTTACCTGTAAAAACCGATGTGCCCCAAGGTGTATCGAGATTGTGTCCTGGTGCG
CGGAGGTCTTTTTTCTGCAGAATGAAGCGAAAAGTCGAAGGGATACGTTGCGGGGAAT
TGCAAGTGAAGGAGGAGTCCGAGTCTGCGAGTGGATGTCATCGACGAAAAAAACAAC
AAAACGTTGACTGGAAGTACTGTGTAATTCAATGCACTGGATATTTAAAGTCGTGGGCG
CCCGCAAAAATCGACCTCGAAGAACACGAAGGTGATGGAGACGGGGAGGCTTGCAAT
TTGTCGTGTTTGGTAGCTGTTGGTCGGTTGCAATCCACGATTCCGACATCTTTACCTAA
GAAGCCCCGTTTGAGATCCATCAAGTTCGTTTCGCGACATGCGATGGACGGTAAATTCC
TCTTCGTCGACCAAAG

>novel_circ_001500
ACACGCCTCTGTCACCAATGGGGCCGTCGTCGCCGAGTCAGGAGGGTCTATTCACGGA
TCTTCGCCTTTTGTCCTTGGAGAAGCAGCTCAATATCGAACTCAAGGTGAAACAGGGG
GCGGAGAACATGATCCAGAGTTTGACGAGCGGAAGGGACAAAAAGTTGCTGCAGGA
GGCTCAACAAATGCTCGACGATTCTCGCGCTAAAATCGAGTTCCTACGTATGCGAATAA
TGAAAGTGCGGCAAGCCCGCCAGCAGCAACACGCGCGAGGCGATGCACCACCGCCG
AACGGCGAGGCGACTAGCAACAAAG

>novel_circ_001501
ACACGCCTCTGTCACCAATGGGGCCGTCGTCGCCGAGTCAGGAGGGTCTATTCACGGA
TCTTCGCCTTTTGTCCTTGGAGAAGCAGCTCAATATCGAACTCAAGGTGAAACAGGGG
GCGGAGAACATGATCCAGAGTTTGACGAGCGGAAGGGACAAAAAGTTGCTGCAGGA
GGCTCAACAAATGCTCGACGATTCTCGCGCTAAAATCGAGTTCCTACGTATGCGAATAA
TGAAAGTGCGGCAAGCCCGCCAGCAGCAACACGCGCGAGGCGATGCACCACCGCCG
AACGGCGAGGCGACTAGCAACAAAGACAGGTACGAGCCTAGTTTGGAGTTGGCGTTG
GAGGAGAGGGTGGAGGAGCTGCGACATCGTTTGCGGATCGAAGCCGCGGTTGTGGAA
GGGGCCAAGAACGTGATACGTCTTTTACAAAGTGCCAAAGTCGCCGATAAGAAGGCCT
TAACGGAG

>novel_circ_001502
ACGGCGTTGCACTGGGCGGCCAAGCACGGCGACGAGAATATCGTCAAGCTGATCGCC
GGCACCTACAAGGATTACATCAAGAGCGTCAATGAAACTTCGAACGGG

>novel_circ_001503
GGTGAGAAATATTTAGTCCTCAAGAAGAAGTATCGTCAACTTCCTATAGATCTGGGGAT
TTCGGACCAAATTACATCACCCATAAATACTCCTGATATATCAACTCCGCTTTCTCCTTTA

CGAGTTCCGCCACCTTACCGACCACCACCTCCCGCGCCCCTTAGTCCACCTACCAATAA
TACGAACCGCGAAGAATCGTGTTCGAATTTTGACCGTGAAGAGGCCGCTATTAATGCG
CGGAAAAATGGCGTTGAAAGTGTCGAGGCAGGATTTTCGACACCCTCACCTCCTGTAC
CACCGCGTCGTAAAAGTCAAGATAAAGTCAAGATGGAAAATAAGGAGAATGTCGACA
GAAATAGGGGAGGATCGGAGGCAGTTATCAAG

>novel_circ_001504
ATACGGCGCCGGATACGATTTGGCAGCGCGGCGGAAGAACGCGACGAGGGAGTCGAC
GGCCACGCTGAAGGCATGGCTGAACGAGCACAAGAAGAATCCTTATCCGACCAAGGG
CGAGAAGATCATGCTTGCGATCATCACGAAGATGACACTGACGCAGGTATCCACTTGG
TTCGCGAACGCGCGGCGGCGTCTGAAAAAAGAGAACAAGATGACCTGGGAGCCGAA
AAACAAAACCGACGATGACGATGACGCGGTTCTCACGGACTCGGAGGACAACAAGGA
GAAGGACGATCTCGCGGGTGACAATCAAGGCGACAGAGTCGGGGAGGAGGCGAGGA
GAGGCCTCGAGGAAAATG

>novel_circ_001505
AGCCGATGAGGCACGTGAAAGCGGAGCACTTGCAGCACGACAAGGACCTGGACGAG
GAGGACGACCTCGATCTCGAGGAGGATCCCCGGCGGGGCGAGCATCCGTTCCACCAC
GCGATGCAGCACCACCACCACCACCACCAAGCGTACCCCGGCGAGGACCACCTCAAG
GACGAGGGGATCGTGAAAACGGACTGCAGCGCGGCAGGCGTGCCGATACCCGCGACC
AAGCCGAAAATCTGGTCGCTGGCCGACACGGCCGCGTGCAAGACGCCCCCGCCCCCC
ACCCACCCCCATCACCAGCAGTACCACCACCTCCACCACCACCAGCAGCACTACGGCC
AGCAGCAGCAGCAGCAGCAGCAGCAGCACCAGCACCACCACATGCCCAGCCAAGGC
CACCACCATCACTCGCAGCAGCCTTGGCTGGGCGGCGCGGGGGGCGGGGGTAACCTG
AGCTCGTTCGCGCTTCCGTCCTCGGCGTCGATGAGCCCGTCCGCAGCCGCGACAGCGC
CTTACTCGAGCGCCGCCACGAGGTACGGCGGCTTCCTCTCGTCCTCGTCAGGCGGCCA
ACTCCACTACAACCCGAACTCGTCGTCCGGCTCGAGTTCCTCGGCGTCCTCCTCGGCG
GCTGCGGGGTTTCCCGAGGTTGGTACGGACACCCCACCCCAGACACCGCCCAACATG
AAGGTGGCTACACCGAATGGAGTGATCCAGGCACCAACGGCCGGTTACTGCACCGGG
AACGGGAACGGGAACGCGGCCAGCAACGGTAATCCAAATCCGTACGGGCAGAGCCAT
CCGGCGGCCGCCGGCTATCTGTCGACCAGTTCCGGCTCGGCAAGCAGCGGCTCCTCGT
TCAGCTCGAGGCTGCAGGCGTCCCCGCACAAGGACTACTCGCCCGCGGGCCAGAATT
CTATTCTGCACCAGCACCAGACCACTGCCAGCTTACCGCCCACGGAGGCCACCACCGC
GTTCAAACCGTTCTACAAGGGCTCGCAGTCCATGGGTAGCGGATTCGTGTCGCCGGTC
TAAGCAACTCGATGGATCGATTCTGGATCAACGGTCGGATTCCTCCAG

>novel_circ_001506
TTACGGGATGGATCTGAACGGGGCAAGACGAAAGAACGCGACGCGGGAGACGACGA
GCACGTTGAAGGCCTGGCTTAACGATCATAAGAAGAATCCTTATCCAACGAAGGGCGA
GAAGATCATGTTGGCGATCATCACTAAGATGACTCTGACCCAAGTGTCCACGTGGTTCG
CGAACGCGCGGAGACGTTTGAAGAAGGAGAACAAGATGACGTGGGAGCCGAGGAAC
AGGGTCGAGGACGAGGATAATAATAACGAGGATGACGACAGTGGAAGAAAGAGCGTG
GACGAGAAAGATCGGTTAGACTCAAAAGACTCGGGCACCGGATCGAGCGAGGACGGC
GAGCGGCCGGCGCACCGGATGGATCTGCTCGGCACGAGCGGCGGTTCCACCGGTGTC

CAAGGCAGAACCGAGAGCGAATGGAGCGAGTCCCGAGCAGACAGCGGCCCAGACAG
CCCGGAGTGCCTCTACGACCAAAGAGAACCTCCCAGGCATCCCTTGCAACTGCAACAT
CCCGCGTATCTCGCCTCCTCTCACGGCCGACTGTTGCGCCACCCGTCACCCGAGAGCA
CGCCACCTAGCAGCCACCACGTCCCGCCAAGCACGACGACAGCGTCGTCGACGGGCA
GCGGCGTAACCACGAAGCCTAGGATCTGGTCCCTTGCAGACATGGCGAGCAAAGATG
GCGAACAGCAGAGCCCGATACCGACCACTATGACAGGTCTCTCATCACCTTACGGTGG
AAGCGGCGGAGCCGGCGGAGGAGTGGCGGGCGGGGGTGGGGGCGGGGGCAAGTTGA
TGAGCCCTTTGGCCAGTCGTCTGCCGCCTCATCATCCGCTCGCGATGCACTCCGGCGCG
CAATTTGTCCGACACCATCCGGATTTCTACCGCAACCTGTACGGGGCCTCCCATCTCGG
ATCGGGAGACATGTCGCTGTTGGAGACTTACTCGAGGACCCTGGGCGGACTGAGCGG
CGTCATGCCCCCCTCCACGGCGCCCAGTATACTGACGTCGTCCGCGTCTGCCGTCTCGG
CCAGTGGCAAACCTTTCTCGATCAACGGTGGCAGCACTGCTCCAGGTGGCGCTGTGCT
CCTCACCACAGCGACATCTGGCCTGTCTCCCTCGTCCTCGTCCACGGCGTCCTCGGGC
GGGTCGGACCAGTCCCCTCACCCGAGCCTGTCGTCCACGCAGGAGCTCAAGTCGCCT
GGCCGAGTGTGATTACGAACGG

>novel_circ_001507
CCGGTGGTGAAGAATCTCCGAAAGAATCACCTTTGCAACCAGCTGCAAGTACCAATGA
TGTAAACGCAGAAGACGATCGTCAAGATTTAAGCTCTATTGGAGATATAACGATCGATA
GCGTGAAGGATCATTTGCTTAACAGTTCACTTTTCAGAAATGCAACGTCTTCCGAAGA
ACTTCACAATCGTCTTGCAGGACTTAAAATGGAGGTGACCTCACGAACTTCGGAAATC
TCTACTCAAAATCAATCGCTAGTTTTAAAGACAACTACCGTTGTTTCACAAACAACCAA
CAATACCACTGCTAATGGAGAGATTGAAACAAGTCAAACAAATGAAACTCAACAAGT
GAACA

>novel_circ_001510
ATAATCCAAATGCCAGTCCAGTACGCGAATCTTTTGGAAATCGCCATCTCGAAAGCTCC
TTGGTATCAGATGGTCGTCGTAGGTCGTAGATTTTGCCGCGACGTTTCTTCCGACCAGT
TGCACGTTAGCCGTTTCGTACGATGGGCGTGCATTTGCCTTTTCTTCCGTGTTTCTTTTC
GTACATCTGTCTTCCTACTTTTTCTTTATTTTCTCCTACCTAATTGAGTGAAAGAGCGTTA
CGAAAATAATCAAATTAGTTACGTATATTGTGCATATGTGATATATTTCTTTTTTCTTCTTT
TTAATATCAAGACTCTTTTTACGATCGAATTATTAGTGAAATTTTACAAGTTAAAACGAT
GTTTTAAAAGCAGATTAACGAAAATCATAAATTTCTCGACCTGAATTCGAAAAGAGAA
ATTTATAACCTATAACCTCTAATTTGTTGTCGAATCGCTAATAATATTGATTGGTAAATTAT
CGTGCTCTGACGTTCGAAACAATTCAAAAGACGAAAAAAGACGAGGAAAGATCGTTG
GGAAATACACGATATAGAAATCTAAAGTGACAAGAGACAATCGATAAATTAAAACGGA
GAAAGGACAATGTTGTCGTACATGCTACCGACCGAAGACAAGCCACCACCCGGAGAC
TCGAATCAGGGGAATTACCGCGCTAATAAGTTGCAGAGGAGCCCGAAGGAACGTGCAT
CCGCGGTTGCGAAACCGGTGGGCGGCAAAGTGATCACGATCTCCCCGAAAAAATACG
TGGACAGTTCGTCGAAAATGATCGTTTCGACGAAGATCGAGGAGCGCGACGGCGACG
ATCAGCGCCATCTGCAGGACGAACAGCGGCAACAGCATCAGGGGGTGTTGAGTGAAA
GTCCGAAGCACAACGGTACAGTGGTGAAAAAATCGACTTTGCACACCTCGAAGGTCA
CTAAAGATGACAGCCTGTACAGTATAAACAGCGACGCGAGCACGGTTGGAAGTGATCG
AAAGAGTAAAATTTTTAACGGGGCGAAGCACACTAGTCTGAAAAG

>novel_circ_001511
ATAAATTAAAACGGAGAAAGGACAATGTTGTCGTACATGCTACCGACCGAAGACAAGC
CACCACCCGGAGACTCGAATCAGGGGAATTACCGCGCTAATAAGTTGCAGAGGAGCCC
GAAGGAACGTGCATCCGCGGTTGCGAAACCGGTGGGCGGCAAAGTGATCACGATCTC
CCCGAAAAAATACGTGGACAGTTCGTCGAAAATGATCGTTTCGACGAAGATCGAGGAG
CGCGACGGCGACGATCAGCGCCATCTGCAGGACGAACAGCGGCAACAGCATCAGGGG
GTGTTGAGTGAAAGTCCGAAGCACAACGGTACAGTGGTGAAAAAATCGACTTTGCAC
ACCTCGAAGGTCACTAAAGATGACAGCCTGTACAGTATAAACAGCGACGCGAGCACG
GTTGGAAGTGATCGAAAGAGTAAAATTTTTAACGGGGCGAAGCACACTAGTCTGAAA
AGAGTATCCTTTGGCTCAAGCAAAGGATCAATGGTAGAAACTCTAGTGTATGAGACAC
CTGTTCAAGAAGAGCCGGAAATCAATCGTTTTATGGATCACAACGGACGCATACCCTC
CATGATTACATCTGTACCTGATACCGA

>novel_circ_001513
GGTTATTGGAACTTGCACATAGAGAATATCAAGCAGGCGATTACGAAAATGCTGAAAG
ACATTGTATGCAACTTTGGAGGCAAGAAACAAATAATACTGGAGTTCTTCTATTATTATC
TTCTATACATTTTCAATGCAGAAGACTAGAAAAATCTGCTCATTATAGCAGTCTAGCAAT
TAAACAAAATCCCTTATTGGCAGAGGCTTATAGCAATCTTGGAAATGTTTTCAAGGAAC
GTGGTCAGCTACAAGAAGCTTTGGAAAACTACAGACATGCTGTTAGATTGAAACCAGA
TTTCATTGATGGTTACATCAATTTAGCAGCAGCATTAGTAGCTGCAGGAGACATGGAGC
AAGCAGTTCAAGCATATGTAACTGCTCTACAATACAACCCTGATCTTTACTGTGTGAGA
AGTGATCTTGGTAATCTTTTGAAAGCATTAGCAAGACTTGATGAAGCTAAGGCTTGCTA
TCTAAAGGCTATCGAAACGCGTCCAGACTTCGCAGTTGCATGGAGCAATCTTGGATGT
GTTTTCAATGCACAAGGCGAGATATGGTTGGCGATTCATCATTTTGAGAAGGCCGTAGC
ATTGGATCCAAATTTTTTGGATGCTTACATCAATCTTGGTAATGTCCTCAAGGAAGCCA
GGATTTTTGATAG

>novel_circ_001515
GTGAGCGGGCTACGGTTTGAGACGCAGCTGCGTACGCTGAATCAATTTCCGGACACGC
TCCTTGGCGATCCGCATAGGCGTATCCGATACTTCGATCCGCTTCGCAACGAGTACTTCT
TCGATCGGAATCGGCCCTCCTTCGACGCGATACTTTACTATTACCAGAGTGGTGGCAGG
CTCCGTCGCCCGGTCAACGTTCCTCTCGATGTCTTCTCCGAGGAGATCAAGTTCTACGA
GCTGGGCGAGCTGGCCACCAACAAATTCAGGGAGGACGAGGGGTTCATCAAGGAGGA
GGAGAAGCCGCTGCCGTCGCACGAGTTGCAGAGGAAGGTCTGGCTGCTGTTCGAGTA
CCCGGAGAGCTCGCAAGGCGCCCGGGTAGTGGCCATAATATCCGTGATCGTGATCCTCC
TCTCGATCGTGATATTCTGCCTGGAGACCCTGCCCGAGTTCAAACACTACAAGGTCTTC
AACACGACCACGAACGGCACGAAGATCGAGGAGGACGAAGTGCCGGATATCACGGAC
CCGTTCTTCCTAATAGAGACTATTTGTATCATCTGGTTCACGTTCGAGTTATCGGTGCGC
TTCCTCGCCTGTCCAAATAAACTGAACTTCTTCCGTGACGTAATGAACTTCATCGACAT
CATCGCGATCATTCCTTACTTCATCACCCTCGGCACAGTGATGGCCGAGGAGGAGGAG
ACGCTTGACCTACCAAAGGCGCCGGTAAGCCCGCAGGACAAGAGCACGAACCAGGCG
ATGTCCCTGGCGATACTCAGGGTCATCAGGCTCGTAAGGGTGTTCCGGATATTCAAGCT
GTCCAGGCATAGTAAAGGCCTTCAGATCCTCGGCCGTACCCTCAAGGCCTCCATGAGG

GAGCTCGGCTTACTCATCTTTTTCCTATTCATCG

>novel_circ_001516
CCATATTGACGCTTTATCTAAAGAATCAACTGATGTACAGTTCTAATACATCTACGGTAA
TATATCATGTCTTCTCGATGATGGTGTACTTTTTCCCTTTATTTGGTGCGATGCTGGCAGA
TTCGTTATTGGGAAAATTTCGCACGATATTCTATTTAAGTATTGTCTACGCCATAGGACA
AATTCTATTATCTTTAAGTGCGGCACCTCCCTTGGGATTACCAAGCAGAGAATGTTCATT
ACTTAGCCTACTTCTTATAGCCATTGGCACAGGGGGTATAAAACCTTGTGTGGCTGCAT
TCGGTGGTGATCAATTCATGCTACCGCAACAGGAACGATATTTGTCCACATTTTTCTCCT
TGTTTTACTTTTCGATCAATTCTGGATCGTTGATCTCGAGCTTCTTGATGCCCATAATACG
TAATGATATCGCATGTTTTGGTGAAAATACTTGTTATTCTTTAGCATTTTTTGTGCCAGCC
GTACTTATGATTTTATCAATCATATTATTTATCTTTGGTAAACCTTTGTACAAAATAGTAA
AACCTACAGGTAATGTTGTGTTAAATGTTTCCAAATGTATATCGCATGCTATTTATAAAA
AGATAACATCAAAAAATGTTAAAAAGGATTATTGGCTCGATTATGCAGATGACAAGTAT
GACAAATTGTTGATAAATGACATAAAAGCAGCTCTACAGGTAATGATATTATTTATTCCG
ATACCGATTTTTTGGGCACTTTTCGATCAGCAAGGATCTCGATGGACGTTTCAAGCGAC
CAGAATGAACGGTGAAATTGGAAACTTCCTACTTCAACCAGATCAGATGCAAGTGTTT
AATCCTTTTTTGGTTCTTGCGTTCATACCTTTGTTTGAAACTTGTCTTTATCCAATCATGA
TGAAATTTGGCTTTCGAACTCCGTTAAGAATAATGGTAATCGGTGGCTTTTTAGCTGCTT
TATCTTTCGTTATCGCAGCTATCGTTGAATTTCAACTCGAGTCAACGTATCCAGTTTTAC
CTTCAAAAGATTTTGCGCAATTACGCATTTTCAATACTTTAAATTGTTCTATAGAGGGAA
AATTACGAGACGAGAATGAATTATCGCAAGAATTGTCCTTTATCGTCAAAAGTATGGAT
ATGTGGGAGAATAAATATATTAAAGCAAATGGCCAAAAAAATTTAGTTATGAAGTAAA
TTTCAAGACATGCAATGAAGCCGGAATTACGAACATATCTTCAAATAAAATAAATGGAA
GCATTTTTGTAATTGAAGCCGAAGCAAATTCTTGGATTATCACGCCAGAAGGTTTTGTT
GATTATTATAAAGATTCGATAGACAAATCAAGAACAGGTGATCCTTTAGTGCGTGGTCTT
ATTTTTGTAAACACATTAAAATCTATTTCCTTAGAATTGATGAAGGATGGTACATCTTTTC
TTAAATTAATGATGAATGGCACGTTTGACAAAAATGAAGAATTAAATTCCATCAAACCT
AATATATATGATATTCATTTAAATGGTAAAAAAGTACAAAGTAATATTCCCTTCAAACTTG
GTGGAGTTTATACAATAGTAGGATCTGTTATTGAAAATAAAACGAATGCTGCAATGGTA
ACGGTGACTGATCCAAATTCAGTACATATATTATGGTTGATTCCTCAGTATGTTATTATTA
CGATGAGTGAAGTCATGTTCTCTGTGACTGGATTAGAATTTGCATTTACCCAAGCACCT
AGCAGTATGAAATCTCTTCTACAAGCTTGCTGGCTATTAACAGTAGCTTTTGGTAATCTC
ATTGTAGTCATTGTTGCTGAAGTTTCCATCTTTAATCGACAA

>novel_circ_001517
AGAATGTTCATTACTTAGCCTACTTCTTATAGCCATTGGCACAGGGGGTATAAAACCTTG
TGTGGCTGCATTCGGTGGTGATCAATTCATGCTACCGCAACAGGAACGATATTTGTCCA
CATTTTTCTCCTTGTTTTACTTTTCGATCAATTCTGGATCGTTGATCTCGAGCTTCTTGAT
GCCCATAATACGTAATGATATCGCATGTTTTGGTGAAAATACTTGTTATTCTTTAGCATTT
TTTGTGCCAGCCGTACTTATGATTTTATCAATCATATTATTTATCTTTGGTAAACCTTTGTA
CAAAATAGTAAAACCTACAGGTAATGTTGTGTTAAATGTTTCCAAATGTATATCGCATGC
TATTTATAAAAAGATAACATCAAAAAATGTTAAAAAGGATTATTGGCTCGATTATGCAGA
TGACAAGTATGACAAATTGTTGATAAATGACATAAAAGCAGCTCTACAGGTAATGATAT

TATTTATTCCGATACCGATTTTTTGGGCACTTTTCGATCAGCAAGGATCTCGATGGACGT
TTCAAGCGACCAGAATGAACGGTGAAATTGGAAACTTCCTACTTCAACCAGATCAGAT
GCAAGTGTTTAATCCTTTTTTGGTTCTTGCGTTCATACCTTTGTTTGAAACTTGTCTTTAT
CCAATCATGATGAAATTTGGCTTTCGAACTCCGTTAAGAATAATGGTAATCGGTGGCTTT
TTAGCTGCTTTATCTTTCGTTATCGCAGCTATCGTTGAATTTCAACTCGAGTCAACGTAT
CCAGTTTTACCTTCAAAAGATTTTGCGCAATTACGCATTTTCAATACTTTAAATTGTTCTA
TAGAGGGAAAATTACGAGACGAGAATGAATTATCGCAAGAATTGTCCTTTATCGTCAAA
AGTATGGATATGTGGGAGAATAAATATATTAAAGCAAATGGCCAAAAAAATTTTAGTTAT
GAAGTAAATTTCAAGACATGCAATGAAGCCGGAATTACGAACATATCTTCAAATAAAAT
AAATG

>novel_circ_001518
CAACTGAAAGATGAGCTGGGTGAAGTGGTGGCTGAAATGGAGGCGATGGAGGGCGGT
GGTCTCGCCGTCGATGAAACCAAGCCATCGAATAAGGCTAAGCAGACGTCGATCGGCC
GTAAGAAGTTCAACATGGACCCTAAGAAGGGCATCGAATACCTGATCGAGCACAATCT
GCTAGCGCCGACGCCCGAGGACGTCGCCCAATTCCTCTACAAGGGCGAAGGTCTTAAC
AAAACGGCGATTGGCGATTACCTTGGTGAAAGACACGACTTCAACGAGAGGGTGTTG
AGGGCATTCGTCGAATTGCATGATTTCACCGATTTGATCCTCGTACAAGCTCTTCGACA
ATTTCTTTGGTCGTTCCGATTGCCCGGCGAGGCGCAAAAGATCGATAGGATGATGGAAT
GCTTCGCACAGAGGTACTGCCAGTTGAATCCGAATATATTTACGAATACGGACACTTGT
TACGTGCTCAGTTTCGCTATCATCATGTTGAATACGTCCTTGCACAATCCGAGCGTCAA
GGATAAACCTAGCGTGGAACAGTTCATATCCATGAACCGTGGAATTAACAATGGCGGC
GACTTGCCTCGAGAACTTCTTGTG

>novel_circ_001519
TTGGCGGCATGAATCGGCCGTCCAAAGCTGTGACCCAAGCGAAGAAAGTGGCACCAC
CAGCTGTACCCGATGTATTTCGACATTCTGGCTCTTCCTTCGGTTCTGCTGGATACGCAA
GCAGTGAAGATAGTTGTTTCCTGCCCGGAAGTGGACCAGGCGGAACAACCGACGAAA
GTTCTTACGGTGTGCCATCTGGGAAAAGTCCGGGGCCTATTTTTACCCATCCTGGGTTT
GCCTTTCCACCAGTTGTTGGCAAATATGCCCATGCCGAGGATCAAGGAATCGATATGAC
TCAAAGTCCTGGAAGAGATAGCCCAGGTAGTTCAGGATCAGGTTCAGGTTCTAGGCAT
TCTACCGCTTCATTAGATTCTGGAAGAGCTTCTGGATATCATTTAGGTCCTAGAGGACCT
GGTGCTCTTGCTTCATCTCCCAGATGTTCTGTAAGTTCACTTGGCAGTCATCCCGATAG
ACCAGCGGATCTTGATGTTGTACATGCTTGGTTGACTGAACTTCAATTCGAAGAATACT
TTCCTTTATTTGCTTCCGCTGGTTATGATTTAGCTACTATTACACGTATGACGCCAGAAGA
TTTGACAGCAATAGGAATTAAGAAACCTAATCATCGAAAACGCTTGAAAGCGGAAATA
GATAATTTAAATATAGGAGATGGTTTACCAGAGCATATTCCTGGTTCATTAGAAGAATGG
CTTAGACTTTTACGACTCGAAGAATATCTTGGAGCTCTTCATCAACAGGGTATGCGTTC
AGTTGAAGATGTAACAACTCTCACTTGGGAAGATCTTGAAGATATTGGTATTGTGCGTC
TCGGCCATCAGAAAAAAATTATTATTGGCAATTAAAAGAGTTAAAGATATTCGCGCTGGT
AAACGTATACAACCCCTTGATCTTGCACGATTACCACCACATCCTGGACAAACACAGGA
TGTAGTTATTCAACGAGGAGGTCCTGACTTGCCATCTCCTGATGAAGATTGTTCTTCAC
CTGTTTTGAGGTCTTTCCAGAGAGGAGGAAGTGATACATCTGGAACTACATGGAGAAG
TATGTATGCAGCTTTACCAGCAGATTATAATATTGTTGGTCGAACTGGTTCACGAGGAA

AATCTTTAGAAAGTTTAGAGGATGCACCTCTTGGATATCCTCCATCACCAGCACCTTCT
GCACATCCACAACCAATTGAATGGCGTCCACGTAGTTTTGAAGATGGCGATCTAACTCC
TACAAATGATACTTCCGTAGTGGATGCAGGTGGAGGAACTCTTCCACGACCAAGACAT
TGTCTTGTTCGTCCACGACCAGTAGCAAAGGTCACTGCAACTCCAGGTCAATTTAAAT
CATTACCAAGAGATTTTGATAATAAATATCAATTGACTTATGGTCTTGAAAGTAGTCCAC
ATCTTCCAAAACGTTGTCCTCCATCTCCTCCAAGACGTCAAAGTTCCAGAGATAATGCT
TCTGGTGTAGGTGTTGGAGGATCAGGAGTTGGCGATGTTGTGATAGATTGCAGCGGAC
CAGTTCCAACTGCTTCTTGCGAGGAACATCATATACATCATCATCAACATCATCATTTGA
TTCATCATCCTTCTCCTCCACCACCCGCACCCGCACCAGTGCCATCAACTCCGCCACAG
ATAAATAGACCATCTTCGTCTATGTCTCGTTCTTGGGGTAGTGTCAGCGTCAATGTTAAT
GAAGAACATGAATTAATAGCTTCTCTTGCATTGCAGCATCGTAATGGTTCTGATGCCAG
TTTTAAG

>novel_circ_001520
ATTTCGTGTTTTATGTCGATTATTGGTCGATCAAAGATGGTTTGATAACGTAGTACTTTTT
TTTATCGGTCTCAATTGTATTACATTGGCAATGGAACGGCCAAATATACCACCTGATAGT
GGAGAAAGACTTTTTTTATCAACTGCGAATTATATTTTTACCGGGGTATTTGCAATTGAA
ATGTTTATTAAGGTTGTTGCATCTGGTATGCTGTATGGCTCCGACGCTTATTTTACTTCAG
GTTGGAATATTATGGATGGAGTTCTTGTAATCATTTCTATAATTGATTTATCAATGTCCTTA
CTGTCATCGAGTAGTCCAAGAATATTTGGAATATTGAGG

>novel_circ_001521
GTATCAATAACTGCTATGGAAGAATCAAGGATATTATTATGGCATAGAGACAAATTAAAA
TTAAGTATAATTAGTGATCAATTTCTGCAGGCAGTGTTTGATCATATTTTGGGAAGGGAT
GTAGTCAAAAAATTGATGCAGGTTAGTGAAACAATGGCTGCTAGCAGTCATCAACAGC
AAAATGGACAAGTAATTGGTTTAGGTGGTATTGGAGCCTTAGAAAATGATCCTGACACC
AAACTTTTTGTTGTGAAAAAGACTGGTGATAGTCAAGGTATCACTGCTCTCATCAGTCG
TCAACTTCAAG

>novel_circ_001523
GCATGATGGATTCGCAACCAAATAATGATGTATCATCGATTTACGTGGCGAAATATAATC
GTTTTGTGGACAATATTTTAGGAAGGATTAATAAAATCTTGCGTAGTAATTACGATCCAG
TTACTGTGAAATTAACTAACGCAAATGCTAAAACGAAATCAAACAAACACAAGAACAA
GAGTAAAACAAAGAAAAATAACAAAAGTGGAACGAAAAAGAGCAATTTGGGAATAGT
TGTGGTATCCACAGAAAAAACCATACAAGAAATTCCCGAAATCACACAGAAAACAATA
GAAACCACTGAAAATACAATCATGTCTGTTACAAATATTGAAAAAGAAGAGCCTCATA
CTACCATATCTGAATTTGCAAATATGGAAATTACTACAAACAGACAAGTACTTAATTCAA
GTACCAAGAAATCAAAGAATATGCAAAATACAAAATCTACAAACAAACGAGTATCCAA
CTTAACAAATCAAACTAGCAAGAAATCGAACAAAACTCGTGGTGGAACGAAGAATCA
GAAAAAAACAAACAAACAGAATAACAACAACAAAAATAAGATAAAGAAAACCAAAA
AACCAAAAGCAAAAGCCACACTTTATGGACTTGCAAGCCTTAAAAGATCAGGAGACG
TAACAGTAAACATGATGAGCGATCACACTATGATCAAAACGATGTTTACCCTTGGTCCT
CTTGTATTGAAAGTCGAAAAGGAATTTGGTAGGGCTGCAAAAAAAGAATTGAGAAGC
GCGACTGCAACCACTGCAGAAATGTCAGGAAAATTAAGCCTTCGAATTCTTCATGGTG

GTGCTGCTACCCTTAATTCCATTCGAGTTTACAACCTAAACAA

>novel_circ_001524
GACCGAAAGAATTCAACTATAGAGGGCACAACTATTTCTACAGCGGACATATTCCCGCT
CATGCAAATCAAAGGGTGGATTGGTTAGATGCTAGAAACATTTGTAGAGAATATTGTAT
GGATCTTGTTTCAATGGAAACGCAAGATGAAAATAACTTAATCTTCAGACTCATTCAAC
AAA

>novel_circ_001525
TGATGAATCAAAAACGCGTATTCAAGAAATCTTTTCACAAATTGAACAATTGAAAATAG
AAGAAAAATTATTGTTGTACCTGAAGCTTCCATTATCATTGACTAATACTGGAGGAACTA
TTGATCCTTTGAGACAACCATTAAATCCATTAGGAAATCGTTATGAAATTCATCAAACTA
TCATGTGGATTAAAACACATTTGGAAGAAGATCCAGATGTTTCTTTACCAAAACAAGA
AGTTTATGATGAATATAATATGTATTGTATGCGAAATTCTATGAAACCTCTATCAACTGCT
GATTTTGGCAAAGTTATGAAACAAGTATATCCAAGAGTTCGACCACGTCGACTAGGCA
CACGTGGAAATTCACGTTATTGTTATGCTGGAATGCGCAAAAGAGTTAAATTGGATTCT
CCGACATTGCCTAATATATCTGGCACTCAAACT

>novel_circ_001526
AATGGCTGAAAAGTCTGGATGTGGCGAATTACAACAATTAGTTCAAGAAGAAGAATGT
GAAGAACCATTGAGTCAAGGTGGAGAAGCTACACCACCTTCTACACCAGCAAGATCTC
ATCATCCAAGTAAAAATGATACACGTAATTCTCATATATCGCAACAAATATTATGGGAAA
CAAATATACAAACATCACCTATTGTTAGTAAAGGAAGTTCTGGTCAAGCAACAAGTCA
AGGATCTATGGTTTCTGGTCGAGCCGTGAATCTTTCAAATCATAGTAATCAATCTCGATT
AATGTATATGAATGAAAAGGAAAAACAAAAACCACATCGACCAGAATTATCAAAAATT
AATCGTCAACATAGAGCTATGGCACCATGCAAACATCATTGCTTTTTGTCAGAGGTTCC
TGATGTTCGACGTATGGAACAAGCTTTATTACAACTTTTAGAAGATTTTCATAGTGGTAA
TTTACGAGCATTTGGAAAAGATTGTAGTATGGAACAAATGACAGAAATACGAGAACAA
CAAGAGCAATTAGCCAAACTTCATTTTGAGTTAGGTCAACGACAAGGAATTGGTGGAG
ATCAATCAGGTCTCAGACATTCAAGTGCAAATATGGATAATTTATTGGAATGTTTGCAAG
AACTTAGTGTTTGTATTGAAAAATTACATAGCAA

>novel_circ_001527
AATGGCTGAAAAGTCTGGATGTGGCGAATTACAACAATTAGTTCAAGAAGAAGAATGT
GAAGAACCATTGAGTCAAGGTGGAGAAGCTACACCACCTTCTACACCAGCAAGATCTC
ATCATCCAAGTAAAAATGATACACGTAATTCTCATATATCGCAACAAATATTATGGGAAA
CAAATATACAAACATCACCTATTGTTAGTAAAGGAAGTTCTGGTCAAGCAACAAGTCA
AGGATCTATGGTTTCTGGTCGAGCCGTGAATCTTTCAAATCATAGTAATCAATCTCGATT
AATGTATATGAATGAAAAGGAAAAACAAAAACCACATCGACCAGAATTATCAAAAATT
AATCGTCAACATAGAGCTATGGCACCATGCAAACATCATTGCTTTTTGTCAGAGGTTCC
TGATGTTCGACGTATGGAACAAGCTTTATTACAACTTTTAGAAGATTTTCATAGTGGTAA
TTTACGAGCATTTGGAAAAGATTGTAGTATGGAACAAATGACAGAAATACGAGAACAA
CAAGAGCAATTAGCCAAACTTCATTTTGAGTTAGGTCAACGACAAGGAATTGGTGGAG
ATCAATCAGGTCTCAGACATTCAAGTGCAAATATGGATAATTTATTGGAATGTTTGCAAG

AACTTAGT

>novel_circ_001530
CTGGTGGAGAGGCTGCAAGAGGAGAAGAGGCTGGAACAAATACGAAGAAAGGAAAG
AACAGAAGCATACTTGTATATAACCGTCAACGTCCTTCTTGAGGATAATTTTGACGGTC
ACCAAGGGAATGACTTGTATGATCCAGAACATGCTTTGTATCGTGTATTTCGCGTACGTA
AGCAGTGTACCTTGCACGAGTTTCTCGAATTGCTGAGTGATAGCTTGAAATATCCAATA
GAACAAATTCGTCTATGGCCACTGAATGTACGTTCAAACCAAACTTGTAGACCAATGCC
AATTGAATTAGAAAATGATCTTCAAAAATCTATTTATCAATGCGCGGAGAACCCAAATG
TTTGGAATGTATTTGTTGAACTTGTTCCTCCAGATTCTGATTTAACAGCATTGCCACCTT
TCGATAAAGATACTGATGTTCTCTTATTTTTAAATTATATGATCCTAAAAATAAAAAGAT
TCATTATTGTGGACATCATTATATGCCTGTCACAGCTAAAGTCCAGGAACTTATACCAAT
TTTAAATGAGAGAGCTGGTTTTCCACCTGATACGGAATTAGCTCTTTACGAAGAAATTA
AACCAAATTTGGTAGAAAAAATAGATAATCTAACAGAACCATTAGAAAAAGTTCTTGA
AGAATTAATGGACGGAGATATTATTGTTTTTCAAAAAGAAGGAGACAATCAAATGTATG
AGCTTCCAACATGTAGAGAATATTTTAAAGATCTATTTCATAGAGTAGAAGTTACATTTT
GTGATAAAACGATTCCTAATGATACTGGTTTTACAATGGAACTTTCATTAAGAATGACAT
ATGACCAAATGGCAAGAGCTGTGGCACAGAGACTTGGTACAGATCCTTATCTTTTGCA
GTTCTTTAAATGTCAAACTTACAAAGATTCACCTGGACATCCATTAAAATGTACATTTGA
AGGTTCATTGAAAGATTTGGTTTCTTATTGTAAACCTAAAGCGAAAAAATTATATTATCA
ACAGCTTAGTATTAGAGTAAATGAACTTGAAAATAAAAAGCAATTCAAATGTATATGGG
TTGGTCCATCTCTTAAAGAAGAAAAAGAAATTATTCTTTATCCTAACAAAAACGGAACA
GTAGCAACATTACTTGAAGAAGCTAAAAAACAAGTAGAATTATCAGAAAATGGATCCG
GAAAATTAAGGATATTAGAAATTAATTCTAGTAAAGTCTCACCTGGTCCAAGAGAAGAT
GTACCTTTAGATAACTTAAATACATCTGGTACAAAATTATACAGGATAGAAGAAATTCCA
AATGATGAATTAAATTTGGCAGATGATGAAATGTTGGTTCCTGTTGCACATTTCCATAAG
GATATTTTTTCAACATTTGGTATTCCCTTTTTCTTTAAAATCAAACATGGTGAACCTTTTC
CAAAAATGAAGGAAAGACTATTGAAGAAATTGGGAGTACAGGAAAAAGAATTTGAAA
AGTTTAAGTTCGCGGTGGTAACAATGGGTAAACCACACTTTATTATGGATTCGCCAGAA
TATTGCATGGATCTTGCTGATTTTCGTATTCATCCAAATCAAATTTATCCACTTTTAAATG
CAGGCACATCGCCACTTAGGCCTTGGCTTGGCCTAGAACACGTCAACAAAGCGCCAA
AGCGCTCTCGTATCAACTACCTCGAAAAGGCCATTAAAATTTACAATTAAATTTTCATCA
TACGAAGAAGAATAAAATTAGGCAGCCACTCATATAATTTATACGACTCTATAGTAAGA
AAGCAACGTCTGATATACGATGGTTGGAAATGCGTTTGTATTCGGTTCTTCAGCCAGAT
AGTACACAGTTTTAATATTACGATCATGTTAACGAAAGGTAAATTTACGCTTTGTATACG
ATGGATTAAGAGACTGTAATGACCCTGCAATGATACTTTATGCATCATGGAAGTTCTCCC
TTTCGAATCGACAGGGATTTTACGTAATGGCTGGATCGATGGTACTACATTGATTCCACT
ATGCTCCAACAATGGATTAGACACTTCTCTCTACGAAATCAATTTTTATGTTTGTAAATA
TTTTGCTGATCAATGGCAATTATTTAACAAATATTTTTGAAAACACTGTTTTCATTATATT
CGAGTCTATCAAGAACAAGAATTTATTTAAATACATATTTTATTACTAAATAAAGAAATA
CGGATAACAACTGCAGAATGAAGAAAACTCGTATCAGGAAACAAAAAG

>novel_circ_001533
GTGTACAAAGGCCTCGACATCGTGACCGCGAAAGTAACGAAAGAGGAACAAGCAAA

GGCGGCTCACCACATGTTGGACATCGTAGACCCTCTGAACCCTAGCTACACGGTGGTA
CAATTCAGGGATGCTGCCATACCTATTATCAACGATCTTCTCGCGAGGAGGAAGCTCCC
GATCATCGTCGGTGGCACCAACTATTACATAGAGTCCATCCTTTGGGAAGTTCTGATGA
ACGACTCGAGGATCGGCAAGGACGATGATGACCGGTTGGACGAGAATGTGTCGCGTA
CGAAGAGGATGAAGATCGAGTTGGATCGATCGATGACAAAGGATAACCAGGAGCTTTA
CGAAGAGTTGGTTAAAGTGGATCCGGAAATGGCGAAGAGATTTCATCCGAACAATAGG
AGGAAGATCATTCG

>novel_circ_001534
AGGAGGAAGAGATCGACGTGGTGACCTTCGAGAAACCGTGCAGACCGGCGGCATTAC
CAACGTACCCGAGCCTGGCCGACCAACAGCACTTCCAATTAACGGTGAACACAGCGTT
CAAAGAGAAAGCTCCTGGAACCAGACCACGTGGCCGACCTCCCTCGAATCCAGCTCG
GAAAAGGACCGCTCAATCGGAGCCGAAGCCGGCGAAACGGGCCCGGCACAGGACTT
ACCAGAGGAGGACCAAGGTGGGACGATGCGTATCGTCGTCGCCCAAGAGCGTGTCGA
GAAGCTCCTCCGACGACGAGCTGGACACCGAGAAGAGGAGTCTGCACAACAACATGG
AACGCCAGAGGCGGATAGAGCTGAGAAACGCGTTCGAGGATCTGCGTATCCTCGTGCC
AGCTGTCGAGAAGAAGGAGAAGGCGCCGAAAGTCGCGATCTTGAGGCAAGCGGCTG
TCTACTGTGATACATTGAACGAGATGAACCAGATCTCGATGGCCCAGGTGGCGGATCTG
AGGCGGCGACAAGAGAGGCTTCGAACGAGGCTCAGTGTTCTAAGGAGGAGTCTCGCC
ATGACGCGCTGAGGGAGAGACTCGGGAGAATCGTGTCAGATTCAGCCCACGAACACG
CGAACCACACCAACCACTTGCTCGAAACGTACGAGTCGAATTTGTTGTTACTCCCTCG
GAGAGAAGAGAATGGAGGACGATGGATGGATGGATGGATGGACGAGCGACAAATCTC
TACTCGACGTTTCGAGACCGTAGGGACGATTTTTTTTCAAACACGCCTGCATGCGAAC
GAGGAAGACTGCCTGACATTGTTTAATTTGACACTCTGACACTTGTATCTGCCTTCGGT
CGCACAGCATAGGGGAAAGAAACGCTTCGAACTAGAGCAAGATGGCGCCGTTGAGTA
AGAAGCGTCGGGCATTTTTAGTCCTGATCAACGAGAAGAGAGATGAACGTGTTGCGAT
TCTCCGATCCTAATCGAAGTTCTCGGAACTTGGTCGGATATGGATAAATCCATATCCGTT
TTATCGTATAAGAGAAACGAATATATATATACATATAATATTTATATATATATATGTATATATA
TTTATATATATGCACACGTAATGTCAAAAGCGTCCCAAGTTTCGTTTGCTTCGAGTTAAG
GGGAGAATCGATGAAGGATCGATGATATTAAATGATGATTCGTTCTGTCGAACGTGTAC
CAAGAACGAAGAGAGAAGAGATGAAATATCGTTCGCATATAATCGACACCAACAAACA
TTGGAAGAGATCGAAGAAACCGCCAAAAAAAAGGAAAATAGCTTCGAAGAGGATATT
TATATATACGTATATTTGTAGAAGAGTTTCTTCGAATAGCGAACTGCAAAAAAGGTGAA
GGGAAAGAATTGATAGAAGATCGCTTTGGCCGGAGTTCGCAGAAG

>novel_circ_001535
GCGAAATGCCGGTGCCAGTTTGGGATTGCTCTGACCTAGGCCTGTTGGACGAGCCTCT
GGCTACCACGACAGTGGTATCCGATGACATATGGAAGAAATTCGATTTTGACTTCCCTC
TGGACCGTTTGGACGCCGTCCATACGGATGTGTATCACGAGCGCACGTTCGAGGATAG
CGTTCTGCCGGATTTGCTGTACGGCAAAATGAGCTGCCTTGCGTCACGAAAGATACGT
CATCATGATTGCATGTGGGCTGGGCTGTGCATCAGTAAAGAGCATAACAGGACGTTGC
CCGCGAAGAAAAACTCACAGATGCAGAAAAAGGTGCCGGCCGGTCGTAGTGTGTTGA
TATCGCGGGCAGGCATAACTCATATAGGAAATTCTTTGGTCAATCAGCAACAACAGTGC
CGTACAAGAAATCTGGAGAGTGACGGAGATTCAACTAGACCGGAAACACCGAGCTCG

AGCTCCGATACGGAGACGGAGGACGAGGGCCCGTTCTTCAGACACGATCAGATCAATA
TACACGAGAAATTGTCCGAGTGCATGTCAGACGCGGTTACCAAGGCTGTCCCAGTGAG
CGAGGTCACTGGCCAGCTGGTCAGGTTCCATGACAGGAGGAAAGAGGAGGAGGAAA
ATCAGTGTCAAGTGCATAGAGAGACCAATATCAGGAACACTCTGAGCGACCATTGCTAT
CATCTGAATCAGCCTATTGGGAAGAACCTGGAACATCTTGGTGTACAGACACCTTCTGA
TTCCG

>novel_circ_001536
ATGCCTTTCGTGTTGTAAATAATACTGTGAAAAGAAACCTCAGACCGAAGATGGGCAA
GCTTTTGAGCCTTCTGGCTCGAGATGAGTCTACCTGTTGCACCCCTCAAAAGTACGAC
GTCTTTTTGGATTTCGAAAATGCACAACCTTCCGATATAGAACGGGAGACCTTTGAAGC
GGTGCAAAGAGTTTTGAAAAATTCAGAATCTATTTTAGAGGAGATTCAATGCTACAAA
GGTGCCGGAAAAGAAATCAGGGAGGCGATTTCGGCTCCCACGGAAGAGTGTCAACGA
AAAGCTTACCTGACTGTTGCACCTCTAGTCGCCAAGCTGAAAAGATTCTATGAATTTTC
ATTGGAACTTGAGAAGGTAGTACCAAAAATCTTAGGCCAACTGTGCTCCGGTAATCTCT
CCCCGACCCAACATCTCGAGACTCAACAGGCATTGGTGAAACAGCTGGCGGAAATTCT
GGAATTCGTCTTGAAATTCGACGAGCACAAGATGAAGACACCCGCTATTCAAAATGAT
TTTAGTTATTACAGAAGAACGTTGACCAGAGCATCTCTGGCGCGACAAGAAAGCGCTG
AAAAGGACCTCGTGGTCGGGAACGAGCTCGCCAACCGAATGTCCTTGTTCTACGCCCA
CGCGACACCCATGCTTCGTGTTCTGAGTCACGCGACCATTACTTTCTTGATGGAC

>novel_circ_001537
GAAAGTGACTTTCCACGGCGATTGATACCATCCCTCGAAGAGACCGCAGAGGGTCCGT
TGGAACGAGGACTCGTGGAGTTGGGGGGAGGGGAGCGAGACCACGGGTCTTTCGAG
ACCCTCGTTAAAACCTATCCCCGAACGACCGAGTTGGAAATAGTTGGAAAAGATGTAC
CTCTCGTTGCTGAAACAATCCCCCGTGGAACTTTATTGTTGATTATTCAGACGAATTTAT
CGCGACTCGTTTGTTGTTGATCCCCCGCGCCCGTTAAACTTGTTGATCCTCGCGAGTGT
GCGCCGCCATCGAACGCGTCTCCTCGTGGAATTCACCGTGGAAACTGAAAACGGATAA
CACTGTACGCTCGCTCGTGCGGCGTTCGAGCGTGTCGAGTGGAGGAGGAACGAGCCG
TCGCTTGTTGTGTTATGAAAACGGATACGGACGAACGTGATCGAAGAAGAGGAAGAAT
AGTTGTTTTGTTCGCTCGTTCGGTTGTAAAAAGGGTGGAGGGAGAGAGGGAGGGAGA
TTGTTAGCGTCTCGAAGAAGATTGTTGTTTCTTCGTTTCTCTCTTCGGGAGGGGAAAGT
ATTTTTTATGGAATTGAAAAGAAGTGAATCACCGACGATCATCGTATTTTCATCGTGTAC
GAAAAAAAACCAGTTGCAAAGTTTTTCTCGACAATTCTTCTTTATCTTAAATAAAAATT
CAAAGTGAGTAAAAGAGAAATACATAGTAAATGAAACGGAGTTTTTGCGATCGGATCA
GTATTAAGGATCTTCCCCGTGCGATAAGAAGAAAGAAAGAAAGAAAGAAAGAAGGAA
AGAGGATGAAATATACAAATGGAGATACGAGCGACACCGATTTAACTTGATTAAAAATT
GGAAAAAGGATCGTTTTACACCGCGCTTTTAACACCGAATGTGCTTGGAAATTACGAA
GAAGATTAAGAAATTCCCCAAAGTTCCCCGAAAGGAGTTTGTCCCCAAGGAAACGCTC
TTGTCCACGATAAAATTTCTTGGAGAAATTTCGAAAAGTGGATCTTCCGCGAAATTTTC
GAAATCGAACCAGCGATCAAGAAGAATCGTTTTGTGGAAAGTTGTGTAATTGTAGGGA
GTGGATGACGAGTCTCGGCTAAACACGAGCAAACAGGTCTCCTCGAGGAGGTTCTCC
CTTCGAGGGAAACTGGAAAGCTTGGTGAATTGTTGGAGCGGAAGCTGAGCCGAGGGA
GCGTCGAATCGAGCATCGAGTCGAAGGTGGACGGGATCGGATCGGGGGAAGGGAGAG

GAAGGGAGAGAAAGAAATTCGAAACGGCTGCTCGACGAAAGTGGGTGTGGTGTGGAT
CGAAGGATCGATCTCGTTGGGAAATCGATCCGGACAACTCTGCCAGATGACTTTAAGG
AGGGTCCACGAGGACTCGGCTGACACGTCGTCGATATTCGAGTACGAGGACGTGATAA
AGTTATAATAACAGGAGGGAGCCTTAAGTTTGAAAAATCGTTGAGAGAGAAATATATCT
AGTTAGTACCCGCTCCTATTTCCCTTAAAAAAAAAAAAAAGAAAAGAGAAATAAAAAA
GAGAGAGAGATATATATATATTCATATACCTAGATATATATGTATACATAAATGATATATTGA
TGTTAATCGAAGTTTATTGATCGATCGTTTTCTTCCCACTCCCGTTCTGTTGAAAGATA
AAACCGATTTGGCGACGATTCCTAGGGGTGGGGTGGGGGTGACACGGAAAATTGGCG
AGGAAACACGAGGGGAAAAATGGCGATGGTAATATCGCCCCGTAGCAAGATCGTGACA
TTGTCGTTGTTGATCGGTATTATCACGGTGGTGGCGTTCATAGTGACTGTGATCGGGATA
TGGCACAAGAGCGAGAAACCTGGCAAGACTGGGACGAAGGTGGTCAGCAGCAAGCT
TCAACAGATCACCGGATACACTGCCACCAAACAC

>novel_circ_001538
GGTCCGCCAGGGAAGAAAGGAGAACCCGGCACGTGCACCTGCAATGCAACAGCCCTG
ATGGCATCCTTTACGATGCCAAAGATGATCCAGGGACCGAAAGGAGAACAAGGGGTG
CCGGGGCAAGAAGGAAAACAAGGCCAAATGGGGTTGACGGGTGTTGCTGGGCCACC
AGGAGAGAGAGGACTAGAAGGACCACAGGGGCCCAAGGGAGATAAAGGTGACGTGG
GAATACCGGGACCGGAAGGTCCTCAAGGGCAAAAAGGAGAACCTGGCCGCGATGGAA
TACCAGGGGAAAAGGGCGCTCAAGGACCACCAGGCCCCCCAGGAAAAGGAGAATTCT
CCGGATACGAT

>novel_circ_001539
GGTGTTGCTGGGCCACCAGGAGAGAGAGGACTAGAAGGACCACAGGGGCCCAAGGG
AGATAAAGGTGACGTGGGAATACCGGGACCGGAAGGTCCTCAAGGGCAAAAAGGAG
AACCTGGCCGCGATGGAATACCAGGGGAAAAGGGCGCTCAAGGACCACCAGGCCCCC
CAGGAAAAGGAGAATTCTCCGGATACGATCCCAGTTGGAAACCTCGGGGCATTTATAG
GACGGAAGGCATCACGATGAGACCAGGACTACCAGGGCAGAAAGGCGAGGCAGGCC
TTCCAGGAAGTCCAGGACCAAAAGGAGAGACGGGAATCGCTGGTGCCAAAGGTAACA
AAGGCGAGCCAGGTCACAAAGGCGCGAAAGGAGATCACGGGAATGAGGGTGCTCGA
GGAATTCAAGGATCGAAGGGTGAACCTGGTGCACCGGGAGCGCCGGGTCTCCCTGGT
GCGCCAGGTGAGAATGGAAGGCCAGCCGAAAAGGGCGATAAAGGAGACACAGGACC
AGAAGGGAAGCCAGGCCCCCCGGGAGCGCCTGGACCGCCAGGATTGCCTGGATTAAG
CGGTCCTGGAGGGGTAAACGTTGGAGAATCGATGTTAAGGGAAAAAGGCGACAAAGG
AGAGGGTGGTGCGCGTGGTTACAAAGGGGACAAGGGCACCAAAGGCGAGAAGGGAG
ATAAAGGTGATTCTGGACCAGCCGGAATTCCCGGTGTAAATGGTATTCAAGGACCGCA
AGGGAACAAAGGCGAGCCAGGCAAGGATGGAGTTGCCGGTGTTCAGGGAATCGCGG
GTGCAAAAGGTGAAAAAGGTGAGAGAGGTCCGCCAGGAGCCACCGCTATAGCGAGTT
CCGGAGACTACATCACGATCAAGGGTGAGAAGGGCGCGGAGGGGAAGAGGGGGAGA
AGAGGACGCCCTGGACCACCGGGACCCGTCGGCCCACCTGGAAAGCCGGGAGCGATG
GGAGAAATTGGGTTGCCTGGATGGGTGAACACCATGAAAGGTCGTCCTGGAACTCCC
GGACTTCCTGGACCTGTTGGACCAGCGGGGCCCAAGGGAGAAAAAGGAGAACCGGG
CACACCGAGCCCTTATGGAGTCTCTGTCGGC

>novel_circ_001540
GCGAAGGGGGACAATATTTCGCGTGAGGCCGAGAAACAGCTCTGTCCAGGAAGGCC
GTGACGTAACAATATCTTGCGAAGTTGGAAACAGAGTTGGGATCGTGCAGTGGGTGAA
AGATGGCTTCGCCTATGTCATACAATCGAATGGAGAAATCGTGGGGCATCCGCGATTGA
GATTAATAGGCGATCAGAGCACAGGAGTTTACAATCTTCAGATCACGCAAGCTTCTTTG
ACCGACGATGGGGAATATCAGTGCCAAGTTGGACCGTATTTGCGCATTAAATCGATCCG
TGCCAACGCTCATCTCACCGTCATCT

>novel_circ_001541
GTAGCAAACACGTAGAATGGTAGCCTCCCAACACGACGGTACCAGAAGTGTCGGTGG
CGGCGGAGACGGCGGAGGCGGCGGCGGCGGCGGCGGCGGCGATGAGACCGTGT
ACACGGTGACCGTGAGTGGTGTCGGTTACAGGAACGAGGGTTACAAGGACGACGGTA
CCGACGGTATACCTCCCGAGCCGCAGAAACCGCCGCAATTATCGTCGACGGACGACGA
CACCCAGTCGAGAAAGTTCAAACTGTCCCGCGGCGAGAAATGGCGTATCTTGAAGAA
CATTAGCACCGTGTCCGTCGCGTTCATGGTGCAGTTCACCGCGTTTCAAGGGACGGCC
AATCTCCAGTCGTCGATCAACGCCAGTGACGGTTTAGGCACCGTTTCCCTCTCCGCTAT
TTACGCTGCTTTGGTGCTGTCGTGCATCTTCGTCCCCACCTTCGTTATCAAACGGCTCA
CCGTTAAATGGACACTGTGCATCTCGATGCTGTGTTACGCGCCCTACATCGGGTCCCAG
TTCTACCCGAAATTTTACACCCTGGTGCCGGCTGGCGTTCTCCTCGGTTTGGGGGCCGC
GCCTATGTGGGCAGCGCAGGCCACCTATCTCACGCAGGTCGGCGGTGTGTACGCGAAA
CTCACCGATCAGCCGGTAGACGCCATCGTGGTCAGGTTCTTCGGCTTCTTCTTCCTTGC
ATGGCAGACCGCTGAACTCTGGGGGAATCTCATTTCTTCGCTTGTGCTCAGCGGGGGA
GAGTTCGGCAGCGGGAGCGAGAACAGCACCACCAATTCGAACAAGATCAAGCACTGC
GGCGCAAATTTCTGCGTCCTTGGGAACGGCGGTCACGAGACCTTGGAACGTCCCCCG
GAATCGGAGATCTATGAAATATCCGCGATCTATCTTTCTTGTGTAATCGTTGCCGTCATC
ATCGTCGCCCTGTTCGTCGATCCGCTCTCGAG

>novel_circ_001542
GCATGCCCCCTGAACCCTAACCCCCAGCCGTTGACCGGCCAGCAAGAGTTGCTGCAAG
ATTTCTCGAAGAGGTTCTCGCCCGCGATCCGCGGCGTGGTCGAATTCGCGAAACGCAT
CCCGGGATTCAGCCTGTTGGCGCAGGACGATCAGGTGACGCTGCTGAAGGCGGGCGT
TTTCGAGGTGTTGCTCGTCCGGCTCGCTTGCATGTTCGACGCCCAGACGAACAGCATG
ATATGCCTGAACGGGCAGGTGTTGAAGCGAGAGTCCATCCACAACAGCAGCAACGCC
CGTTTCCTCATGGACTCGATGTTCGACTTCGCCGAGAGGGTGAACTCGTTGCGGTTGT
CGGACGCAGAACTCGGCCTCTTCTGTTCCGTTGTCGTGATCGCCGCCGACAGGCCGGG
ATTGCGCAACACCGAGCTGGTCGAGCGCATGCACAACAAGTTGCGGAACGCCCTCCA
GACAGTCCTCGCGCAGAACCACCCCCAACACCCGGACATCCTGAGGGAGCTGCTGAA
AAAGATCCCTGACCTGAGAACCTTGAACACCCTCCACTCGGAGAAACTGTTGGCGTTC
AAGATGACCGAGCAACAGCAACAGATGCAGGCGCAGCAGCAGCACCAGCAACAGCA
ACAGCAAACGCAGCACGTGATCAACGCTCAACAACCGCAGCAGCAGCAGCAACAAC
AACAGCAACAACAACAACAACAGCAGCAGCAACAGCAACAGCAGCACTGGCCTATG
GAGGAGGAGCCGGCGGCGTCCTGGGGCTCAGCCTCGGACGTTACCCTGGACGAGGCT
GTGAAGAGTCCTCTGGGCAGCGTGTCGAGCACCGAGAGCACCTGCAGCGGGGAAGTC
GCCTCCCTGACGGAGTATCACCACGTGGCCCCGCCCAGCGGGCACCACGCGTCCAGC

GCCCCCCTTCTTGCCGCCACCCTGGCGGGCGGCCTCTGCCCCCATCGCCGCCGAGCGA
ATTCGGGCAGCACGAGCTCAGGGGACGACGAGTTGCACCGCGCCTCCCTCTCGAAAA
CCCCCCAACCGCCCCAATGCCCCCGATTCCGCAAGCTCGACTCGCCGAGCGACAGCGG
GATCGAGTCAGGGACCGAGAAACCGGACAAACCGGCGAGCAGCAGCGCGAGCAGCG
CGCCGACCTCGGTCTGCTCCAGCCCCCGCTCCGAGGACAAGGAGGTGGAGGACATGC
CGGTGCTGAAACGGGTGCTGCAAGCGCCACCCCTGTACGACACCAACTCGCTGATGG
ACGAGGCGTACAAGCCCCACAAGAAGTTCCGCGCCCTCCGCCAGAAGGACAGCGCGG
AGGCCGAGCCGGCCGTGATCGTGCAGCACACGCAGTCCCAGCTACATCTCCACTTGAC
CTCGCCGCCGGCACGGAGCCCGTCGAGCCAGGCGCAGGCGAGCCAATGCCCCCAGAC
GGCAAGCCTGTTGAGCAGCACCCACTCGACCCTCGCGAGAAGCCTGATGGAGGGGCC
GCGGATGACGGCCGAGCAGTTGAAACGCACCGATATCATTCACAATTACATAATGCGG
GGCGAGGCGAGCCCGAGGTCGCCGAACGCGTCGCCCTCGCCCGCGGAGCAATGCGCC
TCCACCACCACCATCACCGCGCGATCACCCCAAGGATCTCAAGGACTGCTGCAGTGCG
CGACCAGCAACTACTCGACGACCAGGTGGCCGGCCACGTCCGTCATCACGACGACGA
CGGGCGCCCGGCAGCAGCAGCAGCAGCAGCAACAGCAGCAGCAACAGCAGCAGCAG
CAACAACAGTCTTCCTCGGACTACCTCATGGTCGGGAACTCGCCGGCCTCGTCGCCAA
GGTACCTGTCCGCGGCGGCGACGAGTAGTACGAGCACGAGTCCACGGCCCGCCTCGA
GCACGGCTGCGACGCTCGTGTTGTCCGGCTGCCCGAGCAACATGATGGAGCTACAGGT
GGACATAGCGGACAGTCAACAACCGTTGAACCTGTCGAAGAAGTCGCCGTCCCCGTC
GCCAAGGCCGTTGGTGGGCCCGTGCAAGGCGTTGTCCCTCGAGGCGTAGTGGTCTGAT
GGACGGGCCGTATGAACGTGTCTTGTCGCTCGTGACATGGGTGATGATGATGATGATGA
TGATGATGATGATGATGATGATCGTTGGTTCGTTGTATATTCGTTGTTAACACCCCGCAC
GATAACAACATCCCTGGACGACAGAGCGCGGCGAGAAAAAAAGAATTGTATATCGGCG
GTGCGGCGATTAACGGATGCATGCACGGTGTGTTTCGTCGCGAGGGGAGAAAGAAGG
GAGAAGGGGAAGGGAGGAAAAGTCGGAGAGATCAACGACTTTTGGTTGATTGGTGGT
GTGAAAATGAAAAAAAGTTGGTGTGTCGCGCGCCACGTCGTTCCCCCTTCCAGGAGA
CACGGAGTGAGAATGGAATGGGAGAAGGAGGAATTTAAAAAAGAAGAAGAAAAAAA
AGAAAAAGAAAAAAAAAAGAAGGAAAAGTGCCAGGACGCTCGGAAGCGCGTGATAA
ACCAAATAATTTATTAATTTAAACTATTAACTATCGATATAAATGAAATTTGCGCGAGAC
ACACGCGAGGGAGTATGTATGTATAACGCAATGGTGACCAGATGGCGGAACCGTGAAA
GTTCTGGACATTTTGGGACACCCGGTGCACGAGACACGCGCGTGTTGACGCGCGATCG
GGAGGAATGGAAAGAGGAATAGGAGGAGGCGGAGTGTGCTAAGTGTATGGGAGGATG
CTATCTGTTTTCGTTTCCTTTTTTTTTTTTTTTTTTTTTCCTTCTCTGACAATGATCTCCG
CCCCCGGAAAGACGCGTGTCGTGATCGATGGACCGAAGCGGATGGATGGATGGATGGA
TCGAACACACGTGATCGGGAAGAGTGTGCACGATCTCGAGAGAAAAGAAATTTAAAA
AAAAAAAGAAAAAAAACGGGGAAACGGAGAGAGTGCCGTGCTTTGTTTTTTTTTTTT
TTTTTGTCCCCCCCTTTTTTCGGGGAAGAATCACCATCAAGTGTAGTACATCACTTTGA
AACGGAAGCTAAACCTACCCATCTACCTTACCTACCACCTATCGACGAACAACTCGAA
GAACAACAGCCACAAACAGGAAG

>novel_circ_001544
GGTTCAATTAATTAGAGAATAAATATAAAAGTTTAATTAATGGAATATTAGAAGTCTTTG
AGTGAACAGCAAGTTACATAGCAGTTACACTCAACAGTTAAGTGAACTTCATAAACTT
CTACAGCGTCTAATTGCATTTATTGGCCCTTTGTCCCGTGAAAATTGAATTGGTCCCGCC

ATTAAGCACGACAGCTGTTCATTCGATATCCGGTAACTTACCCGCGAATTTTAAAATTCC
TTATCAAATATATATATATATATATAAATTTCTATCAATATTTGATATTCCATTCATAATGATA
GAAACATTCATTTTAATGAAATTCTTAATTATATTAAATTAATTCTAAAATATATTATTTTTT
AGAAAGAGATTAAGATTGTCTATTTTTCAAAAATACTTCAATATATTTATCCACTTCTATA
AAGAATAACGAAAACTTTAACCAAAACTCTTCAAGAGCCTCTTAAAATCGTTTTCATAC
GCTTGCAACAATAGAAAGTAATGGCACCATCGGGAAATTCGATGAACTCGTGAATCCC
AAAGGATTTCTAGGAGAGAACGAAAGAAG

>novel_circ_001546
GCTTATTCCACAAGGCTATCGCGCAGAGCGGAGTGGCGATTAATCCTTGGGCTATAATA
ACGAAAGAGCCGAGCAAGTACGCGTACGAGTTGGCGGCAAAACTTGGCGAGAAATCG
ACTGATCCTAAAACCGTGCTCGAGTTCTTGAGGACCGTGGACGCGCAGAAACTTGCAT
TTACCGAGACACAGATTTTAAAGACAAACCAACCTTATGTAACATTTGGAACATTTGCA
CCTTGTATTGACAACAAATCCCCGAATCCATTTATGCCTCGACATCCCTCGGAAATGATG
AAAACAGGTATCAAAGTACCCTTACTGATTGGTTATAACGAGAACGAGGGAAGCAGCG
TTATTAACTTTT

>novel_circ_001547
AAAAAATTAAAAATAAGTAAAGAAGATAATTCTAAATTAGAAAAAGCAAAACGAGAA
GGAAATTTACATGAAGCTTTATTAGATAGAAGAAGCAAAATGAAAGCAGATCGATATTG
TAAATAAATTTTGTATACTATTATATTATGTATCTTATAGTTAATTATTATTTCATAAAATGTA
TATTTATTAAAAATATAAAAAAATGATTATAAGCTATAAACAAAAA

>novel_circ_001548
TAGATGTTGCCGTCCCTGGCGAAGCACGTCAACTCGGCGGCGAGAGGCACACGGCAG
TGGCAGCAACGAAGACAGCCGCAGTGCCACGCGCGGTCCGCAGCCCTCAGGTACCAT
CGCTCGGCTATCTCCCTTCCGCAACCCCCGCAACCGAGGACCCCGTCGGTGTTGTTGTT
CTCGCCGCCTCCGAGCTGCCGGTTGCTGACGGCACCATCCCCTGGCGGGGGTGTGCTG
CTCCCGCACCCGACTTCCTTCAGCATCTCGAGACGGACCGCCGCGGCCACCGGCCCAT
TCTGCCGCACGCAAATCTCCTCAGATCTTTGCCGAAATTTCGCCAAG

>novel_circ_001549
CCGGCCAGCTCGCAGGAGCGTGGCAGCGGCTTGGCTGTGGGGCGTCAAGGGGGGCCC
GGCAGTGCGGGGGGGCCCGGAGCAGTGGACCAGGCGAGGGACCTAAGTCCCTGCCTC
CCTGCGTCTATGGGCGACGCTGCACGACCCACCACCTTACCGTGCAGTCCTCCTGCCG
CCCACCATCACGGGCCCCATCATACACCCCTACATCAGACCCACTGCGGAACCAACAAT
AATTGCTACGACGGACCCACCACCCCCTACGACTCTAGAGACTACGATTCCCCCGGAG
GTGGGCAAGAGTACAGGAACAGCAGCAGCAACTTCGAGAACCCGAGCGACCTGAGC
ATGCCCCCGCAAACGACCCACCACCACCATCATCACCACCATCACTCGCATCTGCACC
CGCAGACTCACTCGCAGGAACAGCAGACCACGGAACACTTGCACGCCATCCCGAAAC
TGGAGCCGCCGCCCACCCCGCCCGCGCAGTCGGACGACGTGCCGGGGGTGGTTTGCG
CCGGATGCGGCCTCCGGATATCCGACAGGTTTTACCTTCAGGCGGTGGACAGGCGGTG
GCACGCGGCCTGCCTTCAGTGCTCCCACTGCCGCCAGGGCCTCGACGGCGAAGTCAC
CTGTTTCAGCAGGGACGGAAACATTTACTGCAAGAAGGACTATTATCG

>novel_circ_001550
CAGCCGGCCAGCTCGCAGGAGCGTGGCAGCGGCTTGGCTGTGGGGCGTCAAGGGGG
GCCCGGCAGTGCGGGGGGGCCCGGAGCAGTGGACCAGGCGAGGGACCTAAGTCCCT
GCCTCCCTGCGTCTATGGGCGACGCTGCACGACCCACCACCTTACCGTGCAGTCCTCC
TGCCGCCCACCATCACGGGCCCCATCATACACCCCTACATCAGACCCACTGCGGAACC
AACAATAATTGCTACGACGGACCCACCACCCCCTACGACTCTAGAGACTACGATTCCCC
CGGAGGTGGGCAAGAGTACAGGAACAGCAGCAGCAACTTCGAGAACCCGAGCGACC
TGAGCATGCCCCCGCAAACGACCCACCACCACCATCATCACCACCATCACTCGCATCT
GCACCCGCAGACTCACTCGCAGGAACAGCAGACCACGGAACACTTGCACGCCATCCC
GAAACTGGAGCCGCCGCCCACCCCGCCCGCGCAGTCGGACGACGTGCCGGGGGTGGT
TTGCGCCGGATGCGGCCTCCGGATATCCGACAGGTTTTACCTTCAGGCGGTGGACAGG
CGGTGGCACGCGGCCTGCCTTCAGTGCTCCCACTGCCGCCAGGGCCTCGACGGCGAA
GTCACCTGTTTCAGCAGGGACGGAAACATTTACTGCAAGAAGGACTATTATCG

>novel_circ_001551
TAAAGTTAGAAAAATTAAAAGTGATTTTCCTTCTACGAAAAATATATGTATATAAGTCTC
TTTAGTTAGCCTTTGAAATCTGCGTCCTTCGAAATATGAATAAAGTAGCAAGCAGGAAT
AAGAATCTTTCCACTTAAGAAGATGAAGTTTTTAAGGGGAAAATTGTTCAC

>novel_circ_001552
GCAACTCTTCCGCGGATGGCAAGGTGCAATTGCAGATCATCTGTCAACCGGAGCAGCA
GCACAGGGCTCGTTACCAGACGGAAGGCTCGAGAGGGGCGGTGAAGGATCGTACCGG
GAATGGATTCCCGATCGTGCGGCTGGTCGGCTACGACAAGCCGACCACGCTCCAAGTC
TTCATCGGGACGGATCTCGGCCGCGTGGCGCCCCACATGTTTTATCAGGCGTGCCGCGT
GAGCGGGAAAAATTCGACGCCGTGCGTGGAGCGGAAGATCGACGGGACGATCGTGAT
CGAGGTGGACATGGATCCGGCCAAGGACATGATAGTGACGTGCGATTGCGTGGGCATT
TTGAAGGAGCGTAACGTGGACGTGGAGCACAGATTCCCGCAGGAGGCTGGGGTGCTT
CAGGGGCGCAGCAAGAAGAAGTCGACCCGTTGCCGTATGGTTTTTCGCACGACCATCA
CCCATCCCGACGGAACGACGGAAACATTGCAAGTTTGCTCGCAGCCGATAGTATGCAC
CCAACCGCCGGGAATACCGGAGATCTGCAAGAAATCGCTCACGTCGTGTCCGTGTACC
GGCGGATTGGAGTTATTTATACTTGGGAAGAACTTTCTGAAAGACACGCGGGTCGTATT
TCAACTGGACAACGACAATCTGTCGAGCAGTTTGGAACCTCACTGGGAATGCGCTGTT
CTGCCCGACAAGGAGTTCCTGCAGCAAACGCATCTGGTCTGCGTGGTGCCCGCGTACA
GGCGACAAGATCTGGCCCCCTCCGAAACGGTTAGCGTTAAATTGTACGCGGTCTCCTC
GGGGAAGACTAGCGAGCCTCACACGTTTCTCTACACCGCCGCGTCTACGCCACCCGAG
CCATCTGTGGGTAAAGTCGAGCCGATAACACCGCCTCTGTCCACCACGAATGGCGAGG
TTGCGCTAGCAACGTCTCCTGTAGCGGTACCCCTGACCAAAGGTGTATCACCGAACA

>novel_circ_001553
ATCGATCGTAGACGAGCATAAAAATTTAACGAATCGAGATCGAGGGGATGGCAACGTT
TTTCTTTAATCATCCGTGACCCGAAATCCAAGGTATTTGAGAAGTATCGTTCGAATTGCA
CGGATGGAAAGGAACGGCCGAGCCACGGATGGAAACGGGCACCGTGGAGAATGGAA
AACGGGCACAACAACTCGCGACCCAAAAATACTTGATAAAAATCACCACGGTCGTTAC

GAGGAGCGCAGCTGCAAGCATCACCAAAGAAAACACACGGAAATAGAGCGGACACG
AAGCTAGAGCCGTACACTTTGAGCGCGCGCGAGCGAGCCTGAGCCGCTCGTGTCACC
GATCGAGTGGATCTAGAAGGAGGAAGCGAG

>novel_circ_001555
TGTCGCCCAACTGAAGGAAGCGATCGAGAGGGAATGCGGCGTGGTCTCGGCGCACCA
GGTGCTGCTGATGAGCGGAGGCGAAAGCCTGGAACCAACCGCGCGTGTGTGCTCCTA
CTCGGCCGGAACCGACACGAATCCGATCTATTTGTTCAGCAAGGCCGCCATTGAGAGC
CACCTTCCACCCACGCCGAGCATAGATTACGGTTCCG

>novel_circ_001556
AGGAAGTGCTGGGCTCCCCGGTCGCATTCTGGATCTCGCCCGACGGACGACACCTTGC
TTTCGCCACGTTCAACGACACCAACGTCCGCGATATCGTGATATCTAAATACGGCTCCC
CTGGAAACTCGAGGGATCAATATCCGAACGAGATCAGGATAAAATATCCGAAAGCGGG
CACCACGAACCCATTCGTGTCCCTGAGCGTGATCGACTTGCACGATCCCTCCTCGAAAT
TGATCGATCTTCCGCCGCCTGTCGATGTCGTTGGAGC

>novel_circ_001557
GTAATACACTCGAAAAGAATGAAAATAAAAAATTTGGAAGAACAGAAAATTTGGATCT
AATTGTGGGTTCAAATGTAAAAGTTCTTTTGGATTCTGATGTACATTATGGGATTATTCG
TTGGATTGGAACCCCTCCTGGTATTACTTCTGGTAAATTGATTGCAGCTGTTGAATTGGT
ATGTATAACAAAAAAACAAATTCTAGAAAATTAATAAAAATACTAATTATTTTTTTATA
GGATGATAGTCATCCATCAGGTACTGATGGAACATTTAAAGATGTAAGATATTTCCATTG
TCGGCCATATAAAGCTATTTTTACAGATCTCGAACAATGTTCACGATATGATGCCATGGT
AATTGCTATTATTTCATGTATGCTTTTTAAATTCGTATAAAAAAAAATATATATATATATATT
TTTTTTATAGACAAAAGTTGATGATTCAACAACATTGATAAATACAGATAATTTCGGAAA
TATAGAAAGTTCCGTTATTGTTGGTATGGTACGTCCTATCTCTGTAAAAGGAAACTTGGA
AAGTATTTGTGGAAAATATAGAGGAATTCAAGGTCATCACAATTCGTGTTATTTGGATG
CCACTCTTTTTAGCATGTTTACATTTACTAGTGTTTTTGATAATTTATTATTTAGGTATTAA
TTAAATATTTATTCTCTTATTCTTTATTTTTTATTGAAAATAAAATTAAAAATAAATGATAT
TTTATTTTTCAGACCACCAAATGAAAAAGATTGTCCGCAATACGAAGAAGTACAAAGA
GTATTACGTGAAGAAATTGTAAATCCACTTAGAAAAAATATGTTTGTAAGAGCAGATCG
AGTGATGAAACTCCGAACTTTACTTGAAAAATTATCATCTGTATCCGGACTTACTAGCG
AAGAAAAAGGTATTTCATTAAAAATATATTTCGATATTTTAAGAATTAAAATTTTAAATTA
TTATTATTATTATTACTATTATTATTATTTAAATCTGTACAGATCCTGAAGAATTTTTAACTT
CTTTGGTTGCACAAATTTTGAATGCTGAACCATTTCTTAAATTAAGTTCTGGTCAAGAT
GCGTATCATTATCAACTTTTTGTTGAAAAAGATGATCATTTAAATCTTCCAACTGTGCAA
CAATTATTAGAACAAAGTTTTCTTACAAGTAACATAAGATTGAAAGAAGTTCCTTCATG
TCTTATTATACAAATGCCACGATTTGGAAAATCATTTAAGATGTATCAAAAAATTCAACC
TACATTGTTACTTGATGTAACAGACATAATTGAGGATTGTAGGTATTGTTATAGTATATAT
ATTTATATATTATTAATATGATATATTTCAATTATTTCTTTATTATTATCTTTTATATACAGCTC
CAAGACAATGTACTGTTTGCGGAAAATTAGCAGAATACGAATGTAAAGAATGTTTTGG
ACAATGTGGTGTTGGACTTGAAAGTATTGCATTTTGTTTGCAATGTTTAGAAAAGGTAA
AATAAAGAATATTTAAAAAGTATAATAGATAAAAAGAAAATTATTTAAAAAATTCTTTT

AGGTACATCGACATGAACGAAGAACAAATCATGAACCAAAGAAGCTTGTAGTACCAAT
GGAATTCGCTATTTTACAAGAACATTGTCCAGTTCCACGATTGTATCTTGAATTGTCGGC
AGTAGTTTGTATCGAAACCTCCCATTATGTATGTTTTGTAAAATGTGGTTCTGGCTCTGA
AGCTCCATGGTGTTTTTTTGATTCCATGGCAGATAGAAAAGGTTTCAATCACATTTAATA
AATTCATATATTGTATCATTATTATTAATTTATAAAATTTTTCTTCTACTTTTAGGAGAACA
AAACGGTTACAATATACCGGAAATGGTTCCATGTCCGGATTTTCCATATTGGTTAAGCGA
AGAAGGTGGAAATTATTTAACTGAATTAACAGACGATCGTCATCTACCTGAACATGCAA
AACGACTGTTATGTGATGCATATATGTGCATGTATCAATCTCCAGATGTTATGATGTACAA
ATAAATTAAAATGCCTGATAATATAGGTAAGTTAGGTTTAAAATTATTATTTTTAATAAAA
ATAACATAATGTTTGTGCTTATTGCTTTATTTTATGTAAATTTATATAATAATAGTAAAATTT
ATTCACAAAAATTTTATACTATTAAAGATAAAAGGAAAAGAAAAAATTGAAAATTTGAT
CAGCTCATAAAGTTTATTCAAATGCAGAAAAACATGTAATCTTAAGGGAACTAAGGTTA
TACATATATATTATACAAATTATATGAAAATCACGATTTTCAATCAAAAACTAGTAATCTA
ACTCGGGTATAGTACTGGTTTTTAAATTCACAAGATATATTCATTCATATATATATATATTTA
TCAGTGACATAAAATTAATATTTTTCATATTATACGAAAATTATTGGAAAATTTACTATAC
TATTGTAAAATTTATACACATATTGCACGATCTAATTAATCATCAAATTTTATCAGTTTGTT
GAAAATGAATTCAAAATATTGAATTAATATCTTGATAATTTATAAAACTGAATATTGTATT
GATACATTAAGAATATATATTTGTCAAAAGTCATATATGAGGTATAAAATTTTATACGAAA
AGCTTATCAAAATGTTATCATATGACTGATTTTTATAGCAAAGAGCACAAACAATTAATA
AATCTTTTATAACTTAAAGATATAATGACTAAGATTAAGAAATTCTGCACTTTTAGATCTA
TTGTTGCTCTAATAATTTTTGTACTCCCATTTTATAATATTAGATTAAATCTGTTTTTTAAT
TTAATTATTTAATAATTATAATAATCTTAAAGAAATCTAATAGAGCAACTTAGCACAACA
ACAACAACAATTTAAAATCCACTAAGTTCATTACGTTTTTATATAAATAATGATAAAGAT
CTTTTTGATATTCTAAACTTGAATATTTTACATAGAATGAAAATCTAGATCTTAGATTTGT
ACAAATTTAATTAATGTGTAGCAGATTTATATACTTAATGCATCTCTGATAGTAAATATGT
ATATGAAAATTATAAAGTCACAAAGAAAAATTGTTTCAAAATCTTACAAAATTTTCAAA
ACTATTAGATTTTTTCTTGACTACAATTACTTCAATATCATATAAAAAAAAGATAAAAAA
AGAATTTTTGTTAGACTTAAGTCTGAAAGAAATATTACTACAAGTGAAAATAAACGAAA
TAATTATGAAAAATTTGAGATGTATCATTGTACATAAGTTATGTAATAGATAAAATTTAAT
GTTCATTCATGCCTTTGTGGCAAAACTAATAAAGATAGTGACGAAGTTTGTATAAACAT
GTATTATGAATTTAGTATCTGTCTTCATCTTCATCCATGGCAAAGTCACTGCCAACACCA
GAATCTAATACGTAGAGAGGACTGTAAACATATACATAAAAATAATAATAACTAAGTAA
ACAAATGTGATATTGAAATATAAATACTTACTTTATTGCTCTGCATGGAAAAAACACAAT
GCGTTTTAGCTTTTTATTATAGAAGAAATAATTAAAAGACCATAAGCATCCATCTTCTCC
AAATGGATCAGATGCAAAATCTGGATTGTAACTAAAAATTTTCATATTCTCGATTTATTT
AATATATTTTGCAACTTAATAACTTCTATAAAATTAGAATAACAAATATATTATATGTTGAG
TATAAAATGAATTCAAGAATTTACCTATAAATATCACATTCGTTTAATGATATTTCGTCAT
CAATGGCAGCCCAAAGTGCCGTTCGTAAAGTACGATAATGGTCTCCCGCAGTTGCACT
CAAATTACTGTCTACAGCATTCATGACCCATTGCAAGCTTGGCTCTTTACTGAATTCATG
ACTTTTAGCATCAGAAAAATCATAATCTGGATGAAAAGCAGAATTCAAAGTAGCAATCA
AATAGAATAGTGTTTTTCTACTGATTGTATCACATAATGGACCATCTTCATCACCAGAAA
TACTGCGACTATAGCAAAAATATATCTATTAAATCAATGTACAGATAAGGTATTATCAAA
AATATTAATGTATTTTGTAAGGTATTCTAGATTTTTATAACTTTTTACATATATTTAAACAT
GAATTTTATTCCATATTAAAGAAGATAATATTCATATTTTTAGATTTATTGTTTTATTACATC

TCATTTTTAATTTTTAATACCTAAAGTATCCTTGAGCAGGTGATGTTCCTAATGATGTTTG
TGGTGGTGATAAAGCTTGAAGATCATGTGGAGTAAAACCTTGTTCTGCATTGAAACGTT
TATATAGCTGTTTATCATTTCCAGCCATTTGCAAGAGTAGCTCTCTATTCTGTGGATGAA
AAAAAATATAATGTTTAGGATTATTTTAAAGGATAGAACAGTTCAATAAATATTGATCCA
ATTATCTTACCTGCCTATTATTTTGCTGTCTCCAGTCTTGATGGACAAGGCACTGTTTATG
GCTTCAAAACGTGTACTCTCCAGCAATTTCATCTTGTCAGCAGTAGTTTACTTGTACGT
TGTGTAAGAACAGTGTCTCTGTACTGGATACTCTTGGTGCATCTCTCAGTGAAACACTT
TTGTCATTAATACTCCATTAGATAGGAGTTGATGACCTTTAATGTATTTTTCGACTAAACT
TGACCTCGTTTCTCAGTCGGACGGCCAAGGGATGTTCAATATAATCGAAAATTTAAAAA
ACTTCTGCGACATCGAAGACTTATTGAGTCTTTGTACATCTTTTTTATATATATGAAAATT
CCTTTCCTCTTTGAGTGTACGAAATTAATCTTGGACGTAATGATTTTTCGCGCGGCGCCG
AGCTTGTACATCACAGATCTCTAATTAAAGATATCGAGACGAATATACGTCCGTGCGCA
ATCCGATTTCAGCAATCGGTTCTTAGATTAAACTTCAACTGTGATCAGTGTTGGCTTCTC
GTGCTACGCAAATGACGTGCAAGTTAAAGAGAGGGAAAACGGAGTAAAGGATAGGCG
ACTACCCTTGAGAAACAAAAGACGCCGTTTTTTAGGTCAAAAACTCGCGCTAAACAGA
AAGTTGATGGCAAACGTGCTATAAAGTCGACAGAAAACAATGAATCGGACTTGTTTAT
TGGAAATCAGCTAAACAAAGTGACTAAGAAATGTTATATGTCCGCTACTGATGAAAAC
AATCCATAATTACAGACATTGTTGTCACTATCACTCGAATTATCACGGTCACTGTTAAAC
AAAAGCACCCAAAATTCAATTAAACTCCGATGCAATAATCAAAAATCGACAAACTTCA
ACTCTTTTCGCTCCTATTTCGCACCGTTTACTAATTATTTGCGTTTCGATATTAGTACATCT
ATATGCAACAACGAATATATGCGTTCTACGTGACAGAAATAGCACAGCAAAGCGTCTTG
AATCCTTCTCTCTCAGATTCTGTTTCTCTTTCTTTTACTCTTTTATATCACTTCCCCTCGCT
TCACCCCTTAAACCATATATGTTTTTAGTGTGGATATGTACATGAATTTTATTCAGCCAAT
CAAAAGCCAACGATAAAGATGTGTATGTTAGGATACGATGCAACGAGTCGTATACTACC
TTTGGATATGTATAGAGTTGCGTATATGATTCAATTCTAGTTCGCGTATTCGGTTTGATTC
TAACCTTGCAGAATGCTGATAGGAATTCGAATTTGTTGGTAAAATGTCAAATCGTTGGG
GCAGCGACTATGTGGTTACCGAGCACCGTGATTTACAGGCTAATACTATGGCCGTGGAT
GCGACGGGTAATTATGTATTACTTGCCGGACGCAGATGTTTTGCGGTAAAACACCTTGA
CGAAAGTCTTGATATATTAAAAAAATTTCAAAGACAGAGTAAATATGAAGTTGGCTCTG
CAGAATGGAATCCTACAAGTATAAATTGCCATCTTTGCGCTGTATCGAGCAATACACGTA
TTGAAATATTATCTCTTATTGGAACTGGTAGTTATGACTTACAAACAACAAATAGTTTAA
AAGCACATACAAGAGTTGTGAGTGATTTAAACTGGCATCCTAAAGAACAAGATATCATT
GCTTCTTGTAGTATTGATACATTTATACATATATGGGATATAAGAGATCAAAGACGGCCTT
GTTTATCTTTATCTGCAGTTGCTGGTTCTTCTCAAGTAAGATGGAACACTCTTTCTCCTA
ACATATTGGCAACAGCTCATGATGGAGATATTAAAATATGGGATCAAAGAAAAGGAAAT
AGTCCTATGCAATATATAGCTGCTCACCTAACAAAAATACATGGTTTAGATTGGTGTCCA
TTTCAACAAAATCAATTGGCAACATCTAGTCAAGATTGTACTGTGAAGATTTTTGATATC
AGTAATCCACGAAGAGCTGAAAGCATTTTAACAACAAATTCACCAGTATGGAGGGCAA
GATATACGCCATTTGGAGAAGGACTGGTAACAATAGTAGTACCACAATTACGACGAGG
AGAAAATAGTTTATTATTGTGGAATACAACAAATCTCAATGCTCCTATTTATACTTTTGTA
GGACATACAGATGTTGTTCTTGAATTTCAATGGAGACATCAAAAATTAGAAAATAGTGA
TTTTGAACTAATCACATGGTCAAAAGACCAATGTTTAAGGATATATAAAATTGATCCATT
TTTAAAAAAATTATGTGGACATGGTATAGATGATAGTACATCTATTTATACTCAGTATTCT
GAAGATAATAATTTGAGAACATTACAATCTGTCCAACAATTACAGCTTAATGATAGTCA

AACTGATCGCGAAATGAATTGTATGACAACAAAAGTAGATGAACCTATATTAAATTCTG
AAGCAGATGTATATATTGAAAATAATAAAGAAATATCATCTCCTACACAACCAAAGACT
CTGCAACAAGAATTTTCATTAATAAATATGAACATACCAAATATAGAGGTCAATGCAATG
GATGTTGTAGAACGTAGTTGTACAGTAACAGCATCTAATAAAAGTTATAATGTGATATTG
AAAGTAAATTTCCCTCCAAATTATCCATACAGTGCACAACCTACATTTCAATTTTGTCCT
GGGACAACAATTGATAATGCAACATTGGCAAAATTGTTGAAAGTTTTAAAACAAACTG
CTCAACAACGTGTTAAAAAAAACAGATCATGTTTAGAACCTTGTCTTAGACAATTAATT
ACAACATTGGAACAAACATGTAAAAAAGATGAAAATGAAAGTACTCATTTAGGATATG
ACATTCAAGACAATGCAAATTTTTTAAATTCTTCTAATATGTATACGAATTATCAAGATGC
CTATATACCATTTCCTAGAACATCTGGTGCAAAGTTTTGTTGTGTAGGTATACTTGTATGT
TTTGGTCGTGCTTCATATACGAGAAGATCATCTATGAAGCCTGAGTGTACAACACCTAG
AGCACTTTCTGCATTAGGAAATGGAATAGGCAGTGCAGAACATTTTATACATATGTATCC
AAATTCTTATGTACAGTCAAATGATAACATTCCAATTAGTTCCTTTTATTTTCAAGACCG
AAAAATGAATCGTGGATTACATAATACAAATAGAATGATCTATAGATCATGTTGTAAAAA
TTATCACTTTATGGTGATGATATATGATGCCTCTTCATTATTTTTCGTTAATAAAGAACTTG
CCGAAAAATATGTTATTAATATCACCGATATTCCGGCAATGTGTCAATATAATGCAAATAT
TGCTGCATCATTAGAACGTGCTGATTTAGTTCAAGCATGGTGTTTAGCTGCTCTTGTGAT
ATCGCAACCAATTTCAAATGTTGGACAAAATTCATGTCAGTCGCCTGACATTGATGCTC
CATGGCATCTGCATCCTTTTGGTCAAAATTTAATTCATTCTTTAATACAGCATTATGCTAA
ACAATCAGATATTCAAATGGCAGGAATGCTGAGTTGTGCTTTTAGTTATCGTTCGGAAA
ATCCTGACGTTGCTCAAACACGAATAAGTAGCAAATCCATTAACATCAGT

>novel_circ_001558
AGAAAGTGTTGATAACCAGGCTGACTACGCAAAAGTGGTTGGACGTCGGGCCAAATT
GGACCGAGGACGAGTACAGGAGCGCCCATTCGAAGATGGTGTACGAGTACAGGGTGA
CTTGCGTGGCGCATTATTACGGGAAGGGTTGCGAGAACCTGTGCAGGCCGAGGGACG
ACAACTTCGGTCATTACAGCTGTAGCCCGAGCGGGGAGCGCGTATGCCTCTCTGGATG
GAAGGGCGACTACTGCAATACCC

>novel_circ_001559
GTGATCTTTTACGACAGCGACTGGAATCCAACAGTAGATCAGCAAGCTATGGATCGTGC
TCATAGACTAGGTCAAACGAAACAGGTTACGGTATATCGATTAATTTGCAAAGGAACAA
TCGAGGAAAGGATATTGCAACGTGCTCGAGAAAAAAGCGAGATACAACGCATGGTAAT
TAGTGGAGGGAATTTTAAACCAGACACTTTGAAGCCTAAGGAAGTTGTTTCCCTTTTAT
TAGATGACGAGGAAATCGAAGCGAAATATAGTCAACGGAGCGAGGAGAGAAAACAGC
ACGCGGAGGATGCCCGCTTCGAGTCGAACTTATACCACAAGGAGAGGGAGCGGAAAA
GGAAGTTAACCGCGCTTCCGGTTAAGGGCGATGTAAAGAAGCCTTGCCTATCTGATGC
AAATGGAGATAAGATTGGGGATATTATTCAAATTAATTCTAATGAAAGTAGTCAAGCTAA
TAGCGGTATTCAATCATATCAGATTAACAATCATCAGCAAATTCTCGATAATGCAGACGA
TTACAGTGTGCCAACCAGTCCTGTTAAATCAGAGGATGAGACGAGTAACGATGGTCTG
GTTGTCGATGTCGATGGTCCAGTAGGAGCATCCTCTGGTGATACTCGACAATCACGAGC
GGGTGGTCAGTTTGGAGATCCAGCCAAAGTCAGTCGTCTCGATCATCATATGTCTTTCA
CTAGTGGATCTAGTGTTCGGATAAGGCCTACAATTCGTGGTACCAGCAAAAGAGGCAG
ACCTCGTGGTTCTCGCAGAGGAGGGCCCGTTGGTGGGAAAGGTAGAGGACTCCTTCT

GCTTCATCCACCATCTACGGGTCTTGGAAGTAGTCCACAGTCACCGGCATCTTTATCTC
CGACCAGTACAACTTTAAACGATCAAACTTTACAACATG

>novel_circ_001560
AGGAAAGAATAAATAAACTGAGACTCTATAATATGACAAGTGTGGGAGAAGAACGACA
TTGGTTGCAGGATATTTTATTAAGTGATTCGTCAGATAGTTCTGCTTCAGGGTCTGATAC
AGATAATCCCATTACAGAAGAAGATTTCAAGAAATGTTGAAGTATCATATACTTAGGA
AAAAATATCAAGCACGTTTTTATCAAAAACCAGAAAACATTCAGTACCAATATTATAGT
GCTGGTTTGCTATCCAATTATGACAGATTTTTAGAACATCAAAAATTAATAGTTGGAAAT
AAAAAGAAAAAAGAAAAGAAACCAGAAAAGAAAGTTATGAAAATAAAAAAAGAAAA
GATATCGCGTCATAGATCTTCTGAAGATTATCCT

>novel_circ_001561
TATGGTAGGGACAAGGGTCTATGTCGGTGGGCTTCCATACGGCACCAGGGAAAGAGAC
CTTGAGAGATTTTTCAGAGGCTACGGTCGATTCCGTGATGTCCTCATCAAGAACGGTTA
CGGTTTTGTTATCTTGACACAATAACTTTCAAGTTTTATTATATTTTATTATTTTATTGCTG
ACCAAAATGTGTAGAGGGTAAAGTGTTTTACTTAAAAAATAACTTGAACTCGTGAGTT
GATCTGACCTACCTTGAGCCCTGAACACATTGGTACTGTGGCTGCTTAATGGAACTGTG
TGCTGGTTTTCATCGATTAATCTACAAAATATTT

>novel_circ_001562
TTGTCTCCTGGACGAGACGGATCAGATACAGACATGGAACCTACTGCGTCACAGTCTG
CGGATTGTGAACCTACGCCGCCAGTACAAAGACATGGTTCTAAAAGCAATTTCTTCCTG
CCGCCTGTGGAGAATAGTGAATCTCCCAGGCATCCTCCACCATCGCAACATCGCCATCC
AATGCATCACACTAGAGGCAGCCCTCATCCAAGGCCAAGGTATATTATCAATACCTATA
GGGGAATGAATGGCTCCCGAAGCCATACGCCCGATCCATTATCGCCAGAAGCAAGTGG
GTCACCAGCTACAGCTTCTCAAAGAAGCCAAGGTCAGGGGGGTGCTAGTCCAACAAC
ACAACGGATTAAAGCTATTGGAGTACCAACTCCACTTGCCATTTCCAGTCCAATTCGCA
G

>novel_circ_001563
GATTCTTTTGGGTGCTAAGGATTACCAGACTTTCCTCAAAACTGCTGCATGGGCCCGTG
TCCATGTTAATGAAGGACAATTTCTTAAGGCATTTGTTGCTGCTGTATTAACTCGTCAAG
ATACTCAAGGTGTTATTTTCCCACCAGTTTATGAAATTTTACCTCAACATCACTTGGATT
CACGAGTTATTCAAGAAGCTCAAAATATTGCTATTCAAAACACTCAAGGAAAGAACAA
TCAGCAAAACATTCTTATTCCTGTTAACTATTCTGCACTTTTATCTCATGATGAACAACA
GTTATCTTATTTCACTCAAGATATTGGTCTTGCTGCATACTATGCCCAAGTTAATCTTGCT
GGATATATTCAAGAACAGAATCAACAACAACAACAGCAACAGCCTTTAACTCAGCAAC
AATATCAACAACAGATTGTAGGAAAATATTTGCAACAACAAGCGGGCCAACAGGATCA
ACAAGCAAATATTGGTCGCGGAGCTCAATATCTGTACCTTCATCAACAACTTTTGGCAC
GTTATGAACTTAATCGTCTTTCTAACGGACTTGGTCCTATTAAGGATATTGACTACGAAA
ATGTACAAAGTCTCTATCAACCACATCTTCGCGGTCTCAATGGACTTGAATTCGCAGGC
CGTCCTCAGAATCTTCAACTTCAATCTCAAAGGAACCAGCTTATCCAATATGTTGCCAC
TTTAGAGAAAAGACTGAGAGATGCTATTGATTCTGGAAATGTGATTACCCCACAAGGC

GTCTTTCTTTCTCTGTATCAACCACAAGGTATGAACATTCTTGGTGATCTGATTGAAGG
AACTGGTAGAAGTGTTAATCCAAGGTATTATGGAAGTCTGCAAGCTGCTGCTCGCAAA
CTTCTTGGAAATGCTCCTGAAGTTGAAAACATTTGGGACTACACACCATCATCACTTGA
ACTTGGAGAAGTTGCCGTCCACGACCCAGTTTTCTATCAATTGTACAAGAAAGTTATGA
ACCTTTATCAACAGTATCAACAATCTTTACCTGTCTATCAATACAATGATCTTATTCTTCC
TGGAGTGACCATTCAGAACGTTGATGTCAGTCAACTTGTAACTCTTTTCACCGATTTCT
ACGTCGATCTCGATGCCGTTACTGGTCACCAATCCCAACAGCAACAAGAAGAACAAAC
TCAATCTCGTGTTAGAGCTCATCTCAAGAGATTGGATCATCAACCTTATCAATATAAAAT
TGCAGTTCACAGCGAACAGAACGTGCCAGGCGCAGTTGTCCGTGTCTTCCTTGGACCC
AAACACGACCATCAAGGCCGACCAATTAGTATTTCTAAGAATCAACATCTCTTTGTTGA
ACTTGATCAATTTATCCAGAACCTCCATGCTGGTGAAAATACCATTATTAGAAATTCTCA
ACAAGCTCCTGGACAGAGCCCGGATTGGCCGTCTACTTCTCAAATTCAACGAGGAGTC
AATGCTGCTATCCGATCCCAGGAACCATTCTACATCACTGAGCCACATCAAATCTTCAG
TTTCCCTGCTAGATTGTCTCTTCCCAAAGGTCAGCCACAAGGTTTCCCTCTTCAATTCTT
AGTTGTGATCTCTTCGTCAAATCCACTTAACGTCCCATACGGGCCAGTGATCCCTGAAC
AAAGTTTGACTTATCAAGATCAACAGTACCAGGTCGTAAGCGTTGAGCAATATCAACA
ACTGAAAGAGCAAGGTCAAATTTCTCAAGTGGGCGGAGGAATTCAGCAAAATGTAGA
GGTTCTCCCTGAGAATTTGGTCAATGCTCAGCAACAAGTTCAAGCTGTTAGAAACTATT
ACGCCAATCTTTACACTAAATACCACGGACAATACCCGAACACCCAGATCCAGAATCCT
GTAGGCCAGGGACAAGACATGACATACTCCGTTCAAGGTGTTGGAGTAGTCAATGCAG
GCGGTTGGTTAGGACAGCAAGGTAATAGTTGGAGCCAGCAACAAGTGCAACAAGCCC
AACAAGTCCAACAACAAATGCAAGCAGCAATGGCAGCTGTACAACAGAGTCAACAGA
GGCATCAGCATGCTGCTCAAATGATCTATGGACACCAACAATCTCATCATGGATTGCAC
ATTAACTCCTCCCCATCCTCTGTACAATCTGGACAACAACAACAATCCGTACTGCAAGG
ATTAGGTGTTCAAGGTGTGCAACAAGGTGTCCAAGGTGTCCAAACACCTCAGGGCGTC
CAAGGTGTCCAAGGTGTCCAGGGTGTCCAAGGCGTCCAAGGCGTCCAAGGCGTCCAA
GGCGTCCCAGGCCTTCTGCAAGGAGTGCAACAAGTATTTGGACAAGGTGTCCAAGGA
ATGAACGTCCCCTATGGCATGCAAAGAGGCCAAAGTGGTGGTCAAACGTGGTCAAACT
CCCAAGTCCAAGGCGTAGCAGTCCCGGGAAGTGGCATCGTCGCTAGTGGACAGCAAC
ACGCTGGTGGGTGGCAAAGTATCTACGCTCAACCTCAAACGGTTCAGGACCAGATTGT
CAGCGAATACTACCAGAACAAGCCGATCTCGGAGGTGATTGGCGGTGCCATTTCCCTT
GACGGCAAACCTCTCGGTTTCCCCCTGGACAGACCGTTGTCCCTCGGTGCACTCAGCG
TTCCTAACATTTTCGTCAAGGATGTGCTCGTCTTCCACCAAGGTCAACCCACCAATGAC
ATCACTCAGTAAATATTCCTACGGTGAAGCACGCAAACAGTTGTTAGTAATGGGAATGA
TTTATATGAGAGACTTAGAGATATTGTTAATTAAAAAGAAAAAAAATTTATCGATGTGAA
TTAC

>novel_circ_001564
GCGTCCAAGGCGTCCAAGGCGTCCAAGGCGTCCCAGGCCTTCTGCAAGGAGTGCAAC
AAGTATTTGGACAAGGTGTCCAAGGAATGAACGTCCCCTATGGCATGCAAAGAGGCCA
AAGTGGTGGTCAAACGTGGTCAAACTCCCAAGTCCAAGGCGTAGCAGTCCCGGGAAG
TGGCATCGTCGCTAGTGGACAGCAACACGCTGGTGGGTGGCAAAGTATCTACGCTCAA
CCTCAAACGGTTCAGGACCAGATTGTCAGCGAATACTACCAGAACAAGCCGATCTCGG
AGGTGATTGGCGGTGCCATTTCCCTTGACGGCAAACCTCTCG

>novel_circ_001565
GTAATAGTTGGAGCCAGCAACAAGTGCAACAAGCCCAACAAGTCCAACAACAAATGC
AAGCAGCAATGGCAGCTGTACAACAGAGTCAACAGAGGCATCAGCATGCTGCTCAAA
TGATCTATGGACACCAACAATCTCATCATGGATTGCACATTAACTCCTCCCCATCCTCTG
TACAATCTGGACAACAACAACAATCCGTACTGCAAGGATTAGGTGTTCAAGGTGTGCA
ACAAGGTGTCCAAGGTGTCCAAACACCTCAGGGCGTCCAAGGTGTCCAAG

>novel_circ_001566
GTAATAGTTGGAGCCAGCAACAAGTGCAACAAGCCCAACAAGTCCAACAACAAATGC
AAGCAGCAATGGCAGCTGTACAACAGAGTCAACAGAGGCATCAGCATGCTGCTCAAA
TGATCTATGGACACCAACAATCTCATCATGGATTGCACATTAACTCCTCCCCATCCTCTG
TACAATCTGGACAACAACAACAATCCGTACTGCAAGGATTAGGTGTTCAAGGTGTGCA
ACAAGGTGTCCAAG

>novel_circ_001567
TCCAACAACAAATGCAAGCAGCAATGGCAGCTGTACAACAGAGTCAACAGAGGCATC
AGCATGCTGCTCAAATGATCTATGGACACCAACAATCTCATCATGGATTGCACATTAAC
TCCTCCCCATCCTCTGTACAATCTGGACAACAACAACAATCCGTACTGCAAGGATTAGG
TGTTCAAGGTGTGCAACAAG

>novel_circ_001568
CCCAACAAGTCCAACAACAAATGCAAGCAGCAATGGCAGCTGTACAACAGAGTCAAC
AGAGGCATCAGCATGCTGCTCAAATGATCTATGGACACCAACAATCTCATCATGGATTG
CACATTAACTCCTCCCCATCCTCTGTACAATCTGGACAACAACAACAATCCGTACTGCA
AGGATTAGGTGTTCAAGGTGTGCAACAAG

>novel_circ_001569
TGCAACAAGCCCAACAAGTCCAACAACAAATGCAAGCAGCAATGGCAGCTGTACAAC
AGAGTCAACAGAGGCATCAGCATGCTGCTCAAATGATCTATGGACACCAACAATCTCA
TCATGGATTGCACATTAACTCCTCCCCATCCTCTGTACAATCTGGACAACAACAACAAT
CCGTACTGCAAGGATTAGGTGTTCAAGGTGTGCAACAAG

>novel_circ_001570
GTAATAGTTGGAGCCAGCAACAAGTGCAACAAGCCCAACAAGTCCAACAACAAATGC
AAGCAGCAATGGCAGCTGTACAACAGAGTCAACAGAGGCATCAGCATGCTGCTCAAA
TGATCTATGGACACCAACAATCTCATCATGGATTGCACATTAACTCCTCCCCATCCTCTG
TACAATCTGGACAACAACAACAATCCGTACTGCAAGGATTAGGTGTTCAAGGTGTGCA
ACAAG

>novel_circ_001571
GTAATAGTTGGAGCCAGCAACAAGTGCAACAAGCCCAACAAGTCCAACAACAAATGC
AAGCAGCAATGGCAGCTGTACAACAGAGTCAACAGAGGCATCAGCATGCTGCTCAAA
TGATCTATGGACACCAACAATCTCATCATGGATTGCACATTAACTCCTCCCCATCCTCTG

TACAATCTGGACAACAACAACAATCCGTACTGCAAGGATTAGGTGTTCAAG

>novel_circ_001572
GTGTTGGAGTAGTCAATGCAGGCGGTTGGTTAGGACAGCAAGGTAATAGTTGGAGCCA
GCAACAAGTGCAACAAGCCCAACAAGTCCAACAACAAATGCAAGCAGCAATGGCAG
CTGTACAACAGAGTCAACAGAGGCATCAGCATGCTGCTCAAATGATCTATGGACACCA
ACAATCTCATCATGGATTGCACATTAACTCCTCCCCATCCTCTGTACAATCTGGACAACA
ACAACAATCCGTACTGCAAGGATTAGGTGTTCAAG

>novel_circ_001573
CGAACACCCAGATCCAGAATCCTGTAGGCCAGGGACAAGACATGACATACTCCGTTCA
AGGTGTTGGAGTAGTCAATGCAGGCGGTTGGTTAGGACAGCAAGGTAATAGTTGGAGC
CAGCAACAAGTGCAACAAGCCCAACAAGTCCAACAACAAATGCAAGCAGCAATGGC
AGCTGTACAACAGAGTCAACAGAGGCATCAGCATGCTGCTCAAATGATCTA

>novel_circ_001574
CCACATCAAATCTTCAGTTTCCCTGCTAGATTGTCTCTTCCCAAAGGTCAGCCACAAGG
TTTCCCTCTTCAATTCTTAGTTGTGATCTCTTCGTCAAATCCACTTAACGTCCCATACGG
GCCAGTGATCCCTGAACAAAGTTTGACTTATCAAGATCAACAGTACCAGGTCGTAAGC
GTTGAGCAATATCAACAACTGAAAGAGCAAGGTCAAATTTCTCAAGTGGGCGGAGGA
ATTCAGCAAAATGTAGAGGTTCTCCCTGAGAATTTGGTCAATGCTCAGCAACAAGTTC
AAG

>novel_circ_001575
GAAGAGATCACAACTAAGAATTGAAGAGGGAAACCTTGTGGCTGACCTTTGGGAAGA
GACAATCTAGCAGGGAAACTGAAGATTTGATGTGGCTGTAAAAATATTATTAGATTATTA
CAAAATTTATATAATAATCATTAATCATTTTCTTTAAGAAAAACAAGTTTGTCAACCTCA
GTGATGTAGAATGGTTCCTGGGATCGGATAGCAGCATTGACTCCTCGTTGAATTTGAGA
AGTAGACGGCCAATCCGGGCTCTGTCCAGGAGCTTGTTGAGAATTTCTAATAATGGTAT
TTTCACCAGCATGGACTAAATATACAAAATAATATTAATCATAAACAAATTGATCGGATAT
AAATAATTTATTAGAAGAAGAAACAATTGAAACTTACGGTTCTGGATAAATTGATCAAG
TTCAACAAAGAGATGTTGATTCTTAGAAATACTAATTGGTCGGCCTTGATGGTCGTGTT
TGGGTCCAAGGAAGACACGGACAACTGCGCCTGGCACGTTCTGTTCGCTGTGAACTG
CAATTTT

>novel_circ_001576
CACAGCGAACAGAACGTGCCAGGCGCAGTTGTCCGTGTCTTCCTTGGACCCAAACAC
GACCATCAAGGCCGACCAATTAGTATTTCTAAGAATCAACATCTCTTTGTTGAACTTGA
TCAATTTATCCAGAACCTCCATGCTGGTGAAAATACCATTATTAGAAATTCTCAACAAG
CTCCTGGACAGAGCCCGGATTGGCCGTCTACTTCTCAAATTCAACGAGGAGTCAATGC
TGCTATCCGATCCCAGGAACCATTCTAC

>novel_circ_001577
GTTGATGATCCAATCTCTTGAGATGAGCTCTAACACGAGATTGAGTTTGTTCTTCTTGTT

GCTGTTGGGATTGGTGACCAGTAACGGCATCGAGATCGACGTAGAAATCGGTGAAAAG
AGTTACAAGTTGACTGACATCAACGTTCTGAATGGTCACTCCAGGAAGAATAAGATCA
TTGTATTGATAGACAGGTAAAGATTGTTGATACT

>novel_circ_001578
GTTGATGATCCAATCTCTTGAGATGAGCTCTAACACGAGATTGAGTTTGTTCTTCTTGTT
GCTGTTGGGATTGGTGACCAGTAACGGCATCGAGATCGACGTAGAAATCGGTGAAAAG
AGTTACAAGTTGACTGACATCAACGTTCTGAATGGTCACTCCAGGAAGAATAAGATCA
TTGTATTGATAGACAGGTAAAGATTGTTGATACTGTTGATAAAG

>novel_circ_001579
CTCCTGAAGTTGAAAACATTTGGGACTACACACCATCATCACTTGAACTTGGAGAAGT
TGCCGTCCACGACCCAGTTTTCTATCAATTGTACAAGAAAGTTATGAACCTTTATCAAC
AGTATCAACAATCTTTACCTGTCTATCAATACAATGATCTTATTC

>novel_circ_001580
ATTGTTGATACTGTTGATAAAGGTTCATAACTTTCTTGTACAATTGATAGAAAACTGGGT
CGTGGACGGCAACTTCTCCAAGTTCAAGTGATGATGGTGTGTAGTCCCAAATGTTTTCA
ACTTCAGGAGCATTTCCAAGAAGTTTGCGAGCAGCAGCTTGCAGACTTCCATAATACCT
ATTTAAAGTGAAGAAACGAGTAATAGAAACATAATTCTATAATTTTTAATTATAGTTATA
TATTTTTTACTGACCTTGGATTAACACTTCTACCAGTTCCTTCAATCAGATCACCAAGAA
TGTTCATACCTTGTGGTTGATACAGAGAAAGAAAGACGCCTTGTGGGGTAATCACATTT
CCAGAATCAATAGCATCTCTCAGTCTTTTCTCTAAAGTGGCAACATATTGGATAAGCTG
GTTCCTTTGAGATTGAAGTTGAAGATTCTGAGGACGGCCTGCGAATTCAAGTCCATTG
AGACCGCGAAGATGTGGTTGATAGAGACTTTGTACATTTTCGTAGTCAATATCCTTAATA
GGACCAAGTCCGTTAGAAAGACGATTAAGTTCATAACGTGCCAAAAGTTGTTGATGAA
GGTACAGATATTGAGCTCCGCGACCAATATTTGCTTGTTGATCCTGTTGGCCCGCTTGTT
GTTGCAAATATTTTCCTACAATCT

>novel_circ_001581
TTTTCAACTTCAGGAGCATTTCCAAGAAGTTTGCGAGCAGCAGCTTGCAGACTTCCAT
AATACCTATTTAAAGTGAAGAAACGAGTAATAGAAACATAATTCTATAATTTTTAATTAT
AGTTATATATTTTTTACTGACCTTGGATTAACACTTCTACCAGTTCCTTCAATCAGATCAC
CAAGAATGTTCATACCTTGTGGTTGATACAGAGAAAGAAAGACGCCTTGTGGGGTAAT
CACATTTCCAGAATCAATAGCATCTCTCAGTCTTTTCTCTAAAGTGGCAACATATTGGAT
AAGCTGGTTCC

>novel_circ_001583
AGACGATTAAGTTCATAACGTGCCAAAAGTTGTTGATGAAGGTACAGATATTGAGCTCC
GCGACCAATATTTGCTTGTTGATCCTGTTGGCCCGCTTGTTGTTGCAAATATTTTCCTAC
AATCTGTTGTTGATATTGTTGCTGAGTTAAAGGCTGTTGCTGTTGTTGTTGTTGATTCTG
CTATTTAGAGAAATTGATTACATTAATTTATAATGTTTTCCAAAATTTGATTACATTATTTA
TATATTTATTTATATATTATATACTTCTTGAATATATCCAGCAAGATTAACTTGGGCATAGTA
TGCAGCAAGACCAATATCTTGAGTGAAATAAGATAACTGTTGTTCATCATGAGATAAAA

GTGCAGAATAGTTAACAGGAATAAGAATGTTTTGCTGATTGTTCTTTCCTTGAGT

>novel_circ_001584
CCTTAAGAAATTGTCCTTCATTAACATGGACACGGGCCCATGCAGCAGTTTTGAGGAA
AGTCTGGTAATCCTTAGCACCCAAAAGAATCCTATAGAGGAGGGAAACTTCTTTGCGA
AGTTGGCTGATTGAATTGCTAAAAGTTGTACCTTGAGGTTGAACTAAACCTGCTTTTAC
AGCACCTGCATAGTACATG

>novel_circ_001585
CCTTAAGAAATTGTCCTTCATTAACATGGACACGGGCCCATGCAGCAGTTTTGAGGAA
AGTCTGGTAATCCTTAGCACCCAAAAGAATCCTATAGAGGAGGGAAACTTCTTTGCGA
AGTTGGCTGATTGAATTGCTAAAAGTTGTACCTTGAGGTTGAACTAAACCTGCTTTTAC
AGCACCTGCATAGTACATGACAATG

>novel_circ_001586
TCAACTTCTACAAAAAATTTCCCAACCAATCCCAAATCAGGAACTTCAAAATCTTGGTG
CTAGTTATGACATTGAGAGTAATTCTCATCAGTATAAGAACCCAATCATTGTCATGTACT
ATGCAGGTGCTGTAAAAGCAGGTTTAGTTCAACCTCAAGGTACAACTTTTAGCAATTC
AATCAG

>novel_circ_001587
GTCATTCAACTTCTACAAAAAATTTCCCAACCAATCCCAAATCAGGAACTTCAAAATCT
TGGTGCTAGTTATGACATTGAGAGTAATTCTCATCAGTATAAGAACCCAATCATTGTCAT
GTACTATGCAGGTGCTGTAAAAGCAGGTTTAGTTCAACCTCAAGGTACAACTTTTAGCA
ATTCA

>novel_circ_001588
GATCTCCTTAACAAACAACAAGATGTCATTCAACTTCTACAAAAAATTTCCCAACCAAT
CCCAAATCAGGAACTTCAAAATCTTGGTGCTAGTTATGACATTGAGAGTAATTCTCATC
AGTATAAGAACCCAATCATTGTCATGTACTATGCAGGTGCTGTAAAAGCAGGTTTAGTT
CAACCTCAAG

>novel_circ_001589
GATCTCCTTAACAAACAACAAGATGTCATTCAACTTCTACAAAAAATTTCCCAACCAAT
CCCAAATCAGGAACTTCAAAATCTTGGTGCTAGTTATGACATTGAGAGTAATTCTCATC
AGTATAAGAACCCAATCATTGTCATGTACTATGCAGGTGCTGTAAAAGCAG

>novel_circ_001590
AAAAAAGAAAAAAAAACATGCCACGACGTGGACGTGCTGCAAATCCTCCACCAAGGA
CGTAAGTACTTTTTCTTCGAATTAGATTTATACGCATAATTTTTTGTAAACTATCTTTAATT
ATTCAATGACGTTGCAGATTTTACTTGACGATATTCATAATCAAATTATATGATCGCTTTT
TTTTTTATTTTTTTTATAATTATACATAATTTTATAATTGTGAAAAATAATTTTATTTTTTAG
GGTTAGACGAACATCGGCTCCAGTCGCAACTCCTGCACAACCTCCTGCACATGCTCCT
GCAGCAACACCAATGATGGCGCAACCTCAGCAGCCATCCCTCATGGGACAAATGGCTG

CTACTGCTGGAGGTGTCGCTATAGGTTCTGCTGTTGGTCATACTATTGGACATGCAGTA
ACTGGTCTTTTCAGTGGAGGATCTAGTGAATCTGCTGTTGCACCTGCAGCACCTGCAG
CACCTGCTGTAGCACAAAGTGCACCAGCTTCTGCTGCACCTATTGGTGGAGCTTGTGC
TTGGGAAGTAAAACAATTTTTGGAATGTGCTAATAATCAATCTGATCTTACACTGTGCG
AAGGATTCAATGAAGCTCTCCGTCAGTGCAAAGCAGCTAATAGTAAGTTTAAATTATTA
TTATTATTATATTACTTGTATTATATTAATGTATTATATATATTTTATGTACATTATGTTCTTTA
ATTTCAGATGTAGTTTAAACGAGCATCTTTCCAAAAACATTTGGATGATAGAAGAAATT
TAGGAGCTCAGCATCATGTGAATTAATTTAATAAATCTTATAAAAATTTGGATTTAATTTA
CAATAAATAAAAATTATGTTAAAATTATTATTAATATGAATTAGACAATATCTTTGTATGTA
TAGAAGTAAAATAAATTGACTTCTATTTACA

>novel_circ_001591
ATGTCACAGAATCGGATAAGGTGAAGTTCGAAGAATCAAGAGATTCGACGAGCGGATC
GATTGGGGCGGCCGGCGGTACCAACAAACGTGCTAAAAACAAGAAGAGACGCGAAA
CGCAACAAGAATTCACGCATTCTTGGGTGGTCGGTGCCCTCAAAGGGCACACGGGGC
CCGTCCTCGACATGAATTTTTCCAGTAATGGAAAATTCATCGCATCTTGTGCTGAAG

>novel_circ_001592
GGACCGTTCGTCTGCCTGAACAACGCCAGATACGGAATCGCTTGGGGCGCTTTGGGCG
CGGCCGAGGCCTGCCTGCACATAAGCAGAGCGTACAGTCTGCACAGGAAACAGTTCA
AAAGGCCGTTGGCCGCTAATCAATTGATACAGAAGAAGTTGGTCGAAATGATGTGCGA
CATCTCGTTTGGCCTTCAAGCTTGTCTGAGGGTGGGCAGGCTGAAGGACGAGAACAA

>novel_circ_001593
AAAGCGCACTTTTCTGGAAAAATTGATCGAACCTGGGCGAGCGATAAAGTTGGGAGTT
GGGTGCTCGATGATTGTATTTTAACAAGGAAATTGTCACGGCTCGGTGACCACGAGCC
ACCTGCTCCACCACTGATAAGGAACCTACGCGCGCGCGCACAGAGCCTGTCCTTCCTC
CTCCTTCCTTTTTCTTCGCATGTATATTCAATTACAATTCCGCAAAATGGGATGAATATCC
ATTAGATGGCTTATCAATTTTTTTTTTTTCCAACTGAGATAAACGATTCTTATCGCTCTTT
TTCCTCGTTGTACATAGAAATTTGACGTCTCAGACGTCTCCAACGCAGTTTCGCACCCC
CCGGTTCTCAATCAATCCCCTCGACGGATTCTTCATTTCTCCGTTTCACACCCCCCTGCG
CTCGCATCATCCCGCTGTTGCGTGGCGCGCAACCGTGTCCTTTTTTCCCCCCTTTTCGT
GCGCGGAACGACCGGATCGGAGCACAGTTGCGCCCCGACCTCGATCAGGACCGCTGT
CCGCTCTCCCCCTTTTGCAAAATTTCATCTCCCAGAGTGGAAAAAAAGGAAAAGGAGT
AAACAAACAAACTGTTCGTGAAGGTGAACGAAAAAGGAGATTGTTTCCAACGAGAGA
GAGAGATAGAGATAGAGATAAGATTTGGTAAAGCGGGAAGGAGAGTGGGAGGCAGGG
AGGGAGAGATATTCGCCATGACAACGGACGGAGTGACGGCGGAGGACGCGGGACGG
AAGAGATCGCGCGATCTCGAGCACGCGAGGAAGGAGCTCGCCTCGGTGATGGAGAGG
ATCGAGGGGACGGGCGGCCTGGCCGGCCGGCCCATCAAATATTTCGGGCGCGGCGGT
TACTCGCGCGCTCGGCGAGGCGCGCTCGGTCGAGCCGCCTCCTCCCACTCCTCCCAGC
GACGGCCGGCAAACACCTGTGGATCCTAGCCCCGATCCTCGCCTCGATCCTCGGCCGA
GCGGCGTGGAATTACGGGCAGGCCGCGCGCCACCACAAG

>novel_circ_001594

GTGAGAATCTTGGTTGTTTCGGTGGCAGCAAGAAGAAGAAGGACGGACAACCTGTGC
AAAAAGAGACGACCGCGTCGCGCACAACGACCAGTCCGAGAACCGAACCCGTCAGA
GGCATGGAGCTCCTCTGGCAACCGGAGGTCGTCCAATCCTATCTGAAGCTGCTGCAGA
CGTGCTCGAATCCTGAGACCCTCGAGGCGGCGGCAGGCGCGCTTCAGAACCTCGCAG
CCTGTTATTGGCAGCCCAGTATCGAGATTCGAGCCGCTGTGAGAAAGGAGAAAGGGCT
TCCCATTTTGGTGGAGCTGCTGCGGATGGAGGTGGACCGCGTGGTCTGCGCCGTGGCC
ACGGCTCTCAGGAATCTCGCGATAGATCAACGGAACAAGGAGCTGATAGGCAAGTACG
CGATGCGGGATCTTATTCAAAAGTTACCCTCGGGGAATAATCAGCACGACCAGGGAAC
CAGCGACGATACAATCGCCGCTGTCCTCGCCACGTTGAACGAGGTGATCAAGAAGAAC
GCCGAGTTCTCGCGATCTCTATTGGACGCTGGGGGTGTCGACAGACTGATGAACATCA
CCAGGCAACGCCAAAAGTACACGCCCCGTGTTCTTAAATTTGCAGGACAAGTCTTGTT
CACGATGTGGCAGCACCAAGAGTTGAGAGACGTTTACAAGAAGCACGGTTGGAAGGA
GCAAGATTTTGTAACGAAAACGGTGGCTGCGCGAAATTCAGGGCCTAATTCACCCAAC
AACGCGAATAGCTATGATTGTAGCACGTTGAATCGACCGATGGCGAGTCAAGGGAGCA
CCAGATACGAGGACAGAACGATCCAGAGAGCGAACATGAACTCGAATAACGTTGGTC
GACCTACCATTTATCAGCCT

>novel_circ_001595
ACGCGTTCTCCAGGAGAAGGGCCCCGACATGCCGCAATACACCGGACAGAGTGATGC
AGATTACCACGGAAGTAACGGCCAGGCGGATACCCATTCGTTGCATTCGTCTCATTTAT
CTGTCCAGGAGGATCCATTACTCATCCGGAGCCATAAGCAGCAAACTACTCAACAAGT
AACAAACGTCTCGAAGGTGGTGCGCGAGGTGTCACACATGGAGCCGGATCCGGGCGC
GGTGAGCTACATGTCGGTGCCGTTGATGTCCCAGGACTACCAGCACGCCGACCGACGG
TACCCGGCCGACTCCTACATGGTCGGTTTCGAGCATTACGAGCCCTACCTCGGCTATCC
GCCCCAACCAGGGTATCCAGGCCCTCATGGAATCTACATGCCACGCTCGCACTCCCCTC
ACAGCCCCCACAGTCCTTCGGAGCACAGTCGTGCCTCGCCTCCGCATGAATACCTGCG
CAAGGCGGCGCCGTACGTGGAAGGAGGTTACAACGACATCGATCCAGGTTTGAATCCC
GCGTTGCAAGATCATTATCGTATTACTCCTAGTCCTGGTGGCCCTGGAGATCAATATGAT
CAAATCAGCAACTCTTGGAATATGGATGACTCCGGCGAACCCTCTCAGCATCCTCATGA
CGAGAACAAAGTGGCCTACGGTTACGTGTCCCCGTCCCCGTACGGGCCGGTCGGATAC
GGCCCTGGCGTCGGCGTGGGTCCGGTCCAGGTTCCCAGCGACGTGCCCGGCTACGAC
GAGGGCCACCCGGTCCCGCTGCCCGCGGGCGTGCCGGCCGGCATGTTCGACGACGAG
GTCCACCTGCAACGGCTGCAGTCGCGCCACCCTGTCGTGGCCGGGATGGCGAGCCCG
TTGGACGACGACCAGAAGTCGATGAGATGGCGGGACCCGAATCTGTCGGAGGTGATC
GGCTTCCTCAGCAACCCGAACAACGTAATCAAGGCGAACGCCGCCGCTTACCTGCAAC
ACCTGTGCTACATGGACGACCCGAACAAGCAGAAGACGCGTAGCCTGGGCGGTATACC
GCCCCTGGTTCAGTTGCTCGACCACGACAATCCGGACGTGTACAGGAACGCGTGCGGC
GCGTTGAGGAACTTGTCGTACGGGAGGCAGAACGACGAGAACAAGAGGGCCATCAA
GAACGCGGGCGGTGTCCCCGCGTTGATCAATCTGCTTAGGAGAACGTCCGACGCGGAC
GTCAAGGAGCTGGTCACGGGGGTCCTGTGGAATTTGTCCTCGTGCGAGGACCTGAAG
AAATCCATCATCGACGACGGCGTGACGATGGTGGTGAACAACATAATAATCCCTCACA
GCGGCTGGGATCCGAGCTCCTCGAGCGGGGAGACGTGCTGGTCGACCGTGTTCAGAA
ACGCTTCCGGGGTGTTGAGAAACGTGTCGAGCGCCGGCGAATACGCGAGGAAGAAGC
TGCGAGAGTGCGACGGTTTGGTGGACGCCCTCCTCTACGTGGTCCGATCCGCCATCGA

GAAATCGAACATCGGGAACAAGATAGTGGAGAATTGCGTGTGCATCCTGCGAAACCTG
AGCTATCGTTGCCAGGAGGTGGAGGATCCGAATTACGACAAACACCCGATCCAATCGA
CGGTTCAGAACAGGGTAGCCGCGCCCGCCAAAG

>novel_circ_001596
ATTCGGGTCCCGCCATCTCATCGACTTCTGGTCGTCGTCCAACGGGCTCGCCATCCCGG
CCACGACAGGGTGGCGCGACTGCAGCCGTTGCAGGTGGACCTCGTCGTCGAACATGC
CGGCCGGCACGCCCGCGGGCAGCGGGACCGGGTGGCCCTCGTCGTAGCCGGGCACGT
CGCTGGGAACCTGGACCGGACCCACGCCGACGCCAGGGCCGTATCCGACCGGCCCGT
ACGGGGACGGGGACACGTAACC

>novel_circ_001597
AGGTGCTAAACGGTATCTGCAACGCGAGCAAGGAGGAGGAGAGGCGGCGAAAAGGT
GCAGGCTACCACGAACTATATAACGGTGTTGTCTGCAATGACACGATACTAGCGACAG
GTGTCTTCAGCTCGAAGGAGCTTTGGATGCTTTCAAAAGCGTCCATACTGCATGACCTC
AGCGACATGCAGCCTCAAATAAACGCGATCAAAATAGAGCACCCGCCGTACTACTGCA
GCAGCTACACCCCTCCGTCCGCAGCCACGGCCGCGGATCATGCTCAGCCAGCGGCTAG
CTTCA

>novel_circ_001598
AGGTGCTAAACGGTATCTGCAACGCGAGCAAGGAGGAGGAGAGGCGGCGAAAAGGT
GCAGGCTACCACGAACTATATAACGGTGTTGTCTGCAATGACACGATACTAGCGACAG
GTGTCTTCAGCTCGAAGGAGCTTTGGATGCTTTCAAAAG

>novel_circ_001599
AACGAGAAGCTGGAGGTGAAACAGAAATGGGCCTCGCGGCTTTTGGGCAAGTTGCGA
AAGGATATCACGCAGGAGTACAGAGAGGACTTCGTGCTGAGCATAACGGGGAAGAGG
CCGGCCATGTGCGTCCTTAGTAATCCCGATCAGAAGGGTAAAATGCGCCGTATCGACTG
CCTGCGTCAAGCCGATAAGGTATGGAGGTTGGACCTAGTAATGGTGATCCTCTTCAAGG
CAATTCCGTTGGAGTCGACGGACGGGGAACGGCTGGAGAAGACTGCGGAATGCGCTC
AGCCGGGCCTCTGCGTCAACCCCTATCACATCAACGTCTCCGTCCGGGAACTCGATCTC
TACCTCGCCAATTTTATCACCAGCCACG

>novel_circ_001600
CTCCTCCAGGCATGAATCCTATGAATCCAAGAATGAATCCTCCACGAGGACCAGGAAT
GGGTCCAATGGGACCAGGAAGTTACGGACCTGGAATGAGAGGGCCACCTCCTAATAGT
AGTTTAGGTCCAGGAGGTCCTGGAGGTCCTGGTATGCCACCAATGAGTATGGCTGGAC
CAGGTGGTAGACAACAGTGGCAACCTAATACATCTACGCCGATGAATTATTCGTCATCA
TCACCGGGTAATTATGGAGGACCACCTGGTTCAACAGGTCCTCCCGGTCCAGGTACAC
CAATTATGCCCAGCCCACAAGATAGTAGTAATAGCGGTGGTGAAAATATGTACACCATG
ATGAAACCTGTACCTGGAGGAAATATGCCTGGAGATTTTCCAATGAGTGGTGGACCAG
AAGGTGGTCCAATGGGTCCTATGGGTCCAAATACAATGGGTCCTGTTCTAAATGGTGAC
GGCCTTGATGGAATGAAAAATAGTCCAGCTAATGGTGGCCCTGGAACACCACGAGAAG
ATAGTGGAAGTGGAATGGGTGACTATAATCTGGGTGGTTTTGGAGGTCCTGGAGAAAA

T

>novel_circ_001601
GCTATTCGAGAGGGGAAGTGTTCAGCCGGCGAGAAACAAGAAGTAACGGGGAGCATA
GAAGTGGCGAGGAGGGAAAATAGGACGAGCAACGTCTTCTCCTCGAGTAGCCTCGAC
CATTCTTTCTCTTTCCAAGAGCGTAATTGCCAGTTTTCGAAATAAAATTTTACTAATATTC
CCCTCTATCGCTGCCAGCTCGCCGTTAATCGTCCATTTTTCGCCTCCGCCCCTCCATCGT
TTTTTAAAGGTCCTATAGATCCATCGATTGCTCTTTTCATCTTTTACACATCCCTATATTTA
TAATCTCAACTTTATTTATAAAACGTACTTTTAAAAATCCTTTAAAAAAATTTCTCTTTCA
ATTGCTCTTTTATCAGTCTGTCAAATCAATCGATCAATCAATTCATTTACAATACAAATTT
TTACAATTTTCTAGATAAATCGAAACATTTATACCAGAGAGAGAAAAAGAGGGAGAAA
GAAAGAATGCGTGTAACTTGTAAATATAACCGAACGAGATAATTTCTCTGGTTCAACGA
CAACACAGAAATTCTCCAATTTATATTCCGACATGGCCCTGTGCTTGCTCGACACATCA
CTGACCGAGGGGGTTGACTGGTCTGGTGGGGATAGAGCAGCTGGGGTGGCTGGAGCT
CGAATGCGAGCCCGCGGTACCTACACGCCGGGTTCTGGGTTTGGCCGGGTCGCGCGGC
GGGTCCTGCGCAACCAAAGAGACACACTCGGACAAAGAGAGAAAAGCACAGAGAGA
GAGAGAAGGAGGGAGGGAGGGAGGGAGAGAGAGGGAGAGCAAGCGTGACAGAAAA
AGAGCGTGGAAAGCAGAACGGCGAAGGCGAGGCGAGGCGAGGCGAGTAAAAGCGA
GCTAGTTTGGCGAGCAAGCATCTAGACAGTCGAGCAAATCTTCAAGCAGGCAAACGA
GACTGGCAGACAG

>novel_circ_001602
ATAAATCGAAACATTTATACCAGAGAGAGAAAAAGAGGGAGAAAGAAAGAATGCGTG
TAACTTGTAAATATAACCGAACGAGATAATTTCTCTGGTTCAACGACAACACAGAAATT
CTCCAATTTATATTCCGACATGGCCCTGTGCTTGCTCGACACATCACTGACCGAGGGGG
TTGACTGGTCTGGTGGGGATAGAGCAGCTGGGGTGGCTGGAGCTCGAATGCGAGCCC
GCGGTACCTACACGCCGGGTTCTGGGTTTGGCCGGGTCGCGCGGCGGGTCCTGCGCAA
CCAAAGAGACACACTCGGACAAAGAGAGAAAAGCACAGAGAGAGAGAGAAGGAGG
GAGGGAGGGAGGGAGAGAGAGGGAGAGCAAGCGTGACAGAAAAAGAGCGTGGAAA
GCAGAACGGCGAAGGCGAGGCGAGGCGAGGCGAGTAAAAGCGAGCTAGTTTGGCGA
GCAAGCATCTAGACAGTCGAGCAAATCTTCAAGCAGGCAAACGAGACTGGCAGACAG
GTAGGCAAGCAGGCAGGCAAGAGCGAGAGCACAACTACCGGGCTTCAGAGCCACCC
CTAGTCGCCTGCCCGCCACCCAAATCGCTCCCCCCACCTTGGATCCACCGCTTGGTACA
CCCCTCCTCGACGTTGCTTCTTCGCACTACTCCTACTCTTTCTCCGGTGCTTTCAGCCGC
CTCGTTCGTTCGTACGGTCGTTCGTCGGCCGCTTCGCTCTCCTCTCCTCGGTGCCCTCG
TCATTCAATCGTGCTCAGAGAGTGTATATCAGTATATCTTTGGGCCTTTAGTTTCGGTCT
TGTTTTTTTTTTTTTTTCCATCGACAAATCGTGTGTGTCGGTTTGGTTCCCGTTGCCGCT
TCTTGTGCAAAAGGATATCTCGTGCGTTTTTTGGAAGCAAAGCGTGGCAGCTTGCAAT
CCTCTGCCAGCAACGCACTGACCTGCCTTTCGTCCCGGCATCACGACACATTCTTTCTT
CTCGTCTCTCCTCGAGCTCTCCTCGTCTCTCCTCGTCTCTCCTCGAGCTCTCCTCGTTTC
CTCGCGCGAGCGAAGGCCGATTCGGTGGGGAAGTAGAGAGGGGGCTCGTATATACAA
CGCGAGAAACCCGATACCCTCCTTGGTGGACGTTTACCGAATTGATTCGACGGTATTGG
TCAACTTTAGAAGGAAGGAAAAAATTCTGAAGGGGAGTCGTTTTATTATATTGTGTTTT
CGGAAGGAGGATGCACAGATGGAATTTTTGGTGAAGGAAAGTGAGGAAGAATATCTG

CGTGACGACAAGTGATGGCGAGATGAGGAGGCGCGACGTCGCAATAGAAGGGAAGAC
GCGGCTGTGAAGCCCGGATAAGCGTGCACGTTGTGTGTCGAGGGACGTTCACACGCG
CGACTCCATCGGTTGCCAAGCCGATTCTCCTCGCACCAGGCGACGAGAACTTCGTACC
TAAGCCGGTCGTTCAACGTGGCTGATCCTTGGACAT

>novel_circ_001605
GTAAAAGATAAAGGAAAAGATATGAAAACGGTATATAAAGGATTAAAATGGAAAGGAC
GATCAAATAGAAGCGATACAAGTTTGAGATTCCATCAACCAAGATCAGCACCTAGTTCA
CCTACATCGGATCGAAGGCCTATCGATTTTTCATCACCTCCAAAATCTCCAAATGGTTTT
ACTGCTACTACTAGTTATAGAAAAGATCTTCGAATACGTAATAGTAATTTCACCGATTCA
AGCAGAAAACAATATTCACCATTAAGTCCTAGTTCACCAGAAGAATCTGATTTTCCACC
AGAAAGAGTAAATATTGATTTGATGCAAGAACTCCGTCATGTAATATTTCCGAACACAC
CTCCTGTTGATAGAACA

>novel_circ_001606
GTGTTCGGTGGCATCGAAGTTCATCGTAGGACAAATATTGCGATGTATATGTGCATATAT
AACTTGTATACCGAAGGATGTGTACGAGTTTAAGCAGAAGATACAAAGGGGACCAGAA
CGGCGAGGAGACGAGAAATCGTGGATGGGCGAGTGGTCGAGAGGGCAGAGGGGTGA
CGATGAGAGTGAGGGGAAAAAGGACTCGGAACTCGTGTCGTAGCGGTACGACGTTCG
GTCGGGTATCTCCGCATCGCGAGCCACACGCTCGGCCGGTGTCGGCTCGCATACGCGC
TCAGTCGTGCACTGGCCTCGCTTGCGAACTAACGTCGGGCGAGAGCTATTCGATACGT
ACAACACGAAATACGTTCACAGTGTTTTCTTCTTCTTTCATGCATACGTATGTGCGTAAG
TGTGTGTACACAACCGGCAAAAGATATACATGTACATTTATATACGTGTATCGTATATAAC
ACACGCAACTTTCCTTCCTATTTCGTCCGTCAATCGTCTCACGTAGAAAGGGTTAATGT
GTCGTGTACGAGTATACCGAGTGCAATCATCAGTAGTGATCTATCGAAACGTTATGTTAT
GACTGACTATCTATATATACATATATACATATACATATATACTAATAGTGTAGTGACAGCAA
TGACGAAGGGACGCCAAGTCTAACGTGCTCCGATTAACTTCGTTCCGTTAGTCAGCCG
AGTGTGTGGTAAAGTTGAGAGTGCCTGCGAGAGTAGGAAGGAAGAACAAAGAAGCG
AGTCCTTTGCCAGTCGGTGGAAGTGAGTGAGGTTTGCGAAGACTGTCTCCTCTTGAAG
TCTCTCTTCTCTGTCTCTTCACTCCTTTCTTTGTGGATCTCAGTGTTCCCTTCAGCTCTTT
CTCTCGCTTTCTTCATCTCTACCATCGACATCGTGTGAACAGCAACGACAAGTTGGTTT
CGTCTCGAG

>novel_circ_001607
CATAGGTGTTCGGTGGCATCGAAGTTCATCGTAGGACAAATATTGCGATGTATATGTGCA
TATATAACTTGTATACCGAAGGATGTGTACGAGTTTAAGCAGAAGATACAAAGGGGACC
AGAACGGCGAGGAGACGAGAAATCGTGGATGGGCGAGTGGTCGAGAGGGCAGAGGG
GTGACGATGAGAGTGAGGGGAAAAAGGACTCGGAACTCGTGTCGTAGCGGTACGACG
TTCGGTCGGGTATCTCCGCATCGCGAGCCACACGCTCGGCCGGTGTCGGCTCGCATAC
GCGCTCAGTCGTGCACTGGCCTCGCTTGCGAACTAACGTCGGGCGAGAGCTATTCGAT
ACGTACAACACGAAATACGTTCACAGTGTTTTCTTCTTCTTTCATGCATACGTATGTGCG
TAAGTGTGTGTACACAACCGGCAAAAGATATACATGTACATTTATATACGTGTATCGTAT
ATAACACACGCAACTTTCCTTCCTATTTCGTCCGTCAATCGTCTCACGTAGAAAGGGTT
AATGTGTCGTGTACGAGTATACCGAGTGCAATCATCAGTAGTGATCTATCGAAACGTTAT

GTTATGACTGACTATCTATATATACATATATACATATACATATATACTAATAGTGTAGTGAC
AGCAATGACGAAGGGACGCCAAGTCTAACGTGCTCCGATTAACTTCGTTCCGTTAGTC
AGCCGAGTGTGTGGTAAAGTTGAGAGTGCCTGCGAGAGTAGGAAGGAAGAACAAAG
AAGCGAGTCCTTTGCCAGTCGGTGGAAGTGAGTGAGGTTTGCGAAGACTGTCTCCTCT
TGAAGTCTCTCTTCTCTGTCTCTTCACTCCTTTCTTTGTGGATCTCAGTGTTCCCTTCAG
CTCTTTCTCTCGCTTTCTTCATCTCTACCATCGACATCGTGTGAACAGCAACGACAAGT
TGGTTTCGTCTCGAG

>novel_circ_001608
TGACAAAGAAGATACTTCACACAATATGCCACCAGATAGACCAGATGCATCTGATGTGG
AAACTGGTATAGGACTAAGTACACAAGTTTTCAACTTTGGAGATACAGTATATACAATG
AAAGAAAAAGAAAATGCAAGTGTTTTTGTTGAAACAAATTCAACTGATGAACTTTCTA
TGTCTGAAAAATCTATTGAAAAGAATAGCCAGTCAGATGATGATCTGACTTCTTTAAGT
TGGCTTCATCAACAGAATCTATTAAAGGGTTTAGATATTTCAAATCCTACTAAAGAAATG
AAAAATGAAAATGTTTTAAATAATAATGTTTGTGATGATATGGCAGACTATTCTGAAAAT
ACTAATTCTATATCAAGTTTAGATGATGGTTATTGTCCAGGAGATAACAATAGCAAGATA
AATAATACAACATCACATGGTAATAATCAAAACTATCAAGTCTCAAGTAAAAATGGTCA
AAAATCAATGCAAATATTTCAGGAATCAGTAAAAAATCATTATAGTATTTCACAAAATAA
TCAGACGAAGATTTCTTTAAACAACAATAATTTGCCAGTCTCAAATCGCAACAAACAC
CCAACTCATATACCATATGATCCTCATTTACATAGAAACAGTAAACCTCCATATTCATTTT
CTTGTTTAATTTTCATGGCTATTGAAGATAGCCCTGTAAAAGCTCTACCAGTCAAGGAA
GTGTATGCTTGGATTTTGGATCACTTTCCATATTTTAGGAATGCTCCAACTGGGTGGAAA
AATTCTGTTAGACATAATCTAAGCCTAAATAAGTGCTTTCGTAAAGTAGAGAAAGCACC
AAATTTGGGCAAAGGTTCACTGTGGATGGTAGATGCCCAATATCGTCCAAACCTGATAC
AAGCGTTATCTCGTGCTCCTTTTCCTCCTCCAACTGTTCAAACTTTGTCTTCACCCGAA
AAACCACAAAAAAAGAATATCAGTACACGTCTACCTGATCCTACACTTTTTCCATATCT
TTCCAAAAGACTGGCATCGAGCAACATTATTGACAACACAGATACTGAAGTAGATAGC
GATGTAGATGCAGCTGCAGCTGCTATGCTTTCTTTTAAACATGGACCTATTATTTTAAAT
CATAATAAAGATCGTAAGCGAAAAGTACCAGAATCAGAAATGTTGGTTCCTGTAATTAC
TAGGAGTTCTAGTGAAGATCACACTTACAGTTGTATTACCTCAGTAAGACAAGAAAG

>novel_circ_001609
TGACAAAGAAGATACTTCACACAATATGCCACCAGATAGACCAGATGCATCTGATGTGG
AAACTGGTATAGGACTAAGTACACAAGTTTTCAACTTTGGAGATACAGTATATACAATG
AAAGAAAAAGAAAATGCAAGTGTTTTTGTTGAAACAAATTCAACTGATGAACTTTCTA
TGTCTGAAAAATCTATTGAAAAGAATAGCCAGTCAGATGATGATCTGACTTCTTTAAGT
TGGCTTCATCAACAGAATCTATTAAAGGGTTTAGATATTTCAAATCCTACTAAAGAAATG
AAAAATGAAAATGTTTTAAATAATAATGTTTGTGATGATATGGCAGACTATTCTGAAAAT
ACTAATTCTATATCAAGTTTAGATGATGGTTATTGTCCAGGAGATAACAATAGCAAGATA
AATAATACAACATCACATGGTAATAATCAAAACTATCAAGTCTCAAGTAAAAATGGTCA
AAAATCAATGCAAATATTTCAGGAATCAGTAAAAAATCATTATAGTATTTCACAAAATAA
TCAGACGAAGATTTCTTTAAACAACAATAATTTGCCAGTCTCAAATCGCAACAAACAC
CCAACTCATATACCATATGATCCTCATTTACATAGAAACAGTAAACCTCCATATTCATTTT
CTTGTTTAATTTTCATGGCTATTGAAGATAGCCCTGTAAAAGCTCTACCAGTCAAGGAA

GTGTATGCTTGGATTTTGGATCACTTTCCATATTTAGGAATGCTCCAACTGGGTGGAAA
AATTCTGTTAGACATAATCTAAGCCTAAATAAGTGCTTTCGTAAAGTAGAGAAAGCACC
AAATTTGGGCAAAGGTTCACTGTGGATGGTAGATGCCCAATATCGTCCAAACCTGATAC
AAGCGTTATCTCGTGCTCCTTTTCCTCCTCCAACTGTTCAAACTTTGTCTTCACCCGAA
AAACCACAAAAAAAGAATATCAGTACACGTCTACCTGATCCTACACTTTTTCCATATCT
TTCCAAAAGACTGGCATCGAGCAACATTATTGACAACACAGATACTGAAGTAGATAGC
GATGTAGATGCAGCTGCAGCTGCTATGCTTTCTTTTAAACATGGACCTATTATTTTAAAT
CATAATAAAGATCGTAAGCGAAAAGTACCAGAATCAGAAATGTTGGTTCCTGTAATTAC
TAGGAGTTCTAGTGAAGATCACACTTACAGTTGTATTACCTCAGTAAGACAAGAAAGTA
AATATCCAAGGAAAGAAACAAATACCGATTTTGATGAACAACGGAAAATAGCAGAAGG
TGCAGATGCACTTTTAAATTTAGCAGGTGTTACTACACCACTTAACCACAGTAGGGCAC
ATCATATACAAAGTATTAATTCGACATCAAAATCCGAGAGTTCATCAAAACTAAAAAAA
CGTTCAGCTTCGAACGATTATTCGAATATGTCTGAGAAAAGACGTAAGCATTGGCCAAA
ATGGAGTGAAGGAAAACG

>novel_circ_001610
TCGTGTCACTTGTGTGACTACACGCAACTGACTAACTGACTACACACAGTCCAATTAAT
AAGTGCAAGATGGCAAGCGAGGTAAATGAAATTGCTGCAGAAGCGAGTGAAAATAAT
GAAGCGATTACGGGAAGAATATCTTCGACATCTGAGGGAATGGCTCTTGCTTATGGAAG
CTTGATAATTATGGCTATTTTACCTATCTTCTTTGGTAGTTACCAAGCTGTTAAACACCAT
AAGGAGCAGCAGCAACAATGTAAAAAAAGTGGAGAACAGCCAGATACAATGTCTCGC
AAAGAAGCAGCAATATTTCCTTTTATTTCCAGTATTACATTAGTTGGACTGTATGTATTAT
ATAAG

>novel_circ_001611
CATCATCGAGGGCTGAGCTATCGAAAAATATGGCGTCAGGAATACCGAGCGTTCATCAG
AGGAAGCTTGGACCAGCGCCGATAGCATCCTTCGAAGACCTGTCCGACGAGGTGGAA
AACGGGGAACCGGCTGCGCGAATGCCAGGCATCGTCGAGGTCACCACGCCGGCAACG
CCTGGTAGTTCGCTTAAGACACCCAGCACGCCGAGAATTGACATCAGCAGGGCGAGC
AGTTCCTCCCACCATGAGGACAGTAACTCCAGGGATTCCACGCCCGAGAGGGAACTCC
TCGCAG

>novel_circ_001612
AATGCAGTAAACATGCCGTCATTGTTGGAGGCGCTGGAGCTTAAGTACGGCAGTTCAA
CGACCGACTGCTCCCTCACGGACGAGGAGACCGAGTCGAGCTCGCCGAAGGCCGCCC
TCTCGGTCAGCATCTTCATCCCGAAGAAGTCGCCCCGGCACACCGTGCCGGCGTTGCT
CGTGCTGCAGGACTGCGACATCGAGTCGGCCGGGAACGACGCGGAGAAACTGAGCA
AGAAATGCAGGAACGTGGAGGAGCTCGATCTCGCGCAAAACAAACTGTCCCAATGGA
CCGAGGTGTTCGGTATTCTGCAGCACATGCCCAAGATCAAATTCGTGAATCTCAGCTTC
AATTGTCTGGCCGAGGTTCTCGAGATCAAGCACGGTAGCTACGACATGTTGAAGAATC
TCGTGTTGAACGGAACCAGGGTCACCTGGTCAACGGTCCAGGGATTGATACGTCTGCT
TCGTAATCTCGAGGAGCTTCATCTTTCGTTGAACGAGTACAAGACGGTCGACCTGGAC
TATCAATTGCCCGAGAACAAGAACGTGTCCGTGAAAAAGCTCCATTTCACCGGGAACC
CCGTCGAGGTGTGGAACGAAATTTCCAAGCTTGGCTACGTATTCCCCAATCTCGAGAG

CCTAGTGCTAGCCGAGTGCCCGATAAGGTCGTTGGCTCTCGTCGACAATAGAAACTCG
AACGAGGAGCCCTGCACGAAGAAGGGGGCGGGGAAGCTGAGCGAGGACAACGAGAA
TATGAACAGTCTGAGGGGGGAGGATGAGGAGGAGGAGGAGGAGGAGGACGAGGAGG
AGGGGACATCGGTGGTGGACGAGAAGGGGAACAGGATATTCAGCGAGGGAAAATTGA
ATTACGATAGATCGGAGTCGGAATCGGAATCTAGTGGCACTACCATCAGGTCGTCTCAC
GATCCTTTCAGAAAGCTAAGGTTTCTGAACGTGAACGGCACCCTGTTGTCCACGTGGG
ACGATGTCGAGCGACTTGCCAGGTTTCCAGCGCTTAAATCGTTGAGGATTCAGGGCTG
CCCCCTTTTCGAGAGTCCTCGAGAATACACCGAGCACGAGAGGCGGCAACTGTTAATC
GCCAGACTTCCGAATGTCGAGACATTGAACGGCGGCGGTGTGATATCCTCCCAGGAGC
GCGAGGATGCCGAAAGGGCCTTCATTCGATATTACATGGACAAACCGGAAGCGGACAG
GCCGGAAAGGTACTCCGAGCTGGTGGCTATTCACGGAAAGCTGGATCCTCTGGTACAC
GTCGACCTGACACCTGAGAAGAGGGTCAAGGTCACATTCACTTACGGAGACCTAGTC
GAG

>novel_circ_001613
AGCCGCAATTCGCGCTGGGACTGCAAAACGGATCCTGGAGAAGATCGTTCCTGGTGG
GCGTTCTAGTGCTGGTTTTGCTGATCTCGGCCGGCGCGTTCTACGCTCGCTGCTGGCTG
CCACTGGTTCTGCTCTGCCGCGACAAATTCGCCCCTCTCGAGGAGAACGATGGAAAGG
AAAGCGACGCCCTGGTCTGCTACCACGAGAAAGACTCGAACCTGGCCATCGGCATAGT
TATACCAACGCTGGAGTCGAGGCATCGGTACAAGTGCACGTCGCTCGAGCTCTCCCAA
CTGAATCAGAATT

>novel_circ_001614
GGGAGAAATCGAGCGGATACACGGGGAAGCCGCTGCCAACGTACAGGCCAGCCTCGC
AGTTGGTTCCGCTGGGAGGAGCTGCGAGATTGTTTTGCGAGGCGTATCTCGGCAAGGT
GGAGCTGCCGGACGCGAAAAATTCGGTGACATGGTCGAAGAGCGACAGCAACGTGAC
GTTGCCGAGTCACGGAAGGATCGCCCAGCACAAAGTGTCCAGGGAAAACAATCAGAT
CATTGGCTCTTACTTGGAGATCGAGGACATCACTCTGGAAGATTACGGGGAGTACAAAT
GCGAAGTTAGCAACGGAGTAGACGAGGAGATCACGTTACCAGCTCACGTTTATCGACA
AGAGCCGCAATTCGCGCTGGGACTGCAAAACGGATCCTGGAGAAGATCGTTCCTGGT
GGGCGTTCTAGTGCTGGTTTTGCTGATCTCGGCCGGCGCGTTCTACGCTCGCTGCTGGC
TGCCACTGGTTCTGCTCTGCCGCGACAAATTCGCCCCTCTCGAGGAGAACGATGGAAA
GGAAAGCGACGCCCTGGTCTGCTACCACGAGAAAGACTCGAACCTGGCCATCGGCAT
AGTTATACCAACGCTGGAGTCGAGGCATCGGTACAAGTGCACGTCGCTCGAGCTCTCC
CAACTGAATCAGAATT

>novel_circ_001615
GGAAAACAATCAGATCATTGGCTCTTACTTGGAGATCGAGGACATCACTCTGGAAGATT
ACGGGGAGTACAAATGCGAAGTTAGCAACGGAGTAGACGAGGAGATCACGTTACCAG
CTCACGTTTATCGACAAGAGCCGCAATTCGCGCTGGGACTGCAAAACGGATCCTGGAG
AAGATCGTTCCTGGTGGGCGTTCTAGTGCTGGTTTTGCTGATCTCGGCCGGCGCGTTCT
ACGCTCGCTGCTGGCTGCCACTGGTTCTGCTCTGCCGCGACAAATTCGCCCCTCTCGA
GGAGAACG

>novel_circ_001616
ATATGCGAAATACAGCAATGCCTGATGAACGAGCTGTGATGACCTACGTTTCTTCCTAC
TACCACTGTTTTAGCGGTGCGCAAAAGGCAGAGACAGCGGCGAATAGAATATGCAAAG
TTCTGAAAGTGAATCAAGAGAACGAAAGGCTGATGGAAGAGTATGAACGTCTGGCAT
CTGATTTGTTGGAGTGGATAAGGCGAACAATGCCTTGGTTGGCTAGTCGACAAACAGA
TAACTCCCTTGCTGGCTGTCAAAAGAAATTAGAAGAATATAGAACGTATCGTCGTAAGC
ACAAACCACCCCGGGTCGAGCAGAAAGCTAAGCTGGAAACAAATTTCAATACTTTGC
AAACGAAATTGAGATTGAGCAATCGACCTGCTTACATGCCAACAGAAGGAAAAATGGT
ATCCGATATAAACAAGGCTTGGAAAGGTTTGGAGTTGGCAGAGAAATCATTTGAAGAG
TGGCTGTTGTCGGAAATGATGCGTCTAGAACGCTTGGAACATTTGGCTCAGAAATTTAA
GCACAAAGCTGATGCGCATGAAGAATGGACAGCAGGAAAGGAGGAGATGCTTACCTC
GCAACACTTCCGCCAATGTAAGCTTAACGAGCTCAAAGCTTTGAAGAAAAAGCACGA
AGCTTTCGAATCGGATCTTGCTGCTCATCAGGATCGGGTAGAACAGATAGCCGCTATCG
CTCAAGAGCTCAA

>novel_circ_001617
GTGAATTCCTCGAAGAATACGGTAACATCTCGTACACCAGGAAATTTGTCGTCGACCA
GATTGAGAAAACCCACTGCATTCACTTTGACGCCATTGCAAAGCAAAACTACACAGCA
AAACACATTCGCGAATCATCGAAAGACTCATCTGACCGATGGAAAATTACAAACCGTT
GTTAACGAAACATTGGATACCGTGGATGAAAGAACGGAAACTTCGAAACCTGAGAAA
GATATCGATCAAAATAGTAGAGCGCAAGAAATCGCGGTCACATTGGCGGATCCACCGT
CCGTGCAATCATCAACCACCG

>novel_circ_001619
GTTTGCTTTCCTAAGGGAAGAGGAGGGGCGAAGAAGGATGTCGAACGACGAGGAGA
AACCGTTCGCGGTGGAGAGGGCGAAAAGTGGGCGGGCCAAATGCAAGAAGTGCAAG
TGTCCAATAGAGAAGGACACGGTGAGGATCGCCAAATTGGTCGCGAATCCGTTCACGG
ATGGCAAGATGAAAGCGTGGCATCACCTGACCTGCCTATTCGAGGTATTCGCTAAACA
GAGAGCCAGTACCAAGCGAATAGACGATCCCGCGGAGGACGTGAGCGGGTGGGAAGA
TCTGAGCAAGGAGGACAGAGCGATCGTGCTTCGTATGATCGAAGAGTTCGAAAAGGAT
TCGAAAAATCACACTCCTAAGGGGAAAGCAGTGGAAGCGAGAAAGCGCAAAAGTGA
CATTGGCACGAAATCCGATCCTTCTCCGAGCAAAAAAACTAAGGAGAAAGAGAAGGA
GAATGAGAAGAAGAAGAAGAAGAACGATTCGATATCCTCGAGCGAGAAGGAAGAAA
CGGATAGCTCCTCGGCGACCAGGAGCCCAGAGAAAAGAGCGACGAGAGACGATTCGT
TCAAGGAATTTCAACGGGTTTGCAACGATGTGGCAAAAGTGGACGCGTATACCGAAAA
AACGGCCGTCATGAAAAGCTTGTTCAGCAAAGGATCCGAAGCTG

>novel_circ_001620
CGATAAAAACAGGACCGAGGGAATGACGGGAAGGTCAAGCGCGAAACGTAGGACAG
CCCCGTGTCTGTTGGCACTGTTGTTGTTGTTGTTCTTCCATCTCTCACCGTCGTCCGTCG
GCGGGGAACCGATACCGTCCGAGTTCTACGGCAAAACCTCGGACAACGATTTCATATT
GCTCAACAAGCTGAAGGAGTTGTTCGAGGAGAAACAGTACGTGGCAGAACGGGAGA
AAGAGTTGGACAAGGAACGTATGAAGATCCAGTCGATAATAATGGAGGGGAAGGAATC
GGAGATACAATCGGACTCGGATTACTACGCTGAGAAATTACCGACACCGAATGCCGTG

GTCAGACAAGAGCCGCAGCATTCCGGCAAACGTACCATGATCAGCT

>novel_circ_001622
GAAGATCGACAAGTGGAGCGCGCGATGGCGGACGATCAAGAAGCAAACAACACGTG
CGAGGATTCCCTCGGGCCCGGTGATGCAAACACCGCATTCGCTAAGAAAATCCGAGGA
CGAAAAGGAGCCCTGAAGAAAAAGAATGTCTACGTGGTCAAGAATCACAGTTTCATG
CCTCGTTTCTTCAAACAACCAACGTTCTGCAGTCATTGCAAGGACTTTATATGTTTCAG
GGGCTTCGGGAAGCAAGGATACCAATGTCAAGTCTGCAGTTTCGTTGTACACAAACGA
TGCCACGAGTACGTGACCTTCACGTGCCCCGGTGCTGACAAAGGAGCTGATTCCGACG
ACACGCGGACGAAGCACCAATTCAAGCAACACACCTACAACAGCCCCACCTTCTGTG
ACCACTGCGGTAGTCTTCTCTATGGTGTCATCCACCAGGGCATGAAGTGCCAAGATGA
GCACCGCACACAAATGGGAGGTTTTCACGTACCTGACGCCGACCTTCTGCGACCACTG
CGGCTCGATGCTTCACGGTATCGCGCATCAGGGACTTAAATGTTCAG

>novel_circ_001623
GTGTTCGCGGCTAAGTGTCGCGAGTTCGGCACCGTTTCCAACAGTAGCTGCGAGAGCG
ACGCGACGCGTCACACTTGGCCGGCCGTTCGCTTTCCTTCGCAACCACCTTCGCAACC
ACCTCCGTGGGGAAATTCGATCGAGTTTCTCGAGGACGACGATTTAGATAGATCCAAAT
GTCCAAGAACGGGATCCCCGTGGACCGCAAGTCCGATCTCAAGAAACCTCCGGAGGT
GTTCGAGACCCAAGTTCCCGCGCCATCCGGCTCGCTTCTGATCACGGGATCGTTGTTAA
TCATCGTTATCGGTGTACCACGAAACCGTCAACCGATTCATAGCGACGAGAAACCGATA
TTATTGTCGCACAGTGAGTGTTGGGTAGCGCCCGAACAAGTATTTTGGAAGAATCATCG
CGGAAGTATCATCTTTGTCGCCGTCGTCTTCATTGTCGTCGTCATCGTGCAAACTAAGA
GCCTGGTGTTTGCCGAAAGATTGCGCGCAACAGTGTTGATTTTCCCCAACTACCAATAA
GAAGAACATAGCGAACCACTCTATATCTCTGTGTTGAAAGCGGCACGCAAGTATACACT
TATACACACAGTGTCTTCTCTTCGCTTCGCGACAACCTACTCGAGGCAGGGGATCCTCC
AATCGGAAATTGCAGAAACTTGGTATTTCCTTCTACATTTTCATTTAGCGTTCGTATTCG
TAGCGATTCACGAATAACGAACTAACGGATATATCGTTTCCGTATCGTGTCGCGTGTGC
GTAGAGCGCGTGTCAAGCGTGCAATGAACGGCAACGGCAGACCTCATGGAGAGAATA
GAGGACGAAATGGCCACGTGCTTGTGTGGGAACAACCTGGCCTGGAGGAAGAGGAAT
CACCTGCGCGAATGCGTAAAAACCTCGAAATTCCGCAACTTTCTGTCCGAGTTTCTGCT
TATCTCCACTCTCGAGGTCGTATATATCTCTGTCATAGATCGAAACTAGTTTACGAAACT
TGATGTATCCTATAAACCACGCGTCGAACCACGTGCTCCCGCCGTCATTCTTCGCTCTCT
TCCTCATTTACGCCGAGCATTGGTGGGGTCGCGTCACATAGTGACTCTGATGCTGTTCT
TCGGTATGGCGAATGCCTATGTGATGAGAACTAATATGTCGGTGGCGATCGTCGCTATG
GTCAATCACACTTCCATCACGGAGAGCAAGGAACCTGTGGTGAACGAGTGCGGCCAC
GATGGGAATGCGAGCAACGATTCTTCCACCTTGGCCAACCATGACGGAGAGTTTCATT
GGGACAGCACCATGCAGGGTTACTTGCTCAGCTCGTTCTTCTACGGTTACGTCATCACG
CAAATACCATTCGGAATACTCGCGAAACGTTACGGATCGAAATACTTTCTTGGCATCGG
TATGCTGATCAATTCGATGTTTGGACTGCTGGTCCCTGTGTCCGCACGTGCCGGTTACC
ATTGGCTGATGATCGTTCGATTTATTCAAGGTTTGGGCGAG

>novel_circ_001624
CATTGGTGGGGTCGCGTCACATAGTGACTCTGATGCTGTTCTTCGGTATGGCGAATGCC

TATGTGATGAGAACTAATATGTCGGTGGCGATCGTCGCTATGGTCAATCACACTTCCATC
ACGGAGAGCAAGGAACCTGTGGTGAACGAGTGCGGCCACGATGGGAATGCGAGCAA
CGATTCTTCCACCTTGGCCAACCATGACGGAGAGTTTCATTGGGACAGCACCATGCAG
GGTTACTTGCTCAGCTCGTTCTTCTACGGTTACGTCATCACGCAAATACCATTCGGAATA
CTCGCGAAACGTTACGGATCGAAATACTTTCTTGGCATCGGTATGCTGATCAATTCGAT
GTTTGGACTGCTGGTCCCTGTGTCCGCACGTGCCGGTTACCATTGGCTGATGATCGTTC
GATTTATTCAAGGTTTGGGCGAG

>novel_circ_001625
AACCATGACGGAGAGTTTCATTGGGACAGCACCATGCAGGGTTACTTGCTCAGCTCGT
TCTTCTACGGTTACGTCATCACGCAAATACCATTCGGAATACTCGCGAAACGTTACGGA
TCGAAATACTTTCTTGGCATCGGTATGCTGATCAATTCGATGTTTGGACTGCTGGTCCCT
GTGTCCGCACGTGCCGGTTACCATTGGCTGATGATCGTTCGATTTATTCAAGGTTTGGG
CGAGGGTCCTATTGTGCCTTGCACACACGCGATGCTGGCCAAGTGGATACCGCCTAAC
GAACGTAGTAGAATGGGAGCCTTCGTATACGCTG

>novel_circ_001626
GAAACGCATGCGACCAAAAGAGAAGAAAGTTACAACGTTGCAGCGTTGACGGTAAAT
GCAGGGTTCGGTGAATGTTGGAGGAAACAGAATGTCCAGCGAGCAAGCGCGAGCAAG
CGCCATCGCGACGAAAGCGCCGCTCGGCTGATCGATACATTCGAGTTTTAGGGTGGTG
CGACGGAGCGAGGAAGAGAACGTAGGAAATGGTTTAAGAAGGGGTGACCTTGTCTCC
TATTTTCGGAAGGTAGCTTTCTCGTGATTCCCGATAATACTCTCTTTCTCTCTCTCTTTTT
CTCTCTTATAGATTTGTATTCTAAGAGATACGGGGATCGTCGAAGCCGCATTATGAACAA
CGAAAGAAACTGAAAGATAATAGGGAAAGAAAAGACGCGGTAATAACGAGAAAAGA
AACGTGGATATCGATATCGAATTTAAATGAAAAAAGTTTGATCGATTGAAAACGATCGT
TCATTAGAGAAACCACCTGCAACTGATTTTCAGAGGTCTCAACGTTTCACTCTGTTTAT
TGCTTTTTCTTTTCTTGTATTGCCTCATTTTTTCAAAAGAAAAAAAAAAAAATTGTATTC
TGTAATCTGGGAATAAAACGCATCGGCCGAATACGTTATTAGCGATATTATCGCGATTTT
TAATCGCGGATGTACGTATATAAAATTGAAAACTCGGCATTTGTTGTTATCGTTTTTCTTT
TTTCTTGACGAATAAATTACTCGCTGTGTTTAAAATAGTTTGTAATACCGAACAATACCG
ATTCGGTAACGTAAAACCGAAACCTGTTTTTATCGAACATCAAACGAGAAAATAAAA
GGAAAATCAAATTTGCGATAAAGATAAAACGCGTCGGGCAAAAAAATTCGCGTTTATT
CGCCTGCGCGATTTTCCGAGCGAAAAGAGAACGAGAGGTAAAATGGGATGAAAAGAA
CAGAAGAGACAAAGAAAAGAATGGCAACTGGGCCTAGTACTAGACGGCGCTATGCGA
CGCGACGAAAACAATCGAGCAACGCAGACGAGTCCAAATTTTAACGGTACGTGGTAG
TCGGTGGTAAAACGAAGGAACGTGGAAAGGACGAAGAGAGACGATCGATGTAGCAG
CGGAGAGGGGAGGAGCGCGCAAAGGCAGCAGGAAGACGGGCAGAAGGGCAACGAT
TGCGAATAGGAGGATGCGGTGAAGAAGCGACGGCTGTTGCTAGAGCGGTAGAGAAAC
TGGTGGGTGGCGGTGAGGAGACGAACGTAGCGAAGCAGTTTTACAATTACGTAGCCG
GCTCGCGCTCAGGCGAAAAGTACAAATGTGCTTCGAACGCAACAATACCGGGTCTCTA
TAAATAACCGAGATTCGCGAGTCTCACGAGGCAGGGCGCTGTCATCGTGCGCGCGCGA
CACCCCCCCAGAGAATGCGAGAGAATCGCGAAAGAAACAGTAGCGCGCATCGCGCGC
GCAGTTCTCTTTCGTTATCCGTTACGTTTTCGCGCGCGCGTGCCCATGTTCCAACGAATT
CGCAATTTATACCTTTATATTTGCGAGTTGTTCGGACGACTCTACTGTTATAATTCGTTT

ATCGTTGACGTGCCGATTCCTTCGAATCGATGGCTGCTTGTCTTCGATAAAACGCAAGA
CGTTATTTTTCTCGCTTATAATGTTTTCCAACTGATTCGCGATTAAATTCATCGGCTACCA
TTGGAATCGAATGGTGTTTGTTGGTGCACAGTAAGTGATATAAGTGCGCAGTGATGCAC
ATTGGTGACTAAATCACGTTCTAAATCTTATCAGTTGGTGTCAGCATTTAATTAGACCTA
CAAATATACCGACGTGATGTATGTTCTTCGAGAATGACGAACGAACGAACGAACAAAC
GAACGGAATTCGCTTTAAACGAGCGAGTGTATTCTAGTAACGCGTCGTTGCGCATTTAA
TCACACGCGTATTTTTTCTCTTTCTTTGTTATCGCGATTCGACATTTGCCTCGCATCTTTC
TCTTCCTCTTCATTTCACCATTGATTCTTCTTTGTTCATCGAAATCTTTTATTCTCCGGTT
TTCTATGTAATATTCGACGATCGAATTCACTCTTCATCGATCATTGGCTACGAACGTCGT
AATATATCGTAAATTATCGAAACGACCAACGTTCTCTAACATTCTTATTTTTATCTCATCA
TAATTCAATGTATACTCGAGTCTCAAAATTCCAATTATTTTTACCATTTTTATATACCTTTT
TTAATCCATATTCGACAATTTTTCTCAATCGATAATCCACGTTTTTCTATTCGAATCGTCT
CGATTTCGTGTCGTGATTCAAAAGCCAATTTTGGATCAACCGGACACCTGTTGTACTTG
GCGTCGTTTGTGTAACTTCGAGAGTATATATCGACGCGTTAATATTTCTTCTTCGTTCTCT
TATTCATTTTTTTTAATTTACGATGCGCATCCAAGTTTTTACATGGACTCTCAACAGTTTC
TCAATTTCATAAATAATAACGCTATCGTTTCTTTCATGCAAGCTTTGCATTCTTCTCGATG
CTTGTTTTCCTGTCAACCGGTAAATGGCATAAACGACGAGAAAAGAAGAGAAAACAA
AGATATTGACGCGTACGCAATATAATCACGTCTGTACGGAGAAAGACCAGTCGGACTA
GTTTTCGCAAATGGTTTTAAATACCATTTACGAAAGAGGGGAAGCAATAAGAGGGACG
AACTTTGTCATATGGATGGGCAAGGAAAAATTCACATTCGAGATGCCAACACCGGGTC
ACGCGTTCGCACGCACGCACGTGCGCGCGCCACGATTCATCGTTTTACCACGGTAGAC
TGACATTTGCCGGGATATCGCGGATGACGACACACGTCAGCGCATGCGCACATTGCCC
GTACAAATTCGACTGTTGAATTCGCGATTCTTCTCGATACGAGAAAAAGTTCGATACAA
ATTTTTCTCGTCGCGATATACGATATTCTCGAAACGGATTCGCGAATACATCGCAACAAA
GAGGCAGAAAGTGAACAAGCTTGACGTCACTGGAATCATTCGCGCCACTTAGACCAA
GTCACGAGTTCAATTTAAAATGCGAACGGTCAAGTACCGTGGTTCGATCGGCCGTTTC
GTTCCGTAACCTGTAAAATGTTGGAATCGTTTCATGTTGGGAGAATAATCTGTTCGTACA
TGTACGAAAAAAAATATTCACTTGGAATCATCGTGGACTTTGAAAAGTCAATGGTATGA
ATATAGCGTGGAAAGGGGGAAAACAGAGAGAGAAAGAGTCTAGTCTAGACCCAAATG
CGACCGAGTCGATTCGAGTTATACCGATATCACCACCATTATGAGGTCATTGGTCTTCCT
TCCTCTTGCTTACTCTGAACTCGTGGAAATACATACGTACGTAGCCGTGTATACGTATAC
ACGCCTACACGTCAGACCAGCGCGATCTCGTTACTTCACCTCGGTTGTTTTTCGAATTA
AATCATTCTTCGTCACGATTTTTTCGTTCGTTTTGTCGTTCGTTCGCACAATTTTTTTTT
CCTATCACACGTAGAGCGTCGCAAATCGACGCGACAAAATATATGAAACTCGATCTAAT
TGCCGAATCTCCGATACGTTTTCCGCGAAGAAAAACGATGATTGAAAAAAGAACGAAG
GGACAAACGGAGAAAGTAACGTGTGCGCGTATAGGTGTGACGGGGTAGAGAGGAAGA
GGACTAGAAGGAGACGCGTGTACAGTCGCGTTTAGTAGGTGCGTATATAAATGTCATCC
TATATATAGCGAACGTTCGTCTAATCTTAGCTACTCGGCCACCGGCCAGGAACACGAGT
AAAAACTTACTTTGCCTCTGTTTGCTCGTCCTCCTGTCTCGCTCGCACCAACTATCCTTC
GGTGAACCGACGCGACGCAATCGAGTTTCCGCTCTCGACTTTTCTCCCACGCGTTCTC
TCTCGGATCCTTAGATTCTCTCTCGCGATACGCTATCTCGCGGATTCGGGATTGGATGAC
GGTCGATCGTTGTCGCGCGTTGTCGTCATTTTTCCATCGAAAAGTGTGTATATATATATAT
ATATATAAATATACACGTTAGTGGAGATACGACGAATTCGTATTTCAAGACACGAAGAC
CACCGGTAGAATACGAACCACTGGTTTCGCTGAACCGACCGTGGTCACCTTTTTCTCTT

TCTACATCAACTACTCGGCTGACCTACACACCGACATTCGGTCGCTAATAATAACGGTA
CGCTGTGGCGAGGCGTCGATATCACGATTCTCTTTTAATCCGATTCGATTCGACTCGGCT
CGATTCCATTCATATCTCGTTTTGCCTCGATATGAAAAACGACCTATTCGCGTATCGATC
GATCGATCAATCGATGCGAATCGTAATCGTGTTTCGGCTAATCGCTCGAATTGAAATCG
AAAACGTTTTTTTTTTTTTCTCTCACGATTTCGACTCGATTAGAATCGAATTTCGAGAGA
GCTAGCCTCTCGCAAAGAGAATGGAATTTTTCGAACGCGTTCAAAAAAAGCGTGGCG
GATAGAGAACGAGTTTCGCCGTAAATAATTATTACCGATTAATAAATATCGATAAATATC
GATAAATCGAGGCACGTATTTCCGGTTGGAACGAGCGAACGATTGTAATTGCGATCCCT
ACCTTTCGGTTGGGGTAACAGGAGCACGGAGGAAAGAGAGGTGAACAAGAGCGGCA
AAGCAAACCCGTCGAGGAACGGCCCTCCTCCACGATTAACCTTGGACTTGACTCGAAG
GAATTCTCGCAGCGTACAGTGACCTCTGCTCGCCCGCTAACTCTCGACGCTCGTCGAG
CCTCGATCCGTTCGCGCTCGCGCTCATCTTCGGCCCGTTCCTCTCTCTCTCTCGTTCGCC
CCATCCGTTCCCAACCGATTCCAACCGATTCCAACCGATTCCAACCGATCGATCCCATT
CAAATCGAGAGAATCGAGTGCTGGCCGCGCACCGCTCTGCGCCTGCGCTGTAACGACC
CTTCCTTCCCTCCTCGGTCGGTACGAATCGAGCGAGCGAGCAAGCTTTCCCGATAAAA
AAACTTTGGTGTTTATCTCTTTTCATATACCAAACTCTTCTCTCTCTCTCTCTCTCTCT
TTCTTTCTCTCTCTGTTTCAGAAGACGTGGGTTACAGAAGTGTCGAGATCGCGTCCGCG
TTTACGCATCTACCGCAGAAAGAACGCGAGAAAAAGAGAAGATAAGAGATGGCCAGG
GACACACCAGGCTCCCCGATGTCCAGACCGAGAGACTTGGTCGGCGGAGTTGGCGCT
ACTACGACCACCTTGACATCGAGCGGTTATCACACCGGCTCCGCGGATCCATCCACCCT
TCACGGCCACGTGTTGCAGCAGAAGATACTCGAGCTACAACAGCATCATCAACTTCAA
CAACAAATTCTCCGGCAACAATATCAGGCGCAAGAACGACAATTGGCCGAACTGCACG
AGCAACAGATGCATCAGTTGAAGCTTTGGGAGCAGCAGAAACAACTGGAGGAGCAAA
GGAGAGAGAAGGAGAGGCTCGAAGCGTTGAGGAAGAAGGACAAACACGATCACAGC
GCGATCGCTTCGACGGAGGTGAAGCAACGACTTCAG

>novel_circ_001627
GAAACGCATGCGACCAAAAGAGAAGAAAGTTACAACGTTGCAGCGTTGACGGTAAAT
GCAGGGTTCGGTGAATGTTGGAGGAAACAGAATGTCCAGCGAGCAAGCGCGAGCAAG
CGCCATCGCGACGAAAGCGCCGCTCGGCTGATCGATACATTCGAGTTTTAGGGTGGTG
CGACGGAGCGAGGAAGAGAACGTAGGAAATGGTTTAAGAAGGGGTGACCTTGTCTCC
TATTTTCGGAAGGTAGCTTTCTCGTGATTCCCGATAATACTCTCTTTCTCTCTCTCTTTTT
CTCTCTTATAGATTTGTATTCTAAGAGATACGGGGATCGTCGAAGCCGCATTATGAACAA
CGAAAGAAACTGAAAGATAATAGGGAAAGAAAAGACGCGGTAATAACGAGAAAAGA
AACGTGGATATCGATATCGAATTTAAATGAAAAAAGTTTGATCGATTGAAAACGATCGT
TCATTAGAGAAACCACCTGCAACTGATTTTCAGAGGTCTCAACGTTTCACTCTGTTTAT
TGCTTTTTCTTTTCTTGTATTGCCTCATTTTTTCAAAAGAAAAAAAAAAAAATTGTATTC
TGTAATCTGGGAATAAAACGCATCGGCCGAATACGTTATTAGCGATATTATCGCGATTTT
TAATCGCGGATGTACGTATATAAAATTGAAAACTCGGCATTTGTTGTTATCGTTTTTCTTT
TTTCTTGACGAATAAATTACTCGCTGTGTTTAAAATAGTTTGTAATACCGAACAATACCG
ATTCGGTAACGTAAAACCGAAACCTGTTTTTATCGAACATCAAACGAGAAAATAAAA
GGAAAATCAAATTTGCGATAAAGATAAAACGCGTCGGGCAAAAAAATTCGCGTTTATT
CGCCTGCGCGATTTTCCGAGCGAAAAGAGAACGAGAGGTAAAATGGGATGAAAAGAA
CAGAAGAGACAAAGAAAAGAATGGCAACTGGGCCTAGTACTAGACGGCGCTATGCGA

CGCGACGAAAACAATCGAGCAACGCAGACGAGTCCAAATTTTAACGGTACGTGGTAG
TCGGTGGTAAAACGAAGGAACGTGGAAAGGACGAAGAGAGACGATCGATGTAGCAG
CGGAGAGGGGAGGAGCGCGCAAAGGCAGCAGGAAGACGGGCAGAAGGGCAACGAT
TGCGAATAGGAGGATGCGGTGAAGAAGCGACGGCTGTTGCTAGAGCGGTAGAGAAAC
TGGTGGGTGGCGGTGAGGAGACGAACGTAGCGAAGCAGTTTTACAATTACGTAGCCG
GCTCGCGCTCAGGCGAAAAGTACAAATGTGCTTCGAACGCAACAATACCGGGTCTCTA
TAAATAACCGAGATTCGCGAGTCTCACGAGGCAGGGCGCTGTCATCGTGCGCGCGCGA
CACCCCCCCAGAGAATGCGAGAGAATCGCGAAAGAAACAGTAGCGCGCATCGCGCGC
GCAGTTCTCTTTCGTTATCCGTTACGTTTTCGCGCGCGCGTGCCCATGTTCCAACGAATT
CGCAATTTATACCTTTATATTTTGCGAGTTGTTCGGACGACTCTACTGTTATAATTCGTTT
ATCGTTGACGTGCCGATTCCTTCGAATCGATGGCTGCTTGTCTTCGATAAAACGCAAGA
CGTTATTTTTCTCGCTTATAATGTTTTCCAACTGATTCGCGATTAAATTCATCGGCTACCA
TTGGAATCGAATGGTGTTTGTTGGTGCACAGTAAGTGATATAAGTGCGCAGTGATGCAC
ATTGGTGACTAAATCACGTTCTAAATCTTATCAGTTGGTGTCAGCATTTAATTAGACCTA
CAAATATACCGACGTGATGTATGTTCTTCGAGAATGACGAACGAACGAACGAACAAAC
GAACGGAATTCGCTTTAAACGAGCGAGTGTATTCTAGTAACGCGTCGTTGCGCATTTAA
TCACACGCGTATTTTTTCTCTTTCTTTGTTATCGCGATTCGACATTTGCCTCGCATCTTTC
TCTTCCTCTTCATTTCACCATTGATTCTTCTTTGTTCATCGAAATCTTTTATTCTCCGGTT
TTCTATGTAATATTCGACGATCGAATTCACTCTTCATCGATCATTGGCTACGAACGTCGT
AATATATCGTAAATTATCGAAACGACCAACGTTCTCTAACATTCTTATTTTTATCTCATCA
TAATTCAATGTATACTCGAGTCTCAAAATTCCAATTATTTTTACCATTTTTATATACCTTTT
TTAATCCATATTCGACAATTTTTCTCAATCGATAATCCACGTTTTTCTATTCGAATCGTCT
CGATTTCGTGTCGTGATTCAAAAGCCAATTTTGGATCAACCGGACACCTGTTGTACTTG
GCGTCGTTTGTGTAACTTCGAGAGTATATATCGACGCGTTAATATTTCTTCTTCGTTCTCT
TATTCATTTTTTTTAATTTACGATGCGCATCCAAGTTTTTACATGGACTCTCAACAGTTTC
TCAATTTCATAAATAATAACGCTATCGTTTCTTTCATGCAAGCTTTGCATTCTTCTCGATG
CTTGTTTTCCTGTCAACCGGTAAATGGCATAAACGACGAGAAAAGAAGAGAAAACAA
AGATATTGACGCGTACGCAATATAATCACGTCTGTACGGAGAAAGACCAGTCGGACTA
GTTTTCGCAAATGGTTTTAAATACCATTTACGAAAGAGGGGAAGCAATAAGAGGGACG
AACTTTGTCATATGGATGGGCAAGGAAAAAATTCACATTCGAGATGCCAACACCGGGTC
ACGCGTTCGCACGCACGCACGTGCGCGCGCCACGATTCATCGTTTTACCACGGTAGAC
TGACATTTGCCGGGATATCGCGGATGACGACACACGTCAGCGCATGCGCACATTGCCC
GTACAAATTCGACTGTTGAATTCGCGATTCTTCTCGATACGAGAAAAAGTTCGATACAA
ATTTTTCTCGTCGCGATATACGATATTCTCGAAACGGATTCGCGAATACATCGCAACAAA
GAGGCAGAAAGTGAACAAGCTTGACGTCACTGGAATCATTCGCGCCACTTAGACCAA
GTCACGAGTTCAATTTAAAATGCGAACGGTCAAGTACCGTGGTTCGATCGGCCGTTTC
GTTCCGTAACCTGTAAAATGTTGGAATCGTTTCATGTTGGGAGAATAATCTGTTCGTACA
TGTACGAAAAAAAATATTCACTTGGAATCATCGTGGACTTTGAAAAGTCAATGGTATGA
ATATAGCGTGGAAAGGGGGAAAACAGAGAGAGAAAGAGTCTAGTCTAGACCCAAATG
CGACCGAGTCGATTCGAGTTATACCGATATCACCACCATTATGAGGTCATTGGTCTTCCT
TCCTCTTGCTTACTCTGAACTCGTGGAAATACATACGTACGTAGCCGTGTATACGTATAC
ACGCCTACACGTCAGACCAGCGCGATCTCGTTACTTCACCTCGGTTGTTTTTCGAATTA
AATCATTCTTCGTCACGATTTTTTCGTTCGTTTTGTCGTTCGTTCGCACAATTTTTTTTT
CCTATCACACGTAGAGCGTCGCAAATCGACGCGACAAAATATATGAAACTCGATCTAAT

TGCCGAATCTCCGATACGTTTTCCGCGAAGAAAAACGATGATTGAAAAAAGAACGAAG
GGACAAACGGAGAAAGTAACGTGTGCGCGTATAGGTGTGACGGGGTAGAGAGGAAGA
GGACTAGAAGGAGACGCGTGTACAGTCGCGTTTAGTAGGTGCGTATATAAATGTCATCC
TATATATAGCGAACGTTCGTCTAATCTTAGCTACTCGGCCACCGGCCAGGAACACGAGT
AAAAACTTACTTTGCCTCTGTTTGCTCGTCCTCCTGTCTCGCTCGCACCAACTATCCTTC
GGTGAACCGACGCGACGCAATCGAGTTTCCGCTCTCGACTTTTCTCCCACGCGTTCTC
TCTCGGATCCTTAGATTCTCTCTCGCGATACGCTATCTCGCGGATTCGGGATTGGATGAC
GGTCGATCGTTGTCGCGCGTTGTCGTCATTTTTCCATCGAAAAGTGTGTATATATATATAT
ATATATAAATATACACGTTAGTGGAGATACGACGAATTCGTATTTCAAGACACGAAGAC
CACCGGTAGAATACGAACCACTGGTTTCGCTGAACCGACCGTGGTCACCTTTTTCTCTT
TCTACATCAACTACTCGGCTGACCTACACACCGACATTCGGTCGCTAATAATAACGGTA
CGCTGTGGCGAGGCGTCGATATCACGATTCTCTTTTAATCCGATTCGATTCGACTCGGCT
CGATTCCATTCATATCTCGTTTTGCCTCGATATGAAAAACGACCTATTCGCGTATCGATC
GATCGATCAATCGATGCGAATCGTAATCGTGTTTCGGCTAATCGCTCGAATTGAAATCG
AAAACGTTTTTTTTTTTTTCTCTCACGATTTCGACTCGATTAGAATCGAATTTCGAGAGA
GCTAGCCTCTCGCAAAGAGAATGGAATTTTTCGAACGCGTTCAAAAAAAGCGTGGCG
GATAGAGAACGAGTTTCGCCGTAAATAATTATTACCGATTAATAAATATCGATAAATATC
GATAAATCGAGGCACGTATTTCCGGTTGGAACGAGCGAACGATTGTAATTGCGATCCCT
ACCTTTCGGTTGGGGTAACAGGAGCACGGAGGAAAGAGAGGTGAACAAGAGCGGCA
AAGCAAACCCGTCGAGGAACGGCCCTCCTCCACGATTAACCTTGGACTTGACTCGAAG
GAATTCTCGCAGCGTACAGTGACCTCTGCTCGCCCGCTAACTCTCGACGCTCGTCGAG
CCTCGATCCGTTCGCGCTCGCGCTCATCTTCGGCCCGTTCCTCTCTCTCTCTCGTTCGCC
CCATCCGTTCCCAACCGATTCCAACCGATTCCAACCGATTCCAACCGATCGATCCCATT
CAAATCGAGAGAATCGAGTGCTGGCCGCGCACCGCTCTGCGCCTGCGCTGTAACGACC
CTTCCTTCCCTCCTCGGTCGGTACGAATCGAGCGAGCGAGCAAGCTTTCCCGATAAAA
AAACTTTGGTGTTTATCTCTTTTCATATACCAAACTCTTCTCTCTCTCTCTCTCTCTCT
TTCTTTCTCTCTCTGTTTCAGAAGACGTGGGTTACAGAAGTGTCGAGATCGCGTCCGCG
TTTACGCATCTACCGCAGAAAGAACGCGAGAAAAAGAGAAGATAAGAGATGGCCAGG
GACACACCAGGCTCCCCGATGTCCAGACCGAGAGACTTGGTCGGCGGAGTTGGCGCT
ACTACGACCACCTTGACATCGAGCGGTTATCACACCGGCTCCGCGGATCCATCCACCCT
TCACGGCCACGTGTTGCAGCAGAAGATACTCGAGCTACAACAGCATCATCAACTTCAA
CAACAAATTCTCCGGCAACAATATCAGGCGCAAGAACGACAATTGGCCGAACTGCACG
AGCAACAGATGCATCAGTTGAAGCTTTGGGAGCAGCAGAAACAACTGGAGGAGCAAA
GGAGAGAGAAGGAGAGGCTCGAAGCGTTGAGGAAGAAGGACAAACACGATCACAGC
GCGATCGCTTCGACGGAGGTGAAGCAACGACTTCAGAGCTTCCTCGTGAACAAGAAG
CAAAGGGAGGCCGCCGCGGCTGCTAACGGGGCCGTACCTGGCACACCCGGATACAGA
AGCTGGTTGCAGCCGCAATCGGCGGAATCGGCGGGCGCAGCGAACGCCTCGCATCCGT
ACAGGATGCCGCAGATGTTGCAGGAAAAATTCGCCGATGATTTCCCTCTACGGAAAAC
AG

>novel_circ_001628
AGATGGCCAGGGACACACCAGGCTCCCCGATGTCCAGACCGAGAGACTTGGTCGGCG
GAGTTGGCGCTACTACGACCACCTTGACATCGAGCGGTTATCACACCGGCTCCGCGGA
TCCATCCACCCTTCACGGCCACGTGTTGCAGCAGAAGATACTCGAGCTACAACAGCAT

CATCAACTTCAACAACAAATTCTCCGGCAACAATATCAGGCGCAAGAACGACAATTGG
CCGAACTGCACGAGCAACAGATGCATCAGTTGAAGCTTTGGGAGCAGCAGAAACAAC
TGGAGGAGCAAAGGAGAGAGAAGGAGAGGCTCGAAGCGTTGAGGAAGAAGGACAA
ACACGATCACAGCGCGATCGCTTCGACGGAGGTGAAGCAACGACTTCAGAGCTTCCT
CGTGAACAAGAAGCAAAGGGAGGCCGCCGCGGCTGCTAACGGGGCCGTACCTGGCAC
ACCCGGATACAGAAGCTG

>novel_circ_001629
AGATGGCCAGGGACACACCAGGCTCCCCGATGTCCAGACCGAGAGACTTGGTCGGCG
GAGTTGGCGCTACTACGACCACCTTGACATCGAGCGGTTATCACACCGGCTCCGCGGA
TCCATCCACCCTTCACGGCCACGTGTTGCAGCAGAAGATACTCGAGCTACAACAGCAT
CATCAACTTCAACAACAAATTCTCCGGCAACAATATCAGGCGCAAGAACGACAATTGG
CCGAACTGCACGAGCAACAGATGCATCAGTTGAAGCTTTGGGAGCAGCAGAAACAAC
TGGAGGAGCAAAGGAGAGAGAAGGAGAGGCTCGAAGCGTTGAGGAAGAAGGACAA
ACACGATCACAGCGCGATCGCTTCGACGGAGGTGAAGCAACGACTTCAGAGCTTCCT
CGTGAACAAGAAGCAAAGGGAGGCCGCCGCGGCTGCTAACGGGGCCGTACCTGGCAC
ACCCGGATACAGAAGCTGGTTGCAGCCGCAATCGGCGGAATCGGCGGGCGCAGCGAA
CGCCTCGCATCCGTACAGGATGCCGCAGATGTTGCAGGAAAAATTCGCCGATGATTTCC
CTCTACGGAAAACAGCCTCGGAGCCGAACCTGCTCAAGGTGCGACTAAAGCAACGCG
TGGAGAGGAACATGGCCGCGTCGAGAAATTCACCCCTGATGGCACGACGGAAGGATC
GGCTGCTGTCGCACCTCAAGCGGAAATCATTGCTAGCAAATTCTAGTAGTAATCCAGAG
TCCGGGCCTAATTCACCACCGACCGTGAACAATTCTCAAGCGAGCCCCACAGCGGGTG
GAAACGCAGCGGCGATCCAAGAAGAGACGGAGAACACCGGTTACGGGGGTCCGTTG
ACCAGCAGCAGTCAACAAGGTAGCCTATCGGATCTTTCGTTGTTCAGTTCACCGTCCAT
GCCCAATATCTCGTTGGGACGACCTCACGTTCCATCCGGTTCCAACACGACCGGTACG
AAATTGGCCACGGTCTCGGAGGCAGAGGTACGCGCGGCGTTCACCGCACGACTAGGA
ATGCCGCTCACGGGACAAATGTTGCCCGGCACCTTGCCTTTTTATCCATCGTTGACGGT
AATCGAGGGAGGAGAGGCGACGTCGACCGGTGGCTACGTTCACAAGCAGATGCAGCA
AAATTTGGAACATCCATCGGTAACGAATCGGCAACATCCACCGTCCGCGGTCTATCACG
GTAGCACGACGGCAAATCCCATTACGGACACGCAAGTAGCGCATGCGAGGCTGCAAA
AGGCTGGTCACCGGCCTTTAGGTAGTAGGACTCAATCCGCGCCGTTGCCGTTGGGCCA
TCCGATGTTGCAAGGTGGCATGATGGCGCCCCAGACCCATTACGAAGAGTACCTGGCA
GAGAAACAATTGCACGACCAGCAACAGGCGCACAATTACCTGAAACAGCAGATACGT
CAAACGGTGTTGACGCGAGTCGGATCTAGGGGGCAGGCGAATCAATTGGACGAAGCG
CCTGAAACCGAGGAATCTGCCGAGGTGATAGATCTTACAGGAGGGAAGAAAGACATG
TCGGAAGAGAGCGAAATCTCGAAACAGCAACGGGATCGTGAACAATTCCTCCAACAG
CAGAGAGATCTGATGATGAGACACACGTTGCAGATCTCGAACGAATCTTCGACGGCCT
ATGGCGGCAGCGGGGGCGGTAGTGGGAGCGGAAGCAGAAACAATCAACCTAGCGCA
AGACCGTTATCCAGAGCTCTTTCCAGCCCATTGGTTCATTTGG

>novel_circ_001631
AAACATCGAATTCAAGAATCATAGATGGGGAACATGTACAGTCAAGAATTCGTAGCAA
CAGCGCAGAATCATGGGAGTCGACGAGTGCGAATAACATGTTAGAAACTGCTAGGCAT
CATTGGTCACGAAGACAGGACGACGCTGAACGAAAGCGTAATATAAATGGAAATGACG

ATGGCATTTATGTGGATACAACTAGAACTAGCGACGAAAATGCGATAATTGTCGAATCG
AAAGGATCTAGAAGTCAACAACAACAAGATGATTACACGCAAATCATTAAAACTGAAC
ATGGTATGCTACCATCGGATAATGATACGCACAATAACCAAGTTGTATTCAGACGACAA
AAGAATACGCAAATCGCAGACGAAGATCGTGCCACGAGAAGAGTGTCTTACATAAAA
GCAACTTGGGGTGAACGACTTCACGTTGACAGTGATTTGGAAAGCGATTACGAGCCTG
TTATTCATACTTCTAGAAG

>novel_circ_001632
GGTGCGTCATCCAGCTTAGTGGTAAAGTTCGCAGATACTGAGAAAGAGAGACAACTAA
GACGCATGCAGCAAATGGCCGGGAACATGAGCCTCCTTAACCCTTTCAACGTCTTCAA
TCAGTTCGGCGCTTACGGCGCTTACGCTCAGCAGCAAGCAGCTCTGATGGCTGCGGCA
ACGGCACAAGGGACGTATATCAATCCAATGGCGGCATTGGCACACGTTGGCGCTGGCC
AACTGCCGCACGCGTTGAACGGCATGCCAAACCCCGTCGTTCCACCGACTTCCG

>novel_circ_001633
GGGCGACCGGGAGAGAAAGGGCAGAAAGGAGAGCTTGGCAGTCCCGGGTTCGACGT
CTTTTCCGCAGTAAAGGTCAATGCACAGGGATTAGGGTTAAAGAGGTCGGTGACGACG
CTCCGCGGCGGGACGCTTGGCTATGCGGAAATCGTCGCTCTGAAGGGACTTCAGGAGC
AAGGGCATAATATCTCAGCGCAGACCATCATACAACTGAAAGGAGAGCCAGGCGAGCC
TGGACCACCGGGGCCACCCGGTCCTCCGGGTGCCGAGGGTCTTCCCGGCCACGAGGG
AAGGCAAGGAGCTCCGGGTGACGTTGGGCCGCCGGGTGAAAAGGGAACACCGGGAT
CGAGTGGGCCGATTGGTCCGCCTGGTACACCGGGACTTCCGGGTCCTAAGGGTGACAA
GGGTGATAGAGGCGACCGAGGTATAACAACGTCTTTGAACGGGGAACCATTTGCAACT
GGAGTATTCGAAGGACCCCCTGGTCCACCAGGACCACCTG

>novel_circ_001634
GTCCTCCCGGTGAGCCTGGAAAACGAGGGAAAAAAGGGAAAAAGGGCGACCCTGGG
GAGCCAGGCCCGCAGGGTGTGGCCGGGACGCCGGGCAAGAACGGTTTTCCGGGACCT
ATTGGGTTAGATGGACCAAAAGGTGAGCCGGGGCGACCGGGAGAGAAAGGGCAGAA
AGGAGAGCTTGGCAGTCCCGGGTTCGACGTCTTTTCCGCAGTAAAGGTCAATGCACAG
GGATTAGGGTTAAAGAGGTCGGTGACGACGCTCCGCGGCGGGACGCTTGGCTATGCGG
AAATCGTCGCTCTGAAGGGACTTCAGGAGCAAGGGCATAATATCTCAGCGCAGACCAT
CATACAACTGAAAGGAGAGCCAGGCGAGCCTGGACCACCGGGGCCACCCGGTCCTCC
GGGTGCCGAGGGTCTTCCCGGCCACGAGGGAAGGCAAGGAGCTCCGGGTGACGTTGG
GCCGCCGGGTGAAAAGGGAACACCGGGATCGAGTGGGCCGATTGGTCCGCCTGGTAC
ACCGGGACTTCCGGGTCCTAAG

>novel_circ_001636
ATCCGAATTCACAGGACGATTCAGTTCGAGTTGGGTGCTCGGATTACGTGTTGACGGG
GGAAAGATTGAGAGAGATCAGATCCGAGTTCATGTACTGGTTCTTCGACAAGGGGGGT
GACGATGACGAGGGCGATTATCTGAAGGAAATTCAAGCCTCGACACCTCAGACCAATA
AGAATTTCAATTTTCAACTGCCCTTTTTCGGATTCAGATTCAATCAAACCAGAGTATCGA
TAAACGGTTACTTGGAGTTCACCGACCCCCCAGAACGTTACACGTACCCTCTCGTGTTC
CCTGTCAAATGTTGGCCGAAGGAGAATGACCCCAGTTTCATAGGGATATTTTTCAGCAA

ATGTCGTATAGGGGAAATATGGTCGACGGACAGAGATCGGAGGAAGCCAGGTGTATAC
TTTAGGATCGAGCGAGATTTGCGAACGAGGAAAGACCAACTCGGAGTGGAGATGCGC
GAGCGTCTCAAGTGGGACGTTCGGGAGGGTATGAGCGGGGCGGAAGGTTTCGTGCCG
AAACACGCGATCACCGTCACGTGGAAGAACGTATCGTTCACGGGTGGCATCGACAATT
CCCTTTACACCACCAACACTTTCCAGATGGTCCTAGCGACCGACGAGGCGAACACGTA
CGCGATGTTCAATTATCCGCGCGTTGAGTGGACCAGCCACACGGAGGCAGGTGGCGAC
ACTGTGCACGGCGATGGCGGTATTTCAGCATTCGTTGGTTTCAACGCAGGAAACGGCA
CAGGAAGCTACGAGTACAAGCCTTTCTCTCAAACGCCGCACATACGTGATCTGACCAG
AGCAGGATGGGTAAACGGTTTTCCAGGACGACACATGTTCAAGATAGACGAGAAAATA
ATTGCTGCAACTTGCAGCTAAGACATTCCATGCCT

>novel_circ_001637
ATCCGAATTCACAGGACGATTCAGTTCGAGTTGGGTGCTCGGATTACGTGTTGACGGG
GGAAAGATTGAGAGAGATCAGATCCGAGTTCATGTACTGGTTCTTCGACAAGGGGGGT
GACGATGACGAGGGCGATTATCTGAAGGAAATTCAAGCCTCGACACCTCAGACCAATA
AGAATTTCAATTTTCAACTGCCCTTTTTCGGATTCAGATTCAATCAAACCAGAGTATCGA
TAAACGGTTACTTGGAGTTCACCGACCCCCCAGAACGTTACACGTACCCTCTCGTGTTC
CCTGTCAAATGTTGGCCGAAGGAGAATGACCCCAGTTTCATAGGGATATTTTTCAGCAA
ATGTCGTATAGGGGAAATATGGTCGACGGACAGAGATCGGAGGAAGCCAGGTGTATAC
TTTAGGATCGAGCGAGATTTGCGAACGAGGAAAGACCAACTCGGAGTGGAGATGCGC
GAGCGTCTCAAGTGGGACGTTCGGGAGGGTATGAGCGGGGCGGAAGGTTTCGTGCCG
AAACACGCGATCACCGTCACGTGGAAGAACGTATCGTTCACGGGTGGCATCGACAATT
CCCTTTACACCACCAACACTTTCCAGATGGTCCTAGCGACCGACGAGGCGAACACGTA
CGCGATGTTCAATTATCCGCGCGTTGAGTGGACCAGCCACACGGAGGCAGGTGGCGAC
ACTGTGCACGGCGATGGCGGTATTTCAGCATTCGTTGGTTTCAACGCAGGAAACGGCA
CAGGAAGCTACGAGTACAAGCCTTTCTCTCAAACGCCGCACATACGTGATCTGACCAG
AGCAGGATGGGTAAACGGTTTTCCAGGACGACACATGTTCAAGATAGACGAGAAAATA
ATTGCTGCAACTTGCAGCTAAGACATTCCATGCCTGTAAATAATAA

>novel_circ_001638
TGCAAGGACTGAACACACCTGGGAGCGATTGAAAAGCACATCCCACAGACCCCTCCA
ACTAAAATCACTCGCCTAACCTTCCCCCTGCCCACCTTATCCTGCCTCTTATCCTTCCCC
TCCCCCTTCAAATCTCAAGAAGGTCTTTCTCTCTCTCTCTCTCTCTCTTTCTCTCTATC
TATCTCTTTCTTTCTTTCTCTCTCTCTGTCTCTCTCTTTCTCTTTTTCTCTCTCTCTCTC
TCTCTATCTGAACCACAGGAATCTGTGACTCCGTTATGGACAGTATGGATTCGAAACCA
GTTTTGAACGATGACCCCTCGGTCACTCTGACCATTCGCCTGATTATGCAAGGCAAGGA
GGTCGGGAGCATCATTGGGAAGAAAGGAGAGATCGTCAAAAGGTTCCGTGAAGAGTC
TGGCGCGAAGATCAACATCTCCGACGGTTCGTGCCCGGAACGAATAGTGACGGTGACC
GGGCCGACGAACTCGATCTTCAAAGCGTTCACCCTGATCTGCAAAAAGTTCGAGGAAT
GGTGTTCCCAGTTCCACGACATCCAGGGGAGCGGTGGCGTGCCCAGGCCGCCCATCAC
CCTCAGGCTGATCGTGCCCGCGTCCCAGTGCGGCTCCCTGATCGGCAAGGGTGGTTCC
AAGATCAAGGAGATCCGCGAGGTGACCGGCGCCTCGATCCAGGTCGCCTCCGACATGC
TGCCTAACTCGACGGAGCGTGCGGTCACCATATCGGGCACCAGCGAGGCCATCACGCA
GTGCATATATCACATCTGCTGTGTTATGCTAGAGTCCCCTCCCAAAGGTGCCACGATCCC

TTATCGCCCCAAACCCCAAGTTGGTGGGCCAGTGATCCTGGCTGGTGGGCAAGCCTAC
ACCATCCAGGGTAATTACGCGGTGCCCGCCCACTCGGACGTAAGTACAGTATTGCCATT
GCCCGCGCCCGCTGCCGGGCCCACCCCGCCCTCGCTGAACACCCAAACTTTGACGACT
TCGCCCTTCGCGGCCCACCCCTCATCCTCGGCCTTCGCCGCTTCTCACGGGCATCCGCT
CCTATCCACGGCCCACCTTACAACACCCCATCATCCCCTCCACCCGAGCCCCTTACACG
CCAGCGTGGCAGGCCTCGCCGACCCCCTGCTGAAGAGTGGACACTTGCAGGCAGCCC
TCCCGCCCGCACACCTCGTGGCAGACGCCATGGGCAAACTTGGTAGCAATCCGTTGGC
TGGGCTCGCGGCTCTTGGCCTCGGAGGCCTTGCCACTCCTGCCAACACTGGTGGACTA
AACCCTGCAGCATTAGCAGCACTTGCTGGTAGTCAACTACGTACCAGCAATACGAATA
GGCAACAACCAGCTGCAAACAATCAGACGCACGAGATGACCGTGCCAAACGAGCTGA
TCGGTTGTATCATCGGCAAAGGTGGCACCAAGATCGCTGAGATTCGTCAAATCTCCGGT
GCCATGATCAGAATCAGCAACTGCGAGGAAAGGGAGGGTGGCGCCACGGATCGTACA
ATCACCATTACTGGAAATCCGGACGCTGTGTCATTGGCACAATATCTCATCAGTATGAG

>novel_circ_001639
GAATCTGTGACTCCGTTATGGACAGTATGGATTCGAAACCAGTTTTGAACGATGACCCC
TCGGTCACTCTGACCATTCGCCTGATTATGCAAGGCAAGGAGGTCGGGAGCATCATTG
GGAAGAAAGGAGAGATCGTCAAAAGGTTCCGTGAAGAGTCTGGCGCGAAGATCAACA
TCTCCGACGGTTCGTGCCCGGAACGAATAGTGACGGTGACCGGGCCGACGAACTCGAT
CTTCAAAGCGTTCACCCTGATCTGCAAAAAGTTCGAGGAATGGTGTTCCCAGTTCCAC
GACATCCAGGGGAGCGGTGGCGTGCCCAGGCCGCCCATCACCCTCAGGCTGATCGTGC
CCGCGTCCCAGTGCGGCTCCCTGATCGGCAAGGGTGGTTCCAAGATCAAGGAGATCCG
CGAGGTGACCGGCGCCTCGATCCAGGTCGCCTCCGACATGCTGCCTAACTCGACGGAG
CGTGCGGTCACCATATCGGGCACCAGCGAGGCCATCACGCAGTGCATATATCACATCTG
CTGTGTTATGCTAGAGTCCCCTCCCAAAGGTGCCACGATCCCTTATCGCCCCAAACCCC
AAGTTGGTGGGCCAGTGATCCTGGCTGGTGGGCAAGCCTACACCATCCAGGGTAATTA
CGCGGTGCCCGCCCACTCGGAC

>novel_circ_001640
GAATCTGTGACTCCGTTATGGACAGTATGGATTCGAAACCAGTTTTGAACGATGACCCC
TCGGTCACTCTGACCATTCGCCTGATTATGCAAGGCAAGGAGGTCGGGAGCATCATTG
GGAAGAAAGGAGAGATCGTCAAAAGGTTCCGTGAAGAGTCTGGCGCGAAGATCAACA
TCTCCGACGGTTCGTGCCCGGAACGAATAGTGACGGTGACCGGGCCGACGAACTCGAT
CTTCAAAGCGTTCACCCTGATCTGCAAAAAGTTCGAGGAATGGTGTTCCCAGTTCCAC
GACATCCAGGGGAGCGGTGGCGTGCCCAGGCCGCCCATCACCCTCAGGCTGATCGTGC
CCGCGTCCCAGTGCGGCTCCCTGATCGGCAAGGGTGGTTCCAAGATCAAGGAGATCCG
CGAGGTGACCGGCGCCTCGATCCAGGTCGCCTCCGACATGCTGCCTAACTCGACGGAG
CGTGCGGTCACCATATCGGGCACCAGCGAGGCCATCACGCAGTGCATATATCACATCTG
CTGTGTTATGCTAGAGTCCCCTCCCAAAGGTGCCACGATCCCTTATCGCCCCAAACCCC
AAGTTGGTGGGCCAGTGATCCTGGCTGGTGGGCAAGCCTACACCATCCAGGGTAATTA
CGCGGTGCCCGCCCACTCGGACGTAAGTACAGTATTGCCATTGCCCGCGCCCGCTGCC
GGGCCCACCCCGCCCTCGCTGAACACCCAAACTTTGACGACTTCGCCCTTCGCGGCCC
ACCCCTCATCCTCGGCCTTCGCCGCTTCTCACGGGCATCCGCTCCTATCCACGGCCCAC
CTTACAACACCCCATCATCCCCTCCACCCGAGCCCCTTACACGCCAGCGTGGCAGGCC

TCGCCGACCCCCTGCTGAAGAGTGGACACTTGCAGGCAGCCCTCCCGCCCGCACACC
TCGTGGCAGACGCCATGGGCAAACTTGGTAGCAATCCGTTGGCTGGGCTCGCGGCTCT
TGGCCTCGGAGGCCTTGCCACTCCTGCCAACACTGGTGGACTAAACCCTGCAGCATTA
GCAGCACTTGCTGGTAGTCAACTACGTACCAGCAATACGAATAGGCAACAACCAGCTG
CAAACAATCAGACGCACGAGATGACCGTGCCAAACGAGCTGATCGGTTGTATCATCGG
CAAAGGTGGCACCAAGATCGCTGAGATTCGTCAAATCTCCGGTGCCATGATCAGAATC
AGCAACTGCGAGGAAAGGGAGGGTGGCGCCACGGATCGTACAATCACCATTACTGGA
AATCCGGACGCTGTGTCATTGGCACAATATCTCATCAGTATGAGTGTTGAACTGCAGAA
AGCTAACCTAGAGGCCCAAAATACCCAAACCCCTGGCAGCGGTACCACTCCCGGGGCA
TCCGGGGCCAGTGCTTCTCCCTCTACCACCACTACCACTGCCTCTCCCTTGGCCAGCGC
CATTCCCTTGGCTCAGCTGCTAAGCAAGCCAGGCGCCCTCAACGCCCTCTCTAGCCTC
ACCGCCCTCGGCGGACTCACGGAATTGCTAGGTGGCGCTGCTGGCGCGGCTGCATCCG
CGCTACCTGTCCAGACAACCGGCGTGCACCGGTCGCACAAGTTCACGCCGAGGCTGC
GTAGCCCAGGCGCGCCCGGGCCAACCGACAGCGGAAAATTCAAATCCGAGCGCACCA
AGTACAACCCGTACTGAGCAAGTGATGGACAACAGGTGGAGAGACGCGAGATCAGGG
AGATCATTACACGTACACGCATTACTCATCACTACCACCACTACCGCCACAATTATTACC
AACACCACTGTAATCGCAACCACAACCACCAAGATCATGATATGTACACGGAACACGA
GATTTACATCGGAGAAAAGTGCGTACGGACGCCAGGGTAGCGCGATATCCGGATATTCT
TCAAGACTTGGCTAAGGATCGGACCAACATATGCTATATAGTTTCTCACAGGATTATTAT
ACAGCGTTGCCACGCTGAATGTAACAAATGCGTACATATTCGTAGGGGTAAGACAAGG
CGCGACGACAAGGATCGAACAATTTCTTTTTTTTTTTCTTTTTTTAAATATTTGTCACGA
TCGTTCATTCTTTTTTTTTCTTTTTTTTTCTAACTCTTACTAATTATTTTTCGAAGAGAAAT
TTGAAAATAATAACGCGAGGCGAGATCCGATGCTAAGTCTTCGGACCTTCACACTAAG
TGCGACACGCCCTCCGAGTTTGATGAATCTTTTATCCTCTCGTATACCTTATTGCTAAGG
AACTGATTTTAATTTTTTTTTCTTTTTTTTTCTTTTTTTGTTTCTTATATTTTTGCCGTTTTCA
TAAATATAATATTATATTTAGTTTTCGAGAACTGTAATATCGTAACGATATGGACAATTGA
AAATAATGATGTTAGTATCCCGTGTATCCGCCAGATGGCGACTCGGAGGACGCGACCGC
TTGTGCACCGTCGCAAGACAGCCTTTTTATATGTATATGTATACATATATATATATATATAT
ATATATATATATATATATATATATATATAATATAATTACGATGAATTATTAATTGATTAATGTTT
GAAGCATTAAAAAAAAAAGGCTCTTCTTATTATAGATTTAGGTAATTCCGATGTAATTTT
TTCTTGTAGTTGTATAGTCGACCACTTTTTGAATTGAGAGAAAGGGAGAGAGAGAGAG
CGAGAGACAGTATGAATGTGCGAGCGGACGATTTTTAGCAATGAAACGAAAGAAAAT
GATTAAAAAAAAAAAAAAAAATTTAGAAAGGCGTGTACGAGATTAATTATATATAATTA
TGATTTCTTTTTTTTTTTTTTTTTCTTTTTTCGATTATATCTTTATCTTTACACGAAGAAAT
GCAGAAGAAAAAGAGTATATGTGTGCGTGTGCATCCGTGTGAATGCGAATGATGATTG
GAATGAAAGAAGATAAAAATGATTAAGCGAAACGAAATAGAATGTTCAGATATACATC
GTGTCCCGTAATCATGTAACCGTGCTTTAAAATCAACGCCGGACATTTTCTCCGGAAAT
AGAAATATGGTCTTATCGCGACCGAATGAGAAAGAATGGTAAAAGAGAGAAAAAAAA
AAAAAGGGAAGGGAGAAGAGAATAATAATCACAGAGAGCTTATTTGCACCTCTGCTTA
TACGAGGTAGCGCACCTCATCGAGTGTTGTGTCGGTGAGCATTAAAATAACATTGTATT
CGCTAGATGAGAGAGAAAGAAAAAAAAAAAATAGAACACATACAAAGTAATCAATCG
ATTTACGATAGTGGTCGTTTTATTGTAACGATAATGATGGTAATTATATACAAATAAAGCA
GAACGTAATCATATTTCACGAGATCACGCTCGATTTATATATGTATAGATATAAATACGTAT
ATAATTATATATATAAATACACATATATATATATATATAATGTATATATATATATATGTATATATA

TATACATATTAATATGGAAATAGAGAAGAAAGTAACGATTCCCCATGCGAGTTCAGAATT
AATTAGCATTAACTAACATTTTAGATACGTATACGTATACATTTACTTATTTACACTTACA
CACTACTATATACACACGTATACAGGTACATACAAGGATGAAAATATAAAAAAGAAAAT
AAGAGATCGTGTTTTATCGAAGAAATATCGAGTTATTCGCTGTAAGAATCGATCGTCTTT
TCTTCTTCGATCGGAACCTGAGGGAGATAGAATAAAAAAAAAAAAAAAAAAAACTGAG
AAGTGAAAACGAAGAATGAATTTTTACTTATTGCTCGAGATGGGAATATAAAAAATATC
GATTTAATCGATATGTAACGCGGCTGGAAACGGAGAATCAAGGAAGAAAAAGAAGAA
AAGACACGGCGAACGTACATCGATTTTTAATGACGATCGACTGCATTAGGAGCTCCCA
GATCAATGTTGTGTGCTCAATTTCTAGTTTGTAGCGAGAGAAAGAGGAAGAGAGAGAA
GATAATATACCATGCATAGATCGAACAG

>novel_circ_001641
TCTGGCGCGAAGATCAACATCTCCGACGGTTCGTGCCCGGAACGAATAGTGACGGTGA
CCGGGCCGACGAACTCGATCTTCAAAGCGTTCACCCTGATCTGCAAAAAGTTCGAGGA
ATGGTGTTCCCAGTTCCACGACATCCAGGGGAGCGGTGGCGTGCCCAGGCCGCCCATC
ACCCTCAGGCTGATCGTGCCCGCGTCCCAGTGCGGCTCCCTGATCGGCAAGGGTGGTT
CCAAGATCAAGGAGATCCGCGAGGTGACCGGCGCCTCGATCCAGGTCGCCTCCGACAT
GCTGCCTAACTCGACGGAGCGTGCGGTCACCATATCGGGCACCAGCGAGGCCATCACG
CAGTGCATATATCACATCTGCTGTGTTATGCTAGAGTCCCCTCCCAAAGGTGCCACGAT
CCCTTATCGCCCCAAACCCCAAGTTGGTGGGCCAGTGATCCTGGCTGGTGGGCAAGCC
TACACCATCCAGGGTAATTACGCGGTGCCCGCCCACTCGGAC

>novel_circ_001642
ATGGGCAAACTTGGTAGCAATCCGTTGGCTGGGCTCGCGGCTCTTGGCCTCGGAGGCC
TTGCCACTCCTGCCAACACTGGTGGACTAAACCCTGCAGCATTAGCAGCACTTGCTGG
TAGTCAACTACGTACCAGCAATACGAATAGGCAACAACCAGCTGCAAACAATCAGACG
CACGAGATGACCGTGCCAAACGAGCTGATCGGTTGTATCATCGGCAAAGGTGGCACCA
AGATCGCTGAGATTCGTCAAATCTCCGGTGCCATGATCAGAATCAGCAACTGCGAGGA
AAGGGAGGGTGGCGCCACGGATCGTACAATCACCATTACTGGAAATCCGGACGCTGTG
TCATTGGCACAATATCTCATCAGTATGAG

>novel_circ_001643
GTGATAAGACGGACGGAAGTTAAAGGATGCCGGTGCTGTACGCTGCCATATGTCATTCT
TCTTGGATACAAAGCGGCGAACGCGTGCTGAGATCGCGTCAGCACGACCGCGGAACC
AGCCAGGAAAGGCGTTACAAGCGGAGCCATCGCCGATCGCCGAGCAGCGGCCGACAA
CACGGTGGTCATCGTGATCGCGATCGTGATCGTCTGGCGCATCCCACTTCCTCTAGACG
TGCAAGGATCTACCGTCATCATGCCTCGTCGTCGACCAGCCAGGACCGGCATAGGCAT
CGCTCGCCATCCTCAAGAAGTCAG

>novel_circ_001644
ATGCCACGAAGCAGGAGGCGTCACCGTACTCGCAGCCATTCAAGAGCACGTTCAAATT
CTGGAGGCTCTCTTCAATATGAACACAAGAGACGCCGCATAGATTATGAAAGTCCAAG
AAGTAAAGGAACATATAGAGTCGTTGTCGGTGCGTTCGAGCTTCCACATTGTTGCCCG
GGAAGGTCTTGACTTCAAGATTTACCGGACATAATTGATTGAGTAGGAAATAGTCTTTG

GCTATTATCTGAAAAAGCGATTTGAAAAATATACGTGAGTGCTGATGTGGGTTTTATCCG
AAATGATTTCATTGGGAAAAAGTTGCAAACGTGCGAAAGGTATAAATGAGTCGAGGTG
ATAAGACGGACGGAAGTTAAAGGATGCCGGTGCTGTACGCTGCCATATGTCATTCTTCT
TGGATACAAAGCGGCGAACGCGTGCTGAGATCGCGTCAGCACGACCGCGGAACCAGC
CAGGAAAGGCGTTACAAGCGGAGCCATCGCCGATCGCCGAGCAGCGGCCGACAACAC
GGTGGTCATCGTGATCGCGATCGTGATCGTCTGGCGCATCCCACTTCCTCTAGACGTGC
AAGGATCTACCGTCATCATGCCTCGTCGTCGACCAGCCAGGACCGGCATAGGCATCGC
TCGCCATCCTCAAGAAGTCAG

>novel_circ_001645
AATTTTTTGAGCTGATGTCTACGTCGTTGTCAAAAAATCCTGTCATACTACAAGCATCAT
CTTCACGTTCCTGGATATCATCCATCCTCAGTTTCATTCGGGATCAAGTGAGCTTGTTTC
GTCTTCCTAATATGCCACGAAGCAGGAGGCGTCACCGTACTCGCAGCCATTCAAGAGC
ACGTTCAAATTCTGGAGGCTCTCTTCAATATGAACACAAGAGACGCCGCATAGATTATG
AAAGTCCAAGAAGTAAAGGAACATATAGAGTCGTTGTCGGTGCGTTCGAGCTTCCACA
TTGTTGCCCGGGAAGGTCTTGACTTCAAGATTTACCGGACATAATTGATTGAGTAGGAA
ATAGTCTTTGGCTATTATCTGAAAAAGCGATTTGAAAAATATACGTGAGTGCTGATGTG
GGTTTTATCCGAAATGATTTCATTGGGAAAAAGTTGCAAACGTGCGAAAGGTATAAATG
AGTCGAGGTGATAAGACGGACGGAAGTTAAAGGATGCCGGTGCTGTACGCTGCCATAT
GTCATTCTTCTTGGATACAAAGCGGCGAACGCGTGCTGAGATCGCGTCAGCACGACCG
CGGAACCAGCCAGGAAAGGCGTTACAAGCGGAGCCATCGCCGATCGCCGAGCAGCGG
CCGACAACACGGTGGTCATCGTGATCGCGATCGTGATCGTCTGGCGCATCCCACTTCCT
CTAGACGTGCAAGGATCTACCGTCATCATGCCTCGTCGTCGACCAGCCAGGACCGGCA
TAGGCATCGCTCGCCATCCTCAAGAAGTCAG

>novel_circ_001646
ATAAGACTAGTGTTTCTCTTGGCGACAGTATGGATTACCCTGACGAGATCTCAAGATCC
AGAGATCTCCTCGACAAATTTAGATGTGACCCAACCACCGATAAAACTGGATAAAAAT
CTTCGTCGAGCTTTGTTGAAAGCCCTGACCGACCTAGAAGCCGAATCAGCCGAGCAAT
ACAAAGATGAATCGGAAACCATCTCTCAAAGGGACACGAGCGCGTTCGAGGGTATAG
CAAAAAAGAATTTGAATGACAATCTAGGTGTACAAAAGACCACATTCTCTTTCAAGAG
CTTCCCAGGCGACGATGACGTACCAAGCGAAGATAAATTACAGAATTCCAGTTTTGTC
GAAGGTATTCGTTACGTAACACTTAATACACCTTCGATGAATATCGAGAAAGCGACACA
AGAAGACGCCAGAAGCTTTCACGTTCAGAACGTGATATTGCCAGAGAAAAAGTCAAA
TTTCAGAAGCGAATCGAAGGAACAGCGTGAGAAGACACCTGCCACGATGAATATCTTC
CCTGTAAATAAAATGGAAAGTTTAACACAGAAACCAGCGGATGTTTCCGAGAGTCTAA
CCAGAAGTGCTTCAAATAGCATTGCAAACGCGTTAGTCAGTCCTAAACCAACCGTAGT
TACTCCTACAGTTTCAACGAAAAATAACGTGACAATAACGGAATTAGATGATTCTAAAG
ACAAAACGGAGGAGGTTAAAATCTTCCAGGCGCCTTTGGTTGCAGCATTCACTGTGCA
ACAAGACGAACAAGGCGTACCCAAGAGCGTCGTTCCGATCTTCAGATCGCCTAACGAT
GGACAAGCTCTGACTCTTCAAGAGCAACTAGAATTTAAACAACAACTTTTGGAAAAAC
AATTGGCAGAGCTTCAACAGCAACAGTTTCAACAGACGCAGTTTCTAGTGAGGCAGC
AGCAACTATACGAACAACAATTGAGACAGAAACAACAACAACAATACTATCTTCAGGA
ACAAGTCAGGATCAAACAGTTAGAGGAACAGACCAAGCTTAAACAACTGGAAGAACA

AAGGCTCAAGCAGTTGGAGGAGCAGAGGTTGAATCGATTCCAACTTCCTCCTGTACCA
CAGAAGCAGTTCTTCTTCGATCAGAGCAACAACTTCTTGACGTTCCAGCCTCCTGTCG
AGTCCAACGTTCACCTACAACCTAGCCTGACTTTAGAAGTGCCAAATGTAGCTGCGCC
TCCAACCTTCCAACCGATCTTCCAACAAGACTCATTGCGTCTGCAACCCTTCCGGCAA
CAGCAGCAATCACAATACCTCCATCAGCAGCAACAGATTCAACCGCAACAGTTGCAAC
AATCGCAACAACAACACCAGAGACTTCAGCAGAGCTTCGGCTCGTTCTCGACCGATTT
CCAGCCTTCGGTATCCACGAGATTCAATCGTCAGGAGGCTTTTAATTTGGTGGGAAATT
TTGGTTTCAACGGAGACAATAAGATCCCAGCCAACAGAGGCAACTTTGGCTTCGTTCC
ACCGCACAGAACTCCAGCGAATTTCTACAATCCTTATGCCCAGTTCAAGCAATCGAAG
CCTCCCACGCCGGCAAGGCAGATTCAACATCTGCTGTACCAATCTGGAATCGCTGGCG
ATCCGAATAACGTGCAAGGAATTGGAAATCCGGAAGATTTGAATATCGTTTCGAAGGTT
CTGGCTCTGAACGTGGGCGCAGTGCCGAATAAGAATCTCCAGTTCGCGACGAATCCGG
GTTCCAGGTCGTTGGGAAAAAATTCCGCGTGAAGAAAGAAGATGGAGGATGGAGTTA
GAGACGAGTCATGTAAATCGGTGCGGAGGACGATATTAGTAAG

>novel_circ_001650
AAAAAGACTACCGAATATACCTCCAATTAAAAATCCTTTATTAAGATTAAGTGCAACTA
CAATAGCAAAAAAAATCAGAAATGGAGATCTTAAAAGCGAGACAATAGTGAAAATATA
TATCGATCGTATCCAAGAAGTAAATCCTTTTATAAACGCAGTAATAGAGGACAGGTTCG
AGCTAGCTATAAATGAAGCAAAATTATACGATGAACAGTTAAAAAGTGGAAAATTCACT
ATACACATATTGGAAAAGGAAAAACCACTTTATGGTGTACCAATCACTATTAAAGAAAG
TTGTTGTCTATCAGGAATGAGTTATACCGGAGGTTCCTTATTAAGAAAAGGAATAAAGG
CCTTAGTAGATGGACCTACAGTAAAAATAATAAAAGATGCTGGAGCCATTCCTTTATTA
GTTAGTAATACCTCTGAATTCTGTACAAGTCTACATAGTTATAATTTTCTTTATGGTCATA
CATTGAATCCATATGATAGAAGAAGAACACCAGGAGGATCATCAGGAGGTGAGGCCGC
ATTACTTGGTGCAGGAGCATCACTCATGGGACTTGGTTCGGATATAGCTGGTTCCATAA
GGATACCATCTCTCTTCTGTGGTATTTTTGGTCATAAACCTACTGCAG

>novel_circ_001651
ACACCATTATCTTTCACGCAACAACTGCAACAATGCTCGAGGGGAGCCAAGAGTCGTC
CACTCAGCAAACTTTTATCACCTCTGAAAAGTGAACCGCAAAGTCCTATCTACGCTTCA
CCACCTCATTCGCCGTTAAGTGACAGCCTGATACCTTATTCGCCAACAAACACAAGTCC
ACCTAGTGCTCAGACCGCAGCGAATGTGATACAGAATCAACTTGGAGTATCATTGTTAA
CTGGAAATCAAAATATACCATATAAAGTGCAGATCGGAGGATATAATCATACATCGGCTA
TTTACGAACCGATTAATATAAAAACCGAGATCATCGATAAAAATGAAATTAGCAAACCA
TTTGAATTAACGAACGAAATGTTAGGTTTAAGTGTAACAAAAGATAAAAAAAATGGAC
TTGATGAAAAAAGAAATTCAGCCGACACTCATTTCACTAGAGATACTCATATGAGAATA
ATATTGGGTAATAGTGGTGCTGGTGTTGGCAGAAGCGTAAAACATGCTTCAGATCATAC
ACCTGCTAGTGCAAGTAATGCAAAACCAAAGCGTAATGGTTTGGTTTCCAATCCAGCC
ATGAGATTAGTAGCAGGTGTTAGGAATGGAATTGAAAATGCAACTAATAGAAACAAAC
CTATCACAACGCGAGTTAATGGATTTCAGTGGAAGGCTGAGGCACGAAAGGAAACAC
CTTTGTAGGGACAG

>novel_circ_001652

GCAGTGGTTAAAAATATGTCAGAACACATGTCATCAACTGAAAGTCACGAATTTGAAC
AAATAAGAGTTTTAACTCTAAATAATGGAGAACATCTTGATGGTATATTATATAGATTAA
AGAAAGGATGGAGAGTAGAATTTCGTTTAGGCGCTTCTCTTCTTGGAAGAACTGTTGA
TCTTTTTATAAATTATCCTTTAGGAGAGAATAAAAAATTTGAAAGACATACATATTATCTC
TTAGAATGGATTAATGAAACAGCTAATATATGTTTATCTCTAGCTGGTTCATTTCATTATT
ATCTTATAGATCCTAATTCTGAAGGTATGAAGCCTATTGCTTCTGGATATCTCCTTGTAGA
TCCAGAACTTAAAATAGGGGAACATGGGAATGATTTACCATTAGATTGTATTCAATGTC
AAACTGTTCTTGCAAAATGTTTAGGATCATTTTCTACCTGGGAAGATAAATTACTAGTCG
CTAAAAATTCTGGATATAACATGATTCATTTACACCAATTCAGGAATTGGGATATTCTA
AATCTTCTTATAGTCTTAGTGATCAATTAAAATTAAATCCTTCTTTTAATGATGATAATAA
ATCAATTACATATGATGATATTGAAAAATTAATTAATAAAATGCGCAATGAATGGAATATG
TTAAGCATCTGTGATATTGTTTTGAATCATACAGCAAATGAAAGTCCTTTTTTGGTATCT
CATCCAGAATGTACATATAATTGTTTGAATAGTCCTCATTTACGTCCTGCATATATTTTGG
ATGCTGCATTATTTGAATTAACAGTGCAAGTGGCTGCTGGAGAATGGGAATTTAAAGGC
ATCCCTTTGATAGTTGAAACTGAAGAACATTTAAATTCAATTCGTCATGCACTTCACAC
ATATTTTTTGCCGCTTATAAAAATACACGAATTATATATAATAGATATTAATGAAACTATTG
CTGAGTTTTTGAATTTAGCACGAAATCAAATGCCTCAAGACATCATTGAATCATCAAAA
ACATCAGAAGATATTAAAATAATACAAGATTCAAAATTTCGCAGATTAAAAGCAACTAT
AAATATGCAACTTGCTTTACAAAAATATAACATATATAGAACTGATTGTTTTGATGAAGA
GACTCGTTTGAAACGTTGTGCTGAAGATTTAAGAAAAAAATTGCAGGAACTTAATAATA
TAATCATTAATGAAATACAAAATCATTTAAATGCTGCAGTTGAAAATACAATTGCTGGTA
TAAGATATTTTAGAGTGCAAGTTGATGGACCTAAACTTAAAGAAATAAGTGAAAGAAA
TCCACTAGTTCCTAGATATTTTACTGATTATGGAACACCTAAAACATTAACTGAAAGAG
AAGCAACAATGTATTCAGATGCAGGATGTTATCTTATGGCTCATAATGGATGGGTAATGA
ATGGCGATCCTTTAAAAAATTTTGCAGATTCTGATTCTAATGTTTATATAAGAAGAGAAC
TTATTGCTTGGGGTGATAGTGTAAAATTGAGATATGGAGATAAACCAGAAGATTGTCCT
TTTCTATGGCAGCATATTAGTACATATGTTGAACAAACAGCTAAAATTTTTGATGGTATT
CGATTAGACAATTGTCATTCAACTCCTATTCCAGTTGCAGAG

>novel_circ_001654
AGTTATCGAAAGATCGCCCGACAAGTAAGTACGCCGGATAGCGCGCCGGCAGGCTTAG
GCAGCCCACAGACTGGAATAGGAGGGCTGCAACAGCATCAGCAACAAGCGCAACAGC
AATCGCAACAAACGCTTCATCAGCCGGATCCCATGCAGCAATCCAAGGACAATCTGAA
TCCGGCGGCGAACCAGCAAACCGGGGCACTTAGGAACGGAAATCCTCCGCCTCCCGT
CGTTTCTATA

>novel_circ_001656
TCCCCGTGATGCCACCGCCGCCGATCGTTTGGAAACGTTAGCAACGGGCCCCGAAAAA
TGGCCTCGATCTTGGGAGACGGGACGGACCCAGGGGGCTCGGGGCCCTCCCGATGCC
TGTTGCTCCTGCAAAGGTCCCTCGCGATCCAGCTGACCAGGGGCCCGCCTCCGCAGGT
TCTCAACGCAAGCCAACGCGAGAGTCCCAAGGAGCATCCAGCCGACGAGATGTGGAT
GTACGATAAAGGTTACAATTTGTTTCAGAGTTTCTTGGAGGCGAACTCGAAATGCTGGT
GGAACGCAGCCCTGGTGGACGCAACCCGGCAACTCAGGTACAAGGGCCACGTGTCAC
CCGGCGTGCTGATGGTCGGCGGACCACCCTGTGCCCTCGAGGTCTTGAGGGCAGCCTG

GGCTAGGAATGTCCTACGACCGCCTGCCGATCACGCGATAACTTGCCTCG

>novel_circ_001657
ATTAAACCAATAAACATTCTAGACGGATGATACCATAAGGGTATGTACGGGAACCAGAA
ATAGCTTTACGTTGCCAATTAGCCCAGGAAACTTCATTTGAAGTAGAGGTACTTGGAAA
CGAAAAGATGGAAACCCGTATGGTTCGCTGTTTGAGTACAATCCGCAAGCCAGAAAAT
TTATCTTCTGGAACTCAATTCAGACTAATCTGAG

>novel_circ_001658
AAAAAAAAGAACAAGACGTACTCAAAAATTTCAACGTGCTATTGTAGGTGCATCTCTT
ACAGATATTTTAGCAAAACGTAATATGAAACCAGAAATTCGTAAAGCACAGAGAGAAC
AAGCTATTAAAATGTCTTAAAATAAATGAATTGCTTCACCCAGAATTGTTATATTCATTG
CACTGTCTGGCTTTTGCGGGTTCATACAAATCTTATACATATAGATATTATTATATGTACA
CATCTTATGTTATTATATTTATTAAAATATATATATGTTAAATAAATTCTATTTTAGAGCTGC
AAAGGAACAAAAGAAAGCTGCAAAAGCAACAAAGAAAGCAGTTGTACCTCCTAAAA
CAAAGGCTATTCCAAAACATAAAGCTGCAAAAATAACACAAAAATCAGCTCCACGTGT
TGGAGGAAAACGTTAAATTTTAGCATTGTATAAATAAAAAGATACTTTATATTTAAA

>novel_circ_001660
AGAGGTACGTACAGTCGTGCTCAACAAAGGCTCCTCCGGACTGGGTTTCAATATCGTC
GGTGGCGAAGATGGCGAGGGAATTTTTATTTCCTTCATCCTAGCCGGAGGCCCGGCCG
ATCTCAGCGGTGAGCTACGTCGTGGCGATCAGATACTCAGTGTCAATGGCATTAATCTT
CGGACAGCCACCCATGAAGAGGCCGCCGCGGCCCTCAAGGGCACCGGGCAGACAGT
GACGATCGTGGTACAGTACAAGCCCGAAGACTACAACAGATTCGAGGCGAAGATCCA
CGATCTTAAACAGCAGATCTCTCAACAGAATTTGATGACAGGCACCCTAATGAGGACT
TCGCAAAAGAAATCGCTTTACGTTAG

>novel_circ_001661
CAGGGCGACAAGAACCTGGAGAACGTGACGCACGAGGAAGCGGTGGCGACGCTCAA
GGCGACCCAGGATCGCGTGGTTCTTCTCGTGGCGAAACCGGAAACCGGAATCGTGCC
GCCGCCGCCACCCCCAATAGCCTCGGACAATTCGCTCTCGCCGCAACCACGAAAACAA
AATGGTTCCGTGTCAGCGCTGGAGAACAACTCGACGCTGCCGTATTCACAGGAATCAC
GTCACGCGTCTTCTCTTGCTCTCCATGGAGCGGCCACGCCACGAGCAGTTTCTCAGGA
GGACGTATCACG

>novel_circ_001662
GTCAACGGAGACGATGACTGGGAGTACGAGGAGATCGTGCTCGAAAGGGGTGGCGCC
GGGCTTGGTTTCAGCATCGCTGGGGGCACGGACAATCCACACTTCGGCAACGACACTG
CTATTTACATCACGAAACTTATACTCGGCGGTGCCGCGTCCGCTGACGGCCGTTTACGC
GTCAACGACACGATACTCCAGGTGAACGACGTTTCCGTAGTCGACGTACCGCACGCGG
CTGCCGTCGACGCGCTTAAACGGGCGGGGAACACCGTTAAACTATACGTGCGCCGACG
GAGACACACGCAGCTGATCGAAATCGAGCTAATTAAGGGTAACAAGGGACTCGGTTTC
AGTATCGCCGGTGGTATCGGCAATCAGCATATACCTGGCGACAATGGGATCTACGTAAC
GAAAATTATGGAGGGCGGTGCCGCACAAGTGGATGGCCGTCTTGTCGTTGGGGACAAA

TTGGTGGCCGTCAGGAATGCTCTGCAGGGCGACAAGAACCTGGAGAACGTGACGCAC
GAGGAAGCGGTGGCGACGCTCAAGGCGACCCAGGATCGCGTGGTTCTTCTCGTGGCG
AAACCGGAAACCGGAATCGTGCCGCCGCCGCCACCCCCAATAGCCTCGGACAATTCGC
TCTCGCCGCAACCACGAAAACAAAATGGTTCCGTGTCAGCGCTGGAGAACAACTCGA
CGCTGCCGTATTCACAGGAATCACGTCACGCGTCTTCTCTTGCTCTCCATGGAGCGGCC
ACGCCACGAGCAGTTTCTCAGGAGGACGTATCACG

>novel_circ_001664
GTCAACGGAGACGATGACTGGGAGTACGAGGAGATCGTGCTCGAAAGGGGTGGCGCC
GGGCTTGGTTTCAGCATCGCTGGGGGCACGGACAATCCACACTTCGGCAACGACACTG
CTATTTACATCACGAAACTTATACTCGGCGGTGCCGCGTCCGCTGACGGCCGTTTACGC
GTCAACGACACGATACTCCAGGTGAACGACGTTTCCGTAGTCGACGTACCGCACGCGG
CTGCCGTCGACGCGCTTAAACGGGCGGGGAACACCGTTAAACTATACGTGCGCCGACG
GAGACACACGCAGCTGATCGAAATCGAGCTAATTAAGGGTAACAAGGGACTCGGTTTC
AGTATCGCCGGTGGTATCGGCAATCAGCATATACCTGGCGACAATGGGATCTACGTAAC
GAAAATTATGGAGGGCGGTGCCGCACAAGTGGATGGCCGTCTTGTCGTTGGGGACAAA
TTGGTGGCCGTCAGGAATGCTCTG

>novel_circ_001665
GTCAACGGAGACGATGACTGGGAGTACGAGGAGATCGTGCTCGAAAGGGGTGGCGCC
GGGCTTGGTTTCAGCATCGCTGGGGGCACGGACAATCCACACTTCGGCAACGACACTG
CTATTTACATCACGAAACTTATACTCGGCGGTGCCGCGTCCGCTGACGGCCGTTTACGC
GTCAACGACACGATACTCCAGGTGAACGACGTTTCCGTAGTCGACGTACCGCACGCGG
CTGCCGTCGACGCGCTTAAACGGGCGGGGAACACCGTTAAACTA

>novel_circ_001667
GTTCACGTTAAATGCGTTCGATCGATCGTAAAGAAACCGAGAAGGGAGAGAGAGAGA
TGGAAAGAAATCGCGGGTAGACAGGGTGGACAGGCAAAGAAACAGAGATGTCGTAAT
TTAGGTCGAGATAAGAAACGTGCCGGGTGTTTTCTTGGTTATCCGCAGTTTTCATTTATA
CCGTGGAACGGGCCGGACAGATTAAATATTGTTGGGAAAAGAATGGCCCCGACAGAAT
CGAAAACAATACGAAAGTCTGTCGATAGAGCGAGTGATACCACCACGGCTTCTAAAAG
GACGGTTTTCCTACCTTTCTTTTTCGCTCTCGACATGCGCTTTCTTTCCGCTCTGTCCAA
GTTTAAACTTGGAACGATATTGTACTGCCACTTGTGTGTATATCGATATTTCTGTCAAAC
AAGATCGAACATTAAACGAGAAACTGTTTCGAGCTGGAATCCCGTATCTGTCTATATAC
TCTCTCCACGCTCGAAAAATATACACTTGTAAATTTCACGCGGCAATCGTTCTGTTTCG
CTATTTCAAACATACATACGTGCATATGTAGTTATAAAGAGAACTGGTTCGGAATATTATA
TGATGATGCATGGTAATTCTATGAAAAATTGAAAGAAAAATAGAATAGCATGGGAAACG
TTAAACCCGTGGAACAAAAAAATATCATGAAAATTTGAAAATTTGGGTCAAACAAAAT
GTCGATATTTCGAAAGAAATGGAATATATTATCGCAAAAAACACGATATTTTTGTAAAAA
TCGAACAAATCTTGTAAATTCCTATGATAATATCGAATAATATAAGGATTCAAATTTGAGT
TCATTTAAATTTCTAATATTTATTCTATCCAAATTTTCTCTCTCTTATAATCCAAATCTGAT
TTTAGATGAGATATTTTTAACGTGAAGAGGCTGGTTCAATGGCTGTCAAATTTCTTTCCT
CTTCTTCCATGGGATTTACTTCCACGAAATATAATTCCCCGTATGATAATCGCAAAGCTT
TCAGATTATTGTGCATCGATGCAACGATTTGTCTGGGCGCAGTTAGTTATAACAGAGCG

CGTAGCATCGGAACGAAATCGATGAACGAAACGACTCTGGGTTGGATTAAACCACGCA
TATCTGGCATCCATTGCAGGTTGCTGCGCTGCCAAATATTTCTGTCGACAACGAGCAGT
GGGAAGACACTGAAATATGACCTGTGGCGGCCGCGCTACTGGATTGCGAAATCTGGAT
ATATCATGCGCGAATAAATGACCCAGGCTACAAGTATTATCAAAACTGGTGTGGAACGA
AAAAACGAGAACTGGAACGTTGATGAAAATCGTGACAGATACGAAAATAATTCGAAA
CGGTGGAAAACGTCAATTAATATTAACGAGAACGATCGACAAAG

>novel_circ_001668
ATATCCAGGAGTTTTACGAGCTGACGTTGCTCGATGAGTCGAAATCGGTGCAGCAGAA
AACGGCGGAGACGATCCGCATCGCCGACAAATGGGAAGCCAGCGATGGGCCAATTAC
GCCGACCTTCGCTCAAAATGACGGTGGTCCAGACTTCCCGGTGAACCAGACTCTGTCG
AACATTTATGGCCGTAAGTCGGCCAGTCCAGAGGCTCCAGACCACGTAGACGAAACTC
AGAAACACAGTGACACAATGATCGATTGGGTGTCTATCGTGCGTCATTCGCGACGTCG
TTTCAGCGACTATATGTACGGCGTACCTATTAAGGTACGCAAACGAAGAACAAAGAGA
ACCTGTCACTTCGGGGAACACGTAAATCGTAGCGTTGTCACGCGTGCCTCGAAACGAT
CGACCAGTTTTTGCGGATGCTGTTGGAATCACAACGATTTTGAAAAAATGATTTTTCTA
AACGTGCCGAAAGTTAGACCGAGCGATATCGTGAAAGTGCAACAACAATCGCAACAA
CGAACTAACGCGGAATTAGAACACGAGCAATCGTGTGTGAAACGTAAATCATCAAGAT
CGGCCAACTTGCGACGGAATAAATTGTTTCGAATGATTTTCGATGGTCTGCACAAGACC
CGTTACGCTGGTGGAGGAGTAGAGGAGCAGCTGCCACCACCGCCGTCGCCACCCCAG
TTGAAAGAGGCCAGCCAGCTGGATGGAGTTCCTAGACCGAATTCGGTCGATCGTATCC
TACATCCCGACGGTAGCCAGCATATCCTAACTAGCCACGATACTCTGAACAAG

>novel_circ_001669
ATATTTCTGTCAAACAAGATCGAACATTAAACGAGAAACTGTTTCGAGCTGGAATCCC
GTATCTGTCTATATACTCTCTCCACGCTCGAAAAATATACACTTGTAAATTTCACGCGGC
AATCGTTCTGTTTCGCTATTTCAAACATACATACGTGCATATGTAGTTATAAAGAGAACT
GGTTCGGAATATTATATGATGATGCATGGTAATTCTATGAAAAATTGAAAGAAAAATAGA
ATAGCATGGGAAACGTTAAACCCGTGGAACAAAAAAATATCATGAAAATTTGAAAATT
TGGGTCAAACAAAATGTCGATATTTCGAAAGAAATGGAATATATTATCGCAAAAAACAC
GATATTTTTGTAAAAATCGAACAAATCTTGTAAATTCCTATGATAATATCGAATAATATAA
GGATTCAAATTTGAGTTCATTTAAATTTCTAATATTTATTCTATCCAAATTTTCTCTCTCTT
ATAATCCAAATCTGATTTAGATGAGATATTTTTAACGTGAAGAGGCTGGTTCAATGGCT
GTCAAATTTCTTTCCTCTTCTTCCATGGGATTTACTTCCACGAAATATAATTCCCCGTATG
ATAATCGCAAAGCTTTCAGATTATTGTGCATCGATGCAACGATTTGTCTGGGCGCAGTT
AGTTATAACAGAGCGCGTAGCATCGGAACGAAATCGATGAACGAAACGACTCTGGGTT
GGATTAAACCACGCATATCTGGCATCCATTGCAGGTTGCTGCGCTGCCAAATATTTCTGT
CGACAACGAGCAGTGGGAAGACACTGAAATATGACCTGTGGCGGCCGCGCTACTGGA
TTGCGAAATCTGGATATATCATGCGCGAATAAATGACCCAGGCTACAAGTATTATCAAA
ACTGGTGTGGAACGAAAAAACGAGAACTGGAACGTTGATGAAAATCGTGACAGATAC
GAAAATAATTCGAAACGGTGGAAAACGTCAATTAATATTAACGAGAACGATCGACAAA
G

>novel_circ_001670

GTGCTGCGATTCTCTCCGAATCGAAAAAAGAGAGAGAGAGAGAAAAAATCGGGGAAA
AAAAACAAAACAAAAATACGAGAGATGGATAATAAAAATCCCGCTATCTGGAGAGAAC
GATGCACTCGAGCACGAATGCTGCTCATCTCTCCACCACCGATCCACCGTTTTGCCGAA
ATTTACGATATGGCCGTGGGCATCGCGCATAAAACCAACTTTAAAACGGTTCGATCGGC
AAACTACACAGAGAGAGAGAGAGAGAGAGACGAAGGAATGGCCGTGGCCAAAACAATC
GGGAATCGGAGAAAGATAAACAAGGAACGAATGGGAACGAAAGGAAGAGGGGGGGG
GGGGGAACGCCGTCGAGGGAGTAATGGCCGAGGCCGTACACGCGCGATATTTCCCCTG
TTTGTTTTCCATTACTCCGCGGAAGGCAGCTGTCCGTTGCCGGCGTGGAATATAGCGCG
GCAATGATAAGGGAAGAGGGTTCGGTGTACATAATGGAGAGAATTGTGCCTTCACGAC
TCGACGAGCCCTTTGGTGGCGCGAAACTCTGTGCCATCCTTCTGCTGGTTTCACCCTGT
TCCATAGATTGCACCAGCCACTTGAACGGTGCCCGATTTCCCGCGGGATCGCGCGTGG
AATCGATAAAAAAGCTTCCTTTTATTTTTCGTTGGGATGGTAGAACCGAAAGAATCGAG
CTCGTTCGAGGGGGTTGTAGCTGTGGGGTGGTCGAGTGGTGCACGCCTTGGACGGGA
CGGGATTAAAG

>novel_circ_001671
AGGCTCACAGAGCTCTAGAGCTCCTCGAAGATTATCACGCGAAACTGACGAGGCCTCA
GGACAAGCAGCTGCGATTGGCCATCGAGCGTGTCATCCGTATCTTCAAAAGCAGGCTG
TTCCAGGCGTTATTGGGTGAGTTGTTTCGTTAAAAGTATTTTGTCGAACAATCTTAAAA
AGCGTTTTCCGCTCGTTCGACTTCCATCCCGGTAAAATCACTTTTCTTATATCCCGCGCG
ATATTTTATAAAATCCGAAAAATTCCTGGCGAATTCATGGAAGATTCAAAGAGATTCTG
CACCTCATCCCATTCTCTATGCTCGGGCTCAATCTCATCTTATTTATCGGAACTGGCACG
TTGCCTCTCGAACGTTTCTCGCGCCATTGTTATTCGGAATAAAGCGCTCTTACCGCGCG
ATTCATTCCGTTCGCATCCTCTTTCAGATGAAAACTCGTCCGAAATTTTGACCGAGGCT
TTGTACGATCGGCGGAGGAAAAGGGAGAAAAAGAGAGTGGAGGAGGGAAGGGGAGG
GGCAATATCGAGCCTTGTTTTTACTTCAATCCAAGGAATCGAGGATTCTTGTGAACACA
GGAGGGAGGTTTTCATCGTATTCTTTGAGT

>novel_circ_001672
ATTGATATTGGCAAAGATAGTGGGATTTGACTTGACAGTTTTGCCAGAGGATTTTTATAT
GGTCTTTTTACTTTGTGGAACAGACAAGGATGAGGGTGCAGTCAACGTTGGGACCGGT
AGCCCAAAAGACACACGCGGATCTCACGCGTTCTCTTCACACCCCTCTCGTCAAGCAC
CCCCTTCTGACACCTCTCACATCTTCGAACGGTCGTACGGCCCTGAAAGAAACGTGGC
GTGATTCGAACTACGTATTTTCTCTTTCTCTCTCTCTCTCTTTCTCTTTCTCTCTCCCTTC
TTAGGACACGCTCCAAGTCCTGAGGCAAACGAACGTCAGGAGCCGTATACATCGGCCA
TGGTGCTCGTTTACCGGGCCGAGTGGTGGGTGTGTGATAAAAATATAATGCTGGTGACG
CGCGTGACACGATACAACGCGATATGACAGTTCCTTCGGCTCTTTCGTCGTCGTACATA
CCCTGCTGCGTATGGATTCGCGTGTGACCAGACGATAAACGCAATGTTGTGACGCGTG
ACACACCGCGCACCGGTCGTCCGCGTTCGCAATGCTCAGGGACGTTGCCACTGTTCCT
TCGCCTCCTCTTCTCTCGCGCACATCCGAGCATGGATGATAAATGCTCGAGCGAACGGT
AAATGCCAACCCGGGACCCGAATTTATCGATTTCGTACACACGCGTATCATTTGATCG
GTATATGTGATTTATTCTACGCTACGGATAATCGGTGTGCTGCGTTTTCGTTCCGTGTAAC
GTTTCTCGTTGTTCACCAGGATTTTCCATTTCCACACCGTGTCACAGGTTCTCGCAGAA
ATATGACGCTACCGCTACGCGCCTTTTTGAGGACAATGACCCCGTGCACCTTGCTTTCT

TCCTAGTGTGTCTGACGTTCGTTGCTGATTTTAACGTTTTCACCTATTTTTCACTTTTCG
CTCCATTTGTTCGAACAAAGGACATTTTTTTCACAAAGGAGATTATTTTCGAATATTTCT
ATTATACTGGGAAACGAATAATTTTACGTATACGTTACACAAGATTTCGGGTTTAATACG
ATGCGTGTACGGAAAAATCGTACGTGTATCTTCCAATCGTACCGCGACATTTAACAACA
AGAACTTTATGCGATACTTTCAATAATGCCACACGGTATAGTGACACTAAAGTCATTAGT
AACGTTCCAACTAGACCGAATGCGGTTTTTGCGGTACTTCAATTTCAATGGAAACATAC
GCAATGATGCGTGTTTATAGACGTACCGTTGAAATAACATCACAATTCTTTTTTGTTTTT
TTTTCTTTCGATTGTTTGCTTTGCATAATTCCTACTACAATCTACAACGTATATAAGCAGA
CAGTATAAGAGATAATATTTTTATCCAATCTCTACCAAGTATTTCGTCTTTCTTTTTTTCTT
TTTTTCCTTTACAACGTAAGTATCATGATCGTCTGATCGTAAAATTGATTATTTCTCTCCC
GCAATTTGATTTCATCTTATGAAATCTTACGCGTATGTGTGCCACCTACGTTTCCCTATTG
TGATTTCCCTCTCTCGTCGCATGAGCATATTAATCAGTGTTGTGGTTTACTACAATGCAT
TCCAACATGCATAACACTGAAATATTCTCCTTTATGTTCTCCCCTTCTTCCATCATTCTTT
TACTCTGTGTCACTTCTCTGCCTCAAGAGCAATCCTCTACTCTTGTCGAGTCTACTCCA
ACGTATCCCTGTGTACAGGCTAACGTAAGAATGAAAGGTTAAAAGCATTACGAGCCGT
GCAGCTCTCCCCGACGAAAGCGCGCAAGCAAATCGCACGCTTGCAAAATTTGGAGGA
TTATCGATCGAACATATTTCTGCCAAGTTTGTTACAAAGGATAATCGATGTTACTACGGA
TTGGTGAAATTTAAAAAGGAATTGGAAAAACGAGAACAGAGTTAAGTCCGTGTAGCA
CGTTGGTTGGTGGATCCCGTGGTGGCAACGAGTGAGATTCCCCCGCAGACCGATAAAG
TGCAGCTTTTTATTTTCCACTTTTTAGGACGAGCATTCAAAGTGGAAAAAGATTGTGTC
GGACTAATCCGCGATTTCTGTATGGCGGTAAAAAAAAAAAAAAGAGCAACAATGAAAAT
CCGTGGAACTATGTGTGTTCGATGAGAGATTTGGCTGAGAAATCTCCACGTTGAATCGT
GTTTTTTTTTTCCTTTTTTTTTTTTTTTTGGGGGGGGGGGTAACACGCATTGATTTTTATTT
CTATGTTACGCTGTTGCGTGTCATCGCGCGAGTGAAATTTTGCGATCAAGAGATTTGGA
GTTTGGTCGAGATCGATTGATCCGATAATTATTTGGAAATGGACCGTTGTTTCGCCGTG
GAAAAGAAGGATGGAGAAGAGAAATTCGACGTTTTCGAGGAGAGGCGATCGTTCTTT
CAATTTTTGTGATCATCGGCTCAACGCATCGCTCGACCATGAATACTTGGAATTTGAAG
AGGTGTCGTTTCTTTGTGATGAAGTGAACAACGTTCTCGAAAATGATCGTCGATCGAG
TTGCTTTGACAAGAATTCGTATATAAATATTTGTACAGAAATCGAATGAACAAACCGAG
TGTTCGAAATATGCATCGTGAAAAAGAATTAAAATTCTCGATGGGATTCTTCCGTATCCG
GATCTTTAATCGAAGAAGATTGTATTATATGGAAGATCGTCATGTTTGAAAGACATTATA
TTTACTCTTAACGAAATCGAATTTAAACAAGATTCCAAAATTTTGGGAGAAAAAACATG
GACAATAACCATGGAATTGGCGAAAAGAACACGAAGGGTGTGAGGATGCTGGAACGA
CAGGATTCCACTTGCACCGGTTAAAATGAATCGCAGGGAAGACCCGCTGTTGCGGCAG
CATCGCAACCGCTTGCTGCAATTAGCGGAGGTGACAAACATCAAGCCTGGCCGTGGCA
GCGGGAAAAGACGAGGCCAGAGGCAATCGTACGGTTTCGGACCCAGCAATGGCGGTT
CGAGTACCGGTAGCCGCATCACCCTTCAGGATATCGTGAGAAGCGATCCTGGATACAC
GCCAAACATCGAGCATTTAGTTTTCCCGGAGCGCAAAAGCAGTAATCCGAATCTGGCC
AAAACAGCCGAGGAATCGCCGCCAAACAAATCCAAAGTCAATGCAACCACCTCGGGG
AGATCCAGGCTCGCTGAAGTGTTCTCCAGGCATTATTCGAGAGGCTCGTCCCAAGACT
CTTCTGTTACGTCCAGAAAGAATCACTTAAACAGCACGTCGCTGGACAGCGGAGGGA
CGGTGGTGGTGTCCCATCAGGCAACAGGGTCGAGCGTATCAACGGTGGTGACGAGAG
GCGGCGGCGGCGGCGGTCGCCGTTGGTTGTCGCAGAGTTCGCAATCCTCGAGGCAAC
ATTCGGCCGAGGACGGGGTACGCGGGGGCAACGTGGGCGTCATAGGGCCCGTTTCGA

CCTCGTCCTCGAGCCAGGCCCCTGCTCCCGCCCTGCCACCTCGTAGAGTCAGTCCAGC
CGCGAATTCTGCCGACATCTCGAATACGGCCACTGGCTCCCGCGGCGACAGAAGATCC
CGCAGACGATGTCGGTTAAAAGAAGTCAAAACGTGTCGATCCTGCATCTGCGCAGCAC
CTCGGATCCCTGGGAAGTCGGTTCTCAGGGTTCTTCGTACAGCGTCGGTAGCAATAAA
AGTCAAAGCGGAGGCAAAGGAAAATCAAGTGGAAACGCCCGGGGCTCGTATACACGA
GGCACTTCAATACCATCGTTGAAACGGCACGATACAACGGCATCGGCAACGGCATCGA
CTTCCAGCCTAAAGCAACATCGGCAGGACAGCCATAGCCAGGTGACGAGTTCTCGTCG
TCGAGAATCCTCGAATTCGTTCCTGTCGGGTAACAGCGGCACTTCCGGCGAACTTTTCA
TCAGCGGAGATTGCAGCAGGGAATTGTCGCCGGTCCGATGGTGCGACAGAGAAGTGG
ACGGCGTGTACCTGGGTCGATCCGGTTGGGTTCAGGTGCAGCAACGATCCCTGGACGA
GAATCGGCGCACGAGCTACGAGAGTAGCCTGGTGACGGCTGCCACGGGCACTCTCCC
GTTACCGCGGCGAGCCACGGTAAAATTGGCCGATTATCATTGCAACAGCGAGCCAGGC
AAATGCCCGGACGAATTTCTTCGATTGGACAGCCAGAGGCCGGACTATTTGGCCCTTC
ACGGTCAAGATTTCGAATCCGTGGGGAGCAGCGCGTGCACCTCGCCCCACAGTATCCC
GGAATCGTATTCGCCGCCCTCGATCACACCGATAATCTCCCCGCCACCGGCGTTCCAGG
ATGCCGTCGGGAAGAAAACAACCTCGTCCAAGCATTCGTCGGGGCGCAACAATTACG
GGAAACCGCCTTTCCTCCCCCGTTCCAACGCCATAGTGGACAGCGATATCATCAGCCCT
CCACCTTCGCCCCCGCACCAAGTCAATTGGAGTTCCTTGCCGCCCACGTCCCGAAAAT
CCTCGAGCACGCCGTCCAGAGCTCGGAGGCAGAACAGCGGCGGTCAACAGCAGCAG
CAGCAACAACAGCAACAGCAACAACAGTACAGAATGGCGCAAGCCAAGTCGTTGGA
GGATCAGTCTTCGTCGAGGAGGTCGCAATTCGTGCAACGTTATCTCGAATCGTCGAGCT
CGTCCTCGTCGTCGGTCGGTTTCAGAAGTCTGGATAGCTGCGTGACGAGATCTACCATG
CCCAGATTGGCGGAGAACACGGATTCCTCGGTGGAAGGATACGACGACGGAGACGAC
GAGGACGACAACCCGAGCTCCTCGTTGAACCTAAGCCTCGTCTCCTCCTCGATAATAG
CGTTGAACTCGTCCACGGAATCTATCCGCGCGAACGGGGAGAGGATATCGCCGAACAG
GCAGAGCCGACATCACAGGAGTCAACAGGGATTAAGAAGATCGCCCGGATCGTCGGA
ATCCGGCAAGTTGTCGTTCAACTCGCCGTCCTCCTCCTCCTCGTCGTCGAGCAGCACG
GACAGACAGGGCAGATCGCCGACACCGAACCCTTTCAGGCGCAGCAACGCTTCCAGG
CAGCATCAGAGCGCGAGAACCACGCAAGCCTCTCCGGATTCTCATCAGTCGCGGGTGA
GAAGGTCCAGAAGTCTTCAATTACCGGAGAAAAGGTCCCCGAGCGGCGCAGCGCCAT
TGAATAGGGAACACTCGAGGGAATCCAACGAGCCCCATAGATTGGTTGTGAAAATTTC
CAGCGATCGAGGCAACGAGAGAAAACGTCATGCTCTTCAGAACAGAAAAGCGCAGTC
GACCGAGAACGCGTTAAACGAGGAGTTGCTTCGTGAGACCGAGGCTGTAACCGAGTT
CCTTTACGGTACGAGGAGCCGCGCGATAGCCAGAAGCCTGCTGTCGTGCCGGTTCGAG
CGTCGCGAGGATGGCCAGGAGCAAAGGCAAAGGAGTAATCCCAACACTTACGATGTTT
ACTTTATCTCCTCTAAACACCAGCAATCTTCCAAACCTTCCACCGGTTCGTCCATGCAA
CAACAGTCGCAGCAGCAGCAGCAGCAGCAAACGCAACAACAACAACAACAACAACA
ACAGCAGCAGCAGCAGCAGCAGCAGCAACAGTCGAGGCGACCTAAAACGCTTCAAA
GGGGAGCAACGACCCCGAATGCGAATCCCGCGAACCACGACTTCTGCATCAGTCCCG
ACACGAAGCTGCGCTGCAACAGCAGCACCTGCGATTTCTGGCCGCATTGCTCGCAGAG
AGACACTCTTTACTCGCCCAACCAAGCTCCGGTATTTATGAAATTATCCCAGAGCTACC
CGGCTCATCAGAGGCTGGCCACGGACGCTGGAGGCAGCCACGCGAGCAGAGGCAGC
GCCAGCTCGTCCCCGGCATCGTTGGAGCGAATGGACGATCGTGAGTTTCGCGATCGTG
ACGCGTCCCGAAAGAATCGGGCAGCTCGTGATCGGAGCAACAGCAGCGCGGCCAAAT

ATCAGGAGAGGCAGCCGAAGAGCGTGTTGAAGCAGTCGAGCCGATGGTCGTCCCTGG
AAGGATCGAACGGCGGGGGGAACGGTTCGAGCGTGGATAAGAGGGAATATCATAGGTA
TTATCAGAAAGCTGAATTCAGTTCGGCCAGTTTTGAGGGGAGTTGTGAGAACAAGGAT
AGAAAGGTGTCCCCGGTTCAAGGGAAGAGCGTAGCGAGTAGATCGCCGAGCGGCCAC
GCAGCATCCTCTTCCACCACCTCGTCATCCTCCAGTAGCGACATTTGGGTGACCACGTC
CGATCGAACCGTGACCAAGAGTCCGAGGAATGCGAAGAGCTCGGGCGCATCAACTCC
CATGGAGGATGCGGTGATTGGCTCTCTGAAGACGTTGATGGAGCCGCCGAAAGATGGA
ATGCTGTCCAGGCCTGGAAGCGCTCCAACGAGGGGCGAGGATACTCTGTCGGCGGAC
GGATCCTTGGATCCTCATCAGCGGTCACTATCGTTGCCTAAGTCCTTCTTGACGCACAA
TGG

>novel_circ_001674
GTGCTGGAGGAATCGGATACGTCGTCGGAAATAAGTTGCAGGAAGGATGCTTTGAATC
TCGAGAATGGCGACTTCAAAACGTCCATCGAGAAGGCGATCACGAAACTTCCCAGGAT
ACAACACTTGTGTCACGAAGCGGAGAAACCACCGCCCCCTTCGTCTCAAGAATCCAG
ATGGGAGTTTCCTAGAGATAAGCTTAGGTTACAGACGGTCCTCGGCCAAGGAAACTTC
GGCCAGGTCTGGAAGGCTGAAGCCGATGATTTGACAGGTCATCAAGGGACGACCCGT
TTAGTGGCCGTGAAAACGGTGAAAGAGGGTGCTTCCGACCGTGAGAAGGAGGATCTG
GTTCGAGAACTGGAAATCATGCAGCAGCTTGGCAGCCATCCGAACGTTGTCACTCTTC
TGGGTTGCTGTACGGAACAAG

>novel_circ_001677
GTAAACGAACACCGTGGTCGCTAGAAGACACGAAATGAATCCAATAGAAATTAAATCT
ATCCGTAGTCGACTTGAAAAACTTGGAAAACTTTGGAACCACGTGAACGTATATCATAT
TTATTGTAACTTTCTCAAGTTCTATTTCTCCATCTTCGAGGGATCGATATTCATTGTTCCG
TAACTTGTACAGCCGTAAACGAAACAAAAGAAACAGGAAAAGGAAGAAATATATATAT
ATATATAAATATACATATAGATCTATATCCTAAAAAAAAAAAAAAAAAAAAAAGGATAAA
GAAGATTGAAGCAAAGCCAAAGATAAAAACCGAAGAGAAGGAAAAAAGAGAGGAAA
AGAATTCTAAACAATAGAAAGATTTTTTTTTTAAAAAAGCAACGTCATTAGGGATTGT
CCAGGGGAAAAAAAGGAGGGAATAATTGATTAGGATTATTTTGTTCAATTGAAACGGT
GGCAAAAACGTCAGTGAGAAAAAGCGTCAAAATGAGCACAATAAGCGACAACCAATG
TACGTCAGTGAGAGATTTCTACAAGGACAGATCTATCTTCATCACCGGTGGTACCGGAT
TTATGGGCAAAGTTCTCGTTGAGAAATTACTCAGATCTTGTCCCGGAATAAAGAACATT
TATATTCTGATGAGACCTAAGAAGAGTCAGGATATTCAACAGAGGTTGCAGAAATTGTT
AGATGTGCCGCTGTTCGATAAATTGCGTCGGGACACTCCTGATGAACTTTTGAAGATAA
TCCCTATAGCTGGAGACGTGACGGAGCATGAATTGGGCATCTCAGAAGCTGATCAAAA
TGTTATAATAAGAGACGTGTCGATCGTTTTTCATTCTGCTGCCACTGTGAAATTCGACG
AACCGTTGAAGCGTTCGGTTCACATCAATATGATCGGCACTAAACAACTGTTAAACTTG
TGCCATCGTATGCACAATTTAGAG

>novel_circ_001678
CTGTTCGATAAATTGCGTCGGGACACTCCTGATGAACTTTTGAAGATAATCCCTATAGCT
GGAGACGTGACGGAGCATGAATTGGGCATCTCAGAAGCTGATCAAAATGTTATAATAA
GAGACGTGTCGATCGTTTTTCATTCTGCTGCCACTGTGAAATTCGACGAACCGTTGAA

GCGTTCGGTTCACATCAATATGATCGGCACTAAACAACTGTTAAACTTGTGCCATCGTA
TGCACAATTTAGAGGCTCTGATTCATGTTTCAACGGCATATTGTAATTGTGACCGCTACG
ATGTCGCTGAGGAGATTTATCCTGTGTCGGCGGAACCAGAAGAAATAATGGCTTTAAC
GAAATTAATGGATAGCCAGATGATTGATAATATAACACCGACTTTGATCGGTAATCGGCC
GAATACATATACGTTTACCAAAGCTCTGACTGAAAGGATGTTGCAATCCGAATGTGGCC
ACTTGCCGATTGCAATTGTAAGACCCTCTATCGTTCTTTCCTCGTTTAGAGAACCAGTAT
CTGGATGGGTGGACAATTTAAATGGACCAACTGGAATTGTTGCCGCTGCTGGCAAAGG
TTTCTTCAG

>novel_circ_001679
GGATCGTTTCCATGCAAGAAAGATGGCTTCCTCACTCTTGGCCACGAATTCGCAGGCA
CGGTAGACGCGATTGGCTCCTCGGTGAAGAACTTCAAAGTCGGGCAAAGAGTGGCTG
TCGATCCTAACAGTGGATGTAATACGTGCAACTACTGTCACGATGGTTCTTATCAGCAC
TGTAGCGCCGGTGGTATAAACAGCACGATAGGGATTTATAAAGACGGTGGATTTTCCAC
TCACGCTATCGTACCCGAAAGTCAGGTGTATTTAATACCAGACGACGTCGAATTGCATC
AAGCCGTCCTCGTCGAGCCTCTGTCGTGTTTGGCTCACGGATGGAAGAAACTTAATAG
CGTCAACGTTGGTAGCAACGTGCTCGTAATTGGCGCCGGAATCATTGGTCTTTTGTGGG
CTTGTATGTTACATTTGCACGGGCTTAGGAAATCCGTGACGATCAGCGAGCCTCAGGAA
AAACGGCGGAAACTGGTGACGAAACTTG

>novel_circ_001680
AGAACATATGGAAAATGGTGGGATAAAGCACCTTCAAGATTAATAGATTATATGCCACA
GAACAATATAAATATTACTAGCCAGGATTATATAGATATTTTTAACTTATTGGAATATTATA
CTGATACCATTAATACTTATGATATATGAAAAATGGAAATAAATTAGAATATTTTTATATT
ATCAAGATCTAGAATATTATCAAGAGGTATATCCAATTAGAGTATCAATATATGAAACTTA
CAATCCTGGAAGTGTAATTGGGATTTGGGCACAAAATTCCGAAGGCAAGTGGTATCAA
TTATGGAATGGATTTCCTCAAGTTGTGCCACACAAGCCGCGAATATTTTCTCCATATTTG
CAGCTATGTAATTTTAAGACAAAAGTAATAAGACTAGAATTTAATCATACTTTATTAGAC
TATTATACAGAATTAGATGCTGTATTACTTATTGGTACATCAGAATTAATTGTACCTAATA
ACTTGCACAATCAAAATTTAAATGATCTTTCACAAGAATTGGGGTATCTTAAACAAAGT
GATGATGATATTTATAATTTAACACCTGATTATTTAAAAGCAAATCAAGATTTAATAATTC
TTAAAAAAACACTTTCTAAACACTGTAAACTTTTTAAAAGTAAAGTAATAGATAATGTA
TCCAAGGGTAAATTAATATCTAAAATAGGTCAACATTATCAATCTGTTCCTCCTATAGAA
GAAGCATTCAACAGTTTACAACAATTTTTACAAGAAGATTTTCCAAAACTTATTAGAGA
TATTCATCATTCAATACCAAATACTACTGAACAAAATAATTCTCTCCAGAAAAAATTTTT
AGTATCTTCAACTGATTTTGAAAATCAATCATATTGCAATTTTTCAATACTTCCGGATGA
AACTGTATTAAAAATTTTAAAAAATCTAGATTTAAGATCATTATGTTGTTTATGCAGAGT
AAATAAACATTTTAATAACATTGCAAGAGATGCTTTGTTGTATACAAGTCTTAATTTAAA
ACCTTACTGGTATTGTTTAGATACAGCTGCATTAAATAGTTTAGCACCTAGGTGTCATTA
TTTGCAACAATTAGATCTTTCATGGTGTGGAAATTATAATATGATTAAGTATCAAGATTTT
ATATATTTTCTTCGTATATCTGGAACTGTTTTGACACATTTAAGACTAAATTGTTGTCAAT
TTGTAAATGATCCTATTATTCTTGAAATTTCGAAAATATGTAAGAATTTAAAAG

>novel_circ_001681

CAAAAAATAAAAATGAGAAGAAAAAAACAGTATGTGTTTACCAGCAAGATTAAAAG
GGAGACAAACTAAACATAGCAAATTTGTCAGAGACTTAATTCGTGAAGTAACTGGTCA
TGCACCATATGAAAAGCGTGCTATGGAATTATTAAAAGTTTCCAAAGATAAACGTGCAT
TGAAATTTTTAAAAAGAAGGTTGGGTACACATATCAGAGCTAAAAGGAAGCGTGAAGA
ACTTGGAAATATTCTTGTCCAAATGAGGAAAGCTGCTGCACATCATTAAATAATATATTA
TTAAATTTTTATAATAAGTGTATAATAAAAACTATACATAAAAAAAAAAATTTTTTACTTT
ACA

>novel_circ_001682
TATCCTCGAGCTGCGCAAGGAGAAGAGCAGGGACGCTGCCAGGTCTCGAAGGGGGAA
AGAGAACTTCGAGTTCTACGAGTTAGCGAAAATGTTGCCATTACCGGCGGCTATTACCT
CCCAGTTGGACAAAGCCTCTATAATAAGGCTAACCATCTCGTACCTCAAACTTCGGGAG
TTCTCGGGCCACGGAGACCCGCCATGGAATCGGGATGGGCCACCGCCAAATAAATCTG
GCAAAGGTGCGAATCGAGTTAGGTCATCGGCATCCGCTGCTATGGACCATTTCGATGTA
CATCAGGGTACCCATATTCTTCAGTCGCTGGACGGGTTCGCGATGGCGGTCGCCGCTGA
TGGACGGTTCCTGTACATATCCGAGACCGTCTCAATTTACCTTGGACTCTCACAG

>novel_circ_001684
GCGGGTTCCTGGGCGACATCGCTCTGCCCAACATCAAATACGAGACCGAATGGAGGCA
GCAGAAGCTCAACAAGAGTTTCTTGGAGGAGCTGGAGAAATATCGGGACGAGGTGTT
GAAAGAGGGTCTCCAGGTCGAGGAGGAGGGTCTTACAGAGATTCTCCAATTCAAAAA
TGGGCACGAGGCTAACGAGCCGCATCAGGAGAACGAGGAGATGGTGGCTTCTCAAGA
GAAATCCGAACCAATTATGGTGGACCGGAATCAATACAGCATGGACGGCGAGCTCGAG
GGTGGATTCAGTTTTAACGCTAGGAACGAGGATGCCAGGCCTGGGGTGAGGGACGAA
GGCGTTGAATTCAGGCTAGAGTCAACGACCCCACCAACGAAGAAGAGGCATTCCGCT
CGAAAACAAGGACGTGAAGCCTCGCAAACGGCGTCGAACGCTACAAAATCGATGCAA
GCATTTTTGAGAACGTCTGAAGAGTTGAACGTCGCGTTTCCCACGAGCACGGAGGGC
ACCAATCAAATGAGAAATTTGAACGCGGAAAACATCGAGCAAGTGTTCACCATCCAAC
CGGAAAGCAGCACGCGGAACACGTCCAAGCAACGTCATCGTCGTCATCATCGTAGGA
G

>novel_circ_001685
GCGGGTTCCTGGGCGACATCGCTCTGCCCAACATCAAATACGAGACCGAATGGAGGCA
GCAGAAGCTCAACAAGAGTTTCTTGGAGGAGCTGGAGAAATATCGGGACGAGGTGTT
GAAAGAGGGTCTCCAGGTCGAGGAGGAGGGTCTTACAGAGATTCTCCAATTCAAAAA
TGGGCACGAGGCTAACGAGCCGCATCAGGAGAACGAGGAGATGGTGGCTTCTCAAGA
GAAATCCGAACCAATTATGGTGGACCGGAATCAATACAGCATGGACGGCGAGCTCGAG
GGTGGATTCAGTTTTAACGCTAGGAACGAGGATGCCAGGCCTGGGGTGAGGGACGAA
GGCGTTGAATTCAG

>novel_circ_001686
GATATCCCGGCTGTCCAGGTTTTCCTAGTGTTCCCGGTTGGCCAGGATATCCAGGTTGT
GATGTTCCAGGTATACTAGATATTCCAGGTGTTCCTGGGGTTCCAG

>novel_circ_001687
GAAAATTTGCTGACTGATGGTGAGGGTAGCACAGGAGAAGTTGATGATCAGGAAGTC
ACAGATGAATGTGCTATGTTAATTCACACAGATCATATGCACAAACATACACATGTCAA
GAGTGGACAAGATAAAAGTACAGGTATTTGTGGTGATGGTTTGACGGTTCCTGTAAGT
TCTCCTCGGGGAAAAGACACAACCCCCATAGCAACACCGGTTCCAGGCCCGGATTCAT
TAACTTCCCCCTCTCCACCAGTAGTTCATGTAAAGAAAGGAAAATCATTACAACCCACG
CAAATGCCTGCTAGTACACGAGCAAGTATTGTTTTTCCATTGCTTGCAAGTGGTGGGTT
ATTCAATCAAACTCGGGAACCAAATAACATAA

>novel_circ_001688
GTATTTTCATACTGATGATTTTGGGATGCCTCGGTCTTGCAACCGGTTGCATTCTCTTCG
TGTTTCAACCGTACGAGTTACTTTTTAAACTGAAAATAATCTTCAGCCCCAACAGCGAG
ATATTCGAGTTATGGCGGAAACCGGATGTTGAACTTTATCTCAAGGTCTACCTTTTCAA
CGTGACCAATCACGAAGAATTTTTGTCCGGCAAGGAGAGCAAGTTAAAATTCCAAGAA
GTTGGTCCTTACGTTTACAAAGAATCATTCGAACATGCGAATGTTCTATTCAATGATAAC
AGCACGGTGACGGCGCGGCTGCTTCATCCATTGGAATACGTACCGAAAATGAGCAACG
GCACGGAAGAGGACGAAATGATATTACCAAACATCGCGTTATTC

>novel_circ_001689
CATCGAAATCGAACCTGGTCGTGGACATCGAGCCGAAACGACCGACTGCCGTCCGATT
GGGCGAGAGCCTGCAAATTTTGTGCAGAGTTGGTAGACCGCTTCGGGTGTGCCGTGTC
GAGATACCTGGCGAGGAAGGTGGTATAGTTCTGTCGAAGGGGCAACCGCCCGAAGAC
GGCATCGAGTATTACGGGGAGGGAACGGAGGCGGGCCAGTGCGGTGTCCGCATAGCC
AAGATCAAGGAAAGCCACGATGGAATATTCAAATGCACCCTGACCACGACCGACAGCC
GATCGGAGGAGCAGGCATCCATGAGGATCATCGTTGCCAAACCGCCGAATAATCCAGA
GCTTCACACTAGTCAAGGATCGGATGGAAGGAATATATACAGGAAGGGAGAAAAATTG
GAAGTGAGTTGCAGCGCGCCAGGCGGGCGTCCAGCGGCCAACGTTTCTTTGTTCCTCG
ACGACGAGCCAATCGGTAACGAGGAAAGGCCAACGATTTACGACTCGAACGTGGACG
ACAATTCACTGGCCGTGCAAAATGCATCGCGCGCCTTGGACTGGACCGACAACGGGA
AAGTTCTTCGATGCGTGGCCGGTCACATCGCCCTCGACCGGCCTAAAGAAACCACTAT
GCAACTGCAGGTCTACTATCCTCCTCAACCTCAGTCAACGATCGAACGATTTGGATACG
TAATTGGACGGCAGGGAATCGTGAACGTGACCGTTTACGCAAATCCTAGGCCGCGTTT
TTTATGGCGTGTGAACAATGAGATAATCAACGAGGGACGTCCAGACGAGAGCAATCGG
CTCGAAACCTCGACCGCCGTGGATCTG

>novel_circ_001690
AACCGCCGAATAATCCAGAGCTTCACACTAGTCAAGGATCGGATGGAAGGAATATATAC
AGGAAGGGAGAAAAATTGGAAGTGAGTTGCAGCGCGCCAGGCGGGCGTCCAGCGGC
CAACGTTTCTTTGTTCCTCGACGACGAGCCAATCGGTAACGAGGAAAGGCCAACGATT
TACGACTCGAACGTGGACGACAATTCACTGGCCGTGCAAAATGCATCGCGCGCCTTGG
ACTGGACCGACAACGGGAAAGTTCTTCGATGCGTGGCCGGTCACATCGCCCTCGACCG
GCCTAAAGAAACCACTATGCAACTGCAGGTCTACT

>novel_circ_001691

TGTCGCCTCCTTTGCCGCCTGTGGTCGAAACAGCGTGGGACAGAAACCGGGAACGTG
AACGAGAGAGGGTGCGAGAGAGGAGCACGGAGAACGAGCACAGGGACAGGCAGCT
AGACAGAGAATCGGGGATAAGGGAGACGAGGGATCGGGAGAAGGAGCGGGATCGCG
ACAGGGAGAGGCAAAGGGACAGGGATAGGGAGCGGGACGTGTCGGTCCGCATGATG
GAACCGCTCGATCCCGTCTTCGTCTCCCCCTCGGCGGCCCTCCTCGCGTTGCAGAGGAT
AAAGGAAACGTCCCTGGTGTTCCACAAGCGTCCAGCGCTGGCGAGCGGAATAGGGAT
CGGCGGAAGGGTGAACGCCGGTGTCGGAGGGAACGGCGGCAGCCTCGCGATGGACA
ACGAATGCGGCGACGCGTTGAAGAGGCGCAAAGTTCACAGATGCGACGTGGCCGGTT
GCGACAAGGTGTACACCAAGAGCTCCCACCTCAAGGCTCACAAGAGGACCCACACCG

>novel_circ_001692
GCGGCGGGACCGCCGGTGCAGCTGGAACCAGTGGATCTGTCCGTGAAAACTCCCGTG
GTGTTGCAGGTGCCCCGGTACAGTCCAGCGGCTATAATTACCGCCACGGCTAGAAGAA
TCCCTTCACCTTCCACGACGCCTACGCCACCGCCGCTCTCCGCATCTACCG

>novel_circ_001693
GTGACGTACGCCGAACGGGCCAGTATTCATGCCAGTATGCCAATCAGATGCCAGTAGA
GAGCCAGTATCTGTATCTCGTCGCCACCGATAGCCAATTAATAAGTTACTTAGATAGTTA
AATTAATAGGCAATACAAAAGGGAAAAAAAAAAAAAAAAGAAAAAGGAAAAAAAAA
CGTCAACGTTTTTTTTGAATAATTGTCGTTCAGAAGGCGTCGTTCAATAAGCGTGACGG
ATATATATATATATATATATATATATATATATATATATTACGTGTATTCAATCATACACGTAGTC
TACGTATATCGATATATATAAATATAATTAAACGTAAAAAAAATATATATATATACAATATAT
AATATATATATATAATACAATCGAGAGAAGCGATAAGTGTCGGGAATCGTGCGAGCAATT
ATTTTTCGCATCGTACTTTTTTCTCTCTCTCTCCTCCCCTCCCATCCTCTCCCCTCTCTTC
TTCGACCATATTTTTCTCCGCTCGGTTGATCGGTTTTCTTTCTTTCTTTCTTCTTTTTTTTT
TTTTTTGAATTTATATCGGTGACCGCGTATATGATTCCCCGCGGAGAGTATATCGACAAG
ATTGGACAGAGAGTGGAATCGAGTTGTGATTATCGAAGTGGACCGACCGATCGATTTC
TCGGCTGTGAAGTGAATTAATATCCCAGGAAAAAGGATTGAAAACGGAGAAAGGAAG
TGAGTACAAGCAGCGAGTACACGGGCGAGCAACGGGTGGCTGGAAACGGTGACTTAC
ATCGAGAGCGCCAACCTGCAATCAAGGTTCATGGTTTTCATG

>novel_circ_001694
GATGATGTCGAACAACGTGTATGATTCGACCGGGTGGATAGAGAGGCCTGTGCAAAGT
CAACGCGATACTGGAATCGCTTGGAACGACGGTCTGCGAACGGGCTAGGTCCTTGTCT
CTCTGATACTCGCTTATACATCCGCCAGCTTCTAAATTTGCAGCCGAGGTAGGGGTGGA
ACCGCAACCGCAACACATCACCGAGCATGCTATCGTTTTCCACTGAAAACGATGCGAT
TTATGGCGTGATTCTCGATTATGTAAGAACACTTATCACTTATCCCTAACTCATTCTTTTT
TTAGGGATTTTATTAACATAAAATTGATAGAATTTAAACAGCGATAACGAATCATCACTC
TGATTATAATAATTGTAACATTTTTATCAATTTCGCAATAACAATCAAATTCTATCAATTTT
ATGAATCGAATATTCGATAAAGTAAGATAAGAGTAATGATATTCTATTTGGTTTTTTCAA
AGAATTATATAGGAATTCAATACGTTCCAGTTCTTCCTACAATAAAGTACGTACCTTAGG
ATCGACGCTTCTTTTTATAGCATTGATAAGATCAGCTCTTAAGCTCTCCATCTGTCTAAG
TCCTATCCTAGGTACAACGTCTTTTCCAACTACTACGGATGTAATAAATTCCTGCGAGTA
TTGCTGAGCGGGCATGCTCAAGAGACCACCCGGTGGAGCGAACGAGAAGCACACCAG

GTCAGGATAATCTTGCTTAAGCAAAATCGCCAAGATAGCTGCCGTACCAGCTCCCAAA
GAATGACCAACAAGTGCCAGACCGAATTGATGGGTTCCCCTCGATGTATCCTGTATACA

>novel_circ_001695
ACACAAAAAGACAAAATATGTGGATCCAAATGTAGAAGTGTACAAGATGAATCACAAA
CGAAGAGGCACAGCGTTAATTTTCAATAACGTAAAGATATATCAAGAAAGAAATAATAC
GGAGGCATGTGCGAGGAATCTGTCGCAAACGTTTCAGATGATGGGCTTTGACGTGGAA
CTCATCGTCGACGCAACCTTGCAAGTGATAATAGAAAAATTGAAGAGTGTTGCTTCAC
AGGATCATACGAATAACGACTGCCTGGCCGTGGCCGTTTTGTCTACCGGGACGTCGGG
TTATCTGAAAGCAGACAACATGTTTTATCCAGCCCAAATGCTCTGGATGCCTTTCCTGC
CCGATTCGTGTCCAGGCTTGATCGACAAGCCAAAGATATTCTTCCTCCAGACTTACCAT
GATTCATCGTACAACGGAATACACGGTTTTGACCAATCGATCAATTTCTCTTTCCCAGAT
ATTATGGTCGTACACTTATTTATAATT

>novel_circ_001696
TTGCTTCACAGGATCATACGAATAACGACTGCCTGGCCGTGGCCGTTTTGTCTACCGGG
ACGTCGGGTTATCTGAAAGCAGACAACATGTTTTATCCAGCCCAAATGCTCTGGATGCC
TTTCCTGCCCGATTCGTGTCCAGGCTTGATCGACAAGCCAAAGATATTCTTCCTCCAGA
CTTACCATGATTCATCGTACAACGGAATACACGGTTTTGACCAATCGATCAATTTCTCTT
TCCCAGATATTATGGTCGTACACTTATTTATAATT

>novel_circ_001697
TACTTCATCCTCAAAGGTAATCGTGAACAACTTCCCAGCTCAGGCCAGAATAGAAGATA
TCGAAGCTTTATTTTCGAACTGTGGACAAGTGCAGTCCGTTGAAAAGTTATCCTCCCGA
GACCCGAACACTCAAACCGTCCTAATCAGTTATGAGACGCAGGAACAAGCACAACAG
GCTGTGAACCAATTTAATGGCCACGAATACGAAGGTAGCCCACTAAAAGTGGAAATGT
CTACGGTGGAGAACCGACGAAGAGGCCGCAGCCAGCGAAGCGGCGTAGCTTATTCCG
GAGTTTCGGGTTCTGGGCGGCAAGCAGACTTCCCGCTTCGTATTCTCGTCCAGTCGGA
AATGGTAGGAGCCATAATTGGTCGTCAAGGATCAACCATACGTCAGATCACCCAGATGA
CGCGCGCGCGAGTGGATGTTCATCGAAAGGACAGTCTCGGCGCGGCGGAGAAAGCCA
TCACTATCTACGGCAATCCAGAGAATTGCACGAACGCCTGCAAGAAAATCATGGAAGT
CACGCAGCAAGAGGCTTACGGTTTGAGCAAAGGTGAGATATCATTAAGGATCCTCGCG
CACAACAATTTGATCGGGCGAATCATCGGAAAGGGTGGCACCACCATCAAAAAGATCA
TGCAAGACACCGACACCAAGATAACCGTAAGCAGTATCAACGACATCAACAATTTCAA
TCTCGAACGCATCATCACGGTCAAAGGCAGTATCGATAACATGAGCAAAGCCGAATCA
ATGATCTCCAACAAACTGCGTCAAAGTTACGAGAACGACTTGCAGGCAATGGCTCCCC
AGAGTTTGATGTTCCCGGGACTCCATCCTATGGCGATGATGTCCACTGCTGGCATGGGA
TACAGTTCACGTGGTCCCGGTTTGTACGGTTCTGGCCCAGCTCCCTATCCGTATCAGGC
TAGTTTGCCGACTCAACAGGGTATTCCGATAGGCGATACCCAAGAGACTGCTTTCCTTT
ATATTCCAAATACAAGCGTAGGTGCCATCATAGGAAGCAAGGGTTCTCATATCAGAAAC
ATTATCAGATTCTCCGGCGCCAGTGTGAAAATCGCGCCTATCGAGCAGGATAAACCTGT
GGATCAACAAAATGACAGGAAAGTAACCATCGTCGGGTCTCCCGAATCTCAGTGGAAG

>novel_circ_001698

AGGATAAGGAGAGTGGCGTAGATTACGGTCGTAAGCTGGATTAAGGGCACGGACGTCA
TGGAGCGGTACGAGGAGGAAGGCGAGGACAGCGACAACGAGACAGACCCGGAACAA
CTGTTGAACGAATGGCTCGGCGAGTTGGACAGCCTGACCGTGGTGTGCCTCCTACGTT
CTGTTGTGCAACGCAGAGGAAGTGGAACGAGCCGTCTACCGAAAGAAGAAGGAACTT
TGAACTTGAAAAAAAAAAAAAATTGATCATTTCAAACGAAATTACGTTTTTGGTAAG
ATAAACGTATTAAATATCATCGAATAAAGAATCGTTTAACGTTGTGCGAAAATGTATTCA
TCGAAAAAAAGAAGAAGAGAAAGAAATTGTAAAAGAAGCAATATTTACGTGTTTACA
AAAGACGAAAAAGAGATGGTTGGTTTGGAAATACAAGATTGGGATCCACGGTATGGG
AGAGGAACGAAAAGGAGAGGATTTTGAGAAACACTGCGTATCTGTGCCGAGCGCCAC
GCGACCAGGTCGTCCGGTCGGGCTGGGTGCCGGGTGTTGTTGAAATCAACGTGGTGG
GGTTCCTTCGACGACCACAACCGGAGCCTTATGCCTTCAGTCGACTCTCGGCTCTCACT
GCGACTCGAGCTGCCTGAGATTTCCGTTAATTAACCAACTACCGATCCGCGACAGCGG
CTGGCCAACCACATCGCAGCCGCTCCAGATACGAATTCACCTTTCCTCTCGCCGGAATC
CGGAGTTGTCGTCCGCCAAAAAAGAAAAGAAAAGAAAGAGAACATCTCGAGAAGAA
GAGGCGAAGTCTGTTCAATCCCAATCCATTCCAATTGCGCGACGTCTCGTCCCGGAGG
ATGAATCGATCGAAGTCGGGCGAGGATCGACGAGGCCTACCGTTTTAAAAGCGCGAGT
GAGCGAGCGAGCGAGCGACTGTGAGGAACGATCGGCCGATCCCGGAGATCCGATCCG
ACCAGTTCCTTTTACCGGTCGATCACCTCGATCCCGCGTCACAATCGCTCGTGGAGCGG
GACGTGCACGTCGCGTACGCGTCGTTGGCTGTTAATTAGTACGTGGGTGCCGTGATAGA
GTGGGAAGAGACGAAAGAGGGAGAGGGAGAGAGAGGGAGAGAGAAGAGTGAGTGG
ATGCGAAGAGATGCCGAAAGCGGAGGGAGGACGATTGAGAGGAGCGGAGGAAGAGA
GGATCGAGAAGTAAAAGAGGCGAGAAAGAGGGTTGGAGGTTAAAGCCGGAGTTAAAT
AGATGCAGGAATCTGTACGCGGCGGCGCCATGCTGGTGTAGCGAGCGAGAAAAAATAT
TCGGTTTCCCGGTGTGTATCTCCTCTCCGTGTGAACGTGGCCGTGCGGATGGAACGTAG
GAATCCCAGGAGCGTAACAGTGAACGCGTTCGCGGCCGGTTGAAGGGAGCGTGAATT
CGTTCAGGAGGACCAGTTCGCCGCTGCACAGGCGTTAAGAACCCGTTGAGACGTTGA
GAGGAGGAAAAGAGGTGAACGGTTCGGGAGAATTGTCTCGAGAGGAGATAACAGTG
GACGATTCTTCTCGGTGAAAACGAATTTTATTATAAACGAGTTGGAAAAGGATCGATGA
TCCTCGATCAGTGATTATTTTAACTTAATTTTTCTTTTCTTTTCTTCTTTCTCTCTCTCTCT
CTCTCTCTCTCTCTTTCTTCTCGTGTTGTACGAACGAGTATAAAGGAATATACGCGAACG
CGCGAGGAACATGTTGTGCGGCACGTTCAGGAAGAAGAGAAGGCATCCAGGGGATGA
GTATCGATTGGTGAGATCGAACACGATGCCTAGAATCCTGTCGGCGAAGGAAGGTAGC
CTGGTCACGGTCCGGAGAACAAAGTCCTCGAGGGTGGCGGCTCGTTCCCCTCATCTCA
GGGATCTTCTGCACATG

>novel_circ_001699
AGGATAAGGAGAGTGGCGTAGATTACGGTCGTAAGCTGGATTAAGGGCACGGACGTCA
TGGAGCGGTACGAGGAGGAAGGCGAGGACAGCGACAACGAGACAGACCCGGAACAA
CTGTTGAACGAATGGCTCGGCGAGTTGGACAGCCTGACCGTGGTGTGCCTCCTACGTT
CTGTTGTGCAACGCAGAGGAAGTGGAACGAGCCGTCTACCGAAAGAAGAAGGAACTT
TGAACTTGAAAAAAAAAAAAAATTGATCATTTCAAACGAAATTACGTTTTTGGTAAG
ATAAACGTATTAAATATCATCGAATAAAGAATCGTTTAACGTTGTGCGAAAATGTATTCA
TCGAAAAAAAGAAGAAGAGAAAGAAATTGTAAAAGAAGCAATATTTACGTGTTTACA
AAAGACGAAAAAGAGATGGTTGGTTTGGAAATACAAGATTGGGATCCACGGTATGGG

AGAGGAACGAAAAGGAGAGGATTTGAGAAACACTGCGTATCTGTGCCGAGCGCCAC
GCGACCAGGTCGTCCGGTCGGGCTGGGTGCCGGGTGTTGTTGAAATCAACGTGGTGG
GGTTCCTTCGACGACCACAACCGGAGCCTTATGCCTTCAGTCGACTCTCGGCTCTCACT
GCGACTCGAGCTGCCTGAGATTTCCGTTAATTAACCAACTACCGATCCGCGACAGCGG
CTGGCCAACCACATCGCAGCCGCTCCAGATACGAATTCACCTTTCCTCTCGCCGGAATC
CGGAGTTGTCGTCCGCCAAAAAAGAAAAGAAAAGAAAGAGAACATCTCGAGAAGAA
GAGGCGAAGTCTGTTCAATCCCAATCCATTCCAATTGCGCGACGTCTCGTCCCGGAGG
ATGAATCGATCGAAGTCGGGCGAGGATCGACGAGGCCTACCGTTTTAAAAGCGCGAGT
GAGCGAGCGAGCGAGCGACTGTGAGGAACGATCGGCCGATCCCGGAGATCCGATCCG
ACCAGTTCCTTTTACCGGTCGATCACCTCGATCCCGCGTCACAATCGCTCGTGGAGCGG
GACGTGCACGTCGCGTACGCGTCGTTGGCTGTTAATTAGTACGTGGGTGCCGTGATAGA
GTGGGAAGAGACGAAAGAGGGAGAGGGAGAGAGAGGGAGAGAGAAGAGTGAGTGG
ATGCGAAGAGATGCCGAAAGCGGAGGGAGGACGATTGAGAGGAGCGGAGGAAGAGA
GGATCGAGAAGTAAAAGAGGCGAGAAAGAGGGTTGGAGGTTAAAGCCGGAGTTAAAT
AGATGCAGGAATCTGTACGCGGCGGCGCCATGCTGGTGTAGCGAGCGAGAAAAAATAT
TCGGTTTCCCGGTGTGTATCTCCTCTCCGTGTGAACGTGGCCGTGCGGATGGAACGTAG
GAATCCCAGGAGCGTAACAGTGAACGCGTTCGCGGCCGGTTGAAGGGAGCGTGAATT
CGTTCAGGAGGACCAGTTCGCCGCTGCACAGGCGTTAAGAACCCGTTGAGACGTTGA
GAGGAGGAAAAGAGGTGAACGGTTCGGGAGAATTGTCTCGAGAGGAGATAACAGTG
GACGATTCTTCTCGGTGAAAACGAATTTTATTATAAACGAGTTGGAAAAGGATCGATGA
TCCTCGATCAGTGATTATTTTAACTTAATTTTTCTTTTCTTTTCTTCTTTCTCTCTCTCTCT
CTCTCTCTCTCTCTTTCTTCTCGTGTTGTACGAACGAGTATAAAGGAATATACGCGAACG
CGCGAGGAACATGTTGTGCGGCACGTTCAGGAAGAAGAGAAGGCATCCAGGGGATGA
GTATCGATTGGTGAGATCGAACACGATGCCTAGAATCCTGTCGGCGAAGGAAGGTAGC
CTGGTCACGGTCCGGAGAACAAAGTCCTCGAGGGTGGCGGCTCGTTCCCCTCATCTCA
GGGATCTTCTGCACATGGTAAGCCATAAACGCGATCTCGCGCGCCGGTGATTCCACGGT
ATATTTCCTATCCTCGCGATAAATATCGCGATATCGGCCAAGGACTCTCGAGAAAAGAA
ATATGGCCGATTACCGTTTCGTTGTCCTTTCGAGAGATCGGCCGGTGAAACAAAGAGA
AAACGTTTTTGCGATTTTATCTTTTTTTCCTTATCGTTCTTTCCTTTCCTTCGATCGATCG
AGAAAATCGATTTCGATAATATTACCGAATGTATTTCGTATTCGGTTCGCGTTTCGAAGC
AGCTGTTTGAACACGGACAAACGATTTCGACCATGTAACGCAGCTGCTAGAAGATTAG
ATCGAGAAATTTAATAAAATCATCTCTCGTCTATCGATAAAGCAACGTCCTCAACGTTTT
TAAGAATAATTTTTTCAATCGAATTCTGTTGCTCGCGAAAAAGTTTCAGATTCACTCGAT
TCTCTCGAAGAAACTGAGCGAAACGAAAATAATTCGAAGAAAAGGTCGATCGTGCGG
CATATTTTGCCCCGTATGGCAACGAAAGGGAGGGGAGGGGGGACTTGGAGCCGGCCC
AATCGATTCTTCCTCGAACGACTCTCCAATCGTCTCTCGAACACGCGAATCCGAATGCT
TCGTGCCGTTCCCATATATCCGCGTGTGCACGCGATCGCACAGTCCAGCGGGCGTTGCA
TAATTTACGACACAGCTCGTCGAACGCTCGGAGCGGATTCGGGTGGCGACCGATCCGA
TTCGGCTAATCGCGCTATTACCGATAATACCCTCTTGATGCGATATCGATACCCTCTTCGC
CTATCTCGTTGTACCTGTTTCGGCGGCGGTTACACGAGACACCTGTTCAATTTAATCGC
AACGACACGTTCTTCCATTACCTTGGAATCGGGCTGAAAACTAGTAATTAAACTAATGT
GAATATATTTGCTCCTCAAAAGAGGAAATTCGAAGATTAACTCAATCTAGGAGTATATAA
TGAAAGCGAGTCTGCAAAGGAAGCGAGAAAATGGAGAGGAGGCGTCGTGCAGAAAT
TCGGCACGTCTTCCAAGAAGCTCGACGAATTCCCCCGGTTACACGATCACGTTGGGGA

CACGTATCGATCGGTGGATCGAGGGGGGCCGGTCACGATCATCCTCTTTGTCCTCGCCT
TTCCGAAACTCCTCCTCGCTCTTTCTTTTACGGTCTAAATCCGGCCACGGAGGCGCCTG
TGACCGCTCGCAGAAGTCGCCACGAGATACCCACGTGAGAGGGAGAGAGAGAGAGA
GAGAGCAAAAGGAAGCACGCGAGCCACCTTCTCGAGATGTTGGCCTTATAAGGCCTCC
TCCGGGTGCTGCTCGGTCTTCCATTAGAAATCCTTTGTCGTCCCGACCTCTTTTTCCGG
ACGGATTCTCGAGGGCTTTTCGTCGCCGCGGGGATAGAATCGTCGCGGGCCCAACGGA
TAGCGAAAGGAGGGGAGATCGTTAGGAGGAACGTTTCGTTCGTGTCGTGGGACAACA
CGGTGGAGAAGAGTTTTTAAGATAATGAGAAACCGAGCCTTCCTTCTTCCTTTCTTTCT
TCGTCCGCTCGAGAGAATTACCAGAGAAAGGGATGACGTCGAAAGGGACGCGATCTG
GATCGGCCTTTCTAACGCTGTAAAACCGGGATTCTTGGCCGGACGAGCCTTTTTTACCG
GAACGAATCTTTGCCCGCGCTCCCGGGGACCGACCACCTCGGCTCCCTTTGTCGAGGA
CACCCGGTAACTGGTGTAAACCCTTTAGTTAACGGATCCCTCCTTCGCTCGAGCTGTTG
ATTCCCACTTCTCCTTCCTATTTATATAAAGGAAGAATGACTTTTCCAACTCGCGTAACG
ATAAGCGATAAAAATCGTGCGTGCAACGAGATCGATCACGTTAGAAGAAACGTTAGAT
TCTTGGTAAAAACGCGAGAGCGATACAGCGTGAAAATTTGTCGTTCCAGTTGTCCATC
TCGCGATCGTTTTGCGTTTTACAGCCGCTGGTTTCACCTCGGCTCCTTTGCCTCTCTTCC
TCCTCGACATGGCCGGTAACGCGCATAAAAACGATCTCGACTCGATTTCTATAATTGCC
GTGGTACTTTTTCAACGTCCTTCGTTGCCGAGTAATCACCACCGCTGGCCTTTCACTATT
CATTCGTCGGAGCGAGTTTTCAATACCTCGTCCGATCGGGAACGCGCGAGAAGCAGGA
AATTAGATTCGAATCCGATGGCGTTGGCGAGCGATCGGGGAGGAGAGGGCGGATTTTT
TTCCATCCCCTCGAGGGGATGAAACGGGGATGGGAGAAGAGGAGGCGATCTCATCTCG
GTTGGCAATTTGTGTACCCGGTCAGCCCGGTCCGTTCCCTTTTGCGCGCTTTTGTTCCC
TCTCCCCTCCTCTTTTTGCTCGTCCCATTCTTCCATCCCTCTCCTTCTTCTTCTTCTTCTT
CTTCGGCTTGGTCTTCTCTCGTTGGAATTCCAATTCTCCCTCCCCCACTCCAGGACTCG
CGAAATATCTCGACGCATTCCTGCGCACCGTGCAGGCTCCGATTTGTGCCCTCTTGTCG
TCGCTCTTAAGGACCTGTTACGACTCGAAGCGTCCACCTTCGATTCCTCGAAATTAACG
CGACGGAAAAAAAACACCGATACATTAAAATCGATGCACCGATGATAAGTAGTAAACA
GTATTACCGATTGATATTTTTCCTTGAAAAGAGGAGGAACGCGCGTTGTTTCTATCCAA
CAAGCCGAGTATTAATAATGGATTAACTCGTTAATGCAGAAATATTTCTAGATGCCACAA
CCGCTATTTCTATCCATCTAAGGATGGAAGGCAGCTTGGTAATCCTCCTCGTAACTGTTA
CCATCGATCCGAACCGCCTTTTAGAGAACCCGCTTTTCTTCCGGCAACCTTCCATCGTC
TGACGACAGGTCTGGACGATGAAAAATTCCCACGAAAATTGCGCAATCGCGGCGAAAT
CGTTGTATAAAAGCGGCGGTGACGAGAGGACGCCAAAGGAGTAGCTCTCTCGGTTCA
AAGAACTTGTATCGTCGCGCCCTATATTTCGTTATCTCGGTGTCGTTAAACCACGCTTTA
ACTCGCCGGTTTTCTTCTCGGTCGATAACGCGATACCGCGGCTCAATTGCGAGTCGAGA
ATTACGCCTCTCAAACGGACGCTTCTTTTGTTTTTCTTTTTTCTTTTTTTTTTTTGCGAGT
ATGGAAAACGAGGATTCCTTTGTCGTTGGAAAAAAAAAAATTGTTCGAGGCCGAGAC
GAGAGGATGGGATTATTTTTAGGGATAGGATTATTTTTCGGGTAATTAGCACGACTTAAA
TTGATTTCAGACTCGGTCGTGGCTCCGCGATGGATAGAACGGTTCGCGCACAGCTCCG
GTTGAGGAAGAGAAGGAACAGGTTGAAAGGTTTTAGAAAGTCATCCTGACTTATTGA
GCTTTACTCTTTTATTAGAAAGTTTGCTGATACTGTGATTCTTAACGGAAGACCAGAAA
GCAATTTACAGTTAACAATTGGTGAAATCAAACGTATTGTTCAATTTCCTACATACGGGT
CTTTATTATTTGCAATAACTTTTATTAGTTTTATAAATATGTGTATATATATATGTATATATGT
ACATATATATATATACATATATATATATATATATATATATATTATTTACTTTTACTTTATCGATC

GTTGATGAGGTTGAATAGAAGGGAGAAATTTGAGAAGAGAGGAAGAGAGGAGGAAA
GAAAGAGTAAAAAAAGGCGTCGCATTGGAACGCAATTTATTAACGGGACGGGGATTC
GTTTGACTCGATTCGATCCGTTGGAGCGTTCTTTTTACCTCCTCCTCCTCCTCCGGATTG
GTTTACGCCGGTTTCAATTTGTGGCGCCGTCCGCGAAGCTTCTTCTAATTACCGCTAATT
AGTGCCGATGCTGTAAGTGGCGGCTGTTCTCTTCTCGGATCGTTGAAAGGAATTCCCCT
CGACTATTGGCGTCGCCTAGTTTCCAGGAACGTTACTTTATCCAGAAACGGGACAGAG
ACTCTGAAAGCGATACCGGCGCTTCTTCTTGCGTTACATCCTCGTTAAGAGACTAGGCA
TTTTGCCTAGGTATCAAAAATTTTAATCTTTTCGAATTGGATATTCCGCGGATATTTTTTT
AATTCCACCTATATATTTCCAATTTCAACTCTTCTTCTTCCTTGCATTAATTAAACGCGTA
TTTATGTAATTAATACTTTTTTTTTTTTTTTAAGTGTTACAATCGATTTATCGCATTCCAAC
AGATTCACAAGCTCTTTGAGTCGGAAGAATGATTAAAAATGGCGGAAGAGCGTTGTTA
CGAGGAAACTCGGTCGCGAGAAACAATGGTGTAACTCTATTAGGATCGTGCAAAGGTT
GGCGGGCGAATATGACGATTCTAGGAGAGGTTAAATCAGGCCCACATGCGCATTTCACT
GGGCGTGGCCGGCCACTGTACTCCACTTAATCCGAACGACAACGTCGACGAAACACG
CTTCCCTTCGCTGCAGAATTCTCCAAAACGATTGAATTTCCTGGTTCAGTTGGATCGAT
AAACGGATAAGGGATAATGAAAGGAAAGAAAAATCCAG

>novel_circ_001700
AGGATAAGGAGAGTGGCGTAGATTACGGTCGTAAGCTGGATTAAGGGCACGGACGTCA
TGGAGCGGTACGAGGAGGAAGGCGAGGACAGCGACAACGAGACAGACCCGGAACAA
CTGTTGAACGAATGGCTCGGCGAGTTGGACAGCCTGACCGTGGTGTGCCTCCTACGTT
CTGTTGTGCAACGCAGAGGAAGTGGAACGAGCCGTCTACCGAAAGAAGAAGGAACTT
TGAACTTGAAAAAAAAAAAAAATTGATCATTTCAAACGAAATTACGTTTTTTGGTAAG
ATAAACGTATTAAATATCATCGAATAAAGAATCGTTTAACGTTGTGCGAAAATGTATTCA
TCGAAAAAAAGAAGAAGAGAAAGAAATTGTAAAAGAAGCAATATTTACGTGTTTACA
AAAGACGAAAAAGAGATGGTTGGTTTGGAAATACAAGATTGGGATCCACGGTATGGG
AGAGGAACGAAAAGGAGAGGATTTTGAGAAACACTGCGTATCTGTGCCGAGCGCCAC
GCGACCAGGTCGTCCGGTCGGGCTGGGTGCCGGGTGTTGTTGAAATCAACGTGGTGG
GGTTCCTTCGACGACCACAACCGGAGCCTTATGCCTTCAGTCGACTCTCGGCTCTCACT
GCGACTCGAGCTGCCTGAGATTTCCGTTAATTAACCAACTACCGATCCGCGACAGCGG
CTGGCCAACCACATCGCAGCCGCTCCAGATACGAATTCACCTTTCCTCTCGCCGGAATC
CGGAGTTGTCGTCCGCCAAAAAAGAAAAGAAAAGAAAGAGAACATCTCGAGAAGAA
GAGGCGAAGTCTGTTCAATCCCAATCCATTCCAATTGCGCGACGTCTCGTCCCGGAGG
ATGAATCGATCGAAGTCGGGCGAGGATCGACGAGGCCTACCGTTTTAAAAGCGCGAGT
GAGCGAGCGAGCGAGCGACTGTGAGGAACGATCGGCCGATCCCGGAGATCCGATCCG
ACCAGTTCCTTTTACCGGTCGATCACCTCGATCCCGCGTCACAATCGCTCGTGGAGCGG
GACGTGCACGTCGCGTACGCGTCGTTGGCTGTTAATTAGTACGTGGGTGCCGTGATAGA
GTGGGAAGAGACGAAAGAGGGAGAGGGAGAGAGAGGGAGAGAGAAGAGTGAGTGG
ATGCGAAGAGATGCCGAAAGCGGAGGGAGGACGATTGAGAGGAGCGGAGGAAGAGA
GGATCGAGAAGTAAAAGAGGCGAGAAAGAGGGTTGGAGGTTAAAGCCGGAGTTAAAT
AGATGCAGGAATCTGTACGCGGCGGCGCCATGCTGGTGTAGCGAGCGAGAAAAAATAT
TCGGTTTCCCGGTGTGTATCTCCTCTCCGTGTGAACGTGGCCGTGCGGATGGAACGTAG
GAATCCCAGGAGCGTAACAGTGAACGCGTTCGCGGCCGGTTGAAGGGAGCGTGAATT
CGTTCAGGAGGACCAGTTCGCCGCTGCACAGGCGTTAAGAACCCGTTGAGACGTTGA

GAGGAGGAAAAGAGGTGAACGGTTCGGGAGAATTGTCTCGAGAGGAGATAACAGTG
GACGATTCTTCTCGGTGAAAACGAATTTTATTATAAACGAGTTGGAAAAGGATCGATGA
TCCTCGATCAGTGATTATTTTAACTTAATTTTTCTTTTCTTTTCTTCTTTCTCTCTCTCTCT
CTCTCTCTCTCTCTTTCTTCTCGTGTTGTACGAACGAGTATAAAGGAATATACGCGAACG
CGCGAGGAACATGTTGTGCGGCACGTTCAGGAAGAAGAGAAGGCATCCAGGGGATGA
GTATCGATTGGTGAGATCGAACACGATGCCTAGAATCCTGTCGGCGAAGGAAGGTAGC
CTGGTCACGGTCCGGAGAACAAAGTCCTCGAGGGTGGCGGCTCGTTCCCCTCATCTCA
GGGATCTTCTGCACATGTGGAGAAATAGAGTCGAGCTAAAGCGACGAGGGAAGCTGT
AACGAAATACGTATAAAGAGCGCATATATAATATATGCGACGGAAGAAAGATAGAGCGA
GATAGAGTTGGCAAAGAAACAGAAACGAACGAGAGGAACGAAAGAACGAAGAGGG
AAAACAGGAGGCTAACAAAGGGTGGGAGGAAGACGGAGTGAAGGTAGCGCGATTGA
GGCTAACGTACAGAGAACAGAGAGGGTACTAGAGAGTTGCGGGGAGAGTCGTAGAGT
GGAACCAAGATGGGATGACGAAACTACGAGGAGGAGAAAGAAGAAATTAAAGGGAG
AGAGAGAAGATTGAACGAGAGGAAGATAAACGGTAGGTCTGACTGAATCATCGAAGA
AGGATGGAAGATACGATGCGGTTGTGAGTAACGGGGAAAGAACGACGAGAGAGAGGT
AGTGAAAAGGTGACGAGTGCGAGAGAGACGGAGGAGCGGCGGACGATTCAGTGAAA
AGAGCGAGAGTGAGCGACAAACGGAGCTTTCGAGGGTACGAGAGAGAAAGTATTACT
GTTCCCTGTCCGTGGAAAAGAGAGGGAGAGGGCAGATGAAGCACGTCTGGACAAGA
GAAAGAGAGAGTGTGAGCGTGGAAGGGAAAGAGCAGGGACGAGACAGAGTGCGCC
CACCGAGCCGCCTCCATACCACCTCCAGCATTCCCGGCCTGCACACACAGTTGCAGCT
GCACCGCCGCCGTGGTTCGCTCGTGTGCACATGCGAGACTGCGCGCGCGTCCATCTCT
CGCTCGCTCGCAATCATCTCTTCGCCGGTACGCTGTGTGTATTAACGACGCGCTCTCTT
CTCGATATCGAACCGTATAGCGTGTAAGTTTTTCCTCTCGACAGCAGAGTGTCGTGTAC
CAAGTTCGAGCAGCCAGTTTTCCTCGTGACTGTCGCGCAAAGAGGATCGTGATCGTAT
CGATCGGATTATTCGCCGTCAATGTTCGCGAACGAAATTTCGAAAAACTTTCAAACTTT
TCGAAAAATCGCATCGATATTGTTCTCCAACGAAATTATCTTCATCCATAGTGAGATATC
CAGTGAACAAATTTTTTCCAAATAAAATTTCTCGGAAGCATATACGCGATTGGTGCACG
TGCATTCGTGGTCCCGGTAACACGTCGAAATTTCGACATCTTGTTAATTCCAGGAATATA
TTACACGATCGATCAGAAAAACTTTGACATTTGGCAAATGTCAGCGGTAGTCTCTGGC
GGAGTCTCTTCCGTATAATCGAATCGTGGCTCGTGATAAACGATAAGAAAAAGCGGTTA
CAGCGGTCGAGTGTTTCTCTGTTCTTTCGATACACACGTACGCACGCACACACGAGTAT
CGCGGTTGTTTCCTTCGCTGTTGTGATATCCGATCGATTTCAAGGGAGACATGTGATCTC
GCGTCGAAACGAGTAAGGAATAGGATCGAGAAGAAGCATCAGCTTGACCGGTCGCTC
AGAGGCTCGAAAGGGATTTGAAGATGGATTGGATGAGAAGGCAGGACATTAAGGAGG
AGTCACCGGAAACTCCTACCGCTCTGACACCGGATGAGCCGGACGAGTCCTACTTCGA
GGAAGACACCTCGATAGACGAGGGCATGCTCGCGGATCACCATCCAGTGTTCGCAATA
CCACGCCAGTAGTCGCCGCTCCTCCGCTCCCAACCTCGATCGTCCTTTCTCGCGCTCCT
TCCCTCCTCGAGATCTCCAACGACGAAACGTCATCTCATCTCATCGTAGTGAAAGAAA
AGAAATTACGAAGAATTCTTTAGAATACAAAGCGTTTTTTTTTTTTTTTTTTTTACAAA
AATTTGTGCGATCTCGAGTCGGCGAAAACGATGCTGTCGGCAGTTTCCCTCTCTTAGAT
CCGGAAGGAGGAGGAGTCAGGTTGGAAGGATTACGAAGTGAGATCGATGTGATCGAT
TCCTGTACGGGCTGTCCCGCCGCCGGAAATCGCGCGCGCCGTCGCCACGCGGGAGGA
AATCCGAGCGGGCCGATGATTTCATTAGGCCGGTCCTCGCTCGAGAGAGGAGGCGGTT
GAACGTTGTTGAGAACGTTCAGGCCCATGACCGCGATGACTATCGTCGTCGCCAATAA

ACGATCCCTCGGCCAGGTTCAAGAGCGACGCGGTGTTTCACCCTCCCCCTCGTATCATC
ATCCTCTCCTATCCATTCCAGCCTCGATCGAGGAGGAGGGATCGATTCGAAGCGGGAA
AGATCTCGTATCAATCTCAAGTCGATGGAATTTTTCCAAGATCGATCTCTTCTCACGATC
GTTCCTCTCGCCATGTCGGGGATAATCCATGTGGTGGATCGTTACTCGGCCGTCCGACC
GATTTTTCTGAACGACGATCACCGAGGAGGAGGGGATTATCGAATTTGGAAGATGTGA
AGAAGAGGCAAAGGCCGCCAAATGGACAGGTTTTTCCGATGGAAAAGTCACAACAGG
GCGCAGGAGGACGGTCTGGACAATGTGAGCTCAGCGACCCTCAGGCCTTTCAACTCG
GACATCAACACGCCGAGGATCGACAGCTATAGGTTCTCCATGGCGAATCTGGAAG

>novel_circ_001701
AACTCTTTAGGAACGCAGCAGAGGCTGGACCTCTGGACATCGTGAAGCTTTGCAAAG
AGGACAAACTGTTGAACATTTCGACTAATTTGCCGGCGAACACACCCGATTCTCCCTAT
GTCCTTCAGATCGTCGGAGCTCATCCGGCCTCGTCGGGGGTGATCGAGGCGGAAGTGC
TGTGGGCCCTCGAGAGGCGTGTGGCGGCCTTGGAACGGCAACTTAGGGAGCATCAAG
AAGCTCTGTACGCATCGCCTTCGTTCGCTCTTCGCGAACTGAAGCGGCAGGTCGACAG
TTTCAAGAACAAGTTGGAGCACAACGACCAACTCAGTTGGCTGAGTTTCTACAAACA
GCTTCCAGAGCCCCTCTACGCGGATTCCTGCCGCAGATTGCAGTATCGGCGGAAGAGC
GACAGCATGAAGCGGAAGGTGCGAGAAAAGTTCCTCAATATTTG

>novel_circ_001702
AACTCTTTAGGAACGCAGCAGAGGCTGGACCTCTGGACATCGTGAAGCTTTGCAAAG
AGGACAAACTGTTGAACATTTCGACTAATTTGCCGGCGAACACACCCGATTCTCCCTAT
GTCCTTCAGATCGTCGGAGCTCATCCGGCCTCGTCGGGGGTGATCGAGGCGGAAGTGC
TGTGGGCCCTCGAGAGGCGTGTGGCGGCCTTGGAACGGCAACTTAGGGAGCATCAAG
AAGCTCTGTACGCATCGCCTTCGTTCGCTCTTCGCGAACTGAAGCGGCAGGTCGACAG
TTTCAAGAACAAGTTGGAGCACAACGACCAACTCAGTTGGCTGA

>novel_circ_001703
ATCGTGAAAGGAGCGGGGAAAGCATGGCCTTCGATTCGACGGGGAATAAAATCGTACG
CGCCGTCTGAGATTCTCGGCCCAAGCGTTCCCACGAAAAAGGTATTAGTTCCTCGAAC
CGCGTTGTTGGTTTGCCTTCAAAGTTGTTCGAGTGGCGAGCAAAGAGGAAGAGGAGG
AAGAGAGCGAAGGGGACGACGAGCAAACAAAAAGGGAAGAATCTTGCCAATTACTG
CCGCTCTGAAATAAG

>novel_circ_001704
TTGAACGTTCACCAAAGACCATTGGGGCAAATTTGTCCAACTGGTGGAAGTGTTCCAC
AAGGGCAGCAGAGCAGCGCAGGTTCCGTCGGCTCGGCACCTAGTCCGGCATCGCCAA
GACCAACTCCTCAACCACGGCCACCCGTTGCATCGACCGCTTCGCAACCCTCGAGAAC
GTTGGACGATGACAGATCGACGGACGCTGTAGGTACTGGGACCGCAGCGGAAGATTC
CGACGATGGCGAGAGCATAGCGCAATACGTGAGCACAAATTGCGTCGTCTTCACCCAT
TATCGAGGGGATGCCGCGTCCGAAGTGGAGGAACACTTTCAAAGGGCATTAGCGCAC
GACAAACCGAAAG

>novel_circ_001705

AATGTTGGCAGCTGGTAATAAAATCCAAGAACATACCATCAATGGTGAAAGCCAAGGA
CACCGATTACGTGATCCTGGACTGCGACTACGATCTCGAGGACACGCCGAGCAAGGGA
TTAGTCGTAAAATGGTTCTTCAACGCCAACGAAGTGGCTTACCAATGGATATACGGTAG
GGACCCTTTGGCCGGGGACATCACGCGGAAGTACGTCGACCTGAAGTACAAAGCCAG
CGACGATCCGTACACCACGTATCGAGCCATGAAATTGAATAAGCCCGGTATCGATCTTA
CTGGCGAGTACAAGTGCGTCATATCCACTTATGCGGATGAGCAGTCCGCGAGTTCCTCC
ATGGTTGTGTATTCAACGGAGGACAAATTCGAACTTCTATATAAAAAGAAAACTATAGA
TGATAAGGATGGAGTAGAGATAACATGCATAGCAGAAGGGTTATATCCAATACCCACTC
TGGATATATCTATCGAGGGTGTCCTGGAAAAACAAGCAGCAAAGCCCACCGTTACGCT
GCGTGCCGATGGACTGTACAATATCTTATCACGGACGGCCTTACTGGACGAAGATTTAC
CGGAAACAGCGATTGTCAAATGCTTGCTCGGTATACCAAAGGTGAACTACAATGTTTCC
CGCAAAACCGTCTACTATCCCG

>novel_circ_001706
AATTAGCAGATCCTATTTGACCACTTGGACCCATTTGTGCGCCTGGACCGCCAGATCCC
ATTTGATTATTTGAACCATTTCCTGGACCCATTTGTCCACCTGGCCCATTTCCTGGACCA
ATTTGACCACTAGGTCCACTTCCTGATGTCATTGGATTTCCAGAAGGATTTCCAGGTCC
CATTTGATTTCCACCACTTCCAGGTCCTATTTGTCCTCCAGTATTATTTCCTGGGGTTATT
TGTCCTCCACTACTTGATCCAATTTGTCCACCAG

>novel_circ_001707
AAATGGATCAGAATGGTGGAGATTGCGGAAAGAATTTCAAAAAGTTTCTAGTAAACCT
CAAGATGTGATTAATTATTTAAAAGAAACAGATTGTGTTATCCAGGAATTTGTTGAGTTA
TGTAACAATGAAAAATTTGCAGATTTTTTACCACTTTTATCACGTTTATTTCTTGAATTG
ACATGTTTGGTTGTTTTTGATATAAGACTGAATAGTTTTTCAAAAGAAGAAAGATGTGA
AAATTCTATAAGTTCAAAGTTAATTAAAGCTGCCTTTGCAACAAATAGTGCAATTTTAA
AGTTAGATAATGGATTGCAATTATGGCGTTTATTTGAAACACCATTATATAGAAAATTAC
GTAAGGCACAAACATATATGGAAACGATTGCATTAGAATTGGTATCTCGTAAAAAAAAC
AATATGAAAATACGATATAATAAATCATTTCTAGATGCTTATTTAGAAAATCCTGTTTTGG
ACATTAAGGACATTGTGGGCATGGCTTGTGATATGTTACTTGCTGGAATAGATACT

>novel_circ_001709
CTTTAAAACCAAAGGCAATCTCACGAAGCATATGAAGTCGAAAGCTCATTACAAAAAA
TGCGTGGAATTAGGCGTTGTCCCAGTACCTACAGCAGTCTGCGATGAAAACATCGATA
AAGACGCTATAGCCCGGTTAGCAGCAGGTGGCAATATCGAGGAGTCCTCGGAGGAAG
AGGAAGAGAGCGAAGGAGAGGAGAGCGAGGAATCGAGGAGCGAGGAGCAAGAAGC
TGCACAGAGTTTGCTAAGTCTGTCGCAACGTAATACGAATAGATATCCAGGATTATTACC
ATTTGGACGACCTACGACTTATCCTTACACTCTCACCTTCCCGACAACTTCCACATTCG
CAAGCGTATCCACCACGATAAGCAACAATACTTGTCTGACTAGCCAATCTGTCACTATG
CAAAAAACCACTCTCGTTCGAAACGAATTGTCCCAACGCTACTATTTTTCGAGTCGAAT
CGGGCCCGAAGACTCGAGGACGAGCGTAATCCAATCATCGAAGAAAGAAGAATGCCC
TGATGTCGAAGTGAACGAGATCGTCGATATCGATTCAGCAAATAGTTTGCCGCAGCCCA
TAGATCTGACAACGAAGCCAGCCTTGCAAACATTGCCCTCTTCCATACCGCAAAGAGC
CAGACCAGCAGACATTTTAACTCCCGTTTCGGAACCGGTCTTGTTACAAACGATCGTTC

AAACTATGGAAAGACTGCCGATACAGGGGAGAGAGTGGAAACCGGATGCCGAGGGAC
ACATGTTGCAAGCGTATCTCACTGAAAGGCATGTTATGGATAGCAAGATTAAACAACAG
TATCGGGTGGGGAACATTAAGAAACAAGTGAAGGAGAGGGATATTTATCCGCAACAAA
ACTCTCCGAAATCGAGAAGCATTGAATTAATGGAAAGTATTCCAGCAACGACTTATACA
GATTCCACCAAGATGCAAATGCAGGCAGTGATAGATTCCAGAATCAAGCATACATCGA
AACATACCGCGGAAAATATCGGTTTAACGAGTCCTGTCAATTCGAAATTGAATCACGAC
ATACGTTTGAGCCAAGATATGCCTTCCTCGAATAACATGGAGACTGCCAGACCAGTCAA
CATAGATAACAGACCTCCGAGCAGAGATATGATTTTATTGTCGGATAACAGACACCATG
CCCAATCTCAAGAATCGAGAGTTAATTTTGAAAAGTTGCCATTAATGATGCACGAATCC
TCTAAAATACATGAAGTCTCGAGACCGCAGAACATTGATATGAGAAACATGGATCGTAT
TGAAATGAATCATACGGATATAACGAAACAAAGCATTGCGGAAAGTATTAAACATACAG
TTGCGCGGAAAATGGTTGTTGGAGGGCCAGGCTTCAGATCACCCTTTCCTAATGCATCA
ACCACCAAACCACAAGCTGAATTCTTGCAGCCTTCGAATGGACCTGCCCCTAATTACG
TCAGTTACAGTGTGACTGAGGATGGAAGAAGCGTGTGCGGAATTTGTAATAAGGTGTT
CAGCAAACCTAGTCAGTTACGGTTGCATATCAACATTCATTACTTCGAGAGACCATTTA
GATGTGAAAGTTGCGCTGTCTCTTTTCGAACCAAGGGTCATTTGACAAAACATGAGAG
ATCTGTTTCTCACCATAATAAG

>novel_circ_001710
AGTTGCTCTTTGGATTCTGTGACTCATGATGTGATTTGCGAATGTTTCGACGGATACACC
GGTCCTCGTTGCGAAAGCTGTGCGGAAAATTACTTTGGCCATCCTGAGATTCCTGGTG
GCAGCTGTGAGTTTTGTAACTGCAATAACAATACGGACCTTCGTCTAGCTGGAAATTGT
GATCCTCATACTGGACGTTGCCTACAATGTTTGTACAACACTGATGGATCAAACTGTCA
GATATGCAAACCAGGATTTTACGGAAACGCTCTTGAACAAGATTGTCAGGATTGCCAG
TGTGATGTTTTGGGTACAGATAGAAATGCTGGACCTTGTGATCATCGAACAGGACAATG
TCCTTGCTTACCTCATGTGATCG

>novel_circ_001711
CTCCCCCACCTGGACCCACAACAAGTTCTTCTGTTGGAGCAGCTGGAGCAACTGGAA
GCAGTGTTGTAGCTTCTAGTTCTTTAGGGCTTGTTTCCACACAACAAACACACACTCCT
CCTCAACCTCCTGAACTGCCAATGTTTTCATGTCCTCGGAGACCAAATATAGGACGTGA
AGGCAGGCCAATTGTCTTAAGAGCTAATCACTTCCAAATAACAATGCCACGTGGTTATG
TGCACCATTATGATATTAATATTCAACCTGATAAATGTCCTCGTAAAGTTAATAGAGAAAT
AATAGAAACAATGGTTCATGCATATACCAAAATATTTGGAACTCTTAAACCTGTATTTGA
TGGAAGAAATAATTTATATACAAGGGACCCCTTGCCTATTGGTACTGATAAAATAGAATT
AGAAGTAACACTGCCTGGTGAGGGTAAAGATAGAGTTTTCAGAGTTGTGATAAAGTGG
TTGGCTCAAGTTTCACTATTTGCATTAGAAGAAGCTTTAGAAGGACGAACAAGACAAA
TTCCATATGATGCTATACTTGCTTTAGATGTTGTAATGAGGCATTTACCATCTATGACATAT
ACTCCAGTAGGTAGATCATTCTTTAGTACACCAGATGGATATTATCATCCATTAGGTGGT
GGAAGAGAAGTTTGGTTTGGTTTTCATCAATCAGTTAGGCCATCTCAATGGAAAATGAT
GTTAAATATTGATGTATCTGCAACTGCATTTTATAAAGCACAACCTGTCATAGAATTTATG
TGTGAAGTATTAGACATTCGAGATATAGGAGACCAAAAAAGACCTTTAACAGATTCTCA
ACGAGTTAAATTTACCAAAGAAATTAAAGGACTTAAAATTGAAATCACACATTGTGGA
ACAATGAGACGAAAATACAGAGTGTGTAATGTTACACGTAAACCTGCTCAAATGCAAT

CATTCCCGTTACAATTAGAAAATGGACAAACAGTTGAATGTACAGTTGCAAAATATTTC
TTAGATAAGTATAAAATGAAGTTACGTCATCCATATCTTCCATGTCTTCAAGTTGGACAG
GAACATAAACATACATATTTGCCACTTGAG

>novel_circ_001712
TGTTTTCATGTCCTCGGAGACCAAATATAGGACGTGAAGGCAGGCCAATTGTCTTAAGA
GCTAATCACTTCCAAATAACAATGCCACGTGGTTATGTGCACCATTATGATATTAATATTC
AACCTGATAAATGTCCTCGTAAAGTTAATAGAGAAATAATAGAAACAATGGTTCATGCA
TATACCAAAATATTTGGAACTCTTAAACCTGTATTTGATGGAAGAAATAATTTATATACA
AGGGACCCCTTGCCTATTGGTACTGATAAAATAGAATTAGAAGTAACACTGCCTGGTGA
GGGTAAAGATAGAGTTTTCAGAGTTGTGATAAAGTGGTTGGCTCAAGTTTCACTATTTG
CATTAGAAGAAGCTTTAGAAGGACGAACAAGACAAATTCCATATGATGCTATACTTGCT
TTAGATGTTGTAATGAGGCATTTACCATCTATGACATATACTCCAGTAGGTAGATCATTCT
TTAGTACACCAGATGGATATTATCATCCATTAGGTGGTGGAAGAGAAGTTTGGTTTGGT
TTTCATCAATCAGTTAGGCCATCTCAATGGAAAATGATGTTAAATATTGATGTATCTGCA
ACTGCATTTTATAAAGCACAACCTGTCATAGAATTTATGTGTGAAGTATTAGACATTCGA
GATATAGGAGACCAAAAAAGACCTTTAACAGATTCTCAACGAGTTAAATTTACCAAAG
AAATTAAAGGACTTAAAATTGAAATCACACATTGTGGAACAATGAGACGAAAATACAG
AGTGTGTAATGTTACACGTAAACCTGCTCAAATGCAATCATTCCCGTTACAATTAGAAA
ATGGACAAACAGTTGAATGTACAGTTGCAAAATATTTCTTAGATAAGTATAAAATGAAG
TTACGTCATCCATATCTTCCATGTCTTCAAGTTGGACAGGAACATAAACATACATATTTG
CCACTTGAGGTCTGCAATATTGTTGCTGGGCAACGTTGTATCAAAAAATTGACTGATAT
GCAGACTTCTACTATGATAAAAGCTACAGCTCGTTCTGCTCCAGATCGCGAAAGAGAA
ATAAATAATTTAGTTAGAAGAGCTGATTTTAATAATGATTCATACGTTCAAGAATTTGGT
TTGACAATATCAAATAATATGATGGAAGTTAGAGGTCGTGTTCTACCTCCACCCAAACT
TCAGTACGGAGGGCGCGTAAGTTCCCTCAGTGGACAGACAAAACAACAAGCTATACCT
AATGGTGGTGTTTGGGACATGCGTGGAAAACAATTTTTCACGGGTGTGGAAATTAGAG
TTTGGGCAATTGCCTGCTTTGCTCCACAAAGAACTGTGCGAGATGACGCAATACGTAAT
TTTATAGCACAATTACAAAGAATAAGCAATGATGCTGGAATGCCAATTATTGGGCAACC
ATGTTTTTGTAAATATGCTACTGGCCCAGATCAAGTAGAGCCCATGTTTAGATATTTAAA
AGCAACATTTTCATCATTGCAACTTGTTTGTGTTATATTACCTGGAAAAACGCCTGTATA
TGCTGAAGTTAAAAGAGTAGGAGATACATTATTGGGTATGGCAACGCAATGTGTACAA
GCAAAAAATGTTAATAAAACATCACCACAAACGCTATCAAATTTATGTTTGAAAATTAA
CGTAAAATTAGGTGGTATTAATAGTATTCTAGTACCCACTATTAGACCAAAAGTATTTGA
TGAACCTGTTATATTTTTTGGAGCTGATGTGACTCATCCACCTGCTGGAGATAATAAAA
AACCTAGCATAGCTGCAGTAGTTGCCAGTATGGATGCTCATCCATCTCGATATGCAGCTA
CTGTTAGAGTTCAACAACATAGACAAGAAATTATTCAGGAACTTAGTTCAATGGTGAG

>novel_circ_001714
GGGAGGACGGAGTCGAAGGCTCAGGGCCCGGCGCGGACCTCCAGGGGGAGGAAGGC
GGCGATTGCGCGGAGGATGACGGCCCAATTAGTCCTAGCGAGAGAGGTTGCGCAACG
GTGGCCAAGGAGGAGGAAGATGGGGACGCGACCGACGAGGAGGACGACGAGGACG
GATCGCCGCCCGCCCCATCGCCTTCCGCGACAGCGAGACTGAACCCCGCCATTCTGAG
GGACAGTGGACCGCCGGACAG

>novel_circ_001715
GTAAGTAGTGGTCTCCGATCGTTTGAAAAGCGAGCGGCCCGCAGCCGCCGCACCGGA
CGGGAGACATGCGCAGCAAGCAGCAGCCGAGGCCTTCCTTTCGGTGGCCGCAGCAAC
GCGGCGATAATCTTACCGGGGAGGACGGAGTCGAAGGCTCAGGGCCCGGCGCGGACC
TCCAGGGGGAGGAAGGCGGCGATTGCGCGGAGGATGACGGCCCAATTAGTCCTAGCG
AGAGAGGTTGCGCAACGGTGGCCAAGGAGGAGGAAGATGGGGACGCGACCGACGAG
GAGGACGACGAGGACGGATCGCCGCCCGCCCCATCGCCTTCCGCGACAGCGAGACTG
AACCCCGCCATTCTGAGGGACAGTGGACCGCCGGACAG

>novel_circ_001716
GTGTGGGTAGATTCTCCCGTAGGAGCCATGAGTTATCAAATGTCATCGAATCAATCTAG
ACCAATGTCTCACGGAAATTATCAAGGTTTGGGTGATTTACCCATGGGTTCTGCCAAAG
AACAAACTTTAATGTGGCAACAAAACTCTTATCTGGGTGACTCCGGAATTCATTCTGGT
GCCGTCACCCAAGCACCATCTTTATCGGGCAAAGAAGACGATGAGATGGAAGGTGATC
AATTGATGTTTGATTTGGATCAGGGATTTGCTCAAGGTTTTACGCAAGATCAAGTCGAT
GAAATGAACCAACAATTGAATCATACAAGATCTCAGCGTGTTCGGGCGGCAATGTTTC
CAGAGACATTGGAAGAAGGTATAGAGATTCCCTCCACTCAGTACGATCCAGCTCAACC
TACCGCTGTTCAAAGATTGGCAGAACCAAGTCAAATGTTGAAGCACGCAGTTGTCAAT
TTGATAAATTATCAGGATGACGCAGATCTTGCAACGCGCGCGATACCGGAATTAATAAA
GTTGTTAAACGACGAGGATCAAGTCGTTGTGTCTAAGGCAGCGATGGTAGTCCATCAA
CTTTCCAAGAAGGAAGCTTCTCGCCACGCCATAATGAATAGCTCGCAAATGGTGGCTG
CCCTGGTCCGTGCCATTTCAAACAGTGACGACCTCGAATCGACGAAGGCAGCGGTCG
GCACGTTGCACAATTTGTCTCATCACAGGCAAGGTCTGCTGGCTATTTTCAAGAGCGG
CGGTATACCAGCTCTCGTGAAGCTTTTGAGTTCTCCGATGGAATCGGTCCTCTTTTACG
CGATCACGACACTGCATAATTTACTCTTGCATCAGGATGGTTCTAAAATGGCTGTACGA
CTCGCAGGAGGGTTACAAAAGATGGTTGCCCTGCTTCAACGGGACAACGTGAAATTCT
TAGCCATAGTAACCGATTGCTTGCAAATTCTTGCGTACGGTAATCAAGAGAGCAAATTA
ATCATATTAGCTTCCCAAGGGCCGATAGAACTGGTACGCATTATGCGTTCCTACGATTAC
GAGAAGCTTCTATGGACGACATCGAGAGCATTGAAAGTGTTGTCCGTTTGCCTTAGTAA
TAAACCGGTGATAGTCGAGGCTGGTGGCATGCAAGCGCTTGCCATGCATCTCGGTAATC
CGAGTCAAAGGCTCGTCCAAAATTGTTTGTGGACGTTACGAAATTTATCGGACGCTGG
CACCAAGGTCGACGGATTGGAGGGTTTATTACAAAGTCTTGTACAAGTTCTTAGCTCTA
CCGACGTGAATGTCGTCACATGTGCCGCTGGTATTTTGTCTAACCTGACTTGCAATAAT
CAACGTAACAAAGTAACGGTATGTCAAGTGGGAGGTGTTGACGCTCTTGTGCGTACGA
TCATCTATGCTGATAGCCGGGAGGAGATCAGCGAGCCAGCGGTATGCGCGCTCCGTCA
CTTAACGTCACGCCACGTGGAGGCAGAAATGGCACAGAATTCTGTCCGCCTAAATTAT
GGAATTCAGGTTATAGTAAAACTTTTACACCCGCCTTCGCGTTGGCCGTTGGTTAAAGC
GGTGATAGGATTAATTCGCAATCTGGCGCTTTGCCCCGCGAATCACGGCCCGCTGCGCG
ATCACGGCGCGATCCATCATCTGGTCAGGCTTCTGATGCGCGCTTTCCCCGAAACGCAA
CGGCAGCAACGCTCGTCCGTGGCAAGCACCGGAAGCCAGCAAGCGTCAGGCGCGTAC
GCAGACGGTGGTGTACGAATGGAGGAGATAGTGGAGGGTACTGTGGGCGCGCTTCATA
TCCTCGCAAGGGAATCTCACAATAGGGTCATAATTAGATCTCAGAACGTGATTCCGATT
TTCGTACAGTTATTGTTCAACGAGATTGAGAATATTCAACGTGTCGCTGCAGGCGTGCT

CTGCGAGCTCGCCGCGGATAAGGAAGGTGCCGAAATGATCGAGCAAGAAGGTGCTAC
AGCTCCTTTAACAGAACTGCTTCACTCAAGGAACGAAGGTGTCGCGACATACGCGGCA
GCTGTCCTGTTCCGCATGAGCGAGGATAAACCACAGGAATACAAGAAACGGCTGTCCA
TGGAACTTACCAATTCTTTGTTGCGCGAAGATACGAATCTTTGGAATAACGCTGACTTT
GGGATGGGTCCAGATCTACAG

>novel_circ_001718
GGGTAAATACGCGACAGTGAAGAGATGTCGAGAGAGGTCCAGTGGACGACAATGGGC
CGCAAAATTTCTACGGAAGAGGAGACGAGCGCAGGAATTGAGAGCGGAAGCGCTTCA
CGAAGTCGCGGTGCTGGATGCTGCGGCCAATTGTTCCCGTCTGGTATCCTTACACCAAG
TCTTCGAAACGAACACGGAGATGGTCCTGGTTTTGGAATTGGCACCCGGCGGTGAATT
ACAAATGATATTGGATAGAGACGAAGTTCCGGAAGAACGACAAGTGGCCAGATTGTTG
AAGCAGATTTTAGACGGAATAGCGTTCCTGCATTCGTTGAACGTGGCTCATCTCGACAT
CAAG

>novel_circ_001720
GATGGCGCCAAAGAAAATCGAGGAACCGGAACGGAAACCGCTTATTGGCCGTGTCGG
GACCAATTTGAAAGTTGGTATAGTTGGAATACCTAACGTGGGGAAATCGACCTTCTTCA
ACGTGTTGACCAAGAGTCAAGCGGCCGCTGAGAACTTCCCCTTCTGCACCATCGATCC
CAATGAAAATATTATAATTGAATAATTAAATCCTATCGACTCGTTGAATTTCGAAAGAAA
CGTATCGATCTTGCGCGAATCGATGAAAGAGGCGCGCGTCGTTTGTTCGAATTTTGCTC
GAAAGGGATTTTCAAAGATAACCTGAAAAATGAAAATATGTGCGACAAGAAACGCATT
GCACATTTACGGAAGAAGAAGAAGAAGAGGGAAGAGTTCGACGAGTTCGACACGGA
CGCATCGTTCATCTTGAAGAAAAGTCACTTCAAACACGTTAAAGGTTACCCCCGCGAG
GAATACGCCCCCGAATGGCCCAAGCCTCTCCCCCACCTGTACCCGAAAGCGAACAATT
ACTACGCGTTTCACGAGGACGCGAACCCGTTCACCAGATTCTCCGACTCGTGTTTGTC
GTCCAACGACCACGAGAACACTCCGCACTTCCCCCCCTCCGAGGAGCCGTACGCGAG
TCGGGACCCGTATCTGGACACCGACGCGAGAGCGAAATACCTGGAGAAGGAGCCGAC
CTGTTTCGTGATGCTCGGCAAACCCGACCTCAACACCATGAAACTCGCGAGCATGATC
GCCACCACGTGGAAATGCATACTGATCTGCCCCGTTTACCTGATCAAACTCGAGATCGA
GGAGGGTAGCGAGAAAGGCCGATTGATGGCTAACATATTGAAGCTGGGCGGTTACGTA
GGCCCAGATATAATCATGAACCTGATACTGAACCGTCTCAACAAACGGGACATCCGTTA
CAAAGGTTACATCGTGGAAGGGTTGCCGCTCATCCCTAACAAGAAGCTCGACTACTCC
TCCTATCTGCCCATGGACGAGGCGGCGATCAACGAAGCGTACAGCAACGCCATGAAAT
ATTTCAACCCGTCGCTCGAGATGACGTGCGGGGAGGCGAACCGCGGGGACTGGAAGG
GTGGCGGGTGCGCGGTCGAGGCGAGTTACGAGGAGTTCGTCTCGAGCCAGATCGAGG
ACATATTCTCCAACTGGCCGTTGAAACCGACCCTCATCATATACCTAATGTGCCCGGAC
GTGGACCACATGAAGAAACGGAACCACTTCCGTTTGAATCCGTTGATCGGCCGTATCA
TCGACACGAGTTTCCTCGAGATGACGGACAAGATCCAAATGTTGTTCAACCACGAGAA
GACCAGACACTACTTGAACGCGAGCTACGAATTGTACAAAGAGTTGATGGACGAGGA
GAGGATATTGGACGAGGATCACGGCAAGTATCTGTTGAAACGGATGTCGGACGAGCCG
TCCAACGTGAAGATCCAATGCGAGTTGTACAAACGTTTGTTCATACCCGTCATCGACAA
ACGCATCCTGCTGCACAATCCTCAGAACGTGATCCGCCTCGACGGCCGCTCGCCCCTC
GCGTTCATGTTCCGCACCCTGAGCGCACGGTTGCGCACGTTGCCGGTGCGACGCGTCA

TCCTGCCGTCGAGGATGACGGAGAAGCTCGAGGATGAGTCGTCGCCCGGGGACGAGT
TCGAGGAGAGATCGAACGAGGAGGCTTATCGCGACTTGGCCAACAGGGAGACCGTCT
CCCCGTTGTTCCCTTGGAGATTGTCCACCTGGAATTTCCTCTGCCCGGTCGAGTTGACC
AGAGGAAGGACAAGGGTAGGGAGCCCTAGGCACAGCGTCCGTTTCATGAACAAGATA
TTCTTCCTCTCGTCGAGCAAGGCGGTCGATCTGTTCGTCGAGAATCCGCGAACCTTTCT
CCACCCGTTCTCCCCGCGGCCCACGTGCAGAGTCGTCGTGTTCGGCCCCGATTACTCG
GGCAAGTCCGACCTCTGCCGCCGGTTGGCCCGAGAGTTGAACGGGGTCGTGATAAAC
GTGAAGGAGATCAAGAGATCCTGGATCCCCTTCGATCCAGAGGAGGAGCAGGAAGAG
GAGGAGGAGGAGGAGCTGTATCTGACGAGCGGCGTTTCGTTCAGGAACGAGGCGGAC
GCGATTATCAAACGGATAAGAGATATACCCGGGGAGAGGATCGACGAGAAGGTGTGGA
GGAACGGTGGCTACGTGGTGGACGGCATGTACCCGAGCGTCGACTCTTGGAACATGAT
ACGGGATTCGGGGATCGTGTTCGAGGACGCGATCCTCCTGGTCGACGAGGAGCCCTAC
GATCACCTTACGTCCAAGTGGCGCGACATCTTCGAGGTTAAAAACGAGACGTTCGATC
GTTCTAGAGAAGAGGAGGAGGAGGAGGAGGACGAGGAGGAGGAGTACAAGGAAGG
ACTGTTCGAATACGTAAGGCGCGTGCAACAGTTTTACCTCGATTGGAAGGCGATGGAG
GAGATGGTCGCCGACAGTTGCGAGACGTTGATCCCCTGCAACGTCGGGAAACTCGTC
GACCCGTTCGCCCACGCGATCCAACGGATCAAGGATCGTTACACGGACAAAGCTAGGA
TCATGACCGAGGAGGAGAAGGAGAGGGAGAAATTTCTGGCCGAGTACGTGGGCATGG
CCGACGTGACCGTGGATATGGAAGAGGAAGAGGAGGAGGAGGAGGAGGTGGAGGGG
GAGGGAAAGGAGGTGACGACAGGGGAGCAGAAGCAGAGCGGCAATCTCCGTTTCGG
CGACACGGCCAATTACTGCCCGGTCGCGTTGATGAGGTACAACGTGTTCTGGAGAGGG
AAGGAAGAGTTCGCCGCCATTTTCATGGACAAGATATACCACTGCTCCAACGACCGAG
CCCTCGAGGAATTCTTGCGCGACCCGGCGAGACTCAATCTCCCCTTGAGGAAACCGTT
GCCCACCCTGCCACCCCTCCGCGTGAGCGTGATCGGCCCAACCGGGAGCGGGAGGAG
CACGTTGTCGGACGCGTTGTCGCGTGAGTGCGGCCTCGTTCACGTGGATTACCTCAAC
TGCTTCGCCGCCTATATGAGGTCGCGCGCGATGCCACCCATCACCGACAAACACGCGAT
AATCGTGACGAACAGGGGAGAAGGTCTGCTGGAACCGGTCGAGCTGCCCGACGATCT
CGACGACGAGAGGTACAACAGCGACCCGAGGACCGTGCAAACGTTCGTCCGCCGTTA
CTGGAGCGAGGGTGGCCACCTGCCGTCCAGGATGCGCAAGGAGTGCCTGTTGGATTAC
TTCAACGGGCTGTTCAACCGAACTGGACTGGTGCTCGACCAATTTCCAAGCTGCCCCG
AAGACGTGGAGACCGCGTTGAGGGATTACGCGGTGCCCGAGATAGTGTTGGAGCTTC
GCTGCGGCAAGGAGACGGCTACCGAGAGGATGATGGGCAACCTGTTCTCGTTGTGGG
AGAAGAACTTGGAGGCGGAGAAGGAGGCCGAGGGGGAGCGGTACGCGATGGAGATG
AAGCATTACGAGAGCAGGAGGGACGATTGGATCAGGGAGACGTTGGAGAGGGAGGCC
GGGTTATTCGATCCACGGGAGGTGGAGGAGGAGGAGGAGGAGGAGGAGATCGAATCG
AGGAGGTACGAGTTGGAGGAGATGTGGTATCAGGAGAATCCCGAGCCTGTGTTGTTCA
CCGAGTGGGAGGATTTCGAGACGGCGAGAATGAGGATCGAGGAAGAGTTTTCGAGGA
GGTACGACAACGAGGCCGAGAGGGTGAGCGCCGTGAGGGAGAGTTTGGCCAACGAG
TCGATACCGTACGCGGTGATCGACGGTGAGAAGAGTCCCAGGGACGTTTTCCTCCAAG
CGATGCTCGTCGTCGAGCCGTACGCCCGCCGCACCTCGTCCACCCTCGAGCAGATATC
CACCGTGGACAGCGAAACGGCCGACAATCTCCTCGACTGCGGCTACTATTTCCTCAGC
TCGTTCGGCCGTTGGTGCCCGGTCCAGTTGCGCCGCGGCGAGATCCCCCTCCAAATGT
TCCTCCCGTTGGAGATCAGGGAGGACGTGTACCCGGTCATCCACCGCCAATACGTGTA
CTTCCTCGGCGGCAAGGAAGCGCAGGAGGAATTCGTCAAGGATCCGTACAAGTATCTC

GAGCAAGACTCTTGCGCGCCGGTCATCCAGTTTCGAATCTCCGTGATCGGGCCGCCTA
AGTGCGGCAAGACCACCCTCGCCGAACGATTCGCCAAGAAGTACAAGCTGAAGGTGA
TCACCCGCGGCTCGGCCCTGCGCTACATCCTCAAATACTTCCCCTGGGTGGAATCGGCC
GAGTCGGTCGAGTCCCAGCTACGCATGGGCCGGCTAGCCCCCATGGAGCAGATGAAAC
GAGCCATCGAGATGTACTCCATCGATCCGAGCATAATCTCCCAGGGTTACGTACTGGAC
GGTTTCCCGATCAGTCAAAAGGAATACGAACAACTCACTTATCTCGGCATGCAACCGA
TGGTAATCCTCGATCTGAGATCCGACCTAGCCTTCTCCGTCGAATGCTTGTCGCGGATC
GGGGACGACGACGTGGTGACGAAACCGCCCACGTTTTCCAGCAGATTTTTGGCCCAC
CTTTACGACGTGTGGCGGATCGATCAGGCCGATTATCGCCGCTGGTTGAGAAGTTTCAA
TCAGAACGTGGTCGAGCTGGACGCGACCAGGTGCATGTGGCACGTGTGGACCCGTGC
CGAGCAGAGCGTGTGCTCGATATACCTCGGGATCAGGAAGTATTTCCGCGAATGCGACT
ACGAGAAGGTCCACTGTTTGAGATACATGAGCGTGTCCCCGTACGAGTTCAGGAGCAG
GCAAAGCTCCAGTTTCCAGTCGTATTGTCCCGTGTGCTTGTACCGCGAGGACGCGATG
ACGAGGAGCGACCCTGTACCCGATCGTCGAGGAACGGTTCAATTCAGGGAACACTTGT
ACTGGATCTGTCCAAGCCACATGGACGAGTTCGTGCAGGATCCGGAGGGATTCCTTCC
ACCGGCAAACGCCAAGCGTCTCCCGGACGAGCGTCCACGAGTGTTGACGGAGACGAT
CGATCTGGAGCATCCGTGTTGGGTGAAACGGTTGCGGGTGAAAGGGTTGTGCTCGGTC
ACGTACGTGGACCATCTGCCCGACCGGAAAATCGTGCGCGGAAGATTGGACGTGGGC
GTGTTGTACAGGGACAACGTGTACCTGTTCTGCTCGAGCGAGTGCCGCGACAAATTCA
TGGAGAGGCCCGAAAGGTACGCGGACTTCGTCATCAACTTCACCCGCACTTTGGCGCC
GATCGACCCGAGGACGTTGCCGAACCTCGGCTACCTGGAACAGACGGTCGCTTCGTCG
CGTACCCGTCCCTGACGCAAGATTCGATTATCTCTGCGACTATTTCAAACCGGCCAGCA
AAGTTCCCGCTTTCTTGAACGTCGTGGACATCGCTGGATTGGTCAAGGGCGCGGCGGA
GGGGCAAGGATTGGGCAACAGTTTCCTCTCGCACATCAACGCTTGCGACGGAATTTTC
CATCTTTGTCGGGCGTTCGATGACGACGACGTTACTCACGTGGAGGGGGACGTGAACC
CCGTGAGGGACCTCGAGATCATCAGCGAGGAGTTACGGTTGAAGGACATCGAATTCTT
GAACGGCCATCTCGAGAAATTGGAGAAACTTGTTGTTCGAGGAAACGACAAGAAGCT
CAAGCCTGAATAC

>novel_circ_001722
GCTCGCAACAAACCAGCAGATGGAGTGGGCCACGTGAACCAAAAGGGCTCGACAGG
TCAAAAAGCGCCGCTGACAGCCGGGCAGAAGGTGGCGCCCGGTGCGAAAGTTGCGG
CGAAACCAGCTCAGAAACCCACTGCTCAGAAGGGGCAAGTAAAGGTTACGCCTAAGG
CGGCTTCCGTCAAGACTGGGAAGAATAAGAAGAAGGGGCAGAGGCAGCAGTACGAC
CTAATCGTGACCATCAATTTGAAGTTCGAACGGGCGAAGGAAGTCCTCCGCCACGCGG
TGACGTTGCCTGGCTCGCCAGCTCATAAATAATATTATTTGTGTTAGCCGTGCTAGTCGA
GTTTCTTCCGATTGAGAGAAGAAGGCAGCGGCAACGATTCTCTTAGTGCGACTCTTCG
AGGCTGAGCTTATTTATTAAAAGGGAAGAGGAGAGGAGAGAAAAGTCTCTCGCCTCGT
GAGAAAAATTGGTGAATGTAAATTTCGGATTTGTGAGTGAAGCGATATGGAAACGGAG
AAGAAATACACGACCGCCTATGGGAAATTGCGAAGATTGACGAGCACCATAGACGGCC
AGATTCGACAGATAAGCTCTGAATATGGACTCACGGAGGATCAAGTGGCGGAGTTCAA
GGAGGCGTTCATGCTGTTCGACAAGGACGAGGACGGCACGATCACGATGGCGGAGCT
CGGCGTGGTGATGAGATCTTTAGGGCAAAGGCCGTCCGAGACGGAATTGCGAGATATG
GTGAACGAGGTGGATCAGGATGGGAACGGTACCATCGAGTTCAACGAGTTCTTGCAAA

TGATGTCGAAAAAGATGAAGGGCGCCGACGGGGAGGACGAGTTGCGCGAGGCGTTCA
GAGTGTTCGACAAGAACAAGGACGGGCTGATCTCGTCGAAGGAGTTGCGACACGTGA
TGACGAACCTTGGGGAGAAATTGTCGGAGGAGGAGGTGGACGATATGATCAAGGAGG
CGGATCTGGACGGCGATGGAATGGTGAATTATGAAGAATTCGTGACAATCCTGACGTC
GAAGAATTAGTTGAGTGGCCACGGGTCGAACTAGAAGCTATATCCAGCTGCCGGAAGT
CTTCGTCGAAGAACAGAGAAACTCAAACCCGTCGTCGTCAAGTTCCTCCCCAAATATC
GTAGCAAGATGGGAACCCCTTCCCTCGAACCCCCACTTTGATCGAGAAACACCGATGA
AGAAAGTAGCGAGCGAGAGAATCAGAGAAAATTGGAAAAGAATCGAAGAAAATAAA
AAAACCGCTTGAAGAGGATCGATCGATCGATCGTTCGATTTATGGCGTAAGG

>novel_circ_001723
ATAACGATTTCGAGCCATGAGGACGCATCAGCTGCGGCTCGCATCGCCTGGAATGTAGT
CGTTCGAGAGGAGGAGAAAGATCCGCAAAAGGAGACAAAAGAGGGAGAGGGAGAG
AGGGAGGAGGCGTTCACACGTGGAGGAACCGAACGTGGCGAGGGAGGAATCGAGCG
GAAATAAGGGTGACAGGCAGGGACATGCAAATGTCCCCGTCGAGCTTCCGTGAAATCT
ACCCACGAGAGCCGAGAGGAAACAGAGACAGCGACGCGAGACGTCGCTCGACCGCT
GTCACGATTCCGCGATAAAAAACTCCCTCGTAGAGCGAGGGATACCCGGATCTCGATC
AAAGCCCCCTGGCTGTGTTTACTTACCCTAAAAAAAAAAAAAAAAAAAAAAAAATCGT
AATCGATAGTGTTCAAACGATCTCGTCTCTTCGGTCGACGTATGCACATTTCGAGGATT
CATAGGAAGAAACGACACTGATACGGTGAGAAGAGAGGGGTGGTGGTGGATCGAGAA
GAGACGAGGGTCAATATATAGTTATAGTCTAGCAGATATATGGGGAGAAGAGGGAGGG
AGAAGAGGAGGAATCATCCAGATATATCCGCGTAGTACAAACGTAGGAAAGAAGAAG
AAGAAGAAGAAGAAGATATTCAAAATTACAAGCACACACACACACACTCGTCGTATGT
CAATCGTATCGATACGTATCGTAGCTAAGTAAAAGAAACTTCCAATAATAACCCTCGATA
TAATATCAACGATAATCCGTGTGATCGAGAAGCAATAAAAGGATAGCGAAGAAAAAAA
ATAAAAAGAGAGGAAAGAATCTCTCGAAACTCGTATTCGTAAGTATATTCGCGTAAGA
GAATCCCGCGTATTTATATATTTCGTTCGGCTGTGGTATCGTATATCTCGCGTGTGTCTAT
CGCGGGGGAAGAAAGAAGAGAAAGGGGGAAAAAAAGTCGTATCGTGAATCTCGAAG
GGAGCTGCCCGAACGGGGGGGGAGTGCATCGACAGGATGGCGTTCGCCGCGGCGAGA
ATGATCCTGAAAGACAAGAGGCGGAAGACAAGCAAGGAGGATTTCGGAGGACCAAG
GAATAGGAGCAAG

>novel_circ_001724
TGCTCACCTCTATGCCGGGGCTGACCTTGAACTCTCTGAACACCCAGTCAGCGAAGAA
GCTGTTCACCTGCCAGCTGTGCGGCAAGGTGCTATGCAGCAAAGCATCCCTGAAACGC
CACGTTGCCGACAAGCACGCCGAGCGACAGGAGGAGTACAGATGCGTCATCTGCGAG
CGGGTATATTGCTCGCGTAACTCCCTGATGACCCACATATATACGTATCATAAGAGCAGA
CCCGGCGACATTGACATTAAGTTCTTTTGAAGGTTTAGATAAG

>novel_circ_001725
AGAGAATAATCGTGCGCCCTTCTCGATTAGTCGCGCACAAGTTGCACCGTCACCCGAC
CAAGCCGTGCTCAAACCGTCTTTTTATTTATCTATTTTATTTTTTTTATTTTTTTTTTTTTG
TTTTATATATATATACTTTTTTTTTTTTTTGCGTTACTTTGGAACCACGTAATTATGCTTGC
AGGTGCTACCACATCATGTATGTATATAGCATCTTCATCGTACGCTCGTTCTCTACTTTTT

ATCTTTTTATTACTTATTAGTTTTTTTTTATTATTTCTTTGTTACACACGTTTATATACGTGG
TACGGCGTGCGCGCTTTCCGGGGAGAAAGTATCACCCTTCTACGTTTTCTTTTCACCAT
TGTCATTGTTTCTTTCTTTCTTTCTTTTCTTTTTCTTTTCTCCATAATCTAAACCTCTCCTC
GAAGCACACGGTTCCGTCAAAATTCTACGCAACGATTAGAGAAGAAGATTTCACGAAG
ATCGTTCTTTCGATTTACTGCATGAAGAATATCAATATCTTTAAATTGATAATATTACGCG
TATAATTTCACATTAAATTTGTGTCGTGATTCGTATTAATGACAATGGCCACAGAATGGA
ATCGCAGAAGGGTCTATTCTTTTTTCCGTGTCGGAAGGCTACGTACTGCCGTACCATTT
CGATATTCGTGATTCGTCGTCATGCTCTTTTTTTTTTTTTTTTTATATTTTTTTTCTTACTTT
TCTATTACTTTTCTAGCGCTTACGATTAACGATAGTGGAGTGCAAGCAATTCCAAGATTC
CAGTGAACGATTTCTGTGAGCGATGACGTTAGTGTTTTTTCAGAGCACCAGAGATGTAA
TTCCATGTCGATGCGTATTATTTACAGCAAGACAGAGCAGAGTAGATTCTTGTGGTGGT
ATACAAATGTGTATATAAATATTATGTATATATATATATACAAGAGTAGTGATATTTTGTCTA
ACGGGATGATGCGATGAAGCGAAACACTTGATTATTGATCTCTCTGTGTATTTTCATACG
TGCGTAATAATACTCGTAAAACGAGAACCCTCCGCCTATATATATATATATATATATATATAT
ATATATATATAAAATTCGTAAAATTTTCCCATCGTAACGACTACGTTCCGGATATTTTTATT
ATCATTATATTTTTGGTATATTTTGTCAGTGAAACAACAAATTTTTCATTCCATCGAATTC
TCTTTTATTTCTCTCGATTCAATTCTCTAACGACATTCGCTATGGAGCAACAATTACGTT
CTTTTTTTTTTTCTTTTTTCTTTTTTCTTTGCGCGCGTTTTGCCTTAACTATACTATTACTT
ATATACTCGCGATATAACGTCGTTCCTCTTCTTCTCGCACGAACAAGATCGACACAAGT
TTGTCGGCGAAACGTTATGGAAAATCCGATTGATCGAGCCAAGCGTGAGAAAGGTGCA
GACAGAGAGAGAGAGAGAGAGAGAGATATGAGCGGACGGAACGATAAAAAAGATAA
GGAGGAAGGGGGTGAGAAATTAGTCTAGTGAACGGTATCGAAAGATTGTTCTTTTTAA
ACGCCTGATAGGACGGGATTTGTATTTGCCGACCTGCATGGATAAACAGATTCGTACGA
TCACGTGGTAATATTCGGGGGAGGAAAAGCTCTGTCGGCAAAAATACGTTCGTGCAGT
CAGAGATTCAAGGAAAGAGAGAAAAAGAGAGATCGCACTATGCATCCGTGCTGCACC
ACCGGGTGGTGTCGCTGATCGACGGGGAAACAAGGTTGACTTTATCGTCGGGTAAAGC
ACGCAAGGGTAGAACGAGCTAGCTACTACCCTTGGCTCGCTCTCTAGGGTGGATGAGG
AAGAGCACGGTCGTACCGGAGGAGAGTGGTATCGATTTTGCCGCCGGGAAGATTTGCT
CGCTCGTCAACTTCCGTCGCGAAACTAGATGGCGGAACTTATTTGGGAGGAAAGGGTC
GCGCGTGAAACGACCTCTTTGGCGTGGTTCGAGGTAATCGGTAAAATCGGTTGTTGTA
CCATGAGGAAGAAAAAAAAAAAAAAAAGAAAAAAACGGACTAGGATCGACGGTAACG
GAAATTATATACTTTCGCGAATAATATCACGCATAAAATTGGCTAGAACGATTGTCCCTT
CGTCGCTTTCTCGGCCAATCCCCTGCGCTCGTTCCTTCTTCTTCCTTCGGATGGATCTCC
GCGGGCGGGGCAAGGCGGAACCTCTTGCTCGCATCCAAGACTAGACCATCGGTGTCTG
CCAATACTTCGCCTCGCACTGTTCCTCGTGCTCCTTCCGCTCTTTCTTCGCGTGATACTG
GCTCTTATGGCTGACCAGACTGTTCCTCGACTTGTAACTCCTGCCGCACTGGTCGCAGC
AGAGCGACTGCGGCACGTAAAAATGCGTGTCCGCCAGATGTCTCTGCAGGGACCAGG
CGCGGGAGAAAGTCTTCTGGCAAAAGCTACAGTAAGTGCCGCTGATCGAGCGGCCCG
TGTACTTGAGATCCTGACCCTCGGCCTGTGCCTGTGCCCTTCCGTCGCCACGGACGTGC
TTCGAGGACGGGGCCTGCGAGTTCCCGTTTTGCATCAACCAGCGAAGATCGGCGAAC
GGCACGCTCTCGTTCGTCGAGGCGACCGTCCTCGGCTGCTGCTGTTGCTGTTGCTGCT
GTTGCTGTTGCCTGGGCTGCTGGCGCATCTGTTGCCCGTTACCTGGGGCATTGTTATTG
CAGGCCGAGTCCCGAGTTTCCGACTGCAGATCGATCTGCTCGGTGAGCAAACAGGACT
CGAGCTCGTCTATCTTGGGCGACAGAGGTTCCGACTTGAGCACGATCTGCATCTGATCG

TTAATGTCGTCTAAGAGATCGATAATGGGTAGGTCCGCAGGATGGTGTTGCTGATGCTC
CTGCATCATCTCGCACTGACTAGTTCCCGTACTATGGCTCGATTGGCCGGTTTGCCGTTG
ACCGTCTCCGTTGGTAGGGGACTGCCAACCCCCGTCCTCCTCGGCGACCTCCCGGTCT
GCAACCAAAGAGGAGGGGGCTGAGTCCATCGAGATGTTCACGCCGGATTTAAACCCG
TGTCGGCCGCGGCACCGATCTCATCTCGGGGGAGGATCTCGCGGTGGTCTTGGTCTCG
GCGTCGGAAGAAAGGCAACGTGAATTCGAATGAGAGAGAGGAGAGGGGAAAGGCTT
CGGTTAAATGGATAACGGGCGCGTCGTTAATCGTCGCGCATCGCCCGTTAAAAGGAGA
GAAGAATCGCGGGCGAAAAGACGGAGAAGAGAAGAGGAGAGGAGAGGAGAATAGG
GGCGGAAAGGAGTATAGGGGTTGAAAGGTGGGCTAAGAGGGAAGAGATAGGGCGAG
TGTACATTCATCGTCTCGTAAAAGCCGGTACAACGTCCGCGAGTATGAGATGTCGATAC
AACAACGAGAGAGCAACGGCGGGTAAGAGTAAGGAGCAAGGAGTAAGGAGCAAGAG
GAGCAAGAGCGGGAACGGCATAACGGCGATGCTTCTTCTTTGCGGGATTTTATCCAGC
CGGTTACCTTAGCAACCGAACCCCCCCACGTACGACGCTCCTTCCCCCGTTTTTCCGTG
GTGCCGGGAAGGGGATATATACCTAGGATGCGTATTATTCTTAATAACCCCCGTATGCCG
TTGCGCTGTATTATGACTGATGTTGACTCTAAGCGGAGAGCCACGGCGATCGATAGCTC
GCTACCACCGCGACTATGAAACCGAGAGAGAGAGAGAGAGAAAGACAGAGAGAGAG
AGAGCACGACTAAGGGAGGGCCAAGGGGTTGTAAAGAGGAGAAAGAGAGAGAGAG
AGTGAGAGAGAAGGGTGGAGGAGGATAGGGCCGAGGGGATCGGCGGGGAGAGAAAA
ATCTAAGTAGCTTCCGGATTGGTTACGCGAGTCTTACGAACTTTTGAAAGAGTTACGTG
GACAGTGGTGGGGGCGGAAGAAGCGAGGAGGACGCAACCAAGAACTACCCTCCCCT
CCGACCACGGCCCTTTTTCCATTCGATGCGGTTGCGCGTTTCGCGAAATCACCGCGATC
AAACGATAAGTCTTCCCCGGATATTGGATCGTGTGTATTTAGCCGTTCACGGGGAGGAA
TCGTTTCTGAAACGAGATAAAGAGAGAGAGAGAGAAAGGATATAAAGCATATACTCCC
CCTTCTTTCCCCAATTAACCGAATCGAAACGTTTATTAGAGATTCGGGTACAGATCTCTC
TAGTTCGAGATGCAACGAATCTACGAGTGCAACGACGACGGCCTTTGGAGAGCGCGT
GACGACGACGACGACGACTATGCTTTCTTCTTCTTCTTTTTTTCTTCCTCTCTCTCTCTC
TTTTTTATGATACGTCGAATCGCGTTAATCGGTCGCGATAAGAGGAAAAAAACGGAGG
AGGGGAGGAGGAACGAATTCGTTCGCGGAGAGAGAGAGAGAGAGAGAGAGAGAGAT
CGGTGCACGCGCGCTGGTGGAGACTGGCGGCGATGCTCGAAACTAAAATGTCTCAATG
AATTAAGCACCGCAACCAAGTAGAGAGACCTTCACCTTGTCTCGAACCGTGTTACGAG
ACGCCTCCTCACGGAGATCGCGATTCGACGCGATACGTAAACAAGTTTCTCGTCCAAA
TTTCCAATCCTCCTCCCTCCCTCCCTCGACTAATTTTTAACGCGACCTCCGATCGCCGAT
CGTAACCGGTCCTTCGCCTCTCCTCTTACGTAACACCTCTCACGAACAACCTCGGCGCG
TTCGAATCGATCTCGCGATTCGAAAAGCGAAAGAAAAGAACAGAAGGGAATGCAACC
GAGGTATGGAAAGATCGGGACGGGTGAAATTTGTCGGAAGAGGCGGCATATCGTTTTG
GAAAGTTGAAAGAAAAGAGAAAAAGAGGAGAGGAGAGGAGAGGAAAAGAGAAGAA
AAAAAGGAGAGAGAGAGAGAGATGGAGCGACGACGGCGAACGATATAGAGCGAAAGTG
TAAACGCCCCTCTCTCTTTCGCTCTCCTGCCCCTCCCTCGCGCGTTCCTAGCCCCCCTTC
CCTTGTCTCGCTCTTTCGTCCCGTCATATTCTCGTCTCCGTCACACCCGCGCTCACTGTT
GCGCAGTTTTCGAGGTCACCTCAAGTTCATCATCGATAATAGCACAAGGCGAAAACAA
GCAGCTAGACCTGGGCGTCCCCGATAAGTACATACTACAGATAAAAAATTCCATCGGAT
TTGGTTCAGAGCGAACTTTCGTTTGGCCTTGGGAGGCGCCGCGTACGCGGCCCAAAGT
AAATTGCCAACGCACGGATCGTTGGCTACCGAAACGAGAGGGACGGCGGCACGGTGG
TGGAGGGAAGGGGGCAGGAAGGAGAGGGCCGACGGAGGAGAGAGAAAGAAAGAGA

GAAAGAGAGAGAGGGAACGAGCGAGATGAGTAAGTATCGAGTAAATAGACGTATGCA
AGGAGGACTCGCGGACGTCACGTAAGCAAGAGGCAGACACTTTTGCACACACCATCG
GAATCGACAACGTCGACAGATGGCAGCATGAATTCCGTGTTTCGCAAACACGGGGCCA
TTAGCGAGCCCATTACGTAACCCTCCGAATTTCTTCTCTCCTAATTTTTTTTCGACTCCG
AACCGTTTCCTCCGCCCGAAAAATCGCCATCCTTCGAATCCTACTCGTCGTCCAACACG
AGAAGGGAAAAAGAAAGAAAATGAAAAAAAGAAATAAATCCTAGGACCAGAGAGAC
TAGGAATTTCCCTTCCCTTCCCGTAATCGCGTCGTGGAACGCGCTCTCTCGCAAAAATT
CGGTCGGCTCGGTTAAAGTAGAAAAGAAAAAAGAAAAAAAGAAGAGAGAAAAAAGA
AAGATAGAACGGGGGTGAGAGGGAGAAAGAAGCGGGGAAGGGGCGGAGCGCGGTAT
GTCCACCGCGCTGGGAAATACGTCCCGTGAAATAAATATTTGCTCGATGCGCTACAACG
GTTCTCCCTGATACAAAGAGACAGAGAAAGAGGATAAAGTGCTCGACTCCAGAGCCC
GGCACCCGCTATTTTTATGCGGCCGGGTTTTACAACAGGTTACCGCGGATGCAGGCGTT
CGCGGAAAAGAGCAGAGAACGAGAGGAAATAAAGGAGGAGAGAGAGAGAGAGGGA
GAGAGGGAGGAGAAAGAGAAAAGGGAATCGCGACGGTTGCCACTGTCCCGCCCGCC
GCCGACACCTCGCGCGCACGGTATCGGGTTTTTTAAACAATTTTAGTGGCCGAGCGGG
AAACGGTGTAGGCTAAGAATATCTAGGCCCGGGGAAAGAATTTCTCCTCTCTTTGCGAT
AATGACATCGAAACCGGCGAGTGAAACGCGGGCAAAGGGGAGGGGGGAGGGCGGGA
AGGGGAGCGGGATGAATAGATTTAAAATTAGACCGCGATAAACGCATCTTTCTTCCTCC
CCCCTCCCTCTCCCTCGGCTCCCGCCTCTATGCAACCGATCCTCCTTGAAAAAGTAAAC
GCATCGATCACGCACACGTACAATTATTTTAATTTCAAATGTCACGGTGCGAAATTCATC
GTCTCGTTTTTTTTCGGTAAACAACCGAAGCAAATGTGAAAGGTTAACGACACGATCG
ACCGTATAATTTTCATTGTTTTCGCGCAATTTCCTCTCTCTCTTTTTTTATATATATATATAT
ATATAGATGTATATATATATATATATATATATAGAAAAATTCGCGTATTCCTAAAGACAAATTC
TCGAATAGAAAAAAGATATTACAGAAAATTCGAGCAAATTTCGTTCCAACATTTTTTTC
TTTCCTCTCTCTCTCTCTCTCTCTCTTTCGACGCCAAGAACGAATCTGTCTAGAGAAAT
ACCGCAGACTGCCATATGCTCGCAGCCGGCTCCGTCGGAATAAAAAGATATTAAACCG
AGAGAGAGAGAGAGAGAGAGAGAAAGGGAGAGAGAGAGAGAGGAGAGCGAAGGA
ACAAAGAAACTCGTGCGCTCTAAGGCCCCCCGATCGTTGCGACAAGTGGTCGTACGTT
GTACTTTCTATCTGGCACGATTGTTTTTTACGTAACCGGCCATTACGAATCAGAGATGAT
TAAAGAACGGGCGGAGCGTGCTATTTATTGACAAGAATAAAATTTCACTTTATCGGCGT
ATATCGAACGATAACGAGCTTTCTAAATTAAATCTACGAGAAGAGAGACTTCTAGACCT
TGCTCTTTTCCCGATAAAATCACCACTATTAAACCTCACTATCGTCACTATCATTATCGTC
TCGAGTACTCGACGTACGGGACGGCCTTCTTTCCCAATAGAAAAATATCATCCCGATCG
AACAGCCCACCACTACTAACTAGACAGAGAATAGAAGGAAAAGAGAGGGAGAATCGA
AAATCTACTCGCGCCTTTAGCTTTAATTTTAACGAAACGGTTAAGGCGGAAAGTTTCGC
GGGACCGACCGACGACCCTGCAATCCTATCTCCCTACGTCGCCTGTTCGACGTCTGAA
ACCTTGCCAAAATCGCGTCGTTGCAGTTGGTTGGATCCGAAAGAAAAGAGACAGAGA
GAGAGAAGAGAGAAAAAAAAATCGCGAGCGAGCAAACGCGCGCGCGACCGCGATAGA
TAGGAGACGAGCGCAAGAGCTTCGTCGAGGAATCTCTTGGCCGAGATAAAAATGCGA
AAAAACAAACGGTCGTTCGCTGCAAATGACCGATTAGAGGAGGTGTCCCTGGCCAGG
CGCTTTTTTTCTTTTCTTTTTTTTCTCCTCTTCTTTTTTTTTTCTTTCCTCTTCTTCTTCTTT
CCCAACCGAGTATAATTTTCACGGTCAAGAATGATGAGTATCGTTTTTAATGGCCGTCTC
TACTCGAGCATTACCCGTCTATTTTCGCACGCCGACCGTAACCGTCCTCCTGCTGTTTCC
TCGCTCTCGCACGAATGGAGGAAACCTACCATGCGTAAAGAGAAGACAGAGACAGAG

AGAGAGAGAGAGAGAGGGAGAGGGAGAGAGGCGACGACTTTTTCGAAAAGAAAAG
GAGGGGGAGGGTGACGGTGGAGAAAGAGGAGAAGGAGGAGGAGGAGGAGGAG
GGAGGAGCAGACAGAGAATACAGCGAATTAGGCTGCGGGACGAACAGTTTCGCGGAC
AAGTATAATTTAATGCGTCTAATGATTCGCAGCGCGCCGTAAAGAACGTGAGTGGCGTA
CCAAGGTCGTGGTATTTTTAGTTTGGCCAATGTTTAGTTGGCCACGTTCGTCAGAGAGA
TAGAGGGACCTTGAATTTCGCGGATAACATCTGCGGCCTTATCGACGGCCCCTGATAAC
TTTTTCAGTACTAACCTAGTATAAGCCAGCCGTTCAAATATTAAATCGGAGGAGGAGGA
GGCGACGATTGGCCGCACCTGAATTTACTTAACGATCTTACTTACTACCAGGGCGCGTT
TGTTAAGAAGAGAAGAAAAGAAAAAAAAAGTAAAAAAGAAACGCCCGGTCCACTTAC
GTCTCGATCGGAAAAAAAAAAAAAAAATAAGAGAAAACACGACACGAATTTCCTTTCT
CGAATCCCCGAGATTTTTTATATTTTTTTTGTCCACTGGAGGAATCCTATTACCGCACGG
AGGACTCTCGAGTGATAATCGCGATAAAAAAACGCGTAAAAATTATATTCGCAAAATCG
TGCGATACGCGTCCGCACGGATCGACGAACGAAAGAAAAGTGAAAGCCTATTGTATTG
TATTCGCCCTACGGTACGCCGAATCCAAACAAGACCGTACAATCGAAACACGAGCGCG
TACGTACGCGCCGTATAATCCCAAACATATACAAAGTCGAAACTTCGAAATCGTTTATC
GTTTATTAAGCGAGCGTTTAACCCTTCTTTCCTCCTCTCCCTCCCTCTCGCCCCCTTTTT
TCTCCCGATGCGCGCGTTGCGCCACCCCCGAATGTGAACATACGAACGCACGAGTTAC
GAATAATAAAAAAAGAGAAAAAGAGCGAGGAAATTCTTCTCGTTCGAAAAACGAGAG
GAGAAAGAAAGAAAAAGAGAAAGAGAAAGAGAAAGAAGAAGAAAAAGAATAAGTC
GAGAGATATACCGGAATATAATTTTACCATATGGTACACGCACGTCGTACCGGGGTCAA
ATGTCGTTCGAATAAAAACGTAAAAAAAAGGAAAAAAAGCGTGACAAAACAACGACC
TATCACATCCTCTTTCGTAATTGGAAACGAGCGGGCTGCAAAAATGTCTCGTGTCAAAA
CTTCTTCTTTTTTTTTTTTATAAATTGTAAATTTTACGCACAATTAAAATACGGTGATCGA
TTGTAATCGATTATTGATTACGTCTTGTACAAAATTATAATAAAAAGGAAATATATGTATA
TATATACATATATACATTTAAATAAAAATCGATGCGTCGATCGTTTCGCTGTATTTTTTACG
TATATGTATATATATATATATATATATATAAATGGTATATAAAATTATAAGCAATATCGTAATG
CTGGCCGTATCGGCGATATCGCGGTAGCGGCGTGTTTTCCGTTACCAAACGTTAAACGG
GACGACAGCACTGATATAAAAAACTTAAAAGAAGAAGAAAAAAAAAAAAAAAAGAAA
AAAACGACGAATTGGACAATATATACGGCACGTATATGAAACGTGGAGTTTTAAACGA
GGGATCAATCGTCGATCAAAAAAAAAAAAAAAGAAAAACGACACTTGACGCGACTCGG
AGAAATTTTAAGAAGCGTTTTTTAAAATAAATTCCTTTTCATTTTTGTAAAATACTCGGC
GGAGACAATTTATCATTATCAGGTATTTATCAGACAACAAGGAGAACGACAGTCTGTCA
CTTCTGCTGTCGTTTCACTGTCGTCTCACTTCTTCTGCGCTTTCTTTCCCTACGTCTTTC
GTTCGTTTCGTTCTTTTTTTTTTTTTTTTTTTTATTATAATTAATATTCCTCGATCATTTTCCA
AGGGGTTCTTTTTCGATTTTTCTTTTTTTTTTTTTTTTCTTTTCCTCTTTTCCATCAGAGA
CCGTGAGATTTGTGGAGATTTTTTTTTTTTTTTTGCGGCAGATACTTTGTACAGTGATGC
CAAACAGTGATGATTGCATTCATCGCTTTGTGCCAATTAGAATGAATCGGATCGAATTC
GACGACTTCGAAGGAACGAACGTATCCGCTGTATTCCTTTCTTCTTTGCCCGCCAGTTA
TCCGCAGTTACACGACGAGCGAGTATATATATCTATCTATAAAACGATATCGATCGATTC
CGCGATTCCTCTGTATACGTGTGTGTGTGTACATATACGCGTGTATGTTCTTTTCCTTTCC
ATTTTGCGCCAAGAAATTCGCGATCACGAAAGAGGAAGATCCGATGGATCAGCAGATA
AATTCATTCCAGTCGAATCGCGAACGATCGATCCGCGATTGATAATCGCTCGAACGAGT
TGTTATGACATTCGAATGAAATGTGTATGCAAGGCGAATTCGAATAAATGGTATTTTCAT
AAATGATGATATCGATGATTTTTTTGTAAACGCATGCTTAATTAGAAGAGGTTTTTAATC

GTCCAAGCAGACAAGTAATGCTCTTTCACCGCAACCAAATAAAAAAAAAATAAATAAA
TATATATATATATATAAAATATGTGTGTATGTATGTATGTATGTATGCAATATATATATATGGT
ATATACCTATATTCTTATACAATATTTATATGATTTTTTTGTTAGTGTTTGATATAACTGCA
CAAGTCCTTCTTTTTTCCTTTTTTTTTTTTTTAGTTACTTTTTTTTCCTTTCCTTTCCTTTT
TTTTTTTTTTTTTTTTTTTTTTTTAATTCAAGAGCGAACACCGCCACACCGCGGGCAATT
GCGGGCCACGATGTGATCGACCGTGATGCGATCGATACAAGAGACATGTAATGTAATAT
AATATAATGGAACACAGCATAGAAGAAGAGTTTGCCCTTTTTTTTCTTTTTTTCTTTTTT
TTTTTTTTTTGGTGAAAAATCAAAAGAAATCAAGCTTGTTCAAGTCAACATCCCCCGA
TCAACAAATCGGAAATCGGTAAAAAAACTCGTTCAAATAATCACGAGAACGCGCATAT
ATAGAACAAGTATACATATATATACACAGGGGGAGAGGGGCGGGGGTTGTGTTCCAACT
CGTGTTCCCTCGAGTTCCTCGATCTCGTTGATTCTAATGATTTTACATTACAGGGCAGAG
CCAG

>novel_circ_001727
GTGAATAGATATAACGATGGTAGATACGCAACACTTTTGTCTGCGATGGAACAATTACC
AAAGCAGTATAACGTCGGCATTTGAGAACTTGAGGGACGATGAGGATTTTGTGGACGT
CACATTGGCCTGCGATGGCAGAAGCCTCAAAGCGCACCGCGTCGTACTTTCCGCTTGC
AGCCCCTATTTTAGAGAATTGCTCAAGAGCACACCGTGTAAACACCCGGTGATAGTACT
TCAGGACGTAGCGTTCAGCGACTTGCACGCATTGGTCGAGTTCATCTATCACGGGGAG
GTGAACGTGCATCAGCGTTCCCTTAGCAGTTTTTTGAAGACTGCCGAAGTCCTCAGGG
TATCGGGGCTTACGCAACAGGCAGACCAAACGGACAGGGACGAGCTGTCCCACGTGC
GCGCGTTGGCCGCCGGCGGGAATCACCTTCCTTTCCACGAGAAATTGGAGGAGAGTTT
TCCCAGGGGTGGCTCGCCACCGACGCCTGTCACCCCGACACCGACCACCGTGCAGCA
ATTGTTGCGCAGAGCGCAGATACGCAGGAACGAGAGACGGACGCCCGATCCCCACGA
TGAGACGGCGAAGAAGCCGAGGGTGCTGTCGCCTCCGCTCAACAACAACGACGCTAC
GCCCACCGATTTCTCGATGGGGGTGAAGAACAACCACGTGTCGAGCAAGGTGGAGGG
GAACGGGGTCCACGAGGAGAACAGCCCCCTCGAGGACAATATCAAGTGCGAGCCGTT
GGAGCTGACAGGTGGCAACAGCGGTAACGCGGCGGGGAACAACGAGGACTCGTCGG
ATTCCGGCGCGGCGGCGTCCGATCGGCCGCCCGCGTCGGCGAGCAGTAACGAGCACG
AGGCCGAGTCCGAGCACACGTCCACCCCCAATTTTTTGTCCGAGACGAAAATCTTCCC
GCCCACGCCGGGAAGCTTTAATTTCAGTATGGCGGCGCTGGCCACGGAGCATACACCG
TTGTCAGTGAAATTTCCAGGAATGGGCCACGGGTTGCAACCACCGGACTTGGCAGGA
ACTTCGCAAG

>novel_circ_001728
GTGAATAGATATAACGATGGTAGATACGCAACACTTTTGTCTGCGATGGAACAATTACC
AAAGCAGTATAACGTCGGCATTTGAGAACTTGAGGGACGATGAGGATTTTGTGGACGT
CACATTGGCCTGCGATGGCAGAAGCCTCAAAGCGCACCGCGTCGTACTTTCCGCTTGC
AGCCCCTATTTTAGAGAATTGCTCAAGAGCACACCGTGTAAACACCCGGTGATAGTACT
TCAGGACGTAGCGTTCAGCGACTTGCACGCATTGGTCGAGTTCATCTATCACGGGGAG
GTGAACGTGCATCAGCGTTCCCTTAGCAGTTTTTTGAAGACTGCCGAAGTCCTCAGGG
TATCGGGGCTTACGCAACAGGCAGACCAAACGGACAGGGACGAGCTGTCCCACGTGC
GCGCGTTGGCCGCCGGCGGGAATCACCTTCCTTTCCACGAGAAATTGGAGGAGAGTTT
TCCCAGGGGTGGCTCGCCACCGACGCCTGTCACCCCGACACCGACCACCGTGCAGCA

ATTGTTGCGCAGAGCGCAGATACGCAGGAACGAGAGACGGACGCCCGATCCCCACGA
TGAGACGGCGAAGAAGCCGAGGGTGCTGTCGCCTCCGCTCAACAACAACGACGCTAC
GCCCACCGATTTCTCGATGGGGGTGAAGAACAACCACGTGTCGAGCAAGGTGGAGGG
GAACGGGGTCCACGAGGAGAACAGCCCCCTCGAGGACAATATCAAGTGCGAGCCGTT
GGAGCTGACAGGTGGCAACAGCGGTAACGCGGCGGGGAACAACGAGGACTCGTCGG
ATTCCGGCGCGGCGGCGTCCGATCGGCCGCCCGCGTCGGCGAGCAGTAACGAGCACG
AGGCCGAGTCCGAGCACACGTCCACCCCCAATTTTTGTCCGAGACGAAAATCTTCCC
GCCCACGCCGGGAAGCTTTAATTTCAGTATGGCGGCGCTGGCCACGGAGCATACACCG
TTGTCAG

>novel_circ_001729
GTGAATAGATATAACGATGGTAGATACGCAACACTTTTGTCTGCGATGGAACAATTACC
AAAGCAGTATAACGTCGGCATTTGAGAACTTGAGGGACGATGAGGATTTTGTGGACGT
CACATTGGCCTGCGATGGCAGAAGCCTCAAAGCGCACCGCGTCGTACTTTCCGCTTGC
AGCCCCTATTTTAGAGAATTGCTCAAGAGCACACCGTGTAAACACCCGGTGATAGTACT
TCAGGACGTAGCGTTCAGCGACTTGCACGCATTGGTCGAGTTCATCTATCACGGGGAG
GTGAACGTGCATCAGCGTTCCCTTAGCAGTTTTTTGAAGACTGCCGAAGTCCTCAGGG
TATCGGGGCTTACGCAACAGGCAGACCAAACGGACAGGGACGAG

>novel_circ_001730
CTGTTTCAGAACCGAGTAGCGACATGGAGGCAAAGGAGAGGGAGAAGCCGCTGGTTA
AAGACTTGAACGAACATATCGTATGTCCTCTGTGCAGAGGCTACCTCATAGATGCAACG
ACTCTCGTGGAATGTCTGCATTCTTTTTGCAGAGGTTGTATAGTGAGACGGTTGAGCAG
CGGTGCCAGAGCGTGTCCCGTGTGCAATGTCGCAACGTCTCCTCCACTCTTGCCGGAC
GTAAAGCTGCAACGACTTGTATATCTTATGGTACCCGGCCTCTTCCGGTCGGAGCTTGA
ACGAAGGCGTCATTTCCGGCTGGTCAATCCACAATGCCCACCTTTGGCCTTGCCCTTGG
GCGCCCTAAACTTAACCTTGGACGATTTCGTCAGCCTGAGTTTATGCGAGCTCGACGA
ACCGGAAGGAGAGGCGGCCGTCCACGAGGCGGAAAACCGTGCCGGGTCACGATACC
GGGCGAACGCCGATCAAACGAAACAGGAAGACACGATTCAG

>novel_circ_001732
ACGTGGCGGCCTCGGTGGAGGTTCACTTCGAGAACAAAGATGGCGGATTTTTCCTGAA
GCACATACCGAGGCAGTACTATCCCGTGAGGGGGGACTCGATCTCTGCGATATCCCCGT
CGACGGGGACGCTGTCGAACAACGGGGGGGCCCCGTCGCCTGCACCGGGCTCGGTCC
TCTCCATAGACAAATTCACCGTGTTTCAGACGAGCGAGCCCGTCTCGGTGCGGGCCAC
TTACGGCCCATTTAGCACGAAGCAAACCGTGCCCGCCCGTTACATAGTGCCGGATCCAT
TGGACGCGCTCCCCGGCCCGGAAACGAGGCGGAATCTCACGGCTGCGACGATTCTGG
ACGCGCAGGAGCTGGGCGCCCGTCACCTGGACATGTCCGCCCATCTGGTCGGGAGCA
GCGTGCCTCGCGACTCGCCCGTGCTCAGAGTCCTGTTCCACGCCGGAAGCGAGGCGG
GCGGAAGGCGCCAGTTGCTCCTTGCCCGCCATCAGAGAGTGTGCGTGGTCCTTCACGC
GAGCCTTGCCGCCCCGTCGAGGAGCACCGTTAACAGGAACAACGGGAGGGCTTACTC
CTCTTCGTCTTCCCCATCCTCCTCCTCCTCCTCCTCCTCCGCTTCCTCCTTCTCGAG
CTCGTCGTCCACTTCCGCTCTAACGGCAGCGTGCAGCCCCGACGGGGAGAACGGAGT
TTGCCTGGCGCAGATCACCATACCCGCCGATTGGTGGGCACCGTTACCACCGCCGGAC

GCTTCCGGCCGGGTCAAGGTCGCCAAGAGCCTGCCCAGGCTCGTGCAGGTCGCCTACT
CCGTGCTCGAGCCACGCACGGAGGAGGCAGGCTCGGCCGACAACGGGGCAGGCTTCT
GCAGGCCGAGGGTCCAGATCCAACCCGTCACGCCGCTCGGTCAGGTGCCCCTCGCTCC
GAACCGGGCCGATTACAAGGAGCTCAAGGCCGACGATTCGCTCACTCTTCTGGTGCCG
CACGGCCCCCTCTACCCGAGGTCCCGGCTGCACGTCCCGGCGTTCCTTCATCCGTCCA
AGACCACCGATCCGAGGCAGGAGAGGGCACCGCTCGTCATCGCAGTATACCTTAG

>novel_circ_001733
ACGTGGCGGCCTCGGTGGAGGTTCACTTCGAGAACAAAGATGGCGGATTTTTCCTGAA
GCACATACCGAGGCAGTACTATCCCGTGAGGGGGGACTCGATCTCTGCGATATCCCCGT
CGACGGGGACGCTGTCGAACAACGGGGGGGCCCCGTCGCCTGCACCGGGCTCGGTCC
TCTCCATAGACAAATTCACCGTGTTTCAGACGAGCGAGCCCGTCTCGGTGCGGGCCAC
TTACGGCCCATTTAGCACGAAGCAAACCGTGCCCGCCCGTTACATAGTGCCGGATCCAT
TGGACGCGCTCCCCGGCCCGGAAACGAGGCGGAATCTCACGGCTGCGACGATTCTGG
ACGCGCAGGAGCTGGGCGCCCGTCACCTGGACATGTCCGCCCATCTGGTCGGGAGCA
GCGTGCCTCGCGACTCGCCCGTGCTCAGAGTCCTGTTCCACGCCGGAAGCGAGGCGG
GCGGAAGGCGCCAGTTGCTCCTTGCCCGCCATCAGAGAGTGTGCGTGGTCCTTCACGC
GAGCCTTGCCGCCCCGTCGAGGAGCACCGTTAACAGGAACAACGGGAGGGCTTACTC
CTCTTCGTCTTCCCCATCCTCCTCCTCCTCCTCCTCCTCCTCCGCTTCCTCCTTCTCGAG
CTCGTCGTCCACTTCCGCTCTAACGGCAGCGTGCAGCCCCGACGGGGAGAACGGAGT
TTGCCTGGCGCAGATCACCATACCCGCCGATTGGTGGGCACCGTTACCACCGCCGGAC
GCTTCCGGCCGGGTCAAGGTCGCCAAGAGCCTGCCCAGGCTCGTGCAGGTCGCCTACT
CCGTGCTCGAGCCACGCACGGAGGAGGCAGGCTCGGCCGACAACGGGGCAGGCTTCT
GCAGGCCGAGGGTCCAGATCCAACCCGTCACGCCGCTCGGTCAGGTGCCCCTCGCTCC
GAACCGGGCCGATTACAAGGAGCTCAAGGCCGACGATTCGCTCACTCTTCTGGTGCCG
CACGGCCCCCTCTACCCGAGGTCCCGGCTGCACGTCCCGGCGTTCCTTCATCCGTCCA
AGACCACCGATCCGAGGCAGGAGAGGGCACCGCTCGTCATCGCAGTATACCTTAGGGC
CAGGGTGAAATCCGGCGTGAAAATCTTGGACGCATCGTCCAGCAACCCGGACTGGATC
GTCGACAGCAAGATCAACGCGAAGAACACGATCGCTACGTTCACCGCGAGGCGAAAG
GAGAATGCCTCCCGATTACCAAGCGC

>novel_circ_001734
ACGTGGCGGCCTCGGTGGAGGTTCACTTCGAGAACAAAGATGGCGGATTTTTCCTGAA
GCACATACCGAGGCAGTACTATCCCGTGAGGGGGGACTCGATCTCTGCGATATCCCCGT
CGACGGGGACGCTGTCGAACAACGGGGGGGCCCCGTCGCCTGCACCGGGCTCGGTCC
TCTCCATAGACAAATTCACCGTGTTTCAGACGAGCGAGCCCGTCTCGGTGCGGGCCAC
TTACGGCCCATTTAGCACGAAGCAAACCGTGCCCGCCCGTTACATAGTGCCGGATCCAT
TGGACGCGCTCCCCGGCCCGGAAACGAGGCGGAATCTCACGGCTGCGACGATTCTGG
ACGCGCAGGAGCTGGGCGCCCGTCACCTGGACATGTCCGCCCATCTGGTCGGGAGCA
GCGTGCCTCGCGACTCGCCCGTGCTCAGAGTCCTGTTCCACGCCGGAAGCGAGGCGG
GCGGAAGGCGCCAGTTGCTCCTTGCCCGCCATCAGAGAGTGTGCGTGGTCCTTCACGC
GAGCCTTGCCGCCCCGTCGAGGAGCACCGTTAACAGGAACAACGGGAGGGCTTACTC
CTCTTCGTCTTCCCCATCCTCCTCCTCCTCCTCCTCCTCCTCCGCTTCCTCCTTCTCGAG
CTCGTCGTCCACTTCCGCTCTAACGGCAGCGTGCAGCCCCGACGGGGAGAACGGAGT

TTGCCTGGCGCAGATCACCATACCCGCCGATTGGTGGGCACCGTTACCACCGCCGGAC
GCTTCCGGCCGGGTCAAGGTCGCCAAGAGCCTGCCCAGGCTCGTGCAGGTCGCCTACT
CCGTGCTCGAGCCACGCACGGAGGAGGCAGGCTCGGCCGACAACGGGGCAGGCTTCT
GCAGGCCGAGGGTCCAGATCCAACCCGTCACGCCGCTCGGTCAGGTGCCCCTCGCTCC
GAACCGGGCCGATTACAAGGAGCTCAAGGCCGACGATTCGCTCACTCTTCTGGTGCCG
CACGGCCCCCTCTACCCGAGGTCCCGGCTGCACGTCCCGGCGTTCCTTCATCCGTCCA
AGACCACCGATCCGAGGCAGGAGAGGGCACCGCTCGTCATCGCAGTATACCTTAGGGC
CAGGGTGAAATCCGGCGTGAAAATCTTGGACGCATCGTCCAGCAACCCGGACTGGATC
GTCGACAGCAAGATCAACGCGAAGAACACGATCGCTACGTTCACCGCGAGGCGAAAG
GAGAATGCCTCCCGATTACCAAGCGCATCGGCGGAGGAGATCCTGACGATGCTTCTCG
AGGCGAGCGAGGAGGGGATGGAGGGGAATTGGGACGGTGGTCGGATCGTGTGGAGC
GTGCGTTACGCTTTCGACAACGGGTCCCTGTTGAACGGGATGGATCAGCTGCAGCAGA
GATTGCAGCAGAGGCAGATGCAGCAACATCATCATCATCATCACGCCGAGAGGCGGAA
GCTCCAGGTTCGTCTTGAGATACAGAAGGACGATATACAGGCTGTCCTGCCTATATCCA
AG

>novel_circ_001735
ATCGGCGGAGGAGATCCTGACGATGCTTCTCGAGGCGAGCGAGGAGGGGATGGAGGG
GAATTGGGACGGTGGTCGGATCGTGTGGAGCGTGCGTTACGCTTTCGACAACGGGTCC
CTGTTGAACGGGATGGATCAGCTGCAGCAGAGATTGCAGCAGAGGCAGATGCAGCAA
CATCATCATCATCATCACGCCGAGAGGCGGAAGCTCCAGGTTCGTCTTGAGATACAGA
AGGACGATATACAGGCTGTCCTGCCTATATCCAAG

>novel_circ_001736
AACTGGGAGGTGATGAACACGGCGGTGTTGACCGGGCGGCAAGTGTCGCAGGGGATG
AAGGTGTTCATCGTGAGCCAAGCGGGAACCGTGGCCGACGTCACGCTGCAATCCTCCT
GCCACTCGGAGGACGAGAGTGTTCTCAAGGTGTCGTCCTCGTGTAGCAGCGTGTACGT
GGACGGAACGGAGATACGGGGCTCGAGCAACGCTTCCGTCCTCGTCAAATACGGCAC
GTACACGGGCCTGGCTAAATTCACAGTTTGGATGCCGGAATTCCCTTTGGAGATGACC
GTGGCAGATACGAGATTGAGCCAGATCAAAGGGTGGAAAGTGGCCGAGGAGCACGTA
GCCGGGGCGAAGAGCAAACGCAGTTTGAACTCGACGTTCTCGCGGCCGGACGAGCCG
ATCCCCCCCCTCCCCAGGACGAGAAAGAGGAGCGCCGCGGAATTGGAGGACACCTCG
ATGGATATCGAGGACGCGGACGTGATCTTGGAGGAGGACGAGCAGGAGGAGAGATTT
CGTGGGAACGATAACTGGGATGCTATTAACTCCATCGACAGGGGGCCGTCGACCAATT
GCAGGCTCCGTTTCCAGCAGAGCCCGGTCGAGGTGCACGCGAGATTCCTGGCCACCG
ACCACGATTCCGGCCGAGTATCCTATTTCGTGAATCGTCGAACTTGGCTACGAGTGACC
GATCTGGTGAACGGGATGCTCCGGGTTTCGGATCCAAGGATCGCCGACCTGTTGCAGG
GAAGGATCGTGCAGGGTCGTGGGGTGGGGAGGACGGAAGTGCAGGTTCTTTCGCCGA
TCACGAGCAAAGTGATCGGCGCGAAGGAAGTGCGAGTGGGCAACGATCGAGTGGCGA
TCACCAGGTTGTCCGTCAGAGTGGTGTCCGGTTTACAACTGACCATCAGCCCCGATAC
CGCTATAGAGAATGGATACGTGGCGGAAACGTCGGTCACGAGGAAGTTGACCGCTCAA
TATCAGGAGGGTCTGTTGGATATAGACGTGGAATTCTCGGACGGTACGAGAACACCGC
TGAGAGAGATCGCCGTGAACGACTATCATTTGTTGGTGGAGAGTTTAGATCCGGAAGT
GGTGGCGTTCGCCCCGATGGTCGCCTCTCATCATCCGCGAGTGATCGCAGTGGGCGAG

GGGCGAGGCGATTTGTTACGGGTCAGTCTTCAACTGGCGGATGCCTGCCGCCTGACCG
GCAGGAGGTCCGGGAAGGTGTCTCAAAGGGCGACCGCCGCCACCCTCGCCACCGCTT
CAGCCAACGTCGAGGTGGACTTCGCCTCGAGCGATCTCCCCAACCGACCCGAATTCGT
GCAGAACGACGGAGGCGGAACGGTCGGCTCCCATCATCACAGGGAGAGGAAGACAA
GCAGGGGAGAGATGGCGCCCGATTTGCACGACATCTTAATCG

>novel_circ_001737
ATCTAGAAAACAGCTGGCACGAGCGAAGGTCACCGGCCATGGAATATAATTAGTCCGA
CACTTATGGAACGTCAGGCCAGCTATGGTTCGCAGTGGCCGCAACGAGGTAGACGAGG
TAGTGGTCTAGGCACTTGGAGATCGGCCTTTGCTCGACACGTGTGCCCATGGACAACG
TTATTTCCTTCTCGAATACTGGGTCCTTGGTCCGAATCGTATGCCACTCGCACCGCCATA
AAAGGAACGATCGGGAAATGGGCAGCTGATGATCAATATGATCGGGGGGGGAAGGGA
AAAAAATATTTCAAGAGTAATCGGCTATTTTTCAGAAGAAGCAGAAACTTCCTAGCGA
ACCTCCTCTCTCTCCTCCATTATGGTATTAACTCGGGACTACACTCGGGATGGATCGATC
ATTCAAGGAAGCCTTGGACGAATCTGTGGATTTTCCAAATAGGAGGCAACCGATGGTG
AGACTACGTGGTAATTGCGACAGTTGTAATTACCAGTGTATTAACCGTTGCAAATGTCG
AAAGAGCCAACCTCGAGCCCGCAAGGTTTCCCCCGTAAATAGGGGAAGAAGATTGTC
CAATTAACTGTCTCGTTTAATTGAATCTTATTTCCTTTTCACTTGGTCCAGTTTCATTGGG
GCTTCTGCGCTTTATCGCGTGAGTCGAATGTAATGCACAGCGAGGTCGGAAGCTTGGC
CGAAGAACAACCCACGACAAAAGAGGGATCGAGTTGAACGTCGCGGGATATTTTTGG
CCATACTCCCGCCCACTCCAGCCTTCGACAGCTCGCAAATTGTCGTCGTCGAGTGGCG
CGTGAATATCGTGGAAACGGACTACCGTTCTACTCGTTTAATGCCCGTTTAGGCGCGCA
GGGTATTCTTTCAAATTTTTTTCAGACAATTCGTTTTGCTGCCGGTGGAACGAGGAAGA
GGAGCAAGGGATGAAGATGGAGGTGGGAGGGGGTGGGAGAGAGCTCGAGGAGAGAA
AGGAGGATAGGAAGGAGCAATTCCTCGGGACGAGACGAGACGGGACGAGACGAGAC
GAGACGAGACGGAGGTGAAGGTATTCTTTTTAAGGCGGAACACTTTACTGCTGCTCGT
TCCACGCTGGTAATGGAGAATCCCCCAGCCGCCCCAACGAATTAATTCGGGGCTAACG
CCGAGACACTCCCCGATATCGAGTCACTTCGTTCCACGGGCCAGT

>novel_circ_001738
ATCTAGAAAACAGCTGGCACGAGCGAAGGTCACCGGCCATGGAATATAATTAGTCCGA
CACTTATGGAACGTCAGGCCAGCTATGGTTCGCAGTGGCCGCAACGAGGTAGACGAGG
TAGTGGTCTAGGCACTTGGAGATCGGCCTTTGCTCGACACGTGTGCCCATGGACAACG
TTATTTCCTTCTCGAATACTGGGTCCTTGGTCCGAATCGTATGCCACTCGCACCGCCATA
AAAGGAACGATCGGGAAATGGGCAGCTGATGATCAATATGATCGGGGGGGGAAGGGA
AAAAAATATTTCAAGAGTAATCGGCTATTTTTCAGAAGAAGCAGAAACTTCCTAGCGA
ACCTCCTCTCTCTCCTCCATTATGGTATTAACTCGGGACTACACTCGGGATGGATCGATC
ATTCAAGGAAGCCTTGGACGAATCTGTGGATTTTCCAAATAGGAGGCAACCGATGGTG
AGACTACGTG

>novel_circ_001739
ATCTAGAAAACAGCTGGCACGAGCGAAGGTCACCGGCCATGGAATATAATTAGTCCGA
CACTTATGGAACGTCAGGCCAGCTATGGTTCGCAGTGGCCGCAACGAGGTAGACGAGG
TAGTGGTCTAGGCACTTGGAGATCGGCCTTTGCTCGACACGTGTGCCCATGGACAACG

TTATTTCCTTCTCGAATACTGGGTCCTTGGTCCGAATCGTATGCCACTCGCACCGCCATA
AAAGGAACGATCGGGAAATGGCAGCTGATGATCAATATGATCGGGGGGGGAAGGGA
AAAAAATATTTCAAGAGTAATCGGCTATTTTTCAGAAGAAGCAGAAACTTCCTAGCGA
ACCTCCTCTCTCTCCTCCATTATGGTATTAACTCGGGACTACACTCGGGATGGATCGATC
ATTCAAGGAAGCCTTGGACGAATCTGTGGATTTTCCAAATAGGAGGCAACCGATG

>novel_circ_001740
GTTCTGCGATATTAATTTAACTCATCATCATTTTCTGGGACGAAGTTTAACGAAGTTACG
AGCAACAGGCTGACGAAAGTTTAAGGATCGCTATCGGAGAGCAGAGTTTGGAAGTTC
GTTCGAGGAAAAAAAAAAAGGGAAAAGGGAAATCTCTTCGTCCAATCCTTGGAAAAAT
TTCTCTCGCTCGAATACACTTTCGCTGAAAGAACTGCGAGAAAAACTTTTATTAACGGC
TACACAATTTGTTGCACGTCCACCGTCTCTGGCACGTGGCTATATAATAACCAACAAAC
GAATCCTCTCACCTCTTGTCTACACTAGTCGCTTGTCGACTCGAAAAAAAAAAACTTCT
TTTCCCATTTTTTTTCTTTTTTTTTGCCATCTCATCGGAGAGAATTAAGTTAAACGAAAA
CTCTCGTACTCGGCAAAGTATATCGAGATAACGGCTGGCAAAATAATTAATCCACGTGG
CAAACGAGCCATCTTTTACGACGCAACTGCTCGTCAAATTGATGTAAACGTCGTCATCG
TGATCTCGAAATAGCGAGACGAAGACACGAGCGAGGCACTGTGTGGATCGTCACTGT
GGTTGACGACGATTAGAGAATCGAGTTATTCGATTTTTTTGGAAAGGGATAGAAGAAC
GAATATTCGATAAAGATCGATAAAGAAAGAAAGGAAAAGGAAAAGCGGAAGAGATCA
GGTATTGATCGAGCGGTGGTGTCGATCAAATTCGGGACGATTTTACGAGGTAAACCAA
GTCACATTTTGCTCATCAACACTGAGTGGTTCCAATTGACGGTCGGGTCGACCCTCCA
GCGTAGACACTATTGGTCAGCCTTTTAATACAGGCGGTCGCGATTGGATCAGATACCAA
GATCGTTCTTAGACGGAAGGCATCGCTGGAGCGGATTCGGTACAAAACTTTTAACGTC
GCCAGTGTCGCGCAATTTGCTGCCTGCCCGTCGTCGTGCGCAAGGGTCACACAGATCC
ACAAGAGACATTCGCACATCCAGTTTGTCTCAGTCGGTTTAAGTCGGTCAACGATTGC
GCCGAGCTTGTAAAAGATAATCAACCAGAGCATATACAGAAGGAAAGAATTGGAAATC
AGGAAATCGAAACCGTTTTTTCGTGCTCTGCTCACTATCTTCGAAAGAAGATATTCTATT
CTCAAATCGTTGGAAAAAGAGGGGAAAAAGAAAGGACAATCGAAGGAAATAATCGAA
TCCCAACGTTTGATAACTCGAACAACATCGTGGAAAGTTTCGATTGACCAGTTGAATG
GCTCATATTTATTGGCTCTTATTATTCGAAAGATCTTGATCGTTACGTTGCACTGCCTACT
TGGAAAGAAAGGAGAATCGAGCTTAGAGAGGAGATTCGTGTTTTTTTTTTTAAGTAGT
ACGACGACGAAGAAGACATTTCTGGCTGACTTCTTCGCGAGACTCGACTTTCCTTAGG
GTGGAAAGAAGTTTGAGGAACATCGAGGAACGAAACAAATCCCATTTGGTTTTACGCC
ACGAAGCCTCGTTTTTCCCCCCCCTTCTGGATCAGGTTTATCTTTCCGTCGGCATCATGG
AACGAAGGATCCTGAAAATGTGGTGACGACGTGATAGGCGAAGGCCTTCGATTATCGA
GACACTATCAAGTGGTGTGAGCCATAGTCGTTAGAAAGAAAATATATTTTTACGTGACA
CCCTCCCCAAGTTAAGAGCGACAATCGTTTTTAACGTATAATATATACATATACATATATA
CATATATATATATATAAAAAAAAGAAACGAATTTTTATCGAGAATAATAAAATAGAAATA
GAATTTCTAGAATTCAAATCCTGATCTAAGTTTCATCTTAACTAACAAGTGACAAACCT
CCATCCCATCGGACATTTTTTAATATATTCGGATAATCGAGAATAATATAATAGGAAGAA
GAGAGAAAGAGTGAGAGAGAGAGAGAGGAAGAGAGAGAGAGAGAGAGGGAGAGA
GAGAAAAAGAGTAAGAAATAGCCGCCGTTCTTTCGCGAAACTCGTAGATTTTCAAGAG
AAAAAATAGTGACAAAATGCGTTGCAGCCAAGGTGTGGCGATGGTATTCGAGGACGC
GTTCGTCGTCTTTTCGCGGTTTCTTTCCCTTAAGTCACGGGGTTGCGCACTCCTAGAAA

CGCATCGGCAACGCGAAACAACTGGCATCAAGAAATTGTACAATAGCTTCTTCGTAAT
GTTCTAAGGATAACTCTTCCTTAAGGAAGTTCGGCCCTTTATTAACCGGTGGTCGAGTG
ATGCACCGTGCACCTCCGAGATCATTGTCGATCCCGATCGAGGAAAATACGCGGAGAA
AACGCACAAAAGAAGGAGGACGTTTTGGTTAGTAAACGAAAGAAAAGGAACGAGCT
GAGCGGAGGAAGCCGAGAAGCGGCGAAAACAAAGAGGAAAAAAAAAAGAAAAAAA
ATAGAAAAAGGGGAAAGGACTGGAAAGGAAAATTATCCGATGTGACTGAAGAATGTT
ACGTTAATGTTTAGAAAAGGAGAAGTCCCTAAGTCCCTTAAGTTGATGCTAAGAAATTT
CATCGTGGACGTTCTAACTAGAACTTGTAGAACTGGACTTATAGCCTTCTCTTCCAAAA
CGGGCCACCAAAGCGTTCTCCAAAGAATCTTTGAGCACTCTCTGAACTTGGTAAGGGC
GAATAATATAATCGTGATTAGGATCAAAACAATTGGTATTCGAAAAGGTAGTAGTGTGT
AGTAGCCTGAAAAGTAGAAAGCCTAACAACGAGTGCTTCGAGATTTAAAGTCGTTGAA
TGGAGCTCCAATTCATCATGGACGGCAGTGCAGATGCTTTGAATGGAACGATATGGTTT
CCCAATACACCGTCACCTCCGTACGAACAGCTGTCCCCAGTTCGTCAGAACAGATGTG
ATCCCATCGCCACTCAACACGCGATGGGATATCACAGTGCAGAAAATTCTTTACGGCAG
GATAAATACAACTCACCGAAGGATTCCCCGGAGGACGGTGTCCAAATACGTTCGAGCA
GCGAATCGCCCCAACATCTCAGCCCGTCTTCCGACAATTCCCCCAATTGCCAGCAGAG
CACCCTGAAACGTCGAGCCGGAGAGCCGTTGTCGCAAATTACAGAACTCGAGAAGAA
GCATGCCAAGAGAGATG

>novel_circ_001741
GGAACGAAACTATGGATTATTATGGAGTACTTGGGTGGCGGCTCGGCCCTGGACTTGAT
GAAGGCCGGTAACTTCGAGGAAATGCATATCGCGGTGATATTGCGCGAGGTGCTCAAA
GGGTTGGATTATCTTCACAGCGAACGGAAGCTCCATCGCGATATTAAAGCGGCGAACG
TTTTGCTGAGCGAGATGGGCGACGTCAAGTTGGCCGATTTTGGCGTTGCCGGACAGTT
GACCAACACGACCAGCAAACGGAACACTTTCGTCGGGACGCCGTTCTGGATGGCACC
GGAAGTCATCAAGCAAGCTTCCTACGATTCCAAGGCTGACATTTGGTCGCTAGGCATC
ACGGCGATCGAATTGGCCAAGGGAGAACCGCCGAACAGCGAGCTGCATCCTATGAGG
GTCCTGTTTCTCATACCGAAGAACAATCCGCCCCAATTAACCGGAAATTATACCAAGCA
ATTCAAGGAGTTCGTCGAGGCCTGCCTCAACAAGGACCCTGAAAACAGGCCGACGGC
GAAAGAACTATTGAAGTTCCAGTTCATCAGGAAGGCGAAGAAAAACTCGTACCTGATC
GACCTCATAGACAGATACAAAAAGTGGAAGTCGCAGAGAAGCGAGGAATCCGAGACG
GAGAGCGAAAATTCGGACTCGGAAGAGTCGAAGCAGGATAGCGACATGGACGATCCA
TGGATAATGACGGTGAAAGGTCCACACGTGGTCAAAGGTCCCGTGGTTCCTATGCTGC
CGTTGGACGAGCAGCCACCCTCGTTGACCCTCACACACACGCCGAATCACAGATCGGC
GAATCACAATCAACAAGCGAAGCCACACGCGAACGGTACCTCGAGATCTCCGCCAAA
CGATTCCACGTTGAATCATTACAGGAGCGAGTCGAGGGATTCGGTTCGCGATGGCCAA
GCGAAGCATACGCCGTCCCGACCGCACGAGGCTGCGCCACCGCCGCCCGTGGACACG
CGAGAAAAACGAGAGGACTACCGGGATTTCAATCGGGATTCCTCGTTGAGGGAATTGA
AGGAGATGCGCGAGAGCAGGGAGAGCAGGGACAGGGAGCGGGACAGAGAAGGGCG
GGAAAGGGAAAGAGACAGAGAAAGGGAGTTTAGCGCGAAAGAGAAGAGGAACAGT
AAACCGGACGTACCTGTCGTACCTATGGTGGACCATAGGCCCGGTGAAGACAAGCAGA
GGCCAAGGAGCTCGCAGGAACTGAAGCATTCTCGAAGCGCGGCCCTTTCCGGTGTTG
TTTTACCTCTCCTGTCCGAGCTTCAAAGGAAACATCAATATTCGGCAAGAGACAATAAT
GGTGAAGGCGGAAGCGGCATTGGCAAGAACGGAGGCGCGATAGAGGAACTGCGCAG

CGCGTTCGAAACAGCGGAACGCACCTCGCCGGGAATGACCGAGGCCCTTATCAGGGA
ACTCCTCAAGTCTTTACTTCCTGCCAATCATTCGGAGGGTTGCCTGTTCATGCTGATGG
AAAAAGTTATCCTGAG

>novel_circ_001742
AAGGTGGACCCCGAGCTGATATTCACGAAACAAGAACGAATTGGGAAGGGTAGTTTC
GGAGAGGTGTTCAAAGGCATCGACAATAGGACCCAGCAGGTTGTCGCCATCAAGATCA
TCGATCTCGAGGAGGCGGAGGATGAGATCGAGGATATACAGCAGGAGATCATGGTGCT
GTCTCAATGCGACAGCCCATACGTCACCAAGTATTACGGGTCCTATCTCAAGGGAACG
AAACTATGGATTATTATGGAGTACTTGGGTGGCGGCTCGGCCCTGGACTTGATGAAGGC
CGGTAACTTCGAGGAAATGCATATCGCGGTGATATTGCGCGAGGTGCTCAAAGGGTTG
GATTATCTTCACAGCGAACGGAAGCTCCATCGCGATATTAAAGCGGCGAACGTTTTGCT
GAGCGAGATGGGCGACGTCAAGTTGGCCGATTTTGGCGTTGCCGGACAGTTGACCAA
CACGACCAGCAAACGGAACACTTTCGTCGGGACGCCGTTCTGGATGGCACCGGAAGT
CATCAAGCAAGCTTCCTACGATTCCAAGGCTGACATTTGGTCGCTAGGCATCACGGCGA
TCGAATTGGCCAAGGGAGAACCGCCGAACAGCGAGCTGCATCCTATGAGGGTCCTGTT
TCTCATACCGAAGAACAATCCGCCCCAATTAACCGGAAATTATACCAAGCAATTCAAGG
AGTTCGTCGAGGCCTGCCTCAACAAGGACCCTGAAAACAGGCCGACGGCGAAAGAA
CTATTGAAGTTCCAGTTCATCAGGAAGGCGAAGAAAAACTCGTACCTGATCGACCTCA
TAGACAGATACAAAAAGTGGAAGTCGCAGAGAAGCGAGGAATCCGAGACGGAGAGC
GAAAATTCGGACTCGGAAGAGTCGAAGCAGGATAGCGACATGGACGATCCATGGATAA
TGACGGTGAAAGGTCCACACGTGGTCAAAGGTCCCGTGGTTCCTATGCTGCCGTTGGA
CGAGCAGCCACCCTCGTTGACCCTCACACACACGCCGAATCACAGATCGGCGAATCAC
AATCAACAAGCGAAGCCACACGCGAACGGTACCTCGAGATCTCCGCCAAACGATTCC
ACGTTGAATCATTACAGGAGCGAGTCGAGGGATTCGGTTCGCGATGGCCAAGCGAAGC
ATACGCCGTCCCGACCGCACGAGGCTGCGCCACCGCCGCCCGTGGACACGCGAGAAA
AACGAGAGGACTACCGGGATTTCAATCGGGATTCCTCGTTGAGGGAATTGAAGGAGAT
GCGCGAGAGCAGGGAGAGCAGGGACAGGGAGCGGGACAGAGAAGGGCGGGAAAGG
GAAAGAGACAGAGAAAGGGAGTTTAGCGCGAAAGAGAAGAGGAACAGTAAACCGGA
CGTACCTGTCGTACCTATGGTGGACCATAGGCCCGGTGAAGACAAGCAGAGGCCAAGG
AGCTCGCAGGAACTGAAGCATTCTCGAAGCGCGGCCCTTTCCGGTGTTGTTTTACCTC
TCCTGTCCGAGCTTCAAAGGAAACATCAATATTCGGCAAGAGACAATAATGGTGAAGG
CGGAAGCGGCATTGGCAAGAACGGAGGCGCGATAGAGGAACTGCGCAGCGCGTTCGA
AACAGCGGAACGCACCTCGCCGGGAATGACCGAGGCCCTTATCAGGGAACTCCTCAA
GTCTTTACTTCCTGCCAATCATTCGGAGGGTTGCCTGTTCATGCTGATGGAAAAAGTTA
TCCTGAG

>novel_circ_001743
GGAACGAAACTATGGATTATTATGGAGTACTTGGGTGGCGGCTCGGCCCTGGACTTGAT
GAAGGCCGGTAACTTCGAGGAAATGCATATCGCGGTGATATTGCGCGAGGTGCTCAAA
GGGTTGGATTATCTTCACAGCGAACGGAAGCTCCATCGCGATATTAAAGCGGCGAACG
TTTTGCTGAGCGAGATGGGCGACGTCAAGTTGGCCGATTTTGGCGTTGCCGGACAGTT
GACCAACACGACCAGCAAACGGAACACTTTCGTCGGGACGCCGTTCTGGATGGCACC
GGAAGTCATCAAGCAAGCTTCCTACGATTCCAAG

>novel_circ_001744
TATTGCAGGACTAGGTTGCTTGCAGTACTGAATCAAAATGGAATCAATGGATGCGCGCC
TTGTGGCGCAAGCATTGAACTACCATGGTCAACAGTTGCAGAAAGTGTGGGAAGGAG
AACGCAACGAGAATGAGCTGGCCATGCTCAATCTCAAGGAACCAAATTTTGAGATCTA
TCAACAACGTCAAAAGACTTTGAGTTTTGGTGATAGAGGAAAAAGGCTAAAGCTACA
GCAGTTCTTAGCAAAGAAAGCAGATGCCCTTTATGATAAATCAAATTTGGAGAAAACA
GTTGAACCTATTAAGCAAGAATTAGGAGATGAAGAATTTTATGCAACAATGCCAGGCCT
TGATACTTTTGTCACCATGGAAAAATCTCAACGTATTCGTAATTTTTGGAGAGTTTGGT
CATTGGAGATGTTATTTACGCGCAAGTGATGGGTAAAAGTGCTGCTGGACTTCTTCTGA
AGGTGCTTTGCAACTGCAGCGATTGCCCCAGAGTTGTTACTGATTTAGGAGTTAAGGCT
CTAATATTGAACACGGCCACAGTGCCGGCAGTGGACAAGAAAGGTGTAACAAGAGGC
TACATGGCAAATGATCTCATCTGCGTCGTAGTCAGTGAAGTTAACGTTGAAGCCGAGC
GAGTTGTTGCAGTTATGAACGTACCTGCCCGTGAAGGGCAAGCACCACACCCACCTAT
GGGACTTATCCATTCTGATGATCTTCCAGAAGCTTATAA

>novel_circ_001746
GATTGTGCACAATTTATCCTCGACTTCTTAATCCTACTATTAACGATGCCATCACTATTAA
TTGGAATGGTGTCATTCAATTTATCAGATCAGACGAATGGATCTTGATGTCTGGTGATCA
CATACATCATACGTATAGCATCTTTGTGAAATTAGTACAAAATAAGAAATTGGCTTTAAG
AGTTTATATCGGTGAACATGAAATGGAAATTATGCCTCTTGAGGCAAAAGTATCTGTGA
AAATAGATAATAAAATTATTGATGATTATCAAAAAGGTATTACAGTATCCGAGAACGAGT
CACATTCTTATCCTATGAGATTAACCACTGCCTATAACTATGTAGTAATTGATTCACAAAC
GATACCAGTTCATATTTTCTATAAAGTAGACAGTGTGACTGTTTCTCTGCATACTGATCT
TCAGAGTCGAGTAACTGGAATCTGTGGTAACATGGATGTTATGCATAAAGATGAAGTTC
CAGACGTACATAGTGTTTCATATCTCTAATAAAATAAAATGAAATCATCTAATCATATTTT
CCATCTTATGAAATATGTTTAGTGTCATTATTTAGCAGCAGTTATTGATTTTAATATTATAA
TTATATTAACAATCAATAATTTCTTTTTAGGATAATATGGAATGATGATGTA

>novel_circ_001747
ATTCATTAGTGGAAGCTGCTTTAGCATTTAAATTTCCACGAATATCGATTCTCTTACTGC
GTAAAAGATCCATTTTTTGTGTCTCGTGAAAATCTTGAATGCTAACTTTTCCAATTAAGT
CGATCTAATGAAAAAAAAAAAAAAAAATATTAAAAGAAAAATACATGCGTATTCTGATCG
GTTATTATAAAACGTGAAAGAATTTTTTCAATTTACCACCGAAGTAGGATAAATGATACT
TGGTTCGACTTTATAAACGAGAGAACAACTTTTTCCTTGTCCCAAAATTATATC

>novel_circ_001749
GAGGAGCAGGTGACAGCAATCGCGCTGGAGCTTTAAGAACCAAGGGGTGGAGAAGG
CTGGAGAACGCATTTCCCGTCGGATACTTGGATCAGCCGAGGAAGGAGGATCGTTTTC
AAGGAAGCTCTCGTGCAACCTGTGCACGTTCTCCTCCTTCTTCGTCGTCGTCTGTGGTA
ACGAGCAAGAAAGACTTAAAAGAGAGAGAGGGTGAGGGCGAGGATGAGGCAAGTTG
TCGTGTTGGAGCCGGCTGGCAGCGTTCTGGTGATCAGGCAGACTTCCCTCGGTAGTAA
CGTCACTACCGGTAACACCAATTCCAATGCTGGATCCAACAACGCTCACGAGACCCAT
GGGCTGGCCAATCACGCCGCCGTGTCCACCATTGCCTCCAACGATCACAATCAGAATC

GTATCGTGGTCGTACGCTCGTCCTCCTCGACCTGCATGACTACCACG

>novel_circ_001750
GAGGAGCAGGTGACAGCAATCGCGCTGGAGCTTTAAGAACCAAGGGGTGGAGAAGG
CTGGAGAACGCATTTCCCGTCGGATACTTGGATCAGCCGAGGAAGGAGGATCGTTTTC
AAGGAAGCTCTCGTGCAACCTGTGCACGTTCTCCTCCTTCTTCGTCGTCGTCTGTGGTA
ACGAGCAAGAAAGACTTAAAAGAGAGAGAGGGTGAGGGCGAGGATGAGGCAAGTTG
TCGTGTTGGAGCCGGCTGGCAGCGTTCTGGTGATCAGGCAGACTTCCCTCGGTAGTAA
CGTCACTACCGGTAACACCAATTCCAATGCTGGATCCAACAACGCTCACGAGACCCAT
GGGCTGGCCAATCACGCCGCCGTGTCCACCATTGCCTCCAACGATCACAATCAGAATC
GTATCGTGGTCGTACGCTCGTCCTCCTCGACCTGCATGACTACCACGAAAAGTTCTCGC
GATGTAACGACAAGGTTCCATAGAAGACATCCAACATTTTTCACCAGGCTGACAATCAT
TCATCCGCGAACGGTAACAACATCAATATTAACGGCAACCTGAAGGGGAACCCGGGGG
CAATCGGTAGCAAAACGTCGACGAAGGTTGTGGTTGGTGGCGGAGTCTCCAAGTCGA
ACGAGAGAAACGATCATCTTAGAAACGATCACAAGAGCCAAGATGGGATCGGTGAATC
GAATCCTGGATGTCGTCCAAGGACGAGCAAGGAAGCCGCACACGAGAACGTTCCCTT
AGACAGGAAGCTCGATGGCGCGGATCCCATCTCCG

>novel_circ_001751
GCTGACAATCATTCATCCGCGAACGGTAACAACATCAATATTAACGGCAACCTGAAGG
GGAACCCGGGGGCAATCGGTAGCAAAACGTCGACGAAGGTTGTGGTTGGTGGCGGAG
TCTCCAAGTCGAACGAGAGAAACGATCATCTTAGAAACGATCACAAGAGCCAAGATG
GGATCGGTGAATCGAATCCTGGATGTCGTCCAAGGACGAGCAAGGAAGCCGCACACG
AGAACGTTCCCTTAGACAGGAAGCTCGATGGCGCGGATCCCATCTCCGGGACGGAGGT
CGAGAGTCGCGGGTTGGCTCACTCTGCGGCTCCCGCCGCTCGGGAGCAGACACGCGG
GGCCAAAGACTAATTTGAACCTATTTTATTAACAAAACAAAAACATACATATTTGTAAAT
AACACTAAATAACACGAAGTAATACACACGTCTCCTAAAGTCTCTTTATCTACTCTAAAT
AAAAAAAAAAAAGAATAAAGGAAACTCTTAGACCTCTGTAAAAAAAAGAAAAAGCG
AACAAACAAAAAAGAACAAAAGGAACGCAAGCGACAAAGTTAATACTATTTCCAGTC
CCTAAGCGAATCGTTACGAAGCCTATAACGCGACGGAAACGAGGATACGTGTGAGGAT
AAAGCGAGGGAAAGAGAGTGGCCTAGAGAGAAAGAGAGAGAGAGAGAGAGAAAGG
GGGAGGCTATCGGTGGTGTCGGCAAGAGAAAGGCGCGCGTGCGAGAAAGAGAGAAA
GATAGAGAAGAAAGGAAGGAAGGAAGGAAGGAAGGAAGGAAGGAAGGAAGAGCGG
AAGAAGAGAGAGACGAACGAGAAGGCAAAGGATCGAACGTGGTTAAAGGCGATCGTAA
AACGCGTCAGCTGTGGGCGAATCTCTTTTTAAGTTCTCTCTGTACTGTGTGACCCTTAC
ATTATTTTGTCCCTCCAGGGACAGGTTCAGTGTTGTAACTTAGGATATAAGAAGAATAA
GAGCTCCTGGAACGCACAACAAGAAAGAAAATTAATAAAAGGGAAGAGTTGAAGAA
AGAACGAGAGAAAGAGAGTGTGTGCTTGTGAGAGAGAGCGAGAGAGAGAGAGAGA
GAGAGAGAGAGCGCTTGCGTGCGTGCGTGTGTGTGTGTGTGTGTCAGGAAGAGAAAG
AGAGAGAGAGAGAGAAAGAGAGATTCGAGAAAGGACTGAGACTGAGAAAGAAAGA
GAACCAAGGTCGAAGAGGTACAGAGAACACTAAGAGATAGAGGTAGACGAAGACGG
GAGAAGAGAAGAAAACGAAGAGAAGATTGTCGTCGAGAAAATTGTTCGTTGAATTAA
TCTACGAGAATAAAATGGGGCTCCAGTTTTTTGTACTAACGCGAACAAGTATTTGAAAA
ACGAAGAAGAGGAAGAGTACGGAAGAGAGAGTATATAATATTATATAGATCGAGAAGA

AGAAGAAGAAGAAGAAGAAGAAGAGGTTCGATCTTTATCACGGTGTCGGGACAGTTT
CTATCTCCACGTGTGTTGTCCATTACACGACGTCGCATCGAGAGAGGGAGGGTCGTCTT
CAACGTTTGATCGTGAATCGTTTATCTGTCTGCTGTGACTCTTGATTCCTTTAGGGGAG
AAGCGGGCGCGGAAATAAATTCGATTGACTTTTGAAACGAGGGTAGAATAATTCGAAC
GAACAACCTGGCTCGCGTACACGATTCTTTGGGGGATATGTCCTGCCGTTCTAAGGGG
AATCACCACCCACGTGCACGAGCCTCACGCAGAGGGCAACGTCAAGCGAGTCGTGTT
TCCATCCTAGAACCCGGCAGCAAGCTCTCCTGACAAAAAGAGTGCCGTCGTAGCTTGT
CACGGTCTAGAATCACCGCGAGCACCGACCATTGTCGAGTCGCCATATCAGCCGCACA
CTCGTCCAAATTCACGAGGAGCCTGGGGATGTCTGCGAGGGCACAGAAACGGTCGCG
CTACGCGTACGTTCAGGGACGATGACGATCGGCCACGATCGGTAAGATCCAAGAAGCG
GTCGCTCTGACGAGCGCCACGGAAATGGCGGGCTACAATCATTCGAATCCACAGCGTG
TCGTCTATCATGCGACCTACCCGACGCCACTCGCACCAAATGGAGATCCGATAAAACTT
CCGGAGAATTTGGAGACCTTGCCACGAGCTGAACATTTTCCAACTCAGAGGCACCGAT
GGAACACCAACGAG

>novel_circ_001752
CTGTCAAAGCGCCGGATATTGGTGAACGAACGAGTGGGTACCGCTTGGGGCCCCCATA
GAGAGGGCCCCCAACCGGAACAGGCCGCCGAAAGGCGGTCCATCTATCATCAGACGG
TTCCGAGGGAATAGCGGCGAGTGTTCATGAATGGATCGATGGGAGCGTAGAGTGTGTC
GCGTGTGAATACAGCATTTTGTGCACGTTTTTTTAATAAAGTTTCTTCGTTCGAGGAG
AGGGGAAAAACTTTCGGCCAAAGTTTCTTCTTCGGCAAACAAGGCGACGCGGAGTTT
TCGTCGATAATAGGGACTTTTGTTTCTTTCTTTCTTTTCTTGTCTTCTCTTTTCTTTTTTCT
TCATTTATTCCTCCTCCCTGTGCTCCGTTTTTATCGTTCGACGAGGAGAGAGAGAGAGA
GAGAGAGAGTACTCGGATTTATTTTATGCCGGCGCTTTGAAACTTGGTTTCAAGGAAG
AAGGAGAGAGTTTACCGTATAATTCTCTGGGCATGGAGCAGTTCGAACAACTGCTAAC
GTGCGCGATATGCCTGGACCGGTACAGGAACCCCAAGTTATTGCCATGTCAGCACAGC
TTCTGCATGGAACCGTGTATGGACGGCCTCGTCGACTACGTGCGTCGACAGGTGAAAT
GCCCGGAATGCCGTGCGGAGCATCGTATACCCTACCAGGGTGTCCAAGCGTTCCCGAC
CAACGTTACCCTGCAACGATTCCTGGAGCTGCACATCGAGATCACGGGAGAGCTGCCG
GACCCGACCAGCGGCCAAACCATGGAACGTTGCGGCGTTTGCTCCGAGAAAAGCTAC
TGCAGCCTCTGCGTCCACTGCGAGAAAAAATGTTGCCCCGAGTGCAAGGACGCTCAC
ATGGACATCCTGAGGCGCGAAATAACGCGCATCAATTCCCAGATTCGCAGGGGGCTGC
ACAGGTTGCAAGACGCGCTCACCCTGGTGGAGAAAAACACATTGGGTCTGCAAACAA
ACTGCGCCTCGGTCACGGAAGAGGTGGACGAGATTTATCGGAGGCTGAGCAAGGCTT
TGAAAGACCGTACGGAACATCTGCGTAACGAGGTCGATCGATACCTGAGCACCGAGCT
CAGAGGCTTGATTCAACTCAAAGAGAATCTCGAATTGGAGATCGCGAATATTCAGAGC
AACTGCGATCTGGCGGAGGCTCACATCAACGAGAACGTGCCCTGGGACGACTCGGAA
CTCCTCGATACAAAAGAACTCTTCCTGCGTACGGTGGAATTCATCAGGAACTTCGAGTA
CGAGGCAGGGGATTACAGTCGGCGGGTGCGTTTCGTGATGGCGCACGATCCGAATCAG
CTGGTCCTACACGTAGCCGGTTACGGCGAATTGAATATCAAGCCGGAGACCGGAAGTG
GAGGATTGCTGGGCAGCTCGAGCAGTCTGGCACCACCGGGGGGATCACCGGGCCTGA
TGAGAAGCAAAAGCGACCACCGCTTGGCCTCCCAGTACAGGCAGCAAGAGGAGGAG
CGGCTGGCGAGAAATCGATACGTCCCCGAATACGAGTACGACGCGCCGGAATACGAGG
TGCCGAGGAACAAGTCGAGGTACAGAAGTCGATTCATGCGGCATCGGGACGGCGACG

ACTCCGACGGCGATTCGAGATCCACCGTGCGATTCACATCCACCCCCCAGGAGTCGTC
GGGGCTCCGCGAGCGTGTCCTCGACACGGAGGACGCGGCGCGCGGCCCGCTTTCCGG
CATTTTTCGGCTCACCGACTCGCCACGCGTGATGAGAAAGCTTCAGGAGTACGAGAGG
GCCGGGAAAAGGAAGAAGGAGGAACCGCCGCCCCATCCTGTTCAACAACCTCAACCG
CCCAAGCCTCAG

>novel_circ_001753
GCATGGAGCAGTTCGAACAACTGCTAACGTGCGCGATATGCCTGGACCGGTACAGGAA
CCCCAAGTTATTGCCATGTCAGCACAGCTTCTGCATGGAACCGTGTATGGACGGCCTCG
TCGACTACGTGCGTCGACAGGTGAAATGCCCGGAATGCCGTGCGGAGCATCGTATACC
CTACCAGGGTGTCCAAGCGTTCCCGACCAACGTTACCCTGCAACGATTCCTGGAGCTG
CACATCGAGATCACGGGAGAGCTGCCGGACCCGACCAGCGGCCAAACCATGGAACGT
TGCGGCGTTTGCTCCGAGAAAAGCTACTGCAGCCTCTGCGTCCACTGCGAGAAAAAAT
GTTGCCCCGAGTGCAAGGACGCTCACATGGACATCCTGAGGCGCGAAATAACGCGCAT
CAATTCCCAGATTCGCAGGGGGCTGCACAGGTTGCAAGACGCGCTCACCCTGGTGGA
GAAAAACACATTGGGTCTGCAAACAAACTGCGCCTCGGTCACGGAAGAGGTGGACGA
GATTTATCGGAGGCTGAGCAAGGCTTTGAAAGACCGTACGGAACATCTGCGTAACGAG
GTCGATCGATACCTGAGCACCGAGCTCAGAGGCTTGATTCAACTCAAAGAGAATCTCG
AATTGGAGATCGCGAATATTCAGAGCAACTGCGATCTGGCGGAGGCTCACATCAACGA
GAACGTGCCCTGGGACGACTCGGAACTCCTCGATACAAAAGAACTCTTCCTGCGTACG
GTGGAATTCATCAG

>novel_circ_001755
GCATGGAGCAGTTCGAACAACTGCTAACGTGCGCGATATGCCTGGACCGGTACAGGAA
CCCCAAGTTATTGCCATGTCAGCACAGCTTCTGCATGGAACCGTGTATGGACGGCCTCG
TCGACTACGTGCGTCGACAGGTGAAATGCCCGGAATGCCGTGCGGAGCATCGTATACC
CTACCAGGGTGTCCAAGCGTTCCCGACCAACGTTACCCTGCAACGATTCCTGGAGCTG
CACATCGAGATCACGGGAGAGCTGCCGGACCCGACCAGCGGCCAAACCATGGAACGT
TGCGGCGTTTGCTCCGAGAAAAGCTACTGCAGCCTCTGCGTCCACTGCGAGAAAAAAT
GTTGCCCCGAGTGCAAGGACGCTCACATGGACATCCTGAGGCGCGAAATAACGCGCAT
CAATTCCCAG

>novel_circ_001757
ATGCAGTCTATCCTGTATCACAACAGTTCCGGGGCGCAAGCGTTCACCGGAAACTTGA
AGAAAAAAGAAGTTCACCGTGCTATTACGTTGCATCCTGTTAAAAATCCGCCGCACAT
GTACAGATTGCACAATTACATGAGGGGACTTAAGATTCAAGATCTGCAACAAGAGAGA
CTCAATCTTCACAGGGATATTTACACTATGGCGAAGCAATTGGGGATCAGAGTGGAGAA
TCTGAAGAATTACGAGATAGCCGATCGTGTCCCACTGTTTCCAGTCAAGCCTTATTCGA
AACTGTATCCCGGTGACACCGAAATATTGG

>novel_circ_001758
GTTTGAGCGACCGGTGCTCGGTATCGAGAAGGAGGAGCGATGGGATCGTACGAAGAA
TTTCCAGCACTCGCGCTAATCAACACATTGGAATCATTAAAGAAGGGGATCACGGGCG
CTAGAAAGATGAACCCAGCCGGAGATCCACAGACCCCACGAGATCAATGGATGAGGT

GACGTTTCACCCAGTGATGCGTAAACGACGTGGACTGGCCAGCGCCATTCTGGGCCTG
ATAGTGGGCCTTCTCCTAGGTTTCCTCATCCAAAGCTACAAGATACTCTCCTCGAGCAG
CCAACATCAACGGTTAAACGTTTACGCGTCCTCGATGCCTGGCTCGCGGCTGTTAATCA
AACCATCGGCGAGAAACGTTCCGAAATTGGAAGACGACGAGCGGATAAACAGTTCCT
CGACCAGTTTGGTGTTCGTGGGCGTGATGACGGCGAGCAAGTATCTGGATTCGCGAGC
CAAGGCCGTCTACGAGACTTGGGGCAAGGAGCTGCCGGGAAAAATTGCCTTCTTCTCG
TCCGAAAACTCGGTAGTCCCGAACAATTGCCCCGATCTACCGTTGGTGGCGTTGCCCC
GTGTCGACGACACGTATCCACCTCAGAAGAAATCGTTCATGATGCTTCAGTACATGTGG
AACAACTACGGGGACCGTTTCGAGTGGTTTCTCAGGGCGGACGATGACGTTTACGTGA
GGACCGATCGTCTGGAGCAACTGCTACGATCCGTCAATTCCAACAGGGCGATGTACAT
CGGCCAGGCGGGAAGAGGAAACTCGGAGGAGTTCGGCCTCCTCTCGTTGGAGTACGA
CGAGAACTTTTGCATGGGCGGGCCAGGCGTTATTCTGTCCAGGGAGACGTTGAGAAGA
ATCGTGCCCCACATCAAGTATTGCCTCAGACATTTGTACACCACTCACGAGGACGTTGA
ATTGGGAAGATGCGTGCAAAAGTACGCCGGTATACCTTGCACCTGGAGCTACGAGATG
CAGTCTATCCTGTATCACAACAGTTCCGGGGCGCAAGCGTTCACCGGAAACTTGAAGA
AAAAAGAAGTTCACCGTGCTATTACGTTGCATCCTGTTAAAAATCCGCCGCACATGTAC
AGATTGCACAATTACATGAGGGGACTTAAGATTCAAGATCTGCAACAAGAGAGACTCA
ATCTTCACAGGGATATTTACACTATGGCGAAGCAATTGGGGATCAGAGTGGAGAATCTG
AAGAATTACGAGATAGCCGATCGTGTCCCACTGTTTCCAGTCAAGCCTTATTCGAAACT
GTATCCCGGTGACACCGAAATATTGG

>novel_circ_001759
AAAGTTGTTTGTTGGTGGTTTGTCCTGGGAGACAACACAAGAAAATCTTCAACGTTAC
TTCGGCCGTTATGGCGAAGTAATTGACTGCGTTGTTATGAAAAACAGTGAATCTGGTCG
CAGTCGTGGTTTTGGATTTGTAACATTTAGTGATCCAGCTAATGTTCCATTAGTTCTTCA
GAATGGACCACATCAACTTGATGGGCGCACAATTGACCCGAAACCATGTAATCCACGC
ACTCAACAAAAACCAAAACGCAGTGGAGGCTTTCCGAAAGTTTTTCTTGGTGGCTTAC
CAAGTAATGTGACAGAGACCGATTTGAGGTCTTTCTTTACCCGTTTTGGTAAAGTTATG
GAGGTTGTCATAATGTATGACCAAGAGAAGAAGAAATCAAGGGGATTTGGCTTTCTCA
GTTTCGAGGATGAAGATGCAGTGGACCGATGCGTAGCGGAGCATTTCGTCAACTTGAA
TGGAAAACAGGTTGAAATTAAACGCGCAGAACCAAGAGATTCTTCTAGCAAAATGAAT
GATAGTCATCAAGGTCAGTGGGGACCACCTCAACAAGGTGGACCTCCAATGGGAATGG
CTGGGAATATGGGACCTATGGGTGGTCCAAATGGACAGATGAGTGGACCTATGATGGG
AGGTCCAATGGGTCCACCAGGAAATATGATGCAGCAATATCAAGGTTGGGGTACCAGT
CCTCAAACTGGAGGATATGCTGCTGGATATACTCAGTATAATTCTCAGGGCTGGGGTGC
ACCACCTGGCCCTCCTCAGCAACAACAAATTCCACCACCACCACATCATCAATGGGGT
AGTAGTTATAATGTTCAACCTGCAGCAGCTACTCAAGGTTATGGAAGTTATGGTGGGCC
GGCAGCGGCCGCTTCAGGGGGCTATGGGCCCACAGCGGGTACTGGCGGGGCTGCGGG
GGGCGGGCCCGGGGGTTCTTGGAACTCCTGGAATATGCCACAAAATGGGCCCCCTCCT
GGGACTCAGCCACCACCTCAGCCCCCTCAGCCTAATTCCTCGCAGCCCAACTCCAACT
CGAACCCCTCTGGCCCGCAGG

>novel_circ_001760
AAAGTTGTTTGTTGGTGGTTTGTCCTGGGAGACAACACAAGAAAATCTTCAACGTTAC

TTCGGCCGTTATGGCGAAGTAATTGACTGCGTTGTTATGAAAAACAGTGAATCTGGTCG
CAGTCGTGGTTTTGGATTTGTAACATTTAGTGATCCAGCTAATGTTCCATTAGTTCTTCA
GAATGGACCACATCAACTTGATGGGCGCACAATTGACCCGAAACCATGTAATCCACGC
ACTCAACAAAAACCAAAACGCAGTGGAGGCTTTCCGAAAGTTTTTCTTGGTGGCTTAC
CAAGTAATGTGACAGAGACCGATTTGAGGTCTTTCTTTACCCGTTTTGGTAAAGTTATG
GAGGTTGTCATAATGTATGACCAAGAGAAGAAGAAATCAAGGGGATTTGGCTTTCTCA
GTTTCGAGGATGAAGATGCAGTGGACCGATGCGTAGCGGAGCATTTCGTCAACTTGAA
TGGAAAACAGGTTGAAATTAAACGCGCAGAACCAAGAGATTCTTCTAGCAAAATGAAT
GATAGTCATCAAGGTCAGTGGGGACCACCTCAACAAGGTGGACCTCCAATGGGAATGG
CTGGGAATATGGGACCTATGGGTGGTCCAAATGGACAGATGAGTGGACCTATGATGGG
AGGTCCAATGGGTCCACCAGGAAATATGATGCAGCAATATCAAGGTTGGGGTACCAGT
CCTCAAACTGGAGGATATGCTGCTGGATATACTCAGTATAATTCTCAGGGCTGGGGTGC
ACCACCTGGCCCTCCTCAGCAACAACAAATTCCACCACCACCACATCATCAATGGGGT
AGTAGTTATAATGTTCAACCTGCAGCAGCTACTCAAGGTTATGGAAGTTATGGTGGGCC
GGCAGCGGCCGCTTCAGGGGGCTATGGGCCCACAGCGGGTACTGGCGGGGCTGCGGG
GGGCGGGCCCGGGGGTTCTTGGAACTCCTGGAATATGCCACAAAATGGGCCCCCTCCT
GGGACTCAGCCACCACCTCAGCCCCCTCAGCCTAATTCCTCGCAGCCCAACTCCAACT
CGAACCCCTCTGGCCCGCAGGGTGACATGTATTCACGACAGACCACCGGCTCAGGTGC
ACCTGGGTCCAGTAGCAGTTCAGCTAAAACTCCGGATTATACTGGGTACTCTGCATATG
GTAATTACGCTGACACCAGTTATCCTCAGCGTTCGTATCAAGGAGGAGAAAGCAACCA
AG

>novel_circ_001761
GTGGGCCGGCAGCGGCCGCTTCAGGGGGCTATGGGCCCACAGCGGGTACTGGCGGGG
CTGCGGGGGGCGGGCCCGGGGGTTCTTGGAACTCCTGGAATATGCCACAAAATGGGCC
CCCTCCTGGGACTCAGCCACCACCTCAGCCCCCTCAGCCTAATTCCTCGCAGCCCAAC
TCCAACTCGAACCCCTCTGGCCCGCAGGGTGACATGTATTCACGACAGACCACCGGCT
CAGGTGCACCTGGGTCCAGTAGCAGTTCAGCTAAAACTCCGGATTATACTGGGTACTCT
GCATATGGTAATTACGCTGACACCAGTTATCCTCAGCGTTCGTATCAAGGAGGAGAAAG
CAACCAAG

>novel_circ_001762
GGCAGGGATGGCAAGGGAAGTATCTACGTGTGGGCTTCTGGTAATGGAGGTTCGAAAT
CGGACGACTGCGGATGCGACGGATACGTAGGAAGCATTTACACGATCGCAGTTGGCTC
TGCCAGTCAGACCGGAAGATTTCCATGGTACGGTGAAAGCTGTCCCGCGACAATGGCC
ACCACGTACAGCAGCGGAGCTTACTACGATCAAATGATAGTGACGACAGACTTGAAAA
ACACGTGCACCGTTGGACACACTGGTACTTCCGCATCCGCTCCACTAGCTGCCGGTATC
CTGGCCTTAGCTTTGCAAGTAAACAAAAATTTAACCTGGAGAGACGTGCAACATCTCA
TCGTTTGGAGCTCGGAATACAGTCCACTCAGGGAGAATCCTGGCTGGTTCAGAAACTC
GGCTGGATTTTGGTTCAACTCACGTTTCGGCTTCGGACTTATGAACGCGTACTCGTTGG
TCTCGGCAAGTTACAATTGGACCACCGTCCCCGCCAAGGCCATCTGCAAAGTGAACGT
TGCTAAAGGAATCGAGAAGAAGTTGGCTTATGGAAATACGAGGAGGATACGATTCGAG
GCGGAAGATGAGTGTCGTGCGGCGGAGAACGAGATTACGTTTTTGGAACACGTGGAA
ATCGAGGTTAGCCTCGAGTACAGCGTTCGCGGTTCTATTCAGATACACCTAACTGCGCC

ATCAG

>novel_circ_001763
GGAAAAAAATGGTGGATAAATTGGAATGGTTAAATAACGAATTCGTGCAAAGGGTCCT
CCAGTACAATGAGTACGACACCAGCATCAATGTAACGAATATATCCGCGAAACCGGCG
ACCAGCAAAGGTGACAATTATGCCAGCGACATGTACAGGGTGACCGTGAAATACACGC
AGAAGGAGGGCAAGACCAGGGTCAATAAGGAGACGTCGATTGTGTGCAAGTTCGAGC
CGTTGGAGGAGGACGCTCGAAGGGAAGCGATCATGTCAATGGGTATCTTCGAGAACG
AAATCTTCATGATGTCGAATTCGCTACGAAAGATGCAACAAATGTTGGGCACTCGGCTG
GGAGCAAATGTACTTTACTTTAGAATGGAACGGCCGGTTTGTTTGATATTGGAAGACCT
TGCTCGTCTAGGATTTCGTATGGCCGATCGTTTCCGTGGTTTAGATTTCGATCATTCCAA
GCTAACTCTTCAAAGCTTAGGCAAATTTCACGCGGCGTCGGTCGCACTGTGCGAGAAG
GAACCCAAATTAAAGACTGTATATCAGAAAGGGATACTGTACGAAGGAATGCCGACCG
AATTCAAATTCTTCTTCATCTCCGCAATTAAAGCTCTTGGAGACGATTTGGCAAATTGG
CCGCAAGACGTGAAAAG

>novel_circ_001764
CCGCTCAGGCGAAAATCGTGGGTCCGGAAGACGTGTACGTGAAGAAGGGTAGCACGA
TAAGCCTCACGTGCATCGTTAATGTGCAAAGTACACCGCCCAGCAGCGTTTCGTGGCAT
CACGGAGGAGATGTGATGGATTTCGACAGTCCCAGCGTTCACCGTAGGGGCGGGGTTT
CCTTGGACACGGAGAAGACGGAAAGCGGAACGACGAGCAGACTTCTGGTGACACAA
GCCAGATTGACCGACAGCGGAAATTACACCTGCATTCCCAGCAACGCGAATCCAGCCA
GCGTAATGGTCCACGTGCTGAATG

>novel_circ_001765
GGGAACATCCAGCGGCCATGCAACACGGCGGAAGTTGCGGCGTCGCGCCCACCATTTT
ATCGGCGACCCTTACTCTGGTGATCTCGAATCTGCTAAGGTGAGCGGTGTCGTGCTTGT
GAATCGTGCGACTTGGAAGCCATCACACGGGCTGTGGTCGAGCTCGTTTCGTGAACCG
GTGAGACGGTACCCCCGAAGGAAGGAGGAGGGATTGGTGGTTCTTGTTCAAAGGTAA
AAAAGCGCGAACGAAGGAGAAGGCCGCGCGTCCCTCTGCTTTGAGAGAGGAAAGCAT
ACGGCGGAAATCACGTTGCGAGAATAGAAACGTGACTTAGCGGTGCTGCCAACGGTG
CTGCGAAATCATCGGGGTGGTTTTTAAATGGGGGGTGGAACGGCAGGGGGACAGGGA
GAGGAAGGGATTTAGGGGTTGGAACAAAGTGCAGTGGATCTGGACGGCAGTGGTGGT
GAATGACGCAGTGACACGGAAAGTGGAGCTCGACTTTGCACAC

>novel_circ_001766
AAATTCAACGAAATAGGGAATTTTACAAGAACGCCGACGTCAGACCGCCGTTTACATA
TGCATCATTAATTAGACAGTCGATCATCGAGTCGCCGGATAAACAGTTGACCTTGAACG
AGATCTACAACTGGTTCCAGAACACATTCTGCTACTTCCGGCGCAACGCAGCAACGTG
GAAGAACGCAGTGCGACACAACCTGTCTCTCCACAAATGTTTCATGCGAGTCGAAAA
CGTGAAAGGCGCCGTATGGACGGTGGACGAAGTGGAGTTTTACAAGAGACGTCCTCA
ACGCGCTTGCAGCACGACCGG

>novel_circ_001767

ACACTTGAACACGGAGCACACGCTGGACGACAGATCGACGGCGCAGGCTAGGGTCCA
GATGCAAGTGGTCTCGCAACTGGAGATCCAGTTGCAGAAGGAACGGACCGGTTAAC
CGCTATGATGCATCACCTGCACGTGGCGAAACAAATGGCTTCCCCCGAACCGCCAAAG
TCGTCCGAGTCATCGACGGGCTCGAGTATACCAAAGTTAAATCTCTCCACCGCTCTGAT
GAGCCAGCCACCACCGAATTTCGGTGTCTCTCAAGTGTCTCCCGTCTCGATGTCCGCTT
TGGTGTCAGCGGTCAGGTCACCAGCAGGTGGACAATTGCCACCTTCGGCCGGAGCAC
CTATGCCACCCATTCCTAACATGTCCAACATGTCAGGGATGCCTCCGTTGCCGAATATGC
CTGGTAGCATGCCCACCATGCCTACCATGCCCAGCATGGCGGGGCCCATCAGACGACG
TATCAGTGATAAATCCGCGCTTTCCTTGGCAGGAG

>novel_circ_001769
GCTACTTCGCTGTTTGCAGCCCTGAAGCAGCAACAACAGCAACAGCAACCGCGCGAC
ACTGTCCCCTCGTCGAGGGAGCGCGTCGAGAATCGAGATCGAGATCGATTGTCGGTCC
GTAACCGTGAGAACAACCGGAACGAGATAGGTGGTGGCGTGACGGAGCAATCGCAGC
CAGCGCAGCAACAACAACAGCAGCAGCAGCAGCAGCAACAGGAGCTCACGATCGAG
TATCAGAGCAATGGAAAGCTCAGTCCCGCTGGGCACGCAGTGACAGCAGCACCCATG
ACCCAGCAAAAGCAGCCTATCATCACGCAGCAATCGCAACAGCCGAGCTCGGGGGCG
CCTGGTCCCCAACCGAGTCCTCATCAGAGTCCGCAGGCCCCTCAGAGGGGTTCGCCGC
CAAATCCTTCGCAGGGTCCCCCGCCGGGAGGGCCGCCAGGGGCACCACCTTCGCAAA
ATCCCTCGCAGATGATGATCAGCCCGGCTAGCGGCATCCACCAGATGCAACAACTGCT
GCAACAACACATACTCAGCCCCACCCAACTGCAATCCTTCATGCAGCAACACTCGCTC
TATTTACAACAGCAACAACAGCAACATCATCAGGATTCCTCGTCGGAGCATGCGTCCAA
TCAGGAACGATTCGGCTACTTTTCGTCCCTTAAAGACCATCAACACCAGTTCGCCGAG
CTGGGCAGGAAGAAGCTGGAGCAGGCGATACAGCAACTGCAGGAACAGTTGCAGTTG
AACGTGATCCAGCAGACGCATCTGTTGCAAACGGCGGACAAGAAGAAGGCGTCCGCC
CCGCTTCAACAATTGGCGCTTCAACAGCAACGTCTGATACAGCAACTGCAGATCACGC
AGAGCCAATATCTTCTTCAACAGGGTCTGGGTCTTCAGGGTCACAATCCTTCTTCGGGT
CTCCAACCTGGCGAAGGACTACCAATGTGGAAATCGGACACGTCGGATGGTCCGGAAT
CGCATCAGAACTCGAACGTTCCAAAATCCGTGGCTGGACTCAATGGACTTCTCAATTC
GACAGTGTCGAGTCGGCGGTCGGATATGAACGGAACCACTCCATTGGACGAGAAACC
GCTGGACGTTTCCTCCAACGACAAGGTTCATCCCCTGTACGGTCATGGGGTCTGTAAG
TGGCCGGGCTGTGAAGTTATTTGTGAAGACTATCAAGCATTCCTTAA

>novel_circ_001770
GCTACTTCGCTGTTTGCAGCCCTGAAGCAGCAACAACAGCAACAGCAACCGCGCGAC
ACTGTCCCCTCGTCGAGGGAGCGCGTCGAGAATCGAGATCGAGATCGATTGTCGGTCC
GTAACCGTGAGAACAACCGGAACGAGATAGGTGGTGGCGTGACGGAGCAATCGCAGC
CAGCGCAGCAACAACAACAGCAGCAGCAGCAGCAGCAACAGGAGCTCACGATCGAG
TATCAGAGCAATGGAAAGCTCAGTCCCGCTGGGCACGCAGTGACAGCAGCACCCATG
ACCCAGCAAAAGCAGCCTATCATCACGCAGCAATCGCAACAGCCGAGCTCGGGGGCG
CCTGGTCCCCAACCGAGTCCTCATCAGAGTCCGCAGGCCCCTCAGAGGGGTTCGCCGC
CAAATCCTTCGCAGGGTCCCCCGCCGGGAGGGCCGCCAGGGGCACCACCTTCGCAAA
ATCCCTCGCAGATGATGATCAGCCCGGCTAGCGGCATCCACCAGATGCAACAACTGCT
GCAACAACACATACTCAGCCCCACCCAACTGCAATCCTTCATGCAGCAACACTCGCTC

TATTTACAACAGCAACAACAGCAACATCATCAGGATTCCTCGTCGGAGCATGCGTCCAA
TCAGGAACGATTCGGCTACTTTTCGTCCCTTAAAGACCATCAACACCAGTTCGCCGAG
CTGGGCAGGAAGAAGCTGGAGCAGGCGATACAGCAACTGCAGGAACAGTTGCAGTTG
AACGTGATCCAGCAGACGCATCTGTTGCAAACGGCGGACAAGAAGAAGGCGTCCGCC
CCGCTTCAACAATTGGCGCTTCAACAGCAACGTCTGATACAGCAACTGCAGATCACGC
AGAGCCAATATCTTCTTCAACAGGGTCTGGGTCTTCAGGGTCACAATCCTTCTTCGG

>novel_circ_001771
GCTACTTCGCTGTTTGCAGCCCTGAAGCAGCAACAACAGCAACAGCAACCGCGCGAC
ACTGTCCCCTCGTCGAGGGAGCGCGTCGAGAATCGAGATCGAGATCGATTGTCGGTCC
GTAACCGTGAGAACAACCGGAACGAGATAGGTGGTGGCGTGACGGAGCAATCGCAGC
CAGCGCAGCAACAACAACAGCAGCAGCAGCAGCAGCAACAGGAGCTCACGATCGAG
TATCAGAGCAATGGAAAGCTCAGTCCCGCTGGGCACGCAGTGACAGCAGCACCCATG
ACCCAGCAAAAGCAGCCTATCATCACGCAGCAATCGCAACAGCCGAGCTCGGGGGCG
CCTGGTCCCCAACCGAGTCCTCATCAGAGTCCGCAGGCCCCTCAGAGGGGTTCGCCGC
CAAATCCTTCGCAGGGTCCCCCGCCGGGAGGGCCGCCAGGGGCACCACCTTCGCAAA
ATCCCTCGCAGATGATGATCAGCCCGGCTAGCGGCATCCACCAGATGCAACAACTGCT
GCAACAACACATACTCAGCCCCACCCAACTGCAATCCTTCATGCAGCAACACTCGCTC
TATTTACAACAGCAACAACAGCAACATCATCAGGATTCCTCGTCGGAGCATGCGTCCAA
TCAGGAACGATTCGGCTACTTTTCGTCCCTTAAAGAC

>novel_circ_001772
AGGTTGGCGGGGGAAATCTTGGCGGGGACAAGGGGAAAAACTGGAAACAGGCTGTG
CGGCGGTCGGCTTTCTCGGCGCAATAGGGTCGCAGGGGATCAGGGAAATGCAGAAGG
TTACTAAGAGCTAGGGAACTTGTAGCCAACACATAGAAGTTTCGCAAACACACACGGA
CGCGCGTCCATAGACATTCCACACTGGCAAGCTCTCTCATACTCTGGGGCAAGTATCGT
CTGGCTAATGTAAAAAACATGGGTGCACACAACGTGTTGTGTGTTGCTTACATTGCGAC
TCTCCTTATTCCCGTAGGGTTAAAACGGGGGGAGGGAGGGGCTGGTCGCGGGCTGTAG
GAGGCTGGCGAAGCAGGGGAGGAGGGGGGAGTAGGAGGGAGGGAGGCTGTATGATA
CCACGGGGTTAGCATTGTTTGTGCGACGACCACTCAGACTCCTCGTCAAAAGAGCTG
CTAATGTCGGTGCGCGTCTACTGTGACGTCAATAACGCATGAGGCCAAATTGAAAAATT
ATCACACCAAAGCGAGGACCGTTGTTTAGAAAGAGGGTGGCCTTCCTTCGTTCCAAAG
ATCGTCGCTAGTTACGCCAGATAGCCAGACAGCCTAATTTCACCCAATACGGTTAACTC
GATCG

>novel_circ_001773
ACAAAAATGAATTGGAAGCAGCTCAGAATTTTCTAGAGCAAGCAGCAAGGACTAATCT
CCATGAATTCTTGCAAAGACTTAGCAGCGTGCTTGTTACTGCTGCTGCAAGCCCTGTTG
CTCGTATGGCAGCAGGTCTTCAGCTTAAAAATCAACTTACATCCAAAGATCCAGACCTA
AAATATCAATATCAACAACGTTGGCTTGCGATTCCTGTAGAAACAAGAGAATATATTAA
GAAAAATATTTTTGGGGCACTTGGAACAGAGAACAATAGGCCAGGTTCTGCACCACAG
TGTGTTGCATATGTAGCAGTTGCTGAATTACCTGTTCGTGAATGGACCAATGTTATTCAG
CTATTAGTTAATAATGTTGTAAATCCAAACAGTACAGAAATGTTAAAGGAGGCAACTCT
AGAAGCCATTGGTTACATTTGTCAAGATATAGAAAGCGACGTTTTGGTACCTCAGTCTA

ACGAAATTCTCACAGCTATTATTCACGGTATGAAAGGATCTAGTACTTCGCATTATGTTC
GTCTTGCAGCTACGAGTGCACTCTACAATTCATTAGAATTTACCAAAGGAAATTTTGAG
ATAGAGACAGAACGAAACTTCATTATGGAAGTGGTATGCGAGGCGACGCAATCTTTAA
ACACACAAGTTAAAGTAGCAGCATTACAGTGTCTTGTGAAGATTATGTCGTTATATTATC
AATATATGGAACCTTATATGGCTCCAGCACTTTTTCCGATTACTTTAGAAGCTATGAAAT
CAGATATTGATGAAGTTGCACTGCAAGGAATTGAGTTTTGGTCAAACGTATCCGATGAA
GAAGTAGATTTAGCCATGGAAGAAGGTGAAGCCTCCGATGGTGGTCGACCGCCGGTCA
AAGTATCGAGGCATTATGCAAAGGGTGCTTTGCAATACTTGGTACCTGTTTTGATGAAG
AAACTCACTAAACAAGAAGAATTCGATGACGAAGACGATTGGAATCCTTCAAAAGCA
GCTGGA

>novel_circ_001774
ATTAATTAGCGATGTGGGTCTGGAAATTTTGCTCGACAAATTTCATGGCGTACAAATCA
AGCCAAAGTCTCTACAAGGAGAGGAGTTCATACTTGAGAGATATTTCTTTTTTTCTGG
ATAAGATAATTCAATATATAAACCAAGCATTTTACGAGGATCGAAGCTTTCAAAGACAA
CAAATAGCTTTTGCAGCTCCTCCGGTTTGCAGTTGGCCTTGAGCTTCCTCAGATGCAAC
CCCTTGCGCCTCTGCTTCTTGTCCACGGCTGGCAAAGCAGCCTCGACGAGATTCTCCT
CGTCGTCCTCGTCGTCCTCCTCGTCCTCGTCCTCTTCATTCTCGCAGGCGTTGCTCTTGT
TGTCGGGGCCAGCGATCTCCACGTGATACGCCGTGCCCGGCAGACGATGGTTCTCCAG
GGCGCCGCTGTCTTCTCGCTGGCAATATTGCTGAATGTTGGTCACAGCACCGCCCCCGG
ATATCGTGCAACCCTTCTCCACCGGAGAATACGTCCTCGATAAACCGGGCCGCGACTCG
CAGGACATGACCGAAGTGGTACGTTGCTCAGTGTTCTCTTTCCACACTTGTATGTCGGT
GCTGTGGTGGCCACCCTCCTCGTCCACCAAAGAACTTTCGAAGTCCCTGTTCCCGAGC
ACATCGCATCTCATGATGTCGCAGTAATCCGCGAGGCACACCGCATCCTCCGCCTGCGT
TACAATTATTTTAATTATTTTTTATTTATAATTCATTCGATCTCGTGGCGTAACAAACATTT
CGAAATAATTGAGGACGATATTTCTCTGGGAAAAATACGATTGCTTATATTGATATCGAC
GTATAATATCGAGAGAAAAGAATAGAATAAGATGATCGTCAACTATTTCGAATAAATCGT
GAGAAGAGGCGGAAGAAATTTTAATACCGTGTTAACGTTACTCTTGTGTCTGTTATCGA
AGTGGGCATCCAAGTGTTTCTCCGCGTAGAAGGATTTGCCGCACAGGCCGCAGGTCCA
TTGCGATGGCCTGTGCTGATGTTTCGCCTTTTGCTGGGGCCGAAAAATGTCCCGACTTT
CGTGGAACGGACACTCCAGCGGCAATTCCACTTGATGTTTCTTCAGGATTGGCATCCAT
CTCTGAAAAG

>novel_circ_001775
CTTTTGCAGCTCCTCCGGTTTGCAGTTGGCCTTGAGCTTCCTCAGATGCAACCCCTTGC
GCCTCTGCTTCTTGTCCACGGCTGGCAAAGCAGCCTCGACGAGATTCTCCTCGTCGTC
CTCGTCGTCCTCCTCGTCCTCGTCCTCTTCATTCTCGCAGGCGTTGCTCTTGTTGTCGG
GGCCAGCGATCTCCACGTGATACGCCGTGCCCGGCAGACGATGGTTCTCCAGGGCGCC
GCTGTCTTCTCGCTGGCAATATTGCTGAATGTTGGTCACAGCACCGCCCCCGGATATCG
TGCAACCCTTCTCCACCGGAGAATACGTCCTCGATAAACCGGGCCGCGACTCGCAGGA
CATGACCGAAGTGGTACGTTGCTCAGTGTTCTCTTTCCACACTTGTATGTCGGTGCTGT
GGTGGCCACCCTCCTCGTCCACCAAAGAACTTTCGAAGTCCCTGTTCCCGAGCACATC
GCATCTCATGATGTCGCAGTAATCCGCGAGGCACACCGCATCCTCCGCCTGCGTTACAA
TTATTTTAATTATTTTTTATTTATAATTCATTCGATCTCGTGGCGTAACAAACATTTCGAAA

TAATTGAGGACGATATTTCTCTGGGAAAAATACGATTGCTTATATTGATATCGACGTATAA
TATCGAGAGAAAAGAATAGAATAAGATGATCGTCAACTATTTCGAATAAATCGTGAGAA
GAGGCGGAAGAAATTTTAATACCGTGTTAACGTTACTCTTGTGTCTGTTATCGAAGTGG
GCATCCAAGTGTTTCTCCGCGTAGAAGGATTTGCCGCACAGGCCGCAGGTCCATTGCG
ATGGCCTGTGCTGATGTTTCGCCTTTTGCTGGGGCCGAAAAATGTCCCGACTTTCGTGG
AACGGACACTCCAGCGGCAATTCCACTTGATGTTTCTTCAGGATTGGCATCCATCTCTG
AAAAG

>novel_circ_001776
ATAACTGTTTCGATACGTCACGACGTATACGTATACGTGAAAGAAAGTGAAAAGAATG
ACAAGCGTGAAGCAGTTTGTTCGCTTTGCACGTAGACTGGGCCAGATATCAGAGAGTG
GAACAAAAGTTGTAAACCGGTTCGTGAGATATGCGTCTACTCAAGCTCAACACAAATT
CATAGAGGATGAAAATGGGAAAAAAATTTTCCCTTCACCGTTTGGGGATTTCACGCCTT
CCGATACCCTCGTTCATGAATACATATGGAAGAATGTCGAGACGTTCCCTAATCGCATC
GCATTGGTAATATACGTGGCAAAAATAATTTTTCCATCCAAATTATTTTGTCTGTATTTTG
GGAAAAATATAATGTGATGTTATATAACACGAGAACTTGCGAATGTGGGATAACCGGGA
CAAAATACACGTATGCCAAAGCGAGGGACGCGAGTAACTATATCGCGAGAAGTTTGAG
GAATATGGGTTTGAAGAAAGGTGACTTGGTGGCCCTAGTAGCGCCCAATTATCCGGAA
ACGATTTTAGCTGCTGTCGGTGTCTTAGAAGCTGATCTTATTCTCACTACCATGAATCCT
ACCTATACAATCGAGGAAATGAAGAAACAAATAAAGGATTGCGAGGCAAATGCGATCA
TTACAGTTGCAGAAATTGCACATATTGTTCTTGAAGCAAGAAAAAATACTTCCGCGTCA
AGTGGACCTTTCGTGGTTATCGAGGATGGTACCAGATCTATTCCCGAAGGCAGTGTGCC
GTTTAAGGATCTTATCACACGAGGTAAAACATTACCGCCTATAACGCATTATCAAATGAG
TTCGAATGATCTAGCCATATTGCCATATTCCAGTGGAACGACTGGCATGCCGAAAGGTG
TCATGTTGACGCACAAGAATTTAGTAAGTAACATGGAAATGGTGGAATATACGACGAA
AGAAAGGCTGTGGCGACATACGACTGCCGATTTTCAAGAGGTAGTACCTCTAATTATAC
CCTTCTTCCATATTTTCGGATTGAATGCCGCGACGTTGCCTCGTCTCTACAACGGTACAA
AAATAATCACACTTCCAAAATTCGTGCCGGAAGTATTCGTGGACATTTTGACGAAAAA
AAATATAACAGGTTTGTTCGCAGTACCATCTTTGATCACGTTTATAAATATCTGCCCTTT
GCTTAAAAAGGAGATTTTCCAAAATATACATCACATTATCACCGGTGCGACACCATTAC
CGGAGGTGGATGTGGAGAGATTTTACGAAAGATATCAGATAAGCAGCGATGACTTGAA
ATTTTCTCAAGGATACGGGATGACGGAAACTTCACCAGTGATTTGCTTAGATTCGTGGA
GTCGAAAACCATCAAGTATCGGGCAAAATATTGCCGGTTGCGAAATACGATTAGTCGAT
TCGGCAACGAACGAGGATATCTCTGTAGCTGGTCAGAAGGGTGAAATATGGGCAAGGG
GGCCTCACATCATGAAAGGATATTTAAACAACGAGAAGGCGACGAGCGAGATGATCGT
CGACGGTTGGCTGAAAACTGGGGACATTGGTTATTTCGACGACGAGTTTTATTTCTTCG
TCACGGATAGGAAGAAAGATTTGATCAAAGTTAAAGGATTCCAGGTACCACCAGCCGA
GTTGGAAGCTTTGATAAAAAGACATCCAAATGTTATCGAGGCAGCTGTGATTGGTATTC
CAAACGAAAGGTTCGGCGAGATACCTAAGGCTTTCGTAATTCTCAAGGAAGGTAGCAA
AACGACAGATGACGATATCAAAAATTTTGTAAAAGACAAAGTGTCGGAATACAAGCAA
CTCAGAGGAGGAGTGACATTCGTTGACAGTATACCAAAGAACGCATCCGGAAAGATTT
TACGAAACAAATTGAAAAACGAGTACAAATAGTAAAAGTTTATGCTGAAGAGTGAAGA
AGAAAATTTCACGCTCCGAGGCCTCGATTTACATCTCGTACCGTAATGGGATCAAGAAT
TATCGATTAAGAAAGGGTACGATATATCGTTTCTGTATTTCTGATTGGCATAATGAATGGC

ATATGAATTATTCAACTGTTATATTGATGTATGGTGAAAGATATGCTAATAACGTTTTTC
ACTTTGTTTTATGTATAATATTGTTATCTCATAAGTGTTTTACAAGTGCTCTTACTGAAGT
GTCTTATTGATTACGACAATTTTTTTAATATTTTTACATTTATATATACATATATACATATAT
CTATTTTGAAAGAATGGGTTTAGTAAAAACATATATAAATACCGATGTACTTATTAGAAT
AGCGGGAAATAGCGGTAAAAAGAAAACGTGATTACGAAGTACATTTTTAAAATTATATA
CGTATATCCTTTTATTTTTTAATATCATTTAATATACGCAAATATTATCGGTTGTTGTGCAAT
ATTTGACAATGCAATTAATTTTTAAGATTATTTTTTAGATTATTAAATTTTATCGATTTTTT
ATAAAACATTTGTACAAGAAAGTTACAGGGATTTTAAACTTTTAAACAATAAAAATCTT
TTATAGTTTTACGGTTGTGTTATAAATAATTGTTATAAATAAACATTTTTCAACAAGCATG
TCGGATTATTTAAAATTCAATATCTTTACATTCGTTTTAAATAATTCTTAACCACTACTGA
TTTATTTTGAATGTTACATATGTTAATTTATGTCTGTCTATCGTTTCATATTATCTCGATTTC
GATCGATTAAAATCTTGAAATCAATTGAACCATCATTTTTGTTTTATTTATACATCATTAAT
AATTTATCTGATGTTTCATTCGATTAAATATTTACTATCAGAATACTGCATCGCTTCATACC
TTGTAAAACTAATTTTTCCTTTAATTATATTTTATAATTTATGTTCGAAATAGAATAGAGAT
AGATATCGCAAGATTGCAAGTTTTTATTGTCATAGAATAAAGATAAAGATAAATTAAATT
GCATAAAGATTAATTAAATTGCAATCACTTAATAAAATAGATAAAAAAAAAGAACTTAT
CAGATCATACTTTTTTAATTTTAAATTCAAATGAACGATACGATCTATATAACTTCACACA
TAGATAATTTCAATTATATCTATGAAACGGATGTCTATGAAATTTATTACCATCTCACTCA
CGTTTTGCAAAATAAAATTCCTAGAAAAATATAATAAATACATATTTCCTTCTTCGTCTTT
CCTTTGTTTCATTCTCTCCGATCTAACTCTTTAAATTGTTCTTCTTCCTTACTTTATCATCA
TCAGTGTGATAACTATCACGATAAAAGTTAATTCCATTCGAACGATGCGCGATGCGATT
GTTCTCGAGATCTTCGATTCCCATTCTCTATTTCTTCCTATTTACACGCATTCCGTGTGAA
TTGTACGGTAATCGCCAAATCTGCATCCCACCGACTTCCCGCCATCCGAAAACGTTTTC
GAATTAAGGTTCCGCGCGTGAATTGTCTGCGTGCCCGTCATTTAATTCCCTTCGGGATT
GCTACCACCGTTGTTACCACCGTTCTTCGCCTCCCCAAATGCGCCCAAATACGAGTCTC
GAGCTTTCGAGCTTTCCTTTCGAGGCTGGGCCCTCGTGCCAGGGTGAAACAGGTGAA
GGTGGCCGGGCTCGAATCGATGCTTGGAACAGGCGAGGAGGAGGAGGGGGATGAAG
GGTCGAATGGAAACAGACAGAGCTGCTTTCCAACCCCTAAACGCGTGGCTTTGAGTGC
GTGGTTCGATCCCTCGGGGGAAAAGAGATGAACTGTGCTTAATCCTGTTCGTCGGATA
GAAATGCATTCAACAGGGATAAACCGAAGAACCGGGTTGACGAAGCCAATTTCTTATA
TTTACTTTAAACGAAAGTGGAAAGATATTCGAGGGATGAGGAATTAGAGGAAGAGTGG
AGTATAATAAAGTGAACGATGTAGTTAGCGTTTGTTTATTACAACTCTTTCTTTTCTTTTT
TATTATGTTTGATAGGGTTTACCTGTTGATCTCGTGTTTGTTGGAACGAGAAGGCAAATT
AAAATACGTTGGTAATAATACGTTAGCAATAGAATCAAGAAATGGGAAATCTAACTCGC
CTCGAATTGTAATTCTCTCCAATTCCGTTTCATTCCATTTTATTCTATTACAGTTGCGTTT
TTTCCAAAATTCCAAAATTTCTTACACTTCGTGTTCGCGCCTTTAATTATTTCGATTATTT
TTAACTTGTAAATTAGTTACAAAAAAATAACAATTCTTCCTCGCCGCATCGTTTAAATGG
AAATTCTGATCACTGTTTGCCCACCGTTTCGTCATGTATTTTTTTTCATCAAAAATATATT
TCTTCCGTATATTTTTATAAAACACGATATACATTCGATCGAAATTTATGACCATCCCGAT
ATATCCCCCGATATTTCTATTTTCCATTCCTCCCCTTTCTTATGGCAAACATTTCCATCCG
AGGAAATGAAGTTTTTCAAGACAAGTGACAAGCACAAAGTTGTCGCGCGCCCTATCCT
GGATCCTGGAAGTAAAGTTTCCTTCGATTGGAAACGTTCATCGGTGGGAACCCCCGTC
GCGTAAACGTTGGGAATTTATTTCACGGGCGCGTTCAGTTTTCCAAGCGATTCACCGGC
ACGGTTCGCGTTCGATAGAAAATAGTGTTGCCCGGGCGGAAATGGACGCAAACAAATC

AATTGGTTTTCCGAATGAAATCAAACAAGCGCGCGAGACATTCCACCCCATCGACGAG
CGACCATCCTCCTCCTTGTTTCTCTTTTCCACGCCGCCTCGCAGGCAAAGAGAGGATTG
GAAAATGAAAAATAGGAGACAGAGGAGGCTGCACCGTCGTGACACCCATTTTTTCCCT
CTCTAGCTCACTGTTTCTCGCGTTGCCAGTCTTCTTCTTCTTCTTCTTCTTCTTCTTCCAC
GATTTTTATTCTTTTTCCTCTCTCGACAGTAGTCACGCTCTACTAACGATAGATCTCATTG
AATGACAGTGAAAAGTCCACCTCCTTTCTCTTCTTCTTCTTTTCTTCTTCTTCTTCTTCT
TCTTTGCTTTTATTCTGCTTGCACTGGATGACTCGACGCGATTTATCAGTGTCGTTGACG
ATTCGAAAAAAAAAGAATCATTGAAAGGGGAAGGGAGGATGAAAGTAAAGAAATTGCT
TCGAAAATTTTAGTTTCTCTTAGCAATAGATTTTACTTGGATTTAATTGTCAAACGTACA
AATTGTACAAGGATTTCATTTGAAATTGACGAGAAATAAAGAAAAAAGAGAATATAGTT
ATCAAAAGCAAAGTTTTTAAAAATACGCACTTCTCTCTTAGAAGGGAACGTGTCTGTCT
GTCACTCGTGAAATATAGATGACACGCTGTGAGAGGAATTTAGTTGCGAGGTTTCGCG
GTTGAAGAGAGGGATATTCTTCTTGGAACTTTGTCGTGGAGTTGAATCCGTTCCAATGT
TGGACGTTTTACATAGTGCAAAGTGGTGTCAATTAGGCTCGTAATATCGTCTTCTAAAA
GAGTTGCTACTCTCCGTGGCAATGATTGTCTCTATTTCTCACTATCTAAAATTTCATCGA
GTTCTGCTTTCTCCCTCTCCAACTTTACTTTATCCCTAAAAACTTTTCCCTTTCGAACTT
TTATCCCGTAAAAAAAGAAGGATATTCGAACAAATTGTAATTCGACAATCAAAGGGCG
GATTAATTTTAAGAAAATAATAACAATAATAATAAAAAAAGAAAAGGATCATTCATAGA
AAATTTTTTACTAATCAGAAATCTTCACTTTCAGGCGCGATTGAATCCGAGATGAAGCT
TGGCTCGTTCAACCGAATCGAGGTTTCGCACAAACCCCCGTTGGATCGTTGAGTCGAT
GCCGGATAAATTTATTTTAAGTGTCCTTCCGATACCTCGATTCACCATGCTCGGTCGAAA
GGAGTAATAGCAACTGAGAGACTCGTCTGGCAAGTGTTCTCGTTTGTGGTAAGAATTC
TTCGTTTCTTTCTTTTCTCCTCTTTTCTTTTTTCTCTTTCTTTCTTTTTTTTCCAGTGACCC
GACGCTGTGTCAGACGCGATTGAAAAAAGAAAAAAAAAGGAAAAGAAGGTAGAGGC
GCGAGAAGAAAGGGATTTCCGGGCGCATATGAAATGCACGAGTGACGGGAGGGGAGA
AAAAACATCCGTCACTTTTATTTATTTTCTAGAGAGGAAG

>novel_circ_001778
GTGTAGTATGGAGGCCTGTAGGATTGTCACTAGTTTATTTGGCTCTGATGCTATATTCGC
CCCACGTGCCAATACCGGACTCAAAAACAATGGCCGGCCACACGGGCCACTATTTAAA
AACTTGCATCGGCCTGTCTTTTCTCACAACAACCAGCCAAATCACTTTCCACATAGTTT
TACTGGCTCTGCCCACTTACGGACACTTCCTCCACAATTGCGAACCTATGGAAGTGATC
TTCAGACACTTAGGTTTCGTAAGGCTAGATAGCGCGACCGAGTGGGAGATATTCTTCTG
GTTGACACCAGAATTAATCGTCCTACCCACTAGTACAATAATATATTTCATATGTCGTTTC
CTATCGCAAAAAACCATCACCGAAGAAGGAGACGCCTCATTGCATCAAAACATCGAGG
CCTCTAAGAAAATCGTAGATGGAAATATTAAGATCATCAACTTCTTAGGACGCATAGGT
ACTTACGTGGTACTGGCATCGTTATGCATCGCAGCATCTCTGAAACCTTCGATCGAAGG
CGGTTTCTATTTCCTCATCTTCTTGGGAGCTTCGACCTGGTGGGCGTGCAACAAAGAGC
TTCGAAAAGGGTTCGCCAAGCTGTGCAGGATCTTGATGGCGGTCGTGGTACTTCATATC
CTGGTTCTGCTCAGCTACCAGAATCAAATACCTCAAGAATTAATACCCGTGAACAGCAC
TTGGCAACGATACCTTGCCCTGATTCCCGTCTATCGAATCAACTGCTCGAATCCAAGAT
ACGTGGAATACACCGATTATACAGATTGGCTGATTTACGGATACTCCCTGAGACTGTTCT
GGCTTTATTTCGTTCTGGCGTTGCAATCACAGTTCCTCAGCAAAAAGCCG

>novel_circ_001779
GTTGGGTTCCAACAACGTGTTGGGAAGTAAGTTCTTTCAAGCATTCAGCGTTGTCCAG
GATAGGTACATTAGCCTTTTGAAGGATGTTTGGTAAGGTTGGTTTTGCTGTTTTAGAGAT
TGATCCCCATCCAGAGAGAACAGCTTGTCCAGTTTGTTTCTCTCCTTGTTTGGGCAGGG
TAATGGGTTGTACCAAGTTGTTGAATTTCAAGGGGGAGCTCAGGACGAGAAGAGCGAT
ATCGTGTTGCGCAACTCCGCTAAAATAAAATAAAGAAAATTATATTAGTGGAGCACGAG
CGATACCGATTTTATTCCAAGTCAACAATTGAAAGATCGCCTCTCTTCTTATCTCGTCTC
ATTTTTTTTTTTTTCGAGAATGGGAAATGTCGAGACGAGATTACGTATCGTCTTTCTTGA
TCAACGAGAAAGAAAACGACGAAGAAAATATTTGATATATCTTTTAATAATATTTTGA
TTTCTATTTTAGTTTTTTAAACATCATCAATTCCATTTTTCCCTCTCTCTCTTTTTCTTCCT
TTTTTTTTTATTTCAACAAATTGACCATCAAGTTTTAAATTGATTTTATTTTAAACACGAG
ATTGGAATAATTCTCTAATCTAAGATGAAATAAATTTTGCTCACCCTTTGTACCCTTTGT
GAACGATACTCTTGGCCACGTCAACGACTTGTTGAGAAGATTCAGTCTTGTCCAGCTC
GTATTTCCCGGCAATAACTCTAGATTTTCCAACTTTCATAATACAGTGTCCCGCGGTCAG
GACGTATCTTTCGTTCAGGATGGATCCACCACACGAGTGGCTGTATTGAGTTAAGG

>novel_circ_001780
ATTTACGGTAGGAGAGAATATGGTGTACAACGAGACGGAAGATGATTTAACAATCGATA
ATGAAATCAGAGGATTCTCTTTCTCGAACATCGTCGATTCTGTGTTCACCGTGATGGCT
AACGGACTCGATTCCATCGTGACTAGTATCATCGGCACGGAGAAAAGTGGCAAGCCTA
AACCCAACCGAGTGAATTACAAGAATTATCAATTGATCAGATTGTTCCCTAGTTCCCAA
GGCCACGTGAAGGAGTTGAAGGAGCTGAAGGAGACCGAGACCGAGGATATTAAATTT
TGGAGCGAGCCGATGTACAACAAAACAACCGACGTGGTCGTCGCCCCAGATCTTGTCT
CCGAACTGAAAATATTCCTTAGAGACAAAGGAATCGATTTCAAAGTTCTGATAACGAAT
ATTCAGAAAACGATAGCGAATCAAAATCCAAAAATGTCGAAGGAACAACGCGATGATC
TGGTCTCGAGCCAGGGACACAGTATGACTTGGAAACGATATCATCGGTATGGAGAGAT
CGTCAGGTACATGGAGTATCTAACTCTGAAGTATCCTAAACTGGTCGAAGTGATAACGA
TAGGTCACAGTTACGAGGGTCAACCCATTAAAATGGTCAAAATTTCGAACGGGCGAAA
CAAGAAGAATGGATCCAAATCGGCTGTGTGGATAGACGCTG

>novel_circ_001781
TCGCACAATGACTCCGCTTCATCGTGGACCGAGAGGAGCATAAGTCCCGGAAGCGCGG
TGGTGAGCAGCGCGTCGAGGCCTCAAACTCCGGCATTTCCTGTGCATCCCCGGACCCC
GTACGTGAACAACTCGTCGCCCACGGTCACGTTCGCGGCTGATCGCTCATACAGCACG
AACGCCACGTACAACGTGAACAACAACAACAATAATTTGAATAACGTCGAGGCAGCC
GGGAACAACAACAACAACGTCGGGAATTACGCGAGCGAACGAACGATCTACATCGAA
GAACGAAGAGAAGTCTCGAAAGAGACGGGGCTTCCACCCAAGAGTCCGACAACACA
GAG

>novel_circ_001782
TCGCAGTCGAGGGCAGCGCTTTACACATCCGCGCCGGCGAGCCCGTTGCTTCCGCAAC
GGTCGAGCAGCCGGAAGTACAACGGTACCACCGGAACCGGTACCCTGGGACGGCCGC
GGGCCGAGAGCGTGAACGAGCGCCCCTTCATGTCCGTGAAACGGGCATACGAATATCG
CAAATACAGCGACAACCAGAGCCCTCCGACATCCCCCCGCTCAGGCTTAGCAAGCACG

CGACCCTTAGGATTAGCAGTGCAGCTACAAACACCGTTCATTAACACGGACAAGCCTC
AACCACCGAGGCCAGAGTCGCGTAGCGACAGCTTTGCTCGCGCTGACGCTTTGTTGCG
CGAGCACAACAGGCAGCAGCAGCAGCAGCAGCAACAGCAGCAGCAGCAGCAGCAGT
TGGCCGCGAGCAACGCGGAGCAACAGAAACAAGCAACGATTGGCCAAGATTTCGCCA
CGACCATGCCTCGTAACACTCGACCGGAATCGAGAAATCGAATCGTGTGTTTGAGCTC
GACCACGTTGGCCGGGAACGTTGAGAGGACCGCCGACCACGTGGACGGAGGATTGTT
GACGATGGTCGATCAGCAACAATCGAGCACGCGCAACAATCCGGACCCTCTCGTGAGC
CTCATTCAATCATTGGCTGAAATTTCGCCCATTCGATCATCCCATACGGTGACTAACAAC
AACAACAACAACAGCAACAGCAAGACAGACACGAACGATCATCATGCGCCGAGTAGT
ATAGGCGCAGCAACCACGACGAATGCGAGCAGCAACGTTGTTAACGCCATCACTAATT
GCTCCCCTAACCAGTCGCCCAAAGCATGGCAGAATTGCACCCAGTCGCACAATGACTC
CGCTTCATCGTGGACCGAGAGGAGCATAAGTCCCGGAAGCGCGGTGGTGAGCAGCGC
GTCGAGGCCTCAAACTCCGGCATTTCCTGTGCATCCCCGGACCCCGTACGTGAACAAC
TCGTCGCCCACGGTCACGTTCGCGGCTGATCGCTCATACAGCACGAACGCCACGTACA
ACGTGAACAACAACAACAATAATTTGAATAACGTCGAGGCAGCCGGGAACAACAACA
ACAACGTCGGGAATTACGCGAGCGAACGAACGATCTACATCGAAGAACGAAGAGAAG
TCTCGAAAGAGACGGGGCTTCCACCCAAGAGTCCGACAACACAGAG

>novel_circ_001783
ATTATCATGGCTGAGGCAACAGCAGTTGAAACTTGCCGCGAGGAGGGATGAAAGAAA
CCCGGGGAAACTTTGGCGACAAGAAACCGGAAATCGCGTGATCGGCGAGCTGAGAAG
CTTGCAGAGGGGAAGGATGAGGCACGACGGGTACGCCAGCGATTCTGCCGTTCTTGA
CGACGACGAGGACACGTGGCTTGTCAATTCCACTTCCGGCCAGTCGCAGTCGAGGGC
AGCGCTTTACACATCCGCGCCGGCGAGCCCGTTGCTTCCGCAACGGTCGAGCAGCCGG
AAGTACAACGGTACCACCGGAACCGGTACCCTGGGACGGCCGCGGGCCGAGAGCGTG
AACGAGCGCCCCTTCATGTCCGTGAAACGGGCATACGAATATCGCAAATACAGCGACA
ACCAG

>novel_circ_001784
AAGGACGGGAAACTCCATTATCACCGCCACCACCTTCGAGCTCCTCCGTTCAGACGTC
GCCTCGCGGATTGGGTTACACCTGCGGTCCCATAGATGGATCCCTGTACGCTGTGGTAC
ACCAGGGTGCTGCCGGTGGTGCCCGTGGCTCGCCATTGACAGTTTCCATGGACAGCGG
AATTAGCAGCGCCCCGCCGCAACGACCTAGCCCACCCGAGCCGGAGCCGGACAACCA
GGCTCACGGTGATCTCGATAAGCTGCTCCATGATATGATGCTCACCGTCGAGTCGATGC
CGGATCCCCCAACGGAGGATTACAGAACGGACAGGAGCGAGAAGATGTACATTCAAG
AAACTCGCAGCTACAGCAGCCACGCGAGCAGCCATTCCCCGTCCACCTACTCGACGCT
GAAGGATTCGTACAGGAATTACAGCACCGGGAAAGATTCGAGCCCCCCGTACAACTCG
AGCAGCAGTTACAGCACACTGAAGAACGAATACCGCGAGCCGTCAACCAAGATCGAT
CATCGAAGGGACGAGTTCGAGTATCGGGTATCGCCCGACCGGAAGATCACCGAGTCGT
CGACCGATCCGTACAGGAAGCACGGTGATTCGTTCTCGCCACCCGGGAACATCGATTA
CATAGACGAGGATATACCTTATCACGCGAGGCAGACGAGTCAACCGTTCTCTTACGGCG
CCACCACCGACATGCTCAAACATCAGAAACTTTCCTCCCCCTCTTTGGTGAGGAAGGC
GTCCGTACGAGGCGCAGCGCCCGTCGTCGACTTCGAGGACATCCTCGGGGAGAACTC
GAGGAGACCTGTCAGCCCTTTGAGCCCCAACACCCCAGATCCTCCGCCAGAATTCGCC

AACGGGTCTGCTAAGAACGCCTCGGATCACGTGGACGG

>novel_circ_001785
GTACATCAAATATTTCAGCGGACTTTTATCAGGCTCCTTAAGGATCAGCCAATCCCCGCT
CTATTTAACGCATCTCACCGTGCTTGGCGTCCCACTGTTCGAGCCCACGGGTTGCCGGG
CATTCCTCAAGGTCTACGAAGGTCTGAACCCCGTCTACACCTCGGATCTCTACTCCGTG
ACGAATGCACGCGAGTTCACCGTGAATCTCGGTGGTCTACGCCTGAGGGGAGACATTC
TGGTGAAATGTTACCACAGAGTGTACTCGAAGCAGAGCCGGGAAGTAATGTTTTCCCT
GCAGTTCCACACCTGCGTGATCACGGAGAACGTTGTTTCCTTTCCTCGATCGGAGCTC
GACGTCGCCTGCGACGATCCAAGGTGTCCACCTGACAGCGTGGTCACGCTGTACTTCG
CCACGGACGCGAAACGCCAGGACGGGCCTGTACCAGCGCCGACGCCCGCCGTCCCCC
ACGCCTCGGCTCATCACGACCCCATCCTCCACTGGGACAGCTACACGAATCTCGAGAT
CAGCGACGACG

>novel_circ_001786
ATTCAGACGTCCTCGTCGACGACGACACCCGCGAGGACGCCAGGCGTCGGGGCGGAC
GCGACGACGACAGGAAGCGGGAAAGGCGGTGGCCGTGTTACGGAAGTGATGGAGCTT
TGTTACGTCACCGAAAGAATCATCGCGCTCTGGTACAGGGAGGATGCGCAGGGAGTCC
TGGAACACGCCACCGCTCTTCTGCGTGGAAAGCACGCTGATAATTATATGATATTCAAT
CTCTCGTCGCCGGGTCGTGCGGGGGAGCCGAACACGAGGGAAGCGGGATGGCCTCAG
GGTCTGGCGCCCAGTTTGGAAAGATTGTGCGCCCTGTGCAAGGAGCTGGACTCCTGGC
TGAACGGGGCTCCGAATCGCGTGGTCGTCCTCCACGCCAGGGGAAGCAAAGAACGAT
TGGGTGTGGCTGTGTCCGCTTACATGAACTACAGCAGCATCTGCGGCGGACGTGATCA
GGCGTTGGACAGGTTTGCCATGAGGAGATTCCTCGACGACAAGATTGGATCCCTGCAG
GTTCCCTCGCATCGAAGGTACATCAAATATTTCAGCGGACTTTTATCAGGCTCCTTAAG
GATCAGCCAATCCCCGCTCTATTTAACGCATCTCACCGTGCTTGGCGTCCCACTGTTCG
AGCCCACGGGTTGCCGGGCATTCCTCAAGGTCTACGAAGGTCTGAACCCCGTCTACAC
CTCGGATCTCTACTCCGTGACGAATGCACGCGAGTTCACCGTGAATCTCGGTGGTCTAC
GCCTGAGGGGAGACATTCTGGTGAAATGTTACCACAGAGTGTACTCGAAGCAGAGCC
GGGAAGTAATGTTTTCCCTGCAGTTCCACACCTGCGTGATCACGGAGAACGTTGTTTC
CTTTCCTCGATCGGAGCTCGACGTCGCCTGCGACGATCCAAGGTGTCCACCTGACAGC
GTGGTCACGCTGTACTTCGCCACGGACGCGAAACGCCAGGACGGGCCTGTACCAGCG
CCGACGCCCGCCGTCCCCCACGCCTCGGCTCATCACGACCCCATCCTCCACTGGGACA
GCTACACGAATCTCGAGATCAGCGACGACG

>novel_circ_001787
CATAATGGGGGTTACGAGGCGCAACCGAAGAATGTTAACACCATCTCGAGAAGCAAGC
AGCAGCAGCAACAACAGAAAAGCTCCGCTTCGACTCGAGCGCCGAGCAAACCGATGT
CGATTCGTTACGAGATTCCCGTAGGGACGACATGCCGGACAACGTGGTCGTCAGGATC
GTCTACGCGCCGAAATATTCCAAGATTCAGACGTCCTCGTCGACGACGACACCCGCGA
GGACGCCAGGCGTCGGGGCGGACGCGACGACGACAGGAAGCGGGAAAGGCGGTGGC
CGTGTTACGGAAGTGATGGAGCTTTGTTACGTCACCGAAAGAATCATCGCGCTCTGGT
ACAGGGAGGATGCGCAGGGAGTCCTGGAACACGCCACCGCTCTTCTGCGTGGAAAGC
ACGCTGATAATTATATGATATTCAATCTCTCGTCGCCGGGTCGTGCGGGGGAGCCGAAC

ACGAGGGAAGCGGGATGGCCTCAGGGTCTGGCGCCCAGTTTGGAAAGATTGTGCGCC
CTGTGCAAGGAGCTGGACTCCTGGCTGAACGGGGCTCCGAATCGCGTGGTCGTCCTCC
ACGCCAGGGGAAGCAAAGAACGATTGGGTGTGGCTGTGTCCGCTTACATGAACTACA
GCAGCATCTGCGGCGGACGTGATCAGGCGTTGGACAGGTTTGCCATGAGGAGATTCCT
CGACGACAAGATTGGATCCCTGCAGGTTCCCTCGCATCGAAGGTACATCAAATATTTCA
GCGGACTTTTATCAGGCTCCTTAAGGATCAGCCAATCCCCGCTCTATTTAACGCATCTCA
CCGTGCTTGGCGTCCCACTGTTCGAGCCCACGGGTTGCCGGGCATTCCTCAAGGTCTA
CGAAGGTCTGAACCCCGTCTACACCTCGGATCTCTACTCCGTGACGAATGCACGCGAG
TTCACCGTGAATCTCGGTGGTCTACGCCTGAGGGGAGACATTCTGGTGAAATGTTACC
ACAGAGTGTACTCGAAGCAGAGCCGGGAAGTAATGTTTTCCCTGCAGTTCCACACCTG
CGTGATCACGGAGAACGTTGTTTCCTTTCCTCGATCGGAGCTCGACGTCGCCTGCGAC
GATCCAAGGTGTCCACCTGACAGCGTGGTCACGCTGTACTTCGCCACGGACGCGAAAC
GCCAGGACGGGCCTGTACCAGCGCCGACGCCCGCCGTCCCCCACGCCTCGGCTCATCA
CGACCCCATCCTCCACTGGGACAGCTACACGAATCTCGAGATCAGCGACGACG

>novel_circ_001788
GGTGCTAGATTCCGGGACACCTCGCAGCAAATCCACAAGCAATGGCAGGCGGATCCGG
CACGAGCGTTTCCAACAGTTGGCACGGTCGCCACCGCAGGCTCGATCTCACCGACGTC
GGGCGAAATTTCAACCCCGAATTCGACGCCCAGCCCAAGCCCCAGCCCCACTAACAGT
CAACAAGTCGCGCGAGTTCCGTCGGCTTTTGCCATCGAACGTGCCGCGCGTGTCGTCC
TTTCCTCGCGGTTTCGCTTTTCCACGCCTCGCCTTCTTCTCGAAACAAAAAAAAAAAG
GAATAGAAAAGAGTCGAACGAGATTAGGGATAAGTTCAATTCGGGCGATGAACCATGC
ACTTCCGGTCGAACGTTGAAGATGGCTCGATCGATGATACGATCGAGAGTACATGGTGA
TACGAGAATACGAAGATTCCTCGATCGTTGGAGGGGGTGGCGAGGTAGTGAGAGTTGT
GTAGTGTGTTTCGAAAATTGATCGGCAATCAATTGTTTCGTGGGTGACAGCTGTCAACA
TGCTGTCCACCGTTCAGTGCCATGGTGACGTGCATCAACGGAACTATCACGTCGCGTAT
ACGGGCGACGTGTATCGACGAGCTTATCCAAACGAGCATGACAAACTCGAACAGTAGC
TCGCTGTCGCGCGTCTGGCTACGAGCAAATTGCTGCCGTTGCGGTGACACGAGGCAGG
ACACCGTGAACAATAACCTCAAGGATGAGACACCACTGCGGGAGGAGACCGAGACCC
TTTTGCATCGTATCGCATTCACGGTTTGGCACACTGCCCTCCATATAGAGAGCGAGCAT
AATGGGGGTTACGAGGCGCAACCGAAGAATGTTAACACCATCTCGAGAAGCAAGCAG
CAGCAGCAACAACAGAAAAGCTCCGCTTCGACTCGAGCGCCGAGCAAACCGATGTCG
ATTCGTTACGAGATTCCCGTAGGGACGACATGCCGGACAACGTGGTCGTCAGGATCGT
CTACGCGCCGAAATATTCCAAGATTCAGACGTCCTCGTCGACGACGACACCCGCGAGG
ACGCCAGGCGTCGGGGCGGACGCGACGACGACAGGAAGCGGGAAAGGCGGTGGCCG
TGTTACGGAAGTGATGGAGCTTTGTTACGTCACCGAAAGAATCATCGCGCTCTGGTAC
AGGGAGGATGCGCAGGGAGTCCTGGAACACGCCACCGCTCTTCTGCGTGGAAAGCAC
GCTGATAATTATATGATATTCAATCTCTCGTCGCCGGGTCGTGCGGGGGAGCCGAACAC
GAGGGAAGCGGGATGGCCTCAGGGTCTGGCGCCCAGTTTGGAAAGATTGTGCGCCCT
GTGCAAGGAGCTGGACTCCTGGCTGAACGGGGCTCCGAATCGCGTGGTCGTCCTCCA
CGCCAGGGGAAGCAAAGAACGATTGGGTGTGGCTGTGTCCGCTTACATGAACTACAG
CAGCATCTGCGGCGGACGTGATCAGGCGTTGGACAGGTTTGCCATGAGGAGATTCCTC
GACGACAAGATTGGATCCCTGCAGGTTCCCTCGCATCGAAGGTACATCAAATATTTCAG
CGGACTTTTATCAGGCTCCTTAAGGATCAGCCAATCCCCGCTCTATTTAACGCATCTCAC

CGTGCTTGGCGTCCCACTGTTCGAGCCCACGGGTTGCCGGGCATTCCTCAAGGTCTAC
GAAGGTCTGAACCCCGTCTACACCTCGGATCTCTACTCCGTGACGAATGCACGCGAGT
TCACCGTGAATCTCGGTGGTCTACGCCTGAGGGGAGACATTCTGGTGAAATGTTACCA
CAGAGTGTACTCGAAGCAGAGCCGGGAAGTAATGTTTTCCCTGCAGTTCCACACCTGC
GTGATCACGGAGAACGTTGTTTCCTTTCCTCGATCGGAGCTCGACGTCGCCTGCGACG
ATCCAAGGTGTCCACCTGACAGCGTGGTCACGCTGTACTTCGCCACGGACGCGAAACG
CCAGGACGGGCCTGTACCAGCGCCGACGCCCGCCGTCCCCCACGCCTCGGCTCATCAC
GACCCCATCCTCCACTGGGACAGCTACACGAATCTCGAGATCAGCGACGACG

>novel_circ_001789
ATTCAGACGTCCTCGTCGACGACGACACCCGCGAGGACGCCAGGCGTCGGGGCGGAC
GCGACGACGACAGGAAGCGGGAAAGGCGGTGGCCGTGTTACGGAAGTGATGGAGCTT
TGTTACGTCACCGAAAGAATCATCGCGCTCTGGTACAGGGAGGATGCGCAGGGAGTCC
TGGAACACGCCACCGCTCTTCTGCGTGGAAAGCACGCTGATAATTATATGATATTCAAT
CTCTCGTCGCCGGGTCGTGCGGGGGAGCCGAACACGAGGGAAGCGGGATGGCCTCAG
GGTCTGGCGCCCAGTTTGGAAAGATTGTGCGCCCTGTGCAAGGAGCTGGACTCCTGGC
TGAACGGGGCTCCGAATCGCGTGGTCGTCCTCCACGCCAGGGGAAGCAAAGAACGAT
TGGGTGTGGCTGTGTCCGCTTACATGAACTACAGCAGCATCTGCGGCGGACGTGATCA
GGCGTTGGACAGGTTTGCCATGAGGAGATTCCTCGACGACAAGATTGGATCCCTGCAG
GTTCCCTCGCATCGAAG

>novel_circ_001790
ATTCAGACGTCCTCGTCGACGACGACACCCGCGAGGACGCCAGGCGTCGGGGCGGAC
GCGACGACGACAGGAAGCGGGAAAGGCGGTGGCCGTGTTACGGAAGTGATGGAGCTT
TGTTACGTCACCGAAAGAATCATCGCGCTCTGGTACAGGGAGGATGCGCAGGGAGTCC
TGGAACACGCCACCGCTCTTCTGCGTGGAAAGCACGCTGATAATTATATGATATTCAAT
CTCTCGTCGCCGGGTCGTGCGGGGGAGCCGAACACGAGGGAAGCGGGATGGCCTCAG
GGTCTGGCGCCCAGTTTGGAAAGATTGTGCGCCCTGTGCAAGGAGCTGGACTCCTGGC
TGAACGGGGCTCCGAATCGCGTGGTCGTCCTCCACGCCAG

>novel_circ_001792
GCCAATCCATGCCAGCATCCGATCGTGATCCTGAGGGACGTCGCCTCGTCGGACATGG
AGTCGTTGCTGCGCTTCATGTACCACGGAGAGGTGCACGTAGGTCAGGAACAGCTCGC
CGCGTTCCTAAAGACGGCGCAGATGCTTCAGGTTCGAGGGTTGGCCGACGTGAACAG
CGGCGCTGCCAAAATTCCTCCTCCCTCGTCCTCCGGCGGGAACAATGGCAGTGCGCCG
GCGACACCGCGCAATCCTTGGCAAGACAACGGCAGAGGAGAGTTGAACGAGAGCGG
TCTGAGCCCGCCACCAGAGAAGAGACCTAGAAGCTATAGCCCACCGTTGGGTAACCAC
GTGGAGCCCAAGACCGACCTTCAAGAATCGCTTTTGGGCCAGGCTTTGGCGGAGGGA
CCCACGATACACACGACACCCACGAACAACGTTCAGGCGCAATCTACCGGCGAGGAC
TCGAACTCCATGTCGGACAACGAGGAGGAGATGTCGAATAACGACAGCATCCTGAACT
CGGTGAAGACCGAGCCAAGCGACATCCTGAATGATTCTATGGAACATCATCGTAATTCG
TTTCCGGCGCTCTTGGGAATACCAGGACTGATACCAGGACCGTCCGGGATCCACGCGG
CGAATCAGGATCCGAATTACG

>novel_circ_001793
AAGAGGAGAGATCGAGGCGGGTAAATAGGCGAAGTGGGGTGACGAGCAAAAGGGGC
GATCGGAGCAAAAGGGACGATCGAGAAGGTGGAGGAGGAGGAGGAGGAGGTGGAGA
TAAGGGCATGATAAGCAAGGACGTTGGACGAATATCAGGTGACACGGTAGACGATAGC
CAAGAAGGGTTGAGCGGAGGAGGGAGGAAGGTGTATCGACAGATTCGCCGTCTTGTC
ACGCGGCAACGGATGTCGGAATGACGCGTCGATCAAGCCGTGGCTGGGCAGAGGGTG
CAAGGACTTCGTTAAACGGAAGAAAGTGATAGTCGTGGACTCGCTTCGCGGGAGGCG
GAGAGGCGGAGTTCGTTTGGTGAAGGGAGTGGAAGAGGAAGGAAGCGTCGGGGATC
GTCCAACTTGTCAGGTAACGAGATAAGAAGCGATCGCGGCCTCATTGTCTCATCTCACT
CTCCAGCAACGGCGACGGATAAACGTCCTCTCGTCGCTATCACGATGTTGTCGTTATGA
GATTCGTCGTATTTCCTTATACCTGACAGTCAACAATACTGTTCGATTGCTTCTTCCCCG
AGGGGGAAGGTGAGTAAAATAGAAGGGATCTCGAGCGGACCAAGCAAAAAGAAGAA
GAAAAGAAAAAGAAAAAAATCATTTATCGTATAATAAGAGATCGTACAACTGGAAGAA
GAAACGAGACTACTACCAAAGAATTTTGATTCGAGGGAAGAGGGTCAAAGTTAACTG
GCCGAGAGCACACAGTAGCAAAGGAAACAGCGATCTCGTTTAACCTTGGAGAGAAGA
GAGAGAGAGAGAGAGAGAGCATCGTAAATCGCAGAACATCGTAATCGCGCAAGGACA
GGCAACCGACTGTCCGCGTGGCTGTTGCGTTGGAAGGGTGCCGATAAAAGAATAGAG
GGGTGCGTGGTGAAACGCGACGAGAGGGTGAAGTGAAGGTGTCCTCGTGGGGTGCC
AGAGTGAGCACGATAGCACCTCTGGTGCCCTCGGCCGTGGGTCCCGAGGGGACGGAC
GCCATGGCGGCCTCTTCGTCCTCGTCGAGCGGCGAGCAACAGTACTCACTCAGGTGGA
ACGACTTCCACTCGAGCATCCTCAGCTCATTTCGTCATCTCAGGGACGAGGAGGATTTC
GTGGACGTGACCCTGGCATGCGACAGTAGCAGCTTCACCGCGCACAAGGTCGTTCTCT
CTGCGTGCAGCCCTTACTTCCGCCGCCTCCTCAAGGCCAATCCATGCCAGCATCCGATC
GTGATCCTGAGGGACGTCGCCTCGTCGGACATGGAGTCGTTGCTGCGCTTCATGTACC
ACGGAGAGGTGCACGTAGGTCAGGAACAGCTCGCCGCGTTCCTAAAGACGGCGCAGA
TGCTTCAGGTTCGAGGGTTGGCCGACGTGAACAGCGGCGCTGCCAAAATTCCTCCTCC
CTCGTCCTCCGGCGGGAACAATGGCAGTGCGCCGGCGACACCGCGCAATCCTTGGCA
AGACAACGGCAGAGGAGAGTTGAACGAGAGCGGTCTGAGCCCGCCACCAGAGAAGA
GACCTAGAAGCTATAGCCCACCGTTGGGTAACCACGTGGAGCCCAAGACCGACCTTCA
AGAATCGCTTTTGGGCCAGGCTTTGGCGGAGGGACCCACGATACACACGACACCCAC
GAACAACGTTCAGGCGCAATCTACCGGCGAGGACTCGAACTCCATGTCGGACAACGA
GGAGGAGATGTCGAATAACGACAGCATCCTGAACTCGGTGAAGACCGAGCCAAGCGA
CATCCTGAATGATTCTATGGAACATCATCGTAATTCGTTTCCGGCGCTCTTGGGAATACC
AG

>novel_circ_001794
AGGAGAGATCGAGGCGGGTAAATAGGCGAAGTGGGGTGACGAGCAAAAGGGGCGAT
CGGAGCAAAAGGGACGATCGAGAAGGTGGAGGAGGAGGAGGAGGAGGTGGAGATAA
GGGCATGATAAGCAAGGACGTTGGACGAATATCAGGTGACACGGTAGACGATAGCCAA
GAAGGGTTGAGCGGAGGAGGGAGGAAGGTGTATCGACAGATTCGCCGTCTTGTCACG
CGGCAACGGATGTCGGAATGACGCGTCGATCAAGCCGTGGCTGGGCAGAGGGTGCAA
GGACTTCGTTAAACGGAAGAAAGTGATAGTCGTGGACTCGCTTCGCGGGAGGCGGAG
AGGCGGAGTTCGTTTGGTGAAGGGAGTGGAAGAGGAAGGAAGCGTCGGGGATCGTC
CAACTTGTCAGGTAACGAGATAAGAAGCGATCGCGGCCTCATTGTCTCATCTCACTCTC

CAGCAACGGCGACGGATAAACGTCCTCTCGTCGCTATCACGATGTTGTCGTTATGAGAT
TCGTCGTATTTCCTTATACCTGACAGTCAACAATACTGTTCGATTGCTTCTTCCCCGAGG
GGGAAGGTGAGTAAAATAGAAGGGATCTCGAGCGGACCAAGCAAAAAGAAGAAGAA
AAGAAAAAGAAAAAAATCATTTATCGTATAATAAGAGATCGTACAACTGGAAGAAGAA
ACGAGACTACTACCAAAGAATTTTGATTCGAGGGAAGAGGGTCAAAGTTAACTGGCCG
AGAGCACACAGTAGCAAAGGAAACAGCGATCTCGTTTAACCTTGGAGAGAAGAGAGA
GAGAGAGAGAGAGAGCATCGTAAATCGCAGAACATCGTAATCGCGCAAGGACAGGCA
ACCGACTGTCCGCGTGGCTGTTGCGTTGGAAGGGTGCCGATAAAAGAATAGAGGGGT
GCGTGGTGAAACGCGACGAGAGGGTGAAGTGAAGGTGTCCTCGTGGGGTGCCAGAG
TGAGCACGATAGCACCTCTGGTGCCCTCGGCCGTGGGTCCCGAGGGGACGGACGCCAT
GGCGGCCTCTTCGTCCTCGTCGAGCGGCGAGCAACAGTACTCACTCAGGTGGAACGA
CTTCCACTCGAGCATCCTCAGCTCATTTCGTCATCTCAGGGACGAGGAGGATTTCGTGG
ACGTGACCCTGGCATGCGACAGTAGCAGCTTCACCGCGCACAAGGTCGTTCTCTCTGC
GTGCAGCCCTTACTTCCGCCGCCTCCTCAAG

>novel_circ_001795
AAGAGGAGAGATCGAGGCGGGTAAATAGGCGAAGTGGGGTGACGAGCAAAAGGGGC
GATCGGAGCAAAAGGGACGATCGAGAAGGTGGAGGAGGAGGAGGAGGAGGTGGAGA
TAAGGGCATGATAAGCAAGGACGTTGGACGAATATCAGGTGACACGGTAGACGATAGC
CAAGAAGGGTTGAGCGGAGGAGGGAGGAAGGTGTATCGACAGATTCGCCGTCTTGTC
ACGCGGCAACGGATGTCGGAATGACGCGTCGATCAAGCCGTGGCTGGGCAGAGGGTG
CAAGGACTTCGTTAAACGGAAGAAAGTGATAGTCGTGGACTCGCTTCGCGGGAGGCG
GAGAGGCGGAGTTCGTTTGGTGAAGGGAGTGGAAGAGGAAGGAAGCGTCGGGGATC
GTCCAACTTGTCAGGTAACGAGATAAGAAGCGATCGCGGCCTCATTGTCTCATCTCACT
CTCCAGCAACGGCGACGGATAAACGTCCTCTCGTCGCTATCACGATGTTGTCGTTATGA
GATTCGTCGTATTTCCTTATACCTGACAGTCAACAATACTGTTCGATTGCTTCTTCCCCG
AGGGGGAAGGTGAGTAAAATAGAAGGGATCTCGAGCGGACCAAGCAAAAAGAAGAA
GAAAAGAAAAAGAAAAAAATCATTTATCGTATAATAAGAGATCGTACAACTGGAAGAA
GAAACGAGACTACTACCAAAGAATTTTGATTCGAGGGAAGAGGGTCAAAGTTAACTG
GCCGAGAGCACACAGTAGCAAAGGAAACAGCGATCTCGTTTAACCTTGGAGAGAAGA
GAGAGAGAGAGAGAGAGAGCATCGTAAATCGCAGAACATCGTAATCGCGCAAGGACA
GGCAACCGACTGTCCGCGTGGCTGTTGCGTTGGAAGGGTGCCGATAAAAGAATAGAG
GGGTGCGTGGTGAAACGCGACGAGAGGGTGAAGTGAAGGTGTCCTCGTGGGGTGCC
AGAGTGAGCACGATAGCACCTCTGGTGCCCTCGGCCGTGGGTCCCGAGGGGACGGAC
GCCATGGCGGCCTCTTCGTCCTCGTCGAGCGGCGAGCAACAGTACTCACTCAGGTGGA
ACGACTTCCACTCGAGCATCCTCAGCTCATTTCGTCATCTCAGGGACGAGGAGGATTTC
GTGGACGTGACCCTGGCATGCGACAGTAGCAGCTTCACCGCGCACAAGGTCGTTCTCT
CTGCGTGCAGCCCTTACTTCCGCCGCCTCCTCAAG

>novel_circ_001796
GTATCTGTCACACATGCGGTGAAAAAGTGACCGGAGCTGGCCAAGCCTGCCAAGCCAT
GGGCAATCTTTATCACACAAATTGCTTTATATGCTGTTCGTGTGGAAGAGCGTTACGCG
GCAAAGCGTTCTATAATGTTCACGGAAGGGTATACTGTGAGGAAGATTATCTGTATTCT
GGCTTCCAACAAACCGCCGAGAAATGCGCAATCTGCGGTCACCTCATCATGGAAATGA

TATTGCAAGCAATGGGGAAGTCTTATCATCCAGGATGCTTTAGATGTTGCGTTTGCAAC
GAATGTCTTGACGGTGTTCCCTTCACCGTGGATGTGGACAATAAAATTTATTGTGTAAA
CGATTACCACAGAATGTTTGCGCCGAAATGTGCCTCCTGTGGTAAAGGAATCACTCCA
GTCGAA

>novel_circ_001797
TGCCATTATTCGGTGAAATCGTGCGCCGCTGTCTGCGGATTGGGGACGGACAACTGCG
TGATGGTTCCTAGCGACGATCGAGGCCGGATGATTCCTTCGAAACTGGAAGATTTAATT
CTTGAACGCAAGGCCAAAGGACACATCCCCTTCTTCGTGAACGCTACCGCAGGAACG
ACCGTCATTGGAGCGTTCGATCCTATTCCAGAAATCGCTGATATATGCCAGAGACACAA
GCTCTGGCTGCACATCGACGCGGCTTGGGGTGGTGGACTGCTTCTCTCGAGGAAATAC
AGGCATCCTCGAATGACAGGCATCGAACGAGCCGATTCCGTGACCTGGAACCCGCACA
AACTCATGGGGGCGTTGCTTCAATGTTCGACGATTCACTTCAAGGAGGACGGTTTGCT
GATTAGCTGCAATCAAATGTCAGCCGAGTATTTGTTCATGACAGACAAACTGTACGACG
TGAAGTACGATACCGGAGATAAAGTGATTCAATGCGGGCGTCATAATGACATCTTCAAA
CTTTGGTTGCAATGGCGAGCCAAGGGTACAGAAGGATTTGGGAAACACATGGATCGAC
TGATGGAACTTTCGGAGTACATGGTGAGAAGGATCAAGCAGATGCCTGACAAGTATTA
TCTAATCCTAGAACCCGAAATGGTGAACGTCTGCTTCTGGTATTTGCCAACACGCGTTA
GGAACATGCCGCACACTCAGGAGAGAATCAAAATCTTGGCAGATATCTGTCCAATCTT
GAAAGGCCGAATGATGCAAGCCGGCACACTGATGGTAGGTTACCAGCCGGACGATCG
ACGACCCAATTTCTTCAGAAATATCATCTCGAGTGCCGCGGTCACGGAAGCGGACGTC
GATTTTCTGCTGGCGGAAATGGACCGGTTAGGCCACGATCT

>novel_circ_001798
ACCTCTCGCCATTGGGTGACAAGGGTGGCAGCCTGGTCGACGAGCTTGGAGGTGGTG
GCGGCGGTTCCTTGAGGAACGGTAAAGGGAAGAAGATGAGGAAACCACGGACGATTT
ACAGCAGCCTTCAGTTGCAACAGCTCAACAGGCGGTTCCAGAGAACGCAATACCTCG
CGTTACCGGAAAGAGCGGAGCTTGCGGCGAGTCTTGGATTAACGCAAACGCAG

>novel_circ_001799
GTGAGACCGTGGTGAAACGACACACGTGCTCCAGGGATGCGGGAAATCGCCGCCCCA
TTAGAGTTCCATCCCGTGAGGCGAGCGTTGCCGAGCGGAGCGTGAAACGAAGCGTATC
CACCTCCCTTACCGGAAAACCGTGAGAGGATGCGATTCACGGGAAAGCTCGAGGGAT
AATAAAATATCGGAATCCTTCCTTAAACCATTTTCTTAAAACCCCCCCGACCGCTATGAA
GGAGGGAAGGGTAAAATTCTATCGAGGAATCGTCGAATAAATATGAGGAAGGTGAAGA
TTCTCGGGTTATCACGAACCCCTCTCATCGCCGAGAAAAAGAGCACGGACAGTTGGAC
AGGCACGAAGTACCTTTCATTACCGCGTCTCCTCGATCGTCTTAATCCCGGTTGCTCGA
TCAATGCGCACCAAGTTGAAACTGAGGGAAGGGTGTGTTAAGCCGTTGGAATTGGAC
GTCGAGGTAACGATAACCGATCGAACGAGATAGTATCGTGCCTCTTTCGCGCCCCGCCC
GTGTACCGTTTCGAGCCGTGAGAATAACCGTTTCGAGTCGATCGACTCGAACGAGGTG
AATTTTTGTTGTTGGAAAGTGCGTCGAGGAAAGTGTGTGTGCAACAGCAGTAAGTAGC
AAGTGTGAAAAGACAAAAGCTACACGGTTCGTTCGCTTTCGTTTGGATTATCTCATCTT
TCGGTCGGTCTGTCTTGACCGATTTCCATCCCCGTTCCACGATGCCGTTCCTTCCATCGT
AAAAAAATATAACGGCTTATGGGGGACGACGGAGGGACGAAGAGTGAGAGGGAAGC

GGGCCGCGATATATCTGCACACCATTGGACGGTATAATCTCGTAAAAGCTGCACACAAG
TGTCCATCTACGTGAGCCCGTACGTAAAGCCGTCGACGAGGATCACGAGGTCTCTAAC
CAGGAAACGTGGTACGCCGGCCACTTTCGACCACGCTTCGTTCCAACTGTATTCCACG
TGACAGAGGAACGTTTCCTCGAGGAAACGATCCCTCGAAGAATATACTCGGTCACGGA
CGGTGTTGAACCGTTGGGTGATCTCGTAAACCCTATCCTGAACCCTCACCGTCGAGACT
ATATTTTTGGATGGATCTTATAATATATACGAAGTGACAGTAAGTTCTCTGTGATGCTTCG
CTTCGTGAAAGCTTCTGGCTGTTTCGAGCAATTTTATCGTATTTATACGTAAAAAAAAA
AAAAAAGAAAAAGAAAAAGAAGAAACGAATTAGAGGTATATAATATTTTATAATAACT
AGATTTAAAAAAAAAAAAGATAGATTCAATTTTGTTTTTAATTTTGTAAAGGAACGAGAG
TTTGTAAGATTGTAGAAATTTTTACACGAGCGATCGTAAAGTGATTTTTTTAATATATATA
TATAATATTCGTCGTATATTGAATAGAAAGAAGATTGTAGTTTTTGGTAATTAATTGGTAA
TGCGCGAGTGCCACGTTCACGTTATCTGAATCACGGAAGGATGGATCAAGTGCACGTG
AAATCGTCGAGATCGACGTCGAGGAGCAACCCGGCGAAAATGGCGTGCGAGAAGGCG
AGCAAGAGGCTCAGGACGATTCTGGTTAAGGCGTCGCGCCTCGGCGTCTCGACGACG
GAAGTTGCCAAGTTGGCCAGCGCCAAGAAGCTCATTCCGCAGAGAGATCACGGATGG
AAGTTGGCGGTGTTCCTCCTGGTCATGTTCGGCGGCGGAGTGATCGTCGCCTTGGCCT
GCACGGCGAGGAAAGGCTGCTCGAAGCTGGAGGAGATC

>novel_circ_001800
ATACCATTATCAATGATAAGTAAGAAGGTGGAGAAGCACTGGGGCGCCAGATGGGGCA
ATATCACGGTGTGGGCGAGTCTCATTATCGGACAACCACTTTGCATAATGATGTATTACC
ACGATTACGTGATAACTCACTTCGGCGAGAACTTGATCGAGGATTACTCCGTGGTGTAG
GTGCAGAGGAATCGAACTCGAGGCGGACGATCACGCAGGGGTCTGGAAGAAGGAGAT
TCTCCGGGCAAACTCGCCACTCTTCCATCAATTTTCTCCTTTTTCTATTTTCTATTTCCGT
CATCTTTACTCGTGTATACTAACCTATTTTTCAACCGACGTTTACGCGAATTCTTCCACTT
TTTTTTTCTTTTCTTTTCTTATTCTCATTCCAATGGATGGATGAGAGTCGGTCACGTGTTC
GTTATCGCGAGCATTAATTCCTTAAGTATTACGCATTGGCTTTATCCATTTTTTCCTCTTC
CAGTAGGAATTATATATAGATTTTTTTTTCGAAAGAATCGAAATTTCGAGATACAAATT
GCGCGTTTATTCATCGGTGTGTATAGGATAATCAGGTAATTTGTAAAACTTCTGTTAAAT
TTAATACCGGTAACTCGAGGGCTAATTCTGGTAAATGTACAGCGTGTATTTGAACGTTTT
CTCCTAATGTATTTTACTCGTGTTTAAATGGAAGGAATTGTCGTGAAACGTACTAACATA
AATAAGTTCGCGCAGAAATTGAATTAAAATTGAATAAATCCACTAACGTGATACATCGA
TGGAGGAGATCGTATATTTCTGGCACGTAAATCCAGAATAAGCTCTGCGTGCATCATAA
AATTTTACAAATAATTCTCTCTCTCGTAACTCTTTCTCACTCTCTCTCGAGAAGTTGCAG
ATGTAATTCCTACTGGATCGTATCGTGAACGTCAGTGCTCGATATTCGATCGAATTTCCT
TGAACAAAAAAAAAAAAAAGAAAAAAGAAAAAGAAAAAAAAAAAGAAGATTTTTGCAC
GAGGCGGTTAACAAGCTCGATCGAAAAACTCAATCGCTAGATTGCGGATATTTATGCAA
TTATTAAATTCCAGATATGGAAATGAAACGGAAATATGCTTCGCTTAAAAAATATGTATT
TTCGATATTTTGTACATTTTTCACAACTTTTTTAACAAATGCATAAACTTTCGCAGTCTAC
ATCCTATCGTACGTATATATATATATATATATATATATATATATCACTCGTTCTTTTCCCTCGT
ATACGCGTGGGTGGTGGTGGTGATGTTTGTTGATGAATTGTTGCTATTGTAAGCCGTTG
AAATATTCACCCATTCGGTCATTTTTATGCACGTTTTAACCGATAAAGAATTGTTAATCA
CTTTGATATCAGCGTTTTACATAGATTTCTCGTTGAACGAACGCTCGATCATTCCCGTCC
ATCCACGAAGATCGCCGATCCTTTATACGAACCGTCGAAACAATGAGATTGTCACGCAT

CGAGGGGAGGAGGGGGGCATTTACTCTGTCATTAGCACTTATATGTATATTTAATCGAA
ACGATTTTTTACATATGATCACGAGCGCAAAACCCTTGGCATCCCTCCTCCACTTTTACG
AGCAGCGCGTGCCTCGTAAGCGCGGAACGCAGGTAGAGATATTATGCGTGCGTCGAGA
CAATAATTCACTACATACTTTTTATTTTTTTATTTTTTTCCCCCTATCTGACGATGCTTT
ATAGCAGGGGGACCTCGAGCGCATCGAGGGAATATTAGCCGTAATAAATTTATCCATGG
CGCGGAGTGCAGTTGCATTTACAGGGTAAATATTTCACCGGAATCGTTGCGTATCACTG
CGAAGAAACAGTTGGCGAGGATTTAACGGGTACAAGTTGGTGCCAGTTGTGTACGAG
GAGCAGACGATTTTGTAAAATTCGTCCAACGCTCGCGTGCCGCGCGAGCGTCATTCGT
TGTCGAAATAAACGAAATCTTTGTTACAAGATCTCCTCCCCCGTTTAATTCCAGTCCCG
TTCCTTCGAACGATCGTCGGAGATGATGCACCAGGTATCGGTAAATATCTTTCTTCTTCG
ATGTTTCAGGTTTCGTAATTTTTTTTTTTTTTTTGGCGAAATCGATTGACAATCGATAAC
ACGTTGAAATTAACAATGAATTATTGCAGGATTTTTTTTATCGATGCTGCAGGTGGAGA
GATGGAATTTTTTTTTAATTCGACAAACGAATAGTATCGTGTTCTTTTTTTTTTTTTTTT
TGAAAATTTCTCGACGCAATAACGTTCGTAATTTTTAATTAAAACGCAATTTGTATGTTG
AAAATGATATATTTGTATATTGATTATCAAGTGAATGTTAAATTACAACCAATTACAAAAA
TGAAACATATAATAGGAATAAAAATCTCGCGAATTTTACGAGCGTTTGTGTCGAATAAA
ATTTCCGTCCTAGTTCTTGCAAGTCGAAATCGAATCGAATAAAAGAAAAAAAGAAAAA
GATAAAACCTCATCTCCACAAAAGAAAAGAGGAAGGACCAAAGACACGATACGTGAT
CTTTATCGTGCATGCAAAATACCTACCCATTTGGCAAAATAACTTCCTCCTTACCACCCT
GATAATAAGAAACAAGTCATGGAGGGTTTACTGGAACACTCGACATCTTTTGACGGTC
GGCTCATCTTTTTTGAAATTCCTCTCAACGCTGGAAACGATATAATCGGAGGAATTGCC
TGAACGAAACGTACGAAACGATCATTCTCTCCGAAATGCGAGAACAAGAGAGGGACG
AAGAGGGTGGAAGAAAAAAGAAGAGTTTCCAAGGTCCAAAAGATGCTCGTCGAAGA
AGAATGTTTTCTTTTTTTTTTCTCCTCCTTCTTTTCTCTTTTCTCGAGCCATCATCCCTTG
GGGTTTGTTGGGTGTCTGATGAGCCTGGATGTCGACCCGTTTGCCCTGGAGCAGTCTC
GGCGATGGATAAATTAATGATGCGGGGCGCTCACCACCGACAGTTGGACATACGTCTG
CGTCAGAGAGACGCGATGTTATATACCATCCAG

>novel_circ_001801
CTTCCACCGCGAAGCAAAACGGCGAGGAGGTCGACGACTCGAAGCACGAAGTAGAC
GTAGCTGGCACGGTCTTGGTGCAATATCCGGACAACTTGACTTTCCGTGATCTTTACTA
CTTCATCCTGGCGCCTACCCTCTGCTACGAACTGAATTTCCCTAGAACACAAAGGATTC
GGAAAAGATTTCTGATTAAACGGATCCTGGAGGTGGTGGTCGGCTGCCAAGTGGTCAT
GTCGCTCTTCCAACAATGGATGGTCCCGTGTGTGAAGAATTCCCTCGTACCGTTCAGC

>novel_circ_001802
GATATCATAGCTCATGGGCCAGGAATGGTGTTCCACATCGTAAACCTCATCGTGATGGT
ACTGATGCCGATGGTGGTCATCCACGTGAAGGATTCCGGATTCAGTCTGTCGCAGAATC
CGGCCTCGATGCTGATGGAGAGAGAGAGGGAGAGAGAGGGACCATTCATGATTCAAA
GCGCACGCGAATCATAGCGTTGCGGGAATACGAAGTCGTCCCGCCACTAAACTGGATA
AATTTCGAAGACGAGCACGATGGACACGTCGGTGCTATGTACGTGTGCATGCTGTACG
CCGTACTCTTCCTCAAGCTGTGGTCGTACGTGCAGGTGAACATGTGGTGTCGGGTGAA
CAATCGAAGAAAGGCAACGAGCCAGGGGAGGATGCGACGACAATCTCTGTCCTACAA
TAATCTACAGTCTTCCACCGCGAAGCAAAACGGCGAGGAGGTCGACGACTCGAAGCA

CGAAGTAGACGTAGCTGGCACGGTCTTGGTGCAATATCCGGACAACTTGACTTTCCGT
GATCTTTACTACTTCATCCTGGCGCCTACCCTCTGCTACGAACTGAATTTCCCTAGAACA
CAAAGGATTCGGAAAAGATTTCTGATTAAACGGATCCTGGAGGTGGTGGTCGGCTGCC
AAGTGGTCATGTCGCTCTTCCAACAATGGATGGTCCCGTGTGTGAAGAATTCCCTCGTA
CCGTTCAGC

>novel_circ_001804
GATATCATAGCTCATGGGCCAGGAATGGTGTTCCACATCGTAAACCTCATCGTGATGGT
ACTGATGCCGATGGTGGTCATCCACGTGAAGGATTCCGGATTCAGTCTGTCGCAGAATC
CGGCCTCGATGCTGATGGAGAGAGAGAGGGAGAGAGAGGGACCATTCATGATTCAAA
GCGCACGCGAATCATAGCGTTGCGGGAATACGAAGTCGTCCCGCCACTAAACTGGATA
AATTTCGAAGACGAGCACGATGGACACGTCGGTGCTATGTACGTGTGCATGCTGTACG
CCGTACTCTTCCTCAAGCTGTGGTCGTACGTGCAGGTGAACATGTGGTGTCGGGTGAA
CAATCGAAGAAAGGCAACGAGCCAGGGGAGGATGCGACGACAATCTCTGTCCTACAA
TAATCTACAGT

>novel_circ_001805
GATATCATAGCTCATGGGCCAGGAATGGTGTTCCACATCGTAAACCTCATCGTGATGGT
ACTGATGCCGATGGTGGTCATCCACGTGAAGGATTCCGGATTCAGTCTGTCGCAGAATC
CGGCCTCGATGCTGATGGAGAGAGAGAGGGAGAGAGAGGGACCATTCATGATTCAAA
GCGCACGCGAATCATAGCGTTGCGGGAATACGAAGTCGTCCCGCCACTAAACTGGATA
AATTTCGAAGACGAGCACGATGGACACG

>novel_circ_001807
GTTCTTTCGGGAATCAAGATCGCAGACGAATGAGGATTCGTTTGTCCAGCGAAGAAAA
ACAAAAGATGGCCGCTTCCTGCGGTCAAGTACAGCCGCCCTCGAGCACTTTGCTAATA
AGCGTTCGCCGAAAGCGACGCGGGCCGAGGCCAAGGATCGTGTTACATCTCCGTTGA
AATGTTCAAGAGTGGCACTCTCGCCTTTTTAAACAACCTTGTTTCTTCCTCTCTGCGTT
TCCACGAGATTCCTTCGGGCTGCACGTGTCATGCGTTTCCCTCTTGAGCCTAATTTCCC
TCGTACAAGTGCACCAAATAAATCCTCGCCTGTCAATCCTTGAAAATTAATCTTCCAAA
GAAAAAAAAAAAGAAAAATCTTTCTTCTCCTTAAATCTTTTTCTCCTTTAATTACATCG
AGATTGAGATTTAATTTTACTTAACGAAAGACTAATTATTTCTTCAAGAATTGTCTTTTC
TTTTCTTTTCTTTTCTTTCTTTCTTCTTTAAAGATACTGAGAGAATATAACAGATGTCCTT
TTATCAAGAAGGTGCATCTAGCAGTACGTTTAGCAATCTTTCGAGATTGTCGAAGAAAG
ACACGTCGAATGAAAAAATGAAGATCAGCGGAACAGAAGAAGAACAAAGTGATCGA
AGCGAAGGCGATTGGACGGAACGAGAAGGATCGTTTCATATGTCTGCATCCGGTTGGT
CATTTCCGGCACGCGCTGAGACACGTGGCTTGCCCCTTGCGCGCTCTAGCCCCTTCTCC
ACGCAGGGGGGAGTACATATTTTTCTTTTAAGGGCCCACCGAAATTAGGACCCATCTAG
GCCAAGGGTCCGTCTCTAAACATGGAGCAATAGGAAGTATCTTTCGTGCTTGTCGGATG
GGTGCTTCAAATGTAAACGCCTCTCAAACCTTTCCTCTTGATAATTGCCCCGCGCATTGT
ACCCCCCTTCTCTTAACAATTGAGGAGAACAGTAATTAACCGTAAAAGAAACGAATTA
AAAAGAGGCTTTTATTTATACCAGGATCAATTTATTATCAAACGATTATTTTTGGGAAAA
GAATAATGTATATATATATATATTGAATAAAAATAATCGTATGAAATGGTTACAACGGCGA
GGAGTTAATAATAATTTAAAGTTAAAAGAGTTAAATTAATAATAATAAAAGAAAAGGGC

GAGGAAATAACGAGCACAGCTGATTTTAAAGTCCTGGCAAACACTATTGTAGGAGCGC
AGGACTTTAAAAGTTCTTTCCTTCACCGGAGAACCTGGTCCTTTTTCTCTACACTTGTG
CGCCATTTACGCAAGCCAGTACGCACTTGCCGCGCTCCGTTCTTGGACGGCGCGCGTA
GCATGAATGGTCCACGGCCTTGAATAGAGAGGAATTCACAAAGAAGCGGGCAAAAGG
ACCGACAAAGAAAGCCCGGGACTTCTTTTGTCAGAGGGTCTAGCAGTCGAGGTGACA
CACAGGTACGGATTCGGATCCCGAGGAGGCGAACGGCGCAGCCTCCGCTAACCTACGT
GGATCGATCTCCAGCTCCTTCAACCTGCGGAATGCGTCCCTGATCCGTCCGTTTCTCCT
CTTCATGGACGCGAGCACAAGAAAAAGAGGACGAGGACGAAGAAGAAGAAGAAGA
AGAAGAAGAAGACGAAGAAGAAGAAGTGTGTGGACAG

>novel_circ_001808
AGGCAGCCGCCAGCCACCGGAGCGGACTGACTGACAGGTTTAATTCTCAAGGTAGCTA
GAGCGAGTAATCCGCGCCGGGCTGCAAAGTATTAATACAAAATTTTACGATGTTGCTCG
AGATGCATGGCGGCCGACAATGGAGTCGCACGAGATACAAAATGCAGCACAATGTTTC
CGTCCGTTGGTCTCATATTCGGTGGAACGGGTCTGTAGCACGAGGTTGAAATAG

>novel_circ_001810
TTTTCAACGTCGTTCAAAATTAGATTAGCACCTATCATCAACTGCAGATCGTGCTCCAAT
ATTTTGTTTCGAAGGGATGTATAATTAGTAAGTGGAATAGCAATATTCACAGTTGCATGA
CCGAGCAATATCATAAATGAAAGAATAAATTTCTTCATGATTAGTGATTTTTATTTGGTG
AATACTCTAA

>novel_circ_001812
GCAATTATCCACGGTTTTTGCGGCCACGTCTAGACGATGCCTCGTTCGTGTCCGCGTAG
CGACGAAGAAACGAAATGGCAGGTAACGAGGCGCCTGACATCCGTCGAAGAACTGGC
GACCGACGATAACGGTGCTCCAGAAACGATAACCAGTTCGAGCCGTCTGGTTCGGCGA
CCAATAAGGATCATTGACTCCCTGTTATCGCGCCGCAATTCCCAGATGTCTAATACCCGG
CGAGAGAGTATTTTTCGCAAATATTTGACCAAACCGAGGACCGAGAATGGGCAAGAT
ATCGAAGATTTCTACCATGTTATTTCTTGGAAATAA

>novel_circ_001813
GCAATTATCCACGGTTTTTGCGGCCACGTCTAGACGATGCCTCGTTCGTGTCCGCGTAG
CGACGAAGAAACGAAATGGCAGGTAACGAGGCGCCTGACATCCGTCGAAGAACTGGC
GACCGACGATAACGGTGCTCCAGAAACGATAACCAGTTCGAGCCGTCTGGTTCGGCGA
CCAATAAGGATCATTGACTCCCTGTTATCGCGCCGCAATTCCCAGATGTCTAATACCCGG
CGAGAGAGTATTTTTCGCAAATATTTGACCAAACCGAGGACCGAGAATGGGCAAGAT
ATCGAAGATTTCTACCATGTTATTTCTTGGAAATAAAGGAGGCTGTCCTCGCGTTCCGC
ATCTTTCGCGAGGCTTTAATGACCACCACGTGGTGCTATCATCGGAGGTAGTACGTGAG
AAACACGGACGAGTATCATCACGCGGTGACTCGCTAAAATTATCCGTGCTTCTCTTTCG
AGGCAACACCGTAGGATGAGAATTAAGCCGGCGCAAAAAACCAAGGCGACCGTGAAG
ACCAGCGACGGTCTCTTCGGTTTCTCAAAGGACTTCTCCTGGGAAG

>novel_circ_001814
AGCCACCGAGGCGTGAGTGTCTACGCGAGTAACAGTTTTCCGACTCGGTTCCTAAAAC

TTTCACGGTTATTCGCCCGGGCCCGACAACTTCGTGAGAGATTGTCTTCTCGTATATAG
AGAACGAAAGCTGATGAATAGCGAACGGAAGTCGATCGTCTCGTCGGATCGGAAGCT
GTGAACCTAGAACGTTCCCACGATTCTCACCAAGGATATACCGTATAATAATTAGTGCG
AGAAGACAATAAATTTCCATTCGAACAAAAATCGATCGATCAGTGTGTGATCTTCGATG
AAACCAGAGCTTG

>novel_circ_001815
AGCCACCGAGGCGTGAGTGTCTACGCGAGTAACAGTTTTCCGACTCGGTTCCTAAAAC
TTTCACGGTTATTCGCCCGGGCCCGACAACTTCGTGAGAGATTGTCTTCTCGTATATAG
AGAACGAAAGCTGATGAATAGCGAACGGAAGTCGATCGTCTCGTCGGATCGGAAGCT
GTGAACCTAGAACGTTCCCACGATTCTCACCAAGGATATACCGTATAATAATTAGTGCG
AGAAGACAATAAATTTCCATTCGAACAAAAATCGATCGATCAGTGTGTGATCTTCGATG
AAACCAGAGCTTGGTGAAACGATGCGGTGATACCCGAGGAGCCCCGCTTCAACACCC
GTACATATCAATTCATATCTCTGCCCGATAAACATCGCCAAACATCAATCGACCAATCAA
ACGAAGCATATCGAAACGCAATGAAGACCCAACGATGATCGAATCGCGGCCAAGGCAT
ATCAGGCGTCGATCAAGCGTGATCGGCCAGACTGTTCAACCCACTCTATCCTAACCAG
AGACCTGTCCATAAACCTTGCACCACCCCCCGGTCGACGACACCAATTGAATCAACCT
TGGTCCAACCGCCTCTCCCTCCCCCTCCCCCCTCCTCCCCTCCACCACGTCCCATATCA
CCACGCCCAATCGGAAATCAACGGAGAGAAGCAAATAGAAGGAAGGATGTTGCTGAG
GTAACGCGGCCTCTCGAGCCGGGGTTAAGAATCGATCGTTAAATCGAAACCGTGCTCG
AACAACGAAGGGGATACCAGGTGGCGGATTGCATAGTGGATTCGCGTAGGGGAGAGA
GATCCAGAGTGAAGGATGGGTTTCCCCAGGAGGAGATCCCTGTGGTTGAAGGTGGCCA
TCCTGGCCACCGCGGTTTGGGCGACCGTGTGCTTCCTCTTGTACACGGAGGACCGTGC
CAACGCGGCTGTTCAAGGGTTGGCGCCGTCAGGCGTCGCGGCCCCCCAAATGGCCAA
CGGTTTCGTGCCCGCAGCCGCGCCTCTTAGAAAGGAGACGCCGCAACCGAAGCCAAA
GGTGATTCAACCTGGACCCGAGCAAGGTGCTGGTGTGCTTGTTGCTCCCCGTGAACCA
GATGCAAGCGCACCTGGCGAGATGGGAAGGCCAGTGATTCTGCCAACGAATTTGACTG
CCGAAACTAAAAAACTTGTTGACGATGGATGGCTAAACAACGCGTTCAATCAGTACGT
TAGCGATCTGATTTCTGTTCATAGAACACTGCCCGACCCTCGTGATCCATG

>novel_circ_001816
GTGAAACGATGCGGTGATACCCGAGGAGCCCCGCTTCAACACCCGTACATATCAATTCA
TATCTCTGCCCGATAAACATCGCCAAACATCAATCGACCAATCAAACGAAGCATATCGA
AACGCAATGAAGACCCAACGATGATCGAATCGCGGCCAAGGCATATCAGGCGTCGATC
AAGCGTGATCGGCCAGACTGTTCAACCCACTCTATCCTAACCAGAGACCTGTCCATAA
ACCTTGCACCACCCCCCGGTCGACGACACCAATTGAATCAACCTTGGTCCAACCGCCT
CTCCCTCCCCCTCCCCCCTCCTCCCCTCCACCACGTCCCATATCACCACGCCCAATCGG
AAATCAACGGAGAGAAGCAAATAGAAGGAAGGATGTTGCTGAGGTAACGCGGCCTCT
CGAGCCGGGGTTAAGAATCGATCGTTAAATCGAAACCGTGCTCGAACAACGAAGGGG
ATACCAGGTGGCGGATTGCATAGTGGATTCGCGTAGGGGAGAGAGATCCAGAGTGAAG
GATGGGTTTCCCCAGGAGGAGATCCCTGTGGTTGAAGGTGGCCATCCTGGCCACCGCG
GTTTGGGCGACCGTGTGCTTCCTCTTGTACACGGAGGACCGTGCCAACGCGGCTGTTC
AAGGGTTGGCGCCGTCAGGCGTCGCGGCCCCCCAAATGGCCAACGGTTTCGTGCCCG
CAGCCGCGCCTCTTAGAAAGGAGACGCCGCAACCGAAGCCAAAGGTGATTCAACCTG

GACCCGAGCAAG

>novel_circ_001817
GTTAAAACAGAACGGCTCACGCCAGACAATAATTCAACGTCGTCGAGGAGCGTGACG
CCCTCATCGTCCAGCCATCCAGGAACGCCACCAAATGCTACCCCTTTGGGTGGACCCG
AGGGCCCACCACCACCCCATCACCTCAAACACATGGAGCAGATGATGGGTCGGAATTA
TAGCGATTTCATGAGAAGTCTCGCTGCCAAGTACAATAACGCCAATCCTAATGACTACT
TCAGTTCGCCCAGAAACGGCTATCCTCCAGGCCTAGATCCAAGGTTTCCGGCATTCAA
AACTGCCGCCACACCCTTCGTCGGTTTGATGGCGCCGTTGGGTGCGCCACAACCGTCA
ACCGTGTCCACCACCTCCCCTCTATCTAATAAAGATCCAAAAGAGCAGAAGCAGGACA
GTCTGTTCGGTAATCCCGTGTTCCCACCGATGCTGGACATGTCGTCGACGCAAGCACTT
TTGCATATGGTGAGGACTGCGAATGCTGCACAAAATGCTGCAGAATTGGAAACGTATC
TCAAAGGTGCGAACAAAAGAGACACGGGAGTGACGAGTCCTTTGGATCTGTCGAGTC
CAGGTGGATTCGCACCCCGGAAGAGGCAGAGGGCGGAAACGAGGAGATCGGGAAGC
GTGTCTCCGAAACCGAAGCCAACTCCTCGACCGACCACACCACCTCCAGTCAGATGCC
CATCCCTGTGTGGCCACATGCCATGTGGCGATGGGCAAGCTGTCAATCGATGGACCATC
GAGGACGTCGTCAATTACGTGTCGTCGATCGATATTTGCGCAGAGTATGCACAG

>novel_circ_001818
GTGAGGTATCAGGTGAGAACGTGTCCGCGGGTTCTGGACCACGTAGCGGCGATCGTGG
AGAGGGGACTGATTTAGGTACACACACGGAAAATAACGACGGGGCCACCCTGGCCGC
GAACACAGCGGTCGACGCGAGGGGCGGAAGCCCCCAGGGTGCCCACCTTTCCGGTGC
GGCCACCCCCAGACCGCTAAGGGGTAGGAGACGACAAAACCAGAATGGTCTCGACAC
CACTCAAGTTAAAACAGAACGGCTCACGCCAGACAATAATTCAACGTCGTCGAGGAG
CGTGACGCCCTCATCGTCCAGCCATCCAGGAACGCCACCAAATGCTACCCCTTTGGGT
GGACCCGAGGGCCCACCACCACCCCATCACCTCAAACACATGGAGCAGATGATGGGT
CGGAATTATAGCGATTTCATGAGAAGTCTCGCTGCCAAGTACAATAACGCCAATCCTAA
TGACTACTTCAGTTCGCCCAGAAACGGCTATCCTCCAGGCCTAGATCCAAGGTTTCCGG
CATTCAAAACTGCCGCCACACCCTTCGTCGGTTTGATGGCGCCGTTGGGTGCGCCACA
ACCGTCAACCGTGTCCACCACCTCCCCTCTATCTAATAAAGATCCAAAAGAGCAGAAG
CAGGACAGTCTGTTCGGTAATCCCGTGTTCCCACCGATGCTGGACATGTCGTCGACGC
AAGCACTTTTGCATATGGTGAGGACTGCGAATGCTGCACAAAATGCTGCAGAATTGGA
AACGTATCTCAAAGGTGCGAACAAAAGAGACACGGGAGTGACGAGTCCTTTGGATCT
GTCGAGTCCAGGTGGATTCGCACCCCGGAAGAGGCAGAGGGCGGAAACGAGGAGAT
CGGGAAGCGTGTCTCCGAAACCGAAGCCAACTCCTCGACCGACCACACCACCTCCAG
TCAGATGCCCATCCCTGTGTGGCCACATGCCATGTGGCGATGGGCAAGCTGTCAATCGA
TGGACCATCGAGGACGTCGTCAATTACGTGTCGTCGATCGATATTTGCGCAGAGTATGC
ACAG

>novel_circ_001819
GTTAAAACAGAACGGCTCACGCCAGACAATAATTCAACGTCGTCGAGGAGCGTGACG
CCCTCATCGTCCAGCCATCCAGGAACGCCACCAAATGCTACCCCTTTGGGTGGACCCG
AGGGCCCACCACCACCCCATCACCTCAAACACATGGAGCAGATGATGGGTCGGAATTA
TAGCGATTTCATGAGAAGTCTCGCTGCCAAGTACAATAACGCCAATCCTAATGACTACT

TCAGTTCGCCCAGAAACGGCTATCCTCCAGGCCTAGATCCAAGGTTTCCGGCATTCAA
AACTGCCGCCACACCCTTCGTCGGTTTGATGGCGCCGTTGGGTGCGCCACAACCGTCA
ACCGTGTCCACCACCTCCCCTCTATCTAATAAAGATCCAAAAGAGCAGAAGCAGGACA
GTCTGTTCGGTAATCCCGTGTTCCCACCGATGCTGGACATGTCGTCGACGCAAGCACTT
TTGCATATGGTGAGGACTGCGAATGCTGCACAAAATGCTGCAGAATTGGAAACGTATC
TCAAAG

>novel_circ_001820
GTGAGGTATCAGGTGAGAACGTGTCCGCGGGTTCTGGACCACGTAGCGGCGATCGTGG
AGAGGGGACTGATTTAGGTACACACACGGAAAATAACGACGGGGCCACCCTGGCCGC
GAACACAGCGGTCGACGCGAGGGGCGGAAGCCCCCAGGGTGCCCACCTTTCCGGTGC
GGCCACCCCCAGACCGCTAAGGGGTAGGAGACGACAAAACCAGAATGGTCTCGACAC
CACTCAAGTTAAAACAGAACGGCTCACGCCAGACAATAATTCAACGTCGTCGAGGAG
CGTGACGCCCTCATCGTCCAGCCATCCAGGAACGCCACCAAATGCTACCCCTTTGGGT
GGACCCGAGGGCCCACCACCACCCCATCACCTCAAACACATGGAGCAGATGATGGGT
CGGAATTATAGCGATTTCATGAGAAGTCTCGCTGCCAAGTACAATAACGCCAATCCTAA
TGACTACTTCAGTTCGCCCAGAAACGGCTATCCTCCAGGCCTAGATCCAAGGTTTCCGG
CATTCAAAACTGCCGCCACACCCTTCGTCGGTTTGATGGCGCCGTTGGGTGCGCCACA
ACCGTCAACCGTGTCCACCACCTCCCCTCTATCTAATAAAGATCCAAAAGAGCAGAAG
CAGGACAGTCTGTTCGGTAATCCCGTGTTCCCACCGATGCTGGACATGTCGTCGACGC
AAGCACTTTTGCATATGGTGAGGACTGCGAATGCTGCACAAAATGCTGCAGAATTGGA
AACGTATCTCAAAG

>novel_circ_001821
GTGAGGTATCAGGTGAGAACGTGTCCGCGGGTTCTGGACCACGTAGCGGCGATCGTGG
AGAGGGGACTGATTTAGGTACACACACGGAAAATAACGACGGGGCCACCCTGGCCGC
GAACACAGCGGTCGACGCGAGGGGCGGAAGCCCCCAGGGTGCCCACCTTTCCGGTGC
GGCCACCCCCAGACCGCTAAGGGGTAGGAGACGACAAAACCAGAATGGTCTCGACAC
CACTCAAGTTAAAACAGAACGGCTCACGCCAGACAATAATTCAACGTCGTCGAGGAG
CGTGACGCCCTCATCGTCCAGCCATCCAGGAACGCCACCAAATGCTACCCCTTTGGGT
GGACCCGAGGGCCCACCACCACCCCATCACCTCAAACACATGGAGCAGATGATGGGT
CGGAATTATAGCGATTTCATGAGAAGTCTCGCTGCCAAGTACAATAACGCCAATCCTAA
TGA

>novel_circ_001822
TCCAGGAGAAACAAGAGTGAGGAGTAAAAAGTAAAAGTGAAGAAGAATTCGAAATTT
GTGCACGATGTCTCGATGTTCAAAAATTGTTCCTGTACCAAAATTGAAAGAGATGAATG
ATATTCTTCTTGTGCACAGCCGACGGGTAATCTAATTCCCGGTGATAGTGATGTTACTTC
TTATCTACGCTTAATCTAACAATCGAACCATCTTTCTATGAACACTCGACGAGAAGGAA
CATTCGCAAAATGAATTTACGAAACTCTCGAAAATTACTTATCCATTATCGAAAAAATC
GAATCGAACTGAACTAAACTCCCAGCCATTGAGAGTTCTACGAGATATCCTCACGATGC
ATCTGTCTCACGATGTATCGAGCGTGAAAGAGCGTGCCCAGTTCGTTCGCACGTGCAG
TTAACGTGTCGCAGTCATATTGGTCCGGAAGCGTGCGTGCATGCGGTATGTGCGTACGA
TTGAAGGAAACGTTGAACGTATTCGCAGCACTCCGGTTCCGGCGTTCGGTTGTGCTCG

TACGGCTTCGTTTGCATAGAAGTGCGTGCGTAACGTACGCCGGAGGCCACGGCGTAAG
GTGGTGTACACGGGATTCGGATCGTCCCGCCGCTTTGTTTACGCTCGTATCGGGCCGAT
GATCGAGCATCGTGTGTACACACCGCTTCTTATCCGCCGTTGCGTTATCCACCTTCGAA
ATGGCGCGTGTCGCGTGACGAGAGCCTCCTCAGTCGGTTTTCGCGCGTGCAACGCTTC
CGATCGGACCGTCAGACGCGTGTCGACGATCGTCGCCGTCGCCGTCGCCGTCGCCTCC
TACGTTTCTCTCGATCATCCGACGGGGAAGAAGAGCTGCGCTCGCGTGCGCCTCTCTTT
CCATCACGGTCGTGTGCGTACGTGTGCTTGTGTGTGCGCGTCTCTGTGTGTATGTGTGT
TCGTTGTGTCGTCGCGTCGGTGGTCGTGGCACACGTGTGCCTCGTTGTCGGTTACGCG
AATCCAACCTTATTGTGTGAGTTCGTCGTGAGAGTTTGCCGCGTGTGACCTCTTCCGTG
ACTAGGAAAATGAGACGATCCAGTGGCAACGCGTTCTGAGGATCATCGAGGAGGAAC
GGCAACGGGCAACCAGCCTCGCATCGAACCG

>novel_circ_001823
ATTGAACAAACAATCGATGAGTATCGCAACGCTTTGGAGAGCAACGCTTTGGGCACAG
GGAAATCGGCCTCTGCGTTCCTCAAGTTGGTACCTCTAGTCAGATCAGCCTCTCCAGAC
GAGCTATTGAAAATATTAAAAAGTCCCCGATATCGTCAACTGAAACCGCAACTATTGGA
CGTGTATGGCGTGGCCTCAACCGTGGCGAGTCATCAAGCAGCCATGAAGATTCTGAAA
CAGGATGAAAGGGGCGACGAAACCGAAAGATATCTCTGGGCCCTGTCCTTGTCCCCAA
CTCCAGATGCAGATATCGCTAAAAACATTCTGAAACGGTCAGAAGAGACTAATCCAAA
CGACAAAGTAGCAGAGACGTTAGCCCTAACTGCGGCAGCTATGGCACGCCATCTAGGA
TCTCCAGCTGCCATAGAAAAAGCAAGGACTAGCTTAGAAATTGGCCTGGACAGTTGCA
CGGGAGAAGAGTGCAAATTAAAGTTCCTTAGAGCGTTGAGAAATTTAAGAAGCAAAA
CTGCGATCCCTAAATTGTTAAAATTTGCGACAAGTGAGGAGAAGTCGCTCAGCATTGC
TGCCTGGAAAGCTTTAGCTACTCTGCCCAAAGATTCGATCACATCCGAGGTGAAGGAA
GCAGCCAGACGTGTTTTCTATCAAATAGGTGGACCCAAGAGGGACAGCAGCGCCAGA
ACTCTGGCCCTGGATATCATTCTGGAAACAGGCCCGTCGAAGGAAGACGTTGGACACC
TCGTGGAATACTTGGCAGGCACAGATCCTGCGTACGAGGTCCGCAAGTATCTCAGTCA
ACGATTGGAACAGGTTTCTGAAAAAGATCCCCGATTAGCCAAGGATTTGAACGAAGTC
TTAAGCACAAGTGGGAAAGATATCGTTAATTATAACGTGTTTTCCCAAAAAGGTTTAAG
CACAGCGTTCACTAGAAGCTTCTTAAGATCGCCAGACAGCAATGGATCGCTGGTAACC
ATTCAGGAAATCAGCTCAGGATTGATGAAACGTGGGATAGTGGACGTGATTCTCCAAG
TGGAGGACCACGAGGAGGCGTTATTCTCCCTTGGCCTGTTCGCGGGGGGTCTAGGAAG
CTTCGTCTCGACGTCTGAGGAAGAAGATATCCAAGACAACGAACCTGCCACGGCAGG
GATGGAGCTAGATTTCTTGGGAGTTGGCATCAGGCCGTTCGTTTTCTTCTCTGGCCAAG
GGGAGCTCATGGGCCACGTCTGGTCCGGCACAGGGTCTGAACGGACCCCAGCCTTCC
AAGCCTTGTCCTCAATCCACTATCACAACGAATACATCCCACTGGGTTCGGGGATCGTG
ATTGAGATTGACGTTCAGGGCGCGGTCAGCTTCGATTTAGCTGGACAGATTCAACTGA
GTCTGTGGTCCAGAAATGCCCAGTCTCTGGTAGATTTGAAGGCTGGAGTGGTGATCCA
AGGAGGGGCGAAGGTGCGATCCGACTTCGTGCAGAGCATGGCCGAATTCTCCATGACC
ATGGAGCCCAAATTGGAGTTGGCCACCGACGTGGACTTTTCTGGACCTGTCTCTTTGT
GCATGAAACTCAGCCAACCAGAGAACGTGGTCAAGTATCAAGTGTACAAGGTCGAAA
GGGTGGCCGGAAGTAGGCACAAGCTGAGGAAGACTAGGAGGATAAGGCTGCACAATC
CTG

>novel_circ_001824
ATTGAACAAACAATCGATGAGTATCGCAACGCTTTGGAGAGCAACGCTTTGGGCACAG
GGAAATCGGCCTCTGCGTTCCTCAAGTTGGTACCTCTAGTCAGATCAGCCTCTCCAGAC
GAGCTATTGAAAATATTAAAAAGTCCCCGATATCGTCAACTGAAACCGCAACTATTGGA
CGTGTATGGCGTGGCCTCAACCGTGGCGAGTCATCAAGCAGCCATGAAGATTCTGAAA
CAGGATGAAAGGGGCGACGAAACCGAAAGATATCTCTGGGCCCTGTCCTTGTCCCCAA
CTCCAGATGCAGATATCGCTAAAAACATTCTGAAACGGTCAGAAGAGACTAATCCAAA
CGACAAAGTAGCAGAGACGTTAGCCCTAACTGCGGCAGCTATGGCACGCCATCTAGGA
TCTCCAGCTGCCATAGAAAAAGCAAGGACTAGCTTAGAAATTGGCCTGGACAGTTGCA
CGGGAGAAGAGTGCAAATTAAAGTTCCTTAGAGCGTTGAGAAATTTAAGAAGCAAAA
CTGCGATCCCTAAATTGTTAAAATTTGCGACAAGTGAGGAGAAGTCGCTCAGCATTGC
TGCCTGGAAAGCTTTAGCTACTCTGCCCAAAGATTCGATCACATCCGAGGTGAAGGAA
GCAGCCAGACGTGTTTTCTATCAAATAGGTGGACCCAAGAGGGACAGCAGCGCCAGA
ACTCTGGCCCTGGATATCATTCTGGAAACAGGCCCGTCGAAGGAAGACGTTGGACACC
TCGTGGAATACTTGGCAGGCACAGATCCTGCGTACGAGGTCCGCAAGTATCTCAGTCA
ACGATTGGAACAGGTTTCTGAAAAAGATCCCCGATTAGCCAAGGATTTGAACGAAGTC
TTAAGCACAAGTGGGAAAGATATCGTTAATTATAACGTGTTTTCCCAAAAAGGTTTAAG
CACAGCGTTCACTAGAAGCTTCTTAAGATCGCCAGACAGCAATGGATCGCTGGTAACC
ATTCAGGAAATCAGCTCAGGATTGATGAAACGTGGGATAGTGGACGTGATTCTCCAAG
TGGAGGACCACGAGGAGGCGTTATTCTCCCTTGGCCTGTTCGCGGGGGGTCTAGGAAG
CTTCGTCTCGACGTCTGAGGAAGAAGATATCCAAGACAACGAACCTGCCACGGCAGG
GATGGAGCTAGATTTCTTGGGAGTTGGCATCAGGCCGTTCGTTTTCTTCTCTGGCCAAG
GGGAGCTCATGGGCCACGTCTGGTCCGGCACAGGGTCTGAACGGACCCCAGCCTTCC
AAGCCTTGTCCTCAATCCACTATCACAACGAATACATCCCACTGGGTTCGGGGATCGTG
ATTGAGATTGACGTTCAGGGCGCGGTCAGCTTCGATTTAGCTGGACAGATTCAACTGA
GTCTGTGGTCCAGAAATGCCCAGTCTCTGGTAGATTTGAAGGCTGGAGTGGTGATCCA
AGGAGGGGCGAAGGTGCGATCCGACTTCGTGCAGAGCATGGCCGAATTCTCCATGACC
ATGGAGCCCAAATTGGAGTTGGCCACCGACGTGGACTTTTCTGGACCTGTCTCTTTGT
GCATGAAACTCAGCCAACCAGAGAACGTGGTCAAGTATCAAGTGTACAAGGTCGAAA
GGGTGGCCGGAAGTAGGCACAAGCTGAGGAAGACTAGGAGGATAAGGCTGCACAATC
CTGGTAAATCTTATCTGTTGAACAGAAAGAATAACGAAATGTGTTCCAAGATTACTGGT
TGAATTGAAGACTGGTTTTGTTAAAGTTTGCCACGAAACTTTATAAAATATACAAAGAC
AATTGTAAAGGATATCAAATTCTTCCTCCATGTGCAGAAAGAGAAACCATGCGTTACAT
GTGAAACAAAGATATAAGGCGAGAGATTTTTATTATTGT

>novel_circ_001825
GGCACCGACTTGATCAAAGTGTCGAGGTTTTACAAGGATAGCAAGGAGGGAAAGTTC
GTGAGCTTCTTGTACGAGGACACGAGGACTCTGTACGACGGTTTCCGTAAAGGCGCCA
AGGAGTCTAACAACGGTCCTTGTCTTGGCTGGCGGGATGGACCAAACAAACCATATCA
ATGGCTACATTACAACGAGACCCTGCTCAGGGCGAAGAATTTCGGCTCTGGCCTAGTG
TCTCTGGGGCTGATGCCAGGATCGCACACGCTCATCGGTCTCTACAGTCAGAACTGTC
CAGAATGGATCCTCACGGAGCAGGCTTGTTACACGTACTCTTTGGTAGTAGTACCTTTA
TACGACACATTGGGCCCTGACGCGTGCGCCTTCATCATTAATCAAGCCGAGATCAATTT
GGTCGTGGTCGAGAACGATACCAAGTGCAATTTGCTTCTCGACAAAGCGCCAAGATGC

CTACGAAAATTGGTGGTGATAAAGGAGACGAGGCAGACGACGAACCAGAGGGCGAA
GAACCGCGGCGTCGAGCTGCTCAAGTTCGACGACGTTGAGCGGCTGGGCGCCCAGAA
GAATCACCCGGAAGTTCCCCCCAAATCGACCGATCTGTGCACCATATCGTACACGTCTG
GGACCACGGGTAACCCGAAAGGGGTGATGTTGACACACCAAAACGTGATAGCGGGCA
TAAGCGCGGTTCTCGTTCAATTGGGCGAGCACAAGCCCTCGTACAAGGACACGATGAT
CAGCTTCCTGCCACTGGCGCACATGTTGGAACGTTGCTGCGAGAATGGAATGTACATG
GTGGGCGGCTCCGTAGGATTTACAGCGGCGATATCAAAAGGTTGTCCGAAGATATGA
AAGCCTTGAGGCCTACTGTGATGCCCGCTGTGCCCAGGCTGTTGAATCGAATGTACGA
CAAGATCCAAACTGAACTTCAAAACTCATGCTTGAAAAAGCTGGTATTCAGCCTGGGG
ATGCGGGCGAAGGAAGCGGAAATCAAGAAGGGGATAATCAGGAACAACAGCATATGG
GATAAACTGGTCTTCAGCAAGATCAAGGAGTCAACGGGAGGGAGGGTGAGGCTGATG
GTCGTTGGTTCCGCACCTTTGGCGGGAAACGTTCTCACATTCACGAGATGCGCACTTG
GTTGTTTGATCGTCGAAGGATACGGCCAGACTGAGTGCTGTGCACCGATCACTCTTACC
GTTCAG

>novel_circ_001826
ACAACGGTCCTTGTCTTGGCTGGCGGGATGGACCAAACAAACCATATCAATGGCTACA
TTACAACGAGACCCTGCTCAGGGCGAAGAATTTCGGCTCTGGCCTAGTGTCTCTGGGG
CTGATGCCAGGATCGCACACGCTCATCGGTCTCTACAGTCAGAACTGTCCAGAATGGA
TCCTCACGGAGCAGGCTTGTTACACGTACTCTTTGGTAGTAGTACCTTTATACGACACA
TTGGGCCCTGACGCGTGCGCCTTCATCATTAATCAAGCCGAGATCAATTTGGTCGTGGT
CGAGAACGATACCAAGTGCAATTTGCTTCTCGACAAAGCGCCAAGATGCCTACGAAAA
TTGGTGGTGATAAAGGAGACGAGGCAGACGACGAACCAGAGGGCGAAGAACCGCGG
CGTCGAGCTGCTCAAGTTCGACGACGTTGAGCGGCTGGGCGCCCAGAAGAATCACCC
GGAAGTTCCCCCCAAATCGACCGATCTGTGCACCATATCGTACACGTCTGGGACCACG
GGTAACCCGAAAGGGGTGATGTTGACACACCAAAACGTGATAGCGGGCATAAGCGCG
GTTCTCGTTCAATTGGGCGAGCACAAGCCCTCGTACAAGGACACGATGATCAGCTTCC
TGCCACTGGCGCACATGTTGGAACGTTGCTGCGAGAATGGAATGTACATGGTGGGCGG
CTCCGTAGGATTTACAGCGGCGATATCAAAAGGTTGTCCGAAGATATGAAAGCCTTGA
GGCCTACTGTGATGCCCGCTGTGCCCAGGCTGTTGAATCGAATGTACGACAAG

>novel_circ_001828
GGGCCAAAGAACGCGACGAAATATTACATCGCTTGCCAGGCGCCAATGGAGTCGACTG
TCACCGACTTTTGGAGGATGATATGGGAGCAACAGAGCAAAGTGATTATCATGCTTACC
GATCTCGTTGAAAATGGGGTGGAAAAGTGCACGGAGTATATTCCGCCCTCGGAGGTCA
CCGACTGTCGACGTTTGTACGGCGACTTCCAAGTCACCCTGAAGAAGAGGGAAACCA
AAGAAAAATACGCCATCTCCACGCTTCATTTGAACAATTTGGAGAAGAACACGTTCCG
AGAGGTATACCACATTTGGTATTTGTGGCCCGTGAACGGAGTGCAATCGGACGGTGCC
GGGTTGATAGCCGTGCTACTCGAGGCGAGGGCACTTCAAAGGGGTGGCCCTGGACCC
ATTGTAGTCCACTGTAGTCCCGGCACTGGACGCACGGGAACGTTAATAGCTCTTGACCT
AGGAATTAGACAATATGAGATTACGAGGACTGTGGACGTGCCAAGGGTCGTTTACACT
ATCAGACGGGATCGTGCTGGGGCTGTCAAGACGAAAGAACAATACGCTTTCATTTACA
AG

>novel_circ_001830
AACGCATCTGCGAAGGCGAAGGAAGCGACATCGGGATCCGCGACAAACACCTCGAGC
AACGGTTCGAGCGGGAACAATGGGGGACACGCGCCACTTCGCCGCTACACATCCCTG
GTAACAACCCTGATCGAGCAGCACCGTAAACTGGATCGCAGCGCGAGCGAGCCGACC
TCCCGGTACAAGACCGAGTTGTGCAGGCCGTTCGAGGAGAGCGGCACCTGCAAATAC
GGCGACAAGTGTCAGTTCGCTCACGGGTACAGCGAGTTGCGCAACCTCGCCCGCCAC
CCCAAATACAAGACCGAGCTGTGCCGCACGTTTCACACGATCGGTTTCTGCCCGTACG
GGCCCCGTTGCCATTTCATCCACAACTTCGAGGAGGCACGGATCCACAATCAGAAAGT
GAGCGCCCAACTGGGTTCCACCCAGCCCAACATAATAAGCCTGAACCCGTTGTTGAGC
GCAGCGGCGGCCGCGGCCGTGGCCGCCGGTGGAAACGGGTGCAACGGATCCAACGTG
AACTCGAGCAGCAACGTCTCGTTGCTCGAGCAGATCGTCGCCTCCCCCCAGGTGACGG
CGATCGCCGCCGCGGCCACCACCCCCAACCTGATGAGAACCAACTCGTTGAGCCTGTG
CAACTCGGGCAACGTGTACAACCCACCGCCATTGCTCAGAACCAACTCGTTGAGCTTG
ACGACCAGCCAACACCATCACCACCACCACCACCATCACCACCATCACCAGACCGGTT
CCACGAGCGCGAGCTCGGTGATCGGCGCCACCAGGCCCAAGCCGTTGAATCTCAGCC
CGACCTTCTCGCTCGGCAGCACCGGGGACTCGATTTCACCTTCGTCCAGCCTCAGCCA
GTCGCCCACCAACTCGATGGCGAGTTTCTTCAGCGACGACTGTAACCAACAACCGTCC
GTGACCAGCCTGCCCACCACGCCGTTCAGCTTCGGCCAGGATTTCGGCCTTCTCGCGA
CCAGGCAGAGCCCCAGTCCGCGAACCAACAGCGTTTGCAGCCCGTTGGGCTCGGACG
AGCTCGCGCCAAGGACACCGTCCCCGTTGTCGCCCAACGCCGAGTCCAGGCTCCCCG
TGTTCAACAGGATCAGCAGCACTCTCGCCGACTTCGACAATCTTAAGATCTAAAGGGG
GCGCTCTTCTTTCCGACTCCTGGGTCCTTCCACTCTTTG

>novel_circ_001831
GTGCTGGTGTTATTTCTATGGCCAATTCAGGACCAAATAGTAATGGATCACAATTTTTCA
TTACATTAGCACCTACACAATGGCTTGATGGAAAACATACAATTTTTGGTAGAGTTCATT
CTGGTATGGCAATAGTAAAAAGAATTGGATTGGTAGAAACTGACAAAAATGACAGACC
TGTTGATGATATAAAAATTGTTAAAGGTTCCATAAGGAATGCATAACGAAATAAATATCT
TCTTG

>novel_circ_001833
GCAACCATCACCAGGATCCGGCACAACCGGCACGTCCACAGTTTCCTCGAATGCTCCG
GATGGAGAAAATGGCCAAAGCATCAATTTATATCCGGTGGGAAGCGTGGGTCCAACAC
CAACGAACCCAACAACGGCTACACCAATCACGACCACGGCAACAATATCATTGGCCAC
CCCTGGAACCAGAACCCAAGAACCTGGACCCAGTATCACCAGAAGCGTATCAGCACC
CACTGCACCTAGGCCAGTCGTCCAAGTGGCTGCCAGTACCTCCAGCAACAATGGCACA
CAAAAACCGAGATTGAGGAGGACTATGAGTCGCACTGAAGCTATCAGGAACTACATCA
GACGCGAAACAGCGCAATTCTTTGGGGTGGACGAGGAATCTGAGGCTGTGGAGAGAC
AACGATGGCTCGATCGGAGACGGAGAATGGCGTCGAGGAAATACGGTGCCCTTGTTCC
GGAACACAGACCACCGGATCCTGATATCACCAGAGACGTGCCGGATATTACGGATGTG
CCAGAAGGTGTTACATTGAGAAGATGGCAGCAACCTGTAAGACGAAAAGATTCTGTG
GCCAGAATGACGCTTTCGGGGCTTCATTATGTCGTCGAG

>novel_circ_001834

GCAACCATCACCAGGATCCGGCACAACCGGCACGTCCACAGTTTCCTCGAATGCTCCG
GATGGAGAAAATGGCCAAAGCATCAATTTATATCCGGTGGGAAGCGTGGGTCCAACAC
CAACGAACCCAACAACGGCTACACCAATCACGACCACGGCAACAATATCATTGGCCAC
CCCTGGAACCAGAACCCAAGAACCTGGACCCAGTATCACCAGAAGCGTATCAGCACC
CACTGCACCTAGGCCAGTCGTCCAAGTGGCTGCCAGTACCTCCAGCAACAATGGCACA
CAAAAACCGAGATTGAGGAGGACTATGAGTCGCACTGAAGCTATCAGGAACTACATCA
GACGCGAAACAGCGCAATTCTTTGGGGTGGACGAGGAATCTGAGGCTGTGGAGAGAC
AACGATGGCTCGATCGGAGACGGAGAATGGCGTCGAGGAAATACGGTGCCCTTGTTCC
GGAACACAGACCACCGGATCCTGATATCACCAGAGACGTGCCGGATATTACGGATGTG
CCAGAA

>novel_circ_001835
GTTGAAAAAATTGTTACAATCATGGCTAATCCTAGACAATACAAGATTCCCGATTGGTT
TTTGAATAGGCAAAAAGATATTGTAGATGGTAAATATTCACAGCTCACTAGTTCTTATTT
GGATTCAAAACTTCGTGAAGATTTGGAACGAATGAAGAAAATTCGTGCTCATAGAGGT
TTGCGTCATTATTGGGGTTTACGTGTACGTGGTCAGCATACAAAAACTACAGGTCGTCG
TGGACGTACAGTGGGTGTATCGAAAAAGAA

>novel_circ_001836
ATCCGGAGGATGAGCTAAAACGGAGAACGAAATAGAGAAGAGCGGAACGGACGAGT
CATCGACGAATTTTCCTAGGTATGCAGCCAGCAGGGAGGATGCAACGGTCGATGACGT
GAATCGACCGACTTTTCGGTCGATAACTGCCCGCCACGATTGGCAGCCTCTGAATGGCT
GGCCAACCGTCCCGTTCTCACTTCTGTCGGTGGACTGTTGTGCCCCGGTACCGGATAA
CCGGCCTATAAGTGCGTTTCCTCCGAACGAATAATCCTTTATTCCGTTCGTGATATCCCA
TTTCTCTGTATTACCAAATGCTGTTCGTGTACGGTTGAAGTGATCCCCTTGACCGTCTCC
GAGTTTCTTCGAGCCGCTCCCCGCTCCGCGCGATCGTTGGAACGAGCAGTCCCCCCCA
CCACCGCGCCACGAGTTTCGAGAGGAGTGGGGGAAGAGGCGAGCAGAAAGGCGGGA
AGAAGAACAGGGAAGAAGCGGCCTATTCTCTGTTTGGAATAACAGTTTGTCGAAGGTC
GTTGGCGAGCATCTTCCGTCGCGTAATCGAAAAGTTGGAAATAAAAGAGAGAGGAAC
GAAGAGAGAAGGAGTTTGTCGGAAGGTTCTTCTTTTTCTTTTTTCCTTCTCCTCCTCTC
CCTTTCCCCTCACCCGTGGAAGACAAGTGGCCACTTTCTCTCTATCCTCTCCTCAGGGG
TCGGCGGATGAAGGCAAAGTGGACGGCGAGCAACGCGGAACACCGGCTCTTTCGCTA
CGCGCCTCGCCCTTGATTCAGAAGGACGGCCACGTGCACGACGACGGCGCGAGATAAT
CGGTATCACGGGGAATCACGTTCATTTCCCCACCAACTACGGCCACATGACTTGACGAT
CGCCCCACCTACCGCTGTTCGTCGTTGAGCGAAAGCGGCGACAGTGGCAGCAACGTG
ACCGACGTGGGGAGGGCGAGCGCATCACGACGACCACGACGACGATCGGCGACACC
AACAACGGCGACGACGACGACGACGACGACGACGACGACGACGACGGCGACGACGA
CGAGGAAGAAGGAAGAAGAACGGCGGAGGAGTAAGTCATCGGTTACTACTCGCAAGG
CTACTTAACGAGTGACATAGGCCGTTGGTGGTTCAGTTAGCCGCGGACGGTCAACCCA
GGTGCGAGAGTCGTTCGCGCTCTTTTATCCTTTTTTCTTTTTCGTTATCTCCCCCCTCCC
TCTTCCGTTCTTTCTTTTCTCCCCCTTATTCCACCTCTATTTCGCCTTTTTTCTCGAACGA
CTTTTCGGGTGGGTAGAAAAAGATTCGCCAAGATTGGATAGATAGATAGTTAGATAGTT
AGATAGATAGATAGGTAGATAAGTGTTGATCGTGGTGACTGTGCTGGTGCCGTTGTTTG
TTGTCGTTGTTCTTCGGCTCGGGAAGTAGCGGTTTTCAGTGTGGATAGTGCGGATAACA

GTGACGAGTGTGAAACTGGAGCCGCAAGCGGTAGAAGCAGTTGCAGTCGAGAGCTAT
CATGTGCCCGCGGAGGATCTTCGGACTGGCCATCGTCGCGCTGGTGACTTTTGGTGGA
CGAGCGGCAGCCTATACGAATCAGTGGGCAGTGCATATCGAGGGGGGACCGGATGTGG
CCCAGCAGGTCGCGCGAGAGCATGGATTTCAATATCTCGATAAG

>novel_circ_001837
ATAGTGGACGATTGGTACCACATGGTGCACACGTCAGTCGTGAAGAGATCAGCGGAAC
CGCATTTCGGGGTGCAGGGGAGGCTAATAGAGGACCGCAGAGTGCGTCGAGCTGAGC
AACAACGGGTAAAATCGAGAACGAAACGGGATCTAATTATTAAACGCGGTCCGTCCAA
CCTGAGAACAGTTCTCAACGACGAGATGTGGCCTCAGATGTGGTACTTGAACAGGGG
AAAGGGGTTAGATATGAACGTGCAGGAAGCTTGGGCTGAAGGAATTACGGGACGCGG
AGTCGTGGTCACGATTCTTGATGACGGATTGGAGAAGAATCATCCGGATCTTTATAAGA
ACTAC

>novel_circ_001838
ATAGTGGACGATTGGTACCACATGGTGCACACGTCAGTCGTGAAGAGATCAGCGGAAC
CGCATTTCGGGGTGCAGGGGAGGCTAATAGAGGACCGCAGAGTGCGTCGAGCTGAGC
AACAACGGGTAAAATCGAGAACGAAACGGGATCTAATTATTAAACGCGGTCCGTCCAA
CCTGAGAACAGTTCTCAACGACGAGATGTGGCCTCAGATGTGGTACTTGAACAGGGG
AAAGGGGTTAGATATGAACGTGCAGGAAGCTTGGGCTGAAGGAATTACGGGACGCGG
AGTCGTGGTCACGATTCTTGATGACGGATTGGAGAAGAATCATCCGGATCTTTATAAGA
ACTACGACCCACAAGCCAGCTACGATGTCAATAACCATGATGAAGATCCCATGCCACGA
TACGATGTTCTAGACAGTAATCGACATGGCACCAGATGCGCTGGTGAAGTGGCTGCAA
CTGCTAACAATTCCTTGTGCGCGGTAGGAGTTGCATTTGGAGCTGGCGTCGGGGGAGT
GAGAATGTTGGACGGGGACGTGACGGATGCCGTGGAAGCAAGGTCTCTGAGCCTGAA
TCCTCAACACATCGATATTTATAGTGCCTCATGGGGACCTGACGACGATGGAAAAACCG
TCGATGGTCCTGGCGAACTTGCCACTAGAGCTTTCATCGAGGGGATCACAAAG

>novel_circ_001839
ATAGTGGACGATTGGTACCACATGGTGCACACGTCAGTCGTGAAGAGATCAGCGGAAC
CGCATTTCGGGGTGCAGGGGAGGCTAATAGAGGACCGCAGAGTGCGTCGAGCTGAGC
AACAACGGGTAAAATCGAGAACGAAACGGGATCTAATTATTAAACGCGGTCCGTCCAA
CCTGAGAACAGTTCTCAACGACGAGATGTGGCCTCAGATGTGGTACTTGAACAGGGG
AAAGGGGTTAGATATGAACGTGCAGGAAGCTTGGGCTGAAGGAATTACGGGACGCGG
AGTCGTGGTCACGATTCTTGATGACGGATTGGAGAAGAATCATCCGGATCTTTATAAGA
ACTACGACCCACAAGCCAGCTACGATGTCAATAACCATGATGAAGATCCCATGCCACGA
TACGATGTTCTAGACAGTAATCGACATGGCACCAGATGCGCTGGTGAAGTGGCTGCAA
CTGCTAACAATTCCTTGTGCGCGGTAGGAGTTGCATTTGGAGCTGGCGTCGGGGGAGT
GAGAATGTTGGACGGGGACGTGACGGATGCCGTGGAAGCAAGGTCTCTGAGCCTGAA
TCCTCAACACATCGATATTTATAGTGCCTCATGGGGACCTGACGACGATGGAAAAACCG
TCGATGGTCCTGGCGAACTTGCCACTAGAGCTTTCATCGAGGGGATCACAAAGGTTG

>novel_circ_001840
ATAGTGGACGATTGGTACCACATGGTGCACACGTCAGTCGTGAAGAGATCAGCGGAAC

CGCATTTCGGGGTGCAGGGGAGGCTAATAGAGGACCGCAGAGTGCGTCGAGCTGAGC
AACAACGGGTAAAATCGAGAACGAAACGGGATCTAATTATTAAACGCGGTCCGTCCAA
CCTGAGAACAGTTCTCAACGACGAGATGTGGCCTCAGATGTGGTACTTGAACAGGGG
AAAGGGGTTAGATATGAACGTGCAGGAAGCTTGGGCTGAAGGAATTACGGGACGCGG
AGTCGTGGTCACGATTCTTGATGACGGATTGGAGAAGAATCATCCGGATCTTTATAAGA
ACTACGACCCACAAGCCAGCTACGATGTCAATAACCATGATGAAGATCCCATGCCACGA
TACGATGTTCTAGACAGTAATCGACATGGCACCAGATGCGCTGGTGAAGTGGCTGCAA
CTGCTAACAATTCCTTGTGCGCGGTAGGAGTTGCATTTGGAGCTGGCGTCGGGGGAGT
GAGAATGTTGGACGGGGACGTGACGGATGCCGTGGAAGCAAGGTCTCTGAGCCTGAA
TCCTCAACACATCGATATTTATAGTGCCTCATGGGGACCTGACGACGATGGAAAAACCG
TCGATGGTCCTGGCGAACTTGCCACTAGAGCTTTCATCGAGGGGATCACAAAGGGTCG
CAACGGTAGAGGGTCGATTTTCGTCTGGGCGTCGGGGAACGGCGGCAGGGATCACGA
CAACTGCAACTGCGACGGTTACACGAACAGCATCTGGACGTTGTCCATCAGCAGCGCG
ACGGAGAACGGCCAGGTGCCTTGGTACAGCGAGGCGTGCTCGTCCACCCTGGCTACG
ACCTACAGTTCCGGTTCATCGGGGGAGAAACAAGTGGTGACCACTGACCTGCATCACC
TTTGCACGACCAGCCACACGGGCACGTCTGCATCGGCGCCCTTGGCAGCCGGTATATG
CGCCCTCGCCCTCGAGGCCAACAGAGATCTGACTTGGAGGGATATGCAACACATCGTC
GTGAGGACAGCGAAACCTGCCAATCTGAAAGCTATGGATTGGGTGACCAATGGTGTTG
GACGAAACGTGAGCCACAGTTTTGGTTATGGCTTAATGGATGCAGCTGCCATGGTGCG
GTTGGCCAAAAGATGGCGAACAGTACCGGAACAACACAAATGCGAGGTGTCAGCACC
ACACATGGGCAGGCCAATACCACCAAAGAGCCAGCTAACTCTGGAATTGCACGTCAA
GGAGTGTAGCGGCGTTAATTTTCTCGAGCACGTGCAAGCAAAGGTTTCATTGATGGCG
TCGAGAAGAGGGGATCTTCAGATACAACTAACATCTCCACAAGGTACAAAGAGTACCC
TTCTTGCAAAAAGGTCACACGACGTCTCGAAGGCTGGTTTCAATCAATGGCCATTTATG
TCGGTTCACACTTGGGGAGAAAGGCCACACGGCACGTGGAAACTTGAAATCCACAAT
GAGGGTCGATACCAAG

>novel_circ_001841
ATAGTGGACGATTGGTACCACATGGTGCACACGTCAGTCGTGAAGAGATCAGCGGAAC
CGCATTTCGGGGTGCAGGGGAGGCTAATAGAGGACCGCAGAGTGCGTCGAGCTGAGC
AACAACGGGTAAAATCGAGAACGAAACGGGATCTAATTATTAAACGCGGTCCGTCCAA
CCTGAGAACAGTTCTCAACGACGAGATGTGGCCTCAGATGTGGTACTTGAACAGGGG
AAAGGGGTTAGATATGAACGTGCAGGAAGCTTGGGCTGAAGGAATTACGGGACGCGG
AGTCGTGGTCACGATTCTTGATGACGGATTGGAGAAGAATCATCCGGATCTTTATAAGA
ACTACGACCCACAAGCCAGCTACGATGTCAATAACCATGATGAAGATCCCATGCCACGA
TACGATGTTCTAGACAGTAATCGACATGGCACCAGATGCGCTGGTGAAGTGGCTGCAA
CTGCTAACAATTCCTTGTGCGCGGTAGGAGTTGCATTTGGAGCTGGCGTCGGGGGAGT
GAGAATGTTGGACGGGGACGTGACGGATGCCGTGGAAGCAAGGTCTCTGAGCCTGAA
TCCTCAACACATCGATATTTATAGTGCCTCATGGGGACCTGACGACGATGGAAAAACCG
TCGATGGTCCTGGCGAACTTGCCACTAGAGCTTTCATCGAGGGGATCACAAAGGGTCG
CAACGGTAGAGGGTCGATTTTCGTCTGGGCGTCGGGGAACGGCGGCAGGGATCACGA
CAACTGCAACTGCGACGGTTACACGAACAGCATCTGGACGTTGTCCATCAGCAGCGCG
ACGGAGAACGGCCAGGTGCCTTGGTACAGCGAGGCGTGCTCGTCCACCCTGGCTACG
ACCTACAGTTCCGGTTCATCGGGGGAGAAACAAGTGGTGACCACTGACCTGCATCACC

TTTGCACGACCAGCCACACGGGCACGTCTGCATCGGCGCCCTTGGCAGCCGGTATATG
CGCCCTCGCCCTCGAGGCCAACAGAGATCTGACTTGGAGGGATATGCAACACATCGTC
GTGAGGACAGCGAAACCTGCCAATCTGAAAGCTATGGATTGGGTGACCAATGGTGTTG
GACGAAACGTGAGCCACAGTTTTGGTTATGGCTTAATGGATGCAGCTGCCATGGTGCG
GTTGGCCAAAAGATGGCGAACAGTACCGGAACAACACAAATGCGAGGTGTCAGCACC
ACACATGGGCAGGCCAATACCACCAAAGAGCCAGCTAACTCTGGAATTGCACGTCAA
GGAGTGTAGCGGCGTTAATTTCTCGAGCACGTGCAAGCAAAGGTTTCATTGATGGCG
TCGAGAAGAGGGGATCTTCAGATACAACTAACATCTCCACAAGGTACAAAGAGTACCC
TTCTTGCAAAAAGGTCACACGACGTCTCGAAGGCTGGTTTCAATCAATGGCCATTTATG
TCGGTTCACACTTGGGGAGAAAGGCCACACGGCACGTGGAAACTTGAAATCCACAAT
GAGGGTCGATACCAAGGTCGCGCCACGCTTCACGAATGGGCGTTGATATTTTACGGGA
CGTCGACGCTTCCCGATCGCACGGAATACGCGTTGTACAATCCCGCCGGGATGAACATA
CCGAGAAGGCCATTCGTCAAACCGCGCGAGACAGTGTTCTCCAAGGCTCAGAACTCT
CTCACGAGCTCGAAGATCAAGCAGAGCCAGCACAAGTCGGAGAAGCCGGGTAGTCTC
AGCCCGCCTTACGTGGTGAACGCCCAGATACAGTCGCAGAGGAAGAGCAAGGCGCGC
GGCCAGCACAAAAATGGCAAATCGGCCAATAAACCGACCCCGAAGCCGACCGTGCAG
ACGTTGAGGGGGTTGACCGTGGCGAGGGGGCCTAACGTCGCGGGGGAGGTACGCCTG
ATCACGTCCAGGCCACGAGGGAGGAGCACACCAAGCCCTGTCACGACCACTGTCGCG
CACCAAACCAGTCCGCCCACCGCTGTTCCAACCACAAAAGTGGCCAGCAGGCAAAGA
ATCGTGGACGTGGCGACTGCCTCGTCCCTTCAGAGGGGTTTGATCCTCTTGGAGCCAC
CAGCGAAACCTGCCATCAACAACGTGCCAGCCATTTTCCAACAGTATCCCAAGATTCA
ACAGCTCTATCCATTGTATCCTGTCTATCCCGGGGTTAGAGGAGCCGGCCAGCAACACG
CGACCAGGGCCAAGGGGTTGGAATTGCTGCAGGATGAACAGTTCGATTCGAAGAACT
TGGATAAGGACACCCTCGACGAGTTCAAGCTAGTGTTCTACGGCACGGAAACGTCGTT
GGAATTCGACGACGAGTTGGACAAGGACAAACCGTTGCCACCGGTGAACCAGCAGGA
CGTGAGCCAGGACAATACGGCGATAGGCGCGAGGCACAACGTGATGGACACGGAGGG
TGGTCCGTGGACAGGTAGCCAACAGGTGGAGCGCGTGTCCCATCCGGAAGTGCAGAG
GCCCACGACCGAGAACCAGACGAGCGGTTGCAGCGGCATCGACACTGGAAATGGCAG
GTGCCTAG

>novel_circ_001844
GAGCCGCAGCCGCATCCAGGGTGTACGTGGACGCATTGTCTCGATTAGCACGCCAGGC
GCAACTGGGTACATGGGGTGGTTCCAAGGATGTCGGGTTCGCGCTGATGAGGATCGTC
GAGGTGTACAAGGAGATACAGGAGCAGGAGATGAATATCCTGAAAGCGTTCTACGTCG
ACCTACTGGTCCCCCTCGAGACGAATCTGGAGAAAGACACCAAGGTCGTGCAGAGCG
AGCAGAAGAAGTTTCTGCAGCAGCACAAGACCAGGTCCGAAACTTATAGCAAAGCGG
CGGCCACGATGAAGAAGCAGAGGAAAAAGTCGAGGGGCGCGAGCAAGAGTGGACTG
GCCATGGACAAAGAGCTGAAGAATATGCAGATCCTCGAGGAGGAGAAGTCTAAGCTA
GACGCCTTTTGTGAACAGAGTTTGAAGAATGCCATGACTCAGGAACGAAGAAGGTAC
GGCTTTGTTCTCGAACGACAATGCTCGTTGGCGAAACATTATGCGAGTTTTCACGAGGT
CGCGTTAGCTGCTCTTCATCCCTCGGTTGATAAGTGGCGCGAGGTAGCTGCTACCAGGG
AATATTTACCCCAATCCGTGGAAGATATGTTCGCTTCCAGGCTTAGACAAGTATCATTCT
GGCCGGAGGACGAAGAGAACGGTGGATCCGAATTAACGATGAGCTCACAATTGAGAA
AGACCAGAAGCATGGATAGTTCTTGCTTGGAACTTCGACCTGGGCCGCCATCCGGAAA

TGTGCCAAGTACCACTTATCAAGGTCCGACCTCGCATCTTTCATCCGCCCTCTCGAGAG
CAAGATCGGAGGCCAATCTCCACGCTTCGACATTGTCCCTCGATCCAG

>novel_circ_001846
GTTTGGTGAGGATGCGTATCTGGTTTGACCTGATAATTTAACAGTGCGAGGCTGATAAA
TAAGTTGAAGAAAGTAAGAATTAAAAATGATGGATCTGAGATCATTTTGACGAACATTA
AAGGTATATTTGATTGAATTAAAATAGAAAGTTTTTAATTAAAACGTGTAACGTGAAATT
ATCTTCTTTTCTTTTCTTATATTTTCGATAGATATCGTAATGAATGACAGGAGTAATGATT
TCCAAGTCGGCTCATTAATTGAGATATTATAATTGCTTTACAAGCACATTCCAACTTCAA
CATGAGTCGAAGTTTCATTAAAGTGAATCTTTGATGCACGAAAATGATGCATATAATAC
GATTTCACGTGGAAGAGGAGCGAGTTATCGGACAAGGATTTATTAGAATCGTGAAACA
AATAGTCGTTTCTACACATTGTATCACGTTGATTGATTTGTTTCTTCTTGAAATTGATCGT
CGGTGTAAATTTCCGGTATAATCTGATTGCCTGTTTAATGTAAATCACAAATCGTTCACG
ATTTGTTAAGGAAATTAGCTATGAATTAAAATTTGTAAAATTAATAATCGAAAAGTAGAG
CAATTATTTAGTGAAAAGGTAAGATTATATTTTAATAAATATATGTACGGTGAATAGTTTA
ATTTTTTAATCGATAAATTGTTTTTTTTTTAGAAAAATTCCGAATAAAAAGGAAACTGCG
TGCGCAAATGTGTAAATTGCATTTTTTATATTTATATTTATTTAAGGCAAAAGAATTTTAA
TGTAGAAAATGGAGAATATTTTTATTTTAAAACAATCGCAATAAAAAGAAACAAAGGA
GGAAGATAAGAATTGATTTAATTAATTTATCGCATAGAGCGTGGTGGGGATAGTCTAGTT
TAAGACTATTGTTATTCGAGAAAGTCGAATCAGTATGCCAACGGAGGTTCGCGCG

>novel_circ_001847
ATGCAATCCATGGTTCGTGCGATGCATCAAGCCGAACACGGAGAAAGCGCCAATGAAG
TTCGACATGCCTTGCGTCCTCGAGCAACTAAGGTACACAGGGATGTTGGAGACGATTC
GAATTCGAAAAACTGGTTATCCCGTCCGACTTCTCTTTGGACATTTCGTGGACAGATAC
AGATATCTGGTTTCCACGCATCTGCCCAGAGGCGCACCCAACAAGGAACTTTGTCGTAT
CATTTTGGACAAAGCTGCGCCCAAGGACGCGCAAGCCCAGTATCAACTTGGCCTCACC
AGAGTATTTCTGAGAGAATCTTTGGAAAGGACTTTGGAATACAATAGAGCTCTGATTTT
GGAAAGGGCTGCCATAACTGTTCAAAGATACACGAGAGGATTTCTAGCTAGAAGAAGA
TTCCTGAATATTTCACGGAGCACGGTGTTGATTCAAGCTGTTTATCGTGGTTATCGCGA
GAGGAAGAAGTTCAAGTCGTTGAAGAAAGGTGTACTTATGGCGCAGAAGATATACAG
GGGGAAAAAACAGCGTGAGAAGTTCAAAGTATTGAAAGAGGAAATGGCGAAGAGGG
CGGAGATCGAACGAGCGAGCAAAGAAAGAGCCAAGGCGAAACAGCAAAGGGAGGA
GCAAGAAAGAACCTCCAGAGCCGTGGCCGGTGTTAATCATTTGGAAATTCCTGCTGAA
CTTGCCTTCATTTACAGCAAACTCGATG

>novel_circ_001849
ACTTTGTCATAATCATGGCACATGCTTGGATCTGATCATCATCGATCGTCATCGAAAAAC
ATTGATGTCATAGATCATACGTTGCTACTAAATAATCCTTCGCCAGTTTTAACATGGATCT
TCTTGGATCTATCTTCATCTTCTGTAAAAAGGGGTAAGTTGGTTTTTATTTAATATTTTAA
TTCATACAGAAATTAAAAGATTGCCAGTTTATATTGATTTCGAGGTTAGATGAATTTATT
GGACACTGATTTATTGGATAGAATTTTACAAAATTTTTTAATACTTATCTTTTATAGTTCT
TTTCAAAAATTTTATTCAATATTGTGATTGTGTATATATTTTGATGTCTCTGATATTTCTTC
TATTCTATTTTTTTTTATTCGTTTTAAGAAATAGAATTTTTGATAATTGAAATTTAGGTAAT

CATTATTTTAAATAAATATTTTATTAAAATTTCATTCGTTGTACTTCAATTACAATTATGAT
TTTATAATATCCTTTTGAAAAAAAAAAAATAGTTTTTCTCTTTATCAGCAAATGATAATAAC
GAGGAAAATTATTTATTATAAAAATTGGTCATAAACATTCGAAGAAAAAAAAAAGATATT
TCCAAATTTTTCCAAAATCAAAGTCAAAGATACCATTGAAATTATATAGAACGAAGAAT
ACATCGAATGAAAATGAAGAAAAAAATTGTCTTCTTAGGAAAATAACGTTCGATATTAAA
TAAACGAGACGACGATATCATTCGCTGTCCAGAAAAATTTTTTATTTCTTTCTGATGCAA
TAAACAACGAATCTTACATTCGATGGTATCAGTCGTATCTAGAAAATAGTATAATCGTAA
CATGTGTTCTACACTGCGAACGATACATAGTATGTACAGAAGATTCTGTCAGGACCAGA
ACGAAATTCATGCCATTGGAAACAAGAGAAGACATTGCGATCTCGACATGCCATCATC
GGGCTGTACACGATTTAAAAATGAACACACAACGGCACGCGCGAGTCGCATTGTCTTC
TTTGTCGACAATGATGAAAATTTGCACGAAACAACGCAGAATCTTTCGACGCGTGATT
CTCAACGGTGCGAAACAAGCGCGTAACAATGTCTAAAGAATGATACGATCCATTCTTTT
CTAAGATAAACTGACGTCAACTAGAAAATTGGCTAGATTTATTCTTCAGTATTTCCACTC
AGGCTCGGTTCATCATCGGTTGTCGCTCTCTTCCATTCGTTATCTTACGAATTTTATTGA
GGTATTGGTTAACTCACACCAATATCTGGCATGATCTCTGCACGTTCCTCTCTCCTCTTC
TTTCCCTTTCCTCCTTTTAAGTAACTTTATTCGTCGATCGCCATGTCGATCGAAGCGACG
ATCGAAGCGACGATCGAAGCGCCCTCATCTTGACGAATATTTTTTTACGTAAAATCTAA
CAGGAAGATTGGTGTGTTATACTGTTATATCCATTCTCAGCATCGATATCGATAAACACA
CAATTAACTACGAGATCGTGCACGTTTCCTCGAAAATGTGAATTTTCTTCGATGAACGA
ACGCGATTACTGCCCACTGGCGATCTCCTTCATACGGTTTAGAGCTGAACCTGCCTTGA
ACCAGGAAATTTGTTGCTCGTTCATGGTGTGATTTAACGTGATCTTGTCCACTTTCCCAT
CCTTGTGTTTGATTTCTGCTTGAACGGGCTGTAATAGAGGAAAGAAATTAGGATCAGAC
TCGTTAATAAATGACGACACTGTTAGGTGACAGAATAGAACCTTTCGATTGGAAATTTG
GTTGTTAGCCAAGATTTTTTTTATTCTAGAAAGGATATGGATTAAAATCGTTATATAGGA
ACTAAGGATTGCCTCATATCCTTGAATACTTGAATGGAATCCGATGATAGCTATATATCAT
AATGTTAGATATTTTTAATGTTTCTTGATCGACTATATATGGATGACAAATTTAAATTCAA
ATATATCGCGTTGCATATTATAACTCAATAAACTAAAATAAACTAGAAATAAAATAATAAT
CGAAAAGCATAAAGGATACATGGATGTTTGTATGAAAGAATGAATAGAATTTTGAACAA
TTAATTAAATCAATAATACATAAAGGAAAATGTAAGTAGTTGGGTTATTTTTCTCGAGAA
AGATAATTGAATCATTAATGAAAACGTACTTTTCCAGGTGCCAAATCGTTTAATCCAAG
AAGGCTGATCTTATCAGTAGGTTGAATCTTATCGTAATCGCCAGCATTGGCGAAGGTGA
GAGGCAACAATCCTTGTTTCTTCAAGTTGGTCTCGTGGATACGTGCAAAGCTCTTAACG
ATAATGGCACGGCCGCCTAAGTGTCTCGGCTCCAGAGCTGCGTGCTCTCGGGACGAAC
CCTCGCCGTAGTTCTCGTCGCCAACTGCTACCCATTTGACGCCATTTTTCTTGTAATGCC
TGGCAACGTCCGGAACTTTACCCCACTCGCCAGTAAGCTGGTTCTTTATTTTGTTCATC
TCGCCATTTTCTGCATTCACAGCTCTATAAAATTGGTCGAAATTCAATATATTTGTTTAAA
TTGTAGAACAGTCGAATAAGCGAAGAGAAGAAAGATCTTCAATCTTCAAACATTTACC
CTATGAACATATTGTTCGAGATATTGTCCAAGTGGCCACGATATTTCAACCATGGGCCAG
CAGCCGAGATGTGGTCGGTGGTACATTTTCCTTTTACTTTGATTAAAACGGTCAAATCG
GTGAGATCTTTGCCGTCCCATTTGTCGAACGGTTCTAATAATTGCAATCTCTCGCTCTTC
GGGTTCACGTCGACTTTCACGCTGCTGCCATCTGCTGGTGGAGCGTCATAAGTGTCTTG
GCCAGGGTCGAAACCGCGGTTTGGAAGTTCATCACCTGGAATAACATGATTTCTTGGA
AAATTTTTCCAATATTTCCTATATTGTTTCATAATGAAAGAAATTCTTCTTTCATTATGAA
ATTATCAAAAAGTACATTTCCAAGGAAAATAATGTTCGAGAATTGCCAGTATGTAAAAA

ATATATCGTGAAATATTTCATAAACGAGTTTCCTTCGAAAAACACACTTATCGTTGAAAT
AATTGTCTCAAAGATACCGAAGAACTTTGTTCTTTCCATAAAAAAGTGAATAAATAGAT
TCACGAATATTTTATCAATCTCTCGCTGTTTATCCACGAAATAATCTCGTAGAATGAAAT
AATTAACTACATTTTTTCTTCTCGTCATCAAATTAAATAATTTCAAAAACAGAAATTATAT
CAAATATTTCGAAATCTGATTCAATCTTCTACTATTAAATAAATAGTAAAATTAAATGTAA
CGTTGTAAATAACCAACAATTTCATTCAATATGCAGACATATTGATTAACTCATTACAAA
AATAAATATCACAGATTGTGACTCATGTTTAACTGTCTGCAGACTAATAATAATAAAAAA
GAAAAAAGAAATCAAGTAGAATTTCATTTGTATTTGAAGTTATTAATGAAGCCACGATT
TTGAATTATTCTTGAATTAATTCTTGTTCACATAAGTTGTGGTTCAAGTTCAAGATAATC
GAATCATTGTTTAAAATTATCTCGTAAACAATGAAATGTTTGTGATAAAATAATTCACGA
ATATATATACGCGAGTGTACGTCGTATTTACCGAACGGATCTTTAAGAAGGAATTCCTTT
CCATCCTTGGCCTTGAGCTTATCGGTAACCGGATTAAAGTCCAATCGGCCAGCGAGCGA
AAGAGCAGTGACGAGTTCAGGGCTGGTGACGAAAGCGTGTGTCGCCGGATTGGCATC
GTTTCGACCCGTGAAATTACGATTGTACGATGTGACGATCGTGTTCTTATCACCCTTCTT
GATGTCTTTACGATCCCATTGACCGATGCAGGGCCCGCAAGCGTTCGCCAACACGGTG
CCTCCAAATTTCGAAGAATTTCGGCCTAAATTAAACGAGGAAAATCTTAAGTTAAGTA
GTGATATTTTTTTAAATAATTTTATTATACATTAACGATTTCAAAATATTGAAGAATGATT
TTCAAATATCATTTTTCAAAAATCCTCTCCCCTTTGTAATATTTAGAATTTTTCCAATAGG
TTCTAACATTCTTTTTTTAAACTTGGCTAAAAATTTTAAAACGCATCTCTAATTGTACCC
GGATCGATCTGATTGTCATCCATTGATCCCCCGATCACGAGAATCGACAAGTCACTATTT
TTTCCCTTTCCCATTGTCAAAATACATCGAAGCCAAATTAAGAATTTGGCGTGGGTCGC
GTAACTTGTTTCTAAGTGACATGTACCAATGTCTGGCACGGGTTCAAAGTAATCTGTGC
AACTGTTACCGCCCAATCCTTTATACCAAAGCATCTAGTTCTAGAAATATACGTATATATA
CTTACTATTCCGTCACGCTCGATCGTGGCACGAATTTGTTCCGAGCCTGGCGTGACATT
GAACGCCGATTTCGCTTTCAATCCATTATCGATGGCTTGCTTAGCGATATTAGCGCAACG
ACCCATGTCCTCGTAAGAGCTGTTCGTGCAGGAACCGATCAGGCCGACCTTGATCTCG
TTAGGCCAGCCATTCTTCTTGGCAGTTTCACCTGGTTCGAATTAGTGCAGACAATATGA
GCTTTGGCTTTTTCTTCAAACTCTTTACCTCTTTCTCTTGGAATTAAAATTAACCCTCGT
GTAAAATATACATAGCAGGAAATTTTTACTAATCTTCTAATTACCTGAAGTTATTTTATAA
CGAAGAGATTGATCGATATTACAAGAGAATCGATTAAAAGAAATAACTGGATGGAAAA
TCCAATTCAATCGATTACTCACCCAACTTAGAAATGGGATGAGCGAGATCGGGCGTGA
ACGGTCCGTTCACGTGTGGCTCCAACGTAGAAAGATCGAGCTCGATGATCTGATCGTAT
TTAGCGTTGGCGTCGGCAGTGAGAAGGCTCTTCCTGTGCAGATCGGCCGCGTTTGCGA
TCTCGGATCGTCCAGTCGCCCTCAAGTAATCCTGCATACGAAAGTTGTAGGGGAAGAT
GGACGTTGTCGCGCCTATCTCGGCGCCCATGTTGCAGATGGTAGCCATCCCAGTGCAAC
TTATGCTCTCGACACCTGGCCCATAGTATTCGACGATGGCACCGGTTCCTCCTTTCACC
GTCAGGATACCGGCCACCTTGAGAATGATGTCCTTGGGACTCGTCCACCCCTTGAGGG
CCCCCGTCAGCTTCACCCCGATCACCTTGGGGCATTTCAACTCCCACGGTATGTTGGCC
ATCACGTCCACGGCGTCAGCGCCACCTACACCGATGCAGAGACAGCCCAGACCGCCTC
CGTTGGGCGTGTGGGAGTCGGTGCCGATCATCAGCAGACCTGGAAATGCGTAATTCTC
GAGGATGATTTGGTGGATGATACCGGAACCAGGGTTCCAGAAACCGACGCCGTACTTG
GCACCGGCCGTCTTCAGGAAATTGTACACTTCCTTGTTGATGTCCTTGGCCCGTTTCAA
ATCTTGCTCGCCACCCACTTGGGCCTCGATCAAGTGGTCGCAGTGGATCGTCGAGGGC
ACGGCGACTTTTGGCAGTCCTGCGAAGAGAATTCAGAATTTTCG

>novel_circ_001850
CGAGATCTATTGCACGGGAGAGCTGCTGAAGACGATACAGCTGGCTGAAATCTTCCCA
GACAGCAAGACGTTCGTCGATCTTCATCAGATGAACGACCCCGAAATAACTCTCTCCA
ACTTCTACAGTTTGATGAATGAAACGGGGAACAAACCGAGTAAAAGTCAGCTGGCGA
GATATGTTAATGAAAATTTCGCCTCGTCGAACGAGCTCGTGAACTGGACGCTGCCAGAT
TGGACGGAGAGCCCTTCGATCTTGAAACGGATCAACGAGGCCAAGTATCGCGAGTGG
GCGAAACATCTGAACGAAATTTGGAAGGAATTGGCGAGGAAGATCAATCCAGAGGTG
GCCGAGTATCCTGAGAGGCATAGTTTGATATACGTGGACAATGGATTCATCGTTCCTGG
CGGTCGGTTCAAAGAATTTTATTACTGGGACAGTTACTGGGTGATTGAAGGTCTGCTGT
TATCCGACATGTATCAAACTGCCAGGGGAATGATAGACAATTTCTTATACATGGTGAAG
AAATATGGTTTCATACCCAATGGTGGCAGAATTTATTACCTTATGAGAAGCCAGCCACCT
CTATTACATCTGATGGTCTCAAGATATTTGGACTTCACTGGGGATTACGATTATTTACGTT
CGATTATATCCACTCTGGAAACTGAATTTTCTTTCTGGCAGCGAGAGAAGATGATCGAC
GTTGAGAAGGATGGAAAAATATATAAAATGGCGCATTACGTGGTTAATAGTACCAGTCC
TCGACCAGAAAGTTACAG

>novel_circ_001851
CGAGATCTATTGCACGGGAGAGCTGCTGAAGACGATACAGCTGGCTGAAATCTTCCCA
GACAGCAAGACGTTCGTCGATCTTCATCAGATGAACGACCCCGAAATAACTCTCTCCA
ACTTCTACAGTTTGATGAATGAAACGGGGAACAAACCGAGTAAAAGTCAGCTGGCGA
GATATGTTAATGAAAATTTCGCCTCGTCGAACGAGCTCGTGAACTGGACGCTGCCAGAT
TGGACGGAGAGCCCTTCGATCTTGAAACGGATCAACGAGGCCAAGTATCGCGAGTGG
GCGAAACATCTGAACGAAATTTGGAAGGAATTGGCGAGGAAGATCAATCCAGAGGTG
GCCGAGTATCCTGAGAGGCATAGTTTGATATACGTGGACAATGGATTCATCGTTCCTGG
CGGTCGGTTCAAAG

>novel_circ_001852
TTTCAGGACATGGATGGCTCGTCGTCGGAGAACCTGACGGCTTGTCGAATCCTACCGC
AATATAATTGGCCGTTATTCCAGATGTCGTTTTTCCTGATGAGCTACCTGCTGCCACTGA
TGCTGATATGCTTCTTTTACATTTGCATGCTCATCAGGCTCTGGCGCACCGATCGCGTCA
GTGCCGAAAGCCGTCGTGGAAGAAAACGAGTCACGAGGCTGGTCTTCGTCGTCGTTG
GCGTTTTCGCCTTTTGTTGGTGCCCTATACAGGTGATACTAGTGACGAAATCGTTGGAC
GTGTATCCATTGACATCAGCGACGATAATGGTGCAAATAGCGAGCCACATTTTGGCATA
CACGAACAGCTGTGTCAATCCTATCCTTTACGCCTTCCTCAGCGACAGTTTTCGAAAAG
CTTTTCGAAAAATTATTTACTGCAGACCTCGATCGGAGCAAAACAGACAATTGGGACC
ATTGACGAAGACTACGAGAGCTGCCAGTACCGGTGACATCATTTAG

>novel_circ_001854
TACGTCGATCTCGACGAGTTTCTCTCCGAGAACGGTATTCCCGTGGATGGAGTGGCCG
GCGGCGGACAAGGGACGATGCAGAGCGGACAACTGCACAAAATGAACAACACCGAA
ACACCAGGGCACCAAGGCCCAGCCGGCCTCCACTTAGAGCCGGTCACCAAACGCGAG
AGATCACCCTCGCCGAGCGAGTGTTGCTCACCCGACACTATGAACCCTCCCTCGCCCG
CGGATTCCACGCTCTCGATGGCCTCATCGGGGCGAGACTTCGATCCACGCACCCGGGC

CTTCTCCGACGAGGAGCTGAAACCCCAACCGATGATCAAGAAATCACGGAAGCAGTT
CGTTCCGGATGACCTGAAGGATGACAAATACTGGGCGCGTCGTAGGAAGAATAACATG
GCAGCGAAACGATCACGGGATGCACGTCGCATGAAGGAGAACCAGATCGCTCTCAGG
GCCGGCTTCCTCGAGAAAGAAAATATGGGTCTACGACAGGAACTGGACCGGTTAAAG
AACGAGAACATGCTGCTGCGTGACAAGCTGAGCAAATACACGGACGTCTAACAGTGC
GCCGGGCAAGCAACAGCTCGTCGTG

>novel_circ_001855
ATTCGATGACGGTCACGACGGATTTCTCGATTTATCCGAGCTGAAACGCATGATGGAGG
TTTTGGGAGCACCGCAGACCCATCTTGGTTTGAAAGCGATGATACAGGAAGTAGACGA
GGATGGCGATGGACGCATTTCCTTCCGTGAGTTTCTATTGATCTATCGTAAAGCACGAG
CTGGTGAATTAGAACAAGATTCTGGATTGGGGCAGCTTGCACGTCTTACAGAAGTCGA
TGTGGATGAAGTAGGAGTAACAGGAGCTAAACATTTCTTTGAGGCTAAAATCGAGCAA
CTACGGAAGGCGAGCAAATTCGAAGATGAGATCCGCATGGAACAGGAAGAACGAAAG
CGAGAAGAGGAGGAGAAGGCAGAAAGACGAGCGCAATTCAGACAGAAAGCAGCTTT
ATTTGGCAATTGAAA

>novel_circ_001856
TTTTGTATTTTTCATTGATTAATTAATATTTTAAAGTTGTGTTGTAATTACAGTCTTCCTTG
TGATTACAGTATGTTTAATTGGAGTTTGATCTGTTGTTGTTGTTTGTGGATCGGCTACTA
ACCAAGGACTTATATCAGAAACTGCCGAATTATCATCCTGAAACAGAAAAAAAAAATA
TCATTTATACTTTAAATTTTTTATGCATAAAATTTTAATTAAAAATATAAAAATATAAAGA
CATTTAAAAAAAACAAGCACATGTTTGCATACAATTTGCTTAAGCTAAAGCATTTTTAA
ATGAAACAAACCAAAAACTGTTACAGTTACTAATTATCAAGCATTTAAGATTGAGCCAC
ACCTTGTGAGTAAGAGTCGTAGTAGTAGTTTGTATCTCTTCAGTTTTCACGACTCTCGC
TCCATTTTTTTCTATTTCCGCTTCAGCTTTCTTTTCAACCATAGGTTGTATTTCTTTAAAA
GGATTAAGCAAGCCCAAAAATGGATTTGAATCAATAAACGGATTTGTATTTGTTTTTTTT
TCTTCTTCTGTTTTCGTATCTTCCTCTGTATTTTCAGAGATATTATTAGTAATTTCGTTGTT
ATTATCACTAGTAAATGGATTGAATGGATTTTGAGATTGATTTGAAATTTTAGGATTCAG
TTGTGTTGTTTTTTGTTCAATTTGTCCCCAGCTATCTAGAGAAAAGTGTGTTACAGTCAC
GTTGTCGTCAGCAGATGGATTTATCATTGTAGTTCCGCTCACTGAAAGTGTCAGTTTATC
TCCTCCCTATATAAGAAATAACGCGAAAAAGCAAACAAGTCACATCTAAAGCTTAAAA
GCAATAAAACAAACAAAAATAGTAAAAAAATTATTGAAATAAATTATTTATGTAATAATA
CAAAATATATCTAAAAATGCAACATGAAAATAAAAATGAAATTAAAATAAAAATATAAG
TAAAAATATCTCGTATGTACGAATTTTTCAGATCTATTTAAATATTTGTCAGTGACATTT
ACATTTTATTACTACGTAATCCTAATTTAAATCGTAAATATAATCTTTTATAATGTATTTATT
TAAAAAATGTTTATAATGCAAAAATAATTTAATTAATGAATAGCATTCTAAAAATTGGAA
AGAACTTTCAGCTTTTTACTATAATAAATATATTTGAAATTTACTTATATATTTTATACGTT
AACGATATTATAAAAGTTTAGTTTTCTTACCACCGATACAATTGTTGCTGCATCAGAGTG
CTGATTGTTTTTATTAATAACTGTAGGTGTATCCATGGATGTAATTGAAACTAAATTTGAA
TTTTTCCCTTGTTCTAAAGACTTTGCTTTTAATATATTGCATTTTAATGATTCTAGAGGAA
TAGGTGTACGCGGAGGTAGTGAAGAGGTACCACCAACTTGATTATTTGGTACTAAGGAT
GTTTGATTACTTATATTTTCGATAATTAAACTCGTTTTCATTGGTTCTGATTCTTTTTCTGT
ATTAACAATTGTTTGATTTCTAGGTTCTAAAGGTTGCTGGTCTTTGGCCAGAGATTTCAA

TAAGGAATCTACTAAAGGCGAAGGCGTAGGATGTACGCTAGAATCTGAAGATTTTGTAC
TACATTGACTTGATTGACGTTGTAAAGATTCTTCTTCATCTAAAAAAGAAACAAAAAAG
AAAAATTTATTTGGAGAAAATTTCTATTTATATAATTATTCTGAATTTTTAATTATTGAATT
ATTTAAATATTCGTTAACAGAATTAAAATATTTTTATGCATACTAGTAATCACTGGAGCTG
ATTGTGGTTGCTCCACTTGTTGACGCTGACGTTCTCTTAAAACATGACTTTGACGGCGT
GCGTATCTTTGACTTGGTCGTCTTTCAAACTGTACTGTTCTACGTGCTCGATTCAATTGA
GTAGTTTGAAATTCTGTCTTACCACTATAACGAAAACGCGAACCCATACGAAAGAAGTT
TTGACGTCCACTAGCACCTTTAACAGGTGCACGTAATCGAAAGAAAGCATGATGTTCT
ACAGCACATTTCCATAAATGTTTACAAGCCTTTTCATTAACAAGCCTAAATACAAATGTA
TGTTCTTGTTCTCCTCTTCCTTCATCATCTTCTTCTACTACAACTAATGTTAATTTCTTTTT
TTTAAAATCTAATCGACCAATTTTTGGCCAAAAAAATAATCCTATTTTTTGTGTTCCTTC
AAAAACTAGGATTCCAGTTGGCGTTAAACCCAAAGAATATTCACATGCATCTTTACCAA
GAACAGTATGCATATCTACACCATACATATCTAACCATTTCGCTTTACTGAGATAAGCTG
ATTCAGCCTGTGCTGGACTAAGACCAGAACATTTAGTATATTCTTCTAATATTTCAAGTT
CCATTTGCTCTGTTTGACCTGGTACAAATCTAAATTCTGAAACTGTAGCTGCACTATGTA
TGGCAGGATCATAATCACCAAGTTCAGCTACAAATATATACAAAATTTTAAAAAATATTA
AAATATAAATTTTTTTTAACTTACTCTACTTATTTTTTTCTTTTTATTTTTTTAATACATACA
TTGAAGAGCTAAAGCAGCCAACTGTACAGCAACTTGATGAGGACAATGAAGTTTTCCT
TCCAAAACATCTTGTTTAAGTTGTAAAAAGAACTGATATCTTGTTAGTTCTTCACGTAA
AGTATTAGGTTCTGATGAATAAAACTTTACTTTAAGCCGTAAAGTATATGGTGGTCCAAC
TATATAAAATATTGCTTATTAATATTAAAGAAACAAATATAAAAGGTTTTAATAAATAGTT
TAAAAAATGATACATTTATTATTTAATAAATAAAAAAGAAAAACTATATAAGTATGCAAA
AAACATACTTTTTACTTGTTTTTTTATTGGTTTTGTAGGATCTAGCCAATGTTGAACATTA
TTACTATCTGTATATTGCAATCCAAAATAATCTTTTTCTATTAAATCTAAGCTATAAAATAC
TTGTTCATATAAATCACTAGCCAATGCTTTTTTCTGTAACACAAAAATATAATAAAAATAT
CAAAATTCATAATAAAAATCTATATATAAAAAAAATATATAGAAGAAATAAATAAGAAAC
TGGAAAAATATTATTTTTCTTTTTTTTTATTTATATTAACTATATATTCTAACGACAAGAAA
TTAACATTTAATAATACTGATTTCATTATGATGACATTTTTCAAAAATTTATTTTTTAAAA
AATATAAACGATAAAAAAATCTATTATTTTTTAATAAACTATTTTACTTACTGATAATTCTA
TAGGCAAATCTGTGCCATCAAGTAATACAACTCTGCATTGAACAACATTTTTATTACTTA
GTAATCGTTGGTTTTTAATTTGAGAGGACGTAGTTTGTCCACTAACACTACGAGCTCTG
CGTCGCGATAGAAATCTTAACATTTTATTTGACATATGTCAGAATAAATTTGCTGGTAAG
CAATAACGATATATTGACCCAAAATGCTACATTCCTTGTTTTACATAGATATATGTTTTTT
CATGATACGTTGCGTCGGTTAATACGCCCACTTGTACACGAAACACAAGTATAATATATT
AAAACGATTAGAAAAAAAATGTTAGTTAAAGGCACATATTAAAAATAATATTACAAAAA
AAATTTTAAGTTACATTTATATTTGTTGACTAGACATAATAATTCGCGAGTAATCGCGAA
ATTCAAAGAATCCGGAGAAGATACACATCTCTCGGTTACAATAAAGCTACGGATATTTT
TTTTATCGCTAATCAGATGGCGCTAGTCTCAATTTTGAGAATACAAACCATAGACAAAAT
ATAAAATAGATCTACTATCTTAGAGAATTTTGTGTTTAATATTTTGAAATATATAAATATCG
AATAAGAAATCATAAATCATACAAAAAATTATGAAAAGATAAAAATATATTATTTAGAAA
ATTAATTAATTAAGATGAAAATTTATAAATTGAATTAATAATTATAATGTCCTTTACCACAT
AGAAAAGTATTTCTAAAATAATTAATGATAAATTATCAGTACTAAAATTTTTTATAAAGCT
AATAAATATGTAAAATTATAATATTTTACATATTTATTAGAAATTGTTATAATCGATAAATTA
TAAAATTAATCAAATAGAAAATATTAATAGAATATTAGAAATTATAATATTAAAATTTTTC

ATATTTTAAAATATTCGTTTAAAATAATTATGATATAACCATCTTTTATAAATTAGTTCCGG
TTTGATTGTTAAAGTTTTTAATATGTTGAGATTATGTTCTATTCGATTTTGTGTGTAATTTA
AAATTGTGAATAAATATCGTGTTTAAAATATATATTATTCGCTAATATTAATTTCTTTTATTT
TTAAGTATGTATATTATGTATTGTTATTTAAAAGTGCTTTAAATACATTTATTCGTAATAGT
TTCGCACATCTTATTTCGAGTGTCATATACTTCATTATTATTATGAAAAAATGAGAATTTA
TTGCTATATCAATACTAAGTTAATATTCTTTTATGAATGTCTAATTTATTTTTAAAATATAA
ATGTTATTTTTTATTCAGTTTTCATTATTCCAGTTTTAATTATATATTCTATTTTTAATTTAAT
ATTTTATAATGTGCATTAAATATAAATTCGATTTTCATTTCATATAAAGCAAAAATGAGAA
ATTATTTAAATATTTTCGTCAATCAAATTCAAAAAATTGATATGTAATGAATAGAAATTAA
AACTTGAAACTAAATTTTAATAAAAACTAAATTTTAATAAATTTTTAAAGTATAAATTTTA
TACTAATAATGATTTTTTTTATAAAATGACATTTAAAATATTATAACATTATAACAATATTAT
ATAAATACAATATAAATTGAAAAATAATAATATAAATTTTAGTTTTAGTAAAAATATATTG
ATTATTACACATAATATCATAATGAATTATTAAATCTATAAATTAATTATAAAAATTGATTAT
TATAAATCCTTTTGTGTTTATTTTTTACAAATTATTTATTTTTTAAAATGACTTAAAATATT
TTAAGATTGTATTATTTTAACATTGTATAGATAGAAAATTATAATATTAATATTTTATTATTA
TTATTAATTATATAATTAATATTTATATATATCGATAGATTGATTTATAATCGGCAAAGTACA
TATACTTTCGCCGAACTTCGTAACCATTCTTCTTCCATAAATATAAAGCAGGTTGCGTGA
GAACGGTCATTTATCGACGAACTTAATCGAGAGAAGGATGTTACAACATTTTGATTAAA
TTTTTATTTATAATATAAAGAATAATTTTTTTTTGTATTATTTACTCTTATTTAACAACTCTT
ATTTTACATTTAATATTACTATAAATATATTATAGATATAGTATACATATACTATTATACTTAT
TATTATTTAATTTATTTTTTAATTAATATTTAATAATTAATATTTATTTATTATTTAATTAATAT
TTATTTATATATTTATTTTTTTAAACATTTATTTCTTAATTTACTTATTCCTTTAATTATTCAT
GTACTTATTTATTAATTTTACTTATGTTTCATTTATTTATTGATGATTAATTTTAACATTTAT
TTATCATTTATATTATATTTATATTTTAATTTATCATTTATACTTTTTTTACTTTTAATTTACAT
TTTTATTTCCATTTATTTCTTAATTTATATTTTTATTTATTTATTTATTGATGTATAAATTCAT
TTTTTTTATAATGTTATATTTTAATACATAAATTTTATTTTTTGTTATGGGTCTCAATGTAGT
CACCTCCTCGTTGGTGAGACCTGGTAATTGGTATCAATTTTTATATTGTATTAATTAATTA
AAAATACATTTGAAAATTGTATTACAAATATTAGATTGTATTACTGAATTATATAAAAAAA
TTAATTTTGAAAATTGATGCCAAGTTAAGAACTACACTAATTTCTTTTTTTATATTTCAGA
TATATTTTATATAATCTGTAATTTCTATTTAATTTATCTAGATTTTAATAATGAATAACAAAT
AAATAACATAAATTATTCTAATTTCATAAAATTCATAAGCATTTTGAATTATTAAGGATTA
TTTTATAATTCAAAATAGTATTTTCTTAAATAAATGCTAGATAAAAAGAAAAAGAAAAAT
TTTTGAATGTATAATTATTTTTTTTCTATCTTATCCCGTCCTATTAACATAATTCCATTTTAA
GGAAATTTGTAATGTATTAATAGAATCATTAAAACATCATTAATAGAATCATTAAAACATC
ATTAAAACAACAAATATTTGTTGTTGTTAAAGTTTTTTGAATCCTTCAATAAATAACGCG
AAATTAGAGTCAGTATTTGTTCGTTGATAATAATTTAATAAGTTCCGTTAATAGTATTTTT
TTATAAGACATAAGTGAATAGAAAGTGAATATAGATAAGATAAGAATTGAACATGAGCA
CCATCTCTTGCAAGCTAGAAACATCTCTTTAAAAAAAATATTTTTCTAAAGGCACTAAGA
GATGGCAATAATACTTCATTCTTCCTCTACCTTATCCTCTTTGGAGAAAAATCTTATCCTG
TGATTCAAATACTTTTAATTTATAATTATTAACTTTTGTTATATTCCCTTTTTATGTTTTCAT
ACTTATTTATTGTAATTAATATACAATTTTTATTTCAAGTTCAGTTAGATTTAGTTAATTGT
TATGTTACTTTTTTTTTATATATCTCTTCTAATTCAACAACAATTAATATTTATGTTAAATCTT
TTTTTTTATGTTTTAACATTCTTTAAATTTTTTTAAATTTTAGATTAAATAATTTTTCCATG
CGACAATTTTTTTGTATTCTTTTTGTATATTAAAATGTTCAATTCTTTTCTTATATCTTGCA

ACTTTGTTAGATTTAGTTAATTATTATGTTAAATTTCTTTTTTTATGTTTTCTCTTTTTTTTC
TTTATTCAATTAACATTATTGTTGTTAAATTCTTTTTTGATATTTTAATTCTTTTAGATTTTT
TTAACTTTGAGATTAAATTCTTCAACTATGCTTCAATCTTTTTTTACTTTATGGTACTTAAT
ACTTGTGTTGCATTCTCTTCTTATGTGTTTATATATATATTTATGTATTTGCATATTTATTTAT
TTTTATATTATAATTATATTATATATTTATATTAAATTCTTTCACATATTGCAATAACAATTAA
TTAAATCTTATACTGCTGCAACATGTAAAGAAAGAATTTAACATTGTAATACATAAATAA
TGAATAAAACACAAAAAGAGTATATAATATAAAAATTAATTATCATAAAGTAAAAAAAA
TTGAAACATTGGAGAGGAATTTAACCTCAAAGTTAAAAAAAGTCTAAAAGAAATGAAA
TATAAAAAAAGTATTTAATGGAAATATTATTTAAATTAAAGGAAATATTTAAACAAAAAC
AACAACAAAAATTTTATATTTTATATTTTAGATTTCTTTTATATTTTAGATTTTTAGATTTA
GATTTTTTTTTGAAATTAAATTCTTTAATTATGTTTCAGTCTTTTTTTTACTGTATTATAAT
TGACTTTTATATTACATTCTCTTCTTATGTATTTATATATATTTATTTATTCATTATTTATATAT
AAATAAAAAATACTTATATATAAATAAGAATCATTATGATGCATAAATAATAAAAAGGGA
ATATAATATAAGAATTAATTGTTATAAAGTAAAATAAGATTGAAAATAGGAAAAGAATTT
AATCTGAAAGTTAAAAAATCTAAAAGAATTGAAGCATAAAAAAAGATTCAACAGAAAT
ATTATCGAATATATCAAATATTAACATAAATATTTAATATATTAAATATTTATATATTAAATC
AAAAAAAATATTTGAAAAGAAAAAAATAAAGTTATTTATGAAATTTACAATGTTTAATT
TTTTGATTTAAATGTTTTTTCTAATTTAATTAACACTTCTGTTAAATTCTTTTTTGTTTTAG
ATTTTTATATTCTTAGATTAGGTTTTTTTTAACTTTAAAGTTAAATTCTTCAGCTATGTTTC
AATTTTTTTTTTTACTTTATTATAGTTAACTTTTTTATGTTATATTCTCTTCTTATGTTTTTAT
ATTTTATCATTATTTATATATATCATAATAATAAATTCTTTTATATGATATAACATAATAATTA
ATTAAATCTTACAATGCTGCAACATATAAGAAATATATTTTACATTATGATAATACATAAAT
AATGAATAAAATATAAAAAGATATTATAATATAAAAGTTAAATTATCATGAAGTAATAAA
AGATAATTAAAATATTGCAGAAGAATTTAACCTCAAAGTTAAAAAAGTCTAAAAGAATT
GAAATAAAAAACAAGGACAATAATAGAAATATTTAAGAAAAATATTATCTAAATCAAGA
GAAACGTTTAAACAAAACTTAATTAAGCTTTTTTTTTTATATATGTTTTTTCTAATTGAAG
AATTTAACTTATGTTATGCTTAACATTTATATTAAATTCTTTCTTTGATGTTTTAGATTTTT
GGGTTTTTTTTAACTTTGAGAATAAAATTTTTCTCTTATGTTTCGTTTTTATTTTTTATTTT
ATAGTAGTTAATCTTTTGTTTGTGTTACACTCTCTTTTAATTTTTTCATTATTCATTATTTAT
GTACTATATTATTATTAATTTTCTTCTATATACCTATATATATATATATATTTATTCTATATATATAT
ATATATATATATATATATATATATATTTATTCTTCCAATTAAATTAATTTATGTGTGAAATTATA
TTTACACTCTATGTTTTTTTAGATTTATATAACTTTAATAATATTTTTCCTTTATATTTTAAT
TCTGTTCAGATTGTGTTCATCTTAATTTAAAATACCTTACTTGTATTTCAATCTTTCCTAA
TCTCTTTGAATCTTATATTATATATTTTTTCTCGTTTTTTTTCATTTATTATTAATTAATTATG
TTAAATATTCTTTTGTTAATTTTTACGTTGATATTGTTTTTTTTTTTAAATATAATTTAATTC
CTTAAATTTATTTATTTTAAATACAACTTCTGAGTTTTGATTATTTTAAATTCAGTTATTAA
ATTTATCAAATTCAACTTTATATTGTGTTCTCTTTCTATTTTTTTCACTTTTTATGAATTTAT
TTAAGTTTTTTGTTAAATTTCTTTTTTGTTTTTACTTTTGTTATTACTTCTATTTAAGTTTA
ATTAATCTTTTTATTGAATTCCTTCTTTGCATTATAAGTATTTTATTATCTTGTTAAATTAGT
AATTTTATTAATAATTTTTTTTTCCCCAATAAATAGATCTTTACAGGATTCCAATGTTACA
AGCAACAATAATTAATTTATATTATATTTCAATTTTTCGCGCGATTTTTGAATGAAAAAAA
TAAGCATTATGAACCAGCCAATCAGAACATCTCTTAATTCGAAACGCGGGACGAATCG
GTGCGCATTGCAGAGCGCAACGCAATGCGCATCATATTGCGCATGTCGTCAGTCTCTAG
TTGAAAATTGTGTGGAGTGGACGTGTCGTCGCCGAATTCGGATTATTCGGTAAGTTATT

TTACTTTTATTATTACCAAATTTATCAATCATTGCTAATTTAAGTTTTAAATAATCCATTC
AATATAGTGAAAACGAAATTAATTAGTTTTTGCTTATCTTTGTGTCTTTTCCATCCATTG
TTCATTTAGAGTCTATTGCGTCATTAGCGTCTCGTATCGAAATATTTCGAGACAGAATTT
GAAATCATATATTGGTTTTAAATATAATTTAATCGTATACGTTTATTTTACAATTTCTTG
ACCTTTATTTTTTATCATTTCAATCATATTATAATAAAAGAACATTTTTACGGTTTATTACG
TATTCATTTCGTAAAATCGAAGTATTATTCTAATATCGCATATCTTTTCGAATGTATATATAT
TGTGTATAACGTCTTCCCTCGTGTATGTTATTGTTTACTTTTTTTGGTCTATCGCTTATAAT
TTGCTCGAGGCGAGAAACATTTCTTATGATATTTCACTTCCGAGAAATTGGATTTGTGTT
CAAAACATGTTTCGACTTGTCGAATTGTCGAGCGTATCCCCTCTCCATCTTGTTATTCGA
TGCGCGAAATGGAAGCGCAAAGTAACATTGTTCGTCGATTCTTCGTGACAATTAGACG
TTTGAAATTTTCTCGAGACATCTGTTCTATTTATTGCATAGTATTTTAATATTATTTTTTTT
ATACTACTTTTGTTATTATTCGTGCGTTAATTAATACGTCTTTATTCAATGTTTTATTACAA
TTGATAGTTTTTTGACAACTAAATAATTTTGAGTTCCTCGTTTATCATATAATTGGAGTGT
CGAATTACAAGATGGCTTATATCGTGAGTTCTTACTTGATTTGATTGTTTTGATACTTTAA
TATAATCTAATTTCGTACATATTATTCATTTATTTAGAAGTTTTGATTTTTCTCTGCGCAGA
TTTAATCGAGGGGGAAAAAGGCGAGAAAGAGGCGGGAAAAAGGCGGGAAAAAATTCC
AATATGGACGCCATGTCTTTTTTGGTGACTTTCTTTGGTGGTAAAACTTTATTTTATTTCG
TATGTATAAATTTTATTCTGTTTTATTGAATTATTAGTTTAGATTGTTGTTAAGCAATTCAT
AATAAAATTATTAATTCTAATTAAAGGAAAGGGAAAAAATTAAAAATTTATGAAAAATTT
AACGATTTATCATTGGAAATTAAATTTTAAAATCTGATAACGAACATTTTATTAAATTTCA
GATATTAATAAATAATTGATAATATTTTTATACATAATTTATGATCAGTTAAAATAAAAAGG
GAATATTTTCAGAACAAATGCTCGATAAGAAGAAGAAAATGTCAAAAAGATATTACTTT
TTCTTCTATCATTCCCCACCCCTCTACTAATTTAATGTTAATTTCAGAAAATATTTGTGAC
CACGCGCAATGTGGTTAGTAGAATCAGTGTTTGCTTCGTCATAGTATTTTGAATATTTTT
TAGAGCACAGCAGCTCTGATTAAATTTAATTAGAATAATGAAACGCGAAGGTAGAATCA
GTGTTTGTTCGCCGATTATAATATATACGGACGCGTTATTAGTTTCTTATTTTATTTTTTTT
TTTTTTTATTCTCAATATTCGTTTTCGGTACAGAGTAGAATCAGTGCTCCTCTCAAGAGA
ATAGGGCGATCATCCACCACGCGAAGAGGTAAGAAAAATCATTAGATTCTCATTTTTTA
GATTTTAATCTTTTATATTAGAATTTCATACTTTTATTTATATTAAAATTTATTAGAAACTTA
ATTTTTTTTTTATTAATGTTTAAAGTTAAATATAGTTTTATTTTTTATTACTAATCCAAATTT
TAAATAAAGAATATTATATTTTCAAGAAAAAAATTACAATACAATATTTCTCATTTTATTT
CACAGATGCTGATGCTGATGATCTTTATGAAGATTTTATTTGTGACCACGCGCAATGTGG
TTAGTAGAATCAGTGTTTGCTTCGTCATAGTATTTTGAATATTTTTTAGAGCACAACAGC
TCTGATTAAATTTAATTAGAATAATGAAACGCGAAGGTAGAATCAGTGTTTGTTCGCCG
ATTATAATTTATACGGACGCGTTATTAGTTTCTTATTTTTATTTTTTTCTTTTTACAAATATT
CGTTTTCGGTACAGGGTAGAGTCAGTGCTTCTCTCAAGAGAATAAGGCGATCATCCATC
ACGCGAAGAGGTAATTATGCTTTTTTATATATCTAAATTTTTATTATTCTAAATAGAATACT
TTTAGAATACTTTAGTTAACTAGTAATTTCAATATTAAATTAGTTAAAGTAATTTCAATAT
T

>novel_circ_001857
GCTCGGCGATCGAAAAATTATGCGAGTGCGCGACGGAGGACAGCCTAGCTGACCGAG
GCTCCGTGGTGAGGGCCGCGAGATGCCTCCTCGGCTCCGTGACCAAGGTCCTCCTCCT
GGCCGATATCGTCGTGGTGAAGCAGCTCCTCCTCGCGAAGAAGAAGGTCGCCCGTAGC

CTCGGCCGCCTCGAAAGCGTTTCCAACTTCACCGAATTCGTCAAGGCTTTCAGCCAGT
TCGGGGCGGAAATGGTGGAATTGGCGCATTTGACGGGTGACCGCCAAAATGACTTGA
AAGACGAGAGGCGAAGGGCGCAGATGGCAGCGGCTCGCCAAGTTTTGGAGAGGAGT
ACCATGATGCTGCTCACGTCCAGCAAAACCTGCCTTAGGCATCCGGAGTGTCCGTCTG
CCAGAGAGAACAGAGACACCGTTTTCTGCCAGATGCGAAGGGCCATGGACTTGATACA
TTACGTGGTCAAAGACGGTGTCCTCGATTGCAGCGAGTCCCAGTCTTACTCCAATTCCC
AG

>novel_circ_001859
AAACTCGAAAGAACAAAAGACTCCATCGCGCGACCGAAACGGAAGAAAAGAAGAGA
AAACGGAAGAGGAAAAATAAAAAATTCAGAGAGAGAGAGAGAGAGAGGGAGGGAG
GAGAATCACCGGTGAGAATTGCTTCGTTTTTGCCACTGGCATTCATATACAACAAGCTC
GTTACTTAACACCTTCGAAAGAAGAAGGGAGAGGAGAGGGGGAGAAGGCATTAAGTA
AAAGCGGGCGGTGCCACGGAGCGCGGGATGGGAGAGAGAGAGAGAGAGAGAGAGA
GAGAGAGAGAGAGAGAGAGAGAGAGAGGAAGGGAGGAGGGGGACACGGCCCCACT
CATTCCTCGGCCTGTTAACTTTTCAACGAACCTTGCTGCCAGACTCAAAAGCTCTCGGT
AAAGCGTGATTTACACGTCACGGAAACTTACCCCGTATTTTCCCCTCCCCTACGTCCGC
CATTTCCTCGGCCAACCCAACTCGGCTATGATTCTTTCGTCGAGGTAAAAATTCTGCCC
GTTTCCCTCCTCGAAGAAATAATTCGTTCTCATAAAGAGAACTTCGTGCACACGGATGT
ATCGTTTACCGGAAATAATTCAAAATTTGCATTTATCGAGTTTTTTCGGGAAGATGAGGA
TGATGTATCGTTGTTGTTGCTTAGATCGCGAGCGAAGGAATTTTTAAACTGCGAACTTT
CAACGTTTGACATCTCGTTTGATGCGTTGATATGCATGTAGAAGTGGAGAGATTTTTAA
GTTTTACGCAAGTTTTATACGAATGGATTTTCTCTCTCCTTTGATTTAATTTCGTTGGTCT
ATTTTTTTTTAAAACTATCTTTAATTTTGCGCTCGATCCAATAGCGCGATTAATTCGAAAA
CATTTGTGAAGTTAGATTAATATTAAATTGGGGCGAAGACGGGAATTCCGTGAGCTATA
ACGATGCCACATATGAATCTTTGTGCAAGTTTAATATGTTGGTTAGGGATTTGCGTTAGG
GCGTGGCAATTCTAATTTTACCAGGCCACTGGATATTGCGCAAGCTGTGCCCTGTGTGT
ACAAACTCGTAACCAACCACCCAACTAATTTTTAGATTGAAGTTTATGTTATTGAATATA
TCCCATGATAGATGTTGTGAAAGTTATTAGAATGTTGCAAGATATTCCAATTTGGCGGTG
TCTTCCGAGTCCATTTATCCATTAAAAGTATTGTTCTCAATTGATAATCATCAAATTGATT
TTTACACGGTAAAATCGTAATTAAAATATTCAGAAAATCATATCATTCGAAGATCCTTTT
TGTCTCGTTCCCGGTATCTTAATTAATTATCGAAATACAAAGACTTAAAGTATCCATAGA
AATTAAGATTTTACTAATTGACTAAAAACTTAACAATTTTTCTTGGAAATCGCGTTCTTC
CAGGAATCTGGAGTTTGAGATTAGTCAGTGAGAGGTCCGCAAATAACAAAGACGATAA
ACGATCGAAAATTAACTTTCTCTTTACGCGACGATATCTCGGGAACCGGAAATCGTATC
GAGATAAACGAGAAAGCGTTTCAAAGGGCAAGGTTCTGTCCTTCCTTATGATCGTTCA
CTCGTTGCCCGGAAATTGTTATCTTGAGAATTATAGCGGTCACAAAATTCTCCTAATTTT
AACAAGCCTAATTTGCAAAGTCTTTATTAAAAATTACGGATGATATGATTTCGAATGTTG
TATGAATTGAAAAGCATTTCAAAGTTCCGTTCTGTTTCCAATGATGGCTCACTTCGTTG
ACCGGAAGTTACTATGTTACAGTTTTAAAGTTTTCCTAATTTTAATAGGATTTCGCGAGA
CTTAAGGTGATTAAAAATTCAAATTGAATTTTTCCAATTTAAAAGTACAAGGAGAATAA
TGTATATGACGGAGAATACGGATACGGTAAATTTTTAATAGATAGAGGCAGGGATGAAA
AATCGAACGAGCCGCTTTGCATGTTTATTGTCCGCGTGATTAAAGGCAAGAAAGTTTGT
ATTAGCCATTAAAAAGTCGGGGGGGAGGAGGTACGAGTTGCAGAGAGGATGGAGCAA

GTTCGGTTGGACTTTAAATGAGAACAAGTCGGCGGGTTAAAGCGCGATTCGCTCGTTG
TTGCACGTGTTTCGCGTGGGCAATAAGCTTGCGTTAAAATTGATTAAAGGAAATTCGTA
TAACGATTGCGATGCGCGCGAAGACGTTCGAAAATTATTTATTTTATACGTACGGGGG
AGATAACTCTCCAGTTTCGAGTAGTATCGAGTTCTGTTACATATTTAATTAGCACTCTCG
TGATATCGAGAGTGTATAATTTATAATAATCTAGAAATTATTGAAATTCTCTCTCTCTCAC
GGATATTTACAAATAATTAATCTTCGTTTCAAGAATTTTAGACTCGACTCGGTAATTTGTT
ACATGTAAAAAAGAAAAGAATTAATTAATCCAATTTTATTTTATTTATTTATTTTTTTCAA
AGTTTCTCCTTAATAATTTCCTTTAATTAAATAAATTATTAAACGGGGATGATCTGGATTT
AATTTAATTTATAATATCTTGAAACTTACGCTTACTATAACAATTATTTCCATTTGTTTGTT
ATTATTGATCGTTAAAAGCTGTTGCAATAATTTACCCACTAATTTGTTTACATATTTTGAT
TATAATTAATACAATATTGTATTATAACAAAGATGCAAAAGTAATATTACACGCTGTGAAT
TATGAAATAATATAATTTGATTCGTTTCTCACACTATCAACAACAAGGGACGTATTATTTT
CCATCGCCATTATTACTCGCCGTTATTATTATTATTATTATTATTATTATTATTATTATTA
GTCGGAGACTGCAATCATTCGAAAGATACGTCGGATGGGTTTTCTTTTTAAAGGGACA
AAGGAAATGTAAAGGGGGCAATATCGAGAAGGTAACTCTTTATTTTCTTCCGTTCTCTT
GCCTTTTACACAATAACTTTTCGTTTCTTGTCTACTCTCTCTCTCTCTCTCTTTCCTTACG
AGAGAAATAAACGCTTCTTTCTGCAATAACAAGATATAATAACGCTGTATAAGTAGGAT
CGATTGACTGAACAGTGGAGACGTTGAAAATTATCGTTCGATGATATGAAACGTGAGTT
AATAATATATCGTGGGGAGAAAAATGTTTCGTCGTGATATTTAATTGGACAATTAATTTAT
ATTAACATACTTTTTACATAACAATAACATCGTAACAACGAGCCAAACTGATCAACATC
CAAGAATGGTTCGTACAAAAATTTCTATCGATCTCTCAGATCATCTGAGATCGAACAAT
TTTTAAAAGTGTTGATTAGTTTGACTTGTCTACTCGTATTATATATGCCAGTAAAAATTAT
TAAATATACTGCAACACAATTGTTTTTTCTATCGAAACTTCGTGGTCTGGGAGTGAACG
ACCAGGCAAACTACGTGTATTCTCAAGCAATAACAGCGCAAATGTGTGTTAATTCCCCA
AGAATACGCGTCGCCAACCTGGCCTATGAAGTCTATCCTTGATCTTCCTTCTTTCTTCTT
TCTTCTCCTCCTCTTCCCCTCTTTGACAATTTCTTTCTTTTCTTTTCGTCATCGTACTAGC
CGATTCCGATTCCCTCCGTTCTCTGTAAAAATTTCCATCCCAATTGCGGAGAAAATGAA
AATTAAATTATACAATTCGCGATATATTTCGAAGGAGAAATCGTTAAAATCGTTCTTTCG
TTTCTTTCTATTCTATCATTTTTCAAAATAATATAGTTTGGAGTTGATCGGTGAAAATGAA
AATTAATACAATTCACGCGATATATTTCGAAGGAGAAATCGTTAAAATCGTTCTTTCCAT
TCTTTCTATTCTGTTATTTTTCAAAATGATAGTTTGGAATTGATCGGTGAAAATGAAAAT
TAATACAATTCACATGATATATTTTGAAGGAGAAATCGTTAAAATCATTTTTTCCTTTCTT
TCTATTCTCATTTTTCAAAATGATATAGTTTGGAGTTGATCGGTGAAAATGAAAATTAAA
TTACAATTCGCGCGATGTATTTTGAAGGAAAAATCGTTAAAATCGTTTCTTTCTATTCTG
TCATTTTTCAAAATGATATAGTTTGGAGTTGATCGATGACAACTTCCAAATTTATTATTTA
TTACAATGAACGCGGTACGGAATAATTGAATAATAGTAGTAGTATTAATCACTCGAGATG
AAACTATGAAAAAAATAATTAATCAAAATATTGTTTTTTCAACAGCGACCGTATAAAAC
GTAGGCGTGGACAGGGCATAAAACTAATGGAACACCGTTGTAGTCCCGAACAAGGAC
TCCTCTCGTGAAAGGTTCTGCTCCTGATGTACGCGCATATCTGCATATTTCCGCACTCGT
GTGGAAACGCGTGTTCCCCACGTTTCCGCGATTTAACCGTATCCGTATGGCGGAATGGA
CCGTAAAGCGATGAAATACGAATGGATATCACGAACGAAGGAACGAAGATGAATATTAT
ATTGGCGTGATATTTCTTGCGAGTTATAAATATCGAATATCTTTCCAAATCCAAAAATATT
AATTCTCTCCTCCTTTCCATCTTTAGAAAATCGAAATTTGTTATTCCGTTTGATCTTTTTC
ATTTTTCAATGATTATTCAAAGTATTCTAATTTCGAATCCAAAAATATCGATTCTTTTCTG

ATTTTTGAAGAAGAGAGATTTTTAGGGTTTTATGTTTAGAAAATGGAAAATGTATTTCAT
AAATTTCAAAAAATCAAAAAATTGTACGATTTCAATCCAAAATATTCTAATTCCGAGTC
AAAAAATATCGATTCTCCCCTAATTTCTGAAGGATCCATTTTTAGAAAATCGAAAGCATA
TTCCATTTTATCTTTTTCAATCATTGTCCAAATCCAAAATATTCTAATTCCAAATCCAAAA
ATATCGATCTCCTAATTTCTGAAGGATCTTTAGAAAATCGAAATTCCATTTGATCTTTTTC
ATTTCAATTATTGTCTAAATCCAAAAATATCGATCCTCCCTGATTTCTAAACGATCCACG
ATTCCATCTTTACAAAATCGAAATTTGATCTTTTTCATTTCAATCATTGTTCAAATCCAAA
ATATTCTATTTAATTTAATTTTCAAATACAAAAATATCGATTCTCTTCTAATTTCTGAAGGA
TACCATCTTTAGAAAATCGAAATTCCATTTGATCATTTTCATTTCAATCATTGTCCAAATC
CAAAATATTCTAATTCCAAATCCAAAAATATCGATTCTATCCTAATTTCTGAATGATTCCA
TCTTTAGAAAATCGAAATTCATATTCCATTTGATCTTTTTCATTTCAATCATTATCTAAAT
GCAAAAATATCGATCCTCCCTGATTTCTAAAGGATCCACGATTCTATCTTTAGAAAATCG
AAATTCCATTTGATCTTTTTCGTTTCAATCATTGTCCAAAATATTCTAATTCCAAATCCAA
AAATATCGATGCTCCCCTGATTTCCGAAGGATCCATCTTTAGAAAATCGGAAGCGTACT
ATTCCATTTGATCTTTTTCATTTCAAAAAATCAAAAATTGTAGGAACGCGATTTCCAATC
GTCGCCGTGCACGATATTCGTTGTACACAGACGGATTGTTCGGACAAACAAGTCCGCG
AGACCGCGAGAGCGGCAATCGAGAAATTTTTCCATCCGATCGGTTTCACCGGCCGATC
GTTCTTCGATTACAAAACGCGCGGAAACGCCTCGCGTTACAGGCGAGCGCGCGCGATT
ACAGGACAAGCCCCGTGTCGGGGGCGCAATTTCCCCGTGACGCGAACTGATAATAGAA
CGTCACACAACCTGCACGAGCCACGTGGAAATATCGGCGACGATAATGAGAAACGCG
AGTATCGAATGACGGAATCGTTCAATTTTACGAATCAAATTATTCGTTGAGTTCCTTTTT
TCTTTTAACGTGGTTTAGCATGTTAATTGGCTTATTTCGAGGATCGATTCCACGGATATTT
TCAAAATACAAATTTTACAATCCAACAATTTTTAAATTCGTCTCGAAGAAGCCAATTTCT
AGAAAGAATCGAATATCACTACAACTCGCGAATAATTTCCATAAAAGGTGGACGGATTG
TTCGTTACAAACGTGGCCCACATCCCAAATTCATTTCATTATTCTCACTTGTTCCAATGA
TCGAGTGGATCATCGTTCCCCCGCGGATGGCATTACGGTTTTCGATCGGGGCCGTTGTT
CCGATCGAGTGGCCATCCAACGAATGCTCCACTTTGTTATCATCCGGCCCTCGAACGAT
CCCCATCCACCAGCTTTTCTACACCATCGTTGTGGAAATTAGTCGACACGCGCGACAA
GCGTTGCGCATCGCTCTGCCCAACTCGCTCTGCCCCGTTTTCCATTGATTTAATCTTTTC
ATTTCAGCCGCAACGAGCTCCGTGAACGAACGCCAAAGAGACCGTGGATTTCAATTTC
TTTGCGATTTTGCCGGAGGGAGAAAGAAAATGAAGTGAAGGAAGATAGATTGTGTGG
AGAATTTTTGGCAA

>novel_circ_001860
GTCATATAAGGGATATGAAACGTTTCATTGATGAAAGTAGTAGTCATTTATCTATATTTTT
ATTTGATCAATATCTTCAACAAAGTGTATAATTTGTTTATTACAGTTAAAAAGATATATAA
ATTAAAATTTCATGTTTTCAAATTTTCTATATATAAGTTTAATCCATGATAAAGCATACTCC
TTTTTTTAGTTATTCAGAATTTTACATTTAGAATTAATATTGTATATTAATATTATATTTTT

>novel_circ_001861
GCGATGAAGATGAGCAAAGTGGGCAACCAGACCAATTCGCAGGACCCACAGGCGGTG
AATTCCCGGGTTTTCGTCGGGAATCTAAACACGTTCCAGTGCAGCAAGACCGATGTGG
AGCGAATGTTTCAGCGTTACGGCCGTCTGGCAGGAATATCTATGCACAAAGGCTATGCA
TTCGTGCAATTCACAAATCCCTTTGATGCCCGGAGCGCCTGCCTCGGCGAAGATGGCC

GCACCGTTCTTAGCCAGATACTCG

>novel_circ_001863
AGGCGTTGGGGACCGGAAGCTCGTCGTGGCAGAGCGAGGATGGCGGACCAAACTCGC
GGCGAGGCGGCGAGACGCCCTCGTCTTGCGCGACCCCGACGTCGGCCAGTTTCCCGT
CGGAGCCAGAGGTCGACGCCGACGTCGGAGTGAACGCCGACGGGAGCAACGCGACA
GCCCCTTACCCCTGCCAATTCTGCGACCGCACGTTCCCCCGGCTTAGCTATCTCAAGAA
GCACGAACAGAGTCACGGGGACCAAATGCCATACAGGTGCAGCTGGTGTGCCAGATT
GTTCAAACATAAACGCAGCAGAGATCGACACGTGAAGCTTCACACAGGAGACCGAAG
ATATCGATGCACGCACTGCGAGGCTGCTTTCTCCAGGAG

>novel_circ_001864
GCGAAGATTCGGTAATAGATCATCACAAAGGACAAACGCAACTCTGGATTCAGAGGAA
GAAGCGAATCGTTCAGGTGATATGGCGATGTGTGATGAAAACGAAAACGTATCTGGGG
ATTACATGTTTAAAAGATACTCTCAGGATGCAATTAGAAAATCAAATCTTTCGATAAACA
GTTGCGTTTCAATCGGAAGTACGATGAGTTCATATGGTCGTAAAAAACGAAGAGCACC
ACAACCACCACGACGTTCAGAAAATGTAGAAACAAATGAA

>novel_circ_001865
GTTTGTGCGCGGCGTGCATGTTGGCGAGAGTGGAGCAAGAACGTTTGGAGAGTCTCA
AGTACGACAGGGCGACGATTTATATAAAGTGCGTGGAGGATAACGAGGATACGAAGTC
GGAAAACGACAGTGAGATGCCCGCCAAGAGACCGAAGCTGGAGAACACGAGGCCGT
CGAGGACCAGGCGGAACTTGAAGGGGTCCCACGAGCTCAAAGTTTCCTCGGAGATCA
CGTTGAAGGAGCTGAAACTGATG

>novel_circ_001866
GTTTGTGCGCGGCGTGCATGTTGGCGAGAGTGGAGCAAGAACGTTTGGAGAGTCTCA
AGTACGACAGGGCGACGATTTATATAAAGTGCGTGGAGGATAACGAGGATACGAAGTC
GGAAAACGACAGTGAGATGCCCGCCAAGAGACCGAAGCTGGAGAACACGAGGCCGT
CGAGGACCAGGCGGAACTTGAAGGGGTCCCACGAGCTCAAAGTTTCCTCGGAGATCA
CGTTGAAGGAGCTGAAACTGATGACCATGCAAATTTGCGGCGCTGGACCTTACGACCA
ACATCTAATGCTGGGTGAACACGAGCTGACCGATCATAGTCAAAGCCTCGCTGCTCTT
GGTATATTTCCCGGGGCGCTTCTCACCTTGAAG

>novel_circ_001867
GGGTGGCAACGGTCTCGCTAGGAGGTATCGAAGCTGGCGCGTGGGGCGGGAGAGTCA
GCGGTGGTCTGACTCTGGTTCCGGTGGTAGCGGCGGAAACGGAAGCCGCGACAGCAA
CAGATCAGAGAGAACGACTGGAAAAGGCTGGTGCGACCCGCTGGAGAAGGAAATTTG
ATCGTCTTCTATTTTCTACCTTTCCCATCCCCGGTTGTGGAAAATTCGAGAGCGTCTATC
TGCCATCGAAGAAAGCGTCGTCGACGGCGTGACACTGGGCCCCAACCACCTTCGAGC
AAGAAGGCATACGAAATAGGATTAATCCATTCCTGAAAAATCACATTTTAGGCTTTGTT
GGAGGTGTACCACCTCCTCCTCGAGAAACACCCTTTTCCACCCTTCACGGGTAGTCAC
GCTTAGCGCATCATTAGACAGGCATCATGAGCAGCGAGCTCGAATCCCTACGTCAGGA
ATCGGACAAGCTGAAAAATGCCATTCGA

>novel_circ_001869
ACGACCACCTTGATAGGACTATTGAAGACGGCTCGTCTTTTGCGACTCGTCAGAGTAG
CCAGGAAGATCGACAGGTACAGCGAATATGGAGCTGCTGTTTTACTACTTTTGATGGGC
ACCTTTGCTCTTAGTGCCCATTGGATGGCGTGTATATGGTACGCCATAGGGAACGCGGA
GAGACCAACGTTGAAGAGCAAAGTCGGTTGGCTCGATATACTTGCCAACGACACGCAT
CAGTTTTATTTTCACAATAACACCGGTGGGCCGAGTATAAAGAGCCGCTACATCACAGC
ACTGTATTTTACCTTTAGTAGTTTAACATCGGTGGGCTTTGGAAACGTGGCTCCAAACA
CGGACACCGAGAAGATATTTACTATAATCGTTATGCTAATTGGATCTTTGATGTATGCCA
GTATCTTCGGTAACGTCTCGGCGATCATTCAGAGGCTTTACAGCGGCACAGCAAGATAC
CACACGCAGATGCTTCGAGTTCGAGAGTTCATCAGATTCCATCAGATCCCGAATCCGCT
TCGTCAGAGGTTGGAGGAGTACTTTCAACACGCTTGGACGTACACGAACGGAATCGA
CATGAACAGCGTTCTGAAAGGATTCCCCGAGTGCCTTCAGGCGGACATATGTCTTCATC
TGAACAGAAATCTTTTAAACAATTGTAGAGCCTTCGAGGGTGCGAGTCCTGGTTGTTT
GAGGGCGTTATCGTTAAAATTTAAAACCACACACGCGCCACCCGGTGACACGTTGGTT
CATCGAGGAGACGTGTTAACGTCCCTGTACTTCATATCGAGAGGAAGCATAGAGATATT
GAAGGGCGACGTCGTGATGGCCATACTCGCCAAGGACGACATATTCGGGGAGAATCCT
TGCATATATCCGACCGTTGGCAAAGCGTCTTGCAACGTGCGAGCGTTGACCTATTGCGA
TCTGCACAAGATACACAGGGACGATCTGCTCGACGTTCTGGCCCTTTATCCAGAATTCT
CGAATCACTTCAGCCAGAATCTCGAGATCACTTTTAATCTGAGAGACGAGGAACAGGC
TGGTGTCGATCCAATATCAGCAAGGTTTCCTGTCAGTACTCCAACGGACGTCGATGCCG
ACGCTAGGAGATTCGCTTTCCGTCCACCAAGATACCGTCGAGGACCCGGTGGTGGTCC
TGGCACCAATCTTATCCCCACTGGTCGACAAGACTCTTTACAGGGTGATCAGGAAGATT
ACGACAAAGGAAGTGGCCACGGAATTCTGGAGTTCAGCACCGACAAAGCCGGCCAA
GATGTGACGCCATTGAACCTAGAATTCGACGAGCCGAAACAGAGGAGCTCCACGTTG
AACTCCATAACCG

>novel_circ_001870
GTGGTGAAGATGTTGGTGGCCGTGGTGGTGTTGTTCGTCATTTGCTGGGGTCCAATTCT
CGTGGATAACATGCTAACAGCTTATGGATACCTGCCACGCGTAAAAGTTGGCACGTACA
AGCATCTTAACACCGCTTTTCAGCTGATGGCTTATTTTAACAGTTGCATAAATCCAATCG
TTTACGGATTTATGTCGAAACACTTCAGGGAGAGTTTTTTGGCCGCTGCCTGCGGCAGC
TGGTGGATCTGTTGCCCCCGAAGAGGGTACAGGGCGCCGGTGAGGAGGCATCCGAGC
CTAAGCCAGACAAGGACGACCAGTATAAG

>novel_circ_001871
GCGGAGGAATGGCCAACCAATCCAGACGATTTTCTGGACTCTATTTTCAAGCTGGAGG
ATAATCAATTCAACTTGATCGATAATGATTTAGACGCGATCCCTTCTTCCTCGTCGGACA
GCGGGCTGTCGAGCGCGCTGAGTCTCTCTTGCGAGCAACAATTAAGCCCGCTTCTGCC
GATCGAGGAGGATCAAGTGGAGATGAGTAATTCTGAGATGAGCAGTCCTCAGAATATC
ATGGATTTTGATTCGTTGGGCAGTCCTAATCAATCGATGGCCAGTATAGATAGTCCTGTC
AGATCGGTGGTTAGTTCTAATATTGGTTCACCCGCCATGGATGTAGCCGAAGAAATGGA
CGTGGAACACACCCTCGTGGCTGTGGTGAACCCGACTAACACCGTGATAGATGCTAAT
ACGACAATTGTAACGGAGCAACCGGTTATCGATTTAGAGAAAACACCGAAGCCTCAAC

AGATTCACGTTGCATCTCCGGAAAATAACACGATAAATGTGCGCAATATCACGTGCAAT
GGGAAACGGAATATCAGACAATTGATACGCGTTACACCGATGGGTTCCGGTAATCCAA
GATCGATCCTACTGCCAGTGAGCTTGAAAGACATGAAAGAAGTGAGAACCATCAAGAT
CATCAACGCTTCTCAGGCTAGAAATCTCAAAGGGTTCAAGATCAACCAGGCCAACATC
ATTAACAAACCAGTTCAGATGAACATCAAACAAGAATTCAAAGAAGATTCCAGTAGCG
AGAAGGGTTGCAATTCGAGCGACGAGGTCTCGGAATCGGATTCCCCGTATCCGAGGCT
AAAACTGAACGCGGAGGAGAAACGTCTGCTCCAAAAAGAAGGTATCACCCTGCCTAC
ACATTATCCGTTGACGAAGCACGAGGAAAGGGAATTGAAGAGGATCAGGCGCAAGAT
TCGCAATAAAATTTCCGCTCAAGACTCGCGGAAGAGGAAAAAGGAGTACGTGGACGG
GTTGGAGGATCGGGTGAAGCAGTGTACCGAGGAGAATATGACACTGTTGAAACGTATC
AAGGCGCTTCAGTCGCAGAATCAGAGCCTGGCCGGCCAGCTGAAGAGGTTGCAAGCA
CTGTTGCAAAAAGGCAACAAGAGCGCCCAGCCTGCCACTTGTCTAATGGTTTTGTTGC
TCTCGTTGGCCCTGGTCGCTGTGCCGAATCTTCGGCCACACTCCAATTCAAACAATGA
ACTCACGCAAGAGCAGGAACAGCCGGAGAAAATGCCACCTTTGGCGG

>novel_circ_001873
AGCTGTACTCTTTGCAAGGGATGGAGTTCCTGGAGGGTTTCGGGAAGAAAAAACAGC
AGCCTAATCAACAATCGAACAATATATCGTCGGTGCACATCAATCCCCTTACGGCACAG
TCTCTCAACATACACCTGCATGCTCTTTTCGCCGCAGTCGAGCATGGACACCTGGACAA
AGCCAGAACGATTTTAGAGTCGACGGACGTAGACGTGAACAGCGTGAACAGCGACGG
CCTGTCACCCCTGGATGTTGCGGTGCTCAGCAACAATAGGCCGCTTGCAAAAATGCTG
GTCGCGTTCGGCGCACAGGAGGGTAACCAGT

>novel_circ_001874
TTGCCAAACGTCACGTCCGCGTTGAACAATATCACGATGCTGATGAAGGACAACATCG
TGTCGGAAGTCAGCCAGGGTAAAGAGTCGTTTCTGAAAATTCAGAAAGACATTCAATA
CGCCGTGAATCAGACCATCCCAGTCGTCAGCGCGAGCATCCGAAATGCCGGCGATTTT
CTGGCCGACCTCGCGAAAAACATGACAATGCTCATCGATAGGATAAACAACGACATAG
ACAAGGTTTACATGAAACAGATAGACGTTGCACGGACGAACATCGATCAGTATTCACC
GTACAGACAAATGTATTTTCCCTCTAGGTACTATCTAGGACTCGGGATATCGGGCATTTT
GTTAACAGTTCTGATGTGTCTGACTTTCGGTCTTTTCTGTGGCATTTGTGGAAAGCGGC
CCGATGGTTATGGAGACGACTGTTGCAACAAAGGATCCGGTGCACGATTTCTCATGATG
GCTGTTTGGATCATATTCCTTCTGACCAGCATTCTAATGGTGATCACGGTGATACACATG
GTCGTCGGTGTTCTGGCGCAAAGAGCTGTCTGCGAGCCTTTGAAAAATCCACAGGATA
ACAGGATGTTCGCTCTGGTCGACGAGATCGTACAGATAAAGAAGATACTGTATCCGAAT
AAACCGAATGCCGATGTGAACATGAGCTATATCATAACAAACTGCCATCGAAACGAAA
CGTTGTACAAAGTTCTCAAGTTGAATTATCTGTTCGACGTGAACACGTTGCGCGAATAC
ACCGGTCGCTACGACATCAACAACACGATTCAACAGCTTCGGCGCAAGATCAGTCTCT
CACCCGGCGTGGTTATCCTCACGGAAAGCGCAAAGTCGAAATTGAACGATCTCGCGCA
GAGTGGCCTCAGCGATATCAAGTTCTATCAATACGTGGAGATTCTCGCTGATAATATCAC
GAATATCAACTTGGAACATCTCGCGAAACAATTGTTGGACGTGTCAACGGAATTGCCA
AAGGGGCAGGACGACATTCGAGCAAGTTTGGAGAAAAACGCACTGGATTTGGCGTAT
TATCACGAACATTTGGTGAAACCAATGGCGATGCTCAGCGAACAATTGGCAGCCAAGG
CTGTGACCCTCGAGGAGAAGATAAAGTTTAATCATAGCTCGATGGCAGAGGCCATTCA

CAATCTCGTCGACGAGGTGACGAAGGCGCAAAAGTTTTTGAACGAAGATGGGCCAGA
ATACGTGCAACAATTGGCTACTAAATTTGGAAACGCTTTCCTTCGTCAAGTGGACGATT
TCCTCGAGAGAGTTATCGATCATGCTTTGTTCCACGTAGGAAAATGCACGCCCGTTTCG
AACGCGTACAACGCCACGCTGGTCGCTGGATGTAGCAAAATTTTAGATCCATTTAATGG
TTTCTGGGTGAGTGTAGGCTGGTGTTTAATCCTCTTCATCCCGACCATCGTGCTCTGCGT
AAAACTAAGCGCACTCTATCAAAAATCGGATCCCTATCCAGGACCCTTAGTCGAAGC

>novel_circ_001875
CGTTCACGACAAGAAATACGTGTCGCATAGCAGAGATCCTTACGCAAAATACGAAAGC
TACGACGGTCCCGCGGGTGGATACAGCGATCGGGAAAGGGTGCCCGGGGAAGCGCAT
CAGAGCACGTCCCACTATCATCACTATTCCAGATATTCCGACGTGGCGCCGAAGTAATT
CCACTCCCACGTGGCGAAGCCACGAACATACCGCCCCCTCCAAAAAAGAACCTCACA
ATAAAGTCTTTAGCATATCGGTAGAAACGCAAAATTGTGACTTCCCTAACGGTGGTCCA
CCCGGGTATCAGCCAGCTGCCGCAGCTCCTCCACCACTAAGCACGGAATACGAGCGGC
CACCTCCCTATTATTATTATCCTGGGCCAGGGTGAGTCGAAGGGGACACAAATTAGGAA
ACAAGTGCCGTGGCATGCACAAAGGTAGCGCTGAATAGCGTGGCTACCCACAGTCTCA
CCCGCAGTTACAAAAGGACGCTCAAAAAGAAAGAGAAATCCGGATGTAAACGAGACT
ATGTTTAATATGTAGCGTTCCTGTTTGAGCGTGCCACGGACCGTGCGAACGGCAAAATC
AAATTAAAGACCAAGTTTACGATATATACAGACTGAGGTAGGCTGAATGGATCCTTCGG
CGAGCCGCAAGTAATGTGATCAATGT

>novel_circ_001876
AAATTGTGACTTCCCTAACGGTGGTCCACCCGGGTATCAGCCAGCTGCCGCAGCTCCT
CCACCACTAAGCACGGAATACGAGCGGCCACCTCCCTATTATTATTATCCTGGGCCAGG
GTGAGTCGAAGGGGACACAAATTAGGAAACAAGTGCCGTGGCATGCACAAAGGTAGC
GCTGAATAGCGTGGCTACCCACAGTCTCACCCGCAGTTACAAAAGGACGCTCAAAAA
GAAAGAGAAATCCGGATGTAAACGAGACTATGTTTAATATGTAGCGTTCCTGTTTGAGC
GTGCCACGGACCGTGCGAACGGCAAAATCAAATTAAAGACCAAGTTTACGATATATAC
AGACTGAG

>novel_circ_001877
AAATTGTGACTTCCCTAACGGTGGTCCACCCGGGTATCAGCCAGCTGCCGCAGCTCCT
CCACCACTAAGCACGGAATACGAGCGGCCACCTCCCTATTATTATTATCCTGGGCCAGG
GTGAGTCGAAGGGGACACAAATTAGGAAACAAGTGCCGTGGCATGCACAAAGGTAGC
GCTGAATAGCGTGGCTACCCACAGTCTCACCCGCAGTTACAAAAGGACGCTCAAAAA
GAAAGAGAAATCCGGATGTAAACGAGACTATGTTTAATATGTAGCGTTCCTGTTTGAGC
GTGCCACGGACCGTGCGAACGGCAAAATCAAATTAAAGACCAAGTTTACGATATATAC
AGACTGAGGTAGGCTGAATGGATCCTTCGGCGAGCCGCAAGTAATGTGATCAATGT

>novel_circ_001878
GTTTCCAAGGGAACAACAAACTCTGCCCAATCACTTCTACTTCACCGATTTCGAGAGG
CATACGGCCGAGATCGCCGCGTTTCATCTGGACAGGCTGCTTGGATTCAGGAGGGCGA
TGCCGGTTACGGGGAGAACGTTGAACCTAACGACAGAGATTTATCAGATCGCCGACGG
CGAACTGCTGAAGACGTTCTTCGTCAGCCCCGCCGGTAATATCTGCTTCCACGGCAAG

TGCAGTTATTACTGCGATACGGCGCACGCGATTTGCGGAAATCCGGACACCCTTGAAG
GCAGTTTCGCCGCTTTCTTGCCGGATAAATCTTTCGTCGCGAGGAAGGCATGGAGACA
CCCCTGGCGGAGGAGTTATCACAAGAGGAAGAAGGCCCAGTGGGAGCACGATTCGGA
TTACTGTTCCCTCGTGAAAGAGATCCCACCCTATCACGAGGGCAGAAGGTTGCTGGAT
CTAATGGACATGGCTGTCTTAGACTTCCTCATGGGGAACATGGACAGACACCATTACGA
GACGTTCAAGATTTTCGGCAACAACACGTTCCCCCTGCACTTGGACCACGGAAGAGGA
TTCGGCAGACCCTTCCACGACGAGATCTCCATCCTTGCCCCCATCTTACAGTGTTGCAT
GATCAGGCAAACGACTCTAAGCACCCTTCTAAAATTTCACAACGGACCAGTCCCCCTG
AGCGAGGCATTGCGAAAAAGCATGGCCAAGGACCCAGTGGCCCCCGTTCTCTGGGAA
CCCCATCTGGCGGCCCTCGACAGGAGGGTGCGCGTGATTCTTCAAGCGATCAGAGACT
GCGTGAATCGTGAGGATTCGAGTCAGATCGTCCACGAGAAAACGGAGGATACCGGATC
CTAGCCGTTCCTTTCGATTCTCTTAGCGTGAATCGGAGAATTCTGATGAAACGTTCTGA
TCAGACGAAGAGAAGAGGAA

>novel_circ_001879
ATTCGGATTACTGTTCCCTCGTGAAAGAGATCCCACCCTATCACGAGGGCAGAAGGTT
GCTGGATCTAATGGACATGGCTGTCTTAGACTTCCTCATGGGGAACATGGACAGACAC
CATTACGAGACGTTCAAGATTTTCGGCAACAACACGTTCCCCCTGCACTTGGACCACG
GAAGAGGATTCGGCAGACCCTTCCACGACGAGATCTCCATCCTTGCCCCCATCTTACA
GTGTTGCATGATCAGGCAAACGACTCTAAGCACCCTTCTAAA

>novel_circ_001880
GTTTCCAAGGGAACAACAAACTCTGCCCAATCACTTCTACTTCACCGATTTCGAGAGG
CATACGGCCGAGATCGCCGCGTTTCATCTGGACAGGCTGCTTGGATTCAGGAGGGCGA
TGCCGGTTACGGGGAGAACGTTGAACCTAACGACAGAGATTTATCAGATCGCCGACGG
CGAACTGCTGAAGACGTTCTTCGTCAGCCCCGCCGGTAATATCTGCTTCCACGGCAAG
TGCAGTTATTACTGCGATACGGCGCACGCGATTTGCGGAAATCCGGACACCCTTGAAG
GCAGTTTCGCCGCTTTCTTGCCGGATAAATCTTTCGTCGCGAGGAAGGCATGGAGACA
CCCCTGGCGGAGGAGTTATCACAAGAGGAAGAAGGCCCAGTGGGAGCACGATTCGGA
TTACTGTTCCCTCGTGAAAGAGATCCCACCCTATCACGAGGGCAGAAGGTTGCTGGAT
CTAATGGACATGGCTGTCTTAGACTTCCTCATGGGGAACATGGACAGACACCATTACGA
GACGTTCAAGATTTTCGGCAACAACACGTTCCCCCTGCACTTGGACCACGGAAGAGGA
TTCGGCAGACCCTTCCACGACGAGATCTCCATCCTTGCCCCCATCTTACAGTGTTGCAT
GATCAGGCAAACGACTCTAAGCACCCTTCTAAA

>novel_circ_001881
GAAGAACAGTTCGACGTTGGAGAGATTCCATCTGCAAATCTCGAGGATAGAACTGTAT
CCGGAGAGGTCGAAGTACGTGGACCAATTGTTGCACGAGATCGCGACGAGGCCTATAG
TTCACGTCGTTCAAAAAGAGGGCGGCACCCAGCTGAAACTGCAGATAAATTATTCCAA
CATGCAGGCGCTGTTCAAACCCATGAGGTTTCCAAGGGAACAACAAACTCTGCCCAAT
CACTTCTACTTCACCGATTTCGAGAGGCATACGGCCGAGATCGCCGCGTTTCATCTGGA
CAGGCTGCTTGGATTCAGGAGGGCGATGCCGGTTACGGGGAGAACGTTGAACCTAAC
GACAGAGATTTATCAGATCGCCGACGGCGAACTGCTGAAGACGTTCTTCGTCAGCCCC
GCCGGTAATATCTGCTTCCACGGCAAGTGCAGTTATTACTGCGATACGGCGCACGCGAT

TTGCGGAAATCCGGACACCCTTGAAGGCAGTTTCGCCGCTTTCTTGCCGGATAAATCTT
TCGTCGCGAGGAAGGCATGGAGACACCCCTGGCGGAGGAGTTATCACAAGAGGAAGA
AGGCCCAGTGGGAGCACGATTCGGATTACTGTTCCCTCGTGAAAGAGATCCCACCCTA
TCACGAGGGCAGAAGGTTGCTGGATCTAATGGACATGGCTGTCTTAGACTTCCTCATG
GGGAACATGGACAGACACCATTACGAGACGTTCAAGATTTTCGGCAACAACACGTTCC
CCCTGCACTTGGACCACGGAAGAGGATTCGGCAGACCCTTCCACGACGAGATCTCCAT
CCTTGCCCCCATCTTACAGTGTTGCATGATCAGGCAAACGACTCTAAGCACCCTTCTAA
A

>novel_circ_001882
GTTTCCAAGGGAACAACAAACTCTGCCCAATCACTTCTACTTCACCGATTTCGAGAGG
CATACGGCCGAGATCGCCGCGTTTCATCTGGACAGGCTGCTTGGATTCAGGAGGGCGA
TGCCGGTTACGGGGAGAACGTTGAACCTAACGACAGAGATTTATCAGATCGCCGACGG
CGAACTGCTGAAGACGTTCTTCGTCAGCCCCGCCGGTAATATCTGCTTCCACGGCAAG
TGCAGTTATTACTGCGATACGGCGCACGCGATTTGCGGAAATCCGGACACCCTTGAAG
GCAGTTTCGCCGCTTTCTTGCCGGATAAATCTTTCGTCGCGAGGAAGGCATGGAGACA
CCCCTGGCGGAGGAGTTATCACAAGAGGAAGAAGGCCCAGTGGGAGCACG

>novel_circ_001883
TTCAAAAAGAGGGCGGCACCCAGCTGAAACTGCAGATAAATTATTCCAACATGCAGGC
GCTGTTCAAACCCATGAGGTTTCCAAGGGAACAACAAACTCTGCCCAATCACTTCTAC
TTCACCGATTTCGAGAGGCATACGGCCGAGATCGCCGCGTTTCATCTGGACAGGCTGC
TTGGATTCAGGAGGGCGATGCCGGTTACGGGGAGAACGTTGAACCTAACGACAGAGA
TTTATCAGATCGCCGACGGCGAACTGCTGAAGACGTTCTTCGTCAGCCCCGCCGGTAAT
ATCTGCTTCCACGGCAAGTGCAGTTATTACTGCGATACGGCGCACGCGATTTGCGGAAA
TCCGGACACCCTTGAAGGCAGTTTCGCCGCTTTCTTGCCGGATAAATCTTTCGTCGCGA
GGAAGGCATGGAGACACCCCTGGCGGAGGAGTTATCACAAGAGGAAGAAGGCCCAGT
GGGAGCACG

>novel_circ_001884
ATGGAAGCGAAGGGGGAGAGAAAAAGTAATTGCAAAGTGGCGATGATTCCCTACTAA
CAGAGTGTGGAGAATTAACCGAGTTGTTGAAGTGAGAGCCGCATTAACGTGTTATTAA
CGTCATGTAACCGACATCGCGAAAGTAAGAAGCATGGAGAAGGTATGCAGGCCTGCCT
GGAGTAATCCTCGACCCACCAAGG

>novel_circ_001885
GGACGAGGAATGGTGGGATTCCGGGAACGGAAGGGCGGCGGTGTGGATGCCGGAGG
AAAGTGAGAGGAAACGAAGAAACGATGGCAGAAAACAGAACTTTGCTCTCCAACGG
CCGTGAGTCATCGTCGCGTGGCCGCCTCCACCTTCGACTTCATTCATCCTGGGCGAAA
AAAAGTCATCGTCGCTGTTCGTGATTCAATCAACCAAAAGTTGATTTCTTTCTTTCTTTC
TTTTTCTTTCTTTCTTTCTTTCTTTCTTTCCTTCTTTCTTTCCTTCTTTCGCGAAAAGAAA
AACTGGAACTGGAAGAAACGTGGTACAACACGGACAAGTGACTTGAAAAAAAAGTC
GGGGCGAGGTTCGGTTTTGGATTCGAGAGAAAGGAAAGAAGAAAGGTTCATTCGCAC
GCGTGAAGCGAGTGGCATGGAGGAGCCACAGAAGCTGTTCCGTCTTCCCGGCACCAT

CGAGGAGGAGGAGGAGAAGGATAACGAGGACCGAAGGCGGCAACAGCTGCAGGAGA
ATCCGAGCACGGCCGACTCTCCGTTGCTGGAGCAGAGGAACACGGGAACCTTGTCCA
GCCTGCCCAGGTACGTGACCCTGGTCAGGGTCAGCACCCAGAGCAGCAGCATCGGTG
GTGGCGTCGGTCGCCAGGATTCTACCAG

>novel_circ_001886
GAGTCGGTACCGAGCATGGCCAACGCGAAAGTTGCGCCGTCGAGCCGTGCTGAAGAA
CGGGGACTGCAACGTCCTCCAGTCCCGTATCTCTCGGCGTTCCCTGCGCTTCCTGCGA
GACATCTTCACGACCCTGGTGGACACTCAATGGCGATGGATGTTGCTCTGCTTCAGCCT
GAGCTTCGTCCTCTCGTGGCTGGGCTTCGCCGTGATCTGGTGGCTGATCGCGTTCAGCC
ACGGCGACTTCGAGGAGCGCCACCTGCCCCCCTACCAGATCGAGAACAACTGGACGC
CGTGCGTGAACAACATATTCTCGTTCACGAGCTGCTTCCTGTTCAGCATCGAGACCCAG
CACACGATCGGTTACGGGAGCCGCGGCACGACCGAGGAATGCCCCGAGGCGATCTTC
GTCATGTGCATCCAGAGCATCGTCGGGGTGATGATCCAGGCGTTCATGGTGGGAATAGT
GTTCGCCAAGATGAGCAGGCCGAAGCAGAGGACTCAAACGTTGCTGTTCTCGAGGAA
CGCGGTCATCTGTCAGAGGGACGGTGAACTGTGCTTGATGTTTCGGGTAGGCGACATG
AGGAAGAGTCACATAATCGGGGCCGCGATCCGGGCTCAGTTGATAAGGTCGAGGACG
ACGAAGGAGGGGGAGGTGTTGTCGCAGAATCAACAGGAGTTGGCGGTGGGGACGGA
CGGGCAGAACGGGAACCTGTTCTTCATATGGCCGACAACGATCGTCCACAGGATAAAC
GAGGAGTCCCCGTTCTACAACATGTCCGCGGAGGACATGCTGACGGAAAGGTTCGAG
ATCGTGGCCATCCTCGAGGGGACGATAGAGTCGACCGGGCAAACGACCCAGGCGAGA
TCGAGCTACCTGCCCCAGGAGATCCTGTGGGGTTACAGGTTCGAGCCGATGGTCACGT
ACTCGAAGGAGAGGCAGGGCTACGAGGTGGATTACTCGTTGTTCAACAGCACGACGC
AGGTGGGCACCCCGCTGTGCTCGGGCAGGGAGCTGGCCGAATTTTACAAGGCTCAGG
AGGAGCTTCGTCACGGGAACG

>novel_circ_001887
CCGGTGCGGTAGTGATCGTGGCCTCGAAGCGAGTCCTTTTACGGCGGGGACGAGCATC
GAAAGCGGAGGAGAGCAGCCTCCCTCTCCCCCTCCCTCTGTCTTTCTCTTTCTTCCCCC
GACTTTGGCCCACGGCCGCCTTTATCCACCGGTCCAAGACTGGAGAAGATCGCGAGAA
GGAAGGTAGAAGAAGAGAGAAAGAGAGAAGAAAAAGGAGAGATCGAAAGCTAGAC
CAGTGTCCCAGTGCGCTCGAAGTTGGCATGGAGGGATAAAACACGGTGCAGAGAATT
GTGAGAAGAGAAAGGGGGAGAGAGAGAGAGGGAGGAGGAGGAGGAGGAGGATTGG
AAAAATTGGCGGTGGATGGATAGCGATAAATTCGGCATGAGGTAGAGGAGAATTTCGA
AGCTGGTTGGTCGGAGAGAGGAGCGATCGATCGTGGAGGGATAAGGGGGGAAGCTAA
ATGCCAGTTAAAAGAAAGGCGAGGAAGGAGGGTTAGAGGTGGAAAAGTGGTCGGGG
ATAGGAAGAGGCGGGTGGTGAGGAGGGAGAGGAATGGGTGCCGGGATGGGCAGGAG
CGGCACGAGCGGCGGCTTCCTCGAGGCACTTCCCACGGGAACGCCGCTCCACGTGGC
CATGCTGGGCCTCGACAGCGCGGGGAAAACCACCGCGTTGTATCGGCTCAAGTTCGAC
CAATACCTGAACACTGTGCCCACTATCGGCTTCAACTGCGAGAGAATCCGTGGCGGCA
TCGGGAAAGCGAAAGGTGTGAATTTTCTCGTATGGGACGTGGGCGGGCAAGAGAAAT
TACGGCCGTTATGGAAGTCTTACACCAGATGCACGGACGGTATTATCTTCGTGGTCGAC
TCGTGCGATGCGGAACGGCTCGAGGAGGCCAAAATGGAGCTGACCAGAACGGCGAG
GAGCCCGGATAACGCCGGTGTGCCCATTCTGATCCTAGCCAACAAGCAGGATCTGCCA

G

>novel_circ_001888
AAAAAAAATAAATTACGTGATTGGTTACGTGTTGTATCAAAAGACAAAGAAACCTATG
AGTTAAGGTACTTCCAGATTAACAGTCAAGAAGATGATGATGAAGAAGATGCAGAATA
AAATTTTAATTGTATGATGCAATTACAGAATTATCTGTACATATTTCTACTAATAAATAATA
GGAAAAAA

>novel_circ_001889
CAAAGAAAAAAAAAAAGTTAAAGAGGGAAACGAATGAAAAATACGAGAGAGGATAA
TGTCCGTGTATTAATGGAGGAAAACATGTATATCTACGTATATACGTGTATATGTACATAA
ATTATTACGAATTATCGATGTAAGATTGAACTATACATACATACATATATAAATAATATAAC
TTATAAA

>novel_circ_001891
AATGCGTTGAAGATCCGGCCGGTCGAGGAGAATCGAGATGTCCGCCGTAACCAGCCCG
TATGGCCCCACGGAGATGCTTTCCGACTCGGTGTACAACTACGCGACGACCCGGCGGG
GGACCGGCGATCAGGACAGCGACTCCCAGTACGAGGGCCAGGCCCAGCTGCAGATCA
CGAGCATCCAGAAGCCGCGCAACAAGCGCAAAAACTTCAAGCCGATAAGCAGCCGGA
TGGTGGAGGTGTCCGATGAATCCGAGAAGGAGGACGACGAGCTGGACGGGGCAATGG
AGGAGAGCTCGAGCGAGATACTCGAGTACGAGAGGAAGACGATGCTGTTGCCCGCGG
AGGACAGGTCCAAGCAGAGGTTGAACAACAACGAGGTCAGCCCCATGGACCTGTCCG
TCGCCACCAGGCCCCCGTCCTCCGAGGCCGACGACGATAGCGGCGACTCTCTCAGGCA
CAAGTTCATCCTGGAGCAGCTCAGATCTCAGAAACTCTACTCCCCTGGCACCGAGGCC
AGGAGTCCGAACTCGGAATCGTCGGAGAAGAGTGGAGGAAGCGGTGTCCTGGACGC
GGACTCGGGACGGCTGGACGACACCCCGTTCGAGGACGAGCGGGGGCAGGACGGGG
ACGGCGAGGCCGAGTGCGAGGAGAGGAAACCGTTCGAGGGGATGAGGGAGTACGCG
GAGTCGACTATGCAGGAGTTGTTGGCTATCTACGGGCTGACGGGAGGAGAATTGGTCA
AGTCTGTGAGCAGGCAGCTACCGCCGACCTTCTTGAATCCCCCGACTTCGGGCCATCA
GCCTCACG

>novel_circ_001892
CGGAGATGCTTTCCGACTCGGTGTACAACTACGCGACGACCCGGCGGGGGACCGGCG
ATCAGGACAGCGACTCCCAGTACGAGGGCCAGGCCCAGCTGCAGATCACGAGCATCC
AGAAGCCGCGCAACAAGCGCAAAAACTTCAAGCCGATAAGCAGCCGGATGGTGGAGG
TGTCCGATGAATCCGAGAAGGAGGACGACGAGCTGGACGGGGCAATGGAGGAGAGCT
CGAGCGAGATACTCGAGTACGAGAGGAAGACGATGCTGTTGCCCGCGGAGGACAGGT
CCAAGCAGAGGTTGAACAACAACGAGGTCAGCCCCATGGACCTGTCCGTCGCCACCA
GGCCCCCGTCCTCCGAGGCCGACGACGATAGCGGCGACTCTCTCAGGCACAAGTTCAT
CCTGGAGCAGCTCAGATCTCAGAAACTCTACTCCCCTGGCACCGAGGCCAGGAGTCCG
AACTCGGAATCGTCGGAGAAGAGTGGAGGAAGCGGTGTCCTGGACGCGGACTCGGG
ACGGCTGGACGACACCCCGTTCGAGGACGAGCGGGGGCAGGACGGGGACGGCGAGG
CCGAGTGCGAGGAGAGGAAACCGTTCGAGGGGATGAGGGAGTACGCGGAGTCGACTA
TGCAGGAGTTGTTGGCTATCTACGGGCTGACGGGAGGAGAATTGGTCAAGTCTGTGAG

CAGGCAGCTACCGCCGACCTTCTTGAATCCCCCGACTTCGGGCCATCAGCCTCACG

>novel_circ_001893
GGAAGGTAAAAGTAAAAGAGGAAAAGGATGCTTCCTCGATGGCGCCAGGAAGCCCAC
CGACGCCACCGCACACCCCCCAAAGCCCACCTCGACACAATCCACCCCCGGGTAACCT
GTCTCAGTCCTGTCAGACGTCGAACGCCAATTCTCCGAATCTGCAGCAACAGCAACAG
CAGCACCAACAGCACCAACACCATCAACAGCAGCACCAGCAGCAGCAAGAGTCGAAT
CAACAGCAGCAGCAGCAGCAGCAACAACAGCAGCAGCAACAGCAGCATCAGCATCAT
CATCAATCCATTCACGCGATGACCAACACACCCCTGAGCCAGATCTTGGTCCAGAATCC
GGCCCTTGCTCAACTTCTGCAACAGAATCCCGCGATTCTGTCGCAGAATCCCCAGTTG
GCGCAGTTGCTGCAGCAAAATCTTCAAGTTCAGATGGGCAGCGTCCTTTCCAGAACG
TGAGGAGGGAGGAACCTGAGGAATCGAGG

>novel_circ_001894
CATTATTGAGACAAAGCATGTCTCCGGTTGCGGACACCTTGATCGGTAGCTCGAAAAG
CTCCGTGTCACAACCAATAATCGATTACTCCCGATACGTGAGGCGGTACACGTCGGGTC
AAGAATGCGGTAGCTCGTATTGTAAGGAGCTCGGTTGCCGAGAGCATTTTCACTGTCTG
GATTGCAGTGGTAGGGTCTTTGTGAAAAAGGAGGAAATGATCAGGCATTTCAAGTGGC
ACAAAAAGAGGGACGAATCTTTGCAGCACGGCTTCATGAGGTATTCGCCGACGGATGA
TTGCTCGGAGAGACATCCGGGCCGTGCCTGCCCCCACAATAGAAAACAGACGCACTAC
CACTGTATCCACGAGAATTGCGATAAAGTTTACATATCGACGTCGGACGTGCAGATGCA
CGCCAATTACCATCGCAAGGATTCGGCTATCATACAAGAGGGTTTCCAGAGATTTCGAG
CTACCGAGAGCTGTGCTACCGATCACTGTCCCTTCGTTGGCCAGCGGACCACGCACTT
CCATTGCCGGCGACCAGGTTGCCGGTTCACCTTCAAGAACAAGGCTGACATGGACAA
GCACAAATCGTACCACATCAAAGACGAGCAATTGTCCCGCGATGGGTTCAAGAAATTC
ATGAAGTCCGAGGCATGTCCGTTCGAGCAATGCCGTTTCTCGCGCGTGTGCAATCACAT
ACACTGCATACGACCGCACTGTAGCTACGTTCTTCACTCGTCGGGCCAATTGTTCTCCC
ATAAACGAAAACACGAGCGCCACGAGACCGAGTTGGCCTACAGAAAGTACAAGCAGA
TCAACGCGGTCGGCGGTATCCGGCCGCCAGGCTCCTGGCCACCGGACGATCTCACCAC
GCAAAGTCTCTCCCTCAGCCTGAACGGCGAAAGTTCGAACGAGGACAGAGGCTCCCC
CTCCTACTATTCCCTCGACGATTCGTTTCAGAGTGGCAGCGATCTAGCGATAGATCTAAC
CGCGCCGACCACGACCACCGTCACCACGGCAAGCGGGAGTTCCACGGTGACCATGGA
CCAACAGCAGCAGCAGCAGCAACAGCAACAGAGCGGCCAACAGCTCACCGCCATCG
AATTGGCGTATCTGGTGCCCGGTAGCGCCGATTGCCCCTGTCACCTGGGCAGGGAACA
CCATCACTGCGGGTTGGACCGTTGCGGCGCACCTCTCAAGGATCCTCGCGAGGTGAGG
GAACATTTGAAGGAGCACGAGACCCAGGAGCGCATCACGGACGCCTTCTTCGAGGAG
GGTGGATGCGACGACAGTTGCCCGTACGCCGACAAGGAGAAGCATTATCACTGCAACT
GGGAGAATTGCCGGGAGGTGATCCTCTCCACGGACAAGCCGTTCCGCCGACTTCAGC
ACTACAAGATACACGAGTACAGCCGACAGTTGAACCTGTCCTGCACCTCCCACGCCCT
GTCCTCGGACGTGACGTTGACCCATCTGACCAACATCGACGCCATGTTCAGGCGGAAG
AGGGGCAGGCCACCGAAGAACAGAGTGATCGAGATCTGGTCGGGTGGCGACCCGGCC
ACCCACACCCACGACTCCCCCCAAGCGATCTTCACGAGTTTCAAGCTGCCGAAACCTC
AAACCCCTAGTCTCGCGTCGAATCCTATGGCCGAGGATCGCGAGGGGAGCGTCTCGCC
CTCGAACAGCCTCGGCGAGCCCGACGGTTTCTCCACCTATAATCCCGACGGATGCCCA

GATCCGGCTTGCGTGTTGAGATCTACCAGACACCATCATTGCTCCCAGCTTAGGTGCCA
CTTCTCCACGGACAGGCCCGACCAATTACTGATGCACAGCAAAGACTTTCACGATAAC
GTCGACATACCGCCCGGCTTCGCCTTCTTCGATAAGATGGTAGATTGCCGGCTGGTCGG
TTGTCACAGTAATCGCGTTAATCGCCATTTCCATTGCACCAGGCCGAACTGCGGCTACT
CGTTCGTCAGATACTCGACCATGGCGATGCACGAGAAACAGCACGGCAATAGCCACGT
AGGCAACGACAGCCGGTCCAACGTCGAATCGTCGTCGTCGTCGTCGTCCAACCAAGA
GTGTCAGACCCACGGCCCTCAACCAATGGTCCAGGATACGACGAAGCCTAGGATCCAA
GTGAAGAATCCGGCCGAGCTGATCGAAAAACCCGAAGAATGTATGGCGAGTGGTGGT
GGTGGTGGTAGTGGTGGTGGTGGTGGTGATATGGAGAATAGATCGATGATGGACGAGA
AGGAATCGGAGATTCCGAGCCCGGATGTCAACAAGACGACCG

>novel_circ_001896
GACAAGCTGTTGACCAAGCTCGAGAAAAACGAGCCGGAATTAGCCTCCTCTCAAGAA
GACGAGGTGAGTAAACGATTTCTTGCGATTACTTTAAAGATCGATCGAAGGTTGGTAG
GATTCTCGGTTGAATTGGCCTCGGTGGAATCGGATATCTCGATTGGATGAAAAATCACA
GGAGGGAGGGGAAAAGGGAATTACCACTTAATCTTGGAGCGAAAGAAATTTACTCGG
TCGGAAGTCGAGGTTACCCCCCTCGATTTAATTATCATCCCGGTGTTGAGGGTCCCGGC
CAGGCGAG

>novel_circ_001897
GAGAACAAGAAGTTTAAACGCACCTAGTCCGATTCAACAATTTGGGCCATGCGGACGC
ATCATGAAGAATTCTGCGATGGTGATTATGCATATTGCTCGCGCTTGCGCGCCGTCTGAT
TGTTTACCGTTAAAAAACGTAGCGTCGTGTTCGGTCCACGATCAGTTCGAGCGTGGCT
CTCAAACTGGTATCACGTCATGGTTGGAATTATCAGGACCATACAAGATCCAGCCTCCC
AGAGGTTGACGTGGTACAAGCCACCGCTCACTGTTCTCGTTATCAAAAAAGTCCGGGA
TTCCTCGGTACTGCCACCATTTGTACAATTAGTTACATGGTTAATAGAAGAAAAACGGA
TGGTAGTATTCGTAGAGGCATCTGTCCTGGAAGATCCAGCCTTGGCTAGGGATCTCAGA
TTTCAAGAGGTTCGAGATAGGCTACAAACTTTCAGAGATGGCACGGATGATCTACAGG
ATCGTATCGATTTCATTGTCTGTCTGGGAGGCGATGGAACGCTTCTCTACGCAAGTTTG
CTATTCCAGCAATCGGTACCTCCAGTGATGGCATTTCATCTTGGTTCCTTAGGATTCCTC
ACGCCCTTCGAATTCGATAATTTTCAAGAACAAGTAACGAACGTATTAGAAGGTCACG
CGGCTTTGACCCTGAGGAGTCGACTGAAATGTATCATCATGCGAAAAGGAGAGGAGA
ACAAGGAGACAAAACCGCCACGAACGGAATTATTGGTGCTGAACGAGGTCGTGGTGG
ATCGAGGCCCGTCCCCGTATCTCTCAAACATCGACCTCTTCATCGACGGTAAACATGTG
ACCAGTGTACAGGGTGATGGCCTGATCGTAAGCACGCCGACCGGGTCTACGGCGTATG
CTGTTGCGGCTGGGGCCAGCATGATTCATCCTTCAGTACCTGCGATCATGATTACACCG
ATCTGCCCTCACTCTTTGTCCTTCAGGCCGATCGTTGTGCCCGCCGGCGTCGAATTGAA
G

>novel_circ_001898
GTAAGGCCGAGGGATCGAGGAGGAGGCTGCTGTTGGATCGCGCGTCGATAGGCGTCG
AGGGACGAAAGGAGAAAAAGAAGGAAGAAGAAAAGGAAAGAAGTTGCGTCGTCAC
GTCGAAGAAACGATCGGTGGAAGAAAGATACAACGCCCCTGTGATTTTTCGTACCCCT
CTGATTCGCGAGTCGGGAAGAGAATCGCGTGGACGAGGGAAGAAATAGAAGGCGGCC

TAGTTATGTCGAGTGTGGAAGGCTGACCCCGAAGGAAAAAGGCGGAAGGGGAGGAG
AATTGAAGATAGATGGGATGCCGGAGCAGTGAACCGAATGGAAGGATGTATCATCCGG
CGTTGTGACTGCCTATGGATATATTCGGCCGTTGCAACGGCGGCGGTGGCGGAAGCAG
CACGGTATCCGGAAGCGGCGCTGGCTCGGTGAGGGGGTCCGGCAACGAGTCCAGCTG
TGGAACGGCGAGCGGTGCCAGCGAGAGCGGCGGAACGAGCGACAGGATGCAGTTGA
CAGGGGCCTCGGCTCCCGGCGTCGCCGCCTCGCCGGAGTCGGGCGTTTCTCCTTTGAC
CGACGCCTACACCAAGATGACCAGCGACATCCTCGCCGAGAGGACTCTCGGCGACTTT
GTCAGCGAGCATCCCGGAGAACTCGTGCGGACAGGCTCCCCGCACCTGGTGTGCACC
GTTTTACCGGCCCATTGGAGGTCGAACAAGACCCTCCCGGTCGCGTTCAAGGTGGTCG
CTCTCGGCGAGGTCGGGGACGGCACGCTGGTCACCGTTCGCGCGGGCAACGACGAGA
ATTGCTGCGCCGAGTTGAGGAACTCGACCGCCCTCATGAAGAACCAGGTGGCCAAGT
TCAACGATCTGAGGTTCGTCGGGAGAAGCGGGAGAG

>novel_circ_001899
GTCATATCCATCGAGCCGAGATTCCTAAGGGATTTCTGCACGCCCCAGAATCTGGCCAC
TTGGCTTCCGCTGGTAAAGGACACTCTGTTGCCCGTCTTCCTCGCGCTGTTCGACAAAA
TGTTCTGCCGTTACGTGGCCAAGGTCTACACGAAACAGGACCACGCGAGGAGGTTGT
CTCACG

>novel_circ_001900
CCCCTGATAATGGTCCCCAGCGTTGAGGAAGATCGCGTTCGGCCTCTCCTTCAACAAC
CGATTCACAGCCGTGGAAATCCTCGCTATTCCCCCACGCAGCTCGTCTCCTGCCCCTC
TTGACACGTTCCGGATCGTGGATTGGTTTGCTCGAACCTGGAAAAGAAGAGAATAGAA
CACTTCTTGGAAACTTGAAGAGAGAAAATCAATGATCGTTCTCGATCGGATTTTTATTG
TCGTCGAAATCGACGGACAGCGCGTGCAGGGAATGGAAATTTTGTCAGAGCGTGCCG
TCGGTTAACGAGAGTGTTGAGCAACGACGAGGAGAGGAAAAAAAAAAGGGAACACG
GTGGAAGGAAAAGAAGGAGAAGACGATTGACCCGAATCATCGCGCCCCTGACCCTGA
GATTTCGCTTGGCAGGACCTTGGAATCGAATGGCGCATGCCGCGCTTTCCGATTCGATT
GTTCTGTTTACGTAACCGATCGTTACGTCTCTCTCTCTCTTTCTCTTTTTTTTCACGATTG
ATTCCTCGAATCGAATCCAGTTGAAAATCTGAAAAATCTCTCGCGAATTCTCGAGATCA
ACGCTAGGATCTGTCGGATTATTTATTCATGAAAAATTATTAATTAATTTATAATTTATTAA
GAATTAATTAAGAAAGGAAGGAAAAAAAAACACGTGACGATTCCGTTCAAAGATAATT
TCAATAATTTCGCAATAATAATTTGAAAGATAAAGAGATAATTTCTTTCACTTTCACTCA
CTTGAATTGATCGATGAGAAGATAGATTGAAAATTAATCGGATCGCTTTCGATTGGTTAT
CGATCGATCGCGCGAATGTATTATAAATTAATTAAAAAAGTCGCTGTTTTATTTTATTAGA
TTAGATCGCTTATTTTATTAGAAGAGTTTCTTTTTCGAAAATGTTATAGAAGAAATTTTCT
AAAAGATAGAATTTTTAATTTGAGAGAATCAATCAATTCAACATTTCTTTGAATATCGAT
TAATAAGATTATTCATAAGATTATTTATTAAATTCGTAAAAAAAAAAGATTAATCTTCCAT
GCGTAGCAAGAATTTATCGAATTTATGAATTTATCGAATATTTCCAGAACGATCGATACG
ATATTTCGAGCCTCGTCAATATATTTTATTCCTTTTTTAATTTTCGATCATGAAATAGAAG
AATATTTTATATCTTCGAAACTCGAAGACATTTCTACAATTTTTTTAAATAAATCTTCAAT
TCTTTTCAATTCATGCACCGCTATAATAATAACGTCTTGCAATTCGCGCAACCAATTTAA
CGATCGATGGATCAATCCCCTTACTTCATCAATCGCTTTCTCTTAACGAGAAAGAAGTTT
ACAATCCGTCCAACGATTTAGATGGACTGGAAAGATACGCTCGATACAGCATTATTAAA

CGGTTAAACATCATTAAAGGTTAAACGTTTGAGAGGATCGATGAATCTCGAACATTTTT
CTCACCTCGCGTGGAAATCGTTCAGATGTATCACCGACAACTCGAACAAATCCGATCGT
CCCGGATAACGCGCTCCTCTTCCGGTGGATCCGTGGCATATCGGGGCGAGGAGCAACA
AGGGCAACAAGAGCCGATAAATTCGGCCACCGTGGTTGCAATTCATCCGAAA

>novel_circ_001901
ATTCGAGCAGACGTCGAAATTGTCGAGCGTTTGCTCGGCGAAAGAGGCGAAGGAGAA
GAAATGCTACGGGGGATTTGCCAGGTTGGCGACACTGATCCGGCAAGCGCGAAAATCG
TCTGTCCCCTGCCTCTTCTTAAACGCTGGGGACACGTATCAAGGATCCATCTGGTACAA
CGTGTACAAATGGAAGATAGTTGCCAAGTTGATGAATCTTTTGGCTCCGAACGCCACG
AGCTTAGGGAATCACGAGTTCGACGATGGCGTGGACGGTCTGATACCGTTCATCCAGA
ACGCCACGTTCCCAATTGTCACTTCGAATCTAAATTTGAGCAAACAGCCGAACTTGGC
CGCCACCAAGCTGTTGAACAGCACGATATTGACCGTGAACGGGGTAAAGATCGGTATA
ATCGGTTATCTGACCCCTGACACGAGGATAATCTCTAGAACGGACAACGTGATCTTCTT
GGACGAGGTGCAGAGCATACGAAGAGAGGCGAAGGAGTTGAAGCACAAGGGTGTTA
ATGTATTGATCGCCGTCGGCCATTCCGGCTTCAAAACGGATAAAAGGATCGCCCGAGA
GGTGGAGGATATCGATATAGTGATCGGGGGGCACACCAACACTTTTCTCTACAACGGG
AAACAACCGGATAGGGAGATACCGGAAGGGCTTTATCCGACGGAAGTAACGCAGAAA
AGCGGGAGGAAAGTGTACGTGGTTCAAGCTTTCGCTTACACCAAATATCTTGGCAATTT
TACCGTAACATTCAACGATAAGGGAGAAGTAAGCAATATCAGTGGAAATCCTATTCTCG
TGGACAGTAGCATAGAGCAG

>novel_circ_001902
ACTCGGGTGCTAGTATGTGTTCACGGCTCAGTCCTTCGAGTAGCGGTTACAAATCGCAA
AGTCAGCGCAGCGATGCCACGATATCACCGTCTCCGTCGCCATCCCCCGTGGCAACGA
TGACCTGCAGTCATACCCAAGTATCTAGTCAGGATCTCGCCTCCCCGGAATCGAATAAT
CAGACGGTGATGTGGGCGGTCACGAGTATATCGAGCGTGCCGCCTGGTAGCATAATTAT
TAACCCGCAAACGAATCAACCGTACACGAATCCGGATGGTTCGATTTATCGCTTCGATC
CGGATAATCCACCCAAGTTTTATACACCTTCGTTGTCTCACGAGAACGAGAGAAACGA
GAACGTGTCGCCCGCGAAGATCGTAGAAACGTCCGAGCCATCCAAAGTAACGGTTAG
AAACGAGAATAGAAAGAGATCGCAATCCAGCAAGCACAACGGTGGCAACAATGGAAC
GACAGTGACCCACGTGACCAACTCGGCAACGTCGCCGAGTTTGCCGTTCACCCCACC
ACCTCGAGATAATCCAGGACCATTGCGACAGCAACAGCAACAGCCGCAGCCGCAGCA
GCAGCAGCAACAAACTCTAGTCAAATCGTCGTCGACGGTGCAAACTTGTAATCACAAT
CAAGCAGCTCAAACATACGCGACCTACGTACCTACCTCAGAACCGTTCAACCAGGGTG
TCTATCCACCGCCCCATGTGGGACAACAGACTGTGATGATGGCCCCCCATCCGAGTCA
GAGTCAGACGAATGTTCAAAACAATGGACAGAACGATGTGTTTAACCAGAATTCGGTT
TACGCGAATTATGCACCAGTACCTGTACAGCAAGGACCAGTATCGCAGACAACGGAGA
TAACCGAATTGTCCGGTTATTTTATGGGCATGAGCATCTACGATCAACGTGTAACTGGTG
ATAATCATTCAACGCCACCGCACTCTTATCCTCAACCGCAGCAATCCACCGCGAATCAA
ACGAATCTGCAAAATGTGCAGACGATGCCGCAAAATTATTGGCAACCACCACCTAATT
CCGTACTGGTATGAATTATTATATTACGTTATTTTATATATATATTTTTTCAATATAATTTCAA
TTCTTTCATTATTTTTGCATTATTTTCCAGCCGCAACAAACAATGTACTTCGTACCTCCG
CCTGGGGCGGCACTTTCGGTTAGCCAAGGTCCAGCCGACAGACAACAGTTGCACCAG

CAACAAAGATTTCCAACAAATTATTCTTTCAATGCACAAACTATGACTCCTCCGAGCCA
ATCGAATTGTAGGTAGATTTTCTAAGATTTTTCTAAATTATTATTATCGTTACTCGCGTCT
CGCGTGTTTCTAAATTACGTGGTAAATATTATTTGATTTTCCATTCTTTTTTCTTTCAGCT
AATTACGTGGGTACTTATCCAGTCCCTTACAACTCGATGCCCGCTGTGACGCCAGCACC
AGGGGACTACACGTATCAGCCACCTGTTCACATGGTCCCCACGTACTATCCACCGAATC
AAACGACCATTCAACCGCCACCTGTCATGTACAGAGTACCAACACCACCAAATACTCC
AACTTCTAACCAGGTAAAAATAGCCATTGCTTTAAACGGCGGTGTATTAATTTTAAAATA
TTCATTGCCAGATTAGATAAAAGTATAAATATCCTTGTAATCGATCAATAATATAATACAT
AGATGCCTGGGGTGCCATTGATGTATGTCAACTCTGGCAGCTATCCACCACCAACGATG
GTATCCAATGGGACTTTTGGTCATCAGGTTGGACACAATGGCACGTCTCCAGCGCCCCC
TGGAACTTACATGACACCAGCGCTGGTTCCCAATCTCGTGTTTAGGCAAAATGTTCCCG
TAAGTTGGTACTATTGCAAGAGAGAAATGGAGAAAAATTCAACTATTATTATCATATTAT
TATTATTATTCAGGTCGTTGCTGGTGTTCGAGCTTCAACGCCAGGTAATTCTCAGAAAA
CTAGCAGGTCGCCGACTCCAGCACACGAGTTGTTCGGAAGTGGGAGCGGGGACAGAA
GCGCACAACCCCAACCACGCTACCCACTGCCAATGTATCAGGGTGTCCATATTGTCCAA
GGTACGATATATATATATATATATAGAATTTTAGAATTTTTTTATGGGATTCGTTATCTTTTGAT
ATTCCATATTCGCAGGAGATATGAGATTGATGCATCCCGCAGTGCCAGCTAATCCGCGAT
TGCAGTACGCGCCAGTACCATCACCACCTGTTGTTCAAGGTTGTCCACGACCGTATCGA
CCACCTTCTTATTCGTCGAACACCTCAGGCGCTGGTACACCCACATCGTTCGATGGGAG
AAATCAGAAAATTCGTAAACAGAGGTGATTTCAGATAGCATTGCTTTGCGCGATATACG
GTTAAAATGGAAAAAAGAGAGAGAGAGAGCGGTGACGTTAATTTTCCTTTCATTCCAG
GTCCAAAGTAACACCGTTGCCGTTAACAGGCGGTCGGCCCAATCTTTATCAGACTCCTT
CCATGCCGTCGCTCTCGTCTAACGTACCGAAAGATATCAGAGAAG

>novel_circ_001903
ATAGTAGTACGGAATTGTATACGGACAGCACAGGGATAGATCTGGAACATTTCATCGCC
GAGACCATAAATCGTAATCAGAAGGATCGGACGGTGCTGTTGAAAATCGAGAAAGATC
TGATAGAGTTCGCCAGGGACAAGCAGAAAGTCTGTCACAAGTTTCCCAACATGTCCTC
GTACAACAGGATGTTGGTGCACCGCGTGGCCGCGTACTTCGGTATGGAGCACAACGTG
GACCAATCTGGTTCGAGCGTGATAGTGACGAGGACGAAGAACGTGCGAATACCGAAC
ACCCGTTTCAAGGAGCACATAAGGGACGACTTGATACTGTCCGAGGAACCGAGGAGA
AGCATACTGAAGAGGGACTCGAACTCGTTCGAGGACAGTTTCAACTTCAAATCGCCGG
ATAGATTGTCGGGCGATTATTGCCGGCAGAGCAAGAGTTTCGAGGAGAGGGAGGAGG
AGTACGAGCGCGCGAGGCGGAGGATATTCAAGGACAGCAGCGGGGAGAGCAGCGAG
GTTGCGTCCTGGCCTTACTGGTCCTCCTCGGAAAGCTCCGACGCGTCTGCGAGGTATC
GTTTGTTACACCCGTCGGATCACACGATCATCAGGTGAATATCCTGCCGAATCAAAGTT
AGCTATTATTAAACGTGTCGAGTAATAACAGCGAGATCATATTACCTATACTTATATATATA
TATATATGTACATGTATATATATATGATAGGCAAACGAAGCTGTTGAAAGGAGAATCGTT
AGATGGAAGGGAATCCTGTCGGGGTTCGGTACTGAGACCATCCGTTTCCAAGTCCTTT
AGCTTCGGTGGTTATACCAGGGGAATGCTTTCTAGAGGGGACAGTGTTACATCCACTCA
TAGCGCGGGAGCTCGTCTTATGAAGCAAG

>novel_circ_001904
ATAGTAGTACGGAATTGTATACGGACAGCACAGGGATAGATCTGGAACATTTCATCGCC

GAGACCATAAATCGTAATCAGAAGGATCGGACGGTGCTGTTGAAAATCGAGAAAGATC
TGATAGAGTTCGCCAGGGACAAGCAGAAAGTCTGTCACAAGTTTCCCAACATGTCCTC
GTACAACAGGATGTTGGTGCACCGCGTGGCCGCGTACTTCGGTATGGAGCACAACGTG
GACCAATCTGGTTCGAGCGTGATAGTGACGAGGACGAAGAACGTGCGAATACCGAAC
ACCCGTTTCAAGGAGCACATAAGGGACGACTTGATACTGTCCGAGGAACCGAGGAGA
AGCATACTGAAGAGGGACTCGAACTCGTTCGAGGACAGTTTCAACTTCAAATCGCCGG
ATAGATTGTCGGGCGATTATTGCCGGCAGAGCAAGAGTTTCGAGGAGAGGGAGGAGG
AGTACGAGCGCGCGAGGCGGAGGATATTCAAGGACAGCAGCGGGGAGAGCAGCGAG
GTTGCGTCCTGGCCTTACTGGTCCTCCTCGGAAAGCTCCGACGCGTCTGCGAGGTATC
GTTTGTTACACCCGTCGGATCACACGATCATCAG

>novel_circ_001906
AGTGTACGATGTTGGAAGGTGGACGTGCAGTGATTTGGCTCCTTGTATCAGCCCCACG
AGCTGCACGCCACCCGAAAGATTCACGTACTAACCCAGTTGCACCTGTTTGAACCCAC
CAGTTGCAGAGTCTGCACCGTAACCCCATGACGCATTTAACACATGGCCCGCCTGGAG
AGTAAGTGTTTATTCACGCTCACGATCTTACGTCTCTCTCTCTCTCTCTCTATTTATCT
TTCTCTCTTTCTCTCTGCTCCCCTTGGCTTCTCCTTTCAAATCTTGATTCGTTACGCAGA
CGAATTCTTTCCAAGGAATTAATCCGTTTCATCTTGCATACATTGCCATACATTCGGATC
AAGGGAAATTTTATATTGGAAATTCTGTTTTTTTTTTCTTCTTCTTCTTTTTCTCTCCAAA
AATCCGGGCATGTCAAACGAATACGCGAAGGGAATACGCAAGTGAGTGGTGGGTTTTG
TTTGCAGTACCAAGTATAGTGGTGCATAAAGGCAGCAGGTCGCACAGCCGACCTGACA
GTCCCACGGTGGTGGTGCGAGGAGGTATGCCCACGCTGCCGTCCTCCAGGCAAGGCTT
CGTCGCGAGTGGCTCGAACGCGTCGTATCAGAACAGCACGGAGCCGGATATGTTGGAC
GGGAATGCCAATCCGAATGTCGTCTCCGGCTCCTCTCCCGCGTCGAACTCGCACCATC
GTGGCGGCGCGCCCTCCTGCCCTCCCCCCGACAACAAATCGGACGCAGAGAGCAAAA
ACTTTCGCAACAGG

>novel_circ_001907
CTCAAGCTGGTGCCGTCGATGAAGAAACCTTCCTTACAACATTCGAAGATGTGCCATC
CGTAAATTTATTTCCGCAAAAGATCTTGAAGAGCAAATGAAAATTATAAAAGATAATG
TTGGCGATGATAAAAAGGATTGGAAACAAAGGACGGAAAGTATGAAAAAATTAAGAG
CAATCATTATCGCAGGCGGTACGAATTACGAAAACTTTTTAGAAAATTTGAAGAATGTT
CAAAGACCTTTTGAGGTTGCTTGCACGGATCTTAGATCGCAAGTAGTGAGGGAGGCGT
GCATTACTTTAGCATATCTTAGTCAACAGTTAAAAAATAAGTTTGCCAGCTTTGGAGAA
GCGGTCTTACTCACCTTAATGAATCTCATACAAAATAGTGCCAAGGTTGTGGCAACAGC
TGGTGCCGTAGCAGTAAGGTTTATTCTTCAAAATACACATTGCAGTCGTTTTGTGCCAA
TTATCACGTCGTGCTTAAGTCACAAGAGTAAAGACATACGTCGGGCTTCATGCGAATAT
CTAAATCTAATTTTACAAATATGGCCTACTCAAATATTACAAAAACACGTGACTACGTTG
CAAGACACGATCAAGAAAGGTATTGCAGATTCAGATTCGGAAGCGAGAGCTTTTGCTA
GGAAATCGTATTGGGCGTTCAAAGATCATTTCCCTGAACAAGCAGAAGCGTTGCTCAA
TAGCCTTGACACTGCATACAAACGTTCGTTGATGTCTCTCAGCAACAGCGGTAGCATTA
ACAGTTTAAATGTAGTTACGAGATCGGCGAGCGTTAGTCCCCGAACATCGAGACCAGT
CATGAGCGTTACAGGTAGTACGGAAAATTTGCATCAAACTTCGGGTCAACCACATGGC
CCGCTCAGACGAACGCCGTCCTTGCCACGGTCATATCGTCAATCTGGTATCCCAGTCCT

TCAAAGACCCACTGATAGCCATTATCGTGGAACTTCGAGTGTTAGGTCGACTAGTGCGA
TAGATTTGCAAGCCGCGCAAAGAGCTAAAGCTAGAATGATGTATGCGAACATGAGTAG
ACAGAAAGCATCGCTTCCACGTCCTAGCAAATCACCAGACACAACTGCTGTTGCTAGT
CCAGAAAGAACGGCAAGAACAAGAACTCGAATGTCTGGAGTGTCGCAATCTCAGCCT
AGCAGTAGATCAGGATCGCCATCGTCAAGACTGAGTTATGCAACTTATAATCGTGAAGG
AGAATCATTAATTGCAAGACCTAGACGGTTATCTGGACATGGAATCAGAAGTACCGGTA
ATAGTCGTGAGCCAAGCCCACAAAGGTTTGGAATGGACAGAAGTTTTGCAAGCAAAAT
ACGGGGAAGAAGTTTACATATGTCTCCAACGGATAGACCTCAATCCAGACCAGTCATG
GCACAAAAGATGTTGCAACAATCACGCGAAGCAGAATCAGCATTGGCGGATGCGCTTA
CATTTGAAAATATTGATAATTATACAAGAACACCGAGAGGAAAAGGTGATCACAGTGAT
GACAGTGAAACTAGCAGCATATGTTCTGAACGAAGTATGGACAGTTTTAGACGACCAA
ACGAT

>novel_circ_001908
GTCGTCCAGAATGGTTTAGAAATCCTCACGTTTCTAGCGGATCGAATGGGTCACGACTT
CAAACCTTATATTTCCACTATTATTCAACCAACAATAGATCGACTGGGTGACAGCAAAG
ATGCGACGCGAGAAAAAGCGCAGCTAGTGCTTCTTAAAATAATAGAAAAAGGATGTAT
GACTCCTCAGCAACTTTTGGATAGACTGCGGCCGGCCTTTAATCATAAGAATGCAAAAT
TACGGGAAGAAGCTCTTATTTTGTTAACAACAACGTTGAACGAGCACGGCGCTGATGA
AATGATGTTGTCAGGAGTGATTCCAAGTATCGTAAAATTATTATCAGATCCATCTGAAAA
AGTGCGAGAAACTGCTTTGAACACATTAGCGGATATCTACAGACACGTTGGCGAGAGA
TTACGCGTGGATTTACAGAGAAAGCACAATGTACCGCAAGCTAAATTACTGTTGTTGAT
AGAGAAATTCGATCAATTGAAAGCGGCCGGCGACCTGCTGCCTCTAGCAATGTCCTCG
GATG

>novel_circ_001909
GCCCGTCTGATCCGACGTCCAAGGGGTCCACTCGTCTCAGTAGTAACTGAATTTTCATG
GTTGCTGATTGTTCTGCTGGTTGACCACGTGCTGGAGAGTGAAACTCAAGGCCCGAGG
ATCACCGTGAAGCCTCAAGGCTTGTTCCAATCGTTGCTCTTGCAATCGCAACGCTTCCC
CTGTGAACCGTGATGAATTTCTTTGAAATGAAATCGTTCCAATACTCTCTTCCCTGCTGC
TTCGATCGAACGAACGACGACAACAACGATAACAATAATAATCGACGAAAGATGAACG
ACGGAGAGAAAGACTTACCAAGGTCAGGCGGTAAGGTGTCTTGTTGAGACATCTGTT
GGTTCGTGTGGTGTCCAGGCATGCCAGAGGTGCTGCCTCCGTTATGCTGGCCCAAATG
ATGTCTCATGTCCCCACCGACCGCGGAACTGGCAGCCGCCGCTGCTGCTTGCGACACC
TGACGAGCGAAAGAGCGTGTCATACAACGTGTAACGGAGATATATTAAG

>novel_circ_001910
TGCAGAGCAAAGTGAGATGTTCCGAGCAGTGGATGGAAGTCGACATCGTAAGGAGCA
GCCCACAAGCGAGGATATATCTGCAACAAATGAAAGACTTTCCAGACGAAGCTTGCAA
ACCTCGGCTTAGCAACGGTGTCGCCACGTTCCGATTACCTCTCCTAGAAGAGGACATG
TTGCGATGCGGCATCACGAGGGTCGTTAACAAAGTCACGGGTCAGAAGGTGTACTTTC
ATCGGATAATAGTGGAGGAGCTCGCGGATTCCAGCAAGCACACGTTCACCGTAAAATG
TGTTATCACCGAGGTCGTTGTGAAACCTGGAATCTCGATCGCAGCCAATCACACCGTG
GTTCGCAGGAGCGTTCTACCAGCAGGTTTCCAGGAACCCGA

>novel_circ_001911
GAACGGATCGGAGAAGATAGGAGGGAGAGGAACTTGTGGTAGATAGAAAAGGAGAGA
TTGCGCGTACCGGTGAACGAAATGGAAGACGAGGACTTTGGCTTCGCTTCGAAAAGG
GCTGATTCTAAGGAAACGATGAAAAGTGATAAGGATAGCTGCCCCCCGATCGTCGGCG
ATAATTGCTCGGGAGAGAAGACGAGGGAAACGATCGGTGGTGGTGGGTATAGATCAGT
GATGACAGCGCCGTTATTCGGGGCGACCGAGGCCGGAAACGCCGTCCCTGAGCGGGC
AGCGACCGCCTCTTCATCCTCTCCGGCTTCCTCAGTCGTTTCCAACCCGCCGAACATAC
CGAACGTAGTCGAGTATCATCTGAAAGTGAAGCCTGGCCAAACGCGGAGAATTAGCAC
CACTCGACTCGATAATAGTAGC

>novel_circ_001912
GAATCCTGCTGGCAAACGCGATCTCGGCCGAACGTAAGGCAACCAGGCCGAAATTCA
AGATCGCCACCACGTCGACGACCACGACAACGACCACCACAGAGGAGAGCCAGAGG
AGCGAGAACGAGGATGTTGAGGCGACCACCACCGCAACCGTGGCGGCTGGCGGGGA
GGCGAACGCGACCAGCGGCCACGTCCTCACAGGTATCCCGCAGATCGACTACATCTGG
GACCCGAATTTGCCCCGCGAGTTGAACGGCTACAACCTGAGCGATTACCCGTTCTACA
ACAGCATACCCGAGGACATCGACTTCAAATGCGACGGCCTCCACGATGGATTTTACGC
GAGCGTGCCGCACAAATGCCAG

>novel_circ_001913
GAAATGTCACAAATCAGACGAAACAATGCGATACCCAAAAGATGGAGAATCAATGCGA
TGAAAACGAGAGATGCTTTCAAAATTCGAAAGGGGAAAAAGCATATTGCAAATGCAA
GATAGGTTACGATCTGGAGAAAGGCCGATGCGTCCAGGTTACGATTACCACGGTTGCC
AGTGTAACTGATCAACCAAACGTCACGGCCAATTCAGCAGGTAGTTCTGTAGCCGCTG
GCTTGTTAATACCAACTTTTCTCGTAGTAATGAGCGTATTACTGTATTTTGTCGCGAGGC
GATATAAATGGTTGCAACGAGTTCGACAGCTTCGTCCAAACCATTATGGTAATGTCCTA
GTCACAAGAGACGACGATGACGATGACGATCCACCTATAGC

>novel_circ_001914
ATTTTCCCGCAGGAAATGTCACAAATCAGACGAAACAATGCGATACCCAAAAGATGGA
GAATCAATGCGATGAAAACGAGAGATGCTTTCAAAATTCGAAAGGGGAAAAAGCATAT
TGCAAATGCAAGATAGGTTACGATCTGGAGAAAGGCCGATGCGTCCAGGTTACGATTA
CCACGGTTGCCAGTGTAACTGATCAACCAAACGTCACGGCCAATTCAGCAGGTAGTTC
TGTAGCCGCTGGCTTGTTAATACCAACTTTTCTCGTAGTAATGAGCGTATTACTGTATTT
TGTCGCGAGGCGATATAAATGGTTGCAACGAGTTCGACAGCTTCGTCCAAACCATTATG
GTAATGTCCTAGTCACAAGAGACGACGATGACGATGACGATCCACCTATAGC

>novel_circ_001915
GCGAGGCGGCGTCCGCGAGAGATTCGTCGTCGACGAAACGAAGGAGAGGAAAAAAG
AAGAAAGAGAATCGAAGAGAACGAGGAGGAAAAGGAGAGGAAATGCGAGAGATCTC
GGCTGTTCGTTGAGGGCGAAACGGCGAAGAGCAGCTTTGTGCAGCTTTGCGGAGGAA
GAGGGGGGGCGGGGGGGAAAAAGGAAAAAGTCAAAAACGCAGTTTGTACGCAGGCA
TCGGGAGCGGTACTTCCGGTAGAAATAAAATCGAGCCGTGCGGTAGATGCAGCCGCGT

CCACGTGGGCCGCTCGTTCCGTGGAAGCGAACAGGGGCCAAGCGCGGCAACGAGAC
ACGTAACGGCGACGAGATCGTTTGGACGGGCCTAATCGCGGTTCATGGTATAGGAGGA
ATCCGATCGTTGCTTTTCGGGCGGAGAGGGGAGGGGAGGAGCGTGGGGAAGAGGTGA
GTGAGAGCGAGGAAGGGGGCGGGGGGGAGGAGAGAGACGCGTCGCTCCGCGCGTGG
ATCGGCGCGGGCGTGTGGAGGTTGGTTCGCGGGGAGGGCGCGAGGAAGGGGGCGGT
TGGAAGGGTTCCGCGACCTGGAGCAGGAGCAGGGGGAGGAGGGGGAGGAGGGAGA
GGCTAGGACGAGAGGGTGGTTGCGCGTCGTGGTCGCGTGACGCGCGAGGAAAGTGCC
GGGGGACGGGCGGGAGGAGGAGGAGGAGGAGGAGGAGGCATGAGGAGGAGAGAGT
GACGACGCTGCCGGTGCTGCAGCCTCGCACGGATCGTGAGAGGACCGGCGGCCCCAC
GGTGGGGCTGCGATAAGAATGGCCCCCTTCAATATGGCGGGCGTAGCAGGCCTTCTAG
CCACCTGCGCCGCCGCTGCTGCTTCCGCTGCTTACCTTCTGCCGTTGCTGCTGTTGCTG
ATCTGCCCGCGAGGGACCCACGCGGACCCAG

>novel_circ_001916
TGGGTGGAAGAGTCGTTCACCAACTTCGACAAGGGCATCAATTGGCGGAGTTACGTTG
TCTGCGACGTCGCGTACAACAACGTGAACAATTGGCTGTGGACCCCGTTCATAGAGAG
GGGACCGGCGAACCGCATGTACATAGAGATCAAATTCACGACTCGCGACTGCTCCCTG
TTCCCAGGAAACGCGCTCAGTTGCAAAGAAACTTTCAGCCTACTTTACTACGAGTTCG
ACGTGGCCACCAAGGAGCCGCCACCGTGGGAAACGGACAGTTACAAGTTGATCG

>novel_circ_001917
TGGGTGGAAGAGTCGTTCACCAACTTCGACAAGGGCATCAATTGGCGGAGTTACGTTG
TCTGCGACGTCGCGTACAACAACGTGAACAATTGGCTGTGGACCCCGTTCATAGAGAG
GGGACCGGCGAACCGCATGTACATAGAGATCAAATTCACGACTCGCGACTGCTCCCTG
TTCCCAGGAAACGCGCTCAGTTGCAAAGAAACTTTCAGCCTACTTTACTACGAGTTCG
ACGTGGCCACCAAGGAGCCGCCACCGTGGGAAACGGACAGTTACAAGTTGATCGGGC
GCATAGCCGCTGGGGAGGGAAGGTTCAACACGAACACCGAGGTGGTGATAAACACGG
AGGTGAAATCCATCCCCGTGACGAAGAAGGGAGTCTACTTCGCGTTCAGGGACCAGG
GCGCTTGCATCTCGATCCTTGCCATCAAGGTTTATTACATCAGTTGCCCGGAGATTTCCG
TCAACTTCGCCCACTTCCCTGCCACGCCGACCGGCCGCGAGGTGGCGCTGATCGAGCA
AACGATCGGCACCTGCGTGGCGAACGCCGTCGTGATCGAGCAGCCCACCTTCCTCTGC
AAGGGGGACGGGAAATGGTACTTACCGAGCGGCGGGTGCCACTGCAAACCGGGATAT
CAGGCCGACGTGGAGAAGCAGGAGTGCACCGAGTGCCCGATCGGTAAATTCAAACAC
GAGGCGGGGTCGCACAGCTGCGAGGCCTGCCCGGCCCACAGCAAATCCTCCGATTAC
GGATTCACCGAGTGCCGTTGCGATCCCGGCTACTTCAGGGCAGAGAAGGATCCCAAGA
AAATGCCCTGCACCCAGCCACCGTCGGCGCCGCAAAATCTGACGGTGAACTTCGTCGA
CCAGTCCACCGTGTTCCTGTCCTGGAACGCGCCGCACATGCTCGGCGGGAGGACAGA
CACGACTTATAGGGTGGTCTGCGACGCGTGCAGCATGGGTGTCAAATACATTCCCAAC
ACCGAGGTTTTCAACGACACGAAGATTACGATAACTGGATTGAACGCGGTCACCACTT
ATCGATTCCAAGTGTTCGCCGAGAACGGGGTCTCGGCGTTGGCCGGCAAGTCGGAATA
CGTGGACATCACCGTGACCACGGATGCGAGCGTGCCGAGTTTG

>novel_circ_001918
TGGGTGGAAGAGTCGTTCACCAACTTCGACAAGGGCATCAATTGGCGGAGTTACGTTG

TCTGCGACGTCGCGTACAACAACGTGAACAATTGGCTGTGGACCCCGTTCATAGAGAG
GGGACCGGCGAACCGCATGTACATAGAGATCAAATTCACGACTCGCGACTGCTCCCTG
TTCCCAGGAAACGCGCTCAGTTGCAAAGAAACTTTCAGCCTACTTTACTACGAGTTCG
ACGTGGCCACCAAGGAGCCGCCACCGTGGGAAACGGACAGTTACAAGTTGATCGGGC
GCATAGCCGCTGGGGAGGGAAGGTTCAACACGAACACCGAGGTGGTGATAAACACGG
AGGTGAAATCCATCCCCGTGACGAAGAAGGGAGTCTACTTCGCGTTCAGGGACCAGG
GCGCTTGCATCTCGATCCTTGCCATCAAGGTTTATTACATCAGTTGCCCGGAGATTTCCG
TCAACTTCGCCCACTTCCCTGCCACGCCGACCGGCCGCGAGGTGGCGCTGATCGAGCA
AACGATCGGCACCTGCGTGGCGAACGCCGTCGTGATCGAGCAGCCCACCTTCCTCTGC
AAGGGGGACGGGAAATGGTACTTACCGAGCGGCGGGTGCCACTGCAAACCGGGATAT
CAGGCCGACGTGGAGAAGCAGGAGTGCACCGAGTGCCCGATCGGTAAATTCAAACAC
GAGGCGGGGTCGCACAGCTGCGAGGCCTGCCCGGCCCACAGCAAATCCTCCGATTAC
GGATTCACCGAGTGCCGTTGCGATCCCGGCTACTTCAGGGCAGAGAAGGATCCCAAGA
AAATGCCCTGCACCCAGCCACCGTCGGCGCCGCAAAATCTGACGGTGAACTTCGTCGA
CCAGTCCACCGTGTTCCTGTCCTGGAACGCGCCGCACATGCTCGGCGGGAGGACAGA
CACGACTTATAGGGTGGTCTGCGACGCGTGCAGCATGGGTGTCAAATACATTCCCAAC
ACCGAGGTTTTCAACGACACGAAGATTACGATAACTGGATTGAACGCGGTCACCACTT
ATCGATTCCAAGTGTTCGCCGAGAACGGGGTCTCGGCGTTGGCCGGCAAGTCGGAATA
CGTGGACATCACCGTGACCACGGATGCGAGCGTGCCGAGTTTGGTGAGCAACGTGAG
GATTACGAGCGTGAAGAGCTCCGAGTTGAGCATAAGTTGGGACGCGCCGATAACCGAG
ATCGGCGGGGACAGCGACCTGGTGGAAAGATACGAAGTGAGGTGTTACCCGCGTTAC
GACGACGCCACCAACGCAACCGTGATCCAAACCTCGGAATTGTCGGCGACGTTCAAA
GGCCTGAAACCATCCACGGATTACGCGATACAGGTTCGAGCGAAAACTACGCGAGGTT
GGGGCGAGTACACGCCCGTCGTGTACAAGAAGACGCCTCACGCTATGGGATTAGATTA
CGTCGGGGAGGATGACAATATGCAAGTGAGAATCATAGCGGGTGCTATAGTCGCCGTG
GTGGTTCTTCTCGTCATCATCATCATCATGACAGTCTTGATATTGAGAAGCAGGGCCTC
GGACGAATGCAACAAGAAACAGCCGAGCGATTGCGACACTCTGGAGTATAGAAACGG
CGAAG

>novel_circ_001919
TGGGTGGAAGAGTCGTTCACCAACTTCGACAAGGGCATCAATTGGCGGAGTTACGTTG
TCTGCGACGTCGCGTACAACAACGTGAACAATTGGCTGTGGACCCCGTTCATAGAGAG
GGGACCGGCGAACCGCATGTACATAGAGATCAAATTCACGACTCGCGACTGCTCCCTG
TTCCCAGGAAACGCGCTCAGTTGCAAAGAAACTTTCAGCCTACTTTACTACGAGTTCG
ACGTGGCCACCAAGGAGCCGCCACCGTGGGAAACGGACAGTTACAAGTTGATCGGGC
GCATAGCCGCTGGGGAGGGAAGGTTCAACACGAACACCGAGGTGGTGATAAACACGG
AGGTGAAATCCATCCCCGTGACGAAGAAGGGAGTCTACTTCGCGTTCAGGGACCAGG
GCGCTTGCATCTCGATCCTTGCCATCAAGGTTTATTACATCAGTTGCCCGGAGATTTCCG
TCAACTTCGCCCACTTCCCTGCCACGCCGACCGGCCGCGAGGTGGCGCTGATCGAGCA
AACGATCGGCACCTGCGTGGCGAACGCCGTCGTGATCGAGCAGCCCACCTTCCTCTGC
AAGGGGGACGGGAAATGGTACTTACCGAGCGGCGGGTGCCACTGCAAACCGGGATAT
CAGGCCGACGTGGAGAAGCAGGAGTGCACCGAGTGCCCGATCGGTAAATTCAAACAC
GAGGCGGGGTCGCACAGCTGCGAGGCCTGCCCGGCCCACAGCAAATCCTCCGATTAC
GGATTCACCGAGTGCCGTTGCGATCCCGGCTACTTCAGGGCAGAGAAGGATCCCAAGA

AAATGCCCTGCACCCAGCCACCGTCGGCGCCGCAAAATCTGACGGTGAACTTCGTCGA
CCAGTCCACCGTGTTCCTGTCCTGGAACGCGCCGCACATGCTCGGCGGGAGGACAGA
CACGACTTATAGGGTGGTCTGCGACGCGTGCAGCATGGGTGTCAAATACATTCCCAAC
ACCGAGGTTTTCAACGACACGAAGATTACGATAACTGGATTGAACGCGGTCACCACTT
ATCGATTCCAAGTGTTCGCCGAGAACGGGGTCTCGGCGTTGGCCGGCAAGTCGGAATA
CGTGGACATCACCGTGACCACGGATGCGAGCGTGCCGAGTTTGGTGAGCAACGTGAG
GATTACGAGCGTGAAGAGCTCCGAGTTGAGCATAAGTTGGGACGCGCCGATAACCGAG
ATCGGCGGGGACAGCGACCTGGTGGAAAGATACGAAGTGAGGTGTTACCCGCGTTAC
GACGACGCCACCAACGCAACCGTGATCCAAACCTCGGAATTGTCGGCGACGTTCAAA
GGCCTGAAACCATCCACGGATTACGCGATACAGGTTCGAGCGAAAACTACGCGAGGTT
GGGGCGAGTACACGCCCGTCGTGTACAAGAAGACGCCTCACGCTATGGGATTAGATTA
CGTCGGGGAGGATGACAATATGCAAGTGAGAATCATAGCGGGTGCTATAGTCGCCGTG
GTGGTTCTTCTCGTCATCATCATCATCATGACAGTCTTGATATTGAGAAGCAGGGCCTC
GGACGAATGCAACAAGAAACAGCCGAGCGATTGCGACACTCTGGAGTATAGAAACGG
CGAAGGACTAGTTGTGACCTACATGCACTGCAAAATGGACAGTTCACCGATTGTGACA
ACCCACACCAACAACAAGAGCAAGTCCTCGCTGACCACGCCGCTGTTCACACCTGCA
GTGGGAGTTGCCGCCGCGAGTGCTGGTGGCGCGGGTGGCGGCGGGGCGAGAAGTTAC
GTCGATCCTCACACGTACGAGGACCCGAACCAAGCCGTGCGAGAATTCGCGCGGGAG
ATCGACGCGGGATACATCACGATAGAGGCGATCATAGGTGGGGGAGAGTTCGGCGACG
TGTGTCGCGGGAAATTGAAGTTACCGCCGGACGGTCGAACGGAGATCGACGTTGCCAT
CAAAACACTGAAACCCGGCTCAGCGGATAAGGCGCGCAACGACTTCTTGACCGAGGC
ATCGATCATGGGCCAGTTCGAGCATCCGAACGTGATATTCCTGCAGGGTGTTGTCACGA
AGAGCAACCCGGTGATGATCATCACCGAGTTCATGGAGAACGGGAGCCTGGACACTTT
CCTTCGCGCGAACGACGGCAAGTTTCAGGTGCTCCAGCTTGTGGGCATGCTTCGCGGT
ATAGCGAGCGGTATGCAGTATCTGGCCGAGATGAACTACGTGCACCGGGATCTCGCAG
CGAGGAACGTGCTCGTGAACGCCGCCCTCGTTTGCAAGATCGCAGATTTCGGATTGAG
CAGGGAGATCGAGAGCGCGACGGAAGGAGCGTACACGACCAGG

>novel_circ_001920
TGACCACGCCGCTGTTCACACCTGCAGTGGGAGTTGCCGCCGCGAGTGCTGGTGGCG
CGGGTGGCGGCGGGGCGAGAAGTTACGTCGATCCTCACACGTACGAGGACCCGAACC
AAGCCGTGCGAGAATTCGCGCGGGAGATCGACGCGGGATACATCACGATAGAGGCGAT
CATAGGTGGGGGAGAGTTCGGCGACGTGTGTCGCGGGAAATTGAAGTTACCGCCGGA
CGGTCGAACGGAGATCGACGTTGCCATCAAAACACTGAAACCCGGCTCAGCGGATAA
GGCGCGCAACGACTTCTTGACCGAGGCATCGATCATGGGCCAGTTCGAGCATCCGAAC
GTGATATTCCTGCAGGGTGTTGTCACGAAGAGCAACCCGGTGATGATCATCACCGAGT
TCATGGAGAACGGGAGCCTGGACACTTTCCTTCGCGCGAACGACGGCAAGTTTCAGG
TGCTCCAGCTTGTGGGCATGCTTCGCGGTATAGCGAGCGGTATGCAGTATCTGGCCGAG
ATGAACTACGTGCACCGGGATCTCGCAGCGAGGAACGTGCTCGTGAACGCCGCCCTCG
TTTGCAAGATCGCAGATTTCGGATTGAGCAGGGAGATCGAGAGCGCGACGGAAGGAG
CGTACACGACCAGGGGTGGAAAGATCCCGGTGCGATGGACGGCCCCGGAAGCGATAG
CATTCCGAAAATTCACCAGCGCTTCCGACGTGTGGAGCATGGGGATCGTTTGTTGGGA
GGTGATGTCGTACGGCGAGAGGCCGTACTGGAACTGGTCCAATCAGGACGTGATAAAG
TCGATCGAGAAAGGATACAGGCTTCCAGCACCGATGGATTGCCCGGAGGCTATCTATCA

GCTGATGCTCGATTGCTGGCAAAAGGAGCGAACTCATCGCCCCACTTTCGCCAATTTA
ACGCAAACATTGGACAAACTGATACGAAGCCCGGACACGTTGAGGAAAATCGCCCAG
AACAG

>novel_circ_001922
AATAATGCTGAGGTTCGTTTGGTTACTCGGAATAATCGTTCACAACACTTTTGCGACAC
AAGTTCCGGTGAATAATCCCGGTGAATGGTCATGGCAAGTCGATAATCCCGGGGAACC
GGGCAAAGGGAAAAGAGCTGTTTTAGATGGCGATGGAGATGCAAATCCGAACATAGA
AATCGTCAATCCTCCCAACGAGCAACGATCCGTAGAATCGGTGATCGACGAAATTCTC
GTGTCTAATCGGCAGGGTCGCAACGTCGAGGGTTTCGATCAATTATACGCAGACCCGG
AGGTGAAGAATGTCCTTCAATTGGGAAACGAAACGATCGCTCGTACATACATCAGGGA
CAAACTATGCTCTCTAGGATTGATGAACTGCGACAATATCGAGGGACGTCGGCCGTACT
ACTCTCCTCATCGTGGGATATATCCGCAAGATATAATTTACGCTCAACCCGTGACTATCA
AACCTGTCGGCAGACCTCTACCAGCCGTACCTGTGAAACGGCCATACACTCCTGCCGT
AAGACCGCCTGGATTTATTTCCTCCGGACCACCAGCGCCGGAAGTGATCTACGGAGTT
GGTGGTGCGCCTCATCCACCTTCGTTTGTCTCCTCCTCTTCTTATCCCGTGTTCAAGAA
ACCCGGCTATCCCTCATTCTCGGCGAGGCCTGTGTACGAGGCAGGAGAAAATTTCGAA
TTCGCGGGCAACGGTCAATTGATCGACAAAAAGCAGATCGCGTTGAGACCGACGATC
GGCTCGGAAACCGTCCAGCAACACGTTCATCATCACTATCATCACGCGGATGGTGGCG
CGACGATCGGTGGCGTCCCTTCGTCTCTTAGTTACGGTTCTGGAAATATAGGAACGGGT
TACGATATTCACAGCTCTGGAGTGCTCGGTGCCGGTTTCCAGGATTTGGACGATTATAA
AAAGGCGTTCAAAGTAAAAGGAACGACGAACAATGATGGTTCCGTGTCGGCGAGCAG
CTACGCTGAGAGATATCCCGTGTACGAGAAACCGACGCGAGACTTTGATTCGCACGGT
AAGGCGGACGGTTATAAATCTGGCAAATACTTTTCACCGGGCAACTACGTTTCTCCTAA
CGCCGTTCAATCGTCTTCCAACGATTACGTTACCTCTGGATCGACTAGCAGTTATTATTA
CGGAAACAACGAAGGCAATGATTTTTATACGGAGGATTGCGTCTGCGTACCGTACGGA
CAGTGCCCAAGGGAACAAGCAGGTCGTAAGGACGATTTGTTCCTTCCCATCGATCCTC
GAAATCTGGAAAAGAATATCGAGGCCGAATCTACGGAGGAGACAACCGCGATGGTGAT
AGAAAAGAATACGAACGAAACGTTTGCCTTCGGATCGAATGATTTTAAAACTGTTATAC
AAAAGGATGAACAGCGAACTGAGGAAACGAACAAAGCTCGAAGCAAGAGGGAAACG
ACGACGAGTGATGGCGAATCGATAAATATCGAAGGAAGAAAGAAGTCTAACGGTGAA
GGG

>novel_circ_001923
CGCGAGGGAATGAAATCAAACGCATTTATGGGTTAGCAAAGTGGGAATAATTACGATTA
CGGTCACGATCACAGTCACCACGTTCATCAGCACAGTGATATCATCACAGTTTTACCCA
AGATATCAATCGTCCAAGTAACGAAGAATCTCAAGGAATCGAAATTTATCTTCTAAAGG
AAGAATCGCGTTAGTCGATGATTATTCCACGATCGTTTCTTTGCAACAGTGCGAATCGG
CGATAGATTTGGAAATAAGAGGAGAGAGATTTATCCGCATCGAGGAGGAGGAGAGAG
ACGGAAGTGAAAAGAAAAGGTGGTCGCGAGATCGAGCGAAAAGCAAAATCGAGGCG
AAGATCGAGACGTCGGTAATGAGACAGGGAAAGGAAAACTGTTGAGAGAGGCGGTC
GCGAGACAGGGGCAGTGCCGTATCCTGGGGGCCCAGGGTATGCGCAAGATCTATTAAT
AACTTGTAATACGGTAGTAGTGGGGCGCTGCCCGCTAGCGGGTGAAAATAGAAAAGAA
TGTTTTTGGAATCCAATTGGACTTGGTTGGCCGAACGATGGGGCAATATGGCCGACTTG

GCCGAGCGGGTGGACGATCTGATATGCAGTTTCGACTCGGCTGGTGACACGACCATGG
ATACCTTGTCGATGCTCATATTCGGCTGGATGTTGTTCGGATTGATGGTACTGTGCGTCG
GCAAATACGTTTACAATCGGTTCGTTTTGAACGGGCTCAGTTCTGGGACAATAGTTACC
GCCAAGGACGGCCATATCGTCCACGGTGATAGCGTCGGTGTCAGTGGTGGTGGTGGTG
GTGGTGGTGCTGGTGGTGGTGGCGGCAGTGGTGGTGGTGGTACTGGTGGTGGAGGTG
GTAGTGGCGGCAAATTGCTTTTGCTTGGCAAGCAAAAAATCGTTCCATCCTCGGGGGC
CGGGGCAGGTGGTGGCGGCGCCGGTTCCGGATCGCTTGCATCCGTCGGTGGTGCCGCC
GGTCCAACTTCCGCTACCTCGCCTTACGTCCCGCCGACACCTCCTGTCCGCAAACGTCT
CACCAGGAAGACATCCGGCGCTCTGATCAGCCCGGCGAGAAGCAGCAAAAGTTTGCA
TCTACCGACGGCAACTGGAGCCGACCCCGACGCTGTCCGCTGGGTCAACGAGGTTATC
GTTTGGCTCTACTCCGATCCAGCGATTTTGGACGAGCTGTTGGCCGTTTGGGTCGCGTC
TCTCAATCAATTTACCGCCAACTCTGTCGACGAG

>novel_circ_001924
CGAAGTGAGCATATCGTCCGCTCGTGCGTCGGTAATGGTATATGACGACGTTAATAAAA
AATGGGTACCAAGTGGCAGTTCCTCAGGACTCTCCAAAGTTCAGTTATACCATCATCAA
GTGAATAATACATTTCGCGTAGTGGGAAGAAAGTTGCAGGATCACGAAATCGTGATCA
ATTGCGCGATCCTCAAAGGATTAAAATACAATCAGGCGACAGTCACATTTCATCAATGG
CGAGATAATAAACAAGTATACGGTCTGAATTTCTCCAGTAAGGATGACGCCGACGCATT
TGCTAGGGCGATGTTACAAGCACTCGATGTATTGAGCAATGGAAGCAATATATCGAGAA
GTCTTGCACCTGCCTCCGCTACTCAACAACAATCCGTCTTTCAGCAACAGCAACCTACT
AATGCTCAATACGATGAAGATATGGGATACAGAACCATGACCAGAGAAGATGTAGCAA
TTATTCAGGAACGCAGAATGTCTCAACAAAGCCAAGCAACAAATAGTCCGAACGCAAT
TTCTCCAAGTTGTCCTCTCGCATCGCAGCAACAGCAACAGCCGCCTCCACAACAACAA
CAACAACAACCGCAACCACAACCTCAACAATCTATGCAACAACAACAATCGCAGCCG
CCAGCGCCACCACAACCTCCGCAACAGCAGCAACAACAACAACATCAAGTGCAGCAG
CAGCAGCAACAATCGCAGCCATTGCAGCAACAACCACAATCCGGTCATCATAGAACTT
CGTCAGCACCTCCAGCTCCGCAACCTCAGCAACAAACTGTTATACAATCGATGCAGGT
TACCGGTGGCATTGGAACTAGTGCAGCACAAATGACGAGCGGGGGTGCGCCTCCGGTA
CCTCCACCACAGCCACCTATGGCGGCAACAGCTCCGCCGTGTCCACCGGCAATGATAA
ACTTTAACGTCCCGGTACCACCACCTCCAATGATGATGAGCCAATACGCGCAATCCCCA
TCTTCTCAATCAAATTTGCCGAATCAATCGCAAGCACAGTCAATTTATGGTAGTAGTCA
AGGAAATCAGTATGGAGCCGCACCGAACAATAGTCAATACGCGACTGTCGCTGTCGTC
GGGCAAAGTCCATGCCAGTATTCTTCTGGAAATCAGAATAACTTCTACAATAACAACGG
ACAAGTCGCTAACGCGTATTCTAGTGGACAGCCTGGACAAACAAATCAGTATGGCGGC
GGTGGTGGTAGTAGTGGTGCTGGTGGTCCTGGTGGCCCTGGTGGGAATCAGTATGGAA
GTAGTAATTCGATTGGTCAGTATGCCAGTAGCGGAGCTGCGACAACAGGTCAGGGTTAT
GGGGTAGGTGTCGCAGTCAACGTTGGTGTCGGCCAGCCGAATCAGTCTCAGTACAATA
TTGTATCTCAGGCACAGGCACAATATGGTACTAGCCAAATACAAGCAGTTTCTAGCGCT
ACGAATCCTTATGGAACGCCATCAAATTCAGTGAATCAATACAGTAGCACTAGCGGTGT
AGGTGGTAGCGGTGGTACTTGTGGAAATACTGTTGGCAGCGCTAGCGGTGGCCCTGGC
AGTGGACATCACGTACCACCTGTTAATCCTAATGTTTACGCTCCTCTCGGTAATGCTGGT
CCTCAACCACCTCCTATGCTACCAGTGGCTGGGCCTGCCGCTCCACCTCCACCTCCTCC
ACCACCTGCCCCGAAGATCAACGCTGTTAATCTAAATCCACTTTCTGGATCTGGTTCCA

CTGGGTCAGTAAGTTCTGTCACAAATAGCGAGCCATCACCGGATTCCAATTCGTTGGCT
GCAGCAATTCAAGCAACTCGTCTTAAAAAGAAGCAACAGGCATCACAGCAACCAGTA
GAAAACAGTGGTTCTAGTACTAGTAGCAGTGGAAGTGCTGGAAGCGGAAATTATGGTA
CTTTAGGAAGAGGTGGAGGTGGCGGTATGGCTTCCATGATGGATGAAATGGCTAAAAC
TTTAGCCCGTAGACGAGCTGCCGTTGAGAAAAAACAACCCGAGCAACCACAAGAACC
TGAAAGTTCACCCGATAAGAAATGGGATAAGAATAGTTCATCTAACAATAAATTCTCAA
ATGGTGCCGAATCACCAAAATCGGTTCGCAAGAGGTTTGGTTCAGCTTCAGAAGATAC
ACTTTTAAAAGTAAACGGTGTTAACGATGGGGCTGTGCTCACTGCTCAAGAAATGGAA
GCATTTAAAGCAGAGATTATCAAGGAAGTGCGTAAAGAATTTCAAAAGATGAAACAAG
AGATTGTAGATGCTGTCAGAACGGAACTAAGTAGAAGATAAGGATGAGATGCGGACCA
GTTTGAACTAAAACGAAGAAAAAAAAAATGAAGGATTCTACCATTGTGTAGATCAACC
GAAACGATCATCGACAACCGTACACGAATTTTTTTTACTACGTATTTGCGAAAATGGA
ATGCAGTTATACCCCATGTATATCAGACATGTTTGTGT

>novel_circ_001925
TAGTACCAGTGCGAGCAAAATATAATCTCCATAATTATTGAATGATGAAGGAATCATTAG
TAGAAATTATCAAATAAACATGGCATCTCAAATAAAGAATTTAATGGTTAACTATTAGGC
ATATTAAGGATATGCTTTATATCATCAGGAAGGAACTAAATGAATGAATAAAATTATAAG
GAATGATATTCTATTTGAATCTATTGGATGTTAGATAAAGTGAAAAATGTCTGGTGATTC
AGGATGGAATACTGTTATTCACCATCCTTCAGAATTAGAATTACAGAATTGGAATTGGG
AAGAAAACGATGGAGAACAAGAAAGGGAGCTCCCTTCAACTGTTGATATGGATGCTAT
AAAGGGTGGTGGTAGCTGGGATCCAACTACTGAACCTAATGGAACAGATTCGGTTGAT
TCCGAAGAAGATTCAGATTCCGACGACGAAACCTCTGGCGGTACAGAAGGTAGTTGTT
CTTATTCAGATTCGAATTCGGATTCAGACGACGAAAGTAGCGGTGACGAGGAAGAAGA
TACAGGATCCAATTCGGATTGTACATCGGAACATAGCGAACAAACATTTTCCATTCAAA
AAACAAATTTTGAACCGGGAGCGCTTAAATTAAAAATCAGTATGAAAGGTCCAAAAAA
AGAGGATTCGGAAAAAGAAAAAAATAAAAGAAGCATACCAAAAAAAGTTAAAACGAA
TCGAGGAGGAAAGTCATCAGAATGCAGTAGTGATGAGTCTGACTCATCCGAAAATCAT
TCAACTTCACAACATACACAGCAACAACAGCAACAAATGCAACAATCTCAACAACAA
CAACAGCCACAACAGCATCCAACTCTTCAAGAGATTAATCAAGAAGATTTGGCGGCTA
TTTTACCAGATCAAGAGCACGAAGATGCTCCATTTGATGGATTTGAAGATTCAGAAAGT
GGTCG

>novel_circ_001926
TATCGGCGCTCCGATCACCAGCAAGTTCTGCTCCAGAACGTGAGCCTGTACACCAGCG
GGAAATACAAGTGCGAGGTGATCACAGAGGCTCCTAGCTTCAACGCGGTCAACGCGG
AGGCCAAGATGGAGATCGTAGTTCTACCCCAAGAGGGACCGTTGATAACGGGCGAGG
AGAAGATTTACGCGAGCGGCGACATCCTCGGGTTGAACTGCACCAGCGGCAAGTCTCA
TCCCGCCTCCACGTTCAAATGGTTCATCAATGGGAAGCAG

>novel_circ_001927
GTCCACCGACGATCCTGGTGAGGCCACAATCGCAGCAGGTGAAGGCAGGCGGGATAG
CGAGCTTCTACTGCACAGCGGAGGGAGCGCCACCGCCCCAGATTCACTGGCGGAAAA
ACGGAAAGAGAGTTTCCCAATCTCAGTCACGGTATCTGGTGCACAACTACGAGAACGG

AGCTTTGCTGCGGATCGAGCCTGTGAGGCCTGTTCGAGACAACACGATGTACGAGTGC
CTGGCCGAGAATGGAGTCGGCGACGCCGTGTCGGCGGAGGCGCAGCTTCGAGTCTAC
GAAG

>novel_circ_001928
AACCGGGTACCGCACCGCGGAAGGTTCAGGCCCGGCCATTGAGCTCGAGCACGATGG
TCATACAATGGGTCGAGCCAGAGACACCGAACGGGCAAGTGACGGGTTACAAGATATA
TTACACGACGGATCCGAATCAACCGATGGCCTCCTGGAAGTATCAGATGGTGGACAAC
AATCAACTGACCACGATCTCGGATCTGGACACCCACACGATATACACGATTCGAGTGC
AGGCGTTGACAAGCGTCGGTCCCGGTCCATTGAGCACACCTGTCCAGATTAAGACGCA
ACAAGGGGTGCCGAGCCAACCGGAGATGTTGACCGCGATAGATATCGGGGAGACCAC
GGTAACCCTCCAGTGGAATAAACCTATTCATAGCGCGGAAAATATTCTCAGCTATGAAT
TGTATTGGAACGATACGTACGCTCAGGAGAAGCATCATCGCAGGATACCTGTCACCGA
GAACTACACGTTGACCGGCCTCTATCCGAACACCCTGTATTACGTTTGGCTGGCCGCCC
GGAGTCAACGGGGGGAAGGAGCCACCACGATACCGTATCCGGTTCGCACGAAGCAGT
ACG

>novel_circ_001929
AACCGGGTACCGCACCGCGGAAGGTTCAGGCCCGGCCATTGAGCTCGAGCACGATGG
TCATACAATGGGTCGAGCCAGAGACACCGAACGGGCAAGTGACGGGTTACAAGATATA
TTACACGACGGATCCGAATCAACCGATGGCCTCCTGGAAGTATCAGATGGTGGACAAC
AATCAACTGACCACGATCTCGGATCTGGACACCCACACGATATACACGATTCGAGTGC
AGGCGTTGACAAGCGTCGGTCCCGGTCCATTGAGCACACCTGTCCAGATTAAGACGCA
ACAAGGGGTGCCGAGCCAACCGGAGATGTTGACCGCGATAGATATCGGGGAGACCAC
GGTAACCCTCCAGTGGAATAAACCTATTCATAGCGCGGAAAATATTCTCAGCTATGAAT
TGTATTGGAACGATACGTACGCTCAGGAGAAGCATCATCGCAGGATACCTGTCACCGA
GAACTACACGTTGACCGGCCTCTATCCGAACACCCTGTATTACGTTTGGCTGGCCGCCC
GGAGTCAACGGGGGGAAGGAGCCACCACGATACCGTATCCGGTTCGCACGAAGCAGT
ACGTCCCCGGGGCTCCGCCTCGCAATGTAAGCGGAGAAGCGGTCAGCCCGACCTCCAT
ACGGGTAACATGGGAACCACCGCCTGCCGATCGATCGAACGGAAGGATCGCGTACTAC
AAGCTGCAAGTGGTCGAGAGCGGACGCTCCGACTCGGAGGCGAAAGTTATCAAACTG
AATGACACGCAATTCGTGTTGGACGAACTGAAAAAGTGGACCGAGTATCGGATTTGGG
TATTGGCCGGGACCAGCGTAGGTGACGGACCTCCGAGTTATCCTATCTCTGTCAGAACT
CACGAGGACG

>novel_circ_001931
GCTGGTTTCGACACGCGATGTAAATTACTCGAGGGCGGCAATTTTTACATCCGGTTTTG
CCGTGAACGCGGCAACCCCTCCCGATACCCCGACGATTCCAACTTTCGACGCGGAAAA
CACGGTTTCGTTGGACGAGTAGCGGAAACGCGATAACTATTCCCAGATAAAACCTCTA
GGCAGCTTTCGAGGTACATACACGTACATATACGCCTATTCCCCTCCCTCTCTCTCTCTC
TCTCTCTCTCTCTCTCTCTCTCTCTCTCTCCCTCTCCTTACTCCTCGTATACGCGATAAGC
GAAATCGCTTATCATCGATCGAAATCTAATACGAATTTAATATCGGTGGAAATATTAGCC
GTAAAGGTAAAAGACAGAGAGACGTGTACCGTGTATGTGTTATCGAGCGAATTTCGAT
GATGGGAGCGAGAGAAGAAGAAATTGGGAGAGGAGGGGATGGAGGGAGAGGGAGG

GGAGAGGGGGATATGGGGCATAAGCTTGGACAAAGATACGGGAACGAAATAGAAATG
GAATGGAACGAAATGGATACACCAGCGGCGCATTAATTTCGAGGTTCAACGACCTATC
GC

>novel_circ_001933
ACAGTGAGTGTAGATTCCGCAACGACGACGATGTTTAATTCAGCGGCATATTTTAGCGA
AGATCCTGCGGCTGATCGTCTGTTCGTCGTGACTAATCCTTGACCATTGTACATTCGTTA
TGAATATTCTATCATTGTTTTTGCGACACAGAAATTTTTGGGTGAAGTTTTAGCGAAAA
AGAATTTACTACGTGTTACAGTAGGTGAACCTTTGGCTTCTTGTATATTTGAAATTATGA
TATGTATTAATAATAATGTATGATAATTCATTTGATATTATTATTAACACATTATACGTATATA
TTTACACATTATACGTATATATACATGAATGATTATAATATCTATGATTCGGATATCGTATTT
CGTGGAATTTGATTATCTTTATAATAGTTATCATTATCGTTCTCGTCGTTACGAGTATCGTT
GTATCATCATCGTTAGGTATGTATAAGTTAAATATTTTTATGAAAATTTAATTATTTATATT
GATTTTTATTAATACCGATACATACTTACCTACGATGTATTGAGATACGACAACACGAATT
ACGATATCGTTAATATAATCATTAAATTGTTCCTAGGTGAACGAGTGGTAAATCGATAAT
TCTTGCAATTATACGATAATAATATGTGCAGTTTGCAAGATAGAACAGATCTTTTTTTAT
TAATATATTTTCTTTTCCGTTTCTCTATCTTTTATGTAAGTATGTATGTACATACAAATATGT
AAAGTTTGAAATGCATAAGCTCAAGAGAAGAAGCAAGTTGGAACATCTTGTAACATAC
CTGTGATTTTTCGCAATTTTATCACAAATAATTATGCTGTGTATATACAGTTTATCTCAATT
TCAATAATACAAATTTCTGCAAGATTTGTAATCGAAAATTGGAATCGTTAGAACAATTTG
ACATTTTAATGTAAACAATGTTGTATCAAAGAAAACGCGTTAAATAATATTAAAAGCAG
CTGCGCTGCTTGAATTTTTAAATATAGAAAACATACAATACGGTCCCCGTATTTTTTTATT
TTGTAGACTTATAGATGTGTACGCATATAATATTTCGACTCTATGAGACGGAATCGAAAA
CATCGAAAGAAGGCGCGACGCGGCGCGTTCCAGTCAAGACAAATTTAGAAATGGACA
ATCTGAAATCCAACGTATATTAATAAGAACGAATAGTCGTTGACCGAAAAACTTTCTATT
CTTTCTTCGACGAATTATACGGTCGCTCGCATAGTTTTATAGTAAGTAGTCACGTATGGT
TTTATATATACATATATGTATATATATATAAATATATATATTATACATATATTACATTTCGATTT
TTTACAAAAACATGATTGTTTTAGAATATTTTTTACGAGAGAGTTTTCTGATCTTCTTTAT
TCACAAATGATCGATTAACGCATAATAATCGTTATAATAAATGAAAGAAAAGCTTGGATA
GGATATCGAACTTACTTTTATATAATATATATTATATATATATATTATACGTATGGTTTATCTG
TTGTGAATAATTGTATAAAAAAAAATAGAATAATACGACATTATGTGAGATTCGAACAGAT
ATCATAATAAGTTATAATATGTTGTAGTGATGGTAAATTTTCAGGAAAGATTCATCGGTA
CGAAGATACATCATTGGTCATGTACGAATTTAAAAGAACGACGAGTATGTACGTTGAAT
TTATATTTCTGTTTGTTAAAGTTAATCAGTCGTGGATTATTATATAGTATATCGCATATTATT
ATTGATCTCTTCGACAATAGTAACCAAGAGTTTTACATTTTTGACAGAGATGTTGCGGA
TGGACCTTACTTTGGTCGGACACATCCAATCCGTCTGGTTTCTCTAATGGTCTCTGAAA
ATATGTAGTAAAATGTAACGGGATTACGTAATTACGCAATCATGTTGGCGTGAAATGAA
AATGCGGTAAATTTGTGATTACTCGTTGCCAGGCTGGGACAAACAATTCTAATACGAAT
TTGATGCAATAGCAGATTATTAGCAATCAAAG

>novel_circ_001934
GATGACGATCAAGATACTCTTATAAAAGGATTATTCAGTTTAAAAGAGGTGCTGAATGA
AGCAAAAGACTTATCTTGTTTAGATCCAGGCGTTTTTTTGGCTCCATTTTTAGAGATTAT
ACGATCAGAAGAAACAACTGGACCTGTTACAAGTCTTGCATTGTCTGCTGTTAATAAAA

TGATATCTTATAGTCTTATAGATTCAGATCATCCAGCAGTAGCCCAATGCGTAGAAGCTA
TTGCTGATGCTGTTACGCATGCAAGATTTGTTGGGACTGATGCATCTGGAGATGGAGTT
GTTTTAATGAGAATTCTTCAAGTTTTGAGGGCTCTTATGTTAGCACCAGCTGGTGATTAT
CTTTCAAACGAAAGTATTTGTGAAATTATGCTCAGTTGTTTTAGAATATGTTTTGAAACA
CGACTCAGCGAATTATTAAGAAGGACAGCAGAGCATTGTTTAAGAGATATGGTACAAC
ATCTTTTCACACGATTACCTCAATTTGTTGATGATAGTAGAGTTTTATTAAATATGAAAA
AAATGAGAACAAATAGCATGGAAAGTAATCGAGGTAAGAACAGAAGAAATAAAAATC
ATAGTAAACAAAAGACAAAACAAAATGTAGATAATATAGGAGATATTGAAGATCCACTT
CCAAATCCAACTGATAAAGTTAGATCGAGTTGTTTAACGACGTCTGTTAGTTCGACAGG
AAATATTGTTGATATGCAAGGTTCTTTAGATCAAGGTAGCATCGAAAAAATAGAGGAAG
AAAAGAATGATAACAAAAATCATAACAAAAAAGATTTAAATGATGTAAACGAAACTATT
AAGCAGGGAGATATATTTGATTTGAAAAAAGAAAAGGAAGATATAAAAGATTCGAAAG
AGTGTAATAAGCAAAATTGTAGTGAAACAGAGGAGAAAAGAAATGTGATTGATGAAA
AAGAAATTTTATTGAAAGAAGATAAAAATATAGACAATATAGAAATAGATAAACATAAT
GAAAGTAAATCAGTAAATTTATTAAAAGAAGGAAACCAAGAGGACATGAAGAAATCGA
TAAATGATAAAAAAAATACAGATTCAATAAAATCTTTGGCAGGAAGTGTTGAAGATTTG
ATGTCAGTCGATGAAAATATAAATAATGCATACAAAACATCTAAAACCAAAGAATCTGA
ACAAATTGAAGAATATATAAATGCCCAAGGAGTTCGTTTTATGCCACATCAACAATTAA
CACCTTATGGTGCTTTGTGTGTCCGTGAATTATTTAGATTTCTCGTATCTTTATGTAGTCC
TCTCGATAAACAAAATAACGAAGTTATGACACATCTTGGGCTCAATTTATTGCAAGTAG
CTTTAGAAATTGCGGCTGACTATCTTTCCAATTTTCCGTCATTACTGGCACTTGTGAAAG
ATGATCTTTGTAGAAATCTTATTTTGCTTCTTGGCACAGATCGATTGTCGATTCTTGCAG
CGGATTTGCAAGTATCGTTCTTGCTATTTGAATCGCAAAGACAGCATTTGAAATTCCAA
ATGGAGCATCATATAAACAAATTAATGGAAATAGTAAGTTCAGATTCGAATAGGATATCA
TATGATCAGCGTGAATTAGCTCTCGAAGCTATTGTACGATTATGGAGAATATCCGGTTTA
CCAGCAGAATTATATTTGAACTATGATTGCGGCTTGTATTCTTCTAATTTATACGAAGAAT
TAATGAAATTATTGTCAAAGAATGCTTCCGCATTAATGGGTAATATGCAAAATATGCAGT
TCGTTTCTTTGGATGCTATATTCACGTTGATTTCTGGAATGGAAATCAGATGTAAAGGCT
ACAAAGAATTGTGTAAACCATCTCGACATGATGCATCACCAAATCTTCCAACACGAGA
GGAACTGCTTAGTATAAAAGCAAATAAACGGTGGTTGATGCTTGGTACAGAAAAATTC
AATGAAAATCCACGAGAAGGAATTGCTAAACTTACGGAACATAATCTTTTGGGTGGAA
GTCCGGGAAATCCCGATCCAGAGAAAGTAGCTAAATTTTTGAAGGAAAATCCTGGCCT
TGATAAAAAAGCTATCGGGGAATACATTAGTAAAAAAGAAAATAAAAATGTTTTAAATT
GTTTCGTTCATAGTTTTGATTTGAAGGATATGCGAATCGATCAAGCTTTACGACTTTACT
TAGAATCTTTCAGACTTCCTGGCGAAGCGCCACTTATTTCTTTGTTGCTCGAAAAATTT
GCCGAACATTGGCAC

>novel_circ_001935
AGAGGTGTGCGTAGACGAAGGCCAACAATTGTCCTTGGCTCATGGATGTCAATTTTAC
GAGGTTAGTGCGGCGGAAAGTCCTGCGGGAGCAGCACTGGCATTTCAAGCGCTGCTC
CGTGAGGCAAGATCGGTTCAATTGCTCCGAGCACTTCCTATTCGCCGGAAATTGGGCG
TCCACTCGGTCTCGAGGGTGCTCGGCACGATCTTTGGCAAGAATACGACGAAGGACCG
CAAAAAGAGGCCCTCGTTAAGCATATGACGCCTCCTCTGCGCCCTCCTTCTCGGAGGT
CGTGACGAACCTGACAGAAGGACCGATGTGATTGTTCTTACCAGCAACGTCTATTCGA

ATCGTTGACGAGAACATGGAAAACGATTATCTGACCAACGTGGATCAACAGTCGCCGA
TCTTTCGCCTTTTGATGTCTTCTGAAAAAAAAAGTTCCTGGCGTTTTGATTCGGTATCA
AGACTGATATAGAGAAACATTCGGACGTTGTCTGGCGGACAG

>novel_circ_001936
GTTTGAAGCGTATATGATGACCGGTGACCTAATGTTGAACCTGTCGCGCACCCAGCAA
AGCAGCAGCCTGCTCCCGAAGCACCAGAAGAAGGTCGATTCCCTGAGGTACAACAAT
CATCACACCCACCATCACCATAATCACCACAATCACCATCATCATAACTCGGTACCCAC
CAGTCCTAACGAGATGCTGGGCCATCACCGTGCTTACAAGTGTACCGGTTCGAATTCG
GCCAGCACCTCACCGGTCGGGATCAGCAAACGTGAAAGCGGCCAGTGTCCGGGCCAG
AGCGATAATAGCAAACAAAATACCGCGAGTTTTGGTTATTCGAGCGGTTTCGTCCGCAC
CTCGCGCTCCGAGGACCACTTGCAATTCCAAAAGGATCCGTCGATGAGCGCGGTGGAC
ATTGACATTGACGATGACGTCACGTCCAGCCTAAACACCCTGTTGGACACGAGGCCTG
ACAGCGGCCAAGGTTTGTCCAGCGAGAGGATCGTCTGGACGTACAACGCGCCGGTCA
GCTCGCCATCAGCCTCGGAGCGCACCTCCTGCTGCCAGAATGGCAGCTCTTCGCAATC
CTCGTCGAGTAGCTCCTCGACCGAGGGAACTAGTCCCCAAAGATCCTTATCGCCAACT
TCCCCTACCTCGGTCTCGTCCTCCGTCATGTCCTCCAATTCTGGATCCCGTAGGTTCCCA
CCGCCGGTACCCACGGGCACCACACCGTCAGCCCTGGGACCCTCCGGCGACGGAACC
GCATCCTCGGCCTCCGGCCTTCATCCCACCAACGGTGACCTCAGCCAGTCCGAGGCGA
TCAGCAACATGTCCAGTCCCGACTACAACGACGAAGAGACTATGGACATACTGAGTGC
ACGCGATATCATGATGGTCAGTGATCCGAGTGACAGTGACTCGACGATACTCGCCAGC
GAACCACCTCAGAGGAGGTTGAAGGCAGCTGCTGCGGCCACCGTGCAACAAACGTCG
AACGTTTATGCATCAGCACAGGAAAATACCGAGCATAGGATAGTGATACAGGTGAAGG
GGCCGGACAAGGACATTGCGGTGGCGAGGAATACGAGTCCAAGGCAGAACAGGAGA
CGGGGAAATCTCGGTAATCCGGAACTCGTAGCCACGTCCACCGCACAGAACATCGTGC
AACGTCCGCCCAGCAATAACACCGCTGGGAACGTTACGCCTGAATTCGTTGGGTATCA
G

>novel_circ_001937
ATCCTGCAATAAAAGCTCATGTTCGTCGGTTATTGTCACCTGGACAACCGGTATGTGAC
GAAACGGATTCATCGAAGATATCTCTCGAGGCAACAACACCTGCGAATAACCCTAGAA
CAGTGTCGTCTGGCAACGGAGAGGGCACGGAACTAAGGAAACCTGGAACGCCAGAA
CTTCCGGGAGGTTCGCCACAGGAACCAACTGGAGCGACACAATATGCGACCAGAAGC
AATGGATCCTATAGTGATGCCTCTGGCTCCTCCGAGTTAGAAGCACTACTTCCACCAAT
GGATAGTTTCAAAGAAGAGGAACGCGCACAATGGGAGCCGCTTTCCGGTGACAAGTT
TTCCAGACAGAAGAACACGCCTAGGTTTGAAGCGTATATGATGACCGGTGACCTAATG
TTGAACCTGTCGCGCACCCAGCAAAGCAGCAGCCTGCTCCCGAAGCACCAGAAGAAG
GTCGATTCCCTGAGGTACAACAATCATCACACCCACCATCACCATAATCACCACAATCA
CCATCATCATAACTCGGTACCCACCAGTCCTAACGAGATGCTGGGCCATCACCGTGCTT
ACAAGTGTACCGGTTCGAATTCGGCCAGCACCTCACCGGTCGGGATCAGCAAACGTGA
AAGCGGCCAGTGTCCGGGCCAGAGCGATAATAGCAAACAAAATACCGCGAGTTTTGGT
TATTCGAGCGGTTTCGTCCGCACCTCGCGCTCCGAGGACCACTTGCAATTCCAAAAGG
ATCCGTCGATGAGCGCGGTGGACATTGACATTGACGATGACGTCACGTCCAGCCTAAA
CACCCTGTTGGACACGAGGCCTGACAGCGGCCAAGGTTTGTCCAGCGAGAGGATCGT

CTGGACGTACAACGCGCCGGTCAGCTCGCCATCAGCCTCGGAGCGCACCTCCTGCTGC
CAGAATGGCAGCTCTTCGCAATCCTCGTCGAGTAGCTCCTCGACCGAGGGAACTAGTC
CCCAAAGATCCTTATCGCCAACTTCCCCTACCTCGGTCTCGTCCTCCGTCATGTCCTCC
AATTCTGGATCCCGTAGGTTCCCACCGCCGGTACCCACGGGCACCACACCGTCAGCCC
TGGGACCCTCCGGCGACGGAACCGCATCCTCGGCCTCCGGCCTTCATCCCACCAACGG
TGACCTCAGCCAGTCCGAGGCGATCAGCAACATGTCCAGTCCCGACTACAACGACGA
AGAGACTATGGACATACTGAGTGCACGCGATATCATGATGGTCAGTGATCCGAGTGACA
GTGACTCGACGATACTCGCCAGCGAACCACCTCAGAGGAGGTTGAAGGCAGCTGCTG
CGGCCACCGTGCAACAAACGTCGAACGTTTATGCATCAGCACAGGAAAATACCGAGCA
TAGGATAGTGATACAGGTGAAGGGGCCGGACAAGGACATTGCGGTGGCGAGGAATAC
GAGTCCAAGGCAGAACAGGAGACGGGGAAATCTCGGTAATCCGGAACTCGTAGCCAC
GTCCACCGCACAGAACATCGTGCAACGTCCGCCCAGCAATAACACCGCTGGGAACGTT
ACGCCTGAATTCGTTGGGTATCAG

>novel_circ_001939
CTTTCTGTAAGTTGCGATTTATGTTAAATTCTCACTCTCGTCACTTGAAAACTCACTCTC
TCACATTGAAGATCTTCAGCTGAAGATAATGAACTTTGCTCTGAACGGATGGAGTTCTT
TCGGAGTTCAAATGCTTTGCTAAATACTAAAATGCCATAAATCGATTATCGTATCGCTTA
AGATATGTGATTAAAAAGCTTTCGATTGAAATCTTTTCTATTTTGAAACATTAAAAGAAA
GAATTCGGATAAATTCTACATGAAGAAAAGATAAACTATGTTACACAACATACAGTATT
AAACTTGAAACTGAATTAAAATAACCATTCCATCATTTAGAAGATTAATGATAAATAATA
GTTTATATTTAATGCATTTAGAAATCTTAGAAAATTTAGGAATGAATTTCTATTTTTGATT
CCGAATTTTGCTGTCTTTCATTGCTTAAATAGTTTTAAGCATAATAAAATATAATAAATAT
ATATATACAAATAGATATATATATATATATATAATTATAATGTTATAAATACTTAAAAGTCTTAC
TTAAATATTCTATTTATTAGAAAAATATAGTATTTTTTTATATTTCATCATAGTATCTTATTC
CAATTTCCAGTCAATTGTATTTTTAGATTAATTTTTTTATATTAAACTTTACTAGAAATATT
TTAATATTGACTAGTCTTAGATATATTTTAGAATATGTAAATAAATTATTTATTAGATTTATA
AAATTATTATTATAATAGATATAAACTTCTACAAATACAATAAATATTCTTTAAATTGTAAT
ATATATTCTTTAAATTATTATTATTATAACAGATATAAAGTTCTATAAACACAATAAATATT
CTTTAAATTATAATATATAAAATATATTCTTTCGTATATACAATAATTCAGTTTATGATGATA
GATCCAAACTAAATTTTTCTTTATTTATAAAGAAGTTTTATTTATTAAGAAATCTTTATTTA
TAAAGTTTTACATATTTTTATATGTATATAGAGAATTAAGTTTTGAGATATAAAAAATCGG
TCAAGATTTCAATATGCTCATATTGCCAAAAGCAAATTGACTTCGGCAATAATCGGATAC
AATTAGTTTGTACAATTATTAAAAGTGATAGCTTGATAGTATCCGATTCCTTGAAATTGTA
TTGAAACTTTTAGAATTCTATCTTACTCAAATATTTCAAAATTTATCTCGAATGTTCATTT
TCTAATAACATTATCCATTAATTTGTTATTACATATATAGAATTTTTTATTCAAACTTTTTC
AAATCTTTTCTTTCGTTATTATTCGTTCACCAAAAACTTCTACGAATTAATTAATACAAG
AGCAAATTTCAAAAGAAATCAGATCATACTTTCGGAAAATATTTTATATATACAAAAATA
TTAAATTTTTTTTTTTATGTAATTAATTATTGAGGAAAATTTAGTTTTTTGATCATCCTTTG
TATATAAATCATAAATAATCACGAAAGTATAAAAATAAATTCATATCAAGAACATAAGTTT
ATTGAATATAAATCCGTTAAAATAAAGAATATAAAACAGTTTAAACCATCTGACAATAAC
AAACCATCGAAAAAATTAAAGAAAAAAGGAATCGTCGAAAGCAAGAGATCAGGAGG
AAATACGTACATACACATGCGCTTAAGTACATGCATACATACATATCAAGAGCAAATCGA
GTTCTCCAGATGTTCTCGAGACGAGAACATTTTCGCCGTCTGTCTCGAGAAAACAAGA

AAGAGACGGAGCGAACGAATAATTTAAGCGGAGAAAGAGAGAGAAAGAGAGAAAGA
GAGAAAGAGAGAGAGAGAGAGAGAGAAAGACGCGTGCAAAAGTGAAACGGAAGCA
GAAAGAGAAAGAGGATACTGGAAGGAAGGAGAATATGCACCGTTTGCCAGGTATATAC
GCTTACGTCCCGCCTCGTTGTCATTAATTAATCTCACGTGATAAATGTTAAGTCGTCTGC
CAACTTCGAAGATGTTAACGCGATCCCGCGCGATGGCCCAGCTGCGTGCCACGATTGA
ATCAGCAACGGGCGAATACTGAGACACCGGTGGCAGATGTTGCTGCATAAATTTCTGTT
AATCCTTCGTGGCAACGACCGGAATCGTTTGTCCCATGGCGCCCGCGAAAGATCGATC
ATTGTAGCACGTAGATGCGCCGATCGATGAAGATTAGTTTTATTAGATAAATTTTTTCAA
TTTATTTTTTGTTTTGTTATAAACGATTTTCAAAATTTCTTATGTCTTATTACTATTATATAA
TGATAAAATATTGTTGAAAAAATATTTTTAATATTAATCTCATAATAATGTTATTTTTTTCGA
TAAATATTTTATTTTTTAATGACAATTTCTTAAAACTTCTTATTAAAAAATAATAAATTAAT
ATTAACTTTTAAATATTCAATATTTAAATACTTCAATTATTTAAAATTTATTTCAATAATAA
TTAATGAGCTTCGTTCTACGTACTTTAGATACATCTTTTTCTGAAAAGTTCTTATCTCTGT
CATTGAATTGTACTTCCAAACAAAGGGACTAGGATTTAAACTATCTTCTTAAGATCTAAA
TAATCTTCTCTATTATTTCTTTTCTTTTTAAATCTGTTCTCCAACAATTTAACATTTTTTTC
ATCTTTATTCTTCTATATATATAATAGATATGTTCAGATAAATATCAAAAAATTGAGATTAA
AATACATCAGTTATTTGAGAAATTAAGTGATCTAAATTATTGATAATTTCTTTCTCAATTT
CTTATAAATGACATAGAGAAAAGTGTTTATATTTTATTTTACGATGTGTATTTTAAGTTTT
CAAGAAATATATGTCTCAAAAAATTGAAATTGCATTTTTAAGAATTGATAATAATTTCTTT
GAAAATTAAATAAACAAAATATGACAATTAATACACTGTAAGTGCAATTTATTCCATTTC
GAAACATTAAAAGTACAATACAAAATATGACGATAATCTTTCTATCGATAGTTTCACAAT
TTTCAAACTAACTCTTGTATTTTCTCGAGCACTAAGAATTTTTGTTAGATTGAAAAACA
GAACTGATCACCATAAGAAAATAGAATTTCACGATCTCCAAAATCGTGTCCTTCTTTGT
TGAGACAAAAGAGTTAAAAATTCATTTCAAAACTCATTATCATAAATCGAGCCTAATCG
AAAAAAAAAACATAGATGAAAAGAATTTTAAGAAGGAAAAAGAAGAAAGACAATGA
AGAAAGAAAGTAAAAGAACAGTTACCTGTGACTAGGATAGGATCAAATTTAATATAATA
TATCTAAATCCAAATTAAATCTAATGCTAAAGTTGATTCACTTCGAATATTTATTTAATAT
GTATTTCAAATACTTCTAAATATTCGAATATTATTGATTTTTATTAATATTATTATATTAATAT
GAGTCTATTTATTCTTGTCAAGATGTATAAAGATTTGCATTTAATTTAAAAATGTTGATTT
AAAAATCTAATTAAAATGCTAATTTAAAAATTACGTTTCTATTAATCTAAATTTATGTATTA
TATAAATAATATTTATGAAAATTATTTATAACTTAATCTTTATATATATTTTAAAATGTAGAA
ACGAAATAATCTATTGTTTTCTCAATATTTATTTACATCTTTTTATTTTTTATATATTTTTATA
TATTTTTTTAAACTCATATAAATTAGACAAGACTTTTTTTCATTCTCAAAATTTAATCTTC
TTAATCTATCAAGTTTCATTAAATTTCAAGAAATGTATTCTTACAAATTATTCAATTTTTT
TCTAATCTAATCTAACTTATTCGCGATATAGTTAGACCATCGCTACTCAGACCAACCTAA
CCTAGCTCTTTTACGAGGTAGGTACGTTCTCGATGGACTTTTCTCGTAGTCACGAAAAT
AAATTGAATCACGCAGAAAATAGAAGTACAAACACATCCACGCCTACCGATGGCCGTA
ATTCCAAGCTACTTTCCCAGGTTGCGATTGCAGGGAATGGTGGGGGCGGAAAGAGCCG
AGTTAAAGAGGGGAGAGGTCTTCCAACTTGGACATCCTGAAGTATTATCTATCTCGGAA
GTTCGTCCCCGAGGCTGCGGCTGACTCCGTTGACGATGACGAAGACGACGAGAACGG
TCACGCTAATCGCAGCTGGCTGGAGATTGGCTTTTAGCACAGAGTTCGACCAACTTGTA
TCGCCAGGAAATCGCGGGCGCACCCCTCCGCTCAAATTTACAGGGCGATTCCCTCGAC
CGCCCCCTTCGTCGCGAGGGATGGTATTTGCGGCCACGGGGGAAGCATTGATGGAAGG
GGTTACGATCAGGTCGACGTGGTCTCTAGCGGTGATTTAAATTGCTCTAGGCGAGGGCC

TCTGTGCACTTTAGTTTCTGGCTTCTTTTTAGAGATTGTTCTTTAGAAGTTTGGTGGTTG
ATGAGGTCCTTGATGGTATACAGGTTGTAAGTGGTTTTCTTCATTGTTTAGCTTTATACA
TTTTTCTTTCAGGGATGACTCGAGATTGGATTATTAGATTGAATTTGAAATCATGTGAGA
TCTTAACATTTTTTTTTAAATTCAAAAATATTTATTTTTTCTTCTTTCTTTTTTAAGAAAA
AAGATTTTCTTCTTATGTTTATTAATAATAATAATCTTAAAAAAATTTGATTGATCTTTGAA
TTCGCGTTTTTATGATATTATAATAGTAATAATAGTAATAATTTGTTCTTTTGGATTATGAA
AAATTAAGTATTTATTTTAAATGTGATATTATATTAAAATAATATGAGACTAAGCAATTATT
ATAAATCTAAAATCTAAATACAAATTTTTTACTTTTAGTTGATTTTATTTAATAGATTACAT
AGAATCAGTGATATTCTAATCAATGAAGTTATATAATAATGTAATAAGTTATAATAATGTAT
TATAGTATTAATGTATTATGTATTATAGTAAAAAAATTGTAAATATTATAAATGTATTCATA
GATATCAATACTAAATTTTAAAAAATCTCAAATAATATGAGAAAATAAGGAAGAAGTTTA
TAAATCAATTACTACATATTCTAAAAGACATTTTTATTGAAAAGAAAATTTTATTCCAATT
AATATTTTTTTTTTAATGTGTAGAACAATATTTTTAATTGAAATATCATTTTAAAAAGAAC
GAAATCAAGATTCAAAATTTTAATAAATTTTTATATTTTTAAATATAATTTTAATAATATAA
AATGGAAAATTTTAAATTAAATAAAATATTTAGAATTTAGTGTTTATTATAAAAAAATTTC
CAGATAAAATATTATTTGAAATATGAAAAAATATTGAAAAATAAAAGGAATATTGAAAC
GGTCTTACCTTTAAAAAAGACTTCTCATCAATCACAAAATTGCACAAAGTAATATATTTT
ACATAAATATAAATTTTCATTGAAATCAAAATTTTTAAAGAAGATGAATTTTTCTGTAATA
TATTTTCATAAATTATTATATAAGAAAAATAATTAGCAATCAATGAAATTAACTGTCATTT
CATATTTTCGAATTGATATTATTCCTTTTATTTCTACTTCTTTTTATTTCATTTTTTCTTTTC
ATGTATTTCGTAATAAATTATATTTTCATACATAAATTTAAGTAATCTCATGTGATAGATAA
GTAAAAATTGTAATAGGCTGAAAATGTAAAAGTATTTAGAATTTTACAGAACTTTTGAA
TTGTTATAAGAATTTTTTTTAAAGCTATATTCTATATAACTTCAAATGTTAAATTATAGAAT
TATAGAAATTTAAAATATTAAATATAAGATAATACATTAATTTGTTAGAAATTTAAAGAAA
TTGAAAGAAGAAAAAGAATAGATATTTAAAACAGTAATTCTCAACTTTAATTAATATATT
AATTATAATTAAATAATCTGTGATATTCCTGTATATATTTTTATATAAGTTCATTTTAATAAG
ACAATGAAATCATTTTTTTTTTGTTTCTTGAAAATAAATATTTACAATATAAAAAATTCAA
TCAAATAAATAGATATTAAATATTCATGATAAAGTTCCTTATAAAAAAATATCGATTTCTT
AGAAATTTTATAATCTCTTTAAAATTATAATTGTAGATAATAAGATAAGAATATTAATATAA
ATACTGTTTAATATTAATTTAATTAATATTAAATATTCATATATTGAAAACTTCAATTTGCA
ATTAAATATTCTTACACAATTTATTATTTCTATAATATAGAATTTATATTTTTCATATTAATCT
TCTATATTATTAAAATTATTAATTTTAATATTGATACACCAAAGATGAAGAATTTTAATCAA
TCATAAAATTGGAGGAAAGAAAATTTAGCGAAGAAATTAATCAAGAATATTTTACATGG
GAAAACGTAACTTCAGACGTATTGGATAGAGTTTGAGACACCGAAGATGGCATTTTAC
ATACTCCAGACCCGCGTGACTCAGAATGCCAATTTGAATATGGTTCAACTACATTTCCTT
GAGTTAGCATGTTTCCCCGGGAGACTGGTGGAAGGATGTCTACAAGTTGCTACTCATTT
ACATATTCCTCGGACCAAGAAACTCTCCTTCGTCGCCAGCAGGATTATTTGCGAAATTG
TAAACGTTTTCGATTAACGATTCAGCCCTGTTACAGAG

>novel_circ_001940
CTTTCTGTAAGTTGCGATTTATGTTAAATTCTCACTCTCGTCACTTGAAAACTCACTCTC
TCACATTGAAGATCTTCAGCTGAAGATAATGAACTTTGCTCTGAACGGATGGAGTTCTT
TCGGAGTTCAAATGCTTTGCTAAATACTAAAATGCCATAAATCGATTATCGTATCGCTTA
AGATATGTGATTAAAAAGCTTTCGATTGAAATCTTTTCTATTTTGAAACATTAAAAGAAA

GAATTCGGATAAATTCTACATGAAGAAAAGATAAACTATGTTACACAACATACAGTATT
AAACTTGAAACTGAATTAAAATAACCATTCCATCATTTAGAAGATTAATGATAAATAATA
GTTTATATTTAATGCATTTAGAAATCTTAGAAAATTTAGGAATGAATTTCTATTTTTGATT
CCGAATTTTGCTGTCTTTCATTGCTTAAATAGTTTTAAGCATAATAAAATATAATAAATAT
ATATATACAAATAGATATATATATATATATATAATTATAATGTTATAAATACTTAAAAGTCTTAC
TTAAATATTCTATTTATTAGAAAAATATAGTATTTTTTTATATTTCATCATAGTATCTTATTC
CAATTTCCAGTCAATTGTATTTTTAGATTAATTTTTTTATATTAAACTTTACTAGAAATATT
TTAATATTGACTAGTCTTAGATATATTTTAGAATATGTAAATAAATTATTTATTAGATTTATA
AAATTATTATTATAATAGATATAAACTTCTACAAATACAATAAATATTCTTTAAATTGTAAT
ATATATTCTTTAAATTATTATTATTATAACAGATATAAAGTTCTATAAACACAATAAATATT
CTTTAAATTATAATATATAAAATATATTCTTTCGTATATACAATAATTCAGTTTATGATGATA
GATCCAAACTAAATTTTTCTTTATTTATAAAGAAGTTTTATTTATTAAGAAATCTTTATTTA
TAAAGTTTTACATATTTTTATATGTATATAGAGAATTAAGTTTTGAGATATAAAAAATCGG
TCAAGATTTCAATATGCTCATATTGCCAAAAGCAAATTGACTTCGGCAATAATCGGATAC
AATTAGTTTGTACAATTATTAAAAGTGATAGCTTGATAGTATCCGATTCCTTGAAATTGTA
TTGAAACTTTTAGAATTCTATCTTACTCAAATATTTCAAAATTTATCTCGAATGTTCATTT
TCTAATAACATTATCCATTAATTTGTTATTACATATATAGAATTTTTTATTCAAACTTTTTC
AAATCTTTTCTTTCGTTATTATTCGTTCACCAAAAACTTCTACGAATTAATTAATACAAG
AGCAAATTTCAAAAGAAATCAGATCATACTTTCGGAAAATATTTTATATATACAAAAATA
TTAAATTTTTTTTTTTATGTAATTAATTATTGAGGAAAATTTAGTTTTTTGATCATCCTTTG
TATATAAATCATAAATAATCACGAAAGTATAAAAATAAATTCATATCAAGAACATAAGTTT
ATTGAATATAAATCCGTTAAAATAAAGAATATAAAACAGTTTAAACCATCTGACAATAAC
AAACCATCGAAAAAATTAAAGAAAAAAGGAATCGTCGAAAGCAAGAGATCAGGAGG
AAATACGTACATACACATGCGCTTAAGTACATGCATACATACATATCAAGAGCAAATCGA
GTTCTCCAGATGTTCTCGAGACGAGAACATTTTCGCCGTCTGTCTCGAGAAAACAAGA
AAGAGACGGAGCGAACGAATAATTTAAGCGGAGAAAGAGAGAGAAAGAGAGAAAGA
GAGAAAGAGAGAGAGAGAGAGAGAGAGAAAGACGCGTGCAAAAGTGAAACGGAAGCA
GAAAGAGAAAGAGGATACTGGAAGGAAGGAGAATATGCACCGTTTGCCAG

>novel_circ_001941
AGCCTGGAGGAGCGCGAGCGCAAGCACGCCGTGCACAAGGAACAGCTGTCCCGCGA
ACAGAGGTTCCTGAGACGACGGCTGGAGCAGCTGACGAACCAGACGGGCCTGCACG
GGTTACACGGCCTCCACGGCCTCCACGGGCTGAGCTCGAGCGCCCCGACCGGTTCCTC
TTGCGGCCCCGGAGCCGCCGCGGCTGCAGCTCTCCTCTCAAAACGGCGCAGCGTTTCC
GAGTGCTCGCTTGGCACGGCCTCGTCAACCAGCTCCACGGCCAGCTCCAGGAACTCC
GACAGGTCTGCGGGCAGTCCCTCCGTCTCGGAGTCCG

>novel_circ_001942
AATGTCAGCTGTCCATTGAACCGGTTCCACGTCAAGGATGGAGAGACATGGTTACACG
AGAACTTTGTCCATGATATATTTGCGTAACGTTTCACGCCTCGACGAGTTTTCATTCCGT
GGACGCGTGTGTATCGCGTCCGCCGAAGTAAGTTCGGCCGGCCCTTGTACGATTTGCA
CGGATGAAACGGTCCTTGTGTCGGATAATCGAGGTTGGCTGGAACCGAGGTTGTCGCC
GATCCACGACTCGATGCTTGTAATTAATTTCGGTCGTTGTTGCCGCGTGATTTAGTAGCC
TCCGGTGGCCATGGATGGCTGATGGTAAAACTAATTGCAATTACTGTAATTACCATGCGT

TGTTAACTGCGAACCTCTGACGAGGTTTTTTTTCTTTTTATAGGTCTTTTTTATATCTCA
GGGAATTTAAGAAAATTGATTTTGAGATATATATGTATGAATTGAATTAATATGAGAGTG
TTAATTTAATTATTGGGATTTTTATATAATGGTGGAAAATTTGATTTATTGGATTTATTGTC
TTTTTTTTTTAAACAATAAGATTGGTAAGTTTATTAATATTGGAATTGTTATGTATGATTTT
TAAGGGAGTATTAAGGAACTTTAGGTAAAGGAAATTTAACGTAATCTAATTTAATCTCTA
GTGATTTTCACTGCACAGTTTGTATAGGGAATTTGATCGTAAAAGATATTTAGATATTATT
AAAGTTATATAAAAATTAATTTGATTATTGAGATTTTTATGCTAAAAAAATATTATGGAAT
ATTTCTAGAAAATTTTGTTAATCTTCTTTTAAGAAGATTGATTATATATTGCAATTGCAAA
ACTAATGTAATTTAATTTAATCTCTAATAATTTTTGTTTAATAGTTTATTTAAGAAGTTTAA
GAAAATTGAGAAATATTTAAGATTAAATTTATGTAGAGATTAATTTAATTATTGCAATTTT
TATGTATAGAATTAAATTAAATATTGCATTTATTATAATTATTGTGATAATTGAATCAAGTA
ATATAATTTATTATTTATTTTTAAATTAATTGTGTTTAAACAAGTTTTGATTTTTTTTATCAT
TTACTAAATATAAATAGTAAAACAATACAAAATATAATAATAAAACAATATAAATAGTAA
AAACAAAATACAAGTTTTAATTTTAAAGATAATTATGATTAATGTCTATTTAAAATTCATA
TAAATAGAAGCAATTACTATTTAAATAGCATCAAATAAAAATAATATGTTTAATCCTATTT
TCCTTTTTTCAAACATATTTTTGTTTATTTTCTAAATAAATTATAAAATGATTAATAAATAT
TATTTTTAATCTTTTATCATTCTTTATATAAAAATTTATTTCAATATAAATATTTTGTACAAT
TATAAATTTATTTTAAATATATAAAAAAAGATATAATAAATTATTTTTAATTTTTATATATATA
TCGTGATTTATACAAATAATTATTCCAATAAGAAAAATTATAAATAATAACCAAAGTAATT
TTAAATTAATATTTGAAATAAAGAAAAATAGTTTAATTGAATTTAATTTATTATTTGAAAA
ATTTTTCAATATTTTAGAAAATTAACATTTTTAGAAAAAATTTCTTCCCAAATCCATTTTA
TCCAAATTTTTTTCTATTATATAAGAATATAATTAAATTATATTCTACGTACGAAAATCATC
TTTAAACATTCAAAGAATGTAAATTTCCAGGATAATAAAAAACTTAAAATAAAATAATA
AACTTCATCTTTTATTAAACCCTCGTGAAAAATATTAAATTTCATACCAAACTTCGTCAA
ATACAATGTTCCCAAGTATCCACGAGCAGAAAAGGGTCTTCTTTATCAGTTCCCTTGAT
AAAGTCTCGAAACATAAAATTTCTGCCACTCTTATTAGCAAACTTCTCCAAAGTATGTT
ACCCTATAAACTTCAACGAAGAATTATCCCCCAGTTATATCTTATTCCTTAAAAATCGGT
GAAACTTTATTCAAATTATCGAGGAGGACGAAAAGTTGAAGCGAGCCAATTTTTCAAC
CGGATATCCGTACAAACTCGTAATCATCACCTTATTCGGTATTACGGATGGACGATGGAT
TCTTTCAAGGGGAAATTTTCCACGGTTCGAAACGTTTGATTAAATTTTTTTTATTCGACG
AGACGTTGCACGTCACACAGGTATTGCAAATTTCGATCACGTCAACCGTGCGATCCAC
CCCCATAAGATATTCAATTTCCAGCGATCGGATTTTCTCAGATGAACGTTGATCAAACTA
TTTCTGCGCCGGTCAATATCGCACACGATGGATTTTCCTATTTTCGTTTCACACTTTTGG
GATATAATATTTTCTTGGGATATAAAAAATGTAATATCTCTTTTGTTTTACTTCTTTTATGG
TTTATATTCGGATTTGAATATTGTTGATTTAAGATATATTGTTCTATTCTGAACGAGATGA
GTGAATTTATGCAGGATTATAATTTTAATTTTAATAATATCATTTTAAAATTATAAAAGTGT
ATTTTATAAACTTTAGCAGACAAAAGTGTACAGAAGATATCTTTCTATTCTCAAACAATA
AAACGTTTTATTTGTTTTTCTCTATTTTCATTCTTATTTTACGTGTTGACTTTGTAAAAAA
AAATAGTAACTAGACAAAGTTTTATTAGATATTTATGAAATATTTTTAAAAAATCAATTC
AAAACGTCAAAAAAATATAAAAAGTATATACAAAAATATTAGTTGAAGCTTATTTAATAA
GTTACAGGCAATTCATGTTCGCATTAAATGAAACGAGGCGGGAAAAAGTCTTATTTTAT
TTTCAATTTGCTTTCTCTAAAGGAAAGTTCAATAAACCATCGAATAATGAATTAATTTTA
TTGAGACCAACTTTTGTTTTTATTTTTAAAATCGAGTCATTAAATATATCTTACAAATATT
GTATATTGTTAAAAAATGGAACAAATATTTTATATAAATATATGTACATATTATTTGGTATT

GATAGTGACACAATTTGAAAAATTTCAAATTTGCTTTTATTAAACTCAAAGTTACAAATT
TTAATAAATCTTTTCCTAAAATTTCGATCTGTTCCGCCATCAATACAGCTATATGCAAAAT
TGGAATACATTGGTTTCGAAAACACTGAAATTATCTATAAAGTACTGAAACTGTAGAGG
ACTAAACTATCGTTTCGTCCAAGGCTGTACGTCAGAACTAGATTCTTCGTCGTGGCCGC
TTAAACACGGAACCCCTTTTCCTAAATGGCCGTGCATAACAGCGTCCCGCGGGTAGAA
TGCATCCATCCAACCCCGGAAACCGTCCACTCCGCGGCCCGCGAGACATAATACCGTT
TGATGACATCATTAAGCCGGAGCCGGGCTTATTGTTTCTCATAAAATCGTACGTGCAAA
CGCGCATCCTCTCTCCACCGTCTTGTCCGTAATGTTTGGACGACGATGACGTAGCTGGC
CGAGGCGAAGGACACAGGCGTCGTTGTCTCCATTTTCCACGACGGGACAATAAACCG
GGTATAAAGTTATAATTAAATGGCTGTAATGGCGCGTGTTTGCCACACGTGACGCGACA
CGGATTATTATTGCTAGAAGGGAAATAAATCTTCAAGCGTCTGGGACATGCTGTGTAGG
CACGTTGATTTTTTTTGGGGTTGTTTTTTTGTGGATTCTTATTTCGGTTTGTTTTCTTAAT
TTTTAATAGAAACGAACTAAATTTGGATTTGTGAATATCTTATAAATTAAATTAGGTTATA
AAATAAATATTGCACTTTGAGAATTGTTATCGCGGATTTTTAGATGCTTTTTTTAATGGG
AATAAATAATTTTTGATATAAATGAATTAAATTGGATTTGTATAATTTAATTGGAATAGTG
ATGCTATGTCGATTTTTTAGAAAATTTAAGGGATGTTTTTTTGCTGATTTTTAACATTTGA
ATCAGAATTCATGCTTTTTAATTTTTAATGAAAATAAATAATTTTTAACGAAAAGAAATT
AAATTTTAATTTGTATTTTCTTTTCTTTTTGATTTTAAAACTTTTTTGAATAAACATATAAA
TTCTATCAAAATACAAATTTTATAAAAGTTATTATTCCATATTCTTTTAAATATTTATTGAT
CTAAAATTTTATTCGCTGAAATAAAATATAATTTCAAATTCATTCCAAAAAAAAAATGTT
ACAAATTATCTGTTCTGATTATTTATAAAAATTCCATAATCACGATTATTATGTTTTATTGA
AATTTTAAAATTAATAACTTGTAACTTAGAATTAGTAATTAATAGATTAGATTAGGATAGG
TAATTTATCTTAATATTTATTTTATATATATAATGTGCAACTATATTTTTCTTAATAAAATAC
AATATTTTACTAAACACTTCTATTACATCATTTTTAATAATCATAAAAAAACATGTGAAAT
CATTTTAACGAATAATTCTAATGTTAAACAAAAATTATATAAATTGTACTACTTTCAAAA
GTCATTGTCTTACAATTTTTCTACTGATTTCAAATCCTTTCTATTAAAATAAAACTCAAA
AATCTTCAAACAAAAAAAAAACTAATCTAAAACCAACAACAAAATTTCTAATTTTTACT
ATTTTTTAAAATATTGTACTTCAAAATCTTAAACAATAATTGTATAAATTCTATCAGATTAT
CTTTTAGAAATTACTATATGGTTTCCATTCATTTCAAACTCTTTTCAAAATAAAACTTAA
GAATCTTTGAACTATATTCAAAAAAAAAAAAAAAACAATTCTGAGCTTCGACCATTTAC
AAATATTCCACAATCATGACTATGTTTTTATATTATTTAAAAATGATAAAATGATAAGTAA
AAAATACAATAAAAATACAATTTCAAAAACCATCAAAATAAATATCGTATTACTTCAAAA
TCTTAAAATTATTCTACCACTTTCAGAAATCACTATCTCGCATCATTTCCACCGAAATAA
AACTCAAAAACCTTCAAACTAACTATAATCCCCCTCCCCCTCAAAAAAAAGATCAAGC
TGGCGAAATCACCACCACCGACCATCCTCTCTGACCCAAACTAAATCTCTCCAACCGT
CGAAAAAGGAAGCGACCCCGGCTCGCCGCGCGACACTCCCCCACCCAATAAAAATAA
TCGCCATTGGGGTTCCCCGCTTTGGTATTCCGGTTGTATGTTTATCGCGCGATTCGTTCA
ACTAATGGGCCGTGATCGCCGCGATAATCGGGATCTTATCGCTTTCTTCCGGACACTTTC
GCGGCGGCCAACTCTGGCGTGTAGACACTCCATGCGACCCGTGGTGTAAGCAGCCGAT
GGGGATAACCCCGTTCAATTTCGCCGTCTGTTTGCCGGCTGGATTCCGCTCCATTCGTT
GGTGGCCGTATACGTGACGCGAAAAACGAATGCCGCCCGCGTGGTTTCCTTCTGGCCT
GGTGGGTAATTCGAAAACCTTTCCGGAGAAGACGAATCGAGTCCGCGTGAAATTGGCC
GCCATTGCTCGGAACGGTGATATAGGTGTGAGTGGGTAAAATGTTGCACGTTTTGTGCA
CGTTGGTTTCGGATAGTTTTAATAGTTCTTCTGTGGGTATTTATAGTTTGTAGAATCTTTT

TATTGAGAAATTTATTTATTTATTTATTTATTTTAAACGAAGTAACGATTCTAATTTGCTT
CCAATATTTTAAAAATTGTTAAGAATTTTTGTTTTTTCTTTTTGCAAGAGATAAATAAATA
AATAAATAATAAAGATAAATGAAGTATTGAAATAGAGGATCTTGAAATAAGAAGGTTAA
GGACAATTTTTGAAATATTATTATAGTAATGAAGAAAAAATTGAAAGTATATATTTTTTTT
TCAATTAGAAAATAATTTTCCAATTTATTATTTGAGAAGAATTATTGCATGTTTTTAAAGT
ATTGTGAGATAAATAGTAAAATGAAATTTGTGAATATTAATTTTACTATAGATAATAGCAA
TATAATTGAATAATTTGAAAATAAAAATTCTGTAATAAAAATTCTTGGATCTATGATAATT
CTTTTGATGAAATAATTATATTTTCTTTATTTAAATAATTTTTATTTTTGAGTTTTATTATAA
TATATAAATTGCAAAAGATAATAAAAAAATTTTATTATTTTTGTATATATAAATTCTTAGTT
TATAATTTATATAGTTATATAATAATAAAAAATATACATACTTACACTTTATATGTTTAAAAT
AATAGGAATTTTGAAGATTGAATTTATTATAATAGGTAGTTGCCAAGAATAGACATTAAG
ATTTGTAGTTAAAATTAATTGGTCATTATGATTTAACTATAAAATGTATTATACCTTAAGAT
TATGATATGCAGATTATGATATGCAGATTATGATATGTTAAAATTTAATTAAGATTATATTT
TGTATTATTATGTTAAAACTTTAAAAAAAAATTGAACATATTATAATAATCTTTTAATTATA
ATATTAATTTTATTTTTCTAAATTTATATTCATGTTAAACAATTCTAATACTCTTAAACATC
ATGCATATATAAGAGATTAAAAAAAAGGAAATTTTTTACATTTTTAAGAATGAGAAATTT
AAAATTCTTATAATTAGTATTTAGAAATTAAAAAAAAAAATTGTACATTGCTCATGTATT
AAATGTTTGATGCATTTTTTGTTACAGGAGAGCTCACCTCAGAAATTGCCTTGAAAAGT
TGAAGGTGTTGGTTCCGTTAGGTCCGGAAACCTCAAGACATACCACGTTGGGCCTCTT
GACTAAGGCCAAGCGTTTTATCAAG

>novel_circ_001943
ATATATGTGTAATGCAGCAAAACGAAATATAACAATAGAAAATACAAGCAGAATTATAA
AGCGGATAACAATTAATTGTCAACATGGCGGATAGTGATTTTCTCATTACAATTTCAGGT
CCTGCTTTATCATTATTGTTTTACGAAAATGTTCGTAGTAGCGGGGACCAGATAGGCTTT
TTACTAGGAGAAACACTTGAATTTCACATAAAAACTTATACAGATTTAGATAATCAAGT
AGAAACAGTAAAAATATATAATAACATTGAAGCTGTTGTAACTTGTCCATTGCCTCATAC
ATTACATAATTCTCTTGGCAAAATTAATAAAGAGAAGTTAAAGGACTTTTTACGTGACA
AGAGTAAACAAGTGATAGGTTGGTTTCATTTTAGACGAGATGTAAGCTTGGTGCCTACA
TTCAGGGATAAATTATTACATAAAGAATTTGCCTCGTATTTTTGTAATGACAATGGTTCTA
AAAATGAGTTTTTTGTTACTTGTTTGTTAAGTTCTTCAACAAGTAATGAAGGGGGAACT
TATAAATTCAAACATGTCTTCTTAAGATATAAAAGAGGAGCATTTGAACCAGTTCCTTTA
CGGATAAGTAACTTAGGAAGTAATTCATTTGCACATGAAGGTTCAGATTATAAACCTAC
TCCTACCAAAAAATCATCTGATGTTCCAGATATATTTACTAAATTTATAGAATCTTTAAAT
TTGGATTTAACAAAGACATCTGCAGTTGAATCTGCTATCAGTATACAAAAAGCAGCTGA
GCAACATTTGAGTCAATTAATACCAGAATTATGTAAATCTGATCTTGAAGTAGCAGAGC
TTGAAAAACAGGTTAAGGAATTTATGTTTAGCAAAAAGATAAAAATTAATGGTAGTCAA
ACTTATGAAGTTAAAAAAGATGAAATTAATGAAGGAAAAGCAAAATCAGAATCTTCAG
ATGAATCTTCAGATGATACTTATCAGGAATGTCGAACCAGAAATGTAGATCCAGGAATG
TTCAAGGATCTTTCTATTGCAACT

>novel_circ_001944
ATATATGTGTAATGCAGCAAAACGAAATATAACAATAGAAAATACAAGCAGAATTATAA
AGCGGATAACAATTAATTGTCAACATGGCGGATAGTGATTTTCTCATTACAATTTCAGGT

CCTGCTTTATCATTATTGTTTTACGAAAATGTTCGTAGTAGCGGGGACCAGATAGGCTTT
TTACTAGGAGAAACACTTGAATTTCACATAAAAACTTATACAGATTTAGATAATCAAGT
AGAAACAGTAAAAATATATAATAACATTGAAGCTGTTGTAACTTGTCCATTGCCTCATAC
ATTACATAATTCTCTTGGCAAAATTAATAAAGAGAAGTTAAAGGACTTTTTACGTGACA
AGAGTAAACAAGTGATAGGTTGGTTTCATTTTAGACGAGATGTAAGCTTGGTGCCTACA
TTCAGGGATAAATTATTACATAAAGAATTTGCCTCGTATTTTTGTAATGACAATGGTTCTA
AAAATGAGTTTTTTGTTACTTGTTTGTTAAGTTCTTCAACAAGTAATGAAGGGGGAACT
TATAAATTCAAACATGTCTTCTTAAGATATAAAAGAGGAGCATTTGAACCAGTTCCTTTA
CGGATAAGTAACTTAGGAAGTAATTCATTTGCACATGAAGGTTCAGATTATAAACCTAC
TCCTACCAAAAAATCATCTGATGTTCCAGATATATTTACTAAATTTATAGAATCTTTAAAT
TTGGATTTAACAAAGACATCTGCAGTTGAATCTGCTATCAGTATACAAAAAGCAGCTGA
GCAACATTTGAGTCAATTAATACCAGAATTATGTAAATCTGATCTTGAAGTAGCAGAGC
TTGAAAAACAGGTTAAGGAATTTATGTTTAGCAAAAAGATAAAAATTAATGGTAGTCAA
ACTTATGAAGTTAAAAAAGATGAAATTAATGAAGGAAAAGCAAAATCAGAATCTTCAG
ATGAATCTTCAGATGATACTTATCAGGAATGTCGAACCAGAAATGTAGATCCAGGAATG
TTCAAGGATCTTTCTATTGCAACTAGTCAAAGTACTCATAAACTTAGAAATACAATGTAT
ATTGAAAAGAATATTAACCAAAACAAATTAAGAAAATCATCTACCACAAGCATAATAAA
TAGTTCCAGTCAGGAACCATCACCATTTCTAAATTCAGAAATAACAGAAAATGTATTTA
GTAAGAGTAAACGTCTTTCCAATATGGAATCAGAAATTGTAAAAGAATCAAGTTGTAAA
AGTGACACAAATATGAGTGGTATTGGACGAGGTAGAGGCAAATCAATGCACGATGTTT
ATTCAGGATTAAAAAAAGCTAAAAGGACTTCAGGCTCAGAATCGTCTGAAACAAATAG
TGTACAAAATACTTCTTTGCAAACTTATAGTCAAGTTACAAAGAAAAAG

>novel_circ_001946
ATGGACAAGAGCAAGTGGATCCTCAGAAGCAAGGTGTAAAGGATGCAAGTGCTAAAG
CTGCGAAACGTGCACTGAAGAAAGCTAAGAAAGAAGCCAGGAGGAAAATGCTCGGT
GGTGTTTCGCCTGCGAGATCTCCTTGCAATGGATCACCAAG

>novel_circ_001947
AAAAGGTTAAAATCAGTCCATCCTATTGGAACAACAGAGTTGATGAGGGCGATTCATC
GGCTAACGCCACGGAACCAACGGAGGAACGGATGGAATCGACAGATTGTGACAGCAA
TCCATCAATGGCGTATTGCGCCACACCCAGGAGACTTAGTAGTTCGTCCGTGGAAAGC
GCTAAAATACCGGAAGAAGAGGAGAAAAAAACATTCGCGGCAGCTGATTCGACGATG
AGGCCGAGAACGGGTCGTATACCTCGAAAGAAACGATCTGTAGGATCTATGGAAGATT
TGTGGGACGAGACCGTGTTTGAAGATCCAACGAGAACAGCGAGGACCACTCCGGTGA
TTAAGATCTCTTTCGGTGCTCAAGGCGAGGGAACCGTTCTGAAGATACCTTCCAAAAT
CCAGGATCCGGAATATGAGCAGGAAACTGATGATGCTGAGGACGTGCAGACGGAAAC
GGAGAGAGATCCACTAGAATTGCCGAGAAATTTTGATAATGATTATGATTATCGTGAAG
ATGGGGAAGAAGATGGACAAGAGCAAGTGGATCCTCAGAAGCAAGGTGTAAAGGATG
CAAGTGCTAAAGCTGCGAAACGTGCACTGAAGAAAGCTAAGAAAGAAGCCAGGAGG
AAAATGCTCGGTGGTGTTTCGCCTGCGAGATCTCCTTGCAATGGATCACCAAG

>novel_circ_001948
TTATAGAAATGTCGAAGAAAAATAGGAAACGGAAATTAGAAGGAGATGATCAGTCAAT

GGTCGACTGGGAACAAGAGAATATTTTTCCAGAGGAAATAAAAAATGAAATGGAAACT
ATGTGGGAGATTCCACAAATATTTCATTTCCTTCATTTGGCAAAAGAAGCTTTAAACATA
CCCCATTTGTCTATGTATGAAATGGAACGAATGCTTCTGATACCAAGAGCTTCTAAACA
ATTGGCAAATATAATGACATCCTTGTTAAGTTCTCCCATAACAAAAGCAAAATTGAGAA
AAATACCACCAATGCCGTACGAATTTTGGACAAATATCCTTGCTTATAAAATGAAAAGT
TGGTTCAAAATTTACGAAGCTAAACACCAAAACGCAGTAAAAGTTTTAGAAACTATCG
GCGTAGAACCGGAATTTTGGAATGTTTTTCCTGATGCTCCTCTATTGAATGGAAAAGAT
TTTGAAGAACTGACTTTTAAACAAAAAGTTTGGCTTTTGAAAACAGTCTGTGATACAG
TTATG

>novel_circ_001949
TTATAGAAATGTCGAAGAAAAATAGGAAACGGAAATTAGAAGGAGATGATCAGTCAAT
GGTCGACTGGGAACAAGAGAATATTTTTCCAGAGGAAATAAAAAATGAAATGGAAACT
ATGTGGGAGATTCCACAAATATTTCATTTCCTTCATTTGGCAAAAGAAGCTTTAAACATA
CCCCATTTGTCTATGTATGAAATGGAACGAATGCTTCTGATACCAAGAGCTTCTAAACA
ATTGGCAAATATAATGACATCCTTGTTAAGTTCTCCCATAACAAAAGCAAAATTGAGAA
AAATACCACCAATGCCGTACGAATTTTGGACAAATATCCTTGCTTATAAAATGAAAAGT
TGGTTCAAAATTTACGAAGCTAAACACCAAAACGCAGTAAAAGTTTTAGAAACTATCG
GCGTAGAACCGGAATTTTGGAATGTTTTTCCTGATGCTCCTCTATTGAATGGAAAAGAT
TTTGAAGAACTGACTTTTAAACAAAAAGTTTGGCTTTTGAAAACAGTCTGTGATACAG
TTATGCACACTAGAAAAACTGTGCAAGAGGAAATAGCCAAGCAACCATGGGAAAACC
AATTTGAAACTGTTCTAGGCACTGATCGTTATGGAGCAAGATACATTTATTTTCCTCAGT
TTCTTAAAAGTGATTTGAGAATTTATAGACATTGTCTTGATAATAAAATTCTATCCACAGT
AAAGCCAATCAAACCTAAATTAAAAACAGAATTAGAAAACAAAACATTAAATGAAAAT
GATTCTGTGAAAAAGAAAACTAAATATAGAAGAAGAAAATCTCGATGGAGTAATGGAT
TACCTCCAAAATCAAAGAAAAAAATAAGTAAATGTGATGACAAAGTTGTAAATGATTG
CAAGTGCACTAGTGACAGTGCAATAGGCTCTTTGATAAATGAGGATACGAATTTTAGTA
GTACAAGCACTTGTAGTAATAATAATAATAATAGCAATACTAATAATAATAATAATGATAA
TAATAGTAATAATAATAATAATATCAACTTAGATATTATCAATGTCGAAAGATTGAGAAGT
TCGTCGAAGTGTTCGAAAGATGCATCCCAAAAAACATGCTGTAAAAGCACAAAATCAA
GTGGTTACGATACAAGCAATTCTAATCACGTCATTTCTGGTAAGAGATTATCTCAAAAA
ATGTTTAAAGGATTTTCTGACAGTAGTAATAATGTTAAATGTGATATAGAAATAATCAAC
GGAATTTTGTCCAATCTAAAATCGGAAACTAACGAAGAAAAAGTAACAGATGATAATG
ATATATCTTTAACCCATGCTTCGAAAAACAAATTAAAATTAAATCCGACAAGACAACAA
TATTTAGATTCAATGGATCTACAAATAAATAAAGATAATCCTGTAGATAGAACAATTGAT
AATTTATCTACCACTTCAAAAACAGATGATGAAAAACTAAATGAAATTATCAGTGCAGA
AAATTTAAAATTAAACGATAAAAGTTCAAATGATATTCATAATGTAAGAAAAACGCATC
AAATATCGATTGTATCAAAATCTGATGATGAAAAATTAAATGAATCAAGAACTCATACTT
ATAACCAAGGAGATAATGTTAACTTTTCTGAAAAAAAAACAGGAAGCAGAGTTCTTAG
ACAAAATGGAAAATTTAAAGAACAGAAATCAGATAATGAATCAGATCAAACAAGTAAA
ACAAATGAAATAGAATCATTTGATGATAAGGATCTTTCTTTGAGCGAATTAAGAAGCAT
GCTACAGAAGGAAGCAATGGAAGATGATTTTTCTTTCGATGAGAGTAGCGAGAATGGC
AAATATAATCTTAGAAAATTGAATAATAAGAAAACAAATGATTCGAAACAGGAAACAG
ATGATTTTAATGTGATGCTCTTGGAACTTAGTACTTCGAAATTTCAACTTGTAGCTGATA

GTCTTAATTCATTGAGAGATCTTATTTCTTCTTTTTCACAAAAAAGTAATAATCCAAGTA
CTGATACAGATACTGATGCTGAACTTATGCCTCCCTGTGAAGTAAAATTAGTTAAAAAA
ATGACAGAATTATTAACGTCTTTGGAAGAAATAGAACCAGCTTTACGAGATTCAATGAA
AAGAGCAAGAGGAAAACTTCAAAAAGAATGGACTAATTTTAAAGAAGGCAGTGCGGA
GGATCAGGACTCATCTGGGGAGGGTCTCGGCTCTAATTGGTGGGTTCTTGGATCGCAG
GGCTATCAATTGCCAACTTCAGGAGACGCAACGTTACAAACGTTACCGCAACTTACCG
TATCCTCAGCTGGCTCCCAAAACATCTCCAATAAAAACGACGAAGAAATCATTAAATGC
ACAGAACACAGTAAGAATGTCGAACAAGAATGTAAAGAACCCCAAGATGAGAATTCA
CAAAACGAAATACAAAGAGAACAATCTGAAAGAGACGAAAGTAACACAGCTCAAGA
AAACACAGGAACTGAAGGAGAGACAAAAGAAGATTCAAGTGAAGAAGAACATCAAA
CACGAAGAGTCTTACGAGCCCGAGGCGTTTCCTCATACACAGAACAATTTTATTCAGA
CGACGATATGGAGGAGAACGAGCTGGAGGAATGGACCGACTTTGAGGCCGTGTACGC
GGCTCCCAGCACACAGACCAGTGCATCCACTCCACACTTTGGTCCGAAAGTCAGATAT
GCTGACGATAGGACAAACGAGGAGGAGGACTCAGACCAAGATTGGATTCTACCAAGC
TCTCGCAAAAGGAAAAATAAACGTCCGT

>novel_circ_001950
GCATGAGAGAACCGAGAGACGATTGAGGCACAAAAAGGAGGCGAATCCACCCCCCAT
GGAACCACCTCCGCAACTACTGAATAATAATTATTTGTACATCGACTTAAACATGAACTT
CAGTGAGAATACTAAGCAACAGACTACCAAAACAAAAAAGTTTCAATTGACAACAATC
CATTTCCATACTTTCTAAAAGATCCAATTTCTACAAATCCAGTTTTTAATAATCTAACCAC
TTATAATCTCAACTTCAATCCATTTCTTTACTATCCAAAACATCCAACTCTATAAATCAAC
TTTTTACGTAACGTACTACTTACTCGACGAAGTGGAGCCGCAATAAGCACGGGGGGAC
GATACTTGCATGACAAGACCTTGCGGCTCACCACACACTATGTCCAAAGACCAAGACA
CTCATGTTACGCCTATGTGTGTTGTCACGGCGTCGTGGATCCGTCTACGGGGATCAAGA
AGCGCGGAAAACTCGTCTCGAGGTTGGTTAATGCGCGAACAAACACCTTCTTGTGGAG
GGGTACCATAACGCTCCTCCCTTCGTGTCCTCCTTACATTTTTATATGAGCTTTTAGTGC
GGTATCGTGCGTGGGATCGTTAGTGTCGCGAATGATTCCTCAACTTTTTTTGTTTTCAAT
TATTTTATTTTATTTTATTTTTTTCTTTTGTTTCTTCTTGTTCTGATGCTTTCGTATAATACG
CGCGGAAGTGCGTGCGAATGATGGTGGATGTTCGAGGTGGTCGATTGAATTCTTGTAG
ATAAAAAGTTATTTCTTCAATTCTTAGAGATTTCCTATCTATCATACAGACATTTTCATTAT
AGAATAGAATTAGAAATTAAAAGATGTTTCTTCAATTACAATTTCTTTATTTATATAAATA
GGATATTCAAAAGAACATAAAACCACAACATTTTCCTTCATTTATAAATTTTTTATCACA
ATATAATTAGAATCAATGGAATAAATAATATTCTCTAACTACAAGTCTCCCATTCATCAAA
ATACATTCTACATAAACAAAGTATCCAAAAAAATAGTTATCCAAAAGACTTTCAATTGT
CTTTTCAACTGAGACTTATATACATAATATACAACTTATATACAAGTTTTCCATTCATAAC
AAAGATCAGAATTATATATATCCAGAAGATATTCCTTCAATTATAAATCCCCTATTACATAT
GTATGTACTTACATACATGACAAATCATTCAATAGACCATCCACCGAACTTAGAAGGCG
TTCCTTCCCCTATCCATCATAGATATATATCCGTTCAGATAGGACAGACTAGCCGAGAAA
TAGAATAAGAGAAATAGAGGGGGAAACGTGGCGGATTATCCGTGGTGGCCGACCGGAT
CGAACCTAGAAAGGCGGTTGTTCTAAGGTTTCCACGCCGACTGTAGGAAGCCAGCCCG
TGGACTGGCTCTCGAACCCGCCAGAGTGAGAATCGTGACTCGTGGATCGTTAAAAGAT
CAAGAAAATCGACTTGTTCCACTCCTACCCCATTAATCTGGCCCCCTTCTACCCCGGAT
GGATCGGAACGAGAGCGCAAACGAGGGTGGAAGCTCGCCGGACACGGTGATCGCGC

GCTCCTCGTCCGAAGAGGAGCGGAACAGGCTCGCGTGCAGCACTTCCGGCGAGTACG
TGACTGTGGGCGATAGCAGTTCCAGCCTGGACACGGTGACAAGCGTTGGCACGACCA
CCGTCTCGACCACCACCGACGCGACCGCCACAGAGGACAAAAAGAGGGGCAGATTCG
GCGCGTTGAAGAGCAGCTTCCGCAAAGAGGGTGGATTGTTCAAGATTAAGAAGAAGA
GCAGGCAGCTGGACGAGCCGGCTGCCGAGCTTCAGCCGGATGTCGAGGAAAAAGAG
ACGGATCGCAAGCAGAGCCCGGTGGAGGAGGAGGATGATCGCAAGGGTGGTGGCTCG
ACGACGACCTTGAAGAAGAAAAAGAAGAAGTCGAACTCGCTGGTACGCAAATTAAGC
CTGAACAAATTTCGCCTGTCCACCGAGCAAGAGCCTAGGCACACGCCCGAAGGTGGT
GATAACAGCCGTCCACAGAGCCAATCGTCGCAGGAGTCGCCTAGTTTCTCGGACAGTT
CGTCCGGGATCGGGAGGAAAAGGCAGGAGGTCGAGAGGCTCGGCCATCGCGACGGCT
ACGAGGCCGTAGAACCCTCGTACGACGTGGACAGAGAGAAGGGAGAGCCGAGGAAA
CCGGAGAAGCAAGAGAAACTGGTGAGCAGCGTGACCGTAGTACAGAGCAAGTCGCC
GTCGTTTACCATCAGGAGCTTCAATTTGACGAGTGAGCACGATTCCGAGCAGAAGTCA
CCTCGACCGGAAGCGCGGTGCAGCTCGACGCTTCCGTACGCAATTTCAAAATGGTCGG
AGCAAAAGGTCTCGAGATCAACCTCGAACGAGCCGACTAAGAAACCCAAACTGAACA
CGAAATCCGAATCGGAGTCCAGTATATCTAAATCCTCGGTCCGCGAAGTCAGGCGGAA
GTTGTCGTTGTTGGAAGAGAAACGCGCAATTTTTCAACGCCGTTTGTTCGAGACTTCT
CAGGACTCGGATTCCGGCGAGACGGTGATCGCCATTACCCTTGACGACGAAAACAAA
ACGTCGGCCAAGGAGGAGGAAAATCGTGTGGAACAGAACAAGAAGAAGAAGAAGGC
CAGGCATATTAGCGTGAAGAAGTTCGACACATTTTCCACGTTTGAGGGGACGTTCGAC
GAGGAGACAGGGATTGGTTATCTGGCTGGTATCGAGGAGGATTACACGGAGAGTTACG
AGAGGCAGCGCGATAATTACGTGGCCAACATGGGGCCCATGATGGCCGACAACGCGAT
GGAGAAGAGCAAGGTCAGCCAGGAGAGCATG

>novel_circ_001951
GAATTAAAATCGAAACCCGGAAAACTAGTTCCTCTTCAATGCGATCTTTCCAACCAGA
ACGATATCTTGAAAGTCATAGAATGGGTCGAGAAGAACTTAGGAGCTATAGATATTCTC
ATTAATAATGCAACTATTAATATCGATGTCACACTGCAAAATGATGAAGTGTTAGATTGG
AAGAAAATTTTTGACATAAATCTTCTTGGACTGACTTGCATGATCCAAGAAGTTCTGAA
ATTAATGAAGAAAAAAG

>novel_circ_001952
AAAACAAGCACAATTTAATATATCAAGGAATTCGCAATGGAACAAAATTGGATTGATAA
AGTTGCTTTAGTTACTGGAGCTAACTCGGGCATTGGCAAATGTCTAATAGAATGTTTAG
TTGGCAAAGGAATGAAAGTTATTGGAATTGCTCCACAAGTCGATAAAATGAAGACTCT
TGTTGAGGAATTAAAATCGAAACCCGGAAAACTAGTTCCTCTTCAATGCGATCTTTCCA
ACCAGAACGATATCTTGAAAGTCATAGAATGGGTCGAGAAGAACTTAGGAGCTATAGA
TATTCTCATTAATAATGCAACTATTAATATCGATGTCACACTGCAAAATGATGAAGTGTTA
GATTGGAAGAAAATTTTTGACATAAATCTTCTTGGACTGACTTGCATGATCCAAGAAGT
TCTGAAATTAATGAAGAAAAAAG

>novel_circ_001953
AAAACAAGCACAATTTAATATATCAAGGAATTCGCAATGGAACAAAATTGGATTGATAA
AGTTGCTTTAGTTACTGGAGCTAACTCGGGCATTGGCAAATGTCTAATAGAATGTTTAG

TTGGCAAAGGAATGAAAGTTATTGGAATTGCTCCACAAGTCGATAAAATGAAG

>novel_circ_001954
AAAAAAAAGAAAACAAGCACAATTTAATATATCAAGGAATTCGCAATGGAACAAAATT
GGATTGATAAAGTTGCTTTAGTTACTGGAGCTAACTCGGGCATTGGCAAATGTCTAATA
GAATGTTTAGTTGGCAAAGGAATGAAAGTTATTGGAATTGCTCCACAAGTC

>novel_circ_001955
TACAAGTAAAAAAAAGAAAACAAGCACAATTTAATATATCAAGGAATTCGCAATGGAA
CAAAATTGGATTGATAAAGTTGCTTTAGTTACTGGAGCTAACTCGGGCATTGGCAAATG
TCTAATAGAATGTTTAGTTGGCAAAGGAATGAAAGTTATTGGAATTGCTC

>novel_circ_001957
GAATATTCGCTCCAAAAATCTCATTCCTTCGTGTCTTCCGTATCACTACATTGAGGCCCA
TTAATCGTATCGCTTCTTCGGTTTCGTTCTTGCCCTTCTAATTACACTACTCAGATTCGTG
CAGCACCTGGGAACATCACGTAAGTCTCTCTTTCACGAGACTCGATTTACACAAATCTT
TTTTTATAACAATCTATTTACCATTTTCAGGATGAAAAGAAAAGAAAAGATTCTTGAAA
AAACAATCCCAACCTCCAATATTCCTAAACATTTCGAATCGAAATCATTAATTAATCAAT
TAATTGATTAATTAACAATCTCTCGTGTAAAAATTTCCTTTCGAAATATCCAAAAAAAAA
AAAAAAAAGAATCTATAAATGGCGCGAAGAACGAATCCTCGAACGAATCTTTCGCCCG
TTCGAAATCCACGCCCGATAAAAGTGGGCCCGTGCACACGAACAAATCGTTTAATCCG
AGTCGGGGTCCCGATCGAGGTAATGACGGCCATAAACTGCAGCCACGGAAGGGTGCA
CGAAACGAGGCGGAGGAGCGAGCGAGAGAGAGAAGGCGAGGGATTCTGGGCCCGAG
CGGAATTAGGCCTGACAGGCGAAATGGGCCTCGTGGTCCCGGGCCACGTATACCTGAC
TCCTCTTTTCCTCTTCTCCTCCTTCTCTCTCTCTCTCTCTGCTCCTCTCCTCCTTCTCCAA
CCCGTCGCAACGGTCGGTTCGTGTCCCTCCGCGCGCCGTTTCTCTTTCTCTTGTTTTCT
CGGACGGACGTGCAACTCGGATGCCACAAGTCTGCGATCCCAGATGGAGGATCCGGTT
CCAGGAATCGAGCGTGTAAAACATCGGTTAAAGGCGTGCTTTCCGACCGGGAATCGAT
GGGAAAACAATGGGCCGACAGCTCCTAAAGTGTCTGACAAATGTTCCGCGCGTGTTGC
CCATCCGGGGCCTCGAACCTCGCGGATTCTTTTTTTTTTCCTTTTTTCCTAACTTGCGAA
AAATTTGGGTGGCGTTCGAGGATTATATACACGTGGACGATAGTCGAGCCGTGGTGTTT
TTTTTTTTTTTTTTTTTTTTGGAAAGGTGGAAGAGGCTTTGGAAAAGGTTAAGAGTGGA
AATATAAAATTGTGGAAAAATATTGTTCCTTTTAGGAAGCCATTTTTAATTAAGGAAGTT
TTGAGTGAAATTGTTGAACTTACGACTGCTATTATAGGATGAATGTTCTTCAATGTTTCT
TTAATCTGGTTCAATTTTTGAATTTTAGAAATCCTATCGAATCTTGGAATTTCTTCCAAG
ACGCAAAGTTCCAGCATTGGAAAACTTTCGACGTATCGTCATCCGCGATTGGCGTTTCC
AATAAGTCAGCGTCCACTAGTCGGGAGTGTTTGCATTTGAATAACACGCTGGCCGACA
CGAGCTTTTTCCACGAATGTGTGCCGCGAGATAAATGGACAATCGGGACGTGGACCAC
CGAGCAGACAGGGTAAGCAAGCTCGTAAAGTCGAAATTAAAGCTGGATTAACAGGGT
CGATCCATGGATCGAAGAATCGATTTTTCTCTTTTAAAACGTGAAATTTCGCGAGAAGA
AATCTTGATTAATTCTTCGAGCTGCAAAATGAAATGGAAACACGTTTAATTTTTTAATAA
ATGGAATATTATACTTGAAGGACAATATTATAAATTCTTGAAATCTAATTAATTATAGAAT
TAATCTAATCTATTATTAATTAGATATAATTCTTGAATTAATTCTATAAATTCATTTTGTTGT
TTAATGACAAATATAATCCAAAATTTAAATAATCAATCCTTTCAATTTTCCAATTTACCTT

GACTTTTATATTACATCGCATGATCGAAATAATAAATCTCTTGAGCACAAGTTAATTTATA
TTTTGGATTATTATTATCTCTGATATCTTGTTTAATTATCCGATTAAAAAAATTCAACAAAT
CCGTGAGATTTAAAAACGAGGGGAAATTAATCACGCGAGAAGTAAGTCGATATAAGAA
GAAGTTGGAAACGGAAGCCGAGACTTTTCCATTCCCGGAAGCCGACAAGCGTATCAA
GGGTCAAGCGATCCGCTTCGAATCGAGACACAAATCGGGTGTGGAATGGCGGGTCTTT
CACGGTAGGCTGAGCAACATCGAGGAAGACGTTGGCCGCGATATGATTAGAGGCGAG
AAGTTGCGGCAAGATCGATAGCGGGACGTTAACAGTGTGGAAATCGCGGAGGCCTGCT
ATTTTCCATCCTTGCTTCCTGCATCGAAGCGCTACCACTACAGCTTTCCGCTCATCGGCG
CCTGTTGCTCAATGATATCGATATCGAGTAGAGATTTATCGATCCTTCGTCCTTTTTTCCC
TTCTTCTTCTTCTTTTTAACGATTTGTGAAACTCGCGTTGGGGATTATATTCACTCGTTTG
TTGATCAATATTATTTTCGCGCGAGAAAACTGTTATTATTTAACGAAAACTGTGAACAAA
TTTGTGATTAATGAATTCTATCCTCCATTCTTTTTCTCCTATTCTTAAAATCTTTCGATCGA
TATTTTATTTTCATTGATTTATTTATACGTGTTGAATCAACAGCAAGTTTGAAATTGATGG
ATTCGTGTTCGTTTCGATATCGATTAATATTATTTCTGTGTAAAAATTATTTTGAGAAAAA
ATATTGTTATTCGAATTTCAGAAGAAATCGAAACGAGAATAAGTAAGAATTCCTCGTTT
CCTCCATTCCACCCAAAATCGTTTGAAAATCTGGTCTCACAGTCAGTCAGTTCAACAA
ACTTTCAGCAAACATACCCACTCTTTCTCTCTCTCTCTCTTTCACTCCTTTTTCTGTCCT
TCTCTCCTTGTACCCTCGAGCTCTGGCCATCCGGAGGGATCGAAGTCACCGGGGAACA
CAAATTCGCGTGTCCCATCTAATACCATAAATCTGTCATCCGGCGGCGGGGGAACAAGC
TCGTTTGACTCGCGTTGCTTTGTCGGTGTGGAACGCGGCGGAATTCGCAATTTAGGCTC
GTCGCCGGTCCGCGCGTGTAAGAATCAGTCCAATCGAGGGGATCTCCGTGGAGGCGCA
ACGGTCCAACCCGCGAGAAAGAGATAATTCTTTAAAGGGGATCCCCGGTTGTATGCCT
GGTATACTCCTCCTCCTCCTCCTCCCCCCTCCCTCCTTTCTCTGTCTTTCGTTCACTTACC
TCCTTCTGCTCCCTTTCCCCCCCCCCCCCCCTAGTAGTGGCCACAAGGTGTTCCACGGG
CAACGTGGAACTGTCCGACCTTCTGCCCCACACCCTTCTCTTTCCCTCGAACACACCC
CCGACAATATTGTAAACAAAATGGTACCAGTATTTCATTGGTAACCCGACACCCCCTTC
GTTGAACCCACACGCATCGCCTTCTGCCGCCTCCTCGTTTTCCTCTTCCACTTTTTCTCC
TCCTCTCGTACCTTGTTCCTTTTCTATATACTCGTTTTTTCTCCTCCTCCTCCCCCCCTGA
CCGACTTGTTTGGCTCCGGTTTGAGATTTTGCACGATTTCGACGATTTTCGAGGGTTCG
GATATTCACGTACGTTGTATAAGTTTTTAATGTATATATATATATTATTGTATTTAAAATTAG
GATGAAAAATACGAGTACGAAGATGTTAACAATGTTAATAAAGCAAGTACAAAGTGTTT
ACAACGCTTTGCAAAAAATTTAGGAATCCAGGTGCAATTATGTAGTAAACCGATATCGA
GAAATCCTATTGTAGATAATAAGGAACTAGTTTGAAATGTCTATATACGGTTGCCAGAAA
TCGGGCGTGTTTCGTTTTGATCCGAGCATAGAACTAGTTTGGAATAATCATTCAGATCC
AAGATCCCCGTGGTTATCTACGTGAAATTTCACTCCGATCAAAGAAACTAGTTCTCTAG
AAGAATTCGAAAACACGATCACGGAAACTCGATGGTAGAGAATTCCCGTGCTAACACT
AATCTTAACAATCAAACGAACAGTCGAACAACGCTCTCTGTACTTTTTACCAATAATGA
ACTGGCTTTTTCCGCGGGGAAAAGAAACTGGCTCCATCTGTAATGTACCATTCCAACCT
CTAAGTACGCTCCACGGCCAAATAAAACTCATACGTCGCTTCGATCGCAACACGTCAAT
TAACCGTACTTTCGCCGTGGATCCTATCACCTGGTGTTACACGCAAAAACGAGAATTGT
AAAGTCCAGCGTTGAAACCAGCCGTCGAATACAATCTCCACGGTTCACAGGTAAATCG
TTCGACAAGTATGATTACCCGGTTACGGCAAAAAAAGCACGAGCAGAAATCGCATTAA
CAAGCTCTTAATAGCGGCCGCGATCGCCATCGGAACAATCAGACCGTTTCTTCGACTCC
GTGTTATTTTCCTTTAAGAAACCATTCCTTCTTCGAAGTGAAAAAGGAACGAATCGAG

GAAAAATATCGCGGACGGTTGTTCCTGCGGGAGCAACGTTGGTGTACGGTAGAAATGC
AAATTCGAGGGAAATCGAGGCTGGAAGAGGTTAGCTTGGCAAACATATAGTTATGTTG
GCCCAGGTTATGATAGGTTCGCGTGGCTGGGCCACATTAGACAGATCCACGGATCGAG
ATCTCGAATTGTAGAAATTCCGATTCTCGACAGAGAGATGATACTCGGTTATTAGGCGT
GTAACGCTCTTCCAAGTCTCTTTTCCTTCGCTTTCTATACGATCGGCCGGCACGGACTTC
TCTTGTATTACACGATTCGTCTTACGTATATACCGGGCCTGGATCGTTTTCAGATTGTAAT
TCCACGGATTCGATTCTCGACCAGTCGAGAAGAGGGATCGTAAGAGGAGGAGAGAAA
ATTACGGGACATCTTTCTTTCCGGATTCGCGACTGGGACAGGGAGCATTAAGAGAGTT
GGTGGAATTCTTCGATCGGATCGTGTTTCG

>novel_circ_001958
GAAGGATGTCGAAATCGGGAAGTATCAAGGATGGCGAGAAAGGGCCATACGACCCGG
TTCCAAGTACAGACAAAGGCAACGATGAGATCAAGCTCGAGGCAAAAATGTCTTTGTT
GAATGGAATCACCGTGATCGTCGGCTCCATTATTGGTTCTGGCATATTCGTTAGCCCAAC
TGGTGTTCTCAAGTACACGGGAAGCGTGAACGCGAGTCTTTTGGTGTGGACAGCTTCC
GGTATTTTCTCCACGGTAGGGGCTTACTGTTACGCGGAACTTGGATGCATGATCAGGAA
GTCAGGCGCCGATTACGCATATATTATGGAAACTTTCGGACCTTTCCTCGCATTCATCAG
GCTCTGGGTTGAATGTATGATAGTGCGGCCTTGCAGTCAGGCCATCGTGGCCTTGACTT
TCAGCGTTTATGTGCTGAAGCCTGTGTTTCCAGATTGTGCTCCACCAGATGATGCTACG
AGAATACTTGCAGCCTGTTGCATCT

>novel_circ_001959
ATCTAAATGTGTTCCATAGCGATACCAGCTGAATTGCTTCTGGGGCATAGTCCCCCAGT
GTATAGCTCGCTGTTATACAGTCCCTTGGTGGTAACGGCACCGACGATTACAAGCAGGT
CGGTACCACCCAGAATAAGGCCATGCCTGTCATCTGGTTCCTTGGCTATAAACAACATT
CGAAACAGTAATGATGGTAGTAATAAACAGAAGAAAAGAGTGGTGTTTGCCGATGATC
GTGGCCGTCCTCTTACTCAGGTACGTGTGATGTCGGAGCCTAGTAACGTACCACCTTTG
TGGAGCACAGCTTACTTGGCCGGATTAACAGGGCGGCTTTTAAATTCGAAAATTGAGA
ATGAACAATCCATAGAGCTCGTGTCACCTTGGCAAGTAACTTTCCCTCAACCGGCGAG
CGATTATTTAGCCTTCCGTCAGAAACTTGATCAGGAGAATGTCAGCTTGGAAAACGTGA
TAGTGAAAGAGTCGGAACAGTGTCTAGTAGGTACCATCAAGGTACGTAATCTGGCGTAT
GACAAGGAAGTCGTGGTCAGGGTGAGCAACGATTCTTGGAAGACACACGAGGACGTT
CATTGCACTTATGTCGAACAACCGGGCTCTCCGGCCCTGATCCTTTACGATACCTTCCG
CTTTCGATTAACTTTACCATTGAAGTCAAATGTGATAGAATTCTGTGTACGATATCGAAC
CGATGGAAAAGAATATTGGGACAACAACGAGGGAAAGAATTTTATCGTTAGAAAAAA
GTTAGAGCCACGAGCACCACCCAAACGATACGATATTCTGACAACGTGCGATGGCTTT
TCGAAGAATAATAATCGAGACAGTATACCACGAATCACAGATGCGACCAAAGCGAACG
TACGCACGTGGAGCGAGTTCGCATCCTGGCAGCATCTTGCGAATGATGCTCCATACTGG
TGAATCTTCGTCACTGGTCACAAACGATGTACGAATGTCGTACGAATTCTTCCAGTCTA
TTTGGTTTAACGATCACAAGATAGTAGACTATGCGGGACGAGGTGGCAATATATCTACG
CGACGGCATGCATAAGATTGTCGAAAACATTGTCGTGTAATATTTATACGCGATAGCGTA
CAAGTGTATGGACATCAAGATCAAGGGAACGCAGAAGAGAGCAATGACGATAGAAGG
GAAGCGATGGACTGTGATTAAGTTCAAATTATAAAGGACATGCTTATTGAACGGAAG
AAAGAGAGACATAATCAGAAGTCGATAGCGCGGAACAGGCGAGAAAGGAGGCACTAG

TCGCGTTACATTTATG

>novel_circ_001960
AGGCGACGAAGCCGGTCCAGCGCGGGATCTTGGGATCAAGACATTCGGATCAGGACA
CCCTCGAGTCGGCGAACAGGTGAGCGTATCCCCTGTAAGGAAGGTCCTCCTCGCCTAT
GGAGGAGGACCGTTTCTTCCGGTAATGGTGGTGGCCGACGTCCTGCGCGTACACCACG
TGGTGGCCGCCGCCGCTCTCGTGCCCGCTCGCCAGTTTCTTCACGGCCACGATCAACG
AGAGCATCAGGGCCAACGCGGACACCATCAACGCCTTCATGGCCAGCAGACCGAGAG
CGCCGAATCCTAGCGCGGCCATCATCTTGCCTGAAAATCGCAGAAATAAACCAG

>novel_circ_001961
GGAGGACTGAAAGCGGCCATCCTCTCAGACGTTATACAAGGTTTGACCATGATCGGTG
TGTCGCTCGTGATAATCGTGAGAGGCTCCGCTGATATCGGTCCCAGTCAAATTTTAAAC
GTGACGTACGAACGTGGCCGATTGGAATTCTTCGACATGAATTTCGATCCCACACTTCG
AGTGACGACTCTGTCGGCAACTCTGGGTCAATTATTCATGAGCCTGTCCATCTTCGGTT
GTCAGCAGAACTTCGTGCAGAGATATTGTAGCATGACCAGCGAGAGGAAAGTGATCAA

>novel_circ_001962
GTTCAAGTCGAAGCTAGTCAGATGCCTCGCAAGCTTCTCCTACGTGATACGAAGCCTG
CTGAACTTGGCCGTCACGATATTCACGCCTTGCGTAGCTCTTAAGGCTGTGATTGGCCT
GCCTTACTGGGCCAGTATAGTCGGCATTACATCTATATCTGTCGCCTTTTCAATTATGGG
AGGACTGAAAGCGGCCATCCTCTCAGACGTTATACAAGGTTTGACCATGATCGGTGTG
TCGCTCGTGATAATCGTGAGAGGCTCCGCTGATATCGGTCCCAGTCAAATTTTAAACGT
GACGTACGAACGTGGCCGATTGGAATTCTTCGACATGAATTTCGATCCCACACTTCGAG
TGACGACTCTGTCGGCAACTCTGGGTCAATTATTCATGAGCCTGTCCATCTTCGGTTGT
CAGCAGAACTTCGTGCAGAGATATTGTAGCATGACCAGCGAGAGGAAAGTGATCAA

>novel_circ_001963
GCAGCAGCAGTACTCGAGGGTGAAGGAAGAGGCGGCGGAATCCCAAGAGAGCCCGG
GATGACGATCAAACCGTCGTCCATGGTGACCGACTATCTGGTCTTCGTCGGTTGCATCG
TTGCCTCGTTCGTGATACCATTGTGGGGGAAATTTCGCGGCAGGCGGAAGGAGGCGAC
CAAGGCCGATTACGTGTTCGCTACGGGGACCGTATCTATGGGCGCCATGATGCTGTCGA
TCGCGCGCGGCACGCTTGGCGTCAGATCCTTTCTAGGTTATCCGTCGGAGCTGTACTAT
AGAGGGAGCGCCATGTGGGAGGTCATCTACGGTATGATGCTCGCGTATCCCATCGTGTG
CTTCATCTTCGTCCCGGTTTACTACAGCCTCGGGATCACGTCCGTCTATCAGTATCTAGA
TATGAGGTTCAAGTCGAAGCTAGTCAGATGCCTCGCAAGCTTCTCCTACGTGATACGAA
GCCTGCTGAACTTGGCCGTCACGATATTCACGCCTTGCGTAGCTCTTAAGGCTGTGATT
GGCCTGCCTTACTGGGCCAGTATAGTCGGCATTACATCTATATCTGTCGCCTTTTCAATTA
TGGGAGGACTGAAAGCGGCCATCCTCTCAGACGTTATACAAGGTTTGACCATGATCGG
TGTGTCGCTCGTGATAATCGTGAGAGGCTCCGCTGATATCGGTCCCAGTCAAATTTTAA
ACGTGACGTACGAACGTGGCCGATTGGAATTCTTCGACATGAATTTCGATCCCACACTT
CGAGTGACGACTCTGTCGGCAACTCTGGGTCAATTATTCATGAGCCTGTCCATCTTCGG
TTGTCAGCAGAACTTCGTGCAGAGATATTGTAGCATGACCAGCGAGAGGAAAGTGATC
AA

>novel_circ_001964
CAGGCAGCAGCAGTACTCGAGGGTGAAGGAAGAGGCGGCGGAATCCCAAGAGAGCC
CGGGATGACGATCAAACCGTCGTCCATGGTGACCGACTATCTGGTCTTCGTCGGTTGCA
TCGTTGCCTCGTTCGTGATACCATTGTGGGGGAAATTTCGCGGCAGGCGGAAGGAGGC
GACCAAGGCCGATTACGTGTTCGCTACGGGGACCGTATCTATGGGCGCCATGATGCTGT
CGATCGCGCGCGGCACGCTTGGCGTCAGATCCTTTCTAGGTTATCCGTCGGAGCTGTAC
TATAGAGGGAGCGCCATGTGGGAGGTCATCTACGGTATGATGCTCGCGTATCCCATCGT
GTGCTTCATCTTCGTCCCGGTTTACTACAGCCTCGGGATCACGTCCGTCTATCAGTATCT
AGATATGAGGTTCAAGTCGAAGCTAGTCAGATGCCTCGCAAGCTTCTCCTACGTGATAC
GAAGCCTGCTGAACTTGGCCGTCACGATATTCACGCCTTGCGTAGCTCTTAAGGCTGTG
ATTGGCCTGCCTTACTGGGCCAGTATAGTCGGCATTACATCTATATCTGTCGCCTTTTCA
ATTATGGGAGGACTGAAAGCGGCCATCCTCTCAGACGTTATACAAGGTTTGACCATGAT
CGGTGTGTCGCTCGTGATAATCGTGAGAGGCTCCGCTGATATCGGTCCCAGTCAAATTT
TAAACGTGACGTACGAACGTGGCCGATTGGAATTCTTCGACATGAATTTCGATCCCACA
CTTCGAGTGACGACTCTGTCGGCAACTCTGGGTCAATTATTCATGAGCCTGTCCATCTT
CGGTTGTCAGCAGAACTTCGTGCAGAGATATTGTAGCATGACCAGCGAGAGGAAAGTG
ATCAA

>novel_circ_001965
AAACGCGGCGCTCGAGGCACGCGCGAGAGCGAAAGCAATCACGAACGTCCATCTCTA
TTTGCACGCAAAGGATAGAAAGTAGCTAAATACGCGACATCTGTAAACATAGCTCGAC
CAACCTCTTGCTCGTGGCCGTTCCGCGGATAGATTCAAACACGAGGCCGTTAAGGATA
AACGAGTACCACGTCGGTAACATTGTTCGTACGCTCTTGTTTCCCGAAAGCAGGCAGC
AGCAGTACTCGAGGGTGAAGGAAGAGGCGGCGGAATCCCAAGAGAGCCCGGGATGA
CGATCAAACCGTCGTCCATGGTGACCGACTATCTGGTCTTCGTCGGTTGCATCGTTGCC
TCGTTCGTGATACCATTGTGGGGGAAATTTCGCGGCAGGCGGAAGGAGGCGACCAAG
GCCGATTACGTGTTCGCTACGGGGACCGTATCTATGGGCGCCATGATGCTGTCGATCGC
GCGCGGCACGCTTGGCGTCAGATCCTTTCTAGGTTATCCGTCGGAGCTGTACTATAGAG
GGAGCGCCATGTGGGAGGTCATCTACGGTATGATGCTCGCGTATCCCATCGTGTGCTTC
ATCTTCGTCCCGGTTTACTACAGCCTCGGGATCACGTCCGTCTATCAGTATCTAGATATG
AGGTTCAAGTCGAAGCTAGTCAGATGCCTCGCAAGCTTCTCCTACGTGATACGAAGCC
TGCTGAACTTGGCCGTCACGATATTCACGCCTTGCGTAGCTCTTAAGGCTGTGATTGGC
CTGCCTTACTGGGCCAGTATAGTCGGCATTACATCTATATCTGTCGCCTTTTCAATTATGG
GAGGACTGAAAGCGGCCATCCTCTCAGACGTTATACAAGGTTTGACCATGATCGGTGT
GTCGCTCGTGATAATCGTGAGAGGCTCCGCTGATATCGGTCCCAGTCAAATTTTAAACG
TGACGTACGAACGTGGCCGATTGGAATTCTTCGACATGAATTTCGATCCCACACTTCGA
GTGACGACTCTGTCGGCAACTCTGGGTCAATTATTCATGAGCCTGTCCATCTTCGGTTG
TCAGCAGAACTTCGTGCAGAGATATTGTAGCATGACCAGCGAGAGGAAAGTGATCAA

>novel_circ_0019662
TTATATAATGTATATTTTATCTGAGGAGGAGAATATCAATAGGAAGTAATAAAATACAGTT
ATTCCAGTTATACACATTTTGTGTGATTCGAACATTCTAACAGTGAAAATCTTTGACGCT
TCTTTTTAATTACTTAAGATCTTTTTCATTTACTTAAGATCTATAATTAATAATGACTCAAA

TACCAATTCTTACATTTTCTAATGGATATAAGATGCCTGCATTTGGTCTTGGTACTTATCA
GTCACGTCCTGGAGAAGTAGAAAATGCTGTAAAAGAAGCAATAAACTTAGGATATCGT
CACATAGATACAGCATATTTTTATCAAAATGAGAAAGAAATCGGTGAAGCGATTCAAGC
GAAAATTAAAGATGGAACTGTGAAAAGAGAAGATCTTTTCATTACAACTAAACTTTGG
AATAATTTTCACAAACAAGAAAGTGTAGTCCCAATATGTAAGAAATCGTTAGAAAATCT
TGGATTAAGTTACATAGATCTCTATTTAGTTCACTGGCCATTTGCCTTCAAGGAAGGTGA
TGATTTGTTACCCAGAGACGAAAATGGCACTCTTTTGTTATCGGATACTGATTATCTTGA
AACTTGGAAAGGAATGGAAGAATGTGTGCAATTAGGATTGACTCGTAGTATCGGAATT
AGTAATTTCAATCAAGAACAAATCACTCGCTTGCTTAATGCAGCTAAAATTTTGCCTGT
AAATAATCAGGTTGAAGTCAGCATAAATATAAATCAAACGCCATTAATAGAATTTTGTAA
AAAGCATAATATTACAGTAACTGGTTATTCACCATTGGGACAACCTGGAAATAGATCAG
GTTTACCAACTTCCTTAGATCATCCAAAAGTAATAGAACTTTCAAAAAAATATAACAAA
ACATCAGCACAAATTGTTTTAAGATATATT

>novel_circ_001967
ATGACGCCTTCCTGGATTCTTCTTTTCCCTTCTTTATTTTCCTTGGTTCACGGTCAGCTG
GATATACCGTCCTTGAACGAGAATTCCCGCGGTAATTTCGGCAGGCGGCTTCCGCCTAC
GGAGGGTGAGCTGCAAGACATTTTAGCAAGATTGGACTTCCTTGGTACCGAGAGATGC
AGCGCAAATGTGGCTGCGCAATGGTCCTACGAGACGGACGTGAACGAGTACACGCAG
ATACAGGCGCTCGAGGCTCAGAGAATATACGCAGATTTTCAGAATCAGGCTTGGTTCCT
GATTTCGAGGATCGATAAAAATAATATCAGAGATCCTGTGATCGGAAGACGTCTCAGGT
ATTTGTCTGTGGTTGGTCCATCGGCTCTTCCACCGGATCAATTGGACAGGTACAATCGC
GTGATCAACGATATGCTGGCAGTGTACAACGAGGCTACCATTTGCGCGTACAACGATCC
CTTCCGGTGCGGGCTGCGGCTGTATCCAGATATATCGCAGATCATGGCGAAAAGTCGCA
ACTGGGACGAGTTGCAATATGTTTGGACGGAATGGAGAAGACGTAGTGGCATGAGGAT
CAAGGATCTGTATCAACAACTTGTCACATTGAACAATGAGGCGGCTAAACTGAACAAT
TTCACCGACGCGGCAGAATATTGGATGTTCCCTTACGAATCTCCAAATCTGCAACAAGA
TATCGATGAAGTTTGGGAAATGATACGCCCCCTTTACGAAGAATTGCACGCGTATATTC
GAAGGAAATTAAGGGACTTGTACGGGCCCGAGAAGATCGGCTCTCATTCTCTTCTCCC
AGCTCATATTCTTGGGAATATTTGGGCTCAATCGTGGACTAACATTATCGATATCACGAT
TCCATATCCGGGAAAAAATAACCTAGATGTGACGCAGGAAATGCAGGCCCAAGGTTAC
ACGCCTATCGAGATGTTCAGAATCGCCGAGGAATTTTACTTATCCTTGAACCTGAGCGC
CATGCCACCGGAATTCTGGGCCGGAAGTATAATCGTCGACCCTGGAGATCGATCTTTGA
TTTGCCAGGCTTCCGCCTGGGACTTTTGCAACCGTCTAGATTACAGGATCAAGATGTGC
ACCAAAGTCACCATGAAGGATTTAATCACGGTGCATCACGAGATGGCCCATATCGAATA
CTTCTTACGATACAGCGGATTAACTCGTGAATTCAGGGATGGCGCCAATCCAGGATTCC
ACGAAGCGGTGGGTGAAGCCGTGGCCCTCAGCGTCGCAACTCCTCGCCATCTTCAGA
CGCTAAGCTTGGGTAACAAGTACATCGACGAGTTTACAACGGATATCAATTATCTGTTC
ACTCTTGCCATGGATAAATTGGTCATGTTGCCGTTCAGCATCGTAATGGACAGGTGGCG
GTGGGACGTGTTCCGTGGTTACGTCACAAGGGAAGACTACAATTGTCATTGGCACAGG
TTGATGGAGCAATACGCCGGTATCAAGCCCCCCGTTTTGAGATCGGAGGACGATTTCGA
TCCTGGCGCCAAATACCACATTCCTGCGAATATCCCGTACATCCG

>novel_circ_001968

ATGGAAGACGAGGGCAATCACGGCAACGACGACACCAGATGCTTCATCCTATCTACTC
TCGCAGCGCTTCAGTGGTCCAGAGTCTCGTGCGTCCTTTGTCGGGCACCTATGCTCGTG
TTCGACAGGTATCCATTGGTGGACGGCACCTTCTTCTTGTCACCTAGACAGCACTCGTC
CGCCTGTGCAGAGGTTAAAGTGGAAGGTCGCACGCAATTCTTGTCTGCCGTTTGTATG
AGTTGCCTGGAGGGTAGTGGCGGTCAGCCTGTACGTTGTCGTTGCTGTACTCAACCCT
GGGACGGGTCTAGCTTAGTCCTTGGAACCATGTATAGCTATGATATCTTCGCTGCTATGC
CCTGTTGCACCGAAAGACTTAAGTGCAACAGCTGCCAGAAGCCGCTCATCTACCCACA
CCAGCGGCTCAACTTCTATTCGGACTACAGCCGCGTGTTCGCGTGTCCGCATTGCCGTG
CAGTCGACGCCCATTTCGTGAAACCGCTGTCGGTCTGCTTCACCCGCGACCAATTCCA
GCTGTACAGCCAGTGGCCGTAACCATTAGAGAACTTAGCTAACCGGCGTCATCGAATC
ACTGCGACTGAACCGTGCCCCTTCTTCTTCGATCCGCTCATCTCTTTGCTCCGAACGAC
CTTGCTCTAGATCCTTCCATCGTTTTTTTCATTCTTTCTTCTAATATGATTACATAAACAC
AAACACAAACAATCACACACACACACACACACACACACACACACACACACACAC
ACACACACACACACACACACACACACACACACACACACACACACACACACACACAC
ACACACACACACACACACACACACACACACACACAAATACAAATAAAAATACAAATA
AAAATAAAAAAAAATAAAAAAAAAATAAAAATAAAAATACAAACATAAATATAAACAC
AATTACACACAATTACACACACACACACACACACACACAAACACAAATACAAACATAA
ACACACACGCATGCACGCACACACATACACGTACACATGCACATACACATTATGTATGT
CATTAAAGATGATGGAGATGAAAAAGAAAAAGAAAGAAAAAAAAAGAGCAGAAAAA
GCGTAAGGAGAAGGAAATGTTGGAAGGAAGAAGAAGTAAAAAATATAGCGCGGGTAC
TCGCCACTTCGCTCTTTTCCATTCTCATGGAGAAATATCATTTCCCCCCACCCCTTTTTC
TTTCCGTTTAATAATTCGTTAATTAAAATCGTGCATTCAATAAATAGATTCTTTGATGATT
TTCGTTTCTTTTTCCGATTCGAATTAATAGAACAGACGTTCCCCTCCAATGTGTGTCGGA
ACGAATAATTCTAACAATTATTAGGGAAACGAGTAAGCTTTTTTCCCTTGTTTTATTACG
ATTCAAGTATTATCGGATCTTTGAGAGTGGAAAAGAGCGGAGTGCCGAGTGCCTGGGA
AGTTTTTTCTTAGAAACCATTTCAAGATTTTTCTTTTTTTTCAATAGTAAGGTTTATATAT
GTTGATACTACATTTAATGCAAGGGACGAAGTGTATTCGATAGACGGACGCTTTGGATG
GTGGGCGTAATATTGGGCACGTTGACACCGAAATCGACGACGTATCGATCGATTTCAAT
CAAAATTGTTTTTTTAAAAAAAACAAAAAATACACAGTCCTTGGCAGGAGCCAAGGAC
CAAATATCATCAGTATTATCCATTTATAAAAAATGGAGGAAAGGAGGAAAGGAAGGGA
GTTTTTTTTTTATCTTTTTACGTTACGTTTTTTCATTTATTTCTTTTTGTTTAAAAGTTACG
AAGGACCCGATGAATTAACTTCGTATCTTATATTTTTTTTTCTGTTTTCTTTTTTTTGTCTT
TTGTTAGTTTTTTTTTTTTTTTTGTTTCGTTTCTTATTGTGATTTTTCATTCGGCGAAGAA
CCGATTTAGTCGAATTTAAATGCGAGTCAAATTATTTAGGATAGTTATAAATGATGAAAA
GAAAAAGAAAAGGGACGTTTAGTCGTTCTAGAGAGAAATAAGAGAGAAATATTTGAG
AAAATATTATAGGTTTATAAAAATGAAAGGGAGGAAGGAAGAATCATCGTGGAAATAC
GTCGATACGTGGTCGATTCGCGTGGTAATCCGAAAAGTTTTAATCGTCCCTCGCATTTTC
TATATAGTCGAATTGCGTTGATCGAAGGGATAAAAGAAAGATGACGAATCGTGGATAAA
TATGAAATCCGAGATACGCAGAATTATAAAAATAAACAAAAAAAAAAAAAAGTAATAAT
GGTCAAACTTCCCTGTATATTAAGTAGGTATTGTGTTAAGGAAGATGAAAAGAAAAAA
AGGAAAAAAAAAAAAAGAGAAAAATAAGAGAGAGAAAAAAAAAAAAGAATGATAAAA
GGCGATTTCGTACGAAAAGGGGGGAGAAAAGATATGAGAACTATCGTTTGTTACTTAG
ATAATCGAACGTTGTTTTTTTTACATTTTATTAGTTTATACGTTTACGGCGGCCATGTTGA
TTTTGTTTAAGGATCTACAACTCATTCGAATGAGTTTCACGTTGATCGGCAGGACGCCC

AGCGATCTTCCACCCTCGAGATCAATCCCCCTTACGATTATATTTGAGAATAAAATTCC
TCTGGAAATATATATATATATATTTTTCACGGCCAGATATCGTGTTTCGAGAATAATTATCC
TGAAAAACTAATTTTCGGATAATAATTTCTCGATACAATGAAAAATGACCAGATAAATTC
TCGATAGTTTTACCCTCGACCGTATCCTCATATTCCTTACCCCTCAAGTCCATCCCTCTCT
CACTCTCTCTCTCTTTTTTTTCTTTTTTTCGTGAAACAGAGGACATTCGGTTTACCACAC
GAAAACGGGAGAAAACAAATCCTCGATTTACTTTCCTCTCGATCCCGGATCCCTTTTTC
CACCCCATTTTCGTTTCAATTTCGCAGCAACGGCGTGTTCCAAGTTGGAAGTATACTCG
AGGGTTGGTCGAAAGCCACGGGCGTCCGAAGAAGCCAGTGTAAATACGTAATTAAGC
GGGAGAGGAATAAAGGGAGTAGTTGGAAGGATGGCGGAAGGAACCGCAGGGTTCTTT
TAAAAAAGATGGCGGAAGAGTGAAAGCACGGGTCGTACGATACGTTTTTCTCCGTAAG
GAAGATGGAGAATATGGGAATGGATATCGCGAGAGAAGAAAGAAAAGGAAGAGAAA
GAAAGAAAGAAAGAGAGAAAGAAAAGGAAAGAAAAAAAAGATCTTCGTTTTTCTG
GCGATGCTGGTCGTTGCTATCCATCAATAGCGGCCGTCCCACAAACAGAAGACCGCCC
ACCGTTTTCCCAAG

>novel_circ_001969
ATGGAAGACGAGGGCAATCACGGCAACGACGACACCAGATGCTTCATCCTATCTACTC
TCGCAGCGCTTCAGTGGTCCAGAGTCTCGTGCGTCCTTTGTCGGGCACCTATGCTCGTG
TTCGACAGGTATCCATTGGTGGACGGCACCTTCTTCTTGTCACCTAGACAGCACTCGTC
CGCCTGTGCAGAGGTTAAAGTGGAAGGTCGCACGCAATTCTTGTCTGCCGTTTGTATG
AGTTGCCTGGAGGGTAGTGGCGGTCAGCCTGTACGTTGTCGTTGCTGTACTCAACCCT
GGGACGGGTCTAGCTTAGTCCTTGGAACCATGTATAGCTATGATATCTTCGCTGCTATGC
CCTGTTGCACCGAAAGACTTAAG

>novel_circ_001971
ATGGACTCCCCCGAACGCGAGATATACCGCGACCCGGACGTGGTGAGAGCCGACGAC
AGGGTGAACGAGGTGGTGCACACGGACACAGCGAGAAAGATGCTGTCCATCTTCCGG
CAGATGGAGGAGAACGCGTGCAAGAAGGAATTGCCGAACGGGCCGAAACCTTTGAA
GTGCTTCACGCCGCCCCCGGAGGACAAGTACGCGAAGGCGACCGCGTCGGACTCCGA
GCAGGACGAGGACGAGGATGGCGAGGAGACGGAGGGTGAGGACAGCGGCGAGGAA
AGGGATCCTAACTACGTGCGAGCTTCCGATAAGGTTGAGGACGAGTTTTTGAAGCAAG
CGCAGAACGCGGCCCGCGCAAAAACCTTGTGCGCGAAATTCGAGCACTGGGAGGAGA
CGGATGGGAAGACGACGACGAGCAACCAGCACATCGCCGAGATGGAAATCGCTCAAA
ATTCGTCCGGGGAACAGCTGAGCATAGAGTCTGCGAGCAGTCTGAGGGCCCGTTTCGA
GTCCCTCGGCTCGCAAACCAACGAATCGCCGCGCACACCTAAGGTCAAAGTCAATCGA
TTCGTG

>novel_circ_001973
CCTCAGACAAACCGGACCTGGGCCTCGAGGAGTTGTCGAGCCTGAACGTGAAGTCCC
GGTTCCAAGTGTTCGAGAAGGCCGGCGCCGAGACTAACGAGATCGAAAGGTCGCCGT
CGCAGATCGCCGTGAAGAGGTCCCCGAGTATCTTGAGCAAACTCGCCAAATTCCAGGC
GAAAGGCATGGATATCGGAGTTGCGGACGAGTCGCTGAATGGGATCCCTTACGAGGAG
TCGAGCGAGAGCGAGGAGGAGGAAGAGGCGGAGGAGACGGAGGAGGTGGACAGCG
AGATCGTGAAGGCTAAACGCGCGACCCGCGAGCGACCGATCAGCTTCACGAAAATGG

ACGACATAAAGAATCGTTGGGAGACGACCAGCCAGCAGGGGAGACGGGAGGTTCAAC
GGGAGGCCAGGAAGGAGGAGATAGCCGGGATTCGTTCGAGATTGTTCATGGGCAAAC
AGGGTAAAATGAAGGAGATGTATCAGCAAGCGGTAGCCGGAAGCGAGCGCGTGACGA
AGATCAATGCGGCGGAGGAGATCCAACACTCGACCCACGCTCGTTCCATCAAAGAGA
GATTCGAACGTGGCGAGCCCATAGCCGCGTCGGACGACGAGACCGACAGCAAACCGA
AACCGGAGAAGGCTGACGAGGAAGTGATCGCAGCAG

>novel_circ_001974
ATTCCAGGCGAAAGGCATGGATATCGGAGTTGCGGACGAGTCGCTGAATGGGATCCCT
TACGAGGAGTCGAGCGAGAGCGAGGAGGAGGAAGAGGCGGAGGAGACGGAGGAGG
TGGACAGCGAGATCGTGAAGGCTAAACGCGCGACCCGCGAGCGACCGATCAGCTTCA
CGAAAATGGACGACATAAAGAATCGTTGGGAGACGACCAGCCAGCAGGGGAGACGG
GAGGTTCAACGGGAGGCCAGGAAGGAGGAGATAGCCGGGATTCGTTCGAGATTGTTC
ATG

>novel_circ_001977
GTGGGTCAGAACGGCGACACGAACCCGAACTGCCGCAGCTGTGGCAAGGTCGTCTTC
CAAATGGAACAGACAAAGGCCGAGGGTCTGGTCTGGCACAAGAATTGCTTCCGGTGC
GTCCAGTGTAGCAAGCAGCTTAACGTGGACAATTACGAGAGCCACGAGAGCACGCTTT
ACTGTAAGCCACACTTCAAGGAACTCTTCCAACCGAAACCGGTTGAGGAGTCGGACC
AGCCTGTGCGACCTCGAAAGCCGGAGCTGATCATACGGGAGAACGAGCCGAAAGAGT
TGCCGCCCGATGTGGTCAGAGCCTCAGACAAACCGGACCTGGGCCTCGAGGAGTTGT
CGAGCCTGAACGTGAAGTCCCGGTTCCAAGTGTTCGAGAAGGCCGGCGCCGAGACTA
ACGAGATCGAAAGGTCGCCGTCGCAGATCGCCGTGAAGAGGTCCCCGAGTATCTTGAG
CAAACTCGCCAA

>novel_circ_001978
AAGTCGAGCTTCAATAAATTCGACGCCCTCAGCAAGAAGACGGTGCTGCATGTGCGCT
CGATCGATGCCGGCAAAGTGCAACAGCAGCTGAACGCCGTGGGTCAGAACGGCGACA
CGAACCCGAACTGCCGCAGCTGTGGCAAGGTCGTCTTCCAAATGGAACAGACAAAGG
CCGAGGGTCTGGTCTGGCACAAGAATTGCTTCCGGTGCGTCCAGTGTAGCAAGCAGCT
TAACGTGGACAATTACGAGAGCCACGAGAGCACGCTTTACTGTAAGCCACACTTCAAG
GAACTCTTCCAACCGAAACCGGTTGAGGAGTCGGACCAGCCTG

>novel_circ_001979
AGCGAAAGCCGAGAGGAAACGACGAGCGTGACAACGGTGAGCTCGCAGAGCGCGAA
GGGTGGGACCAAGACGAAGAAGAAGAAGATCGAGGGCGTCGCCGGTAATGATGCCTC
GCCCAATCTCACTCCCCGCACAAAGAAGACCAAAAAGAGCAAGAAGAACGAGCTCG
AGAAGGAAAACATTTCTGTGAACTGTAACTTGCAAGGGACGAGCATGCACCCTGGTCC
CCGCCAGATGAACTCGGAAACCCTTGATTTCTCGGACGAGGACGATCTGTCGAACGTC
AACGTGAAATCTCTG

>novel_circ_001980
GTGTCCACGGTGCGACTGTCCGACGATCCAGGCGCGCCATAATGGCGGAGGTGGCGAC

CAAGAAATCGATCAATGTAGTAGTAAGTAGCAAAACTAGCGAGGTGTGTAGCGTTCAA
TCTAACAACGGTTTGATCCAGTTGGATAACATAACCGCGAGTAACAAGCAACTAATATC
GTTGTGCGAGAGCAACGACGACGAGGACGATCTAATCCTGACTAACAACACCCGTAAC
GAATTGGACACGGACGACAAAAGCCTTCAGGAGTTGATCGAGAGCGAGCTGGCGCTG
AGAATTTGCTCCAACGCCGAGCAGAAGGAGGAGGAGGAGGAGGAGGAAGAGGAAAA
CGAAACCGGTATAGAGATGGAAGGGGGGAAGGAGGCAGAAGCGGTGAGGAGGATCG
TGCTCGACAGGCAGCAGGAGGAGCGTGTGGACGCGAACGCGGAATTCATCGCCGCGG
AAACGGAACACTCGGCCTCCGTCGGGGAACCGATTTACAGCTATCAGAACGGCCACGT
GTCGCCCGACGCGGCGCTCGTCGACGCGCCCTGCTACGAGGGGGGGAGCGCGGAAGC
GGTCGGCGAGGGGAAGGAGGAGCAAGTGTTCGAGGAGCGGGCGGAAGTTGGCAGCG
GGACGGCGATCGAAGAAACAACGACGCGCGAGCTCGAGGACGGGGCGCGTGAGCAG
GCGGCGGACGATGGCGGATGCCCGCGGATGCCCGAGGAGGACAACGAAGAACCGTGT
CCCGATTATCCCGCCCTTCGGCCCTTCGGCGAGGACAGACTGCCGGATGCGATGAGCC
AGCGACCGTTCGAGTCGTCGTGCGAGGAGGCCGAGGAGGAGCGGGTGAAAGCAGCC
GACAAGTATCGGGAGGAAATAGAG

>novel_circ_001981
GCGAGGACACGCAGTGCGGTACCGAGGAGATTGTCGCCACCAGCCAGGGAGGCGACC
AACTCCAGAGGATGACGATGGACGGCATGGAAGTGGTGGGCTCGCAACCCCACGCGG
CCACCAGCCCGTTCTTGCTCTCGCCGCCACCCCTTGGACACCACCATCATCATCATCAT
CCACACGCCACCTTCAACCTGCAAACGGTGCAGCTCAGTTTCGACGCGTGTCTACCGT
ACAACTCGGCGACGTCCTCGAATTCGATCGTATGCGGCGTTGGCGGCACGTCAACACC
GGCCGCCACCACTTGCACCTCGTCCACCAGCTGCAACAAGACGATACCGAGCCTGTCG
CTGGCCCCTTGCGCGCCCCTGCAAGCCCAGCATCAAAACAGTGCCGATACCACGGATC
ATCATGGCCGGCATCATCAACACCACCATCAACGGCTCAACGATCACCAGCATCACCG
GCACGTCGACGCTTCCTCGCCCTCCAGCGACTCGACCGACGGAAACGACGTTTGCA
GTCCGTCAACGAGGAGAAGGACTGCTCCAGGGAGTGCAGAGACGATCTTCAGGAGTC
TAACGATAAGGATAAAACAG

>novel_circ_001982
TCAGGATCTGCTGACGGTGTGCAGGGAACTGAATCTGGAGAATTCGGTGGAGGAGTTA
ATGAGAGAATTGGGGGCGGACGAGCACGGTCGCATTTCCTATCAAGAGTTCCTGAGGT
GCCGGTTGGCCCTACGACCGGAGATCGAGGCCCTCAGGTCCGGCAAGCACAGAACTA
GCCCCCATCACACGCACACGCCCGAATACTTGCCGACGAGCAGCGACAACAGCCTCG
GTACCATATCCGGGCGGCATGAACGCTGGGAGTTCGATAGCGGGGCGCGCGACCTCTC
GCCGGAGCCGCACACTCTGCAGAAACTGGTGGAAGCTGCCGCGGGAGGGACTGGCA
ACATGCTGGACCTGGCAAACAAG

>novel_circ_001983
GGACAGGATCGATCGAGAGGGCGGAGAGGAGGTGCCGATCGTAGGAGTCGAGGGGG
AAAAGGTTGAAAGCGTGGGAGGAGGGTCGAAAGGTTGAGCGATCCGCGGAGGAGGA
AGAAGAGAAAGAGGACGTGGCGGAAGCACGTTCGCTGGCGGTGGAAGAGGAAGAGG
AAGAGGAAGAGGAGGAGGAGGAGGAGGGAGAGGGAGAGAAAGAAGATCGAAAAAG
GGAAGGAGACGGTGGTCGAGGGAGGAAGAGAGATGAGCCTGGTGGGTGACTGGCAA

AGTGCGGTGGTCGGCTGCTCGACGAAAGGCAGATAGCGCCATTAGTTATTGCTGGGCC
GGAGAACGTCGTCGAGCCGATGCGCGCTACGGCCCTGGCCGTTGACGAAGAAGGGGA
AGAGGAGGAGGGGGAGGAGGAGGAGGAGGAGGAGGCGGCGGAGGGAGGAGAGGG
AGGAGAGGAGGAGAGGAAGGGGGCCCAAAGGGCCGCGATGCATCGCTTCGGACCTA
GGCAACGTTATCCCCGAATCGGAGAATCCCTGACTTTCCTTCTGTCGCTCGGTTGCCTT
CTGATCGTCGCCGTTGCCTCGCCTCTTCATCCCGCGCCACCGCACAGAG

>novel_circ_001984
GGACAGGATCGATCGAGAGGGCGGAGAGGAGGTGCCGATCGTAGGAGTCGAGGGGG
AAAAGGTTGAAAGCGTGGGAGGAGGGTCGAAAGGTTGAGCGATCCGCGGAGGAGGA
AGAAGAGAAAGAGGACGTGGCGGAAGCACGTTCGCTGGCGGTGGAAGAGGAAGAGG
AAGAGGAAGAGGAGGAGGAGGAGGAGGGAGAGGGAGAGAAAGAAGATCGAAAAAG
GGAAGGAGACGGTGGTCGAGGGAGGAAGAGAGATGAGCCTGGTGGGTGACTGGCAA
AGTGCGGTGGTCGGCTGCTCGACGAAAGGCAGATAGCGCCATTAGTTATTGCTGGGCC
GGAGAACGTCGTCGAGCCGATGCGCGCTACGGCCCTGGCCGTTGACGAAGAAGGGGA
AGAGGAGGAGGGGGAGGAGGAGGAGGAGGAGGAGGCGGCGGAGGGAGGAGAGGG
AGGAGAGGAGGAGAGGAAGGGGGCCCAAAGGGCCGCGATGCATCGCTTCGGACCTA
GGCAACGTTATCCCCGAATCGGAGAATCCCTGACTTTCCTTCTGTCGCTCGGTTGCCTT
CTGATCGTCGCCGTTGCCTCGCCTCTTCATCCCGCGCCACCGCACAGAGGTATCACGCA
CGGTTCTTACATCGGTTATTACGAGGTGGCCTCCTATGATACCGCGGCCTTGAGACAAC
ATCGTAACCGGATGCGCCGCGATGTCTCCAACGATGTGGTCGACAATCAGCCTTTGCTG
CTGCAGTTGAAAGCGCTCGACAAACAATGGACATTACGGTTGGTTCGAGACAGGAGC
CTGTTCAGCCAGGACGCGACGTTCGAGAACACGAACGGCCCCATCGACTTCGACGCG
TCGCACTCTTACGTGGGCACAGTGTTGG

>novel_circ_001985
ACCTATCTGGGCTGACTCTGCAATCGGGAGTAGGCGTAGGACCAGGTGGTAGATTGGT
CAAAGATGAATATTCAGTTGGTGGTGGGAGCAGTGCAACAGCTGGAGCAGTAGGATCT
GCAATGGATGTTGATGGAGAAATGAACCAAACTATTCAGCATCATCCACCTACTCAACC
TACTTCATCTAATAACACTCAGCCATCTCAACAGACTTTTATACCAGGTCTAGCACCACC
TAATTCAACTAGTGGTGAGGGTGTGTTTGGCAGTAATGGGGGAGGGAATAATGGTGGT
AATCCACATAATAAATTGGATGACAATAATTGCCCCAGACAAACCTGGATTCCTACACC
TCATCATCCTACAACACGTAACATTCATCATC

>novel_circ_001987
GTAGAATTGGATCGAGTGGTTGGCAGAAAAAGGGTAGACGGGCGAAGGGATCGGAGG
GGTGAAAGGCGCGTGAAAGGCGAAATCGGTGGAAGCGAAAAGAGGAAGAAGCCGAA
AGAGGAAGAAGAAAGAAGGTAGAAGAGGAGGAGGAGGAGAAGGAGGAGGAGGAGG
AGGAGGAGGAGGAGGAGGAGGAGGAGGAGGAGGAGGAAGAAGAAGAAGAGGAAG
AGGCAGGATTTGCCGGCGGCGACAGACTGCACTCTCGCACCACCGTTCTTCAGGGCG
CAAGCGTGCACGTATGTTGTGCGTTTCCGTGCCCTAACCCTGTCTCAACACGACGAGA
GAGTCGTCGTTTCTCGGCGTTCTCATCCCTACTCCGCCAACATCGCCGCCGTTGCCGCC
ACCGTTATCGCCACCGCCACCGCCGCCGTTACCGCTACCGCTACCGCCACCGTCACCG
ACGCTACTATCGGCTCACCATCCCTCCAACGTTCAACGCGAACAAGCACCACCGCCAT

AGCCGACGTAACGACGACGACGACGAGGACGACGACGACGACGACAACGAAGACGA
AGAAGAAGACGAGGGCCGAGAAGACGAGAACGAAGAAGGCGACCACGATCACCACC
ACCACCACCACCACCACCACCACCGCCGCTACCACCACCGCCGCCACGACCACCTCCT
CTACGACCACTACCACCACCACCGCCGCCACCGCCGCCGCCGCCACCACCACTACCAC
CACCACCACCACTACCACCGCTATCGCCATCACCGTGAACGATAGAAGGAGAAGGAGG
ACGAGAAGGAGGATACCGATCACGAGGACGAGGACGAGGACGAGGACGAGGACGAC
GACAACGACGTCGACGACGACGACGACGACGACGAAGACGAGGACGAGGACGAGG
ACGGAGGACGGTCGGTGGCGTGGGGGGTGGACATCCACGCGGGAACTCGAGGCAGG
CGCACCCGGCCGGCACGCACACACATTCATTCTAAGATGTGATTTCAAAAATGGCGGA
TTGCCCCTATGCCCGTTGCATACAGGAACGCAGACACATCCGACGGGAGCTGCTGCGA
TGGACAAAGAATATGGTCTTCGTAGTCG

>novel_circ_001988
TTCGGAAGATGGCGATCGCCGCGACTCTTTCTTGGTTCGTTTGGTTGGCGAGGGGATG
GTTCCCCAAAGAGGCGAGGGGAGGAGGGGGCGTTGGTTTCTCGCTGATTGAAAACGC
TGATTGGCCGGGAAAGTCGTTTGAAACAGGATCACGGTGATCGTTTTGAGAGACGATA
AACGTTGGCAATTATAAGCTACATCGAAGCTAAGGAAACGCGGCGACGAGGGTGAAA
AAAGGGCTGGAAATGCCCGGGCAAACGGTTACAAGTATTTCCATTGACGGAACGTCGC
CGCATCCCTGCTTCGTAG

>novel_circ_001989
TTAAAAAAAAAGTATGATAAGAATTAAGAGAACGACGATGCGAAAAATCAACATCCTG
TACCTCTTCTGTATAATTGTTACATTATACACTGAAATTATTATTAAGCGAAATTTAATTGT
ATTATCGTGTAATTACTAATTATTGTCGTAATATAGGTTTCTTGGTGTAAAAAATTGACCT
AACTCTTAAGATTTAATGGTAAA

>novel_circ_001990
AAAAAGTATGATAAGAATTAAGAGAACGACGATGCGAAAAATCAACATCCTGTACCTC
TTCTGTATAATTGTTACATTATACACTGAAATTATTATTAAGCGAAATTTAATTGTATTATC
GTGTAATTACTAATTATTGTCGTAATATAGGTTTCTTGGTGTAAAAAATTGACCTAACTCT
TAAGATTTAATGGTAAAGTGATGTATTCAG

>novel_circ_001992
GTGCAACAACAGCTGGAGGTGGTATCACAACTGGTTGTCCAGGTATTGGTTGAATTATT
GCAGGAGGTCGAGTTAATGTTTGCGGTATAGCTATACCAGGAGGAGGTATAACAGGTG
CTACTGTTGCTATAGTTGGTGGTGCCATCATTGTTGCAGGAGCAATACTGGGTCTTACTA
TCCCAGGTAAAGCTATATGTATATAAAAAATTATATATTCATTTCTATAAATCTTATAAAAA
ACTTAAAAATATAAAATTTTTTCAATTATTTTTAATTCATACCTTGATTTAAAATCGGCGG
TGCAGCTGCTCCAAGCTTAGTCAAACCAAGAGCAACTGCATTACTGGCTACCGCATCC
ATCGCTTGAATTTTTGCAGTTGCAGCTGCAGCTGCAACTGCTGCAGCAGTAGGCATCAT
ACTAGTTCCACTTGGCGGACCCATAAGAGCATTTGGTGGCGTAATAGCCCTGCCTACTC

>novel_circ_001993
ATGACATAGAATGGGTTGGGTGGCGCTAGGGCGGTGTGCGGGTGGTGGCGGCCGCCTC

TCGTCCGTCCTCTCGCTGTCTCTCACCTCCCTCGCAAGCTCCCTCATCTTCACCATCCTC
TGTATCCTCACACTTGCCCTCACTCTTGTCACTCTCGTGCGTGCCGAGGACATCTTCGA
GGACGGCAAGTCGGACAAGGAGATCCTGGACAACCTGCTGCTGTCGACCCGTTACGA
CAAGCGGCTTCTACCCCCGGTCCAAGGTACACTGAGGCCCCCGCCCCACACCCGCCAA
GACGACTACACCCCTGACCACCTCGTACCCAATGACCCTCATCATCCAGAGCACCTCG
AACACCACCGGCACCGCTACCACCATACAGCACCACACAACCACAGCTCACTGCTCAC
CCCTCGTCAAAAGGAACAAATTACTCGTGATATTATCCTACGAGTACGAATTAATATCGA
AAGAAAAATATATAATATTATTCATTTATGTCGTGAAAGTCCGTACAGTCTATTAATAAGC
AATGCGACGAACAGCTCGAAGCGTCACGTGACACCGATCAATTAATAAACTAATATTTC
GACGCAAGTGATTTTTTATTTTTTCTTTTTTTTTTTTTCTTTTTAAAGCGGCTATCCTAGG
AAAGGCAACGTTTCATCTTTTCAGAGAGAAACTCGTGACAAAAGAATCGTTCCCCTAT
TGGAGGAAACGTGGAAAGTTGCCACGACGCCCTCGAGCGCCGGCACCTCGTTTGTTC
ACGGGGCGGACGCTAGAAGGAAAGCGAGGTTTCGAAATCGCGTGTGTGCATCGTCGG
CAATCTCCCTCCTCGCTTTCGTCCGCCACGGAACCGTTTTCCAATTGAGGCGACAAAA
GAGGCGCCACTGTCGTCGATTGCTTGTTTCTCGCTAACCACACTCTCTTCACTCCGGGA
TGAAACGCGAGACTTGTTATTATTTACGTGGCAAAGAAGGAAGTAACGAACGAGGGA
AAAGTAAGGAATCGTGGATAGAAACGAGTGTATGGAGTGAAGAACGTCGTCGACAAA
CGAACGAGGCACGAACTCGCAACTGTAGTTGACTTTACTTCGGTATGCGGATTTCTGCT
GCGGAATGCGATGGCCAGGTGACGCCACCGGCCTGTCGAACCGGTCGTCGACCTCCTC
TAACGACCCCAAGAACCAGTACAAGAACCAGAACAATAACCACTACACGTCGCACCA
ACACCTTCGCACCCACCTTCGCGCGCTATGTGATTCTCAACCTGAAATGCTTGAAAATT
GTGCATTCCTTTATAAACAGTGGTTTCATTTAAATCAAACTTTGAATATTGCGGAACCT
TGACCGTGAACGTGAGCGTGCTGTTGCTAAGTTTGGCTTCCCCAGACGAGTCCAGTTT
GAAATACGAGGTGGAGTTCTTGCTGCAACAACAATGGTACGACCCGCGATTACGATAC
AGCAACCGGTCCCAATATGAATTCTTGAACGCTATTCATCATTACGATGATATCTGGTTG
CCGGACACGTACTTCATAATGCACGGAGACTTCAAGGACCCGCTCATACCTGTTCACTT
CGCCCTACGTATATATCGCAACGGCACGGTCAACTATCTTATGAGGCGTCATCTCATCCT
GTCCTGCCAGGGTAGACTCAATATCTTCCCCTTCGATGACCCACTGTGTTCATTTGCGAT
CGAGAGCATATCGTACGAACAAACGGCGATCACGTACGTGTGGAAGAATGACGAGGGT
ACGTTGCGGAAGAGTCCTAGTCTAACGTCGTTGAACGCATACCTCATTAAGAACCAGA
CAATCACGTGTCCGATCAAAGTAAGCTGGAGAGCTGACGGACAGATCATGGTCGACTA
CGAGGACGAGTTCGATGAGTTTGGGGACTCAAAGTGTTCGCTGTGCCAGCGCAGATTT
GAGGAGCAAGGTAACTACAGCTGCCTGAAGGTGGATCTGATTTTTACACGAGATCGTG
CCTTCTACTTCACAACAGTCTTCATCCCGGGTATTATTTTGGTGACCAGCTCGTTCATTA
CCTTCTGGCTTGAGTGGAACGCGGTACCGGCGCGAGTAATGATCGGTGTGACGACGAT
GCTCAACTTCTTCACGACATCGAACGGTTTCCGCTCTACGCTGCCCGTCGTGTCAAATT
TAACTGCCATGAACGTGTGGGACGGCGTGTGTATGTGCTTCATCTACGCCAGTTTGCTC
GAATTCGTCTGCGTGAATTACGTTGGCCGTAAACGGCCGATGCACAATGTCGTCTATCG
CCCCGGGGAGAATCCTGTCACCCAG

>novel_circ_001995
CTGACGGACAGATCATGGTCGACTACGAGGACGAGTTCGATGAGTTTGGGGACTCAAA
GTGTTCGCTGTGCCAGCGCAGATTTGAGGAGCAAGGTAACTACAGCTGCCTGAAGGTG
GATCTGATTTTTACACGAGATCGTGCCTTCTACTTCACAACAGTCTTCATCCCGGGTATT

ATTTTGGTGACCAGCTCGTTCATTACCTTCTGGCTTGAGTGGAACGCGGTACCGGCGCG
AGTAATGATCGGTGTGACGACGATGCTCAACTTCTTCACGACATCGAACGGTTTCCGCT
CTACGCTGCCCGTCGTGTCAAATTTAACTGCCATGAACGTGTGGGACGGCGTGTGTATG
TGCTTCATCTACGCCAGTTTGCTCGAATTCGTCTGCGTGAATTACGTTGGCCGTAAACG
GCCGATGCACAATGTCGTCTATCGCCCCGGGGAGAATCCTGTCACCCAG

>novel_circ_001996
GTAACTACAGCTGCCTGAAGGTGGATCTGATTTTTACACGAGATCGTGCCTTCTACTTC
ACAACAGTCTTCATCCCGGGTATTATTTTGGTGACCAGCTCGTTCATTACCTTCTGGCTT
GAGTGGAACGCGGTACCGGCGCGAGTAATGATCGGTGTGACGACGATGCTCAACTTCT
TCACGACATCGAACGGTTTCCGCTCTACGCTGCCCGTCGTGTCAAATTTAACTGCCATG
AACGTGTGGGACGGCGTGTGTATGTGCTTCATCTACGCCAGTTTGCTCGAATTCGTCTG
CGTGAATTACGTTGGCCGTAAACGGCCGATGCACAATGTCGTCTATCGCCCCGGGGAG
AATCCTGTCACCCAG

>novel_circ_001997
CGAACGCGACCAGATTGAGGCATTCGAGACACCTGGATTTGAGATCTCAGTTCTCCAA
CGACGAAGAATTTGCGATGCAGCAGGATGAAAATCACAATCACAAGCCTGTGTTCTCG
AATTGTTCGAATTACGCGCCAGTTGTGAAGGAGGAGGAACCGGTGGGCACAGTTGTGA
TCCAAGTACACGCCAAGGACAGGGACAATCCGGACAACGGAGGTACCATCACTTACA
GTTTCGTGACCGCGCCTGGCGAGAAGTTGAAATTCGAGATCAACAATAGAACCGGACT
GATCAGGACGACGCAGGTGTTGGACAGGGATGAACCGGCTCGTGAAAAAGAGGCCTA
CCTGACCGTTCTCGCGACCGACAATGGAAGACCGCAGCTCGACGACGTGTGCACGTT
CAAAGTCACCATCGAGGACGTGAACGACAATTCCCCCGTTTTTGACAAAGTG

>novel_circ_001999
GATATATTGGAGAACGAGCAGATCAAATTGGATCGCGTGTTCCGTGGATCGTTGATCCA
CGATATCGTGCGAGGGATGGTGTATCTTCACGCGTCCGAGGTGAAGAGCCATGGAAAT
CTGAAATCGTCCAACTGCGTGGTCGATTCACGCTTCGTCCTCAAGATCGCCGACTTTGG
ATTGCACGAGTTGAGGAGGCCTGCTTATTGCGGGGCGGAAGTCGACAAGAACAATTAC
GCGTTTTGGAGGGGGCAGCTGTGGACGGCACCGGAATTATTGCGAATGGAAAGGCGG
CCACCCGAGGGTACTCAGAAGGGTGATGTGTACAGTTTCGCTATAATCGTTCACGAGAT
CGTGATACGCCAGGGGCCATTTTACTTGGGCGACGATTACGACTTTTCCCCGCAAG

>novel_circ_002000
TTCCAATTATACCAGAGTTTCTGTACGATATCAAGCATCCGAATGCAACGTTGAGCCAA
CATTTCGAACAAAATGGACGAGCAACGGTGACCACTGTTCATCCAACGAGTATCAATA
TCGTGACCTCCACGAGCCCGAGCGTGACCACCACGCCGAAATGCCTCTGCACGTCGG
CCAAGACCAATGAATCTCAATTGGAATTTCTTTACCCGAGCACACCGTCCACCGTCGA
GTCGACAACGGGTTTGCTCGGGACAACGAGCAGTAGCGAAACGGGTTCAACGGCAGA
ATCGGAAGAGAAACAACGACATCGCGAGTTACTTCAAGAAACGGTCGCCGTTGGTATA
ATGTTCGCCTCGAAGGCGTTCGTTCAATTGCTGGCAAATCCGATTGTCGGCCCCCTCAC
CCACAAGATCGGTTACAGCATACCCATGTTCACCGGTTTTATTATTATGTTCATTTCCACT
CTGATATTCGCGTTTGGCCGAAGTTACGGCATTCTTTTCCTAGCAAGAGCGCTTCAAGG

CGTTGGTTCGTCGTGCTCGAGCGTATCTGGTATGGGCATGCTTGCTGAAAGATATCAAG
ATGACAAAGAGCGTGGAAACGCAATGGGTATCGCGCTCGGTGGATTGGCCCTTGGTGT
TCTCATCGGCCCACCATTCGGCGGTGTAATGTACGAATTCGTTGGGAAATCGGCACCGT
TCTTGATACTATCCGCCTTGGCACTTGGTGATGGAATTTTACAACTTCTGGTGCTTCAAC
CGTCCGTCGTGTACACTGAAGCGGATCCACCATCGTTAAAAACACTAATCACTGATCCT
TATATCGTTTTGGCTGCTGGTGCAATAACATTCGCCAATATGGGTATCGCTATGCTCGAG
CCCAGTCTTCCAATTTGGATGATGGATACGATGTGTGCAAGTAGATGGGAACAAGGTG
CTACGTTTCTGCCTGCAAGCATTAGTTATTTAATTGGAACTAATCTTTTTGGACCGCTTG
GGCATAGAATGGGCAG

>novel_circ_002001
GTGAAACGGGAGGTTGCGGCATCACCGACGACGTGGTGGAGTGGATGGACAGGATAC
AAAATTCCGCGGAACCGGACGAGGAACGTCCGGTGACCGAGAAGAAATCCGAGCCGC
CGCCGGTGGCGAAACCGTGGAACGGTGATCAGGAGTCGCCCGGGCACAAGTATTCGA
GGGAAGCTAACGAGCCTGGCCACAGGAGGACGCGGAGGGCGACCAGGCCGAAGGAG
GACAAGAAGAACAACACTTGTTCCCTTTTCATTCAAACCGATCCCCTGATATGGAGAC
ACATATCGGAACAGTTACACCACGACGCGGAGAAGACCAGGGAGGAGATATTGTCTCT
GATCGCGCATCACGTCACCGCGGTGAATTACATTTACAGAGACACCAGATTCGACGGG
AGGATCAAGCATAGAAACATCAAGTTCGAGGTGCAACGGATAAAG

>novel_circ_002002
CTCGCCGTTTGAACGAATATATCCGCCACTACGAACCACTCTCTTATCCAACCGAAGAG
GTCCACCGAGGTCACTTGAGGGCCAAGAGGTCGGTTACCCGCGACAATTCCGTGACTC
TGAAGTTTCGCTCGCACGGAAGGGACTTCCACATTCGGTTGAAGCGGGATCTGGCCAC
CTTTAGCAATAATTTAATTATCGAAGGGCCGTCCGGTCAAACGGAAGACCTCGATACGT
CCCATATTTACCAGGGCCACTTAGTCGGCGAGCCCGGCAGCCACGTGTTCGGAAGTAT
CAGCGACGGCGTTTTCCACGGGAAAATCATATCGCCCCGAGGTGGCGCGTGGTACGTG
GAAAAGGCGTACTATTACTTCCCGCCGCACGAGATAAATGACACGTTGCATTCCGTCAT
CTATCATGAGAATAACGTGGACGATCCGTACGCCCATCTTCGAAAAG

>novel_circ_002004
CGATCGATCAGAGGACGGTGCCCGAGGAGCTTCCTGTATCCGGCGACCCGTACAACGA
GACCACGCTGGAGCTGATCTTTCCCGGAAGGCAGCCACGTTTCAAATTGAACGGGAA
GAGGCTGCAGCTGCTCGAACCTTTGGACAGGGACGACGAGAATCTATCGCACGTGGTT
TTTCAGTTGAGCTGCACGGTAAAACAGACGAACAAGAAACGGACTATACCGGTGATCG
TTCGAGTGTCGGACATAAACGACAACGCGCCCAAATTCATCAATACGCCGTACGAGAC
CACCGTGCCCGAG

>novel_circ_002005
TTCAACGGTTTCTCCAGAAGTTCGAACGAATTCGGAATATTGTTCCTGCATGGTCAGGT
CAGCTTTCGAAGAATATTCGAGGATCGTTCGATACTGGTTTCAAAAATTCAGACGTGGA
AACAGGAATTTTGAAAATATATTAAACGAAGCCAACAAATTGGAAGCCAATCCACGAG
CAAACACACTCCAACGACATTTGGATGGATGTAAACACGGATAAAGTGAAAAAAATTG
AAGGATCAAAAGTTGCTCGAAACGAATCCATTTTTGAACAGAATTACATAACCTGAGA

AATGGATGCCCGATACAATGGTTGGATGCTAACGAAAATTGACTAAACGAAACCATCGT
GGCCTGTAAAGAGAATAATTCATTATTCTTTTAAGGGTAGCATCCTGTTCTGCCGAGAA
ATTCGATCGACGAAACCATAAAAGTTAGCTGAAATCTCGACCCATCCATCCGATATTTC
ACCGACCGATCATCGTTTACAATTATCTCTTGAAAGAATAAAATACTCGACGAGAGAAG
CTGTATTCTATCAG

>novel_circ_002007
GTGCTTTTATAATTCCGTTCGTTTTAATGCTGATAACCATGGGATTACCGATATTTTTTT
GGAACTGGCTGTTGGACAGTATTCCGGGCTCGGCCCTAACGAGGCGTTCAAACGTATG
GCGCCGGCACTCGAGGGCCTCGGTTATTGCACCCTGATCGTCATTCTGCTCGTAATGGT
CTACTACATGGTGATCGTTGCCTGGACACTCTTCTACACGTTCCTCTCGTTCCTGCCGA
AGCTCAGTTGGGCCTACTGCGACAACGATTTCAACACGAACC

>novel_circ_002008
GTGCTTTTATAATTCCGTTCGTTTTAATGCTGATAACCATGGGATTACCGATATTTTTTT
GGAACTGGCTGTTGGACAGTATTCCGGGCTCGGCCCTAACGAGGCGTTCAAACGTATG
GCGCCGGCACTCGAGGGCCTCGGTTATTGCACCCTGATCGTCATTCTGCTCGTAATGGT
CTACTACATGGTGATCGTTGCCTGGACACTCTTCTACACGTTCCTCTCGTTCCTGCCGA
AGCTCAGTTGGGCCTACTGCGACAACGATTTCAACACGAACCATTGTTACAGCGGGCT
TCAAGAGATTCAATGTCAAACGGACGATCCGGAAACGATATTTTACAACAAGAGTTGC
ATATCGGCCAGCCACGTATGCCGGAGCTTCGATTTCGACGATGGGAACATCACGCATTG
TTTCAAAGCTGACAAAGTGGAACTCCTTCGAAACCTTTACACACGGATCCTTTCGTCG
GAGGAATATTTCAACGACTATGTGCTTGGTATACGAGGAGCTACGTGGGAACACTTCGG
AGGCGTGAGATGGGAATTGTTAGGATGTCTGACCCTCGCTTGGATAATCTGCTTCTTGT
GTCTGATGCGCGGCGTTCAATCCATTGGAAAAATCGTCTACTTCACCGCCCTTTTCCCC
TACGCCATGCTCATAGCGTTGCTGATACGAGGTGTGACGTTGGAAGGTGCCCTGGACG
GCTCTCTTTGGTTCATCATGCCAAAATGGTCCACCTTGCAATCCACCAATGTCTGGGCG
GATGCTGCTTCTCAAGTTTTCTATTCCCTCGGTATCGGTTGCGGTTCCCTGATCACGCTC
TCGAGCTACAGTAACTTCAACAATAATTGTCACAG

>novel_circ_002009
GTGCTTTTATAATTCCGTTCGTTTTAATGCTGATAACCATGGGATTACCGATATTTTTTT
GGAACTGGCTGTTGGACAGTATTCCGGGCTCGGCCCTAACGAGGCGTTCAAACGTATG
GCGCCGGCACTCGAGGGCCTCGGTTATTGCACCCTGATCGTCATTCTGCTCGTAATGGT
CTACTACATGGTGATCGTTGCCTGGACACTCTTCTACACGTTCCTCTCGTTCCTGCCGA
AGCTCAGTTGGGCCTACTGCGACAACGATTTCAACACGAACCATTGTTACAGCGGGCT
TCAAGAGATTCAATGTCAAACGGACGATCCGGAAACGATATTTTACAACAAGAGTTGC
ATATCGGCCAGCCACGTATGCCGGAGCTTCGATTTCGACGATGGGAACATCACGCATTG
TTTCAAAGCTGACAAAGTGGAACTCCTTCGAAACCTTTACACACGGATCCTTTCGTCG
GAGGAATATTTCAACGACTATGTGCTTGGTATACGAGGAGCTACGTGGGAACACTTCGG
AGGCGTGAGATGGGAATTGTTAGGATGTCTGACCCTCGCTTGGATAATCTGCTTCTTGT
GTCTGATGCGCGGCGTTCAATCCATTGGAAAAATCGTCTACTTCACCGCCCTTTTCCCC
TACGCCATGCTCATAGCGTTGCTGATACGAGGTGTGACGTTGGAAGGTGCCCTGGACG
GCTCTCTTTGGTTCATCATGCCAAAATGGTCCACCTTGCAATCCACCAATGTCTGGGCG

GATGCTGCTTCTCAAGTTTTCTATTCCCTCGGTATCGGTTGCGGTTCCCTGATCACGCTC
TCGAGCTACAGTAACTTCAACAATAATTGTCACAGAGACGCGATATTCGTAACTTTCAC
GAACTTAGCCACGTCGATTTTCGCCGGTTTCGTCATCTTCTCGATCATGGGATTCCTCGC
CCGTCAAATGGGGGTTTCGGTCGAGGAAGTGATACAGAGTGGAGCCGCGCTTGCATTC
ATCGCGTATCCGGAAGCGGTGGTGCGGATGCCGTTGCCCAACGTGTGGGCCGTCCTGT
TCTTCGTCATGCTGTTCATCCTGGGTATCGGGAGCCAA

>novel_circ_002011
ATTGTTACAGCGGGCTTCAAGAGATTCAATGTCAAACGGACGATCCGGAAACGATATTT
TACAACAAGAGTTGCATATCGGCCAGCCACGTATGCCGGAGCTTCGATTTCGACGATGG
GAACATCACGCATTGTTTCAAAGCTGACAAAGTGGAACTCCTTCGAAACCTTTACACA
CGGATCCTTTCGTCGGAGGAATATTTCAACGACTATGTGCTTGGTATACGAGGAGCTAC
GTGGGAACACTTCGGAGGCGTGAGATGGGAATTGTTAGGATGTCTGACCCTCGCTTGG
ATAATCTGCTTCTTGTGTCTGATGCGCGGCGTTCAATCCATTGGAAAAATCGTCTACTTC
ACCGCCCTTTTCCCCTACGCCATGCTCATAGCGTTGCTGATACGAGGTGTGACGTTGGA
AGGTGCCCTGGACGGCTCTCTTTGGTTCATCATGCCAAAATGGTCCACCTTGCAATCCA
CCAATGTCTGGGCGGATGCTGCTTCTCAAGTTTTCTATTCCCTCGGTATCGGTTGCGGTT
CCCTGATCACGCTCTCGAGCTACAGTAACTTCAACAATAATTGTCACAG

>novel_circ_002012
ATTGTTACAGCGGGCTTCAAGAGATTCAATGTCAAACGGACGATCCGGAAACGATATTT
TACAACAAGAGTTGCATATCGGCCAGCCACGTATGCCGGAGCTTCGATTTCGACGATGG
GAACATCACGCATTGTTTCAAAGCTGACAAAGTGGAACTCCTTCGAAACCTTTACACA
CGGATCCTTTCGTCGGAGGAATATTTCAACGACTATGTGCTTGGTATACGAGGAGCTAC
GTGGGAACACTTCGGAGGCGTGAGATGGGAATTGTTAGGATGTCTGACCCTCGCTTGG
ATAATCTGCTTCTTGTGTCTGATGCGCGGCGTTCAATCCATTGGAAAAATCGTCTACTTC
ACCGCCCTTTTCCCCTACGCCATGCTCATAGCGTTGCTGATACGAGGTGTGACGTTGGA
AGGTGCCCTGGACGGCTCTCTTTGGTTCATCATGCCAAAATGGTCCACCTTGCAATCCA
CCAATGTCTGGGCGGATGCTGCTTCTCAAGTTTTCTATTCCCTCGGTATCGGTTGCGGTT
CCCTGATCACGCTCTCGAGCTACAGTAACTTCAACAATAATTGTCACAGAGACGCGATA
TTCGTAACTTTCACGAACTTAGCCACGTCGATTTTCGCCGGTTTCGTCATCTTCTCGATC
ATGGGATTCCTCGCCCGTCAAATGGGGGTTTCGGTCGAGGAAGTGATACAGAGTGGAG
CCGCGCTTGCATTCATCGCGTATCCGGAAGCGGTGGTGCGGATGCCGTTGCCCAACGT
GTGGGCCGTCCTGTTCTTCGTCATGCTGTTCATCCTGGGTATCGGGAGCCAA

>novel_circ_002014
CGACTATGTGCTTGGTATACGAGGAGCTACGTGGGAACACTTCGGAGGCGTGAGATGG
GAATTGTTAGGATGTCTGACCCTCGCTTGGATAATCTGCTTCTTGTGTCTGATGCGCGG
CGTTCAATCCATTGGAAAAATCGTCTACTTCACCGCCCTTTTCCCCTACGCCATGCTCAT
AGCGTTGCTGATACGAGGTGTGACGTTGGAAGGTGCCCTGGACGGCTCTCTTTGGTTC
ATCATGCCAAAATGGTCCACCTTGCAATCCACCAATGTCTGGGCGGATGCTGCTTCTCA
AGTTTTCTATTCCCTCGGTATCGGTTGCGGTTCCCTGATCACGCTCTCGAGCTACAGTAA
CTTCAACAATAATTGTCACAG

>novel_circ_002015
GTGTGACGTTGGAAGGTGCCCTGGACGGCTCTCTTTGGTTCATCATGCCAAAATGGTC
CACCTTGCAATCCACCAATGTCTGGGCGGATGCTGCTTCTCAAGTTTTCTATTCCCTCG
GTATCGGTTGCGGTTCCCTGATCACGCTCTCGAGCTACAGTAACTTCAACAATAATTGT
CACAGAGACGCGATATTCGTAACTTTCACGAACTTAGCCACGTCGATTTTCGCCGGTTT
CGTCATCTTCTCGATCATGGGATTCCTCGCCCGTCAAATGGGGGTTTCGGTCGAGGAAG
TGATACAGAGTGGAGCCGCGCTTGCATTCATCGCGTATCCGGAAGCGGTGGTGCGGAT
GCCGTTGCCCAACGTGTGGGCCGTCCTGTTCTTCGTCATGCTGTTCATCCTGGGTATCG
GGAGCCAA

>novel_circ_002016
AAATTAAAATGCCGATCGATTTTTATCAACTTCCCGGAAGTCCTCCGTGCCGCGCAGTT
GCGTTGACGGCTGCCGCTCTCGACATCGAGATGAACTTCAAACAGGTCAATTTGATGA
ACGGGGAACACTTGAAGCCTGAATTTTTAAAGATAAACCCGCAACACACGATACCCAC
GATTGATGACAATGGTTTCCGTCTGTGGGAAAGTCGAGCCATCATGACGTACTTGGCG
GACCAATACGGCAAAAATGACACTCTGTATCCGAAAGATCTGAAGAAACGTGCGATCG
TCAATCAAAGATTGTACTTCGACATGTGCAGTTTATACAAATCGTTCATGGATTATTAC

>novel_circ_002017
GGCCGATCGTGACGGCGAGGGCTGTTCTTCAAGGTACGGCGGAATTGCCATGCGACAT
TCGTCCCCCTCGACAAAATGATTCCGCGATACTAGTGGTTTGGTACAAGAGCGACATCA
CGCCTATATACAGTTACGACATGAGGGGTAAACAGTCGGAAAAGGATTCGCACTGGAA
CGATAAGAATCACTTGAACGATCGTGCCTTCTTCAGAACGGTTACCGAACCGGCGACG
TTAAACATCAATCACATCGAAGAAAGGGACGGGGGTGAATACAGGTGTCGGGTAGATT
TTGCCAAGAGCCCAACCAGAAATTCCAGGATTCATCTGACGGTTATAGTACCGCCGCAT
AAACCGAATATCATCGACGAGCATGGAACGATTGTACAAACGATAACAGGTCCGTACG
AAGAGAACGAGGATATGAAATTGCAGTGTCTCGTGTCAGGGGGTCGGCCAGAACCGA
AAGTAAAATGGTGGCGTGGAGAAATGCTTCTCGACTCGACCGACGAGCCGGGAGAAT
TCCCATCCCTTCGTAGAAACACTTTAATAGTAAAACAACTTTCAAGGGCTGATCTTCAC
GCTGTATTTATTTGCCAAGCATCCAACAACAATATCAGTCAACCTGTTTCAGCGTCCGTT
ACCATTGAAATGCATCTGAAGCCAATGTCCGTAACGATATTGAGCAACGATACACCACT
TAGCGCTGGCCGAAAGTACGATATAAATTGTATAACCGTTGGATCGAGGCCACCGGCCA
AGTTATCTTGGTACATGAATGGAAAGAAATTGAATAATCACACGGAGAAGGTGTCTCA
AGATGGAAACACGACCAGTTCCACGTTAATCCTGAAACCAACTTTATCCGATCACGATA
AAATGATAACTTGTCGAGCGGAAAACTCTAAGATTCAACGAAGTATAATGGAAGATAC
GTGGAAATTAAACGTATTTTTCGTTCCAATTTTACATCTCAAGCTAGGATCGAATATGAA
TCCGGACGACATCGAAGAAGGCGATGACGTGTATTTCGAATGCAAGGTCCATGCAAAT
CCTAATGCTTACAAGGTCATATGGAAACATAACGGAAACGTTATTCAGAACAATGCGAA
AAACGGTGTGATAGTGCAGGAGTACAGCTTGGCTCTGAGGAAAGTGAATCGTTCTCAA
GCAGGCAATTACACTTGTATCGCTAGCAACGTCGAGGGAGACGGTTACAGCAATACCG
TCGAACTTAAAATTATGT

>novel_circ_002018
GGTCTTTTCATATGTACAACTATGAATCGGGTCTCGGTACGCGCATCAGCAACACCGAG

CTTTCCAAATCCGGGAGAGTAGCGCCGCTCGCGGGAATAGCCTACCAGCGGAGCGGTA
CTCGCTCTGCCCGTTTCGTGCGCCAACGAAATATCTTCGACTAGACTGAAATCTTCTTC
GGGCACCCAATATAAAAATATGGCGCCTATGTTGCTAGAAATATCTATTAATGTATTGTTT
TACATATATGTATGCGAGTTGGGACAGATTAAAAACTTTTTTTGCAATATTTGTATAATAT
TTTTATATTAAGTGTGTATATATGTATACATGAGACATGCGTATAGCAATTATTCGTGAAAT
GTACAAATTCCGTCTTCATTTTGTTCTCCTTCTACTTGTTATCTAACCTTATATTACGCAC
AGATTTGATCTCCACTGGCATGGAGCTAGGAGCACATTGATAGTGATAGTCTACAAGAT
CTTGGTTTCTATGCATTGCCTGCCAAAGAAGAACTGAGCCCAATGTTTCCACACAATTC
TAGTTCACATGAGATTTCTACAGAAACAAATGCCTTCGTACAAAATTCCATG

>novel_circ_002020
ATCATAAGATTGTAATAAGAGCAAAAATAAAACGGATGACAAGCTAAGAAAAAATGCA
CAATCAGTTGGATAACCTAATAAATCAAATGCAACGGCTCATCCAGGATAAAATATGTT
GAATAGATCCATAATTCTGCAATCAACACATCTGAAGGCTATTCAGATTGATAAAGTTTA
ATACAAAGATAATGGCAATATCAATAGGGCTATTAATTTGGAAAATGGTAAGTAAAAATT
TCTAGATTTGTTTTACGATTGATTTATATATATATATATATATATGATCTTGTACTGCTTATCA
TGTTTTCGAGAAGTAAACAAGGATGCATAGTCCTTCCATACGAATGCTATATAGCAGGT
CAAATTCATACGATACTAGCAATTGATAATAAGAAAATTTAAATTCAAAAATTTTAATCT
TACTAATTTAAATAAAAACTAATGAAAACAATGAGTGCGCTTTTTAGCGAATCTCATTCT
CAACGGAATTTTCTACAGTTTTCATACAAGCCTCAAACTGACAAAGAAAATTAATAACG
AAGCGTAGTGAGGAGGTAATAACAGTAGAGCTTTATAGAACTCATTTCTATTCCCCCTC
TTATGTTAAAATTTTTAGAAGAAAATCCTAACGTCACGAATGAAGTCGATTTTTATTCTG
AATTAAAATTGAAATGGGAAAAATAGACTCGGAAAGATCTTTCCGGGAGGAATAAAAA
TATCAAGCCAGCCGAAAAAATGATGGACTCACTTAAAACCTATTCCAGGAAACGTAAA
AAACCCATCTATCGAATACAGGCAAATGGGATATTCATATTAGAAAACATTAATTCTAAA
GTTCAAACCGAGGACTCTCTCATCGAAACTTGTTAAAAACGTCAATTCAAGGATTGAG
ACACGGAAAAAATAATCCAG

>novel_circ_002021
GATGCAGTGAAAAATATTGTTTATTAGTGGAATTCACATCCGTGGAATAATTAAAACTGC
AAATTCAATATGCATTTGATCGCATTAATGGAATGCAAAAGATAATATAAAATTCGATGC
AATATAGTAAATAGATTGCAAAATTGCCTCGACGACAATGATAATCACATAGAATAAATA
TTTTTACTAAAAATGATTCGAAAATGGATCAATTCTTTGAGTTCGTATAAAAAATAGAAA
TTCATTAAATGAAAGTAAGTATTAAAAAATATAATTTTAAAATATTTTCAATATACTAAAA
ATAATCTAATGATTCCAGAATTTTCATAATATATAAGAATTTTTCATTCAATTTTTACTGCT
TCAAGGGAAAAAATTCAACATTATCATATGTGGAGAACGAAGAGGCCATATGTGAAAA
AATATGCATTTTTTGTGACCGATGATGTGGAGCATATATAAAAT

>novel_circ_002022
GATGCAGTGAAAAATATTGTTTATTAGTGGAATTCACATCCGTGGAATAATTAAAACTGC
AAATTCAATATGCATTTGATCGCATTAATGGAATGCAAAAGATAATATAAAATTCGATGC
AATATAGTAAATAGATTGCAAAATTGCCTCGACGACAATGATAATCACATAGAATAAATA
TTTTTACTAAAAATGATTCGAAAATGGATCAATTCTTTGAGTTCGTATAAAAAATAGAAA
TTCATTAAATGAAAGTAAGTATTAAAAAATATAATTTTAAAATATTTTCAATATACTAAAA

ATAATCTAATGATTCCAGAATTTTCATAATATATAAGAATTTTTCATTCAATTTTTACTGCT
TCAAGGGAAAAAATTCAACATTATCATATGTGGAGAACGAAGAGGCCATATGTGAAAA
AATATGCATTTTTTGTGACCGATGATGTGGAGCATATATAAAATGTAATGTAAAAATATAA
AATATACATAAATAACATATAATAGCCATCATATAATTGCAAATATATCGCATTCATTATCG
AG

>novel_circ_002023
ATGTCTGAGGAGAATCCGGAGATGCGGGAAATGGCAGCGCGTATTTGTCGCGATTATCT
TCATGGAGTTTGGAAACACGTTACCGCGGAAAACATAATATTGAAACGTATTAGTGGTG
GATTAAGCAATTGGTTATACAATGTCCAATTACCAGATGAAACTGTCCCGATTCGAGGT
GAGCCTCGGCAAGTTTTGTTACGATTGTATGGTCAAATACATGGAGAAAGAGCTTTGG
AAGGATTGATCACCGAATCGGTGATCTTCACATTATTGTCGGAAAGGCGACTTGGACCC
AAATTACACGGTATATTTCCTGGCGGTCGAATAGAAGAATATATACCGGCAAGACCGTT
ACTTACCAAAGAATTGGCGGATCCGACTTTGAGCTGTATGATCGCTGAAAAAATGGCG
CAGATTCATTGCATGCAAGTTCCCATAAGCAAAGAGCCAACTTGGCTTTGGGATACTAT
GGCTAAATGGTTAGATACGACTAGAGATATTTTAGAGAACATCGAAGATATAGATGTCC
GGCACTTGAAGAATGTGAACATGATACGAATAATTGATTTAGATCACGAGATTAAATGG
TTCAG

>novel_circ_002024
GCATCCTGATCATTCTACCACTTTGCAGCCGAGGAATGTAGGCGACGAAAGTACAGATC
AGAATAATAATATAACTATGATGAGTGAAGATCTGCTGCAGCCTGGCCATGTGGTCAAA
GATCGCTGGAAAGTTGTCAAGAGAATAGGAGGTGGTGGATTTGGTGAAATTTATGAAG
GGGTAGATTTTATGACTAGAGAACAGGTTGCATTGAAGGTAGAATCAGCACGTCAATC
AAAACAAGTTCTTAAAATGGAAGTGGCAGTATTGAAAAAACTTCAGGGTCGGGAACA
CGTATGCAGGTTTATAGGTTGTGGTCGTAATGATCGATTCAATTATGTTGTGATGCAATT
ACAAGGAAAAAATTTGGCTGAATTACGACGTGCTCAGCCTCGAGGTGCTTTTTCATTG
TCTACCACTCTACGTTTAGGACTTCAAATTCTTAAAGCAATTGAAAGTATACATCGTGTA
GGCTTTCTTCATAGAGATATTAAACCTATGAATAAAATTCATGTACACTATTTAAAAGTC
AAACTTTTCAATGGGACGACTTCCACATACCTCGAAGTTGGTTTATATGTTAGATTTTGG
TTTGGCTCGTCAATTTACGACGGGTACTGGTGAAGTTAGACCACCTCGTAGTGCGGCA
GGATTTAGAGGAACCGTCCGGTACGCATCTGTGAATGCGCATAAAAATAAAGAAATGG
GCAGACATGACGATTTGTGGTCATTGTTTATATGCTAGTAGAATTTGTTAATGGTCAAC
TTCCATGGCGAAAAATAAAGGATAAAGAACAGGTTGGTCTTATGAAAGAAAAGTACGA
TCATCGTTTACTTTTAAAACATCTTCCAGCGGATCTTCGTGTATTTCTGGAACATATTCA
GAGTTTGGGATATGCAGATAAACCAGATTATGCAATGCTGGCAGGTTTATTTGAACGTT
GCTTGAAACGTAGAGGAGTAAAAGCTACTGATCCCTATGATTGGGAGAAACCATTTATA
TCGAATGAAAGTTTGCCAACTACTAGATCTCTACCTAGTAATCATCAGGTCGCTATGCCT
CCAGTATTGACCACAGCAACTACGATTAATCAACCACCAACTACACCAAATGTTGACA
CAAATAATCAAGAAAATGTTGAACCTGATAACAGAAAAGAATTAGACGCACAAATGGA
TTATGAAAGTATATGTAGAAGAAATAAACAACGAGGAGTAGAAGGATCTTCGGTGCCG
TTTACATCAGCTATCAGCAATCATGGCCGAGATAAAAATTGTAACGCGACGATACCTAC
GATGGACAATACTCATCAACAAGCTGGACAACAAGCTCAAGATTCACCTAAAAAGAG
GCGTGCAGTTTCAATGGCAGCAGAACCACAAACAGGGGTGGTAGACAATGAAGAAGA

TGCATTAATGGCTTCTGCTCGTTCTGCTTCAGAAGTTCCTCGCAATAAAGTTGGAGTCA
ATGCTGCAGCACCATTAAGAAAATCTGCAAGTGGTGCAACATTTGCTAGATTACGAGTC
ACACCTGCAGTTGAAACAGCACCTGGCGAACCTCCCAGTCCTCGAGTACAAACTGAA
GTTGATGCTTCTTACTGTTCCTATGATGTTACCAATCCAAAATATGTTGGCAATCAGAGT
CCTGTTACGGAACAGAGAGAACCATTAAAAAGAGAACCACGAAGAAGAACAGAAGG
ACGTCAACAGACAAGAACTAATCGTGCTGCAATAAGAGATTATTCCATTACACAATTTG
CACAGATAGATGATGATAATGTATCAGCATTACAACAAGTAACTAAAGGTGGTGGTGGT
TTGACACTAGCTTCTCAATGGAAATCACAATTCGATGATTCTGAAGAGACTGATAATGA
ATGGAAAGGAGAAAATTTACAAAGTCCTGAACATAAAGGAATTACTATGCCAATTATGG
TTCCACAGTCAACAGTTGGTAGTGATGCTGTAACTATCGCAACTTCAGTGCCTGCATCT
ATCGATGCGAAAACGACAGTAATACAATCAACTATGTCGATTGCGCCTCAACATTCGAT
AATACCAACTGCTCTCTCTCGAATAAGTGAAGATTCTACAAAGCCTACAGTTCCTCACA
GGTGTTTAAACATTGCTGGTATTGAAAAATATCCAGATCTCCAAGGCGCACTTCCTCGC
GCTTGGAGCGTTCCTGCATTGGCACCGCATGTTCGTGCTTATCTCGAAGCACCATTGGT
TCAACAAGCTGCATTTGATGATTTACTATATGAAGTCGATGTTATGAGAAATATAGCAAC
ACGTTATGATGAACCGAGACAAAGTCCTCCGTTACGAAGGGCAAGCGTTCCAGCAGTT
GCTTTTTCTCCACCCAACACAATACCTAGTACACCTGGAGGAGAAGAAGAAGAGGAA
GCAGTAGCAGGAAGATTGGAAATTAGAGTAGTGGATGCAACAGGTGGTGCAACTGTTG
TACAAACAACTGCAGATAGAGAACCATTGCAAAGAAAAATGACAAACGGTACAGTAG
ATAGCCCAGCTAGTCCAAAACCAGAAAGAGAAGAATCATCGATATTCGATACTGCTGA
AAATCGAGCATCTGTTGATGATGTTATGCATAAGGAAAACAGCACTATCACTGCCTCGA
CAACCATACCGAATATATTACCACTTCGTGAATATAAGGATGATAAAGAAAAGGAGGCT
TCTAATAGGACTCATAATACTGTCAGTAAAATTCCAATTCCGGTTAAAAGTCCTAGATGT
TCGTTATCGGAGTCAACTCCAGAAACGAATACACAAAATACCCGGAGTGGTGTGGGGA
ATGAAATAGAAAAATCATCTACACCTGTGGCTATAACCAGGGAAAAAGATGCTA

>novel_circ_002025
TCAGAACTTATCAATTGCCGAGATCCGACATTGAAGATCAAACGAAGCAATGGCTACA
AACAACGAAGATTCCTGCACTTTGTTCGAGACGCAGTGTACGCAGCTGCTTACGCGCT
TCACAATATGCAAAGGAATGTATGCGGTGAAAACTGGACGAGCCTCTGTGACAAGATG
CGGCCCCCGAACAAGGATATCTTCTTTCAATATTTGGCCAACGTGTCTTTCAAAGACGA
AGCCGGGAATGGATTCAGATTCGTGGACGCGGTTGACGGACCACCAAGATATTCCATC
TTGAATTATCAACGAAAAGTGAACGGTTCGTACCATTGGACAGTGATCGGGAACTACA
CGT

>novel_circ_002026
AAAAGGAAGCAAACGTTTTTGTTCGCCCACTTGTCGGATTACGCGGTGTATAAGATCA
CAAACACCAGGAAGAGATACGGAGCACCCTTCCCGTGGGGCATCTGCCTGAGGCCGA
CCAGCACCGGCCAACTCAAAACCATCGAGTCTGGAGAAGCCGGACTCAAGGTCATCG
CTTTCCACAGTGAAAAGTCTCGCGCCTGTTGGCTCACGGCCATGAGACTCGCCAAGTA
TGGCAAGCAACTGAGAGAAAACTACAGAGCCTTTAAGAATAAACAGTGCGAACAAAC
TGACAGCCCAAAGGATCGATACGTCAACTACAACGTGCCCAAC

>novel_circ_002028

AACTGTACAGATTGACTGGCGAAGTTCTTGGCGAAGGTGCTTACGCCTCGGTTCAGAC
TTGCAGGTCGCTTTACACAGACCTGGAATATGCTGTCAAAATTATTGATAAAATTCCTG
GTCATGCACGGGCACGTGTATTCAAAGAAGTCGAAACATTTCATCATTGTCAAGGACA
CCCAAACATTATTCAATTAATCGAGTTTTTCGAAGACGAAGAAAAGTTTTATTTGGTATT
CGAGAAAGTAAACGGTGGTCAATTGTTGAGCCGGATACAAGAAAGAATTCATTTCAGT
GAACGAGAAGCAAGTCAGATAATTCAAGAGATCGCAAGCGCGTTAAATTTCCTTCATA
AGAAAGGTATCGCTCACAGGGATCTGAAACCCGAAAACATCTTGTGCGTGTATCCGGA
TAAGTTAACACCAATTAAAGTGTGCGATTTTGATCTTGGCTCAGGCATAAAATTCAACA
ACTCATTGTCAAGTCCTGTAGCTACTCCGCAACTTTTAACACCAGTCGGCAGTGCCGAT
TTCATGGCTCCGGAAGTTGTGGAAGCTTTTATCGGAGAAGCGAATTATTATGACAAACG
TTGTGATCTCTGGAGCCTTGGCGTGATTATGTACATTCTCCTTTGTGGTTATCCACCTTT
CTATGGGAATTGCGGCTCCGACTGTGGCTGGGAGAGAGGCGAGAATTGTCAAGCTTGC
CAAGAGCTTCTATTTACAAGTATTCAGGAGGGCAGGTATGAGTTCCCGGACAACGAGT
GGAGGTGTATTTCGGAAGACGCGAAAGATTTGATCAGAGGCCTGCTGGTAAAGGAAG
CTCATCAAAGGCTTAGCGCCGAGAGTATTCTGAAACATCCGTGGATTAATCCTGGTCCA
AGTTCCGTTGAAAATACCGAAAAATCTCTCACAACTCCTCATATTATCAGAAGAAACAA
TTCTGCCAGAGAACTCTCGGCGTTTGCTGAATCGGCTATGGCTGTGAATCGTGTTGTAC
TTCAACATTTCTCCGTGAATTTGGAGGAATTAGCGGAGAAGAGGGAACCGAGATTGTC
TACTTCGTCCACGGATGAGGATAACCATCCTTACGGGCACATGTCCGATTCAAGCAGCG
AATTATCGGAGCACA

>novel_circ_002029
AAGATGCAAATTGCAGAGATACCGTGAATTCCACGGGCGAATCTATGAGCGCGGTGCA
AGCTCGACAAGAAGAAGCTAGGCGCAAAAGACGCAAGAAGAAACGTTCTGGATCATC
CTTAATGTCTTCTTGCTTCCAAGAACTGTACAGATTGACTGGCGAAGTTCTTGGCGAAG
GTGCTTACGCCTCGGTTCAGACTTGCAGGTCGCTTTACACAGACCTGGAATATGCTGTC
AAAATTATTGATAAAATTCCTGGTCATGCACGGGCACGTGTATTCAAAGAAGTCGAAAC
ATTTCATCATTGTCAAGGACACCCAAACATTATTCAATTAATCGAGTTTTTCGAAGACG
AAGAAAAGTTTTATTTGGTATTCGAGAAAGTAAACGGTGGTCAATTGTTGAGCCGGAT
ACAAGAAAGAATTCATTTCAGTGAACGAGAAGCAAGTCAGATAATTCAAGAGATCGCA
AGCGCGTTAAATTTCCTTCATAAGAAAGGTATCGCTCACAGGGATCTGAAACCCGAAA
ACATCTTGTGCGTGTATCCGGATAAGTTAACACCAATTAAAGTGTGCGATTTTGATCTTG
GCTCAGGCATAAAATTCAACAACTCATTGTCAAGTCCTGTAGCTACTCCGCAACTTTTA
ACACCAGTCGGCAGTGCCGATTTCATGGCTCCGGAAGTTGTGGAAGCTTTTATCGGAG
AAGCGAATTATTATGACAAACGTTGTGATCTCTGGAGCCTTGGCGTGATTATGTACATTC
TCCTTTGTGGTTATCCACCTTTCTATGGGAATTGCGGCTCCGACTGTGGCTGGGAGAGA
GGCGAGAATTGTCAAGCTTGCCAAGAGCTTCTATTTACAAGTATTCAGGAGGGCAGGT
ATGAGTTCCCGGACAACGAGTGGAGGTGTATTTCGGAAGACGCGAAAGATTTGATCAG
AGGCCTGCTGGTAAAGGAAGCTCATCAAAGGCTTAGCGCCGAGAGTATTCTGAAACAT
CCGTGGATTAATCCTGGTCCAAGTTCCGTTGAAAATACCGAAAAATCTCTCACAACTCC
TCATATTATCAGAAGAAACAATTCTGCCAGAGAACTCTCGGCGTTTGCTGAATCGGCTA
TGGCTGTGAATCGTGTTGTACTTCAACATTTCTCCGTGAATTTGGAGGAATTAGCGGAG
AAGAGGGAACCGAGATTGTCTACTTCGTCCACGGATGAGGATAACCATCCTTACGGGC
ACATGTCCGATTCAAGCAGCGAATTATCGGAGCACA

>novel_circ_002030
TGAAATTTATCTTTTAATTCAACTCCTTTAGCAACTGTAACACCATCTTTAGTAATTTTTG
GACTACCCCAACTTTGTTCTAAGATAACATTACGTCCTTTTGGACCCATTGTTACTGCAA
CTGCATCTGCTAAAATATCAACACCTTGCAACATAAGTGCTCTAACTTCTGCACCAAAA
CGTACATCTTTAGCATATGAACGAGTTTGTAATTGGCGCAAGGCTGCACTTCGCAATAT
GGTTGGTAATCTGT

>novel_circ_002031
CAGGATTGGCACCTTTACTTATTTTTTCAAAACCTTCTTTAGCAATGGCTCTTGCCAAAA
CTGTAGCTGTAGTTGTACCATCACCTGCCTCTTCATTCGTATTATTTGCTACATCTTGTAC
TAATTTTGCTCCAATATTCTGAAATTTATCTTTTAATTCAACTCCTTTAGCAACTGTAACA
CCATCTTTAGTAATTTTTG

>novel_circ_002032
GTGTTATGTTAGCTGTTGATAAAGTTAAAGATGAATTAAAAGCCTTAAGTAAGCCAGTA
ACAACTCCTGAAGAAATTGCACAAGTAGCAACTATTTCAGCTAATGGAGATAAAGCAA
TTGGTAATCTTATTTCTGATGCTATGAAAAAAGTTGGAAAAGAAG

>novel_circ_002033
GGCAAAGGAAAAAAAAGTGATATTGATCATAGAGCAGACGTAATTAGAGATCAGATTG
CAAATACAACATCTGACTATGAAAAAGAAAAGTTACAAGAACGTTTGGCAAGGTTAGC
ATCAGGAGTTGCAGTTCTCAGAGTTGGTGGAAGTAGTGAAGTTGAA

>novel_circ_002034
AGAAATCTCCTTCCGTATTTCGAGAACGCTCGAAGAGTTAGAAGAGAGATGCGCCAAC
TTCCGTCGCTGCGGATGTTCCACGTGCTGCTCGAGTTCATCGACGCGTTGTCGCACTGC
GCCCCGTGCTAGGGCCATGTCCTAGCGGCACACTTTCGACCCACCAAACCCACCCACT
GCCAGAAACAGAGAGACTCCCACGAATAAAATAGCTCAAAAATTCTTATTAAATGTGA
TCCCTCGTCGTCCAAGAGCCATCCACGCCCGATCGAACCAGATCCCTGAGATGCCGGG
CGAGGACGGAGCCGGTGGTGGTCGCCTCGAGTGTCTTCCTCGTCGAGTGTCGGCGTCA
TCCCTTTTTTGAGCCGCGCGGAGGCCGGCCAAACAAAAAGACTGATATACCGATCCGT
GTGATGCGGCGCGCGACCTGCTCAACGTCCCCGCAATAAAATGTTCGGCAAGGTGCCA
CCCTCGAACACGCCGGGCCCACCCACGGCGAACGATGGAGCGTCGAAATCGTGCGCC
AGGCCGACCACGGCCGCCACCACCTGCCTTCCTTGTTTCTCGGTCGACGAGCCCCCCC
CCATCCTCCCCGACGAGAAGTCGAAGCCTTCTTGGAGGGCCAAGGAGAACGTGCCCG
TGGTCGTCTCCGAGGCGCCCTCCACGGGACTCGAGGAGAAGCTGTGGAGATCGAAGG
AGGAGCCACCACCCTGCATCGCGGAGGAGCTGAGGCCCAAGAGGGCGAAGGAGGGA
GGGTCCTCGAGCGAGGAGAAGTCGAAGAACGAGCAACAAGCATCGGACGAGAAATC
GAAGGGTTGGGGCGTCGATCATTGCTCGCAACCGACCTCGTCCAATTTCTCTCCTCAGT
TCGAGGCGAGCAGAGGCTCCAAGAATTGTTCCAAGTACTCGGCGAAGGAGGCAGGGC
ACGGGAGAAGCCAGGCGGCGGGTTCGAAGGGTGTTCACAAGTGCTCGTCGAACTCGA
CGAGTTGCGCCAACTTGGCTAGGTCCGCCAAGTCGCAAAGGGTGCTTTGTTCCACCAG
CCTGAGCAATCTGAACGTGGACAACGGGGGGGCGAGGGATTGCTCGAAGCCGGGGAA

GAAGCACGGGTGCCCGAGCCGGGAGTTTCTCAAGGAGCAGCATGGAATGCCTGGAAA
GTCCGTTTCCCAGAGGTCGCAGGGAGTCGCGAGCAAGTCTGGCGGCAGATCCTCCGAT
CCCAACGACTGCCAGCCTGCCAACCCGTCCATGGATTACAACACCGCCATCTCCGTGA
TCCAGTCGACCGACGAGCCATGCCAGAAGTCCAAGTACCACGTGGAGAACATGGACA
TCGTGGACATGGTCGCCACGGCCACCTCCGCCAACAATTGCGAGCAAAGGAACTGCA
GGGGGGATTTCGCCACCTCGACACCTGCCAAGGATTGGATGAAGTCTAACGGAGACGC
GTTGCAGCAGAGCTGCGTCTCCTGTCTGCCGCAGGATCTTCCCACGGAGCTGCAACTC
TCCTCGCCGAGGAATTACAGGGAGAAGTGCCGCGACCCGTGGCGGCCCACCGAGATG
GAGAGCAATATCTGCAGCCTGAACGTGTGCCCCGGCGACCAGTATCAGGATACAGGGA
CGAAGAGGAGGACGGGCGCTTCCTGTCAGGCATTGAGGCATGCGGTCGCCTCGTTGA
ACCGATTGGACGATTTTTATATGGAGAAGATCGGCGCTGGCTTCTTCTCGGAGGTCTTC
AAG

>novel_circ_002035
AGGGGAAGAGTTACGTATCGAGGAAGAGGATCGAATTGTTTGGATCGGAGGAGAGGA
GGAGGGAGCCGATGTTTGGTAAAAACAGGAAAAAATGTCGGGTGAGACAGAGGGATA
GTTTCGTGGCAAGTTCAAGGCTGAGAAGATTTGCGCGGTCGGCAAACTGGATTCATTG
AGCGTGGACAAATTGAGTGTCGAGAGTGCGTGCTCGCCTGGATGAAGTTGCACCGAG
TTGCTACTGGATGATAGAGAGATAGAGGAATCGTTACGATAGACAGGTGGCTCGGAAG
AGAGAGAGAGAGAGAGAGAGAGAGAGAGAGAGAAAGAGGAAAAGAAGAGGTTTTCAA
AAGTGCGGGGAGAGACCGAGACGTCTCTGTATCGGATTCACTTTACTCCAGCCTTTAA
CTGTCAACCGATCTACAGAACGAAATTCGACAG

>novel_circ_002036
CAAGCTCCCCGGAGCCGCGTACGTGCCCATACTTGGGCAAATTTACGGTGACTGGGGT
GAACCGGAACCAGAGGAACATTCGTGAGAGCCGCAGGAGCCAGCACCAGGAGGGGG
CCGTCGTGAGGCGTGACAGGGAGAAAGTTCGACACGCCCAGGAACGTATCCAGGAGG
ATCGACGGGGCAGGAAGCTCGACTTCGACGTGGAGAAACGCAGGGTGGCCAACAAC
GAGTACCTCGTCCACGAGCGTATGGGCAGGCGTGCCAGGTCGAACCGCAACAACAGA
AACCATAGGGAGCACAGCCTGGAGGAGGCGTCGTCGAAGAGGAGCCTGGACATCGAG
GATTTGTTGCGGGATCGTGCGAGATCGAAGGTGAGAAGGTCCGAGGAGCAAGGCTCG
ACGAGGTCGAAGGTGAGAAGGGTGCGCGAGATCGGGGTGAACAGGTCGAGGAGAAA
GATGGAGGACTTTGGGATGGACGCGGATAATTTCGCGCGAATCAGGCAATTCTGGACG
ACCGATTTCGACGACAGTGACGGGTCCAGCATGCTCGAGGTGCAGGAGGATTATTTCG
GCGATTACCCGGACACCGCCGACCTGCCCAAGAAGAGGTCGGGCAGGGGAGGGAATA
TTTTGACGAGGAGGAGGAGGAGGAGGAAGAGGAGGAGGAGAAACGTGGAGGAGCT
GGATTCCGGCAATCGGTCGTTCGAAAAAAGGGAGAAGAGGGAGGAGGATGTGTCCAG
ATGCAGCTCGGAGGTTACCACCATCACTATCGGGTGCTCCACCGCGGATAGAATGGAAT
TCCAGACGGATTGCGTCGACGACAATGCTATTGCTTACTCCTGCCACGGTAGGTGGTTC
GACGCCGAGGGAACCCAATTCGTGATCGCTACCCAACTGGCACCTGGAACTGGCTGGT
CGAACGAGAACAACAGGCCCCAACCGTACAGGAACAGCCGTAGACTGTGCTTCATGT
ACAAAGAGAGCGGCGGTGTTGTCAGTTTGACTGCCAGCGAGGTGGCCTGCCAGAGGG
GAATACCCCCACCCCCGCCAATGTTGGCCTTCAATGCTACCAGTACCG

>novel_circ_002037
GTGGGGATGAAGCGCGTGTCGCGAGGGCGAGAGGAAGCGGACGCGGAGCATTACCG
AGCCTCATCCTCGCTAATGGAACGCCCTTGTCGAGAGTGTCACCACCTCGCGCGGGCT
GGCCAGTGCGGATCAACGAGGCGACACTTACTACCCCGAGTCAGTCAATTCATTATCA
GCAGCAACAACAACATCACCACCACCAGAATCAGCATACATCGAGTCCAAGCACACA
GAGAGCTGTCGAACAATGGGCTAGCGAGCGGGCAACATCGTCGGTTTTGTCGGTTGGT
CGTTCCGCATCACCATCCTCGCCAGAAACAGCAAGGAGACCGGGAACAGGATCGTCTA
GAAGAGTTTTCTCGTCGTCGAGTCGAGTTAGGCCAGGAGGCGGAGGTGGGGGTGACG
GAAGGGGAGCAGAAAGAAGCACAGAGATCGGTAACGGTATCAATTTCACGAATCACT
GGCAGGAGGAAGAAGAGCTGGAGGCGAAGAAGAAGGAGGAGCACGGACGGAAGCG
TTGCGTGGACACAGCGGCGCATTGTCAAGCCGAGCATCTTGAACTGAGAGGTCGGCC
GATGTCGCAAAACAATGCGATCGCGCCGCTCTCGCGGCCACCCTCCTCGCAGACGAAC
GTAAATGACACCACCGATGCCGTAGCGAACAGCAACGTTGACGATGGAGAAGCGCATT
CCGTTCAAAATGTCACCACCACTGGTCGCGTCCATGATCACAATCGGCCGTTTCCTCCG
CCAAGATTGCCGACCGTGCACGTTGCCACTGCCTCGACAGCGCCCCCTCTCGCGGCAT
CGTCGAATCCGAGAGTGTTGGTCACAGCTCCGCTCAGTCCACTCTCGAACACCTTCCG
AAGCAGACCGAGAAACGTGGATACATCGACGGGCGAGACGAATGCGCGGGGATTGGT
ATCGAACGACACGCCACCTAGAAGTCCTATAAGCGAAGTGAGTTTGACCGTGCCCAGG
CCAGACGGAGAGAGGGCGATCAACGAATACGTCGAGGCGCCGTTCAAACATAGCAAC
CAACAGGAGCGACGGGTCTTCGAGGATGAGAACAAACGGGGGATCGGTGCCGATTGC
CCGAAGATCGACAGGAGGCATCGGCAGAAAGGTGGTGATGCGTCCGTCGCGGCGACG
CATCGGCTGCACGCGAGCAGGACTCAAGCGTTTCGTGGTTTGATCGCGAGCAGTGGG
ACGAATGCGACCGCGTCCGCAGGCTCGATCGCCGCGAACACCGCGACACCGAATGCG
GTGACCAAGCAGCCGGTCTCGTTCACCAAAGAACCGGCTAATAGCCTCGCTTCTAGGA
CTTATGATCCGGCCGGTTTGTCGATAATGTGCAATTTCTGCGGTAGATGTCGTTGCGAGT
CTTGTCGGGAACCGCCTCCATTGCCCAGCAAATGGCTGTGCGACAACAAGTGTTTCTG
TTCCGCCGATACGATTCTCGATTACGCTTCTTGCCTGTGCTGCGTGAAAGGACTGTTTTA
TCATTGCGTAGACGGGAATGGGTCAGGTGGAATCGATGGGGAGGCGGCTGCTAGTTGT
GCGGACGAGCCGTGCTCCTGTACGGGAAATAGGAGAGTCTCCAGGTGGGCCTGCCTC
GGCGCCTTGACACTCGTCATGCCCTGCTTATTGTGCTACTGGCCGTTCAAAGGATGTGT
CGCGGCATGTGAGGTGTGTTATGCACGGCACGCAGCCCAGGGATGCAGATGTAATCCG
AACACGACTGGGAACGCCGGAAACAGACATCATCCGATCGCGAACGATATCGGGGATT
CGAGGGATCCGGAGAAAAGGTTACTCGACCCTGTCACGCCGGAACTCTAATTGGAAG
AGATACGACAACGCAGTAATACCAATACCACCCGCAGTGTTATCTCTCACGTAGGGGG
GGACACAAAAATTGCTCTTCTACGCACAGAGATTGTCCGACAGGAGAGGGGAGCAAA
AACGAAAATTGCATTAAGTACAAGAGTTGCATTTAAGTCGAGAAACGATAATCGAGAA
TGACGAATTTGTACGTCTAACGTCGCGAGTGTTTTATTTATTATTCTTCCATGCGCGCGC
GAAAATGCGAAGCGAGCGAGAGAGATTGAGAGAGCAAGAAAGAAAGAACGAATGGA
AGAACGCGAGATCGTGTCACGGGGGAGGGAGATGATGCTAAAAAGGATATAGTGGCGT
AGGAAAGATGATAGAATGATAGCGAGGAGAAAGGGCGAGAAAGATGACAAGGTACGC
GAGCTTGCGTATTAACGCAGAAACATACCACTTACGTATAGATAGGTCTATACACGATAG
AATATATAAATAGTACCGCGCACACACAGAGAACGAGTGCGAGCGTGACCAAGCGAAT
GTGTGGAACGTGAACGTGAGAGAGTGAGAGAGTGAATAGAAGAACCACTAAAGTATA
TTAAACGCGAGCACGTGTACGCGCGTATACACGCGTTTCTGCTGTTACTTCTTGCTTGA

AGCGCGTGTATGCGCGCATCTACACTTAGATTATAGATATGTATATTGTATATTATATTTGT
ATAAAGAAAAATCTTTTCTACGGTTAATTTATTCGTGGCTGAATTGCCAAGTAACACATA
TATAAAGTTTCGCAGTAAGAGGAGCACGACACGGAGAAAAGAGGCGAGACGAGAGA
AGGCCGAGCGAAGACTGGGATTATTTGCAAAAATTATTTCCAGTCTCTCACCGCTGCGA
TAGGAAACGCATAAAGAGTATCGGCCGTAAAGAGGAAGAAAGAGGGGAAAGAAAGC
AGGAAAGGGCGGGGCAGGGGGAAGGAAAGCTCTGAAAAGAAGCATTGTCCGTATTTC
CGAATGTTTTCCTCGGCATATGATGTATACGCTTTTATACTCGTTAACAGTGGAATTGTAT
CGAGCGCGCGAGGCCCTGTCGAGCGTTAGAGAGCGTGATATGACACGAGATCGAGATA
ATGCACGCGCGTTCGGGTTGCGTGAGAGCGAGAGAACTGTAAATCTGTAAGAATGAAC
GTTTGCGTTTATGTGTGTATGTTAAGTATGTATGCGTATGTGGGTGTGCTTCATGGGGTC
GACGAGAATGCGGGCAGATGGAAAAAGGACAATTGCGTGGGGGGCATAAGCGCGAGG
AGAAGAAAACACAGATCAGGAGGAGGGAGTGGGGAGAAAGAGCAACGCACGCCTGA
AATCTCTTCTAC

>novel_circ_002038
ATCGCGAGAAAACGGATGATGACCGTGCGACGACCCCGGAGTCGCTTCGCAGTAGCA
GCCCGGCCTCGTCTCCACCCCCTCAATGGCAATCGACGAACACACCGAGTAACACGCC
GCCCAGACGATTTATCTCGAGTATCCTCGGTGGGGATATCCCGTACGGAAGCAGGGGTC
ACGTTCTAACCAGGGCGGAGCGCAAGGAGTACAGCAATCCCCCAATCGCGCCCCCGTC
CAGTGATGCTACGCAATTCCTCGCTAAATCGGAAAAATTGGTGTTGCCCAGACCAACC
ACCCCTCCGAAAGCGCCCAGGGTCGAGCCGCCCACACGGGTCTCGGTCATTCAAAGG
GTCCCTCCTTCCCAAAGTCAATCGAGGAGGGAAGACAAGATCGAGGTGCCACAAACT
CAAGAGCCGGAACAAGAGCAGCCTATCGATTACGCGGTGCCGAAGAGAAAGGAAGA
GGACGAGGAGCGAGGCCGCGAAGGCGCAAAAGTGTCACGCGCCGCGATAGGCAACT
CGATTGCCAGGCCTCTGCTGGCCATGAGACTGTCCGGTCCCCAAGTGGTCCACGCGGC
CGCAGGCCACGGAAGATCGTCGAATTCCGGCGGGAGCGGCGGTTCCGGGTCCGGCAA
CACCTCCAGCGGCGGCGGCGGCAACTCGGGAGGCGGTTCCTCCTCCTCCTCCTCCTCG
AATTCGGGCGGAAGTGGCGGTTACTGCGGGGGAGGAGCGGCCACTGGCGGTGGAGGT
GGTGGTGGAGGTGGTGGCGGAGGTGCCGTGGGCGGAGGTGCGGGCGGCGGGATGAA
TCCCGGAGGAAACGGGGGCCGGGGAAATTACGGGCCAAGCTCGCCGCCCACCGGCTC
CCTGCCGCCGTTCTACGAGTCGCTCAAAGGCGGCAATAATCTGGCGAATTTCGCGAAT
CAGTACAACACCACTCAAGGAAATGGATACCTGACACCATCGCCAACGGTGGGGATGG
AATGCGACACCGGTCAGCAGGACGTGAACTCGCAGCATTCCCAATACAACGCTCAAG
AAGGGAAGCAATACTCCCTGTTGCAAAATGTCTGCGCCTCGTACGGTTTAACGTTGAA
GGAGGAAGAAGATTTGTCCCCGTACAAGATCCAAGCGAACGATCTGCTGTCCGGACAA
TATGGCGCATACGACATGACCGATACGGGAATGATGGTGGACATGGTGACTGGCGCTGT
TGTGGATCCTCTTCAATTCACCGCCACTCTAACGTTCAGCTCACCTTCCGATCATACCG
CCCTCTTGGAAAGCCTGAGCGACGCTGCCGACCTATTCCTGCCGAGATTACCGGCCGA
AGATGGTAGCAACGATCTTCTCGAAGAATCGTTGCATTCGCCTGCTTCGGCTGGGAGC
GGGATCGGCCAGGATAACGGGCAAATGACCACTCCTGTAGAGCCGAGCGTCGATCCTT
TCCCAGAGCATAGCATGGCCTTGACTAGAAGCTTCGATACGTCGAGGCACTATACGGCA
GCTCCTCAACATTTCAGCACTTCGAAATTAGGACTAACTTATGCTACAGGCGAATCGAG
TTATCAATCCGTTTCAAAAGAACGGCCCGAACTCGCGCTTCACATTAATCAGAATCATC
AACACCAATCCGAACCACAGCTGCAACAATTGCAGATACAGGTGCAGTTGCAGCAAC

AACAACAGCAACAACAAACAACGTCCACTTCACCTCATCAACAACAACATCAAGGATT
ACTCAGCCCGGGATTAAGCTTCACCGGTAGTGGTCTGGAACTAGATTCAGGTAGTAGC
GTGGGTGGAAGTTTACCAAGTCCCGGAGCAGCGAGCTGTTCGTTAGACGCTGCTTCTA
CTAGTACGTCGCCATCTTGCGCTTTGATGGAGCATGCGCCTAGCCCAGCAGCCACTGTA
TCTTCGGCATCGGTAAATACTGTACAATCGACAGGTCCAGCCGGAGAACCACCACTGA
CGCAACGGGTCGGTGTACTGCAGCAAAGGCTGGGATTACCTGGTGACTGTCAATTGGA
GTTCGTGAACGGTGGCCACGGAATTAAAAATCCACTCGCGATTGAGGGTCAGAGACA
GGCAGCGGCTAATCGCGAGGAAGAGAGAGCAGCTCGACCTCCGCCTGGCAAGGACGA
CGATCCAAATCGTTTTACGTGCCGTGTGTGCAGCAAAAATTTCAGTCTGCAACGATTGT
TGAATCGTCACATGAAATGCCACAGTGATGTGAAACGTTATTTATGCACATTCTGCGGT
AAAGGCTTCAATGATACATTTGATTTGAAGAGGCATACTAGAACGCACACTGGTGTTCG
ACCATATAAATGCAATCTTTGTGAAAAGAGCTTTACCCAGAGGTGTTCCTTGGAAAGTC
ATTGTCTTAAAGTTCATGGTGTTCAACATCAATATGCCTATAAAGAACGTCGAACAAAG
GTATACGTGTGTGAAGAATGTGGTCATACGACACAGGAACCGGAGGTTCACTACCTAC
ATTTGAAAGATAAGCATCCATACAGCCCGGCTTTGTTGAAATTTTACGATAAGCGACAT
TTCAAGTTTACCAACAGCAATTTCGCCAACATGCTGCTCCAG

>novel_circ_002039
CCATTTACAATTGGCGGAGAGACACACGATATGGTCTCCCTTTGGAAATTGGAGAAAC
GGTGCAGATTCTGGAGGAATGCGCAGGCTGGTATAGAGGCTTCTCGATCAAGAATCGC
GCAGTCAAAGGAATCTTTCCCAGCACATACGTCCATCTGAAACCATGCAAAATCGAGA
ATGAGGGTTTGTTCGAGTCGGTGATACCTCTGGAGGATCCGGTAGTACGAGAAGTCAC
CCTCGTGCTTCGAGAATGGGGCGGAATATGGAAGAGGCTCTATGTGGAAAGGGAGACC
TACAAATTCAACACATTGCGCAAAGTAATGCGGGAATTGCTGGAATGGCGGCGTCAAC
TTTTGGCCGGTACTCTTACCACCGATCAAACACGGGAGCTCAAGTTGCGAATAATCAA
CAAAGTGGACTGGGGAAATCG

>novel_circ_002041
ATAAATAATGCCAGAAGAAAAATCTATAAAGATGATTCCGCAAGAAAAAAAATCTTGA
AAATGGCCAAAGTGACTTTAACCAAATAAAAATCAATTCGAAAATGAATGAGAAGAGG
ATGGAATAAAAACAATGGATGACAAACGGAGGATAGACCGATTCGTGGTTTCGCGAGA
CGAAGCCTAATAAAAACGTTAGAAACCTACCGTGAGATGTTTCAAGTTTCGAGCCGGC
TGGTGGCGCCAGGCTACGCTACATTCTGTCCAGGGGTAGCTCGGTCTATATGCCGATCT
CGGTGTGGAGGGGAGGCTTGTGCCGGGATATCGCACACAGACACTGTAAAATCAAATT
CTACACCGACTCACATTTGTAGGGATGGCGCAGCTCGAACTGGTCACCGAGCGGAAGG
AGAAAAGGATTATAGGGACACAATGGAAGAGCGAGCGAGATAAGTGGACGAGGATGA
ACCGAGGAGAGGGGTAGGTTGACCCTGCCGGGTCAAGAGAGAGACAGTCGTATCGTA
GGAAAAGGGACAGCTACAAAGGTGTAATTCGATTCCGTTCTCAGATAACAAAGCGAGC
TGCATGGAGTTAGAAAAGGAAAAGGACGCAATGAGCGAAGCAGGTTGATTGCGGGAG
AAAGATGCAAGGAGGGAGGGGTTGGCCGACCCTGAGCAAGTGGACGAATACCAATCG
CCAG

>novel_circ_002042
GAGCGAACGGAATGCGCAGGCGCGGCCGCCAGACCTATACGCGCTACCAGACGCTCG

AACTGGAGAAGGAATTCCACACGAACCACTACCTCACCAGGCGGAGGCGGATCGAGA
TGGCACACTCGCTCTGCCTGACGGAACGGCAGATCAAGATCTGGTTCCAGAATCGGCG
GATGAAGCTGAAGAAGGAGATACAGGCGATCAAGGAGCTGAACGAACAGGAGAAGC
AGGCGCAGGCGCAGAAGGCAGCGGCAGCAGCGGCCGCGGCTGCGCATCAGCAGCAA
GCGGCCGGTGGGGGACCGGAGGGGGCCAACTAGGGGTACGCCCTGCACCGGAGCCCC
CCTGACCACCCCACCACCAATCCACACCACCCTCTGCTAGCACCGCAATGTACACAGC
TATATTAAAG

>novel_circ_002043
GTTAGATAGAGACCTGTTACGTGTTTTGGTTCTCTACATGTAAATTTCTTTGTTACGTCT
TTGTTTGGAGAGATAAATTATTGTTAAGAGTGTTATTACAATGTTAATGAATCATGCTCA
AGTATATTTCAAGTATATTTCGTGGATATGAAGTGTGGATAAGATGCTTGATAAAAAGAT
GCTTCGCTGGAGAATAATATAGCTGCAAATACCTTTTTTCTTTTTTTTCTTTCATACACAG
TTAGAGTAGAGTTTTCTAATCTTAAGAGTGAATCGCATCAGATACCGCTTTAAATTAGAA
CATTTTTAACCTTAAGGTGGGTAGAATAATTTCCATGTTAACGTCAATCGATGACCATGT
AACCATGCATTACTTAAAACTGCTTCAACGTATGTTACACACAGGATACGTTTCAACTT
TCATACAATGCTAACCTAACATTAACGAGGATGTCAAAGATGGAAGGCCACTTGATCTT
GAAGAAGTAAATGTCACAGATGAAGAAGTAGGATGTCTAGCTTTAGGGTTACGCAGTG
TTGTACATCCTCCCCTTGGCCGTCTAATTTGCATCCACAGCGATTAACATAATCTGTATC
CCGAATATTTATTTGGAATTAGCCTTGGCTTGCTCGTAACTCATGCAGGAATTAGTTAAG
CTCAAGGATTTCGGGCTTACCAATCCCTCCCTCTTTCAAGCTGCTGCCTTCACCTTCTC
CTCCTCCTTCTCCCACCGTTATCGCGGAATCCTTTCACCTCCTGTTAACCCTTCACCCCG
CCACAGGATCCCGTGACGCGTTTTAAACAAACAAACAGTCAACAGAGATAACTCTGG
GACGTTAAATCATCGGTATATACCTTCCGGCTTCACCGGTTCCCCTCCATGCTTTTTCAC
CAAGCCTGGCTAGGAGTGATCCTTAATTGCCCGGCCAATATGGGAGTCGACGGCGCAA
AAACTTGTTACTCATCGAGGTAAATGGCGAGATCTCTCGTTCCAACTCTCCCTTATTTAT
TATATCAATTTATCGACTGATCTTTCAAACGTTTGGAATAATTCAATTTATTATTTCCCATC
CAGAGTAGCGAAGCGAGAAGAGGAAAATTTCGAATTTCTTTCTAACCTTTAAAATTCG
AATCTACGTATCTTTAACGCACAACCAAGTTATTCTGTATTTATCGCCATTGTGGTGTCA
CCGATCACGAAATTCGAATGCGAAAACGAAGAAGCAAATTCGTCCTGCTTTGCGGATC
GAAGGGGGTTTGGCAAGAAGTTTTGGCCAGGCATTGATCCCATATAAACCCATCCCGC
GGATTACACGGGTGTGTGCGCGTGTTTTGGTCGGAGGCGAGGCAAAAAGGGCCCCAA
GGCAA

>novel_circ_002044
GTGCGGCACAACTTCGTTAGCAGGGTGGACAGAGGAACGGAACGTGTCTGGAGCGGC
TTGGCGTTTTGAACGAAGGTACCAAAAAACGAACGAAGGAGAACTCTGAGATATCAC
CGTCTTTAACCAACCCCCCTCGTGTACAAGTTTCAAAACAACAACTCGATCATTCTCCC
AAAATTCATAACTCGCTCTCATTATTAATAGGTTCTCTAGTAGTAGTAGTGCTCCAGTTT
CTTTCTTTTCGTTTCTGTAAACGAATAAAAATAAATGGGATATCCGAGTGTGATATAAAA
CAACGGGCAGGAGAAAAAGAAAGGAGTGGGAGGGAGAAGGGGAAGAGAGGGACAC
GAAGGAAGGAGTAATAATAATAATAATAATAATAATAATAATAATAATTCTACAAGTGATT
TCCAAGTGATGTCGGTGTGATACGAGTGTTTGCGTGTTGTACGAAAGTGTGTCGTGGA
GGGGGGAGGAGGAGGAGGAGATGTTTCGTGTATAAATAGAAGAGGAGAGAAAAAAA

AAAATCACCCATGTATACGTAAATAAGGACGTGTGTGTCGCAGTGTGTCCGAGTATTCG
AGTACAAAAGAAGAATATTACGATAAAAATAAATAAAACCAACACATAGAAAACCACG
ATAAGAAAGAAACGAGAGAGAGAGAGAGAGAGAGAAAGAAACCGAGCAAGAGTCG
TTATAGGGGGGGGAGAGAAAAGAGAGGGGAGATCCCTCGAAGCGTGAAAGACTCGA
AGGAAGGAGAAAGAGAGGAAGACTCGTGACTCGTGATAAACAATAATAGAAGAAAGA
AGAAGAAAAAAAAAAGGGGGGGCCGAGGTGGAATCAGAAGGTGGGCCAGGTGGGA
CAGTAGCATGAGTTCGTATTTCGCGAATTCGTACATCCCGGACCTGCGTAATGGCGGGG
TGGAACACCCGCATCAGCATCAGCAGCACTACGGTGCGGCCGTCCAGGTGCCCCAGC
AGACGCAGTCGGTGCAGCAACAGTCCCAGCAGGCCGGGGACCCGTGCGACCCGAGC
CTGCTACGCCAGGGCGTGCCCGGCCACCATTACGGGGCGGCGGGCAGCCAGCAAGAC
ATGCCTTATCCGAGGTTCCCGCCCTACAACCGGATGGACATGCGTAACGCGACGTATTA
TCAGCACCAACAGGACCACGGGAGCGGGATGGACGGGATGGGTGGTTACAGGTCGGC
GTCGCCGAGCCCTGGCATGGGCCACATGGGGCACACGCCGACCCCTAACGGGCACCC
GTCCACCCCGATCGTGTACGCGAGCTGCAAGCTGCAGGCGGCGGCGGTCGATCACCA
GGGGAGCGTGCTCGACGGGCCGGACAGCCCGCCGCTGGTCGAGTCGCAGATGCACCA
CCAAATGCACACGCAACACCCCCACATGCAGCCGCAGCAGGGCCAGCACCAGTCGCA
AGCACAGCAGCAGCATCTTCAGGCGCAGCAGCAGCACATGATGTACCAGCAGCAGCA
GCAGTCGCAGGCGGCCTCGCAACAGTCGCAGCCAGGCATGCACCCGCAACAGCAGCA
GCAAGCTCAGCAACACCAAGGGGTGGTCACGTCGCCGCTAAGCCAGCAGCAACAGGC
CGCGCCTCAGGGCGCGGCAAGCGCCAACCTACCGAGCCCTCTGTACCCGTGGATGAG
AAGTCAATTCG

>novel_circ_002045
GGAGGCGGCGGCACGTGCGCCGGAGCTGCGGGCCAGGTGGTGGAGCTCGTGCCGCTG
TTGAGGGGACAGCGGCGGGGGTGGGAGGCTGCGCGGCGGGCGGAGGGGCGGGCGAG
GGCAGGAGGAAGAGGAAGGGAGGATGCGAGGAGGCGGGAGGGGGGGCGAGCGCGG
CCGAGGAGGTTAGTCGCTCGCCGGTGGCGGATGGCGACGAGGCCTGCCGCGGCGACA
GCGCCACCATCGACTGCCGCCACCGCCGTCCTCACCTCCACCGCGCGCACCACCGTCG
CATGCACGCCCACCGTCAACAACACCGGGAACAACAGGGATCGCCGGATCGATGGGA
GGAGGAGGAGGAGGAGGAGCGGAGGGACGAGGCGGGTAGCGGGAGCGAGGAGCGG
CAGCTCGTCGCCGACGAGGAAGCCGTCGCCTCCCCCCCTCGCCGCCGGGAGATCCATC
GCCACCATTGCCACCGCTTGCGCCACCACTGCTACCACCACCACCATCACAACAACAA
CAACAACAACAACAACAATCATCGTCGCCGCCGTCGCCAACACCACCATCTTCGCCAC
CATCACCGTTGCCGCCACCACCACCACCACCACCACCACTACCACCACCATCACCACC
ATCACCACCACCACCATCACCATCCCCATCTCCACCACCGGCCCCGGGACGACAGACC
CCCCTGCGAACGGGACCAGCGGTGCGTGGACCGAGCACTGGCCCCTGCGCCCAGGGA
CGAGCGCAATAAAGCCGATGCCACCGACGAAAAATACCCTGCCACCGTCACTGCCATG
ACGAGGGGATGCAGGATCCCGCCCTACCTCGCCACGCTACTCATCCTGTCTTACACTCT
CTTCGGTATAGCAGACGCCTGTTCATCGCGGTCGACGCCGAAACCGCGGCCACCGACG
CCGACGCCTCGACCGAATATCACATTCCACATGTACACGTGTCCACCGGATTACGCCGA
GTACTACTGCTTGAACGGTGCCACGTGTTTCACTGTCAAAATCGTCGATTCCCTCCTTT
ACAATTGTTT

>novel_circ_002046

GGAGGCGGCGGCACGTGCGCCGGAGCTGCGGGCCAGGTGGTGGAGCTCGTGCCGCTG
TTGAGGGGACAGCGGCGGGGGTGGGAGGCTGCGCGGCGGGCGGAGGGGCGGGCGAG
GGCAGGAGGAAGAGGAAGGGAGGATGCGAGGAGGCGGGAGGGGGGGCGAGCGCGG
CCGAGGAGGTTAGTCGCTCGCCGGTGGCGGATGGCGACGAGGCCTGCCGCGGCGACA
GCGCCACCATCGACTGCCGCCACCGCCGTCCTCACCTCCACCGCGCGCACCACCGTCG
CATGCACGCCCACCGTCAACAACACCGGGAACAACAGGGATCGCCGGATCGATGGGA
GGAGGAGGAGGAGGAGGAGCGGAGGGACGAGGCGGGTAGCGGGAGCGAGGAGCGG
CAGCTCGTCGCCGACGAGGAAGCCGTCGCCTCCCCCCCTCGCCGCCGGGAGATCCATC
GCCACCATTGCCACCGCTTGCGCCACCACTGCTACCACCACCACCATCACAACAACAA
CAACAACAACAACAACAATCATCGTCGCCGCCGTCGCCAACACCACCATCTTCGCCAC
CATCACCGTTGCCGCCACCACCACCACCACCACCACTACCACCACCATCACCACC
ATCACCACCACCACCATCACCATCCCCATCTCCACCACCGGCCCCGGGACGACAGACC
CCCCTGCGAACGGGACCAGCGGTGCGTGGACCGAGCACTGGCCCCTGCGCCCAGGGA
CGAGCGCAATAAAGCCGATGCCACCGACGAAAAATACCCTGCCACCGTCACTGCCATG
ACGAGGGGATGCAGGATCCCGCCCTACCTCGCCACGCTACTCATCCTGTCTTACACTCT
CTTCGGTATAGCAG

>novel_circ_002047
GATATGCAAATGCAAAAATTTATAAATGTGATAATGAGAAATGTCCAAGACCAGGATGT
TATATATCTGGAGGATCAAGCAAAGATGATTCATTTCCTTGTTTACGACCAGTTTGCTCT
GGACGATTTCAACTTGTACGTCATGTATCCTTTGTTGATTGTCCAGGTCATGATATTCTTA
TGGCAACTATGTTGAATGGAGCAGCTGTCATGGATGCGGCATTACTTTTAATTGCTGGC
AATGAATCATGTCCTCAGCCTCAAACATCAGAACATTTGGCTGCTATTGAAATTATGAA
ACTAAAACATATTGTAATTTTACAAAATAAAATAGATTTAGTAAAGGAAGCTCAAGCAA
AAGAGCAATATGAACAAATTTTAAAATTTGTACAAGGTACAGTAGCTGAAGGAGCACC
TGTAATACCAATTTCCGCACAACTTAAATATAATATTGAAGTCTTATGTGAATATATTACA
AAGAAAATTCCAGTACCTTTAAGAGATTTCACTTCAGAACCGAGATTAATTGTAATTCG
ATCATTTGACGTAAATAAGCCTGGTTGTGAAGTAGATGATTTGAAAGGTGGTGTTGCTG
GAGGAAGTATTTTAAGAGGAGTATTAAAAGTGGGTATGGAAATTGAAGTCCGCCCTGG
ATTAGTATCGAAAGATAGTGAAGGAAAATTGACTTGTCGACCCATTTTTCACGGATAG
TGTCATTATTTGCTGAACAAAATGAACTTCAATTTGCTGTTCCTGGAGGTCTTATTG

>novel_circ_002048
GTGAATCAAATGTTTACACATTAGGCAATATTGGTGCACATAGAATTGTATGCACAAAA
CTACCAACTGTAGGTCATACAAGAGAAGCAATGACTGCAGCAGGAAATACAACTACCA
GATTATTAGGTACGTTTCAAAAGGTTGATTTTGTTTTCTTAATTGGAATTGGAGGAGGTG
TACCACATTACACAGATTATAATAAACATGTACGACTTGGTGATGTGGTTATATCTTATCC
TACTCCTTTAAATAATAAATATATCTATGTTTATTGTGAAAGTGCAAAGACAAATGAAAA
TGGTGATTATCATTTTGAAACCAAAGAATATTGTCCACCAAATTTATGCCTTCAAGAAAT
CGCTGCTAATCTTAAACATCAGTCGGAAAATGAATCAAATCCTCCATGGCAAACATACT
TGAAAGAAGGTTTAGATAATTTGACTAATCAAACAGAGCATGATTTTAAATCACCTCCA
CCAGAATCTGATAAATTATACATGGCTATTGGTGAAAGAGACGTTATTGAAGTTGCACA
TCCTACTGCACCTTCAGATGCGATATATAAAAGAATAAATGGTTGTCCACGAATTCATTT
AGCACCTATAGCATCTGGTAGATATATTGCACGTGATGATCAATTGAGACAGAAATTTGC

AGTTCGTTTTGGAGCTCTTGCATTTGATACAGAAATGGATGCTGTGGTCGAAAGTATTT
TAG

>novel_circ_002049
GTTAATCCAGGCAGTAAAGCGGCTCAACAGGGGGTGAGAGAGGGTGATTTAATTAGCA
GCATAAATGGAAGAACTACCAGGGATTTAACGAACAGTGAGGCGCATGCACTCTTACG
AAACGCCGGTGAACATTTGAAACTCGGTTTGAATCAGGAAAATATTGGCTCTCCGAAG
AGACGAATTTATAGAAGTAGTTTGCAGGAAAACACCACCACTGAAATTCTCACTAAGA
CGACGACGACGACGAGAACGACCACGACCATATCGAACACACGGATCCCATCCACAG
ACTCGAAGAAGAACGAAGCAA

>novel_circ_002050
GCGTTTGGTCACCTGGAAGCGAGCCACCCCCGAAGGAGCCGAGCCCCGAGCGGAAA
GAATCTGAAAAGGATGGTGGTATACCACCCATCTGGACACCTTCCAGCGCTGGAGCCA
GCCCAATCCCCGAGAAAAAGGAATTTCGACCGGTGCAATTCGAGAGCCCTACCCTGAG
CCGTAAGAAGCTAGCGCAGCAAGAAGGTACGAAGGAAACTCCACCACCTTGGGAGAC
GGAAGTGGAAAAGAAGGAAATAACAAGAAGTAGTTATGAGAGCACTAGTACCAGTTC
GCGAATTGTCAATTCCCACTCAGCACCGTCTCAGGGATTAAATTCATTAAGTTCAACGC
CTCGACTGCCACGTGCTCAGAATCCAACTATAACACTGCTTCAAAAAGCAAGAGAAGG
ACAATTGCCAAAGGGAGCAGCCTATCTAGAAGAGAACGAGGCAGAGAAGAAGCATCC
ATCCAGCGACGAGAAAGGCATAATTAGTCCAGGAGAGATCATATACACGGTGAAGAAA
GAATACGAGAGCGAGCCGGAAACGGAAAACGAACCGCCGAAGAAGATGGCCGACTT
GGGTCCTCGAAAATTCGAGGGTATCGGCCCAACGTCTAAGGAGGGTATTCCCCTTGTTT
TGAGATCGGAAGTCAAGGAGAATAATCAGACAAAGTGGTATAAGAAAATGTACGACTC
GTTGCATCGTGCAGACAGAAGCGATGACTACGTAACCATTCGTTACAAACAGAGAAGA
GGGACGAGATACGGTTACGGAAGTGGATATTTAAGCGAACCGGAACACCGGTTGTACT
CGGATCGATCCGCCACTTTGGACAATCGTCGACGCCTTCGTAACAAGGAAAATGATTTC
TTCACGTCGACTATGCCTAGAAATTTGAGGAATGGTACGTTGAAGTACAGTTCAGAAG
TTTATAAAAATCAGCCTGGCCGTATCGAGGACTACGAACCTGGACGATCTTCAATCGCC
GAAAAGGAAGCCAAGGAGTGGTGGGACGAGGTCATGGACATATTTGACGGGTGGTTG
AACGACAATGGACATCCTCAGGGTACAGAGATGGAGGATCTTGGCGACGCGCAGCTG
AGCCACCGCGCTCTCAGCCTCTCCTACCGACCGGAGACCGGTCGCAATCCGTTCGACC
AGCACAAAAATCGTCCCCCGCAAAGATTGAAACCGTACATGTCACACGCTCTCAAAGA
ATCGGGATACGAGAGTGATTCGACGCTGGTGTTCCGTCGGAAGGAGGACATCGGTCCG
TTGAGTGAACTCGAGCAAAGATTGGCCTACAAAACGGTCCAAAGTGGTGGCGACGTG
CCGCTTCATGGCCTTCGTAAACCAGCCCCAGAACGACCGAAGG

>novel_circ_002051
GGACGAGATACGGTTACGGAAGTGGATATTTAAGCGAACCGGAACACCGGTTGTACTC
GGATCGATCCGCCACTTTGGACAATCGTCGACGCCTTCGTAACAAGGAAAATGATTTCT
TCACGTCGACTATGCCTAGAAATTTGAGGAATGGTACGTTGAAGTACAGTTCAGAAGT
TTATAAAAATCAGCCTGGCCGTATCGAGGACTACGAACCTGGACGATCTTCAATCGCCG
AAAAGGAAGCCAAGGAGTGGTGGGACGAGGTCATGGACATATTTGACGGGTGGTTGA
ACGACAATGGACATCCTCAGGGTACAGAGATGGAGGATCTTGGCGACGCGCAGCTGA

GCCACCGCGCTCTCAGCCTCTCCTACCGACCGGAGACCGGTCGCAATCCGTTCGACCA
GCACAAAAATCGTCCCCCGCAAAGATTGAAACCGTACATGTCACACGCTCTCAAAGAA
TCGGGATACGAGAGTGATTCGACGCTGGTGTTCCGTCGGAAGGAGGACATCGGTCCGT
TGAGTGAACTCGAGCAAAGATTGGCCTACAAAACGGTCCAAAGTGGTGGCGACGTGC
CGCTTCATGGCCTTCGTAAACCAGCCCCAGAACGACCGAAGG

>novel_circ_002053
CCGTTCGACCAGCACAAAAATCGTCCCCCGCAAAGATTGAAACCGTACATGTCACACG
CTCTCAAAGAATCGGGATACGAGAGTGATTCGACGCTGGTGTTCCGTCGGAAGGAGGA
CATCGGTCCGTTGAGTGAACTCGAGCAAAGATTGGCCTACAAAACGGTCCAAAGTGGT
GGCGACGTGCCGCTTCATGGCCTTCGTAAACCAGCCCCAGAACGACCGAAGG

>novel_circ_002054
AATCACCGAGGCGTTATGTGGAGGGCGAGGTGACGATTCACTATCGTTCACCAGTGCG
TACAGAGGCGAAAGAGCCATTGAGCGAGGAAGAGCTCGCTCGTCGAAGCGCGGAGAA
TATGCGACGCGTTTATCAAGAGGAACGGCGTCGCAAATATCTCCAGGAGCTCCACGAC
ATCGATTCGAGGAGGCACACCGACAATTTCATACCATCGCAGAAATCACCGATCCCTTT
GAACCGTTACGACGATTTCGTGGATGATCTGAGCCACAGAAGCCGATCCCAGGAGCAA
ACGCCGGAACCTCGGCTCGTGGCAAGGGCGCTTTACAACTTTATCGGCCAATCTTGCC
GGGAATTGAATTTCCGTCGCGGTGACATAATATTCGTGCGTCGCCAGGTAGACAAGAA
CTGGTACGAGGGTGAGCATAATGCTATGATCGGCCTGTTCCCATCGAATTACGTCGAGA
TTTTGCCCTACGACGGTATGCGAACCACACCAAAGAAACCATACGAGGGACAAGCAC
GAGCTAAATTCAACTTCGTTGCTCAAACAAATCTTGAGTTATCTTTGGCCAAAGGAGA
ACTTGTCGTCTTGACGAGAAGAGTCGATGAAAACTGGTATGAGGGGCGTATCGGAAAC
AGAAAAGGGATATTCCCCATCTCTTACGTCGAAGTTATCACAGAGCCTGGCCTCAGATC
AGAAACGCCAACACAAAACAAACCAGTTGCCGCTCCAGCTGCGCATAGCCTTTTGGC
AAATGGATCAGCTGGAGGGAAAATGAGTATGGGACCTCATCATTATATGCCGTCCATAC
CAGTTAATATGAACACAACTCAGCCACACTATAATTCACTGCCACGAATGGGAGGAAA
CAAATTACATGTTTCCGAAGCACTACACATCGACACACACTCTGAACCAATACCATATC
GAGCTCTCTATAATTATAGACCACAAAACGAAGATGAACTTGAATTGAAAGAAGGCGA
CACGGTATACGTAATGGAGAAGTGCGACGACGGTTG

>novel_circ_002055
CTGTGTCAGATATTATGAACTCTCTGGATCCAAAATTTCCACTGTATAGAATTGGTAATT
TACCAGATAAAAAGACAAATTTAAATGATAATGATTTGGCTGTCCTTATGGCTAATGTTT
CTTCGGCTCCACCACTTCTTCAAGAAAAAGGTTCTTATAATGATCAATTAGTGTATATTT
TCACTAGTGGTACAACTGGCCTTCCAAAAGCAGCTGTTATAACAAATTCTAGGTTTATG
TTTATGGCAGTCGGTATATTTATGTTAGCAAAGTTTAAAACTTCAGATAGGATTTATACG
CCATTACCCTTATATCATACTGCTGGTGGTGTAATGTCTATTGGTGCAGCCCTTCTTCATG
GAGCTACAGTTGTAATAAGAAGAAAATTTTCAGCAAGCGCTTATTTCATTGATTGCATC
AAATATAAGTGTACAATTGGTCAATATATTGGAGAAATGTGTAGATATATTTTAGCTGTAC
CTCCAAAACCGGAAGACAAACAACACAATATCAGGATAATGTTTGGAAATGGTTTGAG
GCCACAAATATGGCCCGAATTCGTAGAACGATTCAATATTCCACAAATTGCTGAATTCTA
TGGAGCAACAGAAGGCAATGCTAACATTGTAAATGTCGATAATACTGTTGGAGCTATTG

GTTTTGTATCTCGAATAATTCCATCAGTTTATCCTATTTCCATTATAAAAGTTGATGCTGA
TGGAGAACCTGTAAGGAATGCAAAAGGTTTATGTCAAATATGTGAACCAAATGAACCA
GGTGTTTTTGTTGGAAAAATTATACCGAACAATCCATTTAGAGCATATTTGGGATATGTG
GATCAGAAAGCATCTGAAAAAAAAATAGTTCGCGATGTATTTGCTAAAGGCGATTCAG
CATTTATATCTGGTGACATTTTAATAGCAGATGAGTTCGGTAATTTATTTTTTAAAGATAG
AACTGGGGATACATTTAGATGGAAAGGAGAAAATGTATCTACATCTGAAATTGAAGCTA
TTATTAGTAATCTTATTAATTATAGAGATTGTATTGTATATGGAGTTGAGATTCCAGGTCTT
GAAGGTAAAGCTGGAATGGCAGCAATATATGATGAAAAAGCTACATTAGATGTCCATCA
ATTATCAATTGATTTAAAAGAACATTTAGCAAGTTACGCAGTACCTAGGTTTATTAGGAT
TTTATCAAAGATTGATCTCACAGGCACATTTAAATTAAAAAAAAAAGATCTTGTAGCTG
AAGGATATAATCCAAATATAATAGGAGATAAAGTTTACTATTTAAATGAAAAGTCTGGAT
ACCAATTGTTAACTGCTGAAATTTATGAGCAGATTCAACAAGGAAATATTCGTCTTTAAT
ATGTTTTGAAGTAAAAAAAATTCATGGGAACAATTTTTGCTGTAATTCTTTACATGCACA
TCAATAAAGTGAAGAAGCTTTTTATCTTCATTATATTAAATCAAAAATTACTTTATATTGA
G

>novel_circ_002058
ATCCGGAACCGGTCACATCCATCCAAGCCATAGACATCAACGACACGAGGATCACGCT
CACTTGGGATATACCCCGTGGACAGTACGACGCTTTCGAGGTTCAATACATAAACACGG
AGGGGAATTACATTCAGAACATCACCACGGTGAACTCGATAACTATCTCCGATTTGAAA
CCGCATAGAAATTACACTTTCACATTGGTGGTCCGTTCCGGGACCGCATCCTCTTATCTG
AGAATATCGAATCCTCTGAGCGCGAGTTTCACCACGAGCGAGTCCTATCCTGGAAAGG
TGGAGAAGTTCCATCCCACGGATATTCAACCGAGCGACATCAGCTTCGAGTGGTCGCT
ACCCAGCCAAGAGCAGAACGGCATCATAATTAAATACACCATCACCTACGGTTTGGAC
GGCTCGAACCACACTCAGATGCAGGATTTCAAGCCGAACGAGTACCGCGGCGTGATCA
AGTCTCTGATACCTGGAAAGACGTACATGTTCAGGATCCAGGCGCAGACGAGGATAGG
GTTCGGGCCCGAGGCGGTGTGGAAGCAGAAGATGCCCATCCTCGCTCCTCCTAAACCG
CCCACGCAGGTGGTGCCGACCGAGGTGTGCAGGAGCAGCACGACGATACAAATACGG
TTCAGGAAGAATTACTTCAGCGAGCAGAACGGAGCCGTCACGTCCTACACGATCATAG
TCGCCGAGGACGACAGCAAGAACGCGTCTGGCTTGGAGATGCCCAGCTGGAGGGATG
TTCAGGCTTACAGTATCTGGCCACCGTATCAGGTGATGGAGCCTTACTATCCTTTCAAA
AACGGGTCCGTGGAGGATTTCACGATCGGCAGCGAGAATTGCGACAACAAGATCGGAT
ACTGCAACGGCCCGTTGAAATCTGGATCCACGTACAGAGTAAAAGTTCGCGCGTTCAC
CGCCCCGGACAAATTCACGGACACGAGTTACAGTTTCCCGATTCAAACAG

>novel_circ_002059
GGCTCGAACCACACTCAGATGCAGGATTTCAAGCCGAACGAGTACCGCGGCGTGATCA
AGTCTCTGATACCTGGAAAGACGTACATGTTCAGGATCCAGGCGCAGACGAGGATAGG
GTTCGGGCCCGAGGCGGTGTGGAAGCAGAAGATGCCCATCCTCGCTCCTCCTAAACCG
CCCACGCAGGTGGTGCCGACCGAGGTGTGCAGGAGCAGCACGACGATACAAATACGG
TTCAGGAAGAATTACTTCAGCGAGCAGAACGGAGCCGTCACGTCCTACACGATCATAG
TCGCCGAGGACGACAGCAAGAACGCGTCTGGCTTGGAGATGCCCAGCTGGAGGGATG
TTCAGGCTTACAGTATCTGGCCACCGTATCAGGTGATGGAGCCTTACTATCCTTTCAAA
AACGGGTCCGTGGAGGATTTCACGATCGGCAGCGAGAATTGCGACAACAAGATCGGAT

ACTGCAACGGCCCGTTGAAATCTGGATCCACGTACAGAGTAAAAGTTCGCGCGTTCAC
CGCCCCGGACAAATTCACGGACACGAGTTACAGTTTCCCGATTCAAACAG

>novel_circ_002060
GGCTCGAACCACACTCAGATGCAGGATTTCAAGCCGAACGAGTACCGCGGCGTGATCA
AGTCTCTGATACCTGGAAAGACGTACATGTTCAGGATCCAGGCGCAGACGAGGATAGG
GTTCGGGCCCGAGGCGGTGTGGAAGCAGAAGATGCCCATCCTCGCTCCTCCTAAACCG
CCCACGCAGGTGGTGCCGACCGAGGTGTGCAGGAGCAGCACGACGATACAAATACGG
TTCAGGAAGAATTACTTCAGCGAGCAGAACGGAGCCGTCACGTCCTACACGATCATAG
TCGCCGAGGACGACAGCAAGAACGCGTCTGGCTTGGAGATGCCCAGCTGGAGGGATG
TTCAGGCTTACAGTATCTGGCCACCGTATCAGGTGATGGAGCCTTACTATCCTTTCAAA
AACGGGTCCGTGGAGGATTTCACGATCGGCAGCGAGAATTGCGACAACAAGATCGGAT
ACTGCAACGGCCCGTTGAAATCTGGATCCACGTACAGAGTAAAAGTTCGCGCGTTCAC
CGCCCCGGACAAATTCACGGACACGAGTTACAGTTTCCCGATTCAAACAGGATTGCTG
CTGGCAGATAAGGACAATACGGCTATCATAGTTGGAGTCACGGTCCCGATAGTACTTTT
ACTGGCTTTACTTGGTATCGGGTTATTGGTCAGGCGGAGAAGAAGTCAGGGACGGAAG
ACGACCGAAACGAGGACCACCGACAATTTGTCCCTACCTGACAGCGTGATCGACACG
AGTCGGCCGATCAAGATAGAAGATTTTTCTGAACATTATCGGACGATGTCGGCCGATTC
CGATTTCCGTTTCTCGGAGGAGTTCGAAGAGTTGAAGCACGTAGGAAGGGATCAACCT
TGCACAGCCGCCGATCTACCGTGCAACAGGCCGAAGAATCGTTTCACCAATATTTTACC
CTACGATCACAGCAGATTCAAATTGCAACCCGTGGACGACGAGGAGGGTTCCGATTAC
ATCAACGCCAATTACGTCCCCGGGCACAATTCTCCGAGAGAGTTCATCGTCACCCAAG
GTCCTTTGCACTCGACGCGTGACGATTTCTGGAGAATGGTCTGGGAAAGCAACAGCAG
GGCGATCGTAATGCTGACCAGGTGTATCGAGAAGGGAAGAGAGAAGTGTGACCATTAT
TGGCCAGTCGACACTCATCCCGTCTATTACGGTGACATTTGCGTAACTATATTGAACGA
GACGCATTATCCCGATTGGAGCATCACCGAATTTATGCTGTGCAGAGGAGACGCGAAA
AGGGTGATCCAACACTTCCATTTCACGACGTGGCCGGACTTTGGCGTTCCAAATCCTC
CTCAAACGTTGGCCAGATTCGTTCGGGCGTTTAGGGAGAGAGTGAGGCCGGATCAGA
GGCCGATCGTGGTCCACTGCAGCGCGGGAGTCGGCCGCAGCGGTACGTTCATCACGTT
GGACAGAATACTACAGCAGATCCTGGTGTCCAAGTACGTGGACATATTTGGGATAGTGT
GGGCGATGAGGAAGGAGCGCGTGTGGATGGTCCAGACTGAGCAACAGTACATTTGCAT
ACACCAGTGTCTTCTGGCGGTGTTGGAGGGCCAGGACATGACTGGCCCGCCGCGAGA
GATTCACGACAATCAGGGATTCGAAG

>novel_circ_002061
ATAAGGACAATACGGCTATCATAGTTGGAGTCACGGTCCCGATAGTACTTTTACTGGCTT
TACTTGGTATCGGGTTATTGGTCAGGCGGAGAAGAAGTCAGGGACGGAAGACGACCG
AAACGAGGACCACCGACAATTTGTCCCTACCTGACAGCGTGATCGACACGAGTCGGCC
GATCAAGATAGAAGATTTTTCTGAACATTATCGGACGATGTCGGCCGATTCCGATTTCC
GTTTCTCGGAGGAGTTCGAAGAGTTGAAGCACGTAGGAAGGGATCAACCTTGCACAG
CCGCCGATCTACCGTGCAACAGGCCGAAGAATCGTTTCACCAATATTTTACCCTACGAT
CACAGCAGATTCAAATTGCAACCCGTGGACGACGAGGAGGGTTCCGATTACATCAACG
CCAATTACGTCCCCGGGCACAATTCTCCGAGAGAGTTCATCGTCACCCAAGGTCCTTTG
CACTCGACGCGTGACGATTTCTGGAGAATGGTCTGGGAAAGCAACAGCAGGGCGATC

GTAATGCTGACCAGGTGTATCGAGAAGGGAAGAGAGAAGTGTGACCATTATTGGCCAG
TCGACACTCATCCCGTCTATTACGGTGACATTTGCGTAACTATATTGAACGAGACGCATT
ATCCCGATTGGAGCATCACCGAATTTATGCTGTGCAGAGGAGACGCGAAAAGGGTGAT
CCAACACTTCCATTTCACGACGTGGCCGGACTTTGGCGTTCCAAATCCTCCTCAAACG
TTGGCCAGATTCGTTCGGGCGTTTAGGGAGAGAGTGAGGCCGGATCAGAGGCCGATCG
TGGTCCACTGCAGCGCGGGAGTCGGCCGCAGCGGTACGTTCATCACGTTGGACAGAAT
ACTACAGCAGATCCTGGTGTCCAAGTACGTGGACATATTTGGGATAGTGTGGGCGATGA
GGAAGGAGCGCGTGTGGATGGTCCAGACTGAGCAACAGTACATTTGCATACACCAGTG
TCTTCTGGCGGTGTTGGAGGGCCAGGACATGACTGGCCCGCCGCGAGAGATTCACGA
CAATCAGGGATTCGAAG

>novel_circ_002062
ATAGAGCAACGATTATCCAGCAAATAAATAATCACGCGACGTAAATGAGCGAAAACTCA
TCGGAGTGGAAGATCATCGAGAACGAAATCCTGATAATCGGCCTTAACCGATCGAGAA
AGTTAATAATTTCCATCCCGTGGTAGAAAATAAGAGGAAACGATCGATGAGAGAAAAA
AAATAGATCGAGAAAGGGGGAATCGATTTCAACTCAGCAAGAGGGGGAGAGGAGAGG
AGAGGGAGGGGGGGAGAAAAAGAAGAAAAAGAAGAAAGAGAGGAAAACGATGCCG
GTTCTAACCATGTTCTAGGAAACGAAAAAACATTTGTGTTGCATACTCGGGAACAATGT
TCGGCCAAGTGACGTGAAAAAGATACAATACACTTTCAAATATATATACACGTCTTAGC
ACACATCGTGGATTAACTGTAACAAAGATTTCTAAGTGGCGATAAACAGAGCACGAATT
ATCCAATTATCCTTAATTGACGTGAATTTTAACAGTGAAACACACGGGAGGGGGAAGC
GATTTCAGCAACATGCGTTATCTCCTCGTGTGATCTCCAACGTGATGTTCGTTATAATATT
AACTCGTGTAACTCTCTGATATTGAATATACATGCGTGTTGGGAAATCTTGAAAGGAAG
GAGGAAAAAAAAGAAGAAGAACGAAGAAACAAGGAAAGAAAGAAGAAGAAGAAG
AAAGAAAAAAGAAAGAAGTCACGTTCACAACGTGGTTGATCACAATTGTTACGAAATT
AAAAGTGGACGGACCGTTCGTAAAGGATCAGGTTGTTGTTGATTGACGGGCAAATTGT
GCGAACACGATGAAATTCATAGGATCGTTGTTGGGGATCAGCTCGCCCAAGAGGATGG
GCAAGGAGTACATGAAGGTGGGGAGGAACGCCCTGTTCGGCGTAAGGACGAGCCAAC
ACGACGGCCTCTACACGGACAAGGTCCCTAGACCTTTGTCCTTCCTCGTTTCTAAGAAT
GGGAAACTGAAACACG

>novel_circ_002063
GTACATAGACACGCAGCGTGGCCGCACACACGATAAGGCTGATTTCGTTGTCCTCGAG
GGAAGAATGAAAAGGAGACGAAGAAGGAGCCGGAGAAAACGAGAACTAATGAGCGG
CGCGGCTGATCCCGCAGTGGCGAGGTCGCGAGGGATCGTGGCGTGAGACGAGACAAG
AGGAGAGGGAGGGAGAGGTATAGAGAAGAGAAATTTGTCGAGGGTGGAAGGAAGAA
AGAAAAAGGCGGAAAGGAAGGAAGGAAGGAAGGAAGGAAGGCGTGACGAAAGAGG
AGAGCGAGTAAAGCGGAACGGGGCGGAAGAGAGAGGAGATTCCTGGATCGTGAGGC
GGGCGCGTGTGCAACAGGCGATTTCTGACCGATTCATCTTGGCAGGTTTGCAGGTGTT
CGCGTGCGTCCTTCGTGGCCTCGTTAACGCGGGCTTCCTCTTTCTCTAGCCTGGCCCCT
AGCTCCTTTTATCGTTTCCTTCGACCTTGGTCCGGATCCGGGGAAAATAATAGGCAACG
GTTCCGAATAGAGACGAAAGAGACGAATACAACGGTATACGCACGCGTATATATACATA
TATACACACATATATATATAAAAGGAAAGAAGAGCGAGGTTGTTGCGAAGGCGAGGAAT
AGAAGAGGAGGAGAAGAAAGGAGAGAAGAGGAAAAGAAGGAGGAAAGTCGAGGG

GAAGAAGAAGAAGAAGAAGAAGAGAAAACGTGGGGAGACGGAGAGAGAGAGAGAG
AGAGAGAGAGGAAAATCGAGCGAAAGAGGGAAAGGAGGAAGGAAGGTGGGACAGG
TAGAGAGAGAGAGGAGAGGGGGGGAGCAGCGCGAAAGAGGAGAGGGAGAAGAAGA
GGCGCGTGGTGGTCGTGGTGGGTGCTCGGTAGTGGACGGACGAGAGCGAGCGAGCAT
CCAGATCTGTCGCGTGCTGCGTCGCTCTGGGATGGGACGGAATCGGGACGACACGACC
ACGACCACGACGATCGCAACCACGACGATCGCAACAGCGACGACAATAACGACGACG
AGGAGGTCGACGACGACGACGACGACGACGACGACGGCGACGAGGACGGGACGAG
ACGAGACGAAACGGGACGAAACGGGACGACGACGACGACGGATACCACTACACGCA
CTCCGGGATGGGATGGATGTTGCGGCCCCGTGTGAGTAGCAGCGAGTCGAGTCGATCC
CTGGTGCGCGCTGGTATTGGTTTCACTCTCGGCTGCTGCCGGCCCGTGTGCTGGTGTGT
CGATCTCTCTACGAGAGCGAACCGAGGAGCGTGGCCGAACCGAGCCGGAGAAGAGA
ACCGCGGCACTCGTTCAGTAGTTTCTCGGACGCCGCACCGGCACCATCCGACCTACCA
GATAACCCCGTCGACTTACCGCCGACTACCCCCGACCGATCGACCGACCACGCAACAT
CGCACACCTTCCCACCACCACCTTCGATAATATATAATACATATATATATGTGTATGTATAT
ATTTTATATATACGTGTATATATATATATATAGAGAGAGAGAATAATTTCGATCCGATTTTC
GGCCTCTCGACGCGTCTCGAAGTGGAGGAGGAGGAGAAATATCGTCGGTTGTGGAAG
ATCGTATCGGCCACGAGGGGGATTGGAAAAAGTAATCGCGATACGGCGACGGTCGCGT
GACGACGACGACAACGACGACGACGACTCTCCACCTTGGCCTTTTTTTTTTTTTTCCC
CTCTCCTGCCGATATTTAACGCGTGCCTATGTACACATAAATACGTACACGCGCGTATAC
GCATGTACATACGTATACACATATACGTAATAGCGGTGTGGTGTGATTTTTTTCCTTCCCC
CCTTCGAAGCTCCAGAAGAGTGTGACACGCGCGCACCTCGTTTTCTTTTTTTCCCTTTC
CTTTTCTTTTCTCTCTCTGTCTCGTCGTCGTGTCGCGCTCGTTCGATTGTTTATCAGCAG
GCCCGGCCCCCGGTGTGGTGTGCAGCGCGCGGCGGAGGGAAGGATCTCGCGGTGAAT
AGCGGATCTTTGCCAGGTGTTTTCTCTTCGCGCGTTTATATACGCAGTGAAACGGACCG
TGTGTGTAGTACGCGTGCCTTGTGCGTACAAGTAAGTGTGCCTTCGCGAACGTAATTTA
CCGCGCCGATTCCCCGGTTCGCGCGAGGGGGAACCAAGTGGTGGAACAAGAAGAAG
AAGAAGAAGAAGAAGAAGAAGAAAGTAATAAAGGAGAAGAAGGAAGAGAGAA
GAGGAGACGGAAGAGGAGAAAGGAAGAAAGAGGAGGAGTAGTTAGCCGATAGTAGT
TACACAGTTCGGCGCAGTGTTCGTTCCTCGAAGATTAGTGGGTGATATCTGCCTCCTTT
CTTTCTTTCTTTCTTTCTTTTTTTTTTCTTTCTCTTTCCCGCCCCCCTCCCCTCCCAACTC
GAGGATATTTCGCGCGATCGCGACCCTTCGCCTCGACTCCGGAAGGATACTCCGCGGT
CTCCCACGGACGGCAGGAGAGAGAGAGGGAGAAAGAGGCAAGTGAAAGAGAAATCG
TGATATAGACGAAGACAAGGAGAAAAAGAGGGAAGAGGAGGTGGTGGTGGCGGCGG
TGGTGGTGGAAGAGAGGGAGAAAGAGAGAGATAGAGAGAGAGAAACCCGTTAAGGC
GGTACGAGTAACGTGTCGGTGGAAGTTCTTGCGATTGATAGAGGGACTGAGAAAGAA
AGGAAGGAAGAGAGGGAGAGAGAGAGAGAGAAAGGGACGGAGCGAGGTCGACGAA
GGTAATCGCGGTGGCATCGAGCTGCGTGCCTTCGACAAAACCGATTAAAGACGATCGA
GGCGAAGGATCGATCTCCATCTATCGCCCTTTCGCCGACTCTCGCGGCCGAAACGACG
ACGACTACGACGACGCGTTCCCGGCGACGTTTCCACGGCTCCGCCACCTCGATGCTGC
CCCCTCGTAAACCGATCCCGTGGTTCCCCATCTTCGCTAACAAACCGTTCTGACTGCCG
CCCCCCGCGCAACAACCGCCTTCCGAACCGCGCGTCCGCACGACGAAAACGAGGAGG
ACGACGCGTTGCGAAGAAAGAATCAGGCCCCGTTGATAGCCCCGCAATAATGCGAGAC
TCGAGCGGGAAACGCGGTGCATGGACGATAAAGGCGGGCCGGCCGGTGCATCCTCCT
CGCTGCTGCGCATCCGTCCACGGTATCTCGTTCAAGGCGAAAGTGGAAATCGAGGATG

AGGTGGACTCGTATCCAGCCAGCGAATAATAATAATAATAATAATAATAATAATAATAATA
ATAACGATAACGACAACGGTAATAATAAGGATAATAATAATAATAACAATAAGAAGAAG
AAGAAGAACGACGATAATATCTCGTTGCCCCGCGATCGGCCGCGATCCGTTTCTACGAA
TCGTCGTCGAATCAGCAACGTCCCCGACGCGTTTTGACCGGCCTTCCCCCACCCGGCC
CCCGTCCCCAGGACGGAAGAAGGAAGGAAGGAAGGAAGGGAGGAGGGAAGAGAGG
GAGCGAGAGCGAGCGAAAGGAGAGATCAGTGTAGGGAGATCGGCGAGGAAGATCGG
CGGCAGGCGGTGGGAGGAAGCGGAGCGTGGATAGATAAGAAGAAAGGAGGGCTCGG
AAAGAGGAGTGTGGATGAGAGCGTCATGCCCGCTGAGATCGACACGGGCGGGCCAAG
GGTGCTGCACTGTGATTACCGTGCCGACTGCCCGATACAGAGGATCGTAGTAGCTTCCG
CCATCGAGCCTTCCTCTTCCTTCCGCCCCGAGGAGAAGAGGAAACGGAAACAGCTCG
TTCCAGGGGGGGCCCGGATCGTGCCCAGATCTTGCAACGCCCGCGAAAACCACGAGC
CACGCGATCCTCGTGATCCTTGCTCCCCTGCCTCCTCCACCTCCTCTCCCTCCTCTCGCC
CGATCGCGTGCCACGCCGCGCTCCAATGCGATCCTTGCCTTCGTCGTCGTCGTCGTCGT
CGTCGTCGTCGTCGTGGAATAATTCTCGACGACGGCGACGACGAGCGGAACCGCCGCC
GTCATCCCTCACCGTCCGACCACCACCGATCATCGTCACCCGATAACGCGACCACGTCT
GCGATTCGATGCGAGGTATCGTCGTCGGATCGCCGTCGCCCACTCCGTTCGTATCATCG
TCCTCGTCGTCCTCCTCGACGGCGAGATGCCGTCGCGTTCGAGTCCATCCGTCGTCGA
GATCCTTCGGTAACGGGGGAGAGGAGGAGGAGGAGGAGTTCGTTGGATCGAGGCGAA
ACGAAGCGGAGGAGACTCGAGGAAGAGAAGCAAGAGGAGGAGGAGAAGGAGGAAT
CGAGGATCTTGTTCTGTTCGGGGAAACGGGAAAACGAGGCGACGACGGGTCAGGGTG
GTCGATACTGGCATCCGGAGGAGGAAGAAGAGGAAGAAGAAGAAGAAGTGGAAGTG
GAAGAGGAAGAAGGAGAAGGAAGGTGGTGCTCGAAAGGACGCGAGGAGCCGGCGT
GGAAGAGCAGTAGTGGTGGTTGTAGTGGTGGTGTTGGTGGTGATGGTGGTGGTGGTG
GTGGTGTGGTCGCGAACCGTGGTGCACGGTTCTCTAGTCACTGGAACGAGGCTTGGAC
GTACGAGGAGGAGAAGGAGGAGGTGGAGGAGGAGGAGGCGGCGAGGAACGGCACC
GCGTTTCTTCCGTCCTTGGCCGATGAAACGTGGAAGGAAGTGGAAGTCGCGACGAATT
CCTCGAGCGGTGAGTCGCGCGTCGCCAAGGTCGTGGCCGATCCCCGCCAGACCGACT
TTCCTTCGACGTTGTGGAAGCACGAGCACGGGTCCTTGACGACCACGACGACGACCA
CGACGACGACGACGACGAAGACGACGAAGACGAAGACGACGACGACGACGACGAC
GACGAGGAGCGAGCGTAGAACGAGGAAGAAGAAGAAGAAGAAGAAGAGACGCGCG
TGCACCGCGGCCAACAACCGTTCCACGTACATTCAGTTAGCGTTGCTCCTGTTCCTCGT
CGCGTTCGGCCAGTGCGCACTTCGTCGATCGGCCGGTGTGTTGAACAACAATAATTGC
AGTGCGAGACCGAGTACGATCGGGTTTAGTGTGTTGGCGATCGGGACGGTTATCAGTG
CAGTGAGTGCCGCGCCCCTCGATTTGACGGGCGACGCCGGGGTGAGGGCGGAACGAT
CGGCCAATTTGTCCCACATCACGGGCGCCTCGAGGAAAATCCAGATGTATATCAAGAAT
CGCCACTTGCAGATCCTGCCCGATGGCACGGTCAACGGTTCCAACGACGACACTTCGG
ATTACA

>novel_circ_002064
CGCGAGTACACGGACGATTGCGTGTTCAACGAGACCCTGGAGCAGCACAACTACAAC
ACGTACAGCTCGGCGAAATGGTCGACGGCCAAGAAAACGTTGTACCTCGGATTGAAC
CGTCACGGCCAGCCGAGGAGGGTACAGGCCAAGGGTCACAATCTCGGCAGGCTGTCC
GCCTACGCGCGGGTGTTGACTCAGGTCGCGCCCCTGGACCGGGTGGAGACCCTCCAA
AGGCGGATGCTCGGCGCCCAGCACAACGTCCGCCACCGTCACAACACCCACCGCGGC

GACCTCATCCAGCAGTCCCTCTGCCCCGCCCTCCCGGCCCAGGAGAAGGACGGCCGG
GACAAGTTCAGGTGTCGCAAGCGGAAAAAGAGGAAGAAGAGGAAGCGGAGGTGCAG
GCCGGGCGAGAAGCCGGGCCCCCAGTGCCAGATAGCCGAGTCGAGCAAGCCGGTCCC
CCCGGCTAGGAACCAGAGCCTAGCCGCGAACGGGACGTCCCCGATCCAGTCGAAGAG
ATCGTGCGAGGGTTTGGCGAGCGAGGAGGCCTGTCGAAGGGAGGCCCTCTCGGTCCC
CTCCAAGAAACGGAAGCTGCGGGTCGAGGAGATTGCGCCCGACGGAGAAGAGGAGA
CAGAAGGAAGAGAAGGTGAAAGAGGAGAAGGAGGAGGAGGAGAGAGAGGAGGAA
AAGGAAAAGGAGGAGGAGGGAGGAACGTCAGCGGGAGCAGCAACG

>novel_circ_002065
CGAGTCTTATCGTAATGCACTACAAGCTTTCATAGATCGAAGACGATCTTCCAACGACG
TGGAGATTGAGACATCGAACAGTCCTGATAAATTAAGAGCCGATCTTTCATCGAAAAAT
AATAACAATAAATGCGATAATAAATGGAAATCACAAGAATTGAAATCAATTATACCAGG
TGTACCACATATAGCAGCTCCTGAAATTTTTGAAATTGGATGGATAGCCGGCACAGAAG
ATCGATATCTAGAATTAATTTTTGCCGAATGGCAAAATACTCGAGTTTCACTGAAGAGG
CATTCACATCCGGAATGCAAAGATGCTGTGAAAGCAGATTTAGATATACTTTCAGAAAT
TCGACACCCTAACGTGCTATTGTTAATGGCCACCACCTTTACTGACGATCATGGTTTAGT
CTCTATTTTTGAATCTGTCGATTGTACCCTTTATCATTATATGCACGATCAGGGTGAACGA
ATATCCATGCAAGGTATTGCAAAATGCGGGGGAAGACTATCCGATGCTTTAAGACATTC
TCATATGCGCGGCTATGTGCATACTGCCATCAGTTCGCATTGCGTCTATCTGGCTTCTAAT
GGTATCATAAAACTAGGTGGATGGGAACTGGCCATGCATATCAACAATCCAAAACCAA
AGAGAGAATACGAGGAACGTTTACGTGCCGAAGTTTTCCGCTGGCAAGCGCCAGAAC
TTTTTCATGGTTACGAACCATATAAGGAGAGCGATGTCTATGGATTGGCTTTATTAATTT
GGGAAATGTGTACAA

>novel_circ_002067
GTGCAAACGTGAGATCACCCGGGAAAACCTCCCCGGCGGGGATGATTGGAAGCAGAG
AGGGAACAGGAATTGGTAGAACGATGAAAGAATACGAAGATCAATTGGAGGCTCTTAA
AAAGGAGAACTTCAACCTTAAATTACGGATCTATTTTCTCGAGGAGCGAATGGGGATTA
CGTCGGCCGATGAGAACGCGATAAAGAAAAATATTGAACTGAAGGTCGAAATTGAATC
GTTGAGGAAAGAGTTGGTCGAGAAACAGGAACTTCTCAGCCAGGCAGCGAAAGCGTT
TGAGTTAATCGAGGAACAGAAGGAAGTTTCATCGCGGAATCAAGCTCAGTATCAACAA
TCGCTTGAAAATGAACGGGAAAAAATCAGAAAACTCGAGAAGGAATTGGCCGAATAT
CAGGAAAAAATGGTTGATGCGTCCATCTATTACAAGGAAGCTTTCGGAATTACGCCGG
AAAAGGCGTTGGAAAACGAGGAGAAATTGCATCAGATGGAGGAGCTCGTTGCATCTCT
AGAAGCTGAGGTGAAACAGGTGACGAGCAGTTTGGACGAGGAACGCGTGTGGGCGC
AAGAACTGGAGAGCGAACGGGATGAATTCAGGGAACGTTTGGAGGCTGAGACCCGTT
TGAAGGAGAATTTGGACGCGGAGAGGCAGGAGGACATCGGGGCGCTTCGGTTGAGAG
TAAAAGAACTGGAGGAACATCTGTTGAAAAGGGACACTGTCGTGCAGCAGTGCAAAA
ACGAGCTGCTCGAGAAAGAAAGAGTGATTAAGGAAAAAAATTTACAGCTGGAGGAGA
GGTGTCGGCTGTACGAAGAGTTGAACGCCGTTTCGGAGAAAAGGAAGAAGCAAGTCG
ATCAGTTGAGGGTTTCTATAAAAACCAGGGACGATGCCCTCACCGACTTGAACAACAA
ACATCGCGCCCTCCTCTCTCAATTCGAGAATGGATATGCGAAAAGATCATCACCCCCTA
ATAGTCCGTCGACCGTGAATCCAAACGAAGATCCCTTGCAAATGAGAATGGGACAAAA

GGTAACTGTGCAAGGAATGACGAAGAGAGTGAACACTTGTCTCGATTGGGAACCAAA
TCGAGAAAGGTCAACTAGAATGAAGTCGCCGGTGCATGGTTTAAGTGGAGAGATCAA
AGACGTGAGAGATTTAATCAAGGAAATAGAAGATAAAGAAAGCGAATTGAAACGTCA
AGAGGAGACGAGGAAGCAGTTGGTGTTGAAATTGTGCAACATGCAGAAGCACGTAGA
GACGACCGATTACAAGTTGAAAAAATTGGAGGGCGAACACGAGAAAGCGATCAAGAC
TATTCAAGGTTTTATGGAGAGGCAGCAGCAATTGGAGAACACGAAGCTGAGGAAGGA
GCAGAAAATCATGGAGTTGGAGATCGAGTTGAACAGGCTGAGGGAGTGCGAGAGCTT
GAAAGCCTCGAGGGATGGCCACAATCAATTCGTTCGAAGAGACTTCAGCACCGACATG
ACGGATGATCCAGAGCGTGATCCCAGCACACAG

>novel_circ_002068
CACTGGCAGATCCCGTTGGGGAGAAGATTCAGATCGTTAAAGCTTTGGTTCGTCTTGA
GGATGTACGGCGTGGAGAATCTTCAACGATACATCAGAAAACACGTGGAGCAGGCTCG
TGAATTCGAGGCGATGATCCTATCCGATCCACGATTCGAGATCGTGGCCGAGGTCGTTC
TAGGGCTCGTCTGTTTCCGGTTAAAG

>novel_circ_002070
ATAGTCGGATGCGGGCTCGCACGGTAGGCCAGGTTCTCCCGGTATACCCGGAACACCG
ACCGGTCCTTGAAGGCCCGGCAAACCAGGCGCGCCGACAGGTCCCGGCAAACCTGGC
AAACCGACGGGCCCTGGCGGCCCTTGAAGCCCTATCGCACCTTTCACTCCGTTCGATC
CCGGGAACCCTGGCGGGCCTGGAATTCCTTTCTCTCCCGGTGGTCCTGGCGGGCCGAC
CGGTCCTGCGCGAAAACCGAACGGATGCAAAATCTCGATCCGAGATGATCCGTCGTTT
CTTATTATTTTTTTATTAACATAACGTACCGGGGAAACCGCGATCTCCTTTCGGTCCCAT
CGGTGGCTCGATGCAGTCGTCACCCTTGTCGCCTTTCTGACCGACGTATCCAACCACGC
CCATCAATCCCACCGGACCTGGATACCCTCTGTCACCTTTCTCTCCGACCGGACCCGGC
AACCCGCTCACGCCATCCCAACCTCTATCGCCCTTTTCGCCGGGAATTCCATCTATTCCG
CTCGGCCCTGTATCATTAGGTATTGCTCGAGTATTATTAAAGTTTCCAGCAATTCGTGTAT
CTATTTTATAATCTATTACGCGCGATTCTTAATCAATATACGAATTTTTCTTTTCATTTACC
AGGGAATCCAGGTTCTCCCTTCTGCCCAATGGGACCGGGTGGTCCACGTAAACCAGCC
TCTCCTTTCTCGCCTCTCTCGCCCGGCAAACCGGGGAATCCTTTGACGCCTGGTTGGCC
ATCGCGACCCGTTAAACCGGGAGGACCCATGAGACCCATATCACCCTTTTCGCCCGGC
AACCCGACTGGACCTGGCGGCCCTCGTTCTCCCTTGTGTCCCGGTAAACCTGGCAATC
CTTGCTCCCCTTTCTCTCCCGGTCGTCCCGGTATTCCTTCCCTTCCATGCTTTCCAGGCA
TACCCGGCAAACCTTTCTCGCCCTTGATCGTCAATCCAGGGACTCCCGTAGGCCCGCGT
GGACCTTCGTCTCCTGAAACGATATTTGTCGCGATCAAGTAACGAAAAGAACGAAAGA
CGAAAGCGTTGATCGTTGGAAACGAGTATTTGAGTATCGTCACCTTTAGCACCGGGGA
GACCGGATGGACCTGGATTTCCAACGGGACCAGGCGCGCCGCTATCCCCTTTCTCGCC
GTCGTAGCCACTCAGACCTTCCAACCCGCGATCTCCCTTTTCTCCAGGCGCACCTCGTA
AACCGGGAATACCCGGTTCGCCTTTTTCACCTTTGATCGAAATGGTCGGGCCGGGCGG
TCCACGATCGCCTTTCTCGCCGATCGCCCCGGGAATACCAGGAAGTCCAACGGCGCCT
TAACGTCACATATATATGTCATTATATACGCTCGAATAGAAGTTAATCTTCTCTTTTTCGA
CTCGTTTACCTGGAATACCGCGATCCCCTTTGGCGCCGGGGATTCCAGGATGTCCCGGT
GCCCCCATTTCACCTTCCAAACCGGGCAACCCAGGAACTCCGGGTGGACCGACGTCG
CCTTGTTCACCTGGCAATCCGTTGATTCCGGGCAGTCCAGGCTCGCCGGGAGCCCCGA

TCGGTCCCTGAAAATCGAAACAGAAACATCGATCGATTTCTCGGAACTTGGCTCGAAA
ACGAGAGAAATAGTGATCCAGCGAAAGGGACGTACAATGTCTCCTTTAAAACCGGGG
AAACCTCTCACGCCAGCGTCACCTTTCAGTCCTTGGAATCCTGGCAAACCGTCACGAC
CTGGCGGCCCGGGCTCTCCCTTGGGACCAGGCAGACCATCCCGGCCGATGCCTGGCAA
CCCAGGCTCTCCTTTCGCCCCGTCCACGCCATTTCTGCCAGGGATGCCGGGAGGACCG
CGGATCCCAGGCATTCCCGGCTCACCCTTCTGCCCTTTCTCCGATACCTGACCCGGTTC
ACCTTTACGTCCCTGGATTAACCATCATCACAGATGTGAAAAATCCGTAACTTTTATTAA
AACGTGAAACTTCCACTAAATTTTTACTAAATCGTGAAACTTAGCAGCTTACCGGTAAT
CCCGGCGGACCTCGATCACCTTTGAATCCTTCGTATCCTATAACACCTGGGAGGCCCGG
AGCGCCGGGAGGACCCAAGTCCCCTTTCATCCCGGGTGGTCCAGGACGACCGGGTTT
CCCTTCGATCCCGGGCAATCCGGGGAACCCGGCCGACCCTTTCTCGCCACGTTCTCCTT
TCATACCAGGCGCACCTTCCAGGCCGGGTCTACCAGTCGCGCCCGGCAACCCACGCCT
TCCCGGATAACCTTTCGATCCGTCGATGCCCGGCTCGCCTCGATCACCCTTCACGCCTG
GAAATCCCGGAAGACCCGCCCTTCCACCTGCCCCCGGCAATCCCGGTTCACCTACGCA
GAAATATTACGTTTACATCTCGTAAATTAAAACTTTTTCTTTAAGTTTACCTTTGTTTCCG
TCTTTGCCGGGTAATCCCGCGATACCGGGAGGACCAGGAGGCCCGATGGGACCGGTAT
CTCCTTTCTCCCCTGGCGTGCCGGGCAACCCGCTTATCCCCGGCTCACCCGTGTCTCCC
TTCTCCCCCACGAATCCCGGCAAACCTTGCAAACCGATAGGACCGTCTTTTCCCGGCG
GCCCGGGAAGACCTTTCTCGCCCTTGTCGCCCGGTGGACCAGGCAAGCCAGGGCTAC
CCTTCACCGATATCCCAGGCTCGCCCTTTTCTCCTTTCTGCGCTGGAGCGCCCGGGAAT
CCTCTGGGTCCTTCGCGACCAGGTTCCCCGGGGAAACCCATCGGACCCGTGTTACCCT
TGTCGCCGCGCGGACCCGGCAACCCTCTCGGCCCTACCGGCCCAGGCGGACCGATTTG
TCCAGCGGGACCAGGCGGACCCTATATCATAATGTGATGTTTTTACATTCCTTTGTGTCT
TGGAAGGAAGATTAGGTAAGTAAACAATAATAATGGAATCGATTCGTACCGCGAATCCT
TGTAAACCTTTGTCACCGCTCAACCCGTCCCTGCCCGGTGGGCCTGGCTCTCCCTTCTC
GCCAGGCGCGCCTCTGTCTCCCTTGCTGTTGATCAAATCGAGCGGTGGACACTGGCCT
ATTTATTTAAAGATAATTTAGAAGTAATGCAAGAAGATATTTGGAGATGTAGAATATATAT
AATATTGGATAAGAGAGGATGAGGGGTATTTACCGGGTTCTCCTTTTTGTCCGTACGAA
CCAGGTTCACCCTTCAAACCTATTTGACCCGAACGGCCCGGTTCTCCAGGCTCCCCTTT
CACGCTCAGACCACGCTCTCCTTTTCGACCTGGAATACCTGGCCTTCCAGGAACCCCG
TCTTGGCCCGGAAATCCTCTGTCACCCTGTTGCGACAACAAGTGCGTACAAACATGAA
GAACGTTGGAGGAACGAGCGAAAATAGGAGAAGAGACTAGCGTTTATTACTTACAGC
GGTACCCTTTGGACCCGGGAAACCAGCAAATCCTATAGCGCCCTTCTCCCCGACTGGA
CCTTGGGGACCCGGTGGACCTGGCCTTCCCACCTCTCCCCTTACACCTTTGTCGCCCTT
CTCCGTTTTAATCATGTTGGAC

>novel_circ_002071
GATTCCCCGGTTTGCCGGGCGAGAGAGGCGAGAAAGGAGAGGCTGGTTTACGTGGAC
CACCCGGTCCCATTGGGCAGAAGGGAGAACCTGGATTCCCTGGGCCGAGCGGAATAG
ATGGAATTCCCGGCGAAAAGGGCGATAGAGGTTGGGATGGCGTGAGCGGGTTGCCGG
GTCCGGTCGGAGAGAAAGGTGACAGAGGGTATCCAGGTCCGGTGGGATTGATGGGCG
TGGTTGGATACGTCGGTCAGAAAGGCGACAAGG

>novel_circ_002073

GGTTTCCGTGGCTCTCCTGGATTGCCGGGTATGCAAGGTGACGTGGGCGACACGGTGT
ACGGTATAAAGGGCGCGGAGGGTGAGCAAGGAGACAGGGGCGAACCTGGGCCTCCG
AGTAACGGTGAGTTCAAGGAAAAAACGAAGGTGACGCCGGTGAAGGGCGCCACCGG
CCCCAAGGGAATGCGGGGAGACGCGGGGGAGCGAGGAAGAAAGGGAGAAATGGGCG
CCAAAGGACCTATCGGTCGGGCAGGATTCTTCGGTATCAAAGGTACGAAGGGGGAGCA
AGGTGACGACGGACCAAGGGGTAAGCAAGGTGCGGAGGGTCCGCCAGGACCCGCGG
GAGAGAAGGGAGAGAAGGGAGCGCCGGGATACGCTGGAAAACCGGGACCGGACGGA
TTTGACGGAGAACTGGGAGATGGGGGAAATCAAGGACCACCGGGTAAACAAGGAGCT
CCTGGAGAACCAGGAAAATACATACCGGAATTGGACGAAATAATTCAAGGAAATATCG
GGATTCAAGGAGATTTAGGCCCACCCGGTAATCCAGGAGAACCAGGAACACCAGGTTT
ACCCGGCAAAGTAGGTTATATAGGGCCGCCTGGTCCTCCAGGACCCCCCGGCTCCTCT
GGGTTACCAGGATTGAAAGGATCTTCCGTAAAAGGAGAGCCAGGAGACGATGGTTTTA
CCGGAGAAATGGGTCTGCAAGGTCCCCCGGGTGCGCCGGGTATACCTGGAACCGTTGG
GCTTAAAGGATACCCTGGATTAACTATAGTTGGACCACCAGGTCCCGATGGAAAACCTG
GGTATCCAGGTGTGCCTGGATTGCCTGGAGATCGGGGTGATCCCGGTGTCCAAGGTCC
AAAAGGTTTTCCTGGCAAGGGGTCGAGGAATCCGGGTCCTCCGGGTGAGCGTGGTTT
GCCAGGTATACCAGGACTTCCTGGACCCGATGGGTGGCCAGGTTTACCAGGTCTGAAA
GGCGTGAAAGGCTTTAAAGGAGACGATTGCAATTGTCCGCCAG

>novel_circ_002074
GAAACGCTAGCAGTACTTCGGAATTTGTAAGGCAAGAATTGAGAAGAGCGATCGTTGG
GGCGCGAACGCAACAACAGCAGCAACAAAGGATACCCAACAACATTCAGAACAACTT
ATCCGGACAAGTGTCGCAAGATGATCTCGAAGCACTCGGTTTAACGTTTGAAATGTCTT
CTGCAGGTGAGGCTGTGGTTAGCGATGGCCCTGCAAAGAGCTGGGCCATTGGGAGTAC
CGGAAGTGCCCCCTCGTCCTCCAGG

>novel_circ_002076
ATTCGGCACATTAGGGAACAGGAAGGCTCGAATAGTCATGCCGTGCAACAGGGGGAA
GTATCTTCTTCGAATCCTAACATATTGTCCAACGATCAAGTTGGTCCTATTTTACTTGAG
GCGCTAGATGGTTTTTTGTTCGTTGTAAATACGGAAGGACGAGTGGAATATGTAACAGA
TAACATAACGCAGTATATAAATTATACGAAGGATGATGTTCTTGGCAAGGATATTTATAAT
ATTATTCATCATGGAGATCATAACACCTTTATGCCGAGTTTGTTGCCTATGCAATTAGGCT
GGACGAACGAGCAACAGCCCCAAAGAAACCGCACGTTCAATTGTCGCTTCTTGGTGA
AGCCTCCTGATGATAAAGAAGAGACTATGGAAGAGAAGCAGCAACGAGTATCGAAATA
CGAATCTATGCAAATCTGTTCAGCTCTTTTGCCAAATAATAGCGATCGTCTGGAGAGCG
GTGACGTATCTTCCGAATCTTCGGACAACGGTCCTTGCGTAATGTGCGTGGCTCGTAGG
ATACCCCCGAACGAAAAGCCCATTGGTACGCCCATCGAGCAATTTACCGTCAAGTTGG
ACACCACGGGGAAGATTATCGCGGTTGATGTTATCTGGTTGTCGTCTCCTTACTCTGAG
TACCTAAGCAAGGTAAGCAAGGAGCTGATTGGCACTGCGATAAAGGATTTGTGCCACC
CTCATGATCTCAATAAATTAACAGCACATTTGAACGATACGCTTCAAGTCGGTGAGAGT
ACCAGTGGTGTATACCGATTACGCGTTAGTCCTGATAAGTTCCTTAACATTCAAACAAA
GTCAAAACTTTTCAAAGCAAATGTGATGAATACACTTGTTACCGACTTCATTATGGCTA
CCAATACTATTATTGG

>novel_circ_002077
TAACAAGTGCCTTAACGAAAAAAGACGACGGAACCAGGAGAACCTGTTTATCGATGA
GCTTGCCGAGCTGATTTCCGCCACGGACATGAGCTCTGGCAAGACTGACAAATGCCAG
ATCCTTCAGAGAACCGTCGACCAGGTCACGTGACCTTGAATGACGTCACAGAGGCCAA
TAGGAAACCTTCCAAACGGCGTTATAACCCGAACTACCGGAGCAAGAGTTCGTGTGAC
GTCATCTGCGCAGTCGAGCCATGCGCATCCGTCTGTGTCCAACGACGTGACCGAGATT
CGGCACATTAGGGAACAGGAAGGCTCGAATAGTCATGCCGTGCAACAGGGGGAAGTA
TCTTCTTCGAATCCTAACATATTGTCCAACGATCAAGTTGGTCCTATTTTACTTGAGGCG
CTAGATGGTTTTTTGTTCGTTGTAAATACGGAAGGACGAGTGGAATATGTAACAGATAA
CATAACGCAGTATATAAATTATACGAAGGATGATGTTCTTGGCAAGGATATTTATAATATT
ATTCATCATGGAGATCATAACACCTTTATGCCGAGTTTGTTGCCTATGCAATTAGGCTGG
ACGAACGAGCAACAGCCCCAAAGAAACCGCACGTTCAATTGTCGCTTCTTGGTGAAG
CCTCCTGATGATAAAGAAGAGACTATGGAAGAGAAGCAGCAACGAGTATCGAAATACG
AATCTATGCAAATCTGTTCAGCTCTTTTGCCAAATAATAGCGATCGTCTGGAGAGCGGT
GACGTATCTTCCGAATCTTCGGACAACGGTCCTTGCGTAATGTGCGTGGCTCGTAGGAT
ACCCCCGAACGAAAAGCCCATTGGTACGCCCATCGAGCAATTTACCGTCAAGTTGGAC
ACCACGGGGAAGATTATCGCGGTTGATGTTATCTGGTTGTCGTCTCCTTACTCTGAGTA
CCTAAGCAAGGTAAGCAAGGAGCTGATTGGCACTGCGATAAAGGATTTGTGCCACCCT
CATGATCTCAATAAATTAACAGCACATTTGAACGATACGCTTCAAGTCGGTGAGAGTAC
CAGTGGTGTATACCGATTACGCGTTAGTCCTGATAAGTTCCTTAACATTCAAACAAAGT
CAAAACTTTTCAAAGCAAATGTGATGAATACACTTGTTACCGACTTCATTATGGCTACC
AATACTATTATTGG

>novel_circ_002078
GATCGGCAAGATTACGAAGAACTGTAACAAACTTCTAGAAGGATGAAAAAAATTGTGG
AAAACTCAAAATTCCAGCAAGACGATTCCCAAAAAATATAATAACCATCAGATAATTGG
CAATTGAGATGTCCTCTGCTTAGTATCTTGGTGAGAATTGCCTGTCATTGAATATTTATA
AGCGATTCCAGATTTGGAGGATAAACAGAAAACCTGAGATCGTTCCTGAATAGTAGCA
ACGACAAGTTAAAAAAATAAAATATCTAGGATTTCTATTTTTCGCCAGTAGATTACGCAT
ACGGTTAACAAATCTGTCTATATGTACGTGAATAAGTGAGAAAGTGGCGTGTCGAAAA
GTGAATCTGATGTTCGAGGGCGAAAAGGAAACGACGACAGTGACGGTGCTCGGTCGA
TAAATAGAGTTATATAAAGTGCAGAAAGAAGATCAAAGAAGAAAAATGTGTACTTAAT
CTTGATTTTTTTCGCGAGTGATTTTACGTTGGTGACGTGAACAGTGTCTATTGTAAAGA
ACATCCCGAATGTCGGTAAATAAAGAAGCATACGATTGAACGATGCAATTAAGAACGC
GTGTTCGATCAGTGAGTGTCGGTCGAGAAAAAAAAACCTATTCGTTTTGATTTGATTTT
TTTCTTATTTCGCATAGATTCAAACGGGTGACGAAACAAGAAATAATTTAGAGTGAAAT
GAATTATTATTTTTTCCATTCGTCATTTTGTAAACTCCCGTTATGTACTTGATTTTGATCAT
AGTTTGGAATTTGACGTAAAAAGAAAAGAAAAGAAAAAAAAGAAGCAAAGTTTTCGC
GTTGAACGGAAGAAAGGAAAGATTTCGAAAATTTGTCGCATTGGAAAAGTGTTAAGTT
GGTGATAATAGTGGGTGACGTTTATTGATTCGCAGTGGTGATATCTTTATTATTTGATATC
CATATTATTGGTTGTAAAGGTATGGTGAAGAAAACAGGTTAGTGGTGATAGTGGCATAT
CGCGTAGAAAGAGAACACTCGACTGTGACTCTTAACTCATTACCACGGATTTCTCCCG
GCAGTTTGACACAACAGCCATCCTCGATGAGAGGAGGCACATGTCCATTGCTGCCGCT
GAAAATGCAGGGCTCGTTCGAGAACGATGCACGGGCCTTGGACTGGAAGTTCGCTAC

GAGTCTCTACGCGGCGGCGGCGGCGGCCGGTCGATAATCGTAAGGAGGAAAGGAGCG
AAGGGCAGCGATCGCGTATGCGGTTGGATTTGAACAGAGGAAACACGCGACGGTGGC
GTATGCGGCGGTGGTGAGCGATGATCGCGAAGAGAGGGAAGTTGGATGCGAGCGCGC
GCAGTGAGTGGCGCTGCGTTCTGGAGAAGATAGGAAAGGAGAGATGGAAGAACGGA
CGGGCAGCGCGTATTGGCAACGAGGAGAAGAGTGGAGAGGAGCATTGGGTCCGTGAC
AACTGGGTGACGGCTGCGTGGATACACGACGGTGGCAGTGATGCAAGTGTTCTTCTAC
GCGATAAGTGGTGACGTCGGCAGAAACGAGCACCGGCTTCTTGCCGCGCTGCGGGTG
TGCTCCTTTGCCTCTCTCGTCTTCCTCCTCCCTTCTCTCCTCTCTTTTCACTTCCGTTTTA
CCTTGGTCCGTCTCGTCCGTTCGATCGACCCGCTCTCTTCCTTCCACTAGTTTCGCGTC
GTCTATCTCGCGCGCGAGTGCTTGTGATAAATCGTTTCGCGGATACGATGATGTGCGTTT
AACGAGGAATATCGAGGAATGGAAAGTTGGTTTCTAGGTGTCGTCCACACGTCTTGTT
GGAGTCTGTCGTGGGACCTCCGTGAGGATGCTTCTCGGAGAAAACTTTCCATTGAATTA
TATTTTATTTTTATCTTATCGTGCCTATCCATTCCGATATCTTTTCACCGTGATCGTAAGAT
ACATAAATTATTAAACGTTTTATTCGAAGAAATGGCGTTATTACGACAACACGCTATCGG
CACCAGGCCTAGCCCGCGTGAATTACAGGACCCGCTTTGGGTCAAAATGAGTGCGATT
ACTGGCAGCATCAGCAAAAAAAGAAAGAAATCAGATGCCAAGCCTCAGTCGCAAATT
AACAAGTGCCTTAACGAAAAAAGACGACGGAACCAGGAGAACCTGTTTATCGATGAG
CTTGCCGAGCTGATTTCCGCCACGGACATGAGCTCTGGCAAGACTGACAAATGCCAGA
TCCTTCAGAGAACCGTCGACCAGGTCACGTGACCTTGAATGACGTCACAGAGGCCAAT
AGGAAACCTTCCAAACGGCGTTATAACCCGAACTACCGGAGCAAGAGTTCGTGTGAC
GTCATCTGCGCAGTCGAGCCATGCGCATCCGTCTGTGTCCAACGACGTGACCGAGATT
CGGCACATTAGGGAACAGGAAGGCTCGAATAGTCATGCCGTGCAACAGGGGGAAGTA
TCTTCTTCGAATCCTAACATATTGTCCAACGATCAAGTTGGTCCTATTTTACTTGAGGCG
CTAGATGGTTTTTTGTTCGTTGTAAATACGGAAGGACGAGTGGAATATGTAACAGATAA
CATAACGCAGTATATAAATTATACGAAGGATGATGTTCTTGGCAAGGATATTTATAATATT
ATTCATCATGGAGATCATAACACCTTTATGCCGAGTTTGTTGCCTATGCAATTAGGCTGG
ACGAACGAGCAACAGCCCCAAAGAAACCGCACGTTCAATTGTCGCTTCTTGGTGAAG
CCTCCTGATGATAAAGAAGAGACTATGGAAGAGAAGCAGCAACGAGTATCGAAATACG
AATCTATGCAAATCTGTTCAGCTCTTTTGCCAAATAATAGCGATCGTCTGGAGAGCGGT
GACGTATCTTCCGAATCTTCGGACAACGGTCCTTGCGTAATGTGCGTGGCTCGTAGGAT
ACCCCCGAACGAAAAGCCCATTGGTACGCCCATCGAGCAATTTACCGTCAAGTTGGAC
ACCACGGGGAAGATTATCGCGGTTGATGTTATCTGGTTGTCGTCTCCTTACTCTGAGTA
CCTAAGCAAGGTAAGCAAGGAGCTGATTGGCACTGCGATAAAGGATTTGTGCCACCCT
CATGATCTCAATAAATTAACAGCACATTTGAACGATACGCTTCAAGTCGGTGAGAGTAC
CAGTGGTGTATACCGATTACGCGTTAGTCCTGATAAGTTCCTTAACATTCAAACAAAGT
CAAAACTTTTCAAAGCAAATGTGATGAATACACTTGTTACCGACTTCATTATGGCTACC
AATACTATTATTGG

>novel_circ_002079
ATTCGGCACATTAGGGAACAGGAAGGCTCGAATAGTCATGCCGTGCAACAGGGGGAA
GTATCTTCTTCGAATCCTAACATATTGTCCAACGATCAAGTTGGTCCTATTTTACTTGAG
GCGCTAGATGGTTTTTTGTTCGTTGTAAATACGGAAGGACGAGTGGAATATGTAACAGA
TAACATAACGCAGTATATAAATTATACGAAGGATGATGTTCTTGGCAAGGATATTTATAAT
ATTATTCATCATGGAGATCATAACACCTTTATGCCGAGTTTGTTGCCTATGCAATTAG

>novel_circ_002081
GCCTAGCCCGCGTGAATTACAGGACCCGCTTTGGGTCAAAATGAGTGCGATTACTGGC
AGCATCAGCAAAAAAAGAAAGAAATCAGATGCCAAGCCTCAGTCGCAAATTAACAAG
TGCCTTAACGAAAAAAGACGACGGAACCAGGAGAACCTGTTTATCGATGAGCTTGCC
GAGCTGATTTCCGCCACGGACATGAGCTCTGGCAAGACTGACAAATGCCAGATCCTTC
AGAGAACCGTCGACCAG

>novel_circ_002082
ATTATTCATAAATTACGAATGCCTCACATTGGTGACGAATTACATCATTCTGGTTGGAATA
TTTGTAGCAGTTGTCATAATAAACAACAAAAACGAGATACTCTGATACTTCCATGTCTA
ATGTCCGATCGTGTTTACTTCATTGATACAAGTTCTGAACGAACTCCATCAATCAAAAA
GGTATTAAGCTCGATAGAAGTAAATCAATATGGGATATCGACTTTACATACAAGTCATTG
CTTGCCAACTGGTGAAATTTTGATTTCTGCAATGGGTAAACCAAATGGAGATGCTATAG
GTGAATTTCTTTGCGTCAATGCAGAAACACTCGAAATGAAAGGTACCTGGACCATAGG
AGAGAAAAAAGCAGCATTCGGTTACGATTTTTGGTATCAACCATATCATGACGTACTCG
TTGCATCTGAATGGGCCCTACCTAGAGTTTTCAAAAAAGGCTATGCAACAACTGATATA
GCCGATCCTGCAATATATGGAAGAAGTTTAAATTTTTATTCATGGAACGAAAGAAAATT
GAAACAAGTTTTAAATTTGGGTGAAGATGGAATTGCGCCACTTGAAGTACGATTTCTTC
ATAATCCAAAATCTAGCGAAGGATTTGTAGGATGTGCGGTTACTTCAAATGTATATAAAT
TTTATAAAACATCAAATGGAGAATGGGCTGCAAAAAAAGTGATTCAAATTCTTAATAAA
CAAGTGGAAGGATGGATTACTCCACAAATGCCAG
